# Inferring the Association between the Risk of COVID-19 Case Fatality and N501Y Substitution in SARS-CoV-2

**DOI:** 10.3390/v13040638

**Published:** 2021-04-08

**Authors:** Shi Zhao, Jingzhi Lou, Marc K. C. Chong, Lirong Cao, Hong Zheng, Zigui Chen, Renee W. Y. Chan, Benny C. Y. Zee, Paul K. S. Chan, Maggie H. Wang

**Affiliations:** 1 JC School of Public Health and Primary Care, Chinese University of Hong Kong, Hong Kong, China; jzl@bethbio.com (J.L.); marc@cuhk.edu.hk (M.K.C.C.); caolr@link.cuhk.edu.hk (L.C.); hongzheng@cuhk.edu.hk (H.Z.); bzee@cuhk.edu.hk (B.C.Y.Z.); 2 CUHK Shenzhen Research Institute, Shenzhen 518000, China; 3 Department of Microbiology, Chinese University of Hong Kong, Hong Kong, China; zigui.chen@cuhk.edu.hk (Z.C.); paulkschan@cuhk.edu.hk (P.K.S.C.); 4 Department of Pediatrics, Chinese University of Hong Kong, Hong Kong, China; reneewy@cuhk.edu.hk; 5 Hong Kong Hub of Pediatric Excellence, Chinese University of Hong Kong, Shatin, N.T., Hong Kong, China; 6 CUHK-UMCU Joint Research Laboratory of Respiratory Virus & Immunobiology, Chinese University of Hong Kong, Shatin, N.T., Hong Kong, China; 7 Li Ka Shing Institute of Health Sciences, Faculty of Medicine, Chinese University of Hong Kong, Shatin, N.T., Hong Kong, China

**Keywords:** COVID-19, SARS-CoV-2, N501Y substitution, B.1.1.7 lineage, case fatality, statistical modelling

## Abstract

As COVID-19 is posing a serious threat to global health, the emerging mutation in SARS-CoV-2 genomes, for example, N501Y substitution, is one of the major challenges against control of the pandemic. Characterizing the relationship between mutation activities and the risk of severe clinical outcomes is of public health importance for informing the healthcare decision-making process. Using a likelihood-based approach, we developed a statistical framework to reconstruct a time-varying and variant-specific case fatality ratio (CFR), and to estimate changes in CFR associated with a single mutation empirically. For illustration, the statistical framework is implemented to the COVID-19 surveillance data in the United Kingdom (UK). The reconstructed instantaneous CFR gradually increased from 1.0% in September to 2.2% in November 2020 and stabilized at this level thereafter, which monitors the mortality risk of COVID-19 on a real-time basis. We identified a link between the SARS-CoV-2 mutation activity at molecular scale and COVID-19 mortality risk at population scale, and found that the 501Y variants may slightly but not significantly increase 18% of fatality risk than the preceding 501N variants. We found no statistically significant evidence of change in COVID-19 mortality risk associated with 501Y variants, and highlighted the real-time estimating potentials of the modelling framework.

## 1. Introduction

The coronavirus disease 2019 (COVID-19), whose etiological agent is the severe acute respiratory syndrome coronavirus 2 (SARS-CoV-2) [1], poses a serious threat to global health and spread to over 200 countries globally [2]. As of 28 February 2021, over 110 million COVID-19 cases were confirmed in the world with over 2.5 million associated deaths [3]. The emerging mutation in SARS-CoV-2 genomes is one of the major challenges against the control of pandemic. Around September 2020, genetic variants carrying the N501Y substitution on the spike (S) protein of SARS-CoV-2 were first detected in the United Kingdom (UK) [4], then spread to elsewhere globally, and trended to reach fixation rapidly in many places, for example, South Africa [5], Brazil [6], and the U.S. [7]. In the UK, besides other variants, the 501Y variants, as well as Δ69/Δ70 deletion, were clustered into the B.1.1.7 lineage by COVID-19 Genomics Consortium UK, which is also known as the variant of concern (VoC) 202012/01 [8]. These emerging new variants are of public health concerns to affect the epidemiological characteristics of COVID-19 [9,10], as well as the effectiveness of vaccines [11,12,13,14]. 

Recent analyses reported evidence that the 501Y variants were associated with an increase in transmissibility of SARS-CoV-2 at population scale [15,16,17], and similar findings were also reported for the D614G substitution in previous studies [18,19,20,21]. By contrast, whether the N501Y substitution leads to a difference in disease severity among SARS-CoV-2 infections remains largely unassessed. On one hand, exploring the relationship between the mutation activities and the severity of COVID-19 outcomes is of importance to assess the risks posed by the evolution of SARS-CoV-2, which may inform case management strategies and the healthcare decision-making process. On the other hand, together with some recent scientific preprints, the relevant peer-reviewed literature found either no significant evidence or a weakly positive association between clinical severity and N501Y [17,22,23,24]. Concerning the heterogeneities in epidemiological settings and analytical schemes of these studies, the existing evidence appears inconclusive.

Classic epidemiological investigations, for example, cohort or case-control studies, using individual patients’ information with a large sample size may explore the evidence about the association between severity of COVID-19 and N501Y directly. A recent study adopted individual patients’ data from more than 2 million SARS-CoV-2 infections in the UK, and found significant positive association of B.1.1.7 lineage (which is dominant by N501Y substitution) and the fatality risk in terms of the hazard and absolute risks [25]. However, these samples of patients, as required for classic epidemiological study design, are financially expensive and difficult to collect in a short period, and commonly unavailable during the emerging stage of exposure variables, for example, the 501Y variant in this study. In this study, we adopt a statistical inference framework to assess the risk of COVID-19 case fatality using the disease surveillance data on a real-time basis. We reconstruct the real-time and variant-specific case fatality ratio (CFR) of COVID-19 empirically, and infer the change in fatality risk associated with N501Y substitution in SARS-CoV-2. 

## 2. Methods 

### 2.1. SARS-CoV-2 Sequencing Data and COVID-19 Surveillance Data

The SARS-CoV-2 strains were obtained via the global initiative on sharing all influenza data (GISAID) with collection dates ranging from 1 September 2020 to 31 January 2021 in the UK [26]. A total of 182,982 complete human SARS-CoV-2 strains were retrieved. All SARS-CoV-2 strains used for analysis are provided in the appendix. Multiple sequence alignment was performed using MAFFT version 7 [27], and the “Wuhan-Hu-1” genome (GenBank: “NC_045512.2”, GISAID: “EPI_ISL_402125”) is considered as the reference sequence. We excluded sequences with more than 5% ambiguous amino acids during the alignment, and a total of 149,789 sequences were included for further analysis. The acknowledgment table for the sequencing data used can be found in the Supplementary Materials.

**Figure 1:**
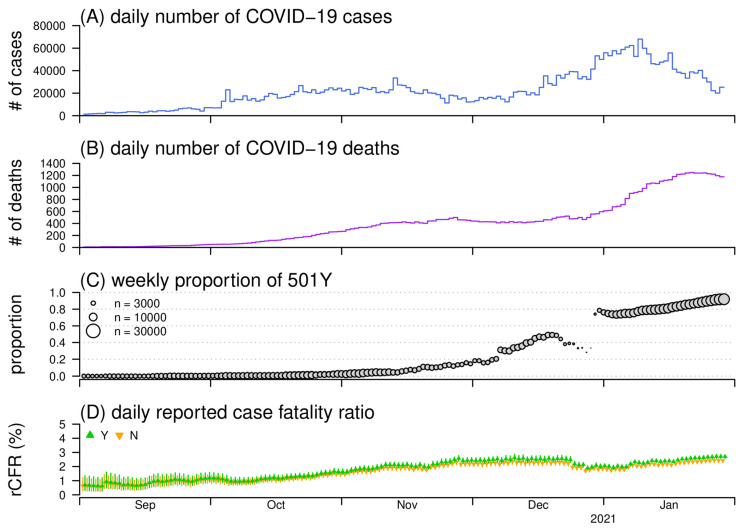
The daily number of COVID-19 cases (**A**) and deaths (**B**), proportion of the 501Y SARS-CoV-2 variants (**C**), and the reconstructed reported case fatality ratios (rCFR) (**D**). Panel (**A**) and (**B**) show the daily number of COVID-19 cases and deaths time series in the UK, respectively. Panel (**C**) shows the observed proportion of 501Y variants among all samples of SARS-CoV-2 strains, where the size of the dot indicates the weekly sample size. Panel (**D**) shows the estimated rCFR of 501N (in orange) and rCFR of 501Y (in green). where the dots are the point estimates and bars are the 95%CIs.

The surveillance data of COVID-19 cases and deaths in the UK were collected from the World Health Organization (WHO) COVID-19 surveillance platform [3]. Especially, to adjust for the weekly cycle in the COVID-19 deaths time series, the 7-day moving average was adopted to smooth the time series for further analysis. The COVID-19 outbreaks in the UK can be considered as two epidemics waves, and the N501Y substitution is observed during the second epidemic wave, which started in September 2020. To avoid the under-ascertainment due to reporting delays, we drop the observations in February 2021. Hence, the surveillance data of COVID-19 cases from 1 September 2020 to 31 January 2021 are included in the analysis, which matches the period of the SARS-CoV-2 sequencing data. The COVID-19 cases and deaths time series are shown in Figure 1A,B, respectively. 

### 2.2. Statistical Parameterization

#### 2.2.1. Reconstruction of the Instantaneous Case Fatality Ratio

We consider the time delay between the ascertainment of COVID-19 case and death. For an individual who deceased due to COVID-19, we used *f* to denote the probability distribution function (PDF) of the time interval (denoted by *g*) between being reported as a case and the death. To formulate *f*(*g*), we denoted the time interval between symptom onset and death by *s* following the PDF of *h*(*s*), and the time interval between symptom onset and being reported by *q* following the PDF of *δ*(*q*). Following the previous study [28,29], the *g* is the difference of *s* minus *q*, i.e., *g* = *s* − *q*, and we remark that *g* is not necessarily positive. Then, the PDF of *g*, that is, *f*(*g*), is formulated as in Equation (1).

(1) $$f\left(g\right)=\int^{\text{​}}h\left(g+q\right)\cdot \delta \left(q\right)dq.$$

For COVID-19, we set *h*(*s*) as a Gamma distribution with mean (±SD) at 20 days (±10) referring to [30,31], and *δ*(*q*) as another Gamma distribution with mean (±SD) at 7 days (±4) referring to [28]. Although the analytical formula in Equation (1) is difficult to derive, we approach *f*(*g*) by applying Monte Carlo methods, which is also adopted in [32]. Slight changes or similar alternative settings in *h*(*s*) or *δ*(*q*) will not affect the main results. 

Since each disease-related death is diagnosed as a case in the first place, each individual case is considered as a “source” (or “pool”) of the death, that is, subjects at mortality risk. We consider all reported cases as the pool to generate deaths, and we model this candidate pool as a time-varying function denoted by Φ(*t*) at calendar time *t*. In other words, if one case is reported at time *τ* who dies eventually, the value of *f*(*g*) is considered as the relative likelihood of death at calendar time (*τ* + *g*). Similar ideation was adopted to quantify the relative likelihood of transmission in [33]. Thus, the *i*-th case (out of all cases), who is reported at calendar time *τ_i_*, contributes *f*(*g* = *t* − *τ_i_*) to Φ(*t*) at calendar time *t*. For the contribution from all reported cases, that is, for all *i*s, the Φ(*t*) is summated as follows Equation (2).

(2) $$\Phi \left(t\right)=\sum_{i}f\left(g=t-\tau_{i}\right).$$

The information of *τ_i_* for all is can be obtained from the epidemic curve shown in Figure 1A. Hence, the instantaneous (or real-time) reported case fatality ratio (rCFR), that is, fatality ratio among reported cases, at calendar time *t* can be calculated by the ratio rCFR*_t_* = *d_t_*/Φ*_t_*. Here, the *d_t_* is the observed number of deaths, and Φ*_t_* is the discretized Φ(*t*) at calendar date *t*, which means $\Phi_{t}=\int_{\mathrm{day}\;t}\Phi \left(x\right)dx$. The value of *d_t_* can be obtained from the number of COVID-19 deaths time series as shown in Figure 1B. We remark that different SARS-CoV-2 variants are not distinguished at this stage, and thus the rCFR here is the overall (or average) case fatality ratio for all variants. 

#### 2.2.2. Variant-Specific Case Fatality Ratio

To incorporate the information of SARS-CoV-2 variants, we denote the proportion (or prevalence) of the variant of concern, for example, 501Y variant in this study, at calendar time *t* by *ρ*(*t*), which is time-varying. The *ρ*(*t*) can be calculated by using the SARS-CoV-2 empirically, which is shown in Figure 1C. Then, the relative likelihood of death due to the *j*-th variant of concern at calendar time *t* is formulated in Equation (3).

(3) $$\Phi_{j}\left(t\right)=\sum_{i}f\left(g=t-\tau_{i}\right)\cdot \rho_{j}\left(t=\tau_{i}\right).$$

Here, the index *j* = 0 for 501N variant, and *j* = 1 for 501Y variant. Straightforwardly, $\sum_{j}\rho_{j}\left(t\right)=1$, and $\sum_{j}\Phi_{j}\left(t\right)=\Phi \left(t\right)$ for all *t*s. 

We denote the variant-specific instantaneous case fatality ratio of the *j*-th SARS-CoV-2 variant by rCFR*_j_*_,*t*_, and then the expected number of deaths due to the *j*-th variant, E[*d_j_*_,*t*_], at calendar date *t* is modelled in Equation (4).

(4) $$E\left[d_{j,t}\right]={\mathrm{rCFR}}_{j,t}\Phi_{j,t}.$$

Here, the Φ*_j_*(*t*) is be discretized into Φ*_j_*_,*t*_ with the same fashion as for Φ(*t*). 

#### 2.2.3. Risk Ratio of Case Fatality Associated with N501Y Substitution

By definition, the case fatality ratio is a measurement that quantifies the mortality risk due to SARS-CoV-2 infection. Then, the difference in the variant-specific case fatality ratios characterizing the change of mortality risk associated with N501Y substitution in SARS-CoV-2. Thus, the risk ratio (RR) of N501Y substitution, denoted by *ζ* (≥0), can be defined as *ζ* = rCFR*_j_*_= 1,*t*_/rCFR*_j_*_= 0,*t*_. We consider *δ* as a constant, which reflects the intrinsic nature of the new SARS-CoV-2 variant and thus is invariant with time. Hence, we have rCFR*_j_*_= 1,*t*_ = *ζ* × rCFR*_j_*_= 0,*t*_ for all calendar date *t*, which subject to 0 ≤ rCFR*_j_*_= 1,*t*_ ≤ 1. Straightforwardly, *ζ* > 1 indicates that N501Y substitution is associated with an increase in case fatality risk, *ζ* < 1 indicates that N501Y substitution is associated with a decrease in case fatality risk, and *ζ* = 1 indicates that N501Y substitution is not associated with any increase or decrease in case fatality risk. 

Similar to Equation (4), we calculate the expected number of COVID-19 deaths, E[*d_t_*], at calendar date *t* in Equation (5).

(5) $$E\left[d_{t}\right]={\mathrm{rCFR}}_{j=0,t}\Phi_{j=0,t}+\zeta \cdot {\mathrm{rCFR}}_{j=0,t}\Phi_{j=1,t}={\mathrm{rCFR}}_{j=0,t}\cdot \left(\Phi_{j=0,t}+\zeta \cdot \Phi_{j=1,t}\right).$$

We remark that as defined, the condition for term *ζ* × rCFR*_j_*_= 0,*t*_ = rCFR*_j_*_= 1,*t*_ ≤ 1 should always be guaranteed. 

### 2.3. Likelihood-Based Inference Framework

To construct the likelihood profile, we model the number of deaths due to the *j*-th variant, *d_j_*_,*t*_, as a binomial process with sizes at Φ*_j_*_,*t*_ (rounding to the closest integer) and successful probabilities at rCFR*_j_*_,*t*_ for each independent Bernoulli trial. Then, the number of COVID-19 deaths, *d_t_*, follows a Poisson binomial process, *π_t_*(∙), which contains Φ*_j_*_= 0,*t*_ independent Bernoulli trials with probability of success at rCFR*_j_*_= 0,*t*_ and Φ*_j_*_= 1,*t*_ independent Bernoulli trials with probability of success at (rCFR*_j_*_= 1,*t*_ =) *ζ* × rCFR*_j_*_= 0,*t*_ for all *t*s. The expectation of *d_t_* is given in Equation (5). As such, for calendar date *t*, we construct the loglikelihood function *ℓ_t_* of the observed daily number of COVID-19 deaths *d_t_* using the Poisson binomial framework in Equation (6).

(6) $$\ell_{t}\left(d_{t}|\zeta ,{\mathrm{rCFR}}_{j=0,t},\Phi_{j=0,t},\Phi_{j=1,t}\right)=\log\left[\pi_{t}\left(d_{t}|\zeta ,{\mathrm{rCFR}}_{j=0,t},\Phi_{j=0,t},\Phi_{j=1,t}\right)\right].\;$$

The detailed formulation of Poisson binomial distribution *π*(∙) can be found in [34] as well as otherwhere in statistical literature, which is relatively lengthy and thus is omitted in this study. 

With Equation (6), we reconstruct the rCFR*_j_*_= 0,*t*_ time series, denoted by $\left\{{\mathrm{rCFR}}_{j=0,t}\right\}$, and estimate *ζ* using the overall loglikelihood function (*ℓ*) defined in Equation (7).

(7) $$\ell \left(\left\{{\mathrm{rCFR}}_{j=0,t}\right\},\zeta |\left\{d_{t}\right\},\left\{\Phi_{j=0,t}\right\},\left\{\Phi_{j=1,t}\right\}\right)=\sum_{t}\ell_{t}.$$

Hence, by fitting to the daily number of deaths time series, the rCFR and *ζ* can be estimated by using the maximum likelihood estimation approach. The 95% confidence intervals (95% CI) are calculated by using the profile likelihood estimation framework with a cutoff threshold determined by a Chi-square quantile [35], as well as previously adopted in [36,37,38,39,40,41,42]. 

### 2.4. Sensitivity Analysis 

Sensitivity analysis was carried out on the robustness and significance of the association between rCFR of COVID-19 and SARS-CoV-2 mutation activity. We examined the consistency of both the sign (or comparing with 1 for RR) and the 95%CI of the effect in case fatality risk associated with N501Y substitution under the alternative settings. We consider two sensitivity checking schemes as follows. 

For the first scheme, we repeat the estimating process of *ζ* with alternative PDF of and *δ*(*q*), which is introduced in Section 2.2.1. We consider short and long versions of the time interval between symptom onset and being reported (*q*) with means at 5 and 9 days, respectively. 

For the second scheme, we consider a univariate logistic regression model between rCFR*_t_* (see Section 2.2.1) as response and *ρ_j_*_= 1,*t*_ (see Section 2.2.2) as regressor. The regression coefficient of *ρ*, is evaluated as the effect size of interest. 

## 3. Results and Discussion

For the second epidemic wave in the UK as shown in Figure 1A, the epidemic curve grew gradually from September to December 2020, peaked in early January 2021 with the daily number of COVID-19 cases over 60,000, and declined thereafter. The number of COVID-19 deaths time series presents a similar trend as the cases curve with a lag accounting for the progression of COVID-19 (see Figure 1B). The 501Y variants emerged around September, and maintained at a relatively low prevalence below 5% until November 2020 (see Figure 1C). Then, the 501Y variants rapidly increased and dominated with prevalence at 50% by the end of December 2020, and trended to reach fixation after January 2021. 

We estimate the rCFR at 2.0% (95% CI: 1.8, 2.1) for all reported COVID-19 cases in the UK. In previous studies, the fatality ratio estimates vary across a wide range due to different concerns of “case”, which range from 0.7% to 1.3% among all SARS-CoV-2 infections [31,43], from 1.4% to 2.6% among clinically diagnosable cases [30,31,43,44], from 3.9% to 8.4% among reported cases [28,45,46,47,48], at 14% among hospitalized cases [49]. Most of these CFR estimates are calculated in the early outbreaks or during the first half of 2020, when the detecting rate of SARS-CoV-2 infections might not be as high as that after September 2020 (our study period). Thus, given that the case ascertainment efforts were largely improved in the UK, we consider that each clinically diagnosable case was likely reported. As such, the rCFR estimates in this work should be interpreted as the fatality ratio among those clinically diagnosable COVID-19 cases, which is largely in line with the existing estimates [30,31,43,44]. 

We reconstruct the daily instantaneous rCFR of 501N and 501Y SARS-CoV-2 variants, respectively (see Figure 1D). The overall trends of rCFR gradually increased from 1.0% in September to 2.2% in November 2020, and stabilized at this level thereafter. We suspect that the increase trend of rCFR between September and November might associate with the decreasing air temperature during the same period in the UK, about which the evidence is found in previous studies for COVID-19 [50,51,52,53,54]. The instantaneous CFR estimates may also have the potential to monitor the mortality risk on a real-time basis, and to further examine the associations with its potential determinants, for example, pathogenic evolution, supply of critical care resources [55] and exposure to environmental factors [56]. 

For the variant-specific change in CFR, we infer *ζ* at 1.18 (95% CI: 0.40, 3.28) indicating that the 501Y variants may increase 18% of fatality risk among the reported cases compared with the original 501N variants. Hence, in Figure 1D, the rCFR of the 501Y variant appears higher than that of the 501N variant. By rapidly screening recent peer-reviewed literature, we identified 4 relevant studies that investigated the risk of clinical severity associated with N501Y, or alternatively, B.1.1.7, variants [17,22,23,24], and they detected either no statistically significant evidence (3 out of 4) or weakly positive association (1 out of 4). Together with our estimates, the concern is that the new 501Y variants might slightly increase the risks of mortality among COVID-19 patients, and more solid evidence is recently affirmed by [25]. For sensitivity checking, we find that the *ζ* estimates are consistently but significantly larger than 1 in similar scales as the main estimates (data not shown), which validates our findings. 

A similar phenomenon was also reported regarding the previous D614G substitution. Consistent evidence of the transmission advantage of 614G variants was reported both statistically [18,21,57] and experimentally [19,58,59,60,61], but less evidence of clinical severity linked with 614G was found [62]. The 614G variants rapidly increased since March 2020, and replaced preceding 614D variants by reaching fixation after June 2020. The D614G substitution is believed to increase the intensity of the COVID-19 pandemic during the same period, which includes increasing COVID-19 deaths globally. In the same sense, provided the transmission advantage of 501Y [15,16,17], the increasing intensity of COVID-19 related mortality is of public health concern as 501Y variants are replacing their predecessor. The real-time assessment of the change in mortality risk may inform the case-management strategies and healthcare planning against the consequences from mutated SARS-CoV-2 strains. As such, we highlight the importance of our analytical framework, and the public health risks related to viral mutations may be controllable with early preparedness.

This study has the following limitations. First, we presume that COVID-19 induced deaths and their time of death are correctly reported. This setting is practically reasonable since the case facility is one of the most serious clinical outcomes, which is under more rigorous surveillance, and thus is unlikely mis-ascertained. Second, for the variant-specific rCFR in Section 2.2.2, Equation (2) holds when the COVID-19 cases and SARS-CoV-2 strains match along the same timeline. If one considers a constant reporting lag, *q*, the reported cases time series will have the same trends as the case time series by onset but shifted for the reporting lag. Considering the similar reporting delay also occurred for the SARS-CoV-2 sequencing data, the effects of the two reporting lags may be counteracted. Given the *ζ* estimates consistently hold in sensitivity analysis, that is, for a different distribution of *q*, we remark that this limitation is unlikely to affect the main conclusions in this study. Furthermore, with detailed reporting lag information of each individual case, adjustment for reporting delay can surely be carried out based on the current analytical framework. Third, the reconstruction of rCFR*_t_* relies on the settings of *h*(*s*) and *δ*(*q*) (see Section 2.2.1). We model both *h*(*s*) and *δ*(*q*) of COVID-19 as two fixed Gamma distributions, which follow previous studies [28,30,31]. In the real-world situation, the distribution of *s* or *q* might be time-varying [63], which may affect the reconstruction of rCFR*_t_*. However, the overall trends of rCFR*_t_* estimates are unlikely changed by a slight variation in *h*(*s*) or *δ*(*q*), and similar situations have been studied for other epidemiological measurements, for example, reproduction number [63]. Thus, we consider the impact of this limitation on the inference of variant-specific change in mortality risk may be negligible, and our model can be extended to a more complex time-varying context about the model settings. Fourth, the distribution of *s* or *q* might be altered by the mutated variants in theory. However, by screening the literature of COVID-19, we find no evidence that *h*(*s*) or *δ*(*q*) is varied associated with the N501Y substitution, and thus we adopted fixed distributions to govern the process. Fifth, this study focuses on exploring the effects on changing the case fatality risk associated with a single mutation, that is, N501Y, but the intrinsic biological mechanisms are commonly more complex and remain uncovered. As an example of the influenza virus, on one hand, the R384G substitution in H3N2 enhances the ability of in-host immune-escape [64], which indicates an increase in infectivity [65], but this substitution appears detrimental. On the other hand, the co-mutations of R384G in nucleoprotein (NP) could improve and compensate the viral fitness or functionality of [66,67], such that the mutated strains reached fixation rapidly in the 1993–1994 flu season. Future studies are needed for exploring the relationship of how the 501Y SARS-CoV-2 variant affects the mortality risk of COVID-19. Sixth, there are co-mutations of N501Y, for example, Δ69/Δ70 deletion and E484K [4,11,15], and we remark that the independent effects of each co-mutation cannot be disentangled in this study, where the interaction among these co-mutations is unassessed. Seventh, due to the lack of individual patients’ information, time-series data was used in this work, which means information loss from the data aggregation. Thus, estimating the effects of more types of variants could encounter identification issues that the samples might fail to inform each estimate. Lastly, as a data-driven study, the estimated association should be interpreted with caution. With an ecological setting, the findings in this study cannot guarantee causality, which needs further biomedical experiments in more sophisticated contexts. 

## Data Availability

All data used in this work are publicly available, and please see the data section for details.

## Supplementary Materials

The following are available online at https://www.mdpi.com/article/10.3390/v13040638/s1.
