# Supplementary material for: Inferring the Association between the Risk of COVID-19 Case Fatality and N501Y Substitution in SARS-CoV-2
Source: Viruses. 2021 Apr 8;13(4):638. doi: 10.3390/v13040638 (PMC8070306; doi:10.3390/v13040638)
Supplement: Supplementary file 1 [file viruses-13-00638-s001.zip › gisaid_hcov-19_UKAT_201114-201121.pdf]

We gratefully acknowledge the following Authors from the Originating laboratories responsible for obtaining the specimens, as well as the Submitting laboratories where the genome data were generated and shared via GISAID, on which this research is based.

All Submitters of data may be contacted directly via www.gisaid.org

Authors are sorted alphabetically.

Accession ID	Originating Laboratory	Submitting Laboratory	Authors
EPI_ISL_1104320	Liverpool Clinical Laboratories	COVID-19 Genomics UK (COG-UK) Consortium	Sam Haldenby, Anita Lucaci, Steve Paterson, Julian Hiscox, Alistair Darby, M Almsaud, A Alrezaihi, Muhannad Alruwaili, Stuart D Armstrong, Jones Benjamin, Eleanor G Bentley, Anu Chawla, Jordan J Clark, Angela Cowell, Richard Eccles, Isabel García-Dorival, Matthew Gemmell, Alessandro Gerada, PKF Gilmore, Richard Gregory, Ximeng Han, Catherine Hartley, Margaret Hughes, Miren Iturriza-Gomara, James Johnson, L Luu, Jenifer Manson, Charlotte Nelson, Elaine O'Toole, Cassie Olateju, Rebekah Penrice-Randal , Lucille Rainbow, N.P Randle, Trevor Ian Robinson, Parul Sharma, Ghada T Shawli, James P Stewart, Neil Swainston, Ecaterina Vamos, Joanne Watts, Mark Whitehead
EPI_ISL_1104841, EPI_ISL_1104842, EPI_ISL_1104854, EPI_ISL_1104868, EPI_ISL_1104869, EPI_ISL_1104877, EPI_ISL_1104878, EPI_ISL_1104891, EPI_ISL_1104896, EPI_ISL_1104897, EPI_ISL_1104898, EPI_ISL_1104907, EPI_ISL_1104914, EPI_ISL_1104915, EPI_ISL_1104928, EPI_ISL_1104935, EPI_ISL_1104948, EPI_ISL_1104968, EPI_ISL_1105058, EPI_ISL_1105083			
see above	University College London Hospital	COVID-19 Genomics UK (COG-UK) Consortium	Judith Heaney, Matthew Byott, Catherine Houlihan, Dan Frampton, Stuart Kirk, Moira Spyer and Eleni Nastouli
EPI_ISL_1107755	Centre for Enzyme Innovation, University of Portsmouth / Translational Research Laboratory, Portsmouth Hospitals NHS Trust	COVID-19 Genomics UK (COG-UK) Consortium	Angela Beckett,Salman Goudarzi,Christopher Fearn,Kate Cook,Katie Loveson,Sharon Glaysher,Scott Elliott,Samuel Robson
EPI_ISL_1177824, EPI_ISL_1177825, EPI_ISL_1177826, EPI_ISL_1177827	Liverpool Clinical Laboratories	COVID-19 Genomics UK (COG-UK) Consortium	Sam Haldenby, Alistair Darby, Steve Paterson, Anita Lucaci, Julian Hiscox, M Almsaud, A Alrezaihi, Muhannad Alruwaili, Stuart D Armstrong, Jones Benjamin, Eleanor G Bentley, Anu Chawla, Jordan J Clark, Angela Cowell, Richard Eccles, Isabel García-Dorival, Matthew Gemmell, Alessandro Gerada, PKF Gilmore, Richard Gregory, Ximeng Han, Catherine Hartley, Margaret Hughes, Miren Iturriza-Gomara, James Johnson, L Luu, Jenifer Manson, Charlotte Nelson, Elaine O'Toole, Cassie Olateju, Rebekah Penrice-Randal , Lucille Rainbow, N.P Randle, Trevor Ian Robinson, Parul Sharma, Ghada T Shawli, James P Stewart, Neil Swainston, Ecaterina Vamos, Joanne Watts, Mark Whitehead, Hermione Webster
EPI_ISL_1247540, EPI_ISL_1247542, EPI_ISL_1247544, EPI_ISL_1247545, EPI_ISL_1247546, EPI_ISL_1247547	University of Exeter	COVID-19 Genomics UK (COG-UK) Consortium	Ben Temperton,Aaron Jeffries,Michelle Michelsen,Joanna Warwick-Dugdale,Audrey Farbos,Robyn Manley,Stephen Michell,Jane Masoli
EPI_ISL_1474800	University College London, Great Ormond Street Hospital for Children NHS Foundation Trust, Imperial College Healthcare NHS Trust	COVID-19 Genomics UK (COG-UK) Consortium	Sergi Castellano, Rachel Williams, Mark Kristiansen, Paola Resende Silva, Sunando Roy, Tony Brooks, Helena Tutill, Paola Niola, Patricia Dyal, Charlotte Williams, Leysa Forrest, Yasmin Panchbhaya, Jacqueline Findlay, Samuel Weeks, Julianne Brown, Kathryn Harris, Paul Randell, James Price, Alison Holmes, Judith Breuer
EPI_ISL_1476752	Bioinformatics and Biostatistics Lab, Advanced Sequencing Facility	COVID-19 Genomics UK (COG-UK) Consortium	Aengus Stewart,Jerome Nicod,Chelsea Sawyer,Laura Cubitt,Harshil Patel,Margaret Crawford
EPI_ISL_645133, EPI_ISL_645134, EPI_ISL_645135, EPI_ISL_645136, EPI_ISL_649108, EPI_ISL_649110, EPI_ISL_649891, EPI_ISL_649892, EPI_ISL_649936, EPI_ISL_649937	Respiratory Virus Unit, Microbiology Services Colindale, Public Health England	COVID-19 Genomics UK (COG-UK) Consortium	PHE Covid Sequencing Team
EPI_ISL_650178, EPI_ISL_650187	Queens Medical Centre, Clinical Microbiology Department / DeepSeq Nottingham	COVID-19 Genomics UK (COG-UK) Consortium	Gemma Clark, Wendy Smith, Manjinder Khakh, Vicki M Fleming, Michelle M Lister, Hannah Howson-Wells, Jonathan Ball, Patrick McClure, Joseph Chappell, Theocharis Tsoleridis, Nadine Holmes, Matthew Carlisle, Christopher Moore, Fei Sang, Johnny Debebe, Victoria Wright, Matthew Loose
EPI_ISL_650193	Quadram Institute Bioscience	COVID-19 Genomics UK (COG-UK) Consortium	Dave J. Baker, Gemma L. Kay, Alp Aydin, Thanh Le-Viet, Steven Rudder, Ana P. Tedim, Anastasia Kolyva, Maria Diaz, Leonardo de Oliveira Martins, Nabil-Fareed Alikhan, Lizzie Meadows, Rachael Stanley, Ngozi Elumogo, Muhammed Yasir, Nicholas M. Thomson, Alexander J Trotter, Rachel Gilroy, Samuel Bloomfield, Claire Stuart, Andrew Bell, Reenesh Prakash, Samir Dervisevic, Alison E. Mather, John Wain, Mark Webber, Andrew J. Page, Justin O'Grady
EPI_ISL_650238, EPI_ISL_650266, EPI_ISL_650276	Queens Medical Centre, Clinical Microbiology Department / DeepSeq Nottingham	COVID-19 Genomics UK (COG-UK) Consortium	Gemma Clark, Wendy Smith, Manjinder Khakh, Vicki M Fleming, Michelle M Lister, Hannah Howson-Wells, Jonathan Ball, Patrick McClure, Joseph Chappell, Theocharis Tsoleridis, Nadine Holmes, Matthew Carlisle, Christopher Moore, Fei Sang, Johnny Debebe, Victoria Wright, Matthew Loose
EPI_ISL_650289	Quadram Institute Bioscience	COVID-19 Genomics UK (COG-UK) Consortium	Dave J. Baker, Gemma L. Kay, Alp Aydin, Thanh Le-Viet, Steven Rudder, Ana P. Tedim, Anastasia Kolyva, Maria Diaz, Leonardo de Oliveira Martins, Nabil-Fareed Alikhan, Lizzie Meadows, Rachael Stanley, Ngozi Elumogo, Muhammed Yasir, Nicholas M. Thomson, Alexander J Trotter, Rachel Gilroy, Samuel Bloomfield, Claire Stuart, Andrew Bell, Reenesh Prakash, Samir Dervisevic, Alison E. Mather, John Wain, Mark Webber, Andrew J. Page, Justin O'Grady
EPI_ISL_650291, EPI_ISL_650314, EPI_ISL_650443	Queens Medical Centre, Clinical Microbiology Department / DeepSeq Nottingham	COVID-19 Genomics UK (COG-UK) Consortium	Gemma Clark, Wendy Smith, Manjinder Khakh, Vicki M Fleming, Michelle M Lister, Hannah Howson-Wells, Jonathan Ball, Patrick McClure, Joseph Chappell, Theocharis Tsoleridis, Nadine Holmes, Matthew Carlisle, Christopher Moore, Fei Sang, Johnny Debebe, Victoria Wright, Matthew Loose
EPI_ISL_650459, EPI_ISL_650469	Quadram Institute Bioscience	COVID-19 Genomics UK (COG-UK) Consortium	Dave J. Baker, Gemma L. Kay, Alp Aydin, Thanh Le-Viet, Steven Rudder, Ana P. Tedim, Anastasia Kolyva, Maria Diaz, Leonardo de Oliveira Martins, Nabil-Fareed Alikhan, Lizzie Meadows, Rachael Stanley, Ngozi Elumogo, Muhammed Yasir, Nicholas M. Thomson, Alexander J Trotter, Rachel Gilroy, Samuel Bloomfield, Claire Stuart, Andrew Bell, Reenesh Prakash, Samir Dervisevic, Alison E. Mather, John Wain, Mark Webber, Andrew J. Page, Justin O'Grady
EPI_ISL_650471	Queens Medical Centre, Clinical Microbiology Department / DeepSeq Nottingham	COVID-19 Genomics UK (COG-UK) Consortium	Gemma Clark, Wendy Smith, Manjinder Khakh, Vicki M Fleming, Michelle M Lister, Hannah Howson-Wells, Jonathan Ball, Patrick McClure, Joseph Chappell, Theocharis Tsoleridis, Nadine Holmes, Matthew Carlisle, Christopher Moore, Fei Sang, Johnny Debebe, Victoria Wright, Matthew Loose
EPI_ISL_650546	Quadram Institute Bioscience	COVID-19 Genomics UK (COG-UK) Consortium	Dave J. Baker, Gemma L. Kay, Alp Aydin, Thanh Le-Viet, Steven Rudder, Ana P. Tedim, Anastasia Kolyva, Maria Diaz, Leonardo de Oliveira Martins, Nabil-Fareed Alikhan, Lizzie Meadows, Rachael Stanley, Ngozi Elumogo, Muhammed Yasir, Nicholas M. Thomson, Alexander J Trotter, Rachel Gilroy, Samuel Bloomfield, Claire Stuart, Andrew Bell, Reenesh Prakash, Samir Dervisevic, Alison E. Mather, John Wain, Mark Webber, Andrew J. Page, Justin O'Grady
EPI_ISL_650599	Queens Medical Centre, Clinical Microbiology Department / DeepSeq Nottingham	COVID-19 Genomics UK (COG-UK) Consortium	Gemma Clark, Wendy Smith, Manjinder Khakh, Vicki M Fleming, Michelle M Lister, Hannah Howson-Wells, Jonathan Ball, Patrick McClure, Joseph Chappell, Theocharis Tsoleridis, Nadine Holmes, Matthew Carlisle, Christopher Moore, Fei Sang, Johnny Debebe, Victoria Wright, Matthew Loose
EPI_ISL_650677	Quadram Institute Bioscience	COVID-19 Genomics UK (COG-UK) Consortium	Dave J. Baker, Gemma L. Kay, Alp Aydin, Thanh Le-Viet, Steven Rudder, Ana P. Tedim, Anastasia Kolyva, Maria Diaz, Leonardo de Oliveira Martins, Nabil-Fareed Alikhan, Lizzie Meadows, Rachael Stanley, Ngozi Elumogo, Muhammed Yasir, Nicholas M. Thomson, Alexander J Trotter, Rachel Gilroy, Samuel Bloomfield, Claire Stuart, Andrew Bell, Reenesh Prakash, Samir Dervisevic, Alison E. Mather, John Wain, Mark Webber, Andrew J. Page, Justin O'Grady
EPI_ISL_650786, EPI_ISL_650821, EPI_ISL_650830, EPI_ISL_650835, EPI_ISL_650979, EPI_ISL_650987, EPI_ISL_651065, EPI_ISL_651107	Queens Medical Centre, Clinical Microbiology Department / DeepSeq Nottingham	COVID-19 Genomics UK (COG-UK) Consortium	Gemma Clark, Wendy Smith, Manjinder Khakh, Vicki M Fleming, Michelle M Lister, Hannah Howson-Wells, Jonathan Ball, Patrick McClure, Joseph Chappell, Theocharis Tsoleridis, Nadine Holmes, Matthew Carlisle, Christopher Moore, Fei Sang, Johnny Debebe, Victoria Wright, Matthew Loose
EPI_ISL_651111	Quadram Institute Bioscience	COVID-19 Genomics UK (COG-UK) Consortium	Dave J. Baker, Gemma L. Kay, Alp Aydin, Thanh Le-Viet, Steven Rudder, Ana P. Tedim, Anastasia Kolyva, Maria Diaz, Leonardo de Oliveira Martins, Nabil-Fareed Alikhan, Lizzie Meadows, Rachael Stanley, Ngozi Elumogo, Muhammed Yasir, Nicholas M. Thomson, Alexander J Trotter, Rachel Gilroy,

			Samuel Bloomfield, Claire Stuart, Andrew Bell, Reenesh Prakash, Samir Dervisevic, Alison E. Mather, John Wain, Mark Webber, Andrew J. Page, Justin O'Grady
EPI_ISL_651189	Queens Medical Centre, Clinical Microbiology Department / DeepSeq Nottingham	COVID-19 Genomics UK (COG-UK) Consortium	Gemma Clark, Wendy Smith, Manjinder Khakh, Vicki M Fleming, Michelle M Lister, Hannah Howson-Wells, Jonathan Ball, Patrick McClure, Joseph Chappell, Theocharis Tsoleridis, Nadine Holmes, Matthew Carlisle, Christopher Moore, Fei Sang, Johnny Debebe, Victoria Wright, Matthew Loose
EPI_ISL_651237	Quadram Institute Bioscience	COVID-19 Genomics UK (COG-UK) Consortium	Dave J. Baker, Gemma L. Kay, Alp Aydin, Thanh Le-Viet, Steven Rudder, Ana P. Tedim, Anastasia Kolyva, Maria Diaz, Leonardo de Oliveira Martins, Nabil-Fareed Alikhan, Lizzie Meadows, Rachael Stanley, Ngozi Elumogo, Muhammed Yasir, Nicholas M. Thomson, Alexander J Trotter, Rachel Gilroy, Samuel Bloomfield, Claire Stuart, Andrew Bell, Reenesh Prakash, Samir Dervisevic, Alison E. Mather, John Wain, Mark Webber, Andrew J. Page, Justin O'Grady
EPI_ISL_651260, EPI_ISL_651286, EPI_ISL_651308, EPI_ISL_651313	Queens Medical Centre, Clinical Microbiology Department / DeepSeq Nottingham	COVID-19 Genomics UK (COG-UK) Consortium	Gemma Clark, Wendy Smith, Manjinder Khakh, Vicki M Fleming, Michelle M Lister, Hannah Howson-Wells, Jonathan Ball, Patrick McClure, Joseph Chappell, Theocharis Tsoleridis, Nadine Holmes, Matthew Carlisle, Christopher Moore, Fei Sang, Johnny Debebe, Victoria Wright, Matthew Loose
EPI_ISL_651399	Quadram Institute Bioscience	COVID-19 Genomics UK (COG-UK) Consortium	Dave J. Baker, Gemma L. Kay, Alp Aydin, Thanh Le-Viet, Steven Rudder, Ana P. Tedim, Anastasia Kolyva, Maria Diaz, Leonardo de Oliveira Martins, Nabil-Fareed Alikhan, Lizzie Meadows, Rachael Stanley, Ngozi Elumogo, Muhammed Yasir, Nicholas M. Thomson, Alexander J Trotter, Rachel Gilroy, Samuel Bloomfield, Claire Stuart, Andrew Bell, Reenesh Prakash, Samir Dervisevic, Alison E. Mather, John Wain, Mark Webber, Andrew J. Page, Justin O'Grady
EPI_ISL_651401, EPI_ISL_651408, EPI_ISL_652389, EPI_ISL_652390, EPI_ISL_652391, EPI_ISL_652392, EPI_ISL_652393, EPI_ISL_652394, EPI_ISL_652395, EPI_ISL_652396, EPI_ISL_652397, EPI_ISL_652398, EPI_ISL_652399, EPI_ISL_652400, EPI_ISL_652401, EPI_ISL_652402, EPI_ISL_652403, EPI_ISL_652404, EPI_ISL_652405, EPI_ISL_652406, EPI_ISL_652407, EPI_ISL_652408, EPI_ISL_652409, EPI_ISL_652410, EPI_ISL_652411, EPI_ISL_652412, EPI_ISL_652413			
see above	Queens Medical Centre, Clinical Microbiology Department / DeepSeq Nottingham	COVID-19 Genomics UK (COG-UK) Consortium	Gemma Clark, Wendy Smith, Manjinder Khakh, Vicki M Fleming, Michelle M Lister, Hannah Howson-Wells, Jonathan Ball, Patrick McClure, Joseph Chappell, Theocharis Tsoleridis, Nadine Holmes, Matthew Carlisle, Christopher Moore, Fei Sang, Johnny Debebe, Victoria Wright, Matthew Loose
EPI_ISL_660794, EPI_ISL_661175	Respiratory Virus Unit, Microbiology Services Colindale, Public Health England	COVID-19 Genomics UK (COG-UK) Consortium	PHE Covid Sequencing Team
EPI_ISL_661632, EPI_ISL_661633, EPI_ISL_661634, EPI_ISL_661635, EPI_ISL_661636, EPI_ISL_661637, EPI_ISL_661639, EPI_ISL_661642, EPI_ISL_661643, EPI_ISL_661644, EPI_ISL_661645, EPI_ISL_661646, EPI_ISL_661648, EPI_ISL_661649, EPI_ISL_661650, EPI_ISL_661655, EPI_ISL_661657, EPI_ISL_661658, EPI_ISL_661659, EPI_ISL_661660, EPI_ISL_661661, EPI_ISL_661662, EPI_ISL_661663, EPI_ISL_661665, EPI_ISL_661666, EPI_ISL_661667, EPI_ISL_661668, EPI_ISL_661669, EPI_ISL_661670, EPI_ISL_661671, EPI_ISL_661672, EPI_ISL_661673, EPI_ISL_661674, EPI_ISL_661676, EPI_ISL_661677, EPI_ISL_661678, EPI_ISL_661681, EPI_ISL_661682, EPI_ISL_661683, EPI_ISL_661685, EPI_ISL_661686, EPI_ISL_661688, EPI_ISL_661689, EPI_ISL_661690, EPI_ISL_661692, EPI_ISL_661694, EPI_ISL_661698, EPI_ISL_661699, EPI_ISL_661701, EPI_ISL_661703, EPI_ISL_661704, EPI_ISL_661705, EPI_ISL_661706, EPI_ISL_661707, EPI_ISL_661708, EPI_ISL_661709, EPI_ISL_661714, EPI_ISL_661715, EPI_ISL_661716, EPI_ISL_661718, EPI_ISL_661719, EPI_ISL_661720, EPI_ISL_661722, EPI_ISL_661723, EPI_ISL_661724, EPI_ISL_661725, EPI_ISL_661727, EPI_ISL_661728, EPI_ISL_661730, EPI_ISL_661731, EPI_ISL_661733, EPI_ISL_661735, EPI_ISL_661736, EPI_ISL_661737, EPI_ISL_661738, EPI_ISL_661739, EPI_ISL_661740, EPI_ISL_661744, EPI_ISL_661745, EPI_ISL_661746, EPI_ISL_661748, EPI_ISL_661750, EPI_ISL_661753, EPI_ISL_661754, EPI_ISL_661755, EPI_ISL_661756, EPI_ISL_661757, EPI_ISL_661758, EPI_ISL_661760, EPI_ISL_661762, EPI_ISL_661763, EPI_ISL_661764, EPI_ISL_661765, EPI_ISL_661766, EPI_ISL_661767, EPI_ISL_661768, EPI_ISL_661769, EPI_ISL_661770, EPI_ISL_661771, EPI_ISL_661772, EPI_ISL_661773, EPI_ISL_661774, EPI_ISL_661776, EPI_ISL_661777, EPI_ISL_661780, EPI_ISL_661782, EPI_ISL_661784, EPI_ISL_661786, EPI_ISL_661787, EPI_ISL_661788, EPI_ISL_661790, EPI_ISL_661792, EPI_ISL_661793, EPI_ISL_661794, EPI_ISL_661795, EPI_ISL_661796, EPI_ISL_661798, EPI_ISL_661800, EPI_ISL_661802, EPI_ISL_661803, EPI_ISL_661804, EPI_ISL_661806, EPI_ISL_661807, EPI_ISL_661808, EPI_ISL_661809, EPI_ISL_661810, EPI_ISL_661812, EPI_ISL_661813, EPI_ISL_661814, EPI_ISL_661816, EPI_ISL_661818, EPI_ISL_661819, EPI_ISL_661820, EPI_ISL_661821, EPI_ISL_661822, EPI_ISL_661823, EPI_ISL_661824, EPI_ISL_661825, EPI_ISL_661826, EPI_ISL_661827, EPI_ISL_661828, EPI_ISL_661829, EPI_ISL_661831, EPI_ISL_661832, EPI_ISL_661835, EPI_ISL_661837, EPI_ISL_661838, EPI_ISL_661839, EPI_ISL_661840, EPI_ISL_661841, EPI_ISL_661842, EPI_ISL_661843, EPI_ISL_661846, EPI_ISL_661847, EPI_ISL_661848, EPI_ISL_661851, EPI_ISL_661852, EPI_ISL_661855, EPI_ISL_661857, EPI_ISL_661858, EPI_ISL_661859, EPI_ISL_661860, EPI_ISL_661861, EPI_ISL_661862, EPI_ISL_661864, EPI_ISL_661865, EPI_ISL_661866, EPI_ISL_661867, EPI_ISL_661868, EPI_ISL_661870, EPI_ISL_661871, EPI_ISL_661872, EPI_ISL_661875, EPI_ISL_661877, EPI_ISL_661878, EPI_ISL_661879, EPI_ISL_661880, EPI_ISL_661882, EPI_ISL_661884, EPI_ISL_661885, EPI_ISL_661888, EPI_ISL_661889, EPI_ISL_661890, EPI_ISL_661891, EPI_ISL_661892, EPI_ISL_661893, EPI_ISL_661894, EPI_ISL_661896, EPI_ISL_661898, EPI_ISL_661899, EPI_ISL_661901, EPI_ISL_661904, EPI_ISL_661905, EPI_ISL_661906, EPI_ISL_661907, EPI_ISL_661908, EPI_ISL_661909, EPI_ISL_661910, EPI_ISL_661911, EPI_ISL_661912, EPI_ISL_661913, EPI_ISL_661914, EPI_ISL_661915, EPI_ISL_661916, EPI_ISL_661919, EPI_ISL_661920, EPI_ISL_661921, EPI_ISL_661922, EPI_ISL_661923, EPI_ISL_661924, EPI_ISL_661927, EPI_ISL_661929, EPI_ISL_661931, EPI_ISL_661932, EPI_ISL_661933, EPI_ISL_661934, EPI_ISL_661935, EPI_ISL_661936, EPI_ISL_661937, EPI_ISL_661938, EPI_ISL_661939, EPI_ISL_661940, EPI_ISL_661942, EPI_ISL_661943, EPI_ISL_661945, EPI_ISL_661946, EPI_ISL_661948, EPI_ISL_661949, EPI_ISL_661950, EPI_ISL_661952, EPI_ISL_661955			
see above	Lighthouse Lab in Glasgow	Wellcome Sanger Institute for the COVID-19 Genomics UK (COG-UK) Consortium	Harper VanSteenhouse, Yumi Kasai, David Gray, Carol Clugston, Anna Dominiczak and Alex Alderton, Roberto Amato, Sonia Goncalves, Ewan Harrison, David K. Jackson, Ian Johnston, Dominic Kwiatkowski, Cordelia Langford, John Sillitoe on behalf of the Wellcome Sanger Institute COVID-19 Surveillance Team
EPI_ISL_664113	Liverpool Clinical Laboratories	COVID-19 Genomics UK (COG-UK) Consortium	Sam Haldenby, Anita Lucaci, Steve Paterson, Julian Hiscox, Alistair Darby, M Almsaud, A Alrezaihi, Muhannad Alruwaili, Stuart D Armstrong, Jones Benjamin, Eleanor G Bentley, Anu Chawla, Jordan J Clark, Angela Cowell, Richard Eccles, Isabel García-Dorival, Matthew Gemmell, Alessandro Gerada, PKF Gilmore, Richard Gregory, Ximeng Han, Catherine Hartley, Margaret Hughes, Miren Iturriza-Gomara, James Johnson, L Luu, Jenifer Manson, Charlotte Nelson, Elaine O'Toole, Cassie Olateju, Rebekah Penrice-Randal , Lucille Rainbow, N.P Randle, Trevor Ian Robinson, Parul Sharma, Ghada T Shawli, James P Stewart, Neil Swainston, Ecaterina Vamos, Joanne Watts, Mark Whitehead
EPI_ISL_664120, EPI_ISL_664139, EPI_ISL_664140, EPI_ISL_664149, EPI_ISL_664155	Department of Pathology, University of Cambridge	COVID-19 Genomics UK (COG-UK) Consortium	Aminu S. Jahun, Yasmin Chaudhry, Grant Hall, Iliana Georgana, Myra Hosmillo, Martin D. Curran, Malte Pinckert, Surendra Parmar, Ian Goodfellow
EPI_ISL_664163	Liverpool Clinical Laboratories	COVID-19 Genomics UK (COG-UK) Consortium	Sam Haldenby, Anita Lucaci, Steve Paterson, Julian Hiscox, Alistair Darby, M Almsaud, A Alrezaihi, Muhannad Alruwaili, Stuart D Armstrong, Jones Benjamin, Eleanor G Bentley, Anu Chawla, Jordan J Clark, Angela Cowell, Richard Eccles, Isabel García-Dorival, Matthew Gemmell, Alessandro Gerada, PKF Gilmore, Richard Gregory, Ximeng Han, Catherine Hartley, Margaret Hughes, Miren Iturriza-Gomara, James Johnson, L Luu, Jenifer Manson, Charlotte Nelson, Elaine O'Toole, Cassie Olateju, Rebekah Penrice-Randal , Lucille Rainbow, N.P Randle, Trevor Ian Robinson, Parul Sharma, Ghada T Shawli, James P Stewart, Neil Swainston, Ecaterina Vamos, Joanne Watts, Mark Whitehead
EPI_ISL_664199, EPI_ISL_664200, EPI_ISL_664234, EPI_ISL_664291	Department of Pathology, University of Cambridge	COVID-19 Genomics UK (COG-UK) Consortium	Aminu S. Jahun, Yasmin Chaudhry, Grant Hall, Iliana Georgana, Myra Hosmillo, Martin D. Curran, Malte Pinckert, Surendra Parmar, Ian Goodfellow
EPI_ISL_664299	Liverpool Clinical Laboratories	COVID-19 Genomics UK (COG-UK) Consortium	Sam Haldenby, Anita Lucaci, Steve Paterson, Julian Hiscox, Alistair Darby, M Almsaud, A Alrezaihi, Muhannad Alruwaili, Stuart D Armstrong, Jones Benjamin, Eleanor G Bentley, Anu Chawla, Jordan J Clark, Angela Cowell, Richard Eccles, Isabel García-Dorival, Matthew Gemmell, Alessandro Gerada, PKF Gilmore, Richard Gregory, Ximeng Han, Catherine Hartley, Margaret Hughes, Miren Iturriza-Gomara, James Johnson, L Luu, Jenifer Manson, Charlotte Nelson, Elaine O'Toole, Cassie Olateju, Rebekah Penrice-Randal , Lucille Rainbow, N.P Randle, Trevor Ian Robinson, Parul Sharma, Ghada T Shawli, James P Stewart, Neil Swainston, Ecaterina Vamos, Joanne Watts, Mark Whitehead
EPI_ISL_664304, EPI_ISL_664316, EPI_ISL_664317, EPI_ISL_664326	Department of Pathology, University of Cambridge	COVID-19 Genomics UK (COG-UK) Consortium	Aminu S. Jahun, Yasmin Chaudhry, Grant Hall, Iliana Georgana, Myra Hosmillo, Martin D. Curran, Malte Pinckert, Surendra Parmar, Ian Goodfellow
EPI_ISL_664332, EPI_ISL_664337	Liverpool Clinical Laboratories	COVID-19 Genomics UK (COG-UK) Consortium	Sam Haldenby, Anita Lucaci, Steve Paterson, Julian Hiscox, Alistair Darby, M Almsaud, A Alrezaihi, Muhannad Alruwaili, Stuart D Armstrong, Jones Benjamin, Eleanor G Bentley, Anu Chawla, Jordan J Clark, Angela Cowell, Richard Eccles, Isabel García-Dorival, Matthew Gemmell, Alessandro Gerada, PKF Gilmore, Richard Gregory, Ximeng Han, Catherine Hartley, Margaret Hughes, Miren Iturriza-Gomara, James Johnson, L Luu, Jenifer Manson, Charlotte Nelson, Elaine O'Toole, Cassie Olateju, Rebekah Penrice-Randal , Lucille Rainbow, N.P Randle, Trevor Ian Robinson, Parul Sharma, Ghada T Shawli, James P Stewart, Neil Swainston, Ecaterina Vamos, Joanne Watts, Mark Whitehead
EPI_ISL_664346	Department of Pathology, University of Cambridge	COVID-19 Genomics UK (COG-UK) Consortium	Aminu S. Jahun, Yasmin Chaudhry, Grant Hall, Iliana Georgana, Myra Hosmillo, Martin D. Curran, Malte Pinckert, Surendra Parmar, Ian Goodfellow
EPI_ISL_664350, EPI_ISL_664363	Liverpool Clinical Laboratories	COVID-19 Genomics UK (COG-UK) Consortium	Sam Haldenby, Anita Lucaci, Steve Paterson, Julian Hiscox, Alistair Darby, M Almsaud, A Alrezaihi, Muhannad Alruwaili, Stuart D Armstrong, Jones Benjamin, Eleanor G Bentley, Anu Chawla, Jordan J Clark, Angela Cowell, Richard Eccles, Isabel García-Dorival, Matthew Gemmell, Alessandro Gerada, PKF Gilmore, Richard Gregory, Ximeng Han, Catherine Hartley, Margaret Hughes, Miren Iturriza-Gomara, James Johnson, L Luu, Jenifer Manson, Charlotte Nelson, Elaine O'Toole, Cassie Olateju, Rebekah Penrice-Randal , Lucille Rainbow, N.P Randle, Trevor Ian Robinson, Parul Sharma, Ghada T Shawli, James P Stewart, Neil Swainston, Ecaterina Vamos, Joanne Watts, Mark Whitehead
EPI_ISL_664380, EPI_ISL_664381, EPI_ISL_664382, EPI_ISL_664383, EPI_ISL_664384	Department of Pathology, University of Cambridge	COVID-19 Genomics UK (COG-UK) Consortium	Aminu S. Jahun, Yasmin Chaudhry, Grant Hall, Iliana Georgana, Myra Hosmillo, Martin D. Curran, Malte Pinckert, Surendra Parmar, Ian Goodfellow
EPI_ISL_664430	Liverpool Clinical Laboratories	COVID-19 Genomics UK (COG-UK) Consortium	Sam Haldenby, Anita Lucaci, Steve Paterson, Julian Hiscox, Alistair Darby, M Almsaud, A Alrezaihi, Muhannad Alruwaili, Stuart D Armstrong, Jones Benjamin, Eleanor G Bentley, Anu Chawla, Jordan J Clark, Angela Cowell, Richard Eccles, Isabel García-Dorival, Matthew Gemmell, Alessandro Gerada, PKF Gilmore, Richard Gregory, Ximeng Han, Catherine Hartley, Margaret Hughes, Miren Iturriza-Gomara, James Johnson, L Luu, Jenifer Manson, Charlotte Nelson, Elaine O'Toole, Cassie Olateju, Rebekah Penrice-Randal , Lucille Rainbow, N.P Randle, Trevor Ian Robinson, Parul Sharma, Ghada T Shawli, James P Stewart, Neil Swainston, Ecaterina Vamos, Joanne Watts, Mark Whitehead

EPI_ISL_664446, EPI_ISL_664455, EPI_ISL_664470	Department of Pathology, University of Cambridge	COVID-19 Genomics UK (COG-UK) Consortium	Aminu S. Jahun, Yasmin Chaudhry, Grant Hall, Iliana Georgana, Myra Hosmillo, Martin D. Curran, Malte Pinckert, Surendra Parmar, Ian Goodfellow
EPI_ISL_664478, EPI_ISL_664480	Liverpool Clinical Laboratories	COVID-19 Genomics UK (COG-UK) Consortium	Sam Haldenby, Anita Lucaci, Steve Paterson, Julian Hiscox, Alistair Darby, M Almsaud, A Alrezaihi, Muhannad Alruwaili, Stuart D Armstrong, Jones Benjamin, Eleanor G Bentley, Anu Chawla, Jordan J Clark, Angela Cowell, Richard Eccles, Isabel García-Dorival, Matthew Gemmell, Alessandro Gerada, PKF Gilmore, Richard Gregory, Ximeng Han, Catherine Hartley, Margaret Hughes, Miren Iturriza-Gomara, James Johnson, L Luu, Jenifer Manson, Charlotte Nelson, Elaine O'Toole, Cassie Olateju, Rebekah Penrice-Randal , Lucille Rainbow, N.P Randle, Trevor Ian Robinson, Parul Sharma, Ghada T Shawli, James P Stewart, Neil Swainston, Ecaterina Vamos, Joanne Watts, Mark Whitehead
EPI_ISL_664516, EPI_ISL_664527, EPI_ISL_664529, EPI_ISL_664535, EPI_ISL_664536, EPI_ISL_664537, EPI_ISL_664538, EPI_ISL_664543, EPI_ISL_664546, EPI_ISL_664555, EPI_ISL_664588, EPI_ISL_664589, EPI_ISL_664590, EPI_ISL_664591, EPI_ISL_664601			
see above	Department of Pathology, University of Cambridge	COVID-19 Genomics UK (COG-UK) Consortium	Aminu S. Jahun, Yasmin Chaudhry, Grant Hall, Iliana Georgana, Myra Hosmillo, Martin D. Curran, Malte Pinckert, Surendra Parmar, Ian Goodfellow
EPI_ISL_664607	Liverpool Clinical Laboratories	COVID-19 Genomics UK (COG-UK) Consortium	Sam Haldenby, Anita Lucaci, Steve Paterson, Julian Hiscox, Alistair Darby, M Almsaud, A Alrezaihi, Muhannad Alruwaili, Stuart D Armstrong, Jones Benjamin, Eleanor G Bentley, Anu Chawla, Jordan J Clark, Angela Cowell, Richard Eccles, Isabel García-Dorival, Matthew Gemmell, Alessandro Gerada, PKF Gilmore, Richard Gregory, Ximeng Han, Catherine Hartley, Margaret Hughes, Miren Iturriza-Gomara, James Johnson, L Luu, Jenifer Manson, Charlotte Nelson, Elaine O'Toole, Cassie Olateju, Rebekah Penrice-Randal , Lucille Rainbow, N.P Randle, Trevor Ian Robinson, Parul Sharma, Ghada T Shawli, James P Stewart, Neil Swainston, Ecaterina Vamos, Joanne Watts, Mark Whitehead
EPI_ISL_664610	Department of Pathology, University of Cambridge	COVID-19 Genomics UK (COG-UK) Consortium	Aminu S. Jahun, Yasmin Chaudhry, Grant Hall, Iliana Georgana, Myra Hosmillo, Martin D. Curran, Malte Pinckert, Surendra Parmar, Ian Goodfellow
EPI_ISL_664613	Liverpool Clinical Laboratories	COVID-19 Genomics UK (COG-UK) Consortium	Sam Haldenby, Anita Lucaci, Steve Paterson, Julian Hiscox, Alistair Darby, M Almsaud, A Alrezaihi, Muhannad Alruwaili, Stuart D Armstrong, Jones Benjamin, Eleanor G Bentley, Anu Chawla, Jordan J Clark, Angela Cowell, Richard Eccles, Isabel García-Dorival, Matthew Gemmell, Alessandro Gerada, PKF Gilmore, Richard Gregory, Ximeng Han, Catherine Hartley, Margaret Hughes, Miren Iturriza-Gomara, James Johnson, L Luu, Jenifer Manson, Charlotte Nelson, Elaine O'Toole, Cassie Olateju, Rebekah Penrice-Randal , Lucille Rainbow, N.P Randle, Trevor Ian Robinson, Parul Sharma, Ghada T Shawli, James P Stewart, Neil Swainston, Ecaterina Vamos, Joanne Watts, Mark Whitehead
EPI_ISL_664616	Department of Pathology, University of Cambridge	COVID-19 Genomics UK (COG-UK) Consortium	Aminu S. Jahun, Yasmin Chaudhry, Grant Hall, Iliana Georgana, Myra Hosmillo, Martin D. Curran, Malte Pinckert, Surendra Parmar, Ian Goodfellow
EPI_ISL_664633	Liverpool Clinical Laboratories	COVID-19 Genomics UK (COG-UK) Consortium	Sam Haldenby, Anita Lucaci, Steve Paterson, Julian Hiscox, Alistair Darby, M Almsaud, A Alrezaihi, Muhannad Alruwaili, Stuart D Armstrong, Jones Benjamin, Eleanor G Bentley, Anu Chawla, Jordan J Clark, Angela Cowell, Richard Eccles, Isabel García-Dorival, Matthew Gemmell, Alessandro Gerada, PKF Gilmore, Richard Gregory, Ximeng Han, Catherine Hartley, Margaret Hughes, Miren Iturriza-Gomara, James Johnson, L Luu, Jenifer Manson, Charlotte Nelson, Elaine O'Toole, Cassie Olateju, Rebekah Penrice-Randal , Lucille Rainbow, N.P Randle, Trevor Ian Robinson, Parul Sharma, Ghada T Shawli, James P Stewart, Neil Swainston, Ecaterina Vamos, Joanne Watts, Mark Whitehead
EPI_ISL_664635, EPI_ISL_664656	Department of Pathology, University of Cambridge	COVID-19 Genomics UK (COG-UK) Consortium	Aminu S. Jahun, Yasmin Chaudhry, Grant Hall, Iliana Georgana, Myra Hosmillo, Martin D. Curran, Malte Pinckert, Surendra Parmar, Ian Goodfellow
EPI_ISL_664678	Liverpool Clinical Laboratories	COVID-19 Genomics UK (COG-UK) Consortium	Sam Haldenby, Anita Lucaci, Steve Paterson, Julian Hiscox, Alistair Darby, M Almsaud, A Alrezaihi, Muhannad Alruwaili, Stuart D Armstrong, Jones Benjamin, Eleanor G Bentley, Anu Chawla, Jordan J Clark, Angela Cowell, Richard Eccles, Isabel García-Dorival, Matthew Gemmell, Alessandro Gerada, PKF Gilmore, Richard Gregory, Ximeng Han, Catherine Hartley, Margaret Hughes, Miren Iturriza-Gomara, James Johnson, L Luu, Jenifer Manson, Charlotte Nelson, Elaine O'Toole, Cassie Olateju, Rebekah Penrice-Randal , Lucille Rainbow, N.P Randle, Trevor Ian Robinson, Parul Sharma, Ghada T Shawli, James P Stewart, Neil Swainston, Ecaterina Vamos, Joanne Watts, Mark Whitehead
EPI_ISL_664731	Department of Pathology, University of Cambridge	COVID-19 Genomics UK (COG-UK) Consortium	Aminu S. Jahun, Yasmin Chaudhry, Grant Hall, Iliana Georgana, Myra Hosmillo, Martin D. Curran, Malte Pinckert, Surendra Parmar, Ian Goodfellow
EPI_ISL_664738	Liverpool Clinical Laboratories	COVID-19 Genomics UK (COG-UK) Consortium	Sam Haldenby, Anita Lucaci, Steve Paterson, Julian Hiscox, Alistair Darby, M Almsaud, A Alrezaihi, Muhannad Alruwaili, Stuart D Armstrong, Jones Benjamin, Eleanor G Bentley, Anu Chawla, Jordan J Clark, Angela Cowell, Richard Eccles, Isabel García-Dorival, Matthew Gemmell, Alessandro Gerada, PKF Gilmore, Richard Gregory, Ximeng Han, Catherine Hartley, Margaret Hughes, Miren Iturriza-Gomara, James Johnson, L Luu, Jenifer Manson, Charlotte Nelson, Elaine O'Toole, Cassie Olateju, Rebekah Penrice-Randal , Lucille Rainbow, N.P Randle, Trevor Ian Robinson, Parul Sharma, Ghada T Shawli, James P Stewart, Neil Swainston, Ecaterina Vamos, Joanne Watts, Mark Whitehead
EPI_ISL_664748, EPI_ISL_664749, EPI_ISL_664980, EPI_ISL_664982, EPI_ISL_664986, EPI_ISL_664994, EPI_ISL_664995, EPI_ISL_664996, EPI_ISL_664997, EPI_ISL_665005, EPI_ISL_665006, EPI_ISL_665007, EPI_ISL_665008, EPI_ISL_665009, EPI_ISL_665010, EPI_ISL_665011, EPI_ISL_665012, EPI_ISL_665014, EPI_ISL_665015, EPI_ISL_665016, EPI_ISL_665017, EPI_ISL_665018, EPI_ISL_665019, EPI_ISL_665020, EPI_ISL_665021, EPI_ISL_665022			
see above	Department of Pathology, University of Cambridge	COVID-19 Genomics UK (COG-UK) Consortium	Aminu S. Jahun, Yasmin Chaudhry, Grant Hall, Iliana Georgana, Myra Hosmillo, Martin D. Curran, Malte Pinckert, Surendra Parmar, Ian Goodfellow
EPI_ISL_665121, EPI_ISL_665122, EPI_ISL_665127	Liverpool Clinical Laboratories	COVID-19 Genomics UK (COG-UK) Consortium	Sam Haldenby, Anita Lucaci, Steve Paterson, Julian Hiscox, Alistair Darby, M Almsaud, A Alrezaihi, Muhannad Alruwaili, Stuart D Armstrong, Jones Benjamin, Eleanor G Bentley, Anu Chawla, Jordan J Clark, Angela Cowell, Richard Eccles, Isabel García-Dorival, Matthew Gemmell, Alessandro Gerada, PKF Gilmore, Richard Gregory, Ximeng Han, Catherine Hartley, Margaret Hughes, Miren Iturriza-Gomara, James Johnson, L Luu, Jenifer Manson, Charlotte Nelson, Elaine O'Toole, Cassie Olateju, Rebekah Penrice-Randal , Lucille Rainbow, N.P Randle, Trevor Ian Robinson, Parul Sharma, Ghada T Shawli, James P Stewart, Neil Swainston, Ecaterina Vamos, Joanne Watts, Mark Whitehead
EPI_ISL_665268	Quadram Institute Bioscience	COVID-19 Genomics UK (COG-UK) Consortium	Dave J. Baker, Gemma L. Kay, Alp Aydin, Thanh Le-Viet, Steven Rudder, Ana P. Tedim, Anastasia Kolyva, Maria Diaz, Leonardo de Oliveira Martins, Nabil-Fareed Alikhan, Lizzie Meadows, Rachael Stanley, Ngozi Elumogo, Muhammed Yasir, Nicholas M. Thomson, Alexander J Trotter, Rachel Gilroy, Samuel Bloomfield, Claire Stuart, Andrew Bell, Reenesh Prakash, Samir Dervisevic, Alison E. Mather, John Wain, Mark Webber, Andrew J. Page, Justin O'Grady
EPI_ISL_665286	West of Scotland Specialist Virology Centre, NHSGGC / MRC-University of Glasgow Centre for Virus Research	COVID-19 Genomics UK (COG-UK) Consortium	Ana da Silva Filipe, Natasha Johnson, Kathy Smollett, Daniel Mair, Stephen Carmichael, Alice Broos, Lily Tong, Jenna Nichols, Kyriaki Nomikou; Sarah McDonald; Richard Orton, Joseph Hughes, Sreenu Vattipally, David L Robertson; Alasdair MacLean, Rory Gunson; Sharif Shaaban, Matthew Holden; Rachel Blacow, Guy Mollett, Kathy Li, James Shepherd, Antonia Ho, Emma Thomson
EPI_ISL_665287	University College London Hospital	COVID-19 Genomics UK (COG-UK) Consortium	Judith Heaney, Matthew Byott, Catherine Houlihan, Dan Frampton, Stuart Kirk, Moira Spyer and Eleni Nastouli
EPI_ISL_665290, EPI_ISL_665291	Queens Medical Centre, Clinical Microbiology Department / DeepSeq Nottingham	COVID-19 Genomics UK (COG-UK) Consortium	Gemma Clark, Wendy Smith, Manjinder Khakh, Vicki M Fleming, Michelle M Lister, Hannah Howson-Wells, Jonathan Ball, Patrick McClure, Joseph Chappell, Theocharis Tsoleridis, Nadine Holmes, Matthew Carlisle, Christopher Moore, Fei Sang, Johnny Debebe, Victoria Wright, Matthew Loose
EPI_ISL_665316	Quadram Institute Bioscience	COVID-19 Genomics UK (COG-UK) Consortium	Dave J. Baker, Gemma L. Kay, Alp Aydin, Thanh Le-Viet, Steven Rudder, Ana P. Tedim, Anastasia Kolyva, Maria Diaz, Leonardo de Oliveira Martins, Nabil-Fareed Alikhan, Lizzie Meadows, Rachael Stanley, Ngozi Elumogo, Muhammed Yasir, Nicholas M. Thomson, Alexander J Trotter, Rachel Gilroy, Samuel Bloomfield, Claire Stuart, Andrew Bell, Reenesh Prakash, Samir Dervisevic, Alison E. Mather, John Wain, Mark Webber, Andrew J. Page, Justin O'Grady
EPI_ISL_665320	University College London Hospital	COVID-19 Genomics UK (COG-UK) Consortium	Judith Heaney, Matthew Byott, Catherine Houlihan, Dan Frampton, Stuart Kirk, Moira Spyer and Eleni Nastouli
EPI_ISL_665336	Quadram Institute Bioscience	COVID-19 Genomics UK (COG-UK) Consortium	Dave J. Baker, Gemma L. Kay, Alp Aydin, Thanh Le-Viet, Steven Rudder, Ana P. Tedim, Anastasia Kolyva, Maria Diaz, Leonardo de Oliveira Martins, Nabil-Fareed Alikhan, Lizzie Meadows, Rachael Stanley, Ngozi Elumogo, Muhammed Yasir, Nicholas M. Thomson, Alexander J Trotter, Rachel Gilroy, Samuel Bloomfield, Claire Stuart, Andrew Bell, Reenesh Prakash, Samir Dervisevic, Alison E. Mather, John Wain, Mark Webber, Andrew J. Page, Justin O'Grady
EPI_ISL_665345	Queens Medical Centre, Clinical Microbiology Department / DeepSeq Nottingham	COVID-19 Genomics UK (COG-UK) Consortium	Gemma Clark, Wendy Smith, Manjinder Khakh, Vicki M Fleming, Michelle M Lister, Hannah Howson-Wells, Jonathan Ball, Patrick McClure, Joseph Chappell, Theocharis Tsoleridis, Nadine Holmes, Matthew Carlisle, Christopher Moore, Fei Sang, Johnny Debebe, Victoria Wright, Matthew Loose
EPI_ISL_665362	Quadram Institute Bioscience	COVID-19 Genomics UK (COG-UK) Consortium	Dave J. Baker, Gemma L. Kay, Alp Aydin, Thanh Le-Viet, Steven Rudder, Ana P. Tedim, Anastasia Kolyva, Maria Diaz, Leonardo de Oliveira Martins, Nabil-Fareed Alikhan, Lizzie Meadows, Rachael Stanley, Ngozi Elumogo, Muhammed Yasir, Nicholas M. Thomson, Alexander J Trotter, Rachel Gilroy, Samuel Bloomfield, Claire Stuart, Andrew Bell, Reenesh Prakash, Samir Dervisevic, Alison E. Mather, John Wain, Mark Webber, Andrew J. Page, Justin O'Grady
EPI_ISL_665363	West of Scotland Specialist Virology Centre, NHSGGC / MRC-University of Glasgow Centre for Virus Research	COVID-19 Genomics UK (COG-UK) Consortium	Ana da Silva Filipe, Natasha Johnson, Kathy Smollett, Daniel Mair, Stephen Carmichael, Alice Broos, Lily Tong, Jenna Nichols, Kyriaki Nomikou; Sarah McDonald; Richard Orton, Joseph Hughes, Sreenu Vattipally, David L Robertson; Alasdair MacLean, Rory Gunson; Sharif Shaaban, Matthew Holden; Rachel Blacow, Guy Mollett, Kathy Li, James Shepherd, Antonia Ho, Emma Thomson

EPI_ISL_665369, EPI_ISL_665370, EPI_ISL_665377, EPI_ISL_665380, EPI_ISL_665381	Quadram Institute Bioscience	COVID-19 Genomics UK (COG-UK) Consortium	Dave J. Baker, Gemma L. Kay, Alp Aydin, Thanh Le-Viet, Steven Rudder, Ana P. Tedim, Anastasia Kolyva, Maria Diaz, Leonardo de Oliveira Martins, Nabil-Fareed Alikhan, Lizzie Meadows, Rachael Stanley, Ngozi Elumogo, Muhammed Yasir, Nicholas M. Thomson, Alexander J Trotter, Rachel Gilroy, Samuel Bloomfield, Claire Stuart, Andrew Bell, Reenesh Prakash, Samir Dervisevic, Alison E. Mather, John Wain, Mark Webber, Andrew J. Page, Justin O'Grady
EPI_ISL_665415	Queens Medical Centre, Clinical Microbiology Department / DeepSeq Nottingham	COVID-19 Genomics UK (COG-UK) Consortium	Gemma Clark, Wendy Smith, Manjinder Khakh, Vicki M Fleming, Michelle M Lister, Hannah Howson-Wells, Jonathan Ball, Patrick McClure, Joseph Chappell, Theocharis Tsoleridis, Nadine Holmes, Matthew Carlisle, Christopher Moore, Fei Sang, Johnny Debebe, Victoria Wright, Matthew Loose
EPI_ISL_665416	West of Scotland Specialist Virology Centre, NHSGGC / MRC-University of Glasgow Centre for Virus Research	COVID-19 Genomics UK (COG-UK) Consortium	Ana da Silva Filipe, Natasha Johnson, Kathy Smollett, Daniel Mair, Stephen Carmichael, Alice Broos, Lily Tong, Jenna Nichols, Kyriaki Nomikou; Sarah McDonald; Richard Orton, Joseph Hughes, Sreenu Vattipally, David L Robertson; Alasdair MacLean, Rory Gunson; Sharif Shaaban, Matthew Holden; Rachel Blacow, Guy Mollett, Kathy Li, James Shepherd, Antonia Ho, Emma Thomson
EPI_ISL_665420, EPI_ISL_665421	Queens Medical Centre, Clinical Microbiology Department / DeepSeq Nottingham	COVID-19 Genomics UK (COG-UK) Consortium	Gemma Clark, Wendy Smith, Manjinder Khakh, Vicki M Fleming, Michelle M Lister, Hannah Howson-Wells, Jonathan Ball, Patrick McClure, Joseph Chappell, Theocharis Tsoleridis, Nadine Holmes, Matthew Carlisle, Christopher Moore, Fei Sang, Johnny Debebe, Victoria Wright, Matthew Loose
EPI_ISL_665423	Quadram Institute Bioscience	COVID-19 Genomics UK (COG-UK) Consortium	Dave J. Baker, Gemma L. Kay, Alp Aydin, Thanh Le-Viet, Steven Rudder, Ana P. Tedim, Anastasia Kolyva, Maria Diaz, Leonardo de Oliveira Martins, Nabil-Fareed Alikhan, Lizzie Meadows, Rachael Stanley, Ngozi Elumogo, Muhammed Yasir, Nicholas M. Thomson, Alexander J Trotter, Rachel Gilroy, Samuel Bloomfield, Claire Stuart, Andrew Bell, Reenesh Prakash, Samir Dervisevic, Alison E. Mather, John Wain, Mark Webber, Andrew J. Page, Justin O'Grady
EPI_ISL_665424	Queens Medical Centre, Clinical Microbiology Department / DeepSeq Nottingham	COVID-19 Genomics UK (COG-UK) Consortium	Gemma Clark, Wendy Smith, Manjinder Khakh, Vicki M Fleming, Michelle M Lister, Hannah Howson-Wells, Jonathan Ball, Patrick McClure, Joseph Chappell, Theocharis Tsoleridis, Nadine Holmes, Matthew Carlisle, Christopher Moore, Fei Sang, Johnny Debebe, Victoria Wright, Matthew Loose
EPI_ISL_665425	West of Scotland Specialist Virology Centre, NHSGGC / MRC-University of Glasgow Centre for Virus Research	COVID-19 Genomics UK (COG-UK) Consortium	Ana da Silva Filipe, Natasha Johnson, Kathy Smollett, Daniel Mair, Stephen Carmichael, Alice Broos, Lily Tong, Jenna Nichols, Kyriaki Nomikou; Sarah McDonald; Richard Orton, Joseph Hughes, Sreenu Vattipally, David L Robertson; Alasdair MacLean, Rory Gunson; Sharif Shaaban, Matthew Holden; Rachel Blacow, Guy Mollett, Kathy Li, James Shepherd, Antonia Ho, Emma Thomson
EPI_ISL_665426	Queens Medical Centre, Clinical Microbiology Department / DeepSeq Nottingham	COVID-19 Genomics UK (COG-UK) Consortium	Gemma Clark, Wendy Smith, Manjinder Khakh, Vicki M Fleming, Michelle M Lister, Hannah Howson-Wells, Jonathan Ball, Patrick McClure, Joseph Chappell, Theocharis Tsoleridis, Nadine Holmes, Matthew Carlisle, Christopher Moore, Fei Sang, Johnny Debebe, Victoria Wright, Matthew Loose
EPI_ISL_665427, EPI_ISL_665429	Quadram Institute Bioscience	COVID-19 Genomics UK (COG-UK) Consortium	Dave J. Baker, Gemma L. Kay, Alp Aydin, Thanh Le-Viet, Steven Rudder, Ana P. Tedim, Anastasia Kolyva, Maria Diaz, Leonardo de Oliveira Martins, Nabil-Fareed Alikhan, Lizzie Meadows, Rachael Stanley, Ngozi Elumogo, Muhammed Yasir, Nicholas M. Thomson, Alexander J Trotter, Rachel Gilroy, Samuel Bloomfield, Claire Stuart, Andrew Bell, Reenesh Prakash, Samir Dervisevic, Alison E. Mather, John Wain, Mark Webber, Andrew J. Page, Justin O'Grady
EPI_ISL_665430	Queens Medical Centre, Clinical Microbiology Department / DeepSeq Nottingham	COVID-19 Genomics UK (COG-UK) Consortium	Gemma Clark, Wendy Smith, Manjinder Khakh, Vicki M Fleming, Michelle M Lister, Hannah Howson-Wells, Jonathan Ball, Patrick McClure, Joseph Chappell, Theocharis Tsoleridis, Nadine Holmes, Matthew Carlisle, Christopher Moore, Fei Sang, Johnny Debebe, Victoria Wright, Matthew Loose
EPI_ISL_665436	West of Scotland Specialist Virology Centre, NHSGGC / MRC-University of Glasgow Centre for Virus Research	COVID-19 Genomics UK (COG-UK) Consortium	Ana da Silva Filipe, Natasha Johnson, Kathy Smollett, Daniel Mair, Stephen Carmichael, Alice Broos, Lily Tong, Jenna Nichols, Kyriaki Nomikou; Sarah McDonald; Richard Orton, Joseph Hughes, Sreenu Vattipally, David L Robertson; Alasdair MacLean, Rory Gunson; Sharif Shaaban, Matthew Holden; Rachel Blacow, Guy Mollett, Kathy Li, James Shepherd, Antonia Ho, Emma Thomson
EPI_ISL_665438	Quadram Institute Bioscience	COVID-19 Genomics UK (COG-UK) Consortium	Dave J. Baker, Gemma L. Kay, Alp Aydin, Thanh Le-Viet, Steven Rudder, Ana P. Tedim, Anastasia Kolyva, Maria Diaz, Leonardo de Oliveira Martins, Nabil-Fareed Alikhan, Lizzie Meadows, Rachael Stanley, Ngozi Elumogo, Muhammed Yasir, Nicholas M. Thomson, Alexander J Trotter, Rachel Gilroy, Samuel Bloomfield, Claire Stuart, Andrew Bell, Reenesh Prakash, Samir Dervisevic, Alison E. Mather, John Wain, Mark Webber, Andrew J. Page, Justin O'Grady
EPI_ISL_665439	West of Scotland Specialist Virology Centre, NHSGGC / MRC-University of Glasgow Centre for Virus Research	COVID-19 Genomics UK (COG-UK) Consortium	Ana da Silva Filipe, Natasha Johnson, Kathy Smollett, Daniel Mair, Stephen Carmichael, Alice Broos, Lily Tong, Jenna Nichols, Kyriaki Nomikou; Sarah McDonald; Richard Orton, Joseph Hughes, Sreenu Vattipally, David L Robertson; Alasdair MacLean, Rory Gunson; Sharif Shaaban, Matthew Holden; Rachel Blacow, Guy Mollett, Kathy Li, James Shepherd, Antonia Ho, Emma Thomson
EPI_ISL_665440	Quadram Institute Bioscience	COVID-19 Genomics UK (COG-UK) Consortium	Dave J. Baker, Gemma L. Kay, Alp Aydin, Thanh Le-Viet, Steven Rudder, Ana P. Tedim, Anastasia Kolyva, Maria Diaz, Leonardo de Oliveira Martins, Nabil-Fareed Alikhan, Lizzie Meadows, Rachael Stanley, Ngozi Elumogo, Muhammed Yasir, Nicholas M. Thomson, Alexander J Trotter, Rachel Gilroy, Samuel Bloomfield, Claire Stuart, Andrew Bell, Reenesh Prakash, Samir Dervisevic, Alison E. Mather, John Wain, Mark Webber, Andrew J. Page, Justin O'Grady
EPI_ISL_665443	Queens Medical Centre, Clinical Microbiology Department / DeepSeq Nottingham	COVID-19 Genomics UK (COG-UK) Consortium	Gemma Clark, Wendy Smith, Manjinder Khakh, Vicki M Fleming, Michelle M Lister, Hannah Howson-Wells, Jonathan Ball, Patrick McClure, Joseph Chappell, Theocharis Tsoleridis, Nadine Holmes, Matthew Carlisle, Christopher Moore, Fei Sang, Johnny Debebe, Victoria Wright, Matthew Loose
EPI_ISL_665450	Quadram Institute Bioscience	COVID-19 Genomics UK (COG-UK) Consortium	Dave J. Baker, Gemma L. Kay, Alp Aydin, Thanh Le-Viet, Steven Rudder, Ana P. Tedim, Anastasia Kolyva, Maria Diaz, Leonardo de Oliveira Martins, Nabil-Fareed Alikhan, Lizzie Meadows, Rachael Stanley, Ngozi Elumogo, Muhammed Yasir, Nicholas M. Thomson, Alexander J Trotter, Rachel Gilroy, Samuel Bloomfield, Claire Stuart, Andrew Bell, Reenesh Prakash, Samir Dervisevic, Alison E. Mather, John Wain, Mark Webber, Andrew J. Page, Justin O'Grady
EPI_ISL_665452	Queens Medical Centre, Clinical Microbiology Department / DeepSeq Nottingham	COVID-19 Genomics UK (COG-UK) Consortium	Gemma Clark, Wendy Smith, Manjinder Khakh, Vicki M Fleming, Michelle M Lister, Hannah Howson-Wells, Jonathan Ball, Patrick McClure, Joseph Chappell, Theocharis Tsoleridis, Nadine Holmes, Matthew Carlisle, Christopher Moore, Fei Sang, Johnny Debebe, Victoria Wright, Matthew Loose
EPI_ISL_665453, EPI_ISL_665463, EPI_ISL_665465, EPI_ISL_665475, EPI_ISL_665508	Quadram Institute Bioscience	COVID-19 Genomics UK (COG-UK) Consortium	Dave J. Baker, Gemma L. Kay, Alp Aydin, Thanh Le-Viet, Steven Rudder, Ana P. Tedim, Anastasia Kolyva, Maria Diaz, Leonardo de Oliveira Martins, Nabil-Fareed Alikhan, Lizzie Meadows, Rachael Stanley, Ngozi Elumogo, Muhammed Yasir, Nicholas M. Thomson, Alexander J Trotter, Rachel Gilroy, Samuel Bloomfield, Claire Stuart, Andrew Bell, Reenesh Prakash, Samir Dervisevic, Alison E. Mather, John Wain, Mark Webber, Andrew J. Page, Justin O'Grady
EPI_ISL_665512	Queens Medical Centre, Clinical Microbiology Department / DeepSeq Nottingham	COVID-19 Genomics UK (COG-UK) Consortium	Gemma Clark, Wendy Smith, Manjinder Khakh, Vicki M Fleming, Michelle M Lister, Hannah Howson-Wells, Jonathan Ball, Patrick McClure, Joseph Chappell, Theocharis Tsoleridis, Nadine Holmes, Matthew Carlisle, Christopher Moore, Fei Sang, Johnny Debebe, Victoria Wright, Matthew Loose
EPI_ISL_665518, EPI_ISL_665519	Quadram Institute Bioscience	COVID-19 Genomics UK (COG-UK) Consortium	Dave J. Baker, Gemma L. Kay, Alp Aydin, Thanh Le-Viet, Steven Rudder, Ana P. Tedim, Anastasia Kolyva, Maria Diaz, Leonardo de Oliveira Martins, Nabil-Fareed Alikhan, Lizzie Meadows, Rachael Stanley, Ngozi Elumogo, Muhammed Yasir, Nicholas M. Thomson, Alexander J Trotter, Rachel Gilroy, Samuel Bloomfield, Claire Stuart, Andrew Bell, Reenesh Prakash, Samir Dervisevic, Alison E. Mather, John Wain, Mark Webber, Andrew J. Page, Justin O'Grady
EPI_ISL_665521, EPI_ISL_665524	West of Scotland Specialist Virology Centre, NHSGGC / MRC-University of Glasgow Centre for Virus Research	COVID-19 Genomics UK (COG-UK) Consortium	Ana da Silva Filipe, Natasha Johnson, Kathy Smollett, Daniel Mair, Stephen Carmichael, Alice Broos, Lily Tong, Jenna Nichols, Kyriaki Nomikou; Sarah McDonald; Richard Orton, Joseph Hughes, Sreenu Vattipally, David L Robertson; Alasdair MacLean, Rory Gunson; Sharif Shaaban, Matthew Holden; Rachel Blacow, Guy Mollett, Kathy Li, James Shepherd, Antonia Ho, Emma Thomson
EPI_ISL_665528	Queens Medical Centre, Clinical Microbiology Department / DeepSeq Nottingham	COVID-19 Genomics UK (COG-UK) Consortium	Gemma Clark, Wendy Smith, Manjinder Khakh, Vicki M Fleming, Michelle M Lister, Hannah Howson-Wells, Jonathan Ball, Patrick McClure, Joseph Chappell, Theocharis Tsoleridis, Nadine Holmes, Matthew Carlisle, Christopher Moore, Fei Sang, Johnny Debebe, Victoria Wright, Matthew Loose
EPI_ISL_665531, EPI_ISL_665532	West of Scotland Specialist Virology Centre, NHSGGC / MRC-University of Glasgow Centre for Virus Research	COVID-19 Genomics UK (COG-UK) Consortium	Ana da Silva Filipe, Natasha Johnson, Kathy Smollett, Daniel Mair, Stephen Carmichael, Alice Broos, Lily Tong, Jenna Nichols, Kyriaki Nomikou; Sarah McDonald; Richard Orton, Joseph Hughes, Sreenu Vattipally, David L Robertson; Alasdair MacLean, Rory Gunson; Sharif Shaaban, Matthew Holden; Rachel Blacow, Guy Mollett, Kathy Li, James Shepherd, Antonia Ho, Emma Thomson
EPI_ISL_665534	Quadram Institute Bioscience	COVID-19 Genomics UK (COG-UK) Consortium	Dave J. Baker, Gemma L. Kay, Alp Aydin, Thanh Le-Viet, Steven Rudder, Ana P. Tedim, Anastasia Kolyva, Maria Diaz, Leonardo de Oliveira Martins, Nabil-Fareed Alikhan, Lizzie Meadows, Rachael Stanley, Ngozi Elumogo, Muhammed Yasir, Nicholas M. Thomson, Alexander J Trotter, Rachel Gilroy, Samuel Bloomfield, Claire Stuart, Andrew Bell, Reenesh Prakash, Samir Dervisevic, Alison E. Mather, John Wain, Mark Webber, Andrew J. Page, Justin O'Grady
EPI_ISL_665538	West of Scotland Specialist Virology Centre, NHSGGC / MRC-University of Glasgow Centre for Virus Research	COVID-19 Genomics UK (COG-UK) Consortium	Ana da Silva Filipe, Natasha Johnson, Kathy Smollett, Daniel Mair, Stephen Carmichael, Alice Broos, Lily Tong, Jenna Nichols, Kyriaki Nomikou; Sarah McDonald; Richard Orton, Joseph Hughes, Sreenu Vattipally, David L Robertson; Alasdair MacLean, Rory Gunson; Sharif Shaaban, Matthew Holden; Rachel Blacow, Guy Mollett, Kathy Li, James Shepherd, Antonia Ho, Emma Thomson

EPI_ISL_665543, EPI_ISL_665544, EPI_ISL_665550, EPI_ISL_665551	Queens Medical Centre, Clinical Microbiology Department / DeepSeq Nottingham	COVID-19 Genomics UK (COG-UK) Consortium	Gemma Clark, Wendy Smith, Manjinder Khakh, Vicki M Fleming, Michelle M Lister, Hannah Howson-Wells, Jonathan Ball, Patrick McClure, Joseph Chappell, Theocharis Tsoleridis, Nadine Holmes, Matthew Carlisle, Christopher Moore, Fei Sang, Johnny Debebe, Victoria Wright, Matthew Loose
EPI_ISL_665560	Quadram Institute Bioscience	COVID-19 Genomics UK (COG-UK) Consortium	Dave J. Baker, Gemma L. Kay, Alp Aydin, Thanh Le-Viet, Steven Rudder, Ana P. Tedim, Anastasia Kolyva, Maria Diaz, Leonardo de Oliveira Martins, Nabil-Fareed Alikhan, Lizzie Meadows, Rachael Stanley, Ngozi Elumogo, Muhammed Yasir, Nicholas M. Thomson, Alexander J Trotter, Rachel Gilroy, Samuel Bloomfield, Claire Stuart, Andrew Bell, Reenesh Prakash, Samir Dervisevic, Alison E. Mather, John Wain, Mark Webber, Andrew J. Page, Justin O'Grady
EPI_ISL_665561, EPI_ISL_665562	Queens Medical Centre, Clinical Microbiology Department / DeepSeq Nottingham	COVID-19 Genomics UK (COG-UK) Consortium	Gemma Clark, Wendy Smith, Manjinder Khakh, Vicki M Fleming, Michelle M Lister, Hannah Howson-Wells, Jonathan Ball, Patrick McClure, Joseph Chappell, Theocharis Tsoleridis, Nadine Holmes, Matthew Carlisle, Christopher Moore, Fei Sang, Johnny Debebe, Victoria Wright, Matthew Loose
EPI_ISL_665574	Quadram Institute Bioscience	COVID-19 Genomics UK (COG-UK) Consortium	Dave J. Baker, Gemma L. Kay, Alp Aydin, Thanh Le-Viet, Steven Rudder, Ana P. Tedim, Anastasia Kolyva, Maria Diaz, Leonardo de Oliveira Martins, Nabil-Fareed Alikhan, Lizzie Meadows, Rachael Stanley, Ngozi Elumogo, Muhammed Yasir, Nicholas M. Thomson, Alexander J Trotter, Rachel Gilroy, Samuel Bloomfield, Claire Stuart, Andrew Bell, Reenesh Prakash, Samir Dervisevic, Alison E. Mather, John Wain, Mark Webber, Andrew J. Page, Justin O'Grady
EPI_ISL_665579	West of Scotland Specialist Virology Centre, NHSGGC / MRC-University of Glasgow Centre for Virus Research	COVID-19 Genomics UK (COG-UK) Consortium	Ana da Silva Filipe, Natasha Johnson, Kathy Smollett, Daniel Mair, Stephen Carmichael, Alice Broos, Lily Tong, Jenna Nichols, Kyriaki Nomikou; Sarah McDonald; Richard Orton, Joseph Hughes, Sreenu Vattipally, David L Robertson; Alasdair MacLean, Rory Gunson; Sharif Shaaban, Matthew Holden; Rachel Blacow, Guy Mollett, Kathy Li, James Shepherd, Antonia Ho, Emma Thomson
EPI_ISL_665594, EPI_ISL_665596, EPI_ISL_665599	Quadram Institute Bioscience	COVID-19 Genomics UK (COG-UK) Consortium	Dave J. Baker, Gemma L. Kay, Alp Aydin, Thanh Le-Viet, Steven Rudder, Ana P. Tedim, Anastasia Kolyva, Maria Diaz, Leonardo de Oliveira Martins, Nabil-Fareed Alikhan, Lizzie Meadows, Rachael Stanley, Ngozi Elumogo, Muhammed Yasir, Nicholas M. Thomson, Alexander J Trotter, Rachel Gilroy, Samuel Bloomfield, Claire Stuart, Andrew Bell, Reenesh Prakash, Samir Dervisevic, Alison E. Mather, John Wain, Mark Webber, Andrew J. Page, Justin O'Grady
EPI_ISL_665601	Queens Medical Centre, Clinical Microbiology Department / DeepSeq Nottingham	COVID-19 Genomics UK (COG-UK) Consortium	Gemma Clark, Wendy Smith, Manjinder Khakh, Vicki M Fleming, Michelle M Lister, Hannah Howson-Wells, Jonathan Ball, Patrick McClure, Joseph Chappell, Theocharis Tsoleridis, Nadine Holmes, Matthew Carlisle, Christopher Moore, Fei Sang, Johnny Debebe, Victoria Wright, Matthew Loose
EPI_ISL_665603	Quadram Institute Bioscience	COVID-19 Genomics UK (COG-UK) Consortium	Dave J. Baker, Gemma L. Kay, Alp Aydin, Thanh Le-Viet, Steven Rudder, Ana P. Tedim, Anastasia Kolyva, Maria Diaz, Leonardo de Oliveira Martins, Nabil-Fareed Alikhan, Lizzie Meadows, Rachael Stanley, Ngozi Elumogo, Muhammed Yasir, Nicholas M. Thomson, Alexander J Trotter, Rachel Gilroy, Samuel Bloomfield, Claire Stuart, Andrew Bell, Reenesh Prakash, Samir Dervisevic, Alison E. Mather, John Wain, Mark Webber, Andrew J. Page, Justin O'Grady
EPI_ISL_665609	West of Scotland Specialist Virology Centre, NHSGGC / MRC-University of Glasgow Centre for Virus Research	COVID-19 Genomics UK (COG-UK) Consortium	Ana da Silva Filipe, Natasha Johnson, Kathy Smollett, Daniel Mair, Stephen Carmichael, Alice Broos, Lily Tong, Jenna Nichols, Kyriaki Nomikou; Sarah McDonald; Richard Orton, Joseph Hughes, Sreenu Vattipally, David L Robertson; Alasdair MacLean, Rory Gunson; Sharif Shaaban, Matthew Holden; Rachel Blacow, Guy Mollett, Kathy Li, James Shepherd, Antonia Ho, Emma Thomson
EPI_ISL_665612	Queens Medical Centre, Clinical Microbiology Department / DeepSeq Nottingham	COVID-19 Genomics UK (COG-UK) Consortium	Gemma Clark, Wendy Smith, Manjinder Khakh, Vicki M Fleming, Michelle M Lister, Hannah Howson-Wells, Jonathan Ball, Patrick McClure, Joseph Chappell, Theocharis Tsoleridis, Nadine Holmes, Matthew Carlisle, Christopher Moore, Fei Sang, Johnny Debebe, Victoria Wright, Matthew Loose
EPI_ISL_665616	Centre for Enzyme Innovation, University of Portsmouth / Translational Research Laboratory, Portsmouth Hospitals NHS Trust	COVID-19 Genomics UK (COG-UK) Consortium	Angela Beckett,Yann Bourgeois,Garry Scarlett,Sharon Glaysher,Scott Elliott,Kelly Bicknell,Robert Impey,Allyson Lloyd,Sarah Wyllie,Ethan Butcher,Anoop Chauhan,Samuel Robson
EPI_ISL_665622	Queens Medical Centre, Clinical Microbiology Department / DeepSeq Nottingham	COVID-19 Genomics UK (COG-UK) Consortium	Gemma Clark, Wendy Smith, Manjinder Khakh, Vicki M Fleming, Michelle M Lister, Hannah Howson-Wells, Jonathan Ball, Patrick McClure, Joseph Chappell, Theocharis Tsoleridis, Nadine Holmes, Matthew Carlisle, Christopher Moore, Fei Sang, Johnny Debebe, Victoria Wright, Matthew Loose
EPI_ISL_665625	Centre for Enzyme Innovation, University of Portsmouth / Translational Research Laboratory, Portsmouth Hospitals NHS Trust	COVID-19 Genomics UK (COG-UK) Consortium	Angela Beckett,Yann Bourgeois,Garry Scarlett,Sharon Glaysher,Scott Elliott,Kelly Bicknell,Robert Impey,Allyson Lloyd,Sarah Wyllie,Ethan Butcher,Anoop Chauhan,Samuel Robson
EPI_ISL_665636, EPI_ISL_665637	University College London Hospital	COVID-19 Genomics UK (COG-UK) Consortium	Judith Heaney, Matthew Byott, Catherine Houlihan, Dan Frampton, Stuart Kirk, Moira Spyer and Eleni Nastouli
EPI_ISL_665638, EPI_ISL_665639	Quadram Institute Bioscience	COVID-19 Genomics UK (COG-UK) Consortium	Dave J. Baker, Gemma L. Kay, Alp Aydin, Thanh Le-Viet, Steven Rudder, Ana P. Tedim, Anastasia Kolyva, Maria Diaz, Leonardo de Oliveira Martins, Nabil-Fareed Alikhan, Lizzie Meadows, Rachael Stanley, Ngozi Elumogo, Muhammed Yasir, Nicholas M. Thomson, Alexander J Trotter, Rachel Gilroy, Samuel Bloomfield, Claire Stuart, Andrew Bell, Reenesh Prakash, Samir Dervisevic, Alison E. Mather, John Wain, Mark Webber, Andrew J. Page, Justin O'Grady
EPI_ISL_665641	West of Scotland Specialist Virology Centre, NHSGGC / MRC-University of Glasgow Centre for Virus Research	COVID-19 Genomics UK (COG-UK) Consortium	Ana da Silva Filipe, Natasha Johnson, Kathy Smollett, Daniel Mair, Stephen Carmichael, Alice Broos, Lily Tong, Jenna Nichols, Kyriaki Nomikou; Sarah McDonald; Richard Orton, Joseph Hughes, Sreenu Vattipally, David L Robertson; Alasdair MacLean, Rory Gunson; Sharif Shaaban, Matthew Holden; Rachel Blacow, Guy Mollett, Kathy Li, James Shepherd, Antonia Ho, Emma Thomson
EPI_ISL_665643, EPI_ISL_665645, EPI_ISL_665648	University College London Hospital	COVID-19 Genomics UK (COG-UK) Consortium	Judith Heaney, Matthew Byott, Catherine Houlihan, Dan Frampton, Stuart Kirk, Moira Spyer and Eleni Nastouli
EPI_ISL_665665, EPI_ISL_665668, EPI_ISL_665678	Quadram Institute Bioscience	COVID-19 Genomics UK (COG-UK) Consortium	Dave J. Baker, Gemma L. Kay, Alp Aydin, Thanh Le-Viet, Steven Rudder, Ana P. Tedim, Anastasia Kolyva, Maria Diaz, Leonardo de Oliveira Martins, Nabil-Fareed Alikhan, Lizzie Meadows, Rachael Stanley, Ngozi Elumogo, Muhammed Yasir, Nicholas M. Thomson, Alexander J Trotter, Rachel Gilroy, Samuel Bloomfield, Claire Stuart, Andrew Bell, Reenesh Prakash, Samir Dervisevic, Alison E. Mather, John Wain, Mark Webber, Andrew J. Page, Justin O'Grady
EPI_ISL_665685	West of Scotland Specialist Virology Centre, NHSGGC / MRC-University of Glasgow Centre for Virus Research	COVID-19 Genomics UK (COG-UK) Consortium	Ana da Silva Filipe, Natasha Johnson, Kathy Smollett, Daniel Mair, Stephen Carmichael, Alice Broos, Lily Tong, Jenna Nichols, Kyriaki Nomikou; Sarah McDonald; Richard Orton, Joseph Hughes, Sreenu Vattipally, David L Robertson; Alasdair MacLean, Rory Gunson; Sharif Shaaban, Matthew Holden; Rachel Blacow, Guy Mollett, Kathy Li, James Shepherd, Antonia Ho, Emma Thomson
EPI_ISL_665689, EPI_ISL_665692, EPI_ISL_665693, EPI_ISL_665697, EPI_ISL_665712, EPI_ISL_665714, EPI_ISL_665730, EPI_ISL_665737	Quadram Institute Bioscience	COVID-19 Genomics UK (COG-UK) Consortium	Dave J. Baker, Gemma L. Kay, Alp Aydin, Thanh Le-Viet, Steven Rudder, Ana P. Tedim, Anastasia Kolyva, Maria Diaz, Leonardo de Oliveira Martins, Nabil-Fareed Alikhan, Lizzie Meadows, Rachael Stanley, Ngozi Elumogo, Muhammed Yasir, Nicholas M. Thomson, Alexander J Trotter, Rachel Gilroy, Samuel Bloomfield, Claire Stuart, Andrew Bell, Reenesh Prakash, Samir Dervisevic, Alison E. Mather, John Wain, Mark Webber, Andrew J. Page, Justin O'Grady
EPI_ISL_665740	Queens Medical Centre, Clinical Microbiology Department / DeepSeq Nottingham	COVID-19 Genomics UK (COG-UK) Consortium	Gemma Clark, Wendy Smith, Manjinder Khakh, Vicki M Fleming, Michelle M Lister, Hannah Howson-Wells, Jonathan Ball, Patrick McClure, Joseph Chappell, Theocharis Tsoleridis, Nadine Holmes, Matthew Carlisle, Christopher Moore, Fei Sang, Johnny Debebe, Victoria Wright, Matthew Loose
EPI_ISL_665746, EPI_ISL_665749	Quadram Institute Bioscience	COVID-19 Genomics UK (COG-UK) Consortium	Dave J. Baker, Gemma L. Kay, Alp Aydin, Thanh Le-Viet, Steven Rudder, Ana P. Tedim, Anastasia Kolyva, Maria Diaz, Leonardo de Oliveira Martins, Nabil-Fareed Alikhan, Lizzie Meadows, Rachael Stanley, Ngozi Elumogo, Muhammed Yasir, Nicholas M. Thomson, Alexander J Trotter, Rachel Gilroy, Samuel Bloomfield, Claire Stuart, Andrew Bell, Reenesh Prakash, Samir Dervisevic, Alison E. Mather, John Wain, Mark Webber, Andrew J. Page, Justin O'Grady
EPI_ISL_665757, EPI_ISL_665758, EPI_ISL_665786	University College London Hospital	COVID-19 Genomics UK (COG-UK) Consortium	Judith Heaney, Matthew Byott, Catherine Houlihan, Dan Frampton, Stuart Kirk, Moira Spyer and Eleni Nastouli
EPI_ISL_665789	Queens Medical Centre, Clinical Microbiology Department / DeepSeq Nottingham	COVID-19 Genomics UK (COG-UK) Consortium	Gemma Clark, Wendy Smith, Manjinder Khakh, Vicki M Fleming, Michelle M Lister, Hannah Howson-Wells, Jonathan Ball, Patrick McClure, Joseph Chappell, Theocharis Tsoleridis, Nadine Holmes, Matthew Carlisle, Christopher Moore, Fei Sang, Johnny Debebe, Victoria Wright, Matthew Loose
EPI_ISL_665820, EPI_ISL_665821, EPI_ISL_665824, EPI_ISL_665825, EPI_ISL_665832, EPI_ISL_665833, EPI_ISL_665834, EPI_ISL_665835, EPI_ISL_665836, EPI_ISL_665837, EPI_ISL_665838, EPI_ISL_665839, EPI_ISL_665840, EPI_ISL_665841, EPI_ISL_665845, EPI_ISL_665846, EPI_ISL_665847, EPI_ISL_665848			
see above	University College London Hospital	COVID-19 Genomics UK (COG-UK) Consortium	Judith Heaney, Matthew Byott, Catherine Houlihan, Dan Frampton, Stuart Kirk, Moira Spyer and Eleni Nastouli
EPI_ISL_665876, EPI_ISL_665897	Quadram Institute Bioscience	COVID-19 Genomics UK (COG-UK) Consortium	Dave J. Baker, Gemma L. Kay, Alp Aydin, Thanh Le-Viet, Steven Rudder, Ana P. Tedim, Anastasia Kolyva, Maria Diaz, Leonardo de Oliveira Martins, Nabil-Fareed Alikhan, Lizzie Meadows, Rachael Stanley, Ngozi Elumogo, Muhammed Yasir, Nicholas M. Thomson, Alexander J Trotter, Rachel Gilroy, Samuel Bloomfield, Claire Stuart, Andrew Bell, Reenesh Prakash, Samir Dervisevic, Alison E. Mather, John Wain, Mark Webber, Andrew J. Page, Justin

			O'Grady
EPI_ISL_665901, EPI_ISL_665902, EPI_ISL_665903, EPI_ISL_665904, EPI_ISL_665905, EPI_ISL_665906, EPI_ISL_665907, EPI_ISL_665908, EPI_ISL_665909, EPI_ISL_665910, EPI_ISL_665911, EPI_ISL_665912, EPI_ISL_665913, EPI_ISL_665914, EPI_ISL_665915, EPI_ISL_665916, EPI_ISL_665917, EPI_ISL_665918, EPI_ISL_665919, EPI_ISL_665920, EPI_ISL_665921, EPI_ISL_665922, EPI_ISL_665923, EPI_ISL_665924, EPI_ISL_665925, EPI_ISL_665926, EPI_ISL_665942			
see above	Queens Medical Centre, Clinical Microbiology Department / DeepSeq Nottingham	COVID-19 Genomics UK (COG-UK) Consortium	Gemma Clark, Wendy Smith, Manjinder Khakh, Vicki M Fleming, Michelle M Lister, Hannah Howson-Wells, Jonathan Ball, Patrick McClure, Joseph Chappell, Theocharis Tsoleridis, Nadine Holmes, Matthew Carlisle, Christopher Moore, Fei Sang, Johnny Debebe, Victoria Wright, Matthew Loose
EPI_ISL_666036, EPI_ISL_666038, EPI_ISL_666039, EPI_ISL_666040, EPI_ISL_666041, EPI_ISL_666042, EPI_ISL_666043, EPI_ISL_666044, EPI_ISL_666045, EPI_ISL_666046, EPI_ISL_666047, EPI_ISL_666048, EPI_ISL_666049, EPI_ISL_666050, EPI_ISL_666051, EPI_ISL_666052, EPI_ISL_666053, EPI_ISL_666054, EPI_ISL_666055, EPI_ISL_666056, EPI_ISL_666057, EPI_ISL_666058, EPI_ISL_666059			
see above	West of Scotland Specialist Virology Centre, NHSGGC / MRC-University of Glasgow Centre for Virus Research	COVID-19 Genomics UK (COG-UK) Consortium	Ana da Silva Filipe, Natasha Johnson, Kathy Smollett, Daniel Mair, Stephen Carmichael, Alice Broos, Lily Tong, Jenna Nichols, Kyriaki Nomikou; Sarah McDonald; Richard Orton, Joseph Hughes, Sreenu Vattipally, David L Robertson; Alasdair MacLean, Rory Gunson; Sharif Shaaban, Matthew Holden; Rachel Blacow, Guy Mollett, Kathy Li, James Shepherd, Antonia Ho, Emma Thomson
EPI_ISL_667814, EPI_ISL_667816, EPI_ISL_667822, EPI_ISL_667824, EPI_ISL_667825, EPI_ISL_667828, EPI_ISL_667831, EPI_ISL_667832, EPI_ISL_667836, EPI_ISL_667838, EPI_ISL_667843, EPI_ISL_667844, EPI_ISL_667847, EPI_ISL_667849, EPI_ISL_667850, EPI_ISL_667851, EPI_ISL_667855, EPI_ISL_667857, EPI_ISL_667859, EPI_ISL_667860, EPI_ISL_667863, EPI_ISL_667864, EPI_ISL_667866, EPI_ISL_667867, EPI_ISL_667868, EPI_ISL_667872, EPI_ISL_667873, EPI_ISL_667876, EPI_ISL_667879, EPI_ISL_667881, EPI_ISL_667883, EPI_ISL_667885, EPI_ISL_667887, EPI_ISL_667888, EPI_ISL_667889, EPI_ISL_667891, EPI_ISL_667893, EPI_ISL_667895, EPI_ISL_667898, EPI_ISL_667900, EPI_ISL_667904, EPI_ISL_667907, EPI_ISL_667912, EPI_ISL_667915, EPI_ISL_667917, EPI_ISL_667918, EPI_ISL_667919, EPI_ISL_667920, EPI_ISL_667922, EPI_ISL_667924, EPI_ISL_667925, EPI_ISL_667927, EPI_ISL_667928, EPI_ISL_667929, EPI_ISL_667930, EPI_ISL_667931, EPI_ISL_667938, EPI_ISL_667939, EPI_ISL_667940, EPI_ISL_667941, EPI_ISL_667943, EPI_ISL_667947, EPI_ISL_667948, EPI_ISL_667950, EPI_ISL_667952, EPI_ISL_667957, EPI_ISL_667958, EPI_ISL_667959, EPI_ISL_667960, EPI_ISL_667962, EPI_ISL_667963, EPI_ISL_667966, EPI_ISL_667967, EPI_ISL_667969, EPI_ISL_667970, EPI_ISL_667972, EPI_ISL_667973, EPI_ISL_667975, EPI_ISL_667976, EPI_ISL_667977, EPI_ISL_667978, EPI_ISL_667980, EPI_ISL_667982, EPI_ISL_667983, EPI_ISL_667986, EPI_ISL_667987, EPI_ISL_667989, EPI_ISL_667990, EPI_ISL_667992, EPI_ISL_667996, EPI_ISL_667997, EPI_ISL_667998, EPI_ISL_668000, EPI_ISL_668001, EPI_ISL_668004, EPI_ISL_668005, EPI_ISL_668006, EPI_ISL_668010, EPI_ISL_668013, EPI_ISL_668020, EPI_ISL_668021, EPI_ISL_668022, EPI_ISL_668024, EPI_ISL_668028, EPI_ISL_668029, EPI_ISL_668030, EPI_ISL_668031, EPI_ISL_668032, EPI_ISL_668033, EPI_ISL_668034, EPI_ISL_668035, EPI_ISL_668036, EPI_ISL_668038, EPI_ISL_668044, EPI_ISL_668045, EPI_ISL_668049, EPI_ISL_668050, EPI_ISL_668052, EPI_ISL_668053, EPI_ISL_668054, EPI_ISL_668055, EPI_ISL_668056, EPI_ISL_668057, EPI_ISL_668060, EPI_ISL_668062, EPI_ISL_668063, EPI_ISL_668064, EPI_ISL_668066, EPI_ISL_668067, EPI_ISL_668073, EPI_ISL_668074, EPI_ISL_668075, EPI_ISL_668078, EPI_ISL_668082, EPI_ISL_668083, EPI_ISL_668084, EPI_ISL_668093, EPI_ISL_668094, EPI_ISL_668095, EPI_ISL_668096, EPI_ISL_668098, EPI_ISL_668102, EPI_ISL_668105, EPI_ISL_668106, EPI_ISL_668107, EPI_ISL_668111, EPI_ISL_668113, EPI_ISL_668114, EPI_ISL_668116, EPI_ISL_668117, EPI_ISL_668118, EPI_ISL_668119, EPI_ISL_668120, EPI_ISL_668121, EPI_ISL_668124, EPI_ISL_668125, EPI_ISL_668126, EPI_ISL_668129, EPI_ISL_668131, EPI_ISL_668132, EPI_ISL_668134, EPI_ISL_668135, EPI_ISL_668139, EPI_ISL_668142, EPI_ISL_668143, EPI_ISL_668144, EPI_ISL_668145, EPI_ISL_668146, EPI_ISL_668147, EPI_ISL_668150, EPI_ISL_668151, EPI_ISL_668152, EPI_ISL_668156, EPI_ISL_668157, EPI_ISL_668158, EPI_ISL_668159, EPI_ISL_668160, EPI_ISL_668162, EPI_ISL_668164, EPI_ISL_668165, EPI_ISL_668167, EPI_ISL_668168, EPI_ISL_668170, EPI_ISL_668173, EPI_ISL_668174, EPI_ISL_668175, EPI_ISL_668176, EPI_ISL_668177, EPI_ISL_668178, EPI_ISL_668179, EPI_ISL_668181, EPI_ISL_668182, EPI_ISL_668184, EPI_ISL_668186, EPI_ISL_668187, EPI_ISL_668189, EPI_ISL_668191, EPI_ISL_668193, EPI_ISL_668195, EPI_ISL_668196, EPI_ISL_668198, EPI_ISL_668202, EPI_ISL_668203, EPI_ISL_668204, EPI_ISL_668205, EPI_ISL_668206, EPI_ISL_668207, EPI_ISL_668208, EPI_ISL_668209, EPI_ISL_668210, EPI_ISL_668213, EPI_ISL_668214, EPI_ISL_668215, EPI_ISL_668216, EPI_ISL_668220, EPI_ISL_668221, EPI_ISL_668223, EPI_ISL_668225, EPI_ISL_668226, EPI_ISL_668229, EPI_ISL_668230, EPI_ISL_668231, EPI_ISL_668233, EPI_ISL_668234, EPI_ISL_668235, EPI_ISL_668236, EPI_ISL_668238, EPI_ISL_668241, EPI_ISL_668242, EPI_ISL_668243, EPI_ISL_668245, EPI_ISL_668246, EPI_ISL_668247, EPI_ISL_668248, EPI_ISL_668250, EPI_ISL_668253, EPI_ISL_668256, EPI_ISL_668257, EPI_ISL_668258, EPI_ISL_668259, EPI_ISL_668260, EPI_ISL_668263, EPI_ISL_668264, EPI_ISL_668266, EPI_ISL_668268, EPI_ISL_668269, EPI_ISL_668270, EPI_ISL_668271, EPI_ISL_668272, EPI_ISL_668273, EPI_ISL_668274, EPI_ISL_668276, EPI_ISL_668277, EPI_ISL_668278, EPI_ISL_668279, EPI_ISL_668282, EPI_ISL_668284, EPI_ISL_668285, EPI_ISL_668286, EPI_ISL_668287, EPI_ISL_668288, EPI_ISL_668290, EPI_ISL_668291, EPI_ISL_668292, EPI_ISL_668293, EPI_ISL_668295, EPI_ISL_668300, EPI_ISL_668301, EPI_ISL_668302, EPI_ISL_668303, EPI_ISL_668304, EPI_ISL_668305, EPI_ISL_668306, EPI_ISL_668308, EPI_ISL_668309, EPI_ISL_668310			
see above	Lighthouse Lab in Glasgow	Wellcome Sanger Institute for the COVID-19 Genomics UK (COG-UK) Consortium	Harper VanSteenhouse, Yumi Kasai, David Gray, Carol Clugston, Anna Dominiczak and Alex Alderton, Roberto Amato, Sonia Goncalves, Ewan Harrison, David K. Jackson, Ian Johnston, Dominic Kwiatkowski, Cordelia Langford, John Sillitoe on behalf of the Wellcome Sanger Institute COVID-19 Surveillance Team
EPI_ISL_668311, EPI_ISL_668312, EPI_ISL_668314, EPI_ISL_668318, EPI_ISL_668320, EPI_ISL_668323, EPI_ISL_668324, EPI_ISL_668325, EPI_ISL_668326, EPI_ISL_668327, EPI_ISL_668328, EPI_ISL_668330, EPI_ISL_668331, EPI_ISL_668332, EPI_ISL_668333, EPI_ISL_668334, EPI_ISL_668335, EPI_ISL_668337, EPI_ISL_668338, EPI_ISL_668339, EPI_ISL_668340, EPI_ISL_668343, EPI_ISL_668346, EPI_ISL_668347, EPI_ISL_668349, EPI_ISL_668350, EPI_ISL_668351, EPI_ISL_668352, EPI_ISL_668354, EPI_ISL_668356, EPI_ISL_668357, EPI_ISL_668358, EPI_ISL_668359, EPI_ISL_668360, EPI_ISL_668361, EPI_ISL_668362, EPI_ISL_668363, EPI_ISL_668364, EPI_ISL_668366, EPI_ISL_668367, EPI_ISL_668370, EPI_ISL_668371, EPI_ISL_668372, EPI_ISL_668375, EPI_ISL_668377, EPI_ISL_668378, EPI_ISL_668380, EPI_ISL_668381, EPI_ISL_668384			
see above	Lighthouse Lab in Glasgow	Wellcome Sanger Institute for the COVID-19 Genomics UK (COG-UK) Consortium	Harper VanSteenhouse, Yumi Kasai, David Gray, Carol Clugston, Anna Dominiczak and Alex Alderton, Roberto Amato, Sonia Goncalves, Ewan Harrison, David K. Jackson, Ian Johnston, Dominic Kwiatkowski, Cordelia Langford, John Sillitoe on behalf of the Wellcome Sanger Institute COVID-19 Surveillance Team (http://www.sanger.ac.uk/covid-team)
EPI_ISL_672752, EPI_ISL_672753, EPI_ISL_672754, EPI_ISL_672755, EPI_ISL_672756, EPI_ISL_672757, EPI_ISL_672758, EPI_ISL_672759, EPI_ISL_672760, EPI_ISL_672761, EPI_ISL_672762, EPI_ISL_672763, EPI_ISL_672764, EPI_ISL_672765, EPI_ISL_672766, EPI_ISL_672767, EPI_ISL_672768, EPI_ISL_672769, EPI_ISL_672770, EPI_ISL_672771, EPI_ISL_672772, EPI_ISL_672773, EPI_ISL_672774, EPI_ISL_672775, EPI_ISL_672776, EPI_ISL_672777, EPI_ISL_672778, EPI_ISL_672779, EPI_ISL_672780, EPI_ISL_672781, EPI_ISL_672782, EPI_ISL_672783, EPI_ISL_672784, EPI_ISL_672785, EPI_ISL_672786, EPI_ISL_672787, EPI_ISL_672788, EPI_ISL_672789, EPI_ISL_672790, EPI_ISL_672791, EPI_ISL_672792, EPI_ISL_672793, EPI_ISL_672794, EPI_ISL_672795, EPI_ISL_672796, EPI_ISL_672797, EPI_ISL_672798, EPI_ISL_672799, EPI_ISL_672800, EPI_ISL_672801, EPI_ISL_672802, EPI_ISL_672803, EPI_ISL_672804, EPI_ISL_672805, EPI_ISL_672806, EPI_ISL_672807, EPI_ISL_672808, EPI_ISL_672809, EPI_ISL_672810, EPI_ISL_672811, EPI_ISL_672812, EPI_ISL_672813, EPI_ISL_672814, EPI_ISL_672815, EPI_ISL_672816, EPI_ISL_672817, EPI_ISL_672818, EPI_ISL_672819, EPI_ISL_672820, EPI_ISL_672821, EPI_ISL_672822, EPI_ISL_672823, EPI_ISL_672824, EPI_ISL_672825, EPI_ISL_672826, EPI_ISL_672827, EPI_ISL_672828, EPI_ISL_672829, EPI_ISL_672830, EPI_ISL_672831, EPI_ISL_672832, EPI_ISL_672833, EPI_ISL_672834, EPI_ISL_672835, EPI_ISL_672836, EPI_ISL_672837, EPI_ISL_672838, EPI_ISL_672839, EPI_ISL_672840, EPI_ISL_672841, EPI_ISL_672842, EPI_ISL_672843, EPI_ISL_672844, EPI_ISL_672845, EPI_ISL_672846, EPI_ISL_672847, EPI_ISL_672848, EPI_ISL_672849, EPI_ISL_672850, EPI_ISL_672851, EPI_ISL_672852, EPI_ISL_672853, EPI_ISL_672854, EPI_ISL_672855, EPI_ISL_672856, EPI_ISL_672857, EPI_ISL_672858, EPI_ISL_672859, EPI_ISL_672860, EPI_ISL_672861, EPI_ISL_672862, EPI_ISL_672863, EPI_ISL_672864, EPI_ISL_672865, EPI_ISL_672866, EPI_ISL_672867, EPI_ISL_672868, EPI_ISL_672869, EPI_ISL_672870, EPI_ISL_672871, EPI_ISL_672872, EPI_ISL_672873, EPI_ISL_672874, EPI_ISL_672875, EPI_ISL_672876, EPI_ISL_672877, EPI_ISL_672878, EPI_ISL_672879, EPI_ISL_672880, EPI_ISL_672881, EPI_ISL_672882, EPI_ISL_672883, EPI_ISL_672884, EPI_ISL_672885, EPI_ISL_672886, EPI_ISL_672887, EPI_ISL_672888, EPI_ISL_672889, EPI_ISL_672890, EPI_ISL_672891, EPI_ISL_672892, EPI_ISL_672893, EPI_ISL_672894, EPI_ISL_672895, EPI_ISL_672896, EPI_ISL_672897, EPI_ISL_672898, EPI_ISL_672899, EPI_ISL_672900, EPI_ISL_672901, EPI_ISL_672902, EPI_ISL_672903, EPI_ISL_672904, EPI_ISL_672905, EPI_ISL_672906, EPI_ISL_672907, EPI_ISL_672908, EPI_ISL_672909, EPI_ISL_672910, EPI_ISL_672911			
see above	Lighthouse Lab in Glasgow	Wellcome Sanger Institute for the COVID-19 Genomics UK (COG-UK) Consortium	Harper VanSteenhouse, Yumi Kasai, David Gray, Carol Clugston, Anna Dominiczak and Alex Alderton, Roberto Amato, Sonia Goncalves, Ewan Harrison, David K. Jackson, Ian Johnston, Dominic Kwiatkowski, Cordelia Langford, John Sillitoe on behalf of the Wellcome Sanger Institute COVID-19 Surveillance Team
EPI_ISL_673236, EPI_ISL_673237, EPI_ISL_673238, EPI_ISL_673239, EPI_ISL_673240, EPI_ISL_673242, EPI_ISL_673243, EPI_ISL_673244, EPI_ISL_673245, EPI_ISL_673246, EPI_ISL_673247, EPI_ISL_673248, EPI_ISL_673249, EPI_ISL_673250, EPI_ISL_673251, EPI_ISL_673252, EPI_ISL_673253, EPI_ISL_673254, EPI_ISL_673255, EPI_ISL_673256, EPI_ISL_673257, EPI_ISL_673258, EPI_ISL_673259, EPI_ISL_673260, EPI_ISL_673261, EPI_ISL_673262, EPI_ISL_673263, EPI_ISL_673264, EPI_ISL_673265, EPI_ISL_673266, EPI_ISL_673267, EPI_ISL_673268, EPI_ISL_673269, EPI_ISL_673270, EPI_ISL_673271, EPI_ISL_673272, EPI_ISL_673273, EPI_ISL_673274, EPI_ISL_673275, EPI_ISL_673276, EPI_ISL_673277, EPI_ISL_673278, EPI_ISL_673279, EPI_ISL_673280, EPI_ISL_673281, EPI_ISL_673282, EPI_ISL_673283, EPI_ISL_673284, EPI_ISL_673285, EPI_ISL_673286, EPI_ISL_673287, EPI_ISL_673288, EPI_ISL_673289, EPI_ISL_673290, EPI_ISL_673291, EPI_ISL_673292, EPI_ISL_673293, EPI_ISL_673294, EPI_ISL_673295, EPI_ISL_673296, EPI_ISL_673297, EPI_ISL_673299, EPI_ISL_673300, EPI_ISL_673301, EPI_ISL_673302, EPI_ISL_673303, EPI_ISL_673304, EPI_ISL_673305, EPI_ISL_673306, EPI_ISL_673307, EPI_ISL_673308, EPI_ISL_673309, EPI_ISL_673311, EPI_ISL_673312, EPI_ISL_673313, EPI_ISL_673314, EPI_ISL_673315, EPI_ISL_673316, EPI_ISL_673317, EPI_ISL_673318, EPI_ISL_673319, EPI_ISL_673320, EPI_ISL_673321, EPI_ISL_673322, EPI_ISL_673323, EPI_ISL_673324, EPI_ISL_673325, EPI_ISL_673326, EPI_ISL_673329, EPI_ISL_673330, EPI_ISL_673331, EPI_ISL_673332, EPI_ISL_673333, EPI_ISL_673334, EPI_ISL_673335, EPI_ISL_673336, EPI_ISL_673338, EPI_ISL_673339, EPI_ISL_673340, EPI_ISL_673341, EPI_ISL_673342, EPI_ISL_673343, EPI_ISL_673344, EPI_ISL_673345, EPI_ISL_673346, EPI_ISL_673347, EPI_ISL_673348, EPI_ISL_673349, EPI_ISL_673350, EPI_ISL_673351, EPI_ISL_673352, EPI_ISL_673353, EPI_ISL_673354, EPI_ISL_673355, EPI_ISL_673356, EPI_ISL_673357, EPI_ISL_673358, EPI_ISL_673359, EPI_ISL_673360, EPI_ISL_673361, EPI_ISL_673362, EPI_ISL_673363, EPI_ISL_673365, EPI_ISL_673366, EPI_ISL_673368, EPI_ISL_673369, EPI_ISL_673370, EPI_ISL_673371, EPI_ISL_673372, EPI_ISL_673373, EPI_ISL_673374, EPI_ISL_673375, EPI_ISL_673376, EPI_ISL_673377, EPI_ISL_673378, EPI_ISL_673379, EPI_ISL_673380, EPI_ISL_673381, EPI_ISL_673382, EPI_ISL_673383, EPI_ISL_673384, EPI_ISL_673386, EPI_ISL_673387, EPI_ISL_673388, EPI_ISL_673390, EPI_ISL_673391, EPI_ISL_673392, EPI_ISL_673393, EPI_ISL_673394, EPI_ISL_673395, EPI_ISL_673396, EPI_ISL_673397, EPI_ISL_673398, EPI_ISL_673399, EPI_ISL_673400, EPI_ISL_673401, EPI_ISL_673402, EPI_ISL_673403, EPI_ISL_673404, EPI_ISL_673405, EPI_ISL_673406, EPI_ISL_673407, EPI_ISL_673409, EPI_ISL_673410, EPI_ISL_673412, EPI_ISL_673413, EPI_ISL_673414, EPI_ISL_673415, EPI_ISL_673416, EPI_ISL_673417, EPI_ISL_673418, EPI_ISL_673419, EPI_ISL_673420, EPI_ISL_673421, EPI_ISL_673422, EPI_ISL_673423, EPI_ISL_673424, EPI_ISL_673425, EPI_ISL_673427, EPI_ISL_673428, EPI_ISL_673429, EPI_ISL_673430, EPI_ISL_673431, EPI_ISL_673432, EPI_ISL_673433, EPI_ISL_673434, EPI_ISL_673435, EPI_ISL_673436, EPI_ISL_673437, EPI_ISL_673438, EPI_ISL_673439, EPI_ISL_673440, EPI_ISL_673442, EPI_ISL_673443, EPI_ISL_673444, EPI_ISL_673445, EPI_ISL_673446, EPI_ISL_673449, EPI_ISL_673450, EPI_ISL_673451, EPI_ISL_673452, EPI_ISL_673453, EPI_ISL_673454, EPI_ISL_673455, EPI_ISL_673456, EPI_ISL_673457, EPI_ISL_673458, EPI_ISL_673459, EPI_ISL_673460, EPI_ISL_673461, EPI_ISL_673462, EPI_ISL_673463, EPI_ISL_673464, EPI_ISL_673465, EPI_ISL_673466, EPI_ISL_673467, EPI_ISL_673468, EPI_ISL_673469, EPI_ISL_673470, EPI_ISL_673471, EPI_ISL_673472, EPI_ISL_673473, EPI_ISL_673474, EPI_ISL_673475, EPI_ISL_673476, EPI_ISL_673477, EPI_ISL_673478, EPI_ISL_673479, EPI_ISL_673480, EPI_ISL_673481, EPI_ISL_673482, EPI_ISL_673483, EPI_ISL_673484, EPI_ISL_673485, EPI_ISL_673486, EPI_ISL_673487, EPI_ISL_673488, EPI_ISL_673489, EPI_ISL_673490, EPI_ISL_673491, EPI_ISL_673492, EPI_ISL_673493, EPI_ISL_673494, EPI_ISL_673495, EPI_ISL_673496, EPI_ISL_673497, EPI_ISL_673498, EPI_ISL_673499, EPI_ISL_673500, EPI_ISL_673501, EPI_ISL_673502, EPI_ISL_673503, EPI_ISL_673504, EPI_ISL_673505, EPI_ISL_673506, EPI_ISL_673507, EPI_ISL_673508, EPI_ISL_673509, EPI_ISL_673510, EPI_ISL_673511, EPI_ISL_673512, EPI_ISL_673513, EPI_ISL_673514, EPI_ISL_673515, EPI_ISL_673516, EPI_ISL_673517, EPI_ISL_673518, EPI_ISL_673519, EPI_ISL_673520, EPI_ISL_673521, EPI_ISL_673522, EPI_ISL_673523, EPI_ISL_673524, EPI_ISL_673525, EPI_ISL_673526, EPI_ISL_673527, EPI_ISL_673528, EPI_ISL_673529, EPI_ISL_673530, EPI_ISL_673531, EPI_ISL_673532, EPI_ISL_673533, EPI_ISL_673534, EPI_ISL_673535, EPI_ISL_673536, EPI_ISL_673537, EPI_ISL_673538, EPI_ISL_673539, EPI_ISL_673540, EPI_ISL_673541, EPI_ISL_673542, EPI_ISL_673543, EPI_ISL_673544, EPI_ISL_673545, EPI_ISL_673546, EPI_ISL_673547, EPI_ISL_673548, EPI_ISL_673549, EPI_ISL_673550, EPI_ISL_673551, EPI_ISL_673552, EPI_ISL_673553, EPI_ISL_673554, EPI_ISL_673555, EPI_ISL_673556, EPI_ISL_673557, EPI_ISL_673558, EPI_ISL_673559, EPI_ISL_673560, EPI_ISL_673561, EPI_ISL_673562, EPI_ISL_673563, EPI_ISL_673564, EPI_ISL_673565, EPI_ISL_673567, EPI_ISL_673568, EPI_ISL_673569, EPI_ISL_673570, EPI_ISL_673571, EPI_ISL_673572, EPI_ISL_673573, EPI_ISL_673574, EPI_ISL_673575, EPI_ISL_673576, EPI_ISL_673578, EPI_ISL_673579, EPI_ISL_673580, EPI_ISL_673581, EPI_ISL_673582, EPI_ISL_673583, EPI_ISL_673584, EPI_ISL_673585, EPI_ISL_673586, EPI_ISL_673587, EPI_ISL_673588, EPI_ISL_673589, EPI_ISL_673590, EPI_ISL_673591, EPI_ISL_673593			
see above	Lighthouse Lab in Cambridge	Wellcome Sanger Institute for the COVID-19 Genomics UK (COG-UK) Consortium	Rob Howes, The Lighthouse Lab in Cambridge and Alex Alderton, Roberto Amato, Sonia Goncalves, Ewan Harrison, David K. Jackson, Ian Johnston, Dominic Kwiatkowski, Cordelia Langford, John Sillitoe on behalf of the Wellcome Sanger Institute COVID-19 Surveillance Team
EPI_ISL_673594	Lighthouse Lab in Milton Keynes	Wellcome Sanger Institute for the COVID-19 Genomics UK (COG-UK) Consortium	The Lighthouse Lab in Milton Keynes and Alex Alderton, Roberto Amato, Sonia Goncalves, Ewan Harrison, David K. Jackson, Ian Johnston, Dominic Kwiatkowski, Cordelia Langford, John Sillitoe on behalf of the Wellcome Sanger Institute COVID-19 Surveillance Team
EPI_ISL_673595, EPI_ISL_673596, EPI_ISL_673597, EPI_ISL_673598, EPI_ISL_673600, EPI_ISL_673602, EPI_ISL_673604, EPI_ISL_673605, EPI_ISL_673606, EPI_ISL_673607, EPI_ISL_673608, EPI_ISL_673609, EPI_ISL_673610, EPI_ISL_673611, EPI_ISL_673612, EPI_ISL_673613, EPI_ISL_673614, EPI_ISL_673615, EPI_ISL_673617, EPI_ISL_673618, EPI_ISL_673619, EPI_ISL_673620			
see above	Lighthouse Lab in Cambridge	Wellcome Sanger Institute for the COVID-19 Genomics UK (COG-UK) Consortium	Rob Howes, The Lighthouse Lab in Cambridge and Alex Alderton, Roberto Amato, Sonia Goncalves, Ewan Harrison, David K. Jackson, Ian Johnston, Dominic Kwiatkowski, Cordelia Langford, John Sillitoe on behalf of the Wellcome Sanger Institute COVID-19 Surveillance Team
EPI_ISL_673621	Lighthouse Lab in Milton Keynes	Wellcome Sanger Institute for the COVID-19 Genomics UK (COG-UK) Consortium	The Lighthouse Lab in Milton Keynes and Alex Alderton, Roberto Amato, Sonia Goncalves, Ewan Harrison, David K. Jackson, Ian Johnston, Dominic Kwiatkowski, Cordelia Langford, John Sillitoe on behalf of the Wellcome Sanger Institute COVID-19 Surveillance Team
EPI_ISL_673622, EPI_ISL_673623, EPI_ISL_673624, EPI_ISL_673625, EPI_ISL_673626, EPI_ISL_673627, EPI_ISL_673628, EPI_ISL_673629, EPI_ISL_673630, EPI_ISL_673631, EPI_ISL_673632			
see above	Lighthouse Lab in Cambridge	Wellcome Sanger Institute for the COVID-19 Genomics UK	Rob Howes, The Lighthouse Lab in Cambridge and Alex Alderton, Roberto Amato, Sonia Goncalves, Ewan Harrison, David K. Jackson, Ian Johnston,

		(COG-UK) Consortium	Dominic Kwiatkowski, Cordelia Langford, John Sillitoe on behalf of the Wellcome Sanger Institute COVID-19 Surveillance Team
EPI_ISL_673634	Lighthouse Lab in Milton Keynes	Wellcome Sanger Institute for the COVID-19 Genomics UK (COG-UK) Consortium	The Lighthouse Lab in Milton Keynes and Alex Alderton, Roberto Amato, Sonia Goncalves, Ewan Harrison, David K. Jackson, Ian Johnston, Dominic Kwiatkowski, Cordelia Langford, John Sillitoe on behalf of the Wellcome Sanger Institute COVID-19 Surveillance Team
EPI_ISL_673635, EPI_ISL_673636, EPI_ISL_673637, EPI_ISL_673638, EPI_ISL_673639, EPI_ISL_673640, EPI_ISL_673641, EPI_ISL_673643, EPI_ISL_673644	Lighthouse Lab in Cambridge	Wellcome Sanger Institute for the COVID-19 Genomics UK (COG-UK) Consortium	Rob Howes, The Lighthouse Lab in Cambridge and Alex Alderton, Roberto Amato, Sonia Goncalves, Ewan Harrison, David K. Jackson, Ian Johnston, Dominic Kwiatkowski, Cordelia Langford, John Sillitoe on behalf of the Wellcome Sanger Institute COVID-19 Surveillance Team
EPI_ISL_673646	Lighthouse Lab in Milton Keynes	Wellcome Sanger Institute for the COVID-19 Genomics UK (COG-UK) Consortium	The Lighthouse Lab in Milton Keynes and Alex Alderton, Roberto Amato, Sonia Goncalves, Ewan Harrison, David K. Jackson, Ian Johnston, Dominic Kwiatkowski, Cordelia Langford, John Sillitoe on behalf of the Wellcome Sanger Institute COVID-19 Surveillance Team
EPI_ISL_673647, EPI_ISL_673648, EPI_ISL_673649, EPI_ISL_673650, EPI_ISL_673651, EPI_ISL_673653, EPI_ISL_673654, EPI_ISL_673656, EPI_ISL_673658, EPI_ISL_673660, EPI_ISL_673661, EPI_ISL_673662, EPI_ISL_673664, EPI_ISL_673665, EPI_ISL_673666, EPI_ISL_673667, EPI_ISL_673668, EPI_ISL_673669, EPI_ISL_673670, EPI_ISL_673671, EPI_ISL_673672, EPI_ISL_673674, EPI_ISL_673675			
see above	Lighthouse Lab in Cambridge	Wellcome Sanger Institute for the COVID-19 Genomics UK (COG-UK) Consortium	Rob Howes, The Lighthouse Lab in Cambridge and Alex Alderton, Roberto Amato, Sonia Goncalves, Ewan Harrison, David K. Jackson, Ian Johnston, Dominic Kwiatkowski, Cordelia Langford, John Sillitoe on behalf of the Wellcome Sanger Institute COVID-19 Surveillance Team
EPI_ISL_673676	Lighthouse Lab in Milton Keynes	Wellcome Sanger Institute for the COVID-19 Genomics UK (COG-UK) Consortium	The Lighthouse Lab in Milton Keynes and Alex Alderton, Roberto Amato, Sonia Goncalves, Ewan Harrison, David K. Jackson, Ian Johnston, Dominic Kwiatkowski, Cordelia Langford, John Sillitoe on behalf of the Wellcome Sanger Institute COVID-19 Surveillance Team
EPI_ISL_673677, EPI_ISL_673678, EPI_ISL_673679, EPI_ISL_673680, EPI_ISL_673681, EPI_ISL_673682, EPI_ISL_673683, EPI_ISL_673684, EPI_ISL_673685	Lighthouse Lab in Cambridge	Wellcome Sanger Institute for the COVID-19 Genomics UK (COG-UK) Consortium	Rob Howes, The Lighthouse Lab in Cambridge and Alex Alderton, Roberto Amato, Sonia Goncalves, Ewan Harrison, David K. Jackson, Ian Johnston, Dominic Kwiatkowski, Cordelia Langford, John Sillitoe on behalf of the Wellcome Sanger Institute COVID-19 Surveillance Team
EPI_ISL_673686	Lighthouse Lab in Milton Keynes	Wellcome Sanger Institute for the COVID-19 Genomics UK (COG-UK) Consortium	The Lighthouse Lab in Milton Keynes and Alex Alderton, Roberto Amato, Sonia Goncalves, Ewan Harrison, David K. Jackson, Ian Johnston, Dominic Kwiatkowski, Cordelia Langford, John Sillitoe on behalf of the Wellcome Sanger Institute COVID-19 Surveillance Team
EPI_ISL_673687	Lighthouse Lab in Cambridge	Wellcome Sanger Institute for the COVID-19 Genomics UK (COG-UK) Consortium	Rob Howes, The Lighthouse Lab in Cambridge and Alex Alderton, Roberto Amato, Sonia Goncalves, Ewan Harrison, David K. Jackson, Ian Johnston, Dominic Kwiatkowski, Cordelia Langford, John Sillitoe on behalf of the Wellcome Sanger Institute COVID-19 Surveillance Team
EPI_ISL_673688	Lighthouse Lab in Milton Keynes	Wellcome Sanger Institute for the COVID-19 Genomics UK (COG-UK) Consortium	The Lighthouse Lab in Milton Keynes and Alex Alderton, Roberto Amato, Sonia Goncalves, Ewan Harrison, David K. Jackson, Ian Johnston, Dominic Kwiatkowski, Cordelia Langford, John Sillitoe on behalf of the Wellcome Sanger Institute COVID-19 Surveillance Team
EPI_ISL_673689, EPI_ISL_673690	Lighthouse Lab in Cambridge	Wellcome Sanger Institute for the COVID-19 Genomics UK (COG-UK) Consortium	Rob Howes, The Lighthouse Lab in Cambridge and Alex Alderton, Roberto Amato, Sonia Goncalves, Ewan Harrison, David K. Jackson, Ian Johnston, Dominic Kwiatkowski, Cordelia Langford, John Sillitoe on behalf of the Wellcome Sanger Institute COVID-19 Surveillance Team
EPI_ISL_673691	Lighthouse Lab in Milton Keynes	Wellcome Sanger Institute for the COVID-19 Genomics UK (COG-UK) Consortium	The Lighthouse Lab in Milton Keynes and Alex Alderton, Roberto Amato, Sonia Goncalves, Ewan Harrison, David K. Jackson, Ian Johnston, Dominic Kwiatkowski, Cordelia Langford, John Sillitoe on behalf of the Wellcome Sanger Institute COVID-19 Surveillance Team
EPI_ISL_673692, EPI_ISL_673693, EPI_ISL_673694, EPI_ISL_673695, EPI_ISL_673697, EPI_ISL_673698, EPI_ISL_673699, EPI_ISL_673700, EPI_ISL_673701	Lighthouse Lab in Cambridge	Wellcome Sanger Institute for the COVID-19 Genomics UK (COG-UK) Consortium	Rob Howes, The Lighthouse Lab in Cambridge and Alex Alderton, Roberto Amato, Sonia Goncalves, Ewan Harrison, David K. Jackson, Ian Johnston, Dominic Kwiatkowski, Cordelia Langford, John Sillitoe on behalf of the Wellcome Sanger Institute COVID-19 Surveillance Team
EPI_ISL_673702	Lighthouse Lab in Milton Keynes	Wellcome Sanger Institute for the COVID-19 Genomics UK (COG-UK) Consortium	The Lighthouse Lab in Milton Keynes and Alex Alderton, Roberto Amato, Sonia Goncalves, Ewan Harrison, David K. Jackson, Ian Johnston, Dominic Kwiatkowski, Cordelia Langford, John Sillitoe on behalf of the Wellcome Sanger Institute COVID-19 Surveillance Team
EPI_ISL_673703, EPI_ISL_673704, EPI_ISL_673705, EPI_ISL_673706, EPI_ISL_673707, EPI_ISL_673710, EPI_ISL_673711, EPI_ISL_673712, EPI_ISL_673713, EPI_ISL_673714, EPI_ISL_673716, EPI_ISL_673717, EPI_ISL_673718, EPI_ISL_673719, EPI_ISL_673720, EPI_ISL_673721, EPI_ISL_673723, EPI_ISL_673724, EPI_ISL_673725			
see above	Lighthouse Lab in Cambridge	Wellcome Sanger Institute for the COVID-19 Genomics UK (COG-UK) Consortium	Rob Howes, The Lighthouse Lab in Cambridge and Alex Alderton, Roberto Amato, Sonia Goncalves, Ewan Harrison, David K. Jackson, Ian Johnston, Dominic Kwiatkowski, Cordelia Langford, John Sillitoe on behalf of the Wellcome Sanger Institute COVID-19 Surveillance Team
EPI_ISL_673726	Lighthouse Lab in Milton Keynes	Wellcome Sanger Institute for the COVID-19 Genomics UK (COG-UK) Consortium	The Lighthouse Lab in Milton Keynes and Alex Alderton, Roberto Amato, Sonia Goncalves, Ewan Harrison, David K. Jackson, Ian Johnston, Dominic Kwiatkowski, Cordelia Langford, John Sillitoe on behalf of the Wellcome Sanger Institute COVID-19 Surveillance Team
EPI_ISL_673727, EPI_ISL_673728, EPI_ISL_673731, EPI_ISL_673732, EPI_ISL_673734, EPI_ISL_673735, EPI_ISL_673736, EPI_ISL_673737, EPI_ISL_673739, EPI_ISL_673740, EPI_ISL_673741, EPI_ISL_673742, EPI_ISL_673743, EPI_ISL_673744, EPI_ISL_673747, EPI_ISL_673748, EPI_ISL_673749			
see above	Lighthouse Lab in Cambridge	Wellcome Sanger Institute for the COVID-19 Genomics UK (COG-UK) Consortium	Rob Howes, The Lighthouse Lab in Cambridge and Alex Alderton, Roberto Amato, Sonia Goncalves, Ewan Harrison, David K. Jackson, Ian Johnston, Dominic Kwiatkowski, Cordelia Langford, John Sillitoe on behalf of the Wellcome Sanger Institute COVID-19 Surveillance Team
EPI_ISL_673750	Lighthouse Lab in Milton Keynes	Wellcome Sanger Institute for the COVID-19 Genomics UK (COG-UK) Consortium	The Lighthouse Lab in Milton Keynes and Alex Alderton, Roberto Amato, Sonia Goncalves, Ewan Harrison, David K. Jackson, Ian Johnston, Dominic Kwiatkowski, Cordelia Langford, John Sillitoe on behalf of the Wellcome Sanger Institute COVID-19 Surveillance Team
EPI_ISL_673751, EPI_ISL_673752, EPI_ISL_673753, EPI_ISL_673754, EPI_ISL_673755, EPI_ISL_673756, EPI_ISL_673757, EPI_ISL_673758, EPI_ISL_673759, EPI_ISL_673761, EPI_ISL_673762, EPI_ISL_673763, EPI_ISL_673766, EPI_ISL_673768, EPI_ISL_673769, EPI_ISL_673771, EPI_ISL_673772, EPI_ISL_673773, EPI_ISL_673774, EPI_ISL_673775, EPI_ISL_673776, EPI_ISL_673777, EPI_ISL_673779, EPI_ISL_673780, EPI_ISL_673781, EPI_ISL_673782, EPI_ISL_673783, EPI_ISL_673784, EPI_ISL_673786, EPI_ISL_673788, EPI_ISL_673789			
see above	Lighthouse Lab in Cambridge	Wellcome Sanger Institute for the COVID-19 Genomics UK (COG-UK) Consortium	Rob Howes, The Lighthouse Lab in Cambridge and Alex Alderton, Roberto Amato, Sonia Goncalves, Ewan Harrison, David K. Jackson, Ian Johnston, Dominic Kwiatkowski, Cordelia Langford, John Sillitoe on behalf of the Wellcome Sanger Institute COVID-19 Surveillance Team
EPI_ISL_673790	Lighthouse Lab in Milton Keynes	Wellcome Sanger Institute for the COVID-19 Genomics UK (COG-UK) Consortium	The Lighthouse Lab in Milton Keynes and Alex Alderton, Roberto Amato, Sonia Goncalves, Ewan Harrison, David K. Jackson, Ian Johnston, Dominic Kwiatkowski, Cordelia Langford, John Sillitoe on behalf of the Wellcome Sanger Institute COVID-19 Surveillance Team
EPI_ISL_673791, EPI_ISL_673792, EPI_ISL_673793, EPI_ISL_673794, EPI_ISL_673795, EPI_ISL_673799, EPI_ISL_673800, EPI_ISL_673802, EPI_ISL_673804, EPI_ISL_673805, EPI_ISL_673806, EPI_ISL_673807, EPI_ISL_673808, EPI_ISL_673809, EPI_ISL_673810, EPI_ISL_673811, EPI_ISL_673815, EPI_ISL_673816, EPI_ISL_673817, EPI_ISL_673818, EPI_ISL_673819, EPI_ISL_673820			
see above	Lighthouse Lab in Cambridge	Wellcome Sanger Institute for the COVID-19 Genomics UK (COG-UK) Consortium	Rob Howes, The Lighthouse Lab in Cambridge and Alex Alderton, Roberto Amato, Sonia Goncalves, Ewan Harrison, David K. Jackson, Ian Johnston, Dominic Kwiatkowski, Cordelia Langford, John Sillitoe on behalf of the Wellcome Sanger Institute COVID-19 Surveillance Team
EPI_ISL_673821	Lighthouse Lab in Milton Keynes	Wellcome Sanger Institute for the COVID-19 Genomics UK (COG-UK) Consortium	The Lighthouse Lab in Milton Keynes and Alex Alderton, Roberto Amato, Sonia Goncalves, Ewan Harrison, David K. Jackson, Ian Johnston, Dominic Kwiatkowski, Cordelia Langford, John Sillitoe on behalf of the Wellcome Sanger Institute COVID-19 Surveillance Team
EPI_ISL_673823, EPI_ISL_673824, EPI_ISL_673825, EPI_ISL_673826	Lighthouse Lab in Cambridge	Wellcome Sanger Institute for the COVID-19 Genomics UK (COG-UK) Consortium	Rob Howes, The Lighthouse Lab in Cambridge and Alex Alderton, Roberto Amato, Sonia Goncalves, Ewan Harrison, David K. Jackson, Ian Johnston, Dominic Kwiatkowski, Cordelia Langford, John Sillitoe on behalf of the Wellcome Sanger Institute COVID-19 Surveillance Team
EPI_ISL_673828, EPI_ISL_673829	Lighthouse Lab in Milton Keynes	Wellcome Sanger Institute for the COVID-19 Genomics UK (COG-UK) Consortium	The Lighthouse Lab in Milton Keynes and Alex Alderton, Roberto Amato, Sonia Goncalves, Ewan Harrison, David K. Jackson, Ian Johnston, Dominic Kwiatkowski, Cordelia Langford, John Sillitoe on behalf of the Wellcome Sanger Institute COVID-19 Surveillance Team
EPI_ISL_673830, EPI_ISL_673831	Lighthouse Lab in Cambridge	Wellcome Sanger Institute for the COVID-19 Genomics UK (COG-UK) Consortium	Rob Howes, The Lighthouse Lab in Cambridge and Alex Alderton, Roberto Amato, Sonia Goncalves, Ewan Harrison, David K. Jackson, Ian Johnston, Dominic Kwiatkowski, Cordelia Langford, John Sillitoe on behalf of the Wellcome Sanger Institute COVID-19 Surveillance Team
EPI_ISL_673833, EPI_ISL_673835	Lighthouse Lab in Milton Keynes	Wellcome Sanger Institute for the COVID-19 Genomics UK (COG-UK) Consortium	The Lighthouse Lab in Milton Keynes and Alex Alderton, Roberto Amato, Sonia Goncalves, Ewan Harrison, David K. Jackson, Ian Johnston, Dominic Kwiatkowski, Cordelia Langford, John Sillitoe on behalf of the Wellcome Sanger Institute COVID-19 Surveillance Team
EPI_ISL_673836, EPI_ISL_673837, EPI_ISL_673838, EPI_ISL_673839	Lighthouse Lab in Cambridge	Wellcome Sanger Institute for the COVID-19 Genomics UK (COG-UK) Consortium	Rob Howes, The Lighthouse Lab in Cambridge and Alex Alderton, Roberto Amato, Sonia Goncalves, Ewan Harrison, David K. Jackson, Ian Johnston, Dominic Kwiatkowski, Cordelia Langford, John Sillitoe on behalf of the Wellcome Sanger Institute COVID-19 Surveillance Team
EPI_ISL_673840	Lighthouse Lab in Milton Keynes	Wellcome Sanger Institute for the COVID-19 Genomics UK	The Lighthouse Lab in Milton Keynes and Alex Alderton, Roberto Amato, Sonia Goncalves, Ewan Harrison, David K. Jackson, Ian Johnston, Dominic

		(COG-UK) Consortium	Kwiatkowski, Cordelia Langford, John Sillitoe on behalf of the Wellcome Sanger Institute COVID-19 Surveillance Team
EPI_ISL_673841, EPI_ISL_673842, EPI_ISL_673843, EPI_ISL_673844, EPI_ISL_673845, EPI_ISL_673846, EPI_ISL_673847	Lighthouse Lab in Cambridge	Wellcome Sanger Institute for the COVID-19 Genomics UK (COG-UK) Consortium	Rob Howes, The Lighthouse Lab in Cambridge and Alex Alderton, Roberto Amato, Sonia Goncalves, Ewan Harrison, David K. Jackson, Ian Johnston, Dominic Kwiatkowski, Cordelia Langford, John Sillitoe on behalf of the Wellcome Sanger Institute COVID-19 Surveillance Team
EPI_ISL_673848	Lighthouse Lab in Milton Keynes	Wellcome Sanger Institute for the COVID-19 Genomics UK (COG-UK) Consortium	The Lighthouse Lab in Milton Keynes and Alex Alderton, Roberto Amato, Sonia Goncalves, Ewan Harrison, David K. Jackson, Ian Johnston, Dominic Kwiatkowski, Cordelia Langford, John Sillitoe on behalf of the Wellcome Sanger Institute COVID-19 Surveillance Team
EPI_ISL_673849, EPI_ISL_673850, EPI_ISL_673852, EPI_ISL_673853, EPI_ISL_673854, EPI_ISL_673855	Lighthouse Lab in Cambridge	Wellcome Sanger Institute for the COVID-19 Genomics UK (COG-UK) Consortium	Rob Howes, The Lighthouse Lab in Cambridge and Alex Alderton, Roberto Amato, Sonia Goncalves, Ewan Harrison, David K. Jackson, Ian Johnston, Dominic Kwiatkowski, Cordelia Langford, John Sillitoe on behalf of the Wellcome Sanger Institute COVID-19 Surveillance Team
EPI_ISL_673857	Lighthouse Lab in Milton Keynes	Wellcome Sanger Institute for the COVID-19 Genomics UK (COG-UK) Consortium	The Lighthouse Lab in Milton Keynes and Alex Alderton, Roberto Amato, Sonia Goncalves, Ewan Harrison, David K. Jackson, Ian Johnston, Dominic Kwiatkowski, Cordelia Langford, John Sillitoe on behalf of the Wellcome Sanger Institute COVID-19 Surveillance Team
EPI_ISL_673858, EPI_ISL_673860, EPI_ISL_673861, EPI_ISL_673862, EPI_ISL_673863, EPI_ISL_673864, EPI_ISL_673866, EPI_ISL_673868, EPI_ISL_673869, EPI_ISL_673870	Lighthouse Lab in Cambridge	Wellcome Sanger Institute for the COVID-19 Genomics UK (COG-UK) Consortium	Rob Howes, The Lighthouse Lab in Cambridge and Alex Alderton, Roberto Amato, Sonia Goncalves, Ewan Harrison, David K. Jackson, Ian Johnston, Dominic Kwiatkowski, Cordelia Langford, John Sillitoe on behalf of the Wellcome Sanger Institute COVID-19 Surveillance Team
EPI_ISL_673921, EPI_ISL_673936, EPI_ISL_673954, EPI_ISL_673959, EPI_ISL_674005, EPI_ISL_674009, EPI_ISL_674013, EPI_ISL_674028, EPI_ISL_674034, EPI_ISL_674063, EPI_ISL_674097			
see above	Lighthouse Lab in Milton Keynes	Wellcome Sanger Institute for the COVID-19 Genomics UK (COG-UK) Consortium	The Lighthouse Lab in Milton Keynes and Alex Alderton, Roberto Amato, Sonia Goncalves, Ewan Harrison, David K. Jackson, Ian Johnston, Dominic Kwiatkowski, Cordelia Langford, John Sillitoe on behalf of the Wellcome Sanger Institute COVID-19 Surveillance Team
EPI_ISL_674159, EPI_ISL_674160, EPI_ISL_674161, EPI_ISL_674162, EPI_ISL_674164, EPI_ISL_674165, EPI_ISL_674168, EPI_ISL_674169, EPI_ISL_674170, EPI_ISL_674171, EPI_ISL_674172, EPI_ISL_674174, EPI_ISL_674175, EPI_ISL_674176, EPI_ISL_674177, EPI_ISL_674179, EPI_ISL_674181, EPI_ISL_674182, EPI_ISL_674186, EPI_ISL_674188, EPI_ISL_674190, EPI_ISL_674192, EPI_ISL_674193, EPI_ISL_674194, EPI_ISL_674195, EPI_ISL_674197, EPI_ISL_674199, EPI_ISL_674201, EPI_ISL_674203, EPI_ISL_674204, EPI_ISL_674206, EPI_ISL_674207, EPI_ISL_674208, EPI_ISL_674211			
see above	Lighthouse Lab in Alderley Park	Wellcome Sanger Institute for the COVID-19 Genomics UK (COG-UK) Consortium	Jacquelyn Wynn, Mairead Hyland, The Lighthouse Lab in Alderley Park and Alex Alderton, Roberto Amato, Sonia Goncalves, Ewan Harrison, David K. Jackson, Ian Johnston, Dominic Kwiatkowski, Cordelia Langford, John Sillitoe on behalf of the Wellcome Sanger Institute COVID-19 Surveillance Team
EPI_ISL_674212	Lighthouse Lab in Milton Keynes	Wellcome Sanger Institute for the COVID-19 Genomics UK (COG-UK) Consortium	The Lighthouse Lab in Milton Keynes and Alex Alderton, Roberto Amato, Sonia Goncalves, Ewan Harrison, David K. Jackson, Ian Johnston, Dominic Kwiatkowski, Cordelia Langford, John Sillitoe on behalf of the Wellcome Sanger Institute COVID-19 Surveillance Team
EPI_ISL_674217, EPI_ISL_674222, EPI_ISL_674224, EPI_ISL_674226, EPI_ISL_674227, EPI_ISL_674228, EPI_ISL_674229, EPI_ISL_674231	Lighthouse Lab in Alderley Park	Wellcome Sanger Institute for the COVID-19 Genomics UK (COG-UK) Consortium	Jacquelyn Wynn, Mairead Hyland, The Lighthouse Lab in Alderley Park and Alex Alderton, Roberto Amato, Sonia Goncalves, Ewan Harrison, David K. Jackson, Ian Johnston, Dominic Kwiatkowski, Cordelia Langford, John Sillitoe on behalf of the Wellcome Sanger Institute COVID-19 Surveillance Team
EPI_ISL_674232	Lighthouse Lab in Milton Keynes	Wellcome Sanger Institute for the COVID-19 Genomics UK (COG-UK) Consortium	The Lighthouse Lab in Milton Keynes and Alex Alderton, Roberto Amato, Sonia Goncalves, Ewan Harrison, David K. Jackson, Ian Johnston, Dominic Kwiatkowski, Cordelia Langford, John Sillitoe on behalf of the Wellcome Sanger Institute COVID-19 Surveillance Team
EPI_ISL_674233, EPI_ISL_674234	Lighthouse Lab in Alderley Park	Wellcome Sanger Institute for the COVID-19 Genomics UK (COG-UK) Consortium	Jacquelyn Wynn, Mairead Hyland, The Lighthouse Lab in Alderley Park and Alex Alderton, Roberto Amato, Sonia Goncalves, Ewan Harrison, David K. Jackson, Ian Johnston, Dominic Kwiatkowski, Cordelia Langford, John Sillitoe on behalf of the Wellcome Sanger Institute COVID-19 Surveillance Team
EPI_ISL_674238, EPI_ISL_674239	Lighthouse Lab in Milton Keynes	Wellcome Sanger Institute for the COVID-19 Genomics UK (COG-UK) Consortium	The Lighthouse Lab in Milton Keynes and Alex Alderton, Roberto Amato, Sonia Goncalves, Ewan Harrison, David K. Jackson, Ian Johnston, Dominic Kwiatkowski, Cordelia Langford, John Sillitoe on behalf of the Wellcome Sanger Institute COVID-19 Surveillance Team
EPI_ISL_674240, EPI_ISL_674241, EPI_ISL_674242, EPI_ISL_674243, EPI_ISL_674245, EPI_ISL_674249, EPI_ISL_674253, EPI_ISL_674254, EPI_ISL_674255, EPI_ISL_674260, EPI_ISL_674261, EPI_ISL_674264, EPI_ISL_674267, EPI_ISL_674268			
see above	Lighthouse Lab in Alderley Park	Wellcome Sanger Institute for the COVID-19 Genomics UK (COG-UK) Consortium	Jacquelyn Wynn, Mairead Hyland, The Lighthouse Lab in Alderley Park and Alex Alderton, Roberto Amato, Sonia Goncalves, Ewan Harrison, David K. Jackson, Ian Johnston, Dominic Kwiatkowski, Cordelia Langford, John Sillitoe on behalf of the Wellcome Sanger Institute COVID-19 Surveillance Team
EPI_ISL_674269, EPI_ISL_674271	Lighthouse Lab in Milton Keynes	Wellcome Sanger Institute for the COVID-19 Genomics UK (COG-UK) Consortium	The Lighthouse Lab in Milton Keynes and Alex Alderton, Roberto Amato, Sonia Goncalves, Ewan Harrison, David K. Jackson, Ian Johnston, Dominic Kwiatkowski, Cordelia Langford, John Sillitoe on behalf of the Wellcome Sanger Institute COVID-19 Surveillance Team
EPI_ISL_674272	Lighthouse Lab in Alderley Park	Wellcome Sanger Institute for the COVID-19 Genomics UK (COG-UK) Consortium	Jacquelyn Wynn, Mairead Hyland, The Lighthouse Lab in Alderley Park and Alex Alderton, Roberto Amato, Sonia Goncalves, Ewan Harrison, David K. Jackson, Ian Johnston, Dominic Kwiatkowski, Cordelia Langford, John Sillitoe on behalf of the Wellcome Sanger Institute COVID-19 Surveillance Team
EPI_ISL_674275	Lighthouse Lab in Milton Keynes	Wellcome Sanger Institute for the COVID-19 Genomics UK (COG-UK) Consortium	The Lighthouse Lab in Milton Keynes and Alex Alderton, Roberto Amato, Sonia Goncalves, Ewan Harrison, David K. Jackson, Ian Johnston, Dominic Kwiatkowski, Cordelia Langford, John Sillitoe on behalf of the Wellcome Sanger Institute COVID-19 Surveillance Team
EPI_ISL_674276, EPI_ISL_674277	Lighthouse Lab in Alderley Park	Wellcome Sanger Institute for the COVID-19 Genomics UK (COG-UK) Consortium	Jacquelyn Wynn, Mairead Hyland, The Lighthouse Lab in Alderley Park and Alex Alderton, Roberto Amato, Sonia Goncalves, Ewan Harrison, David K. Jackson, Ian Johnston, Dominic Kwiatkowski, Cordelia Langford, John Sillitoe on behalf of the Wellcome Sanger Institute COVID-19 Surveillance Team
EPI_ISL_674280	Lighthouse Lab in Milton Keynes	Wellcome Sanger Institute for the COVID-19 Genomics UK (COG-UK) Consortium	The Lighthouse Lab in Milton Keynes and Alex Alderton, Roberto Amato, Sonia Goncalves, Ewan Harrison, David K. Jackson, Ian Johnston, Dominic Kwiatkowski, Cordelia Langford, John Sillitoe on behalf of the Wellcome Sanger Institute COVID-19 Surveillance Team
EPI_ISL_674281, EPI_ISL_674283, EPI_ISL_674284, EPI_ISL_674285, EPI_ISL_674286, EPI_ISL_674287, EPI_ISL_674292, EPI_ISL_674294, EPI_ISL_674295, EPI_ISL_674296, EPI_ISL_674297, EPI_ISL_674299, EPI_ISL_674300, EPI_ISL_674301, EPI_ISL_674305			
see above	Lighthouse Lab in Alderley Park	Wellcome Sanger Institute for the COVID-19 Genomics UK (COG-UK) Consortium	Jacquelyn Wynn, Mairead Hyland, The Lighthouse Lab in Alderley Park and Alex Alderton, Roberto Amato, Sonia Goncalves, Ewan Harrison, David K. Jackson, Ian Johnston, Dominic Kwiatkowski, Cordelia Langford, John Sillitoe on behalf of the Wellcome Sanger Institute COVID-19 Surveillance Team
EPI_ISL_674306, EPI_ISL_674307	Lighthouse Lab in Milton Keynes	Wellcome Sanger Institute for the COVID-19 Genomics UK (COG-UK) Consortium	The Lighthouse Lab in Milton Keynes and Alex Alderton, Roberto Amato, Sonia Goncalves, Ewan Harrison, David K. Jackson, Ian Johnston, Dominic Kwiatkowski, Cordelia Langford, John Sillitoe on behalf of the Wellcome Sanger Institute COVID-19 Surveillance Team
EPI_ISL_674308, EPI_ISL_674310, EPI_ISL_674311, EPI_ISL_674313	Lighthouse Lab in Alderley Park	Wellcome Sanger Institute for the COVID-19 Genomics UK (COG-UK) Consortium	Jacquelyn Wynn, Mairead Hyland, The Lighthouse Lab in Alderley Park and Alex Alderton, Roberto Amato, Sonia Goncalves, Ewan Harrison, David K. Jackson, Ian Johnston, Dominic Kwiatkowski, Cordelia Langford, John Sillitoe on behalf of the Wellcome Sanger Institute COVID-19 Surveillance Team
EPI_ISL_674315	Lighthouse Lab in Milton Keynes	Wellcome Sanger Institute for the COVID-19 Genomics UK (COG-UK) Consortium	The Lighthouse Lab in Milton Keynes and Alex Alderton, Roberto Amato, Sonia Goncalves, Ewan Harrison, David K. Jackson, Ian Johnston, Dominic Kwiatkowski, Cordelia Langford, John Sillitoe on behalf of the Wellcome Sanger Institute COVID-19 Surveillance Team
EPI_ISL_674317, EPI_ISL_674318, EPI_ISL_674319, EPI_ISL_674322, EPI_ISL_674323, EPI_ISL_674324	Lighthouse Lab in Alderley Park	Wellcome Sanger Institute for the COVID-19 Genomics UK (COG-UK) Consortium	Jacquelyn Wynn, Mairead Hyland, The Lighthouse Lab in Alderley Park and Alex Alderton, Roberto Amato, Sonia Goncalves, Ewan Harrison, David K. Jackson, Ian Johnston, Dominic Kwiatkowski, Cordelia Langford, John Sillitoe on behalf of the Wellcome Sanger Institute COVID-19 Surveillance Team
EPI_ISL_674325	Lighthouse Lab in Milton Keynes	Wellcome Sanger Institute for the COVID-19 Genomics UK (COG-UK) Consortium	The Lighthouse Lab in Milton Keynes and Alex Alderton, Roberto Amato, Sonia Goncalves, Ewan Harrison, David K. Jackson, Ian Johnston, Dominic Kwiatkowski, Cordelia Langford, John Sillitoe on behalf of the Wellcome Sanger Institute COVID-19 Surveillance Team
EPI_ISL_674328, EPI_ISL_674329, EPI_ISL_674332, EPI_ISL_674334, EPI_ISL_674337, EPI_ISL_674339, EPI_ISL_674340, EPI_ISL_674341	Lighthouse Lab in Alderley Park	Wellcome Sanger Institute for the COVID-19 Genomics UK (COG-UK) Consortium	Jacquelyn Wynn, Mairead Hyland, The Lighthouse Lab in Alderley Park and Alex Alderton, Roberto Amato, Sonia Goncalves, Ewan Harrison, David K. Jackson, Ian Johnston, Dominic Kwiatkowski, Cordelia Langford, John Sillitoe on behalf of the Wellcome Sanger Institute COVID-19 Surveillance Team
EPI_ISL_674342	Lighthouse Lab in Milton Keynes	Wellcome Sanger Institute for the COVID-19 Genomics UK (COG-UK) Consortium	The Lighthouse Lab in Milton Keynes and Alex Alderton, Roberto Amato, Sonia Goncalves, Ewan Harrison, David K. Jackson, Ian Johnston, Dominic Kwiatkowski, Cordelia Langford, John Sillitoe on behalf of the Wellcome Sanger Institute COVID-19 Surveillance Team
EPI_ISL_674343, EPI_ISL_674345, EPI_ISL_674346, EPI_ISL_674347, EPI_ISL_674351, EPI_ISL_674352,	Lighthouse Lab in Alderley Park	Wellcome Sanger Institute for the COVID-19 Genomics UK (COG-UK) Consortium	Jacquelyn Wynn, Mairead Hyland, The Lighthouse Lab in Alderley Park and Alex Alderton, Roberto Amato, Sonia Goncalves, Ewan Harrison, David K. Jackson, Ian Johnston, Dominic Kwiatkowski, Cordelia Langford, John Sillitoe on behalf of the Wellcome Sanger Institute COVID-19 Surveillance Team

EPI_ISL_674354, EPI_ISL_674357			
EPI_ISL_674359	Lighthouse Lab in Milton Keynes	Wellcome Sanger Institute for the COVID-19 Genomics UK (COG-UK) Consortium	The Lighthouse Lab in Milton Keynes and Alex Alderton, Roberto Amato, Sonia Goncalves, Ewan Harrison, David K. Jackson, Ian Johnston, Dominic Kwiatkowski, Cordelia Langford, John Sillitoe on behalf of the Wellcome Sanger Institute COVID-19 Surveillance Team
EPI_ISL_674360, EPI_ISL_674361, EPI_ISL_674362, EPI_ISL_674364	Lighthouse Lab in Alderley Park	Wellcome Sanger Institute for the COVID-19 Genomics UK (COG-UK) Consortium	Jacquelyn Wynn, Mairead Hyland, The Lighthouse Lab in Alderley Park and Alex Alderton, Roberto Amato, Sonia Goncalves, Ewan Harrison, David K. Jackson, Ian Johnston, Dominic Kwiatkowski, Cordelia Langford, John Sillitoe on behalf of the Wellcome Sanger Institute COVID-19 Surveillance Team
EPI_ISL_674365	Lighthouse Lab in Milton Keynes	Wellcome Sanger Institute for the COVID-19 Genomics UK (COG-UK) Consortium	The Lighthouse Lab in Milton Keynes and Alex Alderton, Roberto Amato, Sonia Goncalves, Ewan Harrison, David K. Jackson, Ian Johnston, Dominic Kwiatkowski, Cordelia Langford, John Sillitoe on behalf of the Wellcome Sanger Institute COVID-19 Surveillance Team
EPI_ISL_674366, EPI_ISL_674367, EPI_ISL_674368, EPI_ISL_674369, EPI_ISL_674370, EPI_ISL_674372, EPI_ISL_674374, EPI_ISL_674375, EPI_ISL_674376, EPI_ISL_674380, EPI_ISL_674383, EPI_ISL_674385, EPI_ISL_674389, EPI_ISL_674390, EPI_ISL_674391, EPI_ISL_674392, EPI_ISL_674394, EPI_ISL_674396, EPI_ISL_674397, EPI_ISL_674399, EPI_ISL_674400, EPI_ISL_674402, EPI_ISL_674404, EPI_ISL_674406, EPI_ISL_674408, EPI_ISL_674409, EPI_ISL_674411, EPI_ISL_674412, EPI_ISL_674413			
see above	Lighthouse Lab in Alderley Park	Wellcome Sanger Institute for the COVID-19 Genomics UK (COG-UK) Consortium	Jacquelyn Wynn, Mairead Hyland, The Lighthouse Lab in Alderley Park and Alex Alderton, Roberto Amato, Sonia Goncalves, Ewan Harrison, David K. Jackson, Ian Johnston, Dominic Kwiatkowski, Cordelia Langford, John Sillitoe on behalf of the Wellcome Sanger Institute COVID-19 Surveillance Team
EPI_ISL_674415	Lighthouse Lab in Milton Keynes	Wellcome Sanger Institute for the COVID-19 Genomics UK (COG-UK) Consortium	The Lighthouse Lab in Milton Keynes and Alex Alderton, Roberto Amato, Sonia Goncalves, Ewan Harrison, David K. Jackson, Ian Johnston, Dominic Kwiatkowski, Cordelia Langford, John Sillitoe on behalf of the Wellcome Sanger Institute COVID-19 Surveillance Team
EPI_ISL_674416, EPI_ISL_674419, EPI_ISL_674420, EPI_ISL_674421	Lighthouse Lab in Alderley Park	Wellcome Sanger Institute for the COVID-19 Genomics UK (COG-UK) Consortium	Jacquelyn Wynn, Mairead Hyland, The Lighthouse Lab in Alderley Park and Alex Alderton, Roberto Amato, Sonia Goncalves, Ewan Harrison, David K. Jackson, Ian Johnston, Dominic Kwiatkowski, Cordelia Langford, John Sillitoe on behalf of the Wellcome Sanger Institute COVID-19 Surveillance Team
EPI_ISL_674423	Lighthouse Lab in Milton Keynes	Wellcome Sanger Institute for the COVID-19 Genomics UK (COG-UK) Consortium	The Lighthouse Lab in Milton Keynes and Alex Alderton, Roberto Amato, Sonia Goncalves, Ewan Harrison, David K. Jackson, Ian Johnston, Dominic Kwiatkowski, Cordelia Langford, John Sillitoe on behalf of the Wellcome Sanger Institute COVID-19 Surveillance Team
EPI_ISL_674424, EPI_ISL_674428, EPI_ISL_674429	Lighthouse Lab in Alderley Park	Wellcome Sanger Institute for the COVID-19 Genomics UK (COG-UK) Consortium	Jacquelyn Wynn, Mairead Hyland, The Lighthouse Lab in Alderley Park and Alex Alderton, Roberto Amato, Sonia Goncalves, Ewan Harrison, David K. Jackson, Ian Johnston, Dominic Kwiatkowski, Cordelia Langford, John Sillitoe on behalf of the Wellcome Sanger Institute COVID-19 Surveillance Team
EPI_ISL_674430	Lighthouse Lab in Milton Keynes	Wellcome Sanger Institute for the COVID-19 Genomics UK (COG-UK) Consortium	The Lighthouse Lab in Milton Keynes and Alex Alderton, Roberto Amato, Sonia Goncalves, Ewan Harrison, David K. Jackson, Ian Johnston, Dominic Kwiatkowski, Cordelia Langford, John Sillitoe on behalf of the Wellcome Sanger Institute COVID-19 Surveillance Team
EPI_ISL_674431, EPI_ISL_674432, EPI_ISL_674434, EPI_ISL_674435, EPI_ISL_674436, EPI_ISL_674438, EPI_ISL_674439, EPI_ISL_674442	Lighthouse Lab in Alderley Park	Wellcome Sanger Institute for the COVID-19 Genomics UK (COG-UK) Consortium	Jacquelyn Wynn, Mairead Hyland, The Lighthouse Lab in Alderley Park and Alex Alderton, Roberto Amato, Sonia Goncalves, Ewan Harrison, David K. Jackson, Ian Johnston, Dominic Kwiatkowski, Cordelia Langford, John Sillitoe on behalf of the Wellcome Sanger Institute COVID-19 Surveillance Team
EPI_ISL_674443	Lighthouse Lab in Milton Keynes	Wellcome Sanger Institute for the COVID-19 Genomics UK (COG-UK) Consortium	The Lighthouse Lab in Milton Keynes and Alex Alderton, Roberto Amato, Sonia Goncalves, Ewan Harrison, David K. Jackson, Ian Johnston, Dominic Kwiatkowski, Cordelia Langford, John Sillitoe on behalf of the Wellcome Sanger Institute COVID-19 Surveillance Team
EPI_ISL_674445, EPI_ISL_674446, EPI_ISL_674447	Lighthouse Lab in Alderley Park	Wellcome Sanger Institute for the COVID-19 Genomics UK (COG-UK) Consortium	Jacquelyn Wynn, Mairead Hyland, The Lighthouse Lab in Alderley Park and Alex Alderton, Roberto Amato, Sonia Goncalves, Ewan Harrison, David K. Jackson, Ian Johnston, Dominic Kwiatkowski, Cordelia Langford, John Sillitoe on behalf of the Wellcome Sanger Institute COVID-19 Surveillance Team
EPI_ISL_674450	Lighthouse Lab in Milton Keynes	Wellcome Sanger Institute for the COVID-19 Genomics UK (COG-UK) Consortium	The Lighthouse Lab in Milton Keynes and Alex Alderton, Roberto Amato, Sonia Goncalves, Ewan Harrison, David K. Jackson, Ian Johnston, Dominic Kwiatkowski, Cordelia Langford, John Sillitoe on behalf of the Wellcome Sanger Institute COVID-19 Surveillance Team
EPI_ISL_674451, EPI_ISL_674452, EPI_ISL_674454, EPI_ISL_674455, EPI_ISL_674456, EPI_ISL_674459, EPI_ISL_674462	Lighthouse Lab in Alderley Park	Wellcome Sanger Institute for the COVID-19 Genomics UK (COG-UK) Consortium	Jacquelyn Wynn, Mairead Hyland, The Lighthouse Lab in Alderley Park and Alex Alderton, Roberto Amato, Sonia Goncalves, Ewan Harrison, David K. Jackson, Ian Johnston, Dominic Kwiatkowski, Cordelia Langford, John Sillitoe on behalf of the Wellcome Sanger Institute COVID-19 Surveillance Team
EPI_ISL_674464	Lighthouse Lab in Milton Keynes	Wellcome Sanger Institute for the COVID-19 Genomics UK (COG-UK) Consortium	The Lighthouse Lab in Milton Keynes and Alex Alderton, Roberto Amato, Sonia Goncalves, Ewan Harrison, David K. Jackson, Ian Johnston, Dominic Kwiatkowski, Cordelia Langford, John Sillitoe on behalf of the Wellcome Sanger Institute COVID-19 Surveillance Team
EPI_ISL_674465, EPI_ISL_674466, EPI_ISL_674467, EPI_ISL_674469, EPI_ISL_674470, EPI_ISL_674471, EPI_ISL_674472, EPI_ISL_674474, EPI_ISL_674476, EPI_ISL_674477, EPI_ISL_674478, EPI_ISL_674479			
see above	Lighthouse Lab in Alderley Park	Wellcome Sanger Institute for the COVID-19 Genomics UK (COG-UK) Consortium	Jacquelyn Wynn, Mairead Hyland, The Lighthouse Lab in Alderley Park and Alex Alderton, Roberto Amato, Sonia Goncalves, Ewan Harrison, David K. Jackson, Ian Johnston, Dominic Kwiatkowski, Cordelia Langford, John Sillitoe on behalf of the Wellcome Sanger Institute COVID-19 Surveillance Team
EPI_ISL_674483	Lighthouse Lab in Milton Keynes	Wellcome Sanger Institute for the COVID-19 Genomics UK (COG-UK) Consortium	The Lighthouse Lab in Milton Keynes and Alex Alderton, Roberto Amato, Sonia Goncalves, Ewan Harrison, David K. Jackson, Ian Johnston, Dominic Kwiatkowski, Cordelia Langford, John Sillitoe on behalf of the Wellcome Sanger Institute COVID-19 Surveillance Team
EPI_ISL_674484, EPI_ISL_674485, EPI_ISL_674486, EPI_ISL_674487, EPI_ISL_674488, EPI_ISL_674490, EPI_ISL_674493, EPI_ISL_674495	Lighthouse Lab in Alderley Park	Wellcome Sanger Institute for the COVID-19 Genomics UK (COG-UK) Consortium	Jacquelyn Wynn, Mairead Hyland, The Lighthouse Lab in Alderley Park and Alex Alderton, Roberto Amato, Sonia Goncalves, Ewan Harrison, David K. Jackson, Ian Johnston, Dominic Kwiatkowski, Cordelia Langford, John Sillitoe on behalf of the Wellcome Sanger Institute COVID-19 Surveillance Team
EPI_ISL_674498, EPI_ISL_674499, EPI_ISL_674501, EPI_ISL_674504, EPI_ISL_674514, EPI_ISL_674516, EPI_ISL_674522, EPI_ISL_674527, EPI_ISL_674528, EPI_ISL_674530, EPI_ISL_674543, EPI_ISL_674544, EPI_ISL_674545, EPI_ISL_674550, EPI_ISL_674558, EPI_ISL_674563, EPI_ISL_674565, EPI_ISL_674569, EPI_ISL_674585, EPI_ISL_674589, EPI_ISL_674590, EPI_ISL_674592, EPI_ISL_674595, EPI_ISL_674603, EPI_ISL_674605, EPI_ISL_674609, EPI_ISL_674613, EPI_ISL_674625, EPI_ISL_674629, EPI_ISL_674635, EPI_ISL_674652, EPI_ISL_674653, EPI_ISL_674655, EPI_ISL_674657, EPI_ISL_674658, EPI_ISL_674661, EPI_ISL_674662, EPI_ISL_674667, EPI_ISL_674670, EPI_ISL_674674, EPI_ISL_674678, EPI_ISL_674685, EPI_ISL_674690, EPI_ISL_674691, EPI_ISL_674692, EPI_ISL_674698, EPI_ISL_674707, EPI_ISL_674714, EPI_ISL_674716, EPI_ISL_674717, EPI_ISL_674722, EPI_ISL_674723, EPI_ISL_674724, EPI_ISL_674725, EPI_ISL_674736, EPI_ISL_674740, EPI_ISL_674743, EPI_ISL_674744, EPI_ISL_674745, EPI_ISL_674746, EPI_ISL_674747, EPI_ISL_674749, EPI_ISL_674751, EPI_ISL_674752, EPI_ISL_674756, EPI_ISL_674758, EPI_ISL_674762, EPI_ISL_674763, EPI_ISL_674772, EPI_ISL_674773, EPI_ISL_674779, EPI_ISL_674781, EPI_ISL_674783, EPI_ISL_674786, EPI_ISL_674791, EPI_ISL_674792, EPI_ISL_674794, EPI_ISL_674795, EPI_ISL_674804, EPI_ISL_674808, EPI_ISL_674809			
see above	Lighthouse Lab in Glasgow	Wellcome Sanger Institute for the COVID-19 Genomics UK (COG-UK) Consortium	Harper VanSteenhouse, Yumi Kasai, David Gray, Carol Clugston, Anna Dominiczak and Alex Alderton, Roberto Amato, Sonia Goncalves, Ewan Harrison, David K. Jackson, Ian Johnston, Dominic Kwiatkowski, Cordelia Langford, John Sillitoe on behalf of the Wellcome Sanger Institute COVID-19 Surveillance Team
EPI_ISL_674817, EPI_ISL_674818, EPI_ISL_674819, EPI_ISL_674820, EPI_ISL_674822, EPI_ISL_674823, EPI_ISL_674824, EPI_ISL_674825	Lighthouse Lab in Milton Keynes	Wellcome Sanger Institute for the COVID-19 Genomics UK (COG-UK) Consortium	The Lighthouse Lab in Milton Keynes and Alex Alderton, Roberto Amato, Sonia Goncalves, Ewan Harrison, David K. Jackson, Ian Johnston, Dominic Kwiatkowski, Cordelia Langford, John Sillitoe on behalf of the Wellcome Sanger Institute COVID-19 Surveillance Team
EPI_ISL_674826, EPI_ISL_674828	Lighthouse Lab in Cambridge	Wellcome Sanger Institute for the COVID-19 Genomics UK (COG-UK) Consortium	Rob Howes, The Lighthouse Lab in Cambridge and Alex Alderton, Roberto Amato, Sonia Goncalves, Ewan Harrison, David K. Jackson, Ian Johnston, Dominic Kwiatkowski, Cordelia Langford, John Sillitoe on behalf of the Wellcome Sanger Institute COVID-19 Surveillance Team
EPI_ISL_674830	Lighthouse Lab in Milton Keynes	Wellcome Sanger Institute for the COVID-19 Genomics UK (COG-UK) Consortium	The Lighthouse Lab in Milton Keynes and Alex Alderton, Roberto Amato, Sonia Goncalves, Ewan Harrison, David K. Jackson, Ian Johnston, Dominic Kwiatkowski, Cordelia Langford, John Sillitoe on behalf of the Wellcome Sanger Institute COVID-19 Surveillance Team
EPI_ISL_674834	Lighthouse Lab in Cambridge	Wellcome Sanger Institute for the COVID-19 Genomics UK (COG-UK) Consortium	Rob Howes, The Lighthouse Lab in Cambridge and Alex Alderton, Roberto Amato, Sonia Goncalves, Ewan Harrison, David K. Jackson, Ian Johnston, Dominic Kwiatkowski, Cordelia Langford, John Sillitoe on behalf of the Wellcome Sanger Institute COVID-19 Surveillance Team
EPI_ISL_674835, EPI_ISL_674836	Lighthouse Lab in Milton Keynes	Wellcome Sanger Institute for the COVID-19 Genomics UK (COG-UK) Consortium	The Lighthouse Lab in Milton Keynes and Alex Alderton, Roberto Amato, Sonia Goncalves, Ewan Harrison, David K. Jackson, Ian Johnston, Dominic Kwiatkowski, Cordelia Langford, John Sillitoe on behalf of the Wellcome Sanger Institute COVID-19 Surveillance Team
EPI_ISL_674837, EPI_ISL_674838	Lighthouse Lab in Cambridge	Wellcome Sanger Institute for the COVID-19 Genomics UK (COG-UK) Consortium	Rob Howes, The Lighthouse Lab in Cambridge and Alex Alderton, Roberto Amato, Sonia Goncalves, Ewan Harrison, David K. Jackson, Ian Johnston, Dominic Kwiatkowski, Cordelia Langford, John Sillitoe on behalf of the Wellcome Sanger Institute COVID-19 Surveillance Team
EPI_ISL_674839	Lighthouse Lab in Milton Keynes	Wellcome Sanger Institute for the COVID-19 Genomics UK (COG-UK) Consortium	The Lighthouse Lab in Milton Keynes and Alex Alderton, Roberto Amato, Sonia Goncalves, Ewan Harrison, David K. Jackson, Ian Johnston, Dominic Kwiatkowski, Cordelia Langford, John Sillitoe on behalf of the Wellcome Sanger Institute COVID-19 Surveillance Team
EPI_ISL_674840	Lighthouse Lab in Cambridge	Wellcome Sanger Institute for the COVID-19 Genomics UK (COG-UK) Consortium	Rob Howes, The Lighthouse Lab in Cambridge and Alex Alderton, Roberto Amato, Sonia Goncalves, Ewan Harrison, David K. Jackson, Ian Johnston, Dominic Kwiatkowski, Cordelia Langford, John Sillitoe on behalf of the Wellcome Sanger Institute COVID-19 Surveillance Team

EPI_ISL_674841, EPI_ISL_674842, EPI_ISL_674843, EPI_ISL_674844	Lighthouse Lab in Milton Keynes	Wellcome Sanger Institute for the COVID-19 Genomics UK (COG-UK) Consortium	The Lighthouse Lab in Milton Keynes and Alex Alderton, Roberto Amato, Sonia Goncalves, Ewan Harrison, David K. Jackson, Ian Johnston, Dominic Kwiatkowski, Cordelia Langford, John Sillitoe on behalf of the Wellcome Sanger Institute COVID-19 Surveillance Team
EPI_ISL_674845, EPI_ISL_674846, EPI_ISL_674847, EPI_ISL_674849, EPI_ISL_674850, EPI_ISL_674851	Lighthouse Lab in Cambridge	Wellcome Sanger Institute for the COVID-19 Genomics UK (COG-UK) Consortium	Rob Howes, The Lighthouse Lab in Cambridge and Alex Alderton, Roberto Amato, Sonia Goncalves, Ewan Harrison, David K. Jackson, Ian Johnston, Dominic Kwiatkowski, Cordelia Langford, John Sillitoe on behalf of the Wellcome Sanger Institute COVID-19 Surveillance Team
EPI_ISL_674852, EPI_ISL_674854	Lighthouse Lab in Milton Keynes	Wellcome Sanger Institute for the COVID-19 Genomics UK (COG-UK) Consortium	The Lighthouse Lab in Milton Keynes and Alex Alderton, Roberto Amato, Sonia Goncalves, Ewan Harrison, David K. Jackson, Ian Johnston, Dominic Kwiatkowski, Cordelia Langford, John Sillitoe on behalf of the Wellcome Sanger Institute COVID-19 Surveillance Team
EPI_ISL_674859	Lighthouse Lab in Cambridge	Wellcome Sanger Institute for the COVID-19 Genomics UK (COG-UK) Consortium	Rob Howes, The Lighthouse Lab in Cambridge and Alex Alderton, Roberto Amato, Sonia Goncalves, Ewan Harrison, David K. Jackson, Ian Johnston, Dominic Kwiatkowski, Cordelia Langford, John Sillitoe on behalf of the Wellcome Sanger Institute COVID-19 Surveillance Team
EPI_ISL_674860, EPI_ISL_674861	Lighthouse Lab in Milton Keynes	Wellcome Sanger Institute for the COVID-19 Genomics UK (COG-UK) Consortium	The Lighthouse Lab in Milton Keynes and Alex Alderton, Roberto Amato, Sonia Goncalves, Ewan Harrison, David K. Jackson, Ian Johnston, Dominic Kwiatkowski, Cordelia Langford, John Sillitoe on behalf of the Wellcome Sanger Institute COVID-19 Surveillance Team
EPI_ISL_674862	Lighthouse Lab in Cambridge	Wellcome Sanger Institute for the COVID-19 Genomics UK (COG-UK) Consortium	Rob Howes, The Lighthouse Lab in Cambridge and Alex Alderton, Roberto Amato, Sonia Goncalves, Ewan Harrison, David K. Jackson, Ian Johnston, Dominic Kwiatkowski, Cordelia Langford, John Sillitoe on behalf of the Wellcome Sanger Institute COVID-19 Surveillance Team
EPI_ISL_674863	Lighthouse Lab in Milton Keynes	Wellcome Sanger Institute for the COVID-19 Genomics UK (COG-UK) Consortium	The Lighthouse Lab in Milton Keynes and Alex Alderton, Roberto Amato, Sonia Goncalves, Ewan Harrison, David K. Jackson, Ian Johnston, Dominic Kwiatkowski, Cordelia Langford, John Sillitoe on behalf of the Wellcome Sanger Institute COVID-19 Surveillance Team
EPI_ISL_674867, EPI_ISL_674868	Lighthouse Lab in Cambridge	Wellcome Sanger Institute for the COVID-19 Genomics UK (COG-UK) Consortium	Rob Howes, The Lighthouse Lab in Cambridge and Alex Alderton, Roberto Amato, Sonia Goncalves, Ewan Harrison, David K. Jackson, Ian Johnston, Dominic Kwiatkowski, Cordelia Langford, John Sillitoe on behalf of the Wellcome Sanger Institute COVID-19 Surveillance Team
EPI_ISL_674870	Lighthouse Lab in Milton Keynes	Wellcome Sanger Institute for the COVID-19 Genomics UK (COG-UK) Consortium	The Lighthouse Lab in Milton Keynes and Alex Alderton, Roberto Amato, Sonia Goncalves, Ewan Harrison, David K. Jackson, Ian Johnston, Dominic Kwiatkowski, Cordelia Langford, John Sillitoe on behalf of the Wellcome Sanger Institute COVID-19 Surveillance Team
EPI_ISL_674871	Lighthouse Lab in Cambridge	Wellcome Sanger Institute for the COVID-19 Genomics UK (COG-UK) Consortium	Rob Howes, The Lighthouse Lab in Cambridge and Alex Alderton, Roberto Amato, Sonia Goncalves, Ewan Harrison, David K. Jackson, Ian Johnston, Dominic Kwiatkowski, Cordelia Langford, John Sillitoe on behalf of the Wellcome Sanger Institute COVID-19 Surveillance Team
EPI_ISL_674872	Lighthouse Lab in Milton Keynes	Wellcome Sanger Institute for the COVID-19 Genomics UK (COG-UK) Consortium	The Lighthouse Lab in Milton Keynes and Alex Alderton, Roberto Amato, Sonia Goncalves, Ewan Harrison, David K. Jackson, Ian Johnston, Dominic Kwiatkowski, Cordelia Langford, John Sillitoe on behalf of the Wellcome Sanger Institute COVID-19 Surveillance Team
EPI_ISL_674873	Lighthouse Lab in Cambridge	Wellcome Sanger Institute for the COVID-19 Genomics UK (COG-UK) Consortium	Rob Howes, The Lighthouse Lab in Cambridge and Alex Alderton, Roberto Amato, Sonia Goncalves, Ewan Harrison, David K. Jackson, Ian Johnston, Dominic Kwiatkowski, Cordelia Langford, John Sillitoe on behalf of the Wellcome Sanger Institute COVID-19 Surveillance Team
EPI_ISL_674874, EPI_ISL_674875	Lighthouse Lab in Milton Keynes	Wellcome Sanger Institute for the COVID-19 Genomics UK (COG-UK) Consortium	The Lighthouse Lab in Milton Keynes and Alex Alderton, Roberto Amato, Sonia Goncalves, Ewan Harrison, David K. Jackson, Ian Johnston, Dominic Kwiatkowski, Cordelia Langford, John Sillitoe on behalf of the Wellcome Sanger Institute COVID-19 Surveillance Team
EPI_ISL_674876	Lighthouse Lab in Cambridge	Wellcome Sanger Institute for the COVID-19 Genomics UK (COG-UK) Consortium	Rob Howes, The Lighthouse Lab in Cambridge and Alex Alderton, Roberto Amato, Sonia Goncalves, Ewan Harrison, David K. Jackson, Ian Johnston, Dominic Kwiatkowski, Cordelia Langford, John Sillitoe on behalf of the Wellcome Sanger Institute COVID-19 Surveillance Team
EPI_ISL_674879, EPI_ISL_674880, EPI_ISL_674881, EPI_ISL_674882	Lighthouse Lab in Milton Keynes	Wellcome Sanger Institute for the COVID-19 Genomics UK (COG-UK) Consortium	The Lighthouse Lab in Milton Keynes and Alex Alderton, Roberto Amato, Sonia Goncalves, Ewan Harrison, David K. Jackson, Ian Johnston, Dominic Kwiatkowski, Cordelia Langford, John Sillitoe on behalf of the Wellcome Sanger Institute COVID-19 Surveillance Team
EPI_ISL_674883	Lighthouse Lab in Cambridge	Wellcome Sanger Institute for the COVID-19 Genomics UK (COG-UK) Consortium	Rob Howes, The Lighthouse Lab in Cambridge and Alex Alderton, Roberto Amato, Sonia Goncalves, Ewan Harrison, David K. Jackson, Ian Johnston, Dominic Kwiatkowski, Cordelia Langford, John Sillitoe on behalf of the Wellcome Sanger Institute COVID-19 Surveillance Team
EPI_ISL_674884, EPI_ISL_674886, EPI_ISL_674887, EPI_ISL_674888, EPI_ISL_674890, EPI_ISL_674891	Lighthouse Lab in Milton Keynes	Wellcome Sanger Institute for the COVID-19 Genomics UK (COG-UK) Consortium	The Lighthouse Lab in Milton Keynes and Alex Alderton, Roberto Amato, Sonia Goncalves, Ewan Harrison, David K. Jackson, Ian Johnston, Dominic Kwiatkowski, Cordelia Langford, John Sillitoe on behalf of the Wellcome Sanger Institute COVID-19 Surveillance Team
EPI_ISL_674893, EPI_ISL_674896	Lighthouse Lab in Cambridge	Wellcome Sanger Institute for the COVID-19 Genomics UK (COG-UK) Consortium	Rob Howes, The Lighthouse Lab in Cambridge and Alex Alderton, Roberto Amato, Sonia Goncalves, Ewan Harrison, David K. Jackson, Ian Johnston, Dominic Kwiatkowski, Cordelia Langford, John Sillitoe on behalf of the Wellcome Sanger Institute COVID-19 Surveillance Team
EPI_ISL_674898	Lighthouse Lab in Milton Keynes	Wellcome Sanger Institute for the COVID-19 Genomics UK (COG-UK) Consortium	The Lighthouse Lab in Milton Keynes and Alex Alderton, Roberto Amato, Sonia Goncalves, Ewan Harrison, David K. Jackson, Ian Johnston, Dominic Kwiatkowski, Cordelia Langford, John Sillitoe on behalf of the Wellcome Sanger Institute COVID-19 Surveillance Team
EPI_ISL_674899	Lighthouse Lab in Cambridge	Wellcome Sanger Institute for the COVID-19 Genomics UK (COG-UK) Consortium	Rob Howes, The Lighthouse Lab in Cambridge and Alex Alderton, Roberto Amato, Sonia Goncalves, Ewan Harrison, David K. Jackson, Ian Johnston, Dominic Kwiatkowski, Cordelia Langford, John Sillitoe on behalf of the Wellcome Sanger Institute COVID-19 Surveillance Team
EPI_ISL_674900, EPI_ISL_674901, EPI_ISL_674902, EPI_ISL_674903, EPI_ISL_674904	Lighthouse Lab in Milton Keynes	Wellcome Sanger Institute for the COVID-19 Genomics UK (COG-UK) Consortium	The Lighthouse Lab in Milton Keynes and Alex Alderton, Roberto Amato, Sonia Goncalves, Ewan Harrison, David K. Jackson, Ian Johnston, Dominic Kwiatkowski, Cordelia Langford, John Sillitoe on behalf of the Wellcome Sanger Institute COVID-19 Surveillance Team
EPI_ISL_674906	Lighthouse Lab in Cambridge	Wellcome Sanger Institute for the COVID-19 Genomics UK (COG-UK) Consortium	Rob Howes, The Lighthouse Lab in Cambridge and Alex Alderton, Roberto Amato, Sonia Goncalves, Ewan Harrison, David K. Jackson, Ian Johnston, Dominic Kwiatkowski, Cordelia Langford, John Sillitoe on behalf of the Wellcome Sanger Institute COVID-19 Surveillance Team
EPI_ISL_674907	Lighthouse Lab in Milton Keynes	Wellcome Sanger Institute for the COVID-19 Genomics UK (COG-UK) Consortium	The Lighthouse Lab in Milton Keynes and Alex Alderton, Roberto Amato, Sonia Goncalves, Ewan Harrison, David K. Jackson, Ian Johnston, Dominic Kwiatkowski, Cordelia Langford, John Sillitoe on behalf of the Wellcome Sanger Institute COVID-19 Surveillance Team
EPI_ISL_674908, EPI_ISL_674909, EPI_ISL_674910	Lighthouse Lab in Cambridge	Wellcome Sanger Institute for the COVID-19 Genomics UK (COG-UK) Consortium	Rob Howes, The Lighthouse Lab in Cambridge and Alex Alderton, Roberto Amato, Sonia Goncalves, Ewan Harrison, David K. Jackson, Ian Johnston, Dominic Kwiatkowski, Cordelia Langford, John Sillitoe on behalf of the Wellcome Sanger Institute COVID-19 Surveillance Team
EPI_ISL_674912	Lighthouse Lab in Milton Keynes	Wellcome Sanger Institute for the COVID-19 Genomics UK (COG-UK) Consortium	The Lighthouse Lab in Milton Keynes and Alex Alderton, Roberto Amato, Sonia Goncalves, Ewan Harrison, David K. Jackson, Ian Johnston, Dominic Kwiatkowski, Cordelia Langford, John Sillitoe on behalf of the Wellcome Sanger Institute COVID-19 Surveillance Team
EPI_ISL_674913	Lighthouse Lab in Cambridge	Wellcome Sanger Institute for the COVID-19 Genomics UK (COG-UK) Consortium	Rob Howes, The Lighthouse Lab in Cambridge and Alex Alderton, Roberto Amato, Sonia Goncalves, Ewan Harrison, David K. Jackson, Ian Johnston, Dominic Kwiatkowski, Cordelia Langford, John Sillitoe on behalf of the Wellcome Sanger Institute COVID-19 Surveillance Team
EPI_ISL_674914	Lighthouse Lab in Milton Keynes	Wellcome Sanger Institute for the COVID-19 Genomics UK (COG-UK) Consortium	The Lighthouse Lab in Milton Keynes and Alex Alderton, Roberto Amato, Sonia Goncalves, Ewan Harrison, David K. Jackson, Ian Johnston, Dominic Kwiatkowski, Cordelia Langford, John Sillitoe on behalf of the Wellcome Sanger Institute COVID-19 Surveillance Team
EPI_ISL_674917	Lighthouse Lab in Cambridge	Wellcome Sanger Institute for the COVID-19 Genomics UK (COG-UK) Consortium	Rob Howes, The Lighthouse Lab in Cambridge and Alex Alderton, Roberto Amato, Sonia Goncalves, Ewan Harrison, David K. Jackson, Ian Johnston, Dominic Kwiatkowski, Cordelia Langford, John Sillitoe on behalf of the Wellcome Sanger Institute COVID-19 Surveillance Team
EPI_ISL_674918	Lighthouse Lab in Milton Keynes	Wellcome Sanger Institute for the COVID-19 Genomics UK (COG-UK) Consortium	The Lighthouse Lab in Milton Keynes and Alex Alderton, Roberto Amato, Sonia Goncalves, Ewan Harrison, David K. Jackson, Ian Johnston, Dominic Kwiatkowski, Cordelia Langford, John Sillitoe on behalf of the Wellcome Sanger Institute COVID-19 Surveillance Team
EPI_ISL_674919, EPI_ISL_674920	Lighthouse Lab in Cambridge	Wellcome Sanger Institute for the COVID-19 Genomics UK (COG-UK) Consortium	Rob Howes, The Lighthouse Lab in Cambridge and Alex Alderton, Roberto Amato, Sonia Goncalves, Ewan Harrison, David K. Jackson, Ian Johnston, Dominic Kwiatkowski, Cordelia Langford, John Sillitoe on behalf of the Wellcome Sanger Institute COVID-19 Surveillance Team
EPI_ISL_674921	Lighthouse Lab in Milton Keynes	Wellcome Sanger Institute for the COVID-19 Genomics UK (COG-UK) Consortium	The Lighthouse Lab in Milton Keynes and Alex Alderton, Roberto Amato, Sonia Goncalves, Ewan Harrison, David K. Jackson, Ian Johnston, Dominic Kwiatkowski, Cordelia Langford, John Sillitoe on behalf of the Wellcome Sanger Institute COVID-19 Surveillance Team
EPI_ISL_674922, EPI_ISL_674923, EPI_ISL_674924, EPI_ISL_674926	Lighthouse Lab in Cambridge	Wellcome Sanger Institute for the COVID-19 Genomics UK (COG-UK) Consortium	Rob Howes, The Lighthouse Lab in Cambridge and Alex Alderton, Roberto Amato, Sonia Goncalves, Ewan Harrison, David K. Jackson, Ian Johnston, Dominic Kwiatkowski, Cordelia Langford, John Sillitoe on behalf of the Wellcome Sanger Institute COVID-19 Surveillance Team
EPI_ISL_674927, EPI_ISL_674928, EPI_ISL_674929, EPI_ISL_674930, EPI_ISL_674931, EPI_ISL_674932	Lighthouse Lab in Milton Keynes	Wellcome Sanger Institute for the COVID-19 Genomics UK (COG-UK) Consortium	The Lighthouse Lab in Milton Keynes and Alex Alderton, Roberto Amato, Sonia Goncalves, Ewan Harrison, David K. Jackson, Ian Johnston, Dominic Kwiatkowski, Cordelia Langford, John Sillitoe on behalf of the Wellcome Sanger Institute COVID-19 Surveillance Team
EPI_ISL_674933	Lighthouse Lab in Cambridge	Wellcome Sanger Institute for the COVID-19 Genomics UK	Rob Howes, The Lighthouse Lab in Cambridge and Alex Alderton, Roberto Amato, Sonia Goncalves, Ewan Harrison, David K. Jackson, Ian Johnston,

		(COG-UK) Consortium	Dominic Kwiatkowski, Cordelia Langford, John Sillitoe on behalf of the Wellcome Sanger Institute COVID-19 Surveillance Team
EPI_ISL_674935	Lighthouse Lab in Milton Keynes	Wellcome Sanger Institute for the COVID-19 Genomics UK (COG-UK) Consortium	The Lighthouse Lab in Milton Keynes and Alex Alderton, Roberto Amato, Sonia Goncalves, Ewan Harrison, David K. Jackson, Ian Johnston, Dominic Kwiatkowski, Cordelia Langford, John Sillitoe on behalf of the Wellcome Sanger Institute COVID-19 Surveillance Team
EPI_ISL_674936	Lighthouse Lab in Cambridge	Wellcome Sanger Institute for the COVID-19 Genomics UK (COG-UK) Consortium	Rob Howes, The Lighthouse Lab in Cambridge and Alex Alderton, Roberto Amato, Sonia Goncalves, Ewan Harrison, David K. Jackson, Ian Johnston, Dominic Kwiatkowski, Cordelia Langford, John Sillitoe on behalf of the Wellcome Sanger Institute COVID-19 Surveillance Team
EPI_ISL_674937	Lighthouse Lab in Milton Keynes	Wellcome Sanger Institute for the COVID-19 Genomics UK (COG-UK) Consortium	The Lighthouse Lab in Milton Keynes and Alex Alderton, Roberto Amato, Sonia Goncalves, Ewan Harrison, David K. Jackson, Ian Johnston, Dominic Kwiatkowski, Cordelia Langford, John Sillitoe on behalf of the Wellcome Sanger Institute COVID-19 Surveillance Team
EPI_ISL_674939, EPI_ISL_674940	Lighthouse Lab in Cambridge	Wellcome Sanger Institute for the COVID-19 Genomics UK (COG-UK) Consortium	Rob Howes, The Lighthouse Lab in Cambridge and Alex Alderton, Roberto Amato, Sonia Goncalves, Ewan Harrison, David K. Jackson, Ian Johnston, Dominic Kwiatkowski, Cordelia Langford, John Sillitoe on behalf of the Wellcome Sanger Institute COVID-19 Surveillance Team
EPI_ISL_674943	Lighthouse Lab in Milton Keynes	Wellcome Sanger Institute for the COVID-19 Genomics UK (COG-UK) Consortium	The Lighthouse Lab in Milton Keynes and Alex Alderton, Roberto Amato, Sonia Goncalves, Ewan Harrison, David K. Jackson, Ian Johnston, Dominic Kwiatkowski, Cordelia Langford, John Sillitoe on behalf of the Wellcome Sanger Institute COVID-19 Surveillance Team
EPI_ISL_674944, EPI_ISL_674946	Lighthouse Lab in Cambridge	Wellcome Sanger Institute for the COVID-19 Genomics UK (COG-UK) Consortium	Rob Howes, The Lighthouse Lab in Cambridge and Alex Alderton, Roberto Amato, Sonia Goncalves, Ewan Harrison, David K. Jackson, Ian Johnston, Dominic Kwiatkowski, Cordelia Langford, John Sillitoe on behalf of the Wellcome Sanger Institute COVID-19 Surveillance Team
EPI_ISL_674947	Lighthouse Lab in Milton Keynes	Wellcome Sanger Institute for the COVID-19 Genomics UK (COG-UK) Consortium	The Lighthouse Lab in Milton Keynes and Alex Alderton, Roberto Amato, Sonia Goncalves, Ewan Harrison, David K. Jackson, Ian Johnston, Dominic Kwiatkowski, Cordelia Langford, John Sillitoe on behalf of the Wellcome Sanger Institute COVID-19 Surveillance Team
EPI_ISL_674949, EPI_ISL_674950, EPI_ISL_674951, EPI_ISL_674952, EPI_ISL_674953	Lighthouse Lab in Cambridge	Wellcome Sanger Institute for the COVID-19 Genomics UK (COG-UK) Consortium	Rob Howes, The Lighthouse Lab in Cambridge and Alex Alderton, Roberto Amato, Sonia Goncalves, Ewan Harrison, David K. Jackson, Ian Johnston, Dominic Kwiatkowski, Cordelia Langford, John Sillitoe on behalf of the Wellcome Sanger Institute COVID-19 Surveillance Team
EPI_ISL_674954, EPI_ISL_674955, EPI_ISL_674956	Lighthouse Lab in Milton Keynes	Wellcome Sanger Institute for the COVID-19 Genomics UK (COG-UK) Consortium	The Lighthouse Lab in Milton Keynes and Alex Alderton, Roberto Amato, Sonia Goncalves, Ewan Harrison, David K. Jackson, Ian Johnston, Dominic Kwiatkowski, Cordelia Langford, John Sillitoe on behalf of the Wellcome Sanger Institute COVID-19 Surveillance Team
EPI_ISL_674957	Lighthouse Lab in Cambridge	Wellcome Sanger Institute for the COVID-19 Genomics UK (COG-UK) Consortium	Rob Howes, The Lighthouse Lab in Cambridge and Alex Alderton, Roberto Amato, Sonia Goncalves, Ewan Harrison, David K. Jackson, Ian Johnston, Dominic Kwiatkowski, Cordelia Langford, John Sillitoe on behalf of the Wellcome Sanger Institute COVID-19 Surveillance Team
EPI_ISL_674958	Lighthouse Lab in Milton Keynes	Wellcome Sanger Institute for the COVID-19 Genomics UK (COG-UK) Consortium	The Lighthouse Lab in Milton Keynes and Alex Alderton, Roberto Amato, Sonia Goncalves, Ewan Harrison, David K. Jackson, Ian Johnston, Dominic Kwiatkowski, Cordelia Langford, John Sillitoe on behalf of the Wellcome Sanger Institute COVID-19 Surveillance Team
EPI_ISL_674959	Lighthouse Lab in Cambridge	Wellcome Sanger Institute for the COVID-19 Genomics UK (COG-UK) Consortium	Rob Howes, The Lighthouse Lab in Cambridge and Alex Alderton, Roberto Amato, Sonia Goncalves, Ewan Harrison, David K. Jackson, Ian Johnston, Dominic Kwiatkowski, Cordelia Langford, John Sillitoe on behalf of the Wellcome Sanger Institute COVID-19 Surveillance Team
EPI_ISL_674961	Lighthouse Lab in Milton Keynes	Wellcome Sanger Institute for the COVID-19 Genomics UK (COG-UK) Consortium	The Lighthouse Lab in Milton Keynes and Alex Alderton, Roberto Amato, Sonia Goncalves, Ewan Harrison, David K. Jackson, Ian Johnston, Dominic Kwiatkowski, Cordelia Langford, John Sillitoe on behalf of the Wellcome Sanger Institute COVID-19 Surveillance Team
EPI_ISL_674962	Lighthouse Lab in Cambridge	Wellcome Sanger Institute for the COVID-19 Genomics UK (COG-UK) Consortium	Rob Howes, The Lighthouse Lab in Cambridge and Alex Alderton, Roberto Amato, Sonia Goncalves, Ewan Harrison, David K. Jackson, Ian Johnston, Dominic Kwiatkowski, Cordelia Langford, John Sillitoe on behalf of the Wellcome Sanger Institute COVID-19 Surveillance Team
EPI_ISL_674964, EPI_ISL_674969	Lighthouse Lab in Milton Keynes	Wellcome Sanger Institute for the COVID-19 Genomics UK (COG-UK) Consortium	The Lighthouse Lab in Milton Keynes and Alex Alderton, Roberto Amato, Sonia Goncalves, Ewan Harrison, David K. Jackson, Ian Johnston, Dominic Kwiatkowski, Cordelia Langford, John Sillitoe on behalf of the Wellcome Sanger Institute COVID-19 Surveillance Team
EPI_ISL_674972, EPI_ISL_674973, EPI_ISL_674977	Lighthouse Lab in Cambridge	Wellcome Sanger Institute for the COVID-19 Genomics UK (COG-UK) Consortium	Rob Howes, The Lighthouse Lab in Cambridge and Alex Alderton, Roberto Amato, Sonia Goncalves, Ewan Harrison, David K. Jackson, Ian Johnston, Dominic Kwiatkowski, Cordelia Langford, John Sillitoe on behalf of the Wellcome Sanger Institute COVID-19 Surveillance Team
EPI_ISL_674981, EPI_ISL_674982	Lighthouse Lab in Milton Keynes	Wellcome Sanger Institute for the COVID-19 Genomics UK (COG-UK) Consortium	The Lighthouse Lab in Milton Keynes and Alex Alderton, Roberto Amato, Sonia Goncalves, Ewan Harrison, David K. Jackson, Ian Johnston, Dominic Kwiatkowski, Cordelia Langford, John Sillitoe on behalf of the Wellcome Sanger Institute COVID-19 Surveillance Team
EPI_ISL_674983	Lighthouse Lab in Cambridge	Wellcome Sanger Institute for the COVID-19 Genomics UK (COG-UK) Consortium	Rob Howes, The Lighthouse Lab in Cambridge and Alex Alderton, Roberto Amato, Sonia Goncalves, Ewan Harrison, David K. Jackson, Ian Johnston, Dominic Kwiatkowski, Cordelia Langford, John Sillitoe on behalf of the Wellcome Sanger Institute COVID-19 Surveillance Team
EPI_ISL_674987	Lighthouse Lab in Milton Keynes	Wellcome Sanger Institute for the COVID-19 Genomics UK (COG-UK) Consortium	The Lighthouse Lab in Milton Keynes and Alex Alderton, Roberto Amato, Sonia Goncalves, Ewan Harrison, David K. Jackson, Ian Johnston, Dominic Kwiatkowski, Cordelia Langford, John Sillitoe on behalf of the Wellcome Sanger Institute COVID-19 Surveillance Team
EPI_ISL_674988	Lighthouse Lab in Cambridge	Wellcome Sanger Institute for the COVID-19 Genomics UK (COG-UK) Consortium	Rob Howes, The Lighthouse Lab in Cambridge and Alex Alderton, Roberto Amato, Sonia Goncalves, Ewan Harrison, David K. Jackson, Ian Johnston, Dominic Kwiatkowski, Cordelia Langford, John Sillitoe on behalf of the Wellcome Sanger Institute COVID-19 Surveillance Team
EPI_ISL_674989, EPI_ISL_674991	Lighthouse Lab in Milton Keynes	Wellcome Sanger Institute for the COVID-19 Genomics UK (COG-UK) Consortium	The Lighthouse Lab in Milton Keynes and Alex Alderton, Roberto Amato, Sonia Goncalves, Ewan Harrison, David K. Jackson, Ian Johnston, Dominic Kwiatkowski, Cordelia Langford, John Sillitoe on behalf of the Wellcome Sanger Institute COVID-19 Surveillance Team
EPI_ISL_674992, EPI_ISL_674995	Lighthouse Lab in Cambridge	Wellcome Sanger Institute for the COVID-19 Genomics UK (COG-UK) Consortium	Rob Howes, The Lighthouse Lab in Cambridge and Alex Alderton, Roberto Amato, Sonia Goncalves, Ewan Harrison, David K. Jackson, Ian Johnston, Dominic Kwiatkowski, Cordelia Langford, John Sillitoe on behalf of the Wellcome Sanger Institute COVID-19 Surveillance Team
EPI_ISL_674996, EPI_ISL_674997	Lighthouse Lab in Milton Keynes	Wellcome Sanger Institute for the COVID-19 Genomics UK (COG-UK) Consortium	The Lighthouse Lab in Milton Keynes and Alex Alderton, Roberto Amato, Sonia Goncalves, Ewan Harrison, David K. Jackson, Ian Johnston, Dominic Kwiatkowski, Cordelia Langford, John Sillitoe on behalf of the Wellcome Sanger Institute COVID-19 Surveillance Team
EPI_ISL_674998	Lighthouse Lab in Cambridge	Wellcome Sanger Institute for the COVID-19 Genomics UK (COG-UK) Consortium	Rob Howes, The Lighthouse Lab in Cambridge and Alex Alderton, Roberto Amato, Sonia Goncalves, Ewan Harrison, David K. Jackson, Ian Johnston, Dominic Kwiatkowski, Cordelia Langford, John Sillitoe on behalf of the Wellcome Sanger Institute COVID-19 Surveillance Team
EPI_ISL_674999, EPI_ISL_675001, EPI_ISL_675004	Lighthouse Lab in Milton Keynes	Wellcome Sanger Institute for the COVID-19 Genomics UK (COG-UK) Consortium	The Lighthouse Lab in Milton Keynes and Alex Alderton, Roberto Amato, Sonia Goncalves, Ewan Harrison, David K. Jackson, Ian Johnston, Dominic Kwiatkowski, Cordelia Langford, John Sillitoe on behalf of the Wellcome Sanger Institute COVID-19 Surveillance Team
EPI_ISL_675006, EPI_ISL_675008, EPI_ISL_675009	Lighthouse Lab in Cambridge	Wellcome Sanger Institute for the COVID-19 Genomics UK (COG-UK) Consortium	Rob Howes, The Lighthouse Lab in Cambridge and Alex Alderton, Roberto Amato, Sonia Goncalves, Ewan Harrison, David K. Jackson, Ian Johnston, Dominic Kwiatkowski, Cordelia Langford, John Sillitoe on behalf of the Wellcome Sanger Institute COVID-19 Surveillance Team
EPI_ISL_675010, EPI_ISL_675011, EPI_ISL_675013, EPI_ISL_675015	Lighthouse Lab in Milton Keynes	Wellcome Sanger Institute for the COVID-19 Genomics UK (COG-UK) Consortium	The Lighthouse Lab in Milton Keynes and Alex Alderton, Roberto Amato, Sonia Goncalves, Ewan Harrison, David K. Jackson, Ian Johnston, Dominic Kwiatkowski, Cordelia Langford, John Sillitoe on behalf of the Wellcome Sanger Institute COVID-19 Surveillance Team
EPI_ISL_675018	Lighthouse Lab in Cambridge	Wellcome Sanger Institute for the COVID-19 Genomics UK (COG-UK) Consortium	Rob Howes, The Lighthouse Lab in Cambridge and Alex Alderton, Roberto Amato, Sonia Goncalves, Ewan Harrison, David K. Jackson, Ian Johnston, Dominic Kwiatkowski, Cordelia Langford, John Sillitoe on behalf of the Wellcome Sanger Institute COVID-19 Surveillance Team
EPI_ISL_675020, EPI_ISL_675022	Lighthouse Lab in Milton Keynes	Wellcome Sanger Institute for the COVID-19 Genomics UK (COG-UK) Consortium	The Lighthouse Lab in Milton Keynes and Alex Alderton, Roberto Amato, Sonia Goncalves, Ewan Harrison, David K. Jackson, Ian Johnston, Dominic Kwiatkowski, Cordelia Langford, John Sillitoe on behalf of the Wellcome Sanger Institute COVID-19 Surveillance Team
EPI_ISL_675024	Lighthouse Lab in Cambridge	Wellcome Sanger Institute for the COVID-19 Genomics UK (COG-UK) Consortium	Rob Howes, The Lighthouse Lab in Cambridge and Alex Alderton, Roberto Amato, Sonia Goncalves, Ewan Harrison, David K. Jackson, Ian Johnston, Dominic Kwiatkowski, Cordelia Langford, John Sillitoe on behalf of the Wellcome Sanger Institute COVID-19 Surveillance Team
EPI_ISL_675025	Lighthouse Lab in Milton Keynes	Wellcome Sanger Institute for the COVID-19 Genomics UK (COG-UK) Consortium	The Lighthouse Lab in Milton Keynes and Alex Alderton, Roberto Amato, Sonia Goncalves, Ewan Harrison, David K. Jackson, Ian Johnston, Dominic Kwiatkowski, Cordelia Langford, John Sillitoe on behalf of the Wellcome Sanger Institute COVID-19 Surveillance Team
EPI_ISL_675026, EPI_ISL_675027	Lighthouse Lab in Cambridge	Wellcome Sanger Institute for the COVID-19 Genomics UK (COG-UK) Consortium	Rob Howes, The Lighthouse Lab in Cambridge and Alex Alderton, Roberto Amato, Sonia Goncalves, Ewan Harrison, David K. Jackson, Ian Johnston, Dominic Kwiatkowski, Cordelia Langford, John Sillitoe on behalf of the Wellcome Sanger Institute COVID-19 Surveillance Team
EPI_ISL_675031, EPI_ISL_675032	Lighthouse Lab in Milton Keynes	Wellcome Sanger Institute for the COVID-19 Genomics UK (COG-UK) Consortium	The Lighthouse Lab in Milton Keynes and Alex Alderton, Roberto Amato, Sonia Goncalves, Ewan Harrison, David K. Jackson, Ian Johnston, Dominic Kwiatkowski, Cordelia Langford, John Sillitoe on behalf of the Wellcome Sanger Institute COVID-19 Surveillance Team
EPI_ISL_675033	Lighthouse Lab in Cambridge	Wellcome Sanger Institute for the COVID-19 Genomics UK (COG-UK) Consortium	Rob Howes, The Lighthouse Lab in Cambridge and Alex Alderton, Roberto Amato, Sonia Goncalves, Ewan Harrison, David K. Jackson, Ian Johnston, Dominic Kwiatkowski, Cordelia Langford, John Sillitoe on behalf of the Wellcome Sanger Institute COVID-19 Surveillance Team

EPI_ISL_675035, EPI_ISL_675037	Lighthouse Lab in Milton Keynes	Wellcome Sanger Institute for the COVID-19 Genomics UK (COG-UK) Consortium	The Lighthouse Lab in Milton Keynes and Alex Alderton, Roberto Amato, Sonia Goncalves, Ewan Harrison, David K. Jackson, Ian Johnston, Dominic Kwiatkowski, Cordelia Langford, John Sillitoe on behalf of the Wellcome Sanger Institute COVID-19 Surveillance Team
EPI_ISL_675038, EPI_ISL_675039, EPI_ISL_675040	Lighthouse Lab in Cambridge	Wellcome Sanger Institute for the COVID-19 Genomics UK (COG-UK) Consortium	Rob Howes, The Lighthouse Lab in Cambridge and Alex Alderton, Roberto Amato, Sonia Goncalves, Ewan Harrison, David K. Jackson, Ian Johnston, Dominic Kwiatkowski, Cordelia Langford, John Sillitoe on behalf of the Wellcome Sanger Institute COVID-19 Surveillance Team
EPI_ISL_675041	Lighthouse Lab in Milton Keynes	Wellcome Sanger Institute for the COVID-19 Genomics UK (COG-UK) Consortium	The Lighthouse Lab in Milton Keynes and Alex Alderton, Roberto Amato, Sonia Goncalves, Ewan Harrison, David K. Jackson, Ian Johnston, Dominic Kwiatkowski, Cordelia Langford, John Sillitoe on behalf of the Wellcome Sanger Institute COVID-19 Surveillance Team
EPI_ISL_675042	Lighthouse Lab in Cambridge	Wellcome Sanger Institute for the COVID-19 Genomics UK (COG-UK) Consortium	Rob Howes, The Lighthouse Lab in Cambridge and Alex Alderton, Roberto Amato, Sonia Goncalves, Ewan Harrison, David K. Jackson, Ian Johnston, Dominic Kwiatkowski, Cordelia Langford, John Sillitoe on behalf of the Wellcome Sanger Institute COVID-19 Surveillance Team
EPI_ISL_675045	Lighthouse Lab in Milton Keynes	Wellcome Sanger Institute for the COVID-19 Genomics UK (COG-UK) Consortium	The Lighthouse Lab in Milton Keynes and Alex Alderton, Roberto Amato, Sonia Goncalves, Ewan Harrison, David K. Jackson, Ian Johnston, Dominic Kwiatkowski, Cordelia Langford, John Sillitoe on behalf of the Wellcome Sanger Institute COVID-19 Surveillance Team
EPI_ISL_675046	Lighthouse Lab in Cambridge	Wellcome Sanger Institute for the COVID-19 Genomics UK (COG-UK) Consortium	Rob Howes, The Lighthouse Lab in Cambridge and Alex Alderton, Roberto Amato, Sonia Goncalves, Ewan Harrison, David K. Jackson, Ian Johnston, Dominic Kwiatkowski, Cordelia Langford, John Sillitoe on behalf of the Wellcome Sanger Institute COVID-19 Surveillance Team
EPI_ISL_675049, EPI_ISL_675052	Lighthouse Lab in Milton Keynes	Wellcome Sanger Institute for the COVID-19 Genomics UK (COG-UK) Consortium	The Lighthouse Lab in Milton Keynes and Alex Alderton, Roberto Amato, Sonia Goncalves, Ewan Harrison, David K. Jackson, Ian Johnston, Dominic Kwiatkowski, Cordelia Langford, John Sillitoe on behalf of the Wellcome Sanger Institute COVID-19 Surveillance Team
EPI_ISL_675053	Lighthouse Lab in Cambridge	Wellcome Sanger Institute for the COVID-19 Genomics UK (COG-UK) Consortium	Rob Howes, The Lighthouse Lab in Cambridge and Alex Alderton, Roberto Amato, Sonia Goncalves, Ewan Harrison, David K. Jackson, Ian Johnston, Dominic Kwiatkowski, Cordelia Langford, John Sillitoe on behalf of the Wellcome Sanger Institute COVID-19 Surveillance Team
EPI_ISL_675054, EPI_ISL_675055	Lighthouse Lab in Milton Keynes	Wellcome Sanger Institute for the COVID-19 Genomics UK (COG-UK) Consortium	The Lighthouse Lab in Milton Keynes and Alex Alderton, Roberto Amato, Sonia Goncalves, Ewan Harrison, David K. Jackson, Ian Johnston, Dominic Kwiatkowski, Cordelia Langford, John Sillitoe on behalf of the Wellcome Sanger Institute COVID-19 Surveillance Team
EPI_ISL_675056	Lighthouse Lab in Cambridge	Wellcome Sanger Institute for the COVID-19 Genomics UK (COG-UK) Consortium	Rob Howes, The Lighthouse Lab in Cambridge and Alex Alderton, Roberto Amato, Sonia Goncalves, Ewan Harrison, David K. Jackson, Ian Johnston, Dominic Kwiatkowski, Cordelia Langford, John Sillitoe on behalf of the Wellcome Sanger Institute COVID-19 Surveillance Team
EPI_ISL_675057, EPI_ISL_675058	Lighthouse Lab in Milton Keynes	Wellcome Sanger Institute for the COVID-19 Genomics UK (COG-UK) Consortium	The Lighthouse Lab in Milton Keynes and Alex Alderton, Roberto Amato, Sonia Goncalves, Ewan Harrison, David K. Jackson, Ian Johnston, Dominic Kwiatkowski, Cordelia Langford, John Sillitoe on behalf of the Wellcome Sanger Institute COVID-19 Surveillance Team
EPI_ISL_675064	Lighthouse Lab in Cambridge	Wellcome Sanger Institute for the COVID-19 Genomics UK (COG-UK) Consortium	Rob Howes, The Lighthouse Lab in Cambridge and Alex Alderton, Roberto Amato, Sonia Goncalves, Ewan Harrison, David K. Jackson, Ian Johnston, Dominic Kwiatkowski, Cordelia Langford, John Sillitoe on behalf of the Wellcome Sanger Institute COVID-19 Surveillance Team
EPI_ISL_675065, EPI_ISL_675068, EPI_ISL_675069, EPI_ISL_675070	Lighthouse Lab in Milton Keynes	Wellcome Sanger Institute for the COVID-19 Genomics UK (COG-UK) Consortium	The Lighthouse Lab in Milton Keynes and Alex Alderton, Roberto Amato, Sonia Goncalves, Ewan Harrison, David K. Jackson, Ian Johnston, Dominic Kwiatkowski, Cordelia Langford, John Sillitoe on behalf of the Wellcome Sanger Institute COVID-19 Surveillance Team
EPI_ISL_675071	Lighthouse Lab in Cambridge	Wellcome Sanger Institute for the COVID-19 Genomics UK (COG-UK) Consortium	Rob Howes, The Lighthouse Lab in Cambridge and Alex Alderton, Roberto Amato, Sonia Goncalves, Ewan Harrison, David K. Jackson, Ian Johnston, Dominic Kwiatkowski, Cordelia Langford, John Sillitoe on behalf of the Wellcome Sanger Institute COVID-19 Surveillance Team
EPI_ISL_675072, EPI_ISL_675074	Lighthouse Lab in Milton Keynes	Wellcome Sanger Institute for the COVID-19 Genomics UK (COG-UK) Consortium	The Lighthouse Lab in Milton Keynes and Alex Alderton, Roberto Amato, Sonia Goncalves, Ewan Harrison, David K. Jackson, Ian Johnston, Dominic Kwiatkowski, Cordelia Langford, John Sillitoe on behalf of the Wellcome Sanger Institute COVID-19 Surveillance Team
EPI_ISL_675075	Lighthouse Lab in Cambridge	Wellcome Sanger Institute for the COVID-19 Genomics UK (COG-UK) Consortium	Rob Howes, The Lighthouse Lab in Cambridge and Alex Alderton, Roberto Amato, Sonia Goncalves, Ewan Harrison, David K. Jackson, Ian Johnston, Dominic Kwiatkowski, Cordelia Langford, John Sillitoe on behalf of the Wellcome Sanger Institute COVID-19 Surveillance Team
EPI_ISL_675079	Lighthouse Lab in Milton Keynes	Wellcome Sanger Institute for the COVID-19 Genomics UK (COG-UK) Consortium	The Lighthouse Lab in Milton Keynes and Alex Alderton, Roberto Amato, Sonia Goncalves, Ewan Harrison, David K. Jackson, Ian Johnston, Dominic Kwiatkowski, Cordelia Langford, John Sillitoe on behalf of the Wellcome Sanger Institute COVID-19 Surveillance Team
EPI_ISL_675080	Lighthouse Lab in Cambridge	Wellcome Sanger Institute for the COVID-19 Genomics UK (COG-UK) Consortium	Rob Howes, The Lighthouse Lab in Cambridge and Alex Alderton, Roberto Amato, Sonia Goncalves, Ewan Harrison, David K. Jackson, Ian Johnston, Dominic Kwiatkowski, Cordelia Langford, John Sillitoe on behalf of the Wellcome Sanger Institute COVID-19 Surveillance Team
EPI_ISL_675081	Lighthouse Lab in Milton Keynes	Wellcome Sanger Institute for the COVID-19 Genomics UK (COG-UK) Consortium	The Lighthouse Lab in Milton Keynes and Alex Alderton, Roberto Amato, Sonia Goncalves, Ewan Harrison, David K. Jackson, Ian Johnston, Dominic Kwiatkowski, Cordelia Langford, John Sillitoe on behalf of the Wellcome Sanger Institute COVID-19 Surveillance Team
EPI_ISL_675082	Lighthouse Lab in Cambridge	Wellcome Sanger Institute for the COVID-19 Genomics UK (COG-UK) Consortium	Rob Howes, The Lighthouse Lab in Cambridge and Alex Alderton, Roberto Amato, Sonia Goncalves, Ewan Harrison, David K. Jackson, Ian Johnston, Dominic Kwiatkowski, Cordelia Langford, John Sillitoe on behalf of the Wellcome Sanger Institute COVID-19 Surveillance Team
EPI_ISL_675084, EPI_ISL_675085	Lighthouse Lab in Milton Keynes	Wellcome Sanger Institute for the COVID-19 Genomics UK (COG-UK) Consortium	The Lighthouse Lab in Milton Keynes and Alex Alderton, Roberto Amato, Sonia Goncalves, Ewan Harrison, David K. Jackson, Ian Johnston, Dominic Kwiatkowski, Cordelia Langford, John Sillitoe on behalf of the Wellcome Sanger Institute COVID-19 Surveillance Team
EPI_ISL_675087	Lighthouse Lab in Cambridge	Wellcome Sanger Institute for the COVID-19 Genomics UK (COG-UK) Consortium	Rob Howes, The Lighthouse Lab in Cambridge and Alex Alderton, Roberto Amato, Sonia Goncalves, Ewan Harrison, David K. Jackson, Ian Johnston, Dominic Kwiatkowski, Cordelia Langford, John Sillitoe on behalf of the Wellcome Sanger Institute COVID-19 Surveillance Team
EPI_ISL_675090, EPI_ISL_675091	Lighthouse Lab in Milton Keynes	Wellcome Sanger Institute for the COVID-19 Genomics UK (COG-UK) Consortium	The Lighthouse Lab in Milton Keynes and Alex Alderton, Roberto Amato, Sonia Goncalves, Ewan Harrison, David K. Jackson, Ian Johnston, Dominic Kwiatkowski, Cordelia Langford, John Sillitoe on behalf of the Wellcome Sanger Institute COVID-19 Surveillance Team
EPI_ISL_675094	Lighthouse Lab in Cambridge	Wellcome Sanger Institute for the COVID-19 Genomics UK (COG-UK) Consortium	Rob Howes, The Lighthouse Lab in Cambridge and Alex Alderton, Roberto Amato, Sonia Goncalves, Ewan Harrison, David K. Jackson, Ian Johnston, Dominic Kwiatkowski, Cordelia Langford, John Sillitoe on behalf of the Wellcome Sanger Institute COVID-19 Surveillance Team
EPI_ISL_675095, EPI_ISL_675099, EPI_ISL_675100, EPI_ISL_675101	Lighthouse Lab in Milton Keynes	Wellcome Sanger Institute for the COVID-19 Genomics UK (COG-UK) Consortium	The Lighthouse Lab in Milton Keynes and Alex Alderton, Roberto Amato, Sonia Goncalves, Ewan Harrison, David K. Jackson, Ian Johnston, Dominic Kwiatkowski, Cordelia Langford, John Sillitoe on behalf of the Wellcome Sanger Institute COVID-19 Surveillance Team
EPI_ISL_675104	Lighthouse Lab in Cambridge	Wellcome Sanger Institute for the COVID-19 Genomics UK (COG-UK) Consortium	Rob Howes, The Lighthouse Lab in Cambridge and Alex Alderton, Roberto Amato, Sonia Goncalves, Ewan Harrison, David K. Jackson, Ian Johnston, Dominic Kwiatkowski, Cordelia Langford, John Sillitoe on behalf of the Wellcome Sanger Institute COVID-19 Surveillance Team
EPI_ISL_675106	Lighthouse Lab in Milton Keynes	Wellcome Sanger Institute for the COVID-19 Genomics UK (COG-UK) Consortium	The Lighthouse Lab in Milton Keynes and Alex Alderton, Roberto Amato, Sonia Goncalves, Ewan Harrison, David K. Jackson, Ian Johnston, Dominic Kwiatkowski, Cordelia Langford, John Sillitoe on behalf of the Wellcome Sanger Institute COVID-19 Surveillance Team
EPI_ISL_675107	Lighthouse Lab in Cambridge	Wellcome Sanger Institute for the COVID-19 Genomics UK (COG-UK) Consortium	Rob Howes, The Lighthouse Lab in Cambridge and Alex Alderton, Roberto Amato, Sonia Goncalves, Ewan Harrison, David K. Jackson, Ian Johnston, Dominic Kwiatkowski, Cordelia Langford, John Sillitoe on behalf of the Wellcome Sanger Institute COVID-19 Surveillance Team
EPI_ISL_675109	Lighthouse Lab in Milton Keynes	Wellcome Sanger Institute for the COVID-19 Genomics UK (COG-UK) Consortium	The Lighthouse Lab in Milton Keynes and Alex Alderton, Roberto Amato, Sonia Goncalves, Ewan Harrison, David K. Jackson, Ian Johnston, Dominic Kwiatkowski, Cordelia Langford, John Sillitoe on behalf of the Wellcome Sanger Institute COVID-19 Surveillance Team
EPI_ISL_675110	Lighthouse Lab in Cambridge	Wellcome Sanger Institute for the COVID-19 Genomics UK (COG-UK) Consortium	Rob Howes, The Lighthouse Lab in Cambridge and Alex Alderton, Roberto Amato, Sonia Goncalves, Ewan Harrison, David K. Jackson, Ian Johnston, Dominic Kwiatkowski, Cordelia Langford, John Sillitoe on behalf of the Wellcome Sanger Institute COVID-19 Surveillance Team
EPI_ISL_675114	Lighthouse Lab in Milton Keynes	Wellcome Sanger Institute for the COVID-19 Genomics UK (COG-UK) Consortium	The Lighthouse Lab in Milton Keynes and Alex Alderton, Roberto Amato, Sonia Goncalves, Ewan Harrison, David K. Jackson, Ian Johnston, Dominic Kwiatkowski, Cordelia Langford, John Sillitoe on behalf of the Wellcome Sanger Institute COVID-19 Surveillance Team
EPI_ISL_675117	Lighthouse Lab in Cambridge	Wellcome Sanger Institute for the COVID-19 Genomics UK (COG-UK) Consortium	Rob Howes, The Lighthouse Lab in Cambridge and Alex Alderton, Roberto Amato, Sonia Goncalves, Ewan Harrison, David K. Jackson, Ian Johnston, Dominic Kwiatkowski, Cordelia Langford, John Sillitoe on behalf of the Wellcome Sanger Institute COVID-19 Surveillance Team
EPI_ISL_675118, EPI_ISL_675119	Lighthouse Lab in Milton Keynes	Wellcome Sanger Institute for the COVID-19 Genomics UK (COG-UK) Consortium	The Lighthouse Lab in Milton Keynes and Alex Alderton, Roberto Amato, Sonia Goncalves, Ewan Harrison, David K. Jackson, Ian Johnston, Dominic Kwiatkowski, Cordelia Langford, John Sillitoe on behalf of the Wellcome Sanger Institute COVID-19 Surveillance Team
EPI_ISL_675120	Lighthouse Lab in Cambridge	Wellcome Sanger Institute for the COVID-19 Genomics UK (COG-UK) Consortium	Rob Howes, The Lighthouse Lab in Cambridge and Alex Alderton, Roberto Amato, Sonia Goncalves, Ewan Harrison, David K. Jackson, Ian Johnston, Dominic Kwiatkowski, Cordelia Langford, John Sillitoe on behalf of the Wellcome Sanger Institute COVID-19 Surveillance Team
EPI_ISL_675123, EPI_ISL_675124	Lighthouse Lab in Milton Keynes	Wellcome Sanger Institute for the COVID-19 Genomics UK (COG-UK) Consortium	The Lighthouse Lab in Milton Keynes and Alex Alderton, Roberto Amato, Sonia Goncalves, Ewan Harrison, David K. Jackson, Ian Johnston, Dominic Kwiatkowski, Cordelia Langford, John Sillitoe on behalf of the Wellcome Sanger Institute COVID-19 Surveillance Team

EPI_ISL_675126	Lighthouse Lab in Cambridge	Wellcome Sanger Institute for the COVID-19 Genomics UK (COG-UK) Consortium	Rob Howes, The Lighthouse Lab in Cambridge and Alex Alderton, Roberto Amato, Sonia Goncalves, Ewan Harrison, David K. Jackson, Ian Johnston, Dominic Kwiatkowski, Cordelia Langford, John Sillitoe on behalf of the Wellcome Sanger Institute COVID-19 Surveillance Team
EPI_ISL_675128, EPI_ISL_675129	Lighthouse Lab in Milton Keynes	Wellcome Sanger Institute for the COVID-19 Genomics UK (COG-UK) Consortium	The Lighthouse Lab in Milton Keynes and Alex Alderton, Roberto Amato, Sonia Goncalves, Ewan Harrison, David K. Jackson, Ian Johnston, Dominic Kwiatkowski, Cordelia Langford, John Sillitoe on behalf of the Wellcome Sanger Institute COVID-19 Surveillance Team
EPI_ISL_675130, EPI_ISL_675133, EPI_ISL_675135, EPI_ISL_675136, EPI_ISL_675137, EPI_ISL_675138, EPI_ISL_675139, EPI_ISL_675140, EPI_ISL_675141, EPI_ISL_675142, EPI_ISL_675143, EPI_ISL_675144, EPI_ISL_675146, EPI_ISL_675147, EPI_ISL_675148, EPI_ISL_675149, EPI_ISL_675150, EPI_ISL_675151, EPI_ISL_675152, EPI_ISL_675154, EPI_ISL_675155, EPI_ISL_675156, EPI_ISL_675157, EPI_ISL_675158, EPI_ISL_675159, EPI_ISL_675161, EPI_ISL_675162, EPI_ISL_675163, EPI_ISL_675164, EPI_ISL_675166, EPI_ISL_675167, EPI_ISL_675168, EPI_ISL_675169, EPI_ISL_675171, EPI_ISL_675172, EPI_ISL_675173, EPI_ISL_675174, EPI_ISL_675175, EPI_ISL_675176, EPI_ISL_675177, EPI_ISL_675180, EPI_ISL_675181, EPI_ISL_675182, EPI_ISL_675184, EPI_ISL_675186, EPI_ISL_675187, EPI_ISL_675189, EPI_ISL_675190, EPI_ISL_675191, EPI_ISL_675192, EPI_ISL_675193, EPI_ISL_675194, EPI_ISL_675195, EPI_ISL_675196, EPI_ISL_675197, EPI_ISL_675198, EPI_ISL_675199, EPI_ISL_675200, EPI_ISL_675201, EPI_ISL_675202, EPI_ISL_675203, EPI_ISL_675204, EPI_ISL_675205, EPI_ISL_675206, EPI_ISL_675207, EPI_ISL_675208, EPI_ISL_675209, EPI_ISL_675211, EPI_ISL_675213, EPI_ISL_675214, EPI_ISL_675215, EPI_ISL_675216, EPI_ISL_675218, EPI_ISL_675220, EPI_ISL_675221, EPI_ISL_675222, EPI_ISL_675223, EPI_ISL_675225, EPI_ISL_675226, EPI_ISL_675229, EPI_ISL_675230, EPI_ISL_675231, EPI_ISL_675232, EPI_ISL_675233, EPI_ISL_675234, EPI_ISL_675235, EPI_ISL_675237, EPI_ISL_675239, EPI_ISL_675240, EPI_ISL_675242, EPI_ISL_675245, EPI_ISL_675246, EPI_ISL_675247, EPI_ISL_675248, EPI_ISL_675249, EPI_ISL_675250, EPI_ISL_675251, EPI_ISL_675252, EPI_ISL_675253, EPI_ISL_675254, EPI_ISL_675255, EPI_ISL_675256, EPI_ISL_675257, EPI_ISL_675258, EPI_ISL_675259, EPI_ISL_675261, EPI_ISL_675262, EPI_ISL_675263, EPI_ISL_675265, EPI_ISL_675266, EPI_ISL_675267, EPI_ISL_675268, EPI_ISL_675269, EPI_ISL_675270, EPI_ISL_675271, EPI_ISL_675272, EPI_ISL_675273, EPI_ISL_675274, EPI_ISL_675278, EPI_ISL_675279, EPI_ISL_675280, EPI_ISL_675281, EPI_ISL_675283, EPI_ISL_675287, EPI_ISL_675288, EPI_ISL_675289, EPI_ISL_675290, EPI_ISL_675291, EPI_ISL_675293, EPI_ISL_675294, EPI_ISL_675295, EPI_ISL_675296, EPI_ISL_675297, EPI_ISL_675298, EPI_ISL_675300, EPI_ISL_675302, EPI_ISL_675303, EPI_ISL_675304, EPI_ISL_675310, EPI_ISL_675312, EPI_ISL_675314, EPI_ISL_675315, EPI_ISL_675316, EPI_ISL_675321, EPI_ISL_675322, EPI_ISL_675323, EPI_ISL_675324, EPI_ISL_675325, EPI_ISL_675327, EPI_ISL_675328, EPI_ISL_675329, EPI_ISL_675330, EPI_ISL_675333, EPI_ISL_675334, EPI_ISL_675335, EPI_ISL_675336, EPI_ISL_675337, EPI_ISL_675338, EPI_ISL_675339, EPI_ISL_675341, EPI_ISL_675342, EPI_ISL_675343, EPI_ISL_675344, EPI_ISL_675345, EPI_ISL_675346, EPI_ISL_675347, EPI_ISL_675348, EPI_ISL_675349, EPI_ISL_675350, EPI_ISL_675351, EPI_ISL_675352, EPI_ISL_675354, EPI_ISL_675355, EPI_ISL_675356, EPI_ISL_675358, EPI_ISL_675359, EPI_ISL_675360, EPI_ISL_675361, EPI_ISL_675362, EPI_ISL_675363, EPI_ISL_675365, EPI_ISL_675366, EPI_ISL_675367, EPI_ISL_675369, EPI_ISL_675370, EPI_ISL_675372, EPI_ISL_675373, EPI_ISL_675374, EPI_ISL_675375, EPI_ISL_675376, EPI_ISL_675377, EPI_ISL_675378, EPI_ISL_675379, EPI_ISL_675380, EPI_ISL_675381, EPI_ISL_675382, EPI_ISL_675383, EPI_ISL_675384, EPI_ISL_675385, EPI_ISL_675387, EPI_ISL_675388, EPI_ISL_675390, EPI_ISL_675391, EPI_ISL_675392, EPI_ISL_675394, EPI_ISL_675395, EPI_ISL_675396, EPI_ISL_675398, EPI_ISL_675401, EPI_ISL_675402, EPI_ISL_675403, EPI_ISL_675405, EPI_ISL_675406, EPI_ISL_675407, EPI_ISL_675408, EPI_ISL_675409, EPI_ISL_675410, EPI_ISL_675411, EPI_ISL_675412, EPI_ISL_675413, EPI_ISL_675414, EPI_ISL_675415, EPI_ISL_675416, EPI_ISL_675418, EPI_ISL_675419, EPI_ISL_675421, EPI_ISL_675422, EPI_ISL_675425, EPI_ISL_675426, EPI_ISL_675427, EPI_ISL_675428, EPI_ISL_675429, EPI_ISL_675430, EPI_ISL_675433, EPI_ISL_675434, EPI_ISL_675435, EPI_ISL_675437, EPI_ISL_675438, EPI_ISL_675440, EPI_ISL_675441, EPI_ISL_675442, EPI_ISL_675445, EPI_ISL_675447, EPI_ISL_675448, EPI_ISL_675449, EPI_ISL_675450, EPI_ISL_675451, EPI_ISL_675452, EPI_ISL_675453, EPI_ISL_675454, EPI_ISL_675455, EPI_ISL_675456, EPI_ISL_675457, EPI_ISL_675458, EPI_ISL_675459, EPI_ISL_675460, EPI_ISL_675461, EPI_ISL_675463, EPI_ISL_675464, EPI_ISL_675465, EPI_ISL_675466, EPI_ISL_675468, EPI_ISL_675469, EPI_ISL_675470, EPI_ISL_675471, EPI_ISL_675473, EPI_ISL_675474, EPI_ISL_675476, EPI_ISL_675477, EPI_ISL_675478, EPI_ISL_675479, EPI_ISL_675481, EPI_ISL_675482, EPI_ISL_675484, EPI_ISL_675487, EPI_ISL_675488, EPI_ISL_675492, EPI_ISL_675493, EPI_ISL_675497, EPI_ISL_675498, EPI_ISL_675501, EPI_ISL_675502, EPI_ISL_675508, EPI_ISL_675509, EPI_ISL_675510, EPI_ISL_675511, EPI_ISL_675512, EPI_ISL_675515, EPI_ISL_675517, EPI_ISL_675519, EPI_ISL_675520, EPI_ISL_675521, EPI_ISL_675522, EPI_ISL_675523, EPI_ISL_675524, EPI_ISL_675525, EPI_ISL_675526, EPI_ISL_675527, EPI_ISL_675529, EPI_ISL_675530, EPI_ISL_675531, EPI_ISL_675532, EPI_ISL_675534, EPI_ISL_675536, EPI_ISL_675539, EPI_ISL_675541, EPI_ISL_675543, EPI_ISL_675544, EPI_ISL_675545, EPI_ISL_675548, EPI_ISL_675549, EPI_ISL_675551, EPI_ISL_675553, EPI_ISL_675554, EPI_ISL_675557, EPI_ISL_675561, EPI_ISL_675562, EPI_ISL_675563, EPI_ISL_675567, EPI_ISL_675568, EPI_ISL_675570, EPI_ISL_675571, EPI_ISL_675573, EPI_ISL_675574, EPI_ISL_675575, EPI_ISL_675577, EPI_ISL_675579, EPI_ISL_675580, EPI_ISL_675581, EPI_ISL_675582, EPI_ISL_675583, EPI_ISL_675584, EPI_ISL_675585, EPI_ISL_675587, EPI_ISL_675588, EPI_ISL_675590, EPI_ISL_675591, EPI_ISL_675592, EPI_ISL_675593, EPI_ISL_675594, EPI_ISL_675597, EPI_ISL_675598, EPI_ISL_675599, EPI_ISL_675600, EPI_ISL_675603, EPI_ISL_675604, EPI_ISL_675607, EPI_ISL_675608, EPI_ISL_675609, EPI_ISL_675610, EPI_ISL_675611, EPI_ISL_675612, EPI_ISL_675613, EPI_ISL_675614, EPI_ISL_675615, EPI_ISL_675616, EPI_ISL_675617, EPI_ISL_675618, EPI_ISL_675619, EPI_ISL_675620, EPI_ISL_675622, EPI_ISL_675623, EPI_ISL_675624, EPI_ISL_675625, EPI_ISL_675626, EPI_ISL_675627, EPI_ISL_675628, EPI_ISL_675629, EPI_ISL_675630, EPI_ISL_675632, EPI_ISL_675635, EPI_ISL_675636, EPI_ISL_675637, EPI_ISL_675638, EPI_ISL_675639, EPI_ISL_675640, EPI_ISL_675641, EPI_ISL_675645, EPI_ISL_675646, EPI_ISL_675647, EPI_ISL_675648, EPI_ISL_675649, EPI_ISL_675650, EPI_ISL_675653, EPI_ISL_675654, EPI_ISL_675655, EPI_ISL_675657, EPI_ISL_675658, EPI_ISL_675659, EPI_ISL_675662, EPI_ISL_675664, EPI_ISL_675665, EPI_ISL_675666, EPI_ISL_675668, EPI_ISL_675669, EPI_ISL_675670, EPI_ISL_675671, EPI_ISL_675672, EPI_ISL_675673, EPI_ISL_675674, EPI_ISL_675675, EPI_ISL_675677, EPI_ISL_675678, EPI_ISL_675679, EPI_ISL_675680, EPI_ISL_675683, EPI_ISL_675684, EPI_ISL_675686, EPI_ISL_675687, EPI_ISL_675688, EPI_ISL_675689, EPI_ISL_675692, EPI_ISL_675696, EPI_ISL_675697, EPI_ISL_675699, EPI_ISL_675700, EPI_ISL_675701, EPI_ISL_675702, EPI_ISL_675703, EPI_ISL_675705, EPI_ISL_675706, EPI_ISL_675707, EPI_ISL_675708, EPI_ISL_675710			
see above	Lighthouse Lab in Alderley Park	Wellcome Sanger Institute for the COVID-19 Genomics UK (COG-UK) Consortium	Jacquelyn Wynn, Mairead Hyland, The Lighthouse Lab in Alderley Park and Alex Alderton, Roberto Amato, Sonia Goncalves, Ewan Harrison, David K. Jackson, Ian Johnston, Dominic Kwiatkowski, Cordelia Langford, John Sillitoe on behalf of the Wellcome Sanger Institute COVID-19 Surveillance Team
EPI_ISL_675713	Lighthouse Lab in Milton Keynes	Wellcome Sanger Institute for the COVID-19 Genomics UK (COG-UK) Consortium	The Lighthouse Lab in Milton Keynes and Alex Alderton, Roberto Amato, Sonia Goncalves, Ewan Harrison, David K. Jackson, Ian Johnston, Dominic Kwiatkowski, Cordelia Langford, John Sillitoe on behalf of the Wellcome Sanger Institute COVID-19 Surveillance Team
EPI_ISL_675715, EPI_ISL_675716, EPI_ISL_675719	Lighthouse Lab in Alderley Park	Wellcome Sanger Institute for the COVID-19 Genomics UK (COG-UK) Consortium	Jacquelyn Wynn, Mairead Hyland, The Lighthouse Lab in Alderley Park and Alex Alderton, Roberto Amato, Sonia Goncalves, Ewan Harrison, David K. Jackson, Ian Johnston, Dominic Kwiatkowski, Cordelia Langford, John Sillitoe on behalf of the Wellcome Sanger Institute COVID-19 Surveillance Team
EPI_ISL_675728, EPI_ISL_675730	Lighthouse Lab in Milton Keynes	Wellcome Sanger Institute for the COVID-19 Genomics UK (COG-UK) Consortium	The Lighthouse Lab in Milton Keynes and Alex Alderton, Roberto Amato, Sonia Goncalves, Ewan Harrison, David K. Jackson, Ian Johnston, Dominic Kwiatkowski, Cordelia Langford, John Sillitoe on behalf of the Wellcome Sanger Institute COVID-19 Surveillance Team
EPI_ISL_675735	Lighthouse Lab in Alderley Park	Wellcome Sanger Institute for the COVID-19 Genomics UK (COG-UK) Consortium	Jacquelyn Wynn, Mairead Hyland, The Lighthouse Lab in Alderley Park and Alex Alderton, Roberto Amato, Sonia Goncalves, Ewan Harrison, David K. Jackson, Ian Johnston, Dominic Kwiatkowski, Cordelia Langford, John Sillitoe on behalf of the Wellcome Sanger Institute COVID-19 Surveillance Team
EPI_ISL_675736, EPI_ISL_675738	Lighthouse Lab in Milton Keynes	Wellcome Sanger Institute for the COVID-19 Genomics UK (COG-UK) Consortium	The Lighthouse Lab in Milton Keynes and Alex Alderton, Roberto Amato, Sonia Goncalves, Ewan Harrison, David K. Jackson, Ian Johnston, Dominic Kwiatkowski, Cordelia Langford, John Sillitoe on behalf of the Wellcome Sanger Institute COVID-19 Surveillance Team
EPI_ISL_675739, EPI_ISL_675740	Lighthouse Lab in Alderley Park	Wellcome Sanger Institute for the COVID-19 Genomics UK (COG-UK) Consortium	Jacquelyn Wynn, Mairead Hyland, The Lighthouse Lab in Alderley Park and Alex Alderton, Roberto Amato, Sonia Goncalves, Ewan Harrison, David K. Jackson, Ian Johnston, Dominic Kwiatkowski, Cordelia Langford, John Sillitoe on behalf of the Wellcome Sanger Institute COVID-19 Surveillance Team
EPI_ISL_675741, EPI_ISL_675747, EPI_ISL_675748, EPI_ISL_675749	Lighthouse Lab in Milton Keynes	Wellcome Sanger Institute for the COVID-19 Genomics UK (COG-UK) Consortium	The Lighthouse Lab in Milton Keynes and Alex Alderton, Roberto Amato, Sonia Goncalves, Ewan Harrison, David K. Jackson, Ian Johnston, Dominic Kwiatkowski, Cordelia Langford, John Sillitoe on behalf of the Wellcome Sanger Institute COVID-19 Surveillance Team
EPI_ISL_675750, EPI_ISL_675751, EPI_ISL_675756, EPI_ISL_675757, EPI_ISL_675759, EPI_ISL_675760, EPI_ISL_675766	Lighthouse Lab in Alderley Park	Wellcome Sanger Institute for the COVID-19 Genomics UK (COG-UK) Consortium	Jacquelyn Wynn, Mairead Hyland, The Lighthouse Lab in Alderley Park and Alex Alderton, Roberto Amato, Sonia Goncalves, Ewan Harrison, David K. Jackson, Ian Johnston, Dominic Kwiatkowski, Cordelia Langford, John Sillitoe on behalf of the Wellcome Sanger Institute COVID-19 Surveillance Team
EPI_ISL_675768	Lighthouse Lab in Milton Keynes	Wellcome Sanger Institute for the COVID-19 Genomics UK (COG-UK) Consortium	The Lighthouse Lab in Milton Keynes and Alex Alderton, Roberto Amato, Sonia Goncalves, Ewan Harrison, David K. Jackson, Ian Johnston, Dominic Kwiatkowski, Cordelia Langford, John Sillitoe on behalf of the Wellcome Sanger Institute COVID-19 Surveillance Team
EPI_ISL_675770, EPI_ISL_675771, EPI_ISL_675774, EPI_ISL_675775, EPI_ISL_675776, EPI_ISL_675777	Lighthouse Lab in Alderley Park	Wellcome Sanger Institute for the COVID-19 Genomics UK (COG-UK) Consortium	Jacquelyn Wynn, Mairead Hyland, The Lighthouse Lab in Alderley Park and Alex Alderton, Roberto Amato, Sonia Goncalves, Ewan Harrison, David K. Jackson, Ian Johnston, Dominic Kwiatkowski, Cordelia Langford, John Sillitoe on behalf of the Wellcome Sanger Institute COVID-19 Surveillance Team
EPI_ISL_675780, EPI_ISL_675793	Lighthouse Lab in Milton Keynes	Wellcome Sanger Institute for the COVID-19 Genomics UK (COG-UK) Consortium	The Lighthouse Lab in Milton Keynes and Alex Alderton, Roberto Amato, Sonia Goncalves, Ewan Harrison, David K. Jackson, Ian Johnston, Dominic Kwiatkowski, Cordelia Langford, John Sillitoe on behalf of the Wellcome Sanger Institute COVID-19 Surveillance Team
EPI_ISL_675794, EPI_ISL_675798, EPI_ISL_675799, EPI_ISL_675800, EPI_ISL_675802, EPI_ISL_675803	Lighthouse Lab in Alderley Park	Wellcome Sanger Institute for the COVID-19 Genomics UK (COG-UK) Consortium	Jacquelyn Wynn, Mairead Hyland, The Lighthouse Lab in Alderley Park and Alex Alderton, Roberto Amato, Sonia Goncalves, Ewan Harrison, David K. Jackson, Ian Johnston, Dominic Kwiatkowski, Cordelia Langford, John Sillitoe on behalf of the Wellcome Sanger Institute COVID-19 Surveillance Team
EPI_ISL_675804	Lighthouse Lab in Milton Keynes	Wellcome Sanger Institute for the COVID-19 Genomics UK (COG-UK) Consortium	The Lighthouse Lab in Milton Keynes and Alex Alderton, Roberto Amato, Sonia Goncalves, Ewan Harrison, David K. Jackson, Ian Johnston, Dominic Kwiatkowski, Cordelia Langford, John Sillitoe on behalf of the Wellcome Sanger Institute COVID-19 Surveillance Team
EPI_ISL_675806, EPI_ISL_675810, EPI_ISL_675812, EPI_ISL_675813	Lighthouse Lab in Alderley Park	Wellcome Sanger Institute for the COVID-19 Genomics UK (COG-UK) Consortium	Jacquelyn Wynn, Mairead Hyland, The Lighthouse Lab in Alderley Park and Alex Alderton, Roberto Amato, Sonia Goncalves, Ewan Harrison, David K. Jackson, Ian Johnston, Dominic Kwiatkowski, Cordelia Langford, John Sillitoe on behalf of the Wellcome Sanger Institute COVID-19 Surveillance Team
EPI_ISL_675816	Lighthouse Lab in Milton Keynes	Wellcome Sanger Institute for the COVID-19 Genomics UK (COG-UK) Consortium	The Lighthouse Lab in Milton Keynes and Alex Alderton, Roberto Amato, Sonia Goncalves, Ewan Harrison, David K. Jackson, Ian Johnston, Dominic Kwiatkowski, Cordelia Langford, John Sillitoe on behalf of the Wellcome Sanger Institute COVID-19 Surveillance Team
EPI_ISL_675817, EPI_ISL_675818, EPI_ISL_675820, EPI_ISL_675822, EPI_ISL_675823, EPI_ISL_675825, EPI_ISL_675826, EPI_ISL_675829, EPI_ISL_675833	Lighthouse Lab in Alderley Park	Wellcome Sanger Institute for the COVID-19 Genomics UK (COG-UK) Consortium	Jacquelyn Wynn, Mairead Hyland, The Lighthouse Lab in Alderley Park and Alex Alderton, Roberto Amato, Sonia Goncalves, Ewan Harrison, David K. Jackson, Ian Johnston, Dominic Kwiatkowski, Cordelia Langford, John Sillitoe on behalf of the Wellcome Sanger Institute COVID-19 Surveillance Team
EPI_ISL_675836, EPI_ISL_675838, EPI_ISL_675844, EPI_ISL_675846	Lighthouse Lab in Milton Keynes	Wellcome Sanger Institute for the COVID-19 Genomics UK (COG-UK) Consortium	The Lighthouse Lab in Milton Keynes and Alex Alderton, Roberto Amato, Sonia Goncalves, Ewan Harrison, David K. Jackson, Ian Johnston, Dominic Kwiatkowski, Cordelia Langford, John Sillitoe on behalf of the Wellcome Sanger Institute COVID-19 Surveillance Team
EPI_ISL_675847, EPI_ISL_675848	Lighthouse Lab in Alderley Park	Wellcome Sanger Institute for the COVID-19 Genomics UK (COG-UK) Consortium	Jacquelyn Wynn, Mairead Hyland, The Lighthouse Lab in Alderley Park and Alex Alderton, Roberto Amato, Sonia Goncalves, Ewan Harrison, David K. Jackson, Ian Johnston, Dominic Kwiatkowski, Cordelia Langford, John Sillitoe on behalf of the Wellcome Sanger Institute COVID-19 Surveillance Team
EPI_ISL_675851	Lighthouse Lab in Milton Keynes	Wellcome Sanger Institute for the COVID-19 Genomics UK (COG-UK) Consortium	The Lighthouse Lab in Milton Keynes and Alex Alderton, Roberto Amato, Sonia Goncalves, Ewan Harrison, David K. Jackson, Ian Johnston, Dominic Kwiatkowski, Cordelia Langford, John Sillitoe on behalf of the Wellcome Sanger Institute COVID-19 Surveillance Team
EPI_ISL_675852	Lighthouse Lab in Alderley Park	Wellcome Sanger Institute for the COVID-19 Genomics UK	Jacquelyn Wynn, Mairead Hyland, The Lighthouse Lab in Alderley Park and Alex Alderton, Roberto Amato, Sonia Goncalves, Ewan Harrison, David K.

		(COG-UK) Consortium	Jackson, Ian Johnston, Dominic Kwiatkowski, Cordelia Langford, John Sillitoe on behalf of the Wellcome Sanger Institute COVID-19 Surveillance Team
EPI_ISL_675856, EPI_ISL_675857, EPI_ISL_675862, EPI_ISL_675867	Lighthouse Lab in Milton Keynes	Wellcome Sanger Institute for the COVID-19 Genomics UK (COG-UK) Consortium	The Lighthouse Lab in Milton Keynes and Alex Alderton, Roberto Amato, Sonia Goncalves, Ewan Harrison, David K. Jackson, Ian Johnston, Dominic Kwiatkowski, Cordelia Langford, John Sillitoe on behalf of the Wellcome Sanger Institute COVID-19 Surveillance Team
EPI_ISL_675868, EPI_ISL_675878, EPI_ISL_675880, EPI_ISL_675882	Lighthouse Lab in Alderley Park	Wellcome Sanger Institute for the COVID-19 Genomics UK (COG-UK) Consortium	Jacquelyn Wynn, Mairead Hyland, The Lighthouse Lab in Alderley Park and Alex Alderton, Roberto Amato, Sonia Goncalves, Ewan Harrison, David K. Jackson, Ian Johnston, Dominic Kwiatkowski, Cordelia Langford, John Sillitoe on behalf of the Wellcome Sanger Institute COVID-19 Surveillance Team
EPI_ISL_675884, EPI_ISL_675885, EPI_ISL_675888, EPI_ISL_675889	Lighthouse Lab in Milton Keynes	Wellcome Sanger Institute for the COVID-19 Genomics UK (COG-UK) Consortium	The Lighthouse Lab in Milton Keynes and Alex Alderton, Roberto Amato, Sonia Goncalves, Ewan Harrison, David K. Jackson, Ian Johnston, Dominic Kwiatkowski, Cordelia Langford, John Sillitoe on behalf of the Wellcome Sanger Institute COVID-19 Surveillance Team
EPI_ISL_675890, EPI_ISL_675892	Lighthouse Lab in Alderley Park	Wellcome Sanger Institute for the COVID-19 Genomics UK (COG-UK) Consortium	Jacquelyn Wynn, Mairead Hyland, The Lighthouse Lab in Alderley Park and Alex Alderton, Roberto Amato, Sonia Goncalves, Ewan Harrison, David K. Jackson, Ian Johnston, Dominic Kwiatkowski, Cordelia Langford, John Sillitoe on behalf of the Wellcome Sanger Institute COVID-19 Surveillance Team
EPI_ISL_675893	Lighthouse Lab in Milton Keynes	Wellcome Sanger Institute for the COVID-19 Genomics UK (COG-UK) Consortium	The Lighthouse Lab in Milton Keynes and Alex Alderton, Roberto Amato, Sonia Goncalves, Ewan Harrison, David K. Jackson, Ian Johnston, Dominic Kwiatkowski, Cordelia Langford, John Sillitoe on behalf of the Wellcome Sanger Institute COVID-19 Surveillance Team
EPI_ISL_675894, EPI_ISL_675897, EPI_ISL_675901, EPI_ISL_675902, EPI_ISL_675903, EPI_ISL_675908, EPI_ISL_675913, EPI_ISL_675916, EPI_ISL_675920	Lighthouse Lab in Alderley Park	Wellcome Sanger Institute for the COVID-19 Genomics UK (COG-UK) Consortium	Jacquelyn Wynn, Mairead Hyland, The Lighthouse Lab in Alderley Park and Alex Alderton, Roberto Amato, Sonia Goncalves, Ewan Harrison, David K. Jackson, Ian Johnston, Dominic Kwiatkowski, Cordelia Langford, John Sillitoe on behalf of the Wellcome Sanger Institute COVID-19 Surveillance Team
EPI_ISL_675921	Lighthouse Lab in Milton Keynes	Wellcome Sanger Institute for the COVID-19 Genomics UK (COG-UK) Consortium	The Lighthouse Lab in Milton Keynes and Alex Alderton, Roberto Amato, Sonia Goncalves, Ewan Harrison, David K. Jackson, Ian Johnston, Dominic Kwiatkowski, Cordelia Langford, John Sillitoe on behalf of the Wellcome Sanger Institute COVID-19 Surveillance Team
EPI_ISL_675923, EPI_ISL_675924	Lighthouse Lab in Alderley Park	Wellcome Sanger Institute for the COVID-19 Genomics UK (COG-UK) Consortium	Jacquelyn Wynn, Mairead Hyland, The Lighthouse Lab in Alderley Park and Alex Alderton, Roberto Amato, Sonia Goncalves, Ewan Harrison, David K. Jackson, Ian Johnston, Dominic Kwiatkowski, Cordelia Langford, John Sillitoe on behalf of the Wellcome Sanger Institute COVID-19 Surveillance Team
EPI_ISL_675926	Lighthouse Lab in Milton Keynes	Wellcome Sanger Institute for the COVID-19 Genomics UK (COG-UK) Consortium	The Lighthouse Lab in Milton Keynes and Alex Alderton, Roberto Amato, Sonia Goncalves, Ewan Harrison, David K. Jackson, Ian Johnston, Dominic Kwiatkowski, Cordelia Langford, John Sillitoe on behalf of the Wellcome Sanger Institute COVID-19 Surveillance Team
EPI_ISL_675929, EPI_ISL_675930, EPI_ISL_675934, EPI_ISL_675937, EPI_ISL_675939	Lighthouse Lab in Alderley Park	Wellcome Sanger Institute for the COVID-19 Genomics UK (COG-UK) Consortium	Jacquelyn Wynn, Mairead Hyland, The Lighthouse Lab in Alderley Park and Alex Alderton, Roberto Amato, Sonia Goncalves, Ewan Harrison, David K. Jackson, Ian Johnston, Dominic Kwiatkowski, Cordelia Langford, John Sillitoe on behalf of the Wellcome Sanger Institute COVID-19 Surveillance Team
EPI_ISL_675940	Lighthouse Lab in Milton Keynes	Wellcome Sanger Institute for the COVID-19 Genomics UK (COG-UK) Consortium	The Lighthouse Lab in Milton Keynes and Alex Alderton, Roberto Amato, Sonia Goncalves, Ewan Harrison, David K. Jackson, Ian Johnston, Dominic Kwiatkowski, Cordelia Langford, John Sillitoe on behalf of the Wellcome Sanger Institute COVID-19 Surveillance Team
EPI_ISL_675942, EPI_ISL_675944	Lighthouse Lab in Alderley Park	Wellcome Sanger Institute for the COVID-19 Genomics UK (COG-UK) Consortium	Jacquelyn Wynn, Mairead Hyland, The Lighthouse Lab in Alderley Park and Alex Alderton, Roberto Amato, Sonia Goncalves, Ewan Harrison, David K. Jackson, Ian Johnston, Dominic Kwiatkowski, Cordelia Langford, John Sillitoe on behalf of the Wellcome Sanger Institute COVID-19 Surveillance Team
EPI_ISL_675947, EPI_ISL_675949, EPI_ISL_675950	Lighthouse Lab in Milton Keynes	Wellcome Sanger Institute for the COVID-19 Genomics UK (COG-UK) Consortium	The Lighthouse Lab in Milton Keynes and Alex Alderton, Roberto Amato, Sonia Goncalves, Ewan Harrison, David K. Jackson, Ian Johnston, Dominic Kwiatkowski, Cordelia Langford, John Sillitoe on behalf of the Wellcome Sanger Institute COVID-19 Surveillance Team
EPI_ISL_675952, EPI_ISL_675954, EPI_ISL_675962	Lighthouse Lab in Alderley Park	Wellcome Sanger Institute for the COVID-19 Genomics UK (COG-UK) Consortium	Jacquelyn Wynn, Mairead Hyland, The Lighthouse Lab in Alderley Park and Alex Alderton, Roberto Amato, Sonia Goncalves, Ewan Harrison, David K. Jackson, Ian Johnston, Dominic Kwiatkowski, Cordelia Langford, John Sillitoe on behalf of the Wellcome Sanger Institute COVID-19 Surveillance Team
EPI_ISL_675967	Lighthouse Lab in Milton Keynes	Wellcome Sanger Institute for the COVID-19 Genomics UK (COG-UK) Consortium	The Lighthouse Lab in Milton Keynes and Alex Alderton, Roberto Amato, Sonia Goncalves, Ewan Harrison, David K. Jackson, Ian Johnston, Dominic Kwiatkowski, Cordelia Langford, John Sillitoe on behalf of the Wellcome Sanger Institute COVID-19 Surveillance Team
EPI_ISL_675968	Lighthouse Lab in Alderley Park	Wellcome Sanger Institute for the COVID-19 Genomics UK (COG-UK) Consortium	Jacquelyn Wynn, Mairead Hyland, The Lighthouse Lab in Alderley Park and Alex Alderton, Roberto Amato, Sonia Goncalves, Ewan Harrison, David K. Jackson, Ian Johnston, Dominic Kwiatkowski, Cordelia Langford, John Sillitoe on behalf of the Wellcome Sanger Institute COVID-19 Surveillance Team
EPI_ISL_675971	Lighthouse Lab in Milton Keynes	Wellcome Sanger Institute for the COVID-19 Genomics UK (COG-UK) Consortium	The Lighthouse Lab in Milton Keynes and Alex Alderton, Roberto Amato, Sonia Goncalves, Ewan Harrison, David K. Jackson, Ian Johnston, Dominic Kwiatkowski, Cordelia Langford, John Sillitoe on behalf of the Wellcome Sanger Institute COVID-19 Surveillance Team
EPI_ISL_675973, EPI_ISL_675974, EPI_ISL_675976	Lighthouse Lab in Alderley Park	Wellcome Sanger Institute for the COVID-19 Genomics UK (COG-UK) Consortium	Jacquelyn Wynn, Mairead Hyland, The Lighthouse Lab in Alderley Park and Alex Alderton, Roberto Amato, Sonia Goncalves, Ewan Harrison, David K. Jackson, Ian Johnston, Dominic Kwiatkowski, Cordelia Langford, John Sillitoe on behalf of the Wellcome Sanger Institute COVID-19 Surveillance Team
EPI_ISL_675977	Lighthouse Lab in Milton Keynes	Wellcome Sanger Institute for the COVID-19 Genomics UK (COG-UK) Consortium	The Lighthouse Lab in Milton Keynes and Alex Alderton, Roberto Amato, Sonia Goncalves, Ewan Harrison, David K. Jackson, Ian Johnston, Dominic Kwiatkowski, Cordelia Langford, John Sillitoe on behalf of the Wellcome Sanger Institute COVID-19 Surveillance Team
EPI_ISL_675978, EPI_ISL_675980, EPI_ISL_675982	Lighthouse Lab in Alderley Park	Wellcome Sanger Institute for the COVID-19 Genomics UK (COG-UK) Consortium	Jacquelyn Wynn, Mairead Hyland, The Lighthouse Lab in Alderley Park and Alex Alderton, Roberto Amato, Sonia Goncalves, Ewan Harrison, David K. Jackson, Ian Johnston, Dominic Kwiatkowski, Cordelia Langford, John Sillitoe on behalf of the Wellcome Sanger Institute COVID-19 Surveillance Team
EPI_ISL_675984	Lighthouse Lab in Milton Keynes	Wellcome Sanger Institute for the COVID-19 Genomics UK (COG-UK) Consortium	The Lighthouse Lab in Milton Keynes and Alex Alderton, Roberto Amato, Sonia Goncalves, Ewan Harrison, David K. Jackson, Ian Johnston, Dominic Kwiatkowski, Cordelia Langford, John Sillitoe on behalf of the Wellcome Sanger Institute COVID-19 Surveillance Team
EPI_ISL_675986, EPI_ISL_675987, EPI_ISL_675988, EPI_ISL_675993	Lighthouse Lab in Alderley Park	Wellcome Sanger Institute for the COVID-19 Genomics UK (COG-UK) Consortium	Jacquelyn Wynn, Mairead Hyland, The Lighthouse Lab in Alderley Park and Alex Alderton, Roberto Amato, Sonia Goncalves, Ewan Harrison, David K. Jackson, Ian Johnston, Dominic Kwiatkowski, Cordelia Langford, John Sillitoe on behalf of the Wellcome Sanger Institute COVID-19 Surveillance Team
EPI_ISL_675995	Lighthouse Lab in Milton Keynes	Wellcome Sanger Institute for the COVID-19 Genomics UK (COG-UK) Consortium	The Lighthouse Lab in Milton Keynes and Alex Alderton, Roberto Amato, Sonia Goncalves, Ewan Harrison, David K. Jackson, Ian Johnston, Dominic Kwiatkowski, Cordelia Langford, John Sillitoe on behalf of the Wellcome Sanger Institute COVID-19 Surveillance Team
EPI_ISL_676000, EPI_ISL_676002, EPI_ISL_676006	Lighthouse Lab in Alderley Park	Wellcome Sanger Institute for the COVID-19 Genomics UK (COG-UK) Consortium	Jacquelyn Wynn, Mairead Hyland, The Lighthouse Lab in Alderley Park and Alex Alderton, Roberto Amato, Sonia Goncalves, Ewan Harrison, David K. Jackson, Ian Johnston, Dominic Kwiatkowski, Cordelia Langford, John Sillitoe on behalf of the Wellcome Sanger Institute COVID-19 Surveillance Team
EPI_ISL_676009	Lighthouse Lab in Milton Keynes	Wellcome Sanger Institute for the COVID-19 Genomics UK (COG-UK) Consortium	The Lighthouse Lab in Milton Keynes and Alex Alderton, Roberto Amato, Sonia Goncalves, Ewan Harrison, David K. Jackson, Ian Johnston, Dominic Kwiatkowski, Cordelia Langford, John Sillitoe on behalf of the Wellcome Sanger Institute COVID-19 Surveillance Team
EPI_ISL_676010	Lighthouse Lab in Alderley Park	Wellcome Sanger Institute for the COVID-19 Genomics UK (COG-UK) Consortium	Jacquelyn Wynn, Mairead Hyland, The Lighthouse Lab in Alderley Park and Alex Alderton, Roberto Amato, Sonia Goncalves, Ewan Harrison, David K. Jackson, Ian Johnston, Dominic Kwiatkowski, Cordelia Langford, John Sillitoe on behalf of the Wellcome Sanger Institute COVID-19 Surveillance Team
EPI_ISL_676011, EPI_ISL_676013, EPI_ISL_676018, EPI_ISL_676031, EPI_ISL_676033, EPI_ISL_676038, EPI_ISL_676042, EPI_ISL_676043, EPI_ISL_676044, EPI_ISL_676045, EPI_ISL_676046, EPI_ISL_676051, EPI_ISL_676052, EPI_ISL_676053, EPI_ISL_676058, EPI_ISL_676069, EPI_ISL_676070, EPI_ISL_676072, EPI_ISL_676075, EPI_ISL_676079, EPI_ISL_676084, EPI_ISL_676087, EPI_ISL_676091, EPI_ISL_676093, EPI_ISL_676097, EPI_ISL_676101, EPI_ISL_676104, EPI_ISL_676109, EPI_ISL_676116, EPI_ISL_676120, EPI_ISL_676126, EPI_ISL_676134, EPI_ISL_676138, EPI_ISL_676139, EPI_ISL_676142, EPI_ISL_676146, EPI_ISL_676149, EPI_ISL_676150, EPI_ISL_676151, EPI_ISL_676160, EPI_ISL_676166, EPI_ISL_676169, EPI_ISL_676179, EPI_ISL_676184, EPI_ISL_676186, EPI_ISL_676187, EPI_ISL_676188, EPI_ISL_676191, EPI_ISL_676193, EPI_ISL_676198, EPI_ISL_676199, EPI_ISL_676200, EPI_ISL_676203, EPI_ISL_676204, EPI_ISL_676205, EPI_ISL_676213, EPI_ISL_676215, EPI_ISL_676226, EPI_ISL_676227, EPI_ISL_676228, EPI_ISL_676234, EPI_ISL_676235, EPI_ISL_676236, EPI_ISL_676248, EPI_ISL_676250, EPI_ISL_676254, EPI_ISL_676258, EPI_ISL_676259, EPI_ISL_676262, EPI_ISL_676263, EPI_ISL_676266, EPI_ISL_676268, EPI_ISL_676271, EPI_ISL_676276, EPI_ISL_676277, EPI_ISL_676281, EPI_ISL_676284, EPI_ISL_676286, EPI_ISL_676288, EPI_ISL_676289, EPI_ISL_676292, EPI_ISL_676295, EPI_ISL_676298, EPI_ISL_676300, EPI_ISL_676302, EPI_ISL_676303, EPI_ISL_676307			
see above	Lighthouse Lab in Milton Keynes	Wellcome Sanger Institute for the COVID-19 Genomics UK (COG-UK) Consortium	The Lighthouse Lab in Milton Keynes and Alex Alderton, Roberto Amato, Sonia Goncalves, Ewan Harrison, David K. Jackson, Ian Johnston, Dominic Kwiatkowski, Cordelia Langford, John Sillitoe on behalf of the Wellcome Sanger Institute COVID-19 Surveillance Team
EPI_ISL_676353, EPI_ISL_676354, EPI_ISL_676355, EPI_ISL_676356, EPI_ISL_676357, EPI_ISL_676358	Lighthouse Lab in Glasgow	Wellcome Sanger Institute for the COVID-19 Genomics UK (COG-UK) Consortium	Harper VanSteenhouse, Yumi Kasai, David Gray, Carol Clugston, Anna Dominiczak and Alex Alderton, Roberto Amato, Sonia Goncalves, Ewan Harrison, David K. Jackson, Ian Johnston, Dominic Kwiatkowski, Cordelia Langford, John Sillitoe on behalf of the Wellcome Sanger Institute COVID-19 Surveillance Team
EPI_ISL_676511, EPI_ISL_678706, EPI_ISL_678708, EPI_ISL_678709, EPI_ISL_678710, EPI_ISL_678711, EPI_ISL_678712, EPI_ISL_678713, EPI_ISL_678714, EPI_ISL_678715, EPI_ISL_678716, EPI_ISL_678717, EPI_ISL_678718, EPI_ISL_678719, EPI_ISL_678720, EPI_ISL_678721, EPI_ISL_678722, EPI_ISL_678723, EPI_ISL_678724, EPI_ISL_678725, EPI_ISL_678726, EPI_ISL_678727, EPI_ISL_678728, EPI_ISL_678729, EPI_ISL_678730, EPI_ISL_678731, EPI_ISL_678732, EPI_ISL_678733, EPI_ISL_678734, EPI_ISL_678735, EPI_ISL_678736, EPI_ISL_678737, EPI_ISL_678738, EPI_ISL_678739, EPI_ISL_678740, EPI_ISL_678741, EPI_ISL_678742, EPI_ISL_678743, EPI_ISL_678744, EPI_ISL_678745, EPI_ISL_678746, EPI_ISL_678747, EPI_ISL_678748, EPI_ISL_678749, EPI_ISL_678750, EPI_ISL_678752, EPI_ISL_678753, EPI_ISL_678754, EPI_ISL_678755, EPI_ISL_678756, EPI_ISL_678757, EPI_ISL_678758, EPI_ISL_678759, EPI_ISL_678760, EPI_ISL_678761, EPI_ISL_678762, EPI_ISL_678763, EPI_ISL_678764, EPI_ISL_678765, EPI_ISL_678767, EPI_ISL_678768, EPI_ISL_678769, EPI_ISL_678774, EPI_ISL_678776			
see above	Respiratory Virus Unit, Microbiology Services Colindale,	COVID-19 Genomics UK (COG-UK) Consortium	PHE Covid Sequencing Team

	Public Health England		
EPI_ISL_679104, EPI_ISL_679105, EPI_ISL_679106, EPI_ISL_679107, EPI_ISL_679108, EPI_ISL_679109, EPI_ISL_679117, EPI_ISL_679118, EPI_ISL_679119, EPI_ISL_679120, EPI_ISL_679121, EPI_ISL_679122, EPI_ISL_679123, EPI_ISL_679124, EPI_ISL_679127, EPI_ISL_679128			
see above	University of Birmingham	COVID-19 Genomics UK (COG-UK) Consortium	Institute of Microbiology, University of Birmingham: Claire McMurray, Joanne Stockton, Samuel Nicholls, Radoslaw Poplawski, Will Rowe, Josh Quick, Nicholas Loman. University of Birmingham Testing Laboratory: Celina M Whalley, Andrew Bosworth, Charlotte Poxon, Kasun Wanigasooriya, Oliver Pickles, Mike Kidd, Alex Richter, Andrew D Beggs PHE Heartlands Lab: Husam Osman, Andrew Bosworth. Queen Elizabeth Hospital: Anna Casey
EPI_ISL_679156, EPI_ISL_679159, EPI_ISL_679160, EPI_ISL_679161, EPI_ISL_679162, EPI_ISL_679163, EPI_ISL_679164, EPI_ISL_679165, EPI_ISL_679166, EPI_ISL_679167, EPI_ISL_679168, EPI_ISL_679169, EPI_ISL_679170, EPI_ISL_679171, EPI_ISL_679172, EPI_ISL_679173, EPI_ISL_679174, EPI_ISL_679175, EPI_ISL_679176, EPI_ISL_679177, EPI_ISL_679178, EPI_ISL_679179, EPI_ISL_679181, EPI_ISL_679183, EPI_ISL_679185, EPI_ISL_679187, EPI_ISL_679189, EPI_ISL_679190, EPI_ISL_679191, EPI_ISL_679192, EPI_ISL_679193, EPI_ISL_679194, EPI_ISL_679195, EPI_ISL_679196, EPI_ISL_679197, EPI_ISL_679198, EPI_ISL_679199, EPI_ISL_679200, EPI_ISL_679201, EPI_ISL_679202, EPI_ISL_679203, EPI_ISL_679204, EPI_ISL_679205, EPI_ISL_679206, EPI_ISL_679207, EPI_ISL_679208, EPI_ISL_679209, EPI_ISL_679210, EPI_ISL_679211, EPI_ISL_679212, EPI_ISL_679213, EPI_ISL_679214, EPI_ISL_679215, EPI_ISL_679216, EPI_ISL_679217, EPI_ISL_679218, EPI_ISL_679220, EPI_ISL_679221, EPI_ISL_679223, EPI_ISL_679224, EPI_ISL_679226, EPI_ISL_679228, EPI_ISL_679230, EPI_ISL_679232, EPI_ISL_679234, EPI_ISL_679236, EPI_ISL_679238, EPI_ISL_679240, EPI_ISL_679242, EPI_ISL_679244, EPI_ISL_679246, EPI_ISL_679248			
see above	Department of Pathology, University of Cambridge	COVID-19 Genomics UK (COG-UK) Consortium	Aminu S. Jahun, Yasmin Chaudhry, Grant Hall, Iliana Georgana, Myra Hosmillo, Martin D. Curran, Malte Pinckert, Surendra Parmar, Ian Goodfellow
EPI_ISL_679249, EPI_ISL_679250, EPI_ISL_679251, EPI_ISL_679252, EPI_ISL_679253, EPI_ISL_679254, EPI_ISL_679255, EPI_ISL_679256, EPI_ISL_679257, EPI_ISL_679258, EPI_ISL_679259, EPI_ISL_679260, EPI_ISL_679261, EPI_ISL_679262, EPI_ISL_679263, EPI_ISL_679264, EPI_ISL_679265, EPI_ISL_679266, EPI_ISL_679267, EPI_ISL_679273, EPI_ISL_679274, EPI_ISL_679275, EPI_ISL_679276, EPI_ISL_679277, EPI_ISL_679278, EPI_ISL_679279, EPI_ISL_679280, EPI_ISL_679281, EPI_ISL_679282, EPI_ISL_679283, EPI_ISL_679284, EPI_ISL_679285, EPI_ISL_679286, EPI_ISL_679287, EPI_ISL_679288, EPI_ISL_679289, EPI_ISL_679290, EPI_ISL_679291, EPI_ISL_679292, EPI_ISL_679293, EPI_ISL_679294, EPI_ISL_679295, EPI_ISL_679296, EPI_ISL_679297, EPI_ISL_679298, EPI_ISL_679300, EPI_ISL_679301, EPI_ISL_679302, EPI_ISL_679305, EPI_ISL_679308, EPI_ISL_679309, EPI_ISL_679310, EPI_ISL_679311, EPI_ISL_679312			
see above	Queens Medical Centre, Clinical Microbiology Department / DeepSeq Nottingham	COVID-19 Genomics UK (COG-UK) Consortium	Gemma Clark, Wendy Smith, Manjinder Khakh, Vicki M Fleming, Michelle M Lister, Hannah Howson-Wells, Jonathan Ball, Patrick McClure, Joseph Chappell, Theocharis Tsoleridis, Nadine Holmes, Matthew Carlisle, Christopher Moore, Fei Sang, Johnny Debebe, Victoria Wright, Matthew Loose
EPI_ISL_679393, EPI_ISL_679394, EPI_ISL_679398, EPI_ISL_679400	Liverpool Clinical Laboratories	COVID-19 Genomics UK (COG-UK) Consortium	Sam Haldenby, Anita Lucaci, Steve Paterson, Julian Hiscox, Alistair Darby, M Almsaud, A Alrezaihi, Muhannad Alruwaili, Stuart D Armstrong, Jones Benjamin, Eleanor G Bentley, Anu Chawla, Jordan J Clark, Angela Cowell, Richard Eccles, Isabel García-Dorival, Matthew Gemmell, Alessandro Gerada, PKF Gilmore, Richard Gregory, Ximeng Han, Catherine Hartley, Margaret Hughes, Miren Iturriza-Gomara, James Johnson, L Luu, Jenifer Manson, Charlotte Nelson, Elaine O'Toole, Cassie Olateju, Rebekah Penrice-Randal , Lucille Rainbow, N.P Randle, Trevor Ian Robinson, Parul Sharma, Ghada T Shawli, James P Stewart, Neil Swainston, Ecaterina Vamos, Joanne Watts, Mark Whitehead
EPI_ISL_679406, EPI_ISL_679407, EPI_ISL_679416, EPI_ISL_679417, EPI_ISL_679418, EPI_ISL_679419, EPI_ISL_679420, EPI_ISL_679421, EPI_ISL_679422, EPI_ISL_679423, EPI_ISL_679424, EPI_ISL_679425, EPI_ISL_679426, EPI_ISL_679429, EPI_ISL_679430, EPI_ISL_679431, EPI_ISL_679432, EPI_ISL_679433, EPI_ISL_679434, EPI_ISL_679435, EPI_ISL_679436, EPI_ISL_679437, EPI_ISL_679438, EPI_ISL_679439, EPI_ISL_679440, EPI_ISL_679441, EPI_ISL_679442, EPI_ISL_679443, EPI_ISL_679444, EPI_ISL_679445, EPI_ISL_679446			
see above	University College London Hospital	COVID-19 Genomics UK (COG-UK) Consortium	Judith Heaney, Matthew Byott, Catherine Houlihan, Dan Frampton, Stuart Kirk, Moira Spyer and Eleni Nastouli
EPI_ISL_679506, EPI_ISL_679507, EPI_ISL_679508, EPI_ISL_679509, EPI_ISL_679510, EPI_ISL_679511, EPI_ISL_679512, EPI_ISL_679513, EPI_ISL_679514, EPI_ISL_679515, EPI_ISL_679516, EPI_ISL_679517, EPI_ISL_679518, EPI_ISL_679519, EPI_ISL_679520, EPI_ISL_679521, EPI_ISL_679522, EPI_ISL_679523, EPI_ISL_679524, EPI_ISL_679525, EPI_ISL_679526, EPI_ISL_679527, EPI_ISL_679528, EPI_ISL_679529, EPI_ISL_679531, EPI_ISL_679532, EPI_ISL_679533, EPI_ISL_679534, EPI_ISL_679535, EPI_ISL_679536, EPI_ISL_679537, EPI_ISL_679538, EPI_ISL_679540, EPI_ISL_679541, EPI_ISL_679542, EPI_ISL_679543, EPI_ISL_679544, EPI_ISL_679545, EPI_ISL_679546, EPI_ISL_679547, EPI_ISL_679548, EPI_ISL_679549, EPI_ISL_679575, EPI_ISL_679576, EPI_ISL_679577, EPI_ISL_679578, EPI_ISL_679579, EPI_ISL_679580, EPI_ISL_679581, EPI_ISL_679582, EPI_ISL_679583, EPI_ISL_679584, EPI_ISL_679585, EPI_ISL_679586, EPI_ISL_679587, EPI_ISL_679588, EPI_ISL_679589, EPI_ISL_679590, EPI_ISL_679591, EPI_ISL_679592, EPI_ISL_679594, EPI_ISL_679595, EPI_ISL_679596, EPI_ISL_679597			
see above	University College London, Great Ormond Street Hospital for Children NHS Foundation Trust, Imperial College Healthcare NHS Trust	COVID-19 Genomics UK (COG-UK) Consortium	Sergi Castellano, Rachel Williams, Mark Kristiansen, Paola Resende Silva, Sunando Roy, Tony Brooks, Helena Tutill, Paola Niola, Patricia Dyal, Charlotte Williams, Leysa Forrest, Yasmin Panchbhaya, Jacqueline Findlay, Samuel Weeks, Julianne Brown, Kathryn Harris, Paul Randell, James Price, Alison Holmes, Judith Breuer
EPI_ISL_679933	Queens Medical Centre, Clinical Microbiology Department / DeepSeq Nottingham	COVID-19 Genomics UK (COG-UK) Consortium	Gemma Clark, Wendy Smith, Manjinder Khakh, Vicki M Fleming, Michelle M Lister, Hannah Howson-Wells, Jonathan Ball, Patrick McClure, Joseph Chappell, Theocharis Tsoleridis, Nadine Holmes, Matthew Carlisle, Christopher Moore, Fei Sang, Johnny Debebe, Victoria Wright, Matthew Loose
EPI_ISL_680102, EPI_ISL_680103, EPI_ISL_680104, EPI_ISL_680105, EPI_ISL_680106, EPI_ISL_680107, EPI_ISL_680108, EPI_ISL_680109, EPI_ISL_680111, EPI_ISL_680113, EPI_ISL_680114, EPI_ISL_680119, EPI_ISL_680128, EPI_ISL_680135, EPI_ISL_680136, EPI_ISL_680137, EPI_ISL_680138, EPI_ISL_680144, EPI_ISL_680147, EPI_ISL_680150, EPI_ISL_680154, EPI_ISL_680155, EPI_ISL_680158, EPI_ISL_680164, EPI_ISL_680174, EPI_ISL_680178, EPI_ISL_680183, EPI_ISL_680185, EPI_ISL_680187, EPI_ISL_680192			
see above	Virology Department, Sheffield Teaching Hospitals NHS Foundation Trust/Department of Infection, Immunity and Cardiovascular Disease, The Medical School, University of Sheffield	COVID-19 Genomics UK (COG-UK) Consortium	Thushan de Silva, Matthew Parker, Nikki Smith, Adri Angyal, Rebecca Brown, Luke Green, Rachel Tucker, Paul Parsons, Danielle Groves, Katie Johnson, Laura Carrilero, Alex Keeley, Dave Partridge, Matthew Wyles, Benjamin Lindsey, Mehmet Yavuz, Mohammad Raza, Cariad Evans
EPI_ISL_680449, EPI_ISL_680450, EPI_ISL_680451, EPI_ISL_680452	West of Scotland Specialist Virology Centre, NHSGGC / MRC-University of Glasgow Centre for Virus Research	COVID-19 Genomics UK (COG-UK) Consortium	Ana da Silva Filipe, Natasha Johnson, Kathy Smollett, Daniel Mair, Stephen Carmichael, Alice Broos, Lily Tong, Jenna Nichols, Kyriaki Nomikou; Sarah McDonald; Richard Orton, Joseph Hughes, Sreenu Vattipally, David L Robertson; Alasdair MacLean, Rory Gunson; Sharif Shaaban, Matthew Holden; Rachel Blacow, Guy Mollett, Kathy Li, James Shepherd, Antonia Ho, Emma Thomson
EPI_ISL_680461, EPI_ISL_680462, EPI_ISL_680463, EPI_ISL_680464, EPI_ISL_680465, EPI_ISL_680466, EPI_ISL_680467, EPI_ISL_680468, EPI_ISL_680486, EPI_ISL_680487, EPI_ISL_680488, EPI_ISL_680489, EPI_ISL_680490, EPI_ISL_680540, EPI_ISL_680541, EPI_ISL_680542, EPI_ISL_680543, EPI_ISL_680548, EPI_ISL_680549, EPI_ISL_680550, EPI_ISL_680551, EPI_ISL_680552, EPI_ISL_680553, EPI_ISL_680554, EPI_ISL_680555			
see above	Virology Department, Royal Infirmary of Edinburgh, NHS Lothian / School of Biological Sciences, University of Edinburgh / Institute of Genetics and Molecular Medicine, University of Edinburgh	COVID-19 Genomics UK (COG-UK) Consortium	McHugh M, Dewar R, Rooke S, Gallagher M, Balcaza C, O'Toole Á, Scher E, Hill V, McCrone JT, Colquhoun R, Yu X, Jackson B, Rambaut A, Williams TC, Templeton K
EPI_ISL_680591, EPI_ISL_680592, EPI_ISL_680593, EPI_ISL_680599, EPI_ISL_680600, EPI_ISL_680601, EPI_ISL_680602, EPI_ISL_680603, EPI_ISL_680604, EPI_ISL_680605, EPI_ISL_680608, EPI_ISL_680609, EPI_ISL_680610, EPI_ISL_680649, EPI_ISL_680650, EPI_ISL_680651, EPI_ISL_680652, EPI_ISL_680653, EPI_ISL_680654, EPI_ISL_680655, EPI_ISL_680656, EPI_ISL_680657, EPI_ISL_680658, EPI_ISL_680659, EPI_ISL_680660, EPI_ISL_680661, EPI_ISL_680662, EPI_ISL_680663, EPI_ISL_680664, EPI_ISL_680665, EPI_ISL_680666, EPI_ISL_680667, EPI_ISL_680668, EPI_ISL_680669, EPI_ISL_680670, EPI_ISL_680712, EPI_ISL_680948, EPI_ISL_680949, EPI_ISL_680950, EPI_ISL_680951, EPI_ISL_680952, EPI_ISL_681019, EPI_ISL_681020			
see above	Wales Specialist Virology Centre Sequencing lab: Pathogen Genomics Unit	COVID-19 Genomics UK (COG-UK) Consortium	Catherine Moore, Johnathan Evans, Laura Gifford, Malorie Perry, Simon Cottrell, Angela Marchbank, Alec Birchley, Alexander Adams, Amy Gaskin, Bree Gatica-Wilcox, Jason Coombes, Joel Southgate, Lauren Gilbert, Lee Graham, Nicole Pacchiarini, Sara Kumziene-Summerhayes, Sarah Taylor, Sophie Jones, Sara Rey, Matthew Bull, Joanne Watkins, Sally Corden, Tom Connor
EPI_ISL_681325, EPI_ISL_681326, EPI_ISL_681327, EPI_ISL_681328, EPI_ISL_681329, EPI_ISL_681330, EPI_ISL_681331, EPI_ISL_681332, EPI_ISL_681333, EPI_ISL_681334, EPI_ISL_681335, EPI_ISL_681336, EPI_ISL_681337, EPI_ISL_681338, EPI_ISL_681339, EPI_ISL_681340, EPI_ISL_681341, EPI_ISL_681342, EPI_ISL_681343, EPI_ISL_681344, EPI_ISL_681345, EPI_ISL_681346, EPI_ISL_681347, EPI_ISL_681348, EPI_ISL_681349, EPI_ISL_681350, EPI_ISL_681351, EPI_ISL_681352, EPI_ISL_681353, EPI_ISL_681354, EPI_ISL_681355, EPI_ISL_681356, EPI_ISL_681357, EPI_ISL_681358, EPI_ISL_681359, EPI_ISL_681360, EPI_ISL_681361, EPI_ISL_681362, EPI_ISL_681363, EPI_ISL_681364, EPI_ISL_681365, EPI_ISL_681366, EPI_ISL_681367, EPI_ISL_681368, EPI_ISL_681369, EPI_ISL_681370, EPI_ISL_681371, EPI_ISL_681372, EPI_ISL_681373, EPI_ISL_681374, EPI_ISL_681375, EPI_ISL_681376, EPI_ISL_681377, EPI_ISL_681378, EPI_ISL_681379, EPI_ISL_681380, EPI_ISL_681381, EPI_ISL_681382, EPI_ISL_681383, EPI_ISL_681384, EPI_ISL_681385, EPI_ISL_681386, EPI_ISL_681387, EPI_ISL_681388, EPI_ISL_681389, EPI_ISL_681390, EPI_ISL_681391, EPI_ISL_681392, EPI_ISL_681393, EPI_ISL_681394, EPI_ISL_681395, EPI_ISL_681396, EPI_ISL_681397, EPI_ISL_681398, EPI_ISL_681399, EPI_ISL_681400, EPI_ISL_681401, EPI_ISL_681402, EPI_ISL_681403, EPI_ISL_681404, EPI_ISL_681405, EPI_ISL_681406, EPI_ISL_681407, EPI_ISL_681408, EPI_ISL_681409, EPI_ISL_681410, EPI_ISL_681411, EPI_ISL_681412, EPI_ISL_681413, EPI_ISL_681414, EPI_ISL_681415, EPI_ISL_681416, EPI_ISL_681417, EPI_ISL_681418, EPI_ISL_681419, EPI_ISL_681420, EPI_ISL_681421, EPI_ISL_681422, EPI_ISL_681423, EPI_ISL_681424, EPI_ISL_681425, EPI_ISL_681426, EPI_ISL_681427, EPI_ISL_681428, EPI_ISL_681429, EPI_ISL_681430, EPI_ISL_681431, EPI_ISL_681432, EPI_ISL_681433, EPI_ISL_681434, EPI_ISL_681435, EPI_ISL_681436, EPI_ISL_681437, EPI_ISL_681438, EPI_ISL_681439, EPI_ISL_681440, EPI_ISL_681441, EPI_ISL_681442, EPI_ISL_681443, EPI_ISL_681444, EPI_ISL_681445, EPI_ISL_681446, EPI_ISL_681447, EPI_ISL_681448, EPI_ISL_681449, EPI_ISL_681450, EPI_ISL_681451, EPI_ISL_681452, EPI_ISL_681453, EPI_ISL_681454, EPI_ISL_681455, EPI_ISL_681456, EPI_ISL_681457, EPI_ISL_681458, EPI_ISL_681459, EPI_ISL_681460, EPI_ISL_681461, EPI_ISL_681462, EPI_ISL_681463, EPI_ISL_681464, EPI_ISL_681465, EPI_ISL_681466, EPI_ISL_681467, EPI_ISL_681468, EPI_ISL_681469, EPI_ISL_681470, EPI_ISL_681471, EPI_ISL_681472, EPI_ISL_681473, EPI_ISL_681474, EPI_ISL_681475, EPI_ISL_681476, EPI_ISL_681477, EPI_ISL_681478, EPI_ISL_681479, EPI_ISL_681480, EPI_ISL_681481, EPI_ISL_681482, EPI_ISL_681483, EPI_ISL_681484, EPI_ISL_681485, EPI_ISL_681486, EPI_ISL_681487, EPI_ISL_681488, EPI_ISL_681489, EPI_ISL_681490, EPI_ISL_681491, EPI_ISL_681492, EPI_ISL_681493, EPI_ISL_681494, EPI_ISL_681495, EPI_ISL_681496, EPI_ISL_681497, EPI_ISL_681498, EPI_ISL_681499, EPI_ISL_681500, EPI_ISL_681501, EPI_ISL_681502, EPI_ISL_681503, EPI_ISL_681504, EPI_ISL_681505, EPI_ISL_681506, EPI_ISL_681507, EPI_ISL_681508, EPI_ISL_681509, EPI_ISL_681510, EPI_ISL_681511, EPI_ISL_681512, EPI_ISL_681513, EPI_ISL_681514, EPI_ISL_681515, EPI_ISL_681516, EPI_ISL_681517, EPI_ISL_681518, EPI_ISL_681519, EPI_ISL_681520, EPI_ISL_681521, EPI_ISL_681522, EPI_ISL_681523, EPI_ISL_681524, EPI_ISL_681525, EPI_ISL_681526, EPI_ISL_681527, EPI_ISL_681528, EPI_ISL_681529, EPI_ISL_681530, EPI_ISL_681531, EPI_ISL_681532, EPI_ISL_681533, EPI_ISL_681534, EPI_ISL_681535, EPI_ISL_681536, EPI_ISL_681537, EPI_ISL_681538, EPI_ISL_681539, EPI_ISL_681540, EPI_ISL_681541, EPI_ISL_681542, EPI_ISL_681543, EPI_ISL_681544, EPI_ISL_681545, EPI_ISL_681546, EPI_ISL_681547, EPI_ISL_681548, EPI_ISL_681549, EPI_ISL_681550, EPI_ISL_681551, EPI_ISL_681552, EPI_ISL_681553, EPI_ISL_681554, EPI_ISL_681555, EPI_ISL_681556, EPI_ISL_681557, EPI_ISL_681558, EPI_ISL_681559, EPI_ISL_681560, EPI_ISL_681561, EPI_ISL_681562, EPI_ISL_681563, EPI_ISL_681564, EPI_ISL_681565, EPI_ISL_681566, EPI_ISL_681567, EPI_ISL_681568, EPI_ISL_681569, EPI_ISL_681570, EPI_ISL_681571, EPI_ISL_681572, EPI_ISL_681573, EPI_ISL_681574, EPI_ISL_681575, EPI_ISL_681576, EPI_ISL_681577, EPI_ISL_681578, EPI_ISL_681579, EPI_ISL_681580, EPI_ISL_681581, EPI_ISL_681582, EPI_ISL_681583, EPI_ISL_681584, EPI_ISL_681585, EPI_ISL_681586, EPI_ISL_681587, EPI_ISL_681588, EPI_ISL_681589, EPI_ISL_681590, EPI_ISL_681591, EPI_ISL_681592, EPI_ISL_681593, EPI_ISL_681594, EPI_ISL_681595, EPI_ISL_681596, EPI_ISL_681597, EPI_ISL_681598, EPI_ISL_681599, EPI_ISL_681600, EPI_ISL_681601, EPI_ISL_681602, EPI_ISL_681603, EPI_ISL_681604, EPI_ISL_681605, EPI_ISL_681606, EPI_ISL_681607, EPI_ISL_681608, EPI_ISL_681609, EPI_ISL_681610, EPI_ISL_681611, EPI_ISL_681612, EPI_ISL_681613, EPI_ISL_681614, EPI_ISL_681615, EPI_ISL_681616, EPI_ISL_681617, EPI_ISL_681618, EPI_ISL_681619, EPI_ISL_681620, EPI_ISL_681621, EPI_ISL_681622, EPI_ISL_681623, EPI_ISL_681624, EPI_ISL_681625, EPI_ISL_681626, EPI_ISL_681627, EPI_ISL_681628, EPI_ISL_681629, EPI_ISL_681630, EPI_ISL_681631, EPI_ISL_681632, EPI_ISL_681633, EPI_ISL_681634, EPI_ISL_681635, EPI_ISL_681636, EPI_ISL_681637, EPI_ISL_681638, EPI_ISL_681639, EPI_ISL_681640, EPI_ISL_681641, EPI_ISL_681642, EPI_ISL_681643, EPI_ISL_681644, EPI_ISL_681645, EPI_ISL_681646, EPI_ISL_681647, EPI_ISL_681648, EPI_ISL_681649, EPI_ISL_681650, EPI_ISL_681651, EPI_ISL_681652, EPI_ISL_681653, EPI_ISL_681654, EPI_ISL_681655			
see above	Lighthouse Lab in Milton Keynes	Wellcome Sanger Institute for the COVID-19 Genomics UK (COG-UK) Consortium	The Lighthouse Lab in Milton Keynes and Alex Alderton, Roberto Amato, Sonia Goncalves, Ewan Harrison, David K. Jackson, Ian Johnston, Dominic Kwiatkowski, Cordelia Langford, John Sillitoe on behalf of the Wellcome Sanger Institute COVID-19 Surveillance Team
EPI_ISL_681658, EPI_ISL_681659	Lighthouse Lab in Cambridge	Wellcome Sanger Institute for the COVID-19 Genomics UK (COG-UK) Consortium	Rob Howes, The Lighthouse Lab in Cambridge and Alex Alderton, Roberto Amato, Sonia Goncalves, Ewan Harrison, David K. Jackson, Ian Johnston, Dominic Kwiatkowski, Cordelia Langford, John Sillitoe on behalf of the Wellcome Sanger Institute COVID-19 Surveillance Team

EPI_ISL_681661, EPI_ISL_681662, EPI_ISL_681665	Lighthouse Lab in Alderley Park	Wellcome Sanger Institute for the COVID-19 Genomics UK (COG-UK) Consortium	Jacquelyn Wynn, Mairead Hyland, The Lighthouse Lab in Alderley Park and Alex Alderton, Roberto Amato, Sonia Goncalves, Ewan Harrison, David K. Jackson, Ian Johnston, Dominic Kwiatkowski, Cordelia Langford, John Sillitoe on behalf of the Wellcome Sanger Institute COVID-19 Surveillance Team
EPI_ISL_681668, EPI_ISL_681669	Lighthouse Lab in Milton Keynes	Wellcome Sanger Institute for the COVID-19 Genomics UK (COG-UK) Consortium	The Lighthouse Lab in Milton Keynes and Alex Alderton, Roberto Amato, Sonia Goncalves, Ewan Harrison, David K. Jackson, Ian Johnston, Dominic Kwiatkowski, Cordelia Langford, John Sillitoe on behalf of the Wellcome Sanger Institute COVID-19 Surveillance Team
EPI_ISL_683457, EPI_ISL_683461, EPI_ISL_683475, EPI_ISL_683476, EPI_ISL_683478, EPI_ISL_683482, EPI_ISL_683484, EPI_ISL_683489, EPI_ISL_683490, EPI_ISL_683534, EPI_ISL_683535, EPI_ISL_683536, EPI_ISL_683537, EPI_ISL_683538, EPI_ISL_683539, EPI_ISL_683540, EPI_ISL_683541, EPI_ISL_683542, EPI_ISL_683543, EPI_ISL_683544, EPI_ISL_683545, EPI_ISL_683546, EPI_ISL_683547, EPI_ISL_683548, EPI_ISL_683549, EPI_ISL_683550, EPI_ISL_683551, EPI_ISL_683552, EPI_ISL_683553, EPI_ISL_683554, EPI_ISL_683555, EPI_ISL_683556, EPI_ISL_683557, EPI_ISL_683558, EPI_ISL_683559, EPI_ISL_683560, EPI_ISL_683561, EPI_ISL_683562, EPI_ISL_683563, EPI_ISL_683564, EPI_ISL_683565, EPI_ISL_683566, EPI_ISL_683567, EPI_ISL_683568, EPI_ISL_683569, EPI_ISL_683570, EPI_ISL_683571, EPI_ISL_683572, EPI_ISL_686584, EPI_ISL_686585, EPI_ISL_686586, EPI_ISL_686587, EPI_ISL_686593, EPI_ISL_686596, EPI_ISL_686598, EPI_ISL_686601, EPI_ISL_686602, EPI_ISL_686603, EPI_ISL_686604, EPI_ISL_686605, EPI_ISL_686606, EPI_ISL_686607, EPI_ISL_686608, EPI_ISL_686609, EPI_ISL_686610			
see above	Respiratory Virus Unit, Microbiology Services Colindale, Public Health England	COVID-19 Genomics UK (COG-UK) Consortium	PHE Covid Sequencing Team
EPI_ISL_693332, EPI_ISL_693333	Lighthouse Lab in Alderley Park	Wellcome Sanger Institute for the COVID-19 Genomics UK (COG-UK) Consortium	Jacquelyn Wynn, Mairead Hyland, The Lighthouse Lab in Alderley Park and Alex Alderton, Roberto Amato, Sonia Goncalves, Ewan Harrison, David K. Jackson, Ian Johnston, Dominic Kwiatkowski, Cordelia Langford, John Sillitoe on behalf of the Wellcome Sanger Institute COVID-19 Surveillance Team
EPI_ISL_693395, EPI_ISL_693397, EPI_ISL_693402, EPI_ISL_693403, EPI_ISL_693404, EPI_ISL_693406, EPI_ISL_693407, EPI_ISL_693434, EPI_ISL_693436, EPI_ISL_693440, EPI_ISL_693441, EPI_ISL_693442, EPI_ISL_693444, EPI_ISL_693447, EPI_ISL_693449, EPI_ISL_693450, EPI_ISL_693451, EPI_ISL_693452, EPI_ISL_693453, EPI_ISL_693454, EPI_ISL_693455, EPI_ISL_693456, EPI_ISL_693457, EPI_ISL_693458, EPI_ISL_693459, EPI_ISL_693460, EPI_ISL_693461, EPI_ISL_693462, EPI_ISL_693463, EPI_ISL_693464, EPI_ISL_693465, EPI_ISL_693466, EPI_ISL_693468, EPI_ISL_693469, EPI_ISL_693470			
see above	Respiratory Virus Unit, Microbiology Services Colindale, Public Health England	COVID-19 Genomics UK (COG-UK) Consortium	PHE Covid Sequencing Team
EPI_ISL_701673, EPI_ISL_701674, EPI_ISL_701675, EPI_ISL_701676, EPI_ISL_701677, EPI_ISL_701678, EPI_ISL_701679, EPI_ISL_701680, EPI_ISL_701681, EPI_ISL_701682, EPI_ISL_701683, EPI_ISL_701684, EPI_ISL_701685, EPI_ISL_701686, EPI_ISL_701687, EPI_ISL_701688, EPI_ISL_701689, EPI_ISL_701690, EPI_ISL_701691, EPI_ISL_701692, EPI_ISL_701693, EPI_ISL_701694, EPI_ISL_701695, EPI_ISL_701696, EPI_ISL_701697, EPI_ISL_701698, EPI_ISL_701699, EPI_ISL_701700, EPI_ISL_701701, EPI_ISL_701702, EPI_ISL_701703, EPI_ISL_701704, EPI_ISL_701705, EPI_ISL_701706, EPI_ISL_701707, EPI_ISL_701708, EPI_ISL_701709, EPI_ISL_701710, EPI_ISL_701711, EPI_ISL_701712, EPI_ISL_701713, EPI_ISL_701714, EPI_ISL_701715, EPI_ISL_701716, EPI_ISL_701717, EPI_ISL_701718, EPI_ISL_701719, EPI_ISL_701720, EPI_ISL_701721, EPI_ISL_701722, EPI_ISL_701723, EPI_ISL_701725, EPI_ISL_701726, EPI_ISL_701727, EPI_ISL_701728, EPI_ISL_701729, EPI_ISL_701730, EPI_ISL_701731, EPI_ISL_701732, EPI_ISL_701733, EPI_ISL_701734, EPI_ISL_701735, EPI_ISL_701736, EPI_ISL_701737, EPI_ISL_701738, EPI_ISL_701739, EPI_ISL_701740, EPI_ISL_701741, EPI_ISL_701742, EPI_ISL_701743, EPI_ISL_701744, EPI_ISL_701745, EPI_ISL_701746, EPI_ISL_701747, EPI_ISL_701748, EPI_ISL_701749, EPI_ISL_701750, EPI_ISL_701751, EPI_ISL_701752, EPI_ISL_701753, EPI_ISL_701754, EPI_ISL_701755, EPI_ISL_701756, EPI_ISL_701757, EPI_ISL_701758, EPI_ISL_701759, EPI_ISL_701760, EPI_ISL_701761, EPI_ISL_701762, EPI_ISL_701763, EPI_ISL_701764, EPI_ISL_701765, EPI_ISL_701766, EPI_ISL_701767, EPI_ISL_701768, EPI_ISL_701769, EPI_ISL_701770, EPI_ISL_701771, EPI_ISL_701772, EPI_ISL_701773, EPI_ISL_701774, EPI_ISL_701775, EPI_ISL_701776, EPI_ISL_701777, EPI_ISL_701778, EPI_ISL_701779, EPI_ISL_701780, EPI_ISL_701781, EPI_ISL_701782, EPI_ISL_701783, EPI_ISL_701784, EPI_ISL_701785, EPI_ISL_701786, EPI_ISL_701787, EPI_ISL_701788, EPI_ISL_701789, EPI_ISL_701790, EPI_ISL_701791, EPI_ISL_701792, EPI_ISL_701793, EPI_ISL_701794, EPI_ISL_701795, EPI_ISL_701797, EPI_ISL_701798, EPI_ISL_701799, EPI_ISL_701800, EPI_ISL_701801, EPI_ISL_701802, EPI_ISL_701803, EPI_ISL_701804, EPI_ISL_701805, EPI_ISL_701806, EPI_ISL_701807, EPI_ISL_701808, EPI_ISL_701809, EPI_ISL_701810, EPI_ISL_701811, EPI_ISL_701812, EPI_ISL_701813, EPI_ISL_701814, EPI_ISL_701815, EPI_ISL_701816, EPI_ISL_701817, EPI_ISL_701818, EPI_ISL_701819, EPI_ISL_701820, EPI_ISL_701821, EPI_ISL_701822, EPI_ISL_701823, EPI_ISL_701824, EPI_ISL_701825, EPI_ISL_701826, EPI_ISL_701827, EPI_ISL_701828, EPI_ISL_701829, EPI_ISL_701830, EPI_ISL_701831, EPI_ISL_701832, EPI_ISL_701833, EPI_ISL_701834, EPI_ISL_701835, EPI_ISL_701836, EPI_ISL_701837, EPI_ISL_701838, EPI_ISL_701839, EPI_ISL_701840, EPI_ISL_701841, EPI_ISL_701842, EPI_ISL_701843, EPI_ISL_701844, EPI_ISL_701845, EPI_ISL_701846, EPI_ISL_701847, EPI_ISL_701848, EPI_ISL_701849, EPI_ISL_701850, EPI_ISL_701851, EPI_ISL_701852, EPI_ISL_701853, EPI_ISL_701854, EPI_ISL_701855, EPI_ISL_701856, EPI_ISL_701857, EPI_ISL_701858, EPI_ISL_701859, EPI_ISL_701860, EPI_ISL_701861, EPI_ISL_701862, EPI_ISL_701863, EPI_ISL_701864, EPI_ISL_701865, EPI_ISL_701866, EPI_ISL_701867, EPI_ISL_701868, EPI_ISL_701869, EPI_ISL_701870, EPI_ISL_701871, EPI_ISL_701872, EPI_ISL_701873, EPI_ISL_701874, EPI_ISL_701875, EPI_ISL_701876, EPI_ISL_701877, EPI_ISL_701878, EPI_ISL_701879, EPI_ISL_701880, EPI_ISL_701881, EPI_ISL_701882, EPI_ISL_701883, EPI_ISL_701884, EPI_ISL_701885, EPI_ISL_701886, EPI_ISL_701887, EPI_ISL_701888, EPI_ISL_701889, EPI_ISL_701890, EPI_ISL_701891, EPI_ISL_701892, EPI_ISL_701893, EPI_ISL_701894, EPI_ISL_701895, EPI_ISL_701896, EPI_ISL_701897, EPI_ISL_701898, EPI_ISL_701899, EPI_ISL_701900, EPI_ISL_701901, EPI_ISL_701902, EPI_ISL_701903, EPI_ISL_701904, EPI_ISL_701905, EPI_ISL_701906, EPI_ISL_701907, EPI_ISL_701908, EPI_ISL_701909, EPI_ISL_701910, EPI_ISL_701911, EPI_ISL_701912, EPI_ISL_701913, EPI_ISL_701914, EPI_ISL_701915, EPI_ISL_701916, EPI_ISL_701917, EPI_ISL_701918, EPI_ISL_701919, EPI_ISL_701920, EPI_ISL_701921, EPI_ISL_701922, EPI_ISL_701923, EPI_ISL_701924, EPI_ISL_701925, EPI_ISL_701926, EPI_ISL_701927, EPI_ISL_701928, EPI_ISL_701929, EPI_ISL_701930, EPI_ISL_701931, EPI_ISL_701932, EPI_ISL_701933, EPI_ISL_701934, EPI_ISL_701935, EPI_ISL_701936, EPI_ISL_701937, EPI_ISL_701938, EPI_ISL_701939, EPI_ISL_701940, EPI_ISL_701941, EPI_ISL_701942, EPI_ISL_701943, EPI_ISL_701944, EPI_ISL_701945, EPI_ISL_701946, EPI_ISL_701947, EPI_ISL_701948, EPI_ISL_701949, EPI_ISL_701950, EPI_ISL_701951, EPI_ISL_701952, EPI_ISL_701953, EPI_ISL_701954, EPI_ISL_701955, EPI_ISL_701956, EPI_ISL_701957, EPI_ISL_701958, EPI_ISL_701959, EPI_ISL_701960, EPI_ISL_701962, EPI_ISL_701963, EPI_ISL_701964, EPI_ISL_701965, EPI_ISL_701966, EPI_ISL_701967, EPI_ISL_701968, EPI_ISL_701969, EPI_ISL_701970, EPI_ISL_701971, EPI_ISL_701972, EPI_ISL_701973, EPI_ISL_701974, EPI_ISL_701975, EPI_ISL_701976, EPI_ISL_701977, EPI_ISL_701978, EPI_ISL_701979, EPI_ISL_701980, EPI_ISL_701981, EPI_ISL_701982, EPI_ISL_701983, EPI_ISL_701984, EPI_ISL_701985, EPI_ISL_701986, EPI_ISL_701987, EPI_ISL_701988, EPI_ISL_701989, EPI_ISL_701990, EPI_ISL_701991, EPI_ISL_701992, EPI_ISL_701993, EPI_ISL_701994, EPI_ISL_701995, EPI_ISL_701996, EPI_ISL_701997, EPI_ISL_701998, EPI_ISL_701999, EPI_ISL_702000, EPI_ISL_702001, EPI_ISL_702002, EPI_ISL_702003, EPI_ISL_702004, EPI_ISL_702005, EPI_ISL_702006, EPI_ISL_702007, EPI_ISL_702008, EPI_ISL_702009, EPI_ISL_702010, EPI_ISL_702011, EPI_ISL_702012, EPI_ISL_702013, EPI_ISL_702014, EPI_ISL_702015, EPI_ISL_702016, EPI_ISL_702017, EPI_ISL_702018, EPI_ISL_702019, EPI_ISL_702020, EPI_ISL_702021, EPI_ISL_702022, EPI_ISL_702370, EPI_ISL_702371, EPI_ISL_702372, EPI_ISL_702373, EPI_ISL_702374, EPI_ISL_702375, EPI_ISL_702376, EPI_ISL_702377, EPI_ISL_702378, EPI_ISL_702379, EPI_ISL_702380, EPI_ISL_702381, EPI_ISL_702382, EPI_ISL_702383, EPI_ISL_702384, EPI_ISL_702386, EPI_ISL_702387, EPI_ISL_702388, EPI_ISL_702389, EPI_ISL_702390, EPI_ISL_702391, EPI_ISL_702392, EPI_ISL_702393, EPI_ISL_702394, EPI_ISL_702395, EPI_ISL_702396, EPI_ISL_702397, EPI_ISL_702398, EPI_ISL_702399, EPI_ISL_702400, EPI_ISL_702401, EPI_ISL_702402, EPI_ISL_702403, EPI_ISL_702404, EPI_ISL_702405, EPI_ISL_702406, EPI_ISL_702407, EPI_ISL_702408, EPI_ISL_702409, EPI_ISL_702410, EPI_ISL_702412, EPI_ISL_702413, EPI_ISL_702414, EPI_ISL_702415, EPI_ISL_702416, EPI_ISL_702417, EPI_ISL_702418, EPI_ISL_702419, EPI_ISL_702420, EPI_ISL_702421, EPI_ISL_702422, EPI_ISL_702423, EPI_ISL_702424, EPI_ISL_702425, EPI_ISL_702426, EPI_ISL_702427, EPI_ISL_702428, EPI_ISL_702429, EPI_ISL_702430, EPI_ISL_702431, EPI_ISL_702432, EPI_ISL_702433, EPI_ISL_702434, EPI_ISL_702435, EPI_ISL_702436, EPI_ISL_702437, EPI_ISL_702439, EPI_ISL_702440, EPI_ISL_702441, EPI_ISL_702442, EPI_ISL_702443, EPI_ISL_702444, EPI_ISL_702445, EPI_ISL_702446, EPI_ISL_702447, EPI_ISL_702448, EPI_ISL_702449, EPI_ISL_702450, EPI_ISL_702451, EPI_ISL_702452, EPI_ISL_702453, EPI_ISL_702454, EPI_ISL_702455, EPI_ISL_702456, EPI_ISL_702457, EPI_ISL_702458, EPI_ISL_702459, EPI_ISL_702460, EPI_ISL_702461, EPI_ISL_702462, EPI_ISL_702463, EPI_ISL_702465, EPI_ISL_702466, EPI_ISL_702467, EPI_ISL_702468, EPI_ISL_702470, EPI_ISL_702472, EPI_ISL_702473, EPI_ISL_702475, EPI_ISL_702477, EPI_ISL_702479, EPI_ISL_702480, EPI_ISL_702482, EPI_ISL_702483, EPI_ISL_702485, EPI_ISL_702486, EPI_ISL_702488, EPI_ISL_702490, EPI_ISL_702491, EPI_ISL_702493, EPI_ISL_702494			
see above	Lighthouse Lab in Alderley Park	Wellcome Sanger Institute for the COVID-19 Genomics UK (COG-UK) Consortium	Jacquelyn Wynn, Mairead Hyland, The Lighthouse Lab in Alderley Park and Alex Alderton, Roberto Amato, Sonia Goncalves, Ewan Harrison, David K. Jackson, Ian Johnston, Dominic Kwiatkowski, Cordelia Langford, John Sillitoe on behalf of the Wellcome Sanger Institute COVID-19 Surveillance Team
EPI_ISL_702495	Northumbria University / South Tees Hospitals NHS Foundation Trust / North Cumbria Integrated Care NHS Foundation Trust / North Tees and Hartlepool NHS Foundation Trust / Newcastle Hospitals NHS Foundation Trust	COVID-19 Genomics UK (COG-UK) Consortium	Darren L Smith,Andrew Nelson,Matthew Bashton,Greg R Young,Joshua Loh,John Allan,Mohammad A Tariq,Giles S Holt,Gary Black,Wen C Yew,Lynn Dover,Paul Baker,Steve Liggett,Sarah Essex,Jane Greenaway,Debra Padgett,Clive Graham,Garren Scott,Edward Barton,Emma Swindells,Brendan Payne,Jennifer Collins,Yusri Taha,Gary Eltringham
EPI_ISL_702496, EPI_ISL_702497, EPI_ISL_702499, EPI_ISL_702501, EPI_ISL_702502, EPI_ISL_702503, EPI_ISL_702506, EPI_ISL_702508, EPI_ISL_702509	Lighthouse Lab in Alderley Park	Wellcome Sanger Institute for the COVID-19 Genomics UK (COG-UK) Consortium	Jacquelyn Wynn, Mairead Hyland, The Lighthouse Lab in Alderley Park and Alex Alderton, Roberto Amato, Sonia Goncalves, Ewan Harrison, David K. Jackson, Ian Johnston, Dominic Kwiatkowski, Cordelia Langford, John Sillitoe on behalf of the Wellcome Sanger Institute COVID-19 Surveillance Team
EPI_ISL_702510	Liverpool Clinical Laboratories	COVID-19 Genomics UK (COG-UK) Consortium	Sam Haldenby, Anita Lucaci, Steve Paterson, Julian Hiscox, Alistair Darby, M Almsaud, A Alrezaihi, Muhannad Alruwaili, Stuart D Armstrong, Jones Benjamin, Eleanor G Bentley, Anu Chawla, Jordan J Clark, Angela Cowell, Richard Eccles, Isabel García-Dorival, Matthew Gemmell, Alessandro Gerada, PKF Gilmore, Richard Gregory, Ximeng Han, Catherine Hartley, Margaret Hughes, Miren Iturriza-Gomara, James Johnson, L Luu, Jenifer Manson, Charlotte Nelson, Elaine O'Toole, Cassie Olateju, Rebekah Penrice-Randal , Lucille Rainbow, N.P Randle, Trevor Ian Robinson, Parul Sharma, Ghada T Shawli, James P Stewart, Neil Swainston, Ecaterina Vamos, Joanne Watts, Mark Whitehead
EPI_ISL_702511, EPI_ISL_702513, EPI_ISL_702514, EPI_ISL_702516, EPI_ISL_702517, EPI_ISL_702519, EPI_ISL_702521, EPI_ISL_702522, EPI_ISL_702524, EPI_ISL_702526, EPI_ISL_702527, EPI_ISL_702529, EPI_ISL_702530, EPI_ISL_702532			
see above	Lighthouse Lab in Alderley Park	Wellcome Sanger Institute for the COVID-19 Genomics UK (COG-UK) Consortium	Jacquelyn Wynn, Mairead Hyland, The Lighthouse Lab in Alderley Park and Alex Alderton, Roberto Amato, Sonia Goncalves, Ewan Harrison, David K. Jackson, Ian Johnston, Dominic Kwiatkowski, Cordelia Langford, John Sillitoe on behalf of the Wellcome Sanger Institute COVID-19 Surveillance Team
EPI_ISL_702533	Northumbria University / South Tees Hospitals NHS Foundation Trust / North Cumbria Integrated Care NHS Foundation Trust / North Tees and Hartlepool NHS Foundation Trust / Newcastle Hospitals NHS Foundation Trust	COVID-19 Genomics UK (COG-UK) Consortium	Darren L Smith,Andrew Nelson,Matthew Bashton,Greg R Young,Joshua Loh,John Allan,Mohammad A Tariq,Giles S Holt,Gary Black,Wen C Yew,Lynn Dover,Paul Baker,Steve Liggett,Sarah Essex,Jane Greenaway,Debra Padgett,Clive Graham,Garren Scott,Edward Barton,Emma Swindells,Brendan Payne,Jennifer Collins,Yusri Taha,Gary Eltringham
EPI_ISL_702534, EPI_ISL_702535, EPI_ISL_702537, EPI_ISL_702538	Lighthouse Lab in Alderley Park	Wellcome Sanger Institute for the COVID-19 Genomics UK (COG-UK) Consortium	Jacquelyn Wynn, Mairead Hyland, The Lighthouse Lab in Alderley Park and Alex Alderton, Roberto Amato, Sonia Goncalves, Ewan Harrison, David K. Jackson, Ian Johnston, Dominic Kwiatkowski, Cordelia Langford, John Sillitoe on behalf of the Wellcome Sanger Institute COVID-19 Surveillance Team
EPI_ISL_702539	Northumbria University / South Tees Hospitals NHS Foundation Trust / North Cumbria Integrated Care NHS Foundation Trust / North Tees and Hartlepool NHS Foundation Trust / Newcastle Hospitals NHS Foundation Trust	COVID-19 Genomics UK (COG-UK) Consortium	Darren L Smith,Andrew Nelson,Matthew Bashton,Greg R Young,Joshua Loh,John Allan,Mohammad A Tariq,Giles S Holt,Gary Black,Wen C Yew,Lynn Dover,Paul Baker,Steve Liggett,Sarah Essex,Jane Greenaway,Debra Padgett,Clive Graham,Garren Scott,Edward Barton,Emma Swindells,Brendan Payne,Jennifer Collins,Yusri Taha,Gary Eltringham
EPI_ISL_702540, EPI_ISL_702542, EPI_ISL_702543	Lighthouse Lab in Alderley Park	Wellcome Sanger Institute for the COVID-19 Genomics UK (COG-UK) Consortium	Jacquelyn Wynn, Mairead Hyland, The Lighthouse Lab in Alderley Park and Alex Alderton, Roberto Amato, Sonia Goncalves, Ewan Harrison, David K. Jackson, Ian Johnston, Dominic Kwiatkowski, Cordelia Langford, John Sillitoe on behalf of the Wellcome Sanger Institute COVID-19 Surveillance Team
EPI_ISL_702544	University College London, Great Ormond Street Hospital for Children NHS Foundation Trust, Imperial College Healthcare NHS Trust	COVID-19 Genomics UK (COG-UK) Consortium	Sergi Castellano, Rachel Williams, Mark Kristiansen, Paola Resende Silva, Sunando Roy, Tony Brooks, Helena Tutill, Paola Niola, Patricia Dyal, Charlotte Williams, Leysa Forrest, Yasmin Panchbhaya, Jacqueline Findlay, Samuel Weeks, Julianne Brown, Kathryn Harris, Paul Randell, James Price, Alison Holmes, Judith Breuer
EPI_ISL_702545, EPI_ISL_702546	Lighthouse Lab in Alderley Park	Wellcome Sanger Institute for the COVID-19 Genomics UK (COG-UK) Consortium	Jacquelyn Wynn, Mairead Hyland, The Lighthouse Lab in Alderley Park and Alex Alderton, Roberto Amato, Sonia Goncalves, Ewan Harrison, David K. Jackson, Ian Johnston, Dominic Kwiatkowski, Cordelia Langford, John Sillitoe on behalf of the Wellcome Sanger Institute COVID-19 Surveillance Team

EPI_ISL_702547	Northumbria University / South Tees Hospitals NHS Foundation Trust / North Cumbria Integrated Care NHS Foundation Trust / North Tees and Hartlepool NHS Foundation Trust / Newcastle Hospitals NHS Foundation Trust	COVID-19 Genomics UK (COG-UK) Consortium	Darren L Smith,Andrew Nelson,Matthew Bashton,Greg R Young,Joshua Loh,John Allan,Mohammad A Tariq,Giles S Holt,Gary Black,Wen C Yew,Lynn Dover,Paul Baker,Steve Liggett,Sarah Essex,Jane Greenaway,Debra Padgett,Clive Graham,Garren Scott,Edward Barton,Emma Swindells,Brendan Payne,Jennifer Collins,Yusri Taha,Gary Eltringham
EPI_ISL_702548, EPI_ISL_702551, EPI_ISL_702552	Lighthouse Lab in Alderley Park	Wellcome Sanger Institute for the COVID-19 Genomics UK (COG-UK) Consortium	Jacquelyn Wynn, Mairead Hyland, The Lighthouse Lab in Alderley Park and Alex Alderton, Roberto Amato, Sonia Goncalves, Ewan Harrison, David K. Jackson, Ian Johnston, Dominic Kwiatkowski, Cordelia Langford, John Sillitoe on behalf of the Wellcome Sanger Institute COVID-19 Surveillance Team
EPI_ISL_702553	Wales Specialist Virology Centre Sequencing lab: Pathogen Genomics Unit	COVID-19 Genomics UK (COG-UK) Consortium	Catherine Moore, Johnathan Evans, Laura Gifford, Malorie Perry, Simon Cottrell, Angela Marchbank, Alec Birchley, Alexander Adams, Amy Gaskin, Bree Gatica-Wilcox, Jason Coombes, Joel Southgate, Lauren Gilbert, Lee Graham, Nicole Pacchiarini, Sara Kumziene-Summerhayes, Sarah Taylor, Sophie Jones, Sara Rey, Matthew Bull, Joanne Watkins, Sally Corden, Tom Connor
EPI_ISL_702554, EPI_ISL_702555, EPI_ISL_702557, EPI_ISL_702559	Lighthouse Lab in Alderley Park	Wellcome Sanger Institute for the COVID-19 Genomics UK (COG-UK) Consortium	Jacquelyn Wynn, Mairead Hyland, The Lighthouse Lab in Alderley Park and Alex Alderton, Roberto Amato, Sonia Goncalves, Ewan Harrison, David K. Jackson, Ian Johnston, Dominic Kwiatkowski, Cordelia Langford, John Sillitoe on behalf of the Wellcome Sanger Institute COVID-19 Surveillance Team
EPI_ISL_702561	Department of Pathology, University of Cambridge	COVID-19 Genomics UK (COG-UK) Consortium	Aminu S. Jahun, Yasmin Chaudhry, Grant Hall, Iliana Georgana, Myra Hosmillo, Martin D. Curran, Malte Pinckert, Surendra Parmar, Ian Goodfellow
EPI_ISL_702562	University of Exeter	COVID-19 Genomics UK (COG-UK) Consortium	Ben Temperton,Aaron Jeffries,Michelle Michelsen,Joanna Warwick-Dugdale,Audrey Farbos,Robyn Manley,Stephen Michell,Jane Masoli
EPI_ISL_702563, EPI_ISL_702567, EPI_ISL_702568, EPI_ISL_702570	Lighthouse Lab in Alderley Park	Wellcome Sanger Institute for the COVID-19 Genomics UK (COG-UK) Consortium	Jacquelyn Wynn, Mairead Hyland, The Lighthouse Lab in Alderley Park and Alex Alderton, Roberto Amato, Sonia Goncalves, Ewan Harrison, David K. Jackson, Ian Johnston, Dominic Kwiatkowski, Cordelia Langford, John Sillitoe on behalf of the Wellcome Sanger Institute COVID-19 Surveillance Team
EPI_ISL_702571	Liverpool Clinical Laboratories	COVID-19 Genomics UK (COG-UK) Consortium	Sam Haldenby, Anita Lucaci, Steve Paterson, Julian Hiscox, Alistair Darby, M Almsaud, A Alrezaihi, Muhannad Alruwaili, Stuart D Armstrong, Jones Benjamin, Eleanor G Bentley, Anu Chawla, Jordan J Clark, Angela Cowell, Richard Eccles, Isabel García-Dorival, Matthew Gemmell, Alessandro Gerada, PKF Gilmore, Richard Gregory, Ximeng Han, Catherine Hartley, Margaret Hughes, Miren Iturriza-Gomara, James Johnson, L Luu, Jenifer Manson, Charlotte Nelson, Elaine O'Toole, Cassie Olateju, Rebekah Penrice-Randal , Lucille Rainbow, N.P Randle, Trevor Ian Robinson, Parul Sharma, Ghada T Shawli, James P Stewart, Neil Swainston, Ecaterina Vamos, Joanne Watts, Mark Whitehead
EPI_ISL_702572, EPI_ISL_702573, EPI_ISL_702575, EPI_ISL_702576, EPI_ISL_702578, EPI_ISL_702580, EPI_ISL_702581, EPI_ISL_702583, EPI_ISL_702584, EPI_ISL_702586, EPI_ISL_702588, EPI_ISL_702589			
see above	Lighthouse Lab in Alderley Park	Wellcome Sanger Institute for the COVID-19 Genomics UK (COG-UK) Consortium	Jacquelyn Wynn, Mairead Hyland, The Lighthouse Lab in Alderley Park and Alex Alderton, Roberto Amato, Sonia Goncalves, Ewan Harrison, David K. Jackson, Ian Johnston, Dominic Kwiatkowski, Cordelia Langford, John Sillitoe on behalf of the Wellcome Sanger Institute COVID-19 Surveillance Team
EPI_ISL_702591	Liverpool Clinical Laboratories	COVID-19 Genomics UK (COG-UK) Consortium	Sam Haldenby, Anita Lucaci, Steve Paterson, Julian Hiscox, Alistair Darby, M Almsaud, A Alrezaihi, Muhannad Alruwaili, Stuart D Armstrong, Jones Benjamin, Eleanor G Bentley, Anu Chawla, Jordan J Clark, Angela Cowell, Richard Eccles, Isabel García-Dorival, Matthew Gemmell, Alessandro Gerada, PKF Gilmore, Richard Gregory, Ximeng Han, Catherine Hartley, Margaret Hughes, Miren Iturriza-Gomara, James Johnson, L Luu, Jenifer Manson, Charlotte Nelson, Elaine O'Toole, Cassie Olateju, Rebekah Penrice-Randal , Lucille Rainbow, N.P Randle, Trevor Ian Robinson, Parul Sharma, Ghada T Shawli, James P Stewart, Neil Swainston, Ecaterina Vamos, Joanne Watts, Mark Whitehead
EPI_ISL_702592, EPI_ISL_702593, EPI_ISL_702595, EPI_ISL_702596, EPI_ISL_702598, EPI_ISL_702599, EPI_ISL_702601, EPI_ISL_702603, EPI_ISL_702604, EPI_ISL_702606, EPI_ISL_702608, EPI_ISL_702609			
see above	Lighthouse Lab in Alderley Park	Wellcome Sanger Institute for the COVID-19 Genomics UK (COG-UK) Consortium	Jacquelyn Wynn, Mairead Hyland, The Lighthouse Lab in Alderley Park and Alex Alderton, Roberto Amato, Sonia Goncalves, Ewan Harrison, David K. Jackson, Ian Johnston, Dominic Kwiatkowski, Cordelia Langford, John Sillitoe on behalf of the Wellcome Sanger Institute COVID-19 Surveillance Team
EPI_ISL_702610	University College London, Great Ormond Street Hospital for Children NHS Foundation Trust, Imperial College Healthcare NHS Trust	COVID-19 Genomics UK (COG-UK) Consortium	Sergi Castellano, Rachel Williams, Mark Kristiansen, Paola Resende Silva, Sunando Roy, Tony Brooks, Helena Tutill, Paola Niola, Patricia Dyal, Charlotte Williams, Leysa Forrest, Yasmin Panchbhaya, Jacqueline Findlay, Samuel Weeks, Julianne Brown, Kathryn Harris, Paul Randell, James Price, Alison Holmes, Judith Breuer
EPI_ISL_702611	Lighthouse Lab in Alderley Park	Wellcome Sanger Institute for the COVID-19 Genomics UK (COG-UK) Consortium	Jacquelyn Wynn, Mairead Hyland, The Lighthouse Lab in Alderley Park and Alex Alderton, Roberto Amato, Sonia Goncalves, Ewan Harrison, David K. Jackson, Ian Johnston, Dominic Kwiatkowski, Cordelia Langford, John Sillitoe on behalf of the Wellcome Sanger Institute COVID-19 Surveillance Team
EPI_ISL_702612	Liverpool Clinical Laboratories	COVID-19 Genomics UK (COG-UK) Consortium	Sam Haldenby, Anita Lucaci, Steve Paterson, Julian Hiscox, Alistair Darby, M Almsaud, A Alrezaihi, Muhannad Alruwaili, Stuart D Armstrong, Jones Benjamin, Eleanor G Bentley, Anu Chawla, Jordan J Clark, Angela Cowell, Richard Eccles, Isabel García-Dorival, Matthew Gemmell, Alessandro Gerada, PKF Gilmore, Richard Gregory, Ximeng Han, Catherine Hartley, Margaret Hughes, Miren Iturriza-Gomara, James Johnson, L Luu, Jenifer Manson, Charlotte Nelson, Elaine O'Toole, Cassie Olateju, Rebekah Penrice-Randal , Lucille Rainbow, N.P Randle, Trevor Ian Robinson, Parul Sharma, Ghada T Shawli, James P Stewart, Neil Swainston, Ecaterina Vamos, Joanne Watts, Mark Whitehead
EPI_ISL_702613, EPI_ISL_702614	Lighthouse Lab in Alderley Park	Wellcome Sanger Institute for the COVID-19 Genomics UK (COG-UK) Consortium	Jacquelyn Wynn, Mairead Hyland, The Lighthouse Lab in Alderley Park and Alex Alderton, Roberto Amato, Sonia Goncalves, Ewan Harrison, David K. Jackson, Ian Johnston, Dominic Kwiatkowski, Cordelia Langford, John Sillitoe on behalf of the Wellcome Sanger Institute COVID-19 Surveillance Team
EPI_ISL_702615	Liverpool Clinical Laboratories	COVID-19 Genomics UK (COG-UK) Consortium	Sam Haldenby, Anita Lucaci, Steve Paterson, Julian Hiscox, Alistair Darby, M Almsaud, A Alrezaihi, Muhannad Alruwaili, Stuart D Armstrong, Jones Benjamin, Eleanor G Bentley, Anu Chawla, Jordan J Clark, Angela Cowell, Richard Eccles, Isabel García-Dorival, Matthew Gemmell, Alessandro Gerada, PKF Gilmore, Richard Gregory, Ximeng Han, Catherine Hartley, Margaret Hughes, Miren Iturriza-Gomara, James Johnson, L Luu, Jenifer Manson, Charlotte Nelson, Elaine O'Toole, Cassie Olateju, Rebekah Penrice-Randal , Lucille Rainbow, N.P Randle, Trevor Ian Robinson, Parul Sharma, Ghada T Shawli, James P Stewart, Neil Swainston, Ecaterina Vamos, Joanne Watts, Mark Whitehead
EPI_ISL_702616, EPI_ISL_702617, EPI_ISL_702619, EPI_ISL_702621, EPI_ISL_702622	Lighthouse Lab in Alderley Park	Wellcome Sanger Institute for the COVID-19 Genomics UK (COG-UK) Consortium	Jacquelyn Wynn, Mairead Hyland, The Lighthouse Lab in Alderley Park and Alex Alderton, Roberto Amato, Sonia Goncalves, Ewan Harrison, David K. Jackson, Ian Johnston, Dominic Kwiatkowski, Cordelia Langford, John Sillitoe on behalf of the Wellcome Sanger Institute COVID-19 Surveillance Team
EPI_ISL_702623	Liverpool Clinical Laboratories	COVID-19 Genomics UK (COG-UK) Consortium	Sam Haldenby, Anita Lucaci, Steve Paterson, Julian Hiscox, Alistair Darby, M Almsaud, A Alrezaihi, Muhannad Alruwaili, Stuart D Armstrong, Jones Benjamin, Eleanor G Bentley, Anu Chawla, Jordan J Clark, Angela Cowell, Richard Eccles, Isabel García-Dorival, Matthew Gemmell, Alessandro Gerada, PKF Gilmore, Richard Gregory, Ximeng Han, Catherine Hartley, Margaret Hughes, Miren Iturriza-Gomara, James Johnson, L Luu, Jenifer Manson, Charlotte Nelson, Elaine O'Toole, Cassie Olateju, Rebekah Penrice-Randal , Lucille Rainbow, N.P Randle, Trevor Ian Robinson, Parul Sharma, Ghada T Shawli, James P Stewart, Neil Swainston, Ecaterina Vamos, Joanne Watts, Mark Whitehead
EPI_ISL_702624, EPI_ISL_702625, EPI_ISL_702627	Lighthouse Lab in Alderley Park	Wellcome Sanger Institute for the COVID-19 Genomics UK (COG-UK) Consortium	Jacquelyn Wynn, Mairead Hyland, The Lighthouse Lab in Alderley Park and Alex Alderton, Roberto Amato, Sonia Goncalves, Ewan Harrison, David K. Jackson, Ian Johnston, Dominic Kwiatkowski, Cordelia Langford, John Sillitoe on behalf of the Wellcome Sanger Institute COVID-19 Surveillance Team
EPI_ISL_702628	Liverpool Clinical Laboratories	COVID-19 Genomics UK (COG-UK) Consortium	Sam Haldenby, Anita Lucaci, Steve Paterson, Julian Hiscox, Alistair Darby, M Almsaud, A Alrezaihi, Muhannad Alruwaili, Stuart D Armstrong, Jones Benjamin, Eleanor G Bentley, Anu Chawla, Jordan J Clark, Angela Cowell, Richard Eccles, Isabel García-Dorival, Matthew Gemmell, Alessandro Gerada, PKF Gilmore, Richard Gregory, Ximeng Han, Catherine Hartley, Margaret Hughes, Miren Iturriza-Gomara, James Johnson, L Luu, Jenifer Manson, Charlotte Nelson, Elaine O'Toole, Cassie Olateju, Rebekah Penrice-Randal , Lucille Rainbow, N.P Randle, Trevor Ian Robinson, Parul Sharma, Ghada T Shawli, James P Stewart, Neil Swainston, Ecaterina Vamos, Joanne Watts, Mark Whitehead
EPI_ISL_702629, EPI_ISL_702630	Lighthouse Lab in Alderley Park	Wellcome Sanger Institute for the COVID-19 Genomics UK (COG-UK) Consortium	Jacquelyn Wynn, Mairead Hyland, The Lighthouse Lab in Alderley Park and Alex Alderton, Roberto Amato, Sonia Goncalves, Ewan Harrison, David K. Jackson, Ian Johnston, Dominic Kwiatkowski, Cordelia Langford, John Sillitoe on behalf of the Wellcome Sanger Institute COVID-19 Surveillance Team
EPI_ISL_702631	Liverpool Clinical Laboratories	COVID-19 Genomics UK (COG-UK) Consortium	Sam Haldenby, Anita Lucaci, Steve Paterson, Julian Hiscox, Alistair Darby, M Almsaud, A Alrezaihi, Muhannad Alruwaili, Stuart D Armstrong, Jones Benjamin, Eleanor G Bentley, Anu Chawla, Jordan J Clark, Angela Cowell, Richard Eccles, Isabel García-Dorival, Matthew Gemmell, Alessandro Gerada, PKF Gilmore, Richard Gregory, Ximeng Han, Catherine Hartley, Margaret Hughes, Miren Iturriza-Gomara, James Johnson, L Luu, Jenifer Manson, Charlotte Nelson, Elaine O'Toole, Cassie Olateju, Rebekah Penrice-Randal , Lucille Rainbow, N.P Randle, Trevor Ian Robinson, Parul Sharma, Ghada T Shawli, James P Stewart, Neil Swainston, Ecaterina Vamos, Joanne Watts, Mark Whitehead
EPI_ISL_702632, EPI_ISL_702634, EPI_ISL_702635, EPI_ISL_702637, EPI_ISL_702638	Lighthouse Lab in Alderley Park	Wellcome Sanger Institute for the COVID-19 Genomics UK (COG-UK) Consortium	Jacquelyn Wynn, Mairead Hyland, The Lighthouse Lab in Alderley Park and Alex Alderton, Roberto Amato, Sonia Goncalves, Ewan Harrison, David K. Jackson, Ian Johnston, Dominic Kwiatkowski, Cordelia Langford, John Sillitoe on behalf of the Wellcome Sanger Institute COVID-19 Surveillance Team
EPI_ISL_702639	Liverpool Clinical Laboratories	COVID-19 Genomics UK (COG-UK) Consortium	Sam Haldenby, Anita Lucaci, Steve Paterson, Julian Hiscox, Alistair Darby, M Almsaud, A Alrezaihi, Muhannad Alruwaili, Stuart D Armstrong, Jones Benjamin, Eleanor G Bentley, Anu Chawla, Jordan J Clark, Angela Cowell, Richard Eccles, Isabel García-Dorival, Matthew Gemmell, Alessandro Gerada,

			PKF Gilmore, Richard Gregory, Ximeng Han, Catherine Hartley, Margaret Hughes, Miren Iturriza-Gomara, James Johnson, L Luu, Jenifer Manson, Charlotte Nelson, Elaine O'Toole, Cassie Olateju, Rebekah Penrice-Randal , Lucille Rainbow, N.P Randle, Trevor Ian Robinson, Parul Sharma, Ghada T Shawli, James P Stewart, Neil Swainston, Ecaterina Vamos, Joanne Watts, Mark Whitehead
EPI_ISL_702640, EPI_ISL_702641	Lighthouse Lab in Alderley Park	Wellcome Sanger Institute for the COVID-19 Genomics UK (COG-UK) Consortium	Jacquelyn Wynn, Mairead Hyland, The Lighthouse Lab in Alderley Park and Alex Alderton, Roberto Amato, Sonia Goncalves, Ewan Harrison, David K. Jackson, Ian Johnston, Dominic Kwiatkowski, Cordelia Langford, John Sillitoe on behalf of the Wellcome Sanger Institute COVID-19 Surveillance Team
EPI_ISL_702642	Liverpool Clinical Laboratories	COVID-19 Genomics UK (COG-UK) Consortium	Sam Haldenby, Anita Lucaci, Steve Paterson, Julian Hiscox, Alistair Darby, M Almsaud, A Alrezaihi, Muhannad Alruwaili, Stuart D Armstrong, Jones Benjamin, Eleanor G Bentley, Anu Chawla, Jordan J Clark, Angela Cowell, Richard Eccles, Isabel García-Dorival, Matthew Gemmell, Alessandro Gerada, PKF Gilmore, Richard Gregory, Ximeng Han, Catherine Hartley, Margaret Hughes, Miren Iturriza-Gomara, James Johnson, L Luu, Jenifer Manson, Charlotte Nelson, Elaine O'Toole, Cassie Olateju, Rebekah Penrice-Randal , Lucille Rainbow, N.P Randle, Trevor Ian Robinson, Parul Sharma, Ghada T Shawli, James P Stewart, Neil Swainston, Ecaterina Vamos, Joanne Watts, Mark Whitehead
EPI_ISL_702643, EPI_ISL_702645, EPI_ISL_702646, EPI_ISL_702648	Lighthouse Lab in Alderley Park	Wellcome Sanger Institute for the COVID-19 Genomics UK (COG-UK) Consortium	Jacquelyn Wynn, Mairead Hyland, The Lighthouse Lab in Alderley Park and Alex Alderton, Roberto Amato, Sonia Goncalves, Ewan Harrison, David K. Jackson, Ian Johnston, Dominic Kwiatkowski, Cordelia Langford, John Sillitoe on behalf of the Wellcome Sanger Institute COVID-19 Surveillance Team
EPI_ISL_702649	Liverpool Clinical Laboratories	COVID-19 Genomics UK (COG-UK) Consortium	Sam Haldenby, Anita Lucaci, Steve Paterson, Julian Hiscox, Alistair Darby, M Almsaud, A Alrezaihi, Muhannad Alruwaili, Stuart D Armstrong, Jones Benjamin, Eleanor G Bentley, Anu Chawla, Jordan J Clark, Angela Cowell, Richard Eccles, Isabel García-Dorival, Matthew Gemmell, Alessandro Gerada, PKF Gilmore, Richard Gregory, Ximeng Han, Catherine Hartley, Margaret Hughes, Miren Iturriza-Gomara, James Johnson, L Luu, Jenifer Manson, Charlotte Nelson, Elaine O'Toole, Cassie Olateju, Rebekah Penrice-Randal , Lucille Rainbow, N.P Randle, Trevor Ian Robinson, Parul Sharma, Ghada T Shawli, James P Stewart, Neil Swainston, Ecaterina Vamos, Joanne Watts, Mark Whitehead
EPI_ISL_702650, EPI_ISL_702651, EPI_ISL_702653, EPI_ISL_702654, EPI_ISL_702657, EPI_ISL_702658	Lighthouse Lab in Alderley Park	Wellcome Sanger Institute for the COVID-19 Genomics UK (COG-UK) Consortium	Jacquelyn Wynn, Mairead Hyland, The Lighthouse Lab in Alderley Park and Alex Alderton, Roberto Amato, Sonia Goncalves, Ewan Harrison, David K. Jackson, Ian Johnston, Dominic Kwiatkowski, Cordelia Langford, John Sillitoe on behalf of the Wellcome Sanger Institute COVID-19 Surveillance Team
EPI_ISL_702659	West of Scotland Specialist Virology Centre, NHSGGC / MRC-University of Glasgow Centre for Virus Research	COVID-19 Genomics UK (COG-UK) Consortium	Ana da Silva Filipe, Natasha Johnson, Kathy Smollett, Daniel Mair, Stephen Carmichael, Alice Broos, Lily Tong, Jenna Nichols, Kyriaki Nomikou; Sarah McDonald; Richard Orton, Joseph Hughes, Sreenu Vattipally, David L Robertson; Alasdair MacLean, Rory Gunson; Sharif Shaaban, Matthew Holden; Rachel Blacow, Guy Mollett, Kathy Li, James Shepherd, Antonia Ho, Emma Thomson
EPI_ISL_702660, EPI_ISL_702662, EPI_ISL_702663, EPI_ISL_702665, EPI_ISL_702666, EPI_ISL_702668, EPI_ISL_702670, EPI_ISL_702671, EPI_ISL_702673, EPI_ISL_702675, EPI_ISL_702676, EPI_ISL_702678, EPI_ISL_702680, EPI_ISL_702681, EPI_ISL_702683, EPI_ISL_702685, EPI_ISL_702686, EPI_ISL_702688, EPI_ISL_702689, EPI_ISL_702691			
see above	Lighthouse Lab in Alderley Park	Wellcome Sanger Institute for the COVID-19 Genomics UK (COG-UK) Consortium	Jacquelyn Wynn, Mairead Hyland, The Lighthouse Lab in Alderley Park and Alex Alderton, Roberto Amato, Sonia Goncalves, Ewan Harrison, David K. Jackson, Ian Johnston, Dominic Kwiatkowski, Cordelia Langford, John Sillitoe on behalf of the Wellcome Sanger Institute COVID-19 Surveillance Team
EPI_ISL_702692	Wales Specialist Virology Centre Sequencing lab: Pathogen Genomics Unit	COVID-19 Genomics UK (COG-UK) Consortium	Catherine Moore, Johnathan Evans, Laura Gifford, Malorie Perry, Simon Cottrell, Angela Marchbank, Alec Birchley, Alexander Adams, Amy Gaskin, Bree Gatica-Wilcox, Jason Coombes, Joel Southgate, Lauren Gilbert, Lee Graham, Nicole Pacchiarini, Sara Kumziene-Summerhayes, Sarah Taylor, Sophie Jones, Sara Rey, Matthew Bull, Joanne Watkins, Sally Corden, Tom Connor
EPI_ISL_702693, EPI_ISL_702695, EPI_ISL_702696, EPI_ISL_702699, EPI_ISL_702701, EPI_ISL_702702, EPI_ISL_702704, EPI_ISL_702706, EPI_ISL_702707, EPI_ISL_702709, EPI_ISL_702711, EPI_ISL_702712			
see above	Lighthouse Lab in Alderley Park	Wellcome Sanger Institute for the COVID-19 Genomics UK (COG-UK) Consortium	Jacquelyn Wynn, Mairead Hyland, The Lighthouse Lab in Alderley Park and Alex Alderton, Roberto Amato, Sonia Goncalves, Ewan Harrison, David K. Jackson, Ian Johnston, Dominic Kwiatkowski, Cordelia Langford, John Sillitoe on behalf of the Wellcome Sanger Institute COVID-19 Surveillance Team
EPI_ISL_702713	Wales Specialist Virology Centre Sequencing lab: Pathogen Genomics Unit	COVID-19 Genomics UK (COG-UK) Consortium	Catherine Moore, Johnathan Evans, Laura Gifford, Malorie Perry, Simon Cottrell, Angela Marchbank, Alec Birchley, Alexander Adams, Amy Gaskin, Bree Gatica-Wilcox, Jason Coombes, Joel Southgate, Lauren Gilbert, Lee Graham, Nicole Pacchiarini, Sara Kumziene-Summerhayes, Sarah Taylor, Sophie Jones, Sara Rey, Matthew Bull, Joanne Watkins, Sally Corden, Tom Connor
EPI_ISL_702714, EPI_ISL_702715, EPI_ISL_702717, EPI_ISL_702719, EPI_ISL_702720, EPI_ISL_702722, EPI_ISL_702724, EPI_ISL_702725, EPI_ISL_702727, EPI_ISL_702728	Lighthouse Lab in Alderley Park	Wellcome Sanger Institute for the COVID-19 Genomics UK (COG-UK) Consortium	Jacquelyn Wynn, Mairead Hyland, The Lighthouse Lab in Alderley Park and Alex Alderton, Roberto Amato, Sonia Goncalves, Ewan Harrison, David K. Jackson, Ian Johnston, Dominic Kwiatkowski, Cordelia Langford, John Sillitoe on behalf of the Wellcome Sanger Institute COVID-19 Surveillance Team
EPI_ISL_702729	Liverpool Clinical Laboratories	COVID-19 Genomics UK (COG-UK) Consortium	Sam Haldenby, Anita Lucaci, Steve Paterson, Julian Hiscox, Alistair Darby, M Almsaud, A Alrezaihi, Muhannad Alruwaili, Stuart D Armstrong, Jones Benjamin, Eleanor G Bentley, Anu Chawla, Jordan J Clark, Angela Cowell, Richard Eccles, Isabel García-Dorival, Matthew Gemmell, Alessandro Gerada, PKF Gilmore, Richard Gregory, Ximeng Han, Catherine Hartley, Margaret Hughes, Miren Iturriza-Gomara, James Johnson, L Luu, Jenifer Manson, Charlotte Nelson, Elaine O'Toole, Cassie Olateju, Rebekah Penrice-Randal , Lucille Rainbow, N.P Randle, Trevor Ian Robinson, Parul Sharma, Ghada T Shawli, James P Stewart, Neil Swainston, Ecaterina Vamos, Joanne Watts, Mark Whitehead
EPI_ISL_702730	Lighthouse Lab in Alderley Park	Wellcome Sanger Institute for the COVID-19 Genomics UK (COG-UK) Consortium	Jacquelyn Wynn, Mairead Hyland, The Lighthouse Lab in Alderley Park and Alex Alderton, Roberto Amato, Sonia Goncalves, Ewan Harrison, David K. Jackson, Ian Johnston, Dominic Kwiatkowski, Cordelia Langford, John Sillitoe on behalf of the Wellcome Sanger Institute COVID-19 Surveillance Team
EPI_ISL_702731	University College London, Great Ormond Street Hospital for Children NHS Foundation Trust, Imperial College Healthcare NHS Trust	COVID-19 Genomics UK (COG-UK) Consortium	Sergi Castellano, Rachel Williams, Mark Kristiansen, Paola Resende Silva, Sunando Roy, Tony Brooks, Helena Tutill, Paola Niola, Patricia Dyal, Charlotte Williams, Leysa Forrest, Yasmin Panchbhaya, Jacqueline Findlay, Samuel Weeks, Julianne Brown, Kathryn Harris, Paul Randell, James Price, Alison Holmes, Judith Breuer
EPI_ISL_702732, EPI_ISL_702733, EPI_ISL_702735, EPI_ISL_702737, EPI_ISL_702738, EPI_ISL_702740, EPI_ISL_702741	Lighthouse Lab in Alderley Park	Wellcome Sanger Institute for the COVID-19 Genomics UK (COG-UK) Consortium	Jacquelyn Wynn, Mairead Hyland, The Lighthouse Lab in Alderley Park and Alex Alderton, Roberto Amato, Sonia Goncalves, Ewan Harrison, David K. Jackson, Ian Johnston, Dominic Kwiatkowski, Cordelia Langford, John Sillitoe on behalf of the Wellcome Sanger Institute COVID-19 Surveillance Team
EPI_ISL_702742	Wales Specialist Virology Centre Sequencing lab: Pathogen Genomics Unit	COVID-19 Genomics UK (COG-UK) Consortium	Catherine Moore, Johnathan Evans, Laura Gifford, Malorie Perry, Simon Cottrell, Angela Marchbank, Alec Birchley, Alexander Adams, Amy Gaskin, Bree Gatica-Wilcox, Jason Coombes, Joel Southgate, Lauren Gilbert, Lee Graham, Nicole Pacchiarini, Sara Kumziene-Summerhayes, Sarah Taylor, Sophie Jones, Sara Rey, Matthew Bull, Joanne Watkins, Sally Corden, Tom Connor
EPI_ISL_702743, EPI_ISL_702745, EPI_ISL_702746	Lighthouse Lab in Alderley Park	Wellcome Sanger Institute for the COVID-19 Genomics UK (COG-UK) Consortium	Jacquelyn Wynn, Mairead Hyland, The Lighthouse Lab in Alderley Park and Alex Alderton, Roberto Amato, Sonia Goncalves, Ewan Harrison, David K. Jackson, Ian Johnston, Dominic Kwiatkowski, Cordelia Langford, John Sillitoe on behalf of the Wellcome Sanger Institute COVID-19 Surveillance Team
EPI_ISL_702747	Northumbria University / South Tees Hospitals NHS Foundation Trust / North Cumbria Integrated Care NHS Foundation Trust / North Tees and Hartlepool NHS Foundation Trust / Newcastle Hospitals NHS Foundation Trust	COVID-19 Genomics UK (COG-UK) Consortium	Darren L Smith,Andrew Nelson,Matthew Bashton,Greg R Young,Joshua Loh,John Allan,Mohammad A Tariq,Giles S Holt,Gary Black,Wen C Yew,Lynn Dover,Paul Baker,Steve Liggett,Sarah Essex,Jane Greenaway,Debra Padgett,Clive Graham,Garren Scott,Edward Barton,Emma Swindells,Brendan Payne,Jennifer Collins,Yusri Taha,Gary Eltringham
EPI_ISL_702748, EPI_ISL_702750, EPI_ISL_702752, EPI_ISL_702754, EPI_ISL_702755, EPI_ISL_702757, EPI_ISL_702759, EPI_ISL_702760	Lighthouse Lab in Alderley Park	Wellcome Sanger Institute for the COVID-19 Genomics UK (COG-UK) Consortium	Jacquelyn Wynn, Mairead Hyland, The Lighthouse Lab in Alderley Park and Alex Alderton, Roberto Amato, Sonia Goncalves, Ewan Harrison, David K. Jackson, Ian Johnston, Dominic Kwiatkowski, Cordelia Langford, John Sillitoe on behalf of the Wellcome Sanger Institute COVID-19 Surveillance Team
EPI_ISL_702761	Virology Department, Sheffield Teaching Hospitals NHS Foundation Trust/Department of Infection, Immunity and Cardiovascular Disease, The Medical School, University of Sheffield	COVID-19 Genomics UK (COG-UK) Consortium	Thushan de Silva, Matthew Parker, Nikki Smith, Adri Angyal, Rebecca Brown, Luke Green, Rachel Tucker, Paul Parsons, Danielle Groves, Katie Johnson, Laura Carrilero, Alex Keeley, Dave Partridge, Matthew Wyles, Benjamin Lindsey, Mehmet Yavuz, Mohammad Raza, Cariad Evans
EPI_ISL_702762, EPI_ISL_702764, EPI_ISL_702765, EPI_ISL_702767, EPI_ISL_702768	Lighthouse Lab in Alderley Park	Wellcome Sanger Institute for the COVID-19 Genomics UK (COG-UK) Consortium	Jacquelyn Wynn, Mairead Hyland, The Lighthouse Lab in Alderley Park and Alex Alderton, Roberto Amato, Sonia Goncalves, Ewan Harrison, David K. Jackson, Ian Johnston, Dominic Kwiatkowski, Cordelia Langford, John Sillitoe on behalf of the Wellcome Sanger Institute COVID-19 Surveillance Team

EPI_ISL_702769	Liverpool Clinical Laboratories	COVID-19 Genomics UK (COG-UK) Consortium	Sam Haldenby, Anita Lucaci, Steve Paterson, Julian Hiscox, Alistair Darby, M Almsaud, A Alrezaihi, Muhannad Alruwaili, Stuart D Armstrong, Jones Benjamin, Eleanor G Bentley, Anu Chawla, Jordan J Clark, Angela Cowell, Richard Eccles, Isabel García-Dorival, Matthew Gemmell, Alessandro Gerada, PKF Gilmore, Richard Gregory, Ximeng Han, Catherine Hartley, Margaret Hughes, Miren Iturriza-Gomara, James Johnson, L Luu, Jenifer Manson, Charlotte Nelson, Elaine O'Toole, Cassie Olateju, Rebekah Penrice-Randal , Lucille Rainbow, N.P Randle, Trevor Ian Robinson, Parul Sharma, Ghada T Shawli, James P Stewart, Neil Swainston, Ecaterina Vamos, Joanne Watts, Mark Whitehead
EPI_ISL_702770, EPI_ISL_702772, EPI_ISL_702773, EPI_ISL_702775, EPI_ISL_702777, EPI_ISL_702778, EPI_ISL_702780, EPI_ISL_702781, EPI_ISL_702783, EPI_ISL_702785, EPI_ISL_702786, EPI_ISL_702788, EPI_ISL_702789, EPI_ISL_702791, EPI_ISL_702793, EPI_ISL_702796, EPI_ISL_702797, EPI_ISL_702799, EPI_ISL_702800			
see above	Lighthouse Lab in Alderley Park	Wellcome Sanger Institute for the COVID-19 Genomics UK (COG-UK) Consortium	Jacquelyn Wynn, Mairead Hyland, The Lighthouse Lab in Alderley Park and Alex Alderton, Roberto Amato, Sonia Goncalves, Ewan Harrison, David K. Jackson, Ian Johnston, Dominic Kwiatkowski, Cordelia Langford, John Sillitoe on behalf of the Wellcome Sanger Institute COVID-19 Surveillance Team
EPI_ISL_702801	Liverpool Clinical Laboratories	COVID-19 Genomics UK (COG-UK) Consortium	Sam Haldenby, Anita Lucaci, Steve Paterson, Julian Hiscox, Alistair Darby, M Almsaud, A Alrezaihi, Muhannad Alruwaili, Stuart D Armstrong, Jones Benjamin, Eleanor G Bentley, Anu Chawla, Jordan J Clark, Angela Cowell, Richard Eccles, Isabel García-Dorival, Matthew Gemmell, Alessandro Gerada, PKF Gilmore, Richard Gregory, Ximeng Han, Catherine Hartley, Margaret Hughes, Miren Iturriza-Gomara, James Johnson, L Luu, Jenifer Manson, Charlotte Nelson, Elaine O'Toole, Cassie Olateju, Rebekah Penrice-Randal , Lucille Rainbow, N.P Randle, Trevor Ian Robinson, Parul Sharma, Ghada T Shawli, James P Stewart, Neil Swainston, Ecaterina Vamos, Joanne Watts, Mark Whitehead
EPI_ISL_702802, EPI_ISL_702804, EPI_ISL_702805, EPI_ISL_702807, EPI_ISL_702808, EPI_ISL_702810, EPI_ISL_702811	Lighthouse Lab in Alderley Park	Wellcome Sanger Institute for the COVID-19 Genomics UK (COG-UK) Consortium	Jacquelyn Wynn, Mairead Hyland, The Lighthouse Lab in Alderley Park and Alex Alderton, Roberto Amato, Sonia Goncalves, Ewan Harrison, David K. Jackson, Ian Johnston, Dominic Kwiatkowski, Cordelia Langford, John Sillitoe on behalf of the Wellcome Sanger Institute COVID-19 Surveillance Team
EPI_ISL_702812	Northumbria University / South Tees Hospitals NHS Foundation Trust / North Cumbria Integrated Care NHS Foundation Trust / North Tees and Hartlepool NHS Foundation Trust / Newcastle Hospitals NHS Foundation Trust	COVID-19 Genomics UK (COG-UK) Consortium	Darren L Smith,Andrew Nelson,Matthew Bashton,Greg R Young,Joshua Loh,John Allan,Mohammad A Tariq,Giles S Holt,Gary Black,Wen C Yew,Lynn Dover,Paul Baker,Steve Liggett,Sarah Essex,Jane Greenaway,Debra Padgett,Clive Graham,Garren Scott,Edward Barton,Emma Swindells,Brendan Payne,Jennifer Collins,Yusri Taha,Gary Eltringham
EPI_ISL_702813, EPI_ISL_702814	Lighthouse Lab in Alderley Park	Wellcome Sanger Institute for the COVID-19 Genomics UK (COG-UK) Consortium	Jacquelyn Wynn, Mairead Hyland, The Lighthouse Lab in Alderley Park and Alex Alderton, Roberto Amato, Sonia Goncalves, Ewan Harrison, David K. Jackson, Ian Johnston, Dominic Kwiatkowski, Cordelia Langford, John Sillitoe on behalf of the Wellcome Sanger Institute COVID-19 Surveillance Team
EPI_ISL_702815	Northumbria University / South Tees Hospitals NHS Foundation Trust / North Cumbria Integrated Care NHS Foundation Trust / North Tees and Hartlepool NHS Foundation Trust / Newcastle Hospitals NHS Foundation Trust	COVID-19 Genomics UK (COG-UK) Consortium	Darren L Smith,Andrew Nelson,Matthew Bashton,Greg R Young,Joshua Loh,John Allan,Mohammad A Tariq,Giles S Holt,Gary Black,Wen C Yew,Lynn Dover,Paul Baker,Steve Liggett,Sarah Essex,Jane Greenaway,Debra Padgett,Clive Graham,Garren Scott,Edward Barton,Emma Swindells,Brendan Payne,Jennifer Collins,Yusri Taha,Gary Eltringham
EPI_ISL_702816	Lighthouse Lab in Alderley Park	Wellcome Sanger Institute for the COVID-19 Genomics UK (COG-UK) Consortium	Jacquelyn Wynn, Mairead Hyland, The Lighthouse Lab in Alderley Park and Alex Alderton, Roberto Amato, Sonia Goncalves, Ewan Harrison, David K. Jackson, Ian Johnston, Dominic Kwiatkowski, Cordelia Langford, John Sillitoe on behalf of the Wellcome Sanger Institute COVID-19 Surveillance Team
EPI_ISL_702817	Wales Specialist Virology Centre Sequencing lab: Pathogen Genomics Unit	COVID-19 Genomics UK (COG-UK) Consortium	Catherine Moore, Johnathan Evans, Laura Gifford, Malorie Perry, Simon Cottrell, Angela Marchbank, Alec Birchley, Alexander Adams, Amy Gaskin, Bree Gatica-Wilcox, Jason Coombes, Joel Southgate, Lauren Gilbert, Lee Graham, Nicole Pacchiarini, Sara Kumziene-Summerhayes, Sarah Taylor, Sophie Jones, Sara Rey, Matthew Bull, Joanne Watkins, Sally Corden, Tom Connor
EPI_ISL_702818, EPI_ISL_702819, EPI_ISL_702821, EPI_ISL_702822	Lighthouse Lab in Alderley Park	Wellcome Sanger Institute for the COVID-19 Genomics UK (COG-UK) Consortium	Jacquelyn Wynn, Mairead Hyland, The Lighthouse Lab in Alderley Park and Alex Alderton, Roberto Amato, Sonia Goncalves, Ewan Harrison, David K. Jackson, Ian Johnston, Dominic Kwiatkowski, Cordelia Langford, John Sillitoe on behalf of the Wellcome Sanger Institute COVID-19 Surveillance Team
EPI_ISL_702823	Northumbria University / South Tees Hospitals NHS Foundation Trust / North Cumbria Integrated Care NHS Foundation Trust / North Tees and Hartlepool NHS Foundation Trust / Newcastle Hospitals NHS Foundation Trust	COVID-19 Genomics UK (COG-UK) Consortium	Darren L Smith,Andrew Nelson,Matthew Bashton,Greg R Young,Joshua Loh,John Allan,Mohammad A Tariq,Giles S Holt,Gary Black,Wen C Yew,Lynn Dover,Paul Baker,Steve Liggett,Sarah Essex,Jane Greenaway,Debra Padgett,Clive Graham,Garren Scott,Edward Barton,Emma Swindells,Brendan Payne,Jennifer Collins,Yusri Taha,Gary Eltringham
EPI_ISL_702826	Liverpool Clinical Laboratories	COVID-19 Genomics UK (COG-UK) Consortium	Sam Haldenby, Anita Lucaci, Steve Paterson, Julian Hiscox, Alistair Darby, M Almsaud, A Alrezaihi, Muhannad Alruwaili, Stuart D Armstrong, Jones Benjamin, Eleanor G Bentley, Anu Chawla, Jordan J Clark, Angela Cowell, Richard Eccles, Isabel García-Dorival, Matthew Gemmell, Alessandro Gerada, PKF Gilmore, Richard Gregory, Ximeng Han, Catherine Hartley, Margaret Hughes, Miren Iturriza-Gomara, James Johnson, L Luu, Jenifer Manson, Charlotte Nelson, Elaine O'Toole, Cassie Olateju, Rebekah Penrice-Randal , Lucille Rainbow, N.P Randle, Trevor Ian Robinson, Parul Sharma, Ghada T Shawli, James P Stewart, Neil Swainston, Ecaterina Vamos, Joanne Watts, Mark Whitehead
EPI_ISL_702827	Northumbria University / South Tees Hospitals NHS Foundation Trust / North Cumbria Integrated Care NHS Foundation Trust / North Tees and Hartlepool NHS Foundation Trust / Newcastle Hospitals NHS Foundation Trust	COVID-19 Genomics UK (COG-UK) Consortium	Darren L Smith,Andrew Nelson,Matthew Bashton,Greg R Young,Joshua Loh,John Allan,Mohammad A Tariq,Giles S Holt,Gary Black,Wen C Yew,Lynn Dover,Paul Baker,Steve Liggett,Sarah Essex,Jane Greenaway,Debra Padgett,Clive Graham,Garren Scott,Edward Barton,Emma Swindells,Brendan Payne,Jennifer Collins,Yusri Taha,Gary Eltringham
EPI_ISL_702838	Liverpool Clinical Laboratories	COVID-19 Genomics UK (COG-UK) Consortium	Sam Haldenby, Anita Lucaci, Steve Paterson, Julian Hiscox, Alistair Darby, M Almsaud, A Alrezaihi, Muhannad Alruwaili, Stuart D Armstrong, Jones Benjamin, Eleanor G Bentley, Anu Chawla, Jordan J Clark, Angela Cowell, Richard Eccles, Isabel García-Dorival, Matthew Gemmell, Alessandro Gerada, PKF Gilmore, Richard Gregory, Ximeng Han, Catherine Hartley, Margaret Hughes, Miren Iturriza-Gomara, James Johnson, L Luu, Jenifer Manson, Charlotte Nelson, Elaine O'Toole, Cassie Olateju, Rebekah Penrice-Randal , Lucille Rainbow, N.P Randle, Trevor Ian Robinson, Parul Sharma, Ghada T Shawli, James P Stewart, Neil Swainston, Ecaterina Vamos, Joanne Watts, Mark Whitehead
EPI_ISL_702844	University of Exeter	COVID-19 Genomics UK (COG-UK) Consortium	Ben Temperton,Aaron Jeffries,Michelle Michelsen,Joanna Warwick-Dugdale,Audrey Farbos,Robyn Manley,Stephen Michell,Jane Masoli
EPI_ISL_702845	Liverpool Clinical Laboratories	COVID-19 Genomics UK (COG-UK) Consortium	Sam Haldenby, Anita Lucaci, Steve Paterson, Julian Hiscox, Alistair Darby, M Almsaud, A Alrezaihi, Muhannad Alruwaili, Stuart D Armstrong, Jones Benjamin, Eleanor G Bentley, Anu Chawla, Jordan J Clark, Angela Cowell, Richard Eccles, Isabel García-Dorival, Matthew Gemmell, Alessandro Gerada, PKF Gilmore, Richard Gregory, Ximeng Han, Catherine Hartley, Margaret Hughes, Miren Iturriza-Gomara, James Johnson, L Luu, Jenifer Manson, Charlotte Nelson, Elaine O'Toole, Cassie Olateju, Rebekah Penrice-Randal , Lucille Rainbow, N.P Randle, Trevor Ian Robinson, Parul Sharma, Ghada T Shawli, James P Stewart, Neil Swainston, Ecaterina Vamos, Joanne Watts, Mark Whitehead
EPI_ISL_702855	Northumbria University / South Tees Hospitals NHS Foundation Trust / North Cumbria Integrated Care NHS Foundation Trust / North Tees and Hartlepool NHS Foundation Trust / Newcastle Hospitals NHS Foundation Trust	COVID-19 Genomics UK (COG-UK) Consortium	Darren L Smith,Andrew Nelson,Matthew Bashton,Greg R Young,Joshua Loh,John Allan,Mohammad A Tariq,Giles S Holt,Gary Black,Wen C Yew,Lynn Dover,Paul Baker,Steve Liggett,Sarah Essex,Jane Greenaway,Debra Padgett,Clive Graham,Garren Scott,Edward Barton,Emma Swindells,Brendan Payne,Jennifer Collins,Yusri Taha,Gary Eltringham
EPI_ISL_702857, EPI_ISL_702859, EPI_ISL_702863, EPI_ISL_702864	Wales Specialist Virology Centre Sequencing lab: Pathogen Genomics Unit	COVID-19 Genomics UK (COG-UK) Consortium	Catherine Moore, Johnathan Evans, Laura Gifford, Malorie Perry, Simon Cottrell, Angela Marchbank, Alec Birchley, Alexander Adams, Amy Gaskin, Bree Gatica-Wilcox, Jason Coombes, Joel Southgate, Lauren Gilbert, Lee Graham, Nicole Pacchiarini, Sara Kumziene-Summerhayes, Sarah Taylor, Sophie Jones, Sara Rey, Matthew Bull, Joanne Watkins, Sally Corden, Tom Connor
EPI_ISL_702867	West of Scotland Specialist Virology Centre, NHSGGC / MRC-University of Glasgow Centre for Virus Research	COVID-19 Genomics UK (COG-UK) Consortium	Ana da Silva Filipe, Natasha Johnson, Kathy Smollett, Daniel Mair, Stephen Carmichael, Alice Broos, Lily Tong, Jenna Nichols, Kyriaki Nomikou; Sarah McDonald; Richard Orton, Joseph Hughes, Sreenu Vattipally, David L Robertson; Alasdair MacLean, Rory Gunson; Sharif Shaaban, Matthew Holden; Rachel Blacow, Guy Mollett, Kathy Li, James Shepherd, Antonia Ho, Emma Thomson
EPI_ISL_702876	Department of Pathology, University of Cambridge	COVID-19 Genomics UK (COG-UK) Consortium	Aminu S. Jahun, Yasmin Chaudhry, Grant Hall, Iliana Georgana, Myra Hosmillo, Martin D. Curran, Malte Pinckert, Surendra Parmar, Ian Goodfellow
EPI_ISL_702882	Quadram Institute Bioscience	COVID-19 Genomics UK (COG-UK) Consortium	Dave J. Baker, Gemma L. Kay, Alp Aydin, Thanh Le-Viet, Steven Rudder, Ana P. Tedim, Anastasia Kolyva, Maria Diaz, Leonardo de Oliveira Martins, Nabil-Fareed Alikhan, Lizzie Meadows, Rachael Stanley, Ngozi Elumogo, Muhammed Yasir, Nicholas M. Thomson, Alexander J Trotter, Rachel Gilroy, Samuel Bloomfield, Claire Stuart, Andrew Bell, Reenesh Prakash, Samir Dervisevic, Alison E. Mather, John Wain, Mark Webber, Andrew J. Page, Justin O'Grady
EPI_ISL_702891	Department of Pathology, University of Cambridge	COVID-19 Genomics UK (COG-UK) Consortium	Aminu S. Jahun, Yasmin Chaudhry, Grant Hall, Iliana Georgana, Myra Hosmillo, Martin D. Curran, Malte Pinckert, Surendra Parmar, Ian Goodfellow
EPI_ISL_702895	Wales Specialist Virology Centre Sequencing lab: Pathogen	COVID-19 Genomics UK (COG-UK) Consortium	Catherine Moore, Johnathan Evans, Laura Gifford, Malorie Perry, Simon Cottrell, Angela Marchbank, Alec Birchley, Alexander Adams, Amy Gaskin, Bree

	Genomics Unit		Gatica-Wilcox, Jason Coombes, Joel Southgate, Lauren Gilbert, Lee Graham, Nicole Pacchiarini, Sara Kumziene-Summerhayes, Sarah Taylor, Sophie Jones, Sara Rey, Matthew Bull, Joanne Watkins, Sally Corden, Tom Connor
EPI_ISL_702898, EPI_ISL_702899, EPI_ISL_702901, EPI_ISL_702903, EPI_ISL_702904	Lighthouse Lab in Alderley Park	Wellcome Sanger Institute for the COVID-19 Genomics UK (COG-UK) Consortium	Jacquelyn Wynn, Mairead Hyland, The Lighthouse Lab in Alderley Park and Alex Alderton, Roberto Amato, Sonia Goncalves, Ewan Harrison, David K. Jackson, Ian Johnston, Dominic Kwiatkowski, Cordelia Langford, John Sillitoe on behalf of the Wellcome Sanger Institute COVID-19 Surveillance Team
EPI_ISL_702905	Wales Specialist Virology Centre Sequencing lab: Pathogen Genomics Unit	COVID-19 Genomics UK (COG-UK) Consortium	Catherine Moore, Johnathan Evans, Laura Gifford, Malorie Perry, Simon Cottrell, Angela Marchbank, Alec Birchley, Alexander Adams, Amy Gaskin, Bree Gatica-Wilcox, Jason Coombes, Joel Southgate, Lauren Gilbert, Lee Graham, Nicole Pacchiarini, Sara Kumziene-Summerhayes, Sarah Taylor, Sophie Jones, Sara Rey, Matthew Bull, Joanne Watkins, Sally Corden, Tom Connor
EPI_ISL_702906	Lighthouse Lab in Alderley Park	Wellcome Sanger Institute for the COVID-19 Genomics UK (COG-UK) Consortium	Jacquelyn Wynn, Mairead Hyland, The Lighthouse Lab in Alderley Park and Alex Alderton, Roberto Amato, Sonia Goncalves, Ewan Harrison, David K. Jackson, Ian Johnston, Dominic Kwiatkowski, Cordelia Langford, John Sillitoe on behalf of the Wellcome Sanger Institute COVID-19 Surveillance Team
EPI_ISL_702907	Liverpool Clinical Laboratories	COVID-19 Genomics UK (COG-UK) Consortium	Sam Haldenby, Anita Lucaci, Steve Paterson, Julian Hiscox, Alistair Darby, M Almsaud, A Alrezaihi, Muhannad Alruwaili, Stuart D Armstrong, Jones Benjamin, Eleanor G Bentley, Anu Chawla, Jordan J Clark, Angela Cowell, Richard Eccles, Isabel García-Dorival, Matthew Gemmell, Alessandro Gerada, PKF Gilmore, Richard Gregory, Ximeng Han, Catherine Hartley, Margaret Hughes, Miren Iturriza-Gomara, James Johnson, L Luu, Jenifer Manson, Charlotte Nelson, Elaine O'Toole, Cassie Olateju, Rebekah Penrice-Randal , Lucille Rainbow, N.P Randle, Trevor Ian Robinson, Parul Sharma, Ghada T Shawli, James P Stewart, Neil Swainston, Ecaterina Vamos, Joanne Watts, Mark Whitehead
EPI_ISL_702908, EPI_ISL_702909, EPI_ISL_702912, EPI_ISL_702914, EPI_ISL_702916, EPI_ISL_702918, EPI_ISL_702919, EPI_ISL_702921, EPI_ISL_702923, EPI_ISL_702924, EPI_ISL_702926, EPI_ISL_702927, EPI_ISL_702929			
see above	Lighthouse Lab in Alderley Park	Wellcome Sanger Institute for the COVID-19 Genomics UK (COG-UK) Consortium	Jacquelyn Wynn, Mairead Hyland, The Lighthouse Lab in Alderley Park and Alex Alderton, Roberto Amato, Sonia Goncalves, Ewan Harrison, David K. Jackson, Ian Johnston, Dominic Kwiatkowski, Cordelia Langford, John Sillitoe on behalf of the Wellcome Sanger Institute COVID-19 Surveillance Team
EPI_ISL_702930	University College London, Great Ormond Street Hospital for Children NHS Foundation Trust, Imperial College Healthcare NHS Trust	COVID-19 Genomics UK (COG-UK) Consortium	Sergi Castellano, Rachel Williams, Mark Kristiansen, Paola Resende Silva, Sunando Roy, Tony Brooks, Helena Tutill, Paola Niola, Patricia Dyal, Charlotte Williams, Leysa Forrest, Yasmin Panchbhaya, Jacqueline Findlay, Samuel Weeks, Julianne Brown, Kathryn Harris, Paul Randell, James Price, Alison Holmes, Judith Breuer
EPI_ISL_702931, EPI_ISL_702933, EPI_ISL_702934	Lighthouse Lab in Alderley Park	Wellcome Sanger Institute for the COVID-19 Genomics UK (COG-UK) Consortium	Jacquelyn Wynn, Mairead Hyland, The Lighthouse Lab in Alderley Park and Alex Alderton, Roberto Amato, Sonia Goncalves, Ewan Harrison, David K. Jackson, Ian Johnston, Dominic Kwiatkowski, Cordelia Langford, John Sillitoe on behalf of the Wellcome Sanger Institute COVID-19 Surveillance Team
EPI_ISL_702935	Liverpool Clinical Laboratories	COVID-19 Genomics UK (COG-UK) Consortium	Sam Haldenby, Anita Lucaci, Steve Paterson, Julian Hiscox, Alistair Darby, M Almsaud, A Alrezaihi, Muhannad Alruwaili, Stuart D Armstrong, Jones Benjamin, Eleanor G Bentley, Anu Chawla, Jordan J Clark, Angela Cowell, Richard Eccles, Isabel García-Dorival, Matthew Gemmell, Alessandro Gerada, PKF Gilmore, Richard Gregory, Ximeng Han, Catherine Hartley, Margaret Hughes, Miren Iturriza-Gomara, James Johnson, L Luu, Jenifer Manson, Charlotte Nelson, Elaine O'Toole, Cassie Olateju, Rebekah Penrice-Randal , Lucille Rainbow, N.P Randle, Trevor Ian Robinson, Parul Sharma, Ghada T Shawli, James P Stewart, Neil Swainston, Ecaterina Vamos, Joanne Watts, Mark Whitehead
EPI_ISL_702936, EPI_ISL_702938, EPI_ISL_702940, EPI_ISL_702942, EPI_ISL_702943, EPI_ISL_702945, EPI_ISL_702946	Lighthouse Lab in Alderley Park	Wellcome Sanger Institute for the COVID-19 Genomics UK (COG-UK) Consortium	Jacquelyn Wynn, Mairead Hyland, The Lighthouse Lab in Alderley Park and Alex Alderton, Roberto Amato, Sonia Goncalves, Ewan Harrison, David K. Jackson, Ian Johnston, Dominic Kwiatkowski, Cordelia Langford, John Sillitoe on behalf of the Wellcome Sanger Institute COVID-19 Surveillance Team
EPI_ISL_702947	Liverpool Clinical Laboratories	COVID-19 Genomics UK (COG-UK) Consortium	Sam Haldenby, Anita Lucaci, Steve Paterson, Julian Hiscox, Alistair Darby, M Almsaud, A Alrezaihi, Muhannad Alruwaili, Stuart D Armstrong, Jones Benjamin, Eleanor G Bentley, Anu Chawla, Jordan J Clark, Angela Cowell, Richard Eccles, Isabel García-Dorival, Matthew Gemmell, Alessandro Gerada, PKF Gilmore, Richard Gregory, Ximeng Han, Catherine Hartley, Margaret Hughes, Miren Iturriza-Gomara, James Johnson, L Luu, Jenifer Manson, Charlotte Nelson, Elaine O'Toole, Cassie Olateju, Rebekah Penrice-Randal , Lucille Rainbow, N.P Randle, Trevor Ian Robinson, Parul Sharma, Ghada T Shawli, James P Stewart, Neil Swainston, Ecaterina Vamos, Joanne Watts, Mark Whitehead
EPI_ISL_702948, EPI_ISL_702951, EPI_ISL_702952, EPI_ISL_702954, EPI_ISL_702955, EPI_ISL_702957, EPI_ISL_702958	Lighthouse Lab in Alderley Park	Wellcome Sanger Institute for the COVID-19 Genomics UK (COG-UK) Consortium	Jacquelyn Wynn, Mairead Hyland, The Lighthouse Lab in Alderley Park and Alex Alderton, Roberto Amato, Sonia Goncalves, Ewan Harrison, David K. Jackson, Ian Johnston, Dominic Kwiatkowski, Cordelia Langford, John Sillitoe on behalf of the Wellcome Sanger Institute COVID-19 Surveillance Team
EPI_ISL_702959	Northumbria University / South Tees Hospitals NHS Foundation Trust / North Cumbria Integrated Care NHS Foundation Trust / North Tees and Hartlepool NHS Foundation Trust / Newcastle Hospitals NHS Foundation Trust	COVID-19 Genomics UK (COG-UK) Consortium	Darren L Smith,Andrew Nelson,Matthew Bashton,Greg R Young,Joshua Loh,John Allan,Mohammad A Tariq,Giles S Holt,Gary Black,Wen C Yew,Lynn Dover,Paul Baker,Steve Liggett,Sarah Essex,Jane Greenaway,Debra Padgett,Clive Graham,Garren Scott,Edward Barton,Emma Swindells,Brendan Payne,Jennifer Collins,Yusri Taha,Gary Eltringham
EPI_ISL_702960, EPI_ISL_702962, EPI_ISL_702963	Lighthouse Lab in Alderley Park	Wellcome Sanger Institute for the COVID-19 Genomics UK (COG-UK) Consortium	Jacquelyn Wynn, Mairead Hyland, The Lighthouse Lab in Alderley Park and Alex Alderton, Roberto Amato, Sonia Goncalves, Ewan Harrison, David K. Jackson, Ian Johnston, Dominic Kwiatkowski, Cordelia Langford, John Sillitoe on behalf of the Wellcome Sanger Institute COVID-19 Surveillance Team
EPI_ISL_702964	Virology Department, Sheffield Teaching Hospitals NHS Foundation Trust/Department of Infection, Immunity and Cardiovascular Disease, The Medical School, University of Sheffield	COVID-19 Genomics UK (COG-UK) Consortium	Thushan de Silva, Matthew Parker, Nikki Smith, Adri Angyal, Rebecca Brown, Luke Green, Rachel Tucker, Paul Parsons, Danielle Groves, Katie Johnson, Laura Carrilero, Alex Keeley, Dave Partridge, Matthew Wyles, Benjamin Lindsey, Mehmet Yavuz, Mohammad Raza, Cariad Evans
EPI_ISL_702965, EPI_ISL_702967, EPI_ISL_702968, EPI_ISL_702970, EPI_ISL_702972, EPI_ISL_702973, EPI_ISL_702975, EPI_ISL_702976, EPI_ISL_702978, EPI_ISL_702979	Lighthouse Lab in Alderley Park	Wellcome Sanger Institute for the COVID-19 Genomics UK (COG-UK) Consortium	Jacquelyn Wynn, Mairead Hyland, The Lighthouse Lab in Alderley Park and Alex Alderton, Roberto Amato, Sonia Goncalves, Ewan Harrison, David K. Jackson, Ian Johnston, Dominic Kwiatkowski, Cordelia Langford, John Sillitoe on behalf of the Wellcome Sanger Institute COVID-19 Surveillance Team
EPI_ISL_702980	Liverpool Clinical Laboratories	COVID-19 Genomics UK (COG-UK) Consortium	Sam Haldenby, Anita Lucaci, Steve Paterson, Julian Hiscox, Alistair Darby, M Almsaud, A Alrezaihi, Muhannad Alruwaili, Stuart D Armstrong, Jones Benjamin, Eleanor G Bentley, Anu Chawla, Jordan J Clark, Angela Cowell, Richard Eccles, Isabel García-Dorival, Matthew Gemmell, Alessandro Gerada, PKF Gilmore, Richard Gregory, Ximeng Han, Catherine Hartley, Margaret Hughes, Miren Iturriza-Gomara, James Johnson, L Luu, Jenifer Manson, Charlotte Nelson, Elaine O'Toole, Cassie Olateju, Rebekah Penrice-Randal , Lucille Rainbow, N.P Randle, Trevor Ian Robinson, Parul Sharma, Ghada T Shawli, James P Stewart, Neil Swainston, Ecaterina Vamos, Joanne Watts, Mark Whitehead
EPI_ISL_702981, EPI_ISL_702983, EPI_ISL_702984, EPI_ISL_702986, EPI_ISL_702988, EPI_ISL_702989, EPI_ISL_702991, EPI_ISL_702993, EPI_ISL_702995	Lighthouse Lab in Alderley Park	Wellcome Sanger Institute for the COVID-19 Genomics UK (COG-UK) Consortium	Jacquelyn Wynn, Mairead Hyland, The Lighthouse Lab in Alderley Park and Alex Alderton, Roberto Amato, Sonia Goncalves, Ewan Harrison, David K. Jackson, Ian Johnston, Dominic Kwiatkowski, Cordelia Langford, John Sillitoe on behalf of the Wellcome Sanger Institute COVID-19 Surveillance Team
EPI_ISL_702996	Liverpool Clinical Laboratories	COVID-19 Genomics UK (COG-UK) Consortium	Sam Haldenby, Anita Lucaci, Steve Paterson, Julian Hiscox, Alistair Darby, M Almsaud, A Alrezaihi, Muhannad Alruwaili, Stuart D Armstrong, Jones Benjamin, Eleanor G Bentley, Anu Chawla, Jordan J Clark, Angela Cowell, Richard Eccles, Isabel García-Dorival, Matthew Gemmell, Alessandro Gerada, PKF Gilmore, Richard Gregory, Ximeng Han, Catherine Hartley, Margaret Hughes, Miren Iturriza-Gomara, James Johnson, L Luu, Jenifer Manson, Charlotte Nelson, Elaine O'Toole, Cassie Olateju, Rebekah Penrice-Randal , Lucille Rainbow, N.P Randle, Trevor Ian Robinson, Parul Sharma, Ghada T Shawli, James P Stewart, Neil Swainston, Ecaterina Vamos, Joanne Watts, Mark Whitehead
EPI_ISL_702997, EPI_ISL_702998	Lighthouse Lab in Alderley Park	Wellcome Sanger Institute for the COVID-19 Genomics UK (COG-UK) Consortium	Jacquelyn Wynn, Mairead Hyland, The Lighthouse Lab in Alderley Park and Alex Alderton, Roberto Amato, Sonia Goncalves, Ewan Harrison, David K. Jackson, Ian Johnston, Dominic Kwiatkowski, Cordelia Langford, John Sillitoe on behalf of the Wellcome Sanger Institute COVID-19 Surveillance Team
EPI_ISL_702999	University College London, Great Ormond Street Hospital for Children NHS Foundation Trust, Imperial College Healthcare NHS Trust	COVID-19 Genomics UK (COG-UK) Consortium	Sergi Castellano, Rachel Williams, Mark Kristiansen, Paola Resende Silva, Sunando Roy, Tony Brooks, Helena Tutill, Paola Niola, Patricia Dyal, Charlotte Williams, Leysa Forrest, Yasmin Panchbhaya, Jacqueline Findlay, Samuel Weeks, Julianne Brown, Kathryn Harris, Paul Randell, James Price, Alison Holmes, Judith Breuer

EPI_ISL_703000, EPI_ISL_703001, EPI_ISL_703003, EPI_ISL_703004, EPI_ISL_703006, EPI_ISL_703008, EPI_ISL_703009	Lighthouse Lab in Alderley Park	Wellcome Sanger Institute for the COVID-19 Genomics UK (COG-UK) Consortium	Jacquelyn Wynn, Mairead Hyland, The Lighthouse Lab in Alderley Park and Alex Alderton, Roberto Amato, Sonia Goncalves, Ewan Harrison, David K. Jackson, Ian Johnston, Dominic Kwiatkowski, Cordelia Langford, John Sillitoe on behalf of the Wellcome Sanger Institute COVID-19 Surveillance Team
EPI_ISL_703010	Northumbria University / South Tees Hospitals NHS Foundation Trust / North Cumbria Integrated Care NHS Foundation Trust / North Tees and Hartlepool NHS Foundation Trust / Newcastle Hospitals NHS Foundation Trust	COVID-19 Genomics UK (COG-UK) Consortium	Darren L Smith,Andrew Nelson,Matthew Bashton,Greg R Young,Joshua Loh,John Allan,Mohammad A Tariq,Giles S Holt,Gary Black,Wen C Yew,Lynn Dover,Paul Baker,Steve Liggett,Sarah Essex,Jane Greenaway,Debra Padgett,Clive Graham,Garren Scott,Edward Barton,Emma Swindells,Brendan Payne,Jennifer Collins,Yusri Taha,Gary Eltringham
EPI_ISL_703011, EPI_ISL_703012	Lighthouse Lab in Alderley Park	Wellcome Sanger Institute for the COVID-19 Genomics UK (COG-UK) Consortium	Jacquelyn Wynn, Mairead Hyland, The Lighthouse Lab in Alderley Park and Alex Alderton, Roberto Amato, Sonia Goncalves, Ewan Harrison, David K. Jackson, Ian Johnston, Dominic Kwiatkowski, Cordelia Langford, John Sillitoe on behalf of the Wellcome Sanger Institute COVID-19 Surveillance Team
EPI_ISL_703013	Northumbria University / South Tees Hospitals NHS Foundation Trust / North Cumbria Integrated Care NHS Foundation Trust / North Tees and Hartlepool NHS Foundation Trust / Newcastle Hospitals NHS Foundation Trust	COVID-19 Genomics UK (COG-UK) Consortium	Darren L Smith,Andrew Nelson,Matthew Bashton,Greg R Young,Joshua Loh,John Allan,Mohammad A Tariq,Giles S Holt,Gary Black,Wen C Yew,Lynn Dover,Paul Baker,Steve Liggett,Sarah Essex,Jane Greenaway,Debra Padgett,Clive Graham,Garren Scott,Edward Barton,Emma Swindells,Brendan Payne,Jennifer Collins,Yusri Taha,Gary Eltringham
EPI_ISL_703014, EPI_ISL_703016, EPI_ISL_703017, EPI_ISL_703019	Lighthouse Lab in Alderley Park	Wellcome Sanger Institute for the COVID-19 Genomics UK (COG-UK) Consortium	Jacquelyn Wynn, Mairead Hyland, The Lighthouse Lab in Alderley Park and Alex Alderton, Roberto Amato, Sonia Goncalves, Ewan Harrison, David K. Jackson, Ian Johnston, Dominic Kwiatkowski, Cordelia Langford, John Sillitoe on behalf of the Wellcome Sanger Institute COVID-19 Surveillance Team
EPI_ISL_703020	Virology Department, Sheffield Teaching Hospitals NHS Foundation Trust/Department of Infection, Immunity and Cardiovascular Disease, The Medical School, University of Sheffield	COVID-19 Genomics UK (COG-UK) Consortium	Thushan de Silva, Matthew Parker, Nikki Smith, Adri Angyal, Rebecca Brown, Luke Green, Rachel Tucker, Paul Parsons, Danielle Groves, Katie Johnson, Laura Carrilero, Alex Keeley, Dave Partridge, Matthew Wyles, Benjamin Lindsey, Mehmet Yavuz, Mohammad Raza, Cariad Evans
EPI_ISL_703021, EPI_ISL_703022, EPI_ISL_703024, EPI_ISL_703025	Lighthouse Lab in Alderley Park	Wellcome Sanger Institute for the COVID-19 Genomics UK (COG-UK) Consortium	Jacquelyn Wynn, Mairead Hyland, The Lighthouse Lab in Alderley Park and Alex Alderton, Roberto Amato, Sonia Goncalves, Ewan Harrison, David K. Jackson, Ian Johnston, Dominic Kwiatkowski, Cordelia Langford, John Sillitoe on behalf of the Wellcome Sanger Institute COVID-19 Surveillance Team
EPI_ISL_703026	Virology Department, Sheffield Teaching Hospitals NHS Foundation Trust/Department of Infection, Immunity and Cardiovascular Disease, The Medical School, University of Sheffield	COVID-19 Genomics UK (COG-UK) Consortium	Thushan de Silva, Matthew Parker, Nikki Smith, Adri Angyal, Rebecca Brown, Luke Green, Rachel Tucker, Paul Parsons, Danielle Groves, Katie Johnson, Laura Carrilero, Alex Keeley, Dave Partridge, Matthew Wyles, Benjamin Lindsey, Mehmet Yavuz, Mohammad Raza, Cariad Evans
EPI_ISL_703027, EPI_ISL_703028, EPI_ISL_703030, EPI_ISL_703031, EPI_ISL_703033	Lighthouse Lab in Alderley Park	Wellcome Sanger Institute for the COVID-19 Genomics UK (COG-UK) Consortium	Jacquelyn Wynn, Mairead Hyland, The Lighthouse Lab in Alderley Park and Alex Alderton, Roberto Amato, Sonia Goncalves, Ewan Harrison, David K. Jackson, Ian Johnston, Dominic Kwiatkowski, Cordelia Langford, John Sillitoe on behalf of the Wellcome Sanger Institute COVID-19 Surveillance Team
EPI_ISL_703034	Northumbria University / South Tees Hospitals NHS Foundation Trust / North Cumbria Integrated Care NHS Foundation Trust / North Tees and Hartlepool NHS Foundation Trust / Newcastle Hospitals NHS Foundation Trust	COVID-19 Genomics UK (COG-UK) Consortium	Darren L Smith,Andrew Nelson,Matthew Bashton,Greg R Young,Joshua Loh,John Allan,Mohammad A Tariq,Giles S Holt,Gary Black,Wen C Yew,Lynn Dover,Paul Baker,Steve Liggett,Sarah Essex,Jane Greenaway,Debra Padgett,Clive Graham,Garren Scott,Edward Barton,Emma Swindells,Brendan Payne,Jennifer Collins,Yusri Taha,Gary Eltringham
EPI_ISL_703035, EPI_ISL_703037, EPI_ISL_703039, EPI_ISL_703041, EPI_ISL_703042, EPI_ISL_703044, EPI_ISL_703045, EPI_ISL_703047, EPI_ISL_703049, EPI_ISL_703050	Lighthouse Lab in Alderley Park	Wellcome Sanger Institute for the COVID-19 Genomics UK (COG-UK) Consortium	Jacquelyn Wynn, Mairead Hyland, The Lighthouse Lab in Alderley Park and Alex Alderton, Roberto Amato, Sonia Goncalves, Ewan Harrison, David K. Jackson, Ian Johnston, Dominic Kwiatkowski, Cordelia Langford, John Sillitoe on behalf of the Wellcome Sanger Institute COVID-19 Surveillance Team
EPI_ISL_703051	Liverpool Clinical Laboratories	COVID-19 Genomics UK (COG-UK) Consortium	Sam Haldenby, Anita Lucaci, Steve Paterson, Julian Hiscox, Alistair Darby, M Almsaud, A Alrezaihi, Muhannad Alruwaili, Stuart D Armstrong, Jones Benjamin, Eleanor G Bentley, Anu Chawla, Jordan J Clark, Angela Cowell, Richard Eccles, Isabel García-Dorival, Matthew Gemmell, Alessandro Gerada, PKF Gilmore, Richard Gregory, Ximeng Han, Catherine Hartley, Margaret Hughes, Miren Iturriza-Gomara, James Johnson, L Luu, Jenifer Manson, Charlotte Nelson, Elaine O'Toole, Cassie Olateju, Rebekah Penrice-Randal , Lucille Rainbow, N.P Randle, Trevor Ian Robinson, Parul Sharma, Ghada T Shawli, James P Stewart, Neil Swainston, Ecaterina Vamos, Joanne Watts, Mark Whitehead
EPI_ISL_703052, EPI_ISL_703054, EPI_ISL_703055, EPI_ISL_703057, EPI_ISL_703058, EPI_ISL_703060	Lighthouse Lab in Alderley Park	Wellcome Sanger Institute for the COVID-19 Genomics UK (COG-UK) Consortium	Jacquelyn Wynn, Mairead Hyland, The Lighthouse Lab in Alderley Park and Alex Alderton, Roberto Amato, Sonia Goncalves, Ewan Harrison, David K. Jackson, Ian Johnston, Dominic Kwiatkowski, Cordelia Langford, John Sillitoe on behalf of the Wellcome Sanger Institute COVID-19 Surveillance Team
EPI_ISL_703061	Liverpool Clinical Laboratories	COVID-19 Genomics UK (COG-UK) Consortium	Sam Haldenby, Anita Lucaci, Steve Paterson, Julian Hiscox, Alistair Darby, M Almsaud, A Alrezaihi, Muhannad Alruwaili, Stuart D Armstrong, Jones Benjamin, Eleanor G Bentley, Anu Chawla, Jordan J Clark, Angela Cowell, Richard Eccles, Isabel García-Dorival, Matthew Gemmell, Alessandro Gerada, PKF Gilmore, Richard Gregory, Ximeng Han, Catherine Hartley, Margaret Hughes, Miren Iturriza-Gomara, James Johnson, L Luu, Jenifer Manson, Charlotte Nelson, Elaine O'Toole, Cassie Olateju, Rebekah Penrice-Randal , Lucille Rainbow, N.P Randle, Trevor Ian Robinson, Parul Sharma, Ghada T Shawli, James P Stewart, Neil Swainston, Ecaterina Vamos, Joanne Watts, Mark Whitehead
EPI_ISL_703062, EPI_ISL_703063	Lighthouse Lab in Alderley Park	Wellcome Sanger Institute for the COVID-19 Genomics UK (COG-UK) Consortium	Jacquelyn Wynn, Mairead Hyland, The Lighthouse Lab in Alderley Park and Alex Alderton, Roberto Amato, Sonia Goncalves, Ewan Harrison, David K. Jackson, Ian Johnston, Dominic Kwiatkowski, Cordelia Langford, John Sillitoe on behalf of the Wellcome Sanger Institute COVID-19 Surveillance Team
EPI_ISL_703064	Northumbria University / South Tees Hospitals NHS Foundation Trust / North Cumbria Integrated Care NHS Foundation Trust / North Tees and Hartlepool NHS Foundation Trust / Newcastle Hospitals NHS Foundation Trust	COVID-19 Genomics UK (COG-UK) Consortium	Darren L Smith,Andrew Nelson,Matthew Bashton,Greg R Young,Joshua Loh,John Allan,Mohammad A Tariq,Giles S Holt,Gary Black,Wen C Yew,Lynn Dover,Paul Baker,Steve Liggett,Sarah Essex,Jane Greenaway,Debra Padgett,Clive Graham,Garren Scott,Edward Barton,Emma Swindells,Brendan Payne,Jennifer Collins,Yusri Taha,Gary Eltringham
EPI_ISL_703065	Lighthouse Lab in Alderley Park	Wellcome Sanger Institute for the COVID-19 Genomics UK (COG-UK) Consortium	Jacquelyn Wynn, Mairead Hyland, The Lighthouse Lab in Alderley Park and Alex Alderton, Roberto Amato, Sonia Goncalves, Ewan Harrison, David K. Jackson, Ian Johnston, Dominic Kwiatkowski, Cordelia Langford, John Sillitoe on behalf of the Wellcome Sanger Institute COVID-19 Surveillance Team
EPI_ISL_703066	Wales Specialist Virology Centre Sequencing lab: Pathogen Genomics Unit	COVID-19 Genomics UK (COG-UK) Consortium	Catherine Moore, Johnathan Evans, Laura Gifford, Malorie Perry, Simon Cottrell, Angela Marchbank, Alec Birchley, Alexander Adams, Amy Gaskin, Bree Gatica-Wilcox, Jason Coombes, Joel Southgate, Lauren Gilbert, Lee Graham, Nicole Pacchiarini, Sara Kumziene-Summerhayes, Sarah Taylor, Sophie Jones, Sara Rey, Matthew Bull, Joanne Watkins, Sally Corden, Tom Connor
EPI_ISL_703067, EPI_ISL_703068, EPI_ISL_703070, EPI_ISL_703072, EPI_ISL_703073	Lighthouse Lab in Alderley Park	Wellcome Sanger Institute for the COVID-19 Genomics UK (COG-UK) Consortium	Jacquelyn Wynn, Mairead Hyland, The Lighthouse Lab in Alderley Park and Alex Alderton, Roberto Amato, Sonia Goncalves, Ewan Harrison, David K. Jackson, Ian Johnston, Dominic Kwiatkowski, Cordelia Langford, John Sillitoe on behalf of the Wellcome Sanger Institute COVID-19 Surveillance Team
EPI_ISL_703074	Wales Specialist Virology Centre Sequencing lab: Pathogen Genomics Unit	COVID-19 Genomics UK (COG-UK) Consortium	Catherine Moore, Johnathan Evans, Laura Gifford, Malorie Perry, Simon Cottrell, Angela Marchbank, Alec Birchley, Alexander Adams, Amy Gaskin, Bree Gatica-Wilcox, Jason Coombes, Joel Southgate, Lauren Gilbert, Lee Graham, Nicole Pacchiarini, Sara Kumziene-Summerhayes, Sarah Taylor, Sophie Jones, Sara Rey, Matthew Bull, Joanne Watkins, Sally Corden, Tom Connor
EPI_ISL_703075	Lighthouse Lab in Alderley Park	Wellcome Sanger Institute for the COVID-19 Genomics UK (COG-UK) Consortium	Jacquelyn Wynn, Mairead Hyland, The Lighthouse Lab in Alderley Park and Alex Alderton, Roberto Amato, Sonia Goncalves, Ewan Harrison, David K. Jackson, Ian Johnston, Dominic Kwiatkowski, Cordelia Langford, John Sillitoe on behalf of the Wellcome Sanger Institute COVID-19 Surveillance Team
EPI_ISL_703076	Department of Pathology, University of Cambridge	COVID-19 Genomics UK (COG-UK) Consortium	Aminu S. Jahun, Yasmin Chaudhry, Grant Hall, Iliana Georgana, Myra Hosmillo, Martin D. Curran, Malte Pinckert, Surendra Parmar, Ian Goodfellow
EPI_ISL_703078, EPI_ISL_703079, EPI_ISL_703081, EPI_ISL_703083, EPI_ISL_703084, EPI_ISL_703086, EPI_ISL_703087, EPI_ISL_703089, EPI_ISL_703091, EPI_ISL_703092, EPI_ISL_703094, EPI_ISL_703095, EPI_ISL_703097, EPI_ISL_703098, EPI_ISL_703100, EPI_ISL_703101, EPI_ISL_703103, EPI_ISL_703104			
see above	Lighthouse Lab in Alderley Park	Wellcome Sanger Institute for the COVID-19 Genomics UK (COG-UK) Consortium	Jacquelyn Wynn, Mairead Hyland, The Lighthouse Lab in Alderley Park and Alex Alderton, Roberto Amato, Sonia Goncalves, Ewan Harrison, David K. Jackson, Ian Johnston, Dominic Kwiatkowski, Cordelia Langford, John Sillitoe on behalf of the Wellcome Sanger Institute COVID-19 Surveillance Team
EPI_ISL_703105	Northumbria University / South Tees Hospitals NHS	COVID-19 Genomics UK (COG-UK) Consortium	Darren L Smith,Andrew Nelson,Matthew Bashton,Greg R Young,Joshua Loh,John Allan,Mohammad A Tariq,Giles S Holt,Gary Black,Wen C Yew,Lynn

	Foundation Trust / North Cumbria Integrated Care NHS Foundation Trust / North Tees and Hartlepool NHS Foundation Trust / Newcastle Hospitals NHS Foundation Trust		Dover,Paul Baker,Steve Liggett,Sarah Essex,Jane Greenaway,Debra Padgett,Clive Graham,Garren Scott,Edward Barton,Emma Swindells,Brendan Payne,Jennifer Collins,Yusri Taha,Gary Eltringham
EPI_ISL_703106, EPI_ISL_703108, EPI_ISL_703109, EPI_ISL_703111, EPI_ISL_703112, EPI_ISL_703114, EPI_ISL_703116, EPI_ISL_703117, EPI_ISL_703119, EPI_ISL_703120, EPI_ISL_703122, EPI_ISL_703123, EPI_ISL_703125, EPI_ISL_703126, EPI_ISL_703128, EPI_ISL_703130, EPI_ISL_703131, EPI_ISL_703134, EPI_ISL_703135, EPI_ISL_703137, EPI_ISL_703138, EPI_ISL_703140, EPI_ISL_703142, EPI_ISL_703143, EPI_ISL_703145, EPI_ISL_703146, EPI_ISL_703148, EPI_ISL_703149, EPI_ISL_703151, EPI_ISL_703153, EPI_ISL_703154, EPI_ISL_703156, EPI_ISL_703157, EPI_ISL_703159, EPI_ISL_703160, EPI_ISL_703161			
see above	Lighthouse Lab in Alderley Park	Wellcome Sanger Institute for the COVID-19 Genomics UK (COG-UK) Consortium	Jacquelyn Wynn, Mairead Hyland, The Lighthouse Lab in Alderley Park and Alex Alderton, Roberto Amato, Sonia Goncalves, Ewan Harrison, David K. Jackson, Ian Johnston, Dominic Kwiatkowski, Cordelia Langford, John Sillitoe on behalf of the Wellcome Sanger Institute COVID-19 Surveillance Team
EPI_ISL_703162	Liverpool Clinical Laboratories	COVID-19 Genomics UK (COG-UK) Consortium	Sam Haldenby, Anita Lucaci, Steve Paterson, Julian Hiscox, Alistair Darby, M Almsaud, A Alrezaihi, Muhannad Alruwaili, Stuart D Armstrong, Jones Benjamin, Eleanor G Bentley, Anu Chawla, Jordan J Clark, Angela Cowell, Richard Eccles, Isabel García-Dorival, Matthew Gemmell, Alessandro Gerada, PKF Gilmore, Richard Gregory, Ximeng Han, Catherine Hartley, Margaret Hughes, Miren Iturriza-Gomara, James Johnson, L Luu, Jenifer Manson, Charlotte Nelson, Elaine O'Toole, Cassie Olateju, Rebekah Penrice-Randal , Lucille Rainbow, N.P Randle, Trevor Ian Robinson, Parul Sharma, Ghada T Shawli, James P Stewart, Neil Swainston, Ecaterina Vamos, Joanne Watts, Mark Whitehead
EPI_ISL_703163, EPI_ISL_703164, EPI_ISL_703166, EPI_ISL_703168, EPI_ISL_703169, EPI_ISL_703171, EPI_ISL_703173	Lighthouse Lab in Alderley Park	Wellcome Sanger Institute for the COVID-19 Genomics UK (COG-UK) Consortium	Jacquelyn Wynn, Mairead Hyland, The Lighthouse Lab in Alderley Park and Alex Alderton, Roberto Amato, Sonia Goncalves, Ewan Harrison, David K. Jackson, Ian Johnston, Dominic Kwiatkowski, Cordelia Langford, John Sillitoe on behalf of the Wellcome Sanger Institute COVID-19 Surveillance Team
EPI_ISL_703174	Liverpool Clinical Laboratories	COVID-19 Genomics UK (COG-UK) Consortium	Sam Haldenby, Anita Lucaci, Steve Paterson, Julian Hiscox, Alistair Darby, M Almsaud, A Alrezaihi, Muhannad Alruwaili, Stuart D Armstrong, Jones Benjamin, Eleanor G Bentley, Anu Chawla, Jordan J Clark, Angela Cowell, Richard Eccles, Isabel García-Dorival, Matthew Gemmell, Alessandro Gerada, PKF Gilmore, Richard Gregory, Ximeng Han, Catherine Hartley, Margaret Hughes, Miren Iturriza-Gomara, James Johnson, L Luu, Jenifer Manson, Charlotte Nelson, Elaine O'Toole, Cassie Olateju, Rebekah Penrice-Randal , Lucille Rainbow, N.P Randle, Trevor Ian Robinson, Parul Sharma, Ghada T Shawli, James P Stewart, Neil Swainston, Ecaterina Vamos, Joanne Watts, Mark Whitehead
EPI_ISL_703175	Lighthouse Lab in Alderley Park	Wellcome Sanger Institute for the COVID-19 Genomics UK (COG-UK) Consortium	Jacquelyn Wynn, Mairead Hyland, The Lighthouse Lab in Alderley Park and Alex Alderton, Roberto Amato, Sonia Goncalves, Ewan Harrison, David K. Jackson, Ian Johnston, Dominic Kwiatkowski, Cordelia Langford, John Sillitoe on behalf of the Wellcome Sanger Institute COVID-19 Surveillance Team
EPI_ISL_703176	Liverpool Clinical Laboratories	COVID-19 Genomics UK (COG-UK) Consortium	Sam Haldenby, Anita Lucaci, Steve Paterson, Julian Hiscox, Alistair Darby, M Almsaud, A Alrezaihi, Muhannad Alruwaili, Stuart D Armstrong, Jones Benjamin, Eleanor G Bentley, Anu Chawla, Jordan J Clark, Angela Cowell, Richard Eccles, Isabel García-Dorival, Matthew Gemmell, Alessandro Gerada, PKF Gilmore, Richard Gregory, Ximeng Han, Catherine Hartley, Margaret Hughes, Miren Iturriza-Gomara, James Johnson, L Luu, Jenifer Manson, Charlotte Nelson, Elaine O'Toole, Cassie Olateju, Rebekah Penrice-Randal , Lucille Rainbow, N.P Randle, Trevor Ian Robinson, Parul Sharma, Ghada T Shawli, James P Stewart, Neil Swainston, Ecaterina Vamos, Joanne Watts, Mark Whitehead
EPI_ISL_703177, EPI_ISL_703178, EPI_ISL_703180, EPI_ISL_703181	Lighthouse Lab in Alderley Park	Wellcome Sanger Institute for the COVID-19 Genomics UK (COG-UK) Consortium	Jacquelyn Wynn, Mairead Hyland, The Lighthouse Lab in Alderley Park and Alex Alderton, Roberto Amato, Sonia Goncalves, Ewan Harrison, David K. Jackson, Ian Johnston, Dominic Kwiatkowski, Cordelia Langford, John Sillitoe on behalf of the Wellcome Sanger Institute COVID-19 Surveillance Team
EPI_ISL_703182	Liverpool Clinical Laboratories	COVID-19 Genomics UK (COG-UK) Consortium	Sam Haldenby, Anita Lucaci, Steve Paterson, Julian Hiscox, Alistair Darby, M Almsaud, A Alrezaihi, Muhannad Alruwaili, Stuart D Armstrong, Jones Benjamin, Eleanor G Bentley, Anu Chawla, Jordan J Clark, Angela Cowell, Richard Eccles, Isabel García-Dorival, Matthew Gemmell, Alessandro Gerada, PKF Gilmore, Richard Gregory, Ximeng Han, Catherine Hartley, Margaret Hughes, Miren Iturriza-Gomara, James Johnson, L Luu, Jenifer Manson, Charlotte Nelson, Elaine O'Toole, Cassie Olateju, Rebekah Penrice-Randal , Lucille Rainbow, N.P Randle, Trevor Ian Robinson, Parul Sharma, Ghada T Shawli, James P Stewart, Neil Swainston, Ecaterina Vamos, Joanne Watts, Mark Whitehead
EPI_ISL_703183, EPI_ISL_703185, EPI_ISL_703186, EPI_ISL_703188, EPI_ISL_703189, EPI_ISL_703191, EPI_ISL_703193, EPI_ISL_703194, EPI_ISL_703196, EPI_ISL_703197, EPI_ISL_703199, EPI_ISL_703201, EPI_ISL_703202, EPI_ISL_703204, EPI_ISL_703205, EPI_ISL_703207, EPI_ISL_703209, EPI_ISL_703210, EPI_ISL_703212, EPI_ISL_703214, EPI_ISL_703215, EPI_ISL_703217, EPI_ISL_703218			
see above	Lighthouse Lab in Alderley Park	Wellcome Sanger Institute for the COVID-19 Genomics UK (COG-UK) Consortium	Jacquelyn Wynn, Mairead Hyland, The Lighthouse Lab in Alderley Park and Alex Alderton, Roberto Amato, Sonia Goncalves, Ewan Harrison, David K. Jackson, Ian Johnston, Dominic Kwiatkowski, Cordelia Langford, John Sillitoe on behalf of the Wellcome Sanger Institute COVID-19 Surveillance Team
EPI_ISL_703219	Liverpool Clinical Laboratories	COVID-19 Genomics UK (COG-UK) Consortium	Sam Haldenby, Anita Lucaci, Steve Paterson, Julian Hiscox, Alistair Darby, M Almsaud, A Alrezaihi, Muhannad Alruwaili, Stuart D Armstrong, Jones Benjamin, Eleanor G Bentley, Anu Chawla, Jordan J Clark, Angela Cowell, Richard Eccles, Isabel García-Dorival, Matthew Gemmell, Alessandro Gerada, PKF Gilmore, Richard Gregory, Ximeng Han, Catherine Hartley, Margaret Hughes, Miren Iturriza-Gomara, James Johnson, L Luu, Jenifer Manson, Charlotte Nelson, Elaine O'Toole, Cassie Olateju, Rebekah Penrice-Randal , Lucille Rainbow, N.P Randle, Trevor Ian Robinson, Parul Sharma, Ghada T Shawli, James P Stewart, Neil Swainston, Ecaterina Vamos, Joanne Watts, Mark Whitehead
EPI_ISL_703220, EPI_ISL_703221, EPI_ISL_703223, EPI_ISL_703224, EPI_ISL_703226, EPI_ISL_703228, EPI_ISL_703229, EPI_ISL_703231, EPI_ISL_703232, EPI_ISL_703234	Lighthouse Lab in Alderley Park	Wellcome Sanger Institute for the COVID-19 Genomics UK (COG-UK) Consortium	Jacquelyn Wynn, Mairead Hyland, The Lighthouse Lab in Alderley Park and Alex Alderton, Roberto Amato, Sonia Goncalves, Ewan Harrison, David K. Jackson, Ian Johnston, Dominic Kwiatkowski, Cordelia Langford, John Sillitoe on behalf of the Wellcome Sanger Institute COVID-19 Surveillance Team
EPI_ISL_703235	Liverpool Clinical Laboratories	COVID-19 Genomics UK (COG-UK) Consortium	Sam Haldenby, Anita Lucaci, Steve Paterson, Julian Hiscox, Alistair Darby, M Almsaud, A Alrezaihi, Muhannad Alruwaili, Stuart D Armstrong, Jones Benjamin, Eleanor G Bentley, Anu Chawla, Jordan J Clark, Angela Cowell, Richard Eccles, Isabel García-Dorival, Matthew Gemmell, Alessandro Gerada, PKF Gilmore, Richard Gregory, Ximeng Han, Catherine Hartley, Margaret Hughes, Miren Iturriza-Gomara, James Johnson, L Luu, Jenifer Manson, Charlotte Nelson, Elaine O'Toole, Cassie Olateju, Rebekah Penrice-Randal , Lucille Rainbow, N.P Randle, Trevor Ian Robinson, Parul Sharma, Ghada T Shawli, James P Stewart, Neil Swainston, Ecaterina Vamos, Joanne Watts, Mark Whitehead
EPI_ISL_703236, EPI_ISL_703237, EPI_ISL_703239	Lighthouse Lab in Alderley Park	Wellcome Sanger Institute for the COVID-19 Genomics UK (COG-UK) Consortium	Jacquelyn Wynn, Mairead Hyland, The Lighthouse Lab in Alderley Park and Alex Alderton, Roberto Amato, Sonia Goncalves, Ewan Harrison, David K. Jackson, Ian Johnston, Dominic Kwiatkowski, Cordelia Langford, John Sillitoe on behalf of the Wellcome Sanger Institute COVID-19 Surveillance Team
EPI_ISL_703240	West of Scotland Specialist Virology Centre, NHSGGC / MRC-University of Glasgow Centre for Virus Research	COVID-19 Genomics UK (COG-UK) Consortium	Ana da Silva Filipe, Natasha Johnson, Kathy Smollett, Daniel Mair, Stephen Carmichael, Alice Broos, Lily Tong, Jenna Nichols, Kyriaki Nomikou; Sarah McDonald; Richard Orton, Joseph Hughes, Sreenu Vattipally, David L Robertson; Alasdair MacLean, Rory Gunson; Sharif Shaaban, Matthew Holden; Rachel Blacow, Guy Mollett, Kathy Li, James Shepherd, Antonia Ho, Emma Thomson
EPI_ISL_703241, EPI_ISL_703242	Lighthouse Lab in Alderley Park	Wellcome Sanger Institute for the COVID-19 Genomics UK (COG-UK) Consortium	Jacquelyn Wynn, Mairead Hyland, The Lighthouse Lab in Alderley Park and Alex Alderton, Roberto Amato, Sonia Goncalves, Ewan Harrison, David K. Jackson, Ian Johnston, Dominic Kwiatkowski, Cordelia Langford, John Sillitoe on behalf of the Wellcome Sanger Institute COVID-19 Surveillance Team
EPI_ISL_703243	West of Scotland Specialist Virology Centre, NHSGGC / MRC-University of Glasgow Centre for Virus Research	COVID-19 Genomics UK (COG-UK) Consortium	Ana da Silva Filipe, Natasha Johnson, Kathy Smollett, Daniel Mair, Stephen Carmichael, Alice Broos, Lily Tong, Jenna Nichols, Kyriaki Nomikou; Sarah McDonald; Richard Orton, Joseph Hughes, Sreenu Vattipally, David L Robertson; Alasdair MacLean, Rory Gunson; Sharif Shaaban, Matthew Holden; Rachel Blacow, Guy Mollett, Kathy Li, James Shepherd, Antonia Ho, Emma Thomson
EPI_ISL_703244	Lighthouse Lab in Alderley Park	Wellcome Sanger Institute for the COVID-19 Genomics UK (COG-UK) Consortium	Jacquelyn Wynn, Mairead Hyland, The Lighthouse Lab in Alderley Park and Alex Alderton, Roberto Amato, Sonia Goncalves, Ewan Harrison, David K. Jackson, Ian Johnston, Dominic Kwiatkowski, Cordelia Langford, John Sillitoe on behalf of the Wellcome Sanger Institute COVID-19 Surveillance Team
EPI_ISL_703246	West of Scotland Specialist Virology Centre, NHSGGC / MRC-University of Glasgow Centre for Virus Research	COVID-19 Genomics UK (COG-UK) Consortium	Ana da Silva Filipe, Natasha Johnson, Kathy Smollett, Daniel Mair, Stephen Carmichael, Alice Broos, Lily Tong, Jenna Nichols, Kyriaki Nomikou; Sarah McDonald; Richard Orton, Joseph Hughes, Sreenu Vattipally, David L Robertson; Alasdair MacLean, Rory Gunson; Sharif Shaaban, Matthew Holden; Rachel Blacow, Guy Mollett, Kathy Li, James Shepherd, Antonia Ho, Emma Thomson
EPI_ISL_703251, EPI_ISL_703259	Wales Specialist Virology Centre Sequencing lab: Pathogen Genomics Unit	COVID-19 Genomics UK (COG-UK) Consortium	Catherine Moore, Johnathan Evans, Laura Gifford, Malorie Perry, Simon Cottrell, Angela Marchbank, Alec Birchley, Alexander Adams, Amy Gaskin, Bree Gatica-Wilcox, Jason Coombes, Joel Southgate, Lauren Gilbert, Lee Graham, Nicole Pacchiarini, Sara Kumziene-Summerhayes, Sarah Taylor, Sophie Jones, Sara Rey, Matthew Bull, Joanne Watkins, Sally Corden, Tom Connor
EPI_ISL_703269	West of Scotland Specialist Virology Centre, NHSGGC / MRC-University of Glasgow Centre for Virus Research	COVID-19 Genomics UK (COG-UK) Consortium	Ana da Silva Filipe, Natasha Johnson, Kathy Smollett, Daniel Mair, Stephen Carmichael, Alice Broos, Lily Tong, Jenna Nichols, Kyriaki Nomikou; Sarah McDonald; Richard Orton, Joseph Hughes, Sreenu Vattipally, David L Robertson; Alasdair MacLean, Rory Gunson; Sharif Shaaban, Matthew Holden; Rachel Blacow, Guy Mollett, Kathy Li, James Shepherd, Antonia Ho, Emma Thomson
EPI_ISL_703272	Liverpool Clinical Laboratories	COVID-19 Genomics UK (COG-UK) Consortium	Sam Haldenby, Anita Lucaci, Steve Paterson, Julian Hiscox, Alistair Darby, M Almsaud, A Alrezaihi, Muhannad Alruwaili, Stuart D Armstrong, Jones

			Benjamin, Eleanor G Bentley, Anu Chawla, Jordan J Clark, Angela Cowell, Richard Eccles, Isabel García-Dorival, Matthew Gemmell, Alessandro Gerada, PKF Gilmore, Richard Gregory, Ximeng Han, Catherine Hartley, Margaret Hughes, Miren Iturriza-Gomara, James Johnson, L Luu, Jenifer Manson, Charlotte Nelson, Elaine O'Toole, Cassie Olateju, Rebekah Penrice-Randal , Lucille Rainbow, N.P Randle, Trevor Ian Robinson, Parul Sharma, Ghada T Shawli, James P Stewart, Neil Swainston, Ecaterina Vamos, Joanne Watts, Mark Whitehead
EPI_ISL_703278	Wales Specialist Virology Centre Sequencing lab: Pathogen Genomics Unit	COVID-19 Genomics UK (COG-UK) Consortium	Catherine Moore, Johnathan Evans, Laura Gifford, Malorie Perry, Simon Cottrell, Angela Marchbank, Alec Birchley, Alexander Adams, Amy Gaskin, Bree Gatica-Wilcox, Jason Coombes, Joel Southgate, Lauren Gilbert, Lee Graham, Nicole Pacchiarini, Sara Kumziene-Summerhayes, Sarah Taylor, Sophie Jones, Sara Rey, Matthew Bull, Joanne Watkins, Sally Corden, Tom Connor
EPI_ISL_703289	Liverpool Clinical Laboratories	COVID-19 Genomics UK (COG-UK) Consortium	Sam Haldenby, Anita Lucaci, Steve Paterson, Julian Hiscox, Alistair Darby, M Almsaud, A Alrezaihi, Muhannad Alruwaili, Stuart D Armstrong, Jones Benjamin, Eleanor G Bentley, Anu Chawla, Jordan J Clark, Angela Cowell, Richard Eccles, Isabel García-Dorival, Matthew Gemmell, Alessandro Gerada, PKF Gilmore, Richard Gregory, Ximeng Han, Catherine Hartley, Margaret Hughes, Miren Iturriza-Gomara, James Johnson, L Luu, Jenifer Manson, Charlotte Nelson, Elaine O'Toole, Cassie Olateju, Rebekah Penrice-Randal , Lucille Rainbow, N.P Randle, Trevor Ian Robinson, Parul Sharma, Ghada T Shawli, James P Stewart, Neil Swainston, Ecaterina Vamos, Joanne Watts, Mark Whitehead
EPI_ISL_703302	Wales Specialist Virology Centre Sequencing lab: Pathogen Genomics Unit	COVID-19 Genomics UK (COG-UK) Consortium	Catherine Moore, Johnathan Evans, Laura Gifford, Malorie Perry, Simon Cottrell, Angela Marchbank, Alec Birchley, Alexander Adams, Amy Gaskin, Bree Gatica-Wilcox, Jason Coombes, Joel Southgate, Lauren Gilbert, Lee Graham, Nicole Pacchiarini, Sara Kumziene-Summerhayes, Sarah Taylor, Sophie Jones, Sara Rey, Matthew Bull, Joanne Watkins, Sally Corden, Tom Connor
EPI_ISL_703319	University College London, Great Ormond Street Hospital for Children NHS Foundation Trust, Imperial College Healthcare NHS Trust	COVID-19 Genomics UK (COG-UK) Consortium	Sergi Castellano, Rachel Williams, Mark Kristiansen, Paola Resende Silva, Sunando Roy, Tony Brooks, Helena Tutill, Paola Niola, Patricia Dyal, Charlotte Williams, Leysa Forrest, Yasmin Panchbhaya, Jacqueline Findlay, Samuel Weeks, Julianne Brown, Kathryn Harris, Paul Randell, James Price, Alison Holmes, Judith Breuer
EPI_ISL_703321	Wales Specialist Virology Centre Sequencing lab: Pathogen Genomics Unit	COVID-19 Genomics UK (COG-UK) Consortium	Catherine Moore, Johnathan Evans, Laura Gifford, Malorie Perry, Simon Cottrell, Angela Marchbank, Alec Birchley, Alexander Adams, Amy Gaskin, Bree Gatica-Wilcox, Jason Coombes, Joel Southgate, Lauren Gilbert, Lee Graham, Nicole Pacchiarini, Sara Kumziene-Summerhayes, Sarah Taylor, Sophie Jones, Sara Rey, Matthew Bull, Joanne Watkins, Sally Corden, Tom Connor
EPI_ISL_703324	Liverpool Clinical Laboratories	COVID-19 Genomics UK (COG-UK) Consortium	Sam Haldenby, Anita Lucaci, Steve Paterson, Julian Hiscox, Alistair Darby, M Almsaud, A Alrezaihi, Muhannad Alruwaili, Stuart D Armstrong, Jones Benjamin, Eleanor G Bentley, Anu Chawla, Jordan J Clark, Angela Cowell, Richard Eccles, Isabel García-Dorival, Matthew Gemmell, Alessandro Gerada, PKF Gilmore, Richard Gregory, Ximeng Han, Catherine Hartley, Margaret Hughes, Miren Iturriza-Gomara, James Johnson, L Luu, Jenifer Manson, Charlotte Nelson, Elaine O'Toole, Cassie Olateju, Rebekah Penrice-Randal , Lucille Rainbow, N.P Randle, Trevor Ian Robinson, Parul Sharma, Ghada T Shawli, James P Stewart, Neil Swainston, Ecaterina Vamos, Joanne Watts, Mark Whitehead
EPI_ISL_703330	Wales Specialist Virology Centre Sequencing lab: Pathogen Genomics Unit	COVID-19 Genomics UK (COG-UK) Consortium	Catherine Moore, Johnathan Evans, Laura Gifford, Malorie Perry, Simon Cottrell, Angela Marchbank, Alec Birchley, Alexander Adams, Amy Gaskin, Bree Gatica-Wilcox, Jason Coombes, Joel Southgate, Lauren Gilbert, Lee Graham, Nicole Pacchiarini, Sara Kumziene-Summerhayes, Sarah Taylor, Sophie Jones, Sara Rey, Matthew Bull, Joanne Watkins, Sally Corden, Tom Connor
EPI_ISL_703335	University College London, Great Ormond Street Hospital for Children NHS Foundation Trust, Imperial College Healthcare NHS Trust	COVID-19 Genomics UK (COG-UK) Consortium	Sergi Castellano, Rachel Williams, Mark Kristiansen, Paola Resende Silva, Sunando Roy, Tony Brooks, Helena Tutill, Paola Niola, Patricia Dyal, Charlotte Williams, Leysa Forrest, Yasmin Panchbhaya, Jacqueline Findlay, Samuel Weeks, Julianne Brown, Kathryn Harris, Paul Randell, James Price, Alison Holmes, Judith Breuer
EPI_ISL_703343	Wales Specialist Virology Centre Sequencing lab: Pathogen Genomics Unit	COVID-19 Genomics UK (COG-UK) Consortium	Catherine Moore, Johnathan Evans, Laura Gifford, Malorie Perry, Simon Cottrell, Angela Marchbank, Alec Birchley, Alexander Adams, Amy Gaskin, Bree Gatica-Wilcox, Jason Coombes, Joel Southgate, Lauren Gilbert, Lee Graham, Nicole Pacchiarini, Sara Kumziene-Summerhayes, Sarah Taylor, Sophie Jones, Sara Rey, Matthew Bull, Joanne Watkins, Sally Corden, Tom Connor
EPI_ISL_703353	Liverpool Clinical Laboratories	COVID-19 Genomics UK (COG-UK) Consortium	Sam Haldenby, Anita Lucaci, Steve Paterson, Julian Hiscox, Alistair Darby, M Almsaud, A Alrezaihi, Muhannad Alruwaili, Stuart D Armstrong, Jones Benjamin, Eleanor G Bentley, Anu Chawla, Jordan J Clark, Angela Cowell, Richard Eccles, Isabel García-Dorival, Matthew Gemmell, Alessandro Gerada, PKF Gilmore, Richard Gregory, Ximeng Han, Catherine Hartley, Margaret Hughes, Miren Iturriza-Gomara, James Johnson, L Luu, Jenifer Manson, Charlotte Nelson, Elaine O'Toole, Cassie Olateju, Rebekah Penrice-Randal , Lucille Rainbow, N.P Randle, Trevor Ian Robinson, Parul Sharma, Ghada T Shawli, James P Stewart, Neil Swainston, Ecaterina Vamos, Joanne Watts, Mark Whitehead
EPI_ISL_703359	Wales Specialist Virology Centre Sequencing lab: Pathogen Genomics Unit	COVID-19 Genomics UK (COG-UK) Consortium	Catherine Moore, Johnathan Evans, Laura Gifford, Malorie Perry, Simon Cottrell, Angela Marchbank, Alec Birchley, Alexander Adams, Amy Gaskin, Bree Gatica-Wilcox, Jason Coombes, Joel Southgate, Lauren Gilbert, Lee Graham, Nicole Pacchiarini, Sara Kumziene-Summerhayes, Sarah Taylor, Sophie Jones, Sara Rey, Matthew Bull, Joanne Watkins, Sally Corden, Tom Connor
EPI_ISL_703364, EPI_ISL_703367, EPI_ISL_703372, EPI_ISL_703375	Liverpool Clinical Laboratories	COVID-19 Genomics UK (COG-UK) Consortium	Sam Haldenby, Anita Lucaci, Steve Paterson, Julian Hiscox, Alistair Darby, M Almsaud, A Alrezaihi, Muhannad Alruwaili, Stuart D Armstrong, Jones Benjamin, Eleanor G Bentley, Anu Chawla, Jordan J Clark, Angela Cowell, Richard Eccles, Isabel García-Dorival, Matthew Gemmell, Alessandro Gerada, PKF Gilmore, Richard Gregory, Ximeng Han, Catherine Hartley, Margaret Hughes, Miren Iturriza-Gomara, James Johnson, L Luu, Jenifer Manson, Charlotte Nelson, Elaine O'Toole, Cassie Olateju, Rebekah Penrice-Randal , Lucille Rainbow, N.P Randle, Trevor Ian Robinson, Parul Sharma, Ghada T Shawli, James P Stewart, Neil Swainston, Ecaterina Vamos, Joanne Watts, Mark Whitehead
EPI_ISL_703377	Northumbria University / South Tees Hospitals NHS Foundation Trust / North Cumbria Integrated Care NHS Foundation Trust / North Tees and Hartlepool NHS Foundation Trust / Newcastle Hospitals NHS Foundation Trust	COVID-19 Genomics UK (COG-UK) Consortium	Darren L Smith,Andrew Nelson,Matthew Bashton,Greg R Young,Joshua Loh,John Allan,Mohammad A Tariq,Giles S Holt,Gary Black,Wen C Yew,Lynn Dover,Paul Baker,Steve Liggett,Sarah Essex,Jane Greenaway,Debra Padgett,Clive Graham,Garren Scott,Edward Barton,Emma Swindells,Brendan Payne,Jennifer Collins,Yusri Taha,Gary Eltringham
EPI_ISL_703383, EPI_ISL_703385	University College London, Great Ormond Street Hospital for Children NHS Foundation Trust, Imperial College Healthcare NHS Trust	COVID-19 Genomics UK (COG-UK) Consortium	Sergi Castellano, Rachel Williams, Mark Kristiansen, Paola Resende Silva, Sunando Roy, Tony Brooks, Helena Tutill, Paola Niola, Patricia Dyal, Charlotte Williams, Leysa Forrest, Yasmin Panchbhaya, Jacqueline Findlay, Samuel Weeks, Julianne Brown, Kathryn Harris, Paul Randell, James Price, Alison Holmes, Judith Breuer
EPI_ISL_703387	Liverpool Clinical Laboratories	COVID-19 Genomics UK (COG-UK) Consortium	Sam Haldenby, Anita Lucaci, Steve Paterson, Julian Hiscox, Alistair Darby, M Almsaud, A Alrezaihi, Muhannad Alruwaili, Stuart D Armstrong, Jones Benjamin, Eleanor G Bentley, Anu Chawla, Jordan J Clark, Angela Cowell, Richard Eccles, Isabel García-Dorival, Matthew Gemmell, Alessandro Gerada, PKF Gilmore, Richard Gregory, Ximeng Han, Catherine Hartley, Margaret Hughes, Miren Iturriza-Gomara, James Johnson, L Luu, Jenifer Manson, Charlotte Nelson, Elaine O'Toole, Cassie Olateju, Rebekah Penrice-Randal , Lucille Rainbow, N.P Randle, Trevor Ian Robinson, Parul Sharma, Ghada T Shawli, James P Stewart, Neil Swainston, Ecaterina Vamos, Joanne Watts, Mark Whitehead
EPI_ISL_703390	Northumbria University / South Tees Hospitals NHS Foundation Trust / North Cumbria Integrated Care NHS Foundation Trust / North Tees and Hartlepool NHS Foundation Trust / Newcastle Hospitals NHS Foundation Trust	COVID-19 Genomics UK (COG-UK) Consortium	Darren L Smith,Andrew Nelson,Matthew Bashton,Greg R Young,Joshua Loh,John Allan,Mohammad A Tariq,Giles S Holt,Gary Black,Wen C Yew,Lynn Dover,Paul Baker,Steve Liggett,Sarah Essex,Jane Greenaway,Debra Padgett,Clive Graham,Garren Scott,Edward Barton,Emma Swindells,Brendan Payne,Jennifer Collins,Yusri Taha,Gary Eltringham
EPI_ISL_703395, EPI_ISL_703398	Wales Specialist Virology Centre Sequencing lab: Pathogen Genomics Unit	COVID-19 Genomics UK (COG-UK) Consortium	Catherine Moore, Johnathan Evans, Laura Gifford, Malorie Perry, Simon Cottrell, Angela Marchbank, Alec Birchley, Alexander Adams, Amy Gaskin, Bree Gatica-Wilcox, Jason Coombes, Joel Southgate, Lauren Gilbert, Lee Graham, Nicole Pacchiarini, Sara Kumziene-Summerhayes, Sarah Taylor, Sophie Jones, Sara Rey, Matthew Bull, Joanne Watkins, Sally Corden, Tom Connor
EPI_ISL_703457	Department of Pathology, University of Cambridge	COVID-19 Genomics UK (COG-UK) Consortium	Aminu S. Jahun, Yasmin Chaudhry, Grant Hall, Iliana Georgana, Myra Hosmillo, Martin D. Curran, Malte Pinckert, Surendra Parmar, Ian Goodfellow
EPI_ISL_703466	Lighthouse Lab in Glasgow	Wellcome Sanger Institute for the COVID-19 Genomics UK (COG-UK) Consortium	Harper VanSteenhouse, Yumi Kasai, David Gray, Carol Clugston, Anna Dominiczak and Alex Alderton, Roberto Amato, Sonia Goncalves, Ewan Harrison, David K. Jackson, Ian Johnston, Dominic Kwiatkowski, Cordelia Langford, John Sillitoe on behalf of the Wellcome Sanger Institute COVID-19 Surveillance Team
EPI_ISL_703484	Northumbria University / South Tees Hospitals NHS Foundation Trust / North Cumbria Integrated Care NHS Foundation Trust / North Tees and Hartlepool NHS Foundation Trust / Newcastle Hospitals NHS Foundation Trust	COVID-19 Genomics UK (COG-UK) Consortium	Darren L Smith,Andrew Nelson,Matthew Bashton,Greg R Young,Joshua Loh,John Allan,Mohammad A Tariq,Giles S Holt,Gary Black,Wen C Yew,Lynn Dover,Paul Baker,Steve Liggett,Sarah Essex,Jane Greenaway,Debra Padgett,Clive Graham,Garren Scott,Edward Barton,Emma Swindells,Brendan Payne,Jennifer Collins,Yusri Taha,Gary Eltringham
EPI_ISL_703486	Lighthouse Lab in Glasgow	Wellcome Sanger Institute for the COVID-19 Genomics UK (COG-UK) Consortium	Harper VanSteenhouse, Yumi Kasai, David Gray, Carol Clugston, Anna Dominiczak and Alex Alderton, Roberto Amato, Sonia Goncalves, Ewan Harrison, David K. Jackson, Ian Johnston, Dominic Kwiatkowski, Cordelia Langford, John Sillitoe on behalf of the Wellcome Sanger Institute COVID-19 Surveillance

			Team
EPI_ISL_703527	Liverpool Clinical Laboratories	COVID-19 Genomics UK (COG-UK) Consortium	Sam Haldenby, Anita Lucaci, Steve Paterson, Julian Hiscox, Alistair Darby, M Almsaud, A Alrezaihi, Muhannad Alruwaili, Stuart D Armstrong, Jones Benjamin, Eleanor G Bentley, Anu Chawla, Jordan J Clark, Angela Cowell, Richard Eccles, Isabel García-Dorival, Matthew Gemmell, Alessandro Gerada, PKF Gilmore, Richard Gregory, Ximeng Han, Catherine Hartley, Margaret Hughes, Miren Iturriza-Gomara, James Johnson, L Luu, Jenifer Manson, Charlotte Nelson, Elaine O'Toole, Cassie Olateju, Rebekah Penrice-Randal , Lucille Rainbow, N.P Randle, Trevor Ian Robinson, Parul Sharma, Ghada T Shawli, James P Stewart, Neil Swainston, Ecaterina Vamos, Joanne Watts, Mark Whitehead
EPI_ISL_703540, EPI_ISL_703542, EPI_ISL_703545	Northumbria University / South Tees Hospitals NHS Foundation Trust / North Cumbria Integrated Care NHS Foundation Trust / North Tees and Hartlepool NHS Foundation Trust / Newcastle Hospitals NHS Foundation Trust	COVID-19 Genomics UK (COG-UK) Consortium	Darren L Smith,Andrew Nelson,Matthew Bashton,Greg R Young,Joshua Loh,John Allan,Mohammad A Tariq,Giles S Holt,Gary Black,Wen C Yew,Lynn Dover,Paul Baker,Steve Liggett,Sarah Essex,Jane Greenaway,Debra Padgett,Clive Graham,Garren Scott,Edward Barton,Emma Swindells,Brendan Payne,Jennifer Collins,Yusri Taha,Gary Eltringham
EPI_ISL_703575	Virology Department, Sheffield Teaching Hospitals NHS Foundation Trust/Department of Infection, Immunity and Cardiovascular Disease, The Medical School, University of Sheffield	COVID-19 Genomics UK (COG-UK) Consortium	Thushan de Silva, Matthew Parker, Nikki Smith, Adri Angyal, Rebecca Brown, Luke Green, Rachel Tucker, Paul Parsons, Danielle Groves, Katie Johnson, Laura Carrilero, Alex Keeley, Dave Partridge, Matthew Wyles, Benjamin Lindsey, Mehmet Yavuz, Mohammad Raza, Cariad Evans
EPI_ISL_703577	Liverpool Clinical Laboratories	COVID-19 Genomics UK (COG-UK) Consortium	Sam Haldenby, Anita Lucaci, Steve Paterson, Julian Hiscox, Alistair Darby, M Almsaud, A Alrezaihi, Muhannad Alruwaili, Stuart D Armstrong, Jones Benjamin, Eleanor G Bentley, Anu Chawla, Jordan J Clark, Angela Cowell, Richard Eccles, Isabel García-Dorival, Matthew Gemmell, Alessandro Gerada, PKF Gilmore, Richard Gregory, Ximeng Han, Catherine Hartley, Margaret Hughes, Miren Iturriza-Gomara, James Johnson, L Luu, Jenifer Manson, Charlotte Nelson, Elaine O'Toole, Cassie Olateju, Rebekah Penrice-Randal , Lucille Rainbow, N.P Randle, Trevor Ian Robinson, Parul Sharma, Ghada T Shawli, James P Stewart, Neil Swainston, Ecaterina Vamos, Joanne Watts, Mark Whitehead
EPI_ISL_703585, EPI_ISL_703593	Virology Department, Sheffield Teaching Hospitals NHS Foundation Trust/Department of Infection, Immunity and Cardiovascular Disease, The Medical School, University of Sheffield	COVID-19 Genomics UK (COG-UK) Consortium	Thushan de Silva, Matthew Parker, Nikki Smith, Adri Angyal, Rebecca Brown, Luke Green, Rachel Tucker, Paul Parsons, Danielle Groves, Katie Johnson, Laura Carrilero, Alex Keeley, Dave Partridge, Matthew Wyles, Benjamin Lindsey, Mehmet Yavuz, Mohammad Raza, Cariad Evans
EPI_ISL_703599	Northumbria University / South Tees Hospitals NHS Foundation Trust / North Cumbria Integrated Care NHS Foundation Trust / North Tees and Hartlepool NHS Foundation Trust / Newcastle Hospitals NHS Foundation Trust	COVID-19 Genomics UK (COG-UK) Consortium	Darren L Smith,Andrew Nelson,Matthew Bashton,Greg R Young,Joshua Loh,John Allan,Mohammad A Tariq,Giles S Holt,Gary Black,Wen C Yew,Lynn Dover,Paul Baker,Steve Liggett,Sarah Essex,Jane Greenaway,Debra Padgett,Clive Graham,Garren Scott,Edward Barton,Emma Swindells,Brendan Payne,Jennifer Collins,Yusri Taha,Gary Eltringham
EPI_ISL_703614	Wales Specialist Virology Centre Sequencing lab: Pathogen Genomics Unit	COVID-19 Genomics UK (COG-UK) Consortium	Catherine Moore, Johnathan Evans, Laura Gifford, Malorie Perry, Simon Cottrell, Angela Marchbank, Alec Birchley, Alexander Adams, Amy Gaskin, Bree Gatica-Wilcox, Jason Coombes, Joel Southgate, Lauren Gilbert, Lee Graham, Nicole Pacchiarini, Sara Kumziene-Summerhayes, Sarah Taylor, Sophie Jones, Sara Rey, Matthew Bull, Joanne Watkins, Sally Corden, Tom Connor
EPI_ISL_703619	Department of Pathology, University of Cambridge	COVID-19 Genomics UK (COG-UK) Consortium	Aminu S. Jahun, Yasmin Chaudhry, Grant Hall, Iliana Georgana, Myra Hosmillo, Martin D. Curran, Malte Pinckert, Surendra Parmar, Ian Goodfellow
EPI_ISL_703624	Liverpool Clinical Laboratories	COVID-19 Genomics UK (COG-UK) Consortium	Sam Haldenby, Anita Lucaci, Steve Paterson, Julian Hiscox, Alistair Darby, M Almsaud, A Alrezaihi, Muhannad Alruwaili, Stuart D Armstrong, Jones Benjamin, Eleanor G Bentley, Anu Chawla, Jordan J Clark, Angela Cowell, Richard Eccles, Isabel García-Dorival, Matthew Gemmell, Alessandro Gerada, PKF Gilmore, Richard Gregory, Ximeng Han, Catherine Hartley, Margaret Hughes, Miren Iturriza-Gomara, James Johnson, L Luu, Jenifer Manson, Charlotte Nelson, Elaine O'Toole, Cassie Olateju, Rebekah Penrice-Randal , Lucille Rainbow, N.P Randle, Trevor Ian Robinson, Parul Sharma, Ghada T Shawli, James P Stewart, Neil Swainston, Ecaterina Vamos, Joanne Watts, Mark Whitehead
EPI_ISL_703629, EPI_ISL_703679, EPI_ISL_703693	Department of Pathology, University of Cambridge	COVID-19 Genomics UK (COG-UK) Consortium	Aminu S. Jahun, Yasmin Chaudhry, Grant Hall, Iliana Georgana, Myra Hosmillo, Martin D. Curran, Malte Pinckert, Surendra Parmar, Ian Goodfellow
EPI_ISL_703698	Liverpool Clinical Laboratories	COVID-19 Genomics UK (COG-UK) Consortium	Sam Haldenby, Anita Lucaci, Steve Paterson, Julian Hiscox, Alistair Darby, M Almsaud, A Alrezaihi, Muhannad Alruwaili, Stuart D Armstrong, Jones Benjamin, Eleanor G Bentley, Anu Chawla, Jordan J Clark, Angela Cowell, Richard Eccles, Isabel García-Dorival, Matthew Gemmell, Alessandro Gerada, PKF Gilmore, Richard Gregory, Ximeng Han, Catherine Hartley, Margaret Hughes, Miren Iturriza-Gomara, James Johnson, L Luu, Jenifer Manson, Charlotte Nelson, Elaine O'Toole, Cassie Olateju, Rebekah Penrice-Randal , Lucille Rainbow, N.P Randle, Trevor Ian Robinson, Parul Sharma, Ghada T Shawli, James P Stewart, Neil Swainston, Ecaterina Vamos, Joanne Watts, Mark Whitehead
EPI_ISL_703704	Northumbria University / South Tees Hospitals NHS Foundation Trust / North Cumbria Integrated Care NHS Foundation Trust / North Tees and Hartlepool NHS Foundation Trust / Newcastle Hospitals NHS Foundation Trust	COVID-19 Genomics UK (COG-UK) Consortium	Darren L Smith,Andrew Nelson,Matthew Bashton,Greg R Young,Joshua Loh,John Allan,Mohammad A Tariq,Giles S Holt,Gary Black,Wen C Yew,Lynn Dover,Paul Baker,Steve Liggett,Sarah Essex,Jane Greenaway,Debra Padgett,Clive Graham,Garren Scott,Edward Barton,Emma Swindells,Brendan Payne,Jennifer Collins,Yusri Taha,Gary Eltringham
EPI_ISL_703706	University College London, Great Ormond Street Hospital for Children NHS Foundation Trust, Imperial College Healthcare NHS Trust	COVID-19 Genomics UK (COG-UK) Consortium	Sergi Castellano, Rachel Williams, Mark Kristiansen, Paola Resende Silva, Sunando Roy, Tony Brooks, Helena Tutill, Paola Niola, Patricia Dyal, Charlotte Williams, Leysa Forrest, Yasmin Panchbhaya, Jacqueline Findlay, Samuel Weeks, Julianne Brown, Kathryn Harris, Paul Randell, James Price, Alison Holmes, Judith Breuer
EPI_ISL_703717	West of Scotland Specialist Virology Centre, NHSGGC / MRC-University of Glasgow Centre for Virus Research	COVID-19 Genomics UK (COG-UK) Consortium	Ana da Silva Filipe, Natasha Johnson, Kathy Smollett, Daniel Mair, Stephen Carmichael, Alice Broos, Lily Tong, Jenna Nichols, Kyriaki Nomikou; Sarah McDonald; Richard Orton, Joseph Hughes, Sreenu Vattipally, David L Robertson; Alasdair MacLean, Rory Gunson; Sharif Shaaban, Matthew Holden; Rachel Blacow, Guy Mollett, Kathy Li, James Shepherd, Antonia Ho, Emma Thomson
EPI_ISL_703722	Wales Specialist Virology Centre Sequencing lab: Pathogen Genomics Unit	COVID-19 Genomics UK (COG-UK) Consortium	Catherine Moore, Johnathan Evans, Laura Gifford, Malorie Perry, Simon Cottrell, Angela Marchbank, Alec Birchley, Alexander Adams, Amy Gaskin, Bree Gatica-Wilcox, Jason Coombes, Joel Southgate, Lauren Gilbert, Lee Graham, Nicole Pacchiarini, Sara Kumziene-Summerhayes, Sarah Taylor, Sophie Jones, Sara Rey, Matthew Bull, Joanne Watkins, Sally Corden, Tom Connor
EPI_ISL_703728	Northumbria University / South Tees Hospitals NHS Foundation Trust / North Cumbria Integrated Care NHS Foundation Trust / North Tees and Hartlepool NHS Foundation Trust / Newcastle Hospitals NHS Foundation Trust	COVID-19 Genomics UK (COG-UK) Consortium	Darren L Smith,Andrew Nelson,Matthew Bashton,Greg R Young,Joshua Loh,John Allan,Mohammad A Tariq,Giles S Holt,Gary Black,Wen C Yew,Lynn Dover,Paul Baker,Steve Liggett,Sarah Essex,Jane Greenaway,Debra Padgett,Clive Graham,Garren Scott,Edward Barton,Emma Swindells,Brendan Payne,Jennifer Collins,Yusri Taha,Gary Eltringham
EPI_ISL_703732	Liverpool Clinical Laboratories	COVID-19 Genomics UK (COG-UK) Consortium	Sam Haldenby, Anita Lucaci, Steve Paterson, Julian Hiscox, Alistair Darby, M Almsaud, A Alrezaihi, Muhannad Alruwaili, Stuart D Armstrong, Jones Benjamin, Eleanor G Bentley, Anu Chawla, Jordan J Clark, Angela Cowell, Richard Eccles, Isabel García-Dorival, Matthew Gemmell, Alessandro Gerada, PKF Gilmore, Richard Gregory, Ximeng Han, Catherine Hartley, Margaret Hughes, Miren Iturriza-Gomara, James Johnson, L Luu, Jenifer Manson, Charlotte Nelson, Elaine O'Toole, Cassie Olateju, Rebekah Penrice-Randal , Lucille Rainbow, N.P Randle, Trevor Ian Robinson, Parul Sharma, Ghada T Shawli, James P Stewart, Neil Swainston, Ecaterina Vamos, Joanne Watts, Mark Whitehead
EPI_ISL_703739	University College London, Great Ormond Street Hospital for Children NHS Foundation Trust, Imperial College Healthcare NHS Trust	COVID-19 Genomics UK (COG-UK) Consortium	Sergi Castellano, Rachel Williams, Mark Kristiansen, Paola Resende Silva, Sunando Roy, Tony Brooks, Helena Tutill, Paola Niola, Patricia Dyal, Charlotte Williams, Leysa Forrest, Yasmin Panchbhaya, Jacqueline Findlay, Samuel Weeks, Julianne Brown, Kathryn Harris, Paul Randell, James Price, Alison Holmes, Judith Breuer
EPI_ISL_703741	Department of Pathology, University of Cambridge	COVID-19 Genomics UK (COG-UK) Consortium	Aminu S. Jahun, Yasmin Chaudhry, Grant Hall, Iliana Georgana, Myra Hosmillo, Martin D. Curran, Malte Pinckert, Surendra Parmar, Ian Goodfellow
EPI_ISL_703743	West of Scotland Specialist Virology Centre, NHSGGC / MRC-University of Glasgow Centre for Virus Research	COVID-19 Genomics UK (COG-UK) Consortium	Ana da Silva Filipe, Natasha Johnson, Kathy Smollett, Daniel Mair, Stephen Carmichael, Alice Broos, Lily Tong, Jenna Nichols, Kyriaki Nomikou; Sarah McDonald; Richard Orton, Joseph Hughes, Sreenu Vattipally, David L Robertson; Alasdair MacLean, Rory Gunson; Sharif Shaaban, Matthew Holden; Rachel Blacow, Guy Mollett, Kathy Li, James Shepherd, Antonia Ho, Emma Thomson
EPI_ISL_703746	Wales Specialist Virology Centre Sequencing lab: Pathogen Genomics Unit	COVID-19 Genomics UK (COG-UK) Consortium	Catherine Moore, Johnathan Evans, Laura Gifford, Malorie Perry, Simon Cottrell, Angela Marchbank, Alec Birchley, Alexander Adams, Amy Gaskin, Bree Gatica-Wilcox, Jason Coombes, Joel Southgate, Lauren Gilbert, Lee Graham, Nicole Pacchiarini, Sara Kumziene-Summerhayes, Sarah Taylor, Sophie Jones, Sara Rey, Matthew Bull, Joanne Watkins, Sally Corden, Tom Connor
EPI_ISL_703747	Department of Pathology, University of Cambridge	COVID-19 Genomics UK (COG-UK) Consortium	Aminu S. Jahun, Yasmin Chaudhry, Grant Hall, Iliana Georgana, Myra Hosmillo, Martin D. Curran, Malte Pinckert, Surendra Parmar, Ian Goodfellow

EPI_ISL_703748, EPI_ISL_703749, EPI_ISL_703750, EPI_ISL_703751, EPI_ISL_703756	West of Scotland Specialist Virology Centre, NHSGGC / MRC-University of Glasgow Centre for Virus Research	COVID-19 Genomics UK (COG-UK) Consortium	Ana da Silva Filipe, Natasha Johnson, Kathy Smollett, Daniel Mair, Stephen Carmichael, Alice Broos, Lily Tong, Jenna Nichols, Kyriaki Nomikou; Sarah McDonald; Richard Orton, Joseph Hughes, Sreenu Vattipally, David L Robertson; Alasdair MacLean, Rory Gunson; Sharif Shaaban, Matthew Holden; Rachel Blacow, Guy Mollett, Kathy Li, James Shepherd, Antonia Ho, Emma Thomson
EPI_ISL_703760, EPI_ISL_703761	Wales Specialist Virology Centre Sequencing lab: Pathogen Genomics Unit	COVID-19 Genomics UK (COG-UK) Consortium	Catherine Moore, Johnathan Evans, Laura Gifford, Malorie Perry, Simon Cottrell, Angela Marchbank, Alec Birchley, Alexander Adams, Amy Gaskin, Bree Gatica-Wilcox, Jason Coombes, Joel Southgate, Lauren Gilbert, Lee Graham, Nicole Pacchiarini, Sara Kumziene-Summerhayes, Sarah Taylor, Sophie Jones, Sara Rey, Matthew Bull, Joanne Watkins, Sally Corden, Tom Connor
EPI_ISL_703767	University College London, Great Ormond Street Hospital for Children NHS Foundation Trust, Imperial College Healthcare NHS Trust	COVID-19 Genomics UK (COG-UK) Consortium	Sergi Castellano, Rachel Williams, Mark Kristiansen, Paola Resende Silva, Sunando Roy, Tony Brooks, Helena Tutill, Paola Niola, Patricia Dyal, Charlotte Williams, Leysa Forrest, Yasmin Panchbhaya, Jacqueline Findlay, Samuel Weeks, Julianne Brown, Kathryn Harris, Paul Randell, James Price, Alison Holmes, Judith Breuer
EPI_ISL_703768	Wales Specialist Virology Centre Sequencing lab: Pathogen Genomics Unit	COVID-19 Genomics UK (COG-UK) Consortium	Catherine Moore, Johnathan Evans, Laura Gifford, Malorie Perry, Simon Cottrell, Angela Marchbank, Alec Birchley, Alexander Adams, Amy Gaskin, Bree Gatica-Wilcox, Jason Coombes, Joel Southgate, Lauren Gilbert, Lee Graham, Nicole Pacchiarini, Sara Kumziene-Summerhayes, Sarah Taylor, Sophie Jones, Sara Rey, Matthew Bull, Joanne Watkins, Sally Corden, Tom Connor
EPI_ISL_703770	Liverpool Clinical Laboratories	COVID-19 Genomics UK (COG-UK) Consortium	Sam Haldenby, Anita Lucaci, Steve Paterson, Julian Hiscox, Alistair Darby, M Almsaud, A Alrezaihi, Muhannad Alruwaili, Stuart D Armstrong, Jones Benjamin, Eleanor G Bentley, Anu Chawla, Jordan J Clark, Angela Cowell, Richard Eccles, Isabel García-Dorival, Matthew Gemmell, Alessandro Gerada, PKF Gilmore, Richard Gregory, Ximeng Han, Catherine Hartley, Margaret Hughes, Miren Iturriza-Gomara, James Johnson, L Luu, Jenifer Manson, Charlotte Nelson, Elaine O'Toole, Cassie Olateju, Rebekah Penrice-Randal , Lucille Rainbow, N.P Randle, Trevor Ian Robinson, Parul Sharma, Ghada T Shawli, James P Stewart, Neil Swainston, Ecaterina Vamos, Joanne Watts, Mark Whitehead
EPI_ISL_703773	West of Scotland Specialist Virology Centre, NHSGGC / MRC-University of Glasgow Centre for Virus Research	COVID-19 Genomics UK (COG-UK) Consortium	Ana da Silva Filipe, Natasha Johnson, Kathy Smollett, Daniel Mair, Stephen Carmichael, Alice Broos, Lily Tong, Jenna Nichols, Kyriaki Nomikou; Sarah McDonald; Richard Orton, Joseph Hughes, Sreenu Vattipally, David L Robertson; Alasdair MacLean, Rory Gunson; Sharif Shaaban, Matthew Holden; Rachel Blacow, Guy Mollett, Kathy Li, James Shepherd, Antonia Ho, Emma Thomson
EPI_ISL_703810	Virology Department, Royal Infirmary of Edinburgh, NHS Lothian / School of Biological Sciences, University of Edinburgh / Institute of Genetics and Molecular Medicine, University of Edinburgh	COVID-19 Genomics UK (COG-UK) Consortium	McHugh M, Dewar R, Rooke S, Gallagher M, Balcaza C, O'Toole Á, Scher E, Hill V, McCrone JT, Colquhoun R, Yu X, Jackson B, Rambaut A, Williams TC, Templeton K
EPI_ISL_703821, EPI_ISL_703823, EPI_ISL_703826	Liverpool Clinical Laboratories	COVID-19 Genomics UK (COG-UK) Consortium	Sam Haldenby, Anita Lucaci, Steve Paterson, Julian Hiscox, Alistair Darby, M Almsaud, A Alrezaihi, Muhannad Alruwaili, Stuart D Armstrong, Jones Benjamin, Eleanor G Bentley, Anu Chawla, Jordan J Clark, Angela Cowell, Richard Eccles, Isabel García-Dorival, Matthew Gemmell, Alessandro Gerada, PKF Gilmore, Richard Gregory, Ximeng Han, Catherine Hartley, Margaret Hughes, Miren Iturriza-Gomara, James Johnson, L Luu, Jenifer Manson, Charlotte Nelson, Elaine O'Toole, Cassie Olateju, Rebekah Penrice-Randal , Lucille Rainbow, N.P Randle, Trevor Ian Robinson, Parul Sharma, Ghada T Shawli, James P Stewart, Neil Swainston, Ecaterina Vamos, Joanne Watts, Mark Whitehead
EPI_ISL_703858, EPI_ISL_703860	Wales Specialist Virology Centre Sequencing lab: Pathogen Genomics Unit	COVID-19 Genomics UK (COG-UK) Consortium	Catherine Moore, Johnathan Evans, Laura Gifford, Malorie Perry, Simon Cottrell, Angela Marchbank, Alec Birchley, Alexander Adams, Amy Gaskin, Bree Gatica-Wilcox, Jason Coombes, Joel Southgate, Lauren Gilbert, Lee Graham, Nicole Pacchiarini, Sara Kumziene-Summerhayes, Sarah Taylor, Sophie Jones, Sara Rey, Matthew Bull, Joanne Watkins, Sally Corden, Tom Connor
EPI_ISL_703865	University College London, Great Ormond Street Hospital for Children NHS Foundation Trust, Imperial College Healthcare NHS Trust	COVID-19 Genomics UK (COG-UK) Consortium	Sergi Castellano, Rachel Williams, Mark Kristiansen, Paola Resende Silva, Sunando Roy, Tony Brooks, Helena Tutill, Paola Niola, Patricia Dyal, Charlotte Williams, Leysa Forrest, Yasmin Panchbhaya, Jacqueline Findlay, Samuel Weeks, Julianne Brown, Kathryn Harris, Paul Randell, James Price, Alison Holmes, Judith Breuer
EPI_ISL_703870, EPI_ISL_703873	Wales Specialist Virology Centre Sequencing lab: Pathogen Genomics Unit	COVID-19 Genomics UK (COG-UK) Consortium	Catherine Moore, Johnathan Evans, Laura Gifford, Malorie Perry, Simon Cottrell, Angela Marchbank, Alec Birchley, Alexander Adams, Amy Gaskin, Bree Gatica-Wilcox, Jason Coombes, Joel Southgate, Lauren Gilbert, Lee Graham, Nicole Pacchiarini, Sara Kumziene-Summerhayes, Sarah Taylor, Sophie Jones, Sara Rey, Matthew Bull, Joanne Watkins, Sally Corden, Tom Connor
EPI_ISL_703879	University College London, Great Ormond Street Hospital for Children NHS Foundation Trust, Imperial College Healthcare NHS Trust	COVID-19 Genomics UK (COG-UK) Consortium	Sergi Castellano, Rachel Williams, Mark Kristiansen, Paola Resende Silva, Sunando Roy, Tony Brooks, Helena Tutill, Paola Niola, Patricia Dyal, Charlotte Williams, Leysa Forrest, Yasmin Panchbhaya, Jacqueline Findlay, Samuel Weeks, Julianne Brown, Kathryn Harris, Paul Randell, James Price, Alison Holmes, Judith Breuer
EPI_ISL_703884, EPI_ISL_703891, EPI_ISL_703897	Liverpool Clinical Laboratories	COVID-19 Genomics UK (COG-UK) Consortium	Sam Haldenby, Anita Lucaci, Steve Paterson, Julian Hiscox, Alistair Darby, M Almsaud, A Alrezaihi, Muhannad Alruwaili, Stuart D Armstrong, Jones Benjamin, Eleanor G Bentley, Anu Chawla, Jordan J Clark, Angela Cowell, Richard Eccles, Isabel García-Dorival, Matthew Gemmell, Alessandro Gerada, PKF Gilmore, Richard Gregory, Ximeng Han, Catherine Hartley, Margaret Hughes, Miren Iturriza-Gomara, James Johnson, L Luu, Jenifer Manson, Charlotte Nelson, Elaine O'Toole, Cassie Olateju, Rebekah Penrice-Randal , Lucille Rainbow, N.P Randle, Trevor Ian Robinson, Parul Sharma, Ghada T Shawli, James P Stewart, Neil Swainston, Ecaterina Vamos, Joanne Watts, Mark Whitehead
EPI_ISL_703911	West of Scotland Specialist Virology Centre, NHSGGC / MRC-University of Glasgow Centre for Virus Research	COVID-19 Genomics UK (COG-UK) Consortium	Ana da Silva Filipe, Natasha Johnson, Kathy Smollett, Daniel Mair, Stephen Carmichael, Alice Broos, Lily Tong, Jenna Nichols, Kyriaki Nomikou; Sarah McDonald; Richard Orton, Joseph Hughes, Sreenu Vattipally, David L Robertson; Alasdair MacLean, Rory Gunson; Sharif Shaaban, Matthew Holden; Rachel Blacow, Guy Mollett, Kathy Li, James Shepherd, Antonia Ho, Emma Thomson
EPI_ISL_703913	Liverpool Clinical Laboratories	COVID-19 Genomics UK (COG-UK) Consortium	Sam Haldenby, Anita Lucaci, Steve Paterson, Julian Hiscox, Alistair Darby, M Almsaud, A Alrezaihi, Muhannad Alruwaili, Stuart D Armstrong, Jones Benjamin, Eleanor G Bentley, Anu Chawla, Jordan J Clark, Angela Cowell, Richard Eccles, Isabel García-Dorival, Matthew Gemmell, Alessandro Gerada, PKF Gilmore, Richard Gregory, Ximeng Han, Catherine Hartley, Margaret Hughes, Miren Iturriza-Gomara, James Johnson, L Luu, Jenifer Manson, Charlotte Nelson, Elaine O'Toole, Cassie Olateju, Rebekah Penrice-Randal , Lucille Rainbow, N.P Randle, Trevor Ian Robinson, Parul Sharma, Ghada T Shawli, James P Stewart, Neil Swainston, Ecaterina Vamos, Joanne Watts, Mark Whitehead
EPI_ISL_703918	Wales Specialist Virology Centre Sequencing lab: Pathogen Genomics Unit	COVID-19 Genomics UK (COG-UK) Consortium	Catherine Moore, Johnathan Evans, Laura Gifford, Malorie Perry, Simon Cottrell, Angela Marchbank, Alec Birchley, Alexander Adams, Amy Gaskin, Bree Gatica-Wilcox, Jason Coombes, Joel Southgate, Lauren Gilbert, Lee Graham, Nicole Pacchiarini, Sara Kumziene-Summerhayes, Sarah Taylor, Sophie Jones, Sara Rey, Matthew Bull, Joanne Watkins, Sally Corden, Tom Connor
EPI_ISL_703937	Liverpool Clinical Laboratories	COVID-19 Genomics UK (COG-UK) Consortium	Sam Haldenby, Anita Lucaci, Steve Paterson, Julian Hiscox, Alistair Darby, M Almsaud, A Alrezaihi, Muhannad Alruwaili, Stuart D Armstrong, Jones Benjamin, Eleanor G Bentley, Anu Chawla, Jordan J Clark, Angela Cowell, Richard Eccles, Isabel García-Dorival, Matthew Gemmell, Alessandro Gerada, PKF Gilmore, Richard Gregory, Ximeng Han, Catherine Hartley, Margaret Hughes, Miren Iturriza-Gomara, James Johnson, L Luu, Jenifer Manson, Charlotte Nelson, Elaine O'Toole, Cassie Olateju, Rebekah Penrice-Randal , Lucille Rainbow, N.P Randle, Trevor Ian Robinson, Parul Sharma, Ghada T Shawli, James P Stewart, Neil Swainston, Ecaterina Vamos, Joanne Watts, Mark Whitehead
EPI_ISL_703942	Wales Specialist Virology Centre Sequencing lab: Pathogen Genomics Unit	COVID-19 Genomics UK (COG-UK) Consortium	Catherine Moore, Johnathan Evans, Laura Gifford, Malorie Perry, Simon Cottrell, Angela Marchbank, Alec Birchley, Alexander Adams, Amy Gaskin, Bree Gatica-Wilcox, Jason Coombes, Joel Southgate, Lauren Gilbert, Lee Graham, Nicole Pacchiarini, Sara Kumziene-Summerhayes, Sarah Taylor, Sophie Jones, Sara Rey, Matthew Bull, Joanne Watkins, Sally Corden, Tom Connor
EPI_ISL_703952	Liverpool Clinical Laboratories	COVID-19 Genomics UK (COG-UK) Consortium	Sam Haldenby, Anita Lucaci, Steve Paterson, Julian Hiscox, Alistair Darby, M Almsaud, A Alrezaihi, Muhannad Alruwaili, Stuart D Armstrong, Jones Benjamin, Eleanor G Bentley, Anu Chawla, Jordan J Clark, Angela Cowell, Richard Eccles, Isabel García-Dorival, Matthew Gemmell, Alessandro Gerada, PKF Gilmore, Richard Gregory, Ximeng Han, Catherine Hartley, Margaret Hughes, Miren Iturriza-Gomara, James Johnson, L Luu, Jenifer Manson, Charlotte Nelson, Elaine O'Toole, Cassie Olateju, Rebekah Penrice-Randal , Lucille Rainbow, N.P Randle, Trevor Ian Robinson, Parul Sharma, Ghada T Shawli, James P Stewart, Neil Swainston, Ecaterina Vamos, Joanne Watts, Mark Whitehead
EPI_ISL_703955	Wales Specialist Virology Centre Sequencing lab: Pathogen Genomics Unit	COVID-19 Genomics UK (COG-UK) Consortium	Catherine Moore, Johnathan Evans, Laura Gifford, Malorie Perry, Simon Cottrell, Angela Marchbank, Alec Birchley, Alexander Adams, Amy Gaskin, Bree Gatica-Wilcox, Jason Coombes, Joel Southgate, Lauren Gilbert, Lee Graham, Nicole Pacchiarini, Sara Kumziene-Summerhayes, Sarah Taylor, Sophie Jones, Sara Rey, Matthew Bull, Joanne Watkins, Sally Corden, Tom Connor
EPI_ISL_703961, EPI_ISL_703977, EPI_ISL_703995	Liverpool Clinical Laboratories	COVID-19 Genomics UK (COG-UK) Consortium	Sam Haldenby, Anita Lucaci, Steve Paterson, Julian Hiscox, Alistair Darby, M Almsaud, A Alrezaihi, Muhannad Alruwaili, Stuart D Armstrong, Jones Benjamin, Eleanor G Bentley, Anu Chawla, Jordan J Clark, Angela Cowell, Richard Eccles, Isabel García-Dorival, Matthew Gemmell, Alessandro Gerada,

			PKF Gilmore, Richard Gregory, Ximeng Han, Catherine Hartley, Margaret Hughes, Miren Iturriza-Gomara, James Johnson, L Luu, Jenifer Manson, Charlotte Nelson, Elaine O'Toole, Cassie Olateju, Rebekah Penrice-Randal , Lucille Rainbow, N.P Randle, Trevor Ian Robinson, Parul Sharma, Ghada T Shawli, James P Stewart, Neil Swainston, Ecaterina Vamos, Joanne Watts, Mark Whitehead
EPI_ISL_704001, EPI_ISL_704014	Wales Specialist Virology Centre Sequencing lab: Pathogen Genomics Unit	COVID-19 Genomics UK (COG-UK) Consortium	Catherine Moore, Johnathan Evans, Laura Gifford, Malorie Perry, Simon Cottrell, Angela Marchbank, Alec Birchley, Alexander Adams, Amy Gaskin, Bree Gatica-Wilcox, Jason Coombes, Joel Southgate, Lauren Gilbert, Lee Graham, Nicole Pacchiarini, Sara Kumziene-Summerhayes, Sarah Taylor, Sophie Jones, Sara Rey, Matthew Bull, Joanne Watkins, Sally Corden, Tom Connor
EPI_ISL_704022	Liverpool Clinical Laboratories	COVID-19 Genomics UK (COG-UK) Consortium	Sam Haldenby, Anita Lucaci, Steve Paterson, Julian Hiscox, Alistair Darby, M Almsaud, A Alrezaihi, Muhannad Alruwaili, Stuart D Armstrong, Jones Benjamin, Eleanor G Bentley, Anu Chawla, Jordan J Clark, Angela Cowell, Richard Eccles, Isabel García-Dorival, Matthew Gemmell, Alessandro Gerada, PKF Gilmore, Richard Gregory, Ximeng Han, Catherine Hartley, Margaret Hughes, Miren Iturriza-Gomara, James Johnson, L Luu, Jenifer Manson, Charlotte Nelson, Elaine O'Toole, Cassie Olateju, Rebekah Penrice-Randal , Lucille Rainbow, N.P Randle, Trevor Ian Robinson, Parul Sharma, Ghada T Shawli, James P Stewart, Neil Swainston, Ecaterina Vamos, Joanne Watts, Mark Whitehead
EPI_ISL_704025	University College London, Great Ormond Street Hospital for Children NHS Foundation Trust, Imperial College Healthcare NHS Trust	COVID-19 Genomics UK (COG-UK) Consortium	Sergi Castellano, Rachel Williams, Mark Kristiansen, Paola Resende Silva, Sunando Roy, Tony Brooks, Helena Tutill, Paola Niola, Patricia Dyal, Charlotte Williams, Leysa Forrest, Yasmin Panchbhaya, Jacqueline Findlay, Samuel Weeks, Julianne Brown, Kathryn Harris, Paul Randell, James Price, Alison Holmes, Judith Breuer
EPI_ISL_704030	Wales Specialist Virology Centre Sequencing lab: Pathogen Genomics Unit	COVID-19 Genomics UK (COG-UK) Consortium	Catherine Moore, Johnathan Evans, Laura Gifford, Malorie Perry, Simon Cottrell, Angela Marchbank, Alec Birchley, Alexander Adams, Amy Gaskin, Bree Gatica-Wilcox, Jason Coombes, Joel Southgate, Lauren Gilbert, Lee Graham, Nicole Pacchiarini, Sara Kumziene-Summerhayes, Sarah Taylor, Sophie Jones, Sara Rey, Matthew Bull, Joanne Watkins, Sally Corden, Tom Connor
EPI_ISL_704033	Liverpool Clinical Laboratories	COVID-19 Genomics UK (COG-UK) Consortium	Sam Haldenby, Anita Lucaci, Steve Paterson, Julian Hiscox, Alistair Darby, M Almsaud, A Alrezaihi, Muhannad Alruwaili, Stuart D Armstrong, Jones Benjamin, Eleanor G Bentley, Anu Chawla, Jordan J Clark, Angela Cowell, Richard Eccles, Isabel García-Dorival, Matthew Gemmell, Alessandro Gerada, PKF Gilmore, Richard Gregory, Ximeng Han, Catherine Hartley, Margaret Hughes, Miren Iturriza-Gomara, James Johnson, L Luu, Jenifer Manson, Charlotte Nelson, Elaine O'Toole, Cassie Olateju, Rebekah Penrice-Randal , Lucille Rainbow, N.P Randle, Trevor Ian Robinson, Parul Sharma, Ghada T Shawli, James P Stewart, Neil Swainston, Ecaterina Vamos, Joanne Watts, Mark Whitehead
EPI_ISL_704036	University College London, Great Ormond Street Hospital for Children NHS Foundation Trust, Imperial College Healthcare NHS Trust	COVID-19 Genomics UK (COG-UK) Consortium	Sergi Castellano, Rachel Williams, Mark Kristiansen, Paola Resende Silva, Sunando Roy, Tony Brooks, Helena Tutill, Paola Niola, Patricia Dyal, Charlotte Williams, Leysa Forrest, Yasmin Panchbhaya, Jacqueline Findlay, Samuel Weeks, Julianne Brown, Kathryn Harris, Paul Randell, James Price, Alison Holmes, Judith Breuer
EPI_ISL_704038, EPI_ISL_704044	Wales Specialist Virology Centre Sequencing lab: Pathogen Genomics Unit	COVID-19 Genomics UK (COG-UK) Consortium	Catherine Moore, Johnathan Evans, Laura Gifford, Malorie Perry, Simon Cottrell, Angela Marchbank, Alec Birchley, Alexander Adams, Amy Gaskin, Bree Gatica-Wilcox, Jason Coombes, Joel Southgate, Lauren Gilbert, Lee Graham, Nicole Pacchiarini, Sara Kumziene-Summerhayes, Sarah Taylor, Sophie Jones, Sara Rey, Matthew Bull, Joanne Watkins, Sally Corden, Tom Connor
EPI_ISL_704049	Liverpool Clinical Laboratories	COVID-19 Genomics UK (COG-UK) Consortium	Sam Haldenby, Anita Lucaci, Steve Paterson, Julian Hiscox, Alistair Darby, M Almsaud, A Alrezaihi, Muhannad Alruwaili, Stuart D Armstrong, Jones Benjamin, Eleanor G Bentley, Anu Chawla, Jordan J Clark, Angela Cowell, Richard Eccles, Isabel García-Dorival, Matthew Gemmell, Alessandro Gerada, PKF Gilmore, Richard Gregory, Ximeng Han, Catherine Hartley, Margaret Hughes, Miren Iturriza-Gomara, James Johnson, L Luu, Jenifer Manson, Charlotte Nelson, Elaine O'Toole, Cassie Olateju, Rebekah Penrice-Randal , Lucille Rainbow, N.P Randle, Trevor Ian Robinson, Parul Sharma, Ghada T Shawli, James P Stewart, Neil Swainston, Ecaterina Vamos, Joanne Watts, Mark Whitehead
EPI_ISL_704086, EPI_ISL_704092	University College London, Great Ormond Street Hospital for Children NHS Foundation Trust, Imperial College Healthcare NHS Trust	COVID-19 Genomics UK (COG-UK) Consortium	Sergi Castellano, Rachel Williams, Mark Kristiansen, Paola Resende Silva, Sunando Roy, Tony Brooks, Helena Tutill, Paola Niola, Patricia Dyal, Charlotte Williams, Leysa Forrest, Yasmin Panchbhaya, Jacqueline Findlay, Samuel Weeks, Julianne Brown, Kathryn Harris, Paul Randell, James Price, Alison Holmes, Judith Breuer
EPI_ISL_704095	Wales Specialist Virology Centre Sequencing lab: Pathogen Genomics Unit	COVID-19 Genomics UK (COG-UK) Consortium	Catherine Moore, Johnathan Evans, Laura Gifford, Malorie Perry, Simon Cottrell, Angela Marchbank, Alec Birchley, Alexander Adams, Amy Gaskin, Bree Gatica-Wilcox, Jason Coombes, Joel Southgate, Lauren Gilbert, Lee Graham, Nicole Pacchiarini, Sara Kumziene-Summerhayes, Sarah Taylor, Sophie Jones, Sara Rey, Matthew Bull, Joanne Watkins, Sally Corden, Tom Connor
EPI_ISL_704100	Quadram Institute Bioscience	COVID-19 Genomics UK (COG-UK) Consortium	Dave J. Baker, Gemma L. Kay, Alp Aydin, Thanh Le-Viet, Steven Rudder, Ana P. Tedim, Anastasia Kolyva, Maria Diaz, Leonardo de Oliveira Martins, Nabil-Fareed Alikhan, Lizzie Meadows, Rachael Stanley, Ngozi Elumogo, Muhammed Yasir, Nicholas M. Thomson, Alexander J Trotter, Rachel Gilroy, Samuel Bloomfield, Claire Stuart, Andrew Bell, Reenesh Prakash, Samir Dervisevic, Alison E. Mather, John Wain, Mark Webber, Andrew J. Page, Justin O'Grady
EPI_ISL_704111, EPI_ISL_704126	Wales Specialist Virology Centre Sequencing lab: Pathogen Genomics Unit	COVID-19 Genomics UK (COG-UK) Consortium	Catherine Moore, Johnathan Evans, Laura Gifford, Malorie Perry, Simon Cottrell, Angela Marchbank, Alec Birchley, Alexander Adams, Amy Gaskin, Bree Gatica-Wilcox, Jason Coombes, Joel Southgate, Lauren Gilbert, Lee Graham, Nicole Pacchiarini, Sara Kumziene-Summerhayes, Sarah Taylor, Sophie Jones, Sara Rey, Matthew Bull, Joanne Watkins, Sally Corden, Tom Connor
EPI_ISL_704131, EPI_ISL_704137	Liverpool Clinical Laboratories	COVID-19 Genomics UK (COG-UK) Consortium	Sam Haldenby, Anita Lucaci, Steve Paterson, Julian Hiscox, Alistair Darby, M Almsaud, A Alrezaihi, Muhannad Alruwaili, Stuart D Armstrong, Jones Benjamin, Eleanor G Bentley, Anu Chawla, Jordan J Clark, Angela Cowell, Richard Eccles, Isabel García-Dorival, Matthew Gemmell, Alessandro Gerada, PKF Gilmore, Richard Gregory, Ximeng Han, Catherine Hartley, Margaret Hughes, Miren Iturriza-Gomara, James Johnson, L Luu, Jenifer Manson, Charlotte Nelson, Elaine O'Toole, Cassie Olateju, Rebekah Penrice-Randal , Lucille Rainbow, N.P Randle, Trevor Ian Robinson, Parul Sharma, Ghada T Shawli, James P Stewart, Neil Swainston, Ecaterina Vamos, Joanne Watts, Mark Whitehead
EPI_ISL_704161	Wales Specialist Virology Centre Sequencing lab: Pathogen Genomics Unit	COVID-19 Genomics UK (COG-UK) Consortium	Catherine Moore, Johnathan Evans, Laura Gifford, Malorie Perry, Simon Cottrell, Angela Marchbank, Alec Birchley, Alexander Adams, Amy Gaskin, Bree Gatica-Wilcox, Jason Coombes, Joel Southgate, Lauren Gilbert, Lee Graham, Nicole Pacchiarini, Sara Kumziene-Summerhayes, Sarah Taylor, Sophie Jones, Sara Rey, Matthew Bull, Joanne Watkins, Sally Corden, Tom Connor
EPI_ISL_704168	Liverpool Clinical Laboratories	COVID-19 Genomics UK (COG-UK) Consortium	Sam Haldenby, Anita Lucaci, Steve Paterson, Julian Hiscox, Alistair Darby, M Almsaud, A Alrezaihi, Muhannad Alruwaili, Stuart D Armstrong, Jones Benjamin, Eleanor G Bentley, Anu Chawla, Jordan J Clark, Angela Cowell, Richard Eccles, Isabel García-Dorival, Matthew Gemmell, Alessandro Gerada, PKF Gilmore, Richard Gregory, Ximeng Han, Catherine Hartley, Margaret Hughes, Miren Iturriza-Gomara, James Johnson, L Luu, Jenifer Manson, Charlotte Nelson, Elaine O'Toole, Cassie Olateju, Rebekah Penrice-Randal , Lucille Rainbow, N.P Randle, Trevor Ian Robinson, Parul Sharma, Ghada T Shawli, James P Stewart, Neil Swainston, Ecaterina Vamos, Joanne Watts, Mark Whitehead
EPI_ISL_704179	University College London, Great Ormond Street Hospital for Children NHS Foundation Trust, Imperial College Healthcare NHS Trust	COVID-19 Genomics UK (COG-UK) Consortium	Sergi Castellano, Rachel Williams, Mark Kristiansen, Paola Resende Silva, Sunando Roy, Tony Brooks, Helena Tutill, Paola Niola, Patricia Dyal, Charlotte Williams, Leysa Forrest, Yasmin Panchbhaya, Jacqueline Findlay, Samuel Weeks, Julianne Brown, Kathryn Harris, Paul Randell, James Price, Alison Holmes, Judith Breuer
EPI_ISL_704190	Virology Department, Sheffield Teaching Hospitals NHS Foundation Trust/Department of Infection, Immunity and Cardiovascular Disease, The Medical School, University of Sheffield	COVID-19 Genomics UK (COG-UK) Consortium	Thushan de Silva, Matthew Parker, Nikki Smith, Adri Angyal, Rebecca Brown, Luke Green, Rachel Tucker, Paul Parsons, Danielle Groves, Katie Johnson, Laura Carrilero, Alex Keeley, Dave Partridge, Matthew Wyles, Benjamin Lindsey, Mehmet Yavuz, Mohammad Raza, Cariad Evans
EPI_ISL_704209	Liverpool Clinical Laboratories	COVID-19 Genomics UK (COG-UK) Consortium	Sam Haldenby, Anita Lucaci, Steve Paterson, Julian Hiscox, Alistair Darby, M Almsaud, A Alrezaihi, Muhannad Alruwaili, Stuart D Armstrong, Jones Benjamin, Eleanor G Bentley, Anu Chawla, Jordan J Clark, Angela Cowell, Richard Eccles, Isabel García-Dorival, Matthew Gemmell, Alessandro Gerada, PKF Gilmore, Richard Gregory, Ximeng Han, Catherine Hartley, Margaret Hughes, Miren Iturriza-Gomara, James Johnson, L Luu, Jenifer Manson, Charlotte Nelson, Elaine O'Toole, Cassie Olateju, Rebekah Penrice-Randal , Lucille Rainbow, N.P Randle, Trevor Ian Robinson, Parul Sharma, Ghada T Shawli, James P Stewart, Neil Swainston, Ecaterina Vamos, Joanne Watts, Mark Whitehead
EPI_ISL_704228	West of Scotland Specialist Virology Centre, NHSGGC / MRC-University of Glasgow Centre for Virus Research	COVID-19 Genomics UK (COG-UK) Consortium	Ana da Silva Filipe, Natasha Johnson, Kathy Smollett, Daniel Mair, Stephen Carmichael, Alice Broos, Lily Tong, Jenna Nichols, Kyriaki Nomikou; Sarah McDonald; Richard Orton, Joseph Hughes, Sreenu Vattipally, David L Robertson; Alasdair MacLean, Rory Gunson; Sharif Shaaban, Matthew Holden; Rachel Blacow, Guy Mollett, Kathy Li, James Shepherd, Antonia Ho, Emma Thomson
EPI_ISL_704294, EPI_ISL_704297, EPI_ISL_704299	Department of Pathology, University of Cambridge	COVID-19 Genomics UK (COG-UK) Consortium	Aminu S. Jahun, Yasmin Chaudhry, Grant Hall, Iliana Georgana, Myra Hosmillo, Martin D. Curran, Malte Pinckert, Surendra Parmar, Ian Goodfellow
EPI_ISL_704312	University College London, Great Ormond Street Hospital for	COVID-19 Genomics UK (COG-UK) Consortium	Sergi Castellano, Rachel Williams, Mark Kristiansen, Paola Resende Silva, Sunando Roy, Tony Brooks, Helena Tutill, Paola Niola, Patricia Dyal, Charlotte

	Children NHS Foundation Trust, Imperial College Healthcare NHS Trust		Williams, Leysa Forrest, Yasmin Panchbhaya, Jacqueline Findlay, Samuel Weeks, Julianne Brown, Kathryn Harris, Paul Randell, James Price, Alison Holmes, Judith Breuer
EPI_ISL_704339, EPI_ISL_704341, EPI_ISL_704344	Department of Pathology, University of Cambridge	COVID-19 Genomics UK (COG-UK) Consortium	Aminu S. Jahun, Yasmin Chaudhry, Grant Hall, Iliana Georgana, Myra Hosmillo, Martin D. Curran, Malte Pinckert, Surendra Parmar, Ian Goodfellow
EPI_ISL_704372	University College London, Great Ormond Street Hospital for Children NHS Foundation Trust, Imperial College Healthcare NHS Trust	COVID-19 Genomics UK (COG-UK) Consortium	Sergi Castellano, Rachel Williams, Mark Kristiansen, Paola Resende Silva, Sunando Roy, Tony Brooks, Helena Tutill, Paola Niola, Patricia Dyal, Charlotte Williams, Leysa Forrest, Yasmin Panchbhaya, Jacqueline Findlay, Samuel Weeks, Julianne Brown, Kathryn Harris, Paul Randell, James Price, Alison Holmes, Judith Breuer
EPI_ISL_704382	Department of Pathology, University of Cambridge	COVID-19 Genomics UK (COG-UK) Consortium	Aminu S. Jahun, Yasmin Chaudhry, Grant Hall, Iliana Georgana, Myra Hosmillo, Martin D. Curran, Malte Pinckert, Surendra Parmar, Ian Goodfellow
EPI_ISL_704387, EPI_ISL_704422, EPI_ISL_704425	Northumbria University / South Tees Hospitals NHS Foundation Trust / North Cumbria Integrated Care NHS Foundation Trust / North Tees and Hartlepool NHS Foundation Trust / Newcastle Hospitals NHS Foundation Trust	COVID-19 Genomics UK (COG-UK) Consortium	Darren L Smith,Andrew Nelson,Matthew Bashton,Greg R Young,Joshua Loh,John Allan,Mohammad A Tariq,Giles S Holt,Gary Black,Wen C Yew,Lynn Dover,Paul Baker,Steve Liggett,Sarah Essex,Jane Greenaway,Debra Padgett,Clive Graham,Garren Scott,Edward Barton,Emma Swindells,Brendan Payne,Jennifer Collins,Yusri Taha,Gary Eltringham
EPI_ISL_704481, EPI_ISL_704484, EPI_ISL_704486, EPI_ISL_704489, EPI_ISL_704494, EPI_ISL_704497, EPI_ISL_704499, EPI_ISL_704502, EPI_ISL_704505, EPI_ISL_704507, EPI_ISL_704516, EPI_ISL_704535, EPI_ISL_704537, EPI_ISL_704557			
see above	Department of Pathology, University of Cambridge	COVID-19 Genomics UK (COG-UK) Consortium	Aminu S. Jahun, Yasmin Chaudhry, Grant Hall, Iliana Georgana, Myra Hosmillo, Martin D. Curran, Malte Pinckert, Surendra Parmar, Ian Goodfellow
EPI_ISL_704562	Liverpool Clinical Laboratories	COVID-19 Genomics UK (COG-UK) Consortium	Sam Haldenby, Anita Lucaci, Steve Paterson, Julian Hiscox, Alistair Darby, M Almsaud, A Alrezaihi, Muhannad Alruwaili, Stuart D Armstrong, Jones Benjamin, Eleanor G Bentley, Anu Chawla, Jordan J Clark, Angela Cowell, Richard Eccles, Isabel García-Dorival, Matthew Gemmell, Alessandro Gerada, PKF Gilmore, Richard Gregory, Ximeng Han, Catherine Hartley, Margaret Hughes, Miren Iturriza-Gomara, James Johnson, L Luu, Jenifer Manson, Charlotte Nelson, Elaine O'Toole, Cassie Olateju, Rebekah Penrice-Randal , Lucille Rainbow, N.P Randle, Trevor Ian Robinson, Parul Sharma, Ghada T Shawli, James P Stewart, Neil Swainston, Ecaterina Vamos, Joanne Watts, Mark Whitehead
EPI_ISL_704570, EPI_ISL_704572, EPI_ISL_704573, EPI_ISL_704574, EPI_ISL_704576, EPI_ISL_704577, EPI_ISL_704578	Northumbria University / South Tees Hospitals NHS Foundation Trust / North Cumbria Integrated Care NHS Foundation Trust / North Tees and Hartlepool NHS Foundation Trust / Newcastle Hospitals NHS Foundation Trust	COVID-19 Genomics UK (COG-UK) Consortium	Darren L Smith,Andrew Nelson,Matthew Bashton,Greg R Young,Joshua Loh,John Allan,Mohammad A Tariq,Giles S Holt,Gary Black,Wen C Yew,Lynn Dover,Paul Baker,Steve Liggett,Sarah Essex,Jane Greenaway,Debra Padgett,Clive Graham,Garren Scott,Edward Barton,Emma Swindells,Brendan Payne,Jennifer Collins,Yusri Taha,Gary Eltringham
EPI_ISL_704584	Wales Specialist Virology Centre Sequencing lab: Pathogen Genomics Unit	COVID-19 Genomics UK (COG-UK) Consortium	Catherine Moore, Johnathan Evans, Laura Gifford, Malorie Perry, Simon Cottrell, Angela Marchbank, Alec Birchley, Alexander Adams, Amy Gaskin, Bree Gatica-Wilcox, Jason Coombes, Joel Southgate, Lauren Gilbert, Lee Graham, Nicole Pacchiarini, Sara Kumziene-Summerhayes, Sarah Taylor, Sophie Jones, Sara Rey, Matthew Bull, Joanne Watkins, Sally Corden, Tom Connor
EPI_ISL_704595	West of Scotland Specialist Virology Centre, NHSGGC / MRC-University of Glasgow Centre for Virus Research	COVID-19 Genomics UK (COG-UK) Consortium	Ana da Silva Filipe, Natasha Johnson, Kathy Smollett, Daniel Mair, Stephen Carmichael, Alice Broos, Lily Tong, Jenna Nichols, Kyriaki Nomikou; Sarah McDonald; Richard Orton, Joseph Hughes, Sreenu Vattipally, David L Robertson; Alasdair MacLean, Rory Gunson; Sharif Shaaban, Matthew Holden; Rachel Blacow, Guy Mollett, Kathy Li, James Shepherd, Antonia Ho, Emma Thomson
EPI_ISL_704598, EPI_ISL_704599	Quadram Institute Bioscience	COVID-19 Genomics UK (COG-UK) Consortium	Dave J. Baker, Gemma L. Kay, Alp Aydin, Thanh Le-Viet, Steven Rudder, Ana P. Tedim, Anastasia Kolyva, Maria Diaz, Leonardo de Oliveira Martins, Nabil-Fareed Alikhan, Lizzie Meadows, Rachael Stanley, Ngozi Elumogo, Muhammed Yasir, Nicholas M. Thomson, Alexander J Trotter, Rachel Gilroy, Samuel Bloomfield, Claire Stuart, Andrew Bell, Reenesh Prakash, Samir Dervisevic, Alison E. Mather, John Wain, Mark Webber, Andrew J. Page, Justin O'Grady
EPI_ISL_704603, EPI_ISL_704605	Virology Department, Sheffield Teaching Hospitals NHS Foundation Trust/Department of Infection, Immunity and Cardiovascular Disease, The Medical School, University of Sheffield	COVID-19 Genomics UK (COG-UK) Consortium	Thushan de Silva, Matthew Parker, Nikki Smith, Adri Angyal, Rebecca Brown, Luke Green, Rachel Tucker, Paul Parsons, Danielle Groves, Katie Johnson, Laura Carrilero, Alex Keeley, Dave Partridge, Matthew Wyles, Benjamin Lindsey, Mehmet Yavuz, Mohammad Raza, Cariad Evans
EPI_ISL_704619, EPI_ISL_704620, EPI_ISL_704621, EPI_ISL_704622, EPI_ISL_704623, EPI_ISL_704624, EPI_ISL_704625, EPI_ISL_704626, EPI_ISL_704627, EPI_ISL_704628, EPI_ISL_704633, EPI_ISL_704634, EPI_ISL_704635			
see above	Quadram Institute Bioscience	COVID-19 Genomics UK (COG-UK) Consortium	Dave J. Baker, Gemma L. Kay, Alp Aydin, Thanh Le-Viet, Steven Rudder, Ana P. Tedim, Anastasia Kolyva, Maria Diaz, Leonardo de Oliveira Martins, Nabil-Fareed Alikhan, Lizzie Meadows, Rachael Stanley, Ngozi Elumogo, Muhammed Yasir, Nicholas M. Thomson, Alexander J Trotter, Rachel Gilroy, Samuel Bloomfield, Claire Stuart, Andrew Bell, Reenesh Prakash, Samir Dervisevic, Alison E. Mather, John Wain, Mark Webber, Andrew J. Page, Justin O'Grady
EPI_ISL_704637, EPI_ISL_704638	Virology Department, Sheffield Teaching Hospitals NHS Foundation Trust/Department of Infection, Immunity and Cardiovascular Disease, The Medical School, University of Sheffield	COVID-19 Genomics UK (COG-UK) Consortium	Thushan de Silva, Matthew Parker, Nikki Smith, Adri Angyal, Rebecca Brown, Luke Green, Rachel Tucker, Paul Parsons, Danielle Groves, Katie Johnson, Laura Carrilero, Alex Keeley, Dave Partridge, Matthew Wyles, Benjamin Lindsey, Mehmet Yavuz, Mohammad Raza, Cariad Evans
EPI_ISL_704713, EPI_ISL_704750, EPI_ISL_704761, EPI_ISL_704779, EPI_ISL_704801, EPI_ISL_704803	Lighthouse Lab in Cambridge	Wellcome Sanger Institute for the COVID-19 Genomics UK (COG-UK) Consortium	Rob Howes, The Lighthouse Lab in Cambridge and Alex Alderton, Roberto Amato, Sonia Goncalves, Ewan Harrison, David K. Jackson, Ian Johnston, Dominic Kwiatkowski, Cordelia Langford, John Sillitoe on behalf of the Wellcome Sanger Institute COVID-19 Surveillance Team
EPI_ISL_704820, EPI_ISL_704823	Quadram Institute Bioscience	COVID-19 Genomics UK (COG-UK) Consortium	Dave J. Baker, Gemma L. Kay, Alp Aydin, Thanh Le-Viet, Steven Rudder, Ana P. Tedim, Anastasia Kolyva, Maria Diaz, Leonardo de Oliveira Martins, Nabil-Fareed Alikhan, Lizzie Meadows, Rachael Stanley, Ngozi Elumogo, Muhammed Yasir, Nicholas M. Thomson, Alexander J Trotter, Rachel Gilroy, Samuel Bloomfield, Claire Stuart, Andrew Bell, Reenesh Prakash, Samir Dervisevic, Alison E. Mather, John Wain, Mark Webber, Andrew J. Page, Justin O'Grady
EPI_ISL_704827, EPI_ISL_704830	Lighthouse Lab in Cambridge	Wellcome Sanger Institute for the COVID-19 Genomics UK (COG-UK) Consortium	Rob Howes, The Lighthouse Lab in Cambridge and Alex Alderton, Roberto Amato, Sonia Goncalves, Ewan Harrison, David K. Jackson, Ian Johnston, Dominic Kwiatkowski, Cordelia Langford, John Sillitoe on behalf of the Wellcome Sanger Institute COVID-19 Surveillance Team
EPI_ISL_704831	Quadram Institute Bioscience	COVID-19 Genomics UK (COG-UK) Consortium	Dave J. Baker, Gemma L. Kay, Alp Aydin, Thanh Le-Viet, Steven Rudder, Ana P. Tedim, Anastasia Kolyva, Maria Diaz, Leonardo de Oliveira Martins, Nabil-Fareed Alikhan, Lizzie Meadows, Rachael Stanley, Ngozi Elumogo, Muhammed Yasir, Nicholas M. Thomson, Alexander J Trotter, Rachel Gilroy, Samuel Bloomfield, Claire Stuart, Andrew Bell, Reenesh Prakash, Samir Dervisevic, Alison E. Mather, John Wain, Mark Webber, Andrew J. Page, Justin O'Grady
EPI_ISL_704833, EPI_ISL_704841, EPI_ISL_704867	Lighthouse Lab in Cambridge	Wellcome Sanger Institute for the COVID-19 Genomics UK (COG-UK) Consortium	Rob Howes, The Lighthouse Lab in Cambridge and Alex Alderton, Roberto Amato, Sonia Goncalves, Ewan Harrison, David K. Jackson, Ian Johnston, Dominic Kwiatkowski, Cordelia Langford, John Sillitoe on behalf of the Wellcome Sanger Institute COVID-19 Surveillance Team
EPI_ISL_704903, EPI_ISL_704905	Liverpool Clinical Laboratories	COVID-19 Genomics UK (COG-UK) Consortium	Sam Haldenby, Anita Lucaci, Steve Paterson, Julian Hiscox, Alistair Darby, M Almsaud, A Alrezaihi, Muhannad Alruwaili, Stuart D Armstrong, Jones Benjamin, Eleanor G Bentley, Anu Chawla, Jordan J Clark, Angela Cowell, Richard Eccles, Isabel García-Dorival, Matthew Gemmell, Alessandro Gerada, PKF Gilmore, Richard Gregory, Ximeng Han, Catherine Hartley, Margaret Hughes, Miren Iturriza-Gomara, James Johnson, L Luu, Jenifer Manson, Charlotte Nelson, Elaine O'Toole, Cassie Olateju, Rebekah Penrice-Randal , Lucille Rainbow, N.P Randle, Trevor Ian Robinson, Parul Sharma, Ghada T Shawli, James P Stewart, Neil Swainston, Ecaterina Vamos, Joanne Watts, Mark Whitehead
EPI_ISL_704928	Lighthouse Lab in Cambridge	Wellcome Sanger Institute for the COVID-19 Genomics UK (COG-UK) Consortium	Rob Howes, The Lighthouse Lab in Cambridge and Alex Alderton, Roberto Amato, Sonia Goncalves, Ewan Harrison, David K. Jackson, Ian Johnston, Dominic Kwiatkowski, Cordelia Langford, John Sillitoe on behalf of the Wellcome Sanger Institute COVID-19 Surveillance Team
EPI_ISL_704931	Wales Specialist Virology Centre Sequencing lab: Pathogen Genomics Unit	COVID-19 Genomics UK (COG-UK) Consortium	Catherine Moore, Johnathan Evans, Laura Gifford, Malorie Perry, Simon Cottrell, Angela Marchbank, Alec Birchley, Alexander Adams, Amy Gaskin, Bree Gatica-Wilcox, Jason Coombes, Joel Southgate, Lauren Gilbert, Lee Graham, Nicole Pacchiarini, Sara Kumziene-Summerhayes, Sarah Taylor, Sophie Jones, Sara Rey, Matthew Bull, Joanne Watkins, Sally Corden, Tom Connor
EPI_ISL_704932	Lighthouse Lab in Cambridge	Wellcome Sanger Institute for the COVID-19 Genomics UK (COG-UK) Consortium	Rob Howes, The Lighthouse Lab in Cambridge and Alex Alderton, Roberto Amato, Sonia Goncalves, Ewan Harrison, David K. Jackson, Ian Johnston, Dominic Kwiatkowski, Cordelia Langford, John Sillitoe on behalf of the Wellcome Sanger Institute COVID-19 Surveillance Team
EPI_ISL_704950	Liverpool Clinical Laboratories	COVID-19 Genomics UK (COG-UK) Consortium	Sam Haldenby, Anita Lucaci, Steve Paterson, Julian Hiscox, Alistair Darby, M Almsaud, A Alrezaihi, Muhannad Alruwaili, Stuart D Armstrong, Jones Benjamin, Eleanor G Bentley, Anu Chawla, Jordan J Clark, Angela Cowell, Richard Eccles, Isabel García-Dorival, Matthew Gemmell, Alessandro Gerada,

			PKF Gilmore, Richard Gregory, Ximeng Han, Catherine Hartley, Margaret Hughes, Miren Iturriza-Gomara, James Johnson, L Luu, Jenifer Manson, Charlotte Nelson, Elaine O'Toole, Cassie Olateju, Rebekah Penrice-Randal , Lucille Rainbow, N.P Randle, Trevor Ian Robinson, Parul Sharma, Ghada T Shawli, James P Stewart, Neil Swainston, Ecaterina Vamos, Joanne Watts, Mark Whitehead
EPI_ISL_704951	Lighthouse Lab in Cambridge	Wellcome Sanger Institute for the COVID-19 Genomics UK (COG-UK) Consortium	Rob Howes, The Lighthouse Lab in Cambridge and Alex Alderton, Roberto Amato, Sonia Goncalves, Ewan Harrison, David K. Jackson, Ian Johnston, Dominic Kwiatkowski, Cordelia Langford, John Sillitoe on behalf of the Wellcome Sanger Institute COVID-19 Surveillance Team
EPI_ISL_704955, EPI_ISL_704965	University College London, Great Ormond Street Hospital for Children NHS Foundation Trust, Imperial College Healthcare NHS Trust	COVID-19 Genomics UK (COG-UK) Consortium	Sergi Castellano, Rachel Williams, Mark Kristiansen, Paola Resende Silva, Sunando Roy, Tony Brooks, Helena Tutill, Paola Niola, Patricia Dyal, Charlotte Williams, Leysa Forrest, Yasmin Panchbhaya, Jacqueline Findlay, Samuel Weeks, Julianne Brown, Kathryn Harris, Paul Randell, James Price, Alison Holmes, Judith Breuer
EPI_ISL_704968, EPI_ISL_704973	Liverpool Clinical Laboratories	COVID-19 Genomics UK (COG-UK) Consortium	Sam Haldenby, Anita Lucaci, Steve Paterson, Julian Hiscox, Alistair Darby, M Almsaud, A Alrezaihi, Muhannad Alruwaili, Stuart D Armstrong, Jones Benjamin, Eleanor G Bentley, Anu Chawla, Jordan J Clark, Angela Cowell, Richard Eccles, Isabel García-Dorival, Matthew Gemmell, Alessandro Gerada, PKF Gilmore, Richard Gregory, Ximeng Han, Catherine Hartley, Margaret Hughes, Miren Iturriza-Gomara, James Johnson, L Luu, Jenifer Manson, Charlotte Nelson, Elaine O'Toole, Cassie Olateju, Rebekah Penrice-Randal , Lucille Rainbow, N.P Randle, Trevor Ian Robinson, Parul Sharma, Ghada T Shawli, James P Stewart, Neil Swainston, Ecaterina Vamos, Joanne Watts, Mark Whitehead
EPI_ISL_704976	Northumbria University / South Tees Hospitals NHS Foundation Trust / North Cumbria Integrated Care NHS Foundation Trust / North Tees and Hartlepool NHS Foundation Trust / Newcastle Hospitals NHS Foundation Trust	COVID-19 Genomics UK (COG-UK) Consortium	Darren L Smith,Andrew Nelson,Matthew Bashton,Greg R Young,Joshua Loh,John Allan,Mohammad A Tariq,Giles S Holt,Gary Black,Wen C Yew,Lynn Dover,Paul Baker,Steve Liggett,Sarah Essex,Jane Greenaway,Debra Padgett,Clive Graham,Garren Scott,Edward Barton,Emma Swindells,Brendan Payne,Jennifer Collins,Yusri Taha,Gary Eltringham
EPI_ISL_704977	Lighthouse Lab in Cambridge	Wellcome Sanger Institute for the COVID-19 Genomics UK (COG-UK) Consortium	Rob Howes, The Lighthouse Lab in Cambridge and Alex Alderton, Roberto Amato, Sonia Goncalves, Ewan Harrison, David K. Jackson, Ian Johnston, Dominic Kwiatkowski, Cordelia Langford, John Sillitoe on behalf of the Wellcome Sanger Institute COVID-19 Surveillance Team
EPI_ISL_704981	Wales Specialist Virology Centre Sequencing lab: Pathogen Genomics Unit	COVID-19 Genomics UK (COG-UK) Consortium	Catherine Moore, Johnathan Evans, Laura Gifford, Malorie Perry, Simon Cottrell, Angela Marchbank, Alec Birchley, Alexander Adams, Amy Gaskin, Bree Gatica-Wilcox, Jason Coombes, Joel Southgate, Lauren Gilbert, Lee Graham, Nicole Pacchiarini, Sara Kumziene-Summerhayes, Sarah Taylor, Sophie Jones, Sara Rey, Matthew Bull, Joanne Watkins, Sally Corden, Tom Connor
EPI_ISL_704983	Liverpool Clinical Laboratories	COVID-19 Genomics UK (COG-UK) Consortium	Sam Haldenby, Anita Lucaci, Steve Paterson, Julian Hiscox, Alistair Darby, M Almsaud, A Alrezaihi, Muhannad Alruwaili, Stuart D Armstrong, Jones Benjamin, Eleanor G Bentley, Anu Chawla, Jordan J Clark, Angela Cowell, Richard Eccles, Isabel García-Dorival, Matthew Gemmell, Alessandro Gerada, PKF Gilmore, Richard Gregory, Ximeng Han, Catherine Hartley, Margaret Hughes, Miren Iturriza-Gomara, James Johnson, L Luu, Jenifer Manson, Charlotte Nelson, Elaine O'Toole, Cassie Olateju, Rebekah Penrice-Randal , Lucille Rainbow, N.P Randle, Trevor Ian Robinson, Parul Sharma, Ghada T Shawli, James P Stewart, Neil Swainston, Ecaterina Vamos, Joanne Watts, Mark Whitehead
EPI_ISL_704987	Lighthouse Lab in Cambridge	Wellcome Sanger Institute for the COVID-19 Genomics UK (COG-UK) Consortium	Rob Howes, The Lighthouse Lab in Cambridge and Alex Alderton, Roberto Amato, Sonia Goncalves, Ewan Harrison, David K. Jackson, Ian Johnston, Dominic Kwiatkowski, Cordelia Langford, John Sillitoe on behalf of the Wellcome Sanger Institute COVID-19 Surveillance Team
EPI_ISL_704989	Liverpool Clinical Laboratories	COVID-19 Genomics UK (COG-UK) Consortium	Sam Haldenby, Anita Lucaci, Steve Paterson, Julian Hiscox, Alistair Darby, M Almsaud, A Alrezaihi, Muhannad Alruwaili, Stuart D Armstrong, Jones Benjamin, Eleanor G Bentley, Anu Chawla, Jordan J Clark, Angela Cowell, Richard Eccles, Isabel García-Dorival, Matthew Gemmell, Alessandro Gerada, PKF Gilmore, Richard Gregory, Ximeng Han, Catherine Hartley, Margaret Hughes, Miren Iturriza-Gomara, James Johnson, L Luu, Jenifer Manson, Charlotte Nelson, Elaine O'Toole, Cassie Olateju, Rebekah Penrice-Randal , Lucille Rainbow, N.P Randle, Trevor Ian Robinson, Parul Sharma, Ghada T Shawli, James P Stewart, Neil Swainston, Ecaterina Vamos, Joanne Watts, Mark Whitehead
EPI_ISL_704997, EPI_ISL_704999	University College London, Great Ormond Street Hospital for Children NHS Foundation Trust, Imperial College Healthcare NHS Trust	COVID-19 Genomics UK (COG-UK) Consortium	Sergi Castellano, Rachel Williams, Mark Kristiansen, Paola Resende Silva, Sunando Roy, Tony Brooks, Helena Tutill, Paola Niola, Patricia Dyal, Charlotte Williams, Leysa Forrest, Yasmin Panchbhaya, Jacqueline Findlay, Samuel Weeks, Julianne Brown, Kathryn Harris, Paul Randell, James Price, Alison Holmes, Judith Breuer
EPI_ISL_705003, EPI_ISL_705004	Lighthouse Lab in Cambridge	Wellcome Sanger Institute for the COVID-19 Genomics UK (COG-UK) Consortium	Rob Howes, The Lighthouse Lab in Cambridge and Alex Alderton, Roberto Amato, Sonia Goncalves, Ewan Harrison, David K. Jackson, Ian Johnston, Dominic Kwiatkowski, Cordelia Langford, John Sillitoe on behalf of the Wellcome Sanger Institute COVID-19 Surveillance Team
EPI_ISL_705010	Northumbria University / South Tees Hospitals NHS Foundation Trust / North Cumbria Integrated Care NHS Foundation Trust / North Tees and Hartlepool NHS Foundation Trust / Newcastle Hospitals NHS Foundation Trust	COVID-19 Genomics UK (COG-UK) Consortium	Darren L Smith,Andrew Nelson,Matthew Bashton,Greg R Young,Joshua Loh,John Allan,Mohammad A Tariq,Giles S Holt,Gary Black,Wen C Yew,Lynn Dover,Paul Baker,Steve Liggett,Sarah Essex,Jane Greenaway,Debra Padgett,Clive Graham,Garren Scott,Edward Barton,Emma Swindells,Brendan Payne,Jennifer Collins,Yusri Taha,Gary Eltringham
EPI_ISL_705012	Lighthouse Lab in Cambridge	Wellcome Sanger Institute for the COVID-19 Genomics UK (COG-UK) Consortium	Rob Howes, The Lighthouse Lab in Cambridge and Alex Alderton, Roberto Amato, Sonia Goncalves, Ewan Harrison, David K. Jackson, Ian Johnston, Dominic Kwiatkowski, Cordelia Langford, John Sillitoe on behalf of the Wellcome Sanger Institute COVID-19 Surveillance Team
EPI_ISL_705018	University College London, Great Ormond Street Hospital for Children NHS Foundation Trust, Imperial College Healthcare NHS Trust	COVID-19 Genomics UK (COG-UK) Consortium	Sergi Castellano, Rachel Williams, Mark Kristiansen, Paola Resende Silva, Sunando Roy, Tony Brooks, Helena Tutill, Paola Niola, Patricia Dyal, Charlotte Williams, Leysa Forrest, Yasmin Panchbhaya, Jacqueline Findlay, Samuel Weeks, Julianne Brown, Kathryn Harris, Paul Randell, James Price, Alison Holmes, Judith Breuer
EPI_ISL_705023, EPI_ISL_705031, EPI_ISL_705047, EPI_ISL_705053	Lighthouse Lab in Cambridge	Wellcome Sanger Institute for the COVID-19 Genomics UK (COG-UK) Consortium	Rob Howes, The Lighthouse Lab in Cambridge and Alex Alderton, Roberto Amato, Sonia Goncalves, Ewan Harrison, David K. Jackson, Ian Johnston, Dominic Kwiatkowski, Cordelia Langford, John Sillitoe on behalf of the Wellcome Sanger Institute COVID-19 Surveillance Team
EPI_ISL_705054	Liverpool Clinical Laboratories	COVID-19 Genomics UK (COG-UK) Consortium	Sam Haldenby, Anita Lucaci, Steve Paterson, Julian Hiscox, Alistair Darby, M Almsaud, A Alrezaihi, Muhannad Alruwaili, Stuart D Armstrong, Jones Benjamin, Eleanor G Bentley, Anu Chawla, Jordan J Clark, Angela Cowell, Richard Eccles, Isabel García-Dorival, Matthew Gemmell, Alessandro Gerada, PKF Gilmore, Richard Gregory, Ximeng Han, Catherine Hartley, Margaret Hughes, Miren Iturriza-Gomara, James Johnson, L Luu, Jenifer Manson, Charlotte Nelson, Elaine O'Toole, Cassie Olateju, Rebekah Penrice-Randal , Lucille Rainbow, N.P Randle, Trevor Ian Robinson, Parul Sharma, Ghada T Shawli, James P Stewart, Neil Swainston, Ecaterina Vamos, Joanne Watts, Mark Whitehead
EPI_ISL_705061, EPI_ISL_705066	Lighthouse Lab in Cambridge	Wellcome Sanger Institute for the COVID-19 Genomics UK (COG-UK) Consortium	Rob Howes, The Lighthouse Lab in Cambridge and Alex Alderton, Roberto Amato, Sonia Goncalves, Ewan Harrison, David K. Jackson, Ian Johnston, Dominic Kwiatkowski, Cordelia Langford, John Sillitoe on behalf of the Wellcome Sanger Institute COVID-19 Surveillance Team
EPI_ISL_705067	Northumbria University / South Tees Hospitals NHS Foundation Trust / North Cumbria Integrated Care NHS Foundation Trust / North Tees and Hartlepool NHS Foundation Trust / Newcastle Hospitals NHS Foundation Trust	COVID-19 Genomics UK (COG-UK) Consortium	Darren L Smith,Andrew Nelson,Matthew Bashton,Greg R Young,Joshua Loh,John Allan,Mohammad A Tariq,Giles S Holt,Gary Black,Wen C Yew,Lynn Dover,Paul Baker,Steve Liggett,Sarah Essex,Jane Greenaway,Debra Padgett,Clive Graham,Garren Scott,Edward Barton,Emma Swindells,Brendan Payne,Jennifer Collins,Yusri Taha,Gary Eltringham
EPI_ISL_705069	Lighthouse Lab in Cambridge	Wellcome Sanger Institute for the COVID-19 Genomics UK (COG-UK) Consortium	Rob Howes, The Lighthouse Lab in Cambridge and Alex Alderton, Roberto Amato, Sonia Goncalves, Ewan Harrison, David K. Jackson, Ian Johnston, Dominic Kwiatkowski, Cordelia Langford, John Sillitoe on behalf of the Wellcome Sanger Institute COVID-19 Surveillance Team
EPI_ISL_705072	University College London, Great Ormond Street Hospital for Children NHS Foundation Trust, Imperial College Healthcare NHS Trust	COVID-19 Genomics UK (COG-UK) Consortium	Sergi Castellano, Rachel Williams, Mark Kristiansen, Paola Resende Silva, Sunando Roy, Tony Brooks, Helena Tutill, Paola Niola, Patricia Dyal, Charlotte Williams, Leysa Forrest, Yasmin Panchbhaya, Jacqueline Findlay, Samuel Weeks, Julianne Brown, Kathryn Harris, Paul Randell, James Price, Alison Holmes, Judith Breuer
EPI_ISL_705086	Liverpool Clinical Laboratories	COVID-19 Genomics UK (COG-UK) Consortium	Sam Haldenby, Anita Lucaci, Steve Paterson, Julian Hiscox, Alistair Darby, M Almsaud, A Alrezaihi, Muhannad Alruwaili, Stuart D Armstrong, Jones Benjamin, Eleanor G Bentley, Anu Chawla, Jordan J Clark, Angela Cowell, Richard Eccles, Isabel García-Dorival, Matthew Gemmell, Alessandro Gerada, PKF Gilmore, Richard Gregory, Ximeng Han, Catherine Hartley, Margaret Hughes, Miren Iturriza-Gomara, James Johnson, L Luu, Jenifer Manson, Charlotte Nelson, Elaine O'Toole, Cassie Olateju, Rebekah Penrice-Randal , Lucille Rainbow, N.P Randle, Trevor Ian Robinson, Parul Sharma, Ghada T Shawli, James P Stewart, Neil Swainston, Ecaterina Vamos, Joanne Watts, Mark Whitehead
EPI_ISL_705090, EPI_ISL_705095, EPI_ISL_705104, EPI_ISL_705128, EPI_ISL_705131, EPI_ISL_705133	Lighthouse Lab in Cambridge	Wellcome Sanger Institute for the COVID-19 Genomics UK (COG-UK) Consortium	Rob Howes, The Lighthouse Lab in Cambridge and Alex Alderton, Roberto Amato, Sonia Goncalves, Ewan Harrison, David K. Jackson, Ian Johnston, Dominic Kwiatkowski, Cordelia Langford, John Sillitoe on behalf of the Wellcome Sanger Institute COVID-19 Surveillance Team
EPI_ISL_705134	University of Exeter	COVID-19 Genomics UK (COG-UK) Consortium	Ben Temperton,Aaron Jeffries,Michelle Michelsen,Joanna Warwick-Dugdale,Audrey Farbos,Robyn Manley,Stephen Michell,Jane Masoli

EPI_ISL_705137	Northumbria University / South Tees Hospitals NHS Foundation Trust / North Cumbria Integrated Care NHS Foundation Trust / North Tees and Hartlepool NHS Foundation Trust / Newcastle Hospitals NHS Foundation Trust	COVID-19 Genomics UK (COG-UK) Consortium	Darren L Smith,Andrew Nelson,Matthew Bashton,Greg R Young,Joshua Loh,John Allan,Mohammad A Tariq,Giles S Holt,Gary Black,Wen C Yew,Lynn Dover,Paul Baker,Steve Liggett,Sarah Essex,Jane Greenaway,Debra Padgett,Clive Graham,Garren Scott,Edward Barton,Emma Swindells,Brendan Payne,Jennifer Collins,Yusri Taha,Gary Eltringham
EPI_ISL_705141	Lighthouse Lab in Cambridge	Wellcome Sanger Institute for the COVID-19 Genomics UK (COG-UK) Consortium	Rob Howes, The Lighthouse Lab in Cambridge and Alex Alderton, Roberto Amato, Sonia Goncalves, Ewan Harrison, David K. Jackson, Ian Johnston, Dominic Kwiatkowski, Cordelia Langford, John Sillitoe on behalf of the Wellcome Sanger Institute COVID-19 Surveillance Team
EPI_ISL_705143	University College London, Great Ormond Street Hospital for Children NHS Foundation Trust, Imperial College Healthcare NHS Trust	COVID-19 Genomics UK (COG-UK) Consortium	Sergi Castellano, Rachel Williams, Mark Kristiansen, Paola Resende Silva, Sunando Roy, Tony Brooks, Helena Tutill, Paola Niola, Patricia Dyal, Charlotte Williams, Leysa Forrest, Yasmin Panchbhaya, Jacqueline Findlay, Samuel Weeks, Julianne Brown, Kathryn Harris, Paul Randell, James Price, Alison Holmes, Judith Breuer
EPI_ISL_705144, EPI_ISL_705146	Lighthouse Lab in Cambridge	Wellcome Sanger Institute for the COVID-19 Genomics UK (COG-UK) Consortium	Rob Howes, The Lighthouse Lab in Cambridge and Alex Alderton, Roberto Amato, Sonia Goncalves, Ewan Harrison, David K. Jackson, Ian Johnston, Dominic Kwiatkowski, Cordelia Langford, John Sillitoe on behalf of the Wellcome Sanger Institute COVID-19 Surveillance Team
EPI_ISL_705148	Northumbria University / South Tees Hospitals NHS Foundation Trust / North Cumbria Integrated Care NHS Foundation Trust / North Tees and Hartlepool NHS Foundation Trust / Newcastle Hospitals NHS Foundation Trust	COVID-19 Genomics UK (COG-UK) Consortium	Darren L Smith,Andrew Nelson,Matthew Bashton,Greg R Young,Joshua Loh,John Allan,Mohammad A Tariq,Giles S Holt,Gary Black,Wen C Yew,Lynn Dover,Paul Baker,Steve Liggett,Sarah Essex,Jane Greenaway,Debra Padgett,Clive Graham,Garren Scott,Edward Barton,Emma Swindells,Brendan Payne,Jennifer Collins,Yusri Taha,Gary Eltringham
EPI_ISL_705150	Liverpool Clinical Laboratories	COVID-19 Genomics UK (COG-UK) Consortium	Sam Haldenby, Anita Lucaci, Steve Paterson, Julian Hiscox, Alistair Darby, M Almsaud, A Alrezaihi, Muhannad Alruwaili, Stuart D Armstrong, Jones Benjamin, Eleanor G Bentley, Anu Chawla, Jordan J Clark, Angela Cowell, Richard Eccles, Isabel García-Dorival, Matthew Gemmell, Alessandro Gerada, PKF Gilmore, Richard Gregory, Ximeng Han, Catherine Hartley, Margaret Hughes, Miren Iturriza-Gomara, James Johnson, L Luu, Jenifer Manson, Charlotte Nelson, Elaine O'Toole, Cassie Olateju, Rebekah Penrice-Randal , Lucille Rainbow, N.P Randle, Trevor Ian Robinson, Parul Sharma, Ghada T Shawli, James P Stewart, Neil Swainston, Ecaterina Vamos, Joanne Watts, Mark Whitehead
EPI_ISL_705159	Lighthouse Lab in Cambridge	Wellcome Sanger Institute for the COVID-19 Genomics UK (COG-UK) Consortium	Rob Howes, The Lighthouse Lab in Cambridge and Alex Alderton, Roberto Amato, Sonia Goncalves, Ewan Harrison, David K. Jackson, Ian Johnston, Dominic Kwiatkowski, Cordelia Langford, John Sillitoe on behalf of the Wellcome Sanger Institute COVID-19 Surveillance Team
EPI_ISL_705163	Virology Department, Sheffield Teaching Hospitals NHS Foundation Trust/Department of Infection, Immunity and Cardiovascular Disease, The Medical School, University of Sheffield	COVID-19 Genomics UK (COG-UK) Consortium	Thushan de Silva, Matthew Parker, Nikki Smith, Adri Angyal, Rebecca Brown, Luke Green, Rachel Tucker, Paul Parsons, Danielle Groves, Katie Johnson, Laura Carrilero, Alex Keeley, Dave Partridge, Matthew Wyles, Benjamin Lindsey, Mehmet Yavuz, Mohammad Raza, Cariad Evans
EPI_ISL_705168, EPI_ISL_705176	Liverpool Clinical Laboratories	COVID-19 Genomics UK (COG-UK) Consortium	Sam Haldenby, Anita Lucaci, Steve Paterson, Julian Hiscox, Alistair Darby, M Almsaud, A Alrezaihi, Muhannad Alruwaili, Stuart D Armstrong, Jones Benjamin, Eleanor G Bentley, Anu Chawla, Jordan J Clark, Angela Cowell, Richard Eccles, Isabel García-Dorival, Matthew Gemmell, Alessandro Gerada, PKF Gilmore, Richard Gregory, Ximeng Han, Catherine Hartley, Margaret Hughes, Miren Iturriza-Gomara, James Johnson, L Luu, Jenifer Manson, Charlotte Nelson, Elaine O'Toole, Cassie Olateju, Rebekah Penrice-Randal , Lucille Rainbow, N.P Randle, Trevor Ian Robinson, Parul Sharma, Ghada T Shawli, James P Stewart, Neil Swainston, Ecaterina Vamos, Joanne Watts, Mark Whitehead
EPI_ISL_705177	Lighthouse Lab in Cambridge	Wellcome Sanger Institute for the COVID-19 Genomics UK (COG-UK) Consortium	Rob Howes, The Lighthouse Lab in Cambridge and Alex Alderton, Roberto Amato, Sonia Goncalves, Ewan Harrison, David K. Jackson, Ian Johnston, Dominic Kwiatkowski, Cordelia Langford, John Sillitoe on behalf of the Wellcome Sanger Institute COVID-19 Surveillance Team
EPI_ISL_705179	Liverpool Clinical Laboratories	COVID-19 Genomics UK (COG-UK) Consortium	Sam Haldenby, Anita Lucaci, Steve Paterson, Julian Hiscox, Alistair Darby, M Almsaud, A Alrezaihi, Muhannad Alruwaili, Stuart D Armstrong, Jones Benjamin, Eleanor G Bentley, Anu Chawla, Jordan J Clark, Angela Cowell, Richard Eccles, Isabel García-Dorival, Matthew Gemmell, Alessandro Gerada, PKF Gilmore, Richard Gregory, Ximeng Han, Catherine Hartley, Margaret Hughes, Miren Iturriza-Gomara, James Johnson, L Luu, Jenifer Manson, Charlotte Nelson, Elaine O'Toole, Cassie Olateju, Rebekah Penrice-Randal , Lucille Rainbow, N.P Randle, Trevor Ian Robinson, Parul Sharma, Ghada T Shawli, James P Stewart, Neil Swainston, Ecaterina Vamos, Joanne Watts, Mark Whitehead
EPI_ISL_705191, EPI_ISL_705192	Lighthouse Lab in Milton Keynes	Wellcome Sanger Institute for the COVID-19 Genomics UK (COG-UK) Consortium	The Lighthouse Lab in Milton Keynes and Alex Alderton, Roberto Amato, Sonia Goncalves, Ewan Harrison, David K. Jackson, Ian Johnston, Dominic Kwiatkowski, Cordelia Langford, John Sillitoe on behalf of the Wellcome Sanger Institute COVID-19 Surveillance Team
EPI_ISL_705193	Liverpool Clinical Laboratories	COVID-19 Genomics UK (COG-UK) Consortium	Sam Haldenby, Anita Lucaci, Steve Paterson, Julian Hiscox, Alistair Darby, M Almsaud, A Alrezaihi, Muhannad Alruwaili, Stuart D Armstrong, Jones Benjamin, Eleanor G Bentley, Anu Chawla, Jordan J Clark, Angela Cowell, Richard Eccles, Isabel García-Dorival, Matthew Gemmell, Alessandro Gerada, PKF Gilmore, Richard Gregory, Ximeng Han, Catherine Hartley, Margaret Hughes, Miren Iturriza-Gomara, James Johnson, L Luu, Jenifer Manson, Charlotte Nelson, Elaine O'Toole, Cassie Olateju, Rebekah Penrice-Randal , Lucille Rainbow, N.P Randle, Trevor Ian Robinson, Parul Sharma, Ghada T Shawli, James P Stewart, Neil Swainston, Ecaterina Vamos, Joanne Watts, Mark Whitehead
EPI_ISL_705194, EPI_ISL_705196, EPI_ISL_705197, EPI_ISL_705199, EPI_ISL_705200, EPI_ISL_705202	Lighthouse Lab in Milton Keynes	Wellcome Sanger Institute for the COVID-19 Genomics UK (COG-UK) Consortium	The Lighthouse Lab in Milton Keynes and Alex Alderton, Roberto Amato, Sonia Goncalves, Ewan Harrison, David K. Jackson, Ian Johnston, Dominic Kwiatkowski, Cordelia Langford, John Sillitoe on behalf of the Wellcome Sanger Institute COVID-19 Surveillance Team
EPI_ISL_705203, EPI_ISL_705208	Liverpool Clinical Laboratories	COVID-19 Genomics UK (COG-UK) Consortium	Sam Haldenby, Anita Lucaci, Steve Paterson, Julian Hiscox, Alistair Darby, M Almsaud, A Alrezaihi, Muhannad Alruwaili, Stuart D Armstrong, Jones Benjamin, Eleanor G Bentley, Anu Chawla, Jordan J Clark, Angela Cowell, Richard Eccles, Isabel García-Dorival, Matthew Gemmell, Alessandro Gerada, PKF Gilmore, Richard Gregory, Ximeng Han, Catherine Hartley, Margaret Hughes, Miren Iturriza-Gomara, James Johnson, L Luu, Jenifer Manson, Charlotte Nelson, Elaine O'Toole, Cassie Olateju, Rebekah Penrice-Randal , Lucille Rainbow, N.P Randle, Trevor Ian Robinson, Parul Sharma, Ghada T Shawli, James P Stewart, Neil Swainston, Ecaterina Vamos, Joanne Watts, Mark Whitehead
EPI_ISL_705209	University College London, Great Ormond Street Hospital for Children NHS Foundation Trust, Imperial College Healthcare NHS Trust	COVID-19 Genomics UK (COG-UK) Consortium	Sergi Castellano, Rachel Williams, Mark Kristiansen, Paola Resende Silva, Sunando Roy, Tony Brooks, Helena Tutill, Paola Niola, Patricia Dyal, Charlotte Williams, Leysa Forrest, Yasmin Panchbhaya, Jacqueline Findlay, Samuel Weeks, Julianne Brown, Kathryn Harris, Paul Randell, James Price, Alison Holmes, Judith Breuer
EPI_ISL_705211	Quadram Institute Bioscience	COVID-19 Genomics UK (COG-UK) Consortium	Dave J. Baker, Gemma L. Kay, Alp Aydin, Thanh Le-Viet, Steven Rudder, Ana P. Tedim, Anastasia Kolyva, Maria Diaz, Leonardo de Oliveira Martins, Nabil-Fareed Alikhan, Lizzie Meadows, Rachael Stanley, Ngozi Elumogo, Muhammed Yasir, Nicholas M. Thomson, Alexander J Trotter, Rachel Gilroy, Samuel Bloomfield, Claire Stuart, Andrew Bell, Reenesh Prakash, Samir Dervisevic, Alison E. Mather, John Wain, Mark Webber, Andrew J. Page, Justin O'Grady
EPI_ISL_705214	Northumbria University / South Tees Hospitals NHS Foundation Trust / North Cumbria Integrated Care NHS Foundation Trust / North Tees and Hartlepool NHS Foundation Trust / Newcastle Hospitals NHS Foundation Trust	COVID-19 Genomics UK (COG-UK) Consortium	Darren L Smith,Andrew Nelson,Matthew Bashton,Greg R Young,Joshua Loh,John Allan,Mohammad A Tariq,Giles S Holt,Gary Black,Wen C Yew,Lynn Dover,Paul Baker,Steve Liggett,Sarah Essex,Jane Greenaway,Debra Padgett,Clive Graham,Garren Scott,Edward Barton,Emma Swindells,Brendan Payne,Jennifer Collins,Yusri Taha,Gary Eltringham
EPI_ISL_705215, EPI_ISL_705216	University College London, Great Ormond Street Hospital for Children NHS Foundation Trust, Imperial College Healthcare NHS Trust	COVID-19 Genomics UK (COG-UK) Consortium	Sergi Castellano, Rachel Williams, Mark Kristiansen, Paola Resende Silva, Sunando Roy, Tony Brooks, Helena Tutill, Paola Niola, Patricia Dyal, Charlotte Williams, Leysa Forrest, Yasmin Panchbhaya, Jacqueline Findlay, Samuel Weeks, Julianne Brown, Kathryn Harris, Paul Randell, James Price, Alison Holmes, Judith Breuer
EPI_ISL_705227, EPI_ISL_705231	Liverpool Clinical Laboratories	COVID-19 Genomics UK (COG-UK) Consortium	Sam Haldenby, Anita Lucaci, Steve Paterson, Julian Hiscox, Alistair Darby, M Almsaud, A Alrezaihi, Muhannad Alruwaili, Stuart D Armstrong, Jones Benjamin, Eleanor G Bentley, Anu Chawla, Jordan J Clark, Angela Cowell, Richard Eccles, Isabel García-Dorival, Matthew Gemmell, Alessandro Gerada, PKF Gilmore, Richard Gregory, Ximeng Han, Catherine Hartley, Margaret Hughes, Miren Iturriza-Gomara, James Johnson, L Luu, Jenifer Manson, Charlotte Nelson, Elaine O'Toole, Cassie Olateju, Rebekah Penrice-Randal , Lucille Rainbow, N.P Randle, Trevor Ian Robinson, Parul Sharma, Ghada T Shawli, James P Stewart, Neil Swainston, Ecaterina Vamos, Joanne Watts, Mark Whitehead
EPI_ISL_705237	University of Exeter	COVID-19 Genomics UK (COG-UK) Consortium	Ben Temperton,Aaron Jeffries,Michelle Michelsen,Joanna Warwick-Dugdale,Audrey Farbos,Robyn Manley,Stephen Michell,Jane Masoli
EPI_ISL_705239	Wales Specialist Virology Centre Sequencing lab: Pathogen Genomics Unit	COVID-19 Genomics UK (COG-UK) Consortium	Catherine Moore, Johnathan Evans, Laura Gifford, Malorie Perry, Simon Cottrell, Angela Marchbank, Alec Birchley, Alexander Adams, Amy Gaskin, Bree Gatica-Wilcox, Jason Coombes, Joel Southgate, Lauren Gilbert, Lee Graham, Nicole Pacchiarini, Sara Kumziene-Summerhayes, Sarah Taylor, Sophie Jones, Sara Rey, Matthew Bull, Joanne Watkins, Sally Corden, Tom Connor

EPI_ISL_705240, EPI_ISL_705241	Liverpool Clinical Laboratories	COVID-19 Genomics UK (COG-UK) Consortium	Sam Haldenby, Anita Lucaci, Steve Paterson, Julian Hiscox, Alistair Darby, M Almsaud, A Alrezaihi, Muhannad Alruwaili, Stuart D Armstrong, Jones Benjamin, Eleanor G Bentley, Anu Chawla, Jordan J Clark, Angela Cowell, Richard Eccles, Isabel García-Dorival, Matthew Gemmell, Alessandro Gerada, PKF Gilmore, Richard Gregory, Ximeng Han, Catherine Hartley, Margaret Hughes, Miren Iturriza-Gomara, James Johnson, L Luu, Jenifer Manson, Charlotte Nelson, Elaine O'Toole, Cassie Olateju, Rebekah Penrice-Randal , Lucille Rainbow, N.P Randle, Trevor Ian Robinson, Parul Sharma, Ghada T Shawli, James P Stewart, Neil Swainston, Ecaterina Vamos, Joanne Watts, Mark Whitehead
EPI_ISL_705244	Wales Specialist Virology Centre Sequencing lab: Pathogen Genomics Unit	COVID-19 Genomics UK (COG-UK) Consortium	Catherine Moore, Johnathan Evans, Laura Gifford, Malorie Perry, Simon Cottrell, Angela Marchbank, Alec Birchley, Alexander Adams, Amy Gaskin, Bree Gatica-Wilcox, Jason Coombes, Joel Southgate, Lauren Gilbert, Lee Graham, Nicole Pacchiarini, Sara Kumziene-Summerhayes, Sarah Taylor, Sophie Jones, Sara Rey, Matthew Bull, Joanne Watkins, Sally Corden, Tom Connor
EPI_ISL_705248	Liverpool Clinical Laboratories	COVID-19 Genomics UK (COG-UK) Consortium	Sam Haldenby, Anita Lucaci, Steve Paterson, Julian Hiscox, Alistair Darby, M Almsaud, A Alrezaihi, Muhannad Alruwaili, Stuart D Armstrong, Jones Benjamin, Eleanor G Bentley, Anu Chawla, Jordan J Clark, Angela Cowell, Richard Eccles, Isabel García-Dorival, Matthew Gemmell, Alessandro Gerada, PKF Gilmore, Richard Gregory, Ximeng Han, Catherine Hartley, Margaret Hughes, Miren Iturriza-Gomara, James Johnson, L Luu, Jenifer Manson, Charlotte Nelson, Elaine O'Toole, Cassie Olateju, Rebekah Penrice-Randal , Lucille Rainbow, N.P Randle, Trevor Ian Robinson, Parul Sharma, Ghada T Shawli, James P Stewart, Neil Swainston, Ecaterina Vamos, Joanne Watts, Mark Whitehead
EPI_ISL_705266	Northumbria University / South Tees Hospitals NHS Foundation Trust / North Cumbria Integrated Care NHS Foundation Trust / North Tees and Hartlepool NHS Foundation Trust / Newcastle Hospitals NHS Foundation Trust	COVID-19 Genomics UK (COG-UK) Consortium	Darren L Smith,Andrew Nelson,Matthew Bashton,Greg R Young,Joshua Loh,John Allan,Mohammad A Tariq,Giles S Holt,Gary Black,Wen C Yew,Lynn Dover,Paul Baker,Steve Liggett,Sarah Essex,Jane Greenaway,Debra Padgett,Clive Graham,Garren Scott,Edward Barton,Emma Swindells,Brendan Payne,Jennifer Collins,Yusri Taha,Gary Eltringham
EPI_ISL_705269	Liverpool Clinical Laboratories	COVID-19 Genomics UK (COG-UK) Consortium	Sam Haldenby, Anita Lucaci, Steve Paterson, Julian Hiscox, Alistair Darby, M Almsaud, A Alrezaihi, Muhannad Alruwaili, Stuart D Armstrong, Jones Benjamin, Eleanor G Bentley, Anu Chawla, Jordan J Clark, Angela Cowell, Richard Eccles, Isabel García-Dorival, Matthew Gemmell, Alessandro Gerada, PKF Gilmore, Richard Gregory, Ximeng Han, Catherine Hartley, Margaret Hughes, Miren Iturriza-Gomara, James Johnson, L Luu, Jenifer Manson, Charlotte Nelson, Elaine O'Toole, Cassie Olateju, Rebekah Penrice-Randal , Lucille Rainbow, N.P Randle, Trevor Ian Robinson, Parul Sharma, Ghada T Shawli, James P Stewart, Neil Swainston, Ecaterina Vamos, Joanne Watts, Mark Whitehead
EPI_ISL_705270	Virology Department, Sheffield Teaching Hospitals NHS Foundation Trust/Department of Infection, Immunity and Cardiovascular Disease, The Medical School, University of Sheffield	COVID-19 Genomics UK (COG-UK) Consortium	Thushan de Silva, Matthew Parker, Nikki Smith, Adri Angyal, Rebecca Brown, Luke Green, Rachel Tucker, Paul Parsons, Danielle Groves, Katie Johnson, Laura Carrilero, Alex Keeley, Dave Partridge, Matthew Wyles, Benjamin Lindsey, Mehmet Yavuz, Mohammad Raza, Cariad Evans
EPI_ISL_705271, EPI_ISL_705274	Liverpool Clinical Laboratories	COVID-19 Genomics UK (COG-UK) Consortium	Sam Haldenby, Anita Lucaci, Steve Paterson, Julian Hiscox, Alistair Darby, M Almsaud, A Alrezaihi, Muhannad Alruwaili, Stuart D Armstrong, Jones Benjamin, Eleanor G Bentley, Anu Chawla, Jordan J Clark, Angela Cowell, Richard Eccles, Isabel García-Dorival, Matthew Gemmell, Alessandro Gerada, PKF Gilmore, Richard Gregory, Ximeng Han, Catherine Hartley, Margaret Hughes, Miren Iturriza-Gomara, James Johnson, L Luu, Jenifer Manson, Charlotte Nelson, Elaine O'Toole, Cassie Olateju, Rebekah Penrice-Randal , Lucille Rainbow, N.P Randle, Trevor Ian Robinson, Parul Sharma, Ghada T Shawli, James P Stewart, Neil Swainston, Ecaterina Vamos, Joanne Watts, Mark Whitehead
EPI_ISL_705276	Virology Department, Sheffield Teaching Hospitals NHS Foundation Trust/Department of Infection, Immunity and Cardiovascular Disease, The Medical School, University of Sheffield	COVID-19 Genomics UK (COG-UK) Consortium	Thushan de Silva, Matthew Parker, Nikki Smith, Adri Angyal, Rebecca Brown, Luke Green, Rachel Tucker, Paul Parsons, Danielle Groves, Katie Johnson, Laura Carrilero, Alex Keeley, Dave Partridge, Matthew Wyles, Benjamin Lindsey, Mehmet Yavuz, Mohammad Raza, Cariad Evans
EPI_ISL_705278	University College London, Great Ormond Street Hospital for Children NHS Foundation Trust, Imperial College Healthcare NHS Trust	COVID-19 Genomics UK (COG-UK) Consortium	Sergi Castellano, Rachel Williams, Mark Kristiansen, Paola Resende Silva, Sunando Roy, Tony Brooks, Helena Tutill, Paola Niola, Patricia Dyal, Charlotte Williams, Leysa Forrest, Yasmin Panchbhaya, Jacqueline Findlay, Samuel Weeks, Julianne Brown, Kathryn Harris, Paul Randell, James Price, Alison Holmes, Judith Breuer
EPI_ISL_705282	Northumbria University / South Tees Hospitals NHS Foundation Trust / North Cumbria Integrated Care NHS Foundation Trust / North Tees and Hartlepool NHS Foundation Trust / Newcastle Hospitals NHS Foundation Trust	COVID-19 Genomics UK (COG-UK) Consortium	Darren L Smith,Andrew Nelson,Matthew Bashton,Greg R Young,Joshua Loh,John Allan,Mohammad A Tariq,Giles S Holt,Gary Black,Wen C Yew,Lynn Dover,Paul Baker,Steve Liggett,Sarah Essex,Jane Greenaway,Debra Padgett,Clive Graham,Garren Scott,Edward Barton,Emma Swindells,Brendan Payne,Jennifer Collins,Yusri Taha,Gary Eltringham
EPI_ISL_705287	Liverpool Clinical Laboratories	COVID-19 Genomics UK (COG-UK) Consortium	Sam Haldenby, Anita Lucaci, Steve Paterson, Julian Hiscox, Alistair Darby, M Almsaud, A Alrezaihi, Muhannad Alruwaili, Stuart D Armstrong, Jones Benjamin, Eleanor G Bentley, Anu Chawla, Jordan J Clark, Angela Cowell, Richard Eccles, Isabel García-Dorival, Matthew Gemmell, Alessandro Gerada, PKF Gilmore, Richard Gregory, Ximeng Han, Catherine Hartley, Margaret Hughes, Miren Iturriza-Gomara, James Johnson, L Luu, Jenifer Manson, Charlotte Nelson, Elaine O'Toole, Cassie Olateju, Rebekah Penrice-Randal , Lucille Rainbow, N.P Randle, Trevor Ian Robinson, Parul Sharma, Ghada T Shawli, James P Stewart, Neil Swainston, Ecaterina Vamos, Joanne Watts, Mark Whitehead
EPI_ISL_705295	Northumbria University / South Tees Hospitals NHS Foundation Trust / North Cumbria Integrated Care NHS Foundation Trust / North Tees and Hartlepool NHS Foundation Trust / Newcastle Hospitals NHS Foundation Trust	COVID-19 Genomics UK (COG-UK) Consortium	Darren L Smith,Andrew Nelson,Matthew Bashton,Greg R Young,Joshua Loh,John Allan,Mohammad A Tariq,Giles S Holt,Gary Black,Wen C Yew,Lynn Dover,Paul Baker,Steve Liggett,Sarah Essex,Jane Greenaway,Debra Padgett,Clive Graham,Garren Scott,Edward Barton,Emma Swindells,Brendan Payne,Jennifer Collins,Yusri Taha,Gary Eltringham
EPI_ISL_705299	Liverpool Clinical Laboratories	COVID-19 Genomics UK (COG-UK) Consortium	Sam Haldenby, Anita Lucaci, Steve Paterson, Julian Hiscox, Alistair Darby, M Almsaud, A Alrezaihi, Muhannad Alruwaili, Stuart D Armstrong, Jones Benjamin, Eleanor G Bentley, Anu Chawla, Jordan J Clark, Angela Cowell, Richard Eccles, Isabel García-Dorival, Matthew Gemmell, Alessandro Gerada, PKF Gilmore, Richard Gregory, Ximeng Han, Catherine Hartley, Margaret Hughes, Miren Iturriza-Gomara, James Johnson, L Luu, Jenifer Manson, Charlotte Nelson, Elaine O'Toole, Cassie Olateju, Rebekah Penrice-Randal , Lucille Rainbow, N.P Randle, Trevor Ian Robinson, Parul Sharma, Ghada T Shawli, James P Stewart, Neil Swainston, Ecaterina Vamos, Joanne Watts, Mark Whitehead
EPI_ISL_705300	West of Scotland Specialist Virology Centre, NHSGGC / MRC-University of Glasgow Centre for Virus Research	COVID-19 Genomics UK (COG-UK) Consortium	Ana da Silva Filipe, Natasha Johnson, Kathy Smollett, Daniel Mair, Stephen Carmichael, Alice Broos, Lily Tong, Jenna Nichols, Kyriaki Nomikou; Sarah McDonald; Richard Orton, Joseph Hughes, Sreenu Vattipally, David L Robertson; Alasdair MacLean, Rory Gunson; Sharif Shaaban, Matthew Holden; Rachel Blacow, Guy Mollett, Kathy Li, James Shepherd, Antonia Ho, Emma Thomson
EPI_ISL_705310	University College London, Great Ormond Street Hospital for Children NHS Foundation Trust, Imperial College Healthcare NHS Trust	COVID-19 Genomics UK (COG-UK) Consortium	Sergi Castellano, Rachel Williams, Mark Kristiansen, Paola Resende Silva, Sunando Roy, Tony Brooks, Helena Tutill, Paola Niola, Patricia Dyal, Charlotte Williams, Leysa Forrest, Yasmin Panchbhaya, Jacqueline Findlay, Samuel Weeks, Julianne Brown, Kathryn Harris, Paul Randell, James Price, Alison Holmes, Judith Breuer
EPI_ISL_705317	Virology Department, Sheffield Teaching Hospitals NHS Foundation Trust/Department of Infection, Immunity and Cardiovascular Disease, The Medical School, University of Sheffield	COVID-19 Genomics UK (COG-UK) Consortium	Thushan de Silva, Matthew Parker, Nikki Smith, Adri Angyal, Rebecca Brown, Luke Green, Rachel Tucker, Paul Parsons, Danielle Groves, Katie Johnson, Laura Carrilero, Alex Keeley, Dave Partridge, Matthew Wyles, Benjamin Lindsey, Mehmet Yavuz, Mohammad Raza, Cariad Evans
EPI_ISL_705326	Liverpool Clinical Laboratories	COVID-19 Genomics UK (COG-UK) Consortium	Sam Haldenby, Anita Lucaci, Steve Paterson, Julian Hiscox, Alistair Darby, M Almsaud, A Alrezaihi, Muhannad Alruwaili, Stuart D Armstrong, Jones Benjamin, Eleanor G Bentley, Anu Chawla, Jordan J Clark, Angela Cowell, Richard Eccles, Isabel García-Dorival, Matthew Gemmell, Alessandro Gerada, PKF Gilmore, Richard Gregory, Ximeng Han, Catherine Hartley, Margaret Hughes, Miren Iturriza-Gomara, James Johnson, L Luu, Jenifer Manson, Charlotte Nelson, Elaine O'Toole, Cassie Olateju, Rebekah Penrice-Randal , Lucille Rainbow, N.P Randle, Trevor Ian Robinson, Parul Sharma, Ghada T Shawli, James P Stewart, Neil Swainston, Ecaterina Vamos, Joanne Watts, Mark Whitehead
EPI_ISL_705327	Wales Specialist Virology Centre Sequencing lab: Pathogen Genomics Unit	COVID-19 Genomics UK (COG-UK) Consortium	Catherine Moore, Johnathan Evans, Laura Gifford, Malorie Perry, Simon Cottrell, Angela Marchbank, Alec Birchley, Alexander Adams, Amy Gaskin, Bree Gatica-Wilcox, Jason Coombes, Joel Southgate, Lauren Gilbert, Lee Graham, Nicole Pacchiarini, Sara Kumziene-Summerhayes, Sarah Taylor, Sophie Jones, Sara Rey, Matthew Bull, Joanne Watkins, Sally Corden, Tom Connor
EPI_ISL_705329, EPI_ISL_705334	Liverpool Clinical Laboratories	COVID-19 Genomics UK (COG-UK) Consortium	Sam Haldenby, Anita Lucaci, Steve Paterson, Julian Hiscox, Alistair Darby, M Almsaud, A Alrezaihi, Muhannad Alruwaili, Stuart D Armstrong, Jones Benjamin, Eleanor G Bentley, Anu Chawla, Jordan J Clark, Angela Cowell, Richard Eccles, Isabel García-Dorival, Matthew Gemmell, Alessandro Gerada,

			PKF Gilmore, Richard Gregory, Ximeng Han, Catherine Hartley, Margaret Hughes, Miren Iturriza-Gomara, James Johnson, L Luu, Jenifer Manson, Charlotte Nelson, Elaine O'Toole, Cassie Olateju, Rebekah Penrice-Randal , Lucille Rainbow, N.P Randle, Trevor Ian Robinson, Parul Sharma, Ghada T Shawli, James P Stewart, Neil Swainston, Ecaterina Vamos, Joanne Watts, Mark Whitehead
EPI_ISL_705342	University College London, Great Ormond Street Hospital for Children NHS Foundation Trust, Imperial College Healthcare NHS Trust	COVID-19 Genomics UK (COG-UK) Consortium	Sergi Castellano, Rachel Williams, Mark Kristiansen, Paola Resende Silva, Sunando Roy, Tony Brooks, Helena Tutill, Paola Niola, Patricia Dyal, Charlotte Williams, Leysa Forrest, Yasmin Panchbhaya, Jacqueline Findlay, Samuel Weeks, Julianne Brown, Kathryn Harris, Paul Randell, James Price, Alison Holmes, Judith Breuer
EPI_ISL_705343, EPI_ISL_705353	Northumbria University / South Tees Hospitals NHS Foundation Trust / North Cumbria Integrated Care NHS Foundation Trust / North Tees and Hartlepool NHS Foundation Trust / Newcastle Hospitals NHS Foundation Trust	COVID-19 Genomics UK (COG-UK) Consortium	Darren L Smith,Andrew Nelson,Matthew Bashton,Greg R Young,Joshua Loh,John Allan,Mohammad A Tariq,Giles S Holt,Gary Black,Wen C Yew,Lynn Dover,Paul Baker,Steve Liggett,Sarah Essex,Jane Greenaway,Debra Padgett,Clive Graham,Garren Scott,Edward Barton,Emma Swindells,Brendan Payne,Jennifer Collins,Yusri Taha,Gary Eltringham
EPI_ISL_705358	Liverpool Clinical Laboratories	COVID-19 Genomics UK (COG-UK) Consortium	Sam Haldenby, Anita Lucaci, Steve Paterson, Julian Hiscox, Alistair Darby, M Almsaud, A Alrezaihi, Muhannad Alruwaili, Stuart D Armstrong, Jones Benjamin, Eleanor G Bentley, Anu Chawla, Jordan J Clark, Angela Cowell, Richard Eccles, Isabel García-Dorival, Matthew Gemmell, Alessandro Gerada, PKF Gilmore, Richard Gregory, Ximeng Han, Catherine Hartley, Margaret Hughes, Miren Iturriza-Gomara, James Johnson, L Luu, Jenifer Manson, Charlotte Nelson, Elaine O'Toole, Cassie Olateju, Rebekah Penrice-Randal , Lucille Rainbow, N.P Randle, Trevor Ian Robinson, Parul Sharma, Ghada T Shawli, James P Stewart, Neil Swainston, Ecaterina Vamos, Joanne Watts, Mark Whitehead
EPI_ISL_705361, EPI_ISL_705364, EPI_ISL_705367	Northumbria University / South Tees Hospitals NHS Foundation Trust / North Cumbria Integrated Care NHS Foundation Trust / North Tees and Hartlepool NHS Foundation Trust / Newcastle Hospitals NHS Foundation Trust	COVID-19 Genomics UK (COG-UK) Consortium	Darren L Smith,Andrew Nelson,Matthew Bashton,Greg R Young,Joshua Loh,John Allan,Mohammad A Tariq,Giles S Holt,Gary Black,Wen C Yew,Lynn Dover,Paul Baker,Steve Liggett,Sarah Essex,Jane Greenaway,Debra Padgett,Clive Graham,Garren Scott,Edward Barton,Emma Swindells,Brendan Payne,Jennifer Collins,Yusri Taha,Gary Eltringham
EPI_ISL_705368	University College London, Great Ormond Street Hospital for Children NHS Foundation Trust, Imperial College Healthcare NHS Trust	COVID-19 Genomics UK (COG-UK) Consortium	Sergi Castellano, Rachel Williams, Mark Kristiansen, Paola Resende Silva, Sunando Roy, Tony Brooks, Helena Tutill, Paola Niola, Patricia Dyal, Charlotte Williams, Leysa Forrest, Yasmin Panchbhaya, Jacqueline Findlay, Samuel Weeks, Julianne Brown, Kathryn Harris, Paul Randell, James Price, Alison Holmes, Judith Breuer
EPI_ISL_705383	University of Exeter	COVID-19 Genomics UK (COG-UK) Consortium	Ben Temperton,Aaron Jeffries,Michelle Michelsen,Joanna Warwick-Dugdale,Audrey Farbos,Robyn Manley,Stephen Michell,Jane Masoli
EPI_ISL_705384	Wales Specialist Virology Centre Sequencing lab: Pathogen Genomics Unit	COVID-19 Genomics UK (COG-UK) Consortium	Catherine Moore, Johnathan Evans, Laura Gifford, Malorie Perry, Simon Cottrell, Angela Marchbank, Alec Birchley, Alexander Adams, Amy Gaskin, Bree Gatica-Wilcox, Jason Coombes, Joel Southgate, Lauren Gilbert, Lee Graham, Nicole Pacchiarini, Sara Kumziene-Summerhayes, Sarah Taylor, Sophie Jones, Sara Rey, Matthew Bull, Joanne Watkins, Sally Corden, Tom Connor
EPI_ISL_705388	Liverpool Clinical Laboratories	COVID-19 Genomics UK (COG-UK) Consortium	Sam Haldenby, Anita Lucaci, Steve Paterson, Julian Hiscox, Alistair Darby, M Almsaud, A Alrezaihi, Muhannad Alruwaili, Stuart D Armstrong, Jones Benjamin, Eleanor G Bentley, Anu Chawla, Jordan J Clark, Angela Cowell, Richard Eccles, Isabel García-Dorival, Matthew Gemmell, Alessandro Gerada, PKF Gilmore, Richard Gregory, Ximeng Han, Catherine Hartley, Margaret Hughes, Miren Iturriza-Gomara, James Johnson, L Luu, Jenifer Manson, Charlotte Nelson, Elaine O'Toole, Cassie Olateju, Rebekah Penrice-Randal , Lucille Rainbow, N.P Randle, Trevor Ian Robinson, Parul Sharma, Ghada T Shawli, James P Stewart, Neil Swainston, Ecaterina Vamos, Joanne Watts, Mark Whitehead
EPI_ISL_705397	University College London, Great Ormond Street Hospital for Children NHS Foundation Trust, Imperial College Healthcare NHS Trust	COVID-19 Genomics UK (COG-UK) Consortium	Sergi Castellano, Rachel Williams, Mark Kristiansen, Paola Resende Silva, Sunando Roy, Tony Brooks, Helena Tutill, Paola Niola, Patricia Dyal, Charlotte Williams, Leysa Forrest, Yasmin Panchbhaya, Jacqueline Findlay, Samuel Weeks, Julianne Brown, Kathryn Harris, Paul Randell, James Price, Alison Holmes, Judith Breuer
EPI_ISL_705398, EPI_ISL_705408	Liverpool Clinical Laboratories	COVID-19 Genomics UK (COG-UK) Consortium	Sam Haldenby, Anita Lucaci, Steve Paterson, Julian Hiscox, Alistair Darby, M Almsaud, A Alrezaihi, Muhannad Alruwaili, Stuart D Armstrong, Jones Benjamin, Eleanor G Bentley, Anu Chawla, Jordan J Clark, Angela Cowell, Richard Eccles, Isabel García-Dorival, Matthew Gemmell, Alessandro Gerada, PKF Gilmore, Richard Gregory, Ximeng Han, Catherine Hartley, Margaret Hughes, Miren Iturriza-Gomara, James Johnson, L Luu, Jenifer Manson, Charlotte Nelson, Elaine O'Toole, Cassie Olateju, Rebekah Penrice-Randal , Lucille Rainbow, N.P Randle, Trevor Ian Robinson, Parul Sharma, Ghada T Shawli, James P Stewart, Neil Swainston, Ecaterina Vamos, Joanne Watts, Mark Whitehead
EPI_ISL_705412, EPI_ISL_705413	Wales Specialist Virology Centre Sequencing lab: Pathogen Genomics Unit	COVID-19 Genomics UK (COG-UK) Consortium	Catherine Moore, Johnathan Evans, Laura Gifford, Malorie Perry, Simon Cottrell, Angela Marchbank, Alec Birchley, Alexander Adams, Amy Gaskin, Bree Gatica-Wilcox, Jason Coombes, Joel Southgate, Lauren Gilbert, Lee Graham, Nicole Pacchiarini, Sara Kumziene-Summerhayes, Sarah Taylor, Sophie Jones, Sara Rey, Matthew Bull, Joanne Watkins, Sally Corden, Tom Connor
EPI_ISL_705422	Quadram Institute Bioscience	COVID-19 Genomics UK (COG-UK) Consortium	Dave J. Baker, Gemma L. Kay, Alp Aydin, Thanh Le-Viet, Steven Rudder, Ana P. Tedim, Anastasia Kolyva, Maria Diaz, Leonardo de Oliveira Martins, Nabil-Fareed Alikhan, Lizzie Meadows, Rachael Stanley, Ngozi Elumogo, Muhammed Yasir, Nicholas M. Thomson, Alexander J Trotter, Rachel Gilroy, Samuel Bloomfield, Claire Stuart, Andrew Bell, Reenesh Prakash, Samir Dervisevic, Alison E. Mather, John Wain, Mark Webber, Andrew J. Page, Justin O'Grady
EPI_ISL_705434, EPI_ISL_705435, EPI_ISL_705440, EPI_ISL_705441, EPI_ISL_705442, EPI_ISL_705445	Wales Specialist Virology Centre Sequencing lab: Pathogen Genomics Unit	COVID-19 Genomics UK (COG-UK) Consortium	Catherine Moore, Johnathan Evans, Laura Gifford, Malorie Perry, Simon Cottrell, Angela Marchbank, Alec Birchley, Alexander Adams, Amy Gaskin, Bree Gatica-Wilcox, Jason Coombes, Joel Southgate, Lauren Gilbert, Lee Graham, Nicole Pacchiarini, Sara Kumziene-Summerhayes, Sarah Taylor, Sophie Jones, Sara Rey, Matthew Bull, Joanne Watkins, Sally Corden, Tom Connor
EPI_ISL_705458, EPI_ISL_705486	Northumbria University / South Tees Hospitals NHS Foundation Trust / North Cumbria Integrated Care NHS Foundation Trust / North Tees and Hartlepool NHS Foundation Trust / Newcastle Hospitals NHS Foundation Trust	COVID-19 Genomics UK (COG-UK) Consortium	Darren L Smith,Andrew Nelson,Matthew Bashton,Greg R Young,Joshua Loh,John Allan,Mohammad A Tariq,Giles S Holt,Gary Black,Wen C Yew,Lynn Dover,Paul Baker,Steve Liggett,Sarah Essex,Jane Greenaway,Debra Padgett,Clive Graham,Garren Scott,Edward Barton,Emma Swindells,Brendan Payne,Jennifer Collins,Yusri Taha,Gary Eltringham
EPI_ISL_705490	West of Scotland Specialist Virology Centre, NHSGGC / MRC-University of Glasgow Centre for Virus Research	COVID-19 Genomics UK (COG-UK) Consortium	Ana da Silva Filipe, Natasha Johnson, Kathy Smollett, Daniel Mair, Stephen Carmichael, Alice Broos, Lily Tong, Jenna Nichols, Kyriaki Nomikou; Sarah McDonald; Richard Orton, Joseph Hughes, Sreenu Vattipally, David L Robertson; Alasdair MacLean, Rory Gunson; Sharif Shaaban, Matthew Holden; Rachel Blacow, Guy Mollett, Kathy Li, James Shepherd, Antonia Ho, Emma Thomson
EPI_ISL_705491	Quadram Institute Bioscience	COVID-19 Genomics UK (COG-UK) Consortium	Dave J. Baker, Gemma L. Kay, Alp Aydin, Thanh Le-Viet, Steven Rudder, Ana P. Tedim, Anastasia Kolyva, Maria Diaz, Leonardo de Oliveira Martins, Nabil-Fareed Alikhan, Lizzie Meadows, Rachael Stanley, Ngozi Elumogo, Muhammed Yasir, Nicholas M. Thomson, Alexander J Trotter, Rachel Gilroy, Samuel Bloomfield, Claire Stuart, Andrew Bell, Reenesh Prakash, Samir Dervisevic, Alison E. Mather, John Wain, Mark Webber, Andrew J. Page, Justin O'Grady
EPI_ISL_705496	Northumbria University / South Tees Hospitals NHS Foundation Trust / North Cumbria Integrated Care NHS Foundation Trust / North Tees and Hartlepool NHS Foundation Trust / Newcastle Hospitals NHS Foundation Trust	COVID-19 Genomics UK (COG-UK) Consortium	Darren L Smith,Andrew Nelson,Matthew Bashton,Greg R Young,Joshua Loh,John Allan,Mohammad A Tariq,Giles S Holt,Gary Black,Wen C Yew,Lynn Dover,Paul Baker,Steve Liggett,Sarah Essex,Jane Greenaway,Debra Padgett,Clive Graham,Garren Scott,Edward Barton,Emma Swindells,Brendan Payne,Jennifer Collins,Yusri Taha,Gary Eltringham
EPI_ISL_705528	West of Scotland Specialist Virology Centre, NHSGGC / MRC-University of Glasgow Centre for Virus Research	COVID-19 Genomics UK (COG-UK) Consortium	Ana da Silva Filipe, Natasha Johnson, Kathy Smollett, Daniel Mair, Stephen Carmichael, Alice Broos, Lily Tong, Jenna Nichols, Kyriaki Nomikou; Sarah McDonald; Richard Orton, Joseph Hughes, Sreenu Vattipally, David L Robertson; Alasdair MacLean, Rory Gunson; Sharif Shaaban, Matthew Holden; Rachel Blacow, Guy Mollett, Kathy Li, James Shepherd, Antonia Ho, Emma Thomson
EPI_ISL_705559, EPI_ISL_705560, EPI_ISL_705561, EPI_ISL_705563	Department of Pathology, University of Cambridge	COVID-19 Genomics UK (COG-UK) Consortium	Aminu S. Jahun, Yasmin Chaudhry, Grant Hall, Iliana Georgana, Myra Hosmillo, Martin D. Curran, Malte Pinckert, Surendra Parmar, Ian Goodfellow
EPI_ISL_705567, EPI_ISL_705568, EPI_ISL_705569, EPI_ISL_705570, EPI_ISL_705571, EPI_ISL_705572, EPI_ISL_705573, EPI_ISL_705574, EPI_ISL_705575, EPI_ISL_705576, EPI_ISL_705577, EPI_ISL_705578, EPI_ISL_705579, EPI_ISL_705580, EPI_ISL_705581, EPI_ISL_705582, EPI_ISL_705583, EPI_ISL_705584, EPI_ISL_705585, EPI_ISL_705593, EPI_ISL_705594, EPI_ISL_705595, EPI_ISL_705596, EPI_ISL_705597, EPI_ISL_705598, EPI_ISL_705599, EPI_ISL_705600, EPI_ISL_705601, EPI_ISL_705602, EPI_ISL_705603, EPI_ISL_705605, EPI_ISL_705607, EPI_ISL_705608, EPI_ISL_705609, EPI_ISL_705610, EPI_ISL_705611, EPI_ISL_705612			
see above	Quadram Institute Bioscience	COVID-19 Genomics UK (COG-UK) Consortium	Dave J. Baker, Gemma L. Kay, Alp Aydin, Thanh Le-Viet, Steven Rudder, Ana P. Tedim, Anastasia Kolyva, Maria Diaz, Leonardo de Oliveira Martins, Nabil-Fareed Alikhan, Lizzie Meadows, Rachael Stanley, Ngozi Elumogo, Muhammed Yasir, Nicholas M. Thomson, Alexander J Trotter, Rachel Gilroy, Samuel Bloomfield, Claire Stuart, Andrew Bell, Reenesh Prakash, Samir Dervisevic, Alison E. Mather, John Wain, Mark Webber, Andrew J. Page, Justin O'Grady

EPI_ISL_705613, EPI_ISL_705614	Queens Medical Centre, Clinical Microbiology Department / DeepSeq Nottingham	COVID-19 Genomics UK (COG-UK) Consortium	Gemma Clark, Wendy Smith, Manjinder Khakh, Vicki M Fleming, Michelle M Lister, Hannah Howson-Wells, Jonathan Ball, Patrick McClure, Joseph Chappell, Theocharis Tsoleridis, Nadine Holmes, Matthew Carlisle, Christopher Moore, Fei Sang, Johnny Debebe, Victoria Wright, Matthew Loose
EPI_ISL_705619, EPI_ISL_705620, EPI_ISL_705621, EPI_ISL_705622, EPI_ISL_705623, EPI_ISL_705624, EPI_ISL_705625, EPI_ISL_705626, EPI_ISL_705627, EPI_ISL_705628	Virology Department, Sheffield Teaching Hospitals NHS Foundation Trust/Department of Infection, Immunity and Cardiovascular Disease, The Medical School, University of Sheffield	COVID-19 Genomics UK (COG-UK) Consortium	Thushan de Silva, Matthew Parker, Nikki Smith, Adri Angyal, Rebecca Brown, Luke Green, Rachel Tucker, Paul Parsons, Danielle Groves, Katie Johnson, Laura Carrilero, Alex Keeley, Dave Partridge, Matthew Wyles, Benjamin Lindsey, Mehmet Yavuz, Mohammad Raza, Cariad Evans
EPI_ISL_705641, EPI_ISL_705642, EPI_ISL_705643, EPI_ISL_705644, EPI_ISL_705645, EPI_ISL_705646, EPI_ISL_705647, EPI_ISL_705648, EPI_ISL_705649, EPI_ISL_705650, EPI_ISL_705651, EPI_ISL_705652, EPI_ISL_705653, EPI_ISL_705654, EPI_ISL_705655, EPI_ISL_705656, EPI_ISL_705657, EPI_ISL_705658, EPI_ISL_705659, EPI_ISL_705660, EPI_ISL_705661, EPI_ISL_705662, EPI_ISL_705663, EPI_ISL_705664, EPI_ISL_705665, EPI_ISL_705666, EPI_ISL_705667, EPI_ISL_705668, EPI_ISL_705669, EPI_ISL_705670, EPI_ISL_705671, EPI_ISL_705672, EPI_ISL_705673, EPI_ISL_705674, EPI_ISL_705675, EPI_ISL_705676, EPI_ISL_705677, EPI_ISL_705678, EPI_ISL_705679, EPI_ISL_705680, EPI_ISL_705681, EPI_ISL_705708, EPI_ISL_705709, EPI_ISL_705710, EPI_ISL_705711, EPI_ISL_705730			
see above	West of Scotland Specialist Virology Centre, NHSGGC / MRC-University of Glasgow Centre for Virus Research	COVID-19 Genomics UK (COG-UK) Consortium	Ana da Silva Filipe, Natasha Johnson, Kathy Smollett, Daniel Mair, Stephen Carmichael, Alice Broos, Lily Tong, Jenna Nichols, Kyriaki Nomikou; Sarah McDonald; Richard Orton, Joseph Hughes, Sreenu Vattipally, David L Robertson; Alasdair MacLean, Rory Gunson; Sharif Shaaban, Matthew Holden; Rachel Blacow, Guy Mollett, Kathy Li, James Shepherd, Antonia Ho, Emma Thomson
EPI_ISL_705803, EPI_ISL_705804	Virology Department, Royal Infirmary of Edinburgh, NHS Lothian / School of Biological Sciences, University of Edinburgh / Institute of Genetics and Molecular Medicine, University of Edinburgh	COVID-19 Genomics UK (COG-UK) Consortium	McHugh M, Dewar R, Rooke S, Gallagher M, Balcaza C, O'Toole Á, Scher E, Hill V, McCrone JT, Colquhoun R, Yu X, Jackson B, Rambaut A, Williams TC, Templeton K
EPI_ISL_705809, EPI_ISL_705810, EPI_ISL_705811, EPI_ISL_705812, EPI_ISL_705813, EPI_ISL_705814, EPI_ISL_705815, EPI_ISL_705816, EPI_ISL_705817, EPI_ISL_705818, EPI_ISL_705819, EPI_ISL_705820, EPI_ISL_705821, EPI_ISL_705822, EPI_ISL_705823, EPI_ISL_705859, EPI_ISL_705861, EPI_ISL_705875, EPI_ISL_705886, EPI_ISL_705887, EPI_ISL_705889, EPI_ISL_705890, EPI_ISL_705892, EPI_ISL_705894, EPI_ISL_705929, EPI_ISL_705930, EPI_ISL_705931, EPI_ISL_705932, EPI_ISL_705933, EPI_ISL_705934, EPI_ISL_705935, EPI_ISL_705936, EPI_ISL_705937, EPI_ISL_705938, EPI_ISL_705939, EPI_ISL_705940			
see above	Liverpool Clinical Laboratories	COVID-19 Genomics UK (COG-UK) Consortium	Sam Haldenby, Anita Lucaci, Steve Paterson, Julian Hiscox, Alistair Darby, M Almsaud, A Alrezaihi, Muhannad Alruwaili, Stuart D Armstrong, Jones Benjamin, Eleanor G Bentley, Anu Chawla, Jordan J Clark, Angela Cowell, Richard Eccles, Isabel García-Dorival, Matthew Gemmell, Alessandro Gerada, PKF Gilmore, Richard Gregory, Ximeng Han, Catherine Hartley, Margaret Hughes, Miren Iturriza-Gomara, James Johnson, L Luu, Jenifer Manson, Charlotte Nelson, Elaine O'Toole, Cassie Olateju, Rebekah Penrice-Randal , Lucille Rainbow, N.P Randle, Trevor Ian Robinson, Parul Sharma, Ghada T Shawli, James P Stewart, Neil Swainston, Ecaterina Vamos, Joanne Watts, Mark Whitehead
EPI_ISL_705949	University College London Hospital	COVID-19 Genomics UK (COG-UK) Consortium	Judith Heaney, Matthew Byott, Catherine Houlihan, Dan Frampton, Stuart Kirk, Moira Spyer and Eleni Nastouli
EPI_ISL_705961, EPI_ISL_705962, EPI_ISL_705963, EPI_ISL_705964, EPI_ISL_705965, EPI_ISL_705966, EPI_ISL_705967, EPI_ISL_705968, EPI_ISL_705969, EPI_ISL_705970, EPI_ISL_705971			
see above	University College London, Great Ormond Street Hospital for Children NHS Foundation Trust, Imperial College Healthcare NHS Trust	COVID-19 Genomics UK (COG-UK) Consortium	Sergi Castellano, Rachel Williams, Mark Kristiansen, Paola Resende Silva, Sunando Roy, Tony Brooks, Helena Tutill, Paola Niola, Patricia Dyal, Charlotte Williams, Leysa Forrest, Yasmin Panchbhaya, Jacqueline Findlay, Samuel Weeks, Julianne Brown, Kathryn Harris, Paul Randell, James Price, Alison Holmes, Judith Breuer
EPI_ISL_706279, EPI_ISL_706301, EPI_ISL_706302, EPI_ISL_706303, EPI_ISL_706306, EPI_ISL_706307, EPI_ISL_706308, EPI_ISL_706309, EPI_ISL_706310, EPI_ISL_706311, EPI_ISL_706312, EPI_ISL_706313, EPI_ISL_706314, EPI_ISL_706316, EPI_ISL_706317, EPI_ISL_706318, EPI_ISL_706319, EPI_ISL_706320, EPI_ISL_706321, EPI_ISL_706322, EPI_ISL_706323, EPI_ISL_706324, EPI_ISL_706325, EPI_ISL_706326, EPI_ISL_706330, EPI_ISL_706332, EPI_ISL_706333, EPI_ISL_706334, EPI_ISL_706335, EPI_ISL_706336, EPI_ISL_706337, EPI_ISL_706338, EPI_ISL_706339, EPI_ISL_706341, EPI_ISL_706342, EPI_ISL_706343, EPI_ISL_706344, EPI_ISL_706345, EPI_ISL_706346, EPI_ISL_706347, EPI_ISL_706348, EPI_ISL_706349, EPI_ISL_706350, EPI_ISL_706351, EPI_ISL_706352			
see above	Northumbria University / South Tees Hospitals NHS Foundation Trust / North Cumbria Integrated Care NHS Foundation Trust / North Tees and Hartlepool NHS Foundation Trust / Newcastle Hospitals NHS Foundation Trust	COVID-19 Genomics UK (COG-UK) Consortium	Darren L Smith,Andrew Nelson,Matthew Bashton,Greg R Young,Joshua Loh,John Allan,Mohammad A Tariq,Giles S Holt,Gary Black,Wen C Yew,Lynn Dover,Paul Baker,Steve Liggett,Sarah Essex,Jane Greenaway,Debra Padgett,Clive Graham,Garren Scott,Edward Barton,Emma Swindells,Brendan Payne,Jennifer Collins,Yusri Taha,Gary Eltringham
EPI_ISL_706420	Queens Medical Centre, Clinical Microbiology Department / DeepSeq Nottingham	COVID-19 Genomics UK (COG-UK) Consortium	Gemma Clark, Wendy Smith, Manjinder Khakh, Vicki M Fleming, Michelle M Lister, Hannah Howson-Wells, Jonathan Ball, Patrick McClure, Joseph Chappell, Theocharis Tsoleridis, Nadine Holmes, Matthew Carlisle, Christopher Moore, Fei Sang, Johnny Debebe, Victoria Wright, Matthew Loose
EPI_ISL_706440, EPI_ISL_706444, EPI_ISL_706445, EPI_ISL_706446, EPI_ISL_706447, EPI_ISL_706448, EPI_ISL_706449, EPI_ISL_706451, EPI_ISL_706452, EPI_ISL_706453, EPI_ISL_706454, EPI_ISL_706455, EPI_ISL_706456, EPI_ISL_706457, EPI_ISL_706458, EPI_ISL_706459, EPI_ISL_706460, EPI_ISL_706461, EPI_ISL_706462, EPI_ISL_706463, EPI_ISL_706506, EPI_ISL_706514, EPI_ISL_706515, EPI_ISL_706516, EPI_ISL_706517, EPI_ISL_706518, EPI_ISL_706519, EPI_ISL_706520, EPI_ISL_706521, EPI_ISL_706522, EPI_ISL_706523, EPI_ISL_706524, EPI_ISL_706525, EPI_ISL_706526, EPI_ISL_706527, EPI_ISL_706528, EPI_ISL_706529, EPI_ISL_706530, EPI_ISL_706531, EPI_ISL_706532, EPI_ISL_706533, EPI_ISL_706534, EPI_ISL_706535, EPI_ISL_706536, EPI_ISL_706537, EPI_ISL_706538, EPI_ISL_706539, EPI_ISL_706540, EPI_ISL_706541, EPI_ISL_706542, EPI_ISL_706543, EPI_ISL_706544, EPI_ISL_706545, EPI_ISL_706546, EPI_ISL_706547, EPI_ISL_706548, EPI_ISL_706549, EPI_ISL_706550, EPI_ISL_706551, EPI_ISL_706552, EPI_ISL_706553, EPI_ISL_706554, EPI_ISL_706555, EPI_ISL_706556, EPI_ISL_706557, EPI_ISL_706558, EPI_ISL_706559, EPI_ISL_706560, EPI_ISL_706561, EPI_ISL_706562, EPI_ISL_706563, EPI_ISL_706564, EPI_ISL_706565, EPI_ISL_706566, EPI_ISL_706567, EPI_ISL_706568, EPI_ISL_706569, EPI_ISL_706570, EPI_ISL_706571, EPI_ISL_706572, EPI_ISL_706573, EPI_ISL_706574, EPI_ISL_706575, EPI_ISL_706576, EPI_ISL_706577, EPI_ISL_706578, EPI_ISL_706579, EPI_ISL_706580, EPI_ISL_706581, EPI_ISL_706582, EPI_ISL_706583, EPI_ISL_706584, EPI_ISL_706585, EPI_ISL_706586, EPI_ISL_706587, EPI_ISL_706588, EPI_ISL_706596, EPI_ISL_706597, EPI_ISL_706598, EPI_ISL_706599, EPI_ISL_706600, EPI_ISL_706601, EPI_ISL_706602, EPI_ISL_706607, EPI_ISL_706608, EPI_ISL_706609, EPI_ISL_706610, EPI_ISL_706611, EPI_ISL_706612, EPI_ISL_706613, EPI_ISL_706620, EPI_ISL_706621, EPI_ISL_706622, EPI_ISL_706623, EPI_ISL_706624, EPI_ISL_706625, EPI_ISL_706626, EPI_ISL_706627, EPI_ISL_706628, EPI_ISL_706629, EPI_ISL_706630, EPI_ISL_706631, EPI_ISL_706632, EPI_ISL_706633, EPI_ISL_706634, EPI_ISL_706635, EPI_ISL_706639, EPI_ISL_706640, EPI_ISL_706641, EPI_ISL_706642, EPI_ISL_706643, EPI_ISL_706644, EPI_ISL_706645, EPI_ISL_706646, EPI_ISL_706647, EPI_ISL_706648, EPI_ISL_706649, EPI_ISL_706650, EPI_ISL_706651, EPI_ISL_706652, EPI_ISL_706653, EPI_ISL_706654, EPI_ISL_706655, EPI_ISL_706656, EPI_ISL_706657, EPI_ISL_706662, EPI_ISL_706776, EPI_ISL_706781, EPI_ISL_706782, EPI_ISL_706783, EPI_ISL_706784, EPI_ISL_706785, EPI_ISL_706786, EPI_ISL_706787, EPI_ISL_706788, EPI_ISL_706789, EPI_ISL_706790, EPI_ISL_706791, EPI_ISL_706792, EPI_ISL_706793, EPI_ISL_706794, EPI_ISL_706795, EPI_ISL_706796, EPI_ISL_706797, EPI_ISL_706798, EPI_ISL_706799, EPI_ISL_706800, EPI_ISL_706801, EPI_ISL_706802, EPI_ISL_706803, EPI_ISL_706804, EPI_ISL_706805, EPI_ISL_706806, EPI_ISL_706807, EPI_ISL_706808, EPI_ISL_706809, EPI_ISL_706810, EPI_ISL_706811, EPI_ISL_706812, EPI_ISL_706813, EPI_ISL_706814, EPI_ISL_706815, EPI_ISL_706816, EPI_ISL_706817, EPI_ISL_706818, EPI_ISL_706819, EPI_ISL_706820, EPI_ISL_706821, EPI_ISL_706822, EPI_ISL_706823, EPI_ISL_706824, EPI_ISL_706825, EPI_ISL_706826, EPI_ISL_706827, EPI_ISL_706828, EPI_ISL_706829, EPI_ISL_706830, EPI_ISL_706831, EPI_ISL_706832, EPI_ISL_706833, EPI_ISL_706834, EPI_ISL_706835, EPI_ISL_706836, EPI_ISL_706837, EPI_ISL_706838, EPI_ISL_706839, EPI_ISL_706840, EPI_ISL_706841, EPI_ISL_706842, EPI_ISL_706843, EPI_ISL_706844, EPI_ISL_706845, EPI_ISL_706846, EPI_ISL_706847, EPI_ISL_706848, EPI_ISL_706849, EPI_ISL_706850, EPI_ISL_706851, EPI_ISL_706852, EPI_ISL_706853, EPI_ISL_706854, EPI_ISL_706855, EPI_ISL_706856, EPI_ISL_706857, EPI_ISL_706858, EPI_ISL_706859, EPI_ISL_706860, EPI_ISL_706861, EPI_ISL_706862, EPI_ISL_706863			
see above	Wales Specialist Virology Centre Sequencing lab: Pathogen Genomics Unit	COVID-19 Genomics UK (COG-UK) Consortium	Catherine Moore, Johnathan Evans, Laura Gifford, Malorie Perry, Simon Cottrell, Angela Marchbank, Alec Birchley, Alexander Adams, Amy Gaskin, Bree Gatica-Wilcox, Jason Coombes, Joel Southgate, Lauren Gilbert, Lee Graham, Nicole Pacchiarini, Sara Kumziene-Summerhayes, Sarah Taylor, Sophie Jones, Sara Rey, Matthew Bull, Joanne Watkins, Sally Corden, Tom Connor
EPI_ISL_706988, EPI_ISL_706991	Virology Department, Sheffield Teaching Hospitals NHS Foundation Trust/Department of Infection, Immunity and Cardiovascular Disease, The Medical School, University of Sheffield	COVID-19 Genomics UK (COG-UK) Consortium	Thushan de Silva, Matthew Parker, Nikki Smith, Adri Angyal, Rebecca Brown, Luke Green, Rachel Tucker, Paul Parsons, Danielle Groves, Katie Johnson, Laura Carrilero, Alex Keeley, Dave Partridge, Matthew Wyles, Benjamin Lindsey, Mehmet Yavuz, Mohammad Raza, Cariad Evans
EPI_ISL_709882, EPI_ISL_709883, EPI_ISL_709884, EPI_ISL_709894, EPI_ISL_709896, EPI_ISL_709897	Lighthouse Lab in Milton Keynes	Wellcome Sanger Institute for the COVID-19 Genomics UK (COG-UK) Consortium	The Lighthouse Lab in Milton Keynes and Alex Alderton, Roberto Amato, Sonia Goncalves, Ewan Harrison, David K. Jackson, Ian Johnston, Dominic Kwiatkowski, Cordelia Langford, John Sillitoe on behalf of the Wellcome Sanger Institute COVID-19 Surveillance Team
EPI_ISL_710699, EPI_ISL_710700, EPI_ISL_710701, EPI_ISL_710702, EPI_ISL_710703, EPI_ISL_710704, EPI_ISL_710705, EPI_ISL_710706, EPI_ISL_710707, EPI_ISL_710708, EPI_ISL_710709, EPI_ISL_710710, EPI_ISL_710711, EPI_ISL_710712, EPI_ISL_710713, EPI_ISL_710714, EPI_ISL_710715, EPI_ISL_710716, EPI_ISL_710717, EPI_ISL_710718, EPI_ISL_710719, EPI_ISL_710720, EPI_ISL_710721, EPI_ISL_710722, EPI_ISL_710723, EPI_ISL_710724, EPI_ISL_710725, EPI_ISL_710726, EPI_ISL_710727, EPI_ISL_710728, EPI_ISL_710729, EPI_ISL_710730, EPI_ISL_710731, EPI_ISL_710732, EPI_ISL_710733, EPI_ISL_710734, EPI_ISL_710735, EPI_ISL_710736, EPI_ISL_710737, EPI_ISL_710738, EPI_ISL_710739, EPI_ISL_710740, EPI_ISL_710741, EPI_ISL_710742, EPI_ISL_710743, EPI_ISL_710744			
see above	Lighthouse Lab in Alderley Park	Wellcome Sanger Institute for the COVID-19 Genomics UK (COG-UK) Consortium	Jacquelyn Wynn, Mairead Hyland, The Lighthouse Lab in Alderley Park and Alex Alderton, Roberto Amato, Sonia Goncalves, Ewan Harrison, David K. Jackson, Ian Johnston, Dominic Kwiatkowski, Cordelia Langford, John Sillitoe on behalf of the Wellcome Sanger Institute COVID-19 Surveillance Team
EPI_ISL_710821, EPI_ISL_710822, EPI_ISL_710824, EPI_ISL_710826, EPI_ISL_710829	Lighthouse Lab in Cambridge	Wellcome Sanger Institute for the COVID-19 Genomics UK (COG-UK) Consortium	Rob Howes, The Lighthouse Lab in Cambridge and Alex Alderton, Roberto Amato, Sonia Goncalves, Ewan Harrison, David K. Jackson, Ian Johnston, Dominic Kwiatkowski, Cordelia Langford, John Sillitoe on behalf of the Wellcome Sanger Institute COVID-19 Surveillance Team
EPI_ISL_710830, EPI_ISL_710831, EPI_ISL_710832, EPI_ISL_710833, EPI_ISL_710834	Lighthouse Lab in Glasgow	Wellcome Sanger Institute for the COVID-19 Genomics UK (COG-UK) Consortium	Harper VanSteenhouse, Yumi Kasai, David Gray, Carol Clugston, Anna Dominiczak and Alex Alderton, Roberto Amato, Sonia Goncalves, Ewan Harrison, David K. Jackson, Ian Johnston, Dominic Kwiatkowski, Cordelia Langford, John Sillitoe on behalf of the Wellcome Sanger Institute COVID-19 Surveillance Team
EPI_ISL_710837	Lighthouse Lab in Alderley Park	Wellcome Sanger Institute for the COVID-19 Genomics UK (COG-UK) Consortium	Jacquelyn Wynn, Mairead Hyland, The Lighthouse Lab in Alderley Park and Alex Alderton, Roberto Amato, Sonia Goncalves, Ewan Harrison, David K. Jackson, Ian Johnston, Dominic Kwiatkowski, Cordelia Langford, John Sillitoe on behalf of the Wellcome Sanger Institute COVID-19 Surveillance Team
EPI_ISL_710838, EPI_ISL_710839, EPI_ISL_710840, EPI_ISL_710841, EPI_ISL_710842, EPI_ISL_710844, EPI_ISL_710845, EPI_ISL_710846, EPI_ISL_710848, EPI_ISL_710849, EPI_ISL_710850, EPI_ISL_710851, EPI_ISL_710852, EPI_ISL_710853, EPI_ISL_710854, EPI_ISL_710855, EPI_ISL_710857, EPI_ISL_710858, EPI_ISL_710859, EPI_ISL_710860, EPI_ISL_710861, EPI_ISL_719605, EPI_ISL_719606, EPI_ISL_719607, EPI_ISL_719608, EPI_ISL_719609, EPI_ISL_719610, EPI_ISL_719611, EPI_ISL_719612, EPI_ISL_719613, EPI_ISL_719614, EPI_ISL_719615, EPI_ISL_719616, EPI_ISL_719617, EPI_ISL_719618, EPI_ISL_719619,			

EPI_ISL_719620, EPI_ISL_719621, EPI_ISL_719622, EPI_ISL_719623, EPI_ISL_719624, EPI_ISL_719625, EPI_ISL_719626, EPI_ISL_719627, EPI_ISL_719628, EPI_ISL_719629, EPI_ISL_719630, EPI_ISL_719631, EPI_ISL_719632, EPI_ISL_719633, EPI_ISL_719634, EPI_ISL_719635, EPI_ISL_719636, EPI_ISL_719637,
EPI_ISL_719638, EPI_ISL_719639, EPI_ISL_719640, EPI_ISL_719641, EPI_ISL_719642, EPI_ISL_719643, EPI_ISL_719644, EPI_ISL_719645, EPI_ISL_719646, EPI_ISL_719647, EPI_ISL_719648, EPI_ISL_719649, EPI_ISL_719650, EPI_ISL_719651, EPI_ISL_719652, EPI_ISL_719653, EPI_ISL_719654, EPI_ISL_719655,
EPI_ISL_719656, EPI_ISL_719657, EPI_ISL_719658, EPI_ISL_719659, EPI_ISL_719660, EPI_ISL_719661, EPI_ISL_719662, EPI_ISL_719663, EPI_ISL_719664, EPI_ISL_719665, EPI_ISL_719666, EPI_ISL_719667, EPI_ISL_719668, EPI_ISL_719669, EPI_ISL_719670, EPI_ISL_719671, EPI_ISL_719672, EPI_ISL_719673,
EPI_ISL_719674, EPI_ISL_719675, EPI_ISL_719676, EPI_ISL_719677, EPI_ISL_719678, EPI_ISL_719679, EPI_ISL_719680, EPI_ISL_719681, EPI_ISL_719682, EPI_ISL_719683, EPI_ISL_719684, EPI_ISL_719685, EPI_ISL_719686, EPI_ISL_719687, EPI_ISL_719688, EPI_ISL_719689, EPI_ISL_719690, EPI_ISL_719691,
EPI_ISL_719692, EPI_ISL_719693, EPI_ISL_719694, EPI_ISL_719695, EPI_ISL_719696, EPI_ISL_719697, EPI_ISL_719698, EPI_ISL_719699, EPI_ISL_719700, EPI_ISL_719701, EPI_ISL_719702, EPI_ISL_719703, EPI_ISL_719704, EPI_ISL_719705, EPI_ISL_719706, EPI_ISL_719707, EPI_ISL_719708, EPI_ISL_719709,
EPI_ISL_719710, EPI_ISL_719711, EPI_ISL_719712, EPI_ISL_719713, EPI_ISL_719714, EPI_ISL_719715, EPI_ISL_719716, EPI_ISL_719717, EPI_ISL_719719, EPI_ISL_719720, EPI_ISL_719721, EPI_ISL_719722, EPI_ISL_719723, EPI_ISL_719724, EPI_ISL_719725, EPI_ISL_719726, EPI_ISL_719727, EPI_ISL_719728,
EPI_ISL_719729, EPI_ISL_719730, EPI_ISL_719731, EPI_ISL_719732, EPI_ISL_719733, EPI_ISL_719734, EPI_ISL_719735, EPI_ISL_719736, EPI_ISL_719737, EPI_ISL_719738, EPI_ISL_719739, EPI_ISL_719740, EPI_ISL_719741, EPI_ISL_719742, EPI_ISL_719743, EPI_ISL_719744, EPI_ISL_719745, EPI_ISL_719746,
EPI_ISL_719747, EPI_ISL_719748, EPI_ISL_719749, EPI_ISL_719750, EPI_ISL_719751, EPI_ISL_719752, EPI_ISL_719753, EPI_ISL_719754, EPI_ISL_719755, EPI_ISL_719756, EPI_ISL_719757, EPI_ISL_719758, EPI_ISL_719759, EPI_ISL_719760, EPI_ISL_719761, EPI_ISL_719762, EPI_ISL_719763, EPI_ISL_719764,
EPI_ISL_719765, EPI_ISL_719766, EPI_ISL_719767, EPI_ISL_719768, EPI_ISL_719769, EPI_ISL_719770, EPI_ISL_719771, EPI_ISL_719772, EPI_ISL_719773, EPI_ISL_719774, EPI_ISL_719775, EPI_ISL_719776, EPI_ISL_719777, EPI_ISL_719778, EPI_ISL_719779, EPI_ISL_719780, EPI_ISL_719781, EPI_ISL_719782,
EPI_ISL_719783, EPI_ISL_719784, EPI_ISL_719785, EPI_ISL_719786, EPI_ISL_719787, EPI_ISL_719788, EPI_ISL_719789, EPI_ISL_719790, EPI_ISL_719791, EPI_ISL_719792, EPI_ISL_719793, EPI_ISL_719794, EPI_ISL_719795, EPI_ISL_719796, EPI_ISL_719797, EPI_ISL_719798, EPI_ISL_719799, EPI_ISL_719800,
EPI_ISL_719801, EPI_ISL_719802, EPI_ISL_719803, EPI_ISL_719804, EPI_ISL_719805, EPI_ISL_719806, EPI_ISL_719807, EPI_ISL_719808, EPI_ISL_719809, EPI_ISL_719810, EPI_ISL_719811, EPI_ISL_719812, EPI_ISL_719813, EPI_ISL_719814, EPI_ISL_719815, EPI_ISL_719816, EPI_ISL_719817, EPI_ISL_719818,
EPI_ISL_719819, EPI_ISL_719820, EPI_ISL_719821, EPI_ISL_719822, EPI_ISL_719823, EPI_ISL_719824, EPI_ISL_719825, EPI_ISL_719826, EPI_ISL_719827, EPI_ISL_719828, EPI_ISL_719829, EPI_ISL_719830, EPI_ISL_719831, EPI_ISL_719832, EPI_ISL_719833, EPI_ISL_719834, EPI_ISL_719835, EPI_ISL_719836,
EPI_ISL_719837, EPI_ISL_719838, EPI_ISL_719839, EPI_ISL_719840, EPI_ISL_719841, EPI_ISL_719842, EPI_ISL_719843, EPI_ISL_719844, EPI_ISL_719845, EPI_ISL_719846, EPI_ISL_719847, EPI_ISL_719848, EPI_ISL_719849, EPI_ISL_719850, EPI_ISL_719851, EPI_ISL_719852, EPI_ISL_719853, EPI_ISL_719854,
EPI_ISL_719855, EPI_ISL_719856, EPI_ISL_719857, EPI_ISL_719858, EPI_ISL_719859, EPI_ISL_719860, EPI_ISL_719861, EPI_ISL_719862, EPI_ISL_719863, EPI_ISL_719864, EPI_ISL_719865, EPI_ISL_719866, EPI_ISL_719867, EPI_ISL_719868, EPI_ISL_719869, EPI_ISL_719870, EPI_ISL_719871, EPI_ISL_719872,
EPI_ISL_719873, EPI_ISL_719874, EPI_ISL_719875, EPI_ISL_719876, EPI_ISL_719877, EPI_ISL_719878, EPI_ISL_719879, EPI_ISL_719880, EPI_ISL_719881, EPI_ISL_719882, EPI_ISL_719883, EPI_ISL_719884, EPI_ISL_719885, EPI_ISL_719886, EPI_ISL_719887, EPI_ISL_719888, EPI_ISL_719889, EPI_ISL_719890,
EPI_ISL_719891, EPI_ISL_719892, EPI_ISL_719893, EPI_ISL_719894, EPI_ISL_719895, EPI_ISL_719896, EPI_ISL_719897, EPI_ISL_719898, EPI_ISL_719899, EPI_ISL_719900, EPI_ISL_719901, EPI_ISL_719902, EPI_ISL_719903, EPI_ISL_719904, EPI_ISL_719905, EPI_ISL_719906, EPI_ISL_719907, EPI_ISL_719908,
EPI_ISL_719909, EPI_ISL_719910, EPI_ISL_719911, EPI_ISL_719912, EPI_ISL_719913, EPI_ISL_719914, EPI_ISL_719915, EPI_ISL_719916, EPI_ISL_719917, EPI_ISL_719918, EPI_ISL_719919, EPI_ISL_719920, EPI_ISL_719921, EPI_ISL_719922, EPI_ISL_719923, EPI_ISL_719924, EPI_ISL_719925, EPI_ISL_719926,
EPI_ISL_719927, EPI_ISL_719928, EPI_ISL_719929, EPI_ISL_719930, EPI_ISL_719931, EPI_ISL_719932, EPI_ISL_719933, EPI_ISL_719934, EPI_ISL_719935, EPI_ISL_719936, EPI_ISL_719937, EPI_ISL_719938, EPI_ISL_719939, EPI_ISL_719940, EPI_ISL_719941, EPI_ISL_719942, EPI_ISL_719943, EPI_ISL_719944,
EPI_ISL_719945, EPI_ISL_719946, EPI_ISL_719947, EPI_ISL_719948, EPI_ISL_719949, EPI_ISL_719950, EPI_ISL_719951, EPI_ISL_719952, EPI_ISL_719953, EPI_ISL_719954, EPI_ISL_719955, EPI_ISL_719956, EPI_ISL_719957, EPI_ISL_719958, EPI_ISL_719959, EPI_ISL_719960, EPI_ISL_719961, EPI_ISL_719962,
EPI_ISL_719963, EPI_ISL_719964, EPI_ISL_719965, EPI_ISL_719966, EPI_ISL_719967, EPI_ISL_719968, EPI_ISL_719969, EPI_ISL_719970, EPI_ISL_719971, EPI_ISL_719972, EPI_ISL_719973, EPI_ISL_719974, EPI_ISL_719975, EPI_ISL_719976, EPI_ISL_719977, EPI_ISL_719978, EPI_ISL_719979, EPI_ISL_719980,
EPI_ISL_719981, EPI_ISL_719982, EPI_ISL_719983, EPI_ISL_719984, EPI_ISL_719985, EPI_ISL_719986

see above	Lighthouse Lab in Glasgow	Wellcome Sanger Institute for the COVID-19 Genomics UK (COG-UK) Consortium	Harper VanSteenhouse, Yumi Kasai, David Gray, Carol Clugston, Anna Dominiczak and Alex Alderton, Roberto Amato, Sonia Goncalves, Ewan Harrison, David K. Jackson, Ian Johnston, Dominic Kwiatkowski, Cordelia Langford, John Sillitoe on behalf of the Wellcome Sanger Institute COVID-19 Surveillance Team

EPI_ISL_719987, EPI_ISL_719988, EPI_ISL_719989, EPI_ISL_719990, EPI_ISL_719991, EPI_ISL_719992, EPI_ISL_719993, EPI_ISL_719994, EPI_ISL_719995, EPI_ISL_719996, EPI_ISL_719997, EPI_ISL_719998, EPI_ISL_719999, EPI_ISL_720000, EPI_ISL_720001, EPI_ISL_720002, EPI_ISL_720003, EPI_ISL_720004,
EPI_ISL_720005, EPI_ISL_720006, EPI_ISL_720007, EPI_ISL_720008, EPI_ISL_720009, EPI_ISL_720010, EPI_ISL_720011, EPI_ISL_720012, EPI_ISL_720013, EPI_ISL_720014, EPI_ISL_720015, EPI_ISL_720016, EPI_ISL_720017, EPI_ISL_720018, EPI_ISL_720019, EPI_ISL_720020, EPI_ISL_720021, EPI_ISL_720022,
EPI_ISL_720023, EPI_ISL_720024, EPI_ISL_720025, EPI_ISL_720026, EPI_ISL_720027, EPI_ISL_720028, EPI_ISL_720029, EPI_ISL_720030, EPI_ISL_720031, EPI_ISL_720032, EPI_ISL_720033, EPI_ISL_720034, EPI_ISL_720035, EPI_ISL_720036, EPI_ISL_720037, EPI_ISL_720038, EPI_ISL_720039, EPI_ISL_720040,
EPI_ISL_720041, EPI_ISL_720042, EPI_ISL_720043, EPI_ISL_720044, EPI_ISL_720045, EPI_ISL_720046, EPI_ISL_720047, EPI_ISL_720048, EPI_ISL_720049, EPI_ISL_720050, EPI_ISL_720051, EPI_ISL_720052, EPI_ISL_720053, EPI_ISL_720054, EPI_ISL_720055, EPI_ISL_720056, EPI_ISL_720057, EPI_ISL_720058,
EPI_ISL_720059, EPI_ISL_720060, EPI_ISL_720061, EPI_ISL_720062, EPI_ISL_720063, EPI_ISL_720064, EPI_ISL_720065, EPI_ISL_720066, EPI_ISL_720067, EPI_ISL_720068, EPI_ISL_720069, EPI_ISL_720070, EPI_ISL_720071, EPI_ISL_720072, EPI_ISL_720073, EPI_ISL_720074, EPI_ISL_720075, EPI_ISL_720076,
EPI_ISL_720077, EPI_ISL_720078, EPI_ISL_720079, EPI_ISL_720080, EPI_ISL_720081, EPI_ISL_720082, EPI_ISL_720083, EPI_ISL_720084, EPI_ISL_720085, EPI_ISL_720086, EPI_ISL_720087, EPI_ISL_720088, EPI_ISL_720089, EPI_ISL_720090, EPI_ISL_720091, EPI_ISL_720092, EPI_ISL_720093, EPI_ISL_720094,
EPI_ISL_720095, EPI_ISL_720096, EPI_ISL_720097, EPI_ISL_720098, EPI_ISL_720099, EPI_ISL_720100, EPI_ISL_720101, EPI_ISL_720102, EPI_ISL_720103, EPI_ISL_720104, EPI_ISL_720105, EPI_ISL_720106, EPI_ISL_720107, EPI_ISL_720108, EPI_ISL_720109, EPI_ISL_720110, EPI_ISL_720111, EPI_ISL_720112,
EPI_ISL_720113, EPI_ISL_720114, EPI_ISL_720115, EPI_ISL_720116, EPI_ISL_720117, EPI_ISL_720118, EPI_ISL_720119, EPI_ISL_720120, EPI_ISL_720121, EPI_ISL_720122, EPI_ISL_720123, EPI_ISL_720124, EPI_ISL_720125, EPI_ISL_720126, EPI_ISL_720127, EPI_ISL_720128, EPI_ISL_720129, EPI_ISL_720130,
EPI_ISL_720131, EPI_ISL_720132, EPI_ISL_720133, EPI_ISL_720134, EPI_ISL_720135, EPI_ISL_720136, EPI_ISL_720137, EPI_ISL_720138, EPI_ISL_720139, EPI_ISL_720140, EPI_ISL_720141, EPI_ISL_720142, EPI_ISL_720143, EPI_ISL_720144, EPI_ISL_720145, EPI_ISL_720147, EPI_ISL_720148, EPI_ISL_720149,
EPI_ISL_720150, EPI_ISL_720151, EPI_ISL_720152, EPI_ISL_720153, EPI_ISL_720154, EPI_ISL_720155, EPI_ISL_720156, EPI_ISL_720157, EPI_ISL_720158, EPI_ISL_720159, EPI_ISL_720160, EPI_ISL_720161, EPI_ISL_720162, EPI_ISL_720163, EPI_ISL_720164, EPI_ISL_720165, EPI_ISL_720166, EPI_ISL_720167,
EPI_ISL_720168, EPI_ISL_720169, EPI_ISL_720170, EPI_ISL_720171, EPI_ISL_720172, EPI_ISL_720173, EPI_ISL_720174, EPI_ISL_720175, EPI_ISL_720176, EPI_ISL_720177, EPI_ISL_720178, EPI_ISL_720179, EPI_ISL_720180, EPI_ISL_720181, EPI_ISL_720182, EPI_ISL_720183, EPI_ISL_720184, EPI_ISL_720185,
EPI_ISL_720186, EPI_ISL_720187, EPI_ISL_720188, EPI_ISL_720189, EPI_ISL_720190, EPI_ISL_720191, EPI_ISL_720192, EPI_ISL_720193, EPI_ISL_720194, EPI_ISL_720195, EPI_ISL_720196, EPI_ISL_720197, EPI_ISL_720198, EPI_ISL_720199, EPI_ISL_720200, EPI_ISL_720201, EPI_ISL_720202, EPI_ISL_720203,
EPI_ISL_720204, EPI_ISL_720205, EPI_ISL_720206, EPI_ISL_720207, EPI_ISL_720208, EPI_ISL_720209, EPI_ISL_720210, EPI_ISL_720211, EPI_ISL_720212, EPI_ISL_720213, EPI_ISL_720214, EPI_ISL_720215, EPI_ISL_720216, EPI_ISL_720217, EPI_ISL_720218, EPI_ISL_720219, EPI_ISL_720220, EPI_ISL_720221,
EPI_ISL_720222, EPI_ISL_720223, EPI_ISL_720224, EPI_ISL_720225, EPI_ISL_720226, EPI_ISL_720227, EPI_ISL_720228, EPI_ISL_720229, EPI_ISL_720230, EPI_ISL_720231, EPI_ISL_720232, EPI_ISL_720233, EPI_ISL_720234, EPI_ISL_720235, EPI_ISL_720236, EPI_ISL_720237, EPI_ISL_720238, EPI_ISL_720239,
EPI_ISL_720240, EPI_ISL_720241, EPI_ISL_720242, EPI_ISL_720243, EPI_ISL_720244, EPI_ISL_720245, EPI_ISL_720246, EPI_ISL_720247, EPI_ISL_720248, EPI_ISL_720249, EPI_ISL_720250, EPI_ISL_720251, EPI_ISL_720252, EPI_ISL_720253, EPI_ISL_720254, EPI_ISL_720255, EPI_ISL_720256, EPI_ISL_720257,
EPI_ISL_720258, EPI_ISL_720259, EPI_ISL_720260, EPI_ISL_720261, EPI_ISL_720262, EPI_ISL_720263, EPI_ISL_720264, EPI_ISL_720265, EPI_ISL_720266, EPI_ISL_720267, EPI_ISL_720268, EPI_ISL_720269, EPI_ISL_720270, EPI_ISL_720271, EPI_ISL_720272, EPI_ISL_720273, EPI_ISL_720274, EPI_ISL_720275,
EPI_ISL_720276, EPI_ISL_720277, EPI_ISL_720278, EPI_ISL_720279, EPI_ISL_720280, EPI_ISL_720281, EPI_ISL_720282, EPI_ISL_720283, EPI_ISL_720284, EPI_ISL_720285, EPI_ISL_720286, EPI_ISL_720287, EPI_ISL_720288, EPI_ISL_720289, EPI_ISL_720290, EPI_ISL_720291, EPI_ISL_720292, EPI_ISL_720293,
EPI_ISL_720294, EPI_ISL_720295, EPI_ISL_720296, EPI_ISL_720297, EPI_ISL_720298, EPI_ISL_720299, EPI_ISL_720300, EPI_ISL_720301, EPI_ISL_720302, EPI_ISL_720303, EPI_ISL_720304, EPI_ISL_720305, EPI_ISL_720306, EPI_ISL_720307, EPI_ISL_720308, EPI_ISL_720309, EPI_ISL_720310, EPI_ISL_720311,
EPI_ISL_720312, EPI_ISL_720313, EPI_ISL_720314, EPI_ISL_720315, EPI_ISL_720316, EPI_ISL_720317, EPI_ISL_720318, EPI_ISL_720319, EPI_ISL_720320, EPI_ISL_720321, EPI_ISL_720322, EPI_ISL_720323, EPI_ISL_720324, EPI_ISL_720325, EPI_ISL_720326, EPI_ISL_720327, EPI_ISL_720328, EPI_ISL_720329,
EPI_ISL_720330, EPI_ISL_720331, EPI_ISL_720332, EPI_ISL_720333, EPI_ISL_720334, EPI_ISL_720335, EPI_ISL_720336, EPI_ISL_720337, EPI_ISL_720338, EPI_ISL_720339, EPI_ISL_720340, EPI_ISL_720341, EPI_ISL_720342, EPI_ISL_720343, EPI_ISL_720344, EPI_ISL_720345, EPI_ISL_720346, EPI_ISL_720347,
EPI_ISL_720348, EPI_ISL_720349, EPI_ISL_720350, EPI_ISL_720351, EPI_ISL_720352, EPI_ISL_720353, EPI_ISL_720354, EPI_ISL_720355, EPI_ISL_720356, EPI_ISL_720357, EPI_ISL_720358, EPI_ISL_720359, EPI_ISL_720360, EPI_ISL_720361, EPI_ISL_720362, EPI_ISL_720363, EPI_ISL_720364, EPI_ISL_720365,
EPI_ISL_720366, EPI_ISL_720367, EPI_ISL_720368, EPI_ISL_720369, EPI_ISL_720370, EPI_ISL_720371, EPI_ISL_720372, EPI_ISL_720373, EPI_ISL_720374, EPI_ISL_720375, EPI_ISL_720376, EPI_ISL_720377, EPI_ISL_720378, EPI_ISL_720379, EPI_ISL_720380, EPI_ISL_720381, EPI_ISL_720382, EPI_ISL_720383,
EPI_ISL_720384, EPI_ISL_720385, EPI_ISL_720386, EPI_ISL_720387, EPI_ISL_720388, EPI_ISL_720389, EPI_ISL_720390, EPI_ISL_720391, EPI_ISL_720392, EPI_ISL_720393, EPI_ISL_720394, EPI_ISL_720395, EPI_ISL_720396, EPI_ISL_720397, EPI_ISL_720398, EPI_ISL_720399, EPI_ISL_720400, EPI_ISL_720401,
EPI_ISL_720402, EPI_ISL_720403, EPI_ISL_720404, EPI_ISL_720405, EPI_ISL_720406, EPI_ISL_720407, EPI_ISL_720408, EPI_ISL_720409, EPI_ISL_720410, EPI_ISL_720411, EPI_ISL_720412, EPI_ISL_720413, EPI_ISL_720414, EPI_ISL_720415, EPI_ISL_720416, EPI_ISL_720417, EPI_ISL_720418, EPI_ISL_720419,
EPI_ISL_720420, EPI_ISL_720421, EPI_ISL_720422, EPI_ISL_720423, EPI_ISL_720424, EPI_ISL_720425, EPI_ISL_720426, EPI_ISL_720427, EPI_ISL_720428, EPI_ISL_720429, EPI_ISL_720430, EPI_ISL_720431, EPI_ISL_720432, EPI_ISL_720433, EPI_ISL_720434, EPI_ISL_720435, EPI_ISL_720436, EPI_ISL_720437,
EPI_ISL_720438, EPI_ISL_720439, EPI_ISL_720440, EPI_ISL_720441, EPI_ISL_720442, EPI_ISL_720443, EPI_ISL_720444, EPI_ISL_720445, EPI_ISL_720446, EPI_ISL_720447, EPI_ISL_720448, EPI_ISL_720449, EPI_ISL_720450, EPI_ISL_720451, EPI_ISL_720452, EPI_ISL_720453, EPI_ISL_720454, EPI_ISL_720455,
EPI_ISL_720456, EPI_ISL_720457, EPI_ISL_720458, EPI_ISL_720459, EPI_ISL_720460, EPI_ISL_720461, EPI_ISL_720462, EPI_ISL_720463, EPI_ISL_720464, EPI_ISL_720465, EPI_ISL_720466, EPI_ISL_720467, EPI_ISL_720468, EPI_ISL_720469, EPI_ISL_720470, EPI_ISL_720471, EPI_ISL_720472, EPI_ISL_720473,
EPI_ISL_720474, EPI_ISL_720475, EPI_ISL_720476, EPI_ISL_720477, EPI_ISL_720478, EPI_ISL_720479, EPI_ISL_720480, EPI_ISL_720481, EPI_ISL_720482, EPI_ISL_720483, EPI_ISL_720484, EPI_ISL_720485, EPI_ISL_720486, EPI_ISL_720487, EPI_ISL_720488, EPI_ISL_720489, EPI_ISL_720490, EPI_ISL_720491,
EPI_ISL_720492, EPI_ISL_720493, EPI_ISL_720494, EPI_ISL_720495, EPI_ISL_720496, EPI_ISL_720497, EPI_ISL_720498, EPI_ISL_720499, EPI_ISL_720500, EPI_ISL_720501, EPI_ISL_720502, EPI_ISL_720503, EPI_ISL_720504, EPI_ISL_720505, EPI_ISL_720506, EPI_ISL_720507, EPI_ISL_720508, EPI_ISL_720509,
EPI_ISL_720510, EPI_ISL_720511, EPI_ISL_720512, EPI_ISL_720513, EPI_ISL_720514, EPI_ISL_720515, EPI_ISL_720516, EPI_ISL_720517, EPI_ISL_720518, EPI_ISL_720519, EPI_ISL_720520, EPI_ISL_720521, EPI_ISL_720522, EPI_ISL_720523, EPI_ISL_720524, EPI_ISL_720525, EPI_ISL_720526, EPI_ISL_720527,
EPI_ISL_720528, EPI_ISL_720529, EPI_ISL_720530, EPI_ISL_720531, EPI_ISL_720532, EPI_ISL_720533, EPI_ISL_720534, EPI_ISL_720535, EPI_ISL_720536, EPI_ISL_720537, EPI_ISL_720538, EPI_ISL_720539, EPI_ISL_720540, EPI_ISL_720541, EPI_ISL_720542, EPI_ISL_720543, EPI_ISL_720544, EPI_ISL_720545,
EPI_ISL_720546, EPI_ISL_720547, EPI_ISL_720548, EPI_ISL_720549, EPI_ISL_720550, EPI_ISL_720551, EPI_ISL_720552, EPI_ISL_720553, EPI_ISL_720554, EPI_ISL_720555, EPI_ISL_720556, EPI_ISL_720557, EPI_ISL_720558, EPI_ISL_720559, EPI_ISL_720560, EPI_ISL_720561, EPI_ISL_720562, EPI_ISL_720563,
EPI_ISL_720564, EPI_ISL_720565, EPI_ISL_720566, EPI_ISL_720567, EPI_ISL_720568, EPI_ISL_720569, EPI_ISL_720570, EPI_ISL_720571, EPI_ISL_720572, EPI_ISL_720573, EPI_ISL_720574, EPI_ISL_720575, EPI_ISL_720576, EPI_ISL_720577, EPI_ISL_720578, EPI_ISL_720579, EPI_ISL_720580, EPI_ISL_720581,
EPI_ISL_720582, EPI_ISL_720583, EPI_ISL_720584, EPI_ISL_720585, EPI_ISL_720586, EPI_ISL_720587, EPI_ISL_720588, EPI_ISL_720589, EPI_ISL_720590, EPI_ISL_720591, EPI_ISL_720592, EPI_ISL_720593, EPI_ISL_720594, EPI_ISL_720595, EPI_ISL_720596, EPI_ISL_720597, EPI_ISL_720598, EPI_ISL_720599,
EPI_ISL_720600, EPI_ISL_720601, EPI_ISL_720602, EPI_ISL_720603, EPI_ISL_720604, EPI_ISL_720605, EPI_ISL_720606, EPI_ISL_720607, EPI_ISL_720608, EPI_ISL_720609, EPI_ISL_720610, EPI_ISL_720611, EPI_ISL_720612, EPI_ISL_720613, EPI_ISL_720614, EPI_ISL_720615, EPI_ISL_720616, EPI_ISL_720617,
EPI_ISL_720618, EPI_ISL_720619, EPI_ISL_720620, EPI_ISL_720621, EPI_ISL_720622, EPI_ISL_720623, EPI_ISL_720624, EPI_ISL_720625, EPI_ISL_720626, EPI_ISL_720627, EPI_ISL_720628, EPI_ISL_720629, EPI_ISL_720630, EPI_ISL_720631, EPI_ISL_720632, EPI_ISL_720633, EPI_ISL_720634, EPI_ISL_720635,
EPI_ISL_720636, EPI_ISL_720637, EPI_ISL_720638, EPI_ISL_720639, EPI_ISL_720640, EPI_ISL_720641, EPI_ISL_720642, EPI_ISL_720643, EPI_ISL_720644, EPI_ISL_720645, EPI_ISL_720646, EPI_ISL_720647, EPI_ISL_720648, EPI_ISL_720649, EPI_ISL_720650, EPI_ISL_720651, EPI_ISL_720652, EPI_ISL_720653,
EPI_ISL_720654, EPI_ISL_720655, EPI_ISL_720656, EPI_ISL_720657, EPI_ISL_720658, EPI_ISL_720659, EPI_ISL_720660, EPI_ISL_720661, EPI_ISL_720662, EPI_ISL_720663, EPI_ISL_720664, EPI_ISL_720665, EPI_ISL_720666, EPI_ISL_720667

see above	Lighthouse Lab in Milton Keynes	Wellcome Sanger Institute for the COVID-19 Genomics UK (COG-UK) Consortium	The Lighthouse Lab in Milton Keynes and Alex Alderton, Roberto Amato, Sonia Goncalves, Ewan Harrison, David K. Jackson, Ian Johnston, Dominic Kwiatkowski, Cordelia Langford, John Sillitoe on behalf of the Wellcome Sanger Institute COVID-19 Surveillance Team

EPI_ISL_720909, EPI_ISL_720910, EPI_ISL_720911, EPI_ISL_720912, EPI_ISL_720913, EPI_ISL_720914, EPI_ISL_720915, EPI_ISL_720916, EPI_ISL_720917, EPI_ISL_720918, EPI_ISL_720919, EPI_ISL_720920, EPI_ISL_720921, EPI_ISL_720922, EPI_ISL_720923, EPI_ISL_720924, EPI_ISL_720925, EPI_ISL_720926,
EPI_ISL_720927, EPI_ISL_720928, EPI_ISL_720929, EPI_ISL_720930, EPI_ISL_720931, EPI_ISL_720932, EPI_ISL_720933, EPI_ISL_720934, EPI_ISL_720935, EPI_ISL_720936, EPI_ISL_720937, EPI_ISL_720938, EPI_ISL_720939, EPI_ISL_720940, EPI_ISL_720941, EPI_ISL_720942, EPI_ISL_720943, EPI_ISL_720944,
EPI_ISL_720945, EPI_ISL_720946, EPI_ISL_720947, EPI_ISL_720948, EPI_ISL_720949, EPI_ISL_720950, EPI_ISL_720951, EPI_ISL_720952, EPI_ISL_720953, EPI_ISL_720954, EPI_ISL_720955, EPI_ISL_720956, EPI_ISL_720957, EPI_ISL_720958, EPI_ISL_720959, EPI_ISL_720960, EPI_ISL_720961, EPI_ISL_720962,
EPI_ISL_720963, EPI_ISL_720964, EPI_ISL_720965, EPI_ISL_720966, EPI_ISL_720967, EPI_ISL_720968, EPI_ISL_720969, EPI_ISL_720970, EPI_ISL_720971, EPI_ISL_720972, EPI_ISL_720973, EPI_ISL_720974, EPI_ISL_720975, EPI_ISL_720976, EPI_ISL_720977, EPI_ISL_720978, EPI_ISL_720979, EPI_ISL_720980,
EPI_ISL_720981, EPI_ISL_720982, EPI_ISL_720983, EPI_ISL_720984, EPI_ISL_720985, EPI_ISL_720986, EPI_ISL_720987, EPI_ISL_720988, EPI_ISL_720989, EPI_ISL_720990, EPI_ISL_720991, EPI_ISL_720992, EPI_ISL_720993, EPI_ISL_720994, EPI_ISL_720995, EPI_ISL_720996, EPI_ISL_720997, EPI_ISL_720998,
EPI_ISL_720999, EPI_ISL_721000, EPI_ISL_721001, EPI_ISL_721002, EPI_ISL_721003, EPI_ISL_721004, EPI_ISL_721005, EPI_ISL_721006, EPI_ISL_721007, EPI_ISL_721008, EPI_ISL_721009, EPI_ISL_721010, EPI_ISL_721011, EPI_ISL_721012, EPI_ISL_721013, EPI_ISL_721014, EPI_ISL_721015, EPI_ISL_721016,
EPI_ISL_721017, EPI_ISL_721018, EPI_ISL_721019, EPI_ISL_721020, EPI_ISL_721021, EPI_ISL_721022, EPI_ISL_721023, EPI_ISL_721024, EPI_ISL_721025, EPI_ISL_721026, EPI_ISL_721027, EPI_ISL_721028, EPI_ISL_721029, EPI_ISL_721030, EPI_ISL_721031, EPI_ISL_721032, EPI_ISL_721033, EPI_ISL_721034,
EPI_ISL_721035, EPI_ISL_721036, EPI_ISL_721037, EPI_ISL_721038, EPI_ISL_721039, EPI_ISL_721040, EPI_ISL_721041, EPI_ISL_721042, EPI_ISL_721043, EPI_ISL_721044, EPI_ISL_721045, EPI_ISL_721046, EPI_ISL_721047, EPI_ISL_721048, EPI_ISL_721049, EPI_ISL_721050, EPI_ISL_721051, EPI_ISL_721052,
EPI_ISL_721053, EPI_ISL_721054, EPI_ISL_721055, EPI_ISL_721056, EPI_ISL_721057, EPI_ISL_721058, EPI_ISL_721059, EPI_ISL_721060, EPI_ISL_721061, EPI_ISL_721062, EPI_ISL_721063, EPI_ISL_721064, EPI_ISL_721065, EPI_ISL_721066, EPI_ISL_721067, EPI_ISL_721068, EPI_ISL_721069, EPI_ISL_721070,
EPI_ISL_721071, EPI_ISL_721072, EPI_ISL_721073, EPI_ISL_721074, EPI_ISL_721075, EPI_ISL_721076, EPI_ISL_721077, EPI_ISL_721078, EPI_ISL_721079, EPI_ISL_721080, EPI_ISL_721081, EPI_ISL_721082, EPI_ISL_721083, EPI_ISL_721084, EPI_ISL_721085, EPI_ISL_721086, EPI_ISL_721087, EPI_ISL_721088,
EPI_ISL_721089, EPI_ISL_721090, EPI_ISL_721091, EPI_ISL_721092, EPI_ISL_721093, EPI_ISL_721094, EPI_ISL_721095, EPI_ISL_721096, EPI_ISL_721097, EPI_ISL_721098, EPI_ISL_721099, EPI_ISL_721100, EPI_ISL_721101, EPI_ISL_721102, EPI_ISL_721103, EPI_ISL_721104, EPI_ISL_721105, EPI_ISL_721106,
EPI_ISL_721107, EPI_ISL_721108, EPI_ISL_721109, EPI_ISL_721110, EPI_ISL_721111, EPI_ISL_721112, EPI_ISL_721113, EPI_ISL_721114, EPI_ISL_721115, EPI_ISL_721116, EPI_ISL_721117, EPI_ISL_721118, EPI_ISL_721119, EPI_ISL_721120, EPI_ISL_721121, EPI_ISL_721122, EPI_ISL_721123, EPI_ISL_721124,
EPI_ISL_721125, EPI_ISL_721126, EPI_ISL_721127, EPI_ISL_721128, EPI_ISL_721129, EPI_ISL_721130, EPI_ISL_721131, EPI_ISL_721132, EPI_ISL_721133, EPI_ISL_721134, EPI_ISL_721135, EPI_ISL_721136, EPI_ISL_721137, EPI_ISL_721138, EPI_ISL_721139, EPI_ISL_721140, EPI_ISL_721141, EPI_ISL_721142,
EPI_ISL_721143, EPI_ISL_721144, EPI_ISL_721145, EPI_ISL_721146, EPI_ISL_721147, EPI_ISL_721148, EPI_ISL_721149, EPI_ISL_721150, EPI_ISL_721151, EPI_ISL_721152, EPI_ISL_721153, EPI_ISL_721154, EPI_ISL_721155, EPI_ISL_721156, EPI_ISL_721157, EPI_ISL_721158, EPI_ISL_721159, EPI_ISL_721160,
EPI_ISL_721161, EPI_ISL_721162, EPI_ISL_721163, EPI_ISL_721164, EPI_ISL_721165, EPI_ISL_721166, EPI_ISL_721167, EPI_ISL_721168, EPI_ISL_721169, EPI_ISL_721170, EPI_ISL_721171, EPI_ISL_721172, EPI_ISL_721173, EPI_ISL_721174, EPI_ISL_721175, EPI_ISL_721176, EPI_ISL_721177, EPI_ISL_721178,
EPI_ISL_721179, EPI_ISL_721180, EPI_ISL_721181, EPI_ISL_721182, EPI_ISL_721183, EPI_ISL_721184

see above	Lighthouse Lab in Glasgow	Wellcome Sanger Institute for the COVID-19 Genomics UK (COG-UK) Consortium	Harper VanSteenhouse, Yumi Kasai, David Gray, Carol Clugston, Anna Dominiczak and Alex Alderton, Roberto Amato, Sonia Goncalves, Ewan Harrison, David K. Jackson, Ian Johnston, Dominic Kwiatkowski, Cordelia Langford, John Sillitoe on behalf of the Wellcome Sanger Institute COVID-19 Surveillance Team

EPI_ISL_721187, EPI_ISL_721199, EPI_ISL_721203, EPI_ISL_721204, EPI_ISL_721210, EPI_ISL_721213, EPI_ISL_721223, EPI_ISL_721225, EPI_ISL_721243, EPI_ISL_721247, EPI_ISL_721255, EPI_ISL_721268, EPI_ISL_721270, EPI_ISL_721271, EPI_ISL_721273, EPI_ISL_721276, EPI_ISL_721279, EPI_ISL_721280,

EPI_ISL_721281, EPI_ISL_721284, EPI_ISL_721298, EPI_ISL_721299, EPI_ISL_721306, EPI_ISL_721307, EPI_ISL_721331, EPI_ISL_721332, EPI_ISL_721333, EPI_ISL_721339, EPI_ISL_721341, EPI_ISL_721358, EPI_ISL_721359, EPI_ISL_721365, EPI_ISL_721367, EPI_ISL_721370, EPI_ISL_721372, EPI_ISL_721373, EPI_ISL_721374, EPI_ISL_721376, EPI_ISL_721377, EPI_ISL_721390, EPI_ISL_721391, EPI_ISL_721406, EPI_ISL_721419, EPI_ISL_721424, EPI_ISL_721430, EPI_ISL_721443, EPI_ISL_721456, EPI_ISL_721459, EPI_ISL_721460, EPI_ISL_721472, EPI_ISL_721476, EPI_ISL_721481, EPI_ISL_721488, EPI_ISL_721496, EPI_ISL_721504, EPI_ISL_721507, EPI_ISL_721516			
see above	Lighthouse Lab in Alderley Park	Wellcome Sanger Institute for the COVID-19 Genomics UK (COG-UK) Consortium	Jacquelyn Wynn, Mairead Hyland, The Lighthouse Lab in Alderley Park and Alex Alderton, Roberto Amato, Sonia Goncalves, Ewan Harrison, David K. Jackson, Ian Johnston, Dominic Kwiatkowski, Cordelia Langford, John Sillitoe on behalf of the Wellcome Sanger Institute COVID-19 Surveillance Team
EPI_ISL_723855	Department of Pathology, University of Cambridge	COVID-19 Genomics UK (COG-UK) Consortium	Aminu S. Jahun, Yasmin Chaudhry, Grant Hall, Iliana Georgana, Myra Hosmillo, Martin D. Curran, Malte Pinckert, Surendra Parmar, Ian Goodfellow
EPI_ISL_724063, EPI_ISL_724067, EPI_ISL_724068, EPI_ISL_724069, EPI_ISL_724070, EPI_ISL_724071, EPI_ISL_724072, EPI_ISL_724073, EPI_ISL_724074, EPI_ISL_724075, EPI_ISL_724076, EPI_ISL_724077, EPI_ISL_724078, EPI_ISL_724079, EPI_ISL_724080, EPI_ISL_724081, EPI_ISL_724082, EPI_ISL_724083, EPI_ISL_724084, EPI_ISL_724085, EPI_ISL_724086, EPI_ISL_724087, EPI_ISL_724088, EPI_ISL_724089, EPI_ISL_724090, EPI_ISL_724091, EPI_ISL_724092, EPI_ISL_724093, EPI_ISL_724094, EPI_ISL_724095, EPI_ISL_724096, EPI_ISL_724097, EPI_ISL_724098, EPI_ISL_724099, EPI_ISL_724100, EPI_ISL_724101, EPI_ISL_724102, EPI_ISL_724103, EPI_ISL_724104, EPI_ISL_724105, EPI_ISL_724106, EPI_ISL_724107, EPI_ISL_724108, EPI_ISL_724109, EPI_ISL_724110, EPI_ISL_724111, EPI_ISL_724112, EPI_ISL_724113, EPI_ISL_724114, EPI_ISL_724115, EPI_ISL_724116, EPI_ISL_724117, EPI_ISL_724118, EPI_ISL_724119, EPI_ISL_724120, EPI_ISL_724121, EPI_ISL_724122, EPI_ISL_724123			
see above	Northumbria University / South Tees Hospitals NHS Foundation Trust / North Cumbria Integrated Care NHS Foundation Trust / North Tees and Hartlepool NHS Foundation Trust / Newcastle Hospitals NHS Foundation Trust	COVID-19 Genomics UK (COG-UK) Consortium	Darren L Smith,Andrew Nelson,Matthew Bashton,Greg R Young,Joshua Loh,John Allan,Mohammad A Tariq,Giles S Holt,Gary Black,Wen C Yew,Lynn Dover,Paul Baker,Steve Liggett,Sarah Essex,Jane Greenaway,Debra Padgett,Clive Graham,Garren Scott,Edward Barton,Emma Swindells,Brendan Payne,Jennifer Collins,Yusri Taha,Gary Eltringham
EPI_ISL_724124, EPI_ISL_724125, EPI_ISL_724126, EPI_ISL_724127, EPI_ISL_724128, EPI_ISL_724129, EPI_ISL_724130, EPI_ISL_724131, EPI_ISL_724132, EPI_ISL_724133, EPI_ISL_724134, EPI_ISL_724135, EPI_ISL_724136, EPI_ISL_724137, EPI_ISL_724138, EPI_ISL_724139, EPI_ISL_724140, EPI_ISL_724141, EPI_ISL_724142, EPI_ISL_724143, EPI_ISL_724144, EPI_ISL_724145, EPI_ISL_724146, EPI_ISL_724147, EPI_ISL_724148, EPI_ISL_724149, EPI_ISL_724150, EPI_ISL_724151, EPI_ISL_724152, EPI_ISL_724153, EPI_ISL_724154, EPI_ISL_724155, EPI_ISL_724156, EPI_ISL_724157, EPI_ISL_724158, EPI_ISL_724159, EPI_ISL_724160, EPI_ISL_724161, EPI_ISL_724162, EPI_ISL_724163, EPI_ISL_724164, EPI_ISL_724165, EPI_ISL_724166, EPI_ISL_724167, EPI_ISL_724168, EPI_ISL_724169, EPI_ISL_724170, EPI_ISL_724171, EPI_ISL_724172, EPI_ISL_724173, EPI_ISL_724174, EPI_ISL_724175, EPI_ISL_724176, EPI_ISL_724177, EPI_ISL_724178, EPI_ISL_724179, EPI_ISL_724180, EPI_ISL_724181, EPI_ISL_724182, EPI_ISL_724183, EPI_ISL_724184, EPI_ISL_724185, EPI_ISL_724186, EPI_ISL_724187, EPI_ISL_724188, EPI_ISL_724189, EPI_ISL_724190, EPI_ISL_724191, EPI_ISL_724192, EPI_ISL_724193, EPI_ISL_724194, EPI_ISL_724195, EPI_ISL_724196, EPI_ISL_724197, EPI_ISL_724198, EPI_ISL_724199, EPI_ISL_724200, EPI_ISL_724201, EPI_ISL_724202, EPI_ISL_724203, EPI_ISL_724204, EPI_ISL_724205, EPI_ISL_724206, EPI_ISL_724207, EPI_ISL_724208, EPI_ISL_724209			
see above	Quadram Institute Bioscience	COVID-19 Genomics UK (COG-UK) Consortium	Dave J. Baker, Gemma L. Kay, Alp Aydin, Thanh Le-Viet, Steven Rudder, Ana P. Tedim, Anastasia Kolyva, Maria Diaz, Leonardo de Oliveira Martins, Nabil-Fareed Alikhan, Lizzie Meadows, Rachael Stanley, Ngozi Elumogo, Muhammed Yasir, Nicholas M. Thomson, Alexander J Trotter, Rachel Gilroy, Samuel Bloomfield, Claire Stuart, Andrew Bell, Reenesh Prakash, Samir Dervisevic, Alison E. Mather, John Wain, Mark Webber, Andrew J. Page, Justin O'Grady
EPI_ISL_724212	West of Scotland Specialist Virology Centre, NHSGGC / MRC-University of Glasgow Centre for Virus Research	COVID-19 Genomics UK (COG-UK) Consortium	Ana da Silva Filipe, Natasha Johnson, Kathy Smollett, Daniel Mair, Stephen Carmichael, Alice Broos, Lily Tong, Jenna Nichols, Kyriaki Nomikou; Sarah McDonald; Richard Orton, Joseph Hughes, Sreenu Vattipally, David L Robertson; Alasdair MacLean, Rory Gunson; Sharif Shaaban, Matthew Holden; Rachel Blacow, Guy Mollett, Kathy Li, James Shepherd, Antonia Ho, Emma Thomson
EPI_ISL_724541, EPI_ISL_724549, EPI_ISL_724575, EPI_ISL_724610, EPI_ISL_724612, EPI_ISL_724616, EPI_ISL_724619, EPI_ISL_724622, EPI_ISL_724624, EPI_ISL_724627, EPI_ISL_724634, EPI_ISL_724641, EPI_ISL_724647, EPI_ISL_724653, EPI_ISL_724654, EPI_ISL_724657, EPI_ISL_724663, EPI_ISL_724667, EPI_ISL_724668, EPI_ISL_724669, EPI_ISL_724670, EPI_ISL_724688, EPI_ISL_724701, EPI_ISL_724703, EPI_ISL_724704, EPI_ISL_724706, EPI_ISL_724711, EPI_ISL_724712, EPI_ISL_724714, EPI_ISL_724721, EPI_ISL_724726, EPI_ISL_724727, EPI_ISL_724728, EPI_ISL_724729, EPI_ISL_724730, EPI_ISL_724731, EPI_ISL_724732, EPI_ISL_724733, EPI_ISL_724767, EPI_ISL_724768, EPI_ISL_724769, EPI_ISL_724770, EPI_ISL_724772, EPI_ISL_724774, EPI_ISL_724775			
see above	University College London, Great Ormond Street Hospital for Children NHS Foundation Trust, Imperial College Healthcare NHS Trust	COVID-19 Genomics UK (COG-UK) Consortium	Sergi Castellano, Rachel Williams, Mark Kristiansen, Paola Resende Silva, Sunando Roy, Tony Brooks, Helena Tutill, Paola Niola, Patricia Dyal, Charlotte Williams, Leysa Forrest, Yasmin Panchbhaya, Jacqueline Findlay, Samuel Weeks, Julianne Brown, Kathryn Harris, Paul Randell, James Price, Alison Holmes, Judith Breuer
EPI_ISL_724924, EPI_ISL_724925, EPI_ISL_724926, EPI_ISL_724927, EPI_ISL_724928, EPI_ISL_724929, EPI_ISL_724930, EPI_ISL_724931, EPI_ISL_724932, EPI_ISL_724933, EPI_ISL_724934, EPI_ISL_724935, EPI_ISL_724936, EPI_ISL_724937, EPI_ISL_724938, EPI_ISL_724939, EPI_ISL_724940, EPI_ISL_724941, EPI_ISL_724970, EPI_ISL_725003, EPI_ISL_725004, EPI_ISL_725005, EPI_ISL_725006, EPI_ISL_725007, EPI_ISL_725008, EPI_ISL_725009, EPI_ISL_725010, EPI_ISL_725011, EPI_ISL_725012, EPI_ISL_725013, EPI_ISL_725014, EPI_ISL_725015			
see above	Northumbria University / South Tees Hospitals NHS Foundation Trust / North Cumbria Integrated Care NHS Foundation Trust / North Tees and Hartlepool NHS Foundation Trust / Newcastle Hospitals NHS Foundation Trust	COVID-19 Genomics UK (COG-UK) Consortium	Darren L Smith,Andrew Nelson,Matthew Bashton,Greg R Young,Joshua Loh,John Allan,Mohammad A Tariq,Giles S Holt,Gary Black,Wen C Yew,Lynn Dover,Paul Baker,Steve Liggett,Sarah Essex,Jane Greenaway,Debra Padgett,Clive Graham,Garren Scott,Edward Barton,Emma Swindells,Brendan Payne,Jennifer Collins,Yusri Taha,Gary Eltringham
EPI_ISL_725114, EPI_ISL_725123, EPI_ISL_725125, EPI_ISL_725127, EPI_ISL_725160, EPI_ISL_725164, EPI_ISL_725171, EPI_ISL_725174, EPI_ISL_725176, EPI_ISL_725177, EPI_ISL_725178, EPI_ISL_725179, EPI_ISL_725180, EPI_ISL_725181, EPI_ISL_725183, EPI_ISL_725184, EPI_ISL_725186, EPI_ISL_725188, EPI_ISL_725189, EPI_ISL_725192, EPI_ISL_725194, EPI_ISL_725195, EPI_ISL_725196, EPI_ISL_725197, EPI_ISL_725198, EPI_ISL_725199, EPI_ISL_725200, EPI_ISL_725201, EPI_ISL_725202, EPI_ISL_725203, EPI_ISL_725204, EPI_ISL_725205, EPI_ISL_725206, EPI_ISL_725207, EPI_ISL_725208, EPI_ISL_725209, EPI_ISL_725210, EPI_ISL_725211, EPI_ISL_725212, EPI_ISL_725213, EPI_ISL_725214, EPI_ISL_725215, EPI_ISL_725216, EPI_ISL_725217, EPI_ISL_725218, EPI_ISL_725219, EPI_ISL_725220, EPI_ISL_725221, EPI_ISL_725222, EPI_ISL_725223, EPI_ISL_725224, EPI_ISL_725225, EPI_ISL_725226, EPI_ISL_725227, EPI_ISL_725228, EPI_ISL_725229, EPI_ISL_725230, EPI_ISL_725231, EPI_ISL_725233, EPI_ISL_725234, EPI_ISL_725235, EPI_ISL_725237, EPI_ISL_725238, EPI_ISL_725239, EPI_ISL_725240, EPI_ISL_725241, EPI_ISL_725242, EPI_ISL_725244, EPI_ISL_725245, EPI_ISL_725247, EPI_ISL_725248, EPI_ISL_725249, EPI_ISL_725250, EPI_ISL_725251, EPI_ISL_725252, EPI_ISL_725253, EPI_ISL_725254, EPI_ISL_725255, EPI_ISL_725256, EPI_ISL_725257, EPI_ISL_725258, EPI_ISL_725260, EPI_ISL_725261, EPI_ISL_725262, EPI_ISL_725263, EPI_ISL_725265, EPI_ISL_725268, EPI_ISL_725269, EPI_ISL_725276, EPI_ISL_725281, EPI_ISL_725283, EPI_ISL_725290, EPI_ISL_725292			
see above	Quadram Institute Bioscience	COVID-19 Genomics UK (COG-UK) Consortium	Dave J. Baker, Gemma L. Kay, Alp Aydin, Thanh Le-Viet, Steven Rudder, Ana P. Tedim, Anastasia Kolyva, Maria Diaz, Leonardo de Oliveira Martins, Nabil-Fareed Alikhan, Lizzie Meadows, Rachael Stanley, Ngozi Elumogo, Muhammed Yasir, Nicholas M. Thomson, Alexander J Trotter, Rachel Gilroy, Samuel Bloomfield, Claire Stuart, Andrew Bell, Reenesh Prakash, Samir Dervisevic, Alison E. Mather, John Wain, Mark Webber, Andrew J. Page, Justin O'Grady
EPI_ISL_725519	Queens Medical Centre, Clinical Microbiology Department / DeepSeq Nottingham	COVID-19 Genomics UK (COG-UK) Consortium	Gemma Clark, Wendy Smith, Manjinder Khakh, Vicki M Fleming, Michelle M Lister, Hannah Howson-Wells, Jonathan Ball, Patrick McClure, Joseph Chappell, Theocharis Tsoleridis, Nadine Holmes, Matthew Carlisle, Christopher Moore, Fei Sang, Johnny Debebe, Victoria Wright, Matthew Loose
EPI_ISL_725679, EPI_ISL_725680, EPI_ISL_725681, EPI_ISL_725716, EPI_ISL_725718, EPI_ISL_725719, EPI_ISL_725720, EPI_ISL_725721, EPI_ISL_725722, EPI_ISL_725723, EPI_ISL_725724, EPI_ISL_725725, EPI_ISL_725727, EPI_ISL_725728, EPI_ISL_725729, EPI_ISL_725730, EPI_ISL_725731, EPI_ISL_725733, EPI_ISL_725982, EPI_ISL_725983, EPI_ISL_725984, EPI_ISL_725992, EPI_ISL_725993, EPI_ISL_725994, EPI_ISL_725995, EPI_ISL_725996, EPI_ISL_725997, EPI_ISL_725998, EPI_ISL_726000, EPI_ISL_726001, EPI_ISL_726002, EPI_ISL_726003, EPI_ISL_726004, EPI_ISL_726005, EPI_ISL_726006, EPI_ISL_726007, EPI_ISL_726008, EPI_ISL_726009, EPI_ISL_726010, EPI_ISL_726011, EPI_ISL_726012, EPI_ISL_726013, EPI_ISL_726014, EPI_ISL_726015, EPI_ISL_726016, EPI_ISL_726021, EPI_ISL_726022, EPI_ISL_726023, EPI_ISL_726024, EPI_ISL_726025, EPI_ISL_726026, EPI_ISL_726027, EPI_ISL_726028, EPI_ISL_726029, EPI_ISL_726030, EPI_ISL_726031, EPI_ISL_726032, EPI_ISL_726053, EPI_ISL_726057, EPI_ISL_726058, EPI_ISL_726059, EPI_ISL_726060, EPI_ISL_726061, EPI_ISL_726062, EPI_ISL_726063, EPI_ISL_726064, EPI_ISL_726065, EPI_ISL_726066, EPI_ISL_726067, EPI_ISL_726068, EPI_ISL_726069, EPI_ISL_726070, EPI_ISL_726071, EPI_ISL_726072, EPI_ISL_726073, EPI_ISL_726074, EPI_ISL_726075, EPI_ISL_726076, EPI_ISL_726077, EPI_ISL_726078, EPI_ISL_726079, EPI_ISL_726080, EPI_ISL_726081, EPI_ISL_726082, EPI_ISL_726083, EPI_ISL_726084, EPI_ISL_726085, EPI_ISL_726086, EPI_ISL_726087, EPI_ISL_726088, EPI_ISL_726089, EPI_ISL_726090, EPI_ISL_726091, EPI_ISL_726092, EPI_ISL_726093, EPI_ISL_726094, EPI_ISL_726095, EPI_ISL_726096, EPI_ISL_726097, EPI_ISL_726098, EPI_ISL_726099, EPI_ISL_726100, EPI_ISL_726101, EPI_ISL_726102, EPI_ISL_726103, EPI_ISL_726104, EPI_ISL_726105, EPI_ISL_726106, EPI_ISL_726107, EPI_ISL_726108, EPI_ISL_726109, EPI_ISL_726110, EPI_ISL_726111, EPI_ISL_726112, EPI_ISL_726113, EPI_ISL_726114, EPI_ISL_726115, EPI_ISL_726116, EPI_ISL_726117, EPI_ISL_726118, EPI_ISL_726119, EPI_ISL_726120, EPI_ISL_726121, EPI_ISL_726122, EPI_ISL_726123, EPI_ISL_726124, EPI_ISL_726125, EPI_ISL_726126, EPI_ISL_726127, EPI_ISL_726128, EPI_ISL_726129, EPI_ISL_726130, EPI_ISL_726131, EPI_ISL_726132, EPI_ISL_726133, EPI_ISL_726134, EPI_ISL_726135, EPI_ISL_726136, EPI_ISL_726137, EPI_ISL_726138, EPI_ISL_726139, EPI_ISL_726140, EPI_ISL_726141, EPI_ISL_726142, EPI_ISL_726143, EPI_ISL_726144, EPI_ISL_726145, EPI_ISL_726146, EPI_ISL_726147, EPI_ISL_726148, EPI_ISL_726149, EPI_ISL_726150, EPI_ISL_726151, EPI_ISL_726152, EPI_ISL_726153, EPI_ISL_726154, EPI_ISL_726155, EPI_ISL_726156, EPI_ISL_726157, EPI_ISL_726158, EPI_ISL_726159, EPI_ISL_726160, EPI_ISL_726161, EPI_ISL_726162, EPI_ISL_726163, EPI_ISL_726164, EPI_ISL_726165, EPI_ISL_726166, EPI_ISL_726167, EPI_ISL_726168, EPI_ISL_726169, EPI_ISL_726170, EPI_ISL_726171, EPI_ISL_726172, EPI_ISL_726173, EPI_ISL_726174, EPI_ISL_726175, EPI_ISL_726176, EPI_ISL_726177, EPI_ISL_726178, EPI_ISL_726179, EPI_ISL_726180, EPI_ISL_726181, EPI_ISL_726182, EPI_ISL_726183, EPI_ISL_726184, EPI_ISL_726185, EPI_ISL_726186, EPI_ISL_726187, EPI_ISL_726188, EPI_ISL_726189, EPI_ISL_726190, EPI_ISL_726191, EPI_ISL_726192, EPI_ISL_726193, EPI_ISL_726194, EPI_ISL_726195, EPI_ISL_726197, EPI_ISL_726198, EPI_ISL_726208, EPI_ISL_726209, EPI_ISL_726210, EPI_ISL_726213, EPI_ISL_726218, EPI_ISL_726222, EPI_ISL_726223, EPI_ISL_726224, EPI_ISL_726225, EPI_ISL_726226, EPI_ISL_726227, EPI_ISL_726242, EPI_ISL_726243, EPI_ISL_726253, EPI_ISL_726254, EPI_ISL_726255, EPI_ISL_726256, EPI_ISL_726257, EPI_ISL_726258, EPI_ISL_726259, EPI_ISL_726260, EPI_ISL_726261, EPI_ISL_726262, EPI_ISL_726263, EPI_ISL_726264, EPI_ISL_726268, EPI_ISL_726277, EPI_ISL_726278, EPI_ISL_726279, EPI_ISL_726280, EPI_ISL_726305, EPI_ISL_726306, EPI_ISL_726307, EPI_ISL_726308, EPI_ISL_726309, EPI_ISL_726310, EPI_ISL_726312, EPI_ISL_726314, EPI_ISL_726319, EPI_ISL_726320, EPI_ISL_726321, EPI_ISL_726322, EPI_ISL_726323, EPI_ISL_726324, EPI_ISL_726325, EPI_ISL_726326, EPI_ISL_726327, EPI_ISL_726328, EPI_ISL_726329, EPI_ISL_726330, EPI_ISL_726331, EPI_ISL_726332, EPI_ISL_726333, EPI_ISL_726334, EPI_ISL_726335, EPI_ISL_726336, EPI_ISL_726337, EPI_ISL_726338, EPI_ISL_726339, EPI_ISL_726340, EPI_ISL_726341, EPI_ISL_726342, EPI_ISL_726344, EPI_ISL_726345, EPI_ISL_726346, EPI_ISL_726347, EPI_ISL_726348, EPI_ISL_726349, EPI_ISL_726350, EPI_ISL_726500, EPI_ISL_726501, EPI_ISL_726522, EPI_ISL_726526, EPI_ISL_726537, EPI_ISL_726538, EPI_ISL_726539, EPI_ISL_726540, EPI_ISL_726541, EPI_ISL_726546, EPI_ISL_726547, EPI_ISL_726567, EPI_ISL_726568, EPI_ISL_726570, EPI_ISL_726571, EPI_ISL_726572, EPI_ISL_726573, EPI_ISL_726574, EPI_ISL_726575, EPI_ISL_726576, EPI_ISL_726577, EPI_ISL_726578, EPI_ISL_726579, EPI_ISL_726580, EPI_ISL_726581, EPI_ISL_726582, EPI_ISL_726583, EPI_ISL_726584, EPI_ISL_726585, EPI_ISL_726587, EPI_ISL_726589, EPI_ISL_726591, EPI_ISL_726594, EPI_ISL_726599, EPI_ISL_726601, EPI_ISL_726603, EPI_ISL_726605, EPI_ISL_726606, EPI_ISL_726607, EPI_ISL_726608, EPI_ISL_726609, EPI_ISL_726610, EPI_ISL_726611, EPI_ISL_726612, EPI_ISL_726613, EPI_ISL_726614, EPI_ISL_726615, EPI_ISL_726616, EPI_ISL_726617, EPI_ISL_726618, EPI_ISL_726619, EPI_ISL_726620, EPI_ISL_726621, EPI_ISL_726622, EPI_ISL_726623, EPI_ISL_726624, EPI_ISL_726625, EPI_ISL_726626, EPI_ISL_726627, EPI_ISL_726628, EPI_ISL_726629, EPI_ISL_726630, EPI_ISL_726631, EPI_ISL_726632, EPI_ISL_726633, EPI_ISL_726634, EPI_ISL_726635, EPI_ISL_726636, EPI_ISL_726639, EPI_ISL_726640, EPI_ISL_726641, EPI_ISL_726642, EPI_ISL_726643, EPI_ISL_726644, EPI_ISL_726645, EPI_ISL_726646, EPI_ISL_726648, EPI_ISL_726649, EPI_ISL_726650, EPI_ISL_726651, EPI_ISL_726652, EPI_ISL_726654, EPI_ISL_726655, EPI_ISL_726656, EPI_ISL_726865, EPI_ISL_726866, EPI_ISL_726867, EPI_ISL_726868, EPI_ISL_726869, EPI_ISL_726870, EPI_ISL_726871, EPI_ISL_726872, EPI_ISL_726873, EPI_ISL_726874, EPI_ISL_726875, EPI_ISL_726876			
see above	Wales Specialist Virology Centre Sequencing lab: Pathogen Genomics Unit	COVID-19 Genomics UK (COG-UK) Consortium	Catherine Moore, Johnathan Evans, Laura Gifford, Malorie Perry, Simon Cottrell, Angela Marchbank, Alec Birchley, Alexander Adams, Amy Gaskin, Bree Gatica-Wilcox, Jason Coombes, Joel Southgate, Lauren Gilbert, Lee Graham, Nicole Pacchiarini, Sara Kumziene-Summerhayes, Sarah Taylor, Sophie Jones, Sara Rey, Matthew Bull, Joanne Watkins, Sally Corden, Tom Connor
EPI_ISL_727734	Centre for Enzyme Innovation, University of Portsmouth / Translational Research Laboratory, Portsmouth Hospitals NHS Trust	COVID-19 Genomics UK (COG-UK) Consortium	Angela Beckett,Yann Bourgeois,Garry Scarlett,Sharon Glaysher,Scott Elliott,Kelly Bicknell,Robert Impey,Allyson Lloyd,Sarah Wyllie,Ethan Butcher,Anoop Chauhan,Samuel Robson
EPI_ISL_727807, EPI_ISL_727808, EPI_ISL_727809, EPI_ISL_727810, EPI_ISL_727811, EPI_ISL_727812, EPI_ISL_727813, EPI_ISL_727814, EPI_ISL_727815, EPI_ISL_727816, EPI_ISL_727817, EPI_ISL_727818, EPI_ISL_727819, EPI_ISL_727820, EPI_ISL_727821, EPI_ISL_727822, EPI_ISL_727823, EPI_ISL_727824,			

EPI_ISL_727825, EPI_ISL_727826, EPI_ISL_727827, EPI_ISL_727828, EPI_ISL_727829, EPI_ISL_727830, EPI_ISL_727831, EPI_ISL_727832, EPI_ISL_727833, EPI_ISL_727834, EPI_ISL_727835, EPI_ISL_727836, EPI_ISL_727837, EPI_ISL_727838, EPI_ISL_727839, EPI_ISL_727840, EPI_ISL_727841, EPI_ISL_727842, EPI_ISL_727843, EPI_ISL_727844, EPI_ISL_727845, EPI_ISL_727846, EPI_ISL_727847, EPI_ISL_727848, EPI_ISL_727849, EPI_ISL_727850, EPI_ISL_727851, EPI_ISL_727852, EPI_ISL_727853, EPI_ISL_727854, EPI_ISL_727855, EPI_ISL_727856, EPI_ISL_727857, EPI_ISL_727858, EPI_ISL_727859, EPI_ISL_727860			
see above	Oxford Viromics, NDM, University of Oxford; Oxford University Hospitals; Basingstoke and North Hampshire Hospital	COVID-19 Genomics UK (COG-UK) Consortium	Tanya Golubchik, David Bonsall, George Macintyre, Amy Trebes, Mariateresa de Cesare, Catrin Moore, Alex Mobbs, Anita Justice, Robert Shaw, Monique Andersson, Timothy Peto, Emma Wise, Nathan Moore, Jessica Lynch, Nick Cortes, Matilde Mori, Stephen Kidd, David Buck, John Todd, Christophe Fraser
EPI_ISL_727991	Virology Department, Sheffield Teaching Hospitals NHS Foundation Trust/Department of Infection, Immunity and Cardiovascular Disease, The Medical School, University of Sheffield	COVID-19 Genomics UK (COG-UK) Consortium	Thushan de Silva, Matthew Parker, Nikki Smith, Adri Angyal, Rebecca Brown, Luke Green, Rachel Tucker, Paul Parsons, Danielle Groves, Katie Johnson, Laura Carrilero, Alex Keeley, Dave Partridge, Matthew Wyles, Benjamin Lindsey, Mehmet Yavuz, Mohammad Raza, Cariad Evans
EPI_ISL_728300	Lighthouse Lab in Glasgow	Wellcome Sanger Institute for the COVID-19 Genomics UK (COG-UK) Consortium	Harper VanSteenhouse, Yumi Kasai, David Gray, Carol Clugston, Anna Dominiczak and Alex Alderton, Roberto Amato, Sonia Goncalves, Ewan Harrison, David K. Jackson, Ian Johnston, Dominic Kwiatkowski, Cordelia Langford, John Sillitoe on behalf of the Wellcome Sanger Institute COVID-19 Surveillance Team
EPI_ISL_731251, EPI_ISL_731252, EPI_ISL_731253	Lighthouse Lab in Milton Keynes	Wellcome Sanger Institute for the COVID-19 Genomics UK (COG-UK) Consortium	The Lighthouse Lab in Milton Keynes and Alex Alderton, Roberto Amato, Sonia Goncalves, Ewan Harrison, David K. Jackson, Ian Johnston, Dominic Kwiatkowski, Cordelia Langford, John Sillitoe on behalf of the Wellcome Sanger Institute COVID-19 Surveillance Team
EPI_ISL_731894	Lighthouse Lab in Glasgow	Wellcome Sanger Institute for the COVID-19 Genomics UK (COG-UK) Consortium	Harper VanSteenhouse, Yumi Kasai, David Gray, Carol Clugston, Anna Dominiczak and Alex Alderton, Roberto Amato, Sonia Goncalves, Ewan Harrison, David K. Jackson, Ian Johnston, Dominic Kwiatkowski, Cordelia Langford, John Sillitoe on behalf of the Wellcome Sanger Institute COVID-19 Surveillance Team
EPI_ISL_741516	Quadram Institute Bioscience	COVID-19 Genomics UK (COG-UK) Consortium	Dave J. Baker, Gemma L. Kay, Alp Aydin, Thanh Le-Viet, Steven Rudder, Ana P. Tedim, Anastasia Kolyva, Maria Diaz, Leonardo de Oliveira Martins, Nabil-Fareed Alikhan, Lizzie Meadows, Rachael Stanley, Ngozi Elumogo, Muhammed Yasir, Nicholas M. Thomson, Alexander J Trotter, Rachel Gilroy, Samuel Bloomfield, Claire Stuart, Andrew Bell, Reenesh Prakash, Samir Dervisevic, Alison E. Mather, John Wain, Mark Webber, Andrew J. Page, Justin O'Grady
EPI_ISL_741716, EPI_ISL_741717, EPI_ISL_741718, EPI_ISL_741719, EPI_ISL_741720, EPI_ISL_741721, EPI_ISL_741722, EPI_ISL_741740, EPI_ISL_741741, EPI_ISL_741743, EPI_ISL_741759			
see above	Centre for Enzyme Innovation, University of Portsmouth / Translational Research Laboratory, Portsmouth Hospitals NHS Trust	COVID-19 Genomics UK (COG-UK) Consortium	Angela Beckett,Yann Bourgeois,Garry Scarlett,Sharon Glaysher,Scott Elliott,Kelly Bicknell,Robert Impey,Allyson Lloyd,Sarah Wyllie,Ethan Butcher,Anoop Chauhan,Samuel Robson
EPI_ISL_741777, EPI_ISL_741778, EPI_ISL_741779, EPI_ISL_741780, EPI_ISL_741781, EPI_ISL_741782, EPI_ISL_741783, EPI_ISL_741784, EPI_ISL_741785, EPI_ISL_741786, EPI_ISL_741787, EPI_ISL_741788, EPI_ISL_741789, EPI_ISL_741790, EPI_ISL_741791, EPI_ISL_741792, EPI_ISL_741793, EPI_ISL_741794, EPI_ISL_741795, EPI_ISL_741796, EPI_ISL_741797, EPI_ISL_741798, EPI_ISL_741799, EPI_ISL_741800, EPI_ISL_741801, EPI_ISL_741802, EPI_ISL_741803, EPI_ISL_741804, EPI_ISL_741805, EPI_ISL_741806, EPI_ISL_741807, EPI_ISL_741808, EPI_ISL_741809, EPI_ISL_741810, EPI_ISL_741811, EPI_ISL_741812, EPI_ISL_741813, EPI_ISL_741814, EPI_ISL_741815, EPI_ISL_741816, EPI_ISL_741817, EPI_ISL_741818, EPI_ISL_741819, EPI_ISL_741820, EPI_ISL_741821, EPI_ISL_741822, EPI_ISL_741823, EPI_ISL_741824, EPI_ISL_741825, EPI_ISL_741827, EPI_ISL_741828, EPI_ISL_741829, EPI_ISL_741830, EPI_ISL_741831, EPI_ISL_741832, EPI_ISL_741833, EPI_ISL_741834, EPI_ISL_741835, EPI_ISL_741836, EPI_ISL_741837, EPI_ISL_741838, EPI_ISL_741839, EPI_ISL_741840, EPI_ISL_741841, EPI_ISL_741842, EPI_ISL_741843, EPI_ISL_741844, EPI_ISL_741845, EPI_ISL_741846, EPI_ISL_741847, EPI_ISL_741848, EPI_ISL_741849, EPI_ISL_741850, EPI_ISL_741851, EPI_ISL_741852, EPI_ISL_741853, EPI_ISL_741854, EPI_ISL_741855, EPI_ISL_741856, EPI_ISL_741857, EPI_ISL_741858, EPI_ISL_741859, EPI_ISL_741860, EPI_ISL_741861, EPI_ISL_741862, EPI_ISL_741863, EPI_ISL_741864, EPI_ISL_741865, EPI_ISL_741866, EPI_ISL_741867, EPI_ISL_741868, EPI_ISL_741869, EPI_ISL_741870, EPI_ISL_741871, EPI_ISL_741872, EPI_ISL_741873, EPI_ISL_741874, EPI_ISL_741875, EPI_ISL_741876, EPI_ISL_741877, EPI_ISL_741878, EPI_ISL_741879, EPI_ISL_741880, EPI_ISL_741881, EPI_ISL_741882, EPI_ISL_741883, EPI_ISL_741884, EPI_ISL_741885, EPI_ISL_741886, EPI_ISL_741887, EPI_ISL_741888, EPI_ISL_741889, EPI_ISL_741890, EPI_ISL_741891, EPI_ISL_741892, EPI_ISL_741893, EPI_ISL_741894, EPI_ISL_741895, EPI_ISL_741896, EPI_ISL_741897, EPI_ISL_741898, EPI_ISL_741899, EPI_ISL_741900, EPI_ISL_741901, EPI_ISL_741902, EPI_ISL_741903, EPI_ISL_741904, EPI_ISL_741905, EPI_ISL_741906, EPI_ISL_741907, EPI_ISL_741908, EPI_ISL_741909, EPI_ISL_741910, EPI_ISL_741911, EPI_ISL_741912, EPI_ISL_741913, EPI_ISL_741914, EPI_ISL_741915, EPI_ISL_741916, EPI_ISL_741917, EPI_ISL_741918, EPI_ISL_741919, EPI_ISL_741920, EPI_ISL_741934			
see above	Oxford Viromics, NDM, University of Oxford; Oxford University Hospitals; Basingstoke and North Hampshire Hospital	COVID-19 Genomics UK (COG-UK) Consortium	Tanya Golubchik, David Bonsall, George Macintyre, Amy Trebes, Mariateresa de Cesare, Catrin Moore, Alex Mobbs, Anita Justice, Robert Shaw, Monique Andersson, Timothy Peto, Emma Wise, Nathan Moore, Jessica Lynch, Nick Cortes, Matilde Mori, Stephen Kidd, David Buck, John Todd, Christophe Fraser
EPI_ISL_742174	Regional Virus Laboratory, Belfast Health and Social Care Trust	COVID-19 Genomics UK (COG-UK) Consortium	Conall McCaughey, James McKenna, Tanya Curran, Susan Feeney, Alison Watt, Ciara Cox, Mairead Connor, Zoltan Molnar, David Simpson, Derek Fairley
EPI_ISL_742247, EPI_ISL_742248, EPI_ISL_742249, EPI_ISL_742250	Oxford Viromics, NDM, University of Oxford; Oxford University Hospitals; Basingstoke and North Hampshire Hospital	COVID-19 Genomics UK (COG-UK) Consortium	Tanya Golubchik, David Bonsall, George Macintyre, Amy Trebes, Mariateresa de Cesare, Catrin Moore, Alex Mobbs, Anita Justice, Robert Shaw, Monique Andersson, Timothy Peto, Emma Wise, Nathan Moore, Jessica Lynch, Nick Cortes, Matilde Mori, Stephen Kidd, David Buck, John Todd, Christophe Fraser
EPI_ISL_742344, EPI_ISL_742346, EPI_ISL_742347, EPI_ISL_742348, EPI_ISL_742349, EPI_ISL_742350, EPI_ISL_742541, EPI_ISL_742542, EPI_ISL_742543, EPI_ISL_742544, EPI_ISL_742547, EPI_ISL_742548, EPI_ISL_742549, EPI_ISL_742559, EPI_ISL_742561, EPI_ISL_742563, EPI_ISL_742564, EPI_ISL_742585, EPI_ISL_742586, EPI_ISL_742587, EPI_ISL_742588, EPI_ISL_742589, EPI_ISL_742590, EPI_ISL_742591, EPI_ISL_742592, EPI_ISL_742593, EPI_ISL_742594, EPI_ISL_742595, EPI_ISL_742596, EPI_ISL_742597, EPI_ISL_742598, EPI_ISL_742599, EPI_ISL_742600, EPI_ISL_742601, EPI_ISL_742602, EPI_ISL_742603, EPI_ISL_742604, EPI_ISL_742605, EPI_ISL_742606, EPI_ISL_742607, EPI_ISL_742608, EPI_ISL_742609, EPI_ISL_742610, EPI_ISL_742611, EPI_ISL_742612, EPI_ISL_742613, EPI_ISL_742614, EPI_ISL_742615, EPI_ISL_742616, EPI_ISL_742617, EPI_ISL_742618, EPI_ISL_742619, EPI_ISL_742620, EPI_ISL_742621, EPI_ISL_742622, EPI_ISL_742623, EPI_ISL_742624, EPI_ISL_742625, EPI_ISL_742626, EPI_ISL_742627, EPI_ISL_742628, EPI_ISL_742629, EPI_ISL_742630, EPI_ISL_742631, EPI_ISL_742632, EPI_ISL_742633, EPI_ISL_742634, EPI_ISL_742635, EPI_ISL_742636			
see above	Wales Specialist Virology Centre Sequencing lab: Pathogen Genomics Unit	COVID-19 Genomics UK (COG-UK) Consortium	Catherine Moore, Johnathan Evans, Laura Gifford, Malorie Perry, Simon Cottrell, Angela Marchbank, Alec Birchley, Alexander Adams, Amy Gaskin, Bree Gatica-Wilcox, Jason Coombes, Joel Southgate, Lauren Gilbert, Lee Graham, Nicole Pacchiarini, Sara Kumziene-Summerhayes, Sarah Taylor, Sophie Jones, Sara Rey, Matthew Bull, Joanne Watkins, Sally Corden, Tom Connor
EPI_ISL_757187, EPI_ISL_757188, EPI_ISL_757189, EPI_ISL_757190, EPI_ISL_757191, EPI_ISL_757192, EPI_ISL_757193, EPI_ISL_757194, EPI_ISL_757195, EPI_ISL_757196, EPI_ISL_757197, EPI_ISL_757198, EPI_ISL_757199, EPI_ISL_757200, EPI_ISL_757201, EPI_ISL_757202, EPI_ISL_757203, EPI_ISL_757204, EPI_ISL_757205, EPI_ISL_757206, EPI_ISL_757207, EPI_ISL_757208, EPI_ISL_757209, EPI_ISL_757210, EPI_ISL_757211, EPI_ISL_757212, EPI_ISL_757213, EPI_ISL_757214, EPI_ISL_757215, EPI_ISL_757216, EPI_ISL_757217, EPI_ISL_757218, EPI_ISL_757219, EPI_ISL_757220, EPI_ISL_757221, EPI_ISL_757222, EPI_ISL_757223, EPI_ISL_757224, EPI_ISL_757273, EPI_ISL_757274, EPI_ISL_757275, EPI_ISL_757276, EPI_ISL_757277, EPI_ISL_757278, EPI_ISL_757279, EPI_ISL_757280, EPI_ISL_757281, EPI_ISL_757282, EPI_ISL_757283, EPI_ISL_757284, EPI_ISL_760490			
see above	Lighthouse Lab in Glasgow	Wellcome Sanger Institute for the COVID-19 Genomics UK (COG-UK) Consortium	Harper VanSteenhouse, Yumi Kasai, David Gray, Carol Clugston, Anna Dominiczak and Alex Alderton, Roberto Amato, Sonia Goncalves, Ewan Harrison, David K. Jackson, Ian Johnston, Dominic Kwiatkowski, Cordelia Langford, John Sillitoe on behalf of the Wellcome Sanger Institute COVID-19 Surveillance Team
EPI_ISL_764360	Virology Department, Royal Infirmary of Edinburgh, NHS Lothian / School of Biological Sciences, University of Edinburgh / Institute of Genetics and Molecular Medicine, University of Edinburgh	COVID-19 Genomics UK (COG-UK) Consortium	McHugh M, Dewar R, Rooke S, Gallagher M, Balcaza C, O'Toole Á, Scher E, Hill V, McCrone JT, Colquhoun R, Yu X, Jackson B, Rambaut A, Williams TC, Templeton K
EPI_ISL_764437	Regional Virus Laboratory, Belfast Health and Social Care Trust	COVID-19 Genomics UK (COG-UK) Consortium	Conall McCaughey, James McKenna, Tanya Curran, Susan Feeney, Alison Watt, Ciara Cox, Mairead Connor, Zoltan Molnar, David Simpson, Derek Fairley
EPI_ISL_766071, EPI_ISL_766077, EPI_ISL_766094, EPI_ISL_766098, EPI_ISL_766268, EPI_ISL_766270, EPI_ISL_766271, EPI_ISL_766272, EPI_ISL_766273, EPI_ISL_766274, EPI_ISL_766275, EPI_ISL_766276			
see above	Respiratory Virus Unit, National Infection Service, Public Health England	COVID-19 Genomics UK (COG-UK) Consortium	PHE Covid Sequencing Team
EPI_ISL_767239	Lighthouse Lab in Milton Keynes	Wellcome Sanger Institute for the COVID-19 Genomics UK (COG-UK) Consortium	The Lighthouse Lab in Milton Keynes and Alex Alderton, Roberto Amato, Sonia Goncalves, Ewan Harrison, David K. Jackson, Ian Johnston, Dominic Kwiatkowski, Cordelia Langford, John Sillitoe on behalf of the Wellcome Sanger Institute COVID-19 Surveillance Team
EPI_ISL_767322, EPI_ISL_767324, EPI_ISL_767326, EPI_ISL_767329, EPI_ISL_767331, EPI_ISL_767333	Lighthouse Lab in Alderley Park	Wellcome Sanger Institute for the COVID-19 Genomics UK (COG-UK) Consortium	Jacquelyn Wynn, Mairead Hyland, The Lighthouse Lab in Alderley Park and Alex Alderton, Roberto Amato, Sonia Goncalves, Ewan Harrison, David K. Jackson, Ian Johnston, Dominic Kwiatkowski, Cordelia Langford, John Sillitoe on behalf of the Wellcome Sanger Institute COVID-19 Surveillance Team
EPI_ISL_769865	Respiratory Virus Unit, National Infection Service, Public Health England	COVID-19 Genomics UK (COG-UK) Consortium	PHE Covid Sequencing Team
EPI_ISL_783069, EPI_ISL_783070, EPI_ISL_783071, EPI_ISL_783072, EPI_ISL_783073, EPI_ISL_783074, EPI_ISL_783075, EPI_ISL_783076, EPI_ISL_783077, EPI_ISL_783078, EPI_ISL_783079, EPI_ISL_783080, EPI_ISL_783081, EPI_ISL_783082, EPI_ISL_783083, EPI_ISL_783084, EPI_ISL_783085, EPI_ISL_783086, EPI_ISL_783087, EPI_ISL_783088, EPI_ISL_783089, EPI_ISL_783090, EPI_ISL_783091, EPI_ISL_783092, EPI_ISL_783093, EPI_ISL_783094, EPI_ISL_783095, EPI_ISL_783096, EPI_ISL_783097, EPI_ISL_783098, EPI_ISL_783099, EPI_ISL_783100, EPI_ISL_783101, EPI_ISL_783102, EPI_ISL_783103, EPI_ISL_783104, EPI_ISL_783105, EPI_ISL_783106, EPI_ISL_783107, EPI_ISL_783108, EPI_ISL_783109, EPI_ISL_783110, EPI_ISL_783111, EPI_ISL_783112, EPI_ISL_783113, EPI_ISL_783114, EPI_ISL_783115, EPI_ISL_783116, EPI_ISL_783117, EPI_ISL_783118, EPI_ISL_783119, EPI_ISL_783120, EPI_ISL_783121, EPI_ISL_783122, EPI_ISL_783123, EPI_ISL_783124, EPI_ISL_783125, EPI_ISL_783126, EPI_ISL_783127, EPI_ISL_783128, EPI_ISL_783129, EPI_ISL_783130, EPI_ISL_783131, EPI_ISL_783132, EPI_ISL_783133, EPI_ISL_783134, EPI_ISL_783135, EPI_ISL_783136, EPI_ISL_783137, EPI_ISL_783138, EPI_ISL_783139, EPI_ISL_783140, EPI_ISL_783141, EPI_ISL_783142, EPI_ISL_783143, EPI_ISL_783144, EPI_ISL_783145, EPI_ISL_783146, EPI_ISL_783147, EPI_ISL_783148, EPI_ISL_783149, EPI_ISL_783150, EPI_ISL_783151, EPI_ISL_783152, EPI_ISL_783153, EPI_ISL_783154, EPI_ISL_783155, EPI_ISL_783156, EPI_ISL_783157, EPI_ISL_783158, EPI_ISL_783159, EPI_ISL_783160, EPI_ISL_783161, EPI_ISL_783162, EPI_ISL_783163, EPI_ISL_783164, EPI_ISL_783165, EPI_ISL_783166, EPI_ISL_783167, EPI_ISL_783168, EPI_ISL_783169, EPI_ISL_783170, EPI_ISL_783171, EPI_ISL_783172, EPI_ISL_783173, EPI_ISL_783174, EPI_ISL_783175, EPI_ISL_783176, EPI_ISL_783177, EPI_ISL_783178, EPI_ISL_783179, EPI_ISL_783180, EPI_ISL_783181, EPI_ISL_783182, EPI_ISL_783183, EPI_ISL_783184, EPI_ISL_783185, EPI_ISL_783186, EPI_ISL_783187, EPI_ISL_783188, EPI_ISL_783189, EPI_ISL_783190, EPI_ISL_783191, EPI_ISL_783192, EPI_ISL_783193, EPI_ISL_783194, EPI_ISL_783195, EPI_ISL_783196, EPI_ISL_783197, EPI_ISL_783198, EPI_ISL_783199, EPI_ISL_783200, EPI_ISL_783201, EPI_ISL_783202, EPI_ISL_783203, EPI_ISL_783204, EPI_ISL_783205, EPI_ISL_783206, EPI_ISL_783207, EPI_ISL_783208, EPI_ISL_783209, EPI_ISL_783210, EPI_ISL_783211, EPI_ISL_783212,			

EPI_ISL_783213, EPI_ISL_783214, EPI_ISL_783215, EPI_ISL_783216, EPI_ISL_783217, EPI_ISL_783218, EPI_ISL_783219, EPI_ISL_783220, EPI_ISL_783221, EPI_ISL_783222, EPI_ISL_783223, EPI_ISL_783224, EPI_ISL_783225, EPI_ISL_783226, EPI_ISL_783227, EPI_ISL_783228, EPI_ISL_783229, EPI_ISL_783230, EPI_ISL_783231, EPI_ISL_783232, EPI_ISL_783233, EPI_ISL_783234, EPI_ISL_783235, EPI_ISL_783236, EPI_ISL_783237, EPI_ISL_783238, EPI_ISL_783239, EPI_ISL_783240, EPI_ISL_783241, EPI_ISL_783242, EPI_ISL_783243, EPI_ISL_783244, EPI_ISL_783245, EPI_ISL_783246, EPI_ISL_783247, EPI_ISL_783248, EPI_ISL_783249, EPI_ISL_783250, EPI_ISL_783251, EPI_ISL_783252, EPI_ISL_783253, EPI_ISL_783254, EPI_ISL_783255, EPI_ISL_783256, EPI_ISL_783257, EPI_ISL_783258, EPI_ISL_783259, EPI_ISL_783260, EPI_ISL_783261, EPI_ISL_783262, EPI_ISL_783263, EPI_ISL_783264, EPI_ISL_783265, EPI_ISL_783266, EPI_ISL_783267, EPI_ISL_783268, EPI_ISL_783269, EPI_ISL_783270, EPI_ISL_783271, EPI_ISL_783272, EPI_ISL_783273, EPI_ISL_783274, EPI_ISL_783275, EPI_ISL_783276, EPI_ISL_783277, EPI_ISL_783278, EPI_ISL_783279, EPI_ISL_783280, EPI_ISL_783281, EPI_ISL_783282, EPI_ISL_783283, EPI_ISL_783284, EPI_ISL_783285, EPI_ISL_783286, EPI_ISL_783287, EPI_ISL_783288, EPI_ISL_783289, EPI_ISL_783290, EPI_ISL_783291, EPI_ISL_783292, EPI_ISL_783293, EPI_ISL_783294, EPI_ISL_783295, EPI_ISL_783296, EPI_ISL_783297, EPI_ISL_783298, EPI_ISL_783299, EPI_ISL_783300, EPI_ISL_783301, EPI_ISL_783302, EPI_ISL_783303, EPI_ISL_783304, EPI_ISL_783305, EPI_ISL_783306, EPI_ISL_783307, EPI_ISL_783308, EPI_ISL_783309, EPI_ISL_783310, EPI_ISL_783311, EPI_ISL_783312, EPI_ISL_783313, EPI_ISL_783314, EPI_ISL_783315, EPI_ISL_783316, EPI_ISL_783317, EPI_ISL_783318, EPI_ISL_783319, EPI_ISL_783320, EPI_ISL_783321, EPI_ISL_783322, EPI_ISL_783323, EPI_ISL_783324, EPI_ISL_783325, EPI_ISL_783326, EPI_ISL_783327, EPI_ISL_783328, EPI_ISL_783329, EPI_ISL_783330, EPI_ISL_783331, EPI_ISL_783332, EPI_ISL_783333, EPI_ISL_783334, EPI_ISL_783335, EPI_ISL_783336, EPI_ISL_783337, EPI_ISL_783338, EPI_ISL_783339, EPI_ISL_783340, EPI_ISL_783341, EPI_ISL_783342, EPI_ISL_783343, EPI_ISL_783344, EPI_ISL_783345, EPI_ISL_783346, EPI_ISL_783347, EPI_ISL_783348, EPI_ISL_783349, EPI_ISL_783350, EPI_ISL_783351, EPI_ISL_783352, EPI_ISL_783353, EPI_ISL_783354, EPI_ISL_783355, EPI_ISL_783356, EPI_ISL_783357, EPI_ISL_783358, EPI_ISL_783359, EPI_ISL_783360, EPI_ISL_783361, EPI_ISL_783362, EPI_ISL_783363, EPI_ISL_783364, EPI_ISL_783365, EPI_ISL_783366			
see above	Lighthouse Lab in Milton Keynes	Wellcome Sanger Institute for the COVID-19 Genomics UK (COG-UK) Consortium	The Lighthouse Lab in Milton Keynes and Alex Alderton, Roberto Amato, Sonia Goncalves, Ewan Harrison, David K. Jackson, Ian Johnston, Dominic Kwiatkowski, Cordelia Langford, John Sillitoe on behalf of the Wellcome Sanger Institute COVID-19 Surveillance Team
EPI_ISL_800887, EPI_ISL_800893, EPI_ISL_800897, EPI_ISL_800907, EPI_ISL_800914, EPI_ISL_800918, EPI_ISL_800920, EPI_ISL_800924, EPI_ISL_800936, EPI_ISL_800943, EPI_ISL_800948, EPI_ISL_800956, EPI_ISL_800960, EPI_ISL_800967, EPI_ISL_800968, EPI_ISL_800971, EPI_ISL_800983, EPI_ISL_800995, EPI_ISL_800999, EPI_ISL_801002, EPI_ISL_801026, EPI_ISL_801038, EPI_ISL_801039, EPI_ISL_801055, EPI_ISL_801061, EPI_ISL_801063, EPI_ISL_801064, EPI_ISL_801088, EPI_ISL_801089, EPI_ISL_801098, EPI_ISL_801108, EPI_ISL_801110, EPI_ISL_801125, EPI_ISL_801137, EPI_ISL_801142, EPI_ISL_801144, EPI_ISL_801145, EPI_ISL_801148, EPI_ISL_801150, EPI_ISL_801157, EPI_ISL_801161, EPI_ISL_801169, EPI_ISL_801172, EPI_ISL_801174, EPI_ISL_801176			
see above	Lighthouse Lab in Cambridge	Wellcome Sanger Institute for the COVID-19 Genomics UK (COG-UK) Consortium	Rob Howes, The Lighthouse Lab in Cambridge and Alex Alderton, Roberto Amato, Sonia Goncalves, Ewan Harrison, David K. Jackson, Ian Johnston, Dominic Kwiatkowski, Cordelia Langford, John Sillitoe on behalf of the Wellcome Sanger Institute COVID-19 Surveillance Team
EPI_ISL_801182, EPI_ISL_801183, EPI_ISL_801184, EPI_ISL_801185, EPI_ISL_801186, EPI_ISL_801187, EPI_ISL_801188, EPI_ISL_801189, EPI_ISL_801190, EPI_ISL_801191, EPI_ISL_801192, EPI_ISL_801193, EPI_ISL_801194, EPI_ISL_801195, EPI_ISL_801196, EPI_ISL_801197, EPI_ISL_801198, EPI_ISL_801199, EPI_ISL_801200, EPI_ISL_801201, EPI_ISL_801202, EPI_ISL_801203, EPI_ISL_801204, EPI_ISL_801205, EPI_ISL_801206, EPI_ISL_801207, EPI_ISL_801208, EPI_ISL_801209, EPI_ISL_801210, EPI_ISL_801211, EPI_ISL_801212, EPI_ISL_801213, EPI_ISL_801214, EPI_ISL_801215, EPI_ISL_801216, EPI_ISL_801217, EPI_ISL_801218, EPI_ISL_801219, EPI_ISL_801220, EPI_ISL_801221, EPI_ISL_801222, EPI_ISL_801223, EPI_ISL_801224, EPI_ISL_801225, EPI_ISL_801226, EPI_ISL_801227, EPI_ISL_801228, EPI_ISL_801229, EPI_ISL_801230			
see above	Lighthouse Lab in Milton Keynes	Wellcome Sanger Institute for the COVID-19 Genomics UK (COG-UK) Consortium	The Lighthouse Lab in Milton Keynes and Alex Alderton, Roberto Amato, Sonia Goncalves, Ewan Harrison, David K. Jackson, Ian Johnston, Dominic Kwiatkowski, Cordelia Langford, John Sillitoe on behalf of the Wellcome Sanger Institute COVID-19 Surveillance Team
EPI_ISL_801231, EPI_ISL_801232, EPI_ISL_801233, EPI_ISL_801234, EPI_ISL_801235, EPI_ISL_801236, EPI_ISL_801237, EPI_ISL_801238, EPI_ISL_801239, EPI_ISL_801240, EPI_ISL_801241, EPI_ISL_801242, EPI_ISL_801243, EPI_ISL_801244, EPI_ISL_801245, EPI_ISL_801246, EPI_ISL_801247, EPI_ISL_801248, EPI_ISL_801249, EPI_ISL_801250, EPI_ISL_801251, EPI_ISL_801252, EPI_ISL_801253, EPI_ISL_801254, EPI_ISL_801255, EPI_ISL_801256, EPI_ISL_801257, EPI_ISL_801258, EPI_ISL_801259, EPI_ISL_801260, EPI_ISL_801261, EPI_ISL_801262, EPI_ISL_801263, EPI_ISL_801264, EPI_ISL_801265, EPI_ISL_801266, EPI_ISL_801267, EPI_ISL_801268, EPI_ISL_801269, EPI_ISL_801270, EPI_ISL_801271, EPI_ISL_801272, EPI_ISL_801273, EPI_ISL_801274, EPI_ISL_801275, EPI_ISL_801276, EPI_ISL_801277, EPI_ISL_801278, EPI_ISL_801279, EPI_ISL_801280, EPI_ISL_801281, EPI_ISL_801282, EPI_ISL_801283, EPI_ISL_801284, EPI_ISL_801285, EPI_ISL_801286, EPI_ISL_801287, EPI_ISL_801288, EPI_ISL_801289, EPI_ISL_801290, EPI_ISL_801291, EPI_ISL_801292, EPI_ISL_801293, EPI_ISL_801294, EPI_ISL_801295, EPI_ISL_801297, EPI_ISL_801298, EPI_ISL_801299, EPI_ISL_801300, EPI_ISL_801301, EPI_ISL_801302, EPI_ISL_801303, EPI_ISL_801304, EPI_ISL_801305, EPI_ISL_801306, EPI_ISL_801307, EPI_ISL_801308, EPI_ISL_801309, EPI_ISL_801310, EPI_ISL_801311, EPI_ISL_801312, EPI_ISL_801314, EPI_ISL_801315, EPI_ISL_801316, EPI_ISL_801317, EPI_ISL_801318, EPI_ISL_801319, EPI_ISL_801320, EPI_ISL_801321, EPI_ISL_801322, EPI_ISL_801323, EPI_ISL_801324, EPI_ISL_801325, EPI_ISL_801326, EPI_ISL_801327, EPI_ISL_801328, EPI_ISL_801329, EPI_ISL_801330, EPI_ISL_801331, EPI_ISL_801332, EPI_ISL_801333, EPI_ISL_801334, EPI_ISL_801335, EPI_ISL_801336, EPI_ISL_801337, EPI_ISL_801338, EPI_ISL_801339, EPI_ISL_801340, EPI_ISL_801341, EPI_ISL_801342, EPI_ISL_801343, EPI_ISL_801344, EPI_ISL_801345, EPI_ISL_801346, EPI_ISL_801347, EPI_ISL_801348, EPI_ISL_801349, EPI_ISL_801350, EPI_ISL_801351, EPI_ISL_801352, EPI_ISL_801353, EPI_ISL_801354, EPI_ISL_801355, EPI_ISL_801356, EPI_ISL_801357, EPI_ISL_801358, EPI_ISL_801359, EPI_ISL_801360, EPI_ISL_801361, EPI_ISL_801362, EPI_ISL_801363, EPI_ISL_801364, EPI_ISL_801365, EPI_ISL_801366, EPI_ISL_801367, EPI_ISL_801368, EPI_ISL_801369, EPI_ISL_801370, EPI_ISL_801371, EPI_ISL_801372, EPI_ISL_801373, EPI_ISL_801374, EPI_ISL_801375, EPI_ISL_801376, EPI_ISL_801377, EPI_ISL_801378, EPI_ISL_801379, EPI_ISL_801380, EPI_ISL_801381, EPI_ISL_801382, EPI_ISL_801383, EPI_ISL_801384, EPI_ISL_801385			
see above	Lighthouse Lab in Glasgow	Wellcome Sanger Institute for the COVID-19 Genomics UK (COG-UK) Consortium	Harper VanSteenhouse, Yumi Kasai, David Gray, Carol Clugston, Anna Dominiczak and Alex Alderton, Roberto Amato, Sonia Goncalves, Ewan Harrison, David K. Jackson, Ian Johnston, Dominic Kwiatkowski, Cordelia Langford, John Sillitoe on behalf of the Wellcome Sanger Institute COVID-19 Surveillance Team
EPI_ISL_813751	Liverpool Clinical Laboratories	COVID-19 Genomics UK (COG-UK) Consortium	Sam Haldenby, Anita Lucaci, Steve Paterson, Julian Hiscox, Alistair Darby, M Almsaud, A Alrezaihi, Muhannad Alruwaili, Stuart D Armstrong, Jones Benjamin, Eleanor G Bentley, Anu Chawla, Jordan J Clark, Angela Cowell, Richard Eccles, Isabel García-Dorival, Matthew Gemmell, Alessandro Gerada, PKF Gilmore, Richard Gregory, Ximeng Han, Catherine Hartley, Margaret Hughes, Miren Iturriza-Gomara, James Johnson, L Luu, Jenifer Manson, Charlotte Nelson, Elaine O'Toole, Cassie Olateju, Rebekah Penrice-Randal , Lucille Rainbow, N.P Randle, Trevor Ian Robinson, Parul Sharma, Ghada T Shawli, James P Stewart, Neil Swainston, Ecaterina Vamos, Joanne Watts, Mark Whitehead
EPI_ISL_814321, EPI_ISL_814343	Wales Specialist Virology Centre Sequencing lab: Pathogen Genomics Unit	COVID-19 Genomics UK (COG-UK) Consortium	Catherine Moore, Johnathan Evans, Laura Gifford, Malorie Perry, Simon Cottrell, Angela Marchbank, Alec Birchley, Alexander Adams, Amy Gaskin, Bree Gatica-Wilcox, Jason Coombes, Joel Southgate, Lauren Gilbert, Lee Graham, Nicole Pacchiarini, Sara Kumziene-Summerhayes, Sarah Taylor, Sophie Jones, Sara Rey, Matthew Bull, Joanne Watkins, Sally Corden, Tom Connor
EPI_ISL_814534, EPI_ISL_814571	Virology Department, Royal Infirmary of Edinburgh, NHS Lothian / School of Biological Sciences, University of Edinburgh / Institute of Genetics and Molecular Medicine, University of Edinburgh	COVID-19 Genomics UK (COG-UK) Consortium	McHugh M, Dewar R, Rooke S, Gallagher M, Balcaza C, O'Toole Á, Scher E, Hill V, McCrone JT, Colquhoun R, Yu X, Jackson B, Rambaut A, Williams TC, Templeton K
EPI_ISL_814695, EPI_ISL_814764, EPI_ISL_814765, EPI_ISL_814796, EPI_ISL_814797	Wales Specialist Virology Centre Sequencing lab: Pathogen Genomics Unit	COVID-19 Genomics UK (COG-UK) Consortium	Catherine Moore, Johnathan Evans, Laura Gifford, Malorie Perry, Simon Cottrell, Angela Marchbank, Alec Birchley, Alexander Adams, Amy Gaskin, Bree Gatica-Wilcox, Jason Coombes, Joel Southgate, Lauren Gilbert, Lee Graham, Nicole Pacchiarini, Sara Kumziene-Summerhayes, Sarah Taylor, Sophie Jones, Sara Rey, Matthew Bull, Joanne Watkins, Sally Corden, Tom Connor
EPI_ISL_816324, EPI_ISL_816453	Virology Department, Sheffield Teaching Hospitals NHS Foundation Trust/Department of Infection, Immunity and Cardiovascular Disease, The Medical School, University of Sheffield	COVID-19 Genomics UK (COG-UK) Consortium	Thushan de Silva, Matthew Parker, Nikki Smith, Adri Angyal, Rebecca Brown, Luke Green, Rachel Tucker, Paul Parsons, Danielle Groves, Katie Johnson, Laura Carrilero, Alex Keeley, Dave Partridge, Matthew Wyles, Benjamin Lindsey, Mehmet Yavuz, Mohammad Raza, Cariad Evans
EPI_ISL_819462, EPI_ISL_819463	University College London, Great Ormond Street Hospital for Children NHS Foundation Trust, Imperial College Healthcare NHS Trust	COVID-19 Genomics UK (COG-UK) Consortium	Sergi Castellano, Rachel Williams, Mark Kristiansen, Paola Resende Silva, Sunando Roy, Tony Brooks, Helena Tutill, Paola Niola, Patricia Dyal, Charlotte Williams, Leysa Forrest, Yasmin Panchbhaya, Jacqueline Findlay, Samuel Weeks, Julianne Brown, Kathryn Harris, Paul Randell, James Price, Alison Holmes, Judith Breuer
EPI_ISL_825597, EPI_ISL_825598, EPI_ISL_825599, EPI_ISL_825600, EPI_ISL_825601, EPI_ISL_825602, EPI_ISL_825603, EPI_ISL_825604	Respiratory Virus Unit, National Infection Service, Public Health England	COVID-19 Genomics UK (COG-UK) Consortium	PHE Covid Sequencing Team
EPI_ISL_838281	Virology Department, Royal Infirmary of Edinburgh, NHS Lothian / School of Biological Sciences, University of Edinburgh / Institute of Genetics and Molecular Medicine, University of Edinburgh	COVID-19 Genomics UK (COG-UK) Consortium	McHugh M, Dewar R, Rooke S, Gallagher M, Balcaza C, O'Toole Á, Scher E, Hill V, McCrone JT, Colquhoun R, Yu X, Jackson B, Rambaut A, Williams TC, Templeton K
EPI_ISL_838344, EPI_ISL_838345, EPI_ISL_838349	Queens Medical Centre, Clinical Microbiology Department / DeepSeq Nottingham	COVID-19 Genomics UK (COG-UK) Consortium	Gemma Clark, Wendy Smith, Manjinder Khakh, Vicki M Fleming, Michelle M Lister, Hannah Howson-Wells, Jonathan Ball, Patrick McClure, Joseph Chappell, Theocharis Tsoleridis, Nadine Holmes, Matthew Carlisle, Christopher Moore, Fei Sang, Johnny Debebe, Victoria Wright, Matthew Loose
EPI_ISL_840186, EPI_ISL_840187, EPI_ISL_840188, EPI_ISL_840189, EPI_ISL_840190, EPI_ISL_840191, EPI_ISL_840192, EPI_ISL_840193, EPI_ISL_840194, EPI_ISL_840195, EPI_ISL_840196, EPI_ISL_840197, EPI_ISL_840198, EPI_ISL_840199, EPI_ISL_840204, EPI_ISL_840205, EPI_ISL_840206, EPI_ISL_840207			
see above	Oxford Viromics, NDM, University of Oxford; Oxford University Hospitals; Basingstoke and North Hampshire Hospital	COVID-19 Genomics UK (COG-UK) Consortium	Tanya Golubchik, David Bonsall, George Macintyre, Amy Trebes, Mariateresa de Cesare, Catrin Moore, Alex Mobbs, Anita Justice, Robert Shaw, Monique Andersson, Timothy Peto, Emma Wise, Nathan Moore, Jessica Lynch, Nick Cortes, Matilde Mori, Stephen Kidd, David Buck, John Todd, Christophe Fraser
EPI_ISL_842204	Virology Department, Sheffield Teaching Hospitals NHS Foundation Trust/Department of Infection, Immunity and Cardiovascular Disease, The Medical School, University of Sheffield	COVID-19 Genomics UK (COG-UK) Consortium	Thushan de Silva, Matthew Parker, Nikki Smith, Adri Angyal, Rebecca Brown, Luke Green, Rachel Tucker, Paul Parsons, Danielle Groves, Katie Johnson, Laura Carrilero, Alex Keeley, Dave Partridge, Matthew Wyles, Benjamin Lindsey, Mehmet Yavuz, Mohammad Raza, Cariad Evans
EPI_ISL_842910, EPI_ISL_842911, EPI_ISL_842912, EPI_ISL_842913, EPI_ISL_842914, EPI_ISL_842915, EPI_ISL_842916, EPI_ISL_842923, EPI_ISL_842924, EPI_ISL_842925, EPI_ISL_842926, EPI_ISL_842927, EPI_ISL_842928, EPI_ISL_842929, EPI_ISL_842930, EPI_ISL_842931, EPI_ISL_842943, EPI_ISL_842944, EPI_ISL_842945, EPI_ISL_842946, EPI_ISL_842947, EPI_ISL_842948, EPI_ISL_842949, EPI_ISL_842963			
see above	Barts Health NHS Trust	COVID-19 Genomics UK (COG-UK) Consortium	CUTINO-MOGUEL, Maria-Teresa; HARRINGTON, David; OWOYEMI, Dola; SHYLINI, Raghavendran; BROAD, Claire; KELE, Beatrix
EPI_ISL_859555, EPI_ISL_859557,	Lighthouse Lab in Cambridge	Wellcome Sanger Institute for the COVID-19 Genomics UK	Rob Howes, The Lighthouse Lab in Cambridge and Alex Alderton, Roberto Amato, Sonia Goncalves, Ewan Harrison, David K. Jackson, Ian Johnston,

EPI_ISL_859560		(COG-UK) Consortium	Dominic Kwiatkowski, Cordelia Langford, John Sillitoe on behalf of the Wellcome Sanger Institute COVID-19 Surveillance Team
EPI_ISL_865172, EPI_ISL_865173	Liverpool Clinical Laboratories	COVID-19 Genomics UK (COG-UK) Consortium	Sam Haldenby, Anita Lucaci, Steve Paterson, Julian Hiscox, Alistair Darby, M Almsaud, A Alrezaihi, Muhannad Alruwaili, Stuart D Armstrong, Jones Benjamin, Eleanor G Bentley, Anu Chawla, Jordan J Clark, Angela Cowell, Richard Eccles, Isabel García-Dorival, Matthew Gemmell, Alessandro Gerada, PKF Gilmore, Richard Gregory, Ximeng Han, Catherine Hartley, Margaret Hughes, Miren Iturriza-Gomara, James Johnson, L Luu, Jenifer Manson, Charlotte Nelson, Elaine O'Toole, Cassie Olateju, Rebekah Penrice-Randal , Lucille Rainbow, N.P Randle, Trevor Ian Robinson, Parul Sharma, Ghada T Shawli, James P Stewart, Neil Swainston, Ecaterina Vamos, Joanne Watts, Mark Whitehead
EPI_ISL_865683, EPI_ISL_865685, EPI_ISL_865697, EPI_ISL_865698, EPI_ISL_865699, EPI_ISL_865700, EPI_ISL_865701	University College London, Great Ormond Street Hospital for Children NHS Foundation Trust, Imperial College Healthcare NHS Trust	COVID-19 Genomics UK (COG-UK) Consortium	Sergi Castellano, Rachel Williams, Mark Kristiansen, Paola Resende Silva, Sunando Roy, Tony Brooks, Helena Tutill, Paola Niola, Patricia Dyal, Charlotte Williams, Leysa Forrest, Yasmin Panchbhaya, Jacqueline Findlay, Samuel Weeks, Julianne Brown, Kathryn Harris, Paul Randell, James Price, Alison Holmes, Judith Breuer
EPI_ISL_866054	University College London Hospital	COVID-19 Genomics UK (COG-UK) Consortium	Judith Heaney, Matthew Byott, Catherine Houlihan, Dan Frampton, Stuart Kirk, Moira Spyer and Eleni Nastouli
EPI_ISL_866805	Quadram Institute Bioscience	COVID-19 Genomics UK (COG-UK) Consortium	Dave J. Baker, Gemma L. Kay, Alp Aydin, Thanh Le-Viet, Steven Rudder, Ana P. Tedim, Anastasia Kolyva, Maria Diaz, Leonardo de Oliveira Martins, Nabil-Fareed Alikhan, Lizzie Meadows, Rachael Stanley, Ngozi Elumogo, Muhammed Yasir, Nicholas M. Thomson, Alexander J Trotter, Rachel Gilroy, Samuel Bloomfield, Claire Stuart, Andrew Bell, Reenesh Prakash, Samir Dervisevic, Alison E. Mather, John Wain, Mark Webber, Andrew J. Page, Justin O'Grady
EPI_ISL_868758, EPI_ISL_868759, EPI_ISL_868761, EPI_ISL_868762, EPI_ISL_868764, EPI_ISL_868765, EPI_ISL_868767, EPI_ISL_868768, EPI_ISL_868769, EPI_ISL_868771, EPI_ISL_868774, EPI_ISL_868775, EPI_ISL_868777, EPI_ISL_868778, EPI_ISL_868779, EPI_ISL_868785, EPI_ISL_868787			
see above	Bioinformatics and Biostatistics Lab, Advanced Sequencing Facility	COVID-19 Genomics UK (COG-UK) Consortium	Aengus Stewart,Jerome Nicod,Chelsea Sawyer,Laura Cubitt,Harshil Patel,Margaret Crawford
EPI_ISL_915440, EPI_ISL_915443, EPI_ISL_915445, EPI_ISL_915451, EPI_ISL_915453, EPI_ISL_915465, EPI_ISL_915469, EPI_ISL_915470, EPI_ISL_915480, EPI_ISL_915485, EPI_ISL_915488, EPI_ISL_915495, EPI_ISL_915500, EPI_ISL_915512, EPI_ISL_915515, EPI_ISL_915528, EPI_ISL_915529, EPI_ISL_915533, EPI_ISL_915534, EPI_ISL_915539, EPI_ISL_915543, EPI_ISL_915558, EPI_ISL_915565, EPI_ISL_915574, EPI_ISL_915587, EPI_ISL_915589, EPI_ISL_915595, EPI_ISL_915603, EPI_ISL_915619, EPI_ISL_915621, EPI_ISL_915624, EPI_ISL_915625, EPI_ISL_915633, EPI_ISL_915635, EPI_ISL_915649, EPI_ISL_915650, EPI_ISL_915656, EPI_ISL_915657, EPI_ISL_915658, EPI_ISL_915659, EPI_ISL_915666, EPI_ISL_915669, EPI_ISL_915671, EPI_ISL_915675, EPI_ISL_915682, EPI_ISL_915684, EPI_ISL_915686, EPI_ISL_915688, EPI_ISL_915691, EPI_ISL_915692, EPI_ISL_915693, EPI_ISL_915702, EPI_ISL_915706, EPI_ISL_915724, EPI_ISL_915735, EPI_ISL_915737, EPI_ISL_915740, EPI_ISL_915741, EPI_ISL_915744, EPI_ISL_915745, EPI_ISL_915747, EPI_ISL_915759, EPI_ISL_915773, EPI_ISL_915775, EPI_ISL_915776, EPI_ISL_915779, EPI_ISL_915783, EPI_ISL_915784, EPI_ISL_915788, EPI_ISL_915789, EPI_ISL_915792, EPI_ISL_915793, EPI_ISL_915794, EPI_ISL_915799, EPI_ISL_915801, EPI_ISL_915806, EPI_ISL_915807, EPI_ISL_915812, EPI_ISL_915816, EPI_ISL_915819, EPI_ISL_915822, EPI_ISL_915827, EPI_ISL_915828, EPI_ISL_915834, EPI_ISL_915835, EPI_ISL_915837, EPI_ISL_915838, EPI_ISL_915839, EPI_ISL_915841, EPI_ISL_915842, EPI_ISL_915844, EPI_ISL_915845, EPI_ISL_915852, EPI_ISL_915856, EPI_ISL_915857, EPI_ISL_915858, EPI_ISL_915860, EPI_ISL_915866, EPI_ISL_915875, EPI_ISL_915877, EPI_ISL_915880, EPI_ISL_915890, EPI_ISL_915896, EPI_ISL_915898, EPI_ISL_915900, EPI_ISL_915901, EPI_ISL_915907, EPI_ISL_915908, EPI_ISL_915910, EPI_ISL_915912, EPI_ISL_915913, EPI_ISL_915914, EPI_ISL_915915, EPI_ISL_915916, EPI_ISL_915919, EPI_ISL_915921, EPI_ISL_915926, EPI_ISL_915927, EPI_ISL_915931, EPI_ISL_915932, EPI_ISL_915933, EPI_ISL_915934, EPI_ISL_915937, EPI_ISL_915940, EPI_ISL_915941, EPI_ISL_915944, EPI_ISL_915946, EPI_ISL_915954, EPI_ISL_915955, EPI_ISL_915956, EPI_ISL_915960, EPI_ISL_915968, EPI_ISL_915970, EPI_ISL_915971, EPI_ISL_915973, EPI_ISL_915974, EPI_ISL_915976, EPI_ISL_915978, EPI_ISL_915980, EPI_ISL_915982, EPI_ISL_915984, EPI_ISL_915985, EPI_ISL_915986, EPI_ISL_915987, EPI_ISL_915988, EPI_ISL_915990, EPI_ISL_915991, EPI_ISL_915996, EPI_ISL_916000, EPI_ISL_916011, EPI_ISL_916012, EPI_ISL_916016, EPI_ISL_916017, EPI_ISL_916019, EPI_ISL_916023, EPI_ISL_916034, EPI_ISL_916036, EPI_ISL_916044, EPI_ISL_916047, EPI_ISL_916050, EPI_ISL_916051, EPI_ISL_916055, EPI_ISL_916058, EPI_ISL_916060, EPI_ISL_916067, EPI_ISL_916068, EPI_ISL_916069, EPI_ISL_916070, EPI_ISL_916071, EPI_ISL_916072, EPI_ISL_916073, EPI_ISL_916074, EPI_ISL_916075, EPI_ISL_916076, EPI_ISL_916077, EPI_ISL_916078, EPI_ISL_916079, EPI_ISL_916080, EPI_ISL_916081, EPI_ISL_916082, EPI_ISL_916083, EPI_ISL_916084, EPI_ISL_916085, EPI_ISL_916086, EPI_ISL_916087, EPI_ISL_916088, EPI_ISL_916089, EPI_ISL_916090, EPI_ISL_916091, EPI_ISL_916092, EPI_ISL_916093, EPI_ISL_916094, EPI_ISL_916095, EPI_ISL_916096, EPI_ISL_916097, EPI_ISL_916098, EPI_ISL_916099, EPI_ISL_916100, EPI_ISL_916101, EPI_ISL_916102, EPI_ISL_916103, EPI_ISL_916104, EPI_ISL_916105, EPI_ISL_916106, EPI_ISL_916107, EPI_ISL_916108, EPI_ISL_916109, EPI_ISL_916110, EPI_ISL_916111, EPI_ISL_916112, EPI_ISL_916113, EPI_ISL_916114, EPI_ISL_916115, EPI_ISL_916116, EPI_ISL_916117, EPI_ISL_916118, EPI_ISL_916120, EPI_ISL_916121, EPI_ISL_916122, EPI_ISL_916123, EPI_ISL_916124, EPI_ISL_916125, EPI_ISL_916126, EPI_ISL_916127, EPI_ISL_916128, EPI_ISL_916129, EPI_ISL_916130, EPI_ISL_916131, EPI_ISL_916132, EPI_ISL_916133, EPI_ISL_916134, EPI_ISL_916135, EPI_ISL_916136, EPI_ISL_916137, EPI_ISL_916138, EPI_ISL_916139, EPI_ISL_916140, EPI_ISL_916141, EPI_ISL_916142, EPI_ISL_916143, EPI_ISL_916144, EPI_ISL_916145, EPI_ISL_916146, EPI_ISL_916147, EPI_ISL_916148, EPI_ISL_916149, EPI_ISL_916150, EPI_ISL_916152, EPI_ISL_916153, EPI_ISL_916154, EPI_ISL_916155, EPI_ISL_916156, EPI_ISL_916157, EPI_ISL_916158, EPI_ISL_916159, EPI_ISL_916160, EPI_ISL_916161, EPI_ISL_916162, EPI_ISL_916163, EPI_ISL_916165, EPI_ISL_916166, EPI_ISL_916167, EPI_ISL_916168, EPI_ISL_916169, EPI_ISL_916170, EPI_ISL_916171, EPI_ISL_916172, EPI_ISL_916173, EPI_ISL_916174, EPI_ISL_916175, EPI_ISL_916176, EPI_ISL_916177, EPI_ISL_916178, EPI_ISL_916179			
see above	Lighthouse Lab in Alderley Park	Wellcome Sanger Institute for the COVID-19 Genomics UK (COG-UK) Consortium	Jacquelyn Wynn, Mairead Hyland, The Lighthouse Lab in Alderley Park and Alex Alderton, Roberto Amato, Sonia Goncalves, Ewan Harrison, David K. Jackson, Ian Johnston, Dominic Kwiatkowski, Cordelia Langford, John Sillitoe on behalf of the Wellcome Sanger Institute COVID-19 Surveillance Team
EPI_ISL_918564, EPI_ISL_918565, EPI_ISL_918566, EPI_ISL_918567, EPI_ISL_918568, EPI_ISL_918585, EPI_ISL_918586, EPI_ISL_918587, EPI_ISL_918588, EPI_ISL_918589, EPI_ISL_918590, EPI_ISL_918591, EPI_ISL_918592, EPI_ISL_918593, EPI_ISL_918665			
see above	University of Birmingham	COVID-19 Genomics UK (COG-UK) Consortium	Institute of Microbiology, University of Birmingham: Claire McMurray, Joanne Stockton, Samuel Nicholls, Radoslaw Poplawski, Will Rowe, Josh Quick, Nicholas Loman. University of Birmingham Testing Laboratory: Celina M Whalley, Andrew Bosworth, Charlotte Poxon, Kasun Wanigasooriya, Oliver Pickles, Mike Kidd, Alex Richter, Andrew D Beggs PHE Heartlands Lab: Husam Osman, Andrew Bosworth. Queen Elizabeth Hospital: Anna Casey
EPI_ISL_920268, EPI_ISL_920278, EPI_ISL_920355, EPI_ISL_920364, EPI_ISL_920376, EPI_ISL_920482, EPI_ISL_920517, EPI_ISL_920577	University College London Hospital	COVID-19 Genomics UK (COG-UK) Consortium	Judith Heaney, Matthew Byott, Catherine Houlihan, Dan Frampton, Stuart Kirk, Moira Spyer and Eleni Nastouli
EPI_ISL_924784, EPI_ISL_924785, EPI_ISL_924786, EPI_ISL_924789, EPI_ISL_924791	Bioinformatics and Biostatistics Lab, Advanced Sequencing Facility	COVID-19 Genomics UK (COG-UK) Consortium	Aengus Stewart,Jerome Nicod,Chelsea Sawyer,Laura Cubitt,Harshil Patel,Margaret Crawford
EPI_ISL_933495	Lighthouse Lab in Milton Keynes	Wellcome Sanger Institute for the COVID-19 Genomics UK (COG-UK) Consortium	The Lighthouse Lab in Milton Keynes and Alex Alderton, Roberto Amato, Sonia Goncalves, Ewan Harrison, David K. Jackson, Ian Johnston, Dominic Kwiatkowski, Cordelia Langford, John Sillitoe on behalf of the Wellcome Sanger Institute COVID-19 Surveillance Team
EPI_ISL_949347, EPI_ISL_949348, EPI_ISL_949349, EPI_ISL_949350, EPI_ISL_949351, EPI_ISL_949352, EPI_ISL_949353, EPI_ISL_949354, EPI_ISL_949355, EPI_ISL_949356, EPI_ISL_949357, EPI_ISL_949358, EPI_ISL_949359, EPI_ISL_949360, EPI_ISL_949361, EPI_ISL_949362, EPI_ISL_949363, EPI_ISL_949364, EPI_ISL_949365, EPI_ISL_949366, EPI_ISL_949367, EPI_ISL_949368, EPI_ISL_949369, EPI_ISL_949370, EPI_ISL_949371, EPI_ISL_949372, EPI_ISL_949373, EPI_ISL_949374, EPI_ISL_949375, EPI_ISL_949376, EPI_ISL_949377, EPI_ISL_949378, EPI_ISL_949379, EPI_ISL_949380, EPI_ISL_949381, EPI_ISL_949382, EPI_ISL_949383, EPI_ISL_949384, EPI_ISL_949385, EPI_ISL_949386, EPI_ISL_949387, EPI_ISL_949388, EPI_ISL_949389, EPI_ISL_949390			
see above	University of Birmingham	COVID-19 Genomics UK (COG-UK) Consortium	Institute of Microbiology, University of Birmingham: Claire McMurray, Joanne Stockton, Samuel Nicholls, Radoslaw Poplawski, Will Rowe, Josh Quick, Nicholas Loman. University of Birmingham Testing Laboratory: Celina M Whalley, Andrew Bosworth, Charlotte Poxon, Kasun Wanigasooriya, Oliver Pickles, Mike Kidd, Alex Richter, Andrew D Beggs PHE Heartlands Lab: Husam Osman, Andrew Bosworth. Queen Elizabeth Hospital: Anna Casey
EPI_ISL_952428, EPI_ISL_952431	Centre for Enzyme Innovation, University of Portsmouth / Translational Research Laboratory, Portsmouth Hospitals NHS Trust	COVID-19 Genomics UK (COG-UK) Consortium	Angela Beckett,Salman Goudarzi,Christopher Fearn,Kate Cook,Katie Loveson,Sharon Glaysher,Scott Elliott,Samuel Robson
EPI_ISL_991311, EPI_ISL_991315, EPI_ISL_991317, EPI_ISL_991320, EPI_ISL_991324, EPI_ISL_991325, EPI_ISL_991326, EPI_ISL_991329, EPI_ISL_991334, EPI_ISL_991336, EPI_ISL_991337, EPI_ISL_991342			
see above	Lighthouse Lab in Alderley Park	Wellcome Sanger Institute for the COVID-19 Genomics UK (COG-UK) Consortium	Jacquelyn Wynn, Mairead Hyland, The Lighthouse Lab in Alderley Park and Alex Alderton, Roberto Amato, Sonia Goncalves, Ewan Harrison, David K. Jackson, Ian Johnston, Dominic Kwiatkowski, Cordelia Langford, John Sillitoe on behalf of the Wellcome Sanger Institute COVID-19 Surveillance Team
EPI_ISL_997313, EPI_ISL_997314, EPI_ISL_997320, EPI_ISL_997321	University of Exeter	COVID-19 Genomics UK (COG-UK) Consortium	Ben Temperton,Aaron Jeffries,Michelle Michelsen,Joanna Warwick-Dugdale,Audrey Farbos,Robyn Manley,Stephen Michell,Jane Masoli
