# Supplementary material for: Inferring the Association between the Risk of COVID-19 Case Fatality and N501Y Substitution in SARS-CoV-2
Source: Viruses. 2021 Apr 8;13(4):638. doi: 10.3390/v13040638 (PMC8070306; doi:10.3390/v13040638)
Supplement: Supplementary file 1 [file viruses-13-00638-s001.zip › gisaid_hcov-19_UKAT_201228-210101.pdf]

We gratefully acknowledge the following Authors from the Originating laboratories responsible for obtaining the specimens, as well as the Submitting laboratories where the genome data were generated and shared via GISAID, on which this research is based.

All Submitters of data may be contacted directly via www.gisaid.org

Authors are sorted alphabetically.

Accession ID	Originating Laboratory	Submitting Laboratory	Authors
EPI_ISL_1000023	Wales Specialist Virology Centre Sequencing lab: Pathogen Genomics Unit	Public Health Wales Microbiology Cardiff Wales Specialist Virology Centre	Catherine Moore, Johnathan Evans, Laura Gifford, Malorie Perry, Simon Cottrell, Angela Marchbank, Alec Birchley, Alexander Adams, Amy Gaskin, Bree Gatica-Wilcox, Jason Coombes, Joel Southgate, Lauren Gilbert, Lee Graham, Nicole Pacchiarini, Sara Kumziene-Summerhayes, Sarah Taylor, Sophie Jones, Sara Rey, Matthew Bull, Joanne Watkins, Sally Corden, Tom Connor
EPI_ISL_1000237, EPI_ISL_1000238, EPI_ISL_1000239, EPI_ISL_1000255, EPI_ISL_1000264, EPI_ISL_1000266, EPI_ISL_1000275, EPI_ISL_1000276, EPI_ISL_1000279, EPI_ISL_1000283, EPI_ISL_1000284, EPI_ISL_1000285, EPI_ISL_1000286, EPI_ISL_1000287, EPI_ISL_1000325, EPI_ISL_1000326, EPI_ISL_1000327, EPI_ISL_1000328, EPI_ISL_1000329, EPI_ISL_1000330, EPI_ISL_1000332, EPI_ISL_1000333, EPI_ISL_1000334, EPI_ISL_1000335, EPI_ISL_1000337, EPI_ISL_1000338, EPI_ISL_1000339, EPI_ISL_1000340, EPI_ISL_1000341, EPI_ISL_1000342, EPI_ISL_1000343, EPI_ISL_1000344, EPI_ISL_1000345, EPI_ISL_1000346, EPI_ISL_1000347, EPI_ISL_1000348, EPI_ISL_1000349, EPI_ISL_1000350, EPI_ISL_1000351, EPI_ISL_1000352, EPI_ISL_1000353, EPI_ISL_1000354, EPI_ISL_1000355, EPI_ISL_1000356, EPI_ISL_1000357, EPI_ISL_1000358, EPI_ISL_1000359, EPI_ISL_1000360, EPI_ISL_1000361, EPI_ISL_1000362, EPI_ISL_1000363, EPI_ISL_1000364, EPI_ISL_1000365, EPI_ISL_1000366, EPI_ISL_1000367, EPI_ISL_1000368, EPI_ISL_1000369, EPI_ISL_1000370, EPI_ISL_1000371, EPI_ISL_1000372, EPI_ISL_1000373, EPI_ISL_1000374, EPI_ISL_1000375, EPI_ISL_1000376, EPI_ISL_1000377, EPI_ISL_1000378, EPI_ISL_1000379, EPI_ISL_1000380, EPI_ISL_1000381, EPI_ISL_1000382, EPI_ISL_1000383, EPI_ISL_1000384, EPI_ISL_1000385, EPI_ISL_1000386, EPI_ISL_1000387, EPI_ISL_1000388, EPI_ISL_1000389, EPI_ISL_1000390, EPI_ISL_1000391, EPI_ISL_1000392, EPI_ISL_1000393, EPI_ISL_1000394, EPI_ISL_1000395, EPI_ISL_1000396, EPI_ISL_1000397, EPI_ISL_1000398, EPI_ISL_1000399, EPI_ISL_1000400, EPI_ISL_1000401, EPI_ISL_1000402, EPI_ISL_1000403, EPI_ISL_1000478, EPI_ISL_1000479, EPI_ISL_1000480, EPI_ISL_1000481, EPI_ISL_1000484, EPI_ISL_1000485, EPI_ISL_1000486, EPI_ISL_1000487, EPI_ISL_1000488, EPI_ISL_1000489, EPI_ISL_1000490, EPI_ISL_1000491, EPI_ISL_1000492, EPI_ISL_1000493, EPI_ISL_1000494, EPI_ISL_1000495, EPI_ISL_1000496, EPI_ISL_1000497, EPI_ISL_1000498, EPI_ISL_1000499, EPI_ISL_1000500, EPI_ISL_1000501, EPI_ISL_1000502, EPI_ISL_1000503, EPI_ISL_1000504, EPI_ISL_1000505, EPI_ISL_1000506, EPI_ISL_1000507, EPI_ISL_1000508, EPI_ISL_1000509, EPI_ISL_1000510, EPI_ISL_1000511, EPI_ISL_1000512, EPI_ISL_1000513, EPI_ISL_1000514, EPI_ISL_1000515, EPI_ISL_1000516, EPI_ISL_1000517, EPI_ISL_1000518, EPI_ISL_1000520, EPI_ISL_1000521, EPI_ISL_1000522, EPI_ISL_1000523, EPI_ISL_1000524, EPI_ISL_1000526, EPI_ISL_1000527, EPI_ISL_1000528, EPI_ISL_1000529, EPI_ISL_1000530, EPI_ISL_1000531, EPI_ISL_1000532, EPI_ISL_1000534, EPI_ISL_1000535, EPI_ISL_1000536, EPI_ISL_1000537, EPI_ISL_1000538, EPI_ISL_1000539, EPI_ISL_1000540, EPI_ISL_1000541, EPI_ISL_1000543, EPI_ISL_1000544, EPI_ISL_1000545, EPI_ISL_1000547, EPI_ISL_1000548, EPI_ISL_1000549, EPI_ISL_1000550, EPI_ISL_1000551, EPI_ISL_1000552, EPI_ISL_1000553, EPI_ISL_1000554, EPI_ISL_1000555, EPI_ISL_1000556, EPI_ISL_1000557, EPI_ISL_1000558, EPI_ISL_1000559, EPI_ISL_1000560, EPI_ISL_1000561, EPI_ISL_1000562, EPI_ISL_1000563, EPI_ISL_1000564, EPI_ISL_1000565, EPI_ISL_1000566, EPI_ISL_1000567, EPI_ISL_1000568, EPI_ISL_1000569, EPI_ISL_1000570, EPI_ISL_1000571, EPI_ISL_1000572, EPI_ISL_1000574, EPI_ISL_1000575, EPI_ISL_1000577, EPI_ISL_1000578, EPI_ISL_1000579, EPI_ISL_1000580, EPI_ISL_1000581, EPI_ISL_1000582, EPI_ISL_1000583, EPI_ISL_1000584, EPI_ISL_1000585, EPI_ISL_1000586, EPI_ISL_1000587, EPI_ISL_1000588, EPI_ISL_1000589, EPI_ISL_1000590, EPI_ISL_1000591, EPI_ISL_1000592, EPI_ISL_1000594			
see above	Centre for Enzyme Innovation, University of Portsmouth / Translational Research Laboratory, Portsmouth Hospitals NHS Trust	COVID-19 Genomics UK (COG-UK) Consortium	Angela Beckett,Salman Goudarzi,Christopher Fearn,Kate Cook,Katie Loveson,Sharon Glaysher,Scott Elliott,Samuel Robson
EPI_ISL_1047015, EPI_ISL_1047065	University of Birmingham	COVID-19 Genomics UK (COG-UK) Consortium	Institute of Microbiology, University of Birmingham: Claire McMurray, Joanne Stockton, Samuel Nicholls, Radoslaw Poplawski, Will Rowe, Josh Quick, Nicholas Loman. University of Birmingham Testing Laboratory: Celina M Whalley, Andrew Bosworth, Charlotte Poxon, Kasun Wanigasooriya, Oliver Pickles, Mike Kidd, Alex Richter, Andrew D Beggs PHE Heartlands Lab: Husam Osman, Andrew Bosworth. Queen Elizabeth Hospital: Anna Casey
EPI_ISL_1047071, EPI_ISL_1047078, EPI_ISL_1047079, EPI_ISL_1047085, EPI_ISL_1047089, EPI_ISL_1047099	Department of Pathology, University of Cambridge	COVID-19 Genomics UK (COG-UK) Consortium	Aminu S. Jahun, Yasmin Chaudhry, Iliana Georgana, Myra Hosmillo, Rhys Izuagbe, William L. Hamilton, Martin D. Curran, Surendra Parmar, Ian Goodfellow
EPI_ISL_1047930, EPI_ISL_1047932, EPI_ISL_1047933, EPI_ISL_1047934, EPI_ISL_1047935, EPI_ISL_1047936, EPI_ISL_1047937, EPI_ISL_1047950	University Hospitals Of Leicester NHS Trust and DeepSeq Nottingham	COVID-19 Genomics UK (COG-UK) Consortium	Christopher Holmes, Paul Bird, Thomas Helmer, Karlie Fallon, Julian Tang, Jonathan Ball, Patrick McClure, Joeseph Chappell, Nadine Holmes, Matthew Carlisle, Christopher Moore, Fei Sang, Johnny Debebe, Victoria Wright, Matthew Loose
EPI_ISL_1048050	University College London, Great Ormond Street Hospital for Children NHS Foundation Trust, Imperial College Healthcare NHS Trust	COVID-19 Genomics UK (COG-UK) Consortium	Sergi Castellano, Rachel Williams, Mark Kristiansen, Paola Resende Silva, Sunando Roy, Tony Brooks, Helena Tutill, Paola Niola, Patricia Dyal, Charlotte Williams, Leysa Forrest, Yasmin Panchbhaya, Jacqueline Findlay, Samuel Weeks, Julianne Brown, Kathryn Harris, Paul Randell, James Price, Alison Holmes, Judith Breuer
EPI_ISL_1048067	Barts Health NHS Trust	COVID-19 Genomics UK (COG-UK) Consortium	CUTINO-MOGUEL, Maria-Teresa; HARRINGTON, David; OWOYEMI, Dola; KULASEGARAN-SHYLINI, Raghavendran; BROAD, Claire; KELE, Beatrix
EPI_ISL_1050245, EPI_ISL_1050246, EPI_ISL_1050247, EPI_ISL_1050248, EPI_ISL_1050249, EPI_ISL_1050250, EPI_ISL_1050251, EPI_ISL_1050252, EPI_ISL_1050253, EPI_ISL_1050254, EPI_ISL_1050255, EPI_ISL_1050256, EPI_ISL_1050257, EPI_ISL_1050258, EPI_ISL_1050259, EPI_ISL_1050260, EPI_ISL_1050261, EPI_ISL_1050262, EPI_ISL_1050263, EPI_ISL_1050264, EPI_ISL_1050265, EPI_ISL_1050266, EPI_ISL_1050267, EPI_ISL_1050268, EPI_ISL_1050269, EPI_ISL_1050270, EPI_ISL_1050271, EPI_ISL_1050272, EPI_ISL_1050273, EPI_ISL_1050274, EPI_ISL_1050275, EPI_ISL_1050276, EPI_ISL_1050277, EPI_ISL_1050278, EPI_ISL_1050279, EPI_ISL_1050280, EPI_ISL_1050281, EPI_ISL_1050282, EPI_ISL_1050283, EPI_ISL_1050284, EPI_ISL_1050285, EPI_ISL_1050286, EPI_ISL_1050287, EPI_ISL_1050288, EPI_ISL_1050289, EPI_ISL_1050290, EPI_ISL_1050291, EPI_ISL_1050292, EPI_ISL_1050307, EPI_ISL_1050308, EPI_ISL_1050309, EPI_ISL_1050310, EPI_ISL_1050311, EPI_ISL_1050312, EPI_ISL_1050313, EPI_ISL_1050314, EPI_ISL_1050315, EPI_ISL_1050316, EPI_ISL_1050317, EPI_ISL_1050318, EPI_ISL_1050319, EPI_ISL_1050320, EPI_ISL_1050321, EPI_ISL_1050322, EPI_ISL_1050325, EPI_ISL_1050348, EPI_ISL_1050349, EPI_ISL_1050350			
see above	University of Exeter	COVID-19 Genomics UK (COG-UK) Consortium	Ben Temperton,Aaron Jeffries,Michelle Michelsen,Joanna Warwick-Dugdale,Audrey Farbos,Robyn Manley,Stephen Michell,Jane Masoli
EPI_ISL_1050920, EPI_ISL_1050921, EPI_ISL_1050923, EPI_ISL_1050924, EPI_ISL_1050926, EPI_ISL_1050927, EPI_ISL_1050929, EPI_ISL_1050930, EPI_ISL_1050932, EPI_ISL_1050933, EPI_ISL_1050934, EPI_ISL_1050935, EPI_ISL_1050936, EPI_ISL_1050937, EPI_ISL_1050938, EPI_ISL_1050939, EPI_ISL_1050940, EPI_ISL_1050942, EPI_ISL_1050947, EPI_ISL_1050948, EPI_ISL_1050967, EPI_ISL_1050971, EPI_ISL_1050972, EPI_ISL_1050973, EPI_ISL_1050975, EPI_ISL_1050976, EPI_ISL_1050977, EPI_ISL_1050978, EPI_ISL_1050979, EPI_ISL_1050980, EPI_ISL_1050981, EPI_ISL_1050982, EPI_ISL_1050983, EPI_ISL_1050984, EPI_ISL_1050985, EPI_ISL_1050986, EPI_ISL_1050987, EPI_ISL_1050988, EPI_ISL_1050989, EPI_ISL_1050996, EPI_ISL_1050997, EPI_ISL_1050998, EPI_ISL_1050999, EPI_ISL_1051000, EPI_ISL_1051006, EPI_ISL_1051018, EPI_ISL_1051020, EPI_ISL_1051021, EPI_ISL_1051028, EPI_ISL_1051029, EPI_ISL_1051030, EPI_ISL_1051039			
see above	Northumbria University / South Tees Hospitals NHS Foundation Trust / North Cumbria Integrated Care NHS Foundation Trust / North Tees and Hartlepool NHS Foundation Trust / Newcastle Hospitals NHS Foundation Trust	COVID-19 Genomics UK (COG-UK) Consortium	Darren L Smith,Andrew Nelson,Matthew Bashton,Greg R Young,Joshua Loh,John Allan,Mohammad A Tariq,Giles S Holt,Gary Black,Wen C Yew,Lynn Dover,Paul Baker,Steve Liggett,Sarah Essex,Jane Greenaway,Debra Padgett,Clive Graham,Garren Scott,Edward Barton,Emma Swindells,Brendan Payne,Jennifer Collins,Yusri Taha,Gary Eltringham
EPI_ISL_1051440, EPI_ISL_1051446, EPI_ISL_1051470, EPI_ISL_1051498, EPI_ISL_1051505, EPI_ISL_1051508, EPI_ISL_1051525, EPI_ISL_1051526, EPI_ISL_1051529	Quadram Institute Bioscience	COVID-19 Genomics UK (COG-UK) Consortium	Dave J. Baker, Gemma L. Kay, Alp Aydin, Thanh Le-Viet, Steven Rudder, Ana P. Tedim, Anastasia Kolyva, Maria Diaz, Leonardo de Oliveira Martins, Nabil-Fareed Alikhan, Lizzie Meadows, Rachael Stanley, Ngozi Elumogo, Muhammed Yasir, Nicholas M. Thomson, Alexander J Trotter, Rachel Gilroy, Samuel Bloomfield, Claire Stuart, Andrew Bell, Reenesh Prakash, Samir Dervisevic, Alison E. Mather, John Wain, Mark Webber, Andrew J. Page, Justin O'Grady
EPI_ISL_1053526	Originating lab: Wales Specialist Virology Centre Sequencing lab: Pathogen Genomics Unit	Public Health Wales Microbiology Cardiff Wales Specialist Virology Centre	Catherine Moore, Johnathan Evans, Laura Gifford, Malorie Perry, Simon Cottrell, Angela Marchbank, Alec Birchley, Alexander Adams, Amy Gaskin, Bree Gatica-Wilcox, Jason Coombes, Joel Southgate, Lauren Gilbert, Lee Graham, Nicole Pacchiarini, Sara Kumziene-Summerhayes, Sarah Taylor, Sophie Jones, Sara Rey, Matthew Bull, Joanne Watkins, Sally Corden, Tom Connor
EPI_ISL_1053754, EPI_ISL_1053755, EPI_ISL_1053756, EPI_ISL_1053757, EPI_ISL_1053758, EPI_ISL_1053759, EPI_ISL_1053760, EPI_ISL_1053761, EPI_ISL_1053762, EPI_ISL_1053763, EPI_ISL_1053764, EPI_ISL_1053765, EPI_ISL_1053766, EPI_ISL_1053767, EPI_ISL_1053768, EPI_ISL_1053769, EPI_ISL_1053770, EPI_ISL_1053771, EPI_ISL_1053772, EPI_ISL_1053773, EPI_ISL_1053774, EPI_ISL_1053775, EPI_ISL_1053776, EPI_ISL_1053777, EPI_ISL_1053778, EPI_ISL_1053779, EPI_ISL_1053780, EPI_ISL_1053781, EPI_ISL_1053782, EPI_ISL_1053783, EPI_ISL_1053784, EPI_ISL_1053785, EPI_ISL_1053786, EPI_ISL_1053787, EPI_ISL_1053788, EPI_ISL_1053789, EPI_ISL_1053790, EPI_ISL_1053791, EPI_ISL_1053792, EPI_ISL_1053793, EPI_ISL_1053794, EPI_ISL_1053795, EPI_ISL_1053796, EPI_ISL_1053797, EPI_ISL_1053798, EPI_ISL_1053799, EPI_ISL_1053800, EPI_ISL_1053801, EPI_ISL_1053802, EPI_ISL_1053803, EPI_ISL_1053804, EPI_ISL_1053805, EPI_ISL_1053806, EPI_ISL_1053807, EPI_ISL_1053808, EPI_ISL_1053809, EPI_ISL_1053810, EPI_ISL_1053811, EPI_ISL_1053812, EPI_ISL_1053813, EPI_ISL_1053814, EPI_ISL_1053815, EPI_ISL_1053816, EPI_ISL_1053903, EPI_ISL_1053904, EPI_ISL_1053905, EPI_ISL_1053908, EPI_ISL_1053909, EPI_ISL_1053910, EPI_ISL_1053911, EPI_ISL_1053912, EPI_ISL_1053913, EPI_ISL_1053914, EPI_ISL_1053917, EPI_ISL_1053918, EPI_ISL_1053919, EPI_ISL_1053920, EPI_ISL_1053921, EPI_ISL_1053924, EPI_ISL_1053925, EPI_ISL_1053928, EPI_ISL_1053929, EPI_ISL_1053930, EPI_ISL_1053931			
see above	Centre for Enzyme Innovation, University of Portsmouth / Translational Research Laboratory, Portsmouth Hospitals NHS Trust	COVID-19 Genomics UK (COG-UK) Consortium	Angela Beckett,Salman Goudarzi,Christopher Fearn,Kate Cook,Katie Loveson,Sharon Glaysher,Scott Elliott,Samuel Robson
EPI_ISL_1103878, EPI_ISL_1103879, EPI_ISL_1103880, EPI_ISL_1103881, EPI_ISL_1103882, EPI_ISL_1103886, EPI_ISL_1103887, EPI_ISL_1103889	Department of Pathology, University of Cambridge	COVID-19 Genomics UK (COG-UK) Consortium	Aminu S. Jahun, Yasmin Chaudhry, Iliana Georgana, Myra Hosmillo, Rhys Izuagbe, William L. Hamilton, Martin D. Curran, Surendra Parmar, Ian Goodfellow
EPI_ISL_1104579, EPI_ISL_1104580, EPI_ISL_1104581, EPI_ISL_1104582, EPI_ISL_1104583, EPI_ISL_1104584, EPI_ISL_1104585, EPI_ISL_1104586, EPI_ISL_1104587, EPI_ISL_1104588, EPI_ISL_1104589, EPI_ISL_1104590, EPI_ISL_1104591, EPI_ISL_1104592, EPI_ISL_1104593, EPI_ISL_1104594, EPI_ISL_1104595, EPI_ISL_1104596, EPI_ISL_1104597, EPI_ISL_1104598, EPI_ISL_1104599			

see above	University College London, Great Ormond Street Hospital for Children NHS Foundation Trust, Imperial College Healthcare NHS Trust	COVID-19 Genomics UK (COG-UK) Consortium	Sergi Castellano, Rachel Williams, Mark Kristiansen, Paola Resende Silva, Sunando Roy, Tony Brooks, Helena Tutill, Paola Niola, Patricia Dyal, Charlotte Williams, Leysa Forrest, Yasmin Panchbhaya, Jacqueline Findlay, Samuel Weeks, Julianne Brown, Kathryn Harris, Paul Randell, James Price, Alison Holmes, Judith Breuer
EPI_ISL_1104931, EPI_ISL_1104937, EPI_ISL_1104938, EPI_ISL_1104949, EPI_ISL_1104972, EPI_ISL_1104973, EPI_ISL_1104975, EPI_ISL_1104976, EPI_ISL_1104978, EPI_ISL_1104981, EPI_ISL_1104982, EPI_ISL_1104986, EPI_ISL_1104988, EPI_ISL_1104990, EPI_ISL_1104991, EPI_ISL_1104992, EPI_ISL_1104997, EPI_ISL_1104998, EPI_ISL_1104999, EPI_ISL_1105000, EPI_ISL_1105001, EPI_ISL_1105005, EPI_ISL_1105009, EPI_ISL_1105010, EPI_ISL_1105011, EPI_ISL_1105018, EPI_ISL_1105026, EPI_ISL_1105027, EPI_ISL_1105034, EPI_ISL_1105035, EPI_ISL_1105069, EPI_ISL_1105169, EPI_ISL_1105255, EPI_ISL_1105283, EPI_ISL_1105301, EPI_ISL_1105302, EPI_ISL_1105310			
see above	University College London Hospital	COVID-19 Genomics UK (COG-UK) Consortium	Judith Heaney, Matthew Byott, Catherine Houlihan, Dan Frampton, Stuart Kirk, Moira Spyer and Eleni Nastouli
EPI_ISL_1105450	University College London, Great Ormond Street Hospital for Children NHS Foundation Trust, Imperial College Healthcare NHS Trust	COVID-19 Genomics UK (COG-UK) Consortium	Sergi Castellano, Rachel Williams, Mark Kristiansen, Paola Resende Silva, Sunando Roy, Tony Brooks, Helena Tutill, Paola Niola, Patricia Dyal, Charlotte Williams, Leysa Forrest, Yasmin Panchbhaya, Jacqueline Findlay, Samuel Weeks, Julianne Brown, Kathryn Harris, Paul Randell, James Price, Alison Holmes, Judith Breuer
EPI_ISL_1105657, EPI_ISL_1105711, EPI_ISL_1105712, EPI_ISL_1105716, EPI_ISL_1105717, EPI_ISL_1105720, EPI_ISL_1105724, EPI_ISL_1105725, EPI_ISL_1105727, EPI_ISL_1105728	Northumbria University / South Tees Hospitals NHS Foundation Trust / North Cumbria Integrated Care NHS Foundation Trust / North Tees and Hartlepool NHS Foundation Trust / Newcastle Hospitals NHS Foundation Trust	COVID-19 Genomics UK (COG-UK) Consortium	Darren L Smith,Andrew Nelson,Matthew Bashton,Greg R Young,Joshua Loh,John Allan,Mohammad A Tariq,Giles S Holt,Gary Black,Wen C Yew,Lynn Dover,Paul Baker,Steve Liggett,Sarah Essex,Jane Greenaway,Debra Padgett,Clive Graham,Garren Scott,Edward Barton,Emma Swindells,Brendan Payne,Jennifer Collins,Yusri Taha,Gary Eltringham
EPI_ISL_1106644	Originating lab: Wales Specialist Virology Centre Sequencing lab: Pathogen Genomics Unit	Public Health Wales Microbiology Cardiff Wales Specialist Virology Centre	Catherine Moore, Johnathan Evans, Laura Gifford, Malorie Perry, Simon Cottrell, Angela Marchbank, Alec Birchley, Alexander Adams, Amy Gaskin, Bree Gatica-Wilcox, Jason Coombes, Joel Southgate, Lauren Gilbert, Lee Graham, Nicole Pacchiarini, Sara Kumziene-Summerhayes, Sarah Taylor, Sophie Jones, Sara Rey, Matthew Bull, Joanne Watkins, Sally Corden, Tom Connor
EPI_ISL_1107544, EPI_ISL_1107545, EPI_ISL_1107546, EPI_ISL_1107547, EPI_ISL_1107548, EPI_ISL_1107549, EPI_ISL_1107550, EPI_ISL_1107551, EPI_ISL_1107552, EPI_ISL_1107553, EPI_ISL_1107554, EPI_ISL_1107555, EPI_ISL_1107556, EPI_ISL_1107557, EPI_ISL_1107558, EPI_ISL_1107559, EPI_ISL_1107560, EPI_ISL_1107561, EPI_ISL_1107562, EPI_ISL_1107563, EPI_ISL_1107564, EPI_ISL_1107565, EPI_ISL_1107566, EPI_ISL_1107567, EPI_ISL_1107568, EPI_ISL_1107569, EPI_ISL_1107570, EPI_ISL_1107571, EPI_ISL_1107572, EPI_ISL_1107573, EPI_ISL_1107574, EPI_ISL_1107575, EPI_ISL_1107576, EPI_ISL_1107577, EPI_ISL_1107578, EPI_ISL_1107579, EPI_ISL_1107580, EPI_ISL_1107581, EPI_ISL_1107582, EPI_ISL_1107583, EPI_ISL_1107584, EPI_ISL_1107585, EPI_ISL_1107588, EPI_ISL_1107589, EPI_ISL_1107590, EPI_ISL_1107591, EPI_ISL_1107592, EPI_ISL_1107593, EPI_ISL_1107594, EPI_ISL_1107595, EPI_ISL_1107596, EPI_ISL_1107597, EPI_ISL_1107598, EPI_ISL_1107599, EPI_ISL_1107600, EPI_ISL_1107601, EPI_ISL_1107602, EPI_ISL_1107603, EPI_ISL_1107604, EPI_ISL_1107605, EPI_ISL_1107606, EPI_ISL_1107607, EPI_ISL_1107608, EPI_ISL_1107609, EPI_ISL_1107610, EPI_ISL_1107611, EPI_ISL_1107612, EPI_ISL_1107614, EPI_ISL_1107615, EPI_ISL_1107616, EPI_ISL_1107617, EPI_ISL_1107618, EPI_ISL_1107619, EPI_ISL_1107620, EPI_ISL_1107621, EPI_ISL_1107622, EPI_ISL_1107623, EPI_ISL_1107624, EPI_ISL_1107625, EPI_ISL_1107626, EPI_ISL_1107627, EPI_ISL_1107628, EPI_ISL_1107629, EPI_ISL_1107630, EPI_ISL_1107700, EPI_ISL_1107701, EPI_ISL_1107702, EPI_ISL_1107703, EPI_ISL_1107704, EPI_ISL_1107705, EPI_ISL_1107706, EPI_ISL_1107707, EPI_ISL_1107709, EPI_ISL_1107710, EPI_ISL_1107711, EPI_ISL_1107712			
see above	Centre for Enzyme Innovation, University of Portsmouth / Translational Research Laboratory, Portsmouth Hospitals NHS Trust	COVID-19 Genomics UK (COG-UK) Consortium	Angela Beckett,Salman Goudarzi,Christopher Fearn,Kate Cook,Katie Loveson,Sharon Glaysher,Scott Elliott,Samuel Robson
EPI_ISL_1177643, EPI_ISL_1177644, EPI_ISL_1177645, EPI_ISL_1177646, EPI_ISL_1177648, EPI_ISL_1177650, EPI_ISL_1177676	Department of Pathology, University of Cambridge	COVID-19 Genomics UK (COG-UK) Consortium	Aminu S. Jahun, Yasmin Chaudhry, Iliana Georgana, Myra Hosmillo, Rhys Izuagbe, William L. Hamilton, Martin D. Curran, Surendra Parmar, Ian Goodfellow
EPI_ISL_1177785	University of Exeter	COVID-19 Genomics UK (COG-UK) Consortium	Ben Temperton,Aaron Jeffries,Michelle Michelsen,Joanna Warwick-Dugdale,Audrey Farbos,Robyn Manley,Stephen Michell,Jane Masoli
EPI_ISL_1179680, EPI_ISL_1179733, EPI_ISL_1179734, EPI_ISL_1179736, EPI_ISL_1179763, EPI_ISL_1179779, EPI_ISL_1179781, EPI_ISL_1179786, EPI_ISL_1179799, EPI_ISL_1179801, EPI_ISL_1179805, EPI_ISL_1179807, EPI_ISL_1179810, EPI_ISL_1179826, EPI_ISL_1179835, EPI_ISL_1179840, EPI_ISL_1179841, EPI_ISL_1179842, EPI_ISL_1179844, EPI_ISL_1179845, EPI_ISL_1179846, EPI_ISL_1179847, EPI_ISL_1179848, EPI_ISL_1179849, EPI_ISL_1179850, EPI_ISL_1179851			
see above	Centre for Enzyme Innovation, University of Portsmouth / Translational Research Laboratory, Portsmouth Hospitals NHS Trust	COVID-19 Genomics UK (COG-UK) Consortium	Angela Beckett,Salman Goudarzi,Christopher Fearn,Kate Cook,Katie Loveson,Sharon Glaysher,Scott Elliott,Samuel Robson
EPI_ISL_1247369, EPI_ISL_1247370, EPI_ISL_1247371, EPI_ISL_1247374, EPI_ISL_1247375, EPI_ISL_1247377, EPI_ISL_1247378, EPI_ISL_1247379, EPI_ISL_1247380, EPI_ISL_1247382, EPI_ISL_1247383, EPI_ISL_1247384, EPI_ISL_1247385, EPI_ISL_1247387, EPI_ISL_1247388, EPI_ISL_1247389, EPI_ISL_1247390, EPI_ISL_1247392, EPI_ISL_1247393, EPI_ISL_1247396, EPI_ISL_1247397, EPI_ISL_1247398, EPI_ISL_1247399, EPI_ISL_1247400, EPI_ISL_1247401, EPI_ISL_1247402, EPI_ISL_1247403, EPI_ISL_1247404			
see above	Quadram Institute Bioscience	COVID-19 Genomics UK (COG-UK) Consortium	Dave J. Baker, Gemma L. Kay, Alp Aydin, Thanh Le-Viet, Steven Rudder, Ana P. Tedim, Anastasia Kolyva, Maria Diaz, Leonardo de Oliveira Martins, Nabil-Fareed Alikhan, Lizzie Meadows, Rachael Stanley, Ngozi Elumogo, Muhammed Yasir, Nicholas M. Thomson, Alexander J Trotter, Rachel Gilroy, Samuel Bloomfield, Claire Stuart, Andrew Bell, Reenesh Prakash, Samir Dervisevic, Alison E. Mather, John Wain, Mark Webber, Andrew J. Page, Justin O'Grady
EPI_ISL_1247405, EPI_ISL_1247406, EPI_ISL_1247407, EPI_ISL_1247408, EPI_ISL_1247409, EPI_ISL_1247410, EPI_ISL_1247411, EPI_ISL_1247412, EPI_ISL_1247413, EPI_ISL_1247414, EPI_ISL_1247416, EPI_ISL_1247417, EPI_ISL_1247418, EPI_ISL_1247419, EPI_ISL_1247420, EPI_ISL_1247421, EPI_ISL_1247422, EPI_ISL_1247423, EPI_ISL_1247424, EPI_ISL_1247425, EPI_ISL_1247426, EPI_ISL_1247427, EPI_ISL_1247428, EPI_ISL_1247429, EPI_ISL_1247431, EPI_ISL_1247432			
see above	University of Birmingham	COVID-19 Genomics UK (COG-UK) Consortium	Institute of Microbiology, University of Birmingham: Claire McMurray, Joanne Stockton, Samuel Nicholls, Radoslaw Poplawski, Will Rowe, Josh Quick, Nicholas Loman. University of Birmingham Testing Laboratory: Celina M Whalley, Andrew Bosworth, Charlotte Poxon, Kasun Wanigasooriya, Oliver Pickles, Mike Kidd, Alex Richter, Andrew D Beggs PHE Heartlands Lab: Husam Osman, Andrew Bosworth. Queen Elizabeth Hospital: Anna Casey
EPI_ISL_1247572, EPI_ISL_1247575, EPI_ISL_1247577, EPI_ISL_1247579, EPI_ISL_1247593, EPI_ISL_1247602, EPI_ISL_1247604, EPI_ISL_1247606, EPI_ISL_1247607, EPI_ISL_1247609, EPI_ISL_1247610, EPI_ISL_1247623, EPI_ISL_1247630			
see above	Department of Pathology, University of Cambridge	COVID-19 Genomics UK (COG-UK) Consortium	Aminu S. Jahun, Yasmin Chaudhry, Iliana Georgana, Myra Hosmillo, Rhys Izuagbe, William L. Hamilton, Martin D. Curran, Surendra Parmar, Ian Goodfellow
EPI_ISL_1247997, EPI_ISL_1247998, EPI_ISL_1247999, EPI_ISL_1248001, EPI_ISL_1248002, EPI_ISL_1248003, EPI_ISL_1248004, EPI_ISL_1248005, EPI_ISL_1248006, EPI_ISL_1248007, EPI_ISL_1248008, EPI_ISL_1248009, EPI_ISL_1248010, EPI_ISL_1248011, EPI_ISL_1248012, EPI_ISL_1248013			
see above	University College London, Great Ormond Street Hospital for Children NHS Foundation Trust, Imperial College Healthcare NHS Trust	COVID-19 Genomics UK (COG-UK) Consortium	Sergi Castellano, Rachel Williams, Mark Kristiansen, Paola Resende Silva, Sunando Roy, Tony Brooks, Helena Tutill, Paola Niola, Patricia Dyal, Charlotte Williams, Leysa Forrest, Yasmin Panchbhaya, Jacqueline Findlay, Samuel Weeks, Julianne Brown, Kathryn Harris, Paul Randell, James Price, Alison Holmes, Judith Breuer
EPI_ISL_1248377, EPI_ISL_1248379, EPI_ISL_1248387, EPI_ISL_1248398, EPI_ISL_1248399, EPI_ISL_1248410, EPI_ISL_1248421, EPI_ISL_1248431, EPI_ISL_1248435, EPI_ISL_1248443, EPI_ISL_1248448, EPI_ISL_1248455, EPI_ISL_1248491			
see above	University College London Hospital	COVID-19 Genomics UK (COG-UK) Consortium	Dr Judith Heaney, Matthew Byott, Dr Catherine Houlihan, Dr Daniel Frampton, Stuart Kirk, Dr Moira Spyer, Dr Paul Grant and Dr Eleni Nastouli
EPI_ISL_1249888, EPI_ISL_1249889, EPI_ISL_1249890, EPI_ISL_1249891, EPI_ISL_1249892, EPI_ISL_1249893, EPI_ISL_1249894, EPI_ISL_1249895, EPI_ISL_1249896, EPI_ISL_1249897, EPI_ISL_1249898, EPI_ISL_1249899, EPI_ISL_1249900, EPI_ISL_1249901, EPI_ISL_1249902, EPI_ISL_1249903, EPI_ISL_1249904, EPI_ISL_1249905, EPI_ISL_1249906, EPI_ISL_1249907, EPI_ISL_1249908, EPI_ISL_1249909, EPI_ISL_1249910, EPI_ISL_1249911, EPI_ISL_1249912, EPI_ISL_1249913, EPI_ISL_1249914, EPI_ISL_1249915, EPI_ISL_1249916, EPI_ISL_1249917, EPI_ISL_1249918, EPI_ISL_1249920, EPI_ISL_1249921, EPI_ISL_1249922, EPI_ISL_1249923, EPI_ISL_1249924, EPI_ISL_1249925, EPI_ISL_1249926, EPI_ISL_1249927, EPI_ISL_1249928, EPI_ISL_1249929, EPI_ISL_1249930, EPI_ISL_1249931, EPI_ISL_1249932, EPI_ISL_1249933, EPI_ISL_1249934, EPI_ISL_1249935, EPI_ISL_1249936, EPI_ISL_1249937, EPI_ISL_1249938, EPI_ISL_1249939, EPI_ISL_1249940, EPI_ISL_1249941, EPI_ISL_1249942, EPI_ISL_1249943, EPI_ISL_1249944, EPI_ISL_1249945, EPI_ISL_1249946, EPI_ISL_1249947, EPI_ISL_1249948, EPI_ISL_1249949, EPI_ISL_1249951, EPI_ISL_1249952, EPI_ISL_1249953, EPI_ISL_1249954, EPI_ISL_1249955, EPI_ISL_1249956, EPI_ISL_1249957, EPI_ISL_1249958, EPI_ISL_1249960, EPI_ISL_1249961, EPI_ISL_1249962, EPI_ISL_1249963, EPI_ISL_1249964, EPI_ISL_1249965, EPI_ISL_1249966, EPI_ISL_1250031, EPI_ISL_1250032, EPI_ISL_1250033, EPI_ISL_1250034, EPI_ISL_1250035, EPI_ISL_1250036, EPI_ISL_1250037, EPI_ISL_1250038, EPI_ISL_1250039, EPI_ISL_1250040, EPI_ISL_1250041, EPI_ISL_1250042, EPI_ISL_1250043, EPI_ISL_1250044, EPI_ISL_1250045, EPI_ISL_1250046, EPI_ISL_1250047, EPI_ISL_1250048, EPI_ISL_1250049, EPI_ISL_1250050, EPI_ISL_1250051, EPI_ISL_1250052, EPI_ISL_1250053, EPI_ISL_1250054, EPI_ISL_1250055, EPI_ISL_1250057, EPI_ISL_1250058, EPI_ISL_1250059, EPI_ISL_1250060, EPI_ISL_1250062, EPI_ISL_1250063, EPI_ISL_1250064, EPI_ISL_1250065, EPI_ISL_1250066, EPI_ISL_1250067, EPI_ISL_1250068, EPI_ISL_1250069, EPI_ISL_1250070, EPI_ISL_1250071, EPI_ISL_1250072, EPI_ISL_1250073, EPI_ISL_1250074, EPI_ISL_1250075, EPI_ISL_1250076, EPI_ISL_1250077, EPI_ISL_1250078, EPI_ISL_1250079, EPI_ISL_1250080, EPI_ISL_1250081, EPI_ISL_1250082, EPI_ISL_1250083, EPI_ISL_1250084, EPI_ISL_1250085, EPI_ISL_1250086, EPI_ISL_1250087, EPI_ISL_1250088, EPI_ISL_1250089, EPI_ISL_1250090, EPI_ISL_1250091, EPI_ISL_1250092, EPI_ISL_1250093, EPI_ISL_1250096, EPI_ISL_1250097, EPI_ISL_1250098, EPI_ISL_1250099, EPI_ISL_1250100, EPI_ISL_1250101, EPI_ISL_1250102, EPI_ISL_1250103, EPI_ISL_1250104, EPI_ISL_1250106, EPI_ISL_1250107, EPI_ISL_1250108, EPI_ISL_1250120, EPI_ISL_1250121, EPI_ISL_1250122			
see above	Centre for Enzyme Innovation, University of Portsmouth / Translational Research Laboratory, Portsmouth Hospitals NHS Trust	COVID-19 Genomics UK (COG-UK) Consortium	Angela Beckett,Salman Goudarzi,Christopher Fearn,Kate Cook,Katie Loveson,Sharon Glaysher,Scott Elliott,Samuel Robson
EPI_ISL_1296494, EPI_ISL_1296500	Respiratory Virus Unit, National Infection Service, Public Health England	COVID-19 Genomics UK (COG-UK) Consortium	PHE Covid Sequencing Team
EPI_ISL_1308448, EPI_ISL_1308449, EPI_ISL_1308450, EPI_ISL_1308452, EPI_ISL_1308453, EPI_ISL_1308454, EPI_ISL_1308455, EPI_ISL_1308456, EPI_ISL_1308457, EPI_ISL_1308458, EPI_ISL_1308459, EPI_ISL_1308460, EPI_ISL_1308461, EPI_ISL_1308462, EPI_ISL_1308463, EPI_ISL_1308464, EPI_ISL_1308465, EPI_ISL_1308466, EPI_ISL_1308467, EPI_ISL_1308468, EPI_ISL_1308469, EPI_ISL_1308470, EPI_ISL_1308480			
see above	University of Birmingham	COVID-19 Genomics UK (COG-UK) Consortium	Institute of Microbiology, University of Birmingham: Claire McMurray, Joanne Stockton, Samuel Nicholls, Radoslaw Poplawski, Will Rowe, Josh Quick,

			Nicholas Loman. University of Birmingham Testing Laboratory: Celina M Whalley, Andrew Bosworth, Charlotte Poxon, Kasun Wanigasooriya, Oliver Pickles, Mike Kidd, Alex Richter, Andrew D Beggs PHE Heartlands Lab: Husam Osman, Andrew Bosworth. Queen Elizabeth Hospital: Anna Casey
EPI_ISL_1308580, EPI_ISL_1308597, EPI_ISL_1308599	University of Exeter	COVID-19 Genomics UK (COG-UK) Consortium	Ben Temperton,Aaron Jeffries,Michelle Michelsen,Joanna Warwick-Dugdale,Audrey Farbos,Robyn Manley,Stephen Michell,Jane Masoli
EPI_ISL_1308698	Department of Pathology, University of Cambridge	COVID-19 Genomics UK (COG-UK) Consortium	Aminu S. Jahun, Yasmin Chaudhry, Iliana Georgana, Myra Hosmillo, Rhys Izuagbe, William L. Hamilton, Martin D. Curran, Surendra Parmar, Ian Goodfellow
EPI_ISL_1308832, EPI_ISL_1308833, EPI_ISL_1308834, EPI_ISL_1308835	Virology Department, Royal Infirmary of Edinburgh, NHS Lothian / School of Biological Sciences, University of Edinburgh	COVID-19 Genomics UK (COG-UK) Consortium	McHugh M, Dewar R, Cotton S, Rooke S, O'Toole Á, Scher E, Hill V, McCrone JT, Colquhoun R, Yu X, Jackson B, Rambaut A, Templeton K
EPI_ISL_1308975, EPI_ISL_1308976, EPI_ISL_1308979, EPI_ISL_1308980, EPI_ISL_1308989, EPI_ISL_1309005, EPI_ISL_1309007, EPI_ISL_1309008, EPI_ISL_1309009, EPI_ISL_1309010, EPI_ISL_1309011, EPI_ISL_1309012, EPI_ISL_1309013, EPI_ISL_1309014, EPI_ISL_1309015, EPI_ISL_1309016, EPI_ISL_1309017, EPI_ISL_1309018, EPI_ISL_1309019, EPI_ISL_1309020, EPI_ISL_1309021, EPI_ISL_1309031, EPI_ISL_1309032, EPI_ISL_1309036, EPI_ISL_1309041, EPI_ISL_1309044, EPI_ISL_1309046, EPI_ISL_1309048, EPI_ISL_1309049, EPI_ISL_1309054, EPI_ISL_1309092, EPI_ISL_1309093, EPI_ISL_1309094, EPI_ISL_1309121, EPI_ISL_1309124			
see above	University College London, Great Ormond Street Hospital for Children NHS Foundation Trust, Imperial College Healthcare NHS Trust	COVID-19 Genomics UK (COG-UK) Consortium	Sergi Castellano, Rachel Williams, Mark Kristiansen, Paola Resende Silva, Sunando Roy, Tony Brooks, Helena Tutill, Paola Niola, Patricia Dyal, Charlotte Williams, Leysa Forrest, Yasmin Panchbhaya, Jacqueline Findlay, Samuel Weeks, Julianne Brown, Kathryn Harris, Paul Randell, James Price, Alison Holmes, Judith Breuer
EPI_ISL_1309434, EPI_ISL_1309436, EPI_ISL_1309437, EPI_ISL_1309438, EPI_ISL_1309439, EPI_ISL_1309440, EPI_ISL_1309441, EPI_ISL_1309442, EPI_ISL_1309443, EPI_ISL_1309444, EPI_ISL_1309447, EPI_ISL_1309448, EPI_ISL_1309449, EPI_ISL_1309457, EPI_ISL_1309458, EPI_ISL_1309479, EPI_ISL_1309486, EPI_ISL_1309487, EPI_ISL_1309556, EPI_ISL_1309557, EPI_ISL_1309558, EPI_ISL_1309562, EPI_ISL_1309563, EPI_ISL_1309564, EPI_ISL_1309567, EPI_ISL_1309568, EPI_ISL_1309569, EPI_ISL_1309570, EPI_ISL_1309572, EPI_ISL_1309573, EPI_ISL_1309574, EPI_ISL_1309575, EPI_ISL_1309576, EPI_ISL_1309577, EPI_ISL_1309578, EPI_ISL_1309579, EPI_ISL_1309580, EPI_ISL_1309581, EPI_ISL_1309582, EPI_ISL_1309583, EPI_ISL_1309584, EPI_ISL_1309585, EPI_ISL_1309586, EPI_ISL_1309587, EPI_ISL_1309588, EPI_ISL_1309589, EPI_ISL_1309590, EPI_ISL_1309591, EPI_ISL_1309592, EPI_ISL_1309593, EPI_ISL_1309594, EPI_ISL_1309595, EPI_ISL_1309596, EPI_ISL_1309599, EPI_ISL_1309600, EPI_ISL_1309601, EPI_ISL_1309602, EPI_ISL_1309603, EPI_ISL_1309604, EPI_ISL_1309605, EPI_ISL_1309606, EPI_ISL_1309607, EPI_ISL_1309609, EPI_ISL_1309611, EPI_ISL_1309634			
see above	Northumbria University / South Tees Hospitals NHS Foundation Trust / North Cumbria Integrated Care NHS Foundation Trust / North Tees and Hartlepool NHS Foundation Trust / Newcastle Hospitals NHS Foundation Trust	COVID-19 Genomics UK (COG-UK) Consortium	Darren L Smith,Andrew Nelson,Matthew Bashton,Greg R Young,Joshua Loh,John Allan,Mohammad A Tariq,Giles S Holt,Gary Black,Wen C Yew,Lynn Dover,Paul Baker,Steve Liggett,Sarah Essex,Jane Greenaway,Debra Padgett,Clive Graham,Garren Scott,Edward Barton,Emma Swindells,Brendan Payne,Jennifer Collins,Yusri Taha,Gary Eltringham
EPI_ISL_1310289, EPI_ISL_1310290, EPI_ISL_1310327, EPI_ISL_1310362, EPI_ISL_1310363, EPI_ISL_1310364, EPI_ISL_1310365, EPI_ISL_1310403, EPI_ISL_1310404, EPI_ISL_1310405, EPI_ISL_1310406, EPI_ISL_1310407, EPI_ISL_1310408, EPI_ISL_1310409, EPI_ISL_1310410, EPI_ISL_1310411, EPI_ISL_1310412, EPI_ISL_1310413, EPI_ISL_1310414, EPI_ISL_1310415, EPI_ISL_1310416, EPI_ISL_1310417, EPI_ISL_1310418, EPI_ISL_1310419, EPI_ISL_1310420, EPI_ISL_1310421, EPI_ISL_1310422, EPI_ISL_1310423, EPI_ISL_1310424, EPI_ISL_1310425, EPI_ISL_1310426, EPI_ISL_1310427, EPI_ISL_1310428, EPI_ISL_1310429, EPI_ISL_1310430, EPI_ISL_1310431, EPI_ISL_1310432, EPI_ISL_1310433, EPI_ISL_1310434, EPI_ISL_1310435, EPI_ISL_1310436, EPI_ISL_1310437			
see above	Centre for Enzyme Innovation, University of Portsmouth / Translational Research Laboratory, Portsmouth Hospitals NHS Trust	COVID-19 Genomics UK (COG-UK) Consortium	Angela Beckett,Salman Goudarzi,Christopher Fearn,Kate Cook,Katie Loveson,Sharon Glaysher,Scott Elliott,Samuel Robson
EPI_ISL_1386412, EPI_ISL_1386414, EPI_ISL_1386415, EPI_ISL_1386420, EPI_ISL_1386421, EPI_ISL_1386422, EPI_ISL_1386423, EPI_ISL_1386425, EPI_ISL_1386428, EPI_ISL_1386429, EPI_ISL_1386430, EPI_ISL_1386431, EPI_ISL_1386432, EPI_ISL_1386435, EPI_ISL_1386436, EPI_ISL_1386437, EPI_ISL_1386438, EPI_ISL_1386442, EPI_ISL_1386443, EPI_ISL_1386444, EPI_ISL_1386445, EPI_ISL_1386446, EPI_ISL_1386447, EPI_ISL_1386448, EPI_ISL_1386449, EPI_ISL_1386452, EPI_ISL_1386454, EPI_ISL_1386457, EPI_ISL_1386459, EPI_ISL_1386462, EPI_ISL_1386463, EPI_ISL_1386465, EPI_ISL_1386466, EPI_ISL_1386467, EPI_ISL_1386469, EPI_ISL_1386470, EPI_ISL_1386471, EPI_ISL_1386474, EPI_ISL_1386475			
see above	Quadram Institute Bioscience	COVID-19 Genomics UK (COG-UK) Consortium	Dave J. Baker, Gemma L. Kay, Alp Aydin, Thanh Le-Viet, Steven Rudder, Ana P. Tedim, Anastasia Kolyva, Maria Diaz, Leonardo de Oliveira Martins, Nabil-Fareed Alikhan, Lizzie Meadows, Rachael Stanley, Ngozi Elumogo, Muhammed Yasir, Nicholas M. Thomson, Alexander J Trotter, Rachel Gilroy, Samuel Bloomfield, Claire Stuart, Andrew Bell, Reenesh Prakash, Samir Dervisevic, Alison E. Mather, John Wain, Mark Webber, Andrew J. Page, Justin O'Grady
EPI_ISL_1386523, EPI_ISL_1386529, EPI_ISL_1386534, EPI_ISL_1386535, EPI_ISL_1386536, EPI_ISL_1386537, EPI_ISL_1386539	University of Birmingham	COVID-19 Genomics UK (COG-UK) Consortium	Institute of Microbiology, University of Birmingham: Claire McMurray, Joanne Stockton, Samuel Nicholls, Radoslaw Poplawski, Will Rowe, Josh Quick, Nicholas Loman. University of Birmingham Testing Laboratory: Celina M Whalley, Andrew Bosworth, Charlotte Poxon, Kasun Wanigasooriya, Oliver Pickles, Mike Kidd, Alex Richter, Andrew D Beggs PHE Heartlands Lab: Husam Osman, Andrew Bosworth. Queen Elizabeth Hospital: Anna Casey
EPI_ISL_1386576, EPI_ISL_1386582, EPI_ISL_1386584, EPI_ISL_1386585, EPI_ISL_1386587	Department of Pathology, University of Cambridge	COVID-19 Genomics UK (COG-UK) Consortium	Aminu S. Jahun, Yasmin Chaudhry, Iliana Georgana, Myra Hosmillo, Rhys Izuagbe, William L. Hamilton, Martin D. Curran, Surendra Parmar, Ian Goodfellow
EPI_ISL_1386901, EPI_ISL_1386909	University College London, Great Ormond Street Hospital for Children NHS Foundation Trust, Imperial College Healthcare NHS Trust	COVID-19 Genomics UK (COG-UK) Consortium	Sergi Castellano, Rachel Williams, Mark Kristiansen, Paola Resende Silva, Sunando Roy, Tony Brooks, Helena Tutill, Paola Niola, Patricia Dyal, Charlotte Williams, Leysa Forrest, Yasmin Panchbhaya, Jacqueline Findlay, Samuel Weeks, Julianne Brown, Kathryn Harris, Paul Randell, James Price, Alison Holmes, Judith Breuer
EPI_ISL_1387053, EPI_ISL_1387055, EPI_ISL_1387056, EPI_ISL_1387057, EPI_ISL_1387058, EPI_ISL_1387059, EPI_ISL_1387060, EPI_ISL_1387061, EPI_ISL_1387076, EPI_ISL_1387086, EPI_ISL_1387087, EPI_ISL_1387089, EPI_ISL_1387092, EPI_ISL_1387093, EPI_ISL_1387101, EPI_ISL_1387102, EPI_ISL_1387103, EPI_ISL_1387104, EPI_ISL_1387105, EPI_ISL_1387106, EPI_ISL_1387107, EPI_ISL_1387109, EPI_ISL_1387110, EPI_ISL_1387111, EPI_ISL_1387112			
see above	Northumbria University / South Tees Hospitals NHS Foundation Trust / North Cumbria Integrated Care NHS Foundation Trust / North Tees and Hartlepool NHS Foundation Trust / Newcastle Hospitals NHS Foundation Trust	COVID-19 Genomics UK (COG-UK) Consortium	Darren L Smith,Andrew Nelson,Matthew Bashton,Greg R Young,Joshua Loh,John Allan,Mohammad A Tariq,Giles S Holt,Gary Black,Wen C Yew,Lynn Dover,Paul Baker,Steve Liggett,Sarah Essex,Jane Greenaway,Debra Padgett,Clive Graham,Garren Scott,Edward Barton,Emma Swindells,Brendan Payne,Jennifer Collins,Yusri Taha,Gary Eltringham
EPI_ISL_1388654, EPI_ISL_1388656, EPI_ISL_1388658, EPI_ISL_1388741, EPI_ISL_1388762, EPI_ISL_1388811, EPI_ISL_1388814, EPI_ISL_1388816, EPI_ISL_1388822, EPI_ISL_1388824, EPI_ISL_1388826, EPI_ISL_1388828, EPI_ISL_1388831, EPI_ISL_1388832, EPI_ISL_1388835, EPI_ISL_1388837, EPI_ISL_1388839, EPI_ISL_1388841, EPI_ISL_1388843, EPI_ISL_1388846, EPI_ISL_1388848, EPI_ISL_1388849, EPI_ISL_1388851, EPI_ISL_1388853, EPI_ISL_1388855, EPI_ISL_1388857, EPI_ISL_1388859, EPI_ISL_1388861			
see above	Centre for Enzyme Innovation, University of Portsmouth / Translational Research Laboratory, Portsmouth Hospitals NHS Trust	COVID-19 Genomics UK (COG-UK) Consortium	Angela Beckett,Salman Goudarzi,Christopher Fearn,Kate Cook,Katie Loveson,Sharon Glaysher,Scott Elliott,Samuel Robson
EPI_ISL_1474397, EPI_ISL_1474441, EPI_ISL_1474443, EPI_ISL_1474445, EPI_ISL_1474455, EPI_ISL_1474463	University of Birmingham	COVID-19 Genomics UK (COG-UK) Consortium	Institute of Microbiology, University of Birmingham: Claire McMurray, Joanne Stockton, Samuel Nicholls, Radoslaw Poplawski, Will Rowe, Josh Quick, Nicholas Loman. University of Birmingham Testing Laboratory: Celina M Whalley, Andrew Bosworth, Charlotte Poxon, Kasun Wanigasooriya, Oliver Pickles, Mike Kidd, Alex Richter, Andrew D Beggs PHE Heartlands Lab: Husam Osman, Andrew Bosworth. Queen Elizabeth Hospital: Anna Casey
EPI_ISL_1474644	University of Exeter	COVID-19 Genomics UK (COG-UK) Consortium	Ben Temperton,Aaron Jeffries,Michelle Michelsen,Joanna Warwick-Dugdale,Audrey Farbos,Robyn Manley,Stephen Michell,Jane Masoli
EPI_ISL_1474655	Liverpool Clinical Laboratories	COVID-19 Genomics UK (COG-UK) Consortium	Sam Haldenby, Alistair Darby, Steve Paterson, Anita Lucaci, Julian Hiscox, M Almsaud, A Alrezaihi, Muhannad Alruwaili, Stuart D Armstrong, Jones Benjamin, Eleanor G Bentley, Anu Chawla, Jordan J Clark, Angela Cowell, Richard Eccles, Isabel García-Dorival, Matthew Gemmell, Alessandro Gerada, PKF Gilmore, Richard Gregory, Ximeng Han, Catherine Hartley, Margaret Hughes, Miren Iturriza-Gomara, James Johnson, L Luu, Jenifer Manson, Charlotte Nelson, Elaine O'Toole, Cassie Olateju, Rebekah Penrice-Randal , Lucille Rainbow, N.P Randle, Trevor Ian Robinson, Parul Sharma, Ghada T Shawli, James P Stewart, Neil Swainston, Ecaterina Vamos, Joanne Watts, Mark Whitehead, Hermione Webster
EPI_ISL_1474836, EPI_ISL_1474837, EPI_ISL_1474848, EPI_ISL_1474850, EPI_ISL_1474855	University College London, Great Ormond Street Hospital for Children NHS Foundation Trust, Imperial College Healthcare NHS Trust	COVID-19 Genomics UK (COG-UK) Consortium	Sergi Castellano, Rachel Williams, Mark Kristiansen, Paola Resende Silva, Sunando Roy, Tony Brooks, Helena Tutill, Paola Niola, Patricia Dyal, Charlotte Williams, Leysa Forrest, Yasmin Panchbhaya, Jacqueline Findlay, Samuel Weeks, Julianne Brown, Kathryn Harris, Paul Randell, James Price, Alison Holmes, Judith Breuer
EPI_ISL_1475188	Regional Virus Laboratory, Belfast Health and Social Care Trust	COVID-19 Genomics UK (COG-UK) Consortium	Conall McCaughey, James McKenna, Tanya Curran, Susan Feeney, Alison Watt, Ciara Cox, Mairead Connor, Zoltan Molnar, David Simpson, Derek Fairley
EPI_ISL_1475190, EPI_ISL_1475194, EPI_ISL_1475195, EPI_ISL_1475197, EPI_ISL_1475198, EPI_ISL_1475199, EPI_ISL_1475200, EPI_ISL_1475201, EPI_ISL_1475202, EPI_ISL_1475203, EPI_ISL_1475205, EPI_ISL_1475223, EPI_ISL_1475224, EPI_ISL_1475225, EPI_ISL_1475226, EPI_ISL_1475227, EPI_ISL_1475228, EPI_ISL_1475229, EPI_ISL_1475230, EPI_ISL_1475231, EPI_ISL_1475232, EPI_ISL_1475233, EPI_ISL_1475234, EPI_ISL_1475235, EPI_ISL_1475236, EPI_ISL_1475237, EPI_ISL_1475238, EPI_ISL_1475240, EPI_ISL_1475241, EPI_ISL_1475242, EPI_ISL_1475243, EPI_ISL_1475244, EPI_ISL_1475245, EPI_ISL_1475246, EPI_ISL_1475247, EPI_ISL_1475248, EPI_ISL_1475249, EPI_ISL_1475250, EPI_ISL_1475251, EPI_ISL_1475252, EPI_ISL_1475253, EPI_ISL_1475254, EPI_ISL_1475255, EPI_ISL_1475256, EPI_ISL_1475257, EPI_ISL_1475258, EPI_ISL_1475259, EPI_ISL_1475260, EPI_ISL_1475261, EPI_ISL_1475262, EPI_ISL_1475263, EPI_ISL_1475264, EPI_ISL_1475265, EPI_ISL_1475266, EPI_ISL_1475267, EPI_ISL_1475268, EPI_ISL_1475269, EPI_ISL_1475270, EPI_ISL_1475271, EPI_ISL_1475272, EPI_ISL_1475273, EPI_ISL_1475274, EPI_ISL_1475275, EPI_ISL_1475276, EPI_ISL_1475277, EPI_ISL_1475278, EPI_ISL_1475279, EPI_ISL_1475280,			

EPI_ISL_1475281, EPI_ISL_1475282, EPI_ISL_1475283, EPI_ISL_1475284, EPI_ISL_1475285, EPI_ISL_1475286, EPI_ISL_1475287, EPI_ISL_1475288, EPI_ISL_1475289, EPI_ISL_1475290, EPI_ISL_1475291, EPI_ISL_1475292, EPI_ISL_1475293, EPI_ISL_1475294, EPI_ISL_1475295, EPI_ISL_1475296, EPI_ISL_1475297, EPI_ISL_1475298, EPI_ISL_1475299, EPI_ISL_1475300, EPI_ISL_1475301, EPI_ISL_1475302, EPI_ISL_1475303, EPI_ISL_1475304, EPI_ISL_1475305, EPI_ISL_1475387			
see above	Northumbria University / South Tees Hospitals NHS Foundation Trust / North Cumbria Integrated Care NHS Foundation Trust / North Tees and Hartlepool NHS Foundation Trust / Newcastle Hospitals NHS Foundation Trust	COVID-19 Genomics UK (COG-UK) Consortium	Darren L Smith,Andrew Nelson,Matthew Bashton,Greg R Young,Joshua Loh,John Allan,Mohammad A Tariq,Giles S Holt,Gary Black,Wen C Yew,Lynn Dover,Paul Baker,Steve Liggett,Sarah Essex,Jane Greenaway,Debra Padgett,Clive Graham,Garren Scott,Edward Barton,Emma Swindells,Brendan Payne,Jennifer Collins,Yusri Taha,Gary Eltringham
EPI_ISL_1476517, EPI_ISL_1476519	Originating lab: Wales Specialist Virology Centre Sequencing lab: Pathogen Genomics Unit	Public Health Wales Microbiology Cardiff Wales Specialist Virology Centre	Catherine Moore, Johnathan Evans, Laura Gifford, Malorie Perry, Simon Cottrell, Angela Marchbank, Alec Birchley, Alexander Adams, Amy Gaskin, Bree Gatica-Wilcox, Jason Coombes, Joel Southgate, Lauren Gilbert, Lee Graham, Nicole Pacchiarini, Sara Kumziene-Summerhayes, Sarah Taylor, Sophie Jones, Sara Rey, Matthew Bull, Joanne Watkins, Sally Corden, Tom Connor
EPI_ISL_1476566, EPI_ISL_1476567, EPI_ISL_1476568, EPI_ISL_1476569, EPI_ISL_1476570, EPI_ISL_1476571, EPI_ISL_1476572, EPI_ISL_1476573, EPI_ISL_1476574, EPI_ISL_1476575, EPI_ISL_1476576, EPI_ISL_1476577, EPI_ISL_1476578, EPI_ISL_1476579, EPI_ISL_1476580, EPI_ISL_1476581, EPI_ISL_1476582, EPI_ISL_1476583, EPI_ISL_1476584, EPI_ISL_1476585, EPI_ISL_1476586, EPI_ISL_1476587, EPI_ISL_1476588, EPI_ISL_1476589, EPI_ISL_1476590, EPI_ISL_1476591, EPI_ISL_1476592, EPI_ISL_1476593, EPI_ISL_1476594, EPI_ISL_1476595, EPI_ISL_1476596, EPI_ISL_1476597, EPI_ISL_1476598, EPI_ISL_1476599, EPI_ISL_1476600, EPI_ISL_1476601, EPI_ISL_1476602, EPI_ISL_1476603, EPI_ISL_1476604, EPI_ISL_1476605, EPI_ISL_1476606, EPI_ISL_1476607, EPI_ISL_1476608, EPI_ISL_1476609, EPI_ISL_1476610, EPI_ISL_1476611, EPI_ISL_1476612, EPI_ISL_1476613, EPI_ISL_1476614, EPI_ISL_1476615, EPI_ISL_1476616, EPI_ISL_1476617, EPI_ISL_1476618, EPI_ISL_1476619, EPI_ISL_1476620, EPI_ISL_1476621, EPI_ISL_1476622, EPI_ISL_1476623, EPI_ISL_1476624, EPI_ISL_1476625, EPI_ISL_1476630, EPI_ISL_1476631, EPI_ISL_1476632, EPI_ISL_1476633, EPI_ISL_1476634, EPI_ISL_1476635, EPI_ISL_1476636, EPI_ISL_1476637, EPI_ISL_1476638, EPI_ISL_1476639, EPI_ISL_1476640, EPI_ISL_1476641, EPI_ISL_1476642, EPI_ISL_1476643, EPI_ISL_1476644, EPI_ISL_1476645, EPI_ISL_1476646, EPI_ISL_1476647, EPI_ISL_1476648, EPI_ISL_1476649, EPI_ISL_1476651, EPI_ISL_1476652, EPI_ISL_1476653, EPI_ISL_1476654, EPI_ISL_1476655, EPI_ISL_1476656, EPI_ISL_1476657, EPI_ISL_1476658, EPI_ISL_1476659, EPI_ISL_1476660, EPI_ISL_1476661, EPI_ISL_1476662, EPI_ISL_1476663, EPI_ISL_1476664, EPI_ISL_1476666, EPI_ISL_1476667, EPI_ISL_1476668, EPI_ISL_1476669, EPI_ISL_1476670, EPI_ISL_1476671			
see above	Centre for Enzyme Innovation, University of Portsmouth / Translational Research Laboratory, Portsmouth Hospitals NHS Trust	COVID-19 Genomics UK (COG-UK) Consortium	Angela Beckett,Salman Goudarzi,Christopher Fearn,Kate Cook,Katie Loveson,Sharon Glaysher,Scott Elliott,Samuel Robson
EPI_ISL_754302	Respiratory Virus Unit, National Infection Service, Public Health England	COVID-19 Genomics UK (COG-UK) Consortium	PHE Covid Sequencing Team
EPI_ISL_763769	Quadram Institute Bioscience	COVID-19 Genomics UK (COG-UK) Consortium	Dave J. Baker, Gemma L. Kay, Alp Aydin, Thanh Le-Viet, Steven Rudder, Ana P. Tedim, Anastasia Kolyva, Maria Diaz, Leonardo de Oliveira Martins, Nabil-Fareed Alikhan, Lizzie Meadows, Rachael Stanley, Ngozi Elumogo, Muhammed Yasir, Nicholas M. Thomson, Alexander J Trotter, Rachel Gilroy, Samuel Bloomfield, Claire Stuart, Andrew Bell, Reenesh Prakash, Samir Dervisevic, Alison E. Mather, John Wain, Mark Webber, Andrew J. Page, Justin O'Grady
EPI_ISL_766221, EPI_ISL_770462, EPI_ISL_770463, EPI_ISL_770464, EPI_ISL_770465, EPI_ISL_770466, EPI_ISL_770467, EPI_ISL_770468, EPI_ISL_770469, EPI_ISL_791193, EPI_ISL_791195, EPI_ISL_791196, EPI_ISL_791199, EPI_ISL_791200, EPI_ISL_791201, EPI_ISL_791202, EPI_ISL_791203, EPI_ISL_791209, EPI_ISL_791222, EPI_ISL_791249, EPI_ISL_791250, EPI_ISL_791251, EPI_ISL_791252, EPI_ISL_791253, EPI_ISL_791269, EPI_ISL_791270, EPI_ISL_791271, EPI_ISL_791272, EPI_ISL_791273, EPI_ISL_791274, EPI_ISL_791275, EPI_ISL_791276, EPI_ISL_791277, EPI_ISL_791278			
see above	Respiratory Virus Unit, National Infection Service, Public Health England	COVID-19 Genomics UK (COG-UK) Consortium	PHE Covid Sequencing Team
EPI_ISL_796784, EPI_ISL_796785, EPI_ISL_796786, EPI_ISL_796787, EPI_ISL_796788, EPI_ISL_796789, EPI_ISL_796790, EPI_ISL_796791, EPI_ISL_796792, EPI_ISL_796793, EPI_ISL_796794, EPI_ISL_796796, EPI_ISL_796797, EPI_ISL_796798, EPI_ISL_796799, EPI_ISL_796800, EPI_ISL_796801, EPI_ISL_796802, EPI_ISL_796803, EPI_ISL_796804, EPI_ISL_796805, EPI_ISL_796806, EPI_ISL_796807, EPI_ISL_796808, EPI_ISL_796809, EPI_ISL_796810, EPI_ISL_796811, EPI_ISL_796812, EPI_ISL_796813, EPI_ISL_796814, EPI_ISL_796815, EPI_ISL_796816, EPI_ISL_796817, EPI_ISL_796818, EPI_ISL_796819, EPI_ISL_796820, EPI_ISL_796821, EPI_ISL_796822, EPI_ISL_796823, EPI_ISL_796824, EPI_ISL_796825, EPI_ISL_796826, EPI_ISL_796827, EPI_ISL_796828, EPI_ISL_796829, EPI_ISL_796830, EPI_ISL_796831, EPI_ISL_796832, EPI_ISL_796833, EPI_ISL_796834, EPI_ISL_796835, EPI_ISL_796836, EPI_ISL_796837, EPI_ISL_796838, EPI_ISL_796839, EPI_ISL_796840, EPI_ISL_796841, EPI_ISL_796842, EPI_ISL_796843, EPI_ISL_796844, EPI_ISL_796845, EPI_ISL_796846, EPI_ISL_796847, EPI_ISL_796848, EPI_ISL_796849, EPI_ISL_796850, EPI_ISL_796851, EPI_ISL_796852, EPI_ISL_796853, EPI_ISL_796854, EPI_ISL_796855, EPI_ISL_796856, EPI_ISL_796857, EPI_ISL_796858, EPI_ISL_796859, EPI_ISL_796860, EPI_ISL_796861, EPI_ISL_796862, EPI_ISL_796863, EPI_ISL_796864, EPI_ISL_796865, EPI_ISL_796866, EPI_ISL_796867, EPI_ISL_796868, EPI_ISL_796869, EPI_ISL_796870, EPI_ISL_796871, EPI_ISL_796872, EPI_ISL_796873, EPI_ISL_796874, EPI_ISL_796875, EPI_ISL_796876, EPI_ISL_796877, EPI_ISL_796878, EPI_ISL_796879, EPI_ISL_796880, EPI_ISL_796881, EPI_ISL_796882, EPI_ISL_796883, EPI_ISL_796884, EPI_ISL_796885, EPI_ISL_796886, EPI_ISL_796887, EPI_ISL_796888, EPI_ISL_796889, EPI_ISL_796890, EPI_ISL_796891, EPI_ISL_796892, EPI_ISL_796893, EPI_ISL_796894, EPI_ISL_796895, EPI_ISL_796896, EPI_ISL_796897, EPI_ISL_796898, EPI_ISL_796899, EPI_ISL_796900, EPI_ISL_796901, EPI_ISL_796902, EPI_ISL_796903, EPI_ISL_796904, EPI_ISL_796905, EPI_ISL_796906, EPI_ISL_796907, EPI_ISL_796908, EPI_ISL_796909, EPI_ISL_796910, EPI_ISL_796911, EPI_ISL_796912, EPI_ISL_796913, EPI_ISL_796914, EPI_ISL_796915, EPI_ISL_796916, EPI_ISL_796917, EPI_ISL_796918, EPI_ISL_796919, EPI_ISL_796920, EPI_ISL_796921, EPI_ISL_796922, EPI_ISL_796923, EPI_ISL_796924, EPI_ISL_796925, EPI_ISL_796926, EPI_ISL_796927, EPI_ISL_796928, EPI_ISL_796929, EPI_ISL_796930, EPI_ISL_796931, EPI_ISL_796932, EPI_ISL_796933, EPI_ISL_796934, EPI_ISL_796935, EPI_ISL_796936, EPI_ISL_796937, EPI_ISL_796938, EPI_ISL_796939, EPI_ISL_796940, EPI_ISL_796941, EPI_ISL_796942, EPI_ISL_796943, EPI_ISL_796944, EPI_ISL_796945, EPI_ISL_796946, EPI_ISL_796947, EPI_ISL_796948, EPI_ISL_796949, EPI_ISL_796950, EPI_ISL_796951, EPI_ISL_796952, EPI_ISL_796953, EPI_ISL_796954, EPI_ISL_796955, EPI_ISL_796956, EPI_ISL_796957, EPI_ISL_796958, EPI_ISL_796959, EPI_ISL_796960, EPI_ISL_796961, EPI_ISL_796962, EPI_ISL_796963, EPI_ISL_796964, EPI_ISL_796965, EPI_ISL_796966, EPI_ISL_796967, EPI_ISL_796968, EPI_ISL_796969, EPI_ISL_796970, EPI_ISL_796971, EPI_ISL_796972, EPI_ISL_796973, EPI_ISL_796974, EPI_ISL_796975, EPI_ISL_796976, EPI_ISL_796977, EPI_ISL_796978, EPI_ISL_796979, EPI_ISL_796980, EPI_ISL_796981, EPI_ISL_796982, EPI_ISL_796983, EPI_ISL_796984, EPI_ISL_796985, EPI_ISL_796986, EPI_ISL_796987, EPI_ISL_796988, EPI_ISL_796989, EPI_ISL_796990, EPI_ISL_796991, EPI_ISL_796992, EPI_ISL_796993, EPI_ISL_796994, EPI_ISL_796995, EPI_ISL_796996, EPI_ISL_796997, EPI_ISL_796998, EPI_ISL_796999, EPI_ISL_797000, EPI_ISL_797001, EPI_ISL_797002, EPI_ISL_797003, EPI_ISL_797004, EPI_ISL_797005, EPI_ISL_797006, EPI_ISL_797007, EPI_ISL_797008, EPI_ISL_797009, EPI_ISL_797010, EPI_ISL_797011, EPI_ISL_797012, EPI_ISL_797013, EPI_ISL_797014, EPI_ISL_797015, EPI_ISL_797016, EPI_ISL_797017, EPI_ISL_797018, EPI_ISL_797019, EPI_ISL_797020, EPI_ISL_797021, EPI_ISL_797022, EPI_ISL_797023, EPI_ISL_797024, EPI_ISL_797025, EPI_ISL_797026, EPI_ISL_797027, EPI_ISL_797028, EPI_ISL_797029, EPI_ISL_797030, EPI_ISL_797031, EPI_ISL_797032, EPI_ISL_797033, EPI_ISL_797034, EPI_ISL_797035, EPI_ISL_797036, EPI_ISL_797037, EPI_ISL_797038, EPI_ISL_797039, EPI_ISL_797040, EPI_ISL_797041, EPI_ISL_797042, EPI_ISL_797043, EPI_ISL_797044, EPI_ISL_797045, EPI_ISL_797046, EPI_ISL_797047, EPI_ISL_797048, EPI_ISL_797049, EPI_ISL_797050, EPI_ISL_797051, EPI_ISL_797052, EPI_ISL_797053, EPI_ISL_797054, EPI_ISL_797055, EPI_ISL_797056, EPI_ISL_797057, EPI_ISL_797058, EPI_ISL_797059, EPI_ISL_797060, EPI_ISL_797061, EPI_ISL_797062, EPI_ISL_797063, EPI_ISL_797064, EPI_ISL_797065, EPI_ISL_797066, EPI_ISL_797067, EPI_ISL_797068, EPI_ISL_797069, EPI_ISL_797070, EPI_ISL_797071, EPI_ISL_797072, EPI_ISL_797073, EPI_ISL_797074, EPI_ISL_797075, EPI_ISL_797076, EPI_ISL_797077, EPI_ISL_797078, EPI_ISL_797079, EPI_ISL_797080, EPI_ISL_797081, EPI_ISL_797082, EPI_ISL_797083, EPI_ISL_797084, EPI_ISL_797085, EPI_ISL_797086, EPI_ISL_797088, EPI_ISL_797089, EPI_ISL_797090, EPI_ISL_797091, EPI_ISL_797092, EPI_ISL_797093, EPI_ISL_797094, EPI_ISL_797095, EPI_ISL_797096, EPI_ISL_797097, EPI_ISL_797098, EPI_ISL_797099, EPI_ISL_797100, EPI_ISL_797101, EPI_ISL_797102, EPI_ISL_797103, EPI_ISL_797104, EPI_ISL_797105, EPI_ISL_797106, EPI_ISL_797107, EPI_ISL_797108, EPI_ISL_797109, EPI_ISL_797110, EPI_ISL_797111, EPI_ISL_797112, EPI_ISL_797113, EPI_ISL_797114, EPI_ISL_797115, EPI_ISL_797116, EPI_ISL_797117			
see above	Lighthouse Lab in Alderley Park	Wellcome Sanger Institute for the COVID-19 Genomics UK (COG-UK) Consortium	Jacquelyn Wynn, Mairead Hyland, The Lighthouse Lab in Alderley Park and Alex Alderton, Roberto Amato, Sonia Goncalves, Ewan Harrison, David K. Jackson, Ian Johnston, Dominic Kwiatkowski, Cordelia Langford, John Sillitoe on behalf of the Wellcome Sanger Institute COVID-19 Surveillance Team
EPI_ISL_797118, EPI_ISL_797120, EPI_ISL_797121, EPI_ISL_797122, EPI_ISL_797123, EPI_ISL_797124, EPI_ISL_797125, EPI_ISL_797126, EPI_ISL_797127, EPI_ISL_797128, EPI_ISL_797129, EPI_ISL_797131, EPI_ISL_797133, EPI_ISL_797134, EPI_ISL_797135, EPI_ISL_797136, EPI_ISL_797137, EPI_ISL_797138, EPI_ISL_797139, EPI_ISL_797140, EPI_ISL_797141, EPI_ISL_797142, EPI_ISL_797143, EPI_ISL_797144, EPI_ISL_797147, EPI_ISL_797148, EPI_ISL_797149, EPI_ISL_797151, EPI_ISL_797153, EPI_ISL_797154, EPI_ISL_797155, EPI_ISL_797156, EPI_ISL_797157, EPI_ISL_797159, EPI_ISL_797160, EPI_ISL_797161, EPI_ISL_797163, EPI_ISL_797164, EPI_ISL_797165, EPI_ISL_797166, EPI_ISL_797167, EPI_ISL_797168, EPI_ISL_797169, EPI_ISL_797171, EPI_ISL_797172, EPI_ISL_797173, EPI_ISL_797174, EPI_ISL_797175, EPI_ISL_797176, EPI_ISL_797177, EPI_ISL_797178, EPI_ISL_797179, EPI_ISL_797181, EPI_ISL_797182, EPI_ISL_797185, EPI_ISL_797187, EPI_ISL_797190, EPI_ISL_797191, EPI_ISL_797194, EPI_ISL_797195, EPI_ISL_797196, EPI_ISL_797198, EPI_ISL_797200, EPI_ISL_797202, EPI_ISL_797203, EPI_ISL_797204, EPI_ISL_797205, EPI_ISL_797206, EPI_ISL_797207, EPI_ISL_797208, EPI_ISL_797210, EPI_ISL_797212, EPI_ISL_797215, EPI_ISL_797216, EPI_ISL_797217, EPI_ISL_797218, EPI_ISL_797220, EPI_ISL_797222, EPI_ISL_797225, EPI_ISL_797226, EPI_ISL_797227, EPI_ISL_797228, EPI_ISL_797229, EPI_ISL_797231, EPI_ISL_797232, EPI_ISL_797234, EPI_ISL_797238, EPI_ISL_797239, EPI_ISL_797240, EPI_ISL_797242, EPI_ISL_797243, EPI_ISL_797246, EPI_ISL_797247, EPI_ISL_797248, EPI_ISL_797249, EPI_ISL_797251, EPI_ISL_797252, EPI_ISL_797253, EPI_ISL_797254, EPI_ISL_797256, EPI_ISL_797258, EPI_ISL_797259, EPI_ISL_797260, EPI_ISL_797261, EPI_ISL_797262, EPI_ISL_797263, EPI_ISL_797264, EPI_ISL_797265, EPI_ISL_797266, EPI_ISL_797267, EPI_ISL_797269, EPI_ISL_797272, EPI_ISL_797275, EPI_ISL_797276, EPI_ISL_797277, EPI_ISL_797278, EPI_ISL_797279, EPI_ISL_797281, EPI_ISL_797282, EPI_ISL_797283, EPI_ISL_797284, EPI_ISL_797285, EPI_ISL_797286, EPI_ISL_797289, EPI_ISL_797290, EPI_ISL_797292, EPI_ISL_797294, EPI_ISL_797295, EPI_ISL_797296, EPI_ISL_797297, EPI_ISL_797299, EPI_ISL_797301, EPI_ISL_797304, EPI_ISL_797305, EPI_ISL_797306, EPI_ISL_797307, EPI_ISL_797310, EPI_ISL_797312, EPI_ISL_797313, EPI_ISL_797314, EPI_ISL_797315, EPI_ISL_797317, EPI_ISL_797318, EPI_ISL_797319, EPI_ISL_797323, EPI_ISL_797324, EPI_ISL_797325, EPI_ISL_797326, EPI_ISL_797330, EPI_ISL_797331, EPI_ISL_797332, EPI_ISL_797334, EPI_ISL_797335, EPI_ISL_797337, EPI_ISL_797338, EPI_ISL_797339, EPI_ISL_797340, EPI_ISL_797342, EPI_ISL_797345, EPI_ISL_797346, EPI_ISL_797349, EPI_ISL_797352, EPI_ISL_797357, EPI_ISL_797359, EPI_ISL_797360, EPI_ISL_797362, EPI_ISL_797363, EPI_ISL_797364, EPI_ISL_797365, EPI_ISL_797366, EPI_ISL_797367, EPI_ISL_797369, EPI_ISL_797371, EPI_ISL_797373, EPI_ISL_797374, EPI_ISL_797376, EPI_ISL_797377, EPI_ISL_797378, EPI_ISL_797379, EPI_ISL_797380, EPI_ISL_797381, EPI_ISL_797383, EPI_ISL_797384, EPI_ISL_797385, EPI_ISL_797387, EPI_ISL_797389, EPI_ISL_797390, EPI_ISL_797391, EPI_ISL_797392, EPI_ISL_797394, EPI_ISL_797395, EPI_ISL_797396			
see above	Lighthouse Lab in Milton Keynes	Wellcome Sanger Institute for the COVID-19 Genomics UK (COG-UK) Consortium	The Lighthouse Lab in Milton Keynes and Alex Alderton, Roberto Amato, Sonia Goncalves, Ewan Harrison, David K. Jackson, Ian Johnston, Dominic Kwiatkowski, Cordelia Langford, John Sillitoe on behalf of the Wellcome Sanger Institute COVID-19 Surveillance Team
EPI_ISL_797397, EPI_ISL_797398	Lighthouse Lab in Cambridge	Wellcome Sanger Institute for the COVID-19 Genomics UK (COG-UK) Consortium	Rob Howes, The Lighthouse Lab in Cambridge and Alex Alderton, Roberto Amato, Sonia Goncalves, Ewan Harrison, David K. Jackson, Ian Johnston, Dominic Kwiatkowski, Cordelia Langford, John Sillitoe on behalf of the Wellcome Sanger Institute COVID-19 Surveillance Team
EPI_ISL_797399	Lighthouse Lab in Alderley Park	Wellcome Sanger Institute for the COVID-19 Genomics UK (COG-UK) Consortium	Jacquelyn Wynn, Mairead Hyland, The Lighthouse Lab in Alderley Park and Alex Alderton, Roberto Amato, Sonia Goncalves, Ewan Harrison, David K. Jackson, Ian Johnston, Dominic Kwiatkowski, Cordelia Langford, John Sillitoe on behalf of the Wellcome Sanger Institute COVID-19 Surveillance Team
EPI_ISL_797400, EPI_ISL_797401	Lighthouse Lab in Cambridge	Wellcome Sanger Institute for the COVID-19 Genomics UK (COG-UK) Consortium	Rob Howes, The Lighthouse Lab in Cambridge and Alex Alderton, Roberto Amato, Sonia Goncalves, Ewan Harrison, David K. Jackson, Ian Johnston, Dominic Kwiatkowski, Cordelia Langford, John Sillitoe on behalf of the Wellcome Sanger Institute COVID-19 Surveillance Team
EPI_ISL_797402	Lighthouse Lab in Alderley Park	Wellcome Sanger Institute for the COVID-19 Genomics UK (COG-UK) Consortium	Jacquelyn Wynn, Mairead Hyland, The Lighthouse Lab in Alderley Park and Alex Alderton, Roberto Amato, Sonia Goncalves, Ewan Harrison, David K. Jackson, Ian Johnston, Dominic Kwiatkowski, Cordelia Langford, John Sillitoe on behalf of the Wellcome Sanger Institute COVID-19 Surveillance Team
EPI_ISL_797403, EPI_ISL_797404, EPI_ISL_797406, EPI_ISL_797407	Lighthouse Lab in Cambridge	Wellcome Sanger Institute for the COVID-19 Genomics UK (COG-UK) Consortium	Rob Howes, The Lighthouse Lab in Cambridge and Alex Alderton, Roberto Amato, Sonia Goncalves, Ewan Harrison, David K. Jackson, Ian Johnston, Dominic Kwiatkowski, Cordelia Langford, John Sillitoe on behalf of the Wellcome Sanger Institute COVID-19 Surveillance Team
EPI_ISL_797409	Lighthouse Lab in Alderley Park	Wellcome Sanger Institute for the COVID-19 Genomics UK (COG-UK) Consortium	Jacquelyn Wynn, Mairead Hyland, The Lighthouse Lab in Alderley Park and Alex Alderton, Roberto Amato, Sonia Goncalves, Ewan Harrison, David K. Jackson, Ian Johnston, Dominic Kwiatkowski, Cordelia Langford, John Sillitoe on behalf of the Wellcome Sanger Institute COVID-19 Surveillance Team
EPI_ISL_797410	Lighthouse Lab in Cambridge	Wellcome Sanger Institute for the COVID-19 Genomics UK (COG-UK) Consortium	Rob Howes, The Lighthouse Lab in Cambridge and Alex Alderton, Roberto Amato, Sonia Goncalves, Ewan Harrison, David K. Jackson, Ian Johnston, Dominic Kwiatkowski, Cordelia Langford, John Sillitoe on behalf of the Wellcome Sanger Institute COVID-19 Surveillance Team
EPI_ISL_797411	Lighthouse Lab in Alderley Park	Wellcome Sanger Institute for the COVID-19 Genomics UK	Jacquelyn Wynn, Mairead Hyland, The Lighthouse Lab in Alderley Park and Alex Alderton, Roberto Amato, Sonia Goncalves, Ewan Harrison, David K.

		(COG-UK) Consortium	Jackson, Ian Johnston, Dominic Kwiatkowski, Cordelia Langford, John Sillitoe on behalf of the Wellcome Sanger Institute COVID-19 Surveillance Team
EPI_ISL_797412	Lighthouse Lab in Cambridge	Wellcome Sanger Institute for the COVID-19 Genomics UK (COG-UK) Consortium	Rob Howes, The Lighthouse Lab in Cambridge and Alex Alderton, Roberto Amato, Sonia Goncalves, Ewan Harrison, David K. Jackson, Ian Johnston, Dominic Kwiatkowski, Cordelia Langford, John Sillitoe on behalf of the Wellcome Sanger Institute COVID-19 Surveillance Team
EPI_ISL_797413	Lighthouse Lab in Alderley Park	Wellcome Sanger Institute for the COVID-19 Genomics UK (COG-UK) Consortium	Jacquelyn Wynn, Mairead Hyland, The Lighthouse Lab in Alderley Park and Alex Alderton, Roberto Amato, Sonia Goncalves, Ewan Harrison, David K. Jackson, Ian Johnston, Dominic Kwiatkowski, Cordelia Langford, John Sillitoe on behalf of the Wellcome Sanger Institute COVID-19 Surveillance Team
EPI_ISL_797414, EPI_ISL_797415	Lighthouse Lab in Cambridge	Wellcome Sanger Institute for the COVID-19 Genomics UK (COG-UK) Consortium	Rob Howes, The Lighthouse Lab in Cambridge and Alex Alderton, Roberto Amato, Sonia Goncalves, Ewan Harrison, David K. Jackson, Ian Johnston, Dominic Kwiatkowski, Cordelia Langford, John Sillitoe on behalf of the Wellcome Sanger Institute COVID-19 Surveillance Team
EPI_ISL_797416	Lighthouse Lab in Alderley Park	Wellcome Sanger Institute for the COVID-19 Genomics UK (COG-UK) Consortium	Jacquelyn Wynn, Mairead Hyland, The Lighthouse Lab in Alderley Park and Alex Alderton, Roberto Amato, Sonia Goncalves, Ewan Harrison, David K. Jackson, Ian Johnston, Dominic Kwiatkowski, Cordelia Langford, John Sillitoe on behalf of the Wellcome Sanger Institute COVID-19 Surveillance Team
EPI_ISL_797417, EPI_ISL_797418, EPI_ISL_797419	Lighthouse Lab in Cambridge	Wellcome Sanger Institute for the COVID-19 Genomics UK (COG-UK) Consortium	Rob Howes, The Lighthouse Lab in Cambridge and Alex Alderton, Roberto Amato, Sonia Goncalves, Ewan Harrison, David K. Jackson, Ian Johnston, Dominic Kwiatkowski, Cordelia Langford, John Sillitoe on behalf of the Wellcome Sanger Institute COVID-19 Surveillance Team
EPI_ISL_797420, EPI_ISL_797421	Lighthouse Lab in Alderley Park	Wellcome Sanger Institute for the COVID-19 Genomics UK (COG-UK) Consortium	Jacquelyn Wynn, Mairead Hyland, The Lighthouse Lab in Alderley Park and Alex Alderton, Roberto Amato, Sonia Goncalves, Ewan Harrison, David K. Jackson, Ian Johnston, Dominic Kwiatkowski, Cordelia Langford, John Sillitoe on behalf of the Wellcome Sanger Institute COVID-19 Surveillance Team
EPI_ISL_797422, EPI_ISL_797423	Lighthouse Lab in Cambridge	Wellcome Sanger Institute for the COVID-19 Genomics UK (COG-UK) Consortium	Rob Howes, The Lighthouse Lab in Cambridge and Alex Alderton, Roberto Amato, Sonia Goncalves, Ewan Harrison, David K. Jackson, Ian Johnston, Dominic Kwiatkowski, Cordelia Langford, John Sillitoe on behalf of the Wellcome Sanger Institute COVID-19 Surveillance Team
EPI_ISL_797424	Lighthouse Lab in Alderley Park	Wellcome Sanger Institute for the COVID-19 Genomics UK (COG-UK) Consortium	Jacquelyn Wynn, Mairead Hyland, The Lighthouse Lab in Alderley Park and Alex Alderton, Roberto Amato, Sonia Goncalves, Ewan Harrison, David K. Jackson, Ian Johnston, Dominic Kwiatkowski, Cordelia Langford, John Sillitoe on behalf of the Wellcome Sanger Institute COVID-19 Surveillance Team
EPI_ISL_797425, EPI_ISL_797426, EPI_ISL_797427	Lighthouse Lab in Cambridge	Wellcome Sanger Institute for the COVID-19 Genomics UK (COG-UK) Consortium	Rob Howes, The Lighthouse Lab in Cambridge and Alex Alderton, Roberto Amato, Sonia Goncalves, Ewan Harrison, David K. Jackson, Ian Johnston, Dominic Kwiatkowski, Cordelia Langford, John Sillitoe on behalf of the Wellcome Sanger Institute COVID-19 Surveillance Team
EPI_ISL_797428	Lighthouse Lab in Alderley Park	Wellcome Sanger Institute for the COVID-19 Genomics UK (COG-UK) Consortium	Jacquelyn Wynn, Mairead Hyland, The Lighthouse Lab in Alderley Park and Alex Alderton, Roberto Amato, Sonia Goncalves, Ewan Harrison, David K. Jackson, Ian Johnston, Dominic Kwiatkowski, Cordelia Langford, John Sillitoe on behalf of the Wellcome Sanger Institute COVID-19 Surveillance Team
EPI_ISL_797429	Lighthouse Lab in Cambridge	Wellcome Sanger Institute for the COVID-19 Genomics UK (COG-UK) Consortium	Rob Howes, The Lighthouse Lab in Cambridge and Alex Alderton, Roberto Amato, Sonia Goncalves, Ewan Harrison, David K. Jackson, Ian Johnston, Dominic Kwiatkowski, Cordelia Langford, John Sillitoe on behalf of the Wellcome Sanger Institute COVID-19 Surveillance Team
EPI_ISL_797430	Lighthouse Lab in Alderley Park	Wellcome Sanger Institute for the COVID-19 Genomics UK (COG-UK) Consortium	Jacquelyn Wynn, Mairead Hyland, The Lighthouse Lab in Alderley Park and Alex Alderton, Roberto Amato, Sonia Goncalves, Ewan Harrison, David K. Jackson, Ian Johnston, Dominic Kwiatkowski, Cordelia Langford, John Sillitoe on behalf of the Wellcome Sanger Institute COVID-19 Surveillance Team
EPI_ISL_797431, EPI_ISL_797433, EPI_ISL_797434	Lighthouse Lab in Cambridge	Wellcome Sanger Institute for the COVID-19 Genomics UK (COG-UK) Consortium	Rob Howes, The Lighthouse Lab in Cambridge and Alex Alderton, Roberto Amato, Sonia Goncalves, Ewan Harrison, David K. Jackson, Ian Johnston, Dominic Kwiatkowski, Cordelia Langford, John Sillitoe on behalf of the Wellcome Sanger Institute COVID-19 Surveillance Team
EPI_ISL_797435	Lighthouse Lab in Alderley Park	Wellcome Sanger Institute for the COVID-19 Genomics UK (COG-UK) Consortium	Jacquelyn Wynn, Mairead Hyland, The Lighthouse Lab in Alderley Park and Alex Alderton, Roberto Amato, Sonia Goncalves, Ewan Harrison, David K. Jackson, Ian Johnston, Dominic Kwiatkowski, Cordelia Langford, John Sillitoe on behalf of the Wellcome Sanger Institute COVID-19 Surveillance Team
EPI_ISL_797436, EPI_ISL_797437	Lighthouse Lab in Cambridge	Wellcome Sanger Institute for the COVID-19 Genomics UK (COG-UK) Consortium	Rob Howes, The Lighthouse Lab in Cambridge and Alex Alderton, Roberto Amato, Sonia Goncalves, Ewan Harrison, David K. Jackson, Ian Johnston, Dominic Kwiatkowski, Cordelia Langford, John Sillitoe on behalf of the Wellcome Sanger Institute COVID-19 Surveillance Team
EPI_ISL_797438	Lighthouse Lab in Alderley Park	Wellcome Sanger Institute for the COVID-19 Genomics UK (COG-UK) Consortium	Jacquelyn Wynn, Mairead Hyland, The Lighthouse Lab in Alderley Park and Alex Alderton, Roberto Amato, Sonia Goncalves, Ewan Harrison, David K. Jackson, Ian Johnston, Dominic Kwiatkowski, Cordelia Langford, John Sillitoe on behalf of the Wellcome Sanger Institute COVID-19 Surveillance Team
EPI_ISL_797439, EPI_ISL_797440	Lighthouse Lab in Cambridge	Wellcome Sanger Institute for the COVID-19 Genomics UK (COG-UK) Consortium	Rob Howes, The Lighthouse Lab in Cambridge and Alex Alderton, Roberto Amato, Sonia Goncalves, Ewan Harrison, David K. Jackson, Ian Johnston, Dominic Kwiatkowski, Cordelia Langford, John Sillitoe on behalf of the Wellcome Sanger Institute COVID-19 Surveillance Team
EPI_ISL_797441	Lighthouse Lab in Alderley Park	Wellcome Sanger Institute for the COVID-19 Genomics UK (COG-UK) Consortium	Jacquelyn Wynn, Mairead Hyland, The Lighthouse Lab in Alderley Park and Alex Alderton, Roberto Amato, Sonia Goncalves, Ewan Harrison, David K. Jackson, Ian Johnston, Dominic Kwiatkowski, Cordelia Langford, John Sillitoe on behalf of the Wellcome Sanger Institute COVID-19 Surveillance Team
EPI_ISL_797442, EPI_ISL_797443, EPI_ISL_797444, EPI_ISL_797446, EPI_ISL_797447, EPI_ISL_797448, EPI_ISL_797449, EPI_ISL_797450, EPI_ISL_797452, EPI_ISL_797453, EPI_ISL_797454, EPI_ISL_797455			
see above	Lighthouse Lab in Cambridge	Wellcome Sanger Institute for the COVID-19 Genomics UK (COG-UK) Consortium	Rob Howes, The Lighthouse Lab in Cambridge and Alex Alderton, Roberto Amato, Sonia Goncalves, Ewan Harrison, David K. Jackson, Ian Johnston, Dominic Kwiatkowski, Cordelia Langford, John Sillitoe on behalf of the Wellcome Sanger Institute COVID-19 Surveillance Team
EPI_ISL_797456	Lighthouse Lab in Alderley Park	Wellcome Sanger Institute for the COVID-19 Genomics UK (COG-UK) Consortium	Jacquelyn Wynn, Mairead Hyland, The Lighthouse Lab in Alderley Park and Alex Alderton, Roberto Amato, Sonia Goncalves, Ewan Harrison, David K. Jackson, Ian Johnston, Dominic Kwiatkowski, Cordelia Langford, John Sillitoe on behalf of the Wellcome Sanger Institute COVID-19 Surveillance Team
EPI_ISL_797457, EPI_ISL_797458, EPI_ISL_797460	Lighthouse Lab in Cambridge	Wellcome Sanger Institute for the COVID-19 Genomics UK (COG-UK) Consortium	Rob Howes, The Lighthouse Lab in Cambridge and Alex Alderton, Roberto Amato, Sonia Goncalves, Ewan Harrison, David K. Jackson, Ian Johnston, Dominic Kwiatkowski, Cordelia Langford, John Sillitoe on behalf of the Wellcome Sanger Institute COVID-19 Surveillance Team
EPI_ISL_797461, EPI_ISL_797462	Lighthouse Lab in Alderley Park	Wellcome Sanger Institute for the COVID-19 Genomics UK (COG-UK) Consortium	Jacquelyn Wynn, Mairead Hyland, The Lighthouse Lab in Alderley Park and Alex Alderton, Roberto Amato, Sonia Goncalves, Ewan Harrison, David K. Jackson, Ian Johnston, Dominic Kwiatkowski, Cordelia Langford, John Sillitoe on behalf of the Wellcome Sanger Institute COVID-19 Surveillance Team
EPI_ISL_797463, EPI_ISL_797464, EPI_ISL_797465, EPI_ISL_797466, EPI_ISL_797467	Lighthouse Lab in Cambridge	Wellcome Sanger Institute for the COVID-19 Genomics UK (COG-UK) Consortium	Rob Howes, The Lighthouse Lab in Cambridge and Alex Alderton, Roberto Amato, Sonia Goncalves, Ewan Harrison, David K. Jackson, Ian Johnston, Dominic Kwiatkowski, Cordelia Langford, John Sillitoe on behalf of the Wellcome Sanger Institute COVID-19 Surveillance Team
EPI_ISL_797468	Lighthouse Lab in Alderley Park	Wellcome Sanger Institute for the COVID-19 Genomics UK (COG-UK) Consortium	Jacquelyn Wynn, Mairead Hyland, The Lighthouse Lab in Alderley Park and Alex Alderton, Roberto Amato, Sonia Goncalves, Ewan Harrison, David K. Jackson, Ian Johnston, Dominic Kwiatkowski, Cordelia Langford, John Sillitoe on behalf of the Wellcome Sanger Institute COVID-19 Surveillance Team
EPI_ISL_797469, EPI_ISL_797470, EPI_ISL_797471, EPI_ISL_797472, EPI_ISL_797473, EPI_ISL_797474	Lighthouse Lab in Cambridge	Wellcome Sanger Institute for the COVID-19 Genomics UK (COG-UK) Consortium	Rob Howes, The Lighthouse Lab in Cambridge and Alex Alderton, Roberto Amato, Sonia Goncalves, Ewan Harrison, David K. Jackson, Ian Johnston, Dominic Kwiatkowski, Cordelia Langford, John Sillitoe on behalf of the Wellcome Sanger Institute COVID-19 Surveillance Team
EPI_ISL_797475	Lighthouse Lab in Alderley Park	Wellcome Sanger Institute for the COVID-19 Genomics UK (COG-UK) Consortium	Jacquelyn Wynn, Mairead Hyland, The Lighthouse Lab in Alderley Park and Alex Alderton, Roberto Amato, Sonia Goncalves, Ewan Harrison, David K. Jackson, Ian Johnston, Dominic Kwiatkowski, Cordelia Langford, John Sillitoe on behalf of the Wellcome Sanger Institute COVID-19 Surveillance Team
EPI_ISL_797476, EPI_ISL_797477, EPI_ISL_797478, EPI_ISL_797479	Lighthouse Lab in Cambridge	Wellcome Sanger Institute for the COVID-19 Genomics UK (COG-UK) Consortium	Rob Howes, The Lighthouse Lab in Cambridge and Alex Alderton, Roberto Amato, Sonia Goncalves, Ewan Harrison, David K. Jackson, Ian Johnston, Dominic Kwiatkowski, Cordelia Langford, John Sillitoe on behalf of the Wellcome Sanger Institute COVID-19 Surveillance Team
EPI_ISL_797480	Lighthouse Lab in Alderley Park	Wellcome Sanger Institute for the COVID-19 Genomics UK (COG-UK) Consortium	Jacquelyn Wynn, Mairead Hyland, The Lighthouse Lab in Alderley Park and Alex Alderton, Roberto Amato, Sonia Goncalves, Ewan Harrison, David K. Jackson, Ian Johnston, Dominic Kwiatkowski, Cordelia Langford, John Sillitoe on behalf of the Wellcome Sanger Institute COVID-19 Surveillance Team
EPI_ISL_797481, EPI_ISL_797482, EPI_ISL_797483	Lighthouse Lab in Cambridge	Wellcome Sanger Institute for the COVID-19 Genomics UK (COG-UK) Consortium	Rob Howes, The Lighthouse Lab in Cambridge and Alex Alderton, Roberto Amato, Sonia Goncalves, Ewan Harrison, David K. Jackson, Ian Johnston, Dominic Kwiatkowski, Cordelia Langford, John Sillitoe on behalf of the Wellcome Sanger Institute COVID-19 Surveillance Team
EPI_ISL_797484	Lighthouse Lab in Alderley Park	Wellcome Sanger Institute for the COVID-19 Genomics UK (COG-UK) Consortium	Jacquelyn Wynn, Mairead Hyland, The Lighthouse Lab in Alderley Park and Alex Alderton, Roberto Amato, Sonia Goncalves, Ewan Harrison, David K. Jackson, Ian Johnston, Dominic Kwiatkowski, Cordelia Langford, John Sillitoe on behalf of the Wellcome Sanger Institute COVID-19 Surveillance Team
EPI_ISL_797485, EPI_ISL_797486, EPI_ISL_797487, EPI_ISL_797488	Lighthouse Lab in Cambridge	Wellcome Sanger Institute for the COVID-19 Genomics UK (COG-UK) Consortium	Rob Howes, The Lighthouse Lab in Cambridge and Alex Alderton, Roberto Amato, Sonia Goncalves, Ewan Harrison, David K. Jackson, Ian Johnston, Dominic Kwiatkowski, Cordelia Langford, John Sillitoe on behalf of the Wellcome Sanger Institute COVID-19 Surveillance Team
EPI_ISL_797489	Lighthouse Lab in Alderley Park	Wellcome Sanger Institute for the COVID-19 Genomics UK (COG-UK) Consortium	Jacquelyn Wynn, Mairead Hyland, The Lighthouse Lab in Alderley Park and Alex Alderton, Roberto Amato, Sonia Goncalves, Ewan Harrison, David K. Jackson, Ian Johnston, Dominic Kwiatkowski, Cordelia Langford, John Sillitoe on behalf of the Wellcome Sanger Institute COVID-19 Surveillance Team
EPI_ISL_797490	Lighthouse Lab in Cambridge	Wellcome Sanger Institute for the COVID-19 Genomics UK (COG-UK) Consortium	Rob Howes, The Lighthouse Lab in Cambridge and Alex Alderton, Roberto Amato, Sonia Goncalves, Ewan Harrison, David K. Jackson, Ian Johnston, Dominic Kwiatkowski, Cordelia Langford, John Sillitoe on behalf of the Wellcome Sanger Institute COVID-19 Surveillance Team

EPI_ISL_797491	Lighthouse Lab in Alderley Park	Wellcome Sanger Institute for the COVID-19 Genomics UK (COG-UK) Consortium	Jacquelyn Wynn, Mairead Hyland, The Lighthouse Lab in Alderley Park and Alex Alderton, Roberto Amato, Sonia Goncalves, Ewan Harrison, David K. Jackson, Ian Johnston, Dominic Kwiatkowski, Cordelia Langford, John Sillitoe on behalf of the Wellcome Sanger Institute COVID-19 Surveillance Team
EPI_ISL_797492, EPI_ISL_797493, EPI_ISL_797494, EPI_ISL_797495, EPI_ISL_797496, EPI_ISL_797497, EPI_ISL_797498	Lighthouse Lab in Cambridge	Wellcome Sanger Institute for the COVID-19 Genomics UK (COG-UK) Consortium	Rob Howes, The Lighthouse Lab in Cambridge and Alex Alderton, Roberto Amato, Sonia Goncalves, Ewan Harrison, David K. Jackson, Ian Johnston, Dominic Kwiatkowski, Cordelia Langford, John Sillitoe on behalf of the Wellcome Sanger Institute COVID-19 Surveillance Team
EPI_ISL_797499	Lighthouse Lab in Alderley Park	Wellcome Sanger Institute for the COVID-19 Genomics UK (COG-UK) Consortium	Jacquelyn Wynn, Mairead Hyland, The Lighthouse Lab in Alderley Park and Alex Alderton, Roberto Amato, Sonia Goncalves, Ewan Harrison, David K. Jackson, Ian Johnston, Dominic Kwiatkowski, Cordelia Langford, John Sillitoe on behalf of the Wellcome Sanger Institute COVID-19 Surveillance Team
EPI_ISL_797501, EPI_ISL_797502, EPI_ISL_797503	Lighthouse Lab in Cambridge	Wellcome Sanger Institute for the COVID-19 Genomics UK (COG-UK) Consortium	Rob Howes, The Lighthouse Lab in Cambridge and Alex Alderton, Roberto Amato, Sonia Goncalves, Ewan Harrison, David K. Jackson, Ian Johnston, Dominic Kwiatkowski, Cordelia Langford, John Sillitoe on behalf of the Wellcome Sanger Institute COVID-19 Surveillance Team
EPI_ISL_797504	Lighthouse Lab in Alderley Park	Wellcome Sanger Institute for the COVID-19 Genomics UK (COG-UK) Consortium	Jacquelyn Wynn, Mairead Hyland, The Lighthouse Lab in Alderley Park and Alex Alderton, Roberto Amato, Sonia Goncalves, Ewan Harrison, David K. Jackson, Ian Johnston, Dominic Kwiatkowski, Cordelia Langford, John Sillitoe on behalf of the Wellcome Sanger Institute COVID-19 Surveillance Team
EPI_ISL_797505, EPI_ISL_797506, EPI_ISL_797507, EPI_ISL_797508	Lighthouse Lab in Cambridge	Wellcome Sanger Institute for the COVID-19 Genomics UK (COG-UK) Consortium	Rob Howes, The Lighthouse Lab in Cambridge and Alex Alderton, Roberto Amato, Sonia Goncalves, Ewan Harrison, David K. Jackson, Ian Johnston, Dominic Kwiatkowski, Cordelia Langford, John Sillitoe on behalf of the Wellcome Sanger Institute COVID-19 Surveillance Team
EPI_ISL_797509	Lighthouse Lab in Alderley Park	Wellcome Sanger Institute for the COVID-19 Genomics UK (COG-UK) Consortium	Jacquelyn Wynn, Mairead Hyland, The Lighthouse Lab in Alderley Park and Alex Alderton, Roberto Amato, Sonia Goncalves, Ewan Harrison, David K. Jackson, Ian Johnston, Dominic Kwiatkowski, Cordelia Langford, John Sillitoe on behalf of the Wellcome Sanger Institute COVID-19 Surveillance Team
EPI_ISL_797510, EPI_ISL_797511	Lighthouse Lab in Cambridge	Wellcome Sanger Institute for the COVID-19 Genomics UK (COG-UK) Consortium	Rob Howes, The Lighthouse Lab in Cambridge and Alex Alderton, Roberto Amato, Sonia Goncalves, Ewan Harrison, David K. Jackson, Ian Johnston, Dominic Kwiatkowski, Cordelia Langford, John Sillitoe on behalf of the Wellcome Sanger Institute COVID-19 Surveillance Team
EPI_ISL_797512	Lighthouse Lab in Alderley Park	Wellcome Sanger Institute for the COVID-19 Genomics UK (COG-UK) Consortium	Jacquelyn Wynn, Mairead Hyland, The Lighthouse Lab in Alderley Park and Alex Alderton, Roberto Amato, Sonia Goncalves, Ewan Harrison, David K. Jackson, Ian Johnston, Dominic Kwiatkowski, Cordelia Langford, John Sillitoe on behalf of the Wellcome Sanger Institute COVID-19 Surveillance Team
EPI_ISL_797513, EPI_ISL_797515, EPI_ISL_797516, EPI_ISL_797517, EPI_ISL_797518, EPI_ISL_797519, EPI_ISL_797520, EPI_ISL_797521, EPI_ISL_797522, EPI_ISL_797523, EPI_ISL_797524, EPI_ISL_797525, EPI_ISL_797526, EPI_ISL_797527, EPI_ISL_797528, EPI_ISL_797529, EPI_ISL_797530, EPI_ISL_797532			
see above	Lighthouse Lab in Cambridge	Wellcome Sanger Institute for the COVID-19 Genomics UK (COG-UK) Consortium	Rob Howes, The Lighthouse Lab in Cambridge and Alex Alderton, Roberto Amato, Sonia Goncalves, Ewan Harrison, David K. Jackson, Ian Johnston, Dominic Kwiatkowski, Cordelia Langford, John Sillitoe on behalf of the Wellcome Sanger Institute COVID-19 Surveillance Team
EPI_ISL_797533	Lighthouse Lab in Alderley Park	Wellcome Sanger Institute for the COVID-19 Genomics UK (COG-UK) Consortium	Jacquelyn Wynn, Mairead Hyland, The Lighthouse Lab in Alderley Park and Alex Alderton, Roberto Amato, Sonia Goncalves, Ewan Harrison, David K. Jackson, Ian Johnston, Dominic Kwiatkowski, Cordelia Langford, John Sillitoe on behalf of the Wellcome Sanger Institute COVID-19 Surveillance Team
EPI_ISL_797534	Lighthouse Lab in Cambridge	Wellcome Sanger Institute for the COVID-19 Genomics UK (COG-UK) Consortium	Rob Howes, The Lighthouse Lab in Cambridge and Alex Alderton, Roberto Amato, Sonia Goncalves, Ewan Harrison, David K. Jackson, Ian Johnston, Dominic Kwiatkowski, Cordelia Langford, John Sillitoe on behalf of the Wellcome Sanger Institute COVID-19 Surveillance Team
EPI_ISL_797535	Lighthouse Lab in Alderley Park	Wellcome Sanger Institute for the COVID-19 Genomics UK (COG-UK) Consortium	Jacquelyn Wynn, Mairead Hyland, The Lighthouse Lab in Alderley Park and Alex Alderton, Roberto Amato, Sonia Goncalves, Ewan Harrison, David K. Jackson, Ian Johnston, Dominic Kwiatkowski, Cordelia Langford, John Sillitoe on behalf of the Wellcome Sanger Institute COVID-19 Surveillance Team
EPI_ISL_797536, EPI_ISL_797537, EPI_ISL_797538, EPI_ISL_797539	Lighthouse Lab in Cambridge	Wellcome Sanger Institute for the COVID-19 Genomics UK (COG-UK) Consortium	Rob Howes, The Lighthouse Lab in Cambridge and Alex Alderton, Roberto Amato, Sonia Goncalves, Ewan Harrison, David K. Jackson, Ian Johnston, Dominic Kwiatkowski, Cordelia Langford, John Sillitoe on behalf of the Wellcome Sanger Institute COVID-19 Surveillance Team
EPI_ISL_797540, EPI_ISL_797541	Lighthouse Lab in Alderley Park	Wellcome Sanger Institute for the COVID-19 Genomics UK (COG-UK) Consortium	Jacquelyn Wynn, Mairead Hyland, The Lighthouse Lab in Alderley Park and Alex Alderton, Roberto Amato, Sonia Goncalves, Ewan Harrison, David K. Jackson, Ian Johnston, Dominic Kwiatkowski, Cordelia Langford, John Sillitoe on behalf of the Wellcome Sanger Institute COVID-19 Surveillance Team
EPI_ISL_797542	Lighthouse Lab in Cambridge	Wellcome Sanger Institute for the COVID-19 Genomics UK (COG-UK) Consortium	Rob Howes, The Lighthouse Lab in Cambridge and Alex Alderton, Roberto Amato, Sonia Goncalves, Ewan Harrison, David K. Jackson, Ian Johnston, Dominic Kwiatkowski, Cordelia Langford, John Sillitoe on behalf of the Wellcome Sanger Institute COVID-19 Surveillance Team
EPI_ISL_797543	Lighthouse Lab in Alderley Park	Wellcome Sanger Institute for the COVID-19 Genomics UK (COG-UK) Consortium	Jacquelyn Wynn, Mairead Hyland, The Lighthouse Lab in Alderley Park and Alex Alderton, Roberto Amato, Sonia Goncalves, Ewan Harrison, David K. Jackson, Ian Johnston, Dominic Kwiatkowski, Cordelia Langford, John Sillitoe on behalf of the Wellcome Sanger Institute COVID-19 Surveillance Team
EPI_ISL_797544, EPI_ISL_797545, EPI_ISL_797546	Lighthouse Lab in Cambridge	Wellcome Sanger Institute for the COVID-19 Genomics UK (COG-UK) Consortium	Rob Howes, The Lighthouse Lab in Cambridge and Alex Alderton, Roberto Amato, Sonia Goncalves, Ewan Harrison, David K. Jackson, Ian Johnston, Dominic Kwiatkowski, Cordelia Langford, John Sillitoe on behalf of the Wellcome Sanger Institute COVID-19 Surveillance Team
EPI_ISL_797547	Lighthouse Lab in Alderley Park	Wellcome Sanger Institute for the COVID-19 Genomics UK (COG-UK) Consortium	Jacquelyn Wynn, Mairead Hyland, The Lighthouse Lab in Alderley Park and Alex Alderton, Roberto Amato, Sonia Goncalves, Ewan Harrison, David K. Jackson, Ian Johnston, Dominic Kwiatkowski, Cordelia Langford, John Sillitoe on behalf of the Wellcome Sanger Institute COVID-19 Surveillance Team
EPI_ISL_797548	Lighthouse Lab in Cambridge	Wellcome Sanger Institute for the COVID-19 Genomics UK (COG-UK) Consortium	Rob Howes, The Lighthouse Lab in Cambridge and Alex Alderton, Roberto Amato, Sonia Goncalves, Ewan Harrison, David K. Jackson, Ian Johnston, Dominic Kwiatkowski, Cordelia Langford, John Sillitoe on behalf of the Wellcome Sanger Institute COVID-19 Surveillance Team
EPI_ISL_797549	Lighthouse Lab in Alderley Park	Wellcome Sanger Institute for the COVID-19 Genomics UK (COG-UK) Consortium	Jacquelyn Wynn, Mairead Hyland, The Lighthouse Lab in Alderley Park and Alex Alderton, Roberto Amato, Sonia Goncalves, Ewan Harrison, David K. Jackson, Ian Johnston, Dominic Kwiatkowski, Cordelia Langford, John Sillitoe on behalf of the Wellcome Sanger Institute COVID-19 Surveillance Team
EPI_ISL_797550, EPI_ISL_797551, EPI_ISL_797552	Lighthouse Lab in Cambridge	Wellcome Sanger Institute for the COVID-19 Genomics UK (COG-UK) Consortium	Rob Howes, The Lighthouse Lab in Cambridge and Alex Alderton, Roberto Amato, Sonia Goncalves, Ewan Harrison, David K. Jackson, Ian Johnston, Dominic Kwiatkowski, Cordelia Langford, John Sillitoe on behalf of the Wellcome Sanger Institute COVID-19 Surveillance Team
EPI_ISL_797553	Lighthouse Lab in Alderley Park	Wellcome Sanger Institute for the COVID-19 Genomics UK (COG-UK) Consortium	Jacquelyn Wynn, Mairead Hyland, The Lighthouse Lab in Alderley Park and Alex Alderton, Roberto Amato, Sonia Goncalves, Ewan Harrison, David K. Jackson, Ian Johnston, Dominic Kwiatkowski, Cordelia Langford, John Sillitoe on behalf of the Wellcome Sanger Institute COVID-19 Surveillance Team
EPI_ISL_797555, EPI_ISL_797556, EPI_ISL_797557, EPI_ISL_797558	Lighthouse Lab in Cambridge	Wellcome Sanger Institute for the COVID-19 Genomics UK (COG-UK) Consortium	Rob Howes, The Lighthouse Lab in Cambridge and Alex Alderton, Roberto Amato, Sonia Goncalves, Ewan Harrison, David K. Jackson, Ian Johnston, Dominic Kwiatkowski, Cordelia Langford, John Sillitoe on behalf of the Wellcome Sanger Institute COVID-19 Surveillance Team
EPI_ISL_797559, EPI_ISL_797560	Lighthouse Lab in Alderley Park	Wellcome Sanger Institute for the COVID-19 Genomics UK (COG-UK) Consortium	Jacquelyn Wynn, Mairead Hyland, The Lighthouse Lab in Alderley Park and Alex Alderton, Roberto Amato, Sonia Goncalves, Ewan Harrison, David K. Jackson, Ian Johnston, Dominic Kwiatkowski, Cordelia Langford, John Sillitoe on behalf of the Wellcome Sanger Institute COVID-19 Surveillance Team
EPI_ISL_797561, EPI_ISL_797562, EPI_ISL_797565, EPI_ISL_797566, EPI_ISL_797567	Lighthouse Lab in Cambridge	Wellcome Sanger Institute for the COVID-19 Genomics UK (COG-UK) Consortium	Rob Howes, The Lighthouse Lab in Cambridge and Alex Alderton, Roberto Amato, Sonia Goncalves, Ewan Harrison, David K. Jackson, Ian Johnston, Dominic Kwiatkowski, Cordelia Langford, John Sillitoe on behalf of the Wellcome Sanger Institute COVID-19 Surveillance Team
EPI_ISL_797568	Lighthouse Lab in Alderley Park	Wellcome Sanger Institute for the COVID-19 Genomics UK (COG-UK) Consortium	Jacquelyn Wynn, Mairead Hyland, The Lighthouse Lab in Alderley Park and Alex Alderton, Roberto Amato, Sonia Goncalves, Ewan Harrison, David K. Jackson, Ian Johnston, Dominic Kwiatkowski, Cordelia Langford, John Sillitoe on behalf of the Wellcome Sanger Institute COVID-19 Surveillance Team
EPI_ISL_797569, EPI_ISL_797570, EPI_ISL_797571, EPI_ISL_797572, EPI_ISL_797573, EPI_ISL_797574	Lighthouse Lab in Cambridge	Wellcome Sanger Institute for the COVID-19 Genomics UK (COG-UK) Consortium	Rob Howes, The Lighthouse Lab in Cambridge and Alex Alderton, Roberto Amato, Sonia Goncalves, Ewan Harrison, David K. Jackson, Ian Johnston, Dominic Kwiatkowski, Cordelia Langford, John Sillitoe on behalf of the Wellcome Sanger Institute COVID-19 Surveillance Team
EPI_ISL_797575, EPI_ISL_797576	Lighthouse Lab in Alderley Park	Wellcome Sanger Institute for the COVID-19 Genomics UK (COG-UK) Consortium	Jacquelyn Wynn, Mairead Hyland, The Lighthouse Lab in Alderley Park and Alex Alderton, Roberto Amato, Sonia Goncalves, Ewan Harrison, David K. Jackson, Ian Johnston, Dominic Kwiatkowski, Cordelia Langford, John Sillitoe on behalf of the Wellcome Sanger Institute COVID-19 Surveillance Team
EPI_ISL_797577, EPI_ISL_797578, EPI_ISL_797579, EPI_ISL_797580, EPI_ISL_797581, EPI_ISL_797582, EPI_ISL_797583, EPI_ISL_797584, EPI_ISL_797585	Lighthouse Lab in Cambridge	Wellcome Sanger Institute for the COVID-19 Genomics UK (COG-UK) Consortium	Rob Howes, The Lighthouse Lab in Cambridge and Alex Alderton, Roberto Amato, Sonia Goncalves, Ewan Harrison, David K. Jackson, Ian Johnston, Dominic Kwiatkowski, Cordelia Langford, John Sillitoe on behalf of the Wellcome Sanger Institute COVID-19 Surveillance Team
EPI_ISL_797586, EPI_ISL_797587	Lighthouse Lab in Alderley Park	Wellcome Sanger Institute for the COVID-19 Genomics UK (COG-UK) Consortium	Jacquelyn Wynn, Mairead Hyland, The Lighthouse Lab in Alderley Park and Alex Alderton, Roberto Amato, Sonia Goncalves, Ewan Harrison, David K. Jackson, Ian Johnston, Dominic Kwiatkowski, Cordelia Langford, John Sillitoe on behalf of the Wellcome Sanger Institute COVID-19 Surveillance Team
EPI_ISL_797588, EPI_ISL_797589, EPI_ISL_797590, EPI_ISL_797592,	Lighthouse Lab in Cambridge	Wellcome Sanger Institute for the COVID-19 Genomics UK (COG-UK) Consortium	Rob Howes, The Lighthouse Lab in Cambridge and Alex Alderton, Roberto Amato, Sonia Goncalves, Ewan Harrison, David K. Jackson, Ian Johnston, Dominic Kwiatkowski, Cordelia Langford, John Sillitoe on behalf of the Wellcome Sanger Institute COVID-19 Surveillance Team

EPI_ISL_797593, EPI_ISL_797594, EPI_ISL_797595, EPI_ISL_797596, EPI_ISL_797597, EPI_ISL_797598			
EPI_ISL_797599	Lighthouse Lab in Alderley Park	Wellcome Sanger Institute for the COVID-19 Genomics UK (COG-UK) Consortium	Jacquelyn Wynn, Mairead Hyland, The Lighthouse Lab in Alderley Park and Alex Alderton, Roberto Amato, Sonia Goncalves, Ewan Harrison, David K. Jackson, Ian Johnston, Dominic Kwiatkowski, Cordelia Langford, John Sillitoe on behalf of the Wellcome Sanger Institute COVID-19 Surveillance Team
EPI_ISL_797600, EPI_ISL_797601, EPI_ISL_797602, EPI_ISL_797603, EPI_ISL_797604	Lighthouse Lab in Cambridge	Wellcome Sanger Institute for the COVID-19 Genomics UK (COG-UK) Consortium	Rob Howes, The Lighthouse Lab in Cambridge and Alex Alderton, Roberto Amato, Sonia Goncalves, Ewan Harrison, David K. Jackson, Ian Johnston, Dominic Kwiatkowski, Cordelia Langford, John Sillitoe on behalf of the Wellcome Sanger Institute COVID-19 Surveillance Team
EPI_ISL_797605, EPI_ISL_797606	Lighthouse Lab in Alderley Park	Wellcome Sanger Institute for the COVID-19 Genomics UK (COG-UK) Consortium	Jacquelyn Wynn, Mairead Hyland, The Lighthouse Lab in Alderley Park and Alex Alderton, Roberto Amato, Sonia Goncalves, Ewan Harrison, David K. Jackson, Ian Johnston, Dominic Kwiatkowski, Cordelia Langford, John Sillitoe on behalf of the Wellcome Sanger Institute COVID-19 Surveillance Team
EPI_ISL_797607, EPI_ISL_797608, EPI_ISL_797609	Lighthouse Lab in Cambridge	Wellcome Sanger Institute for the COVID-19 Genomics UK (COG-UK) Consortium	Rob Howes, The Lighthouse Lab in Cambridge and Alex Alderton, Roberto Amato, Sonia Goncalves, Ewan Harrison, David K. Jackson, Ian Johnston, Dominic Kwiatkowski, Cordelia Langford, John Sillitoe on behalf of the Wellcome Sanger Institute COVID-19 Surveillance Team
EPI_ISL_797610	Lighthouse Lab in Alderley Park	Wellcome Sanger Institute for the COVID-19 Genomics UK (COG-UK) Consortium	Jacquelyn Wynn, Mairead Hyland, The Lighthouse Lab in Alderley Park and Alex Alderton, Roberto Amato, Sonia Goncalves, Ewan Harrison, David K. Jackson, Ian Johnston, Dominic Kwiatkowski, Cordelia Langford, John Sillitoe on behalf of the Wellcome Sanger Institute COVID-19 Surveillance Team
EPI_ISL_797611, EPI_ISL_797612	Lighthouse Lab in Cambridge	Wellcome Sanger Institute for the COVID-19 Genomics UK (COG-UK) Consortium	Rob Howes, The Lighthouse Lab in Cambridge and Alex Alderton, Roberto Amato, Sonia Goncalves, Ewan Harrison, David K. Jackson, Ian Johnston, Dominic Kwiatkowski, Cordelia Langford, John Sillitoe on behalf of the Wellcome Sanger Institute COVID-19 Surveillance Team
EPI_ISL_797613	Lighthouse Lab in Alderley Park	Wellcome Sanger Institute for the COVID-19 Genomics UK (COG-UK) Consortium	Jacquelyn Wynn, Mairead Hyland, The Lighthouse Lab in Alderley Park and Alex Alderton, Roberto Amato, Sonia Goncalves, Ewan Harrison, David K. Jackson, Ian Johnston, Dominic Kwiatkowski, Cordelia Langford, John Sillitoe on behalf of the Wellcome Sanger Institute COVID-19 Surveillance Team
EPI_ISL_797614	Lighthouse Lab in Cambridge	Wellcome Sanger Institute for the COVID-19 Genomics UK (COG-UK) Consortium	Rob Howes, The Lighthouse Lab in Cambridge and Alex Alderton, Roberto Amato, Sonia Goncalves, Ewan Harrison, David K. Jackson, Ian Johnston, Dominic Kwiatkowski, Cordelia Langford, John Sillitoe on behalf of the Wellcome Sanger Institute COVID-19 Surveillance Team
EPI_ISL_797616	Lighthouse Lab in Alderley Park	Wellcome Sanger Institute for the COVID-19 Genomics UK (COG-UK) Consortium	Jacquelyn Wynn, Mairead Hyland, The Lighthouse Lab in Alderley Park and Alex Alderton, Roberto Amato, Sonia Goncalves, Ewan Harrison, David K. Jackson, Ian Johnston, Dominic Kwiatkowski, Cordelia Langford, John Sillitoe on behalf of the Wellcome Sanger Institute COVID-19 Surveillance Team
EPI_ISL_797617, EPI_ISL_797618	Lighthouse Lab in Cambridge	Wellcome Sanger Institute for the COVID-19 Genomics UK (COG-UK) Consortium	Rob Howes, The Lighthouse Lab in Cambridge and Alex Alderton, Roberto Amato, Sonia Goncalves, Ewan Harrison, David K. Jackson, Ian Johnston, Dominic Kwiatkowski, Cordelia Langford, John Sillitoe on behalf of the Wellcome Sanger Institute COVID-19 Surveillance Team
EPI_ISL_797619	Lighthouse Lab in Alderley Park	Wellcome Sanger Institute for the COVID-19 Genomics UK (COG-UK) Consortium	Jacquelyn Wynn, Mairead Hyland, The Lighthouse Lab in Alderley Park and Alex Alderton, Roberto Amato, Sonia Goncalves, Ewan Harrison, David K. Jackson, Ian Johnston, Dominic Kwiatkowski, Cordelia Langford, John Sillitoe on behalf of the Wellcome Sanger Institute COVID-19 Surveillance Team
EPI_ISL_797621, EPI_ISL_797622, EPI_ISL_797623, EPI_ISL_797625, EPI_ISL_797626, EPI_ISL_797628, EPI_ISL_797629, EPI_ISL_797630, EPI_ISL_797631, EPI_ISL_797633, EPI_ISL_797634			
see above	Lighthouse Lab in Cambridge	Wellcome Sanger Institute for the COVID-19 Genomics UK (COG-UK) Consortium	Rob Howes, The Lighthouse Lab in Cambridge and Alex Alderton, Roberto Amato, Sonia Goncalves, Ewan Harrison, David K. Jackson, Ian Johnston, Dominic Kwiatkowski, Cordelia Langford, John Sillitoe on behalf of the Wellcome Sanger Institute COVID-19 Surveillance Team
EPI_ISL_797635	Lighthouse Lab in Alderley Park	Wellcome Sanger Institute for the COVID-19 Genomics UK (COG-UK) Consortium	Jacquelyn Wynn, Mairead Hyland, The Lighthouse Lab in Alderley Park and Alex Alderton, Roberto Amato, Sonia Goncalves, Ewan Harrison, David K. Jackson, Ian Johnston, Dominic Kwiatkowski, Cordelia Langford, John Sillitoe on behalf of the Wellcome Sanger Institute COVID-19 Surveillance Team
EPI_ISL_797636	Lighthouse Lab in Cambridge	Wellcome Sanger Institute for the COVID-19 Genomics UK (COG-UK) Consortium	Rob Howes, The Lighthouse Lab in Cambridge and Alex Alderton, Roberto Amato, Sonia Goncalves, Ewan Harrison, David K. Jackson, Ian Johnston, Dominic Kwiatkowski, Cordelia Langford, John Sillitoe on behalf of the Wellcome Sanger Institute COVID-19 Surveillance Team
EPI_ISL_797637	Lighthouse Lab in Alderley Park	Wellcome Sanger Institute for the COVID-19 Genomics UK (COG-UK) Consortium	Jacquelyn Wynn, Mairead Hyland, The Lighthouse Lab in Alderley Park and Alex Alderton, Roberto Amato, Sonia Goncalves, Ewan Harrison, David K. Jackson, Ian Johnston, Dominic Kwiatkowski, Cordelia Langford, John Sillitoe on behalf of the Wellcome Sanger Institute COVID-19 Surveillance Team
EPI_ISL_797638, EPI_ISL_797639, EPI_ISL_797640	Lighthouse Lab in Cambridge	Wellcome Sanger Institute for the COVID-19 Genomics UK (COG-UK) Consortium	Rob Howes, The Lighthouse Lab in Cambridge and Alex Alderton, Roberto Amato, Sonia Goncalves, Ewan Harrison, David K. Jackson, Ian Johnston, Dominic Kwiatkowski, Cordelia Langford, John Sillitoe on behalf of the Wellcome Sanger Institute COVID-19 Surveillance Team
EPI_ISL_797641, EPI_ISL_797642	Lighthouse Lab in Alderley Park	Wellcome Sanger Institute for the COVID-19 Genomics UK (COG-UK) Consortium	Jacquelyn Wynn, Mairead Hyland, The Lighthouse Lab in Alderley Park and Alex Alderton, Roberto Amato, Sonia Goncalves, Ewan Harrison, David K. Jackson, Ian Johnston, Dominic Kwiatkowski, Cordelia Langford, John Sillitoe on behalf of the Wellcome Sanger Institute COVID-19 Surveillance Team
EPI_ISL_797643	Lighthouse Lab in Cambridge	Wellcome Sanger Institute for the COVID-19 Genomics UK (COG-UK) Consortium	Rob Howes, The Lighthouse Lab in Cambridge and Alex Alderton, Roberto Amato, Sonia Goncalves, Ewan Harrison, David K. Jackson, Ian Johnston, Dominic Kwiatkowski, Cordelia Langford, John Sillitoe on behalf of the Wellcome Sanger Institute COVID-19 Surveillance Team
EPI_ISL_797644	Lighthouse Lab in Alderley Park	Wellcome Sanger Institute for the COVID-19 Genomics UK (COG-UK) Consortium	Jacquelyn Wynn, Mairead Hyland, The Lighthouse Lab in Alderley Park and Alex Alderton, Roberto Amato, Sonia Goncalves, Ewan Harrison, David K. Jackson, Ian Johnston, Dominic Kwiatkowski, Cordelia Langford, John Sillitoe on behalf of the Wellcome Sanger Institute COVID-19 Surveillance Team
EPI_ISL_797645, EPI_ISL_797646, EPI_ISL_797647, EPI_ISL_797648, EPI_ISL_797649, EPI_ISL_797650, EPI_ISL_797651, EPI_ISL_797652, EPI_ISL_797653	Lighthouse Lab in Cambridge	Wellcome Sanger Institute for the COVID-19 Genomics UK (COG-UK) Consortium	Rob Howes, The Lighthouse Lab in Cambridge and Alex Alderton, Roberto Amato, Sonia Goncalves, Ewan Harrison, David K. Jackson, Ian Johnston, Dominic Kwiatkowski, Cordelia Langford, John Sillitoe on behalf of the Wellcome Sanger Institute COVID-19 Surveillance Team
EPI_ISL_797654	Lighthouse Lab in Alderley Park	Wellcome Sanger Institute for the COVID-19 Genomics UK (COG-UK) Consortium	Jacquelyn Wynn, Mairead Hyland, The Lighthouse Lab in Alderley Park and Alex Alderton, Roberto Amato, Sonia Goncalves, Ewan Harrison, David K. Jackson, Ian Johnston, Dominic Kwiatkowski, Cordelia Langford, John Sillitoe on behalf of the Wellcome Sanger Institute COVID-19 Surveillance Team
EPI_ISL_797655, EPI_ISL_797656, EPI_ISL_797657, EPI_ISL_797658, EPI_ISL_797659	Lighthouse Lab in Cambridge	Wellcome Sanger Institute for the COVID-19 Genomics UK (COG-UK) Consortium	Rob Howes, The Lighthouse Lab in Cambridge and Alex Alderton, Roberto Amato, Sonia Goncalves, Ewan Harrison, David K. Jackson, Ian Johnston, Dominic Kwiatkowski, Cordelia Langford, John Sillitoe on behalf of the Wellcome Sanger Institute COVID-19 Surveillance Team
EPI_ISL_797660	Lighthouse Lab in Alderley Park	Wellcome Sanger Institute for the COVID-19 Genomics UK (COG-UK) Consortium	Jacquelyn Wynn, Mairead Hyland, The Lighthouse Lab in Alderley Park and Alex Alderton, Roberto Amato, Sonia Goncalves, Ewan Harrison, David K. Jackson, Ian Johnston, Dominic Kwiatkowski, Cordelia Langford, John Sillitoe on behalf of the Wellcome Sanger Institute COVID-19 Surveillance Team
EPI_ISL_797661, EPI_ISL_797662	Lighthouse Lab in Cambridge	Wellcome Sanger Institute for the COVID-19 Genomics UK (COG-UK) Consortium	Rob Howes, The Lighthouse Lab in Cambridge and Alex Alderton, Roberto Amato, Sonia Goncalves, Ewan Harrison, David K. Jackson, Ian Johnston, Dominic Kwiatkowski, Cordelia Langford, John Sillitoe on behalf of the Wellcome Sanger Institute COVID-19 Surveillance Team
EPI_ISL_797663	Lighthouse Lab in Alderley Park	Wellcome Sanger Institute for the COVID-19 Genomics UK (COG-UK) Consortium	Jacquelyn Wynn, Mairead Hyland, The Lighthouse Lab in Alderley Park and Alex Alderton, Roberto Amato, Sonia Goncalves, Ewan Harrison, David K. Jackson, Ian Johnston, Dominic Kwiatkowski, Cordelia Langford, John Sillitoe on behalf of the Wellcome Sanger Institute COVID-19 Surveillance Team
EPI_ISL_797664, EPI_ISL_797665	Lighthouse Lab in Cambridge	Wellcome Sanger Institute for the COVID-19 Genomics UK (COG-UK) Consortium	Rob Howes, The Lighthouse Lab in Cambridge and Alex Alderton, Roberto Amato, Sonia Goncalves, Ewan Harrison, David K. Jackson, Ian Johnston, Dominic Kwiatkowski, Cordelia Langford, John Sillitoe on behalf of the Wellcome Sanger Institute COVID-19 Surveillance Team
EPI_ISL_797666	Lighthouse Lab in Alderley Park	Wellcome Sanger Institute for the COVID-19 Genomics UK (COG-UK) Consortium	Jacquelyn Wynn, Mairead Hyland, The Lighthouse Lab in Alderley Park and Alex Alderton, Roberto Amato, Sonia Goncalves, Ewan Harrison, David K. Jackson, Ian Johnston, Dominic Kwiatkowski, Cordelia Langford, John Sillitoe on behalf of the Wellcome Sanger Institute COVID-19 Surveillance Team
EPI_ISL_797667, EPI_ISL_797668, EPI_ISL_797669, EPI_ISL_797670, EPI_ISL_797671, EPI_ISL_797672	Lighthouse Lab in Cambridge	Wellcome Sanger Institute for the COVID-19 Genomics UK (COG-UK) Consortium	Rob Howes, The Lighthouse Lab in Cambridge and Alex Alderton, Roberto Amato, Sonia Goncalves, Ewan Harrison, David K. Jackson, Ian Johnston, Dominic Kwiatkowski, Cordelia Langford, John Sillitoe on behalf of the Wellcome Sanger Institute COVID-19 Surveillance Team
EPI_ISL_797673	Lighthouse Lab in Alderley Park	Wellcome Sanger Institute for the COVID-19 Genomics UK (COG-UK) Consortium	Jacquelyn Wynn, Mairead Hyland, The Lighthouse Lab in Alderley Park and Alex Alderton, Roberto Amato, Sonia Goncalves, Ewan Harrison, David K. Jackson, Ian Johnston, Dominic Kwiatkowski, Cordelia Langford, John Sillitoe on behalf of the Wellcome Sanger Institute COVID-19 Surveillance Team
EPI_ISL_797674, EPI_ISL_797675, EPI_ISL_797676, EPI_ISL_797679,	Lighthouse Lab in Cambridge	Wellcome Sanger Institute for the COVID-19 Genomics UK (COG-UK) Consortium	Rob Howes, The Lighthouse Lab in Cambridge and Alex Alderton, Roberto Amato, Sonia Goncalves, Ewan Harrison, David K. Jackson, Ian Johnston, Dominic Kwiatkowski, Cordelia Langford, John Sillitoe on behalf of the Wellcome Sanger Institute COVID-19 Surveillance Team

EPI_ISL_797680, EPI_ISL_797681, EPI_ISL_797682, EPI_ISL_797683, EPI_ISL_797684			
EPI_ISL_797686	Lighthouse Lab in Alderley Park	Wellcome Sanger Institute for the COVID-19 Genomics UK (COG-UK) Consortium	Jacquelyn Wynn, Mairead Hyland, The Lighthouse Lab in Alderley Park and Alex Alderton, Roberto Amato, Sonia Goncalves, Ewan Harrison, David K. Jackson, Ian Johnston, Dominic Kwiatkowski, Cordelia Langford, John Sillitoe on behalf of the Wellcome Sanger Institute COVID-19 Surveillance Team
EPI_ISL_797687, EPI_ISL_797688, EPI_ISL_797689, EPI_ISL_797690, EPI_ISL_797691, EPI_ISL_797692, EPI_ISL_797693, EPI_ISL_797694, EPI_ISL_797695, EPI_ISL_797696, EPI_ISL_797697			
see above	Lighthouse Lab in Cambridge	Wellcome Sanger Institute for the COVID-19 Genomics UK (COG-UK) Consortium	Rob Howes, The Lighthouse Lab in Cambridge and Alex Alderton, Roberto Amato, Sonia Goncalves, Ewan Harrison, David K. Jackson, Ian Johnston, Dominic Kwiatkowski, Cordelia Langford, John Sillitoe on behalf of the Wellcome Sanger Institute COVID-19 Surveillance Team
EPI_ISL_797699	Lighthouse Lab in Alderley Park	Wellcome Sanger Institute for the COVID-19 Genomics UK (COG-UK) Consortium	Jacquelyn Wynn, Mairead Hyland, The Lighthouse Lab in Alderley Park and Alex Alderton, Roberto Amato, Sonia Goncalves, Ewan Harrison, David K. Jackson, Ian Johnston, Dominic Kwiatkowski, Cordelia Langford, John Sillitoe on behalf of the Wellcome Sanger Institute COVID-19 Surveillance Team
EPI_ISL_797700	Lighthouse Lab in Cambridge	Wellcome Sanger Institute for the COVID-19 Genomics UK (COG-UK) Consortium	Rob Howes, The Lighthouse Lab in Cambridge and Alex Alderton, Roberto Amato, Sonia Goncalves, Ewan Harrison, David K. Jackson, Ian Johnston, Dominic Kwiatkowski, Cordelia Langford, John Sillitoe on behalf of the Wellcome Sanger Institute COVID-19 Surveillance Team
EPI_ISL_797701	Lighthouse Lab in Alderley Park	Wellcome Sanger Institute for the COVID-19 Genomics UK (COG-UK) Consortium	Jacquelyn Wynn, Mairead Hyland, The Lighthouse Lab in Alderley Park and Alex Alderton, Roberto Amato, Sonia Goncalves, Ewan Harrison, David K. Jackson, Ian Johnston, Dominic Kwiatkowski, Cordelia Langford, John Sillitoe on behalf of the Wellcome Sanger Institute COVID-19 Surveillance Team
EPI_ISL_797702, EPI_ISL_797703, EPI_ISL_797705, EPI_ISL_797706, EPI_ISL_797707, EPI_ISL_797708	Lighthouse Lab in Cambridge	Wellcome Sanger Institute for the COVID-19 Genomics UK (COG-UK) Consortium	Rob Howes, The Lighthouse Lab in Cambridge and Alex Alderton, Roberto Amato, Sonia Goncalves, Ewan Harrison, David K. Jackson, Ian Johnston, Dominic Kwiatkowski, Cordelia Langford, John Sillitoe on behalf of the Wellcome Sanger Institute COVID-19 Surveillance Team
EPI_ISL_797709	Lighthouse Lab in Alderley Park	Wellcome Sanger Institute for the COVID-19 Genomics UK (COG-UK) Consortium	Jacquelyn Wynn, Mairead Hyland, The Lighthouse Lab in Alderley Park and Alex Alderton, Roberto Amato, Sonia Goncalves, Ewan Harrison, David K. Jackson, Ian Johnston, Dominic Kwiatkowski, Cordelia Langford, John Sillitoe on behalf of the Wellcome Sanger Institute COVID-19 Surveillance Team
EPI_ISL_797710, EPI_ISL_797711	Lighthouse Lab in Cambridge	Wellcome Sanger Institute for the COVID-19 Genomics UK (COG-UK) Consortium	Rob Howes, The Lighthouse Lab in Cambridge and Alex Alderton, Roberto Amato, Sonia Goncalves, Ewan Harrison, David K. Jackson, Ian Johnston, Dominic Kwiatkowski, Cordelia Langford, John Sillitoe on behalf of the Wellcome Sanger Institute COVID-19 Surveillance Team
EPI_ISL_797712, EPI_ISL_797714, EPI_ISL_797715, EPI_ISL_797717	Lighthouse Lab in Alderley Park	Wellcome Sanger Institute for the COVID-19 Genomics UK (COG-UK) Consortium	Jacquelyn Wynn, Mairead Hyland, The Lighthouse Lab in Alderley Park and Alex Alderton, Roberto Amato, Sonia Goncalves, Ewan Harrison, David K. Jackson, Ian Johnston, Dominic Kwiatkowski, Cordelia Langford, John Sillitoe on behalf of the Wellcome Sanger Institute COVID-19 Surveillance Team
EPI_ISL_797718	Lighthouse Lab in Cambridge	Wellcome Sanger Institute for the COVID-19 Genomics UK (COG-UK) Consortium	Rob Howes, The Lighthouse Lab in Cambridge and Alex Alderton, Roberto Amato, Sonia Goncalves, Ewan Harrison, David K. Jackson, Ian Johnston, Dominic Kwiatkowski, Cordelia Langford, John Sillitoe on behalf of the Wellcome Sanger Institute COVID-19 Surveillance Team
EPI_ISL_797719, EPI_ISL_797720, EPI_ISL_797721, EPI_ISL_797722, EPI_ISL_797723, EPI_ISL_797724, EPI_ISL_797725, EPI_ISL_797726, EPI_ISL_797727, EPI_ISL_797728, EPI_ISL_797729, EPI_ISL_797730, EPI_ISL_797731, EPI_ISL_797732, EPI_ISL_797733, EPI_ISL_797734, EPI_ISL_797735, EPI_ISL_797736, EPI_ISL_797737, EPI_ISL_797738, EPI_ISL_797740, EPI_ISL_797741, EPI_ISL_797742, EPI_ISL_797743, EPI_ISL_797744, EPI_ISL_797745, EPI_ISL_797746, EPI_ISL_797747, EPI_ISL_797748, EPI_ISL_797749, EPI_ISL_797750, EPI_ISL_797751, EPI_ISL_797752, EPI_ISL_797753, EPI_ISL_797754, EPI_ISL_797755, EPI_ISL_797756, EPI_ISL_797757, EPI_ISL_797758, EPI_ISL_797759, EPI_ISL_797760, EPI_ISL_797761, EPI_ISL_797762, EPI_ISL_797763, EPI_ISL_797764, EPI_ISL_797765, EPI_ISL_797767, EPI_ISL_797768, EPI_ISL_797769, EPI_ISL_797770, EPI_ISL_797771, EPI_ISL_797772, EPI_ISL_797773, EPI_ISL_797774, EPI_ISL_797776, EPI_ISL_797777, EPI_ISL_797778, EPI_ISL_797779, EPI_ISL_797780, EPI_ISL_797781, EPI_ISL_797782, EPI_ISL_797783, EPI_ISL_797784, EPI_ISL_797785, EPI_ISL_797786, EPI_ISL_797787, EPI_ISL_797788, EPI_ISL_797790, EPI_ISL_797791, EPI_ISL_797792, EPI_ISL_797793, EPI_ISL_797794, EPI_ISL_797795, EPI_ISL_797796, EPI_ISL_797797, EPI_ISL_797798, EPI_ISL_797799, EPI_ISL_797800, EPI_ISL_797801, EPI_ISL_797802, EPI_ISL_797803, EPI_ISL_797804, EPI_ISL_797805, EPI_ISL_797806, EPI_ISL_797807, EPI_ISL_797808, EPI_ISL_797809, EPI_ISL_797810, EPI_ISL_797811, EPI_ISL_797812, EPI_ISL_797813, EPI_ISL_797814, EPI_ISL_797815, EPI_ISL_797816, EPI_ISL_797817, EPI_ISL_797818, EPI_ISL_797819, EPI_ISL_797820, EPI_ISL_797821, EPI_ISL_797822, EPI_ISL_797823, EPI_ISL_797824, EPI_ISL_797826, EPI_ISL_797827, EPI_ISL_797828, EPI_ISL_797831, EPI_ISL_797832, EPI_ISL_797833, EPI_ISL_797834, EPI_ISL_797835, EPI_ISL_797836, EPI_ISL_797837, EPI_ISL_797838, EPI_ISL_797839, EPI_ISL_797840, EPI_ISL_797841, EPI_ISL_797842, EPI_ISL_797843, EPI_ISL_797844, EPI_ISL_797845, EPI_ISL_797846, EPI_ISL_797848, EPI_ISL_797849, EPI_ISL_797851, EPI_ISL_797852, EPI_ISL_797854, EPI_ISL_797855, EPI_ISL_797856, EPI_ISL_797857, EPI_ISL_797858, EPI_ISL_797859, EPI_ISL_797860, EPI_ISL_797861, EPI_ISL_797862, EPI_ISL_797863, EPI_ISL_797864, EPI_ISL_797865, EPI_ISL_797866, EPI_ISL_797868, EPI_ISL_797869, EPI_ISL_797871, EPI_ISL_797872, EPI_ISL_797873, EPI_ISL_797874, EPI_ISL_797875, EPI_ISL_797876, EPI_ISL_797877, EPI_ISL_797878, EPI_ISL_797879, EPI_ISL_797880, EPI_ISL_797881, EPI_ISL_797882, EPI_ISL_797883, EPI_ISL_797884, EPI_ISL_797886, EPI_ISL_797887, EPI_ISL_797888, EPI_ISL_797889, EPI_ISL_797890, EPI_ISL_797891, EPI_ISL_797892, EPI_ISL_797893, EPI_ISL_797894, EPI_ISL_797895, EPI_ISL_797896, EPI_ISL_797897, EPI_ISL_797898, EPI_ISL_797899, EPI_ISL_797900, EPI_ISL_797901, EPI_ISL_797902, EPI_ISL_797903, EPI_ISL_797904, EPI_ISL_797905, EPI_ISL_797906, EPI_ISL_797907, EPI_ISL_797908, EPI_ISL_797909, EPI_ISL_797910, EPI_ISL_797911, EPI_ISL_797912, EPI_ISL_797913, EPI_ISL_797915, EPI_ISL_797916, EPI_ISL_797917, EPI_ISL_797918, EPI_ISL_797919, EPI_ISL_797920, EPI_ISL_797922, EPI_ISL_797923, EPI_ISL_797924, EPI_ISL_797925, EPI_ISL_797926, EPI_ISL_797927, EPI_ISL_797928, EPI_ISL_797930, EPI_ISL_797931, EPI_ISL_797932, EPI_ISL_797933, EPI_ISL_797934, EPI_ISL_797935, EPI_ISL_797936, EPI_ISL_797938, EPI_ISL_797939, EPI_ISL_797940, EPI_ISL_797941, EPI_ISL_797942, EPI_ISL_797943, EPI_ISL_797944, EPI_ISL_797946, EPI_ISL_797947, EPI_ISL_797948, EPI_ISL_797949, EPI_ISL_797950, EPI_ISL_797951, EPI_ISL_797952, EPI_ISL_797953, EPI_ISL_797954, EPI_ISL_797955, EPI_ISL_797956, EPI_ISL_797957, EPI_ISL_797958, EPI_ISL_797959, EPI_ISL_797960, EPI_ISL_797962, EPI_ISL_797963, EPI_ISL_797964, EPI_ISL_797965, EPI_ISL_797966, EPI_ISL_797967, EPI_ISL_797968, EPI_ISL_797969, EPI_ISL_797971, EPI_ISL_797972, EPI_ISL_797973, EPI_ISL_797974, EPI_ISL_797975, EPI_ISL_797976, EPI_ISL_797977, EPI_ISL_797978, EPI_ISL_797979, EPI_ISL_797981, EPI_ISL_797982, EPI_ISL_797983, EPI_ISL_797984, EPI_ISL_797986, EPI_ISL_797987, EPI_ISL_797988, EPI_ISL_797989, EPI_ISL_797990, EPI_ISL_797991, EPI_ISL_797992, EPI_ISL_797993, EPI_ISL_797995, EPI_ISL_797997, EPI_ISL_797998			
see above	Lighthouse Lab in Glasgow	Wellcome Sanger Institute for the COVID-19 Genomics UK (COG-UK) Consortium	Harper VanSteenhouse, Yumi Kasai, David Gray, Carol Clugston, Anna Dominiczak and Alex Alderton, Roberto Amato, Sonia Goncalves, Ewan Harrison, David K. Jackson, Ian Johnston, Dominic Kwiatkowski, Cordelia Langford, John Sillitoe on behalf of the Wellcome Sanger Institute COVID-19 Surveillance Team
EPI_ISL_797999	Lighthouse Lab in Alderley Park	Wellcome Sanger Institute for the COVID-19 Genomics UK (COG-UK) Consortium	Jacquelyn Wynn, Mairead Hyland, The Lighthouse Lab in Alderley Park and Alex Alderton, Roberto Amato, Sonia Goncalves, Ewan Harrison, David K. Jackson, Ian Johnston, Dominic Kwiatkowski, Cordelia Langford, John Sillitoe on behalf of the Wellcome Sanger Institute COVID-19 Surveillance Team
EPI_ISL_798001	Lighthouse Lab in Glasgow	Wellcome Sanger Institute for the COVID-19 Genomics UK (COG-UK) Consortium	Harper VanSteenhouse, Yumi Kasai, David Gray, Carol Clugston, Anna Dominiczak and Alex Alderton, Roberto Amato, Sonia Goncalves, Ewan Harrison, David K. Jackson, Ian Johnston, Dominic Kwiatkowski, Cordelia Langford, John Sillitoe on behalf of the Wellcome Sanger Institute COVID-19 Surveillance Team
EPI_ISL_798002	Lighthouse Lab in Alderley Park	Wellcome Sanger Institute for the COVID-19 Genomics UK (COG-UK) Consortium	Jacquelyn Wynn, Mairead Hyland, The Lighthouse Lab in Alderley Park and Alex Alderton, Roberto Amato, Sonia Goncalves, Ewan Harrison, David K. Jackson, Ian Johnston, Dominic Kwiatkowski, Cordelia Langford, John Sillitoe on behalf of the Wellcome Sanger Institute COVID-19 Surveillance Team
EPI_ISL_798004, EPI_ISL_798005, EPI_ISL_798006	Lighthouse Lab in Glasgow	Wellcome Sanger Institute for the COVID-19 Genomics UK (COG-UK) Consortium	Harper VanSteenhouse, Yumi Kasai, David Gray, Carol Clugston, Anna Dominiczak and Alex Alderton, Roberto Amato, Sonia Goncalves, Ewan Harrison, David K. Jackson, Ian Johnston, Dominic Kwiatkowski, Cordelia Langford, John Sillitoe on behalf of the Wellcome Sanger Institute COVID-19 Surveillance Team
EPI_ISL_798007	Lighthouse Lab in Alderley Park	Wellcome Sanger Institute for the COVID-19 Genomics UK (COG-UK) Consortium	Jacquelyn Wynn, Mairead Hyland, The Lighthouse Lab in Alderley Park and Alex Alderton, Roberto Amato, Sonia Goncalves, Ewan Harrison, David K. Jackson, Ian Johnston, Dominic Kwiatkowski, Cordelia Langford, John Sillitoe on behalf of the Wellcome Sanger Institute COVID-19 Surveillance Team
EPI_ISL_798008	Lighthouse Lab in Glasgow	Wellcome Sanger Institute for the COVID-19 Genomics UK (COG-UK) Consortium	Harper VanSteenhouse, Yumi Kasai, David Gray, Carol Clugston, Anna Dominiczak and Alex Alderton, Roberto Amato, Sonia Goncalves, Ewan Harrison, David K. Jackson, Ian Johnston, Dominic Kwiatkowski, Cordelia Langford, John Sillitoe on behalf of the Wellcome Sanger Institute COVID-19 Surveillance Team
EPI_ISL_798009	Lighthouse Lab in Alderley Park	Wellcome Sanger Institute for the COVID-19 Genomics UK (COG-UK) Consortium	Jacquelyn Wynn, Mairead Hyland, The Lighthouse Lab in Alderley Park and Alex Alderton, Roberto Amato, Sonia Goncalves, Ewan Harrison, David K. Jackson, Ian Johnston, Dominic Kwiatkowski, Cordelia Langford, John Sillitoe on behalf of the Wellcome Sanger Institute COVID-19 Surveillance Team
EPI_ISL_798010	Lighthouse Lab in Glasgow	Wellcome Sanger Institute for the COVID-19 Genomics UK (COG-UK) Consortium	Harper VanSteenhouse, Yumi Kasai, David Gray, Carol Clugston, Anna Dominiczak and Alex Alderton, Roberto Amato, Sonia Goncalves, Ewan Harrison, David K. Jackson, Ian Johnston, Dominic Kwiatkowski, Cordelia Langford, John Sillitoe on behalf of the Wellcome Sanger Institute COVID-19 Surveillance Team
EPI_ISL_798011	Lighthouse Lab in Alderley Park	Wellcome Sanger Institute for the COVID-19 Genomics UK (COG-UK) Consortium	Jacquelyn Wynn, Mairead Hyland, The Lighthouse Lab in Alderley Park and Alex Alderton, Roberto Amato, Sonia Goncalves, Ewan Harrison, David K. Jackson, Ian Johnston, Dominic Kwiatkowski, Cordelia Langford, John Sillitoe on behalf of the Wellcome Sanger Institute COVID-19 Surveillance Team
EPI_ISL_798012	Lighthouse Lab in Glasgow	Wellcome Sanger Institute for the COVID-19 Genomics UK (COG-UK) Consortium	Harper VanSteenhouse, Yumi Kasai, David Gray, Carol Clugston, Anna Dominiczak and Alex Alderton, Roberto Amato, Sonia Goncalves, Ewan Harrison, David K. Jackson, Ian Johnston, Dominic Kwiatkowski, Cordelia Langford, John Sillitoe on behalf of the Wellcome Sanger Institute COVID-19 Surveillance Team
EPI_ISL_798013	Lighthouse Lab in Alderley Park	Wellcome Sanger Institute for the COVID-19 Genomics UK (COG-UK) Consortium	Jacquelyn Wynn, Mairead Hyland, The Lighthouse Lab in Alderley Park and Alex Alderton, Roberto Amato, Sonia Goncalves, Ewan Harrison, David K. Jackson, Ian Johnston, Dominic Kwiatkowski, Cordelia Langford, John Sillitoe on behalf of the Wellcome Sanger Institute COVID-19 Surveillance Team
EPI_ISL_798014	Lighthouse Lab in Glasgow	Wellcome Sanger Institute for the COVID-19 Genomics UK (COG-UK) Consortium	Harper VanSteenhouse, Yumi Kasai, David Gray, Carol Clugston, Anna Dominiczak and Alex Alderton, Roberto Amato, Sonia Goncalves, Ewan Harrison, David K. Jackson, Ian Johnston, Dominic Kwiatkowski, Cordelia Langford, John Sillitoe on behalf of the Wellcome Sanger Institute COVID-19 Surveillance Team
EPI_ISL_798015	Lighthouse Lab in Alderley Park	Wellcome Sanger Institute for the COVID-19 Genomics UK	Jacquelyn Wynn, Mairead Hyland, The Lighthouse Lab in Alderley Park and Alex Alderton, Roberto Amato, Sonia Goncalves, Ewan Harrison, David K.

		(COG-UK) Consortium	Jackson, Ian Johnston, Dominic Kwiatkowski, Cordelia Langford, John Sillitoe on behalf of the Wellcome Sanger Institute COVID-19 Surveillance Team
EPI_ISL_798016, EPI_ISL_798017, EPI_ISL_798019, EPI_ISL_798020, EPI_ISL_798021, EPI_ISL_798023, EPI_ISL_798024	Lighthouse Lab in Glasgow	Wellcome Sanger Institute for the COVID-19 Genomics UK (COG-UK) Consortium	Harper VanSteenhouse, Yumi Kasai, David Gray, Carol Clugston, Anna Dominiczak and Alex Alderton, Roberto Amato, Sonia Goncalves, Ewan Harrison, David K. Jackson, Ian Johnston, Dominic Kwiatkowski, Cordelia Langford, John Sillitoe on behalf of the Wellcome Sanger Institute COVID-19 Surveillance Team
EPI_ISL_798025	Lighthouse Lab in Alderley Park	Wellcome Sanger Institute for the COVID-19 Genomics UK (COG-UK) Consortium	Jacquelyn Wynn, Mairead Hyland, The Lighthouse Lab in Alderley Park and Alex Alderton, Roberto Amato, Sonia Goncalves, Ewan Harrison, David K. Jackson, Ian Johnston, Dominic Kwiatkowski, Cordelia Langford, John Sillitoe on behalf of the Wellcome Sanger Institute COVID-19 Surveillance Team
EPI_ISL_798026	Lighthouse Lab in Glasgow	Wellcome Sanger Institute for the COVID-19 Genomics UK (COG-UK) Consortium	Harper VanSteenhouse, Yumi Kasai, David Gray, Carol Clugston, Anna Dominiczak and Alex Alderton, Roberto Amato, Sonia Goncalves, Ewan Harrison, David K. Jackson, Ian Johnston, Dominic Kwiatkowski, Cordelia Langford, John Sillitoe on behalf of the Wellcome Sanger Institute COVID-19 Surveillance Team
EPI_ISL_798027	Lighthouse Lab in Alderley Park	Wellcome Sanger Institute for the COVID-19 Genomics UK (COG-UK) Consortium	Jacquelyn Wynn, Mairead Hyland, The Lighthouse Lab in Alderley Park and Alex Alderton, Roberto Amato, Sonia Goncalves, Ewan Harrison, David K. Jackson, Ian Johnston, Dominic Kwiatkowski, Cordelia Langford, John Sillitoe on behalf of the Wellcome Sanger Institute COVID-19 Surveillance Team
EPI_ISL_798028	Lighthouse Lab in Glasgow	Wellcome Sanger Institute for the COVID-19 Genomics UK (COG-UK) Consortium	Harper VanSteenhouse, Yumi Kasai, David Gray, Carol Clugston, Anna Dominiczak and Alex Alderton, Roberto Amato, Sonia Goncalves, Ewan Harrison, David K. Jackson, Ian Johnston, Dominic Kwiatkowski, Cordelia Langford, John Sillitoe on behalf of the Wellcome Sanger Institute COVID-19 Surveillance Team
EPI_ISL_798029	Lighthouse Lab in Alderley Park	Wellcome Sanger Institute for the COVID-19 Genomics UK (COG-UK) Consortium	Jacquelyn Wynn, Mairead Hyland, The Lighthouse Lab in Alderley Park and Alex Alderton, Roberto Amato, Sonia Goncalves, Ewan Harrison, David K. Jackson, Ian Johnston, Dominic Kwiatkowski, Cordelia Langford, John Sillitoe on behalf of the Wellcome Sanger Institute COVID-19 Surveillance Team
EPI_ISL_798030, EPI_ISL_798031, EPI_ISL_798033, EPI_ISL_798034	Lighthouse Lab in Glasgow	Wellcome Sanger Institute for the COVID-19 Genomics UK (COG-UK) Consortium	Harper VanSteenhouse, Yumi Kasai, David Gray, Carol Clugston, Anna Dominiczak and Alex Alderton, Roberto Amato, Sonia Goncalves, Ewan Harrison, David K. Jackson, Ian Johnston, Dominic Kwiatkowski, Cordelia Langford, John Sillitoe on behalf of the Wellcome Sanger Institute COVID-19 Surveillance Team
EPI_ISL_798035	Lighthouse Lab in Alderley Park	Wellcome Sanger Institute for the COVID-19 Genomics UK (COG-UK) Consortium	Jacquelyn Wynn, Mairead Hyland, The Lighthouse Lab in Alderley Park and Alex Alderton, Roberto Amato, Sonia Goncalves, Ewan Harrison, David K. Jackson, Ian Johnston, Dominic Kwiatkowski, Cordelia Langford, John Sillitoe on behalf of the Wellcome Sanger Institute COVID-19 Surveillance Team
EPI_ISL_798036, EPI_ISL_798037, EPI_ISL_798039, EPI_ISL_798041, EPI_ISL_798042, EPI_ISL_798044, EPI_ISL_798045, EPI_ISL_798047, EPI_ISL_798049, EPI_ISL_798050, EPI_ISL_798051, EPI_ISL_798052			
see above	Lighthouse Lab in Glasgow	Wellcome Sanger Institute for the COVID-19 Genomics UK (COG-UK) Consortium	Harper VanSteenhouse, Yumi Kasai, David Gray, Carol Clugston, Anna Dominiczak and Alex Alderton, Roberto Amato, Sonia Goncalves, Ewan Harrison, David K. Jackson, Ian Johnston, Dominic Kwiatkowski, Cordelia Langford, John Sillitoe on behalf of the Wellcome Sanger Institute COVID-19 Surveillance Team
EPI_ISL_798054	Lighthouse Lab in Alderley Park	Wellcome Sanger Institute for the COVID-19 Genomics UK (COG-UK) Consortium	Jacquelyn Wynn, Mairead Hyland, The Lighthouse Lab in Alderley Park and Alex Alderton, Roberto Amato, Sonia Goncalves, Ewan Harrison, David K. Jackson, Ian Johnston, Dominic Kwiatkowski, Cordelia Langford, John Sillitoe on behalf of the Wellcome Sanger Institute COVID-19 Surveillance Team
EPI_ISL_798056, EPI_ISL_798057, EPI_ISL_798059	Lighthouse Lab in Glasgow	Wellcome Sanger Institute for the COVID-19 Genomics UK (COG-UK) Consortium	Harper VanSteenhouse, Yumi Kasai, David Gray, Carol Clugston, Anna Dominiczak and Alex Alderton, Roberto Amato, Sonia Goncalves, Ewan Harrison, David K. Jackson, Ian Johnston, Dominic Kwiatkowski, Cordelia Langford, John Sillitoe on behalf of the Wellcome Sanger Institute COVID-19 Surveillance Team
EPI_ISL_798061, EPI_ISL_798062	Lighthouse Lab in Alderley Park	Wellcome Sanger Institute for the COVID-19 Genomics UK (COG-UK) Consortium	Jacquelyn Wynn, Mairead Hyland, The Lighthouse Lab in Alderley Park and Alex Alderton, Roberto Amato, Sonia Goncalves, Ewan Harrison, David K. Jackson, Ian Johnston, Dominic Kwiatkowski, Cordelia Langford, John Sillitoe on behalf of the Wellcome Sanger Institute COVID-19 Surveillance Team
EPI_ISL_798063	Lighthouse Lab in Glasgow	Wellcome Sanger Institute for the COVID-19 Genomics UK (COG-UK) Consortium	Harper VanSteenhouse, Yumi Kasai, David Gray, Carol Clugston, Anna Dominiczak and Alex Alderton, Roberto Amato, Sonia Goncalves, Ewan Harrison, David K. Jackson, Ian Johnston, Dominic Kwiatkowski, Cordelia Langford, John Sillitoe on behalf of the Wellcome Sanger Institute COVID-19 Surveillance Team
EPI_ISL_798064	Lighthouse Lab in Alderley Park	Wellcome Sanger Institute for the COVID-19 Genomics UK (COG-UK) Consortium	Jacquelyn Wynn, Mairead Hyland, The Lighthouse Lab in Alderley Park and Alex Alderton, Roberto Amato, Sonia Goncalves, Ewan Harrison, David K. Jackson, Ian Johnston, Dominic Kwiatkowski, Cordelia Langford, John Sillitoe on behalf of the Wellcome Sanger Institute COVID-19 Surveillance Team
EPI_ISL_798065	Lighthouse Lab in Glasgow	Wellcome Sanger Institute for the COVID-19 Genomics UK (COG-UK) Consortium	Harper VanSteenhouse, Yumi Kasai, David Gray, Carol Clugston, Anna Dominiczak and Alex Alderton, Roberto Amato, Sonia Goncalves, Ewan Harrison, David K. Jackson, Ian Johnston, Dominic Kwiatkowski, Cordelia Langford, John Sillitoe on behalf of the Wellcome Sanger Institute COVID-19 Surveillance Team
EPI_ISL_798066	Lighthouse Lab in Alderley Park	Wellcome Sanger Institute for the COVID-19 Genomics UK (COG-UK) Consortium	Jacquelyn Wynn, Mairead Hyland, The Lighthouse Lab in Alderley Park and Alex Alderton, Roberto Amato, Sonia Goncalves, Ewan Harrison, David K. Jackson, Ian Johnston, Dominic Kwiatkowski, Cordelia Langford, John Sillitoe on behalf of the Wellcome Sanger Institute COVID-19 Surveillance Team
EPI_ISL_798067	Lighthouse Lab in Glasgow	Wellcome Sanger Institute for the COVID-19 Genomics UK (COG-UK) Consortium	Harper VanSteenhouse, Yumi Kasai, David Gray, Carol Clugston, Anna Dominiczak and Alex Alderton, Roberto Amato, Sonia Goncalves, Ewan Harrison, David K. Jackson, Ian Johnston, Dominic Kwiatkowski, Cordelia Langford, John Sillitoe on behalf of the Wellcome Sanger Institute COVID-19 Surveillance Team
EPI_ISL_798068	Lighthouse Lab in Alderley Park	Wellcome Sanger Institute for the COVID-19 Genomics UK (COG-UK) Consortium	Jacquelyn Wynn, Mairead Hyland, The Lighthouse Lab in Alderley Park and Alex Alderton, Roberto Amato, Sonia Goncalves, Ewan Harrison, David K. Jackson, Ian Johnston, Dominic Kwiatkowski, Cordelia Langford, John Sillitoe on behalf of the Wellcome Sanger Institute COVID-19 Surveillance Team
EPI_ISL_798070, EPI_ISL_798071	Lighthouse Lab in Glasgow	Wellcome Sanger Institute for the COVID-19 Genomics UK (COG-UK) Consortium	Harper VanSteenhouse, Yumi Kasai, David Gray, Carol Clugston, Anna Dominiczak and Alex Alderton, Roberto Amato, Sonia Goncalves, Ewan Harrison, David K. Jackson, Ian Johnston, Dominic Kwiatkowski, Cordelia Langford, John Sillitoe on behalf of the Wellcome Sanger Institute COVID-19 Surveillance Team
EPI_ISL_798073	Lighthouse Lab in Alderley Park	Wellcome Sanger Institute for the COVID-19 Genomics UK (COG-UK) Consortium	Jacquelyn Wynn, Mairead Hyland, The Lighthouse Lab in Alderley Park and Alex Alderton, Roberto Amato, Sonia Goncalves, Ewan Harrison, David K. Jackson, Ian Johnston, Dominic Kwiatkowski, Cordelia Langford, John Sillitoe on behalf of the Wellcome Sanger Institute COVID-19 Surveillance Team
EPI_ISL_798074, EPI_ISL_798075, EPI_ISL_798080, EPI_ISL_798081, EPI_ISL_798082	Lighthouse Lab in Glasgow	Wellcome Sanger Institute for the COVID-19 Genomics UK (COG-UK) Consortium	Harper VanSteenhouse, Yumi Kasai, David Gray, Carol Clugston, Anna Dominiczak and Alex Alderton, Roberto Amato, Sonia Goncalves, Ewan Harrison, David K. Jackson, Ian Johnston, Dominic Kwiatkowski, Cordelia Langford, John Sillitoe on behalf of the Wellcome Sanger Institute COVID-19 Surveillance Team
EPI_ISL_798083, EPI_ISL_798084	Lighthouse Lab in Alderley Park	Wellcome Sanger Institute for the COVID-19 Genomics UK (COG-UK) Consortium	Jacquelyn Wynn, Mairead Hyland, The Lighthouse Lab in Alderley Park and Alex Alderton, Roberto Amato, Sonia Goncalves, Ewan Harrison, David K. Jackson, Ian Johnston, Dominic Kwiatkowski, Cordelia Langford, John Sillitoe on behalf of the Wellcome Sanger Institute COVID-19 Surveillance Team
EPI_ISL_798085, EPI_ISL_798087, EPI_ISL_798089, EPI_ISL_798090, EPI_ISL_798091, EPI_ISL_798092	Lighthouse Lab in Glasgow	Wellcome Sanger Institute for the COVID-19 Genomics UK (COG-UK) Consortium	Harper VanSteenhouse, Yumi Kasai, David Gray, Carol Clugston, Anna Dominiczak and Alex Alderton, Roberto Amato, Sonia Goncalves, Ewan Harrison, David K. Jackson, Ian Johnston, Dominic Kwiatkowski, Cordelia Langford, John Sillitoe on behalf of the Wellcome Sanger Institute COVID-19 Surveillance Team
EPI_ISL_798096	Lighthouse Lab in Alderley Park	Wellcome Sanger Institute for the COVID-19 Genomics UK (COG-UK) Consortium	Jacquelyn Wynn, Mairead Hyland, The Lighthouse Lab in Alderley Park and Alex Alderton, Roberto Amato, Sonia Goncalves, Ewan Harrison, David K. Jackson, Ian Johnston, Dominic Kwiatkowski, Cordelia Langford, John Sillitoe on behalf of the Wellcome Sanger Institute COVID-19 Surveillance Team
EPI_ISL_798098, EPI_ISL_798099, EPI_ISL_798100	Lighthouse Lab in Glasgow	Wellcome Sanger Institute for the COVID-19 Genomics UK (COG-UK) Consortium	Harper VanSteenhouse, Yumi Kasai, David Gray, Carol Clugston, Anna Dominiczak and Alex Alderton, Roberto Amato, Sonia Goncalves, Ewan Harrison, David K. Jackson, Ian Johnston, Dominic Kwiatkowski, Cordelia Langford, John Sillitoe on behalf of the Wellcome Sanger Institute COVID-19 Surveillance Team
EPI_ISL_798101	Lighthouse Lab in Alderley Park	Wellcome Sanger Institute for the COVID-19 Genomics UK (COG-UK) Consortium	Jacquelyn Wynn, Mairead Hyland, The Lighthouse Lab in Alderley Park and Alex Alderton, Roberto Amato, Sonia Goncalves, Ewan Harrison, David K. Jackson, Ian Johnston, Dominic Kwiatkowski, Cordelia Langford, John Sillitoe on behalf of the Wellcome Sanger Institute COVID-19 Surveillance Team
EPI_ISL_798102	Lighthouse Lab in Glasgow	Wellcome Sanger Institute for the COVID-19 Genomics UK (COG-UK) Consortium	Harper VanSteenhouse, Yumi Kasai, David Gray, Carol Clugston, Anna Dominiczak and Alex Alderton, Roberto Amato, Sonia Goncalves, Ewan Harrison, David K. Jackson, Ian Johnston, Dominic Kwiatkowski, Cordelia Langford, John Sillitoe on behalf of the Wellcome Sanger Institute COVID-19 Surveillance Team
EPI_ISL_798103	Lighthouse Lab in Alderley Park	Wellcome Sanger Institute for the COVID-19 Genomics UK (COG-UK) Consortium	Jacquelyn Wynn, Mairead Hyland, The Lighthouse Lab in Alderley Park and Alex Alderton, Roberto Amato, Sonia Goncalves, Ewan Harrison, David K. Jackson, Ian Johnston, Dominic Kwiatkowski, Cordelia Langford, John Sillitoe on behalf of the Wellcome Sanger Institute COVID-19 Surveillance Team

EPI_ISL_798104	Lighthouse Lab in Glasgow	Wellcome Sanger Institute for the COVID-19 Genomics UK (COG-UK) Consortium	Harper VanSteenhouse, Yumi Kasai, David Gray, Carol Clugston, Anna Dominiczak and Alex Alderton, Roberto Amato, Sonia Goncalves, Ewan Harrison, David K. Jackson, Ian Johnston, Dominic Kwiatkowski, Cordelia Langford, John Sillitoe on behalf of the Wellcome Sanger Institute COVID-19 Surveillance Team
EPI_ISL_798106	Lighthouse Lab in Alderley Park	Wellcome Sanger Institute for the COVID-19 Genomics UK (COG-UK) Consortium	Jacquelyn Wynn, Mairead Hyland, The Lighthouse Lab in Alderley Park and Alex Alderton, Roberto Amato, Sonia Goncalves, Ewan Harrison, David K. Jackson, Ian Johnston, Dominic Kwiatkowski, Cordelia Langford, John Sillitoe on behalf of the Wellcome Sanger Institute COVID-19 Surveillance Team
EPI_ISL_798107	Lighthouse Lab in Glasgow	Wellcome Sanger Institute for the COVID-19 Genomics UK (COG-UK) Consortium	Harper VanSteenhouse, Yumi Kasai, David Gray, Carol Clugston, Anna Dominiczak and Alex Alderton, Roberto Amato, Sonia Goncalves, Ewan Harrison, David K. Jackson, Ian Johnston, Dominic Kwiatkowski, Cordelia Langford, John Sillitoe on behalf of the Wellcome Sanger Institute COVID-19 Surveillance Team
EPI_ISL_798108	Lighthouse Lab in Alderley Park	Wellcome Sanger Institute for the COVID-19 Genomics UK (COG-UK) Consortium	Jacquelyn Wynn, Mairead Hyland, The Lighthouse Lab in Alderley Park and Alex Alderton, Roberto Amato, Sonia Goncalves, Ewan Harrison, David K. Jackson, Ian Johnston, Dominic Kwiatkowski, Cordelia Langford, John Sillitoe on behalf of the Wellcome Sanger Institute COVID-19 Surveillance Team
EPI_ISL_798109, EPI_ISL_798114, EPI_ISL_798116, EPI_ISL_798118	Lighthouse Lab in Glasgow	Wellcome Sanger Institute for the COVID-19 Genomics UK (COG-UK) Consortium	Harper VanSteenhouse, Yumi Kasai, David Gray, Carol Clugston, Anna Dominiczak and Alex Alderton, Roberto Amato, Sonia Goncalves, Ewan Harrison, David K. Jackson, Ian Johnston, Dominic Kwiatkowski, Cordelia Langford, John Sillitoe on behalf of the Wellcome Sanger Institute COVID-19 Surveillance Team
EPI_ISL_798119	Lighthouse Lab in Alderley Park	Wellcome Sanger Institute for the COVID-19 Genomics UK (COG-UK) Consortium	Jacquelyn Wynn, Mairead Hyland, The Lighthouse Lab in Alderley Park and Alex Alderton, Roberto Amato, Sonia Goncalves, Ewan Harrison, David K. Jackson, Ian Johnston, Dominic Kwiatkowski, Cordelia Langford, John Sillitoe on behalf of the Wellcome Sanger Institute COVID-19 Surveillance Team
EPI_ISL_798120	Lighthouse Lab in Glasgow	Wellcome Sanger Institute for the COVID-19 Genomics UK (COG-UK) Consortium	Harper VanSteenhouse, Yumi Kasai, David Gray, Carol Clugston, Anna Dominiczak and Alex Alderton, Roberto Amato, Sonia Goncalves, Ewan Harrison, David K. Jackson, Ian Johnston, Dominic Kwiatkowski, Cordelia Langford, John Sillitoe on behalf of the Wellcome Sanger Institute COVID-19 Surveillance Team
EPI_ISL_798122, EPI_ISL_798123, EPI_ISL_798124, EPI_ISL_798125, EPI_ISL_798127	Lighthouse Lab in Alderley Park	Wellcome Sanger Institute for the COVID-19 Genomics UK (COG-UK) Consortium	Jacquelyn Wynn, Mairead Hyland, The Lighthouse Lab in Alderley Park and Alex Alderton, Roberto Amato, Sonia Goncalves, Ewan Harrison, David K. Jackson, Ian Johnston, Dominic Kwiatkowski, Cordelia Langford, John Sillitoe on behalf of the Wellcome Sanger Institute COVID-19 Surveillance Team
EPI_ISL_798129	Lighthouse Lab in Glasgow	Wellcome Sanger Institute for the COVID-19 Genomics UK (COG-UK) Consortium	Harper VanSteenhouse, Yumi Kasai, David Gray, Carol Clugston, Anna Dominiczak and Alex Alderton, Roberto Amato, Sonia Goncalves, Ewan Harrison, David K. Jackson, Ian Johnston, Dominic Kwiatkowski, Cordelia Langford, John Sillitoe on behalf of the Wellcome Sanger Institute COVID-19 Surveillance Team
EPI_ISL_798131, EPI_ISL_798133, EPI_ISL_798134	Lighthouse Lab in Alderley Park	Wellcome Sanger Institute for the COVID-19 Genomics UK (COG-UK) Consortium	Jacquelyn Wynn, Mairead Hyland, The Lighthouse Lab in Alderley Park and Alex Alderton, Roberto Amato, Sonia Goncalves, Ewan Harrison, David K. Jackson, Ian Johnston, Dominic Kwiatkowski, Cordelia Langford, John Sillitoe on behalf of the Wellcome Sanger Institute COVID-19 Surveillance Team
EPI_ISL_798135, EPI_ISL_798136, EPI_ISL_798138	Lighthouse Lab in Glasgow	Wellcome Sanger Institute for the COVID-19 Genomics UK (COG-UK) Consortium	Harper VanSteenhouse, Yumi Kasai, David Gray, Carol Clugston, Anna Dominiczak and Alex Alderton, Roberto Amato, Sonia Goncalves, Ewan Harrison, David K. Jackson, Ian Johnston, Dominic Kwiatkowski, Cordelia Langford, John Sillitoe on behalf of the Wellcome Sanger Institute COVID-19 Surveillance Team
EPI_ISL_798139	Lighthouse Lab in Alderley Park	Wellcome Sanger Institute for the COVID-19 Genomics UK (COG-UK) Consortium	Jacquelyn Wynn, Mairead Hyland, The Lighthouse Lab in Alderley Park and Alex Alderton, Roberto Amato, Sonia Goncalves, Ewan Harrison, David K. Jackson, Ian Johnston, Dominic Kwiatkowski, Cordelia Langford, John Sillitoe on behalf of the Wellcome Sanger Institute COVID-19 Surveillance Team
EPI_ISL_798141, EPI_ISL_798142, EPI_ISL_798144, EPI_ISL_798146, EPI_ISL_798147, EPI_ISL_798150	Lighthouse Lab in Glasgow	Wellcome Sanger Institute for the COVID-19 Genomics UK (COG-UK) Consortium	Harper VanSteenhouse, Yumi Kasai, David Gray, Carol Clugston, Anna Dominiczak and Alex Alderton, Roberto Amato, Sonia Goncalves, Ewan Harrison, David K. Jackson, Ian Johnston, Dominic Kwiatkowski, Cordelia Langford, John Sillitoe on behalf of the Wellcome Sanger Institute COVID-19 Surveillance Team
EPI_ISL_798152, EPI_ISL_798154	Lighthouse Lab in Alderley Park	Wellcome Sanger Institute for the COVID-19 Genomics UK (COG-UK) Consortium	Jacquelyn Wynn, Mairead Hyland, The Lighthouse Lab in Alderley Park and Alex Alderton, Roberto Amato, Sonia Goncalves, Ewan Harrison, David K. Jackson, Ian Johnston, Dominic Kwiatkowski, Cordelia Langford, John Sillitoe on behalf of the Wellcome Sanger Institute COVID-19 Surveillance Team
EPI_ISL_798155, EPI_ISL_798156, EPI_ISL_798157, EPI_ISL_798158, EPI_ISL_798159, EPI_ISL_798162, EPI_ISL_798163, EPI_ISL_798164, EPI_ISL_798165	Lighthouse Lab in Glasgow	Wellcome Sanger Institute for the COVID-19 Genomics UK (COG-UK) Consortium	Harper VanSteenhouse, Yumi Kasai, David Gray, Carol Clugston, Anna Dominiczak and Alex Alderton, Roberto Amato, Sonia Goncalves, Ewan Harrison, David K. Jackson, Ian Johnston, Dominic Kwiatkowski, Cordelia Langford, John Sillitoe on behalf of the Wellcome Sanger Institute COVID-19 Surveillance Team
EPI_ISL_798169, EPI_ISL_798170	Lighthouse Lab in Alderley Park	Wellcome Sanger Institute for the COVID-19 Genomics UK (COG-UK) Consortium	Jacquelyn Wynn, Mairead Hyland, The Lighthouse Lab in Alderley Park and Alex Alderton, Roberto Amato, Sonia Goncalves, Ewan Harrison, David K. Jackson, Ian Johnston, Dominic Kwiatkowski, Cordelia Langford, John Sillitoe on behalf of the Wellcome Sanger Institute COVID-19 Surveillance Team
EPI_ISL_798171, EPI_ISL_798172	Lighthouse Lab in Glasgow	Wellcome Sanger Institute for the COVID-19 Genomics UK (COG-UK) Consortium	Harper VanSteenhouse, Yumi Kasai, David Gray, Carol Clugston, Anna Dominiczak and Alex Alderton, Roberto Amato, Sonia Goncalves, Ewan Harrison, David K. Jackson, Ian Johnston, Dominic Kwiatkowski, Cordelia Langford, John Sillitoe on behalf of the Wellcome Sanger Institute COVID-19 Surveillance Team
EPI_ISL_798173, EPI_ISL_798174, EPI_ISL_798175, EPI_ISL_798176	Lighthouse Lab in Alderley Park	Wellcome Sanger Institute for the COVID-19 Genomics UK (COG-UK) Consortium	Jacquelyn Wynn, Mairead Hyland, The Lighthouse Lab in Alderley Park and Alex Alderton, Roberto Amato, Sonia Goncalves, Ewan Harrison, David K. Jackson, Ian Johnston, Dominic Kwiatkowski, Cordelia Langford, John Sillitoe on behalf of the Wellcome Sanger Institute COVID-19 Surveillance Team
EPI_ISL_798177, EPI_ISL_798178, EPI_ISL_798179, EPI_ISL_798180	Lighthouse Lab in Glasgow	Wellcome Sanger Institute for the COVID-19 Genomics UK (COG-UK) Consortium	Harper VanSteenhouse, Yumi Kasai, David Gray, Carol Clugston, Anna Dominiczak and Alex Alderton, Roberto Amato, Sonia Goncalves, Ewan Harrison, David K. Jackson, Ian Johnston, Dominic Kwiatkowski, Cordelia Langford, John Sillitoe on behalf of the Wellcome Sanger Institute COVID-19 Surveillance Team
EPI_ISL_798181, EPI_ISL_798182	Lighthouse Lab in Alderley Park	Wellcome Sanger Institute for the COVID-19 Genomics UK (COG-UK) Consortium	Jacquelyn Wynn, Mairead Hyland, The Lighthouse Lab in Alderley Park and Alex Alderton, Roberto Amato, Sonia Goncalves, Ewan Harrison, David K. Jackson, Ian Johnston, Dominic Kwiatkowski, Cordelia Langford, John Sillitoe on behalf of the Wellcome Sanger Institute COVID-19 Surveillance Team
EPI_ISL_798184, EPI_ISL_798187, EPI_ISL_798188, EPI_ISL_798189, EPI_ISL_798190, EPI_ISL_798191, EPI_ISL_798192, EPI_ISL_798194, EPI_ISL_798195, EPI_ISL_798196, EPI_ISL_798198, EPI_ISL_798200			
see above	Lighthouse Lab in Glasgow	Wellcome Sanger Institute for the COVID-19 Genomics UK (COG-UK) Consortium	Harper VanSteenhouse, Yumi Kasai, David Gray, Carol Clugston, Anna Dominiczak and Alex Alderton, Roberto Amato, Sonia Goncalves, Ewan Harrison, David K. Jackson, Ian Johnston, Dominic Kwiatkowski, Cordelia Langford, John Sillitoe on behalf of the Wellcome Sanger Institute COVID-19 Surveillance Team
EPI_ISL_798202, EPI_ISL_798204	Lighthouse Lab in Alderley Park	Wellcome Sanger Institute for the COVID-19 Genomics UK (COG-UK) Consortium	Jacquelyn Wynn, Mairead Hyland, The Lighthouse Lab in Alderley Park and Alex Alderton, Roberto Amato, Sonia Goncalves, Ewan Harrison, David K. Jackson, Ian Johnston, Dominic Kwiatkowski, Cordelia Langford, John Sillitoe on behalf of the Wellcome Sanger Institute COVID-19 Surveillance Team
EPI_ISL_798205, EPI_ISL_798206, EPI_ISL_798207	Lighthouse Lab in Glasgow	Wellcome Sanger Institute for the COVID-19 Genomics UK (COG-UK) Consortium	Harper VanSteenhouse, Yumi Kasai, David Gray, Carol Clugston, Anna Dominiczak and Alex Alderton, Roberto Amato, Sonia Goncalves, Ewan Harrison, David K. Jackson, Ian Johnston, Dominic Kwiatkowski, Cordelia Langford, John Sillitoe on behalf of the Wellcome Sanger Institute COVID-19 Surveillance Team
EPI_ISL_798208	Lighthouse Lab in Alderley Park	Wellcome Sanger Institute for the COVID-19 Genomics UK (COG-UK) Consortium	Jacquelyn Wynn, Mairead Hyland, The Lighthouse Lab in Alderley Park and Alex Alderton, Roberto Amato, Sonia Goncalves, Ewan Harrison, David K. Jackson, Ian Johnston, Dominic Kwiatkowski, Cordelia Langford, John Sillitoe on behalf of the Wellcome Sanger Institute COVID-19 Surveillance Team
EPI_ISL_798209, EPI_ISL_798210, EPI_ISL_798212, EPI_ISL_798215, EPI_ISL_798219	Lighthouse Lab in Glasgow	Wellcome Sanger Institute for the COVID-19 Genomics UK (COG-UK) Consortium	Harper VanSteenhouse, Yumi Kasai, David Gray, Carol Clugston, Anna Dominiczak and Alex Alderton, Roberto Amato, Sonia Goncalves, Ewan Harrison, David K. Jackson, Ian Johnston, Dominic Kwiatkowski, Cordelia Langford, John Sillitoe on behalf of the Wellcome Sanger Institute COVID-19 Surveillance Team
EPI_ISL_798220, EPI_ISL_798221, EPI_ISL_798222, EPI_ISL_798225	Lighthouse Lab in Alderley Park	Wellcome Sanger Institute for the COVID-19 Genomics UK (COG-UK) Consortium	Jacquelyn Wynn, Mairead Hyland, The Lighthouse Lab in Alderley Park and Alex Alderton, Roberto Amato, Sonia Goncalves, Ewan Harrison, David K. Jackson, Ian Johnston, Dominic Kwiatkowski, Cordelia Langford, John Sillitoe on behalf of the Wellcome Sanger Institute COVID-19 Surveillance Team
EPI_ISL_798226, EPI_ISL_798227, EPI_ISL_798228	Lighthouse Lab in Glasgow	Wellcome Sanger Institute for the COVID-19 Genomics UK (COG-UK) Consortium	Harper VanSteenhouse, Yumi Kasai, David Gray, Carol Clugston, Anna Dominiczak and Alex Alderton, Roberto Amato, Sonia Goncalves, Ewan Harrison, David K. Jackson, Ian Johnston, Dominic Kwiatkowski, Cordelia Langford, John Sillitoe on behalf of the Wellcome Sanger Institute COVID-19 Surveillance Team
EPI_ISL_798229	Lighthouse Lab in Alderley Park	Wellcome Sanger Institute for the COVID-19 Genomics UK	Jacquelyn Wynn, Mairead Hyland, The Lighthouse Lab in Alderley Park and Alex Alderton, Roberto Amato, Sonia Goncalves, Ewan Harrison, David K.

		(COG-UK) Consortium	Jackson, Ian Johnston, Dominic Kwiatkowski, Cordelia Langford, John Sillitoe on behalf of the Wellcome Sanger Institute COVID-19 Surveillance Team
EPI_ISL_798231, EPI_ISL_798232, EPI_ISL_798233	Lighthouse Lab in Glasgow	Wellcome Sanger Institute for the COVID-19 Genomics UK (COG-UK) Consortium	Harper VanSteenhouse, Yumi Kasai, David Gray, Carol Clugston, Anna Dominiczak and Alex Alderton, Roberto Amato, Sonia Goncalves, Ewan Harrison, David K. Jackson, Ian Johnston, Dominic Kwiatkowski, Cordelia Langford, John Sillitoe on behalf of the Wellcome Sanger Institute COVID-19 Surveillance Team
EPI_ISL_798234, EPI_ISL_798235, EPI_ISL_798236	Lighthouse Lab in Alderley Park	Wellcome Sanger Institute for the COVID-19 Genomics UK (COG-UK) Consortium	Jacquelyn Wynn, Mairead Hyland, The Lighthouse Lab in Alderley Park and Alex Alderton, Roberto Amato, Sonia Goncalves, Ewan Harrison, David K. Jackson, Ian Johnston, Dominic Kwiatkowski, Cordelia Langford, John Sillitoe on behalf of the Wellcome Sanger Institute COVID-19 Surveillance Team
EPI_ISL_798237, EPI_ISL_798238	Lighthouse Lab in Glasgow	Wellcome Sanger Institute for the COVID-19 Genomics UK (COG-UK) Consortium	Harper VanSteenhouse, Yumi Kasai, David Gray, Carol Clugston, Anna Dominiczak and Alex Alderton, Roberto Amato, Sonia Goncalves, Ewan Harrison, David K. Jackson, Ian Johnston, Dominic Kwiatkowski, Cordelia Langford, John Sillitoe on behalf of the Wellcome Sanger Institute COVID-19 Surveillance Team
EPI_ISL_798239	Lighthouse Lab in Alderley Park	Wellcome Sanger Institute for the COVID-19 Genomics UK (COG-UK) Consortium	Jacquelyn Wynn, Mairead Hyland, The Lighthouse Lab in Alderley Park and Alex Alderton, Roberto Amato, Sonia Goncalves, Ewan Harrison, David K. Jackson, Ian Johnston, Dominic Kwiatkowski, Cordelia Langford, John Sillitoe on behalf of the Wellcome Sanger Institute COVID-19 Surveillance Team
EPI_ISL_798240	Lighthouse Lab in Glasgow	Wellcome Sanger Institute for the COVID-19 Genomics UK (COG-UK) Consortium	Harper VanSteenhouse, Yumi Kasai, David Gray, Carol Clugston, Anna Dominiczak and Alex Alderton, Roberto Amato, Sonia Goncalves, Ewan Harrison, David K. Jackson, Ian Johnston, Dominic Kwiatkowski, Cordelia Langford, John Sillitoe on behalf of the Wellcome Sanger Institute COVID-19 Surveillance Team
EPI_ISL_798241, EPI_ISL_798242, EPI_ISL_798245, EPI_ISL_798246	Lighthouse Lab in Alderley Park	Wellcome Sanger Institute for the COVID-19 Genomics UK (COG-UK) Consortium	Jacquelyn Wynn, Mairead Hyland, The Lighthouse Lab in Alderley Park and Alex Alderton, Roberto Amato, Sonia Goncalves, Ewan Harrison, David K. Jackson, Ian Johnston, Dominic Kwiatkowski, Cordelia Langford, John Sillitoe on behalf of the Wellcome Sanger Institute COVID-19 Surveillance Team
EPI_ISL_798247	Lighthouse Lab in Glasgow	Wellcome Sanger Institute for the COVID-19 Genomics UK (COG-UK) Consortium	Harper VanSteenhouse, Yumi Kasai, David Gray, Carol Clugston, Anna Dominiczak and Alex Alderton, Roberto Amato, Sonia Goncalves, Ewan Harrison, David K. Jackson, Ian Johnston, Dominic Kwiatkowski, Cordelia Langford, John Sillitoe on behalf of the Wellcome Sanger Institute COVID-19 Surveillance Team
EPI_ISL_798248	Lighthouse Lab in Alderley Park	Wellcome Sanger Institute for the COVID-19 Genomics UK (COG-UK) Consortium	Jacquelyn Wynn, Mairead Hyland, The Lighthouse Lab in Alderley Park and Alex Alderton, Roberto Amato, Sonia Goncalves, Ewan Harrison, David K. Jackson, Ian Johnston, Dominic Kwiatkowski, Cordelia Langford, John Sillitoe on behalf of the Wellcome Sanger Institute COVID-19 Surveillance Team
EPI_ISL_798249	Lighthouse Lab in Glasgow	Wellcome Sanger Institute for the COVID-19 Genomics UK (COG-UK) Consortium	Harper VanSteenhouse, Yumi Kasai, David Gray, Carol Clugston, Anna Dominiczak and Alex Alderton, Roberto Amato, Sonia Goncalves, Ewan Harrison, David K. Jackson, Ian Johnston, Dominic Kwiatkowski, Cordelia Langford, John Sillitoe on behalf of the Wellcome Sanger Institute COVID-19 Surveillance Team
EPI_ISL_798250, EPI_ISL_798255	Lighthouse Lab in Alderley Park	Wellcome Sanger Institute for the COVID-19 Genomics UK (COG-UK) Consortium	Jacquelyn Wynn, Mairead Hyland, The Lighthouse Lab in Alderley Park and Alex Alderton, Roberto Amato, Sonia Goncalves, Ewan Harrison, David K. Jackson, Ian Johnston, Dominic Kwiatkowski, Cordelia Langford, John Sillitoe on behalf of the Wellcome Sanger Institute COVID-19 Surveillance Team
EPI_ISL_798257, EPI_ISL_798259, EPI_ISL_798260, EPI_ISL_798262, EPI_ISL_798263, EPI_ISL_798264, EPI_ISL_798265, EPI_ISL_798267	Lighthouse Lab in Glasgow	Wellcome Sanger Institute for the COVID-19 Genomics UK (COG-UK) Consortium	Harper VanSteenhouse, Yumi Kasai, David Gray, Carol Clugston, Anna Dominiczak and Alex Alderton, Roberto Amato, Sonia Goncalves, Ewan Harrison, David K. Jackson, Ian Johnston, Dominic Kwiatkowski, Cordelia Langford, John Sillitoe on behalf of the Wellcome Sanger Institute COVID-19 Surveillance Team
EPI_ISL_798268	Lighthouse Lab in Alderley Park	Wellcome Sanger Institute for the COVID-19 Genomics UK (COG-UK) Consortium	Jacquelyn Wynn, Mairead Hyland, The Lighthouse Lab in Alderley Park and Alex Alderton, Roberto Amato, Sonia Goncalves, Ewan Harrison, David K. Jackson, Ian Johnston, Dominic Kwiatkowski, Cordelia Langford, John Sillitoe on behalf of the Wellcome Sanger Institute COVID-19 Surveillance Team
EPI_ISL_798269	Lighthouse Lab in Glasgow	Wellcome Sanger Institute for the COVID-19 Genomics UK (COG-UK) Consortium	Harper VanSteenhouse, Yumi Kasai, David Gray, Carol Clugston, Anna Dominiczak and Alex Alderton, Roberto Amato, Sonia Goncalves, Ewan Harrison, David K. Jackson, Ian Johnston, Dominic Kwiatkowski, Cordelia Langford, John Sillitoe on behalf of the Wellcome Sanger Institute COVID-19 Surveillance Team
EPI_ISL_798274, EPI_ISL_798275	Lighthouse Lab in Alderley Park	Wellcome Sanger Institute for the COVID-19 Genomics UK (COG-UK) Consortium	Jacquelyn Wynn, Mairead Hyland, The Lighthouse Lab in Alderley Park and Alex Alderton, Roberto Amato, Sonia Goncalves, Ewan Harrison, David K. Jackson, Ian Johnston, Dominic Kwiatkowski, Cordelia Langford, John Sillitoe on behalf of the Wellcome Sanger Institute COVID-19 Surveillance Team
EPI_ISL_798276	Lighthouse Lab in Glasgow	Wellcome Sanger Institute for the COVID-19 Genomics UK (COG-UK) Consortium	Harper VanSteenhouse, Yumi Kasai, David Gray, Carol Clugston, Anna Dominiczak and Alex Alderton, Roberto Amato, Sonia Goncalves, Ewan Harrison, David K. Jackson, Ian Johnston, Dominic Kwiatkowski, Cordelia Langford, John Sillitoe on behalf of the Wellcome Sanger Institute COVID-19 Surveillance Team
EPI_ISL_798277	Lighthouse Lab in Alderley Park	Wellcome Sanger Institute for the COVID-19 Genomics UK (COG-UK) Consortium	Jacquelyn Wynn, Mairead Hyland, The Lighthouse Lab in Alderley Park and Alex Alderton, Roberto Amato, Sonia Goncalves, Ewan Harrison, David K. Jackson, Ian Johnston, Dominic Kwiatkowski, Cordelia Langford, John Sillitoe on behalf of the Wellcome Sanger Institute COVID-19 Surveillance Team
EPI_ISL_798278, EPI_ISL_798279	Lighthouse Lab in Glasgow	Wellcome Sanger Institute for the COVID-19 Genomics UK (COG-UK) Consortium	Harper VanSteenhouse, Yumi Kasai, David Gray, Carol Clugston, Anna Dominiczak and Alex Alderton, Roberto Amato, Sonia Goncalves, Ewan Harrison, David K. Jackson, Ian Johnston, Dominic Kwiatkowski, Cordelia Langford, John Sillitoe on behalf of the Wellcome Sanger Institute COVID-19 Surveillance Team
EPI_ISL_798280, EPI_ISL_798282, EPI_ISL_798283, EPI_ISL_798284, EPI_ISL_798285	Lighthouse Lab in Alderley Park	Wellcome Sanger Institute for the COVID-19 Genomics UK (COG-UK) Consortium	Jacquelyn Wynn, Mairead Hyland, The Lighthouse Lab in Alderley Park and Alex Alderton, Roberto Amato, Sonia Goncalves, Ewan Harrison, David K. Jackson, Ian Johnston, Dominic Kwiatkowski, Cordelia Langford, John Sillitoe on behalf of the Wellcome Sanger Institute COVID-19 Surveillance Team
EPI_ISL_798286, EPI_ISL_798287, EPI_ISL_798290, EPI_ISL_798291	Lighthouse Lab in Glasgow	Wellcome Sanger Institute for the COVID-19 Genomics UK (COG-UK) Consortium	Harper VanSteenhouse, Yumi Kasai, David Gray, Carol Clugston, Anna Dominiczak and Alex Alderton, Roberto Amato, Sonia Goncalves, Ewan Harrison, David K. Jackson, Ian Johnston, Dominic Kwiatkowski, Cordelia Langford, John Sillitoe on behalf of the Wellcome Sanger Institute COVID-19 Surveillance Team
EPI_ISL_798292	Lighthouse Lab in Alderley Park	Wellcome Sanger Institute for the COVID-19 Genomics UK (COG-UK) Consortium	Jacquelyn Wynn, Mairead Hyland, The Lighthouse Lab in Alderley Park and Alex Alderton, Roberto Amato, Sonia Goncalves, Ewan Harrison, David K. Jackson, Ian Johnston, Dominic Kwiatkowski, Cordelia Langford, John Sillitoe on behalf of the Wellcome Sanger Institute COVID-19 Surveillance Team
EPI_ISL_798293	Lighthouse Lab in Glasgow	Wellcome Sanger Institute for the COVID-19 Genomics UK (COG-UK) Consortium	Harper VanSteenhouse, Yumi Kasai, David Gray, Carol Clugston, Anna Dominiczak and Alex Alderton, Roberto Amato, Sonia Goncalves, Ewan Harrison, David K. Jackson, Ian Johnston, Dominic Kwiatkowski, Cordelia Langford, John Sillitoe on behalf of the Wellcome Sanger Institute COVID-19 Surveillance Team
EPI_ISL_798295, EPI_ISL_798296	Lighthouse Lab in Alderley Park	Wellcome Sanger Institute for the COVID-19 Genomics UK (COG-UK) Consortium	Jacquelyn Wynn, Mairead Hyland, The Lighthouse Lab in Alderley Park and Alex Alderton, Roberto Amato, Sonia Goncalves, Ewan Harrison, David K. Jackson, Ian Johnston, Dominic Kwiatkowski, Cordelia Langford, John Sillitoe on behalf of the Wellcome Sanger Institute COVID-19 Surveillance Team
EPI_ISL_798297	Lighthouse Lab in Glasgow	Wellcome Sanger Institute for the COVID-19 Genomics UK (COG-UK) Consortium	Harper VanSteenhouse, Yumi Kasai, David Gray, Carol Clugston, Anna Dominiczak and Alex Alderton, Roberto Amato, Sonia Goncalves, Ewan Harrison, David K. Jackson, Ian Johnston, Dominic Kwiatkowski, Cordelia Langford, John Sillitoe on behalf of the Wellcome Sanger Institute COVID-19 Surveillance Team
EPI_ISL_798299	Lighthouse Lab in Alderley Park	Wellcome Sanger Institute for the COVID-19 Genomics UK (COG-UK) Consortium	Jacquelyn Wynn, Mairead Hyland, The Lighthouse Lab in Alderley Park and Alex Alderton, Roberto Amato, Sonia Goncalves, Ewan Harrison, David K. Jackson, Ian Johnston, Dominic Kwiatkowski, Cordelia Langford, John Sillitoe on behalf of the Wellcome Sanger Institute COVID-19 Surveillance Team
EPI_ISL_798301, EPI_ISL_798302, EPI_ISL_798303, EPI_ISL_798304	Lighthouse Lab in Glasgow	Wellcome Sanger Institute for the COVID-19 Genomics UK (COG-UK) Consortium	Harper VanSteenhouse, Yumi Kasai, David Gray, Carol Clugston, Anna Dominiczak and Alex Alderton, Roberto Amato, Sonia Goncalves, Ewan Harrison, David K. Jackson, Ian Johnston, Dominic Kwiatkowski, Cordelia Langford, John Sillitoe on behalf of the Wellcome Sanger Institute COVID-19 Surveillance Team
EPI_ISL_798305	Lighthouse Lab in Alderley Park	Wellcome Sanger Institute for the COVID-19 Genomics UK (COG-UK) Consortium	Jacquelyn Wynn, Mairead Hyland, The Lighthouse Lab in Alderley Park and Alex Alderton, Roberto Amato, Sonia Goncalves, Ewan Harrison, David K. Jackson, Ian Johnston, Dominic Kwiatkowski, Cordelia Langford, John Sillitoe on behalf of the Wellcome Sanger Institute COVID-19 Surveillance Team
EPI_ISL_798306, EPI_ISL_798309	Lighthouse Lab in Glasgow	Wellcome Sanger Institute for the COVID-19 Genomics UK (COG-UK) Consortium	Harper VanSteenhouse, Yumi Kasai, David Gray, Carol Clugston, Anna Dominiczak and Alex Alderton, Roberto Amato, Sonia Goncalves, Ewan Harrison, David K. Jackson, Ian Johnston, Dominic Kwiatkowski, Cordelia Langford, John Sillitoe on behalf of the Wellcome Sanger Institute COVID-19 Surveillance Team
EPI_ISL_798310	Lighthouse Lab in Alderley Park	Wellcome Sanger Institute for the COVID-19 Genomics UK (COG-UK) Consortium	Jacquelyn Wynn, Mairead Hyland, The Lighthouse Lab in Alderley Park and Alex Alderton, Roberto Amato, Sonia Goncalves, Ewan Harrison, David K. Jackson, Ian Johnston, Dominic Kwiatkowski, Cordelia Langford, John Sillitoe on behalf of the Wellcome Sanger Institute COVID-19 Surveillance Team

EPI_ISL_798313	Lighthouse Lab in Glasgow	Wellcome Sanger Institute for the COVID-19 Genomics UK (COG-UK) Consortium	Harper VanSteenhouse, Yumi Kasai, David Gray, Carol Clugston, Anna Dominiczak and Alex Alderton, Roberto Amato, Sonia Goncalves, Ewan Harrison, David K. Jackson, Ian Johnston, Dominic Kwiatkowski, Cordelia Langford, John Sillitoe on behalf of the Wellcome Sanger Institute COVID-19 Surveillance Team
EPI_ISL_798314	Lighthouse Lab in Alderley Park	Wellcome Sanger Institute for the COVID-19 Genomics UK (COG-UK) Consortium	Jacquelyn Wynn, Mairead Hyland, The Lighthouse Lab in Alderley Park and Alex Alderton, Roberto Amato, Sonia Goncalves, Ewan Harrison, David K. Jackson, Ian Johnston, Dominic Kwiatkowski, Cordelia Langford, John Sillitoe on behalf of the Wellcome Sanger Institute COVID-19 Surveillance Team
EPI_ISL_798317, EPI_ISL_798318, EPI_ISL_798320	Lighthouse Lab in Glasgow	Wellcome Sanger Institute for the COVID-19 Genomics UK (COG-UK) Consortium	Harper VanSteenhouse, Yumi Kasai, David Gray, Carol Clugston, Anna Dominiczak and Alex Alderton, Roberto Amato, Sonia Goncalves, Ewan Harrison, David K. Jackson, Ian Johnston, Dominic Kwiatkowski, Cordelia Langford, John Sillitoe on behalf of the Wellcome Sanger Institute COVID-19 Surveillance Team
EPI_ISL_798321, EPI_ISL_798323, EPI_ISL_798324	Lighthouse Lab in Alderley Park	Wellcome Sanger Institute for the COVID-19 Genomics UK (COG-UK) Consortium	Jacquelyn Wynn, Mairead Hyland, The Lighthouse Lab in Alderley Park and Alex Alderton, Roberto Amato, Sonia Goncalves, Ewan Harrison, David K. Jackson, Ian Johnston, Dominic Kwiatkowski, Cordelia Langford, John Sillitoe on behalf of the Wellcome Sanger Institute COVID-19 Surveillance Team
EPI_ISL_798325	Lighthouse Lab in Glasgow	Wellcome Sanger Institute for the COVID-19 Genomics UK (COG-UK) Consortium	Harper VanSteenhouse, Yumi Kasai, David Gray, Carol Clugston, Anna Dominiczak and Alex Alderton, Roberto Amato, Sonia Goncalves, Ewan Harrison, David K. Jackson, Ian Johnston, Dominic Kwiatkowski, Cordelia Langford, John Sillitoe on behalf of the Wellcome Sanger Institute COVID-19 Surveillance Team
EPI_ISL_798328, EPI_ISL_798329	Lighthouse Lab in Alderley Park	Wellcome Sanger Institute for the COVID-19 Genomics UK (COG-UK) Consortium	Jacquelyn Wynn, Mairead Hyland, The Lighthouse Lab in Alderley Park and Alex Alderton, Roberto Amato, Sonia Goncalves, Ewan Harrison, David K. Jackson, Ian Johnston, Dominic Kwiatkowski, Cordelia Langford, John Sillitoe on behalf of the Wellcome Sanger Institute COVID-19 Surveillance Team
EPI_ISL_798330, EPI_ISL_798331, EPI_ISL_799006, EPI_ISL_799009	Lighthouse Lab in Glasgow	Wellcome Sanger Institute for the COVID-19 Genomics UK (COG-UK) Consortium	Harper VanSteenhouse, Yumi Kasai, David Gray, Carol Clugston, Anna Dominiczak and Alex Alderton, Roberto Amato, Sonia Goncalves, Ewan Harrison, David K. Jackson, Ian Johnston, Dominic Kwiatkowski, Cordelia Langford, John Sillitoe on behalf of the Wellcome Sanger Institute COVID-19 Surveillance Team
EPI_ISL_799010	Lighthouse Lab in Alderley Park	Wellcome Sanger Institute for the COVID-19 Genomics UK (COG-UK) Consortium	Jacquelyn Wynn, Mairead Hyland, The Lighthouse Lab in Alderley Park and Alex Alderton, Roberto Amato, Sonia Goncalves, Ewan Harrison, David K. Jackson, Ian Johnston, Dominic Kwiatkowski, Cordelia Langford, John Sillitoe on behalf of the Wellcome Sanger Institute COVID-19 Surveillance Team
EPI_ISL_799011, EPI_ISL_799012, EPI_ISL_799014, EPI_ISL_799015, EPI_ISL_799016, EPI_ISL_799019	Lighthouse Lab in Glasgow	Wellcome Sanger Institute for the COVID-19 Genomics UK (COG-UK) Consortium	Harper VanSteenhouse, Yumi Kasai, David Gray, Carol Clugston, Anna Dominiczak and Alex Alderton, Roberto Amato, Sonia Goncalves, Ewan Harrison, David K. Jackson, Ian Johnston, Dominic Kwiatkowski, Cordelia Langford, John Sillitoe on behalf of the Wellcome Sanger Institute COVID-19 Surveillance Team
EPI_ISL_799021, EPI_ISL_799022	Lighthouse Lab in Alderley Park	Wellcome Sanger Institute for the COVID-19 Genomics UK (COG-UK) Consortium	Jacquelyn Wynn, Mairead Hyland, The Lighthouse Lab in Alderley Park and Alex Alderton, Roberto Amato, Sonia Goncalves, Ewan Harrison, David K. Jackson, Ian Johnston, Dominic Kwiatkowski, Cordelia Langford, John Sillitoe on behalf of the Wellcome Sanger Institute COVID-19 Surveillance Team
EPI_ISL_799024	Lighthouse Lab in Glasgow	Wellcome Sanger Institute for the COVID-19 Genomics UK (COG-UK) Consortium	Harper VanSteenhouse, Yumi Kasai, David Gray, Carol Clugston, Anna Dominiczak and Alex Alderton, Roberto Amato, Sonia Goncalves, Ewan Harrison, David K. Jackson, Ian Johnston, Dominic Kwiatkowski, Cordelia Langford, John Sillitoe on behalf of the Wellcome Sanger Institute COVID-19 Surveillance Team
EPI_ISL_799025, EPI_ISL_799026	Lighthouse Lab in Alderley Park	Wellcome Sanger Institute for the COVID-19 Genomics UK (COG-UK) Consortium	Jacquelyn Wynn, Mairead Hyland, The Lighthouse Lab in Alderley Park and Alex Alderton, Roberto Amato, Sonia Goncalves, Ewan Harrison, David K. Jackson, Ian Johnston, Dominic Kwiatkowski, Cordelia Langford, John Sillitoe on behalf of the Wellcome Sanger Institute COVID-19 Surveillance Team
EPI_ISL_799027, EPI_ISL_799028	Lighthouse Lab in Glasgow	Wellcome Sanger Institute for the COVID-19 Genomics UK (COG-UK) Consortium	Harper VanSteenhouse, Yumi Kasai, David Gray, Carol Clugston, Anna Dominiczak and Alex Alderton, Roberto Amato, Sonia Goncalves, Ewan Harrison, David K. Jackson, Ian Johnston, Dominic Kwiatkowski, Cordelia Langford, John Sillitoe on behalf of the Wellcome Sanger Institute COVID-19 Surveillance Team
EPI_ISL_799029	Lighthouse Lab in Alderley Park	Wellcome Sanger Institute for the COVID-19 Genomics UK (COG-UK) Consortium	Jacquelyn Wynn, Mairead Hyland, The Lighthouse Lab in Alderley Park and Alex Alderton, Roberto Amato, Sonia Goncalves, Ewan Harrison, David K. Jackson, Ian Johnston, Dominic Kwiatkowski, Cordelia Langford, John Sillitoe on behalf of the Wellcome Sanger Institute COVID-19 Surveillance Team
EPI_ISL_799030	Lighthouse Lab in Glasgow	Wellcome Sanger Institute for the COVID-19 Genomics UK (COG-UK) Consortium	Harper VanSteenhouse, Yumi Kasai, David Gray, Carol Clugston, Anna Dominiczak and Alex Alderton, Roberto Amato, Sonia Goncalves, Ewan Harrison, David K. Jackson, Ian Johnston, Dominic Kwiatkowski, Cordelia Langford, John Sillitoe on behalf of the Wellcome Sanger Institute COVID-19 Surveillance Team
EPI_ISL_799031	Lighthouse Lab in Cambridge	Wellcome Sanger Institute for the COVID-19 Genomics UK (COG-UK) Consortium	Rob Howes, The Lighthouse Lab in Cambridge and Alex Alderton, Roberto Amato, Sonia Goncalves, Ewan Harrison, David K. Jackson, Ian Johnston, Dominic Kwiatkowski, Cordelia Langford, John Sillitoe on behalf of the Wellcome Sanger Institute COVID-19 Surveillance Team
EPI_ISL_799033, EPI_ISL_799034, EPI_ISL_799038	Lighthouse Lab in Glasgow	Wellcome Sanger Institute for the COVID-19 Genomics UK (COG-UK) Consortium	Harper VanSteenhouse, Yumi Kasai, David Gray, Carol Clugston, Anna Dominiczak and Alex Alderton, Roberto Amato, Sonia Goncalves, Ewan Harrison, David K. Jackson, Ian Johnston, Dominic Kwiatkowski, Cordelia Langford, John Sillitoe on behalf of the Wellcome Sanger Institute COVID-19 Surveillance Team
EPI_ISL_799040	Lighthouse Lab in Alderley Park	Wellcome Sanger Institute for the COVID-19 Genomics UK (COG-UK) Consortium	Jacquelyn Wynn, Mairead Hyland, The Lighthouse Lab in Alderley Park and Alex Alderton, Roberto Amato, Sonia Goncalves, Ewan Harrison, David K. Jackson, Ian Johnston, Dominic Kwiatkowski, Cordelia Langford, John Sillitoe on behalf of the Wellcome Sanger Institute COVID-19 Surveillance Team
EPI_ISL_799041, EPI_ISL_799042, EPI_ISL_799043, EPI_ISL_799044	Lighthouse Lab in Glasgow	Wellcome Sanger Institute for the COVID-19 Genomics UK (COG-UK) Consortium	Harper VanSteenhouse, Yumi Kasai, David Gray, Carol Clugston, Anna Dominiczak and Alex Alderton, Roberto Amato, Sonia Goncalves, Ewan Harrison, David K. Jackson, Ian Johnston, Dominic Kwiatkowski, Cordelia Langford, John Sillitoe on behalf of the Wellcome Sanger Institute COVID-19 Surveillance Team
EPI_ISL_799045	Lighthouse Lab in Alderley Park	Wellcome Sanger Institute for the COVID-19 Genomics UK (COG-UK) Consortium	Jacquelyn Wynn, Mairead Hyland, The Lighthouse Lab in Alderley Park and Alex Alderton, Roberto Amato, Sonia Goncalves, Ewan Harrison, David K. Jackson, Ian Johnston, Dominic Kwiatkowski, Cordelia Langford, John Sillitoe on behalf of the Wellcome Sanger Institute COVID-19 Surveillance Team
EPI_ISL_799046	Lighthouse Lab in Glasgow	Wellcome Sanger Institute for the COVID-19 Genomics UK (COG-UK) Consortium	Harper VanSteenhouse, Yumi Kasai, David Gray, Carol Clugston, Anna Dominiczak and Alex Alderton, Roberto Amato, Sonia Goncalves, Ewan Harrison, David K. Jackson, Ian Johnston, Dominic Kwiatkowski, Cordelia Langford, John Sillitoe on behalf of the Wellcome Sanger Institute COVID-19 Surveillance Team
EPI_ISL_799047	Lighthouse Lab in Alderley Park	Wellcome Sanger Institute for the COVID-19 Genomics UK (COG-UK) Consortium	Jacquelyn Wynn, Mairead Hyland, The Lighthouse Lab in Alderley Park and Alex Alderton, Roberto Amato, Sonia Goncalves, Ewan Harrison, David K. Jackson, Ian Johnston, Dominic Kwiatkowski, Cordelia Langford, John Sillitoe on behalf of the Wellcome Sanger Institute COVID-19 Surveillance Team
EPI_ISL_799049, EPI_ISL_799050, EPI_ISL_799051	Lighthouse Lab in Glasgow	Wellcome Sanger Institute for the COVID-19 Genomics UK (COG-UK) Consortium	Harper VanSteenhouse, Yumi Kasai, David Gray, Carol Clugston, Anna Dominiczak and Alex Alderton, Roberto Amato, Sonia Goncalves, Ewan Harrison, David K. Jackson, Ian Johnston, Dominic Kwiatkowski, Cordelia Langford, John Sillitoe on behalf of the Wellcome Sanger Institute COVID-19 Surveillance Team
EPI_ISL_799053	Lighthouse Lab in Alderley Park	Wellcome Sanger Institute for the COVID-19 Genomics UK (COG-UK) Consortium	Jacquelyn Wynn, Mairead Hyland, The Lighthouse Lab in Alderley Park and Alex Alderton, Roberto Amato, Sonia Goncalves, Ewan Harrison, David K. Jackson, Ian Johnston, Dominic Kwiatkowski, Cordelia Langford, John Sillitoe on behalf of the Wellcome Sanger Institute COVID-19 Surveillance Team
EPI_ISL_799054	Lighthouse Lab in Glasgow	Wellcome Sanger Institute for the COVID-19 Genomics UK (COG-UK) Consortium	Harper VanSteenhouse, Yumi Kasai, David Gray, Carol Clugston, Anna Dominiczak and Alex Alderton, Roberto Amato, Sonia Goncalves, Ewan Harrison, David K. Jackson, Ian Johnston, Dominic Kwiatkowski, Cordelia Langford, John Sillitoe on behalf of the Wellcome Sanger Institute COVID-19 Surveillance Team
EPI_ISL_799055	Lighthouse Lab in Alderley Park	Wellcome Sanger Institute for the COVID-19 Genomics UK (COG-UK) Consortium	Jacquelyn Wynn, Mairead Hyland, The Lighthouse Lab in Alderley Park and Alex Alderton, Roberto Amato, Sonia Goncalves, Ewan Harrison, David K. Jackson, Ian Johnston, Dominic Kwiatkowski, Cordelia Langford, John Sillitoe on behalf of the Wellcome Sanger Institute COVID-19 Surveillance Team
EPI_ISL_799056	Lighthouse Lab in Glasgow	Wellcome Sanger Institute for the COVID-19 Genomics UK (COG-UK) Consortium	Harper VanSteenhouse, Yumi Kasai, David Gray, Carol Clugston, Anna Dominiczak and Alex Alderton, Roberto Amato, Sonia Goncalves, Ewan Harrison, David K. Jackson, Ian Johnston, Dominic Kwiatkowski, Cordelia Langford, John Sillitoe on behalf of the Wellcome Sanger Institute COVID-19 Surveillance Team
EPI_ISL_799057, EPI_ISL_799059	Lighthouse Lab in Alderley Park	Wellcome Sanger Institute for the COVID-19 Genomics UK (COG-UK) Consortium	Jacquelyn Wynn, Mairead Hyland, The Lighthouse Lab in Alderley Park and Alex Alderton, Roberto Amato, Sonia Goncalves, Ewan Harrison, David K. Jackson, Ian Johnston, Dominic Kwiatkowski, Cordelia Langford, John Sillitoe on behalf of the Wellcome Sanger Institute COVID-19 Surveillance Team
EPI_ISL_799060, EPI_ISL_799062, EPI_ISL_799063, EPI_ISL_799064, EPI_ISL_799065, EPI_ISL_799066,	Lighthouse Lab in Glasgow	Wellcome Sanger Institute for the COVID-19 Genomics UK (COG-UK) Consortium	Harper VanSteenhouse, Yumi Kasai, David Gray, Carol Clugston, Anna Dominiczak and Alex Alderton, Roberto Amato, Sonia Goncalves, Ewan Harrison, David K. Jackson, Ian Johnston, Dominic Kwiatkowski, Cordelia Langford, John Sillitoe on behalf of the Wellcome Sanger Institute COVID-19 Surveillance Team

EPI_ISL_799067, EPI_ISL_799068			
EPI_ISL_799069, EPI_ISL_799070	Lighthouse Lab in Alderley Park	Wellcome Sanger Institute for the COVID-19 Genomics UK (COG-UK) Consortium	Jacquelyn Wynn, Mairead Hyland, The Lighthouse Lab in Alderley Park and Alex Alderton, Roberto Amato, Sonia Goncalves, Ewan Harrison, David K. Jackson, Ian Johnston, Dominic Kwiatkowski, Cordelia Langford, John Sillitoe on behalf of the Wellcome Sanger Institute COVID-19 Surveillance Team
EPI_ISL_799071, EPI_ISL_799072, EPI_ISL_799073, EPI_ISL_799075, EPI_ISL_799077	Lighthouse Lab in Glasgow	Wellcome Sanger Institute for the COVID-19 Genomics UK (COG-UK) Consortium	Harper VanSteenhouse, Yumi Kasai, David Gray, Carol Clugston, Anna Dominiczak and Alex Alderton, Roberto Amato, Sonia Goncalves, Ewan Harrison, David K. Jackson, Ian Johnston, Dominic Kwiatkowski, Cordelia Langford, John Sillitoe on behalf of the Wellcome Sanger Institute COVID-19 Surveillance Team
EPI_ISL_799079	Lighthouse Lab in Alderley Park	Wellcome Sanger Institute for the COVID-19 Genomics UK (COG-UK) Consortium	Jacquelyn Wynn, Mairead Hyland, The Lighthouse Lab in Alderley Park and Alex Alderton, Roberto Amato, Sonia Goncalves, Ewan Harrison, David K. Jackson, Ian Johnston, Dominic Kwiatkowski, Cordelia Langford, John Sillitoe on behalf of the Wellcome Sanger Institute COVID-19 Surveillance Team
EPI_ISL_799080, EPI_ISL_799081, EPI_ISL_799082, EPI_ISL_799084	Lighthouse Lab in Glasgow	Wellcome Sanger Institute for the COVID-19 Genomics UK (COG-UK) Consortium	Harper VanSteenhouse, Yumi Kasai, David Gray, Carol Clugston, Anna Dominiczak and Alex Alderton, Roberto Amato, Sonia Goncalves, Ewan Harrison, David K. Jackson, Ian Johnston, Dominic Kwiatkowski, Cordelia Langford, John Sillitoe on behalf of the Wellcome Sanger Institute COVID-19 Surveillance Team
EPI_ISL_799085, EPI_ISL_799086	Lighthouse Lab in Alderley Park	Wellcome Sanger Institute for the COVID-19 Genomics UK (COG-UK) Consortium	Jacquelyn Wynn, Mairead Hyland, The Lighthouse Lab in Alderley Park and Alex Alderton, Roberto Amato, Sonia Goncalves, Ewan Harrison, David K. Jackson, Ian Johnston, Dominic Kwiatkowski, Cordelia Langford, John Sillitoe on behalf of the Wellcome Sanger Institute COVID-19 Surveillance Team
EPI_ISL_799087	Lighthouse Lab in Cambridge	Wellcome Sanger Institute for the COVID-19 Genomics UK (COG-UK) Consortium	Rob Howes, The Lighthouse Lab in Cambridge and Alex Alderton, Roberto Amato, Sonia Goncalves, Ewan Harrison, David K. Jackson, Ian Johnston, Dominic Kwiatkowski, Cordelia Langford, John Sillitoe on behalf of the Wellcome Sanger Institute COVID-19 Surveillance Team
EPI_ISL_799089, EPI_ISL_799090, EPI_ISL_799096, EPI_ISL_799098	Lighthouse Lab in Glasgow	Wellcome Sanger Institute for the COVID-19 Genomics UK (COG-UK) Consortium	Harper VanSteenhouse, Yumi Kasai, David Gray, Carol Clugston, Anna Dominiczak and Alex Alderton, Roberto Amato, Sonia Goncalves, Ewan Harrison, David K. Jackson, Ian Johnston, Dominic Kwiatkowski, Cordelia Langford, John Sillitoe on behalf of the Wellcome Sanger Institute COVID-19 Surveillance Team
EPI_ISL_799099	Lighthouse Lab in Alderley Park	Wellcome Sanger Institute for the COVID-19 Genomics UK (COG-UK) Consortium	Jacquelyn Wynn, Mairead Hyland, The Lighthouse Lab in Alderley Park and Alex Alderton, Roberto Amato, Sonia Goncalves, Ewan Harrison, David K. Jackson, Ian Johnston, Dominic Kwiatkowski, Cordelia Langford, John Sillitoe on behalf of the Wellcome Sanger Institute COVID-19 Surveillance Team
EPI_ISL_799100, EPI_ISL_799101, EPI_ISL_799102	Lighthouse Lab in Glasgow	Wellcome Sanger Institute for the COVID-19 Genomics UK (COG-UK) Consortium	Harper VanSteenhouse, Yumi Kasai, David Gray, Carol Clugston, Anna Dominiczak and Alex Alderton, Roberto Amato, Sonia Goncalves, Ewan Harrison, David K. Jackson, Ian Johnston, Dominic Kwiatkowski, Cordelia Langford, John Sillitoe on behalf of the Wellcome Sanger Institute COVID-19 Surveillance Team
EPI_ISL_799104, EPI_ISL_799105, EPI_ISL_799106	Lighthouse Lab in Alderley Park	Wellcome Sanger Institute for the COVID-19 Genomics UK (COG-UK) Consortium	Jacquelyn Wynn, Mairead Hyland, The Lighthouse Lab in Alderley Park and Alex Alderton, Roberto Amato, Sonia Goncalves, Ewan Harrison, David K. Jackson, Ian Johnston, Dominic Kwiatkowski, Cordelia Langford, John Sillitoe on behalf of the Wellcome Sanger Institute COVID-19 Surveillance Team
EPI_ISL_799107, EPI_ISL_799108	Lighthouse Lab in Glasgow	Wellcome Sanger Institute for the COVID-19 Genomics UK (COG-UK) Consortium	Harper VanSteenhouse, Yumi Kasai, David Gray, Carol Clugston, Anna Dominiczak and Alex Alderton, Roberto Amato, Sonia Goncalves, Ewan Harrison, David K. Jackson, Ian Johnston, Dominic Kwiatkowski, Cordelia Langford, John Sillitoe on behalf of the Wellcome Sanger Institute COVID-19 Surveillance Team
EPI_ISL_799111	Lighthouse Lab in Alderley Park	Wellcome Sanger Institute for the COVID-19 Genomics UK (COG-UK) Consortium	Jacquelyn Wynn, Mairead Hyland, The Lighthouse Lab in Alderley Park and Alex Alderton, Roberto Amato, Sonia Goncalves, Ewan Harrison, David K. Jackson, Ian Johnston, Dominic Kwiatkowski, Cordelia Langford, John Sillitoe on behalf of the Wellcome Sanger Institute COVID-19 Surveillance Team
EPI_ISL_799112	Lighthouse Lab in Glasgow	Wellcome Sanger Institute for the COVID-19 Genomics UK (COG-UK) Consortium	Harper VanSteenhouse, Yumi Kasai, David Gray, Carol Clugston, Anna Dominiczak and Alex Alderton, Roberto Amato, Sonia Goncalves, Ewan Harrison, David K. Jackson, Ian Johnston, Dominic Kwiatkowski, Cordelia Langford, John Sillitoe on behalf of the Wellcome Sanger Institute COVID-19 Surveillance Team
EPI_ISL_799113, EPI_ISL_799114	Lighthouse Lab in Alderley Park	Wellcome Sanger Institute for the COVID-19 Genomics UK (COG-UK) Consortium	Jacquelyn Wynn, Mairead Hyland, The Lighthouse Lab in Alderley Park and Alex Alderton, Roberto Amato, Sonia Goncalves, Ewan Harrison, David K. Jackson, Ian Johnston, Dominic Kwiatkowski, Cordelia Langford, John Sillitoe on behalf of the Wellcome Sanger Institute COVID-19 Surveillance Team
EPI_ISL_799115, EPI_ISL_799118, EPI_ISL_799120	Lighthouse Lab in Glasgow	Wellcome Sanger Institute for the COVID-19 Genomics UK (COG-UK) Consortium	Harper VanSteenhouse, Yumi Kasai, David Gray, Carol Clugston, Anna Dominiczak and Alex Alderton, Roberto Amato, Sonia Goncalves, Ewan Harrison, David K. Jackson, Ian Johnston, Dominic Kwiatkowski, Cordelia Langford, John Sillitoe on behalf of the Wellcome Sanger Institute COVID-19 Surveillance Team
EPI_ISL_799122	Lighthouse Lab in Alderley Park	Wellcome Sanger Institute for the COVID-19 Genomics UK (COG-UK) Consortium	Jacquelyn Wynn, Mairead Hyland, The Lighthouse Lab in Alderley Park and Alex Alderton, Roberto Amato, Sonia Goncalves, Ewan Harrison, David K. Jackson, Ian Johnston, Dominic Kwiatkowski, Cordelia Langford, John Sillitoe on behalf of the Wellcome Sanger Institute COVID-19 Surveillance Team
EPI_ISL_799123	Lighthouse Lab in Glasgow	Wellcome Sanger Institute for the COVID-19 Genomics UK (COG-UK) Consortium	Harper VanSteenhouse, Yumi Kasai, David Gray, Carol Clugston, Anna Dominiczak and Alex Alderton, Roberto Amato, Sonia Goncalves, Ewan Harrison, David K. Jackson, Ian Johnston, Dominic Kwiatkowski, Cordelia Langford, John Sillitoe on behalf of the Wellcome Sanger Institute COVID-19 Surveillance Team
EPI_ISL_799124	Lighthouse Lab in Alderley Park	Wellcome Sanger Institute for the COVID-19 Genomics UK (COG-UK) Consortium	Jacquelyn Wynn, Mairead Hyland, The Lighthouse Lab in Alderley Park and Alex Alderton, Roberto Amato, Sonia Goncalves, Ewan Harrison, David K. Jackson, Ian Johnston, Dominic Kwiatkowski, Cordelia Langford, John Sillitoe on behalf of the Wellcome Sanger Institute COVID-19 Surveillance Team
EPI_ISL_799125, EPI_ISL_799126, EPI_ISL_799127, EPI_ISL_799129, EPI_ISL_799130, EPI_ISL_799131, EPI_ISL_799132, EPI_ISL_799134	Lighthouse Lab in Glasgow	Wellcome Sanger Institute for the COVID-19 Genomics UK (COG-UK) Consortium	Harper VanSteenhouse, Yumi Kasai, David Gray, Carol Clugston, Anna Dominiczak and Alex Alderton, Roberto Amato, Sonia Goncalves, Ewan Harrison, David K. Jackson, Ian Johnston, Dominic Kwiatkowski, Cordelia Langford, John Sillitoe on behalf of the Wellcome Sanger Institute COVID-19 Surveillance Team
EPI_ISL_799137, EPI_ISL_799138, EPI_ISL_799139	Lighthouse Lab in Alderley Park	Wellcome Sanger Institute for the COVID-19 Genomics UK (COG-UK) Consortium	Jacquelyn Wynn, Mairead Hyland, The Lighthouse Lab in Alderley Park and Alex Alderton, Roberto Amato, Sonia Goncalves, Ewan Harrison, David K. Jackson, Ian Johnston, Dominic Kwiatkowski, Cordelia Langford, John Sillitoe on behalf of the Wellcome Sanger Institute COVID-19 Surveillance Team
EPI_ISL_799140	Lighthouse Lab in Glasgow	Wellcome Sanger Institute for the COVID-19 Genomics UK (COG-UK) Consortium	Harper VanSteenhouse, Yumi Kasai, David Gray, Carol Clugston, Anna Dominiczak and Alex Alderton, Roberto Amato, Sonia Goncalves, Ewan Harrison, David K. Jackson, Ian Johnston, Dominic Kwiatkowski, Cordelia Langford, John Sillitoe on behalf of the Wellcome Sanger Institute COVID-19 Surveillance Team
EPI_ISL_799143	Lighthouse Lab in Alderley Park	Wellcome Sanger Institute for the COVID-19 Genomics UK (COG-UK) Consortium	Jacquelyn Wynn, Mairead Hyland, The Lighthouse Lab in Alderley Park and Alex Alderton, Roberto Amato, Sonia Goncalves, Ewan Harrison, David K. Jackson, Ian Johnston, Dominic Kwiatkowski, Cordelia Langford, John Sillitoe on behalf of the Wellcome Sanger Institute COVID-19 Surveillance Team
EPI_ISL_799144	Lighthouse Lab in Glasgow	Wellcome Sanger Institute for the COVID-19 Genomics UK (COG-UK) Consortium	Harper VanSteenhouse, Yumi Kasai, David Gray, Carol Clugston, Anna Dominiczak and Alex Alderton, Roberto Amato, Sonia Goncalves, Ewan Harrison, David K. Jackson, Ian Johnston, Dominic Kwiatkowski, Cordelia Langford, John Sillitoe on behalf of the Wellcome Sanger Institute COVID-19 Surveillance Team
EPI_ISL_799145	Lighthouse Lab in Alderley Park	Wellcome Sanger Institute for the COVID-19 Genomics UK (COG-UK) Consortium	Jacquelyn Wynn, Mairead Hyland, The Lighthouse Lab in Alderley Park and Alex Alderton, Roberto Amato, Sonia Goncalves, Ewan Harrison, David K. Jackson, Ian Johnston, Dominic Kwiatkowski, Cordelia Langford, John Sillitoe on behalf of the Wellcome Sanger Institute COVID-19 Surveillance Team
EPI_ISL_799147	Lighthouse Lab in Glasgow	Wellcome Sanger Institute for the COVID-19 Genomics UK (COG-UK) Consortium	Harper VanSteenhouse, Yumi Kasai, David Gray, Carol Clugston, Anna Dominiczak and Alex Alderton, Roberto Amato, Sonia Goncalves, Ewan Harrison, David K. Jackson, Ian Johnston, Dominic Kwiatkowski, Cordelia Langford, John Sillitoe on behalf of the Wellcome Sanger Institute COVID-19 Surveillance Team
EPI_ISL_799149	Lighthouse Lab in Alderley Park	Wellcome Sanger Institute for the COVID-19 Genomics UK (COG-UK) Consortium	Jacquelyn Wynn, Mairead Hyland, The Lighthouse Lab in Alderley Park and Alex Alderton, Roberto Amato, Sonia Goncalves, Ewan Harrison, David K. Jackson, Ian Johnston, Dominic Kwiatkowski, Cordelia Langford, John Sillitoe on behalf of the Wellcome Sanger Institute COVID-19 Surveillance Team
EPI_ISL_799150, EPI_ISL_799151	Lighthouse Lab in Glasgow	Wellcome Sanger Institute for the COVID-19 Genomics UK (COG-UK) Consortium	Harper VanSteenhouse, Yumi Kasai, David Gray, Carol Clugston, Anna Dominiczak and Alex Alderton, Roberto Amato, Sonia Goncalves, Ewan Harrison, David K. Jackson, Ian Johnston, Dominic Kwiatkowski, Cordelia Langford, John Sillitoe on behalf of the Wellcome Sanger Institute COVID-19 Surveillance Team
EPI_ISL_799152	Lighthouse Lab in Cambridge	Wellcome Sanger Institute for the COVID-19 Genomics UK (COG-UK) Consortium	Rob Howes, The Lighthouse Lab in Cambridge and Alex Alderton, Roberto Amato, Sonia Goncalves, Ewan Harrison, David K. Jackson, Ian Johnston, Dominic Kwiatkowski, Cordelia Langford, John Sillitoe on behalf of the Wellcome Sanger Institute COVID-19 Surveillance Team
EPI_ISL_799154	Lighthouse Lab in Alderley Park	Wellcome Sanger Institute for the COVID-19 Genomics UK (COG-UK) Consortium	Jacquelyn Wynn, Mairead Hyland, The Lighthouse Lab in Alderley Park and Alex Alderton, Roberto Amato, Sonia Goncalves, Ewan Harrison, David K. Jackson, Ian Johnston, Dominic Kwiatkowski, Cordelia Langford, John Sillitoe on behalf of the Wellcome Sanger Institute COVID-19 Surveillance Team

EPI_ISL_799155, EPI_ISL_799156, EPI_ISL_799157, EPI_ISL_799158	Lighthouse Lab in Glasgow	Wellcome Sanger Institute for the COVID-19 Genomics UK (COG-UK) Consortium	Harper VanSteenhouse, Yumi Kasai, David Gray, Carol Clugston, Anna Dominiczak and Alex Alderton, Roberto Amato, Sonia Goncalves, Ewan Harrison, David K. Jackson, Ian Johnston, Dominic Kwiatkowski, Cordelia Langford, John Sillitoe on behalf of the Wellcome Sanger Institute COVID-19 Surveillance Team
EPI_ISL_799160, EPI_ISL_799161	Lighthouse Lab in Alderley Park	Wellcome Sanger Institute for the COVID-19 Genomics UK (COG-UK) Consortium	Jacquelyn Wynn, Mairead Hyland, The Lighthouse Lab in Alderley Park and Alex Alderton, Roberto Amato, Sonia Goncalves, Ewan Harrison, David K. Jackson, Ian Johnston, Dominic Kwiatkowski, Cordelia Langford, John Sillitoe on behalf of the Wellcome Sanger Institute COVID-19 Surveillance Team
EPI_ISL_799162, EPI_ISL_799163, EPI_ISL_799164	Lighthouse Lab in Glasgow	Wellcome Sanger Institute for the COVID-19 Genomics UK (COG-UK) Consortium	Harper VanSteenhouse, Yumi Kasai, David Gray, Carol Clugston, Anna Dominiczak and Alex Alderton, Roberto Amato, Sonia Goncalves, Ewan Harrison, David K. Jackson, Ian Johnston, Dominic Kwiatkowski, Cordelia Langford, John Sillitoe on behalf of the Wellcome Sanger Institute COVID-19 Surveillance Team
EPI_ISL_799165, EPI_ISL_799166	Lighthouse Lab in Alderley Park	Wellcome Sanger Institute for the COVID-19 Genomics UK (COG-UK) Consortium	Jacquelyn Wynn, Mairead Hyland, The Lighthouse Lab in Alderley Park and Alex Alderton, Roberto Amato, Sonia Goncalves, Ewan Harrison, David K. Jackson, Ian Johnston, Dominic Kwiatkowski, Cordelia Langford, John Sillitoe on behalf of the Wellcome Sanger Institute COVID-19 Surveillance Team
EPI_ISL_799167, EPI_ISL_799169	Lighthouse Lab in Glasgow	Wellcome Sanger Institute for the COVID-19 Genomics UK (COG-UK) Consortium	Harper VanSteenhouse, Yumi Kasai, David Gray, Carol Clugston, Anna Dominiczak and Alex Alderton, Roberto Amato, Sonia Goncalves, Ewan Harrison, David K. Jackson, Ian Johnston, Dominic Kwiatkowski, Cordelia Langford, John Sillitoe on behalf of the Wellcome Sanger Institute COVID-19 Surveillance Team
EPI_ISL_799170	Lighthouse Lab in Alderley Park	Wellcome Sanger Institute for the COVID-19 Genomics UK (COG-UK) Consortium	Jacquelyn Wynn, Mairead Hyland, The Lighthouse Lab in Alderley Park and Alex Alderton, Roberto Amato, Sonia Goncalves, Ewan Harrison, David K. Jackson, Ian Johnston, Dominic Kwiatkowski, Cordelia Langford, John Sillitoe on behalf of the Wellcome Sanger Institute COVID-19 Surveillance Team
EPI_ISL_799172, EPI_ISL_799175, EPI_ISL_799176	Lighthouse Lab in Glasgow	Wellcome Sanger Institute for the COVID-19 Genomics UK (COG-UK) Consortium	Harper VanSteenhouse, Yumi Kasai, David Gray, Carol Clugston, Anna Dominiczak and Alex Alderton, Roberto Amato, Sonia Goncalves, Ewan Harrison, David K. Jackson, Ian Johnston, Dominic Kwiatkowski, Cordelia Langford, John Sillitoe on behalf of the Wellcome Sanger Institute COVID-19 Surveillance Team
EPI_ISL_799177, EPI_ISL_799178, EPI_ISL_799179	Lighthouse Lab in Alderley Park	Wellcome Sanger Institute for the COVID-19 Genomics UK (COG-UK) Consortium	Jacquelyn Wynn, Mairead Hyland, The Lighthouse Lab in Alderley Park and Alex Alderton, Roberto Amato, Sonia Goncalves, Ewan Harrison, David K. Jackson, Ian Johnston, Dominic Kwiatkowski, Cordelia Langford, John Sillitoe on behalf of the Wellcome Sanger Institute COVID-19 Surveillance Team
EPI_ISL_799180	Lighthouse Lab in Glasgow	Wellcome Sanger Institute for the COVID-19 Genomics UK (COG-UK) Consortium	Harper VanSteenhouse, Yumi Kasai, David Gray, Carol Clugston, Anna Dominiczak and Alex Alderton, Roberto Amato, Sonia Goncalves, Ewan Harrison, David K. Jackson, Ian Johnston, Dominic Kwiatkowski, Cordelia Langford, John Sillitoe on behalf of the Wellcome Sanger Institute COVID-19 Surveillance Team
EPI_ISL_799182	Lighthouse Lab in Alderley Park	Wellcome Sanger Institute for the COVID-19 Genomics UK (COG-UK) Consortium	Jacquelyn Wynn, Mairead Hyland, The Lighthouse Lab in Alderley Park and Alex Alderton, Roberto Amato, Sonia Goncalves, Ewan Harrison, David K. Jackson, Ian Johnston, Dominic Kwiatkowski, Cordelia Langford, John Sillitoe on behalf of the Wellcome Sanger Institute COVID-19 Surveillance Team
EPI_ISL_799183	Lighthouse Lab in Glasgow	Wellcome Sanger Institute for the COVID-19 Genomics UK (COG-UK) Consortium	Harper VanSteenhouse, Yumi Kasai, David Gray, Carol Clugston, Anna Dominiczak and Alex Alderton, Roberto Amato, Sonia Goncalves, Ewan Harrison, David K. Jackson, Ian Johnston, Dominic Kwiatkowski, Cordelia Langford, John Sillitoe on behalf of the Wellcome Sanger Institute COVID-19 Surveillance Team
EPI_ISL_799188	Lighthouse Lab in Alderley Park	Wellcome Sanger Institute for the COVID-19 Genomics UK (COG-UK) Consortium	Jacquelyn Wynn, Mairead Hyland, The Lighthouse Lab in Alderley Park and Alex Alderton, Roberto Amato, Sonia Goncalves, Ewan Harrison, David K. Jackson, Ian Johnston, Dominic Kwiatkowski, Cordelia Langford, John Sillitoe on behalf of the Wellcome Sanger Institute COVID-19 Surveillance Team
EPI_ISL_799189, EPI_ISL_799190	Lighthouse Lab in Glasgow	Wellcome Sanger Institute for the COVID-19 Genomics UK (COG-UK) Consortium	Harper VanSteenhouse, Yumi Kasai, David Gray, Carol Clugston, Anna Dominiczak and Alex Alderton, Roberto Amato, Sonia Goncalves, Ewan Harrison, David K. Jackson, Ian Johnston, Dominic Kwiatkowski, Cordelia Langford, John Sillitoe on behalf of the Wellcome Sanger Institute COVID-19 Surveillance Team
EPI_ISL_799191	Lighthouse Lab in Alderley Park	Wellcome Sanger Institute for the COVID-19 Genomics UK (COG-UK) Consortium	Jacquelyn Wynn, Mairead Hyland, The Lighthouse Lab in Alderley Park and Alex Alderton, Roberto Amato, Sonia Goncalves, Ewan Harrison, David K. Jackson, Ian Johnston, Dominic Kwiatkowski, Cordelia Langford, John Sillitoe on behalf of the Wellcome Sanger Institute COVID-19 Surveillance Team
EPI_ISL_799192, EPI_ISL_799193, EPI_ISL_799194, EPI_ISL_799196, EPI_ISL_799197	Lighthouse Lab in Glasgow	Wellcome Sanger Institute for the COVID-19 Genomics UK (COG-UK) Consortium	Harper VanSteenhouse, Yumi Kasai, David Gray, Carol Clugston, Anna Dominiczak and Alex Alderton, Roberto Amato, Sonia Goncalves, Ewan Harrison, David K. Jackson, Ian Johnston, Dominic Kwiatkowski, Cordelia Langford, John Sillitoe on behalf of the Wellcome Sanger Institute COVID-19 Surveillance Team
EPI_ISL_799198, EPI_ISL_799200	Lighthouse Lab in Alderley Park	Wellcome Sanger Institute for the COVID-19 Genomics UK (COG-UK) Consortium	Jacquelyn Wynn, Mairead Hyland, The Lighthouse Lab in Alderley Park and Alex Alderton, Roberto Amato, Sonia Goncalves, Ewan Harrison, David K. Jackson, Ian Johnston, Dominic Kwiatkowski, Cordelia Langford, John Sillitoe on behalf of the Wellcome Sanger Institute COVID-19 Surveillance Team
EPI_ISL_799201	Lighthouse Lab in Glasgow	Wellcome Sanger Institute for the COVID-19 Genomics UK (COG-UK) Consortium	Harper VanSteenhouse, Yumi Kasai, David Gray, Carol Clugston, Anna Dominiczak and Alex Alderton, Roberto Amato, Sonia Goncalves, Ewan Harrison, David K. Jackson, Ian Johnston, Dominic Kwiatkowski, Cordelia Langford, John Sillitoe on behalf of the Wellcome Sanger Institute COVID-19 Surveillance Team
EPI_ISL_799202	Lighthouse Lab in Alderley Park	Wellcome Sanger Institute for the COVID-19 Genomics UK (COG-UK) Consortium	Jacquelyn Wynn, Mairead Hyland, The Lighthouse Lab in Alderley Park and Alex Alderton, Roberto Amato, Sonia Goncalves, Ewan Harrison, David K. Jackson, Ian Johnston, Dominic Kwiatkowski, Cordelia Langford, John Sillitoe on behalf of the Wellcome Sanger Institute COVID-19 Surveillance Team
EPI_ISL_799203, EPI_ISL_799204, EPI_ISL_799205, EPI_ISL_799207, EPI_ISL_799209	Lighthouse Lab in Glasgow	Wellcome Sanger Institute for the COVID-19 Genomics UK (COG-UK) Consortium	Harper VanSteenhouse, Yumi Kasai, David Gray, Carol Clugston, Anna Dominiczak and Alex Alderton, Roberto Amato, Sonia Goncalves, Ewan Harrison, David K. Jackson, Ian Johnston, Dominic Kwiatkowski, Cordelia Langford, John Sillitoe on behalf of the Wellcome Sanger Institute COVID-19 Surveillance Team
EPI_ISL_799213, EPI_ISL_799214	Lighthouse Lab in Alderley Park	Wellcome Sanger Institute for the COVID-19 Genomics UK (COG-UK) Consortium	Jacquelyn Wynn, Mairead Hyland, The Lighthouse Lab in Alderley Park and Alex Alderton, Roberto Amato, Sonia Goncalves, Ewan Harrison, David K. Jackson, Ian Johnston, Dominic Kwiatkowski, Cordelia Langford, John Sillitoe on behalf of the Wellcome Sanger Institute COVID-19 Surveillance Team
EPI_ISL_799215, EPI_ISL_799216, EPI_ISL_799218	Lighthouse Lab in Glasgow	Wellcome Sanger Institute for the COVID-19 Genomics UK (COG-UK) Consortium	Harper VanSteenhouse, Yumi Kasai, David Gray, Carol Clugston, Anna Dominiczak and Alex Alderton, Roberto Amato, Sonia Goncalves, Ewan Harrison, David K. Jackson, Ian Johnston, Dominic Kwiatkowski, Cordelia Langford, John Sillitoe on behalf of the Wellcome Sanger Institute COVID-19 Surveillance Team
EPI_ISL_799219	Lighthouse Lab in Alderley Park	Wellcome Sanger Institute for the COVID-19 Genomics UK (COG-UK) Consortium	Jacquelyn Wynn, Mairead Hyland, The Lighthouse Lab in Alderley Park and Alex Alderton, Roberto Amato, Sonia Goncalves, Ewan Harrison, David K. Jackson, Ian Johnston, Dominic Kwiatkowski, Cordelia Langford, John Sillitoe on behalf of the Wellcome Sanger Institute COVID-19 Surveillance Team
EPI_ISL_799221, EPI_ISL_799222, EPI_ISL_799224	Lighthouse Lab in Glasgow	Wellcome Sanger Institute for the COVID-19 Genomics UK (COG-UK) Consortium	Harper VanSteenhouse, Yumi Kasai, David Gray, Carol Clugston, Anna Dominiczak and Alex Alderton, Roberto Amato, Sonia Goncalves, Ewan Harrison, David K. Jackson, Ian Johnston, Dominic Kwiatkowski, Cordelia Langford, John Sillitoe on behalf of the Wellcome Sanger Institute COVID-19 Surveillance Team
EPI_ISL_799227, EPI_ISL_799228, EPI_ISL_799229	Lighthouse Lab in Alderley Park	Wellcome Sanger Institute for the COVID-19 Genomics UK (COG-UK) Consortium	Jacquelyn Wynn, Mairead Hyland, The Lighthouse Lab in Alderley Park and Alex Alderton, Roberto Amato, Sonia Goncalves, Ewan Harrison, David K. Jackson, Ian Johnston, Dominic Kwiatkowski, Cordelia Langford, John Sillitoe on behalf of the Wellcome Sanger Institute COVID-19 Surveillance Team
EPI_ISL_799230	Lighthouse Lab in Glasgow	Wellcome Sanger Institute for the COVID-19 Genomics UK (COG-UK) Consortium	Harper VanSteenhouse, Yumi Kasai, David Gray, Carol Clugston, Anna Dominiczak and Alex Alderton, Roberto Amato, Sonia Goncalves, Ewan Harrison, David K. Jackson, Ian Johnston, Dominic Kwiatkowski, Cordelia Langford, John Sillitoe on behalf of the Wellcome Sanger Institute COVID-19 Surveillance Team
EPI_ISL_799231	Lighthouse Lab in Alderley Park	Wellcome Sanger Institute for the COVID-19 Genomics UK (COG-UK) Consortium	Jacquelyn Wynn, Mairead Hyland, The Lighthouse Lab in Alderley Park and Alex Alderton, Roberto Amato, Sonia Goncalves, Ewan Harrison, David K. Jackson, Ian Johnston, Dominic Kwiatkowski, Cordelia Langford, John Sillitoe on behalf of the Wellcome Sanger Institute COVID-19 Surveillance Team
EPI_ISL_799232	Lighthouse Lab in Glasgow	Wellcome Sanger Institute for the COVID-19 Genomics UK (COG-UK) Consortium	Harper VanSteenhouse, Yumi Kasai, David Gray, Carol Clugston, Anna Dominiczak and Alex Alderton, Roberto Amato, Sonia Goncalves, Ewan Harrison, David K. Jackson, Ian Johnston, Dominic Kwiatkowski, Cordelia Langford, John Sillitoe on behalf of the Wellcome Sanger Institute COVID-19 Surveillance Team
EPI_ISL_799233, EPI_ISL_799234, EPI_ISL_799235	Lighthouse Lab in Alderley Park	Wellcome Sanger Institute for the COVID-19 Genomics UK (COG-UK) Consortium	Jacquelyn Wynn, Mairead Hyland, The Lighthouse Lab in Alderley Park and Alex Alderton, Roberto Amato, Sonia Goncalves, Ewan Harrison, David K. Jackson, Ian Johnston, Dominic Kwiatkowski, Cordelia Langford, John Sillitoe on behalf of the Wellcome Sanger Institute COVID-19 Surveillance Team
EPI_ISL_799236	Lighthouse Lab in Glasgow	Wellcome Sanger Institute for the COVID-19 Genomics UK (COG-UK) Consortium	Harper VanSteenhouse, Yumi Kasai, David Gray, Carol Clugston, Anna Dominiczak and Alex Alderton, Roberto Amato, Sonia Goncalves, Ewan Harrison, David K. Jackson, Ian Johnston, Dominic Kwiatkowski, Cordelia Langford, John Sillitoe on behalf of the Wellcome Sanger Institute COVID-19 Surveillance Team

EPI_ISL_799237	Lighthouse Lab in Alderley Park	Wellcome Sanger Institute for the COVID-19 Genomics UK (COG-UK) Consortium	Jacquelyn Wynn, Mairead Hyland, The Lighthouse Lab in Alderley Park and Alex Alderton, Roberto Amato, Sonia Goncalves, Ewan Harrison, David K. Jackson, Ian Johnston, Dominic Kwiatkowski, Cordelia Langford, John Sillitoe on behalf of the Wellcome Sanger Institute COVID-19 Surveillance Team
EPI_ISL_799240, EPI_ISL_799243	Lighthouse Lab in Glasgow	Wellcome Sanger Institute for the COVID-19 Genomics UK (COG-UK) Consortium	Harper VanSteenhouse, Yumi Kasai, David Gray, Carol Clugston, Anna Dominiczak and Alex Alderton, Roberto Amato, Sonia Goncalves, Ewan Harrison, David K. Jackson, Ian Johnston, Dominic Kwiatkowski, Cordelia Langford, John Sillitoe on behalf of the Wellcome Sanger Institute COVID-19 Surveillance Team
EPI_ISL_799244	Lighthouse Lab in Alderley Park	Wellcome Sanger Institute for the COVID-19 Genomics UK (COG-UK) Consortium	Jacquelyn Wynn, Mairead Hyland, The Lighthouse Lab in Alderley Park and Alex Alderton, Roberto Amato, Sonia Goncalves, Ewan Harrison, David K. Jackson, Ian Johnston, Dominic Kwiatkowski, Cordelia Langford, John Sillitoe on behalf of the Wellcome Sanger Institute COVID-19 Surveillance Team
EPI_ISL_799245, EPI_ISL_799246	Lighthouse Lab in Glasgow	Wellcome Sanger Institute for the COVID-19 Genomics UK (COG-UK) Consortium	Harper VanSteenhouse, Yumi Kasai, David Gray, Carol Clugston, Anna Dominiczak and Alex Alderton, Roberto Amato, Sonia Goncalves, Ewan Harrison, David K. Jackson, Ian Johnston, Dominic Kwiatkowski, Cordelia Langford, John Sillitoe on behalf of the Wellcome Sanger Institute COVID-19 Surveillance Team
EPI_ISL_799248	Lighthouse Lab in Alderley Park	Wellcome Sanger Institute for the COVID-19 Genomics UK (COG-UK) Consortium	Jacquelyn Wynn, Mairead Hyland, The Lighthouse Lab in Alderley Park and Alex Alderton, Roberto Amato, Sonia Goncalves, Ewan Harrison, David K. Jackson, Ian Johnston, Dominic Kwiatkowski, Cordelia Langford, John Sillitoe on behalf of the Wellcome Sanger Institute COVID-19 Surveillance Team
EPI_ISL_799249, EPI_ISL_799250	Lighthouse Lab in Glasgow	Wellcome Sanger Institute for the COVID-19 Genomics UK (COG-UK) Consortium	Harper VanSteenhouse, Yumi Kasai, David Gray, Carol Clugston, Anna Dominiczak and Alex Alderton, Roberto Amato, Sonia Goncalves, Ewan Harrison, David K. Jackson, Ian Johnston, Dominic Kwiatkowski, Cordelia Langford, John Sillitoe on behalf of the Wellcome Sanger Institute COVID-19 Surveillance Team
EPI_ISL_799252	Lighthouse Lab in Alderley Park	Wellcome Sanger Institute for the COVID-19 Genomics UK (COG-UK) Consortium	Jacquelyn Wynn, Mairead Hyland, The Lighthouse Lab in Alderley Park and Alex Alderton, Roberto Amato, Sonia Goncalves, Ewan Harrison, David K. Jackson, Ian Johnston, Dominic Kwiatkowski, Cordelia Langford, John Sillitoe on behalf of the Wellcome Sanger Institute COVID-19 Surveillance Team
EPI_ISL_799255, EPI_ISL_799256	Lighthouse Lab in Glasgow	Wellcome Sanger Institute for the COVID-19 Genomics UK (COG-UK) Consortium	Harper VanSteenhouse, Yumi Kasai, David Gray, Carol Clugston, Anna Dominiczak and Alex Alderton, Roberto Amato, Sonia Goncalves, Ewan Harrison, David K. Jackson, Ian Johnston, Dominic Kwiatkowski, Cordelia Langford, John Sillitoe on behalf of the Wellcome Sanger Institute COVID-19 Surveillance Team
EPI_ISL_799257	Lighthouse Lab in Alderley Park	Wellcome Sanger Institute for the COVID-19 Genomics UK (COG-UK) Consortium	Jacquelyn Wynn, Mairead Hyland, The Lighthouse Lab in Alderley Park and Alex Alderton, Roberto Amato, Sonia Goncalves, Ewan Harrison, David K. Jackson, Ian Johnston, Dominic Kwiatkowski, Cordelia Langford, John Sillitoe on behalf of the Wellcome Sanger Institute COVID-19 Surveillance Team
EPI_ISL_799258, EPI_ISL_799260	Lighthouse Lab in Glasgow	Wellcome Sanger Institute for the COVID-19 Genomics UK (COG-UK) Consortium	Harper VanSteenhouse, Yumi Kasai, David Gray, Carol Clugston, Anna Dominiczak and Alex Alderton, Roberto Amato, Sonia Goncalves, Ewan Harrison, David K. Jackson, Ian Johnston, Dominic Kwiatkowski, Cordelia Langford, John Sillitoe on behalf of the Wellcome Sanger Institute COVID-19 Surveillance Team
EPI_ISL_799261	Lighthouse Lab in Alderley Park	Wellcome Sanger Institute for the COVID-19 Genomics UK (COG-UK) Consortium	Jacquelyn Wynn, Mairead Hyland, The Lighthouse Lab in Alderley Park and Alex Alderton, Roberto Amato, Sonia Goncalves, Ewan Harrison, David K. Jackson, Ian Johnston, Dominic Kwiatkowski, Cordelia Langford, John Sillitoe on behalf of the Wellcome Sanger Institute COVID-19 Surveillance Team
EPI_ISL_799262, EPI_ISL_799263, EPI_ISL_799264	Lighthouse Lab in Glasgow	Wellcome Sanger Institute for the COVID-19 Genomics UK (COG-UK) Consortium	Harper VanSteenhouse, Yumi Kasai, David Gray, Carol Clugston, Anna Dominiczak and Alex Alderton, Roberto Amato, Sonia Goncalves, Ewan Harrison, David K. Jackson, Ian Johnston, Dominic Kwiatkowski, Cordelia Langford, John Sillitoe on behalf of the Wellcome Sanger Institute COVID-19 Surveillance Team
EPI_ISL_799265	Lighthouse Lab in Alderley Park	Wellcome Sanger Institute for the COVID-19 Genomics UK (COG-UK) Consortium	Jacquelyn Wynn, Mairead Hyland, The Lighthouse Lab in Alderley Park and Alex Alderton, Roberto Amato, Sonia Goncalves, Ewan Harrison, David K. Jackson, Ian Johnston, Dominic Kwiatkowski, Cordelia Langford, John Sillitoe on behalf of the Wellcome Sanger Institute COVID-19 Surveillance Team
EPI_ISL_799267, EPI_ISL_799268	Lighthouse Lab in Glasgow	Wellcome Sanger Institute for the COVID-19 Genomics UK (COG-UK) Consortium	Harper VanSteenhouse, Yumi Kasai, David Gray, Carol Clugston, Anna Dominiczak and Alex Alderton, Roberto Amato, Sonia Goncalves, Ewan Harrison, David K. Jackson, Ian Johnston, Dominic Kwiatkowski, Cordelia Langford, John Sillitoe on behalf of the Wellcome Sanger Institute COVID-19 Surveillance Team
EPI_ISL_799269	Lighthouse Lab in Alderley Park	Wellcome Sanger Institute for the COVID-19 Genomics UK (COG-UK) Consortium	Jacquelyn Wynn, Mairead Hyland, The Lighthouse Lab in Alderley Park and Alex Alderton, Roberto Amato, Sonia Goncalves, Ewan Harrison, David K. Jackson, Ian Johnston, Dominic Kwiatkowski, Cordelia Langford, John Sillitoe on behalf of the Wellcome Sanger Institute COVID-19 Surveillance Team
EPI_ISL_799270	Lighthouse Lab in Glasgow	Wellcome Sanger Institute for the COVID-19 Genomics UK (COG-UK) Consortium	Harper VanSteenhouse, Yumi Kasai, David Gray, Carol Clugston, Anna Dominiczak and Alex Alderton, Roberto Amato, Sonia Goncalves, Ewan Harrison, David K. Jackson, Ian Johnston, Dominic Kwiatkowski, Cordelia Langford, John Sillitoe on behalf of the Wellcome Sanger Institute COVID-19 Surveillance Team
EPI_ISL_799271	Lighthouse Lab in Alderley Park	Wellcome Sanger Institute for the COVID-19 Genomics UK (COG-UK) Consortium	Jacquelyn Wynn, Mairead Hyland, The Lighthouse Lab in Alderley Park and Alex Alderton, Roberto Amato, Sonia Goncalves, Ewan Harrison, David K. Jackson, Ian Johnston, Dominic Kwiatkowski, Cordelia Langford, John Sillitoe on behalf of the Wellcome Sanger Institute COVID-19 Surveillance Team
EPI_ISL_799272, EPI_ISL_799273, EPI_ISL_799274, EPI_ISL_799275	Lighthouse Lab in Glasgow	Wellcome Sanger Institute for the COVID-19 Genomics UK (COG-UK) Consortium	Harper VanSteenhouse, Yumi Kasai, David Gray, Carol Clugston, Anna Dominiczak and Alex Alderton, Roberto Amato, Sonia Goncalves, Ewan Harrison, David K. Jackson, Ian Johnston, Dominic Kwiatkowski, Cordelia Langford, John Sillitoe on behalf of the Wellcome Sanger Institute COVID-19 Surveillance Team
EPI_ISL_799276	Lighthouse Lab in Alderley Park	Wellcome Sanger Institute for the COVID-19 Genomics UK (COG-UK) Consortium	Jacquelyn Wynn, Mairead Hyland, The Lighthouse Lab in Alderley Park and Alex Alderton, Roberto Amato, Sonia Goncalves, Ewan Harrison, David K. Jackson, Ian Johnston, Dominic Kwiatkowski, Cordelia Langford, John Sillitoe on behalf of the Wellcome Sanger Institute COVID-19 Surveillance Team
EPI_ISL_799277	Lighthouse Lab in Glasgow	Wellcome Sanger Institute for the COVID-19 Genomics UK (COG-UK) Consortium	Harper VanSteenhouse, Yumi Kasai, David Gray, Carol Clugston, Anna Dominiczak and Alex Alderton, Roberto Amato, Sonia Goncalves, Ewan Harrison, David K. Jackson, Ian Johnston, Dominic Kwiatkowski, Cordelia Langford, John Sillitoe on behalf of the Wellcome Sanger Institute COVID-19 Surveillance Team
EPI_ISL_799278	Lighthouse Lab in Alderley Park	Wellcome Sanger Institute for the COVID-19 Genomics UK (COG-UK) Consortium	Jacquelyn Wynn, Mairead Hyland, The Lighthouse Lab in Alderley Park and Alex Alderton, Roberto Amato, Sonia Goncalves, Ewan Harrison, David K. Jackson, Ian Johnston, Dominic Kwiatkowski, Cordelia Langford, John Sillitoe on behalf of the Wellcome Sanger Institute COVID-19 Surveillance Team
EPI_ISL_799279, EPI_ISL_799280	Lighthouse Lab in Glasgow	Wellcome Sanger Institute for the COVID-19 Genomics UK (COG-UK) Consortium	Harper VanSteenhouse, Yumi Kasai, David Gray, Carol Clugston, Anna Dominiczak and Alex Alderton, Roberto Amato, Sonia Goncalves, Ewan Harrison, David K. Jackson, Ian Johnston, Dominic Kwiatkowski, Cordelia Langford, John Sillitoe on behalf of the Wellcome Sanger Institute COVID-19 Surveillance Team
EPI_ISL_799281	Lighthouse Lab in Alderley Park	Wellcome Sanger Institute for the COVID-19 Genomics UK (COG-UK) Consortium	Jacquelyn Wynn, Mairead Hyland, The Lighthouse Lab in Alderley Park and Alex Alderton, Roberto Amato, Sonia Goncalves, Ewan Harrison, David K. Jackson, Ian Johnston, Dominic Kwiatkowski, Cordelia Langford, John Sillitoe on behalf of the Wellcome Sanger Institute COVID-19 Surveillance Team
EPI_ISL_799282, EPI_ISL_799283, EPI_ISL_799285, EPI_ISL_799286, EPI_ISL_799287, EPI_ISL_799288, EPI_ISL_799289, EPI_ISL_799290	Lighthouse Lab in Glasgow	Wellcome Sanger Institute for the COVID-19 Genomics UK (COG-UK) Consortium	Harper VanSteenhouse, Yumi Kasai, David Gray, Carol Clugston, Anna Dominiczak and Alex Alderton, Roberto Amato, Sonia Goncalves, Ewan Harrison, David K. Jackson, Ian Johnston, Dominic Kwiatkowski, Cordelia Langford, John Sillitoe on behalf of the Wellcome Sanger Institute COVID-19 Surveillance Team
EPI_ISL_799292	Lighthouse Lab in Cambridge	Wellcome Sanger Institute for the COVID-19 Genomics UK (COG-UK) Consortium	Rob Howes, The Lighthouse Lab in Cambridge and Alex Alderton, Roberto Amato, Sonia Goncalves, Ewan Harrison, David K. Jackson, Ian Johnston, Dominic Kwiatkowski, Cordelia Langford, John Sillitoe on behalf of the Wellcome Sanger Institute COVID-19 Surveillance Team
EPI_ISL_799294	Lighthouse Lab in Glasgow	Wellcome Sanger Institute for the COVID-19 Genomics UK (COG-UK) Consortium	Harper VanSteenhouse, Yumi Kasai, David Gray, Carol Clugston, Anna Dominiczak and Alex Alderton, Roberto Amato, Sonia Goncalves, Ewan Harrison, David K. Jackson, Ian Johnston, Dominic Kwiatkowski, Cordelia Langford, John Sillitoe on behalf of the Wellcome Sanger Institute COVID-19 Surveillance Team
EPI_ISL_804222, EPI_ISL_804223, EPI_ISL_804224, EPI_ISL_804242, EPI_ISL_804243, EPI_ISL_804244, EPI_ISL_804245, EPI_ISL_804246, EPI_ISL_804247, EPI_ISL_804248, EPI_ISL_804251, EPI_ISL_804252, EPI_ISL_804253, EPI_ISL_804260, EPI_ISL_804274, EPI_ISL_804275, EPI_ISL_804277, EPI_ISL_804278, EPI_ISL_804279, EPI_ISL_804280, EPI_ISL_804281, EPI_ISL_804282, EPI_ISL_804283, EPI_ISL_804284, EPI_ISL_804285, EPI_ISL_804286, EPI_ISL_804287, EPI_ISL_804288, EPI_ISL_804289, EPI_ISL_804290, EPI_ISL_804291, EPI_ISL_804292, EPI_ISL_804293, EPI_ISL_811040, EPI_ISL_811041, EPI_ISL_811042, EPI_ISL_811043, EPI_ISL_811044, EPI_ISL_811045, EPI_ISL_811046, EPI_ISL_811047, EPI_ISL_811048, EPI_ISL_811049, EPI_ISL_811050, EPI_ISL_811051, EPI_ISL_811052, EPI_ISL_811053, EPI_ISL_811054, EPI_ISL_811055, EPI_ISL_811056, EPI_ISL_811057, EPI_ISL_811058, EPI_ISL_811059, EPI_ISL_811060, EPI_ISL_811061, EPI_ISL_811062, EPI_ISL_811063, EPI_ISL_811064, EPI_ISL_811065, EPI_ISL_811066, EPI_ISL_811067, EPI_ISL_811068, EPI_ISL_811069, EPI_ISL_811070, EPI_ISL_811071, EPI_ISL_811072, EPI_ISL_811073, EPI_ISL_811074, EPI_ISL_811075, EPI_ISL_811076, EPI_ISL_811077, EPI_ISL_811078, EPI_ISL_811079, EPI_ISL_811080, EPI_ISL_811081, EPI_ISL_811082, EPI_ISL_811083, EPI_ISL_811084, EPI_ISL_811085, EPI_ISL_811086, EPI_ISL_811087, EPI_ISL_811088, EPI_ISL_811089, EPI_ISL_811090, EPI_ISL_811091, EPI_ISL_811092, EPI_ISL_811093, EPI_ISL_811094, EPI_ISL_811095, EPI_ISL_811096, EPI_ISL_811097, EPI_ISL_811098, EPI_ISL_811099, EPI_ISL_811100, EPI_ISL_811101, EPI_ISL_811102, EPI_ISL_811103, EPI_ISL_811104, EPI_ISL_811105, EPI_ISL_811106, EPI_ISL_811115, EPI_ISL_811117, EPI_ISL_811118, EPI_ISL_811119, EPI_ISL_811122			
see above	Respiratory Virus Unit, National Infection Service, Public Health England	COVID-19 Genomics UK (COG-UK) Consortium	PHE Covid Sequencing Team

EPI_ISL_811207, EPI_ISL_811211, EPI_ISL_811214, EPI_ISL_811215, EPI_ISL_811217, EPI_ISL_811229, EPI_ISL_811234, EPI_ISL_811236, EPI_ISL_811241, EPI_ISL_811247, EPI_ISL_811248, EPI_ISL_811262, EPI_ISL_811275, EPI_ISL_811280, EPI_ISL_811284, EPI_ISL_811293, EPI_ISL_811309, EPI_ISL_811310, EPI_ISL_811312, EPI_ISL_811321, EPI_ISL_811325, EPI_ISL_811335, EPI_ISL_811344, EPI_ISL_811345, EPI_ISL_811350, EPI_ISL_811353, EPI_ISL_811362, EPI_ISL_811366, EPI_ISL_811377, EPI_ISL_811380, EPI_ISL_811381, EPI_ISL_811382, EPI_ISL_811385, EPI_ISL_811389, EPI_ISL_811392, EPI_ISL_811394, EPI_ISL_811395, EPI_ISL_811400, EPI_ISL_811404, EPI_ISL_811406, EPI_ISL_811410, EPI_ISL_811414, EPI_ISL_811420, EPI_ISL_811422, EPI_ISL_811433, EPI_ISL_811436, EPI_ISL_811454, EPI_ISL_811472, EPI_ISL_811478, EPI_ISL_811482, EPI_ISL_811490, EPI_ISL_811494, EPI_ISL_811500, EPI_ISL_811502, EPI_ISL_811516, EPI_ISL_811519, EPI_ISL_811524, EPI_ISL_811530, EPI_ISL_811538, EPI_ISL_811548, EPI_ISL_811563, EPI_ISL_811565, EPI_ISL_811573, EPI_ISL_811580, EPI_ISL_811584, EPI_ISL_811594			
see above	Lighthouse Lab in Milton Keynes	Wellcome Sanger Institute for the COVID-19 Genomics UK (COG-UK) Consortium	The Lighthouse Lab in Milton Keynes and Alex Alderton, Roberto Amato, Sonia Goncalves, Ewan Harrison, David K. Jackson, Ian Johnston, Dominic Kwiatkowski, Cordelia Langford, John Sillitoe on behalf of the Wellcome Sanger Institute COVID-19 Surveillance Team
EPI_ISL_811595	Lighthouse Lab in Cambridge	Wellcome Sanger Institute for the COVID-19 Genomics UK (COG-UK) Consortium	Rob Howes, The Lighthouse Lab in Cambridge and Alex Alderton, Roberto Amato, Sonia Goncalves, Ewan Harrison, David K. Jackson, Ian Johnston, Dominic Kwiatkowski, Cordelia Langford, John Sillitoe on behalf of the Wellcome Sanger Institute COVID-19 Surveillance Team
EPI_ISL_811606, EPI_ISL_811610, EPI_ISL_811617, EPI_ISL_811618, EPI_ISL_811620, EPI_ISL_811621, EPI_ISL_811626, EPI_ISL_811627, EPI_ISL_811634, EPI_ISL_811645, EPI_ISL_811650, EPI_ISL_811651, EPI_ISL_811660, EPI_ISL_811661, EPI_ISL_811664, EPI_ISL_811666, EPI_ISL_811669, EPI_ISL_811670, EPI_ISL_811671, EPI_ISL_811672, EPI_ISL_811677, EPI_ISL_811678			
see above	Lighthouse Lab in Milton Keynes	Wellcome Sanger Institute for the COVID-19 Genomics UK (COG-UK) Consortium	The Lighthouse Lab in Milton Keynes and Alex Alderton, Roberto Amato, Sonia Goncalves, Ewan Harrison, David K. Jackson, Ian Johnston, Dominic Kwiatkowski, Cordelia Langford, John Sillitoe on behalf of the Wellcome Sanger Institute COVID-19 Surveillance Team
EPI_ISL_811681	Lighthouse Lab in Cambridge	Wellcome Sanger Institute for the COVID-19 Genomics UK (COG-UK) Consortium	Rob Howes, The Lighthouse Lab in Cambridge and Alex Alderton, Roberto Amato, Sonia Goncalves, Ewan Harrison, David K. Jackson, Ian Johnston, Dominic Kwiatkowski, Cordelia Langford, John Sillitoe on behalf of the Wellcome Sanger Institute COVID-19 Surveillance Team
EPI_ISL_811682, EPI_ISL_811688, EPI_ISL_811698, EPI_ISL_811701, EPI_ISL_811702, EPI_ISL_811703, EPI_ISL_811708	Lighthouse Lab in Milton Keynes	Wellcome Sanger Institute for the COVID-19 Genomics UK (COG-UK) Consortium	The Lighthouse Lab in Milton Keynes and Alex Alderton, Roberto Amato, Sonia Goncalves, Ewan Harrison, David K. Jackson, Ian Johnston, Dominic Kwiatkowski, Cordelia Langford, John Sillitoe on behalf of the Wellcome Sanger Institute COVID-19 Surveillance Team
EPI_ISL_811713	Lighthouse Lab in Cambridge	Wellcome Sanger Institute for the COVID-19 Genomics UK (COG-UK) Consortium	Rob Howes, The Lighthouse Lab in Cambridge and Alex Alderton, Roberto Amato, Sonia Goncalves, Ewan Harrison, David K. Jackson, Ian Johnston, Dominic Kwiatkowski, Cordelia Langford, John Sillitoe on behalf of the Wellcome Sanger Institute COVID-19 Surveillance Team
EPI_ISL_811720, EPI_ISL_811726, EPI_ISL_811727, EPI_ISL_811736, EPI_ISL_811740, EPI_ISL_811750, EPI_ISL_811753, EPI_ISL_811756, EPI_ISL_811759, EPI_ISL_811767, EPI_ISL_811776, EPI_ISL_811785, EPI_ISL_811802, EPI_ISL_811808, EPI_ISL_811809, EPI_ISL_811810, EPI_ISL_811811, EPI_ISL_811812, EPI_ISL_811813, EPI_ISL_811814, EPI_ISL_811815, EPI_ISL_811816, EPI_ISL_811817, EPI_ISL_811818, EPI_ISL_811819, EPI_ISL_811820, EPI_ISL_811821, EPI_ISL_811822, EPI_ISL_811823, EPI_ISL_811824, EPI_ISL_811825, EPI_ISL_811826, EPI_ISL_811827, EPI_ISL_811828, EPI_ISL_811829, EPI_ISL_811830, EPI_ISL_811831, EPI_ISL_811832, EPI_ISL_811833, EPI_ISL_811834, EPI_ISL_811835, EPI_ISL_811836, EPI_ISL_811837, EPI_ISL_811838, EPI_ISL_811839, EPI_ISL_811840, EPI_ISL_811841, EPI_ISL_811842, EPI_ISL_811843, EPI_ISL_811844, EPI_ISL_811845, EPI_ISL_811846, EPI_ISL_811847, EPI_ISL_811848, EPI_ISL_811849, EPI_ISL_811850, EPI_ISL_811851, EPI_ISL_811852, EPI_ISL_811853, EPI_ISL_811854, EPI_ISL_811855, EPI_ISL_811856, EPI_ISL_811857, EPI_ISL_811858, EPI_ISL_811859, EPI_ISL_811860, EPI_ISL_811861, EPI_ISL_811862, EPI_ISL_811863, EPI_ISL_811864, EPI_ISL_811865, EPI_ISL_811866, EPI_ISL_811867, EPI_ISL_811868, EPI_ISL_811869, EPI_ISL_811870, EPI_ISL_811871, EPI_ISL_811872, EPI_ISL_811873, EPI_ISL_811874, EPI_ISL_811875, EPI_ISL_811876, EPI_ISL_811877, EPI_ISL_811878, EPI_ISL_811879, EPI_ISL_811880, EPI_ISL_811881, EPI_ISL_811882, EPI_ISL_811883, EPI_ISL_811884, EPI_ISL_811885, EPI_ISL_811886, EPI_ISL_811887, EPI_ISL_811888, EPI_ISL_811889, EPI_ISL_811890, EPI_ISL_811891, EPI_ISL_811892, EPI_ISL_811893, EPI_ISL_811894, EPI_ISL_811895, EPI_ISL_811896, EPI_ISL_811897, EPI_ISL_811898, EPI_ISL_811899, EPI_ISL_811900, EPI_ISL_811901, EPI_ISL_811902, EPI_ISL_811903, EPI_ISL_811904, EPI_ISL_811905, EPI_ISL_811906, EPI_ISL_811907, EPI_ISL_811908, EPI_ISL_811909, EPI_ISL_811910, EPI_ISL_811911, EPI_ISL_811912, EPI_ISL_811913, EPI_ISL_811914, EPI_ISL_811915, EPI_ISL_811916, EPI_ISL_811917, EPI_ISL_811918, EPI_ISL_811919, EPI_ISL_811920, EPI_ISL_811921, EPI_ISL_811922, EPI_ISL_811923, EPI_ISL_811924, EPI_ISL_811925, EPI_ISL_811926, EPI_ISL_811927, EPI_ISL_811928, EPI_ISL_811929, EPI_ISL_811930, EPI_ISL_811931, EPI_ISL_811932, EPI_ISL_811933, EPI_ISL_811934, EPI_ISL_811935, EPI_ISL_811936, EPI_ISL_811937, EPI_ISL_811938, EPI_ISL_811939, EPI_ISL_811940, EPI_ISL_811941, EPI_ISL_811942, EPI_ISL_811943, EPI_ISL_811944, EPI_ISL_811945, EPI_ISL_811946, EPI_ISL_811947, EPI_ISL_811948, EPI_ISL_811949, EPI_ISL_811950, EPI_ISL_811951, EPI_ISL_811952, EPI_ISL_811953, EPI_ISL_811954, EPI_ISL_811955, EPI_ISL_811956, EPI_ISL_811957, EPI_ISL_811958, EPI_ISL_811959, EPI_ISL_811960, EPI_ISL_811961, EPI_ISL_811962, EPI_ISL_811963, EPI_ISL_811964, EPI_ISL_811965, EPI_ISL_811966, EPI_ISL_811967, EPI_ISL_811968, EPI_ISL_811969, EPI_ISL_811970, EPI_ISL_811971, EPI_ISL_811972, EPI_ISL_811973, EPI_ISL_811974, EPI_ISL_811975, EPI_ISL_811976, EPI_ISL_811977, EPI_ISL_811978, EPI_ISL_811979, EPI_ISL_811980, EPI_ISL_811981, EPI_ISL_811982, EPI_ISL_811983, EPI_ISL_811984, EPI_ISL_811985, EPI_ISL_811986, EPI_ISL_811987, EPI_ISL_811988, EPI_ISL_811989, EPI_ISL_811990, EPI_ISL_811991, EPI_ISL_811992, EPI_ISL_811993, EPI_ISL_811994, EPI_ISL_811995, EPI_ISL_811996, EPI_ISL_811997, EPI_ISL_811998, EPI_ISL_811999, EPI_ISL_812000, EPI_ISL_812001, EPI_ISL_812002, EPI_ISL_812003, EPI_ISL_812004, EPI_ISL_812005, EPI_ISL_812006, EPI_ISL_812007, EPI_ISL_812008, EPI_ISL_812009, EPI_ISL_812010, EPI_ISL_812011, EPI_ISL_812012, EPI_ISL_812013, EPI_ISL_812014, EPI_ISL_812015, EPI_ISL_812016, EPI_ISL_812017, EPI_ISL_812018, EPI_ISL_812019, EPI_ISL_812020, EPI_ISL_812021, EPI_ISL_812022, EPI_ISL_812023, EPI_ISL_812024, EPI_ISL_812025, EPI_ISL_812026, EPI_ISL_812027, EPI_ISL_812028, EPI_ISL_812029, EPI_ISL_812030, EPI_ISL_812031, EPI_ISL_812032, EPI_ISL_812033, EPI_ISL_812034, EPI_ISL_812035, EPI_ISL_812036, EPI_ISL_812037, EPI_ISL_812038, EPI_ISL_812039, EPI_ISL_812040, EPI_ISL_812041, EPI_ISL_812042, EPI_ISL_812043, EPI_ISL_812044, EPI_ISL_812045, EPI_ISL_812046, EPI_ISL_812047, EPI_ISL_812048, EPI_ISL_812049, EPI_ISL_812050, EPI_ISL_812051, EPI_ISL_812052, EPI_ISL_812053, EPI_ISL_812054, EPI_ISL_812055, EPI_ISL_812056, EPI_ISL_812057, EPI_ISL_812058, EPI_ISL_812059, EPI_ISL_812060, EPI_ISL_812061, EPI_ISL_812062, EPI_ISL_812063, EPI_ISL_812064, EPI_ISL_812065, EPI_ISL_812066, EPI_ISL_812067, EPI_ISL_812068, EPI_ISL_812069, EPI_ISL_812070, EPI_ISL_812071, EPI_ISL_812072, EPI_ISL_812073, EPI_ISL_812074, EPI_ISL_812075, EPI_ISL_812076, EPI_ISL_812077, EPI_ISL_812078, EPI_ISL_812079, EPI_ISL_812080, EPI_ISL_812081, EPI_ISL_812082, EPI_ISL_812083, EPI_ISL_812084, EPI_ISL_812085, EPI_ISL_812086, EPI_ISL_812087, EPI_ISL_812088, EPI_ISL_812089, EPI_ISL_812090, EPI_ISL_812091, EPI_ISL_812092, EPI_ISL_812093, EPI_ISL_812094, EPI_ISL_812095, EPI_ISL_812096, EPI_ISL_812097, EPI_ISL_812098, EPI_ISL_812099, EPI_ISL_812100, EPI_ISL_812101, EPI_ISL_812102, EPI_ISL_812103, EPI_ISL_812104, EPI_ISL_812105, EPI_ISL_812106, EPI_ISL_812107, EPI_ISL_812108, EPI_ISL_812109, EPI_ISL_812110, EPI_ISL_812111, EPI_ISL_812112, EPI_ISL_812113			
see above	Lighthouse Lab in Milton Keynes	Wellcome Sanger Institute for the COVID-19 Genomics UK (COG-UK) Consortium	The Lighthouse Lab in Milton Keynes and Alex Alderton, Roberto Amato, Sonia Goncalves, Ewan Harrison, David K. Jackson, Ian Johnston, Dominic Kwiatkowski, Cordelia Langford, John Sillitoe on behalf of the Wellcome Sanger Institute COVID-19 Surveillance Team
EPI_ISL_813091, EPI_ISL_813092, EPI_ISL_813093, EPI_ISL_813116	University of Birmingham	COVID-19 Genomics UK (COG-UK) Consortium	Institute of Microbiology, University of Birmingham: Claire McMurray, Joanne Stockton, Samuel Nicholls, Radoslaw Poplawski, Will Rowe, Josh Quick, Nicholas Loman. University of Birmingham Testing Laboratory: Celina M Whalley, Andrew Bosworth, Charlotte Poxon, Kasun Wanigasooriya, Oliver Pickles, Mike Kidd, Alex Richter, Andrew D Beggs PHE Heartlands Lab: Husam Osman, Andrew Bosworth. Queen Elizabeth Hospital: Anna Casey
EPI_ISL_813171, EPI_ISL_813198, EPI_ISL_813199, EPI_ISL_813201, EPI_ISL_813202, EPI_ISL_813203, EPI_ISL_813204, EPI_ISL_813205, EPI_ISL_813206, EPI_ISL_813207, EPI_ISL_813209, EPI_ISL_813210, EPI_ISL_813211, EPI_ISL_813212, EPI_ISL_813213, EPI_ISL_813214, EPI_ISL_813215, EPI_ISL_813216, EPI_ISL_813217, EPI_ISL_813219, EPI_ISL_813220, EPI_ISL_813221, EPI_ISL_813222, EPI_ISL_813223, EPI_ISL_813224, EPI_ISL_813227, EPI_ISL_813256, EPI_ISL_813267, EPI_ISL_813268, EPI_ISL_813269, EPI_ISL_813278, EPI_ISL_813281, EPI_ISL_813283, EPI_ISL_813285, EPI_ISL_813294			
see above	Department of Pathology, University of Cambridge	COVID-19 Genomics UK (COG-UK) Consortium	Aminu S. Jahun, Yasmin Chaudhry, Grant Hall, Iliana Georgana, Myra Hosmillo, Martin D. Curran, Malte Pinckert, Surendra Parmar, Ian Goodfellow
EPI_ISL_813581, EPI_ISL_813582, EPI_ISL_813587, EPI_ISL_813588, EPI_ISL_813589, EPI_ISL_813590, EPI_ISL_813591, EPI_ISL_813592, EPI_ISL_813593, EPI_ISL_813594, EPI_ISL_813595, EPI_ISL_813597, EPI_ISL_813600, EPI_ISL_813605, EPI_ISL_813607, EPI_ISL_813608, EPI_ISL_813609, EPI_ISL_813610, EPI_ISL_813611, EPI_ISL_813612, EPI_ISL_813613, EPI_ISL_813614, EPI_ISL_813616, EPI_ISL_813619, EPI_ISL_813623, EPI_ISL_813624, EPI_ISL_813625, EPI_ISL_813626, EPI_ISL_813627, EPI_ISL_813628, EPI_ISL_813629, EPI_ISL_813630, EPI_ISL_813631, EPI_ISL_813632, EPI_ISL_813633, EPI_ISL_813634, EPI_ISL_813635, EPI_ISL_813636, EPI_ISL_813637, EPI_ISL_813638, EPI_ISL_813639, EPI_ISL_813640, EPI_ISL_813641, EPI_ISL_813642, EPI_ISL_813643, EPI_ISL_813644, EPI_ISL_813645, EPI_ISL_813646, EPI_ISL_813647, EPI_ISL_813648, EPI_ISL_813649, EPI_ISL_813650, EPI_ISL_813651, EPI_ISL_813652, EPI_ISL_813653, EPI_ISL_813654			
see above	University of Exeter	COVID-19 Genomics UK (COG-UK) Consortium	Ben Temperton,Aaron Jeffries,Michelle Michelsen,Joanna Warwick-Dugdale,Audrey Farbos,Robyn Manley,Stephen Michell,Jane Masoli
EPI_ISL_813773, EPI_ISL_813776, EPI_ISL_813778, EPI_ISL_813779, EPI_ISL_813782, EPI_ISL_813810, EPI_ISL_813811, EPI_ISL_813812, EPI_ISL_813813, EPI_ISL_813814, EPI_ISL_813815			
see above	Liverpool Clinical Laboratories	COVID-19 Genomics UK (COG-UK) Consortium	Sam Haldenby, Anita Lucaci, Steve Paterson, Julian Hiscox, Alistair Darby, M Almsaud, A Alrezaihi, Muhannad Alruwaili, Stuart D Armstrong, Jones Benjamin, Eleanor G Bentley, Anu Chawla, Jordan J Clark, Angela Cowell, Richard Eccles, Isabel García-Dorival, Matthew Gemmell, Alessandro Gerada, PKF Gilmore, Richard Gregory, Ximeng Han, Catherine Hartley, Margaret Hughes, Miren Iturriza-Gomara, James Johnson, L Luu, Jenifer Manson, Charlotte Nelson, Elaine O'Toole, Cassie Olateju, Rebekah Penrice-Randal , Lucille Rainbow, N.P Randle, Trevor Ian Robinson, Parul Sharma, Ghada T Shawli, James P Stewart, Neil Swainston, Ecaterina Vamos, Joanne Watts, Mark Whitehead
EPI_ISL_813900, EPI_ISL_813956, EPI_ISL_813958	University College London, Great Ormond Street Hospital for Children NHS Foundation Trust, Imperial College Healthcare NHS Trust	COVID-19 Genomics UK (COG-UK) Consortium	Sergi Castellano, Rachel Williams, Mark Kristiansen, Paola Resende Silva, Sunando Roy, Tony Brooks, Helena Tutill, Paola Niola, Patricia Dyal, Charlotte Williams, Leysa Forrest, Yasmin Panchbhaya, Jacqueline Findlay, Samuel Weeks, Julianne Brown, Kathryn Harris, Paul Randell, James Price, Alison Holmes, Judith Breuer
EPI_ISL_814350	Wales Specialist Virology Centre Sequencing lab: Pathogen Genomics Unit	COVID-19 Genomics UK (COG-UK) Consortium	Catherine Moore, Johnathan Evans, Laura Gifford, Malorie Perry, Simon Cottrell, Angela Marchbank, Alec Birchley, Alexander Adams, Amy Gaskin, Bree Gatica-Wilcox, Jason Coombes, Joel Southgate, Lauren Gilbert, Lee Graham, Nicole Pacchiarini, Sara Kumziene-Summerhayes, Sarah Taylor, Sophie Jones, Sara Rey, Matthew Bull, Joanne Watkins, Sally Corden, Tom Connor
EPI_ISL_814358, EPI_ISL_814429, EPI_ISL_814433, EPI_ISL_814434	Bioinformatics and Biostatistics Lab, Advanced Sequencing Facility	COVID-19 Genomics UK (COG-UK) Consortium	Aengus Stewart,Jerome Nicod,Chelsea Sawyer,Laura Cubitt,Harshil Patel,Margaret Crawford
EPI_ISL_814510, EPI_ISL_815071, EPI_ISL_815240, EPI_ISL_815241, EPI_ISL_815242, EPI_ISL_815243	Wales Specialist Virology Centre Sequencing lab: Pathogen Genomics Unit	COVID-19 Genomics UK (COG-UK) Consortium	Catherine Moore, Johnathan Evans, Laura Gifford, Malorie Perry, Simon Cottrell, Angela Marchbank, Alec Birchley, Alexander Adams, Amy Gaskin, Bree Gatica-Wilcox, Jason Coombes, Joel Southgate, Lauren Gilbert, Lee Graham, Nicole Pacchiarini, Sara Kumziene-Summerhayes, Sarah Taylor, Sophie Jones, Sara Rey, Matthew Bull, Joanne Watkins, Sally Corden, Tom Connor
EPI_ISL_816194, EPI_ISL_816195, EPI_ISL_816203, EPI_ISL_816212, EPI_ISL_816213, EPI_ISL_816214, EPI_ISL_816215, EPI_ISL_816217, EPI_ISL_816218, EPI_ISL_816220, EPI_ISL_816221, EPI_ISL_816222, EPI_ISL_816223, EPI_ISL_816224			
see above	Centre for Enzyme Innovation, University of Portsmouth / Translational Research Laboratory, Portsmouth Hospitals NHS Trust	COVID-19 Genomics UK (COG-UK) Consortium	Angela Beckett,Yann Bourgeois,Garry Scarlett,Sharon Glaysher,Scott Elliott,Kelly Bicknell,Robert Impey,Allyson Lloyd,Sarah Wyllie,Ethan Butcher,Anoop Chauhan,Samuel Robson
EPI_ISL_816226, EPI_ISL_816227, EPI_ISL_816228, EPI_ISL_816229, EPI_ISL_816232, EPI_ISL_816233, EPI_ISL_816235, EPI_ISL_816239, EPI_ISL_816243, EPI_ISL_816246, EPI_ISL_816248, EPI_ISL_816255, EPI_ISL_816256, EPI_ISL_816261, EPI_ISL_816275, EPI_ISL_816276, EPI_ISL_816277, EPI_ISL_816285, EPI_ISL_816290, EPI_ISL_816291, EPI_ISL_816293, EPI_ISL_816294, EPI_ISL_816296, EPI_ISL_816297, EPI_ISL_816305, EPI_ISL_816311, EPI_ISL_816314, EPI_ISL_816316, EPI_ISL_816318, EPI_ISL_816319, EPI_ISL_816320, EPI_ISL_816323, EPI_ISL_816326, EPI_ISL_816327, EPI_ISL_816331, EPI_ISL_816348, EPI_ISL_816350, EPI_ISL_816353, EPI_ISL_816355, EPI_ISL_816357, EPI_ISL_816364, EPI_ISL_816365, EPI_ISL_816368, EPI_ISL_816382, EPI_ISL_816383, EPI_ISL_816390, EPI_ISL_816392, EPI_ISL_816396, EPI_ISL_816405, EPI_ISL_816406, EPI_ISL_816407, EPI_ISL_816409, EPI_ISL_816415, EPI_ISL_816421, EPI_ISL_816423, EPI_ISL_816425, EPI_ISL_816426, EPI_ISL_816427, EPI_ISL_816430, EPI_ISL_816431, EPI_ISL_816434, EPI_ISL_816436, EPI_ISL_816437, EPI_ISL_816438, EPI_ISL_816441, EPI_ISL_816442, EPI_ISL_816444, EPI_ISL_816448, EPI_ISL_816449, EPI_ISL_816458, EPI_ISL_816462, EPI_ISL_816469,			

EPI_ISL_816472, EPI_ISL_816475, EPI_ISL_816477, EPI_ISL_816492, EPI_ISL_816494, EPI_ISL_816495, EPI_ISL_816496, EPI_ISL_816501, EPI_ISL_816505, EPI_ISL_816508, EPI_ISL_816517, EPI_ISL_816525, EPI_ISL_816536, EPI_ISL_816537, EPI_ISL_816539, EPI_ISL_816546, EPI_ISL_816555, EPI_ISL_816560, EPI_ISL_816561, EPI_ISL_816566, EPI_ISL_816571, EPI_ISL_816576, EPI_ISL_816581, EPI_ISL_816582, EPI_ISL_816584, EPI_ISL_816585, EPI_ISL_816600, EPI_ISL_816601, EPI_ISL_816605, EPI_ISL_816606, EPI_ISL_816607, EPI_ISL_816608, EPI_ISL_816611, EPI_ISL_816613, EPI_ISL_816616, EPI_ISL_816620, EPI_ISL_816621, EPI_ISL_816629, EPI_ISL_816632, EPI_ISL_816633, EPI_ISL_816634, EPI_ISL_816635, EPI_ISL_816641, EPI_ISL_816642, EPI_ISL_816646			
see above	Virology Department, Sheffield Teaching Hospitals NHS Foundation Trust/Department of Infection, Immunity and Cardiovascular Disease, The Medical School, University of Sheffield	COVID-19 Genomics UK (COG-UK) Consortium	Thushan de Silva, Matthew Parker, Nikki Smith, Adri Angyal, Rebecca Brown, Luke Green, Rachel Tucker, Paul Parsons, Danielle Groves, Katie Johnson, Laura Carrilero, Alex Keeley, Dave Partridge, Matthew Wyles, Benjamin Lindsey, Mehmet Yavuz, Mohammad Raza, Cariad Evans
EPI_ISL_816825, EPI_ISL_816826, EPI_ISL_816827, EPI_ISL_816828, EPI_ISL_816829, EPI_ISL_816830, EPI_ISL_816831, EPI_ISL_816832, EPI_ISL_816833, EPI_ISL_816834, EPI_ISL_816835, EPI_ISL_816836, EPI_ISL_816837, EPI_ISL_816838, EPI_ISL_816839, EPI_ISL_816840, EPI_ISL_816841, EPI_ISL_816842, EPI_ISL_816843, EPI_ISL_816844, EPI_ISL_816845, EPI_ISL_816846, EPI_ISL_816847, EPI_ISL_816848, EPI_ISL_816849, EPI_ISL_816850, EPI_ISL_816851, EPI_ISL_816852, EPI_ISL_816853, EPI_ISL_816854, EPI_ISL_816855, EPI_ISL_816856, EPI_ISL_816857, EPI_ISL_816858, EPI_ISL_816859, EPI_ISL_816860, EPI_ISL_816892, EPI_ISL_816897, EPI_ISL_816898, EPI_ISL_816903, EPI_ISL_816959, EPI_ISL_816964, EPI_ISL_816965, EPI_ISL_816970, EPI_ISL_817039			
see above	Bioinformatics and Biostatistics Lab, Advanced Sequencing Facility	COVID-19 Genomics UK (COG-UK) Consortium	Aengus Stewart,Jerome Nicod,Chelsea Sawyer,Laura Cubitt,Harshil Patel,Margaret Crawford
EPI_ISL_819370, EPI_ISL_819378, EPI_ISL_819387, EPI_ISL_819388, EPI_ISL_819389, EPI_ISL_819390, EPI_ISL_819398, EPI_ISL_819399, EPI_ISL_819400, EPI_ISL_819401, EPI_ISL_819402, EPI_ISL_819403, EPI_ISL_819404, EPI_ISL_819428, EPI_ISL_819431, EPI_ISL_819433, EPI_ISL_819437, EPI_ISL_819438, EPI_ISL_819439			
see above	Quadram Institute Bioscience	COVID-19 Genomics UK (COG-UK) Consortium	Dave J. Baker, Gemma L. Kay, Alp Aydin, Thanh Le-Viet, Steven Rudder, Ana P. Tedim, Anastasia Kolyva, Maria Diaz, Leonardo de Oliveira Martins, Nabil-Fareed Alikhan, Lizzie Meadows, Rachael Stanley, Ngozi Elumogo, Muhammed Yasir, Nicholas M. Thomson, Alexander J Trotter, Rachel Gilroy, Samuel Bloomfield, Claire Stuart, Andrew Bell, Reenesh Prakash, Samir Dervisevic, Alison E. Mather, John Wain, Mark Webber, Andrew J. Page, Justin O'Grady
EPI_ISL_820246, EPI_ISL_820249, EPI_ISL_820251	University College London, Great Ormond Street Hospital for Children NHS Foundation Trust, Imperial College Healthcare NHS Trust	COVID-19 Genomics UK (COG-UK) Consortium	Sergi Castellano, Rachel Williams, Mark Kristiansen, Paola Resende Silva, Sunando Roy, Tony Brooks, Helena Tutill, Paola Niola, Patricia Dyal, Charlotte Williams, Leysa Forrest, Yasmin Panchbhaya, Jacqueline Findlay, Samuel Weeks, Julianne Brown, Kathryn Harris, Paul Randell, James Price, Alison Holmes, Judith Breuer
EPI_ISL_820253	Quadram Institute Bioscience	COVID-19 Genomics UK (COG-UK) Consortium	Dave J. Baker, Gemma L. Kay, Alp Aydin, Thanh Le-Viet, Steven Rudder, Ana P. Tedim, Anastasia Kolyva, Maria Diaz, Leonardo de Oliveira Martins, Nabil-Fareed Alikhan, Lizzie Meadows, Rachael Stanley, Ngozi Elumogo, Muhammed Yasir, Nicholas M. Thomson, Alexander J Trotter, Rachel Gilroy, Samuel Bloomfield, Claire Stuart, Andrew Bell, Reenesh Prakash, Samir Dervisevic, Alison E. Mather, John Wain, Mark Webber, Andrew J. Page, Justin O'Grady
EPI_ISL_820256, EPI_ISL_820259	University College London, Great Ormond Street Hospital for Children NHS Foundation Trust, Imperial College Healthcare NHS Trust	COVID-19 Genomics UK (COG-UK) Consortium	Sergi Castellano, Rachel Williams, Mark Kristiansen, Paola Resende Silva, Sunando Roy, Tony Brooks, Helena Tutill, Paola Niola, Patricia Dyal, Charlotte Williams, Leysa Forrest, Yasmin Panchbhaya, Jacqueline Findlay, Samuel Weeks, Julianne Brown, Kathryn Harris, Paul Randell, James Price, Alison Holmes, Judith Breuer
EPI_ISL_820261	Quadram Institute Bioscience	COVID-19 Genomics UK (COG-UK) Consortium	Dave J. Baker, Gemma L. Kay, Alp Aydin, Thanh Le-Viet, Steven Rudder, Ana P. Tedim, Anastasia Kolyva, Maria Diaz, Leonardo de Oliveira Martins, Nabil-Fareed Alikhan, Lizzie Meadows, Rachael Stanley, Ngozi Elumogo, Muhammed Yasir, Nicholas M. Thomson, Alexander J Trotter, Rachel Gilroy, Samuel Bloomfield, Claire Stuart, Andrew Bell, Reenesh Prakash, Samir Dervisevic, Alison E. Mather, John Wain, Mark Webber, Andrew J. Page, Justin O'Grady
EPI_ISL_820264, EPI_ISL_820266, EPI_ISL_820268, EPI_ISL_820271, EPI_ISL_820273, EPI_ISL_820276	University College London, Great Ormond Street Hospital for Children NHS Foundation Trust, Imperial College Healthcare NHS Trust	COVID-19 Genomics UK (COG-UK) Consortium	Sergi Castellano, Rachel Williams, Mark Kristiansen, Paola Resende Silva, Sunando Roy, Tony Brooks, Helena Tutill, Paola Niola, Patricia Dyal, Charlotte Williams, Leysa Forrest, Yasmin Panchbhaya, Jacqueline Findlay, Samuel Weeks, Julianne Brown, Kathryn Harris, Paul Randell, James Price, Alison Holmes, Judith Breuer
EPI_ISL_820279	Quadram Institute Bioscience	COVID-19 Genomics UK (COG-UK) Consortium	Dave J. Baker, Gemma L. Kay, Alp Aydin, Thanh Le-Viet, Steven Rudder, Ana P. Tedim, Anastasia Kolyva, Maria Diaz, Leonardo de Oliveira Martins, Nabil-Fareed Alikhan, Lizzie Meadows, Rachael Stanley, Ngozi Elumogo, Muhammed Yasir, Nicholas M. Thomson, Alexander J Trotter, Rachel Gilroy, Samuel Bloomfield, Claire Stuart, Andrew Bell, Reenesh Prakash, Samir Dervisevic, Alison E. Mather, John Wain, Mark Webber, Andrew J. Page, Justin O'Grady
EPI_ISL_820282	University College London, Great Ormond Street Hospital for Children NHS Foundation Trust, Imperial College Healthcare NHS Trust	COVID-19 Genomics UK (COG-UK) Consortium	Sergi Castellano, Rachel Williams, Mark Kristiansen, Paola Resende Silva, Sunando Roy, Tony Brooks, Helena Tutill, Paola Niola, Patricia Dyal, Charlotte Williams, Leysa Forrest, Yasmin Panchbhaya, Jacqueline Findlay, Samuel Weeks, Julianne Brown, Kathryn Harris, Paul Randell, James Price, Alison Holmes, Judith Breuer
EPI_ISL_820284, EPI_ISL_820287, EPI_ISL_820289, EPI_ISL_820292, EPI_ISL_820294	Quadram Institute Bioscience	COVID-19 Genomics UK (COG-UK) Consortium	Dave J. Baker, Gemma L. Kay, Alp Aydin, Thanh Le-Viet, Steven Rudder, Ana P. Tedim, Anastasia Kolyva, Maria Diaz, Leonardo de Oliveira Martins, Nabil-Fareed Alikhan, Lizzie Meadows, Rachael Stanley, Ngozi Elumogo, Muhammed Yasir, Nicholas M. Thomson, Alexander J Trotter, Rachel Gilroy, Samuel Bloomfield, Claire Stuart, Andrew Bell, Reenesh Prakash, Samir Dervisevic, Alison E. Mather, John Wain, Mark Webber, Andrew J. Page, Justin O'Grady
EPI_ISL_820297	Queens Medical Centre, Clinical Microbiology Department / DeepSeq Nottingham	COVID-19 Genomics UK (COG-UK) Consortium	Gemma Clark, Wendy Smith, Manjinder Khakh, Vicki M Fleming, Michelle M Lister, Hannah Howson-Wells, Jonathan Ball, Patrick McClure, Joseph Chappell, Theocharis Tsoleridis, Nadine Holmes, Matthew Carlisle, Christopher Moore, Fei Sang, Johnny Debebe, Victoria Wright, Matthew Loose
EPI_ISL_820300, EPI_ISL_820302, EPI_ISL_820304, EPI_ISL_820306	Quadram Institute Bioscience	COVID-19 Genomics UK (COG-UK) Consortium	Dave J. Baker, Gemma L. Kay, Alp Aydin, Thanh Le-Viet, Steven Rudder, Ana P. Tedim, Anastasia Kolyva, Maria Diaz, Leonardo de Oliveira Martins, Nabil-Fareed Alikhan, Lizzie Meadows, Rachael Stanley, Ngozi Elumogo, Muhammed Yasir, Nicholas M. Thomson, Alexander J Trotter, Rachel Gilroy, Samuel Bloomfield, Claire Stuart, Andrew Bell, Reenesh Prakash, Samir Dervisevic, Alison E. Mather, John Wain, Mark Webber, Andrew J. Page, Justin O'Grady
EPI_ISL_820309	University College London, Great Ormond Street Hospital for Children NHS Foundation Trust, Imperial College Healthcare NHS Trust	COVID-19 Genomics UK (COG-UK) Consortium	Sergi Castellano, Rachel Williams, Mark Kristiansen, Paola Resende Silva, Sunando Roy, Tony Brooks, Helena Tutill, Paola Niola, Patricia Dyal, Charlotte Williams, Leysa Forrest, Yasmin Panchbhaya, Jacqueline Findlay, Samuel Weeks, Julianne Brown, Kathryn Harris, Paul Randell, James Price, Alison Holmes, Judith Breuer
EPI_ISL_820311	Quadram Institute Bioscience	COVID-19 Genomics UK (COG-UK) Consortium	Dave J. Baker, Gemma L. Kay, Alp Aydin, Thanh Le-Viet, Steven Rudder, Ana P. Tedim, Anastasia Kolyva, Maria Diaz, Leonardo de Oliveira Martins, Nabil-Fareed Alikhan, Lizzie Meadows, Rachael Stanley, Ngozi Elumogo, Muhammed Yasir, Nicholas M. Thomson, Alexander J Trotter, Rachel Gilroy, Samuel Bloomfield, Claire Stuart, Andrew Bell, Reenesh Prakash, Samir Dervisevic, Alison E. Mather, John Wain, Mark Webber, Andrew J. Page, Justin O'Grady
EPI_ISL_820314, EPI_ISL_820316, EPI_ISL_820318, EPI_ISL_820321	University College London, Great Ormond Street Hospital for Children NHS Foundation Trust, Imperial College Healthcare NHS Trust	COVID-19 Genomics UK (COG-UK) Consortium	Sergi Castellano, Rachel Williams, Mark Kristiansen, Paola Resende Silva, Sunando Roy, Tony Brooks, Helena Tutill, Paola Niola, Patricia Dyal, Charlotte Williams, Leysa Forrest, Yasmin Panchbhaya, Jacqueline Findlay, Samuel Weeks, Julianne Brown, Kathryn Harris, Paul Randell, James Price, Alison Holmes, Judith Breuer
EPI_ISL_820323	Quadram Institute Bioscience	COVID-19 Genomics UK (COG-UK) Consortium	Dave J. Baker, Gemma L. Kay, Alp Aydin, Thanh Le-Viet, Steven Rudder, Ana P. Tedim, Anastasia Kolyva, Maria Diaz, Leonardo de Oliveira Martins, Nabil-Fareed Alikhan, Lizzie Meadows, Rachael Stanley, Ngozi Elumogo, Muhammed Yasir, Nicholas M. Thomson, Alexander J Trotter, Rachel Gilroy, Samuel Bloomfield, Claire Stuart, Andrew Bell, Reenesh Prakash, Samir Dervisevic, Alison E. Mather, John Wain, Mark Webber, Andrew J. Page, Justin O'Grady
EPI_ISL_820326, EPI_ISL_820328, EPI_ISL_820331, EPI_ISL_820333, EPI_ISL_820335, EPI_ISL_820337, EPI_ISL_820340, EPI_ISL_820343, EPI_ISL_820345, EPI_ISL_820347, EPI_ISL_820350			
see above	University College London, Great Ormond Street Hospital for Children NHS Foundation Trust, Imperial College Healthcare NHS Trust	COVID-19 Genomics UK (COG-UK) Consortium	Sergi Castellano, Rachel Williams, Mark Kristiansen, Paola Resende Silva, Sunando Roy, Tony Brooks, Helena Tutill, Paola Niola, Patricia Dyal, Charlotte Williams, Leysa Forrest, Yasmin Panchbhaya, Jacqueline Findlay, Samuel Weeks, Julianne Brown, Kathryn Harris, Paul Randell, James Price, Alison Holmes, Judith Breuer
EPI_ISL_820353, EPI_ISL_820355, EPI_ISL_820358, EPI_ISL_820360, EPI_ISL_820363, EPI_ISL_820365, EPI_ISL_820368, EPI_ISL_820371	Quadram Institute Bioscience	COVID-19 Genomics UK (COG-UK) Consortium	Dave J. Baker, Gemma L. Kay, Alp Aydin, Thanh Le-Viet, Steven Rudder, Ana P. Tedim, Anastasia Kolyva, Maria Diaz, Leonardo de Oliveira Martins, Nabil-Fareed Alikhan, Lizzie Meadows, Rachael Stanley, Ngozi Elumogo, Muhammed Yasir, Nicholas M. Thomson, Alexander J Trotter, Rachel Gilroy, Samuel Bloomfield, Claire Stuart, Andrew Bell, Reenesh Prakash, Samir Dervisevic, Alison E. Mather, John Wain, Mark Webber, Andrew J. Page, Justin O'Grady
EPI_ISL_820373, EPI_ISL_820376	University College London, Great Ormond Street Hospital for Children NHS Foundation Trust, Imperial College Healthcare	COVID-19 Genomics UK (COG-UK) Consortium	Sergi Castellano, Rachel Williams, Mark Kristiansen, Paola Resende Silva, Sunando Roy, Tony Brooks, Helena Tutill, Paola Niola, Patricia Dyal, Charlotte Williams, Leysa Forrest, Yasmin Panchbhaya, Jacqueline Findlay, Samuel Weeks, Julianne Brown, Kathryn Harris, Paul Randell, James Price, Alison

	NHS Trust		Holmes, Judith Breuer
EPI_ISL_820378	Quadram Institute Bioscience	COVID-19 Genomics UK (COG-UK) Consortium	Dave J. Baker, Gemma L. Kay, Alp Aydin, Thanh Le-Viet, Steven Rudder, Ana P. Tedim, Anastasia Kolyva, Maria Diaz, Leonardo de Oliveira Martins, Nabil-Fareed Alikhan, Lizzie Meadows, Rachael Stanley, Ngozi Elumogo, Muhammed Yasir, Nicholas M. Thomson, Alexander J Trotter, Rachel Gilroy, Samuel Bloomfield, Claire Stuart, Andrew Bell, Reenesh Prakash, Samir Dervisevic, Alison E. Mather, John Wain, Mark Webber, Andrew J. Page, Justin O'Grady
EPI_ISL_820381, EPI_ISL_820383, EPI_ISL_820385	University College London, Great Ormond Street Hospital for Children NHS Foundation Trust, Imperial College Healthcare NHS Trust	COVID-19 Genomics UK (COG-UK) Consortium	Sergi Castellano, Rachel Williams, Mark Kristiansen, Paola Resende Silva, Sunando Roy, Tony Brooks, Helena Tutill, Paola Niola, Patricia Dyal, Charlotte Williams, Leysa Forrest, Yasmin Panchbhaya, Jacqueline Findlay, Samuel Weeks, Julianne Brown, Kathryn Harris, Paul Randell, James Price, Alison Holmes, Judith Breuer
EPI_ISL_820388, EPI_ISL_820391	Quadram Institute Bioscience	COVID-19 Genomics UK (COG-UK) Consortium	Dave J. Baker, Gemma L. Kay, Alp Aydin, Thanh Le-Viet, Steven Rudder, Ana P. Tedim, Anastasia Kolyva, Maria Diaz, Leonardo de Oliveira Martins, Nabil-Fareed Alikhan, Lizzie Meadows, Rachael Stanley, Ngozi Elumogo, Muhammed Yasir, Nicholas M. Thomson, Alexander J Trotter, Rachel Gilroy, Samuel Bloomfield, Claire Stuart, Andrew Bell, Reenesh Prakash, Samir Dervisevic, Alison E. Mather, John Wain, Mark Webber, Andrew J. Page, Justin O'Grady
EPI_ISL_820394	Queens Medical Centre, Clinical Microbiology Department / DeepSeq Nottingham	COVID-19 Genomics UK (COG-UK) Consortium	Gemma Clark, Wendy Smith, Manjinder Khakh, Vicki M Fleming, Michelle M Lister, Hannah Howson-Wells, Jonathan Ball, Patrick McClure, Joseph Chappell, Theocharis Tsoleridis, Nadine Holmes, Matthew Carlisle, Christopher Moore, Fei Sang, Johnny Debebe, Victoria Wright, Matthew Loose
EPI_ISL_820396	University College London, Great Ormond Street Hospital for Children NHS Foundation Trust, Imperial College Healthcare NHS Trust	COVID-19 Genomics UK (COG-UK) Consortium	Sergi Castellano, Rachel Williams, Mark Kristiansen, Paola Resende Silva, Sunando Roy, Tony Brooks, Helena Tutill, Paola Niola, Patricia Dyal, Charlotte Williams, Leysa Forrest, Yasmin Panchbhaya, Jacqueline Findlay, Samuel Weeks, Julianne Brown, Kathryn Harris, Paul Randell, James Price, Alison Holmes, Judith Breuer
EPI_ISL_820399	Quadram Institute Bioscience	COVID-19 Genomics UK (COG-UK) Consortium	Dave J. Baker, Gemma L. Kay, Alp Aydin, Thanh Le-Viet, Steven Rudder, Ana P. Tedim, Anastasia Kolyva, Maria Diaz, Leonardo de Oliveira Martins, Nabil-Fareed Alikhan, Lizzie Meadows, Rachael Stanley, Ngozi Elumogo, Muhammed Yasir, Nicholas M. Thomson, Alexander J Trotter, Rachel Gilroy, Samuel Bloomfield, Claire Stuart, Andrew Bell, Reenesh Prakash, Samir Dervisevic, Alison E. Mather, John Wain, Mark Webber, Andrew J. Page, Justin O'Grady
EPI_ISL_820402	Queens Medical Centre, Clinical Microbiology Department / DeepSeq Nottingham	COVID-19 Genomics UK (COG-UK) Consortium	Gemma Clark, Wendy Smith, Manjinder Khakh, Vicki M Fleming, Michelle M Lister, Hannah Howson-Wells, Jonathan Ball, Patrick McClure, Joseph Chappell, Theocharis Tsoleridis, Nadine Holmes, Matthew Carlisle, Christopher Moore, Fei Sang, Johnny Debebe, Victoria Wright, Matthew Loose
EPI_ISL_820404, EPI_ISL_820407, EPI_ISL_820410, EPI_ISL_820412, EPI_ISL_820415	Quadram Institute Bioscience	COVID-19 Genomics UK (COG-UK) Consortium	Dave J. Baker, Gemma L. Kay, Alp Aydin, Thanh Le-Viet, Steven Rudder, Ana P. Tedim, Anastasia Kolyva, Maria Diaz, Leonardo de Oliveira Martins, Nabil-Fareed Alikhan, Lizzie Meadows, Rachael Stanley, Ngozi Elumogo, Muhammed Yasir, Nicholas M. Thomson, Alexander J Trotter, Rachel Gilroy, Samuel Bloomfield, Claire Stuart, Andrew Bell, Reenesh Prakash, Samir Dervisevic, Alison E. Mather, John Wain, Mark Webber, Andrew J. Page, Justin O'Grady
EPI_ISL_820418	Queens Medical Centre, Clinical Microbiology Department / DeepSeq Nottingham	COVID-19 Genomics UK (COG-UK) Consortium	Gemma Clark, Wendy Smith, Manjinder Khakh, Vicki M Fleming, Michelle M Lister, Hannah Howson-Wells, Jonathan Ball, Patrick McClure, Joseph Chappell, Theocharis Tsoleridis, Nadine Holmes, Matthew Carlisle, Christopher Moore, Fei Sang, Johnny Debebe, Victoria Wright, Matthew Loose
EPI_ISL_820420	University College London, Great Ormond Street Hospital for Children NHS Foundation Trust, Imperial College Healthcare NHS Trust	COVID-19 Genomics UK (COG-UK) Consortium	Sergi Castellano, Rachel Williams, Mark Kristiansen, Paola Resende Silva, Sunando Roy, Tony Brooks, Helena Tutill, Paola Niola, Patricia Dyal, Charlotte Williams, Leysa Forrest, Yasmin Panchbhaya, Jacqueline Findlay, Samuel Weeks, Julianne Brown, Kathryn Harris, Paul Randell, James Price, Alison Holmes, Judith Breuer
EPI_ISL_820423	Queens Medical Centre, Clinical Microbiology Department / DeepSeq Nottingham	COVID-19 Genomics UK (COG-UK) Consortium	Gemma Clark, Wendy Smith, Manjinder Khakh, Vicki M Fleming, Michelle M Lister, Hannah Howson-Wells, Jonathan Ball, Patrick McClure, Joseph Chappell, Theocharis Tsoleridis, Nadine Holmes, Matthew Carlisle, Christopher Moore, Fei Sang, Johnny Debebe, Victoria Wright, Matthew Loose
EPI_ISL_820426	Quadram Institute Bioscience	COVID-19 Genomics UK (COG-UK) Consortium	Dave J. Baker, Gemma L. Kay, Alp Aydin, Thanh Le-Viet, Steven Rudder, Ana P. Tedim, Anastasia Kolyva, Maria Diaz, Leonardo de Oliveira Martins, Nabil-Fareed Alikhan, Lizzie Meadows, Rachael Stanley, Ngozi Elumogo, Muhammed Yasir, Nicholas M. Thomson, Alexander J Trotter, Rachel Gilroy, Samuel Bloomfield, Claire Stuart, Andrew Bell, Reenesh Prakash, Samir Dervisevic, Alison E. Mather, John Wain, Mark Webber, Andrew J. Page, Justin O'Grady
EPI_ISL_820428, EPI_ISL_820431, EPI_ISL_820433	Queens Medical Centre, Clinical Microbiology Department / DeepSeq Nottingham	COVID-19 Genomics UK (COG-UK) Consortium	Gemma Clark, Wendy Smith, Manjinder Khakh, Vicki M Fleming, Michelle M Lister, Hannah Howson-Wells, Jonathan Ball, Patrick McClure, Joseph Chappell, Theocharis Tsoleridis, Nadine Holmes, Matthew Carlisle, Christopher Moore, Fei Sang, Johnny Debebe, Victoria Wright, Matthew Loose
EPI_ISL_820436, EPI_ISL_820439, EPI_ISL_820441, EPI_ISL_820444, EPI_ISL_820446, EPI_ISL_820449, EPI_ISL_820451, EPI_ISL_820454, EPI_ISL_820456, EPI_ISL_820459, EPI_ISL_820462, EPI_ISL_820464, EPI_ISL_820467, EPI_ISL_820470, EPI_ISL_820472, EPI_ISL_820475, EPI_ISL_820478, EPI_ISL_820480, EPI_ISL_820483, EPI_ISL_820485, EPI_ISL_820488, EPI_ISL_820490, EPI_ISL_820493, EPI_ISL_820496, EPI_ISL_820498, EPI_ISL_820501, EPI_ISL_820504, EPI_ISL_820506, EPI_ISL_820509, EPI_ISL_820511, EPI_ISL_820514, EPI_ISL_820516, EPI_ISL_820519, EPI_ISL_820521, EPI_ISL_820524, EPI_ISL_820526, EPI_ISL_820528, EPI_ISL_820531, EPI_ISL_820533, EPI_ISL_820536, EPI_ISL_820539, EPI_ISL_820541, EPI_ISL_820543, EPI_ISL_820546, EPI_ISL_820549, EPI_ISL_820552, EPI_ISL_820554, EPI_ISL_820557			
see above	Quadram Institute Bioscience	COVID-19 Genomics UK (COG-UK) Consortium	Dave J. Baker, Gemma L. Kay, Alp Aydin, Thanh Le-Viet, Steven Rudder, Ana P. Tedim, Anastasia Kolyva, Maria Diaz, Leonardo de Oliveira Martins, Nabil-Fareed Alikhan, Lizzie Meadows, Rachael Stanley, Ngozi Elumogo, Muhammed Yasir, Nicholas M. Thomson, Alexander J Trotter, Rachel Gilroy, Samuel Bloomfield, Claire Stuart, Andrew Bell, Reenesh Prakash, Samir Dervisevic, Alison E. Mather, John Wain, Mark Webber, Andrew J. Page, Justin O'Grady
EPI_ISL_820560	Queens Medical Centre, Clinical Microbiology Department / DeepSeq Nottingham	COVID-19 Genomics UK (COG-UK) Consortium	Gemma Clark, Wendy Smith, Manjinder Khakh, Vicki M Fleming, Michelle M Lister, Hannah Howson-Wells, Jonathan Ball, Patrick McClure, Joseph Chappell, Theocharis Tsoleridis, Nadine Holmes, Matthew Carlisle, Christopher Moore, Fei Sang, Johnny Debebe, Victoria Wright, Matthew Loose
EPI_ISL_820563, EPI_ISL_820565, EPI_ISL_820568, EPI_ISL_820570	Quadram Institute Bioscience	COVID-19 Genomics UK (COG-UK) Consortium	Dave J. Baker, Gemma L. Kay, Alp Aydin, Thanh Le-Viet, Steven Rudder, Ana P. Tedim, Anastasia Kolyva, Maria Diaz, Leonardo de Oliveira Martins, Nabil-Fareed Alikhan, Lizzie Meadows, Rachael Stanley, Ngozi Elumogo, Muhammed Yasir, Nicholas M. Thomson, Alexander J Trotter, Rachel Gilroy, Samuel Bloomfield, Claire Stuart, Andrew Bell, Reenesh Prakash, Samir Dervisevic, Alison E. Mather, John Wain, Mark Webber, Andrew J. Page, Justin O'Grady
EPI_ISL_821003, EPI_ISL_821005, EPI_ISL_821010, EPI_ISL_821011, EPI_ISL_821012, EPI_ISL_821013, EPI_ISL_821018, EPI_ISL_821020, EPI_ISL_821021, EPI_ISL_821022, EPI_ISL_821025, EPI_ISL_821026, EPI_ISL_821028, EPI_ISL_821030, EPI_ISL_821035, EPI_ISL_821036, EPI_ISL_821038, EPI_ISL_821039, EPI_ISL_821042, EPI_ISL_821046, EPI_ISL_821047, EPI_ISL_821050, EPI_ISL_821055, EPI_ISL_821060, EPI_ISL_821063, EPI_ISL_821066, EPI_ISL_821068, EPI_ISL_821073, EPI_ISL_821074, EPI_ISL_821078, EPI_ISL_821082, EPI_ISL_821084, EPI_ISL_821085, EPI_ISL_821090, EPI_ISL_821091, EPI_ISL_821093, EPI_ISL_821095, EPI_ISL_821099, EPI_ISL_821102, EPI_ISL_821104, EPI_ISL_821109, EPI_ISL_821110, EPI_ISL_821111, EPI_ISL_821117, EPI_ISL_821124, EPI_ISL_821125, EPI_ISL_821126, EPI_ISL_821127, EPI_ISL_821128, EPI_ISL_821131, EPI_ISL_821133, EPI_ISL_821134, EPI_ISL_821136, EPI_ISL_821137, EPI_ISL_821140, EPI_ISL_821144, EPI_ISL_821149, EPI_ISL_821151, EPI_ISL_821152, EPI_ISL_821156, EPI_ISL_821158, EPI_ISL_821159, EPI_ISL_821163, EPI_ISL_821165, EPI_ISL_821166, EPI_ISL_821169, EPI_ISL_821170, EPI_ISL_821172, EPI_ISL_821173, EPI_ISL_821174, EPI_ISL_821175, EPI_ISL_821178, EPI_ISL_821180, EPI_ISL_821181, EPI_ISL_821187, EPI_ISL_821190, EPI_ISL_821191, EPI_ISL_821194, EPI_ISL_821197, EPI_ISL_821199, EPI_ISL_821200, EPI_ISL_821201, EPI_ISL_821206, EPI_ISL_821208, EPI_ISL_821214, EPI_ISL_821215, EPI_ISL_821220, EPI_ISL_821222, EPI_ISL_821223, EPI_ISL_821228, EPI_ISL_821230, EPI_ISL_821231, EPI_ISL_821235, EPI_ISL_821243, EPI_ISL_821244, EPI_ISL_821248, EPI_ISL_821250, EPI_ISL_821251, EPI_ISL_821253, EPI_ISL_821254, EPI_ISL_821255, EPI_ISL_821261, EPI_ISL_821263, EPI_ISL_821264, EPI_ISL_821270, EPI_ISL_821271			
see above	Lighthouse Lab in Alderley Park	Wellcome Sanger Institute for the COVID-19 Genomics UK (COG-UK) Consortium	Jacquelyn Wynn, Mairead Hyland, The Lighthouse Lab in Alderley Park and Alex Alderton, Roberto Amato, Sonia Goncalves, Ewan Harrison, David K. Jackson, Ian Johnston, Dominic Kwiatkowski, Cordelia Langford, John Sillitoe on behalf of the Wellcome Sanger Institute COVID-19 Surveillance Team
EPI_ISL_821275, EPI_ISL_821277	Lighthouse Lab in Milton Keynes	Wellcome Sanger Institute for the COVID-19 Genomics UK (COG-UK) Consortium	The Lighthouse Lab in Milton Keynes and Alex Alderton, Roberto Amato, Sonia Goncalves, Ewan Harrison, David K. Jackson, Ian Johnston, Dominic Kwiatkowski, Cordelia Langford, John Sillitoe on behalf of the Wellcome Sanger Institute COVID-19 Surveillance Team
EPI_ISL_821278	Lighthouse Lab in Cambridge	Wellcome Sanger Institute for the COVID-19 Genomics UK (COG-UK) Consortium	Rob Howes, The Lighthouse Lab in Cambridge and Alex Alderton, Roberto Amato, Sonia Goncalves, Ewan Harrison, David K. Jackson, Ian Johnston, Dominic Kwiatkowski, Cordelia Langford, John Sillitoe on behalf of the Wellcome Sanger Institute COVID-19 Surveillance Team
EPI_ISL_821280	Lighthouse Lab in Milton Keynes	Wellcome Sanger Institute for the COVID-19 Genomics UK (COG-UK) Consortium	The Lighthouse Lab in Milton Keynes and Alex Alderton, Roberto Amato, Sonia Goncalves, Ewan Harrison, David K. Jackson, Ian Johnston, Dominic Kwiatkowski, Cordelia Langford, John Sillitoe on behalf of the Wellcome Sanger Institute COVID-19 Surveillance Team
EPI_ISL_821285	Lighthouse Lab in Alderley Park	Wellcome Sanger Institute for the COVID-19 Genomics UK (COG-UK) Consortium	Jacquelyn Wynn, Mairead Hyland, The Lighthouse Lab in Alderley Park and Alex Alderton, Roberto Amato, Sonia Goncalves, Ewan Harrison, David K. Jackson, Ian Johnston, Dominic Kwiatkowski, Cordelia Langford, John Sillitoe on behalf of the Wellcome Sanger Institute COVID-19 Surveillance Team
EPI_ISL_821286	Lighthouse Lab in Cambridge	Wellcome Sanger Institute for the COVID-19 Genomics UK (COG-UK) Consortium	Rob Howes, The Lighthouse Lab in Cambridge and Alex Alderton, Roberto Amato, Sonia Goncalves, Ewan Harrison, David K. Jackson, Ian Johnston, Dominic Kwiatkowski, Cordelia Langford, John Sillitoe on behalf of the Wellcome Sanger Institute COVID-19 Surveillance Team
EPI_ISL_821287, EPI_ISL_821291	Lighthouse Lab in Milton Keynes	Wellcome Sanger Institute for the COVID-19 Genomics UK (COG-UK) Consortium	The Lighthouse Lab in Milton Keynes and Alex Alderton, Roberto Amato, Sonia Goncalves, Ewan Harrison, David K. Jackson, Ian Johnston, Dominic Kwiatkowski, Cordelia Langford, John Sillitoe on behalf of the Wellcome Sanger Institute COVID-19 Surveillance Team
EPI_ISL_821292	Lighthouse Lab in Alderley Park	Wellcome Sanger Institute for the COVID-19 Genomics UK	Jacquelyn Wynn, Mairead Hyland, The Lighthouse Lab in Alderley Park and Alex Alderton, Roberto Amato, Sonia Goncalves, Ewan Harrison, David K.

		(COG-UK) Consortium	Jackson, Ian Johnston, Dominic Kwiatkowski, Cordelia Langford, John Sillitoe on behalf of the Wellcome Sanger Institute COVID-19 Surveillance Team
EPI_ISL_821293	Lighthouse Lab in Milton Keynes	Wellcome Sanger Institute for the COVID-19 Genomics UK (COG-UK) Consortium	The Lighthouse Lab in Milton Keynes and Alex Alderton, Roberto Amato, Sonia Goncalves, Ewan Harrison, David K. Jackson, Ian Johnston, Dominic Kwiatkowski, Cordelia Langford, John Sillitoe on behalf of the Wellcome Sanger Institute COVID-19 Surveillance Team
EPI_ISL_821294	Lighthouse Lab in Alderley Park	Wellcome Sanger Institute for the COVID-19 Genomics UK (COG-UK) Consortium	Jacquelyn Wynn, Mairead Hyland, The Lighthouse Lab in Alderley Park and Alex Alderton, Roberto Amato, Sonia Goncalves, Ewan Harrison, David K. Jackson, Ian Johnston, Dominic Kwiatkowski, Cordelia Langford, John Sillitoe on behalf of the Wellcome Sanger Institute COVID-19 Surveillance Team
EPI_ISL_821295	Lighthouse Lab in Cambridge	Wellcome Sanger Institute for the COVID-19 Genomics UK (COG-UK) Consortium	Rob Howes, The Lighthouse Lab in Cambridge and Alex Alderton, Roberto Amato, Sonia Goncalves, Ewan Harrison, David K. Jackson, Ian Johnston, Dominic Kwiatkowski, Cordelia Langford, John Sillitoe on behalf of the Wellcome Sanger Institute COVID-19 Surveillance Team
EPI_ISL_821298	Lighthouse Lab in Alderley Park	Wellcome Sanger Institute for the COVID-19 Genomics UK (COG-UK) Consortium	Jacquelyn Wynn, Mairead Hyland, The Lighthouse Lab in Alderley Park and Alex Alderton, Roberto Amato, Sonia Goncalves, Ewan Harrison, David K. Jackson, Ian Johnston, Dominic Kwiatkowski, Cordelia Langford, John Sillitoe on behalf of the Wellcome Sanger Institute COVID-19 Surveillance Team
EPI_ISL_821299, EPI_ISL_821300, EPI_ISL_821302	Lighthouse Lab in Milton Keynes	Wellcome Sanger Institute for the COVID-19 Genomics UK (COG-UK) Consortium	The Lighthouse Lab in Milton Keynes and Alex Alderton, Roberto Amato, Sonia Goncalves, Ewan Harrison, David K. Jackson, Ian Johnston, Dominic Kwiatkowski, Cordelia Langford, John Sillitoe on behalf of the Wellcome Sanger Institute COVID-19 Surveillance Team
EPI_ISL_821303	Lighthouse Lab in Alderley Park	Wellcome Sanger Institute for the COVID-19 Genomics UK (COG-UK) Consortium	Jacquelyn Wynn, Mairead Hyland, The Lighthouse Lab in Alderley Park and Alex Alderton, Roberto Amato, Sonia Goncalves, Ewan Harrison, David K. Jackson, Ian Johnston, Dominic Kwiatkowski, Cordelia Langford, John Sillitoe on behalf of the Wellcome Sanger Institute COVID-19 Surveillance Team
EPI_ISL_821304, EPI_ISL_821305, EPI_ISL_821307	Lighthouse Lab in Milton Keynes	Wellcome Sanger Institute for the COVID-19 Genomics UK (COG-UK) Consortium	The Lighthouse Lab in Milton Keynes and Alex Alderton, Roberto Amato, Sonia Goncalves, Ewan Harrison, David K. Jackson, Ian Johnston, Dominic Kwiatkowski, Cordelia Langford, John Sillitoe on behalf of the Wellcome Sanger Institute COVID-19 Surveillance Team
EPI_ISL_821308	Lighthouse Lab in Alderley Park	Wellcome Sanger Institute for the COVID-19 Genomics UK (COG-UK) Consortium	Jacquelyn Wynn, Mairead Hyland, The Lighthouse Lab in Alderley Park and Alex Alderton, Roberto Amato, Sonia Goncalves, Ewan Harrison, David K. Jackson, Ian Johnston, Dominic Kwiatkowski, Cordelia Langford, John Sillitoe on behalf of the Wellcome Sanger Institute COVID-19 Surveillance Team
EPI_ISL_821311	Lighthouse Lab in Cambridge	Wellcome Sanger Institute for the COVID-19 Genomics UK (COG-UK) Consortium	Rob Howes, The Lighthouse Lab in Cambridge and Alex Alderton, Roberto Amato, Sonia Goncalves, Ewan Harrison, David K. Jackson, Ian Johnston, Dominic Kwiatkowski, Cordelia Langford, John Sillitoe on behalf of the Wellcome Sanger Institute COVID-19 Surveillance Team
EPI_ISL_821312, EPI_ISL_821313, EPI_ISL_821315	Lighthouse Lab in Milton Keynes	Wellcome Sanger Institute for the COVID-19 Genomics UK (COG-UK) Consortium	The Lighthouse Lab in Milton Keynes and Alex Alderton, Roberto Amato, Sonia Goncalves, Ewan Harrison, David K. Jackson, Ian Johnston, Dominic Kwiatkowski, Cordelia Langford, John Sillitoe on behalf of the Wellcome Sanger Institute COVID-19 Surveillance Team
EPI_ISL_821316	Lighthouse Lab in Cambridge	Wellcome Sanger Institute for the COVID-19 Genomics UK (COG-UK) Consortium	Rob Howes, The Lighthouse Lab in Cambridge and Alex Alderton, Roberto Amato, Sonia Goncalves, Ewan Harrison, David K. Jackson, Ian Johnston, Dominic Kwiatkowski, Cordelia Langford, John Sillitoe on behalf of the Wellcome Sanger Institute COVID-19 Surveillance Team
EPI_ISL_821318	Lighthouse Lab in Alderley Park	Wellcome Sanger Institute for the COVID-19 Genomics UK (COG-UK) Consortium	Jacquelyn Wynn, Mairead Hyland, The Lighthouse Lab in Alderley Park and Alex Alderton, Roberto Amato, Sonia Goncalves, Ewan Harrison, David K. Jackson, Ian Johnston, Dominic Kwiatkowski, Cordelia Langford, John Sillitoe on behalf of the Wellcome Sanger Institute COVID-19 Surveillance Team
EPI_ISL_821320	Lighthouse Lab in Cambridge	Wellcome Sanger Institute for the COVID-19 Genomics UK (COG-UK) Consortium	Rob Howes, The Lighthouse Lab in Cambridge and Alex Alderton, Roberto Amato, Sonia Goncalves, Ewan Harrison, David K. Jackson, Ian Johnston, Dominic Kwiatkowski, Cordelia Langford, John Sillitoe on behalf of the Wellcome Sanger Institute COVID-19 Surveillance Team
EPI_ISL_821321, EPI_ISL_821322, EPI_ISL_821325, EPI_ISL_821326	Lighthouse Lab in Milton Keynes	Wellcome Sanger Institute for the COVID-19 Genomics UK (COG-UK) Consortium	The Lighthouse Lab in Milton Keynes and Alex Alderton, Roberto Amato, Sonia Goncalves, Ewan Harrison, David K. Jackson, Ian Johnston, Dominic Kwiatkowski, Cordelia Langford, John Sillitoe on behalf of the Wellcome Sanger Institute COVID-19 Surveillance Team
EPI_ISL_821327, EPI_ISL_821329	Lighthouse Lab in Alderley Park	Wellcome Sanger Institute for the COVID-19 Genomics UK (COG-UK) Consortium	Jacquelyn Wynn, Mairead Hyland, The Lighthouse Lab in Alderley Park and Alex Alderton, Roberto Amato, Sonia Goncalves, Ewan Harrison, David K. Jackson, Ian Johnston, Dominic Kwiatkowski, Cordelia Langford, John Sillitoe on behalf of the Wellcome Sanger Institute COVID-19 Surveillance Team
EPI_ISL_821330, EPI_ISL_821332	Lighthouse Lab in Milton Keynes	Wellcome Sanger Institute for the COVID-19 Genomics UK (COG-UK) Consortium	The Lighthouse Lab in Milton Keynes and Alex Alderton, Roberto Amato, Sonia Goncalves, Ewan Harrison, David K. Jackson, Ian Johnston, Dominic Kwiatkowski, Cordelia Langford, John Sillitoe on behalf of the Wellcome Sanger Institute COVID-19 Surveillance Team
EPI_ISL_821335	Lighthouse Lab in Alderley Park	Wellcome Sanger Institute for the COVID-19 Genomics UK (COG-UK) Consortium	Jacquelyn Wynn, Mairead Hyland, The Lighthouse Lab in Alderley Park and Alex Alderton, Roberto Amato, Sonia Goncalves, Ewan Harrison, David K. Jackson, Ian Johnston, Dominic Kwiatkowski, Cordelia Langford, John Sillitoe on behalf of the Wellcome Sanger Institute COVID-19 Surveillance Team
EPI_ISL_821337	Lighthouse Lab in Milton Keynes	Wellcome Sanger Institute for the COVID-19 Genomics UK (COG-UK) Consortium	The Lighthouse Lab in Milton Keynes and Alex Alderton, Roberto Amato, Sonia Goncalves, Ewan Harrison, David K. Jackson, Ian Johnston, Dominic Kwiatkowski, Cordelia Langford, John Sillitoe on behalf of the Wellcome Sanger Institute COVID-19 Surveillance Team
EPI_ISL_821339	Lighthouse Lab in Cambridge	Wellcome Sanger Institute for the COVID-19 Genomics UK (COG-UK) Consortium	Rob Howes, The Lighthouse Lab in Cambridge and Alex Alderton, Roberto Amato, Sonia Goncalves, Ewan Harrison, David K. Jackson, Ian Johnston, Dominic Kwiatkowski, Cordelia Langford, John Sillitoe on behalf of the Wellcome Sanger Institute COVID-19 Surveillance Team
EPI_ISL_821345	Lighthouse Lab in Milton Keynes	Wellcome Sanger Institute for the COVID-19 Genomics UK (COG-UK) Consortium	The Lighthouse Lab in Milton Keynes and Alex Alderton, Roberto Amato, Sonia Goncalves, Ewan Harrison, David K. Jackson, Ian Johnston, Dominic Kwiatkowski, Cordelia Langford, John Sillitoe on behalf of the Wellcome Sanger Institute COVID-19 Surveillance Team
EPI_ISL_821347	Lighthouse Lab in Alderley Park	Wellcome Sanger Institute for the COVID-19 Genomics UK (COG-UK) Consortium	Jacquelyn Wynn, Mairead Hyland, The Lighthouse Lab in Alderley Park and Alex Alderton, Roberto Amato, Sonia Goncalves, Ewan Harrison, David K. Jackson, Ian Johnston, Dominic Kwiatkowski, Cordelia Langford, John Sillitoe on behalf of the Wellcome Sanger Institute COVID-19 Surveillance Team
EPI_ISL_821349, EPI_ISL_821350, EPI_ISL_821355, EPI_ISL_821356, EPI_ISL_821357	Lighthouse Lab in Milton Keynes	Wellcome Sanger Institute for the COVID-19 Genomics UK (COG-UK) Consortium	The Lighthouse Lab in Milton Keynes and Alex Alderton, Roberto Amato, Sonia Goncalves, Ewan Harrison, David K. Jackson, Ian Johnston, Dominic Kwiatkowski, Cordelia Langford, John Sillitoe on behalf of the Wellcome Sanger Institute COVID-19 Surveillance Team
EPI_ISL_821360	Lighthouse Lab in Cambridge	Wellcome Sanger Institute for the COVID-19 Genomics UK (COG-UK) Consortium	Rob Howes, The Lighthouse Lab in Cambridge and Alex Alderton, Roberto Amato, Sonia Goncalves, Ewan Harrison, David K. Jackson, Ian Johnston, Dominic Kwiatkowski, Cordelia Langford, John Sillitoe on behalf of the Wellcome Sanger Institute COVID-19 Surveillance Team
EPI_ISL_821361, EPI_ISL_821364	Lighthouse Lab in Milton Keynes	Wellcome Sanger Institute for the COVID-19 Genomics UK (COG-UK) Consortium	The Lighthouse Lab in Milton Keynes and Alex Alderton, Roberto Amato, Sonia Goncalves, Ewan Harrison, David K. Jackson, Ian Johnston, Dominic Kwiatkowski, Cordelia Langford, John Sillitoe on behalf of the Wellcome Sanger Institute COVID-19 Surveillance Team
EPI_ISL_821368	Lighthouse Lab in Cambridge	Wellcome Sanger Institute for the COVID-19 Genomics UK (COG-UK) Consortium	Rob Howes, The Lighthouse Lab in Cambridge and Alex Alderton, Roberto Amato, Sonia Goncalves, Ewan Harrison, David K. Jackson, Ian Johnston, Dominic Kwiatkowski, Cordelia Langford, John Sillitoe on behalf of the Wellcome Sanger Institute COVID-19 Surveillance Team
EPI_ISL_821369	Lighthouse Lab in Milton Keynes	Wellcome Sanger Institute for the COVID-19 Genomics UK (COG-UK) Consortium	The Lighthouse Lab in Milton Keynes and Alex Alderton, Roberto Amato, Sonia Goncalves, Ewan Harrison, David K. Jackson, Ian Johnston, Dominic Kwiatkowski, Cordelia Langford, John Sillitoe on behalf of the Wellcome Sanger Institute COVID-19 Surveillance Team
EPI_ISL_821370, EPI_ISL_821371	Lighthouse Lab in Alderley Park	Wellcome Sanger Institute for the COVID-19 Genomics UK (COG-UK) Consortium	Jacquelyn Wynn, Mairead Hyland, The Lighthouse Lab in Alderley Park and Alex Alderton, Roberto Amato, Sonia Goncalves, Ewan Harrison, David K. Jackson, Ian Johnston, Dominic Kwiatkowski, Cordelia Langford, John Sillitoe on behalf of the Wellcome Sanger Institute COVID-19 Surveillance Team
EPI_ISL_821374, EPI_ISL_821376	Lighthouse Lab in Cambridge	Wellcome Sanger Institute for the COVID-19 Genomics UK (COG-UK) Consortium	Rob Howes, The Lighthouse Lab in Cambridge and Alex Alderton, Roberto Amato, Sonia Goncalves, Ewan Harrison, David K. Jackson, Ian Johnston, Dominic Kwiatkowski, Cordelia Langford, John Sillitoe on behalf of the Wellcome Sanger Institute COVID-19 Surveillance Team
EPI_ISL_821382, EPI_ISL_821384	Lighthouse Lab in Alderley Park	Wellcome Sanger Institute for the COVID-19 Genomics UK (COG-UK) Consortium	Jacquelyn Wynn, Mairead Hyland, The Lighthouse Lab in Alderley Park and Alex Alderton, Roberto Amato, Sonia Goncalves, Ewan Harrison, David K. Jackson, Ian Johnston, Dominic Kwiatkowski, Cordelia Langford, John Sillitoe on behalf of the Wellcome Sanger Institute COVID-19 Surveillance Team
EPI_ISL_821385	Lighthouse Lab in Milton Keynes	Wellcome Sanger Institute for the COVID-19 Genomics UK (COG-UK) Consortium	The Lighthouse Lab in Milton Keynes and Alex Alderton, Roberto Amato, Sonia Goncalves, Ewan Harrison, David K. Jackson, Ian Johnston, Dominic Kwiatkowski, Cordelia Langford, John Sillitoe on behalf of the Wellcome Sanger Institute COVID-19 Surveillance Team
EPI_ISL_821392, EPI_ISL_821393	Lighthouse Lab in Cambridge	Wellcome Sanger Institute for the COVID-19 Genomics UK (COG-UK) Consortium	Rob Howes, The Lighthouse Lab in Cambridge and Alex Alderton, Roberto Amato, Sonia Goncalves, Ewan Harrison, David K. Jackson, Ian Johnston, Dominic Kwiatkowski, Cordelia Langford, John Sillitoe on behalf of the Wellcome Sanger Institute COVID-19 Surveillance Team
EPI_ISL_821396	Lighthouse Lab in Milton Keynes	Wellcome Sanger Institute for the COVID-19 Genomics UK (COG-UK) Consortium	The Lighthouse Lab in Milton Keynes and Alex Alderton, Roberto Amato, Sonia Goncalves, Ewan Harrison, David K. Jackson, Ian Johnston, Dominic Kwiatkowski, Cordelia Langford, John Sillitoe on behalf of the Wellcome Sanger Institute COVID-19 Surveillance Team
EPI_ISL_821399	Lighthouse Lab in Cambridge	Wellcome Sanger Institute for the COVID-19 Genomics UK (COG-UK) Consortium	Rob Howes, The Lighthouse Lab in Cambridge and Alex Alderton, Roberto Amato, Sonia Goncalves, Ewan Harrison, David K. Jackson, Ian Johnston, Dominic Kwiatkowski, Cordelia Langford, John Sillitoe on behalf of the Wellcome Sanger Institute COVID-19 Surveillance Team
EPI_ISL_821406, EPI_ISL_821407	Lighthouse Lab in Milton Keynes	Wellcome Sanger Institute for the COVID-19 Genomics UK (COG-UK) Consortium	The Lighthouse Lab in Milton Keynes and Alex Alderton, Roberto Amato, Sonia Goncalves, Ewan Harrison, David K. Jackson, Ian Johnston, Dominic Kwiatkowski, Cordelia Langford, John Sillitoe on behalf of the Wellcome Sanger Institute COVID-19 Surveillance Team

EPI_ISL_821412	Lighthouse Lab in Alderley Park	Wellcome Sanger Institute for the COVID-19 Genomics UK (COG-UK) Consortium	Jacquelyn Wynn, Mairead Hyland, The Lighthouse Lab in Alderley Park and Alex Alderton, Roberto Amato, Sonia Goncalves, Ewan Harrison, David K. Jackson, Ian Johnston, Dominic Kwiatkowski, Cordelia Langford, John Sillitoe on behalf of the Wellcome Sanger Institute COVID-19 Surveillance Team
EPI_ISL_821413	Lighthouse Lab in Milton Keynes	Wellcome Sanger Institute for the COVID-19 Genomics UK (COG-UK) Consortium	The Lighthouse Lab in Milton Keynes and Alex Alderton, Roberto Amato, Sonia Goncalves, Ewan Harrison, David K. Jackson, Ian Johnston, Dominic Kwiatkowski, Cordelia Langford, John Sillitoe on behalf of the Wellcome Sanger Institute COVID-19 Surveillance Team
EPI_ISL_821414, EPI_ISL_821415	Lighthouse Lab in Alderley Park	Wellcome Sanger Institute for the COVID-19 Genomics UK (COG-UK) Consortium	Jacquelyn Wynn, Mairead Hyland, The Lighthouse Lab in Alderley Park and Alex Alderton, Roberto Amato, Sonia Goncalves, Ewan Harrison, David K. Jackson, Ian Johnston, Dominic Kwiatkowski, Cordelia Langford, John Sillitoe on behalf of the Wellcome Sanger Institute COVID-19 Surveillance Team
EPI_ISL_821417, EPI_ISL_821420	Lighthouse Lab in Milton Keynes	Wellcome Sanger Institute for the COVID-19 Genomics UK (COG-UK) Consortium	The Lighthouse Lab in Milton Keynes and Alex Alderton, Roberto Amato, Sonia Goncalves, Ewan Harrison, David K. Jackson, Ian Johnston, Dominic Kwiatkowski, Cordelia Langford, John Sillitoe on behalf of the Wellcome Sanger Institute COVID-19 Surveillance Team
EPI_ISL_821421	Lighthouse Lab in Cambridge	Wellcome Sanger Institute for the COVID-19 Genomics UK (COG-UK) Consortium	Rob Howes, The Lighthouse Lab in Cambridge and Alex Alderton, Roberto Amato, Sonia Goncalves, Ewan Harrison, David K. Jackson, Ian Johnston, Dominic Kwiatkowski, Cordelia Langford, John Sillitoe on behalf of the Wellcome Sanger Institute COVID-19 Surveillance Team
EPI_ISL_821422, EPI_ISL_821423, EPI_ISL_821424	Lighthouse Lab in Milton Keynes	Wellcome Sanger Institute for the COVID-19 Genomics UK (COG-UK) Consortium	The Lighthouse Lab in Milton Keynes and Alex Alderton, Roberto Amato, Sonia Goncalves, Ewan Harrison, David K. Jackson, Ian Johnston, Dominic Kwiatkowski, Cordelia Langford, John Sillitoe on behalf of the Wellcome Sanger Institute COVID-19 Surveillance Team
EPI_ISL_821427	Lighthouse Lab in Cambridge	Wellcome Sanger Institute for the COVID-19 Genomics UK (COG-UK) Consortium	Rob Howes, The Lighthouse Lab in Cambridge and Alex Alderton, Roberto Amato, Sonia Goncalves, Ewan Harrison, David K. Jackson, Ian Johnston, Dominic Kwiatkowski, Cordelia Langford, John Sillitoe on behalf of the Wellcome Sanger Institute COVID-19 Surveillance Team
EPI_ISL_821428, EPI_ISL_821429, EPI_ISL_821430	Lighthouse Lab in Milton Keynes	Wellcome Sanger Institute for the COVID-19 Genomics UK (COG-UK) Consortium	The Lighthouse Lab in Milton Keynes and Alex Alderton, Roberto Amato, Sonia Goncalves, Ewan Harrison, David K. Jackson, Ian Johnston, Dominic Kwiatkowski, Cordelia Langford, John Sillitoe on behalf of the Wellcome Sanger Institute COVID-19 Surveillance Team
EPI_ISL_821433, EPI_ISL_821436, EPI_ISL_821437	Lighthouse Lab in Alderley Park	Wellcome Sanger Institute for the COVID-19 Genomics UK (COG-UK) Consortium	Jacquelyn Wynn, Mairead Hyland, The Lighthouse Lab in Alderley Park and Alex Alderton, Roberto Amato, Sonia Goncalves, Ewan Harrison, David K. Jackson, Ian Johnston, Dominic Kwiatkowski, Cordelia Langford, John Sillitoe on behalf of the Wellcome Sanger Institute COVID-19 Surveillance Team
EPI_ISL_821438	Lighthouse Lab in Milton Keynes	Wellcome Sanger Institute for the COVID-19 Genomics UK (COG-UK) Consortium	The Lighthouse Lab in Milton Keynes and Alex Alderton, Roberto Amato, Sonia Goncalves, Ewan Harrison, David K. Jackson, Ian Johnston, Dominic Kwiatkowski, Cordelia Langford, John Sillitoe on behalf of the Wellcome Sanger Institute COVID-19 Surveillance Team
EPI_ISL_821441, EPI_ISL_821442, EPI_ISL_821444	Lighthouse Lab in Cambridge	Wellcome Sanger Institute for the COVID-19 Genomics UK (COG-UK) Consortium	Rob Howes, The Lighthouse Lab in Cambridge and Alex Alderton, Roberto Amato, Sonia Goncalves, Ewan Harrison, David K. Jackson, Ian Johnston, Dominic Kwiatkowski, Cordelia Langford, John Sillitoe on behalf of the Wellcome Sanger Institute COVID-19 Surveillance Team
EPI_ISL_821446	Lighthouse Lab in Alderley Park	Wellcome Sanger Institute for the COVID-19 Genomics UK (COG-UK) Consortium	Jacquelyn Wynn, Mairead Hyland, The Lighthouse Lab in Alderley Park and Alex Alderton, Roberto Amato, Sonia Goncalves, Ewan Harrison, David K. Jackson, Ian Johnston, Dominic Kwiatkowski, Cordelia Langford, John Sillitoe on behalf of the Wellcome Sanger Institute COVID-19 Surveillance Team
EPI_ISL_821447	Lighthouse Lab in Cambridge	Wellcome Sanger Institute for the COVID-19 Genomics UK (COG-UK) Consortium	Rob Howes, The Lighthouse Lab in Cambridge and Alex Alderton, Roberto Amato, Sonia Goncalves, Ewan Harrison, David K. Jackson, Ian Johnston, Dominic Kwiatkowski, Cordelia Langford, John Sillitoe on behalf of the Wellcome Sanger Institute COVID-19 Surveillance Team
EPI_ISL_821450, EPI_ISL_821452	Lighthouse Lab in Milton Keynes	Wellcome Sanger Institute for the COVID-19 Genomics UK (COG-UK) Consortium	The Lighthouse Lab in Milton Keynes and Alex Alderton, Roberto Amato, Sonia Goncalves, Ewan Harrison, David K. Jackson, Ian Johnston, Dominic Kwiatkowski, Cordelia Langford, John Sillitoe on behalf of the Wellcome Sanger Institute COVID-19 Surveillance Team
EPI_ISL_821454	Lighthouse Lab in Cambridge	Wellcome Sanger Institute for the COVID-19 Genomics UK (COG-UK) Consortium	Rob Howes, The Lighthouse Lab in Cambridge and Alex Alderton, Roberto Amato, Sonia Goncalves, Ewan Harrison, David K. Jackson, Ian Johnston, Dominic Kwiatkowski, Cordelia Langford, John Sillitoe on behalf of the Wellcome Sanger Institute COVID-19 Surveillance Team
EPI_ISL_821455	Lighthouse Lab in Milton Keynes	Wellcome Sanger Institute for the COVID-19 Genomics UK (COG-UK) Consortium	The Lighthouse Lab in Milton Keynes and Alex Alderton, Roberto Amato, Sonia Goncalves, Ewan Harrison, David K. Jackson, Ian Johnston, Dominic Kwiatkowski, Cordelia Langford, John Sillitoe on behalf of the Wellcome Sanger Institute COVID-19 Surveillance Team
EPI_ISL_821456	Lighthouse Lab in Alderley Park	Wellcome Sanger Institute for the COVID-19 Genomics UK (COG-UK) Consortium	Jacquelyn Wynn, Mairead Hyland, The Lighthouse Lab in Alderley Park and Alex Alderton, Roberto Amato, Sonia Goncalves, Ewan Harrison, David K. Jackson, Ian Johnston, Dominic Kwiatkowski, Cordelia Langford, John Sillitoe on behalf of the Wellcome Sanger Institute COVID-19 Surveillance Team
EPI_ISL_821457, EPI_ISL_821458	Lighthouse Lab in Milton Keynes	Wellcome Sanger Institute for the COVID-19 Genomics UK (COG-UK) Consortium	The Lighthouse Lab in Milton Keynes and Alex Alderton, Roberto Amato, Sonia Goncalves, Ewan Harrison, David K. Jackson, Ian Johnston, Dominic Kwiatkowski, Cordelia Langford, John Sillitoe on behalf of the Wellcome Sanger Institute COVID-19 Surveillance Team
EPI_ISL_821463	Lighthouse Lab in Cambridge	Wellcome Sanger Institute for the COVID-19 Genomics UK (COG-UK) Consortium	Rob Howes, The Lighthouse Lab in Cambridge and Alex Alderton, Roberto Amato, Sonia Goncalves, Ewan Harrison, David K. Jackson, Ian Johnston, Dominic Kwiatkowski, Cordelia Langford, John Sillitoe on behalf of the Wellcome Sanger Institute COVID-19 Surveillance Team
EPI_ISL_821464, EPI_ISL_821467	Lighthouse Lab in Milton Keynes	Wellcome Sanger Institute for the COVID-19 Genomics UK (COG-UK) Consortium	The Lighthouse Lab in Milton Keynes and Alex Alderton, Roberto Amato, Sonia Goncalves, Ewan Harrison, David K. Jackson, Ian Johnston, Dominic Kwiatkowski, Cordelia Langford, John Sillitoe on behalf of the Wellcome Sanger Institute COVID-19 Surveillance Team
EPI_ISL_821469	Lighthouse Lab in Alderley Park	Wellcome Sanger Institute for the COVID-19 Genomics UK (COG-UK) Consortium	Jacquelyn Wynn, Mairead Hyland, The Lighthouse Lab in Alderley Park and Alex Alderton, Roberto Amato, Sonia Goncalves, Ewan Harrison, David K. Jackson, Ian Johnston, Dominic Kwiatkowski, Cordelia Langford, John Sillitoe on behalf of the Wellcome Sanger Institute COVID-19 Surveillance Team
EPI_ISL_821471	Lighthouse Lab in Milton Keynes	Wellcome Sanger Institute for the COVID-19 Genomics UK (COG-UK) Consortium	The Lighthouse Lab in Milton Keynes and Alex Alderton, Roberto Amato, Sonia Goncalves, Ewan Harrison, David K. Jackson, Ian Johnston, Dominic Kwiatkowski, Cordelia Langford, John Sillitoe on behalf of the Wellcome Sanger Institute COVID-19 Surveillance Team
EPI_ISL_821473, EPI_ISL_821479	Lighthouse Lab in Cambridge	Wellcome Sanger Institute for the COVID-19 Genomics UK (COG-UK) Consortium	Rob Howes, The Lighthouse Lab in Cambridge and Alex Alderton, Roberto Amato, Sonia Goncalves, Ewan Harrison, David K. Jackson, Ian Johnston, Dominic Kwiatkowski, Cordelia Langford, John Sillitoe on behalf of the Wellcome Sanger Institute COVID-19 Surveillance Team
EPI_ISL_821481	Lighthouse Lab in Milton Keynes	Wellcome Sanger Institute for the COVID-19 Genomics UK (COG-UK) Consortium	The Lighthouse Lab in Milton Keynes and Alex Alderton, Roberto Amato, Sonia Goncalves, Ewan Harrison, David K. Jackson, Ian Johnston, Dominic Kwiatkowski, Cordelia Langford, John Sillitoe on behalf of the Wellcome Sanger Institute COVID-19 Surveillance Team
EPI_ISL_821482, EPI_ISL_821483	Lighthouse Lab in Alderley Park	Wellcome Sanger Institute for the COVID-19 Genomics UK (COG-UK) Consortium	Jacquelyn Wynn, Mairead Hyland, The Lighthouse Lab in Alderley Park and Alex Alderton, Roberto Amato, Sonia Goncalves, Ewan Harrison, David K. Jackson, Ian Johnston, Dominic Kwiatkowski, Cordelia Langford, John Sillitoe on behalf of the Wellcome Sanger Institute COVID-19 Surveillance Team
EPI_ISL_821485	Lighthouse Lab in Cambridge	Wellcome Sanger Institute for the COVID-19 Genomics UK (COG-UK) Consortium	Rob Howes, The Lighthouse Lab in Cambridge and Alex Alderton, Roberto Amato, Sonia Goncalves, Ewan Harrison, David K. Jackson, Ian Johnston, Dominic Kwiatkowski, Cordelia Langford, John Sillitoe on behalf of the Wellcome Sanger Institute COVID-19 Surveillance Team
EPI_ISL_821487, EPI_ISL_821489	Lighthouse Lab in Milton Keynes	Wellcome Sanger Institute for the COVID-19 Genomics UK (COG-UK) Consortium	The Lighthouse Lab in Milton Keynes and Alex Alderton, Roberto Amato, Sonia Goncalves, Ewan Harrison, David K. Jackson, Ian Johnston, Dominic Kwiatkowski, Cordelia Langford, John Sillitoe on behalf of the Wellcome Sanger Institute COVID-19 Surveillance Team
EPI_ISL_821490	Lighthouse Lab in Alderley Park	Wellcome Sanger Institute for the COVID-19 Genomics UK (COG-UK) Consortium	Jacquelyn Wynn, Mairead Hyland, The Lighthouse Lab in Alderley Park and Alex Alderton, Roberto Amato, Sonia Goncalves, Ewan Harrison, David K. Jackson, Ian Johnston, Dominic Kwiatkowski, Cordelia Langford, John Sillitoe on behalf of the Wellcome Sanger Institute COVID-19 Surveillance Team
EPI_ISL_821492	Lighthouse Lab in Cambridge	Wellcome Sanger Institute for the COVID-19 Genomics UK (COG-UK) Consortium	Rob Howes, The Lighthouse Lab in Cambridge and Alex Alderton, Roberto Amato, Sonia Goncalves, Ewan Harrison, David K. Jackson, Ian Johnston, Dominic Kwiatkowski, Cordelia Langford, John Sillitoe on behalf of the Wellcome Sanger Institute COVID-19 Surveillance Team
EPI_ISL_821493	Lighthouse Lab in Alderley Park	Wellcome Sanger Institute for the COVID-19 Genomics UK (COG-UK) Consortium	Jacquelyn Wynn, Mairead Hyland, The Lighthouse Lab in Alderley Park and Alex Alderton, Roberto Amato, Sonia Goncalves, Ewan Harrison, David K. Jackson, Ian Johnston, Dominic Kwiatkowski, Cordelia Langford, John Sillitoe on behalf of the Wellcome Sanger Institute COVID-19 Surveillance Team
EPI_ISL_821494, EPI_ISL_821495	Lighthouse Lab in Milton Keynes	Wellcome Sanger Institute for the COVID-19 Genomics UK (COG-UK) Consortium	The Lighthouse Lab in Milton Keynes and Alex Alderton, Roberto Amato, Sonia Goncalves, Ewan Harrison, David K. Jackson, Ian Johnston, Dominic Kwiatkowski, Cordelia Langford, John Sillitoe on behalf of the Wellcome Sanger Institute COVID-19 Surveillance Team
EPI_ISL_821496	Lighthouse Lab in Alderley Park	Wellcome Sanger Institute for the COVID-19 Genomics UK (COG-UK) Consortium	Jacquelyn Wynn, Mairead Hyland, The Lighthouse Lab in Alderley Park and Alex Alderton, Roberto Amato, Sonia Goncalves, Ewan Harrison, David K. Jackson, Ian Johnston, Dominic Kwiatkowski, Cordelia Langford, John Sillitoe on behalf of the Wellcome Sanger Institute COVID-19 Surveillance Team
EPI_ISL_821499	Lighthouse Lab in Milton Keynes	Wellcome Sanger Institute for the COVID-19 Genomics UK (COG-UK) Consortium	The Lighthouse Lab in Milton Keynes and Alex Alderton, Roberto Amato, Sonia Goncalves, Ewan Harrison, David K. Jackson, Ian Johnston, Dominic Kwiatkowski, Cordelia Langford, John Sillitoe on behalf of the Wellcome Sanger Institute COVID-19 Surveillance Team
EPI_ISL_821500, EPI_ISL_821503	Lighthouse Lab in Alderley Park	Wellcome Sanger Institute for the COVID-19 Genomics UK (COG-UK) Consortium	Jacquelyn Wynn, Mairead Hyland, The Lighthouse Lab in Alderley Park and Alex Alderton, Roberto Amato, Sonia Goncalves, Ewan Harrison, David K. Jackson, Ian Johnston, Dominic Kwiatkowski, Cordelia Langford, John Sillitoe on behalf of the Wellcome Sanger Institute COVID-19 Surveillance Team
EPI_ISL_821505	Lighthouse Lab in Milton Keynes	Wellcome Sanger Institute for the COVID-19 Genomics UK (COG-UK) Consortium	The Lighthouse Lab in Milton Keynes and Alex Alderton, Roberto Amato, Sonia Goncalves, Ewan Harrison, David K. Jackson, Ian Johnston, Dominic Kwiatkowski, Cordelia Langford, John Sillitoe on behalf of the Wellcome Sanger Institute COVID-19 Surveillance Team

EPI_ISL_821506	Lighthouse Lab in Cambridge	Wellcome Sanger Institute for the COVID-19 Genomics UK (COG-UK) Consortium	Rob Howes, The Lighthouse Lab in Cambridge and Alex Alderton, Roberto Amato, Sonia Goncalves, Ewan Harrison, David K. Jackson, Ian Johnston, Dominic Kwiatkowski, Cordelia Langford, John Sillitoe on behalf of the Wellcome Sanger Institute COVID-19 Surveillance Team
EPI_ISL_821508, EPI_ISL_821512	Lighthouse Lab in Milton Keynes	Wellcome Sanger Institute for the COVID-19 Genomics UK (COG-UK) Consortium	The Lighthouse Lab in Milton Keynes and Alex Alderton, Roberto Amato, Sonia Goncalves, Ewan Harrison, David K. Jackson, Ian Johnston, Dominic Kwiatkowski, Cordelia Langford, John Sillitoe on behalf of the Wellcome Sanger Institute COVID-19 Surveillance Team
EPI_ISL_821513	Lighthouse Lab in Cambridge	Wellcome Sanger Institute for the COVID-19 Genomics UK (COG-UK) Consortium	Rob Howes, The Lighthouse Lab in Cambridge and Alex Alderton, Roberto Amato, Sonia Goncalves, Ewan Harrison, David K. Jackson, Ian Johnston, Dominic Kwiatkowski, Cordelia Langford, John Sillitoe on behalf of the Wellcome Sanger Institute COVID-19 Surveillance Team
EPI_ISL_821525, EPI_ISL_821526, EPI_ISL_821528	Lighthouse Lab in Alderley Park	Wellcome Sanger Institute for the COVID-19 Genomics UK (COG-UK) Consortium	Jacquelyn Wynn, Mairead Hyland, The Lighthouse Lab in Alderley Park and Alex Alderton, Roberto Amato, Sonia Goncalves, Ewan Harrison, David K. Jackson, Ian Johnston, Dominic Kwiatkowski, Cordelia Langford, John Sillitoe on behalf of the Wellcome Sanger Institute COVID-19 Surveillance Team
EPI_ISL_821530, EPI_ISL_821531, EPI_ISL_821532	Lighthouse Lab in Milton Keynes	Wellcome Sanger Institute for the COVID-19 Genomics UK (COG-UK) Consortium	The Lighthouse Lab in Milton Keynes and Alex Alderton, Roberto Amato, Sonia Goncalves, Ewan Harrison, David K. Jackson, Ian Johnston, Dominic Kwiatkowski, Cordelia Langford, John Sillitoe on behalf of the Wellcome Sanger Institute COVID-19 Surveillance Team
EPI_ISL_821534	Lighthouse Lab in Alderley Park	Wellcome Sanger Institute for the COVID-19 Genomics UK (COG-UK) Consortium	Jacquelyn Wynn, Mairead Hyland, The Lighthouse Lab in Alderley Park and Alex Alderton, Roberto Amato, Sonia Goncalves, Ewan Harrison, David K. Jackson, Ian Johnston, Dominic Kwiatkowski, Cordelia Langford, John Sillitoe on behalf of the Wellcome Sanger Institute COVID-19 Surveillance Team
EPI_ISL_821535	Lighthouse Lab in Milton Keynes	Wellcome Sanger Institute for the COVID-19 Genomics UK (COG-UK) Consortium	The Lighthouse Lab in Milton Keynes and Alex Alderton, Roberto Amato, Sonia Goncalves, Ewan Harrison, David K. Jackson, Ian Johnston, Dominic Kwiatkowski, Cordelia Langford, John Sillitoe on behalf of the Wellcome Sanger Institute COVID-19 Surveillance Team
EPI_ISL_821544, EPI_ISL_821546, EPI_ISL_821549	Lighthouse Lab in Cambridge	Wellcome Sanger Institute for the COVID-19 Genomics UK (COG-UK) Consortium	Rob Howes, The Lighthouse Lab in Cambridge and Alex Alderton, Roberto Amato, Sonia Goncalves, Ewan Harrison, David K. Jackson, Ian Johnston, Dominic Kwiatkowski, Cordelia Langford, John Sillitoe on behalf of the Wellcome Sanger Institute COVID-19 Surveillance Team
EPI_ISL_821550, EPI_ISL_821551	Lighthouse Lab in Alderley Park	Wellcome Sanger Institute for the COVID-19 Genomics UK (COG-UK) Consortium	Jacquelyn Wynn, Mairead Hyland, The Lighthouse Lab in Alderley Park and Alex Alderton, Roberto Amato, Sonia Goncalves, Ewan Harrison, David K. Jackson, Ian Johnston, Dominic Kwiatkowski, Cordelia Langford, John Sillitoe on behalf of the Wellcome Sanger Institute COVID-19 Surveillance Team
EPI_ISL_821554	Lighthouse Lab in Milton Keynes	Wellcome Sanger Institute for the COVID-19 Genomics UK (COG-UK) Consortium	The Lighthouse Lab in Milton Keynes and Alex Alderton, Roberto Amato, Sonia Goncalves, Ewan Harrison, David K. Jackson, Ian Johnston, Dominic Kwiatkowski, Cordelia Langford, John Sillitoe on behalf of the Wellcome Sanger Institute COVID-19 Surveillance Team
EPI_ISL_821556	Lighthouse Lab in Alderley Park	Wellcome Sanger Institute for the COVID-19 Genomics UK (COG-UK) Consortium	Jacquelyn Wynn, Mairead Hyland, The Lighthouse Lab in Alderley Park and Alex Alderton, Roberto Amato, Sonia Goncalves, Ewan Harrison, David K. Jackson, Ian Johnston, Dominic Kwiatkowski, Cordelia Langford, John Sillitoe on behalf of the Wellcome Sanger Institute COVID-19 Surveillance Team
EPI_ISL_821557	Lighthouse Lab in Milton Keynes	Wellcome Sanger Institute for the COVID-19 Genomics UK (COG-UK) Consortium	The Lighthouse Lab in Milton Keynes and Alex Alderton, Roberto Amato, Sonia Goncalves, Ewan Harrison, David K. Jackson, Ian Johnston, Dominic Kwiatkowski, Cordelia Langford, John Sillitoe on behalf of the Wellcome Sanger Institute COVID-19 Surveillance Team
EPI_ISL_821563, EPI_ISL_821564	Lighthouse Lab in Cambridge	Wellcome Sanger Institute for the COVID-19 Genomics UK (COG-UK) Consortium	Rob Howes, The Lighthouse Lab in Cambridge and Alex Alderton, Roberto Amato, Sonia Goncalves, Ewan Harrison, David K. Jackson, Ian Johnston, Dominic Kwiatkowski, Cordelia Langford, John Sillitoe on behalf of the Wellcome Sanger Institute COVID-19 Surveillance Team
EPI_ISL_821567	Lighthouse Lab in Milton Keynes	Wellcome Sanger Institute for the COVID-19 Genomics UK (COG-UK) Consortium	The Lighthouse Lab in Milton Keynes and Alex Alderton, Roberto Amato, Sonia Goncalves, Ewan Harrison, David K. Jackson, Ian Johnston, Dominic Kwiatkowski, Cordelia Langford, John Sillitoe on behalf of the Wellcome Sanger Institute COVID-19 Surveillance Team
EPI_ISL_821577	Lighthouse Lab in Cambridge	Wellcome Sanger Institute for the COVID-19 Genomics UK (COG-UK) Consortium	Rob Howes, The Lighthouse Lab in Cambridge and Alex Alderton, Roberto Amato, Sonia Goncalves, Ewan Harrison, David K. Jackson, Ian Johnston, Dominic Kwiatkowski, Cordelia Langford, John Sillitoe on behalf of the Wellcome Sanger Institute COVID-19 Surveillance Team
EPI_ISL_821578	Lighthouse Lab in Milton Keynes	Wellcome Sanger Institute for the COVID-19 Genomics UK (COG-UK) Consortium	The Lighthouse Lab in Milton Keynes and Alex Alderton, Roberto Amato, Sonia Goncalves, Ewan Harrison, David K. Jackson, Ian Johnston, Dominic Kwiatkowski, Cordelia Langford, John Sillitoe on behalf of the Wellcome Sanger Institute COVID-19 Surveillance Team
EPI_ISL_821579	Lighthouse Lab in Cambridge	Wellcome Sanger Institute for the COVID-19 Genomics UK (COG-UK) Consortium	Rob Howes, The Lighthouse Lab in Cambridge and Alex Alderton, Roberto Amato, Sonia Goncalves, Ewan Harrison, David K. Jackson, Ian Johnston, Dominic Kwiatkowski, Cordelia Langford, John Sillitoe on behalf of the Wellcome Sanger Institute COVID-19 Surveillance Team
EPI_ISL_821581	Lighthouse Lab in Milton Keynes	Wellcome Sanger Institute for the COVID-19 Genomics UK (COG-UK) Consortium	The Lighthouse Lab in Milton Keynes and Alex Alderton, Roberto Amato, Sonia Goncalves, Ewan Harrison, David K. Jackson, Ian Johnston, Dominic Kwiatkowski, Cordelia Langford, John Sillitoe on behalf of the Wellcome Sanger Institute COVID-19 Surveillance Team
EPI_ISL_821583	Lighthouse Lab in Cambridge	Wellcome Sanger Institute for the COVID-19 Genomics UK (COG-UK) Consortium	Rob Howes, The Lighthouse Lab in Cambridge and Alex Alderton, Roberto Amato, Sonia Goncalves, Ewan Harrison, David K. Jackson, Ian Johnston, Dominic Kwiatkowski, Cordelia Langford, John Sillitoe on behalf of the Wellcome Sanger Institute COVID-19 Surveillance Team
EPI_ISL_821587, EPI_ISL_821589	Lighthouse Lab in Milton Keynes	Wellcome Sanger Institute for the COVID-19 Genomics UK (COG-UK) Consortium	The Lighthouse Lab in Milton Keynes and Alex Alderton, Roberto Amato, Sonia Goncalves, Ewan Harrison, David K. Jackson, Ian Johnston, Dominic Kwiatkowski, Cordelia Langford, John Sillitoe on behalf of the Wellcome Sanger Institute COVID-19 Surveillance Team
EPI_ISL_821590	Lighthouse Lab in Cambridge	Wellcome Sanger Institute for the COVID-19 Genomics UK (COG-UK) Consortium	Rob Howes, The Lighthouse Lab in Cambridge and Alex Alderton, Roberto Amato, Sonia Goncalves, Ewan Harrison, David K. Jackson, Ian Johnston, Dominic Kwiatkowski, Cordelia Langford, John Sillitoe on behalf of the Wellcome Sanger Institute COVID-19 Surveillance Team
EPI_ISL_821591, EPI_ISL_821594, EPI_ISL_821598	Lighthouse Lab in Milton Keynes	Wellcome Sanger Institute for the COVID-19 Genomics UK (COG-UK) Consortium	The Lighthouse Lab in Milton Keynes and Alex Alderton, Roberto Amato, Sonia Goncalves, Ewan Harrison, David K. Jackson, Ian Johnston, Dominic Kwiatkowski, Cordelia Langford, John Sillitoe on behalf of the Wellcome Sanger Institute COVID-19 Surveillance Team
EPI_ISL_821600	Lighthouse Lab in Alderley Park	Wellcome Sanger Institute for the COVID-19 Genomics UK (COG-UK) Consortium	Jacquelyn Wynn, Mairead Hyland, The Lighthouse Lab in Alderley Park and Alex Alderton, Roberto Amato, Sonia Goncalves, Ewan Harrison, David K. Jackson, Ian Johnston, Dominic Kwiatkowski, Cordelia Langford, John Sillitoe on behalf of the Wellcome Sanger Institute COVID-19 Surveillance Team
EPI_ISL_821602, EPI_ISL_821604, EPI_ISL_821605	Lighthouse Lab in Cambridge	Wellcome Sanger Institute for the COVID-19 Genomics UK (COG-UK) Consortium	Rob Howes, The Lighthouse Lab in Cambridge and Alex Alderton, Roberto Amato, Sonia Goncalves, Ewan Harrison, David K. Jackson, Ian Johnston, Dominic Kwiatkowski, Cordelia Langford, John Sillitoe on behalf of the Wellcome Sanger Institute COVID-19 Surveillance Team
EPI_ISL_821606	Lighthouse Lab in Alderley Park	Wellcome Sanger Institute for the COVID-19 Genomics UK (COG-UK) Consortium	Jacquelyn Wynn, Mairead Hyland, The Lighthouse Lab in Alderley Park and Alex Alderton, Roberto Amato, Sonia Goncalves, Ewan Harrison, David K. Jackson, Ian Johnston, Dominic Kwiatkowski, Cordelia Langford, John Sillitoe on behalf of the Wellcome Sanger Institute COVID-19 Surveillance Team
EPI_ISL_821610, EPI_ISL_821611	Lighthouse Lab in Milton Keynes	Wellcome Sanger Institute for the COVID-19 Genomics UK (COG-UK) Consortium	The Lighthouse Lab in Milton Keynes and Alex Alderton, Roberto Amato, Sonia Goncalves, Ewan Harrison, David K. Jackson, Ian Johnston, Dominic Kwiatkowski, Cordelia Langford, John Sillitoe on behalf of the Wellcome Sanger Institute COVID-19 Surveillance Team
EPI_ISL_821613	Lighthouse Lab in Cambridge	Wellcome Sanger Institute for the COVID-19 Genomics UK (COG-UK) Consortium	Rob Howes, The Lighthouse Lab in Cambridge and Alex Alderton, Roberto Amato, Sonia Goncalves, Ewan Harrison, David K. Jackson, Ian Johnston, Dominic Kwiatkowski, Cordelia Langford, John Sillitoe on behalf of the Wellcome Sanger Institute COVID-19 Surveillance Team
EPI_ISL_821615	Lighthouse Lab in Milton Keynes	Wellcome Sanger Institute for the COVID-19 Genomics UK (COG-UK) Consortium	The Lighthouse Lab in Milton Keynes and Alex Alderton, Roberto Amato, Sonia Goncalves, Ewan Harrison, David K. Jackson, Ian Johnston, Dominic Kwiatkowski, Cordelia Langford, John Sillitoe on behalf of the Wellcome Sanger Institute COVID-19 Surveillance Team
EPI_ISL_821617	Lighthouse Lab in Alderley Park	Wellcome Sanger Institute for the COVID-19 Genomics UK (COG-UK) Consortium	Jacquelyn Wynn, Mairead Hyland, The Lighthouse Lab in Alderley Park and Alex Alderton, Roberto Amato, Sonia Goncalves, Ewan Harrison, David K. Jackson, Ian Johnston, Dominic Kwiatkowski, Cordelia Langford, John Sillitoe on behalf of the Wellcome Sanger Institute COVID-19 Surveillance Team
EPI_ISL_821619	Lighthouse Lab in Milton Keynes	Wellcome Sanger Institute for the COVID-19 Genomics UK (COG-UK) Consortium	The Lighthouse Lab in Milton Keynes and Alex Alderton, Roberto Amato, Sonia Goncalves, Ewan Harrison, David K. Jackson, Ian Johnston, Dominic Kwiatkowski, Cordelia Langford, John Sillitoe on behalf of the Wellcome Sanger Institute COVID-19 Surveillance Team
EPI_ISL_821626, EPI_ISL_821627, EPI_ISL_821628	Lighthouse Lab in Alderley Park	Wellcome Sanger Institute for the COVID-19 Genomics UK (COG-UK) Consortium	Jacquelyn Wynn, Mairead Hyland, The Lighthouse Lab in Alderley Park and Alex Alderton, Roberto Amato, Sonia Goncalves, Ewan Harrison, David K. Jackson, Ian Johnston, Dominic Kwiatkowski, Cordelia Langford, John Sillitoe on behalf of the Wellcome Sanger Institute COVID-19 Surveillance Team
EPI_ISL_821629, EPI_ISL_821630, EPI_ISL_821631, EPI_ISL_821632, EPI_ISL_821634, EPI_ISL_821635, EPI_ISL_821636, EPI_ISL_821638, EPI_ISL_821639, EPI_ISL_821640, EPI_ISL_821641, EPI_ISL_821642, EPI_ISL_821643, EPI_ISL_821644, EPI_ISL_821645, EPI_ISL_821646, EPI_ISL_821647, EPI_ISL_821648, EPI_ISL_821649, EPI_ISL_821650, EPI_ISL_821651, EPI_ISL_821652, EPI_ISL_821653, EPI_ISL_821654, EPI_ISL_821655, EPI_ISL_821656, EPI_ISL_821657, EPI_ISL_821658			
see above	Lighthouse Lab in Milton Keynes	Wellcome Sanger Institute for the COVID-19 Genomics UK (COG-UK) Consortium	The Lighthouse Lab in Milton Keynes and Alex Alderton, Roberto Amato, Sonia Goncalves, Ewan Harrison, David K. Jackson, Ian Johnston, Dominic Kwiatkowski, Cordelia Langford, John Sillitoe on behalf of the Wellcome Sanger Institute COVID-19 Surveillance Team
EPI_ISL_821659, EPI_ISL_821660	Lighthouse Lab in Glasgow	Wellcome Sanger Institute for the COVID-19 Genomics UK (COG-UK) Consortium	Harper VanSteenhouse, Yumi Kasai, David Gray, Carol Clugston, Anna Dominiczak and Alex Alderton, Roberto Amato, Sonia Goncalves, Ewan Harrison, David K. Jackson, Ian Johnston, Dominic Kwiatkowski, Cordelia Langford, John Sillitoe on behalf of the Wellcome Sanger Institute COVID-19 Surveillance Team
EPI_ISL_821661, EPI_ISL_821662, EPI_ISL_821663, EPI_ISL_821664, EPI_ISL_821665, EPI_ISL_821666, EPI_ISL_821667, EPI_ISL_821668, EPI_ISL_821669, EPI_ISL_821670, EPI_ISL_821671, EPI_ISL_821672, EPI_ISL_821673, EPI_ISL_821674, EPI_ISL_821676, EPI_ISL_821677, EPI_ISL_821678, EPI_ISL_821679, EPI_ISL_821680, EPI_ISL_821681, EPI_ISL_821682, EPI_ISL_821683, EPI_ISL_821684, EPI_ISL_821685, EPI_ISL_821686, EPI_ISL_821687, EPI_ISL_821688, EPI_ISL_821689, EPI_ISL_821690, EPI_ISL_821691, EPI_ISL_821692, EPI_ISL_821693, EPI_ISL_821694, EPI_ISL_821695, EPI_ISL_821696, EPI_ISL_821697,			

EPI_ISL_821698, EPI_ISL_821699, EPI_ISL_821700, EPI_ISL_821701, EPI_ISL_821702, EPI_ISL_821703, EPI_ISL_821704, EPI_ISL_821705, EPI_ISL_821706, EPI_ISL_821707, EPI_ISL_821708, EPI_ISL_821709, EPI_ISL_821710, EPI_ISL_821711, EPI_ISL_821712, EPI_ISL_821713, EPI_ISL_821714, EPI_ISL_821715, EPI_ISL_821716, EPI_ISL_821717, EPI_ISL_821718, EPI_ISL_821719, EPI_ISL_821720, EPI_ISL_821721, EPI_ISL_821722, EPI_ISL_821723, EPI_ISL_821724, EPI_ISL_821725, EPI_ISL_821726, EPI_ISL_821727, EPI_ISL_821728, EPI_ISL_821729, EPI_ISL_821730, EPI_ISL_821731, EPI_ISL_821732, EPI_ISL_821733, EPI_ISL_821734, EPI_ISL_821735, EPI_ISL_821736, EPI_ISL_821737, EPI_ISL_821738, EPI_ISL_821739, EPI_ISL_821740, EPI_ISL_821741, EPI_ISL_821742, EPI_ISL_821743, EPI_ISL_821744, EPI_ISL_821745, EPI_ISL_821746, EPI_ISL_821747, EPI_ISL_821748, EPI_ISL_821749, EPI_ISL_821750, EPI_ISL_821751, EPI_ISL_821752, EPI_ISL_821753, EPI_ISL_821754, EPI_ISL_821755, EPI_ISL_821756, EPI_ISL_821757, EPI_ISL_821758, EPI_ISL_821759, EPI_ISL_821760, EPI_ISL_821761, EPI_ISL_821762, EPI_ISL_821763, EPI_ISL_821764, EPI_ISL_821765, EPI_ISL_821766, EPI_ISL_821767, EPI_ISL_821768, EPI_ISL_821769, EPI_ISL_821770, EPI_ISL_821771, EPI_ISL_821772, EPI_ISL_821773, EPI_ISL_821774, EPI_ISL_821775, EPI_ISL_821776, EPI_ISL_821777, EPI_ISL_821778, EPI_ISL_821779, EPI_ISL_821780, EPI_ISL_821781, EPI_ISL_821782, EPI_ISL_821783, EPI_ISL_821784, EPI_ISL_821785, EPI_ISL_821786, EPI_ISL_821787, EPI_ISL_821788, EPI_ISL_821789, EPI_ISL_821790, EPI_ISL_821791, EPI_ISL_821792, EPI_ISL_821793, EPI_ISL_821794, EPI_ISL_821795, EPI_ISL_821796, EPI_ISL_821797, EPI_ISL_821798, EPI_ISL_821799, EPI_ISL_821800, EPI_ISL_821801, EPI_ISL_821802, EPI_ISL_821803, EPI_ISL_821804, EPI_ISL_821805, EPI_ISL_821806, EPI_ISL_821807, EPI_ISL_821808, EPI_ISL_821809, EPI_ISL_821810, EPI_ISL_821811, EPI_ISL_821812, EPI_ISL_821813, EPI_ISL_821814, EPI_ISL_821815, EPI_ISL_821816, EPI_ISL_821817, EPI_ISL_821818, EPI_ISL_821819, EPI_ISL_821820, EPI_ISL_821821, EPI_ISL_821822, EPI_ISL_821823, EPI_ISL_821824, EPI_ISL_821825, EPI_ISL_821826, EPI_ISL_821827, EPI_ISL_821828, EPI_ISL_821829, EPI_ISL_821830, EPI_ISL_821831, EPI_ISL_821832, EPI_ISL_821833, EPI_ISL_821834, EPI_ISL_821835, EPI_ISL_821836, EPI_ISL_821837, EPI_ISL_821838, EPI_ISL_821839, EPI_ISL_821840, EPI_ISL_821841, EPI_ISL_821842, EPI_ISL_821843, EPI_ISL_821844, EPI_ISL_821845, EPI_ISL_821846, EPI_ISL_821847, EPI_ISL_821848, EPI_ISL_821849, EPI_ISL_821850, EPI_ISL_821851, EPI_ISL_821852, EPI_ISL_821853, EPI_ISL_821854, EPI_ISL_821855, EPI_ISL_821856, EPI_ISL_821857, EPI_ISL_821858, EPI_ISL_821859, EPI_ISL_821860, EPI_ISL_821861, EPI_ISL_821862, EPI_ISL_821863, EPI_ISL_821864, EPI_ISL_821865, EPI_ISL_821866, EPI_ISL_821867, EPI_ISL_821868, EPI_ISL_821869, EPI_ISL_821870, EPI_ISL_821871, EPI_ISL_821872, EPI_ISL_821873, EPI_ISL_821874, EPI_ISL_821875, EPI_ISL_821876, EPI_ISL_821877, EPI_ISL_821878, EPI_ISL_821879, EPI_ISL_821880, EPI_ISL_821881, EPI_ISL_821882, EPI_ISL_821883, EPI_ISL_821885, EPI_ISL_821886, EPI_ISL_821887, EPI_ISL_821888, EPI_ISL_821889, EPI_ISL_821890, EPI_ISL_821891, EPI_ISL_821892, EPI_ISL_821893, EPI_ISL_821894, EPI_ISL_821895, EPI_ISL_821896, EPI_ISL_821897, EPI_ISL_821898, EPI_ISL_821899, EPI_ISL_821900, EPI_ISL_821901, EPI_ISL_821902, EPI_ISL_821903, EPI_ISL_821904, EPI_ISL_821905, EPI_ISL_821906, EPI_ISL_821907, EPI_ISL_821908, EPI_ISL_821909, EPI_ISL_821910, EPI_ISL_821911, EPI_ISL_821912, EPI_ISL_821913, EPI_ISL_821914, EPI_ISL_821915, EPI_ISL_821916, EPI_ISL_821917, EPI_ISL_821918, EPI_ISL_821919, EPI_ISL_821920, EPI_ISL_821921, EPI_ISL_821922, EPI_ISL_821923, EPI_ISL_821924, EPI_ISL_821925, EPI_ISL_821926, EPI_ISL_821927, EPI_ISL_821928, EPI_ISL_821929, EPI_ISL_821930, EPI_ISL_821931, EPI_ISL_821932, EPI_ISL_821933, EPI_ISL_821934, EPI_ISL_821935, EPI_ISL_821936, EPI_ISL_821937, EPI_ISL_821938, EPI_ISL_821939, EPI_ISL_821940, EPI_ISL_821941, EPI_ISL_821942, EPI_ISL_821943, EPI_ISL_821944, EPI_ISL_821945, EPI_ISL_821946, EPI_ISL_821947, EPI_ISL_821948, EPI_ISL_821949, EPI_ISL_821950, EPI_ISL_821951, EPI_ISL_821952, EPI_ISL_821953, EPI_ISL_821954, EPI_ISL_821955, EPI_ISL_821956, EPI_ISL_821957, EPI_ISL_821958, EPI_ISL_821959, EPI_ISL_821960, EPI_ISL_821961, EPI_ISL_821962, EPI_ISL_821963, EPI_ISL_821964, EPI_ISL_821965, EPI_ISL_821966, EPI_ISL_821968, EPI_ISL_821969, EPI_ISL_821970			
see above	Lighthouse Lab in Cambridge	Wellcome Sanger Institute for the COVID-19 Genomics UK (COG-UK) Consortium	Rob Howes, The Lighthouse Lab in Cambridge and Alex Alderton, Roberto Amato, Sonia Goncalves, Ewan Harrison, David K. Jackson, Ian Johnston, Dominic Kwiatkowski, Cordelia Langford, John Sillitoe on behalf of the Wellcome Sanger Institute COVID-19 Surveillance Team
EPI_ISL_821971, EPI_ISL_821972, EPI_ISL_821973, EPI_ISL_821974, EPI_ISL_821976, EPI_ISL_821977, EPI_ISL_821978, EPI_ISL_821979, EPI_ISL_821980, EPI_ISL_821981, EPI_ISL_821982, EPI_ISL_821983, EPI_ISL_821984, EPI_ISL_821985, EPI_ISL_821986, EPI_ISL_821989, EPI_ISL_821990, EPI_ISL_821991, EPI_ISL_821992, EPI_ISL_821994, EPI_ISL_821996, EPI_ISL_821997, EPI_ISL_821998, EPI_ISL_821999, EPI_ISL_822000, EPI_ISL_822002, EPI_ISL_822003, EPI_ISL_822007, EPI_ISL_822008, EPI_ISL_822009, EPI_ISL_822010, EPI_ISL_822012, EPI_ISL_822013, EPI_ISL_822014, EPI_ISL_822015, EPI_ISL_822016, EPI_ISL_822017, EPI_ISL_822018, EPI_ISL_822019, EPI_ISL_822020, EPI_ISL_822021, EPI_ISL_822024, EPI_ISL_822025, EPI_ISL_822026, EPI_ISL_822027, EPI_ISL_822028, EPI_ISL_822030, EPI_ISL_822031, EPI_ISL_822032, EPI_ISL_822034, EPI_ISL_822036, EPI_ISL_822037, EPI_ISL_822038, EPI_ISL_822039, EPI_ISL_822042, EPI_ISL_822043, EPI_ISL_822045, EPI_ISL_822046, EPI_ISL_822047, EPI_ISL_822049, EPI_ISL_822050, EPI_ISL_822052, EPI_ISL_822054, EPI_ISL_822055, EPI_ISL_822056, EPI_ISL_822057, EPI_ISL_822058, EPI_ISL_822060, EPI_ISL_822061, EPI_ISL_822062, EPI_ISL_822064, EPI_ISL_822066, EPI_ISL_822068, EPI_ISL_822070, EPI_ISL_822071, EPI_ISL_822073, EPI_ISL_822074, EPI_ISL_822076, EPI_ISL_822077, EPI_ISL_822079, EPI_ISL_822081, EPI_ISL_822082, EPI_ISL_822084, EPI_ISL_822085, EPI_ISL_822087, EPI_ISL_822088, EPI_ISL_822089, EPI_ISL_822090, EPI_ISL_822091, EPI_ISL_822092, EPI_ISL_822094, EPI_ISL_822095, EPI_ISL_822096, EPI_ISL_822097, EPI_ISL_822100, EPI_ISL_822101, EPI_ISL_822102, EPI_ISL_822103, EPI_ISL_822104, EPI_ISL_822105, EPI_ISL_822106, EPI_ISL_822107, EPI_ISL_822108, EPI_ISL_822109, EPI_ISL_822110, EPI_ISL_822111, EPI_ISL_822112, EPI_ISL_822114, EPI_ISL_822115, EPI_ISL_822116, EPI_ISL_822118, EPI_ISL_822120, EPI_ISL_822121, EPI_ISL_822123, EPI_ISL_822124, EPI_ISL_822125, EPI_ISL_822126, EPI_ISL_822128, EPI_ISL_822129, EPI_ISL_822131, EPI_ISL_822132, EPI_ISL_822133, EPI_ISL_822134, EPI_ISL_822135, EPI_ISL_822136, EPI_ISL_822137, EPI_ISL_822138, EPI_ISL_822139, EPI_ISL_822141, EPI_ISL_822142, EPI_ISL_822143, EPI_ISL_822144, EPI_ISL_822145, EPI_ISL_822146, EPI_ISL_822147, EPI_ISL_822149, EPI_ISL_822150, EPI_ISL_822151, EPI_ISL_822152, EPI_ISL_822154, EPI_ISL_822155, EPI_ISL_822157, EPI_ISL_822160, EPI_ISL_822161, EPI_ISL_822162, EPI_ISL_822163, EPI_ISL_822164, EPI_ISL_822166, EPI_ISL_822167, EPI_ISL_822168, EPI_ISL_822171, EPI_ISL_822172, EPI_ISL_822173, EPI_ISL_822174, EPI_ISL_822175, EPI_ISL_822176, EPI_ISL_822177, EPI_ISL_822179, EPI_ISL_822180, EPI_ISL_822181, EPI_ISL_822183, EPI_ISL_822184, EPI_ISL_822185, EPI_ISL_822186, EPI_ISL_822187, EPI_ISL_822188, EPI_ISL_822189, EPI_ISL_822190, EPI_ISL_822191, EPI_ISL_822192, EPI_ISL_822194, EPI_ISL_822195, EPI_ISL_822196, EPI_ISL_822197, EPI_ISL_822198, EPI_ISL_822199, EPI_ISL_822200, EPI_ISL_822202, EPI_ISL_822203, EPI_ISL_822204, EPI_ISL_822205, EPI_ISL_822206, EPI_ISL_822207, EPI_ISL_822208, EPI_ISL_822210, EPI_ISL_822211, EPI_ISL_822212, EPI_ISL_822213, EPI_ISL_822215, EPI_ISL_822216, EPI_ISL_822217, EPI_ISL_822218, EPI_ISL_822219, EPI_ISL_822220, EPI_ISL_822221, EPI_ISL_822222, EPI_ISL_822224, EPI_ISL_822225, EPI_ISL_822226, EPI_ISL_822227, EPI_ISL_822228, EPI_ISL_822229, EPI_ISL_822231, EPI_ISL_822232, EPI_ISL_822233, EPI_ISL_822234, EPI_ISL_822235, EPI_ISL_822236, EPI_ISL_822238, EPI_ISL_822239, EPI_ISL_822241, EPI_ISL_822246, EPI_ISL_822248, EPI_ISL_822250, EPI_ISL_822251, EPI_ISL_822252, EPI_ISL_822254, EPI_ISL_822255, EPI_ISL_822257, EPI_ISL_822258, EPI_ISL_822259, EPI_ISL_822261, EPI_ISL_822262, EPI_ISL_822263, EPI_ISL_822266, EPI_ISL_822269, EPI_ISL_822270, EPI_ISL_822271, EPI_ISL_822273, EPI_ISL_822274, EPI_ISL_822276, EPI_ISL_822277, EPI_ISL_822278, EPI_ISL_822279, EPI_ISL_822280, EPI_ISL_822283, EPI_ISL_822284, EPI_ISL_822285, EPI_ISL_822286, EPI_ISL_822287, EPI_ISL_822289, EPI_ISL_822290, EPI_ISL_822291			
see above	Lighthouse Lab in Alderley Park	Wellcome Sanger Institute for the COVID-19 Genomics UK (COG-UK) Consortium	Jacquelyn Wynn, Mairead Hyland, The Lighthouse Lab in Alderley Park and Alex Alderton, Roberto Amato, Sonia Goncalves, Ewan Harrison, David K. Jackson, Ian Johnston, Dominic Kwiatkowski, Cordelia Langford, John Sillitoe on behalf of the Wellcome Sanger Institute COVID-19 Surveillance Team
EPI_ISL_822292, EPI_ISL_822293	Lighthouse Lab in Glasgow	Wellcome Sanger Institute for the COVID-19 Genomics UK (COG-UK) Consortium	Harper VanSteenhouse, Yumi Kasai, David Gray, Carol Clugston, Anna Dominiczak and Alex Alderton, Roberto Amato, Sonia Goncalves, Ewan Harrison, David K. Jackson, Ian Johnston, Dominic Kwiatkowski, Cordelia Langford, John Sillitoe on behalf of the Wellcome Sanger Institute COVID-19 Surveillance Team
EPI_ISL_822294	Lighthouse Lab in Alderley Park	Wellcome Sanger Institute for the COVID-19 Genomics UK (COG-UK) Consortium	Jacquelyn Wynn, Mairead Hyland, The Lighthouse Lab in Alderley Park and Alex Alderton, Roberto Amato, Sonia Goncalves, Ewan Harrison, David K. Jackson, Ian Johnston, Dominic Kwiatkowski, Cordelia Langford, John Sillitoe on behalf of the Wellcome Sanger Institute COVID-19 Surveillance Team
EPI_ISL_822295, EPI_ISL_822296	Lighthouse Lab in Glasgow	Wellcome Sanger Institute for the COVID-19 Genomics UK (COG-UK) Consortium	Harper VanSteenhouse, Yumi Kasai, David Gray, Carol Clugston, Anna Dominiczak and Alex Alderton, Roberto Amato, Sonia Goncalves, Ewan Harrison, David K. Jackson, Ian Johnston, Dominic Kwiatkowski, Cordelia Langford, John Sillitoe on behalf of the Wellcome Sanger Institute COVID-19 Surveillance Team
EPI_ISL_822297, EPI_ISL_822298	Lighthouse Lab in Alderley Park	Wellcome Sanger Institute for the COVID-19 Genomics UK (COG-UK) Consortium	Jacquelyn Wynn, Mairead Hyland, The Lighthouse Lab in Alderley Park and Alex Alderton, Roberto Amato, Sonia Goncalves, Ewan Harrison, David K. Jackson, Ian Johnston, Dominic Kwiatkowski, Cordelia Langford, John Sillitoe on behalf of the Wellcome Sanger Institute COVID-19 Surveillance Team
EPI_ISL_822302	Lighthouse Lab in Glasgow	Wellcome Sanger Institute for the COVID-19 Genomics UK (COG-UK) Consortium	Harper VanSteenhouse, Yumi Kasai, David Gray, Carol Clugston, Anna Dominiczak and Alex Alderton, Roberto Amato, Sonia Goncalves, Ewan Harrison, David K. Jackson, Ian Johnston, Dominic Kwiatkowski, Cordelia Langford, John Sillitoe on behalf of the Wellcome Sanger Institute COVID-19 Surveillance Team
EPI_ISL_822303	Lighthouse Lab in Alderley Park	Wellcome Sanger Institute for the COVID-19 Genomics UK (COG-UK) Consortium	Jacquelyn Wynn, Mairead Hyland, The Lighthouse Lab in Alderley Park and Alex Alderton, Roberto Amato, Sonia Goncalves, Ewan Harrison, David K. Jackson, Ian Johnston, Dominic Kwiatkowski, Cordelia Langford, John Sillitoe on behalf of the Wellcome Sanger Institute COVID-19 Surveillance Team
EPI_ISL_822304	Lighthouse Lab in Glasgow	Wellcome Sanger Institute for the COVID-19 Genomics UK (COG-UK) Consortium	Harper VanSteenhouse, Yumi Kasai, David Gray, Carol Clugston, Anna Dominiczak and Alex Alderton, Roberto Amato, Sonia Goncalves, Ewan Harrison, David K. Jackson, Ian Johnston, Dominic Kwiatkowski, Cordelia Langford, John Sillitoe on behalf of the Wellcome Sanger Institute COVID-19 Surveillance Team
EPI_ISL_822612, EPI_ISL_822613, EPI_ISL_822614, EPI_ISL_822615, EPI_ISL_822616, EPI_ISL_822617, EPI_ISL_822618, EPI_ISL_822619, EPI_ISL_822620, EPI_ISL_822621, EPI_ISL_822622, EPI_ISL_822623, EPI_ISL_822624, EPI_ISL_822625, EPI_ISL_822626, EPI_ISL_822627, EPI_ISL_822628, EPI_ISL_822629, EPI_ISL_822630, EPI_ISL_822631, EPI_ISL_822632, EPI_ISL_822633, EPI_ISL_822634, EPI_ISL_822635, EPI_ISL_822636, EPI_ISL_822637, EPI_ISL_822638, EPI_ISL_822639, EPI_ISL_822640, EPI_ISL_822641, EPI_ISL_822642, EPI_ISL_822643, EPI_ISL_822644, EPI_ISL_822645, EPI_ISL_822646, EPI_ISL_822647, EPI_ISL_822648, EPI_ISL_822649, EPI_ISL_822650, EPI_ISL_822651, EPI_ISL_822652, EPI_ISL_822653, EPI_ISL_822654, EPI_ISL_822655, EPI_ISL_822656, EPI_ISL_822657, EPI_ISL_822658, EPI_ISL_822659, EPI_ISL_822660, EPI_ISL_822661, EPI_ISL_822662, EPI_ISL_822663, EPI_ISL_822664, EPI_ISL_822665, EPI_ISL_822666, EPI_ISL_822667, EPI_ISL_822668, EPI_ISL_822669, EPI_ISL_822670, EPI_ISL_822671, EPI_ISL_822672, EPI_ISL_822673, EPI_ISL_822674, EPI_ISL_822675, EPI_ISL_822676, EPI_ISL_822677, EPI_ISL_822678, EPI_ISL_822679, EPI_ISL_822680, EPI_ISL_822681, EPI_ISL_822682, EPI_ISL_822683, EPI_ISL_822684, EPI_ISL_822685, EPI_ISL_822686, EPI_ISL_822687, EPI_ISL_822688, EPI_ISL_822689, EPI_ISL_822690, EPI_ISL_822691, EPI_ISL_822692, EPI_ISL_822693, EPI_ISL_822694, EPI_ISL_822695, EPI_ISL_822696, EPI_ISL_822697, EPI_ISL_822698, EPI_ISL_822699, EPI_ISL_822700, EPI_ISL_822701, EPI_ISL_822702, EPI_ISL_822703, EPI_ISL_822704, EPI_ISL_822705, EPI_ISL_822706, EPI_ISL_822707, EPI_ISL_822708, EPI_ISL_822709, EPI_ISL_822710, EPI_ISL_822711, EPI_ISL_822712, EPI_ISL_822713, EPI_ISL_822714, EPI_ISL_822715, EPI_ISL_822716, EPI_ISL_822717, EPI_ISL_822718, EPI_ISL_822719, EPI_ISL_822720, EPI_ISL_822721, EPI_ISL_822722, EPI_ISL_822723, EPI_ISL_822724, EPI_ISL_822725, EPI_ISL_822726, EPI_ISL_822727, EPI_ISL_822728, EPI_ISL_822729, EPI_ISL_822730, EPI_ISL_822731, EPI_ISL_822732, EPI_ISL_822733, EPI_ISL_822734, EPI_ISL_822735, EPI_ISL_822736, EPI_ISL_822737, EPI_ISL_822738, EPI_ISL_822739, EPI_ISL_822740, EPI_ISL_822741, EPI_ISL_822742, EPI_ISL_822743, EPI_ISL_822744, EPI_ISL_822745, EPI_ISL_822746, EPI_ISL_822747, EPI_ISL_822748, EPI_ISL_822749, EPI_ISL_822750, EPI_ISL_822751, EPI_ISL_822752, EPI_ISL_822753, EPI_ISL_822754, EPI_ISL_822755, EPI_ISL_822756, EPI_ISL_822757, EPI_ISL_822758, EPI_ISL_822759, EPI_ISL_822760, EPI_ISL_822761, EPI_ISL_822762, EPI_ISL_822763, EPI_ISL_822764, EPI_ISL_822765, EPI_ISL_822766, EPI_ISL_822767, EPI_ISL_822768, EPI_ISL_822769, EPI_ISL_822770, EPI_ISL_822771, EPI_ISL_822772, EPI_ISL_822773, EPI_ISL_822774, EPI_ISL_822775, EPI_ISL_822776, EPI_ISL_822777, EPI_ISL_822778, EPI_ISL_822779, EPI_ISL_822780, EPI_ISL_822781, EPI_ISL_822782, EPI_ISL_822783, EPI_ISL_822784, EPI_ISL_822785, EPI_ISL_822786, EPI_ISL_822787, EPI_ISL_822788, EPI_ISL_822789, EPI_ISL_822790, EPI_ISL_822791, EPI_ISL_822792, EPI_ISL_822793, EPI_ISL_822794, EPI_ISL_822795, EPI_ISL_822796, EPI_ISL_822797, EPI_ISL_822798, EPI_ISL_822799, EPI_ISL_822800, EPI_ISL_822801, EPI_ISL_822802, EPI_ISL_822803, EPI_ISL_822804, EPI_ISL_822805, EPI_ISL_822806, EPI_ISL_822807, EPI_ISL_822808, EPI_ISL_822809, EPI_ISL_822810, EPI_ISL_822811, EPI_ISL_822812, EPI_ISL_822813, EPI_ISL_822814, EPI_ISL_822815, EPI_ISL_822839, EPI_ISL_822844, EPI_ISL_822865, EPI_ISL_822880, EPI_ISL_822887, EPI_ISL_822925, EPI_ISL_822932, EPI_ISL_822940, EPI_ISL_822947, EPI_ISL_822949, EPI_ISL_822951, EPI_ISL_822952, EPI_ISL_822954, EPI_ISL_822955, EPI_ISL_822957, EPI_ISL_822971, EPI_ISL_822978, EPI_ISL_823003, EPI_ISL_823019, EPI_ISL_823066, EPI_ISL_823093, EPI_ISL_823094, EPI_ISL_823095, EPI_ISL_823096, EPI_ISL_823097, EPI_ISL_823098, EPI_ISL_823099, EPI_ISL_823100, EPI_ISL_823101, EPI_ISL_823102, EPI_ISL_823103, EPI_ISL_823104, EPI_ISL_823106, EPI_ISL_823107, EPI_ISL_823112, EPI_ISL_823141, EPI_ISL_823149, EPI_ISL_823152, EPI_ISL_823153, EPI_ISL_823154, EPI_ISL_823155, EPI_ISL_823643, EPI_ISL_823644, EPI_ISL_823645, EPI_ISL_823646, EPI_ISL_823647, EPI_ISL_823648, EPI_ISL_823649, EPI_ISL_823769, EPI_ISL_823770, EPI_ISL_823771, EPI_ISL_823772, EPI_ISL_823773, EPI_ISL_823774, EPI_ISL_823775, EPI_ISL_823776, EPI_ISL_823777, EPI_ISL_823778, EPI_ISL_823779, EPI_ISL_823780, EPI_ISL_823781, EPI_ISL_823782, EPI_ISL_823783, EPI_ISL_823784, EPI_ISL_823785, EPI_ISL_823786, EPI_ISL_823787, EPI_ISL_823788, EPI_ISL_823789, EPI_ISL_823790, EPI_ISL_823791, EPI_ISL_823792, EPI_ISL_823793, EPI_ISL_823794, EPI_ISL_823795, EPI_ISL_823803			
see above	Wales Specialist Virology Centre Sequencing lab: Pathogen Genomics Unit	COVID-19 Genomics UK (COG-UK) Consortium	Catherine Moore, Johnathan Evans, Laura Gifford, Malorie Perry, Simon Cottrell, Angela Marchbank, Alec Birchley, Alexander Adams, Amy Gaskin, Bree Gatica-Wilcox, Jason Coombes, Joel Southgate, Lauren Gilbert, Lee Graham, Nicole Pacchiarini, Sara Kumziene-Summerhayes, Sarah Taylor, Sophie Jones, Sara Rey, Matthew Bull, Joanne Watkins, Sally Corden, Tom Connor
EPI_ISL_825499, EPI_ISL_825500, EPI_ISL_825504, EPI_ISL_825534, EPI_ISL_825535, EPI_ISL_825536, EPI_ISL_825537, EPI_ISL_825538, EPI_ISL_825539, EPI_ISL_825540, EPI_ISL_825541, EPI_ISL_825542, EPI_ISL_825543, EPI_ISL_825544, EPI_ISL_825545, EPI_ISL_825568, EPI_ISL_825569, EPI_ISL_825570, EPI_ISL_825610, EPI_ISL_825611, EPI_ISL_825612, EPI_ISL_825613, EPI_ISL_825614, EPI_ISL_825615			
see above	Respiratory Virus Unit, National Infection Service, Public Health England	COVID-19 Genomics UK (COG-UK) Consortium	PHE Covid Sequencing Team
EPI_ISL_826467, EPI_ISL_826472	Lighthouse Lab in Alderley Park	Wellcome Sanger Institute for the COVID-19 Genomics UK (COG-UK) Consortium	Jacquelyn Wynn, Mairead Hyland, The Lighthouse Lab in Alderley Park and Alex Alderton, Roberto Amato, Sonia Goncalves, Ewan Harrison, David K. Jackson, Ian Johnston, Dominic Kwiatkowski, Cordelia Langford, John Sillitoe on behalf of the Wellcome Sanger Institute COVID-19 Surveillance Team
EPI_ISL_826473, EPI_ISL_826475	Lighthouse Lab in Cambridge	Wellcome Sanger Institute for the COVID-19 Genomics UK (COG-UK) Consortium	Rob Howes, The Lighthouse Lab in Cambridge and Alex Alderton, Roberto Amato, Sonia Goncalves, Ewan Harrison, David K. Jackson, Ian Johnston, Dominic Kwiatkowski, Cordelia Langford, John Sillitoe on behalf of the Wellcome Sanger Institute COVID-19 Surveillance Team

EPI_ISL_826476, EPI_ISL_834146, EPI_ISL_834211, EPI_ISL_834212, EPI_ISL_834223, EPI_ISL_834226, EPI_ISL_834228, EPI_ISL_834230, EPI_ISL_834231, EPI_ISL_834233, EPI_ISL_834235, EPI_ISL_834236, EPI_ISL_834237, EPI_ISL_834246, EPI_ISL_834248, EPI_ISL_834250, EPI_ISL_834256, EPI_ISL_834263, EPI_ISL_834264, EPI_ISL_834271, EPI_ISL_834274, EPI_ISL_834280, EPI_ISL_834283, EPI_ISL_834288, EPI_ISL_834290, EPI_ISL_834293, EPI_ISL_834298, EPI_ISL_834299, EPI_ISL_834311, EPI_ISL_834312, EPI_ISL_834313, EPI_ISL_834344, EPI_ISL_834352, EPI_ISL_834353, EPI_ISL_834360, EPI_ISL_834368, EPI_ISL_834370, EPI_ISL_834373, EPI_ISL_834376, EPI_ISL_834377, EPI_ISL_834378, EPI_ISL_834380, EPI_ISL_834381, EPI_ISL_834382, EPI_ISL_834388, EPI_ISL_834402, EPI_ISL_834404, EPI_ISL_834407, EPI_ISL_834409, EPI_ISL_834417, EPI_ISL_834423, EPI_ISL_834427, EPI_ISL_834430, EPI_ISL_834433, EPI_ISL_834436, EPI_ISL_834440, EPI_ISL_834443, EPI_ISL_834445, EPI_ISL_834448, EPI_ISL_834449, EPI_ISL_834456, EPI_ISL_834459, EPI_ISL_834460, EPI_ISL_834466, EPI_ISL_834467, EPI_ISL_834468, EPI_ISL_834484, EPI_ISL_834485, EPI_ISL_834486, EPI_ISL_834488, EPI_ISL_834492, EPI_ISL_834494, EPI_ISL_834497, EPI_ISL_834499, EPI_ISL_834500, EPI_ISL_834503, EPI_ISL_834507, EPI_ISL_834511, EPI_ISL_834517, EPI_ISL_834519, EPI_ISL_834524, EPI_ISL_834529, EPI_ISL_834541, EPI_ISL_834545			
see above	Lighthouse Lab in Alderley Park	Wellcome Sanger Institute for the COVID-19 Genomics UK (COG-UK) Consortium	Jacquelyn Wynn, Mairead Hyland, The Lighthouse Lab in Alderley Park and Alex Alderton, Roberto Amato, Sonia Goncalves, Ewan Harrison, David K. Jackson, Ian Johnston, Dominic Kwiatkowski, Cordelia Langford, John Sillitoe on behalf of the Wellcome Sanger Institute COVID-19 Surveillance Team
EPI_ISL_834578	Lighthouse Lab in Glasgow	Wellcome Sanger Institute for the COVID-19 Genomics UK (COG-UK) Consortium	Harper VanSteenhouse, Yumi Kasai, David Gray, Carol Clugston, Anna Dominiczak and Alex Alderton, Roberto Amato, Sonia Goncalves, Ewan Harrison, David K. Jackson, Ian Johnston, Dominic Kwiatkowski, Cordelia Langford, John Sillitoe on behalf of the Wellcome Sanger Institute COVID-19 Surveillance Team
EPI_ISL_834804, EPI_ISL_834814, EPI_ISL_834823, EPI_ISL_834825, EPI_ISL_834826, EPI_ISL_834833, EPI_ISL_834834, EPI_ISL_834837, EPI_ISL_834848, EPI_ISL_834865, EPI_ISL_834867, EPI_ISL_834870, EPI_ISL_834874, EPI_ISL_834875, EPI_ISL_834886, EPI_ISL_834889, EPI_ISL_834901, EPI_ISL_834903, EPI_ISL_834909, EPI_ISL_834910, EPI_ISL_834920, EPI_ISL_834921, EPI_ISL_834925, EPI_ISL_834931, EPI_ISL_834933, EPI_ISL_834947, EPI_ISL_834949, EPI_ISL_834959, EPI_ISL_834963, EPI_ISL_834972, EPI_ISL_834979, EPI_ISL_834981, EPI_ISL_834982, EPI_ISL_834983, EPI_ISL_834989, EPI_ISL_834991, EPI_ISL_835001, EPI_ISL_835004, EPI_ISL_835008, EPI_ISL_835017, EPI_ISL_835031, EPI_ISL_835038, EPI_ISL_835043, EPI_ISL_835047, EPI_ISL_835055, EPI_ISL_835058, EPI_ISL_835060, EPI_ISL_835067, EPI_ISL_835091, EPI_ISL_835094, EPI_ISL_835112, EPI_ISL_835114, EPI_ISL_835115, EPI_ISL_835127, EPI_ISL_835130, EPI_ISL_835132, EPI_ISL_835140, EPI_ISL_835483, EPI_ISL_835492, EPI_ISL_835495, EPI_ISL_835504, EPI_ISL_835532, EPI_ISL_835546, EPI_ISL_835550, EPI_ISL_835562, EPI_ISL_835615, EPI_ISL_835639, EPI_ISL_835655, EPI_ISL_835680, EPI_ISL_835698			
see above	Lighthouse Lab in Alderley Park	Wellcome Sanger Institute for the COVID-19 Genomics UK (COG-UK) Consortium	Jacquelyn Wynn, Mairead Hyland, The Lighthouse Lab in Alderley Park and Alex Alderton, Roberto Amato, Sonia Goncalves, Ewan Harrison, David K. Jackson, Ian Johnston, Dominic Kwiatkowski, Cordelia Langford, John Sillitoe on behalf of the Wellcome Sanger Institute COVID-19 Surveillance Team
EPI_ISL_837041, EPI_ISL_837042, EPI_ISL_837043, EPI_ISL_837044, EPI_ISL_837045, EPI_ISL_837046, EPI_ISL_837047, EPI_ISL_837048, EPI_ISL_837049, EPI_ISL_837050, EPI_ISL_837051, EPI_ISL_837052, EPI_ISL_837064, EPI_ISL_837067, EPI_ISL_837070, EPI_ISL_837077, EPI_ISL_837080, EPI_ISL_837086, EPI_ISL_837092, EPI_ISL_837093, EPI_ISL_837094, EPI_ISL_837096, EPI_ISL_837135, EPI_ISL_837136, EPI_ISL_837137, EPI_ISL_837138, EPI_ISL_837139, EPI_ISL_837140, EPI_ISL_837141, EPI_ISL_837142, EPI_ISL_837143, EPI_ISL_837144, EPI_ISL_837145, EPI_ISL_837146, EPI_ISL_837147, EPI_ISL_837148, EPI_ISL_837149, EPI_ISL_837150, EPI_ISL_837151, EPI_ISL_837152, EPI_ISL_837153, EPI_ISL_837154, EPI_ISL_837155, EPI_ISL_837156, EPI_ISL_837157, EPI_ISL_837158, EPI_ISL_837159, EPI_ISL_837160, EPI_ISL_837161, EPI_ISL_837162, EPI_ISL_837163, EPI_ISL_837164, EPI_ISL_837165, EPI_ISL_837166, EPI_ISL_837167, EPI_ISL_837168, EPI_ISL_837169, EPI_ISL_837170, EPI_ISL_837171, EPI_ISL_837172, EPI_ISL_837173, EPI_ISL_837174, EPI_ISL_837175, EPI_ISL_837184, EPI_ISL_837188, EPI_ISL_837190, EPI_ISL_837191, EPI_ISL_837192, EPI_ISL_837193, EPI_ISL_837194, EPI_ISL_837226, EPI_ISL_837227, EPI_ISL_837228, EPI_ISL_837229, EPI_ISL_837230, EPI_ISL_837231, EPI_ISL_837232, EPI_ISL_837233, EPI_ISL_837234, EPI_ISL_837235, EPI_ISL_837236, EPI_ISL_837237, EPI_ISL_837238, EPI_ISL_837239			
see above	Respiratory Virus Unit, National Infection Service, Public Health England	COVID-19 Genomics UK (COG-UK) Consortium	PHE Covid Sequencing Team
EPI_ISL_837849, EPI_ISL_837851, EPI_ISL_837853, EPI_ISL_837855, EPI_ISL_837858, EPI_ISL_837860, EPI_ISL_837862, EPI_ISL_837864, EPI_ISL_837866, EPI_ISL_837867, EPI_ISL_837868, EPI_ISL_837870, EPI_ISL_837872, EPI_ISL_837873, EPI_ISL_837874, EPI_ISL_837875, EPI_ISL_837878, EPI_ISL_837880, EPI_ISL_837881, EPI_ISL_837883, EPI_ISL_837884, EPI_ISL_837885, EPI_ISL_837888, EPI_ISL_837889, EPI_ISL_837890, EPI_ISL_837891, EPI_ISL_837893, EPI_ISL_837894, EPI_ISL_837895, EPI_ISL_837897, EPI_ISL_837899, EPI_ISL_837900, EPI_ISL_837926, EPI_ISL_837928			
see above	Department of Pathology, University of Cambridge	COVID-19 Genomics UK (COG-UK) Consortium	Aminu S. Jahun, Yasmin Chaudhry, Grant Hall, Iliana Georgana, Myra Hosmillo, Martin D. Curran, Malte Pinckert, Surendra Parmar, Ian Goodfellow
EPI_ISL_838192, EPI_ISL_838195	West of Scotland Specialist Virology Centre, NHSGGC / MRC-University of Glasgow Centre for Virus Research	COVID-19 Genomics UK (COG-UK) Consortium	Ana da Silva Filipe, Natasha Johnson, Kathy Smollett, Daniel Mair, Stephen Carmichael, Alice Broos, Lily Tong, Jenna Nichols, Kyriaki Nomikou; Sarah McDonald; Richard Orton, Joseph Hughes, Sreenu Vattipally, David L Robertson; Alasdair MacLean, Rory Gunson; Sharif Shaaban, Matthew Holden; Rachel Blacow, Guy Mollett, Kathy Li, James Shepherd, Antonia Ho, Emma Thomson
EPI_ISL_838268, EPI_ISL_838269, EPI_ISL_838270, EPI_ISL_838271, EPI_ISL_838273, EPI_ISL_838277, EPI_ISL_838278, EPI_ISL_838279	Virology Department, Royal Infirmary of Edinburgh, NHS Lothian / School of Biological Sciences, University of Edinburgh / Institute of Genetics and Molecular Medicine, University of Edinburgh	COVID-19 Genomics UK (COG-UK) Consortium	McHugh M, Dewar R, Rooke S, Gallagher M, Balcaza C, O'Toole Á, Scher E, Hill V, McCrone JT, Colquhoun R, Yu X, Jackson B, Rambaut A, Williams TC, Templeton K
EPI_ISL_838330, EPI_ISL_838332, EPI_ISL_838333, EPI_ISL_838338	University of Exeter	COVID-19 Genomics UK (COG-UK) Consortium	Ben Temperton,Aaron Jeffries,Michelle Michelsen,Joanna Warwick-Dugdale,Audrey Farbos,Robyn Manley,Stephen Michell,Jane Masoli
EPI_ISL_838580, EPI_ISL_838581, EPI_ISL_838582, EPI_ISL_838583, EPI_ISL_838584, EPI_ISL_838589, EPI_ISL_838590, EPI_ISL_838591, EPI_ISL_838594, EPI_ISL_838595, EPI_ISL_838596, EPI_ISL_838598, EPI_ISL_838601, EPI_ISL_838604, EPI_ISL_838606, EPI_ISL_838607, EPI_ISL_838608, EPI_ISL_838609, EPI_ISL_838610, EPI_ISL_838611, EPI_ISL_838612, EPI_ISL_838614, EPI_ISL_838615, EPI_ISL_838616, EPI_ISL_838618, EPI_ISL_838621, EPI_ISL_838622, EPI_ISL_838623, EPI_ISL_838624, EPI_ISL_838629, EPI_ISL_838630, EPI_ISL_838631, EPI_ISL_838632, EPI_ISL_838633, EPI_ISL_838634, EPI_ISL_838635, EPI_ISL_838636, EPI_ISL_838637, EPI_ISL_838639, EPI_ISL_838640, EPI_ISL_838641, EPI_ISL_838642, EPI_ISL_838643, EPI_ISL_838644, EPI_ISL_838647, EPI_ISL_838648, EPI_ISL_838649, EPI_ISL_838650, EPI_ISL_838651, EPI_ISL_838652, EPI_ISL_838653, EPI_ISL_838654, EPI_ISL_838656, EPI_ISL_838657, EPI_ISL_838658, EPI_ISL_838659, EPI_ISL_838660, EPI_ISL_838661, EPI_ISL_838663, EPI_ISL_838667, EPI_ISL_838668			
see above	Liverpool Clinical Laboratories	COVID-19 Genomics UK (COG-UK) Consortium	Sam Haldenby, Anita Lucaci, Steve Paterson, Julian Hiscox, Alistair Darby, M Almsaud, A Alrezaihi, Muhannad Alruwaili, Stuart D Armstrong, Jones Benjamin, Eleanor G Bentley, Anu Chawla, Jordan J Clark, Angela Cowell, Richard Eccles, Isabel García-Dorival, Matthew Gemmell, Alessandro Gerada, PKF Gilmore, Richard Gregory, Ximeng Han, Catherine Hartley, Margaret Hughes, Miren Iturriza-Gomara, James Johnson, L Luu, Jenifer Manson, Charlotte Nelson, Elaine O'Toole, Cassie Olateju, Rebekah Penrice-Randal , Lucille Rainbow, N.P Randle, Trevor Ian Robinson, Parul Sharma, Ghada T Shawli, James P Stewart, Neil Swainston, Ecaterina Vamos, Joanne Watts, Mark Whitehead
EPI_ISL_838765, EPI_ISL_838766, EPI_ISL_838767, EPI_ISL_838768, EPI_ISL_838769, EPI_ISL_838770, EPI_ISL_838771, EPI_ISL_838772, EPI_ISL_838773, EPI_ISL_838774, EPI_ISL_838775, EPI_ISL_838776, EPI_ISL_838777, EPI_ISL_838778, EPI_ISL_838779, EPI_ISL_838781, EPI_ISL_838782, EPI_ISL_838783, EPI_ISL_838784, EPI_ISL_838785, EPI_ISL_838786, EPI_ISL_838787, EPI_ISL_838788, EPI_ISL_838789, EPI_ISL_838790, EPI_ISL_838791, EPI_ISL_838792, EPI_ISL_838793, EPI_ISL_838794, EPI_ISL_838795, EPI_ISL_838796, EPI_ISL_838797, EPI_ISL_838798, EPI_ISL_838799, EPI_ISL_838800, EPI_ISL_838801, EPI_ISL_838802, EPI_ISL_838803, EPI_ISL_838804, EPI_ISL_838805, EPI_ISL_838806, EPI_ISL_838808, EPI_ISL_838809, EPI_ISL_838810, EPI_ISL_838811, EPI_ISL_838812, EPI_ISL_838813, EPI_ISL_838814, EPI_ISL_838815, EPI_ISL_838816, EPI_ISL_838817, EPI_ISL_838818, EPI_ISL_838819, EPI_ISL_838820, EPI_ISL_838821, EPI_ISL_838822, EPI_ISL_838823, EPI_ISL_838824, EPI_ISL_838825, EPI_ISL_838826, EPI_ISL_838827, EPI_ISL_838828, EPI_ISL_838829, EPI_ISL_838830, EPI_ISL_838831, EPI_ISL_838832, EPI_ISL_838833, EPI_ISL_838834, EPI_ISL_838838, EPI_ISL_838839, EPI_ISL_838840, EPI_ISL_838841, EPI_ISL_839041, EPI_ISL_839042, EPI_ISL_839043, EPI_ISL_839054, EPI_ISL_839060, EPI_ISL_839063, EPI_ISL_839064, EPI_ISL_839065, EPI_ISL_839066, EPI_ISL_839067, EPI_ISL_839068, EPI_ISL_839069, EPI_ISL_839074, EPI_ISL_839075, EPI_ISL_839080, EPI_ISL_839081, EPI_ISL_839082, EPI_ISL_839083, EPI_ISL_839084, EPI_ISL_839085, EPI_ISL_839086, EPI_ISL_839087, EPI_ISL_839088, EPI_ISL_839089, EPI_ISL_839090, EPI_ISL_839091, EPI_ISL_839092, EPI_ISL_839093, EPI_ISL_839094, EPI_ISL_839095, EPI_ISL_839096, EPI_ISL_839097, EPI_ISL_839098, EPI_ISL_839099, EPI_ISL_839100, EPI_ISL_839101, EPI_ISL_839102, EPI_ISL_839103, EPI_ISL_839104, EPI_ISL_839105, EPI_ISL_839106, EPI_ISL_839107, EPI_ISL_839108, EPI_ISL_839109, EPI_ISL_839110, EPI_ISL_839111, EPI_ISL_839112, EPI_ISL_839113, EPI_ISL_839114, EPI_ISL_839116, EPI_ISL_839209, EPI_ISL_839215, EPI_ISL_839218, EPI_ISL_839219, EPI_ISL_839220, EPI_ISL_839221, EPI_ISL_839222, EPI_ISL_839223, EPI_ISL_839224, EPI_ISL_839225, EPI_ISL_839226, EPI_ISL_839227, EPI_ISL_839232, EPI_ISL_839305, EPI_ISL_839306, EPI_ISL_839307, EPI_ISL_839308, EPI_ISL_839310, EPI_ISL_839311, EPI_ISL_839316, EPI_ISL_839317, EPI_ISL_839326, EPI_ISL_839327, EPI_ISL_839330			
see above	University College London, Great Ormond Street Hospital for Children NHS Foundation Trust, Imperial College Healthcare NHS Trust	COVID-19 Genomics UK (COG-UK) Consortium	Sergi Castellano, Rachel Williams, Mark Kristiansen, Paola Resende Silva, Sunando Roy, Tony Brooks, Helena Tutill, Paola Niola, Patricia Dyal, Charlotte Williams, Leysa Forrest, Yasmin Panchbhaya, Jacqueline Findlay, Samuel Weeks, Julianne Brown, Kathryn Harris, Paul Randell, James Price, Alison Holmes, Judith Breuer
EPI_ISL_839683, EPI_ISL_839684, EPI_ISL_839685, EPI_ISL_839686, EPI_ISL_839687, EPI_ISL_839688, EPI_ISL_839689, EPI_ISL_839690, EPI_ISL_839691, EPI_ISL_839692, EPI_ISL_839693, EPI_ISL_839694, EPI_ISL_839695, EPI_ISL_839696, EPI_ISL_839697, EPI_ISL_839698, EPI_ISL_839699, EPI_ISL_839700, EPI_ISL_839701, EPI_ISL_839702, EPI_ISL_839703, EPI_ISL_839704, EPI_ISL_839705, EPI_ISL_839706, EPI_ISL_839707, EPI_ISL_839708, EPI_ISL_839709, EPI_ISL_839710, EPI_ISL_839711, EPI_ISL_839712, EPI_ISL_839713, EPI_ISL_839714, EPI_ISL_839715, EPI_ISL_839716, EPI_ISL_839717, EPI_ISL_839718, EPI_ISL_839719, EPI_ISL_839720, EPI_ISL_839721, EPI_ISL_839722, EPI_ISL_839723, EPI_ISL_839724, EPI_ISL_839725, EPI_ISL_839726, EPI_ISL_839727, EPI_ISL_839728, EPI_ISL_839729, EPI_ISL_839730			
see above	Northumbria University / South Tees Hospitals NHS Foundation Trust / North Cumbria Integrated Care NHS Foundation Trust / North Tees and Hartlepool NHS Foundation Trust / Newcastle Hospitals NHS Foundation Trust	COVID-19 Genomics UK (COG-UK) Consortium	Darren L Smith,Andrew Nelson,Matthew Bashton,Greg R Young,Joshua Loh,John Allan,Mohammad A Tariq,Giles S Holt,Gary Black,Wen C Yew,Lynn Dover,Paul Baker,Steve Liggett,Sarah Essex,Jane Greenaway,Debra Padgett,Clive Graham,Garren Scott,Edward Barton,Emma Swindells,Brendan Payne,Jennifer Collins,Yusri Taha,Gary Eltringham
EPI_ISL_839994	Queens Medical Centre, Clinical Microbiology Department / DeepSeq Nottingham	COVID-19 Genomics UK (COG-UK) Consortium	Gemma Clark, Wendy Smith, Manjinder Khakh, Vicki M Fleming, Michelle M Lister, Hannah Howson-Wells, Jonathan Ball, Patrick McClure, Joseph Chappell, Theocharis Tsoleridis, Nadine Holmes, Matthew Carlisle, Christopher Moore, Fei Sang, Johnny Debebe, Victoria Wright, Matthew Loose
EPI_ISL_840108, EPI_ISL_840109, EPI_ISL_840110, EPI_ISL_840111, EPI_ISL_840112, EPI_ISL_840113, EPI_ISL_840114, EPI_ISL_840115, EPI_ISL_840116, EPI_ISL_840117, EPI_ISL_840118, EPI_ISL_840119, EPI_ISL_840120, EPI_ISL_840121, EPI_ISL_840122, EPI_ISL_840123, EPI_ISL_840124, EPI_ISL_840126			
see above	Lincolnshire Hospitals and DeepSeq Nottingham	COVID-19 Genomics UK (COG-UK) Consortium	Nichola Duckworth, Tim Sloan, Sarah Walsh, Jonathan Ball, Patrick McClure, Joeseph Chappell, Nadine Holmes, Matthew Carlisle, Christopher Moore, Fei Sang, Johnny Debebe, Victoria Wright, Matthew Loose
EPI_ISL_840332, EPI_ISL_840333	Oxford Viromics, NDM, University of Oxford; Oxford University Hospitals; Basingstoke and North Hampshire Hospital	COVID-19 Genomics UK (COG-UK) Consortium	Tanya Golubchik, David Bonsall, George Macintyre, Amy Trebes, Mariateresa de Cesare, Catrin Moore, Alex Mobbs, Anita Justice, Robert Shaw, Monique Andersson, Timothy Peto, Emma Wise, Nathan Moore, Jessica Lynch, Nick Cortes, Matilde Mori, Stephen Kidd, David Buck, John Todd, Christophe Fraser
EPI_ISL_840371, EPI_ISL_840372, EPI_ISL_840389, EPI_ISL_840390, EPI_ISL_840391, EPI_ISL_840392, EPI_ISL_840393, EPI_ISL_840394, EPI_ISL_840395, EPI_ISL_840396, EPI_ISL_840397, EPI_ISL_840399, EPI_ISL_840513, EPI_ISL_840514, EPI_ISL_840581, EPI_ISL_840582, EPI_ISL_840583, EPI_ISL_840585, EPI_ISL_840587, EPI_ISL_840588, EPI_ISL_840589, EPI_ISL_840591, EPI_ISL_840592, EPI_ISL_840593, EPI_ISL_840594, EPI_ISL_840595, EPI_ISL_840599, EPI_ISL_840600, EPI_ISL_840603, EPI_ISL_840604, EPI_ISL_840606, EPI_ISL_840609, EPI_ISL_840610, EPI_ISL_840611, EPI_ISL_840612, EPI_ISL_840615, EPI_ISL_840617, EPI_ISL_840618, EPI_ISL_840619, EPI_ISL_840620, EPI_ISL_840621, EPI_ISL_840622, EPI_ISL_840623, EPI_ISL_840624, EPI_ISL_840625, EPI_ISL_840626, EPI_ISL_840627, EPI_ISL_840628, EPI_ISL_840629, EPI_ISL_840630, EPI_ISL_840631, EPI_ISL_840632, EPI_ISL_840633, EPI_ISL_840634, EPI_ISL_840635, EPI_ISL_840636, EPI_ISL_840638, EPI_ISL_840639, EPI_ISL_840640, EPI_ISL_840641, EPI_ISL_840643, EPI_ISL_840644, EPI_ISL_840645, EPI_ISL_840646, EPI_ISL_840647, EPI_ISL_840648, EPI_ISL_840649, EPI_ISL_840651, EPI_ISL_840652, EPI_ISL_840653, EPI_ISL_840655, EPI_ISL_840656,			

EPI_ISL_840657, EPI_ISL_840658, EPI_ISL_840671, EPI_ISL_840672, EPI_ISL_840673, EPI_ISL_840674, EPI_ISL_840675, EPI_ISL_840676, EPI_ISL_840695, EPI_ISL_840696, EPI_ISL_840697, EPI_ISL_840698, EPI_ISL_840699, EPI_ISL_840700, EPI_ISL_840701, EPI_ISL_840702, EPI_ISL_840703, EPI_ISL_840774, EPI_ISL_840775, EPI_ISL_840776, EPI_ISL_840777, EPI_ISL_840778, EPI_ISL_840779, EPI_ISL_840781, EPI_ISL_840782, EPI_ISL_840783, EPI_ISL_840785, EPI_ISL_840787, EPI_ISL_840790, EPI_ISL_840794, EPI_ISL_840795, EPI_ISL_840796, EPI_ISL_840798, EPI_ISL_840799, EPI_ISL_840801, EPI_ISL_840802, EPI_ISL_840803, EPI_ISL_840804, EPI_ISL_840805, EPI_ISL_840806, EPI_ISL_840808, EPI_ISL_840810, EPI_ISL_840811, EPI_ISL_840812, EPI_ISL_840813, EPI_ISL_840814, EPI_ISL_840815			
see above	Originating lab: Wales Specialist Virology Centre Sequencing lab: Pathogen Genomics Unit	Public Health Wales Microbiology Cardiff Wales Specialist Virology Centre	Catherine Moore, Johnathan Evans, Laura Gifford, Malorie Perry, Simon Cottrell, Angela Marchbank, Alec Birchley, Alexander Adams, Amy Gaskin, Bree Gatica-Wilcox, Jason Coombes, Joel Southgate, Lauren Gilbert, Lee Graham, Nicole Pacchiarini, Sara Kumziene-Summerhayes, Sarah Taylor, Sophie Jones, Sara Rey, Matthew Bull, Joanne Watkins, Sally Corden, Tom Connor
EPI_ISL_840816, EPI_ISL_840819, EPI_ISL_840821, EPI_ISL_840837, EPI_ISL_840839, EPI_ISL_840841, EPI_ISL_840895, EPI_ISL_840897, EPI_ISL_840898, EPI_ISL_840899, EPI_ISL_840900, EPI_ISL_840916, EPI_ISL_840917, EPI_ISL_840918, EPI_ISL_840919, EPI_ISL_841057, EPI_ISL_841058, EPI_ISL_841083, EPI_ISL_841087, EPI_ISL_841088, EPI_ISL_841089, EPI_ISL_841090, EPI_ISL_841091, EPI_ISL_841092, EPI_ISL_841093, EPI_ISL_841094, EPI_ISL_841095, EPI_ISL_841096, EPI_ISL_841097, EPI_ISL_841099, EPI_ISL_841100, EPI_ISL_841101, EPI_ISL_841102, EPI_ISL_841103, EPI_ISL_841104, EPI_ISL_841105, EPI_ISL_841106, EPI_ISL_841107, EPI_ISL_841109, EPI_ISL_841110, EPI_ISL_841111, EPI_ISL_841112, EPI_ISL_841113, EPI_ISL_841114, EPI_ISL_841115, EPI_ISL_841117, EPI_ISL_841118, EPI_ISL_841119, EPI_ISL_841123, EPI_ISL_841302, EPI_ISL_841303, EPI_ISL_841304, EPI_ISL_841305			
see above	Wales Specialist Virology Centre Sequencing lab: Pathogen Genomics Unit	Public Health Wales Microbiology Cardiff Wales Specialist Virology Centre	Catherine Moore, Johnathan Evans, Laura Gifford, Malorie Perry, Simon Cottrell, Angela Marchbank, Alec Birchley, Alexander Adams, Amy Gaskin, Bree Gatica-Wilcox, Jason Coombes, Joel Southgate, Lauren Gilbert, Lee Graham, Nicole Pacchiarini, Sara Kumziene-Summerhayes, Sarah Taylor, Sophie Jones, Sara Rey, Matthew Bull, Joanne Watkins, Sally Corden, Tom Connor
EPI_ISL_841321, EPI_ISL_841355, EPI_ISL_841359, EPI_ISL_841360, EPI_ISL_841361, EPI_ISL_841362, EPI_ISL_841363, EPI_ISL_841364, EPI_ISL_841365, EPI_ISL_841366, EPI_ISL_841367, EPI_ISL_841368, EPI_ISL_841369, EPI_ISL_841370, EPI_ISL_841508, EPI_ISL_841509, EPI_ISL_841521, EPI_ISL_841522, EPI_ISL_841580, EPI_ISL_841581, EPI_ISL_841584, EPI_ISL_841587, EPI_ISL_841591, EPI_ISL_841608			
see above	Originating lab: Wales Specialist Virology Centre Sequencing lab: Pathogen Genomics Unit	Public Health Wales Microbiology Cardiff Wales Specialist Virology Centre	Catherine Moore, Johnathan Evans, Laura Gifford, Malorie Perry, Simon Cottrell, Angela Marchbank, Alec Birchley, Alexander Adams, Amy Gaskin, Bree Gatica-Wilcox, Jason Coombes, Joel Southgate, Lauren Gilbert, Lee Graham, Nicole Pacchiarini, Sara Kumziene-Summerhayes, Sarah Taylor, Sophie Jones, Sara Rey, Matthew Bull, Joanne Watkins, Sally Corden, Tom Connor
EPI_ISL_841978, EPI_ISL_842004, EPI_ISL_842014, EPI_ISL_842015	Centre for Enzyme Innovation, University of Portsmouth / Translational Research Laboratory, Portsmouth Hospitals NHS Trust	COVID-19 Genomics UK (COG-UK) Consortium	Angela Beckett,Yann Bourgeois,Garry Scarlett,Sharon Glaysher,Scott Elliott,Kelly Bicknell,Robert Impey,Allyson Lloyd,Sarah Wyllie,Ethan Butcher,Anoop Chauhan,Samuel Robson
EPI_ISL_842209, EPI_ISL_842265, EPI_ISL_842270, EPI_ISL_842273, EPI_ISL_842293, EPI_ISL_842312, EPI_ISL_842325, EPI_ISL_842338, EPI_ISL_842341	Virology Department, Sheffield Teaching Hospitals NHS Foundation Trust/Department of Infection, Immunity and Cardiovascular Disease, The Medical School, University of Sheffield	COVID-19 Genomics UK (COG-UK) Consortium	Thushan de Silva, Matthew Parker, Nikki Smith, Adri Angyal, Rebecca Brown, Luke Green, Rachel Tucker, Paul Parsons, Danielle Groves, Katie Johnson, Laura Carrilero, Alex Keeley, Dave Partridge, Matthew Wyles, Benjamin Lindsey, Mehmet Yavuz, Mohammad Raza, Cariad Evans
EPI_ISL_842356, EPI_ISL_842361, EPI_ISL_842362, EPI_ISL_842367, EPI_ISL_842422, EPI_ISL_842423, EPI_ISL_842424, EPI_ISL_842425, EPI_ISL_842426, EPI_ISL_842427, EPI_ISL_842428, EPI_ISL_842429, EPI_ISL_842430, EPI_ISL_842431, EPI_ISL_842432, EPI_ISL_842433, EPI_ISL_842434, EPI_ISL_842435, EPI_ISL_842436, EPI_ISL_842437, EPI_ISL_842438, EPI_ISL_842439, EPI_ISL_842440, EPI_ISL_842441, EPI_ISL_842442, EPI_ISL_842443, EPI_ISL_842444, EPI_ISL_842445, EPI_ISL_842447, EPI_ISL_842448, EPI_ISL_842449, EPI_ISL_842450, EPI_ISL_842451, EPI_ISL_842452, EPI_ISL_842453, EPI_ISL_842454, EPI_ISL_842455, EPI_ISL_842456, EPI_ISL_842457, EPI_ISL_842458, EPI_ISL_842459, EPI_ISL_842460, EPI_ISL_842461, EPI_ISL_842462, EPI_ISL_842463, EPI_ISL_842464, EPI_ISL_842465, EPI_ISL_842466, EPI_ISL_842467, EPI_ISL_842468, EPI_ISL_842469, EPI_ISL_842470, EPI_ISL_842472, EPI_ISL_842473, EPI_ISL_842474, EPI_ISL_842475, EPI_ISL_842476, EPI_ISL_842477, EPI_ISL_842478, EPI_ISL_842479, EPI_ISL_842480, EPI_ISL_842481, EPI_ISL_842482, EPI_ISL_842483, EPI_ISL_842484, EPI_ISL_842485, EPI_ISL_842486, EPI_ISL_842487, EPI_ISL_842488, EPI_ISL_842489, EPI_ISL_842490, EPI_ISL_842491, EPI_ISL_842492, EPI_ISL_842493, EPI_ISL_842494, EPI_ISL_842496, EPI_ISL_842497, EPI_ISL_842498, EPI_ISL_842499, EPI_ISL_842500, EPI_ISL_842501, EPI_ISL_842502, EPI_ISL_842503, EPI_ISL_842504, EPI_ISL_842505, EPI_ISL_842506, EPI_ISL_842507, EPI_ISL_842508, EPI_ISL_842509, EPI_ISL_842510, EPI_ISL_842511, EPI_ISL_842513, EPI_ISL_842583, EPI_ISL_842585, EPI_ISL_842586, EPI_ISL_842587, EPI_ISL_842588, EPI_ISL_842589, EPI_ISL_842590, EPI_ISL_842591, EPI_ISL_842592, EPI_ISL_842593, EPI_ISL_842594, EPI_ISL_842596, EPI_ISL_842598, EPI_ISL_842599, EPI_ISL_842600, EPI_ISL_842601, EPI_ISL_842602, EPI_ISL_842603, EPI_ISL_842604, EPI_ISL_842605, EPI_ISL_842606, EPI_ISL_842607, EPI_ISL_842608			
see above	Bioinformatics and Biostatistics Lab, Advanced Sequencing Facility	COVID-19 Genomics UK (COG-UK) Consortium	Aengus Stewart,Jerome Nicod,Chelsea Sawyer,Laura Cubitt,Harshil Patel,Margaret Crawford
EPI_ISL_843145, EPI_ISL_843146, EPI_ISL_843147, EPI_ISL_843148, EPI_ISL_843149, EPI_ISL_843150	Barts Health NHS Trust	COVID-19 Genomics UK (COG-UK) Consortium	CUTINO-MOGUEL, Maria-Teresa; HARRINGTON, David; OWOYEMI, Dola; SHYLINI, Raghavendran; BROAD, Claire; KELE, Beatrix
EPI_ISL_843151, EPI_ISL_843152, EPI_ISL_843153, EPI_ISL_843154, EPI_ISL_843155, EPI_ISL_843156, EPI_ISL_843157, EPI_ISL_843158, EPI_ISL_843159, EPI_ISL_843160, EPI_ISL_843161, EPI_ISL_843163, EPI_ISL_843164, EPI_ISL_843165, EPI_ISL_843166			
see above	Regional Virus Laboratory, Belfast Health and Social Care Trust	COVID-19 Genomics UK (COG-UK) Consortium	Conall McCaughey, James McKenna, Tanya Curran, Susan Feeney, Alison Watt, Ciara Cox, Mairead Connor, Zoltan Molnar, David Simpson, Derek Fairley
EPI_ISL_846526, EPI_ISL_846536	Lighthouse Lab in Alderley Park	Wellcome Sanger Institute for the COVID-19 Genomics UK (COG-UK) Consortium	Jacquelyn Wynn, Mairead Hyland, The Lighthouse Lab in Alderley Park and Alex Alderton, Roberto Amato, Sonia Goncalves, Ewan Harrison, David K. Jackson, Ian Johnston, Dominic Kwiatkowski, Cordelia Langford, John Sillitoe on behalf of the Wellcome Sanger Institute COVID-19 Surveillance Team
EPI_ISL_852017	Lighthouse Lab in Milton Keynes	Wellcome Sanger Institute for the COVID-19 Genomics UK (COG-UK) Consortium	The Lighthouse Lab in Milton Keynes and Alex Alderton, Roberto Amato, Sonia Goncalves, Ewan Harrison, David K. Jackson, Ian Johnston, Dominic Kwiatkowski, Cordelia Langford, John Sillitoe on behalf of the Wellcome Sanger Institute COVID-19 Surveillance Team
EPI_ISL_852191	Lighthouse Lab in Alderley Park	Wellcome Sanger Institute for the COVID-19 Genomics UK (COG-UK) Consortium	Jacquelyn Wynn, Mairead Hyland, The Lighthouse Lab in Alderley Park and Alex Alderton, Roberto Amato, Sonia Goncalves, Ewan Harrison, David K. Jackson, Ian Johnston, Dominic Kwiatkowski, Cordelia Langford, John Sillitoe on behalf of the Wellcome Sanger Institute COVID-19 Surveillance Team
EPI_ISL_858274, EPI_ISL_858313	Lighthouse Lab in Glasgow	Wellcome Sanger Institute for the COVID-19 Genomics UK (COG-UK) Consortium	Harper VanSteenhouse, Yumi Kasai, David Gray, Carol Clugston, Anna Dominiczak and Alex Alderton, Roberto Amato, Sonia Goncalves, Ewan Harrison, David K. Jackson, Ian Johnston, Dominic Kwiatkowski, Cordelia Langford, John Sillitoe on behalf of the Wellcome Sanger Institute COVID-19 Surveillance Team
EPI_ISL_858573, EPI_ISL_858575, EPI_ISL_858577, EPI_ISL_858578, EPI_ISL_858579, EPI_ISL_858580, EPI_ISL_858582	Lighthouse Lab in Alderley Park	Wellcome Sanger Institute for the COVID-19 Genomics UK (COG-UK) Consortium	Jacquelyn Wynn, Mairead Hyland, The Lighthouse Lab in Alderley Park and Alex Alderton, Roberto Amato, Sonia Goncalves, Ewan Harrison, David K. Jackson, Ian Johnston, Dominic Kwiatkowski, Cordelia Langford, John Sillitoe on behalf of the Wellcome Sanger Institute COVID-19 Surveillance Team
EPI_ISL_858583, EPI_ISL_858584, EPI_ISL_858586, EPI_ISL_858589, EPI_ISL_858590, EPI_ISL_858591, EPI_ISL_858593, EPI_ISL_858595, EPI_ISL_858596, EPI_ISL_858597, EPI_ISL_858599, EPI_ISL_858602, EPI_ISL_858603, EPI_ISL_858605, EPI_ISL_858606, EPI_ISL_858611, EPI_ISL_858614, EPI_ISL_858617, EPI_ISL_858619, EPI_ISL_858620, EPI_ISL_858621, EPI_ISL_858622, EPI_ISL_858624, EPI_ISL_858625, EPI_ISL_858629, EPI_ISL_858631, EPI_ISL_858632, EPI_ISL_858633, EPI_ISL_858634, EPI_ISL_858636, EPI_ISL_858639			
see above	Lighthouse Lab in Glasgow	Wellcome Sanger Institute for the COVID-19 Genomics UK (COG-UK) Consortium	Harper VanSteenhouse, Yumi Kasai, David Gray, Carol Clugston, Anna Dominiczak and Alex Alderton, Roberto Amato, Sonia Goncalves, Ewan Harrison, David K. Jackson, Ian Johnston, Dominic Kwiatkowski, Cordelia Langford, John Sillitoe on behalf of the Wellcome Sanger Institute COVID-19 Surveillance Team
EPI_ISL_858644, EPI_ISL_858645, EPI_ISL_858647, EPI_ISL_858648, EPI_ISL_858649, EPI_ISL_858653, EPI_ISL_858654, EPI_ISL_858655, EPI_ISL_858662, EPI_ISL_858663, EPI_ISL_858666, EPI_ISL_858667, EPI_ISL_858669, EPI_ISL_858670, EPI_ISL_858671, EPI_ISL_858672, EPI_ISL_858673, EPI_ISL_858674, EPI_ISL_858675, EPI_ISL_858676, EPI_ISL_858677, EPI_ISL_858678, EPI_ISL_858679, EPI_ISL_858680, EPI_ISL_858681, EPI_ISL_858682, EPI_ISL_858683, EPI_ISL_858684, EPI_ISL_858685, EPI_ISL_858686, EPI_ISL_858687, EPI_ISL_858688, EPI_ISL_858689, EPI_ISL_858690, EPI_ISL_858691, EPI_ISL_858692, EPI_ISL_858693, EPI_ISL_858694, EPI_ISL_858695, EPI_ISL_858696, EPI_ISL_858697, EPI_ISL_858698, EPI_ISL_858699, EPI_ISL_858700			
see above	Lighthouse Lab in Alderley Park	Wellcome Sanger Institute for the COVID-19 Genomics UK (COG-UK) Consortium	Jacquelyn Wynn, Mairead Hyland, The Lighthouse Lab in Alderley Park and Alex Alderton, Roberto Amato, Sonia Goncalves, Ewan Harrison, David K. Jackson, Ian Johnston, Dominic Kwiatkowski, Cordelia Langford, John Sillitoe on behalf of the Wellcome Sanger Institute COVID-19 Surveillance Team
EPI_ISL_858711, EPI_ISL_858712	Lighthouse Lab in Glasgow	Wellcome Sanger Institute for the COVID-19 Genomics UK (COG-UK) Consortium	Harper VanSteenhouse, Yumi Kasai, David Gray, Carol Clugston, Anna Dominiczak and Alex Alderton, Roberto Amato, Sonia Goncalves, Ewan Harrison, David K. Jackson, Ian Johnston, Dominic Kwiatkowski, Cordelia Langford, John Sillitoe on behalf of the Wellcome Sanger Institute COVID-19 Surveillance Team
EPI_ISL_858713, EPI_ISL_858714	Lighthouse Lab in Alderley Park	Wellcome Sanger Institute for the COVID-19 Genomics UK (COG-UK) Consortium	Jacquelyn Wynn, Mairead Hyland, The Lighthouse Lab in Alderley Park and Alex Alderton, Roberto Amato, Sonia Goncalves, Ewan Harrison, David K. Jackson, Ian Johnston, Dominic Kwiatkowski, Cordelia Langford, John Sillitoe on behalf of the Wellcome Sanger Institute COVID-19 Surveillance Team
EPI_ISL_858715, EPI_ISL_858716, EPI_ISL_858717, EPI_ISL_858718	Lighthouse Lab in Glasgow	Wellcome Sanger Institute for the COVID-19 Genomics UK (COG-UK) Consortium	Harper VanSteenhouse, Yumi Kasai, David Gray, Carol Clugston, Anna Dominiczak and Alex Alderton, Roberto Amato, Sonia Goncalves, Ewan Harrison, David K. Jackson, Ian Johnston, Dominic Kwiatkowski, Cordelia Langford, John Sillitoe on behalf of the Wellcome Sanger Institute COVID-19 Surveillance Team
EPI_ISL_858719, EPI_ISL_858720	Lighthouse Lab in Alderley Park	Wellcome Sanger Institute for the COVID-19 Genomics UK (COG-UK) Consortium	Jacquelyn Wynn, Mairead Hyland, The Lighthouse Lab in Alderley Park and Alex Alderton, Roberto Amato, Sonia Goncalves, Ewan Harrison, David K. Jackson, Ian Johnston, Dominic Kwiatkowski, Cordelia Langford, John Sillitoe on behalf of the Wellcome Sanger Institute COVID-19 Surveillance Team
EPI_ISL_858721, EPI_ISL_858722,	Lighthouse Lab in Glasgow	Wellcome Sanger Institute for the COVID-19 Genomics UK	Harper VanSteenhouse, Yumi Kasai, David Gray, Carol Clugston, Anna Dominiczak and Alex Alderton, Roberto Amato, Sonia Goncalves, Ewan Harrison,

EPI_ISL_858723, EPI_ISL_858724		(COG-UK) Consortium	David K. Jackson, Ian Johnston, Dominic Kwiatkowski, Cordelia Langford, John Sillitoe on behalf of the Wellcome Sanger Institute COVID-19 Surveillance Team
EPI_ISL_858725	Lighthouse Lab in Alderley Park	Wellcome Sanger Institute for the COVID-19 Genomics UK (COG-UK) Consortium	Jacquelyn Wynn, Mairead Hyland, The Lighthouse Lab in Alderley Park and Alex Alderton, Roberto Amato, Sonia Goncalves, Ewan Harrison, David K. Jackson, Ian Johnston, Dominic Kwiatkowski, Cordelia Langford, John Sillitoe on behalf of the Wellcome Sanger Institute COVID-19 Surveillance Team
EPI_ISL_858726, EPI_ISL_858727, EPI_ISL_858728, EPI_ISL_858729, EPI_ISL_858730, EPI_ISL_858731	Lighthouse Lab in Glasgow	Wellcome Sanger Institute for the COVID-19 Genomics UK (COG-UK) Consortium	Harper VanSteenhouse, Yumi Kasai, David Gray, Carol Clugston, Anna Dominiczak and Alex Alderton, Roberto Amato, Sonia Goncalves, Ewan Harrison, David K. Jackson, Ian Johnston, Dominic Kwiatkowski, Cordelia Langford, John Sillitoe on behalf of the Wellcome Sanger Institute COVID-19 Surveillance Team
EPI_ISL_858732	Lighthouse Lab in Alderley Park	Wellcome Sanger Institute for the COVID-19 Genomics UK (COG-UK) Consortium	Jacquelyn Wynn, Mairead Hyland, The Lighthouse Lab in Alderley Park and Alex Alderton, Roberto Amato, Sonia Goncalves, Ewan Harrison, David K. Jackson, Ian Johnston, Dominic Kwiatkowski, Cordelia Langford, John Sillitoe on behalf of the Wellcome Sanger Institute COVID-19 Surveillance Team
EPI_ISL_858733, EPI_ISL_858734, EPI_ISL_858735, EPI_ISL_858736	Lighthouse Lab in Glasgow	Wellcome Sanger Institute for the COVID-19 Genomics UK (COG-UK) Consortium	Harper VanSteenhouse, Yumi Kasai, David Gray, Carol Clugston, Anna Dominiczak and Alex Alderton, Roberto Amato, Sonia Goncalves, Ewan Harrison, David K. Jackson, Ian Johnston, Dominic Kwiatkowski, Cordelia Langford, John Sillitoe on behalf of the Wellcome Sanger Institute COVID-19 Surveillance Team
EPI_ISL_858737	Lighthouse Lab in Alderley Park	Wellcome Sanger Institute for the COVID-19 Genomics UK (COG-UK) Consortium	Jacquelyn Wynn, Mairead Hyland, The Lighthouse Lab in Alderley Park and Alex Alderton, Roberto Amato, Sonia Goncalves, Ewan Harrison, David K. Jackson, Ian Johnston, Dominic Kwiatkowski, Cordelia Langford, John Sillitoe on behalf of the Wellcome Sanger Institute COVID-19 Surveillance Team
EPI_ISL_858738	Lighthouse Lab in Glasgow	Wellcome Sanger Institute for the COVID-19 Genomics UK (COG-UK) Consortium	Harper VanSteenhouse, Yumi Kasai, David Gray, Carol Clugston, Anna Dominiczak and Alex Alderton, Roberto Amato, Sonia Goncalves, Ewan Harrison, David K. Jackson, Ian Johnston, Dominic Kwiatkowski, Cordelia Langford, John Sillitoe on behalf of the Wellcome Sanger Institute COVID-19 Surveillance Team
EPI_ISL_858739	Lighthouse Lab in Alderley Park	Wellcome Sanger Institute for the COVID-19 Genomics UK (COG-UK) Consortium	Jacquelyn Wynn, Mairead Hyland, The Lighthouse Lab in Alderley Park and Alex Alderton, Roberto Amato, Sonia Goncalves, Ewan Harrison, David K. Jackson, Ian Johnston, Dominic Kwiatkowski, Cordelia Langford, John Sillitoe on behalf of the Wellcome Sanger Institute COVID-19 Surveillance Team
EPI_ISL_858740, EPI_ISL_858741, EPI_ISL_858742, EPI_ISL_858743, EPI_ISL_858744, EPI_ISL_858745, EPI_ISL_858746, EPI_ISL_858747	Lighthouse Lab in Glasgow	Wellcome Sanger Institute for the COVID-19 Genomics UK (COG-UK) Consortium	Harper VanSteenhouse, Yumi Kasai, David Gray, Carol Clugston, Anna Dominiczak and Alex Alderton, Roberto Amato, Sonia Goncalves, Ewan Harrison, David K. Jackson, Ian Johnston, Dominic Kwiatkowski, Cordelia Langford, John Sillitoe on behalf of the Wellcome Sanger Institute COVID-19 Surveillance Team
EPI_ISL_858748	Lighthouse Lab in Alderley Park	Wellcome Sanger Institute for the COVID-19 Genomics UK (COG-UK) Consortium	Jacquelyn Wynn, Mairead Hyland, The Lighthouse Lab in Alderley Park and Alex Alderton, Roberto Amato, Sonia Goncalves, Ewan Harrison, David K. Jackson, Ian Johnston, Dominic Kwiatkowski, Cordelia Langford, John Sillitoe on behalf of the Wellcome Sanger Institute COVID-19 Surveillance Team
EPI_ISL_858749, EPI_ISL_858750, EPI_ISL_858751	Lighthouse Lab in Glasgow	Wellcome Sanger Institute for the COVID-19 Genomics UK (COG-UK) Consortium	Harper VanSteenhouse, Yumi Kasai, David Gray, Carol Clugston, Anna Dominiczak and Alex Alderton, Roberto Amato, Sonia Goncalves, Ewan Harrison, David K. Jackson, Ian Johnston, Dominic Kwiatkowski, Cordelia Langford, John Sillitoe on behalf of the Wellcome Sanger Institute COVID-19 Surveillance Team
EPI_ISL_858752, EPI_ISL_858753	Lighthouse Lab in Alderley Park	Wellcome Sanger Institute for the COVID-19 Genomics UK (COG-UK) Consortium	Jacquelyn Wynn, Mairead Hyland, The Lighthouse Lab in Alderley Park and Alex Alderton, Roberto Amato, Sonia Goncalves, Ewan Harrison, David K. Jackson, Ian Johnston, Dominic Kwiatkowski, Cordelia Langford, John Sillitoe on behalf of the Wellcome Sanger Institute COVID-19 Surveillance Team
EPI_ISL_858754, EPI_ISL_858755, EPI_ISL_858756, EPI_ISL_858757, EPI_ISL_858758, EPI_ISL_858759, EPI_ISL_858760, EPI_ISL_858761, EPI_ISL_858762, EPI_ISL_858763, EPI_ISL_858764, EPI_ISL_858765, EPI_ISL_858766, EPI_ISL_858767, EPI_ISL_858768, EPI_ISL_858769, EPI_ISL_858770, EPI_ISL_858771, EPI_ISL_858772, EPI_ISL_858773, EPI_ISL_858774, EPI_ISL_858775, EPI_ISL_858776, EPI_ISL_858777, EPI_ISL_858778			
see above	Lighthouse Lab in Glasgow	Wellcome Sanger Institute for the COVID-19 Genomics UK (COG-UK) Consortium	Harper VanSteenhouse, Yumi Kasai, David Gray, Carol Clugston, Anna Dominiczak and Alex Alderton, Roberto Amato, Sonia Goncalves, Ewan Harrison, David K. Jackson, Ian Johnston, Dominic Kwiatkowski, Cordelia Langford, John Sillitoe on behalf of the Wellcome Sanger Institute COVID-19 Surveillance Team
EPI_ISL_858779, EPI_ISL_858780	Lighthouse Lab in Alderley Park	Wellcome Sanger Institute for the COVID-19 Genomics UK (COG-UK) Consortium	Jacquelyn Wynn, Mairead Hyland, The Lighthouse Lab in Alderley Park and Alex Alderton, Roberto Amato, Sonia Goncalves, Ewan Harrison, David K. Jackson, Ian Johnston, Dominic Kwiatkowski, Cordelia Langford, John Sillitoe on behalf of the Wellcome Sanger Institute COVID-19 Surveillance Team
EPI_ISL_858781, EPI_ISL_858782, EPI_ISL_858783	Lighthouse Lab in Glasgow	Wellcome Sanger Institute for the COVID-19 Genomics UK (COG-UK) Consortium	Harper VanSteenhouse, Yumi Kasai, David Gray, Carol Clugston, Anna Dominiczak and Alex Alderton, Roberto Amato, Sonia Goncalves, Ewan Harrison, David K. Jackson, Ian Johnston, Dominic Kwiatkowski, Cordelia Langford, John Sillitoe on behalf of the Wellcome Sanger Institute COVID-19 Surveillance Team
EPI_ISL_860642, EPI_ISL_860643, EPI_ISL_860644, EPI_ISL_860645, EPI_ISL_860646, EPI_ISL_860647, EPI_ISL_860648, EPI_ISL_860649, EPI_ISL_860650, EPI_ISL_860651, EPI_ISL_860653, EPI_ISL_860654, EPI_ISL_860655, EPI_ISL_860656, EPI_ISL_860657, EPI_ISL_860658, EPI_ISL_860659, EPI_ISL_860660, EPI_ISL_860661, EPI_ISL_860662, EPI_ISL_860663, EPI_ISL_860664, EPI_ISL_860665, EPI_ISL_860782			
see above	Respiratory Virus Unit, National Infection Service, Public Health England	COVID-19 Genomics UK (COG-UK) Consortium	PHE Covid Sequencing Team
EPI_ISL_863532, EPI_ISL_863590, EPI_ISL_863600, EPI_ISL_863631, EPI_ISL_863635, EPI_ISL_863639, EPI_ISL_863645, EPI_ISL_863651, EPI_ISL_863707, EPI_ISL_863721, EPI_ISL_863729, EPI_ISL_863746, EPI_ISL_863803, EPI_ISL_863811, EPI_ISL_863830, EPI_ISL_863841, EPI_ISL_863853			
see above	Lighthouse Lab in Glasgow	Wellcome Sanger Institute for the COVID-19 Genomics UK (COG-UK) Consortium	Harper VanSteenhouse, Yumi Kasai, David Gray, Carol Clugston, Anna Dominiczak and Alex Alderton, Roberto Amato, Sonia Goncalves, Ewan Harrison, David K. Jackson, Ian Johnston, Dominic Kwiatkowski, Cordelia Langford, John Sillitoe on behalf of the Wellcome Sanger Institute COVID-19 Surveillance Team
EPI_ISL_864738, EPI_ISL_864739, EPI_ISL_864740, EPI_ISL_864741, EPI_ISL_864742, EPI_ISL_864743, EPI_ISL_864744, EPI_ISL_864745, EPI_ISL_864746, EPI_ISL_864747, EPI_ISL_864749, EPI_ISL_864750, EPI_ISL_864752, EPI_ISL_864753, EPI_ISL_864757, EPI_ISL_864759, EPI_ISL_864761, EPI_ISL_864763, EPI_ISL_864765, EPI_ISL_864767, EPI_ISL_864769, EPI_ISL_864770, EPI_ISL_864772, EPI_ISL_864785, EPI_ISL_864787, EPI_ISL_864790, EPI_ISL_864795, EPI_ISL_864801, EPI_ISL_864812, EPI_ISL_864816, EPI_ISL_864817, EPI_ISL_864820, EPI_ISL_864822, EPI_ISL_864823, EPI_ISL_864824, EPI_ISL_864825, EPI_ISL_864826, EPI_ISL_864827, EPI_ISL_864830, EPI_ISL_864831, EPI_ISL_864836, EPI_ISL_864848, EPI_ISL_864853, EPI_ISL_864855, EPI_ISL_864858, EPI_ISL_864860, EPI_ISL_864869, EPI_ISL_864871, EPI_ISL_864873, EPI_ISL_864877, EPI_ISL_864879, EPI_ISL_864881, EPI_ISL_864885, EPI_ISL_864887, EPI_ISL_864891, EPI_ISL_864895, EPI_ISL_864917, EPI_ISL_864918, EPI_ISL_864925, EPI_ISL_864947, EPI_ISL_864948			
see above	Department of Pathology, University of Cambridge	COVID-19 Genomics UK (COG-UK) Consortium	Aminu S. Jahun, Yasmin Chaudhry, Grant Hall, Iliana Georgana, Myra Hosmillo, Martin D. Curran, Malte Pinckert, Surendra Parmar, Ian Goodfellow
EPI_ISL_864961, EPI_ISL_864962, EPI_ISL_864963, EPI_ISL_864964, EPI_ISL_864965, EPI_ISL_864966, EPI_ISL_864967, EPI_ISL_864968, EPI_ISL_864969, EPI_ISL_864970, EPI_ISL_864971, EPI_ISL_864972, EPI_ISL_864973, EPI_ISL_864974, EPI_ISL_864975, EPI_ISL_864976, EPI_ISL_864977, EPI_ISL_864978, EPI_ISL_864979, EPI_ISL_864980, EPI_ISL_864981, EPI_ISL_864982, EPI_ISL_864983, EPI_ISL_864992, EPI_ISL_864993			
see above	West of Scotland Specialist Virology Centre, NHSGGC / MRC-University of Glasgow Centre for Virus Research	COVID-19 Genomics UK (COG-UK) Consortium	Ana da Silva Filipe, Natasha Johnson, Kathy Smollett, Daniel Mair, Stephen Carmichael, Alice Broos, Lily Tong, Jenna Nichols, Kyriaki Nomikou; Sarah McDonald; Richard Orton, Joseph Hughes, Sreenu Vattipally, David L Robertson; Alasdair MacLean, Rory Gunson; Sharif Shaaban, Matthew Holden; Rachel Blacow, Guy Mollett, Kathy Li, James Shepherd, Antonia Ho, Emma Thomson
EPI_ISL_865000, EPI_ISL_865001, EPI_ISL_865002, EPI_ISL_865003, EPI_ISL_865004, EPI_ISL_865005, EPI_ISL_865006, EPI_ISL_865008	Lighthouse Lab in Glasgow / MRC-University of Glasgow Centre for Virus Research	COVID-19 Genomics UK (COG-UK) Consortium	Ana da Silva Filipe, Natasha Johnson, Kathy Smollett, Daniel Mair, Stephen Carmichael, Alice Broos, Lily Tong, Jenna Nichols, Kyriaki Nomikou; Sarah McDonald; Harper VanSteenhouse, Yumi Kasai, David Gray, Carol Clugston, Anna Dominiczak; Alasdair MacLean, Rory Gunson; Richard Orton, Joseph Hughes, Sreenu Vattipally, David L Robertson; Sharif Shaaban, Matthew Holden; Kathy Li, James Shepherd, Antonia Ho, Emma Thomson
EPI_ISL_865073, EPI_ISL_865079, EPI_ISL_865080, EPI_ISL_865081, EPI_ISL_865082, EPI_ISL_865083, EPI_ISL_865084, EPI_ISL_865085, EPI_ISL_865086, EPI_ISL_865087, EPI_ISL_865088			
see above	Virology Department, Royal Infirmary of Edinburgh, NHS Lothian / School of Biological Sciences, University of Edinburgh / Institute of Genetics and Molecular Medicine, University of Edinburgh	COVID-19 Genomics UK (COG-UK) Consortium	McHugh M, Dewar R, Rooke S, Gallagher M, Balcaza C, O'Toole Á, Scher E, Hill V, McCrone JT, Colquhoun R, Yu X, Jackson B, Rambaut A, Williams TC, Templeton K
EPI_ISL_865177, EPI_ISL_865354, EPI_ISL_865355, EPI_ISL_865356, EPI_ISL_865357, EPI_ISL_865358, EPI_ISL_865359, EPI_ISL_865360, EPI_ISL_865361, EPI_ISL_865362, EPI_ISL_865363, EPI_ISL_865364, EPI_ISL_865367, EPI_ISL_865368, EPI_ISL_865371, EPI_ISL_865372, EPI_ISL_865373, EPI_ISL_865374, EPI_ISL_865375, EPI_ISL_865376, EPI_ISL_865377, EPI_ISL_865378, EPI_ISL_865379, EPI_ISL_865380, EPI_ISL_865381, EPI_ISL_865382, EPI_ISL_865386, EPI_ISL_865389, EPI_ISL_865398, EPI_ISL_865405			
see above	Liverpool Clinical Laboratories	COVID-19 Genomics UK (COG-UK) Consortium	Sam Haldenby, Anita Lucaci, Steve Paterson, Julian Hiscox, Alistair Darby, M Almsaud, A Alrezaihi, Muhannad Alruwaili, Stuart D Armstrong, Jones Benjamin, Eleanor G Bentley, Anu Chawla, Jordan J Clark, Angela Cowell, Richard Eccles, Isabel García-Dorival, Matthew Gemmell, Alessandro Gerada,

			PKF Gilmore, Richard Gregory, Ximeng Han, Catherine Hartley, Margaret Hughes, Miren Iturriza-Gomara, James Johnson, L Luu, Jenifer Manson, Charlotte Nelson, Elaine O'Toole, Cassie Olateju, Rebekah Penrice-Randal , Lucille Rainbow, N.P Randle, Trevor Ian Robinson, Parul Sharma, Ghada T Shawli, James P Stewart, Neil Swainston, Ecaterina Vamos, Joanne Watts, Mark Whitehead
EPI_ISL_865489, EPI_ISL_865496, EPI_ISL_865497, EPI_ISL_865498, EPI_ISL_865501, EPI_ISL_865502, EPI_ISL_865516, EPI_ISL_865517, EPI_ISL_865518, EPI_ISL_865519, EPI_ISL_865520, EPI_ISL_865521, EPI_ISL_865522, EPI_ISL_865523, EPI_ISL_865524, EPI_ISL_865525, EPI_ISL_865526, EPI_ISL_865527, EPI_ISL_865528, EPI_ISL_865529, EPI_ISL_865530, EPI_ISL_865531, EPI_ISL_865532, EPI_ISL_865533, EPI_ISL_865534, EPI_ISL_865535, EPI_ISL_865536, EPI_ISL_865537, EPI_ISL_865538, EPI_ISL_865539, EPI_ISL_865540, EPI_ISL_865541, EPI_ISL_865542, EPI_ISL_865546, EPI_ISL_865547, EPI_ISL_865548, EPI_ISL_865549, EPI_ISL_865550, EPI_ISL_865551, EPI_ISL_865552, EPI_ISL_865553, EPI_ISL_865554, EPI_ISL_865555, EPI_ISL_865556, EPI_ISL_865557, EPI_ISL_865558, EPI_ISL_865561, EPI_ISL_865562, EPI_ISL_865564, EPI_ISL_865567, EPI_ISL_865568, EPI_ISL_865569, EPI_ISL_865570, EPI_ISL_865572, EPI_ISL_865573, EPI_ISL_865579, EPI_ISL_865580, EPI_ISL_865581, EPI_ISL_865582, EPI_ISL_865583			
see above	Barts Health NHS Trust	COVID-19 Genomics UK (COG-UK) Consortium	CUTINO-MOGUEL, Maria-Teresa; HARRINGTON, David; OWOYEMI, Dola; KULASEGARAN-SHYLINI, Raghavendran; BROAD, Claire; KELE, Beatrix
EPI_ISL_865682, EPI_ISL_865689, EPI_ISL_865691, EPI_ISL_865692, EPI_ISL_865693, EPI_ISL_865694, EPI_ISL_865806, EPI_ISL_865807, EPI_ISL_865809, EPI_ISL_865810, EPI_ISL_865811, EPI_ISL_865812, EPI_ISL_865813, EPI_ISL_865814, EPI_ISL_865816, EPI_ISL_865817, EPI_ISL_865818, EPI_ISL_865819, EPI_ISL_865820, EPI_ISL_865821, EPI_ISL_865822, EPI_ISL_865823, EPI_ISL_865824, EPI_ISL_865825, EPI_ISL_865826, EPI_ISL_865827, EPI_ISL_865828, EPI_ISL_865829, EPI_ISL_865830, EPI_ISL_865831, EPI_ISL_865832, EPI_ISL_865833, EPI_ISL_865834, EPI_ISL_865835, EPI_ISL_865836, EPI_ISL_865837, EPI_ISL_865838, EPI_ISL_865839, EPI_ISL_865840, EPI_ISL_865841, EPI_ISL_865842, EPI_ISL_865843, EPI_ISL_865844, EPI_ISL_865845, EPI_ISL_865846			
see above	University College London, Great Ormond Street Hospital for Children NHS Foundation Trust, Imperial College Healthcare NHS Trust	COVID-19 Genomics UK (COG-UK) Consortium	Sergi Castellano, Rachel Williams, Mark Kristiansen, Paola Resende Silva, Sunando Roy, Tony Brooks, Helena Tutill, Paola Niola, Patricia Dyal, Charlotte Williams, Leysa Forrest, Yasmin Panchbhaya, Jacqueline Findlay, Samuel Weeks, Julianne Brown, Kathryn Harris, Paul Randell, James Price, Alison Holmes, Judith Breuer
EPI_ISL_866071, EPI_ISL_866072, EPI_ISL_866073, EPI_ISL_866074, EPI_ISL_866115, EPI_ISL_866122, EPI_ISL_866127, EPI_ISL_866135, EPI_ISL_866140, EPI_ISL_866162, EPI_ISL_866168			
see above	University College London Hospital	COVID-19 Genomics UK (COG-UK) Consortium	Judith Heaney, Matthew Byott, Catherine Houlihan, Dan Frampton, Stuart Kirk, Moira Spyer and Eleni Nastouli
EPI_ISL_866346, EPI_ISL_866347, EPI_ISL_866348, EPI_ISL_866349, EPI_ISL_866350, EPI_ISL_866351, EPI_ISL_866352, EPI_ISL_866364, EPI_ISL_866365, EPI_ISL_866372	Regional Virus Laboratory, Belfast Health and Social Care Trust	COVID-19 Genomics UK (COG-UK) Consortium	Conall McCaughey, James McKenna, Tanya Curran, Susan Feeney, Alison Watt, Ciara Cox, Mairead Connor, Zoltan Molnar, David Simpson, Derek Fairley
EPI_ISL_866486, EPI_ISL_866487, EPI_ISL_866488, EPI_ISL_866489, EPI_ISL_866490, EPI_ISL_866491, EPI_ISL_866492, EPI_ISL_866493, EPI_ISL_866494, EPI_ISL_866495, EPI_ISL_866496, EPI_ISL_866497, EPI_ISL_866498, EPI_ISL_866499, EPI_ISL_866500, EPI_ISL_866501, EPI_ISL_866502, EPI_ISL_866503, EPI_ISL_866504, EPI_ISL_866505, EPI_ISL_866506, EPI_ISL_866507, EPI_ISL_866508, EPI_ISL_866509, EPI_ISL_866510, EPI_ISL_866511, EPI_ISL_866512, EPI_ISL_866513, EPI_ISL_866514, EPI_ISL_866515, EPI_ISL_866516, EPI_ISL_866517, EPI_ISL_866518, EPI_ISL_866519, EPI_ISL_866520, EPI_ISL_866521, EPI_ISL_866522, EPI_ISL_866523, EPI_ISL_866524, EPI_ISL_866525, EPI_ISL_866526, EPI_ISL_866535			
see above	Northumbria University / South Tees Hospitals NHS Foundation Trust / North Cumbria Integrated Care NHS Foundation Trust / North Tees and Hartlepool NHS Foundation Trust / Newcastle Hospitals NHS Foundation Trust	COVID-19 Genomics UK (COG-UK) Consortium	Darren L Smith,Andrew Nelson,Matthew Bashton,Greg R Young,Joshua Loh,John Allan,Mohammad A Tariq,Giles S Holt,Gary Black,Wen C Yew,Lynn Dover,Paul Baker,Steve Liggett,Sarah Essex,Jane Greenaway,Debra Padgett,Clive Graham,Garren Scott,Edward Barton,Emma Swindells,Brendan Payne,Jennifer Collins,Yusri Taha,Gary Eltringham
EPI_ISL_866601	Quadram Institute Bioscience	COVID-19 Genomics UK (COG-UK) Consortium	Dave J. Baker, Gemma L. Kay, Alp Aydin, Thanh Le-Viet, Steven Rudder, Ana P. Tedim, Anastasia Kolyva, Maria Diaz, Leonardo de Oliveira Martins, Nabil-Fareed Alikhan, Lizzie Meadows, Rachael Stanley, Ngozi Elumogo, Muhammed Yasir, Nicholas M. Thomson, Alexander J Trotter, Rachel Gilroy, Samuel Bloomfield, Claire Stuart, Andrew Bell, Reenesh Prakash, Samir Dervisevic, Alison E. Mather, John Wain, Mark Webber, Andrew J. Page, Justin O'Grady
EPI_ISL_866905	Queens Medical Centre, Clinical Microbiology Department / DeepSeq Nottingham	COVID-19 Genomics UK (COG-UK) Consortium	Gemma Clark, Wendy Smith, Manjinder Khakh, Vicki M Fleming, Michelle M Lister, Hannah Howson-Wells, Jonathan Ball, Patrick McClure, Joseph Chappell, Theocharis Tsoleridis, Nadine Holmes, Matthew Carlisle, Christopher Moore, Fei Sang, Johnny Debebe, Victoria Wright, Matthew Loose
EPI_ISL_866992, EPI_ISL_866993, EPI_ISL_866994, EPI_ISL_866995, EPI_ISL_866996, EPI_ISL_866997, EPI_ISL_866998, EPI_ISL_866999, EPI_ISL_867000, EPI_ISL_867001, EPI_ISL_867002, EPI_ISL_867003, EPI_ISL_867004, EPI_ISL_867005, EPI_ISL_867006, EPI_ISL_867007, EPI_ISL_867008, EPI_ISL_867009, EPI_ISL_867010, EPI_ISL_867011, EPI_ISL_867012, EPI_ISL_867013, EPI_ISL_867014, EPI_ISL_867015, EPI_ISL_867016, EPI_ISL_867017			
see above	Lincolnshire Hospitals and DeepSeq Nottingham	COVID-19 Genomics UK (COG-UK) Consortium	Nichola Duckworth, Tim Sloan, Sarah Walsh, Jonathan Ball, Patrick McClure, Joeseph Chappell, Nadine Holmes, Matthew Carlisle, Christopher Moore, Fei Sang, Johnny Debebe, Victoria Wright, Matthew Loose
EPI_ISL_867038, EPI_ISL_867039, EPI_ISL_867040, EPI_ISL_867041, EPI_ISL_867044, EPI_ISL_867045, EPI_ISL_867046, EPI_ISL_867047, EPI_ISL_867048, EPI_ISL_867049, EPI_ISL_867050, EPI_ISL_867052, EPI_ISL_867053, EPI_ISL_867054, EPI_ISL_867055, EPI_ISL_867056, EPI_ISL_867057, EPI_ISL_867058, EPI_ISL_867059, EPI_ISL_867060, EPI_ISL_867061, EPI_ISL_867062, EPI_ISL_867063, EPI_ISL_867064, EPI_ISL_867065, EPI_ISL_867066, EPI_ISL_867067, EPI_ISL_867068, EPI_ISL_867069, EPI_ISL_867071, EPI_ISL_867072, EPI_ISL_867073, EPI_ISL_867074, EPI_ISL_867075, EPI_ISL_867077, EPI_ISL_867078, EPI_ISL_867079, EPI_ISL_867080, EPI_ISL_867081, EPI_ISL_867082, EPI_ISL_867084, EPI_ISL_867087, EPI_ISL_867088, EPI_ISL_867089, EPI_ISL_867090, EPI_ISL_867093, EPI_ISL_867094, EPI_ISL_867095, EPI_ISL_867096, EPI_ISL_867097, EPI_ISL_867098, EPI_ISL_867130, EPI_ISL_867155, EPI_ISL_867159, EPI_ISL_867161, EPI_ISL_867162, EPI_ISL_867168, EPI_ISL_867169, EPI_ISL_867170, EPI_ISL_867171, EPI_ISL_867181			
see above	Oxford Viromics, NDM, University of Oxford; Oxford University Hospitals; Basingstoke and North Hampshire Hospital	COVID-19 Genomics UK (COG-UK) Consortium	Tanya Golubchik, David Bonsall, George Macintyre, Amy Trebes, Mariateresa de Cesare, Catrin Moore, Alex Mobbs, Anita Justice, Robert Shaw, Monique Andersson, Timothy Peto, Emma Wise, Nathan Moore, Jessica Lynch, Nick Cortes, Matilde Mori, Stephen Kidd, David Buck, John Todd, Christophe Fraser
EPI_ISL_867216, EPI_ISL_867217, EPI_ISL_867218, EPI_ISL_867431, EPI_ISL_867437, EPI_ISL_867438, EPI_ISL_867439, EPI_ISL_867440, EPI_ISL_867441, EPI_ISL_867442, EPI_ISL_867443, EPI_ISL_867486			
see above	Originating lab: Wales Specialist Virology Centre Sequencing lab: Pathogen Genomics Unit	Public Health Wales Microbiology Cardiff Wales Specialist Virology Centre	Catherine Moore, Johnathan Evans, Laura Gifford, Malorie Perry, Simon Cottrell, Angela Marchbank, Alec Birchley, Alexander Adams, Amy Gaskin, Bree Gatica-Wilcox, Jason Coombes, Joel Southgate, Lauren Gilbert, Lee Graham, Nicole Pacchiarini, Sara Kumziene-Summerhayes, Sarah Taylor, Sophie Jones, Sara Rey, Matthew Bull, Joanne Watkins, Sally Corden, Tom Connor
EPI_ISL_867982, EPI_ISL_867983, EPI_ISL_867985, EPI_ISL_867986, EPI_ISL_867987, EPI_ISL_867988, EPI_ISL_867989, EPI_ISL_867991, EPI_ISL_867992, EPI_ISL_867994, EPI_ISL_867995, EPI_ISL_867996, EPI_ISL_867997, EPI_ISL_867998, EPI_ISL_868000, EPI_ISL_868002, EPI_ISL_868003, EPI_ISL_868004, EPI_ISL_868005, EPI_ISL_868006, EPI_ISL_868007, EPI_ISL_868009, EPI_ISL_868010, EPI_ISL_868011, EPI_ISL_868012, EPI_ISL_868013, EPI_ISL_868014, EPI_ISL_868015, EPI_ISL_868017, EPI_ISL_868018, EPI_ISL_868019, EPI_ISL_868020, EPI_ISL_868021, EPI_ISL_868022, EPI_ISL_868024, EPI_ISL_868025, EPI_ISL_868026, EPI_ISL_868027, EPI_ISL_868028, EPI_ISL_868029, EPI_ISL_868030, EPI_ISL_868031, EPI_ISL_868032, EPI_ISL_868033, EPI_ISL_868035, EPI_ISL_868036, EPI_ISL_868037, EPI_ISL_868038, EPI_ISL_868039, EPI_ISL_868040, EPI_ISL_868041, EPI_ISL_868042, EPI_ISL_868043, EPI_ISL_868044, EPI_ISL_868046, EPI_ISL_868047, EPI_ISL_868048, EPI_ISL_868049, EPI_ISL_868050, EPI_ISL_868051, EPI_ISL_868052, EPI_ISL_868053, EPI_ISL_868054, EPI_ISL_868055, EPI_ISL_868056, EPI_ISL_868057, EPI_ISL_868058, EPI_ISL_868059, EPI_ISL_868060, EPI_ISL_868061, EPI_ISL_868062, EPI_ISL_868063, EPI_ISL_868064, EPI_ISL_868066, EPI_ISL_868067, EPI_ISL_868068, EPI_ISL_868069, EPI_ISL_868070, EPI_ISL_868071, EPI_ISL_868072, EPI_ISL_868073, EPI_ISL_868074, EPI_ISL_868076, EPI_ISL_868077, EPI_ISL_868078, EPI_ISL_868079, EPI_ISL_868080, EPI_ISL_868082, EPI_ISL_868083, EPI_ISL_868084, EPI_ISL_868085, EPI_ISL_868086, EPI_ISL_868087, EPI_ISL_868088, EPI_ISL_868089, EPI_ISL_868090, EPI_ISL_868091, EPI_ISL_868092, EPI_ISL_868093, EPI_ISL_868094, EPI_ISL_868095, EPI_ISL_868096, EPI_ISL_868097, EPI_ISL_868098, EPI_ISL_868099, EPI_ISL_868100, EPI_ISL_868101, EPI_ISL_868103, EPI_ISL_868104, EPI_ISL_868105, EPI_ISL_868106, EPI_ISL_868107, EPI_ISL_868108, EPI_ISL_868109, EPI_ISL_868110, EPI_ISL_868111, EPI_ISL_868112, EPI_ISL_868113, EPI_ISL_868114, EPI_ISL_868115, EPI_ISL_868116, EPI_ISL_868117, EPI_ISL_868118, EPI_ISL_868119, EPI_ISL_868120, EPI_ISL_868121, EPI_ISL_868122, EPI_ISL_868123, EPI_ISL_868124, EPI_ISL_868125, EPI_ISL_868126, EPI_ISL_868127, EPI_ISL_868128, EPI_ISL_868129, EPI_ISL_868130, EPI_ISL_868131, EPI_ISL_868133, EPI_ISL_868134, EPI_ISL_868135, EPI_ISL_868136, EPI_ISL_868137, EPI_ISL_868138, EPI_ISL_868139, EPI_ISL_868140, EPI_ISL_868259, EPI_ISL_868260, EPI_ISL_868261, EPI_ISL_868262, EPI_ISL_868263, EPI_ISL_868264, EPI_ISL_868265, EPI_ISL_868266, EPI_ISL_868267, EPI_ISL_868268, EPI_ISL_868269, EPI_ISL_868270, EPI_ISL_868271, EPI_ISL_868272, EPI_ISL_868273, EPI_ISL_868274, EPI_ISL_868275, EPI_ISL_868276, EPI_ISL_868277, EPI_ISL_868278, EPI_ISL_868279, EPI_ISL_868280, EPI_ISL_868281, EPI_ISL_868282, EPI_ISL_868283, EPI_ISL_868284, EPI_ISL_868285, EPI_ISL_868286, EPI_ISL_868287, EPI_ISL_868288, EPI_ISL_868289, EPI_ISL_868290, EPI_ISL_868291, EPI_ISL_868292, EPI_ISL_868293, EPI_ISL_868294, EPI_ISL_868295, EPI_ISL_868296, EPI_ISL_868297, EPI_ISL_868298, EPI_ISL_868299, EPI_ISL_868300, EPI_ISL_868301, EPI_ISL_868302, EPI_ISL_868303, EPI_ISL_868304, EPI_ISL_868305, EPI_ISL_868306, EPI_ISL_868307, EPI_ISL_868308, EPI_ISL_868318, EPI_ISL_868320			
see above	Centre for Enzyme Innovation, University of Portsmouth / Translational Research Laboratory, Portsmouth Hospitals NHS Trust	COVID-19 Genomics UK (COG-UK) Consortium	Angela Beckett,Yann Bourgeois,Garry Scarlett,Sharon Glaysher,Scott Elliott,Kelly Bicknell,Robert Impey,Allyson Lloyd,Sarah Wyllie,Ethan Butcher,Anoop Chauhan,Samuel Robson
EPI_ISL_868372, EPI_ISL_868420, EPI_ISL_868423, EPI_ISL_868485, EPI_ISL_868559, EPI_ISL_868570, EPI_ISL_868576, EPI_ISL_868629	Virology Department, Sheffield Teaching Hospitals NHS Foundation Trust/Department of Infection, Immunity and Cardiovascular Disease, The Medical School, University of Sheffield	COVID-19 Genomics UK (COG-UK) Consortium	Thushan de Silva, Matthew Parker, Nikki Smith, Adri Angyal, Rebecca Brown, Luke Green, Rachel Tucker, Paul Parsons, Danielle Groves, Katie Johnson, Laura Carrilero, Alex Keeley, Dave Partridge, Matthew Wyles, Benjamin Lindsey, Mehmet Yavuz, Mohammad Raza, Cariad Evans
EPI_ISL_868716, EPI_ISL_868717, EPI_ISL_868718, EPI_ISL_868719, EPI_ISL_868720, EPI_ISL_868721, EPI_ISL_868722, EPI_ISL_868723, EPI_ISL_868724, EPI_ISL_868725, EPI_ISL_868726, EPI_ISL_868727, EPI_ISL_868728, EPI_ISL_868729, EPI_ISL_868730, EPI_ISL_868731, EPI_ISL_868732, EPI_ISL_868733, EPI_ISL_868734, EPI_ISL_868735, EPI_ISL_868736, EPI_ISL_868737, EPI_ISL_868738, EPI_ISL_868739, EPI_ISL_868740, EPI_ISL_868741, EPI_ISL_868742, EPI_ISL_868743, EPI_ISL_868744, EPI_ISL_868745, EPI_ISL_868746, EPI_ISL_868747, EPI_ISL_868748, EPI_ISL_868749, EPI_ISL_868750, EPI_ISL_868751, EPI_ISL_868752, EPI_ISL_868753, EPI_ISL_868754, EPI_ISL_868755, EPI_ISL_868756, EPI_ISL_868757, EPI_ISL_868842, EPI_ISL_868846, EPI_ISL_868847, EPI_ISL_868849, EPI_ISL_868852, EPI_ISL_868857, EPI_ISL_868858, EPI_ISL_868862, EPI_ISL_868870, EPI_ISL_868871, EPI_ISL_868874, EPI_ISL_868876, EPI_ISL_868877, EPI_ISL_868901, EPI_ISL_868902, EPI_ISL_868903, EPI_ISL_868904, EPI_ISL_868905, EPI_ISL_868906, EPI_ISL_868907, EPI_ISL_868908, EPI_ISL_868909, EPI_ISL_868910, EPI_ISL_868911, EPI_ISL_868912, EPI_ISL_868913, EPI_ISL_868914, EPI_ISL_868915, EPI_ISL_868916, EPI_ISL_868917, EPI_ISL_868918, EPI_ISL_868919, EPI_ISL_868920, EPI_ISL_868921, EPI_ISL_868922, EPI_ISL_868923, EPI_ISL_868924, EPI_ISL_868925, EPI_ISL_868926, EPI_ISL_868927, EPI_ISL_868928, EPI_ISL_868929, EPI_ISL_868930, EPI_ISL_868931, EPI_ISL_868932, EPI_ISL_868933, EPI_ISL_868934, EPI_ISL_868935, EPI_ISL_868936, EPI_ISL_868937, EPI_ISL_869014, EPI_ISL_869015, EPI_ISL_869016, EPI_ISL_869017, EPI_ISL_869018, EPI_ISL_869019, EPI_ISL_869020, EPI_ISL_869021, EPI_ISL_869022, EPI_ISL_869023, EPI_ISL_869024, EPI_ISL_869025, EPI_ISL_869027, EPI_ISL_869028, EPI_ISL_869029, EPI_ISL_869030, EPI_ISL_869031, EPI_ISL_869032, EPI_ISL_869033, EPI_ISL_869034, EPI_ISL_869035, EPI_ISL_869036, EPI_ISL_869037, EPI_ISL_869038, EPI_ISL_869039, EPI_ISL_869040, EPI_ISL_869041, EPI_ISL_869042, EPI_ISL_869043, EPI_ISL_869044, EPI_ISL_869045, EPI_ISL_869046, EPI_ISL_869048, EPI_ISL_869049, EPI_ISL_869050, EPI_ISL_869051, EPI_ISL_869052, EPI_ISL_869053, EPI_ISL_869054, EPI_ISL_869055, EPI_ISL_869056, EPI_ISL_869057, EPI_ISL_869058, EPI_ISL_869059, EPI_ISL_869060, EPI_ISL_869061, EPI_ISL_869062, EPI_ISL_869063, EPI_ISL_869064, EPI_ISL_869065, EPI_ISL_869066, EPI_ISL_869067, EPI_ISL_869068, EPI_ISL_869069, EPI_ISL_869070, EPI_ISL_869071, EPI_ISL_869072, EPI_ISL_869073, EPI_ISL_869074, EPI_ISL_869075, EPI_ISL_869076, EPI_ISL_869077, EPI_ISL_869078			
see above	Bioinformatics and Biostatistics Lab, Advanced Sequencing	COVID-19 Genomics UK (COG-UK) Consortium	Aengus Stewart,Jerome Nicod,Chelsea Sawyer,Laura Cubitt,Harshil Patel,Margaret Crawford

	Facility		
EPI_ISL_882303, EPI_ISL_882305, EPI_ISL_882307, EPI_ISL_882308, EPI_ISL_882309, EPI_ISL_882314, EPI_ISL_882316, EPI_ISL_882319, EPI_ISL_882320, EPI_ISL_882321, EPI_ISL_882323, EPI_ISL_882327, EPI_ISL_882332, EPI_ISL_882339, EPI_ISL_882341, EPI_ISL_882343, EPI_ISL_882344, EPI_ISL_882352, EPI_ISL_882360, EPI_ISL_882361, EPI_ISL_882363, EPI_ISL_882364, EPI_ISL_882365, EPI_ISL_882371, EPI_ISL_882372, EPI_ISL_882375, EPI_ISL_882376, EPI_ISL_882377, EPI_ISL_882379, EPI_ISL_882382, EPI_ISL_882385, EPI_ISL_882388, EPI_ISL_882390, EPI_ISL_882393, EPI_ISL_882397, EPI_ISL_882398, EPI_ISL_882402, EPI_ISL_882404, EPI_ISL_882409, EPI_ISL_882413, EPI_ISL_882415, EPI_ISL_882418, EPI_ISL_882420, EPI_ISL_882421, EPI_ISL_882427, EPI_ISL_882428, EPI_ISL_882429, EPI_ISL_882434, EPI_ISL_882437, EPI_ISL_882438, EPI_ISL_882440, EPI_ISL_882441, EPI_ISL_882442, EPI_ISL_882445, EPI_ISL_882452, EPI_ISL_882471, EPI_ISL_882473, EPI_ISL_882475, EPI_ISL_882476, EPI_ISL_882480, EPI_ISL_882481, EPI_ISL_882482, EPI_ISL_882484, EPI_ISL_882491, EPI_ISL_882494, EPI_ISL_882505, EPI_ISL_882506, EPI_ISL_882512, EPI_ISL_882518, EPI_ISL_882520, EPI_ISL_882524, EPI_ISL_882525, EPI_ISL_882534, EPI_ISL_882537, EPI_ISL_882538, EPI_ISL_882539, EPI_ISL_882542, EPI_ISL_882544, EPI_ISL_882548, EPI_ISL_882549, EPI_ISL_882550, EPI_ISL_882559, EPI_ISL_882562, EPI_ISL_882563, EPI_ISL_882564, EPI_ISL_882565, EPI_ISL_882566, EPI_ISL_882568, EPI_ISL_882571, EPI_ISL_882576, EPI_ISL_882579, EPI_ISL_882580, EPI_ISL_882585, EPI_ISL_882586, EPI_ISL_882588, EPI_ISL_882589, EPI_ISL_882593, EPI_ISL_882595, EPI_ISL_882600, EPI_ISL_882601, EPI_ISL_882602, EPI_ISL_892212			
see above	Lighthouse Lab in Alderley Park	Wellcome Sanger Institute for the COVID-19 Genomics UK (COG-UK) Consortium	Jacquelyn Wynn, Mairead Hyland, The Lighthouse Lab in Alderley Park and Alex Alderton, Roberto Amato, Sonia Goncalves, Ewan Harrison, David K. Jackson, Ian Johnston, Dominic Kwiatkowski, Cordelia Langford, John Sillitoe on behalf of the Wellcome Sanger Institute COVID-19 Surveillance Team
EPI_ISL_918748, EPI_ISL_918749, EPI_ISL_918750, EPI_ISL_918751, EPI_ISL_918752, EPI_ISL_918753, EPI_ISL_918754, EPI_ISL_918755, EPI_ISL_918756, EPI_ISL_918757, EPI_ISL_918760, EPI_ISL_918761, EPI_ISL_918762, EPI_ISL_918763, EPI_ISL_918940, EPI_ISL_918941, EPI_ISL_918942, EPI_ISL_918943, EPI_ISL_918944, EPI_ISL_918945, EPI_ISL_918946, EPI_ISL_918947, EPI_ISL_918948, EPI_ISL_918949, EPI_ISL_918950, EPI_ISL_918951, EPI_ISL_918952, EPI_ISL_918953, EPI_ISL_918954, EPI_ISL_918955, EPI_ISL_918958, EPI_ISL_918959, EPI_ISL_918960, EPI_ISL_918961, EPI_ISL_918962, EPI_ISL_918963, EPI_ISL_918964, EPI_ISL_918965, EPI_ISL_918966, EPI_ISL_918967, EPI_ISL_918968			
see above	University of Birmingham	COVID-19 Genomics UK (COG-UK) Consortium	Institute of Microbiology, University of Birmingham: Claire McMurray, Joanne Stockton, Samuel Nicholls, Radoslaw Poplawski, Will Rowe, Josh Quick, Nicholas Loman. University of Birmingham Testing Laboratory: Celina M Whalley, Andrew Bosworth, Charlotte Poxon, Kasun Wanigasooriya, Oliver Pickles, Mike Kidd, Alex Richter, Andrew D Beggs PHE Heartlands Lab: Husam Osman, Andrew Bosworth. Queen Elizabeth Hospital: Anna Casey
EPI_ISL_918984, EPI_ISL_918997, EPI_ISL_919005, EPI_ISL_919018, EPI_ISL_919019, EPI_ISL_919020, EPI_ISL_919021, EPI_ISL_919022, EPI_ISL_919024, EPI_ISL_919025, EPI_ISL_919026, EPI_ISL_919027, EPI_ISL_919028, EPI_ISL_919030, EPI_ISL_919031, EPI_ISL_919032, EPI_ISL_919033, EPI_ISL_919034, EPI_ISL_919035, EPI_ISL_919036, EPI_ISL_919038, EPI_ISL_919040, EPI_ISL_919042, EPI_ISL_919053, EPI_ISL_919054, EPI_ISL_919055, EPI_ISL_919058, EPI_ISL_919062, EPI_ISL_919063, EPI_ISL_919067, EPI_ISL_919070, EPI_ISL_919074, EPI_ISL_919075, EPI_ISL_919079, EPI_ISL_919080, EPI_ISL_919082, EPI_ISL_919084, EPI_ISL_919087, EPI_ISL_919091, EPI_ISL_919092, EPI_ISL_919093, EPI_ISL_919095, EPI_ISL_919097, EPI_ISL_919099, EPI_ISL_919101, EPI_ISL_919102, EPI_ISL_919103, EPI_ISL_919106, EPI_ISL_919107, EPI_ISL_919114, EPI_ISL_919119, EPI_ISL_919134, EPI_ISL_919136, EPI_ISL_919139, EPI_ISL_919143			
see above	Department of Pathology, University of Cambridge	COVID-19 Genomics UK (COG-UK) Consortium	Aminu S. Jahun, Yasmin Chaudhry, Iliana Georgana, Myra Hosmillo, Rhys Izu, Martin D. Curran, Surendra Parmar, Ian Goodfellow
EPI_ISL_919257	West of Scotland Specialist Virology Centre, NHSGGC / MRC-University of Glasgow Centre for Virus Research	COVID-19 Genomics UK (COG-UK) Consortium	Ana da Silva Filipe, Natasha Johnson, Kathy Smollett, Daniel Mair, Stephen Carmichael, Alice Broos, Lily Tong, Jenna Nichols, Kyriaki Nomikou; Sarah McDonald; Richard Orton, Joseph Hughes, Sreenu Vattipally, David L Robertson; Alasdair MacLean, Rory Gunson; Sharif Shaaban, Matthew Holden; Rachel Blacow, Guy Mollett, Kathy Li, James Shepherd, Antonia Ho, Emma Thomson
EPI_ISL_919321, EPI_ISL_919322, EPI_ISL_919323, EPI_ISL_919324, EPI_ISL_919325, EPI_ISL_919326, EPI_ISL_919327, EPI_ISL_919328, EPI_ISL_919329, EPI_ISL_919330, EPI_ISL_919331, EPI_ISL_919332, EPI_ISL_919333, EPI_ISL_919334, EPI_ISL_919335, EPI_ISL_919336			
see above	Virology Department, Royal Infirmary of Edinburgh, NHS Lothian / School of Biological Sciences, University of Edinburgh / Institute of Genetics and Molecular Medicine, University of Edinburgh	COVID-19 Genomics UK (COG-UK) Consortium	McHugh M, Dewar R, Rooke S, Gallagher M, Balcaza C, O'Toole Á, Scher E, Hill V, McCrone JT, Colquhoun R, Yu X, Jackson B, Rambaut A, Williams TC, Templeton K
EPI_ISL_919407, EPI_ISL_919408, EPI_ISL_919409, EPI_ISL_919410, EPI_ISL_919411, EPI_ISL_919412, EPI_ISL_919413, EPI_ISL_919414, EPI_ISL_919415, EPI_ISL_919416, EPI_ISL_919417, EPI_ISL_919418, EPI_ISL_919419, EPI_ISL_919420, EPI_ISL_919421, EPI_ISL_919422, EPI_ISL_919423, EPI_ISL_919424, EPI_ISL_919425, EPI_ISL_919426, EPI_ISL_919427, EPI_ISL_919428, EPI_ISL_919429, EPI_ISL_919430, EPI_ISL_919431, EPI_ISL_919432, EPI_ISL_919433, EPI_ISL_919434, EPI_ISL_919435, EPI_ISL_919436, EPI_ISL_919437, EPI_ISL_919438, EPI_ISL_919439, EPI_ISL_919440, EPI_ISL_919441, EPI_ISL_919442, EPI_ISL_919443, EPI_ISL_919444			
see above	University of Exeter	COVID-19 Genomics UK (COG-UK) Consortium	Ben Temperton,Aaron Jeffries,Michelle Michelsen,Joanna Warwick-Dugdale,Audrey Farbos,Robyn Manley,Stephen Michell,Jane Masoli
EPI_ISL_919452, EPI_ISL_919453, EPI_ISL_919476	Liverpool Clinical Laboratories	COVID-19 Genomics UK (COG-UK) Consortium	Sam Haldenby, Anita Lucaci, Steve Paterson, Julian Hiscox, Alistair Darby, M Almsaud, A Alrezaihi, Muhannad Alruwaili, Stuart D Armstrong, Jones Benjamin, Eleanor G Bentley, Anu Chawla, Jordan J Clark, Angela Cowell, Richard Eccles, Isabel García-Dorival, Matthew Gemmell, Alessandro Gerada, PKF Gilmore, Richard Gregory, Ximeng Han, Catherine Hartley, Margaret Hughes, Miren Iturriza-Gomara, James Johnson, L Luu, Jenifer Manson, Charlotte Nelson, Elaine O'Toole, Cassie Olateju, Rebekah Penrice-Randal , Lucille Rainbow, N.P Randle, Trevor Ian Robinson, Parul Sharma, Ghada T Shawli, James P Stewart, Neil Swainston, Ecaterina Vamos, Joanne Watts, Mark Whitehead
EPI_ISL_919781, EPI_ISL_919782, EPI_ISL_919783, EPI_ISL_919784, EPI_ISL_919785, EPI_ISL_919786, EPI_ISL_919787, EPI_ISL_919788, EPI_ISL_919789, EPI_ISL_919790, EPI_ISL_919791, EPI_ISL_919792, EPI_ISL_919793, EPI_ISL_919794, EPI_ISL_919795, EPI_ISL_919796			
see above	Barts Health NHS Trust	COVID-19 Genomics UK (COG-UK) Consortium	CUTINO-MOGUEL, Maria-Teresa; HARRINGTON, David; OWOYEMI, Dola; KULASEGARAN-SHYLINI, Raghavendran; BROAD, Claire; KELE, Beatrix
EPI_ISL_919961, EPI_ISL_919964, EPI_ISL_919966	University College London, Great Ormond Street Hospital for Children NHS Foundation Trust, Imperial College Healthcare NHS Trust	COVID-19 Genomics UK (COG-UK) Consortium	Sergi Castellano, Rachel Williams, Mark Kristiansen, Paola Resende Silva, Sunando Roy, Tony Brooks, Helena Tutill, Paola Niola, Patricia Dyal, Charlotte Williams, Leysa Forrest, Yasmin Panchbhaya, Jacqueline Findlay, Samuel Weeks, Julianne Brown, Kathryn Harris, Paul Randell, James Price, Alison Holmes, Judith Breuer
EPI_ISL_920319, EPI_ISL_920344, EPI_ISL_920354, EPI_ISL_920457, EPI_ISL_920499, EPI_ISL_920629, EPI_ISL_920653	University College London Hospital	COVID-19 Genomics UK (COG-UK) Consortium	Judith Heaney, Matthew Byott, Catherine Houlihan, Dan Frampton, Stuart Kirk, Moira Spyer and Eleni Nastouli
EPI_ISL_920841, EPI_ISL_920842, EPI_ISL_920843, EPI_ISL_920844, EPI_ISL_920845, EPI_ISL_920846, EPI_ISL_920847, EPI_ISL_920854, EPI_ISL_920855	University College London, Great Ormond Street Hospital for Children NHS Foundation Trust, Imperial College Healthcare NHS Trust	COVID-19 Genomics UK (COG-UK) Consortium	Sergi Castellano, Rachel Williams, Mark Kristiansen, Paola Resende Silva, Sunando Roy, Tony Brooks, Helena Tutill, Paola Niola, Patricia Dyal, Charlotte Williams, Leysa Forrest, Yasmin Panchbhaya, Jacqueline Findlay, Samuel Weeks, Julianne Brown, Kathryn Harris, Paul Randell, James Price, Alison Holmes, Judith Breuer
EPI_ISL_921022, EPI_ISL_921027, EPI_ISL_921028, EPI_ISL_921029, EPI_ISL_921030, EPI_ISL_921031, EPI_ISL_921032, EPI_ISL_921033, EPI_ISL_921034, EPI_ISL_921035, EPI_ISL_921036, EPI_ISL_921038, EPI_ISL_921039, EPI_ISL_921041, EPI_ISL_921042, EPI_ISL_921043, EPI_ISL_921044, EPI_ISL_921045, EPI_ISL_921046, EPI_ISL_921072, EPI_ISL_921073, EPI_ISL_921074, EPI_ISL_921075, EPI_ISL_921076, EPI_ISL_921077, EPI_ISL_921085, EPI_ISL_921087, EPI_ISL_921088, EPI_ISL_921089, EPI_ISL_921090, EPI_ISL_921118, EPI_ISL_921119, EPI_ISL_921120, EPI_ISL_921133, EPI_ISL_921135, EPI_ISL_921136			
see above	Regional Virus Laboratory, Belfast Health and Social Care Trust	COVID-19 Genomics UK (COG-UK) Consortium	Conall McCaughey, James McKenna, Tanya Curran, Susan Feeney, Alison Watt, Ciara Cox, Mairead Connor, Zoltan Molnar, David Simpson, Derek Fairley
EPI_ISL_921231, EPI_ISL_921233, EPI_ISL_921234, EPI_ISL_921235, EPI_ISL_921236, EPI_ISL_921237, EPI_ISL_921242, EPI_ISL_921249, EPI_ISL_921253, EPI_ISL_921254, EPI_ISL_921255, EPI_ISL_921258, EPI_ISL_921259, EPI_ISL_921260, EPI_ISL_921261, EPI_ISL_921262, EPI_ISL_921264, EPI_ISL_921265, EPI_ISL_921268, EPI_ISL_921271, EPI_ISL_921272, EPI_ISL_921273, EPI_ISL_921274, EPI_ISL_921275, EPI_ISL_921276, EPI_ISL_921278, EPI_ISL_921280, EPI_ISL_921281, EPI_ISL_921282, EPI_ISL_921283, EPI_ISL_921284, EPI_ISL_921286, EPI_ISL_921288, EPI_ISL_921295, EPI_ISL_921297, EPI_ISL_921298, EPI_ISL_921301, EPI_ISL_921303, EPI_ISL_921305, EPI_ISL_921313, EPI_ISL_921314, EPI_ISL_921317, EPI_ISL_921318, EPI_ISL_921364, EPI_ISL_921365, EPI_ISL_921366, EPI_ISL_921367, EPI_ISL_921368, EPI_ISL_921369, EPI_ISL_921370, EPI_ISL_921371, EPI_ISL_921372, EPI_ISL_921373, EPI_ISL_921374, EPI_ISL_921375, EPI_ISL_921376, EPI_ISL_921377, EPI_ISL_921378, EPI_ISL_921379, EPI_ISL_921380, EPI_ISL_921381, EPI_ISL_921382, EPI_ISL_921383, EPI_ISL_921384, EPI_ISL_921385, EPI_ISL_921386, EPI_ISL_921387, EPI_ISL_921388, EPI_ISL_921389, EPI_ISL_921390, EPI_ISL_921391, EPI_ISL_921392, EPI_ISL_921393, EPI_ISL_921394, EPI_ISL_921395, EPI_ISL_921396, EPI_ISL_921397, EPI_ISL_921398, EPI_ISL_921399, EPI_ISL_921400, EPI_ISL_921401, EPI_ISL_921402, EPI_ISL_921403, EPI_ISL_921404, EPI_ISL_921405, EPI_ISL_921406, EPI_ISL_921414, EPI_ISL_921415, EPI_ISL_921416, EPI_ISL_921417, EPI_ISL_921418, EPI_ISL_921420, EPI_ISL_921421, EPI_ISL_921422, EPI_ISL_921423, EPI_ISL_921424, EPI_ISL_921425, EPI_ISL_921426, EPI_ISL_921432, EPI_ISL_921433, EPI_ISL_921665, EPI_ISL_921666, EPI_ISL_921667, EPI_ISL_921668, EPI_ISL_921669, EPI_ISL_921670, EPI_ISL_921671, EPI_ISL_921672, EPI_ISL_921673, EPI_ISL_921674, EPI_ISL_921675			
see above	Northumbria University / South Tees Hospitals NHS Foundation Trust / North Cumbria Integrated Care NHS Foundation Trust / North Tees and Hartlepool NHS Foundation Trust / Newcastle Hospitals NHS Foundation Trust	COVID-19 Genomics UK (COG-UK) Consortium	Darren L Smith,Andrew Nelson,Matthew Bashton,Greg R Young,Joshua Loh,John Allan,Mohammad A Tariq,Giles S Holt,Gary Black,Wen C Yew,Lynn Dover,Paul Baker,Steve Liggett,Sarah Essex,Jane Greenaway,Debra Padgett,Clive Graham,Garren Scott,Edward Barton,Emma Swindells,Brendan Payne,Jennifer Collins,Yusri Taha,Gary Eltringham
EPI_ISL_921786, EPI_ISL_921787, EPI_ISL_921788, EPI_ISL_921789, EPI_ISL_921790, EPI_ISL_921791, EPI_ISL_921792, EPI_ISL_921793, EPI_ISL_921796, EPI_ISL_921798, EPI_ISL_921799, EPI_ISL_921800, EPI_ISL_921801, EPI_ISL_921802, EPI_ISL_921805, EPI_ISL_921808, EPI_ISL_921809, EPI_ISL_921810, EPI_ISL_921811, EPI_ISL_921813, EPI_ISL_921815, EPI_ISL_921819, EPI_ISL_921820, EPI_ISL_921821, EPI_ISL_921824, EPI_ISL_921825, EPI_ISL_921826, EPI_ISL_921828, EPI_ISL_921839, EPI_ISL_921841, EPI_ISL_921842, EPI_ISL_921846, EPI_ISL_921847, EPI_ISL_921848, EPI_ISL_921849			
see above	Quadram Institute Bioscience	COVID-19 Genomics UK (COG-UK) Consortium	Dave J. Baker, Gemma L. Kay, Alp Aydin, Thanh Le-Viet, Steven Rudder, Ana P. Tedim, Anastasia Kolyva, Maria Diaz, Leonardo de Oliveira Martins, Nabil-Fareed Alikhan, Lizzie Meadows, Rachael Stanley, Ngozi Elumogo, Muhammed Yasir, Nicholas M. Thomson, Alexander J Trotter, Rachel Gilroy, Samuel Bloomfield, Claire Stuart, Andrew Bell, Reenesh Prakash, Samir Dervisevic, Alison E. Mather, John Wain, Mark Webber, Andrew J. Page, Justin O'Grady
EPI_ISL_922241, EPI_ISL_922260, EPI_ISL_922261, EPI_ISL_922262, EPI_ISL_922263, EPI_ISL_922264, EPI_ISL_922267, EPI_ISL_922268, EPI_ISL_922270, EPI_ISL_922271, EPI_ISL_922272, EPI_ISL_922274, EPI_ISL_922275, EPI_ISL_922276, EPI_ISL_922277, EPI_ISL_922279, EPI_ISL_922281, EPI_ISL_922282, EPI_ISL_922287, EPI_ISL_922288, EPI_ISL_922289, EPI_ISL_922290, EPI_ISL_922291, EPI_ISL_922292, EPI_ISL_922293, EPI_ISL_922296, EPI_ISL_922297, EPI_ISL_922299, EPI_ISL_922300, EPI_ISL_922302, EPI_ISL_922305, EPI_ISL_922313, EPI_ISL_922314			
see above	Oxford Viromics, NDM, University of Oxford; Oxford University	COVID-19 Genomics UK (COG-UK) Consortium	Tanya Golubchik, David Bonsall, George Macintyre, Amy Trebes, Mariateresa de Cesare, Catrin Moore, Alex Mobbs, Anita Justice, Robert Shaw, Monique

	Hospitals; Basingstoke and North Hampshire Hospital		Andersson, Timothy Peto, Emma Wise, Nathan Moore, Jessica Lynch, Nick Cortes, Matilde Mori, Stephen Kidd, David Buck, John Todd, Christophe Fraser
EPI_ISL_923242, EPI_ISL_923244, EPI_ISL_923258, EPI_ISL_923265, EPI_ISL_923267, EPI_ISL_923268, EPI_ISL_923295, EPI_ISL_923425, EPI_ISL_923426, EPI_ISL_923427, EPI_ISL_923429, EPI_ISL_923431, EPI_ISL_923432, EPI_ISL_923433, EPI_ISL_923434, EPI_ISL_923435, EPI_ISL_923437, EPI_ISL_923439, EPI_ISL_923440, EPI_ISL_923441, EPI_ISL_923442, EPI_ISL_923444, EPI_ISL_923445, EPI_ISL_923446, EPI_ISL_923447, EPI_ISL_923448, EPI_ISL_923449, EPI_ISL_923450, EPI_ISL_923451, EPI_ISL_923452, EPI_ISL_923453, EPI_ISL_923454, EPI_ISL_923455, EPI_ISL_923456, EPI_ISL_923457, EPI_ISL_923459, EPI_ISL_923460, EPI_ISL_923461, EPI_ISL_923462, EPI_ISL_923463, EPI_ISL_923464, EPI_ISL_923465, EPI_ISL_923466, EPI_ISL_923467, EPI_ISL_923469, EPI_ISL_923470, EPI_ISL_923471, EPI_ISL_923472, EPI_ISL_923473, EPI_ISL_923475, EPI_ISL_923476, EPI_ISL_923477, EPI_ISL_923478, EPI_ISL_923479, EPI_ISL_923480, EPI_ISL_923481, EPI_ISL_923482, EPI_ISL_923483, EPI_ISL_923484, EPI_ISL_923485, EPI_ISL_923486, EPI_ISL_923488, EPI_ISL_923489, EPI_ISL_923490, EPI_ISL_923491, EPI_ISL_923492, EPI_ISL_923493, EPI_ISL_923494, EPI_ISL_923495, EPI_ISL_923496, EPI_ISL_923497, EPI_ISL_923498, EPI_ISL_923499, EPI_ISL_923500, EPI_ISL_923501, EPI_ISL_923502, EPI_ISL_923503, EPI_ISL_923504, EPI_ISL_923505, EPI_ISL_923506, EPI_ISL_923507, EPI_ISL_923508, EPI_ISL_923509, EPI_ISL_923510, EPI_ISL_923511, EPI_ISL_923512, EPI_ISL_923514, EPI_ISL_923515, EPI_ISL_923516, EPI_ISL_923517, EPI_ISL_923520, EPI_ISL_923522, EPI_ISL_923523, EPI_ISL_923524, EPI_ISL_923525, EPI_ISL_923526, EPI_ISL_923527, EPI_ISL_923528, EPI_ISL_923529, EPI_ISL_923530, EPI_ISL_923532, EPI_ISL_923533, EPI_ISL_923535, EPI_ISL_923536, EPI_ISL_923537, EPI_ISL_923538, EPI_ISL_923540, EPI_ISL_923541, EPI_ISL_923542, EPI_ISL_923543, EPI_ISL_923544, EPI_ISL_923545, EPI_ISL_923547, EPI_ISL_923548, EPI_ISL_923549, EPI_ISL_923550, EPI_ISL_923551, EPI_ISL_923553, EPI_ISL_923554, EPI_ISL_923556, EPI_ISL_923557, EPI_ISL_923558, EPI_ISL_923559, EPI_ISL_923560, EPI_ISL_923561, EPI_ISL_923562, EPI_ISL_923563, EPI_ISL_923564, EPI_ISL_923565, EPI_ISL_923566, EPI_ISL_923567, EPI_ISL_923568, EPI_ISL_923569, EPI_ISL_923570, EPI_ISL_923571, EPI_ISL_923572, EPI_ISL_923573, EPI_ISL_923574, EPI_ISL_923575, EPI_ISL_923576, EPI_ISL_923578, EPI_ISL_923579, EPI_ISL_923581, EPI_ISL_923583, EPI_ISL_923584, EPI_ISL_923585, EPI_ISL_923586, EPI_ISL_923599, EPI_ISL_923600, EPI_ISL_923601, EPI_ISL_923602, EPI_ISL_923603, EPI_ISL_923604, EPI_ISL_923606, EPI_ISL_923607, EPI_ISL_923608, EPI_ISL_923609, EPI_ISL_923610, EPI_ISL_923612, EPI_ISL_923614, EPI_ISL_923615, EPI_ISL_923616, EPI_ISL_923617, EPI_ISL_923618, EPI_ISL_923619, EPI_ISL_923620, EPI_ISL_923621, EPI_ISL_923622, EPI_ISL_923623, EPI_ISL_923624, EPI_ISL_923625, EPI_ISL_923626, EPI_ISL_923627, EPI_ISL_923629, EPI_ISL_923630, EPI_ISL_923631, EPI_ISL_923632, EPI_ISL_923633, EPI_ISL_923634, EPI_ISL_923635, EPI_ISL_923636, EPI_ISL_923637, EPI_ISL_923638, EPI_ISL_923639, EPI_ISL_923640, EPI_ISL_923641, EPI_ISL_923642, EPI_ISL_923643, EPI_ISL_923644, EPI_ISL_923645, EPI_ISL_923646, EPI_ISL_923647, EPI_ISL_923648, EPI_ISL_923649, EPI_ISL_923650, EPI_ISL_923651, EPI_ISL_923652, EPI_ISL_923653, EPI_ISL_923654, EPI_ISL_923655, EPI_ISL_923656, EPI_ISL_923657, EPI_ISL_923658, EPI_ISL_923659, EPI_ISL_923661, EPI_ISL_923662			
see above	Centre for Enzyme Innovation, University of Portsmouth / Translational Research Laboratory, Portsmouth Hospitals NHS Trust	COVID-19 Genomics UK (COG-UK) Consortium	Angela Beckett,Salman Goudarzi,Christopher Fearn,Kate Cook,Katie Loveson,Sharon Glaysher,Scott Elliott,Samuel Robson
EPI_ISL_924094, EPI_ISL_924168, EPI_ISL_924213, EPI_ISL_924237, EPI_ISL_924250, EPI_ISL_924280, EPI_ISL_924283, EPI_ISL_924402	Virology Department, Sheffield Teaching Hospitals NHS Foundation Trust/Department of Infection, Immunity and Cardiovascular Disease, The Medical School, University of Sheffield	COVID-19 Genomics UK (COG-UK) Consortium	Thushan de Silva, Matthew Parker, Nikki Smith, Adri Angyal, Rebecca Brown, Luke Green, Rachel Tucker, Paul Parsons, Danielle Groves, Katie Johnson, Laura Carrilero, Alex Keeley, Dave Partridge, Matthew Wyles, Benjamin Lindsey, Mehmet Yavuz, Mohammad Raza, Cariad Evans
EPI_ISL_924430, EPI_ISL_924431, EPI_ISL_924432, EPI_ISL_924433, EPI_ISL_924434, EPI_ISL_924435, EPI_ISL_924436, EPI_ISL_924437, EPI_ISL_924438, EPI_ISL_924439, EPI_ISL_924440, EPI_ISL_924441, EPI_ISL_924443, EPI_ISL_924444, EPI_ISL_924445, EPI_ISL_924446, EPI_ISL_924447, EPI_ISL_924448, EPI_ISL_924449, EPI_ISL_924450, EPI_ISL_924451, EPI_ISL_924452, EPI_ISL_924453, EPI_ISL_924454, EPI_ISL_924455, EPI_ISL_924456, EPI_ISL_924457, EPI_ISL_924458, EPI_ISL_924459, EPI_ISL_924460, EPI_ISL_924461, EPI_ISL_924462, EPI_ISL_924464, EPI_ISL_924465, EPI_ISL_924466, EPI_ISL_924467, EPI_ISL_924468, EPI_ISL_924469, EPI_ISL_924470, EPI_ISL_924471, EPI_ISL_924472, EPI_ISL_924473, EPI_ISL_924474, EPI_ISL_924475, EPI_ISL_924476, EPI_ISL_924477, EPI_ISL_924478, EPI_ISL_924479, EPI_ISL_924480, EPI_ISL_924481, EPI_ISL_924482, EPI_ISL_924483, EPI_ISL_924484, EPI_ISL_924485, EPI_ISL_924486, EPI_ISL_924487, EPI_ISL_924488, EPI_ISL_924489, EPI_ISL_924490, EPI_ISL_924491, EPI_ISL_924492, EPI_ISL_924493, EPI_ISL_924494, EPI_ISL_924495, EPI_ISL_924497, EPI_ISL_924498, EPI_ISL_924500, EPI_ISL_924501, EPI_ISL_924502, EPI_ISL_924503, EPI_ISL_924504, EPI_ISL_924505, EPI_ISL_924506, EPI_ISL_924507, EPI_ISL_924508, EPI_ISL_924509, EPI_ISL_924510, EPI_ISL_924511, EPI_ISL_924512, EPI_ISL_924513, EPI_ISL_924514, EPI_ISL_924515, EPI_ISL_924516, EPI_ISL_924517, EPI_ISL_924518, EPI_ISL_924519, EPI_ISL_924520, EPI_ISL_924521, EPI_ISL_924522, EPI_ISL_924523, EPI_ISL_924524, EPI_ISL_924525, EPI_ISL_924526, EPI_ISL_924527, EPI_ISL_924528, EPI_ISL_924529, EPI_ISL_924530, EPI_ISL_924531, EPI_ISL_924532, EPI_ISL_924533, EPI_ISL_924534, EPI_ISL_924535, EPI_ISL_924537, EPI_ISL_924538, EPI_ISL_924539, EPI_ISL_924540, EPI_ISL_924542, EPI_ISL_924544, EPI_ISL_924545, EPI_ISL_924547, EPI_ISL_924548, EPI_ISL_924549, EPI_ISL_924550, EPI_ISL_924551, EPI_ISL_924552, EPI_ISL_924553, EPI_ISL_924554, EPI_ISL_924555, EPI_ISL_924556, EPI_ISL_924557, EPI_ISL_924558, EPI_ISL_924559, EPI_ISL_924560, EPI_ISL_924561, EPI_ISL_924562, EPI_ISL_924563, EPI_ISL_924564, EPI_ISL_924565, EPI_ISL_924566, EPI_ISL_924567, EPI_ISL_924568, EPI_ISL_924569, EPI_ISL_924570, EPI_ISL_924571, EPI_ISL_924572, EPI_ISL_924573, EPI_ISL_924574, EPI_ISL_924575, EPI_ISL_924576, EPI_ISL_924577, EPI_ISL_924578, EPI_ISL_924579, EPI_ISL_924580, EPI_ISL_924581, EPI_ISL_924582, EPI_ISL_924583, EPI_ISL_924584, EPI_ISL_924588, EPI_ISL_924591, EPI_ISL_924599, EPI_ISL_924602			
see above	Bioinformatics and Biostatistics Lab, Advanced Sequencing Facility	COVID-19 Genomics UK (COG-UK) Consortium	Aengus Stewart,Jerome Nicod,Chelsea Sawyer,Laura Cubitt,Harshil Patel,Margaret Crawford
EPI_ISL_933426, EPI_ISL_933427, EPI_ISL_933428, EPI_ISL_933429, EPI_ISL_933432, EPI_ISL_933433, EPI_ISL_933436, EPI_ISL_933437, EPI_ISL_933438, EPI_ISL_933441, EPI_ISL_933442, EPI_ISL_933443, EPI_ISL_933444, EPI_ISL_933445, EPI_ISL_933446, EPI_ISL_933447, EPI_ISL_933448, EPI_ISL_933449, EPI_ISL_933450, EPI_ISL_933451, EPI_ISL_933452, EPI_ISL_933453, EPI_ISL_933454, EPI_ISL_933455, EPI_ISL_933456, EPI_ISL_933457, EPI_ISL_933458, EPI_ISL_933459, EPI_ISL_933460, EPI_ISL_933461, EPI_ISL_933462, EPI_ISL_933463, EPI_ISL_933464, EPI_ISL_933465, EPI_ISL_933466, EPI_ISL_933467, EPI_ISL_933468, EPI_ISL_933469, EPI_ISL_933470, EPI_ISL_933471, EPI_ISL_933472, EPI_ISL_933473, EPI_ISL_933474, EPI_ISL_933475, EPI_ISL_933476, EPI_ISL_933477, EPI_ISL_933478			
see above	Lighthouse Lab in Milton Keynes	Wellcome Sanger Institute for the COVID-19 Genomics UK (COG-UK) Consortium	The Lighthouse Lab in Milton Keynes and Alex Alderton, Roberto Amato, Sonia Goncalves, Ewan Harrison, David K. Jackson, Ian Johnston, Dominic Kwiatkowski, Cordelia Langford, John Sillitoe on behalf of the Wellcome Sanger Institute COVID-19 Surveillance Team
EPI_ISL_945065	Lighthouse Lab in Glasgow	Wellcome Sanger Institute for the COVID-19 Genomics UK (COG-UK) Consortium	Harper VanSteenhouse, Yumi Kasai, David Gray, Carol Clugston, Anna Dominiczak and Alex Alderton, Roberto Amato, Sonia Goncalves, Ewan Harrison, David K. Jackson, Ian Johnston, Dominic Kwiatkowski, Cordelia Langford, John Sillitoe on behalf of the Wellcome Sanger Institute COVID-19 Surveillance Team
EPI_ISL_945068	Lighthouse Lab in Cambridge	Wellcome Sanger Institute for the COVID-19 Genomics UK (COG-UK) Consortium	Rob Howes, The Lighthouse Lab in Cambridge and Alex Alderton, Roberto Amato, Sonia Goncalves, Ewan Harrison, David K. Jackson, Ian Johnston, Dominic Kwiatkowski, Cordelia Langford, John Sillitoe on behalf of the Wellcome Sanger Institute COVID-19 Surveillance Team
EPI_ISL_945126, EPI_ISL_945136	Lighthouse Lab in Glasgow	Wellcome Sanger Institute for the COVID-19 Genomics UK (COG-UK) Consortium	Harper VanSteenhouse, Yumi Kasai, David Gray, Carol Clugston, Anna Dominiczak and Alex Alderton, Roberto Amato, Sonia Goncalves, Ewan Harrison, David K. Jackson, Ian Johnston, Dominic Kwiatkowski, Cordelia Langford, John Sillitoe on behalf of the Wellcome Sanger Institute COVID-19 Surveillance Team
EPI_ISL_945273	Lighthouse Lab in Milton Keynes	Wellcome Sanger Institute for the COVID-19 Genomics UK (COG-UK) Consortium	The Lighthouse Lab in Milton Keynes and Alex Alderton, Roberto Amato, Sonia Goncalves, Ewan Harrison, David K. Jackson, Ian Johnston, Dominic Kwiatkowski, Cordelia Langford, John Sillitoe on behalf of the Wellcome Sanger Institute COVID-19 Surveillance Team
EPI_ISL_945301	Lighthouse Lab in Glasgow	Wellcome Sanger Institute for the COVID-19 Genomics UK (COG-UK) Consortium	Harper VanSteenhouse, Yumi Kasai, David Gray, Carol Clugston, Anna Dominiczak and Alex Alderton, Roberto Amato, Sonia Goncalves, Ewan Harrison, David K. Jackson, Ian Johnston, Dominic Kwiatkowski, Cordelia Langford, John Sillitoe on behalf of the Wellcome Sanger Institute COVID-19 Surveillance Team
EPI_ISL_945339	Lighthouse Lab in Milton Keynes	Wellcome Sanger Institute for the COVID-19 Genomics UK (COG-UK) Consortium	The Lighthouse Lab in Milton Keynes and Alex Alderton, Roberto Amato, Sonia Goncalves, Ewan Harrison, David K. Jackson, Ian Johnston, Dominic Kwiatkowski, Cordelia Langford, John Sillitoe on behalf of the Wellcome Sanger Institute COVID-19 Surveillance Team
EPI_ISL_949430, EPI_ISL_949433, EPI_ISL_949479, EPI_ISL_949485, EPI_ISL_949487, EPI_ISL_949490, EPI_ISL_949500, EPI_ISL_949502, EPI_ISL_949508, EPI_ISL_949510, EPI_ISL_949512, EPI_ISL_949514, EPI_ISL_949516, EPI_ISL_949518, EPI_ISL_949519, EPI_ISL_949520, EPI_ISL_949521, EPI_ISL_949541			
see above	Department of Pathology, University of Cambridge	COVID-19 Genomics UK (COG-UK) Consortium	Aminu S. Jahun, Yasmin Chaudhry, Iliana Georgana, Myra Hosmillo, Rhys Izu, Martin D. Curran, Surendra Parmar, Ian Goodfellow
EPI_ISL_949761	Barts Health NHS Trust	COVID-19 Genomics UK (COG-UK) Consortium	CUTINO-MOGUEL, Maria-Teresa; HARRINGTON, David; OWOYEMI, Dola; KULASEGARAN-SHYLINI, Raghavendran; BROAD, Claire; KELE, Beatrix
EPI_ISL_949959, EPI_ISL_950105, EPI_ISL_950121, EPI_ISL_950131, EPI_ISL_950132, EPI_ISL_950137, EPI_ISL_950138, EPI_ISL_950139, EPI_ISL_950140, EPI_ISL_950141, EPI_ISL_950143, EPI_ISL_950144, EPI_ISL_950145, EPI_ISL_950149, EPI_ISL_950167			
see above	University College London, Great Ormond Street Hospital for Children NHS Foundation Trust, Imperial College Healthcare NHS Trust	COVID-19 Genomics UK (COG-UK) Consortium	Sergi Castellano, Rachel Williams, Mark Kristiansen, Paola Resende Silva, Sunando Roy, Tony Brooks, Helena Tutill, Paola Niola, Patricia Dyal, Charlotte Williams, Leysa Forrest, Yasmin Panchbhaya, Jacqueline Findlay, Samuel Weeks, Julianne Brown, Kathryn Harris, Paul Randell, James Price, Alison Holmes, Judith Breuer
EPI_ISL_950233, EPI_ISL_950234, EPI_ISL_950235, EPI_ISL_950236, EPI_ISL_950237, EPI_ISL_950238, EPI_ISL_950239, EPI_ISL_950240, EPI_ISL_950241, EPI_ISL_950242, EPI_ISL_950243, EPI_ISL_950244, EPI_ISL_950245, EPI_ISL_950246, EPI_ISL_950247, EPI_ISL_950248, EPI_ISL_950249, EPI_ISL_950250, EPI_ISL_950251, EPI_ISL_950252, EPI_ISL_950253, EPI_ISL_950256, EPI_ISL_950271, EPI_ISL_950273, EPI_ISL_950274, EPI_ISL_950275, EPI_ISL_950276, EPI_ISL_950277, EPI_ISL_950278, EPI_ISL_950279, EPI_ISL_950281, EPI_ISL_950286, EPI_ISL_950287, EPI_ISL_950295, EPI_ISL_950296, EPI_ISL_950299, EPI_ISL_950300, EPI_ISL_950301, EPI_ISL_950302, EPI_ISL_950303, EPI_ISL_950313, EPI_ISL_950314, EPI_ISL_950315, EPI_ISL_950316, EPI_ISL_950318, EPI_ISL_950319, EPI_ISL_950320, EPI_ISL_950322, EPI_ISL_950351, EPI_ISL_950352, EPI_ISL_950353, EPI_ISL_950354, EPI_ISL_950355, EPI_ISL_950356, EPI_ISL_950357, EPI_ISL_950358, EPI_ISL_950359, EPI_ISL_950360, EPI_ISL_950361, EPI_ISL_950362, EPI_ISL_950363, EPI_ISL_950364, EPI_ISL_950365, EPI_ISL_950366, EPI_ISL_950367, EPI_ISL_950368, EPI_ISL_950369, EPI_ISL_950370, EPI_ISL_950371, EPI_ISL_950372, EPI_ISL_950374, EPI_ISL_950375, EPI_ISL_950377, EPI_ISL_950378, EPI_ISL_950380, EPI_ISL_950381, EPI_ISL_950383, EPI_ISL_950384, EPI_ISL_950385, EPI_ISL_950386, EPI_ISL_950387, EPI_ISL_950388, EPI_ISL_950389, EPI_ISL_950390, EPI_ISL_950416, EPI_ISL_950417, EPI_ISL_950418, EPI_ISL_950419, EPI_ISL_950420, EPI_ISL_950421, EPI_ISL_950422, EPI_ISL_950423, EPI_ISL_950436, EPI_ISL_950437, EPI_ISL_950438, EPI_ISL_950439, EPI_ISL_950440, EPI_ISL_950441, EPI_ISL_950442, EPI_ISL_950443, EPI_ISL_950444, EPI_ISL_950445, EPI_ISL_950446, EPI_ISL_950447, EPI_ISL_950448, EPI_ISL_950449, EPI_ISL_950450, EPI_ISL_950451, EPI_ISL_950452, EPI_ISL_950453, EPI_ISL_950454, EPI_ISL_950455, EPI_ISL_950456, EPI_ISL_950457, EPI_ISL_950458			
see above	Northumbria University / South Tees Hospitals NHS Foundation Trust / North Cumbria Integrated Care NHS Foundation Trust / North Tees and Hartlepool NHS Foundation Trust / Newcastle Hospitals NHS Foundation Trust	COVID-19 Genomics UK (COG-UK) Consortium	Darren L Smith,Andrew Nelson,Matthew Bashton,Greg R Young,Joshua Loh,John Allan,Mohammad A Tariq,Giles S Holt,Gary Black,Wen C Yew,Lynn Dover,Paul Baker,Steve Liggett,Sarah Essex,Jane Greenaway,Debra Padgett,Clive Graham,Garren Scott,Edward Barton,Emma Swindells,Brendan Payne,Jennifer Collins,Yusri Taha,Gary Eltringham
EPI_ISL_950555, EPI_ISL_950560, EPI_ISL_950562, EPI_ISL_950579, EPI_ISL_950584, EPI_ISL_950589	Quadram Institute Bioscience	COVID-19 Genomics UK (COG-UK) Consortium	Dave J. Baker, Gemma L. Kay, Alp Aydin, Thanh Le-Viet, Steven Rudder, Ana P. Tedim, Anastasia Kolyva, Maria Diaz, Leonardo de Oliveira Martins, Nabil-Fareed Alikhan, Lizzie Meadows, Rachael Stanley, Ngozi Elumogo, Muhammed Yasir, Nicholas M. Thomson, Alexander J Trotter, Rachel Gilroy, Samuel Bloomfield, Claire Stuart, Andrew Bell, Reenesh Prakash, Samir Dervisevic, Alison E. Mather, John Wain, Mark Webber, Andrew J. Page, Justin O'Grady
EPI_ISL_950709, EPI_ISL_950710, EPI_ISL_950711	Lincolnshire Hospitals and DeepSeq Nottingham	COVID-19 Genomics UK (COG-UK) Consortium	Nichola Duckworth, Tim Sloan, Sarah Walsh, Jonathan Ball, Patrick McClure, Joeseph Chappell, Nadine Holmes, Matthew Carlisle, Christopher Moore, Fei Sang, Johnny Debebe, Victoria Wright, Matthew Loose
EPI_ISL_950849, EPI_ISL_950851, EPI_ISL_950852, EPI_ISL_950854, EPI_ISL_950855, EPI_ISL_950856, EPI_ISL_950857, EPI_ISL_950871, EPI_ISL_950953, EPI_ISL_950954, EPI_ISL_950955, EPI_ISL_950956, EPI_ISL_950957, EPI_ISL_950958, EPI_ISL_950959, EPI_ISL_950961, EPI_ISL_950963, EPI_ISL_950964,			

EPI_ISL_950965, EPI_ISL_950966, EPI_ISL_950968, EPI_ISL_950970, EPI_ISL_950971, EPI_ISL_950972, EPI_ISL_950973, EPI_ISL_950974, EPI_ISL_950976, EPI_ISL_950979, EPI_ISL_950980, EPI_ISL_950981, EPI_ISL_950982, EPI_ISL_950983, EPI_ISL_950984, EPI_ISL_950985, EPI_ISL_950987, EPI_ISL_950988, EPI_ISL_950989, EPI_ISL_950990, EPI_ISL_950991, EPI_ISL_950992, EPI_ISL_950993, EPI_ISL_950994, EPI_ISL_950995, EPI_ISL_950996, EPI_ISL_950997, EPI_ISL_950999, EPI_ISL_951000, EPI_ISL_951002, EPI_ISL_951003, EPI_ISL_951004, EPI_ISL_951005, EPI_ISL_951006, EPI_ISL_951007, EPI_ISL_951008, EPI_ISL_951009, EPI_ISL_951010, EPI_ISL_951011, EPI_ISL_951012, EPI_ISL_951013, EPI_ISL_951014, EPI_ISL_951016, EPI_ISL_951018, EPI_ISL_951020, EPI_ISL_951021, EPI_ISL_951023, EPI_ISL_951024, EPI_ISL_951025, EPI_ISL_951026, EPI_ISL_951027, EPI_ISL_951028, EPI_ISL_951029, EPI_ISL_951030, EPI_ISL_951031, EPI_ISL_951032, EPI_ISL_951033, EPI_ISL_951034, EPI_ISL_951036, EPI_ISL_951037, EPI_ISL_951038, EPI_ISL_951042, EPI_ISL_951043, EPI_ISL_951044, EPI_ISL_951045, EPI_ISL_951046, EPI_ISL_951049, EPI_ISL_951050, EPI_ISL_951052, EPI_ISL_951053, EPI_ISL_951054, EPI_ISL_951055, EPI_ISL_951056, EPI_ISL_951057, EPI_ISL_951058, EPI_ISL_951059, EPI_ISL_951061, EPI_ISL_951062, EPI_ISL_951064, EPI_ISL_951066, EPI_ISL_951079, EPI_ISL_951080, EPI_ISL_951081, EPI_ISL_951083, EPI_ISL_951094, EPI_ISL_951097, EPI_ISL_951098, EPI_ISL_951163, EPI_ISL_951164, EPI_ISL_951165, EPI_ISL_951166, EPI_ISL_951169, EPI_ISL_951170, EPI_ISL_951171, EPI_ISL_951249			
see above	Oxford Viromics, NDM, University of Oxford; Oxford University Hospitals; Basingstoke and North Hampshire Hospital	COVID-19 Genomics UK (COG-UK) Consortium	Tanya Golubchik, David Bonsall, George Macintyre, Amy Trebes, Mariateresa de Cesare, Catrin Moore, Alex Mobbs, Anita Justice, Robert Shaw, Monique Andersson, Timothy Peto, Emma Wise, Nathan Moore, Jessica Lynch, Nick Cortes, Matilde Mori, Stephen Kidd, David Buck, John Todd, Christophe Fraser
EPI_ISL_952430, EPI_ISL_952435, EPI_ISL_952500, EPI_ISL_952501, EPI_ISL_952502, EPI_ISL_952503, EPI_ISL_952504, EPI_ISL_952505, EPI_ISL_952506, EPI_ISL_952508, EPI_ISL_952509, EPI_ISL_952510, EPI_ISL_952511, EPI_ISL_952512, EPI_ISL_952513, EPI_ISL_952514, EPI_ISL_952515, EPI_ISL_952516, EPI_ISL_952517, EPI_ISL_952518, EPI_ISL_952519, EPI_ISL_952520, EPI_ISL_952603, EPI_ISL_952604, EPI_ISL_952605, EPI_ISL_952606, EPI_ISL_952607, EPI_ISL_952608, EPI_ISL_952609, EPI_ISL_952610, EPI_ISL_952611, EPI_ISL_952612, EPI_ISL_952613, EPI_ISL_952614, EPI_ISL_952615, EPI_ISL_952616, EPI_ISL_952617, EPI_ISL_952618, EPI_ISL_952619, EPI_ISL_952620, EPI_ISL_952621, EPI_ISL_952622, EPI_ISL_952623, EPI_ISL_952625, EPI_ISL_952626, EPI_ISL_952627, EPI_ISL_952628, EPI_ISL_952629, EPI_ISL_952630, EPI_ISL_952631, EPI_ISL_952632, EPI_ISL_952633, EPI_ISL_952634, EPI_ISL_952635, EPI_ISL_952637, EPI_ISL_952638, EPI_ISL_952639, EPI_ISL_952640, EPI_ISL_952641, EPI_ISL_952642, EPI_ISL_952643, EPI_ISL_952644, EPI_ISL_952645, EPI_ISL_952646, EPI_ISL_952647, EPI_ISL_952648, EPI_ISL_952649, EPI_ISL_952650, EPI_ISL_952651, EPI_ISL_952652, EPI_ISL_952653, EPI_ISL_952654, EPI_ISL_952655, EPI_ISL_952656, EPI_ISL_952657, EPI_ISL_952658, EPI_ISL_952659, EPI_ISL_952660, EPI_ISL_952661, EPI_ISL_952662, EPI_ISL_952663, EPI_ISL_952664, EPI_ISL_952665, EPI_ISL_952666, EPI_ISL_952667, EPI_ISL_952668, EPI_ISL_952669, EPI_ISL_952670, EPI_ISL_952671, EPI_ISL_952672, EPI_ISL_952674, EPI_ISL_952676, EPI_ISL_952678, EPI_ISL_952679, EPI_ISL_952681, EPI_ISL_952682, EPI_ISL_952683, EPI_ISL_952684, EPI_ISL_952685, EPI_ISL_952686, EPI_ISL_952687, EPI_ISL_952688, EPI_ISL_952690, EPI_ISL_952691, EPI_ISL_952692, EPI_ISL_952693, EPI_ISL_952694, EPI_ISL_952695, EPI_ISL_952696, EPI_ISL_952697, EPI_ISL_952698, EPI_ISL_952699, EPI_ISL_952700, EPI_ISL_952701, EPI_ISL_952702, EPI_ISL_952703, EPI_ISL_952704, EPI_ISL_952705, EPI_ISL_952706, EPI_ISL_952707, EPI_ISL_952708, EPI_ISL_952709, EPI_ISL_952710, EPI_ISL_952711, EPI_ISL_952712, EPI_ISL_952713, EPI_ISL_952714, EPI_ISL_952715, EPI_ISL_952716, EPI_ISL_952717, EPI_ISL_952718, EPI_ISL_952719, EPI_ISL_952720, EPI_ISL_952721, EPI_ISL_952722, EPI_ISL_952723, EPI_ISL_952724, EPI_ISL_952725, EPI_ISL_952726, EPI_ISL_952727, EPI_ISL_952728, EPI_ISL_952729, EPI_ISL_952730, EPI_ISL_952731, EPI_ISL_952732, EPI_ISL_952733, EPI_ISL_952734, EPI_ISL_952735, EPI_ISL_952736, EPI_ISL_952737, EPI_ISL_952738, EPI_ISL_952739, EPI_ISL_952740, EPI_ISL_952741, EPI_ISL_952742, EPI_ISL_952743, EPI_ISL_952744, EPI_ISL_952745, EPI_ISL_952746, EPI_ISL_952747, EPI_ISL_952748, EPI_ISL_952749, EPI_ISL_952750, EPI_ISL_952751, EPI_ISL_952752, EPI_ISL_952753, EPI_ISL_952754, EPI_ISL_952755, EPI_ISL_952756, EPI_ISL_952757, EPI_ISL_952758, EPI_ISL_952759, EPI_ISL_952760, EPI_ISL_952761, EPI_ISL_952762, EPI_ISL_952763, EPI_ISL_952764, EPI_ISL_952765, EPI_ISL_952766, EPI_ISL_952767, EPI_ISL_952768, EPI_ISL_952769, EPI_ISL_952770, EPI_ISL_952771, EPI_ISL_952772, EPI_ISL_952773, EPI_ISL_952774, EPI_ISL_952775, EPI_ISL_952776			
see above	Centre for Enzyme Innovation, University of Portsmouth / Translational Research Laboratory, Portsmouth Hospitals NHS Trust	COVID-19 Genomics UK (COG-UK) Consortium	Angela Beckett,Salman Goudarzi,Christopher Fearn,Kate Cook,Katie Loveson,Sharon Glaysher,Scott Elliott,Samuel Robson
EPI_ISL_996083, EPI_ISL_996084, EPI_ISL_996085, EPI_ISL_996086, EPI_ISL_996087, EPI_ISL_996089, EPI_ISL_996090, EPI_ISL_996091, EPI_ISL_996092, EPI_ISL_996093, EPI_ISL_996094, EPI_ISL_996095, EPI_ISL_996096, EPI_ISL_996097, EPI_ISL_996098, EPI_ISL_996099, EPI_ISL_996100, EPI_ISL_996101, EPI_ISL_996102, EPI_ISL_996103, EPI_ISL_996104, EPI_ISL_996105, EPI_ISL_996106, EPI_ISL_996107, EPI_ISL_996108, EPI_ISL_996109, EPI_ISL_996110, EPI_ISL_996111, EPI_ISL_996112, EPI_ISL_996113, EPI_ISL_996114, EPI_ISL_996115, EPI_ISL_996116, EPI_ISL_996118, EPI_ISL_996119, EPI_ISL_996120, EPI_ISL_996121, EPI_ISL_996122, EPI_ISL_996123, EPI_ISL_996124, EPI_ISL_996125, EPI_ISL_996126, EPI_ISL_996127, EPI_ISL_996128, EPI_ISL_996129, EPI_ISL_996130, EPI_ISL_996131, EPI_ISL_996132, EPI_ISL_996133, EPI_ISL_996134, EPI_ISL_996135, EPI_ISL_996136, EPI_ISL_996137, EPI_ISL_996138, EPI_ISL_996139, EPI_ISL_996140, EPI_ISL_996141, EPI_ISL_996142, EPI_ISL_996143, EPI_ISL_996145, EPI_ISL_996146, EPI_ISL_996147, EPI_ISL_996148, EPI_ISL_996149, EPI_ISL_996150, EPI_ISL_996151, EPI_ISL_996152, EPI_ISL_996153, EPI_ISL_996154, EPI_ISL_996155, EPI_ISL_996156, EPI_ISL_996157, EPI_ISL_996158, EPI_ISL_996159, EPI_ISL_996160, EPI_ISL_996161, EPI_ISL_996162, EPI_ISL_996163, EPI_ISL_996164, EPI_ISL_996165, EPI_ISL_996166, EPI_ISL_996167, EPI_ISL_996168, EPI_ISL_996169, EPI_ISL_996170, EPI_ISL_996171, EPI_ISL_996172, EPI_ISL_996173, EPI_ISL_996174, EPI_ISL_996175, EPI_ISL_996176, EPI_ISL_996177, EPI_ISL_996178, EPI_ISL_996179, EPI_ISL_996180, EPI_ISL_996181, EPI_ISL_996183, EPI_ISL_996185, EPI_ISL_996186, EPI_ISL_996187, EPI_ISL_996188, EPI_ISL_996189, EPI_ISL_996190, EPI_ISL_996191, EPI_ISL_996192, EPI_ISL_996195, EPI_ISL_996196, EPI_ISL_996197, EPI_ISL_996198, EPI_ISL_996199, EPI_ISL_996200, EPI_ISL_996201, EPI_ISL_996202, EPI_ISL_996203, EPI_ISL_996204, EPI_ISL_996205, EPI_ISL_996207, EPI_ISL_996208, EPI_ISL_996209, EPI_ISL_996210, EPI_ISL_996211, EPI_ISL_996212, EPI_ISL_996213, EPI_ISL_996214, EPI_ISL_996215, EPI_ISL_996216, EPI_ISL_996217, EPI_ISL_996218, EPI_ISL_996219, EPI_ISL_996220, EPI_ISL_996221, EPI_ISL_996223, EPI_ISL_996224, EPI_ISL_996225, EPI_ISL_996226, EPI_ISL_996227, EPI_ISL_996228, EPI_ISL_996229, EPI_ISL_996230, EPI_ISL_996231, EPI_ISL_996232, EPI_ISL_996233, EPI_ISL_996234, EPI_ISL_996235, EPI_ISL_996236, EPI_ISL_996237, EPI_ISL_996238, EPI_ISL_996239, EPI_ISL_996240, EPI_ISL_996241, EPI_ISL_996242, EPI_ISL_996243, EPI_ISL_996244, EPI_ISL_996246, EPI_ISL_996247, EPI_ISL_996248, EPI_ISL_996249, EPI_ISL_996250, EPI_ISL_996251, EPI_ISL_996252, EPI_ISL_996253, EPI_ISL_996254, EPI_ISL_996255, EPI_ISL_996256, EPI_ISL_996257, EPI_ISL_996258, EPI_ISL_996259, EPI_ISL_996260, EPI_ISL_996261, EPI_ISL_996262, EPI_ISL_996263, EPI_ISL_996264, EPI_ISL_996265, EPI_ISL_996266, EPI_ISL_996267, EPI_ISL_996268, EPI_ISL_996269, EPI_ISL_996270, EPI_ISL_996271, EPI_ISL_996272, EPI_ISL_996273, EPI_ISL_996274, EPI_ISL_996275, EPI_ISL_996276, EPI_ISL_996277, EPI_ISL_996278, EPI_ISL_996279, EPI_ISL_996280, EPI_ISL_996281, EPI_ISL_996283, EPI_ISL_996284, EPI_ISL_996285, EPI_ISL_996286, EPI_ISL_996287, EPI_ISL_996288, EPI_ISL_996289, EPI_ISL_996290, EPI_ISL_996291, EPI_ISL_996292, EPI_ISL_996293, EPI_ISL_996294, EPI_ISL_996295, EPI_ISL_996296, EPI_ISL_996298, EPI_ISL_996299, EPI_ISL_996300, EPI_ISL_996301, EPI_ISL_996302, EPI_ISL_996303, EPI_ISL_996304, EPI_ISL_996305, EPI_ISL_996306, EPI_ISL_996307, EPI_ISL_996308, EPI_ISL_996309, EPI_ISL_996310, EPI_ISL_996311, EPI_ISL_996312, EPI_ISL_996313, EPI_ISL_996314, EPI_ISL_996315, EPI_ISL_996317, EPI_ISL_996318, EPI_ISL_996319, EPI_ISL_996320, EPI_ISL_996321, EPI_ISL_996323, EPI_ISL_996324, EPI_ISL_996325, EPI_ISL_996326, EPI_ISL_996327, EPI_ISL_996328, EPI_ISL_996329, EPI_ISL_996330, EPI_ISL_996331, EPI_ISL_996332, EPI_ISL_996333, EPI_ISL_996334, EPI_ISL_996336, EPI_ISL_996337, EPI_ISL_996338, EPI_ISL_996339, EPI_ISL_996340, EPI_ISL_996341, EPI_ISL_996343, EPI_ISL_996344, EPI_ISL_996345, EPI_ISL_996346, EPI_ISL_996347, EPI_ISL_996348, EPI_ISL_996349, EPI_ISL_996351, EPI_ISL_996352, EPI_ISL_996353, EPI_ISL_996354, EPI_ISL_996355, EPI_ISL_996356, EPI_ISL_996357, EPI_ISL_996358, EPI_ISL_996359, EPI_ISL_996360, EPI_ISL_996361, EPI_ISL_996362, EPI_ISL_996363, EPI_ISL_996364, EPI_ISL_996365, EPI_ISL_996366, EPI_ISL_996367, EPI_ISL_996368, EPI_ISL_996369, EPI_ISL_996370, EPI_ISL_996371, EPI_ISL_996372, EPI_ISL_996373, EPI_ISL_996374, EPI_ISL_996375, EPI_ISL_996376, EPI_ISL_996377, EPI_ISL_996378, EPI_ISL_996379, EPI_ISL_996380, EPI_ISL_996381, EPI_ISL_996382, EPI_ISL_996383, EPI_ISL_996384, EPI_ISL_996385, EPI_ISL_996386, EPI_ISL_996387, EPI_ISL_996388			
see above	Quadram Institute Bioscience	COVID-19 Genomics UK (COG-UK) Consortium	Dave J. Baker, Gemma L. Kay, Alp Aydin, Thanh Le-Viet, Steven Rudder, Ana P. Tedim, Anastasia Kolyva, Maria Diaz, Leonardo de Oliveira Martins, Nabil-Fareed Alikhan, Lizzie Meadows, Rachael Stanley, Ngozi Elumogo, Muhammed Yasir, Nicholas M. Thomson, Alexander J Trotter, Rachel Gilroy, Samuel Bloomfield, Claire Stuart, Andrew Bell, Reenesh Prakash, Samir Dervisevic, Alison E. Mather, John Wain, Mark Webber, Andrew J. Page, Justin O'Grady
EPI_ISL_996424, EPI_ISL_996441	University of Birmingham	COVID-19 Genomics UK (COG-UK) Consortium	Institute of Microbiology, University of Birmingham: Claire McMurray, Joanne Stockton, Samuel Nicholls, Radoslaw Poplawski, Will Rowe, Josh Quick, Nicholas Loman. University of Birmingham Testing Laboratory: Celina M Whalley, Andrew Bosworth, Charlotte Poxon, Kasun Wanigasooriya, Oliver Pickles, Mike Kidd, Alex Richter, Andrew D Beggs PHE Heartlands Lab: Husam Osman, Andrew Bosworth. Queen Elizabeth Hospital: Anna Casey
EPI_ISL_996483, EPI_ISL_996485, EPI_ISL_996490, EPI_ISL_996492, EPI_ISL_996493, EPI_ISL_996494, EPI_ISL_996495, EPI_ISL_996496, EPI_ISL_996497, EPI_ISL_996498, EPI_ISL_996499, EPI_ISL_996506, EPI_ISL_996507, EPI_ISL_996509, EPI_ISL_996510, EPI_ISL_996512, EPI_ISL_996513			
see above	University of Exeter	COVID-19 Genomics UK (COG-UK) Consortium	Ben Temperton,Aaron Jeffries,Michelle Michelsen,Joanna Warwick-Dugdale,Audrey Farbos,Robyn Manley,Stephen Michell,Jane Masoli
EPI_ISL_996627, EPI_ISL_996630, EPI_ISL_996672, EPI_ISL_996674, EPI_ISL_996678, EPI_ISL_996681, EPI_ISL_996683, EPI_ISL_996684, EPI_ISL_996685, EPI_ISL_996689, EPI_ISL_996690, EPI_ISL_996694, EPI_ISL_996695, EPI_ISL_996696, EPI_ISL_996704, EPI_ISL_996707, EPI_ISL_996708, EPI_ISL_996712, EPI_ISL_996719, EPI_ISL_996724, EPI_ISL_996726, EPI_ISL_996728, EPI_ISL_996730, EPI_ISL_996735, EPI_ISL_996737, EPI_ISL_996741, EPI_ISL_996742, EPI_ISL_996778, EPI_ISL_996791, EPI_ISL_996807, EPI_ISL_996834, EPI_ISL_996852, EPI_ISL_996853			
see above	Department of Pathology, University of Cambridge	COVID-19 Genomics UK (COG-UK) Consortium	Aminu S. Jahun, Yasmin Chaudhry, Iliana Georgana, Myra Hosmillo, Rhys Izuagbe, William L. Hamilton, Martin D. Curran, Surendra Parmar, Ian Goodfellow
EPI_ISL_997118, EPI_ISL_997119	Virology Department, Royal Infirmary of Edinburgh, NHS Lothian / School of Biological Sciences, University of Edinburgh	COVID-19 Genomics UK (COG-UK) Consortium	McHugh M, Dewar R, Cotton S, Rooke S, O'Toole Á, Scher E, Hill V, McCrone JT, Colquhoun R, Yu X, Jackson B, Rambaut A, Templeton K
EPI_ISL_997209, EPI_ISL_997210, EPI_ISL_997211, EPI_ISL_997212, EPI_ISL_997213, EPI_ISL_997214, EPI_ISL_997215, EPI_ISL_997217, EPI_ISL_997218, EPI_ISL_997219, EPI_ISL_997220, EPI_ISL_997221, EPI_ISL_997222, EPI_ISL_997223, EPI_ISL_997224, EPI_ISL_997225, EPI_ISL_997226, EPI_ISL_997227, EPI_ISL_997228, EPI_ISL_997229, EPI_ISL_997230, EPI_ISL_997231, EPI_ISL_997232, EPI_ISL_997233, EPI_ISL_997234, EPI_ISL_997235, EPI_ISL_997236, EPI_ISL_997237, EPI_ISL_997238, EPI_ISL_997240, EPI_ISL_997241, EPI_ISL_997242, EPI_ISL_997243, EPI_ISL_997244, EPI_ISL_997245, EPI_ISL_997246, EPI_ISL_997247, EPI_ISL_997248, EPI_ISL_997249, EPI_ISL_997285, EPI_ISL_997347, EPI_ISL_997366			
see above	University of Exeter	COVID-19 Genomics UK (COG-UK) Consortium	Ben Temperton,Aaron Jeffries,Michelle Michelsen,Joanna Warwick-Dugdale,Audrey Farbos,Robyn Manley,Stephen Michell,Jane Masoli
EPI_ISL_997579, EPI_ISL_997580, EPI_ISL_997581, EPI_ISL_997582, EPI_ISL_997583, EPI_ISL_997584, EPI_ISL_997585, EPI_ISL_997586, EPI_ISL_997587, EPI_ISL_997588, EPI_ISL_997589, EPI_ISL_997590, EPI_ISL_997591, EPI_ISL_997592, EPI_ISL_997593, EPI_ISL_997594, EPI_ISL_997595, EPI_ISL_997596, EPI_ISL_997597, EPI_ISL_997598, EPI_ISL_997599, EPI_ISL_997600, EPI_ISL_997601, EPI_ISL_997602, EPI_ISL_997603, EPI_ISL_997604, EPI_ISL_997605, EPI_ISL_997606, EPI_ISL_997607, EPI_ISL_997608, EPI_ISL_997609, EPI_ISL_997610, EPI_ISL_997611, EPI_ISL_997612, EPI_ISL_997613			
see above	University College London, Great Ormond Street Hospital for Children NHS Foundation Trust, Imperial College Healthcare NHS Trust	COVID-19 Genomics UK (COG-UK) Consortium	Sergi Castellano, Rachel Williams, Mark Kristiansen, Paola Resende Silva, Sunando Roy, Tony Brooks, Helena Tutill, Paola Niola, Patricia Dyal, Charlotte Williams, Leysa Forrest, Yasmin Panchbhaya, Jacqueline Findlay, Samuel Weeks, Julianne Brown, Kathryn Harris, Paul Randell, James Price, Alison Holmes, Judith Breuer
EPI_ISL_998138, EPI_ISL_998139, EPI_ISL_998141, EPI_ISL_998142, EPI_ISL_998143, EPI_ISL_998305, EPI_ISL_998306, EPI_ISL_998307, EPI_ISL_998308	Regional Virus Laboratory, Belfast Health and Social Care Trust	COVID-19 Genomics UK (COG-UK) Consortium	Conall McCaughey, James McKenna, Tanya Curran, Susan Feeney, Alison Watt, Ciara Cox, Mairead Connor, Zoltan Molnar, David Simpson, Derek Fairley
EPI_ISL_998315, EPI_ISL_998322, EPI_ISL_998461, EPI_ISL_998462, EPI_ISL_998463, EPI_ISL_998492, EPI_ISL_998505, EPI_ISL_998506, EPI_ISL_998517, EPI_ISL_998520, EPI_ISL_998525, EPI_ISL_998526, EPI_ISL_998536, EPI_ISL_998537, EPI_ISL_998538, EPI_ISL_998539, EPI_ISL_998540, EPI_ISL_998541, EPI_ISL_998545, EPI_ISL_998547, EPI_ISL_998548, EPI_ISL_998549, EPI_ISL_998550, EPI_ISL_998551			
see above	Northumbria University / South Tees Hospitals NHS Foundation Trust / North Cumbria Integrated Care NHS Foundation Trust / North Tees and Hartlepool NHS Foundation Trust / Newcastle Hospitals NHS Foundation Trust	COVID-19 Genomics UK (COG-UK) Consortium	Darren L Smith,Andrew Nelson,Matthew Bashton,Greg R Young,Joshua Loh,John Allan,Mohammad A Tariq,Giles S Holt,Gary Black,Wen C Yew,Lynn Dover,Paul Baker,Steve Liggett,Sarah Essex,Jane Greenaway,Debra Padgett,Clive Graham,Garren Scott,Edward Barton,Emma Swindells,Brendan Payne,Jennifer Collins,Yusri Taha,Gary Eltringham
EPI_ISL_998926, EPI_ISL_998927, EPI_ISL_998928, EPI_ISL_998929, EPI_ISL_998930, EPI_ISL_998931,	Lincolnshire Hospitals and DeepSeq Nottingham	COVID-19 Genomics UK (COG-UK) Consortium	Nichola Duckworth, Tim Sloan, Sarah Walsh, Jonathan Ball, Patrick McClure, Joeseph Chappell, Nadine Holmes, Matthew Carlisle, Christopher Moore, Fei Sang, Johnny Debebe, Victoria Wright, Matthew Loose

EPI_ISL_998932, EPI_ISL_998933, EPI_ISL_998934			
EPI_ISL_998991	Oxford Viromics, NDM, University of Oxford; Oxford University Hospitals; Basingstoke and North Hampshire Hospital	COVID-19 Genomics UK (COG-UK) Consortium	Tanya Golubchik, David Bonsall, George Macintyre, Amy Trebes, Mariateresa de Cesare, Catrin Moore, Alex Mobbs, Anita Justice, Robert Shaw, Monique Andersson, Timothy Peto, Emma Wise, Nathan Moore, Jessica Lynch, Nick Cortes, Matilde Mori, Stephen Kidd, David Buck, John Todd, Christophe Fraser
