# Supplementary material for: Inferring the Association between the Risk of COVID-19 Case Fatality and N501Y Substitution in SARS-CoV-2
Source: Viruses. 2021 Apr 8;13(4):638. doi: 10.3390/v13040638 (PMC8070306; doi:10.3390/v13040638)
Supplement: Supplementary file 1 [file viruses-13-00638-s001.zip › gisaid_hcov-19_UKAT_210102-210106.pdf]

We gratefully acknowledge the following Authors from the Originating laboratories responsible for obtaining the specimens, as well as the Submitting laboratories where the genome data were generated and shared via GISAID, on which this research is based.

All Submitters of data may be contacted directly via www.gisaid.org

Authors are sorted alphabetically.

Accession ID	Originating Laboratory	Submitting Laboratory	Authors
EPI_ISL_1000747	Virology Department, Sheffield Teaching Hospitals NHS Foundation Trust/Department of Infection, Immunity and Cardiovascular Disease, The Medical School, University of Sheffield	COVID-19 Genomics UK (COG-UK) Consortium	Thushan de Silva, Matthew Parker, Nikki Smith, Adri Angyal, Rebecca Brown, Luke Green, Rachel Tucker, Paul Parsons, Danielle Groves, Katie Johnson, Laura Carrilero, Alex Keeley, Dave Partridge, Matthew Wyles, Benjamin Lindsey, Mehmet Yavuz, Mohammad Raza, Cariad Evans
EPI_ISL_1012629	Lighthouse Lab in Cambridge	Wellcome Sanger Institute for the COVID-19 Genomics UK (COG-UK) Consortium	Rob Howes, The Lighthouse Lab in Cambridge and Alex Alderton, Roberto Amato, Sonia Goncalves, Ewan Harrison, David K. Jackson, Ian Johnston, Dominic Kwiatkowski, Cordelia Langford, John Sillitoe on behalf of the Wellcome Sanger Institute COVID-19 Surveillance Team
EPI_ISL_1046999, EPI_ISL_1047064, EPI_ISL_1047066	University of Birmingham	COVID-19 Genomics UK (COG-UK) Consortium	Institute of Microbiology, University of Birmingham: Claire McMurray, Joanne Stockton, Samuel Nicholls, Radoslaw Poplawski, Will Rowe, Josh Quick, Nicholas Loman. University of Birmingham Testing Laboratory: Celina M Whalley, Andrew Bosworth, Charlotte Poxon, Kasun Wanigasooriya, Oliver Pickles, Mike Kidd, Alex Richter, Andrew D Beggs PHE Heartlands Lab: Husam Osman, Andrew Bosworth. Queen Elizabeth Hospital: Anna Casey
EPI_ISL_1047952	University Hospitals Of Leicester NHS Trust and DeepSeq Nottingham	COVID-19 Genomics UK (COG-UK) Consortium	Christopher Holmes, Paul Bird, Thomas Helmer, Karlie Fallon, Julian Tang, Jonathan Ball, Patrick McClure, Joeseph Chappell, Nadine Holmes, Matthew Carlisle, Christopher Moore, Fei Sang, Johnny Debebe, Victoria Wright, Matthew Loose
EPI_ISL_1050434, EPI_ISL_1050435, EPI_ISL_1050436, EPI_ISL_1050498	University College London, Great Ormond Street Hospital for Children NHS Foundation Trust, Imperial College Healthcare NHS Trust	COVID-19 Genomics UK (COG-UK) Consortium	Sergi Castellano, Rachel Williams, Mark Kristiansen, Paola Resende Silva, Sunando Roy, Tony Brooks, Helena Tutill, Paola Niola, Patricia Dyal, Charlotte Williams, Leysa Forrest, Yasmin Panchbhaya, Jacqueline Findlay, Samuel Weeks, Julianne Brown, Kathryn Harris, Paul Randell, James Price, Alison Holmes, Judith Breuer
EPI_ISL_1050922, EPI_ISL_1050925, EPI_ISL_1050928, EPI_ISL_1050931, EPI_ISL_1050957, EPI_ISL_1050958, EPI_ISL_1050959, EPI_ISL_1051040, EPI_ISL_1051041, EPI_ISL_1051043, EPI_ISL_1051045, EPI_ISL_1051048, EPI_ISL_1051050, EPI_ISL_1051052, EPI_ISL_1051054, EPI_ISL_1051056, EPI_ISL_1051058, EPI_ISL_1051060, EPI_ISL_1051062, EPI_ISL_1051063, EPI_ISL_1051065, EPI_ISL_1051067, EPI_ISL_1051073, EPI_ISL_1051075, EPI_ISL_1051080, EPI_ISL_1051082, EPI_ISL_1051089, EPI_ISL_1051093, EPI_ISL_1051118, EPI_ISL_1051119, EPI_ISL_1051342, EPI_ISL_1051343, EPI_ISL_1051344			
see above	Northumbria University / South Tees Hospitals NHS Foundation Trust / North Cumbria Integrated Care NHS Foundation Trust / North Tees and Hartlepool NHS Foundation Trust / Newcastle Hospitals NHS Foundation Trust	COVID-19 Genomics UK (COG-UK) Consortium	Darren L Smith,Andrew Nelson,Matthew Bashton,Greg R Young,Joshua Loh,John Allan,Mohammad A Tariq,Giles S Holt,Gary Black,Wen C Yew,Lynn Dover,Paul Baker,Steve Liggett,Sarah Essex,Jane Greenaway,Debra Padgett,Clive Graham,Garren Scott,Edward Barton,Emma Swindells,Brendan Payne,Jennifer Collins,Yusri Taha,Gary Eltringham
EPI_ISL_1051429, EPI_ISL_1051432, EPI_ISL_1051434, EPI_ISL_1051439, EPI_ISL_1051442, EPI_ISL_1051444, EPI_ISL_1051448, EPI_ISL_1051453, EPI_ISL_1051457, EPI_ISL_1051458, EPI_ISL_1051460, EPI_ISL_1051461, EPI_ISL_1051463, EPI_ISL_1051464, EPI_ISL_1051472, EPI_ISL_1051480, EPI_ISL_1051483, EPI_ISL_1051487, EPI_ISL_1051492, EPI_ISL_1051496, EPI_ISL_1051499, EPI_ISL_1051502, EPI_ISL_1051509, EPI_ISL_1051511, EPI_ISL_1051512, EPI_ISL_1051513, EPI_ISL_1051518, EPI_ISL_1051524, EPI_ISL_1051527, EPI_ISL_1051528			
see above	Quadram Institute Bioscience	COVID-19 Genomics UK (COG-UK) Consortium	Dave J. Baker, Gemma L. Kay, Alp Aydin, Thanh Le-Viet, Steven Rudder, Ana P. Tedim, Anastasia Kolyva, Maria Diaz, Leonardo de Oliveira Martins, Nabil-Fareed Alikhan, Lizzie Meadows, Rachael Stanley, Ngozi Elumogo, Muhammed Yasir, Nicholas M. Thomson, Alexander J Trotter, Rachel Gilroy, Samuel Bloomfield, Claire Stuart, Andrew Bell, Reenesh Prakash, Samir Dervisevic, Alison E. Mather, John Wain, Mark Webber, Andrew J. Page, Justin O'Grady
EPI_ISL_1051598, EPI_ISL_1051876	Oxford Viromics, NDM, University of Oxford; Oxford University Hospitals; Basingstoke and North Hampshire Hospital	COVID-19 Genomics UK (COG-UK) Consortium	Tanya Golubchik, David Bonsall, George Macintyre, Amy Trebes, Mariateresa de Cesare, Catrin Moore, Alex Mobbs, Anita Justice, Robert Shaw, Monique Andersson, Timothy Peto, Emma Wise, Nathan Moore, Jessica Lynch, Nick Cortes, Matilde Mori, Stephen Kidd, David Buck, John Todd, Christophe Fraser
EPI_ISL_1054760	Bioinformatics and Biostatistics Lab, Advanced Sequencing Facility	COVID-19 Genomics UK (COG-UK) Consortium	Aengus Stewart,Jerome Nicod,Chelsea Sawyer,Laura Cubitt,Harshil Patel,Margaret Crawford
EPI_ISL_1103805	University of Exeter	COVID-19 Genomics UK (COG-UK) Consortium	Ben Temperton,Aaron Jeffries,Michelle Michelsen,Joanna Warwick-Dugdale,Audrey Farbos,Robyn Manley,Stephen Michell,Jane Masoli
EPI_ISL_1104234, EPI_ISL_1104237, EPI_ISL_1104239, EPI_ISL_1104240, EPI_ISL_1104256	Virology Department, Royal Infirmary of Edinburgh, NHS Lothian / School of Biological Sciences, University of Edinburgh	COVID-19 Genomics UK (COG-UK) Consortium	McHugh M, Dewar R, Cotton S, Rooke S, O'Toole Á, Scher E, Hill V, McCrone JT, Colquhoun R, Yu X, Jackson B, Rambaut A, Templeton K
EPI_ISL_1104572	Liverpool Clinical Laboratories	COVID-19 Genomics UK (COG-UK) Consortium	Sam Haldenby, Anita Lucaci, Steve Paterson, Julian Hiscox, Alistair Darby, M Almsaud, A Alrezaihi, Muhannad Alruwaili, Stuart D Armstrong, Jones Benjamin, Eleanor G Bentley, Anu Chawla, Jordan J Clark, Angela Cowell, Richard Eccles, Isabel García-Dorival, Matthew Gemmell, Alessandro Gerada, PKF Gilmore, Richard Gregory, Ximeng Han, Catherine Hartley, Margaret Hughes, Miren Iturriza-Gomara, James Johnson, L Luu, Jenifer Manson, Charlotte Nelson, Elaine O'Toole, Cassie Olateju, Rebekah Penrice-Randal , Lucille Rainbow, N.P Randle, Trevor Ian Robinson, Parul Sharma, Ghada T Shawli, James P Stewart, Neil Swainston, Ecaterina Vamos, Joanne Watts, Mark Whitehead
EPI_ISL_1104600, EPI_ISL_1104601, EPI_ISL_1104602, EPI_ISL_1104603, EPI_ISL_1104604, EPI_ISL_1104605, EPI_ISL_1104606, EPI_ISL_1104607, EPI_ISL_1104608, EPI_ISL_1104609, EPI_ISL_1104610			
see above	University College London, Great Ormond Street Hospital for Children NHS Foundation Trust, Imperial College Healthcare NHS Trust	COVID-19 Genomics UK (COG-UK) Consortium	Sergi Castellano, Rachel Williams, Mark Kristiansen, Paola Resende Silva, Sunando Roy, Tony Brooks, Helena Tutill, Paola Niola, Patricia Dyal, Charlotte Williams, Leysa Forrest, Yasmin Panchbhaya, Jacqueline Findlay, Samuel Weeks, Julianne Brown, Kathryn Harris, Paul Randell, James Price, Alison Holmes, Judith Breuer
EPI_ISL_1104974, EPI_ISL_1105008, EPI_ISL_1105017, EPI_ISL_1105025, EPI_ISL_1105033, EPI_ISL_1105042, EPI_ISL_1105164, EPI_ISL_1105177, EPI_ISL_1105193, EPI_ISL_1105208, EPI_ISL_1105257, EPI_ISL_1105260, EPI_ISL_1105267, EPI_ISL_1105274, EPI_ISL_1105275, EPI_ISL_1105281, EPI_ISL_1105287, EPI_ISL_1105288, EPI_ISL_1105293			
see above	University College London Hospital	COVID-19 Genomics UK (COG-UK) Consortium	Judith Heaney, Matthew Byott, Catherine Houlihan, Dan Frampton, Stuart Kirk, Moira Spyer and Eleni Nastouli
EPI_ISL_1105706, EPI_ISL_1105708, EPI_ISL_1105709, EPI_ISL_1105772, EPI_ISL_1105773, EPI_ISL_1105774, EPI_ISL_1105775, EPI_ISL_1105776, EPI_ISL_1105777, EPI_ISL_1105778, EPI_ISL_1105779, EPI_ISL_1105780, EPI_ISL_1105781, EPI_ISL_1105782, EPI_ISL_1105783, EPI_ISL_1105784, EPI_ISL_1105785, EPI_ISL_1105786, EPI_ISL_1105787, EPI_ISL_1105788, EPI_ISL_1105789, EPI_ISL_1105790, EPI_ISL_1105791, EPI_ISL_1105792, EPI_ISL_1105793, EPI_ISL_1105794, EPI_ISL_1105795, EPI_ISL_1105796, EPI_ISL_1105797, EPI_ISL_1105798, EPI_ISL_1105799, EPI_ISL_1105800, EPI_ISL_1105801, EPI_ISL_1105802, EPI_ISL_1105803, EPI_ISL_1105804, EPI_ISL_1105805, EPI_ISL_1105806, EPI_ISL_1105807, EPI_ISL_1105808, EPI_ISL_1105809, EPI_ISL_1105810, EPI_ISL_1105811			
see above	Northumbria University / South Tees Hospitals NHS Foundation Trust / North Cumbria Integrated Care NHS Foundation Trust / North Tees and Hartlepool NHS Foundation Trust / Newcastle Hospitals NHS Foundation Trust	COVID-19 Genomics UK (COG-UK) Consortium	Darren L Smith,Andrew Nelson,Matthew Bashton,Greg R Young,Joshua Loh,John Allan,Mohammad A Tariq,Giles S Holt,Gary Black,Wen C Yew,Lynn Dover,Paul Baker,Steve Liggett,Sarah Essex,Jane Greenaway,Debra Padgett,Clive Graham,Garren Scott,Edward Barton,Emma Swindells,Brendan Payne,Jennifer Collins,Yusri Taha,Gary Eltringham
EPI_ISL_1177834, EPI_ISL_1177835, EPI_ISL_1177836, EPI_ISL_1177837, EPI_ISL_1177838	Liverpool Clinical Laboratories	COVID-19 Genomics UK (COG-UK) Consortium	Sam Haldenby, Alistair Darby, Steve Paterson, Anita Lucaci, Julian Hiscox, M Almsaud, A Alrezaihi, Muhannad Alruwaili, Stuart D Armstrong, Jones Benjamin, Eleanor G Bentley, Anu Chawla, Jordan J Clark, Angela Cowell, Richard Eccles, Isabel García-Dorival, Matthew Gemmell, Alessandro Gerada, PKF Gilmore, Richard Gregory, Ximeng Han, Catherine Hartley, Margaret Hughes, Miren Iturriza-Gomara, James Johnson, L Luu, Jenifer Manson, Charlotte Nelson, Elaine O'Toole, Cassie Olateju, Rebekah Penrice-Randal , Lucille Rainbow, N.P Randle, Trevor Ian Robinson, Parul Sharma, Ghada T Shawli, James P Stewart, Neil Swainston, Ecaterina Vamos, Joanne Watts, Mark Whitehead, Hermione Webster
EPI_ISL_1178334, EPI_ISL_1178335, EPI_ISL_1178339, EPI_ISL_1178340, EPI_ISL_1178341, EPI_ISL_1178342, EPI_ISL_1178344, EPI_ISL_1178345, EPI_ISL_1178346, EPI_ISL_1178347	Northumbria University / South Tees Hospitals NHS Foundation Trust / North Cumbria Integrated Care NHS Foundation Trust / North Tees and Hartlepool NHS Foundation Trust / Newcastle Hospitals NHS Foundation Trust	COVID-19 Genomics UK (COG-UK) Consortium	Darren L Smith,Andrew Nelson,Matthew Bashton,Greg R Young,Joshua Loh,John Allan,Mohammad A Tariq,Giles S Holt,Gary Black,Wen C Yew,Lynn Dover,Paul Baker,Steve Liggett,Sarah Essex,Jane Greenaway,Debra Padgett,Clive Graham,Garren Scott,Edward Barton,Emma Swindells,Brendan Payne,Jennifer Collins,Yusri Taha,Gary Eltringham
EPI_ISL_1178724	Oxford Viromics, NDM, University of Oxford; Oxford University	COVID-19 Genomics UK (COG-UK) Consortium	Tanya Golubchik, David Bonsall, George Macintyre, Amy Trebes, Mariateresa de Cesare, Catrin Moore, Alex Mobbs, Anita Justice, Robert Shaw, Monique

	Hospitals; Basingstoke and North Hampshire Hospital		Andersson, Timothy Peto, Emma Wise, Nathan Moore, Jessica Lynch, Nick Cortes, Matilde Mori, Stephen Kidd, David Buck, John Todd, Christophe Fraser
EPI_ISL_1179836, EPI_ISL_1179837, EPI_ISL_1179843	Centre for Enzyme Innovation, University of Portsmouth / Translational Research Laboratory, Portsmouth Hospitals NHS Trust	COVID-19 Genomics UK (COG-UK) Consortium	Angela Beckett,Salman Goudarzi,Christopher Fearn,Kate Cook,Katie Loveson,Sharon Glaysher,Scott Elliott,Samuel Robson
EPI_ISL_1247995, EPI_ISL_1247996, EPI_ISL_1248000, EPI_ISL_1248014, EPI_ISL_1248015, EPI_ISL_1248016, EPI_ISL_1248017, EPI_ISL_1248018, EPI_ISL_1248019, EPI_ISL_1248020, EPI_ISL_1248021, EPI_ISL_1248022, EPI_ISL_1248023, EPI_ISL_1248024, EPI_ISL_1248025, EPI_ISL_1248026, EPI_ISL_1248027, EPI_ISL_1248028, EPI_ISL_1248029, EPI_ISL_1248030, EPI_ISL_1248031, EPI_ISL_1248032, EPI_ISL_1248033, EPI_ISL_1248034, EPI_ISL_1248035, EPI_ISL_1248036, EPI_ISL_1248037, EPI_ISL_1248038, EPI_ISL_1248039, EPI_ISL_1248040, EPI_ISL_1248041, EPI_ISL_1248042, EPI_ISL_1248043, EPI_ISL_1248044, EPI_ISL_1248045, EPI_ISL_1248046, EPI_ISL_1248047, EPI_ISL_1248048, EPI_ISL_1248049, EPI_ISL_1248050, EPI_ISL_1248051, EPI_ISL_1248052, EPI_ISL_1248053, EPI_ISL_1248054, EPI_ISL_1248055, EPI_ISL_1248056, EPI_ISL_1248066, EPI_ISL_1248067			
see above	University College London, Great Ormond Street Hospital for Children NHS Foundation Trust, Imperial College Healthcare NHS Trust	COVID-19 Genomics UK (COG-UK) Consortium	Sergi Castellano, Rachel Williams, Mark Kristiansen, Paola Resende Silva, Sunando Roy, Tony Brooks, Helena Tutill, Paola Niola, Patricia Dyal, Charlotte Williams, Leysa Forrest, Yasmin Panchbhaya, Jacqueline Findlay, Samuel Weeks, Julianne Brown, Kathryn Harris, Paul Randell, James Price, Alison Holmes, Judith Breuer
EPI_ISL_1248367, EPI_ISL_1248376, EPI_ISL_1248378, EPI_ISL_1248386, EPI_ISL_1248388, EPI_ISL_1248390, EPI_ISL_1248400, EPI_ISL_1248401, EPI_ISL_1248402, EPI_ISL_1248405, EPI_ISL_1248411, EPI_ISL_1248412, EPI_ISL_1248413, EPI_ISL_1248414, EPI_ISL_1248416, EPI_ISL_1248420, EPI_ISL_1248422, EPI_ISL_1248423, EPI_ISL_1248424, EPI_ISL_1248427, EPI_ISL_1248430, EPI_ISL_1248432, EPI_ISL_1248433, EPI_ISL_1248434, EPI_ISL_1248442, EPI_ISL_1248444, EPI_ISL_1248445, EPI_ISL_1248459, EPI_ISL_1248486, EPI_ISL_1248499			
see above	University College London Hospital	COVID-19 Genomics UK (COG-UK) Consortium	Dr Judith Heaney, Matthew Byott, Dr Catherine Houlihan, Dr Daniel Frampton, Stuart Kirk, Dr Moira Spyer, Dr Paul Grant and Dr Eleni Nastouli
EPI_ISL_1249234, EPI_ISL_1249235, EPI_ISL_1249237	Oxford Viromics, NDM, University of Oxford; Oxford University Hospitals; Basingstoke and North Hampshire Hospital	COVID-19 Genomics UK (COG-UK) Consortium	Tanya Golubchik, David Bonsall, George Macintyre, Amy Trebes, Mariateresa de Cesare, Catrin Moore, Alex Mobbs, Anita Justice, Robert Shaw, Monique Andersson, Timothy Peto, Emma Wise, Nathan Moore, Jessica Lynch, Nick Cortes, Matilde Mori, Stephen Kidd, David Buck, John Todd, Christophe Fraser
EPI_ISL_1296492, EPI_ISL_1296493, EPI_ISL_1296495, EPI_ISL_1296496, EPI_ISL_1296497, EPI_ISL_1296498, EPI_ISL_1296499, EPI_ISL_1296501, EPI_ISL_1296502, EPI_ISL_1296503, EPI_ISL_1296504, EPI_ISL_1296508, EPI_ISL_1296542			
see above	Respiratory Virus Unit, National Infection Service, Public Health England	COVID-19 Genomics UK (COG-UK) Consortium	PHE Covid Sequencing Team
EPI_ISL_1308593, EPI_ISL_1308594, EPI_ISL_1308595, EPI_ISL_1308598	University of Exeter	COVID-19 Genomics UK (COG-UK) Consortium	Ben Temperton,Aaron Jeffries,Michelle Michelsen,Joanna Warwick-Dugdale,Audrey Farbos,Robyn Manley,Stephen Michell,Jane Masoli
EPI_ISL_1308845, EPI_ISL_1308846, EPI_ISL_1308848, EPI_ISL_1308849	Virology Department, Royal Infirmary of Edinburgh, NHS Lothian / School of Biological Sciences, University of Edinburgh	COVID-19 Genomics UK (COG-UK) Consortium	McHugh M, Dewar R, Cotton S, Rooke S, O'Toole Á, Scher E, Hill V, McCrone JT, Colquhoun R, Yu X, Jackson B, Rambaut A, Templeton K
EPI_ISL_1308981, EPI_ISL_1308982, EPI_ISL_1308983, EPI_ISL_1308984, EPI_ISL_1308985, EPI_ISL_1308986, EPI_ISL_1308987, EPI_ISL_1308988, EPI_ISL_1308990, EPI_ISL_1308991, EPI_ISL_1308992, EPI_ISL_1308993, EPI_ISL_1308994, EPI_ISL_1308995, EPI_ISL_1308996, EPI_ISL_1308997, EPI_ISL_1308998, EPI_ISL_1308999, EPI_ISL_1309000, EPI_ISL_1309001, EPI_ISL_1309002, EPI_ISL_1309003, EPI_ISL_1309004, EPI_ISL_1309006, EPI_ISL_1309022, EPI_ISL_1309023, EPI_ISL_1309024, EPI_ISL_1309025, EPI_ISL_1309053, EPI_ISL_1309079, EPI_ISL_1309084, EPI_ISL_1309095, EPI_ISL_1309100, EPI_ISL_1309115, EPI_ISL_1309116, EPI_ISL_1309117, EPI_ISL_1309118, EPI_ISL_1309127			
see above	University College London, Great Ormond Street Hospital for Children NHS Foundation Trust, Imperial College Healthcare NHS Trust	COVID-19 Genomics UK (COG-UK) Consortium	Sergi Castellano, Rachel Williams, Mark Kristiansen, Paola Resende Silva, Sunando Roy, Tony Brooks, Helena Tutill, Paola Niola, Patricia Dyal, Charlotte Williams, Leysa Forrest, Yasmin Panchbhaya, Jacqueline Findlay, Samuel Weeks, Julianne Brown, Kathryn Harris, Paul Randell, James Price, Alison Holmes, Judith Breuer
EPI_ISL_1309550, EPI_ISL_1309551, EPI_ISL_1309552, EPI_ISL_1309553, EPI_ISL_1309554, EPI_ISL_1309555, EPI_ISL_1309559, EPI_ISL_1309560, EPI_ISL_1309561, EPI_ISL_1309565, EPI_ISL_1309566, EPI_ISL_1309571, EPI_ISL_1309597, EPI_ISL_1309598, EPI_ISL_1309608, EPI_ISL_1309610			
see above	Northumbria University / South Tees Hospitals NHS Foundation Trust / North Cumbria Integrated Care NHS Foundation Trust / North Tees and Hartlepool NHS Foundation Trust / Newcastle Hospitals NHS Foundation Trust	COVID-19 Genomics UK (COG-UK) Consortium	Darren L Smith,Andrew Nelson,Matthew Bashton,Greg R Young,Joshua Loh,John Allan,Mohammad A Tariq,Giles S Holt,Gary Black,Wen C Yew,Lynn Dover,Paul Baker,Steve Liggett,Sarah Essex,Jane Greenaway,Debra Padgett,Clive Graham,Garren Scott,Edward Barton,Emma Swindells,Brendan Payne,Jennifer Collins,Yusri Taha,Gary Eltringham
EPI_ISL_1309739, EPI_ISL_1309748	Oxford Viromics, NDM, University of Oxford; Oxford University Hospitals; Basingstoke and North Hampshire Hospital	COVID-19 Genomics UK (COG-UK) Consortium	Tanya Golubchik, David Bonsall, George Macintyre, Amy Trebes, Mariateresa de Cesare, Catrin Moore, Alex Mobbs, Anita Justice, Robert Shaw, Monique Andersson, Timothy Peto, Emma Wise, Nathan Moore, Jessica Lynch, Nick Cortes, Matilde Mori, Stephen Kidd, David Buck, John Todd, Christophe Fraser
EPI_ISL_1386862, EPI_ISL_1386885, EPI_ISL_1386890, EPI_ISL_1386892, EPI_ISL_1386893, EPI_ISL_1386894, EPI_ISL_1386899, EPI_ISL_1386903, EPI_ISL_1386904, EPI_ISL_1386906, EPI_ISL_1386907, EPI_ISL_1386908, EPI_ISL_1386910, EPI_ISL_1386911			
see above	University College London, Great Ormond Street Hospital for Children NHS Foundation Trust, Imperial College Healthcare NHS Trust	COVID-19 Genomics UK (COG-UK) Consortium	Sergi Castellano, Rachel Williams, Mark Kristiansen, Paola Resende Silva, Sunando Roy, Tony Brooks, Helena Tutill, Paola Niola, Patricia Dyal, Charlotte Williams, Leysa Forrest, Yasmin Panchbhaya, Jacqueline Findlay, Samuel Weeks, Julianne Brown, Kathryn Harris, Paul Randell, James Price, Alison Holmes, Judith Breuer
EPI_ISL_1387236	Oxford Viromics, NDM, University of Oxford; Oxford University Hospitals; Basingstoke and North Hampshire Hospital	COVID-19 Genomics UK (COG-UK) Consortium	Tanya Golubchik, David Bonsall, George Macintyre, Amy Trebes, Mariateresa de Cesare, Catrin Moore, Alex Mobbs, Anita Justice, Robert Shaw, Monique Andersson, Timothy Peto, Emma Wise, Nathan Moore, Jessica Lynch, Nick Cortes, Matilde Mori, Stephen Kidd, David Buck, John Todd, Christophe Fraser
EPI_ISL_1474497	University of Birmingham	COVID-19 Genomics UK (COG-UK) Consortium	Institute of Microbiology, University of Birmingham: Claire McMurray, Joanne Stockton, Samuel Nicholls, Radoslaw Poplawski, Will Rowe, Josh Quick, Nicholas Loman. University of Birmingham Testing Laboratory: Celina M Whalley, Andrew Bosworth, Charlotte Poxon, Kasun Wanigasooriya, Oliver Pickles, Mike Kidd, Alex Richter, Andrew D Beggs PHE Heartlands Lab: Husam Osman, Andrew Bosworth. Queen Elizabeth Hospital: Anna Casey
EPI_ISL_1474558	University of Exeter	COVID-19 Genomics UK (COG-UK) Consortium	Ben Temperton,Aaron Jeffries,Michelle Michelsen,Joanna Warwick-Dugdale,Audrey Farbos,Robyn Manley,Stephen Michell,Jane Masoli
EPI_ISL_1474654, EPI_ISL_1474656, EPI_ISL_1474657, EPI_ISL_1474658, EPI_ISL_1474659, EPI_ISL_1474660, EPI_ISL_1474661	Liverpool Clinical Laboratories	COVID-19 Genomics UK (COG-UK) Consortium	Sam Haldenby, Alistair Darby, Steve Paterson, Anita Lucaci, Julian Hiscox, M Almsaud, A Alrezaihi, Muhannad Alruwaili, Stuart D Armstrong, Jones Benjamin, Eleanor G Bentley, Anu Chawla, Jordan J Clark, Angela Cowell, Richard Eccles, Isabel García-Dorival, Matthew Gemmell, Alessandro Gerada, PKF Gilmore, Richard Gregory, Ximeng Han, Catherine Hartley, Margaret Hughes, Miren Iturriza-Gomara, James Johnson, L Luu, Jenifer Manson, Charlotte Nelson, Elaine O'Toole, Cassie Olateju, Rebekah Penrice-Randal , Lucille Rainbow, N.P Randle, Trevor Ian Robinson, Parul Sharma, Ghada T Shawli, James P Stewart, Neil Swainston, Ecaterina Vamos, Joanne Watts, Mark Whitehead, Hermione Webster
EPI_ISL_1474849, EPI_ISL_1474851, EPI_ISL_1474853	University College London, Great Ormond Street Hospital for Children NHS Foundation Trust, Imperial College Healthcare NHS Trust	COVID-19 Genomics UK (COG-UK) Consortium	Sergi Castellano, Rachel Williams, Mark Kristiansen, Paola Resende Silva, Sunando Roy, Tony Brooks, Helena Tutill, Paola Niola, Patricia Dyal, Charlotte Williams, Leysa Forrest, Yasmin Panchbhaya, Jacqueline Findlay, Samuel Weeks, Julianne Brown, Kathryn Harris, Paul Randell, James Price, Alison Holmes, Judith Breuer
EPI_ISL_1474958, EPI_ISL_1474959, EPI_ISL_1474960, EPI_ISL_1474963, EPI_ISL_1474964, EPI_ISL_1474995, EPI_ISL_1475062, EPI_ISL_1475063, EPI_ISL_1475064, EPI_ISL_1475065, EPI_ISL_1475066, EPI_ISL_1475067, EPI_ISL_1475068, EPI_ISL_1475069, EPI_ISL_1475070, EPI_ISL_1475071, EPI_ISL_1475072, EPI_ISL_1475073, EPI_ISL_1475074, EPI_ISL_1475075, EPI_ISL_1475076, EPI_ISL_1475077, EPI_ISL_1475078, EPI_ISL_1475079, EPI_ISL_1475081, EPI_ISL_1475083			
see above	Regional Virus Laboratory, Belfast Health and Social Care Trust	COVID-19 Genomics UK (COG-UK) Consortium	Conall McCaughey, James McKenna, Tanya Curran, Susan Feeney, Alison Watt, Ciara Cox, Mairead Connor, Zoltan Molnar, David Simpson, Derek Fairley
EPI_ISL_1476506, EPI_ISL_1476507, EPI_ISL_1476508, EPI_ISL_1476509, EPI_ISL_1476510, EPI_ISL_1476511, EPI_ISL_1476512, EPI_ISL_1476513, EPI_ISL_1476518, EPI_ISL_1476520, EPI_ISL_1476521, EPI_ISL_1476526, EPI_ISL_1476529, EPI_ISL_1476530, EPI_ISL_1476531, EPI_ISL_1476532, EPI_ISL_1476533, EPI_ISL_1476536, EPI_ISL_1476537, EPI_ISL_1476538, EPI_ISL_1476539, EPI_ISL_1476541, EPI_ISL_1476542, EPI_ISL_1476543, EPI_ISL_1476547			
see above	Originating lab: Wales Specialist Virology Centre Sequencing lab: Pathogen Genomics Unit	Public Health Wales Microbiology Cardiff Wales Specialist Virology Centre	Catherine Moore, Johnathan Evans, Laura Gifford, Malorie Perry, Simon Cottrell, Angela Marchbank, Alec Birchley, Alexander Adams, Amy Gaskin, Bree Gatica-Wilcox, Jason Coombes, Joel Southgate, Lauren Gilbert, Lee Graham, Nicole Pacchiarini, Sara Kumziene-Summerhayes, Sarah Taylor, Sophie Jones, Sara Rey, Matthew Bull, Joanne Watkins, Sally Corden, Tom Connor
EPI_ISL_768815, EPI_ISL_768816, EPI_ISL_791194, EPI_ISL_791197, EPI_ISL_791198, EPI_ISL_791223, EPI_ISL_791224, EPI_ISL_791225, EPI_ISL_791226, EPI_ISL_791227, EPI_ISL_791228, EPI_ISL_791229, EPI_ISL_791230, EPI_ISL_791231, EPI_ISL_791232, EPI_ISL_791233, EPI_ISL_791234, EPI_ISL_791235, EPI_ISL_791236, EPI_ISL_791237, EPI_ISL_791238, EPI_ISL_791239, EPI_ISL_791240, EPI_ISL_791241, EPI_ISL_791242, EPI_ISL_791243, EPI_ISL_791244, EPI_ISL_791245, EPI_ISL_791246, EPI_ISL_791247, EPI_ISL_791248, EPI_ISL_791254, EPI_ISL_791255, EPI_ISL_791256, EPI_ISL_791257, EPI_ISL_791258, EPI_ISL_791259, EPI_ISL_791260, EPI_ISL_791261, EPI_ISL_791262, EPI_ISL_791263, EPI_ISL_791264, EPI_ISL_791265, EPI_ISL_791266, EPI_ISL_791267, EPI_ISL_791268			
see above	Respiratory Virus Unit, National Infection Service, Public Health England	COVID-19 Genomics UK (COG-UK) Consortium	PHE Covid Sequencing Team
EPI_ISL_797405, EPI_ISL_797408, EPI_ISL_797432, EPI_ISL_797445, EPI_ISL_797451, EPI_ISL_797459, EPI_ISL_797500, EPI_ISL_797514, EPI_ISL_797531, EPI_ISL_797554, EPI_ISL_797563, EPI_ISL_797564, EPI_ISL_797615, EPI_ISL_797620, EPI_ISL_797627, EPI_ISL_797632, EPI_ISL_797677, EPI_ISL_797678, EPI_ISL_797685, EPI_ISL_797704, EPI_ISL_797713, EPI_ISL_797716, EPI_ISL_797847, EPI_ISL_797867, EPI_ISL_797870, EPI_ISL_797914, EPI_ISL_797929, EPI_ISL_797945, EPI_ISL_797961, EPI_ISL_797970, EPI_ISL_797980, EPI_ISL_797996			
see above	Lighthouse Lab in Glasgow	Wellcome Sanger Institute for the COVID-19 Genomics UK (COG-UK) Consortium	Harper VanSteenhouse, Yumi Kasai, David Gray, Carol Clugston, Anna Dominiczak and Alex Alderton, Roberto Amato, Sonia Goncalves, Ewan Harrison, David K. Jackson, Ian Johnston, Dominic Kwiatkowski, Cordelia Langford, John Sillitoe on behalf of the Wellcome Sanger Institute COVID-19 Surveillance Team

EPI_ISL_804217, EPI_ISL_804225, EPI_ISL_804249, EPI_ISL_804259, EPI_ISL_804276, EPI_ISL_811107, EPI_ISL_811108, EPI_ISL_811109, EPI_ISL_811110, EPI_ISL_811111, EPI_ISL_811112, EPI_ISL_811113, EPI_ISL_811114, EPI_ISL_811116, EPI_ISL_811121			
see above	Respiratory Virus Unit, National Infection Service, Public Health England	COVID-19 Genomics UK (COG-UK) Consortium	PHE Covid Sequencing Team
EPI_ISL_811194, EPI_ISL_811195, EPI_ISL_811196	Lighthouse Lab in Glasgow	Wellcome Sanger Institute for the COVID-19 Genomics UK (COG-UK) Consortium	Harper VanSteenhouse, Yumi Kasai, David Gray, Carol Clugston, Anna Dominiczak and Alex Alderton, Roberto Amato, Sonia Goncalves, Ewan Harrison, David K. Jackson, Ian Johnston, Dominic Kwiatkowski, Cordelia Langford, John Sillitoe on behalf of the Wellcome Sanger Institute COVID-19 Surveillance Team
EPI_ISL_811197	Lighthouse Lab in Milton Keynes	Wellcome Sanger Institute for the COVID-19 Genomics UK (COG-UK) Consortium	The Lighthouse Lab in Milton Keynes and Alex Alderton, Roberto Amato, Sonia Goncalves, Ewan Harrison, David K. Jackson, Ian Johnston, Dominic Kwiatkowski, Cordelia Langford, John Sillitoe on behalf of the Wellcome Sanger Institute COVID-19 Surveillance Team
EPI_ISL_811198, EPI_ISL_811199, EPI_ISL_811200, EPI_ISL_811201, EPI_ISL_811202, EPI_ISL_811203, EPI_ISL_811204	Lighthouse Lab in Glasgow	Wellcome Sanger Institute for the COVID-19 Genomics UK (COG-UK) Consortium	Harper VanSteenhouse, Yumi Kasai, David Gray, Carol Clugston, Anna Dominiczak and Alex Alderton, Roberto Amato, Sonia Goncalves, Ewan Harrison, David K. Jackson, Ian Johnston, Dominic Kwiatkowski, Cordelia Langford, John Sillitoe on behalf of the Wellcome Sanger Institute COVID-19 Surveillance Team
EPI_ISL_811205	Lighthouse Lab in Milton Keynes	Wellcome Sanger Institute for the COVID-19 Genomics UK (COG-UK) Consortium	The Lighthouse Lab in Milton Keynes and Alex Alderton, Roberto Amato, Sonia Goncalves, Ewan Harrison, David K. Jackson, Ian Johnston, Dominic Kwiatkowski, Cordelia Langford, John Sillitoe on behalf of the Wellcome Sanger Institute COVID-19 Surveillance Team
EPI_ISL_811206, EPI_ISL_811208, EPI_ISL_811209, EPI_ISL_811210	Lighthouse Lab in Glasgow	Wellcome Sanger Institute for the COVID-19 Genomics UK (COG-UK) Consortium	Harper VanSteenhouse, Yumi Kasai, David Gray, Carol Clugston, Anna Dominiczak and Alex Alderton, Roberto Amato, Sonia Goncalves, Ewan Harrison, David K. Jackson, Ian Johnston, Dominic Kwiatkowski, Cordelia Langford, John Sillitoe on behalf of the Wellcome Sanger Institute COVID-19 Surveillance Team
EPI_ISL_811212	Lighthouse Lab in Milton Keynes	Wellcome Sanger Institute for the COVID-19 Genomics UK (COG-UK) Consortium	The Lighthouse Lab in Milton Keynes and Alex Alderton, Roberto Amato, Sonia Goncalves, Ewan Harrison, David K. Jackson, Ian Johnston, Dominic Kwiatkowski, Cordelia Langford, John Sillitoe on behalf of the Wellcome Sanger Institute COVID-19 Surveillance Team
EPI_ISL_811213, EPI_ISL_811216, EPI_ISL_811218, EPI_ISL_811219, EPI_ISL_811220, EPI_ISL_811221, EPI_ISL_811222, EPI_ISL_811223, EPI_ISL_811224, EPI_ISL_811225, EPI_ISL_811226, EPI_ISL_811227, EPI_ISL_811228, EPI_ISL_811230, EPI_ISL_811231, EPI_ISL_811232, EPI_ISL_811233, EPI_ISL_811235, EPI_ISL_811237, EPI_ISL_811238, EPI_ISL_811239, EPI_ISL_811240, EPI_ISL_811242, EPI_ISL_811243, EPI_ISL_811244			
see above	Lighthouse Lab in Glasgow	Wellcome Sanger Institute for the COVID-19 Genomics UK (COG-UK) Consortium	Harper VanSteenhouse, Yumi Kasai, David Gray, Carol Clugston, Anna Dominiczak and Alex Alderton, Roberto Amato, Sonia Goncalves, Ewan Harrison, David K. Jackson, Ian Johnston, Dominic Kwiatkowski, Cordelia Langford, John Sillitoe on behalf of the Wellcome Sanger Institute COVID-19 Surveillance Team
EPI_ISL_811245	Lighthouse Lab in Milton Keynes	Wellcome Sanger Institute for the COVID-19 Genomics UK (COG-UK) Consortium	The Lighthouse Lab in Milton Keynes and Alex Alderton, Roberto Amato, Sonia Goncalves, Ewan Harrison, David K. Jackson, Ian Johnston, Dominic Kwiatkowski, Cordelia Langford, John Sillitoe on behalf of the Wellcome Sanger Institute COVID-19 Surveillance Team
EPI_ISL_811246, EPI_ISL_811249, EPI_ISL_811250	Lighthouse Lab in Glasgow	Wellcome Sanger Institute for the COVID-19 Genomics UK (COG-UK) Consortium	Harper VanSteenhouse, Yumi Kasai, David Gray, Carol Clugston, Anna Dominiczak and Alex Alderton, Roberto Amato, Sonia Goncalves, Ewan Harrison, David K. Jackson, Ian Johnston, Dominic Kwiatkowski, Cordelia Langford, John Sillitoe on behalf of the Wellcome Sanger Institute COVID-19 Surveillance Team
EPI_ISL_811251	Lighthouse Lab in Milton Keynes	Wellcome Sanger Institute for the COVID-19 Genomics UK (COG-UK) Consortium	The Lighthouse Lab in Milton Keynes and Alex Alderton, Roberto Amato, Sonia Goncalves, Ewan Harrison, David K. Jackson, Ian Johnston, Dominic Kwiatkowski, Cordelia Langford, John Sillitoe on behalf of the Wellcome Sanger Institute COVID-19 Surveillance Team
EPI_ISL_811252, EPI_ISL_811253, EPI_ISL_811254	Lighthouse Lab in Glasgow	Wellcome Sanger Institute for the COVID-19 Genomics UK (COG-UK) Consortium	Harper VanSteenhouse, Yumi Kasai, David Gray, Carol Clugston, Anna Dominiczak and Alex Alderton, Roberto Amato, Sonia Goncalves, Ewan Harrison, David K. Jackson, Ian Johnston, Dominic Kwiatkowski, Cordelia Langford, John Sillitoe on behalf of the Wellcome Sanger Institute COVID-19 Surveillance Team
EPI_ISL_811255	Lighthouse Lab in Milton Keynes	Wellcome Sanger Institute for the COVID-19 Genomics UK (COG-UK) Consortium	The Lighthouse Lab in Milton Keynes and Alex Alderton, Roberto Amato, Sonia Goncalves, Ewan Harrison, David K. Jackson, Ian Johnston, Dominic Kwiatkowski, Cordelia Langford, John Sillitoe on behalf of the Wellcome Sanger Institute COVID-19 Surveillance Team
EPI_ISL_811256, EPI_ISL_811257, EPI_ISL_811258, EPI_ISL_811259, EPI_ISL_811260, EPI_ISL_811261, EPI_ISL_811263, EPI_ISL_811264, EPI_ISL_811265, EPI_ISL_811266, EPI_ISL_811267, EPI_ISL_811268			
see above	Lighthouse Lab in Glasgow	Wellcome Sanger Institute for the COVID-19 Genomics UK (COG-UK) Consortium	Harper VanSteenhouse, Yumi Kasai, David Gray, Carol Clugston, Anna Dominiczak and Alex Alderton, Roberto Amato, Sonia Goncalves, Ewan Harrison, David K. Jackson, Ian Johnston, Dominic Kwiatkowski, Cordelia Langford, John Sillitoe on behalf of the Wellcome Sanger Institute COVID-19 Surveillance Team
EPI_ISL_811269	Lighthouse Lab in Milton Keynes	Wellcome Sanger Institute for the COVID-19 Genomics UK (COG-UK) Consortium	The Lighthouse Lab in Milton Keynes and Alex Alderton, Roberto Amato, Sonia Goncalves, Ewan Harrison, David K. Jackson, Ian Johnston, Dominic Kwiatkowski, Cordelia Langford, John Sillitoe on behalf of the Wellcome Sanger Institute COVID-19 Surveillance Team
EPI_ISL_811270, EPI_ISL_811271, EPI_ISL_811272, EPI_ISL_811273, EPI_ISL_811274, EPI_ISL_811276, EPI_ISL_811277, EPI_ISL_811278, EPI_ISL_811279, EPI_ISL_811281	Lighthouse Lab in Glasgow	Wellcome Sanger Institute for the COVID-19 Genomics UK (COG-UK) Consortium	Harper VanSteenhouse, Yumi Kasai, David Gray, Carol Clugston, Anna Dominiczak and Alex Alderton, Roberto Amato, Sonia Goncalves, Ewan Harrison, David K. Jackson, Ian Johnston, Dominic Kwiatkowski, Cordelia Langford, John Sillitoe on behalf of the Wellcome Sanger Institute COVID-19 Surveillance Team
EPI_ISL_811282	Lighthouse Lab in Milton Keynes	Wellcome Sanger Institute for the COVID-19 Genomics UK (COG-UK) Consortium	The Lighthouse Lab in Milton Keynes and Alex Alderton, Roberto Amato, Sonia Goncalves, Ewan Harrison, David K. Jackson, Ian Johnston, Dominic Kwiatkowski, Cordelia Langford, John Sillitoe on behalf of the Wellcome Sanger Institute COVID-19 Surveillance Team
EPI_ISL_811283	Lighthouse Lab in Glasgow	Wellcome Sanger Institute for the COVID-19 Genomics UK (COG-UK) Consortium	Harper VanSteenhouse, Yumi Kasai, David Gray, Carol Clugston, Anna Dominiczak and Alex Alderton, Roberto Amato, Sonia Goncalves, Ewan Harrison, David K. Jackson, Ian Johnston, Dominic Kwiatkowski, Cordelia Langford, John Sillitoe on behalf of the Wellcome Sanger Institute COVID-19 Surveillance Team
EPI_ISL_811285	Lighthouse Lab in Milton Keynes	Wellcome Sanger Institute for the COVID-19 Genomics UK (COG-UK) Consortium	The Lighthouse Lab in Milton Keynes and Alex Alderton, Roberto Amato, Sonia Goncalves, Ewan Harrison, David K. Jackson, Ian Johnston, Dominic Kwiatkowski, Cordelia Langford, John Sillitoe on behalf of the Wellcome Sanger Institute COVID-19 Surveillance Team
EPI_ISL_811286, EPI_ISL_811287, EPI_ISL_811288, EPI_ISL_811289, EPI_ISL_811290, EPI_ISL_811291, EPI_ISL_811292, EPI_ISL_811294, EPI_ISL_811295, EPI_ISL_811296, EPI_ISL_811297, EPI_ISL_811298, EPI_ISL_811299			
see above	Lighthouse Lab in Glasgow	Wellcome Sanger Institute for the COVID-19 Genomics UK (COG-UK) Consortium	Harper VanSteenhouse, Yumi Kasai, David Gray, Carol Clugston, Anna Dominiczak and Alex Alderton, Roberto Amato, Sonia Goncalves, Ewan Harrison, David K. Jackson, Ian Johnston, Dominic Kwiatkowski, Cordelia Langford, John Sillitoe on behalf of the Wellcome Sanger Institute COVID-19 Surveillance Team
EPI_ISL_811300	Lighthouse Lab in Milton Keynes	Wellcome Sanger Institute for the COVID-19 Genomics UK (COG-UK) Consortium	The Lighthouse Lab in Milton Keynes and Alex Alderton, Roberto Amato, Sonia Goncalves, Ewan Harrison, David K. Jackson, Ian Johnston, Dominic Kwiatkowski, Cordelia Langford, John Sillitoe on behalf of the Wellcome Sanger Institute COVID-19 Surveillance Team
EPI_ISL_811301	Lighthouse Lab in Glasgow	Wellcome Sanger Institute for the COVID-19 Genomics UK (COG-UK) Consortium	Harper VanSteenhouse, Yumi Kasai, David Gray, Carol Clugston, Anna Dominiczak and Alex Alderton, Roberto Amato, Sonia Goncalves, Ewan Harrison, David K. Jackson, Ian Johnston, Dominic Kwiatkowski, Cordelia Langford, John Sillitoe on behalf of the Wellcome Sanger Institute COVID-19 Surveillance Team
EPI_ISL_811302	Lighthouse Lab in Milton Keynes	Wellcome Sanger Institute for the COVID-19 Genomics UK (COG-UK) Consortium	The Lighthouse Lab in Milton Keynes and Alex Alderton, Roberto Amato, Sonia Goncalves, Ewan Harrison, David K. Jackson, Ian Johnston, Dominic Kwiatkowski, Cordelia Langford, John Sillitoe on behalf of the Wellcome Sanger Institute COVID-19 Surveillance Team
EPI_ISL_811303	Lighthouse Lab in Glasgow	Wellcome Sanger Institute for the COVID-19 Genomics UK (COG-UK) Consortium	Harper VanSteenhouse, Yumi Kasai, David Gray, Carol Clugston, Anna Dominiczak and Alex Alderton, Roberto Amato, Sonia Goncalves, Ewan Harrison, David K. Jackson, Ian Johnston, Dominic Kwiatkowski, Cordelia Langford, John Sillitoe on behalf of the Wellcome Sanger Institute COVID-19 Surveillance Team
EPI_ISL_811304	Lighthouse Lab in Milton Keynes	Wellcome Sanger Institute for the COVID-19 Genomics UK (COG-UK) Consortium	The Lighthouse Lab in Milton Keynes and Alex Alderton, Roberto Amato, Sonia Goncalves, Ewan Harrison, David K. Jackson, Ian Johnston, Dominic Kwiatkowski, Cordelia Langford, John Sillitoe on behalf of the Wellcome Sanger Institute COVID-19 Surveillance Team
EPI_ISL_811305	Lighthouse Lab in Glasgow	Wellcome Sanger Institute for the COVID-19 Genomics UK (COG-UK) Consortium	Harper VanSteenhouse, Yumi Kasai, David Gray, Carol Clugston, Anna Dominiczak and Alex Alderton, Roberto Amato, Sonia Goncalves, Ewan Harrison, David K. Jackson, Ian Johnston, Dominic Kwiatkowski, Cordelia Langford, John Sillitoe on behalf of the Wellcome Sanger Institute COVID-19 Surveillance Team

EPI_ISL_811306, EPI_ISL_811307	Lighthouse Lab in Milton Keynes	Wellcome Sanger Institute for the COVID-19 Genomics UK (COG-UK) Consortium	The Lighthouse Lab in Milton Keynes and Alex Alderton, Roberto Amato, Sonia Goncalves, Ewan Harrison, David K. Jackson, Ian Johnston, Dominic Kwiatkowski, Cordelia Langford, John Sillitoe on behalf of the Wellcome Sanger Institute COVID-19 Surveillance Team
EPI_ISL_811308	Lighthouse Lab in Glasgow	Wellcome Sanger Institute for the COVID-19 Genomics UK (COG-UK) Consortium	Harper VanSteenhouse, Yumi Kasai, David Gray, Carol Clugston, Anna Dominiczak and Alex Alderton, Roberto Amato, Sonia Goncalves, Ewan Harrison, David K. Jackson, Ian Johnston, Dominic Kwiatkowski, Cordelia Langford, John Sillitoe on behalf of the Wellcome Sanger Institute COVID-19 Surveillance Team
EPI_ISL_811311	Lighthouse Lab in Milton Keynes	Wellcome Sanger Institute for the COVID-19 Genomics UK (COG-UK) Consortium	The Lighthouse Lab in Milton Keynes and Alex Alderton, Roberto Amato, Sonia Goncalves, Ewan Harrison, David K. Jackson, Ian Johnston, Dominic Kwiatkowski, Cordelia Langford, John Sillitoe on behalf of the Wellcome Sanger Institute COVID-19 Surveillance Team
EPI_ISL_811313, EPI_ISL_811314	Lighthouse Lab in Glasgow	Wellcome Sanger Institute for the COVID-19 Genomics UK (COG-UK) Consortium	Harper VanSteenhouse, Yumi Kasai, David Gray, Carol Clugston, Anna Dominiczak and Alex Alderton, Roberto Amato, Sonia Goncalves, Ewan Harrison, David K. Jackson, Ian Johnston, Dominic Kwiatkowski, Cordelia Langford, John Sillitoe on behalf of the Wellcome Sanger Institute COVID-19 Surveillance Team
EPI_ISL_811315	Lighthouse Lab in Milton Keynes	Wellcome Sanger Institute for the COVID-19 Genomics UK (COG-UK) Consortium	The Lighthouse Lab in Milton Keynes and Alex Alderton, Roberto Amato, Sonia Goncalves, Ewan Harrison, David K. Jackson, Ian Johnston, Dominic Kwiatkowski, Cordelia Langford, John Sillitoe on behalf of the Wellcome Sanger Institute COVID-19 Surveillance Team
EPI_ISL_811316, EPI_ISL_811317, EPI_ISL_811318, EPI_ISL_811319, EPI_ISL_811320	Lighthouse Lab in Glasgow	Wellcome Sanger Institute for the COVID-19 Genomics UK (COG-UK) Consortium	Harper VanSteenhouse, Yumi Kasai, David Gray, Carol Clugston, Anna Dominiczak and Alex Alderton, Roberto Amato, Sonia Goncalves, Ewan Harrison, David K. Jackson, Ian Johnston, Dominic Kwiatkowski, Cordelia Langford, John Sillitoe on behalf of the Wellcome Sanger Institute COVID-19 Surveillance Team
EPI_ISL_811322	Lighthouse Lab in Milton Keynes	Wellcome Sanger Institute for the COVID-19 Genomics UK (COG-UK) Consortium	The Lighthouse Lab in Milton Keynes and Alex Alderton, Roberto Amato, Sonia Goncalves, Ewan Harrison, David K. Jackson, Ian Johnston, Dominic Kwiatkowski, Cordelia Langford, John Sillitoe on behalf of the Wellcome Sanger Institute COVID-19 Surveillance Team
EPI_ISL_811323, EPI_ISL_811324, EPI_ISL_811326, EPI_ISL_811327, EPI_ISL_811328	Lighthouse Lab in Glasgow	Wellcome Sanger Institute for the COVID-19 Genomics UK (COG-UK) Consortium	Harper VanSteenhouse, Yumi Kasai, David Gray, Carol Clugston, Anna Dominiczak and Alex Alderton, Roberto Amato, Sonia Goncalves, Ewan Harrison, David K. Jackson, Ian Johnston, Dominic Kwiatkowski, Cordelia Langford, John Sillitoe on behalf of the Wellcome Sanger Institute COVID-19 Surveillance Team
EPI_ISL_811329	Lighthouse Lab in Milton Keynes	Wellcome Sanger Institute for the COVID-19 Genomics UK (COG-UK) Consortium	The Lighthouse Lab in Milton Keynes and Alex Alderton, Roberto Amato, Sonia Goncalves, Ewan Harrison, David K. Jackson, Ian Johnston, Dominic Kwiatkowski, Cordelia Langford, John Sillitoe on behalf of the Wellcome Sanger Institute COVID-19 Surveillance Team
EPI_ISL_811330, EPI_ISL_811331	Lighthouse Lab in Glasgow	Wellcome Sanger Institute for the COVID-19 Genomics UK (COG-UK) Consortium	Harper VanSteenhouse, Yumi Kasai, David Gray, Carol Clugston, Anna Dominiczak and Alex Alderton, Roberto Amato, Sonia Goncalves, Ewan Harrison, David K. Jackson, Ian Johnston, Dominic Kwiatkowski, Cordelia Langford, John Sillitoe on behalf of the Wellcome Sanger Institute COVID-19 Surveillance Team
EPI_ISL_811332	Lighthouse Lab in Milton Keynes	Wellcome Sanger Institute for the COVID-19 Genomics UK (COG-UK) Consortium	The Lighthouse Lab in Milton Keynes and Alex Alderton, Roberto Amato, Sonia Goncalves, Ewan Harrison, David K. Jackson, Ian Johnston, Dominic Kwiatkowski, Cordelia Langford, John Sillitoe on behalf of the Wellcome Sanger Institute COVID-19 Surveillance Team
EPI_ISL_811333	Lighthouse Lab in Glasgow	Wellcome Sanger Institute for the COVID-19 Genomics UK (COG-UK) Consortium	Harper VanSteenhouse, Yumi Kasai, David Gray, Carol Clugston, Anna Dominiczak and Alex Alderton, Roberto Amato, Sonia Goncalves, Ewan Harrison, David K. Jackson, Ian Johnston, Dominic Kwiatkowski, Cordelia Langford, John Sillitoe on behalf of the Wellcome Sanger Institute COVID-19 Surveillance Team
EPI_ISL_811334, EPI_ISL_811336	Lighthouse Lab in Milton Keynes	Wellcome Sanger Institute for the COVID-19 Genomics UK (COG-UK) Consortium	The Lighthouse Lab in Milton Keynes and Alex Alderton, Roberto Amato, Sonia Goncalves, Ewan Harrison, David K. Jackson, Ian Johnston, Dominic Kwiatkowski, Cordelia Langford, John Sillitoe on behalf of the Wellcome Sanger Institute COVID-19 Surveillance Team
EPI_ISL_811337, EPI_ISL_811338	Lighthouse Lab in Glasgow	Wellcome Sanger Institute for the COVID-19 Genomics UK (COG-UK) Consortium	Harper VanSteenhouse, Yumi Kasai, David Gray, Carol Clugston, Anna Dominiczak and Alex Alderton, Roberto Amato, Sonia Goncalves, Ewan Harrison, David K. Jackson, Ian Johnston, Dominic Kwiatkowski, Cordelia Langford, John Sillitoe on behalf of the Wellcome Sanger Institute COVID-19 Surveillance Team
EPI_ISL_811339	Lighthouse Lab in Milton Keynes	Wellcome Sanger Institute for the COVID-19 Genomics UK (COG-UK) Consortium	The Lighthouse Lab in Milton Keynes and Alex Alderton, Roberto Amato, Sonia Goncalves, Ewan Harrison, David K. Jackson, Ian Johnston, Dominic Kwiatkowski, Cordelia Langford, John Sillitoe on behalf of the Wellcome Sanger Institute COVID-19 Surveillance Team
EPI_ISL_811340, EPI_ISL_811341	Lighthouse Lab in Glasgow	Wellcome Sanger Institute for the COVID-19 Genomics UK (COG-UK) Consortium	Harper VanSteenhouse, Yumi Kasai, David Gray, Carol Clugston, Anna Dominiczak and Alex Alderton, Roberto Amato, Sonia Goncalves, Ewan Harrison, David K. Jackson, Ian Johnston, Dominic Kwiatkowski, Cordelia Langford, John Sillitoe on behalf of the Wellcome Sanger Institute COVID-19 Surveillance Team
EPI_ISL_811342	Lighthouse Lab in Milton Keynes	Wellcome Sanger Institute for the COVID-19 Genomics UK (COG-UK) Consortium	The Lighthouse Lab in Milton Keynes and Alex Alderton, Roberto Amato, Sonia Goncalves, Ewan Harrison, David K. Jackson, Ian Johnston, Dominic Kwiatkowski, Cordelia Langford, John Sillitoe on behalf of the Wellcome Sanger Institute COVID-19 Surveillance Team
EPI_ISL_811343, EPI_ISL_811346, EPI_ISL_811347, EPI_ISL_811348, EPI_ISL_811349, EPI_ISL_811351, EPI_ISL_811352, EPI_ISL_811354, EPI_ISL_811355, EPI_ISL_811356, EPI_ISL_811357			
see above	Lighthouse Lab in Glasgow	Wellcome Sanger Institute for the COVID-19 Genomics UK (COG-UK) Consortium	Harper VanSteenhouse, Yumi Kasai, David Gray, Carol Clugston, Anna Dominiczak and Alex Alderton, Roberto Amato, Sonia Goncalves, Ewan Harrison, David K. Jackson, Ian Johnston, Dominic Kwiatkowski, Cordelia Langford, John Sillitoe on behalf of the Wellcome Sanger Institute COVID-19 Surveillance Team
EPI_ISL_811358, EPI_ISL_811359	Lighthouse Lab in Milton Keynes	Wellcome Sanger Institute for the COVID-19 Genomics UK (COG-UK) Consortium	The Lighthouse Lab in Milton Keynes and Alex Alderton, Roberto Amato, Sonia Goncalves, Ewan Harrison, David K. Jackson, Ian Johnston, Dominic Kwiatkowski, Cordelia Langford, John Sillitoe on behalf of the Wellcome Sanger Institute COVID-19 Surveillance Team
EPI_ISL_811360, EPI_ISL_811361, EPI_ISL_811363, EPI_ISL_811364, EPI_ISL_811365	Lighthouse Lab in Glasgow	Wellcome Sanger Institute for the COVID-19 Genomics UK (COG-UK) Consortium	Harper VanSteenhouse, Yumi Kasai, David Gray, Carol Clugston, Anna Dominiczak and Alex Alderton, Roberto Amato, Sonia Goncalves, Ewan Harrison, David K. Jackson, Ian Johnston, Dominic Kwiatkowski, Cordelia Langford, John Sillitoe on behalf of the Wellcome Sanger Institute COVID-19 Surveillance Team
EPI_ISL_811367, EPI_ISL_811368, EPI_ISL_811369	Lighthouse Lab in Milton Keynes	Wellcome Sanger Institute for the COVID-19 Genomics UK (COG-UK) Consortium	The Lighthouse Lab in Milton Keynes and Alex Alderton, Roberto Amato, Sonia Goncalves, Ewan Harrison, David K. Jackson, Ian Johnston, Dominic Kwiatkowski, Cordelia Langford, John Sillitoe on behalf of the Wellcome Sanger Institute COVID-19 Surveillance Team
EPI_ISL_811370, EPI_ISL_811371, EPI_ISL_811372	Lighthouse Lab in Glasgow	Wellcome Sanger Institute for the COVID-19 Genomics UK (COG-UK) Consortium	Harper VanSteenhouse, Yumi Kasai, David Gray, Carol Clugston, Anna Dominiczak and Alex Alderton, Roberto Amato, Sonia Goncalves, Ewan Harrison, David K. Jackson, Ian Johnston, Dominic Kwiatkowski, Cordelia Langford, John Sillitoe on behalf of the Wellcome Sanger Institute COVID-19 Surveillance Team
EPI_ISL_811373	Lighthouse Lab in Milton Keynes	Wellcome Sanger Institute for the COVID-19 Genomics UK (COG-UK) Consortium	The Lighthouse Lab in Milton Keynes and Alex Alderton, Roberto Amato, Sonia Goncalves, Ewan Harrison, David K. Jackson, Ian Johnston, Dominic Kwiatkowski, Cordelia Langford, John Sillitoe on behalf of the Wellcome Sanger Institute COVID-19 Surveillance Team
EPI_ISL_811374, EPI_ISL_811375, EPI_ISL_811376, EPI_ISL_811378, EPI_ISL_811379	Lighthouse Lab in Glasgow	Wellcome Sanger Institute for the COVID-19 Genomics UK (COG-UK) Consortium	Harper VanSteenhouse, Yumi Kasai, David Gray, Carol Clugston, Anna Dominiczak and Alex Alderton, Roberto Amato, Sonia Goncalves, Ewan Harrison, David K. Jackson, Ian Johnston, Dominic Kwiatkowski, Cordelia Langford, John Sillitoe on behalf of the Wellcome Sanger Institute COVID-19 Surveillance Team
EPI_ISL_811383	Lighthouse Lab in Milton Keynes	Wellcome Sanger Institute for the COVID-19 Genomics UK (COG-UK) Consortium	The Lighthouse Lab in Milton Keynes and Alex Alderton, Roberto Amato, Sonia Goncalves, Ewan Harrison, David K. Jackson, Ian Johnston, Dominic Kwiatkowski, Cordelia Langford, John Sillitoe on behalf of the Wellcome Sanger Institute COVID-19 Surveillance Team
EPI_ISL_811384, EPI_ISL_811386, EPI_ISL_811387, EPI_ISL_811388, EPI_ISL_811390, EPI_ISL_811391, EPI_ISL_811393, EPI_ISL_811396	Lighthouse Lab in Glasgow	Wellcome Sanger Institute for the COVID-19 Genomics UK (COG-UK) Consortium	Harper VanSteenhouse, Yumi Kasai, David Gray, Carol Clugston, Anna Dominiczak and Alex Alderton, Roberto Amato, Sonia Goncalves, Ewan Harrison, David K. Jackson, Ian Johnston, Dominic Kwiatkowski, Cordelia Langford, John Sillitoe on behalf of the Wellcome Sanger Institute COVID-19 Surveillance Team
EPI_ISL_811397	Lighthouse Lab in Milton Keynes	Wellcome Sanger Institute for the COVID-19 Genomics UK (COG-UK) Consortium	The Lighthouse Lab in Milton Keynes and Alex Alderton, Roberto Amato, Sonia Goncalves, Ewan Harrison, David K. Jackson, Ian Johnston, Dominic Kwiatkowski, Cordelia Langford, John Sillitoe on behalf of the Wellcome Sanger Institute COVID-19 Surveillance Team
EPI_ISL_811398, EPI_ISL_811399, EPI_ISL_811401, EPI_ISL_811402, EPI_ISL_811403, EPI_ISL_811405, EPI_ISL_811407, EPI_ISL_811408	Lighthouse Lab in Glasgow	Wellcome Sanger Institute for the COVID-19 Genomics UK (COG-UK) Consortium	Harper VanSteenhouse, Yumi Kasai, David Gray, Carol Clugston, Anna Dominiczak and Alex Alderton, Roberto Amato, Sonia Goncalves, Ewan Harrison, David K. Jackson, Ian Johnston, Dominic Kwiatkowski, Cordelia Langford, John Sillitoe on behalf of the Wellcome Sanger Institute COVID-19 Surveillance Team

EPI_ISL_811409	Lighthouse Lab in Milton Keynes	Wellcome Sanger Institute for the COVID-19 Genomics UK (COG-UK) Consortium	The Lighthouse Lab in Milton Keynes and Alex Alderton, Roberto Amato, Sonia Goncalves, Ewan Harrison, David K. Jackson, Ian Johnston, Dominic Kwiatkowski, Cordelia Langford, John Sillitoe on behalf of the Wellcome Sanger Institute COVID-19 Surveillance Team
EPI_ISL_811411, EPI_ISL_811412, EPI_ISL_811413, EPI_ISL_811415, EPI_ISL_811416, EPI_ISL_811417, EPI_ISL_811418, EPI_ISL_811419, EPI_ISL_811421, EPI_ISL_811423, EPI_ISL_811424, EPI_ISL_811425, EPI_ISL_811426, EPI_ISL_811427, EPI_ISL_811428			
see above	Lighthouse Lab in Glasgow	Wellcome Sanger Institute for the COVID-19 Genomics UK (COG-UK) Consortium	Harper VanSteenhouse, Yumi Kasai, David Gray, Carol Clugston, Anna Dominiczak and Alex Alderton, Roberto Amato, Sonia Goncalves, Ewan Harrison, David K. Jackson, Ian Johnston, Dominic Kwiatkowski, Cordelia Langford, John Sillitoe on behalf of the Wellcome Sanger Institute COVID-19 Surveillance Team
EPI_ISL_811429, EPI_ISL_811430, EPI_ISL_811431	Lighthouse Lab in Milton Keynes	Wellcome Sanger Institute for the COVID-19 Genomics UK (COG-UK) Consortium	The Lighthouse Lab in Milton Keynes and Alex Alderton, Roberto Amato, Sonia Goncalves, Ewan Harrison, David K. Jackson, Ian Johnston, Dominic Kwiatkowski, Cordelia Langford, John Sillitoe on behalf of the Wellcome Sanger Institute COVID-19 Surveillance Team
EPI_ISL_811432, EPI_ISL_811434, EPI_ISL_811435, EPI_ISL_811437, EPI_ISL_811438, EPI_ISL_811439, EPI_ISL_811440, EPI_ISL_811441, EPI_ISL_811442, EPI_ISL_811443	Lighthouse Lab in Glasgow	Wellcome Sanger Institute for the COVID-19 Genomics UK (COG-UK) Consortium	Harper VanSteenhouse, Yumi Kasai, David Gray, Carol Clugston, Anna Dominiczak and Alex Alderton, Roberto Amato, Sonia Goncalves, Ewan Harrison, David K. Jackson, Ian Johnston, Dominic Kwiatkowski, Cordelia Langford, John Sillitoe on behalf of the Wellcome Sanger Institute COVID-19 Surveillance Team
EPI_ISL_811444, EPI_ISL_811445	Lighthouse Lab in Milton Keynes	Wellcome Sanger Institute for the COVID-19 Genomics UK (COG-UK) Consortium	The Lighthouse Lab in Milton Keynes and Alex Alderton, Roberto Amato, Sonia Goncalves, Ewan Harrison, David K. Jackson, Ian Johnston, Dominic Kwiatkowski, Cordelia Langford, John Sillitoe on behalf of the Wellcome Sanger Institute COVID-19 Surveillance Team
EPI_ISL_811446, EPI_ISL_811447, EPI_ISL_811448, EPI_ISL_811449, EPI_ISL_811450, EPI_ISL_811451	Lighthouse Lab in Glasgow	Wellcome Sanger Institute for the COVID-19 Genomics UK (COG-UK) Consortium	Harper VanSteenhouse, Yumi Kasai, David Gray, Carol Clugston, Anna Dominiczak and Alex Alderton, Roberto Amato, Sonia Goncalves, Ewan Harrison, David K. Jackson, Ian Johnston, Dominic Kwiatkowski, Cordelia Langford, John Sillitoe on behalf of the Wellcome Sanger Institute COVID-19 Surveillance Team
EPI_ISL_811452	Lighthouse Lab in Milton Keynes	Wellcome Sanger Institute for the COVID-19 Genomics UK (COG-UK) Consortium	The Lighthouse Lab in Milton Keynes and Alex Alderton, Roberto Amato, Sonia Goncalves, Ewan Harrison, David K. Jackson, Ian Johnston, Dominic Kwiatkowski, Cordelia Langford, John Sillitoe on behalf of the Wellcome Sanger Institute COVID-19 Surveillance Team
EPI_ISL_811453	Lighthouse Lab in Glasgow	Wellcome Sanger Institute for the COVID-19 Genomics UK (COG-UK) Consortium	Harper VanSteenhouse, Yumi Kasai, David Gray, Carol Clugston, Anna Dominiczak and Alex Alderton, Roberto Amato, Sonia Goncalves, Ewan Harrison, David K. Jackson, Ian Johnston, Dominic Kwiatkowski, Cordelia Langford, John Sillitoe on behalf of the Wellcome Sanger Institute COVID-19 Surveillance Team
EPI_ISL_811455, EPI_ISL_811456	Lighthouse Lab in Milton Keynes	Wellcome Sanger Institute for the COVID-19 Genomics UK (COG-UK) Consortium	The Lighthouse Lab in Milton Keynes and Alex Alderton, Roberto Amato, Sonia Goncalves, Ewan Harrison, David K. Jackson, Ian Johnston, Dominic Kwiatkowski, Cordelia Langford, John Sillitoe on behalf of the Wellcome Sanger Institute COVID-19 Surveillance Team
EPI_ISL_811457, EPI_ISL_811458, EPI_ISL_811459, EPI_ISL_811460, EPI_ISL_811461, EPI_ISL_811462, EPI_ISL_811463, EPI_ISL_811464, EPI_ISL_811465, EPI_ISL_811466, EPI_ISL_811467, EPI_ISL_811468			
see above	Lighthouse Lab in Cambridge	Wellcome Sanger Institute for the COVID-19 Genomics UK (COG-UK) Consortium	Rob Howes, The Lighthouse Lab in Cambridge and Alex Alderton, Roberto Amato, Sonia Goncalves, Ewan Harrison, David K. Jackson, Ian Johnston, Dominic Kwiatkowski, Cordelia Langford, John Sillitoe on behalf of the Wellcome Sanger Institute COVID-19 Surveillance Team
EPI_ISL_811469	Lighthouse Lab in Milton Keynes	Wellcome Sanger Institute for the COVID-19 Genomics UK (COG-UK) Consortium	The Lighthouse Lab in Milton Keynes and Alex Alderton, Roberto Amato, Sonia Goncalves, Ewan Harrison, David K. Jackson, Ian Johnston, Dominic Kwiatkowski, Cordelia Langford, John Sillitoe on behalf of the Wellcome Sanger Institute COVID-19 Surveillance Team
EPI_ISL_811470, EPI_ISL_811471, EPI_ISL_811473, EPI_ISL_811474, EPI_ISL_811475, EPI_ISL_811476, EPI_ISL_811477, EPI_ISL_811479, EPI_ISL_811480, EPI_ISL_811481, EPI_ISL_811483			
see above	Lighthouse Lab in Cambridge	Wellcome Sanger Institute for the COVID-19 Genomics UK (COG-UK) Consortium	Rob Howes, The Lighthouse Lab in Cambridge and Alex Alderton, Roberto Amato, Sonia Goncalves, Ewan Harrison, David K. Jackson, Ian Johnston, Dominic Kwiatkowski, Cordelia Langford, John Sillitoe on behalf of the Wellcome Sanger Institute COVID-19 Surveillance Team
EPI_ISL_811484	Lighthouse Lab in Milton Keynes	Wellcome Sanger Institute for the COVID-19 Genomics UK (COG-UK) Consortium	The Lighthouse Lab in Milton Keynes and Alex Alderton, Roberto Amato, Sonia Goncalves, Ewan Harrison, David K. Jackson, Ian Johnston, Dominic Kwiatkowski, Cordelia Langford, John Sillitoe on behalf of the Wellcome Sanger Institute COVID-19 Surveillance Team
EPI_ISL_811485, EPI_ISL_811486	Lighthouse Lab in Cambridge	Wellcome Sanger Institute for the COVID-19 Genomics UK (COG-UK) Consortium	Rob Howes, The Lighthouse Lab in Cambridge and Alex Alderton, Roberto Amato, Sonia Goncalves, Ewan Harrison, David K. Jackson, Ian Johnston, Dominic Kwiatkowski, Cordelia Langford, John Sillitoe on behalf of the Wellcome Sanger Institute COVID-19 Surveillance Team
EPI_ISL_811487	Lighthouse Lab in Milton Keynes	Wellcome Sanger Institute for the COVID-19 Genomics UK (COG-UK) Consortium	The Lighthouse Lab in Milton Keynes and Alex Alderton, Roberto Amato, Sonia Goncalves, Ewan Harrison, David K. Jackson, Ian Johnston, Dominic Kwiatkowski, Cordelia Langford, John Sillitoe on behalf of the Wellcome Sanger Institute COVID-19 Surveillance Team
EPI_ISL_811488, EPI_ISL_811489, EPI_ISL_811491, EPI_ISL_811492, EPI_ISL_811493, EPI_ISL_811495, EPI_ISL_811496, EPI_ISL_811497	Lighthouse Lab in Cambridge	Wellcome Sanger Institute for the COVID-19 Genomics UK (COG-UK) Consortium	Rob Howes, The Lighthouse Lab in Cambridge and Alex Alderton, Roberto Amato, Sonia Goncalves, Ewan Harrison, David K. Jackson, Ian Johnston, Dominic Kwiatkowski, Cordelia Langford, John Sillitoe on behalf of the Wellcome Sanger Institute COVID-19 Surveillance Team
EPI_ISL_811498	Lighthouse Lab in Milton Keynes	Wellcome Sanger Institute for the COVID-19 Genomics UK (COG-UK) Consortium	The Lighthouse Lab in Milton Keynes and Alex Alderton, Roberto Amato, Sonia Goncalves, Ewan Harrison, David K. Jackson, Ian Johnston, Dominic Kwiatkowski, Cordelia Langford, John Sillitoe on behalf of the Wellcome Sanger Institute COVID-19 Surveillance Team
EPI_ISL_811499, EPI_ISL_811501, EPI_ISL_811503, EPI_ISL_811504, EPI_ISL_811505, EPI_ISL_811506, EPI_ISL_811507, EPI_ISL_811508, EPI_ISL_811509, EPI_ISL_811510	Lighthouse Lab in Cambridge	Wellcome Sanger Institute for the COVID-19 Genomics UK (COG-UK) Consortium	Rob Howes, The Lighthouse Lab in Cambridge and Alex Alderton, Roberto Amato, Sonia Goncalves, Ewan Harrison, David K. Jackson, Ian Johnston, Dominic Kwiatkowski, Cordelia Langford, John Sillitoe on behalf of the Wellcome Sanger Institute COVID-19 Surveillance Team
EPI_ISL_811511	Lighthouse Lab in Milton Keynes	Wellcome Sanger Institute for the COVID-19 Genomics UK (COG-UK) Consortium	The Lighthouse Lab in Milton Keynes and Alex Alderton, Roberto Amato, Sonia Goncalves, Ewan Harrison, David K. Jackson, Ian Johnston, Dominic Kwiatkowski, Cordelia Langford, John Sillitoe on behalf of the Wellcome Sanger Institute COVID-19 Surveillance Team
EPI_ISL_811512, EPI_ISL_811513, EPI_ISL_811514, EPI_ISL_811515, EPI_ISL_811517, EPI_ISL_811518, EPI_ISL_811520, EPI_ISL_811521, EPI_ISL_811522, EPI_ISL_811523	Lighthouse Lab in Cambridge	Wellcome Sanger Institute for the COVID-19 Genomics UK (COG-UK) Consortium	Rob Howes, The Lighthouse Lab in Cambridge and Alex Alderton, Roberto Amato, Sonia Goncalves, Ewan Harrison, David K. Jackson, Ian Johnston, Dominic Kwiatkowski, Cordelia Langford, John Sillitoe on behalf of the Wellcome Sanger Institute COVID-19 Surveillance Team
EPI_ISL_811525	Lighthouse Lab in Milton Keynes	Wellcome Sanger Institute for the COVID-19 Genomics UK (COG-UK) Consortium	The Lighthouse Lab in Milton Keynes and Alex Alderton, Roberto Amato, Sonia Goncalves, Ewan Harrison, David K. Jackson, Ian Johnston, Dominic Kwiatkowski, Cordelia Langford, John Sillitoe on behalf of the Wellcome Sanger Institute COVID-19 Surveillance Team
EPI_ISL_811526, EPI_ISL_811527, EPI_ISL_811528, EPI_ISL_811529, EPI_ISL_811531, EPI_ISL_811532, EPI_ISL_811533, EPI_ISL_811534, EPI_ISL_811535, EPI_ISL_811536, EPI_ISL_811537, EPI_ISL_811539, EPI_ISL_811540			
see above	Lighthouse Lab in Cambridge	Wellcome Sanger Institute for the COVID-19 Genomics UK (COG-UK) Consortium	Rob Howes, The Lighthouse Lab in Cambridge and Alex Alderton, Roberto Amato, Sonia Goncalves, Ewan Harrison, David K. Jackson, Ian Johnston, Dominic Kwiatkowski, Cordelia Langford, John Sillitoe on behalf of the Wellcome Sanger Institute COVID-19 Surveillance Team
EPI_ISL_811541	Lighthouse Lab in Milton Keynes	Wellcome Sanger Institute for the COVID-19 Genomics UK (COG-UK) Consortium	The Lighthouse Lab in Milton Keynes and Alex Alderton, Roberto Amato, Sonia Goncalves, Ewan Harrison, David K. Jackson, Ian Johnston, Dominic Kwiatkowski, Cordelia Langford, John Sillitoe on behalf of the Wellcome Sanger Institute COVID-19 Surveillance Team
EPI_ISL_811542, EPI_ISL_811543, EPI_ISL_811544	Lighthouse Lab in Cambridge	Wellcome Sanger Institute for the COVID-19 Genomics UK (COG-UK) Consortium	Rob Howes, The Lighthouse Lab in Cambridge and Alex Alderton, Roberto Amato, Sonia Goncalves, Ewan Harrison, David K. Jackson, Ian Johnston, Dominic Kwiatkowski, Cordelia Langford, John Sillitoe on behalf of the Wellcome Sanger Institute COVID-19 Surveillance Team
EPI_ISL_811545	Lighthouse Lab in Milton Keynes	Wellcome Sanger Institute for the COVID-19 Genomics UK (COG-UK) Consortium	The Lighthouse Lab in Milton Keynes and Alex Alderton, Roberto Amato, Sonia Goncalves, Ewan Harrison, David K. Jackson, Ian Johnston, Dominic Kwiatkowski, Cordelia Langford, John Sillitoe on behalf of the Wellcome Sanger Institute COVID-19 Surveillance Team
EPI_ISL_811546, EPI_ISL_811547	Lighthouse Lab in Cambridge	Wellcome Sanger Institute for the COVID-19 Genomics UK (COG-UK) Consortium	Rob Howes, The Lighthouse Lab in Cambridge and Alex Alderton, Roberto Amato, Sonia Goncalves, Ewan Harrison, David K. Jackson, Ian Johnston, Dominic Kwiatkowski, Cordelia Langford, John Sillitoe on behalf of the Wellcome Sanger Institute COVID-19 Surveillance Team
EPI_ISL_811549	Lighthouse Lab in Milton Keynes	Wellcome Sanger Institute for the COVID-19 Genomics UK (COG-UK) Consortium	The Lighthouse Lab in Milton Keynes and Alex Alderton, Roberto Amato, Sonia Goncalves, Ewan Harrison, David K. Jackson, Ian Johnston, Dominic Kwiatkowski, Cordelia Langford, John Sillitoe on behalf of the Wellcome Sanger Institute COVID-19 Surveillance Team

EPI_ISL_811550, EPI_ISL_811551, EPI_ISL_811552, EPI_ISL_811553, EPI_ISL_811554, EPI_ISL_811555	Lighthouse Lab in Cambridge	Wellcome Sanger Institute for the COVID-19 Genomics UK (COG-UK) Consortium	Rob Howes, The Lighthouse Lab in Cambridge and Alex Alderton, Roberto Amato, Sonia Goncalves, Ewan Harrison, David K. Jackson, Ian Johnston, Dominic Kwiatkowski, Cordelia Langford, John Sillitoe on behalf of the Wellcome Sanger Institute COVID-19 Surveillance Team
EPI_ISL_811556	Lighthouse Lab in Milton Keynes	Wellcome Sanger Institute for the COVID-19 Genomics UK (COG-UK) Consortium	The Lighthouse Lab in Milton Keynes and Alex Alderton, Roberto Amato, Sonia Goncalves, Ewan Harrison, David K. Jackson, Ian Johnston, Dominic Kwiatkowski, Cordelia Langford, John Sillitoe on behalf of the Wellcome Sanger Institute COVID-19 Surveillance Team
EPI_ISL_811557, EPI_ISL_811558	Lighthouse Lab in Cambridge	Wellcome Sanger Institute for the COVID-19 Genomics UK (COG-UK) Consortium	Rob Howes, The Lighthouse Lab in Cambridge and Alex Alderton, Roberto Amato, Sonia Goncalves, Ewan Harrison, David K. Jackson, Ian Johnston, Dominic Kwiatkowski, Cordelia Langford, John Sillitoe on behalf of the Wellcome Sanger Institute COVID-19 Surveillance Team
EPI_ISL_811559	Lighthouse Lab in Milton Keynes	Wellcome Sanger Institute for the COVID-19 Genomics UK (COG-UK) Consortium	The Lighthouse Lab in Milton Keynes and Alex Alderton, Roberto Amato, Sonia Goncalves, Ewan Harrison, David K. Jackson, Ian Johnston, Dominic Kwiatkowski, Cordelia Langford, John Sillitoe on behalf of the Wellcome Sanger Institute COVID-19 Surveillance Team
EPI_ISL_811560, EPI_ISL_811561, EPI_ISL_811562, EPI_ISL_811564, EPI_ISL_811566, EPI_ISL_811567, EPI_ISL_811568	Lighthouse Lab in Cambridge	Wellcome Sanger Institute for the COVID-19 Genomics UK (COG-UK) Consortium	Rob Howes, The Lighthouse Lab in Cambridge and Alex Alderton, Roberto Amato, Sonia Goncalves, Ewan Harrison, David K. Jackson, Ian Johnston, Dominic Kwiatkowski, Cordelia Langford, John Sillitoe on behalf of the Wellcome Sanger Institute COVID-19 Surveillance Team
EPI_ISL_811569	Lighthouse Lab in Milton Keynes	Wellcome Sanger Institute for the COVID-19 Genomics UK (COG-UK) Consortium	The Lighthouse Lab in Milton Keynes and Alex Alderton, Roberto Amato, Sonia Goncalves, Ewan Harrison, David K. Jackson, Ian Johnston, Dominic Kwiatkowski, Cordelia Langford, John Sillitoe on behalf of the Wellcome Sanger Institute COVID-19 Surveillance Team
EPI_ISL_811570, EPI_ISL_811571, EPI_ISL_811572, EPI_ISL_811574, EPI_ISL_811575, EPI_ISL_811576, EPI_ISL_811577, EPI_ISL_811578, EPI_ISL_811579, EPI_ISL_811581, EPI_ISL_811582, EPI_ISL_811583, EPI_ISL_811585, EPI_ISL_811586, EPI_ISL_811587, EPI_ISL_811588, EPI_ISL_811589, EPI_ISL_811590			
see above	Lighthouse Lab in Cambridge	Wellcome Sanger Institute for the COVID-19 Genomics UK (COG-UK) Consortium	Rob Howes, The Lighthouse Lab in Cambridge and Alex Alderton, Roberto Amato, Sonia Goncalves, Ewan Harrison, David K. Jackson, Ian Johnston, Dominic Kwiatkowski, Cordelia Langford, John Sillitoe on behalf of the Wellcome Sanger Institute COVID-19 Surveillance Team
EPI_ISL_811591	Lighthouse Lab in Milton Keynes	Wellcome Sanger Institute for the COVID-19 Genomics UK (COG-UK) Consortium	The Lighthouse Lab in Milton Keynes and Alex Alderton, Roberto Amato, Sonia Goncalves, Ewan Harrison, David K. Jackson, Ian Johnston, Dominic Kwiatkowski, Cordelia Langford, John Sillitoe on behalf of the Wellcome Sanger Institute COVID-19 Surveillance Team
EPI_ISL_811592, EPI_ISL_811593, EPI_ISL_811596, EPI_ISL_811597, EPI_ISL_811598, EPI_ISL_811599, EPI_ISL_811600, EPI_ISL_811601, EPI_ISL_811602	Lighthouse Lab in Cambridge	Wellcome Sanger Institute for the COVID-19 Genomics UK (COG-UK) Consortium	Rob Howes, The Lighthouse Lab in Cambridge and Alex Alderton, Roberto Amato, Sonia Goncalves, Ewan Harrison, David K. Jackson, Ian Johnston, Dominic Kwiatkowski, Cordelia Langford, John Sillitoe on behalf of the Wellcome Sanger Institute COVID-19 Surveillance Team
EPI_ISL_811603	Lighthouse Lab in Milton Keynes	Wellcome Sanger Institute for the COVID-19 Genomics UK (COG-UK) Consortium	The Lighthouse Lab in Milton Keynes and Alex Alderton, Roberto Amato, Sonia Goncalves, Ewan Harrison, David K. Jackson, Ian Johnston, Dominic Kwiatkowski, Cordelia Langford, John Sillitoe on behalf of the Wellcome Sanger Institute COVID-19 Surveillance Team
EPI_ISL_811604, EPI_ISL_811605, EPI_ISL_811607, EPI_ISL_811608, EPI_ISL_811609	Lighthouse Lab in Cambridge	Wellcome Sanger Institute for the COVID-19 Genomics UK (COG-UK) Consortium	Rob Howes, The Lighthouse Lab in Cambridge and Alex Alderton, Roberto Amato, Sonia Goncalves, Ewan Harrison, David K. Jackson, Ian Johnston, Dominic Kwiatkowski, Cordelia Langford, John Sillitoe on behalf of the Wellcome Sanger Institute COVID-19 Surveillance Team
EPI_ISL_811611	Lighthouse Lab in Milton Keynes	Wellcome Sanger Institute for the COVID-19 Genomics UK (COG-UK) Consortium	The Lighthouse Lab in Milton Keynes and Alex Alderton, Roberto Amato, Sonia Goncalves, Ewan Harrison, David K. Jackson, Ian Johnston, Dominic Kwiatkowski, Cordelia Langford, John Sillitoe on behalf of the Wellcome Sanger Institute COVID-19 Surveillance Team
EPI_ISL_811612, EPI_ISL_811613, EPI_ISL_811614, EPI_ISL_811615, EPI_ISL_811616, EPI_ISL_811619	Lighthouse Lab in Cambridge	Wellcome Sanger Institute for the COVID-19 Genomics UK (COG-UK) Consortium	Rob Howes, The Lighthouse Lab in Cambridge and Alex Alderton, Roberto Amato, Sonia Goncalves, Ewan Harrison, David K. Jackson, Ian Johnston, Dominic Kwiatkowski, Cordelia Langford, John Sillitoe on behalf of the Wellcome Sanger Institute COVID-19 Surveillance Team
EPI_ISL_811622	Lighthouse Lab in Milton Keynes	Wellcome Sanger Institute for the COVID-19 Genomics UK (COG-UK) Consortium	The Lighthouse Lab in Milton Keynes and Alex Alderton, Roberto Amato, Sonia Goncalves, Ewan Harrison, David K. Jackson, Ian Johnston, Dominic Kwiatkowski, Cordelia Langford, John Sillitoe on behalf of the Wellcome Sanger Institute COVID-19 Surveillance Team
EPI_ISL_811623, EPI_ISL_811624	Lighthouse Lab in Cambridge	Wellcome Sanger Institute for the COVID-19 Genomics UK (COG-UK) Consortium	Rob Howes, The Lighthouse Lab in Cambridge and Alex Alderton, Roberto Amato, Sonia Goncalves, Ewan Harrison, David K. Jackson, Ian Johnston, Dominic Kwiatkowski, Cordelia Langford, John Sillitoe on behalf of the Wellcome Sanger Institute COVID-19 Surveillance Team
EPI_ISL_811625	Lighthouse Lab in Milton Keynes	Wellcome Sanger Institute for the COVID-19 Genomics UK (COG-UK) Consortium	The Lighthouse Lab in Milton Keynes and Alex Alderton, Roberto Amato, Sonia Goncalves, Ewan Harrison, David K. Jackson, Ian Johnston, Dominic Kwiatkowski, Cordelia Langford, John Sillitoe on behalf of the Wellcome Sanger Institute COVID-19 Surveillance Team
EPI_ISL_811628, EPI_ISL_811629, EPI_ISL_811630, EPI_ISL_811631, EPI_ISL_811632, EPI_ISL_811633, EPI_ISL_811635, EPI_ISL_811636, EPI_ISL_811637, EPI_ISL_811638, EPI_ISL_811639, EPI_ISL_811640, EPI_ISL_811641, EPI_ISL_811642, EPI_ISL_811643			
see above	Lighthouse Lab in Cambridge	Wellcome Sanger Institute for the COVID-19 Genomics UK (COG-UK) Consortium	Rob Howes, The Lighthouse Lab in Cambridge and Alex Alderton, Roberto Amato, Sonia Goncalves, Ewan Harrison, David K. Jackson, Ian Johnston, Dominic Kwiatkowski, Cordelia Langford, John Sillitoe on behalf of the Wellcome Sanger Institute COVID-19 Surveillance Team
EPI_ISL_811644	Lighthouse Lab in Milton Keynes	Wellcome Sanger Institute for the COVID-19 Genomics UK (COG-UK) Consortium	The Lighthouse Lab in Milton Keynes and Alex Alderton, Roberto Amato, Sonia Goncalves, Ewan Harrison, David K. Jackson, Ian Johnston, Dominic Kwiatkowski, Cordelia Langford, John Sillitoe on behalf of the Wellcome Sanger Institute COVID-19 Surveillance Team
EPI_ISL_811646, EPI_ISL_811647, EPI_ISL_811648, EPI_ISL_811649, EPI_ISL_811652, EPI_ISL_811653, EPI_ISL_811654, EPI_ISL_811655, EPI_ISL_811656	Lighthouse Lab in Cambridge	Wellcome Sanger Institute for the COVID-19 Genomics UK (COG-UK) Consortium	Rob Howes, The Lighthouse Lab in Cambridge and Alex Alderton, Roberto Amato, Sonia Goncalves, Ewan Harrison, David K. Jackson, Ian Johnston, Dominic Kwiatkowski, Cordelia Langford, John Sillitoe on behalf of the Wellcome Sanger Institute COVID-19 Surveillance Team
EPI_ISL_811657	Lighthouse Lab in Milton Keynes	Wellcome Sanger Institute for the COVID-19 Genomics UK (COG-UK) Consortium	The Lighthouse Lab in Milton Keynes and Alex Alderton, Roberto Amato, Sonia Goncalves, Ewan Harrison, David K. Jackson, Ian Johnston, Dominic Kwiatkowski, Cordelia Langford, John Sillitoe on behalf of the Wellcome Sanger Institute COVID-19 Surveillance Team
EPI_ISL_811658, EPI_ISL_811659, EPI_ISL_811662, EPI_ISL_811663, EPI_ISL_811665, EPI_ISL_811667, EPI_ISL_811668	Lighthouse Lab in Cambridge	Wellcome Sanger Institute for the COVID-19 Genomics UK (COG-UK) Consortium	Rob Howes, The Lighthouse Lab in Cambridge and Alex Alderton, Roberto Amato, Sonia Goncalves, Ewan Harrison, David K. Jackson, Ian Johnston, Dominic Kwiatkowski, Cordelia Langford, John Sillitoe on behalf of the Wellcome Sanger Institute COVID-19 Surveillance Team
EPI_ISL_811673	Lighthouse Lab in Milton Keynes	Wellcome Sanger Institute for the COVID-19 Genomics UK (COG-UK) Consortium	The Lighthouse Lab in Milton Keynes and Alex Alderton, Roberto Amato, Sonia Goncalves, Ewan Harrison, David K. Jackson, Ian Johnston, Dominic Kwiatkowski, Cordelia Langford, John Sillitoe on behalf of the Wellcome Sanger Institute COVID-19 Surveillance Team
EPI_ISL_811674, EPI_ISL_811675, EPI_ISL_811676, EPI_ISL_811679, EPI_ISL_811680, EPI_ISL_811683, EPI_ISL_811684, EPI_ISL_811685, EPI_ISL_811686, EPI_ISL_811687, EPI_ISL_811689, EPI_ISL_811690, EPI_ISL_811691, EPI_ISL_811692, EPI_ISL_811693, EPI_ISL_811694, EPI_ISL_811695			
see above	Lighthouse Lab in Cambridge	Wellcome Sanger Institute for the COVID-19 Genomics UK (COG-UK) Consortium	Rob Howes, The Lighthouse Lab in Cambridge and Alex Alderton, Roberto Amato, Sonia Goncalves, Ewan Harrison, David K. Jackson, Ian Johnston, Dominic Kwiatkowski, Cordelia Langford, John Sillitoe on behalf of the Wellcome Sanger Institute COVID-19 Surveillance Team
EPI_ISL_811696	Lighthouse Lab in Milton Keynes	Wellcome Sanger Institute for the COVID-19 Genomics UK (COG-UK) Consortium	The Lighthouse Lab in Milton Keynes and Alex Alderton, Roberto Amato, Sonia Goncalves, Ewan Harrison, David K. Jackson, Ian Johnston, Dominic Kwiatkowski, Cordelia Langford, John Sillitoe on behalf of the Wellcome Sanger Institute COVID-19 Surveillance Team
EPI_ISL_811697, EPI_ISL_811699, EPI_ISL_811700, EPI_ISL_811704, EPI_ISL_811705, EPI_ISL_811706, EPI_ISL_811707, EPI_ISL_811709, EPI_ISL_811710, EPI_ISL_811711, EPI_ISL_811712, EPI_ISL_811714, EPI_ISL_811715, EPI_ISL_811716, EPI_ISL_811717, EPI_ISL_811718, EPI_ISL_811719, EPI_ISL_811721, EPI_ISL_811722, EPI_ISL_811723, EPI_ISL_811724, EPI_ISL_811725, EPI_ISL_811728, EPI_ISL_811729, EPI_ISL_811730, EPI_ISL_811731, EPI_ISL_811732, EPI_ISL_811733, EPI_ISL_811734, EPI_ISL_811735, EPI_ISL_811737, EPI_ISL_811738, EPI_ISL_811739			
see above	Lighthouse Lab in Cambridge	Wellcome Sanger Institute for the COVID-19 Genomics UK (COG-UK) Consortium	Rob Howes, The Lighthouse Lab in Cambridge and Alex Alderton, Roberto Amato, Sonia Goncalves, Ewan Harrison, David K. Jackson, Ian Johnston, Dominic Kwiatkowski, Cordelia Langford, John Sillitoe on behalf of the Wellcome Sanger Institute COVID-19 Surveillance Team
EPI_ISL_811741	Lighthouse Lab in Milton Keynes	Wellcome Sanger Institute for the COVID-19 Genomics UK (COG-UK) Consortium	The Lighthouse Lab in Milton Keynes and Alex Alderton, Roberto Amato, Sonia Goncalves, Ewan Harrison, David K. Jackson, Ian Johnston, Dominic Kwiatkowski, Cordelia Langford, John Sillitoe on behalf of the Wellcome Sanger Institute COVID-19 Surveillance Team
EPI_ISL_811742, EPI_ISL_811743, EPI_ISL_811744, EPI_ISL_811745, EPI_ISL_811746, EPI_ISL_811747,	Lighthouse Lab in Cambridge	Wellcome Sanger Institute for the COVID-19 Genomics UK (COG-UK) Consortium	Rob Howes, The Lighthouse Lab in Cambridge and Alex Alderton, Roberto Amato, Sonia Goncalves, Ewan Harrison, David K. Jackson, Ian Johnston, Dominic Kwiatkowski, Cordelia Langford, John Sillitoe on behalf of the Wellcome Sanger Institute COVID-19 Surveillance Team

EPI_ISL_811748, EPI_ISL_811749, EPI_ISL_811751, EPI_ISL_811752			
EPI_ISL_811754	Lighthouse Lab in Milton Keynes	Wellcome Sanger Institute for the COVID-19 Genomics UK (COG-UK) Consortium	The Lighthouse Lab in Milton Keynes and Alex Alderton, Roberto Amato, Sonia Goncalves, Ewan Harrison, David K. Jackson, Ian Johnston, Dominic Kwiatkowski, Cordelia Langford, John Sillitoe on behalf of the Wellcome Sanger Institute COVID-19 Surveillance Team
EPI_ISL_811755, EPI_ISL_811757, EPI_ISL_811758, EPI_ISL_811760, EPI_ISL_811761	Lighthouse Lab in Cambridge	Wellcome Sanger Institute for the COVID-19 Genomics UK (COG-UK) Consortium	Rob Howes, The Lighthouse Lab in Cambridge and Alex Alderton, Roberto Amato, Sonia Goncalves, Ewan Harrison, David K. Jackson, Ian Johnston, Dominic Kwiatkowski, Cordelia Langford, John Sillitoe on behalf of the Wellcome Sanger Institute COVID-19 Surveillance Team
EPI_ISL_811762	Lighthouse Lab in Milton Keynes	Wellcome Sanger Institute for the COVID-19 Genomics UK (COG-UK) Consortium	The Lighthouse Lab in Milton Keynes and Alex Alderton, Roberto Amato, Sonia Goncalves, Ewan Harrison, David K. Jackson, Ian Johnston, Dominic Kwiatkowski, Cordelia Langford, John Sillitoe on behalf of the Wellcome Sanger Institute COVID-19 Surveillance Team
EPI_ISL_811763, EPI_ISL_811764, EPI_ISL_811765, EPI_ISL_811766, EPI_ISL_811768, EPI_ISL_811769, EPI_ISL_811770, EPI_ISL_811771, EPI_ISL_811772, EPI_ISL_811773, EPI_ISL_811774, EPI_ISL_811775, EPI_ISL_811777, EPI_ISL_811778, EPI_ISL_811779, EPI_ISL_811780, EPI_ISL_811781, EPI_ISL_811782, EPI_ISL_811783, EPI_ISL_811784, EPI_ISL_811786			
see above	Lighthouse Lab in Cambridge	Wellcome Sanger Institute for the COVID-19 Genomics UK (COG-UK) Consortium	Rob Howes, The Lighthouse Lab in Cambridge and Alex Alderton, Roberto Amato, Sonia Goncalves, Ewan Harrison, David K. Jackson, Ian Johnston, Dominic Kwiatkowski, Cordelia Langford, John Sillitoe on behalf of the Wellcome Sanger Institute COVID-19 Surveillance Team
EPI_ISL_811787	Lighthouse Lab in Milton Keynes	Wellcome Sanger Institute for the COVID-19 Genomics UK (COG-UK) Consortium	The Lighthouse Lab in Milton Keynes and Alex Alderton, Roberto Amato, Sonia Goncalves, Ewan Harrison, David K. Jackson, Ian Johnston, Dominic Kwiatkowski, Cordelia Langford, John Sillitoe on behalf of the Wellcome Sanger Institute COVID-19 Surveillance Team
EPI_ISL_811788, EPI_ISL_811789, EPI_ISL_811790, EPI_ISL_811791, EPI_ISL_811792, EPI_ISL_811793, EPI_ISL_811794, EPI_ISL_811795, EPI_ISL_811796, EPI_ISL_811797, EPI_ISL_811798, EPI_ISL_811799, EPI_ISL_811800, EPI_ISL_811801, EPI_ISL_811803, EPI_ISL_811804, EPI_ISL_811805, EPI_ISL_811806			
see above	Lighthouse Lab in Cambridge	Wellcome Sanger Institute for the COVID-19 Genomics UK (COG-UK) Consortium	Rob Howes, The Lighthouse Lab in Cambridge and Alex Alderton, Roberto Amato, Sonia Goncalves, Ewan Harrison, David K. Jackson, Ian Johnston, Dominic Kwiatkowski, Cordelia Langford, John Sillitoe on behalf of the Wellcome Sanger Institute COVID-19 Surveillance Team
EPI_ISL_811807	Lighthouse Lab in Milton Keynes	Wellcome Sanger Institute for the COVID-19 Genomics UK (COG-UK) Consortium	The Lighthouse Lab in Milton Keynes and Alex Alderton, Roberto Amato, Sonia Goncalves, Ewan Harrison, David K. Jackson, Ian Johnston, Dominic Kwiatkowski, Cordelia Langford, John Sillitoe on behalf of the Wellcome Sanger Institute COVID-19 Surveillance Team
EPI_ISL_813816, EPI_ISL_813817, EPI_ISL_813818, EPI_ISL_813819, EPI_ISL_813820, EPI_ISL_813821, EPI_ISL_813822, EPI_ISL_813823, EPI_ISL_813824, EPI_ISL_813825, EPI_ISL_813826, EPI_ISL_813827, EPI_ISL_813828, EPI_ISL_813829, EPI_ISL_813830, EPI_ISL_813831			
see above	Liverpool Clinical Laboratories	COVID-19 Genomics UK (COG-UK) Consortium	Sam Haldenby, Anita Lucaci, Steve Paterson, Julian Hiscox, Alistair Darby, M Almsaud, A Alrezaihi, Muhannad Alruwaili, Stuart D Armstrong, Jones Benjamin, Eleanor G Bentley, Anu Chawla, Jordan J Clark, Angela Cowell, Richard Eccles, Isabel García-Dorival, Matthew Gemmell, Alessandro Gerada, PKF Gilmore, Richard Gregory, Ximeng Han, Catherine Hartley, Margaret Hughes, Miren Iturriza-Gomara, James Johnson, L Luu, Jenifer Manson, Charlotte Nelson, Elaine O'Toole, Cassie Olateju, Rebekah Penrice-Randal , Lucille Rainbow, N.P Randle, Trevor Ian Robinson, Parul Sharma, Ghada T Shawli, James P Stewart, Neil Swainston, Ecaterina Vamos, Joanne Watts, Mark Whitehead
EPI_ISL_816210, EPI_ISL_816211, EPI_ISL_816216, EPI_ISL_816219	Centre for Enzyme Innovation, University of Portsmouth / Translational Research Laboratory, Portsmouth Hospitals NHS Trust	COVID-19 Genomics UK (COG-UK) Consortium	Angela Beckett,Yann Bourgeois,Garry Scarlett,Sharon Glaysher,Scott Elliott,Kelly Bicknell,Robert Impey,Allyson Lloyd,Sarah Wyllie,Ethan Butcher,Anoop Chauhan,Samuel Robson
EPI_ISL_816237, EPI_ISL_816244, EPI_ISL_816251, EPI_ISL_816253, EPI_ISL_816258, EPI_ISL_816260, EPI_ISL_816264, EPI_ISL_816267, EPI_ISL_816281, EPI_ISL_816284, EPI_ISL_816286, EPI_ISL_816287, EPI_ISL_816299, EPI_ISL_816303, EPI_ISL_816307, EPI_ISL_816309, EPI_ISL_816312, EPI_ISL_816315, EPI_ISL_816322, EPI_ISL_816328, EPI_ISL_816333, EPI_ISL_816334, EPI_ISL_816336, EPI_ISL_816338, EPI_ISL_816341, EPI_ISL_816358, EPI_ISL_816363, EPI_ISL_816367, EPI_ISL_816369, EPI_ISL_816371, EPI_ISL_816373, EPI_ISL_816376, EPI_ISL_816377, EPI_ISL_816378, EPI_ISL_816387, EPI_ISL_816389, EPI_ISL_816391, EPI_ISL_816394, EPI_ISL_816395, EPI_ISL_816399, EPI_ISL_816402, EPI_ISL_816408, EPI_ISL_816413, EPI_ISL_816416, EPI_ISL_816424, EPI_ISL_816433, EPI_ISL_816439, EPI_ISL_816450, EPI_ISL_816451, EPI_ISL_816452, EPI_ISL_816455, EPI_ISL_816463, EPI_ISL_816470, EPI_ISL_816471, EPI_ISL_816479, EPI_ISL_816493, EPI_ISL_816498, EPI_ISL_816502, EPI_ISL_816506, EPI_ISL_816509, EPI_ISL_816518, EPI_ISL_816519, EPI_ISL_816520, EPI_ISL_816523, EPI_ISL_816524, EPI_ISL_816526, EPI_ISL_816527, EPI_ISL_816543, EPI_ISL_816552, EPI_ISL_816559, EPI_ISL_816564, EPI_ISL_816565, EPI_ISL_816572, EPI_ISL_816573, EPI_ISL_816583, EPI_ISL_816588, EPI_ISL_816590, EPI_ISL_816591, EPI_ISL_816596, EPI_ISL_816603, EPI_ISL_816614, EPI_ISL_816618, EPI_ISL_816622, EPI_ISL_816623, EPI_ISL_816624, EPI_ISL_816636, EPI_ISL_816639, EPI_ISL_816648, EPI_ISL_816649, EPI_ISL_816652, EPI_ISL_816653, EPI_ISL_816655			
see above	Virology Department, Sheffield Teaching Hospitals NHS Foundation Trust/Department of Infection, Immunity and Cardiovascular Disease, The Medical School, University of Sheffield	COVID-19 Genomics UK (COG-UK) Consortium	Thushan de Silva, Matthew Parker, Nikki Smith, Adri Angyal, Rebecca Brown, Luke Green, Rachel Tucker, Paul Parsons, Danielle Groves, Katie Johnson, Laura Carrilero, Alex Keeley, Dave Partridge, Matthew Wyles, Benjamin Lindsey, Mehmet Yavuz, Mohammad Raza, Cariad Evans
EPI_ISL_819366, EPI_ISL_819375, EPI_ISL_819377, EPI_ISL_819384, EPI_ISL_819391, EPI_ISL_819392, EPI_ISL_819393, EPI_ISL_819394, EPI_ISL_819395, EPI_ISL_819405, EPI_ISL_819406, EPI_ISL_819407, EPI_ISL_819408, EPI_ISL_819409, EPI_ISL_819410, EPI_ISL_819411, EPI_ISL_819412, EPI_ISL_819413, EPI_ISL_819414, EPI_ISL_819415, EPI_ISL_819416, EPI_ISL_819417, EPI_ISL_819418, EPI_ISL_819419, EPI_ISL_819420, EPI_ISL_819421, EPI_ISL_819424, EPI_ISL_819425, EPI_ISL_819427, EPI_ISL_819429, EPI_ISL_819440, EPI_ISL_819441, EPI_ISL_819442, EPI_ISL_819443, EPI_ISL_819444, EPI_ISL_819445, EPI_ISL_819446			
see above	Quadram Institute Bioscience	COVID-19 Genomics UK (COG-UK) Consortium	Dave J. Baker, Gemma L. Kay, Alp Aydin, Thanh Le-Viet, Steven Rudder, Ana P. Tedim, Anastasia Kolyva, Maria Diaz, Leonardo de Oliveira Martins, Nabil-Fareed Alikhan, Lizzie Meadows, Rachael Stanley, Ngozi Elumogo, Muhammed Yasir, Nicholas M. Thomson, Alexander J Trotter, Rachel Gilroy, Samuel Bloomfield, Claire Stuart, Andrew Bell, Reenesh Prakash, Samir Dervisevic, Alison E. Mather, John Wain, Mark Webber, Andrew J. Page, Justin O'Grady
EPI_ISL_820573, EPI_ISL_820575, EPI_ISL_820578, EPI_ISL_820580, EPI_ISL_820583, EPI_ISL_820586, EPI_ISL_820588, EPI_ISL_820591, EPI_ISL_820593, EPI_ISL_820596, EPI_ISL_820598, EPI_ISL_820601, EPI_ISL_820603, EPI_ISL_820605, EPI_ISL_820607, EPI_ISL_820610			
see above	Queens Medical Centre, Clinical Microbiology Department / DeepSeq Nottingham	COVID-19 Genomics UK (COG-UK) Consortium	Gemma Clark, Wendy Smith, Manjinder Khakh, Vicki M Fleming, Michelle M Lister, Hannah Howson-Wells, Jonathan Ball, Patrick McClure, Joseph Chappell, Theocharis Tsoleridis, Nadine Holmes, Matthew Carlisle, Christopher Moore, Fei Sang, Johnny Debebe, Victoria Wright, Matthew Loose
EPI_ISL_820648, EPI_ISL_820649, EPI_ISL_820650, EPI_ISL_820651, EPI_ISL_820652, EPI_ISL_820653, EPI_ISL_820654, EPI_ISL_820655, EPI_ISL_820656, EPI_ISL_820657, EPI_ISL_820658, EPI_ISL_820659, EPI_ISL_820660, EPI_ISL_820661, EPI_ISL_820662, EPI_ISL_820663, EPI_ISL_820664, EPI_ISL_820665, EPI_ISL_820666, EPI_ISL_820667, EPI_ISL_820668, EPI_ISL_820669, EPI_ISL_820670, EPI_ISL_820671, EPI_ISL_820672, EPI_ISL_820673, EPI_ISL_820674, EPI_ISL_820675, EPI_ISL_820676, EPI_ISL_820677, EPI_ISL_820678, EPI_ISL_820679, EPI_ISL_820680, EPI_ISL_820681, EPI_ISL_820682, EPI_ISL_820683, EPI_ISL_820684, EPI_ISL_820685, EPI_ISL_820686, EPI_ISL_820687, EPI_ISL_820688, EPI_ISL_820689, EPI_ISL_820690, EPI_ISL_820691, EPI_ISL_820692, EPI_ISL_820693, EPI_ISL_820694, EPI_ISL_820695, EPI_ISL_820696, EPI_ISL_820697, EPI_ISL_820698, EPI_ISL_820699, EPI_ISL_820700, EPI_ISL_820701, EPI_ISL_820702, EPI_ISL_820703, EPI_ISL_820704, EPI_ISL_820705, EPI_ISL_820706, EPI_ISL_820707, EPI_ISL_820708, EPI_ISL_820709, EPI_ISL_820710, EPI_ISL_820711, EPI_ISL_820712, EPI_ISL_820713, EPI_ISL_820714, EPI_ISL_820715, EPI_ISL_820716, EPI_ISL_820717, EPI_ISL_820718, EPI_ISL_820719, EPI_ISL_820720, EPI_ISL_820721, EPI_ISL_820722, EPI_ISL_820723, EPI_ISL_820724, EPI_ISL_820725, EPI_ISL_820726, EPI_ISL_820727, EPI_ISL_820728, EPI_ISL_820729, EPI_ISL_820730, EPI_ISL_820731, EPI_ISL_820732, EPI_ISL_820733, EPI_ISL_820734, EPI_ISL_820735, EPI_ISL_820736, EPI_ISL_820737, EPI_ISL_820738, EPI_ISL_820739, EPI_ISL_820740, EPI_ISL_820741, EPI_ISL_820742, EPI_ISL_820743, EPI_ISL_820744, EPI_ISL_820745, EPI_ISL_820746, EPI_ISL_820747, EPI_ISL_820748, EPI_ISL_820749, EPI_ISL_820750, EPI_ISL_820751, EPI_ISL_820752, EPI_ISL_820753, EPI_ISL_820754, EPI_ISL_820755, EPI_ISL_820756, EPI_ISL_820757, EPI_ISL_820758, EPI_ISL_820759, EPI_ISL_820760, EPI_ISL_820761, EPI_ISL_820762, EPI_ISL_820763, EPI_ISL_820764, EPI_ISL_820765, EPI_ISL_820766, EPI_ISL_820767, EPI_ISL_820768, EPI_ISL_820769, EPI_ISL_820770, EPI_ISL_820771, EPI_ISL_820772, EPI_ISL_820773, EPI_ISL_820774, EPI_ISL_820775, EPI_ISL_820776, EPI_ISL_820777, EPI_ISL_820778, EPI_ISL_820779, EPI_ISL_820780, EPI_ISL_820781, EPI_ISL_820782, EPI_ISL_820783, EPI_ISL_820784, EPI_ISL_820785, EPI_ISL_820786, EPI_ISL_820787, EPI_ISL_820788, EPI_ISL_820789, EPI_ISL_820790, EPI_ISL_820791, EPI_ISL_820792, EPI_ISL_820793, EPI_ISL_820794, EPI_ISL_820795, EPI_ISL_820796, EPI_ISL_820797, EPI_ISL_820798, EPI_ISL_820799, EPI_ISL_820800, EPI_ISL_820801, EPI_ISL_820802, EPI_ISL_820803, EPI_ISL_820804, EPI_ISL_820805, EPI_ISL_820806, EPI_ISL_820807, EPI_ISL_820808, EPI_ISL_820809, EPI_ISL_820810, EPI_ISL_820811, EPI_ISL_820812, EPI_ISL_820813, EPI_ISL_820814, EPI_ISL_820815, EPI_ISL_820816, EPI_ISL_820817, EPI_ISL_820818, EPI_ISL_820819, EPI_ISL_820820, EPI_ISL_820821, EPI_ISL_820822, EPI_ISL_820823, EPI_ISL_820824, EPI_ISL_820825, EPI_ISL_820826, EPI_ISL_820827, EPI_ISL_820828, EPI_ISL_820829, EPI_ISL_820830, EPI_ISL_820831, EPI_ISL_820832, EPI_ISL_820833, EPI_ISL_820834, EPI_ISL_820835, EPI_ISL_820836, EPI_ISL_820837, EPI_ISL_820838, EPI_ISL_820839, EPI_ISL_820840, EPI_ISL_820841, EPI_ISL_820842, EPI_ISL_820843, EPI_ISL_820844, EPI_ISL_820845, EPI_ISL_820846, EPI_ISL_820847, EPI_ISL_820848, EPI_ISL_820849, EPI_ISL_820850, EPI_ISL_820851, EPI_ISL_820852, EPI_ISL_820853, EPI_ISL_820854, EPI_ISL_820855, EPI_ISL_820856, EPI_ISL_820857, EPI_ISL_820858, EPI_ISL_820859, EPI_ISL_820860, EPI_ISL_820861, EPI_ISL_820862, EPI_ISL_820863, EPI_ISL_820864, EPI_ISL_820865, EPI_ISL_820866, EPI_ISL_820867, EPI_ISL_820868, EPI_ISL_820869, EPI_ISL_820870, EPI_ISL_820871, EPI_ISL_820872, EPI_ISL_820873, EPI_ISL_820874, EPI_ISL_820875, EPI_ISL_820876, EPI_ISL_820877, EPI_ISL_820878, EPI_ISL_820879, EPI_ISL_820880, EPI_ISL_820881, EPI_ISL_820882, EPI_ISL_820883, EPI_ISL_820884, EPI_ISL_820885, EPI_ISL_820886, EPI_ISL_820887, EPI_ISL_820888, EPI_ISL_820889, EPI_ISL_820890, EPI_ISL_820891, EPI_ISL_820892, EPI_ISL_820893, EPI_ISL_820894, EPI_ISL_820895, EPI_ISL_820896, EPI_ISL_820897, EPI_ISL_820898, EPI_ISL_820899, EPI_ISL_820900, EPI_ISL_820901, EPI_ISL_820902, EPI_ISL_820903, EPI_ISL_820904, EPI_ISL_820905, EPI_ISL_820906, EPI_ISL_820907, EPI_ISL_820908, EPI_ISL_820909, EPI_ISL_820910, EPI_ISL_820911, EPI_ISL_820912, EPI_ISL_820913, EPI_ISL_820914, EPI_ISL_820915, EPI_ISL_820916, EPI_ISL_820917, EPI_ISL_820918, EPI_ISL_820919, EPI_ISL_820920, EPI_ISL_820921, EPI_ISL_820922, EPI_ISL_820923, EPI_ISL_820924, EPI_ISL_820925, EPI_ISL_820926, EPI_ISL_820927, EPI_ISL_820928, EPI_ISL_820929, EPI_ISL_820930, EPI_ISL_820931, EPI_ISL_820932, EPI_ISL_820933, EPI_ISL_820934, EPI_ISL_820935, EPI_ISL_820936, EPI_ISL_820937, EPI_ISL_820938, EPI_ISL_820939, EPI_ISL_820940, EPI_ISL_820941, EPI_ISL_820942, EPI_ISL_820943, EPI_ISL_820944, EPI_ISL_820945, EPI_ISL_820946, EPI_ISL_820947, EPI_ISL_820948, EPI_ISL_820949, EPI_ISL_820950, EPI_ISL_820951, EPI_ISL_820952, EPI_ISL_820953, EPI_ISL_820954, EPI_ISL_820955, EPI_ISL_820956, EPI_ISL_820957, EPI_ISL_820958, EPI_ISL_820959, EPI_ISL_820960, EPI_ISL_820961, EPI_ISL_820962, EPI_ISL_820963, EPI_ISL_820964, EPI_ISL_820965, EPI_ISL_820966, EPI_ISL_820967, EPI_ISL_820968, EPI_ISL_820969, EPI_ISL_820970, EPI_ISL_820971, EPI_ISL_820972, EPI_ISL_820973, EPI_ISL_820974, EPI_ISL_820975, EPI_ISL_820976, EPI_ISL_820977, EPI_ISL_820978, EPI_ISL_820979, EPI_ISL_820980, EPI_ISL_820981, EPI_ISL_820982, EPI_ISL_820983, EPI_ISL_820984, EPI_ISL_820985, EPI_ISL_820986, EPI_ISL_820987, EPI_ISL_820988, EPI_ISL_820989, EPI_ISL_820990, EPI_ISL_820991, EPI_ISL_820992, EPI_ISL_820993, EPI_ISL_820994, EPI_ISL_820995, EPI_ISL_820996, EPI_ISL_820997, EPI_ISL_820998, EPI_ISL_820999, EPI_ISL_821000, EPI_ISL_821001			
see above	Lighthouse Lab in Milton Keynes	Wellcome Sanger Institute for the COVID-19 Genomics UK (COG-UK) Consortium	The Lighthouse Lab in Milton Keynes and Alex Alderton, Roberto Amato, Sonia Goncalves, Ewan Harrison, David K. Jackson, Ian Johnston, Dominic Kwiatkowski, Cordelia Langford, John Sillitoe on behalf of the Wellcome Sanger Institute COVID-19 Surveillance Team
EPI_ISL_821002, EPI_ISL_821004, EPI_ISL_821006, EPI_ISL_821007, EPI_ISL_821008, EPI_ISL_821009, EPI_ISL_821014, EPI_ISL_821015, EPI_ISL_821016, EPI_ISL_821017, EPI_ISL_821019, EPI_ISL_821023, EPI_ISL_821024, EPI_ISL_821027, EPI_ISL_821029, EPI_ISL_821031			
see above	Lighthouse Lab in Alderley Park	Wellcome Sanger Institute for the COVID-19 Genomics UK (COG-UK) Consortium	Jacquelyn Wynn, Mairead Hyland, The Lighthouse Lab in Alderley Park and Alex Alderton, Roberto Amato, Sonia Goncalves, Ewan Harrison, David K. Jackson, Ian Johnston, Dominic Kwiatkowski, Cordelia Langford, John Sillitoe on behalf of the Wellcome Sanger Institute COVID-19 Surveillance Team
EPI_ISL_821032	Lighthouse Lab in Glasgow	Wellcome Sanger Institute for the COVID-19 Genomics UK (COG-UK) Consortium	Harper VanSteenhouse, Yumi Kasai, David Gray, Carol Clugston, Anna Dominiczak and Alex Alderton, Roberto Amato, Sonia Goncalves, Ewan Harrison, David K. Jackson, Ian Johnston, Dominic Kwiatkowski, Cordelia Langford, John Sillitoe on behalf of the Wellcome Sanger Institute COVID-19 Surveillance

			Team
EPI_ISL_821033, EPI_ISL_821034	Lighthouse Lab in Alderley Park	Wellcome Sanger Institute for the COVID-19 Genomics UK (COG-UK) Consortium	Jacquelyn Wynn, Mairead Hyland, The Lighthouse Lab in Alderley Park and Alex Alderton, Roberto Amato, Sonia Goncalves, Ewan Harrison, David K. Jackson, Ian Johnston, Dominic Kwiatkowski, Cordelia Langford, John Sillitoe on behalf of the Wellcome Sanger Institute COVID-19 Surveillance Team
EPI_ISL_821037	Lighthouse Lab in Glasgow	Wellcome Sanger Institute for the COVID-19 Genomics UK (COG-UK) Consortium	Harper VanSteenhouse, Yumi Kasai, David Gray, Carol Clugston, Anna Dominiczak and Alex Alderton, Roberto Amato, Sonia Goncalves, Ewan Harrison, David K. Jackson, Ian Johnston, Dominic Kwiatkowski, Cordelia Langford, John Sillitoe on behalf of the Wellcome Sanger Institute COVID-19 Surveillance Team
EPI_ISL_821040, EPI_ISL_821041, EPI_ISL_821043, EPI_ISL_821044, EPI_ISL_821045, EPI_ISL_821048, EPI_ISL_821049, EPI_ISL_821051, EPI_ISL_821052, EPI_ISL_821053, EPI_ISL_821054			
see above	Lighthouse Lab in Alderley Park	Wellcome Sanger Institute for the COVID-19 Genomics UK (COG-UK) Consortium	Jacquelyn Wynn, Mairead Hyland, The Lighthouse Lab in Alderley Park and Alex Alderton, Roberto Amato, Sonia Goncalves, Ewan Harrison, David K. Jackson, Ian Johnston, Dominic Kwiatkowski, Cordelia Langford, John Sillitoe on behalf of the Wellcome Sanger Institute COVID-19 Surveillance Team
EPI_ISL_821056	Lighthouse Lab in Glasgow	Wellcome Sanger Institute for the COVID-19 Genomics UK (COG-UK) Consortium	Harper VanSteenhouse, Yumi Kasai, David Gray, Carol Clugston, Anna Dominiczak and Alex Alderton, Roberto Amato, Sonia Goncalves, Ewan Harrison, David K. Jackson, Ian Johnston, Dominic Kwiatkowski, Cordelia Langford, John Sillitoe on behalf of the Wellcome Sanger Institute COVID-19 Surveillance Team
EPI_ISL_821057, EPI_ISL_821058, EPI_ISL_821059	Lighthouse Lab in Alderley Park	Wellcome Sanger Institute for the COVID-19 Genomics UK (COG-UK) Consortium	Jacquelyn Wynn, Mairead Hyland, The Lighthouse Lab in Alderley Park and Alex Alderton, Roberto Amato, Sonia Goncalves, Ewan Harrison, David K. Jackson, Ian Johnston, Dominic Kwiatkowski, Cordelia Langford, John Sillitoe on behalf of the Wellcome Sanger Institute COVID-19 Surveillance Team
EPI_ISL_821061, EPI_ISL_821062	Lighthouse Lab in Glasgow	Wellcome Sanger Institute for the COVID-19 Genomics UK (COG-UK) Consortium	Harper VanSteenhouse, Yumi Kasai, David Gray, Carol Clugston, Anna Dominiczak and Alex Alderton, Roberto Amato, Sonia Goncalves, Ewan Harrison, David K. Jackson, Ian Johnston, Dominic Kwiatkowski, Cordelia Langford, John Sillitoe on behalf of the Wellcome Sanger Institute COVID-19 Surveillance Team
EPI_ISL_821064, EPI_ISL_821065, EPI_ISL_821067	Lighthouse Lab in Alderley Park	Wellcome Sanger Institute for the COVID-19 Genomics UK (COG-UK) Consortium	Jacquelyn Wynn, Mairead Hyland, The Lighthouse Lab in Alderley Park and Alex Alderton, Roberto Amato, Sonia Goncalves, Ewan Harrison, David K. Jackson, Ian Johnston, Dominic Kwiatkowski, Cordelia Langford, John Sillitoe on behalf of the Wellcome Sanger Institute COVID-19 Surveillance Team
EPI_ISL_821069	Lighthouse Lab in Glasgow	Wellcome Sanger Institute for the COVID-19 Genomics UK (COG-UK) Consortium	Harper VanSteenhouse, Yumi Kasai, David Gray, Carol Clugston, Anna Dominiczak and Alex Alderton, Roberto Amato, Sonia Goncalves, Ewan Harrison, David K. Jackson, Ian Johnston, Dominic Kwiatkowski, Cordelia Langford, John Sillitoe on behalf of the Wellcome Sanger Institute COVID-19 Surveillance Team
EPI_ISL_821070, EPI_ISL_821071, EPI_ISL_821072, EPI_ISL_821075, EPI_ISL_821076	Lighthouse Lab in Alderley Park	Wellcome Sanger Institute for the COVID-19 Genomics UK (COG-UK) Consortium	Jacquelyn Wynn, Mairead Hyland, The Lighthouse Lab in Alderley Park and Alex Alderton, Roberto Amato, Sonia Goncalves, Ewan Harrison, David K. Jackson, Ian Johnston, Dominic Kwiatkowski, Cordelia Langford, John Sillitoe on behalf of the Wellcome Sanger Institute COVID-19 Surveillance Team
EPI_ISL_821077	Lighthouse Lab in Glasgow	Wellcome Sanger Institute for the COVID-19 Genomics UK (COG-UK) Consortium	Harper VanSteenhouse, Yumi Kasai, David Gray, Carol Clugston, Anna Dominiczak and Alex Alderton, Roberto Amato, Sonia Goncalves, Ewan Harrison, David K. Jackson, Ian Johnston, Dominic Kwiatkowski, Cordelia Langford, John Sillitoe on behalf of the Wellcome Sanger Institute COVID-19 Surveillance Team
EPI_ISL_821079, EPI_ISL_821080, EPI_ISL_821081, EPI_ISL_821083, EPI_ISL_821086, EPI_ISL_821087, EPI_ISL_821088, EPI_ISL_821089, EPI_ISL_821092, EPI_ISL_821094, EPI_ISL_821096, EPI_ISL_821097			
see above	Lighthouse Lab in Alderley Park	Wellcome Sanger Institute for the COVID-19 Genomics UK (COG-UK) Consortium	Jacquelyn Wynn, Mairead Hyland, The Lighthouse Lab in Alderley Park and Alex Alderton, Roberto Amato, Sonia Goncalves, Ewan Harrison, David K. Jackson, Ian Johnston, Dominic Kwiatkowski, Cordelia Langford, John Sillitoe on behalf of the Wellcome Sanger Institute COVID-19 Surveillance Team
EPI_ISL_821098	Lighthouse Lab in Glasgow	Wellcome Sanger Institute for the COVID-19 Genomics UK (COG-UK) Consortium	Harper VanSteenhouse, Yumi Kasai, David Gray, Carol Clugston, Anna Dominiczak and Alex Alderton, Roberto Amato, Sonia Goncalves, Ewan Harrison, David K. Jackson, Ian Johnston, Dominic Kwiatkowski, Cordelia Langford, John Sillitoe on behalf of the Wellcome Sanger Institute COVID-19 Surveillance Team
EPI_ISL_821100, EPI_ISL_821101, EPI_ISL_821103	Lighthouse Lab in Alderley Park	Wellcome Sanger Institute for the COVID-19 Genomics UK (COG-UK) Consortium	Jacquelyn Wynn, Mairead Hyland, The Lighthouse Lab in Alderley Park and Alex Alderton, Roberto Amato, Sonia Goncalves, Ewan Harrison, David K. Jackson, Ian Johnston, Dominic Kwiatkowski, Cordelia Langford, John Sillitoe on behalf of the Wellcome Sanger Institute COVID-19 Surveillance Team
EPI_ISL_821105	Lighthouse Lab in Glasgow	Wellcome Sanger Institute for the COVID-19 Genomics UK (COG-UK) Consortium	Harper VanSteenhouse, Yumi Kasai, David Gray, Carol Clugston, Anna Dominiczak and Alex Alderton, Roberto Amato, Sonia Goncalves, Ewan Harrison, David K. Jackson, Ian Johnston, Dominic Kwiatkowski, Cordelia Langford, John Sillitoe on behalf of the Wellcome Sanger Institute COVID-19 Surveillance Team
EPI_ISL_821106, EPI_ISL_821107, EPI_ISL_821108, EPI_ISL_821112, EPI_ISL_821113, EPI_ISL_821114, EPI_ISL_821115, EPI_ISL_821116, EPI_ISL_821118, EPI_ISL_821119, EPI_ISL_821120, EPI_ISL_821121, EPI_ISL_821122, EPI_ISL_821123, EPI_ISL_821129, EPI_ISL_821130, EPI_ISL_821132, EPI_ISL_821135, EPI_ISL_821138, EPI_ISL_821139, EPI_ISL_821141			
see above	Lighthouse Lab in Alderley Park	Wellcome Sanger Institute for the COVID-19 Genomics UK (COG-UK) Consortium	Jacquelyn Wynn, Mairead Hyland, The Lighthouse Lab in Alderley Park and Alex Alderton, Roberto Amato, Sonia Goncalves, Ewan Harrison, David K. Jackson, Ian Johnston, Dominic Kwiatkowski, Cordelia Langford, John Sillitoe on behalf of the Wellcome Sanger Institute COVID-19 Surveillance Team
EPI_ISL_821142	Lighthouse Lab in Glasgow	Wellcome Sanger Institute for the COVID-19 Genomics UK (COG-UK) Consortium	Harper VanSteenhouse, Yumi Kasai, David Gray, Carol Clugston, Anna Dominiczak and Alex Alderton, Roberto Amato, Sonia Goncalves, Ewan Harrison, David K. Jackson, Ian Johnston, Dominic Kwiatkowski, Cordelia Langford, John Sillitoe on behalf of the Wellcome Sanger Institute COVID-19 Surveillance Team
EPI_ISL_821143, EPI_ISL_821145, EPI_ISL_821146, EPI_ISL_821147, EPI_ISL_821148	Lighthouse Lab in Alderley Park	Wellcome Sanger Institute for the COVID-19 Genomics UK (COG-UK) Consortium	Jacquelyn Wynn, Mairead Hyland, The Lighthouse Lab in Alderley Park and Alex Alderton, Roberto Amato, Sonia Goncalves, Ewan Harrison, David K. Jackson, Ian Johnston, Dominic Kwiatkowski, Cordelia Langford, John Sillitoe on behalf of the Wellcome Sanger Institute COVID-19 Surveillance Team
EPI_ISL_821150	Lighthouse Lab in Glasgow	Wellcome Sanger Institute for the COVID-19 Genomics UK (COG-UK) Consortium	Harper VanSteenhouse, Yumi Kasai, David Gray, Carol Clugston, Anna Dominiczak and Alex Alderton, Roberto Amato, Sonia Goncalves, Ewan Harrison, David K. Jackson, Ian Johnston, Dominic Kwiatkowski, Cordelia Langford, John Sillitoe on behalf of the Wellcome Sanger Institute COVID-19 Surveillance Team
EPI_ISL_821153, EPI_ISL_821154, EPI_ISL_821155, EPI_ISL_821157	Lighthouse Lab in Alderley Park	Wellcome Sanger Institute for the COVID-19 Genomics UK (COG-UK) Consortium	Jacquelyn Wynn, Mairead Hyland, The Lighthouse Lab in Alderley Park and Alex Alderton, Roberto Amato, Sonia Goncalves, Ewan Harrison, David K. Jackson, Ian Johnston, Dominic Kwiatkowski, Cordelia Langford, John Sillitoe on behalf of the Wellcome Sanger Institute COVID-19 Surveillance Team
EPI_ISL_821160	Lighthouse Lab in Glasgow	Wellcome Sanger Institute for the COVID-19 Genomics UK (COG-UK) Consortium	Harper VanSteenhouse, Yumi Kasai, David Gray, Carol Clugston, Anna Dominiczak and Alex Alderton, Roberto Amato, Sonia Goncalves, Ewan Harrison, David K. Jackson, Ian Johnston, Dominic Kwiatkowski, Cordelia Langford, John Sillitoe on behalf of the Wellcome Sanger Institute COVID-19 Surveillance Team
EPI_ISL_821161, EPI_ISL_821162, EPI_ISL_821164, EPI_ISL_821167, EPI_ISL_821168, EPI_ISL_821171, EPI_ISL_821176, EPI_ISL_821177, EPI_ISL_821179, EPI_ISL_821182, EPI_ISL_821183, EPI_ISL_821184, EPI_ISL_821185, EPI_ISL_821186, EPI_ISL_821188, EPI_ISL_821189, EPI_ISL_821192, EPI_ISL_821193			
see above	Lighthouse Lab in Alderley Park	Wellcome Sanger Institute for the COVID-19 Genomics UK (COG-UK) Consortium	Jacquelyn Wynn, Mairead Hyland, The Lighthouse Lab in Alderley Park and Alex Alderton, Roberto Amato, Sonia Goncalves, Ewan Harrison, David K. Jackson, Ian Johnston, Dominic Kwiatkowski, Cordelia Langford, John Sillitoe on behalf of the Wellcome Sanger Institute COVID-19 Surveillance Team
EPI_ISL_821195	Lighthouse Lab in Glasgow	Wellcome Sanger Institute for the COVID-19 Genomics UK (COG-UK) Consortium	Harper VanSteenhouse, Yumi Kasai, David Gray, Carol Clugston, Anna Dominiczak and Alex Alderton, Roberto Amato, Sonia Goncalves, Ewan Harrison, David K. Jackson, Ian Johnston, Dominic Kwiatkowski, Cordelia Langford, John Sillitoe on behalf of the Wellcome Sanger Institute COVID-19 Surveillance Team
EPI_ISL_821196	Lighthouse Lab in Alderley Park	Wellcome Sanger Institute for the COVID-19 Genomics UK (COG-UK) Consortium	Jacquelyn Wynn, Mairead Hyland, The Lighthouse Lab in Alderley Park and Alex Alderton, Roberto Amato, Sonia Goncalves, Ewan Harrison, David K. Jackson, Ian Johnston, Dominic Kwiatkowski, Cordelia Langford, John Sillitoe on behalf of the Wellcome Sanger Institute COVID-19 Surveillance Team
EPI_ISL_821198	Lighthouse Lab in Glasgow	Wellcome Sanger Institute for the COVID-19 Genomics UK (COG-UK) Consortium	Harper VanSteenhouse, Yumi Kasai, David Gray, Carol Clugston, Anna Dominiczak and Alex Alderton, Roberto Amato, Sonia Goncalves, Ewan Harrison, David K. Jackson, Ian Johnston, Dominic Kwiatkowski, Cordelia Langford, John Sillitoe on behalf of the Wellcome Sanger Institute COVID-19 Surveillance Team
EPI_ISL_821202, EPI_ISL_821203, EPI_ISL_821204, EPI_ISL_821205	Lighthouse Lab in Alderley Park	Wellcome Sanger Institute for the COVID-19 Genomics UK (COG-UK) Consortium	Jacquelyn Wynn, Mairead Hyland, The Lighthouse Lab in Alderley Park and Alex Alderton, Roberto Amato, Sonia Goncalves, Ewan Harrison, David K. Jackson, Ian Johnston, Dominic Kwiatkowski, Cordelia Langford, John Sillitoe on behalf of the Wellcome Sanger Institute COVID-19 Surveillance Team
EPI_ISL_821207	Lighthouse Lab in Glasgow	Wellcome Sanger Institute for the COVID-19 Genomics UK (COG-UK) Consortium	Harper VanSteenhouse, Yumi Kasai, David Gray, Carol Clugston, Anna Dominiczak and Alex Alderton, Roberto Amato, Sonia Goncalves, Ewan Harrison, David K. Jackson, Ian Johnston, Dominic Kwiatkowski, Cordelia Langford, John Sillitoe on behalf of the Wellcome Sanger Institute COVID-19 Surveillance Team

EPI_ISL_821209, EPI_ISL_821210, EPI_ISL_821211, EPI_ISL_821212, EPI_ISL_821213, EPI_ISL_821216, EPI_ISL_821217, EPI_ISL_821218, EPI_ISL_821219, EPI_ISL_821221, EPI_ISL_821224

see above	Lighthouse Lab in Alderley Park	Wellcome Sanger Institute for the COVID-19 Genomics UK (COG-UK) Consortium	Jacquelyn Wynn, Mairead Hyland, The Lighthouse Lab in Alderley Park and Alex Alderton, Roberto Amato, Sonia Goncalves, Ewan Harrison, David K. Jackson, Ian Johnston, Dominic Kwiatkowski, Cordelia Langford, John Sillitoe on behalf of the Wellcome Sanger Institute COVID-19 Surveillance Team
EPI_ISL_821225	Lighthouse Lab in Glasgow	Wellcome Sanger Institute for the COVID-19 Genomics UK (COG-UK) Consortium	Harper VanSteenhouse, Yumi Kasai, David Gray, Carol Clugston, Anna Dominiczak and Alex Alderton, Roberto Amato, Sonia Goncalves, Ewan Harrison, David K. Jackson, Ian Johnston, Dominic Kwiatkowski, Cordelia Langford, John Sillitoe on behalf of the Wellcome Sanger Institute COVID-19 Surveillance Team
EPI_ISL_821226	Lighthouse Lab in Alderley Park	Wellcome Sanger Institute for the COVID-19 Genomics UK (COG-UK) Consortium	Jacquelyn Wynn, Mairead Hyland, The Lighthouse Lab in Alderley Park and Alex Alderton, Roberto Amato, Sonia Goncalves, Ewan Harrison, David K. Jackson, Ian Johnston, Dominic Kwiatkowski, Cordelia Langford, John Sillitoe on behalf of the Wellcome Sanger Institute COVID-19 Surveillance Team
EPI_ISL_821227	Lighthouse Lab in Glasgow	Wellcome Sanger Institute for the COVID-19 Genomics UK (COG-UK) Consortium	Harper VanSteenhouse, Yumi Kasai, David Gray, Carol Clugston, Anna Dominiczak and Alex Alderton, Roberto Amato, Sonia Goncalves, Ewan Harrison, David K. Jackson, Ian Johnston, Dominic Kwiatkowski, Cordelia Langford, John Sillitoe on behalf of the Wellcome Sanger Institute COVID-19 Surveillance Team
EPI_ISL_821229, EPI_ISL_821232, EPI_ISL_821233, EPI_ISL_821234, EPI_ISL_821236, EPI_ISL_821237, EPI_ISL_821238, EPI_ISL_821239, EPI_ISL_821240	Lighthouse Lab in Alderley Park	Wellcome Sanger Institute for the COVID-19 Genomics UK (COG-UK) Consortium	Jacquelyn Wynn, Mairead Hyland, The Lighthouse Lab in Alderley Park and Alex Alderton, Roberto Amato, Sonia Goncalves, Ewan Harrison, David K. Jackson, Ian Johnston, Dominic Kwiatkowski, Cordelia Langford, John Sillitoe on behalf of the Wellcome Sanger Institute COVID-19 Surveillance Team
EPI_ISL_821241	Lighthouse Lab in Glasgow	Wellcome Sanger Institute for the COVID-19 Genomics UK (COG-UK) Consortium	Harper VanSteenhouse, Yumi Kasai, David Gray, Carol Clugston, Anna Dominiczak and Alex Alderton, Roberto Amato, Sonia Goncalves, Ewan Harrison, David K. Jackson, Ian Johnston, Dominic Kwiatkowski, Cordelia Langford, John Sillitoe on behalf of the Wellcome Sanger Institute COVID-19 Surveillance Team
EPI_ISL_821242	Lighthouse Lab in Alderley Park	Wellcome Sanger Institute for the COVID-19 Genomics UK (COG-UK) Consortium	Jacquelyn Wynn, Mairead Hyland, The Lighthouse Lab in Alderley Park and Alex Alderton, Roberto Amato, Sonia Goncalves, Ewan Harrison, David K. Jackson, Ian Johnston, Dominic Kwiatkowski, Cordelia Langford, John Sillitoe on behalf of the Wellcome Sanger Institute COVID-19 Surveillance Team
EPI_ISL_821245	Lighthouse Lab in Glasgow	Wellcome Sanger Institute for the COVID-19 Genomics UK (COG-UK) Consortium	Harper VanSteenhouse, Yumi Kasai, David Gray, Carol Clugston, Anna Dominiczak and Alex Alderton, Roberto Amato, Sonia Goncalves, Ewan Harrison, David K. Jackson, Ian Johnston, Dominic Kwiatkowski, Cordelia Langford, John Sillitoe on behalf of the Wellcome Sanger Institute COVID-19 Surveillance Team
EPI_ISL_821246, EPI_ISL_821247	Lighthouse Lab in Alderley Park	Wellcome Sanger Institute for the COVID-19 Genomics UK (COG-UK) Consortium	Jacquelyn Wynn, Mairead Hyland, The Lighthouse Lab in Alderley Park and Alex Alderton, Roberto Amato, Sonia Goncalves, Ewan Harrison, David K. Jackson, Ian Johnston, Dominic Kwiatkowski, Cordelia Langford, John Sillitoe on behalf of the Wellcome Sanger Institute COVID-19 Surveillance Team
EPI_ISL_821249	Lighthouse Lab in Glasgow	Wellcome Sanger Institute for the COVID-19 Genomics UK (COG-UK) Consortium	Harper VanSteenhouse, Yumi Kasai, David Gray, Carol Clugston, Anna Dominiczak and Alex Alderton, Roberto Amato, Sonia Goncalves, Ewan Harrison, David K. Jackson, Ian Johnston, Dominic Kwiatkowski, Cordelia Langford, John Sillitoe on behalf of the Wellcome Sanger Institute COVID-19 Surveillance Team
EPI_ISL_821252, EPI_ISL_821256	Lighthouse Lab in Alderley Park	Wellcome Sanger Institute for the COVID-19 Genomics UK (COG-UK) Consortium	Jacquelyn Wynn, Mairead Hyland, The Lighthouse Lab in Alderley Park and Alex Alderton, Roberto Amato, Sonia Goncalves, Ewan Harrison, David K. Jackson, Ian Johnston, Dominic Kwiatkowski, Cordelia Langford, John Sillitoe on behalf of the Wellcome Sanger Institute COVID-19 Surveillance Team
EPI_ISL_821257, EPI_ISL_821258	Lighthouse Lab in Glasgow	Wellcome Sanger Institute for the COVID-19 Genomics UK (COG-UK) Consortium	Harper VanSteenhouse, Yumi Kasai, David Gray, Carol Clugston, Anna Dominiczak and Alex Alderton, Roberto Amato, Sonia Goncalves, Ewan Harrison, David K. Jackson, Ian Johnston, Dominic Kwiatkowski, Cordelia Langford, John Sillitoe on behalf of the Wellcome Sanger Institute COVID-19 Surveillance Team
EPI_ISL_821259, EPI_ISL_821260, EPI_ISL_821262, EPI_ISL_821265	Lighthouse Lab in Alderley Park	Wellcome Sanger Institute for the COVID-19 Genomics UK (COG-UK) Consortium	Jacquelyn Wynn, Mairead Hyland, The Lighthouse Lab in Alderley Park and Alex Alderton, Roberto Amato, Sonia Goncalves, Ewan Harrison, David K. Jackson, Ian Johnston, Dominic Kwiatkowski, Cordelia Langford, John Sillitoe on behalf of the Wellcome Sanger Institute COVID-19 Surveillance Team
EPI_ISL_821266	Lighthouse Lab in Glasgow	Wellcome Sanger Institute for the COVID-19 Genomics UK (COG-UK) Consortium	Harper VanSteenhouse, Yumi Kasai, David Gray, Carol Clugston, Anna Dominiczak and Alex Alderton, Roberto Amato, Sonia Goncalves, Ewan Harrison, David K. Jackson, Ian Johnston, Dominic Kwiatkowski, Cordelia Langford, John Sillitoe on behalf of the Wellcome Sanger Institute COVID-19 Surveillance Team
EPI_ISL_821267, EPI_ISL_821268	Lighthouse Lab in Alderley Park	Wellcome Sanger Institute for the COVID-19 Genomics UK (COG-UK) Consortium	Jacquelyn Wynn, Mairead Hyland, The Lighthouse Lab in Alderley Park and Alex Alderton, Roberto Amato, Sonia Goncalves, Ewan Harrison, David K. Jackson, Ian Johnston, Dominic Kwiatkowski, Cordelia Langford, John Sillitoe on behalf of the Wellcome Sanger Institute COVID-19 Surveillance Team
EPI_ISL_821269	Lighthouse Lab in Glasgow	Wellcome Sanger Institute for the COVID-19 Genomics UK (COG-UK) Consortium	Harper VanSteenhouse, Yumi Kasai, David Gray, Carol Clugston, Anna Dominiczak and Alex Alderton, Roberto Amato, Sonia Goncalves, Ewan Harrison, David K. Jackson, Ian Johnston, Dominic Kwiatkowski, Cordelia Langford, John Sillitoe on behalf of the Wellcome Sanger Institute COVID-19 Surveillance Team
EPI_ISL_821272, EPI_ISL_821273	Lighthouse Lab in Alderley Park	Wellcome Sanger Institute for the COVID-19 Genomics UK (COG-UK) Consortium	Jacquelyn Wynn, Mairead Hyland, The Lighthouse Lab in Alderley Park and Alex Alderton, Roberto Amato, Sonia Goncalves, Ewan Harrison, David K. Jackson, Ian Johnston, Dominic Kwiatkowski, Cordelia Langford, John Sillitoe on behalf of the Wellcome Sanger Institute COVID-19 Surveillance Team
EPI_ISL_821274	Lighthouse Lab in Milton Keynes	Wellcome Sanger Institute for the COVID-19 Genomics UK (COG-UK) Consortium	The Lighthouse Lab in Milton Keynes and Alex Alderton, Roberto Amato, Sonia Goncalves, Ewan Harrison, David K. Jackson, Ian Johnston, Dominic Kwiatkowski, Cordelia Langford, John Sillitoe on behalf of the Wellcome Sanger Institute COVID-19 Surveillance Team
EPI_ISL_821276, EPI_ISL_821279	Lighthouse Lab in Cambridge	Wellcome Sanger Institute for the COVID-19 Genomics UK (COG-UK) Consortium	Rob Howes, The Lighthouse Lab in Cambridge and Alex Alderton, Roberto Amato, Sonia Goncalves, Ewan Harrison, David K. Jackson, Ian Johnston, Dominic Kwiatkowski, Cordelia Langford, John Sillitoe on behalf of the Wellcome Sanger Institute COVID-19 Surveillance Team
EPI_ISL_821281, EPI_ISL_821282	Lighthouse Lab in Milton Keynes	Wellcome Sanger Institute for the COVID-19 Genomics UK (COG-UK) Consortium	The Lighthouse Lab in Milton Keynes and Alex Alderton, Roberto Amato, Sonia Goncalves, Ewan Harrison, David K. Jackson, Ian Johnston, Dominic Kwiatkowski, Cordelia Langford, John Sillitoe on behalf of the Wellcome Sanger Institute COVID-19 Surveillance Team
EPI_ISL_821283	Lighthouse Lab in Alderley Park	Wellcome Sanger Institute for the COVID-19 Genomics UK (COG-UK) Consortium	Jacquelyn Wynn, Mairead Hyland, The Lighthouse Lab in Alderley Park and Alex Alderton, Roberto Amato, Sonia Goncalves, Ewan Harrison, David K. Jackson, Ian Johnston, Dominic Kwiatkowski, Cordelia Langford, John Sillitoe on behalf of the Wellcome Sanger Institute COVID-19 Surveillance Team
EPI_ISL_821284	Lighthouse Lab in Cambridge	Wellcome Sanger Institute for the COVID-19 Genomics UK (COG-UK) Consortium	Rob Howes, The Lighthouse Lab in Cambridge and Alex Alderton, Roberto Amato, Sonia Goncalves, Ewan Harrison, David K. Jackson, Ian Johnston, Dominic Kwiatkowski, Cordelia Langford, John Sillitoe on behalf of the Wellcome Sanger Institute COVID-19 Surveillance Team
EPI_ISL_821288	Lighthouse Lab in Alderley Park	Wellcome Sanger Institute for the COVID-19 Genomics UK (COG-UK) Consortium	Jacquelyn Wynn, Mairead Hyland, The Lighthouse Lab in Alderley Park and Alex Alderton, Roberto Amato, Sonia Goncalves, Ewan Harrison, David K. Jackson, Ian Johnston, Dominic Kwiatkowski, Cordelia Langford, John Sillitoe on behalf of the Wellcome Sanger Institute COVID-19 Surveillance Team
EPI_ISL_821289, EPI_ISL_821290	Lighthouse Lab in Cambridge	Wellcome Sanger Institute for the COVID-19 Genomics UK (COG-UK) Consortium	Rob Howes, The Lighthouse Lab in Cambridge and Alex Alderton, Roberto Amato, Sonia Goncalves, Ewan Harrison, David K. Jackson, Ian Johnston, Dominic Kwiatkowski, Cordelia Langford, John Sillitoe on behalf of the Wellcome Sanger Institute COVID-19 Surveillance Team
EPI_ISL_821296, EPI_ISL_821297, EPI_ISL_821301, EPI_ISL_821306	Lighthouse Lab in Milton Keynes	Wellcome Sanger Institute for the COVID-19 Genomics UK (COG-UK) Consortium	The Lighthouse Lab in Milton Keynes and Alex Alderton, Roberto Amato, Sonia Goncalves, Ewan Harrison, David K. Jackson, Ian Johnston, Dominic Kwiatkowski, Cordelia Langford, John Sillitoe on behalf of the Wellcome Sanger Institute COVID-19 Surveillance Team
EPI_ISL_821309, EPI_ISL_821310	Lighthouse Lab in Cambridge	Wellcome Sanger Institute for the COVID-19 Genomics UK (COG-UK) Consortium	Rob Howes, The Lighthouse Lab in Cambridge and Alex Alderton, Roberto Amato, Sonia Goncalves, Ewan Harrison, David K. Jackson, Ian Johnston, Dominic Kwiatkowski, Cordelia Langford, John Sillitoe on behalf of the Wellcome Sanger Institute COVID-19 Surveillance Team
EPI_ISL_821314	Lighthouse Lab in Alderley Park	Wellcome Sanger Institute for the COVID-19 Genomics UK (COG-UK) Consortium	Jacquelyn Wynn, Mairead Hyland, The Lighthouse Lab in Alderley Park and Alex Alderton, Roberto Amato, Sonia Goncalves, Ewan Harrison, David K. Jackson, Ian Johnston, Dominic Kwiatkowski, Cordelia Langford, John Sillitoe on behalf of the Wellcome Sanger Institute COVID-19 Surveillance Team
EPI_ISL_821317, EPI_ISL_821319, EPI_ISL_821323, EPI_ISL_821324, EPI_ISL_821328, EPI_ISL_821331	Lighthouse Lab in Milton Keynes	Wellcome Sanger Institute for the COVID-19 Genomics UK (COG-UK) Consortium	The Lighthouse Lab in Milton Keynes and Alex Alderton, Roberto Amato, Sonia Goncalves, Ewan Harrison, David K. Jackson, Ian Johnston, Dominic Kwiatkowski, Cordelia Langford, John Sillitoe on behalf of the Wellcome Sanger Institute COVID-19 Surveillance Team
EPI_ISL_821333	Lighthouse Lab in Cambridge	Wellcome Sanger Institute for the COVID-19 Genomics UK (COG-UK) Consortium	Rob Howes, The Lighthouse Lab in Cambridge and Alex Alderton, Roberto Amato, Sonia Goncalves, Ewan Harrison, David K. Jackson, Ian Johnston, Dominic Kwiatkowski, Cordelia Langford, John Sillitoe on behalf of the Wellcome Sanger Institute COVID-19 Surveillance Team
EPI_ISL_821334, EPI_ISL_821336	Lighthouse Lab in Milton Keynes	Wellcome Sanger Institute for the COVID-19 Genomics UK	The Lighthouse Lab in Milton Keynes and Alex Alderton, Roberto Amato, Sonia Goncalves, Ewan Harrison, David K. Jackson, Ian Johnston, Dominic

		(COG-UK) Consortium	Kwiatkowski, Cordelia Langford, John Sillitoe on behalf of the Wellcome Sanger Institute COVID-19 Surveillance Team
EPI_ISL_821338	Lighthouse Lab in Cambridge	Wellcome Sanger Institute for the COVID-19 Genomics UK (COG-UK) Consortium	Rob Howes, The Lighthouse Lab in Cambridge and Alex Alderton, Roberto Amato, Sonia Goncalves, Ewan Harrison, David K. Jackson, Ian Johnston, Dominic Kwiatkowski, Cordelia Langford, John Sillitoe on behalf of the Wellcome Sanger Institute COVID-19 Surveillance Team
EPI_ISL_821340, EPI_ISL_821341	Lighthouse Lab in Milton Keynes	Wellcome Sanger Institute for the COVID-19 Genomics UK (COG-UK) Consortium	The Lighthouse Lab in Milton Keynes and Alex Alderton, Roberto Amato, Sonia Goncalves, Ewan Harrison, David K. Jackson, Ian Johnston, Dominic Kwiatkowski, Cordelia Langford, John Sillitoe on behalf of the Wellcome Sanger Institute COVID-19 Surveillance Team
EPI_ISL_821342, EPI_ISL_821343	Lighthouse Lab in Cambridge	Wellcome Sanger Institute for the COVID-19 Genomics UK (COG-UK) Consortium	Rob Howes, The Lighthouse Lab in Cambridge and Alex Alderton, Roberto Amato, Sonia Goncalves, Ewan Harrison, David K. Jackson, Ian Johnston, Dominic Kwiatkowski, Cordelia Langford, John Sillitoe on behalf of the Wellcome Sanger Institute COVID-19 Surveillance Team
EPI_ISL_821344, EPI_ISL_821346	Lighthouse Lab in Milton Keynes	Wellcome Sanger Institute for the COVID-19 Genomics UK (COG-UK) Consortium	The Lighthouse Lab in Milton Keynes and Alex Alderton, Roberto Amato, Sonia Goncalves, Ewan Harrison, David K. Jackson, Ian Johnston, Dominic Kwiatkowski, Cordelia Langford, John Sillitoe on behalf of the Wellcome Sanger Institute COVID-19 Surveillance Team
EPI_ISL_821348	Lighthouse Lab in Cambridge	Wellcome Sanger Institute for the COVID-19 Genomics UK (COG-UK) Consortium	Rob Howes, The Lighthouse Lab in Cambridge and Alex Alderton, Roberto Amato, Sonia Goncalves, Ewan Harrison, David K. Jackson, Ian Johnston, Dominic Kwiatkowski, Cordelia Langford, John Sillitoe on behalf of the Wellcome Sanger Institute COVID-19 Surveillance Team
EPI_ISL_821351	Lighthouse Lab in Alderley Park	Wellcome Sanger Institute for the COVID-19 Genomics UK (COG-UK) Consortium	Jacquelyn Wynn, Mairead Hyland, The Lighthouse Lab in Alderley Park and Alex Alderton, Roberto Amato, Sonia Goncalves, Ewan Harrison, David K. Jackson, Ian Johnston, Dominic Kwiatkowski, Cordelia Langford, John Sillitoe on behalf of the Wellcome Sanger Institute COVID-19 Surveillance Team
EPI_ISL_821352, EPI_ISL_821353	Lighthouse Lab in Milton Keynes	Wellcome Sanger Institute for the COVID-19 Genomics UK (COG-UK) Consortium	The Lighthouse Lab in Milton Keynes and Alex Alderton, Roberto Amato, Sonia Goncalves, Ewan Harrison, David K. Jackson, Ian Johnston, Dominic Kwiatkowski, Cordelia Langford, John Sillitoe on behalf of the Wellcome Sanger Institute COVID-19 Surveillance Team
EPI_ISL_821354	Lighthouse Lab in Cambridge	Wellcome Sanger Institute for the COVID-19 Genomics UK (COG-UK) Consortium	Rob Howes, The Lighthouse Lab in Cambridge and Alex Alderton, Roberto Amato, Sonia Goncalves, Ewan Harrison, David K. Jackson, Ian Johnston, Dominic Kwiatkowski, Cordelia Langford, John Sillitoe on behalf of the Wellcome Sanger Institute COVID-19 Surveillance Team
EPI_ISL_821358, EPI_ISL_821359, EPI_ISL_821362	Lighthouse Lab in Milton Keynes	Wellcome Sanger Institute for the COVID-19 Genomics UK (COG-UK) Consortium	The Lighthouse Lab in Milton Keynes and Alex Alderton, Roberto Amato, Sonia Goncalves, Ewan Harrison, David K. Jackson, Ian Johnston, Dominic Kwiatkowski, Cordelia Langford, John Sillitoe on behalf of the Wellcome Sanger Institute COVID-19 Surveillance Team
EPI_ISL_821363, EPI_ISL_821365	Lighthouse Lab in Cambridge	Wellcome Sanger Institute for the COVID-19 Genomics UK (COG-UK) Consortium	Rob Howes, The Lighthouse Lab in Cambridge and Alex Alderton, Roberto Amato, Sonia Goncalves, Ewan Harrison, David K. Jackson, Ian Johnston, Dominic Kwiatkowski, Cordelia Langford, John Sillitoe on behalf of the Wellcome Sanger Institute COVID-19 Surveillance Team
EPI_ISL_821366	Lighthouse Lab in Alderley Park	Wellcome Sanger Institute for the COVID-19 Genomics UK (COG-UK) Consortium	Jacquelyn Wynn, Mairead Hyland, The Lighthouse Lab in Alderley Park and Alex Alderton, Roberto Amato, Sonia Goncalves, Ewan Harrison, David K. Jackson, Ian Johnston, Dominic Kwiatkowski, Cordelia Langford, John Sillitoe on behalf of the Wellcome Sanger Institute COVID-19 Surveillance Team
EPI_ISL_821367	Lighthouse Lab in Cambridge	Wellcome Sanger Institute for the COVID-19 Genomics UK (COG-UK) Consortium	Rob Howes, The Lighthouse Lab in Cambridge and Alex Alderton, Roberto Amato, Sonia Goncalves, Ewan Harrison, David K. Jackson, Ian Johnston, Dominic Kwiatkowski, Cordelia Langford, John Sillitoe on behalf of the Wellcome Sanger Institute COVID-19 Surveillance Team
EPI_ISL_821372	Lighthouse Lab in Alderley Park	Wellcome Sanger Institute for the COVID-19 Genomics UK (COG-UK) Consortium	Jacquelyn Wynn, Mairead Hyland, The Lighthouse Lab in Alderley Park and Alex Alderton, Roberto Amato, Sonia Goncalves, Ewan Harrison, David K. Jackson, Ian Johnston, Dominic Kwiatkowski, Cordelia Langford, John Sillitoe on behalf of the Wellcome Sanger Institute COVID-19 Surveillance Team
EPI_ISL_821373	Lighthouse Lab in Milton Keynes	Wellcome Sanger Institute for the COVID-19 Genomics UK (COG-UK) Consortium	The Lighthouse Lab in Milton Keynes and Alex Alderton, Roberto Amato, Sonia Goncalves, Ewan Harrison, David K. Jackson, Ian Johnston, Dominic Kwiatkowski, Cordelia Langford, John Sillitoe on behalf of the Wellcome Sanger Institute COVID-19 Surveillance Team
EPI_ISL_821375	Lighthouse Lab in Cambridge	Wellcome Sanger Institute for the COVID-19 Genomics UK (COG-UK) Consortium	Rob Howes, The Lighthouse Lab in Cambridge and Alex Alderton, Roberto Amato, Sonia Goncalves, Ewan Harrison, David K. Jackson, Ian Johnston, Dominic Kwiatkowski, Cordelia Langford, John Sillitoe on behalf of the Wellcome Sanger Institute COVID-19 Surveillance Team
EPI_ISL_821377, EPI_ISL_821378, EPI_ISL_821379, EPI_ISL_821380, EPI_ISL_821381, EPI_ISL_821383	Lighthouse Lab in Milton Keynes	Wellcome Sanger Institute for the COVID-19 Genomics UK (COG-UK) Consortium	The Lighthouse Lab in Milton Keynes and Alex Alderton, Roberto Amato, Sonia Goncalves, Ewan Harrison, David K. Jackson, Ian Johnston, Dominic Kwiatkowski, Cordelia Langford, John Sillitoe on behalf of the Wellcome Sanger Institute COVID-19 Surveillance Team
EPI_ISL_821386	Lighthouse Lab in Cambridge	Wellcome Sanger Institute for the COVID-19 Genomics UK (COG-UK) Consortium	Rob Howes, The Lighthouse Lab in Cambridge and Alex Alderton, Roberto Amato, Sonia Goncalves, Ewan Harrison, David K. Jackson, Ian Johnston, Dominic Kwiatkowski, Cordelia Langford, John Sillitoe on behalf of the Wellcome Sanger Institute COVID-19 Surveillance Team
EPI_ISL_821387, EPI_ISL_821388	Lighthouse Lab in Milton Keynes	Wellcome Sanger Institute for the COVID-19 Genomics UK (COG-UK) Consortium	The Lighthouse Lab in Milton Keynes and Alex Alderton, Roberto Amato, Sonia Goncalves, Ewan Harrison, David K. Jackson, Ian Johnston, Dominic Kwiatkowski, Cordelia Langford, John Sillitoe on behalf of the Wellcome Sanger Institute COVID-19 Surveillance Team
EPI_ISL_821389, EPI_ISL_821390	Lighthouse Lab in Alderley Park	Wellcome Sanger Institute for the COVID-19 Genomics UK (COG-UK) Consortium	Jacquelyn Wynn, Mairead Hyland, The Lighthouse Lab in Alderley Park and Alex Alderton, Roberto Amato, Sonia Goncalves, Ewan Harrison, David K. Jackson, Ian Johnston, Dominic Kwiatkowski, Cordelia Langford, John Sillitoe on behalf of the Wellcome Sanger Institute COVID-19 Surveillance Team
EPI_ISL_821391	Lighthouse Lab in Milton Keynes	Wellcome Sanger Institute for the COVID-19 Genomics UK (COG-UK) Consortium	The Lighthouse Lab in Milton Keynes and Alex Alderton, Roberto Amato, Sonia Goncalves, Ewan Harrison, David K. Jackson, Ian Johnston, Dominic Kwiatkowski, Cordelia Langford, John Sillitoe on behalf of the Wellcome Sanger Institute COVID-19 Surveillance Team
EPI_ISL_821394	Lighthouse Lab in Cambridge	Wellcome Sanger Institute for the COVID-19 Genomics UK (COG-UK) Consortium	Rob Howes, The Lighthouse Lab in Cambridge and Alex Alderton, Roberto Amato, Sonia Goncalves, Ewan Harrison, David K. Jackson, Ian Johnston, Dominic Kwiatkowski, Cordelia Langford, John Sillitoe on behalf of the Wellcome Sanger Institute COVID-19 Surveillance Team
EPI_ISL_821395, EPI_ISL_821397	Lighthouse Lab in Milton Keynes	Wellcome Sanger Institute for the COVID-19 Genomics UK (COG-UK) Consortium	The Lighthouse Lab in Milton Keynes and Alex Alderton, Roberto Amato, Sonia Goncalves, Ewan Harrison, David K. Jackson, Ian Johnston, Dominic Kwiatkowski, Cordelia Langford, John Sillitoe on behalf of the Wellcome Sanger Institute COVID-19 Surveillance Team
EPI_ISL_821398	Lighthouse Lab in Alderley Park	Wellcome Sanger Institute for the COVID-19 Genomics UK (COG-UK) Consortium	Jacquelyn Wynn, Mairead Hyland, The Lighthouse Lab in Alderley Park and Alex Alderton, Roberto Amato, Sonia Goncalves, Ewan Harrison, David K. Jackson, Ian Johnston, Dominic Kwiatkowski, Cordelia Langford, John Sillitoe on behalf of the Wellcome Sanger Institute COVID-19 Surveillance Team
EPI_ISL_821400	Lighthouse Lab in Milton Keynes	Wellcome Sanger Institute for the COVID-19 Genomics UK (COG-UK) Consortium	The Lighthouse Lab in Milton Keynes and Alex Alderton, Roberto Amato, Sonia Goncalves, Ewan Harrison, David K. Jackson, Ian Johnston, Dominic Kwiatkowski, Cordelia Langford, John Sillitoe on behalf of the Wellcome Sanger Institute COVID-19 Surveillance Team
EPI_ISL_821401	Lighthouse Lab in Alderley Park	Wellcome Sanger Institute for the COVID-19 Genomics UK (COG-UK) Consortium	Jacquelyn Wynn, Mairead Hyland, The Lighthouse Lab in Alderley Park and Alex Alderton, Roberto Amato, Sonia Goncalves, Ewan Harrison, David K. Jackson, Ian Johnston, Dominic Kwiatkowski, Cordelia Langford, John Sillitoe on behalf of the Wellcome Sanger Institute COVID-19 Surveillance Team
EPI_ISL_821402	Lighthouse Lab in Milton Keynes	Wellcome Sanger Institute for the COVID-19 Genomics UK (COG-UK) Consortium	The Lighthouse Lab in Milton Keynes and Alex Alderton, Roberto Amato, Sonia Goncalves, Ewan Harrison, David K. Jackson, Ian Johnston, Dominic Kwiatkowski, Cordelia Langford, John Sillitoe on behalf of the Wellcome Sanger Institute COVID-19 Surveillance Team
EPI_ISL_821403	Lighthouse Lab in Alderley Park	Wellcome Sanger Institute for the COVID-19 Genomics UK (COG-UK) Consortium	Jacquelyn Wynn, Mairead Hyland, The Lighthouse Lab in Alderley Park and Alex Alderton, Roberto Amato, Sonia Goncalves, Ewan Harrison, David K. Jackson, Ian Johnston, Dominic Kwiatkowski, Cordelia Langford, John Sillitoe on behalf of the Wellcome Sanger Institute COVID-19 Surveillance Team
EPI_ISL_821404	Lighthouse Lab in Cambridge	Wellcome Sanger Institute for the COVID-19 Genomics UK (COG-UK) Consortium	Rob Howes, The Lighthouse Lab in Cambridge and Alex Alderton, Roberto Amato, Sonia Goncalves, Ewan Harrison, David K. Jackson, Ian Johnston, Dominic Kwiatkowski, Cordelia Langford, John Sillitoe on behalf of the Wellcome Sanger Institute COVID-19 Surveillance Team
EPI_ISL_821405	Lighthouse Lab in Milton Keynes	Wellcome Sanger Institute for the COVID-19 Genomics UK (COG-UK) Consortium	The Lighthouse Lab in Milton Keynes and Alex Alderton, Roberto Amato, Sonia Goncalves, Ewan Harrison, David K. Jackson, Ian Johnston, Dominic Kwiatkowski, Cordelia Langford, John Sillitoe on behalf of the Wellcome Sanger Institute COVID-19 Surveillance Team
EPI_ISL_821408	Lighthouse Lab in Cambridge	Wellcome Sanger Institute for the COVID-19 Genomics UK (COG-UK) Consortium	Rob Howes, The Lighthouse Lab in Cambridge and Alex Alderton, Roberto Amato, Sonia Goncalves, Ewan Harrison, David K. Jackson, Ian Johnston, Dominic Kwiatkowski, Cordelia Langford, John Sillitoe on behalf of the Wellcome Sanger Institute COVID-19 Surveillance Team
EPI_ISL_821409, EPI_ISL_821410	Lighthouse Lab in Milton Keynes	Wellcome Sanger Institute for the COVID-19 Genomics UK (COG-UK) Consortium	The Lighthouse Lab in Milton Keynes and Alex Alderton, Roberto Amato, Sonia Goncalves, Ewan Harrison, David K. Jackson, Ian Johnston, Dominic Kwiatkowski, Cordelia Langford, John Sillitoe on behalf of the Wellcome Sanger Institute COVID-19 Surveillance Team
EPI_ISL_821411, EPI_ISL_821416	Lighthouse Lab in Cambridge	Wellcome Sanger Institute for the COVID-19 Genomics UK (COG-UK) Consortium	Rob Howes, The Lighthouse Lab in Cambridge and Alex Alderton, Roberto Amato, Sonia Goncalves, Ewan Harrison, David K. Jackson, Ian Johnston, Dominic Kwiatkowski, Cordelia Langford, John Sillitoe on behalf of the Wellcome Sanger Institute COVID-19 Surveillance Team
EPI_ISL_821418, EPI_ISL_821419, EPI_ISL_821425, EPI_ISL_821426	Lighthouse Lab in Alderley Park	Wellcome Sanger Institute for the COVID-19 Genomics UK (COG-UK) Consortium	Jacquelyn Wynn, Mairead Hyland, The Lighthouse Lab in Alderley Park and Alex Alderton, Roberto Amato, Sonia Goncalves, Ewan Harrison, David K. Jackson, Ian Johnston, Dominic Kwiatkowski, Cordelia Langford, John Sillitoe on behalf of the Wellcome Sanger Institute COVID-19 Surveillance Team
EPI_ISL_821431, EPI_ISL_821432, EPI_ISL_821434	Lighthouse Lab in Milton Keynes	Wellcome Sanger Institute for the COVID-19 Genomics UK (COG-UK) Consortium	The Lighthouse Lab in Milton Keynes and Alex Alderton, Roberto Amato, Sonia Goncalves, Ewan Harrison, David K. Jackson, Ian Johnston, Dominic Kwiatkowski, Cordelia Langford, John Sillitoe on behalf of the Wellcome Sanger Institute COVID-19 Surveillance Team

EPI_ISL_821435, EPI_ISL_821439	Lighthouse Lab in Cambridge	Wellcome Sanger Institute for the COVID-19 Genomics UK (COG-UK) Consortium	Rob Howes, The Lighthouse Lab in Cambridge and Alex Alderton, Roberto Amato, Sonia Goncalves, Ewan Harrison, David K. Jackson, Ian Johnston, Dominic Kwiatkowski, Cordelia Langford, John Sillitoe on behalf of the Wellcome Sanger Institute COVID-19 Surveillance Team
EPI_ISL_821440	Lighthouse Lab in Alderley Park	Wellcome Sanger Institute for the COVID-19 Genomics UK (COG-UK) Consortium	Jacquelyn Wynn, Mairead Hyland, The Lighthouse Lab in Alderley Park and Alex Alderton, Roberto Amato, Sonia Goncalves, Ewan Harrison, David K. Jackson, Ian Johnston, Dominic Kwiatkowski, Cordelia Langford, John Sillitoe on behalf of the Wellcome Sanger Institute COVID-19 Surveillance Team
EPI_ISL_821443, EPI_ISL_821445, EPI_ISL_821448, EPI_ISL_821449	Lighthouse Lab in Milton Keynes	Wellcome Sanger Institute for the COVID-19 Genomics UK (COG-UK) Consortium	The Lighthouse Lab in Milton Keynes and Alex Alderton, Roberto Amato, Sonia Goncalves, Ewan Harrison, David K. Jackson, Ian Johnston, Dominic Kwiatkowski, Cordelia Langford, John Sillitoe on behalf of the Wellcome Sanger Institute COVID-19 Surveillance Team
EPI_ISL_821451, EPI_ISL_821453, EPI_ISL_821459	Lighthouse Lab in Alderley Park	Wellcome Sanger Institute for the COVID-19 Genomics UK (COG-UK) Consortium	Jacquelyn Wynn, Mairead Hyland, The Lighthouse Lab in Alderley Park and Alex Alderton, Roberto Amato, Sonia Goncalves, Ewan Harrison, David K. Jackson, Ian Johnston, Dominic Kwiatkowski, Cordelia Langford, John Sillitoe on behalf of the Wellcome Sanger Institute COVID-19 Surveillance Team
EPI_ISL_821460, EPI_ISL_821461, EPI_ISL_821462	Lighthouse Lab in Milton Keynes	Wellcome Sanger Institute for the COVID-19 Genomics UK (COG-UK) Consortium	The Lighthouse Lab in Milton Keynes and Alex Alderton, Roberto Amato, Sonia Goncalves, Ewan Harrison, David K. Jackson, Ian Johnston, Dominic Kwiatkowski, Cordelia Langford, John Sillitoe on behalf of the Wellcome Sanger Institute COVID-19 Surveillance Team
EPI_ISL_821465, EPI_ISL_821466	Lighthouse Lab in Alderley Park	Wellcome Sanger Institute for the COVID-19 Genomics UK (COG-UK) Consortium	Jacquelyn Wynn, Mairead Hyland, The Lighthouse Lab in Alderley Park and Alex Alderton, Roberto Amato, Sonia Goncalves, Ewan Harrison, David K. Jackson, Ian Johnston, Dominic Kwiatkowski, Cordelia Langford, John Sillitoe on behalf of the Wellcome Sanger Institute COVID-19 Surveillance Team
EPI_ISL_821468	Lighthouse Lab in Milton Keynes	Wellcome Sanger Institute for the COVID-19 Genomics UK (COG-UK) Consortium	The Lighthouse Lab in Milton Keynes and Alex Alderton, Roberto Amato, Sonia Goncalves, Ewan Harrison, David K. Jackson, Ian Johnston, Dominic Kwiatkowski, Cordelia Langford, John Sillitoe on behalf of the Wellcome Sanger Institute COVID-19 Surveillance Team
EPI_ISL_821470	Lighthouse Lab in Alderley Park	Wellcome Sanger Institute for the COVID-19 Genomics UK (COG-UK) Consortium	Jacquelyn Wynn, Mairead Hyland, The Lighthouse Lab in Alderley Park and Alex Alderton, Roberto Amato, Sonia Goncalves, Ewan Harrison, David K. Jackson, Ian Johnston, Dominic Kwiatkowski, Cordelia Langford, John Sillitoe on behalf of the Wellcome Sanger Institute COVID-19 Surveillance Team
EPI_ISL_821472, EPI_ISL_821474, EPI_ISL_821475	Lighthouse Lab in Milton Keynes	Wellcome Sanger Institute for the COVID-19 Genomics UK (COG-UK) Consortium	The Lighthouse Lab in Milton Keynes and Alex Alderton, Roberto Amato, Sonia Goncalves, Ewan Harrison, David K. Jackson, Ian Johnston, Dominic Kwiatkowski, Cordelia Langford, John Sillitoe on behalf of the Wellcome Sanger Institute COVID-19 Surveillance Team
EPI_ISL_821476	Lighthouse Lab in Alderley Park	Wellcome Sanger Institute for the COVID-19 Genomics UK (COG-UK) Consortium	Jacquelyn Wynn, Mairead Hyland, The Lighthouse Lab in Alderley Park and Alex Alderton, Roberto Amato, Sonia Goncalves, Ewan Harrison, David K. Jackson, Ian Johnston, Dominic Kwiatkowski, Cordelia Langford, John Sillitoe on behalf of the Wellcome Sanger Institute COVID-19 Surveillance Team
EPI_ISL_821477, EPI_ISL_821478, EPI_ISL_821480	Lighthouse Lab in Milton Keynes	Wellcome Sanger Institute for the COVID-19 Genomics UK (COG-UK) Consortium	The Lighthouse Lab in Milton Keynes and Alex Alderton, Roberto Amato, Sonia Goncalves, Ewan Harrison, David K. Jackson, Ian Johnston, Dominic Kwiatkowski, Cordelia Langford, John Sillitoe on behalf of the Wellcome Sanger Institute COVID-19 Surveillance Team
EPI_ISL_821484	Lighthouse Lab in Cambridge	Wellcome Sanger Institute for the COVID-19 Genomics UK (COG-UK) Consortium	Rob Howes, The Lighthouse Lab in Cambridge and Alex Alderton, Roberto Amato, Sonia Goncalves, Ewan Harrison, David K. Jackson, Ian Johnston, Dominic Kwiatkowski, Cordelia Langford, John Sillitoe on behalf of the Wellcome Sanger Institute COVID-19 Surveillance Team
EPI_ISL_821486	Lighthouse Lab in Milton Keynes	Wellcome Sanger Institute for the COVID-19 Genomics UK (COG-UK) Consortium	The Lighthouse Lab in Milton Keynes and Alex Alderton, Roberto Amato, Sonia Goncalves, Ewan Harrison, David K. Jackson, Ian Johnston, Dominic Kwiatkowski, Cordelia Langford, John Sillitoe on behalf of the Wellcome Sanger Institute COVID-19 Surveillance Team
EPI_ISL_821488, EPI_ISL_821491	Lighthouse Lab in Cambridge	Wellcome Sanger Institute for the COVID-19 Genomics UK (COG-UK) Consortium	Rob Howes, The Lighthouse Lab in Cambridge and Alex Alderton, Roberto Amato, Sonia Goncalves, Ewan Harrison, David K. Jackson, Ian Johnston, Dominic Kwiatkowski, Cordelia Langford, John Sillitoe on behalf of the Wellcome Sanger Institute COVID-19 Surveillance Team
EPI_ISL_821497	Lighthouse Lab in Alderley Park	Wellcome Sanger Institute for the COVID-19 Genomics UK (COG-UK) Consortium	Jacquelyn Wynn, Mairead Hyland, The Lighthouse Lab in Alderley Park and Alex Alderton, Roberto Amato, Sonia Goncalves, Ewan Harrison, David K. Jackson, Ian Johnston, Dominic Kwiatkowski, Cordelia Langford, John Sillitoe on behalf of the Wellcome Sanger Institute COVID-19 Surveillance Team
EPI_ISL_821498, EPI_ISL_821501	Lighthouse Lab in Milton Keynes	Wellcome Sanger Institute for the COVID-19 Genomics UK (COG-UK) Consortium	The Lighthouse Lab in Milton Keynes and Alex Alderton, Roberto Amato, Sonia Goncalves, Ewan Harrison, David K. Jackson, Ian Johnston, Dominic Kwiatkowski, Cordelia Langford, John Sillitoe on behalf of the Wellcome Sanger Institute COVID-19 Surveillance Team
EPI_ISL_821502	Lighthouse Lab in Alderley Park	Wellcome Sanger Institute for the COVID-19 Genomics UK (COG-UK) Consortium	Jacquelyn Wynn, Mairead Hyland, The Lighthouse Lab in Alderley Park and Alex Alderton, Roberto Amato, Sonia Goncalves, Ewan Harrison, David K. Jackson, Ian Johnston, Dominic Kwiatkowski, Cordelia Langford, John Sillitoe on behalf of the Wellcome Sanger Institute COVID-19 Surveillance Team
EPI_ISL_821504, EPI_ISL_821507, EPI_ISL_821509	Lighthouse Lab in Milton Keynes	Wellcome Sanger Institute for the COVID-19 Genomics UK (COG-UK) Consortium	The Lighthouse Lab in Milton Keynes and Alex Alderton, Roberto Amato, Sonia Goncalves, Ewan Harrison, David K. Jackson, Ian Johnston, Dominic Kwiatkowski, Cordelia Langford, John Sillitoe on behalf of the Wellcome Sanger Institute COVID-19 Surveillance Team
EPI_ISL_821510	Lighthouse Lab in Cambridge	Wellcome Sanger Institute for the COVID-19 Genomics UK (COG-UK) Consortium	Rob Howes, The Lighthouse Lab in Cambridge and Alex Alderton, Roberto Amato, Sonia Goncalves, Ewan Harrison, David K. Jackson, Ian Johnston, Dominic Kwiatkowski, Cordelia Langford, John Sillitoe on behalf of the Wellcome Sanger Institute COVID-19 Surveillance Team
EPI_ISL_821511	Lighthouse Lab in Alderley Park	Wellcome Sanger Institute for the COVID-19 Genomics UK (COG-UK) Consortium	Jacquelyn Wynn, Mairead Hyland, The Lighthouse Lab in Alderley Park and Alex Alderton, Roberto Amato, Sonia Goncalves, Ewan Harrison, David K. Jackson, Ian Johnston, Dominic Kwiatkowski, Cordelia Langford, John Sillitoe on behalf of the Wellcome Sanger Institute COVID-19 Surveillance Team
EPI_ISL_821514, EPI_ISL_821515	Lighthouse Lab in Milton Keynes	Wellcome Sanger Institute for the COVID-19 Genomics UK (COG-UK) Consortium	The Lighthouse Lab in Milton Keynes and Alex Alderton, Roberto Amato, Sonia Goncalves, Ewan Harrison, David K. Jackson, Ian Johnston, Dominic Kwiatkowski, Cordelia Langford, John Sillitoe on behalf of the Wellcome Sanger Institute COVID-19 Surveillance Team
EPI_ISL_821516	Lighthouse Lab in Cambridge	Wellcome Sanger Institute for the COVID-19 Genomics UK (COG-UK) Consortium	Rob Howes, The Lighthouse Lab in Cambridge and Alex Alderton, Roberto Amato, Sonia Goncalves, Ewan Harrison, David K. Jackson, Ian Johnston, Dominic Kwiatkowski, Cordelia Langford, John Sillitoe on behalf of the Wellcome Sanger Institute COVID-19 Surveillance Team
EPI_ISL_821517	Lighthouse Lab in Milton Keynes	Wellcome Sanger Institute for the COVID-19 Genomics UK (COG-UK) Consortium	The Lighthouse Lab in Milton Keynes and Alex Alderton, Roberto Amato, Sonia Goncalves, Ewan Harrison, David K. Jackson, Ian Johnston, Dominic Kwiatkowski, Cordelia Langford, John Sillitoe on behalf of the Wellcome Sanger Institute COVID-19 Surveillance Team
EPI_ISL_821518	Lighthouse Lab in Alderley Park	Wellcome Sanger Institute for the COVID-19 Genomics UK (COG-UK) Consortium	Jacquelyn Wynn, Mairead Hyland, The Lighthouse Lab in Alderley Park and Alex Alderton, Roberto Amato, Sonia Goncalves, Ewan Harrison, David K. Jackson, Ian Johnston, Dominic Kwiatkowski, Cordelia Langford, John Sillitoe on behalf of the Wellcome Sanger Institute COVID-19 Surveillance Team
EPI_ISL_821519	Lighthouse Lab in Cambridge	Wellcome Sanger Institute for the COVID-19 Genomics UK (COG-UK) Consortium	Rob Howes, The Lighthouse Lab in Cambridge and Alex Alderton, Roberto Amato, Sonia Goncalves, Ewan Harrison, David K. Jackson, Ian Johnston, Dominic Kwiatkowski, Cordelia Langford, John Sillitoe on behalf of the Wellcome Sanger Institute COVID-19 Surveillance Team
EPI_ISL_821520, EPI_ISL_821521, EPI_ISL_821522	Lighthouse Lab in Milton Keynes	Wellcome Sanger Institute for the COVID-19 Genomics UK (COG-UK) Consortium	The Lighthouse Lab in Milton Keynes and Alex Alderton, Roberto Amato, Sonia Goncalves, Ewan Harrison, David K. Jackson, Ian Johnston, Dominic Kwiatkowski, Cordelia Langford, John Sillitoe on behalf of the Wellcome Sanger Institute COVID-19 Surveillance Team
EPI_ISL_821523	Lighthouse Lab in Cambridge	Wellcome Sanger Institute for the COVID-19 Genomics UK (COG-UK) Consortium	Rob Howes, The Lighthouse Lab in Cambridge and Alex Alderton, Roberto Amato, Sonia Goncalves, Ewan Harrison, David K. Jackson, Ian Johnston, Dominic Kwiatkowski, Cordelia Langford, John Sillitoe on behalf of the Wellcome Sanger Institute COVID-19 Surveillance Team
EPI_ISL_821524, EPI_ISL_821527	Lighthouse Lab in Milton Keynes	Wellcome Sanger Institute for the COVID-19 Genomics UK (COG-UK) Consortium	The Lighthouse Lab in Milton Keynes and Alex Alderton, Roberto Amato, Sonia Goncalves, Ewan Harrison, David K. Jackson, Ian Johnston, Dominic Kwiatkowski, Cordelia Langford, John Sillitoe on behalf of the Wellcome Sanger Institute COVID-19 Surveillance Team
EPI_ISL_821529	Lighthouse Lab in Cambridge	Wellcome Sanger Institute for the COVID-19 Genomics UK (COG-UK) Consortium	Rob Howes, The Lighthouse Lab in Cambridge and Alex Alderton, Roberto Amato, Sonia Goncalves, Ewan Harrison, David K. Jackson, Ian Johnston, Dominic Kwiatkowski, Cordelia Langford, John Sillitoe on behalf of the Wellcome Sanger Institute COVID-19 Surveillance Team
EPI_ISL_821533	Lighthouse Lab in Alderley Park	Wellcome Sanger Institute for the COVID-19 Genomics UK (COG-UK) Consortium	Jacquelyn Wynn, Mairead Hyland, The Lighthouse Lab in Alderley Park and Alex Alderton, Roberto Amato, Sonia Goncalves, Ewan Harrison, David K. Jackson, Ian Johnston, Dominic Kwiatkowski, Cordelia Langford, John Sillitoe on behalf of the Wellcome Sanger Institute COVID-19 Surveillance Team
EPI_ISL_821536, EPI_ISL_821537	Lighthouse Lab in Milton Keynes	Wellcome Sanger Institute for the COVID-19 Genomics UK (COG-UK) Consortium	The Lighthouse Lab in Milton Keynes and Alex Alderton, Roberto Amato, Sonia Goncalves, Ewan Harrison, David K. Jackson, Ian Johnston, Dominic Kwiatkowski, Cordelia Langford, John Sillitoe on behalf of the Wellcome Sanger Institute COVID-19 Surveillance Team
EPI_ISL_821538	Lighthouse Lab in Alderley Park	Wellcome Sanger Institute for the COVID-19 Genomics UK (COG-UK) Consortium	Jacquelyn Wynn, Mairead Hyland, The Lighthouse Lab in Alderley Park and Alex Alderton, Roberto Amato, Sonia Goncalves, Ewan Harrison, David K. Jackson, Ian Johnston, Dominic Kwiatkowski, Cordelia Langford, John Sillitoe on behalf of the Wellcome Sanger Institute COVID-19 Surveillance Team
EPI_ISL_821539, EPI_ISL_821540, EPI_ISL_821541, EPI_ISL_821542, EPI_ISL_821543, EPI_ISL_821545	Lighthouse Lab in Milton Keynes	Wellcome Sanger Institute for the COVID-19 Genomics UK (COG-UK) Consortium	The Lighthouse Lab in Milton Keynes and Alex Alderton, Roberto Amato, Sonia Goncalves, Ewan Harrison, David K. Jackson, Ian Johnston, Dominic Kwiatkowski, Cordelia Langford, John Sillitoe on behalf of the Wellcome Sanger Institute COVID-19 Surveillance Team
EPI_ISL_821547	Lighthouse Lab in Cambridge	Wellcome Sanger Institute for the COVID-19 Genomics UK (COG-UK) Consortium	Rob Howes, The Lighthouse Lab in Cambridge and Alex Alderton, Roberto Amato, Sonia Goncalves, Ewan Harrison, David K. Jackson, Ian Johnston, Dominic Kwiatkowski, Cordelia Langford, John Sillitoe on behalf of the Wellcome Sanger Institute COVID-19 Surveillance Team
EPI_ISL_821548, EPI_ISL_821552, EPI_ISL_821553	Lighthouse Lab in Alderley Park	Wellcome Sanger Institute for the COVID-19 Genomics UK (COG-UK) Consortium	Jacquelyn Wynn, Mairead Hyland, The Lighthouse Lab in Alderley Park and Alex Alderton, Roberto Amato, Sonia Goncalves, Ewan Harrison, David K. Jackson, Ian Johnston, Dominic Kwiatkowski, Cordelia Langford, John Sillitoe on behalf of the Wellcome Sanger Institute COVID-19 Surveillance Team

EPI_ISL_821555	Lighthouse Lab in Cambridge	Wellcome Sanger Institute for the COVID-19 Genomics UK (COG-UK) Consortium	Rob Howes, The Lighthouse Lab in Cambridge and Alex Alderton, Roberto Amato, Sonia Goncalves, Ewan Harrison, David K. Jackson, Ian Johnston, Dominic Kwiatkowski, Cordelia Langford, John Sillitoe on behalf of the Wellcome Sanger Institute COVID-19 Surveillance Team
EPI_ISL_821558	Lighthouse Lab in Alderley Park	Wellcome Sanger Institute for the COVID-19 Genomics UK (COG-UK) Consortium	Jacquelyn Wynn, Mairead Hyland, The Lighthouse Lab in Alderley Park and Alex Alderton, Roberto Amato, Sonia Goncalves, Ewan Harrison, David K. Jackson, Ian Johnston, Dominic Kwiatkowski, Cordelia Langford, John Sillitoe on behalf of the Wellcome Sanger Institute COVID-19 Surveillance Team
EPI_ISL_821559, EPI_ISL_821560, EPI_ISL_821561, EPI_ISL_821562	Lighthouse Lab in Milton Keynes	Wellcome Sanger Institute for the COVID-19 Genomics UK (COG-UK) Consortium	The Lighthouse Lab in Milton Keynes and Alex Alderton, Roberto Amato, Sonia Goncalves, Ewan Harrison, David K. Jackson, Ian Johnston, Dominic Kwiatkowski, Cordelia Langford, John Sillitoe on behalf of the Wellcome Sanger Institute COVID-19 Surveillance Team
EPI_ISL_821565	Lighthouse Lab in Cambridge	Wellcome Sanger Institute for the COVID-19 Genomics UK (COG-UK) Consortium	Rob Howes, The Lighthouse Lab in Cambridge and Alex Alderton, Roberto Amato, Sonia Goncalves, Ewan Harrison, David K. Jackson, Ian Johnston, Dominic Kwiatkowski, Cordelia Langford, John Sillitoe on behalf of the Wellcome Sanger Institute COVID-19 Surveillance Team
EPI_ISL_821566	Lighthouse Lab in Alderley Park	Wellcome Sanger Institute for the COVID-19 Genomics UK (COG-UK) Consortium	Jacquelyn Wynn, Mairead Hyland, The Lighthouse Lab in Alderley Park and Alex Alderton, Roberto Amato, Sonia Goncalves, Ewan Harrison, David K. Jackson, Ian Johnston, Dominic Kwiatkowski, Cordelia Langford, John Sillitoe on behalf of the Wellcome Sanger Institute COVID-19 Surveillance Team
EPI_ISL_821568	Lighthouse Lab in Cambridge	Wellcome Sanger Institute for the COVID-19 Genomics UK (COG-UK) Consortium	Rob Howes, The Lighthouse Lab in Cambridge and Alex Alderton, Roberto Amato, Sonia Goncalves, Ewan Harrison, David K. Jackson, Ian Johnston, Dominic Kwiatkowski, Cordelia Langford, John Sillitoe on behalf of the Wellcome Sanger Institute COVID-19 Surveillance Team
EPI_ISL_821569	Lighthouse Lab in Milton Keynes	Wellcome Sanger Institute for the COVID-19 Genomics UK (COG-UK) Consortium	The Lighthouse Lab in Milton Keynes and Alex Alderton, Roberto Amato, Sonia Goncalves, Ewan Harrison, David K. Jackson, Ian Johnston, Dominic Kwiatkowski, Cordelia Langford, John Sillitoe on behalf of the Wellcome Sanger Institute COVID-19 Surveillance Team
EPI_ISL_821570	Lighthouse Lab in Cambridge	Wellcome Sanger Institute for the COVID-19 Genomics UK (COG-UK) Consortium	Rob Howes, The Lighthouse Lab in Cambridge and Alex Alderton, Roberto Amato, Sonia Goncalves, Ewan Harrison, David K. Jackson, Ian Johnston, Dominic Kwiatkowski, Cordelia Langford, John Sillitoe on behalf of the Wellcome Sanger Institute COVID-19 Surveillance Team
EPI_ISL_821571, EPI_ISL_821572, EPI_ISL_821573, EPI_ISL_821574, EPI_ISL_821575	Lighthouse Lab in Milton Keynes	Wellcome Sanger Institute for the COVID-19 Genomics UK (COG-UK) Consortium	The Lighthouse Lab in Milton Keynes and Alex Alderton, Roberto Amato, Sonia Goncalves, Ewan Harrison, David K. Jackson, Ian Johnston, Dominic Kwiatkowski, Cordelia Langford, John Sillitoe on behalf of the Wellcome Sanger Institute COVID-19 Surveillance Team
EPI_ISL_821576	Lighthouse Lab in Cambridge	Wellcome Sanger Institute for the COVID-19 Genomics UK (COG-UK) Consortium	Rob Howes, The Lighthouse Lab in Cambridge and Alex Alderton, Roberto Amato, Sonia Goncalves, Ewan Harrison, David K. Jackson, Ian Johnston, Dominic Kwiatkowski, Cordelia Langford, John Sillitoe on behalf of the Wellcome Sanger Institute COVID-19 Surveillance Team
EPI_ISL_821580, EPI_ISL_821582	Lighthouse Lab in Milton Keynes	Wellcome Sanger Institute for the COVID-19 Genomics UK (COG-UK) Consortium	The Lighthouse Lab in Milton Keynes and Alex Alderton, Roberto Amato, Sonia Goncalves, Ewan Harrison, David K. Jackson, Ian Johnston, Dominic Kwiatkowski, Cordelia Langford, John Sillitoe on behalf of the Wellcome Sanger Institute COVID-19 Surveillance Team
EPI_ISL_821584, EPI_ISL_821585, EPI_ISL_821586	Lighthouse Lab in Alderley Park	Wellcome Sanger Institute for the COVID-19 Genomics UK (COG-UK) Consortium	Jacquelyn Wynn, Mairead Hyland, The Lighthouse Lab in Alderley Park and Alex Alderton, Roberto Amato, Sonia Goncalves, Ewan Harrison, David K. Jackson, Ian Johnston, Dominic Kwiatkowski, Cordelia Langford, John Sillitoe on behalf of the Wellcome Sanger Institute COVID-19 Surveillance Team
EPI_ISL_821588, EPI_ISL_821592, EPI_ISL_821593	Lighthouse Lab in Cambridge	Wellcome Sanger Institute for the COVID-19 Genomics UK (COG-UK) Consortium	Rob Howes, The Lighthouse Lab in Cambridge and Alex Alderton, Roberto Amato, Sonia Goncalves, Ewan Harrison, David K. Jackson, Ian Johnston, Dominic Kwiatkowski, Cordelia Langford, John Sillitoe on behalf of the Wellcome Sanger Institute COVID-19 Surveillance Team
EPI_ISL_821595	Lighthouse Lab in Milton Keynes	Wellcome Sanger Institute for the COVID-19 Genomics UK (COG-UK) Consortium	The Lighthouse Lab in Milton Keynes and Alex Alderton, Roberto Amato, Sonia Goncalves, Ewan Harrison, David K. Jackson, Ian Johnston, Dominic Kwiatkowski, Cordelia Langford, John Sillitoe on behalf of the Wellcome Sanger Institute COVID-19 Surveillance Team
EPI_ISL_821596	Lighthouse Lab in Cambridge	Wellcome Sanger Institute for the COVID-19 Genomics UK (COG-UK) Consortium	Rob Howes, The Lighthouse Lab in Cambridge and Alex Alderton, Roberto Amato, Sonia Goncalves, Ewan Harrison, David K. Jackson, Ian Johnston, Dominic Kwiatkowski, Cordelia Langford, John Sillitoe on behalf of the Wellcome Sanger Institute COVID-19 Surveillance Team
EPI_ISL_821597	Lighthouse Lab in Milton Keynes	Wellcome Sanger Institute for the COVID-19 Genomics UK (COG-UK) Consortium	The Lighthouse Lab in Milton Keynes and Alex Alderton, Roberto Amato, Sonia Goncalves, Ewan Harrison, David K. Jackson, Ian Johnston, Dominic Kwiatkowski, Cordelia Langford, John Sillitoe on behalf of the Wellcome Sanger Institute COVID-19 Surveillance Team
EPI_ISL_821599	Lighthouse Lab in Alderley Park	Wellcome Sanger Institute for the COVID-19 Genomics UK (COG-UK) Consortium	Jacquelyn Wynn, Mairead Hyland, The Lighthouse Lab in Alderley Park and Alex Alderton, Roberto Amato, Sonia Goncalves, Ewan Harrison, David K. Jackson, Ian Johnston, Dominic Kwiatkowski, Cordelia Langford, John Sillitoe on behalf of the Wellcome Sanger Institute COVID-19 Surveillance Team
EPI_ISL_821601, EPI_ISL_821603	Lighthouse Lab in Milton Keynes	Wellcome Sanger Institute for the COVID-19 Genomics UK (COG-UK) Consortium	The Lighthouse Lab in Milton Keynes and Alex Alderton, Roberto Amato, Sonia Goncalves, Ewan Harrison, David K. Jackson, Ian Johnston, Dominic Kwiatkowski, Cordelia Langford, John Sillitoe on behalf of the Wellcome Sanger Institute COVID-19 Surveillance Team
EPI_ISL_821607	Lighthouse Lab in Alderley Park	Wellcome Sanger Institute for the COVID-19 Genomics UK (COG-UK) Consortium	Jacquelyn Wynn, Mairead Hyland, The Lighthouse Lab in Alderley Park and Alex Alderton, Roberto Amato, Sonia Goncalves, Ewan Harrison, David K. Jackson, Ian Johnston, Dominic Kwiatkowski, Cordelia Langford, John Sillitoe on behalf of the Wellcome Sanger Institute COVID-19 Surveillance Team
EPI_ISL_821608	Lighthouse Lab in Cambridge	Wellcome Sanger Institute for the COVID-19 Genomics UK (COG-UK) Consortium	Rob Howes, The Lighthouse Lab in Cambridge and Alex Alderton, Roberto Amato, Sonia Goncalves, Ewan Harrison, David K. Jackson, Ian Johnston, Dominic Kwiatkowski, Cordelia Langford, John Sillitoe on behalf of the Wellcome Sanger Institute COVID-19 Surveillance Team
EPI_ISL_821609, EPI_ISL_821612	Lighthouse Lab in Milton Keynes	Wellcome Sanger Institute for the COVID-19 Genomics UK (COG-UK) Consortium	The Lighthouse Lab in Milton Keynes and Alex Alderton, Roberto Amato, Sonia Goncalves, Ewan Harrison, David K. Jackson, Ian Johnston, Dominic Kwiatkowski, Cordelia Langford, John Sillitoe on behalf of the Wellcome Sanger Institute COVID-19 Surveillance Team
EPI_ISL_821614	Lighthouse Lab in Alderley Park	Wellcome Sanger Institute for the COVID-19 Genomics UK (COG-UK) Consortium	Jacquelyn Wynn, Mairead Hyland, The Lighthouse Lab in Alderley Park and Alex Alderton, Roberto Amato, Sonia Goncalves, Ewan Harrison, David K. Jackson, Ian Johnston, Dominic Kwiatkowski, Cordelia Langford, John Sillitoe on behalf of the Wellcome Sanger Institute COVID-19 Surveillance Team
EPI_ISL_821616, EPI_ISL_821618, EPI_ISL_821620	Lighthouse Lab in Cambridge	Wellcome Sanger Institute for the COVID-19 Genomics UK (COG-UK) Consortium	Rob Howes, The Lighthouse Lab in Cambridge and Alex Alderton, Roberto Amato, Sonia Goncalves, Ewan Harrison, David K. Jackson, Ian Johnston, Dominic Kwiatkowski, Cordelia Langford, John Sillitoe on behalf of the Wellcome Sanger Institute COVID-19 Surveillance Team
EPI_ISL_821621	Lighthouse Lab in Glasgow	Wellcome Sanger Institute for the COVID-19 Genomics UK (COG-UK) Consortium	Harper VanSteenhouse, Yumi Kasai, David Gray, Carol Clugston, Anna Dominiczak and Alex Alderton, Roberto Amato, Sonia Goncalves, Ewan Harrison, David K. Jackson, Ian Johnston, Dominic Kwiatkowski, Cordelia Langford, John Sillitoe on behalf of the Wellcome Sanger Institute COVID-19 Surveillance Team
EPI_ISL_821622, EPI_ISL_821623, EPI_ISL_821624	Lighthouse Lab in Cambridge	Wellcome Sanger Institute for the COVID-19 Genomics UK (COG-UK) Consortium	Rob Howes, The Lighthouse Lab in Cambridge and Alex Alderton, Roberto Amato, Sonia Goncalves, Ewan Harrison, David K. Jackson, Ian Johnston, Dominic Kwiatkowski, Cordelia Langford, John Sillitoe on behalf of the Wellcome Sanger Institute COVID-19 Surveillance Team
EPI_ISL_821625	Lighthouse Lab in Milton Keynes	Wellcome Sanger Institute for the COVID-19 Genomics UK (COG-UK) Consortium	The Lighthouse Lab in Milton Keynes and Alex Alderton, Roberto Amato, Sonia Goncalves, Ewan Harrison, David K. Jackson, Ian Johnston, Dominic Kwiatkowski, Cordelia Langford, John Sillitoe on behalf of the Wellcome Sanger Institute COVID-19 Surveillance Team
EPI_ISL_821967	Lighthouse Lab in Cambridge	Wellcome Sanger Institute for the COVID-19 Genomics UK (COG-UK) Consortium	Rob Howes, The Lighthouse Lab in Cambridge and Alex Alderton, Roberto Amato, Sonia Goncalves, Ewan Harrison, David K. Jackson, Ian Johnston, Dominic Kwiatkowski, Cordelia Langford, John Sillitoe on behalf of the Wellcome Sanger Institute COVID-19 Surveillance Team
EPI_ISL_821975	Lighthouse Lab in Glasgow	Wellcome Sanger Institute for the COVID-19 Genomics UK (COG-UK) Consortium	Harper VanSteenhouse, Yumi Kasai, David Gray, Carol Clugston, Anna Dominiczak and Alex Alderton, Roberto Amato, Sonia Goncalves, Ewan Harrison, David K. Jackson, Ian Johnston, Dominic Kwiatkowski, Cordelia Langford, John Sillitoe on behalf of the Wellcome Sanger Institute COVID-19 Surveillance Team
EPI_ISL_821987, EPI_ISL_821988	Lighthouse Lab in Alderley Park	Wellcome Sanger Institute for the COVID-19 Genomics UK (COG-UK) Consortium	Jacquelyn Wynn, Mairead Hyland, The Lighthouse Lab in Alderley Park and Alex Alderton, Roberto Amato, Sonia Goncalves, Ewan Harrison, David K. Jackson, Ian Johnston, Dominic Kwiatkowski, Cordelia Langford, John Sillitoe on behalf of the Wellcome Sanger Institute COVID-19 Surveillance Team
EPI_ISL_821993, EPI_ISL_821995, EPI_ISL_822001, EPI_ISL_822004, EPI_ISL_822005, EPI_ISL_822006, EPI_ISL_822011, EPI_ISL_822022, EPI_ISL_822023, EPI_ISL_822029, EPI_ISL_822033, EPI_ISL_822035, EPI_ISL_822040, EPI_ISL_822041, EPI_ISL_822044, EPI_ISL_822048, EPI_ISL_822051, EPI_ISL_822053, EPI_ISL_822059, EPI_ISL_822063			
see above	Lighthouse Lab in Glasgow	Wellcome Sanger Institute for the COVID-19 Genomics UK (COG-UK) Consortium	Harper VanSteenhouse, Yumi Kasai, David Gray, Carol Clugston, Anna Dominiczak and Alex Alderton, Roberto Amato, Sonia Goncalves, Ewan Harrison, David K. Jackson, Ian Johnston, Dominic Kwiatkowski, Cordelia Langford, John Sillitoe on behalf of the Wellcome Sanger Institute COVID-19 Surveillance Team
EPI_ISL_822065	Lighthouse Lab in Alderley Park	Wellcome Sanger Institute for the COVID-19 Genomics UK (COG-UK) Consortium	Jacquelyn Wynn, Mairead Hyland, The Lighthouse Lab in Alderley Park and Alex Alderton, Roberto Amato, Sonia Goncalves, Ewan Harrison, David K. Jackson, Ian Johnston, Dominic Kwiatkowski, Cordelia Langford, John Sillitoe on behalf of the Wellcome Sanger Institute COVID-19 Surveillance Team
EPI_ISL_822067, EPI_ISL_822069, EPI_ISL_822072, EPI_ISL_822075, EPI_ISL_822078	Lighthouse Lab in Glasgow	Wellcome Sanger Institute for the COVID-19 Genomics UK (COG-UK) Consortium	Harper VanSteenhouse, Yumi Kasai, David Gray, Carol Clugston, Anna Dominiczak and Alex Alderton, Roberto Amato, Sonia Goncalves, Ewan Harrison, David K. Jackson, Ian Johnston, Dominic Kwiatkowski, Cordelia Langford, John Sillitoe on behalf of the Wellcome Sanger Institute COVID-19 Surveillance Team

EPI_ISL_822080	Lighthouse Lab in Alderley Park	Wellcome Sanger Institute for the COVID-19 Genomics UK (COG-UK) Consortium	Jacquelyn Wynn, Mairead Hyland, The Lighthouse Lab in Alderley Park and Alex Alderton, Roberto Amato, Sonia Goncalves, Ewan Harrison, David K. Jackson, Ian Johnston, Dominic Kwiatkowski, Cordelia Langford, John Sillitoe on behalf of the Wellcome Sanger Institute COVID-19 Surveillance Team
EPI_ISL_822083	Lighthouse Lab in Glasgow	Wellcome Sanger Institute for the COVID-19 Genomics UK (COG-UK) Consortium	Harper VanSteenhouse, Yumi Kasai, David Gray, Carol Clugston, Anna Dominiczak and Alex Alderton, Roberto Amato, Sonia Goncalves, Ewan Harrison, David K. Jackson, Ian Johnston, Dominic Kwiatkowski, Cordelia Langford, John Sillitoe on behalf of the Wellcome Sanger Institute COVID-19 Surveillance Team
EPI_ISL_822086	Lighthouse Lab in Alderley Park	Wellcome Sanger Institute for the COVID-19 Genomics UK (COG-UK) Consortium	Jacquelyn Wynn, Mairead Hyland, The Lighthouse Lab in Alderley Park and Alex Alderton, Roberto Amato, Sonia Goncalves, Ewan Harrison, David K. Jackson, Ian Johnston, Dominic Kwiatkowski, Cordelia Langford, John Sillitoe on behalf of the Wellcome Sanger Institute COVID-19 Surveillance Team
EPI_ISL_822098, EPI_ISL_822099, EPI_ISL_822113, EPI_ISL_822117, EPI_ISL_822119, EPI_ISL_822122, EPI_ISL_822127, EPI_ISL_822130, EPI_ISL_822140, EPI_ISL_822148, EPI_ISL_822153, EPI_ISL_822156, EPI_ISL_822158, EPI_ISL_822159, EPI_ISL_822165, EPI_ISL_822169, EPI_ISL_822170, EPI_ISL_822178, EPI_ISL_822182			
see above	Lighthouse Lab in Glasgow	Wellcome Sanger Institute for the COVID-19 Genomics UK (COG-UK) Consortium	Harper VanSteenhouse, Yumi Kasai, David Gray, Carol Clugston, Anna Dominiczak and Alex Alderton, Roberto Amato, Sonia Goncalves, Ewan Harrison, David K. Jackson, Ian Johnston, Dominic Kwiatkowski, Cordelia Langford, John Sillitoe on behalf of the Wellcome Sanger Institute COVID-19 Surveillance Team
EPI_ISL_822193	Lighthouse Lab in Alderley Park	Wellcome Sanger Institute for the COVID-19 Genomics UK (COG-UK) Consortium	Jacquelyn Wynn, Mairead Hyland, The Lighthouse Lab in Alderley Park and Alex Alderton, Roberto Amato, Sonia Goncalves, Ewan Harrison, David K. Jackson, Ian Johnston, Dominic Kwiatkowski, Cordelia Langford, John Sillitoe on behalf of the Wellcome Sanger Institute COVID-19 Surveillance Team
EPI_ISL_822201	Lighthouse Lab in Glasgow	Wellcome Sanger Institute for the COVID-19 Genomics UK (COG-UK) Consortium	Harper VanSteenhouse, Yumi Kasai, David Gray, Carol Clugston, Anna Dominiczak and Alex Alderton, Roberto Amato, Sonia Goncalves, Ewan Harrison, David K. Jackson, Ian Johnston, Dominic Kwiatkowski, Cordelia Langford, John Sillitoe on behalf of the Wellcome Sanger Institute COVID-19 Surveillance Team
EPI_ISL_822209	Lighthouse Lab in Alderley Park	Wellcome Sanger Institute for the COVID-19 Genomics UK (COG-UK) Consortium	Jacquelyn Wynn, Mairead Hyland, The Lighthouse Lab in Alderley Park and Alex Alderton, Roberto Amato, Sonia Goncalves, Ewan Harrison, David K. Jackson, Ian Johnston, Dominic Kwiatkowski, Cordelia Langford, John Sillitoe on behalf of the Wellcome Sanger Institute COVID-19 Surveillance Team
EPI_ISL_822214, EPI_ISL_822223, EPI_ISL_822230, EPI_ISL_822237, EPI_ISL_822240, EPI_ISL_822242, EPI_ISL_822243, EPI_ISL_822244, EPI_ISL_822245, EPI_ISL_822247, EPI_ISL_822249, EPI_ISL_822253, EPI_ISL_822256, EPI_ISL_822260, EPI_ISL_822264, EPI_ISL_822265, EPI_ISL_822267, EPI_ISL_822268, EPI_ISL_822272, EPI_ISL_822275, EPI_ISL_822281, EPI_ISL_822282, EPI_ISL_822288			
see above	Lighthouse Lab in Glasgow	Wellcome Sanger Institute for the COVID-19 Genomics UK (COG-UK) Consortium	Harper VanSteenhouse, Yumi Kasai, David Gray, Carol Clugston, Anna Dominiczak and Alex Alderton, Roberto Amato, Sonia Goncalves, Ewan Harrison, David K. Jackson, Ian Johnston, Dominic Kwiatkowski, Cordelia Langford, John Sillitoe on behalf of the Wellcome Sanger Institute COVID-19 Surveillance Team
EPI_ISL_822816, EPI_ISL_822817, EPI_ISL_822818, EPI_ISL_822819, EPI_ISL_822820, EPI_ISL_822821, EPI_ISL_822822, EPI_ISL_822823, EPI_ISL_822824, EPI_ISL_822825, EPI_ISL_822826, EPI_ISL_822827, EPI_ISL_822828, EPI_ISL_822829, EPI_ISL_822830, EPI_ISL_822831, EPI_ISL_822832, EPI_ISL_822833, EPI_ISL_822834, EPI_ISL_822835, EPI_ISL_822836			
see above	Wales Specialist Virology Centre Sequencing lab: Pathogen Genomics Unit	COVID-19 Genomics UK (COG-UK) Consortium	Catherine Moore, Johnathan Evans, Laura Gifford, Malorie Perry, Simon Cottrell, Angela Marchbank, Alec Birchley, Alexander Adams, Amy Gaskin, Bree Gatica-Wilcox, Jason Coombes, Joel Southgate, Lauren Gilbert, Lee Graham, Nicole Pacchiarini, Sara Kumziene-Summerhayes, Sarah Taylor, Sophie Jones, Sara Rey, Matthew Bull, Joanne Watkins, Sally Corden, Tom Connor
EPI_ISL_826466	Lighthouse Lab in Milton Keynes	Wellcome Sanger Institute for the COVID-19 Genomics UK (COG-UK) Consortium	The Lighthouse Lab in Milton Keynes and Alex Alderton, Roberto Amato, Sonia Goncalves, Ewan Harrison, David K. Jackson, Ian Johnston, Dominic Kwiatkowski, Cordelia Langford, John Sillitoe on behalf of the Wellcome Sanger Institute COVID-19 Surveillance Team
EPI_ISL_826468, EPI_ISL_826469, EPI_ISL_826470, EPI_ISL_826471, EPI_ISL_826474	Lighthouse Lab in Alderley Park	Wellcome Sanger Institute for the COVID-19 Genomics UK (COG-UK) Consortium	Jacquelyn Wynn, Mairead Hyland, The Lighthouse Lab in Alderley Park and Alex Alderton, Roberto Amato, Sonia Goncalves, Ewan Harrison, David K. Jackson, Ian Johnston, Dominic Kwiatkowski, Cordelia Langford, John Sillitoe on behalf of the Wellcome Sanger Institute COVID-19 Surveillance Team
EPI_ISL_826477	Lighthouse Lab in Glasgow	Wellcome Sanger Institute for the COVID-19 Genomics UK (COG-UK) Consortium	Harper VanSteenhouse, Yumi Kasai, David Gray, Carol Clugston, Anna Dominiczak and Alex Alderton, Roberto Amato, Sonia Goncalves, Ewan Harrison, David K. Jackson, Ian Johnston, Dominic Kwiatkowski, Cordelia Langford, John Sillitoe on behalf of the Wellcome Sanger Institute COVID-19 Surveillance Team
EPI_ISL_826478	Lighthouse Lab in Alderley Park	Wellcome Sanger Institute for the COVID-19 Genomics UK (COG-UK) Consortium	Jacquelyn Wynn, Mairead Hyland, The Lighthouse Lab in Alderley Park and Alex Alderton, Roberto Amato, Sonia Goncalves, Ewan Harrison, David K. Jackson, Ian Johnston, Dominic Kwiatkowski, Cordelia Langford, John Sillitoe on behalf of the Wellcome Sanger Institute COVID-19 Surveillance Team
EPI_ISL_826479	Lighthouse Lab in Glasgow	Wellcome Sanger Institute for the COVID-19 Genomics UK (COG-UK) Consortium	Harper VanSteenhouse, Yumi Kasai, David Gray, Carol Clugston, Anna Dominiczak and Alex Alderton, Roberto Amato, Sonia Goncalves, Ewan Harrison, David K. Jackson, Ian Johnston, Dominic Kwiatkowski, Cordelia Langford, John Sillitoe on behalf of the Wellcome Sanger Institute COVID-19 Surveillance Team
EPI_ISL_833586, EPI_ISL_833590, EPI_ISL_833593, EPI_ISL_833596, EPI_ISL_833597, EPI_ISL_833599, EPI_ISL_833602, EPI_ISL_833604, EPI_ISL_833606, EPI_ISL_833607, EPI_ISL_833611, EPI_ISL_833612, EPI_ISL_833614, EPI_ISL_833615, EPI_ISL_833617, EPI_ISL_833618, EPI_ISL_833619, EPI_ISL_833620, EPI_ISL_833621			
see above	Lighthouse Lab in Milton Keynes	Wellcome Sanger Institute for the COVID-19 Genomics UK (COG-UK) Consortium	The Lighthouse Lab in Milton Keynes and Alex Alderton, Roberto Amato, Sonia Goncalves, Ewan Harrison, David K. Jackson, Ian Johnston, Dominic Kwiatkowski, Cordelia Langford, John Sillitoe on behalf of the Wellcome Sanger Institute COVID-19 Surveillance Team
EPI_ISL_833626	Lighthouse Lab in Cambridge	Wellcome Sanger Institute for the COVID-19 Genomics UK (COG-UK) Consortium	Rob Howes, The Lighthouse Lab in Cambridge and Alex Alderton, Roberto Amato, Sonia Goncalves, Ewan Harrison, David K. Jackson, Ian Johnston, Dominic Kwiatkowski, Cordelia Langford, John Sillitoe on behalf of the Wellcome Sanger Institute COVID-19 Surveillance Team
EPI_ISL_833629	Lighthouse Lab in Milton Keynes	Wellcome Sanger Institute for the COVID-19 Genomics UK (COG-UK) Consortium	The Lighthouse Lab in Milton Keynes and Alex Alderton, Roberto Amato, Sonia Goncalves, Ewan Harrison, David K. Jackson, Ian Johnston, Dominic Kwiatkowski, Cordelia Langford, John Sillitoe on behalf of the Wellcome Sanger Institute COVID-19 Surveillance Team
EPI_ISL_833630, EPI_ISL_833631	Lighthouse Lab in Cambridge	Wellcome Sanger Institute for the COVID-19 Genomics UK (COG-UK) Consortium	Rob Howes, The Lighthouse Lab in Cambridge and Alex Alderton, Roberto Amato, Sonia Goncalves, Ewan Harrison, David K. Jackson, Ian Johnston, Dominic Kwiatkowski, Cordelia Langford, John Sillitoe on behalf of the Wellcome Sanger Institute COVID-19 Surveillance Team
EPI_ISL_833632, EPI_ISL_833634, EPI_ISL_833643, EPI_ISL_833647, EPI_ISL_833648, EPI_ISL_833652	Lighthouse Lab in Milton Keynes	Wellcome Sanger Institute for the COVID-19 Genomics UK (COG-UK) Consortium	The Lighthouse Lab in Milton Keynes and Alex Alderton, Roberto Amato, Sonia Goncalves, Ewan Harrison, David K. Jackson, Ian Johnston, Dominic Kwiatkowski, Cordelia Langford, John Sillitoe on behalf of the Wellcome Sanger Institute COVID-19 Surveillance Team
EPI_ISL_833654	Lighthouse Lab in Cambridge	Wellcome Sanger Institute for the COVID-19 Genomics UK (COG-UK) Consortium	Rob Howes, The Lighthouse Lab in Cambridge and Alex Alderton, Roberto Amato, Sonia Goncalves, Ewan Harrison, David K. Jackson, Ian Johnston, Dominic Kwiatkowski, Cordelia Langford, John Sillitoe on behalf of the Wellcome Sanger Institute COVID-19 Surveillance Team
EPI_ISL_833656, EPI_ISL_833658	Lighthouse Lab in Milton Keynes	Wellcome Sanger Institute for the COVID-19 Genomics UK (COG-UK) Consortium	The Lighthouse Lab in Milton Keynes and Alex Alderton, Roberto Amato, Sonia Goncalves, Ewan Harrison, David K. Jackson, Ian Johnston, Dominic Kwiatkowski, Cordelia Langford, John Sillitoe on behalf of the Wellcome Sanger Institute COVID-19 Surveillance Team
EPI_ISL_833662	Lighthouse Lab in Cambridge	Wellcome Sanger Institute for the COVID-19 Genomics UK (COG-UK) Consortium	Rob Howes, The Lighthouse Lab in Cambridge and Alex Alderton, Roberto Amato, Sonia Goncalves, Ewan Harrison, David K. Jackson, Ian Johnston, Dominic Kwiatkowski, Cordelia Langford, John Sillitoe on behalf of the Wellcome Sanger Institute COVID-19 Surveillance Team
EPI_ISL_833667	Lighthouse Lab in Milton Keynes	Wellcome Sanger Institute for the COVID-19 Genomics UK (COG-UK) Consortium	The Lighthouse Lab in Milton Keynes and Alex Alderton, Roberto Amato, Sonia Goncalves, Ewan Harrison, David K. Jackson, Ian Johnston, Dominic Kwiatkowski, Cordelia Langford, John Sillitoe on behalf of the Wellcome Sanger Institute COVID-19 Surveillance Team
EPI_ISL_833671	Lighthouse Lab in Cambridge	Wellcome Sanger Institute for the COVID-19 Genomics UK (COG-UK) Consortium	Rob Howes, The Lighthouse Lab in Cambridge and Alex Alderton, Roberto Amato, Sonia Goncalves, Ewan Harrison, David K. Jackson, Ian Johnston, Dominic Kwiatkowski, Cordelia Langford, John Sillitoe on behalf of the Wellcome Sanger Institute COVID-19 Surveillance Team
EPI_ISL_833676, EPI_ISL_833678	Lighthouse Lab in Milton Keynes	Wellcome Sanger Institute for the COVID-19 Genomics UK (COG-UK) Consortium	The Lighthouse Lab in Milton Keynes and Alex Alderton, Roberto Amato, Sonia Goncalves, Ewan Harrison, David K. Jackson, Ian Johnston, Dominic Kwiatkowski, Cordelia Langford, John Sillitoe on behalf of the Wellcome Sanger Institute COVID-19 Surveillance Team
EPI_ISL_833679, EPI_ISL_833680, EPI_ISL_833683	Lighthouse Lab in Cambridge	Wellcome Sanger Institute for the COVID-19 Genomics UK (COG-UK) Consortium	Rob Howes, The Lighthouse Lab in Cambridge and Alex Alderton, Roberto Amato, Sonia Goncalves, Ewan Harrison, David K. Jackson, Ian Johnston, Dominic Kwiatkowski, Cordelia Langford, John Sillitoe on behalf of the Wellcome Sanger Institute COVID-19 Surveillance Team
EPI_ISL_833685, EPI_ISL_833693, EPI_ISL_833700	Lighthouse Lab in Milton Keynes	Wellcome Sanger Institute for the COVID-19 Genomics UK (COG-UK) Consortium	The Lighthouse Lab in Milton Keynes and Alex Alderton, Roberto Amato, Sonia Goncalves, Ewan Harrison, David K. Jackson, Ian Johnston, Dominic Kwiatkowski, Cordelia Langford, John Sillitoe on behalf of the Wellcome Sanger Institute COVID-19 Surveillance Team
EPI_ISL_833707	Lighthouse Lab in Cambridge	Wellcome Sanger Institute for the COVID-19 Genomics UK	Rob Howes, The Lighthouse Lab in Cambridge and Alex Alderton, Roberto Amato, Sonia Goncalves, Ewan Harrison, David K. Jackson, Ian Johnston,

		(COG-UK) Consortium	Dominic Kwiatkowski, Cordelia Langford, John Sillitoe on behalf of the Wellcome Sanger Institute COVID-19 Surveillance Team
EPI_ISL_833712, EPI_ISL_833716, EPI_ISL_833719, EPI_ISL_833721, EPI_ISL_833725, EPI_ISL_833726, EPI_ISL_833734, EPI_ISL_833735, EPI_ISL_833736, EPI_ISL_833738, EPI_ISL_833743			
see above	Lighthouse Lab in Milton Keynes	Wellcome Sanger Institute for the COVID-19 Genomics UK (COG-UK) Consortium	The Lighthouse Lab in Milton Keynes and Alex Alderton, Roberto Amato, Sonia Goncalves, Ewan Harrison, David K. Jackson, Ian Johnston, Dominic Kwiatkowski, Cordelia Langford, John Sillitoe on behalf of the Wellcome Sanger Institute COVID-19 Surveillance Team
EPI_ISL_833746	Lighthouse Lab in Cambridge	Wellcome Sanger Institute for the COVID-19 Genomics UK (COG-UK) Consortium	Rob Howes, The Lighthouse Lab in Cambridge and Alex Alderton, Roberto Amato, Sonia Goncalves, Ewan Harrison, David K. Jackson, Ian Johnston, Dominic Kwiatkowski, Cordelia Langford, John Sillitoe on behalf of the Wellcome Sanger Institute COVID-19 Surveillance Team
EPI_ISL_833755, EPI_ISL_833756, EPI_ISL_833759, EPI_ISL_833766, EPI_ISL_833767, EPI_ISL_833768	Lighthouse Lab in Milton Keynes	Wellcome Sanger Institute for the COVID-19 Genomics UK (COG-UK) Consortium	The Lighthouse Lab in Milton Keynes and Alex Alderton, Roberto Amato, Sonia Goncalves, Ewan Harrison, David K. Jackson, Ian Johnston, Dominic Kwiatkowski, Cordelia Langford, John Sillitoe on behalf of the Wellcome Sanger Institute COVID-19 Surveillance Team
EPI_ISL_833770	Lighthouse Lab in Cambridge	Wellcome Sanger Institute for the COVID-19 Genomics UK (COG-UK) Consortium	Rob Howes, The Lighthouse Lab in Cambridge and Alex Alderton, Roberto Amato, Sonia Goncalves, Ewan Harrison, David K. Jackson, Ian Johnston, Dominic Kwiatkowski, Cordelia Langford, John Sillitoe on behalf of the Wellcome Sanger Institute COVID-19 Surveillance Team
EPI_ISL_833779, EPI_ISL_833780, EPI_ISL_833789, EPI_ISL_833791, EPI_ISL_833795, EPI_ISL_833796, EPI_ISL_833797, EPI_ISL_833801, EPI_ISL_833803, EPI_ISL_833804, EPI_ISL_833809, EPI_ISL_833814, EPI_ISL_833817, EPI_ISL_833818, EPI_ISL_833821, EPI_ISL_833823, EPI_ISL_833830			
see above	Lighthouse Lab in Milton Keynes	Wellcome Sanger Institute for the COVID-19 Genomics UK (COG-UK) Consortium	The Lighthouse Lab in Milton Keynes and Alex Alderton, Roberto Amato, Sonia Goncalves, Ewan Harrison, David K. Jackson, Ian Johnston, Dominic Kwiatkowski, Cordelia Langford, John Sillitoe on behalf of the Wellcome Sanger Institute COVID-19 Surveillance Team
EPI_ISL_833833	Lighthouse Lab in Cambridge	Wellcome Sanger Institute for the COVID-19 Genomics UK (COG-UK) Consortium	Rob Howes, The Lighthouse Lab in Cambridge and Alex Alderton, Roberto Amato, Sonia Goncalves, Ewan Harrison, David K. Jackson, Ian Johnston, Dominic Kwiatkowski, Cordelia Langford, John Sillitoe on behalf of the Wellcome Sanger Institute COVID-19 Surveillance Team
EPI_ISL_833834, EPI_ISL_833835, EPI_ISL_833841	Lighthouse Lab in Milton Keynes	Wellcome Sanger Institute for the COVID-19 Genomics UK (COG-UK) Consortium	The Lighthouse Lab in Milton Keynes and Alex Alderton, Roberto Amato, Sonia Goncalves, Ewan Harrison, David K. Jackson, Ian Johnston, Dominic Kwiatkowski, Cordelia Langford, John Sillitoe on behalf of the Wellcome Sanger Institute COVID-19 Surveillance Team
EPI_ISL_833842, EPI_ISL_833844	Lighthouse Lab in Cambridge	Wellcome Sanger Institute for the COVID-19 Genomics UK (COG-UK) Consortium	Rob Howes, The Lighthouse Lab in Cambridge and Alex Alderton, Roberto Amato, Sonia Goncalves, Ewan Harrison, David K. Jackson, Ian Johnston, Dominic Kwiatkowski, Cordelia Langford, John Sillitoe on behalf of the Wellcome Sanger Institute COVID-19 Surveillance Team
EPI_ISL_833845, EPI_ISL_833846, EPI_ISL_833848	Lighthouse Lab in Milton Keynes	Wellcome Sanger Institute for the COVID-19 Genomics UK (COG-UK) Consortium	The Lighthouse Lab in Milton Keynes and Alex Alderton, Roberto Amato, Sonia Goncalves, Ewan Harrison, David K. Jackson, Ian Johnston, Dominic Kwiatkowski, Cordelia Langford, John Sillitoe on behalf of the Wellcome Sanger Institute COVID-19 Surveillance Team
EPI_ISL_833849, EPI_ISL_833855	Lighthouse Lab in Cambridge	Wellcome Sanger Institute for the COVID-19 Genomics UK (COG-UK) Consortium	Rob Howes, The Lighthouse Lab in Cambridge and Alex Alderton, Roberto Amato, Sonia Goncalves, Ewan Harrison, David K. Jackson, Ian Johnston, Dominic Kwiatkowski, Cordelia Langford, John Sillitoe on behalf of the Wellcome Sanger Institute COVID-19 Surveillance Team
EPI_ISL_833856	Lighthouse Lab in Milton Keynes	Wellcome Sanger Institute for the COVID-19 Genomics UK (COG-UK) Consortium	The Lighthouse Lab in Milton Keynes and Alex Alderton, Roberto Amato, Sonia Goncalves, Ewan Harrison, David K. Jackson, Ian Johnston, Dominic Kwiatkowski, Cordelia Langford, John Sillitoe on behalf of the Wellcome Sanger Institute COVID-19 Surveillance Team
EPI_ISL_833859, EPI_ISL_833860	Lighthouse Lab in Cambridge	Wellcome Sanger Institute for the COVID-19 Genomics UK (COG-UK) Consortium	Rob Howes, The Lighthouse Lab in Cambridge and Alex Alderton, Roberto Amato, Sonia Goncalves, Ewan Harrison, David K. Jackson, Ian Johnston, Dominic Kwiatkowski, Cordelia Langford, John Sillitoe on behalf of the Wellcome Sanger Institute COVID-19 Surveillance Team
EPI_ISL_833861, EPI_ISL_833865, EPI_ISL_833868, EPI_ISL_833872, EPI_ISL_833879, EPI_ISL_833881, EPI_ISL_833884, EPI_ISL_833889, EPI_ISL_833890, EPI_ISL_833894, EPI_ISL_833895			
see above	Lighthouse Lab in Milton Keynes	Wellcome Sanger Institute for the COVID-19 Genomics UK (COG-UK) Consortium	The Lighthouse Lab in Milton Keynes and Alex Alderton, Roberto Amato, Sonia Goncalves, Ewan Harrison, David K. Jackson, Ian Johnston, Dominic Kwiatkowski, Cordelia Langford, John Sillitoe on behalf of the Wellcome Sanger Institute COVID-19 Surveillance Team
EPI_ISL_833896	Lighthouse Lab in Cambridge	Wellcome Sanger Institute for the COVID-19 Genomics UK (COG-UK) Consortium	Rob Howes, The Lighthouse Lab in Cambridge and Alex Alderton, Roberto Amato, Sonia Goncalves, Ewan Harrison, David K. Jackson, Ian Johnston, Dominic Kwiatkowski, Cordelia Langford, John Sillitoe on behalf of the Wellcome Sanger Institute COVID-19 Surveillance Team
EPI_ISL_833901	Lighthouse Lab in Milton Keynes	Wellcome Sanger Institute for the COVID-19 Genomics UK (COG-UK) Consortium	The Lighthouse Lab in Milton Keynes and Alex Alderton, Roberto Amato, Sonia Goncalves, Ewan Harrison, David K. Jackson, Ian Johnston, Dominic Kwiatkowski, Cordelia Langford, John Sillitoe on behalf of the Wellcome Sanger Institute COVID-19 Surveillance Team
EPI_ISL_833903	Lighthouse Lab in Cambridge	Wellcome Sanger Institute for the COVID-19 Genomics UK (COG-UK) Consortium	Rob Howes, The Lighthouse Lab in Cambridge and Alex Alderton, Roberto Amato, Sonia Goncalves, Ewan Harrison, David K. Jackson, Ian Johnston, Dominic Kwiatkowski, Cordelia Langford, John Sillitoe on behalf of the Wellcome Sanger Institute COVID-19 Surveillance Team
EPI_ISL_833908	Lighthouse Lab in Milton Keynes	Wellcome Sanger Institute for the COVID-19 Genomics UK (COG-UK) Consortium	The Lighthouse Lab in Milton Keynes and Alex Alderton, Roberto Amato, Sonia Goncalves, Ewan Harrison, David K. Jackson, Ian Johnston, Dominic Kwiatkowski, Cordelia Langford, John Sillitoe on behalf of the Wellcome Sanger Institute COVID-19 Surveillance Team
EPI_ISL_833909, EPI_ISL_833910	Lighthouse Lab in Cambridge	Wellcome Sanger Institute for the COVID-19 Genomics UK (COG-UK) Consortium	Rob Howes, The Lighthouse Lab in Cambridge and Alex Alderton, Roberto Amato, Sonia Goncalves, Ewan Harrison, David K. Jackson, Ian Johnston, Dominic Kwiatkowski, Cordelia Langford, John Sillitoe on behalf of the Wellcome Sanger Institute COVID-19 Surveillance Team
EPI_ISL_833916, EPI_ISL_833922, EPI_ISL_833924, EPI_ISL_833930, EPI_ISL_833931	Lighthouse Lab in Milton Keynes	Wellcome Sanger Institute for the COVID-19 Genomics UK (COG-UK) Consortium	The Lighthouse Lab in Milton Keynes and Alex Alderton, Roberto Amato, Sonia Goncalves, Ewan Harrison, David K. Jackson, Ian Johnston, Dominic Kwiatkowski, Cordelia Langford, John Sillitoe on behalf of the Wellcome Sanger Institute COVID-19 Surveillance Team
EPI_ISL_833933, EPI_ISL_833934, EPI_ISL_833935, EPI_ISL_833936, EPI_ISL_833937, EPI_ISL_833938, EPI_ISL_833939, EPI_ISL_833940, EPI_ISL_833941, EPI_ISL_833942, EPI_ISL_833943, EPI_ISL_833944, EPI_ISL_833945, EPI_ISL_833946, EPI_ISL_833947, EPI_ISL_833948, EPI_ISL_833949, EPI_ISL_833950, EPI_ISL_833951, EPI_ISL_833952, EPI_ISL_833953, EPI_ISL_833954, EPI_ISL_833955, EPI_ISL_833956, EPI_ISL_833957, EPI_ISL_833958, EPI_ISL_833959, EPI_ISL_833960, EPI_ISL_833961, EPI_ISL_833962, EPI_ISL_833963, EPI_ISL_833964, EPI_ISL_833965, EPI_ISL_833966, EPI_ISL_833967, EPI_ISL_833968, EPI_ISL_833969, EPI_ISL_833970, EPI_ISL_833971, EPI_ISL_833972, EPI_ISL_833973, EPI_ISL_833974, EPI_ISL_833975, EPI_ISL_833976, EPI_ISL_833977, EPI_ISL_833978, EPI_ISL_833979, EPI_ISL_833980, EPI_ISL_833981, EPI_ISL_833982, EPI_ISL_833983, EPI_ISL_833984, EPI_ISL_833985, EPI_ISL_833986, EPI_ISL_833987, EPI_ISL_833988, EPI_ISL_833989, EPI_ISL_833990, EPI_ISL_833991, EPI_ISL_833992, EPI_ISL_833993, EPI_ISL_833994, EPI_ISL_833995, EPI_ISL_833996, EPI_ISL_833997, EPI_ISL_833998, EPI_ISL_833999, EPI_ISL_834000, EPI_ISL_834001, EPI_ISL_834002, EPI_ISL_834003, EPI_ISL_834004, EPI_ISL_834005, EPI_ISL_834006, EPI_ISL_834007, EPI_ISL_834008, EPI_ISL_834009, EPI_ISL_834010, EPI_ISL_834011, EPI_ISL_834012, EPI_ISL_834013, EPI_ISL_834014, EPI_ISL_834015, EPI_ISL_834016, EPI_ISL_834017, EPI_ISL_834018, EPI_ISL_834019, EPI_ISL_834020, EPI_ISL_834021, EPI_ISL_834022, EPI_ISL_834023, EPI_ISL_834024, EPI_ISL_834025, EPI_ISL_834026, EPI_ISL_834027, EPI_ISL_834028, EPI_ISL_834029, EPI_ISL_834030, EPI_ISL_834031, EPI_ISL_834032, EPI_ISL_834033, EPI_ISL_834034, EPI_ISL_834035, EPI_ISL_834036, EPI_ISL_834037, EPI_ISL_834038, EPI_ISL_834039, EPI_ISL_834040, EPI_ISL_834041, EPI_ISL_834042, EPI_ISL_834043, EPI_ISL_834044, EPI_ISL_834045, EPI_ISL_834046, EPI_ISL_834047, EPI_ISL_834048, EPI_ISL_834049, EPI_ISL_834050, EPI_ISL_834051, EPI_ISL_834052, EPI_ISL_834053, EPI_ISL_834054, EPI_ISL_834055, EPI_ISL_834056, EPI_ISL_834057, EPI_ISL_834058, EPI_ISL_834059, EPI_ISL_834060, EPI_ISL_834061, EPI_ISL_834062, EPI_ISL_834063, EPI_ISL_834064, EPI_ISL_834065, EPI_ISL_834066, EPI_ISL_834067, EPI_ISL_834068, EPI_ISL_834069, EPI_ISL_834070, EPI_ISL_834071, EPI_ISL_834072, EPI_ISL_834073, EPI_ISL_834074, EPI_ISL_834075, EPI_ISL_834076, EPI_ISL_834077, EPI_ISL_834078, EPI_ISL_834079, EPI_ISL_834080, EPI_ISL_834081, EPI_ISL_834082, EPI_ISL_834083, EPI_ISL_834084, EPI_ISL_834085, EPI_ISL_834086, EPI_ISL_834087, EPI_ISL_834088, EPI_ISL_834089, EPI_ISL_834090, EPI_ISL_834091, EPI_ISL_834092, EPI_ISL_834093, EPI_ISL_834094, EPI_ISL_834095, EPI_ISL_834096, EPI_ISL_834097, EPI_ISL_834098, EPI_ISL_834099, EPI_ISL_834100, EPI_ISL_834101, EPI_ISL_834102, EPI_ISL_834103, EPI_ISL_834104, EPI_ISL_834105, EPI_ISL_834106, EPI_ISL_834107, EPI_ISL_834108, EPI_ISL_834109, EPI_ISL_834110, EPI_ISL_834111, EPI_ISL_834112, EPI_ISL_834113, EPI_ISL_834114, EPI_ISL_834115, EPI_ISL_834116, EPI_ISL_834117, EPI_ISL_834118, EPI_ISL_834119, EPI_ISL_834120, EPI_ISL_834121, EPI_ISL_834122, EPI_ISL_834123, EPI_ISL_834124, EPI_ISL_834125, EPI_ISL_834126, EPI_ISL_834127, EPI_ISL_834128, EPI_ISL_834129, EPI_ISL_834130, EPI_ISL_834131, EPI_ISL_834132, EPI_ISL_834133, EPI_ISL_834134, EPI_ISL_834135, EPI_ISL_834136, EPI_ISL_834137, EPI_ISL_834138, EPI_ISL_834139, EPI_ISL_834140, EPI_ISL_834141, EPI_ISL_834142, EPI_ISL_834143, EPI_ISL_834144, EPI_ISL_834145, EPI_ISL_834147, EPI_ISL_834148, EPI_ISL_834149, EPI_ISL_834151, EPI_ISL_834152, EPI_ISL_834153, EPI_ISL_834154, EPI_ISL_834155, EPI_ISL_834156, EPI_ISL_834157, EPI_ISL_834158, EPI_ISL_834159, EPI_ISL_834160, EPI_ISL_834161, EPI_ISL_834162, EPI_ISL_834163, EPI_ISL_834164, EPI_ISL_834165, EPI_ISL_834166, EPI_ISL_834167, EPI_ISL_834168, EPI_ISL_834169, EPI_ISL_834170, EPI_ISL_834171, EPI_ISL_834172, EPI_ISL_834173, EPI_ISL_834174, EPI_ISL_834175, EPI_ISL_834176, EPI_ISL_834177, EPI_ISL_834178, EPI_ISL_834179, EPI_ISL_834180, EPI_ISL_834181, EPI_ISL_834182, EPI_ISL_834183, EPI_ISL_834184, EPI_ISL_834185, EPI_ISL_834186, EPI_ISL_834187, EPI_ISL_834188, EPI_ISL_834189, EPI_ISL_834190, EPI_ISL_834191, EPI_ISL_834192, EPI_ISL_834193, EPI_ISL_834194, EPI_ISL_834195, EPI_ISL_834196, EPI_ISL_834197, EPI_ISL_834198, EPI_ISL_834199, EPI_ISL_834200, EPI_ISL_834201, EPI_ISL_834202, EPI_ISL_834203, EPI_ISL_834204, EPI_ISL_834205, EPI_ISL_834206, EPI_ISL_834207, EPI_ISL_834208, EPI_ISL_834209, EPI_ISL_834210, EPI_ISL_834213, EPI_ISL_834214, EPI_ISL_834215, EPI_ISL_834216, EPI_ISL_834217, EPI_ISL_834218, EPI_ISL_834219, EPI_ISL_834220, EPI_ISL_834221, EPI_ISL_834222, EPI_ISL_834224, EPI_ISL_834225, EPI_ISL_834227, EPI_ISL_834229, EPI_ISL_834232, EPI_ISL_834234, EPI_ISL_834238, EPI_ISL_834239, EPI_ISL_834240, EPI_ISL_834241, EPI_ISL_834242, EPI_ISL_834243, EPI_ISL_834244, EPI_ISL_834245, EPI_ISL_834247, EPI_ISL_834249, EPI_ISL_834251, EPI_ISL_834252, EPI_ISL_834253, EPI_ISL_834254, EPI_ISL_834255, EPI_ISL_834257, EPI_ISL_834258, EPI_ISL_834259, EPI_ISL_834260, EPI_ISL_834261, EPI_ISL_834262, EPI_ISL_834265, EPI_ISL_834266, EPI_ISL_834267, EPI_ISL_834268, EPI_ISL_834269, EPI_ISL_834270, EPI_ISL_834272, EPI_ISL_834273, EPI_ISL_834275, EPI_ISL_834276, EPI_ISL_834277, EPI_ISL_834278, EPI_ISL_834279, EPI_ISL_834281, EPI_ISL_834282, EPI_ISL_834284, EPI_ISL_834285, EPI_ISL_834286, EPI_ISL_834287, EPI_ISL_834289, EPI_ISL_834291, EPI_ISL_834292, EPI_ISL_834294, EPI_ISL_834295, EPI_ISL_834296, EPI_ISL_834297, EPI_ISL_834300, EPI_ISL_834301, EPI_ISL_834302, EPI_ISL_834303, EPI_ISL_834304, EPI_ISL_834305, EPI_ISL_834306, EPI_ISL_834307, EPI_ISL_834308, EPI_ISL_834309, EPI_ISL_834310, EPI_ISL_834314, EPI_ISL_834315, EPI_ISL_834316, EPI_ISL_834317, EPI_ISL_834318, EPI_ISL_834319, EPI_ISL_834320, EPI_ISL_834321, EPI_ISL_834322, EPI_ISL_834323, EPI_ISL_834324, EPI_ISL_834325, EPI_ISL_834326, EPI_ISL_834327, EPI_ISL_834328, EPI_ISL_834329, EPI_ISL_834330, EPI_ISL_834331, EPI_ISL_834332, EPI_ISL_834333, EPI_ISL_834334, EPI_ISL_834335, EPI_ISL_834336, EPI_ISL_834337, EPI_ISL_834338, EPI_ISL_834339, EPI_ISL_834340, EPI_ISL_834341, EPI_ISL_834342, EPI_ISL_834345, EPI_ISL_834346, EPI_ISL_834347, EPI_ISL_834348, EPI_ISL_834349, EPI_ISL_834350, EPI_ISL_834351, EPI_ISL_834354, EPI_ISL_834355, EPI_ISL_834356, EPI_ISL_834357, EPI_ISL_834358, EPI_ISL_834359, EPI_ISL_834361, EPI_ISL_834362, EPI_ISL_834363, EPI_ISL_834364, EPI_ISL_834365, EPI_ISL_834366, EPI_ISL_834367, EPI_ISL_834369, EPI_ISL_834371, EPI_ISL_834372, EPI_ISL_834374, EPI_ISL_834375, EPI_ISL_834379, EPI_ISL_834383, EPI_ISL_834384, EPI_ISL_834385, EPI_ISL_834386, EPI_ISL_834387, EPI_ISL_834389, EPI_ISL_834390, EPI_ISL_834391, EPI_ISL_834392, EPI_ISL_834393, EPI_ISL_834394, EPI_ISL_834395, EPI_ISL_834396, EPI_ISL_834397, EPI_ISL_834398, EPI_ISL_834399, EPI_ISL_834400, EPI_ISL_834401, EPI_ISL_834403, EPI_ISL_834405, EPI_ISL_834406, EPI_ISL_834408, EPI_ISL_834410, EPI_ISL_834411, EPI_ISL_834412, EPI_ISL_834413, EPI_ISL_834414, EPI_ISL_834415, EPI_ISL_834416, EPI_ISL_834418, EPI_ISL_834419, EPI_ISL_834420, EPI_ISL_834421, EPI_ISL_834422, EPI_ISL_834424, EPI_ISL_834425, EPI_ISL_834426, EPI_ISL_834428, EPI_ISL_834429, EPI_ISL_834431, EPI_ISL_834432, EPI_ISL_834434, EPI_ISL_834435, EPI_ISL_834437, EPI_ISL_834438, EPI_ISL_834439, EPI_ISL_834441, EPI_ISL_834442, EPI_ISL_834444, EPI_ISL_834446, EPI_ISL_834447, EPI_ISL_834450, EPI_ISL_834451, EPI_ISL_834452, EPI_ISL_834453, EPI_ISL_834454, EPI_ISL_834455, EPI_ISL_834457, EPI_ISL_834458, EPI_ISL_834461, EPI_ISL_834462, EPI_ISL_834463, EPI_ISL_834464, EPI_ISL_834465, EPI_ISL_834469, EPI_ISL_834470, EPI_ISL_834471, EPI_ISL_834472, EPI_ISL_834473, EPI_ISL_834474, EPI_ISL_834475, EPI_ISL_834476, EPI_ISL_834477, EPI_ISL_834478, EPI_ISL_834479, EPI_ISL_834480, EPI_ISL_834481, EPI_ISL_834482, EPI_ISL_834483, EPI_ISL_834487, EPI_ISL_834489, EPI_ISL_834490, EPI_ISL_834491, EPI_ISL_834493, EPI_ISL_834495, EPI_ISL_834496, EPI_ISL_834498, EPI_ISL_834501, EPI_ISL_834502, EPI_ISL_834504, EPI_ISL_834505, EPI_ISL_834506, EPI_ISL_834508, EPI_ISL_834509, EPI_ISL_834510, EPI_ISL_834512, EPI_ISL_834513, EPI_ISL_834514, EPI_ISL_834515, EPI_ISL_834516, EPI_ISL_834518, EPI_ISL_834520, EPI_ISL_834521, EPI_ISL_834522, EPI_ISL_834523, EPI_ISL_834525, EPI_ISL_834526, EPI_ISL_834527, EPI_ISL_834528, EPI_ISL_834530, EPI_ISL_834531, EPI_ISL_834532, EPI_ISL_834533, EPI_ISL_834534, EPI_ISL_834535, EPI_ISL_834536, EPI_ISL_834537, EPI_ISL_834538, EPI_ISL_834539, EPI_ISL_834540, EPI_ISL_834542, EPI_ISL_834543, EPI_ISL_834544, EPI_ISL_834546, EPI_ISL_834547, EPI_ISL_834548, EPI_ISL_834549, EPI_ISL_834550, EPI_ISL_834551, EPI_ISL_834552			
see above	Lighthouse Lab in Alderley Park	Wellcome Sanger Institute for the COVID-19 Genomics UK	Jacquelyn Wynn, Mairead Hyland, The Lighthouse Lab in Alderley Park and Alex Alderton, Roberto Amato, Sonia Goncalves, Ewan Harrison, David K.

		(COG-UK) Consortium	Jackson, Ian Johnston, Dominic Kwiatkowski, Cordelia Langford, John Sillitoe on behalf of the Wellcome Sanger Institute COVID-19 Surveillance Team
EPI_ISL_834553, EPI_ISL_834554, EPI_ISL_834555, EPI_ISL_834556, EPI_ISL_834557, EPI_ISL_834558, EPI_ISL_834559, EPI_ISL_834560, EPI_ISL_834561, EPI_ISL_834562, EPI_ISL_834563, EPI_ISL_834564, EPI_ISL_834565, EPI_ISL_834566, EPI_ISL_834567, EPI_ISL_834568, EPI_ISL_834569, EPI_ISL_834570, EPI_ISL_834571, EPI_ISL_834572, EPI_ISL_834573, EPI_ISL_834574, EPI_ISL_834575, EPI_ISL_834576, EPI_ISL_834577, EPI_ISL_834579, EPI_ISL_834580, EPI_ISL_834581, EPI_ISL_834582, EPI_ISL_834583, EPI_ISL_834584, EPI_ISL_834585, EPI_ISL_834586, EPI_ISL_834587, EPI_ISL_834588, EPI_ISL_834589, EPI_ISL_834590, EPI_ISL_834591, EPI_ISL_834592, EPI_ISL_834593, EPI_ISL_834594, EPI_ISL_834595, EPI_ISL_834596, EPI_ISL_834597, EPI_ISL_834598, EPI_ISL_834599, EPI_ISL_834600, EPI_ISL_834601, EPI_ISL_834602, EPI_ISL_834603, EPI_ISL_834604, EPI_ISL_834605, EPI_ISL_834606, EPI_ISL_834607, EPI_ISL_834608, EPI_ISL_834609, EPI_ISL_834610, EPI_ISL_834611, EPI_ISL_834612, EPI_ISL_834613, EPI_ISL_834614, EPI_ISL_834615, EPI_ISL_834616, EPI_ISL_834617, EPI_ISL_834618, EPI_ISL_834619, EPI_ISL_834620, EPI_ISL_834621, EPI_ISL_834622, EPI_ISL_834623, EPI_ISL_834624, EPI_ISL_834625, EPI_ISL_834626, EPI_ISL_834627, EPI_ISL_834628, EPI_ISL_834629, EPI_ISL_834630, EPI_ISL_834631, EPI_ISL_834632, EPI_ISL_834633, EPI_ISL_834634, EPI_ISL_834635, EPI_ISL_834636, EPI_ISL_834637, EPI_ISL_834638, EPI_ISL_834639, EPI_ISL_834640, EPI_ISL_834641, EPI_ISL_834642, EPI_ISL_834643, EPI_ISL_834644, EPI_ISL_834645, EPI_ISL_834646, EPI_ISL_834647, EPI_ISL_834648, EPI_ISL_834649, EPI_ISL_834650, EPI_ISL_834651, EPI_ISL_834652, EPI_ISL_834653, EPI_ISL_834654, EPI_ISL_834655, EPI_ISL_834656, EPI_ISL_834657, EPI_ISL_834658, EPI_ISL_834659, EPI_ISL_834660, EPI_ISL_834661, EPI_ISL_834662, EPI_ISL_834663, EPI_ISL_834664, EPI_ISL_834665, EPI_ISL_834666, EPI_ISL_834667, EPI_ISL_834668, EPI_ISL_834669, EPI_ISL_834670, EPI_ISL_834671, EPI_ISL_834672, EPI_ISL_834673, EPI_ISL_834674, EPI_ISL_834675, EPI_ISL_834676, EPI_ISL_834677, EPI_ISL_834678, EPI_ISL_834679, EPI_ISL_834680, EPI_ISL_834681, EPI_ISL_834682, EPI_ISL_834683, EPI_ISL_834684, EPI_ISL_834685, EPI_ISL_834686, EPI_ISL_834687, EPI_ISL_834688, EPI_ISL_834689, EPI_ISL_834690, EPI_ISL_834691, EPI_ISL_834692, EPI_ISL_834693, EPI_ISL_834694, EPI_ISL_834695, EPI_ISL_834696, EPI_ISL_834697, EPI_ISL_834698, EPI_ISL_834699, EPI_ISL_834700, EPI_ISL_834701, EPI_ISL_834702, EPI_ISL_834703, EPI_ISL_834704, EPI_ISL_834705, EPI_ISL_834706, EPI_ISL_834707, EPI_ISL_834708, EPI_ISL_834709, EPI_ISL_834710, EPI_ISL_834711, EPI_ISL_834712, EPI_ISL_834713, EPI_ISL_834714, EPI_ISL_834715, EPI_ISL_834716, EPI_ISL_834717, EPI_ISL_834718, EPI_ISL_834719, EPI_ISL_834720, EPI_ISL_834721, EPI_ISL_834722, EPI_ISL_834723, EPI_ISL_834724, EPI_ISL_834725, EPI_ISL_834726, EPI_ISL_834727, EPI_ISL_834728, EPI_ISL_834729, EPI_ISL_834730, EPI_ISL_834731, EPI_ISL_834732, EPI_ISL_834733, EPI_ISL_834734, EPI_ISL_834735, EPI_ISL_834736, EPI_ISL_834737, EPI_ISL_834738, EPI_ISL_834739, EPI_ISL_834740, EPI_ISL_834741, EPI_ISL_834742, EPI_ISL_834743, EPI_ISL_834744, EPI_ISL_834745, EPI_ISL_834746, EPI_ISL_834747, EPI_ISL_834748, EPI_ISL_834749, EPI_ISL_834750, EPI_ISL_834751, EPI_ISL_834752, EPI_ISL_834753, EPI_ISL_834754, EPI_ISL_834755, EPI_ISL_834756, EPI_ISL_834757, EPI_ISL_834758, EPI_ISL_834759, EPI_ISL_834760, EPI_ISL_834761, EPI_ISL_834762, EPI_ISL_834763, EPI_ISL_834764, EPI_ISL_834765, EPI_ISL_834766, EPI_ISL_834767, EPI_ISL_834768, EPI_ISL_834769, EPI_ISL_834770, EPI_ISL_834771, EPI_ISL_834772, EPI_ISL_834773, EPI_ISL_834774, EPI_ISL_834775, EPI_ISL_834776, EPI_ISL_834777, EPI_ISL_834778, EPI_ISL_834779, EPI_ISL_834780, EPI_ISL_834781, EPI_ISL_834782, EPI_ISL_834783, EPI_ISL_834784, EPI_ISL_834785, EPI_ISL_834786, EPI_ISL_834787, EPI_ISL_834788, EPI_ISL_834789, EPI_ISL_834790, EPI_ISL_834791, EPI_ISL_834792, EPI_ISL_834793, EPI_ISL_834794, EPI_ISL_834795, EPI_ISL_834796, EPI_ISL_834797			
see above	Lighthouse Lab in Glasgow	Wellcome Sanger Institute for the COVID-19 Genomics UK (COG-UK) Consortium	Harper VanSteenhouse, Yumi Kasai, David Gray, Carol Clugston, Anna Dominiczak and Alex Alderton, Roberto Amato, Sonia Goncalves, Ewan Harrison, David K. Jackson, Ian Johnston, Dominic Kwiatkowski, Cordelia Langford, John Sillitoe on behalf of the Wellcome Sanger Institute COVID-19 Surveillance Team
EPI_ISL_834798, EPI_ISL_834799, EPI_ISL_834800, EPI_ISL_834801, EPI_ISL_834802, EPI_ISL_834803, EPI_ISL_834805, EPI_ISL_834806, EPI_ISL_834807, EPI_ISL_834808, EPI_ISL_834809, EPI_ISL_834810, EPI_ISL_834811, EPI_ISL_834812, EPI_ISL_834813, EPI_ISL_834815, EPI_ISL_834816, EPI_ISL_834817, EPI_ISL_834818, EPI_ISL_834819, EPI_ISL_834820, EPI_ISL_834821, EPI_ISL_834822, EPI_ISL_834824, EPI_ISL_834827, EPI_ISL_834828, EPI_ISL_834829, EPI_ISL_834830, EPI_ISL_834831, EPI_ISL_834832, EPI_ISL_834835, EPI_ISL_834836, EPI_ISL_834838, EPI_ISL_834839, EPI_ISL_834840, EPI_ISL_834841, EPI_ISL_834842, EPI_ISL_834843, EPI_ISL_834844, EPI_ISL_834845, EPI_ISL_834846, EPI_ISL_834847, EPI_ISL_834849, EPI_ISL_834850, EPI_ISL_834851, EPI_ISL_834852, EPI_ISL_834853, EPI_ISL_834854, EPI_ISL_834855, EPI_ISL_834856, EPI_ISL_834857, EPI_ISL_834858, EPI_ISL_834859, EPI_ISL_834860, EPI_ISL_834861, EPI_ISL_834862, EPI_ISL_834863, EPI_ISL_834864, EPI_ISL_834866, EPI_ISL_834868, EPI_ISL_834869, EPI_ISL_834871, EPI_ISL_834872, EPI_ISL_834873, EPI_ISL_834876, EPI_ISL_834877, EPI_ISL_834878, EPI_ISL_834879, EPI_ISL_834880, EPI_ISL_834881, EPI_ISL_834882, EPI_ISL_834883, EPI_ISL_834884, EPI_ISL_834885, EPI_ISL_834887, EPI_ISL_834888, EPI_ISL_834890, EPI_ISL_834891, EPI_ISL_834892, EPI_ISL_834893, EPI_ISL_834894, EPI_ISL_834895, EPI_ISL_834896, EPI_ISL_834897, EPI_ISL_834898, EPI_ISL_834899, EPI_ISL_834900, EPI_ISL_834902, EPI_ISL_834904, EPI_ISL_834905, EPI_ISL_834906, EPI_ISL_834907, EPI_ISL_834908, EPI_ISL_834911, EPI_ISL_834912, EPI_ISL_834913, EPI_ISL_834914, EPI_ISL_834915, EPI_ISL_834916, EPI_ISL_834917, EPI_ISL_834918, EPI_ISL_834919, EPI_ISL_834922, EPI_ISL_834923, EPI_ISL_834924, EPI_ISL_834926, EPI_ISL_834927, EPI_ISL_834928, EPI_ISL_834929, EPI_ISL_834930, EPI_ISL_834932, EPI_ISL_834934, EPI_ISL_834935, EPI_ISL_834936, EPI_ISL_834937, EPI_ISL_834938, EPI_ISL_834939, EPI_ISL_834940, EPI_ISL_834941, EPI_ISL_834942, EPI_ISL_834943, EPI_ISL_834944, EPI_ISL_834945, EPI_ISL_834946, EPI_ISL_834948, EPI_ISL_834950, EPI_ISL_834951, EPI_ISL_834952, EPI_ISL_834953, EPI_ISL_834954, EPI_ISL_834955, EPI_ISL_834956, EPI_ISL_834957, EPI_ISL_834958, EPI_ISL_834960, EPI_ISL_834961, EPI_ISL_834962, EPI_ISL_834964, EPI_ISL_834965, EPI_ISL_834966, EPI_ISL_834967, EPI_ISL_834968, EPI_ISL_834969, EPI_ISL_834970, EPI_ISL_834971, EPI_ISL_834973, EPI_ISL_834974, EPI_ISL_834975, EPI_ISL_834976, EPI_ISL_834977, EPI_ISL_834978, EPI_ISL_834980, EPI_ISL_834984, EPI_ISL_834985, EPI_ISL_834986, EPI_ISL_834987, EPI_ISL_834988, EPI_ISL_834990, EPI_ISL_834992, EPI_ISL_834993, EPI_ISL_834994, EPI_ISL_834995, EPI_ISL_834996, EPI_ISL_834997, EPI_ISL_834998, EPI_ISL_834999, EPI_ISL_835000, EPI_ISL_835002, EPI_ISL_835003, EPI_ISL_835005, EPI_ISL_835006, EPI_ISL_835007, EPI_ISL_835009, EPI_ISL_835010, EPI_ISL_835011, EPI_ISL_835012, EPI_ISL_835013, EPI_ISL_835014, EPI_ISL_835015, EPI_ISL_835016, EPI_ISL_835018, EPI_ISL_835019, EPI_ISL_835020, EPI_ISL_835021, EPI_ISL_835022, EPI_ISL_835023, EPI_ISL_835024, EPI_ISL_835025, EPI_ISL_835026, EPI_ISL_835027, EPI_ISL_835028, EPI_ISL_835029, EPI_ISL_835030, EPI_ISL_835032, EPI_ISL_835033, EPI_ISL_835034, EPI_ISL_835035, EPI_ISL_835036, EPI_ISL_835037, EPI_ISL_835039, EPI_ISL_835040, EPI_ISL_835042, EPI_ISL_835044, EPI_ISL_835045, EPI_ISL_835046, EPI_ISL_835048, EPI_ISL_835049, EPI_ISL_835050, EPI_ISL_835051, EPI_ISL_835052, EPI_ISL_835053, EPI_ISL_835054, EPI_ISL_835056, EPI_ISL_835057, EPI_ISL_835059, EPI_ISL_835061, EPI_ISL_835062, EPI_ISL_835063, EPI_ISL_835064, EPI_ISL_835065, EPI_ISL_835066, EPI_ISL_835068, EPI_ISL_835069, EPI_ISL_835070, EPI_ISL_835071, EPI_ISL_835072, EPI_ISL_835073, EPI_ISL_835074, EPI_ISL_835075, EPI_ISL_835076, EPI_ISL_835077, EPI_ISL_835078, EPI_ISL_835079, EPI_ISL_835080, EPI_ISL_835081, EPI_ISL_835082, EPI_ISL_835083, EPI_ISL_835084, EPI_ISL_835085, EPI_ISL_835086, EPI_ISL_835087, EPI_ISL_835088, EPI_ISL_835089, EPI_ISL_835090, EPI_ISL_835092, EPI_ISL_835093, EPI_ISL_835095, EPI_ISL_835096, EPI_ISL_835097, EPI_ISL_835098, EPI_ISL_835099, EPI_ISL_835100, EPI_ISL_835101, EPI_ISL_835102, EPI_ISL_835103, EPI_ISL_835104, EPI_ISL_835105, EPI_ISL_835106, EPI_ISL_835107, EPI_ISL_835108, EPI_ISL_835109, EPI_ISL_835110, EPI_ISL_835111, EPI_ISL_835113, EPI_ISL_835116, EPI_ISL_835117, EPI_ISL_835118, EPI_ISL_835119, EPI_ISL_835120, EPI_ISL_835121, EPI_ISL_835122, EPI_ISL_835123, EPI_ISL_835124, EPI_ISL_835125, EPI_ISL_835126, EPI_ISL_835128, EPI_ISL_835129, EPI_ISL_835131, EPI_ISL_835133, EPI_ISL_835134, EPI_ISL_835135, EPI_ISL_835136, EPI_ISL_835137, EPI_ISL_835138, EPI_ISL_835139, EPI_ISL_835141, EPI_ISL_835142			
see above	Lighthouse Lab in Alderley Park	Wellcome Sanger Institute for the COVID-19 Genomics UK (COG-UK) Consortium	Jacquelyn Wynn, Mairead Hyland, The Lighthouse Lab in Alderley Park and Alex Alderton, Roberto Amato, Sonia Goncalves, Ewan Harrison, David K. Jackson, Ian Johnston, Dominic Kwiatkowski, Cordelia Langford, John Sillitoe on behalf of the Wellcome Sanger Institute COVID-19 Surveillance Team
EPI_ISL_835143, EPI_ISL_835144, EPI_ISL_835145, EPI_ISL_835146, EPI_ISL_835147, EPI_ISL_835148, EPI_ISL_835149, EPI_ISL_835150, EPI_ISL_835151, EPI_ISL_835152, EPI_ISL_835153, EPI_ISL_835154, EPI_ISL_835155, EPI_ISL_835156, EPI_ISL_835157, EPI_ISL_835158, EPI_ISL_835159, EPI_ISL_835160, EPI_ISL_835161, EPI_ISL_835162, EPI_ISL_835163, EPI_ISL_835164, EPI_ISL_835165, EPI_ISL_835166, EPI_ISL_835167, EPI_ISL_835168, EPI_ISL_835169, EPI_ISL_835170, EPI_ISL_835171, EPI_ISL_835172, EPI_ISL_835173, EPI_ISL_835174, EPI_ISL_835175, EPI_ISL_835176, EPI_ISL_835177, EPI_ISL_835178, EPI_ISL_835179, EPI_ISL_835180, EPI_ISL_835181, EPI_ISL_835182, EPI_ISL_835183, EPI_ISL_835184, EPI_ISL_835185, EPI_ISL_835186, EPI_ISL_835187, EPI_ISL_835188, EPI_ISL_835189, EPI_ISL_835190, EPI_ISL_835191, EPI_ISL_835192, EPI_ISL_835193, EPI_ISL_835194, EPI_ISL_835195, EPI_ISL_835196, EPI_ISL_835197, EPI_ISL_835198, EPI_ISL_835199, EPI_ISL_835200, EPI_ISL_835201, EPI_ISL_835202, EPI_ISL_835203, EPI_ISL_835204, EPI_ISL_835205, EPI_ISL_835206, EPI_ISL_835207, EPI_ISL_835208, EPI_ISL_835209, EPI_ISL_835210, EPI_ISL_835211, EPI_ISL_835212, EPI_ISL_835213, EPI_ISL_835214, EPI_ISL_835215, EPI_ISL_835216, EPI_ISL_835217, EPI_ISL_835218, EPI_ISL_835219, EPI_ISL_835220, EPI_ISL_835221, EPI_ISL_835222, EPI_ISL_835223, EPI_ISL_835224, EPI_ISL_835225, EPI_ISL_835226, EPI_ISL_835227, EPI_ISL_835228, EPI_ISL_835229, EPI_ISL_835230, EPI_ISL_835231, EPI_ISL_835232, EPI_ISL_835233, EPI_ISL_835234, EPI_ISL_835235, EPI_ISL_835236, EPI_ISL_835237, EPI_ISL_835238, EPI_ISL_835239, EPI_ISL_835240, EPI_ISL_835241, EPI_ISL_835242, EPI_ISL_835243, EPI_ISL_835244, EPI_ISL_835245, EPI_ISL_835246, EPI_ISL_835247, EPI_ISL_835248, EPI_ISL_835249, EPI_ISL_835250, EPI_ISL_835251, EPI_ISL_835252, EPI_ISL_835253, EPI_ISL_835254, EPI_ISL_835255, EPI_ISL_835256, EPI_ISL_835257, EPI_ISL_835258, EPI_ISL_835259, EPI_ISL_835260, EPI_ISL_835261, EPI_ISL_835262, EPI_ISL_835263, EPI_ISL_835264, EPI_ISL_835265, EPI_ISL_835266, EPI_ISL_835267, EPI_ISL_835268, EPI_ISL_835269, EPI_ISL_835270, EPI_ISL_835271, EPI_ISL_835272, EPI_ISL_835273, EPI_ISL_835274, EPI_ISL_835275, EPI_ISL_835276, EPI_ISL_835277, EPI_ISL_835278, EPI_ISL_835279, EPI_ISL_835280, EPI_ISL_835281, EPI_ISL_835282, EPI_ISL_835283, EPI_ISL_835284, EPI_ISL_835285, EPI_ISL_835286, EPI_ISL_835287, EPI_ISL_835288, EPI_ISL_835289, EPI_ISL_835290, EPI_ISL_835291, EPI_ISL_835292, EPI_ISL_835293, EPI_ISL_835294, EPI_ISL_835295, EPI_ISL_835296, EPI_ISL_835297, EPI_ISL_835298, EPI_ISL_835299, EPI_ISL_835300, EPI_ISL_835301, EPI_ISL_835302, EPI_ISL_835303, EPI_ISL_835304, EPI_ISL_835305, EPI_ISL_835306, EPI_ISL_835307, EPI_ISL_835308, EPI_ISL_835309, EPI_ISL_835310, EPI_ISL_835311, EPI_ISL_835312, EPI_ISL_835313, EPI_ISL_835314, EPI_ISL_835315, EPI_ISL_835316, EPI_ISL_835317, EPI_ISL_835318, EPI_ISL_835319, EPI_ISL_835320, EPI_ISL_835321, EPI_ISL_835322, EPI_ISL_835323, EPI_ISL_835324, EPI_ISL_835325, EPI_ISL_835326, EPI_ISL_835327, EPI_ISL_835328, EPI_ISL_835329, EPI_ISL_835330, EPI_ISL_835331, EPI_ISL_835332, EPI_ISL_835333, EPI_ISL_835334, EPI_ISL_835335, EPI_ISL_835336, EPI_ISL_835337, EPI_ISL_835338, EPI_ISL_835339, EPI_ISL_835340, EPI_ISL_835341, EPI_ISL_835342, EPI_ISL_835343, EPI_ISL_835344, EPI_ISL_835345, EPI_ISL_835346, EPI_ISL_835347, EPI_ISL_835348, EPI_ISL_835349, EPI_ISL_835350, EPI_ISL_835351, EPI_ISL_835352, EPI_ISL_835353, EPI_ISL_835354, EPI_ISL_835355, EPI_ISL_835356, EPI_ISL_835357, EPI_ISL_835358, EPI_ISL_835359, EPI_ISL_835360, EPI_ISL_835361, EPI_ISL_835362, EPI_ISL_835363, EPI_ISL_835364, EPI_ISL_835365, EPI_ISL_835366, EPI_ISL_835367, EPI_ISL_835368, EPI_ISL_835369, EPI_ISL_835370, EPI_ISL_835371, EPI_ISL_835372, EPI_ISL_835373, EPI_ISL_835374, EPI_ISL_835375, EPI_ISL_835376, EPI_ISL_835377, EPI_ISL_835378, EPI_ISL_835379, EPI_ISL_835380, EPI_ISL_835381, EPI_ISL_835382, EPI_ISL_835383, EPI_ISL_835384, EPI_ISL_835385, EPI_ISL_835386, EPI_ISL_835387, EPI_ISL_835388, EPI_ISL_835389, EPI_ISL_835390, EPI_ISL_835391, EPI_ISL_835392, EPI_ISL_835393, EPI_ISL_835394, EPI_ISL_835395, EPI_ISL_835396, EPI_ISL_835397, EPI_ISL_835398, EPI_ISL_835399, EPI_ISL_835400, EPI_ISL_835401, EPI_ISL_835402, EPI_ISL_835403, EPI_ISL_835404, EPI_ISL_835405, EPI_ISL_835406, EPI_ISL_835407, EPI_ISL_835408, EPI_ISL_835409, EPI_ISL_835410, EPI_ISL_835411, EPI_ISL_835412, EPI_ISL_835413, EPI_ISL_835414, EPI_ISL_835415, EPI_ISL_835416, EPI_ISL_835417, EPI_ISL_835418, EPI_ISL_835419, EPI_ISL_835420, EPI_ISL_835421, EPI_ISL_835422, EPI_ISL_835423, EPI_ISL_835424, EPI_ISL_835425			
see above	Lighthouse Lab in Glasgow	Wellcome Sanger Institute for the COVID-19 Genomics UK (COG-UK) Consortium	Harper VanSteenhouse, Yumi Kasai, David Gray, Carol Clugston, Anna Dominiczak and Alex Alderton, Roberto Amato, Sonia Goncalves, Ewan Harrison, David K. Jackson, Ian Johnston, Dominic Kwiatkowski, Cordelia Langford, John Sillitoe on behalf of the Wellcome Sanger Institute COVID-19 Surveillance Team
EPI_ISL_835426, EPI_ISL_835427, EPI_ISL_835428, EPI_ISL_835429, EPI_ISL_835430, EPI_ISL_835431, EPI_ISL_835432	Lighthouse Lab in Alderley Park	Wellcome Sanger Institute for the COVID-19 Genomics UK (COG-UK) Consortium	Jacquelyn Wynn, Mairead Hyland, The Lighthouse Lab in Alderley Park and Alex Alderton, Roberto Amato, Sonia Goncalves, Ewan Harrison, David K. Jackson, Ian Johnston, Dominic Kwiatkowski, Cordelia Langford, John Sillitoe on behalf of the Wellcome Sanger Institute COVID-19 Surveillance Team
EPI_ISL_835433	Lighthouse Lab in Glasgow	Wellcome Sanger Institute for the COVID-19 Genomics UK (COG-UK) Consortium	Harper VanSteenhouse, Yumi Kasai, David Gray, Carol Clugston, Anna Dominiczak and Alex Alderton, Roberto Amato, Sonia Goncalves, Ewan Harrison, David K. Jackson, Ian Johnston, Dominic Kwiatkowski, Cordelia Langford, John Sillitoe on behalf of the Wellcome Sanger Institute COVID-19 Surveillance Team
EPI_ISL_835434, EPI_ISL_835435, EPI_ISL_835436, EPI_ISL_835437, EPI_ISL_835438	Lighthouse Lab in Alderley Park	Wellcome Sanger Institute for the COVID-19 Genomics UK (COG-UK) Consortium	Jacquelyn Wynn, Mairead Hyland, The Lighthouse Lab in Alderley Park and Alex Alderton, Roberto Amato, Sonia Goncalves, Ewan Harrison, David K. Jackson, Ian Johnston, Dominic Kwiatkowski, Cordelia Langford, John Sillitoe on behalf of the Wellcome Sanger Institute COVID-19 Surveillance Team
EPI_ISL_835439	Lighthouse Lab in Glasgow	Wellcome Sanger Institute for the COVID-19 Genomics UK (COG-UK) Consortium	Harper VanSteenhouse, Yumi Kasai, David Gray, Carol Clugston, Anna Dominiczak and Alex Alderton, Roberto Amato, Sonia Goncalves, Ewan Harrison, David K. Jackson, Ian Johnston, Dominic Kwiatkowski, Cordelia Langford, John Sillitoe on behalf of the Wellcome Sanger Institute COVID-19 Surveillance Team
EPI_ISL_835440, EPI_ISL_835441, EPI_ISL_835442, EPI_ISL_835443, EPI_ISL_835444, EPI_ISL_835445, EPI_ISL_835446	Lighthouse Lab in Alderley Park	Wellcome Sanger Institute for the COVID-19 Genomics UK (COG-UK) Consortium	Jacquelyn Wynn, Mairead Hyland, The Lighthouse Lab in Alderley Park and Alex Alderton, Roberto Amato, Sonia Goncalves, Ewan Harrison, David K. Jackson, Ian Johnston, Dominic Kwiatkowski, Cordelia Langford, John Sillitoe on behalf of the Wellcome Sanger Institute COVID-19 Surveillance Team
EPI_ISL_835447, EPI_ISL_835448	Lighthouse Lab in Glasgow	Wellcome Sanger Institute for the COVID-19 Genomics UK (COG-UK) Consortium	Harper VanSteenhouse, Yumi Kasai, David Gray, Carol Clugston, Anna Dominiczak and Alex Alderton, Roberto Amato, Sonia Goncalves, Ewan Harrison, David K. Jackson, Ian Johnston, Dominic Kwiatkowski, Cordelia Langford, John Sillitoe on behalf of the Wellcome Sanger Institute COVID-19 Surveillance Team
EPI_ISL_835449, EPI_ISL_835450, EPI_ISL_835451, EPI_ISL_835452, EPI_ISL_835453	Lighthouse Lab in Alderley Park	Wellcome Sanger Institute for the COVID-19 Genomics UK (COG-UK) Consortium	Jacquelyn Wynn, Mairead Hyland, The Lighthouse Lab in Alderley Park and Alex Alderton, Roberto Amato, Sonia Goncalves, Ewan Harrison, David K. Jackson, Ian Johnston, Dominic Kwiatkowski, Cordelia Langford, John Sillitoe on behalf of the Wellcome Sanger Institute COVID-19 Surveillance Team
EPI_ISL_835454	Lighthouse Lab in Glasgow	Wellcome Sanger Institute for the COVID-19 Genomics UK (COG-UK) Consortium	Harper VanSteenhouse, Yumi Kasai, David Gray, Carol Clugston, Anna Dominiczak and Alex Alderton, Roberto Amato, Sonia Goncalves, Ewan Harrison, David K. Jackson, Ian Johnston, Dominic Kwiatkowski, Cordelia Langford, John Sillitoe on behalf of the Wellcome Sanger Institute COVID-19 Surveillance

			Team
EPI_ISL_835455, EPI_ISL_835456	Lighthouse Lab in Alderley Park	Wellcome Sanger Institute for the COVID-19 Genomics UK (COG-UK) Consortium	Jacquelyn Wynn, Mairead Hyland, The Lighthouse Lab in Alderley Park and Alex Alderton, Roberto Amato, Sonia Goncalves, Ewan Harrison, David K. Jackson, Ian Johnston, Dominic Kwiatkowski, Cordelia Langford, John Sillitoe on behalf of the Wellcome Sanger Institute COVID-19 Surveillance Team
EPI_ISL_835457	Lighthouse Lab in Glasgow	Wellcome Sanger Institute for the COVID-19 Genomics UK (COG-UK) Consortium	Harper VanSteenhouse, Yumi Kasai, David Gray, Carol Clugston, Anna Dominiczak and Alex Alderton, Roberto Amato, Sonia Goncalves, Ewan Harrison, David K. Jackson, Ian Johnston, Dominic Kwiatkowski, Cordelia Langford, John Sillitoe on behalf of the Wellcome Sanger Institute COVID-19 Surveillance Team
EPI_ISL_835458, EPI_ISL_835459, EPI_ISL_835460, EPI_ISL_835461, EPI_ISL_835462, EPI_ISL_835463	Lighthouse Lab in Alderley Park	Wellcome Sanger Institute for the COVID-19 Genomics UK (COG-UK) Consortium	Jacquelyn Wynn, Mairead Hyland, The Lighthouse Lab in Alderley Park and Alex Alderton, Roberto Amato, Sonia Goncalves, Ewan Harrison, David K. Jackson, Ian Johnston, Dominic Kwiatkowski, Cordelia Langford, John Sillitoe on behalf of the Wellcome Sanger Institute COVID-19 Surveillance Team
EPI_ISL_835464, EPI_ISL_835465	Lighthouse Lab in Glasgow	Wellcome Sanger Institute for the COVID-19 Genomics UK (COG-UK) Consortium	Harper VanSteenhouse, Yumi Kasai, David Gray, Carol Clugston, Anna Dominiczak and Alex Alderton, Roberto Amato, Sonia Goncalves, Ewan Harrison, David K. Jackson, Ian Johnston, Dominic Kwiatkowski, Cordelia Langford, John Sillitoe on behalf of the Wellcome Sanger Institute COVID-19 Surveillance Team
EPI_ISL_835466, EPI_ISL_835467, EPI_ISL_835468, EPI_ISL_835469	Lighthouse Lab in Alderley Park	Wellcome Sanger Institute for the COVID-19 Genomics UK (COG-UK) Consortium	Jacquelyn Wynn, Mairead Hyland, The Lighthouse Lab in Alderley Park and Alex Alderton, Roberto Amato, Sonia Goncalves, Ewan Harrison, David K. Jackson, Ian Johnston, Dominic Kwiatkowski, Cordelia Langford, John Sillitoe on behalf of the Wellcome Sanger Institute COVID-19 Surveillance Team
EPI_ISL_835470	Lighthouse Lab in Glasgow	Wellcome Sanger Institute for the COVID-19 Genomics UK (COG-UK) Consortium	Harper VanSteenhouse, Yumi Kasai, David Gray, Carol Clugston, Anna Dominiczak and Alex Alderton, Roberto Amato, Sonia Goncalves, Ewan Harrison, David K. Jackson, Ian Johnston, Dominic Kwiatkowski, Cordelia Langford, John Sillitoe on behalf of the Wellcome Sanger Institute COVID-19 Surveillance Team
EPI_ISL_835471, EPI_ISL_835472, EPI_ISL_835473	Lighthouse Lab in Alderley Park	Wellcome Sanger Institute for the COVID-19 Genomics UK (COG-UK) Consortium	Jacquelyn Wynn, Mairead Hyland, The Lighthouse Lab in Alderley Park and Alex Alderton, Roberto Amato, Sonia Goncalves, Ewan Harrison, David K. Jackson, Ian Johnston, Dominic Kwiatkowski, Cordelia Langford, John Sillitoe on behalf of the Wellcome Sanger Institute COVID-19 Surveillance Team
EPI_ISL_835475	Lighthouse Lab in Glasgow	Wellcome Sanger Institute for the COVID-19 Genomics UK (COG-UK) Consortium	Harper VanSteenhouse, Yumi Kasai, David Gray, Carol Clugston, Anna Dominiczak and Alex Alderton, Roberto Amato, Sonia Goncalves, Ewan Harrison, David K. Jackson, Ian Johnston, Dominic Kwiatkowski, Cordelia Langford, John Sillitoe on behalf of the Wellcome Sanger Institute COVID-19 Surveillance Team
EPI_ISL_835476, EPI_ISL_835477, EPI_ISL_835478, EPI_ISL_835479, EPI_ISL_835480	Lighthouse Lab in Alderley Park	Wellcome Sanger Institute for the COVID-19 Genomics UK (COG-UK) Consortium	Jacquelyn Wynn, Mairead Hyland, The Lighthouse Lab in Alderley Park and Alex Alderton, Roberto Amato, Sonia Goncalves, Ewan Harrison, David K. Jackson, Ian Johnston, Dominic Kwiatkowski, Cordelia Langford, John Sillitoe on behalf of the Wellcome Sanger Institute COVID-19 Surveillance Team
EPI_ISL_835481	Lighthouse Lab in Glasgow	Wellcome Sanger Institute for the COVID-19 Genomics UK (COG-UK) Consortium	Harper VanSteenhouse, Yumi Kasai, David Gray, Carol Clugston, Anna Dominiczak and Alex Alderton, Roberto Amato, Sonia Goncalves, Ewan Harrison, David K. Jackson, Ian Johnston, Dominic Kwiatkowski, Cordelia Langford, John Sillitoe on behalf of the Wellcome Sanger Institute COVID-19 Surveillance Team
EPI_ISL_835482, EPI_ISL_835484, EPI_ISL_835485	Lighthouse Lab in Alderley Park	Wellcome Sanger Institute for the COVID-19 Genomics UK (COG-UK) Consortium	Jacquelyn Wynn, Mairead Hyland, The Lighthouse Lab in Alderley Park and Alex Alderton, Roberto Amato, Sonia Goncalves, Ewan Harrison, David K. Jackson, Ian Johnston, Dominic Kwiatkowski, Cordelia Langford, John Sillitoe on behalf of the Wellcome Sanger Institute COVID-19 Surveillance Team
EPI_ISL_835486, EPI_ISL_835487, EPI_ISL_835488	Lighthouse Lab in Glasgow	Wellcome Sanger Institute for the COVID-19 Genomics UK (COG-UK) Consortium	Harper VanSteenhouse, Yumi Kasai, David Gray, Carol Clugston, Anna Dominiczak and Alex Alderton, Roberto Amato, Sonia Goncalves, Ewan Harrison, David K. Jackson, Ian Johnston, Dominic Kwiatkowski, Cordelia Langford, John Sillitoe on behalf of the Wellcome Sanger Institute COVID-19 Surveillance Team
EPI_ISL_835489, EPI_ISL_835490, EPI_ISL_835491	Lighthouse Lab in Alderley Park	Wellcome Sanger Institute for the COVID-19 Genomics UK (COG-UK) Consortium	Jacquelyn Wynn, Mairead Hyland, The Lighthouse Lab in Alderley Park and Alex Alderton, Roberto Amato, Sonia Goncalves, Ewan Harrison, David K. Jackson, Ian Johnston, Dominic Kwiatkowski, Cordelia Langford, John Sillitoe on behalf of the Wellcome Sanger Institute COVID-19 Surveillance Team
EPI_ISL_835493	Lighthouse Lab in Glasgow	Wellcome Sanger Institute for the COVID-19 Genomics UK (COG-UK) Consortium	Harper VanSteenhouse, Yumi Kasai, David Gray, Carol Clugston, Anna Dominiczak and Alex Alderton, Roberto Amato, Sonia Goncalves, Ewan Harrison, David K. Jackson, Ian Johnston, Dominic Kwiatkowski, Cordelia Langford, John Sillitoe on behalf of the Wellcome Sanger Institute COVID-19 Surveillance Team
EPI_ISL_835494, EPI_ISL_835496	Lighthouse Lab in Alderley Park	Wellcome Sanger Institute for the COVID-19 Genomics UK (COG-UK) Consortium	Jacquelyn Wynn, Mairead Hyland, The Lighthouse Lab in Alderley Park and Alex Alderton, Roberto Amato, Sonia Goncalves, Ewan Harrison, David K. Jackson, Ian Johnston, Dominic Kwiatkowski, Cordelia Langford, John Sillitoe on behalf of the Wellcome Sanger Institute COVID-19 Surveillance Team
EPI_ISL_835497	Lighthouse Lab in Glasgow	Wellcome Sanger Institute for the COVID-19 Genomics UK (COG-UK) Consortium	Harper VanSteenhouse, Yumi Kasai, David Gray, Carol Clugston, Anna Dominiczak and Alex Alderton, Roberto Amato, Sonia Goncalves, Ewan Harrison, David K. Jackson, Ian Johnston, Dominic Kwiatkowski, Cordelia Langford, John Sillitoe on behalf of the Wellcome Sanger Institute COVID-19 Surveillance Team
EPI_ISL_835498	Lighthouse Lab in Alderley Park	Wellcome Sanger Institute for the COVID-19 Genomics UK (COG-UK) Consortium	Jacquelyn Wynn, Mairead Hyland, The Lighthouse Lab in Alderley Park and Alex Alderton, Roberto Amato, Sonia Goncalves, Ewan Harrison, David K. Jackson, Ian Johnston, Dominic Kwiatkowski, Cordelia Langford, John Sillitoe on behalf of the Wellcome Sanger Institute COVID-19 Surveillance Team
EPI_ISL_835499, EPI_ISL_835500	Lighthouse Lab in Glasgow	Wellcome Sanger Institute for the COVID-19 Genomics UK (COG-UK) Consortium	Harper VanSteenhouse, Yumi Kasai, David Gray, Carol Clugston, Anna Dominiczak and Alex Alderton, Roberto Amato, Sonia Goncalves, Ewan Harrison, David K. Jackson, Ian Johnston, Dominic Kwiatkowski, Cordelia Langford, John Sillitoe on behalf of the Wellcome Sanger Institute COVID-19 Surveillance Team
EPI_ISL_835501	Lighthouse Lab in Alderley Park	Wellcome Sanger Institute for the COVID-19 Genomics UK (COG-UK) Consortium	Jacquelyn Wynn, Mairead Hyland, The Lighthouse Lab in Alderley Park and Alex Alderton, Roberto Amato, Sonia Goncalves, Ewan Harrison, David K. Jackson, Ian Johnston, Dominic Kwiatkowski, Cordelia Langford, John Sillitoe on behalf of the Wellcome Sanger Institute COVID-19 Surveillance Team
EPI_ISL_835502, EPI_ISL_835503, EPI_ISL_835505, EPI_ISL_835506, EPI_ISL_835507	Lighthouse Lab in Glasgow	Wellcome Sanger Institute for the COVID-19 Genomics UK (COG-UK) Consortium	Harper VanSteenhouse, Yumi Kasai, David Gray, Carol Clugston, Anna Dominiczak and Alex Alderton, Roberto Amato, Sonia Goncalves, Ewan Harrison, David K. Jackson, Ian Johnston, Dominic Kwiatkowski, Cordelia Langford, John Sillitoe on behalf of the Wellcome Sanger Institute COVID-19 Surveillance Team
EPI_ISL_835508, EPI_ISL_835509, EPI_ISL_835510, EPI_ISL_835511, EPI_ISL_835512, EPI_ISL_835513, EPI_ISL_835514	Lighthouse Lab in Alderley Park	Wellcome Sanger Institute for the COVID-19 Genomics UK (COG-UK) Consortium	Jacquelyn Wynn, Mairead Hyland, The Lighthouse Lab in Alderley Park and Alex Alderton, Roberto Amato, Sonia Goncalves, Ewan Harrison, David K. Jackson, Ian Johnston, Dominic Kwiatkowski, Cordelia Langford, John Sillitoe on behalf of the Wellcome Sanger Institute COVID-19 Surveillance Team
EPI_ISL_835515	Lighthouse Lab in Glasgow	Wellcome Sanger Institute for the COVID-19 Genomics UK (COG-UK) Consortium	Harper VanSteenhouse, Yumi Kasai, David Gray, Carol Clugston, Anna Dominiczak and Alex Alderton, Roberto Amato, Sonia Goncalves, Ewan Harrison, David K. Jackson, Ian Johnston, Dominic Kwiatkowski, Cordelia Langford, John Sillitoe on behalf of the Wellcome Sanger Institute COVID-19 Surveillance Team
EPI_ISL_835516, EPI_ISL_835517	Lighthouse Lab in Alderley Park	Wellcome Sanger Institute for the COVID-19 Genomics UK (COG-UK) Consortium	Jacquelyn Wynn, Mairead Hyland, The Lighthouse Lab in Alderley Park and Alex Alderton, Roberto Amato, Sonia Goncalves, Ewan Harrison, David K. Jackson, Ian Johnston, Dominic Kwiatkowski, Cordelia Langford, John Sillitoe on behalf of the Wellcome Sanger Institute COVID-19 Surveillance Team
EPI_ISL_835518	Lighthouse Lab in Glasgow	Wellcome Sanger Institute for the COVID-19 Genomics UK (COG-UK) Consortium	Harper VanSteenhouse, Yumi Kasai, David Gray, Carol Clugston, Anna Dominiczak and Alex Alderton, Roberto Amato, Sonia Goncalves, Ewan Harrison, David K. Jackson, Ian Johnston, Dominic Kwiatkowski, Cordelia Langford, John Sillitoe on behalf of the Wellcome Sanger Institute COVID-19 Surveillance Team
EPI_ISL_835519, EPI_ISL_835520, EPI_ISL_835521, EPI_ISL_835522, EPI_ISL_835523, EPI_ISL_835524, EPI_ISL_835525, EPI_ISL_835526	Lighthouse Lab in Alderley Park	Wellcome Sanger Institute for the COVID-19 Genomics UK (COG-UK) Consortium	Jacquelyn Wynn, Mairead Hyland, The Lighthouse Lab in Alderley Park and Alex Alderton, Roberto Amato, Sonia Goncalves, Ewan Harrison, David K. Jackson, Ian Johnston, Dominic Kwiatkowski, Cordelia Langford, John Sillitoe on behalf of the Wellcome Sanger Institute COVID-19 Surveillance Team
EPI_ISL_835527	Lighthouse Lab in Glasgow	Wellcome Sanger Institute for the COVID-19 Genomics UK (COG-UK) Consortium	Harper VanSteenhouse, Yumi Kasai, David Gray, Carol Clugston, Anna Dominiczak and Alex Alderton, Roberto Amato, Sonia Goncalves, Ewan Harrison, David K. Jackson, Ian Johnston, Dominic Kwiatkowski, Cordelia Langford, John Sillitoe on behalf of the Wellcome Sanger Institute COVID-19 Surveillance Team
EPI_ISL_835528, EPI_ISL_835529	Lighthouse Lab in Alderley Park	Wellcome Sanger Institute for the COVID-19 Genomics UK (COG-UK) Consortium	Jacquelyn Wynn, Mairead Hyland, The Lighthouse Lab in Alderley Park and Alex Alderton, Roberto Amato, Sonia Goncalves, Ewan Harrison, David K. Jackson, Ian Johnston, Dominic Kwiatkowski, Cordelia Langford, John Sillitoe on behalf of the Wellcome Sanger Institute COVID-19 Surveillance Team

EPI_ISL_835530, EPI_ISL_835531, EPI_ISL_835533	Lighthouse Lab in Glasgow	Wellcome Sanger Institute for the COVID-19 Genomics UK (COG-UK) Consortium	Harper VanSteenhouse, Yumi Kasai, David Gray, Carol Clugston, Anna Dominiczak and Alex Alderton, Roberto Amato, Sonia Goncalves, Ewan Harrison, David K. Jackson, Ian Johnston, Dominic Kwiatkowski, Cordelia Langford, John Sillitoe on behalf of the Wellcome Sanger Institute COVID-19 Surveillance Team
EPI_ISL_835534	Lighthouse Lab in Alderley Park	Wellcome Sanger Institute for the COVID-19 Genomics UK (COG-UK) Consortium	Jacquelyn Wynn, Mairead Hyland, The Lighthouse Lab in Alderley Park and Alex Alderton, Roberto Amato, Sonia Goncalves, Ewan Harrison, David K. Jackson, Ian Johnston, Dominic Kwiatkowski, Cordelia Langford, John Sillitoe on behalf of the Wellcome Sanger Institute COVID-19 Surveillance Team
EPI_ISL_835535	Lighthouse Lab in Glasgow	Wellcome Sanger Institute for the COVID-19 Genomics UK (COG-UK) Consortium	Harper VanSteenhouse, Yumi Kasai, David Gray, Carol Clugston, Anna Dominiczak and Alex Alderton, Roberto Amato, Sonia Goncalves, Ewan Harrison, David K. Jackson, Ian Johnston, Dominic Kwiatkowski, Cordelia Langford, John Sillitoe on behalf of the Wellcome Sanger Institute COVID-19 Surveillance Team
EPI_ISL_835536, EPI_ISL_835537, EPI_ISL_835538	Lighthouse Lab in Alderley Park	Wellcome Sanger Institute for the COVID-19 Genomics UK (COG-UK) Consortium	Jacquelyn Wynn, Mairead Hyland, The Lighthouse Lab in Alderley Park and Alex Alderton, Roberto Amato, Sonia Goncalves, Ewan Harrison, David K. Jackson, Ian Johnston, Dominic Kwiatkowski, Cordelia Langford, John Sillitoe on behalf of the Wellcome Sanger Institute COVID-19 Surveillance Team
EPI_ISL_835539, EPI_ISL_835540, EPI_ISL_835541	Lighthouse Lab in Glasgow	Wellcome Sanger Institute for the COVID-19 Genomics UK (COG-UK) Consortium	Harper VanSteenhouse, Yumi Kasai, David Gray, Carol Clugston, Anna Dominiczak and Alex Alderton, Roberto Amato, Sonia Goncalves, Ewan Harrison, David K. Jackson, Ian Johnston, Dominic Kwiatkowski, Cordelia Langford, John Sillitoe on behalf of the Wellcome Sanger Institute COVID-19 Surveillance Team
EPI_ISL_835542, EPI_ISL_835543, EPI_ISL_835544, EPI_ISL_835545	Lighthouse Lab in Alderley Park	Wellcome Sanger Institute for the COVID-19 Genomics UK (COG-UK) Consortium	Jacquelyn Wynn, Mairead Hyland, The Lighthouse Lab in Alderley Park and Alex Alderton, Roberto Amato, Sonia Goncalves, Ewan Harrison, David K. Jackson, Ian Johnston, Dominic Kwiatkowski, Cordelia Langford, John Sillitoe on behalf of the Wellcome Sanger Institute COVID-19 Surveillance Team
EPI_ISL_835547, EPI_ISL_835548	Lighthouse Lab in Glasgow	Wellcome Sanger Institute for the COVID-19 Genomics UK (COG-UK) Consortium	Harper VanSteenhouse, Yumi Kasai, David Gray, Carol Clugston, Anna Dominiczak and Alex Alderton, Roberto Amato, Sonia Goncalves, Ewan Harrison, David K. Jackson, Ian Johnston, Dominic Kwiatkowski, Cordelia Langford, John Sillitoe on behalf of the Wellcome Sanger Institute COVID-19 Surveillance Team
EPI_ISL_835549, EPI_ISL_835551, EPI_ISL_835552	Lighthouse Lab in Alderley Park	Wellcome Sanger Institute for the COVID-19 Genomics UK (COG-UK) Consortium	Jacquelyn Wynn, Mairead Hyland, The Lighthouse Lab in Alderley Park and Alex Alderton, Roberto Amato, Sonia Goncalves, Ewan Harrison, David K. Jackson, Ian Johnston, Dominic Kwiatkowski, Cordelia Langford, John Sillitoe on behalf of the Wellcome Sanger Institute COVID-19 Surveillance Team
EPI_ISL_835553	Lighthouse Lab in Glasgow	Wellcome Sanger Institute for the COVID-19 Genomics UK (COG-UK) Consortium	Harper VanSteenhouse, Yumi Kasai, David Gray, Carol Clugston, Anna Dominiczak and Alex Alderton, Roberto Amato, Sonia Goncalves, Ewan Harrison, David K. Jackson, Ian Johnston, Dominic Kwiatkowski, Cordelia Langford, John Sillitoe on behalf of the Wellcome Sanger Institute COVID-19 Surveillance Team
EPI_ISL_835554	Lighthouse Lab in Alderley Park	Wellcome Sanger Institute for the COVID-19 Genomics UK (COG-UK) Consortium	Jacquelyn Wynn, Mairead Hyland, The Lighthouse Lab in Alderley Park and Alex Alderton, Roberto Amato, Sonia Goncalves, Ewan Harrison, David K. Jackson, Ian Johnston, Dominic Kwiatkowski, Cordelia Langford, John Sillitoe on behalf of the Wellcome Sanger Institute COVID-19 Surveillance Team
EPI_ISL_835555	Lighthouse Lab in Glasgow	Wellcome Sanger Institute for the COVID-19 Genomics UK (COG-UK) Consortium	Harper VanSteenhouse, Yumi Kasai, David Gray, Carol Clugston, Anna Dominiczak and Alex Alderton, Roberto Amato, Sonia Goncalves, Ewan Harrison, David K. Jackson, Ian Johnston, Dominic Kwiatkowski, Cordelia Langford, John Sillitoe on behalf of the Wellcome Sanger Institute COVID-19 Surveillance Team
EPI_ISL_835556, EPI_ISL_835557, EPI_ISL_835558, EPI_ISL_835559, EPI_ISL_835560, EPI_ISL_835561, EPI_ISL_835563	Lighthouse Lab in Alderley Park	Wellcome Sanger Institute for the COVID-19 Genomics UK (COG-UK) Consortium	Jacquelyn Wynn, Mairead Hyland, The Lighthouse Lab in Alderley Park and Alex Alderton, Roberto Amato, Sonia Goncalves, Ewan Harrison, David K. Jackson, Ian Johnston, Dominic Kwiatkowski, Cordelia Langford, John Sillitoe on behalf of the Wellcome Sanger Institute COVID-19 Surveillance Team
EPI_ISL_835564	Lighthouse Lab in Glasgow	Wellcome Sanger Institute for the COVID-19 Genomics UK (COG-UK) Consortium	Harper VanSteenhouse, Yumi Kasai, David Gray, Carol Clugston, Anna Dominiczak and Alex Alderton, Roberto Amato, Sonia Goncalves, Ewan Harrison, David K. Jackson, Ian Johnston, Dominic Kwiatkowski, Cordelia Langford, John Sillitoe on behalf of the Wellcome Sanger Institute COVID-19 Surveillance Team
EPI_ISL_835565, EPI_ISL_835566, EPI_ISL_835567, EPI_ISL_835568, EPI_ISL_835569	Lighthouse Lab in Alderley Park	Wellcome Sanger Institute for the COVID-19 Genomics UK (COG-UK) Consortium	Jacquelyn Wynn, Mairead Hyland, The Lighthouse Lab in Alderley Park and Alex Alderton, Roberto Amato, Sonia Goncalves, Ewan Harrison, David K. Jackson, Ian Johnston, Dominic Kwiatkowski, Cordelia Langford, John Sillitoe on behalf of the Wellcome Sanger Institute COVID-19 Surveillance Team
EPI_ISL_835570	Lighthouse Lab in Glasgow	Wellcome Sanger Institute for the COVID-19 Genomics UK (COG-UK) Consortium	Harper VanSteenhouse, Yumi Kasai, David Gray, Carol Clugston, Anna Dominiczak and Alex Alderton, Roberto Amato, Sonia Goncalves, Ewan Harrison, David K. Jackson, Ian Johnston, Dominic Kwiatkowski, Cordelia Langford, John Sillitoe on behalf of the Wellcome Sanger Institute COVID-19 Surveillance Team
EPI_ISL_835571, EPI_ISL_835572	Lighthouse Lab in Alderley Park	Wellcome Sanger Institute for the COVID-19 Genomics UK (COG-UK) Consortium	Jacquelyn Wynn, Mairead Hyland, The Lighthouse Lab in Alderley Park and Alex Alderton, Roberto Amato, Sonia Goncalves, Ewan Harrison, David K. Jackson, Ian Johnston, Dominic Kwiatkowski, Cordelia Langford, John Sillitoe on behalf of the Wellcome Sanger Institute COVID-19 Surveillance Team
EPI_ISL_835573, EPI_ISL_835574	Lighthouse Lab in Glasgow	Wellcome Sanger Institute for the COVID-19 Genomics UK (COG-UK) Consortium	Harper VanSteenhouse, Yumi Kasai, David Gray, Carol Clugston, Anna Dominiczak and Alex Alderton, Roberto Amato, Sonia Goncalves, Ewan Harrison, David K. Jackson, Ian Johnston, Dominic Kwiatkowski, Cordelia Langford, John Sillitoe on behalf of the Wellcome Sanger Institute COVID-19 Surveillance Team
EPI_ISL_835575	Lighthouse Lab in Alderley Park	Wellcome Sanger Institute for the COVID-19 Genomics UK (COG-UK) Consortium	Jacquelyn Wynn, Mairead Hyland, The Lighthouse Lab in Alderley Park and Alex Alderton, Roberto Amato, Sonia Goncalves, Ewan Harrison, David K. Jackson, Ian Johnston, Dominic Kwiatkowski, Cordelia Langford, John Sillitoe on behalf of the Wellcome Sanger Institute COVID-19 Surveillance Team
EPI_ISL_835576, EPI_ISL_835577	Lighthouse Lab in Glasgow	Wellcome Sanger Institute for the COVID-19 Genomics UK (COG-UK) Consortium	Harper VanSteenhouse, Yumi Kasai, David Gray, Carol Clugston, Anna Dominiczak and Alex Alderton, Roberto Amato, Sonia Goncalves, Ewan Harrison, David K. Jackson, Ian Johnston, Dominic Kwiatkowski, Cordelia Langford, John Sillitoe on behalf of the Wellcome Sanger Institute COVID-19 Surveillance Team
EPI_ISL_835578	Lighthouse Lab in Alderley Park	Wellcome Sanger Institute for the COVID-19 Genomics UK (COG-UK) Consortium	Jacquelyn Wynn, Mairead Hyland, The Lighthouse Lab in Alderley Park and Alex Alderton, Roberto Amato, Sonia Goncalves, Ewan Harrison, David K. Jackson, Ian Johnston, Dominic Kwiatkowski, Cordelia Langford, John Sillitoe on behalf of the Wellcome Sanger Institute COVID-19 Surveillance Team
EPI_ISL_835579	Lighthouse Lab in Glasgow	Wellcome Sanger Institute for the COVID-19 Genomics UK (COG-UK) Consortium	Harper VanSteenhouse, Yumi Kasai, David Gray, Carol Clugston, Anna Dominiczak and Alex Alderton, Roberto Amato, Sonia Goncalves, Ewan Harrison, David K. Jackson, Ian Johnston, Dominic Kwiatkowski, Cordelia Langford, John Sillitoe on behalf of the Wellcome Sanger Institute COVID-19 Surveillance Team
EPI_ISL_835580, EPI_ISL_835581	Lighthouse Lab in Alderley Park	Wellcome Sanger Institute for the COVID-19 Genomics UK (COG-UK) Consortium	Jacquelyn Wynn, Mairead Hyland, The Lighthouse Lab in Alderley Park and Alex Alderton, Roberto Amato, Sonia Goncalves, Ewan Harrison, David K. Jackson, Ian Johnston, Dominic Kwiatkowski, Cordelia Langford, John Sillitoe on behalf of the Wellcome Sanger Institute COVID-19 Surveillance Team
EPI_ISL_835582	Lighthouse Lab in Glasgow	Wellcome Sanger Institute for the COVID-19 Genomics UK (COG-UK) Consortium	Harper VanSteenhouse, Yumi Kasai, David Gray, Carol Clugston, Anna Dominiczak and Alex Alderton, Roberto Amato, Sonia Goncalves, Ewan Harrison, David K. Jackson, Ian Johnston, Dominic Kwiatkowski, Cordelia Langford, John Sillitoe on behalf of the Wellcome Sanger Institute COVID-19 Surveillance Team
EPI_ISL_835583, EPI_ISL_835584, EPI_ISL_835585, EPI_ISL_835586, EPI_ISL_835587, EPI_ISL_835588	Lighthouse Lab in Alderley Park	Wellcome Sanger Institute for the COVID-19 Genomics UK (COG-UK) Consortium	Jacquelyn Wynn, Mairead Hyland, The Lighthouse Lab in Alderley Park and Alex Alderton, Roberto Amato, Sonia Goncalves, Ewan Harrison, David K. Jackson, Ian Johnston, Dominic Kwiatkowski, Cordelia Langford, John Sillitoe on behalf of the Wellcome Sanger Institute COVID-19 Surveillance Team
EPI_ISL_835589, EPI_ISL_835590	Lighthouse Lab in Glasgow	Wellcome Sanger Institute for the COVID-19 Genomics UK (COG-UK) Consortium	Harper VanSteenhouse, Yumi Kasai, David Gray, Carol Clugston, Anna Dominiczak and Alex Alderton, Roberto Amato, Sonia Goncalves, Ewan Harrison, David K. Jackson, Ian Johnston, Dominic Kwiatkowski, Cordelia Langford, John Sillitoe on behalf of the Wellcome Sanger Institute COVID-19 Surveillance Team
EPI_ISL_835591	Lighthouse Lab in Alderley Park	Wellcome Sanger Institute for the COVID-19 Genomics UK (COG-UK) Consortium	Jacquelyn Wynn, Mairead Hyland, The Lighthouse Lab in Alderley Park and Alex Alderton, Roberto Amato, Sonia Goncalves, Ewan Harrison, David K. Jackson, Ian Johnston, Dominic Kwiatkowski, Cordelia Langford, John Sillitoe on behalf of the Wellcome Sanger Institute COVID-19 Surveillance Team
EPI_ISL_835592	Lighthouse Lab in Glasgow	Wellcome Sanger Institute for the COVID-19 Genomics UK (COG-UK) Consortium	Harper VanSteenhouse, Yumi Kasai, David Gray, Carol Clugston, Anna Dominiczak and Alex Alderton, Roberto Amato, Sonia Goncalves, Ewan Harrison, David K. Jackson, Ian Johnston, Dominic Kwiatkowski, Cordelia Langford, John Sillitoe on behalf of the Wellcome Sanger Institute COVID-19 Surveillance Team
EPI_ISL_835593, EPI_ISL_835594, EPI_ISL_835595, EPI_ISL_835596	Lighthouse Lab in Alderley Park	Wellcome Sanger Institute for the COVID-19 Genomics UK (COG-UK) Consortium	Jacquelyn Wynn, Mairead Hyland, The Lighthouse Lab in Alderley Park and Alex Alderton, Roberto Amato, Sonia Goncalves, Ewan Harrison, David K. Jackson, Ian Johnston, Dominic Kwiatkowski, Cordelia Langford, John Sillitoe on behalf of the Wellcome Sanger Institute COVID-19 Surveillance Team

EPI_ISL_835597	Lighthouse Lab in Glasgow	Wellcome Sanger Institute for the COVID-19 Genomics UK (COG-UK) Consortium	Harper VanSteenhouse, Yumi Kasai, David Gray, Carol Clugston, Anna Dominiczak and Alex Alderton, Roberto Amato, Sonia Goncalves, Ewan Harrison, David K. Jackson, Ian Johnston, Dominic Kwiatkowski, Cordelia Langford, John Sillitoe on behalf of the Wellcome Sanger Institute COVID-19 Surveillance Team
EPI_ISL_835598, EPI_ISL_835599	Lighthouse Lab in Alderley Park	Wellcome Sanger Institute for the COVID-19 Genomics UK (COG-UK) Consortium	Jacquelyn Wynn, Mairead Hyland, The Lighthouse Lab in Alderley Park and Alex Alderton, Roberto Amato, Sonia Goncalves, Ewan Harrison, David K. Jackson, Ian Johnston, Dominic Kwiatkowski, Cordelia Langford, John Sillitoe on behalf of the Wellcome Sanger Institute COVID-19 Surveillance Team
EPI_ISL_835600	Lighthouse Lab in Glasgow	Wellcome Sanger Institute for the COVID-19 Genomics UK (COG-UK) Consortium	Harper VanSteenhouse, Yumi Kasai, David Gray, Carol Clugston, Anna Dominiczak and Alex Alderton, Roberto Amato, Sonia Goncalves, Ewan Harrison, David K. Jackson, Ian Johnston, Dominic Kwiatkowski, Cordelia Langford, John Sillitoe on behalf of the Wellcome Sanger Institute COVID-19 Surveillance Team
EPI_ISL_835601, EPI_ISL_835602	Lighthouse Lab in Alderley Park	Wellcome Sanger Institute for the COVID-19 Genomics UK (COG-UK) Consortium	Jacquelyn Wynn, Mairead Hyland, The Lighthouse Lab in Alderley Park and Alex Alderton, Roberto Amato, Sonia Goncalves, Ewan Harrison, David K. Jackson, Ian Johnston, Dominic Kwiatkowski, Cordelia Langford, John Sillitoe on behalf of the Wellcome Sanger Institute COVID-19 Surveillance Team
EPI_ISL_835603	Lighthouse Lab in Glasgow	Wellcome Sanger Institute for the COVID-19 Genomics UK (COG-UK) Consortium	Harper VanSteenhouse, Yumi Kasai, David Gray, Carol Clugston, Anna Dominiczak and Alex Alderton, Roberto Amato, Sonia Goncalves, Ewan Harrison, David K. Jackson, Ian Johnston, Dominic Kwiatkowski, Cordelia Langford, John Sillitoe on behalf of the Wellcome Sanger Institute COVID-19 Surveillance Team
EPI_ISL_835604, EPI_ISL_835605, EPI_ISL_835606, EPI_ISL_835607, EPI_ISL_835608, EPI_ISL_835609	Lighthouse Lab in Alderley Park	Wellcome Sanger Institute for the COVID-19 Genomics UK (COG-UK) Consortium	Jacquelyn Wynn, Mairead Hyland, The Lighthouse Lab in Alderley Park and Alex Alderton, Roberto Amato, Sonia Goncalves, Ewan Harrison, David K. Jackson, Ian Johnston, Dominic Kwiatkowski, Cordelia Langford, John Sillitoe on behalf of the Wellcome Sanger Institute COVID-19 Surveillance Team
EPI_ISL_835610	Lighthouse Lab in Glasgow	Wellcome Sanger Institute for the COVID-19 Genomics UK (COG-UK) Consortium	Harper VanSteenhouse, Yumi Kasai, David Gray, Carol Clugston, Anna Dominiczak and Alex Alderton, Roberto Amato, Sonia Goncalves, Ewan Harrison, David K. Jackson, Ian Johnston, Dominic Kwiatkowski, Cordelia Langford, John Sillitoe on behalf of the Wellcome Sanger Institute COVID-19 Surveillance Team
EPI_ISL_835611, EPI_ISL_835612, EPI_ISL_835613	Lighthouse Lab in Alderley Park	Wellcome Sanger Institute for the COVID-19 Genomics UK (COG-UK) Consortium	Jacquelyn Wynn, Mairead Hyland, The Lighthouse Lab in Alderley Park and Alex Alderton, Roberto Amato, Sonia Goncalves, Ewan Harrison, David K. Jackson, Ian Johnston, Dominic Kwiatkowski, Cordelia Langford, John Sillitoe on behalf of the Wellcome Sanger Institute COVID-19 Surveillance Team
EPI_ISL_835614, EPI_ISL_835616	Lighthouse Lab in Glasgow	Wellcome Sanger Institute for the COVID-19 Genomics UK (COG-UK) Consortium	Harper VanSteenhouse, Yumi Kasai, David Gray, Carol Clugston, Anna Dominiczak and Alex Alderton, Roberto Amato, Sonia Goncalves, Ewan Harrison, David K. Jackson, Ian Johnston, Dominic Kwiatkowski, Cordelia Langford, John Sillitoe on behalf of the Wellcome Sanger Institute COVID-19 Surveillance Team
EPI_ISL_835617	Lighthouse Lab in Alderley Park	Wellcome Sanger Institute for the COVID-19 Genomics UK (COG-UK) Consortium	Jacquelyn Wynn, Mairead Hyland, The Lighthouse Lab in Alderley Park and Alex Alderton, Roberto Amato, Sonia Goncalves, Ewan Harrison, David K. Jackson, Ian Johnston, Dominic Kwiatkowski, Cordelia Langford, John Sillitoe on behalf of the Wellcome Sanger Institute COVID-19 Surveillance Team
EPI_ISL_835618	Lighthouse Lab in Glasgow	Wellcome Sanger Institute for the COVID-19 Genomics UK (COG-UK) Consortium	Harper VanSteenhouse, Yumi Kasai, David Gray, Carol Clugston, Anna Dominiczak and Alex Alderton, Roberto Amato, Sonia Goncalves, Ewan Harrison, David K. Jackson, Ian Johnston, Dominic Kwiatkowski, Cordelia Langford, John Sillitoe on behalf of the Wellcome Sanger Institute COVID-19 Surveillance Team
EPI_ISL_835619, EPI_ISL_835620, EPI_ISL_835621	Lighthouse Lab in Alderley Park	Wellcome Sanger Institute for the COVID-19 Genomics UK (COG-UK) Consortium	Jacquelyn Wynn, Mairead Hyland, The Lighthouse Lab in Alderley Park and Alex Alderton, Roberto Amato, Sonia Goncalves, Ewan Harrison, David K. Jackson, Ian Johnston, Dominic Kwiatkowski, Cordelia Langford, John Sillitoe on behalf of the Wellcome Sanger Institute COVID-19 Surveillance Team
EPI_ISL_835622, EPI_ISL_835623	Lighthouse Lab in Glasgow	Wellcome Sanger Institute for the COVID-19 Genomics UK (COG-UK) Consortium	Harper VanSteenhouse, Yumi Kasai, David Gray, Carol Clugston, Anna Dominiczak and Alex Alderton, Roberto Amato, Sonia Goncalves, Ewan Harrison, David K. Jackson, Ian Johnston, Dominic Kwiatkowski, Cordelia Langford, John Sillitoe on behalf of the Wellcome Sanger Institute COVID-19 Surveillance Team
EPI_ISL_835624, EPI_ISL_835625, EPI_ISL_835626, EPI_ISL_835627, EPI_ISL_835628, EPI_ISL_835629, EPI_ISL_835630	Lighthouse Lab in Alderley Park	Wellcome Sanger Institute for the COVID-19 Genomics UK (COG-UK) Consortium	Jacquelyn Wynn, Mairead Hyland, The Lighthouse Lab in Alderley Park and Alex Alderton, Roberto Amato, Sonia Goncalves, Ewan Harrison, David K. Jackson, Ian Johnston, Dominic Kwiatkowski, Cordelia Langford, John Sillitoe on behalf of the Wellcome Sanger Institute COVID-19 Surveillance Team
EPI_ISL_835631	Lighthouse Lab in Glasgow	Wellcome Sanger Institute for the COVID-19 Genomics UK (COG-UK) Consortium	Harper VanSteenhouse, Yumi Kasai, David Gray, Carol Clugston, Anna Dominiczak and Alex Alderton, Roberto Amato, Sonia Goncalves, Ewan Harrison, David K. Jackson, Ian Johnston, Dominic Kwiatkowski, Cordelia Langford, John Sillitoe on behalf of the Wellcome Sanger Institute COVID-19 Surveillance Team
EPI_ISL_835632, EPI_ISL_835633, EPI_ISL_835634, EPI_ISL_835635, EPI_ISL_835636, EPI_ISL_835637, EPI_ISL_835638, EPI_ISL_835640, EPI_ISL_835641, EPI_ISL_835642	Lighthouse Lab in Alderley Park	Wellcome Sanger Institute for the COVID-19 Genomics UK (COG-UK) Consortium	Jacquelyn Wynn, Mairead Hyland, The Lighthouse Lab in Alderley Park and Alex Alderton, Roberto Amato, Sonia Goncalves, Ewan Harrison, David K. Jackson, Ian Johnston, Dominic Kwiatkowski, Cordelia Langford, John Sillitoe on behalf of the Wellcome Sanger Institute COVID-19 Surveillance Team
EPI_ISL_835643	Lighthouse Lab in Glasgow	Wellcome Sanger Institute for the COVID-19 Genomics UK (COG-UK) Consortium	Harper VanSteenhouse, Yumi Kasai, David Gray, Carol Clugston, Anna Dominiczak and Alex Alderton, Roberto Amato, Sonia Goncalves, Ewan Harrison, David K. Jackson, Ian Johnston, Dominic Kwiatkowski, Cordelia Langford, John Sillitoe on behalf of the Wellcome Sanger Institute COVID-19 Surveillance Team
EPI_ISL_835644, EPI_ISL_835645	Lighthouse Lab in Alderley Park	Wellcome Sanger Institute for the COVID-19 Genomics UK (COG-UK) Consortium	Jacquelyn Wynn, Mairead Hyland, The Lighthouse Lab in Alderley Park and Alex Alderton, Roberto Amato, Sonia Goncalves, Ewan Harrison, David K. Jackson, Ian Johnston, Dominic Kwiatkowski, Cordelia Langford, John Sillitoe on behalf of the Wellcome Sanger Institute COVID-19 Surveillance Team
EPI_ISL_835646, EPI_ISL_835647	Lighthouse Lab in Glasgow	Wellcome Sanger Institute for the COVID-19 Genomics UK (COG-UK) Consortium	Harper VanSteenhouse, Yumi Kasai, David Gray, Carol Clugston, Anna Dominiczak and Alex Alderton, Roberto Amato, Sonia Goncalves, Ewan Harrison, David K. Jackson, Ian Johnston, Dominic Kwiatkowski, Cordelia Langford, John Sillitoe on behalf of the Wellcome Sanger Institute COVID-19 Surveillance Team
EPI_ISL_835648, EPI_ISL_835649, EPI_ISL_835650	Lighthouse Lab in Alderley Park	Wellcome Sanger Institute for the COVID-19 Genomics UK (COG-UK) Consortium	Jacquelyn Wynn, Mairead Hyland, The Lighthouse Lab in Alderley Park and Alex Alderton, Roberto Amato, Sonia Goncalves, Ewan Harrison, David K. Jackson, Ian Johnston, Dominic Kwiatkowski, Cordelia Langford, John Sillitoe on behalf of the Wellcome Sanger Institute COVID-19 Surveillance Team
EPI_ISL_835651	Lighthouse Lab in Glasgow	Wellcome Sanger Institute for the COVID-19 Genomics UK (COG-UK) Consortium	Harper VanSteenhouse, Yumi Kasai, David Gray, Carol Clugston, Anna Dominiczak and Alex Alderton, Roberto Amato, Sonia Goncalves, Ewan Harrison, David K. Jackson, Ian Johnston, Dominic Kwiatkowski, Cordelia Langford, John Sillitoe on behalf of the Wellcome Sanger Institute COVID-19 Surveillance Team
EPI_ISL_835652, EPI_ISL_835653, EPI_ISL_835654, EPI_ISL_835656, EPI_ISL_835657, EPI_ISL_835658	Lighthouse Lab in Alderley Park	Wellcome Sanger Institute for the COVID-19 Genomics UK (COG-UK) Consortium	Jacquelyn Wynn, Mairead Hyland, The Lighthouse Lab in Alderley Park and Alex Alderton, Roberto Amato, Sonia Goncalves, Ewan Harrison, David K. Jackson, Ian Johnston, Dominic Kwiatkowski, Cordelia Langford, John Sillitoe on behalf of the Wellcome Sanger Institute COVID-19 Surveillance Team
EPI_ISL_835659	Lighthouse Lab in Glasgow	Wellcome Sanger Institute for the COVID-19 Genomics UK (COG-UK) Consortium	Harper VanSteenhouse, Yumi Kasai, David Gray, Carol Clugston, Anna Dominiczak and Alex Alderton, Roberto Amato, Sonia Goncalves, Ewan Harrison, David K. Jackson, Ian Johnston, Dominic Kwiatkowski, Cordelia Langford, John Sillitoe on behalf of the Wellcome Sanger Institute COVID-19 Surveillance Team
EPI_ISL_835660, EPI_ISL_835661, EPI_ISL_835662, EPI_ISL_835663	Lighthouse Lab in Alderley Park	Wellcome Sanger Institute for the COVID-19 Genomics UK (COG-UK) Consortium	Jacquelyn Wynn, Mairead Hyland, The Lighthouse Lab in Alderley Park and Alex Alderton, Roberto Amato, Sonia Goncalves, Ewan Harrison, David K. Jackson, Ian Johnston, Dominic Kwiatkowski, Cordelia Langford, John Sillitoe on behalf of the Wellcome Sanger Institute COVID-19 Surveillance Team
EPI_ISL_835664	Lighthouse Lab in Glasgow	Wellcome Sanger Institute for the COVID-19 Genomics UK (COG-UK) Consortium	Harper VanSteenhouse, Yumi Kasai, David Gray, Carol Clugston, Anna Dominiczak and Alex Alderton, Roberto Amato, Sonia Goncalves, Ewan Harrison, David K. Jackson, Ian Johnston, Dominic Kwiatkowski, Cordelia Langford, John Sillitoe on behalf of the Wellcome Sanger Institute COVID-19 Surveillance Team
EPI_ISL_835665, EPI_ISL_835666, EPI_ISL_835667, EPI_ISL_835668, EPI_ISL_835669, EPI_ISL_835670	Lighthouse Lab in Alderley Park	Wellcome Sanger Institute for the COVID-19 Genomics UK (COG-UK) Consortium	Jacquelyn Wynn, Mairead Hyland, The Lighthouse Lab in Alderley Park and Alex Alderton, Roberto Amato, Sonia Goncalves, Ewan Harrison, David K. Jackson, Ian Johnston, Dominic Kwiatkowski, Cordelia Langford, John Sillitoe on behalf of the Wellcome Sanger Institute COVID-19 Surveillance Team
EPI_ISL_835671	Lighthouse Lab in Glasgow	Wellcome Sanger Institute for the COVID-19 Genomics UK	Harper VanSteenhouse, Yumi Kasai, David Gray, Carol Clugston, Anna Dominiczak and Alex Alderton, Roberto Amato, Sonia Goncalves, Ewan Harrison,

		(COG-UK) Consortium	David K. Jackson, Ian Johnston, Dominic Kwiatkowski, Cordelia Langford, John Sillitoe on behalf of the Wellcome Sanger Institute COVID-19 Surveillance Team
EPI_ISL_835672	Lighthouse Lab in Alderley Park	Wellcome Sanger Institute for the COVID-19 Genomics UK (COG-UK) Consortium	Jacquelyn Wynn, Mairead Hyland, The Lighthouse Lab in Alderley Park and Alex Alderton, Roberto Amato, Sonia Goncalves, Ewan Harrison, David K. Jackson, Ian Johnston, Dominic Kwiatkowski, Cordelia Langford, John Sillitoe on behalf of the Wellcome Sanger Institute COVID-19 Surveillance Team
EPI_ISL_835673	Lighthouse Lab in Glasgow	Wellcome Sanger Institute for the COVID-19 Genomics UK (COG-UK) Consortium	Harper VanSteenhouse, Yumi Kasai, David Gray, Carol Clugston, Anna Dominiczak and Alex Alderton, Roberto Amato, Sonia Goncalves, Ewan Harrison, David K. Jackson, Ian Johnston, Dominic Kwiatkowski, Cordelia Langford, John Sillitoe on behalf of the Wellcome Sanger Institute COVID-19 Surveillance Team
EPI_ISL_835674, EPI_ISL_835675, EPI_ISL_835676	Lighthouse Lab in Alderley Park	Wellcome Sanger Institute for the COVID-19 Genomics UK (COG-UK) Consortium	Jacquelyn Wynn, Mairead Hyland, The Lighthouse Lab in Alderley Park and Alex Alderton, Roberto Amato, Sonia Goncalves, Ewan Harrison, David K. Jackson, Ian Johnston, Dominic Kwiatkowski, Cordelia Langford, John Sillitoe on behalf of the Wellcome Sanger Institute COVID-19 Surveillance Team
EPI_ISL_835677, EPI_ISL_835678	Lighthouse Lab in Glasgow	Wellcome Sanger Institute for the COVID-19 Genomics UK (COG-UK) Consortium	Harper VanSteenhouse, Yumi Kasai, David Gray, Carol Clugston, Anna Dominiczak and Alex Alderton, Roberto Amato, Sonia Goncalves, Ewan Harrison, David K. Jackson, Ian Johnston, Dominic Kwiatkowski, Cordelia Langford, John Sillitoe on behalf of the Wellcome Sanger Institute COVID-19 Surveillance Team
EPI_ISL_835679, EPI_ISL_835681	Lighthouse Lab in Alderley Park	Wellcome Sanger Institute for the COVID-19 Genomics UK (COG-UK) Consortium	Jacquelyn Wynn, Mairead Hyland, The Lighthouse Lab in Alderley Park and Alex Alderton, Roberto Amato, Sonia Goncalves, Ewan Harrison, David K. Jackson, Ian Johnston, Dominic Kwiatkowski, Cordelia Langford, John Sillitoe on behalf of the Wellcome Sanger Institute COVID-19 Surveillance Team
EPI_ISL_835682, EPI_ISL_835683	Lighthouse Lab in Glasgow	Wellcome Sanger Institute for the COVID-19 Genomics UK (COG-UK) Consortium	Harper VanSteenhouse, Yumi Kasai, David Gray, Carol Clugston, Anna Dominiczak and Alex Alderton, Roberto Amato, Sonia Goncalves, Ewan Harrison, David K. Jackson, Ian Johnston, Dominic Kwiatkowski, Cordelia Langford, John Sillitoe on behalf of the Wellcome Sanger Institute COVID-19 Surveillance Team
EPI_ISL_835684, EPI_ISL_835685	Lighthouse Lab in Alderley Park	Wellcome Sanger Institute for the COVID-19 Genomics UK (COG-UK) Consortium	Jacquelyn Wynn, Mairead Hyland, The Lighthouse Lab in Alderley Park and Alex Alderton, Roberto Amato, Sonia Goncalves, Ewan Harrison, David K. Jackson, Ian Johnston, Dominic Kwiatkowski, Cordelia Langford, John Sillitoe on behalf of the Wellcome Sanger Institute COVID-19 Surveillance Team
EPI_ISL_835686, EPI_ISL_835687, EPI_ISL_835688	Lighthouse Lab in Glasgow	Wellcome Sanger Institute for the COVID-19 Genomics UK (COG-UK) Consortium	Harper VanSteenhouse, Yumi Kasai, David Gray, Carol Clugston, Anna Dominiczak and Alex Alderton, Roberto Amato, Sonia Goncalves, Ewan Harrison, David K. Jackson, Ian Johnston, Dominic Kwiatkowski, Cordelia Langford, John Sillitoe on behalf of the Wellcome Sanger Institute COVID-19 Surveillance Team
EPI_ISL_835689, EPI_ISL_835690, EPI_ISL_835691	Lighthouse Lab in Alderley Park	Wellcome Sanger Institute for the COVID-19 Genomics UK (COG-UK) Consortium	Jacquelyn Wynn, Mairead Hyland, The Lighthouse Lab in Alderley Park and Alex Alderton, Roberto Amato, Sonia Goncalves, Ewan Harrison, David K. Jackson, Ian Johnston, Dominic Kwiatkowski, Cordelia Langford, John Sillitoe on behalf of the Wellcome Sanger Institute COVID-19 Surveillance Team
EPI_ISL_835692	Lighthouse Lab in Glasgow	Wellcome Sanger Institute for the COVID-19 Genomics UK (COG-UK) Consortium	Harper VanSteenhouse, Yumi Kasai, David Gray, Carol Clugston, Anna Dominiczak and Alex Alderton, Roberto Amato, Sonia Goncalves, Ewan Harrison, David K. Jackson, Ian Johnston, Dominic Kwiatkowski, Cordelia Langford, John Sillitoe on behalf of the Wellcome Sanger Institute COVID-19 Surveillance Team
EPI_ISL_835693, EPI_ISL_835694	Lighthouse Lab in Alderley Park	Wellcome Sanger Institute for the COVID-19 Genomics UK (COG-UK) Consortium	Jacquelyn Wynn, Mairead Hyland, The Lighthouse Lab in Alderley Park and Alex Alderton, Roberto Amato, Sonia Goncalves, Ewan Harrison, David K. Jackson, Ian Johnston, Dominic Kwiatkowski, Cordelia Langford, John Sillitoe on behalf of the Wellcome Sanger Institute COVID-19 Surveillance Team
EPI_ISL_835695	Lighthouse Lab in Glasgow	Wellcome Sanger Institute for the COVID-19 Genomics UK (COG-UK) Consortium	Harper VanSteenhouse, Yumi Kasai, David Gray, Carol Clugston, Anna Dominiczak and Alex Alderton, Roberto Amato, Sonia Goncalves, Ewan Harrison, David K. Jackson, Ian Johnston, Dominic Kwiatkowski, Cordelia Langford, John Sillitoe on behalf of the Wellcome Sanger Institute COVID-19 Surveillance Team
EPI_ISL_835696, EPI_ISL_835697, EPI_ISL_835699, EPI_ISL_835700	Lighthouse Lab in Alderley Park	Wellcome Sanger Institute for the COVID-19 Genomics UK (COG-UK) Consortium	Jacquelyn Wynn, Mairead Hyland, The Lighthouse Lab in Alderley Park and Alex Alderton, Roberto Amato, Sonia Goncalves, Ewan Harrison, David K. Jackson, Ian Johnston, Dominic Kwiatkowski, Cordelia Langford, John Sillitoe on behalf of the Wellcome Sanger Institute COVID-19 Surveillance Team
EPI_ISL_835701	Lighthouse Lab in Glasgow	Wellcome Sanger Institute for the COVID-19 Genomics UK (COG-UK) Consortium	Harper VanSteenhouse, Yumi Kasai, David Gray, Carol Clugston, Anna Dominiczak and Alex Alderton, Roberto Amato, Sonia Goncalves, Ewan Harrison, David K. Jackson, Ian Johnston, Dominic Kwiatkowski, Cordelia Langford, John Sillitoe on behalf of the Wellcome Sanger Institute COVID-19 Surveillance Team
EPI_ISL_835702	Lighthouse Lab in Alderley Park	Wellcome Sanger Institute for the COVID-19 Genomics UK (COG-UK) Consortium	Jacquelyn Wynn, Mairead Hyland, The Lighthouse Lab in Alderley Park and Alex Alderton, Roberto Amato, Sonia Goncalves, Ewan Harrison, David K. Jackson, Ian Johnston, Dominic Kwiatkowski, Cordelia Langford, John Sillitoe on behalf of the Wellcome Sanger Institute COVID-19 Surveillance Team
EPI_ISL_835703	Lighthouse Lab in Glasgow	Wellcome Sanger Institute for the COVID-19 Genomics UK (COG-UK) Consortium	Harper VanSteenhouse, Yumi Kasai, David Gray, Carol Clugston, Anna Dominiczak and Alex Alderton, Roberto Amato, Sonia Goncalves, Ewan Harrison, David K. Jackson, Ian Johnston, Dominic Kwiatkowski, Cordelia Langford, John Sillitoe on behalf of the Wellcome Sanger Institute COVID-19 Surveillance Team
EPI_ISL_835704, EPI_ISL_835705, EPI_ISL_835706	Lighthouse Lab in Alderley Park	Wellcome Sanger Institute for the COVID-19 Genomics UK (COG-UK) Consortium	Jacquelyn Wynn, Mairead Hyland, The Lighthouse Lab in Alderley Park and Alex Alderton, Roberto Amato, Sonia Goncalves, Ewan Harrison, David K. Jackson, Ian Johnston, Dominic Kwiatkowski, Cordelia Langford, John Sillitoe on behalf of the Wellcome Sanger Institute COVID-19 Surveillance Team
EPI_ISL_835707	Lighthouse Lab in Glasgow	Wellcome Sanger Institute for the COVID-19 Genomics UK (COG-UK) Consortium	Harper VanSteenhouse, Yumi Kasai, David Gray, Carol Clugston, Anna Dominiczak and Alex Alderton, Roberto Amato, Sonia Goncalves, Ewan Harrison, David K. Jackson, Ian Johnston, Dominic Kwiatkowski, Cordelia Langford, John Sillitoe on behalf of the Wellcome Sanger Institute COVID-19 Surveillance Team
EPI_ISL_835708, EPI_ISL_835709	Lighthouse Lab in Alderley Park	Wellcome Sanger Institute for the COVID-19 Genomics UK (COG-UK) Consortium	Jacquelyn Wynn, Mairead Hyland, The Lighthouse Lab in Alderley Park and Alex Alderton, Roberto Amato, Sonia Goncalves, Ewan Harrison, David K. Jackson, Ian Johnston, Dominic Kwiatkowski, Cordelia Langford, John Sillitoe on behalf of the Wellcome Sanger Institute COVID-19 Surveillance Team

EPI_ISL_835710, EPI_ISL_835711, EPI_ISL_835712, EPI_ISL_835713, EPI_ISL_835714, EPI_ISL_835715, EPI_ISL_835716, EPI_ISL_835717, EPI_ISL_835718, EPI_ISL_835719, EPI_ISL_835720, EPI_ISL_835721, EPI_ISL_835722, EPI_ISL_835723, EPI_ISL_835724, EPI_ISL_835725, EPI_ISL_835726, EPI_ISL_835727, EPI_ISL_835728, EPI_ISL_835729, EPI_ISL_835730, EPI_ISL_835731, EPI_ISL_835732, EPI_ISL_835733, EPI_ISL_835734, EPI_ISL_835735, EPI_ISL_835736, EPI_ISL_835737, EPI_ISL_835738, EPI_ISL_835739, EPI_ISL_835740, EPI_ISL_835741, EPI_ISL_835742, EPI_ISL_835743, EPI_ISL_835744, EPI_ISL_835745, EPI_ISL_835746, EPI_ISL_835747, EPI_ISL_835748, EPI_ISL_835749, EPI_ISL_835750, EPI_ISL_835751, EPI_ISL_835752, EPI_ISL_835753, EPI_ISL_835754, EPI_ISL_835755, EPI_ISL_835756, EPI_ISL_835757, EPI_ISL_835758, EPI_ISL_835759, EPI_ISL_835760, EPI_ISL_835761, EPI_ISL_835762, EPI_ISL_835763, EPI_ISL_835764, EPI_ISL_835765, EPI_ISL_835766, EPI_ISL_835767, EPI_ISL_835768, EPI_ISL_835769, EPI_ISL_835770, EPI_ISL_835771, EPI_ISL_835772, EPI_ISL_835773, EPI_ISL_835774, EPI_ISL_835775, EPI_ISL_835776, EPI_ISL_835777, EPI_ISL_835778, EPI_ISL_835779, EPI_ISL_835780, EPI_ISL_835781, EPI_ISL_835782, EPI_ISL_835783, EPI_ISL_835784, EPI_ISL_835785, EPI_ISL_835786, EPI_ISL_835787, EPI_ISL_835788, EPI_ISL_835789, EPI_ISL_835790, EPI_ISL_835791, EPI_ISL_835792, EPI_ISL_835793, EPI_ISL_835794, EPI_ISL_835795, EPI_ISL_835796, EPI_ISL_835797, EPI_ISL_835798, EPI_ISL_835799, EPI_ISL_835800, EPI_ISL_835801, EPI_ISL_835802, EPI_ISL_835803, EPI_ISL_835804, EPI_ISL_835805, EPI_ISL_835806, EPI_ISL_835807, EPI_ISL_835808, EPI_ISL_835809, EPI_ISL_835810, EPI_ISL_835811, EPI_ISL_835812, EPI_ISL_835813, EPI_ISL_835814, EPI_ISL_835815, EPI_ISL_835816, EPI_ISL_835817, EPI_ISL_835818, EPI_ISL_835819, EPI_ISL_835820, EPI_ISL_835821, EPI_ISL_835822, EPI_ISL_835823, EPI_ISL_835824, EPI_ISL_835825, EPI_ISL_835826, EPI_ISL_835827, EPI_ISL_835828, EPI_ISL_835829, EPI_ISL_835830, EPI_ISL_835831, EPI_ISL_835832, EPI_ISL_835833, EPI_ISL_835834, EPI_ISL_835835, EPI_ISL_835836, EPI_ISL_835837, EPI_ISL_835838, EPI_ISL_835839, EPI_ISL_835840, EPI_ISL_835841, EPI_ISL_835842, EPI_ISL_835843, EPI_ISL_835844, EPI_ISL_835845, EPI_ISL_835846, EPI_ISL_835847, EPI_ISL_835848, EPI_ISL_835849, EPI_ISL_835850, EPI_ISL_835851, EPI_ISL_835852, EPI_ISL_835853, EPI_ISL_835854, EPI_ISL_835855, EPI_ISL_835856, EPI_ISL_835857, EPI_ISL_835858, EPI_ISL_835859, EPI_ISL_835860, EPI_ISL_835861, EPI_ISL_835862, EPI_ISL_835863, EPI_ISL_835864, EPI_ISL_835865, EPI_ISL_835866, EPI_ISL_835867, EPI_ISL_835868, EPI_ISL_835869, EPI_ISL_835870, EPI_ISL_835871, EPI_ISL_835872, EPI_ISL_835873, EPI_ISL_835874, EPI_ISL_835875, EPI_ISL_835876, EPI_ISL_835877, EPI_ISL_835878, EPI_ISL_835879, EPI_ISL_835880, EPI_ISL_835881, EPI_ISL_835882, EPI_ISL_835883, EPI_ISL_835884, EPI_ISL_835885, EPI_ISL_835886, EPI_ISL_835887, EPI_ISL_835888, EPI_ISL_835889, EPI_ISL_835890, EPI_ISL_835891, EPI_ISL_835892, EPI_ISL_835893, EPI_ISL_835894, EPI_ISL_835895, EPI_ISL_835896, EPI_ISL_835897, EPI_ISL_835898, EPI_ISL_835899, EPI_ISL_835900, EPI_ISL_835901, EPI_ISL_835902, EPI_ISL_835903, EPI_ISL_835904, EPI_ISL_835905, EPI_ISL_835906, EPI_ISL_835907, EPI_ISL_835908, EPI_ISL_835909, EPI_ISL_835910, EPI_ISL_835911, EPI_ISL_835912, EPI_ISL_835913, EPI_ISL_835914, EPI_ISL_835915, EPI_ISL_835916, EPI_ISL_835917, EPI_ISL_835918, EPI_ISL_835919, EPI_ISL_835920, EPI_ISL_835921, EPI_ISL_835922, EPI_ISL_835923, EPI_ISL_835924, EPI_ISL_835925, EPI_ISL_835926, EPI_ISL_835927, EPI_ISL_835928, EPI_ISL_835929, EPI_ISL_835930, EPI_ISL_835931, EPI_ISL_835932, EPI_ISL_835933, EPI_ISL_835934, EPI_ISL_835935, EPI_ISL_835936, EPI_ISL_835937, EPI_ISL_835938, EPI_ISL_835939, EPI_ISL_835940, EPI_ISL_835941, EPI_ISL_835942, EPI_ISL_835943, EPI_ISL_835944, EPI_ISL_835945, EPI_ISL_835946, EPI_ISL_835947, EPI_ISL_835948, EPI_ISL_835949, EPI_ISL_835950, EPI_ISL_835951, EPI_ISL_835952, EPI_ISL_835953, EPI_ISL_835954, EPI_ISL_835955, EPI_ISL_835956, EPI_ISL_835957, EPI_ISL_835958, EPI_ISL_835959, EPI_ISL_835960, EPI_ISL_835961, EPI_ISL_835962, EPI_ISL_835963, EPI_ISL_835964, EPI_ISL_835965, EPI_ISL_835966, EPI_ISL_835967, EPI_ISL_835968, EPI_ISL_835969, EPI_ISL_835970, EPI_ISL_835971, EPI_ISL_835972, EPI_ISL_835973, EPI_ISL_835974, EPI_ISL_835975, EPI_ISL_835976, EPI_ISL_835977, EPI_ISL_835978, EPI_ISL_835979, EPI_ISL_835980, EPI_ISL_835981, EPI_ISL_835982, EPI_ISL_835983, EPI_ISL_835984, EPI_ISL_835985, EPI_ISL_835986, EPI_ISL_835987, EPI_ISL_835988, EPI_ISL_835989, EPI_ISL_835990, EPI_ISL_835991, EPI_ISL_835992, EPI_ISL_835993, EPI_ISL_835994, EPI_ISL_835995, EPI_ISL_835996, EPI_ISL_835997, EPI_ISL_835998, EPI_ISL_835999, EPI_ISL_836000, EPI_ISL_836001, EPI_ISL_836002, EPI_ISL_836003, EPI_ISL_836004, EPI_ISL_836005, EPI_ISL_836006, EPI_ISL_836007, EPI_ISL_836008, EPI_ISL_836009, EPI_ISL_836010, EPI_ISL_836011, EPI_ISL_836012, EPI_ISL_836013, EPI_ISL_836014, EPI_ISL_836015, EPI_ISL_836016, EPI_ISL_836017, EPI_ISL_836018, EPI_ISL_836019, EPI_ISL_836020, EPI_ISL_836021, EPI_ISL_836022, EPI_ISL_836023, EPI_ISL_836024, EPI_ISL_836025, EPI_ISL_836026, EPI_ISL_836027, EPI_ISL_836028, EPI_ISL_836029, EPI_ISL_836030, EPI_ISL_836031, EPI_ISL_836032, EPI_ISL_836033, EPI_ISL_836034, EPI_ISL_836035, EPI_ISL_836036, EPI_ISL_836037, EPI_ISL_836038, EPI_ISL_836039, EPI_ISL_836040, EPI_ISL_836041, EPI_ISL_836042, EPI_ISL_836043, EPI_ISL_836044, EPI_ISL_836045, EPI_ISL_836046, EPI_ISL_836047, EPI_ISL_836048, EPI_ISL_836049, EPI_ISL_836050, EPI_ISL_836051, EPI_ISL_836052, EPI_ISL_836053, EPI_ISL_836054, EPI_ISL_836055, EPI_ISL_836056, EPI_ISL_836057, EPI_ISL_836058, EPI_ISL_836059, EPI_ISL_836060, EPI_ISL_836061, EPI_ISL_836062, EPI_ISL_836063, EPI_ISL_836064, EPI_ISL_836065, EPI_ISL_836066, EPI_ISL_836067, EPI_ISL_836068, EPI_ISL_836069, EPI_ISL_836070, EPI_ISL_836071, EPI_ISL_836072, EPI_ISL_836073, EPI_ISL_836074, EPI_ISL_836075, EPI_ISL_836076, EPI_ISL_836077, EPI_ISL_836078, EPI_ISL_836079, EPI_ISL_836080, EPI_ISL_836081, EPI_ISL_836082, EPI_ISL_836083, EPI_ISL_836084, EPI_ISL_836085, EPI_ISL_836086, EPI_ISL_836087, EPI_ISL_836088, EPI_ISL_836089, EPI_ISL_836090, EPI_ISL_836091, EPI_ISL_836092, EPI_ISL_836093, EPI_ISL_836094, EPI_ISL_836095, EPI_ISL_836096, EPI_ISL_836097, EPI_ISL_836098, EPI_ISL_836099, EPI_ISL_836100, EPI_ISL_836101, EPI_ISL_836102, EPI_ISL_836103, EPI_ISL_836104, EPI_ISL_836105, EPI_ISL_836106, EPI_ISL_836107, EPI_ISL_836108, EPI_ISL_836109, EPI_ISL_836110, EPI_ISL_836111, EPI_ISL_836112, EPI_ISL_836113, EPI_ISL_836114, EPI_ISL_836115, EPI_ISL_836116, EPI_ISL_836117, EPI_ISL_836118, EPI_ISL_836119, EPI_ISL_836120, EPI_ISL_836121, EPI_ISL_836122, EPI_ISL_836123, EPI_ISL_836124, EPI_ISL_836125, EPI_ISL_836126, EPI_ISL_836127, EPI_ISL_836128, EPI_ISL_836129, EPI_ISL_836130, EPI_ISL_836131, EPI_ISL_836132, EPI_ISL_836133, EPI_ISL_836134, EPI_ISL_836135, EPI_ISL_836136, EPI_ISL_836137, EPI_ISL_836138, EPI_ISL_836139, EPI_ISL_836140, EPI_ISL_836141, EPI_ISL_836142, EPI_ISL_836144, EPI_ISL_836145, EPI_ISL_836146, EPI_ISL_836147, EPI_ISL_836148, EPI_ISL_836149, EPI_ISL_836150, EPI_ISL_836151, EPI_ISL_836152, EPI_ISL_836153, EPI_ISL_836154, EPI_ISL_836155, EPI_ISL_836156, EPI_ISL_836157, EPI_ISL_836158, EPI_ISL_836159, EPI_ISL_836160, EPI_ISL_836161, EPI_ISL_836162, EPI_ISL_836163, EPI_ISL_836164, EPI_ISL_836165, EPI_ISL_836166, EPI_ISL_836167, EPI_ISL_836168, EPI_ISL_836169, EPI_ISL_836170, EPI_ISL_836171, EPI_ISL_836172, EPI_ISL_836173, EPI_ISL_836174, EPI_ISL_836175, EPI_ISL_836176, EPI_ISL_836177, EPI_ISL_836178, EPI_ISL_836179, EPI_ISL_836180, EPI_ISL_836181, EPI_ISL_836182, EPI_ISL_836183, EPI_ISL_836184, EPI_ISL_836185, EPI_ISL_836186, EPI_ISL_836187, EPI_ISL_836188, EPI_ISL_836189, EPI_ISL_836190, EPI_ISL_836191, EPI_ISL_836192, EPI_ISL_836193, EPI_ISL_836194, EPI_ISL_836195, EPI_ISL_836196, EPI_ISL_836197, EPI_ISL_836198, EPI_ISL_836199, EPI_ISL_836200, EPI_ISL_836201, EPI_ISL_836202, EPI_ISL_836203, EPI_ISL_836204, EPI_ISL_836205, EPI_ISL_836206, EPI_ISL_836207, EPI_ISL_836208, EPI_ISL_836209, EPI_ISL_836210, EPI_ISL_836211, EPI_ISL_836212, EPI_ISL_836213, EPI_ISL_836214,

EPI_ISL_836215, EPI_ISL_836216, EPI_ISL_836217, EPI_ISL_836218, EPI_ISL_836219, EPI_ISL_836220, EPI_ISL_836221, EPI_ISL_836222, EPI_ISL_836223, EPI_ISL_836224, EPI_ISL_836225, EPI_ISL_836226, EPI_ISL_836227, EPI_ISL_836228, EPI_ISL_836229, EPI_ISL_836230, EPI_ISL_836231, EPI_ISL_836232, EPI_ISL_836233, EPI_ISL_836234, EPI_ISL_836235, EPI_ISL_836236, EPI_ISL_836237, EPI_ISL_836238, EPI_ISL_836239, EPI_ISL_836240, EPI_ISL_836241, EPI_ISL_836242, EPI_ISL_836243, EPI_ISL_836244, EPI_ISL_836245, EPI_ISL_836246, EPI_ISL_836247, EPI_ISL_836248, EPI_ISL_836249, EPI_ISL_836250, EPI_ISL_836251, EPI_ISL_836252, EPI_ISL_836253, EPI_ISL_836254, EPI_ISL_836255, EPI_ISL_836256, EPI_ISL_836257, EPI_ISL_836258, EPI_ISL_836259, EPI_ISL_836260, EPI_ISL_836261, EPI_ISL_836262, EPI_ISL_836263, EPI_ISL_836264, EPI_ISL_836265, EPI_ISL_836266, EPI_ISL_836267, EPI_ISL_836268, EPI_ISL_836269, EPI_ISL_836270, EPI_ISL_836271, EPI_ISL_836272, EPI_ISL_836273, EPI_ISL_836274, EPI_ISL_836275, EPI_ISL_836276, EPI_ISL_836277, EPI_ISL_836278, EPI_ISL_836279, EPI_ISL_836280, EPI_ISL_836281, EPI_ISL_836282, EPI_ISL_836283, EPI_ISL_836284, EPI_ISL_836285, EPI_ISL_836286, EPI_ISL_836287, EPI_ISL_836288, EPI_ISL_836289, EPI_ISL_836290, EPI_ISL_836291, EPI_ISL_836292, EPI_ISL_836293, EPI_ISL_836294, EPI_ISL_836295, EPI_ISL_836296, EPI_ISL_836297, EPI_ISL_836298, EPI_ISL_836299, EPI_ISL_836300, EPI_ISL_836301, EPI_ISL_836302, EPI_ISL_836303, EPI_ISL_836304, EPI_ISL_836305, EPI_ISL_836306, EPI_ISL_836307, EPI_ISL_836308, EPI_ISL_836309, EPI_ISL_836310, EPI_ISL_836311, EPI_ISL_836312, EPI_ISL_836313, EPI_ISL_836314, EPI_ISL_836315, EPI_ISL_836316, EPI_ISL_836317, EPI_ISL_836318, EPI_ISL_836319, EPI_ISL_836320, EPI_ISL_836321, EPI_ISL_836322, EPI_ISL_836323, EPI_ISL_836324, EPI_ISL_836325, EPI_ISL_836326, EPI_ISL_836327, EPI_ISL_836328, EPI_ISL_836329, EPI_ISL_836330, EPI_ISL_836331, EPI_ISL_836332, EPI_ISL_836333, EPI_ISL_836334, EPI_ISL_836335, EPI_ISL_836336, EPI_ISL_836337, EPI_ISL_836338, EPI_ISL_836339, EPI_ISL_836340, EPI_ISL_836341, EPI_ISL_836342, EPI_ISL_836343, EPI_ISL_836344, EPI_ISL_836345, EPI_ISL_836346, EPI_ISL_836347, EPI_ISL_836348, EPI_ISL_836349, EPI_ISL_836350, EPI_ISL_836351, EPI_ISL_836352, EPI_ISL_836353, EPI_ISL_836354, EPI_ISL_836355, EPI_ISL_836356, EPI_ISL_836357, EPI_ISL_836358, EPI_ISL_836359, EPI_ISL_836360, EPI_ISL_836361, EPI_ISL_836362, EPI_ISL_836363, EPI_ISL_836364, EPI_ISL_836365, EPI_ISL_836366, EPI_ISL_836367, EPI_ISL_836368, EPI_ISL_836369, EPI_ISL_836370, EPI_ISL_836371, EPI_ISL_836372, EPI_ISL_836373, EPI_ISL_836374, EPI_ISL_836375, EPI_ISL_836376, EPI_ISL_836377, EPI_ISL_836378, EPI_ISL_836379, EPI_ISL_836380, EPI_ISL_836381, EPI_ISL_836382, EPI_ISL_836383, EPI_ISL_836384, EPI_ISL_836385, EPI_ISL_836386, EPI_ISL_836387, EPI_ISL_836388, EPI_ISL_836389, EPI_ISL_836390, EPI_ISL_836391, EPI_ISL_836392, EPI_ISL_836393, EPI_ISL_836394, EPI_ISL_836395, EPI_ISL_836396, EPI_ISL_836397, EPI_ISL_836398, EPI_ISL_836399, EPI_ISL_836400, EPI_ISL_836401, EPI_ISL_836402, EPI_ISL_836403, EPI_ISL_836404, EPI_ISL_836405, EPI_ISL_836406, EPI_ISL_836407, EPI_ISL_836408, EPI_ISL_836409, EPI_ISL_836410, EPI_ISL_836411, EPI_ISL_836412, EPI_ISL_836413, EPI_ISL_836414, EPI_ISL_836415, EPI_ISL_836416, EPI_ISL_836417, EPI_ISL_836418, EPI_ISL_836419, EPI_ISL_836420

see above	Lighthouse Lab in Milton Keynes	Wellcome Sanger Institute for the COVID-19 Genomics UK (COG-UK) Consortium	The Lighthouse Lab in Milton Keynes and Alex Alderton, Roberto Amato, Sonia Goncalves, Ewan Harrison, David K. Jackson, Ian Johnston, Dominic Kwiatkowski, Cordelia Langford, John Sillitoe on behalf of the Wellcome Sanger Institute COVID-19 Surveillance Team

EPI_ISL_836421, EPI_ISL_836422, EPI_ISL_836423, EPI_ISL_836424, EPI_ISL_836425, EPI_ISL_836426, EPI_ISL_836427, EPI_ISL_836428, EPI_ISL_836429, EPI_ISL_836430, EPI_ISL_836431, EPI_ISL_836432, EPI_ISL_836433, EPI_ISL_836434, EPI_ISL_836435, EPI_ISL_836436, EPI_ISL_836437, EPI_ISL_836438, EPI_ISL_836439, EPI_ISL_836440, EPI_ISL_836441, EPI_ISL_836442, EPI_ISL_836443, EPI_ISL_836444, EPI_ISL_836445, EPI_ISL_836446, EPI_ISL_836447, EPI_ISL_836448, EPI_ISL_836449, EPI_ISL_836450, EPI_ISL_836451, EPI_ISL_836452, EPI_ISL_836453, EPI_ISL_836454, EPI_ISL_836455, EPI_ISL_836456, EPI_ISL_836457, EPI_ISL_836458, EPI_ISL_836459, EPI_ISL_836460, EPI_ISL_836461, EPI_ISL_836462, EPI_ISL_836463, EPI_ISL_836464, EPI_ISL_836465, EPI_ISL_836466, EPI_ISL_836467, EPI_ISL_836468, EPI_ISL_836469, EPI_ISL_836470, EPI_ISL_836471, EPI_ISL_836472, EPI_ISL_836473, EPI_ISL_836474, EPI_ISL_836475, EPI_ISL_836476, EPI_ISL_836477, EPI_ISL_836478, EPI_ISL_836479, EPI_ISL_836480, EPI_ISL_836481, EPI_ISL_836482, EPI_ISL_836483, EPI_ISL_836484, EPI_ISL_836485, EPI_ISL_836486, EPI_ISL_836487, EPI_ISL_836488, EPI_ISL_836489, EPI_ISL_836490, EPI_ISL_836491, EPI_ISL_836492, EPI_ISL_836493, EPI_ISL_836494, EPI_ISL_836495, EPI_ISL_836496, EPI_ISL_836497, EPI_ISL_836498, EPI_ISL_836499, EPI_ISL_836500, EPI_ISL_836501, EPI_ISL_836502, EPI_ISL_836503, EPI_ISL_836504, EPI_ISL_836505, EPI_ISL_836506, EPI_ISL_836507, EPI_ISL_836508, EPI_ISL_836509, EPI_ISL_836510, EPI_ISL_836511, EPI_ISL_836512, EPI_ISL_836513, EPI_ISL_836514, EPI_ISL_836515, EPI_ISL_836516, EPI_ISL_836517, EPI_ISL_836518, EPI_ISL_836519, EPI_ISL_836520, EPI_ISL_836521, EPI_ISL_836522, EPI_ISL_836523, EPI_ISL_836524, EPI_ISL_836525, EPI_ISL_836526, EPI_ISL_836527, EPI_ISL_836528, EPI_ISL_836529, EPI_ISL_836530, EPI_ISL_836531, EPI_ISL_836532, EPI_ISL_836533, EPI_ISL_836534, EPI_ISL_836535, EPI_ISL_836536, EPI_ISL_836537, EPI_ISL_836538, EPI_ISL_836539, EPI_ISL_836540, EPI_ISL_836541, EPI_ISL_836542, EPI_ISL_836543, EPI_ISL_836544, EPI_ISL_836545, EPI_ISL_836546, EPI_ISL_836547, EPI_ISL_836548, EPI_ISL_836549, EPI_ISL_836550, EPI_ISL_836551, EPI_ISL_836552, EPI_ISL_836553, EPI_ISL_836554, EPI_ISL_836555, EPI_ISL_836556, EPI_ISL_836557, EPI_ISL_836558, EPI_ISL_836559, EPI_ISL_836560, EPI_ISL_836561, EPI_ISL_836562, EPI_ISL_836563, EPI_ISL_836564, EPI_ISL_836565, EPI_ISL_836566, EPI_ISL_836567, EPI_ISL_836568, EPI_ISL_836569, EPI_ISL_836570, EPI_ISL_836571, EPI_ISL_836572, EPI_ISL_836573, EPI_ISL_836574, EPI_ISL_836575, EPI_ISL_836576, EPI_ISL_836577, EPI_ISL_836578, EPI_ISL_836579, EPI_ISL_836580, EPI_ISL_836581, EPI_ISL_836582, EPI_ISL_836583, EPI_ISL_836584, EPI_ISL_836585, EPI_ISL_836586, EPI_ISL_836587, EPI_ISL_836588, EPI_ISL_836589, EPI_ISL_836590, EPI_ISL_836591, EPI_ISL_836592, EPI_ISL_836593, EPI_ISL_836594, EPI_ISL_836595, EPI_ISL_836596, EPI_ISL_836597, EPI_ISL_836598, EPI_ISL_836599, EPI_ISL_836600, EPI_ISL_836601, EPI_ISL_836602, EPI_ISL_836603, EPI_ISL_836604, EPI_ISL_836605, EPI_ISL_836606, EPI_ISL_836607, EPI_ISL_836608, EPI_ISL_836609, EPI_ISL_836610, EPI_ISL_836611, EPI_ISL_836612, EPI_ISL_836613, EPI_ISL_836614, EPI_ISL_836615, EPI_ISL_836616, EPI_ISL_836617, EPI_ISL_836618, EPI_ISL_836619, EPI_ISL_836620, EPI_ISL_836621, EPI_ISL_836622, EPI_ISL_836623, EPI_ISL_836624, EPI_ISL_836625, EPI_ISL_836626, EPI_ISL_836627, EPI_ISL_836628, EPI_ISL_836629, EPI_ISL_836630, EPI_ISL_836631, EPI_ISL_836632, EPI_ISL_836633, EPI_ISL_836634, EPI_ISL_836635, EPI_ISL_836636, EPI_ISL_836637, EPI_ISL_836638, EPI_ISL_836639, EPI_ISL_836640, EPI_ISL_836641, EPI_ISL_836642, EPI_ISL_836643, EPI_ISL_836644, EPI_ISL_836645, EPI_ISL_836646, EPI_ISL_836647, EPI_ISL_836648, EPI_ISL_836649, EPI_ISL_836650, EPI_ISL_836651, EPI_ISL_836652, EPI_ISL_836653, EPI_ISL_836654, EPI_ISL_836655, EPI_ISL_836656, EPI_ISL_836657, EPI_ISL_836658, EPI_ISL_836659, EPI_ISL_836660, EPI_ISL_836661, EPI_ISL_836662, EPI_ISL_836663, EPI_ISL_836664, EPI_ISL_836665, EPI_ISL_836666, EPI_ISL_836667, EPI_ISL_836668, EPI_ISL_836669, EPI_ISL_836670, EPI_ISL_836671, EPI_ISL_836672, EPI_ISL_836673, EPI_ISL_836674, EPI_ISL_836675, EPI_ISL_836676, EPI_ISL_836677, EPI_ISL_836678, EPI_ISL_836679, EPI_ISL_836680, EPI_ISL_836681, EPI_ISL_836682, EPI_ISL_836683, EPI_ISL_836684, EPI_ISL_836685, EPI_ISL_836686, EPI_ISL_836687, EPI_ISL_836688, EPI_ISL_836689, EPI_ISL_836690, EPI_ISL_836691, EPI_ISL_836692, EPI_ISL_836693, EPI_ISL_836694, EPI_ISL_836695, EPI_ISL_836696, EPI_ISL_836697, EPI_ISL_836698, EPI_ISL_836699, EPI_ISL_836700, EPI_ISL_836701, EPI_ISL_836702, EPI_ISL_836703, EPI_ISL_836704, EPI_ISL_836705, EPI_ISL_836706, EPI_ISL_836707, EPI_ISL_836708, EPI_ISL_836709, EPI_ISL_836710, EPI_ISL_836711, EPI_ISL_836712, EPI_ISL_836713, EPI_ISL_836714, EPI_ISL_836715, EPI_ISL_836716, EPI_ISL_836717, EPI_ISL_836718, EPI_ISL_836719, EPI_ISL_836720, EPI_ISL_836721, EPI_ISL_836722, EPI_ISL_836723, EPI_ISL_836724, EPI_ISL_836725, EPI_ISL_836726, EPI_ISL_836727, EPI_ISL_836728, EPI_ISL_836729, EPI_ISL_836730, EPI_ISL_836731, EPI_ISL_836732, EPI_ISL_836733, EPI_ISL_836734, EPI_ISL_836735, EPI_ISL_836736, EPI_ISL_836737, EPI_ISL_836738, EPI_ISL_836739, EPI_ISL_836740, EPI_ISL_836741, EPI_ISL_836742, EPI_ISL_836743, EPI_ISL_836744, EPI_ISL_836745, EPI_ISL_836746, EPI_ISL_836747

see above	Lighthouse Lab in Cambridge	Wellcome Sanger Institute for the COVID-19 Genomics UK (COG-UK) Consortium	Rob Howes, The Lighthouse Lab in Cambridge and Alex Alderton, Roberto Amato, Sonia Goncalves, Ewan Harrison, David K. Jackson, Ian Johnston, Dominic Kwiatkowski, Cordelia Langford, John Sillitoe on behalf of the Wellcome Sanger Institute COVID-19 Surveillance Team
EPI_ISL_836748, EPI_ISL_836749, EPI_ISL_836750, EPI_ISL_836751, EPI_ISL_836752, EPI_ISL_836753	Lighthouse Lab in Glasgow	Wellcome Sanger Institute for the COVID-19 Genomics UK (COG-UK) Consortium	Harper VanSteenhouse, Yumi Kasai, David Gray, Carol Clugston, Anna Dominiczak and Alex Alderton, Roberto Amato, Sonia Goncalves, Ewan Harrison, David K. Jackson, Ian Johnston, Dominic Kwiatkowski, Cordelia Langford, John Sillitoe on behalf of the Wellcome Sanger Institute COVID-19 Surveillance Team
EPI_ISL_836754	Lighthouse Lab in Alderley Park	Wellcome Sanger Institute for the COVID-19 Genomics UK (COG-UK) Consortium	Jacquelyn Wynn, Mairead Hyland, The Lighthouse Lab in Alderley Park and Alex Alderton, Roberto Amato, Sonia Goncalves, Ewan Harrison, David K. Jackson, Ian Johnston, Dominic Kwiatkowski, Cordelia Langford, John Sillitoe on behalf of the Wellcome Sanger Institute COVID-19 Surveillance Team
EPI_ISL_836755, EPI_ISL_836756, EPI_ISL_836757, EPI_ISL_836758, EPI_ISL_836759, EPI_ISL_836760, EPI_ISL_836761, EPI_ISL_836762, EPI_ISL_836763, EPI_ISL_836764	Lighthouse Lab in Glasgow	Wellcome Sanger Institute for the COVID-19 Genomics UK (COG-UK) Consortium	Harper VanSteenhouse, Yumi Kasai, David Gray, Carol Clugston, Anna Dominiczak and Alex Alderton, Roberto Amato, Sonia Goncalves, Ewan Harrison, David K. Jackson, Ian Johnston, Dominic Kwiatkowski, Cordelia Langford, John Sillitoe on behalf of the Wellcome Sanger Institute COVID-19 Surveillance Team
EPI_ISL_836765, EPI_ISL_836766	Lighthouse Lab in Alderley Park	Wellcome Sanger Institute for the COVID-19 Genomics UK (COG-UK) Consortium	Jacquelyn Wynn, Mairead Hyland, The Lighthouse Lab in Alderley Park and Alex Alderton, Roberto Amato, Sonia Goncalves, Ewan Harrison, David K. Jackson, Ian Johnston, Dominic Kwiatkowski, Cordelia Langford, John Sillitoe on behalf of the Wellcome Sanger Institute COVID-19 Surveillance Team
EPI_ISL_836767, EPI_ISL_836768	Lighthouse Lab in Glasgow	Wellcome Sanger Institute for the COVID-19 Genomics UK (COG-UK) Consortium	Harper VanSteenhouse, Yumi Kasai, David Gray, Carol Clugston, Anna Dominiczak and Alex Alderton, Roberto Amato, Sonia Goncalves, Ewan Harrison, David K. Jackson, Ian Johnston, Dominic Kwiatkowski, Cordelia Langford, John Sillitoe on behalf of the Wellcome Sanger Institute COVID-19 Surveillance Team
EPI_ISL_836769	Lighthouse Lab in Alderley Park	Wellcome Sanger Institute for the COVID-19 Genomics UK (COG-UK) Consortium	Jacquelyn Wynn, Mairead Hyland, The Lighthouse Lab in Alderley Park and Alex Alderton, Roberto Amato, Sonia Goncalves, Ewan Harrison, David K. Jackson, Ian Johnston, Dominic Kwiatkowski, Cordelia Langford, John Sillitoe on behalf of the Wellcome Sanger Institute COVID-19 Surveillance Team
EPI_ISL_836770, EPI_ISL_836771	Lighthouse Lab in Glasgow	Wellcome Sanger Institute for the COVID-19 Genomics UK (COG-UK) Consortium	Harper VanSteenhouse, Yumi Kasai, David Gray, Carol Clugston, Anna Dominiczak and Alex Alderton, Roberto Amato, Sonia Goncalves, Ewan Harrison, David K. Jackson, Ian Johnston, Dominic Kwiatkowski, Cordelia Langford, John Sillitoe on behalf of the Wellcome Sanger Institute COVID-19 Surveillance Team
EPI_ISL_836772	Lighthouse Lab in Alderley Park	Wellcome Sanger Institute for the COVID-19 Genomics UK (COG-UK) Consortium	Jacquelyn Wynn, Mairead Hyland, The Lighthouse Lab in Alderley Park and Alex Alderton, Roberto Amato, Sonia Goncalves, Ewan Harrison, David K. Jackson, Ian Johnston, Dominic Kwiatkowski, Cordelia Langford, John Sillitoe on behalf of the Wellcome Sanger Institute COVID-19 Surveillance Team
EPI_ISL_836773, EPI_ISL_836774, EPI_ISL_836775, EPI_ISL_836776	Lighthouse Lab in Glasgow	Wellcome Sanger Institute for the COVID-19 Genomics UK (COG-UK) Consortium	Harper VanSteenhouse, Yumi Kasai, David Gray, Carol Clugston, Anna Dominiczak and Alex Alderton, Roberto Amato, Sonia Goncalves, Ewan Harrison, David K. Jackson, Ian Johnston, Dominic Kwiatkowski, Cordelia Langford, John Sillitoe on behalf of the Wellcome Sanger Institute COVID-19 Surveillance Team
EPI_ISL_836777	Lighthouse Lab in Alderley Park	Wellcome Sanger Institute for the COVID-19 Genomics UK (COG-UK) Consortium	Jacquelyn Wynn, Mairead Hyland, The Lighthouse Lab in Alderley Park and Alex Alderton, Roberto Amato, Sonia Goncalves, Ewan Harrison, David K. Jackson, Ian Johnston, Dominic Kwiatkowski, Cordelia Langford, John Sillitoe on behalf of the Wellcome Sanger Institute COVID-19 Surveillance Team
EPI_ISL_836778, EPI_ISL_836779, EPI_ISL_836780	Lighthouse Lab in Glasgow	Wellcome Sanger Institute for the COVID-19 Genomics UK (COG-UK) Consortium	Harper VanSteenhouse, Yumi Kasai, David Gray, Carol Clugston, Anna Dominiczak and Alex Alderton, Roberto Amato, Sonia Goncalves, Ewan Harrison, David K. Jackson, Ian Johnston, Dominic Kwiatkowski, Cordelia Langford, John Sillitoe on behalf of the Wellcome Sanger Institute COVID-19 Surveillance Team
EPI_ISL_836781	Lighthouse Lab in Alderley Park	Wellcome Sanger Institute for the COVID-19 Genomics UK (COG-UK) Consortium	Jacquelyn Wynn, Mairead Hyland, The Lighthouse Lab in Alderley Park and Alex Alderton, Roberto Amato, Sonia Goncalves, Ewan Harrison, David K. Jackson, Ian Johnston, Dominic Kwiatkowski, Cordelia Langford, John Sillitoe on behalf of the Wellcome Sanger Institute COVID-19 Surveillance Team
EPI_ISL_836782, EPI_ISL_836783, EPI_ISL_836784, EPI_ISL_836785, EPI_ISL_836786, EPI_ISL_836787	Lighthouse Lab in Glasgow	Wellcome Sanger Institute for the COVID-19 Genomics UK (COG-UK) Consortium	Harper VanSteenhouse, Yumi Kasai, David Gray, Carol Clugston, Anna Dominiczak and Alex Alderton, Roberto Amato, Sonia Goncalves, Ewan Harrison, David K. Jackson, Ian Johnston, Dominic Kwiatkowski, Cordelia Langford, John Sillitoe on behalf of the Wellcome Sanger Institute COVID-19 Surveillance Team
EPI_ISL_836788	Lighthouse Lab in Alderley Park	Wellcome Sanger Institute for the COVID-19 Genomics UK (COG-UK) Consortium	Jacquelyn Wynn, Mairead Hyland, The Lighthouse Lab in Alderley Park and Alex Alderton, Roberto Amato, Sonia Goncalves, Ewan Harrison, David K. Jackson, Ian Johnston, Dominic Kwiatkowski, Cordelia Langford, John Sillitoe on behalf of the Wellcome Sanger Institute COVID-19 Surveillance Team
EPI_ISL_836789, EPI_ISL_836790, EPI_ISL_836791	Lighthouse Lab in Glasgow	Wellcome Sanger Institute for the COVID-19 Genomics UK (COG-UK) Consortium	Harper VanSteenhouse, Yumi Kasai, David Gray, Carol Clugston, Anna Dominiczak and Alex Alderton, Roberto Amato, Sonia Goncalves, Ewan Harrison, David K. Jackson, Ian Johnston, Dominic Kwiatkowski, Cordelia Langford, John Sillitoe on behalf of the Wellcome Sanger Institute COVID-19 Surveillance Team
EPI_ISL_836792	Lighthouse Lab in Alderley Park	Wellcome Sanger Institute for the COVID-19 Genomics UK (COG-UK) Consortium	Jacquelyn Wynn, Mairead Hyland, The Lighthouse Lab in Alderley Park and Alex Alderton, Roberto Amato, Sonia Goncalves, Ewan Harrison, David K. Jackson, Ian Johnston, Dominic Kwiatkowski, Cordelia Langford, John Sillitoe on behalf of the Wellcome Sanger Institute COVID-19 Surveillance Team

EPI_ISL_836793, EPI_ISL_836794	Lighthouse Lab in Glasgow	Wellcome Sanger Institute for the COVID-19 Genomics UK (COG-UK) Consortium	Harper VanSteenhouse, Yumi Kasai, David Gray, Carol Clugston, Anna Dominiczak and Alex Alderton, Roberto Amato, Sonia Goncalves, Ewan Harrison, David K. Jackson, Ian Johnston, Dominic Kwiatkowski, Cordelia Langford, John Sillitoe on behalf of the Wellcome Sanger Institute COVID-19 Surveillance Team
EPI_ISL_836795	Lighthouse Lab in Alderley Park	Wellcome Sanger Institute for the COVID-19 Genomics UK (COG-UK) Consortium	Jacquelyn Wynn, Mairead Hyland, The Lighthouse Lab in Alderley Park and Alex Alderton, Roberto Amato, Sonia Goncalves, Ewan Harrison, David K. Jackson, Ian Johnston, Dominic Kwiatkowski, Cordelia Langford, John Sillitoe on behalf of the Wellcome Sanger Institute COVID-19 Surveillance Team
EPI_ISL_836796	Lighthouse Lab in Glasgow	Wellcome Sanger Institute for the COVID-19 Genomics UK (COG-UK) Consortium	Harper VanSteenhouse, Yumi Kasai, David Gray, Carol Clugston, Anna Dominiczak and Alex Alderton, Roberto Amato, Sonia Goncalves, Ewan Harrison, David K. Jackson, Ian Johnston, Dominic Kwiatkowski, Cordelia Langford, John Sillitoe on behalf of the Wellcome Sanger Institute COVID-19 Surveillance Team
EPI_ISL_836797	Lighthouse Lab in Alderley Park	Wellcome Sanger Institute for the COVID-19 Genomics UK (COG-UK) Consortium	Jacquelyn Wynn, Mairead Hyland, The Lighthouse Lab in Alderley Park and Alex Alderton, Roberto Amato, Sonia Goncalves, Ewan Harrison, David K. Jackson, Ian Johnston, Dominic Kwiatkowski, Cordelia Langford, John Sillitoe on behalf of the Wellcome Sanger Institute COVID-19 Surveillance Team
EPI_ISL_836798, EPI_ISL_836799, EPI_ISL_836800, EPI_ISL_836801	Lighthouse Lab in Glasgow	Wellcome Sanger Institute for the COVID-19 Genomics UK (COG-UK) Consortium	Harper VanSteenhouse, Yumi Kasai, David Gray, Carol Clugston, Anna Dominiczak and Alex Alderton, Roberto Amato, Sonia Goncalves, Ewan Harrison, David K. Jackson, Ian Johnston, Dominic Kwiatkowski, Cordelia Langford, John Sillitoe on behalf of the Wellcome Sanger Institute COVID-19 Surveillance Team
EPI_ISL_836802, EPI_ISL_836803	Lighthouse Lab in Alderley Park	Wellcome Sanger Institute for the COVID-19 Genomics UK (COG-UK) Consortium	Jacquelyn Wynn, Mairead Hyland, The Lighthouse Lab in Alderley Park and Alex Alderton, Roberto Amato, Sonia Goncalves, Ewan Harrison, David K. Jackson, Ian Johnston, Dominic Kwiatkowski, Cordelia Langford, John Sillitoe on behalf of the Wellcome Sanger Institute COVID-19 Surveillance Team
EPI_ISL_836804, EPI_ISL_836805	Lighthouse Lab in Glasgow	Wellcome Sanger Institute for the COVID-19 Genomics UK (COG-UK) Consortium	Harper VanSteenhouse, Yumi Kasai, David Gray, Carol Clugston, Anna Dominiczak and Alex Alderton, Roberto Amato, Sonia Goncalves, Ewan Harrison, David K. Jackson, Ian Johnston, Dominic Kwiatkowski, Cordelia Langford, John Sillitoe on behalf of the Wellcome Sanger Institute COVID-19 Surveillance Team
EPI_ISL_836806, EPI_ISL_836807	Lighthouse Lab in Alderley Park	Wellcome Sanger Institute for the COVID-19 Genomics UK (COG-UK) Consortium	Jacquelyn Wynn, Mairead Hyland, The Lighthouse Lab in Alderley Park and Alex Alderton, Roberto Amato, Sonia Goncalves, Ewan Harrison, David K. Jackson, Ian Johnston, Dominic Kwiatkowski, Cordelia Langford, John Sillitoe on behalf of the Wellcome Sanger Institute COVID-19 Surveillance Team
EPI_ISL_836808, EPI_ISL_836809, EPI_ISL_836810	Lighthouse Lab in Glasgow	Wellcome Sanger Institute for the COVID-19 Genomics UK (COG-UK) Consortium	Harper VanSteenhouse, Yumi Kasai, David Gray, Carol Clugston, Anna Dominiczak and Alex Alderton, Roberto Amato, Sonia Goncalves, Ewan Harrison, David K. Jackson, Ian Johnston, Dominic Kwiatkowski, Cordelia Langford, John Sillitoe on behalf of the Wellcome Sanger Institute COVID-19 Surveillance Team
EPI_ISL_836811	Lighthouse Lab in Alderley Park	Wellcome Sanger Institute for the COVID-19 Genomics UK (COG-UK) Consortium	Jacquelyn Wynn, Mairead Hyland, The Lighthouse Lab in Alderley Park and Alex Alderton, Roberto Amato, Sonia Goncalves, Ewan Harrison, David K. Jackson, Ian Johnston, Dominic Kwiatkowski, Cordelia Langford, John Sillitoe on behalf of the Wellcome Sanger Institute COVID-19 Surveillance Team
EPI_ISL_836812	Lighthouse Lab in Glasgow	Wellcome Sanger Institute for the COVID-19 Genomics UK (COG-UK) Consortium	Harper VanSteenhouse, Yumi Kasai, David Gray, Carol Clugston, Anna Dominiczak and Alex Alderton, Roberto Amato, Sonia Goncalves, Ewan Harrison, David K. Jackson, Ian Johnston, Dominic Kwiatkowski, Cordelia Langford, John Sillitoe on behalf of the Wellcome Sanger Institute COVID-19 Surveillance Team
EPI_ISL_836813, EPI_ISL_836814	Lighthouse Lab in Alderley Park	Wellcome Sanger Institute for the COVID-19 Genomics UK (COG-UK) Consortium	Jacquelyn Wynn, Mairead Hyland, The Lighthouse Lab in Alderley Park and Alex Alderton, Roberto Amato, Sonia Goncalves, Ewan Harrison, David K. Jackson, Ian Johnston, Dominic Kwiatkowski, Cordelia Langford, John Sillitoe on behalf of the Wellcome Sanger Institute COVID-19 Surveillance Team
EPI_ISL_836815, EPI_ISL_836816	Lighthouse Lab in Glasgow	Wellcome Sanger Institute for the COVID-19 Genomics UK (COG-UK) Consortium	Harper VanSteenhouse, Yumi Kasai, David Gray, Carol Clugston, Anna Dominiczak and Alex Alderton, Roberto Amato, Sonia Goncalves, Ewan Harrison, David K. Jackson, Ian Johnston, Dominic Kwiatkowski, Cordelia Langford, John Sillitoe on behalf of the Wellcome Sanger Institute COVID-19 Surveillance Team
EPI_ISL_836817	Lighthouse Lab in Alderley Park	Wellcome Sanger Institute for the COVID-19 Genomics UK (COG-UK) Consortium	Jacquelyn Wynn, Mairead Hyland, The Lighthouse Lab in Alderley Park and Alex Alderton, Roberto Amato, Sonia Goncalves, Ewan Harrison, David K. Jackson, Ian Johnston, Dominic Kwiatkowski, Cordelia Langford, John Sillitoe on behalf of the Wellcome Sanger Institute COVID-19 Surveillance Team
EPI_ISL_836818	Lighthouse Lab in Glasgow	Wellcome Sanger Institute for the COVID-19 Genomics UK (COG-UK) Consortium	Harper VanSteenhouse, Yumi Kasai, David Gray, Carol Clugston, Anna Dominiczak and Alex Alderton, Roberto Amato, Sonia Goncalves, Ewan Harrison, David K. Jackson, Ian Johnston, Dominic Kwiatkowski, Cordelia Langford, John Sillitoe on behalf of the Wellcome Sanger Institute COVID-19 Surveillance Team
EPI_ISL_836819	Lighthouse Lab in Alderley Park	Wellcome Sanger Institute for the COVID-19 Genomics UK (COG-UK) Consortium	Jacquelyn Wynn, Mairead Hyland, The Lighthouse Lab in Alderley Park and Alex Alderton, Roberto Amato, Sonia Goncalves, Ewan Harrison, David K. Jackson, Ian Johnston, Dominic Kwiatkowski, Cordelia Langford, John Sillitoe on behalf of the Wellcome Sanger Institute COVID-19 Surveillance Team
EPI_ISL_836820, EPI_ISL_836821	Lighthouse Lab in Glasgow	Wellcome Sanger Institute for the COVID-19 Genomics UK (COG-UK) Consortium	Harper VanSteenhouse, Yumi Kasai, David Gray, Carol Clugston, Anna Dominiczak and Alex Alderton, Roberto Amato, Sonia Goncalves, Ewan Harrison, David K. Jackson, Ian Johnston, Dominic Kwiatkowski, Cordelia Langford, John Sillitoe on behalf of the Wellcome Sanger Institute COVID-19 Surveillance Team
EPI_ISL_836822	Lighthouse Lab in Alderley Park	Wellcome Sanger Institute for the COVID-19 Genomics UK (COG-UK) Consortium	Jacquelyn Wynn, Mairead Hyland, The Lighthouse Lab in Alderley Park and Alex Alderton, Roberto Amato, Sonia Goncalves, Ewan Harrison, David K. Jackson, Ian Johnston, Dominic Kwiatkowski, Cordelia Langford, John Sillitoe on behalf of the Wellcome Sanger Institute COVID-19 Surveillance Team
EPI_ISL_836823, EPI_ISL_836824, EPI_ISL_836825, EPI_ISL_836826, EPI_ISL_836827, EPI_ISL_836828, EPI_ISL_836829, EPI_ISL_836830, EPI_ISL_836831, EPI_ISL_836832, EPI_ISL_836833, EPI_ISL_836834, EPI_ISL_836835			
see above	Lighthouse Lab in Glasgow	Wellcome Sanger Institute for the COVID-19 Genomics UK (COG-UK) Consortium	Harper VanSteenhouse, Yumi Kasai, David Gray, Carol Clugston, Anna Dominiczak and Alex Alderton, Roberto Amato, Sonia Goncalves, Ewan Harrison, David K. Jackson, Ian Johnston, Dominic Kwiatkowski, Cordelia Langford, John Sillitoe on behalf of the Wellcome Sanger Institute COVID-19 Surveillance Team
EPI_ISL_836836, EPI_ISL_836837	Lighthouse Lab in Alderley Park	Wellcome Sanger Institute for the COVID-19 Genomics UK (COG-UK) Consortium	Jacquelyn Wynn, Mairead Hyland, The Lighthouse Lab in Alderley Park and Alex Alderton, Roberto Amato, Sonia Goncalves, Ewan Harrison, David K. Jackson, Ian Johnston, Dominic Kwiatkowski, Cordelia Langford, John Sillitoe on behalf of the Wellcome Sanger Institute COVID-19 Surveillance Team
EPI_ISL_836838, EPI_ISL_836839	Lighthouse Lab in Glasgow	Wellcome Sanger Institute for the COVID-19 Genomics UK (COG-UK) Consortium	Harper VanSteenhouse, Yumi Kasai, David Gray, Carol Clugston, Anna Dominiczak and Alex Alderton, Roberto Amato, Sonia Goncalves, Ewan Harrison, David K. Jackson, Ian Johnston, Dominic Kwiatkowski, Cordelia Langford, John Sillitoe on behalf of the Wellcome Sanger Institute COVID-19 Surveillance Team
EPI_ISL_836840	Lighthouse Lab in Alderley Park	Wellcome Sanger Institute for the COVID-19 Genomics UK (COG-UK) Consortium	Jacquelyn Wynn, Mairead Hyland, The Lighthouse Lab in Alderley Park and Alex Alderton, Roberto Amato, Sonia Goncalves, Ewan Harrison, David K. Jackson, Ian Johnston, Dominic Kwiatkowski, Cordelia Langford, John Sillitoe on behalf of the Wellcome Sanger Institute COVID-19 Surveillance Team
EPI_ISL_836841	Lighthouse Lab in Glasgow	Wellcome Sanger Institute for the COVID-19 Genomics UK (COG-UK) Consortium	Harper VanSteenhouse, Yumi Kasai, David Gray, Carol Clugston, Anna Dominiczak and Alex Alderton, Roberto Amato, Sonia Goncalves, Ewan Harrison, David K. Jackson, Ian Johnston, Dominic Kwiatkowski, Cordelia Langford, John Sillitoe on behalf of the Wellcome Sanger Institute COVID-19 Surveillance Team
EPI_ISL_836842, EPI_ISL_836843	Lighthouse Lab in Alderley Park	Wellcome Sanger Institute for the COVID-19 Genomics UK (COG-UK) Consortium	Jacquelyn Wynn, Mairead Hyland, The Lighthouse Lab in Alderley Park and Alex Alderton, Roberto Amato, Sonia Goncalves, Ewan Harrison, David K. Jackson, Ian Johnston, Dominic Kwiatkowski, Cordelia Langford, John Sillitoe on behalf of the Wellcome Sanger Institute COVID-19 Surveillance Team
EPI_ISL_836844, EPI_ISL_836845, EPI_ISL_836846, EPI_ISL_836847	Lighthouse Lab in Glasgow	Wellcome Sanger Institute for the COVID-19 Genomics UK (COG-UK) Consortium	Harper VanSteenhouse, Yumi Kasai, David Gray, Carol Clugston, Anna Dominiczak and Alex Alderton, Roberto Amato, Sonia Goncalves, Ewan Harrison, David K. Jackson, Ian Johnston, Dominic Kwiatkowski, Cordelia Langford, John Sillitoe on behalf of the Wellcome Sanger Institute COVID-19 Surveillance Team
EPI_ISL_836848	Lighthouse Lab in Alderley Park	Wellcome Sanger Institute for the COVID-19 Genomics UK (COG-UK) Consortium	Jacquelyn Wynn, Mairead Hyland, The Lighthouse Lab in Alderley Park and Alex Alderton, Roberto Amato, Sonia Goncalves, Ewan Harrison, David K. Jackson, Ian Johnston, Dominic Kwiatkowski, Cordelia Langford, John Sillitoe on behalf of the Wellcome Sanger Institute COVID-19 Surveillance Team
EPI_ISL_836849, EPI_ISL_836850	Lighthouse Lab in Glasgow	Wellcome Sanger Institute for the COVID-19 Genomics UK (COG-UK) Consortium	Harper VanSteenhouse, Yumi Kasai, David Gray, Carol Clugston, Anna Dominiczak and Alex Alderton, Roberto Amato, Sonia Goncalves, Ewan Harrison, David K. Jackson, Ian Johnston, Dominic Kwiatkowski, Cordelia Langford, John Sillitoe on behalf of the Wellcome Sanger Institute COVID-19 Surveillance Team
EPI_ISL_836851	Lighthouse Lab in Alderley Park	Wellcome Sanger Institute for the COVID-19 Genomics UK (COG-UK) Consortium	Jacquelyn Wynn, Mairead Hyland, The Lighthouse Lab in Alderley Park and Alex Alderton, Roberto Amato, Sonia Goncalves, Ewan Harrison, David K. Jackson, Ian Johnston, Dominic Kwiatkowski, Cordelia Langford, John Sillitoe on behalf of the Wellcome Sanger Institute COVID-19 Surveillance Team
EPI_ISL_836852, EPI_ISL_836853, EPI_ISL_836854, EPI_ISL_836855,	Lighthouse Lab in Glasgow	Wellcome Sanger Institute for the COVID-19 Genomics UK (COG-UK) Consortium	Harper VanSteenhouse, Yumi Kasai, David Gray, Carol Clugston, Anna Dominiczak and Alex Alderton, Roberto Amato, Sonia Goncalves, Ewan Harrison, David K. Jackson, Ian Johnston, Dominic Kwiatkowski, Cordelia Langford, John Sillitoe on behalf of the Wellcome Sanger Institute COVID-19 Surveillance

EPI_ISL_836856, EPI_ISL_836857			Team
EPI_ISL_836858, EPI_ISL_836859	Lighthouse Lab in Alderley Park	Wellcome Sanger Institute for the COVID-19 Genomics UK (COG-UK) Consortium	Jacquelyn Wynn, Mairead Hyland, The Lighthouse Lab in Alderley Park and Alex Alderton, Roberto Amato, Sonia Goncalves, Ewan Harrison, David K. Jackson, Ian Johnston, Dominic Kwiatkowski, Cordelia Langford, John Sillitoe on behalf of the Wellcome Sanger Institute COVID-19 Surveillance Team
EPI_ISL_836860, EPI_ISL_836861	Lighthouse Lab in Glasgow	Wellcome Sanger Institute for the COVID-19 Genomics UK (COG-UK) Consortium	Harper VanSteenhouse, Yumi Kasai, David Gray, Carol Clugston, Anna Dominiczak and Alex Alderton, Roberto Amato, Sonia Goncalves, Ewan Harrison, David K. Jackson, Ian Johnston, Dominic Kwiatkowski, Cordelia Langford, John Sillitoe on behalf of the Wellcome Sanger Institute COVID-19 Surveillance Team
EPI_ISL_836862, EPI_ISL_836863	Lighthouse Lab in Alderley Park	Wellcome Sanger Institute for the COVID-19 Genomics UK (COG-UK) Consortium	Jacquelyn Wynn, Mairead Hyland, The Lighthouse Lab in Alderley Park and Alex Alderton, Roberto Amato, Sonia Goncalves, Ewan Harrison, David K. Jackson, Ian Johnston, Dominic Kwiatkowski, Cordelia Langford, John Sillitoe on behalf of the Wellcome Sanger Institute COVID-19 Surveillance Team
EPI_ISL_836864, EPI_ISL_836865	Lighthouse Lab in Glasgow	Wellcome Sanger Institute for the COVID-19 Genomics UK (COG-UK) Consortium	Harper VanSteenhouse, Yumi Kasai, David Gray, Carol Clugston, Anna Dominiczak and Alex Alderton, Roberto Amato, Sonia Goncalves, Ewan Harrison, David K. Jackson, Ian Johnston, Dominic Kwiatkowski, Cordelia Langford, John Sillitoe on behalf of the Wellcome Sanger Institute COVID-19 Surveillance Team
EPI_ISL_836866, EPI_ISL_836867	Lighthouse Lab in Alderley Park	Wellcome Sanger Institute for the COVID-19 Genomics UK (COG-UK) Consortium	Jacquelyn Wynn, Mairead Hyland, The Lighthouse Lab in Alderley Park and Alex Alderton, Roberto Amato, Sonia Goncalves, Ewan Harrison, David K. Jackson, Ian Johnston, Dominic Kwiatkowski, Cordelia Langford, John Sillitoe on behalf of the Wellcome Sanger Institute COVID-19 Surveillance Team
EPI_ISL_836868, EPI_ISL_836869, EPI_ISL_836870, EPI_ISL_836871, EPI_ISL_836872, EPI_ISL_836873, EPI_ISL_836874, EPI_ISL_836875	Lighthouse Lab in Glasgow	Wellcome Sanger Institute for the COVID-19 Genomics UK (COG-UK) Consortium	Harper VanSteenhouse, Yumi Kasai, David Gray, Carol Clugston, Anna Dominiczak and Alex Alderton, Roberto Amato, Sonia Goncalves, Ewan Harrison, David K. Jackson, Ian Johnston, Dominic Kwiatkowski, Cordelia Langford, John Sillitoe on behalf of the Wellcome Sanger Institute COVID-19 Surveillance Team
EPI_ISL_836876, EPI_ISL_836877, EPI_ISL_836878	Lighthouse Lab in Alderley Park	Wellcome Sanger Institute for the COVID-19 Genomics UK (COG-UK) Consortium	Jacquelyn Wynn, Mairead Hyland, The Lighthouse Lab in Alderley Park and Alex Alderton, Roberto Amato, Sonia Goncalves, Ewan Harrison, David K. Jackson, Ian Johnston, Dominic Kwiatkowski, Cordelia Langford, John Sillitoe on behalf of the Wellcome Sanger Institute COVID-19 Surveillance Team
EPI_ISL_836879, EPI_ISL_836880, EPI_ISL_836881, EPI_ISL_836882	Lighthouse Lab in Glasgow	Wellcome Sanger Institute for the COVID-19 Genomics UK (COG-UK) Consortium	Harper VanSteenhouse, Yumi Kasai, David Gray, Carol Clugston, Anna Dominiczak and Alex Alderton, Roberto Amato, Sonia Goncalves, Ewan Harrison, David K. Jackson, Ian Johnston, Dominic Kwiatkowski, Cordelia Langford, John Sillitoe on behalf of the Wellcome Sanger Institute COVID-19 Surveillance Team
EPI_ISL_836883	Lighthouse Lab in Alderley Park	Wellcome Sanger Institute for the COVID-19 Genomics UK (COG-UK) Consortium	Jacquelyn Wynn, Mairead Hyland, The Lighthouse Lab in Alderley Park and Alex Alderton, Roberto Amato, Sonia Goncalves, Ewan Harrison, David K. Jackson, Ian Johnston, Dominic Kwiatkowski, Cordelia Langford, John Sillitoe on behalf of the Wellcome Sanger Institute COVID-19 Surveillance Team
EPI_ISL_836884, EPI_ISL_836885, EPI_ISL_836886	Lighthouse Lab in Glasgow	Wellcome Sanger Institute for the COVID-19 Genomics UK (COG-UK) Consortium	Harper VanSteenhouse, Yumi Kasai, David Gray, Carol Clugston, Anna Dominiczak and Alex Alderton, Roberto Amato, Sonia Goncalves, Ewan Harrison, David K. Jackson, Ian Johnston, Dominic Kwiatkowski, Cordelia Langford, John Sillitoe on behalf of the Wellcome Sanger Institute COVID-19 Surveillance Team
EPI_ISL_836887	Lighthouse Lab in Alderley Park	Wellcome Sanger Institute for the COVID-19 Genomics UK (COG-UK) Consortium	Jacquelyn Wynn, Mairead Hyland, The Lighthouse Lab in Alderley Park and Alex Alderton, Roberto Amato, Sonia Goncalves, Ewan Harrison, David K. Jackson, Ian Johnston, Dominic Kwiatkowski, Cordelia Langford, John Sillitoe on behalf of the Wellcome Sanger Institute COVID-19 Surveillance Team
EPI_ISL_836888, EPI_ISL_836889	Lighthouse Lab in Glasgow	Wellcome Sanger Institute for the COVID-19 Genomics UK (COG-UK) Consortium	Harper VanSteenhouse, Yumi Kasai, David Gray, Carol Clugston, Anna Dominiczak and Alex Alderton, Roberto Amato, Sonia Goncalves, Ewan Harrison, David K. Jackson, Ian Johnston, Dominic Kwiatkowski, Cordelia Langford, John Sillitoe on behalf of the Wellcome Sanger Institute COVID-19 Surveillance Team
EPI_ISL_836890, EPI_ISL_836891	Lighthouse Lab in Alderley Park	Wellcome Sanger Institute for the COVID-19 Genomics UK (COG-UK) Consortium	Jacquelyn Wynn, Mairead Hyland, The Lighthouse Lab in Alderley Park and Alex Alderton, Roberto Amato, Sonia Goncalves, Ewan Harrison, David K. Jackson, Ian Johnston, Dominic Kwiatkowski, Cordelia Langford, John Sillitoe on behalf of the Wellcome Sanger Institute COVID-19 Surveillance Team
EPI_ISL_836892, EPI_ISL_836893, EPI_ISL_836894, EPI_ISL_836895, EPI_ISL_836896	Lighthouse Lab in Glasgow	Wellcome Sanger Institute for the COVID-19 Genomics UK (COG-UK) Consortium	Harper VanSteenhouse, Yumi Kasai, David Gray, Carol Clugston, Anna Dominiczak and Alex Alderton, Roberto Amato, Sonia Goncalves, Ewan Harrison, David K. Jackson, Ian Johnston, Dominic Kwiatkowski, Cordelia Langford, John Sillitoe on behalf of the Wellcome Sanger Institute COVID-19 Surveillance Team
EPI_ISL_836897	Lighthouse Lab in Alderley Park	Wellcome Sanger Institute for the COVID-19 Genomics UK (COG-UK) Consortium	Jacquelyn Wynn, Mairead Hyland, The Lighthouse Lab in Alderley Park and Alex Alderton, Roberto Amato, Sonia Goncalves, Ewan Harrison, David K. Jackson, Ian Johnston, Dominic Kwiatkowski, Cordelia Langford, John Sillitoe on behalf of the Wellcome Sanger Institute COVID-19 Surveillance Team
EPI_ISL_836898, EPI_ISL_836899, EPI_ISL_836900, EPI_ISL_836901, EPI_ISL_836902	Lighthouse Lab in Glasgow	Wellcome Sanger Institute for the COVID-19 Genomics UK (COG-UK) Consortium	Harper VanSteenhouse, Yumi Kasai, David Gray, Carol Clugston, Anna Dominiczak and Alex Alderton, Roberto Amato, Sonia Goncalves, Ewan Harrison, David K. Jackson, Ian Johnston, Dominic Kwiatkowski, Cordelia Langford, John Sillitoe on behalf of the Wellcome Sanger Institute COVID-19 Surveillance Team
EPI_ISL_836903	Lighthouse Lab in Alderley Park	Wellcome Sanger Institute for the COVID-19 Genomics UK (COG-UK) Consortium	Jacquelyn Wynn, Mairead Hyland, The Lighthouse Lab in Alderley Park and Alex Alderton, Roberto Amato, Sonia Goncalves, Ewan Harrison, David K. Jackson, Ian Johnston, Dominic Kwiatkowski, Cordelia Langford, John Sillitoe on behalf of the Wellcome Sanger Institute COVID-19 Surveillance Team
EPI_ISL_836904, EPI_ISL_836905	Lighthouse Lab in Glasgow	Wellcome Sanger Institute for the COVID-19 Genomics UK (COG-UK) Consortium	Harper VanSteenhouse, Yumi Kasai, David Gray, Carol Clugston, Anna Dominiczak and Alex Alderton, Roberto Amato, Sonia Goncalves, Ewan Harrison, David K. Jackson, Ian Johnston, Dominic Kwiatkowski, Cordelia Langford, John Sillitoe on behalf of the Wellcome Sanger Institute COVID-19 Surveillance Team
EPI_ISL_836906	Lighthouse Lab in Alderley Park	Wellcome Sanger Institute for the COVID-19 Genomics UK (COG-UK) Consortium	Jacquelyn Wynn, Mairead Hyland, The Lighthouse Lab in Alderley Park and Alex Alderton, Roberto Amato, Sonia Goncalves, Ewan Harrison, David K. Jackson, Ian Johnston, Dominic Kwiatkowski, Cordelia Langford, John Sillitoe on behalf of the Wellcome Sanger Institute COVID-19 Surveillance Team
EPI_ISL_836907, EPI_ISL_836908	Lighthouse Lab in Glasgow	Wellcome Sanger Institute for the COVID-19 Genomics UK (COG-UK) Consortium	Harper VanSteenhouse, Yumi Kasai, David Gray, Carol Clugston, Anna Dominiczak and Alex Alderton, Roberto Amato, Sonia Goncalves, Ewan Harrison, David K. Jackson, Ian Johnston, Dominic Kwiatkowski, Cordelia Langford, John Sillitoe on behalf of the Wellcome Sanger Institute COVID-19 Surveillance Team
EPI_ISL_836909, EPI_ISL_836910	Lighthouse Lab in Alderley Park	Wellcome Sanger Institute for the COVID-19 Genomics UK (COG-UK) Consortium	Jacquelyn Wynn, Mairead Hyland, The Lighthouse Lab in Alderley Park and Alex Alderton, Roberto Amato, Sonia Goncalves, Ewan Harrison, David K. Jackson, Ian Johnston, Dominic Kwiatkowski, Cordelia Langford, John Sillitoe on behalf of the Wellcome Sanger Institute COVID-19 Surveillance Team
EPI_ISL_836911, EPI_ISL_836912	Lighthouse Lab in Glasgow	Wellcome Sanger Institute for the COVID-19 Genomics UK (COG-UK) Consortium	Harper VanSteenhouse, Yumi Kasai, David Gray, Carol Clugston, Anna Dominiczak and Alex Alderton, Roberto Amato, Sonia Goncalves, Ewan Harrison, David K. Jackson, Ian Johnston, Dominic Kwiatkowski, Cordelia Langford, John Sillitoe on behalf of the Wellcome Sanger Institute COVID-19 Surveillance Team
EPI_ISL_836913, EPI_ISL_836914	Lighthouse Lab in Alderley Park	Wellcome Sanger Institute for the COVID-19 Genomics UK (COG-UK) Consortium	Jacquelyn Wynn, Mairead Hyland, The Lighthouse Lab in Alderley Park and Alex Alderton, Roberto Amato, Sonia Goncalves, Ewan Harrison, David K. Jackson, Ian Johnston, Dominic Kwiatkowski, Cordelia Langford, John Sillitoe on behalf of the Wellcome Sanger Institute COVID-19 Surveillance Team
EPI_ISL_836915, EPI_ISL_836916, EPI_ISL_836917, EPI_ISL_836918, EPI_ISL_836919	Lighthouse Lab in Glasgow	Wellcome Sanger Institute for the COVID-19 Genomics UK (COG-UK) Consortium	Harper VanSteenhouse, Yumi Kasai, David Gray, Carol Clugston, Anna Dominiczak and Alex Alderton, Roberto Amato, Sonia Goncalves, Ewan Harrison, David K. Jackson, Ian Johnston, Dominic Kwiatkowski, Cordelia Langford, John Sillitoe on behalf of the Wellcome Sanger Institute COVID-19 Surveillance Team
EPI_ISL_836920, EPI_ISL_836921, EPI_ISL_836922	Lighthouse Lab in Alderley Park	Wellcome Sanger Institute for the COVID-19 Genomics UK (COG-UK) Consortium	Jacquelyn Wynn, Mairead Hyland, The Lighthouse Lab in Alderley Park and Alex Alderton, Roberto Amato, Sonia Goncalves, Ewan Harrison, David K. Jackson, Ian Johnston, Dominic Kwiatkowski, Cordelia Langford, John Sillitoe on behalf of the Wellcome Sanger Institute COVID-19 Surveillance Team
EPI_ISL_836923, EPI_ISL_836924, EPI_ISL_836925	Lighthouse Lab in Glasgow	Wellcome Sanger Institute for the COVID-19 Genomics UK (COG-UK) Consortium	Harper VanSteenhouse, Yumi Kasai, David Gray, Carol Clugston, Anna Dominiczak and Alex Alderton, Roberto Amato, Sonia Goncalves, Ewan Harrison, David K. Jackson, Ian Johnston, Dominic Kwiatkowski, Cordelia Langford, John Sillitoe on behalf of the Wellcome Sanger Institute COVID-19 Surveillance Team
EPI_ISL_836926	Lighthouse Lab in Alderley Park	Wellcome Sanger Institute for the COVID-19 Genomics UK (COG-UK) Consortium	Jacquelyn Wynn, Mairead Hyland, The Lighthouse Lab in Alderley Park and Alex Alderton, Roberto Amato, Sonia Goncalves, Ewan Harrison, David K. Jackson, Ian Johnston, Dominic Kwiatkowski, Cordelia Langford, John Sillitoe on behalf of the Wellcome Sanger Institute COVID-19 Surveillance Team
EPI_ISL_836927	Lighthouse Lab in Glasgow	Wellcome Sanger Institute for the COVID-19 Genomics UK (COG-UK) Consortium	Harper VanSteenhouse, Yumi Kasai, David Gray, Carol Clugston, Anna Dominiczak and Alex Alderton, Roberto Amato, Sonia Goncalves, Ewan Harrison, David K. Jackson, Ian Johnston, Dominic Kwiatkowski, Cordelia Langford, John Sillitoe on behalf of the Wellcome Sanger Institute COVID-19 Surveillance Team
EPI_ISL_836928, EPI_ISL_836929	Lighthouse Lab in Alderley Park	Wellcome Sanger Institute for the COVID-19 Genomics UK	Jacquelyn Wynn, Mairead Hyland, The Lighthouse Lab in Alderley Park and Alex Alderton, Roberto Amato, Sonia Goncalves, Ewan Harrison, David K.

		(COG-UK) Consortium	Jackson, Ian Johnston, Dominic Kwiatkowski, Cordelia Langford, John Sillitoe on behalf of the Wellcome Sanger Institute COVID-19 Surveillance Team
EPI_ISL_836930, EPI_ISL_836931, EPI_ISL_836932, EPI_ISL_836933, EPI_ISL_836934	Lighthouse Lab in Glasgow	Wellcome Sanger Institute for the COVID-19 Genomics UK (COG-UK) Consortium	Harper VanSteenhouse, Yumi Kasai, David Gray, Carol Clugston, Anna Dominiczak and Alex Alderton, Roberto Amato, Sonia Goncalves, Ewan Harrison, David K. Jackson, Ian Johnston, Dominic Kwiatkowski, Cordelia Langford, John Sillitoe on behalf of the Wellcome Sanger Institute COVID-19 Surveillance Team
EPI_ISL_836935, EPI_ISL_836936	Lighthouse Lab in Alderley Park	Wellcome Sanger Institute for the COVID-19 Genomics UK (COG-UK) Consortium	Jacquelyn Wynn, Mairead Hyland, The Lighthouse Lab in Alderley Park and Alex Alderton, Roberto Amato, Sonia Goncalves, Ewan Harrison, David K. Jackson, Ian Johnston, Dominic Kwiatkowski, Cordelia Langford, John Sillitoe on behalf of the Wellcome Sanger Institute COVID-19 Surveillance Team
EPI_ISL_836937, EPI_ISL_836938, EPI_ISL_836939	Lighthouse Lab in Glasgow	Wellcome Sanger Institute for the COVID-19 Genomics UK (COG-UK) Consortium	Harper VanSteenhouse, Yumi Kasai, David Gray, Carol Clugston, Anna Dominiczak and Alex Alderton, Roberto Amato, Sonia Goncalves, Ewan Harrison, David K. Jackson, Ian Johnston, Dominic Kwiatkowski, Cordelia Langford, John Sillitoe on behalf of the Wellcome Sanger Institute COVID-19 Surveillance Team
EPI_ISL_836940	Lighthouse Lab in Alderley Park	Wellcome Sanger Institute for the COVID-19 Genomics UK (COG-UK) Consortium	Jacquelyn Wynn, Mairead Hyland, The Lighthouse Lab in Alderley Park and Alex Alderton, Roberto Amato, Sonia Goncalves, Ewan Harrison, David K. Jackson, Ian Johnston, Dominic Kwiatkowski, Cordelia Langford, John Sillitoe on behalf of the Wellcome Sanger Institute COVID-19 Surveillance Team
EPI_ISL_836941, EPI_ISL_836942, EPI_ISL_836943, EPI_ISL_836944, EPI_ISL_836945	Lighthouse Lab in Glasgow	Wellcome Sanger Institute for the COVID-19 Genomics UK (COG-UK) Consortium	Harper VanSteenhouse, Yumi Kasai, David Gray, Carol Clugston, Anna Dominiczak and Alex Alderton, Roberto Amato, Sonia Goncalves, Ewan Harrison, David K. Jackson, Ian Johnston, Dominic Kwiatkowski, Cordelia Langford, John Sillitoe on behalf of the Wellcome Sanger Institute COVID-19 Surveillance Team
EPI_ISL_836946, EPI_ISL_836947, EPI_ISL_836948	Lighthouse Lab in Alderley Park	Wellcome Sanger Institute for the COVID-19 Genomics UK (COG-UK) Consortium	Jacquelyn Wynn, Mairead Hyland, The Lighthouse Lab in Alderley Park and Alex Alderton, Roberto Amato, Sonia Goncalves, Ewan Harrison, David K. Jackson, Ian Johnston, Dominic Kwiatkowski, Cordelia Langford, John Sillitoe on behalf of the Wellcome Sanger Institute COVID-19 Surveillance Team
EPI_ISL_836949, EPI_ISL_836950, EPI_ISL_836951, EPI_ISL_836952, EPI_ISL_836953, EPI_ISL_836954	Lighthouse Lab in Glasgow	Wellcome Sanger Institute for the COVID-19 Genomics UK (COG-UK) Consortium	Harper VanSteenhouse, Yumi Kasai, David Gray, Carol Clugston, Anna Dominiczak and Alex Alderton, Roberto Amato, Sonia Goncalves, Ewan Harrison, David K. Jackson, Ian Johnston, Dominic Kwiatkowski, Cordelia Langford, John Sillitoe on behalf of the Wellcome Sanger Institute COVID-19 Surveillance Team
EPI_ISL_836955	Lighthouse Lab in Alderley Park	Wellcome Sanger Institute for the COVID-19 Genomics UK (COG-UK) Consortium	Jacquelyn Wynn, Mairead Hyland, The Lighthouse Lab in Alderley Park and Alex Alderton, Roberto Amato, Sonia Goncalves, Ewan Harrison, David K. Jackson, Ian Johnston, Dominic Kwiatkowski, Cordelia Langford, John Sillitoe on behalf of the Wellcome Sanger Institute COVID-19 Surveillance Team
EPI_ISL_836956, EPI_ISL_836957, EPI_ISL_836958, EPI_ISL_836959, EPI_ISL_836960, EPI_ISL_836961	Lighthouse Lab in Glasgow	Wellcome Sanger Institute for the COVID-19 Genomics UK (COG-UK) Consortium	Harper VanSteenhouse, Yumi Kasai, David Gray, Carol Clugston, Anna Dominiczak and Alex Alderton, Roberto Amato, Sonia Goncalves, Ewan Harrison, David K. Jackson, Ian Johnston, Dominic Kwiatkowski, Cordelia Langford, John Sillitoe on behalf of the Wellcome Sanger Institute COVID-19 Surveillance Team
EPI_ISL_836962	Lighthouse Lab in Alderley Park	Wellcome Sanger Institute for the COVID-19 Genomics UK (COG-UK) Consortium	Jacquelyn Wynn, Mairead Hyland, The Lighthouse Lab in Alderley Park and Alex Alderton, Roberto Amato, Sonia Goncalves, Ewan Harrison, David K. Jackson, Ian Johnston, Dominic Kwiatkowski, Cordelia Langford, John Sillitoe on behalf of the Wellcome Sanger Institute COVID-19 Surveillance Team
EPI_ISL_836963, EPI_ISL_836964, EPI_ISL_836965, EPI_ISL_836966, EPI_ISL_836967, EPI_ISL_836968, EPI_ISL_836969, EPI_ISL_836970, EPI_ISL_836971, EPI_ISL_836972, EPI_ISL_836973, EPI_ISL_836974			
see above	Lighthouse Lab in Glasgow	Wellcome Sanger Institute for the COVID-19 Genomics UK (COG-UK) Consortium	Harper VanSteenhouse, Yumi Kasai, David Gray, Carol Clugston, Anna Dominiczak and Alex Alderton, Roberto Amato, Sonia Goncalves, Ewan Harrison, David K. Jackson, Ian Johnston, Dominic Kwiatkowski, Cordelia Langford, John Sillitoe on behalf of the Wellcome Sanger Institute COVID-19 Surveillance Team
EPI_ISL_836975, EPI_ISL_836976	Lighthouse Lab in Alderley Park	Wellcome Sanger Institute for the COVID-19 Genomics UK (COG-UK) Consortium	Jacquelyn Wynn, Mairead Hyland, The Lighthouse Lab in Alderley Park and Alex Alderton, Roberto Amato, Sonia Goncalves, Ewan Harrison, David K. Jackson, Ian Johnston, Dominic Kwiatkowski, Cordelia Langford, John Sillitoe on behalf of the Wellcome Sanger Institute COVID-19 Surveillance Team
EPI_ISL_837240, EPI_ISL_837241, EPI_ISL_837242	Respiratory Virus Unit, National Infection Service, Public Health England	COVID-19 Genomics UK (COG-UK) Consortium	PHE Covid Sequencing Team
EPI_ISL_837843, EPI_ISL_837844, EPI_ISL_837845, EPI_ISL_837846, EPI_ISL_837847, EPI_ISL_837848, EPI_ISL_837850, EPI_ISL_837852, EPI_ISL_837854, EPI_ISL_837856, EPI_ISL_837857, EPI_ISL_837859, EPI_ISL_837861, EPI_ISL_837863, EPI_ISL_837865, EPI_ISL_837869, EPI_ISL_837871, EPI_ISL_837903, EPI_ISL_837905, EPI_ISL_837906, EPI_ISL_837907, EPI_ISL_837908, EPI_ISL_837909, EPI_ISL_837910, EPI_ISL_837911, EPI_ISL_837912, EPI_ISL_837913, EPI_ISL_837914, EPI_ISL_837915, EPI_ISL_837916, EPI_ISL_837917, EPI_ISL_837918, EPI_ISL_837919, EPI_ISL_837920, EPI_ISL_837921, EPI_ISL_837922, EPI_ISL_837923, EPI_ISL_837924, EPI_ISL_837925, EPI_ISL_837927, EPI_ISL_837929, EPI_ISL_837930, EPI_ISL_837931, EPI_ISL_837932, EPI_ISL_837933, EPI_ISL_837934, EPI_ISL_837935, EPI_ISL_837936, EPI_ISL_837937, EPI_ISL_837938, EPI_ISL_837939, EPI_ISL_837940, EPI_ISL_837941, EPI_ISL_837942, EPI_ISL_837943, EPI_ISL_837944, EPI_ISL_837945, EPI_ISL_837946, EPI_ISL_837947, EPI_ISL_837948, EPI_ISL_837949, EPI_ISL_837950, EPI_ISL_837951, EPI_ISL_837952, EPI_ISL_837953, EPI_ISL_837954, EPI_ISL_837955, EPI_ISL_837956, EPI_ISL_837958, EPI_ISL_837960, EPI_ISL_837962, EPI_ISL_837963, EPI_ISL_837964, EPI_ISL_837965, EPI_ISL_837966, EPI_ISL_837967, EPI_ISL_837968, EPI_ISL_837969, EPI_ISL_837970, EPI_ISL_837971			
see above	Department of Pathology, University of Cambridge	COVID-19 Genomics UK (COG-UK) Consortium	Aminu S. Jahun, Yasmin Chaudhry, Grant Hall, Iliana Georgana, Myra Hosmillo, Martin D. Curran, Malte Pinckert, Surendra Parmar, Ian Goodfellow
EPI_ISL_838290, EPI_ISL_838291, EPI_ISL_838293, EPI_ISL_838294, EPI_ISL_838295, EPI_ISL_838296, EPI_ISL_838297, EPI_ISL_838298, EPI_ISL_838299, EPI_ISL_838300, EPI_ISL_838301, EPI_ISL_838302, EPI_ISL_838303, EPI_ISL_838304, EPI_ISL_838305, EPI_ISL_838306, EPI_ISL_838307, EPI_ISL_838308, EPI_ISL_838309, EPI_ISL_838310, EPI_ISL_838311, EPI_ISL_838312			
see above	Virology Department, Royal Infirmary of Edinburgh, NHS Lothian / School of Biological Sciences, University of Edinburgh / Institute of Genetics and Molecular Medicine, University of Edinburgh	COVID-19 Genomics UK (COG-UK) Consortium	McHugh M, Dewar R, Rooke S, Gallagher M, Balcaza C, O'Toole Á, Scher E, Hill V, McCrone JT, Colquhoun R, Yu X, Jackson B, Rambaut A, Williams TC, Templeton K
EPI_ISL_838331, EPI_ISL_838334, EPI_ISL_838335, EPI_ISL_838336, EPI_ISL_838337, EPI_ISL_838339, EPI_ISL_838340, EPI_ISL_838341	University of Exeter	COVID-19 Genomics UK (COG-UK) Consortium	Ben Temperton,Aaron Jeffries,Michelle Michelsen,Joanna Warwick-Dugdale,Audrey Farbos,Robyn Manley,Stephen Michell,Jane Masoli
EPI_ISL_838586, EPI_ISL_838587, EPI_ISL_838592, EPI_ISL_838593, EPI_ISL_838597, EPI_ISL_838600, EPI_ISL_838602, EPI_ISL_838603, EPI_ISL_838605, EPI_ISL_838613, EPI_ISL_838620, EPI_ISL_838628, EPI_ISL_838655, EPI_ISL_838669, EPI_ISL_838670, EPI_ISL_838671			
see above	Liverpool Clinical Laboratories	COVID-19 Genomics UK (COG-UK) Consortium	Sam Haldenby, Anita Lucaci, Steve Paterson, Julian Hiscox, Alistair Darby, M Almsaud, A Alrezaihi, Muhannad Alruwaili, Stuart D Armstrong, Jones Benjamin, Eleanor G Bentley, Anu Chawla, Jordan J Clark, Angela Cowell, Richard Eccles, Isabel García-Dorival, Matthew Gemmell, Alessandro Gerada, PKF Gilmore, Richard Gregory, Ximeng Han, Catherine Hartley, Margaret Hughes, Miren Iturriza-Gomara, James Johnson, L Luu, Jenifer Manson, Charlotte Nelson, Elaine O'Toole, Cassie Olateju, Rebekah Penrice-Randal , Lucille Rainbow, N.P Randle, Trevor Ian Robinson, Parul Sharma, Ghada T Shawli, James P Stewart, Neil Swainston, Ecaterina Vamos, Joanne Watts, Mark Whitehead
EPI_ISL_838807, EPI_ISL_838835, EPI_ISL_838836, EPI_ISL_838837, EPI_ISL_838842, EPI_ISL_838843, EPI_ISL_838844, EPI_ISL_838845, EPI_ISL_838846, EPI_ISL_838847, EPI_ISL_838848, EPI_ISL_838849, EPI_ISL_838850, EPI_ISL_838851, EPI_ISL_838852, EPI_ISL_838853, EPI_ISL_838854, EPI_ISL_838855, EPI_ISL_838856, EPI_ISL_838857, EPI_ISL_838858, EPI_ISL_838859, EPI_ISL_838860, EPI_ISL_838861, EPI_ISL_838862, EPI_ISL_838863, EPI_ISL_838864, EPI_ISL_838865, EPI_ISL_838866, EPI_ISL_838867, EPI_ISL_838868, EPI_ISL_838869, EPI_ISL_838870, EPI_ISL_838871, EPI_ISL_838872, EPI_ISL_838873, EPI_ISL_838874, EPI_ISL_838875, EPI_ISL_838876, EPI_ISL_838877, EPI_ISL_838878, EPI_ISL_838879, EPI_ISL_838880, EPI_ISL_838881, EPI_ISL_838882, EPI_ISL_838883, EPI_ISL_838884, EPI_ISL_838885, EPI_ISL_838886, EPI_ISL_838887, EPI_ISL_838888, EPI_ISL_838889, EPI_ISL_838890, EPI_ISL_838891, EPI_ISL_838892, EPI_ISL_838893, EPI_ISL_838894, EPI_ISL_838895, EPI_ISL_838896, EPI_ISL_838897, EPI_ISL_838898, EPI_ISL_838899, EPI_ISL_838900, EPI_ISL_838901, EPI_ISL_838902, EPI_ISL_838903, EPI_ISL_838904, EPI_ISL_838905, EPI_ISL_838906, EPI_ISL_838907, EPI_ISL_838908, EPI_ISL_838909, EPI_ISL_838910, EPI_ISL_838911, EPI_ISL_838912, EPI_ISL_838913, EPI_ISL_838914, EPI_ISL_838915, EPI_ISL_838916, EPI_ISL_838917, EPI_ISL_838918, EPI_ISL_838919, EPI_ISL_838920, EPI_ISL_838921, EPI_ISL_838922, EPI_ISL_838923, EPI_ISL_838924, EPI_ISL_838925, EPI_ISL_838926, EPI_ISL_838927, EPI_ISL_838928, EPI_ISL_838929, EPI_ISL_838930, EPI_ISL_838931, EPI_ISL_838932, EPI_ISL_838933, EPI_ISL_838934, EPI_ISL_838935, EPI_ISL_838936, EPI_ISL_838950, EPI_ISL_838952, EPI_ISL_838953, EPI_ISL_838954, EPI_ISL_838955, EPI_ISL_838956, EPI_ISL_838957, EPI_ISL_839026, EPI_ISL_839027, EPI_ISL_839028, EPI_ISL_839030, EPI_ISL_839031, EPI_ISL_839032, EPI_ISL_839033, EPI_ISL_839034, EPI_ISL_839035, EPI_ISL_839036, EPI_ISL_839037, EPI_ISL_839038, EPI_ISL_839039, EPI_ISL_839040, EPI_ISL_839044, EPI_ISL_839045, EPI_ISL_839046, EPI_ISL_839047, EPI_ISL_839048, EPI_ISL_839049, EPI_ISL_839050, EPI_ISL_839051, EPI_ISL_839052, EPI_ISL_839053, EPI_ISL_839055, EPI_ISL_839056, EPI_ISL_839057, EPI_ISL_839058, EPI_ISL_839059, EPI_ISL_839061, EPI_ISL_839062, EPI_ISL_839070, EPI_ISL_839071, EPI_ISL_839072, EPI_ISL_839073, EPI_ISL_839076, EPI_ISL_839077, EPI_ISL_839078, EPI_ISL_839079, EPI_ISL_839115, EPI_ISL_839117, EPI_ISL_839118, EPI_ISL_839119, EPI_ISL_839120, EPI_ISL_839121, EPI_ISL_839122, EPI_ISL_839123, EPI_ISL_839124, EPI_ISL_839125, EPI_ISL_839135, EPI_ISL_839137, EPI_ISL_839138, EPI_ISL_839139, EPI_ISL_839140, EPI_ISL_839141, EPI_ISL_839142, EPI_ISL_839143, EPI_ISL_839144, EPI_ISL_839146, EPI_ISL_839147, EPI_ISL_839148, EPI_ISL_839149, EPI_ISL_839150, EPI_ISL_839151, EPI_ISL_839152, EPI_ISL_839153, EPI_ISL_839154, EPI_ISL_839155, EPI_ISL_839156, EPI_ISL_839157, EPI_ISL_839158, EPI_ISL_839159, EPI_ISL_839160, EPI_ISL_839161, EPI_ISL_839162, EPI_ISL_839163, EPI_ISL_839164, EPI_ISL_839165, EPI_ISL_839166, EPI_ISL_839167, EPI_ISL_839168, EPI_ISL_839169, EPI_ISL_839170, EPI_ISL_839171, EPI_ISL_839172, EPI_ISL_839173, EPI_ISL_839174, EPI_ISL_839175, EPI_ISL_839176, EPI_ISL_839177, EPI_ISL_839178, EPI_ISL_839179, EPI_ISL_839180, EPI_ISL_839181, EPI_ISL_839182, EPI_ISL_839183, EPI_ISL_839184, EPI_ISL_839185, EPI_ISL_839186, EPI_ISL_839187, EPI_ISL_839188, EPI_ISL_839189, EPI_ISL_839190, EPI_ISL_839191, EPI_ISL_839192, EPI_ISL_839193, EPI_ISL_839194, EPI_ISL_839195, EPI_ISL_839196, EPI_ISL_839197, EPI_ISL_839198, EPI_ISL_839199, EPI_ISL_839200, EPI_ISL_839201, EPI_ISL_839202, EPI_ISL_839203, EPI_ISL_839204, EPI_ISL_839205, EPI_ISL_839206, EPI_ISL_839207, EPI_ISL_839208, EPI_ISL_839210, EPI_ISL_839211, EPI_ISL_839212, EPI_ISL_839213, EPI_ISL_839214, EPI_ISL_839216, EPI_ISL_839217, EPI_ISL_839228, EPI_ISL_839229, EPI_ISL_839230, EPI_ISL_839231, EPI_ISL_839250, EPI_ISL_839252, EPI_ISL_839260, EPI_ISL_839264, EPI_ISL_839265, EPI_ISL_839273			
see above	University College London, Great Ormond Street Hospital for Children NHS Foundation Trust, Imperial College Healthcare NHS Trust	COVID-19 Genomics UK (COG-UK) Consortium	Sergi Castellano, Rachel Williams, Mark Kristiansen, Paola Resende Silva, Sunando Roy, Tony Brooks, Helena Tutill, Paola Niola, Patricia Dyal, Charlotte Williams, Leysa Forrest, Yasmin Panchbhaya, Jacqueline Findlay, Samuel Weeks, Julianne Brown, Kathryn Harris, Paul Randell, James Price, Alison Holmes, Judith Breuer
EPI_ISL_839733, EPI_ISL_839734	Northumbria University / South Tees Hospitals NHS Foundation Trust / North Cumbria Integrated Care NHS	COVID-19 Genomics UK (COG-UK) Consortium	Darren L Smith,Andrew Nelson,Matthew Bashton,Greg R Young,Joshua Loh,John Allan,Mohammad A Tariq,Giles S Holt,Gary Black,Wen C Yew,Lynn Dover,Paul Baker,Steve Liggett,Sarah Essex,Jane Greenaway,Debra Padgett,Clive Graham,Garren Scott,Edward Barton,Emma Swindells,Brendan

	Foundation Trust / North Tees and Hartlepool NHS Foundation Trust / Newcastle Hospitals NHS Foundation Trust		Payne,Jennifer Collins,Yusri Taha,Gary Eltringham
EPI_ISL_839823, EPI_ISL_839824, EPI_ISL_839825, EPI_ISL_839826, EPI_ISL_839827, EPI_ISL_839828, EPI_ISL_839829, EPI_ISL_839830, EPI_ISL_839831, EPI_ISL_839832, EPI_ISL_839833, EPI_ISL_839834, EPI_ISL_839835, EPI_ISL_839836, EPI_ISL_839837, EPI_ISL_839838, EPI_ISL_839839, EPI_ISL_839840, EPI_ISL_839841, EPI_ISL_839842, EPI_ISL_839843, EPI_ISL_839844, EPI_ISL_839845, EPI_ISL_839846, EPI_ISL_839847, EPI_ISL_839848, EPI_ISL_839849, EPI_ISL_839850, EPI_ISL_839851, EPI_ISL_839852, EPI_ISL_839853, EPI_ISL_839854, EPI_ISL_839855, EPI_ISL_839856, EPI_ISL_839857, EPI_ISL_839858, EPI_ISL_839859, EPI_ISL_839860, EPI_ISL_839861, EPI_ISL_839862, EPI_ISL_839863, EPI_ISL_839864, EPI_ISL_839865, EPI_ISL_839866, EPI_ISL_839867, EPI_ISL_839868, EPI_ISL_839869, EPI_ISL_839870, EPI_ISL_839871, EPI_ISL_839872, EPI_ISL_839873, EPI_ISL_839874, EPI_ISL_839875, EPI_ISL_839876, EPI_ISL_839877, EPI_ISL_839878, EPI_ISL_839879, EPI_ISL_839880, EPI_ISL_839881, EPI_ISL_839882, EPI_ISL_839883, EPI_ISL_839884, EPI_ISL_839885, EPI_ISL_839886, EPI_ISL_839887, EPI_ISL_839888, EPI_ISL_839889, EPI_ISL_839890, EPI_ISL_839891, EPI_ISL_839892, EPI_ISL_839893, EPI_ISL_839894, EPI_ISL_839895, EPI_ISL_839896, EPI_ISL_839897, EPI_ISL_839898, EPI_ISL_839899, EPI_ISL_839900, EPI_ISL_839901, EPI_ISL_839902, EPI_ISL_839903, EPI_ISL_839904, EPI_ISL_839905, EPI_ISL_839906, EPI_ISL_839907, EPI_ISL_839908, EPI_ISL_839909, EPI_ISL_839910, EPI_ISL_839912, EPI_ISL_839913, EPI_ISL_839914, EPI_ISL_839915, EPI_ISL_839916, EPI_ISL_839917, EPI_ISL_839918, EPI_ISL_839919, EPI_ISL_839920, EPI_ISL_839921, EPI_ISL_839922, EPI_ISL_839923, EPI_ISL_839924, EPI_ISL_839925, EPI_ISL_839926, EPI_ISL_839927, EPI_ISL_839928, EPI_ISL_839929, EPI_ISL_839930, EPI_ISL_839931, EPI_ISL_839932, EPI_ISL_839933, EPI_ISL_839934, EPI_ISL_839935, EPI_ISL_839936, EPI_ISL_839937, EPI_ISL_839938, EPI_ISL_839939, EPI_ISL_839940, EPI_ISL_839941, EPI_ISL_839942, EPI_ISL_839943, EPI_ISL_839944, EPI_ISL_839945, EPI_ISL_839946, EPI_ISL_839947, EPI_ISL_839948, EPI_ISL_839949, EPI_ISL_839950, EPI_ISL_839951, EPI_ISL_839952, EPI_ISL_839953, EPI_ISL_839954, EPI_ISL_839955, EPI_ISL_839956, EPI_ISL_839957, EPI_ISL_839958, EPI_ISL_839959, EPI_ISL_839960, EPI_ISL_839961, EPI_ISL_839962, EPI_ISL_839963, EPI_ISL_839964, EPI_ISL_839965, EPI_ISL_839966, EPI_ISL_839967, EPI_ISL_839968, EPI_ISL_839969, EPI_ISL_839970, EPI_ISL_839971, EPI_ISL_839972, EPI_ISL_839973, EPI_ISL_839974, EPI_ISL_839975, EPI_ISL_839976, EPI_ISL_839977, EPI_ISL_839978, EPI_ISL_839979, EPI_ISL_839980			
see above	Quadram Institute Bioscience	COVID-19 Genomics UK (COG-UK) Consortium	Dave J. Baker, Gemma L. Kay, Alp Aydin, Thanh Le-Viet, Steven Rudder, Ana P. Tedim, Anastasia Kolyva, Maria Diaz, Leonardo de Oliveira Martins, Nabil-Fareed Alikhan, Lizzie Meadows, Rachael Stanley, Ngozi Elumogo, Muhammed Yasir, Nicholas M. Thomson, Alexander J Trotter, Rachel Gilroy, Samuel Bloomfield, Claire Stuart, Andrew Bell, Reenesh Prakash, Samir Dervisevic, Alison E. Mather, John Wain, Mark Webber, Andrew J. Page, Justin O'Grady
EPI_ISL_839995, EPI_ISL_839996, EPI_ISL_839997, EPI_ISL_839998, EPI_ISL_839999, EPI_ISL_840000, EPI_ISL_840001, EPI_ISL_840002, EPI_ISL_840003, EPI_ISL_840004, EPI_ISL_840005, EPI_ISL_840006, EPI_ISL_840007, EPI_ISL_840008			
see above	Queens Medical Centre, Clinical Microbiology Department / DeepSeq Nottingham	COVID-19 Genomics UK (COG-UK) Consortium	Gemma Clark, Wendy Smith, Manjinder Khakh, Vicki M Fleming, Michelle M Lister, Hannah Howson-Wells, Jonathan Ball, Patrick McClure, Joseph Chappell, Theocharis Tsoleridis, Nadine Holmes, Matthew Carlisle, Christopher Moore, Fei Sang, Johnny Debebe, Victoria Wright, Matthew Loose
EPI_ISL_840125, EPI_ISL_840127, EPI_ISL_840128, EPI_ISL_840129, EPI_ISL_840130, EPI_ISL_840131, EPI_ISL_840132, EPI_ISL_840133, EPI_ISL_840134, EPI_ISL_840135, EPI_ISL_840136, EPI_ISL_840137, EPI_ISL_840138, EPI_ISL_840139, EPI_ISL_840140, EPI_ISL_840141, EPI_ISL_840142, EPI_ISL_840143, EPI_ISL_840144, EPI_ISL_840145			
see above	Lincolnshire Hospitals and DeepSeq Nottingham	COVID-19 Genomics UK (COG-UK) Consortium	Nichola Duckworth, Tim Sloan, Sarah Walsh, Jonathan Ball, Patrick McClure, Joeseph Chappell, Nadine Holmes, Matthew Carlisle, Christopher Moore, Fei Sang, Johnny Debebe, Victoria Wright, Matthew Loose
EPI_ISL_840650, EPI_ISL_840660, EPI_ISL_840661, EPI_ISL_840663, EPI_ISL_840664, EPI_ISL_840666, EPI_ISL_840677, EPI_ISL_840678, EPI_ISL_840679, EPI_ISL_840680, EPI_ISL_840681, EPI_ISL_840682, EPI_ISL_840683, EPI_ISL_840684, EPI_ISL_840685, EPI_ISL_840686, EPI_ISL_840687, EPI_ISL_840688, EPI_ISL_840689, EPI_ISL_840690, EPI_ISL_840691, EPI_ISL_840692, EPI_ISL_840693, EPI_ISL_840694			
see above	Originating lab: Wales Specialist Virology Centre Sequencing lab: Pathogen Genomics Unit	Public Health Wales Microbiology Cardiff Wales Specialist Virology Centre	Catherine Moore, Johnathan Evans, Laura Gifford, Malorie Perry, Simon Cottrell, Angela Marchbank, Alec Birchley, Alexander Adams, Amy Gaskin, Bree Gatica-Wilcox, Jason Coombes, Joel Southgate, Lauren Gilbert, Lee Graham, Nicole Pacchiarini, Sara Kumziene-Summerhayes, Sarah Taylor, Sophie Jones, Sara Rey, Matthew Bull, Joanne Watkins, Sally Corden, Tom Connor
EPI_ISL_840820, EPI_ISL_840822, EPI_ISL_840824, EPI_ISL_840826, EPI_ISL_840827, EPI_ISL_840840, EPI_ISL_840842, EPI_ISL_840843, EPI_ISL_840845, EPI_ISL_840846, EPI_ISL_840847, EPI_ISL_840848, EPI_ISL_840849, EPI_ISL_840852, EPI_ISL_840853, EPI_ISL_840855, EPI_ISL_840856, EPI_ISL_840857, EPI_ISL_840858, EPI_ISL_840859, EPI_ISL_840861, EPI_ISL_840862, EPI_ISL_840863, EPI_ISL_840864, EPI_ISL_840865, EPI_ISL_840866, EPI_ISL_840867, EPI_ISL_840869, EPI_ISL_840870, EPI_ISL_840871, EPI_ISL_840872, EPI_ISL_840874, EPI_ISL_840875, EPI_ISL_840876, EPI_ISL_840877, EPI_ISL_840878, EPI_ISL_840880, EPI_ISL_840881, EPI_ISL_840882, EPI_ISL_840883, EPI_ISL_840884, EPI_ISL_840907, EPI_ISL_840908, EPI_ISL_840922, EPI_ISL_840925, EPI_ISL_840926, EPI_ISL_840928, EPI_ISL_840929, EPI_ISL_840930, EPI_ISL_840932, EPI_ISL_840933, EPI_ISL_840934, EPI_ISL_840935, EPI_ISL_840936, EPI_ISL_840942, EPI_ISL_840944, EPI_ISL_840945, EPI_ISL_840947, EPI_ISL_840951, EPI_ISL_840952, EPI_ISL_840954, EPI_ISL_840955, EPI_ISL_840956, EPI_ISL_840957, EPI_ISL_840958, EPI_ISL_840959, EPI_ISL_840960, EPI_ISL_840961, EPI_ISL_840962, EPI_ISL_840963, EPI_ISL_840964, EPI_ISL_840965, EPI_ISL_840966, EPI_ISL_840967, EPI_ISL_840968, EPI_ISL_840969, EPI_ISL_840970, EPI_ISL_840971, EPI_ISL_840974, EPI_ISL_840975, EPI_ISL_840976, EPI_ISL_840977, EPI_ISL_840978, EPI_ISL_840979, EPI_ISL_840981, EPI_ISL_840982, EPI_ISL_840983, EPI_ISL_840984, EPI_ISL_840985, EPI_ISL_840989, EPI_ISL_840990, EPI_ISL_840991, EPI_ISL_840992, EPI_ISL_840993, EPI_ISL_840994, EPI_ISL_840995, EPI_ISL_840996, EPI_ISL_840997, EPI_ISL_840998, EPI_ISL_841000, EPI_ISL_841002, EPI_ISL_841004, EPI_ISL_841008, EPI_ISL_841010, EPI_ISL_841012, EPI_ISL_841013, EPI_ISL_841014, EPI_ISL_841015, EPI_ISL_841016, EPI_ISL_841017, EPI_ISL_841018, EPI_ISL_841019, EPI_ISL_841020, EPI_ISL_841021, EPI_ISL_841027, EPI_ISL_841029, EPI_ISL_841030, EPI_ISL_841031, EPI_ISL_841032, EPI_ISL_841036, EPI_ISL_841037, EPI_ISL_841038, EPI_ISL_841040, EPI_ISL_841041, EPI_ISL_841042, EPI_ISL_841043, EPI_ISL_841044, EPI_ISL_841045, EPI_ISL_841046, EPI_ISL_841047, EPI_ISL_841049, EPI_ISL_841050, EPI_ISL_841051, EPI_ISL_841052, EPI_ISL_841053, EPI_ISL_841054, EPI_ISL_841055, EPI_ISL_841056, EPI_ISL_841059, EPI_ISL_841060, EPI_ISL_841062, EPI_ISL_841063, EPI_ISL_841064, EPI_ISL_841067, EPI_ISL_841068, EPI_ISL_841069, EPI_ISL_841070, EPI_ISL_841071, EPI_ISL_841072, EPI_ISL_841073, EPI_ISL_841074, EPI_ISL_841075, EPI_ISL_841076, EPI_ISL_841077, EPI_ISL_841078, EPI_ISL_841079, EPI_ISL_841080, EPI_ISL_841081, EPI_ISL_841086, EPI_ISL_841098, EPI_ISL_841108, EPI_ISL_841116, EPI_ISL_841120, EPI_ISL_841121, EPI_ISL_841122, EPI_ISL_841124, EPI_ISL_841125, EPI_ISL_841126, EPI_ISL_841127, EPI_ISL_841128, EPI_ISL_841129, EPI_ISL_841130, EPI_ISL_841131, EPI_ISL_841133, EPI_ISL_841134, EPI_ISL_841137, EPI_ISL_841138, EPI_ISL_841139, EPI_ISL_841140, EPI_ISL_841141, EPI_ISL_841142, EPI_ISL_841143, EPI_ISL_841144, EPI_ISL_841145, EPI_ISL_841146, EPI_ISL_841147, EPI_ISL_841149, EPI_ISL_841151, EPI_ISL_841152, EPI_ISL_841153, EPI_ISL_841154, EPI_ISL_841155, EPI_ISL_841156, EPI_ISL_841157, EPI_ISL_841158, EPI_ISL_841159, EPI_ISL_841161, EPI_ISL_841162, EPI_ISL_841163, EPI_ISL_841164, EPI_ISL_841165, EPI_ISL_841166, EPI_ISL_841167, EPI_ISL_841168, EPI_ISL_841169, EPI_ISL_841170, EPI_ISL_841172, EPI_ISL_841174, EPI_ISL_841175, EPI_ISL_841176, EPI_ISL_841178, EPI_ISL_841179, EPI_ISL_841182, EPI_ISL_841183, EPI_ISL_841184, EPI_ISL_841185, EPI_ISL_841186, EPI_ISL_841191, EPI_ISL_841192, EPI_ISL_841193, EPI_ISL_841194, EPI_ISL_841195, EPI_ISL_841196, EPI_ISL_841197, EPI_ISL_841200, EPI_ISL_841201, EPI_ISL_841202, EPI_ISL_841203, EPI_ISL_841204, EPI_ISL_841205, EPI_ISL_841206, EPI_ISL_841207, EPI_ISL_841208, EPI_ISL_841209, EPI_ISL_841210, EPI_ISL_841213, EPI_ISL_841214, EPI_ISL_841216, EPI_ISL_841217, EPI_ISL_841220, EPI_ISL_841221, EPI_ISL_841222, EPI_ISL_841223, EPI_ISL_841224, EPI_ISL_841226, EPI_ISL_841227, EPI_ISL_841229, EPI_ISL_841230, EPI_ISL_841231, EPI_ISL_841232, EPI_ISL_841233, EPI_ISL_841234, EPI_ISL_841235, EPI_ISL_841236, EPI_ISL_841237, EPI_ISL_841238, EPI_ISL_841240, EPI_ISL_841242, EPI_ISL_841244, EPI_ISL_841245, EPI_ISL_841246, EPI_ISL_841247, EPI_ISL_841248, EPI_ISL_841250, EPI_ISL_841251, EPI_ISL_841252, EPI_ISL_841253, EPI_ISL_841255, EPI_ISL_841256, EPI_ISL_841257, EPI_ISL_841258, EPI_ISL_841259, EPI_ISL_841261, EPI_ISL_841262, EPI_ISL_841263, EPI_ISL_841264, EPI_ISL_841265, EPI_ISL_841266, EPI_ISL_841267, EPI_ISL_841268, EPI_ISL_841269, EPI_ISL_841270, EPI_ISL_841271, EPI_ISL_841273, EPI_ISL_841275, EPI_ISL_841276, EPI_ISL_841280, EPI_ISL_841281, EPI_ISL_841288, EPI_ISL_841292, EPI_ISL_841293, EPI_ISL_841296, EPI_ISL_841297, EPI_ISL_841299, EPI_ISL_841300, EPI_ISL_841306, EPI_ISL_841307, EPI_ISL_841308, EPI_ISL_841309, EPI_ISL_841310, EPI_ISL_841311, EPI_ISL_841312, EPI_ISL_841313, EPI_ISL_841314, EPI_ISL_841315, EPI_ISL_841316			
see above	Wales Specialist Virology Centre Sequencing lab: Pathogen Genomics Unit	Public Health Wales Microbiology Cardiff Wales Specialist Virology Centre	Catherine Moore, Johnathan Evans, Laura Gifford, Malorie Perry, Simon Cottrell, Angela Marchbank, Alec Birchley, Alexander Adams, Amy Gaskin, Bree Gatica-Wilcox, Jason Coombes, Joel Southgate, Lauren Gilbert, Lee Graham, Nicole Pacchiarini, Sara Kumziene-Summerhayes, Sarah Taylor, Sophie Jones, Sara Rey, Matthew Bull, Joanne Watkins, Sally Corden, Tom Connor
EPI_ISL_841318, EPI_ISL_841319, EPI_ISL_841320, EPI_ISL_841323, EPI_ISL_841332, EPI_ISL_841333, EPI_ISL_841334, EPI_ISL_841335, EPI_ISL_841336, EPI_ISL_841337, EPI_ISL_841338, EPI_ISL_841341, EPI_ISL_841342, EPI_ISL_841358, EPI_ISL_841371, EPI_ISL_841372, EPI_ISL_841374, EPI_ISL_841375, EPI_ISL_841376, EPI_ISL_841377, EPI_ISL_841378, EPI_ISL_841379, EPI_ISL_841380, EPI_ISL_841381, EPI_ISL_841382, EPI_ISL_841383, EPI_ISL_841384, EPI_ISL_841385, EPI_ISL_841386, EPI_ISL_841387, EPI_ISL_841388, EPI_ISL_841389, EPI_ISL_841390, EPI_ISL_841391, EPI_ISL_841392, EPI_ISL_841393, EPI_ISL_841394, EPI_ISL_841395, EPI_ISL_841396, EPI_ISL_841397, EPI_ISL_841399, EPI_ISL_841400, EPI_ISL_841401, EPI_ISL_841402, EPI_ISL_841403, EPI_ISL_841404, EPI_ISL_841405, EPI_ISL_841406, EPI_ISL_841413, EPI_ISL_841414, EPI_ISL_841416, EPI_ISL_841418, EPI_ISL_841419, EPI_ISL_841420, EPI_ISL_841421, EPI_ISL_841422, EPI_ISL_841423, EPI_ISL_841424, EPI_ISL_841425, EPI_ISL_841428, EPI_ISL_841429, EPI_ISL_841432, EPI_ISL_841435, EPI_ISL_841436, EPI_ISL_841498, EPI_ISL_841506, EPI_ISL_841510, EPI_ISL_841511, EPI_ISL_841512, EPI_ISL_841513, EPI_ISL_841514, EPI_ISL_841515, EPI_ISL_841548, EPI_ISL_841586, EPI_ISL_841588, EPI_ISL_841590, EPI_ISL_841594, EPI_ISL_841595, EPI_ISL_841596, EPI_ISL_841597, EPI_ISL_841598, EPI_ISL_841599, EPI_ISL_841600, EPI_ISL_841601, EPI_ISL_841602, EPI_ISL_841603, EPI_ISL_841604, EPI_ISL_841605, EPI_ISL_841606, EPI_ISL_841607, EPI_ISL_841610, EPI_ISL_841611, EPI_ISL_841613, EPI_ISL_841615, EPI_ISL_841616			
see above	Originating lab: Wales Specialist Virology Centre Sequencing lab: Pathogen Genomics Unit	Public Health Wales Microbiology Cardiff Wales Specialist Virology Centre	Catherine Moore, Johnathan Evans, Laura Gifford, Malorie Perry, Simon Cottrell, Angela Marchbank, Alec Birchley, Alexander Adams, Amy Gaskin, Bree Gatica-Wilcox, Jason Coombes, Joel Southgate, Lauren Gilbert, Lee Graham, Nicole Pacchiarini, Sara Kumziene-Summerhayes, Sarah Taylor, Sophie Jones, Sara Rey, Matthew Bull, Joanne Watkins, Sally Corden, Tom Connor
EPI_ISL_841922, EPI_ISL_841927, EPI_ISL_841945, EPI_ISL_842008	Centre for Enzyme Innovation, University of Portsmouth / Translational Research Laboratory, Portsmouth Hospitals NHS Trust	COVID-19 Genomics UK (COG-UK) Consortium	Angela Beckett,Yann Bourgeois,Garry Scarlett,Sharon Glaysher,Scott Elliott,Kelly Bicknell,Robert Impey,Allyson Lloyd,Sarah Wyllie,Ethan Butcher,Anoop Chauhan,Samuel Robson
EPI_ISL_842202, EPI_ISL_842205, EPI_ISL_842206, EPI_ISL_842208, EPI_ISL_842215, EPI_ISL_842216, EPI_ISL_842220, EPI_ISL_842221, EPI_ISL_842224, EPI_ISL_842226, EPI_ISL_842231, EPI_ISL_842239, EPI_ISL_842240, EPI_ISL_842241, EPI_ISL_842244, EPI_ISL_842245, EPI_ISL_842246, EPI_ISL_842251, EPI_ISL_842258, EPI_ISL_842260, EPI_ISL_842261, EPI_ISL_842262, EPI_ISL_842268, EPI_ISL_842279, EPI_ISL_842280, EPI_ISL_842284, EPI_ISL_842285, EPI_ISL_842286, EPI_ISL_842287, EPI_ISL_842288, EPI_ISL_842289, EPI_ISL_842294, EPI_ISL_842299, EPI_ISL_842300, EPI_ISL_842301, EPI_ISL_842303, EPI_ISL_842305, EPI_ISL_842311, EPI_ISL_842315, EPI_ISL_842322, EPI_ISL_842324, EPI_ISL_842329, EPI_ISL_842330, EPI_ISL_842334, EPI_ISL_842340, EPI_ISL_842345, EPI_ISL_842346			
see above	Virology Department, Sheffield Teaching Hospitals NHS Foundation Trust/Department of Infection, Immunity and Cardiovascular Disease, The Medical School, University of Sheffield	COVID-19 Genomics UK (COG-UK) Consortium	Thushan de Silva, Matthew Parker, Nikki Smith, Adri Angyal, Rebecca Brown, Luke Green, Rachel Tucker, Paul Parsons, Danielle Groves, Katie Johnson, Laura Carrilero, Alex Keeley, Dave Partridge, Matthew Wyles, Benjamin Lindsey, Mehmet Yavuz, Mohammad Raza, Cariad Evans
EPI_ISL_845973, EPI_ISL_846022, EPI_ISL_846070	Lighthouse Lab in Cambridge	Wellcome Sanger Institute for the COVID-19 Genomics UK (COG-UK) Consortium	Rob Howes, The Lighthouse Lab in Cambridge and Alex Alderton, Roberto Amato, Sonia Goncalves, Ewan Harrison, David K. Jackson, Ian Johnston, Dominic Kwiatkowski, Cordelia Langford, John Sillitoe on behalf of the Wellcome Sanger Institute COVID-19 Surveillance Team
EPI_ISL_846319, EPI_ISL_846400, EPI_ISL_846510, EPI_ISL_846511, EPI_ISL_846513, EPI_ISL_846514, EPI_ISL_846515, EPI_ISL_846516, EPI_ISL_846517, EPI_ISL_846518, EPI_ISL_846519, EPI_ISL_846520, EPI_ISL_846521, EPI_ISL_846522, EPI_ISL_846523, EPI_ISL_846524, EPI_ISL_846525			
see above	Lighthouse Lab in Alderley Park	Wellcome Sanger Institute for the COVID-19 Genomics UK (COG-UK) Consortium	Jacquelyn Wynn, Mairead Hyland, The Lighthouse Lab in Alderley Park and Alex Alderton, Roberto Amato, Sonia Goncalves, Ewan Harrison, David K. Jackson, Ian Johnston, Dominic Kwiatkowski, Cordelia Langford, John Sillitoe on behalf of the Wellcome Sanger Institute COVID-19 Surveillance Team
EPI_ISL_846527, EPI_ISL_846528, EPI_ISL_846529, EPI_ISL_846530, EPI_ISL_846531, EPI_ISL_846532, EPI_ISL_846533, EPI_ISL_846534, EPI_ISL_846535	Lighthouse Lab in Glasgow	Wellcome Sanger Institute for the COVID-19 Genomics UK (COG-UK) Consortium	Harper VanSteenhouse, Yumi Kasai, David Gray, Carol Clugston, Anna Dominiczak and Alex Alderton, Roberto Amato, Sonia Goncalves, Ewan Harrison, David K. Jackson, Ian Johnston, Dominic Kwiatkowski, Cordelia Langford, John Sillitoe on behalf of the Wellcome Sanger Institute COVID-19 Surveillance Team
EPI_ISL_846537	Lighthouse Lab in Alderley Park	Wellcome Sanger Institute for the COVID-19 Genomics UK	Jacquelyn Wynn, Mairead Hyland, The Lighthouse Lab in Alderley Park and Alex Alderton, Roberto Amato, Sonia Goncalves, Ewan Harrison, David K.

		(COG-UK) Consortium	Jackson, Ian Johnston, Dominic Kwiatkowski, Cordelia Langford, John Sillitoe on behalf of the Wellcome Sanger Institute COVID-19 Surveillance Team
EPI_ISL_846538, EPI_ISL_846539	Lighthouse Lab in Glasgow	Wellcome Sanger Institute for the COVID-19 Genomics UK (COG-UK) Consortium	Harper VanSteenhouse, Yumi Kasai, David Gray, Carol Clugston, Anna Dominiczak and Alex Alderton, Roberto Amato, Sonia Goncalves, Ewan Harrison, David K. Jackson, Ian Johnston, Dominic Kwiatkowski, Cordelia Langford, John Sillitoe on behalf of the Wellcome Sanger Institute COVID-19 Surveillance Team
EPI_ISL_846595, EPI_ISL_846596	Respiratory Virus Unit, National Infection Service, Public Health England	COVID-19 Genomics UK (COG-UK) Consortium	PHE Covid Sequencing Team
EPI_ISL_851079, EPI_ISL_851133, EPI_ISL_851170, EPI_ISL_851251, EPI_ISL_851269, EPI_ISL_851273, EPI_ISL_851298, EPI_ISL_851343	Lighthouse Lab in Cambridge	Wellcome Sanger Institute for the COVID-19 Genomics UK (COG-UK) Consortium	Rob Howes, The Lighthouse Lab in Cambridge and Alex Alderton, Roberto Amato, Sonia Goncalves, Ewan Harrison, David K. Jackson, Ian Johnston, Dominic Kwiatkowski, Cordelia Langford, John Sillitoe on behalf of the Wellcome Sanger Institute COVID-19 Surveillance Team
EPI_ISL_851455, EPI_ISL_851467, EPI_ISL_851559, EPI_ISL_851594, EPI_ISL_851614, EPI_ISL_851648, EPI_ISL_851657	Lighthouse Lab in Glasgow	Wellcome Sanger Institute for the COVID-19 Genomics UK (COG-UK) Consortium	Harper VanSteenhouse, Yumi Kasai, David Gray, Carol Clugston, Anna Dominiczak and Alex Alderton, Roberto Amato, Sonia Goncalves, Ewan Harrison, David K. Jackson, Ian Johnston, Dominic Kwiatkowski, Cordelia Langford, John Sillitoe on behalf of the Wellcome Sanger Institute COVID-19 Surveillance Team
EPI_ISL_851750	Lighthouse Lab in Alderley Park	Wellcome Sanger Institute for the COVID-19 Genomics UK (COG-UK) Consortium	Jacquelyn Wynn, Mairead Hyland, The Lighthouse Lab in Alderley Park and Alex Alderton, Roberto Amato, Sonia Goncalves, Ewan Harrison, David K. Jackson, Ian Johnston, Dominic Kwiatkowski, Cordelia Langford, John Sillitoe on behalf of the Wellcome Sanger Institute COVID-19 Surveillance Team
EPI_ISL_851954	Lighthouse Lab in Glasgow	Wellcome Sanger Institute for the COVID-19 Genomics UK (COG-UK) Consortium	Harper VanSteenhouse, Yumi Kasai, David Gray, Carol Clugston, Anna Dominiczak and Alex Alderton, Roberto Amato, Sonia Goncalves, Ewan Harrison, David K. Jackson, Ian Johnston, Dominic Kwiatkowski, Cordelia Langford, John Sillitoe on behalf of the Wellcome Sanger Institute COVID-19 Surveillance Team
EPI_ISL_851964	Lighthouse Lab in Alderley Park	Wellcome Sanger Institute for the COVID-19 Genomics UK (COG-UK) Consortium	Jacquelyn Wynn, Mairead Hyland, The Lighthouse Lab in Alderley Park and Alex Alderton, Roberto Amato, Sonia Goncalves, Ewan Harrison, David K. Jackson, Ian Johnston, Dominic Kwiatkowski, Cordelia Langford, John Sillitoe on behalf of the Wellcome Sanger Institute COVID-19 Surveillance Team
EPI_ISL_852002	Lighthouse Lab in Milton Keynes	Wellcome Sanger Institute for the COVID-19 Genomics UK (COG-UK) Consortium	The Lighthouse Lab in Milton Keynes and Alex Alderton, Roberto Amato, Sonia Goncalves, Ewan Harrison, David K. Jackson, Ian Johnston, Dominic Kwiatkowski, Cordelia Langford, John Sillitoe on behalf of the Wellcome Sanger Institute COVID-19 Surveillance Team
EPI_ISL_852047	Lighthouse Lab in Alderley Park	Wellcome Sanger Institute for the COVID-19 Genomics UK (COG-UK) Consortium	Jacquelyn Wynn, Mairead Hyland, The Lighthouse Lab in Alderley Park and Alex Alderton, Roberto Amato, Sonia Goncalves, Ewan Harrison, David K. Jackson, Ian Johnston, Dominic Kwiatkowski, Cordelia Langford, John Sillitoe on behalf of the Wellcome Sanger Institute COVID-19 Surveillance Team
EPI_ISL_852190, EPI_ISL_852206	Lighthouse Lab in Milton Keynes	Wellcome Sanger Institute for the COVID-19 Genomics UK (COG-UK) Consortium	The Lighthouse Lab in Milton Keynes and Alex Alderton, Roberto Amato, Sonia Goncalves, Ewan Harrison, David K. Jackson, Ian Johnston, Dominic Kwiatkowski, Cordelia Langford, John Sillitoe on behalf of the Wellcome Sanger Institute COVID-19 Surveillance Team
EPI_ISL_852225	Lighthouse Lab in Alderley Park	Wellcome Sanger Institute for the COVID-19 Genomics UK (COG-UK) Consortium	Jacquelyn Wynn, Mairead Hyland, The Lighthouse Lab in Alderley Park and Alex Alderton, Roberto Amato, Sonia Goncalves, Ewan Harrison, David K. Jackson, Ian Johnston, Dominic Kwiatkowski, Cordelia Langford, John Sillitoe on behalf of the Wellcome Sanger Institute COVID-19 Surveillance Team
EPI_ISL_852256, EPI_ISL_852261, EPI_ISL_852262, EPI_ISL_852263, EPI_ISL_852265, EPI_ISL_852266, EPI_ISL_852267, EPI_ISL_852270, EPI_ISL_852271, EPI_ISL_852272, EPI_ISL_852273, EPI_ISL_852274, EPI_ISL_852277, EPI_ISL_852278, EPI_ISL_852280, EPI_ISL_852282, EPI_ISL_852284, EPI_ISL_852290, EPI_ISL_852291, EPI_ISL_852294, EPI_ISL_852296, EPI_ISL_852297, EPI_ISL_852298, EPI_ISL_852299, EPI_ISL_852302, EPI_ISL_852304, EPI_ISL_852308, EPI_ISL_852309, EPI_ISL_852311, EPI_ISL_852315, EPI_ISL_852317, EPI_ISL_852318, EPI_ISL_852320, EPI_ISL_852323, EPI_ISL_852324, EPI_ISL_852327, EPI_ISL_852329, EPI_ISL_852330, EPI_ISL_852331, EPI_ISL_852332, EPI_ISL_852333, EPI_ISL_852336, EPI_ISL_852337, EPI_ISL_852341, EPI_ISL_852342, EPI_ISL_852346, EPI_ISL_852349, EPI_ISL_852351, EPI_ISL_852352, EPI_ISL_852354, EPI_ISL_852355, EPI_ISL_852356, EPI_ISL_852357, EPI_ISL_852359, EPI_ISL_852362, EPI_ISL_852363, EPI_ISL_852365, EPI_ISL_852366, EPI_ISL_852371, EPI_ISL_852372, EPI_ISL_852373, EPI_ISL_852376, EPI_ISL_852377, EPI_ISL_852379, EPI_ISL_852380, EPI_ISL_852381, EPI_ISL_852382, EPI_ISL_852383, EPI_ISL_852384, EPI_ISL_852385, EPI_ISL_852386, EPI_ISL_852387, EPI_ISL_852388, EPI_ISL_852390, EPI_ISL_852392, EPI_ISL_852394, EPI_ISL_852396, EPI_ISL_852399, EPI_ISL_852400, EPI_ISL_852401, EPI_ISL_852402, EPI_ISL_852403, EPI_ISL_852405, EPI_ISL_852408, EPI_ISL_852409, EPI_ISL_852410, EPI_ISL_852411, EPI_ISL_852418, EPI_ISL_852419, EPI_ISL_852423, EPI_ISL_852427, EPI_ISL_852428, EPI_ISL_852430, EPI_ISL_852431, EPI_ISL_852432, EPI_ISL_852434, EPI_ISL_852436, EPI_ISL_852437, EPI_ISL_852441, EPI_ISL_852442, EPI_ISL_852443, EPI_ISL_852449, EPI_ISL_852450, EPI_ISL_852452, EPI_ISL_852453, EPI_ISL_852455, EPI_ISL_852456, EPI_ISL_852457, EPI_ISL_852458, EPI_ISL_852461, EPI_ISL_852464, EPI_ISL_852465, EPI_ISL_852466, EPI_ISL_852468, EPI_ISL_852469, EPI_ISL_852470, EPI_ISL_852473, EPI_ISL_852477, EPI_ISL_852478, EPI_ISL_852480, EPI_ISL_852482, EPI_ISL_852483, EPI_ISL_852486, EPI_ISL_852488, EPI_ISL_852489, EPI_ISL_852490, EPI_ISL_852492, EPI_ISL_852493, EPI_ISL_852494, EPI_ISL_852495, EPI_ISL_852496, EPI_ISL_852499, EPI_ISL_852501, EPI_ISL_852502, EPI_ISL_852505, EPI_ISL_852507, EPI_ISL_852511, EPI_ISL_852512, EPI_ISL_852513, EPI_ISL_852515, EPI_ISL_852516, EPI_ISL_852518, EPI_ISL_852521, EPI_ISL_852523, EPI_ISL_852525, EPI_ISL_852526, EPI_ISL_852528, EPI_ISL_852538, EPI_ISL_852539, EPI_ISL_852542, EPI_ISL_852543, EPI_ISL_852545, EPI_ISL_852546, EPI_ISL_852547, EPI_ISL_852551, EPI_ISL_852552, EPI_ISL_852553, EPI_ISL_852554, EPI_ISL_852556			
see above	Lighthouse Lab in Glasgow	Wellcome Sanger Institute for the COVID-19 Genomics UK (COG-UK) Consortium	Harper VanSteenhouse, Yumi Kasai, David Gray, Carol Clugston, Anna Dominiczak and Alex Alderton, Roberto Amato, Sonia Goncalves, Ewan Harrison, David K. Jackson, Ian Johnston, Dominic Kwiatkowski, Cordelia Langford, John Sillitoe on behalf of the Wellcome Sanger Institute COVID-19 Surveillance Team
EPI_ISL_855574, EPI_ISL_855575, EPI_ISL_855577, EPI_ISL_855578, EPI_ISL_855579, EPI_ISL_855581, EPI_ISL_855582, EPI_ISL_855584, EPI_ISL_855588, EPI_ISL_855589, EPI_ISL_855590, EPI_ISL_855591, EPI_ISL_855592, EPI_ISL_855593, EPI_ISL_855594, EPI_ISL_855596, EPI_ISL_855597, EPI_ISL_855598, EPI_ISL_855599, EPI_ISL_855600, EPI_ISL_855601, EPI_ISL_855606, EPI_ISL_855607, EPI_ISL_855608, EPI_ISL_855609, EPI_ISL_856703, EPI_ISL_856704, EPI_ISL_856705, EPI_ISL_856706			
see above	Respiratory Virus Unit, National Infection Service, Public Health England	COVID-19 Genomics UK (COG-UK) Consortium	PHE Covid Sequencing Team
EPI_ISL_857546, EPI_ISL_857553, EPI_ISL_857562, EPI_ISL_857584, EPI_ISL_857599, EPI_ISL_857604, EPI_ISL_857642, EPI_ISL_857645, EPI_ISL_857654, EPI_ISL_857682, EPI_ISL_857759, EPI_ISL_857818, EPI_ISL_857819, EPI_ISL_857823, EPI_ISL_857852			
see above	Lighthouse Lab in Cambridge	Wellcome Sanger Institute for the COVID-19 Genomics UK (COG-UK) Consortium	Rob Howes, The Lighthouse Lab in Cambridge and Alex Alderton, Roberto Amato, Sonia Goncalves, Ewan Harrison, David K. Jackson, Ian Johnston, Dominic Kwiatkowski, Cordelia Langford, John Sillitoe on behalf of the Wellcome Sanger Institute COVID-19 Surveillance Team
EPI_ISL_857879, EPI_ISL_857890	Lighthouse Lab in Alderley Park	Wellcome Sanger Institute for the COVID-19 Genomics UK (COG-UK) Consortium	Jacquelyn Wynn, Mairead Hyland, The Lighthouse Lab in Alderley Park and Alex Alderton, Roberto Amato, Sonia Goncalves, Ewan Harrison, David K. Jackson, Ian Johnston, Dominic Kwiatkowski, Cordelia Langford, John Sillitoe on behalf of the Wellcome Sanger Institute COVID-19 Surveillance Team
EPI_ISL_858014, EPI_ISL_858020, EPI_ISL_858028, EPI_ISL_858029, EPI_ISL_858037	Lighthouse Lab in Glasgow	Wellcome Sanger Institute for the COVID-19 Genomics UK (COG-UK) Consortium	Harper VanSteenhouse, Yumi Kasai, David Gray, Carol Clugston, Anna Dominiczak and Alex Alderton, Roberto Amato, Sonia Goncalves, Ewan Harrison, David K. Jackson, Ian Johnston, Dominic Kwiatkowski, Cordelia Langford, John Sillitoe on behalf of the Wellcome Sanger Institute COVID-19 Surveillance Team
EPI_ISL_858064, EPI_ISL_858065, EPI_ISL_858066, EPI_ISL_858067, EPI_ISL_858068, EPI_ISL_858069, EPI_ISL_858070, EPI_ISL_858071, EPI_ISL_858072, EPI_ISL_858073, EPI_ISL_858074, EPI_ISL_858075, EPI_ISL_858076, EPI_ISL_858077, EPI_ISL_858078, EPI_ISL_858079, EPI_ISL_858080, EPI_ISL_858081, EPI_ISL_858082, EPI_ISL_858083, EPI_ISL_858084, EPI_ISL_858085, EPI_ISL_858086, EPI_ISL_858087, EPI_ISL_858088, EPI_ISL_858089, EPI_ISL_858090, EPI_ISL_858091, EPI_ISL_858092, EPI_ISL_858093, EPI_ISL_858094, EPI_ISL_858095, EPI_ISL_858096, EPI_ISL_858097, EPI_ISL_858098, EPI_ISL_858099, EPI_ISL_858100, EPI_ISL_858101, EPI_ISL_858102, EPI_ISL_858103, EPI_ISL_858104, EPI_ISL_858105, EPI_ISL_858106, EPI_ISL_858107, EPI_ISL_858108, EPI_ISL_858109, EPI_ISL_858110, EPI_ISL_858111, EPI_ISL_858112, EPI_ISL_858113, EPI_ISL_858114, EPI_ISL_858115, EPI_ISL_858116, EPI_ISL_858117, EPI_ISL_858118, EPI_ISL_858119, EPI_ISL_858120, EPI_ISL_858121, EPI_ISL_858122, EPI_ISL_858123, EPI_ISL_858124, EPI_ISL_858125, EPI_ISL_858126, EPI_ISL_858127, EPI_ISL_858128, EPI_ISL_858129, EPI_ISL_858130, EPI_ISL_858131, EPI_ISL_858132, EPI_ISL_858133, EPI_ISL_858134, EPI_ISL_858135, EPI_ISL_858136, EPI_ISL_858137, EPI_ISL_858138, EPI_ISL_858139, EPI_ISL_858140, EPI_ISL_858141, EPI_ISL_858142, EPI_ISL_858143, EPI_ISL_858144, EPI_ISL_858145, EPI_ISL_858146, EPI_ISL_858147, EPI_ISL_858148, EPI_ISL_858149, EPI_ISL_858150, EPI_ISL_858151, EPI_ISL_858152, EPI_ISL_858153, EPI_ISL_858154, EPI_ISL_858155, EPI_ISL_858156, EPI_ISL_858157			
see above	Lighthouse Lab in Alderley Park	Wellcome Sanger Institute for the COVID-19 Genomics UK (COG-UK) Consortium	Jacquelyn Wynn, Mairead Hyland, The Lighthouse Lab in Alderley Park and Alex Alderton, Roberto Amato, Sonia Goncalves, Ewan Harrison, David K. Jackson, Ian Johnston, Dominic Kwiatkowski, Cordelia Langford, John Sillitoe on behalf of the Wellcome Sanger Institute COVID-19 Surveillance Team
EPI_ISL_858158, EPI_ISL_858159, EPI_ISL_858160, EPI_ISL_858161, EPI_ISL_858162, EPI_ISL_858163, EPI_ISL_858164, EPI_ISL_858165, EPI_ISL_858166, EPI_ISL_858167, EPI_ISL_858168, EPI_ISL_858169, EPI_ISL_858170, EPI_ISL_858171, EPI_ISL_858172, EPI_ISL_858173, EPI_ISL_858174, EPI_ISL_858175, EPI_ISL_858176, EPI_ISL_858177, EPI_ISL_858178, EPI_ISL_858179, EPI_ISL_858180, EPI_ISL_858181, EPI_ISL_858182, EPI_ISL_858183, EPI_ISL_858184, EPI_ISL_858185, EPI_ISL_858186, EPI_ISL_858187, EPI_ISL_858188, EPI_ISL_858189, EPI_ISL_858190, EPI_ISL_858191, EPI_ISL_858192, EPI_ISL_858193, EPI_ISL_858194, EPI_ISL_858195, EPI_ISL_858196, EPI_ISL_858197, EPI_ISL_858198, EPI_ISL_858199, EPI_ISL_858200, EPI_ISL_858201, EPI_ISL_858202, EPI_ISL_858203, EPI_ISL_858204, EPI_ISL_858205, EPI_ISL_858206			
see above	Lighthouse Lab in Glasgow	Wellcome Sanger Institute for the COVID-19 Genomics UK (COG-UK) Consortium	Harper VanSteenhouse, Yumi Kasai, David Gray, Carol Clugston, Anna Dominiczak and Alex Alderton, Roberto Amato, Sonia Goncalves, Ewan Harrison, David K. Jackson, Ian Johnston, Dominic Kwiatkowski, Cordelia Langford, John Sillitoe on behalf of the Wellcome Sanger Institute COVID-19 Surveillance Team
EPI_ISL_858207, EPI_ISL_858208, EPI_ISL_858209, EPI_ISL_858210, EPI_ISL_858211, EPI_ISL_858212, EPI_ISL_858213, EPI_ISL_858214, EPI_ISL_858215, EPI_ISL_858216, EPI_ISL_858217, EPI_ISL_858218, EPI_ISL_858219, EPI_ISL_858220, EPI_ISL_858221, EPI_ISL_858222			
see above	Lighthouse Lab in Alderley Park	Wellcome Sanger Institute for the COVID-19 Genomics UK (COG-UK) Consortium	Jacquelyn Wynn, Mairead Hyland, The Lighthouse Lab in Alderley Park and Alex Alderton, Roberto Amato, Sonia Goncalves, Ewan Harrison, David K. Jackson, Ian Johnston, Dominic Kwiatkowski, Cordelia Langford, John Sillitoe on behalf of the Wellcome Sanger Institute COVID-19 Surveillance Team
EPI_ISL_858223, EPI_ISL_858224, EPI_ISL_858225, EPI_ISL_858226, EPI_ISL_858227, EPI_ISL_858228, EPI_ISL_858229, EPI_ISL_858230, EPI_ISL_858231, EPI_ISL_858232, EPI_ISL_858233, EPI_ISL_858234, EPI_ISL_858235, EPI_ISL_858236, EPI_ISL_858237, EPI_ISL_858238, EPI_ISL_858239, EPI_ISL_858240, EPI_ISL_858241, EPI_ISL_858242, EPI_ISL_858243, EPI_ISL_858244, EPI_ISL_858245, EPI_ISL_858246, EPI_ISL_858247, EPI_ISL_858248, EPI_ISL_858249, EPI_ISL_858250, EPI_ISL_858251, EPI_ISL_858252, EPI_ISL_858253, EPI_ISL_858254, EPI_ISL_858255, EPI_ISL_858256, EPI_ISL_858257, EPI_ISL_858258, EPI_ISL_858259, EPI_ISL_858260, EPI_ISL_858261, EPI_ISL_858262, EPI_ISL_858263, EPI_ISL_858264, EPI_ISL_858265, EPI_ISL_858266, EPI_ISL_858267			
see above	Lighthouse Lab in Glasgow	Wellcome Sanger Institute for the COVID-19 Genomics UK	Harper VanSteenhouse, Yumi Kasai, David Gray, Carol Clugston, Anna Dominiczak and Alex Alderton, Roberto Amato, Sonia Goncalves, Ewan Harrison,

		(COG-UK) Consortium	David K. Jackson, Ian Johnston, Dominic Kwiatkowski, Cordelia Langford, John Sillitoe on behalf of the Wellcome Sanger Institute COVID-19 Surveillance Team
EPI_ISL_858268	Lighthouse Lab in Milton Keynes	Wellcome Sanger Institute for the COVID-19 Genomics UK (COG-UK) Consortium	The Lighthouse Lab in Milton Keynes and Alex Alderton, Roberto Amato, Sonia Goncalves, Ewan Harrison, David K. Jackson, Ian Johnston, Dominic Kwiatkowski, Cordelia Langford, John Sillitoe on behalf of the Wellcome Sanger Institute COVID-19 Surveillance Team
EPI_ISL_858269, EPI_ISL_858270	Lighthouse Lab in Alderley Park	Wellcome Sanger Institute for the COVID-19 Genomics UK (COG-UK) Consortium	Jacquelyn Wynn, Mairead Hyland, The Lighthouse Lab in Alderley Park and Alex Alderton, Roberto Amato, Sonia Goncalves, Ewan Harrison, David K. Jackson, Ian Johnston, Dominic Kwiatkowski, Cordelia Langford, John Sillitoe on behalf of the Wellcome Sanger Institute COVID-19 Surveillance Team
EPI_ISL_858271, EPI_ISL_858272, EPI_ISL_858273, EPI_ISL_858275, EPI_ISL_858276, EPI_ISL_858277, EPI_ISL_858278, EPI_ISL_858279, EPI_ISL_858280, EPI_ISL_858281, EPI_ISL_858282, EPI_ISL_858283, EPI_ISL_858284, EPI_ISL_858285, EPI_ISL_858286			
see above	Lighthouse Lab in Glasgow	Wellcome Sanger Institute for the COVID-19 Genomics UK (COG-UK) Consortium	Harper VanSteenhouse, Yumi Kasai, David Gray, Carol Clugston, Anna Dominiczak and Alex Alderton, Roberto Amato, Sonia Goncalves, Ewan Harrison, David K. Jackson, Ian Johnston, Dominic Kwiatkowski, Cordelia Langford, John Sillitoe on behalf of the Wellcome Sanger Institute COVID-19 Surveillance Team
EPI_ISL_858287	Lighthouse Lab in Alderley Park	Wellcome Sanger Institute for the COVID-19 Genomics UK (COG-UK) Consortium	Jacquelyn Wynn, Mairead Hyland, The Lighthouse Lab in Alderley Park and Alex Alderton, Roberto Amato, Sonia Goncalves, Ewan Harrison, David K. Jackson, Ian Johnston, Dominic Kwiatkowski, Cordelia Langford, John Sillitoe on behalf of the Wellcome Sanger Institute COVID-19 Surveillance Team
EPI_ISL_858288, EPI_ISL_858289, EPI_ISL_858290, EPI_ISL_858291, EPI_ISL_858292, EPI_ISL_858293, EPI_ISL_858294, EPI_ISL_858295	Lighthouse Lab in Glasgow	Wellcome Sanger Institute for the COVID-19 Genomics UK (COG-UK) Consortium	Harper VanSteenhouse, Yumi Kasai, David Gray, Carol Clugston, Anna Dominiczak and Alex Alderton, Roberto Amato, Sonia Goncalves, Ewan Harrison, David K. Jackson, Ian Johnston, Dominic Kwiatkowski, Cordelia Langford, John Sillitoe on behalf of the Wellcome Sanger Institute COVID-19 Surveillance Team
EPI_ISL_858296	Lighthouse Lab in Alderley Park	Wellcome Sanger Institute for the COVID-19 Genomics UK (COG-UK) Consortium	Jacquelyn Wynn, Mairead Hyland, The Lighthouse Lab in Alderley Park and Alex Alderton, Roberto Amato, Sonia Goncalves, Ewan Harrison, David K. Jackson, Ian Johnston, Dominic Kwiatkowski, Cordelia Langford, John Sillitoe on behalf of the Wellcome Sanger Institute COVID-19 Surveillance Team
EPI_ISL_858297, EPI_ISL_858298, EPI_ISL_858299, EPI_ISL_858300, EPI_ISL_858301, EPI_ISL_858302, EPI_ISL_858303, EPI_ISL_858304, EPI_ISL_858305	Lighthouse Lab in Glasgow	Wellcome Sanger Institute for the COVID-19 Genomics UK (COG-UK) Consortium	Harper VanSteenhouse, Yumi Kasai, David Gray, Carol Clugston, Anna Dominiczak and Alex Alderton, Roberto Amato, Sonia Goncalves, Ewan Harrison, David K. Jackson, Ian Johnston, Dominic Kwiatkowski, Cordelia Langford, John Sillitoe on behalf of the Wellcome Sanger Institute COVID-19 Surveillance Team
EPI_ISL_858306, EPI_ISL_858307, EPI_ISL_858308, EPI_ISL_858309	Lighthouse Lab in Alderley Park	Wellcome Sanger Institute for the COVID-19 Genomics UK (COG-UK) Consortium	Jacquelyn Wynn, Mairead Hyland, The Lighthouse Lab in Alderley Park and Alex Alderton, Roberto Amato, Sonia Goncalves, Ewan Harrison, David K. Jackson, Ian Johnston, Dominic Kwiatkowski, Cordelia Langford, John Sillitoe on behalf of the Wellcome Sanger Institute COVID-19 Surveillance Team
EPI_ISL_858310	Lighthouse Lab in Glasgow	Wellcome Sanger Institute for the COVID-19 Genomics UK (COG-UK) Consortium	Harper VanSteenhouse, Yumi Kasai, David Gray, Carol Clugston, Anna Dominiczak and Alex Alderton, Roberto Amato, Sonia Goncalves, Ewan Harrison, David K. Jackson, Ian Johnston, Dominic Kwiatkowski, Cordelia Langford, John Sillitoe on behalf of the Wellcome Sanger Institute COVID-19 Surveillance Team
EPI_ISL_858311	Lighthouse Lab in Alderley Park	Wellcome Sanger Institute for the COVID-19 Genomics UK (COG-UK) Consortium	Jacquelyn Wynn, Mairead Hyland, The Lighthouse Lab in Alderley Park and Alex Alderton, Roberto Amato, Sonia Goncalves, Ewan Harrison, David K. Jackson, Ian Johnston, Dominic Kwiatkowski, Cordelia Langford, John Sillitoe on behalf of the Wellcome Sanger Institute COVID-19 Surveillance Team
EPI_ISL_858312, EPI_ISL_858314, EPI_ISL_858315, EPI_ISL_858317, EPI_ISL_858318, EPI_ISL_858319, EPI_ISL_858320, EPI_ISL_858321, EPI_ISL_858322, EPI_ISL_858323	Lighthouse Lab in Glasgow	Wellcome Sanger Institute for the COVID-19 Genomics UK (COG-UK) Consortium	Harper VanSteenhouse, Yumi Kasai, David Gray, Carol Clugston, Anna Dominiczak and Alex Alderton, Roberto Amato, Sonia Goncalves, Ewan Harrison, David K. Jackson, Ian Johnston, Dominic Kwiatkowski, Cordelia Langford, John Sillitoe on behalf of the Wellcome Sanger Institute COVID-19 Surveillance Team
EPI_ISL_858324, EPI_ISL_858325, EPI_ISL_858326	Lighthouse Lab in Alderley Park	Wellcome Sanger Institute for the COVID-19 Genomics UK (COG-UK) Consortium	Jacquelyn Wynn, Mairead Hyland, The Lighthouse Lab in Alderley Park and Alex Alderton, Roberto Amato, Sonia Goncalves, Ewan Harrison, David K. Jackson, Ian Johnston, Dominic Kwiatkowski, Cordelia Langford, John Sillitoe on behalf of the Wellcome Sanger Institute COVID-19 Surveillance Team
EPI_ISL_858327	Lighthouse Lab in Glasgow	Wellcome Sanger Institute for the COVID-19 Genomics UK (COG-UK) Consortium	Harper VanSteenhouse, Yumi Kasai, David Gray, Carol Clugston, Anna Dominiczak and Alex Alderton, Roberto Amato, Sonia Goncalves, Ewan Harrison, David K. Jackson, Ian Johnston, Dominic Kwiatkowski, Cordelia Langford, John Sillitoe on behalf of the Wellcome Sanger Institute COVID-19 Surveillance Team
EPI_ISL_858328, EPI_ISL_858329	Lighthouse Lab in Alderley Park	Wellcome Sanger Institute for the COVID-19 Genomics UK (COG-UK) Consortium	Jacquelyn Wynn, Mairead Hyland, The Lighthouse Lab in Alderley Park and Alex Alderton, Roberto Amato, Sonia Goncalves, Ewan Harrison, David K. Jackson, Ian Johnston, Dominic Kwiatkowski, Cordelia Langford, John Sillitoe on behalf of the Wellcome Sanger Institute COVID-19 Surveillance Team
EPI_ISL_858330, EPI_ISL_858331	Lighthouse Lab in Glasgow	Wellcome Sanger Institute for the COVID-19 Genomics UK (COG-UK) Consortium	Harper VanSteenhouse, Yumi Kasai, David Gray, Carol Clugston, Anna Dominiczak and Alex Alderton, Roberto Amato, Sonia Goncalves, Ewan Harrison, David K. Jackson, Ian Johnston, Dominic Kwiatkowski, Cordelia Langford, John Sillitoe on behalf of the Wellcome Sanger Institute COVID-19 Surveillance Team
EPI_ISL_858332	Lighthouse Lab in Alderley Park	Wellcome Sanger Institute for the COVID-19 Genomics UK (COG-UK) Consortium	Jacquelyn Wynn, Mairead Hyland, The Lighthouse Lab in Alderley Park and Alex Alderton, Roberto Amato, Sonia Goncalves, Ewan Harrison, David K. Jackson, Ian Johnston, Dominic Kwiatkowski, Cordelia Langford, John Sillitoe on behalf of the Wellcome Sanger Institute COVID-19 Surveillance Team
EPI_ISL_858333	Lighthouse Lab in Glasgow	Wellcome Sanger Institute for the COVID-19 Genomics UK (COG-UK) Consortium	Harper VanSteenhouse, Yumi Kasai, David Gray, Carol Clugston, Anna Dominiczak and Alex Alderton, Roberto Amato, Sonia Goncalves, Ewan Harrison, David K. Jackson, Ian Johnston, Dominic Kwiatkowski, Cordelia Langford, John Sillitoe on behalf of the Wellcome Sanger Institute COVID-19 Surveillance Team
EPI_ISL_858334	Lighthouse Lab in Alderley Park	Wellcome Sanger Institute for the COVID-19 Genomics UK (COG-UK) Consortium	Jacquelyn Wynn, Mairead Hyland, The Lighthouse Lab in Alderley Park and Alex Alderton, Roberto Amato, Sonia Goncalves, Ewan Harrison, David K. Jackson, Ian Johnston, Dominic Kwiatkowski, Cordelia Langford, John Sillitoe on behalf of the Wellcome Sanger Institute COVID-19 Surveillance Team
EPI_ISL_858335, EPI_ISL_858336, EPI_ISL_858337, EPI_ISL_858338, EPI_ISL_858339, EPI_ISL_858340, EPI_ISL_858341, EPI_ISL_858342	Lighthouse Lab in Glasgow	Wellcome Sanger Institute for the COVID-19 Genomics UK (COG-UK) Consortium	Harper VanSteenhouse, Yumi Kasai, David Gray, Carol Clugston, Anna Dominiczak and Alex Alderton, Roberto Amato, Sonia Goncalves, Ewan Harrison, David K. Jackson, Ian Johnston, Dominic Kwiatkowski, Cordelia Langford, John Sillitoe on behalf of the Wellcome Sanger Institute COVID-19 Surveillance Team
EPI_ISL_858343	Lighthouse Lab in Alderley Park	Wellcome Sanger Institute for the COVID-19 Genomics UK (COG-UK) Consortium	Jacquelyn Wynn, Mairead Hyland, The Lighthouse Lab in Alderley Park and Alex Alderton, Roberto Amato, Sonia Goncalves, Ewan Harrison, David K. Jackson, Ian Johnston, Dominic Kwiatkowski, Cordelia Langford, John Sillitoe on behalf of the Wellcome Sanger Institute COVID-19 Surveillance Team
EPI_ISL_858344, EPI_ISL_858345, EPI_ISL_858346, EPI_ISL_858347, EPI_ISL_858348, EPI_ISL_858349, EPI_ISL_858350, EPI_ISL_858351, EPI_ISL_858352, EPI_ISL_858353	Lighthouse Lab in Glasgow	Wellcome Sanger Institute for the COVID-19 Genomics UK (COG-UK) Consortium	Harper VanSteenhouse, Yumi Kasai, David Gray, Carol Clugston, Anna Dominiczak and Alex Alderton, Roberto Amato, Sonia Goncalves, Ewan Harrison, David K. Jackson, Ian Johnston, Dominic Kwiatkowski, Cordelia Langford, John Sillitoe on behalf of the Wellcome Sanger Institute COVID-19 Surveillance Team
EPI_ISL_858354	Lighthouse Lab in Alderley Park	Wellcome Sanger Institute for the COVID-19 Genomics UK (COG-UK) Consortium	Jacquelyn Wynn, Mairead Hyland, The Lighthouse Lab in Alderley Park and Alex Alderton, Roberto Amato, Sonia Goncalves, Ewan Harrison, David K. Jackson, Ian Johnston, Dominic Kwiatkowski, Cordelia Langford, John Sillitoe on behalf of the Wellcome Sanger Institute COVID-19 Surveillance Team
EPI_ISL_858355, EPI_ISL_858356	Lighthouse Lab in Glasgow	Wellcome Sanger Institute for the COVID-19 Genomics UK (COG-UK) Consortium	Harper VanSteenhouse, Yumi Kasai, David Gray, Carol Clugston, Anna Dominiczak and Alex Alderton, Roberto Amato, Sonia Goncalves, Ewan Harrison, David K. Jackson, Ian Johnston, Dominic Kwiatkowski, Cordelia Langford, John Sillitoe on behalf of the Wellcome Sanger Institute COVID-19 Surveillance Team
EPI_ISL_858357	Lighthouse Lab in Alderley Park	Wellcome Sanger Institute for the COVID-19 Genomics UK (COG-UK) Consortium	Jacquelyn Wynn, Mairead Hyland, The Lighthouse Lab in Alderley Park and Alex Alderton, Roberto Amato, Sonia Goncalves, Ewan Harrison, David K. Jackson, Ian Johnston, Dominic Kwiatkowski, Cordelia Langford, John Sillitoe on behalf of the Wellcome Sanger Institute COVID-19 Surveillance Team
EPI_ISL_858358, EPI_ISL_858359, EPI_ISL_858360, EPI_ISL_858361,	Lighthouse Lab in Glasgow	Wellcome Sanger Institute for the COVID-19 Genomics UK (COG-UK) Consortium	Harper VanSteenhouse, Yumi Kasai, David Gray, Carol Clugston, Anna Dominiczak and Alex Alderton, Roberto Amato, Sonia Goncalves, Ewan Harrison, David K. Jackson, Ian Johnston, Dominic Kwiatkowski, Cordelia Langford, John Sillitoe on behalf of the Wellcome Sanger Institute COVID-19 Surveillance Team

EPI_ISL_858362, EPI_ISL_858363, EPI_ISL_858364, EPI_ISL_858365, EPI_ISL_858366, EPI_ISL_858367			
EPI_ISL_858368	Lighthouse Lab in Alderley Park	Wellcome Sanger Institute for the COVID-19 Genomics UK (COG-UK) Consortium	Jacquelyn Wynn, Mairead Hyland, The Lighthouse Lab in Alderley Park and Alex Alderton, Roberto Amato, Sonia Goncalves, Ewan Harrison, David K. Jackson, Ian Johnston, Dominic Kwiatkowski, Cordelia Langford, John Sillitoe on behalf of the Wellcome Sanger Institute COVID-19 Surveillance Team
EPI_ISL_858369	Lighthouse Lab in Glasgow	Wellcome Sanger Institute for the COVID-19 Genomics UK (COG-UK) Consortium	Harper VanSteenhouse, Yumi Kasai, David Gray, Carol Clugston, Anna Dominiczak and Alex Alderton, Roberto Amato, Sonia Goncalves, Ewan Harrison, David K. Jackson, Ian Johnston, Dominic Kwiatkowski, Cordelia Langford, John Sillitoe on behalf of the Wellcome Sanger Institute COVID-19 Surveillance Team
EPI_ISL_858370	Lighthouse Lab in Alderley Park	Wellcome Sanger Institute for the COVID-19 Genomics UK (COG-UK) Consortium	Jacquelyn Wynn, Mairead Hyland, The Lighthouse Lab in Alderley Park and Alex Alderton, Roberto Amato, Sonia Goncalves, Ewan Harrison, David K. Jackson, Ian Johnston, Dominic Kwiatkowski, Cordelia Langford, John Sillitoe on behalf of the Wellcome Sanger Institute COVID-19 Surveillance Team
EPI_ISL_858371, EPI_ISL_858372, EPI_ISL_858373, EPI_ISL_858374	Lighthouse Lab in Glasgow	Wellcome Sanger Institute for the COVID-19 Genomics UK (COG-UK) Consortium	Harper VanSteenhouse, Yumi Kasai, David Gray, Carol Clugston, Anna Dominiczak and Alex Alderton, Roberto Amato, Sonia Goncalves, Ewan Harrison, David K. Jackson, Ian Johnston, Dominic Kwiatkowski, Cordelia Langford, John Sillitoe on behalf of the Wellcome Sanger Institute COVID-19 Surveillance Team
EPI_ISL_858375	Lighthouse Lab in Alderley Park	Wellcome Sanger Institute for the COVID-19 Genomics UK (COG-UK) Consortium	Jacquelyn Wynn, Mairead Hyland, The Lighthouse Lab in Alderley Park and Alex Alderton, Roberto Amato, Sonia Goncalves, Ewan Harrison, David K. Jackson, Ian Johnston, Dominic Kwiatkowski, Cordelia Langford, John Sillitoe on behalf of the Wellcome Sanger Institute COVID-19 Surveillance Team
EPI_ISL_858376, EPI_ISL_858377, EPI_ISL_858378, EPI_ISL_858379, EPI_ISL_858380	Lighthouse Lab in Glasgow	Wellcome Sanger Institute for the COVID-19 Genomics UK (COG-UK) Consortium	Harper VanSteenhouse, Yumi Kasai, David Gray, Carol Clugston, Anna Dominiczak and Alex Alderton, Roberto Amato, Sonia Goncalves, Ewan Harrison, David K. Jackson, Ian Johnston, Dominic Kwiatkowski, Cordelia Langford, John Sillitoe on behalf of the Wellcome Sanger Institute COVID-19 Surveillance Team
EPI_ISL_858381, EPI_ISL_858382	Lighthouse Lab in Alderley Park	Wellcome Sanger Institute for the COVID-19 Genomics UK (COG-UK) Consortium	Jacquelyn Wynn, Mairead Hyland, The Lighthouse Lab in Alderley Park and Alex Alderton, Roberto Amato, Sonia Goncalves, Ewan Harrison, David K. Jackson, Ian Johnston, Dominic Kwiatkowski, Cordelia Langford, John Sillitoe on behalf of the Wellcome Sanger Institute COVID-19 Surveillance Team
EPI_ISL_858383, EPI_ISL_858384, EPI_ISL_858385, EPI_ISL_858386	Lighthouse Lab in Glasgow	Wellcome Sanger Institute for the COVID-19 Genomics UK (COG-UK) Consortium	Harper VanSteenhouse, Yumi Kasai, David Gray, Carol Clugston, Anna Dominiczak and Alex Alderton, Roberto Amato, Sonia Goncalves, Ewan Harrison, David K. Jackson, Ian Johnston, Dominic Kwiatkowski, Cordelia Langford, John Sillitoe on behalf of the Wellcome Sanger Institute COVID-19 Surveillance Team
EPI_ISL_858387, EPI_ISL_858388, EPI_ISL_858389	Lighthouse Lab in Alderley Park	Wellcome Sanger Institute for the COVID-19 Genomics UK (COG-UK) Consortium	Jacquelyn Wynn, Mairead Hyland, The Lighthouse Lab in Alderley Park and Alex Alderton, Roberto Amato, Sonia Goncalves, Ewan Harrison, David K. Jackson, Ian Johnston, Dominic Kwiatkowski, Cordelia Langford, John Sillitoe on behalf of the Wellcome Sanger Institute COVID-19 Surveillance Team
EPI_ISL_858390, EPI_ISL_858391, EPI_ISL_858392	Lighthouse Lab in Glasgow	Wellcome Sanger Institute for the COVID-19 Genomics UK (COG-UK) Consortium	Harper VanSteenhouse, Yumi Kasai, David Gray, Carol Clugston, Anna Dominiczak and Alex Alderton, Roberto Amato, Sonia Goncalves, Ewan Harrison, David K. Jackson, Ian Johnston, Dominic Kwiatkowski, Cordelia Langford, John Sillitoe on behalf of the Wellcome Sanger Institute COVID-19 Surveillance Team
EPI_ISL_858393	Lighthouse Lab in Alderley Park	Wellcome Sanger Institute for the COVID-19 Genomics UK (COG-UK) Consortium	Jacquelyn Wynn, Mairead Hyland, The Lighthouse Lab in Alderley Park and Alex Alderton, Roberto Amato, Sonia Goncalves, Ewan Harrison, David K. Jackson, Ian Johnston, Dominic Kwiatkowski, Cordelia Langford, John Sillitoe on behalf of the Wellcome Sanger Institute COVID-19 Surveillance Team
EPI_ISL_858394, EPI_ISL_858395, EPI_ISL_858396	Lighthouse Lab in Glasgow	Wellcome Sanger Institute for the COVID-19 Genomics UK (COG-UK) Consortium	Harper VanSteenhouse, Yumi Kasai, David Gray, Carol Clugston, Anna Dominiczak and Alex Alderton, Roberto Amato, Sonia Goncalves, Ewan Harrison, David K. Jackson, Ian Johnston, Dominic Kwiatkowski, Cordelia Langford, John Sillitoe on behalf of the Wellcome Sanger Institute COVID-19 Surveillance Team
EPI_ISL_858397	Lighthouse Lab in Alderley Park	Wellcome Sanger Institute for the COVID-19 Genomics UK (COG-UK) Consortium	Jacquelyn Wynn, Mairead Hyland, The Lighthouse Lab in Alderley Park and Alex Alderton, Roberto Amato, Sonia Goncalves, Ewan Harrison, David K. Jackson, Ian Johnston, Dominic Kwiatkowski, Cordelia Langford, John Sillitoe on behalf of the Wellcome Sanger Institute COVID-19 Surveillance Team
EPI_ISL_858398, EPI_ISL_858399	Lighthouse Lab in Glasgow	Wellcome Sanger Institute for the COVID-19 Genomics UK (COG-UK) Consortium	Harper VanSteenhouse, Yumi Kasai, David Gray, Carol Clugston, Anna Dominiczak and Alex Alderton, Roberto Amato, Sonia Goncalves, Ewan Harrison, David K. Jackson, Ian Johnston, Dominic Kwiatkowski, Cordelia Langford, John Sillitoe on behalf of the Wellcome Sanger Institute COVID-19 Surveillance Team
EPI_ISL_858400	Lighthouse Lab in Alderley Park	Wellcome Sanger Institute for the COVID-19 Genomics UK (COG-UK) Consortium	Jacquelyn Wynn, Mairead Hyland, The Lighthouse Lab in Alderley Park and Alex Alderton, Roberto Amato, Sonia Goncalves, Ewan Harrison, David K. Jackson, Ian Johnston, Dominic Kwiatkowski, Cordelia Langford, John Sillitoe on behalf of the Wellcome Sanger Institute COVID-19 Surveillance Team
EPI_ISL_858401, EPI_ISL_858402	Lighthouse Lab in Glasgow	Wellcome Sanger Institute for the COVID-19 Genomics UK (COG-UK) Consortium	Harper VanSteenhouse, Yumi Kasai, David Gray, Carol Clugston, Anna Dominiczak and Alex Alderton, Roberto Amato, Sonia Goncalves, Ewan Harrison, David K. Jackson, Ian Johnston, Dominic Kwiatkowski, Cordelia Langford, John Sillitoe on behalf of the Wellcome Sanger Institute COVID-19 Surveillance Team
EPI_ISL_858403	Lighthouse Lab in Alderley Park	Wellcome Sanger Institute for the COVID-19 Genomics UK (COG-UK) Consortium	Jacquelyn Wynn, Mairead Hyland, The Lighthouse Lab in Alderley Park and Alex Alderton, Roberto Amato, Sonia Goncalves, Ewan Harrison, David K. Jackson, Ian Johnston, Dominic Kwiatkowski, Cordelia Langford, John Sillitoe on behalf of the Wellcome Sanger Institute COVID-19 Surveillance Team
EPI_ISL_858404, EPI_ISL_858405, EPI_ISL_858406, EPI_ISL_858407, EPI_ISL_858425, EPI_ISL_858426, EPI_ISL_858427, EPI_ISL_858428, EPI_ISL_858429, EPI_ISL_858430, EPI_ISL_858431, EPI_ISL_858432, EPI_ISL_858433, EPI_ISL_858434, EPI_ISL_858435, EPI_ISL_858436, EPI_ISL_858437, EPI_ISL_858438, EPI_ISL_858439, EPI_ISL_858440, EPI_ISL_858441, EPI_ISL_858442, EPI_ISL_858443, EPI_ISL_858444, EPI_ISL_858445, EPI_ISL_858446, EPI_ISL_858447, EPI_ISL_858448, EPI_ISL_858449, EPI_ISL_858450, EPI_ISL_858451, EPI_ISL_858452, EPI_ISL_858453, EPI_ISL_858454, EPI_ISL_858455, EPI_ISL_858456, EPI_ISL_858457, EPI_ISL_858458, EPI_ISL_858459, EPI_ISL_858460, EPI_ISL_858461, EPI_ISL_858462, EPI_ISL_858463, EPI_ISL_858464, EPI_ISL_858465, EPI_ISL_858466, EPI_ISL_858467, EPI_ISL_858468, EPI_ISL_858469, EPI_ISL_858470, EPI_ISL_858471, EPI_ISL_858472, EPI_ISL_858473, EPI_ISL_858474, EPI_ISL_858475, EPI_ISL_858476, EPI_ISL_858477, EPI_ISL_858478, EPI_ISL_858479, EPI_ISL_858480, EPI_ISL_858481, EPI_ISL_858482, EPI_ISL_858483, EPI_ISL_858484, EPI_ISL_858485, EPI_ISL_858486, EPI_ISL_858487, EPI_ISL_858488, EPI_ISL_858489, EPI_ISL_858490, EPI_ISL_858491, EPI_ISL_858492, EPI_ISL_858493, EPI_ISL_858494, EPI_ISL_858495, EPI_ISL_858496, EPI_ISL_858497, EPI_ISL_858498, EPI_ISL_858499, EPI_ISL_858500, EPI_ISL_858501, EPI_ISL_858502, EPI_ISL_858503, EPI_ISL_858504, EPI_ISL_858505, EPI_ISL_858506, EPI_ISL_858507, EPI_ISL_858508, EPI_ISL_858509, EPI_ISL_858510, EPI_ISL_858511, EPI_ISL_858512, EPI_ISL_858513, EPI_ISL_858514, EPI_ISL_858515			
see above	Lighthouse Lab in Glasgow	Wellcome Sanger Institute for the COVID-19 Genomics UK (COG-UK) Consortium	Harper VanSteenhouse, Yumi Kasai, David Gray, Carol Clugston, Anna Dominiczak and Alex Alderton, Roberto Amato, Sonia Goncalves, Ewan Harrison, David K. Jackson, Ian Johnston, Dominic Kwiatkowski, Cordelia Langford, John Sillitoe on behalf of the Wellcome Sanger Institute COVID-19 Surveillance Team
EPI_ISL_858516	Lighthouse Lab in Alderley Park	Wellcome Sanger Institute for the COVID-19 Genomics UK (COG-UK) Consortium	Jacquelyn Wynn, Mairead Hyland, The Lighthouse Lab in Alderley Park and Alex Alderton, Roberto Amato, Sonia Goncalves, Ewan Harrison, David K. Jackson, Ian Johnston, Dominic Kwiatkowski, Cordelia Langford, John Sillitoe on behalf of the Wellcome Sanger Institute COVID-19 Surveillance Team
EPI_ISL_858517, EPI_ISL_858518, EPI_ISL_858519, EPI_ISL_858520	Lighthouse Lab in Glasgow	Wellcome Sanger Institute for the COVID-19 Genomics UK (COG-UK) Consortium	Harper VanSteenhouse, Yumi Kasai, David Gray, Carol Clugston, Anna Dominiczak and Alex Alderton, Roberto Amato, Sonia Goncalves, Ewan Harrison, David K. Jackson, Ian Johnston, Dominic Kwiatkowski, Cordelia Langford, John Sillitoe on behalf of the Wellcome Sanger Institute COVID-19 Surveillance Team
EPI_ISL_858521	Lighthouse Lab in Alderley Park	Wellcome Sanger Institute for the COVID-19 Genomics UK (COG-UK) Consortium	Jacquelyn Wynn, Mairead Hyland, The Lighthouse Lab in Alderley Park and Alex Alderton, Roberto Amato, Sonia Goncalves, Ewan Harrison, David K. Jackson, Ian Johnston, Dominic Kwiatkowski, Cordelia Langford, John Sillitoe on behalf of the Wellcome Sanger Institute COVID-19 Surveillance Team
EPI_ISL_858522	Lighthouse Lab in Glasgow	Wellcome Sanger Institute for the COVID-19 Genomics UK (COG-UK) Consortium	Harper VanSteenhouse, Yumi Kasai, David Gray, Carol Clugston, Anna Dominiczak and Alex Alderton, Roberto Amato, Sonia Goncalves, Ewan Harrison, David K. Jackson, Ian Johnston, Dominic Kwiatkowski, Cordelia Langford, John Sillitoe on behalf of the Wellcome Sanger Institute COVID-19 Surveillance Team
EPI_ISL_858523	Lighthouse Lab in Alderley Park	Wellcome Sanger Institute for the COVID-19 Genomics UK (COG-UK) Consortium	Jacquelyn Wynn, Mairead Hyland, The Lighthouse Lab in Alderley Park and Alex Alderton, Roberto Amato, Sonia Goncalves, Ewan Harrison, David K. Jackson, Ian Johnston, Dominic Kwiatkowski, Cordelia Langford, John Sillitoe on behalf of the Wellcome Sanger Institute COVID-19 Surveillance Team
EPI_ISL_858524, EPI_ISL_858525, EPI_ISL_858526, EPI_ISL_858527, EPI_ISL_858528, EPI_ISL_858529, EPI_ISL_858530	Lighthouse Lab in Glasgow	Wellcome Sanger Institute for the COVID-19 Genomics UK (COG-UK) Consortium	Harper VanSteenhouse, Yumi Kasai, David Gray, Carol Clugston, Anna Dominiczak and Alex Alderton, Roberto Amato, Sonia Goncalves, Ewan Harrison, David K. Jackson, Ian Johnston, Dominic Kwiatkowski, Cordelia Langford, John Sillitoe on behalf of the Wellcome Sanger Institute COVID-19 Surveillance Team
EPI_ISL_858531	Lighthouse Lab in Alderley Park	Wellcome Sanger Institute for the COVID-19 Genomics UK (COG-UK) Consortium	Jacquelyn Wynn, Mairead Hyland, The Lighthouse Lab in Alderley Park and Alex Alderton, Roberto Amato, Sonia Goncalves, Ewan Harrison, David K. Jackson, Ian Johnston, Dominic Kwiatkowski, Cordelia Langford, John Sillitoe on behalf of the Wellcome Sanger Institute COVID-19 Surveillance Team
EPI_ISL_858532, EPI_ISL_858533, EPI_ISL_858534, EPI_ISL_858535, EPI_ISL_858536, EPI_ISL_858537, EPI_ISL_858538, EPI_ISL_858539, EPI_ISL_858540, EPI_ISL_858541, EPI_ISL_858542, EPI_ISL_858543, EPI_ISL_858544, EPI_ISL_858545, EPI_ISL_858546, EPI_ISL_858547, EPI_ISL_858548, EPI_ISL_858549, EPI_ISL_858550, EPI_ISL_858551, EPI_ISL_858552, EPI_ISL_858553, EPI_ISL_858554, EPI_ISL_858555, EPI_ISL_858556			

see above	Lighthouse Lab in Glasgow	Wellcome Sanger Institute for the COVID-19 Genomics UK (COG-UK) Consortium	Harper VanSteenhouse, Yumi Kasai, David Gray, Carol Clugston, Anna Dominiczak and Alex Alderton, Roberto Amato, Sonia Goncalves, Ewan Harrison, David K. Jackson, Ian Johnston, Dominic Kwiatkowski, Cordelia Langford, John Sillitoe on behalf of the Wellcome Sanger Institute COVID-19 Surveillance Team
EPI_ISL_858557	Lighthouse Lab in Alderley Park	Wellcome Sanger Institute for the COVID-19 Genomics UK (COG-UK) Consortium	Jacquelyn Wynn, Mairead Hyland, The Lighthouse Lab in Alderley Park and Alex Alderton, Roberto Amato, Sonia Goncalves, Ewan Harrison, David K. Jackson, Ian Johnston, Dominic Kwiatkowski, Cordelia Langford, John Sillitoe on behalf of the Wellcome Sanger Institute COVID-19 Surveillance Team
EPI_ISL_858558, EPI_ISL_858559, EPI_ISL_858560, EPI_ISL_858561, EPI_ISL_858562	Lighthouse Lab in Glasgow	Wellcome Sanger Institute for the COVID-19 Genomics UK (COG-UK) Consortium	Harper VanSteenhouse, Yumi Kasai, David Gray, Carol Clugston, Anna Dominiczak and Alex Alderton, Roberto Amato, Sonia Goncalves, Ewan Harrison, David K. Jackson, Ian Johnston, Dominic Kwiatkowski, Cordelia Langford, John Sillitoe on behalf of the Wellcome Sanger Institute COVID-19 Surveillance Team
EPI_ISL_858563	Lighthouse Lab in Alderley Park	Wellcome Sanger Institute for the COVID-19 Genomics UK (COG-UK) Consortium	Jacquelyn Wynn, Mairead Hyland, The Lighthouse Lab in Alderley Park and Alex Alderton, Roberto Amato, Sonia Goncalves, Ewan Harrison, David K. Jackson, Ian Johnston, Dominic Kwiatkowski, Cordelia Langford, John Sillitoe on behalf of the Wellcome Sanger Institute COVID-19 Surveillance Team
EPI_ISL_858564, EPI_ISL_858565, EPI_ISL_858566, EPI_ISL_858567, EPI_ISL_858568, EPI_ISL_858569, EPI_ISL_858570, EPI_ISL_858571	Lighthouse Lab in Glasgow	Wellcome Sanger Institute for the COVID-19 Genomics UK (COG-UK) Consortium	Harper VanSteenhouse, Yumi Kasai, David Gray, Carol Clugston, Anna Dominiczak and Alex Alderton, Roberto Amato, Sonia Goncalves, Ewan Harrison, David K. Jackson, Ian Johnston, Dominic Kwiatkowski, Cordelia Langford, John Sillitoe on behalf of the Wellcome Sanger Institute COVID-19 Surveillance Team
EPI_ISL_858572, EPI_ISL_858574, EPI_ISL_858576, EPI_ISL_858581	Lighthouse Lab in Alderley Park	Wellcome Sanger Institute for the COVID-19 Genomics UK (COG-UK) Consortium	Jacquelyn Wynn, Mairead Hyland, The Lighthouse Lab in Alderley Park and Alex Alderton, Roberto Amato, Sonia Goncalves, Ewan Harrison, David K. Jackson, Ian Johnston, Dominic Kwiatkowski, Cordelia Langford, John Sillitoe on behalf of the Wellcome Sanger Institute COVID-19 Surveillance Team
EPI_ISL_858585, EPI_ISL_858587, EPI_ISL_858588, EPI_ISL_858592, EPI_ISL_858594, EPI_ISL_858598, EPI_ISL_858600, EPI_ISL_858601, EPI_ISL_858604, EPI_ISL_858607, EPI_ISL_858608, EPI_ISL_858609, EPI_ISL_858610, EPI_ISL_858612, EPI_ISL_858613, EPI_ISL_858615, EPI_ISL_858616, EPI_ISL_858618, EPI_ISL_858623, EPI_ISL_858626, EPI_ISL_858627, EPI_ISL_858628, EPI_ISL_858630, EPI_ISL_858635, EPI_ISL_858637, EPI_ISL_858638, EPI_ISL_858640, EPI_ISL_858641, EPI_ISL_858642, EPI_ISL_858643, EPI_ISL_858646, EPI_ISL_858650, EPI_ISL_858651, EPI_ISL_858652, EPI_ISL_858656, EPI_ISL_858657, EPI_ISL_858658, EPI_ISL_858659, EPI_ISL_858660, EPI_ISL_858661, EPI_ISL_858664, EPI_ISL_858665, EPI_ISL_858668			
see above	Lighthouse Lab in Glasgow	Wellcome Sanger Institute for the COVID-19 Genomics UK (COG-UK) Consortium	Harper VanSteenhouse, Yumi Kasai, David Gray, Carol Clugston, Anna Dominiczak and Alex Alderton, Roberto Amato, Sonia Goncalves, Ewan Harrison, David K. Jackson, Ian Johnston, Dominic Kwiatkowski, Cordelia Langford, John Sillitoe on behalf of the Wellcome Sanger Institute COVID-19 Surveillance Team
EPI_ISL_860666, EPI_ISL_860667, EPI_ISL_860668, EPI_ISL_860669, EPI_ISL_860670, EPI_ISL_860671, EPI_ISL_860672, EPI_ISL_860673, EPI_ISL_860674, EPI_ISL_860675, EPI_ISL_860676, EPI_ISL_860678, EPI_ISL_860679, EPI_ISL_860680, EPI_ISL_860681, EPI_ISL_860682, EPI_ISL_860683, EPI_ISL_860684			
see above	Respiratory Virus Unit, National Infection Service, Public Health England	COVID-19 Genomics UK (COG-UK) Consortium	PHE Covid Sequencing Team
EPI_ISL_863534, EPI_ISL_863535, EPI_ISL_863536, EPI_ISL_863541, EPI_ISL_863545, EPI_ISL_863548, EPI_ISL_863550, EPI_ISL_863554, EPI_ISL_863559, EPI_ISL_863563, EPI_ISL_863564, EPI_ISL_863568, EPI_ISL_863569, EPI_ISL_863571, EPI_ISL_863573, EPI_ISL_863575, EPI_ISL_863585, EPI_ISL_863596, EPI_ISL_863597, EPI_ISL_863598, EPI_ISL_863602, EPI_ISL_863607, EPI_ISL_863611, EPI_ISL_863613, EPI_ISL_863615, EPI_ISL_863617, EPI_ISL_863620, EPI_ISL_863624, EPI_ISL_863632, EPI_ISL_863634, EPI_ISL_863637, EPI_ISL_863638, EPI_ISL_863642, EPI_ISL_863646, EPI_ISL_863653, EPI_ISL_863657, EPI_ISL_863660, EPI_ISL_863669, EPI_ISL_863677, EPI_ISL_863678, EPI_ISL_863679, EPI_ISL_863681, EPI_ISL_863696, EPI_ISL_863699, EPI_ISL_863700, EPI_ISL_863705, EPI_ISL_863709, EPI_ISL_863711, EPI_ISL_863723, EPI_ISL_863725, EPI_ISL_863726, EPI_ISL_863733, EPI_ISL_863734, EPI_ISL_863741, EPI_ISL_863742, EPI_ISL_863747, EPI_ISL_863759, EPI_ISL_863761, EPI_ISL_863766, EPI_ISL_863773, EPI_ISL_863776, EPI_ISL_863781, EPI_ISL_863784, EPI_ISL_863785, EPI_ISL_863788, EPI_ISL_863789, EPI_ISL_863792, EPI_ISL_863793, EPI_ISL_863795, EPI_ISL_863796, EPI_ISL_863797, EPI_ISL_863802, EPI_ISL_863804, EPI_ISL_863806, EPI_ISL_863809, EPI_ISL_863810, EPI_ISL_863812, EPI_ISL_863838, EPI_ISL_863842, EPI_ISL_863845, EPI_ISL_863847, EPI_ISL_863848, EPI_ISL_863850, EPI_ISL_863851, EPI_ISL_863856, EPI_ISL_863858, EPI_ISL_863860, EPI_ISL_863863, EPI_ISL_863865			
see above	Lighthouse Lab in Milton Keynes	Wellcome Sanger Institute for the COVID-19 Genomics UK (COG-UK) Consortium	The Lighthouse Lab in Milton Keynes and Alex Alderton, Roberto Amato, Sonia Goncalves, Ewan Harrison, David K. Jackson, Ian Johnston, Dominic Kwiatkowski, Cordelia Langford, John Sillitoe on behalf of the Wellcome Sanger Institute COVID-19 Surveillance Team
EPI_ISL_864039	Lighthouse Lab in Alderley Park	Wellcome Sanger Institute for the COVID-19 Genomics UK (COG-UK) Consortium	Jacquelyn Wynn, Mairead Hyland, The Lighthouse Lab in Alderley Park and Alex Alderton, Roberto Amato, Sonia Goncalves, Ewan Harrison, David K. Jackson, Ian Johnston, Dominic Kwiatkowski, Cordelia Langford, John Sillitoe on behalf of the Wellcome Sanger Institute COVID-19 Surveillance Team
EPI_ISL_864204, EPI_ISL_864213, EPI_ISL_864216, EPI_ISL_864217, EPI_ISL_864239, EPI_ISL_864244, EPI_ISL_864255, EPI_ISL_864262, EPI_ISL_864266, EPI_ISL_864274, EPI_ISL_864281, EPI_ISL_864291, EPI_ISL_864297, EPI_ISL_864305, EPI_ISL_864309, EPI_ISL_864311, EPI_ISL_864317, EPI_ISL_864321, EPI_ISL_864324, EPI_ISL_864336, EPI_ISL_864356, EPI_ISL_864361, EPI_ISL_864365, EPI_ISL_864368, EPI_ISL_864383, EPI_ISL_864384, EPI_ISL_864396, EPI_ISL_864399, EPI_ISL_864400, EPI_ISL_864402, EPI_ISL_864403, EPI_ISL_864409, EPI_ISL_864411, EPI_ISL_864414, EPI_ISL_864422, EPI_ISL_864439, EPI_ISL_864440, EPI_ISL_864447, EPI_ISL_864452, EPI_ISL_864461, EPI_ISL_864469, EPI_ISL_864472, EPI_ISL_864475, EPI_ISL_864484, EPI_ISL_864493, EPI_ISL_864515, EPI_ISL_864516, EPI_ISL_864520, EPI_ISL_864527, EPI_ISL_864528, EPI_ISL_864529, EPI_ISL_864534, EPI_ISL_864539, EPI_ISL_864540, EPI_ISL_864542			
see above	Lighthouse Lab in Milton Keynes	Wellcome Sanger Institute for the COVID-19 Genomics UK (COG-UK) Consortium	The Lighthouse Lab in Milton Keynes and Alex Alderton, Roberto Amato, Sonia Goncalves, Ewan Harrison, David K. Jackson, Ian Johnston, Dominic Kwiatkowski, Cordelia Langford, John Sillitoe on behalf of the Wellcome Sanger Institute COVID-19 Surveillance Team
EPI_ISL_864543	Lighthouse Lab in Glasgow	Wellcome Sanger Institute for the COVID-19 Genomics UK (COG-UK) Consortium	Harper VanSteenhouse, Yumi Kasai, David Gray, Carol Clugston, Anna Dominiczak and Alex Alderton, Roberto Amato, Sonia Goncalves, Ewan Harrison, David K. Jackson, Ian Johnston, Dominic Kwiatkowski, Cordelia Langford, John Sillitoe on behalf of the Wellcome Sanger Institute COVID-19 Surveillance Team
EPI_ISL_864748, EPI_ISL_864751, EPI_ISL_864754, EPI_ISL_864755, EPI_ISL_864756, EPI_ISL_864758, EPI_ISL_864760, EPI_ISL_864762, EPI_ISL_864764, EPI_ISL_864766, EPI_ISL_864768, EPI_ISL_864771, EPI_ISL_864773, EPI_ISL_864774, EPI_ISL_864775, EPI_ISL_864776, EPI_ISL_864777, EPI_ISL_864778, EPI_ISL_864779, EPI_ISL_864780, EPI_ISL_864797, EPI_ISL_864799, EPI_ISL_864803, EPI_ISL_864805, EPI_ISL_864807, EPI_ISL_864809, EPI_ISL_864811, EPI_ISL_864813, EPI_ISL_864815, EPI_ISL_864818, EPI_ISL_864819, EPI_ISL_864821, EPI_ISL_864828, EPI_ISL_864829, EPI_ISL_864832, EPI_ISL_864834, EPI_ISL_864838, EPI_ISL_864846, EPI_ISL_864850, EPI_ISL_864856, EPI_ISL_864875, EPI_ISL_864883, EPI_ISL_864889			
see above	Department of Pathology, University of Cambridge	COVID-19 Genomics UK (COG-UK) Consortium	Aminu S. Jahun, Yasmin Chaudhry, Grant Hall, Iliana Georgana, Myra Hosmillo, Martin D. Curran, Malte Pinckert, Surendra Parmar, Ian Goodfellow
EPI_ISL_864984, EPI_ISL_864985, EPI_ISL_864986, EPI_ISL_864987, EPI_ISL_864988, EPI_ISL_864989, EPI_ISL_864990, EPI_ISL_864991, EPI_ISL_864994, EPI_ISL_864995, EPI_ISL_864996, EPI_ISL_864997, EPI_ISL_864998, EPI_ISL_864999			
see above	West of Scotland Specialist Virology Centre, NHSGGC / MRC-University of Glasgow Centre for Virus Research	COVID-19 Genomics UK (COG-UK) Consortium	Ana da Silva Filipe, Natasha Johnson, Kathy Smollett, Daniel Mair, Stephen Carmichael, Alice Broos, Lily Tong, Jenna Nichols, Kyriaki Nomikou; Sarah McDonald; Richard Orton, Joseph Hughes, Sreenu Vattipally, David L Robertson; Alasdair MacLean, Rory Gunson; Sharif Shaaban, Matthew Holden; Rachel Blacow, Guy Mollett, Kathy Li, James Shepherd, Antonia Ho, Emma Thomson
EPI_ISL_865007, EPI_ISL_865009, EPI_ISL_865010, EPI_ISL_865011, EPI_ISL_865012	Lighthouse Lab in Glasgow / MRC-University of Glasgow Centre for Virus Research	COVID-19 Genomics UK (COG-UK) Consortium	Ana da Silva Filipe, Natasha Johnson, Kathy Smollett, Daniel Mair, Stephen Carmichael, Alice Broos, Lily Tong, Jenna Nichols, Kyriaki Nomikou; Sarah McDonald; Harper VanSteenhouse, Yumi Kasai, David Gray, Carol Clugston, Anna Dominiczak; Alasdair MacLean, Rory Gunson; Richard Orton, Joseph Hughes, Sreenu Vattipally, David L Robertson; Sharif Shaaban, Matthew Holden; Kathy Li, James Shepherd, Antonia Ho, Emma Thomson
EPI_ISL_865013, EPI_ISL_865014, EPI_ISL_865041, EPI_ISL_865042, EPI_ISL_865043, EPI_ISL_865044, EPI_ISL_865045, EPI_ISL_865046, EPI_ISL_865047, EPI_ISL_865048, EPI_ISL_865049, EPI_ISL_865051, EPI_ISL_865054			
see above	West of Scotland Specialist Virology Centre, NHSGGC / MRC-University of Glasgow Centre for Virus Research	COVID-19 Genomics UK (COG-UK) Consortium	Ana da Silva Filipe, Natasha Johnson, Kathy Smollett, Daniel Mair, Stephen Carmichael, Alice Broos, Lily Tong, Jenna Nichols, Kyriaki Nomikou; Sarah McDonald; Richard Orton, Joseph Hughes, Sreenu Vattipally, David L Robertson; Alasdair MacLean, Rory Gunson; Sharif Shaaban, Matthew Holden; Rachel Blacow, Guy Mollett, Kathy Li, James Shepherd, Antonia Ho, Emma Thomson
EPI_ISL_865074, EPI_ISL_865075, EPI_ISL_865076, EPI_ISL_865077, EPI_ISL_865094, EPI_ISL_865095, EPI_ISL_865096, EPI_ISL_865097, EPI_ISL_865098, EPI_ISL_865099, EPI_ISL_865100, EPI_ISL_865101, EPI_ISL_865102, EPI_ISL_865103, EPI_ISL_865104, EPI_ISL_865149, EPI_ISL_865150, EPI_ISL_865151, EPI_ISL_865152			
see above	Virology Department, Royal Infirmary of Edinburgh, NHS Lothian / School of Biological Sciences, University of Edinburgh / Institute of Genetics and Molecular Medicine, University of Edinburgh	COVID-19 Genomics UK (COG-UK) Consortium	McHugh M, Dewar R, Rooke S, Gallagher M, Balcaza C, O'Toole Á, Scher E, Hill V, McCrone JT, Colquhoun R, Yu X, Jackson B, Rambaut A, Williams TC, Templeton K
EPI_ISL_865178, EPI_ISL_865179, EPI_ISL_865181, EPI_ISL_865182, EPI_ISL_865183, EPI_ISL_865184, EPI_ISL_865185, EPI_ISL_865186, EPI_ISL_865187, EPI_ISL_865188, EPI_ISL_865189, EPI_ISL_865190, EPI_ISL_865191, EPI_ISL_865192, EPI_ISL_865193, EPI_ISL_865194, EPI_ISL_865196, EPI_ISL_865197, EPI_ISL_865198, EPI_ISL_865199, EPI_ISL_865200, EPI_ISL_865201, EPI_ISL_865202, EPI_ISL_865203, EPI_ISL_865204, EPI_ISL_865205, EPI_ISL_865206, EPI_ISL_865331, EPI_ISL_865332, EPI_ISL_865333, EPI_ISL_865334, EPI_ISL_865335, EPI_ISL_865336, EPI_ISL_865337, EPI_ISL_865338, EPI_ISL_865339, EPI_ISL_865340, EPI_ISL_865365, EPI_ISL_865366, EPI_ISL_865369, EPI_ISL_865370, EPI_ISL_865383, EPI_ISL_865384, EPI_ISL_865385, EPI_ISL_865387, EPI_ISL_865388, EPI_ISL_865390, EPI_ISL_865450, EPI_ISL_865478, EPI_ISL_865479, EPI_ISL_865480, EPI_ISL_865481, EPI_ISL_865482, EPI_ISL_865483, EPI_ISL_865484			
see above	Liverpool Clinical Laboratories	COVID-19 Genomics UK (COG-UK) Consortium	Sam Haldenby, Anita Lucaci, Steve Paterson, Julian Hiscox, Alistair Darby, M Almsaud, A Alrezaihi, Muhannad Alruwaili, Stuart D Armstrong, Jones Benjamin, Eleanor G Bentley, Anu Chawla, Jordan J Clark, Angela Cowell, Richard Eccles, Isabel García-Dorival, Matthew Gemmell, Alessandro Gerada, PKF Gilmore, Richard Gregory, Ximeng Han, Catherine Hartley, Margaret Hughes, Miren Iturriza-Gomara, James Johnson, L Luu, Jenifer Manson, Charlotte Nelson, Elaine O'Toole, Cassie Olateju, Rebekah Penrice-Randal , Lucille Rainbow, N.P Randle, Trevor Ian Robinson, Parul Sharma, Ghada T Shawli, James P Stewart, Neil Swainston, Ecaterina Vamos, Joanne Watts, Mark Whitehead
EPI_ISL_865499, EPI_ISL_865500,	Barts Health NHS Trust	COVID-19 Genomics UK (COG-UK) Consortium	CUTINO-MOGUEL, Maria-Teresa; HARRINGTON, David; OWOYEMI, Dola; KULASEGARAN-SHYLINI, Raghavendran; BROAD, Claire; KELE, Beatrix

EPI_ISL_865571, EPI_ISL_865574, EPI_ISL_865575			
EPI_ISL_866076, EPI_ISL_866077, EPI_ISL_866078, EPI_ISL_866079, EPI_ISL_866109, EPI_ISL_866110, EPI_ISL_866111, EPI_ISL_866147	University College London Hospital	COVID-19 Genomics UK (COG-UK) Consortium	Judith Heaney, Matthew Byott, Catherine Houlihan, Dan Frampton, Stuart Kirk, Moira Spyer and Eleni Nastouli
EPI_ISL_866187, EPI_ISL_866188, EPI_ISL_866189, EPI_ISL_866190, EPI_ISL_866191, EPI_ISL_866192, EPI_ISL_866193	University College London, Great Ormond Street Hospital for Children NHS Foundation Trust, Imperial College Healthcare NHS Trust	COVID-19 Genomics UK (COG-UK) Consortium	Sergi Castellano, Rachel Williams, Mark Kristiansen, Paola Resende Silva, Sunando Roy, Tony Brooks, Helena Tutill, Paola Niola, Patricia Dyal, Charlotte Williams, Leysa Forrest, Yasmin Panchbhaya, Jacqueline Findlay, Samuel Weeks, Julianne Brown, Kathryn Harris, Paul Randell, James Price, Alison Holmes, Judith Breuer
EPI_ISL_866353, EPI_ISL_866354, EPI_ISL_866355, EPI_ISL_866357, EPI_ISL_866358, EPI_ISL_866359, EPI_ISL_866360, EPI_ISL_866361, EPI_ISL_866362, EPI_ISL_866367, EPI_ISL_866370, EPI_ISL_866373, EPI_ISL_866374, EPI_ISL_866375, EPI_ISL_866376, EPI_ISL_866377, EPI_ISL_866378, EPI_ISL_866379, EPI_ISL_866380, EPI_ISL_866381, EPI_ISL_866382, EPI_ISL_866384, EPI_ISL_866385, EPI_ISL_866386, EPI_ISL_866387, EPI_ISL_866388, EPI_ISL_866389, EPI_ISL_866390, EPI_ISL_866391, EPI_ISL_866392, EPI_ISL_866395, EPI_ISL_866396, EPI_ISL_866397, EPI_ISL_866398, EPI_ISL_866399			
see above	Regional Virus Laboratory, Belfast Health and Social Care Trust	COVID-19 Genomics UK (COG-UK) Consortium	Conall McCaughey, James McKenna, Tanya Curran, Susan Feeney, Alison Watt, Ciara Cox, Mairead Connor, Zoltan Molnar, David Simpson, Derek Fairley
EPI_ISL_866527, EPI_ISL_866528, EPI_ISL_866529, EPI_ISL_866530, EPI_ISL_866531, EPI_ISL_866532, EPI_ISL_866533, EPI_ISL_866534, EPI_ISL_866536, EPI_ISL_866537, EPI_ISL_866538, EPI_ISL_866539, EPI_ISL_866540			
see above	Northumbria University / South Tees Hospitals NHS Foundation Trust / North Cumbria Integrated Care NHS Foundation Trust / North Tees and Hartlepool NHS Foundation Trust / Newcastle Hospitals NHS Foundation Trust	COVID-19 Genomics UK (COG-UK) Consortium	Darren L Smith,Andrew Nelson,Matthew Bashton,Greg R Young,Joshua Loh,John Allan,Mohammad A Tariq,Giles S Holt,Gary Black,Wen C Yew,Lynn Dover,Paul Baker,Steve Liggett,Sarah Essex,Jane Greenaway,Debra Padgett,Clive Graham,Garren Scott,Edward Barton,Emma Swindells,Brendan Payne,Jennifer Collins,Yusri Taha,Gary Eltringham
EPI_ISL_866588, EPI_ISL_866589, EPI_ISL_866590, EPI_ISL_866591, EPI_ISL_866592, EPI_ISL_866593, EPI_ISL_866594, EPI_ISL_866595, EPI_ISL_866596, EPI_ISL_866597, EPI_ISL_866598, EPI_ISL_866599, EPI_ISL_866600, EPI_ISL_866602			
see above	Quadram Institute Bioscience	COVID-19 Genomics UK (COG-UK) Consortium	Dave J. Baker, Gemma L. Kay, Alp Aydin, Thanh Le-Viet, Steven Rudder, Ana P. Tedim, Anastasia Kolyva, Maria Diaz, Leonardo de Oliveira Martins, Nabil-Fareed Alikhan, Lizzie Meadows, Rachael Stanley, Ngozi Elumogo, Muhammed Yasir, Nicholas M. Thomson, Alexander J Trotter, Rachel Gilroy, Samuel Bloomfield, Claire Stuart, Andrew Bell, Reenesh Prakash, Samir Dervisevic, Alison E. Mather, John Wain, Mark Webber, Andrew J. Page, Justin O'Grady
EPI_ISL_866906, EPI_ISL_866907	Queens Medical Centre, Clinical Microbiology Department / DeepSeq Nottingham	COVID-19 Genomics UK (COG-UK) Consortium	Gemma Clark, Wendy Smith, Manjinder Khakh, Vicki M Fleming, Michelle M Lister, Hannah Howson-Wells, Jonathan Ball, Patrick McClure, Joseph Chappell, Theocharis Tsoleridis, Nadine Holmes, Matthew Carlisle, Christopher Moore, Fei Sang, Johnny Debebe, Victoria Wright, Matthew Loose
EPI_ISL_867018, EPI_ISL_867019, EPI_ISL_867020, EPI_ISL_867021, EPI_ISL_867022, EPI_ISL_867023, EPI_ISL_867024, EPI_ISL_867025, EPI_ISL_867026, EPI_ISL_867027, EPI_ISL_867028, EPI_ISL_867029, EPI_ISL_867030, EPI_ISL_867031, EPI_ISL_867032, EPI_ISL_867033, EPI_ISL_867034			
see above	Lincolnshire Hospitals and DeepSeq Nottingham	COVID-19 Genomics UK (COG-UK) Consortium	Nichola Duckworth, Tim Sloan, Sarah Walsh, Jonathan Ball, Patrick McClure, Joeseph Chappell, Nadine Holmes, Matthew Carlisle, Christopher Moore, Fei Sang, Johnny Debebe, Victoria Wright, Matthew Loose
EPI_ISL_867091, EPI_ISL_867092, EPI_ISL_867099, EPI_ISL_867100, EPI_ISL_867101, EPI_ISL_867103, EPI_ISL_867105, EPI_ISL_867106, EPI_ISL_867107, EPI_ISL_867108, EPI_ISL_867110, EPI_ISL_867111, EPI_ISL_867112, EPI_ISL_867113, EPI_ISL_867114, EPI_ISL_867118, EPI_ISL_867119, EPI_ISL_867120, EPI_ISL_867121, EPI_ISL_867123, EPI_ISL_867125, EPI_ISL_867127, EPI_ISL_867128, EPI_ISL_867129, EPI_ISL_867131, EPI_ISL_867132, EPI_ISL_867133, EPI_ISL_867134, EPI_ISL_867137, EPI_ISL_867138, EPI_ISL_867139, EPI_ISL_867140, EPI_ISL_867141, EPI_ISL_867142, EPI_ISL_867144, EPI_ISL_867145, EPI_ISL_867147, EPI_ISL_867148, EPI_ISL_867150, EPI_ISL_867151, EPI_ISL_867152, EPI_ISL_867163, EPI_ISL_867164, EPI_ISL_867165, EPI_ISL_867166, EPI_ISL_867167			
see above	Oxford Viromics, NDM, University of Oxford; Oxford University Hospitals; Basingstoke and North Hampshire Hospital	COVID-19 Genomics UK (COG-UK) Consortium	Tanya Golubchik, David Bonsall, George Macintyre, Amy Trebes, Mariateresa de Cesare, Catrin Moore, Alex Mobbs, Anita Justice, Robert Shaw, Monique Andersson, Timothy Peto, Emma Wise, Nathan Moore, Jessica Lynch, Nick Cortes, Matilde Mori, Stephen Kidd, David Buck, John Todd, Christophe Fraser
EPI_ISL_867186, EPI_ISL_867203, EPI_ISL_867204, EPI_ISL_867205, EPI_ISL_867206, EPI_ISL_867207, EPI_ISL_867208, EPI_ISL_867209, EPI_ISL_867210, EPI_ISL_867432, EPI_ISL_867433, EPI_ISL_867434, EPI_ISL_867456, EPI_ISL_867503, EPI_ISL_867512, EPI_ISL_867751, EPI_ISL_867758			
see above	Originating lab: Wales Specialist Virology Centre Sequencing lab: Pathogen Genomics Unit	Public Health Wales Microbiology Cardiff Wales Specialist Virology Centre	Catherine Moore, Johnathan Evans, Laura Gifford, Malorie Perry, Simon Cottrell, Angela Marchbank, Alec Birchley, Alexander Adams, Amy Gaskin, Bree Gatica-Wilcox, Jason Coombes, Joel Southgate, Lauren Gilbert, Lee Graham, Nicole Pacchiarini, Sara Kumziene-Summerhayes, Sarah Taylor, Sophie Jones, Sara Rey, Matthew Bull, Joanne Watkins, Sally Corden, Tom Connor
EPI_ISL_867990, EPI_ISL_868001, EPI_ISL_868008, EPI_ISL_868309, EPI_ISL_868310, EPI_ISL_868311, EPI_ISL_868312, EPI_ISL_868313, EPI_ISL_868314, EPI_ISL_868315, EPI_ISL_868316, EPI_ISL_868317, EPI_ISL_868319, EPI_ISL_868321, EPI_ISL_868322, EPI_ISL_868324, EPI_ISL_868325, EPI_ISL_868326, EPI_ISL_868327, EPI_ISL_868328, EPI_ISL_868343, EPI_ISL_868344, EPI_ISL_868345, EPI_ISL_868346, EPI_ISL_868347, EPI_ISL_868348, EPI_ISL_868349, EPI_ISL_868350, EPI_ISL_868351, EPI_ISL_868352			
see above	Centre for Enzyme Innovation, University of Portsmouth / Translational Research Laboratory, Portsmouth Hospitals NHS Trust	COVID-19 Genomics UK (COG-UK) Consortium	Angela Beckett,Yann Bourgeois,Garry Scarlett,Sharon Glaysher,Scott Elliott,Kelly Bicknell,Robert Impey,Allyson Lloyd,Sarah Wyllie,Ethan Butcher,Anoop Chauhan,Samuel Robson
EPI_ISL_868357, EPI_ISL_868370, EPI_ISL_868390, EPI_ISL_868400, EPI_ISL_868406, EPI_ISL_868416, EPI_ISL_868431, EPI_ISL_868453, EPI_ISL_868455, EPI_ISL_868468, EPI_ISL_868470, EPI_ISL_868479, EPI_ISL_868481, EPI_ISL_868498, EPI_ISL_868499, EPI_ISL_868516, EPI_ISL_868518, EPI_ISL_868531, EPI_ISL_868543, EPI_ISL_868545, EPI_ISL_868561, EPI_ISL_868565, EPI_ISL_868572, EPI_ISL_868590, EPI_ISL_868598, EPI_ISL_868607, EPI_ISL_868609, EPI_ISL_868620, EPI_ISL_868627, EPI_ISL_868659, EPI_ISL_868664, EPI_ISL_868665, EPI_ISL_868675, EPI_ISL_868683, EPI_ISL_868699, EPI_ISL_868704			
see above	Virology Department, Sheffield Teaching Hospitals NHS Foundation Trust/Department of Infection, Immunity and Cardiovascular Disease, The Medical School, University of Sheffield	COVID-19 Genomics UK (COG-UK) Consortium	Thushan de Silva, Matthew Parker, Nikki Smith, Adri Angyal, Rebecca Brown, Luke Green, Rachel Tucker, Paul Parsons, Danielle Groves, Katie Johnson, Laura Carrilero, Alex Keeley, Dave Partridge, Matthew Wyles, Benjamin Lindsey, Mehmet Yavuz, Mohammad Raza, Cariad Evans
EPI_ISL_874170, EPI_ISL_874281, EPI_ISL_882301, EPI_ISL_882302, EPI_ISL_882304, EPI_ISL_882306, EPI_ISL_882310, EPI_ISL_882311, EPI_ISL_882312, EPI_ISL_882313, EPI_ISL_882315, EPI_ISL_882317, EPI_ISL_882318, EPI_ISL_882322, EPI_ISL_882324, EPI_ISL_882325, EPI_ISL_882326, EPI_ISL_882328, EPI_ISL_882329, EPI_ISL_882330, EPI_ISL_882331, EPI_ISL_882334, EPI_ISL_882335, EPI_ISL_882336, EPI_ISL_882337, EPI_ISL_882338, EPI_ISL_882340, EPI_ISL_882342, EPI_ISL_882345, EPI_ISL_882346, EPI_ISL_882347, EPI_ISL_882348, EPI_ISL_882349, EPI_ISL_882350, EPI_ISL_882351, EPI_ISL_882353, EPI_ISL_882354, EPI_ISL_882355, EPI_ISL_882356, EPI_ISL_882357, EPI_ISL_882358, EPI_ISL_882359, EPI_ISL_882362, EPI_ISL_882366, EPI_ISL_882367, EPI_ISL_882368, EPI_ISL_882369, EPI_ISL_882370, EPI_ISL_882373, EPI_ISL_882374, EPI_ISL_882378, EPI_ISL_882380, EPI_ISL_882381, EPI_ISL_882383, EPI_ISL_882384, EPI_ISL_882386, EPI_ISL_882387, EPI_ISL_882389, EPI_ISL_882391, EPI_ISL_882392, EPI_ISL_882394, EPI_ISL_882395, EPI_ISL_882396, EPI_ISL_882399, EPI_ISL_882400, EPI_ISL_882401, EPI_ISL_882403, EPI_ISL_882405, EPI_ISL_882406, EPI_ISL_882407, EPI_ISL_882408, EPI_ISL_882410, EPI_ISL_882411, EPI_ISL_882412, EPI_ISL_882414, EPI_ISL_882416, EPI_ISL_882417, EPI_ISL_882419, EPI_ISL_882422, EPI_ISL_882423, EPI_ISL_882424, EPI_ISL_882425, EPI_ISL_882426, EPI_ISL_882430, EPI_ISL_882431, EPI_ISL_882432, EPI_ISL_882433, EPI_ISL_882435, EPI_ISL_882436, EPI_ISL_882439, EPI_ISL_882443, EPI_ISL_882444, EPI_ISL_882446, EPI_ISL_882447, EPI_ISL_882448, EPI_ISL_882449, EPI_ISL_882450, EPI_ISL_882451, EPI_ISL_882453, EPI_ISL_882454, EPI_ISL_882455, EPI_ISL_882456, EPI_ISL_882457, EPI_ISL_882458, EPI_ISL_882459, EPI_ISL_882460, EPI_ISL_882461, EPI_ISL_882462, EPI_ISL_882463, EPI_ISL_882464, EPI_ISL_882465, EPI_ISL_882466, EPI_ISL_882467, EPI_ISL_882468, EPI_ISL_882469, EPI_ISL_882470, EPI_ISL_882472, EPI_ISL_882474, EPI_ISL_882477, EPI_ISL_882478, EPI_ISL_882479, EPI_ISL_882483, EPI_ISL_882485, EPI_ISL_882486, EPI_ISL_882487, EPI_ISL_882488, EPI_ISL_882489, EPI_ISL_882490, EPI_ISL_882492, EPI_ISL_882493, EPI_ISL_882495, EPI_ISL_882496, EPI_ISL_882497, EPI_ISL_882498, EPI_ISL_882499, EPI_ISL_882500, EPI_ISL_882501, EPI_ISL_882502, EPI_ISL_882503, EPI_ISL_882504, EPI_ISL_882507, EPI_ISL_882508, EPI_ISL_882509, EPI_ISL_882510, EPI_ISL_882511, EPI_ISL_882513, EPI_ISL_882514, EPI_ISL_882515, EPI_ISL_882516, EPI_ISL_882517, EPI_ISL_882519, EPI_ISL_882521, EPI_ISL_882522, EPI_ISL_882523, EPI_ISL_882526, EPI_ISL_882527, EPI_ISL_882528, EPI_ISL_882529, EPI_ISL_882530, EPI_ISL_882531, EPI_ISL_882532, EPI_ISL_882533, EPI_ISL_882535, EPI_ISL_882536, EPI_ISL_882540, EPI_ISL_882541, EPI_ISL_882543, EPI_ISL_882545, EPI_ISL_882546, EPI_ISL_882547, EPI_ISL_882551, EPI_ISL_882552, EPI_ISL_882553, EPI_ISL_882554, EPI_ISL_882555, EPI_ISL_882556, EPI_ISL_882557, EPI_ISL_882558, EPI_ISL_882560, EPI_ISL_882561, EPI_ISL_882567, EPI_ISL_882569, EPI_ISL_882570, EPI_ISL_882572, EPI_ISL_882573, EPI_ISL_882574, EPI_ISL_882575, EPI_ISL_882577, EPI_ISL_882578, EPI_ISL_882581, EPI_ISL_882582, EPI_ISL_882583, EPI_ISL_882584, EPI_ISL_882587, EPI_ISL_882591, EPI_ISL_882592, EPI_ISL_882594, EPI_ISL_882596, EPI_ISL_882597, EPI_ISL_882598, EPI_ISL_882599, EPI_ISL_882603, EPI_ISL_882604, EPI_ISL_892059, EPI_ISL_892060, EPI_ISL_892061, EPI_ISL_892062, EPI_ISL_892063, EPI_ISL_892064, EPI_ISL_892065, EPI_ISL_892066, EPI_ISL_892207, EPI_ISL_892208, EPI_ISL_892209			
see above	Lighthouse Lab in Alderley Park	Wellcome Sanger Institute for the COVID-19 Genomics UK (COG-UK) Consortium	Jacquelyn Wynn, Mairead Hyland, The Lighthouse Lab in Alderley Park and Alex Alderton, Roberto Amato, Sonia Goncalves, Ewan Harrison, David K. Jackson, Ian Johnston, Dominic Kwiatkowski, Cordelia Langford, John Sillitoe on behalf of the Wellcome Sanger Institute COVID-19 Surveillance Team
EPI_ISL_892210, EPI_ISL_892211	Lighthouse Lab in Glasgow	Wellcome Sanger Institute for the COVID-19 Genomics UK (COG-UK) Consortium	Harper VanSteenhouse, Yumi Kasai, David Gray, Carol Clugston, Anna Dominiczak and Alex Alderton, Roberto Amato, Sonia Goncalves, Ewan Harrison, David K. Jackson, Ian Johnston, Dominic Kwiatkowski, Cordelia Langford, John Sillitoe on behalf of the Wellcome Sanger Institute COVID-19 Surveillance Team
EPI_ISL_918584, EPI_ISL_918702, EPI_ISL_918704, EPI_ISL_918705, EPI_ISL_918706, EPI_ISL_918707, EPI_ISL_918712, EPI_ISL_918714, EPI_ISL_918718, EPI_ISL_918720, EPI_ISL_918721, EPI_ISL_918722, EPI_ISL_918723, EPI_ISL_918724, EPI_ISL_918725, EPI_ISL_918726, EPI_ISL_918727, EPI_ISL_918728, EPI_ISL_918729, EPI_ISL_918730, EPI_ISL_918731, EPI_ISL_918735, EPI_ISL_918736, EPI_ISL_918737, EPI_ISL_918738, EPI_ISL_918739, EPI_ISL_918740, EPI_ISL_918741, EPI_ISL_918742, EPI_ISL_918743, EPI_ISL_918744, EPI_ISL_918745, EPI_ISL_918746, EPI_ISL_918747, EPI_ISL_918901, EPI_ISL_918902, EPI_ISL_918903, EPI_ISL_918904, EPI_ISL_918905, EPI_ISL_918906, EPI_ISL_918907, EPI_ISL_918908, EPI_ISL_918909, EPI_ISL_918910, EPI_ISL_918911			
see above	University of Birmingham	COVID-19 Genomics UK (COG-UK) Consortium	Institute of Microbiology, University of Birmingham: Claire McMurray, Joanne Stockton, Samuel Nicholls, Radoslaw Poplawski, Will Rowe, Josh Quick, Nicholas Loman. University of Birmingham Testing Laboratory: Celina M Whalley, Andrew Bosworth, Charlotte Poxon, Kasun Wanigasooriya, Oliver Pickles, Mike Kidd, Alex Richter, Andrew D Beggs PHE Heartlands Lab: Husam Osman, Andrew Bosworth. Queen Elizabeth Hospital: Anna Casey
EPI_ISL_919449, EPI_ISL_919450, EPI_ISL_919451, EPI_ISL_919454, EPI_ISL_919455, EPI_ISL_919459,	Liverpool Clinical Laboratories	COVID-19 Genomics UK (COG-UK) Consortium	Sam Haldenby, Anita Lucaci, Steve Paterson, Julian Hiscox, Alistair Darby, M Almsaud, A Alrezaihi, Muhannad Alruwaili, Stuart D Armstrong, Jones Benjamin, Eleanor G Bentley, Anu Chawla, Jordan J Clark, Angela Cowell, Richard Eccles, Isabel García-Dorival, Matthew Gemmell, Alessandro Gerada, PKF Gilmore, Richard Gregory, Ximeng Han, Catherine Hartley, Margaret Hughes, Miren Iturriza-Gomara, James Johnson, L Luu, Jenifer Manson,

EPI_ISL_919460, EPI_ISL_919461			Charlotte Nelson, Elaine O'Toole, Cassie Olateju, Rebekah Penrice-Randal , Lucille Rainbow, N.P Randle, Trevor Ian Robinson, Parul Sharma, Ghada T Shawli, James P Stewart, Neil Swainston, Ecaterina Vamos, Joanne Watts, Mark Whitehead
EPI_ISL_919779, EPI_ISL_919780, EPI_ISL_919797, EPI_ISL_919798, EPI_ISL_919799, EPI_ISL_919800, EPI_ISL_919801, EPI_ISL_919802, EPI_ISL_919803, EPI_ISL_919804, EPI_ISL_919805, EPI_ISL_919806, EPI_ISL_919807, EPI_ISL_919808, EPI_ISL_919809, EPI_ISL_919810, EPI_ISL_919811, EPI_ISL_919812, EPI_ISL_919813, EPI_ISL_919814, EPI_ISL_919815, EPI_ISL_919816, EPI_ISL_919817, EPI_ISL_919818, EPI_ISL_919819, EPI_ISL_919820, EPI_ISL_919821, EPI_ISL_919822, EPI_ISL_919823, EPI_ISL_919824, EPI_ISL_919825, EPI_ISL_919826, EPI_ISL_919827, EPI_ISL_919828, EPI_ISL_919829, EPI_ISL_919830, EPI_ISL_919831, EPI_ISL_919832, EPI_ISL_919833, EPI_ISL_919834, EPI_ISL_919835, EPI_ISL_919836, EPI_ISL_919837, EPI_ISL_919838, EPI_ISL_919839, EPI_ISL_919840, EPI_ISL_919842, EPI_ISL_919843, EPI_ISL_919844, EPI_ISL_919845, EPI_ISL_919846, EPI_ISL_919847, EPI_ISL_919848, EPI_ISL_919849, EPI_ISL_919850, EPI_ISL_919851			
see above	Barts Health NHS Trust	COVID-19 Genomics UK (COG-UK) Consortium	CUTINO-MOGUEL, Maria-Teresa; HARRINGTON, David; OWOYEMI, Dola; KULASEGARAN-SHYLINI, Raghavendran; BROAD, Claire; KELE, Beatrix
EPI_ISL_919962, EPI_ISL_919963, EPI_ISL_919965, EPI_ISL_919967, EPI_ISL_919968, EPI_ISL_919969, EPI_ISL_919970, EPI_ISL_919971, EPI_ISL_919972, EPI_ISL_919973, EPI_ISL_919974, EPI_ISL_919975, EPI_ISL_919976, EPI_ISL_919977, EPI_ISL_919978, EPI_ISL_919979, EPI_ISL_919980, EPI_ISL_919981, EPI_ISL_919982, EPI_ISL_919983, EPI_ISL_919984, EPI_ISL_919985, EPI_ISL_919986, EPI_ISL_919987, EPI_ISL_919988, EPI_ISL_919989, EPI_ISL_919990, EPI_ISL_919991, EPI_ISL_919992, EPI_ISL_919993, EPI_ISL_919994, EPI_ISL_919996, EPI_ISL_919997, EPI_ISL_919998, EPI_ISL_919999, EPI_ISL_920040, EPI_ISL_920041, EPI_ISL_920046, EPI_ISL_920047, EPI_ISL_920048			
see above	University College London, Great Ormond Street Hospital for Children NHS Foundation Trust, Imperial College Healthcare NHS Trust	COVID-19 Genomics UK (COG-UK) Consortium	Sergi Castellano, Rachel Williams, Mark Kristiansen, Paola Resende Silva, Sunando Roy, Tony Brooks, Helena Tutill, Paola Niola, Patricia Dyal, Charlotte Williams, Leysa Forrest, Yasmin Panchbhaya, Jacqueline Findlay, Samuel Weeks, Julianne Brown, Kathryn Harris, Paul Randell, James Price, Alison Holmes, Judith Breuer
EPI_ISL_920179, EPI_ISL_920288, EPI_ISL_920297, EPI_ISL_920311, EPI_ISL_920689, EPI_ISL_920711	University College London Hospital	COVID-19 Genomics UK (COG-UK) Consortium	Judith Heaney, Matthew Byott, Catherine Houlihan, Dan Frampton, Stuart Kirk, Moira Spyer and Eleni Nastouli
EPI_ISL_920839, EPI_ISL_920840, EPI_ISL_920848, EPI_ISL_920849, EPI_ISL_920850, EPI_ISL_920856, EPI_ISL_920857, EPI_ISL_920858, EPI_ISL_920859	University College London, Great Ormond Street Hospital for Children NHS Foundation Trust, Imperial College Healthcare NHS Trust	COVID-19 Genomics UK (COG-UK) Consortium	Sergi Castellano, Rachel Williams, Mark Kristiansen, Paola Resende Silva, Sunando Roy, Tony Brooks, Helena Tutill, Paola Niola, Patricia Dyal, Charlotte Williams, Leysa Forrest, Yasmin Panchbhaya, Jacqueline Findlay, Samuel Weeks, Julianne Brown, Kathryn Harris, Paul Randell, James Price, Alison Holmes, Judith Breuer
EPI_ISL_920980, EPI_ISL_921019, EPI_ISL_921020, EPI_ISL_921021, EPI_ISL_921023, EPI_ISL_921024, EPI_ISL_921026, EPI_ISL_921040, EPI_ISL_921047, EPI_ISL_921048, EPI_ISL_921049, EPI_ISL_921050, EPI_ISL_921051, EPI_ISL_921052, EPI_ISL_921053, EPI_ISL_921054, EPI_ISL_921055, EPI_ISL_921056, EPI_ISL_921057, EPI_ISL_921058, EPI_ISL_921059, EPI_ISL_921060, EPI_ISL_921061, EPI_ISL_921062, EPI_ISL_921063, EPI_ISL_921064, EPI_ISL_921065, EPI_ISL_921066, EPI_ISL_921067, EPI_ISL_921068, EPI_ISL_921069, EPI_ISL_921070, EPI_ISL_921071, EPI_ISL_921080, EPI_ISL_921081, EPI_ISL_921082, EPI_ISL_921083, EPI_ISL_921084, EPI_ISL_921091, EPI_ISL_921092, EPI_ISL_921093, EPI_ISL_921094, EPI_ISL_921095, EPI_ISL_921098, EPI_ISL_921099, EPI_ISL_921100, EPI_ISL_921102, EPI_ISL_921103, EPI_ISL_921104, EPI_ISL_921106, EPI_ISL_921107, EPI_ISL_921108, EPI_ISL_921109, EPI_ISL_921110, EPI_ISL_921111, EPI_ISL_921112, EPI_ISL_921113, EPI_ISL_921114, EPI_ISL_921115, EPI_ISL_921116, EPI_ISL_921117, EPI_ISL_921123, EPI_ISL_921124, EPI_ISL_921125, EPI_ISL_921126, EPI_ISL_921127, EPI_ISL_921129, EPI_ISL_921130, EPI_ISL_921131, EPI_ISL_921134			
see above	Regional Virus Laboratory, Belfast Health and Social Care Trust	COVID-19 Genomics UK (COG-UK) Consortium	Conall McCaughey, James McKenna, Tanya Curran, Susan Feeney, Alison Watt, Ciara Cox, Mairead Connor, Zoltan Molnar, David Simpson, Derek Fairley
EPI_ISL_921243, EPI_ISL_921244, EPI_ISL_921245, EPI_ISL_921246, EPI_ISL_921247, EPI_ISL_921248, EPI_ISL_921250, EPI_ISL_921251, EPI_ISL_921277, EPI_ISL_921279, EPI_ISL_921285, EPI_ISL_921287, EPI_ISL_921289, EPI_ISL_921290, EPI_ISL_921291, EPI_ISL_921292, EPI_ISL_921293, EPI_ISL_921294, EPI_ISL_921296, EPI_ISL_921299, EPI_ISL_921302, EPI_ISL_921304, EPI_ISL_921306, EPI_ISL_921307, EPI_ISL_921308, EPI_ISL_921309, EPI_ISL_921310, EPI_ISL_921311, EPI_ISL_921312, EPI_ISL_921315, EPI_ISL_921316, EPI_ISL_921319, EPI_ISL_921320, EPI_ISL_921407, EPI_ISL_921408, EPI_ISL_921409, EPI_ISL_921410, EPI_ISL_921411, EPI_ISL_921412, EPI_ISL_921413, EPI_ISL_921419, EPI_ISL_921427, EPI_ISL_921429, EPI_ISL_921430, EPI_ISL_921431, EPI_ISL_921434, EPI_ISL_921435, EPI_ISL_921436, EPI_ISL_921437, EPI_ISL_921438, EPI_ISL_921439, EPI_ISL_921440, EPI_ISL_921441, EPI_ISL_921442, EPI_ISL_921443, EPI_ISL_921444, EPI_ISL_921445, EPI_ISL_921446, EPI_ISL_921447, EPI_ISL_921448, EPI_ISL_921449, EPI_ISL_921450, EPI_ISL_921451, EPI_ISL_921452, EPI_ISL_921453, EPI_ISL_921454, EPI_ISL_921455, EPI_ISL_921456, EPI_ISL_921457, EPI_ISL_921458, EPI_ISL_921459, EPI_ISL_921460, EPI_ISL_921461, EPI_ISL_921462, EPI_ISL_921463, EPI_ISL_921464, EPI_ISL_921465, EPI_ISL_921466, EPI_ISL_921467, EPI_ISL_921468, EPI_ISL_921469, EPI_ISL_921470, EPI_ISL_921471, EPI_ISL_921472, EPI_ISL_921473, EPI_ISL_921474			
see above	Northumbria University / South Tees Hospitals NHS Foundation Trust / North Cumbria Integrated Care NHS Foundation Trust / North Tees and Hartlepool NHS Foundation Trust / Newcastle Hospitals NHS Foundation Trust	COVID-19 Genomics UK (COG-UK) Consortium	Darren L Smith,Andrew Nelson,Matthew Bashton,Greg R Young,Joshua Loh,John Allan,Mohammad A Tariq,Giles S Holt,Gary Black,Wen C Yew,Lynn Dover,Paul Baker,Steve Liggett,Sarah Essex,Jane Greenaway,Debra Padgett,Clive Graham,Garren Scott,Edward Barton,Emma Swindells,Brendan Payne,Jennifer Collins,Yusri Taha,Gary Eltringham
EPI_ISL_922041, EPI_ISL_922042, EPI_ISL_922043, EPI_ISL_922044, EPI_ISL_922045, EPI_ISL_922046, EPI_ISL_922047, EPI_ISL_922048, EPI_ISL_922049, EPI_ISL_922050, EPI_ISL_922051, EPI_ISL_922052, EPI_ISL_922053, EPI_ISL_922054, EPI_ISL_922055, EPI_ISL_922056, EPI_ISL_922057, EPI_ISL_922058, EPI_ISL_922059, EPI_ISL_922061, EPI_ISL_922062			
see above	Lincolnshire Hospitals and DeepSeq Nottingham	COVID-19 Genomics UK (COG-UK) Consortium	Nichola Duckworth, Tim Sloan, Sarah Walsh, Jonathan Ball, Patrick McClure, Joeseph Chappell, Nadine Holmes, Matthew Carlisle, Christopher Moore, Fei Sang, Johnny Debebe, Victoria Wright, Matthew Loose
EPI_ISL_922174, EPI_ISL_922175, EPI_ISL_922177, EPI_ISL_922178, EPI_ISL_922180, EPI_ISL_922182, EPI_ISL_922183, EPI_ISL_922184, EPI_ISL_922185, EPI_ISL_922188, EPI_ISL_922189, EPI_ISL_922190, EPI_ISL_922191, EPI_ISL_922192, EPI_ISL_922198, EPI_ISL_922199, EPI_ISL_922201, EPI_ISL_922242, EPI_ISL_922244, EPI_ISL_922246, EPI_ISL_922249, EPI_ISL_922252, EPI_ISL_922254, EPI_ISL_922255, EPI_ISL_922283, EPI_ISL_922284, EPI_ISL_922285, EPI_ISL_922286, EPI_ISL_922304, EPI_ISL_922308, EPI_ISL_922309, EPI_ISL_922310, EPI_ISL_922311, EPI_ISL_922317, EPI_ISL_922321, EPI_ISL_922323, EPI_ISL_922324, EPI_ISL_922325, EPI_ISL_922326, EPI_ISL_922327, EPI_ISL_922328, EPI_ISL_922329, EPI_ISL_922330, EPI_ISL_922331, EPI_ISL_922332, EPI_ISL_922333, EPI_ISL_922334, EPI_ISL_922335, EPI_ISL_922336, EPI_ISL_922337, EPI_ISL_922338, EPI_ISL_922339, EPI_ISL_922340, EPI_ISL_922341, EPI_ISL_922342, EPI_ISL_922343, EPI_ISL_922345			
see above	Oxford Viromics, NDM, University of Oxford; Oxford University Hospitals; Basingstoke and North Hampshire Hospital	COVID-19 Genomics UK (COG-UK) Consortium	Tanya Golubchik, David Bonsall, George Macintyre, Amy Trebes, Mariateresa de Cesare, Catrin Moore, Alex Mobbs, Anita Justice, Robert Shaw, Monique Andersson, Timothy Peto, Emma Wise, Nathan Moore, Jessica Lynch, Nick Cortes, Matilde Mori, Stephen Kidd, David Buck, John Todd, Christophe Fraser
EPI_ISL_923256, EPI_ISL_923257, EPI_ISL_923261, EPI_ISL_923263, EPI_ISL_923266, EPI_ISL_923379, EPI_ISL_923381, EPI_ISL_923443, EPI_ISL_923577, EPI_ISL_923580, EPI_ISL_923663			
see above	Centre for Enzyme Innovation, University of Portsmouth / Translational Research Laboratory, Portsmouth Hospitals NHS Trust	COVID-19 Genomics UK (COG-UK) Consortium	Angela Beckett,Salman Goudarzi,Christopher Fearn,Kate Cook,Katie Loveson,Sharon Glaysher,Scott Elliott,Samuel Robson
EPI_ISL_924079, EPI_ISL_924107, EPI_ISL_924132, EPI_ISL_924157, EPI_ISL_924292, EPI_ISL_924333, EPI_ISL_924342, EPI_ISL_924383, EPI_ISL_924387, EPI_ISL_924400, EPI_ISL_924412			
see above	Virology Department, Sheffield Teaching Hospitals NHS Foundation Trust/Department of Infection, Immunity and Cardiovascular Disease, The Medical School, University of Sheffield	COVID-19 Genomics UK (COG-UK) Consortium	Thushan de Silva, Matthew Parker, Nikki Smith, Adri Angyal, Rebecca Brown, Luke Green, Rachel Tucker, Paul Parsons, Danielle Groves, Katie Johnson, Laura Carrilero, Alex Keeley, Dave Partridge, Matthew Wyles, Benjamin Lindsey, Mehmet Yavuz, Mohammad Raza, Cariad Evans
EPI_ISL_924585, EPI_ISL_924586, EPI_ISL_924587, EPI_ISL_924589, EPI_ISL_924590, EPI_ISL_924592, EPI_ISL_924593, EPI_ISL_924594, EPI_ISL_924595, EPI_ISL_924596, EPI_ISL_924597, EPI_ISL_924598, EPI_ISL_924600, EPI_ISL_924601, EPI_ISL_924603, EPI_ISL_924604, EPI_ISL_924622, EPI_ISL_924623, EPI_ISL_924625, EPI_ISL_924626, EPI_ISL_924627, EPI_ISL_924628, EPI_ISL_924629, EPI_ISL_924630, EPI_ISL_924631, EPI_ISL_924632, EPI_ISL_924633, EPI_ISL_924634, EPI_ISL_924635, EPI_ISL_924636, EPI_ISL_924637, EPI_ISL_924638, EPI_ISL_924639, EPI_ISL_924640, EPI_ISL_924641, EPI_ISL_924642, EPI_ISL_924644, EPI_ISL_924645, EPI_ISL_924646, EPI_ISL_924647, EPI_ISL_924648, EPI_ISL_924649, EPI_ISL_924650, EPI_ISL_924651, EPI_ISL_924723, EPI_ISL_924726, EPI_ISL_924730, EPI_ISL_924732, EPI_ISL_924734, EPI_ISL_924735, EPI_ISL_924737, EPI_ISL_924738, EPI_ISL_924739, EPI_ISL_924741, EPI_ISL_924743, EPI_ISL_924745, EPI_ISL_924747, EPI_ISL_924757, EPI_ISL_924758, EPI_ISL_924759, EPI_ISL_924760, EPI_ISL_924761, EPI_ISL_924762, EPI_ISL_924763, EPI_ISL_924765, EPI_ISL_924766, EPI_ISL_924768, EPI_ISL_924771, EPI_ISL_924772, EPI_ISL_924773, EPI_ISL_924775, EPI_ISL_924776, EPI_ISL_924777, EPI_ISL_924778, EPI_ISL_924779, EPI_ISL_924780, EPI_ISL_924781, EPI_ISL_924782, EPI_ISL_924893, EPI_ISL_924894, EPI_ISL_924896, EPI_ISL_924900, EPI_ISL_924906			
see above	Bioinformatics and Biostatistics Lab, Advanced Sequencing Facility	COVID-19 Genomics UK (COG-UK) Consortium	Aengus Stewart,Jerome Nicod,Chelsea Sawyer,Laura Cubitt,Harshil Patel,Margaret Crawford
EPI_ISL_931818	Lighthouse Lab in Alderley Park	Wellcome Sanger Institute for the COVID-19 Genomics UK (COG-UK) Consortium	Jacquelyn Wynn, Mairead Hyland, The Lighthouse Lab in Alderley Park and Alex Alderton, Roberto Amato, Sonia Goncalves, Ewan Harrison, David K. Jackson, Ian Johnston, Dominic Kwiatkowski, Cordelia Langford, John Sillitoe on behalf of the Wellcome Sanger Institute COVID-19 Surveillance Team
EPI_ISL_932832, EPI_ISL_933425	Lighthouse Lab in Glasgow	Wellcome Sanger Institute for the COVID-19 Genomics UK (COG-UK) Consortium	Harper VanSteenhouse, Yumi Kasai, David Gray, Carol Clugston, Anna Dominiczak and Alex Alderton, Roberto Amato, Sonia Goncalves, Ewan Harrison, David K. Jackson, Ian Johnston, Dominic Kwiatkowski, Cordelia Langford, John Sillitoe on behalf of the Wellcome Sanger Institute COVID-19 Surveillance Team
EPI_ISL_945050, EPI_ISL_945053, EPI_ISL_945055, EPI_ISL_945057, EPI_ISL_945058, EPI_ISL_945059, EPI_ISL_945060, EPI_ISL_945062	Lighthouse Lab in Milton Keynes	Wellcome Sanger Institute for the COVID-19 Genomics UK (COG-UK) Consortium	The Lighthouse Lab in Milton Keynes and Alex Alderton, Roberto Amato, Sonia Goncalves, Ewan Harrison, David K. Jackson, Ian Johnston, Dominic Kwiatkowski, Cordelia Langford, John Sillitoe on behalf of the Wellcome Sanger Institute COVID-19 Surveillance Team
EPI_ISL_945063	Lighthouse Lab in Cambridge	Wellcome Sanger Institute for the COVID-19 Genomics UK (COG-UK) Consortium	Rob Howes, The Lighthouse Lab in Cambridge and Alex Alderton, Roberto Amato, Sonia Goncalves, Ewan Harrison, David K. Jackson, Ian Johnston, Dominic Kwiatkowski, Cordelia Langford, John Sillitoe on behalf of the Wellcome Sanger Institute COVID-19 Surveillance Team
EPI_ISL_945064, EPI_ISL_945066	Lighthouse Lab in Milton Keynes	Wellcome Sanger Institute for the COVID-19 Genomics UK (COG-UK) Consortium	The Lighthouse Lab in Milton Keynes and Alex Alderton, Roberto Amato, Sonia Goncalves, Ewan Harrison, David K. Jackson, Ian Johnston, Dominic Kwiatkowski, Cordelia Langford, John Sillitoe on behalf of the Wellcome Sanger Institute COVID-19 Surveillance Team

EPI_ISL_945067	Lighthouse Lab in Cambridge	Wellcome Sanger Institute for the COVID-19 Genomics UK (COG-UK) Consortium	Rob Howes, The Lighthouse Lab in Cambridge and Alex Alderton, Roberto Amato, Sonia Goncalves, Ewan Harrison, David K. Jackson, Ian Johnston, Dominic Kwiatkowski, Cordelia Langford, John Sillitoe on behalf of the Wellcome Sanger Institute COVID-19 Surveillance Team
EPI_ISL_945069	Lighthouse Lab in Milton Keynes	Wellcome Sanger Institute for the COVID-19 Genomics UK (COG-UK) Consortium	The Lighthouse Lab in Milton Keynes and Alex Alderton, Roberto Amato, Sonia Goncalves, Ewan Harrison, David K. Jackson, Ian Johnston, Dominic Kwiatkowski, Cordelia Langford, John Sillitoe on behalf of the Wellcome Sanger Institute COVID-19 Surveillance Team
EPI_ISL_945070	Lighthouse Lab in Cambridge	Wellcome Sanger Institute for the COVID-19 Genomics UK (COG-UK) Consortium	Rob Howes, The Lighthouse Lab in Cambridge and Alex Alderton, Roberto Amato, Sonia Goncalves, Ewan Harrison, David K. Jackson, Ian Johnston, Dominic Kwiatkowski, Cordelia Langford, John Sillitoe on behalf of the Wellcome Sanger Institute COVID-19 Surveillance Team
EPI_ISL_945071, EPI_ISL_945074, EPI_ISL_945075	Lighthouse Lab in Milton Keynes	Wellcome Sanger Institute for the COVID-19 Genomics UK (COG-UK) Consortium	The Lighthouse Lab in Milton Keynes and Alex Alderton, Roberto Amato, Sonia Goncalves, Ewan Harrison, David K. Jackson, Ian Johnston, Dominic Kwiatkowski, Cordelia Langford, John Sillitoe on behalf of the Wellcome Sanger Institute COVID-19 Surveillance Team
EPI_ISL_945076	Lighthouse Lab in Cambridge	Wellcome Sanger Institute for the COVID-19 Genomics UK (COG-UK) Consortium	Rob Howes, The Lighthouse Lab in Cambridge and Alex Alderton, Roberto Amato, Sonia Goncalves, Ewan Harrison, David K. Jackson, Ian Johnston, Dominic Kwiatkowski, Cordelia Langford, John Sillitoe on behalf of the Wellcome Sanger Institute COVID-19 Surveillance Team
EPI_ISL_945077, EPI_ISL_945078, EPI_ISL_945079, EPI_ISL_945080, EPI_ISL_945081, EPI_ISL_945082	Lighthouse Lab in Milton Keynes	Wellcome Sanger Institute for the COVID-19 Genomics UK (COG-UK) Consortium	The Lighthouse Lab in Milton Keynes and Alex Alderton, Roberto Amato, Sonia Goncalves, Ewan Harrison, David K. Jackson, Ian Johnston, Dominic Kwiatkowski, Cordelia Langford, John Sillitoe on behalf of the Wellcome Sanger Institute COVID-19 Surveillance Team
EPI_ISL_945083	Lighthouse Lab in Cambridge	Wellcome Sanger Institute for the COVID-19 Genomics UK (COG-UK) Consortium	Rob Howes, The Lighthouse Lab in Cambridge and Alex Alderton, Roberto Amato, Sonia Goncalves, Ewan Harrison, David K. Jackson, Ian Johnston, Dominic Kwiatkowski, Cordelia Langford, John Sillitoe on behalf of the Wellcome Sanger Institute COVID-19 Surveillance Team
EPI_ISL_945086, EPI_ISL_945087, EPI_ISL_945090, EPI_ISL_945091, EPI_ISL_945092, EPI_ISL_945093, EPI_ISL_945094, EPI_ISL_945096, EPI_ISL_945097	Lighthouse Lab in Milton Keynes	Wellcome Sanger Institute for the COVID-19 Genomics UK (COG-UK) Consortium	The Lighthouse Lab in Milton Keynes and Alex Alderton, Roberto Amato, Sonia Goncalves, Ewan Harrison, David K. Jackson, Ian Johnston, Dominic Kwiatkowski, Cordelia Langford, John Sillitoe on behalf of the Wellcome Sanger Institute COVID-19 Surveillance Team
EPI_ISL_945100	Lighthouse Lab in Cambridge	Wellcome Sanger Institute for the COVID-19 Genomics UK (COG-UK) Consortium	Rob Howes, The Lighthouse Lab in Cambridge and Alex Alderton, Roberto Amato, Sonia Goncalves, Ewan Harrison, David K. Jackson, Ian Johnston, Dominic Kwiatkowski, Cordelia Langford, John Sillitoe on behalf of the Wellcome Sanger Institute COVID-19 Surveillance Team
EPI_ISL_945101	Lighthouse Lab in Milton Keynes	Wellcome Sanger Institute for the COVID-19 Genomics UK (COG-UK) Consortium	The Lighthouse Lab in Milton Keynes and Alex Alderton, Roberto Amato, Sonia Goncalves, Ewan Harrison, David K. Jackson, Ian Johnston, Dominic Kwiatkowski, Cordelia Langford, John Sillitoe on behalf of the Wellcome Sanger Institute COVID-19 Surveillance Team
EPI_ISL_945103	Lighthouse Lab in Cambridge	Wellcome Sanger Institute for the COVID-19 Genomics UK (COG-UK) Consortium	Rob Howes, The Lighthouse Lab in Cambridge and Alex Alderton, Roberto Amato, Sonia Goncalves, Ewan Harrison, David K. Jackson, Ian Johnston, Dominic Kwiatkowski, Cordelia Langford, John Sillitoe on behalf of the Wellcome Sanger Institute COVID-19 Surveillance Team
EPI_ISL_945104, EPI_ISL_945105	Lighthouse Lab in Milton Keynes	Wellcome Sanger Institute for the COVID-19 Genomics UK (COG-UK) Consortium	The Lighthouse Lab in Milton Keynes and Alex Alderton, Roberto Amato, Sonia Goncalves, Ewan Harrison, David K. Jackson, Ian Johnston, Dominic Kwiatkowski, Cordelia Langford, John Sillitoe on behalf of the Wellcome Sanger Institute COVID-19 Surveillance Team
EPI_ISL_945106, EPI_ISL_945107	Lighthouse Lab in Cambridge	Wellcome Sanger Institute for the COVID-19 Genomics UK (COG-UK) Consortium	Rob Howes, The Lighthouse Lab in Cambridge and Alex Alderton, Roberto Amato, Sonia Goncalves, Ewan Harrison, David K. Jackson, Ian Johnston, Dominic Kwiatkowski, Cordelia Langford, John Sillitoe on behalf of the Wellcome Sanger Institute COVID-19 Surveillance Team
EPI_ISL_945108, EPI_ISL_945110, EPI_ISL_945112	Lighthouse Lab in Milton Keynes	Wellcome Sanger Institute for the COVID-19 Genomics UK (COG-UK) Consortium	The Lighthouse Lab in Milton Keynes and Alex Alderton, Roberto Amato, Sonia Goncalves, Ewan Harrison, David K. Jackson, Ian Johnston, Dominic Kwiatkowski, Cordelia Langford, John Sillitoe on behalf of the Wellcome Sanger Institute COVID-19 Surveillance Team
EPI_ISL_945116	Lighthouse Lab in Cambridge	Wellcome Sanger Institute for the COVID-19 Genomics UK (COG-UK) Consortium	Rob Howes, The Lighthouse Lab in Cambridge and Alex Alderton, Roberto Amato, Sonia Goncalves, Ewan Harrison, David K. Jackson, Ian Johnston, Dominic Kwiatkowski, Cordelia Langford, John Sillitoe on behalf of the Wellcome Sanger Institute COVID-19 Surveillance Team
EPI_ISL_945118, EPI_ISL_945120, EPI_ISL_945121, EPI_ISL_945124, EPI_ISL_945127, EPI_ISL_945128, EPI_ISL_945129	Lighthouse Lab in Milton Keynes	Wellcome Sanger Institute for the COVID-19 Genomics UK (COG-UK) Consortium	The Lighthouse Lab in Milton Keynes and Alex Alderton, Roberto Amato, Sonia Goncalves, Ewan Harrison, David K. Jackson, Ian Johnston, Dominic Kwiatkowski, Cordelia Langford, John Sillitoe on behalf of the Wellcome Sanger Institute COVID-19 Surveillance Team
EPI_ISL_945131	Lighthouse Lab in Cambridge	Wellcome Sanger Institute for the COVID-19 Genomics UK (COG-UK) Consortium	Rob Howes, The Lighthouse Lab in Cambridge and Alex Alderton, Roberto Amato, Sonia Goncalves, Ewan Harrison, David K. Jackson, Ian Johnston, Dominic Kwiatkowski, Cordelia Langford, John Sillitoe on behalf of the Wellcome Sanger Institute COVID-19 Surveillance Team
EPI_ISL_945132, EPI_ISL_945133, EPI_ISL_945134, EPI_ISL_945135, EPI_ISL_945137	Lighthouse Lab in Milton Keynes	Wellcome Sanger Institute for the COVID-19 Genomics UK (COG-UK) Consortium	The Lighthouse Lab in Milton Keynes and Alex Alderton, Roberto Amato, Sonia Goncalves, Ewan Harrison, David K. Jackson, Ian Johnston, Dominic Kwiatkowski, Cordelia Langford, John Sillitoe on behalf of the Wellcome Sanger Institute COVID-19 Surveillance Team
EPI_ISL_945139	Lighthouse Lab in Cambridge	Wellcome Sanger Institute for the COVID-19 Genomics UK (COG-UK) Consortium	Rob Howes, The Lighthouse Lab in Cambridge and Alex Alderton, Roberto Amato, Sonia Goncalves, Ewan Harrison, David K. Jackson, Ian Johnston, Dominic Kwiatkowski, Cordelia Langford, John Sillitoe on behalf of the Wellcome Sanger Institute COVID-19 Surveillance Team
EPI_ISL_945141	Lighthouse Lab in Milton Keynes	Wellcome Sanger Institute for the COVID-19 Genomics UK (COG-UK) Consortium	The Lighthouse Lab in Milton Keynes and Alex Alderton, Roberto Amato, Sonia Goncalves, Ewan Harrison, David K. Jackson, Ian Johnston, Dominic Kwiatkowski, Cordelia Langford, John Sillitoe on behalf of the Wellcome Sanger Institute COVID-19 Surveillance Team
EPI_ISL_945144	Lighthouse Lab in Glasgow	Wellcome Sanger Institute for the COVID-19 Genomics UK (COG-UK) Consortium	Harper VanSteenhouse, Yumi Kasai, David Gray, Carol Clugston, Anna Dominiczak and Alex Alderton, Roberto Amato, Sonia Goncalves, Ewan Harrison, David K. Jackson, Ian Johnston, Dominic Kwiatkowski, Cordelia Langford, John Sillitoe on behalf of the Wellcome Sanger Institute COVID-19 Surveillance Team
EPI_ISL_945145	Lighthouse Lab in Milton Keynes	Wellcome Sanger Institute for the COVID-19 Genomics UK (COG-UK) Consortium	The Lighthouse Lab in Milton Keynes and Alex Alderton, Roberto Amato, Sonia Goncalves, Ewan Harrison, David K. Jackson, Ian Johnston, Dominic Kwiatkowski, Cordelia Langford, John Sillitoe on behalf of the Wellcome Sanger Institute COVID-19 Surveillance Team
EPI_ISL_945148	Lighthouse Lab in Cambridge	Wellcome Sanger Institute for the COVID-19 Genomics UK (COG-UK) Consortium	Rob Howes, The Lighthouse Lab in Cambridge and Alex Alderton, Roberto Amato, Sonia Goncalves, Ewan Harrison, David K. Jackson, Ian Johnston, Dominic Kwiatkowski, Cordelia Langford, John Sillitoe on behalf of the Wellcome Sanger Institute COVID-19 Surveillance Team
EPI_ISL_945149, EPI_ISL_945150, EPI_ISL_945151, EPI_ISL_945153, EPI_ISL_945154, EPI_ISL_945156	Lighthouse Lab in Milton Keynes	Wellcome Sanger Institute for the COVID-19 Genomics UK (COG-UK) Consortium	The Lighthouse Lab in Milton Keynes and Alex Alderton, Roberto Amato, Sonia Goncalves, Ewan Harrison, David K. Jackson, Ian Johnston, Dominic Kwiatkowski, Cordelia Langford, John Sillitoe on behalf of the Wellcome Sanger Institute COVID-19 Surveillance Team
EPI_ISL_945157	Lighthouse Lab in Cambridge	Wellcome Sanger Institute for the COVID-19 Genomics UK (COG-UK) Consortium	Rob Howes, The Lighthouse Lab in Cambridge and Alex Alderton, Roberto Amato, Sonia Goncalves, Ewan Harrison, David K. Jackson, Ian Johnston, Dominic Kwiatkowski, Cordelia Langford, John Sillitoe on behalf of the Wellcome Sanger Institute COVID-19 Surveillance Team
EPI_ISL_945158, EPI_ISL_945159, EPI_ISL_945161	Lighthouse Lab in Milton Keynes	Wellcome Sanger Institute for the COVID-19 Genomics UK (COG-UK) Consortium	The Lighthouse Lab in Milton Keynes and Alex Alderton, Roberto Amato, Sonia Goncalves, Ewan Harrison, David K. Jackson, Ian Johnston, Dominic Kwiatkowski, Cordelia Langford, John Sillitoe on behalf of the Wellcome Sanger Institute COVID-19 Surveillance Team
EPI_ISL_945162	Lighthouse Lab in Glasgow	Wellcome Sanger Institute for the COVID-19 Genomics UK (COG-UK) Consortium	Harper VanSteenhouse, Yumi Kasai, David Gray, Carol Clugston, Anna Dominiczak and Alex Alderton, Roberto Amato, Sonia Goncalves, Ewan Harrison, David K. Jackson, Ian Johnston, Dominic Kwiatkowski, Cordelia Langford, John Sillitoe on behalf of the Wellcome Sanger Institute COVID-19 Surveillance Team
EPI_ISL_945164, EPI_ISL_945165	Lighthouse Lab in Milton Keynes	Wellcome Sanger Institute for the COVID-19 Genomics UK (COG-UK) Consortium	The Lighthouse Lab in Milton Keynes and Alex Alderton, Roberto Amato, Sonia Goncalves, Ewan Harrison, David K. Jackson, Ian Johnston, Dominic Kwiatkowski, Cordelia Langford, John Sillitoe on behalf of the Wellcome Sanger Institute COVID-19 Surveillance Team
EPI_ISL_945166	Lighthouse Lab in Cambridge	Wellcome Sanger Institute for the COVID-19 Genomics UK (COG-UK) Consortium	Rob Howes, The Lighthouse Lab in Cambridge and Alex Alderton, Roberto Amato, Sonia Goncalves, Ewan Harrison, David K. Jackson, Ian Johnston, Dominic Kwiatkowski, Cordelia Langford, John Sillitoe on behalf of the Wellcome Sanger Institute COVID-19 Surveillance Team
EPI_ISL_945167, EPI_ISL_945168, EPI_ISL_945172, EPI_ISL_945173, EPI_ISL_945174, EPI_ISL_945175	Lighthouse Lab in Milton Keynes	Wellcome Sanger Institute for the COVID-19 Genomics UK (COG-UK) Consortium	The Lighthouse Lab in Milton Keynes and Alex Alderton, Roberto Amato, Sonia Goncalves, Ewan Harrison, David K. Jackson, Ian Johnston, Dominic Kwiatkowski, Cordelia Langford, John Sillitoe on behalf of the Wellcome Sanger Institute COVID-19 Surveillance Team
EPI_ISL_945176	Lighthouse Lab in Cambridge	Wellcome Sanger Institute for the COVID-19 Genomics UK	Rob Howes, The Lighthouse Lab in Cambridge and Alex Alderton, Roberto Amato, Sonia Goncalves, Ewan Harrison, David K. Jackson, Ian Johnston,

		(COG-UK) Consortium	Dominic Kwiatkowski, Cordelia Langford, John Sillitoe on behalf of the Wellcome Sanger Institute COVID-19 Surveillance Team
EPI_ISL_945177, EPI_ISL_945178, EPI_ISL_945179	Lighthouse Lab in Milton Keynes	Wellcome Sanger Institute for the COVID-19 Genomics UK (COG-UK) Consortium	The Lighthouse Lab in Milton Keynes and Alex Alderton, Roberto Amato, Sonia Goncalves, Ewan Harrison, David K. Jackson, Ian Johnston, Dominic Kwiatkowski, Cordelia Langford, John Sillitoe on behalf of the Wellcome Sanger Institute COVID-19 Surveillance Team
EPI_ISL_945180	Lighthouse Lab in Cambridge	Wellcome Sanger Institute for the COVID-19 Genomics UK (COG-UK) Consortium	Rob Howes, The Lighthouse Lab in Cambridge and Alex Alderton, Roberto Amato, Sonia Goncalves, Ewan Harrison, David K. Jackson, Ian Johnston, Dominic Kwiatkowski, Cordelia Langford, John Sillitoe on behalf of the Wellcome Sanger Institute COVID-19 Surveillance Team
EPI_ISL_945182, EPI_ISL_945183, EPI_ISL_945185	Lighthouse Lab in Milton Keynes	Wellcome Sanger Institute for the COVID-19 Genomics UK (COG-UK) Consortium	The Lighthouse Lab in Milton Keynes and Alex Alderton, Roberto Amato, Sonia Goncalves, Ewan Harrison, David K. Jackson, Ian Johnston, Dominic Kwiatkowski, Cordelia Langford, John Sillitoe on behalf of the Wellcome Sanger Institute COVID-19 Surveillance Team
EPI_ISL_945186	Lighthouse Lab in Cambridge	Wellcome Sanger Institute for the COVID-19 Genomics UK (COG-UK) Consortium	Rob Howes, The Lighthouse Lab in Cambridge and Alex Alderton, Roberto Amato, Sonia Goncalves, Ewan Harrison, David K. Jackson, Ian Johnston, Dominic Kwiatkowski, Cordelia Langford, John Sillitoe on behalf of the Wellcome Sanger Institute COVID-19 Surveillance Team
EPI_ISL_945187, EPI_ISL_945189	Lighthouse Lab in Milton Keynes	Wellcome Sanger Institute for the COVID-19 Genomics UK (COG-UK) Consortium	The Lighthouse Lab in Milton Keynes and Alex Alderton, Roberto Amato, Sonia Goncalves, Ewan Harrison, David K. Jackson, Ian Johnston, Dominic Kwiatkowski, Cordelia Langford, John Sillitoe on behalf of the Wellcome Sanger Institute COVID-19 Surveillance Team
EPI_ISL_945191, EPI_ISL_945192	Lighthouse Lab in Cambridge	Wellcome Sanger Institute for the COVID-19 Genomics UK (COG-UK) Consortium	Rob Howes, The Lighthouse Lab in Cambridge and Alex Alderton, Roberto Amato, Sonia Goncalves, Ewan Harrison, David K. Jackson, Ian Johnston, Dominic Kwiatkowski, Cordelia Langford, John Sillitoe on behalf of the Wellcome Sanger Institute COVID-19 Surveillance Team
EPI_ISL_945193, EPI_ISL_945194, EPI_ISL_945196, EPI_ISL_945198	Lighthouse Lab in Milton Keynes	Wellcome Sanger Institute for the COVID-19 Genomics UK (COG-UK) Consortium	The Lighthouse Lab in Milton Keynes and Alex Alderton, Roberto Amato, Sonia Goncalves, Ewan Harrison, David K. Jackson, Ian Johnston, Dominic Kwiatkowski, Cordelia Langford, John Sillitoe on behalf of the Wellcome Sanger Institute COVID-19 Surveillance Team
EPI_ISL_945199	Lighthouse Lab in Cambridge	Wellcome Sanger Institute for the COVID-19 Genomics UK (COG-UK) Consortium	Rob Howes, The Lighthouse Lab in Cambridge and Alex Alderton, Roberto Amato, Sonia Goncalves, Ewan Harrison, David K. Jackson, Ian Johnston, Dominic Kwiatkowski, Cordelia Langford, John Sillitoe on behalf of the Wellcome Sanger Institute COVID-19 Surveillance Team
EPI_ISL_945200	Lighthouse Lab in Milton Keynes	Wellcome Sanger Institute for the COVID-19 Genomics UK (COG-UK) Consortium	The Lighthouse Lab in Milton Keynes and Alex Alderton, Roberto Amato, Sonia Goncalves, Ewan Harrison, David K. Jackson, Ian Johnston, Dominic Kwiatkowski, Cordelia Langford, John Sillitoe on behalf of the Wellcome Sanger Institute COVID-19 Surveillance Team
EPI_ISL_945203	Lighthouse Lab in Cambridge	Wellcome Sanger Institute for the COVID-19 Genomics UK (COG-UK) Consortium	Rob Howes, The Lighthouse Lab in Cambridge and Alex Alderton, Roberto Amato, Sonia Goncalves, Ewan Harrison, David K. Jackson, Ian Johnston, Dominic Kwiatkowski, Cordelia Langford, John Sillitoe on behalf of the Wellcome Sanger Institute COVID-19 Surveillance Team
EPI_ISL_945205, EPI_ISL_945206, EPI_ISL_945210, EPI_ISL_945211, EPI_ISL_945213, EPI_ISL_945214, EPI_ISL_945215, EPI_ISL_945216, EPI_ISL_945218, EPI_ISL_945219, EPI_ISL_945220			
see above	Lighthouse Lab in Milton Keynes	Wellcome Sanger Institute for the COVID-19 Genomics UK (COG-UK) Consortium	The Lighthouse Lab in Milton Keynes and Alex Alderton, Roberto Amato, Sonia Goncalves, Ewan Harrison, David K. Jackson, Ian Johnston, Dominic Kwiatkowski, Cordelia Langford, John Sillitoe on behalf of the Wellcome Sanger Institute COVID-19 Surveillance Team
EPI_ISL_945222	Lighthouse Lab in Cambridge	Wellcome Sanger Institute for the COVID-19 Genomics UK (COG-UK) Consortium	Rob Howes, The Lighthouse Lab in Cambridge and Alex Alderton, Roberto Amato, Sonia Goncalves, Ewan Harrison, David K. Jackson, Ian Johnston, Dominic Kwiatkowski, Cordelia Langford, John Sillitoe on behalf of the Wellcome Sanger Institute COVID-19 Surveillance Team
EPI_ISL_945225, EPI_ISL_945227, EPI_ISL_945228, EPI_ISL_945229, EPI_ISL_945230	Lighthouse Lab in Milton Keynes	Wellcome Sanger Institute for the COVID-19 Genomics UK (COG-UK) Consortium	The Lighthouse Lab in Milton Keynes and Alex Alderton, Roberto Amato, Sonia Goncalves, Ewan Harrison, David K. Jackson, Ian Johnston, Dominic Kwiatkowski, Cordelia Langford, John Sillitoe on behalf of the Wellcome Sanger Institute COVID-19 Surveillance Team
EPI_ISL_945232	Lighthouse Lab in Cambridge	Wellcome Sanger Institute for the COVID-19 Genomics UK (COG-UK) Consortium	Rob Howes, The Lighthouse Lab in Cambridge and Alex Alderton, Roberto Amato, Sonia Goncalves, Ewan Harrison, David K. Jackson, Ian Johnston, Dominic Kwiatkowski, Cordelia Langford, John Sillitoe on behalf of the Wellcome Sanger Institute COVID-19 Surveillance Team
EPI_ISL_945233	Lighthouse Lab in Milton Keynes	Wellcome Sanger Institute for the COVID-19 Genomics UK (COG-UK) Consortium	The Lighthouse Lab in Milton Keynes and Alex Alderton, Roberto Amato, Sonia Goncalves, Ewan Harrison, David K. Jackson, Ian Johnston, Dominic Kwiatkowski, Cordelia Langford, John Sillitoe on behalf of the Wellcome Sanger Institute COVID-19 Surveillance Team
EPI_ISL_945237	Lighthouse Lab in Cambridge	Wellcome Sanger Institute for the COVID-19 Genomics UK (COG-UK) Consortium	Rob Howes, The Lighthouse Lab in Cambridge and Alex Alderton, Roberto Amato, Sonia Goncalves, Ewan Harrison, David K. Jackson, Ian Johnston, Dominic Kwiatkowski, Cordelia Langford, John Sillitoe on behalf of the Wellcome Sanger Institute COVID-19 Surveillance Team
EPI_ISL_945239, EPI_ISL_945242, EPI_ISL_945245, EPI_ISL_945246, EPI_ISL_945251, EPI_ISL_945253, EPI_ISL_945256	Lighthouse Lab in Milton Keynes	Wellcome Sanger Institute for the COVID-19 Genomics UK (COG-UK) Consortium	The Lighthouse Lab in Milton Keynes and Alex Alderton, Roberto Amato, Sonia Goncalves, Ewan Harrison, David K. Jackson, Ian Johnston, Dominic Kwiatkowski, Cordelia Langford, John Sillitoe on behalf of the Wellcome Sanger Institute COVID-19 Surveillance Team
EPI_ISL_945257	Lighthouse Lab in Cambridge	Wellcome Sanger Institute for the COVID-19 Genomics UK (COG-UK) Consortium	Rob Howes, The Lighthouse Lab in Cambridge and Alex Alderton, Roberto Amato, Sonia Goncalves, Ewan Harrison, David K. Jackson, Ian Johnston, Dominic Kwiatkowski, Cordelia Langford, John Sillitoe on behalf of the Wellcome Sanger Institute COVID-19 Surveillance Team
EPI_ISL_945258, EPI_ISL_945259, EPI_ISL_945260	Lighthouse Lab in Milton Keynes	Wellcome Sanger Institute for the COVID-19 Genomics UK (COG-UK) Consortium	The Lighthouse Lab in Milton Keynes and Alex Alderton, Roberto Amato, Sonia Goncalves, Ewan Harrison, David K. Jackson, Ian Johnston, Dominic Kwiatkowski, Cordelia Langford, John Sillitoe on behalf of the Wellcome Sanger Institute COVID-19 Surveillance Team
EPI_ISL_945261	Lighthouse Lab in Cambridge	Wellcome Sanger Institute for the COVID-19 Genomics UK (COG-UK) Consortium	Rob Howes, The Lighthouse Lab in Cambridge and Alex Alderton, Roberto Amato, Sonia Goncalves, Ewan Harrison, David K. Jackson, Ian Johnston, Dominic Kwiatkowski, Cordelia Langford, John Sillitoe on behalf of the Wellcome Sanger Institute COVID-19 Surveillance Team
EPI_ISL_945262, EPI_ISL_945263, EPI_ISL_945264, EPI_ISL_945265, EPI_ISL_945266, EPI_ISL_945267	Lighthouse Lab in Milton Keynes	Wellcome Sanger Institute for the COVID-19 Genomics UK (COG-UK) Consortium	The Lighthouse Lab in Milton Keynes and Alex Alderton, Roberto Amato, Sonia Goncalves, Ewan Harrison, David K. Jackson, Ian Johnston, Dominic Kwiatkowski, Cordelia Langford, John Sillitoe on behalf of the Wellcome Sanger Institute COVID-19 Surveillance Team
EPI_ISL_945268, EPI_ISL_945271	Lighthouse Lab in Cambridge	Wellcome Sanger Institute for the COVID-19 Genomics UK (COG-UK) Consortium	Rob Howes, The Lighthouse Lab in Cambridge and Alex Alderton, Roberto Amato, Sonia Goncalves, Ewan Harrison, David K. Jackson, Ian Johnston, Dominic Kwiatkowski, Cordelia Langford, John Sillitoe on behalf of the Wellcome Sanger Institute COVID-19 Surveillance Team
EPI_ISL_945272, EPI_ISL_945274, EPI_ISL_945276, EPI_ISL_945277, EPI_ISL_945278, EPI_ISL_945280, EPI_ISL_945281, EPI_ISL_945283, EPI_ISL_945284, EPI_ISL_945286, EPI_ISL_945287, EPI_ISL_945290, EPI_ISL_945291, EPI_ISL_945292, EPI_ISL_945294, EPI_ISL_945295, EPI_ISL_945296, EPI_ISL_945298, EPI_ISL_945300, EPI_ISL_945302, EPI_ISL_945303, EPI_ISL_945308, EPI_ISL_945312, EPI_ISL_945313, EPI_ISL_945314, EPI_ISL_945315, EPI_ISL_945316, EPI_ISL_945317, EPI_ISL_945318			
see above	Lighthouse Lab in Milton Keynes	Wellcome Sanger Institute for the COVID-19 Genomics UK (COG-UK) Consortium	The Lighthouse Lab in Milton Keynes and Alex Alderton, Roberto Amato, Sonia Goncalves, Ewan Harrison, David K. Jackson, Ian Johnston, Dominic Kwiatkowski, Cordelia Langford, John Sillitoe on behalf of the Wellcome Sanger Institute COVID-19 Surveillance Team
EPI_ISL_945321	Lighthouse Lab in Cambridge	Wellcome Sanger Institute for the COVID-19 Genomics UK (COG-UK) Consortium	Rob Howes, The Lighthouse Lab in Cambridge and Alex Alderton, Roberto Amato, Sonia Goncalves, Ewan Harrison, David K. Jackson, Ian Johnston, Dominic Kwiatkowski, Cordelia Langford, John Sillitoe on behalf of the Wellcome Sanger Institute COVID-19 Surveillance Team
EPI_ISL_945322, EPI_ISL_945325, EPI_ISL_945326, EPI_ISL_945327, EPI_ISL_945328, EPI_ISL_945329, EPI_ISL_945330, EPI_ISL_945331, EPI_ISL_945333, EPI_ISL_945336	Lighthouse Lab in Milton Keynes	Wellcome Sanger Institute for the COVID-19 Genomics UK (COG-UK) Consortium	The Lighthouse Lab in Milton Keynes and Alex Alderton, Roberto Amato, Sonia Goncalves, Ewan Harrison, David K. Jackson, Ian Johnston, Dominic Kwiatkowski, Cordelia Langford, John Sillitoe on behalf of the Wellcome Sanger Institute COVID-19 Surveillance Team
EPI_ISL_945341	Lighthouse Lab in Cambridge	Wellcome Sanger Institute for the COVID-19 Genomics UK (COG-UK) Consortium	Rob Howes, The Lighthouse Lab in Cambridge and Alex Alderton, Roberto Amato, Sonia Goncalves, Ewan Harrison, David K. Jackson, Ian Johnston, Dominic Kwiatkowski, Cordelia Langford, John Sillitoe on behalf of the Wellcome Sanger Institute COVID-19 Surveillance Team
EPI_ISL_945344	Lighthouse Lab in Milton Keynes	Wellcome Sanger Institute for the COVID-19 Genomics UK (COG-UK) Consortium	The Lighthouse Lab in Milton Keynes and Alex Alderton, Roberto Amato, Sonia Goncalves, Ewan Harrison, David K. Jackson, Ian Johnston, Dominic Kwiatkowski, Cordelia Langford, John Sillitoe on behalf of the Wellcome Sanger Institute COVID-19 Surveillance Team
EPI_ISL_945346	Lighthouse Lab in Cambridge	Wellcome Sanger Institute for the COVID-19 Genomics UK (COG-UK) Consortium	Rob Howes, The Lighthouse Lab in Cambridge and Alex Alderton, Roberto Amato, Sonia Goncalves, Ewan Harrison, David K. Jackson, Ian Johnston, Dominic Kwiatkowski, Cordelia Langford, John Sillitoe on behalf of the Wellcome Sanger Institute COVID-19 Surveillance Team
EPI_ISL_945348, EPI_ISL_945349, EPI_ISL_945352, EPI_ISL_945353, EPI_ISL_945354, EPI_ISL_945355	Lighthouse Lab in Milton Keynes	Wellcome Sanger Institute for the COVID-19 Genomics UK (COG-UK) Consortium	The Lighthouse Lab in Milton Keynes and Alex Alderton, Roberto Amato, Sonia Goncalves, Ewan Harrison, David K. Jackson, Ian Johnston, Dominic Kwiatkowski, Cordelia Langford, John Sillitoe on behalf of the Wellcome Sanger Institute COVID-19 Surveillance Team
EPI_ISL_945356	Lighthouse Lab in Cambridge	Wellcome Sanger Institute for the COVID-19 Genomics UK (COG-UK) Consortium	Rob Howes, The Lighthouse Lab in Cambridge and Alex Alderton, Roberto Amato, Sonia Goncalves, Ewan Harrison, David K. Jackson, Ian Johnston, Dominic Kwiatkowski, Cordelia Langford, John Sillitoe on behalf of the Wellcome Sanger Institute COVID-19 Surveillance Team

EPI_ISL_945359, EPI_ISL_945362	Lighthouse Lab in Milton Keynes	Wellcome Sanger Institute for the COVID-19 Genomics UK (COG-UK) Consortium	The Lighthouse Lab in Milton Keynes and Alex Alderton, Roberto Amato, Sonia Goncalves, Ewan Harrison, David K. Jackson, Ian Johnston, Dominic Kwiatkowski, Cordelia Langford, John Sillitoe on behalf of the Wellcome Sanger Institute COVID-19 Surveillance Team
EPI_ISL_945365	Lighthouse Lab in Cambridge	Wellcome Sanger Institute for the COVID-19 Genomics UK (COG-UK) Consortium	Rob Howes, The Lighthouse Lab in Cambridge and Alex Alderton, Roberto Amato, Sonia Goncalves, Ewan Harrison, David K. Jackson, Ian Johnston, Dominic Kwiatkowski, Cordelia Langford, John Sillitoe on behalf of the Wellcome Sanger Institute COVID-19 Surveillance Team
EPI_ISL_945368, EPI_ISL_945369, EPI_ISL_945370, EPI_ISL_945371, EPI_ISL_945372, EPI_ISL_945373	Lighthouse Lab in Milton Keynes	Wellcome Sanger Institute for the COVID-19 Genomics UK (COG-UK) Consortium	The Lighthouse Lab in Milton Keynes and Alex Alderton, Roberto Amato, Sonia Goncalves, Ewan Harrison, David K. Jackson, Ian Johnston, Dominic Kwiatkowski, Cordelia Langford, John Sillitoe on behalf of the Wellcome Sanger Institute COVID-19 Surveillance Team
EPI_ISL_946746, EPI_ISL_947649	Lighthouse Lab in Alderley Park	Wellcome Sanger Institute for the COVID-19 Genomics UK (COG-UK) Consortium	Jacquelyn Wynn, Mairead Hyland, The Lighthouse Lab in Alderley Park and Alex Alderton, Roberto Amato, Sonia Goncalves, Ewan Harrison, David K. Jackson, Ian Johnston, Dominic Kwiatkowski, Cordelia Langford, John Sillitoe on behalf of the Wellcome Sanger Institute COVID-19 Surveillance Team
EPI_ISL_949406	University of Birmingham	COVID-19 Genomics UK (COG-UK) Consortium	Institute of Microbiology, University of Birmingham: Claire McMurray, Joanne Stockton, Samuel Nicholls, Radoslaw Poplawski, Will Rowe, Josh Quick, Nicholas Loman. University of Birmingham Testing Laboratory: Celina M Whalley, Andrew Bosworth, Charlotte Poxon, Kasun Wanigasooriya, Oliver Pickles, Mike Kidd, Alex Richter, Andrew D Beggs PHE Heartlands Lab: Husam Osman, Andrew Bosworth. Queen Elizabeth Hospital: Anna Casey
EPI_ISL_949748, EPI_ISL_949754, EPI_ISL_949755, EPI_ISL_949756, EPI_ISL_949767, EPI_ISL_949769, EPI_ISL_949775, EPI_ISL_949784	Barts Health NHS Trust	COVID-19 Genomics UK (COG-UK) Consortium	CUTINO-MOGUEL, Maria-Teresa; HARRINGTON, David; OWOYEMI, Dola; KULASEGARAN-SHYLINI, Raghavendran; BROAD, Claire; KELE, Beatrix
EPI_ISL_950048, EPI_ISL_950068, EPI_ISL_950073, EPI_ISL_950076, EPI_ISL_950078, EPI_ISL_950081, EPI_ISL_950082, EPI_ISL_950086, EPI_ISL_950089, EPI_ISL_950091, EPI_ISL_950093, EPI_ISL_950094, EPI_ISL_950095, EPI_ISL_950096, EPI_ISL_950099, EPI_ISL_950103, EPI_ISL_950106, EPI_ISL_950111, EPI_ISL_950113, EPI_ISL_950116, EPI_ISL_950118, EPI_ISL_950122, EPI_ISL_950123, EPI_ISL_950124, EPI_ISL_950127, EPI_ISL_950130, EPI_ISL_950133, EPI_ISL_950134, EPI_ISL_950135, EPI_ISL_950136, EPI_ISL_950142, EPI_ISL_950146, EPI_ISL_950147, EPI_ISL_950148, EPI_ISL_950151, EPI_ISL_950152, EPI_ISL_950153, EPI_ISL_950154, EPI_ISL_950165			
see above	University College London, Great Ormond Street Hospital for Children NHS Foundation Trust, Imperial College Healthcare NHS Trust	COVID-19 Genomics UK (COG-UK) Consortium	Sergi Castellano, Rachel Williams, Mark Kristiansen, Paola Resende Silva, Sunando Roy, Tony Brooks, Helena Tutill, Paola Niola, Patricia Dyal, Charlotte Williams, Leysa Forrest, Yasmin Panchbhaya, Jacqueline Findlay, Samuel Weeks, Julianne Brown, Kathryn Harris, Paul Randell, James Price, Alison Holmes, Judith Breuer
EPI_ISL_950254, EPI_ISL_950255, EPI_ISL_950257, EPI_ISL_950258, EPI_ISL_950259, EPI_ISL_950260, EPI_ISL_950261, EPI_ISL_950262, EPI_ISL_950263, EPI_ISL_950264, EPI_ISL_950265, EPI_ISL_950266, EPI_ISL_950267, EPI_ISL_950268, EPI_ISL_950269, EPI_ISL_950270, EPI_ISL_950288, EPI_ISL_950460, EPI_ISL_950461, EPI_ISL_950462, EPI_ISL_950463, EPI_ISL_950464, EPI_ISL_950465, EPI_ISL_950466, EPI_ISL_950467, EPI_ISL_950468, EPI_ISL_950469, EPI_ISL_950470, EPI_ISL_950471, EPI_ISL_950472, EPI_ISL_950474, EPI_ISL_950475, EPI_ISL_950476, EPI_ISL_950477, EPI_ISL_950478, EPI_ISL_950479, EPI_ISL_950480, EPI_ISL_950481, EPI_ISL_950482, EPI_ISL_950483, EPI_ISL_950484, EPI_ISL_950485, EPI_ISL_950486, EPI_ISL_950487, EPI_ISL_950488, EPI_ISL_950489, EPI_ISL_950490, EPI_ISL_950491, EPI_ISL_950492, EPI_ISL_950493			
see above	Northumbria University / South Tees Hospitals NHS Foundation Trust / North Cumbria Integrated Care NHS Foundation Trust / North Tees and Hartlepool NHS Foundation Trust / Newcastle Hospitals NHS Foundation Trust	COVID-19 Genomics UK (COG-UK) Consortium	Darren L Smith,Andrew Nelson,Matthew Bashton,Greg R Young,Joshua Loh,John Allan,Mohammad A Tariq,Giles S Holt,Gary Black,Wen C Yew,Lynn Dover,Paul Baker,Steve Liggett,Sarah Essex,Jane Greenaway,Debra Padgett,Clive Graham,Garren Scott,Edward Barton,Emma Swindells,Brendan Payne,Jennifer Collins,Yusri Taha,Gary Eltringham
EPI_ISL_950551, EPI_ISL_950582	Quadram Institute Bioscience	COVID-19 Genomics UK (COG-UK) Consortium	Dave J. Baker, Gemma L. Kay, Alp Aydin, Thanh Le-Viet, Steven Rudder, Ana P. Tedim, Anastasia Kolyva, Maria Diaz, Leonardo de Oliveira Martins, Nabil-Fareed Alikhan, Lizzie Meadows, Rachael Stanley, Ngozi Elumogo, Muhammed Yasir, Nicholas M. Thomson, Alexander J Trotter, Rachel Gilroy, Samuel Bloomfield, Claire Stuart, Andrew Bell, Reenesh Prakash, Samir Dervisevic, Alison E. Mather, John Wain, Mark Webber, Andrew J. Page, Justin O'Grady
EPI_ISL_950712	Lincolnshire Hospitals and DeepSeq Nottingham	COVID-19 Genomics UK (COG-UK) Consortium	Nichola Duckworth, Tim Sloan, Sarah Walsh, Jonathan Ball, Patrick McClure, Joeseph Chappell, Nadine Holmes, Matthew Carlisle, Christopher Moore, Fei Sang, Johnny Debebe, Victoria Wright, Matthew Loose
EPI_ISL_951068, EPI_ISL_951069, EPI_ISL_951070, EPI_ISL_951071, EPI_ISL_951072, EPI_ISL_951073, EPI_ISL_951074, EPI_ISL_951075, EPI_ISL_951077, EPI_ISL_951078, EPI_ISL_951082, EPI_ISL_951084, EPI_ISL_951085, EPI_ISL_951087, EPI_ISL_951088, EPI_ISL_951089, EPI_ISL_951090, EPI_ISL_951091, EPI_ISL_951093, EPI_ISL_951101, EPI_ISL_951102, EPI_ISL_951103, EPI_ISL_951104, EPI_ISL_951105, EPI_ISL_951106, EPI_ISL_951107, EPI_ISL_951108, EPI_ISL_951109, EPI_ISL_951110, EPI_ISL_951111, EPI_ISL_951112, EPI_ISL_951114, EPI_ISL_951115, EPI_ISL_951116, EPI_ISL_951117, EPI_ISL_951118, EPI_ISL_951119, EPI_ISL_951120, EPI_ISL_951121, EPI_ISL_951122, EPI_ISL_951125, EPI_ISL_951126, EPI_ISL_951131, EPI_ISL_951132, EPI_ISL_951134, EPI_ISL_951135, EPI_ISL_951136, EPI_ISL_951137, EPI_ISL_951138, EPI_ISL_951139, EPI_ISL_951141, EPI_ISL_951143, EPI_ISL_951144, EPI_ISL_951145, EPI_ISL_951147, EPI_ISL_951148, EPI_ISL_951149, EPI_ISL_951152, EPI_ISL_951153, EPI_ISL_951154, EPI_ISL_951155, EPI_ISL_951158, EPI_ISL_951159, EPI_ISL_951160, EPI_ISL_951161, EPI_ISL_951162, EPI_ISL_951172, EPI_ISL_951173, EPI_ISL_951174, EPI_ISL_951175, EPI_ISL_951176, EPI_ISL_951177, EPI_ISL_951178, EPI_ISL_951180, EPI_ISL_951181, EPI_ISL_951182, EPI_ISL_951183, EPI_ISL_951184, EPI_ISL_951185, EPI_ISL_951186, EPI_ISL_951187, EPI_ISL_951188, EPI_ISL_951189, EPI_ISL_951191, EPI_ISL_951192, EPI_ISL_951193, EPI_ISL_951195, EPI_ISL_951196, EPI_ISL_951197, EPI_ISL_951198, EPI_ISL_951199, EPI_ISL_951200, EPI_ISL_951201, EPI_ISL_951202, EPI_ISL_951204, EPI_ISL_951205, EPI_ISL_951206, EPI_ISL_951207, EPI_ISL_951209, EPI_ISL_951210, EPI_ISL_951211, EPI_ISL_951212, EPI_ISL_951213, EPI_ISL_951214, EPI_ISL_951215, EPI_ISL_951216, EPI_ISL_951218, EPI_ISL_951220, EPI_ISL_951221, EPI_ISL_951223, EPI_ISL_951224, EPI_ISL_951225, EPI_ISL_951226, EPI_ISL_951227, EPI_ISL_951228, EPI_ISL_951229, EPI_ISL_951230, EPI_ISL_951233, EPI_ISL_951234, EPI_ISL_951235, EPI_ISL_951242, EPI_ISL_951319, EPI_ISL_951320, EPI_ISL_951323, EPI_ISL_951324, EPI_ISL_951326, EPI_ISL_951327, EPI_ISL_951328, EPI_ISL_951329, EPI_ISL_951331, EPI_ISL_951333, EPI_ISL_951334, EPI_ISL_951335, EPI_ISL_951336, EPI_ISL_951337, EPI_ISL_951338, EPI_ISL_951339, EPI_ISL_951340, EPI_ISL_951341, EPI_ISL_951342, EPI_ISL_951344, EPI_ISL_951381, EPI_ISL_951382, EPI_ISL_951384, EPI_ISL_951386, EPI_ISL_951397, EPI_ISL_951398, EPI_ISL_951400, EPI_ISL_951401, EPI_ISL_951402, EPI_ISL_951410			
see above	Oxford Viromics, NDM, University of Oxford; Oxford University Hospitals; Basingstoke and North Hampshire Hospital	COVID-19 Genomics UK (COG-UK) Consortium	Tanya Golubchik, David Bonsall, George Macintyre, Amy Trebes, Mariateresa de Cesare, Catrin Moore, Alex Mobbs, Anita Justice, Robert Shaw, Monique Andersson, Timothy Peto, Emma Wise, Nathan Moore, Jessica Lynch, Nick Cortes, Matilde Mori, Stephen Kidd, David Buck, John Todd, Christophe Fraser
EPI_ISL_952407, EPI_ISL_952408, EPI_ISL_952420, EPI_ISL_952423, EPI_ISL_952785, EPI_ISL_952813, EPI_ISL_952814, EPI_ISL_952815	Centre for Enzyme Innovation, University of Portsmouth / Translational Research Laboratory, Portsmouth Hospitals NHS Trust	COVID-19 Genomics UK (COG-UK) Consortium	Angela Beckett,Salman Goudarzi,Christopher Fearn,Kate Cook,Katie Loveson,Sharon Glaysher,Scott Elliott,Samuel Robson
EPI_ISL_953174, EPI_ISL_953175, EPI_ISL_953176, EPI_ISL_953178, EPI_ISL_953179, EPI_ISL_953183, EPI_ISL_953184, EPI_ISL_953186, EPI_ISL_953188, EPI_ISL_953189, EPI_ISL_953190, EPI_ISL_953191, EPI_ISL_953192, EPI_ISL_953194, EPI_ISL_953195, EPI_ISL_953196, EPI_ISL_953197, EPI_ISL_953198, EPI_ISL_953200, EPI_ISL_953201, EPI_ISL_953202, EPI_ISL_953203, EPI_ISL_953204, EPI_ISL_953206, EPI_ISL_953208, EPI_ISL_953209, EPI_ISL_953210, EPI_ISL_953211, EPI_ISL_953212, EPI_ISL_953213, EPI_ISL_953214, EPI_ISL_953215, EPI_ISL_953218, EPI_ISL_953220, EPI_ISL_953221, EPI_ISL_953223, EPI_ISL_953224, EPI_ISL_953225, EPI_ISL_953227, EPI_ISL_953228, EPI_ISL_953229, EPI_ISL_953234, EPI_ISL_953235, EPI_ISL_953237, EPI_ISL_953242, EPI_ISL_953260, EPI_ISL_953273, EPI_ISL_953274, EPI_ISL_953278			
see above	Bioinformatics and Biostatistics Lab, Advanced Sequencing Facility	COVID-19 Genomics UK (COG-UK) Consortium	Aengus Stewart,Jerome Nicod,Chelsea Sawyer,Laura Cubitt,Harshil Patel,Margaret Crawford
EPI_ISL_958564, EPI_ISL_958565	Lighthouse Lab in Milton Keynes	Wellcome Sanger Institute for the COVID-19 Genomics UK (COG-UK) Consortium	The Lighthouse Lab in Milton Keynes and Alex Alderton, Roberto Amato, Sonia Goncalves, Ewan Harrison, David K. Jackson, Ian Johnston, Dominic Kwiatkowski, Cordelia Langford, John Sillitoe on behalf of the Wellcome Sanger Institute COVID-19 Surveillance Team
EPI_ISL_963106, EPI_ISL_963980, EPI_ISL_964107, EPI_ISL_964219, EPI_ISL_964234, EPI_ISL_985788	Lighthouse Lab in Alderley Park	Wellcome Sanger Institute for the COVID-19 Genomics UK (COG-UK) Consortium	Jacquelyn Wynn, Mairead Hyland, The Lighthouse Lab in Alderley Park and Alex Alderton, Roberto Amato, Sonia Goncalves, Ewan Harrison, David K. Jackson, Ian Johnston, Dominic Kwiatkowski, Cordelia Langford, John Sillitoe on behalf of the Wellcome Sanger Institute COVID-19 Surveillance Team
EPI_ISL_986354	Lighthouse Lab in Glasgow	Wellcome Sanger Institute for the COVID-19 Genomics UK (COG-UK) Consortium	Harper VanSteenhouse, Yumi Kasai, David Gray, Carol Clugston, Anna Dominiczak and Alex Alderton, Roberto Amato, Sonia Goncalves, Ewan Harrison, David K. Jackson, Ian Johnston, Dominic Kwiatkowski, Cordelia Langford, John Sillitoe on behalf of the Wellcome Sanger Institute COVID-19 Surveillance Team (http://www.sanger.ac.uk/covid-team)
EPI_ISL_993805, EPI_ISL_993914, EPI_ISL_993916	Lighthouse Lab in Milton Keynes	Wellcome Sanger Institute for the COVID-19 Genomics UK (COG-UK) Consortium	The Lighthouse Lab in Milton Keynes and Alex Alderton, Roberto Amato, Sonia Goncalves, Ewan Harrison, David K. Jackson, Ian Johnston, Dominic Kwiatkowski, Cordelia Langford, John Sillitoe on behalf of the Wellcome Sanger Institute COVID-19 Surveillance Team
EPI_ISL_993955, EPI_ISL_993962, EPI_ISL_993991, EPI_ISL_994034, EPI_ISL_994056	Lighthouse Lab in Milton Keynes	Wellcome Sanger Institute for the COVID-19 Genomics UK (COG-UK) Consortium	The Lighthouse Lab in Milton Keynes and Alex Alderton, Roberto Amato, Sonia Goncalves, Ewan Harrison, David K. Jackson, Ian Johnston, Dominic Kwiatkowski, Cordelia Langford, John Sillitoe on behalf of the Wellcome Sanger Institute COVID-19 Surveillance Team (http://www.sanger.ac.uk/covid-team)
EPI_ISL_994651	Lighthouse Lab in Alderley Park	Wellcome Sanger Institute for the COVID-19 Genomics UK (COG-UK) Consortium	Jacquelyn Wynn, Mairead Hyland, The Lighthouse Lab in Alderley Park and Alex Alderton, Roberto Amato, Sonia Goncalves, Ewan Harrison, David K. Jackson, Ian Johnston, Dominic Kwiatkowski, Cordelia Langford, John Sillitoe on behalf of the Wellcome Sanger Institute COVID-19 Surveillance Team
EPI_ISL_994652	Lighthouse Lab in Milton Keynes	Wellcome Sanger Institute for the COVID-19 Genomics UK (COG-UK) Consortium	The Lighthouse Lab in Milton Keynes and Alex Alderton, Roberto Amato, Sonia Goncalves, Ewan Harrison, David K. Jackson, Ian Johnston, Dominic Kwiatkowski, Cordelia Langford, John Sillitoe on behalf of the Wellcome Sanger Institute COVID-19 Surveillance Team
EPI_ISL_996433, EPI_ISL_996434, EPI_ISL_996435, EPI_ISL_996437, EPI_ISL_996438, EPI_ISL_996454	University of Birmingham	COVID-19 Genomics UK (COG-UK) Consortium	Institute of Microbiology, University of Birmingham: Claire McMurray, Joanne Stockton, Samuel Nicholls, Radoslaw Poplawski, Will Rowe, Josh Quick, Nicholas Loman. University of Birmingham Testing Laboratory: Celina M Whalley, Andrew Bosworth, Charlotte Poxon, Kasun Wanigasooriya, Oliver Pickles, Mike Kidd, Alex Richter, Andrew D Beggs PHE Heartlands Lab: Husam Osman, Andrew Bosworth. Queen Elizabeth Hospital: Anna Casey

EPI_ISL_996482, EPI_ISL_996484, EPI_ISL_996486, EPI_ISL_996487, EPI_ISL_996488, EPI_ISL_996489, EPI_ISL_996491, EPI_ISL_996500, EPI_ISL_996501, EPI_ISL_996502, EPI_ISL_996503, EPI_ISL_996504, EPI_ISL_996505, EPI_ISL_996508, EPI_ISL_996511, EPI_ISL_996527, EPI_ISL_996528, EPI_ISL_996529, EPI_ISL_996570			
see above	University of Exeter	COVID-19 Genomics UK (COG-UK) Consortium	Ben Temperton,Aaron Jeffries,Michelle Michelsen,Joanna Warwick-Dugdale,Audrey Farbos,Robyn Manley,Stephen Michell,Jane Masoli
EPI_ISL_997072, EPI_ISL_997073, EPI_ISL_997120, EPI_ISL_997161, EPI_ISL_997162	Virology Department, Royal Infirmary of Edinburgh, NHS Lothian / School of Biological Sciences, University of Edinburgh	COVID-19 Genomics UK (COG-UK) Consortium	McHugh M, Dewar R, Cotton S, Rooke S, O'Toole Á, Scher E, Hill V, McCrone JT, Colquhoun R, Yu X, Jackson B, Rambaut A, Templeton K
EPI_ISL_997323, EPI_ISL_997324, EPI_ISL_997325	University of Exeter	COVID-19 Genomics UK (COG-UK) Consortium	Ben Temperton,Aaron Jeffries,Michelle Michelsen,Joanna Warwick-Dugdale,Audrey Farbos,Robyn Manley,Stephen Michell,Jane Masoli
EPI_ISL_997614	University College London, Great Ormond Street Hospital for Children NHS Foundation Trust, Imperial College Healthcare NHS Trust	COVID-19 Genomics UK (COG-UK) Consortium	Sergi Castellano, Rachel Williams, Mark Kristiansen, Paola Resende Silva, Sunando Roy, Tony Brooks, Helena Tutill, Paola Niola, Patricia Dyal, Charlotte Williams, Leysa Forrest, Yasmin Panchbhaya, Jacqueline Findlay, Samuel Weeks, Julianne Brown, Kathryn Harris, Paul Randell, James Price, Alison Holmes, Judith Breuer
EPI_ISL_998239	Regional Virus Laboratory, Belfast Health and Social Care Trust	COVID-19 Genomics UK (COG-UK) Consortium	Conall McCaughey, James McKenna, Tanya Curran, Susan Feeney, Alison Watt, Ciara Cox, Mairead Connor, Zoltan Molnar, David Simpson, Derek Fairley
EPI_ISL_998368, EPI_ISL_998369, EPI_ISL_998370, EPI_ISL_998372, EPI_ISL_998737, EPI_ISL_998738, EPI_ISL_998739, EPI_ISL_998740, EPI_ISL_998741, EPI_ISL_998742, EPI_ISL_998743, EPI_ISL_998744, EPI_ISL_998745, EPI_ISL_998746, EPI_ISL_998747, EPI_ISL_998748, EPI_ISL_998750, EPI_ISL_998751, EPI_ISL_998752, EPI_ISL_998753, EPI_ISL_998754, EPI_ISL_998755, EPI_ISL_998756, EPI_ISL_998757, EPI_ISL_998758, EPI_ISL_998759, EPI_ISL_998760, EPI_ISL_998761, EPI_ISL_998762, EPI_ISL_998763, EPI_ISL_998764, EPI_ISL_998765, EPI_ISL_998766, EPI_ISL_998767, EPI_ISL_998768, EPI_ISL_998769, EPI_ISL_998770, EPI_ISL_998771			
see above	Northumbria University / South Tees Hospitals NHS Foundation Trust / North Cumbria Integrated Care NHS Foundation Trust / North Tees and Hartlepool NHS Foundation Trust / Newcastle Hospitals NHS Foundation Trust	COVID-19 Genomics UK (COG-UK) Consortium	Darren L Smith,Andrew Nelson,Matthew Bashton,Greg R Young,Joshua Loh,John Allan,Mohammad A Tariq,Giles S Holt,Gary Black,Wen C Yew,Lynn Dover,Paul Baker,Steve Liggett,Sarah Essex,Jane Greenaway,Debra Padgett,Clive Graham,Garren Scott,Edward Barton,Emma Swindells,Brendan Payne,Jennifer Collins,Yusri Taha,Gary Eltringham
EPI_ISL_998935, EPI_ISL_998936	Lincolnshire Hospitals and DeepSeq Nottingham	COVID-19 Genomics UK (COG-UK) Consortium	Nichola Duckworth, Tim Sloan, Sarah Walsh, Jonathan Ball, Patrick McClure, Joeseph Chappell, Nadine Holmes, Matthew Carlisle, Christopher Moore, Fei Sang, Johnny Debebe, Victoria Wright, Matthew Loose
EPI_ISL_998986, EPI_ISL_998987, EPI_ISL_998988, EPI_ISL_998989, EPI_ISL_998992, EPI_ISL_998996, EPI_ISL_998998, EPI_ISL_999001, EPI_ISL_999002, EPI_ISL_999004, EPI_ISL_999005, EPI_ISL_999006, EPI_ISL_999007, EPI_ISL_999008, EPI_ISL_999009, EPI_ISL_999010, EPI_ISL_999012, EPI_ISL_999013, EPI_ISL_999014, EPI_ISL_999015, EPI_ISL_999017, EPI_ISL_999018, EPI_ISL_999019, EPI_ISL_999020, EPI_ISL_999021, EPI_ISL_999023, EPI_ISL_999024, EPI_ISL_999025, EPI_ISL_999027, EPI_ISL_999028, EPI_ISL_999029, EPI_ISL_999031, EPI_ISL_999051, EPI_ISL_999052, EPI_ISL_999053, EPI_ISL_999054, EPI_ISL_999055, EPI_ISL_999056, EPI_ISL_999057, EPI_ISL_999058, EPI_ISL_999213, EPI_ISL_999215, EPI_ISL_999216, EPI_ISL_999348, EPI_ISL_999349, EPI_ISL_999350, EPI_ISL_999351, EPI_ISL_999352, EPI_ISL_999353, EPI_ISL_999355, EPI_ISL_999356, EPI_ISL_999360, EPI_ISL_999361, EPI_ISL_999362, EPI_ISL_999386, EPI_ISL_999418, EPI_ISL_999424, EPI_ISL_999454, EPI_ISL_999459, EPI_ISL_999465, EPI_ISL_999466, EPI_ISL_999470, EPI_ISL_999509			
see above	Oxford Viromics, NDM, University of Oxford; Oxford University Hospitals; Basingstoke and North Hampshire Hospital	COVID-19 Genomics UK (COG-UK) Consortium	Tanya Golubchik, David Bonsall, George Macintyre, Amy Trebes, Mariateresa de Cesare, Catrin Moore, Alex Mobbs, Anita Justice, Robert Shaw, Monique Andersson, Timothy Peto, Emma Wise, Nathan Moore, Jessica Lynch, Nick Cortes, Matilde Mori, Stephen Kidd, David Buck, John Todd, Christophe Fraser
