# Supplementary material for: Inferring the Association between the Risk of COVID-19 Case Fatality and N501Y Substitution in SARS-CoV-2
Source: Viruses. 2021 Apr 8;13(4):638. doi: 10.3390/v13040638 (PMC8070306; doi:10.3390/v13040638)
Supplement: Supplementary file 1 [file viruses-13-00638-s001.zip › gisaid_hcov-19_UKAT_210107-210111.pdf]

We gratefully acknowledge the following Authors from the Originating laboratories responsible for obtaining the specimens, as well as the Submitting laboratories where the genome data were generated and shared via GISAID, on which this research is based.

All Submitters of data may be contacted directly via www.gisaid.org

Authors are sorted alphabetically.

Accession ID	Originating Laboratory	Submitting Laboratory	Authors
EPI_ISL_1000244, EPI_ISL_1000245, EPI_ISL_1000246, EPI_ISL_1000258, EPI_ISL_1000270, EPI_ISL_1000272	Centre for Enzyme Innovation, University of Portsmouth / Translational Research Laboratory, Portsmouth Hospitals NHS Trust	COVID-19 Genomics UK (COG-UK) Consortium	Angela Beckett,Salman Goudarzi,Christopher Fearn,Kate Cook,Katie Loveson,Sharon Glaysher,Scott Elliott,Samuel Robson
EPI_ISL_1000665	Virology Department, Sheffield Teaching Hospitals NHS Foundation Trust/Department of Infection, Immunity and Cardiovascular Disease, The Medical School, University of Sheffield	COVID-19 Genomics UK (COG-UK) Consortium	Thushan de Silva, Matthew Parker, Nikki Smith, Adri Angyal, Rebecca Brown, Luke Green, Rachel Tucker, Paul Parsons, Danielle Groves, Katie Johnson, Laura Carrilero, Alex Keeley, Dave Partridge, Matthew Wyles, Benjamin Lindsey, Mehmet Yavuz, Mohammad Raza, Cariad Evans
EPI_ISL_1000881, EPI_ISL_1000883, EPI_ISL_1000885, EPI_ISL_1000886, EPI_ISL_1000887, EPI_ISL_1000892, EPI_ISL_1000893, EPI_ISL_1000898, EPI_ISL_1000900, EPI_ISL_1000903, EPI_ISL_1000905, EPI_ISL_1000907, EPI_ISL_1000908			
see above	Bioinformatics and Biostatistics Lab, Advanced Sequencing Facility	COVID-19 Genomics UK (COG-UK) Consortium	Aengus Stewart,Jerome Nicod,Chelsea Sawyer,Laura Cubitt,Harshil Patel,Margaret Crawford
EPI_ISL_1005947, EPI_ISL_1012376, EPI_ISL_1012388	Lighthouse Lab in Cambridge	Wellcome Sanger Institute for the COVID-19 Genomics UK (COG-UK) Consortium	Rob Howes, The Lighthouse Lab in Cambridge and Alex Alderton, Roberto Amato, Sonia Goncalves, Ewan Harrison, David K. Jackson, Ian Johnston, Dominic Kwiatkowski, Cordelia Langford, John Sillitoe on behalf of the Wellcome Sanger Institute COVID-19 Surveillance Team
EPI_ISL_1018924	Lighthouse Lab in Cambridge	Wellcome Sanger Institute for the COVID-19 Genomics UK (COG-UK) Consortium	Rob Howes, The Lighthouse Lab in Cambridge and Alex Alderton, Roberto Amato, Jeffrey Barrett, Sonia Goncalves, Ewan Harrison, David K. Jackson, Ian Johnston, Dominic Kwiatkowski, Cordelia Langford, John Sillitoe on behalf of the Wellcome Sanger Institute COVID-19 Surveillance Team
EPI_ISL_1043207	Lighthouse Lab in Milton Keynes	Wellcome Sanger Institute for the COVID-19 Genomics UK (COG-UK) Consortium	The Lighthouse Lab in Milton Keynes and Alex Alderton, Roberto Amato, Jeffrey Barrett, Sonia Goncalves, Ewan Harrison, David K. Jackson, Ian Johnston, Dominic Kwiatkowski, Cordelia Langford, John Sillitoe on behalf of the Wellcome Sanger Institute COVID-19 Surveillance Team
EPI_ISL_1046997, EPI_ISL_1046998, EPI_ISL_1047067	University of Birmingham	COVID-19 Genomics UK (COG-UK) Consortium	Institute of Microbiology, University of Birmingham: Claire McMurray, Joanne Stockton, Samuel Nicholls, Radoslaw Poplawski, Will Rowe, Josh Quick, Nicholas Loman. University of Birmingham Testing Laboratory: Celina M Whalley, Andrew Bosworth, Charlotte Poxon, Kasun Wanigasooriya, Oliver Pickles, Mike Kidd, Alex Richter, Andrew D Beggs PHE Heartlands Lab: Husam Osman, Andrew Bosworth. Queen Elizabeth Hospital: Anna Casey
EPI_ISL_1047858, EPI_ISL_1047859	Virology Department, Royal Infirmary of Edinburgh, NHS Lothian / School of Biological Sciences, University of Edinburgh	COVID-19 Genomics UK (COG-UK) Consortium	McHugh M, Dewar R, Cotton S, Rooke S, O'Toole Á, Scher E, Hill V, McCrone JT, Colquhoun R, Yu X, Jackson B, Rambaut A, Templeton K
EPI_ISL_1048056, EPI_ISL_1048096	Barts Health NHS Trust	COVID-19 Genomics UK (COG-UK) Consortium	CUTINO-MOGUEL, Maria-Teresa; HARRINGTON, David; OWOYEMI, Dola; KULASEGARAN-SHYLINI, Raghavendran; BROAD, Claire; KELE, Beatrix
EPI_ISL_1050233, EPI_ISL_1050234, EPI_ISL_1050235, EPI_ISL_1050236, EPI_ISL_1050237, EPI_ISL_1050238	West of Scotland Specialist Virology Centre, NHSGGC / MRC-University of Glasgow Centre for Virus Research	COVID-19 Genomics UK (COG-UK) Consortium	Ana da Silva Filipe, Natasha Johnson, Kathy Smollett, Daniel Mair, Stephen Carmichael, Alice Broos, Lily Tong, Jenna Nichols, Kyriaki Nomikou; Sarah McDonald; Richard Orton, Joseph Hughes, Sreenu Vattipally, David L Robertson; Alasdair MacLean, Rory Gunson; Sharif Shaaban, Matthew Holden; Rachel Blacow, Guy Mollett, Kathy Li, James Shepherd, Antonia Ho, Emma Thomson
EPI_ISL_1050431, EPI_ISL_1050432, EPI_ISL_1050433, EPI_ISL_1050437, EPI_ISL_1050438, EPI_ISL_1050449, EPI_ISL_1050452, EPI_ISL_1050453, EPI_ISL_1050454, EPI_ISL_1050455, EPI_ISL_1050456, EPI_ISL_1050463, EPI_ISL_1050467, EPI_ISL_1050468, EPI_ISL_1050469, EPI_ISL_1050470, EPI_ISL_1050471, EPI_ISL_1050472, EPI_ISL_1050473, EPI_ISL_1050474, EPI_ISL_1050475, EPI_ISL_1050476, EPI_ISL_1050477, EPI_ISL_1050478, EPI_ISL_1050479, EPI_ISL_1050480, EPI_ISL_1050481, EPI_ISL_1050482, EPI_ISL_1050483, EPI_ISL_1050484, EPI_ISL_1050485, EPI_ISL_1050486, EPI_ISL_1050487, EPI_ISL_1050488, EPI_ISL_1050489, EPI_ISL_1050490, EPI_ISL_1050491, EPI_ISL_1050492, EPI_ISL_1050493, EPI_ISL_1050494, EPI_ISL_1050495, EPI_ISL_1050496, EPI_ISL_1050497, EPI_ISL_1050499, EPI_ISL_1050500, EPI_ISL_1050501, EPI_ISL_1050503, EPI_ISL_1050504, EPI_ISL_1050505, EPI_ISL_1050506, EPI_ISL_1050507, EPI_ISL_1050509, EPI_ISL_1050512, EPI_ISL_1050513, EPI_ISL_1050514, EPI_ISL_1050515, EPI_ISL_1050518, EPI_ISL_1050522, EPI_ISL_1050523			
see above	University College London, Great Ormond Street Hospital for Children NHS Foundation Trust, Imperial College Healthcare NHS Trust	COVID-19 Genomics UK (COG-UK) Consortium	Sergi Castellano, Rachel Williams, Mark Kristiansen, Paola Resende Silva, Sunando Roy, Tony Brooks, Helena Tutill, Paola Niola, Patricia Dyal, Charlotte Williams, Leysa Forrest, Yasmin Panchbhaya, Jacqueline Findlay, Samuel Weeks, Julianne Brown, Kathryn Harris, Paul Randell, James Price, Alison Holmes, Judith Breuer
EPI_ISL_1051069, EPI_ISL_1051071, EPI_ISL_1051077, EPI_ISL_1051084, EPI_ISL_1051087, EPI_ISL_1051091, EPI_ISL_1051095, EPI_ISL_1051097, EPI_ISL_1051099, EPI_ISL_1051101, EPI_ISL_1051103, EPI_ISL_1051105, EPI_ISL_1051107, EPI_ISL_1051110, EPI_ISL_1051112, EPI_ISL_1051114, EPI_ISL_1051116, EPI_ISL_1051117, EPI_ISL_1051120, EPI_ISL_1051121, EPI_ISL_1051122, EPI_ISL_1051123, EPI_ISL_1051124, EPI_ISL_1051125, EPI_ISL_1051126, EPI_ISL_1051127, EPI_ISL_1051128, EPI_ISL_1051129, EPI_ISL_1051130, EPI_ISL_1051131, EPI_ISL_1051132, EPI_ISL_1051133, EPI_ISL_1051134, EPI_ISL_1051135, EPI_ISL_1051136, EPI_ISL_1051137, EPI_ISL_1051138, EPI_ISL_1051139, EPI_ISL_1051140, EPI_ISL_1051141, EPI_ISL_1051142, EPI_ISL_1051143, EPI_ISL_1051144, EPI_ISL_1051145, EPI_ISL_1051146, EPI_ISL_1051147, EPI_ISL_1051148, EPI_ISL_1051149, EPI_ISL_1051150, EPI_ISL_1051151, EPI_ISL_1051152, EPI_ISL_1051153, EPI_ISL_1051154, EPI_ISL_1051155, EPI_ISL_1051156, EPI_ISL_1051157, EPI_ISL_1051158, EPI_ISL_1051159, EPI_ISL_1051160, EPI_ISL_1051161, EPI_ISL_1051162, EPI_ISL_1051163, EPI_ISL_1051164, EPI_ISL_1051165, EPI_ISL_1051166, EPI_ISL_1051167, EPI_ISL_1051168, EPI_ISL_1051169, EPI_ISL_1051170, EPI_ISL_1051171, EPI_ISL_1051172, EPI_ISL_1051173, EPI_ISL_1051174, EPI_ISL_1051175, EPI_ISL_1051177, EPI_ISL_1051178, EPI_ISL_1051179, EPI_ISL_1051180, EPI_ISL_1051181, EPI_ISL_1051182, EPI_ISL_1051183, EPI_ISL_1051184, EPI_ISL_1051185, EPI_ISL_1051186, EPI_ISL_1051187, EPI_ISL_1051188, EPI_ISL_1051189, EPI_ISL_1051190, EPI_ISL_1051191, EPI_ISL_1051192, EPI_ISL_1051193, EPI_ISL_1051194, EPI_ISL_1051195, EPI_ISL_1051196, EPI_ISL_1051197, EPI_ISL_1051198, EPI_ISL_1051199, EPI_ISL_1051200, EPI_ISL_1051201, EPI_ISL_1051202, EPI_ISL_1051203, EPI_ISL_1051204, EPI_ISL_1051205, EPI_ISL_1051206, EPI_ISL_1051207, EPI_ISL_1051208, EPI_ISL_1051209, EPI_ISL_1051210, EPI_ISL_1051211, EPI_ISL_1051212, EPI_ISL_1051214, EPI_ISL_1051217, EPI_ISL_1051220, EPI_ISL_1051221, EPI_ISL_1051345, EPI_ISL_1051346, EPI_ISL_1051347, EPI_ISL_1051348, EPI_ISL_1051349, EPI_ISL_1051350, EPI_ISL_1051351, EPI_ISL_1051352, EPI_ISL_1051353, EPI_ISL_1051354, EPI_ISL_1051355, EPI_ISL_1051356, EPI_ISL_1051357, EPI_ISL_1051358, EPI_ISL_1051359, EPI_ISL_1051360, EPI_ISL_1051361, EPI_ISL_1051362, EPI_ISL_1051363, EPI_ISL_1051364, EPI_ISL_1051365, EPI_ISL_1051366, EPI_ISL_1051367, EPI_ISL_1051368, EPI_ISL_1051369, EPI_ISL_1051370, EPI_ISL_1051371, EPI_ISL_1051372, EPI_ISL_1051373, EPI_ISL_1051374, EPI_ISL_1051375, EPI_ISL_1051376, EPI_ISL_1051377, EPI_ISL_1051378, EPI_ISL_1051379, EPI_ISL_1051380, EPI_ISL_1051381, EPI_ISL_1051382, EPI_ISL_1051383, EPI_ISL_1051384, EPI_ISL_1051385, EPI_ISL_1051386, EPI_ISL_1051387, EPI_ISL_1051388, EPI_ISL_1051389, EPI_ISL_1051390, EPI_ISL_1051391, EPI_ISL_1051392, EPI_ISL_1051393, EPI_ISL_1051394, EPI_ISL_1051395, EPI_ISL_1051396, EPI_ISL_1051397, EPI_ISL_1051398, EPI_ISL_1051399, EPI_ISL_1051400, EPI_ISL_1051401			
see above	Northumbria University / South Tees Hospitals NHS Foundation Trust / North Cumbria Integrated Care NHS Foundation Trust / North Tees and Hartlepool NHS Foundation Trust / Newcastle Hospitals NHS Foundation Trust	COVID-19 Genomics UK (COG-UK) Consortium	Darren L Smith,Andrew Nelson,Matthew Bashton,Greg R Young,Joshua Loh,John Allan,Mohammad A Tariq,Giles S Holt,Gary Black,Wen C Yew,Lynn Dover,Paul Baker,Steve Liggett,Sarah Essex,Jane Greenaway,Debra Padgett,Clive Graham,Garren Scott,Edward Barton,Emma Swindells,Brendan Payne,Jennifer Collins,Yusri Taha,Gary Eltringham
EPI_ISL_1051450	Quadram Institute Bioscience	COVID-19 Genomics UK (COG-UK) Consortium	Dave J. Baker, Gemma L. Kay, Alp Aydin, Thanh Le-Viet, Steven Rudder, Ana P. Tedim, Anastasia Kolyva, Maria Diaz, Leonardo de Oliveira Martins, Nabil-Fareed Alikhan, Lizzie Meadows, Rachael Stanley, Ngozi Elumogo, Muhammed Yasir, Nicholas M. Thomson, Alexander J Trotter, Rachel Gilroy, Samuel Bloomfield, Claire Stuart, Andrew Bell, Reenesh Prakash, Samir Dervisevic, Alison E. Mather, John Wain, Mark Webber, Andrew J. Page, Justin O'Grady
EPI_ISL_1051789, EPI_ISL_1051791, EPI_ISL_1051792, EPI_ISL_1051810, EPI_ISL_1051811, EPI_ISL_1051812, EPI_ISL_1051828	Oxford Viromics, NDM, University of Oxford; Oxford University Hospitals; Basingstoke and North Hampshire Hospital	COVID-19 Genomics UK (COG-UK) Consortium	Tanya Golubchik, David Bonsall, George Macintyre, Amy Trebes, Mariateresa de Cesare, Catrin Moore, Alex Mobbs, Anita Justice, Robert Shaw, Monique Andersson, Timothy Peto, Emma Wise, Nathan Moore, Jessica Lynch, Nick Cortes, Matilde Mori, Stephen Kidd, David Buck, John Todd, Christophe Fraser
EPI_ISL_1054779, EPI_ISL_1054824	Bioinformatics and Biostatistics Lab, Advanced Sequencing Facility	COVID-19 Genomics UK (COG-UK) Consortium	Aengus Stewart,Jerome Nicod,Chelsea Sawyer,Laura Cubitt,Harshil Patel,Margaret Crawford
EPI_ISL_1103806	University of Exeter	COVID-19 Genomics UK (COG-UK) Consortium	Ben Temperton,Aaron Jeffries,Michelle Michelsen,Joanna Warwick-Dugdale,Audrey Farbos,Robyn Manley,Stephen Michell,Jane Masoli
EPI_ISL_1104197, EPI_ISL_1104220, EPI_ISL_1104221, EPI_ISL_1104231, EPI_ISL_1104235, EPI_ISL_1104236, EPI_ISL_1104242, EPI_ISL_1104243	Virology Department, Royal Infirmary of Edinburgh, NHS Lothian / School of Biological Sciences, University of Edinburgh	COVID-19 Genomics UK (COG-UK) Consortium	McHugh M, Dewar R, Cotton S, Rooke S, O'Toole Á, Scher E, Hill V, McCrone JT, Colquhoun R, Yu X, Jackson B, Rambaut A, Templeton K
EPI_ISL_1104305	University Hospitals Of Leicester NHS Trust and DeepSeq Nottingham	COVID-19 Genomics UK (COG-UK) Consortium	Christopher Holmes, Paul Bird, Thomas Helmer, Karlie Fallon, Julian Tang, Jonathan Ball, Patrick McClure, Joeseph Chappell, Nadine Holmes, Matthew Carlisle, Christopher Moore, Fei Sang, Johnny Debebe, Victoria Wright, Matthew Loose

EPI_ISL_1104346, EPI_ISL_1104347, EPI_ISL_1104348, EPI_ISL_1104359, EPI_ISL_1104360, EPI_ISL_1104361, EPI_ISL_1104380, EPI_ISL_1104381, EPI_ISL_1104382, EPI_ISL_1104383, EPI_ISL_1104384			
see above	Liverpool Clinical Laboratories	COVID-19 Genomics UK (COG-UK) Consortium	Sam Haldenby, Anita Lucaci, Steve Paterson, Julian Hiscox, Alistair Darby, M Almsaud, A Alrezaihi, Muhannad Alruwaili, Stuart D Armstrong, Jones Benjamin, Eleanor G Bentley, Anu Chawla, Jordan J Clark, Angela Cowell, Richard Eccles, Isabel García-Dorival, Matthew Gemmell, Alessandro Gerada, PKF Gilmore, Richard Gregory, Ximeng Han, Catherine Hartley, Margaret Hughes, Miren Iturriza-Gomara, James Johnson, L Luu, Jenifer Manson, Charlotte Nelson, Elaine O'Toole, Cassie Olateju, Rebekah Penrice-Randal , Lucille Rainbow, N.P Randle, Trevor Ian Robinson, Parul Sharma, Ghada T Shawli, James P Stewart, Neil Swainston, Ecaterina Vamos, Joanne Watts, Mark Whitehead
EPI_ISL_1105184, EPI_ISL_1105258, EPI_ISL_1105265, EPI_ISL_1105284, EPI_ISL_1105297, EPI_ISL_1105303	University College London Hospital	COVID-19 Genomics UK (COG-UK) Consortium	Judith Heaney, Matthew Byott, Catherine Houlihan, Dan Frampton, Stuart Kirk, Moira Spyer and Eleni Nastouli
EPI_ISL_1105658, EPI_ISL_1105659, EPI_ISL_1105660, EPI_ISL_1105661, EPI_ISL_1105662, EPI_ISL_1105663, EPI_ISL_1105664, EPI_ISL_1105665, EPI_ISL_1105666, EPI_ISL_1105667, EPI_ISL_1105668, EPI_ISL_1105669, EPI_ISL_1105670, EPI_ISL_1105671, EPI_ISL_1105672, EPI_ISL_1105673, EPI_ISL_1105674, EPI_ISL_1105675, EPI_ISL_1105676, EPI_ISL_1105677, EPI_ISL_1105678, EPI_ISL_1105679, EPI_ISL_1105680, EPI_ISL_1105681, EPI_ISL_1105682, EPI_ISL_1105683, EPI_ISL_1105684, EPI_ISL_1105685, EPI_ISL_1105686, EPI_ISL_1105687, EPI_ISL_1105688, EPI_ISL_1105689, EPI_ISL_1105690, EPI_ISL_1105691, EPI_ISL_1105692, EPI_ISL_1105693, EPI_ISL_1105694, EPI_ISL_1105695, EPI_ISL_1105696, EPI_ISL_1105697, EPI_ISL_1105698, EPI_ISL_1105699, EPI_ISL_1105700, EPI_ISL_1105701, EPI_ISL_1105702, EPI_ISL_1105703, EPI_ISL_1105704, EPI_ISL_1105705, EPI_ISL_1105707			
see above	Northumbria University / South Tees Hospitals NHS Foundation Trust / North Cumbria Integrated Care NHS Foundation Trust / North Tees and Hartlepool NHS Foundation Trust / Newcastle Hospitals NHS Foundation Trust	COVID-19 Genomics UK (COG-UK) Consortium	Darren L Smith,Andrew Nelson,Matthew Bashton,Greg R Young,Joshua Loh,John Allan,Mohammad A Tariq,Giles S Holt,Gary Black,Wen C Yew,Lynn Dover,Paul Baker,Steve Liggett,Sarah Essex,Jane Greenaway,Debra Padgett,Clive Graham,Garren Scott,Edward Barton,Emma Swindells,Brendan Payne,Jennifer Collins,Yusri Taha,Gary Eltringham
EPI_ISL_1107587, EPI_ISL_1107631	Centre for Enzyme Innovation, University of Portsmouth / Translational Research Laboratory, Portsmouth Hospitals NHS Trust	COVID-19 Genomics UK (COG-UK) Consortium	Angela Beckett,Salman Goudarzi,Christopher Fearn,Kate Cook,Katie Loveson,Sharon Glaysher,Scott Elliott,Samuel Robson
EPI_ISL_1177839, EPI_ISL_1177840, EPI_ISL_1177841, EPI_ISL_1177843, EPI_ISL_1177844, EPI_ISL_1177845, EPI_ISL_1177846, EPI_ISL_1177847, EPI_ISL_1177850, EPI_ISL_1177851, EPI_ISL_1177852, EPI_ISL_1177854, EPI_ISL_1177856, EPI_ISL_1177857, EPI_ISL_1177858, EPI_ISL_1177859, EPI_ISL_1177860, EPI_ISL_1177861, EPI_ISL_1177862, EPI_ISL_1177863, EPI_ISL_1177864, EPI_ISL_1177865, EPI_ISL_1177866, EPI_ISL_1177867, EPI_ISL_1177868, EPI_ISL_1177869, EPI_ISL_1177870, EPI_ISL_1177871, EPI_ISL_1177872, EPI_ISL_1177874, EPI_ISL_1177875, EPI_ISL_1177876, EPI_ISL_1177877			
see above	Liverpool Clinical Laboratories	COVID-19 Genomics UK (COG-UK) Consortium	Sam Haldenby, Alistair Darby, Steve Paterson, Anita Lucaci, Julian Hiscox, M Almsaud, A Alrezaihi, Muhannad Alruwaili, Stuart D Armstrong, Jones Benjamin, Eleanor G Bentley, Anu Chawla, Jordan J Clark, Angela Cowell, Richard Eccles, Isabel García-Dorival, Matthew Gemmell, Alessandro Gerada, PKF Gilmore, Richard Gregory, Ximeng Han, Catherine Hartley, Margaret Hughes, Miren Iturriza-Gomara, James Johnson, L Luu, Jenifer Manson, Charlotte Nelson, Elaine O'Toole, Cassie Olateju, Rebekah Penrice-Randal , Lucille Rainbow, N.P Randle, Trevor Ian Robinson, Parul Sharma, Ghada T Shawli, James P Stewart, Neil Swainston, Ecaterina Vamos, Joanne Watts, Mark Whitehead, Hermione Webster
EPI_ISL_1178326, EPI_ISL_1178327, EPI_ISL_1178328, EPI_ISL_1178329, EPI_ISL_1178330, EPI_ISL_1178331, EPI_ISL_1178332, EPI_ISL_1178333, EPI_ISL_1178336, EPI_ISL_1178337, EPI_ISL_1178338, EPI_ISL_1178348, EPI_ISL_1178353, EPI_ISL_1178356, EPI_ISL_1178357, EPI_ISL_1178359, EPI_ISL_1178361, EPI_ISL_1178362, EPI_ISL_1178363, EPI_ISL_1178364, EPI_ISL_1178365, EPI_ISL_1178366, EPI_ISL_1178367, EPI_ISL_1178368, EPI_ISL_1178369, EPI_ISL_1178370, EPI_ISL_1178371, EPI_ISL_1178372, EPI_ISL_1178373, EPI_ISL_1178374, EPI_ISL_1178375, EPI_ISL_1178376, EPI_ISL_1178378, EPI_ISL_1178379, EPI_ISL_1178380, EPI_ISL_1178381, EPI_ISL_1178382, EPI_ISL_1178383, EPI_ISL_1178384, EPI_ISL_1178385, EPI_ISL_1178386, EPI_ISL_1178387, EPI_ISL_1178388, EPI_ISL_1178389, EPI_ISL_1178390, EPI_ISL_1178391, EPI_ISL_1178392, EPI_ISL_1178393, EPI_ISL_1178394, EPI_ISL_1178395, EPI_ISL_1178396, EPI_ISL_1178397, EPI_ISL_1178398, EPI_ISL_1178400, EPI_ISL_1178401, EPI_ISL_1178402, EPI_ISL_1178403, EPI_ISL_1178404, EPI_ISL_1178405, EPI_ISL_1178406, EPI_ISL_1178417, EPI_ISL_1178418, EPI_ISL_1178419, EPI_ISL_1178420, EPI_ISL_1178421			
see above	Northumbria University / South Tees Hospitals NHS Foundation Trust / North Cumbria Integrated Care NHS Foundation Trust / North Tees and Hartlepool NHS Foundation Trust / Newcastle Hospitals NHS Foundation Trust	COVID-19 Genomics UK (COG-UK) Consortium	Darren L Smith,Andrew Nelson,Matthew Bashton,Greg R Young,Joshua Loh,John Allan,Mohammad A Tariq,Giles S Holt,Gary Black,Wen C Yew,Lynn Dover,Paul Baker,Steve Liggett,Sarah Essex,Jane Greenaway,Debra Padgett,Clive Graham,Garren Scott,Edward Barton,Emma Swindells,Brendan Payne,Jennifer Collins,Yusri Taha,Gary Eltringham
EPI_ISL_1178430, EPI_ISL_1178431, EPI_ISL_1178432, EPI_ISL_1178442, EPI_ISL_1178443, EPI_ISL_1178446, EPI_ISL_1178454, EPI_ISL_1178476	Quadram Institute Bioscience	COVID-19 Genomics UK (COG-UK) Consortium	Dave J. Baker, Gemma L. Kay, Alp Aydin, Thanh Le-Viet, Steven Rudder, Ana P. Tedim, Anastasia Kolyva, Maria Diaz, Leonardo de Oliveira Martins, Nabil-Fareed Alikhan, Lizzie Meadows, Rachael Stanley, Ngozi Elumogo, Muhammed Yasir, Nicholas M. Thomson, Alexander J Trotter, Rachel Gilroy, Samuel Bloomfield, Claire Stuart, Andrew Bell, Reenesh Prakash, Samir Dervisevic, Alison E. Mather, John Wain, Mark Webber, Andrew J. Page, Justin O'Grady
EPI_ISL_1178721, EPI_ISL_1178822, EPI_ISL_1178847	Oxford Viromics, NDM, University of Oxford; Oxford University Hospitals; Basingstoke and North Hampshire Hospital	COVID-19 Genomics UK (COG-UK) Consortium	Tanya Golubchik, David Bonsall, George Macintyre, Amy Trebes, Mariateresa de Cesare, Catrin Moore, Alex Mobbs, Anita Justice, Robert Shaw, Monique Andersson, Timothy Peto, Emma Wise, Nathan Moore, Jessica Lynch, Nick Cortes, Matilde Mori, Stephen Kidd, David Buck, John Todd, Christophe Fraser
EPI_ISL_1179796, EPI_ISL_1179822, EPI_ISL_1179823, EPI_ISL_1179830, EPI_ISL_1179838	Centre for Enzyme Innovation, University of Portsmouth / Translational Research Laboratory, Portsmouth Hospitals NHS Trust	COVID-19 Genomics UK (COG-UK) Consortium	Angela Beckett,Salman Goudarzi,Christopher Fearn,Kate Cook,Katie Loveson,Sharon Glaysher,Scott Elliott,Samuel Robson
EPI_ISL_1180108, EPI_ISL_1180126	Bioinformatics and Biostatistics Lab, Advanced Sequencing Facility	COVID-19 Genomics UK (COG-UK) Consortium	Aengus Stewart,Jerome Nicod,Chelsea Sawyer,Laura Cubitt,Harshil Patel,Margaret Crawford
EPI_ISL_1247594	Department of Pathology, University of Cambridge	COVID-19 Genomics UK (COG-UK) Consortium	Aminu S. Jahun, Yasmin Chaudhry, Iliana Georgana, Myra Hosmillo, Rhys Izuagbe, William L. Hamilton, Martin D. Curran, Surendra Parmar, Ian Goodfellow
EPI_ISL_1248057, EPI_ISL_1248058, EPI_ISL_1248059, EPI_ISL_1248060, EPI_ISL_1248061, EPI_ISL_1248062, EPI_ISL_1248063, EPI_ISL_1248064, EPI_ISL_1248065, EPI_ISL_1248068, EPI_ISL_1248069, EPI_ISL_1248070, EPI_ISL_1248071, EPI_ISL_1248072, EPI_ISL_1248073, EPI_ISL_1248074, EPI_ISL_1248075, EPI_ISL_1248076, EPI_ISL_1248077, EPI_ISL_1248078, EPI_ISL_1248079, EPI_ISL_1248080, EPI_ISL_1248081, EPI_ISL_1248082, EPI_ISL_1248083, EPI_ISL_1248084, EPI_ISL_1248085, EPI_ISL_1248086, EPI_ISL_1248087, EPI_ISL_1248088, EPI_ISL_1248089, EPI_ISL_1248091, EPI_ISL_1248092, EPI_ISL_1248093, EPI_ISL_1248094, EPI_ISL_1248095, EPI_ISL_1248096, EPI_ISL_1248097, EPI_ISL_1248098, EPI_ISL_1248099, EPI_ISL_1248100, EPI_ISL_1248101, EPI_ISL_1248102, EPI_ISL_1248103, EPI_ISL_1248104, EPI_ISL_1248105, EPI_ISL_1248106, EPI_ISL_1248113, EPI_ISL_1248117, EPI_ISL_1248118, EPI_ISL_1248119, EPI_ISL_1248120, EPI_ISL_1248121			
see above	University College London, Great Ormond Street Hospital for Children NHS Foundation Trust, Imperial College Healthcare NHS Trust	COVID-19 Genomics UK (COG-UK) Consortium	Sergi Castellano, Rachel Williams, Mark Kristiansen, Paola Resende Silva, Sunando Roy, Tony Brooks, Helena Tutill, Paola Niola, Patricia Dyal, Charlotte Williams, Leysa Forrest, Yasmin Panchbhaya, Jacqueline Findlay, Samuel Weeks, Julianne Brown, Kathryn Harris, Paul Randell, James Price, Alison Holmes, Judith Breuer
EPI_ISL_1248374, EPI_ISL_1248375, EPI_ISL_1248384, EPI_ISL_1248385, EPI_ISL_1248389, EPI_ISL_1248395, EPI_ISL_1248396, EPI_ISL_1248397, EPI_ISL_1248407, EPI_ISL_1248408, EPI_ISL_1248409, EPI_ISL_1248418, EPI_ISL_1248419, EPI_ISL_1248428, EPI_ISL_1248429, EPI_ISL_1248440, EPI_ISL_1248441, EPI_ISL_1248467, EPI_ISL_1248487, EPI_ISL_1248495			
see above	University College London Hospital	COVID-19 Genomics UK (COG-UK) Consortium	Dr Judith Heaney, Matthew Byott, Dr Catherine Houlihan, Dr Daniel Frampton, Stuart Kirk, Dr Moira Spyer, Dr Paul Grant and Dr Eleni Nastouli
EPI_ISL_1249006, EPI_ISL_1249055, EPI_ISL_1249059	Quadram Institute Bioscience	COVID-19 Genomics UK (COG-UK) Consortium	Dave J. Baker, Gemma L. Kay, Alp Aydin, Thanh Le-Viet, Steven Rudder, Ana P. Tedim, Anastasia Kolyva, Maria Diaz, Leonardo de Oliveira Martins, Nabil-Fareed Alikhan, Lizzie Meadows, Rachael Stanley, Ngozi Elumogo, Muhammed Yasir, Nicholas M. Thomson, Alexander J Trotter, Rachel Gilroy, Samuel Bloomfield, Claire Stuart, Andrew Bell, Reenesh Prakash, Samir Dervisevic, Alison E. Mather, John Wain, Mark Webber, Andrew J. Page, Justin O'Grady
EPI_ISL_1249226	Oxford Viromics, NDM, University of Oxford; Oxford University Hospitals; Basingstoke and North Hampshire Hospital	COVID-19 Genomics UK (COG-UK) Consortium	Tanya Golubchik, David Bonsall, George Macintyre, Amy Trebes, Mariateresa de Cesare, Catrin Moore, Alex Mobbs, Anita Justice, Robert Shaw, Monique Andersson, Timothy Peto, Emma Wise, Nathan Moore, Jessica Lynch, Nick Cortes, Matilde Mori, Stephen Kidd, David Buck, John Todd, Christophe Fraser
EPI_ISL_1249959	Centre for Enzyme Innovation, University of Portsmouth / Translational Research Laboratory, Portsmouth Hospitals NHS Trust	COVID-19 Genomics UK (COG-UK) Consortium	Angela Beckett,Salman Goudarzi,Christopher Fearn,Kate Cook,Katie Loveson,Sharon Glaysher,Scott Elliott,Samuel Robson
EPI_ISL_1296505, EPI_ISL_1296506, EPI_ISL_1296507, EPI_ISL_1296509, EPI_ISL_1296510, EPI_ISL_1296511, EPI_ISL_1296512, EPI_ISL_1296513, EPI_ISL_1296514, EPI_ISL_1296515, EPI_ISL_1296516, EPI_ISL_1296517, EPI_ISL_1296518, EPI_ISL_1296519, EPI_ISL_1296520, EPI_ISL_1296521, EPI_ISL_1296522, EPI_ISL_1296523, EPI_ISL_1296525, EPI_ISL_1296526, EPI_ISL_1296527, EPI_ISL_1296530, EPI_ISL_1296531, EPI_ISL_1296532, EPI_ISL_1296536, EPI_ISL_1296537, EPI_ISL_1296549			
see above	Respiratory Virus Unit, National Infection Service, Public Health England	COVID-19 Genomics UK (COG-UK) Consortium	PHE Covid Sequencing Team
EPI_ISL_1308583, EPI_ISL_1308584	University of Exeter	COVID-19 Genomics UK (COG-UK) Consortium	Ben Temperton,Aaron Jeffries,Michelle Michelsen,Joanna Warwick-Dugdale,Audrey Farbos,Robyn Manley,Stephen Michell,Jane Masoli
EPI_ISL_1308703, EPI_ISL_1308704, EPI_ISL_1308705, EPI_ISL_1308838, EPI_ISL_1308839, EPI_ISL_1308842, EPI_ISL_1308843	Virology Department, Royal Infirmary of Edinburgh, NHS Lothian / School of Biological Sciences, University of Edinburgh	COVID-19 Genomics UK (COG-UK) Consortium	McHugh M, Dewar R, Cotton S, Rooke S, O'Toole Á, Scher E, Hill V, McCrone JT, Colquhoun R, Yu X, Jackson B, Rambaut A, Templeton K

EPI_ISL_1309037, EPI_ISL_1309045, EPI_ISL_1309050, EPI_ISL_1309057, EPI_ISL_1309064, EPI_ISL_1309065, EPI_ISL_1309069, EPI_ISL_1309075, EPI_ISL_1309083, EPI_ISL_1309086, EPI_ISL_1309087, EPI_ISL_1309088, EPI_ISL_1309098, EPI_ISL_1309102, EPI_ISL_1309107, EPI_ISL_1309111			
see above	University College London, Great Ormond Street Hospital for Children NHS Foundation Trust, Imperial College Healthcare NHS Trust	COVID-19 Genomics UK (COG-UK) Consortium	Sergi Castellano, Rachel Williams, Mark Kristiansen, Paola Resende Silva, Sunando Roy, Tony Brooks, Helena Tutill, Paola Niola, Patricia Dyal, Charlotte Williams, Leysa Forrest, Yasmin Panchbhaya, Jacqueline Findlay, Samuel Weeks, Julianne Brown, Kathryn Harris, Paul Randell, James Price, Alison Holmes, Judith Breuer
EPI_ISL_1309745, EPI_ISL_1309746, EPI_ISL_1309747	Oxford Viromics, NDM, University of Oxford; Oxford University Hospitals; Basingstoke and North Hampshire Hospital	COVID-19 Genomics UK (COG-UK) Consortium	Tanya Golubchik, David Bonsall, George Macintyre, Amy Trebes, Mariateresa de Cesare, Catrin Moore, Alex Mobbs, Anita Justice, Robert Shaw, Monique Andersson, Timothy Peto, Emma Wise, Nathan Moore, Jessica Lynch, Nick Cortes, Matilde Mori, Stephen Kidd, David Buck, John Todd, Christophe Fraser
EPI_ISL_1346482	Lighthouse Lab in Alderley Park	Wellcome Sanger Institute for the COVID-19 Genomics UK (COG-UK) Consortium	Jacquelyn Wynn, Mairead Hyland, The Lighthouse Lab in Alderley Park and Alex Alderton, Roberto Amato, Jeffrey Barrett, Sonia Goncalves, Ewan Harrison, David K. Jackson, Ian Johnston, Dominic Kwiatkowski, Cordelia Langford, John Sillitoe on behalf of the Wellcome Sanger Institute COVID-19 Surveillance Team
EPI_ISL_1346483	Lighthouse Lab in Milton Keynes	Wellcome Sanger Institute for the COVID-19 Genomics UK (COG-UK) Consortium	The Lighthouse Lab in Milton Keynes and Alex Alderton, Roberto Amato, Jeffrey Barrett, Sonia Goncalves, Ewan Harrison, David K. Jackson, Ian Johnston, Dominic Kwiatkowski, Cordelia Langford, John Sillitoe on behalf of the Wellcome Sanger Institute COVID-19 Surveillance Team
EPI_ISL_1386858, EPI_ISL_1386876, EPI_ISL_1386881, EPI_ISL_1386886, EPI_ISL_1386889, EPI_ISL_1386896, EPI_ISL_1386912, EPI_ISL_1386913	University College London, Great Ormond Street Hospital for Children NHS Foundation Trust, Imperial College Healthcare NHS Trust	COVID-19 Genomics UK (COG-UK) Consortium	Sergi Castellano, Rachel Williams, Mark Kristiansen, Paola Resende Silva, Sunando Roy, Tony Brooks, Helena Tutill, Paola Niola, Patricia Dyal, Charlotte Williams, Leysa Forrest, Yasmin Panchbhaya, Jacqueline Findlay, Samuel Weeks, Julianne Brown, Kathryn Harris, Paul Randell, James Price, Alison Holmes, Judith Breuer
EPI_ISL_1387095	Northumbria University / South Tees Hospitals NHS Foundation Trust / North Cumbria Integrated Care NHS Foundation Trust / North Tees and Hartlepool NHS Foundation Trust / Newcastle Hospitals NHS Foundation Trust	COVID-19 Genomics UK (COG-UK) Consortium	Darren L Smith,Andrew Nelson,Matthew Bashton,Greg R Young,Joshua Loh,John Allan,Mohammad A Tariq,Giles S Holt,Gary Black,Wen C Yew,Lynn Dover,Paul Baker,Steve Liggett,Sarah Essex,Jane Greenaway,Debra Padgett,Clive Graham,Garren Scott,Edward Barton,Emma Swindells,Brendan Payne,Jennifer Collins,Yusri Taha,Gary Eltringham
EPI_ISL_1474961, EPI_ISL_1474962, EPI_ISL_1474965, EPI_ISL_1474966, EPI_ISL_1474967, EPI_ISL_1474968, EPI_ISL_1474969, EPI_ISL_1474970, EPI_ISL_1474971, EPI_ISL_1474972, EPI_ISL_1474974, EPI_ISL_1474975, EPI_ISL_1474976, EPI_ISL_1474977, EPI_ISL_1474978, EPI_ISL_1474979, EPI_ISL_1474980, EPI_ISL_1474981, EPI_ISL_1474982, EPI_ISL_1474984, EPI_ISL_1474985, EPI_ISL_1474991, EPI_ISL_1474992, EPI_ISL_1474993, EPI_ISL_1474994, EPI_ISL_1475004, EPI_ISL_1475005, EPI_ISL_1475006, EPI_ISL_1475082			
see above	Regional Virus Laboratory, Belfast Health and Social Care Trust	COVID-19 Genomics UK (COG-UK) Consortium	Conall McCaughey, James McKenna, Tanya Curran, Susan Feeney, Alison Watt, Ciara Cox, Mairead Connor, Zoltan Molnar, David Simpson, Derek Fairley
EPI_ISL_1476085, EPI_ISL_1476095, EPI_ISL_1476101, EPI_ISL_1476236, EPI_ISL_1476237, EPI_ISL_1476238, EPI_ISL_1476239, EPI_ISL_1476240, EPI_ISL_1476241, EPI_ISL_1476242, EPI_ISL_1476247, EPI_ISL_1476522, EPI_ISL_1476523, EPI_ISL_1476524, EPI_ISL_1476525, EPI_ISL_1476527, EPI_ISL_1476534, EPI_ISL_1476535, EPI_ISL_1476540, EPI_ISL_1476544, EPI_ISL_1476545, EPI_ISL_1476546, EPI_ISL_1476548			
see above	Originating lab: Wales Specialist Virology Centre Sequencing lab: Pathogen Genomics Unit	Public Health Wales Microbiology Cardiff Wales Specialist Virology Centre	Catherine Moore, Johnathan Evans, Laura Gifford, Malorie Perry, Simon Cottrell, Angela Marchbank, Alec Birchley, Alexander Adams, Amy Gaskin, Bree Gatica-Wilcox, Jason Coombes, Joel Southgate, Lauren Gilbert, Lee Graham, Nicole Pacchiarini, Sara Kumziene-Summerhayes, Sarah Taylor, Sophie Jones, Sara Rey, Matthew Bull, Joanne Watkins, Sally Corden, Tom Connor
EPI_ISL_1476665	Centre for Enzyme Innovation, University of Portsmouth / Translational Research Laboratory, Portsmouth Hospitals NHS Trust	COVID-19 Genomics UK (COG-UK) Consortium	Angela Beckett,Salman Goudarzi,Christopher Fearn,Kate Cook,Katie Loveson,Sharon Glaysher,Scott Elliott,Samuel Robson
EPI_ISL_804369, EPI_ISL_811123, EPI_ISL_811124, EPI_ISL_811125, EPI_ISL_811126, EPI_ISL_811150, EPI_ISL_825491, EPI_ISL_825550, EPI_ISL_825551, EPI_ISL_825573, EPI_ISL_825574, EPI_ISL_825616, EPI_ISL_826282			
see above	Respiratory Virus Unit, National Infection Service, Public Health England	COVID-19 Genomics UK (COG-UK) Consortium	PHE Covid Sequencing Team
EPI_ISL_833585, EPI_ISL_833587, EPI_ISL_833588, EPI_ISL_833589, EPI_ISL_833591, EPI_ISL_833592, EPI_ISL_833594, EPI_ISL_833595, EPI_ISL_833598, EPI_ISL_833600, EPI_ISL_833601, EPI_ISL_833603, EPI_ISL_833605, EPI_ISL_833608, EPI_ISL_833609, EPI_ISL_833610, EPI_ISL_833613, EPI_ISL_833616, EPI_ISL_833622, EPI_ISL_833623, EPI_ISL_833624, EPI_ISL_833625, EPI_ISL_833627, EPI_ISL_833628, EPI_ISL_833633, EPI_ISL_833635, EPI_ISL_833636, EPI_ISL_833637, EPI_ISL_833638, EPI_ISL_833639, EPI_ISL_833640, EPI_ISL_833641, EPI_ISL_833642, EPI_ISL_833644, EPI_ISL_833645, EPI_ISL_833646, EPI_ISL_833649, EPI_ISL_833650, EPI_ISL_833651, EPI_ISL_833653, EPI_ISL_833655, EPI_ISL_833657, EPI_ISL_833659, EPI_ISL_833660, EPI_ISL_833661, EPI_ISL_833663, EPI_ISL_833664, EPI_ISL_833665, EPI_ISL_833666, EPI_ISL_833668, EPI_ISL_833669, EPI_ISL_833670, EPI_ISL_833672, EPI_ISL_833673, EPI_ISL_833674, EPI_ISL_833675, EPI_ISL_833677, EPI_ISL_833681, EPI_ISL_833682, EPI_ISL_833684, EPI_ISL_833686, EPI_ISL_833687, EPI_ISL_833688, EPI_ISL_833689, EPI_ISL_833690, EPI_ISL_833691, EPI_ISL_833692, EPI_ISL_833694, EPI_ISL_833695, EPI_ISL_833696, EPI_ISL_833697, EPI_ISL_833698, EPI_ISL_833699, EPI_ISL_833701, EPI_ISL_833702, EPI_ISL_833703, EPI_ISL_833704, EPI_ISL_833705, EPI_ISL_833706, EPI_ISL_833708, EPI_ISL_833709, EPI_ISL_833710, EPI_ISL_833711, EPI_ISL_833713, EPI_ISL_833714, EPI_ISL_833715, EPI_ISL_833717, EPI_ISL_833718, EPI_ISL_833720, EPI_ISL_833722, EPI_ISL_833723, EPI_ISL_833724, EPI_ISL_833727, EPI_ISL_833728, EPI_ISL_833729, EPI_ISL_833730, EPI_ISL_833731, EPI_ISL_833732, EPI_ISL_833733, EPI_ISL_833737, EPI_ISL_833739, EPI_ISL_833740, EPI_ISL_833741, EPI_ISL_833742, EPI_ISL_833744, EPI_ISL_833745, EPI_ISL_833747, EPI_ISL_833748, EPI_ISL_833749, EPI_ISL_833750, EPI_ISL_833751, EPI_ISL_833752, EPI_ISL_833753, EPI_ISL_833754, EPI_ISL_833757, EPI_ISL_833758, EPI_ISL_833760, EPI_ISL_833761, EPI_ISL_833762, EPI_ISL_833763, EPI_ISL_833764, EPI_ISL_833765, EPI_ISL_833769, EPI_ISL_833771, EPI_ISL_833772, EPI_ISL_833773, EPI_ISL_833774, EPI_ISL_833775, EPI_ISL_833776, EPI_ISL_833777, EPI_ISL_833778, EPI_ISL_833781, EPI_ISL_833782, EPI_ISL_833783, EPI_ISL_833784, EPI_ISL_833785, EPI_ISL_833786, EPI_ISL_833787, EPI_ISL_833788, EPI_ISL_833790, EPI_ISL_833792, EPI_ISL_833793, EPI_ISL_833794, EPI_ISL_833798, EPI_ISL_833799, EPI_ISL_833800, EPI_ISL_833802, EPI_ISL_833805, EPI_ISL_833806, EPI_ISL_833807, EPI_ISL_833808, EPI_ISL_833810, EPI_ISL_833811, EPI_ISL_833812, EPI_ISL_833813, EPI_ISL_833815, EPI_ISL_833816, EPI_ISL_833819, EPI_ISL_833820, EPI_ISL_833822, EPI_ISL_833824, EPI_ISL_833825, EPI_ISL_833826, EPI_ISL_833827, EPI_ISL_833828, EPI_ISL_833829, EPI_ISL_833831, EPI_ISL_833832, EPI_ISL_833836, EPI_ISL_833837, EPI_ISL_833838, EPI_ISL_833839, EPI_ISL_833840, EPI_ISL_833843, EPI_ISL_833847, EPI_ISL_833850, EPI_ISL_833851, EPI_ISL_833852, EPI_ISL_833853, EPI_ISL_833854, EPI_ISL_833857, EPI_ISL_833858, EPI_ISL_833862, EPI_ISL_833863, EPI_ISL_833864, EPI_ISL_833866, EPI_ISL_833867, EPI_ISL_833869, EPI_ISL_833870, EPI_ISL_833871, EPI_ISL_833873, EPI_ISL_833874, EPI_ISL_833875, EPI_ISL_833876, EPI_ISL_833877, EPI_ISL_833878, EPI_ISL_833880, EPI_ISL_833882, EPI_ISL_833883, EPI_ISL_833885, EPI_ISL_833886, EPI_ISL_833887, EPI_ISL_833888, EPI_ISL_833891, EPI_ISL_833892, EPI_ISL_833893, EPI_ISL_833897, EPI_ISL_833898, EPI_ISL_833899, EPI_ISL_833900, EPI_ISL_833902, EPI_ISL_833904, EPI_ISL_833905, EPI_ISL_833906, EPI_ISL_833907, EPI_ISL_833911, EPI_ISL_833912, EPI_ISL_833913, EPI_ISL_833914, EPI_ISL_833915, EPI_ISL_833917, EPI_ISL_833918, EPI_ISL_833919, EPI_ISL_833920, EPI_ISL_833921, EPI_ISL_833923, EPI_ISL_833925, EPI_ISL_833926, EPI_ISL_833927, EPI_ISL_833928, EPI_ISL_833929, EPI_ISL_833932			
see above	Lighthouse Lab in Cambridge	Wellcome Sanger Institute for the COVID-19 Genomics UK (COG-UK) Consortium	Rob Howes, The Lighthouse Lab in Cambridge and Alex Alderton, Roberto Amato, Sonia Goncalves, Ewan Harrison, David K. Jackson, Ian Johnston, Dominic Kwiatkowski, Cordelia Langford, John Sillitoe on behalf of the Wellcome Sanger Institute COVID-19 Surveillance Team
EPI_ISL_834150, EPI_ISL_835041	Lighthouse Lab in Alderley Park	Wellcome Sanger Institute for the COVID-19 Genomics UK (COG-UK) Consortium	Jacquelyn Wynn, Mairead Hyland, The Lighthouse Lab in Alderley Park and Alex Alderton, Roberto Amato, Sonia Goncalves, Ewan Harrison, David K. Jackson, Ian Johnston, Dominic Kwiatkowski, Cordelia Langford, John Sillitoe on behalf of the Wellcome Sanger Institute COVID-19 Surveillance Team
EPI_ISL_837055, EPI_ISL_837056, EPI_ISL_837057, EPI_ISL_837059, EPI_ISL_837060, EPI_ISL_837069, EPI_ISL_837176, EPI_ISL_837177, EPI_ISL_837178, EPI_ISL_837179, EPI_ISL_837180, EPI_ISL_837181, EPI_ISL_837182, EPI_ISL_837243, EPI_ISL_837244			
see above	Respiratory Virus Unit, National Infection Service, Public Health England	COVID-19 Genomics UK (COG-UK) Consortium	PHE Covid Sequencing Team
EPI_ISL_837957, EPI_ISL_837959, EPI_ISL_837961, EPI_ISL_837973, EPI_ISL_837974, EPI_ISL_837975, EPI_ISL_837976, EPI_ISL_837977, EPI_ISL_837978, EPI_ISL_837979, EPI_ISL_837980, EPI_ISL_837982, EPI_ISL_837983, EPI_ISL_837984, EPI_ISL_837985, EPI_ISL_837986, EPI_ISL_837987, EPI_ISL_837988, EPI_ISL_837990, EPI_ISL_837991, EPI_ISL_837993, EPI_ISL_837997, EPI_ISL_838000, EPI_ISL_838001, EPI_ISL_838003, EPI_ISL_838004, EPI_ISL_838005, EPI_ISL_838006, EPI_ISL_838007, EPI_ISL_838008, EPI_ISL_838009, EPI_ISL_838010, EPI_ISL_838011, EPI_ISL_838012, EPI_ISL_838013, EPI_ISL_838014, EPI_ISL_838016, EPI_ISL_838019, EPI_ISL_838020, EPI_ISL_838023, EPI_ISL_838024, EPI_ISL_838026, EPI_ISL_838027, EPI_ISL_838028, EPI_ISL_838029, EPI_ISL_838030, EPI_ISL_838031, EPI_ISL_838032, EPI_ISL_838033, EPI_ISL_838036, EPI_ISL_838039, EPI_ISL_838040, EPI_ISL_838041, EPI_ISL_838042, EPI_ISL_838043, EPI_ISL_838044, EPI_ISL_838045, EPI_ISL_838046, EPI_ISL_838047, EPI_ISL_838049, EPI_ISL_838050, EPI_ISL_838051, EPI_ISL_838052, EPI_ISL_838053, EPI_ISL_838054			
see above	Department of Pathology, University of Cambridge	COVID-19 Genomics UK (COG-UK) Consortium	Aminu S. Jahun, Yasmin Chaudhry, Grant Hall, Iliana Georgana, Myra Hosmillo, Martin D. Curran, Malte Pinckert, Surendra Parmar, Ian Goodfellow
EPI_ISL_838315	University of Exeter	COVID-19 Genomics UK (COG-UK) Consortium	Ben Temperton,Aaron Jeffries,Michelle Michelsen,Joanna Warwick-Dugdale,Audrey Farbos,Robyn Manley,Stephen Michell,Jane Masoli
EPI_ISL_838937, EPI_ISL_838938, EPI_ISL_838939, EPI_ISL_838940, EPI_ISL_838941, EPI_ISL_838942, EPI_ISL_838943, EPI_ISL_838944, EPI_ISL_838945, EPI_ISL_838946, EPI_ISL_838947, EPI_ISL_838948, EPI_ISL_838949, EPI_ISL_838951, EPI_ISL_838958, EPI_ISL_838959, EPI_ISL_838960, EPI_ISL_838961, EPI_ISL_838962, EPI_ISL_838963, EPI_ISL_838964, EPI_ISL_838965, EPI_ISL_838966, EPI_ISL_838967, EPI_ISL_838968, EPI_ISL_838969, EPI_ISL_838970, EPI_ISL_838971, EPI_ISL_838972, EPI_ISL_838973, EPI_ISL_838974, EPI_ISL_838975, EPI_ISL_838976, EPI_ISL_838977, EPI_ISL_838978, EPI_ISL_838979, EPI_ISL_838980, EPI_ISL_838981, EPI_ISL_838982, EPI_ISL_838983, EPI_ISL_838984, EPI_ISL_838985, EPI_ISL_838986, EPI_ISL_838987, EPI_ISL_838988, EPI_ISL_838989, EPI_ISL_838990, EPI_ISL_838991, EPI_ISL_838992, EPI_ISL_838993, EPI_ISL_838994, EPI_ISL_838995, EPI_ISL_838996, EPI_ISL_838997, EPI_ISL_838998, EPI_ISL_838999, EPI_ISL_839000, EPI_ISL_839001, EPI_ISL_839002, EPI_ISL_839003, EPI_ISL_839004, EPI_ISL_839005, EPI_ISL_839007, EPI_ISL_839008, EPI_ISL_839009, EPI_ISL_839010, EPI_ISL_839011, EPI_ISL_839012, EPI_ISL_839013, EPI_ISL_839014, EPI_ISL_839015, EPI_ISL_839016, EPI_ISL_839017, EPI_ISL_839018, EPI_ISL_839019, EPI_ISL_839021, EPI_ISL_839022, EPI_ISL_839023, EPI_ISL_839024, EPI_ISL_839025, EPI_ISL_839029, EPI_ISL_839126, EPI_ISL_839127, EPI_ISL_839128, EPI_ISL_839129, EPI_ISL_839130, EPI_ISL_839131, EPI_ISL_839132, EPI_ISL_839133, EPI_ISL_839134, EPI_ISL_839136, EPI_ISL_839145, EPI_ISL_839233, EPI_ISL_839234, EPI_ISL_839235, EPI_ISL_839236, EPI_ISL_839237, EPI_ISL_839238, EPI_ISL_839239, EPI_ISL_839240, EPI_ISL_839241, EPI_ISL_839242, EPI_ISL_839243, EPI_ISL_839244, EPI_ISL_839245, EPI_ISL_839246, EPI_ISL_839247, EPI_ISL_839248, EPI_ISL_839249, EPI_ISL_839251, EPI_ISL_839253, EPI_ISL_839254, EPI_ISL_839255, EPI_ISL_839256, EPI_ISL_839257, EPI_ISL_839258, EPI_ISL_839259, EPI_ISL_839261, EPI_ISL_839262, EPI_ISL_839263, EPI_ISL_839266, EPI_ISL_839267, EPI_ISL_839268, EPI_ISL_839269, EPI_ISL_839270, EPI_ISL_839271, EPI_ISL_839272, EPI_ISL_839274, EPI_ISL_839275, EPI_ISL_839276, EPI_ISL_839277, EPI_ISL_839278, EPI_ISL_839279, EPI_ISL_839280, EPI_ISL_839281, EPI_ISL_839282, EPI_ISL_839283, EPI_ISL_839284, EPI_ISL_839285, EPI_ISL_839286, EPI_ISL_839287, EPI_ISL_839288, EPI_ISL_839289, EPI_ISL_839290, EPI_ISL_839291, EPI_ISL_839292, EPI_ISL_839293, EPI_ISL_839294, EPI_ISL_839295, EPI_ISL_839296, EPI_ISL_839297, EPI_ISL_839298, EPI_ISL_839299, EPI_ISL_839300, EPI_ISL_839301, EPI_ISL_839302, EPI_ISL_839303, EPI_ISL_839304			
see above	University College London, Great Ormond Street Hospital for Children NHS Foundation Trust, Imperial College Healthcare NHS Trust	COVID-19 Genomics UK (COG-UK) Consortium	Sergi Castellano, Rachel Williams, Mark Kristiansen, Paola Resende Silva, Sunando Roy, Tony Brooks, Helena Tutill, Paola Niola, Patricia Dyal, Charlotte Williams, Leysa Forrest, Yasmin Panchbhaya, Jacqueline Findlay, Samuel Weeks, Julianne Brown, Kathryn Harris, Paul Randell, James Price, Alison Holmes, Judith Breuer
EPI_ISL_840009, EPI_ISL_840010, EPI_ISL_840011, EPI_ISL_840012, EPI_ISL_840013, EPI_ISL_840014, EPI_ISL_840015, EPI_ISL_840016, EPI_ISL_840051, EPI_ISL_840052, EPI_ISL_840053, EPI_ISL_840054, EPI_ISL_840055, EPI_ISL_840056, EPI_ISL_840057, EPI_ISL_840058, EPI_ISL_840059, EPI_ISL_840060, EPI_ISL_840061			
see above	Queens Medical Centre, Clinical Microbiology Department /	COVID-19 Genomics UK (COG-UK) Consortium	Gemma Clark, Wendy Smith, Manjinder Khakh, Vicki M Fleming, Michelle M Lister, Hannah Howson-Wells, Jonathan Ball, Patrick McClure, Joseph

	DeepSeq Nottingham		Chappell, Theocharis Tsoleridis, Nadine Holmes, Matthew Carlisle, Christopher Moore, Fei Sang, Johnny Debebe, Victoria Wright, Matthew Loose
EPI_ISL_841039, EPI_ISL_841148, EPI_ISL_841150, EPI_ISL_841277, EPI_ISL_841278, EPI_ISL_841283, EPI_ISL_841284, EPI_ISL_841287, EPI_ISL_841290, EPI_ISL_841291, EPI_ISL_841294, EPI_ISL_841295, EPI_ISL_841298			
see above	Wales Specialist Virology Centre Sequencing lab: Pathogen Genomics Unit	Public Health Wales Microbiology Cardiff Wales Specialist Virology Centre	Catherine Moore, Johnathan Evans, Laura Gifford, Malorie Perry, Simon Cottrell, Angela Marchbank, Alec Birchley, Alexander Adams, Amy Gaskin, Bree Gatica-Wilcox, Jason Coombes, Joel Southgate, Lauren Gilbert, Lee Graham, Nicole Pacchiarini, Sara Kumziene-Summerhayes, Sarah Taylor, Sophie Jones, Sara Rey, Matthew Bull, Joanne Watkins, Sally Corden, Tom Connor
EPI_ISL_841317, EPI_ISL_841322, EPI_ISL_841324, EPI_ISL_841326, EPI_ISL_841327, EPI_ISL_841328, EPI_ISL_841330, EPI_ISL_841331, EPI_ISL_841339, EPI_ISL_841340, EPI_ISL_841343, EPI_ISL_841344, EPI_ISL_841345, EPI_ISL_841346, EPI_ISL_841347, EPI_ISL_841348, EPI_ISL_841349, EPI_ISL_841350, EPI_ISL_841351, EPI_ISL_841352, EPI_ISL_841353, EPI_ISL_841354, EPI_ISL_841417, EPI_ISL_841426, EPI_ISL_841427, EPI_ISL_841430, EPI_ISL_841431, EPI_ISL_841434, EPI_ISL_841437, EPI_ISL_841440, EPI_ISL_841443, EPI_ISL_841444, EPI_ISL_841445, EPI_ISL_841446, EPI_ISL_841448, EPI_ISL_841451, EPI_ISL_841453, EPI_ISL_841454, EPI_ISL_841455, EPI_ISL_841456, EPI_ISL_841457, EPI_ISL_841458, EPI_ISL_841460, EPI_ISL_841461, EPI_ISL_841462, EPI_ISL_841464, EPI_ISL_841465, EPI_ISL_841467, EPI_ISL_841468, EPI_ISL_841469, EPI_ISL_841470, EPI_ISL_841472, EPI_ISL_841473, EPI_ISL_841474, EPI_ISL_841475, EPI_ISL_841476, EPI_ISL_841477, EPI_ISL_841478, EPI_ISL_841480, EPI_ISL_841481, EPI_ISL_841484, EPI_ISL_841487, EPI_ISL_841488, EPI_ISL_841489, EPI_ISL_841492, EPI_ISL_841493, EPI_ISL_841494, EPI_ISL_841495, EPI_ISL_841496, EPI_ISL_841497, EPI_ISL_841502, EPI_ISL_841516, EPI_ISL_841517, EPI_ISL_841518, EPI_ISL_841519, EPI_ISL_841520, EPI_ISL_841523, EPI_ISL_841524, EPI_ISL_841525, EPI_ISL_841526, EPI_ISL_841527, EPI_ISL_841528, EPI_ISL_841530, EPI_ISL_841532, EPI_ISL_841533, EPI_ISL_841534, EPI_ISL_841535, EPI_ISL_841536, EPI_ISL_841537, EPI_ISL_841538, EPI_ISL_841539, EPI_ISL_841540, EPI_ISL_841541, EPI_ISL_841542, EPI_ISL_841543, EPI_ISL_841544, EPI_ISL_841545, EPI_ISL_841546, EPI_ISL_841547, EPI_ISL_841549, EPI_ISL_841550, EPI_ISL_841551, EPI_ISL_841552, EPI_ISL_841553, EPI_ISL_841554, EPI_ISL_841555, EPI_ISL_841556, EPI_ISL_841557, EPI_ISL_841558, EPI_ISL_841614, EPI_ISL_841621, EPI_ISL_841624, EPI_ISL_841625, EPI_ISL_841628, EPI_ISL_841629, EPI_ISL_841631, EPI_ISL_841632, EPI_ISL_841642, EPI_ISL_841644, EPI_ISL_841645, EPI_ISL_841659, EPI_ISL_841661, EPI_ISL_841665, EPI_ISL_841667, EPI_ISL_841669, EPI_ISL_841670, EPI_ISL_841673, EPI_ISL_841676, EPI_ISL_841677, EPI_ISL_841679, EPI_ISL_841684, EPI_ISL_841685, EPI_ISL_841686, EPI_ISL_841687, EPI_ISL_841688, EPI_ISL_841689, EPI_ISL_841690, EPI_ISL_841694, EPI_ISL_841695, EPI_ISL_841700, EPI_ISL_841702, EPI_ISL_841704, EPI_ISL_841705, EPI_ISL_841706, EPI_ISL_841707, EPI_ISL_841709, EPI_ISL_841710, EPI_ISL_841711, EPI_ISL_841712, EPI_ISL_841713, EPI_ISL_841714, EPI_ISL_841715, EPI_ISL_841716, EPI_ISL_841717, EPI_ISL_841718, EPI_ISL_841719, EPI_ISL_841720, EPI_ISL_841721, EPI_ISL_841722, EPI_ISL_841723, EPI_ISL_841724, EPI_ISL_841725, EPI_ISL_841726, EPI_ISL_841727, EPI_ISL_841728, EPI_ISL_841729, EPI_ISL_841730, EPI_ISL_841733, EPI_ISL_841734, EPI_ISL_841735, EPI_ISL_841736, EPI_ISL_841738, EPI_ISL_841739, EPI_ISL_841740, EPI_ISL_841741, EPI_ISL_841742, EPI_ISL_841743, EPI_ISL_841744, EPI_ISL_841745, EPI_ISL_841746, EPI_ISL_841747, EPI_ISL_841748, EPI_ISL_841749, EPI_ISL_841750, EPI_ISL_841751, EPI_ISL_841752, EPI_ISL_841753			
see above	Originating lab: Wales Specialist Virology Centre Sequencing lab: Pathogen Genomics Unit	Public Health Wales Microbiology Cardiff Wales Specialist Virology Centre	Catherine Moore, Johnathan Evans, Laura Gifford, Malorie Perry, Simon Cottrell, Angela Marchbank, Alec Birchley, Alexander Adams, Amy Gaskin, Bree Gatica-Wilcox, Jason Coombes, Joel Southgate, Lauren Gilbert, Lee Graham, Nicole Pacchiarini, Sara Kumziene-Summerhayes, Sarah Taylor, Sophie Jones, Sara Rey, Matthew Bull, Joanne Watkins, Sally Corden, Tom Connor
EPI_ISL_841921	Centre for Enzyme Innovation, University of Portsmouth / Translational Research Laboratory, Portsmouth Hospitals NHS Trust	COVID-19 Genomics UK (COG-UK) Consortium	Angela Beckett,Yann Bourgeois,Garry Scarlett,Sharon Glaysher,Scott Elliott,Kelly Bicknell,Robert Impey,Allyson Lloyd,Sarah Wyllie,Ethan Butcher,Anoop Chauhan,Samuel Robson
EPI_ISL_842201, EPI_ISL_842203, EPI_ISL_842207, EPI_ISL_842210, EPI_ISL_842213, EPI_ISL_842219, EPI_ISL_842222, EPI_ISL_842223, EPI_ISL_842225, EPI_ISL_842227, EPI_ISL_842229, EPI_ISL_842234, EPI_ISL_842237, EPI_ISL_842242, EPI_ISL_842243, EPI_ISL_842247, EPI_ISL_842249, EPI_ISL_842252, EPI_ISL_842253, EPI_ISL_842257, EPI_ISL_842259, EPI_ISL_842267, EPI_ISL_842269, EPI_ISL_842271, EPI_ISL_842275, EPI_ISL_842276, EPI_ISL_842277, EPI_ISL_842290, EPI_ISL_842296, EPI_ISL_842298, EPI_ISL_842302, EPI_ISL_842307, EPI_ISL_842308, EPI_ISL_842309, EPI_ISL_842310, EPI_ISL_842314, EPI_ISL_842320, EPI_ISL_842321, EPI_ISL_842323, EPI_ISL_842326, EPI_ISL_842328, EPI_ISL_842331, EPI_ISL_842332, EPI_ISL_842333, EPI_ISL_842336, EPI_ISL_842337, EPI_ISL_842343, EPI_ISL_842344			
see above	Virology Department, Sheffield Teaching Hospitals NHS Foundation Trust/Department of Infection, Immunity and Cardiovascular Disease, The Medical School, University of Sheffield	COVID-19 Genomics UK (COG-UK) Consortium	Thushan de Silva, Matthew Parker, Nikki Smith, Adri Angyal, Rebecca Brown, Luke Green, Rachel Tucker, Paul Parsons, Danielle Groves, Katie Johnson, Laura Carrilero, Alex Keeley, Dave Partridge, Matthew Wyles, Benjamin Lindsey, Mehmet Yavuz, Mohammad Raza, Cariad Evans
EPI_ISL_845893, EPI_ISL_845894, EPI_ISL_845895, EPI_ISL_845896, EPI_ISL_845897, EPI_ISL_845898, EPI_ISL_845899, EPI_ISL_845900, EPI_ISL_845901, EPI_ISL_845902, EPI_ISL_845903, EPI_ISL_845904, EPI_ISL_845905, EPI_ISL_845906, EPI_ISL_845907, EPI_ISL_845908, EPI_ISL_845909, EPI_ISL_845910, EPI_ISL_845911, EPI_ISL_845912, EPI_ISL_845913, EPI_ISL_845914, EPI_ISL_845915, EPI_ISL_845916, EPI_ISL_845917, EPI_ISL_845918, EPI_ISL_845919, EPI_ISL_845920, EPI_ISL_845921, EPI_ISL_845922, EPI_ISL_845923, EPI_ISL_845924, EPI_ISL_845925, EPI_ISL_845926, EPI_ISL_845927, EPI_ISL_845928, EPI_ISL_845929, EPI_ISL_845930, EPI_ISL_845931, EPI_ISL_845932, EPI_ISL_845933, EPI_ISL_845934, EPI_ISL_845935, EPI_ISL_845936, EPI_ISL_845937, EPI_ISL_845938, EPI_ISL_845939, EPI_ISL_845940, EPI_ISL_845941, EPI_ISL_845942, EPI_ISL_845943, EPI_ISL_845944, EPI_ISL_845945, EPI_ISL_845946, EPI_ISL_845947, EPI_ISL_845948, EPI_ISL_845949, EPI_ISL_845950, EPI_ISL_845951, EPI_ISL_845952, EPI_ISL_845953, EPI_ISL_845954, EPI_ISL_845955, EPI_ISL_845956, EPI_ISL_845957, EPI_ISL_845958, EPI_ISL_845959, EPI_ISL_845960, EPI_ISL_845961, EPI_ISL_845962, EPI_ISL_845963, EPI_ISL_845964, EPI_ISL_845965, EPI_ISL_845966, EPI_ISL_845967, EPI_ISL_845968, EPI_ISL_845969, EPI_ISL_845970, EPI_ISL_845971, EPI_ISL_845972, EPI_ISL_845974, EPI_ISL_845975, EPI_ISL_845976, EPI_ISL_845977, EPI_ISL_845978, EPI_ISL_845979, EPI_ISL_845980, EPI_ISL_845981, EPI_ISL_845982, EPI_ISL_845983, EPI_ISL_845984, EPI_ISL_845985, EPI_ISL_845986, EPI_ISL_845987, EPI_ISL_845988, EPI_ISL_845989, EPI_ISL_845990, EPI_ISL_845991, EPI_ISL_845992, EPI_ISL_845993, EPI_ISL_845994, EPI_ISL_845995, EPI_ISL_845996, EPI_ISL_845997, EPI_ISL_845998, EPI_ISL_845999, EPI_ISL_846000, EPI_ISL_846001, EPI_ISL_846002, EPI_ISL_846003, EPI_ISL_846004, EPI_ISL_846005, EPI_ISL_846006, EPI_ISL_846007, EPI_ISL_846008, EPI_ISL_846009, EPI_ISL_846010, EPI_ISL_846011, EPI_ISL_846012, EPI_ISL_846013, EPI_ISL_846014, EPI_ISL_846015, EPI_ISL_846016, EPI_ISL_846017, EPI_ISL_846018, EPI_ISL_846019, EPI_ISL_846020, EPI_ISL_846021, EPI_ISL_846023, EPI_ISL_846024, EPI_ISL_846025, EPI_ISL_846026, EPI_ISL_846027, EPI_ISL_846028, EPI_ISL_846029, EPI_ISL_846030, EPI_ISL_846031, EPI_ISL_846032, EPI_ISL_846033, EPI_ISL_846034, EPI_ISL_846035, EPI_ISL_846036, EPI_ISL_846037, EPI_ISL_846038, EPI_ISL_846039, EPI_ISL_846040, EPI_ISL_846041, EPI_ISL_846042, EPI_ISL_846043, EPI_ISL_846044, EPI_ISL_846045, EPI_ISL_846046, EPI_ISL_846047, EPI_ISL_846048, EPI_ISL_846049, EPI_ISL_846050, EPI_ISL_846051, EPI_ISL_846052, EPI_ISL_846053, EPI_ISL_846054, EPI_ISL_846055, EPI_ISL_846056, EPI_ISL_846057, EPI_ISL_846058, EPI_ISL_846059, EPI_ISL_846060, EPI_ISL_846061, EPI_ISL_846062, EPI_ISL_846063, EPI_ISL_846064, EPI_ISL_846065, EPI_ISL_846066, EPI_ISL_846067, EPI_ISL_846068, EPI_ISL_846069, EPI_ISL_846071, EPI_ISL_846072, EPI_ISL_846073, EPI_ISL_846074, EPI_ISL_846075, EPI_ISL_846076, EPI_ISL_846077, EPI_ISL_846078, EPI_ISL_846079, EPI_ISL_846080, EPI_ISL_846081, EPI_ISL_846082, EPI_ISL_846083, EPI_ISL_846084, EPI_ISL_846085, EPI_ISL_846086, EPI_ISL_846087, EPI_ISL_846088, EPI_ISL_846089, EPI_ISL_846090, EPI_ISL_846091, EPI_ISL_846092, EPI_ISL_846093, EPI_ISL_846094, EPI_ISL_846095, EPI_ISL_846096, EPI_ISL_846097, EPI_ISL_846098, EPI_ISL_846099, EPI_ISL_846100, EPI_ISL_846101, EPI_ISL_846102, EPI_ISL_846103, EPI_ISL_846104, EPI_ISL_846105, EPI_ISL_846106, EPI_ISL_846107, EPI_ISL_846108, EPI_ISL_846109, EPI_ISL_846110, EPI_ISL_846111, EPI_ISL_846112, EPI_ISL_846113, EPI_ISL_846114, EPI_ISL_846115, EPI_ISL_846116, EPI_ISL_846117, EPI_ISL_846118, EPI_ISL_846119, EPI_ISL_846120, EPI_ISL_846121, EPI_ISL_846122, EPI_ISL_846123, EPI_ISL_846124, EPI_ISL_846125, EPI_ISL_846126, EPI_ISL_846127, EPI_ISL_846128, EPI_ISL_846129, EPI_ISL_846130, EPI_ISL_846131, EPI_ISL_846132, EPI_ISL_846133, EPI_ISL_846134, EPI_ISL_846135, EPI_ISL_846136, EPI_ISL_846137, EPI_ISL_846138, EPI_ISL_846139, EPI_ISL_846140, EPI_ISL_846141, EPI_ISL_846142, EPI_ISL_846143, EPI_ISL_846144, EPI_ISL_846145, EPI_ISL_846146, EPI_ISL_846147, EPI_ISL_846148, EPI_ISL_846149, EPI_ISL_846150, EPI_ISL_846151, EPI_ISL_846152, EPI_ISL_846153, EPI_ISL_846154, EPI_ISL_846155, EPI_ISL_846156, EPI_ISL_846157, EPI_ISL_846158, EPI_ISL_846159, EPI_ISL_846160, EPI_ISL_846161, EPI_ISL_846162, EPI_ISL_846163, EPI_ISL_846164, EPI_ISL_846165, EPI_ISL_846166, EPI_ISL_846167, EPI_ISL_846168, EPI_ISL_846169, EPI_ISL_846170, EPI_ISL_846172, EPI_ISL_846173, EPI_ISL_846174, EPI_ISL_846175, EPI_ISL_846176, EPI_ISL_846177, EPI_ISL_846178, EPI_ISL_846179, EPI_ISL_846180, EPI_ISL_846181, EPI_ISL_846182, EPI_ISL_846183, EPI_ISL_846184, EPI_ISL_846185, EPI_ISL_846186, EPI_ISL_846187, EPI_ISL_846188, EPI_ISL_846189, EPI_ISL_846190, EPI_ISL_846191, EPI_ISL_846192, EPI_ISL_846193, EPI_ISL_846194, EPI_ISL_846195, EPI_ISL_846196, EPI_ISL_846197, EPI_ISL_846198, EPI_ISL_846199			
see above	Lighthouse Lab in Cambridge	Wellcome Sanger Institute for the COVID-19 Genomics UK (COG-UK) Consortium	Rob Howes, The Lighthouse Lab in Cambridge and Alex Alderton, Roberto Amato, Sonia Goncalves, Ewan Harrison, David K. Jackson, Ian Johnston, Dominic Kwiatkowski, Cordelia Langford, John Sillitoe on behalf of the Wellcome Sanger Institute COVID-19 Surveillance Team
EPI_ISL_846200, EPI_ISL_846201, EPI_ISL_846202, EPI_ISL_846203, EPI_ISL_846204, EPI_ISL_846205, EPI_ISL_846206, EPI_ISL_846207, EPI_ISL_846208, EPI_ISL_846209, EPI_ISL_846210, EPI_ISL_846211, EPI_ISL_846212, EPI_ISL_846213, EPI_ISL_846214, EPI_ISL_846215, EPI_ISL_846216, EPI_ISL_846217, EPI_ISL_846218, EPI_ISL_846219, EPI_ISL_846220, EPI_ISL_846221, EPI_ISL_846222, EPI_ISL_846223, EPI_ISL_846224, EPI_ISL_846225, EPI_ISL_846226, EPI_ISL_846227, EPI_ISL_846228, EPI_ISL_846229, EPI_ISL_846230, EPI_ISL_846231, EPI_ISL_846232, EPI_ISL_846233, EPI_ISL_846234, EPI_ISL_846235, EPI_ISL_846236, EPI_ISL_846237, EPI_ISL_846238, EPI_ISL_846239, EPI_ISL_846240, EPI_ISL_846241, EPI_ISL_846242, EPI_ISL_846243, EPI_ISL_846244, EPI_ISL_846245, EPI_ISL_846246, EPI_ISL_846247, EPI_ISL_846248, EPI_ISL_846249, EPI_ISL_846250, EPI_ISL_846251, EPI_ISL_846252, EPI_ISL_846253, EPI_ISL_846254, EPI_ISL_846255, EPI_ISL_846256, EPI_ISL_846257, EPI_ISL_846258, EPI_ISL_846259, EPI_ISL_846260, EPI_ISL_846261, EPI_ISL_846262, EPI_ISL_846263, EPI_ISL_846264, EPI_ISL_846265, EPI_ISL_846266, EPI_ISL_846267, EPI_ISL_846268, EPI_ISL_846269, EPI_ISL_846270, EPI_ISL_846271, EPI_ISL_846272, EPI_ISL_846273, EPI_ISL_846274, EPI_ISL_846275, EPI_ISL_846276, EPI_ISL_846277, EPI_ISL_846278, EPI_ISL_846279, EPI_ISL_846280, EPI_ISL_846281, EPI_ISL_846282, EPI_ISL_846283, EPI_ISL_846284, EPI_ISL_846285, EPI_ISL_846286, EPI_ISL_846287, EPI_ISL_846288, EPI_ISL_846289, EPI_ISL_846290, EPI_ISL_846291, EPI_ISL_846292, EPI_ISL_846293, EPI_ISL_846294, EPI_ISL_846295, EPI_ISL_846296, EPI_ISL_846297, EPI_ISL_846298, EPI_ISL_846299, EPI_ISL_846300, EPI_ISL_846301, EPI_ISL_846302, EPI_ISL_846303, EPI_ISL_846304, EPI_ISL_846305, EPI_ISL_846306, EPI_ISL_846307, EPI_ISL_846308, EPI_ISL_846309, EPI_ISL_846310, EPI_ISL_846311, EPI_ISL_846312, EPI_ISL_846313, EPI_ISL_846314, EPI_ISL_846315, EPI_ISL_846316, EPI_ISL_846317, EPI_ISL_846318, EPI_ISL_846320, EPI_ISL_846321, EPI_ISL_846322, EPI_ISL_846323, EPI_ISL_846324, EPI_ISL_846325, EPI_ISL_846326, EPI_ISL_846327, EPI_ISL_846328, EPI_ISL_846329, EPI_ISL_846330, EPI_ISL_846331, EPI_ISL_846332, EPI_ISL_846333, EPI_ISL_846334, EPI_ISL_846335, EPI_ISL_846336, EPI_ISL_846337, EPI_ISL_846338, EPI_ISL_846339, EPI_ISL_846340, EPI_ISL_846341, EPI_ISL_846342, EPI_ISL_846343, EPI_ISL_846344, EPI_ISL_846345, EPI_ISL_846346, EPI_ISL_846347, EPI_ISL_846348, EPI_ISL_846349, EPI_ISL_846350, EPI_ISL_846351, EPI_ISL_846352, EPI_ISL_846353, EPI_ISL_846354, EPI_ISL_846355, EPI_ISL_846356, EPI_ISL_846357, EPI_ISL_846358, EPI_ISL_846359, EPI_ISL_846360, EPI_ISL_846361, EPI_ISL_846362, EPI_ISL_846363, EPI_ISL_846364, EPI_ISL_846365, EPI_ISL_846366, EPI_ISL_846367, EPI_ISL_846368, EPI_ISL_846369, EPI_ISL_846370, EPI_ISL_846371, EPI_ISL_846372, EPI_ISL_846373, EPI_ISL_846374, EPI_ISL_846375, EPI_ISL_846376, EPI_ISL_846377, EPI_ISL_846378, EPI_ISL_846379, EPI_ISL_846380, EPI_ISL_846381, EPI_ISL_846382, EPI_ISL_846383, EPI_ISL_846384, EPI_ISL_846385, EPI_ISL_846386, EPI_ISL_846387, EPI_ISL_846388, EPI_ISL_846389, EPI_ISL_846390, EPI_ISL_846391, EPI_ISL_846392, EPI_ISL_846393, EPI_ISL_846394, EPI_ISL_846395, EPI_ISL_846396, EPI_ISL_846397, EPI_ISL_846398, EPI_ISL_846399, EPI_ISL_846401, EPI_ISL_846402, EPI_ISL_846403, EPI_ISL_846404, EPI_ISL_846405, EPI_ISL_846406, EPI_ISL_846407, EPI_ISL_846408, EPI_ISL_846409, EPI_ISL_846410, EPI_ISL_846411, EPI_ISL_846412, EPI_ISL_846413, EPI_ISL_846414, EPI_ISL_846415, EPI_ISL_846416, EPI_ISL_846417, EPI_ISL_846418, EPI_ISL_846419, EPI_ISL_846420, EPI_ISL_846421, EPI_ISL_846422, EPI_ISL_846423, EPI_ISL_846424, EPI_ISL_846425, EPI_ISL_846426, EPI_ISL_846427, EPI_ISL_846428, EPI_ISL_846429, EPI_ISL_846430, EPI_ISL_846431, EPI_ISL_846432, EPI_ISL_846433, EPI_ISL_846434, EPI_ISL_846435, EPI_ISL_846436, EPI_ISL_846437, EPI_ISL_846438, EPI_ISL_846439, EPI_ISL_846440, EPI_ISL_846441, EPI_ISL_846442, EPI_ISL_846443, EPI_ISL_846444, EPI_ISL_846445, EPI_ISL_846446, EPI_ISL_846447, EPI_ISL_846448, EPI_ISL_846449, EPI_ISL_846450, EPI_ISL_846451, EPI_ISL_846452, EPI_ISL_846453, EPI_ISL_846454, EPI_ISL_846455, EPI_ISL_846456, EPI_ISL_846457, EPI_ISL_846458, EPI_ISL_846459, EPI_ISL_846460, EPI_ISL_846461, EPI_ISL_846462, EPI_ISL_846463, EPI_ISL_846464, EPI_ISL_846465, EPI_ISL_846466, EPI_ISL_846467, EPI_ISL_846468, EPI_ISL_846469, EPI_ISL_846470, EPI_ISL_846471, EPI_ISL_846472, EPI_ISL_846473, EPI_ISL_846474, EPI_ISL_846475, EPI_ISL_846476, EPI_ISL_846477, EPI_ISL_846478, EPI_ISL_846479, EPI_ISL_846480, EPI_ISL_846481, EPI_ISL_846482, EPI_ISL_846483, EPI_ISL_846484, EPI_ISL_846485, EPI_ISL_846486, EPI_ISL_846487, EPI_ISL_846488, EPI_ISL_846489, EPI_ISL_846490, EPI_ISL_846491, EPI_ISL_846492, EPI_ISL_846493, EPI_ISL_846494, EPI_ISL_846495, EPI_ISL_846496, EPI_ISL_846497, EPI_ISL_846498, EPI_ISL_846499, EPI_ISL_846500, EPI_ISL_846501, EPI_ISL_846502, EPI_ISL_846503, EPI_ISL_846504, EPI_ISL_846505, EPI_ISL_846506, EPI_ISL_846507, EPI_ISL_846508			
see above	Lighthouse Lab in Alderley Park	Wellcome Sanger Institute for the COVID-19 Genomics UK (COG-UK) Consortium	Jacquelyn Wynn, Mairead Hyland, The Lighthouse Lab in Alderley Park and Alex Alderton, Roberto Amato, Sonia Goncalves, Ewan Harrison, David K. Jackson, Ian Johnston, Dominic Kwiatkowski, Cordelia Langford, John Sillitoe on behalf of the Wellcome Sanger Institute COVID-19 Surveillance Team
EPI_ISL_846509	Lighthouse Lab in Cambridge	Wellcome Sanger Institute for the COVID-19 Genomics UK (COG-UK) Consortium	Rob Howes, The Lighthouse Lab in Cambridge and Alex Alderton, Roberto Amato, Sonia Goncalves, Ewan Harrison, David K. Jackson, Ian Johnston, Dominic Kwiatkowski, Cordelia Langford, John Sillitoe on behalf of the Wellcome Sanger Institute COVID-19 Surveillance Team
EPI_ISL_846512	Lighthouse Lab in Alderley Park	Wellcome Sanger Institute for the COVID-19 Genomics UK (COG-UK) Consortium	Jacquelyn Wynn, Mairead Hyland, The Lighthouse Lab in Alderley Park and Alex Alderton, Roberto Amato, Sonia Goncalves, Ewan Harrison, David K. Jackson, Ian Johnston, Dominic Kwiatkowski, Cordelia Langford, John Sillitoe on behalf of the Wellcome Sanger Institute COVID-19 Surveillance Team
EPI_ISL_846597, EPI_ISL_846598	Respiratory Virus Unit, National Infection Service, Public Health England	COVID-19 Genomics UK (COG-UK) Consortium	PHE Covid Sequencing Team
EPI_ISL_851062, EPI_ISL_851063, EPI_ISL_851064, EPI_ISL_851065, EPI_ISL_851066, EPI_ISL_851067, EPI_ISL_851068, EPI_ISL_851069	Lighthouse Lab in Cambridge	Wellcome Sanger Institute for the COVID-19 Genomics UK (COG-UK) Consortium	Rob Howes, The Lighthouse Lab in Cambridge and Alex Alderton, Roberto Amato, Sonia Goncalves, Ewan Harrison, David K. Jackson, Ian Johnston, Dominic Kwiatkowski, Cordelia Langford, John Sillitoe on behalf of the Wellcome Sanger Institute COVID-19 Surveillance Team
EPI_ISL_851070	Lighthouse Lab in Glasgow	Wellcome Sanger Institute for the COVID-19 Genomics UK	Harper VanSteenhouse, Yumi Kasai, David Gray, Carol Clugston, Anna Dominiczak and Alex Alderton, Roberto Amato, Sonia Goncalves, Ewan Harrison,

		(COG-UK) Consortium	David K. Jackson, Ian Johnston, Dominic Kwiatkowski, Cordelia Langford, John Sillitoe on behalf of the Wellcome Sanger Institute COVID-19 Surveillance Team
EPI_ISL_851071, EPI_ISL_851072, EPI_ISL_851073, EPI_ISL_851074, EPI_ISL_851075, EPI_ISL_851076	Lighthouse Lab in Cambridge	Wellcome Sanger Institute for the COVID-19 Genomics UK (COG-UK) Consortium	Rob Howes, The Lighthouse Lab in Cambridge and Alex Alderton, Roberto Amato, Sonia Goncalves, Ewan Harrison, David K. Jackson, Ian Johnston, Dominic Kwiatkowski, Cordelia Langford, John Sillitoe on behalf of the Wellcome Sanger Institute COVID-19 Surveillance Team
EPI_ISL_851077	Lighthouse Lab in Glasgow	Wellcome Sanger Institute for the COVID-19 Genomics UK (COG-UK) Consortium	Harper VanSteenhouse, Yumi Kasai, David Gray, Carol Clugston, Anna Dominiczak and Alex Alderton, Roberto Amato, Sonia Goncalves, Ewan Harrison, David K. Jackson, Ian Johnston, Dominic Kwiatkowski, Cordelia Langford, John Sillitoe on behalf of the Wellcome Sanger Institute COVID-19 Surveillance Team
EPI_ISL_851078, EPI_ISL_851080	Lighthouse Lab in Cambridge	Wellcome Sanger Institute for the COVID-19 Genomics UK (COG-UK) Consortium	Rob Howes, The Lighthouse Lab in Cambridge and Alex Alderton, Roberto Amato, Sonia Goncalves, Ewan Harrison, David K. Jackson, Ian Johnston, Dominic Kwiatkowski, Cordelia Langford, John Sillitoe on behalf of the Wellcome Sanger Institute COVID-19 Surveillance Team
EPI_ISL_851081, EPI_ISL_851082	Lighthouse Lab in Glasgow	Wellcome Sanger Institute for the COVID-19 Genomics UK (COG-UK) Consortium	Harper VanSteenhouse, Yumi Kasai, David Gray, Carol Clugston, Anna Dominiczak and Alex Alderton, Roberto Amato, Sonia Goncalves, Ewan Harrison, David K. Jackson, Ian Johnston, Dominic Kwiatkowski, Cordelia Langford, John Sillitoe on behalf of the Wellcome Sanger Institute COVID-19 Surveillance Team
EPI_ISL_851083, EPI_ISL_851084	Lighthouse Lab in Cambridge	Wellcome Sanger Institute for the COVID-19 Genomics UK (COG-UK) Consortium	Rob Howes, The Lighthouse Lab in Cambridge and Alex Alderton, Roberto Amato, Sonia Goncalves, Ewan Harrison, David K. Jackson, Ian Johnston, Dominic Kwiatkowski, Cordelia Langford, John Sillitoe on behalf of the Wellcome Sanger Institute COVID-19 Surveillance Team
EPI_ISL_851085	Lighthouse Lab in Glasgow	Wellcome Sanger Institute for the COVID-19 Genomics UK (COG-UK) Consortium	Harper VanSteenhouse, Yumi Kasai, David Gray, Carol Clugston, Anna Dominiczak and Alex Alderton, Roberto Amato, Sonia Goncalves, Ewan Harrison, David K. Jackson, Ian Johnston, Dominic Kwiatkowski, Cordelia Langford, John Sillitoe on behalf of the Wellcome Sanger Institute COVID-19 Surveillance Team
EPI_ISL_851086, EPI_ISL_851087, EPI_ISL_851088, EPI_ISL_851089, EPI_ISL_851090, EPI_ISL_851091, EPI_ISL_851092, EPI_ISL_851093	Lighthouse Lab in Cambridge	Wellcome Sanger Institute for the COVID-19 Genomics UK (COG-UK) Consortium	Rob Howes, The Lighthouse Lab in Cambridge and Alex Alderton, Roberto Amato, Sonia Goncalves, Ewan Harrison, David K. Jackson, Ian Johnston, Dominic Kwiatkowski, Cordelia Langford, John Sillitoe on behalf of the Wellcome Sanger Institute COVID-19 Surveillance Team
EPI_ISL_851094, EPI_ISL_851095	Lighthouse Lab in Glasgow	Wellcome Sanger Institute for the COVID-19 Genomics UK (COG-UK) Consortium	Harper VanSteenhouse, Yumi Kasai, David Gray, Carol Clugston, Anna Dominiczak and Alex Alderton, Roberto Amato, Sonia Goncalves, Ewan Harrison, David K. Jackson, Ian Johnston, Dominic Kwiatkowski, Cordelia Langford, John Sillitoe on behalf of the Wellcome Sanger Institute COVID-19 Surveillance Team
EPI_ISL_851096, EPI_ISL_851097, EPI_ISL_851098, EPI_ISL_851099, EPI_ISL_851100	Lighthouse Lab in Cambridge	Wellcome Sanger Institute for the COVID-19 Genomics UK (COG-UK) Consortium	Rob Howes, The Lighthouse Lab in Cambridge and Alex Alderton, Roberto Amato, Sonia Goncalves, Ewan Harrison, David K. Jackson, Ian Johnston, Dominic Kwiatkowski, Cordelia Langford, John Sillitoe on behalf of the Wellcome Sanger Institute COVID-19 Surveillance Team
EPI_ISL_851101, EPI_ISL_851102	Lighthouse Lab in Glasgow	Wellcome Sanger Institute for the COVID-19 Genomics UK (COG-UK) Consortium	Harper VanSteenhouse, Yumi Kasai, David Gray, Carol Clugston, Anna Dominiczak and Alex Alderton, Roberto Amato, Sonia Goncalves, Ewan Harrison, David K. Jackson, Ian Johnston, Dominic Kwiatkowski, Cordelia Langford, John Sillitoe on behalf of the Wellcome Sanger Institute COVID-19 Surveillance Team
EPI_ISL_851103	Lighthouse Lab in Cambridge	Wellcome Sanger Institute for the COVID-19 Genomics UK (COG-UK) Consortium	Rob Howes, The Lighthouse Lab in Cambridge and Alex Alderton, Roberto Amato, Sonia Goncalves, Ewan Harrison, David K. Jackson, Ian Johnston, Dominic Kwiatkowski, Cordelia Langford, John Sillitoe on behalf of the Wellcome Sanger Institute COVID-19 Surveillance Team
EPI_ISL_851104	Lighthouse Lab in Glasgow	Wellcome Sanger Institute for the COVID-19 Genomics UK (COG-UK) Consortium	Harper VanSteenhouse, Yumi Kasai, David Gray, Carol Clugston, Anna Dominiczak and Alex Alderton, Roberto Amato, Sonia Goncalves, Ewan Harrison, David K. Jackson, Ian Johnston, Dominic Kwiatkowski, Cordelia Langford, John Sillitoe on behalf of the Wellcome Sanger Institute COVID-19 Surveillance Team
EPI_ISL_851105, EPI_ISL_851106	Lighthouse Lab in Cambridge	Wellcome Sanger Institute for the COVID-19 Genomics UK (COG-UK) Consortium	Rob Howes, The Lighthouse Lab in Cambridge and Alex Alderton, Roberto Amato, Sonia Goncalves, Ewan Harrison, David K. Jackson, Ian Johnston, Dominic Kwiatkowski, Cordelia Langford, John Sillitoe on behalf of the Wellcome Sanger Institute COVID-19 Surveillance Team
EPI_ISL_851107	Lighthouse Lab in Glasgow	Wellcome Sanger Institute for the COVID-19 Genomics UK (COG-UK) Consortium	Harper VanSteenhouse, Yumi Kasai, David Gray, Carol Clugston, Anna Dominiczak and Alex Alderton, Roberto Amato, Sonia Goncalves, Ewan Harrison, David K. Jackson, Ian Johnston, Dominic Kwiatkowski, Cordelia Langford, John Sillitoe on behalf of the Wellcome Sanger Institute COVID-19 Surveillance Team
EPI_ISL_851108, EPI_ISL_851109, EPI_ISL_851110, EPI_ISL_851111, EPI_ISL_851112, EPI_ISL_851113, EPI_ISL_851114, EPI_ISL_851115	Lighthouse Lab in Cambridge	Wellcome Sanger Institute for the COVID-19 Genomics UK (COG-UK) Consortium	Rob Howes, The Lighthouse Lab in Cambridge and Alex Alderton, Roberto Amato, Sonia Goncalves, Ewan Harrison, David K. Jackson, Ian Johnston, Dominic Kwiatkowski, Cordelia Langford, John Sillitoe on behalf of the Wellcome Sanger Institute COVID-19 Surveillance Team
EPI_ISL_851116	Lighthouse Lab in Glasgow	Wellcome Sanger Institute for the COVID-19 Genomics UK (COG-UK) Consortium	Harper VanSteenhouse, Yumi Kasai, David Gray, Carol Clugston, Anna Dominiczak and Alex Alderton, Roberto Amato, Sonia Goncalves, Ewan Harrison, David K. Jackson, Ian Johnston, Dominic Kwiatkowski, Cordelia Langford, John Sillitoe on behalf of the Wellcome Sanger Institute COVID-19 Surveillance Team
EPI_ISL_851117, EPI_ISL_851118	Lighthouse Lab in Cambridge	Wellcome Sanger Institute for the COVID-19 Genomics UK (COG-UK) Consortium	Rob Howes, The Lighthouse Lab in Cambridge and Alex Alderton, Roberto Amato, Sonia Goncalves, Ewan Harrison, David K. Jackson, Ian Johnston, Dominic Kwiatkowski, Cordelia Langford, John Sillitoe on behalf of the Wellcome Sanger Institute COVID-19 Surveillance Team
EPI_ISL_851119	Lighthouse Lab in Glasgow	Wellcome Sanger Institute for the COVID-19 Genomics UK (COG-UK) Consortium	Harper VanSteenhouse, Yumi Kasai, David Gray, Carol Clugston, Anna Dominiczak and Alex Alderton, Roberto Amato, Sonia Goncalves, Ewan Harrison, David K. Jackson, Ian Johnston, Dominic Kwiatkowski, Cordelia Langford, John Sillitoe on behalf of the Wellcome Sanger Institute COVID-19 Surveillance Team
EPI_ISL_851120, EPI_ISL_851121, EPI_ISL_851122, EPI_ISL_851123	Lighthouse Lab in Cambridge	Wellcome Sanger Institute for the COVID-19 Genomics UK (COG-UK) Consortium	Rob Howes, The Lighthouse Lab in Cambridge and Alex Alderton, Roberto Amato, Sonia Goncalves, Ewan Harrison, David K. Jackson, Ian Johnston, Dominic Kwiatkowski, Cordelia Langford, John Sillitoe on behalf of the Wellcome Sanger Institute COVID-19 Surveillance Team
EPI_ISL_851124, EPI_ISL_851125, EPI_ISL_851126	Lighthouse Lab in Glasgow	Wellcome Sanger Institute for the COVID-19 Genomics UK (COG-UK) Consortium	Harper VanSteenhouse, Yumi Kasai, David Gray, Carol Clugston, Anna Dominiczak and Alex Alderton, Roberto Amato, Sonia Goncalves, Ewan Harrison, David K. Jackson, Ian Johnston, Dominic Kwiatkowski, Cordelia Langford, John Sillitoe on behalf of the Wellcome Sanger Institute COVID-19 Surveillance Team
EPI_ISL_851127, EPI_ISL_851128, EPI_ISL_851129	Lighthouse Lab in Cambridge	Wellcome Sanger Institute for the COVID-19 Genomics UK (COG-UK) Consortium	Rob Howes, The Lighthouse Lab in Cambridge and Alex Alderton, Roberto Amato, Sonia Goncalves, Ewan Harrison, David K. Jackson, Ian Johnston, Dominic Kwiatkowski, Cordelia Langford, John Sillitoe on behalf of the Wellcome Sanger Institute COVID-19 Surveillance Team
EPI_ISL_851130	Lighthouse Lab in Glasgow	Wellcome Sanger Institute for the COVID-19 Genomics UK (COG-UK) Consortium	Harper VanSteenhouse, Yumi Kasai, David Gray, Carol Clugston, Anna Dominiczak and Alex Alderton, Roberto Amato, Sonia Goncalves, Ewan Harrison, David K. Jackson, Ian Johnston, Dominic Kwiatkowski, Cordelia Langford, John Sillitoe on behalf of the Wellcome Sanger Institute COVID-19 Surveillance Team
EPI_ISL_851131, EPI_ISL_851132	Lighthouse Lab in Cambridge	Wellcome Sanger Institute for the COVID-19 Genomics UK (COG-UK) Consortium	Rob Howes, The Lighthouse Lab in Cambridge and Alex Alderton, Roberto Amato, Sonia Goncalves, Ewan Harrison, David K. Jackson, Ian Johnston, Dominic Kwiatkowski, Cordelia Langford, John Sillitoe on behalf of the Wellcome Sanger Institute COVID-19 Surveillance Team
EPI_ISL_851134, EPI_ISL_851135	Lighthouse Lab in Glasgow	Wellcome Sanger Institute for the COVID-19 Genomics UK (COG-UK) Consortium	Harper VanSteenhouse, Yumi Kasai, David Gray, Carol Clugston, Anna Dominiczak and Alex Alderton, Roberto Amato, Sonia Goncalves, Ewan Harrison, David K. Jackson, Ian Johnston, Dominic Kwiatkowski, Cordelia Langford, John Sillitoe on behalf of the Wellcome Sanger Institute COVID-19 Surveillance Team
EPI_ISL_851136	Lighthouse Lab in Cambridge	Wellcome Sanger Institute for the COVID-19 Genomics UK (COG-UK) Consortium	Rob Howes, The Lighthouse Lab in Cambridge and Alex Alderton, Roberto Amato, Sonia Goncalves, Ewan Harrison, David K. Jackson, Ian Johnston, Dominic Kwiatkowski, Cordelia Langford, John Sillitoe on behalf of the Wellcome Sanger Institute COVID-19 Surveillance Team
EPI_ISL_851137	Lighthouse Lab in Glasgow	Wellcome Sanger Institute for the COVID-19 Genomics UK (COG-UK) Consortium	Harper VanSteenhouse, Yumi Kasai, David Gray, Carol Clugston, Anna Dominiczak and Alex Alderton, Roberto Amato, Sonia Goncalves, Ewan Harrison, David K. Jackson, Ian Johnston, Dominic Kwiatkowski, Cordelia Langford, John Sillitoe on behalf of the Wellcome Sanger Institute COVID-19 Surveillance Team
EPI_ISL_851138, EPI_ISL_851139,	Lighthouse Lab in Cambridge	Wellcome Sanger Institute for the COVID-19 Genomics UK	Rob Howes, The Lighthouse Lab in Cambridge and Alex Alderton, Roberto Amato, Sonia Goncalves, Ewan Harrison, David K. Jackson, Ian Johnston,

EPI_ISL_851140, EPI_ISL_851141		(COG-UK) Consortium	Dominic Kwiatkowski, Cordelia Langford, John Sillitoe on behalf of the Wellcome Sanger Institute COVID-19 Surveillance Team
EPI_ISL_851142	Lighthouse Lab in Glasgow	Wellcome Sanger Institute for the COVID-19 Genomics UK (COG-UK) Consortium	Harper VanSteenhouse, Yumi Kasai, David Gray, Carol Clugston, Anna Dominiczak and Alex Alderton, Roberto Amato, Sonia Goncalves, Ewan Harrison, David K. Jackson, Ian Johnston, Dominic Kwiatkowski, Cordelia Langford, John Sillitoe on behalf of the Wellcome Sanger Institute COVID-19 Surveillance Team
EPI_ISL_851143, EPI_ISL_851144, EPI_ISL_851145	Lighthouse Lab in Cambridge	Wellcome Sanger Institute for the COVID-19 Genomics UK (COG-UK) Consortium	Rob Howes, The Lighthouse Lab in Cambridge and Alex Alderton, Roberto Amato, Sonia Goncalves, Ewan Harrison, David K. Jackson, Ian Johnston, Dominic Kwiatkowski, Cordelia Langford, John Sillitoe on behalf of the Wellcome Sanger Institute COVID-19 Surveillance Team
EPI_ISL_851146, EPI_ISL_851147, EPI_ISL_851148	Lighthouse Lab in Glasgow	Wellcome Sanger Institute for the COVID-19 Genomics UK (COG-UK) Consortium	Harper VanSteenhouse, Yumi Kasai, David Gray, Carol Clugston, Anna Dominiczak and Alex Alderton, Roberto Amato, Sonia Goncalves, Ewan Harrison, David K. Jackson, Ian Johnston, Dominic Kwiatkowski, Cordelia Langford, John Sillitoe on behalf of the Wellcome Sanger Institute COVID-19 Surveillance Team
EPI_ISL_851149, EPI_ISL_851150, EPI_ISL_851151, EPI_ISL_851152, EPI_ISL_851153	Lighthouse Lab in Cambridge	Wellcome Sanger Institute for the COVID-19 Genomics UK (COG-UK) Consortium	Rob Howes, The Lighthouse Lab in Cambridge and Alex Alderton, Roberto Amato, Sonia Goncalves, Ewan Harrison, David K. Jackson, Ian Johnston, Dominic Kwiatkowski, Cordelia Langford, John Sillitoe on behalf of the Wellcome Sanger Institute COVID-19 Surveillance Team
EPI_ISL_851154	Lighthouse Lab in Glasgow	Wellcome Sanger Institute for the COVID-19 Genomics UK (COG-UK) Consortium	Harper VanSteenhouse, Yumi Kasai, David Gray, Carol Clugston, Anna Dominiczak and Alex Alderton, Roberto Amato, Sonia Goncalves, Ewan Harrison, David K. Jackson, Ian Johnston, Dominic Kwiatkowski, Cordelia Langford, John Sillitoe on behalf of the Wellcome Sanger Institute COVID-19 Surveillance Team
EPI_ISL_851155, EPI_ISL_851156, EPI_ISL_851157, EPI_ISL_851158, EPI_ISL_851159, EPI_ISL_851160, EPI_ISL_851161, EPI_ISL_851162, EPI_ISL_851163, EPI_ISL_851164, EPI_ISL_851165, EPI_ISL_851166			
see above	Lighthouse Lab in Cambridge	Wellcome Sanger Institute for the COVID-19 Genomics UK (COG-UK) Consortium	Rob Howes, The Lighthouse Lab in Cambridge and Alex Alderton, Roberto Amato, Sonia Goncalves, Ewan Harrison, David K. Jackson, Ian Johnston, Dominic Kwiatkowski, Cordelia Langford, John Sillitoe on behalf of the Wellcome Sanger Institute COVID-19 Surveillance Team
EPI_ISL_851167	Lighthouse Lab in Glasgow	Wellcome Sanger Institute for the COVID-19 Genomics UK (COG-UK) Consortium	Harper VanSteenhouse, Yumi Kasai, David Gray, Carol Clugston, Anna Dominiczak and Alex Alderton, Roberto Amato, Sonia Goncalves, Ewan Harrison, David K. Jackson, Ian Johnston, Dominic Kwiatkowski, Cordelia Langford, John Sillitoe on behalf of the Wellcome Sanger Institute COVID-19 Surveillance Team
EPI_ISL_851168, EPI_ISL_851169, EPI_ISL_851171, EPI_ISL_851172, EPI_ISL_851173, EPI_ISL_851174, EPI_ISL_851175, EPI_ISL_851176, EPI_ISL_851177	Lighthouse Lab in Cambridge	Wellcome Sanger Institute for the COVID-19 Genomics UK (COG-UK) Consortium	Rob Howes, The Lighthouse Lab in Cambridge and Alex Alderton, Roberto Amato, Sonia Goncalves, Ewan Harrison, David K. Jackson, Ian Johnston, Dominic Kwiatkowski, Cordelia Langford, John Sillitoe on behalf of the Wellcome Sanger Institute COVID-19 Surveillance Team
EPI_ISL_851178	Lighthouse Lab in Glasgow	Wellcome Sanger Institute for the COVID-19 Genomics UK (COG-UK) Consortium	Harper VanSteenhouse, Yumi Kasai, David Gray, Carol Clugston, Anna Dominiczak and Alex Alderton, Roberto Amato, Sonia Goncalves, Ewan Harrison, David K. Jackson, Ian Johnston, Dominic Kwiatkowski, Cordelia Langford, John Sillitoe on behalf of the Wellcome Sanger Institute COVID-19 Surveillance Team
EPI_ISL_851179	Lighthouse Lab in Cambridge	Wellcome Sanger Institute for the COVID-19 Genomics UK (COG-UK) Consortium	Rob Howes, The Lighthouse Lab in Cambridge and Alex Alderton, Roberto Amato, Sonia Goncalves, Ewan Harrison, David K. Jackson, Ian Johnston, Dominic Kwiatkowski, Cordelia Langford, John Sillitoe on behalf of the Wellcome Sanger Institute COVID-19 Surveillance Team
EPI_ISL_851180	Lighthouse Lab in Glasgow	Wellcome Sanger Institute for the COVID-19 Genomics UK (COG-UK) Consortium	Harper VanSteenhouse, Yumi Kasai, David Gray, Carol Clugston, Anna Dominiczak and Alex Alderton, Roberto Amato, Sonia Goncalves, Ewan Harrison, David K. Jackson, Ian Johnston, Dominic Kwiatkowski, Cordelia Langford, John Sillitoe on behalf of the Wellcome Sanger Institute COVID-19 Surveillance Team
EPI_ISL_851181	Lighthouse Lab in Cambridge	Wellcome Sanger Institute for the COVID-19 Genomics UK (COG-UK) Consortium	Rob Howes, The Lighthouse Lab in Cambridge and Alex Alderton, Roberto Amato, Sonia Goncalves, Ewan Harrison, David K. Jackson, Ian Johnston, Dominic Kwiatkowski, Cordelia Langford, John Sillitoe on behalf of the Wellcome Sanger Institute COVID-19 Surveillance Team
EPI_ISL_851182	Lighthouse Lab in Glasgow	Wellcome Sanger Institute for the COVID-19 Genomics UK (COG-UK) Consortium	Harper VanSteenhouse, Yumi Kasai, David Gray, Carol Clugston, Anna Dominiczak and Alex Alderton, Roberto Amato, Sonia Goncalves, Ewan Harrison, David K. Jackson, Ian Johnston, Dominic Kwiatkowski, Cordelia Langford, John Sillitoe on behalf of the Wellcome Sanger Institute COVID-19 Surveillance Team
EPI_ISL_851183, EPI_ISL_851184, EPI_ISL_851185, EPI_ISL_851186	Lighthouse Lab in Cambridge	Wellcome Sanger Institute for the COVID-19 Genomics UK (COG-UK) Consortium	Rob Howes, The Lighthouse Lab in Cambridge and Alex Alderton, Roberto Amato, Sonia Goncalves, Ewan Harrison, David K. Jackson, Ian Johnston, Dominic Kwiatkowski, Cordelia Langford, John Sillitoe on behalf of the Wellcome Sanger Institute COVID-19 Surveillance Team
EPI_ISL_851187	Lighthouse Lab in Glasgow	Wellcome Sanger Institute for the COVID-19 Genomics UK (COG-UK) Consortium	Harper VanSteenhouse, Yumi Kasai, David Gray, Carol Clugston, Anna Dominiczak and Alex Alderton, Roberto Amato, Sonia Goncalves, Ewan Harrison, David K. Jackson, Ian Johnston, Dominic Kwiatkowski, Cordelia Langford, John Sillitoe on behalf of the Wellcome Sanger Institute COVID-19 Surveillance Team
EPI_ISL_851188, EPI_ISL_851189	Lighthouse Lab in Cambridge	Wellcome Sanger Institute for the COVID-19 Genomics UK (COG-UK) Consortium	Rob Howes, The Lighthouse Lab in Cambridge and Alex Alderton, Roberto Amato, Sonia Goncalves, Ewan Harrison, David K. Jackson, Ian Johnston, Dominic Kwiatkowski, Cordelia Langford, John Sillitoe on behalf of the Wellcome Sanger Institute COVID-19 Surveillance Team
EPI_ISL_851190	Lighthouse Lab in Glasgow	Wellcome Sanger Institute for the COVID-19 Genomics UK (COG-UK) Consortium	Harper VanSteenhouse, Yumi Kasai, David Gray, Carol Clugston, Anna Dominiczak and Alex Alderton, Roberto Amato, Sonia Goncalves, Ewan Harrison, David K. Jackson, Ian Johnston, Dominic Kwiatkowski, Cordelia Langford, John Sillitoe on behalf of the Wellcome Sanger Institute COVID-19 Surveillance Team
EPI_ISL_851191, EPI_ISL_851192, EPI_ISL_851193, EPI_ISL_851194, EPI_ISL_851195	Lighthouse Lab in Cambridge	Wellcome Sanger Institute for the COVID-19 Genomics UK (COG-UK) Consortium	Rob Howes, The Lighthouse Lab in Cambridge and Alex Alderton, Roberto Amato, Sonia Goncalves, Ewan Harrison, David K. Jackson, Ian Johnston, Dominic Kwiatkowski, Cordelia Langford, John Sillitoe on behalf of the Wellcome Sanger Institute COVID-19 Surveillance Team
EPI_ISL_851196	Lighthouse Lab in Glasgow	Wellcome Sanger Institute for the COVID-19 Genomics UK (COG-UK) Consortium	Harper VanSteenhouse, Yumi Kasai, David Gray, Carol Clugston, Anna Dominiczak and Alex Alderton, Roberto Amato, Sonia Goncalves, Ewan Harrison, David K. Jackson, Ian Johnston, Dominic Kwiatkowski, Cordelia Langford, John Sillitoe on behalf of the Wellcome Sanger Institute COVID-19 Surveillance Team
EPI_ISL_851197, EPI_ISL_851198, EPI_ISL_851199, EPI_ISL_851200, EPI_ISL_851201, EPI_ISL_851202, EPI_ISL_851203, EPI_ISL_851204, EPI_ISL_851205	Lighthouse Lab in Cambridge	Wellcome Sanger Institute for the COVID-19 Genomics UK (COG-UK) Consortium	Rob Howes, The Lighthouse Lab in Cambridge and Alex Alderton, Roberto Amato, Sonia Goncalves, Ewan Harrison, David K. Jackson, Ian Johnston, Dominic Kwiatkowski, Cordelia Langford, John Sillitoe on behalf of the Wellcome Sanger Institute COVID-19 Surveillance Team
EPI_ISL_851206, EPI_ISL_851207	Lighthouse Lab in Glasgow	Wellcome Sanger Institute for the COVID-19 Genomics UK (COG-UK) Consortium	Harper VanSteenhouse, Yumi Kasai, David Gray, Carol Clugston, Anna Dominiczak and Alex Alderton, Roberto Amato, Sonia Goncalves, Ewan Harrison, David K. Jackson, Ian Johnston, Dominic Kwiatkowski, Cordelia Langford, John Sillitoe on behalf of the Wellcome Sanger Institute COVID-19 Surveillance Team
EPI_ISL_851208, EPI_ISL_851209, EPI_ISL_851210, EPI_ISL_851211, EPI_ISL_851212, EPI_ISL_851213, EPI_ISL_851214	Lighthouse Lab in Cambridge	Wellcome Sanger Institute for the COVID-19 Genomics UK (COG-UK) Consortium	Rob Howes, The Lighthouse Lab in Cambridge and Alex Alderton, Roberto Amato, Sonia Goncalves, Ewan Harrison, David K. Jackson, Ian Johnston, Dominic Kwiatkowski, Cordelia Langford, John Sillitoe on behalf of the Wellcome Sanger Institute COVID-19 Surveillance Team
EPI_ISL_851215	Lighthouse Lab in Glasgow	Wellcome Sanger Institute for the COVID-19 Genomics UK (COG-UK) Consortium	Harper VanSteenhouse, Yumi Kasai, David Gray, Carol Clugston, Anna Dominiczak and Alex Alderton, Roberto Amato, Sonia Goncalves, Ewan Harrison, David K. Jackson, Ian Johnston, Dominic Kwiatkowski, Cordelia Langford, John Sillitoe on behalf of the Wellcome Sanger Institute COVID-19 Surveillance Team
EPI_ISL_851216	Lighthouse Lab in Cambridge	Wellcome Sanger Institute for the COVID-19 Genomics UK (COG-UK) Consortium	Rob Howes, The Lighthouse Lab in Cambridge and Alex Alderton, Roberto Amato, Sonia Goncalves, Ewan Harrison, David K. Jackson, Ian Johnston, Dominic Kwiatkowski, Cordelia Langford, John Sillitoe on behalf of the Wellcome Sanger Institute COVID-19 Surveillance Team
EPI_ISL_851217, EPI_ISL_851218	Lighthouse Lab in Glasgow	Wellcome Sanger Institute for the COVID-19 Genomics UK (COG-UK) Consortium	Harper VanSteenhouse, Yumi Kasai, David Gray, Carol Clugston, Anna Dominiczak and Alex Alderton, Roberto Amato, Sonia Goncalves, Ewan Harrison, David K. Jackson, Ian Johnston, Dominic Kwiatkowski, Cordelia Langford, John Sillitoe on behalf of the Wellcome Sanger Institute COVID-19 Surveillance

			Team
EPI_ISL_851219, EPI_ISL_851220, EPI_ISL_851221, EPI_ISL_851222, EPI_ISL_851223, EPI_ISL_851224	Lighthouse Lab in Cambridge	Wellcome Sanger Institute for the COVID-19 Genomics UK (COG-UK) Consortium	Rob Howes, The Lighthouse Lab in Cambridge and Alex Alderton, Roberto Amato, Sonia Goncalves, Ewan Harrison, David K. Jackson, Ian Johnston, Dominic Kwiatkowski, Cordelia Langford, John Sillitoe on behalf of the Wellcome Sanger Institute COVID-19 Surveillance Team
EPI_ISL_851225	Lighthouse Lab in Glasgow	Wellcome Sanger Institute for the COVID-19 Genomics UK (COG-UK) Consortium	Harper VanSteenhouse, Yumi Kasai, David Gray, Carol Clugston, Anna Dominiczak and Alex Alderton, Roberto Amato, Sonia Goncalves, Ewan Harrison, David K. Jackson, Ian Johnston, Dominic Kwiatkowski, Cordelia Langford, John Sillitoe on behalf of the Wellcome Sanger Institute COVID-19 Surveillance Team
EPI_ISL_851226, EPI_ISL_851227, EPI_ISL_851228	Lighthouse Lab in Cambridge	Wellcome Sanger Institute for the COVID-19 Genomics UK (COG-UK) Consortium	Rob Howes, The Lighthouse Lab in Cambridge and Alex Alderton, Roberto Amato, Sonia Goncalves, Ewan Harrison, David K. Jackson, Ian Johnston, Dominic Kwiatkowski, Cordelia Langford, John Sillitoe on behalf of the Wellcome Sanger Institute COVID-19 Surveillance Team
EPI_ISL_851229	Lighthouse Lab in Glasgow	Wellcome Sanger Institute for the COVID-19 Genomics UK (COG-UK) Consortium	Harper VanSteenhouse, Yumi Kasai, David Gray, Carol Clugston, Anna Dominiczak and Alex Alderton, Roberto Amato, Sonia Goncalves, Ewan Harrison, David K. Jackson, Ian Johnston, Dominic Kwiatkowski, Cordelia Langford, John Sillitoe on behalf of the Wellcome Sanger Institute COVID-19 Surveillance Team
EPI_ISL_851230, EPI_ISL_851231, EPI_ISL_851232, EPI_ISL_851233, EPI_ISL_851234, EPI_ISL_851235	Lighthouse Lab in Cambridge	Wellcome Sanger Institute for the COVID-19 Genomics UK (COG-UK) Consortium	Rob Howes, The Lighthouse Lab in Cambridge and Alex Alderton, Roberto Amato, Sonia Goncalves, Ewan Harrison, David K. Jackson, Ian Johnston, Dominic Kwiatkowski, Cordelia Langford, John Sillitoe on behalf of the Wellcome Sanger Institute COVID-19 Surveillance Team
EPI_ISL_851236, EPI_ISL_851237	Lighthouse Lab in Glasgow	Wellcome Sanger Institute for the COVID-19 Genomics UK (COG-UK) Consortium	Harper VanSteenhouse, Yumi Kasai, David Gray, Carol Clugston, Anna Dominiczak and Alex Alderton, Roberto Amato, Sonia Goncalves, Ewan Harrison, David K. Jackson, Ian Johnston, Dominic Kwiatkowski, Cordelia Langford, John Sillitoe on behalf of the Wellcome Sanger Institute COVID-19 Surveillance Team
EPI_ISL_851238, EPI_ISL_851239, EPI_ISL_851240	Lighthouse Lab in Cambridge	Wellcome Sanger Institute for the COVID-19 Genomics UK (COG-UK) Consortium	Rob Howes, The Lighthouse Lab in Cambridge and Alex Alderton, Roberto Amato, Sonia Goncalves, Ewan Harrison, David K. Jackson, Ian Johnston, Dominic Kwiatkowski, Cordelia Langford, John Sillitoe on behalf of the Wellcome Sanger Institute COVID-19 Surveillance Team
EPI_ISL_851241	Lighthouse Lab in Glasgow	Wellcome Sanger Institute for the COVID-19 Genomics UK (COG-UK) Consortium	Harper VanSteenhouse, Yumi Kasai, David Gray, Carol Clugston, Anna Dominiczak and Alex Alderton, Roberto Amato, Sonia Goncalves, Ewan Harrison, David K. Jackson, Ian Johnston, Dominic Kwiatkowski, Cordelia Langford, John Sillitoe on behalf of the Wellcome Sanger Institute COVID-19 Surveillance Team
EPI_ISL_851242, EPI_ISL_851243, EPI_ISL_851244, EPI_ISL_851245	Lighthouse Lab in Cambridge	Wellcome Sanger Institute for the COVID-19 Genomics UK (COG-UK) Consortium	Rob Howes, The Lighthouse Lab in Cambridge and Alex Alderton, Roberto Amato, Sonia Goncalves, Ewan Harrison, David K. Jackson, Ian Johnston, Dominic Kwiatkowski, Cordelia Langford, John Sillitoe on behalf of the Wellcome Sanger Institute COVID-19 Surveillance Team
EPI_ISL_851246	Lighthouse Lab in Glasgow	Wellcome Sanger Institute for the COVID-19 Genomics UK (COG-UK) Consortium	Harper VanSteenhouse, Yumi Kasai, David Gray, Carol Clugston, Anna Dominiczak and Alex Alderton, Roberto Amato, Sonia Goncalves, Ewan Harrison, David K. Jackson, Ian Johnston, Dominic Kwiatkowski, Cordelia Langford, John Sillitoe on behalf of the Wellcome Sanger Institute COVID-19 Surveillance Team
EPI_ISL_851247, EPI_ISL_851248, EPI_ISL_851249, EPI_ISL_851250	Lighthouse Lab in Cambridge	Wellcome Sanger Institute for the COVID-19 Genomics UK (COG-UK) Consortium	Rob Howes, The Lighthouse Lab in Cambridge and Alex Alderton, Roberto Amato, Sonia Goncalves, Ewan Harrison, David K. Jackson, Ian Johnston, Dominic Kwiatkowski, Cordelia Langford, John Sillitoe on behalf of the Wellcome Sanger Institute COVID-19 Surveillance Team
EPI_ISL_851252, EPI_ISL_851253	Lighthouse Lab in Glasgow	Wellcome Sanger Institute for the COVID-19 Genomics UK (COG-UK) Consortium	Harper VanSteenhouse, Yumi Kasai, David Gray, Carol Clugston, Anna Dominiczak and Alex Alderton, Roberto Amato, Sonia Goncalves, Ewan Harrison, David K. Jackson, Ian Johnston, Dominic Kwiatkowski, Cordelia Langford, John Sillitoe on behalf of the Wellcome Sanger Institute COVID-19 Surveillance Team
EPI_ISL_851254, EPI_ISL_851255, EPI_ISL_851256	Lighthouse Lab in Cambridge	Wellcome Sanger Institute for the COVID-19 Genomics UK (COG-UK) Consortium	Rob Howes, The Lighthouse Lab in Cambridge and Alex Alderton, Roberto Amato, Sonia Goncalves, Ewan Harrison, David K. Jackson, Ian Johnston, Dominic Kwiatkowski, Cordelia Langford, John Sillitoe on behalf of the Wellcome Sanger Institute COVID-19 Surveillance Team
EPI_ISL_851257, EPI_ISL_851258, EPI_ISL_851259	Lighthouse Lab in Glasgow	Wellcome Sanger Institute for the COVID-19 Genomics UK (COG-UK) Consortium	Harper VanSteenhouse, Yumi Kasai, David Gray, Carol Clugston, Anna Dominiczak and Alex Alderton, Roberto Amato, Sonia Goncalves, Ewan Harrison, David K. Jackson, Ian Johnston, Dominic Kwiatkowski, Cordelia Langford, John Sillitoe on behalf of the Wellcome Sanger Institute COVID-19 Surveillance Team
EPI_ISL_851260, EPI_ISL_851261, EPI_ISL_851262, EPI_ISL_851263	Lighthouse Lab in Cambridge	Wellcome Sanger Institute for the COVID-19 Genomics UK (COG-UK) Consortium	Rob Howes, The Lighthouse Lab in Cambridge and Alex Alderton, Roberto Amato, Sonia Goncalves, Ewan Harrison, David K. Jackson, Ian Johnston, Dominic Kwiatkowski, Cordelia Langford, John Sillitoe on behalf of the Wellcome Sanger Institute COVID-19 Surveillance Team
EPI_ISL_851264	Lighthouse Lab in Glasgow	Wellcome Sanger Institute for the COVID-19 Genomics UK (COG-UK) Consortium	Harper VanSteenhouse, Yumi Kasai, David Gray, Carol Clugston, Anna Dominiczak and Alex Alderton, Roberto Amato, Sonia Goncalves, Ewan Harrison, David K. Jackson, Ian Johnston, Dominic Kwiatkowski, Cordelia Langford, John Sillitoe on behalf of the Wellcome Sanger Institute COVID-19 Surveillance Team
EPI_ISL_851265, EPI_ISL_851266, EPI_ISL_851267, EPI_ISL_851268	Lighthouse Lab in Cambridge	Wellcome Sanger Institute for the COVID-19 Genomics UK (COG-UK) Consortium	Rob Howes, The Lighthouse Lab in Cambridge and Alex Alderton, Roberto Amato, Sonia Goncalves, Ewan Harrison, David K. Jackson, Ian Johnston, Dominic Kwiatkowski, Cordelia Langford, John Sillitoe on behalf of the Wellcome Sanger Institute COVID-19 Surveillance Team
EPI_ISL_851270	Lighthouse Lab in Glasgow	Wellcome Sanger Institute for the COVID-19 Genomics UK (COG-UK) Consortium	Harper VanSteenhouse, Yumi Kasai, David Gray, Carol Clugston, Anna Dominiczak and Alex Alderton, Roberto Amato, Sonia Goncalves, Ewan Harrison, David K. Jackson, Ian Johnston, Dominic Kwiatkowski, Cordelia Langford, John Sillitoe on behalf of the Wellcome Sanger Institute COVID-19 Surveillance Team
EPI_ISL_851271, EPI_ISL_851272, EPI_ISL_851274, EPI_ISL_851275	Lighthouse Lab in Cambridge	Wellcome Sanger Institute for the COVID-19 Genomics UK (COG-UK) Consortium	Rob Howes, The Lighthouse Lab in Cambridge and Alex Alderton, Roberto Amato, Sonia Goncalves, Ewan Harrison, David K. Jackson, Ian Johnston, Dominic Kwiatkowski, Cordelia Langford, John Sillitoe on behalf of the Wellcome Sanger Institute COVID-19 Surveillance Team
EPI_ISL_851276	Lighthouse Lab in Glasgow	Wellcome Sanger Institute for the COVID-19 Genomics UK (COG-UK) Consortium	Harper VanSteenhouse, Yumi Kasai, David Gray, Carol Clugston, Anna Dominiczak and Alex Alderton, Roberto Amato, Sonia Goncalves, Ewan Harrison, David K. Jackson, Ian Johnston, Dominic Kwiatkowski, Cordelia Langford, John Sillitoe on behalf of the Wellcome Sanger Institute COVID-19 Surveillance Team
EPI_ISL_851277, EPI_ISL_851278, EPI_ISL_851279, EPI_ISL_851280, EPI_ISL_851281, EPI_ISL_851282, EPI_ISL_851283, EPI_ISL_851284	Lighthouse Lab in Cambridge	Wellcome Sanger Institute for the COVID-19 Genomics UK (COG-UK) Consortium	Rob Howes, The Lighthouse Lab in Cambridge and Alex Alderton, Roberto Amato, Sonia Goncalves, Ewan Harrison, David K. Jackson, Ian Johnston, Dominic Kwiatkowski, Cordelia Langford, John Sillitoe on behalf of the Wellcome Sanger Institute COVID-19 Surveillance Team
EPI_ISL_851285	Lighthouse Lab in Glasgow	Wellcome Sanger Institute for the COVID-19 Genomics UK (COG-UK) Consortium	Harper VanSteenhouse, Yumi Kasai, David Gray, Carol Clugston, Anna Dominiczak and Alex Alderton, Roberto Amato, Sonia Goncalves, Ewan Harrison, David K. Jackson, Ian Johnston, Dominic Kwiatkowski, Cordelia Langford, John Sillitoe on behalf of the Wellcome Sanger Institute COVID-19 Surveillance Team
EPI_ISL_851286, EPI_ISL_851287, EPI_ISL_851288, EPI_ISL_851289, EPI_ISL_851290, EPI_ISL_851291, EPI_ISL_851292, EPI_ISL_851293, EPI_ISL_851294, EPI_ISL_851295	Lighthouse Lab in Cambridge	Wellcome Sanger Institute for the COVID-19 Genomics UK (COG-UK) Consortium	Rob Howes, The Lighthouse Lab in Cambridge and Alex Alderton, Roberto Amato, Sonia Goncalves, Ewan Harrison, David K. Jackson, Ian Johnston, Dominic Kwiatkowski, Cordelia Langford, John Sillitoe on behalf of the Wellcome Sanger Institute COVID-19 Surveillance Team
EPI_ISL_851296	Lighthouse Lab in Glasgow	Wellcome Sanger Institute for the COVID-19 Genomics UK (COG-UK) Consortium	Harper VanSteenhouse, Yumi Kasai, David Gray, Carol Clugston, Anna Dominiczak and Alex Alderton, Roberto Amato, Sonia Goncalves, Ewan Harrison, David K. Jackson, Ian Johnston, Dominic Kwiatkowski, Cordelia Langford, John Sillitoe on behalf of the Wellcome Sanger Institute COVID-19 Surveillance Team
EPI_ISL_851297, EPI_ISL_851299, EPI_ISL_851300	Lighthouse Lab in Cambridge	Wellcome Sanger Institute for the COVID-19 Genomics UK (COG-UK) Consortium	Rob Howes, The Lighthouse Lab in Cambridge and Alex Alderton, Roberto Amato, Sonia Goncalves, Ewan Harrison, David K. Jackson, Ian Johnston, Dominic Kwiatkowski, Cordelia Langford, John Sillitoe on behalf of the Wellcome Sanger Institute COVID-19 Surveillance Team
EPI_ISL_851301	Lighthouse Lab in Glasgow	Wellcome Sanger Institute for the COVID-19 Genomics UK (COG-UK) Consortium	Harper VanSteenhouse, Yumi Kasai, David Gray, Carol Clugston, Anna Dominiczak and Alex Alderton, Roberto Amato, Sonia Goncalves, Ewan Harrison, David K. Jackson, Ian Johnston, Dominic Kwiatkowski, Cordelia Langford, John Sillitoe on behalf of the Wellcome Sanger Institute COVID-19 Surveillance Team
EPI_ISL_851302, EPI_ISL_851303,	Lighthouse Lab in Cambridge	Wellcome Sanger Institute for the COVID-19 Genomics UK	Rob Howes, The Lighthouse Lab in Cambridge and Alex Alderton, Roberto Amato, Sonia Goncalves, Ewan Harrison, David K. Jackson, Ian Johnston,

EPI_ISL_851304		(COG-UK) Consortium	Dominic Kwiatkowski, Cordelia Langford, John Sillitoe on behalf of the Wellcome Sanger Institute COVID-19 Surveillance Team
EPI_ISL_851305, EPI_ISL_851306, EPI_ISL_851307	Lighthouse Lab in Glasgow	Wellcome Sanger Institute for the COVID-19 Genomics UK (COG-UK) Consortium	Harper VanSteenhouse, Yumi Kasai, David Gray, Carol Clugston, Anna Dominiczak and Alex Alderton, Roberto Amato, Sonia Goncalves, Ewan Harrison, David K. Jackson, Ian Johnston, Dominic Kwiatkowski, Cordelia Langford, John Sillitoe on behalf of the Wellcome Sanger Institute COVID-19 Surveillance Team
EPI_ISL_851308, EPI_ISL_851309	Lighthouse Lab in Cambridge	Wellcome Sanger Institute for the COVID-19 Genomics UK (COG-UK) Consortium	Rob Howes, The Lighthouse Lab in Cambridge and Alex Alderton, Roberto Amato, Sonia Goncalves, Ewan Harrison, David K. Jackson, Ian Johnston, Dominic Kwiatkowski, Cordelia Langford, John Sillitoe on behalf of the Wellcome Sanger Institute COVID-19 Surveillance Team
EPI_ISL_851310	Lighthouse Lab in Glasgow	Wellcome Sanger Institute for the COVID-19 Genomics UK (COG-UK) Consortium	Harper VanSteenhouse, Yumi Kasai, David Gray, Carol Clugston, Anna Dominiczak and Alex Alderton, Roberto Amato, Sonia Goncalves, Ewan Harrison, David K. Jackson, Ian Johnston, Dominic Kwiatkowski, Cordelia Langford, John Sillitoe on behalf of the Wellcome Sanger Institute COVID-19 Surveillance Team
EPI_ISL_851311, EPI_ISL_851312, EPI_ISL_851313, EPI_ISL_851314, EPI_ISL_851315, EPI_ISL_851316, EPI_ISL_851317, EPI_ISL_851318, EPI_ISL_851319, EPI_ISL_851320, EPI_ISL_851321, EPI_ISL_851322, EPI_ISL_851323, EPI_ISL_851324			
see above	Lighthouse Lab in Cambridge	Wellcome Sanger Institute for the COVID-19 Genomics UK (COG-UK) Consortium	Rob Howes, The Lighthouse Lab in Cambridge and Alex Alderton, Roberto Amato, Sonia Goncalves, Ewan Harrison, David K. Jackson, Ian Johnston, Dominic Kwiatkowski, Cordelia Langford, John Sillitoe on behalf of the Wellcome Sanger Institute COVID-19 Surveillance Team
EPI_ISL_851325	Lighthouse Lab in Glasgow	Wellcome Sanger Institute for the COVID-19 Genomics UK (COG-UK) Consortium	Harper VanSteenhouse, Yumi Kasai, David Gray, Carol Clugston, Anna Dominiczak and Alex Alderton, Roberto Amato, Sonia Goncalves, Ewan Harrison, David K. Jackson, Ian Johnston, Dominic Kwiatkowski, Cordelia Langford, John Sillitoe on behalf of the Wellcome Sanger Institute COVID-19 Surveillance Team
EPI_ISL_851326	Lighthouse Lab in Cambridge	Wellcome Sanger Institute for the COVID-19 Genomics UK (COG-UK) Consortium	Rob Howes, The Lighthouse Lab in Cambridge and Alex Alderton, Roberto Amato, Sonia Goncalves, Ewan Harrison, David K. Jackson, Ian Johnston, Dominic Kwiatkowski, Cordelia Langford, John Sillitoe on behalf of the Wellcome Sanger Institute COVID-19 Surveillance Team
EPI_ISL_851327	Lighthouse Lab in Glasgow	Wellcome Sanger Institute for the COVID-19 Genomics UK (COG-UK) Consortium	Harper VanSteenhouse, Yumi Kasai, David Gray, Carol Clugston, Anna Dominiczak and Alex Alderton, Roberto Amato, Sonia Goncalves, Ewan Harrison, David K. Jackson, Ian Johnston, Dominic Kwiatkowski, Cordelia Langford, John Sillitoe on behalf of the Wellcome Sanger Institute COVID-19 Surveillance Team
EPI_ISL_851328	Lighthouse Lab in Cambridge	Wellcome Sanger Institute for the COVID-19 Genomics UK (COG-UK) Consortium	Rob Howes, The Lighthouse Lab in Cambridge and Alex Alderton, Roberto Amato, Sonia Goncalves, Ewan Harrison, David K. Jackson, Ian Johnston, Dominic Kwiatkowski, Cordelia Langford, John Sillitoe on behalf of the Wellcome Sanger Institute COVID-19 Surveillance Team
EPI_ISL_851329	Lighthouse Lab in Glasgow	Wellcome Sanger Institute for the COVID-19 Genomics UK (COG-UK) Consortium	Harper VanSteenhouse, Yumi Kasai, David Gray, Carol Clugston, Anna Dominiczak and Alex Alderton, Roberto Amato, Sonia Goncalves, Ewan Harrison, David K. Jackson, Ian Johnston, Dominic Kwiatkowski, Cordelia Langford, John Sillitoe on behalf of the Wellcome Sanger Institute COVID-19 Surveillance Team
EPI_ISL_851330	Lighthouse Lab in Cambridge	Wellcome Sanger Institute for the COVID-19 Genomics UK (COG-UK) Consortium	Rob Howes, The Lighthouse Lab in Cambridge and Alex Alderton, Roberto Amato, Sonia Goncalves, Ewan Harrison, David K. Jackson, Ian Johnston, Dominic Kwiatkowski, Cordelia Langford, John Sillitoe on behalf of the Wellcome Sanger Institute COVID-19 Surveillance Team
EPI_ISL_851331	Lighthouse Lab in Glasgow	Wellcome Sanger Institute for the COVID-19 Genomics UK (COG-UK) Consortium	Harper VanSteenhouse, Yumi Kasai, David Gray, Carol Clugston, Anna Dominiczak and Alex Alderton, Roberto Amato, Sonia Goncalves, Ewan Harrison, David K. Jackson, Ian Johnston, Dominic Kwiatkowski, Cordelia Langford, John Sillitoe on behalf of the Wellcome Sanger Institute COVID-19 Surveillance Team
EPI_ISL_851332, EPI_ISL_851333, EPI_ISL_851334	Lighthouse Lab in Cambridge	Wellcome Sanger Institute for the COVID-19 Genomics UK (COG-UK) Consortium	Rob Howes, The Lighthouse Lab in Cambridge and Alex Alderton, Roberto Amato, Sonia Goncalves, Ewan Harrison, David K. Jackson, Ian Johnston, Dominic Kwiatkowski, Cordelia Langford, John Sillitoe on behalf of the Wellcome Sanger Institute COVID-19 Surveillance Team
EPI_ISL_851335	Lighthouse Lab in Glasgow	Wellcome Sanger Institute for the COVID-19 Genomics UK (COG-UK) Consortium	Harper VanSteenhouse, Yumi Kasai, David Gray, Carol Clugston, Anna Dominiczak and Alex Alderton, Roberto Amato, Sonia Goncalves, Ewan Harrison, David K. Jackson, Ian Johnston, Dominic Kwiatkowski, Cordelia Langford, John Sillitoe on behalf of the Wellcome Sanger Institute COVID-19 Surveillance Team
EPI_ISL_851336, EPI_ISL_851337, EPI_ISL_851338, EPI_ISL_851339, EPI_ISL_851340	Lighthouse Lab in Cambridge	Wellcome Sanger Institute for the COVID-19 Genomics UK (COG-UK) Consortium	Rob Howes, The Lighthouse Lab in Cambridge and Alex Alderton, Roberto Amato, Sonia Goncalves, Ewan Harrison, David K. Jackson, Ian Johnston, Dominic Kwiatkowski, Cordelia Langford, John Sillitoe on behalf of the Wellcome Sanger Institute COVID-19 Surveillance Team
EPI_ISL_851341	Lighthouse Lab in Glasgow	Wellcome Sanger Institute for the COVID-19 Genomics UK (COG-UK) Consortium	Harper VanSteenhouse, Yumi Kasai, David Gray, Carol Clugston, Anna Dominiczak and Alex Alderton, Roberto Amato, Sonia Goncalves, Ewan Harrison, David K. Jackson, Ian Johnston, Dominic Kwiatkowski, Cordelia Langford, John Sillitoe on behalf of the Wellcome Sanger Institute COVID-19 Surveillance Team
EPI_ISL_851342, EPI_ISL_851344, EPI_ISL_851345	Lighthouse Lab in Cambridge	Wellcome Sanger Institute for the COVID-19 Genomics UK (COG-UK) Consortium	Rob Howes, The Lighthouse Lab in Cambridge and Alex Alderton, Roberto Amato, Sonia Goncalves, Ewan Harrison, David K. Jackson, Ian Johnston, Dominic Kwiatkowski, Cordelia Langford, John Sillitoe on behalf of the Wellcome Sanger Institute COVID-19 Surveillance Team
EPI_ISL_851346	Lighthouse Lab in Glasgow	Wellcome Sanger Institute for the COVID-19 Genomics UK (COG-UK) Consortium	Harper VanSteenhouse, Yumi Kasai, David Gray, Carol Clugston, Anna Dominiczak and Alex Alderton, Roberto Amato, Sonia Goncalves, Ewan Harrison, David K. Jackson, Ian Johnston, Dominic Kwiatkowski, Cordelia Langford, John Sillitoe on behalf of the Wellcome Sanger Institute COVID-19 Surveillance Team
EPI_ISL_851347, EPI_ISL_851348, EPI_ISL_851349, EPI_ISL_851350	Lighthouse Lab in Cambridge	Wellcome Sanger Institute for the COVID-19 Genomics UK (COG-UK) Consortium	Rob Howes, The Lighthouse Lab in Cambridge and Alex Alderton, Roberto Amato, Sonia Goncalves, Ewan Harrison, David K. Jackson, Ian Johnston, Dominic Kwiatkowski, Cordelia Langford, John Sillitoe on behalf of the Wellcome Sanger Institute COVID-19 Surveillance Team
EPI_ISL_851351	Lighthouse Lab in Glasgow	Wellcome Sanger Institute for the COVID-19 Genomics UK (COG-UK) Consortium	Harper VanSteenhouse, Yumi Kasai, David Gray, Carol Clugston, Anna Dominiczak and Alex Alderton, Roberto Amato, Sonia Goncalves, Ewan Harrison, David K. Jackson, Ian Johnston, Dominic Kwiatkowski, Cordelia Langford, John Sillitoe on behalf of the Wellcome Sanger Institute COVID-19 Surveillance Team
EPI_ISL_851352, EPI_ISL_851353, EPI_ISL_851354, EPI_ISL_851355, EPI_ISL_851356, EPI_ISL_851357	Lighthouse Lab in Cambridge	Wellcome Sanger Institute for the COVID-19 Genomics UK (COG-UK) Consortium	Rob Howes, The Lighthouse Lab in Cambridge and Alex Alderton, Roberto Amato, Sonia Goncalves, Ewan Harrison, David K. Jackson, Ian Johnston, Dominic Kwiatkowski, Cordelia Langford, John Sillitoe on behalf of the Wellcome Sanger Institute COVID-19 Surveillance Team
EPI_ISL_851358	Lighthouse Lab in Glasgow	Wellcome Sanger Institute for the COVID-19 Genomics UK (COG-UK) Consortium	Harper VanSteenhouse, Yumi Kasai, David Gray, Carol Clugston, Anna Dominiczak and Alex Alderton, Roberto Amato, Sonia Goncalves, Ewan Harrison, David K. Jackson, Ian Johnston, Dominic Kwiatkowski, Cordelia Langford, John Sillitoe on behalf of the Wellcome Sanger Institute COVID-19 Surveillance Team
EPI_ISL_851359, EPI_ISL_851360, EPI_ISL_851361, EPI_ISL_851362, EPI_ISL_851363, EPI_ISL_851364	Lighthouse Lab in Cambridge	Wellcome Sanger Institute for the COVID-19 Genomics UK (COG-UK) Consortium	Rob Howes, The Lighthouse Lab in Cambridge and Alex Alderton, Roberto Amato, Sonia Goncalves, Ewan Harrison, David K. Jackson, Ian Johnston, Dominic Kwiatkowski, Cordelia Langford, John Sillitoe on behalf of the Wellcome Sanger Institute COVID-19 Surveillance Team
EPI_ISL_851365	Lighthouse Lab in Glasgow	Wellcome Sanger Institute for the COVID-19 Genomics UK (COG-UK) Consortium	Harper VanSteenhouse, Yumi Kasai, David Gray, Carol Clugston, Anna Dominiczak and Alex Alderton, Roberto Amato, Sonia Goncalves, Ewan Harrison, David K. Jackson, Ian Johnston, Dominic Kwiatkowski, Cordelia Langford, John Sillitoe on behalf of the Wellcome Sanger Institute COVID-19 Surveillance Team
EPI_ISL_851366	Lighthouse Lab in Cambridge	Wellcome Sanger Institute for the COVID-19 Genomics UK (COG-UK) Consortium	Rob Howes, The Lighthouse Lab in Cambridge and Alex Alderton, Roberto Amato, Sonia Goncalves, Ewan Harrison, David K. Jackson, Ian Johnston, Dominic Kwiatkowski, Cordelia Langford, John Sillitoe on behalf of the Wellcome Sanger Institute COVID-19 Surveillance Team
EPI_ISL_851367	Lighthouse Lab in Glasgow	Wellcome Sanger Institute for the COVID-19 Genomics UK (COG-UK) Consortium	Harper VanSteenhouse, Yumi Kasai, David Gray, Carol Clugston, Anna Dominiczak and Alex Alderton, Roberto Amato, Sonia Goncalves, Ewan Harrison, David K. Jackson, Ian Johnston, Dominic Kwiatkowski, Cordelia Langford, John Sillitoe on behalf of the Wellcome Sanger Institute COVID-19 Surveillance Team
EPI_ISL_851368, EPI_ISL_851369, EPI_ISL_851370	Lighthouse Lab in Cambridge	Wellcome Sanger Institute for the COVID-19 Genomics UK (COG-UK) Consortium	Rob Howes, The Lighthouse Lab in Cambridge and Alex Alderton, Roberto Amato, Sonia Goncalves, Ewan Harrison, David K. Jackson, Ian Johnston, Dominic Kwiatkowski, Cordelia Langford, John Sillitoe on behalf of the Wellcome Sanger Institute COVID-19 Surveillance Team
EPI_ISL_851371	Lighthouse Lab in Glasgow	Wellcome Sanger Institute for the COVID-19 Genomics UK (COG-UK) Consortium	Harper VanSteenhouse, Yumi Kasai, David Gray, Carol Clugston, Anna Dominiczak and Alex Alderton, Roberto Amato, Sonia Goncalves, Ewan Harrison, David K. Jackson, Ian Johnston, Dominic Kwiatkowski, Cordelia Langford, John Sillitoe on behalf of the Wellcome Sanger Institute COVID-19 Surveillance Team

EPI_ISL_851372, EPI_ISL_851373	Lighthouse Lab in Cambridge	Wellcome Sanger Institute for the COVID-19 Genomics UK (COG-UK) Consortium	Rob Howes, The Lighthouse Lab in Cambridge and Alex Alderton, Roberto Amato, Sonia Goncalves, Ewan Harrison, David K. Jackson, Ian Johnston, Dominic Kwiatkowski, Cordelia Langford, John Sillitoe on behalf of the Wellcome Sanger Institute COVID-19 Surveillance Team
EPI_ISL_851374	Lighthouse Lab in Glasgow	Wellcome Sanger Institute for the COVID-19 Genomics UK (COG-UK) Consortium	Harper VanSteenhouse, Yumi Kasai, David Gray, Carol Clugston, Anna Dominiczak and Alex Alderton, Roberto Amato, Sonia Goncalves, Ewan Harrison, David K. Jackson, Ian Johnston, Dominic Kwiatkowski, Cordelia Langford, John Sillitoe on behalf of the Wellcome Sanger Institute COVID-19 Surveillance Team
EPI_ISL_851375, EPI_ISL_851376, EPI_ISL_851377, EPI_ISL_851378, EPI_ISL_851379	Lighthouse Lab in Cambridge	Wellcome Sanger Institute for the COVID-19 Genomics UK (COG-UK) Consortium	Rob Howes, The Lighthouse Lab in Cambridge and Alex Alderton, Roberto Amato, Sonia Goncalves, Ewan Harrison, David K. Jackson, Ian Johnston, Dominic Kwiatkowski, Cordelia Langford, John Sillitoe on behalf of the Wellcome Sanger Institute COVID-19 Surveillance Team
EPI_ISL_851380	Lighthouse Lab in Glasgow	Wellcome Sanger Institute for the COVID-19 Genomics UK (COG-UK) Consortium	Harper VanSteenhouse, Yumi Kasai, David Gray, Carol Clugston, Anna Dominiczak and Alex Alderton, Roberto Amato, Sonia Goncalves, Ewan Harrison, David K. Jackson, Ian Johnston, Dominic Kwiatkowski, Cordelia Langford, John Sillitoe on behalf of the Wellcome Sanger Institute COVID-19 Surveillance Team
EPI_ISL_851381	Lighthouse Lab in Cambridge	Wellcome Sanger Institute for the COVID-19 Genomics UK (COG-UK) Consortium	Rob Howes, The Lighthouse Lab in Cambridge and Alex Alderton, Roberto Amato, Sonia Goncalves, Ewan Harrison, David K. Jackson, Ian Johnston, Dominic Kwiatkowski, Cordelia Langford, John Sillitoe on behalf of the Wellcome Sanger Institute COVID-19 Surveillance Team
EPI_ISL_851382, EPI_ISL_851383, EPI_ISL_851384, EPI_ISL_851385, EPI_ISL_851386, EPI_ISL_851387, EPI_ISL_851388, EPI_ISL_851389, EPI_ISL_851390, EPI_ISL_851391, EPI_ISL_851392, EPI_ISL_851393, EPI_ISL_851394, EPI_ISL_851395, EPI_ISL_851396, EPI_ISL_851397, EPI_ISL_851398, EPI_ISL_851399, EPI_ISL_851400, EPI_ISL_851401, EPI_ISL_851402, EPI_ISL_851403, EPI_ISL_851404, EPI_ISL_851405, EPI_ISL_851406, EPI_ISL_851407, EPI_ISL_851408, EPI_ISL_851409, EPI_ISL_851410, EPI_ISL_851411, EPI_ISL_851412, EPI_ISL_851413, EPI_ISL_851414, EPI_ISL_851415, EPI_ISL_851416, EPI_ISL_851417, EPI_ISL_851418, EPI_ISL_851419, EPI_ISL_851420, EPI_ISL_851421, EPI_ISL_851422, EPI_ISL_851423, EPI_ISL_851424, EPI_ISL_851425, EPI_ISL_851426, EPI_ISL_851427, EPI_ISL_851428, EPI_ISL_851429, EPI_ISL_851430, EPI_ISL_851431, EPI_ISL_851432, EPI_ISL_851433, EPI_ISL_851434, EPI_ISL_851435, EPI_ISL_851436, EPI_ISL_851437, EPI_ISL_851438, EPI_ISL_851439, EPI_ISL_851440, EPI_ISL_851441, EPI_ISL_851442, EPI_ISL_851443, EPI_ISL_851444, EPI_ISL_851445, EPI_ISL_851446, EPI_ISL_851447, EPI_ISL_851448, EPI_ISL_851449, EPI_ISL_851450, EPI_ISL_851451, EPI_ISL_851452, EPI_ISL_851453, EPI_ISL_851454, EPI_ISL_851456, EPI_ISL_851457, EPI_ISL_851458, EPI_ISL_851459, EPI_ISL_851460, EPI_ISL_851462, EPI_ISL_851463, EPI_ISL_851464, EPI_ISL_851465, EPI_ISL_851466, EPI_ISL_851468, EPI_ISL_851469, EPI_ISL_851470, EPI_ISL_851471, EPI_ISL_851472, EPI_ISL_851473, EPI_ISL_851474, EPI_ISL_851475, EPI_ISL_851476, EPI_ISL_851477, EPI_ISL_851478, EPI_ISL_851479, EPI_ISL_851480, EPI_ISL_851481, EPI_ISL_851482, EPI_ISL_851483, EPI_ISL_851484, EPI_ISL_851485, EPI_ISL_851486, EPI_ISL_851487, EPI_ISL_851488, EPI_ISL_851489, EPI_ISL_851490, EPI_ISL_851491, EPI_ISL_851492, EPI_ISL_851493, EPI_ISL_851494, EPI_ISL_851495, EPI_ISL_851496, EPI_ISL_851497, EPI_ISL_851498, EPI_ISL_851499, EPI_ISL_851500, EPI_ISL_851501, EPI_ISL_851502, EPI_ISL_851503, EPI_ISL_851504, EPI_ISL_851505, EPI_ISL_851506, EPI_ISL_851507, EPI_ISL_851508, EPI_ISL_851509, EPI_ISL_851510, EPI_ISL_851511, EPI_ISL_851512, EPI_ISL_851513, EPI_ISL_851514, EPI_ISL_851515, EPI_ISL_851516, EPI_ISL_851517, EPI_ISL_851518, EPI_ISL_851520, EPI_ISL_851521, EPI_ISL_851522, EPI_ISL_851523, EPI_ISL_851524, EPI_ISL_851525, EPI_ISL_851526, EPI_ISL_851527, EPI_ISL_851528, EPI_ISL_851529, EPI_ISL_851530, EPI_ISL_851531, EPI_ISL_851532, EPI_ISL_851533, EPI_ISL_851534, EPI_ISL_851535, EPI_ISL_851536, EPI_ISL_851537, EPI_ISL_851538, EPI_ISL_851539, EPI_ISL_851540, EPI_ISL_851541, EPI_ISL_851542, EPI_ISL_851543, EPI_ISL_851544, EPI_ISL_851545, EPI_ISL_851546, EPI_ISL_851547, EPI_ISL_851548, EPI_ISL_851549, EPI_ISL_851550, EPI_ISL_851551, EPI_ISL_851552, EPI_ISL_851553, EPI_ISL_851554, EPI_ISL_851555, EPI_ISL_851556, EPI_ISL_851557, EPI_ISL_851558, EPI_ISL_851560, EPI_ISL_851561, EPI_ISL_851562, EPI_ISL_851563, EPI_ISL_851564, EPI_ISL_851565, EPI_ISL_851566, EPI_ISL_851567, EPI_ISL_851568, EPI_ISL_851569, EPI_ISL_851570, EPI_ISL_851571, EPI_ISL_851572, EPI_ISL_851573, EPI_ISL_851574, EPI_ISL_851575, EPI_ISL_851576, EPI_ISL_851577, EPI_ISL_851578, EPI_ISL_851579, EPI_ISL_851580, EPI_ISL_851581, EPI_ISL_851582, EPI_ISL_851583, EPI_ISL_851584, EPI_ISL_851585, EPI_ISL_851586, EPI_ISL_851587, EPI_ISL_851588, EPI_ISL_851589, EPI_ISL_851590, EPI_ISL_851591, EPI_ISL_851592, EPI_ISL_851593, EPI_ISL_851595, EPI_ISL_851596, EPI_ISL_851597, EPI_ISL_851598, EPI_ISL_851599, EPI_ISL_851600, EPI_ISL_851601, EPI_ISL_851602, EPI_ISL_851603, EPI_ISL_851604, EPI_ISL_851605, EPI_ISL_851606, EPI_ISL_851607, EPI_ISL_851608, EPI_ISL_851609, EPI_ISL_851610, EPI_ISL_851611, EPI_ISL_851612, EPI_ISL_851613, EPI_ISL_851615, EPI_ISL_851616, EPI_ISL_851617, EPI_ISL_851618, EPI_ISL_851619, EPI_ISL_851620, EPI_ISL_851621, EPI_ISL_851622, EPI_ISL_851623, EPI_ISL_851624, EPI_ISL_851625, EPI_ISL_851626, EPI_ISL_851627, EPI_ISL_851629, EPI_ISL_851630, EPI_ISL_851631, EPI_ISL_851632, EPI_ISL_851633, EPI_ISL_851634, EPI_ISL_851635, EPI_ISL_851636, EPI_ISL_851637, EPI_ISL_851638, EPI_ISL_851639, EPI_ISL_851640, EPI_ISL_851641, EPI_ISL_851642, EPI_ISL_851643, EPI_ISL_851644, EPI_ISL_851645, EPI_ISL_851646, EPI_ISL_851647, EPI_ISL_851649, EPI_ISL_851650, EPI_ISL_851651, EPI_ISL_851652, EPI_ISL_851653, EPI_ISL_851654, EPI_ISL_851655, EPI_ISL_851656, EPI_ISL_851658, EPI_ISL_851659, EPI_ISL_851660, EPI_ISL_851661, EPI_ISL_851662, EPI_ISL_851663, EPI_ISL_851664, EPI_ISL_851665, EPI_ISL_851666, EPI_ISL_851667, EPI_ISL_851668, EPI_ISL_851669, EPI_ISL_851670, EPI_ISL_851671, EPI_ISL_851672			
see above	Lighthouse Lab in Glasgow	Wellcome Sanger Institute for the COVID-19 Genomics UK (COG-UK) Consortium	Harper VanSteenhouse, Yumi Kasai, David Gray, Carol Clugston, Anna Dominiczak and Alex Alderton, Roberto Amato, Sonia Goncalves, Ewan Harrison, David K. Jackson, Ian Johnston, Dominic Kwiatkowski, Cordelia Langford, John Sillitoe on behalf of the Wellcome Sanger Institute COVID-19 Surveillance Team
EPI_ISL_851673	Lighthouse Lab in Alderley Park	Wellcome Sanger Institute for the COVID-19 Genomics UK (COG-UK) Consortium	Jacquelyn Wynn, Mairead Hyland, The Lighthouse Lab in Alderley Park and Alex Alderton, Roberto Amato, Sonia Goncalves, Ewan Harrison, David K. Jackson, Ian Johnston, Dominic Kwiatkowski, Cordelia Langford, John Sillitoe on behalf of the Wellcome Sanger Institute COVID-19 Surveillance Team
EPI_ISL_851674	Lighthouse Lab in Glasgow	Wellcome Sanger Institute for the COVID-19 Genomics UK (COG-UK) Consortium	Harper VanSteenhouse, Yumi Kasai, David Gray, Carol Clugston, Anna Dominiczak and Alex Alderton, Roberto Amato, Sonia Goncalves, Ewan Harrison, David K. Jackson, Ian Johnston, Dominic Kwiatkowski, Cordelia Langford, John Sillitoe on behalf of the Wellcome Sanger Institute COVID-19 Surveillance Team
EPI_ISL_851675	Lighthouse Lab in Alderley Park	Wellcome Sanger Institute for the COVID-19 Genomics UK (COG-UK) Consortium	Jacquelyn Wynn, Mairead Hyland, The Lighthouse Lab in Alderley Park and Alex Alderton, Roberto Amato, Sonia Goncalves, Ewan Harrison, David K. Jackson, Ian Johnston, Dominic Kwiatkowski, Cordelia Langford, John Sillitoe on behalf of the Wellcome Sanger Institute COVID-19 Surveillance Team
EPI_ISL_851676	Lighthouse Lab in Glasgow	Wellcome Sanger Institute for the COVID-19 Genomics UK (COG-UK) Consortium	Harper VanSteenhouse, Yumi Kasai, David Gray, Carol Clugston, Anna Dominiczak and Alex Alderton, Roberto Amato, Sonia Goncalves, Ewan Harrison, David K. Jackson, Ian Johnston, Dominic Kwiatkowski, Cordelia Langford, John Sillitoe on behalf of the Wellcome Sanger Institute COVID-19 Surveillance Team
EPI_ISL_851677, EPI_ISL_851678, EPI_ISL_851679, EPI_ISL_851680	Lighthouse Lab in Alderley Park	Wellcome Sanger Institute for the COVID-19 Genomics UK (COG-UK) Consortium	Jacquelyn Wynn, Mairead Hyland, The Lighthouse Lab in Alderley Park and Alex Alderton, Roberto Amato, Sonia Goncalves, Ewan Harrison, David K. Jackson, Ian Johnston, Dominic Kwiatkowski, Cordelia Langford, John Sillitoe on behalf of the Wellcome Sanger Institute COVID-19 Surveillance Team
EPI_ISL_851681	Lighthouse Lab in Glasgow	Wellcome Sanger Institute for the COVID-19 Genomics UK (COG-UK) Consortium	Harper VanSteenhouse, Yumi Kasai, David Gray, Carol Clugston, Anna Dominiczak and Alex Alderton, Roberto Amato, Sonia Goncalves, Ewan Harrison, David K. Jackson, Ian Johnston, Dominic Kwiatkowski, Cordelia Langford, John Sillitoe on behalf of the Wellcome Sanger Institute COVID-19 Surveillance Team
EPI_ISL_851682, EPI_ISL_851683, EPI_ISL_851684	Lighthouse Lab in Alderley Park	Wellcome Sanger Institute for the COVID-19 Genomics UK (COG-UK) Consortium	Jacquelyn Wynn, Mairead Hyland, The Lighthouse Lab in Alderley Park and Alex Alderton, Roberto Amato, Sonia Goncalves, Ewan Harrison, David K. Jackson, Ian Johnston, Dominic Kwiatkowski, Cordelia Langford, John Sillitoe on behalf of the Wellcome Sanger Institute COVID-19 Surveillance Team
EPI_ISL_851685, EPI_ISL_851686, EPI_ISL_851687	Lighthouse Lab in Glasgow	Wellcome Sanger Institute for the COVID-19 Genomics UK (COG-UK) Consortium	Harper VanSteenhouse, Yumi Kasai, David Gray, Carol Clugston, Anna Dominiczak and Alex Alderton, Roberto Amato, Sonia Goncalves, Ewan Harrison, David K. Jackson, Ian Johnston, Dominic Kwiatkowski, Cordelia Langford, John Sillitoe on behalf of the Wellcome Sanger Institute COVID-19 Surveillance Team
EPI_ISL_851688	Lighthouse Lab in Alderley Park	Wellcome Sanger Institute for the COVID-19 Genomics UK (COG-UK) Consortium	Jacquelyn Wynn, Mairead Hyland, The Lighthouse Lab in Alderley Park and Alex Alderton, Roberto Amato, Sonia Goncalves, Ewan Harrison, David K. Jackson, Ian Johnston, Dominic Kwiatkowski, Cordelia Langford, John Sillitoe on behalf of the Wellcome Sanger Institute COVID-19 Surveillance Team
EPI_ISL_851689, EPI_ISL_851690	Lighthouse Lab in Glasgow	Wellcome Sanger Institute for the COVID-19 Genomics UK (COG-UK) Consortium	Harper VanSteenhouse, Yumi Kasai, David Gray, Carol Clugston, Anna Dominiczak and Alex Alderton, Roberto Amato, Sonia Goncalves, Ewan Harrison, David K. Jackson, Ian Johnston, Dominic Kwiatkowski, Cordelia Langford, John Sillitoe on behalf of the Wellcome Sanger Institute COVID-19 Surveillance Team
EPI_ISL_851691, EPI_ISL_851692	Lighthouse Lab in Alderley Park	Wellcome Sanger Institute for the COVID-19 Genomics UK (COG-UK) Consortium	Jacquelyn Wynn, Mairead Hyland, The Lighthouse Lab in Alderley Park and Alex Alderton, Roberto Amato, Sonia Goncalves, Ewan Harrison, David K. Jackson, Ian Johnston, Dominic Kwiatkowski, Cordelia Langford, John Sillitoe on behalf of the Wellcome Sanger Institute COVID-19 Surveillance Team
EPI_ISL_851693	Lighthouse Lab in Glasgow	Wellcome Sanger Institute for the COVID-19 Genomics UK (COG-UK) Consortium	Harper VanSteenhouse, Yumi Kasai, David Gray, Carol Clugston, Anna Dominiczak and Alex Alderton, Roberto Amato, Sonia Goncalves, Ewan Harrison, David K. Jackson, Ian Johnston, Dominic Kwiatkowski, Cordelia Langford, John Sillitoe on behalf of the Wellcome Sanger Institute COVID-19 Surveillance Team
EPI_ISL_851694, EPI_ISL_851695, EPI_ISL_851696, EPI_ISL_851697, EPI_ISL_851698, EPI_ISL_851699, EPI_ISL_851700	Lighthouse Lab in Alderley Park	Wellcome Sanger Institute for the COVID-19 Genomics UK (COG-UK) Consortium	Jacquelyn Wynn, Mairead Hyland, The Lighthouse Lab in Alderley Park and Alex Alderton, Roberto Amato, Sonia Goncalves, Ewan Harrison, David K. Jackson, Ian Johnston, Dominic Kwiatkowski, Cordelia Langford, John Sillitoe on behalf of the Wellcome Sanger Institute COVID-19 Surveillance Team
EPI_ISL_851701	Lighthouse Lab in Glasgow	Wellcome Sanger Institute for the COVID-19 Genomics UK (COG-UK) Consortium	Harper VanSteenhouse, Yumi Kasai, David Gray, Carol Clugston, Anna Dominiczak and Alex Alderton, Roberto Amato, Sonia Goncalves, Ewan Harrison, David K. Jackson, Ian Johnston, Dominic Kwiatkowski, Cordelia Langford, John Sillitoe on behalf of the Wellcome Sanger Institute COVID-19 Surveillance Team
EPI_ISL_851702, EPI_ISL_851703, EPI_ISL_851704, EPI_ISL_851705, EPI_ISL_851706, EPI_ISL_851707, EPI_ISL_851708, EPI_ISL_851709, EPI_ISL_851710, EPI_ISL_851711, EPI_ISL_851712, EPI_ISL_851713			
see above	Lighthouse Lab in Alderley Park	Wellcome Sanger Institute for the COVID-19 Genomics UK (COG-UK) Consortium	Jacquelyn Wynn, Mairead Hyland, The Lighthouse Lab in Alderley Park and Alex Alderton, Roberto Amato, Sonia Goncalves, Ewan Harrison, David K. Jackson, Ian Johnston, Dominic Kwiatkowski, Cordelia Langford, John Sillitoe on behalf of the Wellcome Sanger Institute COVID-19 Surveillance Team
EPI_ISL_851714	Lighthouse Lab in Glasgow	Wellcome Sanger Institute for the COVID-19 Genomics UK (COG-UK) Consortium	Harper VanSteenhouse, Yumi Kasai, David Gray, Carol Clugston, Anna Dominiczak and Alex Alderton, Roberto Amato, Sonia Goncalves, Ewan Harrison, David K. Jackson, Ian Johnston, Dominic Kwiatkowski, Cordelia Langford, John Sillitoe on behalf of the Wellcome Sanger Institute COVID-19 Surveillance Team

EPI_ISL_851715, EPI_ISL_851716, EPI_ISL_851717, EPI_ISL_851718, EPI_ISL_851719	Lighthouse Lab in Alderley Park	Wellcome Sanger Institute for the COVID-19 Genomics UK (COG-UK) Consortium	Jacquelyn Wynn, Mairead Hyland, The Lighthouse Lab in Alderley Park and Alex Alderton, Roberto Amato, Sonia Goncalves, Ewan Harrison, David K. Jackson, Ian Johnston, Dominic Kwiatkowski, Cordelia Langford, John Sillitoe on behalf of the Wellcome Sanger Institute COVID-19 Surveillance Team
EPI_ISL_851720	Lighthouse Lab in Glasgow	Wellcome Sanger Institute for the COVID-19 Genomics UK (COG-UK) Consortium	Harper VanSteenhouse, Yumi Kasai, David Gray, Carol Clugston, Anna Dominiczak and Alex Alderton, Roberto Amato, Sonia Goncalves, Ewan Harrison, David K. Jackson, Ian Johnston, Dominic Kwiatkowski, Cordelia Langford, John Sillitoe on behalf of the Wellcome Sanger Institute COVID-19 Surveillance Team
EPI_ISL_851721, EPI_ISL_851722, EPI_ISL_851723, EPI_ISL_851724	Lighthouse Lab in Alderley Park	Wellcome Sanger Institute for the COVID-19 Genomics UK (COG-UK) Consortium	Jacquelyn Wynn, Mairead Hyland, The Lighthouse Lab in Alderley Park and Alex Alderton, Roberto Amato, Sonia Goncalves, Ewan Harrison, David K. Jackson, Ian Johnston, Dominic Kwiatkowski, Cordelia Langford, John Sillitoe on behalf of the Wellcome Sanger Institute COVID-19 Surveillance Team
EPI_ISL_851725	Lighthouse Lab in Glasgow	Wellcome Sanger Institute for the COVID-19 Genomics UK (COG-UK) Consortium	Harper VanSteenhouse, Yumi Kasai, David Gray, Carol Clugston, Anna Dominiczak and Alex Alderton, Roberto Amato, Sonia Goncalves, Ewan Harrison, David K. Jackson, Ian Johnston, Dominic Kwiatkowski, Cordelia Langford, John Sillitoe on behalf of the Wellcome Sanger Institute COVID-19 Surveillance Team
EPI_ISL_851726	Lighthouse Lab in Alderley Park	Wellcome Sanger Institute for the COVID-19 Genomics UK (COG-UK) Consortium	Jacquelyn Wynn, Mairead Hyland, The Lighthouse Lab in Alderley Park and Alex Alderton, Roberto Amato, Sonia Goncalves, Ewan Harrison, David K. Jackson, Ian Johnston, Dominic Kwiatkowski, Cordelia Langford, John Sillitoe on behalf of the Wellcome Sanger Institute COVID-19 Surveillance Team
EPI_ISL_851727	Lighthouse Lab in Glasgow	Wellcome Sanger Institute for the COVID-19 Genomics UK (COG-UK) Consortium	Harper VanSteenhouse, Yumi Kasai, David Gray, Carol Clugston, Anna Dominiczak and Alex Alderton, Roberto Amato, Sonia Goncalves, Ewan Harrison, David K. Jackson, Ian Johnston, Dominic Kwiatkowski, Cordelia Langford, John Sillitoe on behalf of the Wellcome Sanger Institute COVID-19 Surveillance Team
EPI_ISL_851728	Lighthouse Lab in Alderley Park	Wellcome Sanger Institute for the COVID-19 Genomics UK (COG-UK) Consortium	Jacquelyn Wynn, Mairead Hyland, The Lighthouse Lab in Alderley Park and Alex Alderton, Roberto Amato, Sonia Goncalves, Ewan Harrison, David K. Jackson, Ian Johnston, Dominic Kwiatkowski, Cordelia Langford, John Sillitoe on behalf of the Wellcome Sanger Institute COVID-19 Surveillance Team
EPI_ISL_851729	Lighthouse Lab in Glasgow	Wellcome Sanger Institute for the COVID-19 Genomics UK (COG-UK) Consortium	Harper VanSteenhouse, Yumi Kasai, David Gray, Carol Clugston, Anna Dominiczak and Alex Alderton, Roberto Amato, Sonia Goncalves, Ewan Harrison, David K. Jackson, Ian Johnston, Dominic Kwiatkowski, Cordelia Langford, John Sillitoe on behalf of the Wellcome Sanger Institute COVID-19 Surveillance Team
EPI_ISL_851730, EPI_ISL_851731, EPI_ISL_851732, EPI_ISL_851733	Lighthouse Lab in Alderley Park	Wellcome Sanger Institute for the COVID-19 Genomics UK (COG-UK) Consortium	Jacquelyn Wynn, Mairead Hyland, The Lighthouse Lab in Alderley Park and Alex Alderton, Roberto Amato, Sonia Goncalves, Ewan Harrison, David K. Jackson, Ian Johnston, Dominic Kwiatkowski, Cordelia Langford, John Sillitoe on behalf of the Wellcome Sanger Institute COVID-19 Surveillance Team
EPI_ISL_851734	Lighthouse Lab in Glasgow	Wellcome Sanger Institute for the COVID-19 Genomics UK (COG-UK) Consortium	Harper VanSteenhouse, Yumi Kasai, David Gray, Carol Clugston, Anna Dominiczak and Alex Alderton, Roberto Amato, Sonia Goncalves, Ewan Harrison, David K. Jackson, Ian Johnston, Dominic Kwiatkowski, Cordelia Langford, John Sillitoe on behalf of the Wellcome Sanger Institute COVID-19 Surveillance Team
EPI_ISL_851735, EPI_ISL_851736, EPI_ISL_851737	Lighthouse Lab in Alderley Park	Wellcome Sanger Institute for the COVID-19 Genomics UK (COG-UK) Consortium	Jacquelyn Wynn, Mairead Hyland, The Lighthouse Lab in Alderley Park and Alex Alderton, Roberto Amato, Sonia Goncalves, Ewan Harrison, David K. Jackson, Ian Johnston, Dominic Kwiatkowski, Cordelia Langford, John Sillitoe on behalf of the Wellcome Sanger Institute COVID-19 Surveillance Team
EPI_ISL_851738	Lighthouse Lab in Glasgow	Wellcome Sanger Institute for the COVID-19 Genomics UK (COG-UK) Consortium	Harper VanSteenhouse, Yumi Kasai, David Gray, Carol Clugston, Anna Dominiczak and Alex Alderton, Roberto Amato, Sonia Goncalves, Ewan Harrison, David K. Jackson, Ian Johnston, Dominic Kwiatkowski, Cordelia Langford, John Sillitoe on behalf of the Wellcome Sanger Institute COVID-19 Surveillance Team
EPI_ISL_851739, EPI_ISL_851740, EPI_ISL_851741, EPI_ISL_851742	Lighthouse Lab in Alderley Park	Wellcome Sanger Institute for the COVID-19 Genomics UK (COG-UK) Consortium	Jacquelyn Wynn, Mairead Hyland, The Lighthouse Lab in Alderley Park and Alex Alderton, Roberto Amato, Sonia Goncalves, Ewan Harrison, David K. Jackson, Ian Johnston, Dominic Kwiatkowski, Cordelia Langford, John Sillitoe on behalf of the Wellcome Sanger Institute COVID-19 Surveillance Team
EPI_ISL_851743	Lighthouse Lab in Glasgow	Wellcome Sanger Institute for the COVID-19 Genomics UK (COG-UK) Consortium	Harper VanSteenhouse, Yumi Kasai, David Gray, Carol Clugston, Anna Dominiczak and Alex Alderton, Roberto Amato, Sonia Goncalves, Ewan Harrison, David K. Jackson, Ian Johnston, Dominic Kwiatkowski, Cordelia Langford, John Sillitoe on behalf of the Wellcome Sanger Institute COVID-19 Surveillance Team
EPI_ISL_851744, EPI_ISL_851745, EPI_ISL_851746	Lighthouse Lab in Alderley Park	Wellcome Sanger Institute for the COVID-19 Genomics UK (COG-UK) Consortium	Jacquelyn Wynn, Mairead Hyland, The Lighthouse Lab in Alderley Park and Alex Alderton, Roberto Amato, Sonia Goncalves, Ewan Harrison, David K. Jackson, Ian Johnston, Dominic Kwiatkowski, Cordelia Langford, John Sillitoe on behalf of the Wellcome Sanger Institute COVID-19 Surveillance Team
EPI_ISL_851747	Lighthouse Lab in Glasgow	Wellcome Sanger Institute for the COVID-19 Genomics UK (COG-UK) Consortium	Harper VanSteenhouse, Yumi Kasai, David Gray, Carol Clugston, Anna Dominiczak and Alex Alderton, Roberto Amato, Sonia Goncalves, Ewan Harrison, David K. Jackson, Ian Johnston, Dominic Kwiatkowski, Cordelia Langford, John Sillitoe on behalf of the Wellcome Sanger Institute COVID-19 Surveillance Team
EPI_ISL_851748	Lighthouse Lab in Alderley Park	Wellcome Sanger Institute for the COVID-19 Genomics UK (COG-UK) Consortium	Jacquelyn Wynn, Mairead Hyland, The Lighthouse Lab in Alderley Park and Alex Alderton, Roberto Amato, Sonia Goncalves, Ewan Harrison, David K. Jackson, Ian Johnston, Dominic Kwiatkowski, Cordelia Langford, John Sillitoe on behalf of the Wellcome Sanger Institute COVID-19 Surveillance Team
EPI_ISL_851749	Lighthouse Lab in Glasgow	Wellcome Sanger Institute for the COVID-19 Genomics UK (COG-UK) Consortium	Harper VanSteenhouse, Yumi Kasai, David Gray, Carol Clugston, Anna Dominiczak and Alex Alderton, Roberto Amato, Sonia Goncalves, Ewan Harrison, David K. Jackson, Ian Johnston, Dominic Kwiatkowski, Cordelia Langford, John Sillitoe on behalf of the Wellcome Sanger Institute COVID-19 Surveillance Team
EPI_ISL_851751, EPI_ISL_851752, EPI_ISL_851753	Lighthouse Lab in Alderley Park	Wellcome Sanger Institute for the COVID-19 Genomics UK (COG-UK) Consortium	Jacquelyn Wynn, Mairead Hyland, The Lighthouse Lab in Alderley Park and Alex Alderton, Roberto Amato, Sonia Goncalves, Ewan Harrison, David K. Jackson, Ian Johnston, Dominic Kwiatkowski, Cordelia Langford, John Sillitoe on behalf of the Wellcome Sanger Institute COVID-19 Surveillance Team
EPI_ISL_851754	Lighthouse Lab in Glasgow	Wellcome Sanger Institute for the COVID-19 Genomics UK (COG-UK) Consortium	Harper VanSteenhouse, Yumi Kasai, David Gray, Carol Clugston, Anna Dominiczak and Alex Alderton, Roberto Amato, Sonia Goncalves, Ewan Harrison, David K. Jackson, Ian Johnston, Dominic Kwiatkowski, Cordelia Langford, John Sillitoe on behalf of the Wellcome Sanger Institute COVID-19 Surveillance Team
EPI_ISL_851755, EPI_ISL_851756	Lighthouse Lab in Alderley Park	Wellcome Sanger Institute for the COVID-19 Genomics UK (COG-UK) Consortium	Jacquelyn Wynn, Mairead Hyland, The Lighthouse Lab in Alderley Park and Alex Alderton, Roberto Amato, Sonia Goncalves, Ewan Harrison, David K. Jackson, Ian Johnston, Dominic Kwiatkowski, Cordelia Langford, John Sillitoe on behalf of the Wellcome Sanger Institute COVID-19 Surveillance Team
EPI_ISL_851757	Lighthouse Lab in Glasgow	Wellcome Sanger Institute for the COVID-19 Genomics UK (COG-UK) Consortium	Harper VanSteenhouse, Yumi Kasai, David Gray, Carol Clugston, Anna Dominiczak and Alex Alderton, Roberto Amato, Sonia Goncalves, Ewan Harrison, David K. Jackson, Ian Johnston, Dominic Kwiatkowski, Cordelia Langford, John Sillitoe on behalf of the Wellcome Sanger Institute COVID-19 Surveillance Team
EPI_ISL_851758, EPI_ISL_851759	Lighthouse Lab in Alderley Park	Wellcome Sanger Institute for the COVID-19 Genomics UK (COG-UK) Consortium	Jacquelyn Wynn, Mairead Hyland, The Lighthouse Lab in Alderley Park and Alex Alderton, Roberto Amato, Sonia Goncalves, Ewan Harrison, David K. Jackson, Ian Johnston, Dominic Kwiatkowski, Cordelia Langford, John Sillitoe on behalf of the Wellcome Sanger Institute COVID-19 Surveillance Team
EPI_ISL_851760	Lighthouse Lab in Glasgow	Wellcome Sanger Institute for the COVID-19 Genomics UK (COG-UK) Consortium	Harper VanSteenhouse, Yumi Kasai, David Gray, Carol Clugston, Anna Dominiczak and Alex Alderton, Roberto Amato, Sonia Goncalves, Ewan Harrison, David K. Jackson, Ian Johnston, Dominic Kwiatkowski, Cordelia Langford, John Sillitoe on behalf of the Wellcome Sanger Institute COVID-19 Surveillance Team
EPI_ISL_851761, EPI_ISL_851762	Lighthouse Lab in Alderley Park	Wellcome Sanger Institute for the COVID-19 Genomics UK (COG-UK) Consortium	Jacquelyn Wynn, Mairead Hyland, The Lighthouse Lab in Alderley Park and Alex Alderton, Roberto Amato, Sonia Goncalves, Ewan Harrison, David K. Jackson, Ian Johnston, Dominic Kwiatkowski, Cordelia Langford, John Sillitoe on behalf of the Wellcome Sanger Institute COVID-19 Surveillance Team
EPI_ISL_851763	Lighthouse Lab in Glasgow	Wellcome Sanger Institute for the COVID-19 Genomics UK (COG-UK) Consortium	Harper VanSteenhouse, Yumi Kasai, David Gray, Carol Clugston, Anna Dominiczak and Alex Alderton, Roberto Amato, Sonia Goncalves, Ewan Harrison, David K. Jackson, Ian Johnston, Dominic Kwiatkowski, Cordelia Langford, John Sillitoe on behalf of the Wellcome Sanger Institute COVID-19 Surveillance Team
EPI_ISL_851764, EPI_ISL_851765, EPI_ISL_851766	Lighthouse Lab in Alderley Park	Wellcome Sanger Institute for the COVID-19 Genomics UK (COG-UK) Consortium	Jacquelyn Wynn, Mairead Hyland, The Lighthouse Lab in Alderley Park and Alex Alderton, Roberto Amato, Sonia Goncalves, Ewan Harrison, David K. Jackson, Ian Johnston, Dominic Kwiatkowski, Cordelia Langford, John Sillitoe on behalf of the Wellcome Sanger Institute COVID-19 Surveillance Team
EPI_ISL_851767	Lighthouse Lab in Glasgow	Wellcome Sanger Institute for the COVID-19 Genomics UK (COG-UK) Consortium	Harper VanSteenhouse, Yumi Kasai, David Gray, Carol Clugston, Anna Dominiczak and Alex Alderton, Roberto Amato, Sonia Goncalves, Ewan Harrison, David K. Jackson, Ian Johnston, Dominic Kwiatkowski, Cordelia Langford, John Sillitoe on behalf of the Wellcome Sanger Institute COVID-19 Surveillance Team
EPI_ISL_851768, EPI_ISL_851769	Lighthouse Lab in Alderley Park	Wellcome Sanger Institute for the COVID-19 Genomics UK (COG-UK) Consortium	Jacquelyn Wynn, Mairead Hyland, The Lighthouse Lab in Alderley Park and Alex Alderton, Roberto Amato, Sonia Goncalves, Ewan Harrison, David K. Jackson, Ian Johnston, Dominic Kwiatkowski, Cordelia Langford, John Sillitoe on behalf of the Wellcome Sanger Institute COVID-19 Surveillance Team

EPI_ISL_851770	Lighthouse Lab in Glasgow	Wellcome Sanger Institute for the COVID-19 Genomics UK (COG-UK) Consortium	Harper VanSteenhouse, Yumi Kasai, David Gray, Carol Clugston, Anna Dominiczak and Alex Alderton, Roberto Amato, Sonia Goncalves, Ewan Harrison, David K. Jackson, Ian Johnston, Dominic Kwiatkowski, Cordelia Langford, John Sillitoe on behalf of the Wellcome Sanger Institute COVID-19 Surveillance Team
EPI_ISL_851771	Lighthouse Lab in Alderley Park	Wellcome Sanger Institute for the COVID-19 Genomics UK (COG-UK) Consortium	Jacquelyn Wynn, Mairead Hyland, The Lighthouse Lab in Alderley Park and Alex Alderton, Roberto Amato, Sonia Goncalves, Ewan Harrison, David K. Jackson, Ian Johnston, Dominic Kwiatkowski, Cordelia Langford, John Sillitoe on behalf of the Wellcome Sanger Institute COVID-19 Surveillance Team
EPI_ISL_851772	Lighthouse Lab in Glasgow	Wellcome Sanger Institute for the COVID-19 Genomics UK (COG-UK) Consortium	Harper VanSteenhouse, Yumi Kasai, David Gray, Carol Clugston, Anna Dominiczak and Alex Alderton, Roberto Amato, Sonia Goncalves, Ewan Harrison, David K. Jackson, Ian Johnston, Dominic Kwiatkowski, Cordelia Langford, John Sillitoe on behalf of the Wellcome Sanger Institute COVID-19 Surveillance Team
EPI_ISL_851773	Lighthouse Lab in Alderley Park	Wellcome Sanger Institute for the COVID-19 Genomics UK (COG-UK) Consortium	Jacquelyn Wynn, Mairead Hyland, The Lighthouse Lab in Alderley Park and Alex Alderton, Roberto Amato, Sonia Goncalves, Ewan Harrison, David K. Jackson, Ian Johnston, Dominic Kwiatkowski, Cordelia Langford, John Sillitoe on behalf of the Wellcome Sanger Institute COVID-19 Surveillance Team
EPI_ISL_851774	Lighthouse Lab in Glasgow	Wellcome Sanger Institute for the COVID-19 Genomics UK (COG-UK) Consortium	Harper VanSteenhouse, Yumi Kasai, David Gray, Carol Clugston, Anna Dominiczak and Alex Alderton, Roberto Amato, Sonia Goncalves, Ewan Harrison, David K. Jackson, Ian Johnston, Dominic Kwiatkowski, Cordelia Langford, John Sillitoe on behalf of the Wellcome Sanger Institute COVID-19 Surveillance Team
EPI_ISL_851775, EPI_ISL_851776, EPI_ISL_851777, EPI_ISL_851778, EPI_ISL_851779	Lighthouse Lab in Alderley Park	Wellcome Sanger Institute for the COVID-19 Genomics UK (COG-UK) Consortium	Jacquelyn Wynn, Mairead Hyland, The Lighthouse Lab in Alderley Park and Alex Alderton, Roberto Amato, Sonia Goncalves, Ewan Harrison, David K. Jackson, Ian Johnston, Dominic Kwiatkowski, Cordelia Langford, John Sillitoe on behalf of the Wellcome Sanger Institute COVID-19 Surveillance Team
EPI_ISL_851780, EPI_ISL_851781	Lighthouse Lab in Glasgow	Wellcome Sanger Institute for the COVID-19 Genomics UK (COG-UK) Consortium	Harper VanSteenhouse, Yumi Kasai, David Gray, Carol Clugston, Anna Dominiczak and Alex Alderton, Roberto Amato, Sonia Goncalves, Ewan Harrison, David K. Jackson, Ian Johnston, Dominic Kwiatkowski, Cordelia Langford, John Sillitoe on behalf of the Wellcome Sanger Institute COVID-19 Surveillance Team
EPI_ISL_851782, EPI_ISL_851783, EPI_ISL_851784	Lighthouse Lab in Alderley Park	Wellcome Sanger Institute for the COVID-19 Genomics UK (COG-UK) Consortium	Jacquelyn Wynn, Mairead Hyland, The Lighthouse Lab in Alderley Park and Alex Alderton, Roberto Amato, Sonia Goncalves, Ewan Harrison, David K. Jackson, Ian Johnston, Dominic Kwiatkowski, Cordelia Langford, John Sillitoe on behalf of the Wellcome Sanger Institute COVID-19 Surveillance Team
EPI_ISL_851785	Lighthouse Lab in Glasgow	Wellcome Sanger Institute for the COVID-19 Genomics UK (COG-UK) Consortium	Harper VanSteenhouse, Yumi Kasai, David Gray, Carol Clugston, Anna Dominiczak and Alex Alderton, Roberto Amato, Sonia Goncalves, Ewan Harrison, David K. Jackson, Ian Johnston, Dominic Kwiatkowski, Cordelia Langford, John Sillitoe on behalf of the Wellcome Sanger Institute COVID-19 Surveillance Team
EPI_ISL_851786, EPI_ISL_851787, EPI_ISL_851788, EPI_ISL_851789, EPI_ISL_851790, EPI_ISL_851791	Lighthouse Lab in Alderley Park	Wellcome Sanger Institute for the COVID-19 Genomics UK (COG-UK) Consortium	Jacquelyn Wynn, Mairead Hyland, The Lighthouse Lab in Alderley Park and Alex Alderton, Roberto Amato, Sonia Goncalves, Ewan Harrison, David K. Jackson, Ian Johnston, Dominic Kwiatkowski, Cordelia Langford, John Sillitoe on behalf of the Wellcome Sanger Institute COVID-19 Surveillance Team
EPI_ISL_851792	Lighthouse Lab in Glasgow	Wellcome Sanger Institute for the COVID-19 Genomics UK (COG-UK) Consortium	Harper VanSteenhouse, Yumi Kasai, David Gray, Carol Clugston, Anna Dominiczak and Alex Alderton, Roberto Amato, Sonia Goncalves, Ewan Harrison, David K. Jackson, Ian Johnston, Dominic Kwiatkowski, Cordelia Langford, John Sillitoe on behalf of the Wellcome Sanger Institute COVID-19 Surveillance Team
EPI_ISL_851793	Lighthouse Lab in Alderley Park	Wellcome Sanger Institute for the COVID-19 Genomics UK (COG-UK) Consortium	Jacquelyn Wynn, Mairead Hyland, The Lighthouse Lab in Alderley Park and Alex Alderton, Roberto Amato, Sonia Goncalves, Ewan Harrison, David K. Jackson, Ian Johnston, Dominic Kwiatkowski, Cordelia Langford, John Sillitoe on behalf of the Wellcome Sanger Institute COVID-19 Surveillance Team
EPI_ISL_851794	Lighthouse Lab in Glasgow	Wellcome Sanger Institute for the COVID-19 Genomics UK (COG-UK) Consortium	Harper VanSteenhouse, Yumi Kasai, David Gray, Carol Clugston, Anna Dominiczak and Alex Alderton, Roberto Amato, Sonia Goncalves, Ewan Harrison, David K. Jackson, Ian Johnston, Dominic Kwiatkowski, Cordelia Langford, John Sillitoe on behalf of the Wellcome Sanger Institute COVID-19 Surveillance Team
EPI_ISL_851795	Lighthouse Lab in Alderley Park	Wellcome Sanger Institute for the COVID-19 Genomics UK (COG-UK) Consortium	Jacquelyn Wynn, Mairead Hyland, The Lighthouse Lab in Alderley Park and Alex Alderton, Roberto Amato, Sonia Goncalves, Ewan Harrison, David K. Jackson, Ian Johnston, Dominic Kwiatkowski, Cordelia Langford, John Sillitoe on behalf of the Wellcome Sanger Institute COVID-19 Surveillance Team
EPI_ISL_851796, EPI_ISL_851797, EPI_ISL_851798, EPI_ISL_851799	Lighthouse Lab in Glasgow	Wellcome Sanger Institute for the COVID-19 Genomics UK (COG-UK) Consortium	Harper VanSteenhouse, Yumi Kasai, David Gray, Carol Clugston, Anna Dominiczak and Alex Alderton, Roberto Amato, Sonia Goncalves, Ewan Harrison, David K. Jackson, Ian Johnston, Dominic Kwiatkowski, Cordelia Langford, John Sillitoe on behalf of the Wellcome Sanger Institute COVID-19 Surveillance Team
EPI_ISL_851800, EPI_ISL_851801, EPI_ISL_851802	Lighthouse Lab in Alderley Park	Wellcome Sanger Institute for the COVID-19 Genomics UK (COG-UK) Consortium	Jacquelyn Wynn, Mairead Hyland, The Lighthouse Lab in Alderley Park and Alex Alderton, Roberto Amato, Sonia Goncalves, Ewan Harrison, David K. Jackson, Ian Johnston, Dominic Kwiatkowski, Cordelia Langford, John Sillitoe on behalf of the Wellcome Sanger Institute COVID-19 Surveillance Team
EPI_ISL_851803	Lighthouse Lab in Glasgow	Wellcome Sanger Institute for the COVID-19 Genomics UK (COG-UK) Consortium	Harper VanSteenhouse, Yumi Kasai, David Gray, Carol Clugston, Anna Dominiczak and Alex Alderton, Roberto Amato, Sonia Goncalves, Ewan Harrison, David K. Jackson, Ian Johnston, Dominic Kwiatkowski, Cordelia Langford, John Sillitoe on behalf of the Wellcome Sanger Institute COVID-19 Surveillance Team
EPI_ISL_851804	Lighthouse Lab in Alderley Park	Wellcome Sanger Institute for the COVID-19 Genomics UK (COG-UK) Consortium	Jacquelyn Wynn, Mairead Hyland, The Lighthouse Lab in Alderley Park and Alex Alderton, Roberto Amato, Sonia Goncalves, Ewan Harrison, David K. Jackson, Ian Johnston, Dominic Kwiatkowski, Cordelia Langford, John Sillitoe on behalf of the Wellcome Sanger Institute COVID-19 Surveillance Team
EPI_ISL_851805	Lighthouse Lab in Glasgow	Wellcome Sanger Institute for the COVID-19 Genomics UK (COG-UK) Consortium	Harper VanSteenhouse, Yumi Kasai, David Gray, Carol Clugston, Anna Dominiczak and Alex Alderton, Roberto Amato, Sonia Goncalves, Ewan Harrison, David K. Jackson, Ian Johnston, Dominic Kwiatkowski, Cordelia Langford, John Sillitoe on behalf of the Wellcome Sanger Institute COVID-19 Surveillance Team
EPI_ISL_851806	Lighthouse Lab in Alderley Park	Wellcome Sanger Institute for the COVID-19 Genomics UK (COG-UK) Consortium	Jacquelyn Wynn, Mairead Hyland, The Lighthouse Lab in Alderley Park and Alex Alderton, Roberto Amato, Sonia Goncalves, Ewan Harrison, David K. Jackson, Ian Johnston, Dominic Kwiatkowski, Cordelia Langford, John Sillitoe on behalf of the Wellcome Sanger Institute COVID-19 Surveillance Team
EPI_ISL_851807	Lighthouse Lab in Glasgow	Wellcome Sanger Institute for the COVID-19 Genomics UK (COG-UK) Consortium	Harper VanSteenhouse, Yumi Kasai, David Gray, Carol Clugston, Anna Dominiczak and Alex Alderton, Roberto Amato, Sonia Goncalves, Ewan Harrison, David K. Jackson, Ian Johnston, Dominic Kwiatkowski, Cordelia Langford, John Sillitoe on behalf of the Wellcome Sanger Institute COVID-19 Surveillance Team
EPI_ISL_851808, EPI_ISL_851809, EPI_ISL_851810, EPI_ISL_851811	Lighthouse Lab in Alderley Park	Wellcome Sanger Institute for the COVID-19 Genomics UK (COG-UK) Consortium	Jacquelyn Wynn, Mairead Hyland, The Lighthouse Lab in Alderley Park and Alex Alderton, Roberto Amato, Sonia Goncalves, Ewan Harrison, David K. Jackson, Ian Johnston, Dominic Kwiatkowski, Cordelia Langford, John Sillitoe on behalf of the Wellcome Sanger Institute COVID-19 Surveillance Team
EPI_ISL_851812	Lighthouse Lab in Glasgow	Wellcome Sanger Institute for the COVID-19 Genomics UK (COG-UK) Consortium	Harper VanSteenhouse, Yumi Kasai, David Gray, Carol Clugston, Anna Dominiczak and Alex Alderton, Roberto Amato, Sonia Goncalves, Ewan Harrison, David K. Jackson, Ian Johnston, Dominic Kwiatkowski, Cordelia Langford, John Sillitoe on behalf of the Wellcome Sanger Institute COVID-19 Surveillance Team
EPI_ISL_851813	Lighthouse Lab in Alderley Park	Wellcome Sanger Institute for the COVID-19 Genomics UK (COG-UK) Consortium	Jacquelyn Wynn, Mairead Hyland, The Lighthouse Lab in Alderley Park and Alex Alderton, Roberto Amato, Sonia Goncalves, Ewan Harrison, David K. Jackson, Ian Johnston, Dominic Kwiatkowski, Cordelia Langford, John Sillitoe on behalf of the Wellcome Sanger Institute COVID-19 Surveillance Team
EPI_ISL_851814	Lighthouse Lab in Glasgow	Wellcome Sanger Institute for the COVID-19 Genomics UK (COG-UK) Consortium	Harper VanSteenhouse, Yumi Kasai, David Gray, Carol Clugston, Anna Dominiczak and Alex Alderton, Roberto Amato, Sonia Goncalves, Ewan Harrison, David K. Jackson, Ian Johnston, Dominic Kwiatkowski, Cordelia Langford, John Sillitoe on behalf of the Wellcome Sanger Institute COVID-19 Surveillance Team
EPI_ISL_851815, EPI_ISL_851816, EPI_ISL_851817, EPI_ISL_851818, EPI_ISL_851819, EPI_ISL_851820, EPI_ISL_851821, EPI_ISL_851822, EPI_ISL_851823, EPI_ISL_851824, EPI_ISL_851825, EPI_ISL_851826, EPI_ISL_851827, EPI_ISL_851828			
see above	Lighthouse Lab in Alderley Park	Wellcome Sanger Institute for the COVID-19 Genomics UK (COG-UK) Consortium	Jacquelyn Wynn, Mairead Hyland, The Lighthouse Lab in Alderley Park and Alex Alderton, Roberto Amato, Sonia Goncalves, Ewan Harrison, David K. Jackson, Ian Johnston, Dominic Kwiatkowski, Cordelia Langford, John Sillitoe on behalf of the Wellcome Sanger Institute COVID-19 Surveillance Team
EPI_ISL_851829	Lighthouse Lab in Glasgow	Wellcome Sanger Institute for the COVID-19 Genomics UK (COG-UK) Consortium	Harper VanSteenhouse, Yumi Kasai, David Gray, Carol Clugston, Anna Dominiczak and Alex Alderton, Roberto Amato, Sonia Goncalves, Ewan Harrison, David K. Jackson, Ian Johnston, Dominic Kwiatkowski, Cordelia Langford, John Sillitoe on behalf of the Wellcome Sanger Institute COVID-19 Surveillance Team
EPI_ISL_851830, EPI_ISL_851831, EPI_ISL_851832, EPI_ISL_851833,	Lighthouse Lab in Alderley Park	Wellcome Sanger Institute for the COVID-19 Genomics UK (COG-UK) Consortium	Jacquelyn Wynn, Mairead Hyland, The Lighthouse Lab in Alderley Park and Alex Alderton, Roberto Amato, Sonia Goncalves, Ewan Harrison, David K. Jackson, Ian Johnston, Dominic Kwiatkowski, Cordelia Langford, John Sillitoe on behalf of the Wellcome Sanger Institute COVID-19 Surveillance Team

EPI_ISL_851834, EPI_ISL_851835, EPI_ISL_851836			
EPI_ISL_851837	Lighthouse Lab in Glasgow	Wellcome Sanger Institute for the COVID-19 Genomics UK (COG-UK) Consortium	Harper VanSteenhouse, Yumi Kasai, David Gray, Carol Clugston, Anna Dominiczak and Alex Alderton, Roberto Amato, Sonia Goncalves, Ewan Harrison, David K. Jackson, Ian Johnston, Dominic Kwiatkowski, Cordelia Langford, John Sillitoe on behalf of the Wellcome Sanger Institute COVID-19 Surveillance Team
EPI_ISL_851838, EPI_ISL_851839	Lighthouse Lab in Alderley Park	Wellcome Sanger Institute for the COVID-19 Genomics UK (COG-UK) Consortium	Jacquelyn Wynn, Mairead Hyland, The Lighthouse Lab in Alderley Park and Alex Alderton, Roberto Amato, Sonia Goncalves, Ewan Harrison, David K. Jackson, Ian Johnston, Dominic Kwiatkowski, Cordelia Langford, John Sillitoe on behalf of the Wellcome Sanger Institute COVID-19 Surveillance Team
EPI_ISL_851840	Lighthouse Lab in Glasgow	Wellcome Sanger Institute for the COVID-19 Genomics UK (COG-UK) Consortium	Harper VanSteenhouse, Yumi Kasai, David Gray, Carol Clugston, Anna Dominiczak and Alex Alderton, Roberto Amato, Sonia Goncalves, Ewan Harrison, David K. Jackson, Ian Johnston, Dominic Kwiatkowski, Cordelia Langford, John Sillitoe on behalf of the Wellcome Sanger Institute COVID-19 Surveillance Team
EPI_ISL_851841, EPI_ISL_851842, EPI_ISL_851843, EPI_ISL_851844, EPI_ISL_851845	Lighthouse Lab in Alderley Park	Wellcome Sanger Institute for the COVID-19 Genomics UK (COG-UK) Consortium	Jacquelyn Wynn, Mairead Hyland, The Lighthouse Lab in Alderley Park and Alex Alderton, Roberto Amato, Sonia Goncalves, Ewan Harrison, David K. Jackson, Ian Johnston, Dominic Kwiatkowski, Cordelia Langford, John Sillitoe on behalf of the Wellcome Sanger Institute COVID-19 Surveillance Team
EPI_ISL_851846, EPI_ISL_851847	Lighthouse Lab in Glasgow	Wellcome Sanger Institute for the COVID-19 Genomics UK (COG-UK) Consortium	Harper VanSteenhouse, Yumi Kasai, David Gray, Carol Clugston, Anna Dominiczak and Alex Alderton, Roberto Amato, Sonia Goncalves, Ewan Harrison, David K. Jackson, Ian Johnston, Dominic Kwiatkowski, Cordelia Langford, John Sillitoe on behalf of the Wellcome Sanger Institute COVID-19 Surveillance Team
EPI_ISL_851848, EPI_ISL_851849, EPI_ISL_851850	Lighthouse Lab in Alderley Park	Wellcome Sanger Institute for the COVID-19 Genomics UK (COG-UK) Consortium	Jacquelyn Wynn, Mairead Hyland, The Lighthouse Lab in Alderley Park and Alex Alderton, Roberto Amato, Sonia Goncalves, Ewan Harrison, David K. Jackson, Ian Johnston, Dominic Kwiatkowski, Cordelia Langford, John Sillitoe on behalf of the Wellcome Sanger Institute COVID-19 Surveillance Team
EPI_ISL_851851	Lighthouse Lab in Glasgow	Wellcome Sanger Institute for the COVID-19 Genomics UK (COG-UK) Consortium	Harper VanSteenhouse, Yumi Kasai, David Gray, Carol Clugston, Anna Dominiczak and Alex Alderton, Roberto Amato, Sonia Goncalves, Ewan Harrison, David K. Jackson, Ian Johnston, Dominic Kwiatkowski, Cordelia Langford, John Sillitoe on behalf of the Wellcome Sanger Institute COVID-19 Surveillance Team
EPI_ISL_851852, EPI_ISL_851853, EPI_ISL_851854, EPI_ISL_851855	Lighthouse Lab in Alderley Park	Wellcome Sanger Institute for the COVID-19 Genomics UK (COG-UK) Consortium	Jacquelyn Wynn, Mairead Hyland, The Lighthouse Lab in Alderley Park and Alex Alderton, Roberto Amato, Sonia Goncalves, Ewan Harrison, David K. Jackson, Ian Johnston, Dominic Kwiatkowski, Cordelia Langford, John Sillitoe on behalf of the Wellcome Sanger Institute COVID-19 Surveillance Team
EPI_ISL_851856, EPI_ISL_851857	Lighthouse Lab in Glasgow	Wellcome Sanger Institute for the COVID-19 Genomics UK (COG-UK) Consortium	Harper VanSteenhouse, Yumi Kasai, David Gray, Carol Clugston, Anna Dominiczak and Alex Alderton, Roberto Amato, Sonia Goncalves, Ewan Harrison, David K. Jackson, Ian Johnston, Dominic Kwiatkowski, Cordelia Langford, John Sillitoe on behalf of the Wellcome Sanger Institute COVID-19 Surveillance Team
EPI_ISL_851858	Lighthouse Lab in Alderley Park	Wellcome Sanger Institute for the COVID-19 Genomics UK (COG-UK) Consortium	Jacquelyn Wynn, Mairead Hyland, The Lighthouse Lab in Alderley Park and Alex Alderton, Roberto Amato, Sonia Goncalves, Ewan Harrison, David K. Jackson, Ian Johnston, Dominic Kwiatkowski, Cordelia Langford, John Sillitoe on behalf of the Wellcome Sanger Institute COVID-19 Surveillance Team
EPI_ISL_851859	Lighthouse Lab in Glasgow	Wellcome Sanger Institute for the COVID-19 Genomics UK (COG-UK) Consortium	Harper VanSteenhouse, Yumi Kasai, David Gray, Carol Clugston, Anna Dominiczak and Alex Alderton, Roberto Amato, Sonia Goncalves, Ewan Harrison, David K. Jackson, Ian Johnston, Dominic Kwiatkowski, Cordelia Langford, John Sillitoe on behalf of the Wellcome Sanger Institute COVID-19 Surveillance Team
EPI_ISL_851860, EPI_ISL_851861, EPI_ISL_851862	Lighthouse Lab in Alderley Park	Wellcome Sanger Institute for the COVID-19 Genomics UK (COG-UK) Consortium	Jacquelyn Wynn, Mairead Hyland, The Lighthouse Lab in Alderley Park and Alex Alderton, Roberto Amato, Sonia Goncalves, Ewan Harrison, David K. Jackson, Ian Johnston, Dominic Kwiatkowski, Cordelia Langford, John Sillitoe on behalf of the Wellcome Sanger Institute COVID-19 Surveillance Team
EPI_ISL_851863, EPI_ISL_851864, EPI_ISL_851865, EPI_ISL_851866	Lighthouse Lab in Glasgow	Wellcome Sanger Institute for the COVID-19 Genomics UK (COG-UK) Consortium	Harper VanSteenhouse, Yumi Kasai, David Gray, Carol Clugston, Anna Dominiczak and Alex Alderton, Roberto Amato, Sonia Goncalves, Ewan Harrison, David K. Jackson, Ian Johnston, Dominic Kwiatkowski, Cordelia Langford, John Sillitoe on behalf of the Wellcome Sanger Institute COVID-19 Surveillance Team
EPI_ISL_851867, EPI_ISL_851868, EPI_ISL_851869	Lighthouse Lab in Alderley Park	Wellcome Sanger Institute for the COVID-19 Genomics UK (COG-UK) Consortium	Jacquelyn Wynn, Mairead Hyland, The Lighthouse Lab in Alderley Park and Alex Alderton, Roberto Amato, Sonia Goncalves, Ewan Harrison, David K. Jackson, Ian Johnston, Dominic Kwiatkowski, Cordelia Langford, John Sillitoe on behalf of the Wellcome Sanger Institute COVID-19 Surveillance Team
EPI_ISL_851870, EPI_ISL_851871	Lighthouse Lab in Glasgow	Wellcome Sanger Institute for the COVID-19 Genomics UK (COG-UK) Consortium	Harper VanSteenhouse, Yumi Kasai, David Gray, Carol Clugston, Anna Dominiczak and Alex Alderton, Roberto Amato, Sonia Goncalves, Ewan Harrison, David K. Jackson, Ian Johnston, Dominic Kwiatkowski, Cordelia Langford, John Sillitoe on behalf of the Wellcome Sanger Institute COVID-19 Surveillance Team
EPI_ISL_851872, EPI_ISL_851873, EPI_ISL_851874, EPI_ISL_851875	Lighthouse Lab in Alderley Park	Wellcome Sanger Institute for the COVID-19 Genomics UK (COG-UK) Consortium	Jacquelyn Wynn, Mairead Hyland, The Lighthouse Lab in Alderley Park and Alex Alderton, Roberto Amato, Sonia Goncalves, Ewan Harrison, David K. Jackson, Ian Johnston, Dominic Kwiatkowski, Cordelia Langford, John Sillitoe on behalf of the Wellcome Sanger Institute COVID-19 Surveillance Team
EPI_ISL_851876, EPI_ISL_851877	Lighthouse Lab in Glasgow	Wellcome Sanger Institute for the COVID-19 Genomics UK (COG-UK) Consortium	Harper VanSteenhouse, Yumi Kasai, David Gray, Carol Clugston, Anna Dominiczak and Alex Alderton, Roberto Amato, Sonia Goncalves, Ewan Harrison, David K. Jackson, Ian Johnston, Dominic Kwiatkowski, Cordelia Langford, John Sillitoe on behalf of the Wellcome Sanger Institute COVID-19 Surveillance Team
EPI_ISL_851878, EPI_ISL_851879, EPI_ISL_851880, EPI_ISL_851881, EPI_ISL_851882, EPI_ISL_851883, EPI_ISL_851884	Lighthouse Lab in Alderley Park	Wellcome Sanger Institute for the COVID-19 Genomics UK (COG-UK) Consortium	Jacquelyn Wynn, Mairead Hyland, The Lighthouse Lab in Alderley Park and Alex Alderton, Roberto Amato, Sonia Goncalves, Ewan Harrison, David K. Jackson, Ian Johnston, Dominic Kwiatkowski, Cordelia Langford, John Sillitoe on behalf of the Wellcome Sanger Institute COVID-19 Surveillance Team
EPI_ISL_851885	Lighthouse Lab in Glasgow	Wellcome Sanger Institute for the COVID-19 Genomics UK (COG-UK) Consortium	Harper VanSteenhouse, Yumi Kasai, David Gray, Carol Clugston, Anna Dominiczak and Alex Alderton, Roberto Amato, Sonia Goncalves, Ewan Harrison, David K. Jackson, Ian Johnston, Dominic Kwiatkowski, Cordelia Langford, John Sillitoe on behalf of the Wellcome Sanger Institute COVID-19 Surveillance Team
EPI_ISL_851886, EPI_ISL_851887	Lighthouse Lab in Alderley Park	Wellcome Sanger Institute for the COVID-19 Genomics UK (COG-UK) Consortium	Jacquelyn Wynn, Mairead Hyland, The Lighthouse Lab in Alderley Park and Alex Alderton, Roberto Amato, Sonia Goncalves, Ewan Harrison, David K. Jackson, Ian Johnston, Dominic Kwiatkowski, Cordelia Langford, John Sillitoe on behalf of the Wellcome Sanger Institute COVID-19 Surveillance Team
EPI_ISL_851888	Lighthouse Lab in Glasgow	Wellcome Sanger Institute for the COVID-19 Genomics UK (COG-UK) Consortium	Harper VanSteenhouse, Yumi Kasai, David Gray, Carol Clugston, Anna Dominiczak and Alex Alderton, Roberto Amato, Sonia Goncalves, Ewan Harrison, David K. Jackson, Ian Johnston, Dominic Kwiatkowski, Cordelia Langford, John Sillitoe on behalf of the Wellcome Sanger Institute COVID-19 Surveillance Team
EPI_ISL_851889, EPI_ISL_851890	Lighthouse Lab in Alderley Park	Wellcome Sanger Institute for the COVID-19 Genomics UK (COG-UK) Consortium	Jacquelyn Wynn, Mairead Hyland, The Lighthouse Lab in Alderley Park and Alex Alderton, Roberto Amato, Sonia Goncalves, Ewan Harrison, David K. Jackson, Ian Johnston, Dominic Kwiatkowski, Cordelia Langford, John Sillitoe on behalf of the Wellcome Sanger Institute COVID-19 Surveillance Team
EPI_ISL_851891, EPI_ISL_851892	Lighthouse Lab in Glasgow	Wellcome Sanger Institute for the COVID-19 Genomics UK (COG-UK) Consortium	Harper VanSteenhouse, Yumi Kasai, David Gray, Carol Clugston, Anna Dominiczak and Alex Alderton, Roberto Amato, Sonia Goncalves, Ewan Harrison, David K. Jackson, Ian Johnston, Dominic Kwiatkowski, Cordelia Langford, John Sillitoe on behalf of the Wellcome Sanger Institute COVID-19 Surveillance Team
EPI_ISL_851893, EPI_ISL_851894	Lighthouse Lab in Alderley Park	Wellcome Sanger Institute for the COVID-19 Genomics UK (COG-UK) Consortium	Jacquelyn Wynn, Mairead Hyland, The Lighthouse Lab in Alderley Park and Alex Alderton, Roberto Amato, Sonia Goncalves, Ewan Harrison, David K. Jackson, Ian Johnston, Dominic Kwiatkowski, Cordelia Langford, John Sillitoe on behalf of the Wellcome Sanger Institute COVID-19 Surveillance Team
EPI_ISL_851895	Lighthouse Lab in Glasgow	Wellcome Sanger Institute for the COVID-19 Genomics UK (COG-UK) Consortium	Harper VanSteenhouse, Yumi Kasai, David Gray, Carol Clugston, Anna Dominiczak and Alex Alderton, Roberto Amato, Sonia Goncalves, Ewan Harrison, David K. Jackson, Ian Johnston, Dominic Kwiatkowski, Cordelia Langford, John Sillitoe on behalf of the Wellcome Sanger Institute COVID-19 Surveillance Team
EPI_ISL_851896, EPI_ISL_851897	Lighthouse Lab in Alderley Park	Wellcome Sanger Institute for the COVID-19 Genomics UK (COG-UK) Consortium	Jacquelyn Wynn, Mairead Hyland, The Lighthouse Lab in Alderley Park and Alex Alderton, Roberto Amato, Sonia Goncalves, Ewan Harrison, David K. Jackson, Ian Johnston, Dominic Kwiatkowski, Cordelia Langford, John Sillitoe on behalf of the Wellcome Sanger Institute COVID-19 Surveillance Team
EPI_ISL_851898	Lighthouse Lab in Glasgow	Wellcome Sanger Institute for the COVID-19 Genomics UK (COG-UK) Consortium	Harper VanSteenhouse, Yumi Kasai, David Gray, Carol Clugston, Anna Dominiczak and Alex Alderton, Roberto Amato, Sonia Goncalves, Ewan Harrison, David K. Jackson, Ian Johnston, Dominic Kwiatkowski, Cordelia Langford, John Sillitoe on behalf of the Wellcome Sanger Institute COVID-19 Surveillance Team

EPI_ISL_851899, EPI_ISL_851900	Lighthouse Lab in Alderley Park	Wellcome Sanger Institute for the COVID-19 Genomics UK (COG-UK) Consortium	Jacquelyn Wynn, Mairead Hyland, The Lighthouse Lab in Alderley Park and Alex Alderton, Roberto Amato, Sonia Goncalves, Ewan Harrison, David K. Jackson, Ian Johnston, Dominic Kwiatkowski, Cordelia Langford, John Sillitoe on behalf of the Wellcome Sanger Institute COVID-19 Surveillance Team
EPI_ISL_851901	Lighthouse Lab in Glasgow	Wellcome Sanger Institute for the COVID-19 Genomics UK (COG-UK) Consortium	Harper VanSteenhouse, Yumi Kasai, David Gray, Carol Clugston, Anna Dominiczak and Alex Alderton, Roberto Amato, Sonia Goncalves, Ewan Harrison, David K. Jackson, Ian Johnston, Dominic Kwiatkowski, Cordelia Langford, John Sillitoe on behalf of the Wellcome Sanger Institute COVID-19 Surveillance Team
EPI_ISL_851902, EPI_ISL_851903, EPI_ISL_851904	Lighthouse Lab in Alderley Park	Wellcome Sanger Institute for the COVID-19 Genomics UK (COG-UK) Consortium	Jacquelyn Wynn, Mairead Hyland, The Lighthouse Lab in Alderley Park and Alex Alderton, Roberto Amato, Sonia Goncalves, Ewan Harrison, David K. Jackson, Ian Johnston, Dominic Kwiatkowski, Cordelia Langford, John Sillitoe on behalf of the Wellcome Sanger Institute COVID-19 Surveillance Team
EPI_ISL_851905, EPI_ISL_851906	Lighthouse Lab in Glasgow	Wellcome Sanger Institute for the COVID-19 Genomics UK (COG-UK) Consortium	Harper VanSteenhouse, Yumi Kasai, David Gray, Carol Clugston, Anna Dominiczak and Alex Alderton, Roberto Amato, Sonia Goncalves, Ewan Harrison, David K. Jackson, Ian Johnston, Dominic Kwiatkowski, Cordelia Langford, John Sillitoe on behalf of the Wellcome Sanger Institute COVID-19 Surveillance Team
EPI_ISL_851907, EPI_ISL_851908, EPI_ISL_851909, EPI_ISL_851910, EPI_ISL_851911, EPI_ISL_851912, EPI_ISL_851913, EPI_ISL_851914, EPI_ISL_851915	Lighthouse Lab in Alderley Park	Wellcome Sanger Institute for the COVID-19 Genomics UK (COG-UK) Consortium	Jacquelyn Wynn, Mairead Hyland, The Lighthouse Lab in Alderley Park and Alex Alderton, Roberto Amato, Sonia Goncalves, Ewan Harrison, David K. Jackson, Ian Johnston, Dominic Kwiatkowski, Cordelia Langford, John Sillitoe on behalf of the Wellcome Sanger Institute COVID-19 Surveillance Team
EPI_ISL_851916	Lighthouse Lab in Glasgow	Wellcome Sanger Institute for the COVID-19 Genomics UK (COG-UK) Consortium	Harper VanSteenhouse, Yumi Kasai, David Gray, Carol Clugston, Anna Dominiczak and Alex Alderton, Roberto Amato, Sonia Goncalves, Ewan Harrison, David K. Jackson, Ian Johnston, Dominic Kwiatkowski, Cordelia Langford, John Sillitoe on behalf of the Wellcome Sanger Institute COVID-19 Surveillance Team
EPI_ISL_851917, EPI_ISL_851918, EPI_ISL_851919	Lighthouse Lab in Alderley Park	Wellcome Sanger Institute for the COVID-19 Genomics UK (COG-UK) Consortium	Jacquelyn Wynn, Mairead Hyland, The Lighthouse Lab in Alderley Park and Alex Alderton, Roberto Amato, Sonia Goncalves, Ewan Harrison, David K. Jackson, Ian Johnston, Dominic Kwiatkowski, Cordelia Langford, John Sillitoe on behalf of the Wellcome Sanger Institute COVID-19 Surveillance Team
EPI_ISL_851920	Lighthouse Lab in Glasgow	Wellcome Sanger Institute for the COVID-19 Genomics UK (COG-UK) Consortium	Harper VanSteenhouse, Yumi Kasai, David Gray, Carol Clugston, Anna Dominiczak and Alex Alderton, Roberto Amato, Sonia Goncalves, Ewan Harrison, David K. Jackson, Ian Johnston, Dominic Kwiatkowski, Cordelia Langford, John Sillitoe on behalf of the Wellcome Sanger Institute COVID-19 Surveillance Team
EPI_ISL_851921	Lighthouse Lab in Alderley Park	Wellcome Sanger Institute for the COVID-19 Genomics UK (COG-UK) Consortium	Jacquelyn Wynn, Mairead Hyland, The Lighthouse Lab in Alderley Park and Alex Alderton, Roberto Amato, Sonia Goncalves, Ewan Harrison, David K. Jackson, Ian Johnston, Dominic Kwiatkowski, Cordelia Langford, John Sillitoe on behalf of the Wellcome Sanger Institute COVID-19 Surveillance Team
EPI_ISL_851922	Lighthouse Lab in Glasgow	Wellcome Sanger Institute for the COVID-19 Genomics UK (COG-UK) Consortium	Harper VanSteenhouse, Yumi Kasai, David Gray, Carol Clugston, Anna Dominiczak and Alex Alderton, Roberto Amato, Sonia Goncalves, Ewan Harrison, David K. Jackson, Ian Johnston, Dominic Kwiatkowski, Cordelia Langford, John Sillitoe on behalf of the Wellcome Sanger Institute COVID-19 Surveillance Team
EPI_ISL_851923, EPI_ISL_851924, EPI_ISL_851925, EPI_ISL_851926, EPI_ISL_851927, EPI_ISL_851928	Lighthouse Lab in Alderley Park	Wellcome Sanger Institute for the COVID-19 Genomics UK (COG-UK) Consortium	Jacquelyn Wynn, Mairead Hyland, The Lighthouse Lab in Alderley Park and Alex Alderton, Roberto Amato, Sonia Goncalves, Ewan Harrison, David K. Jackson, Ian Johnston, Dominic Kwiatkowski, Cordelia Langford, John Sillitoe on behalf of the Wellcome Sanger Institute COVID-19 Surveillance Team
EPI_ISL_851929	Lighthouse Lab in Glasgow	Wellcome Sanger Institute for the COVID-19 Genomics UK (COG-UK) Consortium	Harper VanSteenhouse, Yumi Kasai, David Gray, Carol Clugston, Anna Dominiczak and Alex Alderton, Roberto Amato, Sonia Goncalves, Ewan Harrison, David K. Jackson, Ian Johnston, Dominic Kwiatkowski, Cordelia Langford, John Sillitoe on behalf of the Wellcome Sanger Institute COVID-19 Surveillance Team
EPI_ISL_851930, EPI_ISL_851931, EPI_ISL_851932, EPI_ISL_851933	Lighthouse Lab in Alderley Park	Wellcome Sanger Institute for the COVID-19 Genomics UK (COG-UK) Consortium	Jacquelyn Wynn, Mairead Hyland, The Lighthouse Lab in Alderley Park and Alex Alderton, Roberto Amato, Sonia Goncalves, Ewan Harrison, David K. Jackson, Ian Johnston, Dominic Kwiatkowski, Cordelia Langford, John Sillitoe on behalf of the Wellcome Sanger Institute COVID-19 Surveillance Team
EPI_ISL_851934	Lighthouse Lab in Glasgow	Wellcome Sanger Institute for the COVID-19 Genomics UK (COG-UK) Consortium	Harper VanSteenhouse, Yumi Kasai, David Gray, Carol Clugston, Anna Dominiczak and Alex Alderton, Roberto Amato, Sonia Goncalves, Ewan Harrison, David K. Jackson, Ian Johnston, Dominic Kwiatkowski, Cordelia Langford, John Sillitoe on behalf of the Wellcome Sanger Institute COVID-19 Surveillance Team
EPI_ISL_851935	Lighthouse Lab in Alderley Park	Wellcome Sanger Institute for the COVID-19 Genomics UK (COG-UK) Consortium	Jacquelyn Wynn, Mairead Hyland, The Lighthouse Lab in Alderley Park and Alex Alderton, Roberto Amato, Sonia Goncalves, Ewan Harrison, David K. Jackson, Ian Johnston, Dominic Kwiatkowski, Cordelia Langford, John Sillitoe on behalf of the Wellcome Sanger Institute COVID-19 Surveillance Team
EPI_ISL_851936	Lighthouse Lab in Glasgow	Wellcome Sanger Institute for the COVID-19 Genomics UK (COG-UK) Consortium	Harper VanSteenhouse, Yumi Kasai, David Gray, Carol Clugston, Anna Dominiczak and Alex Alderton, Roberto Amato, Sonia Goncalves, Ewan Harrison, David K. Jackson, Ian Johnston, Dominic Kwiatkowski, Cordelia Langford, John Sillitoe on behalf of the Wellcome Sanger Institute COVID-19 Surveillance Team
EPI_ISL_851937, EPI_ISL_851938, EPI_ISL_851939	Lighthouse Lab in Alderley Park	Wellcome Sanger Institute for the COVID-19 Genomics UK (COG-UK) Consortium	Jacquelyn Wynn, Mairead Hyland, The Lighthouse Lab in Alderley Park and Alex Alderton, Roberto Amato, Sonia Goncalves, Ewan Harrison, David K. Jackson, Ian Johnston, Dominic Kwiatkowski, Cordelia Langford, John Sillitoe on behalf of the Wellcome Sanger Institute COVID-19 Surveillance Team
EPI_ISL_851940	Lighthouse Lab in Glasgow	Wellcome Sanger Institute for the COVID-19 Genomics UK (COG-UK) Consortium	Harper VanSteenhouse, Yumi Kasai, David Gray, Carol Clugston, Anna Dominiczak and Alex Alderton, Roberto Amato, Sonia Goncalves, Ewan Harrison, David K. Jackson, Ian Johnston, Dominic Kwiatkowski, Cordelia Langford, John Sillitoe on behalf of the Wellcome Sanger Institute COVID-19 Surveillance Team
EPI_ISL_851941, EPI_ISL_851942, EPI_ISL_851943, EPI_ISL_851944, EPI_ISL_851945, EPI_ISL_851946, EPI_ISL_851947, EPI_ISL_851948, EPI_ISL_851949	Lighthouse Lab in Alderley Park	Wellcome Sanger Institute for the COVID-19 Genomics UK (COG-UK) Consortium	Jacquelyn Wynn, Mairead Hyland, The Lighthouse Lab in Alderley Park and Alex Alderton, Roberto Amato, Sonia Goncalves, Ewan Harrison, David K. Jackson, Ian Johnston, Dominic Kwiatkowski, Cordelia Langford, John Sillitoe on behalf of the Wellcome Sanger Institute COVID-19 Surveillance Team
EPI_ISL_851950	Lighthouse Lab in Glasgow	Wellcome Sanger Institute for the COVID-19 Genomics UK (COG-UK) Consortium	Harper VanSteenhouse, Yumi Kasai, David Gray, Carol Clugston, Anna Dominiczak and Alex Alderton, Roberto Amato, Sonia Goncalves, Ewan Harrison, David K. Jackson, Ian Johnston, Dominic Kwiatkowski, Cordelia Langford, John Sillitoe on behalf of the Wellcome Sanger Institute COVID-19 Surveillance Team
EPI_ISL_851951, EPI_ISL_851952, EPI_ISL_851953	Lighthouse Lab in Alderley Park	Wellcome Sanger Institute for the COVID-19 Genomics UK (COG-UK) Consortium	Jacquelyn Wynn, Mairead Hyland, The Lighthouse Lab in Alderley Park and Alex Alderton, Roberto Amato, Sonia Goncalves, Ewan Harrison, David K. Jackson, Ian Johnston, Dominic Kwiatkowski, Cordelia Langford, John Sillitoe on behalf of the Wellcome Sanger Institute COVID-19 Surveillance Team
EPI_ISL_851955, EPI_ISL_851956	Lighthouse Lab in Glasgow	Wellcome Sanger Institute for the COVID-19 Genomics UK (COG-UK) Consortium	Harper VanSteenhouse, Yumi Kasai, David Gray, Carol Clugston, Anna Dominiczak and Alex Alderton, Roberto Amato, Sonia Goncalves, Ewan Harrison, David K. Jackson, Ian Johnston, Dominic Kwiatkowski, Cordelia Langford, John Sillitoe on behalf of the Wellcome Sanger Institute COVID-19 Surveillance Team
EPI_ISL_851957, EPI_ISL_851958, EPI_ISL_851959, EPI_ISL_851960, EPI_ISL_851961, EPI_ISL_851962, EPI_ISL_851963, EPI_ISL_851965, EPI_ISL_851966, EPI_ISL_851967, EPI_ISL_851968, EPI_ISL_851969, EPI_ISL_851970, EPI_ISL_851971, EPI_ISL_851972, EPI_ISL_851973, EPI_ISL_851974, EPI_ISL_851975, EPI_ISL_851976, EPI_ISL_851977, EPI_ISL_851978, EPI_ISL_851979, EPI_ISL_851980, EPI_ISL_851981, EPI_ISL_851982, EPI_ISL_851983, EPI_ISL_851984, EPI_ISL_851985, EPI_ISL_851986, EPI_ISL_851987, EPI_ISL_851988, EPI_ISL_851990, EPI_ISL_851991, EPI_ISL_851992, EPI_ISL_851993, EPI_ISL_851994, EPI_ISL_851995, EPI_ISL_851996, EPI_ISL_851997, EPI_ISL_851998, EPI_ISL_851999, EPI_ISL_852000, EPI_ISL_852001, EPI_ISL_852003, EPI_ISL_852004, EPI_ISL_852005, EPI_ISL_852006, EPI_ISL_852007, EPI_ISL_852008, EPI_ISL_852009, EPI_ISL_852010, EPI_ISL_852011, EPI_ISL_852012, EPI_ISL_852013, EPI_ISL_852015, EPI_ISL_852016, EPI_ISL_852018, EPI_ISL_852019, EPI_ISL_852020, EPI_ISL_852021, EPI_ISL_852022, EPI_ISL_852023, EPI_ISL_852024			
see above	Lighthouse Lab in Milton Keynes	Wellcome Sanger Institute for the COVID-19 Genomics UK (COG-UK) Consortium	The Lighthouse Lab in Milton Keynes and Alex Alderton, Roberto Amato, Sonia Goncalves, Ewan Harrison, David K. Jackson, Ian Johnston, Dominic Kwiatkowski, Cordelia Langford, John Sillitoe on behalf of the Wellcome Sanger Institute COVID-19 Surveillance Team
EPI_ISL_852025	Lighthouse Lab in Alderley Park	Wellcome Sanger Institute for the COVID-19 Genomics UK (COG-UK) Consortium	Jacquelyn Wynn, Mairead Hyland, The Lighthouse Lab in Alderley Park and Alex Alderton, Roberto Amato, Sonia Goncalves, Ewan Harrison, David K. Jackson, Ian Johnston, Dominic Kwiatkowski, Cordelia Langford, John Sillitoe on behalf of the Wellcome Sanger Institute COVID-19 Surveillance Team
EPI_ISL_852026, EPI_ISL_852027, EPI_ISL_852028, EPI_ISL_852029, EPI_ISL_852030, EPI_ISL_852031, EPI_ISL_852032, EPI_ISL_852033, EPI_ISL_852034, EPI_ISL_852035, EPI_ISL_852036, EPI_ISL_852037, EPI_ISL_852038, EPI_ISL_852039, EPI_ISL_852040, EPI_ISL_852041, EPI_ISL_852042, EPI_ISL_852043, EPI_ISL_852044, EPI_ISL_852045, EPI_ISL_852046, EPI_ISL_852048, EPI_ISL_852049, EPI_ISL_852050, EPI_ISL_852051, EPI_ISL_852052, EPI_ISL_852053, EPI_ISL_852054, EPI_ISL_852055, EPI_ISL_852056, EPI_ISL_852057, EPI_ISL_852058, EPI_ISL_852059, EPI_ISL_852060, EPI_ISL_852061, EPI_ISL_852062, EPI_ISL_852063, EPI_ISL_852064, EPI_ISL_852065, EPI_ISL_852066, EPI_ISL_852067, EPI_ISL_852068, EPI_ISL_852069, EPI_ISL_852070, EPI_ISL_852071, EPI_ISL_852072, EPI_ISL_852073, EPI_ISL_852075, EPI_ISL_852076, EPI_ISL_852077, EPI_ISL_852078, EPI_ISL_852079, EPI_ISL_852080, EPI_ISL_852081, EPI_ISL_852082, EPI_ISL_852084, EPI_ISL_852085, EPI_ISL_852086, EPI_ISL_852087, EPI_ISL_852088, EPI_ISL_852089, EPI_ISL_852090			

see above	Lighthouse Lab in Milton Keynes	Wellcome Sanger Institute for the COVID-19 Genomics UK (COG-UK) Consortium	The Lighthouse Lab in Milton Keynes and Alex Alderton, Roberto Amato, Sonia Goncalves, Ewan Harrison, David K. Jackson, Ian Johnston, Dominic Kwiatkowski, Cordelia Langford, John Sillitoe on behalf of the Wellcome Sanger Institute COVID-19 Surveillance Team
EPI_ISL_852091	Lighthouse Lab in Alderley Park	Wellcome Sanger Institute for the COVID-19 Genomics UK (COG-UK) Consortium	Jacquelyn Wynn, Mairead Hyland, The Lighthouse Lab in Alderley Park and Alex Alderton, Roberto Amato, Sonia Goncalves, Ewan Harrison, David K. Jackson, Ian Johnston, Dominic Kwiatkowski, Cordelia Langford, John Sillitoe on behalf of the Wellcome Sanger Institute COVID-19 Surveillance Team
EPI_ISL_852092, EPI_ISL_852093, EPI_ISL_852094, EPI_ISL_852095, EPI_ISL_852096, EPI_ISL_852097, EPI_ISL_852098, EPI_ISL_852099, EPI_ISL_852100, EPI_ISL_852101, EPI_ISL_852102, EPI_ISL_852103, EPI_ISL_852104, EPI_ISL_852105, EPI_ISL_852106			
see above	Lighthouse Lab in Milton Keynes	Wellcome Sanger Institute for the COVID-19 Genomics UK (COG-UK) Consortium	The Lighthouse Lab in Milton Keynes and Alex Alderton, Roberto Amato, Sonia Goncalves, Ewan Harrison, David K. Jackson, Ian Johnston, Dominic Kwiatkowski, Cordelia Langford, John Sillitoe on behalf of the Wellcome Sanger Institute COVID-19 Surveillance Team
EPI_ISL_852107	Lighthouse Lab in Alderley Park	Wellcome Sanger Institute for the COVID-19 Genomics UK (COG-UK) Consortium	Jacquelyn Wynn, Mairead Hyland, The Lighthouse Lab in Alderley Park and Alex Alderton, Roberto Amato, Sonia Goncalves, Ewan Harrison, David K. Jackson, Ian Johnston, Dominic Kwiatkowski, Cordelia Langford, John Sillitoe on behalf of the Wellcome Sanger Institute COVID-19 Surveillance Team
EPI_ISL_852108, EPI_ISL_852109, EPI_ISL_852110, EPI_ISL_852111, EPI_ISL_852112, EPI_ISL_852113, EPI_ISL_852114, EPI_ISL_852115, EPI_ISL_852116, EPI_ISL_852117, EPI_ISL_852118, EPI_ISL_852119, EPI_ISL_852120, EPI_ISL_852121, EPI_ISL_852122, EPI_ISL_852123, EPI_ISL_852124, EPI_ISL_852125, EPI_ISL_852126, EPI_ISL_852127, EPI_ISL_852128, EPI_ISL_852129, EPI_ISL_852130, EPI_ISL_852131, EPI_ISL_852132, EPI_ISL_852133, EPI_ISL_852134, EPI_ISL_852135, EPI_ISL_852136, EPI_ISL_852137, EPI_ISL_852138, EPI_ISL_852139, EPI_ISL_852140, EPI_ISL_852141, EPI_ISL_852142, EPI_ISL_852143, EPI_ISL_852144, EPI_ISL_852145, EPI_ISL_852146, EPI_ISL_852147, EPI_ISL_852148, EPI_ISL_852149, EPI_ISL_852150, EPI_ISL_852151, EPI_ISL_852152, EPI_ISL_852153, EPI_ISL_852154, EPI_ISL_852155, EPI_ISL_852156, EPI_ISL_852157, EPI_ISL_852158, EPI_ISL_852159, EPI_ISL_852160, EPI_ISL_852161, EPI_ISL_852162, EPI_ISL_852163, EPI_ISL_852164, EPI_ISL_852165, EPI_ISL_852166, EPI_ISL_852167, EPI_ISL_852168, EPI_ISL_852169, EPI_ISL_852170, EPI_ISL_852171, EPI_ISL_852172, EPI_ISL_852173, EPI_ISL_852174, EPI_ISL_852175, EPI_ISL_852176, EPI_ISL_852177, EPI_ISL_852178, EPI_ISL_852180, EPI_ISL_852181, EPI_ISL_852182			
see above	Lighthouse Lab in Milton Keynes	Wellcome Sanger Institute for the COVID-19 Genomics UK (COG-UK) Consortium	The Lighthouse Lab in Milton Keynes and Alex Alderton, Roberto Amato, Sonia Goncalves, Ewan Harrison, David K. Jackson, Ian Johnston, Dominic Kwiatkowski, Cordelia Langford, John Sillitoe on behalf of the Wellcome Sanger Institute COVID-19 Surveillance Team
EPI_ISL_852183	Lighthouse Lab in Alderley Park	Wellcome Sanger Institute for the COVID-19 Genomics UK (COG-UK) Consortium	Jacquelyn Wynn, Mairead Hyland, The Lighthouse Lab in Alderley Park and Alex Alderton, Roberto Amato, Sonia Goncalves, Ewan Harrison, David K. Jackson, Ian Johnston, Dominic Kwiatkowski, Cordelia Langford, John Sillitoe on behalf of the Wellcome Sanger Institute COVID-19 Surveillance Team
EPI_ISL_852184, EPI_ISL_852185, EPI_ISL_852186, EPI_ISL_852187, EPI_ISL_852188, EPI_ISL_852189, EPI_ISL_852192, EPI_ISL_852193, EPI_ISL_852194, EPI_ISL_852195, EPI_ISL_852196, EPI_ISL_852197, EPI_ISL_852198, EPI_ISL_852199, EPI_ISL_852200, EPI_ISL_852201, EPI_ISL_852202, EPI_ISL_852203, EPI_ISL_852204, EPI_ISL_852205, EPI_ISL_852207, EPI_ISL_852208, EPI_ISL_852209, EPI_ISL_852210, EPI_ISL_852211, EPI_ISL_852212, EPI_ISL_852213			
see above	Lighthouse Lab in Milton Keynes	Wellcome Sanger Institute for the COVID-19 Genomics UK (COG-UK) Consortium	The Lighthouse Lab in Milton Keynes and Alex Alderton, Roberto Amato, Sonia Goncalves, Ewan Harrison, David K. Jackson, Ian Johnston, Dominic Kwiatkowski, Cordelia Langford, John Sillitoe on behalf of the Wellcome Sanger Institute COVID-19 Surveillance Team
EPI_ISL_852214	Lighthouse Lab in Alderley Park	Wellcome Sanger Institute for the COVID-19 Genomics UK (COG-UK) Consortium	Jacquelyn Wynn, Mairead Hyland, The Lighthouse Lab in Alderley Park and Alex Alderton, Roberto Amato, Sonia Goncalves, Ewan Harrison, David K. Jackson, Ian Johnston, Dominic Kwiatkowski, Cordelia Langford, John Sillitoe on behalf of the Wellcome Sanger Institute COVID-19 Surveillance Team
EPI_ISL_852215, EPI_ISL_852216, EPI_ISL_852217, EPI_ISL_852218, EPI_ISL_852219, EPI_ISL_852220, EPI_ISL_852221, EPI_ISL_852222, EPI_ISL_852223, EPI_ISL_852224, EPI_ISL_852226, EPI_ISL_852227, EPI_ISL_852228, EPI_ISL_852229, EPI_ISL_852230, EPI_ISL_852231, EPI_ISL_852232, EPI_ISL_852233, EPI_ISL_852234, EPI_ISL_852235, EPI_ISL_852236, EPI_ISL_852237, EPI_ISL_852238, EPI_ISL_852239, EPI_ISL_852240, EPI_ISL_852241, EPI_ISL_852242, EPI_ISL_852243, EPI_ISL_852244, EPI_ISL_852245, EPI_ISL_852246, EPI_ISL_852247, EPI_ISL_852248, EPI_ISL_852249, EPI_ISL_852250, EPI_ISL_852251, EPI_ISL_852252, EPI_ISL_852253, EPI_ISL_852254, EPI_ISL_852255			
see above	Lighthouse Lab in Milton Keynes	Wellcome Sanger Institute for the COVID-19 Genomics UK (COG-UK) Consortium	The Lighthouse Lab in Milton Keynes and Alex Alderton, Roberto Amato, Sonia Goncalves, Ewan Harrison, David K. Jackson, Ian Johnston, Dominic Kwiatkowski, Cordelia Langford, John Sillitoe on behalf of the Wellcome Sanger Institute COVID-19 Surveillance Team
EPI_ISL_852257, EPI_ISL_852258, EPI_ISL_852259, EPI_ISL_852260, EPI_ISL_852264, EPI_ISL_852268, EPI_ISL_852269, EPI_ISL_852275, EPI_ISL_852276, EPI_ISL_852279, EPI_ISL_852281, EPI_ISL_852283, EPI_ISL_852285, EPI_ISL_852286, EPI_ISL_852287, EPI_ISL_852288, EPI_ISL_852289, EPI_ISL_852292, EPI_ISL_852293, EPI_ISL_852295, EPI_ISL_852300, EPI_ISL_852301, EPI_ISL_852303, EPI_ISL_852305, EPI_ISL_852306, EPI_ISL_852307, EPI_ISL_852310, EPI_ISL_852312, EPI_ISL_852313, EPI_ISL_852314, EPI_ISL_852316, EPI_ISL_852319, EPI_ISL_852321, EPI_ISL_852322, EPI_ISL_852325, EPI_ISL_852326, EPI_ISL_852328, EPI_ISL_852334, EPI_ISL_852335, EPI_ISL_852338, EPI_ISL_852339, EPI_ISL_852340, EPI_ISL_852343, EPI_ISL_852344, EPI_ISL_852345, EPI_ISL_852347, EPI_ISL_852348, EPI_ISL_852350, EPI_ISL_852353, EPI_ISL_852358, EPI_ISL_852360, EPI_ISL_852361, EPI_ISL_852364, EPI_ISL_852367, EPI_ISL_852368, EPI_ISL_852369, EPI_ISL_852370, EPI_ISL_852374, EPI_ISL_852375, EPI_ISL_852378, EPI_ISL_852389, EPI_ISL_852391, EPI_ISL_852393, EPI_ISL_852395, EPI_ISL_852397, EPI_ISL_852398, EPI_ISL_852404, EPI_ISL_852406, EPI_ISL_852407, EPI_ISL_852412, EPI_ISL_852413, EPI_ISL_852414, EPI_ISL_852415, EPI_ISL_852416, EPI_ISL_852417, EPI_ISL_852420, EPI_ISL_852421, EPI_ISL_852422, EPI_ISL_852424, EPI_ISL_852425, EPI_ISL_852426, EPI_ISL_852429, EPI_ISL_852433, EPI_ISL_852435, EPI_ISL_852438, EPI_ISL_852439, EPI_ISL_852440, EPI_ISL_852444, EPI_ISL_852445, EPI_ISL_852446, EPI_ISL_852447, EPI_ISL_852448, EPI_ISL_852451, EPI_ISL_852454, EPI_ISL_852459, EPI_ISL_852460, EPI_ISL_852462, EPI_ISL_852463, EPI_ISL_852467, EPI_ISL_852471, EPI_ISL_852472, EPI_ISL_852474, EPI_ISL_852475, EPI_ISL_852476, EPI_ISL_852479, EPI_ISL_852481, EPI_ISL_852484, EPI_ISL_852485, EPI_ISL_852487, EPI_ISL_852491, EPI_ISL_852497, EPI_ISL_852498, EPI_ISL_852500, EPI_ISL_852503, EPI_ISL_852504, EPI_ISL_852506, EPI_ISL_852508, EPI_ISL_852509, EPI_ISL_852510, EPI_ISL_852514, EPI_ISL_852517, EPI_ISL_852519, EPI_ISL_852520, EPI_ISL_852522, EPI_ISL_852524, EPI_ISL_852527, EPI_ISL_852529, EPI_ISL_852530, EPI_ISL_852531, EPI_ISL_852532, EPI_ISL_852533, EPI_ISL_852534, EPI_ISL_852535, EPI_ISL_852536, EPI_ISL_852537, EPI_ISL_852540, EPI_ISL_852541, EPI_ISL_852544, EPI_ISL_852548, EPI_ISL_852549, EPI_ISL_852550, EPI_ISL_852555, EPI_ISL_852557, EPI_ISL_852558, EPI_ISL_852559, EPI_ISL_852560, EPI_ISL_852561, EPI_ISL_852562, EPI_ISL_852563			
see above	Lighthouse Lab in Glasgow	Wellcome Sanger Institute for the COVID-19 Genomics UK (COG-UK) Consortium	Harper VanSteenhouse, Yumi Kasai, David Gray, Carol Clugston, Anna Dominiczak and Alex Alderton, Roberto Amato, Sonia Goncalves, Ewan Harrison, David K. Jackson, Ian Johnston, Dominic Kwiatkowski, Cordelia Langford, John Sillitoe on behalf of the Wellcome Sanger Institute COVID-19 Surveillance Team
EPI_ISL_855595, EPI_ISL_855602, EPI_ISL_855605	Respiratory Virus Unit, National Infection Service, Public Health England	COVID-19 Genomics UK (COG-UK) Consortium	PHE Covid Sequencing Team
EPI_ISL_857542, EPI_ISL_857543, EPI_ISL_857544, EPI_ISL_857545, EPI_ISL_857547, EPI_ISL_857548, EPI_ISL_857549, EPI_ISL_857550, EPI_ISL_857551, EPI_ISL_857552, EPI_ISL_857554, EPI_ISL_857555, EPI_ISL_857556, EPI_ISL_857557, EPI_ISL_857558, EPI_ISL_857559, EPI_ISL_857560, EPI_ISL_857561, EPI_ISL_857563, EPI_ISL_857564, EPI_ISL_857565, EPI_ISL_857566, EPI_ISL_857567, EPI_ISL_857568, EPI_ISL_857569, EPI_ISL_857570, EPI_ISL_857571, EPI_ISL_857572, EPI_ISL_857573, EPI_ISL_857574, EPI_ISL_857575, EPI_ISL_857576, EPI_ISL_857577, EPI_ISL_857578, EPI_ISL_857579, EPI_ISL_857580, EPI_ISL_857581, EPI_ISL_857582, EPI_ISL_857583, EPI_ISL_857585, EPI_ISL_857586, EPI_ISL_857587, EPI_ISL_857588, EPI_ISL_857589, EPI_ISL_857590, EPI_ISL_857591, EPI_ISL_857592, EPI_ISL_857593, EPI_ISL_857594, EPI_ISL_857595, EPI_ISL_857596, EPI_ISL_857597, EPI_ISL_857598, EPI_ISL_857600, EPI_ISL_857601, EPI_ISL_857602, EPI_ISL_857603, EPI_ISL_857605, EPI_ISL_857606, EPI_ISL_857607, EPI_ISL_857608, EPI_ISL_857609, EPI_ISL_857610, EPI_ISL_857611, EPI_ISL_857612, EPI_ISL_857613, EPI_ISL_857614, EPI_ISL_857615, EPI_ISL_857616, EPI_ISL_857617, EPI_ISL_857618, EPI_ISL_857619, EPI_ISL_857620, EPI_ISL_857621, EPI_ISL_857622, EPI_ISL_857623, EPI_ISL_857624, EPI_ISL_857625, EPI_ISL_857626, EPI_ISL_857627, EPI_ISL_857628, EPI_ISL_857629, EPI_ISL_857630, EPI_ISL_857631, EPI_ISL_857632, EPI_ISL_857633, EPI_ISL_857634, EPI_ISL_857635, EPI_ISL_857636, EPI_ISL_857637, EPI_ISL_857638, EPI_ISL_857639, EPI_ISL_857640, EPI_ISL_857641, EPI_ISL_857643, EPI_ISL_857644, EPI_ISL_857646, EPI_ISL_857647, EPI_ISL_857648, EPI_ISL_857649, EPI_ISL_857650, EPI_ISL_857651, EPI_ISL_857652, EPI_ISL_857653, EPI_ISL_857655, EPI_ISL_857656, EPI_ISL_857657, EPI_ISL_857658, EPI_ISL_857659, EPI_ISL_857660, EPI_ISL_857661, EPI_ISL_857662, EPI_ISL_857663, EPI_ISL_857664, EPI_ISL_857665, EPI_ISL_857666, EPI_ISL_857667, EPI_ISL_857668, EPI_ISL_857669, EPI_ISL_857670, EPI_ISL_857671, EPI_ISL_857672, EPI_ISL_857673, EPI_ISL_857674, EPI_ISL_857675, EPI_ISL_857676, EPI_ISL_857677, EPI_ISL_857678, EPI_ISL_857679, EPI_ISL_857680, EPI_ISL_857681, EPI_ISL_857683, EPI_ISL_857684, EPI_ISL_857685, EPI_ISL_857686, EPI_ISL_857687, EPI_ISL_857688, EPI_ISL_857689, EPI_ISL_857690, EPI_ISL_857691, EPI_ISL_857692, EPI_ISL_857693, EPI_ISL_857694, EPI_ISL_857695, EPI_ISL_857696, EPI_ISL_857697, EPI_ISL_857698, EPI_ISL_857699, EPI_ISL_857700, EPI_ISL_857701, EPI_ISL_857702, EPI_ISL_857703, EPI_ISL_857704, EPI_ISL_857705, EPI_ISL_857706, EPI_ISL_857707, EPI_ISL_857708, EPI_ISL_857709, EPI_ISL_857710, EPI_ISL_857711, EPI_ISL_857712, EPI_ISL_857713, EPI_ISL_857714, EPI_ISL_857715, EPI_ISL_857716, EPI_ISL_857717, EPI_ISL_857718, EPI_ISL_857719, EPI_ISL_857720, EPI_ISL_857721, EPI_ISL_857722, EPI_ISL_857723, EPI_ISL_857724, EPI_ISL_857725, EPI_ISL_857726, EPI_ISL_857727, EPI_ISL_857728, EPI_ISL_857729, EPI_ISL_857730, EPI_ISL_857731, EPI_ISL_857732, EPI_ISL_857733, EPI_ISL_857734, EPI_ISL_857735, EPI_ISL_857736, EPI_ISL_857737, EPI_ISL_857738, EPI_ISL_857739, EPI_ISL_857740, EPI_ISL_857741, EPI_ISL_857742, EPI_ISL_857743, EPI_ISL_857744, EPI_ISL_857745, EPI_ISL_857746, EPI_ISL_857747, EPI_ISL_857748, EPI_ISL_857749, EPI_ISL_857750, EPI_ISL_857751, EPI_ISL_857752, EPI_ISL_857753, EPI_ISL_857754, EPI_ISL_857755, EPI_ISL_857756, EPI_ISL_857757, EPI_ISL_857758, EPI_ISL_857760, EPI_ISL_857761, EPI_ISL_857762, EPI_ISL_857763, EPI_ISL_857764, EPI_ISL_857765, EPI_ISL_857766, EPI_ISL_857767, EPI_ISL_857768, EPI_ISL_857769, EPI_ISL_857770, EPI_ISL_857771, EPI_ISL_857772, EPI_ISL_857773, EPI_ISL_857774, EPI_ISL_857775, EPI_ISL_857776, EPI_ISL_857777, EPI_ISL_857778, EPI_ISL_857779, EPI_ISL_857780, EPI_ISL_857781, EPI_ISL_857782, EPI_ISL_857783, EPI_ISL_857784, EPI_ISL_857785, EPI_ISL_857786, EPI_ISL_857787, EPI_ISL_857788, EPI_ISL_857789, EPI_ISL_857790, EPI_ISL_857791, EPI_ISL_857792, EPI_ISL_857793, EPI_ISL_857794, EPI_ISL_857795, EPI_ISL_857796, EPI_ISL_857797, EPI_ISL_857798, EPI_ISL_857799, EPI_ISL_857800, EPI_ISL_857801, EPI_ISL_857802, EPI_ISL_857803, EPI_ISL_857804, EPI_ISL_857805, EPI_ISL_857806, EPI_ISL_857807, EPI_ISL_857808, EPI_ISL_857809, EPI_ISL_857810, EPI_ISL_857811, EPI_ISL_857812, EPI_ISL_857813, EPI_ISL_857814, EPI_ISL_857815, EPI_ISL_857816, EPI_ISL_857817, EPI_ISL_857820, EPI_ISL_857821, EPI_ISL_857822, EPI_ISL_857824, EPI_ISL_857825, EPI_ISL_857826, EPI_ISL_857827, EPI_ISL_857828, EPI_ISL_857829, EPI_ISL_857830, EPI_ISL_857831, EPI_ISL_857832, EPI_ISL_857833, EPI_ISL_857834, EPI_ISL_857835, EPI_ISL_857836, EPI_ISL_857837, EPI_ISL_857838, EPI_ISL_857839, EPI_ISL_857840, EPI_ISL_857841, EPI_ISL_857842, EPI_ISL_857843, EPI_ISL_857844, EPI_ISL_857845, EPI_ISL_857846, EPI_ISL_857847, EPI_ISL_857848, EPI_ISL_857849, EPI_ISL_857850, EPI_ISL_857851, EPI_ISL_857853, EPI_ISL_857854, EPI_ISL_857856, EPI_ISL_857857, EPI_ISL_857858, EPI_ISL_857859, EPI_ISL_857860, EPI_ISL_857861, EPI_ISL_857862, EPI_ISL_857863, EPI_ISL_857864, EPI_ISL_857865, EPI_ISL_857866, EPI_ISL_857867, EPI_ISL_857868, EPI_ISL_857869			
see above	Lighthouse Lab in Cambridge	Wellcome Sanger Institute for the COVID-19 Genomics UK (COG-UK) Consortium	Rob Howes, The Lighthouse Lab in Cambridge and Alex Alderton, Roberto Amato, Sonia Goncalves, Ewan Harrison, David K. Jackson, Ian Johnston, Dominic Kwiatkowski, Cordelia Langford, John Sillitoe on behalf of the Wellcome Sanger Institute COVID-19 Surveillance Team
EPI_ISL_857870, EPI_ISL_857871, EPI_ISL_857872, EPI_ISL_857873	Lighthouse Lab in Glasgow	Wellcome Sanger Institute for the COVID-19 Genomics UK (COG-UK) Consortium	Harper VanSteenhouse, Yumi Kasai, David Gray, Carol Clugston, Anna Dominiczak and Alex Alderton, Roberto Amato, Sonia Goncalves, Ewan Harrison, David K. Jackson, Ian Johnston, Dominic Kwiatkowski, Cordelia Langford, John Sillitoe on behalf of the Wellcome Sanger Institute COVID-19 Surveillance Team
EPI_ISL_857874, EPI_ISL_857875	Lighthouse Lab in Alderley Park	Wellcome Sanger Institute for the COVID-19 Genomics UK (COG-UK) Consortium	Jacquelyn Wynn, Mairead Hyland, The Lighthouse Lab in Alderley Park and Alex Alderton, Roberto Amato, Sonia Goncalves, Ewan Harrison, David K. Jackson, Ian Johnston, Dominic Kwiatkowski, Cordelia Langford, John Sillitoe on behalf of the Wellcome Sanger Institute COVID-19 Surveillance Team
EPI_ISL_857876	Lighthouse Lab in Glasgow	Wellcome Sanger Institute for the COVID-19 Genomics UK (COG-UK) Consortium	Harper VanSteenhouse, Yumi Kasai, David Gray, Carol Clugston, Anna Dominiczak and Alex Alderton, Roberto Amato, Sonia Goncalves, Ewan Harrison, David K. Jackson, Ian Johnston, Dominic Kwiatkowski, Cordelia Langford, John Sillitoe on behalf of the Wellcome Sanger Institute COVID-19 Surveillance Team
EPI_ISL_857877	Lighthouse Lab in Alderley Park	Wellcome Sanger Institute for the COVID-19 Genomics UK (COG-UK) Consortium	Jacquelyn Wynn, Mairead Hyland, The Lighthouse Lab in Alderley Park and Alex Alderton, Roberto Amato, Sonia Goncalves, Ewan Harrison, David K. Jackson, Ian Johnston, Dominic Kwiatkowski, Cordelia Langford, John Sillitoe on behalf of the Wellcome Sanger Institute COVID-19 Surveillance Team
EPI_ISL_857878	Lighthouse Lab in Glasgow	Wellcome Sanger Institute for the COVID-19 Genomics UK (COG-UK) Consortium	Harper VanSteenhouse, Yumi Kasai, David Gray, Carol Clugston, Anna Dominiczak and Alex Alderton, Roberto Amato, Sonia Goncalves, Ewan Harrison, David K. Jackson, Ian Johnston, Dominic Kwiatkowski, Cordelia Langford, John Sillitoe on behalf of the Wellcome Sanger Institute COVID-19 Surveillance Team

EPI_ISL_857880	Lighthouse Lab in Alderley Park	Wellcome Sanger Institute for the COVID-19 Genomics UK (COG-UK) Consortium	Jacquelyn Wynn, Mairead Hyland, The Lighthouse Lab in Alderley Park and Alex Alderton, Roberto Amato, Sonia Goncalves, Ewan Harrison, David K. Jackson, Ian Johnston, Dominic Kwiatkowski, Cordelia Langford, John Sillitoe on behalf of the Wellcome Sanger Institute COVID-19 Surveillance Team
EPI_ISL_857881	Lighthouse Lab in Glasgow	Wellcome Sanger Institute for the COVID-19 Genomics UK (COG-UK) Consortium	Harper VanSteenhouse, Yumi Kasai, David Gray, Carol Clugston, Anna Dominiczak and Alex Alderton, Roberto Amato, Sonia Goncalves, Ewan Harrison, David K. Jackson, Ian Johnston, Dominic Kwiatkowski, Cordelia Langford, John Sillitoe on behalf of the Wellcome Sanger Institute COVID-19 Surveillance Team
EPI_ISL_857882, EPI_ISL_857883, EPI_ISL_857884	Lighthouse Lab in Alderley Park	Wellcome Sanger Institute for the COVID-19 Genomics UK (COG-UK) Consortium	Jacquelyn Wynn, Mairead Hyland, The Lighthouse Lab in Alderley Park and Alex Alderton, Roberto Amato, Sonia Goncalves, Ewan Harrison, David K. Jackson, Ian Johnston, Dominic Kwiatkowski, Cordelia Langford, John Sillitoe on behalf of the Wellcome Sanger Institute COVID-19 Surveillance Team
EPI_ISL_857885	Lighthouse Lab in Glasgow	Wellcome Sanger Institute for the COVID-19 Genomics UK (COG-UK) Consortium	Harper VanSteenhouse, Yumi Kasai, David Gray, Carol Clugston, Anna Dominiczak and Alex Alderton, Roberto Amato, Sonia Goncalves, Ewan Harrison, David K. Jackson, Ian Johnston, Dominic Kwiatkowski, Cordelia Langford, John Sillitoe on behalf of the Wellcome Sanger Institute COVID-19 Surveillance Team
EPI_ISL_857886	Lighthouse Lab in Alderley Park	Wellcome Sanger Institute for the COVID-19 Genomics UK (COG-UK) Consortium	Jacquelyn Wynn, Mairead Hyland, The Lighthouse Lab in Alderley Park and Alex Alderton, Roberto Amato, Sonia Goncalves, Ewan Harrison, David K. Jackson, Ian Johnston, Dominic Kwiatkowski, Cordelia Langford, John Sillitoe on behalf of the Wellcome Sanger Institute COVID-19 Surveillance Team
EPI_ISL_857887, EPI_ISL_857888, EPI_ISL_857889, EPI_ISL_857892, EPI_ISL_857893, EPI_ISL_857894, EPI_ISL_857895, EPI_ISL_857896, EPI_ISL_857897, EPI_ISL_857898, EPI_ISL_857899, EPI_ISL_857900, EPI_ISL_857901, EPI_ISL_857902, EPI_ISL_857903, EPI_ISL_857904, EPI_ISL_857905, EPI_ISL_857906, EPI_ISL_857907, EPI_ISL_857908, EPI_ISL_857909, EPI_ISL_857910, EPI_ISL_857911, EPI_ISL_857912, EPI_ISL_857913, EPI_ISL_857914, EPI_ISL_857915, EPI_ISL_857916, EPI_ISL_857917, EPI_ISL_857918, EPI_ISL_857919, EPI_ISL_857920, EPI_ISL_857921, EPI_ISL_857922, EPI_ISL_857923, EPI_ISL_857924, EPI_ISL_857925, EPI_ISL_857926, EPI_ISL_857927, EPI_ISL_857928, EPI_ISL_857929, EPI_ISL_857930, EPI_ISL_857931, EPI_ISL_857932, EPI_ISL_857933, EPI_ISL_857934, EPI_ISL_857935, EPI_ISL_857936, EPI_ISL_857937, EPI_ISL_857938, EPI_ISL_857939, EPI_ISL_857940, EPI_ISL_857941, EPI_ISL_857942, EPI_ISL_857943, EPI_ISL_857944, EPI_ISL_857945, EPI_ISL_857946, EPI_ISL_857947, EPI_ISL_857948, EPI_ISL_857949, EPI_ISL_857950, EPI_ISL_857951, EPI_ISL_857952, EPI_ISL_857953, EPI_ISL_857954, EPI_ISL_857955, EPI_ISL_857956, EPI_ISL_857957, EPI_ISL_857958, EPI_ISL_857959			
see above	Lighthouse Lab in Glasgow	Wellcome Sanger Institute for the COVID-19 Genomics UK (COG-UK) Consortium	Harper VanSteenhouse, Yumi Kasai, David Gray, Carol Clugston, Anna Dominiczak and Alex Alderton, Roberto Amato, Sonia Goncalves, Ewan Harrison, David K. Jackson, Ian Johnston, Dominic Kwiatkowski, Cordelia Langford, John Sillitoe on behalf of the Wellcome Sanger Institute COVID-19 Surveillance Team
EPI_ISL_857960, EPI_ISL_857961, EPI_ISL_857962, EPI_ISL_857963, EPI_ISL_857964, EPI_ISL_857965, EPI_ISL_857966, EPI_ISL_857967, EPI_ISL_857968, EPI_ISL_857969, EPI_ISL_857970, EPI_ISL_857971, EPI_ISL_857972, EPI_ISL_857973, EPI_ISL_857974, EPI_ISL_857975, EPI_ISL_857976, EPI_ISL_857977, EPI_ISL_857978, EPI_ISL_857979, EPI_ISL_857980, EPI_ISL_857981, EPI_ISL_857982, EPI_ISL_857983, EPI_ISL_857984, EPI_ISL_857985, EPI_ISL_857986, EPI_ISL_857987, EPI_ISL_857988, EPI_ISL_857989, EPI_ISL_857990, EPI_ISL_857991, EPI_ISL_857992, EPI_ISL_857993, EPI_ISL_857994, EPI_ISL_857995, EPI_ISL_857996, EPI_ISL_857997, EPI_ISL_857998, EPI_ISL_857999, EPI_ISL_858000, EPI_ISL_858001, EPI_ISL_858002, EPI_ISL_858003, EPI_ISL_858004, EPI_ISL_858005, EPI_ISL_858006, EPI_ISL_858007, EPI_ISL_858008, EPI_ISL_858009, EPI_ISL_858010			
see above	Lighthouse Lab in Alderley Park	Wellcome Sanger Institute for the COVID-19 Genomics UK (COG-UK) Consortium	Jacquelyn Wynn, Mairead Hyland, The Lighthouse Lab in Alderley Park and Alex Alderton, Roberto Amato, Sonia Goncalves, Ewan Harrison, David K. Jackson, Ian Johnston, Dominic Kwiatkowski, Cordelia Langford, John Sillitoe on behalf of the Wellcome Sanger Institute COVID-19 Surveillance Team
EPI_ISL_858011, EPI_ISL_858012, EPI_ISL_858013, EPI_ISL_858015, EPI_ISL_858016, EPI_ISL_858017, EPI_ISL_858018, EPI_ISL_858019, EPI_ISL_858021, EPI_ISL_858022, EPI_ISL_858023, EPI_ISL_858024, EPI_ISL_858025, EPI_ISL_858026, EPI_ISL_858027, EPI_ISL_858030, EPI_ISL_858031, EPI_ISL_858032, EPI_ISL_858033, EPI_ISL_858034, EPI_ISL_858035, EPI_ISL_858036, EPI_ISL_858038, EPI_ISL_858039, EPI_ISL_858040, EPI_ISL_858041, EPI_ISL_858042, EPI_ISL_858043, EPI_ISL_858044, EPI_ISL_858045, EPI_ISL_858046, EPI_ISL_858047, EPI_ISL_858048, EPI_ISL_858049, EPI_ISL_858050, EPI_ISL_858051, EPI_ISL_858052, EPI_ISL_858053, EPI_ISL_858054, EPI_ISL_858055, EPI_ISL_858056, EPI_ISL_858057, EPI_ISL_858058, EPI_ISL_858059, EPI_ISL_858060, EPI_ISL_858061, EPI_ISL_858062, EPI_ISL_858063, EPI_ISL_858316			
see above	Lighthouse Lab in Glasgow	Wellcome Sanger Institute for the COVID-19 Genomics UK (COG-UK) Consortium	Harper VanSteenhouse, Yumi Kasai, David Gray, Carol Clugston, Anna Dominiczak and Alex Alderton, Roberto Amato, Sonia Goncalves, Ewan Harrison, David K. Jackson, Ian Johnston, Dominic Kwiatkowski, Cordelia Langford, John Sillitoe on behalf of the Wellcome Sanger Institute COVID-19 Surveillance Team
EPI_ISL_860688	Respiratory Virus Unit, National Infection Service, Public Health England	COVID-19 Genomics UK (COG-UK) Consortium	PHE Covid Sequencing Team
EPI_ISL_862886, EPI_ISL_862887, EPI_ISL_862888, EPI_ISL_862889, EPI_ISL_862890, EPI_ISL_862891, EPI_ISL_862892, EPI_ISL_862893, EPI_ISL_862894, EPI_ISL_862895, EPI_ISL_862896, EPI_ISL_862897, EPI_ISL_862898, EPI_ISL_862899, EPI_ISL_862900, EPI_ISL_862901, EPI_ISL_862902, EPI_ISL_862903, EPI_ISL_862904, EPI_ISL_862905, EPI_ISL_862906, EPI_ISL_862907, EPI_ISL_862908, EPI_ISL_862909, EPI_ISL_862910, EPI_ISL_862911, EPI_ISL_862912, EPI_ISL_862913, EPI_ISL_862914, EPI_ISL_862915, EPI_ISL_862916, EPI_ISL_862917, EPI_ISL_862918, EPI_ISL_862919, EPI_ISL_862920, EPI_ISL_862921, EPI_ISL_862922, EPI_ISL_862923, EPI_ISL_862924, EPI_ISL_862925, EPI_ISL_862926, EPI_ISL_862927, EPI_ISL_862928, EPI_ISL_862929, EPI_ISL_862930, EPI_ISL_862931, EPI_ISL_862932, EPI_ISL_862933, EPI_ISL_862934, EPI_ISL_862935, EPI_ISL_862936, EPI_ISL_862937, EPI_ISL_862938, EPI_ISL_862939, EPI_ISL_862940, EPI_ISL_862941, EPI_ISL_862942, EPI_ISL_862943, EPI_ISL_862944, EPI_ISL_862945, EPI_ISL_862946, EPI_ISL_862947, EPI_ISL_862948, EPI_ISL_862949, EPI_ISL_862950, EPI_ISL_862951, EPI_ISL_862952, EPI_ISL_862953, EPI_ISL_862954, EPI_ISL_862955, EPI_ISL_862956, EPI_ISL_862957, EPI_ISL_862958, EPI_ISL_862959, EPI_ISL_862960, EPI_ISL_862961, EPI_ISL_862962, EPI_ISL_862963, EPI_ISL_862964, EPI_ISL_862965, EPI_ISL_862966, EPI_ISL_862967, EPI_ISL_862968, EPI_ISL_862969, EPI_ISL_862970, EPI_ISL_862971, EPI_ISL_862972, EPI_ISL_862973, EPI_ISL_862974, EPI_ISL_862975, EPI_ISL_862976, EPI_ISL_862977, EPI_ISL_862978, EPI_ISL_862979, EPI_ISL_862980, EPI_ISL_862981, EPI_ISL_862982, EPI_ISL_862983, EPI_ISL_862984, EPI_ISL_862985, EPI_ISL_862986, EPI_ISL_862987, EPI_ISL_862988, EPI_ISL_862989, EPI_ISL_862990, EPI_ISL_862991, EPI_ISL_862992, EPI_ISL_862993, EPI_ISL_862994, EPI_ISL_862995, EPI_ISL_862996, EPI_ISL_862997, EPI_ISL_862998, EPI_ISL_862999, EPI_ISL_863000, EPI_ISL_863001, EPI_ISL_863002, EPI_ISL_863003, EPI_ISL_863004, EPI_ISL_863005, EPI_ISL_863006, EPI_ISL_863007, EPI_ISL_863008, EPI_ISL_863009, EPI_ISL_863010, EPI_ISL_863011, EPI_ISL_863012, EPI_ISL_863013, EPI_ISL_863014, EPI_ISL_863015, EPI_ISL_863016, EPI_ISL_863017, EPI_ISL_863018, EPI_ISL_863019, EPI_ISL_863020, EPI_ISL_863021, EPI_ISL_863022, EPI_ISL_863023, EPI_ISL_863024, EPI_ISL_863025, EPI_ISL_863026, EPI_ISL_863027, EPI_ISL_863028, EPI_ISL_863029, EPI_ISL_863030, EPI_ISL_863031, EPI_ISL_863032, EPI_ISL_863033, EPI_ISL_863034, EPI_ISL_863035, EPI_ISL_863036, EPI_ISL_863037, EPI_ISL_863038, EPI_ISL_863039, EPI_ISL_863040, EPI_ISL_863041, EPI_ISL_863042, EPI_ISL_863043, EPI_ISL_863044, EPI_ISL_863045, EPI_ISL_863046, EPI_ISL_863047, EPI_ISL_863048, EPI_ISL_863049, EPI_ISL_863050, EPI_ISL_863051, EPI_ISL_863052, EPI_ISL_863053, EPI_ISL_863054, EPI_ISL_863055, EPI_ISL_863056, EPI_ISL_863057, EPI_ISL_863058, EPI_ISL_863059, EPI_ISL_863060, EPI_ISL_863061, EPI_ISL_863062, EPI_ISL_863063, EPI_ISL_863064, EPI_ISL_863065, EPI_ISL_863066, EPI_ISL_863067, EPI_ISL_863068, EPI_ISL_863069, EPI_ISL_863070, EPI_ISL_863071, EPI_ISL_863072, EPI_ISL_863073, EPI_ISL_863074, EPI_ISL_863075, EPI_ISL_863076, EPI_ISL_863077, EPI_ISL_863078, EPI_ISL_863079, EPI_ISL_863080, EPI_ISL_863081, EPI_ISL_863082, EPI_ISL_863083, EPI_ISL_863084, EPI_ISL_863085, EPI_ISL_863086, EPI_ISL_863087, EPI_ISL_863088, EPI_ISL_863089, EPI_ISL_863090, EPI_ISL_863091, EPI_ISL_863092, EPI_ISL_863093, EPI_ISL_863094, EPI_ISL_863095, EPI_ISL_863096, EPI_ISL_863097, EPI_ISL_863098, EPI_ISL_863099, EPI_ISL_863100, EPI_ISL_863101, EPI_ISL_863102, EPI_ISL_863103, EPI_ISL_863104, EPI_ISL_863105, EPI_ISL_863106, EPI_ISL_863107, EPI_ISL_863108, EPI_ISL_863109, EPI_ISL_863110, EPI_ISL_863111, EPI_ISL_863112, EPI_ISL_863113, EPI_ISL_863114, EPI_ISL_863115, EPI_ISL_863116, EPI_ISL_863117, EPI_ISL_863118, EPI_ISL_863119, EPI_ISL_863120, EPI_ISL_863121, EPI_ISL_863122, EPI_ISL_863123, EPI_ISL_863124, EPI_ISL_863125, EPI_ISL_863126, EPI_ISL_863127, EPI_ISL_863128, EPI_ISL_863129, EPI_ISL_863130, EPI_ISL_863131, EPI_ISL_863132, EPI_ISL_863133, EPI_ISL_863134, EPI_ISL_863135, EPI_ISL_863136, EPI_ISL_863137, EPI_ISL_863138, EPI_ISL_863139, EPI_ISL_863140, EPI_ISL_863141, EPI_ISL_863142, EPI_ISL_863143, EPI_ISL_863144, EPI_ISL_863145, EPI_ISL_863146, EPI_ISL_863147, EPI_ISL_863148, EPI_ISL_863149, EPI_ISL_863150, EPI_ISL_863151, EPI_ISL_863152, EPI_ISL_863153, EPI_ISL_863154, EPI_ISL_863155, EPI_ISL_863156, EPI_ISL_863157, EPI_ISL_863158, EPI_ISL_863159, EPI_ISL_863160, EPI_ISL_863161, EPI_ISL_863162, EPI_ISL_863163, EPI_ISL_863164, EPI_ISL_863165, EPI_ISL_863166, EPI_ISL_863167, EPI_ISL_863168, EPI_ISL_863169, EPI_ISL_863170, EPI_ISL_863171, EPI_ISL_863172, EPI_ISL_863173, EPI_ISL_863174, EPI_ISL_863175, EPI_ISL_863176, EPI_ISL_863177, EPI_ISL_863178, EPI_ISL_863179, EPI_ISL_863180, EPI_ISL_863181, EPI_ISL_863182, EPI_ISL_863183, EPI_ISL_863184, EPI_ISL_863185, EPI_ISL_863186, EPI_ISL_863187, EPI_ISL_863188, EPI_ISL_863189, EPI_ISL_863190, EPI_ISL_863191, EPI_ISL_863192, EPI_ISL_863193, EPI_ISL_863194, EPI_ISL_863195, EPI_ISL_863196, EPI_ISL_863197, EPI_ISL_863198, EPI_ISL_863199, EPI_ISL_863200, EPI_ISL_863201, EPI_ISL_863202, EPI_ISL_863203, EPI_ISL_863204, EPI_ISL_863205, EPI_ISL_863206, EPI_ISL_863207, EPI_ISL_863208, EPI_ISL_863209, EPI_ISL_863210, EPI_ISL_863211, EPI_ISL_863212, EPI_ISL_863213, EPI_ISL_863214, EPI_ISL_863215, EPI_ISL_863216, EPI_ISL_863217, EPI_ISL_863218, EPI_ISL_863219, EPI_ISL_863220, EPI_ISL_863221, EPI_ISL_863222, EPI_ISL_863223, EPI_ISL_863224, EPI_ISL_863225, EPI_ISL_863226, EPI_ISL_863227, EPI_ISL_863228, EPI_ISL_863229, EPI_ISL_863230, EPI_ISL_863231, EPI_ISL_863232, EPI_ISL_863233, EPI_ISL_863234, EPI_ISL_863235, EPI_ISL_863236, EPI_ISL_863237, EPI_ISL_863238, EPI_ISL_863239, EPI_ISL_863240, EPI_ISL_863241, EPI_ISL_863242, EPI_ISL_863243, EPI_ISL_863244, EPI_ISL_863334, EPI_ISL_863396, EPI_ISL_863401, EPI_ISL_863403, EPI_ISL_863424			
see above	Lighthouse Lab in Alderley Park	Wellcome Sanger Institute for the COVID-19 Genomics UK (COG-UK) Consortium	Jacquelyn Wynn, Mairead Hyland, The Lighthouse Lab in Alderley Park and Alex Alderton, Roberto Amato, Sonia Goncalves, Ewan Harrison, David K. Jackson, Ian Johnston, Dominic Kwiatkowski, Cordelia Langford, John Sillitoe on behalf of the Wellcome Sanger Institute COVID-19 Surveillance Team
EPI_ISL_863431	Lighthouse Lab in Glasgow	Wellcome Sanger Institute for the COVID-19 Genomics UK (COG-UK) Consortium	Harper VanSteenhouse, Yumi Kasai, David Gray, Carol Clugston, Anna Dominiczak and Alex Alderton, Roberto Amato, Sonia Goncalves, Ewan Harrison, David K. Jackson, Ian Johnston, Dominic Kwiatkowski, Cordelia Langford, John Sillitoe on behalf of the Wellcome Sanger Institute COVID-19 Surveillance Team
EPI_ISL_863432, EPI_ISL_863467, EPI_ISL_863514, EPI_ISL_863529	Lighthouse Lab in Alderley Park	Wellcome Sanger Institute for the COVID-19 Genomics UK (COG-UK) Consortium	Jacquelyn Wynn, Mairead Hyland, The Lighthouse Lab in Alderley Park and Alex Alderton, Roberto Amato, Sonia Goncalves, Ewan Harrison, David K. Jackson, Ian Johnston, Dominic Kwiatkowski, Cordelia Langford, John Sillitoe on behalf of the Wellcome Sanger Institute COVID-19 Surveillance Team
EPI_ISL_863531, EPI_ISL_863533, EPI_ISL_863537, EPI_ISL_863539, EPI_ISL_863540, EPI_ISL_863542, EPI_ISL_863543, EPI_ISL_863544, EPI_ISL_863546, EPI_ISL_863547, EPI_ISL_863549, EPI_ISL_863551, EPI_ISL_863552, EPI_ISL_863553, EPI_ISL_863555, EPI_ISL_863556, EPI_ISL_863557, EPI_ISL_863558, EPI_ISL_863560, EPI_ISL_863561, EPI_ISL_863562, EPI_ISL_863565, EPI_ISL_863566, EPI_ISL_863567, EPI_ISL_863570, EPI_ISL_863572, EPI_ISL_863574, EPI_ISL_863576, EPI_ISL_863577, EPI_ISL_863578, EPI_ISL_863579, EPI_ISL_863580, EPI_ISL_863581, EPI_ISL_863582, EPI_ISL_863583, EPI_ISL_863584, EPI_ISL_863586, EPI_ISL_863587, EPI_ISL_863588, EPI_ISL_863589, EPI_ISL_863591, EPI_ISL_863592, EPI_ISL_863593, EPI_ISL_863594, EPI_ISL_863595, EPI_ISL_863599, EPI_ISL_863601, EPI_ISL_863603, EPI_ISL_863604, EPI_ISL_863605, EPI_ISL_863606, EPI_ISL_863608, EPI_ISL_863609, EPI_ISL_863610, EPI_ISL_863612, EPI_ISL_863614, EPI_ISL_863616, EPI_ISL_863618, EPI_ISL_863619, EPI_ISL_863621, EPI_ISL_863622, EPI_ISL_863623, EPI_ISL_863625, EPI_ISL_863626, EPI_ISL_863627, EPI_ISL_863628, EPI_ISL_863629, EPI_ISL_863630, EPI_ISL_863633, EPI_ISL_863636, EPI_ISL_863640, EPI_ISL_863641, EPI_ISL_863643, EPI_ISL_863644, EPI_ISL_863647, EPI_ISL_863648, EPI_ISL_863649, EPI_ISL_863650, EPI_ISL_863652, EPI_ISL_863654, EPI_ISL_863655, EPI_ISL_863656, EPI_ISL_863658, EPI_ISL_863659, EPI_ISL_863661, EPI_ISL_863662, EPI_ISL_863663, EPI_ISL_863664, EPI_ISL_863665, EPI_ISL_863666, EPI_ISL_863667, EPI_ISL_863668, EPI_ISL_863670, EPI_ISL_863671, EPI_ISL_863672, EPI_ISL_863673, EPI_ISL_863674, EPI_ISL_863675, EPI_ISL_863676, EPI_ISL_863680, EPI_ISL_863682, EPI_ISL_863683, EPI_ISL_863684, EPI_ISL_863685, EPI_ISL_863686, EPI_ISL_863687, EPI_ISL_863688, EPI_ISL_863689, EPI_ISL_863690, EPI_ISL_863691, EPI_ISL_863692, EPI_ISL_863693, EPI_ISL_863694, EPI_ISL_863695, EPI_ISL_863697, EPI_ISL_863698, EPI_ISL_863701, EPI_ISL_863702, EPI_ISL_863703, EPI_ISL_863704, EPI_ISL_863706, EPI_ISL_863708, EPI_ISL_863710, EPI_ISL_863712, EPI_ISL_863713, EPI_ISL_863714, EPI_ISL_863716, EPI_ISL_863717, EPI_ISL_863718, EPI_ISL_863719, EPI_ISL_863720, EPI_ISL_863722, EPI_ISL_863724, EPI_ISL_863727, EPI_ISL_863728, EPI_ISL_863730, EPI_ISL_863731, EPI_ISL_863735, EPI_ISL_863736, EPI_ISL_863737, EPI_ISL_863738, EPI_ISL_863739, EPI_ISL_863740, EPI_ISL_863743, EPI_ISL_863744, EPI_ISL_863745, EPI_ISL_863748, EPI_ISL_863749, EPI_ISL_863750, EPI_ISL_863751, EPI_ISL_863752, EPI_ISL_863753, EPI_ISL_863754, EPI_ISL_863755, EPI_ISL_863756, EPI_ISL_863757, EPI_ISL_863758, EPI_ISL_863760, EPI_ISL_863763, EPI_ISL_863764, EPI_ISL_863765, EPI_ISL_863767, EPI_ISL_863768, EPI_ISL_863769, EPI_ISL_863770, EPI_ISL_863771, EPI_ISL_863772, EPI_ISL_863774, EPI_ISL_863775, EPI_ISL_863777, EPI_ISL_863778, EPI_ISL_863779, EPI_ISL_863780, EPI_ISL_863782, EPI_ISL_863783, EPI_ISL_863786, EPI_ISL_863787, EPI_ISL_863791, EPI_ISL_863794, EPI_ISL_863798, EPI_ISL_863799, EPI_ISL_863800, EPI_ISL_863801, EPI_ISL_863805, EPI_ISL_863807, EPI_ISL_863808, EPI_ISL_863813, EPI_ISL_863814, EPI_ISL_863815, EPI_ISL_863816, EPI_ISL_863817, EPI_ISL_863818, EPI_ISL_863819, EPI_ISL_863820, EPI_ISL_863821, EPI_ISL_863822, EPI_ISL_863823, EPI_ISL_863824, EPI_ISL_863825, EPI_ISL_863826, EPI_ISL_863827, EPI_ISL_863828, EPI_ISL_863829, EPI_ISL_863831, EPI_ISL_863832, EPI_ISL_863833, EPI_ISL_863834, EPI_ISL_863835, EPI_ISL_863836, EPI_ISL_863837, EPI_ISL_863839, EPI_ISL_863840, EPI_ISL_863843, EPI_ISL_863844, EPI_ISL_863846, EPI_ISL_863849, EPI_ISL_863852, EPI_ISL_863854, EPI_ISL_863855, EPI_ISL_863857, EPI_ISL_863859, EPI_ISL_863861, EPI_ISL_863862, EPI_ISL_863864, EPI_ISL_863866, EPI_ISL_863867, EPI_ISL_863868			
see above	Lighthouse Lab in Milton Keynes	Wellcome Sanger Institute for the COVID-19 Genomics UK (COG-UK) Consortium	The Lighthouse Lab in Milton Keynes and Alex Alderton, Roberto Amato, Sonia Goncalves, Ewan Harrison, David K. Jackson, Ian Johnston, Dominic Kwiatkowski, Cordelia Langford, John Sillitoe on behalf of the Wellcome Sanger Institute COVID-19 Surveillance Team
EPI_ISL_863873	Lighthouse Lab in Alderley Park	Wellcome Sanger Institute for the COVID-19 Genomics UK (COG-UK) Consortium	Jacquelyn Wynn, Mairead Hyland, The Lighthouse Lab in Alderley Park and Alex Alderton, Roberto Amato, Sonia Goncalves, Ewan Harrison, David K. Jackson, Ian Johnston, Dominic Kwiatkowski, Cordelia Langford, John Sillitoe on behalf of the Wellcome Sanger Institute COVID-19 Surveillance Team
EPI_ISL_863899, EPI_ISL_863901,	Lighthouse Lab in Milton Keynes	Wellcome Sanger Institute for the COVID-19 Genomics UK	The Lighthouse Lab in Milton Keynes and Alex Alderton, Roberto Amato, Sonia Goncalves, Ewan Harrison, David K. Jackson, Ian Johnston, Dominic

EPI_ISL_863912, EPI_ISL_863913, EPI_ISL_863921, EPI_ISL_863922		(COG-UK) Consortium	Kwiatkowski, Cordelia Langford, John Sillitoe on behalf of the Wellcome Sanger Institute COVID-19 Surveillance Team
EPI_ISL_863946	Lighthouse Lab in Alderley Park	Wellcome Sanger Institute for the COVID-19 Genomics UK (COG-UK) Consortium	Jacquelyn Wynn, Mairead Hyland, The Lighthouse Lab in Alderley Park and Alex Alderton, Roberto Amato, Sonia Goncalves, Ewan Harrison, David K. Jackson, Ian Johnston, Dominic Kwiatkowski, Cordelia Langford, John Sillitoe on behalf of the Wellcome Sanger Institute COVID-19 Surveillance Team
EPI_ISL_863956, EPI_ISL_863969, EPI_ISL_863996	Lighthouse Lab in Milton Keynes	Wellcome Sanger Institute for the COVID-19 Genomics UK (COG-UK) Consortium	The Lighthouse Lab in Milton Keynes and Alex Alderton, Roberto Amato, Sonia Goncalves, Ewan Harrison, David K. Jackson, Ian Johnston, Dominic Kwiatkowski, Cordelia Langford, John Sillitoe on behalf of the Wellcome Sanger Institute COVID-19 Surveillance Team
EPI_ISL_864004	Lighthouse Lab in Alderley Park	Wellcome Sanger Institute for the COVID-19 Genomics UK (COG-UK) Consortium	Jacquelyn Wynn, Mairead Hyland, The Lighthouse Lab in Alderley Park and Alex Alderton, Roberto Amato, Sonia Goncalves, Ewan Harrison, David K. Jackson, Ian Johnston, Dominic Kwiatkowski, Cordelia Langford, John Sillitoe on behalf of the Wellcome Sanger Institute COVID-19 Surveillance Team
EPI_ISL_864005, EPI_ISL_864009, EPI_ISL_864026, EPI_ISL_864036, EPI_ISL_864043	Lighthouse Lab in Milton Keynes	Wellcome Sanger Institute for the COVID-19 Genomics UK (COG-UK) Consortium	The Lighthouse Lab in Milton Keynes and Alex Alderton, Roberto Amato, Sonia Goncalves, Ewan Harrison, David K. Jackson, Ian Johnston, Dominic Kwiatkowski, Cordelia Langford, John Sillitoe on behalf of the Wellcome Sanger Institute COVID-19 Surveillance Team
EPI_ISL_864064, EPI_ISL_864094	Lighthouse Lab in Alderley Park	Wellcome Sanger Institute for the COVID-19 Genomics UK (COG-UK) Consortium	Jacquelyn Wynn, Mairead Hyland, The Lighthouse Lab in Alderley Park and Alex Alderton, Roberto Amato, Sonia Goncalves, Ewan Harrison, David K. Jackson, Ian Johnston, Dominic Kwiatkowski, Cordelia Langford, John Sillitoe on behalf of the Wellcome Sanger Institute COVID-19 Surveillance Team
EPI_ISL_864098	Lighthouse Lab in Milton Keynes	Wellcome Sanger Institute for the COVID-19 Genomics UK (COG-UK) Consortium	The Lighthouse Lab in Milton Keynes and Alex Alderton, Roberto Amato, Sonia Goncalves, Ewan Harrison, David K. Jackson, Ian Johnston, Dominic Kwiatkowski, Cordelia Langford, John Sillitoe on behalf of the Wellcome Sanger Institute COVID-19 Surveillance Team
EPI_ISL_864110	Lighthouse Lab in Alderley Park	Wellcome Sanger Institute for the COVID-19 Genomics UK (COG-UK) Consortium	Jacquelyn Wynn, Mairead Hyland, The Lighthouse Lab in Alderley Park and Alex Alderton, Roberto Amato, Sonia Goncalves, Ewan Harrison, David K. Jackson, Ian Johnston, Dominic Kwiatkowski, Cordelia Langford, John Sillitoe on behalf of the Wellcome Sanger Institute COVID-19 Surveillance Team
EPI_ISL_864124, EPI_ISL_864133	Lighthouse Lab in Milton Keynes	Wellcome Sanger Institute for the COVID-19 Genomics UK (COG-UK) Consortium	The Lighthouse Lab in Milton Keynes and Alex Alderton, Roberto Amato, Sonia Goncalves, Ewan Harrison, David K. Jackson, Ian Johnston, Dominic Kwiatkowski, Cordelia Langford, John Sillitoe on behalf of the Wellcome Sanger Institute COVID-19 Surveillance Team
EPI_ISL_864142, EPI_ISL_864145	Lighthouse Lab in Alderley Park	Wellcome Sanger Institute for the COVID-19 Genomics UK (COG-UK) Consortium	Jacquelyn Wynn, Mairead Hyland, The Lighthouse Lab in Alderley Park and Alex Alderton, Roberto Amato, Sonia Goncalves, Ewan Harrison, David K. Jackson, Ian Johnston, Dominic Kwiatkowski, Cordelia Langford, John Sillitoe on behalf of the Wellcome Sanger Institute COVID-19 Surveillance Team
EPI_ISL_864165, EPI_ISL_864169, EPI_ISL_864173, EPI_ISL_864175, EPI_ISL_864176	Lighthouse Lab in Milton Keynes	Wellcome Sanger Institute for the COVID-19 Genomics UK (COG-UK) Consortium	The Lighthouse Lab in Milton Keynes and Alex Alderton, Roberto Amato, Sonia Goncalves, Ewan Harrison, David K. Jackson, Ian Johnston, Dominic Kwiatkowski, Cordelia Langford, John Sillitoe on behalf of the Wellcome Sanger Institute COVID-19 Surveillance Team
EPI_ISL_864178	Lighthouse Lab in Alderley Park	Wellcome Sanger Institute for the COVID-19 Genomics UK (COG-UK) Consortium	Jacquelyn Wynn, Mairead Hyland, The Lighthouse Lab in Alderley Park and Alex Alderton, Roberto Amato, Sonia Goncalves, Ewan Harrison, David K. Jackson, Ian Johnston, Dominic Kwiatkowski, Cordelia Langford, John Sillitoe on behalf of the Wellcome Sanger Institute COVID-19 Surveillance Team
EPI_ISL_864191, EPI_ISL_864193, EPI_ISL_864203, EPI_ISL_864205, EPI_ISL_864206, EPI_ISL_864207, EPI_ISL_864208, EPI_ISL_864209, EPI_ISL_864210, EPI_ISL_864211, EPI_ISL_864212, EPI_ISL_864214, EPI_ISL_864215, EPI_ISL_864218, EPI_ISL_864219, EPI_ISL_864220, EPI_ISL_864221, EPI_ISL_864222, EPI_ISL_864223, EPI_ISL_864224, EPI_ISL_864225			
see above	Lighthouse Lab in Milton Keynes	Wellcome Sanger Institute for the COVID-19 Genomics UK (COG-UK) Consortium	The Lighthouse Lab in Milton Keynes and Alex Alderton, Roberto Amato, Sonia Goncalves, Ewan Harrison, David K. Jackson, Ian Johnston, Dominic Kwiatkowski, Cordelia Langford, John Sillitoe on behalf of the Wellcome Sanger Institute COVID-19 Surveillance Team
EPI_ISL_864226	Lighthouse Lab in Alderley Park	Wellcome Sanger Institute for the COVID-19 Genomics UK (COG-UK) Consortium	Jacquelyn Wynn, Mairead Hyland, The Lighthouse Lab in Alderley Park and Alex Alderton, Roberto Amato, Sonia Goncalves, Ewan Harrison, David K. Jackson, Ian Johnston, Dominic Kwiatkowski, Cordelia Langford, John Sillitoe on behalf of the Wellcome Sanger Institute COVID-19 Surveillance Team
EPI_ISL_864227, EPI_ISL_864228, EPI_ISL_864229, EPI_ISL_864230, EPI_ISL_864231, EPI_ISL_864232, EPI_ISL_864233, EPI_ISL_864234, EPI_ISL_864235, EPI_ISL_864236, EPI_ISL_864237, EPI_ISL_864238, EPI_ISL_864240, EPI_ISL_864241, EPI_ISL_864242, EPI_ISL_864243, EPI_ISL_864245, EPI_ISL_864246, EPI_ISL_864247, EPI_ISL_864248, EPI_ISL_864249, EPI_ISL_864250, EPI_ISL_864251, EPI_ISL_864252, EPI_ISL_864253, EPI_ISL_864254, EPI_ISL_864256, EPI_ISL_864257, EPI_ISL_864258, EPI_ISL_864259, EPI_ISL_864260, EPI_ISL_864261, EPI_ISL_864263, EPI_ISL_864264, EPI_ISL_864265			
see above	Lighthouse Lab in Milton Keynes	Wellcome Sanger Institute for the COVID-19 Genomics UK (COG-UK) Consortium	The Lighthouse Lab in Milton Keynes and Alex Alderton, Roberto Amato, Sonia Goncalves, Ewan Harrison, David K. Jackson, Ian Johnston, Dominic Kwiatkowski, Cordelia Langford, John Sillitoe on behalf of the Wellcome Sanger Institute COVID-19 Surveillance Team
EPI_ISL_864267	Lighthouse Lab in Alderley Park	Wellcome Sanger Institute for the COVID-19 Genomics UK (COG-UK) Consortium	Jacquelyn Wynn, Mairead Hyland, The Lighthouse Lab in Alderley Park and Alex Alderton, Roberto Amato, Sonia Goncalves, Ewan Harrison, David K. Jackson, Ian Johnston, Dominic Kwiatkowski, Cordelia Langford, John Sillitoe on behalf of the Wellcome Sanger Institute COVID-19 Surveillance Team
EPI_ISL_864268, EPI_ISL_864269, EPI_ISL_864270, EPI_ISL_864271, EPI_ISL_864272, EPI_ISL_864273, EPI_ISL_864275, EPI_ISL_864276, EPI_ISL_864277, EPI_ISL_864278, EPI_ISL_864279, EPI_ISL_864280, EPI_ISL_864282, EPI_ISL_864283, EPI_ISL_864284, EPI_ISL_864285, EPI_ISL_864286, EPI_ISL_864287, EPI_ISL_864288, EPI_ISL_864289, EPI_ISL_864290, EPI_ISL_864292, EPI_ISL_864293, EPI_ISL_864294, EPI_ISL_864295, EPI_ISL_864296, EPI_ISL_864298, EPI_ISL_864299, EPI_ISL_864300, EPI_ISL_864301, EPI_ISL_864302, EPI_ISL_864303, EPI_ISL_864304, EPI_ISL_864306, EPI_ISL_864307			
see above	Lighthouse Lab in Milton Keynes	Wellcome Sanger Institute for the COVID-19 Genomics UK (COG-UK) Consortium	The Lighthouse Lab in Milton Keynes and Alex Alderton, Roberto Amato, Sonia Goncalves, Ewan Harrison, David K. Jackson, Ian Johnston, Dominic Kwiatkowski, Cordelia Langford, John Sillitoe on behalf of the Wellcome Sanger Institute COVID-19 Surveillance Team
EPI_ISL_864308	Lighthouse Lab in Alderley Park	Wellcome Sanger Institute for the COVID-19 Genomics UK (COG-UK) Consortium	Jacquelyn Wynn, Mairead Hyland, The Lighthouse Lab in Alderley Park and Alex Alderton, Roberto Amato, Sonia Goncalves, Ewan Harrison, David K. Jackson, Ian Johnston, Dominic Kwiatkowski, Cordelia Langford, John Sillitoe on behalf of the Wellcome Sanger Institute COVID-19 Surveillance Team
EPI_ISL_864310, EPI_ISL_864312, EPI_ISL_864313, EPI_ISL_864314, EPI_ISL_864315, EPI_ISL_864316, EPI_ISL_864318, EPI_ISL_864319, EPI_ISL_864320, EPI_ISL_864322, EPI_ISL_864323, EPI_ISL_864325, EPI_ISL_864326, EPI_ISL_864327, EPI_ISL_864328, EPI_ISL_864329, EPI_ISL_864330, EPI_ISL_864331, EPI_ISL_864332, EPI_ISL_864333, EPI_ISL_864334, EPI_ISL_864335, EPI_ISL_864337, EPI_ISL_864338, EPI_ISL_864339, EPI_ISL_864340, EPI_ISL_864341, EPI_ISL_864342, EPI_ISL_864343, EPI_ISL_864344, EPI_ISL_864345, EPI_ISL_864346, EPI_ISL_864347, EPI_ISL_864348, EPI_ISL_864349, EPI_ISL_864350, EPI_ISL_864351, EPI_ISL_864352, EPI_ISL_864353, EPI_ISL_864354, EPI_ISL_864355, EPI_ISL_864357, EPI_ISL_864358, EPI_ISL_864359, EPI_ISL_864360, EPI_ISL_864362, EPI_ISL_864363, EPI_ISL_864364, EPI_ISL_864366, EPI_ISL_864367, EPI_ISL_864369, EPI_ISL_864370, EPI_ISL_864371, EPI_ISL_864372, EPI_ISL_864373, EPI_ISL_864374, EPI_ISL_864375, EPI_ISL_864376, EPI_ISL_864377, EPI_ISL_864378, EPI_ISL_864379, EPI_ISL_864380, EPI_ISL_864381, EPI_ISL_864382, EPI_ISL_864385, EPI_ISL_864386, EPI_ISL_864387, EPI_ISL_864388, EPI_ISL_864389, EPI_ISL_864390, EPI_ISL_864391, EPI_ISL_864392, EPI_ISL_864393, EPI_ISL_864394, EPI_ISL_864395, EPI_ISL_864397, EPI_ISL_864398, EPI_ISL_864401, EPI_ISL_864404, EPI_ISL_864405, EPI_ISL_864406, EPI_ISL_864407, EPI_ISL_864408, EPI_ISL_864410, EPI_ISL_864412, EPI_ISL_864413, EPI_ISL_864415, EPI_ISL_864416, EPI_ISL_864417, EPI_ISL_864418, EPI_ISL_864419, EPI_ISL_864420, EPI_ISL_864421, EPI_ISL_864423, EPI_ISL_864424, EPI_ISL_864425, EPI_ISL_864426, EPI_ISL_864427, EPI_ISL_864428, EPI_ISL_864429, EPI_ISL_864430, EPI_ISL_864431, EPI_ISL_864432, EPI_ISL_864433, EPI_ISL_864434, EPI_ISL_864435, EPI_ISL_864436, EPI_ISL_864437, EPI_ISL_864438, EPI_ISL_864441, EPI_ISL_864442, EPI_ISL_864443, EPI_ISL_864444, EPI_ISL_864445, EPI_ISL_864446, EPI_ISL_864448, EPI_ISL_864449, EPI_ISL_864450, EPI_ISL_864451, EPI_ISL_864453, EPI_ISL_864454, EPI_ISL_864455, EPI_ISL_864456, EPI_ISL_864457, EPI_ISL_864458, EPI_ISL_864459, EPI_ISL_864460, EPI_ISL_864462, EPI_ISL_864463, EPI_ISL_864464, EPI_ISL_864465, EPI_ISL_864466, EPI_ISL_864467, EPI_ISL_864468, EPI_ISL_864470, EPI_ISL_864471, EPI_ISL_864473, EPI_ISL_864474, EPI_ISL_864476, EPI_ISL_864477, EPI_ISL_864478, EPI_ISL_864479, EPI_ISL_864480, EPI_ISL_864481, EPI_ISL_864482, EPI_ISL_864483, EPI_ISL_864485, EPI_ISL_864486, EPI_ISL_864487, EPI_ISL_864488, EPI_ISL_864489, EPI_ISL_864490, EPI_ISL_864491, EPI_ISL_864492, EPI_ISL_864494, EPI_ISL_864495, EPI_ISL_864496, EPI_ISL_864497, EPI_ISL_864498, EPI_ISL_864499, EPI_ISL_864500, EPI_ISL_864501, EPI_ISL_864502, EPI_ISL_864503, EPI_ISL_864504, EPI_ISL_864505, EPI_ISL_864506, EPI_ISL_864507, EPI_ISL_864508, EPI_ISL_864509, EPI_ISL_864510, EPI_ISL_864511, EPI_ISL_864512, EPI_ISL_864513, EPI_ISL_864514, EPI_ISL_864517, EPI_ISL_864518, EPI_ISL_864519, EPI_ISL_864521, EPI_ISL_864522, EPI_ISL_864523, EPI_ISL_864524, EPI_ISL_864525, EPI_ISL_864526, EPI_ISL_864530, EPI_ISL_864531, EPI_ISL_864532, EPI_ISL_864533, EPI_ISL_864535, EPI_ISL_864536, EPI_ISL_864537, EPI_ISL_864538, EPI_ISL_864541			
see above	Lighthouse Lab in Milton Keynes	Wellcome Sanger Institute for the COVID-19 Genomics UK (COG-UK) Consortium	The Lighthouse Lab in Milton Keynes and Alex Alderton, Roberto Amato, Sonia Goncalves, Ewan Harrison, David K. Jackson, Ian Johnston, Dominic Kwiatkowski, Cordelia Langford, John Sillitoe on behalf of the Wellcome Sanger Institute COVID-19 Surveillance Team
EPI_ISL_864810, EPI_ISL_864814, EPI_ISL_864893, EPI_ISL_864926	Department of Pathology, University of Cambridge	COVID-19 Genomics UK (COG-UK) Consortium	Aminu S. Jahun, Yasmin Chaudhry, Grant Hall, Iliana Georgana, Myra Hosmillo, Martin D. Curran, Malte Pinckert, Surendra Parmar, Ian Goodfellow
EPI_ISL_865015, EPI_ISL_865016, EPI_ISL_865017, EPI_ISL_865018, EPI_ISL_865019, EPI_ISL_865020, EPI_ISL_865021, EPI_ISL_865022, EPI_ISL_865023, EPI_ISL_865024, EPI_ISL_865025, EPI_ISL_865026, EPI_ISL_865027, EPI_ISL_865028, EPI_ISL_865029, EPI_ISL_865030, EPI_ISL_865031, EPI_ISL_865032, EPI_ISL_865033, EPI_ISL_865034, EPI_ISL_865035, EPI_ISL_865036, EPI_ISL_865037, EPI_ISL_865038, EPI_ISL_865039, EPI_ISL_865040, EPI_ISL_865050, EPI_ISL_865052, EPI_ISL_865053, EPI_ISL_865055, EPI_ISL_865056, EPI_ISL_865057, EPI_ISL_865058, EPI_ISL_865059, EPI_ISL_865060, EPI_ISL_865061, EPI_ISL_865062, EPI_ISL_865063, EPI_ISL_865064, EPI_ISL_865065, EPI_ISL_865066, EPI_ISL_865067			
see above	West of Scotland Specialist Virology Centre, NHSGGC / MRC-University of Glasgow Centre for Virus Research	COVID-19 Genomics UK (COG-UK) Consortium	Ana da Silva Filipe, Natasha Johnson, Kathy Smollett, Daniel Mair, Stephen Carmichael, Alice Broos, Lily Tong, Jenna Nichols, Kyriaki Nomikou; Sarah McDonald; Richard Orton, Joseph Hughes, Sreenu Vattipally, David L Robertson; Alasdair MacLean, Rory Gunson; Sharif Shaaban, Matthew Holden; Rachel Blacow, Guy Mollett, Kathy Li, James Shepherd, Antonia Ho, Emma Thomson
EPI_ISL_865068	Lighthouse Lab in Glasgow / MRC-University of Glasgow Centre for Virus Research	COVID-19 Genomics UK (COG-UK) Consortium	Ana da Silva Filipe, Natasha Johnson, Kathy Smollett, Daniel Mair, Stephen Carmichael, Alice Broos, Lily Tong, Jenna Nichols, Kyriaki Nomikou; Sarah McDonald; Harper VanSteenhouse, Yumi Kasai, David Gray, Carol Clugston, Anna Dominiczak; Alasdair MacLean, Rory Gunson; Richard Orton, Joseph Hughes, Sreenu Vattipally, David L Robertson; Sharif Shaaban, Matthew Holden; Kathy Li, James Shepherd, Antonia Ho, Emma Thomson
EPI_ISL_865089, EPI_ISL_865111, EPI_ISL_865112, EPI_ISL_865113, EPI_ISL_865114, EPI_ISL_865115, EPI_ISL_865116, EPI_ISL_865117, EPI_ISL_865118, EPI_ISL_865119, EPI_ISL_865120, EPI_ISL_865121, EPI_ISL_865122, EPI_ISL_865123, EPI_ISL_865124, EPI_ISL_865125, EPI_ISL_865126, EPI_ISL_865127, EPI_ISL_865128, EPI_ISL_865129, EPI_ISL_865130, EPI_ISL_865132, EPI_ISL_865133, EPI_ISL_865134, EPI_ISL_865135, EPI_ISL_865140, EPI_ISL_865143, EPI_ISL_865145			
see above	Virology Department, Royal Infirmary of Edinburgh, NHS Lothian / School of Biological Sciences, University of Edinburgh / Institute of Genetics and Molecular Medicine,	COVID-19 Genomics UK (COG-UK) Consortium	McHugh M, Dewar R, Rooke S, Gallagher M, Balcaza C, O'Toole Á, Scher E, Hill V, McCrone JT, Colquhoun R, Yu X, Jackson B, Rambaut A, Williams TC, Templeton K

	University of Edinburgh		
EPI_ISL_865270, EPI_ISL_865271, EPI_ISL_865272, EPI_ISL_865273, EPI_ISL_865274, EPI_ISL_865275, EPI_ISL_865276, EPI_ISL_865277, EPI_ISL_865280, EPI_ISL_865281, EPI_ISL_865282, EPI_ISL_865284, EPI_ISL_865285, EPI_ISL_865286, EPI_ISL_865287, EPI_ISL_865288, EPI_ISL_865289, EPI_ISL_865290, EPI_ISL_865291, EPI_ISL_865292, EPI_ISL_865293, EPI_ISL_865294, EPI_ISL_865295, EPI_ISL_865296, EPI_ISL_865297, EPI_ISL_865298, EPI_ISL_865300, EPI_ISL_865301, EPI_ISL_865302, EPI_ISL_865303, EPI_ISL_865304, EPI_ISL_865305, EPI_ISL_865306, EPI_ISL_865307, EPI_ISL_865308, EPI_ISL_865309, EPI_ISL_865310, EPI_ISL_865311, EPI_ISL_865312, EPI_ISL_865313, EPI_ISL_865314, EPI_ISL_865315, EPI_ISL_865317, EPI_ISL_865318, EPI_ISL_865319, EPI_ISL_865320, EPI_ISL_865321, EPI_ISL_865322, EPI_ISL_865324, EPI_ISL_865326, EPI_ISL_865327, EPI_ISL_865328, EPI_ISL_865341, EPI_ISL_865342, EPI_ISL_865343, EPI_ISL_865344, EPI_ISL_865345, EPI_ISL_865346, EPI_ISL_865347, EPI_ISL_865348, EPI_ISL_865349, EPI_ISL_865350, EPI_ISL_865351, EPI_ISL_865353, EPI_ISL_865449			
see above	Liverpool Clinical Laboratories	COVID-19 Genomics UK (COG-UK) Consortium	Sam Haldenby, Anita Lucaci, Steve Paterson, Julian Hiscox, Alistair Darby, M Almsaud, A Alrezaihi, Muhannad Alruwaili, Stuart D Armstrong, Jones Benjamin, Eleanor G Bentley, Anu Chawla, Jordan J Clark, Angela Cowell, Richard Eccles, Isabel García-Dorival, Matthew Gemmell, Alessandro Gerada, PKF Gilmore, Richard Gregory, Ximeng Han, Catherine Hartley, Margaret Hughes, Miren Iturriza-Gomara, James Johnson, L Luu, Jenifer Manson, Charlotte Nelson, Elaine O'Toole, Cassie Olateju, Rebekah Penrice-Randal , Lucille Rainbow, N.P Randle, Trevor Ian Robinson, Parul Sharma, Ghada T Shawli, James P Stewart, Neil Swainston, Ecaterina Vamos, Joanne Watts, Mark Whitehead
EPI_ISL_865847	University College London, Great Ormond Street Hospital for Children NHS Foundation Trust, Imperial College Healthcare NHS Trust	COVID-19 Genomics UK (COG-UK) Consortium	Sergi Castellano, Rachel Williams, Mark Kristiansen, Paola Resende Silva, Sunando Roy, Tony Brooks, Helena Tutill, Paola Niola, Patricia Dyal, Charlotte Williams, Leysa Forrest, Yasmin Panchbhaya, Jacqueline Findlay, Samuel Weeks, Julianne Brown, Kathryn Harris, Paul Randell, James Price, Alison Holmes, Judith Breuer
EPI_ISL_866080, EPI_ISL_866082, EPI_ISL_866083, EPI_ISL_866085, EPI_ISL_866086, EPI_ISL_866087, EPI_ISL_866088, EPI_ISL_866089, EPI_ISL_866090, EPI_ISL_866091, EPI_ISL_866092, EPI_ISL_866093, EPI_ISL_866094, EPI_ISL_866095, EPI_ISL_866096, EPI_ISL_866097, EPI_ISL_866098, EPI_ISL_866099, EPI_ISL_866100, EPI_ISL_866101, EPI_ISL_866102, EPI_ISL_866103, EPI_ISL_866104, EPI_ISL_866105, EPI_ISL_866112, EPI_ISL_866113, EPI_ISL_866114, EPI_ISL_866117, EPI_ISL_866120, EPI_ISL_866125, EPI_ISL_866126, EPI_ISL_866134, EPI_ISL_866142, EPI_ISL_866143, EPI_ISL_866146, EPI_ISL_866152, EPI_ISL_866154, EPI_ISL_866160, EPI_ISL_866171, EPI_ISL_866174, EPI_ISL_866178, EPI_ISL_866186			
see above	University College London Hospital	COVID-19 Genomics UK (COG-UK) Consortium	Judith Heaney, Matthew Byott, Catherine Houlihan, Dan Frampton, Stuart Kirk, Moira Spyer and Eleni Nastouli
EPI_ISL_866194, EPI_ISL_866195, EPI_ISL_866196, EPI_ISL_866197, EPI_ISL_866199, EPI_ISL_866200, EPI_ISL_866202, EPI_ISL_866203, EPI_ISL_866204, EPI_ISL_866205, EPI_ISL_866206, EPI_ISL_866207, EPI_ISL_866208, EPI_ISL_866209, EPI_ISL_866210, EPI_ISL_866211, EPI_ISL_866212, EPI_ISL_866213, EPI_ISL_866214, EPI_ISL_866216, EPI_ISL_866218, EPI_ISL_866220, EPI_ISL_866222, EPI_ISL_866223, EPI_ISL_866224, EPI_ISL_866225, EPI_ISL_866226, EPI_ISL_866227, EPI_ISL_866228, EPI_ISL_866229, EPI_ISL_866230, EPI_ISL_866232, EPI_ISL_866233, EPI_ISL_866234, EPI_ISL_866235, EPI_ISL_866236, EPI_ISL_866237, EPI_ISL_866238, EPI_ISL_866239, EPI_ISL_866240, EPI_ISL_866241, EPI_ISL_866242, EPI_ISL_866243, EPI_ISL_866244, EPI_ISL_866245, EPI_ISL_866246, EPI_ISL_866247, EPI_ISL_866248, EPI_ISL_866249, EPI_ISL_866250, EPI_ISL_866251, EPI_ISL_866252, EPI_ISL_866253, EPI_ISL_866254, EPI_ISL_866255, EPI_ISL_866256, EPI_ISL_866257, EPI_ISL_866258, EPI_ISL_866259, EPI_ISL_866260, EPI_ISL_866261			
see above	University College London, Great Ormond Street Hospital for Children NHS Foundation Trust, Imperial College Healthcare NHS Trust	COVID-19 Genomics UK (COG-UK) Consortium	Sergi Castellano, Rachel Williams, Mark Kristiansen, Paola Resende Silva, Sunando Roy, Tony Brooks, Helena Tutill, Paola Niola, Patricia Dyal, Charlotte Williams, Leysa Forrest, Yasmin Panchbhaya, Jacqueline Findlay, Samuel Weeks, Julianne Brown, Kathryn Harris, Paul Randell, James Price, Alison Holmes, Judith Breuer
EPI_ISL_866366, EPI_ISL_866393, EPI_ISL_866394	Regional Virus Laboratory, Belfast Health and Social Care Trust	COVID-19 Genomics UK (COG-UK) Consortium	Conall McCaughey, James McKenna, Tanya Curran, Susan Feeney, Alison Watt, Ciara Cox, Mairead Connor, Zoltan Molnar, David Simpson, Derek Fairley
EPI_ISL_866542, EPI_ISL_866543, EPI_ISL_866544, EPI_ISL_866545, EPI_ISL_866546, EPI_ISL_866547, EPI_ISL_866549, EPI_ISL_866550, EPI_ISL_866551, EPI_ISL_866552, EPI_ISL_866553, EPI_ISL_866554, EPI_ISL_866555, EPI_ISL_866557, EPI_ISL_866558, EPI_ISL_866559, EPI_ISL_866561, EPI_ISL_866562, EPI_ISL_866563, EPI_ISL_866564, EPI_ISL_866565, EPI_ISL_866567, EPI_ISL_866568, EPI_ISL_866570, EPI_ISL_866571, EPI_ISL_866572, EPI_ISL_866573, EPI_ISL_866574, EPI_ISL_866575, EPI_ISL_866576, EPI_ISL_866603, EPI_ISL_866604, EPI_ISL_866605, EPI_ISL_866606, EPI_ISL_866607, EPI_ISL_866608, EPI_ISL_866609, EPI_ISL_866610, EPI_ISL_866611, EPI_ISL_866612, EPI_ISL_866613, EPI_ISL_866614, EPI_ISL_866615, EPI_ISL_866616, EPI_ISL_866617, EPI_ISL_866618, EPI_ISL_866620, EPI_ISL_866621, EPI_ISL_866622, EPI_ISL_866623, EPI_ISL_866624, EPI_ISL_866625, EPI_ISL_866626, EPI_ISL_866627, EPI_ISL_866628, EPI_ISL_866629, EPI_ISL_866630, EPI_ISL_866631, EPI_ISL_866632, EPI_ISL_866633, EPI_ISL_866634, EPI_ISL_866635, EPI_ISL_866636, EPI_ISL_866637, EPI_ISL_866638, EPI_ISL_866639, EPI_ISL_866640, EPI_ISL_866641, EPI_ISL_866642, EPI_ISL_866643, EPI_ISL_866644, EPI_ISL_866645, EPI_ISL_866646, EPI_ISL_866647, EPI_ISL_866648, EPI_ISL_866649, EPI_ISL_866650, EPI_ISL_866651, EPI_ISL_866652, EPI_ISL_866653, EPI_ISL_866654, EPI_ISL_866655, EPI_ISL_866656, EPI_ISL_866657, EPI_ISL_866658, EPI_ISL_866659, EPI_ISL_866660, EPI_ISL_866661, EPI_ISL_866662, EPI_ISL_866663, EPI_ISL_866664, EPI_ISL_866665, EPI_ISL_866666, EPI_ISL_866667, EPI_ISL_866668, EPI_ISL_866669, EPI_ISL_866670, EPI_ISL_866671, EPI_ISL_866672, EPI_ISL_866673, EPI_ISL_866674, EPI_ISL_866675, EPI_ISL_866676, EPI_ISL_866677, EPI_ISL_866678, EPI_ISL_866679, EPI_ISL_866680, EPI_ISL_866681, EPI_ISL_866682, EPI_ISL_866683, EPI_ISL_866684, EPI_ISL_866685, EPI_ISL_866686, EPI_ISL_866687, EPI_ISL_866688, EPI_ISL_866689, EPI_ISL_866690, EPI_ISL_866691, EPI_ISL_866692, EPI_ISL_866693, EPI_ISL_866694, EPI_ISL_866695, EPI_ISL_866696, EPI_ISL_866697, EPI_ISL_866698, EPI_ISL_866699, EPI_ISL_866700, EPI_ISL_866701, EPI_ISL_866702, EPI_ISL_866703, EPI_ISL_866704, EPI_ISL_866705, EPI_ISL_866706, EPI_ISL_866707, EPI_ISL_866708, EPI_ISL_866709, EPI_ISL_866710, EPI_ISL_866711, EPI_ISL_866712, EPI_ISL_866713, EPI_ISL_866714, EPI_ISL_866715, EPI_ISL_866716, EPI_ISL_866717, EPI_ISL_866718, EPI_ISL_866719, EPI_ISL_866720, EPI_ISL_866721, EPI_ISL_866722, EPI_ISL_866723, EPI_ISL_866724, EPI_ISL_866725, EPI_ISL_866726, EPI_ISL_866727, EPI_ISL_866728, EPI_ISL_866729, EPI_ISL_866730, EPI_ISL_866731, EPI_ISL_866732, EPI_ISL_866733, EPI_ISL_866734, EPI_ISL_866735, EPI_ISL_866736, EPI_ISL_866737, EPI_ISL_866738, EPI_ISL_866739, EPI_ISL_866740, EPI_ISL_866741, EPI_ISL_866742, EPI_ISL_866744, EPI_ISL_866745, EPI_ISL_866746, EPI_ISL_866747, EPI_ISL_866758, EPI_ISL_866759, EPI_ISL_866761, EPI_ISL_866762, EPI_ISL_866765, EPI_ISL_866766, EPI_ISL_866768, EPI_ISL_866769, EPI_ISL_866771, EPI_ISL_866772, EPI_ISL_866773, EPI_ISL_866776, EPI_ISL_866777, EPI_ISL_866778, EPI_ISL_866779, EPI_ISL_866780, EPI_ISL_866783, EPI_ISL_866784, EPI_ISL_866785, EPI_ISL_866786, EPI_ISL_866787, EPI_ISL_866790, EPI_ISL_866791, EPI_ISL_866792, EPI_ISL_866794, EPI_ISL_866795, EPI_ISL_866796, EPI_ISL_866797, EPI_ISL_866798, EPI_ISL_866799, EPI_ISL_866800, EPI_ISL_866831, EPI_ISL_866832, EPI_ISL_866833, EPI_ISL_866834			
see above	Quadram Institute Bioscience	COVID-19 Genomics UK (COG-UK) Consortium	Dave J. Baker, Gemma L. Kay, Alp Aydin, Thanh Le-Viet, Steven Rudder, Ana P. Tedim, Anastasia Kolyva, Maria Diaz, Leonardo de Oliveira Martins, Nabil-Fareed Alikhan, Lizzie Meadows, Rachael Stanley, Ngozi Elumogo, Muhammed Yasir, Nicholas M. Thomson, Alexander J Trotter, Rachel Gilroy, Samuel Bloomfield, Claire Stuart, Andrew Bell, Reenesh Prakash, Samir Dervisevic, Alison E. Mather, John Wain, Mark Webber, Andrew J. Page, Justin O'Grady
EPI_ISL_866908, EPI_ISL_866909, EPI_ISL_866910, EPI_ISL_866911, EPI_ISL_866912, EPI_ISL_866913, EPI_ISL_866914, EPI_ISL_866915, EPI_ISL_866916, EPI_ISL_866917, EPI_ISL_866918, EPI_ISL_866919, EPI_ISL_866920			
see above	Queens Medical Centre, Clinical Microbiology Department / DeepSeq Nottingham	COVID-19 Genomics UK (COG-UK) Consortium	Gemma Clark, Wendy Smith, Manjinder Khakh, Vicki M Fleming, Michelle M Lister, Hannah Howson-Wells, Jonathan Ball, Patrick McClure, Joseph Chappell, Theocharis Tsoleridis, Nadine Holmes, Matthew Carlisle, Christopher Moore, Fei Sang, Johnny Debebe, Victoria Wright, Matthew Loose
EPI_ISL_867190, EPI_ISL_867191, EPI_ISL_867192, EPI_ISL_867193, EPI_ISL_867194, EPI_ISL_867195, EPI_ISL_867196, EPI_ISL_867197, EPI_ISL_867202, EPI_ISL_867211, EPI_ISL_867212, EPI_ISL_867213, EPI_ISL_867214, EPI_ISL_867215, EPI_ISL_867219, EPI_ISL_867220, EPI_ISL_867221, EPI_ISL_867222, EPI_ISL_867223, EPI_ISL_867224, EPI_ISL_867225, EPI_ISL_867226, EPI_ISL_867227, EPI_ISL_867228, EPI_ISL_867229, EPI_ISL_867230, EPI_ISL_867231, EPI_ISL_867232, EPI_ISL_867233, EPI_ISL_867237, EPI_ISL_867238, EPI_ISL_867239, EPI_ISL_867240, EPI_ISL_867241, EPI_ISL_867242, EPI_ISL_867243, EPI_ISL_867244, EPI_ISL_867245, EPI_ISL_867246, EPI_ISL_867247, EPI_ISL_867248, EPI_ISL_867249, EPI_ISL_867250, EPI_ISL_867251, EPI_ISL_867252, EPI_ISL_867253, EPI_ISL_867254, EPI_ISL_867255, EPI_ISL_867256, EPI_ISL_867257, EPI_ISL_867258, EPI_ISL_867261, EPI_ISL_867269, EPI_ISL_867270, EPI_ISL_867298, EPI_ISL_867299, EPI_ISL_867300, EPI_ISL_867301, EPI_ISL_867305, EPI_ISL_867306, EPI_ISL_867307, EPI_ISL_867308, EPI_ISL_867309, EPI_ISL_867310, EPI_ISL_867311, EPI_ISL_867312, EPI_ISL_867313, EPI_ISL_867314, EPI_ISL_867315, EPI_ISL_867317, EPI_ISL_867318, EPI_ISL_867319, EPI_ISL_867320, EPI_ISL_867321, EPI_ISL_867322, EPI_ISL_867323, EPI_ISL_867324, EPI_ISL_867325, EPI_ISL_867326, EPI_ISL_867327, EPI_ISL_867328, EPI_ISL_867329, EPI_ISL_867330, EPI_ISL_867331, EPI_ISL_867332, EPI_ISL_867333, EPI_ISL_867334, EPI_ISL_867335, EPI_ISL_867336, EPI_ISL_867337, EPI_ISL_867338, EPI_ISL_867339, EPI_ISL_867340, EPI_ISL_867341, EPI_ISL_867342, EPI_ISL_867343, EPI_ISL_867344, EPI_ISL_867345, EPI_ISL_867346, EPI_ISL_867348, EPI_ISL_867349, EPI_ISL_867350, EPI_ISL_867351, EPI_ISL_867352, EPI_ISL_867353, EPI_ISL_867354, EPI_ISL_867355, EPI_ISL_867357, EPI_ISL_867358, EPI_ISL_867359, EPI_ISL_867360, EPI_ISL_867361, EPI_ISL_867362, EPI_ISL_867364, EPI_ISL_867365, EPI_ISL_867366, EPI_ISL_867367, EPI_ISL_867368, EPI_ISL_867369, EPI_ISL_867370, EPI_ISL_867371, EPI_ISL_867372, EPI_ISL_867373, EPI_ISL_867374, EPI_ISL_867375, EPI_ISL_867377, EPI_ISL_867378, EPI_ISL_867379, EPI_ISL_867381, EPI_ISL_867382, EPI_ISL_867383, EPI_ISL_867384, EPI_ISL_867385, EPI_ISL_867386, EPI_ISL_867387, EPI_ISL_867388, EPI_ISL_867390, EPI_ISL_867391, EPI_ISL_867392, EPI_ISL_867393, EPI_ISL_867394, EPI_ISL_867395, EPI_ISL_867396, EPI_ISL_867397, EPI_ISL_867398, EPI_ISL_867399, EPI_ISL_867400, EPI_ISL_867401, EPI_ISL_867403, EPI_ISL_867404, EPI_ISL_867405, EPI_ISL_867416, EPI_ISL_867417, EPI_ISL_867419, EPI_ISL_867424, EPI_ISL_867429, EPI_ISL_867430, EPI_ISL_867450, EPI_ISL_867451, EPI_ISL_867457, EPI_ISL_867458, EPI_ISL_867459, EPI_ISL_867491, EPI_ISL_867492, EPI_ISL_867494, EPI_ISL_867497, EPI_ISL_867498, EPI_ISL_867506, EPI_ISL_867509, EPI_ISL_867510, EPI_ISL_867511, EPI_ISL_867515, EPI_ISL_867518, EPI_ISL_867519, EPI_ISL_867520, EPI_ISL_867521, EPI_ISL_867522, EPI_ISL_867523, EPI_ISL_867525, EPI_ISL_867536, EPI_ISL_867538, EPI_ISL_867539, EPI_ISL_867540, EPI_ISL_867541, EPI_ISL_867542, EPI_ISL_867543, EPI_ISL_867547, EPI_ISL_867550, EPI_ISL_867552, EPI_ISL_867553, EPI_ISL_867556, EPI_ISL_867557, EPI_ISL_867561, EPI_ISL_867562, EPI_ISL_867567, EPI_ISL_867572, EPI_ISL_867573, EPI_ISL_867574, EPI_ISL_867575, EPI_ISL_867579, EPI_ISL_867582, EPI_ISL_867584, EPI_ISL_867586, EPI_ISL_867587, EPI_ISL_867588, EPI_ISL_867589, EPI_ISL_867590, EPI_ISL_867704, EPI_ISL_867705, EPI_ISL_867706, EPI_ISL_867708, EPI_ISL_867716, EPI_ISL_867717, EPI_ISL_867718, EPI_ISL_867719, EPI_ISL_867720, EPI_ISL_867721, EPI_ISL_867722, EPI_ISL_867723, EPI_ISL_867724, EPI_ISL_867725, EPI_ISL_867726, EPI_ISL_867728, EPI_ISL_867730, EPI_ISL_867731, EPI_ISL_867737, EPI_ISL_867738, EPI_ISL_867739, EPI_ISL_867740, EPI_ISL_867741, EPI_ISL_867759, EPI_ISL_867762, EPI_ISL_867763, EPI_ISL_867764, EPI_ISL_867766, EPI_ISL_867768, EPI_ISL_867771, EPI_ISL_867773, EPI_ISL_867776, EPI_ISL_867779, EPI_ISL_867795, EPI_ISL_867796, EPI_ISL_867797, EPI_ISL_867800, EPI_ISL_867801, EPI_ISL_867802, EPI_ISL_867804, EPI_ISL_867805, EPI_ISL_867806, EPI_ISL_867807, EPI_ISL_867808, EPI_ISL_867809, EPI_ISL_867810, EPI_ISL_867811, EPI_ISL_867812, EPI_ISL_867815, EPI_ISL_867816, EPI_ISL_867819, EPI_ISL_867821, EPI_ISL_867823, EPI_ISL_867824, EPI_ISL_867825, EPI_ISL_867828, EPI_ISL_867830, EPI_ISL_867832, EPI_ISL_867833, EPI_ISL_867834, EPI_ISL_867837, EPI_ISL_867838, EPI_ISL_867839, EPI_ISL_867841, EPI_ISL_867842, EPI_ISL_867843, EPI_ISL_867844, EPI_ISL_867845, EPI_ISL_867847, EPI_ISL_867848, EPI_ISL_867849, EPI_ISL_867850, EPI_ISL_867851, EPI_ISL_867853, EPI_ISL_867855, EPI_ISL_867856, EPI_ISL_867859, EPI_ISL_867860, EPI_ISL_867861, EPI_ISL_867862, EPI_ISL_867863, EPI_ISL_867868, EPI_ISL_867873, EPI_ISL_867875, EPI_ISL_867876, EPI_ISL_867878, EPI_ISL_867879, EPI_ISL_867880, EPI_ISL_867881, EPI_ISL_867883, EPI_ISL_867884, EPI_ISL_867885, EPI_ISL_867889, EPI_ISL_867890, EPI_ISL_867893, EPI_ISL_867895, EPI_ISL_867899, EPI_ISL_867900, EPI_ISL_867901, EPI_ISL_867904, EPI_ISL_867906, EPI_ISL_867911, EPI_ISL_867912, EPI_ISL_867913, EPI_ISL_867914, EPI_ISL_867915, EPI_ISL_867916, EPI_ISL_867918, EPI_ISL_867921			
see above	Originating lab: Wales Specialist Virology Centre Sequencing lab: Pathogen Genomics Unit	Public Health Wales Microbiology Cardiff Wales Specialist Virology Centre	Catherine Moore, Johnathan Evans, Laura Gifford, Malorie Perry, Simon Cottrell, Angela Marchbank, Alec Birchley, Alexander Adams, Amy Gaskin, Bree Gatica-Wilcox, Jason Coombes, Joel Southgate, Lauren Gilbert, Lee Graham, Nicole Pacchiarini, Sara Kumziene-Summerhayes, Sarah Taylor, Sophie Jones, Sara Rey, Matthew Bull, Joanne Watkins, Sally Corden, Tom Connor
EPI_ISL_868141, EPI_ISL_868142, EPI_ISL_868143, EPI_ISL_868144, EPI_ISL_868145, EPI_ISL_868146, EPI_ISL_868147, EPI_ISL_868148, EPI_ISL_868150, EPI_ISL_868152, EPI_ISL_868153, EPI_ISL_868154, EPI_ISL_868161, EPI_ISL_868162, EPI_ISL_868163, EPI_ISL_868164, EPI_ISL_868165, EPI_ISL_868166, EPI_ISL_868167, EPI_ISL_868168, EPI_ISL_868169, EPI_ISL_868170, EPI_ISL_868171, EPI_ISL_868172, EPI_ISL_868173, EPI_ISL_868174, EPI_ISL_868177, EPI_ISL_868178, EPI_ISL_868179, EPI_ISL_868181, EPI_ISL_868182, EPI_ISL_868183, EPI_ISL_868184, EPI_ISL_868185, EPI_ISL_868186, EPI_ISL_868187, EPI_ISL_868188, EPI_ISL_868189, EPI_ISL_868190, EPI_ISL_868191, EPI_ISL_868192, EPI_ISL_868193, EPI_ISL_868194, EPI_ISL_868196, EPI_ISL_868197, EPI_ISL_868198, EPI_ISL_868199, EPI_ISL_868200, EPI_ISL_868201, EPI_ISL_868203, EPI_ISL_868204, EPI_ISL_868217, EPI_ISL_868218, EPI_ISL_868323, EPI_ISL_868329, EPI_ISL_868330, EPI_ISL_868331, EPI_ISL_868332, EPI_ISL_868333, EPI_ISL_868334, EPI_ISL_868335, EPI_ISL_868336, EPI_ISL_868337, EPI_ISL_868338, EPI_ISL_868339, EPI_ISL_868340, EPI_ISL_868341, EPI_ISL_868342, EPI_ISL_868353, EPI_ISL_868354, EPI_ISL_868355			
see above	Centre for Enzyme Innovation, University of Portsmouth / Translational Research Laboratory, Portsmouth Hospitals NHS Trust	COVID-19 Genomics UK (COG-UK) Consortium	Angela Beckett,Yann Bourgeois,Garry Scarlett,Sharon Glaysher,Scott Elliott,Kelly Bicknell,Robert Impey,Allyson Lloyd,Sarah Wyllie,Ethan Butcher,Anoop Chauhan,Samuel Robson
EPI_ISL_868358, EPI_ISL_868364, EPI_ISL_868373, EPI_ISL_868381, EPI_ISL_868382, EPI_ISL_868386, EPI_ISL_868388, EPI_ISL_868392, EPI_ISL_868395, EPI_ISL_868399, EPI_ISL_868401, EPI_ISL_868402, EPI_ISL_868408, EPI_ISL_868409, EPI_ISL_868413, EPI_ISL_868415, EPI_ISL_868428, EPI_ISL_868429, EPI_ISL_868430, EPI_ISL_868432, EPI_ISL_868434, EPI_ISL_868442, EPI_ISL_868444, EPI_ISL_868445, EPI_ISL_868446, EPI_ISL_868448, EPI_ISL_868450, EPI_ISL_868454, EPI_ISL_868459, EPI_ISL_868464, EPI_ISL_868465, EPI_ISL_868467, EPI_ISL_868469, EPI_ISL_868471, EPI_ISL_868474, EPI_ISL_868478, EPI_ISL_868480, EPI_ISL_868486, EPI_ISL_868487, EPI_ISL_868491, EPI_ISL_868495, EPI_ISL_868502, EPI_ISL_868503, EPI_ISL_868507, EPI_ISL_868508, EPI_ISL_868513, EPI_ISL_868515, EPI_ISL_868521, EPI_ISL_868522, EPI_ISL_868524, EPI_ISL_868525, EPI_ISL_868533, EPI_ISL_868535, EPI_ISL_868537, EPI_ISL_868541, EPI_ISL_868542, EPI_ISL_868547, EPI_ISL_868552, EPI_ISL_868556, EPI_ISL_868557, EPI_ISL_868560, EPI_ISL_868564, EPI_ISL_868566, EPI_ISL_868573, EPI_ISL_868588, EPI_ISL_868593, EPI_ISL_868594, EPI_ISL_868597, EPI_ISL_868599, EPI_ISL_868601, EPI_ISL_868603, EPI_ISL_868608, EPI_ISL_868612, EPI_ISL_868613, EPI_ISL_868621, EPI_ISL_868622, EPI_ISL_868625, EPI_ISL_868630, EPI_ISL_868631, EPI_ISL_868638, EPI_ISL_868643, EPI_ISL_868644, EPI_ISL_868648, EPI_ISL_868651, EPI_ISL_868654, EPI_ISL_868655, EPI_ISL_868656, EPI_ISL_868660, EPI_ISL_868674, EPI_ISL_868677, EPI_ISL_868678, EPI_ISL_868680, EPI_ISL_868681, EPI_ISL_868684, EPI_ISL_868688, EPI_ISL_868692, EPI_ISL_868693, EPI_ISL_868700, EPI_ISL_868701, EPI_ISL_868715			

see above	Virology Department, Sheffield Teaching Hospitals NHS Foundation Trust/Department of Infection, Immunity and Cardiovascular Disease, The Medical School, University of Sheffield	COVID-19 Genomics UK (COG-UK) Consortium	Thushan de Silva, Matthew Parker, Nikki Smith, Adri Angyal, Rebecca Brown, Luke Green, Rachel Tucker, Paul Parsons, Danielle Groves, Katie Johnson, Laura Carrilero, Alex Keeley, Dave Partridge, Matthew Wyles, Benjamin Lindsey, Mehmet Yavuz, Mohammad Raza, Cariad Evans
EPI_ISL_873279, EPI_ISL_873280	Lighthouse Lab in Milton Keynes	Wellcome Sanger Institute for the COVID-19 Genomics UK (COG-UK) Consortium	The Lighthouse Lab in Milton Keynes and Alex Alderton, Roberto Amato, Sonia Goncalves, Ewan Harrison, David K. Jackson, Ian Johnston, Dominic Kwiatkowski, Cordelia Langford, John Sillitoe on behalf of the Wellcome Sanger Institute COVID-19 Surveillance Team
EPI_ISL_873282	Lighthouse Lab in Cambridge	Wellcome Sanger Institute for the COVID-19 Genomics UK (COG-UK) Consortium	Rob Howes, The Lighthouse Lab in Cambridge and Alex Alderton, Roberto Amato, Sonia Goncalves, Ewan Harrison, David K. Jackson, Ian Johnston, Dominic Kwiatkowski, Cordelia Langford, John Sillitoe on behalf of the Wellcome Sanger Institute COVID-19 Surveillance Team
EPI_ISL_873284, EPI_ISL_873285, EPI_ISL_873287, EPI_ISL_873288, EPI_ISL_873289, EPI_ISL_873290, EPI_ISL_873293	Lighthouse Lab in Milton Keynes	Wellcome Sanger Institute for the COVID-19 Genomics UK (COG-UK) Consortium	The Lighthouse Lab in Milton Keynes and Alex Alderton, Roberto Amato, Sonia Goncalves, Ewan Harrison, David K. Jackson, Ian Johnston, Dominic Kwiatkowski, Cordelia Langford, John Sillitoe on behalf of the Wellcome Sanger Institute COVID-19 Surveillance Team
EPI_ISL_873295	Lighthouse Lab in Cambridge	Wellcome Sanger Institute for the COVID-19 Genomics UK (COG-UK) Consortium	Rob Howes, The Lighthouse Lab in Cambridge and Alex Alderton, Roberto Amato, Sonia Goncalves, Ewan Harrison, David K. Jackson, Ian Johnston, Dominic Kwiatkowski, Cordelia Langford, John Sillitoe on behalf of the Wellcome Sanger Institute COVID-19 Surveillance Team
EPI_ISL_873296, EPI_ISL_873299, EPI_ISL_873300, EPI_ISL_873301, EPI_ISL_873303, EPI_ISL_873305, EPI_ISL_873307, EPI_ISL_873308, EPI_ISL_873309, EPI_ISL_873312, EPI_ISL_873314, EPI_ISL_873315, EPI_ISL_873317, EPI_ISL_873319, EPI_ISL_873322, EPI_ISL_873323, EPI_ISL_873327, EPI_ISL_873328, EPI_ISL_873329, EPI_ISL_873331, EPI_ISL_873332, EPI_ISL_873333, EPI_ISL_873335, EPI_ISL_873338, EPI_ISL_873340, EPI_ISL_873344, EPI_ISL_873345, EPI_ISL_873346, EPI_ISL_873349, EPI_ISL_873351, EPI_ISL_873352, EPI_ISL_873353, EPI_ISL_873355, EPI_ISL_873356, EPI_ISL_873358, EPI_ISL_873360, EPI_ISL_873363			
see above	Lighthouse Lab in Milton Keynes	Wellcome Sanger Institute for the COVID-19 Genomics UK (COG-UK) Consortium	The Lighthouse Lab in Milton Keynes and Alex Alderton, Roberto Amato, Sonia Goncalves, Ewan Harrison, David K. Jackson, Ian Johnston, Dominic Kwiatkowski, Cordelia Langford, John Sillitoe on behalf of the Wellcome Sanger Institute COVID-19 Surveillance Team
EPI_ISL_873365	Lighthouse Lab in Cambridge	Wellcome Sanger Institute for the COVID-19 Genomics UK (COG-UK) Consortium	Rob Howes, The Lighthouse Lab in Cambridge and Alex Alderton, Roberto Amato, Sonia Goncalves, Ewan Harrison, David K. Jackson, Ian Johnston, Dominic Kwiatkowski, Cordelia Langford, John Sillitoe on behalf of the Wellcome Sanger Institute COVID-19 Surveillance Team
EPI_ISL_873366, EPI_ISL_873367	Lighthouse Lab in Milton Keynes	Wellcome Sanger Institute for the COVID-19 Genomics UK (COG-UK) Consortium	The Lighthouse Lab in Milton Keynes and Alex Alderton, Roberto Amato, Sonia Goncalves, Ewan Harrison, David K. Jackson, Ian Johnston, Dominic Kwiatkowski, Cordelia Langford, John Sillitoe on behalf of the Wellcome Sanger Institute COVID-19 Surveillance Team
EPI_ISL_873368	Lighthouse Lab in Cambridge	Wellcome Sanger Institute for the COVID-19 Genomics UK (COG-UK) Consortium	Rob Howes, The Lighthouse Lab in Cambridge and Alex Alderton, Roberto Amato, Sonia Goncalves, Ewan Harrison, David K. Jackson, Ian Johnston, Dominic Kwiatkowski, Cordelia Langford, John Sillitoe on behalf of the Wellcome Sanger Institute COVID-19 Surveillance Team
EPI_ISL_873372, EPI_ISL_873373, EPI_ISL_873375, EPI_ISL_873377, EPI_ISL_873379, EPI_ISL_873380, EPI_ISL_873382, EPI_ISL_873383, EPI_ISL_873384, EPI_ISL_873385, EPI_ISL_873387, EPI_ISL_873390, EPI_ISL_873391, EPI_ISL_873392, EPI_ISL_873393, EPI_ISL_873394, EPI_ISL_873395, EPI_ISL_873397, EPI_ISL_873398, EPI_ISL_873400, EPI_ISL_873401, EPI_ISL_873402, EPI_ISL_873403, EPI_ISL_873404, EPI_ISL_873405, EPI_ISL_873406, EPI_ISL_873407, EPI_ISL_873408, EPI_ISL_873409, EPI_ISL_873410, EPI_ISL_873417			
see above	Lighthouse Lab in Milton Keynes	Wellcome Sanger Institute for the COVID-19 Genomics UK (COG-UK) Consortium	The Lighthouse Lab in Milton Keynes and Alex Alderton, Roberto Amato, Sonia Goncalves, Ewan Harrison, David K. Jackson, Ian Johnston, Dominic Kwiatkowski, Cordelia Langford, John Sillitoe on behalf of the Wellcome Sanger Institute COVID-19 Surveillance Team
EPI_ISL_873418	Lighthouse Lab in Cambridge	Wellcome Sanger Institute for the COVID-19 Genomics UK (COG-UK) Consortium	Rob Howes, The Lighthouse Lab in Cambridge and Alex Alderton, Roberto Amato, Sonia Goncalves, Ewan Harrison, David K. Jackson, Ian Johnston, Dominic Kwiatkowski, Cordelia Langford, John Sillitoe on behalf of the Wellcome Sanger Institute COVID-19 Surveillance Team
EPI_ISL_873419, EPI_ISL_873421, EPI_ISL_873423, EPI_ISL_873424, EPI_ISL_873425, EPI_ISL_873427	Lighthouse Lab in Milton Keynes	Wellcome Sanger Institute for the COVID-19 Genomics UK (COG-UK) Consortium	The Lighthouse Lab in Milton Keynes and Alex Alderton, Roberto Amato, Sonia Goncalves, Ewan Harrison, David K. Jackson, Ian Johnston, Dominic Kwiatkowski, Cordelia Langford, John Sillitoe on behalf of the Wellcome Sanger Institute COVID-19 Surveillance Team
EPI_ISL_873432	Lighthouse Lab in Cambridge	Wellcome Sanger Institute for the COVID-19 Genomics UK (COG-UK) Consortium	Rob Howes, The Lighthouse Lab in Cambridge and Alex Alderton, Roberto Amato, Sonia Goncalves, Ewan Harrison, David K. Jackson, Ian Johnston, Dominic Kwiatkowski, Cordelia Langford, John Sillitoe on behalf of the Wellcome Sanger Institute COVID-19 Surveillance Team
EPI_ISL_873434, EPI_ISL_873436, EPI_ISL_873437, EPI_ISL_873440, EPI_ISL_873441, EPI_ISL_873442, EPI_ISL_873446, EPI_ISL_873447, EPI_ISL_873448, EPI_ISL_873449, EPI_ISL_873452, EPI_ISL_873453, EPI_ISL_873456, EPI_ISL_873458, EPI_ISL_873460, EPI_ISL_873461, EPI_ISL_873463, EPI_ISL_873464, EPI_ISL_873465, EPI_ISL_873467, EPI_ISL_873468, EPI_ISL_873469, EPI_ISL_873470, EPI_ISL_873471, EPI_ISL_873472, EPI_ISL_873474, EPI_ISL_873475, EPI_ISL_873477, EPI_ISL_873478, EPI_ISL_873479, EPI_ISL_873480, EPI_ISL_873483, EPI_ISL_873484, EPI_ISL_873485, EPI_ISL_873486, EPI_ISL_873488			
see above	Lighthouse Lab in Milton Keynes	Wellcome Sanger Institute for the COVID-19 Genomics UK (COG-UK) Consortium	The Lighthouse Lab in Milton Keynes and Alex Alderton, Roberto Amato, Sonia Goncalves, Ewan Harrison, David K. Jackson, Ian Johnston, Dominic Kwiatkowski, Cordelia Langford, John Sillitoe on behalf of the Wellcome Sanger Institute COVID-19 Surveillance Team
EPI_ISL_873489	Lighthouse Lab in Cambridge	Wellcome Sanger Institute for the COVID-19 Genomics UK (COG-UK) Consortium	Rob Howes, The Lighthouse Lab in Cambridge and Alex Alderton, Roberto Amato, Sonia Goncalves, Ewan Harrison, David K. Jackson, Ian Johnston, Dominic Kwiatkowski, Cordelia Langford, John Sillitoe on behalf of the Wellcome Sanger Institute COVID-19 Surveillance Team
EPI_ISL_873490, EPI_ISL_873491, EPI_ISL_873498, EPI_ISL_873502, EPI_ISL_873505, EPI_ISL_873506, EPI_ISL_873507, EPI_ISL_873508, EPI_ISL_873511, EPI_ISL_873512	Lighthouse Lab in Milton Keynes	Wellcome Sanger Institute for the COVID-19 Genomics UK (COG-UK) Consortium	The Lighthouse Lab in Milton Keynes and Alex Alderton, Roberto Amato, Sonia Goncalves, Ewan Harrison, David K. Jackson, Ian Johnston, Dominic Kwiatkowski, Cordelia Langford, John Sillitoe on behalf of the Wellcome Sanger Institute COVID-19 Surveillance Team
EPI_ISL_873513	Lighthouse Lab in Cambridge	Wellcome Sanger Institute for the COVID-19 Genomics UK (COG-UK) Consortium	Rob Howes, The Lighthouse Lab in Cambridge and Alex Alderton, Roberto Amato, Sonia Goncalves, Ewan Harrison, David K. Jackson, Ian Johnston, Dominic Kwiatkowski, Cordelia Langford, John Sillitoe on behalf of the Wellcome Sanger Institute COVID-19 Surveillance Team
EPI_ISL_873514, EPI_ISL_873516, EPI_ISL_873517, EPI_ISL_873519, EPI_ISL_873521, EPI_ISL_873522, EPI_ISL_873523, EPI_ISL_873524, EPI_ISL_873525, EPI_ISL_873526, EPI_ISL_873527, EPI_ISL_873528, EPI_ISL_873529, EPI_ISL_873530, EPI_ISL_873533, EPI_ISL_873534, EPI_ISL_873535, EPI_ISL_873536, EPI_ISL_873538, EPI_ISL_873539, EPI_ISL_873540, EPI_ISL_873541, EPI_ISL_873542, EPI_ISL_873543, EPI_ISL_873544, EPI_ISL_873545, EPI_ISL_873546, EPI_ISL_873547, EPI_ISL_873548, EPI_ISL_873549, EPI_ISL_873550, EPI_ISL_873553, EPI_ISL_873554, EPI_ISL_873555, EPI_ISL_873556, EPI_ISL_873557, EPI_ISL_873558, EPI_ISL_873559, EPI_ISL_873560			
see above	Lighthouse Lab in Milton Keynes	Wellcome Sanger Institute for the COVID-19 Genomics UK (COG-UK) Consortium	The Lighthouse Lab in Milton Keynes and Alex Alderton, Roberto Amato, Sonia Goncalves, Ewan Harrison, David K. Jackson, Ian Johnston, Dominic Kwiatkowski, Cordelia Langford, John Sillitoe on behalf of the Wellcome Sanger Institute COVID-19 Surveillance Team
EPI_ISL_873561	Lighthouse Lab in Cambridge	Wellcome Sanger Institute for the COVID-19 Genomics UK (COG-UK) Consortium	Rob Howes, The Lighthouse Lab in Cambridge and Alex Alderton, Roberto Amato, Sonia Goncalves, Ewan Harrison, David K. Jackson, Ian Johnston, Dominic Kwiatkowski, Cordelia Langford, John Sillitoe on behalf of the Wellcome Sanger Institute COVID-19 Surveillance Team
EPI_ISL_873562, EPI_ISL_873563, EPI_ISL_873564, EPI_ISL_873565, EPI_ISL_873567, EPI_ISL_873568, EPI_ISL_873569, EPI_ISL_873570, EPI_ISL_873572, EPI_ISL_873575, EPI_ISL_873577, EPI_ISL_873578, EPI_ISL_873579, EPI_ISL_873580, EPI_ISL_873581, EPI_ISL_873582, EPI_ISL_873583, EPI_ISL_873584, EPI_ISL_873586, EPI_ISL_873587, EPI_ISL_873588, EPI_ISL_873589, EPI_ISL_873590, EPI_ISL_873591, EPI_ISL_873592, EPI_ISL_873593, EPI_ISL_873594, EPI_ISL_873595, EPI_ISL_873597, EPI_ISL_873598, EPI_ISL_873599, EPI_ISL_873601, EPI_ISL_873602, EPI_ISL_873606, EPI_ISL_873608, EPI_ISL_873609, EPI_ISL_873610			
see above	Lighthouse Lab in Milton Keynes	Wellcome Sanger Institute for the COVID-19 Genomics UK (COG-UK) Consortium	The Lighthouse Lab in Milton Keynes and Alex Alderton, Roberto Amato, Sonia Goncalves, Ewan Harrison, David K. Jackson, Ian Johnston, Dominic Kwiatkowski, Cordelia Langford, John Sillitoe on behalf of the Wellcome Sanger Institute COVID-19 Surveillance Team
EPI_ISL_873611	Lighthouse Lab in Cambridge	Wellcome Sanger Institute for the COVID-19 Genomics UK (COG-UK) Consortium	Rob Howes, The Lighthouse Lab in Cambridge and Alex Alderton, Roberto Amato, Sonia Goncalves, Ewan Harrison, David K. Jackson, Ian Johnston, Dominic Kwiatkowski, Cordelia Langford, John Sillitoe on behalf of the Wellcome Sanger Institute COVID-19 Surveillance Team
EPI_ISL_873612	Lighthouse Lab in Milton Keynes	Wellcome Sanger Institute for the COVID-19 Genomics UK (COG-UK) Consortium	The Lighthouse Lab in Milton Keynes and Alex Alderton, Roberto Amato, Sonia Goncalves, Ewan Harrison, David K. Jackson, Ian Johnston, Dominic Kwiatkowski, Cordelia Langford, John Sillitoe on behalf of the Wellcome Sanger Institute COVID-19 Surveillance Team
EPI_ISL_873613	Lighthouse Lab in Cambridge	Wellcome Sanger Institute for the COVID-19 Genomics UK (COG-UK) Consortium	Rob Howes, The Lighthouse Lab in Cambridge and Alex Alderton, Roberto Amato, Sonia Goncalves, Ewan Harrison, David K. Jackson, Ian Johnston, Dominic Kwiatkowski, Cordelia Langford, John Sillitoe on behalf of the Wellcome Sanger Institute COVID-19 Surveillance Team
EPI_ISL_873615, EPI_ISL_873616, EPI_ISL_873617, EPI_ISL_873618, EPI_ISL_873619, EPI_ISL_873620, EPI_ISL_873622, EPI_ISL_873623	Lighthouse Lab in Milton Keynes	Wellcome Sanger Institute for the COVID-19 Genomics UK (COG-UK) Consortium	The Lighthouse Lab in Milton Keynes and Alex Alderton, Roberto Amato, Sonia Goncalves, Ewan Harrison, David K. Jackson, Ian Johnston, Dominic Kwiatkowski, Cordelia Langford, John Sillitoe on behalf of the Wellcome Sanger Institute COVID-19 Surveillance Team
EPI_ISL_873624	Lighthouse Lab in Cambridge	Wellcome Sanger Institute for the COVID-19 Genomics UK (COG-UK) Consortium	Rob Howes, The Lighthouse Lab in Cambridge and Alex Alderton, Roberto Amato, Sonia Goncalves, Ewan Harrison, David K. Jackson, Ian Johnston, Dominic Kwiatkowski, Cordelia Langford, John Sillitoe on behalf of the Wellcome Sanger Institute COVID-19 Surveillance Team

EPI_ISL_873626, EPI_ISL_873627, EPI_ISL_873628, EPI_ISL_873631, EPI_ISL_873637, EPI_ISL_873641, EPI_ISL_873654, EPI_ISL_873656, EPI_ISL_873657, EPI_ISL_873660, EPI_ISL_873669, EPI_ISL_873672, EPI_ISL_873704, EPI_ISL_873724, EPI_ISL_873725, EPI_ISL_873726, EPI_ISL_873729, EPI_ISL_873730, EPI_ISL_873734, EPI_ISL_873741, EPI_ISL_873743, EPI_ISL_873744			
see above	Lighthouse Lab in Milton Keynes	Wellcome Sanger Institute for the COVID-19 Genomics UK (COG-UK) Consortium	The Lighthouse Lab in Milton Keynes and Alex Alderton, Roberto Amato, Sonia Goncalves, Ewan Harrison, David K. Jackson, Ian Johnston, Dominic Kwiatkowski, Cordelia Langford, John Sillitoe on behalf of the Wellcome Sanger Institute COVID-19 Surveillance Team
EPI_ISL_873746	Lighthouse Lab in Cambridge	Wellcome Sanger Institute for the COVID-19 Genomics UK (COG-UK) Consortium	Rob Howes, The Lighthouse Lab in Cambridge and Alex Alderton, Roberto Amato, Sonia Goncalves, Ewan Harrison, David K. Jackson, Ian Johnston, Dominic Kwiatkowski, Cordelia Langford, John Sillitoe on behalf of the Wellcome Sanger Institute COVID-19 Surveillance Team
EPI_ISL_873749	Lighthouse Lab in Milton Keynes	Wellcome Sanger Institute for the COVID-19 Genomics UK (COG-UK) Consortium	The Lighthouse Lab in Milton Keynes and Alex Alderton, Roberto Amato, Sonia Goncalves, Ewan Harrison, David K. Jackson, Ian Johnston, Dominic Kwiatkowski, Cordelia Langford, John Sillitoe on behalf of the Wellcome Sanger Institute COVID-19 Surveillance Team
EPI_ISL_873750, EPI_ISL_873752	Lighthouse Lab in Cambridge	Wellcome Sanger Institute for the COVID-19 Genomics UK (COG-UK) Consortium	Rob Howes, The Lighthouse Lab in Cambridge and Alex Alderton, Roberto Amato, Sonia Goncalves, Ewan Harrison, David K. Jackson, Ian Johnston, Dominic Kwiatkowski, Cordelia Langford, John Sillitoe on behalf of the Wellcome Sanger Institute COVID-19 Surveillance Team
EPI_ISL_873760, EPI_ISL_873764, EPI_ISL_873769, EPI_ISL_873786, EPI_ISL_873794, EPI_ISL_873806, EPI_ISL_873809, EPI_ISL_873812, EPI_ISL_873817	Lighthouse Lab in Milton Keynes	Wellcome Sanger Institute for the COVID-19 Genomics UK (COG-UK) Consortium	The Lighthouse Lab in Milton Keynes and Alex Alderton, Roberto Amato, Sonia Goncalves, Ewan Harrison, David K. Jackson, Ian Johnston, Dominic Kwiatkowski, Cordelia Langford, John Sillitoe on behalf of the Wellcome Sanger Institute COVID-19 Surveillance Team
EPI_ISL_873825	Lighthouse Lab in Cambridge	Wellcome Sanger Institute for the COVID-19 Genomics UK (COG-UK) Consortium	Rob Howes, The Lighthouse Lab in Cambridge and Alex Alderton, Roberto Amato, Sonia Goncalves, Ewan Harrison, David K. Jackson, Ian Johnston, Dominic Kwiatkowski, Cordelia Langford, John Sillitoe on behalf of the Wellcome Sanger Institute COVID-19 Surveillance Team
EPI_ISL_873833, EPI_ISL_873834, EPI_ISL_873847, EPI_ISL_873850, EPI_ISL_873857, EPI_ISL_873859, EPI_ISL_873865, EPI_ISL_873870, EPI_ISL_873873, EPI_ISL_873875, EPI_ISL_873880			
see above	Lighthouse Lab in Milton Keynes	Wellcome Sanger Institute for the COVID-19 Genomics UK (COG-UK) Consortium	The Lighthouse Lab in Milton Keynes and Alex Alderton, Roberto Amato, Sonia Goncalves, Ewan Harrison, David K. Jackson, Ian Johnston, Dominic Kwiatkowski, Cordelia Langford, John Sillitoe on behalf of the Wellcome Sanger Institute COVID-19 Surveillance Team
EPI_ISL_873882	Lighthouse Lab in Cambridge	Wellcome Sanger Institute for the COVID-19 Genomics UK (COG-UK) Consortium	Rob Howes, The Lighthouse Lab in Cambridge and Alex Alderton, Roberto Amato, Sonia Goncalves, Ewan Harrison, David K. Jackson, Ian Johnston, Dominic Kwiatkowski, Cordelia Langford, John Sillitoe on behalf of the Wellcome Sanger Institute COVID-19 Surveillance Team
EPI_ISL_873884, EPI_ISL_873887, EPI_ISL_873900, EPI_ISL_873904, EPI_ISL_873905, EPI_ISL_873910, EPI_ISL_873913, EPI_ISL_873916, EPI_ISL_873923, EPI_ISL_873924, EPI_ISL_873925, EPI_ISL_873928, EPI_ISL_873934, EPI_ISL_873935, EPI_ISL_873937, EPI_ISL_873943, EPI_ISL_873947, EPI_ISL_873948, EPI_ISL_873954, EPI_ISL_873960			
see above	Lighthouse Lab in Milton Keynes	Wellcome Sanger Institute for the COVID-19 Genomics UK (COG-UK) Consortium	The Lighthouse Lab in Milton Keynes and Alex Alderton, Roberto Amato, Sonia Goncalves, Ewan Harrison, David K. Jackson, Ian Johnston, Dominic Kwiatkowski, Cordelia Langford, John Sillitoe on behalf of the Wellcome Sanger Institute COVID-19 Surveillance Team
EPI_ISL_873961, EPI_ISL_873962, EPI_ISL_873963, EPI_ISL_873966, EPI_ISL_873967, EPI_ISL_873968, EPI_ISL_873969, EPI_ISL_873970, EPI_ISL_873971, EPI_ISL_873972, EPI_ISL_873973, EPI_ISL_873975, EPI_ISL_873977, EPI_ISL_873978, EPI_ISL_873979, EPI_ISL_873980, EPI_ISL_873981, EPI_ISL_873982, EPI_ISL_873983, EPI_ISL_873985, EPI_ISL_873986, EPI_ISL_873988, EPI_ISL_873989, EPI_ISL_873991, EPI_ISL_873992, EPI_ISL_873993, EPI_ISL_873994, EPI_ISL_873995, EPI_ISL_873996, EPI_ISL_873997, EPI_ISL_873998, EPI_ISL_874000, EPI_ISL_874001, EPI_ISL_874002, EPI_ISL_874003, EPI_ISL_874005, EPI_ISL_874006, EPI_ISL_874008, EPI_ISL_874011, EPI_ISL_874013, EPI_ISL_874015, EPI_ISL_874016, EPI_ISL_874018, EPI_ISL_874019, EPI_ISL_874020, EPI_ISL_874023, EPI_ISL_874024, EPI_ISL_874025, EPI_ISL_874026, EPI_ISL_874027, EPI_ISL_874028, EPI_ISL_874029, EPI_ISL_874030, EPI_ISL_874031, EPI_ISL_874033, EPI_ISL_874034, EPI_ISL_874035, EPI_ISL_874036, EPI_ISL_874040, EPI_ISL_874041, EPI_ISL_874042, EPI_ISL_874043, EPI_ISL_874044, EPI_ISL_874045, EPI_ISL_874046, EPI_ISL_874048, EPI_ISL_874049, EPI_ISL_874050, EPI_ISL_874051, EPI_ISL_874052, EPI_ISL_874053, EPI_ISL_874054, EPI_ISL_874056, EPI_ISL_874057, EPI_ISL_874058, EPI_ISL_874059, EPI_ISL_874060, EPI_ISL_874063, EPI_ISL_874065, EPI_ISL_874066, EPI_ISL_874067, EPI_ISL_874068, EPI_ISL_874069, EPI_ISL_874070, EPI_ISL_874072, EPI_ISL_874073, EPI_ISL_874074, EPI_ISL_874075, EPI_ISL_874077, EPI_ISL_874078, EPI_ISL_874079, EPI_ISL_874080, EPI_ISL_874081, EPI_ISL_874082, EPI_ISL_874083, EPI_ISL_874084, EPI_ISL_874086, EPI_ISL_874087, EPI_ISL_874088, EPI_ISL_874089, EPI_ISL_874091, EPI_ISL_874093, EPI_ISL_874094, EPI_ISL_874095, EPI_ISL_874098, EPI_ISL_874099, EPI_ISL_874100, EPI_ISL_874101, EPI_ISL_874102, EPI_ISL_874103, EPI_ISL_874104, EPI_ISL_874105, EPI_ISL_874106, EPI_ISL_874107, EPI_ISL_874108, EPI_ISL_874109, EPI_ISL_874110, EPI_ISL_874111, EPI_ISL_874112, EPI_ISL_874113, EPI_ISL_874114, EPI_ISL_874116, EPI_ISL_874117, EPI_ISL_874119, EPI_ISL_874120, EPI_ISL_874121, EPI_ISL_874122, EPI_ISL_874123, EPI_ISL_874124, EPI_ISL_874125, EPI_ISL_874126, EPI_ISL_874127, EPI_ISL_874128, EPI_ISL_874129, EPI_ISL_874131, EPI_ISL_874132, EPI_ISL_874133, EPI_ISL_874134, EPI_ISL_874136, EPI_ISL_874137, EPI_ISL_874138, EPI_ISL_874139, EPI_ISL_874140, EPI_ISL_874142, EPI_ISL_874144, EPI_ISL_874145, EPI_ISL_874146, EPI_ISL_874147, EPI_ISL_874149, EPI_ISL_874150, EPI_ISL_874151, EPI_ISL_874152, EPI_ISL_874155, EPI_ISL_874156, EPI_ISL_874158, EPI_ISL_874159, EPI_ISL_874161, EPI_ISL_874162, EPI_ISL_874166, EPI_ISL_874167, EPI_ISL_874168, EPI_ISL_874171, EPI_ISL_874172, EPI_ISL_874173, EPI_ISL_874174, EPI_ISL_874175, EPI_ISL_874176, EPI_ISL_874177, EPI_ISL_874178, EPI_ISL_874179, EPI_ISL_874181, EPI_ISL_874182, EPI_ISL_874183, EPI_ISL_874184, EPI_ISL_874185, EPI_ISL_874186, EPI_ISL_874187, EPI_ISL_874189, EPI_ISL_874190, EPI_ISL_874192, EPI_ISL_874194, EPI_ISL_874196, EPI_ISL_874200, EPI_ISL_874201, EPI_ISL_874203, EPI_ISL_874205, EPI_ISL_874206, EPI_ISL_874208, EPI_ISL_874210, EPI_ISL_874212, EPI_ISL_874213, EPI_ISL_874214, EPI_ISL_874216, EPI_ISL_874217, EPI_ISL_874218, EPI_ISL_874219, EPI_ISL_874220, EPI_ISL_874221, EPI_ISL_874222, EPI_ISL_874223, EPI_ISL_874224, EPI_ISL_874225, EPI_ISL_874226, EPI_ISL_874227, EPI_ISL_874228, EPI_ISL_874229, EPI_ISL_874230, EPI_ISL_874231, EPI_ISL_874232, EPI_ISL_874233, EPI_ISL_874234, EPI_ISL_874235, EPI_ISL_874236, EPI_ISL_874237, EPI_ISL_874239, EPI_ISL_874241, EPI_ISL_874242, EPI_ISL_874243, EPI_ISL_874244, EPI_ISL_874245, EPI_ISL_874247, EPI_ISL_874249, EPI_ISL_874250, EPI_ISL_874251, EPI_ISL_874252, EPI_ISL_874253, EPI_ISL_874255, EPI_ISL_874256, EPI_ISL_874257, EPI_ISL_874259, EPI_ISL_874260, EPI_ISL_874261, EPI_ISL_874262, EPI_ISL_874264, EPI_ISL_874265, EPI_ISL_874267, EPI_ISL_874269, EPI_ISL_874270, EPI_ISL_874271, EPI_ISL_874272, EPI_ISL_874273, EPI_ISL_874274, EPI_ISL_874276, EPI_ISL_874277, EPI_ISL_874279, EPI_ISL_874280, EPI_ISL_874282, EPI_ISL_874284, EPI_ISL_874285, EPI_ISL_874286, EPI_ISL_874287, EPI_ISL_874288, EPI_ISL_874289, EPI_ISL_874291, EPI_ISL_874292, EPI_ISL_874294, EPI_ISL_874295, EPI_ISL_874296, EPI_ISL_874298, EPI_ISL_874299, EPI_ISL_874300, EPI_ISL_874302, EPI_ISL_874303, EPI_ISL_874304, EPI_ISL_874305, EPI_ISL_874306, EPI_ISL_874307, EPI_ISL_874308, EPI_ISL_874309, EPI_ISL_874310, EPI_ISL_874315, EPI_ISL_874379, EPI_ISL_874401, EPI_ISL_874404, EPI_ISL_874443, EPI_ISL_874448, EPI_ISL_874469, EPI_ISL_874482, EPI_ISL_874499, EPI_ISL_874502, EPI_ISL_874523, EPI_ISL_874527, EPI_ISL_874528, EPI_ISL_874530, EPI_ISL_874543, EPI_ISL_874558, EPI_ISL_874624, EPI_ISL_874759, EPI_ISL_874816, EPI_ISL_874853, EPI_ISL_874938, EPI_ISL_874956, EPI_ISL_874966			
see above	Lighthouse Lab in Alderley Park	Wellcome Sanger Institute for the COVID-19 Genomics UK (COG-UK) Consortium	Jacquelyn Wynn, Mairead Hyland, The Lighthouse Lab in Alderley Park and Alex Alderton, Roberto Amato, Sonia Goncalves, Ewan Harrison, David K. Jackson, Ian Johnston, Dominic Kwiatkowski, Cordelia Langford, John Sillitoe on behalf of the Wellcome Sanger Institute COVID-19 Surveillance Team
EPI_ISL_874990, EPI_ISL_874991, EPI_ISL_874992, EPI_ISL_874993, EPI_ISL_874994, EPI_ISL_874995, EPI_ISL_874996, EPI_ISL_874997, EPI_ISL_874998, EPI_ISL_874999, EPI_ISL_875000, EPI_ISL_875001, EPI_ISL_875002, EPI_ISL_875003, EPI_ISL_875004, EPI_ISL_875005, EPI_ISL_875006, EPI_ISL_875007, EPI_ISL_875008, EPI_ISL_875009, EPI_ISL_875010, EPI_ISL_875011, EPI_ISL_875012, EPI_ISL_875013, EPI_ISL_875014, EPI_ISL_875015, EPI_ISL_875016, EPI_ISL_875017, EPI_ISL_875018, EPI_ISL_875019, EPI_ISL_875020, EPI_ISL_875021, EPI_ISL_875022, EPI_ISL_875023, EPI_ISL_875024, EPI_ISL_875025, EPI_ISL_875026, EPI_ISL_875027, EPI_ISL_875028, EPI_ISL_875029, EPI_ISL_875030, EPI_ISL_875031, EPI_ISL_875032, EPI_ISL_875033, EPI_ISL_875034, EPI_ISL_875035, EPI_ISL_875036, EPI_ISL_875037, EPI_ISL_875038, EPI_ISL_875039, EPI_ISL_875040, EPI_ISL_875041, EPI_ISL_875042, EPI_ISL_875043, EPI_ISL_875044, EPI_ISL_875045, EPI_ISL_875046, EPI_ISL_875047, EPI_ISL_875048, EPI_ISL_875049, EPI_ISL_875050, EPI_ISL_875051, EPI_ISL_875052, EPI_ISL_875053, EPI_ISL_875054, EPI_ISL_875055, EPI_ISL_875056, EPI_ISL_875057, EPI_ISL_875058, EPI_ISL_875059, EPI_ISL_875060, EPI_ISL_875061, EPI_ISL_875062, EPI_ISL_875063, EPI_ISL_875064, EPI_ISL_875065, EPI_ISL_875066, EPI_ISL_875067, EPI_ISL_875068, EPI_ISL_875069, EPI_ISL_875070, EPI_ISL_875071, EPI_ISL_875072, EPI_ISL_875073, EPI_ISL_875074, EPI_ISL_875075, EPI_ISL_875076, EPI_ISL_875077, EPI_ISL_875078, EPI_ISL_875079, EPI_ISL_875080, EPI_ISL_875081, EPI_ISL_875082, EPI_ISL_875083, EPI_ISL_875084, EPI_ISL_875085, EPI_ISL_875086, EPI_ISL_875087, EPI_ISL_875088, EPI_ISL_875089, EPI_ISL_875090, EPI_ISL_875091, EPI_ISL_875092, EPI_ISL_875093, EPI_ISL_875094, EPI_ISL_875095, EPI_ISL_875096, EPI_ISL_875097, EPI_ISL_875098, EPI_ISL_875099, EPI_ISL_875100, EPI_ISL_875101, EPI_ISL_875102, EPI_ISL_875103, EPI_ISL_875104, EPI_ISL_875105, EPI_ISL_875106, EPI_ISL_875107, EPI_ISL_875108, EPI_ISL_875109, EPI_ISL_875110, EPI_ISL_875111, EPI_ISL_875112, EPI_ISL_875113, EPI_ISL_875114, EPI_ISL_875115, EPI_ISL_875116, EPI_ISL_875117, EPI_ISL_875118, EPI_ISL_875119, EPI_ISL_875120, EPI_ISL_875121, EPI_ISL_875122, EPI_ISL_875123, EPI_ISL_875124, EPI_ISL_875125, EPI_ISL_875126, EPI_ISL_875127, EPI_ISL_875128, EPI_ISL_875129, EPI_ISL_875130, EPI_ISL_875131, EPI_ISL_875132, EPI_ISL_875133, EPI_ISL_875134, EPI_ISL_875135, EPI_ISL_875136, EPI_ISL_875137, EPI_ISL_875138, EPI_ISL_875139, EPI_ISL_875140, EPI_ISL_875141, EPI_ISL_875142, EPI_ISL_875143, EPI_ISL_875144, EPI_ISL_875145, EPI_ISL_875146, EPI_ISL_875147, EPI_ISL_875148, EPI_ISL_875149, EPI_ISL_875150, EPI_ISL_875151, EPI_ISL_875152, EPI_ISL_875153, EPI_ISL_875154, EPI_ISL_875155, EPI_ISL_875156, EPI_ISL_875157, EPI_ISL_875158, EPI_ISL_875159, EPI_ISL_875160, EPI_ISL_875161, EPI_ISL_875162, EPI_ISL_875163, EPI_ISL_875164, EPI_ISL_875165, EPI_ISL_875166, EPI_ISL_875167, EPI_ISL_875168, EPI_ISL_875169, EPI_ISL_875170, EPI_ISL_875171, EPI_ISL_875172, EPI_ISL_875173, EPI_ISL_875174, EPI_ISL_875175, EPI_ISL_875176, EPI_ISL_875177, EPI_ISL_875178, EPI_ISL_875179, EPI_ISL_875180, EPI_ISL_875181, EPI_ISL_875182, EPI_ISL_875183, EPI_ISL_875184, EPI_ISL_875185, EPI_ISL_875186, EPI_ISL_875187, EPI_ISL_875188, EPI_ISL_875189, EPI_ISL_875190, EPI_ISL_875191, EPI_ISL_875192, EPI_ISL_875193, EPI_ISL_875194, EPI_ISL_875195, EPI_ISL_875196, EPI_ISL_875197, EPI_ISL_875198, EPI_ISL_875199, EPI_ISL_875200, EPI_ISL_875201, EPI_ISL_875202, EPI_ISL_875203, EPI_ISL_875204, EPI_ISL_875205, EPI_ISL_875206, EPI_ISL_875207, EPI_ISL_875208, EPI_ISL_875209, EPI_ISL_875210, EPI_ISL_875211, EPI_ISL_875212, EPI_ISL_875213, EPI_ISL_875214, EPI_ISL_875215, EPI_ISL_875216, EPI_ISL_875217, EPI_ISL_875218, EPI_ISL_875219, EPI_ISL_875220, EPI_ISL_875221, EPI_ISL_875222, EPI_ISL_875223, EPI_ISL_875224, EPI_ISL_875225, EPI_ISL_875226, EPI_ISL_875227, EPI_ISL_875228, EPI_ISL_875229, EPI_ISL_875230, EPI_ISL_875231, EPI_ISL_875232, EPI_ISL_875233, EPI_ISL_875234, EPI_ISL_875235, EPI_ISL_875236, EPI_ISL_875237, EPI_ISL_875238, EPI_ISL_875239, EPI_ISL_875240, EPI_ISL_875241, EPI_ISL_875242, EPI_ISL_875243, EPI_ISL_875244, EPI_ISL_875245, EPI_ISL_875246, EPI_ISL_875247, EPI_ISL_875248, EPI_ISL_875249, EPI_ISL_875250, EPI_ISL_875251, EPI_ISL_875252, EPI_ISL_875253, EPI_ISL_875254, EPI_ISL_875255, EPI_ISL_875256, EPI_ISL_875257, EPI_ISL_875258, EPI_ISL_875259, EPI_ISL_875260, EPI_ISL_875261, EPI_ISL_875262, EPI_ISL_875263, EPI_ISL_875264, EPI_ISL_875265, EPI_ISL_875266, EPI_ISL_875267, EPI_ISL_875268, EPI_ISL_875269, EPI_ISL_875270, EPI_ISL_875271, EPI_ISL_875272, EPI_ISL_875273, EPI_ISL_875274, EPI_ISL_875275, EPI_ISL_875276, EPI_ISL_875277, EPI_ISL_875278, EPI_ISL_875279, EPI_ISL_875280, EPI_ISL_875281, EPI_ISL_875282, EPI_ISL_875283, EPI_ISL_875284, EPI_ISL_875285, EPI_ISL_875286, EPI_ISL_875287, EPI_ISL_875288, EPI_ISL_875289, EPI_ISL_875290, EPI_ISL_875291, EPI_ISL_875292, EPI_ISL_875293, EPI_ISL_875294, EPI_ISL_875295, EPI_ISL_875296, EPI_ISL_875297, EPI_ISL_875298, EPI_ISL_875299, EPI_ISL_875300, EPI_ISL_875301, EPI_ISL_875302, EPI_ISL_875303, EPI_ISL_875304, EPI_ISL_875305, EPI_ISL_875306, EPI_ISL_875307, EPI_ISL_875308, EPI_ISL_875309, EPI_ISL_875310, EPI_ISL_875311, EPI_ISL_875312, EPI_ISL_875313, EPI_ISL_875314, EPI_ISL_875315, EPI_ISL_875316, EPI_ISL_875317, EPI_ISL_875318, EPI_ISL_875319, EPI_ISL_875320, EPI_ISL_875321, EPI_ISL_875322, EPI_ISL_875323, EPI_ISL_875324, EPI_ISL_875325, EPI_ISL_875326, EPI_ISL_875327, EPI_ISL_875328, EPI_ISL_875329, EPI_ISL_875330, EPI_ISL_875331, EPI_ISL_875332, EPI_ISL_875333, EPI_ISL_875334, EPI_ISL_875335, EPI_ISL_875336, EPI_ISL_875337, EPI_ISL_875338, EPI_ISL_875339, EPI_ISL_875340, EPI_ISL_875341			
see above	Lighthouse Lab in Milton Keynes	Wellcome Sanger Institute for the COVID-19 Genomics UK (COG-UK) Consortium	The Lighthouse Lab in Milton Keynes and Alex Alderton, Roberto Amato, Sonia Goncalves, Ewan Harrison, David K. Jackson, Ian Johnston, Dominic Kwiatkowski, Cordelia Langford, John Sillitoe on behalf of the Wellcome Sanger Institute COVID-19 Surveillance Team
EPI_ISL_877913, EPI_ISL_877955	Lighthouse Lab in Alderley Park	Wellcome Sanger Institute for the COVID-19 Genomics UK (COG-UK) Consortium	Jacquelyn Wynn, Mairead Hyland, The Lighthouse Lab in Alderley Park and Alex Alderton, Roberto Amato, Sonia Goncalves, Ewan Harrison, David K. Jackson, Ian Johnston, Dominic Kwiatkowski, Cordelia Langford, John Sillitoe on behalf of the Wellcome Sanger Institute COVID-19 Surveillance Team
EPI_ISL_878769, EPI_ISL_878833	Lighthouse Lab in Milton Keynes	Wellcome Sanger Institute for the COVID-19 Genomics UK (COG-UK) Consortium	The Lighthouse Lab in Milton Keynes and Alex Alderton, Roberto Amato, Sonia Goncalves, Ewan Harrison, David K. Jackson, Ian Johnston, Dominic Kwiatkowski, Cordelia Langford, John Sillitoe on behalf of the Wellcome Sanger Institute COVID-19 Surveillance Team
EPI_ISL_878844, EPI_ISL_879274	Lighthouse Lab in Alderley Park	Wellcome Sanger Institute for the COVID-19 Genomics UK (COG-UK) Consortium	Jacquelyn Wynn, Mairead Hyland, The Lighthouse Lab in Alderley Park and Alex Alderton, Roberto Amato, Sonia Goncalves, Ewan Harrison, David K. Jackson, Ian Johnston, Dominic Kwiatkowski, Cordelia Langford, John Sillitoe on behalf of the Wellcome Sanger Institute COVID-19 Surveillance Team
EPI_ISL_879820, EPI_ISL_880038	Lighthouse Lab in Cambridge	Wellcome Sanger Institute for the COVID-19 Genomics UK (COG-UK) Consortium	Rob Howes, The Lighthouse Lab in Cambridge and Alex Alderton, Roberto Amato, Sonia Goncalves, Ewan Harrison, David K. Jackson, Ian Johnston, Dominic Kwiatkowski, Cordelia Langford, John Sillitoe on behalf of the Wellcome Sanger Institute COVID-19 Surveillance Team
EPI_ISL_880586, EPI_ISL_880590, EPI_ISL_880591, EPI_ISL_880595, EPI_ISL_880596, EPI_ISL_880599, EPI_ISL_880601, EPI_ISL_880602, EPI_ISL_880606, EPI_ISL_880608, EPI_ISL_880609, EPI_ISL_880610, EPI_ISL_880611, EPI_ISL_880613, EPI_ISL_880615, EPI_ISL_880616, EPI_ISL_880618, EPI_ISL_880619, EPI_ISL_880620, EPI_ISL_880623, EPI_ISL_880626, EPI_ISL_880627, EPI_ISL_880629, EPI_ISL_880637, EPI_ISL_880639, EPI_ISL_880641, EPI_ISL_880642, EPI_ISL_880647, EPI_ISL_880651, EPI_ISL_880653, EPI_ISL_880654, EPI_ISL_880655, EPI_ISL_880657, EPI_ISL_880660, EPI_ISL_880662, EPI_ISL_880665,			

EPI_ISL_880667, EPI_ISL_880668, EPI_ISL_880669, EPI_ISL_880672, EPI_ISL_880673, EPI_ISL_880674, EPI_ISL_880675, EPI_ISL_880677, EPI_ISL_880679, EPI_ISL_880682, EPI_ISL_880695, EPI_ISL_880701, EPI_ISL_880705, EPI_ISL_880706, EPI_ISL_880708, EPI_ISL_880709, EPI_ISL_880710, EPI_ISL_880713, EPI_ISL_880717, EPI_ISL_880718, EPI_ISL_880721, EPI_ISL_880722, EPI_ISL_880724, EPI_ISL_880725, EPI_ISL_880726, EPI_ISL_880727, EPI_ISL_880729, EPI_ISL_880736, EPI_ISL_880737, EPI_ISL_880738, EPI_ISL_880739, EPI_ISL_880740, EPI_ISL_880745, EPI_ISL_880746, EPI_ISL_880751, EPI_ISL_880752, EPI_ISL_880753, EPI_ISL_880755, EPI_ISL_880759, EPI_ISL_880762, EPI_ISL_880763, EPI_ISL_880766, EPI_ISL_880769, EPI_ISL_880774, EPI_ISL_880775, EPI_ISL_880776, EPI_ISL_880779, EPI_ISL_880781, EPI_ISL_880785, EPI_ISL_880786, EPI_ISL_880789, EPI_ISL_880791, EPI_ISL_880792, EPI_ISL_880797, EPI_ISL_880798, EPI_ISL_880799, EPI_ISL_880800, EPI_ISL_880803, EPI_ISL_880809, EPI_ISL_880812, EPI_ISL_880813, EPI_ISL_880817, EPI_ISL_880818, EPI_ISL_880819, EPI_ISL_880822, EPI_ISL_880824, EPI_ISL_880826, EPI_ISL_880829, EPI_ISL_880830, EPI_ISL_880831, EPI_ISL_880833, EPI_ISL_880835, EPI_ISL_880836, EPI_ISL_880838, EPI_ISL_880839, EPI_ISL_880840, EPI_ISL_880841, EPI_ISL_880843, EPI_ISL_880847, EPI_ISL_880853, EPI_ISL_880854, EPI_ISL_880856, EPI_ISL_880857, EPI_ISL_880858, EPI_ISL_880862, EPI_ISL_880866, EPI_ISL_880868, EPI_ISL_880870, EPI_ISL_880878, EPI_ISL_880879, EPI_ISL_880883, EPI_ISL_880884, EPI_ISL_880886, EPI_ISL_880889, EPI_ISL_880894, EPI_ISL_880895, EPI_ISL_880898, EPI_ISL_880900, EPI_ISL_880901, EPI_ISL_880903, EPI_ISL_880908, EPI_ISL_880909, EPI_ISL_880910, EPI_ISL_880911, EPI_ISL_880914, EPI_ISL_880915, EPI_ISL_880916, EPI_ISL_880921, EPI_ISL_880925, EPI_ISL_880933, EPI_ISL_880934, EPI_ISL_880935			
see above	Lighthouse Lab in Milton Keynes	Wellcome Sanger Institute for the COVID-19 Genomics UK (COG-UK) Consortium	The Lighthouse Lab in Milton Keynes and Alex Alderton, Roberto Amato, Sonia Goncalves, Ewan Harrison, David K. Jackson, Ian Johnston, Dominic Kwiatkowski, Cordelia Langford, John Sillitoe on behalf of the Wellcome Sanger Institute COVID-19 Surveillance Team
EPI_ISL_881277, EPI_ISL_881281, EPI_ISL_881283, EPI_ISL_881289, EPI_ISL_881290, EPI_ISL_881291, EPI_ISL_881293, EPI_ISL_881298, EPI_ISL_881306, EPI_ISL_881307, EPI_ISL_881309, EPI_ISL_881310, EPI_ISL_881315, EPI_ISL_881318, EPI_ISL_881326, EPI_ISL_881341, EPI_ISL_881359, EPI_ISL_881386, EPI_ISL_881388, EPI_ISL_881414, EPI_ISL_881429, EPI_ISL_881455, EPI_ISL_881461, EPI_ISL_881487, EPI_ISL_881494, EPI_ISL_881495, EPI_ISL_881509, EPI_ISL_881518, EPI_ISL_881521, EPI_ISL_881522, EPI_ISL_881531, EPI_ISL_881540, EPI_ISL_881543, EPI_ISL_881546, EPI_ISL_881550, EPI_ISL_881567, EPI_ISL_881570, EPI_ISL_881571, EPI_ISL_881576, EPI_ISL_881580, EPI_ISL_881582, EPI_ISL_881586, EPI_ISL_881588, EPI_ISL_881598, EPI_ISL_881600, EPI_ISL_881607, EPI_ISL_881612			
see above	Lighthouse Lab in Alderley Park	Wellcome Sanger Institute for the COVID-19 Genomics UK (COG-UK) Consortium	Jacquelyn Wynn, Mairead Hyland, The Lighthouse Lab in Alderley Park and Alex Alderton, Roberto Amato, Sonia Goncalves, Ewan Harrison, David K. Jackson, Ian Johnston, Dominic Kwiatkowski, Cordelia Langford, John Sillitoe on behalf of the Wellcome Sanger Institute COVID-19 Surveillance Team
EPI_ISL_881618	Lighthouse Lab in Milton Keynes	Wellcome Sanger Institute for the COVID-19 Genomics UK (COG-UK) Consortium	The Lighthouse Lab in Milton Keynes and Alex Alderton, Roberto Amato, Sonia Goncalves, Ewan Harrison, David K. Jackson, Ian Johnston, Dominic Kwiatkowski, Cordelia Langford, John Sillitoe on behalf of the Wellcome Sanger Institute COVID-19 Surveillance Team
EPI_ISL_881619	Lighthouse Lab in Cambridge	Wellcome Sanger Institute for the COVID-19 Genomics UK (COG-UK) Consortium	Rob Howes, The Lighthouse Lab in Cambridge and Alex Alderton, Roberto Amato, Sonia Goncalves, Ewan Harrison, David K. Jackson, Ian Johnston, Dominic Kwiatkowski, Cordelia Langford, John Sillitoe on behalf of the Wellcome Sanger Institute COVID-19 Surveillance Team
EPI_ISL_881621	Lighthouse Lab in Milton Keynes	Wellcome Sanger Institute for the COVID-19 Genomics UK (COG-UK) Consortium	The Lighthouse Lab in Milton Keynes and Alex Alderton, Roberto Amato, Sonia Goncalves, Ewan Harrison, David K. Jackson, Ian Johnston, Dominic Kwiatkowski, Cordelia Langford, John Sillitoe on behalf of the Wellcome Sanger Institute COVID-19 Surveillance Team
EPI_ISL_881623, EPI_ISL_881624, EPI_ISL_881625, EPI_ISL_881626, EPI_ISL_881627, EPI_ISL_881628, EPI_ISL_881629, EPI_ISL_881630, EPI_ISL_881631, EPI_ISL_881632, EPI_ISL_881633, EPI_ISL_881634, EPI_ISL_881635, EPI_ISL_881636, EPI_ISL_881637, EPI_ISL_881638, EPI_ISL_881639, EPI_ISL_881640, EPI_ISL_881641, EPI_ISL_881642, EPI_ISL_881643, EPI_ISL_881644, EPI_ISL_881645, EPI_ISL_881646, EPI_ISL_881647, EPI_ISL_881648, EPI_ISL_881649, EPI_ISL_881650, EPI_ISL_881651, EPI_ISL_881652, EPI_ISL_881653, EPI_ISL_881654, EPI_ISL_881655, EPI_ISL_881656, EPI_ISL_881657, EPI_ISL_881658, EPI_ISL_881659, EPI_ISL_881660, EPI_ISL_881661, EPI_ISL_881662, EPI_ISL_881663, EPI_ISL_881664, EPI_ISL_881665, EPI_ISL_881666, EPI_ISL_881667, EPI_ISL_881669, EPI_ISL_881670, EPI_ISL_881671, EPI_ISL_881672, EPI_ISL_881673, EPI_ISL_881674, EPI_ISL_881675, EPI_ISL_881676, EPI_ISL_881677, EPI_ISL_881678, EPI_ISL_881679, EPI_ISL_881680, EPI_ISL_881681, EPI_ISL_881682, EPI_ISL_881683, EPI_ISL_881684, EPI_ISL_881685, EPI_ISL_881686, EPI_ISL_881687, EPI_ISL_881688, EPI_ISL_881689, EPI_ISL_881690, EPI_ISL_881691, EPI_ISL_881692, EPI_ISL_881693, EPI_ISL_881694, EPI_ISL_881695, EPI_ISL_881696, EPI_ISL_881697, EPI_ISL_881698, EPI_ISL_881699, EPI_ISL_881700, EPI_ISL_881701, EPI_ISL_881702, EPI_ISL_881703, EPI_ISL_881704, EPI_ISL_881705, EPI_ISL_881706, EPI_ISL_881707, EPI_ISL_881708, EPI_ISL_881709, EPI_ISL_881710, EPI_ISL_881711, EPI_ISL_881712, EPI_ISL_881713, EPI_ISL_881714, EPI_ISL_881715, EPI_ISL_881716, EPI_ISL_881717, EPI_ISL_881718, EPI_ISL_881719, EPI_ISL_881720, EPI_ISL_881721, EPI_ISL_881722, EPI_ISL_881723, EPI_ISL_881724, EPI_ISL_881725, EPI_ISL_881726, EPI_ISL_881727, EPI_ISL_881728, EPI_ISL_881729, EPI_ISL_881730, EPI_ISL_881731, EPI_ISL_881732, EPI_ISL_881733, EPI_ISL_881734, EPI_ISL_881735, EPI_ISL_881736, EPI_ISL_881737, EPI_ISL_881738, EPI_ISL_881739, EPI_ISL_881740, EPI_ISL_881741, EPI_ISL_881742, EPI_ISL_881743, EPI_ISL_881744, EPI_ISL_881745, EPI_ISL_881746, EPI_ISL_881747, EPI_ISL_881748, EPI_ISL_881749, EPI_ISL_881750, EPI_ISL_881751, EPI_ISL_881752, EPI_ISL_881753, EPI_ISL_881754, EPI_ISL_881755, EPI_ISL_881756, EPI_ISL_881757, EPI_ISL_881758, EPI_ISL_881759, EPI_ISL_881760, EPI_ISL_881761, EPI_ISL_881762, EPI_ISL_881763, EPI_ISL_881764, EPI_ISL_881765, EPI_ISL_881766, EPI_ISL_881767, EPI_ISL_881768, EPI_ISL_881769, EPI_ISL_881770, EPI_ISL_881771, EPI_ISL_881772, EPI_ISL_881773, EPI_ISL_881774, EPI_ISL_881775, EPI_ISL_881776, EPI_ISL_881777, EPI_ISL_881778, EPI_ISL_881779, EPI_ISL_881780, EPI_ISL_881781, EPI_ISL_881782, EPI_ISL_881783, EPI_ISL_881784, EPI_ISL_881785, EPI_ISL_881786, EPI_ISL_881787, EPI_ISL_881788, EPI_ISL_881789, EPI_ISL_881790, EPI_ISL_881791, EPI_ISL_881792, EPI_ISL_881793, EPI_ISL_881794, EPI_ISL_881795, EPI_ISL_881796, EPI_ISL_881797, EPI_ISL_881798, EPI_ISL_881799, EPI_ISL_881800, EPI_ISL_881801, EPI_ISL_881802, EPI_ISL_881803, EPI_ISL_881804, EPI_ISL_881805, EPI_ISL_881806, EPI_ISL_881807, EPI_ISL_881808, EPI_ISL_881809, EPI_ISL_881810, EPI_ISL_881811, EPI_ISL_881812, EPI_ISL_881813, EPI_ISL_881814, EPI_ISL_881815, EPI_ISL_881816, EPI_ISL_881817, EPI_ISL_881818, EPI_ISL_881819, EPI_ISL_881820, EPI_ISL_881821, EPI_ISL_881822, EPI_ISL_881823, EPI_ISL_881824, EPI_ISL_881825, EPI_ISL_881826, EPI_ISL_881827, EPI_ISL_881828, EPI_ISL_881829, EPI_ISL_881830, EPI_ISL_881831, EPI_ISL_881832, EPI_ISL_881833, EPI_ISL_881834, EPI_ISL_881835, EPI_ISL_881836, EPI_ISL_881837, EPI_ISL_881838, EPI_ISL_881839, EPI_ISL_881840, EPI_ISL_881841, EPI_ISL_881842, EPI_ISL_881843, EPI_ISL_881844, EPI_ISL_881845, EPI_ISL_881846, EPI_ISL_881847, EPI_ISL_881848, EPI_ISL_881849, EPI_ISL_881850, EPI_ISL_881851, EPI_ISL_881852, EPI_ISL_881853, EPI_ISL_881854, EPI_ISL_881855, EPI_ISL_881856, EPI_ISL_881857, EPI_ISL_881858, EPI_ISL_881859, EPI_ISL_881860, EPI_ISL_881861, EPI_ISL_881862, EPI_ISL_881863, EPI_ISL_881864, EPI_ISL_881865, EPI_ISL_881866, EPI_ISL_881867, EPI_ISL_881868, EPI_ISL_881869, EPI_ISL_881870, EPI_ISL_881871, EPI_ISL_881872, EPI_ISL_881873, EPI_ISL_881874, EPI_ISL_881875, EPI_ISL_881876, EPI_ISL_881877, EPI_ISL_881878, EPI_ISL_881879, EPI_ISL_881880, EPI_ISL_881881, EPI_ISL_881882, EPI_ISL_881883, EPI_ISL_881884, EPI_ISL_881885, EPI_ISL_881886, EPI_ISL_881887, EPI_ISL_881888, EPI_ISL_881889, EPI_ISL_881890, EPI_ISL_881891, EPI_ISL_881892, EPI_ISL_881893, EPI_ISL_881894, EPI_ISL_881895, EPI_ISL_881896, EPI_ISL_881897, EPI_ISL_881898, EPI_ISL_881899, EPI_ISL_881900, EPI_ISL_881901, EPI_ISL_881902, EPI_ISL_881903, EPI_ISL_881904, EPI_ISL_881905, EPI_ISL_881906, EPI_ISL_881907, EPI_ISL_881908, EPI_ISL_881909, EPI_ISL_881910, EPI_ISL_881911, EPI_ISL_881912, EPI_ISL_881913, EPI_ISL_881914, EPI_ISL_881915, EPI_ISL_881916, EPI_ISL_881917, EPI_ISL_881918, EPI_ISL_881919, EPI_ISL_881920, EPI_ISL_881921, EPI_ISL_881922, EPI_ISL_881923, EPI_ISL_881924, EPI_ISL_881925, EPI_ISL_881926, EPI_ISL_881927, EPI_ISL_881928, EPI_ISL_881929, EPI_ISL_881930, EPI_ISL_881931, EPI_ISL_881932, EPI_ISL_881933, EPI_ISL_881934, EPI_ISL_881935, EPI_ISL_881936, EPI_ISL_881937, EPI_ISL_881938, EPI_ISL_881939, EPI_ISL_881940, EPI_ISL_881941, EPI_ISL_881942, EPI_ISL_881943, EPI_ISL_881944, EPI_ISL_881945, EPI_ISL_881946, EPI_ISL_881947, EPI_ISL_881948, EPI_ISL_881949, EPI_ISL_881950, EPI_ISL_881951, EPI_ISL_881952, EPI_ISL_881953, EPI_ISL_881954, EPI_ISL_881955, EPI_ISL_881956, EPI_ISL_881957, EPI_ISL_881958, EPI_ISL_881959, EPI_ISL_881960, EPI_ISL_881961, EPI_ISL_881962, EPI_ISL_881963, EPI_ISL_881964, EPI_ISL_881965, EPI_ISL_881966, EPI_ISL_881967, EPI_ISL_881968, EPI_ISL_881969, EPI_ISL_881970, EPI_ISL_881971, EPI_ISL_881972, EPI_ISL_881973, EPI_ISL_881974, EPI_ISL_881975, EPI_ISL_881976, EPI_ISL_881977, EPI_ISL_881978, EPI_ISL_881979, EPI_ISL_881980, EPI_ISL_881981, EPI_ISL_881982, EPI_ISL_881983, EPI_ISL_881984, EPI_ISL_881985, EPI_ISL_881986, EPI_ISL_881987, EPI_ISL_881988, EPI_ISL_881989, EPI_ISL_881990, EPI_ISL_881991, EPI_ISL_881992, EPI_ISL_881993, EPI_ISL_881994, EPI_ISL_881995, EPI_ISL_881996, EPI_ISL_881997, EPI_ISL_881998, EPI_ISL_881999, EPI_ISL_882000, EPI_ISL_882001, EPI_ISL_882002, EPI_ISL_882003, EPI_ISL_882004, EPI_ISL_882005, EPI_ISL_882006, EPI_ISL_882007, EPI_ISL_882008, EPI_ISL_882009, EPI_ISL_882010, EPI_ISL_882011, EPI_ISL_882012, EPI_ISL_882013, EPI_ISL_882014, EPI_ISL_882015, EPI_ISL_882016, EPI_ISL_882017, EPI_ISL_882018, EPI_ISL_882019, EPI_ISL_882020, EPI_ISL_882021, EPI_ISL_882022, EPI_ISL_882023, EPI_ISL_882024, EPI_ISL_882025, EPI_ISL_882026, EPI_ISL_882027, EPI_ISL_882028, EPI_ISL_882029, EPI_ISL_882030, EPI_ISL_882031, EPI_ISL_882032, EPI_ISL_882033, EPI_ISL_882034, EPI_ISL_882035, EPI_ISL_882036, EPI_ISL_882037, EPI_ISL_882038, EPI_ISL_882039, EPI_ISL_882040, EPI_ISL_882041, EPI_ISL_882042, EPI_ISL_882043, EPI_ISL_882044, EPI_ISL_882045, EPI_ISL_882046, EPI_ISL_882047, EPI_ISL_882048, EPI_ISL_882049, EPI_ISL_882050, EPI_ISL_882051, EPI_ISL_882052, EPI_ISL_882053, EPI_ISL_882054, EPI_ISL_882055, EPI_ISL_882056, EPI_ISL_882057, EPI_ISL_882058, EPI_ISL_882059, EPI_ISL_882060, EPI_ISL_882061, EPI_ISL_882062, EPI_ISL_882063, EPI_ISL_882064, EPI_ISL_882065, EPI_ISL_882066, EPI_ISL_882067, EPI_ISL_882068, EPI_ISL_882069, EPI_ISL_882070, EPI_ISL_882071, EPI_ISL_882072, EPI_ISL_882073, EPI_ISL_882074, EPI_ISL_882075, EPI_ISL_882076, EPI_ISL_882077, EPI_ISL_882078, EPI_ISL_882079, EPI_ISL_882080, EPI_ISL_882081, EPI_ISL_882082, EPI_ISL_882083, EPI_ISL_882084, EPI_ISL_882085, EPI_ISL_882086, EPI_ISL_882087, EPI_ISL_882088, EPI_ISL_882089, EPI_ISL_882090, EPI_ISL_882091, EPI_ISL_882092, EPI_ISL_882093, EPI_ISL_882094, EPI_ISL_882095, EPI_ISL_882096, EPI_ISL_882097, EPI_ISL_882098, EPI_ISL_882099, EPI_ISL_882100, EPI_ISL_882101, EPI_ISL_882102, EPI_ISL_882103, EPI_ISL_882104, EPI_ISL_882105, EPI_ISL_882106, EPI_ISL_882107, EPI_ISL_882108, EPI_ISL_882109, EPI_ISL_882110, EPI_ISL_882111, EPI_ISL_882112, EPI_ISL_882113, EPI_ISL_882114, EPI_ISL_882115, EPI_ISL_882116, EPI_ISL_882117, EPI_ISL_882118, EPI_ISL_882119, EPI_ISL_882120, EPI_ISL_882121, EPI_ISL_882122, EPI_ISL_882123, EPI_ISL_882124, EPI_ISL_882125, EPI_ISL_882126, EPI_ISL_882127, EPI_ISL_882128, EPI_ISL_882129, EPI_ISL_882130, EPI_ISL_882131, EPI_ISL_882132, EPI_ISL_882133, EPI_ISL_882134, EPI_ISL_882135, EPI_ISL_882136, EPI_ISL_882137, EPI_ISL_882138, EPI_ISL_882139, EPI_ISL_882140, EPI_ISL_882141, EPI_ISL_882142, EPI_ISL_882143, EPI_ISL_882144, EPI_ISL_882145, EPI_ISL_882146, EPI_ISL_882147, EPI_ISL_882148, EPI_ISL_882149, EPI_ISL_882150, EPI_ISL_882151, EPI_ISL_882152, EPI_ISL_882153, EPI_ISL_882154, EPI_ISL_882155, EPI_ISL_882156, EPI_ISL_882157, EPI_ISL_882158, EPI_ISL_882159, EPI_ISL_882160, EPI_ISL_882161, EPI_ISL_882162, EPI_ISL_882163, EPI_ISL_882164, EPI_ISL_882165, EPI_ISL_882166, EPI_ISL_882167, EPI_ISL_882168, EPI_ISL_882169, EPI_ISL_882170, EPI_ISL_882171, EPI_ISL_882172, EPI_ISL_882173, EPI_ISL_882174, EPI_ISL_882175, EPI_ISL_882176, EPI_ISL_882177, EPI_ISL_882178, EPI_ISL_882179, EPI_ISL_882180, EPI_ISL_882181, EPI_ISL_882182, EPI_ISL_882183, EPI_ISL_882184, EPI_ISL_882185, EPI_ISL_882186, EPI_ISL_882187, EPI_ISL_882188, EPI_ISL_882189, EPI_ISL_882190, EPI_ISL_882191, EPI_ISL_882192, EPI_ISL_882193, EPI_ISL_882194, EPI_ISL_882195, EPI_ISL_882196, EPI_ISL_882197, EPI_ISL_882198, EPI_ISL_882199, EPI_ISL_882200, EPI_ISL_882201, EPI_ISL_882202, EPI_ISL_882203, EPI_ISL_882204, EPI_ISL_882205, EPI_ISL_882206, EPI_ISL_882207, EPI_ISL_882208, EPI_ISL_882209, EPI_ISL_882210, EPI_ISL_882211, EPI_ISL_882212, EPI_ISL_882213, EPI_ISL_882214, EPI_ISL_882215, EPI_ISL_882216, EPI_ISL_882217, EPI_ISL_882218, EPI_ISL_882219, EPI_ISL_882220, EPI_ISL_882221, EPI_ISL_882222, EPI_ISL_882223, EPI_ISL_882224, EPI_ISL_882225, EPI_ISL_882226, EPI_ISL_882227, EPI_ISL_882228, EPI_ISL_882229, EPI_ISL_882230, EPI_ISL_882231, EPI_ISL_882232, EPI_ISL_882233, EPI_ISL_882234, EPI_ISL_882235, EPI_ISL_882236, EPI_ISL_882237, EPI_ISL_882238, EPI_ISL_882239, EPI_ISL_882240, EPI_ISL_882241, EPI_ISL_882242, EPI_ISL_882243, EPI_ISL_882244, EPI_ISL_882245, EPI_ISL_882246, EPI_ISL_882247, EPI_ISL_882248, EPI_ISL_882249, EPI_ISL_882250, EPI_ISL_882251, EPI_ISL_882252, EPI_ISL_882253, EPI_ISL_882254, EPI_ISL_882255, EPI_ISL_882256, EPI_ISL_882257, EPI_ISL_882258, EPI_ISL_882259, EPI_ISL_882260, EPI_ISL_882261, EPI_ISL_882262, EPI_ISL_882263, EPI_ISL_882264, EPI_ISL_882265, EPI_ISL_882266, EPI_ISL_882267, EPI_ISL_882268, EPI_ISL_882269, EPI_ISL_882270, EPI_ISL_882271, EPI_ISL_882272, EPI_ISL_882273, EPI_ISL_882274, EPI_ISL_882275, EPI_ISL_882276, EPI_ISL_882277, EPI_ISL_882278, EPI_ISL_882279, EPI_ISL_882280, EPI_ISL_882281, EPI_ISL_882282, EPI_ISL_882283, EPI_ISL_882284, EPI_ISL_882285, EPI_ISL_882286, EPI_ISL_882287, EPI_ISL_882288, EPI_ISL_882289, EPI_ISL_882290, EPI_ISL_882291, EPI_ISL_882292, EPI_ISL_882293, EPI_ISL_882294, EPI_ISL_882295, EPI_ISL_882296, EPI_ISL_882297, EPI_ISL_882298, EPI_ISL_882299			
see above	Lighthouse Lab in Alderley Park	Wellcome Sanger Institute for the COVID-19 Genomics UK (COG-UK) Consortium	Jacquelyn Wynn, Mairead Hyland, The Lighthouse Lab in Alderley Park and Alex Alderton, Roberto Amato, Sonia Goncalves, Ewan Harrison, David K. Jackson, Ian Johnston, Dominic Kwiatkowski, Cordelia Langford, John Sillitoe on behalf of the Wellcome Sanger Institute COVID-19 Surveillance Team
EPI_ISL_882610	Lighthouse Lab in Milton Keynes	Wellcome Sanger Institute for the COVID-19 Genomics UK (COG-UK) Consortium	The Lighthouse Lab in Milton Keynes and Alex Alderton, Roberto Amato, Sonia Goncalves, Ewan Harrison, David K. Jackson, Ian Johnston, Dominic Kwiatkowski, Cordelia Langford, John Sillitoe on behalf of the Wellcome Sanger Institute COVID-19 Surveillance Team
EPI_ISL_885240, EPI_ISL_885327, EPI_ISL_885409, EPI_ISL_885514, EPI_ISL_892050, EPI_ISL_892051, EPI_ISL_892052, EPI_ISL_892053, EPI_ISL_892054, EPI_ISL_892055, EPI_ISL_892057, EPI_ISL_892058, EPI_ISL_892067, EPI_ISL_892068, EPI_ISL_892069, EPI_ISL_892070, EPI_ISL_892071, EPI_ISL_892072, EPI_ISL_892073, EPI_ISL_892074, EPI_ISL_892075, EPI_ISL_892076, EPI_ISL_892077, EPI_ISL_892078, EPI_ISL_892079, EPI_ISL_892080, EPI_ISL_892081, EPI_ISL_892082, EPI_ISL_892083, EPI_ISL_892084, EPI_ISL_892085, EPI_ISL_892086, EPI_ISL_892087, EPI_ISL_892088, EPI_ISL_892089, EPI_ISL_892090, EPI_ISL_892091, EPI_ISL_892092, EPI_ISL_892093, EPI_ISL_892094, EPI_ISL_892095, EPI_ISL_892096, EPI_ISL_892097, EPI_ISL_892098, EPI_ISL_892099, EPI_ISL_892100, EPI_ISL_892101, EPI_ISL_892102, EPI_ISL_892103, EPI_ISL_892104, EPI_ISL_892105, EPI_ISL_892106, EPI_ISL_892107, EPI_ISL_892108, EPI_ISL_892109, EPI_ISL_892110, EPI_ISL_892111, EPI_ISL_892112, EPI_ISL_892113, EPI_ISL_892114, EPI_ISL_892115, EPI_ISL_892116, EPI_ISL_892117, EPI_ISL_892118, EPI_ISL_892119, EPI_ISL_892120, EPI_ISL_892121, EPI_ISL_892122, EPI_ISL_892123, EPI_ISL_892124, EPI_ISL_892125, EPI_ISL_892126, EPI_ISL_892179, EPI_ISL_892189, EPI_ISL_892190, EPI_ISL_892191, EPI_ISL_892192, EPI_ISL_892193, EPI_ISL_892194, EPI_ISL_892195, EPI_ISL_892196, EPI_ISL_892197, EPI_ISL_892206			
see above	Lighthouse Lab in Alderley Park	Wellcome Sanger Institute for the COVID-19 Genomics UK (COG-UK) Consortium	Jacquelyn Wynn, Mairead Hyland, The Lighthouse Lab in Alderley Park and Alex Alderton, Roberto Amato, Sonia Goncalves, Ewan Harrison, David K. Jackson, Ian Johnston, Dominic Kwiatkowski, Cordelia Langford, John Sillitoe on behalf of the Wellcome Sanger Institute COVID-19 Surveillance Team
EPI_ISL_907210, EPI_ISL_907750	Lighthouse Lab in Glasgow	Wellcome Sanger Institute for the COVID-19 Genomics UK (COG-UK) Consortium	Harper VanSteenhouse, Yumi Kasai, David Gray, Carol Clugston, Anna Dominiczak and Alex Alderton, Roberto Amato, Sonia Goncalves, Ewan Harrison, David K. Jackson, Ian Johnston, Dominic Kwiatkowski, Cordelia Langford, John Sillitoe on behalf of the Wellcome Sanger Institute COVID-19 Surveillance Team
EPI_ISL_909595, EPI_ISL_909596, EPI_ISL_909597, EPI_ISL_909598, EPI_ISL_909599, EPI_ISL_909600, EPI_ISL_909601, EPI_ISL_909602, EPI_ISL_909603, EPI_ISL_909604, EPI_ISL_909605, EPI_ISL_909606, EPI_ISL_909607, EPI_ISL_909608, EPI_ISL_909609, EPI_ISL_909610, EPI_ISL_909611, EPI_ISL_909612, EPI_ISL_909613, EPI_ISL_909614, EPI_ISL_909615, EPI_ISL_909616, EPI_ISL_909617, EPI_ISL_909618, EPI_ISL_909619, EPI_ISL_909620, EPI_ISL_909621, EPI_ISL_909622, EPI_ISL_909623, EPI_ISL_909624, EPI_ISL_909625, EPI_ISL_909626, EPI_ISL_909627, EPI_ISL_909628, EPI_ISL_909629, EPI_ISL_909630, EPI_ISL_909631, EPI_ISL_909632, EPI_ISL_909633, EPI_ISL_909634, EPI_ISL_909635, EPI_ISL_909636, EPI_ISL_909637, EPI_ISL_909638, EPI_ISL_909639, EPI_ISL_909640, EPI_ISL_909641, EPI_ISL_909642, EPI_ISL_909643, EPI_ISL_909644, EPI_ISL_909645, EPI_ISL_909646, EPI_ISL_909647, EPI_ISL_909648, EPI_ISL_909649, EPI_ISL_909650, EPI_ISL_909651			
see above	Lighthouse Lab in Alderley Park	Wellcome Sanger Institute for the COVID-19 Genomics UK (COG-UK) Consortium	Jacquelyn Wynn, Mairead Hyland, The Lighthouse Lab in Alderley Park and Alex Alderton, Roberto Amato, Sonia Goncalves, Ewan Harrison, David K. Jackson, Ian Johnston, Dominic Kwiatkowski, Cordelia Langford, John Sillitoe on behalf of the Wellcome Sanger Institute COVID-19 Surveillance Team
EPI_ISL_912226	Lighthouse Lab in Alderley Park	Wellcome Sanger Institute for the COVID-19 Genomics UK (COG-UK) Consortium	Jacquelyn Wynn, Mairead Hyland, The Lighthouse Lab in Alderley Park and Alex Alderton, Roberto Amato, Sonia Goncalves, Ewan Harrison, David K. Jackson, Ian Johnston, Dominic Kwiatkowski, Cordelia Langford, John Sillitoe on behalf of the Wellcome Sanger Institute COVID-19 Surveillance Team

			(http://www.sanger.ac.uk/covid-team)
EPI_ISL_916630, EPI_ISL_916637, EPI_ISL_916651, EPI_ISL_916732, EPI_ISL_916746, EPI_ISL_916766, EPI_ISL_916775, EPI_ISL_916779, EPI_ISL_916849, EPI_ISL_916919, EPI_ISL_916934, EPI_ISL_916950, EPI_ISL_917011, EPI_ISL_917060, EPI_ISL_917095, EPI_ISL_917121, EPI_ISL_917792			
see above	Lighthouse Lab in Alderley Park	Wellcome Sanger Institute for the COVID-19 Genomics UK (COG-UK) Consortium	Jacquelyn Wynn, Mairead Hyland, The Lighthouse Lab in Alderley Park and Alex Alderton, Roberto Amato, Sonia Goncalves, Ewan Harrison, David K. Jackson, Ian Johnston, Dominic Kwiatkowski, Cordelia Langford, John Sillitoe on behalf of the Wellcome Sanger Institute COVID-19 Surveillance Team
EPI_ISL_918686, EPI_ISL_918687, EPI_ISL_918688, EPI_ISL_918689, EPI_ISL_918690, EPI_ISL_918691, EPI_ISL_918692, EPI_ISL_918693, EPI_ISL_918694, EPI_ISL_918695, EPI_ISL_918696, EPI_ISL_918697, EPI_ISL_918698, EPI_ISL_918699, EPI_ISL_918700, EPI_ISL_918701, EPI_ISL_918703, EPI_ISL_918708, EPI_ISL_918709, EPI_ISL_918710, EPI_ISL_918713, EPI_ISL_918715, EPI_ISL_918716, EPI_ISL_918717, EPI_ISL_918719, EPI_ISL_918732, EPI_ISL_918733, EPI_ISL_918734, EPI_ISL_918859, EPI_ISL_918860, EPI_ISL_918861, EPI_ISL_918862, EPI_ISL_918863, EPI_ISL_918864, EPI_ISL_918866, EPI_ISL_918867, EPI_ISL_918868, EPI_ISL_918870, EPI_ISL_918871, EPI_ISL_918872, EPI_ISL_918873, EPI_ISL_918879, EPI_ISL_918880, EPI_ISL_918881, EPI_ISL_918884, EPI_ISL_918885, EPI_ISL_918886, EPI_ISL_918887, EPI_ISL_918888, EPI_ISL_918889, EPI_ISL_918890, EPI_ISL_918891, EPI_ISL_918892, EPI_ISL_918893, EPI_ISL_918894, EPI_ISL_918895, EPI_ISL_918896, EPI_ISL_918897, EPI_ISL_918898, EPI_ISL_918899, EPI_ISL_918914, EPI_ISL_918915, EPI_ISL_918916, EPI_ISL_918917, EPI_ISL_918918, EPI_ISL_918919, EPI_ISL_918920, EPI_ISL_918921, EPI_ISL_918922, EPI_ISL_918923, EPI_ISL_918924, EPI_ISL_918925, EPI_ISL_918926, EPI_ISL_918927, EPI_ISL_918928, EPI_ISL_918929, EPI_ISL_918930, EPI_ISL_918931, EPI_ISL_918932, EPI_ISL_918933, EPI_ISL_918934, EPI_ISL_918935, EPI_ISL_918936, EPI_ISL_918937, EPI_ISL_918938, EPI_ISL_918939, EPI_ISL_918973, EPI_ISL_918974, EPI_ISL_918975, EPI_ISL_918976			
see above	University of Birmingham	COVID-19 Genomics UK (COG-UK) Consortium	Institute of Microbiology, University of Birmingham: Claire McMurray, Joanne Stockton, Samuel Nicholls, Radoslaw Poplawski, Will Rowe, Josh Quick, Nicholas Loman. University of Birmingham Testing Laboratory: Celina M Whalley, Andrew Bosworth, Charlotte Poxon, Kasun Wanigasooriya, Oliver Pickles, Mike Kidd, Alex Richter, Andrew D Beggs PHE Heartlands Lab: Husam Osman, Andrew Bosworth. Queen Elizabeth Hospital: Anna Casey
EPI_ISL_919011, EPI_ISL_919012, EPI_ISL_919017, EPI_ISL_919029, EPI_ISL_919037, EPI_ISL_919039, EPI_ISL_919041, EPI_ISL_919043, EPI_ISL_919044, EPI_ISL_919045, EPI_ISL_919046, EPI_ISL_919047, EPI_ISL_919048, EPI_ISL_919049, EPI_ISL_919050, EPI_ISL_919051, EPI_ISL_919052, EPI_ISL_919056, EPI_ISL_919071, EPI_ISL_919073, EPI_ISL_919077, EPI_ISL_919081, EPI_ISL_919089, EPI_ISL_919104, EPI_ISL_919105			
see above	Department of Pathology, University of Cambridge	COVID-19 Genomics UK (COG-UK) Consortium	Aminu S. Jahun, Yasmin Chaudhry, Iliana Georgana, Myra Hosmillo, Rhys Izu, Martin D. Curran, Surendra Parmar, Ian Goodfellow
EPI_ISL_919190, EPI_ISL_919191, EPI_ISL_919192, EPI_ISL_919193, EPI_ISL_919194, EPI_ISL_919195, EPI_ISL_919196, EPI_ISL_919197	West of Scotland Specialist Virology Centre, NHSGGC / MRC-University of Glasgow Centre for Virus Research	COVID-19 Genomics UK (COG-UK) Consortium	Ana da Silva Filipe, Natasha Johnson, Kathy Smollett, Daniel Mair, Stephen Carmichael, Alice Broos, Lily Tong, Jenna Nichols, Kyriaki Nomikou; Sarah McDonald; Richard Orton, Joseph Hughes, Sreenu Vattipally, David L Robertson; Alasdair MacLean, Rory Gunson; Sharif Shaaban, Matthew Holden; Rachel Blacow, Guy Mollett, Kathy Li, James Shepherd, Antonia Ho, Emma Thomson
EPI_ISL_919337, EPI_ISL_919338	Virology Department, Royal Infirmary of Edinburgh, NHS Lothian / School of Biological Sciences, University of Edinburgh / Institute of Genetics and Molecular Medicine, University of Edinburgh	COVID-19 Genomics UK (COG-UK) Consortium	McHugh M, Dewar R, Rooke S, Gallagher M, Balcaza C, O'Toole Á, Scher E, Hill V, McCrone JT, Colquhoun R, Yu X, Jackson B, Rambaut A, Williams TC, Templeton K
EPI_ISL_919462, EPI_ISL_919463, EPI_ISL_919465, EPI_ISL_919467, EPI_ISL_919468, EPI_ISL_919469, EPI_ISL_919470, EPI_ISL_919471, EPI_ISL_919472, EPI_ISL_919473, EPI_ISL_919474, EPI_ISL_919475, EPI_ISL_919478, EPI_ISL_919515, EPI_ISL_919517, EPI_ISL_919591, EPI_ISL_919593, EPI_ISL_919594, EPI_ISL_919595, EPI_ISL_919644			
see above	Liverpool Clinical Laboratories	COVID-19 Genomics UK (COG-UK) Consortium	Sam Haldenby, Anita Lucaci, Steve Paterson, Julian Hiscox, Alistair Darby, M Almsaud, A Alrezaihi, Muhannad Alruwaili, Stuart D Armstrong, Jones Benjamin, Eleanor G Bentley, Anu Chawla, Jordan J Clark, Angela Cowell, Richard Eccles, Isabel García-Dorival, Matthew Gemmell, Alessandro Gerada, PKF Gilmore, Richard Gregory, Ximeng Han, Catherine Hartley, Margaret Hughes, Miren Iturriza-Gomara, James Johnson, L Luu, Jenifer Manson, Charlotte Nelson, Elaine O'Toole, Cassie Olateju, Rebekah Penrice-Randal , Lucille Rainbow, N.P Randle, Trevor Ian Robinson, Parul Sharma, Ghada T Shawli, James P Stewart, Neil Swainston, Ecaterina Vamos, Joanne Watts, Mark Whitehead
EPI_ISL_919841, EPI_ISL_919852	Barts Health NHS Trust	COVID-19 Genomics UK (COG-UK) Consortium	CUTINO-MOGUEL, Maria-Teresa; HARRINGTON, David; OWOYEMI, Dola; KULASEGARAN-SHYLINI, Raghavendran; BROAD, Claire; KELE, Beatrix
EPI_ISL_919995, EPI_ISL_920000, EPI_ISL_920001, EPI_ISL_920002, EPI_ISL_920003, EPI_ISL_920004, EPI_ISL_920005, EPI_ISL_920006, EPI_ISL_920007, EPI_ISL_920008, EPI_ISL_920009, EPI_ISL_920010, EPI_ISL_920011, EPI_ISL_920012, EPI_ISL_920013, EPI_ISL_920014, EPI_ISL_920015, EPI_ISL_920016, EPI_ISL_920017, EPI_ISL_920018, EPI_ISL_920019, EPI_ISL_920020, EPI_ISL_920021, EPI_ISL_920022, EPI_ISL_920023, EPI_ISL_920024, EPI_ISL_920025, EPI_ISL_920027, EPI_ISL_920028, EPI_ISL_920029, EPI_ISL_920034, EPI_ISL_920042, EPI_ISL_920049, EPI_ISL_920050, EPI_ISL_920051			
see above	University College London, Great Ormond Street Hospital for Children NHS Foundation Trust, Imperial College Healthcare NHS Trust	COVID-19 Genomics UK (COG-UK) Consortium	Sergi Castellano, Rachel Williams, Mark Kristiansen, Paola Resende Silva, Sunando Roy, Tony Brooks, Helena Tutill, Paola Niola, Patricia Dyal, Charlotte Williams, Leysa Forrest, Yasmin Panchbhaya, Jacqueline Findlay, Samuel Weeks, Julianne Brown, Kathryn Harris, Paul Randell, James Price, Alison Holmes, Judith Breuer
EPI_ISL_920188, EPI_ISL_920191, EPI_ISL_920193, EPI_ISL_920241, EPI_ISL_920298, EPI_ISL_920299, EPI_ISL_920309, EPI_ISL_920310, EPI_ISL_920318, EPI_ISL_920327, EPI_ISL_920328, EPI_ISL_920329, EPI_ISL_920330, EPI_ISL_920337, EPI_ISL_920338, EPI_ISL_920339, EPI_ISL_920345, EPI_ISL_920353, EPI_ISL_920357, EPI_ISL_920362, EPI_ISL_920363, EPI_ISL_920371, EPI_ISL_920458, EPI_ISL_920466, EPI_ISL_920467, EPI_ISL_920501			
see above	University College London Hospital	COVID-19 Genomics UK (COG-UK) Consortium	Judith Heaney, Matthew Byott, Catherine Houlihan, Dan Frampton, Stuart Kirk, Moira Spyer and Eleni Nastouli
EPI_ISL_920851, EPI_ISL_920852, EPI_ISL_920853, EPI_ISL_920860, EPI_ISL_920861, EPI_ISL_920862, EPI_ISL_920863, EPI_ISL_920864, EPI_ISL_920865, EPI_ISL_920866, EPI_ISL_920867, EPI_ISL_920868, EPI_ISL_920869, EPI_ISL_920870, EPI_ISL_920871, EPI_ISL_920872, EPI_ISL_920873, EPI_ISL_920874, EPI_ISL_920875, EPI_ISL_920876, EPI_ISL_920877, EPI_ISL_920878, EPI_ISL_920879, EPI_ISL_920880, EPI_ISL_920881			
see above	University College London, Great Ormond Street Hospital for Children NHS Foundation Trust, Imperial College Healthcare NHS Trust	COVID-19 Genomics UK (COG-UK) Consortium	Sergi Castellano, Rachel Williams, Mark Kristiansen, Paola Resende Silva, Sunando Roy, Tony Brooks, Helena Tutill, Paola Niola, Patricia Dyal, Charlotte Williams, Leysa Forrest, Yasmin Panchbhaya, Jacqueline Findlay, Samuel Weeks, Julianne Brown, Kathryn Harris, Paul Randell, James Price, Alison Holmes, Judith Breuer
EPI_ISL_920894, EPI_ISL_920895, EPI_ISL_920896, EPI_ISL_920897, EPI_ISL_920898, EPI_ISL_920899, EPI_ISL_920900	Department of Pathology, University of Cambridge	COVID-19 Genomics UK (COG-UK) Consortium	Aminu S. Jahun, Yasmin Chaudhry, Iliana Georgana, Myra Hosmillo, Rhys Izu, Martin D. Curran, Surendra Parmar, Ian Goodfellow
EPI_ISL_920996, EPI_ISL_920997, EPI_ISL_920998, EPI_ISL_920999, EPI_ISL_921000, EPI_ISL_921001, EPI_ISL_921002, EPI_ISL_921003, EPI_ISL_921004, EPI_ISL_921005, EPI_ISL_921006, EPI_ISL_921007, EPI_ISL_921008, EPI_ISL_921009, EPI_ISL_921010, EPI_ISL_921018			
see above	Regional Virus Laboratory, Belfast Health and Social Care Trust	COVID-19 Genomics UK (COG-UK) Consortium	Conall McCaughey, James McKenna, Tanya Curran, Susan Feeney, Alison Watt, Ciara Cox, Mairead Connor, Zoltan Molnar, David Simpson, Derek Fairley
EPI_ISL_921300, EPI_ISL_921475, EPI_ISL_921476, EPI_ISL_921477, EPI_ISL_921478, EPI_ISL_921479, EPI_ISL_921480, EPI_ISL_921481, EPI_ISL_921482, EPI_ISL_921483, EPI_ISL_921484, EPI_ISL_921488, EPI_ISL_921489, EPI_ISL_921490, EPI_ISL_921491, EPI_ISL_921492, EPI_ISL_921493, EPI_ISL_921494, EPI_ISL_921495, EPI_ISL_921496, EPI_ISL_921497, EPI_ISL_921498, EPI_ISL_921499, EPI_ISL_921500, EPI_ISL_921501, EPI_ISL_921503, EPI_ISL_921504, EPI_ISL_921505, EPI_ISL_921506, EPI_ISL_921507, EPI_ISL_921508, EPI_ISL_921509, EPI_ISL_921510, EPI_ISL_921511, EPI_ISL_921512, EPI_ISL_921513, EPI_ISL_921514, EPI_ISL_921515, EPI_ISL_921516, EPI_ISL_921517, EPI_ISL_921518, EPI_ISL_921519, EPI_ISL_921520, EPI_ISL_921541			
see above	Northumbria University / South Tees Hospitals NHS Foundation Trust / North Cumbria Integrated Care NHS Foundation Trust / North Tees and Hartlepool NHS Foundation Trust / Newcastle Hospitals NHS Foundation Trust	COVID-19 Genomics UK (COG-UK) Consortium	Darren L Smith,Andrew Nelson,Matthew Bashton,Greg R Young,Joshua Loh,John Allan,Mohammad A Tariq,Giles S Holt,Gary Black,Wen C Yew,Lynn Dover,Paul Baker,Steve Liggett,Sarah Essex,Jane Greenaway,Debra Padgett,Clive Graham,Garren Scott,Edward Barton,Emma Swindells,Brendan Payne,Jennifer Collins,Yusri Taha,Gary Eltringham
EPI_ISL_922060, EPI_ISL_922063, EPI_ISL_922064, EPI_ISL_922065, EPI_ISL_922066, EPI_ISL_922067, EPI_ISL_922068, EPI_ISL_922069, EPI_ISL_922070, EPI_ISL_922071, EPI_ISL_922072, EPI_ISL_922073, EPI_ISL_922074, EPI_ISL_922075, EPI_ISL_922077, EPI_ISL_922078, EPI_ISL_922079, EPI_ISL_922080, EPI_ISL_922081, EPI_ISL_922082, EPI_ISL_922083, EPI_ISL_922084, EPI_ISL_922085, EPI_ISL_922086, EPI_ISL_922087, EPI_ISL_922088, EPI_ISL_922089, EPI_ISL_922090, EPI_ISL_922091, EPI_ISL_922092, EPI_ISL_922093, EPI_ISL_922094, EPI_ISL_922095, EPI_ISL_922096, EPI_ISL_922097, EPI_ISL_922098, EPI_ISL_922099, EPI_ISL_922100, EPI_ISL_922101, EPI_ISL_922102, EPI_ISL_922103, EPI_ISL_922104, EPI_ISL_922105, EPI_ISL_922106, EPI_ISL_922107, EPI_ISL_922108, EPI_ISL_922109, EPI_ISL_922110, EPI_ISL_922111, EPI_ISL_922112, EPI_ISL_922115, EPI_ISL_922116			
see above	Lincolnshire Hospitals and DeepSeq Nottingham	COVID-19 Genomics UK (COG-UK) Consortium	Nichola Duckworth, Tim Sloan, Sarah Walsh, Jonathan Ball, Patrick McClure, Joeseph Chappell, Nadine Holmes, Matthew Carlisle, Christopher Moore, Fei Sang, Johnny Debebe, Victoria Wright, Matthew Loose
EPI_ISL_922194, EPI_ISL_922197, EPI_ISL_922200, EPI_ISL_922203, EPI_ISL_922206, EPI_ISL_922207, EPI_ISL_922208, EPI_ISL_922209, EPI_ISL_922211, EPI_ISL_922212, EPI_ISL_922213, EPI_ISL_922214, EPI_ISL_922215, EPI_ISL_922216, EPI_ISL_922217, EPI_ISL_922218, EPI_ISL_922219, EPI_ISL_922220, EPI_ISL_922223, EPI_ISL_922224, EPI_ISL_922225, EPI_ISL_922226, EPI_ISL_922227, EPI_ISL_922228, EPI_ISL_922229, EPI_ISL_922230, EPI_ISL_922237, EPI_ISL_922258, EPI_ISL_922259, EPI_ISL_922294, EPI_ISL_922295, EPI_ISL_922346, EPI_ISL_922347, EPI_ISL_922348, EPI_ISL_922349			
see above	Oxford Viromics, NDM, University of Oxford; Oxford University Hospitals; Basingstoke and North Hampshire Hospital	COVID-19 Genomics UK (COG-UK) Consortium	Tanya Golubchik, David Bonsall, George Macintyre, Amy Trebes, Mariateresa de Cesare, Catrin Moore, Alex Mobbs, Anita Justice, Robert Shaw, Monique Andersson, Timothy Peto, Emma Wise, Nathan Moore, Jessica Lynch, Nick Cortes, Matilde Mori, Stephen Kidd, David Buck, John Todd, Christophe Fraser
EPI_ISL_922361, EPI_ISL_922364, EPI_ISL_922380, EPI_ISL_922387, EPI_ISL_922388, EPI_ISL_922393, EPI_ISL_922397, EPI_ISL_922558, EPI_ISL_922588, EPI_ISL_922589, EPI_ISL_922644			
see above	Wales Specialist Virology Centre Sequencing lab: Pathogen Genomics Unit	Public Health Wales Microbiology Cardiff Wales Specialist Virology Centre	Catherine Moore, Johnathan Evans, Laura Gifford, Malorie Perry, Simon Cottrell, Angela Marchbank, Alec Birchley, Alexander Adams, Amy Gaskin, Bree Gatica-Wilcox, Jason Coombes, Joel Southgate, Lauren Gilbert, Lee Graham, Nicole Pacchiarini, Sara Kumziene-Summerhayes, Sarah Taylor, Sophie Jones, Sara Rey, Matthew Bull, Joanne Watkins, Sally Corden, Tom Connor
EPI_ISL_923260, EPI_ISL_923262, EPI_ISL_923275, EPI_ISL_923279, EPI_ISL_923284, EPI_ISL_923293, EPI_ISL_923296, EPI_ISL_923300, EPI_ISL_923301, EPI_ISL_923305, EPI_ISL_923306, EPI_ISL_923372, EPI_ISL_923373, EPI_ISL_923374, EPI_ISL_923375, EPI_ISL_923376, EPI_ISL_923377, EPI_ISL_923378, EPI_ISL_923380, EPI_ISL_923382, EPI_ISL_923383, EPI_ISL_923384, EPI_ISL_923385, EPI_ISL_923386, EPI_ISL_923387, EPI_ISL_923388, EPI_ISL_923390, EPI_ISL_923392, EPI_ISL_923393, EPI_ISL_923394, EPI_ISL_923395, EPI_ISL_923396, EPI_ISL_923397, EPI_ISL_923398, EPI_ISL_923399, EPI_ISL_923401,			

EPI_ISL_923402, EPI_ISL_923403, EPI_ISL_923404, EPI_ISL_923405, EPI_ISL_923406, EPI_ISL_923407, EPI_ISL_923408, EPI_ISL_923409, EPI_ISL_923410, EPI_ISL_923411, EPI_ISL_923412, EPI_ISL_923413, EPI_ISL_923430, EPI_ISL_923539, EPI_ISL_923593, EPI_ISL_923605, EPI_ISL_923611, EPI_ISL_923613, EPI_ISL_923671, EPI_ISL_923678, EPI_ISL_923679			
see above	Centre for Enzyme Innovation, University of Portsmouth / Translational Research Laboratory, Portsmouth Hospitals NHS Trust	COVID-19 Genomics UK (COG-UK) Consortium	Angela Beckett,Salman Goudarzi,Christopher Fearn,Kate Cook,Katie Loveson,Sharon Glaysher,Scott Elliott,Samuel Robson
EPI_ISL_924091, EPI_ISL_924182, EPI_ISL_924270, EPI_ISL_924285, EPI_ISL_924369	Virology Department, Sheffield Teaching Hospitals NHS Foundation Trust/Department of Infection, Immunity and Cardiovascular Disease, The Medical School, University of Sheffield	COVID-19 Genomics UK (COG-UK) Consortium	Thushan de Silva, Matthew Parker, Nikki Smith, Adri Angyal, Rebecca Brown, Luke Green, Rachel Tucker, Paul Parsons, Danielle Groves, Katie Johnson, Laura Carrilero, Alex Keeley, Dave Partridge, Matthew Wyles, Benjamin Lindsey, Mehmet Yavuz, Mohammad Raza, Cariad Evans
EPI_ISL_924724, EPI_ISL_924725, EPI_ISL_924727, EPI_ISL_924728, EPI_ISL_924729, EPI_ISL_924731, EPI_ISL_924733, EPI_ISL_924736, EPI_ISL_924740, EPI_ISL_924742, EPI_ISL_924744, EPI_ISL_924746, EPI_ISL_924748, EPI_ISL_924749, EPI_ISL_924750, EPI_ISL_924752, EPI_ISL_924753, EPI_ISL_924754, EPI_ISL_924755, EPI_ISL_924756, EPI_ISL_924764, EPI_ISL_924769, EPI_ISL_924770, EPI_ISL_924774, EPI_ISL_924794, EPI_ISL_924795, EPI_ISL_924797, EPI_ISL_924798, EPI_ISL_924800, EPI_ISL_924805, EPI_ISL_924807, EPI_ISL_924808, EPI_ISL_924809, EPI_ISL_924816, EPI_ISL_924818, EPI_ISL_924819, EPI_ISL_924827, EPI_ISL_924828, EPI_ISL_924832, EPI_ISL_924836, EPI_ISL_924838, EPI_ISL_924839, EPI_ISL_924846, EPI_ISL_924848, EPI_ISL_924849, EPI_ISL_924850, EPI_ISL_924851, EPI_ISL_924855, EPI_ISL_924860, EPI_ISL_924863, EPI_ISL_924864, EPI_ISL_924865, EPI_ISL_924867, EPI_ISL_924871, EPI_ISL_924877, EPI_ISL_924879, EPI_ISL_924880, EPI_ISL_924883, EPI_ISL_924886, EPI_ISL_924887, EPI_ISL_924890, EPI_ISL_924891, EPI_ISL_924892, EPI_ISL_924895, EPI_ISL_924897, EPI_ISL_924899, EPI_ISL_924901, EPI_ISL_924902, EPI_ISL_924904, EPI_ISL_924905, EPI_ISL_924907, EPI_ISL_924908, EPI_ISL_924909, EPI_ISL_924910, EPI_ISL_924911, EPI_ISL_924912, EPI_ISL_924913, EPI_ISL_924914, EPI_ISL_924915, EPI_ISL_924916, EPI_ISL_924917, EPI_ISL_924918, EPI_ISL_924919, EPI_ISL_924921, EPI_ISL_924922, EPI_ISL_924923, EPI_ISL_924924, EPI_ISL_924925, EPI_ISL_924926, EPI_ISL_924927, EPI_ISL_924928, EPI_ISL_924929, EPI_ISL_924930, EPI_ISL_924931, EPI_ISL_924933, EPI_ISL_924935, EPI_ISL_924936, EPI_ISL_924937, EPI_ISL_924938, EPI_ISL_924939, EPI_ISL_924940, EPI_ISL_924941, EPI_ISL_924942, EPI_ISL_924943, EPI_ISL_924944, EPI_ISL_924945, EPI_ISL_924946, EPI_ISL_924947, EPI_ISL_924950, EPI_ISL_924975, EPI_ISL_924980			
see above	Bioinformatics and Biostatistics Lab, Advanced Sequencing Facility	COVID-19 Genomics UK (COG-UK) Consortium	Aengus Stewart,Jerome Nicod,Chelsea Sawyer,Laura Cubitt,Harshil Patel,Margaret Crawford
EPI_ISL_937818	Lighthouse Lab in Alderley Park	Wellcome Sanger Institute for the COVID-19 Genomics UK (COG-UK) Consortium	Jacquelyn Wynn, Mairead Hyland, The Lighthouse Lab in Alderley Park and Alex Alderton, Roberto Amato, Sonia Goncalves, Ewan Harrison, David K. Jackson, Ian Johnston, Dominic Kwiatkowski, Cordelia Langford, John Sillitoe on behalf of the Wellcome Sanger Institute COVID-19 Surveillance Team
EPI_ISL_945049, EPI_ISL_945056, EPI_ISL_945061, EPI_ISL_945072, EPI_ISL_945084, EPI_ISL_945095, EPI_ISL_945114, EPI_ISL_945115, EPI_ISL_945122, EPI_ISL_945125, EPI_ISL_945130, EPI_ISL_945142, EPI_ISL_945143, EPI_ISL_945146, EPI_ISL_945147, EPI_ISL_945152, EPI_ISL_945155, EPI_ISL_945163, EPI_ISL_945169, EPI_ISL_945181, EPI_ISL_945190, EPI_ISL_945197, EPI_ISL_945207, EPI_ISL_945208, EPI_ISL_945212, EPI_ISL_945217, EPI_ISL_945221, EPI_ISL_945236, EPI_ISL_945238, EPI_ISL_945241, EPI_ISL_945243, EPI_ISL_945244, EPI_ISL_945248, EPI_ISL_945250, EPI_ISL_945254, EPI_ISL_945275, EPI_ISL_945279, EPI_ISL_945285, EPI_ISL_945288, EPI_ISL_945297, EPI_ISL_945304, EPI_ISL_945309, EPI_ISL_945311, EPI_ISL_945319, EPI_ISL_945324, EPI_ISL_945332, EPI_ISL_945334, EPI_ISL_945337, EPI_ISL_945345, EPI_ISL_945347, EPI_ISL_945363, EPI_ISL_945364, EPI_ISL_945366			
see above	Lighthouse Lab in Cambridge	Wellcome Sanger Institute for the COVID-19 Genomics UK (COG-UK) Consortium	Rob Howes, The Lighthouse Lab in Cambridge and Alex Alderton, Roberto Amato, Sonia Goncalves, Ewan Harrison, David K. Jackson, Ian Johnston, Dominic Kwiatkowski, Cordelia Langford, John Sillitoe on behalf of the Wellcome Sanger Institute COVID-19 Surveillance Team
EPI_ISL_946701, EPI_ISL_946780	Lighthouse Lab in Alderley Park	Wellcome Sanger Institute for the COVID-19 Genomics UK (COG-UK) Consortium	Jacquelyn Wynn, Mairead Hyland, The Lighthouse Lab in Alderley Park and Alex Alderton, Roberto Amato, Sonia Goncalves, Ewan Harrison, David K. Jackson, Ian Johnston, Dominic Kwiatkowski, Cordelia Langford, John Sillitoe on behalf of the Wellcome Sanger Institute COVID-19 Surveillance Team
EPI_ISL_949411	University of Birmingham	COVID-19 Genomics UK (COG-UK) Consortium	Institute of Microbiology, University of Birmingham: Claire McMurray, Joanne Stockton, Samuel Nicholls, Radoslaw Poplawski, Will Rowe, Josh Quick, Nicholas Loman. University of Birmingham Testing Laboratory: Celina M Whalley, Andrew Bosworth, Charlotte Poxon, Kasun Wanigasooriya, Oliver Pickles, Mike Kidd, Alex Richter, Andrew D Beggs PHE Heartlands Lab: Husam Osman, Andrew Bosworth. Queen Elizabeth Hospital: Anna Casey
EPI_ISL_949750, EPI_ISL_949757, EPI_ISL_949758, EPI_ISL_949759, EPI_ISL_949760, EPI_ISL_949762, EPI_ISL_949763, EPI_ISL_949764, EPI_ISL_949765, EPI_ISL_949766, EPI_ISL_949768, EPI_ISL_949771, EPI_ISL_949772, EPI_ISL_949773, EPI_ISL_949774, EPI_ISL_949776, EPI_ISL_949779, EPI_ISL_949781, EPI_ISL_949782			
see above	Barts Health NHS Trust	COVID-19 Genomics UK (COG-UK) Consortium	CUTINO-MOGUEL, Maria-Teresa; HARRINGTON, David; OWOYEMI, Dola; KULASEGARAN-SHYLINI, Raghavendran; BROAD, Claire; KELE, Beatrix
EPI_ISL_949788, EPI_ISL_949789, EPI_ISL_949790, EPI_ISL_949927, EPI_ISL_950069, EPI_ISL_950070, EPI_ISL_950071, EPI_ISL_950072, EPI_ISL_950074, EPI_ISL_950075, EPI_ISL_950077, EPI_ISL_950079, EPI_ISL_950080, EPI_ISL_950083, EPI_ISL_950084, EPI_ISL_950085, EPI_ISL_950087, EPI_ISL_950088, EPI_ISL_950090, EPI_ISL_950092, EPI_ISL_950097, EPI_ISL_950098, EPI_ISL_950100, EPI_ISL_950101, EPI_ISL_950102, EPI_ISL_950104, EPI_ISL_950107, EPI_ISL_950108, EPI_ISL_950109, EPI_ISL_950110, EPI_ISL_950112, EPI_ISL_950114, EPI_ISL_950115, EPI_ISL_950117, EPI_ISL_950119, EPI_ISL_950120, EPI_ISL_950125, EPI_ISL_950126, EPI_ISL_950128, EPI_ISL_950129, EPI_ISL_950150, EPI_ISL_950155, EPI_ISL_950162, EPI_ISL_950163, EPI_ISL_950164, EPI_ISL_950169			
see above	University College London, Great Ormond Street Hospital for Children NHS Foundation Trust, Imperial College Healthcare NHS Trust	COVID-19 Genomics UK (COG-UK) Consortium	Sergi Castellano, Rachel Williams, Mark Kristiansen, Paola Resende Silva, Sunando Roy, Tony Brooks, Helena Tutill, Paola Niola, Patricia Dyal, Charlotte Williams, Leysa Forrest, Yasmin Panchbhaya, Jacqueline Findlay, Samuel Weeks, Julianne Brown, Kathryn Harris, Paul Randell, James Price, Alison Holmes, Judith Breuer
EPI_ISL_950272, EPI_ISL_950280, EPI_ISL_950282, EPI_ISL_950283, EPI_ISL_950284, EPI_ISL_950285, EPI_ISL_950289, EPI_ISL_950290, EPI_ISL_950291, EPI_ISL_950292, EPI_ISL_950293, EPI_ISL_950297			
see above	Northumbria University / South Tees Hospitals NHS Foundation Trust / North Cumbria Integrated Care NHS Foundation Trust / North Tees and Hartlepool NHS Foundation Trust / Newcastle Hospitals NHS Foundation Trust	COVID-19 Genomics UK (COG-UK) Consortium	Darren L Smith,Andrew Nelson,Matthew Bashton,Greg R Young,Joshua Loh,John Allan,Mohammad A Tariq,Giles S Holt,Gary Black,Wen C Yew,Lynn Dover,Paul Baker,Steve Liggett,Sarah Essex,Jane Greenaway,Debra Padgett,Clive Graham,Garren Scott,Edward Barton,Emma Swindells,Brendan Payne,Jennifer Collins,Yusri Taha,Gary Eltringham
EPI_ISL_950713	Lincolnshire Hospitals and DeepSeq Nottingham	COVID-19 Genomics UK (COG-UK) Consortium	Nichola Duckworth, Tim Sloan, Sarah Walsh, Jonathan Ball, Patrick McClure, Joeseph Chappell, Nadine Holmes, Matthew Carlisle, Christopher Moore, Fei Sang, Johnny Debebe, Victoria Wright, Matthew Loose
EPI_ISL_951231, EPI_ISL_951232, EPI_ISL_951237, EPI_ISL_951238, EPI_ISL_951239, EPI_ISL_951241, EPI_ISL_951245, EPI_ISL_951246, EPI_ISL_951247, EPI_ISL_951248, EPI_ISL_951252, EPI_ISL_951254, EPI_ISL_951255, EPI_ISL_951256, EPI_ISL_951258, EPI_ISL_951264, EPI_ISL_951265, EPI_ISL_951267, EPI_ISL_951268, EPI_ISL_951269, EPI_ISL_951270, EPI_ISL_951271, EPI_ISL_951272, EPI_ISL_951273, EPI_ISL_951274, EPI_ISL_951275, EPI_ISL_951276, EPI_ISL_951277, EPI_ISL_951281, EPI_ISL_951282, EPI_ISL_951283, EPI_ISL_951286, EPI_ISL_951287, EPI_ISL_951288, EPI_ISL_951289, EPI_ISL_951290, EPI_ISL_951291, EPI_ISL_951293, EPI_ISL_951294, EPI_ISL_951297, EPI_ISL_951299, EPI_ISL_951300, EPI_ISL_951301, EPI_ISL_951304, EPI_ISL_951307, EPI_ISL_951308, EPI_ISL_951309, EPI_ISL_951316, EPI_ISL_951317, EPI_ISL_951345, EPI_ISL_951346, EPI_ISL_951347, EPI_ISL_951348, EPI_ISL_951349, EPI_ISL_951350, EPI_ISL_951351, EPI_ISL_951352, EPI_ISL_951353, EPI_ISL_951354, EPI_ISL_951356, EPI_ISL_951362, EPI_ISL_951363, EPI_ISL_951364, EPI_ISL_951366, EPI_ISL_951367, EPI_ISL_951376, EPI_ISL_951377, EPI_ISL_951389, EPI_ISL_951391, EPI_ISL_951392, EPI_ISL_951393, EPI_ISL_951395, EPI_ISL_951396, EPI_ISL_951404, EPI_ISL_951405, EPI_ISL_951406, EPI_ISL_951407, EPI_ISL_951408, EPI_ISL_951409, EPI_ISL_951412, EPI_ISL_951413, EPI_ISL_951414, EPI_ISL_951415, EPI_ISL_951417, EPI_ISL_951426, EPI_ISL_951427, EPI_ISL_951429, EPI_ISL_951439			
see above	Oxford Viromics, NDM, University of Oxford; Oxford University Hospitals; Basingstoke and North Hampshire Hospital	COVID-19 Genomics UK (COG-UK) Consortium	Tanya Golubchik, David Bonsall, George Macintyre, Amy Trebes, Mariateresa de Cesare, Catrin Moore, Alex Mobbs, Anita Justice, Robert Shaw, Monique Andersson, Timothy Peto, Emma Wise, Nathan Moore, Jessica Lynch, Nick Cortes, Matilde Mori, Stephen Kidd, David Buck, John Todd, Christophe Fraser
EPI_ISL_952381, EPI_ISL_952384, EPI_ISL_952388, EPI_ISL_952396, EPI_ISL_952400, EPI_ISL_952401, EPI_ISL_952402, EPI_ISL_952406, EPI_ISL_952413, EPI_ISL_952415, EPI_ISL_952419, EPI_ISL_952421, EPI_ISL_952425, EPI_ISL_952426, EPI_ISL_952434, EPI_ISL_952559, EPI_ISL_952561, EPI_ISL_952563, EPI_ISL_952565, EPI_ISL_952566, EPI_ISL_952567, EPI_ISL_952568, EPI_ISL_952777, EPI_ISL_952781, EPI_ISL_952782, EPI_ISL_952784, EPI_ISL_952786, EPI_ISL_952787, EPI_ISL_952788, EPI_ISL_952790, EPI_ISL_952791, EPI_ISL_952792, EPI_ISL_952796, EPI_ISL_952797, EPI_ISL_952800, EPI_ISL_952801, EPI_ISL_952802, EPI_ISL_952803, EPI_ISL_952805, EPI_ISL_952806, EPI_ISL_952807, EPI_ISL_952808, EPI_ISL_952809, EPI_ISL_952810, EPI_ISL_952811, EPI_ISL_952812, EPI_ISL_952819			
see above	Centre for Enzyme Innovation, University of Portsmouth / Translational Research Laboratory, Portsmouth Hospitals NHS Trust	COVID-19 Genomics UK (COG-UK) Consortium	Angela Beckett,Salman Goudarzi,Christopher Fearn,Kate Cook,Katie Loveson,Sharon Glaysher,Scott Elliott,Samuel Robson
EPI_ISL_953177, EPI_ISL_953180, EPI_ISL_953181, EPI_ISL_953182, EPI_ISL_953185, EPI_ISL_953187, EPI_ISL_953193, EPI_ISL_953199, EPI_ISL_953205, EPI_ISL_953207, EPI_ISL_953216, EPI_ISL_953217, EPI_ISL_953219, EPI_ISL_953222, EPI_ISL_953226, EPI_ISL_953230, EPI_ISL_953231, EPI_ISL_953232, EPI_ISL_953233, EPI_ISL_953236, EPI_ISL_953238, EPI_ISL_953239			
see above	Bioinformatics and Biostatistics Lab, Advanced Sequencing Facility	COVID-19 Genomics UK (COG-UK) Consortium	Aengus Stewart,Jerome Nicod,Chelsea Sawyer,Laura Cubitt,Harshil Patel,Margaret Crawford
EPI_ISL_959273	Lighthouse Lab in Milton Keynes	Wellcome Sanger Institute for the COVID-19 Genomics UK (COG-UK) Consortium	The Lighthouse Lab in Milton Keynes and Alex Alderton, Roberto Amato, Sonia Goncalves, Ewan Harrison, David K. Jackson, Ian Johnston, Dominic Kwiatkowski, Cordelia Langford, John Sillitoe on behalf of the Wellcome Sanger Institute COVID-19 Surveillance Team
EPI_ISL_963061, EPI_ISL_963296, EPI_ISL_963955	Lighthouse Lab in Alderley Park	Wellcome Sanger Institute for the COVID-19 Genomics UK (COG-UK) Consortium	Jacquelyn Wynn, Mairead Hyland, The Lighthouse Lab in Alderley Park and Alex Alderton, Roberto Amato, Sonia Goncalves, Ewan Harrison, David K. Jackson, Ian Johnston, Dominic Kwiatkowski, Cordelia Langford, John Sillitoe on behalf of the Wellcome Sanger Institute COVID-19 Surveillance Team
EPI_ISL_987284, EPI_ISL_987285, EPI_ISL_987286, EPI_ISL_987297, EPI_ISL_987298, EPI_ISL_987305, EPI_ISL_987329, EPI_ISL_987338, EPI_ISL_987342, EPI_ISL_987343, EPI_ISL_987346, EPI_ISL_987351, EPI_ISL_987352, EPI_ISL_987353, EPI_ISL_987358, EPI_ISL_987364, EPI_ISL_987366, EPI_ISL_987371, EPI_ISL_987374, EPI_ISL_987386, EPI_ISL_987388, EPI_ISL_987396, EPI_ISL_987400, EPI_ISL_987403, EPI_ISL_987423, EPI_ISL_987431, EPI_ISL_987434, EPI_ISL_987442, EPI_ISL_987447, EPI_ISL_987459, EPI_ISL_987460, EPI_ISL_987463, EPI_ISL_987466, EPI_ISL_987468, EPI_ISL_987473, EPI_ISL_987477, EPI_ISL_987487, EPI_ISL_987492, EPI_ISL_987494, EPI_ISL_987495, EPI_ISL_987500, EPI_ISL_987504, EPI_ISL_987512, EPI_ISL_987515, EPI_ISL_987528, EPI_ISL_987531, EPI_ISL_987533, EPI_ISL_987537, EPI_ISL_987538, EPI_ISL_987539, EPI_ISL_987543, EPI_ISL_987551, EPI_ISL_987558, EPI_ISL_987563, EPI_ISL_987564, EPI_ISL_987565, EPI_ISL_987569, EPI_ISL_987580, EPI_ISL_987582, EPI_ISL_987586, EPI_ISL_987588, EPI_ISL_987592, EPI_ISL_987600, EPI_ISL_987601, EPI_ISL_987608, EPI_ISL_987618, EPI_ISL_987626, EPI_ISL_987630, EPI_ISL_987632, EPI_ISL_987634, EPI_ISL_987635			
see above	Lighthouse Lab in Milton Keynes	Wellcome Sanger Institute for the COVID-19 Genomics UK (COG-UK) Consortium	The Lighthouse Lab in Milton Keynes and Alex Alderton, Roberto Amato, Sonia Goncalves, Ewan Harrison, David K. Jackson, Ian Johnston, Dominic Kwiatkowski, Cordelia Langford, John Sillitoe on behalf of the Wellcome Sanger Institute COVID-19 Surveillance Team (http://www.sanger.ac.uk/covid-team)

EPI_ISL_990545, EPI_ISL_994649, EPI_ISL_994650	Lighthouse Lab in Alderley Park	Wellcome Sanger Institute for the COVID-19 Genomics UK (COG-UK) Consortium	Jacquelyn Wynn, Mairead Hyland, The Lighthouse Lab in Alderley Park and Alex Alderton, Roberto Amato, Sonia Goncalves, Ewan Harrison, David K. Jackson, Ian Johnston, Dominic Kwiatkowski, Cordelia Langford, John Sillitoe on behalf of the Wellcome Sanger Institute COVID-19 Surveillance Team
EPI_ISL_996427, EPI_ISL_996428, EPI_ISL_996429, EPI_ISL_996430, EPI_ISL_996436	University of Birmingham	COVID-19 Genomics UK (COG-UK) Consortium	Institute of Microbiology, University of Birmingham: Claire McMurray, Joanne Stockton, Samuel Nicholls, Radoslaw Poplawski, Will Rowe, Josh Quick, Nicholas Loman. University of Birmingham Testing Laboratory: Celina M Whalley, Andrew Bosworth, Charlotte Poxon, Kasun Wanigasooriya, Oliver Pickles, Mike Kidd, Alex Richter, Andrew D Beggs PHE Heartlands Lab: Husam Osman, Andrew Bosworth. Queen Elizabeth Hospital: Anna Casey
EPI_ISL_996514, EPI_ISL_996515, EPI_ISL_996516, EPI_ISL_996517, EPI_ISL_996518, EPI_ISL_996519, EPI_ISL_996520, EPI_ISL_996522, EPI_ISL_996523, EPI_ISL_996524, EPI_ISL_996525, EPI_ISL_996526, EPI_ISL_996531, EPI_ISL_996532, EPI_ISL_996533, EPI_ISL_996534, EPI_ISL_996535, EPI_ISL_996536, EPI_ISL_996537, EPI_ISL_996571, EPI_ISL_996580, EPI_ISL_996581, EPI_ISL_996583, EPI_ISL_996584, EPI_ISL_996592			
see above	University of Exeter	COVID-19 Genomics UK (COG-UK) Consortium	Ben Temperton,Aaron Jeffries,Michelle Michelsen,Joanna Warwick-Dugdale,Audrey Farbos,Robyn Manley,Stephen Michell,Jane Masoli
EPI_ISL_997074	Virology Department, Royal Infirmary of Edinburgh, NHS Lothian / School of Biological Sciences, University of Edinburgh	COVID-19 Genomics UK (COG-UK) Consortium	McHugh M, Dewar R, Cotton S, Rooke S, O'Toole Á, Scher E, Hill V, McCrone JT, Colquhoun R, Yu X, Jackson B, Rambaut A, Templeton K
EPI_ISL_997326, EPI_ISL_997327	University of Exeter	COVID-19 Genomics UK (COG-UK) Consortium	Ben Temperton,Aaron Jeffries,Michelle Michelsen,Joanna Warwick-Dugdale,Audrey Farbos,Robyn Manley,Stephen Michell,Jane Masoli
EPI_ISL_997377	Liverpool Clinical Laboratories	COVID-19 Genomics UK (COG-UK) Consortium	Sam Haldenby, Anita Lucaci, Steve Paterson, Julian Hiscox, Alistair Darby, M Almsaud, A Alrezaihi, Muhannad Alruwaili, Stuart D Armstrong, Jones Benjamin, Eleanor G Bentley, Anu Chawla, Jordan J Clark, Angela Cowell, Richard Eccles, Isabel García-Dorival, Matthew Gemmell, Alessandro Gerada, PKF Gilmore, Richard Gregory, Ximeng Han, Catherine Hartley, Margaret Hughes, Miren Iturriza-Gomara, James Johnson, L Luu, Jenifer Manson, Charlotte Nelson, Elaine O'Toole, Cassie Olateju, Rebekah Penrice-Randal , Lucille Rainbow, N.P Randle, Trevor Ian Robinson, Parul Sharma, Ghada T Shawli, James P Stewart, Neil Swainston, Ecaterina Vamos, Joanne Watts, Mark Whitehead
EPI_ISL_997629, EPI_ISL_997675, EPI_ISL_997676	Barts Health NHS Trust	COVID-19 Genomics UK (COG-UK) Consortium	CUTINO-MOGUEL, Maria-Teresa; HARRINGTON, David; OWOYEMI, Dola; KULASEGARAN-SHYLINI, Raghavendran; BROAD, Claire; KELE, Beatrix
EPI_ISL_997839	University College London, Great Ormond Street Hospital for Children NHS Foundation Trust, Imperial College Healthcare NHS Trust	COVID-19 Genomics UK (COG-UK) Consortium	Sergi Castellano, Rachel Williams, Mark Kristiansen, Paola Resende Silva, Sunando Roy, Tony Brooks, Helena Tutill, Paola Niola, Patricia Dyal, Charlotte Williams, Leysa Forrest, Yasmin Panchbhaya, Jacqueline Findlay, Samuel Weeks, Julianne Brown, Kathryn Harris, Paul Randell, James Price, Alison Holmes, Judith Breuer
EPI_ISL_998136, EPI_ISL_998140, EPI_ISL_998144, EPI_ISL_998154, EPI_ISL_998158, EPI_ISL_998159, EPI_ISL_998163	Regional Virus Laboratory, Belfast Health and Social Care Trust	COVID-19 Genomics UK (COG-UK) Consortium	Conall McCaughey, James McKenna, Tanya Curran, Susan Feeney, Alison Watt, Ciara Cox, Mairead Connor, Zoltan Molnar, David Simpson, Derek Fairley
EPI_ISL_998371, EPI_ISL_998749	Northumbria University / South Tees Hospitals NHS Foundation Trust / North Cumbria Integrated Care NHS Foundation Trust / North Tees and Hartlepool NHS Foundation Trust / Newcastle Hospitals NHS Foundation Trust	COVID-19 Genomics UK (COG-UK) Consortium	Darren L Smith,Andrew Nelson,Matthew Bashton,Greg R Young,Joshua Loh,John Allan,Mohammad A Tariq,Giles S Holt,Gary Black,Wen C Yew,Lynn Dover,Paul Baker,Steve Liggett,Sarah Essex,Jane Greenaway,Debra Padgett,Clive Graham,Garren Scott,Edward Barton,Emma Swindells,Brendan Payne,Jennifer Collins,Yusri Taha,Gary Eltringham
EPI_ISL_998830, EPI_ISL_998832	Quadram Institute Bioscience	COVID-19 Genomics UK (COG-UK) Consortium	Dave J. Baker, Gemma L. Kay, Alp Aydin, Thanh Le-Viet, Steven Rudder, Ana P. Tedim, Anastasia Kolyva, Maria Diaz, Leonardo de Oliveira Martins, Nabil-Fareed Alikhan, Lizzie Meadows, Rachael Stanley, Ngozi Elumogo, Muhammed Yasir, Nicholas M. Thomson, Alexander J Trotter, Rachel Gilroy, Samuel Bloomfield, Claire Stuart, Andrew Bell, Reenesh Prakash, Samir Dervisevic, Alison E. Mather, John Wain, Mark Webber, Andrew J. Page, Justin O'Grady
EPI_ISL_998937, EPI_ISL_998938, EPI_ISL_998939, EPI_ISL_998940, EPI_ISL_998941, EPI_ISL_998942, EPI_ISL_998943, EPI_ISL_998944	Lincolnshire Hospitals and DeepSeq Nottingham	COVID-19 Genomics UK (COG-UK) Consortium	Nichola Duckworth, Tim Sloan, Sarah Walsh, Jonathan Ball, Patrick McClure, Joeseph Chappell, Nadine Holmes, Matthew Carlisle, Christopher Moore, Fei Sang, Johnny Debebe, Victoria Wright, Matthew Loose
EPI_ISL_999033, EPI_ISL_999034, EPI_ISL_999035, EPI_ISL_999036, EPI_ISL_999037, EPI_ISL_999038, EPI_ISL_999039, EPI_ISL_999040, EPI_ISL_999041, EPI_ISL_999042, EPI_ISL_999044, EPI_ISL_999045, EPI_ISL_999048, EPI_ISL_999049, EPI_ISL_999050, EPI_ISL_999059, EPI_ISL_999060, EPI_ISL_999061, EPI_ISL_999062, EPI_ISL_999063, EPI_ISL_999064, EPI_ISL_999065, EPI_ISL_999066, EPI_ISL_999067, EPI_ISL_999076, EPI_ISL_999077, EPI_ISL_999080, EPI_ISL_999088, EPI_ISL_999094, EPI_ISL_999115, EPI_ISL_999128, EPI_ISL_999129, EPI_ISL_999133, EPI_ISL_999199, EPI_ISL_999211, EPI_ISL_999212, EPI_ISL_999364, EPI_ISL_999365, EPI_ISL_999368, EPI_ISL_999369, EPI_ISL_999370, EPI_ISL_999371, EPI_ISL_999372, EPI_ISL_999373, EPI_ISL_999377, EPI_ISL_999378, EPI_ISL_999383, EPI_ISL_999384, EPI_ISL_999388, EPI_ISL_999390, EPI_ISL_999391, EPI_ISL_999392, EPI_ISL_999393, EPI_ISL_999396, EPI_ISL_999399, EPI_ISL_999403, EPI_ISL_999419, EPI_ISL_999420, EPI_ISL_999423, EPI_ISL_999428, EPI_ISL_999431, EPI_ISL_999432, EPI_ISL_999433, EPI_ISL_999434, EPI_ISL_999440, EPI_ISL_999446, EPI_ISL_999447, EPI_ISL_999449, EPI_ISL_999452, EPI_ISL_999455, EPI_ISL_999456, EPI_ISL_999457, EPI_ISL_999458, EPI_ISL_999460, EPI_ISL_999471, EPI_ISL_999472, EPI_ISL_999482, EPI_ISL_999512, EPI_ISL_999513, EPI_ISL_999514			
see above	Oxford Viromics, NDM, University of Oxford; Oxford University Hospitals; Basingstoke and North Hampshire Hospital	COVID-19 Genomics UK (COG-UK) Consortium	Tanya Golubchik, David Bonsall, George Macintyre, Amy Trebes, Mariateresa de Cesare, Catrin Moore, Alex Mobbs, Anita Justice, Robert Shaw, Monique Andersson, Timothy Peto, Emma Wise, Nathan Moore, Jessica Lynch, Nick Cortes, Matilde Mori, Stephen Kidd, David Buck, John Todd, Christophe Fraser
EPI_ISL_999515	Originating lab: Wales Specialist Virology Centre Sequencing lab: Pathogen Genomics Unit	Public Health Wales Microbiology Cardiff Wales Specialist Virology Centre	Catherine Moore, Johnathan Evans, Laura Gifford, Malorie Perry, Simon Cottrell, Angela Marchbank, Alec Birchley, Alexander Adams, Amy Gaskin, Bree Gatica-Wilcox, Jason Coombes, Joel Southgate, Lauren Gilbert, Lee Graham, Nicole Pacchiarini, Sara Kumziene-Summerhayes, Sarah Taylor, Sophie Jones, Sara Rey, Matthew Bull, Joanne Watkins, Sally Corden, Tom Connor
EPI_ISL_999985	Wales Specialist Virology Centre Sequencing lab: Pathogen Genomics Unit	Public Health Wales Microbiology Cardiff Wales Specialist Virology Centre	Catherine Moore, Johnathan Evans, Laura Gifford, Malorie Perry, Simon Cottrell, Angela Marchbank, Alec Birchley, Alexander Adams, Amy Gaskin, Bree Gatica-Wilcox, Jason Coombes, Joel Southgate, Lauren Gilbert, Lee Graham, Nicole Pacchiarini, Sara Kumziene-Summerhayes, Sarah Taylor, Sophie Jones, Sara Rey, Matthew Bull, Joanne Watkins, Sally Corden, Tom Connor
