# Supplementary material for: Inferring the Association between the Risk of COVID-19 Case Fatality and N501Y Substitution in SARS-CoV-2
Source: Viruses. 2021 Apr 8;13(4):638. doi: 10.3390/v13040638 (PMC8070306; doi:10.3390/v13040638)
Supplement: Supplementary file 1 [file viruses-13-00638-s001.zip › gisaid_hcov-19_UKAT_210112-210115.pdf]

We gratefully acknowledge the following Authors from the Originating laboratories responsible for obtaining the specimens, as well as the Submitting laboratories where the genome data were generated and shared via GISAID, on which this research is based.

All Submitters of data may be contacted directly via www.gisaid.org

Authors are sorted alphabetically.

Accession ID	Originating Laboratory	Submitting Laboratory	Authors
EPI_ISL_1000247, EPI_ISL_1000417, EPI_ISL_1000418, EPI_ISL_1000420, EPI_ISL_1000421, EPI_ISL_1000422, EPI_ISL_1000423, EPI_ISL_1000424, EPI_ISL_1000425, EPI_ISL_1000426, EPI_ISL_1000427, EPI_ISL_1000428, EPI_ISL_1000429, EPI_ISL_1000430, EPI_ISL_1000431, EPI_ISL_1000432, EPI_ISL_1000433, EPI_ISL_1000434, EPI_ISL_1000435, EPI_ISL_1000436, EPI_ISL_1000437, EPI_ISL_1000438, EPI_ISL_1000439, EPI_ISL_1000441, EPI_ISL_1000442, EPI_ISL_1000444, EPI_ISL_1000445, EPI_ISL_1000448, EPI_ISL_1000449			
see above	Centre for Enzyme Innovation, University of Portsmouth / Translational Research Laboratory, Portsmouth Hospitals NHS Trust	COVID-19 Genomics UK (COG-UK) Consortium	Angela Beckett,Salman Goudarzi,Christopher Fearn,Kate Cook,Katie Loveson,Sharon Glaysher,Scott Elliott,Samuel Robson
EPI_ISL_1000631, EPI_ISL_1000633, EPI_ISL_1000634, EPI_ISL_1000635, EPI_ISL_1000636, EPI_ISL_1000637, EPI_ISL_1000638, EPI_ISL_1000639, EPI_ISL_1000640	Department of Pathology, University of Cambridge	COVID-19 Genomics UK (COG-UK) Consortium	Aminu S. Jahun, Yasmin Chaudhry, Iliana Georgana, Myra Hosmillo, Rhys Izuagbe, William L. Hamilton, Martin D. Curran, Surendra Parmar, Ian Goodfellow
EPI_ISL_1000811, EPI_ISL_1000884, EPI_ISL_1000889, EPI_ISL_1000890, EPI_ISL_1000899, EPI_ISL_1000901, EPI_ISL_1000904, EPI_ISL_1000912, EPI_ISL_1000936, EPI_ISL_1000938, EPI_ISL_1000939, EPI_ISL_1000941, EPI_ISL_1000942, EPI_ISL_1000944, EPI_ISL_1000946, EPI_ISL_1000948, EPI_ISL_1000949, EPI_ISL_1000950, EPI_ISL_1000951, EPI_ISL_1000952, EPI_ISL_1000953, EPI_ISL_1000954, EPI_ISL_1000955, EPI_ISL_1000956, EPI_ISL_1000957, EPI_ISL_1000958, EPI_ISL_1000959, EPI_ISL_1000961, EPI_ISL_1000962, EPI_ISL_1000965, EPI_ISL_1000966			
see above	Bioinformatics and Biostatistics Lab, Advanced Sequencing Facility	COVID-19 Genomics UK (COG-UK) Consortium	Aengus Stewart,Jerome Nicod,Chelsea Sawyer,Laura Cubitt,Harshil Patel,Margaret Crawford
EPI_ISL_1007433	Lighthouse Lab in Cambridge	Wellcome Sanger Institute for the COVID-19 Genomics UK (COG-UK) Consortium	Rob Howes, The Lighthouse Lab in Cambridge and Alex Alderton, Roberto Amato, Sonia Goncalves, Ewan Harrison, David K. Jackson, Ian Johnston, Dominic Kwiatkowski, Cordelia Langford, John Sillitoe on behalf of the Wellcome Sanger Institute COVID-19 Surveillance Team
EPI_ISL_1007575	Lighthouse Lab in Milton Keynes	Wellcome Sanger Institute for the COVID-19 Genomics UK (COG-UK) Consortium	The Lighthouse Lab in Milton Keynes and Alex Alderton, Roberto Amato, Sonia Goncalves, Ewan Harrison, David K. Jackson, Ian Johnston, Dominic Kwiatkowski, Cordelia Langford, John Sillitoe on behalf of the Wellcome Sanger Institute COVID-19 Surveillance Team
EPI_ISL_1007580, EPI_ISL_1007581, EPI_ISL_1007582	Lighthouse Lab in Alderley Park	Wellcome Sanger Institute for the COVID-19 Genomics UK (COG-UK) Consortium	Jacquelyn Wynn, Mairead Hyland, The Lighthouse Lab in Alderley Park and Alex Alderton, Roberto Amato, Sonia Goncalves, Ewan Harrison, David K. Jackson, Ian Johnston, Dominic Kwiatkowski, Cordelia Langford, John Sillitoe on behalf of the Wellcome Sanger Institute COVID-19 Surveillance Team
EPI_ISL_1019835	Lighthouse Lab in Glasgow	Wellcome Sanger Institute for the COVID-19 Genomics UK (COG-UK) Consortium	Harper VanSteenhouse, Yumi Kasai, David Gray, Carol Clugston, Anna Dominiczak and Alex Alderton, Roberto Amato, Jeffrey Barrett, Sonia Goncalves, Ewan Harrison, David K. Jackson, Ian Johnston, Dominic Kwiatkowski, Cordelia Langford, John Sillitoe on behalf of the Wellcome Sanger Institute COVID-19 Surveillance Team
EPI_ISL_1046996, EPI_ISL_1047060, EPI_ISL_1047063, EPI_ISL_1047068	University of Birmingham	COVID-19 Genomics UK (COG-UK) Consortium	Institute of Microbiology, University of Birmingham: Claire McMurray, Joanne Stockton, Samuel Nicholls, Radoslaw Poplawski, Will Rowe, Josh Quick, Nicholas Loman. University of Birmingham Testing Laboratory: Celina M Whalley, Andrew Bosworth, Charlotte Poxon, Kasun Wanigasooriya, Oliver Pickles, Mike Kidd, Alex Richter, Andrew D Beggs PHE Heartlands Lab: Husam Osman, Andrew Bosworth. Queen Elizabeth Hospital: Anna Casey
EPI_ISL_1047839, EPI_ISL_1047840	West of Scotland Specialist Virology Centre, NHSGGC / MRC-University of Glasgow Centre for Virus Research	COVID-19 Genomics UK (COG-UK) Consortium	Ana da Silva Filipe, Natasha Johnson, Kathy Smollett, Daniel Mair, Stephen Carmichael, Alice Broos, Lily Tong, Jenna Nichols, Kyriaki Nomikou; Sarah McDonald; Richard Orton, Joseph Hughes, Sreenu Vattipally, David L Robertson; Alasdair MacLean, Rory Gunson; Sharif Shaaban, Matthew Holden; Rachel Blacow, Guy Mollett, Kathy Li, James Shepherd, Antonia Ho, Emma Thomson
EPI_ISL_1048051, EPI_ISL_1048052	University College London, Great Ormond Street Hospital for Children NHS Foundation Trust, Imperial College Healthcare NHS Trust	COVID-19 Genomics UK (COG-UK) Consortium	Sergi Castellano, Rachel Williams, Mark Kristiansen, Paola Resende Silva, Sunando Roy, Tony Brooks, Helena Tutill, Paola Niola, Patricia Dyal, Charlotte Williams, Leysa Forrest, Yasmin Panchbhaya, Jacqueline Findlay, Samuel Weeks, Julianne Brown, Kathryn Harris, Paul Randell, James Price, Alison Holmes, Judith Breuer
EPI_ISL_1048071, EPI_ISL_1048088	Barts Health NHS Trust	COVID-19 Genomics UK (COG-UK) Consortium	CUTINO-MOGUEL, Maria-Teresa; HARRINGTON, David; OWOYEMI, Dola; KULASEGARAN-SHYLINI, Raghavendran; BROAD, Claire; KELE, Beatrix
EPI_ISL_1048136, EPI_ISL_1048137, EPI_ISL_1050357, EPI_ISL_1050439, EPI_ISL_1050440, EPI_ISL_1050441, EPI_ISL_1050442, EPI_ISL_1050443, EPI_ISL_1050444, EPI_ISL_1050445, EPI_ISL_1050446, EPI_ISL_1050447, EPI_ISL_1050448, EPI_ISL_1050450, EPI_ISL_1050451, EPI_ISL_1050457, EPI_ISL_1050458, EPI_ISL_1050459, EPI_ISL_1050460, EPI_ISL_1050461, EPI_ISL_1050462, EPI_ISL_1050464, EPI_ISL_1050465, EPI_ISL_1050466, EPI_ISL_1050508, EPI_ISL_1050510, EPI_ISL_1050511, EPI_ISL_1050516, EPI_ISL_1050517, EPI_ISL_1050519, EPI_ISL_1050520, EPI_ISL_1050521, EPI_ISL_1050524, EPI_ISL_1050525			
see above	University College London, Great Ormond Street Hospital for Children NHS Foundation Trust, Imperial College Healthcare NHS Trust	COVID-19 Genomics UK (COG-UK) Consortium	Sergi Castellano, Rachel Williams, Mark Kristiansen, Paola Resende Silva, Sunando Roy, Tony Brooks, Helena Tutill, Paola Niola, Patricia Dyal, Charlotte Williams, Leysa Forrest, Yasmin Panchbhaya, Jacqueline Findlay, Samuel Weeks, Julianne Brown, Kathryn Harris, Paul Randell, James Price, Alison Holmes, Judith Breuer
EPI_ISL_1050949, EPI_ISL_1050951, EPI_ISL_1050952, EPI_ISL_1050953, EPI_ISL_1050954, EPI_ISL_1050955, EPI_ISL_1050956, EPI_ISL_1050960, EPI_ISL_1050992, EPI_ISL_1051213, EPI_ISL_1051215, EPI_ISL_1051216, EPI_ISL_1051218, EPI_ISL_1051219, EPI_ISL_1051411			
see above	Northumbria University / South Tees Hospitals NHS Foundation Trust / North Cumbria Integrated Care NHS Foundation Trust / North Tees and Hartlepool NHS Foundation Trust / Newcastle Hospitals NHS Foundation Trust	COVID-19 Genomics UK (COG-UK) Consortium	Darren L Smith,Andrew Nelson,Matthew Bashton,Greg R Young,Joshua Loh,John Allan,Mohammad A Tariq,Giles S Holt,Gary Black,Wen C Yew,Lynn Dover,Paul Baker,Steve Liggett,Sarah Essex,Jane Greenaway,Debra Padgett,Clive Graham,Garren Scott,Edward Barton,Emma Swindells,Brendan Payne,Jennifer Collins,Yusri Taha,Gary Eltringham
EPI_ISL_1051427, EPI_ISL_1051430, EPI_ISL_1051447, EPI_ISL_1051449, EPI_ISL_1051454, EPI_ISL_1051456, EPI_ISL_1051469, EPI_ISL_1051474, EPI_ISL_1051477, EPI_ISL_1051478, EPI_ISL_1051493, EPI_ISL_1051494, EPI_ISL_1051495, EPI_ISL_1051497, EPI_ISL_1051500, EPI_ISL_1051515			
see above	Quadram Institute Bioscience	COVID-19 Genomics UK (COG-UK) Consortium	Dave J. Baker, Gemma L. Kay, Alp Aydin, Thanh Le-Viet, Steven Rudder, Ana P. Tedim, Anastasia Kolyva, Maria Diaz, Leonardo de Oliveira Martins, Nabil-Fareed Alikhan, Lizzie Meadows, Rachael Stanley, Ngozi Elumogo, Muhammed Yasir, Nicholas M. Thomson, Alexander J Trotter, Rachel Gilroy, Samuel Bloomfield, Claire Stuart, Andrew Bell, Reenesh Prakash, Samir Dervisevic, Alison E. Mather, John Wain, Mark Webber, Andrew J. Page, Justin O'Grady
EPI_ISL_1051788, EPI_ISL_1051790, EPI_ISL_1051794, EPI_ISL_1051797, EPI_ISL_1051798, EPI_ISL_1051799, EPI_ISL_1051800, EPI_ISL_1051801, EPI_ISL_1051802, EPI_ISL_1051803, EPI_ISL_1051805, EPI_ISL_1051806, EPI_ISL_1051807, EPI_ISL_1051808, EPI_ISL_1051809, EPI_ISL_1051813, EPI_ISL_1051814, EPI_ISL_1051816, EPI_ISL_1051822, EPI_ISL_1051824, EPI_ISL_1051825, EPI_ISL_1051826, EPI_ISL_1051827, EPI_ISL_1051829, EPI_ISL_1051832, EPI_ISL_1051838, EPI_ISL_1051840, EPI_ISL_1051841, EPI_ISL_1051843, EPI_ISL_1051844, EPI_ISL_1051845, EPI_ISL_1051849, EPI_ISL_1051851, EPI_ISL_1051852, EPI_ISL_1051853, EPI_ISL_1051854, EPI_ISL_1051855, EPI_ISL_1051856, EPI_ISL_1051858, EPI_ISL_1051873, EPI_ISL_1051874			
see above	Oxford Viromics, NDM, University of Oxford; Oxford University Hospitals; Basingstoke and North Hampshire Hospital	COVID-19 Genomics UK (COG-UK) Consortium	Tanya Golubchik, David Bonsall, George Macintyre, Amy Trebes, Mariateresa de Cesare, Catrin Moore, Alex Mobbs, Anita Justice, Robert Shaw, Monique Andersson, Timothy Peto, Emma Wise, Nathan Moore, Jessica Lynch, Nick Cortes, Matilde Mori, Stephen Kidd, David Buck, John Todd, Christophe Fraser
EPI_ISL_1053817, EPI_ISL_1053818, EPI_ISL_1053819	Centre for Enzyme Innovation, University of Portsmouth / Translational Research Laboratory, Portsmouth Hospitals NHS Trust	COVID-19 Genomics UK (COG-UK) Consortium	Angela Beckett,Salman Goudarzi,Christopher Fearn,Kate Cook,Katie Loveson,Sharon Glaysher,Scott Elliott,Samuel Robson
EPI_ISL_1054792, EPI_ISL_1054794, EPI_ISL_1054796, EPI_ISL_1054798, EPI_ISL_1054801, EPI_ISL_1054803, EPI_ISL_1054804, EPI_ISL_1054805, EPI_ISL_1054806, EPI_ISL_1054807, EPI_ISL_1054809, EPI_ISL_1054810, EPI_ISL_1054811, EPI_ISL_1054812, EPI_ISL_1054813, EPI_ISL_1054814, EPI_ISL_1054816, EPI_ISL_1054817, EPI_ISL_1054819, EPI_ISL_1054821			
see above	Bioinformatics and Biostatistics Lab, Advanced Sequencing Facility	COVID-19 Genomics UK (COG-UK) Consortium	Aengus Stewart,Jerome Nicod,Chelsea Sawyer,Laura Cubitt,Harshil Patel,Margaret Crawford
EPI_ISL_1101110	Lighthouse Lab in Alderley Park	Wellcome Sanger Institute for the COVID-19 Genomics UK (COG-UK) Consortium	Jacquelyn Wynn, Mairead Hyland, The Lighthouse Lab in Alderley Park and Alex Alderton, Roberto Amato, Jeffrey Barrett, Sonia Goncalves, Ewan Harrison, David K. Jackson, Ian Johnston, Dominic Kwiatkowski, Cordelia Langford, John Sillitoe on behalf of the Wellcome Sanger Institute COVID-19 Surveillance Team

EPI_ISL_1104232, EPI_ISL_1104238, EPI_ISL_1104241, EPI_ISL_1104257	Virology Department, Royal Infirmary of Edinburgh, NHS Lothian / School of Biological Sciences, University of Edinburgh	COVID-19 Genomics UK (COG-UK) Consortium	McHugh M, Dewar R, Cotton S, Rooke S, O'Toole Á, Scher E, Hill V, McCrone JT, Colquhoun R, Yu X, Jackson B, Rambaut A, Templeton K
EPI_ISL_1104291, EPI_ISL_1104293, EPI_ISL_1104296	University Hospitals Of Leicester NHS Trust and DeepSeq Nottingham	COVID-19 Genomics UK (COG-UK) Consortium	Christopher Holmes, Paul Bird, Thomas Helmer, Karlie Fallon, Julian Tang, Jonathan Ball, Patrick McClure, Joeseph Chappell, Nadine Holmes, Matthew Carlisle, Christopher Moore, Fei Sang, Johnny Debebe, Victoria Wright, Matthew Loose
EPI_ISL_1104321, EPI_ISL_1104333, EPI_ISL_1104334, EPI_ISL_1104335, EPI_ISL_1104336, EPI_ISL_1104362, EPI_ISL_1104367, EPI_ISL_1104368, EPI_ISL_1104388	Liverpool Clinical Laboratories	COVID-19 Genomics UK (COG-UK) Consortium	Sam Haldenby, Anita Lucaci, Steve Paterson, Julian Hiscox, Alistair Darby, M Almsaud, A Alrezaihi, Muhannad Alruwaili, Stuart D Armstrong, Jones Benjamin, Eleanor G Bentley, Anu Chawla, Jordan J Clark, Angela Cowell, Richard Eccles, Isabel García-Dorival, Matthew Gemmell, Alessandro Gerada, PKF Gilmore, Richard Gregory, Ximeng Han, Catherine Hartley, Margaret Hughes, Miren Iturriza-Gomara, James Johnson, L Luu, Jenifer Manson, Charlotte Nelson, Elaine O'Toole, Cassie Olateju, Rebekah Penrice-Randal , Lucille Rainbow, N.P Randle, Trevor Ian Robinson, Parul Sharma, Ghada T Shawli, James P Stewart, Neil Swainston, Ecaterina Vamos, Joanne Watts, Mark Whitehead
EPI_ISL_1105149, EPI_ISL_1105157, EPI_ISL_1105266, EPI_ISL_1105272, EPI_ISL_1105289, EPI_ISL_1105333	University College London Hospital	COVID-19 Genomics UK (COG-UK) Consortium	Judith Heaney, Matthew Byott, Catherine Houlihan, Dan Frampton, Stuart Kirk, Moira Spyer and Eleni Nastouli
EPI_ISL_1107750	Centre for Enzyme Innovation, University of Portsmouth / Translational Research Laboratory, Portsmouth Hospitals NHS Trust	COVID-19 Genomics UK (COG-UK) Consortium	Angela Beckett,Salman Goudarzi,Christopher Fearn,Kate Cook,Katie Loveson,Sharon Glaysher,Scott Elliott,Samuel Robson
EPI_ISL_1177878	Liverpool Clinical Laboratories	COVID-19 Genomics UK (COG-UK) Consortium	Sam Haldenby, Alistair Darby, Steve Paterson, Anita Lucaci, Julian Hiscox, M Almsaud, A Alrezaihi, Muhannad Alruwaili, Stuart D Armstrong, Jones Benjamin, Eleanor G Bentley, Anu Chawla, Jordan J Clark, Angela Cowell, Richard Eccles, Isabel García-Dorival, Matthew Gemmell, Alessandro Gerada, PKF Gilmore, Richard Gregory, Ximeng Han, Catherine Hartley, Margaret Hughes, Miren Iturriza-Gomara, James Johnson, L Luu, Jenifer Manson, Charlotte Nelson, Elaine O'Toole, Cassie Olateju, Rebekah Penrice-Randal , Lucille Rainbow, N.P Randle, Trevor Ian Robinson, Parul Sharma, Ghada T Shawli, James P Stewart, Neil Swainston, Ecaterina Vamos, Joanne Watts, Mark Whitehead, Hermione Webster
EPI_ISL_1177999	Barts Health NHS Trust	COVID-19 Genomics UK (COG-UK) Consortium	CUTINO-MOGUEL, Maria-Teresa; HARRINGTON, David; OWOYEMI, Dola; KULASEGARAN-SHYLINI, Raghavendran; BROAD, Claire; KELE, Beatrix
EPI_ISL_1178009	University College London, Great Ormond Street Hospital for Children NHS Foundation Trust, Imperial College Healthcare NHS Trust	COVID-19 Genomics UK (COG-UK) Consortium	Sergi Castellano, Rachel Williams, Mark Kristiansen, Paola Resende Silva, Sunando Roy, Tony Brooks, Helena Tutill, Paola Niola, Patricia Dyal, Charlotte Williams, Leysa Forrest, Yasmin Panchbhaya, Jacqueline Findlay, Samuel Weeks, Julianne Brown, Kathryn Harris, Paul Randell, James Price, Alison Holmes, Judith Breuer
EPI_ISL_1178262, EPI_ISL_1178343, EPI_ISL_1178349, EPI_ISL_1178350, EPI_ISL_1178351, EPI_ISL_1178352, EPI_ISL_1178354, EPI_ISL_1178355, EPI_ISL_1178358, EPI_ISL_1178360, EPI_ISL_1178407, EPI_ISL_1178408, EPI_ISL_1178409, EPI_ISL_1178410, EPI_ISL_1178411, EPI_ISL_1178412, EPI_ISL_1178413, EPI_ISL_1178414, EPI_ISL_1178415, EPI_ISL_1178416			
see above	Northumbria University / South Tees Hospitals NHS Foundation Trust / North Cumbria Integrated Care NHS Foundation Trust / North Tees and Hartlepool NHS Foundation Trust / Newcastle Hospitals NHS Foundation Trust	COVID-19 Genomics UK (COG-UK) Consortium	Darren L Smith,Andrew Nelson,Matthew Bashton,Greg R Young,Joshua Loh,John Allan,Mohammad A Tariq,Giles S Holt,Gary Black,Wen C Yew,Lynn Dover,Paul Baker,Steve Liggett,Sarah Essex,Jane Greenaway,Debra Padgett,Clive Graham,Garren Scott,Edward Barton,Emma Swindells,Brendan Payne,Jennifer Collins,Yusri Taha,Gary Eltringham
EPI_ISL_1178590	Queens Medical Centre, Clinical Microbiology Department / DeepSeq Nottingham	COVID-19 Genomics UK (COG-UK) Consortium	Gemma Clark, Wendy Smith, Manjinder Khakh, Vicki M Fleming, Michelle M Lister, Hannah Howson-Wells, Jonathan Ball, Timothy Byaruhanga, Jayasree Dey, Emily Park, Jack Hill, Patrick McClure, Joseph Chappell, Theocharis Tsoleridis, Nadine Holmes, Matthew Carlisle, Christopher Moore, Fei Sang, Johnny Debebe, Victoria Wright, Matthew Loose
EPI_ISL_1178720, EPI_ISL_1178726, EPI_ISL_1178746, EPI_ISL_1178749, EPI_ISL_1178777, EPI_ISL_1178778, EPI_ISL_1178783, EPI_ISL_1178801, EPI_ISL_1178858	Oxford Viromics, NDM, University of Oxford; Oxford University Hospitals; Basingstoke and North Hampshire Hospital	COVID-19 Genomics UK (COG-UK) Consortium	Tanya Golubchik, David Bonsall, George Macintyre, Amy Trebes, Mariateresa de Cesare, Catrin Moore, Alex Mobbs, Anita Justice, Robert Shaw, Monique Andersson, Timothy Peto, Emma Wise, Nathan Moore, Jessica Lynch, Nick Cortes, Matilde Mori, Stephen Kidd, David Buck, John Todd, Christophe Fraser
EPI_ISL_1179792, EPI_ISL_1179825	Centre for Enzyme Innovation, University of Portsmouth / Translational Research Laboratory, Portsmouth Hospitals NHS Trust	COVID-19 Genomics UK (COG-UK) Consortium	Angela Beckett,Salman Goudarzi,Christopher Fearn,Kate Cook,Katie Loveson,Sharon Glaysher,Scott Elliott,Samuel Robson
EPI_ISL_1247571, EPI_ISL_1247573, EPI_ISL_1247574, EPI_ISL_1247576, EPI_ISL_1247578, EPI_ISL_1247580, EPI_ISL_1247581, EPI_ISL_1247582, EPI_ISL_1247583, EPI_ISL_1247584, EPI_ISL_1247585, EPI_ISL_1247586, EPI_ISL_1247587, EPI_ISL_1247588, EPI_ISL_1247589, EPI_ISL_1247590, EPI_ISL_1247591, EPI_ISL_1247592, EPI_ISL_1247595, EPI_ISL_1247596, EPI_ISL_1247597, EPI_ISL_1247598, EPI_ISL_1247599			
see above	Department of Pathology, University of Cambridge	COVID-19 Genomics UK (COG-UK) Consortium	Aminu S. Jahun, Yasmin Chaudhry, Iliana Georgana, Myra Hosmillo, Rhys Izuagbe, William L. Hamilton, Martin D. Curran, Surendra Parmar, Ian Goodfellow
EPI_ISL_1247720, EPI_ISL_1247724	Virology Department, Royal Infirmary of Edinburgh, NHS Lothian / School of Biological Sciences, University of Edinburgh	COVID-19 Genomics UK (COG-UK) Consortium	McHugh M, Dewar R, Cotton S, Rooke S, O'Toole Á, Scher E, Hill V, McCrone JT, Colquhoun R, Yu X, Jackson B, Rambaut A, Templeton K
EPI_ISL_1248107, EPI_ISL_1248108, EPI_ISL_1248109, EPI_ISL_1248110, EPI_ISL_1248111, EPI_ISL_1248112, EPI_ISL_1248114, EPI_ISL_1248115, EPI_ISL_1248116, EPI_ISL_1248122, EPI_ISL_1248123, EPI_ISL_1248124, EPI_ISL_1248125, EPI_ISL_1248126, EPI_ISL_1248127, EPI_ISL_1248128, EPI_ISL_1248129, EPI_ISL_1248130, EPI_ISL_1248131, EPI_ISL_1248132, EPI_ISL_1248133, EPI_ISL_1248134, EPI_ISL_1248135, EPI_ISL_1248136, EPI_ISL_1248137, EPI_ISL_1248138, EPI_ISL_1248139, EPI_ISL_1248140, EPI_ISL_1248141, EPI_ISL_1248142, EPI_ISL_1248143, EPI_ISL_1248144, EPI_ISL_1248145, EPI_ISL_1248146, EPI_ISL_1248147, EPI_ISL_1248148, EPI_ISL_1248151, EPI_ISL_1248152, EPI_ISL_1248156, EPI_ISL_1248158, EPI_ISL_1248164, EPI_ISL_1248165, EPI_ISL_1248173, EPI_ISL_1248174, EPI_ISL_1248175, EPI_ISL_1248181, EPI_ISL_1248182, EPI_ISL_1248184, EPI_ISL_1248191, EPI_ISL_1248193, EPI_ISL_1248203, EPI_ISL_1248205			
see above	University College London, Great Ormond Street Hospital for Children NHS Foundation Trust, Imperial College Healthcare NHS Trust	COVID-19 Genomics UK (COG-UK) Consortium	Sergi Castellano, Rachel Williams, Mark Kristiansen, Paola Resende Silva, Sunando Roy, Tony Brooks, Helena Tutill, Paola Niola, Patricia Dyal, Charlotte Williams, Leysa Forrest, Yasmin Panchbhaya, Jacqueline Findlay, Samuel Weeks, Julianne Brown, Kathryn Harris, Paul Randell, James Price, Alison Holmes, Judith Breuer
EPI_ISL_1248372, EPI_ISL_1248373, EPI_ISL_1248382, EPI_ISL_1248383, EPI_ISL_1248394, EPI_ISL_1248406, EPI_ISL_1248417, EPI_ISL_1248438, EPI_ISL_1248439, EPI_ISL_1248482	University College London Hospital	COVID-19 Genomics UK (COG-UK) Consortium	Dr Judith Heaney, Matthew Byott, Dr Catherine Houlihan, Dr Daniel Frampton, Stuart Kirk, Dr Moira Spyer, Dr Paul Grant and Dr Eleni Nastouli
EPI_ISL_1248990, EPI_ISL_1249057	Quadram Institute Bioscience	COVID-19 Genomics UK (COG-UK) Consortium	Dave J. Baker, Gemma L. Kay, Alp Aydin, Thanh Le-Viet, Steven Rudder, Ana P. Tedim, Anastasia Kolyva, Maria Diaz, Leonardo de Oliveira Martins, Nabil-Fareed Alikhan, Lizzie Meadows, Rachael Stanley, Ngozi Elumogo, Muhammed Yasir, Nicholas M. Thomson, Alexander J Trotter, Rachel Gilroy, Samuel Bloomfield, Claire Stuart, Andrew Bell, Reenesh Prakash, Samir Dervisevic, Alison E. Mather, John Wain, Mark Webber, Andrew J. Page, Justin O'Grady
EPI_ISL_1249242, EPI_ISL_1249244	Oxford Viromics, NDM, University of Oxford; Oxford University Hospitals; Basingstoke and North Hampshire Hospital	COVID-19 Genomics UK (COG-UK) Consortium	Tanya Golubchik, David Bonsall, George Macintyre, Amy Trebes, Mariateresa de Cesare, Catrin Moore, Alex Mobbs, Anita Justice, Robert Shaw, Monique Andersson, Timothy Peto, Emma Wise, Nathan Moore, Jessica Lynch, Nick Cortes, Matilde Mori, Stephen Kidd, David Buck, John Todd, Christophe Fraser
EPI_ISL_1296524, EPI_ISL_1296528, EPI_ISL_1296529, EPI_ISL_1296533, EPI_ISL_1296534, EPI_ISL_1296535, EPI_ISL_1296538, EPI_ISL_1296547, EPI_ISL_1296548, EPI_ISL_1296551, EPI_ISL_1296571			
see above	Respiratory Virus Unit, National Infection Service, Public Health England	COVID-19 Genomics UK (COG-UK) Consortium	PHE Covid Sequencing Team
EPI_ISL_1308578, EPI_ISL_1308587, EPI_ISL_1308589, EPI_ISL_1308590, EPI_ISL_1308591	University of Exeter	COVID-19 Genomics UK (COG-UK) Consortium	Ben Temperton,Aaron Jeffries,Michelle Michelsen,Joanna Warwick-Dugdale,Audrey Farbos,Robyn Manley,Stephen Michell,Jane Masoli
EPI_ISL_1309051, EPI_ISL_1309070, EPI_ISL_1309077, EPI_ISL_1309078, EPI_ISL_1309085, EPI_ISL_1309090, EPI_ISL_1309097, EPI_ISL_1309099, EPI_ISL_1309103, EPI_ISL_1309109, EPI_ISL_1309113, EPI_ISL_1386855, EPI_ISL_1386856, EPI_ISL_1386859, EPI_ISL_1386873, EPI_ISL_1386895			
see above	University College London, Great Ormond Street Hospital for	COVID-19 Genomics UK (COG-UK) Consortium	Sergi Castellano, Rachel Williams, Mark Kristiansen, Paola Resende Silva, Sunando Roy, Tony Brooks, Helena Tutill, Paola Niola, Patricia Dyal, Charlotte

	Children NHS Foundation Trust, Imperial College Healthcare NHS Trust		Williams, Leysa Forrest, Yasmin Panchbhaya, Jacqueline Findlay, Samuel Weeks, Julianne Brown, Kathryn Harris, Paul Randell, James Price, Alison Holmes, Judith Breuer
EPI_ISL_1474382, EPI_ISL_1474383, EPI_ISL_1474385, EPI_ISL_1474386, EPI_ISL_1474387, EPI_ISL_1474388, EPI_ISL_1474389, EPI_ISL_1474390, EPI_ISL_1474391, EPI_ISL_1474392, EPI_ISL_1474393, EPI_ISL_1474394, EPI_ISL_1474395			
see above	University of Birmingham	COVID-19 Genomics UK (COG-UK) Consortium	Institute of Microbiology, University of Birmingham: Claire McMurray, Joanne Stockton, Samuel Nicholls, Radoslaw Poplawski, Will Rowe, Josh Quick, Nicholas Loman. University of Birmingham Testing Laboratory: Celina M Whalley, Andrew Bosworth, Charlotte Poxon, Kasun Wanigasooriya, Oliver Pickles, Mike Kidd, Alex Richter, Andrew D Beggs PHE Heartlands Lab: Husam Osman, Andrew Bosworth. Queen Elizabeth Hospital: Anna Casey
EPI_ISL_1474578, EPI_ISL_1474579, EPI_ISL_1474580	Virology Department, Royal Infirmary of Edinburgh, NHS Lothian / School of Biological Sciences, University of Edinburgh	COVID-19 Genomics UK (COG-UK) Consortium	McHugh M, Dewar R, Cotton S, Rooke S, O'Toole Á, Scher E, Hill V, McCrone JT, Colquhoun R, Yu X, Jackson B, Rambaut A, Templeton K
EPI_ISL_1474857	University College London, Great Ormond Street Hospital for Children NHS Foundation Trust, Imperial College Healthcare NHS Trust	COVID-19 Genomics UK (COG-UK) Consortium	Sergi Castellano, Rachel Williams, Mark Kristiansen, Paola Resende Silva, Sunando Roy, Tony Brooks, Helena Tutill, Paola Niola, Patricia Dyal, Charlotte Williams, Leysa Forrest, Yasmin Panchbhaya, Jacqueline Findlay, Samuel Weeks, Julianne Brown, Kathryn Harris, Paul Randell, James Price, Alison Holmes, Judith Breuer
EPI_ISL_1474986, EPI_ISL_1474987, EPI_ISL_1474988, EPI_ISL_1474989, EPI_ISL_1474990, EPI_ISL_1475003	Regional Virus Laboratory, Belfast Health and Social Care Trust	COVID-19 Genomics UK (COG-UK) Consortium	Conall McCaughey, James McKenna, Tanya Curran, Susan Feeney, Alison Watt, Ciara Cox, Mairead Connor, Zoltan Molnar, David Simpson, Derek Fairley
EPI_ISL_1475239	Northumbria University / South Tees Hospitals NHS Foundation Trust / North Cumbria Integrated Care NHS Foundation Trust / North Tees and Hartlepool NHS Foundation Trust / Newcastle Hospitals NHS Foundation Trust	COVID-19 Genomics UK (COG-UK) Consortium	Darren L Smith,Andrew Nelson,Matthew Bashton,Greg R Young,Joshua Loh,John Allan,Mohammad A Tariq,Giles S Holt,Gary Black,Wen C Yew,Lynn Dover,Paul Baker,Steve Liggett,Sarah Essex,Jane Greenaway,Debra Padgett,Clive Graham,Garren Scott,Edward Barton,Emma Swindells,Brendan Payne,Jennifer Collins,Yusri Taha,Gary Eltringham
EPI_ISL_1476078, EPI_ISL_1476079, EPI_ISL_1476098, EPI_ISL_1476099, EPI_ISL_1476102, EPI_ISL_1476176, EPI_ISL_1476182, EPI_ISL_1476185, EPI_ISL_1476189, EPI_ISL_1476192, EPI_ISL_1476193, EPI_ISL_1476195, EPI_ISL_1476196, EPI_ISL_1476197, EPI_ISL_1476201, EPI_ISL_1476204, EPI_ISL_1476256			
see above	Originating lab: Wales Specialist Virology Centre Sequencing lab: Pathogen Genomics Unit	Public Health Wales Microbiology Cardiff Wales Specialist Virology Centre	Catherine Moore, Johnathan Evans, Laura Gifford, Malorie Perry, Simon Cottrell, Angela Marchbank, Alec Birchley, Alexander Adams, Amy Gaskin, Bree Gatica-Wilcox, Jason Coombes, Joel Southgate, Lauren Gilbert, Lee Graham, Nicole Pacchiarini, Sara Kumziene-Summerhayes, Sarah Taylor, Sophie Jones, Sara Rey, Matthew Bull, Joanne Watkins, Sally Corden, Tom Connor
EPI_ISL_1476563	Centre for Enzyme Innovation, University of Portsmouth / Translational Research Laboratory, Portsmouth Hospitals NHS Trust	COVID-19 Genomics UK (COG-UK) Consortium	Angela Beckett,Salman Goudarzi,Christopher Fearn,Kate Cook,Katie Loveson,Sharon Glaysher,Scott Elliott,Samuel Robson
EPI_ISL_837058, EPI_ISL_837063, EPI_ISL_837065, EPI_ISL_837073, EPI_ISL_837087, EPI_ISL_837183, EPI_ISL_837196, EPI_ISL_837197, EPI_ISL_837245, EPI_ISL_837246	Respiratory Virus Unit, National Infection Service, Public Health England	COVID-19 Genomics UK (COG-UK) Consortium	PHE Covid Sequencing Team
EPI_ISL_838015, EPI_ISL_838017, EPI_ISL_838018, EPI_ISL_838021, EPI_ISL_838022, EPI_ISL_838025, EPI_ISL_838035, EPI_ISL_838038, EPI_ISL_838048	Department of Pathology, University of Cambridge	COVID-19 Genomics UK (COG-UK) Consortium	Aminu S. Jahun, Yasmin Chaudhry, Grant Hall, Iliana Georgana, Myra Hosmillo, Martin D. Curran, Malte Pinckert, Surendra Parmar, Ian Goodfellow
EPI_ISL_839006, EPI_ISL_839020	University College London, Great Ormond Street Hospital for Children NHS Foundation Trust, Imperial College Healthcare NHS Trust	COVID-19 Genomics UK (COG-UK) Consortium	Sergi Castellano, Rachel Williams, Mark Kristiansen, Paola Resende Silva, Sunando Roy, Tony Brooks, Helena Tutill, Paola Niola, Patricia Dyal, Charlotte Williams, Leysa Forrest, Yasmin Panchbhaya, Jacqueline Findlay, Samuel Weeks, Julianne Brown, Kathryn Harris, Paul Randell, James Price, Alison Holmes, Judith Breuer
EPI_ISL_846774	Respiratory Virus Unit, National Infection Service, Public Health England	COVID-19 Genomics UK (COG-UK) Consortium	PHE Covid Sequencing Team
EPI_ISL_852014	Lighthouse Lab in Milton Keynes	Wellcome Sanger Institute for the COVID-19 Genomics UK (COG-UK) Consortium	The Lighthouse Lab in Milton Keynes and Alex Alderton, Roberto Amato, Sonia Goncalves, Ewan Harrison, David K. Jackson, Ian Johnston, Dominic Kwiatkowski, Cordelia Langford, John Sillitoe on behalf of the Wellcome Sanger Institute COVID-19 Surveillance Team
EPI_ISL_855603	Respiratory Virus Unit, National Infection Service, Public Health England	COVID-19 Genomics UK (COG-UK) Consortium	PHE Covid Sequencing Team
EPI_ISL_857855	Lighthouse Lab in Cambridge	Wellcome Sanger Institute for the COVID-19 Genomics UK (COG-UK) Consortium	Rob Howes, The Lighthouse Lab in Cambridge and Alex Alderton, Roberto Amato, Sonia Goncalves, Ewan Harrison, David K. Jackson, Ian Johnston, Dominic Kwiatkowski, Cordelia Langford, John Sillitoe on behalf of the Wellcome Sanger Institute COVID-19 Surveillance Team
EPI_ISL_860686, EPI_ISL_860691, EPI_ISL_860694, EPI_ISL_860695, EPI_ISL_860709, EPI_ISL_862193, EPI_ISL_862259	Respiratory Virus Unit, National Infection Service, Public Health England	COVID-19 Genomics UK (COG-UK) Consortium	PHE Covid Sequencing Team
EPI_ISL_863245, EPI_ISL_863246, EPI_ISL_863247, EPI_ISL_863248, EPI_ISL_863249, EPI_ISL_863250	Lighthouse Lab in Glasgow	Wellcome Sanger Institute for the COVID-19 Genomics UK (COG-UK) Consortium	Harper VanSteenhouse, Yumi Kasai, David Gray, Carol Clugston, Anna Dominiczak and Alex Alderton, Roberto Amato, Sonia Goncalves, Ewan Harrison, David K. Jackson, Ian Johnston, Dominic Kwiatkowski, Cordelia Langford, John Sillitoe on behalf of the Wellcome Sanger Institute COVID-19 Surveillance Team
EPI_ISL_863251	Lighthouse Lab in Alderley Park	Wellcome Sanger Institute for the COVID-19 Genomics UK (COG-UK) Consortium	Jacquelyn Wynn, Mairead Hyland, The Lighthouse Lab in Alderley Park and Alex Alderton, Roberto Amato, Sonia Goncalves, Ewan Harrison, David K. Jackson, Ian Johnston, Dominic Kwiatkowski, Cordelia Langford, John Sillitoe on behalf of the Wellcome Sanger Institute COVID-19 Surveillance Team
EPI_ISL_863252, EPI_ISL_863253	Lighthouse Lab in Glasgow	Wellcome Sanger Institute for the COVID-19 Genomics UK (COG-UK) Consortium	Harper VanSteenhouse, Yumi Kasai, David Gray, Carol Clugston, Anna Dominiczak and Alex Alderton, Roberto Amato, Sonia Goncalves, Ewan Harrison, David K. Jackson, Ian Johnston, Dominic Kwiatkowski, Cordelia Langford, John Sillitoe on behalf of the Wellcome Sanger Institute COVID-19 Surveillance Team
EPI_ISL_863254	Lighthouse Lab in Alderley Park	Wellcome Sanger Institute for the COVID-19 Genomics UK (COG-UK) Consortium	Jacquelyn Wynn, Mairead Hyland, The Lighthouse Lab in Alderley Park and Alex Alderton, Roberto Amato, Sonia Goncalves, Ewan Harrison, David K. Jackson, Ian Johnston, Dominic Kwiatkowski, Cordelia Langford, John Sillitoe on behalf of the Wellcome Sanger Institute COVID-19 Surveillance Team
EPI_ISL_863255, EPI_ISL_863256, EPI_ISL_863257	Lighthouse Lab in Glasgow	Wellcome Sanger Institute for the COVID-19 Genomics UK (COG-UK) Consortium	Harper VanSteenhouse, Yumi Kasai, David Gray, Carol Clugston, Anna Dominiczak and Alex Alderton, Roberto Amato, Sonia Goncalves, Ewan Harrison, David K. Jackson, Ian Johnston, Dominic Kwiatkowski, Cordelia Langford, John Sillitoe on behalf of the Wellcome Sanger Institute COVID-19 Surveillance Team
EPI_ISL_863258	Lighthouse Lab in Alderley Park	Wellcome Sanger Institute for the COVID-19 Genomics UK (COG-UK) Consortium	Jacquelyn Wynn, Mairead Hyland, The Lighthouse Lab in Alderley Park and Alex Alderton, Roberto Amato, Sonia Goncalves, Ewan Harrison, David K. Jackson, Ian Johnston, Dominic Kwiatkowski, Cordelia Langford, John Sillitoe on behalf of the Wellcome Sanger Institute COVID-19 Surveillance Team
EPI_ISL_863259, EPI_ISL_863260, EPI_ISL_863261, EPI_ISL_863262, EPI_ISL_863263, EPI_ISL_863264, EPI_ISL_863265, EPI_ISL_863266, EPI_ISL_863267	Lighthouse Lab in Glasgow	Wellcome Sanger Institute for the COVID-19 Genomics UK (COG-UK) Consortium	Harper VanSteenhouse, Yumi Kasai, David Gray, Carol Clugston, Anna Dominiczak and Alex Alderton, Roberto Amato, Sonia Goncalves, Ewan Harrison, David K. Jackson, Ian Johnston, Dominic Kwiatkowski, Cordelia Langford, John Sillitoe on behalf of the Wellcome Sanger Institute COVID-19 Surveillance Team
EPI_ISL_863268	Lighthouse Lab in Alderley Park	Wellcome Sanger Institute for the COVID-19 Genomics UK (COG-UK) Consortium	Jacquelyn Wynn, Mairead Hyland, The Lighthouse Lab in Alderley Park and Alex Alderton, Roberto Amato, Sonia Goncalves, Ewan Harrison, David K. Jackson, Ian Johnston, Dominic Kwiatkowski, Cordelia Langford, John Sillitoe on behalf of the Wellcome Sanger Institute COVID-19 Surveillance Team
EPI_ISL_863269	Lighthouse Lab in Glasgow	Wellcome Sanger Institute for the COVID-19 Genomics UK	Harper VanSteenhouse, Yumi Kasai, David Gray, Carol Clugston, Anna Dominiczak and Alex Alderton, Roberto Amato, Sonia Goncalves, Ewan Harrison,

		(COG-UK) Consortium	David K. Jackson, Ian Johnston, Dominic Kwiatkowski, Cordelia Langford, John Sillitoe on behalf of the Wellcome Sanger Institute COVID-19 Surveillance Team
EPI_ISL_863270, EPI_ISL_863271, EPI_ISL_863272	Lighthouse Lab in Alderley Park	Wellcome Sanger Institute for the COVID-19 Genomics UK (COG-UK) Consortium	Jacquelyn Wynn, Mairead Hyland, The Lighthouse Lab in Alderley Park and Alex Alderton, Roberto Amato, Sonia Goncalves, Ewan Harrison, David K. Jackson, Ian Johnston, Dominic Kwiatkowski, Cordelia Langford, John Sillitoe on behalf of the Wellcome Sanger Institute COVID-19 Surveillance Team
EPI_ISL_863273	Lighthouse Lab in Glasgow	Wellcome Sanger Institute for the COVID-19 Genomics UK (COG-UK) Consortium	Harper VanSteenhouse, Yumi Kasai, David Gray, Carol Clugston, Anna Dominiczak and Alex Alderton, Roberto Amato, Sonia Goncalves, Ewan Harrison, David K. Jackson, Ian Johnston, Dominic Kwiatkowski, Cordelia Langford, John Sillitoe on behalf of the Wellcome Sanger Institute COVID-19 Surveillance Team
EPI_ISL_863274, EPI_ISL_863275	Lighthouse Lab in Alderley Park	Wellcome Sanger Institute for the COVID-19 Genomics UK (COG-UK) Consortium	Jacquelyn Wynn, Mairead Hyland, The Lighthouse Lab in Alderley Park and Alex Alderton, Roberto Amato, Sonia Goncalves, Ewan Harrison, David K. Jackson, Ian Johnston, Dominic Kwiatkowski, Cordelia Langford, John Sillitoe on behalf of the Wellcome Sanger Institute COVID-19 Surveillance Team
EPI_ISL_863276, EPI_ISL_863277, EPI_ISL_863278, EPI_ISL_863279	Lighthouse Lab in Glasgow	Wellcome Sanger Institute for the COVID-19 Genomics UK (COG-UK) Consortium	Harper VanSteenhouse, Yumi Kasai, David Gray, Carol Clugston, Anna Dominiczak and Alex Alderton, Roberto Amato, Sonia Goncalves, Ewan Harrison, David K. Jackson, Ian Johnston, Dominic Kwiatkowski, Cordelia Langford, John Sillitoe on behalf of the Wellcome Sanger Institute COVID-19 Surveillance Team
EPI_ISL_863280, EPI_ISL_863281	Lighthouse Lab in Alderley Park	Wellcome Sanger Institute for the COVID-19 Genomics UK (COG-UK) Consortium	Jacquelyn Wynn, Mairead Hyland, The Lighthouse Lab in Alderley Park and Alex Alderton, Roberto Amato, Sonia Goncalves, Ewan Harrison, David K. Jackson, Ian Johnston, Dominic Kwiatkowski, Cordelia Langford, John Sillitoe on behalf of the Wellcome Sanger Institute COVID-19 Surveillance Team
EPI_ISL_863282, EPI_ISL_863283, EPI_ISL_863284, EPI_ISL_863285, EPI_ISL_863286, EPI_ISL_863287, EPI_ISL_863288	Lighthouse Lab in Glasgow	Wellcome Sanger Institute for the COVID-19 Genomics UK (COG-UK) Consortium	Harper VanSteenhouse, Yumi Kasai, David Gray, Carol Clugston, Anna Dominiczak and Alex Alderton, Roberto Amato, Sonia Goncalves, Ewan Harrison, David K. Jackson, Ian Johnston, Dominic Kwiatkowski, Cordelia Langford, John Sillitoe on behalf of the Wellcome Sanger Institute COVID-19 Surveillance Team
EPI_ISL_863289, EPI_ISL_863290	Lighthouse Lab in Alderley Park	Wellcome Sanger Institute for the COVID-19 Genomics UK (COG-UK) Consortium	Jacquelyn Wynn, Mairead Hyland, The Lighthouse Lab in Alderley Park and Alex Alderton, Roberto Amato, Sonia Goncalves, Ewan Harrison, David K. Jackson, Ian Johnston, Dominic Kwiatkowski, Cordelia Langford, John Sillitoe on behalf of the Wellcome Sanger Institute COVID-19 Surveillance Team
EPI_ISL_863291, EPI_ISL_863292, EPI_ISL_863293, EPI_ISL_863294, EPI_ISL_863295, EPI_ISL_863296, EPI_ISL_863297, EPI_ISL_863298	Lighthouse Lab in Glasgow	Wellcome Sanger Institute for the COVID-19 Genomics UK (COG-UK) Consortium	Harper VanSteenhouse, Yumi Kasai, David Gray, Carol Clugston, Anna Dominiczak and Alex Alderton, Roberto Amato, Sonia Goncalves, Ewan Harrison, David K. Jackson, Ian Johnston, Dominic Kwiatkowski, Cordelia Langford, John Sillitoe on behalf of the Wellcome Sanger Institute COVID-19 Surveillance Team
EPI_ISL_863299	Lighthouse Lab in Alderley Park	Wellcome Sanger Institute for the COVID-19 Genomics UK (COG-UK) Consortium	Jacquelyn Wynn, Mairead Hyland, The Lighthouse Lab in Alderley Park and Alex Alderton, Roberto Amato, Sonia Goncalves, Ewan Harrison, David K. Jackson, Ian Johnston, Dominic Kwiatkowski, Cordelia Langford, John Sillitoe on behalf of the Wellcome Sanger Institute COVID-19 Surveillance Team
EPI_ISL_863300, EPI_ISL_863301	Lighthouse Lab in Glasgow	Wellcome Sanger Institute for the COVID-19 Genomics UK (COG-UK) Consortium	Harper VanSteenhouse, Yumi Kasai, David Gray, Carol Clugston, Anna Dominiczak and Alex Alderton, Roberto Amato, Sonia Goncalves, Ewan Harrison, David K. Jackson, Ian Johnston, Dominic Kwiatkowski, Cordelia Langford, John Sillitoe on behalf of the Wellcome Sanger Institute COVID-19 Surveillance Team
EPI_ISL_863302	Lighthouse Lab in Alderley Park	Wellcome Sanger Institute for the COVID-19 Genomics UK (COG-UK) Consortium	Jacquelyn Wynn, Mairead Hyland, The Lighthouse Lab in Alderley Park and Alex Alderton, Roberto Amato, Sonia Goncalves, Ewan Harrison, David K. Jackson, Ian Johnston, Dominic Kwiatkowski, Cordelia Langford, John Sillitoe on behalf of the Wellcome Sanger Institute COVID-19 Surveillance Team
EPI_ISL_863303, EPI_ISL_863304, EPI_ISL_863305	Lighthouse Lab in Glasgow	Wellcome Sanger Institute for the COVID-19 Genomics UK (COG-UK) Consortium	Harper VanSteenhouse, Yumi Kasai, David Gray, Carol Clugston, Anna Dominiczak and Alex Alderton, Roberto Amato, Sonia Goncalves, Ewan Harrison, David K. Jackson, Ian Johnston, Dominic Kwiatkowski, Cordelia Langford, John Sillitoe on behalf of the Wellcome Sanger Institute COVID-19 Surveillance Team
EPI_ISL_863306	Lighthouse Lab in Alderley Park	Wellcome Sanger Institute for the COVID-19 Genomics UK (COG-UK) Consortium	Jacquelyn Wynn, Mairead Hyland, The Lighthouse Lab in Alderley Park and Alex Alderton, Roberto Amato, Sonia Goncalves, Ewan Harrison, David K. Jackson, Ian Johnston, Dominic Kwiatkowski, Cordelia Langford, John Sillitoe on behalf of the Wellcome Sanger Institute COVID-19 Surveillance Team
EPI_ISL_863307, EPI_ISL_863308, EPI_ISL_863309	Lighthouse Lab in Glasgow	Wellcome Sanger Institute for the COVID-19 Genomics UK (COG-UK) Consortium	Harper VanSteenhouse, Yumi Kasai, David Gray, Carol Clugston, Anna Dominiczak and Alex Alderton, Roberto Amato, Sonia Goncalves, Ewan Harrison, David K. Jackson, Ian Johnston, Dominic Kwiatkowski, Cordelia Langford, John Sillitoe on behalf of the Wellcome Sanger Institute COVID-19 Surveillance Team
EPI_ISL_863310, EPI_ISL_863311	Lighthouse Lab in Alderley Park	Wellcome Sanger Institute for the COVID-19 Genomics UK (COG-UK) Consortium	Jacquelyn Wynn, Mairead Hyland, The Lighthouse Lab in Alderley Park and Alex Alderton, Roberto Amato, Sonia Goncalves, Ewan Harrison, David K. Jackson, Ian Johnston, Dominic Kwiatkowski, Cordelia Langford, John Sillitoe on behalf of the Wellcome Sanger Institute COVID-19 Surveillance Team
EPI_ISL_863312, EPI_ISL_863313, EPI_ISL_863314, EPI_ISL_863315, EPI_ISL_863316, EPI_ISL_863317	Lighthouse Lab in Glasgow	Wellcome Sanger Institute for the COVID-19 Genomics UK (COG-UK) Consortium	Harper VanSteenhouse, Yumi Kasai, David Gray, Carol Clugston, Anna Dominiczak and Alex Alderton, Roberto Amato, Sonia Goncalves, Ewan Harrison, David K. Jackson, Ian Johnston, Dominic Kwiatkowski, Cordelia Langford, John Sillitoe on behalf of the Wellcome Sanger Institute COVID-19 Surveillance Team
EPI_ISL_863318, EPI_ISL_863319	Lighthouse Lab in Alderley Park	Wellcome Sanger Institute for the COVID-19 Genomics UK (COG-UK) Consortium	Jacquelyn Wynn, Mairead Hyland, The Lighthouse Lab in Alderley Park and Alex Alderton, Roberto Amato, Sonia Goncalves, Ewan Harrison, David K. Jackson, Ian Johnston, Dominic Kwiatkowski, Cordelia Langford, John Sillitoe on behalf of the Wellcome Sanger Institute COVID-19 Surveillance Team
EPI_ISL_863320, EPI_ISL_863321, EPI_ISL_863322, EPI_ISL_863323, EPI_ISL_863324, EPI_ISL_863325	Lighthouse Lab in Glasgow	Wellcome Sanger Institute for the COVID-19 Genomics UK (COG-UK) Consortium	Harper VanSteenhouse, Yumi Kasai, David Gray, Carol Clugston, Anna Dominiczak and Alex Alderton, Roberto Amato, Sonia Goncalves, Ewan Harrison, David K. Jackson, Ian Johnston, Dominic Kwiatkowski, Cordelia Langford, John Sillitoe on behalf of the Wellcome Sanger Institute COVID-19 Surveillance Team
EPI_ISL_863326	Lighthouse Lab in Alderley Park	Wellcome Sanger Institute for the COVID-19 Genomics UK (COG-UK) Consortium	Jacquelyn Wynn, Mairead Hyland, The Lighthouse Lab in Alderley Park and Alex Alderton, Roberto Amato, Sonia Goncalves, Ewan Harrison, David K. Jackson, Ian Johnston, Dominic Kwiatkowski, Cordelia Langford, John Sillitoe on behalf of the Wellcome Sanger Institute COVID-19 Surveillance Team
EPI_ISL_863327, EPI_ISL_863328, EPI_ISL_863329, EPI_ISL_863330, EPI_ISL_863331, EPI_ISL_863332, EPI_ISL_863333, EPI_ISL_863335, EPI_ISL_863336, EPI_ISL_863337	Lighthouse Lab in Glasgow	Wellcome Sanger Institute for the COVID-19 Genomics UK (COG-UK) Consortium	Harper VanSteenhouse, Yumi Kasai, David Gray, Carol Clugston, Anna Dominiczak and Alex Alderton, Roberto Amato, Sonia Goncalves, Ewan Harrison, David K. Jackson, Ian Johnston, Dominic Kwiatkowski, Cordelia Langford, John Sillitoe on behalf of the Wellcome Sanger Institute COVID-19 Surveillance Team
EPI_ISL_863338	Lighthouse Lab in Alderley Park	Wellcome Sanger Institute for the COVID-19 Genomics UK (COG-UK) Consortium	Jacquelyn Wynn, Mairead Hyland, The Lighthouse Lab in Alderley Park and Alex Alderton, Roberto Amato, Sonia Goncalves, Ewan Harrison, David K. Jackson, Ian Johnston, Dominic Kwiatkowski, Cordelia Langford, John Sillitoe on behalf of the Wellcome Sanger Institute COVID-19 Surveillance Team
EPI_ISL_863339, EPI_ISL_863340, EPI_ISL_863341	Lighthouse Lab in Glasgow	Wellcome Sanger Institute for the COVID-19 Genomics UK (COG-UK) Consortium	Harper VanSteenhouse, Yumi Kasai, David Gray, Carol Clugston, Anna Dominiczak and Alex Alderton, Roberto Amato, Sonia Goncalves, Ewan Harrison, David K. Jackson, Ian Johnston, Dominic Kwiatkowski, Cordelia Langford, John Sillitoe on behalf of the Wellcome Sanger Institute COVID-19 Surveillance Team
EPI_ISL_863342, EPI_ISL_863343	Lighthouse Lab in Alderley Park	Wellcome Sanger Institute for the COVID-19 Genomics UK (COG-UK) Consortium	Jacquelyn Wynn, Mairead Hyland, The Lighthouse Lab in Alderley Park and Alex Alderton, Roberto Amato, Sonia Goncalves, Ewan Harrison, David K. Jackson, Ian Johnston, Dominic Kwiatkowski, Cordelia Langford, John Sillitoe on behalf of the Wellcome Sanger Institute COVID-19 Surveillance Team
EPI_ISL_863344, EPI_ISL_863345	Lighthouse Lab in Glasgow	Wellcome Sanger Institute for the COVID-19 Genomics UK (COG-UK) Consortium	Harper VanSteenhouse, Yumi Kasai, David Gray, Carol Clugston, Anna Dominiczak and Alex Alderton, Roberto Amato, Sonia Goncalves, Ewan Harrison, David K. Jackson, Ian Johnston, Dominic Kwiatkowski, Cordelia Langford, John Sillitoe on behalf of the Wellcome Sanger Institute COVID-19 Surveillance Team
EPI_ISL_863346	Lighthouse Lab in Alderley Park	Wellcome Sanger Institute for the COVID-19 Genomics UK (COG-UK) Consortium	Jacquelyn Wynn, Mairead Hyland, The Lighthouse Lab in Alderley Park and Alex Alderton, Roberto Amato, Sonia Goncalves, Ewan Harrison, David K. Jackson, Ian Johnston, Dominic Kwiatkowski, Cordelia Langford, John Sillitoe on behalf of the Wellcome Sanger Institute COVID-19 Surveillance Team
EPI_ISL_863347, EPI_ISL_863348, EPI_ISL_863349, EPI_ISL_863350, EPI_ISL_863351, EPI_ISL_863352, EPI_ISL_863353, EPI_ISL_863354, EPI_ISL_863355	Lighthouse Lab in Glasgow	Wellcome Sanger Institute for the COVID-19 Genomics UK (COG-UK) Consortium	Harper VanSteenhouse, Yumi Kasai, David Gray, Carol Clugston, Anna Dominiczak and Alex Alderton, Roberto Amato, Sonia Goncalves, Ewan Harrison, David K. Jackson, Ian Johnston, Dominic Kwiatkowski, Cordelia Langford, John Sillitoe on behalf of the Wellcome Sanger Institute COVID-19 Surveillance Team
EPI_ISL_863356, EPI_ISL_863357	Lighthouse Lab in Alderley Park	Wellcome Sanger Institute for the COVID-19 Genomics UK	Jacquelyn Wynn, Mairead Hyland, The Lighthouse Lab in Alderley Park and Alex Alderton, Roberto Amato, Sonia Goncalves, Ewan Harrison, David K.

		(COG-UK) Consortium	Jackson, Ian Johnston, Dominic Kwiatkowski, Cordelia Langford, John Sillitoe on behalf of the Wellcome Sanger Institute COVID-19 Surveillance Team
EPI_ISL_863358, EPI_ISL_863359	Lighthouse Lab in Glasgow	Wellcome Sanger Institute for the COVID-19 Genomics UK (COG-UK) Consortium	Harper VanSteenhouse, Yumi Kasai, David Gray, Carol Clugston, Anna Dominiczak and Alex Alderton, Roberto Amato, Sonia Goncalves, Ewan Harrison, David K. Jackson, Ian Johnston, Dominic Kwiatkowski, Cordelia Langford, John Sillitoe on behalf of the Wellcome Sanger Institute COVID-19 Surveillance Team
EPI_ISL_863360	Lighthouse Lab in Alderley Park	Wellcome Sanger Institute for the COVID-19 Genomics UK (COG-UK) Consortium	Jacquelyn Wynn, Mairead Hyland, The Lighthouse Lab in Alderley Park and Alex Alderton, Roberto Amato, Sonia Goncalves, Ewan Harrison, David K. Jackson, Ian Johnston, Dominic Kwiatkowski, Cordelia Langford, John Sillitoe on behalf of the Wellcome Sanger Institute COVID-19 Surveillance Team
EPI_ISL_863361	Lighthouse Lab in Glasgow	Wellcome Sanger Institute for the COVID-19 Genomics UK (COG-UK) Consortium	Harper VanSteenhouse, Yumi Kasai, David Gray, Carol Clugston, Anna Dominiczak and Alex Alderton, Roberto Amato, Sonia Goncalves, Ewan Harrison, David K. Jackson, Ian Johnston, Dominic Kwiatkowski, Cordelia Langford, John Sillitoe on behalf of the Wellcome Sanger Institute COVID-19 Surveillance Team
EPI_ISL_863362, EPI_ISL_863363, EPI_ISL_863364	Lighthouse Lab in Alderley Park	Wellcome Sanger Institute for the COVID-19 Genomics UK (COG-UK) Consortium	Jacquelyn Wynn, Mairead Hyland, The Lighthouse Lab in Alderley Park and Alex Alderton, Roberto Amato, Sonia Goncalves, Ewan Harrison, David K. Jackson, Ian Johnston, Dominic Kwiatkowski, Cordelia Langford, John Sillitoe on behalf of the Wellcome Sanger Institute COVID-19 Surveillance Team
EPI_ISL_863365, EPI_ISL_863366, EPI_ISL_863367, EPI_ISL_863368, EPI_ISL_863369, EPI_ISL_863370, EPI_ISL_863371, EPI_ISL_863372, EPI_ISL_863373, EPI_ISL_863374, EPI_ISL_863375, EPI_ISL_863376			
see above	Lighthouse Lab in Glasgow	Wellcome Sanger Institute for the COVID-19 Genomics UK (COG-UK) Consortium	Harper VanSteenhouse, Yumi Kasai, David Gray, Carol Clugston, Anna Dominiczak and Alex Alderton, Roberto Amato, Sonia Goncalves, Ewan Harrison, David K. Jackson, Ian Johnston, Dominic Kwiatkowski, Cordelia Langford, John Sillitoe on behalf of the Wellcome Sanger Institute COVID-19 Surveillance Team
EPI_ISL_863377	Lighthouse Lab in Alderley Park	Wellcome Sanger Institute for the COVID-19 Genomics UK (COG-UK) Consortium	Jacquelyn Wynn, Mairead Hyland, The Lighthouse Lab in Alderley Park and Alex Alderton, Roberto Amato, Sonia Goncalves, Ewan Harrison, David K. Jackson, Ian Johnston, Dominic Kwiatkowski, Cordelia Langford, John Sillitoe on behalf of the Wellcome Sanger Institute COVID-19 Surveillance Team
EPI_ISL_863378	Lighthouse Lab in Glasgow	Wellcome Sanger Institute for the COVID-19 Genomics UK (COG-UK) Consortium	Harper VanSteenhouse, Yumi Kasai, David Gray, Carol Clugston, Anna Dominiczak and Alex Alderton, Roberto Amato, Sonia Goncalves, Ewan Harrison, David K. Jackson, Ian Johnston, Dominic Kwiatkowski, Cordelia Langford, John Sillitoe on behalf of the Wellcome Sanger Institute COVID-19 Surveillance Team
EPI_ISL_863379	Lighthouse Lab in Alderley Park	Wellcome Sanger Institute for the COVID-19 Genomics UK (COG-UK) Consortium	Jacquelyn Wynn, Mairead Hyland, The Lighthouse Lab in Alderley Park and Alex Alderton, Roberto Amato, Sonia Goncalves, Ewan Harrison, David K. Jackson, Ian Johnston, Dominic Kwiatkowski, Cordelia Langford, John Sillitoe on behalf of the Wellcome Sanger Institute COVID-19 Surveillance Team
EPI_ISL_863380, EPI_ISL_863381, EPI_ISL_863382	Lighthouse Lab in Glasgow	Wellcome Sanger Institute for the COVID-19 Genomics UK (COG-UK) Consortium	Harper VanSteenhouse, Yumi Kasai, David Gray, Carol Clugston, Anna Dominiczak and Alex Alderton, Roberto Amato, Sonia Goncalves, Ewan Harrison, David K. Jackson, Ian Johnston, Dominic Kwiatkowski, Cordelia Langford, John Sillitoe on behalf of the Wellcome Sanger Institute COVID-19 Surveillance Team
EPI_ISL_863383	Lighthouse Lab in Alderley Park	Wellcome Sanger Institute for the COVID-19 Genomics UK (COG-UK) Consortium	Jacquelyn Wynn, Mairead Hyland, The Lighthouse Lab in Alderley Park and Alex Alderton, Roberto Amato, Sonia Goncalves, Ewan Harrison, David K. Jackson, Ian Johnston, Dominic Kwiatkowski, Cordelia Langford, John Sillitoe on behalf of the Wellcome Sanger Institute COVID-19 Surveillance Team
EPI_ISL_863384, EPI_ISL_863385, EPI_ISL_863386	Lighthouse Lab in Glasgow	Wellcome Sanger Institute for the COVID-19 Genomics UK (COG-UK) Consortium	Harper VanSteenhouse, Yumi Kasai, David Gray, Carol Clugston, Anna Dominiczak and Alex Alderton, Roberto Amato, Sonia Goncalves, Ewan Harrison, David K. Jackson, Ian Johnston, Dominic Kwiatkowski, Cordelia Langford, John Sillitoe on behalf of the Wellcome Sanger Institute COVID-19 Surveillance Team
EPI_ISL_863387	Lighthouse Lab in Alderley Park	Wellcome Sanger Institute for the COVID-19 Genomics UK (COG-UK) Consortium	Jacquelyn Wynn, Mairead Hyland, The Lighthouse Lab in Alderley Park and Alex Alderton, Roberto Amato, Sonia Goncalves, Ewan Harrison, David K. Jackson, Ian Johnston, Dominic Kwiatkowski, Cordelia Langford, John Sillitoe on behalf of the Wellcome Sanger Institute COVID-19 Surveillance Team
EPI_ISL_863388, EPI_ISL_863389, EPI_ISL_863390, EPI_ISL_863391, EPI_ISL_863392, EPI_ISL_863393, EPI_ISL_863394	Lighthouse Lab in Glasgow	Wellcome Sanger Institute for the COVID-19 Genomics UK (COG-UK) Consortium	Harper VanSteenhouse, Yumi Kasai, David Gray, Carol Clugston, Anna Dominiczak and Alex Alderton, Roberto Amato, Sonia Goncalves, Ewan Harrison, David K. Jackson, Ian Johnston, Dominic Kwiatkowski, Cordelia Langford, John Sillitoe on behalf of the Wellcome Sanger Institute COVID-19 Surveillance Team
EPI_ISL_863395, EPI_ISL_863397	Lighthouse Lab in Alderley Park	Wellcome Sanger Institute for the COVID-19 Genomics UK (COG-UK) Consortium	Jacquelyn Wynn, Mairead Hyland, The Lighthouse Lab in Alderley Park and Alex Alderton, Roberto Amato, Sonia Goncalves, Ewan Harrison, David K. Jackson, Ian Johnston, Dominic Kwiatkowski, Cordelia Langford, John Sillitoe on behalf of the Wellcome Sanger Institute COVID-19 Surveillance Team
EPI_ISL_863398, EPI_ISL_863399, EPI_ISL_863400, EPI_ISL_863402	Lighthouse Lab in Glasgow	Wellcome Sanger Institute for the COVID-19 Genomics UK (COG-UK) Consortium	Harper VanSteenhouse, Yumi Kasai, David Gray, Carol Clugston, Anna Dominiczak and Alex Alderton, Roberto Amato, Sonia Goncalves, Ewan Harrison, David K. Jackson, Ian Johnston, Dominic Kwiatkowski, Cordelia Langford, John Sillitoe on behalf of the Wellcome Sanger Institute COVID-19 Surveillance Team
EPI_ISL_863404	Lighthouse Lab in Alderley Park	Wellcome Sanger Institute for the COVID-19 Genomics UK (COG-UK) Consortium	Jacquelyn Wynn, Mairead Hyland, The Lighthouse Lab in Alderley Park and Alex Alderton, Roberto Amato, Sonia Goncalves, Ewan Harrison, David K. Jackson, Ian Johnston, Dominic Kwiatkowski, Cordelia Langford, John Sillitoe on behalf of the Wellcome Sanger Institute COVID-19 Surveillance Team
EPI_ISL_863405	Lighthouse Lab in Glasgow	Wellcome Sanger Institute for the COVID-19 Genomics UK (COG-UK) Consortium	Harper VanSteenhouse, Yumi Kasai, David Gray, Carol Clugston, Anna Dominiczak and Alex Alderton, Roberto Amato, Sonia Goncalves, Ewan Harrison, David K. Jackson, Ian Johnston, Dominic Kwiatkowski, Cordelia Langford, John Sillitoe on behalf of the Wellcome Sanger Institute COVID-19 Surveillance Team
EPI_ISL_863406	Lighthouse Lab in Alderley Park	Wellcome Sanger Institute for the COVID-19 Genomics UK (COG-UK) Consortium	Jacquelyn Wynn, Mairead Hyland, The Lighthouse Lab in Alderley Park and Alex Alderton, Roberto Amato, Sonia Goncalves, Ewan Harrison, David K. Jackson, Ian Johnston, Dominic Kwiatkowski, Cordelia Langford, John Sillitoe on behalf of the Wellcome Sanger Institute COVID-19 Surveillance Team
EPI_ISL_863407	Lighthouse Lab in Glasgow	Wellcome Sanger Institute for the COVID-19 Genomics UK (COG-UK) Consortium	Harper VanSteenhouse, Yumi Kasai, David Gray, Carol Clugston, Anna Dominiczak and Alex Alderton, Roberto Amato, Sonia Goncalves, Ewan Harrison, David K. Jackson, Ian Johnston, Dominic Kwiatkowski, Cordelia Langford, John Sillitoe on behalf of the Wellcome Sanger Institute COVID-19 Surveillance Team
EPI_ISL_863408	Lighthouse Lab in Alderley Park	Wellcome Sanger Institute for the COVID-19 Genomics UK (COG-UK) Consortium	Jacquelyn Wynn, Mairead Hyland, The Lighthouse Lab in Alderley Park and Alex Alderton, Roberto Amato, Sonia Goncalves, Ewan Harrison, David K. Jackson, Ian Johnston, Dominic Kwiatkowski, Cordelia Langford, John Sillitoe on behalf of the Wellcome Sanger Institute COVID-19 Surveillance Team
EPI_ISL_863409, EPI_ISL_863410	Lighthouse Lab in Glasgow	Wellcome Sanger Institute for the COVID-19 Genomics UK (COG-UK) Consortium	Harper VanSteenhouse, Yumi Kasai, David Gray, Carol Clugston, Anna Dominiczak and Alex Alderton, Roberto Amato, Sonia Goncalves, Ewan Harrison, David K. Jackson, Ian Johnston, Dominic Kwiatkowski, Cordelia Langford, John Sillitoe on behalf of the Wellcome Sanger Institute COVID-19 Surveillance Team
EPI_ISL_863411	Lighthouse Lab in Alderley Park	Wellcome Sanger Institute for the COVID-19 Genomics UK (COG-UK) Consortium	Jacquelyn Wynn, Mairead Hyland, The Lighthouse Lab in Alderley Park and Alex Alderton, Roberto Amato, Sonia Goncalves, Ewan Harrison, David K. Jackson, Ian Johnston, Dominic Kwiatkowski, Cordelia Langford, John Sillitoe on behalf of the Wellcome Sanger Institute COVID-19 Surveillance Team
EPI_ISL_863412, EPI_ISL_863413	Lighthouse Lab in Glasgow	Wellcome Sanger Institute for the COVID-19 Genomics UK (COG-UK) Consortium	Harper VanSteenhouse, Yumi Kasai, David Gray, Carol Clugston, Anna Dominiczak and Alex Alderton, Roberto Amato, Sonia Goncalves, Ewan Harrison, David K. Jackson, Ian Johnston, Dominic Kwiatkowski, Cordelia Langford, John Sillitoe on behalf of the Wellcome Sanger Institute COVID-19 Surveillance Team
EPI_ISL_863414	Lighthouse Lab in Alderley Park	Wellcome Sanger Institute for the COVID-19 Genomics UK (COG-UK) Consortium	Jacquelyn Wynn, Mairead Hyland, The Lighthouse Lab in Alderley Park and Alex Alderton, Roberto Amato, Sonia Goncalves, Ewan Harrison, David K. Jackson, Ian Johnston, Dominic Kwiatkowski, Cordelia Langford, John Sillitoe on behalf of the Wellcome Sanger Institute COVID-19 Surveillance Team
EPI_ISL_863415	Lighthouse Lab in Glasgow	Wellcome Sanger Institute for the COVID-19 Genomics UK (COG-UK) Consortium	Harper VanSteenhouse, Yumi Kasai, David Gray, Carol Clugston, Anna Dominiczak and Alex Alderton, Roberto Amato, Sonia Goncalves, Ewan Harrison, David K. Jackson, Ian Johnston, Dominic Kwiatkowski, Cordelia Langford, John Sillitoe on behalf of the Wellcome Sanger Institute COVID-19 Surveillance Team
EPI_ISL_863416	Lighthouse Lab in Alderley Park	Wellcome Sanger Institute for the COVID-19 Genomics UK (COG-UK) Consortium	Jacquelyn Wynn, Mairead Hyland, The Lighthouse Lab in Alderley Park and Alex Alderton, Roberto Amato, Sonia Goncalves, Ewan Harrison, David K. Jackson, Ian Johnston, Dominic Kwiatkowski, Cordelia Langford, John Sillitoe on behalf of the Wellcome Sanger Institute COVID-19 Surveillance Team
EPI_ISL_863417, EPI_ISL_863418, EPI_ISL_863419	Lighthouse Lab in Glasgow	Wellcome Sanger Institute for the COVID-19 Genomics UK (COG-UK) Consortium	Harper VanSteenhouse, Yumi Kasai, David Gray, Carol Clugston, Anna Dominiczak and Alex Alderton, Roberto Amato, Sonia Goncalves, Ewan Harrison, David K. Jackson, Ian Johnston, Dominic Kwiatkowski, Cordelia Langford, John Sillitoe on behalf of the Wellcome Sanger Institute COVID-19 Surveillance Team
EPI_ISL_863420, EPI_ISL_863421	Lighthouse Lab in Alderley Park	Wellcome Sanger Institute for the COVID-19 Genomics UK (COG-UK) Consortium	Jacquelyn Wynn, Mairead Hyland, The Lighthouse Lab in Alderley Park and Alex Alderton, Roberto Amato, Sonia Goncalves, Ewan Harrison, David K. Jackson, Ian Johnston, Dominic Kwiatkowski, Cordelia Langford, John Sillitoe on behalf of the Wellcome Sanger Institute COVID-19 Surveillance Team

EPI_ISL_863422	Lighthouse Lab in Glasgow	Wellcome Sanger Institute for the COVID-19 Genomics UK (COG-UK) Consortium	Harper VanSteenhouse, Yumi Kasai, David Gray, Carol Clugston, Anna Dominiczak and Alex Alderton, Roberto Amato, Sonia Goncalves, Ewan Harrison, David K. Jackson, Ian Johnston, Dominic Kwiatkowski, Cordelia Langford, John Sillitoe on behalf of the Wellcome Sanger Institute COVID-19 Surveillance Team
EPI_ISL_863423	Lighthouse Lab in Alderley Park	Wellcome Sanger Institute for the COVID-19 Genomics UK (COG-UK) Consortium	Jacquelyn Wynn, Mairead Hyland, The Lighthouse Lab in Alderley Park and Alex Alderton, Roberto Amato, Sonia Goncalves, Ewan Harrison, David K. Jackson, Ian Johnston, Dominic Kwiatkowski, Cordelia Langford, John Sillitoe on behalf of the Wellcome Sanger Institute COVID-19 Surveillance Team
EPI_ISL_863425	Lighthouse Lab in Glasgow	Wellcome Sanger Institute for the COVID-19 Genomics UK (COG-UK) Consortium	Harper VanSteenhouse, Yumi Kasai, David Gray, Carol Clugston, Anna Dominiczak and Alex Alderton, Roberto Amato, Sonia Goncalves, Ewan Harrison, David K. Jackson, Ian Johnston, Dominic Kwiatkowski, Cordelia Langford, John Sillitoe on behalf of the Wellcome Sanger Institute COVID-19 Surveillance Team
EPI_ISL_863426	Lighthouse Lab in Alderley Park	Wellcome Sanger Institute for the COVID-19 Genomics UK (COG-UK) Consortium	Jacquelyn Wynn, Mairead Hyland, The Lighthouse Lab in Alderley Park and Alex Alderton, Roberto Amato, Sonia Goncalves, Ewan Harrison, David K. Jackson, Ian Johnston, Dominic Kwiatkowski, Cordelia Langford, John Sillitoe on behalf of the Wellcome Sanger Institute COVID-19 Surveillance Team
EPI_ISL_863427, EPI_ISL_863428, EPI_ISL_863429, EPI_ISL_863430, EPI_ISL_863433	Lighthouse Lab in Glasgow	Wellcome Sanger Institute for the COVID-19 Genomics UK (COG-UK) Consortium	Harper VanSteenhouse, Yumi Kasai, David Gray, Carol Clugston, Anna Dominiczak and Alex Alderton, Roberto Amato, Sonia Goncalves, Ewan Harrison, David K. Jackson, Ian Johnston, Dominic Kwiatkowski, Cordelia Langford, John Sillitoe on behalf of the Wellcome Sanger Institute COVID-19 Surveillance Team
EPI_ISL_863434	Lighthouse Lab in Alderley Park	Wellcome Sanger Institute for the COVID-19 Genomics UK (COG-UK) Consortium	Jacquelyn Wynn, Mairead Hyland, The Lighthouse Lab in Alderley Park and Alex Alderton, Roberto Amato, Sonia Goncalves, Ewan Harrison, David K. Jackson, Ian Johnston, Dominic Kwiatkowski, Cordelia Langford, John Sillitoe on behalf of the Wellcome Sanger Institute COVID-19 Surveillance Team
EPI_ISL_863435, EPI_ISL_863436	Lighthouse Lab in Glasgow	Wellcome Sanger Institute for the COVID-19 Genomics UK (COG-UK) Consortium	Harper VanSteenhouse, Yumi Kasai, David Gray, Carol Clugston, Anna Dominiczak and Alex Alderton, Roberto Amato, Sonia Goncalves, Ewan Harrison, David K. Jackson, Ian Johnston, Dominic Kwiatkowski, Cordelia Langford, John Sillitoe on behalf of the Wellcome Sanger Institute COVID-19 Surveillance Team
EPI_ISL_863437	Lighthouse Lab in Alderley Park	Wellcome Sanger Institute for the COVID-19 Genomics UK (COG-UK) Consortium	Jacquelyn Wynn, Mairead Hyland, The Lighthouse Lab in Alderley Park and Alex Alderton, Roberto Amato, Sonia Goncalves, Ewan Harrison, David K. Jackson, Ian Johnston, Dominic Kwiatkowski, Cordelia Langford, John Sillitoe on behalf of the Wellcome Sanger Institute COVID-19 Surveillance Team
EPI_ISL_863438, EPI_ISL_863439, EPI_ISL_863440, EPI_ISL_863441, EPI_ISL_863442	Lighthouse Lab in Glasgow	Wellcome Sanger Institute for the COVID-19 Genomics UK (COG-UK) Consortium	Harper VanSteenhouse, Yumi Kasai, David Gray, Carol Clugston, Anna Dominiczak and Alex Alderton, Roberto Amato, Sonia Goncalves, Ewan Harrison, David K. Jackson, Ian Johnston, Dominic Kwiatkowski, Cordelia Langford, John Sillitoe on behalf of the Wellcome Sanger Institute COVID-19 Surveillance Team
EPI_ISL_863443	Lighthouse Lab in Alderley Park	Wellcome Sanger Institute for the COVID-19 Genomics UK (COG-UK) Consortium	Jacquelyn Wynn, Mairead Hyland, The Lighthouse Lab in Alderley Park and Alex Alderton, Roberto Amato, Sonia Goncalves, Ewan Harrison, David K. Jackson, Ian Johnston, Dominic Kwiatkowski, Cordelia Langford, John Sillitoe on behalf of the Wellcome Sanger Institute COVID-19 Surveillance Team
EPI_ISL_863444, EPI_ISL_863445	Lighthouse Lab in Glasgow	Wellcome Sanger Institute for the COVID-19 Genomics UK (COG-UK) Consortium	Harper VanSteenhouse, Yumi Kasai, David Gray, Carol Clugston, Anna Dominiczak and Alex Alderton, Roberto Amato, Sonia Goncalves, Ewan Harrison, David K. Jackson, Ian Johnston, Dominic Kwiatkowski, Cordelia Langford, John Sillitoe on behalf of the Wellcome Sanger Institute COVID-19 Surveillance Team
EPI_ISL_863446, EPI_ISL_863447, EPI_ISL_863448	Lighthouse Lab in Alderley Park	Wellcome Sanger Institute for the COVID-19 Genomics UK (COG-UK) Consortium	Jacquelyn Wynn, Mairead Hyland, The Lighthouse Lab in Alderley Park and Alex Alderton, Roberto Amato, Sonia Goncalves, Ewan Harrison, David K. Jackson, Ian Johnston, Dominic Kwiatkowski, Cordelia Langford, John Sillitoe on behalf of the Wellcome Sanger Institute COVID-19 Surveillance Team
EPI_ISL_863449, EPI_ISL_863450, EPI_ISL_863451, EPI_ISL_863452, EPI_ISL_863453	Lighthouse Lab in Glasgow	Wellcome Sanger Institute for the COVID-19 Genomics UK (COG-UK) Consortium	Harper VanSteenhouse, Yumi Kasai, David Gray, Carol Clugston, Anna Dominiczak and Alex Alderton, Roberto Amato, Sonia Goncalves, Ewan Harrison, David K. Jackson, Ian Johnston, Dominic Kwiatkowski, Cordelia Langford, John Sillitoe on behalf of the Wellcome Sanger Institute COVID-19 Surveillance Team
EPI_ISL_863454	Lighthouse Lab in Alderley Park	Wellcome Sanger Institute for the COVID-19 Genomics UK (COG-UK) Consortium	Jacquelyn Wynn, Mairead Hyland, The Lighthouse Lab in Alderley Park and Alex Alderton, Roberto Amato, Sonia Goncalves, Ewan Harrison, David K. Jackson, Ian Johnston, Dominic Kwiatkowski, Cordelia Langford, John Sillitoe on behalf of the Wellcome Sanger Institute COVID-19 Surveillance Team
EPI_ISL_863455, EPI_ISL_863456, EPI_ISL_863457, EPI_ISL_863458, EPI_ISL_863459, EPI_ISL_863460	Lighthouse Lab in Glasgow	Wellcome Sanger Institute for the COVID-19 Genomics UK (COG-UK) Consortium	Harper VanSteenhouse, Yumi Kasai, David Gray, Carol Clugston, Anna Dominiczak and Alex Alderton, Roberto Amato, Sonia Goncalves, Ewan Harrison, David K. Jackson, Ian Johnston, Dominic Kwiatkowski, Cordelia Langford, John Sillitoe on behalf of the Wellcome Sanger Institute COVID-19 Surveillance Team
EPI_ISL_863461	Lighthouse Lab in Alderley Park	Wellcome Sanger Institute for the COVID-19 Genomics UK (COG-UK) Consortium	Jacquelyn Wynn, Mairead Hyland, The Lighthouse Lab in Alderley Park and Alex Alderton, Roberto Amato, Sonia Goncalves, Ewan Harrison, David K. Jackson, Ian Johnston, Dominic Kwiatkowski, Cordelia Langford, John Sillitoe on behalf of the Wellcome Sanger Institute COVID-19 Surveillance Team
EPI_ISL_863462, EPI_ISL_863463, EPI_ISL_863464, EPI_ISL_863465, EPI_ISL_863466, EPI_ISL_863468, EPI_ISL_863469	Lighthouse Lab in Glasgow	Wellcome Sanger Institute for the COVID-19 Genomics UK (COG-UK) Consortium	Harper VanSteenhouse, Yumi Kasai, David Gray, Carol Clugston, Anna Dominiczak and Alex Alderton, Roberto Amato, Sonia Goncalves, Ewan Harrison, David K. Jackson, Ian Johnston, Dominic Kwiatkowski, Cordelia Langford, John Sillitoe on behalf of the Wellcome Sanger Institute COVID-19 Surveillance Team
EPI_ISL_863470	Lighthouse Lab in Alderley Park	Wellcome Sanger Institute for the COVID-19 Genomics UK (COG-UK) Consortium	Jacquelyn Wynn, Mairead Hyland, The Lighthouse Lab in Alderley Park and Alex Alderton, Roberto Amato, Sonia Goncalves, Ewan Harrison, David K. Jackson, Ian Johnston, Dominic Kwiatkowski, Cordelia Langford, John Sillitoe on behalf of the Wellcome Sanger Institute COVID-19 Surveillance Team
EPI_ISL_863471, EPI_ISL_863472, EPI_ISL_863473, EPI_ISL_863474, EPI_ISL_863475, EPI_ISL_863476	Lighthouse Lab in Glasgow	Wellcome Sanger Institute for the COVID-19 Genomics UK (COG-UK) Consortium	Harper VanSteenhouse, Yumi Kasai, David Gray, Carol Clugston, Anna Dominiczak and Alex Alderton, Roberto Amato, Sonia Goncalves, Ewan Harrison, David K. Jackson, Ian Johnston, Dominic Kwiatkowski, Cordelia Langford, John Sillitoe on behalf of the Wellcome Sanger Institute COVID-19 Surveillance Team
EPI_ISL_863477	Lighthouse Lab in Alderley Park	Wellcome Sanger Institute for the COVID-19 Genomics UK (COG-UK) Consortium	Jacquelyn Wynn, Mairead Hyland, The Lighthouse Lab in Alderley Park and Alex Alderton, Roberto Amato, Sonia Goncalves, Ewan Harrison, David K. Jackson, Ian Johnston, Dominic Kwiatkowski, Cordelia Langford, John Sillitoe on behalf of the Wellcome Sanger Institute COVID-19 Surveillance Team
EPI_ISL_863478	Lighthouse Lab in Glasgow	Wellcome Sanger Institute for the COVID-19 Genomics UK (COG-UK) Consortium	Harper VanSteenhouse, Yumi Kasai, David Gray, Carol Clugston, Anna Dominiczak and Alex Alderton, Roberto Amato, Sonia Goncalves, Ewan Harrison, David K. Jackson, Ian Johnston, Dominic Kwiatkowski, Cordelia Langford, John Sillitoe on behalf of the Wellcome Sanger Institute COVID-19 Surveillance Team
EPI_ISL_863479	Lighthouse Lab in Alderley Park	Wellcome Sanger Institute for the COVID-19 Genomics UK (COG-UK) Consortium	Jacquelyn Wynn, Mairead Hyland, The Lighthouse Lab in Alderley Park and Alex Alderton, Roberto Amato, Sonia Goncalves, Ewan Harrison, David K. Jackson, Ian Johnston, Dominic Kwiatkowski, Cordelia Langford, John Sillitoe on behalf of the Wellcome Sanger Institute COVID-19 Surveillance Team
EPI_ISL_863480, EPI_ISL_863481, EPI_ISL_863482, EPI_ISL_863483	Lighthouse Lab in Glasgow	Wellcome Sanger Institute for the COVID-19 Genomics UK (COG-UK) Consortium	Harper VanSteenhouse, Yumi Kasai, David Gray, Carol Clugston, Anna Dominiczak and Alex Alderton, Roberto Amato, Sonia Goncalves, Ewan Harrison, David K. Jackson, Ian Johnston, Dominic Kwiatkowski, Cordelia Langford, John Sillitoe on behalf of the Wellcome Sanger Institute COVID-19 Surveillance Team
EPI_ISL_863484	Lighthouse Lab in Alderley Park	Wellcome Sanger Institute for the COVID-19 Genomics UK (COG-UK) Consortium	Jacquelyn Wynn, Mairead Hyland, The Lighthouse Lab in Alderley Park and Alex Alderton, Roberto Amato, Sonia Goncalves, Ewan Harrison, David K. Jackson, Ian Johnston, Dominic Kwiatkowski, Cordelia Langford, John Sillitoe on behalf of the Wellcome Sanger Institute COVID-19 Surveillance Team
EPI_ISL_863485, EPI_ISL_863486, EPI_ISL_863487, EPI_ISL_863488, EPI_ISL_863489, EPI_ISL_863490	Lighthouse Lab in Glasgow	Wellcome Sanger Institute for the COVID-19 Genomics UK (COG-UK) Consortium	Harper VanSteenhouse, Yumi Kasai, David Gray, Carol Clugston, Anna Dominiczak and Alex Alderton, Roberto Amato, Sonia Goncalves, Ewan Harrison, David K. Jackson, Ian Johnston, Dominic Kwiatkowski, Cordelia Langford, John Sillitoe on behalf of the Wellcome Sanger Institute COVID-19 Surveillance Team
EPI_ISL_863491	Lighthouse Lab in Alderley Park	Wellcome Sanger Institute for the COVID-19 Genomics UK (COG-UK) Consortium	Jacquelyn Wynn, Mairead Hyland, The Lighthouse Lab in Alderley Park and Alex Alderton, Roberto Amato, Sonia Goncalves, Ewan Harrison, David K. Jackson, Ian Johnston, Dominic Kwiatkowski, Cordelia Langford, John Sillitoe on behalf of the Wellcome Sanger Institute COVID-19 Surveillance Team
EPI_ISL_863492, EPI_ISL_863493, EPI_ISL_863494, EPI_ISL_863495, EPI_ISL_863496, EPI_ISL_863497, EPI_ISL_863498, EPI_ISL_863499, EPI_ISL_863500, EPI_ISL_863501, EPI_ISL_863502			
see above	Lighthouse Lab in Glasgow	Wellcome Sanger Institute for the COVID-19 Genomics UK (COG-UK) Consortium	Harper VanSteenhouse, Yumi Kasai, David Gray, Carol Clugston, Anna Dominiczak and Alex Alderton, Roberto Amato, Sonia Goncalves, Ewan Harrison, David K. Jackson, Ian Johnston, Dominic Kwiatkowski, Cordelia Langford, John Sillitoe on behalf of the Wellcome Sanger Institute COVID-19 Surveillance Team
EPI_ISL_863503, EPI_ISL_863504	Lighthouse Lab in Alderley Park	Wellcome Sanger Institute for the COVID-19 Genomics UK (COG-UK) Consortium	Jacquelyn Wynn, Mairead Hyland, The Lighthouse Lab in Alderley Park and Alex Alderton, Roberto Amato, Sonia Goncalves, Ewan Harrison, David K. Jackson, Ian Johnston, Dominic Kwiatkowski, Cordelia Langford, John Sillitoe on behalf of the Wellcome Sanger Institute COVID-19 Surveillance Team
EPI_ISL_863505	Lighthouse Lab in Glasgow	Wellcome Sanger Institute for the COVID-19 Genomics UK	Harper VanSteenhouse, Yumi Kasai, David Gray, Carol Clugston, Anna Dominiczak and Alex Alderton, Roberto Amato, Sonia Goncalves, Ewan Harrison,

		(COG-UK) Consortium	David K. Jackson, Ian Johnston, Dominic Kwiatkowski, Cordelia Langford, John Sillitoe on behalf of the Wellcome Sanger Institute COVID-19 Surveillance Team
EPI_ISL_863506	Lighthouse Lab in Alderley Park	Wellcome Sanger Institute for the COVID-19 Genomics UK (COG-UK) Consortium	Jacquelyn Wynn, Mairead Hyland, The Lighthouse Lab in Alderley Park and Alex Alderton, Roberto Amato, Sonia Goncalves, Ewan Harrison, David K. Jackson, Ian Johnston, Dominic Kwiatkowski, Cordelia Langford, John Sillitoe on behalf of the Wellcome Sanger Institute COVID-19 Surveillance Team
EPI_ISL_863507, EPI_ISL_863508, EPI_ISL_863509, EPI_ISL_863510, EPI_ISL_863511, EPI_ISL_863512, EPI_ISL_863513	Lighthouse Lab in Glasgow	Wellcome Sanger Institute for the COVID-19 Genomics UK (COG-UK) Consortium	Harper VanSteenhouse, Yumi Kasai, David Gray, Carol Clugston, Anna Dominiczak and Alex Alderton, Roberto Amato, Sonia Goncalves, Ewan Harrison, David K. Jackson, Ian Johnston, Dominic Kwiatkowski, Cordelia Langford, John Sillitoe on behalf of the Wellcome Sanger Institute COVID-19 Surveillance Team
EPI_ISL_863515, EPI_ISL_863516	Lighthouse Lab in Alderley Park	Wellcome Sanger Institute for the COVID-19 Genomics UK (COG-UK) Consortium	Jacquelyn Wynn, Mairead Hyland, The Lighthouse Lab in Alderley Park and Alex Alderton, Roberto Amato, Sonia Goncalves, Ewan Harrison, David K. Jackson, Ian Johnston, Dominic Kwiatkowski, Cordelia Langford, John Sillitoe on behalf of the Wellcome Sanger Institute COVID-19 Surveillance Team
EPI_ISL_863517, EPI_ISL_863518	Lighthouse Lab in Glasgow	Wellcome Sanger Institute for the COVID-19 Genomics UK (COG-UK) Consortium	Harper VanSteenhouse, Yumi Kasai, David Gray, Carol Clugston, Anna Dominiczak and Alex Alderton, Roberto Amato, Sonia Goncalves, Ewan Harrison, David K. Jackson, Ian Johnston, Dominic Kwiatkowski, Cordelia Langford, John Sillitoe on behalf of the Wellcome Sanger Institute COVID-19 Surveillance Team
EPI_ISL_863519, EPI_ISL_863520	Lighthouse Lab in Alderley Park	Wellcome Sanger Institute for the COVID-19 Genomics UK (COG-UK) Consortium	Jacquelyn Wynn, Mairead Hyland, The Lighthouse Lab in Alderley Park and Alex Alderton, Roberto Amato, Sonia Goncalves, Ewan Harrison, David K. Jackson, Ian Johnston, Dominic Kwiatkowski, Cordelia Langford, John Sillitoe on behalf of the Wellcome Sanger Institute COVID-19 Surveillance Team
EPI_ISL_863521, EPI_ISL_863522, EPI_ISL_863523	Lighthouse Lab in Glasgow	Wellcome Sanger Institute for the COVID-19 Genomics UK (COG-UK) Consortium	Harper VanSteenhouse, Yumi Kasai, David Gray, Carol Clugston, Anna Dominiczak and Alex Alderton, Roberto Amato, Sonia Goncalves, Ewan Harrison, David K. Jackson, Ian Johnston, Dominic Kwiatkowski, Cordelia Langford, John Sillitoe on behalf of the Wellcome Sanger Institute COVID-19 Surveillance Team
EPI_ISL_863524	Lighthouse Lab in Alderley Park	Wellcome Sanger Institute for the COVID-19 Genomics UK (COG-UK) Consortium	Jacquelyn Wynn, Mairead Hyland, The Lighthouse Lab in Alderley Park and Alex Alderton, Roberto Amato, Sonia Goncalves, Ewan Harrison, David K. Jackson, Ian Johnston, Dominic Kwiatkowski, Cordelia Langford, John Sillitoe on behalf of the Wellcome Sanger Institute COVID-19 Surveillance Team
EPI_ISL_863525, EPI_ISL_863526	Lighthouse Lab in Glasgow	Wellcome Sanger Institute for the COVID-19 Genomics UK (COG-UK) Consortium	Harper VanSteenhouse, Yumi Kasai, David Gray, Carol Clugston, Anna Dominiczak and Alex Alderton, Roberto Amato, Sonia Goncalves, Ewan Harrison, David K. Jackson, Ian Johnston, Dominic Kwiatkowski, Cordelia Langford, John Sillitoe on behalf of the Wellcome Sanger Institute COVID-19 Surveillance Team
EPI_ISL_863527	Lighthouse Lab in Alderley Park	Wellcome Sanger Institute for the COVID-19 Genomics UK (COG-UK) Consortium	Jacquelyn Wynn, Mairead Hyland, The Lighthouse Lab in Alderley Park and Alex Alderton, Roberto Amato, Sonia Goncalves, Ewan Harrison, David K. Jackson, Ian Johnston, Dominic Kwiatkowski, Cordelia Langford, John Sillitoe on behalf of the Wellcome Sanger Institute COVID-19 Surveillance Team
EPI_ISL_863528, EPI_ISL_863530	Lighthouse Lab in Glasgow	Wellcome Sanger Institute for the COVID-19 Genomics UK (COG-UK) Consortium	Harper VanSteenhouse, Yumi Kasai, David Gray, Carol Clugston, Anna Dominiczak and Alex Alderton, Roberto Amato, Sonia Goncalves, Ewan Harrison, David K. Jackson, Ian Johnston, Dominic Kwiatkowski, Cordelia Langford, John Sillitoe on behalf of the Wellcome Sanger Institute COVID-19 Surveillance Team
EPI_ISL_863869, EPI_ISL_863870, EPI_ISL_863871, EPI_ISL_863872, EPI_ISL_863874, EPI_ISL_863875, EPI_ISL_863876, EPI_ISL_863877, EPI_ISL_863878, EPI_ISL_863879, EPI_ISL_863880, EPI_ISL_863881, EPI_ISL_863882, EPI_ISL_863883, EPI_ISL_863884, EPI_ISL_863885, EPI_ISL_863886, EPI_ISL_863887, EPI_ISL_863888, EPI_ISL_863889, EPI_ISL_863890, EPI_ISL_863891, EPI_ISL_863892, EPI_ISL_863893, EPI_ISL_863894, EPI_ISL_863895, EPI_ISL_863896, EPI_ISL_863897, EPI_ISL_863898, EPI_ISL_863900, EPI_ISL_863902, EPI_ISL_863903, EPI_ISL_863904, EPI_ISL_863905, EPI_ISL_863906, EPI_ISL_863907, EPI_ISL_863908, EPI_ISL_863909, EPI_ISL_863910, EPI_ISL_863911, EPI_ISL_863914, EPI_ISL_863915, EPI_ISL_863916, EPI_ISL_863917, EPI_ISL_863918, EPI_ISL_863919, EPI_ISL_863920, EPI_ISL_863923, EPI_ISL_863924, EPI_ISL_863925, EPI_ISL_863926, EPI_ISL_863927, EPI_ISL_863928, EPI_ISL_863929, EPI_ISL_863930, EPI_ISL_863931, EPI_ISL_863932, EPI_ISL_863933, EPI_ISL_863934, EPI_ISL_863935, EPI_ISL_863936, EPI_ISL_863937, EPI_ISL_863938, EPI_ISL_863939, EPI_ISL_863940, EPI_ISL_863941, EPI_ISL_863942, EPI_ISL_863943, EPI_ISL_863944, EPI_ISL_863945, EPI_ISL_863947, EPI_ISL_863948, EPI_ISL_863949, EPI_ISL_863950, EPI_ISL_863951, EPI_ISL_863952, EPI_ISL_863953, EPI_ISL_863954, EPI_ISL_863955, EPI_ISL_863957, EPI_ISL_863958, EPI_ISL_863959, EPI_ISL_863960, EPI_ISL_863961, EPI_ISL_863962, EPI_ISL_863963, EPI_ISL_863964, EPI_ISL_863965, EPI_ISL_863966, EPI_ISL_863967, EPI_ISL_863968, EPI_ISL_863970, EPI_ISL_863971, EPI_ISL_863972, EPI_ISL_863973, EPI_ISL_863974, EPI_ISL_863975, EPI_ISL_863976, EPI_ISL_863977, EPI_ISL_863978, EPI_ISL_863979, EPI_ISL_863980, EPI_ISL_863981, EPI_ISL_863982, EPI_ISL_863983, EPI_ISL_863984, EPI_ISL_863985, EPI_ISL_863986, EPI_ISL_863987, EPI_ISL_863988, EPI_ISL_863989, EPI_ISL_863990, EPI_ISL_863991, EPI_ISL_863992, EPI_ISL_863993, EPI_ISL_863994, EPI_ISL_863995, EPI_ISL_863997, EPI_ISL_863998, EPI_ISL_863999, EPI_ISL_864000, EPI_ISL_864001, EPI_ISL_864002, EPI_ISL_864003, EPI_ISL_864006, EPI_ISL_864007, EPI_ISL_864008, EPI_ISL_864010, EPI_ISL_864011, EPI_ISL_864012, EPI_ISL_864013, EPI_ISL_864014, EPI_ISL_864015, EPI_ISL_864016, EPI_ISL_864017, EPI_ISL_864018, EPI_ISL_864019, EPI_ISL_864020, EPI_ISL_864021, EPI_ISL_864022, EPI_ISL_864023, EPI_ISL_864024, EPI_ISL_864025, EPI_ISL_864027, EPI_ISL_864028, EPI_ISL_864029, EPI_ISL_864030, EPI_ISL_864031, EPI_ISL_864032, EPI_ISL_864033, EPI_ISL_864034, EPI_ISL_864035, EPI_ISL_864037, EPI_ISL_864038, EPI_ISL_864040, EPI_ISL_864041, EPI_ISL_864042, EPI_ISL_864044, EPI_ISL_864045, EPI_ISL_864046, EPI_ISL_864047, EPI_ISL_864048, EPI_ISL_864049, EPI_ISL_864050, EPI_ISL_864051, EPI_ISL_864052, EPI_ISL_864053, EPI_ISL_864054, EPI_ISL_864055, EPI_ISL_864056, EPI_ISL_864057, EPI_ISL_864058, EPI_ISL_864059, EPI_ISL_864060, EPI_ISL_864061, EPI_ISL_864062, EPI_ISL_864063, EPI_ISL_864065, EPI_ISL_864066, EPI_ISL_864067, EPI_ISL_864068, EPI_ISL_864069, EPI_ISL_864070, EPI_ISL_864071, EPI_ISL_864073, EPI_ISL_864074, EPI_ISL_864075, EPI_ISL_864076, EPI_ISL_864077, EPI_ISL_864078, EPI_ISL_864079, EPI_ISL_864080, EPI_ISL_864081, EPI_ISL_864082, EPI_ISL_864083, EPI_ISL_864084, EPI_ISL_864085, EPI_ISL_864086, EPI_ISL_864087, EPI_ISL_864088, EPI_ISL_864089, EPI_ISL_864090, EPI_ISL_864091, EPI_ISL_864092, EPI_ISL_864093, EPI_ISL_864095, EPI_ISL_864096, EPI_ISL_864097, EPI_ISL_864099, EPI_ISL_864100, EPI_ISL_864101, EPI_ISL_864102, EPI_ISL_864103, EPI_ISL_864104, EPI_ISL_864105, EPI_ISL_864106, EPI_ISL_864107, EPI_ISL_864108, EPI_ISL_864109, EPI_ISL_864111, EPI_ISL_864112, EPI_ISL_864113, EPI_ISL_864114, EPI_ISL_864115, EPI_ISL_864116, EPI_ISL_864117, EPI_ISL_864118, EPI_ISL_864119, EPI_ISL_864120, EPI_ISL_864121, EPI_ISL_864122, EPI_ISL_864123, EPI_ISL_864125, EPI_ISL_864126, EPI_ISL_864127, EPI_ISL_864128, EPI_ISL_864129, EPI_ISL_864130, EPI_ISL_864131, EPI_ISL_864132, EPI_ISL_864134, EPI_ISL_864135, EPI_ISL_864136, EPI_ISL_864137, EPI_ISL_864138, EPI_ISL_864139, EPI_ISL_864140, EPI_ISL_864141, EPI_ISL_864143, EPI_ISL_864144, EPI_ISL_864146, EPI_ISL_864147, EPI_ISL_864148, EPI_ISL_864149, EPI_ISL_864150, EPI_ISL_864151, EPI_ISL_864152, EPI_ISL_864153, EPI_ISL_864154, EPI_ISL_864155, EPI_ISL_864156, EPI_ISL_864157, EPI_ISL_864158, EPI_ISL_864159, EPI_ISL_864160, EPI_ISL_864161, EPI_ISL_864162, EPI_ISL_864163, EPI_ISL_864164, EPI_ISL_864166, EPI_ISL_864167, EPI_ISL_864168, EPI_ISL_864170, EPI_ISL_864171, EPI_ISL_864172, EPI_ISL_864174, EPI_ISL_864177, EPI_ISL_864179, EPI_ISL_864180, EPI_ISL_864181, EPI_ISL_864182, EPI_ISL_864183, EPI_ISL_864184, EPI_ISL_864185, EPI_ISL_864186, EPI_ISL_864187, EPI_ISL_864188, EPI_ISL_864189, EPI_ISL_864190, EPI_ISL_864192, EPI_ISL_864194, EPI_ISL_864195, EPI_ISL_864196, EPI_ISL_864197, EPI_ISL_864198, EPI_ISL_864199, EPI_ISL_864200, EPI_ISL_864201, EPI_ISL_864202			
see above	Lighthouse Lab in Alderley Park	Wellcome Sanger Institute for the COVID-19 Genomics UK (COG-UK) Consortium	Jacquelyn Wynn, Mairead Hyland, The Lighthouse Lab in Alderley Park and Alex Alderton, Roberto Amato, Sonia Goncalves, Ewan Harrison, David K. Jackson, Ian Johnston, Dominic Kwiatkowski, Cordelia Langford, John Sillitoe on behalf of the Wellcome Sanger Institute COVID-19 Surveillance Team
EPI_ISL_864896, EPI_ISL_864897, EPI_ISL_864898, EPI_ISL_864899, EPI_ISL_864900, EPI_ISL_864901, EPI_ISL_864902, EPI_ISL_864903, EPI_ISL_864904, EPI_ISL_864905, EPI_ISL_864906, EPI_ISL_864907, EPI_ISL_864908, EPI_ISL_864909, EPI_ISL_864910, EPI_ISL_864911, EPI_ISL_864912, EPI_ISL_864913, EPI_ISL_864914, EPI_ISL_864915, EPI_ISL_864916, EPI_ISL_864919, EPI_ISL_864920, EPI_ISL_864921, EPI_ISL_864922, EPI_ISL_864923, EPI_ISL_864924, EPI_ISL_864927, EPI_ISL_864928, EPI_ISL_864930, EPI_ISL_864931, EPI_ISL_864933, EPI_ISL_864934, EPI_ISL_864935, EPI_ISL_864937, EPI_ISL_864938, EPI_ISL_864939, EPI_ISL_864940, EPI_ISL_864941, EPI_ISL_864942, EPI_ISL_864943, EPI_ISL_864944, EPI_ISL_864946, EPI_ISL_864949, EPI_ISL_864950, EPI_ISL_864951, EPI_ISL_864952, EPI_ISL_864953, EPI_ISL_864954, EPI_ISL_864955, EPI_ISL_864956, EPI_ISL_864957, EPI_ISL_864958, EPI_ISL_864959			
see above	Department of Pathology, University of Cambridge	COVID-19 Genomics UK (COG-UK) Consortium	Aminu S. Jahun, Yasmin Chaudhry, Grant Hall, Iliana Georgana, Myra Hosmillo, Martin D. Curran, Malte Pinckert, Surendra Parmar, Ian Goodfellow
EPI_ISL_865092, EPI_ISL_865093, EPI_ISL_865105, EPI_ISL_865106, EPI_ISL_865107, EPI_ISL_865108, EPI_ISL_865109, EPI_ISL_865110, EPI_ISL_865136, EPI_ISL_865138, EPI_ISL_865139, EPI_ISL_865141, EPI_ISL_865144			
see above	Virology Department, Royal Infirmary of Edinburgh, NHS Lothian / School of Biological Sciences, University of Edinburgh / Institute of Genetics and Molecular Medicine, University of Edinburgh	COVID-19 Genomics UK (COG-UK) Consortium	McHugh M, Dewar R, Rooke S, Gallagher M, Balcaza C, O'Toole Á, Scher E, Hill V, McCrone JT, Colquhoun R, Yu X, Jackson B, Rambaut A, Williams TC, Templeton K
EPI_ISL_865472, EPI_ISL_865474, EPI_ISL_865475, EPI_ISL_865476, EPI_ISL_865477	Liverpool Clinical Laboratories	COVID-19 Genomics UK (COG-UK) Consortium	Sam Haldenby, Anita Lucaci, Steve Paterson, Julian Hiscox, Alistair Darby, M Almsaud, A Alrezaihi, Muhannad Alruwaili, Stuart D Armstrong, Jones Benjamin, Eleanor G Bentley, Anu Chawla, Jordan J Clark, Angela Cowell, Richard Eccles, Isabel García-Dorival, Matthew Gemmell, Alessandro Gerada, PKF Gilmore, Richard Gregory, Ximeng Han, Catherine Hartley, Margaret Hughes, Miren Iturriza-Gomara, James Johnson, L Luu, Jenifer Manson, Charlotte Nelson, Elaine O'Toole, Cassie Olateju, Rebekah Penrice-Randal , Lucille Rainbow, N.P Randle, Trevor Ian Robinson, Parul Sharma, Ghada T Shawli, James P Stewart, Neil Swainston, Ecaterina Vamos, Joanne Watts, Mark Whitehead
EPI_ISL_865485, EPI_ISL_865486, EPI_ISL_865487, EPI_ISL_865584, EPI_ISL_865585, EPI_ISL_865586, EPI_ISL_865587, EPI_ISL_865588, EPI_ISL_865589, EPI_ISL_865590, EPI_ISL_865591, EPI_ISL_865592, EPI_ISL_865593, EPI_ISL_865594, EPI_ISL_865595, EPI_ISL_865596, EPI_ISL_865597, EPI_ISL_865598, EPI_ISL_865599, EPI_ISL_865600, EPI_ISL_865601, EPI_ISL_865602, EPI_ISL_865603, EPI_ISL_865604, EPI_ISL_865644, EPI_ISL_865848, EPI_ISL_865849, EPI_ISL_865850, EPI_ISL_865851, EPI_ISL_865852, EPI_ISL_865853, EPI_ISL_865854, EPI_ISL_865855, EPI_ISL_865856, EPI_ISL_865857, EPI_ISL_865858, EPI_ISL_865859, EPI_ISL_865860, EPI_ISL_865861, EPI_ISL_865862, EPI_ISL_865863, EPI_ISL_865864, EPI_ISL_865865, EPI_ISL_865866, EPI_ISL_865867, EPI_ISL_865868, EPI_ISL_865869, EPI_ISL_865870, EPI_ISL_865871, EPI_ISL_865872, EPI_ISL_865873, EPI_ISL_865874, EPI_ISL_865875, EPI_ISL_865876, EPI_ISL_865877, EPI_ISL_865878, EPI_ISL_865879, EPI_ISL_865880, EPI_ISL_865881, EPI_ISL_865882, EPI_ISL_865883, EPI_ISL_865891, EPI_ISL_865895, EPI_ISL_865897, EPI_ISL_865901, EPI_ISL_865905, EPI_ISL_865906, EPI_ISL_865909, EPI_ISL_865910, EPI_ISL_865911, EPI_ISL_865912, EPI_ISL_865913, EPI_ISL_865914, EPI_ISL_865915, EPI_ISL_865916, EPI_ISL_865918, EPI_ISL_865919, EPI_ISL_865921, EPI_ISL_865922, EPI_ISL_865923, EPI_ISL_865924, EPI_ISL_865925, EPI_ISL_865926, EPI_ISL_865927, EPI_ISL_865928, EPI_ISL_865929, EPI_ISL_865930, EPI_ISL_865931, EPI_ISL_865932, EPI_ISL_865933, EPI_ISL_865934, EPI_ISL_865935, EPI_ISL_865938, EPI_ISL_865939, EPI_ISL_865940, EPI_ISL_865941, EPI_ISL_865942, EPI_ISL_865943, EPI_ISL_865944, EPI_ISL_865945, EPI_ISL_865947, EPI_ISL_865948, EPI_ISL_865949, EPI_ISL_865950, EPI_ISL_865952, EPI_ISL_865953, EPI_ISL_865954, EPI_ISL_865955, EPI_ISL_865956, EPI_ISL_865957, EPI_ISL_865958, EPI_ISL_865959, EPI_ISL_865960, EPI_ISL_865961, EPI_ISL_865962, EPI_ISL_865963, EPI_ISL_865964, EPI_ISL_865965, EPI_ISL_865966, EPI_ISL_865968, EPI_ISL_865977, EPI_ISL_865978, EPI_ISL_865986, EPI_ISL_865987, EPI_ISL_865994, EPI_ISL_866000, EPI_ISL_866001, EPI_ISL_866007, EPI_ISL_866009, EPI_ISL_866011, EPI_ISL_866012			
see above	University College London, Great Ormond Street Hospital for Children NHS Foundation Trust, Imperial College Healthcare NHS Trust	COVID-19 Genomics UK (COG-UK) Consortium	Sergi Castellano, Rachel Williams, Mark Kristiansen, Paola Resende Silva, Sunando Roy, Tony Brooks, Helena Tutill, Paola Niola, Patricia Dyal, Charlotte Williams, Leysa Forrest, Yasmin Panchbhaya, Jacqueline Findlay, Samuel Weeks, Julianne Brown, Kathryn Harris, Paul Randell, James Price, Alison Holmes, Judith Breuer
EPI_ISL_866150, EPI_ISL_866153, EPI_ISL_866155, EPI_ISL_866156, EPI_ISL_866158, EPI_ISL_866159, EPI_ISL_866161, EPI_ISL_866163, EPI_ISL_866165, EPI_ISL_866166, EPI_ISL_866167, EPI_ISL_866169, EPI_ISL_866170, EPI_ISL_866172, EPI_ISL_866173, EPI_ISL_866175, EPI_ISL_866176, EPI_ISL_866177, EPI_ISL_866181, EPI_ISL_866182, EPI_ISL_866184, EPI_ISL_866185			
see above	University College London Hospital	COVID-19 Genomics UK (COG-UK) Consortium	Judith Heaney, Matthew Byott, Catherine Houlihan, Dan Frampton, Stuart Kirk, Moira Spyer and Eleni Nastouli
EPI_ISL_866198, EPI_ISL_866201, EPI_ISL_866215, EPI_ISL_866217, EPI_ISL_866219, EPI_ISL_866221, EPI_ISL_866231, EPI_ISL_866262, EPI_ISL_866263, EPI_ISL_866264, EPI_ISL_866265, EPI_ISL_866266, EPI_ISL_866267, EPI_ISL_866268, EPI_ISL_866269, EPI_ISL_866270, EPI_ISL_866271, EPI_ISL_866272,			

EPI_ISL_866273, EPI_ISL_866274, EPI_ISL_866275, EPI_ISL_866276, EPI_ISL_866277, EPI_ISL_866278, EPI_ISL_866279, EPI_ISL_866280, EPI_ISL_866281, EPI_ISL_866282, EPI_ISL_866283, EPI_ISL_866284, EPI_ISL_866285, EPI_ISL_866286, EPI_ISL_866287, EPI_ISL_866288, EPI_ISL_866289, EPI_ISL_866290, EPI_ISL_866291, EPI_ISL_866292, EPI_ISL_866293, EPI_ISL_866294, EPI_ISL_866295, EPI_ISL_866296, EPI_ISL_866297, EPI_ISL_866299, EPI_ISL_866300, EPI_ISL_866301, EPI_ISL_866302, EPI_ISL_866303, EPI_ISL_866304, EPI_ISL_866305, EPI_ISL_866307, EPI_ISL_866308, EPI_ISL_866309, EPI_ISL_866310, EPI_ISL_866313, EPI_ISL_866314, EPI_ISL_866315, EPI_ISL_866316, EPI_ISL_866317, EPI_ISL_866318, EPI_ISL_866319			
see above	University College London, Great Ormond Street Hospital for Children NHS Foundation Trust, Imperial College Healthcare NHS Trust	COVID-19 Genomics UK (COG-UK) Consortium	Sergi Castellano, Rachel Williams, Mark Kristiansen, Paola Resende Silva, Sunando Roy, Tony Brooks, Helena Tutill, Paola Niola, Patricia Dyal, Charlotte Williams, Leysa Forrest, Yasmin Panchbhaya, Jacqueline Findlay, Samuel Weeks, Julianne Brown, Kathryn Harris, Paul Randell, James Price, Alison Holmes, Judith Breuer
EPI_ISL_866541, EPI_ISL_866548, EPI_ISL_866556, EPI_ISL_866566, EPI_ISL_866569, EPI_ISL_866748, EPI_ISL_866749, EPI_ISL_866750, EPI_ISL_866751, EPI_ISL_866752, EPI_ISL_866753, EPI_ISL_866754, EPI_ISL_866755, EPI_ISL_866756, EPI_ISL_866757, EPI_ISL_866760, EPI_ISL_866763, EPI_ISL_866764, EPI_ISL_866767, EPI_ISL_866770, EPI_ISL_866774, EPI_ISL_866775, EPI_ISL_866781, EPI_ISL_866788, EPI_ISL_866789, EPI_ISL_866801, EPI_ISL_866835, EPI_ISL_866836, EPI_ISL_866837, EPI_ISL_866838, EPI_ISL_866839, EPI_ISL_866840, EPI_ISL_866841, EPI_ISL_866842, EPI_ISL_866843, EPI_ISL_866844, EPI_ISL_866845, EPI_ISL_866846, EPI_ISL_866847, EPI_ISL_866848, EPI_ISL_866849, EPI_ISL_866850, EPI_ISL_866851, EPI_ISL_866852, EPI_ISL_866853, EPI_ISL_866854, EPI_ISL_866855, EPI_ISL_866856, EPI_ISL_866857, EPI_ISL_866858, EPI_ISL_866859, EPI_ISL_866860, EPI_ISL_866861, EPI_ISL_866862, EPI_ISL_866863, EPI_ISL_866864, EPI_ISL_866865, EPI_ISL_866866, EPI_ISL_866867, EPI_ISL_866868, EPI_ISL_866869, EPI_ISL_866871, EPI_ISL_866872, EPI_ISL_866873, EPI_ISL_866874, EPI_ISL_866875, EPI_ISL_866876, EPI_ISL_866877, EPI_ISL_866878, EPI_ISL_866879, EPI_ISL_866880, EPI_ISL_866881, EPI_ISL_866882, EPI_ISL_866883, EPI_ISL_866884, EPI_ISL_866885, EPI_ISL_866886, EPI_ISL_866887, EPI_ISL_866888			
see above	Quadram Institute Bioscience	COVID-19 Genomics UK (COG-UK) Consortium	Dave J. Baker, Gemma L. Kay, Alp Aydin, Thanh Le-Viet, Steven Rudder, Ana P. Tedim, Anastasia Kolyva, Maria Diaz, Leonardo de Oliveira Martins, Nabil-Fareed Alikhan, Lizzie Meadows, Rachael Stanley, Ngozi Elumogo, Muhammed Yasir, Nicholas M. Thomson, Alexander J Trotter, Rachel Gilroy, Samuel Bloomfield, Claire Stuart, Andrew Bell, Reenesh Prakash, Samir Dervisevic, Alison E. Mather, John Wain, Mark Webber, Andrew J. Page, Justin O'Grady
EPI_ISL_866921, EPI_ISL_866922, EPI_ISL_866923, EPI_ISL_866924, EPI_ISL_866925, EPI_ISL_866926, EPI_ISL_866927, EPI_ISL_866928, EPI_ISL_866929, EPI_ISL_866930, EPI_ISL_866931, EPI_ISL_866932, EPI_ISL_866933, EPI_ISL_866934, EPI_ISL_866935, EPI_ISL_866936, EPI_ISL_866937, EPI_ISL_866938, EPI_ISL_866939, EPI_ISL_866940, EPI_ISL_866941, EPI_ISL_866942, EPI_ISL_866943, EPI_ISL_866944, EPI_ISL_866945, EPI_ISL_866946, EPI_ISL_866947, EPI_ISL_866948, EPI_ISL_866949, EPI_ISL_866950, EPI_ISL_866971, EPI_ISL_866972, EPI_ISL_866973, EPI_ISL_866974, EPI_ISL_866975, EPI_ISL_866976, EPI_ISL_866977, EPI_ISL_866978, EPI_ISL_866979, EPI_ISL_866980, EPI_ISL_866981, EPI_ISL_866982, EPI_ISL_866983, EPI_ISL_866984, EPI_ISL_866986, EPI_ISL_866987, EPI_ISL_866988, EPI_ISL_866989			
see above	Queens Medical Centre, Clinical Microbiology Department / DeepSeq Nottingham	COVID-19 Genomics UK (COG-UK) Consortium	Gemma Clark, Wendy Smith, Manjinder Khakh, Vicki M Fleming, Michelle M Lister, Hannah Howson-Wells, Jonathan Ball, Patrick McClure, Joseph Chappell, Theocharis Tsoleridis, Nadine Holmes, Matthew Carlisle, Christopher Moore, Fei Sang, Johnny Debebe, Victoria Wright, Matthew Loose
EPI_ISL_867234, EPI_ISL_867235, EPI_ISL_867236, EPI_ISL_867259, EPI_ISL_867260, EPI_ISL_867262, EPI_ISL_867263, EPI_ISL_867264, EPI_ISL_867265, EPI_ISL_867266, EPI_ISL_867267, EPI_ISL_867268, EPI_ISL_867407, EPI_ISL_867408, EPI_ISL_867409, EPI_ISL_867410, EPI_ISL_867411, EPI_ISL_867412, EPI_ISL_867413, EPI_ISL_867414, EPI_ISL_867415, EPI_ISL_867420, EPI_ISL_867421, EPI_ISL_867422, EPI_ISL_867423, EPI_ISL_867425, EPI_ISL_867426, EPI_ISL_867428, EPI_ISL_867436, EPI_ISL_867444, EPI_ISL_867445, EPI_ISL_867446, EPI_ISL_867447, EPI_ISL_867448, EPI_ISL_867449, EPI_ISL_867452, EPI_ISL_867453, EPI_ISL_867454, EPI_ISL_867455, EPI_ISL_867460, EPI_ISL_867462, EPI_ISL_867463, EPI_ISL_867464, EPI_ISL_867465, EPI_ISL_867466, EPI_ISL_867467, EPI_ISL_867468, EPI_ISL_867469, EPI_ISL_867470, EPI_ISL_867471, EPI_ISL_867472, EPI_ISL_867473, EPI_ISL_867474, EPI_ISL_867475, EPI_ISL_867476, EPI_ISL_867477, EPI_ISL_867478, EPI_ISL_867479, EPI_ISL_867480, EPI_ISL_867481, EPI_ISL_867482, EPI_ISL_867483, EPI_ISL_867484, EPI_ISL_867485, EPI_ISL_867487, EPI_ISL_867488, EPI_ISL_867489, EPI_ISL_867495, EPI_ISL_867496, EPI_ISL_867502, EPI_ISL_867549, EPI_ISL_867576, EPI_ISL_867577, EPI_ISL_867585, EPI_ISL_867591, EPI_ISL_867592, EPI_ISL_867594, EPI_ISL_867595, EPI_ISL_867596, EPI_ISL_867597, EPI_ISL_867599, EPI_ISL_867600, EPI_ISL_867601, EPI_ISL_867602, EPI_ISL_867605, EPI_ISL_867607, EPI_ISL_867608, EPI_ISL_867610, EPI_ISL_867611, EPI_ISL_867613, EPI_ISL_867616, EPI_ISL_867617, EPI_ISL_867618, EPI_ISL_867620, EPI_ISL_867621, EPI_ISL_867623, EPI_ISL_867624, EPI_ISL_867625, EPI_ISL_867628, EPI_ISL_867629, EPI_ISL_867630, EPI_ISL_867631, EPI_ISL_867633, EPI_ISL_867634, EPI_ISL_867635, EPI_ISL_867636, EPI_ISL_867637, EPI_ISL_867639, EPI_ISL_867640, EPI_ISL_867641, EPI_ISL_867642, EPI_ISL_867643, EPI_ISL_867644, EPI_ISL_867647, EPI_ISL_867649, EPI_ISL_867650, EPI_ISL_867651, EPI_ISL_867652, EPI_ISL_867653, EPI_ISL_867654, EPI_ISL_867655, EPI_ISL_867656, EPI_ISL_867657, EPI_ISL_867658, EPI_ISL_867659, EPI_ISL_867660, EPI_ISL_867661, EPI_ISL_867662, EPI_ISL_867663, EPI_ISL_867664, EPI_ISL_867665, EPI_ISL_867667, EPI_ISL_867668, EPI_ISL_867670, EPI_ISL_867671, EPI_ISL_867672, EPI_ISL_867673, EPI_ISL_867674, EPI_ISL_867675, EPI_ISL_867676, EPI_ISL_867677, EPI_ISL_867678, EPI_ISL_867679, EPI_ISL_867680, EPI_ISL_867681, EPI_ISL_867682, EPI_ISL_867683, EPI_ISL_867684, EPI_ISL_867686, EPI_ISL_867688, EPI_ISL_867689, EPI_ISL_867690, EPI_ISL_867692, EPI_ISL_867696, EPI_ISL_867697, EPI_ISL_867698, EPI_ISL_867699, EPI_ISL_867700, EPI_ISL_867701, EPI_ISL_867727, EPI_ISL_867732, EPI_ISL_867733, EPI_ISL_867734, EPI_ISL_867735, EPI_ISL_867909, EPI_ISL_867917, EPI_ISL_867922, EPI_ISL_867923, EPI_ISL_867924, EPI_ISL_867926, EPI_ISL_867927, EPI_ISL_867928, EPI_ISL_867929			
see above	Originating lab: Wales Specialist Virology Centre Sequencing lab: Pathogen Genomics Unit	Public Health Wales Microbiology Cardiff Wales Specialist Virology Centre	Catherine Moore, Johnathan Evans, Laura Gifford, Malorie Perry, Simon Cottrell, Angela Marchbank, Alec Birchley, Alexander Adams, Amy Gaskin, Bree Gatica-Wilcox, Jason Coombes, Joel Southgate, Lauren Gilbert, Lee Graham, Nicole Pacchiarini, Sara Kumziene-Summerhayes, Sarah Taylor, Sophie Jones, Sara Rey, Matthew Bull, Joanne Watkins, Sally Corden, Tom Connor
EPI_ISL_868034, EPI_ISL_868102, EPI_ISL_868155, EPI_ISL_868156, EPI_ISL_868157, EPI_ISL_868158, EPI_ISL_868159, EPI_ISL_868160, EPI_ISL_868175, EPI_ISL_868176, EPI_ISL_868180, EPI_ISL_868195, EPI_ISL_868205, EPI_ISL_868206, EPI_ISL_868207, EPI_ISL_868208, EPI_ISL_868209, EPI_ISL_868210, EPI_ISL_868212, EPI_ISL_868213, EPI_ISL_868214, EPI_ISL_868215, EPI_ISL_868216, EPI_ISL_868219, EPI_ISL_868220, EPI_ISL_868221, EPI_ISL_868222, EPI_ISL_868224, EPI_ISL_868225, EPI_ISL_868228, EPI_ISL_868229, EPI_ISL_868230, EPI_ISL_868231, EPI_ISL_868232, EPI_ISL_868233, EPI_ISL_868234, EPI_ISL_868235, EPI_ISL_868236, EPI_ISL_868237, EPI_ISL_868238, EPI_ISL_868240			
see above	Centre for Enzyme Innovation, University of Portsmouth / Translational Research Laboratory, Portsmouth Hospitals NHS Trust	COVID-19 Genomics UK (COG-UK) Consortium	Angela Beckett,Yann Bourgeois,Garry Scarlett,Sharon Glaysher,Scott Elliott,Kelly Bicknell,Robert Impey,Allyson Lloyd,Sarah Wyllie,Ethan Butcher,Anoop Chauhan,Samuel Robson
EPI_ISL_868361, EPI_ISL_868362, EPI_ISL_868365, EPI_ISL_868367, EPI_ISL_868368, EPI_ISL_868378, EPI_ISL_868379, EPI_ISL_868385, EPI_ISL_868387, EPI_ISL_868389, EPI_ISL_868391, EPI_ISL_868397, EPI_ISL_868398, EPI_ISL_868407, EPI_ISL_868424, EPI_ISL_868425, EPI_ISL_868437, EPI_ISL_868439, EPI_ISL_868440, EPI_ISL_868451, EPI_ISL_868476, EPI_ISL_868482, EPI_ISL_868484, EPI_ISL_868492, EPI_ISL_868493, EPI_ISL_868496, EPI_ISL_868500, EPI_ISL_868501, EPI_ISL_868517, EPI_ISL_868523, EPI_ISL_868527, EPI_ISL_868532, EPI_ISL_868536, EPI_ISL_868546, EPI_ISL_868562, EPI_ISL_868569, EPI_ISL_868578, EPI_ISL_868579, EPI_ISL_868581, EPI_ISL_868582, EPI_ISL_868584, EPI_ISL_868592, EPI_ISL_868602, EPI_ISL_868618, EPI_ISL_868623, EPI_ISL_868626, EPI_ISL_868632, EPI_ISL_868634, EPI_ISL_868639, EPI_ISL_868642, EPI_ISL_868661, EPI_ISL_868662, EPI_ISL_868666, EPI_ISL_868679, EPI_ISL_868690, EPI_ISL_868696, EPI_ISL_868698, EPI_ISL_868703, EPI_ISL_868705, EPI_ISL_868708			
see above	Virology Department, Sheffield Teaching Hospitals NHS Foundation Trust/Department of Infection, Immunity and Cardiovascular Disease, The Medical School, University of Sheffield	COVID-19 Genomics UK (COG-UK) Consortium	Thushan de Silva, Matthew Parker, Nikki Smith, Adri Angyal, Rebecca Brown, Luke Green, Rachel Tucker, Paul Parsons, Danielle Groves, Katie Johnson, Laura Carrilero, Alex Keeley, Dave Partridge, Matthew Wyles, Benjamin Lindsey, Mehmet Yavuz, Mohammad Raza, Cariad Evans
EPI_ISL_873281	Lighthouse Lab in Milton Keynes	Wellcome Sanger Institute for the COVID-19 Genomics UK (COG-UK) Consortium	The Lighthouse Lab in Milton Keynes and Alex Alderton, Roberto Amato, Sonia Goncalves, Ewan Harrison, David K. Jackson, Ian Johnston, Dominic Kwiatkowski, Cordelia Langford, John Sillitoe on behalf of the Wellcome Sanger Institute COVID-19 Surveillance Team
EPI_ISL_873283, EPI_ISL_873286, EPI_ISL_873291, EPI_ISL_873292	Lighthouse Lab in Cambridge	Wellcome Sanger Institute for the COVID-19 Genomics UK (COG-UK) Consortium	Rob Howes, The Lighthouse Lab in Cambridge and Alex Alderton, Roberto Amato, Sonia Goncalves, Ewan Harrison, David K. Jackson, Ian Johnston, Dominic Kwiatkowski, Cordelia Langford, John Sillitoe on behalf of the Wellcome Sanger Institute COVID-19 Surveillance Team
EPI_ISL_873294	Lighthouse Lab in Milton Keynes	Wellcome Sanger Institute for the COVID-19 Genomics UK (COG-UK) Consortium	The Lighthouse Lab in Milton Keynes and Alex Alderton, Roberto Amato, Sonia Goncalves, Ewan Harrison, David K. Jackson, Ian Johnston, Dominic Kwiatkowski, Cordelia Langford, John Sillitoe on behalf of the Wellcome Sanger Institute COVID-19 Surveillance Team
EPI_ISL_873297	Lighthouse Lab in Cambridge	Wellcome Sanger Institute for the COVID-19 Genomics UK (COG-UK) Consortium	Rob Howes, The Lighthouse Lab in Cambridge and Alex Alderton, Roberto Amato, Sonia Goncalves, Ewan Harrison, David K. Jackson, Ian Johnston, Dominic Kwiatkowski, Cordelia Langford, John Sillitoe on behalf of the Wellcome Sanger Institute COVID-19 Surveillance Team
EPI_ISL_873298	Lighthouse Lab in Milton Keynes	Wellcome Sanger Institute for the COVID-19 Genomics UK (COG-UK) Consortium	The Lighthouse Lab in Milton Keynes and Alex Alderton, Roberto Amato, Sonia Goncalves, Ewan Harrison, David K. Jackson, Ian Johnston, Dominic Kwiatkowski, Cordelia Langford, John Sillitoe on behalf of the Wellcome Sanger Institute COVID-19 Surveillance Team
EPI_ISL_873302, EPI_ISL_873304, EPI_ISL_873306, EPI_ISL_873310	Lighthouse Lab in Cambridge	Wellcome Sanger Institute for the COVID-19 Genomics UK (COG-UK) Consortium	Rob Howes, The Lighthouse Lab in Cambridge and Alex Alderton, Roberto Amato, Sonia Goncalves, Ewan Harrison, David K. Jackson, Ian Johnston, Dominic Kwiatkowski, Cordelia Langford, John Sillitoe on behalf of the Wellcome Sanger Institute COVID-19 Surveillance Team
EPI_ISL_873311	Lighthouse Lab in Milton Keynes	Wellcome Sanger Institute for the COVID-19 Genomics UK (COG-UK) Consortium	The Lighthouse Lab in Milton Keynes and Alex Alderton, Roberto Amato, Sonia Goncalves, Ewan Harrison, David K. Jackson, Ian Johnston, Dominic Kwiatkowski, Cordelia Langford, John Sillitoe on behalf of the Wellcome Sanger Institute COVID-19 Surveillance Team
EPI_ISL_873313, EPI_ISL_873316, EPI_ISL_873318, EPI_ISL_873320	Lighthouse Lab in Cambridge	Wellcome Sanger Institute for the COVID-19 Genomics UK (COG-UK) Consortium	Rob Howes, The Lighthouse Lab in Cambridge and Alex Alderton, Roberto Amato, Sonia Goncalves, Ewan Harrison, David K. Jackson, Ian Johnston, Dominic Kwiatkowski, Cordelia Langford, John Sillitoe on behalf of the Wellcome Sanger Institute COVID-19 Surveillance Team
EPI_ISL_873321	Lighthouse Lab in Milton Keynes	Wellcome Sanger Institute for the COVID-19 Genomics UK (COG-UK) Consortium	The Lighthouse Lab in Milton Keynes and Alex Alderton, Roberto Amato, Sonia Goncalves, Ewan Harrison, David K. Jackson, Ian Johnston, Dominic Kwiatkowski, Cordelia Langford, John Sillitoe on behalf of the Wellcome Sanger Institute COVID-19 Surveillance Team
EPI_ISL_873324, EPI_ISL_873325, EPI_ISL_873326	Lighthouse Lab in Cambridge	Wellcome Sanger Institute for the COVID-19 Genomics UK (COG-UK) Consortium	Rob Howes, The Lighthouse Lab in Cambridge and Alex Alderton, Roberto Amato, Sonia Goncalves, Ewan Harrison, David K. Jackson, Ian Johnston, Dominic Kwiatkowski, Cordelia Langford, John Sillitoe on behalf of the Wellcome Sanger Institute COVID-19 Surveillance Team
EPI_ISL_873330	Lighthouse Lab in Milton Keynes	Wellcome Sanger Institute for the COVID-19 Genomics UK (COG-UK) Consortium	The Lighthouse Lab in Milton Keynes and Alex Alderton, Roberto Amato, Sonia Goncalves, Ewan Harrison, David K. Jackson, Ian Johnston, Dominic Kwiatkowski, Cordelia Langford, John Sillitoe on behalf of the Wellcome Sanger Institute COVID-19 Surveillance Team
EPI_ISL_873334	Lighthouse Lab in Cambridge	Wellcome Sanger Institute for the COVID-19 Genomics UK (COG-UK) Consortium	Rob Howes, The Lighthouse Lab in Cambridge and Alex Alderton, Roberto Amato, Sonia Goncalves, Ewan Harrison, David K. Jackson, Ian Johnston, Dominic Kwiatkowski, Cordelia Langford, John Sillitoe on behalf of the Wellcome Sanger Institute COVID-19 Surveillance Team
EPI_ISL_873336	Lighthouse Lab in Milton Keynes	Wellcome Sanger Institute for the COVID-19 Genomics UK (COG-UK) Consortium	The Lighthouse Lab in Milton Keynes and Alex Alderton, Roberto Amato, Sonia Goncalves, Ewan Harrison, David K. Jackson, Ian Johnston, Dominic Kwiatkowski, Cordelia Langford, John Sillitoe on behalf of the Wellcome Sanger Institute COVID-19 Surveillance Team
EPI_ISL_873337, EPI_ISL_873339, EPI_ISL_873341, EPI_ISL_873342,	Lighthouse Lab in Cambridge	Wellcome Sanger Institute for the COVID-19 Genomics UK (COG-UK) Consortium	Rob Howes, The Lighthouse Lab in Cambridge and Alex Alderton, Roberto Amato, Sonia Goncalves, Ewan Harrison, David K. Jackson, Ian Johnston, Dominic Kwiatkowski, Cordelia Langford, John Sillitoe on behalf of the Wellcome Sanger Institute COVID-19 Surveillance Team

EPI_ISL_873343, EPI_ISL_873347, EPI_ISL_873348, EPI_ISL_873350			
EPI_ISL_873354	Lighthouse Lab in Milton Keynes	Wellcome Sanger Institute for the COVID-19 Genomics UK (COG-UK) Consortium	The Lighthouse Lab in Milton Keynes and Alex Alderton, Roberto Amato, Sonia Goncalves, Ewan Harrison, David K. Jackson, Ian Johnston, Dominic Kwiatkowski, Cordelia Langford, John Sillitoe on behalf of the Wellcome Sanger Institute COVID-19 Surveillance Team
EPI_ISL_873357, EPI_ISL_873359	Lighthouse Lab in Cambridge	Wellcome Sanger Institute for the COVID-19 Genomics UK (COG-UK) Consortium	Rob Howes, The Lighthouse Lab in Cambridge and Alex Alderton, Roberto Amato, Sonia Goncalves, Ewan Harrison, David K. Jackson, Ian Johnston, Dominic Kwiatkowski, Cordelia Langford, John Sillitoe on behalf of the Wellcome Sanger Institute COVID-19 Surveillance Team
EPI_ISL_873361	Lighthouse Lab in Milton Keynes	Wellcome Sanger Institute for the COVID-19 Genomics UK (COG-UK) Consortium	The Lighthouse Lab in Milton Keynes and Alex Alderton, Roberto Amato, Sonia Goncalves, Ewan Harrison, David K. Jackson, Ian Johnston, Dominic Kwiatkowski, Cordelia Langford, John Sillitoe on behalf of the Wellcome Sanger Institute COVID-19 Surveillance Team
EPI_ISL_873362, EPI_ISL_873364, EPI_ISL_873369, EPI_ISL_873370, EPI_ISL_873371, EPI_ISL_873374	Lighthouse Lab in Cambridge	Wellcome Sanger Institute for the COVID-19 Genomics UK (COG-UK) Consortium	Rob Howes, The Lighthouse Lab in Cambridge and Alex Alderton, Roberto Amato, Sonia Goncalves, Ewan Harrison, David K. Jackson, Ian Johnston, Dominic Kwiatkowski, Cordelia Langford, John Sillitoe on behalf of the Wellcome Sanger Institute COVID-19 Surveillance Team
EPI_ISL_873376, EPI_ISL_873378	Lighthouse Lab in Milton Keynes	Wellcome Sanger Institute for the COVID-19 Genomics UK (COG-UK) Consortium	The Lighthouse Lab in Milton Keynes and Alex Alderton, Roberto Amato, Sonia Goncalves, Ewan Harrison, David K. Jackson, Ian Johnston, Dominic Kwiatkowski, Cordelia Langford, John Sillitoe on behalf of the Wellcome Sanger Institute COVID-19 Surveillance Team
EPI_ISL_873381	Lighthouse Lab in Cambridge	Wellcome Sanger Institute for the COVID-19 Genomics UK (COG-UK) Consortium	Rob Howes, The Lighthouse Lab in Cambridge and Alex Alderton, Roberto Amato, Sonia Goncalves, Ewan Harrison, David K. Jackson, Ian Johnston, Dominic Kwiatkowski, Cordelia Langford, John Sillitoe on behalf of the Wellcome Sanger Institute COVID-19 Surveillance Team
EPI_ISL_873386	Lighthouse Lab in Milton Keynes	Wellcome Sanger Institute for the COVID-19 Genomics UK (COG-UK) Consortium	The Lighthouse Lab in Milton Keynes and Alex Alderton, Roberto Amato, Sonia Goncalves, Ewan Harrison, David K. Jackson, Ian Johnston, Dominic Kwiatkowski, Cordelia Langford, John Sillitoe on behalf of the Wellcome Sanger Institute COVID-19 Surveillance Team
EPI_ISL_873388	Lighthouse Lab in Cambridge	Wellcome Sanger Institute for the COVID-19 Genomics UK (COG-UK) Consortium	Rob Howes, The Lighthouse Lab in Cambridge and Alex Alderton, Roberto Amato, Sonia Goncalves, Ewan Harrison, David K. Jackson, Ian Johnston, Dominic Kwiatkowski, Cordelia Langford, John Sillitoe on behalf of the Wellcome Sanger Institute COVID-19 Surveillance Team
EPI_ISL_873389, EPI_ISL_873396	Lighthouse Lab in Milton Keynes	Wellcome Sanger Institute for the COVID-19 Genomics UK (COG-UK) Consortium	The Lighthouse Lab in Milton Keynes and Alex Alderton, Roberto Amato, Sonia Goncalves, Ewan Harrison, David K. Jackson, Ian Johnston, Dominic Kwiatkowski, Cordelia Langford, John Sillitoe on behalf of the Wellcome Sanger Institute COVID-19 Surveillance Team
EPI_ISL_873399	Lighthouse Lab in Cambridge	Wellcome Sanger Institute for the COVID-19 Genomics UK (COG-UK) Consortium	Rob Howes, The Lighthouse Lab in Cambridge and Alex Alderton, Roberto Amato, Sonia Goncalves, Ewan Harrison, David K. Jackson, Ian Johnston, Dominic Kwiatkowski, Cordelia Langford, John Sillitoe on behalf of the Wellcome Sanger Institute COVID-19 Surveillance Team
EPI_ISL_873411	Lighthouse Lab in Milton Keynes	Wellcome Sanger Institute for the COVID-19 Genomics UK (COG-UK) Consortium	The Lighthouse Lab in Milton Keynes and Alex Alderton, Roberto Amato, Sonia Goncalves, Ewan Harrison, David K. Jackson, Ian Johnston, Dominic Kwiatkowski, Cordelia Langford, John Sillitoe on behalf of the Wellcome Sanger Institute COVID-19 Surveillance Team
EPI_ISL_873412	Lighthouse Lab in Cambridge	Wellcome Sanger Institute for the COVID-19 Genomics UK (COG-UK) Consortium	Rob Howes, The Lighthouse Lab in Cambridge and Alex Alderton, Roberto Amato, Sonia Goncalves, Ewan Harrison, David K. Jackson, Ian Johnston, Dominic Kwiatkowski, Cordelia Langford, John Sillitoe on behalf of the Wellcome Sanger Institute COVID-19 Surveillance Team
EPI_ISL_873413, EPI_ISL_873414, EPI_ISL_873415	Lighthouse Lab in Milton Keynes	Wellcome Sanger Institute for the COVID-19 Genomics UK (COG-UK) Consortium	The Lighthouse Lab in Milton Keynes and Alex Alderton, Roberto Amato, Sonia Goncalves, Ewan Harrison, David K. Jackson, Ian Johnston, Dominic Kwiatkowski, Cordelia Langford, John Sillitoe on behalf of the Wellcome Sanger Institute COVID-19 Surveillance Team
EPI_ISL_873416	Lighthouse Lab in Cambridge	Wellcome Sanger Institute for the COVID-19 Genomics UK (COG-UK) Consortium	Rob Howes, The Lighthouse Lab in Cambridge and Alex Alderton, Roberto Amato, Sonia Goncalves, Ewan Harrison, David K. Jackson, Ian Johnston, Dominic Kwiatkowski, Cordelia Langford, John Sillitoe on behalf of the Wellcome Sanger Institute COVID-19 Surveillance Team
EPI_ISL_873420	Lighthouse Lab in Milton Keynes	Wellcome Sanger Institute for the COVID-19 Genomics UK (COG-UK) Consortium	The Lighthouse Lab in Milton Keynes and Alex Alderton, Roberto Amato, Sonia Goncalves, Ewan Harrison, David K. Jackson, Ian Johnston, Dominic Kwiatkowski, Cordelia Langford, John Sillitoe on behalf of the Wellcome Sanger Institute COVID-19 Surveillance Team
EPI_ISL_873422, EPI_ISL_873426, EPI_ISL_873428	Lighthouse Lab in Cambridge	Wellcome Sanger Institute for the COVID-19 Genomics UK (COG-UK) Consortium	Rob Howes, The Lighthouse Lab in Cambridge and Alex Alderton, Roberto Amato, Sonia Goncalves, Ewan Harrison, David K. Jackson, Ian Johnston, Dominic Kwiatkowski, Cordelia Langford, John Sillitoe on behalf of the Wellcome Sanger Institute COVID-19 Surveillance Team
EPI_ISL_873429, EPI_ISL_873430	Lighthouse Lab in Milton Keynes	Wellcome Sanger Institute for the COVID-19 Genomics UK (COG-UK) Consortium	The Lighthouse Lab in Milton Keynes and Alex Alderton, Roberto Amato, Sonia Goncalves, Ewan Harrison, David K. Jackson, Ian Johnston, Dominic Kwiatkowski, Cordelia Langford, John Sillitoe on behalf of the Wellcome Sanger Institute COVID-19 Surveillance Team
EPI_ISL_873431, EPI_ISL_873433	Lighthouse Lab in Cambridge	Wellcome Sanger Institute for the COVID-19 Genomics UK (COG-UK) Consortium	Rob Howes, The Lighthouse Lab in Cambridge and Alex Alderton, Roberto Amato, Sonia Goncalves, Ewan Harrison, David K. Jackson, Ian Johnston, Dominic Kwiatkowski, Cordelia Langford, John Sillitoe on behalf of the Wellcome Sanger Institute COVID-19 Surveillance Team
EPI_ISL_873435, EPI_ISL_873438, EPI_ISL_873439	Lighthouse Lab in Milton Keynes	Wellcome Sanger Institute for the COVID-19 Genomics UK (COG-UK) Consortium	The Lighthouse Lab in Milton Keynes and Alex Alderton, Roberto Amato, Sonia Goncalves, Ewan Harrison, David K. Jackson, Ian Johnston, Dominic Kwiatkowski, Cordelia Langford, John Sillitoe on behalf of the Wellcome Sanger Institute COVID-19 Surveillance Team
EPI_ISL_873443	Lighthouse Lab in Cambridge	Wellcome Sanger Institute for the COVID-19 Genomics UK (COG-UK) Consortium	Rob Howes, The Lighthouse Lab in Cambridge and Alex Alderton, Roberto Amato, Sonia Goncalves, Ewan Harrison, David K. Jackson, Ian Johnston, Dominic Kwiatkowski, Cordelia Langford, John Sillitoe on behalf of the Wellcome Sanger Institute COVID-19 Surveillance Team
EPI_ISL_873444, EPI_ISL_873445, EPI_ISL_873450	Lighthouse Lab in Milton Keynes	Wellcome Sanger Institute for the COVID-19 Genomics UK (COG-UK) Consortium	The Lighthouse Lab in Milton Keynes and Alex Alderton, Roberto Amato, Sonia Goncalves, Ewan Harrison, David K. Jackson, Ian Johnston, Dominic Kwiatkowski, Cordelia Langford, John Sillitoe on behalf of the Wellcome Sanger Institute COVID-19 Surveillance Team
EPI_ISL_873451, EPI_ISL_873454	Lighthouse Lab in Cambridge	Wellcome Sanger Institute for the COVID-19 Genomics UK (COG-UK) Consortium	Rob Howes, The Lighthouse Lab in Cambridge and Alex Alderton, Roberto Amato, Sonia Goncalves, Ewan Harrison, David K. Jackson, Ian Johnston, Dominic Kwiatkowski, Cordelia Langford, John Sillitoe on behalf of the Wellcome Sanger Institute COVID-19 Surveillance Team
EPI_ISL_873455	Lighthouse Lab in Milton Keynes	Wellcome Sanger Institute for the COVID-19 Genomics UK (COG-UK) Consortium	The Lighthouse Lab in Milton Keynes and Alex Alderton, Roberto Amato, Sonia Goncalves, Ewan Harrison, David K. Jackson, Ian Johnston, Dominic Kwiatkowski, Cordelia Langford, John Sillitoe on behalf of the Wellcome Sanger Institute COVID-19 Surveillance Team
EPI_ISL_873457, EPI_ISL_873459	Lighthouse Lab in Cambridge	Wellcome Sanger Institute for the COVID-19 Genomics UK (COG-UK) Consortium	Rob Howes, The Lighthouse Lab in Cambridge and Alex Alderton, Roberto Amato, Sonia Goncalves, Ewan Harrison, David K. Jackson, Ian Johnston, Dominic Kwiatkowski, Cordelia Langford, John Sillitoe on behalf of the Wellcome Sanger Institute COVID-19 Surveillance Team
EPI_ISL_873462, EPI_ISL_873466, EPI_ISL_873473	Lighthouse Lab in Milton Keynes	Wellcome Sanger Institute for the COVID-19 Genomics UK (COG-UK) Consortium	The Lighthouse Lab in Milton Keynes and Alex Alderton, Roberto Amato, Sonia Goncalves, Ewan Harrison, David K. Jackson, Ian Johnston, Dominic Kwiatkowski, Cordelia Langford, John Sillitoe on behalf of the Wellcome Sanger Institute COVID-19 Surveillance Team
EPI_ISL_873476, EPI_ISL_873481, EPI_ISL_873482	Lighthouse Lab in Cambridge	Wellcome Sanger Institute for the COVID-19 Genomics UK (COG-UK) Consortium	Rob Howes, The Lighthouse Lab in Cambridge and Alex Alderton, Roberto Amato, Sonia Goncalves, Ewan Harrison, David K. Jackson, Ian Johnston, Dominic Kwiatkowski, Cordelia Langford, John Sillitoe on behalf of the Wellcome Sanger Institute COVID-19 Surveillance Team
EPI_ISL_873487	Lighthouse Lab in Milton Keynes	Wellcome Sanger Institute for the COVID-19 Genomics UK (COG-UK) Consortium	The Lighthouse Lab in Milton Keynes and Alex Alderton, Roberto Amato, Sonia Goncalves, Ewan Harrison, David K. Jackson, Ian Johnston, Dominic Kwiatkowski, Cordelia Langford, John Sillitoe on behalf of the Wellcome Sanger Institute COVID-19 Surveillance Team
EPI_ISL_873492	Lighthouse Lab in Cambridge	Wellcome Sanger Institute for the COVID-19 Genomics UK (COG-UK) Consortium	Rob Howes, The Lighthouse Lab in Cambridge and Alex Alderton, Roberto Amato, Sonia Goncalves, Ewan Harrison, David K. Jackson, Ian Johnston, Dominic Kwiatkowski, Cordelia Langford, John Sillitoe on behalf of the Wellcome Sanger Institute COVID-19 Surveillance Team
EPI_ISL_873493	Lighthouse Lab in Milton Keynes	Wellcome Sanger Institute for the COVID-19 Genomics UK (COG-UK) Consortium	The Lighthouse Lab in Milton Keynes and Alex Alderton, Roberto Amato, Sonia Goncalves, Ewan Harrison, David K. Jackson, Ian Johnston, Dominic Kwiatkowski, Cordelia Langford, John Sillitoe on behalf of the Wellcome Sanger Institute COVID-19 Surveillance Team
EPI_ISL_873494, EPI_ISL_873495	Lighthouse Lab in Cambridge	Wellcome Sanger Institute for the COVID-19 Genomics UK (COG-UK) Consortium	Rob Howes, The Lighthouse Lab in Cambridge and Alex Alderton, Roberto Amato, Sonia Goncalves, Ewan Harrison, David K. Jackson, Ian Johnston, Dominic Kwiatkowski, Cordelia Langford, John Sillitoe on behalf of the Wellcome Sanger Institute COVID-19 Surveillance Team
EPI_ISL_873496	Lighthouse Lab in Milton Keynes	Wellcome Sanger Institute for the COVID-19 Genomics UK (COG-UK) Consortium	The Lighthouse Lab in Milton Keynes and Alex Alderton, Roberto Amato, Sonia Goncalves, Ewan Harrison, David K. Jackson, Ian Johnston, Dominic Kwiatkowski, Cordelia Langford, John Sillitoe on behalf of the Wellcome Sanger Institute COVID-19 Surveillance Team
EPI_ISL_873497, EPI_ISL_873499, EPI_ISL_873500, EPI_ISL_873501	Lighthouse Lab in Cambridge	Wellcome Sanger Institute for the COVID-19 Genomics UK (COG-UK) Consortium	Rob Howes, The Lighthouse Lab in Cambridge and Alex Alderton, Roberto Amato, Sonia Goncalves, Ewan Harrison, David K. Jackson, Ian Johnston, Dominic Kwiatkowski, Cordelia Langford, John Sillitoe on behalf of the Wellcome Sanger Institute COVID-19 Surveillance Team
EPI_ISL_873503, EPI_ISL_873504, EPI_ISL_873509	Lighthouse Lab in Milton Keynes	Wellcome Sanger Institute for the COVID-19 Genomics UK (COG-UK) Consortium	The Lighthouse Lab in Milton Keynes and Alex Alderton, Roberto Amato, Sonia Goncalves, Ewan Harrison, David K. Jackson, Ian Johnston, Dominic Kwiatkowski, Cordelia Langford, John Sillitoe on behalf of the Wellcome Sanger Institute COVID-19 Surveillance Team
EPI_ISL_873510	Lighthouse Lab in Cambridge	Wellcome Sanger Institute for the COVID-19 Genomics UK (COG-UK) Consortium	Rob Howes, The Lighthouse Lab in Cambridge and Alex Alderton, Roberto Amato, Sonia Goncalves, Ewan Harrison, David K. Jackson, Ian Johnston, Dominic Kwiatkowski, Cordelia Langford, John Sillitoe on behalf of the Wellcome Sanger Institute COVID-19 Surveillance Team

EPI_ISL_873515	Lighthouse Lab in Milton Keynes	Wellcome Sanger Institute for the COVID-19 Genomics UK (COG-UK) Consortium	The Lighthouse Lab in Milton Keynes and Alex Alderton, Roberto Amato, Sonia Goncalves, Ewan Harrison, David K. Jackson, Ian Johnston, Dominic Kwiatkowski, Cordelia Langford, John Sillitoe on behalf of the Wellcome Sanger Institute COVID-19 Surveillance Team
EPI_ISL_873518	Lighthouse Lab in Cambridge	Wellcome Sanger Institute for the COVID-19 Genomics UK (COG-UK) Consortium	Rob Howes, The Lighthouse Lab in Cambridge and Alex Alderton, Roberto Amato, Sonia Goncalves, Ewan Harrison, David K. Jackson, Ian Johnston, Dominic Kwiatkowski, Cordelia Langford, John Sillitoe on behalf of the Wellcome Sanger Institute COVID-19 Surveillance Team
EPI_ISL_873520, EPI_ISL_873531	Lighthouse Lab in Milton Keynes	Wellcome Sanger Institute for the COVID-19 Genomics UK (COG-UK) Consortium	The Lighthouse Lab in Milton Keynes and Alex Alderton, Roberto Amato, Sonia Goncalves, Ewan Harrison, David K. Jackson, Ian Johnston, Dominic Kwiatkowski, Cordelia Langford, John Sillitoe on behalf of the Wellcome Sanger Institute COVID-19 Surveillance Team
EPI_ISL_873532, EPI_ISL_873537	Lighthouse Lab in Cambridge	Wellcome Sanger Institute for the COVID-19 Genomics UK (COG-UK) Consortium	Rob Howes, The Lighthouse Lab in Cambridge and Alex Alderton, Roberto Amato, Sonia Goncalves, Ewan Harrison, David K. Jackson, Ian Johnston, Dominic Kwiatkowski, Cordelia Langford, John Sillitoe on behalf of the Wellcome Sanger Institute COVID-19 Surveillance Team
EPI_ISL_873551	Lighthouse Lab in Milton Keynes	Wellcome Sanger Institute for the COVID-19 Genomics UK (COG-UK) Consortium	The Lighthouse Lab in Milton Keynes and Alex Alderton, Roberto Amato, Sonia Goncalves, Ewan Harrison, David K. Jackson, Ian Johnston, Dominic Kwiatkowski, Cordelia Langford, John Sillitoe on behalf of the Wellcome Sanger Institute COVID-19 Surveillance Team
EPI_ISL_873552, EPI_ISL_873566, EPI_ISL_873571, EPI_ISL_873573, EPI_ISL_873574, EPI_ISL_873576	Lighthouse Lab in Cambridge	Wellcome Sanger Institute for the COVID-19 Genomics UK (COG-UK) Consortium	Rob Howes, The Lighthouse Lab in Cambridge and Alex Alderton, Roberto Amato, Sonia Goncalves, Ewan Harrison, David K. Jackson, Ian Johnston, Dominic Kwiatkowski, Cordelia Langford, John Sillitoe on behalf of the Wellcome Sanger Institute COVID-19 Surveillance Team
EPI_ISL_873585, EPI_ISL_873596, EPI_ISL_873600, EPI_ISL_873603, EPI_ISL_873604	Lighthouse Lab in Milton Keynes	Wellcome Sanger Institute for the COVID-19 Genomics UK (COG-UK) Consortium	The Lighthouse Lab in Milton Keynes and Alex Alderton, Roberto Amato, Sonia Goncalves, Ewan Harrison, David K. Jackson, Ian Johnston, Dominic Kwiatkowski, Cordelia Langford, John Sillitoe on behalf of the Wellcome Sanger Institute COVID-19 Surveillance Team
EPI_ISL_873605	Lighthouse Lab in Cambridge	Wellcome Sanger Institute for the COVID-19 Genomics UK (COG-UK) Consortium	Rob Howes, The Lighthouse Lab in Cambridge and Alex Alderton, Roberto Amato, Sonia Goncalves, Ewan Harrison, David K. Jackson, Ian Johnston, Dominic Kwiatkowski, Cordelia Langford, John Sillitoe on behalf of the Wellcome Sanger Institute COVID-19 Surveillance Team
EPI_ISL_873607	Lighthouse Lab in Milton Keynes	Wellcome Sanger Institute for the COVID-19 Genomics UK (COG-UK) Consortium	The Lighthouse Lab in Milton Keynes and Alex Alderton, Roberto Amato, Sonia Goncalves, Ewan Harrison, David K. Jackson, Ian Johnston, Dominic Kwiatkowski, Cordelia Langford, John Sillitoe on behalf of the Wellcome Sanger Institute COVID-19 Surveillance Team
EPI_ISL_873614, EPI_ISL_873621, EPI_ISL_873625, EPI_ISL_873629, EPI_ISL_873630, EPI_ISL_873632, EPI_ISL_873633, EPI_ISL_873634, EPI_ISL_873635, EPI_ISL_873636, EPI_ISL_873638, EPI_ISL_873639, EPI_ISL_873640, EPI_ISL_873642, EPI_ISL_873643, EPI_ISL_873644			
see above	Lighthouse Lab in Cambridge	Wellcome Sanger Institute for the COVID-19 Genomics UK (COG-UK) Consortium	Rob Howes, The Lighthouse Lab in Cambridge and Alex Alderton, Roberto Amato, Sonia Goncalves, Ewan Harrison, David K. Jackson, Ian Johnston, Dominic Kwiatkowski, Cordelia Langford, John Sillitoe on behalf of the Wellcome Sanger Institute COVID-19 Surveillance Team
EPI_ISL_873645	Lighthouse Lab in Milton Keynes	Wellcome Sanger Institute for the COVID-19 Genomics UK (COG-UK) Consortium	The Lighthouse Lab in Milton Keynes and Alex Alderton, Roberto Amato, Sonia Goncalves, Ewan Harrison, David K. Jackson, Ian Johnston, Dominic Kwiatkowski, Cordelia Langford, John Sillitoe on behalf of the Wellcome Sanger Institute COVID-19 Surveillance Team
EPI_ISL_873646, EPI_ISL_873647, EPI_ISL_873648, EPI_ISL_873649, EPI_ISL_873650, EPI_ISL_873651	Lighthouse Lab in Cambridge	Wellcome Sanger Institute for the COVID-19 Genomics UK (COG-UK) Consortium	Rob Howes, The Lighthouse Lab in Cambridge and Alex Alderton, Roberto Amato, Sonia Goncalves, Ewan Harrison, David K. Jackson, Ian Johnston, Dominic Kwiatkowski, Cordelia Langford, John Sillitoe on behalf of the Wellcome Sanger Institute COVID-19 Surveillance Team
EPI_ISL_873652	Lighthouse Lab in Milton Keynes	Wellcome Sanger Institute for the COVID-19 Genomics UK (COG-UK) Consortium	The Lighthouse Lab in Milton Keynes and Alex Alderton, Roberto Amato, Sonia Goncalves, Ewan Harrison, David K. Jackson, Ian Johnston, Dominic Kwiatkowski, Cordelia Langford, John Sillitoe on behalf of the Wellcome Sanger Institute COVID-19 Surveillance Team
EPI_ISL_873653, EPI_ISL_873655, EPI_ISL_873658, EPI_ISL_873659, EPI_ISL_873661, EPI_ISL_873662, EPI_ISL_873663, EPI_ISL_873664, EPI_ISL_873665, EPI_ISL_873666, EPI_ISL_873667, EPI_ISL_873668, EPI_ISL_873670, EPI_ISL_873671, EPI_ISL_873673, EPI_ISL_873674, EPI_ISL_873675, EPI_ISL_873676, EPI_ISL_873677, EPI_ISL_873678			
see above	Lighthouse Lab in Cambridge	Wellcome Sanger Institute for the COVID-19 Genomics UK (COG-UK) Consortium	Rob Howes, The Lighthouse Lab in Cambridge and Alex Alderton, Roberto Amato, Sonia Goncalves, Ewan Harrison, David K. Jackson, Ian Johnston, Dominic Kwiatkowski, Cordelia Langford, John Sillitoe on behalf of the Wellcome Sanger Institute COVID-19 Surveillance Team
EPI_ISL_873679	Lighthouse Lab in Milton Keynes	Wellcome Sanger Institute for the COVID-19 Genomics UK (COG-UK) Consortium	The Lighthouse Lab in Milton Keynes and Alex Alderton, Roberto Amato, Sonia Goncalves, Ewan Harrison, David K. Jackson, Ian Johnston, Dominic Kwiatkowski, Cordelia Langford, John Sillitoe on behalf of the Wellcome Sanger Institute COVID-19 Surveillance Team
EPI_ISL_873680, EPI_ISL_873681, EPI_ISL_873682, EPI_ISL_873683	Lighthouse Lab in Cambridge	Wellcome Sanger Institute for the COVID-19 Genomics UK (COG-UK) Consortium	Rob Howes, The Lighthouse Lab in Cambridge and Alex Alderton, Roberto Amato, Sonia Goncalves, Ewan Harrison, David K. Jackson, Ian Johnston, Dominic Kwiatkowski, Cordelia Langford, John Sillitoe on behalf of the Wellcome Sanger Institute COVID-19 Surveillance Team
EPI_ISL_873684	Lighthouse Lab in Milton Keynes	Wellcome Sanger Institute for the COVID-19 Genomics UK (COG-UK) Consortium	The Lighthouse Lab in Milton Keynes and Alex Alderton, Roberto Amato, Sonia Goncalves, Ewan Harrison, David K. Jackson, Ian Johnston, Dominic Kwiatkowski, Cordelia Langford, John Sillitoe on behalf of the Wellcome Sanger Institute COVID-19 Surveillance Team
EPI_ISL_873685, EPI_ISL_873686, EPI_ISL_873687, EPI_ISL_873688, EPI_ISL_873689, EPI_ISL_873690, EPI_ISL_873691, EPI_ISL_873692, EPI_ISL_873693, EPI_ISL_873694, EPI_ISL_873695, EPI_ISL_873696, EPI_ISL_873697, EPI_ISL_873698, EPI_ISL_873699, EPI_ISL_873700, EPI_ISL_873701, EPI_ISL_873702, EPI_ISL_873703, EPI_ISL_873705, EPI_ISL_873706, EPI_ISL_873707, EPI_ISL_873708, EPI_ISL_873709, EPI_ISL_873710, EPI_ISL_873711, EPI_ISL_873712, EPI_ISL_873713, EPI_ISL_873714, EPI_ISL_873715			
see above	Lighthouse Lab in Cambridge	Wellcome Sanger Institute for the COVID-19 Genomics UK (COG-UK) Consortium	Rob Howes, The Lighthouse Lab in Cambridge and Alex Alderton, Roberto Amato, Sonia Goncalves, Ewan Harrison, David K. Jackson, Ian Johnston, Dominic Kwiatkowski, Cordelia Langford, John Sillitoe on behalf of the Wellcome Sanger Institute COVID-19 Surveillance Team
EPI_ISL_873716	Lighthouse Lab in Milton Keynes	Wellcome Sanger Institute for the COVID-19 Genomics UK (COG-UK) Consortium	The Lighthouse Lab in Milton Keynes and Alex Alderton, Roberto Amato, Sonia Goncalves, Ewan Harrison, David K. Jackson, Ian Johnston, Dominic Kwiatkowski, Cordelia Langford, John Sillitoe on behalf of the Wellcome Sanger Institute COVID-19 Surveillance Team
EPI_ISL_873717, EPI_ISL_873718, EPI_ISL_873719, EPI_ISL_873720, EPI_ISL_873721, EPI_ISL_873722, EPI_ISL_873723, EPI_ISL_873727, EPI_ISL_873728, EPI_ISL_873731, EPI_ISL_873732, EPI_ISL_873733, EPI_ISL_873735, EPI_ISL_873736, EPI_ISL_873737, EPI_ISL_873738, EPI_ISL_873739, EPI_ISL_873740, EPI_ISL_873742, EPI_ISL_873745, EPI_ISL_873747			
see above	Lighthouse Lab in Cambridge	Wellcome Sanger Institute for the COVID-19 Genomics UK (COG-UK) Consortium	Rob Howes, The Lighthouse Lab in Cambridge and Alex Alderton, Roberto Amato, Sonia Goncalves, Ewan Harrison, David K. Jackson, Ian Johnston, Dominic Kwiatkowski, Cordelia Langford, John Sillitoe on behalf of the Wellcome Sanger Institute COVID-19 Surveillance Team
EPI_ISL_873748	Lighthouse Lab in Milton Keynes	Wellcome Sanger Institute for the COVID-19 Genomics UK (COG-UK) Consortium	The Lighthouse Lab in Milton Keynes and Alex Alderton, Roberto Amato, Sonia Goncalves, Ewan Harrison, David K. Jackson, Ian Johnston, Dominic Kwiatkowski, Cordelia Langford, John Sillitoe on behalf of the Wellcome Sanger Institute COVID-19 Surveillance Team
EPI_ISL_873751, EPI_ISL_873753, EPI_ISL_873754	Lighthouse Lab in Cambridge	Wellcome Sanger Institute for the COVID-19 Genomics UK (COG-UK) Consortium	Rob Howes, The Lighthouse Lab in Cambridge and Alex Alderton, Roberto Amato, Sonia Goncalves, Ewan Harrison, David K. Jackson, Ian Johnston, Dominic Kwiatkowski, Cordelia Langford, John Sillitoe on behalf of the Wellcome Sanger Institute COVID-19 Surveillance Team
EPI_ISL_873755	Lighthouse Lab in Milton Keynes	Wellcome Sanger Institute for the COVID-19 Genomics UK (COG-UK) Consortium	The Lighthouse Lab in Milton Keynes and Alex Alderton, Roberto Amato, Sonia Goncalves, Ewan Harrison, David K. Jackson, Ian Johnston, Dominic Kwiatkowski, Cordelia Langford, John Sillitoe on behalf of the Wellcome Sanger Institute COVID-19 Surveillance Team
EPI_ISL_873756, EPI_ISL_873757, EPI_ISL_873758, EPI_ISL_873759, EPI_ISL_873761, EPI_ISL_873762, EPI_ISL_873763, EPI_ISL_873765, EPI_ISL_873766, EPI_ISL_873767, EPI_ISL_873768, EPI_ISL_873770, EPI_ISL_873771, EPI_ISL_873772, EPI_ISL_873773, EPI_ISL_873774, EPI_ISL_873775, EPI_ISL_873776, EPI_ISL_873777, EPI_ISL_873778, EPI_ISL_873779, EPI_ISL_873780, EPI_ISL_873781, EPI_ISL_873782			
see above	Lighthouse Lab in Cambridge	Wellcome Sanger Institute for the COVID-19 Genomics UK (COG-UK) Consortium	Rob Howes, The Lighthouse Lab in Cambridge and Alex Alderton, Roberto Amato, Sonia Goncalves, Ewan Harrison, David K. Jackson, Ian Johnston, Dominic Kwiatkowski, Cordelia Langford, John Sillitoe on behalf of the Wellcome Sanger Institute COVID-19 Surveillance Team
EPI_ISL_873783	Lighthouse Lab in Milton Keynes	Wellcome Sanger Institute for the COVID-19 Genomics UK (COG-UK) Consortium	The Lighthouse Lab in Milton Keynes and Alex Alderton, Roberto Amato, Sonia Goncalves, Ewan Harrison, David K. Jackson, Ian Johnston, Dominic Kwiatkowski, Cordelia Langford, John Sillitoe on behalf of the Wellcome Sanger Institute COVID-19 Surveillance Team
EPI_ISL_873784, EPI_ISL_873785, EPI_ISL_873787, EPI_ISL_873788, EPI_ISL_873789, EPI_ISL_873790, EPI_ISL_873791, EPI_ISL_873792, EPI_ISL_873793, EPI_ISL_873795, EPI_ISL_873796			
see above	Lighthouse Lab in Cambridge	Wellcome Sanger Institute for the COVID-19 Genomics UK (COG-UK) Consortium	Rob Howes, The Lighthouse Lab in Cambridge and Alex Alderton, Roberto Amato, Sonia Goncalves, Ewan Harrison, David K. Jackson, Ian Johnston, Dominic Kwiatkowski, Cordelia Langford, John Sillitoe on behalf of the Wellcome Sanger Institute COVID-19 Surveillance Team
EPI_ISL_873797	Lighthouse Lab in Milton Keynes	Wellcome Sanger Institute for the COVID-19 Genomics UK (COG-UK) Consortium	The Lighthouse Lab in Milton Keynes and Alex Alderton, Roberto Amato, Sonia Goncalves, Ewan Harrison, David K. Jackson, Ian Johnston, Dominic Kwiatkowski, Cordelia Langford, John Sillitoe on behalf of the Wellcome Sanger Institute COVID-19 Surveillance Team
EPI_ISL_873798, EPI_ISL_873799, EPI_ISL_873800, EPI_ISL_873801, EPI_ISL_873802, EPI_ISL_873803, EPI_ISL_873804, EPI_ISL_873805, EPI_ISL_873807, EPI_ISL_873808, EPI_ISL_873810, EPI_ISL_873811, EPI_ISL_873813, EPI_ISL_873814, EPI_ISL_873815, EPI_ISL_873816, EPI_ISL_873818, EPI_ISL_873819, EPI_ISL_873820, EPI_ISL_873821			
see above	Lighthouse Lab in Cambridge	Wellcome Sanger Institute for the COVID-19 Genomics UK	Rob Howes, The Lighthouse Lab in Cambridge and Alex Alderton, Roberto Amato, Sonia Goncalves, Ewan Harrison, David K. Jackson, Ian Johnston,

		(COG-UK) Consortium	Dominic Kwiatkowski, Cordelia Langford, John Sillitoe on behalf of the Wellcome Sanger Institute COVID-19 Surveillance Team
EPI_ISL_873822	Lighthouse Lab in Milton Keynes	Wellcome Sanger Institute for the COVID-19 Genomics UK (COG-UK) Consortium	The Lighthouse Lab in Milton Keynes and Alex Alderton, Roberto Amato, Sonia Goncalves, Ewan Harrison, David K. Jackson, Ian Johnston, Dominic Kwiatkowski, Cordelia Langford, John Sillitoe on behalf of the Wellcome Sanger Institute COVID-19 Surveillance Team
EPI_ISL_873823, EPI_ISL_873824, EPI_ISL_873826	Lighthouse Lab in Cambridge	Wellcome Sanger Institute for the COVID-19 Genomics UK (COG-UK) Consortium	Rob Howes, The Lighthouse Lab in Cambridge and Alex Alderton, Roberto Amato, Sonia Goncalves, Ewan Harrison, David K. Jackson, Ian Johnston, Dominic Kwiatkowski, Cordelia Langford, John Sillitoe on behalf of the Wellcome Sanger Institute COVID-19 Surveillance Team
EPI_ISL_873827	Lighthouse Lab in Milton Keynes	Wellcome Sanger Institute for the COVID-19 Genomics UK (COG-UK) Consortium	The Lighthouse Lab in Milton Keynes and Alex Alderton, Roberto Amato, Sonia Goncalves, Ewan Harrison, David K. Jackson, Ian Johnston, Dominic Kwiatkowski, Cordelia Langford, John Sillitoe on behalf of the Wellcome Sanger Institute COVID-19 Surveillance Team
EPI_ISL_873828	Lighthouse Lab in Cambridge	Wellcome Sanger Institute for the COVID-19 Genomics UK (COG-UK) Consortium	Rob Howes, The Lighthouse Lab in Cambridge and Alex Alderton, Roberto Amato, Sonia Goncalves, Ewan Harrison, David K. Jackson, Ian Johnston, Dominic Kwiatkowski, Cordelia Langford, John Sillitoe on behalf of the Wellcome Sanger Institute COVID-19 Surveillance Team
EPI_ISL_873829	Lighthouse Lab in Milton Keynes	Wellcome Sanger Institute for the COVID-19 Genomics UK (COG-UK) Consortium	The Lighthouse Lab in Milton Keynes and Alex Alderton, Roberto Amato, Sonia Goncalves, Ewan Harrison, David K. Jackson, Ian Johnston, Dominic Kwiatkowski, Cordelia Langford, John Sillitoe on behalf of the Wellcome Sanger Institute COVID-19 Surveillance Team
EPI_ISL_873830, EPI_ISL_873831, EPI_ISL_873832, EPI_ISL_873835, EPI_ISL_873836, EPI_ISL_873837, EPI_ISL_873838, EPI_ISL_873839	Lighthouse Lab in Cambridge	Wellcome Sanger Institute for the COVID-19 Genomics UK (COG-UK) Consortium	Rob Howes, The Lighthouse Lab in Cambridge and Alex Alderton, Roberto Amato, Sonia Goncalves, Ewan Harrison, David K. Jackson, Ian Johnston, Dominic Kwiatkowski, Cordelia Langford, John Sillitoe on behalf of the Wellcome Sanger Institute COVID-19 Surveillance Team
EPI_ISL_873840	Lighthouse Lab in Milton Keynes	Wellcome Sanger Institute for the COVID-19 Genomics UK (COG-UK) Consortium	The Lighthouse Lab in Milton Keynes and Alex Alderton, Roberto Amato, Sonia Goncalves, Ewan Harrison, David K. Jackson, Ian Johnston, Dominic Kwiatkowski, Cordelia Langford, John Sillitoe on behalf of the Wellcome Sanger Institute COVID-19 Surveillance Team
EPI_ISL_873841	Lighthouse Lab in Cambridge	Wellcome Sanger Institute for the COVID-19 Genomics UK (COG-UK) Consortium	Rob Howes, The Lighthouse Lab in Cambridge and Alex Alderton, Roberto Amato, Sonia Goncalves, Ewan Harrison, David K. Jackson, Ian Johnston, Dominic Kwiatkowski, Cordelia Langford, John Sillitoe on behalf of the Wellcome Sanger Institute COVID-19 Surveillance Team
EPI_ISL_873842	Lighthouse Lab in Milton Keynes	Wellcome Sanger Institute for the COVID-19 Genomics UK (COG-UK) Consortium	The Lighthouse Lab in Milton Keynes and Alex Alderton, Roberto Amato, Sonia Goncalves, Ewan Harrison, David K. Jackson, Ian Johnston, Dominic Kwiatkowski, Cordelia Langford, John Sillitoe on behalf of the Wellcome Sanger Institute COVID-19 Surveillance Team
EPI_ISL_873843, EPI_ISL_873844, EPI_ISL_873845, EPI_ISL_873846	Lighthouse Lab in Cambridge	Wellcome Sanger Institute for the COVID-19 Genomics UK (COG-UK) Consortium	Rob Howes, The Lighthouse Lab in Cambridge and Alex Alderton, Roberto Amato, Sonia Goncalves, Ewan Harrison, David K. Jackson, Ian Johnston, Dominic Kwiatkowski, Cordelia Langford, John Sillitoe on behalf of the Wellcome Sanger Institute COVID-19 Surveillance Team
EPI_ISL_873848	Lighthouse Lab in Milton Keynes	Wellcome Sanger Institute for the COVID-19 Genomics UK (COG-UK) Consortium	The Lighthouse Lab in Milton Keynes and Alex Alderton, Roberto Amato, Sonia Goncalves, Ewan Harrison, David K. Jackson, Ian Johnston, Dominic Kwiatkowski, Cordelia Langford, John Sillitoe on behalf of the Wellcome Sanger Institute COVID-19 Surveillance Team
EPI_ISL_873849, EPI_ISL_873851, EPI_ISL_873852	Lighthouse Lab in Cambridge	Wellcome Sanger Institute for the COVID-19 Genomics UK (COG-UK) Consortium	Rob Howes, The Lighthouse Lab in Cambridge and Alex Alderton, Roberto Amato, Sonia Goncalves, Ewan Harrison, David K. Jackson, Ian Johnston, Dominic Kwiatkowski, Cordelia Langford, John Sillitoe on behalf of the Wellcome Sanger Institute COVID-19 Surveillance Team
EPI_ISL_873853	Lighthouse Lab in Milton Keynes	Wellcome Sanger Institute for the COVID-19 Genomics UK (COG-UK) Consortium	The Lighthouse Lab in Milton Keynes and Alex Alderton, Roberto Amato, Sonia Goncalves, Ewan Harrison, David K. Jackson, Ian Johnston, Dominic Kwiatkowski, Cordelia Langford, John Sillitoe on behalf of the Wellcome Sanger Institute COVID-19 Surveillance Team
EPI_ISL_873854, EPI_ISL_873855, EPI_ISL_873856, EPI_ISL_873858	Lighthouse Lab in Cambridge	Wellcome Sanger Institute for the COVID-19 Genomics UK (COG-UK) Consortium	Rob Howes, The Lighthouse Lab in Cambridge and Alex Alderton, Roberto Amato, Sonia Goncalves, Ewan Harrison, David K. Jackson, Ian Johnston, Dominic Kwiatkowski, Cordelia Langford, John Sillitoe on behalf of the Wellcome Sanger Institute COVID-19 Surveillance Team
EPI_ISL_873860	Lighthouse Lab in Milton Keynes	Wellcome Sanger Institute for the COVID-19 Genomics UK (COG-UK) Consortium	The Lighthouse Lab in Milton Keynes and Alex Alderton, Roberto Amato, Sonia Goncalves, Ewan Harrison, David K. Jackson, Ian Johnston, Dominic Kwiatkowski, Cordelia Langford, John Sillitoe on behalf of the Wellcome Sanger Institute COVID-19 Surveillance Team
EPI_ISL_873861	Lighthouse Lab in Cambridge	Wellcome Sanger Institute for the COVID-19 Genomics UK (COG-UK) Consortium	Rob Howes, The Lighthouse Lab in Cambridge and Alex Alderton, Roberto Amato, Sonia Goncalves, Ewan Harrison, David K. Jackson, Ian Johnston, Dominic Kwiatkowski, Cordelia Langford, John Sillitoe on behalf of the Wellcome Sanger Institute COVID-19 Surveillance Team
EPI_ISL_873862	Lighthouse Lab in Milton Keynes	Wellcome Sanger Institute for the COVID-19 Genomics UK (COG-UK) Consortium	The Lighthouse Lab in Milton Keynes and Alex Alderton, Roberto Amato, Sonia Goncalves, Ewan Harrison, David K. Jackson, Ian Johnston, Dominic Kwiatkowski, Cordelia Langford, John Sillitoe on behalf of the Wellcome Sanger Institute COVID-19 Surveillance Team
EPI_ISL_873863, EPI_ISL_873864, EPI_ISL_873866, EPI_ISL_873867, EPI_ISL_873868, EPI_ISL_873869, EPI_ISL_873871, EPI_ISL_873872, EPI_ISL_873874, EPI_ISL_873876, EPI_ISL_873877, EPI_ISL_873878, EPI_ISL_873879			
see above	Lighthouse Lab in Cambridge	Wellcome Sanger Institute for the COVID-19 Genomics UK (COG-UK) Consortium	Rob Howes, The Lighthouse Lab in Cambridge and Alex Alderton, Roberto Amato, Sonia Goncalves, Ewan Harrison, David K. Jackson, Ian Johnston, Dominic Kwiatkowski, Cordelia Langford, John Sillitoe on behalf of the Wellcome Sanger Institute COVID-19 Surveillance Team
EPI_ISL_873881	Lighthouse Lab in Milton Keynes	Wellcome Sanger Institute for the COVID-19 Genomics UK (COG-UK) Consortium	The Lighthouse Lab in Milton Keynes and Alex Alderton, Roberto Amato, Sonia Goncalves, Ewan Harrison, David K. Jackson, Ian Johnston, Dominic Kwiatkowski, Cordelia Langford, John Sillitoe on behalf of the Wellcome Sanger Institute COVID-19 Surveillance Team
EPI_ISL_873883, EPI_ISL_873885	Lighthouse Lab in Cambridge	Wellcome Sanger Institute for the COVID-19 Genomics UK (COG-UK) Consortium	Rob Howes, The Lighthouse Lab in Cambridge and Alex Alderton, Roberto Amato, Sonia Goncalves, Ewan Harrison, David K. Jackson, Ian Johnston, Dominic Kwiatkowski, Cordelia Langford, John Sillitoe on behalf of the Wellcome Sanger Institute COVID-19 Surveillance Team
EPI_ISL_873886	Lighthouse Lab in Milton Keynes	Wellcome Sanger Institute for the COVID-19 Genomics UK (COG-UK) Consortium	The Lighthouse Lab in Milton Keynes and Alex Alderton, Roberto Amato, Sonia Goncalves, Ewan Harrison, David K. Jackson, Ian Johnston, Dominic Kwiatkowski, Cordelia Langford, John Sillitoe on behalf of the Wellcome Sanger Institute COVID-19 Surveillance Team
EPI_ISL_873888, EPI_ISL_873889, EPI_ISL_873890, EPI_ISL_873891, EPI_ISL_873892, EPI_ISL_873893, EPI_ISL_873894, EPI_ISL_873895, EPI_ISL_873896, EPI_ISL_873897, EPI_ISL_873898, EPI_ISL_873899, EPI_ISL_873901, EPI_ISL_873902, EPI_ISL_873903, EPI_ISL_873906, EPI_ISL_873907, EPI_ISL_873908, EPI_ISL_873909, EPI_ISL_873911, EPI_ISL_873912, EPI_ISL_873914, EPI_ISL_873915, EPI_ISL_873917, EPI_ISL_873918, EPI_ISL_873919, EPI_ISL_873920			
see above	Lighthouse Lab in Cambridge	Wellcome Sanger Institute for the COVID-19 Genomics UK (COG-UK) Consortium	Rob Howes, The Lighthouse Lab in Cambridge and Alex Alderton, Roberto Amato, Sonia Goncalves, Ewan Harrison, David K. Jackson, Ian Johnston, Dominic Kwiatkowski, Cordelia Langford, John Sillitoe on behalf of the Wellcome Sanger Institute COVID-19 Surveillance Team
EPI_ISL_873921	Lighthouse Lab in Milton Keynes	Wellcome Sanger Institute for the COVID-19 Genomics UK (COG-UK) Consortium	The Lighthouse Lab in Milton Keynes and Alex Alderton, Roberto Amato, Sonia Goncalves, Ewan Harrison, David K. Jackson, Ian Johnston, Dominic Kwiatkowski, Cordelia Langford, John Sillitoe on behalf of the Wellcome Sanger Institute COVID-19 Surveillance Team
EPI_ISL_873922	Lighthouse Lab in Cambridge	Wellcome Sanger Institute for the COVID-19 Genomics UK (COG-UK) Consortium	Rob Howes, The Lighthouse Lab in Cambridge and Alex Alderton, Roberto Amato, Sonia Goncalves, Ewan Harrison, David K. Jackson, Ian Johnston, Dominic Kwiatkowski, Cordelia Langford, John Sillitoe on behalf of the Wellcome Sanger Institute COVID-19 Surveillance Team
EPI_ISL_873926	Lighthouse Lab in Milton Keynes	Wellcome Sanger Institute for the COVID-19 Genomics UK (COG-UK) Consortium	The Lighthouse Lab in Milton Keynes and Alex Alderton, Roberto Amato, Sonia Goncalves, Ewan Harrison, David K. Jackson, Ian Johnston, Dominic Kwiatkowski, Cordelia Langford, John Sillitoe on behalf of the Wellcome Sanger Institute COVID-19 Surveillance Team
EPI_ISL_873927, EPI_ISL_873929, EPI_ISL_873930, EPI_ISL_873931, EPI_ISL_873932, EPI_ISL_873933, EPI_ISL_873936, EPI_ISL_873938, EPI_ISL_873939, EPI_ISL_873940, EPI_ISL_873941			
see above	Lighthouse Lab in Cambridge	Wellcome Sanger Institute for the COVID-19 Genomics UK (COG-UK) Consortium	Rob Howes, The Lighthouse Lab in Cambridge and Alex Alderton, Roberto Amato, Sonia Goncalves, Ewan Harrison, David K. Jackson, Ian Johnston, Dominic Kwiatkowski, Cordelia Langford, John Sillitoe on behalf of the Wellcome Sanger Institute COVID-19 Surveillance Team
EPI_ISL_873942	Lighthouse Lab in Milton Keynes	Wellcome Sanger Institute for the COVID-19 Genomics UK (COG-UK) Consortium	The Lighthouse Lab in Milton Keynes and Alex Alderton, Roberto Amato, Sonia Goncalves, Ewan Harrison, David K. Jackson, Ian Johnston, Dominic Kwiatkowski, Cordelia Langford, John Sillitoe on behalf of the Wellcome Sanger Institute COVID-19 Surveillance Team
EPI_ISL_873944, EPI_ISL_873945, EPI_ISL_873946, EPI_ISL_873949	Lighthouse Lab in Cambridge	Wellcome Sanger Institute for the COVID-19 Genomics UK (COG-UK) Consortium	Rob Howes, The Lighthouse Lab in Cambridge and Alex Alderton, Roberto Amato, Sonia Goncalves, Ewan Harrison, David K. Jackson, Ian Johnston, Dominic Kwiatkowski, Cordelia Langford, John Sillitoe on behalf of the Wellcome Sanger Institute COVID-19 Surveillance Team
EPI_ISL_873950	Lighthouse Lab in Milton Keynes	Wellcome Sanger Institute for the COVID-19 Genomics UK (COG-UK) Consortium	The Lighthouse Lab in Milton Keynes and Alex Alderton, Roberto Amato, Sonia Goncalves, Ewan Harrison, David K. Jackson, Ian Johnston, Dominic Kwiatkowski, Cordelia Langford, John Sillitoe on behalf of the Wellcome Sanger Institute COVID-19 Surveillance Team
EPI_ISL_873951, EPI_ISL_873952, EPI_ISL_873953, EPI_ISL_873955	Lighthouse Lab in Cambridge	Wellcome Sanger Institute for the COVID-19 Genomics UK (COG-UK) Consortium	Rob Howes, The Lighthouse Lab in Cambridge and Alex Alderton, Roberto Amato, Sonia Goncalves, Ewan Harrison, David K. Jackson, Ian Johnston, Dominic Kwiatkowski, Cordelia Langford, John Sillitoe on behalf of the Wellcome Sanger Institute COVID-19 Surveillance Team
EPI_ISL_873956	Lighthouse Lab in Milton Keynes	Wellcome Sanger Institute for the COVID-19 Genomics UK (COG-UK) Consortium	The Lighthouse Lab in Milton Keynes and Alex Alderton, Roberto Amato, Sonia Goncalves, Ewan Harrison, David K. Jackson, Ian Johnston, Dominic Kwiatkowski, Cordelia Langford, John Sillitoe on behalf of the Wellcome Sanger Institute COVID-19 Surveillance Team
EPI_ISL_873957, EPI_ISL_873958,	Lighthouse Lab in Cambridge	Wellcome Sanger Institute for the COVID-19 Genomics UK	Rob Howes, The Lighthouse Lab in Cambridge and Alex Alderton, Roberto Amato, Sonia Goncalves, Ewan Harrison, David K. Jackson, Ian Johnston,

EPI_ISL_873959		(COG-UK) Consortium	Dominic Kwiatkowski, Cordelia Langford, John Sillitoe on behalf of the Wellcome Sanger Institute COVID-19 Surveillance Team
EPI_ISL_873964, EPI_ISL_873965, EPI_ISL_873974, EPI_ISL_873976, EPI_ISL_873984, EPI_ISL_873987, EPI_ISL_873990, EPI_ISL_873999, EPI_ISL_874004, EPI_ISL_874007, EPI_ISL_874009, EPI_ISL_874010, EPI_ISL_874012, EPI_ISL_874014, EPI_ISL_874017, EPI_ISL_874021, EPI_ISL_874022, EPI_ISL_874032, EPI_ISL_874037, EPI_ISL_874038, EPI_ISL_874039, EPI_ISL_874047, EPI_ISL_874055, EPI_ISL_874061, EPI_ISL_874062, EPI_ISL_874064, EPI_ISL_874071, EPI_ISL_874076, EPI_ISL_874085, EPI_ISL_874090, EPI_ISL_874092, EPI_ISL_874096, EPI_ISL_874097, EPI_ISL_874115, EPI_ISL_874118, EPI_ISL_874130, EPI_ISL_874141, EPI_ISL_874143, EPI_ISL_874148, EPI_ISL_874153, EPI_ISL_874154, EPI_ISL_874157, EPI_ISL_874160, EPI_ISL_874163, EPI_ISL_874164, EPI_ISL_874165, EPI_ISL_874169, EPI_ISL_874180, EPI_ISL_874188, EPI_ISL_874191, EPI_ISL_874193, EPI_ISL_874195, EPI_ISL_874197, EPI_ISL_874198, EPI_ISL_874199, EPI_ISL_874202, EPI_ISL_874204, EPI_ISL_874207, EPI_ISL_874209, EPI_ISL_874211, EPI_ISL_874215, EPI_ISL_874238, EPI_ISL_874240, EPI_ISL_874246, EPI_ISL_874248, EPI_ISL_874254, EPI_ISL_874258, EPI_ISL_874263, EPI_ISL_874266, EPI_ISL_874268, EPI_ISL_874275, EPI_ISL_874278, EPI_ISL_874283, EPI_ISL_874290, EPI_ISL_874293, EPI_ISL_874297, EPI_ISL_874301, EPI_ISL_874311, EPI_ISL_874312, EPI_ISL_874313, EPI_ISL_874314, EPI_ISL_874316, EPI_ISL_874317, EPI_ISL_874318, EPI_ISL_874319, EPI_ISL_874320, EPI_ISL_874321, EPI_ISL_874322, EPI_ISL_874323, EPI_ISL_874324, EPI_ISL_874325, EPI_ISL_874326, EPI_ISL_874327, EPI_ISL_874328, EPI_ISL_874329, EPI_ISL_874330, EPI_ISL_874331, EPI_ISL_874332, EPI_ISL_874333, EPI_ISL_874334, EPI_ISL_874335, EPI_ISL_874336, EPI_ISL_874337, EPI_ISL_874338, EPI_ISL_874339, EPI_ISL_874340, EPI_ISL_874341, EPI_ISL_874342, EPI_ISL_874343, EPI_ISL_874344, EPI_ISL_874345, EPI_ISL_874346, EPI_ISL_874347, EPI_ISL_874348, EPI_ISL_874349, EPI_ISL_874350, EPI_ISL_874351, EPI_ISL_874352, EPI_ISL_874353, EPI_ISL_874354, EPI_ISL_874355, EPI_ISL_874356, EPI_ISL_874357, EPI_ISL_874358, EPI_ISL_874359, EPI_ISL_874360, EPI_ISL_874361, EPI_ISL_874362, EPI_ISL_874363, EPI_ISL_874364, EPI_ISL_874365, EPI_ISL_874366, EPI_ISL_874367, EPI_ISL_874368, EPI_ISL_874369, EPI_ISL_874370, EPI_ISL_874371, EPI_ISL_874372, EPI_ISL_874373, EPI_ISL_874374, EPI_ISL_874375, EPI_ISL_874376, EPI_ISL_874377, EPI_ISL_874378, EPI_ISL_874380, EPI_ISL_874381, EPI_ISL_874382, EPI_ISL_874383, EPI_ISL_874384, EPI_ISL_874385, EPI_ISL_874386, EPI_ISL_874387, EPI_ISL_874388, EPI_ISL_874389, EPI_ISL_874390, EPI_ISL_874391, EPI_ISL_874392, EPI_ISL_874393, EPI_ISL_874394, EPI_ISL_874395, EPI_ISL_874396, EPI_ISL_874397, EPI_ISL_874398, EPI_ISL_874399, EPI_ISL_874400, EPI_ISL_874402, EPI_ISL_874403, EPI_ISL_874405, EPI_ISL_874406, EPI_ISL_874407, EPI_ISL_874408, EPI_ISL_874409, EPI_ISL_874410, EPI_ISL_874411, EPI_ISL_874412, EPI_ISL_874413, EPI_ISL_874414, EPI_ISL_874415, EPI_ISL_874416, EPI_ISL_874417, EPI_ISL_874418, EPI_ISL_874419, EPI_ISL_874420, EPI_ISL_874421, EPI_ISL_874422, EPI_ISL_874423, EPI_ISL_874424, EPI_ISL_874425, EPI_ISL_874426, EPI_ISL_874427, EPI_ISL_874428, EPI_ISL_874429, EPI_ISL_874430, EPI_ISL_874431, EPI_ISL_874432, EPI_ISL_874433, EPI_ISL_874434, EPI_ISL_874435, EPI_ISL_874436, EPI_ISL_874437, EPI_ISL_874438, EPI_ISL_874439, EPI_ISL_874440, EPI_ISL_874441, EPI_ISL_874442, EPI_ISL_874444, EPI_ISL_874445, EPI_ISL_874446, EPI_ISL_874447, EPI_ISL_874449, EPI_ISL_874450, EPI_ISL_874451, EPI_ISL_874452, EPI_ISL_874453, EPI_ISL_874454, EPI_ISL_874455, EPI_ISL_874456, EPI_ISL_874457, EPI_ISL_874458, EPI_ISL_874459, EPI_ISL_874460, EPI_ISL_874461, EPI_ISL_874462, EPI_ISL_874463, EPI_ISL_874464, EPI_ISL_874465, EPI_ISL_874466, EPI_ISL_874467, EPI_ISL_874468, EPI_ISL_874470, EPI_ISL_874471, EPI_ISL_874472, EPI_ISL_874473, EPI_ISL_874474, EPI_ISL_874475, EPI_ISL_874476, EPI_ISL_874477, EPI_ISL_874478, EPI_ISL_874479, EPI_ISL_874480, EPI_ISL_874481, EPI_ISL_874483, EPI_ISL_874484, EPI_ISL_874485, EPI_ISL_874486, EPI_ISL_874487, EPI_ISL_874488, EPI_ISL_874489, EPI_ISL_874490, EPI_ISL_874491, EPI_ISL_874492, EPI_ISL_874493, EPI_ISL_874494, EPI_ISL_874495, EPI_ISL_874496, EPI_ISL_874497, EPI_ISL_874498, EPI_ISL_874500, EPI_ISL_874501, EPI_ISL_874503, EPI_ISL_874504, EPI_ISL_874505, EPI_ISL_874506, EPI_ISL_874507, EPI_ISL_874508, EPI_ISL_874509, EPI_ISL_874510, EPI_ISL_874511, EPI_ISL_874512, EPI_ISL_874513, EPI_ISL_874514, EPI_ISL_874515, EPI_ISL_874516, EPI_ISL_874517, EPI_ISL_874518, EPI_ISL_874519, EPI_ISL_874520, EPI_ISL_874521, EPI_ISL_874522, EPI_ISL_874524, EPI_ISL_874525, EPI_ISL_874526, EPI_ISL_874529, EPI_ISL_874531, EPI_ISL_874532, EPI_ISL_874533, EPI_ISL_874534, EPI_ISL_874535, EPI_ISL_874536, EPI_ISL_874537, EPI_ISL_874538, EPI_ISL_874539, EPI_ISL_874540, EPI_ISL_874541, EPI_ISL_874542, EPI_ISL_874544, EPI_ISL_874545, EPI_ISL_874546, EPI_ISL_874547, EPI_ISL_874548, EPI_ISL_874549, EPI_ISL_874550, EPI_ISL_874551, EPI_ISL_874552, EPI_ISL_874553, EPI_ISL_874554, EPI_ISL_874555, EPI_ISL_874556, EPI_ISL_874557, EPI_ISL_874559, EPI_ISL_874560, EPI_ISL_874561, EPI_ISL_874562, EPI_ISL_874563, EPI_ISL_874564, EPI_ISL_874565, EPI_ISL_874566, EPI_ISL_874567, EPI_ISL_874568, EPI_ISL_874569, EPI_ISL_874570, EPI_ISL_874571, EPI_ISL_874572, EPI_ISL_874573, EPI_ISL_874574, EPI_ISL_874575, EPI_ISL_874576, EPI_ISL_874577, EPI_ISL_874578, EPI_ISL_874579, EPI_ISL_874580, EPI_ISL_874581, EPI_ISL_874582, EPI_ISL_874583, EPI_ISL_874584, EPI_ISL_874585, EPI_ISL_874586, EPI_ISL_874587, EPI_ISL_874588, EPI_ISL_874589, EPI_ISL_874590, EPI_ISL_874591, EPI_ISL_874592, EPI_ISL_874593, EPI_ISL_874594, EPI_ISL_874595, EPI_ISL_874596, EPI_ISL_874597, EPI_ISL_874598, EPI_ISL_874599, EPI_ISL_874600, EPI_ISL_874601, EPI_ISL_874602, EPI_ISL_874603, EPI_ISL_874604, EPI_ISL_874605, EPI_ISL_874606, EPI_ISL_874607, EPI_ISL_874608, EPI_ISL_874609, EPI_ISL_874610, EPI_ISL_874611, EPI_ISL_874612, EPI_ISL_874613, EPI_ISL_874614, EPI_ISL_874615, EPI_ISL_874616, EPI_ISL_874617, EPI_ISL_874618, EPI_ISL_874619, EPI_ISL_874620, EPI_ISL_874621, EPI_ISL_874622, EPI_ISL_874623, EPI_ISL_874625, EPI_ISL_874626, EPI_ISL_874627, EPI_ISL_874628, EPI_ISL_874629, EPI_ISL_874630, EPI_ISL_874631, EPI_ISL_874632, EPI_ISL_874633, EPI_ISL_874634, EPI_ISL_874635, EPI_ISL_874636, EPI_ISL_874637, EPI_ISL_874638, EPI_ISL_874639, EPI_ISL_874640, EPI_ISL_874641, EPI_ISL_874642, EPI_ISL_874643, EPI_ISL_874644, EPI_ISL_874645, EPI_ISL_874646, EPI_ISL_874647, EPI_ISL_874648, EPI_ISL_874649, EPI_ISL_874650, EPI_ISL_874651, EPI_ISL_874652, EPI_ISL_874653, EPI_ISL_874654, EPI_ISL_874655			
see above	Lighthouse Lab in Alderley Park	Wellcome Sanger Institute for the COVID-19 Genomics UK (COG-UK) Consortium	Jacquelyn Wynn, Mairead Hyland, The Lighthouse Lab in Alderley Park and Alex Alderton, Roberto Amato, Sonia Goncalves, Ewan Harrison, David K. Jackson, Ian Johnston, Dominic Kwiatkowski, Cordelia Langford, John Sillitoe on behalf of the Wellcome Sanger Institute COVID-19 Surveillance Team
EPI_ISL_874656	Lighthouse Lab in Glasgow	Wellcome Sanger Institute for the COVID-19 Genomics UK (COG-UK) Consortium	Harper VanSteenhouse, Yumi Kasai, David Gray, Carol Clugston, Anna Dominiczak and Alex Alderton, Roberto Amato, Sonia Goncalves, Ewan Harrison, David K. Jackson, Ian Johnston, Dominic Kwiatkowski, Cordelia Langford, John Sillitoe on behalf of the Wellcome Sanger Institute COVID-19 Surveillance Team
EPI_ISL_874657, EPI_ISL_874658, EPI_ISL_874659, EPI_ISL_874660, EPI_ISL_874661, EPI_ISL_874662, EPI_ISL_874663, EPI_ISL_874664, EPI_ISL_874665, EPI_ISL_874666, EPI_ISL_874667, EPI_ISL_874668, EPI_ISL_874669, EPI_ISL_874670, EPI_ISL_874671, EPI_ISL_874672, EPI_ISL_874673, EPI_ISL_874674, EPI_ISL_874675			
see above	Lighthouse Lab in Alderley Park	Wellcome Sanger Institute for the COVID-19 Genomics UK (COG-UK) Consortium	Jacquelyn Wynn, Mairead Hyland, The Lighthouse Lab in Alderley Park and Alex Alderton, Roberto Amato, Sonia Goncalves, Ewan Harrison, David K. Jackson, Ian Johnston, Dominic Kwiatkowski, Cordelia Langford, John Sillitoe on behalf of the Wellcome Sanger Institute COVID-19 Surveillance Team
EPI_ISL_874676	Lighthouse Lab in Glasgow	Wellcome Sanger Institute for the COVID-19 Genomics UK (COG-UK) Consortium	Harper VanSteenhouse, Yumi Kasai, David Gray, Carol Clugston, Anna Dominiczak and Alex Alderton, Roberto Amato, Sonia Goncalves, Ewan Harrison, David K. Jackson, Ian Johnston, Dominic Kwiatkowski, Cordelia Langford, John Sillitoe on behalf of the Wellcome Sanger Institute COVID-19 Surveillance Team
EPI_ISL_874677, EPI_ISL_874678, EPI_ISL_874679, EPI_ISL_874680, EPI_ISL_874681, EPI_ISL_874682, EPI_ISL_874683, EPI_ISL_874684, EPI_ISL_874685, EPI_ISL_874686, EPI_ISL_874687, EPI_ISL_874688, EPI_ISL_874689, EPI_ISL_874690, EPI_ISL_874691, EPI_ISL_874692, EPI_ISL_874693, EPI_ISL_874694, EPI_ISL_874695, EPI_ISL_874696, EPI_ISL_874697, EPI_ISL_874698, EPI_ISL_874699, EPI_ISL_874700, EPI_ISL_874701, EPI_ISL_874702, EPI_ISL_874703, EPI_ISL_874704, EPI_ISL_874705, EPI_ISL_874706, EPI_ISL_874707, EPI_ISL_874708, EPI_ISL_874709, EPI_ISL_874710, EPI_ISL_874711, EPI_ISL_874712, EPI_ISL_874713, EPI_ISL_874714, EPI_ISL_874715, EPI_ISL_874716, EPI_ISL_874717, EPI_ISL_874718, EPI_ISL_874719, EPI_ISL_874720, EPI_ISL_874721, EPI_ISL_874722, EPI_ISL_874723, EPI_ISL_874724, EPI_ISL_874725, EPI_ISL_874726, EPI_ISL_874727, EPI_ISL_874728, EPI_ISL_874729, EPI_ISL_874730, EPI_ISL_874731, EPI_ISL_874732			
see above	Lighthouse Lab in Alderley Park	Wellcome Sanger Institute for the COVID-19 Genomics UK (COG-UK) Consortium	Jacquelyn Wynn, Mairead Hyland, The Lighthouse Lab in Alderley Park and Alex Alderton, Roberto Amato, Sonia Goncalves, Ewan Harrison, David K. Jackson, Ian Johnston, Dominic Kwiatkowski, Cordelia Langford, John Sillitoe on behalf of the Wellcome Sanger Institute COVID-19 Surveillance Team
EPI_ISL_874733	Lighthouse Lab in Glasgow	Wellcome Sanger Institute for the COVID-19 Genomics UK (COG-UK) Consortium	Harper VanSteenhouse, Yumi Kasai, David Gray, Carol Clugston, Anna Dominiczak and Alex Alderton, Roberto Amato, Sonia Goncalves, Ewan Harrison, David K. Jackson, Ian Johnston, Dominic Kwiatkowski, Cordelia Langford, John Sillitoe on behalf of the Wellcome Sanger Institute COVID-19 Surveillance Team
EPI_ISL_874734, EPI_ISL_874735	Lighthouse Lab in Alderley Park	Wellcome Sanger Institute for the COVID-19 Genomics UK (COG-UK) Consortium	Jacquelyn Wynn, Mairead Hyland, The Lighthouse Lab in Alderley Park and Alex Alderton, Roberto Amato, Sonia Goncalves, Ewan Harrison, David K. Jackson, Ian Johnston, Dominic Kwiatkowski, Cordelia Langford, John Sillitoe on behalf of the Wellcome Sanger Institute COVID-19 Surveillance Team
EPI_ISL_874736, EPI_ISL_874737	Lighthouse Lab in Glasgow	Wellcome Sanger Institute for the COVID-19 Genomics UK (COG-UK) Consortium	Harper VanSteenhouse, Yumi Kasai, David Gray, Carol Clugston, Anna Dominiczak and Alex Alderton, Roberto Amato, Sonia Goncalves, Ewan Harrison, David K. Jackson, Ian Johnston, Dominic Kwiatkowski, Cordelia Langford, John Sillitoe on behalf of the Wellcome Sanger Institute COVID-19 Surveillance Team
EPI_ISL_874738	Lighthouse Lab in Alderley Park	Wellcome Sanger Institute for the COVID-19 Genomics UK (COG-UK) Consortium	Jacquelyn Wynn, Mairead Hyland, The Lighthouse Lab in Alderley Park and Alex Alderton, Roberto Amato, Sonia Goncalves, Ewan Harrison, David K. Jackson, Ian Johnston, Dominic Kwiatkowski, Cordelia Langford, John Sillitoe on behalf of the Wellcome Sanger Institute COVID-19 Surveillance Team
EPI_ISL_874739	Lighthouse Lab in Glasgow	Wellcome Sanger Institute for the COVID-19 Genomics UK (COG-UK) Consortium	Harper VanSteenhouse, Yumi Kasai, David Gray, Carol Clugston, Anna Dominiczak and Alex Alderton, Roberto Amato, Sonia Goncalves, Ewan Harrison, David K. Jackson, Ian Johnston, Dominic Kwiatkowski, Cordelia Langford, John Sillitoe on behalf of the Wellcome Sanger Institute COVID-19 Surveillance Team
EPI_ISL_874740, EPI_ISL_874741, EPI_ISL_874742, EPI_ISL_874743, EPI_ISL_874744, EPI_ISL_874745, EPI_ISL_874746, EPI_ISL_874747, EPI_ISL_874748, EPI_ISL_874749, EPI_ISL_874750, EPI_ISL_874751, EPI_ISL_874752			
see above	Lighthouse Lab in Alderley Park	Wellcome Sanger Institute for the COVID-19 Genomics UK (COG-UK) Consortium	Jacquelyn Wynn, Mairead Hyland, The Lighthouse Lab in Alderley Park and Alex Alderton, Roberto Amato, Sonia Goncalves, Ewan Harrison, David K. Jackson, Ian Johnston, Dominic Kwiatkowski, Cordelia Langford, John Sillitoe on behalf of the Wellcome Sanger Institute COVID-19 Surveillance Team
EPI_ISL_874753	Lighthouse Lab in Glasgow	Wellcome Sanger Institute for the COVID-19 Genomics UK (COG-UK) Consortium	Harper VanSteenhouse, Yumi Kasai, David Gray, Carol Clugston, Anna Dominiczak and Alex Alderton, Roberto Amato, Sonia Goncalves, Ewan Harrison, David K. Jackson, Ian Johnston, Dominic Kwiatkowski, Cordelia Langford, John Sillitoe on behalf of the Wellcome Sanger Institute COVID-19 Surveillance Team
EPI_ISL_874754, EPI_ISL_874755, EPI_ISL_874756, EPI_ISL_874757, EPI_ISL_874758, EPI_ISL_874760, EPI_ISL_874761, EPI_ISL_874762, EPI_ISL_874763, EPI_ISL_874764, EPI_ISL_874765, EPI_ISL_874766, EPI_ISL_874767, EPI_ISL_874768, EPI_ISL_874769, EPI_ISL_874770, EPI_ISL_874771, EPI_ISL_874772, EPI_ISL_874773, EPI_ISL_874774			
see above	Lighthouse Lab in Alderley Park	Wellcome Sanger Institute for the COVID-19 Genomics UK (COG-UK) Consortium	Jacquelyn Wynn, Mairead Hyland, The Lighthouse Lab in Alderley Park and Alex Alderton, Roberto Amato, Sonia Goncalves, Ewan Harrison, David K. Jackson, Ian Johnston, Dominic Kwiatkowski, Cordelia Langford, John Sillitoe on behalf of the Wellcome Sanger Institute COVID-19 Surveillance Team
EPI_ISL_874775	Lighthouse Lab in Glasgow	Wellcome Sanger Institute for the COVID-19 Genomics UK (COG-UK) Consortium	Harper VanSteenhouse, Yumi Kasai, David Gray, Carol Clugston, Anna Dominiczak and Alex Alderton, Roberto Amato, Sonia Goncalves, Ewan Harrison, David K. Jackson, Ian Johnston, Dominic Kwiatkowski, Cordelia Langford, John Sillitoe on behalf of the Wellcome Sanger Institute COVID-19 Surveillance Team
EPI_ISL_874776, EPI_ISL_874777, EPI_ISL_874778, EPI_ISL_874779, EPI_ISL_874780, EPI_ISL_874781, EPI_ISL_874782, EPI_ISL_874783, EPI_ISL_874784, EPI_ISL_874785, EPI_ISL_874786, EPI_ISL_874787, EPI_ISL_874788, EPI_ISL_874789, EPI_ISL_874790, EPI_ISL_874791, EPI_ISL_874792, EPI_ISL_874793, EPI_ISL_874794, EPI_ISL_874795, EPI_ISL_874796, EPI_ISL_874797			
see above	Lighthouse Lab in Alderley Park	Wellcome Sanger Institute for the COVID-19 Genomics UK (COG-UK) Consortium	Jacquelyn Wynn, Mairead Hyland, The Lighthouse Lab in Alderley Park and Alex Alderton, Roberto Amato, Sonia Goncalves, Ewan Harrison, David K. Jackson, Ian Johnston, Dominic Kwiatkowski, Cordelia Langford, John Sillitoe on behalf of the Wellcome Sanger Institute COVID-19 Surveillance Team
EPI_ISL_874798	Lighthouse Lab in Glasgow	Wellcome Sanger Institute for the COVID-19 Genomics UK (COG-UK) Consortium	Harper VanSteenhouse, Yumi Kasai, David Gray, Carol Clugston, Anna Dominiczak and Alex Alderton, Roberto Amato, Sonia Goncalves, Ewan Harrison, David K. Jackson, Ian Johnston, Dominic Kwiatkowski, Cordelia Langford, John Sillitoe on behalf of the Wellcome Sanger Institute COVID-19 Surveillance Team
EPI_ISL_874799, EPI_ISL_874800,	Lighthouse Lab in Alderley Park	Wellcome Sanger Institute for the COVID-19 Genomics UK	Jacquelyn Wynn, Mairead Hyland, The Lighthouse Lab in Alderley Park and Alex Alderton, Roberto Amato, Sonia Goncalves, Ewan Harrison, David K.

EPI_ISL_874801, EPI_ISL_874802, EPI_ISL_874803, EPI_ISL_874804, EPI_ISL_874805		(COG-UK) Consortium	Jackson, Ian Johnston, Dominic Kwiatkowski, Cordelia Langford, John Sillitoe on behalf of the Wellcome Sanger Institute COVID-19 Surveillance Team
EPI_ISL_874806	Lighthouse Lab in Glasgow	Wellcome Sanger Institute for the COVID-19 Genomics UK (COG-UK) Consortium	Harper VanSteenhouse, Yumi Kasai, David Gray, Carol Clugston, Anna Dominiczak and Alex Alderton, Roberto Amato, Sonia Goncalves, Ewan Harrison, David K. Jackson, Ian Johnston, Dominic Kwiatkowski, Cordelia Langford, John Sillitoe on behalf of the Wellcome Sanger Institute COVID-19 Surveillance Team
EPI_ISL_874807	Lighthouse Lab in Alderley Park	Wellcome Sanger Institute for the COVID-19 Genomics UK (COG-UK) Consortium	Jacquelyn Wynn, Mairead Hyland, The Lighthouse Lab in Alderley Park and Alex Alderton, Roberto Amato, Sonia Goncalves, Ewan Harrison, David K. Jackson, Ian Johnston, Dominic Kwiatkowski, Cordelia Langford, John Sillitoe on behalf of the Wellcome Sanger Institute COVID-19 Surveillance Team
EPI_ISL_874808	Lighthouse Lab in Glasgow	Wellcome Sanger Institute for the COVID-19 Genomics UK (COG-UK) Consortium	Harper VanSteenhouse, Yumi Kasai, David Gray, Carol Clugston, Anna Dominiczak and Alex Alderton, Roberto Amato, Sonia Goncalves, Ewan Harrison, David K. Jackson, Ian Johnston, Dominic Kwiatkowski, Cordelia Langford, John Sillitoe on behalf of the Wellcome Sanger Institute COVID-19 Surveillance Team
EPI_ISL_874809, EPI_ISL_874810, EPI_ISL_874811, EPI_ISL_874812, EPI_ISL_874813, EPI_ISL_874814, EPI_ISL_874815, EPI_ISL_874817, EPI_ISL_874818, EPI_ISL_874819, EPI_ISL_874820, EPI_ISL_874821, EPI_ISL_874822, EPI_ISL_874823, EPI_ISL_874824, EPI_ISL_874825, EPI_ISL_874826, EPI_ISL_874827, EPI_ISL_874828, EPI_ISL_874829, EPI_ISL_874830, EPI_ISL_874831, EPI_ISL_874832, EPI_ISL_874833, EPI_ISL_874834, EPI_ISL_874835, EPI_ISL_874836, EPI_ISL_874837, EPI_ISL_874838, EPI_ISL_874839, EPI_ISL_874840, EPI_ISL_874841, EPI_ISL_874842, EPI_ISL_874843, EPI_ISL_874844, EPI_ISL_874845, EPI_ISL_874846, EPI_ISL_874847, EPI_ISL_874848, EPI_ISL_874849, EPI_ISL_874850, EPI_ISL_874851, EPI_ISL_874852, EPI_ISL_874854, EPI_ISL_874855, EPI_ISL_874856, EPI_ISL_874857, EPI_ISL_874858, EPI_ISL_874859, EPI_ISL_874860, EPI_ISL_874861, EPI_ISL_874862			
see above	Lighthouse Lab in Alderley Park	Wellcome Sanger Institute for the COVID-19 Genomics UK (COG-UK) Consortium	Jacquelyn Wynn, Mairead Hyland, The Lighthouse Lab in Alderley Park and Alex Alderton, Roberto Amato, Sonia Goncalves, Ewan Harrison, David K. Jackson, Ian Johnston, Dominic Kwiatkowski, Cordelia Langford, John Sillitoe on behalf of the Wellcome Sanger Institute COVID-19 Surveillance Team
EPI_ISL_874863	Lighthouse Lab in Glasgow	Wellcome Sanger Institute for the COVID-19 Genomics UK (COG-UK) Consortium	Harper VanSteenhouse, Yumi Kasai, David Gray, Carol Clugston, Anna Dominiczak and Alex Alderton, Roberto Amato, Sonia Goncalves, Ewan Harrison, David K. Jackson, Ian Johnston, Dominic Kwiatkowski, Cordelia Langford, John Sillitoe on behalf of the Wellcome Sanger Institute COVID-19 Surveillance Team
EPI_ISL_874864, EPI_ISL_874865, EPI_ISL_874866, EPI_ISL_874867, EPI_ISL_874868, EPI_ISL_874869, EPI_ISL_874870, EPI_ISL_874871, EPI_ISL_874872, EPI_ISL_874873, EPI_ISL_874874, EPI_ISL_874875, EPI_ISL_874876, EPI_ISL_874877, EPI_ISL_874878, EPI_ISL_874879, EPI_ISL_874880, EPI_ISL_874881, EPI_ISL_874882, EPI_ISL_874883, EPI_ISL_874884, EPI_ISL_874885, EPI_ISL_874886, EPI_ISL_874887, EPI_ISL_874888, EPI_ISL_874889, EPI_ISL_874890, EPI_ISL_874891, EPI_ISL_874892, EPI_ISL_874893, EPI_ISL_874894, EPI_ISL_874895			
see above	Lighthouse Lab in Alderley Park	Wellcome Sanger Institute for the COVID-19 Genomics UK (COG-UK) Consortium	Jacquelyn Wynn, Mairead Hyland, The Lighthouse Lab in Alderley Park and Alex Alderton, Roberto Amato, Sonia Goncalves, Ewan Harrison, David K. Jackson, Ian Johnston, Dominic Kwiatkowski, Cordelia Langford, John Sillitoe on behalf of the Wellcome Sanger Institute COVID-19 Surveillance Team
EPI_ISL_874896	Lighthouse Lab in Glasgow	Wellcome Sanger Institute for the COVID-19 Genomics UK (COG-UK) Consortium	Harper VanSteenhouse, Yumi Kasai, David Gray, Carol Clugston, Anna Dominiczak and Alex Alderton, Roberto Amato, Sonia Goncalves, Ewan Harrison, David K. Jackson, Ian Johnston, Dominic Kwiatkowski, Cordelia Langford, John Sillitoe on behalf of the Wellcome Sanger Institute COVID-19 Surveillance Team
EPI_ISL_874897	Lighthouse Lab in Alderley Park	Wellcome Sanger Institute for the COVID-19 Genomics UK (COG-UK) Consortium	Jacquelyn Wynn, Mairead Hyland, The Lighthouse Lab in Alderley Park and Alex Alderton, Roberto Amato, Sonia Goncalves, Ewan Harrison, David K. Jackson, Ian Johnston, Dominic Kwiatkowski, Cordelia Langford, John Sillitoe on behalf of the Wellcome Sanger Institute COVID-19 Surveillance Team
EPI_ISL_874898	Lighthouse Lab in Glasgow	Wellcome Sanger Institute for the COVID-19 Genomics UK (COG-UK) Consortium	Harper VanSteenhouse, Yumi Kasai, David Gray, Carol Clugston, Anna Dominiczak and Alex Alderton, Roberto Amato, Sonia Goncalves, Ewan Harrison, David K. Jackson, Ian Johnston, Dominic Kwiatkowski, Cordelia Langford, John Sillitoe on behalf of the Wellcome Sanger Institute COVID-19 Surveillance Team
EPI_ISL_874899, EPI_ISL_874900, EPI_ISL_874901, EPI_ISL_874902, EPI_ISL_874903, EPI_ISL_874904	Lighthouse Lab in Alderley Park	Wellcome Sanger Institute for the COVID-19 Genomics UK (COG-UK) Consortium	Jacquelyn Wynn, Mairead Hyland, The Lighthouse Lab in Alderley Park and Alex Alderton, Roberto Amato, Sonia Goncalves, Ewan Harrison, David K. Jackson, Ian Johnston, Dominic Kwiatkowski, Cordelia Langford, John Sillitoe on behalf of the Wellcome Sanger Institute COVID-19 Surveillance Team
EPI_ISL_874905	Lighthouse Lab in Glasgow	Wellcome Sanger Institute for the COVID-19 Genomics UK (COG-UK) Consortium	Harper VanSteenhouse, Yumi Kasai, David Gray, Carol Clugston, Anna Dominiczak and Alex Alderton, Roberto Amato, Sonia Goncalves, Ewan Harrison, David K. Jackson, Ian Johnston, Dominic Kwiatkowski, Cordelia Langford, John Sillitoe on behalf of the Wellcome Sanger Institute COVID-19 Surveillance Team
EPI_ISL_874906, EPI_ISL_874907, EPI_ISL_874908, EPI_ISL_874909, EPI_ISL_874910, EPI_ISL_874911, EPI_ISL_874912, EPI_ISL_874913, EPI_ISL_874914, EPI_ISL_874915, EPI_ISL_874916, EPI_ISL_874917, EPI_ISL_874918, EPI_ISL_874919, EPI_ISL_874920, EPI_ISL_874921			
see above	Lighthouse Lab in Alderley Park	Wellcome Sanger Institute for the COVID-19 Genomics UK (COG-UK) Consortium	Jacquelyn Wynn, Mairead Hyland, The Lighthouse Lab in Alderley Park and Alex Alderton, Roberto Amato, Sonia Goncalves, Ewan Harrison, David K. Jackson, Ian Johnston, Dominic Kwiatkowski, Cordelia Langford, John Sillitoe on behalf of the Wellcome Sanger Institute COVID-19 Surveillance Team
EPI_ISL_874922	Lighthouse Lab in Glasgow	Wellcome Sanger Institute for the COVID-19 Genomics UK (COG-UK) Consortium	Harper VanSteenhouse, Yumi Kasai, David Gray, Carol Clugston, Anna Dominiczak and Alex Alderton, Roberto Amato, Sonia Goncalves, Ewan Harrison, David K. Jackson, Ian Johnston, Dominic Kwiatkowski, Cordelia Langford, John Sillitoe on behalf of the Wellcome Sanger Institute COVID-19 Surveillance Team
EPI_ISL_874923, EPI_ISL_874924, EPI_ISL_874925, EPI_ISL_874926, EPI_ISL_874927, EPI_ISL_874928, EPI_ISL_874929, EPI_ISL_874930, EPI_ISL_874931, EPI_ISL_874932, EPI_ISL_874933, EPI_ISL_874934			
see above	Lighthouse Lab in Alderley Park	Wellcome Sanger Institute for the COVID-19 Genomics UK (COG-UK) Consortium	Jacquelyn Wynn, Mairead Hyland, The Lighthouse Lab in Alderley Park and Alex Alderton, Roberto Amato, Sonia Goncalves, Ewan Harrison, David K. Jackson, Ian Johnston, Dominic Kwiatkowski, Cordelia Langford, John Sillitoe on behalf of the Wellcome Sanger Institute COVID-19 Surveillance Team
EPI_ISL_874935	Lighthouse Lab in Glasgow	Wellcome Sanger Institute for the COVID-19 Genomics UK (COG-UK) Consortium	Harper VanSteenhouse, Yumi Kasai, David Gray, Carol Clugston, Anna Dominiczak and Alex Alderton, Roberto Amato, Sonia Goncalves, Ewan Harrison, David K. Jackson, Ian Johnston, Dominic Kwiatkowski, Cordelia Langford, John Sillitoe on behalf of the Wellcome Sanger Institute COVID-19 Surveillance Team
EPI_ISL_874936, EPI_ISL_874937, EPI_ISL_874939, EPI_ISL_874940, EPI_ISL_874941, EPI_ISL_874942	Lighthouse Lab in Alderley Park	Wellcome Sanger Institute for the COVID-19 Genomics UK (COG-UK) Consortium	Jacquelyn Wynn, Mairead Hyland, The Lighthouse Lab in Alderley Park and Alex Alderton, Roberto Amato, Sonia Goncalves, Ewan Harrison, David K. Jackson, Ian Johnston, Dominic Kwiatkowski, Cordelia Langford, John Sillitoe on behalf of the Wellcome Sanger Institute COVID-19 Surveillance Team
EPI_ISL_874943	Lighthouse Lab in Glasgow	Wellcome Sanger Institute for the COVID-19 Genomics UK (COG-UK) Consortium	Harper VanSteenhouse, Yumi Kasai, David Gray, Carol Clugston, Anna Dominiczak and Alex Alderton, Roberto Amato, Sonia Goncalves, Ewan Harrison, David K. Jackson, Ian Johnston, Dominic Kwiatkowski, Cordelia Langford, John Sillitoe on behalf of the Wellcome Sanger Institute COVID-19 Surveillance Team
EPI_ISL_874944, EPI_ISL_874945, EPI_ISL_874946, EPI_ISL_874947, EPI_ISL_874948, EPI_ISL_874949	Lighthouse Lab in Alderley Park	Wellcome Sanger Institute for the COVID-19 Genomics UK (COG-UK) Consortium	Jacquelyn Wynn, Mairead Hyland, The Lighthouse Lab in Alderley Park and Alex Alderton, Roberto Amato, Sonia Goncalves, Ewan Harrison, David K. Jackson, Ian Johnston, Dominic Kwiatkowski, Cordelia Langford, John Sillitoe on behalf of the Wellcome Sanger Institute COVID-19 Surveillance Team
EPI_ISL_874950	Lighthouse Lab in Glasgow	Wellcome Sanger Institute for the COVID-19 Genomics UK (COG-UK) Consortium	Harper VanSteenhouse, Yumi Kasai, David Gray, Carol Clugston, Anna Dominiczak and Alex Alderton, Roberto Amato, Sonia Goncalves, Ewan Harrison, David K. Jackson, Ian Johnston, Dominic Kwiatkowski, Cordelia Langford, John Sillitoe on behalf of the Wellcome Sanger Institute COVID-19 Surveillance Team
EPI_ISL_874951, EPI_ISL_874952, EPI_ISL_874953, EPI_ISL_874954, EPI_ISL_874955, EPI_ISL_874957, EPI_ISL_874958, EPI_ISL_874959, EPI_ISL_874960, EPI_ISL_874961, EPI_ISL_874962, EPI_ISL_874963, EPI_ISL_874964			
see above	Lighthouse Lab in Alderley Park	Wellcome Sanger Institute for the COVID-19 Genomics UK (COG-UK) Consortium	Jacquelyn Wynn, Mairead Hyland, The Lighthouse Lab in Alderley Park and Alex Alderton, Roberto Amato, Sonia Goncalves, Ewan Harrison, David K. Jackson, Ian Johnston, Dominic Kwiatkowski, Cordelia Langford, John Sillitoe on behalf of the Wellcome Sanger Institute COVID-19 Surveillance Team
EPI_ISL_874965, EPI_ISL_874967	Lighthouse Lab in Glasgow	Wellcome Sanger Institute for the COVID-19 Genomics UK (COG-UK) Consortium	Harper VanSteenhouse, Yumi Kasai, David Gray, Carol Clugston, Anna Dominiczak and Alex Alderton, Roberto Amato, Sonia Goncalves, Ewan Harrison, David K. Jackson, Ian Johnston, Dominic Kwiatkowski, Cordelia Langford, John Sillitoe on behalf of the Wellcome Sanger Institute COVID-19 Surveillance Team
EPI_ISL_874968, EPI_ISL_874969, EPI_ISL_874970, EPI_ISL_874971, EPI_ISL_874972, EPI_ISL_874973, EPI_ISL_874974, EPI_ISL_874975, EPI_ISL_874976, EPI_ISL_874977, EPI_ISL_874978, EPI_ISL_874979, EPI_ISL_874980, EPI_ISL_874981, EPI_ISL_874982, EPI_ISL_874983, EPI_ISL_874984, EPI_ISL_874985, EPI_ISL_874986, EPI_ISL_874987, EPI_ISL_874988, EPI_ISL_874989, EPI_ISL_877771, EPI_ISL_877781, EPI_ISL_877787, EPI_ISL_877788, EPI_ISL_877796, EPI_ISL_877797, EPI_ISL_877800, EPI_ISL_877802, EPI_ISL_877804, EPI_ISL_877807, EPI_ISL_877809, EPI_ISL_877821, EPI_ISL_877828, EPI_ISL_877833, EPI_ISL_877844, EPI_ISL_877881, EPI_ISL_877884, EPI_ISL_877885, EPI_ISL_877889, EPI_ISL_877899, EPI_ISL_877903, EPI_ISL_877907, EPI_ISL_877918, EPI_ISL_877922, EPI_ISL_877924, EPI_ISL_877930, EPI_ISL_877938, EPI_ISL_877940, EPI_ISL_877948, EPI_ISL_877953, EPI_ISL_877964, EPI_ISL_877969, EPI_ISL_877972, EPI_ISL_877975, EPI_ISL_877981, EPI_ISL_877989, EPI_ISL_877995, EPI_ISL_877997, EPI_ISL_878011, EPI_ISL_878013, EPI_ISL_878021, EPI_ISL_878025, EPI_ISL_878026, EPI_ISL_878032, EPI_ISL_878038, EPI_ISL_878040, EPI_ISL_878041, EPI_ISL_878042, EPI_ISL_878044, EPI_ISL_878053, EPI_ISL_878054, EPI_ISL_878062, EPI_ISL_878064, EPI_ISL_878067, EPI_ISL_878078, EPI_ISL_878083, EPI_ISL_878088, EPI_ISL_878089, EPI_ISL_878098, EPI_ISL_878102, EPI_ISL_878105, EPI_ISL_878108, EPI_ISL_878111, EPI_ISL_878112, EPI_ISL_878113, EPI_ISL_878114, EPI_ISL_878115, EPI_ISL_878116, EPI_ISL_878117, EPI_ISL_878118, EPI_ISL_878119, EPI_ISL_878120, EPI_ISL_878121, EPI_ISL_878122, EPI_ISL_878123, EPI_ISL_878124, EPI_ISL_878125, EPI_ISL_878126, EPI_ISL_878127, EPI_ISL_878128, EPI_ISL_878129, EPI_ISL_878130, EPI_ISL_878131, EPI_ISL_878132, EPI_ISL_878133, EPI_ISL_878135, EPI_ISL_878136, EPI_ISL_878137, EPI_ISL_878138, EPI_ISL_878139, EPI_ISL_878140, EPI_ISL_878141, EPI_ISL_878142, EPI_ISL_878143, EPI_ISL_878144, EPI_ISL_878145, EPI_ISL_878146, EPI_ISL_878147, EPI_ISL_878148, EPI_ISL_878149, EPI_ISL_878150, EPI_ISL_878151, EPI_ISL_878153, EPI_ISL_878154,			

EPI_ISL_878156, EPI_ISL_878160, EPI_ISL_878161, EPI_ISL_878163, EPI_ISL_878164, EPI_ISL_878166, EPI_ISL_878169, EPI_ISL_878171, EPI_ISL_878173, EPI_ISL_878174, EPI_ISL_878176, EPI_ISL_878177, EPI_ISL_878179, EPI_ISL_878181, EPI_ISL_878182, EPI_ISL_878184, EPI_ISL_878185, EPI_ISL_878187, EPI_ISL_878188, EPI_ISL_878190, EPI_ISL_878192, EPI_ISL_878193, EPI_ISL_878195, EPI_ISL_878196, EPI_ISL_878198, EPI_ISL_878200, EPI_ISL_878201, EPI_ISL_878204, EPI_ISL_878206, EPI_ISL_878208, EPI_ISL_878210, EPI_ISL_878213, EPI_ISL_878214, EPI_ISL_878216, EPI_ISL_878218, EPI_ISL_878219, EPI_ISL_878220, EPI_ISL_878222, EPI_ISL_878224, EPI_ISL_878225, EPI_ISL_878227, EPI_ISL_878228, EPI_ISL_878230, EPI_ISL_878231, EPI_ISL_878233, EPI_ISL_878235, EPI_ISL_878236, EPI_ISL_878240, EPI_ISL_878241, EPI_ISL_878243, EPI_ISL_878244, EPI_ISL_878246, EPI_ISL_878247, EPI_ISL_878249, EPI_ISL_878251, EPI_ISL_878252, EPI_ISL_878254, EPI_ISL_878255, EPI_ISL_878257, EPI_ISL_878259, EPI_ISL_878261, EPI_ISL_878262, EPI_ISL_878264, EPI_ISL_878265, EPI_ISL_878267, EPI_ISL_878269, EPI_ISL_878270, EPI_ISL_878272, EPI_ISL_878273, EPI_ISL_878275, EPI_ISL_878277, EPI_ISL_878278, EPI_ISL_878280, EPI_ISL_878282, EPI_ISL_878283, EPI_ISL_878285, EPI_ISL_878286, EPI_ISL_878288, EPI_ISL_878289, EPI_ISL_878291, EPI_ISL_878293, EPI_ISL_878294, EPI_ISL_878296, EPI_ISL_878297, EPI_ISL_878299, EPI_ISL_878301, EPI_ISL_878302, EPI_ISL_878304, EPI_ISL_878306, EPI_ISL_878307, EPI_ISL_878309, EPI_ISL_878310, EPI_ISL_878312, EPI_ISL_878313, EPI_ISL_878315, EPI_ISL_878317, EPI_ISL_878318, EPI_ISL_878320, EPI_ISL_878321, EPI_ISL_878323, EPI_ISL_878324, EPI_ISL_878326, EPI_ISL_878328, EPI_ISL_878331, EPI_ISL_878332, EPI_ISL_878334, EPI_ISL_878335, EPI_ISL_878337, EPI_ISL_878339, EPI_ISL_878340, EPI_ISL_878409, EPI_ISL_878410, EPI_ISL_878412, EPI_ISL_878414, EPI_ISL_878415, EPI_ISL_878417, EPI_ISL_878419, EPI_ISL_878420, EPI_ISL_878422, EPI_ISL_878424, EPI_ISL_878425, EPI_ISL_878427, EPI_ISL_878428, EPI_ISL_878430, EPI_ISL_878432, EPI_ISL_878435, EPI_ISL_878436, EPI_ISL_878438, EPI_ISL_878439, EPI_ISL_878441, EPI_ISL_878442, EPI_ISL_878444, EPI_ISL_878446, EPI_ISL_878449, EPI_ISL_878451, EPI_ISL_878453, EPI_ISL_878454, EPI_ISL_878456, EPI_ISL_878458, EPI_ISL_878459, EPI_ISL_878461, EPI_ISL_878462, EPI_ISL_878464, EPI_ISL_878466, EPI_ISL_878467, EPI_ISL_878469, EPI_ISL_878471, EPI_ISL_878473, EPI_ISL_878475, EPI_ISL_878476, EPI_ISL_878478, EPI_ISL_878481, EPI_ISL_878482, EPI_ISL_878484, EPI_ISL_878486, EPI_ISL_878487, EPI_ISL_878489, EPI_ISL_878490, EPI_ISL_878493, EPI_ISL_878494, EPI_ISL_878496, EPI_ISL_878497, EPI_ISL_878499, EPI_ISL_878501, EPI_ISL_878502, EPI_ISL_878504, EPI_ISL_878505, EPI_ISL_878507, EPI_ISL_878508, EPI_ISL_878510, EPI_ISL_878512, EPI_ISL_878513, EPI_ISL_878515, EPI_ISL_878517, EPI_ISL_878518, EPI_ISL_878520, EPI_ISL_878521, EPI_ISL_878523, EPI_ISL_878525, EPI_ISL_878527, EPI_ISL_878528, EPI_ISL_878530, EPI_ISL_878532, EPI_ISL_878533, EPI_ISL_878535, EPI_ISL_878538, EPI_ISL_878540, EPI_ISL_878542, EPI_ISL_878543, EPI_ISL_878546, EPI_ISL_878548, EPI_ISL_878550, EPI_ISL_878551, EPI_ISL_878555, EPI_ISL_878556, EPI_ISL_878558, EPI_ISL_878559, EPI_ISL_878561, EPI_ISL_878564, EPI_ISL_878567, EPI_ISL_878569, EPI_ISL_878570, EPI_ISL_878572, EPI_ISL_878573, EPI_ISL_878575, EPI_ISL_878577, EPI_ISL_878580, EPI_ISL_878582, EPI_ISL_878583, EPI_ISL_878585, EPI_ISL_878586, EPI_ISL_878588, EPI_ISL_878590, EPI_ISL_878591, EPI_ISL_878593, EPI_ISL_878594, EPI_ISL_878596, EPI_ISL_878598, EPI_ISL_878599, EPI_ISL_878601, EPI_ISL_878602, EPI_ISL_878604, EPI_ISL_878606, EPI_ISL_878607, EPI_ISL_878609, EPI_ISL_878612, EPI_ISL_878614, EPI_ISL_878615, EPI_ISL_878617, EPI_ISL_878618, EPI_ISL_878620, EPI_ISL_878621, EPI_ISL_878625, EPI_ISL_878626, EPI_ISL_878628, EPI_ISL_878630, EPI_ISL_878631, EPI_ISL_878633, EPI_ISL_878634, EPI_ISL_878636, EPI_ISL_878638, EPI_ISL_878639, EPI_ISL_878641, EPI_ISL_878642, EPI_ISL_878644, EPI_ISL_878646, EPI_ISL_878647, EPI_ISL_878649, EPI_ISL_878650, EPI_ISL_878652, EPI_ISL_878654, EPI_ISL_878655, EPI_ISL_878657, EPI_ISL_878659, EPI_ISL_878660, EPI_ISL_878662, EPI_ISL_878663, EPI_ISL_878665, EPI_ISL_878667, EPI_ISL_878668, EPI_ISL_878670, EPI_ISL_878672, EPI_ISL_878673, EPI_ISL_878675, EPI_ISL_878676, EPI_ISL_878678, EPI_ISL_878680, EPI_ISL_878681, EPI_ISL_878683, EPI_ISL_878685, EPI_ISL_878686, EPI_ISL_878688, EPI_ISL_878689, EPI_ISL_878691, EPI_ISL_878692, EPI_ISL_878694, EPI_ISL_878696, EPI_ISL_878698, EPI_ISL_878701, EPI_ISL_878703, EPI_ISL_878704, EPI_ISL_878706, EPI_ISL_878709, EPI_ISL_878711, EPI_ISL_878712, EPI_ISL_878714, EPI_ISL_878715, EPI_ISL_878718, EPI_ISL_878720, EPI_ISL_878722, EPI_ISL_878723, EPI_ISL_878725, EPI_ISL_878727, EPI_ISL_878730			
see above	Lighthouse Lab in Alderley Park	Wellcome Sanger Institute for the COVID-19 Genomics UK (COG-UK) Consortium	Jacquelyn Wynn, Mairead Hyland, The Lighthouse Lab in Alderley Park and Alex Alderton, Roberto Amato, Sonia Goncalves, Ewan Harrison, David K. Jackson, Ian Johnston, Dominic Kwiatkowski, Cordelia Langford, John Sillitoe on behalf of the Wellcome Sanger Institute COVID-19 Surveillance Team
EPI_ISL_878731, EPI_ISL_878740	Lighthouse Lab in Cambridge	Wellcome Sanger Institute for the COVID-19 Genomics UK (COG-UK) Consortium	Rob Howes, The Lighthouse Lab in Cambridge and Alex Alderton, Roberto Amato, Sonia Goncalves, Ewan Harrison, David K. Jackson, Ian Johnston, Dominic Kwiatkowski, Cordelia Langford, John Sillitoe on behalf of the Wellcome Sanger Institute COVID-19 Surveillance Team
EPI_ISL_878751, EPI_ISL_878756	Lighthouse Lab in Milton Keynes	Wellcome Sanger Institute for the COVID-19 Genomics UK (COG-UK) Consortium	The Lighthouse Lab in Milton Keynes and Alex Alderton, Roberto Amato, Sonia Goncalves, Ewan Harrison, David K. Jackson, Ian Johnston, Dominic Kwiatkowski, Cordelia Langford, John Sillitoe on behalf of the Wellcome Sanger Institute COVID-19 Surveillance Team
EPI_ISL_878758	Lighthouse Lab in Cambridge	Wellcome Sanger Institute for the COVID-19 Genomics UK (COG-UK) Consortium	Rob Howes, The Lighthouse Lab in Cambridge and Alex Alderton, Roberto Amato, Sonia Goncalves, Ewan Harrison, David K. Jackson, Ian Johnston, Dominic Kwiatkowski, Cordelia Langford, John Sillitoe on behalf of the Wellcome Sanger Institute COVID-19 Surveillance Team
EPI_ISL_878759	Lighthouse Lab in Milton Keynes	Wellcome Sanger Institute for the COVID-19 Genomics UK (COG-UK) Consortium	The Lighthouse Lab in Milton Keynes and Alex Alderton, Roberto Amato, Sonia Goncalves, Ewan Harrison, David K. Jackson, Ian Johnston, Dominic Kwiatkowski, Cordelia Langford, John Sillitoe on behalf of the Wellcome Sanger Institute COVID-19 Surveillance Team
EPI_ISL_878761, EPI_ISL_878766, EPI_ISL_878770	Lighthouse Lab in Cambridge	Wellcome Sanger Institute for the COVID-19 Genomics UK (COG-UK) Consortium	Rob Howes, The Lighthouse Lab in Cambridge and Alex Alderton, Roberto Amato, Sonia Goncalves, Ewan Harrison, David K. Jackson, Ian Johnston, Dominic Kwiatkowski, Cordelia Langford, John Sillitoe on behalf of the Wellcome Sanger Institute COVID-19 Surveillance Team
EPI_ISL_878772, EPI_ISL_878775, EPI_ISL_878778, EPI_ISL_878780, EPI_ISL_878782	Lighthouse Lab in Milton Keynes	Wellcome Sanger Institute for the COVID-19 Genomics UK (COG-UK) Consortium	The Lighthouse Lab in Milton Keynes and Alex Alderton, Roberto Amato, Sonia Goncalves, Ewan Harrison, David K. Jackson, Ian Johnston, Dominic Kwiatkowski, Cordelia Langford, John Sillitoe on behalf of the Wellcome Sanger Institute COVID-19 Surveillance Team
EPI_ISL_878790	Lighthouse Lab in Alderley Park	Wellcome Sanger Institute for the COVID-19 Genomics UK (COG-UK) Consortium	Jacquelyn Wynn, Mairead Hyland, The Lighthouse Lab in Alderley Park and Alex Alderton, Roberto Amato, Sonia Goncalves, Ewan Harrison, David K. Jackson, Ian Johnston, Dominic Kwiatkowski, Cordelia Langford, John Sillitoe on behalf of the Wellcome Sanger Institute COVID-19 Surveillance Team
EPI_ISL_878792, EPI_ISL_878793	Lighthouse Lab in Milton Keynes	Wellcome Sanger Institute for the COVID-19 Genomics UK (COG-UK) Consortium	The Lighthouse Lab in Milton Keynes and Alex Alderton, Roberto Amato, Sonia Goncalves, Ewan Harrison, David K. Jackson, Ian Johnston, Dominic Kwiatkowski, Cordelia Langford, John Sillitoe on behalf of the Wellcome Sanger Institute COVID-19 Surveillance Team
EPI_ISL_878796	Lighthouse Lab in Cambridge	Wellcome Sanger Institute for the COVID-19 Genomics UK (COG-UK) Consortium	Rob Howes, The Lighthouse Lab in Cambridge and Alex Alderton, Roberto Amato, Sonia Goncalves, Ewan Harrison, David K. Jackson, Ian Johnston, Dominic Kwiatkowski, Cordelia Langford, John Sillitoe on behalf of the Wellcome Sanger Institute COVID-19 Surveillance Team
EPI_ISL_878798, EPI_ISL_878800	Lighthouse Lab in Milton Keynes	Wellcome Sanger Institute for the COVID-19 Genomics UK (COG-UK) Consortium	The Lighthouse Lab in Milton Keynes and Alex Alderton, Roberto Amato, Sonia Goncalves, Ewan Harrison, David K. Jackson, Ian Johnston, Dominic Kwiatkowski, Cordelia Langford, John Sillitoe on behalf of the Wellcome Sanger Institute COVID-19 Surveillance Team
EPI_ISL_878801, EPI_ISL_878802, EPI_ISL_878803	Lighthouse Lab in Cambridge	Wellcome Sanger Institute for the COVID-19 Genomics UK (COG-UK) Consortium	Rob Howes, The Lighthouse Lab in Cambridge and Alex Alderton, Roberto Amato, Sonia Goncalves, Ewan Harrison, David K. Jackson, Ian Johnston, Dominic Kwiatkowski, Cordelia Langford, John Sillitoe on behalf of the Wellcome Sanger Institute COVID-19 Surveillance Team
EPI_ISL_878805	Lighthouse Lab in Alderley Park	Wellcome Sanger Institute for the COVID-19 Genomics UK (COG-UK) Consortium	Jacquelyn Wynn, Mairead Hyland, The Lighthouse Lab in Alderley Park and Alex Alderton, Roberto Amato, Sonia Goncalves, Ewan Harrison, David K. Jackson, Ian Johnston, Dominic Kwiatkowski, Cordelia Langford, John Sillitoe on behalf of the Wellcome Sanger Institute COVID-19 Surveillance Team
EPI_ISL_878806, EPI_ISL_878807	Lighthouse Lab in Milton Keynes	Wellcome Sanger Institute for the COVID-19 Genomics UK (COG-UK) Consortium	The Lighthouse Lab in Milton Keynes and Alex Alderton, Roberto Amato, Sonia Goncalves, Ewan Harrison, David K. Jackson, Ian Johnston, Dominic Kwiatkowski, Cordelia Langford, John Sillitoe on behalf of the Wellcome Sanger Institute COVID-19 Surveillance Team
EPI_ISL_878810	Lighthouse Lab in Cambridge	Wellcome Sanger Institute for the COVID-19 Genomics UK (COG-UK) Consortium	Rob Howes, The Lighthouse Lab in Cambridge and Alex Alderton, Roberto Amato, Sonia Goncalves, Ewan Harrison, David K. Jackson, Ian Johnston, Dominic Kwiatkowski, Cordelia Langford, John Sillitoe on behalf of the Wellcome Sanger Institute COVID-19 Surveillance Team
EPI_ISL_878814	Lighthouse Lab in Milton Keynes	Wellcome Sanger Institute for the COVID-19 Genomics UK (COG-UK) Consortium	The Lighthouse Lab in Milton Keynes and Alex Alderton, Roberto Amato, Sonia Goncalves, Ewan Harrison, David K. Jackson, Ian Johnston, Dominic Kwiatkowski, Cordelia Langford, John Sillitoe on behalf of the Wellcome Sanger Institute COVID-19 Surveillance Team
EPI_ISL_878815	Lighthouse Lab in Cambridge	Wellcome Sanger Institute for the COVID-19 Genomics UK (COG-UK) Consortium	Rob Howes, The Lighthouse Lab in Cambridge and Alex Alderton, Roberto Amato, Sonia Goncalves, Ewan Harrison, David K. Jackson, Ian Johnston, Dominic Kwiatkowski, Cordelia Langford, John Sillitoe on behalf of the Wellcome Sanger Institute COVID-19 Surveillance Team
EPI_ISL_878819	Lighthouse Lab in Alderley Park	Wellcome Sanger Institute for the COVID-19 Genomics UK (COG-UK) Consortium	Jacquelyn Wynn, Mairead Hyland, The Lighthouse Lab in Alderley Park and Alex Alderton, Roberto Amato, Sonia Goncalves, Ewan Harrison, David K. Jackson, Ian Johnston, Dominic Kwiatkowski, Cordelia Langford, John Sillitoe on behalf of the Wellcome Sanger Institute COVID-19 Surveillance Team
EPI_ISL_878824	Lighthouse Lab in Milton Keynes	Wellcome Sanger Institute for the COVID-19 Genomics UK (COG-UK) Consortium	The Lighthouse Lab in Milton Keynes and Alex Alderton, Roberto Amato, Sonia Goncalves, Ewan Harrison, David K. Jackson, Ian Johnston, Dominic Kwiatkowski, Cordelia Langford, John Sillitoe on behalf of the Wellcome Sanger Institute COVID-19 Surveillance Team
EPI_ISL_878825	Lighthouse Lab in Alderley Park	Wellcome Sanger Institute for the COVID-19 Genomics UK (COG-UK) Consortium	Jacquelyn Wynn, Mairead Hyland, The Lighthouse Lab in Alderley Park and Alex Alderton, Roberto Amato, Sonia Goncalves, Ewan Harrison, David K. Jackson, Ian Johnston, Dominic Kwiatkowski, Cordelia Langford, John Sillitoe on behalf of the Wellcome Sanger Institute COVID-19 Surveillance Team
EPI_ISL_878830, EPI_ISL_878831, EPI_ISL_878832, EPI_ISL_878834, EPI_ISL_878839, EPI_ISL_878841	Lighthouse Lab in Cambridge	Wellcome Sanger Institute for the COVID-19 Genomics UK (COG-UK) Consortium	Rob Howes, The Lighthouse Lab in Cambridge and Alex Alderton, Roberto Amato, Sonia Goncalves, Ewan Harrison, David K. Jackson, Ian Johnston, Dominic Kwiatkowski, Cordelia Langford, John Sillitoe on behalf of the Wellcome Sanger Institute COVID-19 Surveillance Team
EPI_ISL_878842, EPI_ISL_878845, EPI_ISL_878846	Lighthouse Lab in Milton Keynes	Wellcome Sanger Institute for the COVID-19 Genomics UK (COG-UK) Consortium	The Lighthouse Lab in Milton Keynes and Alex Alderton, Roberto Amato, Sonia Goncalves, Ewan Harrison, David K. Jackson, Ian Johnston, Dominic Kwiatkowski, Cordelia Langford, John Sillitoe on behalf of the Wellcome Sanger Institute COVID-19 Surveillance Team
EPI_ISL_878847	Lighthouse Lab in Cambridge	Wellcome Sanger Institute for the COVID-19 Genomics UK (COG-UK) Consortium	Rob Howes, The Lighthouse Lab in Cambridge and Alex Alderton, Roberto Amato, Sonia Goncalves, Ewan Harrison, David K. Jackson, Ian Johnston, Dominic Kwiatkowski, Cordelia Langford, John Sillitoe on behalf of the Wellcome Sanger Institute COVID-19 Surveillance Team
EPI_ISL_878848	Lighthouse Lab in Milton Keynes	Wellcome Sanger Institute for the COVID-19 Genomics UK (COG-UK) Consortium	The Lighthouse Lab in Milton Keynes and Alex Alderton, Roberto Amato, Sonia Goncalves, Ewan Harrison, David K. Jackson, Ian Johnston, Dominic Kwiatkowski, Cordelia Langford, John Sillitoe on behalf of the Wellcome Sanger Institute COVID-19 Surveillance Team
EPI_ISL_878850	Lighthouse Lab in Cambridge	Wellcome Sanger Institute for the COVID-19 Genomics UK (COG-UK) Consortium	Rob Howes, The Lighthouse Lab in Cambridge and Alex Alderton, Roberto Amato, Sonia Goncalves, Ewan Harrison, David K. Jackson, Ian Johnston, Dominic Kwiatkowski, Cordelia Langford, John Sillitoe on behalf of the Wellcome Sanger Institute COVID-19 Surveillance Team
EPI_ISL_878851, EPI_ISL_878852, EPI_ISL_878854	Lighthouse Lab in Milton Keynes	Wellcome Sanger Institute for the COVID-19 Genomics UK (COG-UK) Consortium	The Lighthouse Lab in Milton Keynes and Alex Alderton, Roberto Amato, Sonia Goncalves, Ewan Harrison, David K. Jackson, Ian Johnston, Dominic Kwiatkowski, Cordelia Langford, John Sillitoe on behalf of the Wellcome Sanger Institute COVID-19 Surveillance Team
EPI_ISL_878856	Lighthouse Lab in Alderley Park	Wellcome Sanger Institute for the COVID-19 Genomics UK (COG-UK) Consortium	Jacquelyn Wynn, Mairead Hyland, The Lighthouse Lab in Alderley Park and Alex Alderton, Roberto Amato, Sonia Goncalves, Ewan Harrison, David K. Jackson, Ian Johnston, Dominic Kwiatkowski, Cordelia Langford, John Sillitoe on behalf of the Wellcome Sanger Institute COVID-19 Surveillance Team

EPI_ISL_878857	Lighthouse Lab in Cambridge	Wellcome Sanger Institute for the COVID-19 Genomics UK (COG-UK) Consortium	Rob Howes, The Lighthouse Lab in Cambridge and Alex Alderton, Roberto Amato, Sonia Goncalves, Ewan Harrison, David K. Jackson, Ian Johnston, Dominic Kwiatkowski, Cordelia Langford, John Sillitoe on behalf of the Wellcome Sanger Institute COVID-19 Surveillance Team
EPI_ISL_878858, EPI_ISL_878860, EPI_ISL_878861	Lighthouse Lab in Milton Keynes	Wellcome Sanger Institute for the COVID-19 Genomics UK (COG-UK) Consortium	The Lighthouse Lab in Milton Keynes and Alex Alderton, Roberto Amato, Sonia Goncalves, Ewan Harrison, David K. Jackson, Ian Johnston, Dominic Kwiatkowski, Cordelia Langford, John Sillitoe on behalf of the Wellcome Sanger Institute COVID-19 Surveillance Team
EPI_ISL_878862, EPI_ISL_878863, EPI_ISL_878864, EPI_ISL_878869, EPI_ISL_878870, EPI_ISL_878871	Lighthouse Lab in Cambridge	Wellcome Sanger Institute for the COVID-19 Genomics UK (COG-UK) Consortium	Rob Howes, The Lighthouse Lab in Cambridge and Alex Alderton, Roberto Amato, Sonia Goncalves, Ewan Harrison, David K. Jackson, Ian Johnston, Dominic Kwiatkowski, Cordelia Langford, John Sillitoe on behalf of the Wellcome Sanger Institute COVID-19 Surveillance Team
EPI_ISL_878874	Lighthouse Lab in Milton Keynes	Wellcome Sanger Institute for the COVID-19 Genomics UK (COG-UK) Consortium	The Lighthouse Lab in Milton Keynes and Alex Alderton, Roberto Amato, Sonia Goncalves, Ewan Harrison, David K. Jackson, Ian Johnston, Dominic Kwiatkowski, Cordelia Langford, John Sillitoe on behalf of the Wellcome Sanger Institute COVID-19 Surveillance Team
EPI_ISL_878876	Lighthouse Lab in Alderley Park	Wellcome Sanger Institute for the COVID-19 Genomics UK (COG-UK) Consortium	Jacquelyn Wynn, Mairead Hyland, The Lighthouse Lab in Alderley Park and Alex Alderton, Roberto Amato, Sonia Goncalves, Ewan Harrison, David K. Jackson, Ian Johnston, Dominic Kwiatkowski, Cordelia Langford, John Sillitoe on behalf of the Wellcome Sanger Institute COVID-19 Surveillance Team
EPI_ISL_878879, EPI_ISL_878880, EPI_ISL_878882, EPI_ISL_878884	Lighthouse Lab in Milton Keynes	Wellcome Sanger Institute for the COVID-19 Genomics UK (COG-UK) Consortium	The Lighthouse Lab in Milton Keynes and Alex Alderton, Roberto Amato, Sonia Goncalves, Ewan Harrison, David K. Jackson, Ian Johnston, Dominic Kwiatkowski, Cordelia Langford, John Sillitoe on behalf of the Wellcome Sanger Institute COVID-19 Surveillance Team
EPI_ISL_878886	Lighthouse Lab in Alderley Park	Wellcome Sanger Institute for the COVID-19 Genomics UK (COG-UK) Consortium	Jacquelyn Wynn, Mairead Hyland, The Lighthouse Lab in Alderley Park and Alex Alderton, Roberto Amato, Sonia Goncalves, Ewan Harrison, David K. Jackson, Ian Johnston, Dominic Kwiatkowski, Cordelia Langford, John Sillitoe on behalf of the Wellcome Sanger Institute COVID-19 Surveillance Team
EPI_ISL_878889	Lighthouse Lab in Cambridge	Wellcome Sanger Institute for the COVID-19 Genomics UK (COG-UK) Consortium	Rob Howes, The Lighthouse Lab in Cambridge and Alex Alderton, Roberto Amato, Sonia Goncalves, Ewan Harrison, David K. Jackson, Ian Johnston, Dominic Kwiatkowski, Cordelia Langford, John Sillitoe on behalf of the Wellcome Sanger Institute COVID-19 Surveillance Team
EPI_ISL_878892	Lighthouse Lab in Milton Keynes	Wellcome Sanger Institute for the COVID-19 Genomics UK (COG-UK) Consortium	The Lighthouse Lab in Milton Keynes and Alex Alderton, Roberto Amato, Sonia Goncalves, Ewan Harrison, David K. Jackson, Ian Johnston, Dominic Kwiatkowski, Cordelia Langford, John Sillitoe on behalf of the Wellcome Sanger Institute COVID-19 Surveillance Team
EPI_ISL_878894	Lighthouse Lab in Cambridge	Wellcome Sanger Institute for the COVID-19 Genomics UK (COG-UK) Consortium	Rob Howes, The Lighthouse Lab in Cambridge and Alex Alderton, Roberto Amato, Sonia Goncalves, Ewan Harrison, David K. Jackson, Ian Johnston, Dominic Kwiatkowski, Cordelia Langford, John Sillitoe on behalf of the Wellcome Sanger Institute COVID-19 Surveillance Team
EPI_ISL_878896	Lighthouse Lab in Milton Keynes	Wellcome Sanger Institute for the COVID-19 Genomics UK (COG-UK) Consortium	The Lighthouse Lab in Milton Keynes and Alex Alderton, Roberto Amato, Sonia Goncalves, Ewan Harrison, David K. Jackson, Ian Johnston, Dominic Kwiatkowski, Cordelia Langford, John Sillitoe on behalf of the Wellcome Sanger Institute COVID-19 Surveillance Team
EPI_ISL_878899	Lighthouse Lab in Cambridge	Wellcome Sanger Institute for the COVID-19 Genomics UK (COG-UK) Consortium	Rob Howes, The Lighthouse Lab in Cambridge and Alex Alderton, Roberto Amato, Sonia Goncalves, Ewan Harrison, David K. Jackson, Ian Johnston, Dominic Kwiatkowski, Cordelia Langford, John Sillitoe on behalf of the Wellcome Sanger Institute COVID-19 Surveillance Team
EPI_ISL_878902	Lighthouse Lab in Milton Keynes	Wellcome Sanger Institute for the COVID-19 Genomics UK (COG-UK) Consortium	The Lighthouse Lab in Milton Keynes and Alex Alderton, Roberto Amato, Sonia Goncalves, Ewan Harrison, David K. Jackson, Ian Johnston, Dominic Kwiatkowski, Cordelia Langford, John Sillitoe on behalf of the Wellcome Sanger Institute COVID-19 Surveillance Team
EPI_ISL_878903, EPI_ISL_878904, EPI_ISL_878905	Lighthouse Lab in Cambridge	Wellcome Sanger Institute for the COVID-19 Genomics UK (COG-UK) Consortium	Rob Howes, The Lighthouse Lab in Cambridge and Alex Alderton, Roberto Amato, Sonia Goncalves, Ewan Harrison, David K. Jackson, Ian Johnston, Dominic Kwiatkowski, Cordelia Langford, John Sillitoe on behalf of the Wellcome Sanger Institute COVID-19 Surveillance Team
EPI_ISL_878906	Lighthouse Lab in Milton Keynes	Wellcome Sanger Institute for the COVID-19 Genomics UK (COG-UK) Consortium	The Lighthouse Lab in Milton Keynes and Alex Alderton, Roberto Amato, Sonia Goncalves, Ewan Harrison, David K. Jackson, Ian Johnston, Dominic Kwiatkowski, Cordelia Langford, John Sillitoe on behalf of the Wellcome Sanger Institute COVID-19 Surveillance Team
EPI_ISL_878912	Lighthouse Lab in Cambridge	Wellcome Sanger Institute for the COVID-19 Genomics UK (COG-UK) Consortium	Rob Howes, The Lighthouse Lab in Cambridge and Alex Alderton, Roberto Amato, Sonia Goncalves, Ewan Harrison, David K. Jackson, Ian Johnston, Dominic Kwiatkowski, Cordelia Langford, John Sillitoe on behalf of the Wellcome Sanger Institute COVID-19 Surveillance Team
EPI_ISL_878913, EPI_ISL_878915	Lighthouse Lab in Alderley Park	Wellcome Sanger Institute for the COVID-19 Genomics UK (COG-UK) Consortium	Jacquelyn Wynn, Mairead Hyland, The Lighthouse Lab in Alderley Park and Alex Alderton, Roberto Amato, Sonia Goncalves, Ewan Harrison, David K. Jackson, Ian Johnston, Dominic Kwiatkowski, Cordelia Langford, John Sillitoe on behalf of the Wellcome Sanger Institute COVID-19 Surveillance Team
EPI_ISL_878918, EPI_ISL_878919	Lighthouse Lab in Milton Keynes	Wellcome Sanger Institute for the COVID-19 Genomics UK (COG-UK) Consortium	The Lighthouse Lab in Milton Keynes and Alex Alderton, Roberto Amato, Sonia Goncalves, Ewan Harrison, David K. Jackson, Ian Johnston, Dominic Kwiatkowski, Cordelia Langford, John Sillitoe on behalf of the Wellcome Sanger Institute COVID-19 Surveillance Team
EPI_ISL_878924, EPI_ISL_878931	Lighthouse Lab in Cambridge	Wellcome Sanger Institute for the COVID-19 Genomics UK (COG-UK) Consortium	Rob Howes, The Lighthouse Lab in Cambridge and Alex Alderton, Roberto Amato, Sonia Goncalves, Ewan Harrison, David K. Jackson, Ian Johnston, Dominic Kwiatkowski, Cordelia Langford, John Sillitoe on behalf of the Wellcome Sanger Institute COVID-19 Surveillance Team
EPI_ISL_878934	Lighthouse Lab in Milton Keynes	Wellcome Sanger Institute for the COVID-19 Genomics UK (COG-UK) Consortium	The Lighthouse Lab in Milton Keynes and Alex Alderton, Roberto Amato, Sonia Goncalves, Ewan Harrison, David K. Jackson, Ian Johnston, Dominic Kwiatkowski, Cordelia Langford, John Sillitoe on behalf of the Wellcome Sanger Institute COVID-19 Surveillance Team
EPI_ISL_878935	Lighthouse Lab in Cambridge	Wellcome Sanger Institute for the COVID-19 Genomics UK (COG-UK) Consortium	Rob Howes, The Lighthouse Lab in Cambridge and Alex Alderton, Roberto Amato, Sonia Goncalves, Ewan Harrison, David K. Jackson, Ian Johnston, Dominic Kwiatkowski, Cordelia Langford, John Sillitoe on behalf of the Wellcome Sanger Institute COVID-19 Surveillance Team
EPI_ISL_878941, EPI_ISL_878942, EPI_ISL_878943, EPI_ISL_878944, EPI_ISL_878945	Lighthouse Lab in Milton Keynes	Wellcome Sanger Institute for the COVID-19 Genomics UK (COG-UK) Consortium	The Lighthouse Lab in Milton Keynes and Alex Alderton, Roberto Amato, Sonia Goncalves, Ewan Harrison, David K. Jackson, Ian Johnston, Dominic Kwiatkowski, Cordelia Langford, John Sillitoe on behalf of the Wellcome Sanger Institute COVID-19 Surveillance Team
EPI_ISL_878950	Lighthouse Lab in Cambridge	Wellcome Sanger Institute for the COVID-19 Genomics UK (COG-UK) Consortium	Rob Howes, The Lighthouse Lab in Cambridge and Alex Alderton, Roberto Amato, Sonia Goncalves, Ewan Harrison, David K. Jackson, Ian Johnston, Dominic Kwiatkowski, Cordelia Langford, John Sillitoe on behalf of the Wellcome Sanger Institute COVID-19 Surveillance Team
EPI_ISL_878951, EPI_ISL_878952	Lighthouse Lab in Milton Keynes	Wellcome Sanger Institute for the COVID-19 Genomics UK (COG-UK) Consortium	The Lighthouse Lab in Milton Keynes and Alex Alderton, Roberto Amato, Sonia Goncalves, Ewan Harrison, David K. Jackson, Ian Johnston, Dominic Kwiatkowski, Cordelia Langford, John Sillitoe on behalf of the Wellcome Sanger Institute COVID-19 Surveillance Team
EPI_ISL_878959	Lighthouse Lab in Alderley Park	Wellcome Sanger Institute for the COVID-19 Genomics UK (COG-UK) Consortium	Jacquelyn Wynn, Mairead Hyland, The Lighthouse Lab in Alderley Park and Alex Alderton, Roberto Amato, Sonia Goncalves, Ewan Harrison, David K. Jackson, Ian Johnston, Dominic Kwiatkowski, Cordelia Langford, John Sillitoe on behalf of the Wellcome Sanger Institute COVID-19 Surveillance Team
EPI_ISL_878960, EPI_ISL_878962	Lighthouse Lab in Milton Keynes	Wellcome Sanger Institute for the COVID-19 Genomics UK (COG-UK) Consortium	The Lighthouse Lab in Milton Keynes and Alex Alderton, Roberto Amato, Sonia Goncalves, Ewan Harrison, David K. Jackson, Ian Johnston, Dominic Kwiatkowski, Cordelia Langford, John Sillitoe on behalf of the Wellcome Sanger Institute COVID-19 Surveillance Team
EPI_ISL_878964	Lighthouse Lab in Cambridge	Wellcome Sanger Institute for the COVID-19 Genomics UK (COG-UK) Consortium	Rob Howes, The Lighthouse Lab in Cambridge and Alex Alderton, Roberto Amato, Sonia Goncalves, Ewan Harrison, David K. Jackson, Ian Johnston, Dominic Kwiatkowski, Cordelia Langford, John Sillitoe on behalf of the Wellcome Sanger Institute COVID-19 Surveillance Team
EPI_ISL_878966	Lighthouse Lab in Alderley Park	Wellcome Sanger Institute for the COVID-19 Genomics UK (COG-UK) Consortium	Jacquelyn Wynn, Mairead Hyland, The Lighthouse Lab in Alderley Park and Alex Alderton, Roberto Amato, Sonia Goncalves, Ewan Harrison, David K. Jackson, Ian Johnston, Dominic Kwiatkowski, Cordelia Langford, John Sillitoe on behalf of the Wellcome Sanger Institute COVID-19 Surveillance Team
EPI_ISL_878967, EPI_ISL_878971, EPI_ISL_878972	Lighthouse Lab in Milton Keynes	Wellcome Sanger Institute for the COVID-19 Genomics UK (COG-UK) Consortium	The Lighthouse Lab in Milton Keynes and Alex Alderton, Roberto Amato, Sonia Goncalves, Ewan Harrison, David K. Jackson, Ian Johnston, Dominic Kwiatkowski, Cordelia Langford, John Sillitoe on behalf of the Wellcome Sanger Institute COVID-19 Surveillance Team
EPI_ISL_878975, EPI_ISL_878980	Lighthouse Lab in Cambridge	Wellcome Sanger Institute for the COVID-19 Genomics UK (COG-UK) Consortium	Rob Howes, The Lighthouse Lab in Cambridge and Alex Alderton, Roberto Amato, Sonia Goncalves, Ewan Harrison, David K. Jackson, Ian Johnston, Dominic Kwiatkowski, Cordelia Langford, John Sillitoe on behalf of the Wellcome Sanger Institute COVID-19 Surveillance Team
EPI_ISL_878982	Lighthouse Lab in Milton Keynes	Wellcome Sanger Institute for the COVID-19 Genomics UK (COG-UK) Consortium	The Lighthouse Lab in Milton Keynes and Alex Alderton, Roberto Amato, Sonia Goncalves, Ewan Harrison, David K. Jackson, Ian Johnston, Dominic Kwiatkowski, Cordelia Langford, John Sillitoe on behalf of the Wellcome Sanger Institute COVID-19 Surveillance Team
EPI_ISL_878989	Lighthouse Lab in Cambridge	Wellcome Sanger Institute for the COVID-19 Genomics UK (COG-UK) Consortium	Rob Howes, The Lighthouse Lab in Cambridge and Alex Alderton, Roberto Amato, Sonia Goncalves, Ewan Harrison, David K. Jackson, Ian Johnston, Dominic Kwiatkowski, Cordelia Langford, John Sillitoe on behalf of the Wellcome Sanger Institute COVID-19 Surveillance Team
EPI_ISL_878990	Lighthouse Lab in Milton Keynes	Wellcome Sanger Institute for the COVID-19 Genomics UK (COG-UK) Consortium	The Lighthouse Lab in Milton Keynes and Alex Alderton, Roberto Amato, Sonia Goncalves, Ewan Harrison, David K. Jackson, Ian Johnston, Dominic Kwiatkowski, Cordelia Langford, John Sillitoe on behalf of the Wellcome Sanger Institute COVID-19 Surveillance Team
EPI_ISL_878991	Lighthouse Lab in Cambridge	Wellcome Sanger Institute for the COVID-19 Genomics UK (COG-UK) Consortium	Rob Howes, The Lighthouse Lab in Cambridge and Alex Alderton, Roberto Amato, Sonia Goncalves, Ewan Harrison, David K. Jackson, Ian Johnston, Dominic Kwiatkowski, Cordelia Langford, John Sillitoe on behalf of the Wellcome Sanger Institute COVID-19 Surveillance Team
EPI_ISL_878993	Lighthouse Lab in Alderley Park	Wellcome Sanger Institute for the COVID-19 Genomics UK	Jacquelyn Wynn, Mairead Hyland, The Lighthouse Lab in Alderley Park and Alex Alderton, Roberto Amato, Sonia Goncalves, Ewan Harrison, David K.

		(COG-UK) Consortium	Jackson, Ian Johnston, Dominic Kwiatkowski, Cordelia Langford, John Sillitoe on behalf of the Wellcome Sanger Institute COVID-19 Surveillance Team
EPI_ISL_878995, EPI_ISL_878997, EPI_ISL_878998	Lighthouse Lab in Cambridge	Wellcome Sanger Institute for the COVID-19 Genomics UK (COG-UK) Consortium	Rob Howes, The Lighthouse Lab in Cambridge and Alex Alderton, Roberto Amato, Sonia Goncalves, Ewan Harrison, David K. Jackson, Ian Johnston, Dominic Kwiatkowski, Cordelia Langford, John Sillitoe on behalf of the Wellcome Sanger Institute COVID-19 Surveillance Team
EPI_ISL_878999	Lighthouse Lab in Alderley Park	Wellcome Sanger Institute for the COVID-19 Genomics UK (COG-UK) Consortium	Jacquelyn Wynn, Mairead Hyland, The Lighthouse Lab in Alderley Park and Alex Alderton, Roberto Amato, Sonia Goncalves, Ewan Harrison, David K. Jackson, Ian Johnston, Dominic Kwiatkowski, Cordelia Langford, John Sillitoe on behalf of the Wellcome Sanger Institute COVID-19 Surveillance Team
EPI_ISL_879001	Lighthouse Lab in Milton Keynes	Wellcome Sanger Institute for the COVID-19 Genomics UK (COG-UK) Consortium	The Lighthouse Lab in Milton Keynes and Alex Alderton, Roberto Amato, Sonia Goncalves, Ewan Harrison, David K. Jackson, Ian Johnston, Dominic Kwiatkowski, Cordelia Langford, John Sillitoe on behalf of the Wellcome Sanger Institute COVID-19 Surveillance Team
EPI_ISL_879007	Lighthouse Lab in Alderley Park	Wellcome Sanger Institute for the COVID-19 Genomics UK (COG-UK) Consortium	Jacquelyn Wynn, Mairead Hyland, The Lighthouse Lab in Alderley Park and Alex Alderton, Roberto Amato, Sonia Goncalves, Ewan Harrison, David K. Jackson, Ian Johnston, Dominic Kwiatkowski, Cordelia Langford, John Sillitoe on behalf of the Wellcome Sanger Institute COVID-19 Surveillance Team
EPI_ISL_879009	Lighthouse Lab in Milton Keynes	Wellcome Sanger Institute for the COVID-19 Genomics UK (COG-UK) Consortium	The Lighthouse Lab in Milton Keynes and Alex Alderton, Roberto Amato, Sonia Goncalves, Ewan Harrison, David K. Jackson, Ian Johnston, Dominic Kwiatkowski, Cordelia Langford, John Sillitoe on behalf of the Wellcome Sanger Institute COVID-19 Surveillance Team
EPI_ISL_879011, EPI_ISL_879012	Lighthouse Lab in Cambridge	Wellcome Sanger Institute for the COVID-19 Genomics UK (COG-UK) Consortium	Rob Howes, The Lighthouse Lab in Cambridge and Alex Alderton, Roberto Amato, Sonia Goncalves, Ewan Harrison, David K. Jackson, Ian Johnston, Dominic Kwiatkowski, Cordelia Langford, John Sillitoe on behalf of the Wellcome Sanger Institute COVID-19 Surveillance Team
EPI_ISL_879013, EPI_ISL_879014, EPI_ISL_879016, EPI_ISL_879019, EPI_ISL_879021	Lighthouse Lab in Milton Keynes	Wellcome Sanger Institute for the COVID-19 Genomics UK (COG-UK) Consortium	The Lighthouse Lab in Milton Keynes and Alex Alderton, Roberto Amato, Sonia Goncalves, Ewan Harrison, David K. Jackson, Ian Johnston, Dominic Kwiatkowski, Cordelia Langford, John Sillitoe on behalf of the Wellcome Sanger Institute COVID-19 Surveillance Team
EPI_ISL_879022	Lighthouse Lab in Cambridge	Wellcome Sanger Institute for the COVID-19 Genomics UK (COG-UK) Consortium	Rob Howes, The Lighthouse Lab in Cambridge and Alex Alderton, Roberto Amato, Sonia Goncalves, Ewan Harrison, David K. Jackson, Ian Johnston, Dominic Kwiatkowski, Cordelia Langford, John Sillitoe on behalf of the Wellcome Sanger Institute COVID-19 Surveillance Team
EPI_ISL_879023, EPI_ISL_879025, EPI_ISL_879028	Lighthouse Lab in Milton Keynes	Wellcome Sanger Institute for the COVID-19 Genomics UK (COG-UK) Consortium	The Lighthouse Lab in Milton Keynes and Alex Alderton, Roberto Amato, Sonia Goncalves, Ewan Harrison, David K. Jackson, Ian Johnston, Dominic Kwiatkowski, Cordelia Langford, John Sillitoe on behalf of the Wellcome Sanger Institute COVID-19 Surveillance Team
EPI_ISL_879029, EPI_ISL_879031	Lighthouse Lab in Cambridge	Wellcome Sanger Institute for the COVID-19 Genomics UK (COG-UK) Consortium	Rob Howes, The Lighthouse Lab in Cambridge and Alex Alderton, Roberto Amato, Sonia Goncalves, Ewan Harrison, David K. Jackson, Ian Johnston, Dominic Kwiatkowski, Cordelia Langford, John Sillitoe on behalf of the Wellcome Sanger Institute COVID-19 Surveillance Team
EPI_ISL_879032, EPI_ISL_879034, EPI_ISL_879037, EPI_ISL_879038	Lighthouse Lab in Milton Keynes	Wellcome Sanger Institute for the COVID-19 Genomics UK (COG-UK) Consortium	The Lighthouse Lab in Milton Keynes and Alex Alderton, Roberto Amato, Sonia Goncalves, Ewan Harrison, David K. Jackson, Ian Johnston, Dominic Kwiatkowski, Cordelia Langford, John Sillitoe on behalf of the Wellcome Sanger Institute COVID-19 Surveillance Team
EPI_ISL_879040	Lighthouse Lab in Alderley Park	Wellcome Sanger Institute for the COVID-19 Genomics UK (COG-UK) Consortium	Jacquelyn Wynn, Mairead Hyland, The Lighthouse Lab in Alderley Park and Alex Alderton, Roberto Amato, Sonia Goncalves, Ewan Harrison, David K. Jackson, Ian Johnston, Dominic Kwiatkowski, Cordelia Langford, John Sillitoe on behalf of the Wellcome Sanger Institute COVID-19 Surveillance Team
EPI_ISL_879042, EPI_ISL_879043, EPI_ISL_879044	Lighthouse Lab in Milton Keynes	Wellcome Sanger Institute for the COVID-19 Genomics UK (COG-UK) Consortium	The Lighthouse Lab in Milton Keynes and Alex Alderton, Roberto Amato, Sonia Goncalves, Ewan Harrison, David K. Jackson, Ian Johnston, Dominic Kwiatkowski, Cordelia Langford, John Sillitoe on behalf of the Wellcome Sanger Institute COVID-19 Surveillance Team
EPI_ISL_879045	Lighthouse Lab in Cambridge	Wellcome Sanger Institute for the COVID-19 Genomics UK (COG-UK) Consortium	Rob Howes, The Lighthouse Lab in Cambridge and Alex Alderton, Roberto Amato, Sonia Goncalves, Ewan Harrison, David K. Jackson, Ian Johnston, Dominic Kwiatkowski, Cordelia Langford, John Sillitoe on behalf of the Wellcome Sanger Institute COVID-19 Surveillance Team
EPI_ISL_879047, EPI_ISL_879049, EPI_ISL_879050	Lighthouse Lab in Milton Keynes	Wellcome Sanger Institute for the COVID-19 Genomics UK (COG-UK) Consortium	The Lighthouse Lab in Milton Keynes and Alex Alderton, Roberto Amato, Sonia Goncalves, Ewan Harrison, David K. Jackson, Ian Johnston, Dominic Kwiatkowski, Cordelia Langford, John Sillitoe on behalf of the Wellcome Sanger Institute COVID-19 Surveillance Team
EPI_ISL_879051	Lighthouse Lab in Cambridge	Wellcome Sanger Institute for the COVID-19 Genomics UK (COG-UK) Consortium	Rob Howes, The Lighthouse Lab in Cambridge and Alex Alderton, Roberto Amato, Sonia Goncalves, Ewan Harrison, David K. Jackson, Ian Johnston, Dominic Kwiatkowski, Cordelia Langford, John Sillitoe on behalf of the Wellcome Sanger Institute COVID-19 Surveillance Team
EPI_ISL_879052	Lighthouse Lab in Milton Keynes	Wellcome Sanger Institute for the COVID-19 Genomics UK (COG-UK) Consortium	The Lighthouse Lab in Milton Keynes and Alex Alderton, Roberto Amato, Sonia Goncalves, Ewan Harrison, David K. Jackson, Ian Johnston, Dominic Kwiatkowski, Cordelia Langford, John Sillitoe on behalf of the Wellcome Sanger Institute COVID-19 Surveillance Team
EPI_ISL_879054	Lighthouse Lab in Cambridge	Wellcome Sanger Institute for the COVID-19 Genomics UK (COG-UK) Consortium	Rob Howes, The Lighthouse Lab in Cambridge and Alex Alderton, Roberto Amato, Sonia Goncalves, Ewan Harrison, David K. Jackson, Ian Johnston, Dominic Kwiatkowski, Cordelia Langford, John Sillitoe on behalf of the Wellcome Sanger Institute COVID-19 Surveillance Team
EPI_ISL_879055	Lighthouse Lab in Milton Keynes	Wellcome Sanger Institute for the COVID-19 Genomics UK (COG-UK) Consortium	The Lighthouse Lab in Milton Keynes and Alex Alderton, Roberto Amato, Sonia Goncalves, Ewan Harrison, David K. Jackson, Ian Johnston, Dominic Kwiatkowski, Cordelia Langford, John Sillitoe on behalf of the Wellcome Sanger Institute COVID-19 Surveillance Team
EPI_ISL_879056	Lighthouse Lab in Alderley Park	Wellcome Sanger Institute for the COVID-19 Genomics UK (COG-UK) Consortium	Jacquelyn Wynn, Mairead Hyland, The Lighthouse Lab in Alderley Park and Alex Alderton, Roberto Amato, Sonia Goncalves, Ewan Harrison, David K. Jackson, Ian Johnston, Dominic Kwiatkowski, Cordelia Langford, John Sillitoe on behalf of the Wellcome Sanger Institute COVID-19 Surveillance Team
EPI_ISL_879057	Lighthouse Lab in Milton Keynes	Wellcome Sanger Institute for the COVID-19 Genomics UK (COG-UK) Consortium	The Lighthouse Lab in Milton Keynes and Alex Alderton, Roberto Amato, Sonia Goncalves, Ewan Harrison, David K. Jackson, Ian Johnston, Dominic Kwiatkowski, Cordelia Langford, John Sillitoe on behalf of the Wellcome Sanger Institute COVID-19 Surveillance Team
EPI_ISL_879058, EPI_ISL_879062	Lighthouse Lab in Cambridge	Wellcome Sanger Institute for the COVID-19 Genomics UK (COG-UK) Consortium	Rob Howes, The Lighthouse Lab in Cambridge and Alex Alderton, Roberto Amato, Sonia Goncalves, Ewan Harrison, David K. Jackson, Ian Johnston, Dominic Kwiatkowski, Cordelia Langford, John Sillitoe on behalf of the Wellcome Sanger Institute COVID-19 Surveillance Team
EPI_ISL_879063, EPI_ISL_879064, EPI_ISL_879065	Lighthouse Lab in Milton Keynes	Wellcome Sanger Institute for the COVID-19 Genomics UK (COG-UK) Consortium	The Lighthouse Lab in Milton Keynes and Alex Alderton, Roberto Amato, Sonia Goncalves, Ewan Harrison, David K. Jackson, Ian Johnston, Dominic Kwiatkowski, Cordelia Langford, John Sillitoe on behalf of the Wellcome Sanger Institute COVID-19 Surveillance Team
EPI_ISL_879068, EPI_ISL_879069	Lighthouse Lab in Cambridge	Wellcome Sanger Institute for the COVID-19 Genomics UK (COG-UK) Consortium	Rob Howes, The Lighthouse Lab in Cambridge and Alex Alderton, Roberto Amato, Sonia Goncalves, Ewan Harrison, David K. Jackson, Ian Johnston, Dominic Kwiatkowski, Cordelia Langford, John Sillitoe on behalf of the Wellcome Sanger Institute COVID-19 Surveillance Team
EPI_ISL_879070	Lighthouse Lab in Alderley Park	Wellcome Sanger Institute for the COVID-19 Genomics UK (COG-UK) Consortium	Jacquelyn Wynn, Mairead Hyland, The Lighthouse Lab in Alderley Park and Alex Alderton, Roberto Amato, Sonia Goncalves, Ewan Harrison, David K. Jackson, Ian Johnston, Dominic Kwiatkowski, Cordelia Langford, John Sillitoe on behalf of the Wellcome Sanger Institute COVID-19 Surveillance Team
EPI_ISL_879071	Lighthouse Lab in Cambridge	Wellcome Sanger Institute for the COVID-19 Genomics UK (COG-UK) Consortium	Rob Howes, The Lighthouse Lab in Cambridge and Alex Alderton, Roberto Amato, Sonia Goncalves, Ewan Harrison, David K. Jackson, Ian Johnston, Dominic Kwiatkowski, Cordelia Langford, John Sillitoe on behalf of the Wellcome Sanger Institute COVID-19 Surveillance Team
EPI_ISL_879073	Lighthouse Lab in Alderley Park	Wellcome Sanger Institute for the COVID-19 Genomics UK (COG-UK) Consortium	Jacquelyn Wynn, Mairead Hyland, The Lighthouse Lab in Alderley Park and Alex Alderton, Roberto Amato, Sonia Goncalves, Ewan Harrison, David K. Jackson, Ian Johnston, Dominic Kwiatkowski, Cordelia Langford, John Sillitoe on behalf of the Wellcome Sanger Institute COVID-19 Surveillance Team
EPI_ISL_879074	Lighthouse Lab in Cambridge	Wellcome Sanger Institute for the COVID-19 Genomics UK (COG-UK) Consortium	Rob Howes, The Lighthouse Lab in Cambridge and Alex Alderton, Roberto Amato, Sonia Goncalves, Ewan Harrison, David K. Jackson, Ian Johnston, Dominic Kwiatkowski, Cordelia Langford, John Sillitoe on behalf of the Wellcome Sanger Institute COVID-19 Surveillance Team
EPI_ISL_879075	Lighthouse Lab in Milton Keynes	Wellcome Sanger Institute for the COVID-19 Genomics UK (COG-UK) Consortium	The Lighthouse Lab in Milton Keynes and Alex Alderton, Roberto Amato, Sonia Goncalves, Ewan Harrison, David K. Jackson, Ian Johnston, Dominic Kwiatkowski, Cordelia Langford, John Sillitoe on behalf of the Wellcome Sanger Institute COVID-19 Surveillance Team
EPI_ISL_879076, EPI_ISL_879078, EPI_ISL_879082	Lighthouse Lab in Cambridge	Wellcome Sanger Institute for the COVID-19 Genomics UK (COG-UK) Consortium	Rob Howes, The Lighthouse Lab in Cambridge and Alex Alderton, Roberto Amato, Sonia Goncalves, Ewan Harrison, David K. Jackson, Ian Johnston, Dominic Kwiatkowski, Cordelia Langford, John Sillitoe on behalf of the Wellcome Sanger Institute COVID-19 Surveillance Team
EPI_ISL_879086, EPI_ISL_879087, EPI_ISL_879089, EPI_ISL_879092, EPI_ISL_879093, EPI_ISL_879094	Lighthouse Lab in Milton Keynes	Wellcome Sanger Institute for the COVID-19 Genomics UK (COG-UK) Consortium	The Lighthouse Lab in Milton Keynes and Alex Alderton, Roberto Amato, Sonia Goncalves, Ewan Harrison, David K. Jackson, Ian Johnston, Dominic Kwiatkowski, Cordelia Langford, John Sillitoe on behalf of the Wellcome Sanger Institute COVID-19 Surveillance Team
EPI_ISL_879095	Lighthouse Lab in Cambridge	Wellcome Sanger Institute for the COVID-19 Genomics UK (COG-UK) Consortium	Rob Howes, The Lighthouse Lab in Cambridge and Alex Alderton, Roberto Amato, Sonia Goncalves, Ewan Harrison, David K. Jackson, Ian Johnston, Dominic Kwiatkowski, Cordelia Langford, John Sillitoe on behalf of the Wellcome Sanger Institute COVID-19 Surveillance Team
EPI_ISL_879096	Lighthouse Lab in Milton Keynes	Wellcome Sanger Institute for the COVID-19 Genomics UK (COG-UK) Consortium	The Lighthouse Lab in Milton Keynes and Alex Alderton, Roberto Amato, Sonia Goncalves, Ewan Harrison, David K. Jackson, Ian Johnston, Dominic Kwiatkowski, Cordelia Langford, John Sillitoe on behalf of the Wellcome Sanger Institute COVID-19 Surveillance Team

EPI_ISL_879098, EPI_ISL_879100, EPI_ISL_879109, EPI_ISL_879110, EPI_ISL_879116, EPI_ISL_879117, EPI_ISL_879118, EPI_ISL_879120, EPI_ISL_879121, EPI_ISL_879124, EPI_ISL_879134, EPI_ISL_879138, EPI_ISL_879145, EPI_ISL_879149, EPI_ISL_879154, EPI_ISL_879161, EPI_ISL_879164, EPI_ISL_879167, EPI_ISL_879169, EPI_ISL_879171, EPI_ISL_879173, EPI_ISL_879185, EPI_ISL_879187, EPI_ISL_879193, EPI_ISL_879195, EPI_ISL_879201, EPI_ISL_879206, EPI_ISL_879207, EPI_ISL_879214, EPI_ISL_879220, EPI_ISL_879226, EPI_ISL_879227, EPI_ISL_879230, EPI_ISL_879234, EPI_ISL_879235, EPI_ISL_879239,

EPI_ISL_879241, EPI_ISL_879243, EPI_ISL_879247, EPI_ISL_879248, EPI_ISL_879249, EPI_ISL_879253, EPI_ISL_879254, EPI_ISL_879256, EPI_ISL_879258, EPI_ISL_879262, EPI_ISL_879268, EPI_ISL_879270, EPI_ISL_879278, EPI_ISL_879284, EPI_ISL_879286, EPI_ISL_879290, EPI_ISL_879305, EPI_ISL_879314, EPI_ISL_879318, EPI_ISL_879322, EPI_ISL_879329, EPI_ISL_879331, EPI_ISL_879333, EPI_ISL_879338, EPI_ISL_879339, EPI_ISL_879341, EPI_ISL_879343, EPI_ISL_879344, EPI_ISL_879348, EPI_ISL_879354, EPI_ISL_879356, EPI_ISL_879358, EPI_ISL_879363, EPI_ISL_879374, EPI_ISL_879375, EPI_ISL_879377

see above	Lighthouse Lab in Alderley Park	Wellcome Sanger Institute for the COVID-19 Genomics UK (COG-UK) Consortium	Jacquelyn Wynn, Mairead Hyland, The Lighthouse Lab in Alderley Park and Alex Alderton, Roberto Amato, Sonia Goncalves, Ewan Harrison, David K. Jackson, Ian Johnston, Dominic Kwiatkowski, Cordelia Langford, John Sillitoe on behalf of the Wellcome Sanger Institute COVID-19 Surveillance Team
EPI_ISL_879378, EPI_ISL_879379, EPI_ISL_879380	Lighthouse Lab in Milton Keynes	Wellcome Sanger Institute for the COVID-19 Genomics UK (COG-UK) Consortium	The Lighthouse Lab in Milton Keynes and Alex Alderton, Roberto Amato, Sonia Goncalves, Ewan Harrison, David K. Jackson, Ian Johnston, Dominic Kwiatkowski, Cordelia Langford, John Sillitoe on behalf of the Wellcome Sanger Institute COVID-19 Surveillance Team
EPI_ISL_879381	Lighthouse Lab in Cambridge	Wellcome Sanger Institute for the COVID-19 Genomics UK (COG-UK) Consortium	Rob Howes, The Lighthouse Lab in Cambridge and Alex Alderton, Roberto Amato, Sonia Goncalves, Ewan Harrison, David K. Jackson, Ian Johnston, Dominic Kwiatkowski, Cordelia Langford, John Sillitoe on behalf of the Wellcome Sanger Institute COVID-19 Surveillance Team
EPI_ISL_879382	Lighthouse Lab in Alderley Park	Wellcome Sanger Institute for the COVID-19 Genomics UK (COG-UK) Consortium	Jacquelyn Wynn, Mairead Hyland, The Lighthouse Lab in Alderley Park and Alex Alderton, Roberto Amato, Sonia Goncalves, Ewan Harrison, David K. Jackson, Ian Johnston, Dominic Kwiatkowski, Cordelia Langford, John Sillitoe on behalf of the Wellcome Sanger Institute COVID-19 Surveillance Team
EPI_ISL_879383	Lighthouse Lab in Cambridge	Wellcome Sanger Institute for the COVID-19 Genomics UK (COG-UK) Consortium	Rob Howes, The Lighthouse Lab in Cambridge and Alex Alderton, Roberto Amato, Sonia Goncalves, Ewan Harrison, David K. Jackson, Ian Johnston, Dominic Kwiatkowski, Cordelia Langford, John Sillitoe on behalf of the Wellcome Sanger Institute COVID-19 Surveillance Team
EPI_ISL_879384	Lighthouse Lab in Alderley Park	Wellcome Sanger Institute for the COVID-19 Genomics UK (COG-UK) Consortium	Jacquelyn Wynn, Mairead Hyland, The Lighthouse Lab in Alderley Park and Alex Alderton, Roberto Amato, Sonia Goncalves, Ewan Harrison, David K. Jackson, Ian Johnston, Dominic Kwiatkowski, Cordelia Langford, John Sillitoe on behalf of the Wellcome Sanger Institute COVID-19 Surveillance Team
EPI_ISL_879385, EPI_ISL_879387	Lighthouse Lab in Cambridge	Wellcome Sanger Institute for the COVID-19 Genomics UK (COG-UK) Consortium	Rob Howes, The Lighthouse Lab in Cambridge and Alex Alderton, Roberto Amato, Sonia Goncalves, Ewan Harrison, David K. Jackson, Ian Johnston, Dominic Kwiatkowski, Cordelia Langford, John Sillitoe on behalf of the Wellcome Sanger Institute COVID-19 Surveillance Team
EPI_ISL_879388	Lighthouse Lab in Milton Keynes	Wellcome Sanger Institute for the COVID-19 Genomics UK (COG-UK) Consortium	The Lighthouse Lab in Milton Keynes and Alex Alderton, Roberto Amato, Sonia Goncalves, Ewan Harrison, David K. Jackson, Ian Johnston, Dominic Kwiatkowski, Cordelia Langford, John Sillitoe on behalf of the Wellcome Sanger Institute COVID-19 Surveillance Team
EPI_ISL_879389, EPI_ISL_879390, EPI_ISL_879391	Lighthouse Lab in Cambridge	Wellcome Sanger Institute for the COVID-19 Genomics UK (COG-UK) Consortium	Rob Howes, The Lighthouse Lab in Cambridge and Alex Alderton, Roberto Amato, Sonia Goncalves, Ewan Harrison, David K. Jackson, Ian Johnston, Dominic Kwiatkowski, Cordelia Langford, John Sillitoe on behalf of the Wellcome Sanger Institute COVID-19 Surveillance Team
EPI_ISL_879393	Lighthouse Lab in Alderley Park	Wellcome Sanger Institute for the COVID-19 Genomics UK (COG-UK) Consortium	Jacquelyn Wynn, Mairead Hyland, The Lighthouse Lab in Alderley Park and Alex Alderton, Roberto Amato, Sonia Goncalves, Ewan Harrison, David K. Jackson, Ian Johnston, Dominic Kwiatkowski, Cordelia Langford, John Sillitoe on behalf of the Wellcome Sanger Institute COVID-19 Surveillance Team
EPI_ISL_879394	Lighthouse Lab in Cambridge	Wellcome Sanger Institute for the COVID-19 Genomics UK (COG-UK) Consortium	Rob Howes, The Lighthouse Lab in Cambridge and Alex Alderton, Roberto Amato, Sonia Goncalves, Ewan Harrison, David K. Jackson, Ian Johnston, Dominic Kwiatkowski, Cordelia Langford, John Sillitoe on behalf of the Wellcome Sanger Institute COVID-19 Surveillance Team
EPI_ISL_879395, EPI_ISL_879396, EPI_ISL_879397	Lighthouse Lab in Milton Keynes	Wellcome Sanger Institute for the COVID-19 Genomics UK (COG-UK) Consortium	The Lighthouse Lab in Milton Keynes and Alex Alderton, Roberto Amato, Sonia Goncalves, Ewan Harrison, David K. Jackson, Ian Johnston, Dominic Kwiatkowski, Cordelia Langford, John Sillitoe on behalf of the Wellcome Sanger Institute COVID-19 Surveillance Team
EPI_ISL_879398	Lighthouse Lab in Cambridge	Wellcome Sanger Institute for the COVID-19 Genomics UK (COG-UK) Consortium	Rob Howes, The Lighthouse Lab in Cambridge and Alex Alderton, Roberto Amato, Sonia Goncalves, Ewan Harrison, David K. Jackson, Ian Johnston, Dominic Kwiatkowski, Cordelia Langford, John Sillitoe on behalf of the Wellcome Sanger Institute COVID-19 Surveillance Team
EPI_ISL_879399	Lighthouse Lab in Milton Keynes	Wellcome Sanger Institute for the COVID-19 Genomics UK (COG-UK) Consortium	The Lighthouse Lab in Milton Keynes and Alex Alderton, Roberto Amato, Sonia Goncalves, Ewan Harrison, David K. Jackson, Ian Johnston, Dominic Kwiatkowski, Cordelia Langford, John Sillitoe on behalf of the Wellcome Sanger Institute COVID-19 Surveillance Team
EPI_ISL_879400, EPI_ISL_879401, EPI_ISL_879402, EPI_ISL_879403	Lighthouse Lab in Cambridge	Wellcome Sanger Institute for the COVID-19 Genomics UK (COG-UK) Consortium	Rob Howes, The Lighthouse Lab in Cambridge and Alex Alderton, Roberto Amato, Sonia Goncalves, Ewan Harrison, David K. Jackson, Ian Johnston, Dominic Kwiatkowski, Cordelia Langford, John Sillitoe on behalf of the Wellcome Sanger Institute COVID-19 Surveillance Team
EPI_ISL_879404	Lighthouse Lab in Milton Keynes	Wellcome Sanger Institute for the COVID-19 Genomics UK (COG-UK) Consortium	The Lighthouse Lab in Milton Keynes and Alex Alderton, Roberto Amato, Sonia Goncalves, Ewan Harrison, David K. Jackson, Ian Johnston, Dominic Kwiatkowski, Cordelia Langford, John Sillitoe on behalf of the Wellcome Sanger Institute COVID-19 Surveillance Team
EPI_ISL_879406, EPI_ISL_879407, EPI_ISL_879408	Lighthouse Lab in Cambridge	Wellcome Sanger Institute for the COVID-19 Genomics UK (COG-UK) Consortium	Rob Howes, The Lighthouse Lab in Cambridge and Alex Alderton, Roberto Amato, Sonia Goncalves, Ewan Harrison, David K. Jackson, Ian Johnston, Dominic Kwiatkowski, Cordelia Langford, John Sillitoe on behalf of the Wellcome Sanger Institute COVID-19 Surveillance Team
EPI_ISL_879409	Lighthouse Lab in Milton Keynes	Wellcome Sanger Institute for the COVID-19 Genomics UK (COG-UK) Consortium	The Lighthouse Lab in Milton Keynes and Alex Alderton, Roberto Amato, Sonia Goncalves, Ewan Harrison, David K. Jackson, Ian Johnston, Dominic Kwiatkowski, Cordelia Langford, John Sillitoe on behalf of the Wellcome Sanger Institute COVID-19 Surveillance Team
EPI_ISL_879410, EPI_ISL_879411	Lighthouse Lab in Cambridge	Wellcome Sanger Institute for the COVID-19 Genomics UK (COG-UK) Consortium	Rob Howes, The Lighthouse Lab in Cambridge and Alex Alderton, Roberto Amato, Sonia Goncalves, Ewan Harrison, David K. Jackson, Ian Johnston, Dominic Kwiatkowski, Cordelia Langford, John Sillitoe on behalf of the Wellcome Sanger Institute COVID-19 Surveillance Team
EPI_ISL_879412, EPI_ISL_879413	Lighthouse Lab in Milton Keynes	Wellcome Sanger Institute for the COVID-19 Genomics UK (COG-UK) Consortium	The Lighthouse Lab in Milton Keynes and Alex Alderton, Roberto Amato, Sonia Goncalves, Ewan Harrison, David K. Jackson, Ian Johnston, Dominic Kwiatkowski, Cordelia Langford, John Sillitoe on behalf of the Wellcome Sanger Institute COVID-19 Surveillance Team
EPI_ISL_879414	Lighthouse Lab in Cambridge	Wellcome Sanger Institute for the COVID-19 Genomics UK (COG-UK) Consortium	Rob Howes, The Lighthouse Lab in Cambridge and Alex Alderton, Roberto Amato, Sonia Goncalves, Ewan Harrison, David K. Jackson, Ian Johnston, Dominic Kwiatkowski, Cordelia Langford, John Sillitoe on behalf of the Wellcome Sanger Institute COVID-19 Surveillance Team
EPI_ISL_879416, EPI_ISL_879417	Lighthouse Lab in Alderley Park	Wellcome Sanger Institute for the COVID-19 Genomics UK (COG-UK) Consortium	Jacquelyn Wynn, Mairead Hyland, The Lighthouse Lab in Alderley Park and Alex Alderton, Roberto Amato, Sonia Goncalves, Ewan Harrison, David K. Jackson, Ian Johnston, Dominic Kwiatkowski, Cordelia Langford, John Sillitoe on behalf of the Wellcome Sanger Institute COVID-19 Surveillance Team
EPI_ISL_879419, EPI_ISL_879421, EPI_ISL_879422, EPI_ISL_879423	Lighthouse Lab in Cambridge	Wellcome Sanger Institute for the COVID-19 Genomics UK (COG-UK) Consortium	Rob Howes, The Lighthouse Lab in Cambridge and Alex Alderton, Roberto Amato, Sonia Goncalves, Ewan Harrison, David K. Jackson, Ian Johnston, Dominic Kwiatkowski, Cordelia Langford, John Sillitoe on behalf of the Wellcome Sanger Institute COVID-19 Surveillance Team
EPI_ISL_879424, EPI_ISL_879425, EPI_ISL_879426	Lighthouse Lab in Milton Keynes	Wellcome Sanger Institute for the COVID-19 Genomics UK (COG-UK) Consortium	The Lighthouse Lab in Milton Keynes and Alex Alderton, Roberto Amato, Sonia Goncalves, Ewan Harrison, David K. Jackson, Ian Johnston, Dominic Kwiatkowski, Cordelia Langford, John Sillitoe on behalf of the Wellcome Sanger Institute COVID-19 Surveillance Team
EPI_ISL_879428	Lighthouse Lab in Cambridge	Wellcome Sanger Institute for the COVID-19 Genomics UK (COG-UK) Consortium	Rob Howes, The Lighthouse Lab in Cambridge and Alex Alderton, Roberto Amato, Sonia Goncalves, Ewan Harrison, David K. Jackson, Ian Johnston, Dominic Kwiatkowski, Cordelia Langford, John Sillitoe on behalf of the Wellcome Sanger Institute COVID-19 Surveillance Team
EPI_ISL_879429	Lighthouse Lab in Milton Keynes	Wellcome Sanger Institute for the COVID-19 Genomics UK (COG-UK) Consortium	The Lighthouse Lab in Milton Keynes and Alex Alderton, Roberto Amato, Sonia Goncalves, Ewan Harrison, David K. Jackson, Ian Johnston, Dominic Kwiatkowski, Cordelia Langford, John Sillitoe on behalf of the Wellcome Sanger Institute COVID-19 Surveillance Team
EPI_ISL_879430, EPI_ISL_879431, EPI_ISL_879432	Lighthouse Lab in Cambridge	Wellcome Sanger Institute for the COVID-19 Genomics UK (COG-UK) Consortium	Rob Howes, The Lighthouse Lab in Cambridge and Alex Alderton, Roberto Amato, Sonia Goncalves, Ewan Harrison, David K. Jackson, Ian Johnston, Dominic Kwiatkowski, Cordelia Langford, John Sillitoe on behalf of the Wellcome Sanger Institute COVID-19 Surveillance Team
EPI_ISL_879433, EPI_ISL_879434	Lighthouse Lab in Alderley Park	Wellcome Sanger Institute for the COVID-19 Genomics UK (COG-UK) Consortium	Jacquelyn Wynn, Mairead Hyland, The Lighthouse Lab in Alderley Park and Alex Alderton, Roberto Amato, Sonia Goncalves, Ewan Harrison, David K. Jackson, Ian Johnston, Dominic Kwiatkowski, Cordelia Langford, John Sillitoe on behalf of the Wellcome Sanger Institute COVID-19 Surveillance Team
EPI_ISL_879435, EPI_ISL_879436, EPI_ISL_879437, EPI_ISL_879438, EPI_ISL_879439, EPI_ISL_879440, EPI_ISL_879441	Lighthouse Lab in Cambridge	Wellcome Sanger Institute for the COVID-19 Genomics UK (COG-UK) Consortium	Rob Howes, The Lighthouse Lab in Cambridge and Alex Alderton, Roberto Amato, Sonia Goncalves, Ewan Harrison, David K. Jackson, Ian Johnston, Dominic Kwiatkowski, Cordelia Langford, John Sillitoe on behalf of the Wellcome Sanger Institute COVID-19 Surveillance Team
EPI_ISL_879442	Lighthouse Lab in Milton Keynes	Wellcome Sanger Institute for the COVID-19 Genomics UK (COG-UK) Consortium	The Lighthouse Lab in Milton Keynes and Alex Alderton, Roberto Amato, Sonia Goncalves, Ewan Harrison, David K. Jackson, Ian Johnston, Dominic Kwiatkowski, Cordelia Langford, John Sillitoe on behalf of the Wellcome Sanger Institute COVID-19 Surveillance Team
EPI_ISL_879443, EPI_ISL_879445	Lighthouse Lab in Cambridge	Wellcome Sanger Institute for the COVID-19 Genomics UK (COG-UK) Consortium	Rob Howes, The Lighthouse Lab in Cambridge and Alex Alderton, Roberto Amato, Sonia Goncalves, Ewan Harrison, David K. Jackson, Ian Johnston, Dominic Kwiatkowski, Cordelia Langford, John Sillitoe on behalf of the Wellcome Sanger Institute COVID-19 Surveillance Team
EPI_ISL_879448, EPI_ISL_879449, EPI_ISL_879450	Lighthouse Lab in Milton Keynes	Wellcome Sanger Institute for the COVID-19 Genomics UK (COG-UK) Consortium	The Lighthouse Lab in Milton Keynes and Alex Alderton, Roberto Amato, Sonia Goncalves, Ewan Harrison, David K. Jackson, Ian Johnston, Dominic Kwiatkowski, Cordelia Langford, John Sillitoe on behalf of the Wellcome Sanger Institute COVID-19 Surveillance Team
EPI_ISL_879451, EPI_ISL_879452	Lighthouse Lab in Alderley Park	Wellcome Sanger Institute for the COVID-19 Genomics UK (COG-UK) Consortium	Jacquelyn Wynn, Mairead Hyland, The Lighthouse Lab in Alderley Park and Alex Alderton, Roberto Amato, Sonia Goncalves, Ewan Harrison, David K. Jackson, Ian Johnston, Dominic Kwiatkowski, Cordelia Langford, John Sillitoe on behalf of the Wellcome Sanger Institute COVID-19 Surveillance Team
EPI_ISL_879453, EPI_ISL_879454	Lighthouse Lab in Cambridge	Wellcome Sanger Institute for the COVID-19 Genomics UK	Rob Howes, The Lighthouse Lab in Cambridge and Alex Alderton, Roberto Amato, Sonia Goncalves, Ewan Harrison, David K. Jackson, Ian Johnston,

		(COG-UK) Consortium	Dominic Kwiatkowski, Cordelia Langford, John Sillitoe on behalf of the Wellcome Sanger Institute COVID-19 Surveillance Team
EPI_ISL_879455, EPI_ISL_879457, EPI_ISL_879458, EPI_ISL_879459	Lighthouse Lab in Milton Keynes	Wellcome Sanger Institute for the COVID-19 Genomics UK (COG-UK) Consortium	The Lighthouse Lab in Milton Keynes and Alex Alderton, Roberto Amato, Sonia Goncalves, Ewan Harrison, David K. Jackson, Ian Johnston, Dominic Kwiatkowski, Cordelia Langford, John Sillitoe on behalf of the Wellcome Sanger Institute COVID-19 Surveillance Team
EPI_ISL_879460	Lighthouse Lab in Alderley Park	Wellcome Sanger Institute for the COVID-19 Genomics UK (COG-UK) Consortium	Jacquelyn Wynn, Mairead Hyland, The Lighthouse Lab in Alderley Park and Alex Alderton, Roberto Amato, Sonia Goncalves, Ewan Harrison, David K. Jackson, Ian Johnston, Dominic Kwiatkowski, Cordelia Langford, John Sillitoe on behalf of the Wellcome Sanger Institute COVID-19 Surveillance Team
EPI_ISL_879461	Lighthouse Lab in Milton Keynes	Wellcome Sanger Institute for the COVID-19 Genomics UK (COG-UK) Consortium	The Lighthouse Lab in Milton Keynes and Alex Alderton, Roberto Amato, Sonia Goncalves, Ewan Harrison, David K. Jackson, Ian Johnston, Dominic Kwiatkowski, Cordelia Langford, John Sillitoe on behalf of the Wellcome Sanger Institute COVID-19 Surveillance Team
EPI_ISL_879462	Lighthouse Lab in Alderley Park	Wellcome Sanger Institute for the COVID-19 Genomics UK (COG-UK) Consortium	Jacquelyn Wynn, Mairead Hyland, The Lighthouse Lab in Alderley Park and Alex Alderton, Roberto Amato, Sonia Goncalves, Ewan Harrison, David K. Jackson, Ian Johnston, Dominic Kwiatkowski, Cordelia Langford, John Sillitoe on behalf of the Wellcome Sanger Institute COVID-19 Surveillance Team
EPI_ISL_879463	Lighthouse Lab in Cambridge	Wellcome Sanger Institute for the COVID-19 Genomics UK (COG-UK) Consortium	Rob Howes, The Lighthouse Lab in Cambridge and Alex Alderton, Roberto Amato, Sonia Goncalves, Ewan Harrison, David K. Jackson, Ian Johnston, Dominic Kwiatkowski, Cordelia Langford, John Sillitoe on behalf of the Wellcome Sanger Institute COVID-19 Surveillance Team
EPI_ISL_879464	Lighthouse Lab in Milton Keynes	Wellcome Sanger Institute for the COVID-19 Genomics UK (COG-UK) Consortium	The Lighthouse Lab in Milton Keynes and Alex Alderton, Roberto Amato, Sonia Goncalves, Ewan Harrison, David K. Jackson, Ian Johnston, Dominic Kwiatkowski, Cordelia Langford, John Sillitoe on behalf of the Wellcome Sanger Institute COVID-19 Surveillance Team
EPI_ISL_879465	Lighthouse Lab in Cambridge	Wellcome Sanger Institute for the COVID-19 Genomics UK (COG-UK) Consortium	Rob Howes, The Lighthouse Lab in Cambridge and Alex Alderton, Roberto Amato, Sonia Goncalves, Ewan Harrison, David K. Jackson, Ian Johnston, Dominic Kwiatkowski, Cordelia Langford, John Sillitoe on behalf of the Wellcome Sanger Institute COVID-19 Surveillance Team
EPI_ISL_879466	Lighthouse Lab in Alderley Park	Wellcome Sanger Institute for the COVID-19 Genomics UK (COG-UK) Consortium	Jacquelyn Wynn, Mairead Hyland, The Lighthouse Lab in Alderley Park and Alex Alderton, Roberto Amato, Sonia Goncalves, Ewan Harrison, David K. Jackson, Ian Johnston, Dominic Kwiatkowski, Cordelia Langford, John Sillitoe on behalf of the Wellcome Sanger Institute COVID-19 Surveillance Team
EPI_ISL_879467	Lighthouse Lab in Milton Keynes	Wellcome Sanger Institute for the COVID-19 Genomics UK (COG-UK) Consortium	The Lighthouse Lab in Milton Keynes and Alex Alderton, Roberto Amato, Sonia Goncalves, Ewan Harrison, David K. Jackson, Ian Johnston, Dominic Kwiatkowski, Cordelia Langford, John Sillitoe on behalf of the Wellcome Sanger Institute COVID-19 Surveillance Team
EPI_ISL_879468, EPI_ISL_879469, EPI_ISL_879470	Lighthouse Lab in Cambridge	Wellcome Sanger Institute for the COVID-19 Genomics UK (COG-UK) Consortium	Rob Howes, The Lighthouse Lab in Cambridge and Alex Alderton, Roberto Amato, Sonia Goncalves, Ewan Harrison, David K. Jackson, Ian Johnston, Dominic Kwiatkowski, Cordelia Langford, John Sillitoe on behalf of the Wellcome Sanger Institute COVID-19 Surveillance Team
EPI_ISL_879471	Lighthouse Lab in Milton Keynes	Wellcome Sanger Institute for the COVID-19 Genomics UK (COG-UK) Consortium	The Lighthouse Lab in Milton Keynes and Alex Alderton, Roberto Amato, Sonia Goncalves, Ewan Harrison, David K. Jackson, Ian Johnston, Dominic Kwiatkowski, Cordelia Langford, John Sillitoe on behalf of the Wellcome Sanger Institute COVID-19 Surveillance Team
EPI_ISL_879472	Lighthouse Lab in Cambridge	Wellcome Sanger Institute for the COVID-19 Genomics UK (COG-UK) Consortium	Rob Howes, The Lighthouse Lab in Cambridge and Alex Alderton, Roberto Amato, Sonia Goncalves, Ewan Harrison, David K. Jackson, Ian Johnston, Dominic Kwiatkowski, Cordelia Langford, John Sillitoe on behalf of the Wellcome Sanger Institute COVID-19 Surveillance Team
EPI_ISL_879473	Lighthouse Lab in Alderley Park	Wellcome Sanger Institute for the COVID-19 Genomics UK (COG-UK) Consortium	Jacquelyn Wynn, Mairead Hyland, The Lighthouse Lab in Alderley Park and Alex Alderton, Roberto Amato, Sonia Goncalves, Ewan Harrison, David K. Jackson, Ian Johnston, Dominic Kwiatkowski, Cordelia Langford, John Sillitoe on behalf of the Wellcome Sanger Institute COVID-19 Surveillance Team
EPI_ISL_879474	Lighthouse Lab in Cambridge	Wellcome Sanger Institute for the COVID-19 Genomics UK (COG-UK) Consortium	Rob Howes, The Lighthouse Lab in Cambridge and Alex Alderton, Roberto Amato, Sonia Goncalves, Ewan Harrison, David K. Jackson, Ian Johnston, Dominic Kwiatkowski, Cordelia Langford, John Sillitoe on behalf of the Wellcome Sanger Institute COVID-19 Surveillance Team
EPI_ISL_879475	Lighthouse Lab in Alderley Park	Wellcome Sanger Institute for the COVID-19 Genomics UK (COG-UK) Consortium	Jacquelyn Wynn, Mairead Hyland, The Lighthouse Lab in Alderley Park and Alex Alderton, Roberto Amato, Sonia Goncalves, Ewan Harrison, David K. Jackson, Ian Johnston, Dominic Kwiatkowski, Cordelia Langford, John Sillitoe on behalf of the Wellcome Sanger Institute COVID-19 Surveillance Team
EPI_ISL_879477	Lighthouse Lab in Milton Keynes	Wellcome Sanger Institute for the COVID-19 Genomics UK (COG-UK) Consortium	The Lighthouse Lab in Milton Keynes and Alex Alderton, Roberto Amato, Sonia Goncalves, Ewan Harrison, David K. Jackson, Ian Johnston, Dominic Kwiatkowski, Cordelia Langford, John Sillitoe on behalf of the Wellcome Sanger Institute COVID-19 Surveillance Team
EPI_ISL_879478	Lighthouse Lab in Cambridge	Wellcome Sanger Institute for the COVID-19 Genomics UK (COG-UK) Consortium	Rob Howes, The Lighthouse Lab in Cambridge and Alex Alderton, Roberto Amato, Sonia Goncalves, Ewan Harrison, David K. Jackson, Ian Johnston, Dominic Kwiatkowski, Cordelia Langford, John Sillitoe on behalf of the Wellcome Sanger Institute COVID-19 Surveillance Team
EPI_ISL_879479	Lighthouse Lab in Milton Keynes	Wellcome Sanger Institute for the COVID-19 Genomics UK (COG-UK) Consortium	The Lighthouse Lab in Milton Keynes and Alex Alderton, Roberto Amato, Sonia Goncalves, Ewan Harrison, David K. Jackson, Ian Johnston, Dominic Kwiatkowski, Cordelia Langford, John Sillitoe on behalf of the Wellcome Sanger Institute COVID-19 Surveillance Team
EPI_ISL_879480	Lighthouse Lab in Alderley Park	Wellcome Sanger Institute for the COVID-19 Genomics UK (COG-UK) Consortium	Jacquelyn Wynn, Mairead Hyland, The Lighthouse Lab in Alderley Park and Alex Alderton, Roberto Amato, Sonia Goncalves, Ewan Harrison, David K. Jackson, Ian Johnston, Dominic Kwiatkowski, Cordelia Langford, John Sillitoe on behalf of the Wellcome Sanger Institute COVID-19 Surveillance Team
EPI_ISL_879481, EPI_ISL_879482, EPI_ISL_879484, EPI_ISL_879485	Lighthouse Lab in Cambridge	Wellcome Sanger Institute for the COVID-19 Genomics UK (COG-UK) Consortium	Rob Howes, The Lighthouse Lab in Cambridge and Alex Alderton, Roberto Amato, Sonia Goncalves, Ewan Harrison, David K. Jackson, Ian Johnston, Dominic Kwiatkowski, Cordelia Langford, John Sillitoe on behalf of the Wellcome Sanger Institute COVID-19 Surveillance Team
EPI_ISL_879486, EPI_ISL_879487	Lighthouse Lab in Alderley Park	Wellcome Sanger Institute for the COVID-19 Genomics UK (COG-UK) Consortium	Jacquelyn Wynn, Mairead Hyland, The Lighthouse Lab in Alderley Park and Alex Alderton, Roberto Amato, Sonia Goncalves, Ewan Harrison, David K. Jackson, Ian Johnston, Dominic Kwiatkowski, Cordelia Langford, John Sillitoe on behalf of the Wellcome Sanger Institute COVID-19 Surveillance Team
EPI_ISL_879489, EPI_ISL_879490, EPI_ISL_879491	Lighthouse Lab in Cambridge	Wellcome Sanger Institute for the COVID-19 Genomics UK (COG-UK) Consortium	Rob Howes, The Lighthouse Lab in Cambridge and Alex Alderton, Roberto Amato, Sonia Goncalves, Ewan Harrison, David K. Jackson, Ian Johnston, Dominic Kwiatkowski, Cordelia Langford, John Sillitoe on behalf of the Wellcome Sanger Institute COVID-19 Surveillance Team
EPI_ISL_879492	Lighthouse Lab in Milton Keynes	Wellcome Sanger Institute for the COVID-19 Genomics UK (COG-UK) Consortium	The Lighthouse Lab in Milton Keynes and Alex Alderton, Roberto Amato, Sonia Goncalves, Ewan Harrison, David K. Jackson, Ian Johnston, Dominic Kwiatkowski, Cordelia Langford, John Sillitoe on behalf of the Wellcome Sanger Institute COVID-19 Surveillance Team
EPI_ISL_879494	Lighthouse Lab in Cambridge	Wellcome Sanger Institute for the COVID-19 Genomics UK (COG-UK) Consortium	Rob Howes, The Lighthouse Lab in Cambridge and Alex Alderton, Roberto Amato, Sonia Goncalves, Ewan Harrison, David K. Jackson, Ian Johnston, Dominic Kwiatkowski, Cordelia Langford, John Sillitoe on behalf of the Wellcome Sanger Institute COVID-19 Surveillance Team
EPI_ISL_879495	Lighthouse Lab in Milton Keynes	Wellcome Sanger Institute for the COVID-19 Genomics UK (COG-UK) Consortium	The Lighthouse Lab in Milton Keynes and Alex Alderton, Roberto Amato, Sonia Goncalves, Ewan Harrison, David K. Jackson, Ian Johnston, Dominic Kwiatkowski, Cordelia Langford, John Sillitoe on behalf of the Wellcome Sanger Institute COVID-19 Surveillance Team
EPI_ISL_879496, EPI_ISL_879498	Lighthouse Lab in Cambridge	Wellcome Sanger Institute for the COVID-19 Genomics UK (COG-UK) Consortium	Rob Howes, The Lighthouse Lab in Cambridge and Alex Alderton, Roberto Amato, Sonia Goncalves, Ewan Harrison, David K. Jackson, Ian Johnston, Dominic Kwiatkowski, Cordelia Langford, John Sillitoe on behalf of the Wellcome Sanger Institute COVID-19 Surveillance Team
EPI_ISL_879499	Lighthouse Lab in Milton Keynes	Wellcome Sanger Institute for the COVID-19 Genomics UK (COG-UK) Consortium	The Lighthouse Lab in Milton Keynes and Alex Alderton, Roberto Amato, Sonia Goncalves, Ewan Harrison, David K. Jackson, Ian Johnston, Dominic Kwiatkowski, Cordelia Langford, John Sillitoe on behalf of the Wellcome Sanger Institute COVID-19 Surveillance Team
EPI_ISL_879500	Lighthouse Lab in Cambridge	Wellcome Sanger Institute for the COVID-19 Genomics UK (COG-UK) Consortium	Rob Howes, The Lighthouse Lab in Cambridge and Alex Alderton, Roberto Amato, Sonia Goncalves, Ewan Harrison, David K. Jackson, Ian Johnston, Dominic Kwiatkowski, Cordelia Langford, John Sillitoe on behalf of the Wellcome Sanger Institute COVID-19 Surveillance Team
EPI_ISL_879502	Lighthouse Lab in Milton Keynes	Wellcome Sanger Institute for the COVID-19 Genomics UK (COG-UK) Consortium	The Lighthouse Lab in Milton Keynes and Alex Alderton, Roberto Amato, Sonia Goncalves, Ewan Harrison, David K. Jackson, Ian Johnston, Dominic Kwiatkowski, Cordelia Langford, John Sillitoe on behalf of the Wellcome Sanger Institute COVID-19 Surveillance Team
EPI_ISL_879503	Lighthouse Lab in Cambridge	Wellcome Sanger Institute for the COVID-19 Genomics UK (COG-UK) Consortium	Rob Howes, The Lighthouse Lab in Cambridge and Alex Alderton, Roberto Amato, Sonia Goncalves, Ewan Harrison, David K. Jackson, Ian Johnston, Dominic Kwiatkowski, Cordelia Langford, John Sillitoe on behalf of the Wellcome Sanger Institute COVID-19 Surveillance Team
EPI_ISL_879504	Lighthouse Lab in Alderley Park	Wellcome Sanger Institute for the COVID-19 Genomics UK (COG-UK) Consortium	Jacquelyn Wynn, Mairead Hyland, The Lighthouse Lab in Alderley Park and Alex Alderton, Roberto Amato, Sonia Goncalves, Ewan Harrison, David K. Jackson, Ian Johnston, Dominic Kwiatkowski, Cordelia Langford, John Sillitoe on behalf of the Wellcome Sanger Institute COVID-19 Surveillance Team
EPI_ISL_879506, EPI_ISL_879507, EPI_ISL_879508, EPI_ISL_879509	Lighthouse Lab in Cambridge	Wellcome Sanger Institute for the COVID-19 Genomics UK (COG-UK) Consortium	Rob Howes, The Lighthouse Lab in Cambridge and Alex Alderton, Roberto Amato, Sonia Goncalves, Ewan Harrison, David K. Jackson, Ian Johnston, Dominic Kwiatkowski, Cordelia Langford, John Sillitoe on behalf of the Wellcome Sanger Institute COVID-19 Surveillance Team
EPI_ISL_879510	Lighthouse Lab in Alderley Park	Wellcome Sanger Institute for the COVID-19 Genomics UK (COG-UK) Consortium	Jacquelyn Wynn, Mairead Hyland, The Lighthouse Lab in Alderley Park and Alex Alderton, Roberto Amato, Sonia Goncalves, Ewan Harrison, David K. Jackson, Ian Johnston, Dominic Kwiatkowski, Cordelia Langford, John Sillitoe on behalf of the Wellcome Sanger Institute COVID-19 Surveillance Team
EPI_ISL_879511, EPI_ISL_879513	Lighthouse Lab in Cambridge	Wellcome Sanger Institute for the COVID-19 Genomics UK (COG-UK) Consortium	Rob Howes, The Lighthouse Lab in Cambridge and Alex Alderton, Roberto Amato, Sonia Goncalves, Ewan Harrison, David K. Jackson, Ian Johnston, Dominic Kwiatkowski, Cordelia Langford, John Sillitoe on behalf of the Wellcome Sanger Institute COVID-19 Surveillance Team
EPI_ISL_879514	Lighthouse Lab in Milton Keynes	Wellcome Sanger Institute for the COVID-19 Genomics UK	The Lighthouse Lab in Milton Keynes and Alex Alderton, Roberto Amato, Sonia Goncalves, Ewan Harrison, David K. Jackson, Ian Johnston, Dominic

		(COG-UK) Consortium	Kwiatkowski, Cordelia Langford, John Sillitoe on behalf of the Wellcome Sanger Institute COVID-19 Surveillance Team
EPI_ISL_879516, EPI_ISL_879517	Lighthouse Lab in Cambridge	Wellcome Sanger Institute for the COVID-19 Genomics UK (COG-UK) Consortium	Rob Howes, The Lighthouse Lab in Cambridge and Alex Alderton, Roberto Amato, Sonia Goncalves, Ewan Harrison, David K. Jackson, Ian Johnston, Dominic Kwiatkowski, Cordelia Langford, John Sillitoe on behalf of the Wellcome Sanger Institute COVID-19 Surveillance Team
EPI_ISL_879519, EPI_ISL_879520	Lighthouse Lab in Alderley Park	Wellcome Sanger Institute for the COVID-19 Genomics UK (COG-UK) Consortium	Jacquelyn Wynn, Mairead Hyland, The Lighthouse Lab in Alderley Park and Alex Alderton, Roberto Amato, Sonia Goncalves, Ewan Harrison, David K. Jackson, Ian Johnston, Dominic Kwiatkowski, Cordelia Langford, John Sillitoe on behalf of the Wellcome Sanger Institute COVID-19 Surveillance Team
EPI_ISL_879523	Lighthouse Lab in Cambridge	Wellcome Sanger Institute for the COVID-19 Genomics UK (COG-UK) Consortium	Rob Howes, The Lighthouse Lab in Cambridge and Alex Alderton, Roberto Amato, Sonia Goncalves, Ewan Harrison, David K. Jackson, Ian Johnston, Dominic Kwiatkowski, Cordelia Langford, John Sillitoe on behalf of the Wellcome Sanger Institute COVID-19 Surveillance Team
EPI_ISL_879524	Lighthouse Lab in Alderley Park	Wellcome Sanger Institute for the COVID-19 Genomics UK (COG-UK) Consortium	Jacquelyn Wynn, Mairead Hyland, The Lighthouse Lab in Alderley Park and Alex Alderton, Roberto Amato, Sonia Goncalves, Ewan Harrison, David K. Jackson, Ian Johnston, Dominic Kwiatkowski, Cordelia Langford, John Sillitoe on behalf of the Wellcome Sanger Institute COVID-19 Surveillance Team
EPI_ISL_879525	Lighthouse Lab in Cambridge	Wellcome Sanger Institute for the COVID-19 Genomics UK (COG-UK) Consortium	Rob Howes, The Lighthouse Lab in Cambridge and Alex Alderton, Roberto Amato, Sonia Goncalves, Ewan Harrison, David K. Jackson, Ian Johnston, Dominic Kwiatkowski, Cordelia Langford, John Sillitoe on behalf of the Wellcome Sanger Institute COVID-19 Surveillance Team
EPI_ISL_879526	Lighthouse Lab in Milton Keynes	Wellcome Sanger Institute for the COVID-19 Genomics UK (COG-UK) Consortium	The Lighthouse Lab in Milton Keynes and Alex Alderton, Roberto Amato, Sonia Goncalves, Ewan Harrison, David K. Jackson, Ian Johnston, Dominic Kwiatkowski, Cordelia Langford, John Sillitoe on behalf of the Wellcome Sanger Institute COVID-19 Surveillance Team
EPI_ISL_879527, EPI_ISL_879528, EPI_ISL_879529, EPI_ISL_879530, EPI_ISL_879531	Lighthouse Lab in Cambridge	Wellcome Sanger Institute for the COVID-19 Genomics UK (COG-UK) Consortium	Rob Howes, The Lighthouse Lab in Cambridge and Alex Alderton, Roberto Amato, Sonia Goncalves, Ewan Harrison, David K. Jackson, Ian Johnston, Dominic Kwiatkowski, Cordelia Langford, John Sillitoe on behalf of the Wellcome Sanger Institute COVID-19 Surveillance Team
EPI_ISL_879532	Lighthouse Lab in Milton Keynes	Wellcome Sanger Institute for the COVID-19 Genomics UK (COG-UK) Consortium	The Lighthouse Lab in Milton Keynes and Alex Alderton, Roberto Amato, Sonia Goncalves, Ewan Harrison, David K. Jackson, Ian Johnston, Dominic Kwiatkowski, Cordelia Langford, John Sillitoe on behalf of the Wellcome Sanger Institute COVID-19 Surveillance Team
EPI_ISL_879537	Lighthouse Lab in Cambridge	Wellcome Sanger Institute for the COVID-19 Genomics UK (COG-UK) Consortium	Rob Howes, The Lighthouse Lab in Cambridge and Alex Alderton, Roberto Amato, Sonia Goncalves, Ewan Harrison, David K. Jackson, Ian Johnston, Dominic Kwiatkowski, Cordelia Langford, John Sillitoe on behalf of the Wellcome Sanger Institute COVID-19 Surveillance Team
EPI_ISL_879538, EPI_ISL_879539, EPI_ISL_879540	Lighthouse Lab in Milton Keynes	Wellcome Sanger Institute for the COVID-19 Genomics UK (COG-UK) Consortium	The Lighthouse Lab in Milton Keynes and Alex Alderton, Roberto Amato, Sonia Goncalves, Ewan Harrison, David K. Jackson, Ian Johnston, Dominic Kwiatkowski, Cordelia Langford, John Sillitoe on behalf of the Wellcome Sanger Institute COVID-19 Surveillance Team
EPI_ISL_879543, EPI_ISL_879544, EPI_ISL_879545	Lighthouse Lab in Cambridge	Wellcome Sanger Institute for the COVID-19 Genomics UK (COG-UK) Consortium	Rob Howes, The Lighthouse Lab in Cambridge and Alex Alderton, Roberto Amato, Sonia Goncalves, Ewan Harrison, David K. Jackson, Ian Johnston, Dominic Kwiatkowski, Cordelia Langford, John Sillitoe on behalf of the Wellcome Sanger Institute COVID-19 Surveillance Team
EPI_ISL_879546, EPI_ISL_879547	Lighthouse Lab in Alderley Park	Wellcome Sanger Institute for the COVID-19 Genomics UK (COG-UK) Consortium	Jacquelyn Wynn, Mairead Hyland, The Lighthouse Lab in Alderley Park and Alex Alderton, Roberto Amato, Sonia Goncalves, Ewan Harrison, David K. Jackson, Ian Johnston, Dominic Kwiatkowski, Cordelia Langford, John Sillitoe on behalf of the Wellcome Sanger Institute COVID-19 Surveillance Team
EPI_ISL_879548, EPI_ISL_879549	Lighthouse Lab in Milton Keynes	Wellcome Sanger Institute for the COVID-19 Genomics UK (COG-UK) Consortium	The Lighthouse Lab in Milton Keynes and Alex Alderton, Roberto Amato, Sonia Goncalves, Ewan Harrison, David K. Jackson, Ian Johnston, Dominic Kwiatkowski, Cordelia Langford, John Sillitoe on behalf of the Wellcome Sanger Institute COVID-19 Surveillance Team
EPI_ISL_879550	Lighthouse Lab in Alderley Park	Wellcome Sanger Institute for the COVID-19 Genomics UK (COG-UK) Consortium	Jacquelyn Wynn, Mairead Hyland, The Lighthouse Lab in Alderley Park and Alex Alderton, Roberto Amato, Sonia Goncalves, Ewan Harrison, David K. Jackson, Ian Johnston, Dominic Kwiatkowski, Cordelia Langford, John Sillitoe on behalf of the Wellcome Sanger Institute COVID-19 Surveillance Team
EPI_ISL_879551	Lighthouse Lab in Cambridge	Wellcome Sanger Institute for the COVID-19 Genomics UK (COG-UK) Consortium	Rob Howes, The Lighthouse Lab in Cambridge and Alex Alderton, Roberto Amato, Sonia Goncalves, Ewan Harrison, David K. Jackson, Ian Johnston, Dominic Kwiatkowski, Cordelia Langford, John Sillitoe on behalf of the Wellcome Sanger Institute COVID-19 Surveillance Team
EPI_ISL_879552, EPI_ISL_879553, EPI_ISL_879554	Lighthouse Lab in Milton Keynes	Wellcome Sanger Institute for the COVID-19 Genomics UK (COG-UK) Consortium	The Lighthouse Lab in Milton Keynes and Alex Alderton, Roberto Amato, Sonia Goncalves, Ewan Harrison, David K. Jackson, Ian Johnston, Dominic Kwiatkowski, Cordelia Langford, John Sillitoe on behalf of the Wellcome Sanger Institute COVID-19 Surveillance Team
EPI_ISL_879555	Lighthouse Lab in Cambridge	Wellcome Sanger Institute for the COVID-19 Genomics UK (COG-UK) Consortium	Rob Howes, The Lighthouse Lab in Cambridge and Alex Alderton, Roberto Amato, Sonia Goncalves, Ewan Harrison, David K. Jackson, Ian Johnston, Dominic Kwiatkowski, Cordelia Langford, John Sillitoe on behalf of the Wellcome Sanger Institute COVID-19 Surveillance Team
EPI_ISL_879556	Lighthouse Lab in Alderley Park	Wellcome Sanger Institute for the COVID-19 Genomics UK (COG-UK) Consortium	Jacquelyn Wynn, Mairead Hyland, The Lighthouse Lab in Alderley Park and Alex Alderton, Roberto Amato, Sonia Goncalves, Ewan Harrison, David K. Jackson, Ian Johnston, Dominic Kwiatkowski, Cordelia Langford, John Sillitoe on behalf of the Wellcome Sanger Institute COVID-19 Surveillance Team
EPI_ISL_879557, EPI_ISL_879558, EPI_ISL_879559	Lighthouse Lab in Cambridge	Wellcome Sanger Institute for the COVID-19 Genomics UK (COG-UK) Consortium	Rob Howes, The Lighthouse Lab in Cambridge and Alex Alderton, Roberto Amato, Sonia Goncalves, Ewan Harrison, David K. Jackson, Ian Johnston, Dominic Kwiatkowski, Cordelia Langford, John Sillitoe on behalf of the Wellcome Sanger Institute COVID-19 Surveillance Team
EPI_ISL_879560, EPI_ISL_879561	Lighthouse Lab in Milton Keynes	Wellcome Sanger Institute for the COVID-19 Genomics UK (COG-UK) Consortium	The Lighthouse Lab in Milton Keynes and Alex Alderton, Roberto Amato, Sonia Goncalves, Ewan Harrison, David K. Jackson, Ian Johnston, Dominic Kwiatkowski, Cordelia Langford, John Sillitoe on behalf of the Wellcome Sanger Institute COVID-19 Surveillance Team
EPI_ISL_879562	Lighthouse Lab in Cambridge	Wellcome Sanger Institute for the COVID-19 Genomics UK (COG-UK) Consortium	Rob Howes, The Lighthouse Lab in Cambridge and Alex Alderton, Roberto Amato, Sonia Goncalves, Ewan Harrison, David K. Jackson, Ian Johnston, Dominic Kwiatkowski, Cordelia Langford, John Sillitoe on behalf of the Wellcome Sanger Institute COVID-19 Surveillance Team
EPI_ISL_879563, EPI_ISL_879565	Lighthouse Lab in Alderley Park	Wellcome Sanger Institute for the COVID-19 Genomics UK (COG-UK) Consortium	Jacquelyn Wynn, Mairead Hyland, The Lighthouse Lab in Alderley Park and Alex Alderton, Roberto Amato, Sonia Goncalves, Ewan Harrison, David K. Jackson, Ian Johnston, Dominic Kwiatkowski, Cordelia Langford, John Sillitoe on behalf of the Wellcome Sanger Institute COVID-19 Surveillance Team
EPI_ISL_879566	Lighthouse Lab in Milton Keynes	Wellcome Sanger Institute for the COVID-19 Genomics UK (COG-UK) Consortium	The Lighthouse Lab in Milton Keynes and Alex Alderton, Roberto Amato, Sonia Goncalves, Ewan Harrison, David K. Jackson, Ian Johnston, Dominic Kwiatkowski, Cordelia Langford, John Sillitoe on behalf of the Wellcome Sanger Institute COVID-19 Surveillance Team
EPI_ISL_879567, EPI_ISL_879568, EPI_ISL_879569, EPI_ISL_879570	Lighthouse Lab in Cambridge	Wellcome Sanger Institute for the COVID-19 Genomics UK (COG-UK) Consortium	Rob Howes, The Lighthouse Lab in Cambridge and Alex Alderton, Roberto Amato, Sonia Goncalves, Ewan Harrison, David K. Jackson, Ian Johnston, Dominic Kwiatkowski, Cordelia Langford, John Sillitoe on behalf of the Wellcome Sanger Institute COVID-19 Surveillance Team
EPI_ISL_879571, EPI_ISL_879572	Lighthouse Lab in Milton Keynes	Wellcome Sanger Institute for the COVID-19 Genomics UK (COG-UK) Consortium	The Lighthouse Lab in Milton Keynes and Alex Alderton, Roberto Amato, Sonia Goncalves, Ewan Harrison, David K. Jackson, Ian Johnston, Dominic Kwiatkowski, Cordelia Langford, John Sillitoe on behalf of the Wellcome Sanger Institute COVID-19 Surveillance Team
EPI_ISL_879573, EPI_ISL_879574	Lighthouse Lab in Cambridge	Wellcome Sanger Institute for the COVID-19 Genomics UK (COG-UK) Consortium	Rob Howes, The Lighthouse Lab in Cambridge and Alex Alderton, Roberto Amato, Sonia Goncalves, Ewan Harrison, David K. Jackson, Ian Johnston, Dominic Kwiatkowski, Cordelia Langford, John Sillitoe on behalf of the Wellcome Sanger Institute COVID-19 Surveillance Team
EPI_ISL_879575, EPI_ISL_879576	Lighthouse Lab in Milton Keynes	Wellcome Sanger Institute for the COVID-19 Genomics UK (COG-UK) Consortium	The Lighthouse Lab in Milton Keynes and Alex Alderton, Roberto Amato, Sonia Goncalves, Ewan Harrison, David K. Jackson, Ian Johnston, Dominic Kwiatkowski, Cordelia Langford, John Sillitoe on behalf of the Wellcome Sanger Institute COVID-19 Surveillance Team
EPI_ISL_879577, EPI_ISL_879579, EPI_ISL_879580	Lighthouse Lab in Cambridge	Wellcome Sanger Institute for the COVID-19 Genomics UK (COG-UK) Consortium	Rob Howes, The Lighthouse Lab in Cambridge and Alex Alderton, Roberto Amato, Sonia Goncalves, Ewan Harrison, David K. Jackson, Ian Johnston, Dominic Kwiatkowski, Cordelia Langford, John Sillitoe on behalf of the Wellcome Sanger Institute COVID-19 Surveillance Team
EPI_ISL_879581	Lighthouse Lab in Milton Keynes	Wellcome Sanger Institute for the COVID-19 Genomics UK (COG-UK) Consortium	The Lighthouse Lab in Milton Keynes and Alex Alderton, Roberto Amato, Sonia Goncalves, Ewan Harrison, David K. Jackson, Ian Johnston, Dominic Kwiatkowski, Cordelia Langford, John Sillitoe on behalf of the Wellcome Sanger Institute COVID-19 Surveillance Team
EPI_ISL_879583, EPI_ISL_879584	Lighthouse Lab in Cambridge	Wellcome Sanger Institute for the COVID-19 Genomics UK (COG-UK) Consortium	Rob Howes, The Lighthouse Lab in Cambridge and Alex Alderton, Roberto Amato, Sonia Goncalves, Ewan Harrison, David K. Jackson, Ian Johnston, Dominic Kwiatkowski, Cordelia Langford, John Sillitoe on behalf of the Wellcome Sanger Institute COVID-19 Surveillance Team
EPI_ISL_879585, EPI_ISL_879586	Lighthouse Lab in Milton Keynes	Wellcome Sanger Institute for the COVID-19 Genomics UK (COG-UK) Consortium	The Lighthouse Lab in Milton Keynes and Alex Alderton, Roberto Amato, Sonia Goncalves, Ewan Harrison, David K. Jackson, Ian Johnston, Dominic Kwiatkowski, Cordelia Langford, John Sillitoe on behalf of the Wellcome Sanger Institute COVID-19 Surveillance Team
EPI_ISL_879587, EPI_ISL_879588, EPI_ISL_879590	Lighthouse Lab in Cambridge	Wellcome Sanger Institute for the COVID-19 Genomics UK (COG-UK) Consortium	Rob Howes, The Lighthouse Lab in Cambridge and Alex Alderton, Roberto Amato, Sonia Goncalves, Ewan Harrison, David K. Jackson, Ian Johnston, Dominic Kwiatkowski, Cordelia Langford, John Sillitoe on behalf of the Wellcome Sanger Institute COVID-19 Surveillance Team
EPI_ISL_879591, EPI_ISL_879592, EPI_ISL_879594	Lighthouse Lab in Milton Keynes	Wellcome Sanger Institute for the COVID-19 Genomics UK (COG-UK) Consortium	The Lighthouse Lab in Milton Keynes and Alex Alderton, Roberto Amato, Sonia Goncalves, Ewan Harrison, David K. Jackson, Ian Johnston, Dominic Kwiatkowski, Cordelia Langford, John Sillitoe on behalf of the Wellcome Sanger Institute COVID-19 Surveillance Team
EPI_ISL_879595	Lighthouse Lab in Cambridge	Wellcome Sanger Institute for the COVID-19 Genomics UK (COG-UK) Consortium	Rob Howes, The Lighthouse Lab in Cambridge and Alex Alderton, Roberto Amato, Sonia Goncalves, Ewan Harrison, David K. Jackson, Ian Johnston, Dominic Kwiatkowski, Cordelia Langford, John Sillitoe on behalf of the Wellcome Sanger Institute COVID-19 Surveillance Team

EPI_ISL_879596	Lighthouse Lab in Alderley Park	Wellcome Sanger Institute for the COVID-19 Genomics UK (COG-UK) Consortium	Jacquelyn Wynn, Mairead Hyland, The Lighthouse Lab in Alderley Park and Alex Alderton, Roberto Amato, Sonia Goncalves, Ewan Harrison, David K. Jackson, Ian Johnston, Dominic Kwiatkowski, Cordelia Langford, John Sillitoe on behalf of the Wellcome Sanger Institute COVID-19 Surveillance Team
EPI_ISL_879597	Lighthouse Lab in Milton Keynes	Wellcome Sanger Institute for the COVID-19 Genomics UK (COG-UK) Consortium	The Lighthouse Lab in Milton Keynes and Alex Alderton, Roberto Amato, Sonia Goncalves, Ewan Harrison, David K. Jackson, Ian Johnston, Dominic Kwiatkowski, Cordelia Langford, John Sillitoe on behalf of the Wellcome Sanger Institute COVID-19 Surveillance Team
EPI_ISL_879598, EPI_ISL_879599	Lighthouse Lab in Cambridge	Wellcome Sanger Institute for the COVID-19 Genomics UK (COG-UK) Consortium	Rob Howes, The Lighthouse Lab in Cambridge and Alex Alderton, Roberto Amato, Sonia Goncalves, Ewan Harrison, David K. Jackson, Ian Johnston, Dominic Kwiatkowski, Cordelia Langford, John Sillitoe on behalf of the Wellcome Sanger Institute COVID-19 Surveillance Team
EPI_ISL_879600, EPI_ISL_879601	Lighthouse Lab in Milton Keynes	Wellcome Sanger Institute for the COVID-19 Genomics UK (COG-UK) Consortium	The Lighthouse Lab in Milton Keynes and Alex Alderton, Roberto Amato, Sonia Goncalves, Ewan Harrison, David K. Jackson, Ian Johnston, Dominic Kwiatkowski, Cordelia Langford, John Sillitoe on behalf of the Wellcome Sanger Institute COVID-19 Surveillance Team
EPI_ISL_879605, EPI_ISL_879606, EPI_ISL_879607	Lighthouse Lab in Cambridge	Wellcome Sanger Institute for the COVID-19 Genomics UK (COG-UK) Consortium	Rob Howes, The Lighthouse Lab in Cambridge and Alex Alderton, Roberto Amato, Sonia Goncalves, Ewan Harrison, David K. Jackson, Ian Johnston, Dominic Kwiatkowski, Cordelia Langford, John Sillitoe on behalf of the Wellcome Sanger Institute COVID-19 Surveillance Team
EPI_ISL_879608	Lighthouse Lab in Milton Keynes	Wellcome Sanger Institute for the COVID-19 Genomics UK (COG-UK) Consortium	The Lighthouse Lab in Milton Keynes and Alex Alderton, Roberto Amato, Sonia Goncalves, Ewan Harrison, David K. Jackson, Ian Johnston, Dominic Kwiatkowski, Cordelia Langford, John Sillitoe on behalf of the Wellcome Sanger Institute COVID-19 Surveillance Team
EPI_ISL_879609, EPI_ISL_879610	Lighthouse Lab in Cambridge	Wellcome Sanger Institute for the COVID-19 Genomics UK (COG-UK) Consortium	Rob Howes, The Lighthouse Lab in Cambridge and Alex Alderton, Roberto Amato, Sonia Goncalves, Ewan Harrison, David K. Jackson, Ian Johnston, Dominic Kwiatkowski, Cordelia Langford, John Sillitoe on behalf of the Wellcome Sanger Institute COVID-19 Surveillance Team
EPI_ISL_879611	Lighthouse Lab in Milton Keynes	Wellcome Sanger Institute for the COVID-19 Genomics UK (COG-UK) Consortium	The Lighthouse Lab in Milton Keynes and Alex Alderton, Roberto Amato, Sonia Goncalves, Ewan Harrison, David K. Jackson, Ian Johnston, Dominic Kwiatkowski, Cordelia Langford, John Sillitoe on behalf of the Wellcome Sanger Institute COVID-19 Surveillance Team
EPI_ISL_879612	Lighthouse Lab in Cambridge	Wellcome Sanger Institute for the COVID-19 Genomics UK (COG-UK) Consortium	Rob Howes, The Lighthouse Lab in Cambridge and Alex Alderton, Roberto Amato, Sonia Goncalves, Ewan Harrison, David K. Jackson, Ian Johnston, Dominic Kwiatkowski, Cordelia Langford, John Sillitoe on behalf of the Wellcome Sanger Institute COVID-19 Surveillance Team
EPI_ISL_879613	Lighthouse Lab in Milton Keynes	Wellcome Sanger Institute for the COVID-19 Genomics UK (COG-UK) Consortium	The Lighthouse Lab in Milton Keynes and Alex Alderton, Roberto Amato, Sonia Goncalves, Ewan Harrison, David K. Jackson, Ian Johnston, Dominic Kwiatkowski, Cordelia Langford, John Sillitoe on behalf of the Wellcome Sanger Institute COVID-19 Surveillance Team
EPI_ISL_879614	Lighthouse Lab in Alderley Park	Wellcome Sanger Institute for the COVID-19 Genomics UK (COG-UK) Consortium	Jacquelyn Wynn, Mairead Hyland, The Lighthouse Lab in Alderley Park and Alex Alderton, Roberto Amato, Sonia Goncalves, Ewan Harrison, David K. Jackson, Ian Johnston, Dominic Kwiatkowski, Cordelia Langford, John Sillitoe on behalf of the Wellcome Sanger Institute COVID-19 Surveillance Team
EPI_ISL_879615	Lighthouse Lab in Cambridge	Wellcome Sanger Institute for the COVID-19 Genomics UK (COG-UK) Consortium	Rob Howes, The Lighthouse Lab in Cambridge and Alex Alderton, Roberto Amato, Sonia Goncalves, Ewan Harrison, David K. Jackson, Ian Johnston, Dominic Kwiatkowski, Cordelia Langford, John Sillitoe on behalf of the Wellcome Sanger Institute COVID-19 Surveillance Team
EPI_ISL_879617	Lighthouse Lab in Alderley Park	Wellcome Sanger Institute for the COVID-19 Genomics UK (COG-UK) Consortium	Jacquelyn Wynn, Mairead Hyland, The Lighthouse Lab in Alderley Park and Alex Alderton, Roberto Amato, Sonia Goncalves, Ewan Harrison, David K. Jackson, Ian Johnston, Dominic Kwiatkowski, Cordelia Langford, John Sillitoe on behalf of the Wellcome Sanger Institute COVID-19 Surveillance Team
EPI_ISL_879618	Lighthouse Lab in Milton Keynes	Wellcome Sanger Institute for the COVID-19 Genomics UK (COG-UK) Consortium	The Lighthouse Lab in Milton Keynes and Alex Alderton, Roberto Amato, Sonia Goncalves, Ewan Harrison, David K. Jackson, Ian Johnston, Dominic Kwiatkowski, Cordelia Langford, John Sillitoe on behalf of the Wellcome Sanger Institute COVID-19 Surveillance Team
EPI_ISL_879620	Lighthouse Lab in Cambridge	Wellcome Sanger Institute for the COVID-19 Genomics UK (COG-UK) Consortium	Rob Howes, The Lighthouse Lab in Cambridge and Alex Alderton, Roberto Amato, Sonia Goncalves, Ewan Harrison, David K. Jackson, Ian Johnston, Dominic Kwiatkowski, Cordelia Langford, John Sillitoe on behalf of the Wellcome Sanger Institute COVID-19 Surveillance Team
EPI_ISL_879621	Lighthouse Lab in Milton Keynes	Wellcome Sanger Institute for the COVID-19 Genomics UK (COG-UK) Consortium	The Lighthouse Lab in Milton Keynes and Alex Alderton, Roberto Amato, Sonia Goncalves, Ewan Harrison, David K. Jackson, Ian Johnston, Dominic Kwiatkowski, Cordelia Langford, John Sillitoe on behalf of the Wellcome Sanger Institute COVID-19 Surveillance Team
EPI_ISL_879622	Lighthouse Lab in Cambridge	Wellcome Sanger Institute for the COVID-19 Genomics UK (COG-UK) Consortium	Rob Howes, The Lighthouse Lab in Cambridge and Alex Alderton, Roberto Amato, Sonia Goncalves, Ewan Harrison, David K. Jackson, Ian Johnston, Dominic Kwiatkowski, Cordelia Langford, John Sillitoe on behalf of the Wellcome Sanger Institute COVID-19 Surveillance Team
EPI_ISL_879623	Lighthouse Lab in Milton Keynes	Wellcome Sanger Institute for the COVID-19 Genomics UK (COG-UK) Consortium	The Lighthouse Lab in Milton Keynes and Alex Alderton, Roberto Amato, Sonia Goncalves, Ewan Harrison, David K. Jackson, Ian Johnston, Dominic Kwiatkowski, Cordelia Langford, John Sillitoe on behalf of the Wellcome Sanger Institute COVID-19 Surveillance Team
EPI_ISL_879624, EPI_ISL_879625, EPI_ISL_879626	Lighthouse Lab in Cambridge	Wellcome Sanger Institute for the COVID-19 Genomics UK (COG-UK) Consortium	Rob Howes, The Lighthouse Lab in Cambridge and Alex Alderton, Roberto Amato, Sonia Goncalves, Ewan Harrison, David K. Jackson, Ian Johnston, Dominic Kwiatkowski, Cordelia Langford, John Sillitoe on behalf of the Wellcome Sanger Institute COVID-19 Surveillance Team
EPI_ISL_879628	Lighthouse Lab in Milton Keynes	Wellcome Sanger Institute for the COVID-19 Genomics UK (COG-UK) Consortium	The Lighthouse Lab in Milton Keynes and Alex Alderton, Roberto Amato, Sonia Goncalves, Ewan Harrison, David K. Jackson, Ian Johnston, Dominic Kwiatkowski, Cordelia Langford, John Sillitoe on behalf of the Wellcome Sanger Institute COVID-19 Surveillance Team
EPI_ISL_879629	Lighthouse Lab in Alderley Park	Wellcome Sanger Institute for the COVID-19 Genomics UK (COG-UK) Consortium	Jacquelyn Wynn, Mairead Hyland, The Lighthouse Lab in Alderley Park and Alex Alderton, Roberto Amato, Sonia Goncalves, Ewan Harrison, David K. Jackson, Ian Johnston, Dominic Kwiatkowski, Cordelia Langford, John Sillitoe on behalf of the Wellcome Sanger Institute COVID-19 Surveillance Team
EPI_ISL_879630	Lighthouse Lab in Cambridge	Wellcome Sanger Institute for the COVID-19 Genomics UK (COG-UK) Consortium	Rob Howes, The Lighthouse Lab in Cambridge and Alex Alderton, Roberto Amato, Sonia Goncalves, Ewan Harrison, David K. Jackson, Ian Johnston, Dominic Kwiatkowski, Cordelia Langford, John Sillitoe on behalf of the Wellcome Sanger Institute COVID-19 Surveillance Team
EPI_ISL_879631	Lighthouse Lab in Alderley Park	Wellcome Sanger Institute for the COVID-19 Genomics UK (COG-UK) Consortium	Jacquelyn Wynn, Mairead Hyland, The Lighthouse Lab in Alderley Park and Alex Alderton, Roberto Amato, Sonia Goncalves, Ewan Harrison, David K. Jackson, Ian Johnston, Dominic Kwiatkowski, Cordelia Langford, John Sillitoe on behalf of the Wellcome Sanger Institute COVID-19 Surveillance Team
EPI_ISL_879632, EPI_ISL_879633, EPI_ISL_879634, EPI_ISL_879635, EPI_ISL_879636, EPI_ISL_879637, EPI_ISL_879639, EPI_ISL_879640	Lighthouse Lab in Cambridge	Wellcome Sanger Institute for the COVID-19 Genomics UK (COG-UK) Consortium	Rob Howes, The Lighthouse Lab in Cambridge and Alex Alderton, Roberto Amato, Sonia Goncalves, Ewan Harrison, David K. Jackson, Ian Johnston, Dominic Kwiatkowski, Cordelia Langford, John Sillitoe on behalf of the Wellcome Sanger Institute COVID-19 Surveillance Team
EPI_ISL_879642, EPI_ISL_879643	Lighthouse Lab in Milton Keynes	Wellcome Sanger Institute for the COVID-19 Genomics UK (COG-UK) Consortium	The Lighthouse Lab in Milton Keynes and Alex Alderton, Roberto Amato, Sonia Goncalves, Ewan Harrison, David K. Jackson, Ian Johnston, Dominic Kwiatkowski, Cordelia Langford, John Sillitoe on behalf of the Wellcome Sanger Institute COVID-19 Surveillance Team
EPI_ISL_879644, EPI_ISL_879645	Lighthouse Lab in Cambridge	Wellcome Sanger Institute for the COVID-19 Genomics UK (COG-UK) Consortium	Rob Howes, The Lighthouse Lab in Cambridge and Alex Alderton, Roberto Amato, Sonia Goncalves, Ewan Harrison, David K. Jackson, Ian Johnston, Dominic Kwiatkowski, Cordelia Langford, John Sillitoe on behalf of the Wellcome Sanger Institute COVID-19 Surveillance Team
EPI_ISL_879646	Lighthouse Lab in Alderley Park	Wellcome Sanger Institute for the COVID-19 Genomics UK (COG-UK) Consortium	Jacquelyn Wynn, Mairead Hyland, The Lighthouse Lab in Alderley Park and Alex Alderton, Roberto Amato, Sonia Goncalves, Ewan Harrison, David K. Jackson, Ian Johnston, Dominic Kwiatkowski, Cordelia Langford, John Sillitoe on behalf of the Wellcome Sanger Institute COVID-19 Surveillance Team
EPI_ISL_879648, EPI_ISL_879650, EPI_ISL_879651, EPI_ISL_879652	Lighthouse Lab in Cambridge	Wellcome Sanger Institute for the COVID-19 Genomics UK (COG-UK) Consortium	Rob Howes, The Lighthouse Lab in Cambridge and Alex Alderton, Roberto Amato, Sonia Goncalves, Ewan Harrison, David K. Jackson, Ian Johnston, Dominic Kwiatkowski, Cordelia Langford, John Sillitoe on behalf of the Wellcome Sanger Institute COVID-19 Surveillance Team
EPI_ISL_879653, EPI_ISL_879654, EPI_ISL_879655, EPI_ISL_879656	Lighthouse Lab in Milton Keynes	Wellcome Sanger Institute for the COVID-19 Genomics UK (COG-UK) Consortium	The Lighthouse Lab in Milton Keynes and Alex Alderton, Roberto Amato, Sonia Goncalves, Ewan Harrison, David K. Jackson, Ian Johnston, Dominic Kwiatkowski, Cordelia Langford, John Sillitoe on behalf of the Wellcome Sanger Institute COVID-19 Surveillance Team
EPI_ISL_879658, EPI_ISL_879659, EPI_ISL_879660	Lighthouse Lab in Cambridge	Wellcome Sanger Institute for the COVID-19 Genomics UK (COG-UK) Consortium	Rob Howes, The Lighthouse Lab in Cambridge and Alex Alderton, Roberto Amato, Sonia Goncalves, Ewan Harrison, David K. Jackson, Ian Johnston, Dominic Kwiatkowski, Cordelia Langford, John Sillitoe on behalf of the Wellcome Sanger Institute COVID-19 Surveillance Team
EPI_ISL_879663, EPI_ISL_879664	Lighthouse Lab in Milton Keynes	Wellcome Sanger Institute for the COVID-19 Genomics UK (COG-UK) Consortium	The Lighthouse Lab in Milton Keynes and Alex Alderton, Roberto Amato, Sonia Goncalves, Ewan Harrison, David K. Jackson, Ian Johnston, Dominic Kwiatkowski, Cordelia Langford, John Sillitoe on behalf of the Wellcome Sanger Institute COVID-19 Surveillance Team
EPI_ISL_879666	Lighthouse Lab in Alderley Park	Wellcome Sanger Institute for the COVID-19 Genomics UK (COG-UK) Consortium	Jacquelyn Wynn, Mairead Hyland, The Lighthouse Lab in Alderley Park and Alex Alderton, Roberto Amato, Sonia Goncalves, Ewan Harrison, David K. Jackson, Ian Johnston, Dominic Kwiatkowski, Cordelia Langford, John Sillitoe on behalf of the Wellcome Sanger Institute COVID-19 Surveillance Team
EPI_ISL_879667, EPI_ISL_879668, EPI_ISL_879669, EPI_ISL_879670	Lighthouse Lab in Cambridge	Wellcome Sanger Institute for the COVID-19 Genomics UK (COG-UK) Consortium	Rob Howes, The Lighthouse Lab in Cambridge and Alex Alderton, Roberto Amato, Sonia Goncalves, Ewan Harrison, David K. Jackson, Ian Johnston, Dominic Kwiatkowski, Cordelia Langford, John Sillitoe on behalf of the Wellcome Sanger Institute COVID-19 Surveillance Team
EPI_ISL_879672	Lighthouse Lab in Alderley Park	Wellcome Sanger Institute for the COVID-19 Genomics UK (COG-UK) Consortium	Jacquelyn Wynn, Mairead Hyland, The Lighthouse Lab in Alderley Park and Alex Alderton, Roberto Amato, Sonia Goncalves, Ewan Harrison, David K. Jackson, Ian Johnston, Dominic Kwiatkowski, Cordelia Langford, John Sillitoe on behalf of the Wellcome Sanger Institute COVID-19 Surveillance Team
EPI_ISL_879673	Lighthouse Lab in Cambridge	Wellcome Sanger Institute for the COVID-19 Genomics UK	Rob Howes, The Lighthouse Lab in Cambridge and Alex Alderton, Roberto Amato, Sonia Goncalves, Ewan Harrison, David K. Jackson, Ian Johnston,

		(COG-UK) Consortium	Dominic Kwiatkowski, Cordelia Langford, John Sillitoe on behalf of the Wellcome Sanger Institute COVID-19 Surveillance Team
EPI_ISL_879675	Lighthouse Lab in Milton Keynes	Wellcome Sanger Institute for the COVID-19 Genomics UK (COG-UK) Consortium	The Lighthouse Lab in Milton Keynes and Alex Alderton, Roberto Amato, Sonia Goncalves, Ewan Harrison, David K. Jackson, Ian Johnston, Dominic Kwiatkowski, Cordelia Langford, John Sillitoe on behalf of the Wellcome Sanger Institute COVID-19 Surveillance Team
EPI_ISL_879676, EPI_ISL_879677	Lighthouse Lab in Cambridge	Wellcome Sanger Institute for the COVID-19 Genomics UK (COG-UK) Consortium	Rob Howes, The Lighthouse Lab in Cambridge and Alex Alderton, Roberto Amato, Sonia Goncalves, Ewan Harrison, David K. Jackson, Ian Johnston, Dominic Kwiatkowski, Cordelia Langford, John Sillitoe on behalf of the Wellcome Sanger Institute COVID-19 Surveillance Team
EPI_ISL_879678, EPI_ISL_879679	Lighthouse Lab in Milton Keynes	Wellcome Sanger Institute for the COVID-19 Genomics UK (COG-UK) Consortium	The Lighthouse Lab in Milton Keynes and Alex Alderton, Roberto Amato, Sonia Goncalves, Ewan Harrison, David K. Jackson, Ian Johnston, Dominic Kwiatkowski, Cordelia Langford, John Sillitoe on behalf of the Wellcome Sanger Institute COVID-19 Surveillance Team
EPI_ISL_879680	Lighthouse Lab in Cambridge	Wellcome Sanger Institute for the COVID-19 Genomics UK (COG-UK) Consortium	Rob Howes, The Lighthouse Lab in Cambridge and Alex Alderton, Roberto Amato, Sonia Goncalves, Ewan Harrison, David K. Jackson, Ian Johnston, Dominic Kwiatkowski, Cordelia Langford, John Sillitoe on behalf of the Wellcome Sanger Institute COVID-19 Surveillance Team
EPI_ISL_879681	Lighthouse Lab in Milton Keynes	Wellcome Sanger Institute for the COVID-19 Genomics UK (COG-UK) Consortium	The Lighthouse Lab in Milton Keynes and Alex Alderton, Roberto Amato, Sonia Goncalves, Ewan Harrison, David K. Jackson, Ian Johnston, Dominic Kwiatkowski, Cordelia Langford, John Sillitoe on behalf of the Wellcome Sanger Institute COVID-19 Surveillance Team
EPI_ISL_879684, EPI_ISL_879685	Lighthouse Lab in Cambridge	Wellcome Sanger Institute for the COVID-19 Genomics UK (COG-UK) Consortium	Rob Howes, The Lighthouse Lab in Cambridge and Alex Alderton, Roberto Amato, Sonia Goncalves, Ewan Harrison, David K. Jackson, Ian Johnston, Dominic Kwiatkowski, Cordelia Langford, John Sillitoe on behalf of the Wellcome Sanger Institute COVID-19 Surveillance Team
EPI_ISL_879686	Lighthouse Lab in Milton Keynes	Wellcome Sanger Institute for the COVID-19 Genomics UK (COG-UK) Consortium	The Lighthouse Lab in Milton Keynes and Alex Alderton, Roberto Amato, Sonia Goncalves, Ewan Harrison, David K. Jackson, Ian Johnston, Dominic Kwiatkowski, Cordelia Langford, John Sillitoe on behalf of the Wellcome Sanger Institute COVID-19 Surveillance Team
EPI_ISL_879688	Lighthouse Lab in Cambridge	Wellcome Sanger Institute for the COVID-19 Genomics UK (COG-UK) Consortium	Rob Howes, The Lighthouse Lab in Cambridge and Alex Alderton, Roberto Amato, Sonia Goncalves, Ewan Harrison, David K. Jackson, Ian Johnston, Dominic Kwiatkowski, Cordelia Langford, John Sillitoe on behalf of the Wellcome Sanger Institute COVID-19 Surveillance Team
EPI_ISL_879689	Lighthouse Lab in Milton Keynes	Wellcome Sanger Institute for the COVID-19 Genomics UK (COG-UK) Consortium	The Lighthouse Lab in Milton Keynes and Alex Alderton, Roberto Amato, Sonia Goncalves, Ewan Harrison, David K. Jackson, Ian Johnston, Dominic Kwiatkowski, Cordelia Langford, John Sillitoe on behalf of the Wellcome Sanger Institute COVID-19 Surveillance Team
EPI_ISL_879690, EPI_ISL_879691	Lighthouse Lab in Cambridge	Wellcome Sanger Institute for the COVID-19 Genomics UK (COG-UK) Consortium	Rob Howes, The Lighthouse Lab in Cambridge and Alex Alderton, Roberto Amato, Sonia Goncalves, Ewan Harrison, David K. Jackson, Ian Johnston, Dominic Kwiatkowski, Cordelia Langford, John Sillitoe on behalf of the Wellcome Sanger Institute COVID-19 Surveillance Team
EPI_ISL_879692, EPI_ISL_879693	Lighthouse Lab in Alderley Park	Wellcome Sanger Institute for the COVID-19 Genomics UK (COG-UK) Consortium	Jacquelyn Wynn, Mairead Hyland, The Lighthouse Lab in Alderley Park and Alex Alderton, Roberto Amato, Sonia Goncalves, Ewan Harrison, David K. Jackson, Ian Johnston, Dominic Kwiatkowski, Cordelia Langford, John Sillitoe on behalf of the Wellcome Sanger Institute COVID-19 Surveillance Team
EPI_ISL_879694, EPI_ISL_879696, EPI_ISL_879697, EPI_ISL_879698, EPI_ISL_879699	Lighthouse Lab in Cambridge	Wellcome Sanger Institute for the COVID-19 Genomics UK (COG-UK) Consortium	Rob Howes, The Lighthouse Lab in Cambridge and Alex Alderton, Roberto Amato, Sonia Goncalves, Ewan Harrison, David K. Jackson, Ian Johnston, Dominic Kwiatkowski, Cordelia Langford, John Sillitoe on behalf of the Wellcome Sanger Institute COVID-19 Surveillance Team
EPI_ISL_879700	Lighthouse Lab in Alderley Park	Wellcome Sanger Institute for the COVID-19 Genomics UK (COG-UK) Consortium	Jacquelyn Wynn, Mairead Hyland, The Lighthouse Lab in Alderley Park and Alex Alderton, Roberto Amato, Sonia Goncalves, Ewan Harrison, David K. Jackson, Ian Johnston, Dominic Kwiatkowski, Cordelia Langford, John Sillitoe on behalf of the Wellcome Sanger Institute COVID-19 Surveillance Team
EPI_ISL_879701	Lighthouse Lab in Cambridge	Wellcome Sanger Institute for the COVID-19 Genomics UK (COG-UK) Consortium	Rob Howes, The Lighthouse Lab in Cambridge and Alex Alderton, Roberto Amato, Sonia Goncalves, Ewan Harrison, David K. Jackson, Ian Johnston, Dominic Kwiatkowski, Cordelia Langford, John Sillitoe on behalf of the Wellcome Sanger Institute COVID-19 Surveillance Team
EPI_ISL_879703	Lighthouse Lab in Milton Keynes	Wellcome Sanger Institute for the COVID-19 Genomics UK (COG-UK) Consortium	The Lighthouse Lab in Milton Keynes and Alex Alderton, Roberto Amato, Sonia Goncalves, Ewan Harrison, David K. Jackson, Ian Johnston, Dominic Kwiatkowski, Cordelia Langford, John Sillitoe on behalf of the Wellcome Sanger Institute COVID-19 Surveillance Team
EPI_ISL_879704, EPI_ISL_879705, EPI_ISL_879706	Lighthouse Lab in Cambridge	Wellcome Sanger Institute for the COVID-19 Genomics UK (COG-UK) Consortium	Rob Howes, The Lighthouse Lab in Cambridge and Alex Alderton, Roberto Amato, Sonia Goncalves, Ewan Harrison, David K. Jackson, Ian Johnston, Dominic Kwiatkowski, Cordelia Langford, John Sillitoe on behalf of the Wellcome Sanger Institute COVID-19 Surveillance Team
EPI_ISL_879707	Lighthouse Lab in Milton Keynes	Wellcome Sanger Institute for the COVID-19 Genomics UK (COG-UK) Consortium	The Lighthouse Lab in Milton Keynes and Alex Alderton, Roberto Amato, Sonia Goncalves, Ewan Harrison, David K. Jackson, Ian Johnston, Dominic Kwiatkowski, Cordelia Langford, John Sillitoe on behalf of the Wellcome Sanger Institute COVID-19 Surveillance Team
EPI_ISL_879708, EPI_ISL_879709	Lighthouse Lab in Cambridge	Wellcome Sanger Institute for the COVID-19 Genomics UK (COG-UK) Consortium	Rob Howes, The Lighthouse Lab in Cambridge and Alex Alderton, Roberto Amato, Sonia Goncalves, Ewan Harrison, David K. Jackson, Ian Johnston, Dominic Kwiatkowski, Cordelia Langford, John Sillitoe on behalf of the Wellcome Sanger Institute COVID-19 Surveillance Team
EPI_ISL_879710, EPI_ISL_879711, EPI_ISL_879712, EPI_ISL_879713	Lighthouse Lab in Milton Keynes	Wellcome Sanger Institute for the COVID-19 Genomics UK (COG-UK) Consortium	The Lighthouse Lab in Milton Keynes and Alex Alderton, Roberto Amato, Sonia Goncalves, Ewan Harrison, David K. Jackson, Ian Johnston, Dominic Kwiatkowski, Cordelia Langford, John Sillitoe on behalf of the Wellcome Sanger Institute COVID-19 Surveillance Team
EPI_ISL_879714	Lighthouse Lab in Alderley Park	Wellcome Sanger Institute for the COVID-19 Genomics UK (COG-UK) Consortium	Jacquelyn Wynn, Mairead Hyland, The Lighthouse Lab in Alderley Park and Alex Alderton, Roberto Amato, Sonia Goncalves, Ewan Harrison, David K. Jackson, Ian Johnston, Dominic Kwiatkowski, Cordelia Langford, John Sillitoe on behalf of the Wellcome Sanger Institute COVID-19 Surveillance Team
EPI_ISL_879715	Lighthouse Lab in Milton Keynes	Wellcome Sanger Institute for the COVID-19 Genomics UK (COG-UK) Consortium	The Lighthouse Lab in Milton Keynes and Alex Alderton, Roberto Amato, Sonia Goncalves, Ewan Harrison, David K. Jackson, Ian Johnston, Dominic Kwiatkowski, Cordelia Langford, John Sillitoe on behalf of the Wellcome Sanger Institute COVID-19 Surveillance Team
EPI_ISL_879716	Lighthouse Lab in Cambridge	Wellcome Sanger Institute for the COVID-19 Genomics UK (COG-UK) Consortium	Rob Howes, The Lighthouse Lab in Cambridge and Alex Alderton, Roberto Amato, Sonia Goncalves, Ewan Harrison, David K. Jackson, Ian Johnston, Dominic Kwiatkowski, Cordelia Langford, John Sillitoe on behalf of the Wellcome Sanger Institute COVID-19 Surveillance Team
EPI_ISL_879717	Lighthouse Lab in Milton Keynes	Wellcome Sanger Institute for the COVID-19 Genomics UK (COG-UK) Consortium	The Lighthouse Lab in Milton Keynes and Alex Alderton, Roberto Amato, Sonia Goncalves, Ewan Harrison, David K. Jackson, Ian Johnston, Dominic Kwiatkowski, Cordelia Langford, John Sillitoe on behalf of the Wellcome Sanger Institute COVID-19 Surveillance Team
EPI_ISL_879718, EPI_ISL_879719	Lighthouse Lab in Alderley Park	Wellcome Sanger Institute for the COVID-19 Genomics UK (COG-UK) Consortium	Jacquelyn Wynn, Mairead Hyland, The Lighthouse Lab in Alderley Park and Alex Alderton, Roberto Amato, Sonia Goncalves, Ewan Harrison, David K. Jackson, Ian Johnston, Dominic Kwiatkowski, Cordelia Langford, John Sillitoe on behalf of the Wellcome Sanger Institute COVID-19 Surveillance Team
EPI_ISL_879720	Lighthouse Lab in Cambridge	Wellcome Sanger Institute for the COVID-19 Genomics UK (COG-UK) Consortium	Rob Howes, The Lighthouse Lab in Cambridge and Alex Alderton, Roberto Amato, Sonia Goncalves, Ewan Harrison, David K. Jackson, Ian Johnston, Dominic Kwiatkowski, Cordelia Langford, John Sillitoe on behalf of the Wellcome Sanger Institute COVID-19 Surveillance Team
EPI_ISL_879721	Lighthouse Lab in Alderley Park	Wellcome Sanger Institute for the COVID-19 Genomics UK (COG-UK) Consortium	Jacquelyn Wynn, Mairead Hyland, The Lighthouse Lab in Alderley Park and Alex Alderton, Roberto Amato, Sonia Goncalves, Ewan Harrison, David K. Jackson, Ian Johnston, Dominic Kwiatkowski, Cordelia Langford, John Sillitoe on behalf of the Wellcome Sanger Institute COVID-19 Surveillance Team
EPI_ISL_879722, EPI_ISL_879723, EPI_ISL_879724, EPI_ISL_879725	Lighthouse Lab in Cambridge	Wellcome Sanger Institute for the COVID-19 Genomics UK (COG-UK) Consortium	Rob Howes, The Lighthouse Lab in Cambridge and Alex Alderton, Roberto Amato, Sonia Goncalves, Ewan Harrison, David K. Jackson, Ian Johnston, Dominic Kwiatkowski, Cordelia Langford, John Sillitoe on behalf of the Wellcome Sanger Institute COVID-19 Surveillance Team
EPI_ISL_879726	Lighthouse Lab in Alderley Park	Wellcome Sanger Institute for the COVID-19 Genomics UK (COG-UK) Consortium	Jacquelyn Wynn, Mairead Hyland, The Lighthouse Lab in Alderley Park and Alex Alderton, Roberto Amato, Sonia Goncalves, Ewan Harrison, David K. Jackson, Ian Johnston, Dominic Kwiatkowski, Cordelia Langford, John Sillitoe on behalf of the Wellcome Sanger Institute COVID-19 Surveillance Team
EPI_ISL_879727	Lighthouse Lab in Cambridge	Wellcome Sanger Institute for the COVID-19 Genomics UK (COG-UK) Consortium	Rob Howes, The Lighthouse Lab in Cambridge and Alex Alderton, Roberto Amato, Sonia Goncalves, Ewan Harrison, David K. Jackson, Ian Johnston, Dominic Kwiatkowski, Cordelia Langford, John Sillitoe on behalf of the Wellcome Sanger Institute COVID-19 Surveillance Team
EPI_ISL_879728	Lighthouse Lab in Alderley Park	Wellcome Sanger Institute for the COVID-19 Genomics UK (COG-UK) Consortium	Jacquelyn Wynn, Mairead Hyland, The Lighthouse Lab in Alderley Park and Alex Alderton, Roberto Amato, Sonia Goncalves, Ewan Harrison, David K. Jackson, Ian Johnston, Dominic Kwiatkowski, Cordelia Langford, John Sillitoe on behalf of the Wellcome Sanger Institute COVID-19 Surveillance Team
EPI_ISL_879729, EPI_ISL_879730, EPI_ISL_879731, EPI_ISL_879732, EPI_ISL_879733, EPI_ISL_879734, EPI_ISL_879735	Lighthouse Lab in Cambridge	Wellcome Sanger Institute for the COVID-19 Genomics UK (COG-UK) Consortium	Rob Howes, The Lighthouse Lab in Cambridge and Alex Alderton, Roberto Amato, Sonia Goncalves, Ewan Harrison, David K. Jackson, Ian Johnston, Dominic Kwiatkowski, Cordelia Langford, John Sillitoe on behalf of the Wellcome Sanger Institute COVID-19 Surveillance Team
EPI_ISL_879736, EPI_ISL_879737	Lighthouse Lab in Alderley Park	Wellcome Sanger Institute for the COVID-19 Genomics UK (COG-UK) Consortium	Jacquelyn Wynn, Mairead Hyland, The Lighthouse Lab in Alderley Park and Alex Alderton, Roberto Amato, Sonia Goncalves, Ewan Harrison, David K. Jackson, Ian Johnston, Dominic Kwiatkowski, Cordelia Langford, John Sillitoe on behalf of the Wellcome Sanger Institute COVID-19 Surveillance Team
EPI_ISL_879738, EPI_ISL_879739	Lighthouse Lab in Cambridge	Wellcome Sanger Institute for the COVID-19 Genomics UK (COG-UK) Consortium	Rob Howes, The Lighthouse Lab in Cambridge and Alex Alderton, Roberto Amato, Sonia Goncalves, Ewan Harrison, David K. Jackson, Ian Johnston, Dominic Kwiatkowski, Cordelia Langford, John Sillitoe on behalf of the Wellcome Sanger Institute COVID-19 Surveillance Team
EPI_ISL_879740	Lighthouse Lab in Alderley Park	Wellcome Sanger Institute for the COVID-19 Genomics UK	Jacquelyn Wynn, Mairead Hyland, The Lighthouse Lab in Alderley Park and Alex Alderton, Roberto Amato, Sonia Goncalves, Ewan Harrison, David K.

		(COG-UK) Consortium	Jackson, Ian Johnston, Dominic Kwiatkowski, Cordelia Langford, John Sillitoe on behalf of the Wellcome Sanger Institute COVID-19 Surveillance Team
EPI_ISL_879741	Lighthouse Lab in Cambridge	Wellcome Sanger Institute for the COVID-19 Genomics UK (COG-UK) Consortium	Rob Howes, The Lighthouse Lab in Cambridge and Alex Alderton, Roberto Amato, Sonia Goncalves, Ewan Harrison, David K. Jackson, Ian Johnston, Dominic Kwiatkowski, Cordelia Langford, John Sillitoe on behalf of the Wellcome Sanger Institute COVID-19 Surveillance Team
EPI_ISL_879742	Lighthouse Lab in Alderley Park	Wellcome Sanger Institute for the COVID-19 Genomics UK (COG-UK) Consortium	Jacquelyn Wynn, Mairead Hyland, The Lighthouse Lab in Alderley Park and Alex Alderton, Roberto Amato, Sonia Goncalves, Ewan Harrison, David K. Jackson, Ian Johnston, Dominic Kwiatkowski, Cordelia Langford, John Sillitoe on behalf of the Wellcome Sanger Institute COVID-19 Surveillance Team
EPI_ISL_879743	Lighthouse Lab in Cambridge	Wellcome Sanger Institute for the COVID-19 Genomics UK (COG-UK) Consortium	Rob Howes, The Lighthouse Lab in Cambridge and Alex Alderton, Roberto Amato, Sonia Goncalves, Ewan Harrison, David K. Jackson, Ian Johnston, Dominic Kwiatkowski, Cordelia Langford, John Sillitoe on behalf of the Wellcome Sanger Institute COVID-19 Surveillance Team
EPI_ISL_879744	Lighthouse Lab in Alderley Park	Wellcome Sanger Institute for the COVID-19 Genomics UK (COG-UK) Consortium	Jacquelyn Wynn, Mairead Hyland, The Lighthouse Lab in Alderley Park and Alex Alderton, Roberto Amato, Sonia Goncalves, Ewan Harrison, David K. Jackson, Ian Johnston, Dominic Kwiatkowski, Cordelia Langford, John Sillitoe on behalf of the Wellcome Sanger Institute COVID-19 Surveillance Team
EPI_ISL_879745, EPI_ISL_879746	Lighthouse Lab in Cambridge	Wellcome Sanger Institute for the COVID-19 Genomics UK (COG-UK) Consortium	Rob Howes, The Lighthouse Lab in Cambridge and Alex Alderton, Roberto Amato, Sonia Goncalves, Ewan Harrison, David K. Jackson, Ian Johnston, Dominic Kwiatkowski, Cordelia Langford, John Sillitoe on behalf of the Wellcome Sanger Institute COVID-19 Surveillance Team
EPI_ISL_879747, EPI_ISL_879748	Lighthouse Lab in Alderley Park	Wellcome Sanger Institute for the COVID-19 Genomics UK (COG-UK) Consortium	Jacquelyn Wynn, Mairead Hyland, The Lighthouse Lab in Alderley Park and Alex Alderton, Roberto Amato, Sonia Goncalves, Ewan Harrison, David K. Jackson, Ian Johnston, Dominic Kwiatkowski, Cordelia Langford, John Sillitoe on behalf of the Wellcome Sanger Institute COVID-19 Surveillance Team
EPI_ISL_879749	Lighthouse Lab in Cambridge	Wellcome Sanger Institute for the COVID-19 Genomics UK (COG-UK) Consortium	Rob Howes, The Lighthouse Lab in Cambridge and Alex Alderton, Roberto Amato, Sonia Goncalves, Ewan Harrison, David K. Jackson, Ian Johnston, Dominic Kwiatkowski, Cordelia Langford, John Sillitoe on behalf of the Wellcome Sanger Institute COVID-19 Surveillance Team
EPI_ISL_879750, EPI_ISL_879751, EPI_ISL_879752, EPI_ISL_879753	Lighthouse Lab in Alderley Park	Wellcome Sanger Institute for the COVID-19 Genomics UK (COG-UK) Consortium	Jacquelyn Wynn, Mairead Hyland, The Lighthouse Lab in Alderley Park and Alex Alderton, Roberto Amato, Sonia Goncalves, Ewan Harrison, David K. Jackson, Ian Johnston, Dominic Kwiatkowski, Cordelia Langford, John Sillitoe on behalf of the Wellcome Sanger Institute COVID-19 Surveillance Team
EPI_ISL_879754	Lighthouse Lab in Cambridge	Wellcome Sanger Institute for the COVID-19 Genomics UK (COG-UK) Consortium	Rob Howes, The Lighthouse Lab in Cambridge and Alex Alderton, Roberto Amato, Sonia Goncalves, Ewan Harrison, David K. Jackson, Ian Johnston, Dominic Kwiatkowski, Cordelia Langford, John Sillitoe on behalf of the Wellcome Sanger Institute COVID-19 Surveillance Team
EPI_ISL_879755, EPI_ISL_879756, EPI_ISL_879757, EPI_ISL_879758, EPI_ISL_879759, EPI_ISL_879760, EPI_ISL_879761, EPI_ISL_879762	Lighthouse Lab in Alderley Park	Wellcome Sanger Institute for the COVID-19 Genomics UK (COG-UK) Consortium	Jacquelyn Wynn, Mairead Hyland, The Lighthouse Lab in Alderley Park and Alex Alderton, Roberto Amato, Sonia Goncalves, Ewan Harrison, David K. Jackson, Ian Johnston, Dominic Kwiatkowski, Cordelia Langford, John Sillitoe on behalf of the Wellcome Sanger Institute COVID-19 Surveillance Team
EPI_ISL_879763, EPI_ISL_879764	Lighthouse Lab in Cambridge	Wellcome Sanger Institute for the COVID-19 Genomics UK (COG-UK) Consortium	Rob Howes, The Lighthouse Lab in Cambridge and Alex Alderton, Roberto Amato, Sonia Goncalves, Ewan Harrison, David K. Jackson, Ian Johnston, Dominic Kwiatkowski, Cordelia Langford, John Sillitoe on behalf of the Wellcome Sanger Institute COVID-19 Surveillance Team
EPI_ISL_879765, EPI_ISL_879766, EPI_ISL_879767	Lighthouse Lab in Alderley Park	Wellcome Sanger Institute for the COVID-19 Genomics UK (COG-UK) Consortium	Jacquelyn Wynn, Mairead Hyland, The Lighthouse Lab in Alderley Park and Alex Alderton, Roberto Amato, Sonia Goncalves, Ewan Harrison, David K. Jackson, Ian Johnston, Dominic Kwiatkowski, Cordelia Langford, John Sillitoe on behalf of the Wellcome Sanger Institute COVID-19 Surveillance Team
EPI_ISL_879768, EPI_ISL_879769, EPI_ISL_879770, EPI_ISL_879771, EPI_ISL_879772, EPI_ISL_879773, EPI_ISL_879774	Lighthouse Lab in Cambridge	Wellcome Sanger Institute for the COVID-19 Genomics UK (COG-UK) Consortium	Rob Howes, The Lighthouse Lab in Cambridge and Alex Alderton, Roberto Amato, Sonia Goncalves, Ewan Harrison, David K. Jackson, Ian Johnston, Dominic Kwiatkowski, Cordelia Langford, John Sillitoe on behalf of the Wellcome Sanger Institute COVID-19 Surveillance Team
EPI_ISL_879775	Lighthouse Lab in Alderley Park	Wellcome Sanger Institute for the COVID-19 Genomics UK (COG-UK) Consortium	Jacquelyn Wynn, Mairead Hyland, The Lighthouse Lab in Alderley Park and Alex Alderton, Roberto Amato, Sonia Goncalves, Ewan Harrison, David K. Jackson, Ian Johnston, Dominic Kwiatkowski, Cordelia Langford, John Sillitoe on behalf of the Wellcome Sanger Institute COVID-19 Surveillance Team
EPI_ISL_879776, EPI_ISL_879777	Lighthouse Lab in Cambridge	Wellcome Sanger Institute for the COVID-19 Genomics UK (COG-UK) Consortium	Rob Howes, The Lighthouse Lab in Cambridge and Alex Alderton, Roberto Amato, Sonia Goncalves, Ewan Harrison, David K. Jackson, Ian Johnston, Dominic Kwiatkowski, Cordelia Langford, John Sillitoe on behalf of the Wellcome Sanger Institute COVID-19 Surveillance Team
EPI_ISL_879778	Lighthouse Lab in Alderley Park	Wellcome Sanger Institute for the COVID-19 Genomics UK (COG-UK) Consortium	Jacquelyn Wynn, Mairead Hyland, The Lighthouse Lab in Alderley Park and Alex Alderton, Roberto Amato, Sonia Goncalves, Ewan Harrison, David K. Jackson, Ian Johnston, Dominic Kwiatkowski, Cordelia Langford, John Sillitoe on behalf of the Wellcome Sanger Institute COVID-19 Surveillance Team
EPI_ISL_879779	Lighthouse Lab in Cambridge	Wellcome Sanger Institute for the COVID-19 Genomics UK (COG-UK) Consortium	Rob Howes, The Lighthouse Lab in Cambridge and Alex Alderton, Roberto Amato, Sonia Goncalves, Ewan Harrison, David K. Jackson, Ian Johnston, Dominic Kwiatkowski, Cordelia Langford, John Sillitoe on behalf of the Wellcome Sanger Institute COVID-19 Surveillance Team
EPI_ISL_879780	Lighthouse Lab in Alderley Park	Wellcome Sanger Institute for the COVID-19 Genomics UK (COG-UK) Consortium	Jacquelyn Wynn, Mairead Hyland, The Lighthouse Lab in Alderley Park and Alex Alderton, Roberto Amato, Sonia Goncalves, Ewan Harrison, David K. Jackson, Ian Johnston, Dominic Kwiatkowski, Cordelia Langford, John Sillitoe on behalf of the Wellcome Sanger Institute COVID-19 Surveillance Team
EPI_ISL_879781, EPI_ISL_879782	Lighthouse Lab in Cambridge	Wellcome Sanger Institute for the COVID-19 Genomics UK (COG-UK) Consortium	Rob Howes, The Lighthouse Lab in Cambridge and Alex Alderton, Roberto Amato, Sonia Goncalves, Ewan Harrison, David K. Jackson, Ian Johnston, Dominic Kwiatkowski, Cordelia Langford, John Sillitoe on behalf of the Wellcome Sanger Institute COVID-19 Surveillance Team
EPI_ISL_879783, EPI_ISL_879784, EPI_ISL_879785	Lighthouse Lab in Alderley Park	Wellcome Sanger Institute for the COVID-19 Genomics UK (COG-UK) Consortium	Jacquelyn Wynn, Mairead Hyland, The Lighthouse Lab in Alderley Park and Alex Alderton, Roberto Amato, Sonia Goncalves, Ewan Harrison, David K. Jackson, Ian Johnston, Dominic Kwiatkowski, Cordelia Langford, John Sillitoe on behalf of the Wellcome Sanger Institute COVID-19 Surveillance Team
EPI_ISL_879786	Lighthouse Lab in Cambridge	Wellcome Sanger Institute for the COVID-19 Genomics UK (COG-UK) Consortium	Rob Howes, The Lighthouse Lab in Cambridge and Alex Alderton, Roberto Amato, Sonia Goncalves, Ewan Harrison, David K. Jackson, Ian Johnston, Dominic Kwiatkowski, Cordelia Langford, John Sillitoe on behalf of the Wellcome Sanger Institute COVID-19 Surveillance Team
EPI_ISL_879787	Lighthouse Lab in Alderley Park	Wellcome Sanger Institute for the COVID-19 Genomics UK (COG-UK) Consortium	Jacquelyn Wynn, Mairead Hyland, The Lighthouse Lab in Alderley Park and Alex Alderton, Roberto Amato, Sonia Goncalves, Ewan Harrison, David K. Jackson, Ian Johnston, Dominic Kwiatkowski, Cordelia Langford, John Sillitoe on behalf of the Wellcome Sanger Institute COVID-19 Surveillance Team
EPI_ISL_879788	Lighthouse Lab in Cambridge	Wellcome Sanger Institute for the COVID-19 Genomics UK (COG-UK) Consortium	Rob Howes, The Lighthouse Lab in Cambridge and Alex Alderton, Roberto Amato, Sonia Goncalves, Ewan Harrison, David K. Jackson, Ian Johnston, Dominic Kwiatkowski, Cordelia Langford, John Sillitoe on behalf of the Wellcome Sanger Institute COVID-19 Surveillance Team
EPI_ISL_879789	Lighthouse Lab in Alderley Park	Wellcome Sanger Institute for the COVID-19 Genomics UK (COG-UK) Consortium	Jacquelyn Wynn, Mairead Hyland, The Lighthouse Lab in Alderley Park and Alex Alderton, Roberto Amato, Sonia Goncalves, Ewan Harrison, David K. Jackson, Ian Johnston, Dominic Kwiatkowski, Cordelia Langford, John Sillitoe on behalf of the Wellcome Sanger Institute COVID-19 Surveillance Team
EPI_ISL_879790	Lighthouse Lab in Cambridge	Wellcome Sanger Institute for the COVID-19 Genomics UK (COG-UK) Consortium	Rob Howes, The Lighthouse Lab in Cambridge and Alex Alderton, Roberto Amato, Sonia Goncalves, Ewan Harrison, David K. Jackson, Ian Johnston, Dominic Kwiatkowski, Cordelia Langford, John Sillitoe on behalf of the Wellcome Sanger Institute COVID-19 Surveillance Team
EPI_ISL_879791, EPI_ISL_879792, EPI_ISL_879793, EPI_ISL_879794	Lighthouse Lab in Alderley Park	Wellcome Sanger Institute for the COVID-19 Genomics UK (COG-UK) Consortium	Jacquelyn Wynn, Mairead Hyland, The Lighthouse Lab in Alderley Park and Alex Alderton, Roberto Amato, Sonia Goncalves, Ewan Harrison, David K. Jackson, Ian Johnston, Dominic Kwiatkowski, Cordelia Langford, John Sillitoe on behalf of the Wellcome Sanger Institute COVID-19 Surveillance Team
EPI_ISL_879795, EPI_ISL_879796, EPI_ISL_879797, EPI_ISL_879798, EPI_ISL_879799	Lighthouse Lab in Cambridge	Wellcome Sanger Institute for the COVID-19 Genomics UK (COG-UK) Consortium	Rob Howes, The Lighthouse Lab in Cambridge and Alex Alderton, Roberto Amato, Sonia Goncalves, Ewan Harrison, David K. Jackson, Ian Johnston, Dominic Kwiatkowski, Cordelia Langford, John Sillitoe on behalf of the Wellcome Sanger Institute COVID-19 Surveillance Team
EPI_ISL_879800, EPI_ISL_879801, EPI_ISL_879802, EPI_ISL_879803	Lighthouse Lab in Alderley Park	Wellcome Sanger Institute for the COVID-19 Genomics UK (COG-UK) Consortium	Jacquelyn Wynn, Mairead Hyland, The Lighthouse Lab in Alderley Park and Alex Alderton, Roberto Amato, Sonia Goncalves, Ewan Harrison, David K. Jackson, Ian Johnston, Dominic Kwiatkowski, Cordelia Langford, John Sillitoe on behalf of the Wellcome Sanger Institute COVID-19 Surveillance Team
EPI_ISL_879804, EPI_ISL_879805, EPI_ISL_879806, EPI_ISL_879807	Lighthouse Lab in Cambridge	Wellcome Sanger Institute for the COVID-19 Genomics UK (COG-UK) Consortium	Rob Howes, The Lighthouse Lab in Cambridge and Alex Alderton, Roberto Amato, Sonia Goncalves, Ewan Harrison, David K. Jackson, Ian Johnston, Dominic Kwiatkowski, Cordelia Langford, John Sillitoe on behalf of the Wellcome Sanger Institute COVID-19 Surveillance Team
EPI_ISL_879808	Lighthouse Lab in Alderley Park	Wellcome Sanger Institute for the COVID-19 Genomics UK (COG-UK) Consortium	Jacquelyn Wynn, Mairead Hyland, The Lighthouse Lab in Alderley Park and Alex Alderton, Roberto Amato, Sonia Goncalves, Ewan Harrison, David K. Jackson, Ian Johnston, Dominic Kwiatkowski, Cordelia Langford, John Sillitoe on behalf of the Wellcome Sanger Institute COVID-19 Surveillance Team
EPI_ISL_879809	Lighthouse Lab in Cambridge	Wellcome Sanger Institute for the COVID-19 Genomics UK (COG-UK) Consortium	Rob Howes, The Lighthouse Lab in Cambridge and Alex Alderton, Roberto Amato, Sonia Goncalves, Ewan Harrison, David K. Jackson, Ian Johnston, Dominic Kwiatkowski, Cordelia Langford, John Sillitoe on behalf of the Wellcome Sanger Institute COVID-19 Surveillance Team
EPI_ISL_879810, EPI_ISL_879811	Lighthouse Lab in Alderley Park	Wellcome Sanger Institute for the COVID-19 Genomics UK (COG-UK) Consortium	Jacquelyn Wynn, Mairead Hyland, The Lighthouse Lab in Alderley Park and Alex Alderton, Roberto Amato, Sonia Goncalves, Ewan Harrison, David K. Jackson, Ian Johnston, Dominic Kwiatkowski, Cordelia Langford, John Sillitoe on behalf of the Wellcome Sanger Institute COVID-19 Surveillance Team
EPI_ISL_879812	Lighthouse Lab in Cambridge	Wellcome Sanger Institute for the COVID-19 Genomics UK	Rob Howes, The Lighthouse Lab in Cambridge and Alex Alderton, Roberto Amato, Sonia Goncalves, Ewan Harrison, David K. Jackson, Ian Johnston,

		(COG-UK) Consortium	Dominic Kwiatkowski, Cordelia Langford, John Sillitoe on behalf of the Wellcome Sanger Institute COVID-19 Surveillance Team
EPI_ISL_879813, EPI_ISL_879814	Lighthouse Lab in Alderley Park	Wellcome Sanger Institute for the COVID-19 Genomics UK (COG-UK) Consortium	Jacquelyn Wynn, Mairead Hyland, The Lighthouse Lab in Alderley Park and Alex Alderton, Roberto Amato, Sonia Goncalves, Ewan Harrison, David K. Jackson, Ian Johnston, Dominic Kwiatkowski, Cordelia Langford, John Sillitoe on behalf of the Wellcome Sanger Institute COVID-19 Surveillance Team
EPI_ISL_879815, EPI_ISL_879816	Lighthouse Lab in Cambridge	Wellcome Sanger Institute for the COVID-19 Genomics UK (COG-UK) Consortium	Rob Howes, The Lighthouse Lab in Cambridge and Alex Alderton, Roberto Amato, Sonia Goncalves, Ewan Harrison, David K. Jackson, Ian Johnston, Dominic Kwiatkowski, Cordelia Langford, John Sillitoe on behalf of the Wellcome Sanger Institute COVID-19 Surveillance Team
EPI_ISL_879817	Lighthouse Lab in Alderley Park	Wellcome Sanger Institute for the COVID-19 Genomics UK (COG-UK) Consortium	Jacquelyn Wynn, Mairead Hyland, The Lighthouse Lab in Alderley Park and Alex Alderton, Roberto Amato, Sonia Goncalves, Ewan Harrison, David K. Jackson, Ian Johnston, Dominic Kwiatkowski, Cordelia Langford, John Sillitoe on behalf of the Wellcome Sanger Institute COVID-19 Surveillance Team
EPI_ISL_879818	Lighthouse Lab in Cambridge	Wellcome Sanger Institute for the COVID-19 Genomics UK (COG-UK) Consortium	Rob Howes, The Lighthouse Lab in Cambridge and Alex Alderton, Roberto Amato, Sonia Goncalves, Ewan Harrison, David K. Jackson, Ian Johnston, Dominic Kwiatkowski, Cordelia Langford, John Sillitoe on behalf of the Wellcome Sanger Institute COVID-19 Surveillance Team
EPI_ISL_879819, EPI_ISL_879821, EPI_ISL_879822	Lighthouse Lab in Alderley Park	Wellcome Sanger Institute for the COVID-19 Genomics UK (COG-UK) Consortium	Jacquelyn Wynn, Mairead Hyland, The Lighthouse Lab in Alderley Park and Alex Alderton, Roberto Amato, Sonia Goncalves, Ewan Harrison, David K. Jackson, Ian Johnston, Dominic Kwiatkowski, Cordelia Langford, John Sillitoe on behalf of the Wellcome Sanger Institute COVID-19 Surveillance Team
EPI_ISL_879823, EPI_ISL_879824	Lighthouse Lab in Cambridge	Wellcome Sanger Institute for the COVID-19 Genomics UK (COG-UK) Consortium	Rob Howes, The Lighthouse Lab in Cambridge and Alex Alderton, Roberto Amato, Sonia Goncalves, Ewan Harrison, David K. Jackson, Ian Johnston, Dominic Kwiatkowski, Cordelia Langford, John Sillitoe on behalf of the Wellcome Sanger Institute COVID-19 Surveillance Team
EPI_ISL_879825, EPI_ISL_879826, EPI_ISL_879827	Lighthouse Lab in Alderley Park	Wellcome Sanger Institute for the COVID-19 Genomics UK (COG-UK) Consortium	Jacquelyn Wynn, Mairead Hyland, The Lighthouse Lab in Alderley Park and Alex Alderton, Roberto Amato, Sonia Goncalves, Ewan Harrison, David K. Jackson, Ian Johnston, Dominic Kwiatkowski, Cordelia Langford, John Sillitoe on behalf of the Wellcome Sanger Institute COVID-19 Surveillance Team
EPI_ISL_879828	Lighthouse Lab in Cambridge	Wellcome Sanger Institute for the COVID-19 Genomics UK (COG-UK) Consortium	Rob Howes, The Lighthouse Lab in Cambridge and Alex Alderton, Roberto Amato, Sonia Goncalves, Ewan Harrison, David K. Jackson, Ian Johnston, Dominic Kwiatkowski, Cordelia Langford, John Sillitoe on behalf of the Wellcome Sanger Institute COVID-19 Surveillance Team
EPI_ISL_879829	Lighthouse Lab in Alderley Park	Wellcome Sanger Institute for the COVID-19 Genomics UK (COG-UK) Consortium	Jacquelyn Wynn, Mairead Hyland, The Lighthouse Lab in Alderley Park and Alex Alderton, Roberto Amato, Sonia Goncalves, Ewan Harrison, David K. Jackson, Ian Johnston, Dominic Kwiatkowski, Cordelia Langford, John Sillitoe on behalf of the Wellcome Sanger Institute COVID-19 Surveillance Team
EPI_ISL_879830	Lighthouse Lab in Cambridge	Wellcome Sanger Institute for the COVID-19 Genomics UK (COG-UK) Consortium	Rob Howes, The Lighthouse Lab in Cambridge and Alex Alderton, Roberto Amato, Sonia Goncalves, Ewan Harrison, David K. Jackson, Ian Johnston, Dominic Kwiatkowski, Cordelia Langford, John Sillitoe on behalf of the Wellcome Sanger Institute COVID-19 Surveillance Team
EPI_ISL_879831	Lighthouse Lab in Alderley Park	Wellcome Sanger Institute for the COVID-19 Genomics UK (COG-UK) Consortium	Jacquelyn Wynn, Mairead Hyland, The Lighthouse Lab in Alderley Park and Alex Alderton, Roberto Amato, Sonia Goncalves, Ewan Harrison, David K. Jackson, Ian Johnston, Dominic Kwiatkowski, Cordelia Langford, John Sillitoe on behalf of the Wellcome Sanger Institute COVID-19 Surveillance Team
EPI_ISL_879832, EPI_ISL_879833, EPI_ISL_879834, EPI_ISL_879835	Lighthouse Lab in Cambridge	Wellcome Sanger Institute for the COVID-19 Genomics UK (COG-UK) Consortium	Rob Howes, The Lighthouse Lab in Cambridge and Alex Alderton, Roberto Amato, Sonia Goncalves, Ewan Harrison, David K. Jackson, Ian Johnston, Dominic Kwiatkowski, Cordelia Langford, John Sillitoe on behalf of the Wellcome Sanger Institute COVID-19 Surveillance Team
EPI_ISL_879836, EPI_ISL_879837, EPI_ISL_879838	Lighthouse Lab in Alderley Park	Wellcome Sanger Institute for the COVID-19 Genomics UK (COG-UK) Consortium	Jacquelyn Wynn, Mairead Hyland, The Lighthouse Lab in Alderley Park and Alex Alderton, Roberto Amato, Sonia Goncalves, Ewan Harrison, David K. Jackson, Ian Johnston, Dominic Kwiatkowski, Cordelia Langford, John Sillitoe on behalf of the Wellcome Sanger Institute COVID-19 Surveillance Team
EPI_ISL_879839, EPI_ISL_879840	Lighthouse Lab in Cambridge	Wellcome Sanger Institute for the COVID-19 Genomics UK (COG-UK) Consortium	Rob Howes, The Lighthouse Lab in Cambridge and Alex Alderton, Roberto Amato, Sonia Goncalves, Ewan Harrison, David K. Jackson, Ian Johnston, Dominic Kwiatkowski, Cordelia Langford, John Sillitoe on behalf of the Wellcome Sanger Institute COVID-19 Surveillance Team
EPI_ISL_879841, EPI_ISL_879842, EPI_ISL_879843, EPI_ISL_879844	Lighthouse Lab in Alderley Park	Wellcome Sanger Institute for the COVID-19 Genomics UK (COG-UK) Consortium	Jacquelyn Wynn, Mairead Hyland, The Lighthouse Lab in Alderley Park and Alex Alderton, Roberto Amato, Sonia Goncalves, Ewan Harrison, David K. Jackson, Ian Johnston, Dominic Kwiatkowski, Cordelia Langford, John Sillitoe on behalf of the Wellcome Sanger Institute COVID-19 Surveillance Team
EPI_ISL_879845	Lighthouse Lab in Cambridge	Wellcome Sanger Institute for the COVID-19 Genomics UK (COG-UK) Consortium	Rob Howes, The Lighthouse Lab in Cambridge and Alex Alderton, Roberto Amato, Sonia Goncalves, Ewan Harrison, David K. Jackson, Ian Johnston, Dominic Kwiatkowski, Cordelia Langford, John Sillitoe on behalf of the Wellcome Sanger Institute COVID-19 Surveillance Team
EPI_ISL_879846	Lighthouse Lab in Alderley Park	Wellcome Sanger Institute for the COVID-19 Genomics UK (COG-UK) Consortium	Jacquelyn Wynn, Mairead Hyland, The Lighthouse Lab in Alderley Park and Alex Alderton, Roberto Amato, Sonia Goncalves, Ewan Harrison, David K. Jackson, Ian Johnston, Dominic Kwiatkowski, Cordelia Langford, John Sillitoe on behalf of the Wellcome Sanger Institute COVID-19 Surveillance Team
EPI_ISL_879847, EPI_ISL_879848	Lighthouse Lab in Cambridge	Wellcome Sanger Institute for the COVID-19 Genomics UK (COG-UK) Consortium	Rob Howes, The Lighthouse Lab in Cambridge and Alex Alderton, Roberto Amato, Sonia Goncalves, Ewan Harrison, David K. Jackson, Ian Johnston, Dominic Kwiatkowski, Cordelia Langford, John Sillitoe on behalf of the Wellcome Sanger Institute COVID-19 Surveillance Team
EPI_ISL_879849	Lighthouse Lab in Alderley Park	Wellcome Sanger Institute for the COVID-19 Genomics UK (COG-UK) Consortium	Jacquelyn Wynn, Mairead Hyland, The Lighthouse Lab in Alderley Park and Alex Alderton, Roberto Amato, Sonia Goncalves, Ewan Harrison, David K. Jackson, Ian Johnston, Dominic Kwiatkowski, Cordelia Langford, John Sillitoe on behalf of the Wellcome Sanger Institute COVID-19 Surveillance Team
EPI_ISL_879850	Lighthouse Lab in Cambridge	Wellcome Sanger Institute for the COVID-19 Genomics UK (COG-UK) Consortium	Rob Howes, The Lighthouse Lab in Cambridge and Alex Alderton, Roberto Amato, Sonia Goncalves, Ewan Harrison, David K. Jackson, Ian Johnston, Dominic Kwiatkowski, Cordelia Langford, John Sillitoe on behalf of the Wellcome Sanger Institute COVID-19 Surveillance Team
EPI_ISL_879851	Lighthouse Lab in Alderley Park	Wellcome Sanger Institute for the COVID-19 Genomics UK (COG-UK) Consortium	Jacquelyn Wynn, Mairead Hyland, The Lighthouse Lab in Alderley Park and Alex Alderton, Roberto Amato, Sonia Goncalves, Ewan Harrison, David K. Jackson, Ian Johnston, Dominic Kwiatkowski, Cordelia Langford, John Sillitoe on behalf of the Wellcome Sanger Institute COVID-19 Surveillance Team
EPI_ISL_879852, EPI_ISL_879853	Lighthouse Lab in Cambridge	Wellcome Sanger Institute for the COVID-19 Genomics UK (COG-UK) Consortium	Rob Howes, The Lighthouse Lab in Cambridge and Alex Alderton, Roberto Amato, Sonia Goncalves, Ewan Harrison, David K. Jackson, Ian Johnston, Dominic Kwiatkowski, Cordelia Langford, John Sillitoe on behalf of the Wellcome Sanger Institute COVID-19 Surveillance Team
EPI_ISL_879854, EPI_ISL_879855, EPI_ISL_879856	Lighthouse Lab in Alderley Park	Wellcome Sanger Institute for the COVID-19 Genomics UK (COG-UK) Consortium	Jacquelyn Wynn, Mairead Hyland, The Lighthouse Lab in Alderley Park and Alex Alderton, Roberto Amato, Sonia Goncalves, Ewan Harrison, David K. Jackson, Ian Johnston, Dominic Kwiatkowski, Cordelia Langford, John Sillitoe on behalf of the Wellcome Sanger Institute COVID-19 Surveillance Team
EPI_ISL_879857	Lighthouse Lab in Cambridge	Wellcome Sanger Institute for the COVID-19 Genomics UK (COG-UK) Consortium	Rob Howes, The Lighthouse Lab in Cambridge and Alex Alderton, Roberto Amato, Sonia Goncalves, Ewan Harrison, David K. Jackson, Ian Johnston, Dominic Kwiatkowski, Cordelia Langford, John Sillitoe on behalf of the Wellcome Sanger Institute COVID-19 Surveillance Team
EPI_ISL_879858, EPI_ISL_879859	Lighthouse Lab in Alderley Park	Wellcome Sanger Institute for the COVID-19 Genomics UK (COG-UK) Consortium	Jacquelyn Wynn, Mairead Hyland, The Lighthouse Lab in Alderley Park and Alex Alderton, Roberto Amato, Sonia Goncalves, Ewan Harrison, David K. Jackson, Ian Johnston, Dominic Kwiatkowski, Cordelia Langford, John Sillitoe on behalf of the Wellcome Sanger Institute COVID-19 Surveillance Team
EPI_ISL_879860, EPI_ISL_879861, EPI_ISL_879862	Lighthouse Lab in Cambridge	Wellcome Sanger Institute for the COVID-19 Genomics UK (COG-UK) Consortium	Rob Howes, The Lighthouse Lab in Cambridge and Alex Alderton, Roberto Amato, Sonia Goncalves, Ewan Harrison, David K. Jackson, Ian Johnston, Dominic Kwiatkowski, Cordelia Langford, John Sillitoe on behalf of the Wellcome Sanger Institute COVID-19 Surveillance Team
EPI_ISL_879863	Lighthouse Lab in Alderley Park	Wellcome Sanger Institute for the COVID-19 Genomics UK (COG-UK) Consortium	Jacquelyn Wynn, Mairead Hyland, The Lighthouse Lab in Alderley Park and Alex Alderton, Roberto Amato, Sonia Goncalves, Ewan Harrison, David K. Jackson, Ian Johnston, Dominic Kwiatkowski, Cordelia Langford, John Sillitoe on behalf of the Wellcome Sanger Institute COVID-19 Surveillance Team
EPI_ISL_879864	Lighthouse Lab in Cambridge	Wellcome Sanger Institute for the COVID-19 Genomics UK (COG-UK) Consortium	Rob Howes, The Lighthouse Lab in Cambridge and Alex Alderton, Roberto Amato, Sonia Goncalves, Ewan Harrison, David K. Jackson, Ian Johnston, Dominic Kwiatkowski, Cordelia Langford, John Sillitoe on behalf of the Wellcome Sanger Institute COVID-19 Surveillance Team
EPI_ISL_879865, EPI_ISL_879866	Lighthouse Lab in Alderley Park	Wellcome Sanger Institute for the COVID-19 Genomics UK (COG-UK) Consortium	Jacquelyn Wynn, Mairead Hyland, The Lighthouse Lab in Alderley Park and Alex Alderton, Roberto Amato, Sonia Goncalves, Ewan Harrison, David K. Jackson, Ian Johnston, Dominic Kwiatkowski, Cordelia Langford, John Sillitoe on behalf of the Wellcome Sanger Institute COVID-19 Surveillance Team
EPI_ISL_879867	Lighthouse Lab in Cambridge	Wellcome Sanger Institute for the COVID-19 Genomics UK (COG-UK) Consortium	Rob Howes, The Lighthouse Lab in Cambridge and Alex Alderton, Roberto Amato, Sonia Goncalves, Ewan Harrison, David K. Jackson, Ian Johnston, Dominic Kwiatkowski, Cordelia Langford, John Sillitoe on behalf of the Wellcome Sanger Institute COVID-19 Surveillance Team
EPI_ISL_879868, EPI_ISL_879869, EPI_ISL_879870, EPI_ISL_879871, EPI_ISL_879872, EPI_ISL_879873	Lighthouse Lab in Alderley Park	Wellcome Sanger Institute for the COVID-19 Genomics UK (COG-UK) Consortium	Jacquelyn Wynn, Mairead Hyland, The Lighthouse Lab in Alderley Park and Alex Alderton, Roberto Amato, Sonia Goncalves, Ewan Harrison, David K. Jackson, Ian Johnston, Dominic Kwiatkowski, Cordelia Langford, John Sillitoe on behalf of the Wellcome Sanger Institute COVID-19 Surveillance Team
EPI_ISL_879874	Lighthouse Lab in Cambridge	Wellcome Sanger Institute for the COVID-19 Genomics UK (COG-UK) Consortium	Rob Howes, The Lighthouse Lab in Cambridge and Alex Alderton, Roberto Amato, Sonia Goncalves, Ewan Harrison, David K. Jackson, Ian Johnston, Dominic Kwiatkowski, Cordelia Langford, John Sillitoe on behalf of the Wellcome Sanger Institute COVID-19 Surveillance Team
EPI_ISL_879875	Lighthouse Lab in Alderley Park	Wellcome Sanger Institute for the COVID-19 Genomics UK (COG-UK) Consortium	Jacquelyn Wynn, Mairead Hyland, The Lighthouse Lab in Alderley Park and Alex Alderton, Roberto Amato, Sonia Goncalves, Ewan Harrison, David K. Jackson, Ian Johnston, Dominic Kwiatkowski, Cordelia Langford, John Sillitoe on behalf of the Wellcome Sanger Institute COVID-19 Surveillance Team
EPI_ISL_879876, EPI_ISL_879877	Lighthouse Lab in Cambridge	Wellcome Sanger Institute for the COVID-19 Genomics UK (COG-UK) Consortium	Rob Howes, The Lighthouse Lab in Cambridge and Alex Alderton, Roberto Amato, Sonia Goncalves, Ewan Harrison, David K. Jackson, Ian Johnston, Dominic Kwiatkowski, Cordelia Langford, John Sillitoe on behalf of the Wellcome Sanger Institute COVID-19 Surveillance Team

EPI_ISL_879878, EPI_ISL_879879	Lighthouse Lab in Alderley Park	Wellcome Sanger Institute for the COVID-19 Genomics UK (COG-UK) Consortium	Jacquelyn Wynn, Mairead Hyland, The Lighthouse Lab in Alderley Park and Alex Alderton, Roberto Amato, Sonia Goncalves, Ewan Harrison, David K. Jackson, Ian Johnston, Dominic Kwiatkowski, Cordelia Langford, John Sillitoe on behalf of the Wellcome Sanger Institute COVID-19 Surveillance Team
EPI_ISL_879880, EPI_ISL_879881	Lighthouse Lab in Cambridge	Wellcome Sanger Institute for the COVID-19 Genomics UK (COG-UK) Consortium	Rob Howes, The Lighthouse Lab in Cambridge and Alex Alderton, Roberto Amato, Sonia Goncalves, Ewan Harrison, David K. Jackson, Ian Johnston, Dominic Kwiatkowski, Cordelia Langford, John Sillitoe on behalf of the Wellcome Sanger Institute COVID-19 Surveillance Team
EPI_ISL_879882	Lighthouse Lab in Alderley Park	Wellcome Sanger Institute for the COVID-19 Genomics UK (COG-UK) Consortium	Jacquelyn Wynn, Mairead Hyland, The Lighthouse Lab in Alderley Park and Alex Alderton, Roberto Amato, Sonia Goncalves, Ewan Harrison, David K. Jackson, Ian Johnston, Dominic Kwiatkowski, Cordelia Langford, John Sillitoe on behalf of the Wellcome Sanger Institute COVID-19 Surveillance Team
EPI_ISL_879883	Lighthouse Lab in Cambridge	Wellcome Sanger Institute for the COVID-19 Genomics UK (COG-UK) Consortium	Rob Howes, The Lighthouse Lab in Cambridge and Alex Alderton, Roberto Amato, Sonia Goncalves, Ewan Harrison, David K. Jackson, Ian Johnston, Dominic Kwiatkowski, Cordelia Langford, John Sillitoe on behalf of the Wellcome Sanger Institute COVID-19 Surveillance Team
EPI_ISL_879884, EPI_ISL_879885, EPI_ISL_879886, EPI_ISL_879887, EPI_ISL_879888	Lighthouse Lab in Alderley Park	Wellcome Sanger Institute for the COVID-19 Genomics UK (COG-UK) Consortium	Jacquelyn Wynn, Mairead Hyland, The Lighthouse Lab in Alderley Park and Alex Alderton, Roberto Amato, Sonia Goncalves, Ewan Harrison, David K. Jackson, Ian Johnston, Dominic Kwiatkowski, Cordelia Langford, John Sillitoe on behalf of the Wellcome Sanger Institute COVID-19 Surveillance Team
EPI_ISL_879889, EPI_ISL_879890, EPI_ISL_879891, EPI_ISL_879892, EPI_ISL_879893	Lighthouse Lab in Cambridge	Wellcome Sanger Institute for the COVID-19 Genomics UK (COG-UK) Consortium	Rob Howes, The Lighthouse Lab in Cambridge and Alex Alderton, Roberto Amato, Sonia Goncalves, Ewan Harrison, David K. Jackson, Ian Johnston, Dominic Kwiatkowski, Cordelia Langford, John Sillitoe on behalf of the Wellcome Sanger Institute COVID-19 Surveillance Team
EPI_ISL_879894, EPI_ISL_879895	Lighthouse Lab in Alderley Park	Wellcome Sanger Institute for the COVID-19 Genomics UK (COG-UK) Consortium	Jacquelyn Wynn, Mairead Hyland, The Lighthouse Lab in Alderley Park and Alex Alderton, Roberto Amato, Sonia Goncalves, Ewan Harrison, David K. Jackson, Ian Johnston, Dominic Kwiatkowski, Cordelia Langford, John Sillitoe on behalf of the Wellcome Sanger Institute COVID-19 Surveillance Team
EPI_ISL_879896	Lighthouse Lab in Cambridge	Wellcome Sanger Institute for the COVID-19 Genomics UK (COG-UK) Consortium	Rob Howes, The Lighthouse Lab in Cambridge and Alex Alderton, Roberto Amato, Sonia Goncalves, Ewan Harrison, David K. Jackson, Ian Johnston, Dominic Kwiatkowski, Cordelia Langford, John Sillitoe on behalf of the Wellcome Sanger Institute COVID-19 Surveillance Team
EPI_ISL_879897, EPI_ISL_879898	Lighthouse Lab in Alderley Park	Wellcome Sanger Institute for the COVID-19 Genomics UK (COG-UK) Consortium	Jacquelyn Wynn, Mairead Hyland, The Lighthouse Lab in Alderley Park and Alex Alderton, Roberto Amato, Sonia Goncalves, Ewan Harrison, David K. Jackson, Ian Johnston, Dominic Kwiatkowski, Cordelia Langford, John Sillitoe on behalf of the Wellcome Sanger Institute COVID-19 Surveillance Team
EPI_ISL_879899, EPI_ISL_879900	Lighthouse Lab in Cambridge	Wellcome Sanger Institute for the COVID-19 Genomics UK (COG-UK) Consortium	Rob Howes, The Lighthouse Lab in Cambridge and Alex Alderton, Roberto Amato, Sonia Goncalves, Ewan Harrison, David K. Jackson, Ian Johnston, Dominic Kwiatkowski, Cordelia Langford, John Sillitoe on behalf of the Wellcome Sanger Institute COVID-19 Surveillance Team
EPI_ISL_879901	Lighthouse Lab in Alderley Park	Wellcome Sanger Institute for the COVID-19 Genomics UK (COG-UK) Consortium	Jacquelyn Wynn, Mairead Hyland, The Lighthouse Lab in Alderley Park and Alex Alderton, Roberto Amato, Sonia Goncalves, Ewan Harrison, David K. Jackson, Ian Johnston, Dominic Kwiatkowski, Cordelia Langford, John Sillitoe on behalf of the Wellcome Sanger Institute COVID-19 Surveillance Team
EPI_ISL_879902	Lighthouse Lab in Cambridge	Wellcome Sanger Institute for the COVID-19 Genomics UK (COG-UK) Consortium	Rob Howes, The Lighthouse Lab in Cambridge and Alex Alderton, Roberto Amato, Sonia Goncalves, Ewan Harrison, David K. Jackson, Ian Johnston, Dominic Kwiatkowski, Cordelia Langford, John Sillitoe on behalf of the Wellcome Sanger Institute COVID-19 Surveillance Team
EPI_ISL_879903	Lighthouse Lab in Alderley Park	Wellcome Sanger Institute for the COVID-19 Genomics UK (COG-UK) Consortium	Jacquelyn Wynn, Mairead Hyland, The Lighthouse Lab in Alderley Park and Alex Alderton, Roberto Amato, Sonia Goncalves, Ewan Harrison, David K. Jackson, Ian Johnston, Dominic Kwiatkowski, Cordelia Langford, John Sillitoe on behalf of the Wellcome Sanger Institute COVID-19 Surveillance Team
EPI_ISL_879904	Lighthouse Lab in Cambridge	Wellcome Sanger Institute for the COVID-19 Genomics UK (COG-UK) Consortium	Rob Howes, The Lighthouse Lab in Cambridge and Alex Alderton, Roberto Amato, Sonia Goncalves, Ewan Harrison, David K. Jackson, Ian Johnston, Dominic Kwiatkowski, Cordelia Langford, John Sillitoe on behalf of the Wellcome Sanger Institute COVID-19 Surveillance Team
EPI_ISL_879905, EPI_ISL_879906, EPI_ISL_879907	Lighthouse Lab in Alderley Park	Wellcome Sanger Institute for the COVID-19 Genomics UK (COG-UK) Consortium	Jacquelyn Wynn, Mairead Hyland, The Lighthouse Lab in Alderley Park and Alex Alderton, Roberto Amato, Sonia Goncalves, Ewan Harrison, David K. Jackson, Ian Johnston, Dominic Kwiatkowski, Cordelia Langford, John Sillitoe on behalf of the Wellcome Sanger Institute COVID-19 Surveillance Team
EPI_ISL_879908, EPI_ISL_879909, EPI_ISL_879910, EPI_ISL_879911	Lighthouse Lab in Cambridge	Wellcome Sanger Institute for the COVID-19 Genomics UK (COG-UK) Consortium	Rob Howes, The Lighthouse Lab in Cambridge and Alex Alderton, Roberto Amato, Sonia Goncalves, Ewan Harrison, David K. Jackson, Ian Johnston, Dominic Kwiatkowski, Cordelia Langford, John Sillitoe on behalf of the Wellcome Sanger Institute COVID-19 Surveillance Team
EPI_ISL_879912, EPI_ISL_879913, EPI_ISL_879914, EPI_ISL_879915, EPI_ISL_879916	Lighthouse Lab in Alderley Park	Wellcome Sanger Institute for the COVID-19 Genomics UK (COG-UK) Consortium	Jacquelyn Wynn, Mairead Hyland, The Lighthouse Lab in Alderley Park and Alex Alderton, Roberto Amato, Sonia Goncalves, Ewan Harrison, David K. Jackson, Ian Johnston, Dominic Kwiatkowski, Cordelia Langford, John Sillitoe on behalf of the Wellcome Sanger Institute COVID-19 Surveillance Team
EPI_ISL_879917	Lighthouse Lab in Cambridge	Wellcome Sanger Institute for the COVID-19 Genomics UK (COG-UK) Consortium	Rob Howes, The Lighthouse Lab in Cambridge and Alex Alderton, Roberto Amato, Sonia Goncalves, Ewan Harrison, David K. Jackson, Ian Johnston, Dominic Kwiatkowski, Cordelia Langford, John Sillitoe on behalf of the Wellcome Sanger Institute COVID-19 Surveillance Team
EPI_ISL_879918, EPI_ISL_879919	Lighthouse Lab in Alderley Park	Wellcome Sanger Institute for the COVID-19 Genomics UK (COG-UK) Consortium	Jacquelyn Wynn, Mairead Hyland, The Lighthouse Lab in Alderley Park and Alex Alderton, Roberto Amato, Sonia Goncalves, Ewan Harrison, David K. Jackson, Ian Johnston, Dominic Kwiatkowski, Cordelia Langford, John Sillitoe on behalf of the Wellcome Sanger Institute COVID-19 Surveillance Team
EPI_ISL_879920, EPI_ISL_879921	Lighthouse Lab in Cambridge	Wellcome Sanger Institute for the COVID-19 Genomics UK (COG-UK) Consortium	Rob Howes, The Lighthouse Lab in Cambridge and Alex Alderton, Roberto Amato, Sonia Goncalves, Ewan Harrison, David K. Jackson, Ian Johnston, Dominic Kwiatkowski, Cordelia Langford, John Sillitoe on behalf of the Wellcome Sanger Institute COVID-19 Surveillance Team
EPI_ISL_879922, EPI_ISL_879923, EPI_ISL_879925, EPI_ISL_879927	Lighthouse Lab in Alderley Park	Wellcome Sanger Institute for the COVID-19 Genomics UK (COG-UK) Consortium	Jacquelyn Wynn, Mairead Hyland, The Lighthouse Lab in Alderley Park and Alex Alderton, Roberto Amato, Sonia Goncalves, Ewan Harrison, David K. Jackson, Ian Johnston, Dominic Kwiatkowski, Cordelia Langford, John Sillitoe on behalf of the Wellcome Sanger Institute COVID-19 Surveillance Team
EPI_ISL_879928, EPI_ISL_879930	Lighthouse Lab in Cambridge	Wellcome Sanger Institute for the COVID-19 Genomics UK (COG-UK) Consortium	Rob Howes, The Lighthouse Lab in Cambridge and Alex Alderton, Roberto Amato, Sonia Goncalves, Ewan Harrison, David K. Jackson, Ian Johnston, Dominic Kwiatkowski, Cordelia Langford, John Sillitoe on behalf of the Wellcome Sanger Institute COVID-19 Surveillance Team
EPI_ISL_879931, EPI_ISL_879933	Lighthouse Lab in Alderley Park	Wellcome Sanger Institute for the COVID-19 Genomics UK (COG-UK) Consortium	Jacquelyn Wynn, Mairead Hyland, The Lighthouse Lab in Alderley Park and Alex Alderton, Roberto Amato, Sonia Goncalves, Ewan Harrison, David K. Jackson, Ian Johnston, Dominic Kwiatkowski, Cordelia Langford, John Sillitoe on behalf of the Wellcome Sanger Institute COVID-19 Surveillance Team
EPI_ISL_879935	Lighthouse Lab in Cambridge	Wellcome Sanger Institute for the COVID-19 Genomics UK (COG-UK) Consortium	Rob Howes, The Lighthouse Lab in Cambridge and Alex Alderton, Roberto Amato, Sonia Goncalves, Ewan Harrison, David K. Jackson, Ian Johnston, Dominic Kwiatkowski, Cordelia Langford, John Sillitoe on behalf of the Wellcome Sanger Institute COVID-19 Surveillance Team
EPI_ISL_879936, EPI_ISL_879938	Lighthouse Lab in Alderley Park	Wellcome Sanger Institute for the COVID-19 Genomics UK (COG-UK) Consortium	Jacquelyn Wynn, Mairead Hyland, The Lighthouse Lab in Alderley Park and Alex Alderton, Roberto Amato, Sonia Goncalves, Ewan Harrison, David K. Jackson, Ian Johnston, Dominic Kwiatkowski, Cordelia Langford, John Sillitoe on behalf of the Wellcome Sanger Institute COVID-19 Surveillance Team
EPI_ISL_879940, EPI_ISL_879941, EPI_ISL_879943, EPI_ISL_879944, EPI_ISL_879946	Lighthouse Lab in Cambridge	Wellcome Sanger Institute for the COVID-19 Genomics UK (COG-UK) Consortium	Rob Howes, The Lighthouse Lab in Cambridge and Alex Alderton, Roberto Amato, Sonia Goncalves, Ewan Harrison, David K. Jackson, Ian Johnston, Dominic Kwiatkowski, Cordelia Langford, John Sillitoe on behalf of the Wellcome Sanger Institute COVID-19 Surveillance Team
EPI_ISL_879948, EPI_ISL_879949	Lighthouse Lab in Alderley Park	Wellcome Sanger Institute for the COVID-19 Genomics UK (COG-UK) Consortium	Jacquelyn Wynn, Mairead Hyland, The Lighthouse Lab in Alderley Park and Alex Alderton, Roberto Amato, Sonia Goncalves, Ewan Harrison, David K. Jackson, Ian Johnston, Dominic Kwiatkowski, Cordelia Langford, John Sillitoe on behalf of the Wellcome Sanger Institute COVID-19 Surveillance Team
EPI_ISL_879951, EPI_ISL_879952, EPI_ISL_879954	Lighthouse Lab in Cambridge	Wellcome Sanger Institute for the COVID-19 Genomics UK (COG-UK) Consortium	Rob Howes, The Lighthouse Lab in Cambridge and Alex Alderton, Roberto Amato, Sonia Goncalves, Ewan Harrison, David K. Jackson, Ian Johnston, Dominic Kwiatkowski, Cordelia Langford, John Sillitoe on behalf of the Wellcome Sanger Institute COVID-19 Surveillance Team
EPI_ISL_879955, EPI_ISL_879957	Lighthouse Lab in Alderley Park	Wellcome Sanger Institute for the COVID-19 Genomics UK (COG-UK) Consortium	Jacquelyn Wynn, Mairead Hyland, The Lighthouse Lab in Alderley Park and Alex Alderton, Roberto Amato, Sonia Goncalves, Ewan Harrison, David K. Jackson, Ian Johnston, Dominic Kwiatkowski, Cordelia Langford, John Sillitoe on behalf of the Wellcome Sanger Institute COVID-19 Surveillance Team
EPI_ISL_879958, EPI_ISL_879960, EPI_ISL_879962, EPI_ISL_879963	Lighthouse Lab in Cambridge	Wellcome Sanger Institute for the COVID-19 Genomics UK (COG-UK) Consortium	Rob Howes, The Lighthouse Lab in Cambridge and Alex Alderton, Roberto Amato, Sonia Goncalves, Ewan Harrison, David K. Jackson, Ian Johnston, Dominic Kwiatkowski, Cordelia Langford, John Sillitoe on behalf of the Wellcome Sanger Institute COVID-19 Surveillance Team
EPI_ISL_879965, EPI_ISL_879966, EPI_ISL_879968, EPI_ISL_879970, EPI_ISL_879971	Lighthouse Lab in Alderley Park	Wellcome Sanger Institute for the COVID-19 Genomics UK (COG-UK) Consortium	Jacquelyn Wynn, Mairead Hyland, The Lighthouse Lab in Alderley Park and Alex Alderton, Roberto Amato, Sonia Goncalves, Ewan Harrison, David K. Jackson, Ian Johnston, Dominic Kwiatkowski, Cordelia Langford, John Sillitoe on behalf of the Wellcome Sanger Institute COVID-19 Surveillance Team
EPI_ISL_879973	Lighthouse Lab in Cambridge	Wellcome Sanger Institute for the COVID-19 Genomics UK (COG-UK) Consortium	Rob Howes, The Lighthouse Lab in Cambridge and Alex Alderton, Roberto Amato, Sonia Goncalves, Ewan Harrison, David K. Jackson, Ian Johnston, Dominic Kwiatkowski, Cordelia Langford, John Sillitoe on behalf of the Wellcome Sanger Institute COVID-19 Surveillance Team
EPI_ISL_879974, EPI_ISL_879976, EPI_ISL_879978, EPI_ISL_879979, EPI_ISL_879981	Lighthouse Lab in Alderley Park	Wellcome Sanger Institute for the COVID-19 Genomics UK (COG-UK) Consortium	Jacquelyn Wynn, Mairead Hyland, The Lighthouse Lab in Alderley Park and Alex Alderton, Roberto Amato, Sonia Goncalves, Ewan Harrison, David K. Jackson, Ian Johnston, Dominic Kwiatkowski, Cordelia Langford, John Sillitoe on behalf of the Wellcome Sanger Institute COVID-19 Surveillance Team

EPI_ISL_879983	Lighthouse Lab in Cambridge	Wellcome Sanger Institute for the COVID-19 Genomics UK (COG-UK) Consortium	Rob Howes, The Lighthouse Lab in Cambridge and Alex Alderton, Roberto Amato, Sonia Goncalves, Ewan Harrison, David K. Jackson, Ian Johnston, Dominic Kwiatkowski, Cordelia Langford, John Sillitoe on behalf of the Wellcome Sanger Institute COVID-19 Surveillance Team
EPI_ISL_879984, EPI_ISL_879986, EPI_ISL_879988	Lighthouse Lab in Alderley Park	Wellcome Sanger Institute for the COVID-19 Genomics UK (COG-UK) Consortium	Jacquelyn Wynn, Mairead Hyland, The Lighthouse Lab in Alderley Park and Alex Alderton, Roberto Amato, Sonia Goncalves, Ewan Harrison, David K. Jackson, Ian Johnston, Dominic Kwiatkowski, Cordelia Langford, John Sillitoe on behalf of the Wellcome Sanger Institute COVID-19 Surveillance Team
EPI_ISL_879989	Lighthouse Lab in Cambridge	Wellcome Sanger Institute for the COVID-19 Genomics UK (COG-UK) Consortium	Rob Howes, The Lighthouse Lab in Cambridge and Alex Alderton, Roberto Amato, Sonia Goncalves, Ewan Harrison, David K. Jackson, Ian Johnston, Dominic Kwiatkowski, Cordelia Langford, John Sillitoe on behalf of the Wellcome Sanger Institute COVID-19 Surveillance Team
EPI_ISL_879991	Lighthouse Lab in Alderley Park	Wellcome Sanger Institute for the COVID-19 Genomics UK (COG-UK) Consortium	Jacquelyn Wynn, Mairead Hyland, The Lighthouse Lab in Alderley Park and Alex Alderton, Roberto Amato, Sonia Goncalves, Ewan Harrison, David K. Jackson, Ian Johnston, Dominic Kwiatkowski, Cordelia Langford, John Sillitoe on behalf of the Wellcome Sanger Institute COVID-19 Surveillance Team
EPI_ISL_879992, EPI_ISL_879994, EPI_ISL_879996	Lighthouse Lab in Cambridge	Wellcome Sanger Institute for the COVID-19 Genomics UK (COG-UK) Consortium	Rob Howes, The Lighthouse Lab in Cambridge and Alex Alderton, Roberto Amato, Sonia Goncalves, Ewan Harrison, David K. Jackson, Ian Johnston, Dominic Kwiatkowski, Cordelia Langford, John Sillitoe on behalf of the Wellcome Sanger Institute COVID-19 Surveillance Team
EPI_ISL_879997, EPI_ISL_879999	Lighthouse Lab in Alderley Park	Wellcome Sanger Institute for the COVID-19 Genomics UK (COG-UK) Consortium	Jacquelyn Wynn, Mairead Hyland, The Lighthouse Lab in Alderley Park and Alex Alderton, Roberto Amato, Sonia Goncalves, Ewan Harrison, David K. Jackson, Ian Johnston, Dominic Kwiatkowski, Cordelia Langford, John Sillitoe on behalf of the Wellcome Sanger Institute COVID-19 Surveillance Team
EPI_ISL_880001	Lighthouse Lab in Cambridge	Wellcome Sanger Institute for the COVID-19 Genomics UK (COG-UK) Consortium	Rob Howes, The Lighthouse Lab in Cambridge and Alex Alderton, Roberto Amato, Sonia Goncalves, Ewan Harrison, David K. Jackson, Ian Johnston, Dominic Kwiatkowski, Cordelia Langford, John Sillitoe on behalf of the Wellcome Sanger Institute COVID-19 Surveillance Team
EPI_ISL_880002	Lighthouse Lab in Alderley Park	Wellcome Sanger Institute for the COVID-19 Genomics UK (COG-UK) Consortium	Jacquelyn Wynn, Mairead Hyland, The Lighthouse Lab in Alderley Park and Alex Alderton, Roberto Amato, Sonia Goncalves, Ewan Harrison, David K. Jackson, Ian Johnston, Dominic Kwiatkowski, Cordelia Langford, John Sillitoe on behalf of the Wellcome Sanger Institute COVID-19 Surveillance Team
EPI_ISL_880004, EPI_ISL_880005	Lighthouse Lab in Cambridge	Wellcome Sanger Institute for the COVID-19 Genomics UK (COG-UK) Consortium	Rob Howes, The Lighthouse Lab in Cambridge and Alex Alderton, Roberto Amato, Sonia Goncalves, Ewan Harrison, David K. Jackson, Ian Johnston, Dominic Kwiatkowski, Cordelia Langford, John Sillitoe on behalf of the Wellcome Sanger Institute COVID-19 Surveillance Team
EPI_ISL_880007, EPI_ISL_880008, EPI_ISL_880010	Lighthouse Lab in Alderley Park	Wellcome Sanger Institute for the COVID-19 Genomics UK (COG-UK) Consortium	Jacquelyn Wynn, Mairead Hyland, The Lighthouse Lab in Alderley Park and Alex Alderton, Roberto Amato, Sonia Goncalves, Ewan Harrison, David K. Jackson, Ian Johnston, Dominic Kwiatkowski, Cordelia Langford, John Sillitoe on behalf of the Wellcome Sanger Institute COVID-19 Surveillance Team
EPI_ISL_880012, EPI_ISL_880013, EPI_ISL_880015	Lighthouse Lab in Cambridge	Wellcome Sanger Institute for the COVID-19 Genomics UK (COG-UK) Consortium	Rob Howes, The Lighthouse Lab in Cambridge and Alex Alderton, Roberto Amato, Sonia Goncalves, Ewan Harrison, David K. Jackson, Ian Johnston, Dominic Kwiatkowski, Cordelia Langford, John Sillitoe on behalf of the Wellcome Sanger Institute COVID-19 Surveillance Team
EPI_ISL_880016, EPI_ISL_880018	Lighthouse Lab in Alderley Park	Wellcome Sanger Institute for the COVID-19 Genomics UK (COG-UK) Consortium	Jacquelyn Wynn, Mairead Hyland, The Lighthouse Lab in Alderley Park and Alex Alderton, Roberto Amato, Sonia Goncalves, Ewan Harrison, David K. Jackson, Ian Johnston, Dominic Kwiatkowski, Cordelia Langford, John Sillitoe on behalf of the Wellcome Sanger Institute COVID-19 Surveillance Team
EPI_ISL_880020	Lighthouse Lab in Cambridge	Wellcome Sanger Institute for the COVID-19 Genomics UK (COG-UK) Consortium	Rob Howes, The Lighthouse Lab in Cambridge and Alex Alderton, Roberto Amato, Sonia Goncalves, Ewan Harrison, David K. Jackson, Ian Johnston, Dominic Kwiatkowski, Cordelia Langford, John Sillitoe on behalf of the Wellcome Sanger Institute COVID-19 Surveillance Team
EPI_ISL_880021, EPI_ISL_880023	Lighthouse Lab in Alderley Park	Wellcome Sanger Institute for the COVID-19 Genomics UK (COG-UK) Consortium	Jacquelyn Wynn, Mairead Hyland, The Lighthouse Lab in Alderley Park and Alex Alderton, Roberto Amato, Sonia Goncalves, Ewan Harrison, David K. Jackson, Ian Johnston, Dominic Kwiatkowski, Cordelia Langford, John Sillitoe on behalf of the Wellcome Sanger Institute COVID-19 Surveillance Team
EPI_ISL_880025, EPI_ISL_880026	Lighthouse Lab in Cambridge	Wellcome Sanger Institute for the COVID-19 Genomics UK (COG-UK) Consortium	Rob Howes, The Lighthouse Lab in Cambridge and Alex Alderton, Roberto Amato, Sonia Goncalves, Ewan Harrison, David K. Jackson, Ian Johnston, Dominic Kwiatkowski, Cordelia Langford, John Sillitoe on behalf of the Wellcome Sanger Institute COVID-19 Surveillance Team
EPI_ISL_880028, EPI_ISL_880030, EPI_ISL_880031	Lighthouse Lab in Alderley Park	Wellcome Sanger Institute for the COVID-19 Genomics UK (COG-UK) Consortium	Jacquelyn Wynn, Mairead Hyland, The Lighthouse Lab in Alderley Park and Alex Alderton, Roberto Amato, Sonia Goncalves, Ewan Harrison, David K. Jackson, Ian Johnston, Dominic Kwiatkowski, Cordelia Langford, John Sillitoe on behalf of the Wellcome Sanger Institute COVID-19 Surveillance Team
EPI_ISL_880033, EPI_ISL_880035	Lighthouse Lab in Cambridge	Wellcome Sanger Institute for the COVID-19 Genomics UK (COG-UK) Consortium	Rob Howes, The Lighthouse Lab in Cambridge and Alex Alderton, Roberto Amato, Sonia Goncalves, Ewan Harrison, David K. Jackson, Ian Johnston, Dominic Kwiatkowski, Cordelia Langford, John Sillitoe on behalf of the Wellcome Sanger Institute COVID-19 Surveillance Team
EPI_ISL_880036	Lighthouse Lab in Alderley Park	Wellcome Sanger Institute for the COVID-19 Genomics UK (COG-UK) Consortium	Jacquelyn Wynn, Mairead Hyland, The Lighthouse Lab in Alderley Park and Alex Alderton, Roberto Amato, Sonia Goncalves, Ewan Harrison, David K. Jackson, Ian Johnston, Dominic Kwiatkowski, Cordelia Langford, John Sillitoe on behalf of the Wellcome Sanger Institute COVID-19 Surveillance Team
EPI_ISL_880039	Lighthouse Lab in Cambridge	Wellcome Sanger Institute for the COVID-19 Genomics UK (COG-UK) Consortium	Rob Howes, The Lighthouse Lab in Cambridge and Alex Alderton, Roberto Amato, Sonia Goncalves, Ewan Harrison, David K. Jackson, Ian Johnston, Dominic Kwiatkowski, Cordelia Langford, John Sillitoe on behalf of the Wellcome Sanger Institute COVID-19 Surveillance Team
EPI_ISL_880041, EPI_ISL_880043	Lighthouse Lab in Alderley Park	Wellcome Sanger Institute for the COVID-19 Genomics UK (COG-UK) Consortium	Jacquelyn Wynn, Mairead Hyland, The Lighthouse Lab in Alderley Park and Alex Alderton, Roberto Amato, Sonia Goncalves, Ewan Harrison, David K. Jackson, Ian Johnston, Dominic Kwiatkowski, Cordelia Langford, John Sillitoe on behalf of the Wellcome Sanger Institute COVID-19 Surveillance Team
EPI_ISL_880044, EPI_ISL_880046, EPI_ISL_880047, EPI_ISL_880049, EPI_ISL_880051	Lighthouse Lab in Cambridge	Wellcome Sanger Institute for the COVID-19 Genomics UK (COG-UK) Consortium	Rob Howes, The Lighthouse Lab in Cambridge and Alex Alderton, Roberto Amato, Sonia Goncalves, Ewan Harrison, David K. Jackson, Ian Johnston, Dominic Kwiatkowski, Cordelia Langford, John Sillitoe on behalf of the Wellcome Sanger Institute COVID-19 Surveillance Team
EPI_ISL_880052	Lighthouse Lab in Alderley Park	Wellcome Sanger Institute for the COVID-19 Genomics UK (COG-UK) Consortium	Jacquelyn Wynn, Mairead Hyland, The Lighthouse Lab in Alderley Park and Alex Alderton, Roberto Amato, Sonia Goncalves, Ewan Harrison, David K. Jackson, Ian Johnston, Dominic Kwiatkowski, Cordelia Langford, John Sillitoe on behalf of the Wellcome Sanger Institute COVID-19 Surveillance Team
EPI_ISL_880054	Lighthouse Lab in Cambridge	Wellcome Sanger Institute for the COVID-19 Genomics UK (COG-UK) Consortium	Rob Howes, The Lighthouse Lab in Cambridge and Alex Alderton, Roberto Amato, Sonia Goncalves, Ewan Harrison, David K. Jackson, Ian Johnston, Dominic Kwiatkowski, Cordelia Langford, John Sillitoe on behalf of the Wellcome Sanger Institute COVID-19 Surveillance Team
EPI_ISL_880055, EPI_ISL_880057	Lighthouse Lab in Alderley Park	Wellcome Sanger Institute for the COVID-19 Genomics UK (COG-UK) Consortium	Jacquelyn Wynn, Mairead Hyland, The Lighthouse Lab in Alderley Park and Alex Alderton, Roberto Amato, Sonia Goncalves, Ewan Harrison, David K. Jackson, Ian Johnston, Dominic Kwiatkowski, Cordelia Langford, John Sillitoe on behalf of the Wellcome Sanger Institute COVID-19 Surveillance Team
EPI_ISL_880059, EPI_ISL_880060	Lighthouse Lab in Cambridge	Wellcome Sanger Institute for the COVID-19 Genomics UK (COG-UK) Consortium	Rob Howes, The Lighthouse Lab in Cambridge and Alex Alderton, Roberto Amato, Sonia Goncalves, Ewan Harrison, David K. Jackson, Ian Johnston, Dominic Kwiatkowski, Cordelia Langford, John Sillitoe on behalf of the Wellcome Sanger Institute COVID-19 Surveillance Team
EPI_ISL_880062, EPI_ISL_880064	Lighthouse Lab in Alderley Park	Wellcome Sanger Institute for the COVID-19 Genomics UK (COG-UK) Consortium	Jacquelyn Wynn, Mairead Hyland, The Lighthouse Lab in Alderley Park and Alex Alderton, Roberto Amato, Sonia Goncalves, Ewan Harrison, David K. Jackson, Ian Johnston, Dominic Kwiatkowski, Cordelia Langford, John Sillitoe on behalf of the Wellcome Sanger Institute COVID-19 Surveillance Team
EPI_ISL_880065	Lighthouse Lab in Cambridge	Wellcome Sanger Institute for the COVID-19 Genomics UK (COG-UK) Consortium	Rob Howes, The Lighthouse Lab in Cambridge and Alex Alderton, Roberto Amato, Sonia Goncalves, Ewan Harrison, David K. Jackson, Ian Johnston, Dominic Kwiatkowski, Cordelia Langford, John Sillitoe on behalf of the Wellcome Sanger Institute COVID-19 Surveillance Team
EPI_ISL_880067	Lighthouse Lab in Alderley Park	Wellcome Sanger Institute for the COVID-19 Genomics UK (COG-UK) Consortium	Jacquelyn Wynn, Mairead Hyland, The Lighthouse Lab in Alderley Park and Alex Alderton, Roberto Amato, Sonia Goncalves, Ewan Harrison, David K. Jackson, Ian Johnston, Dominic Kwiatkowski, Cordelia Langford, John Sillitoe on behalf of the Wellcome Sanger Institute COVID-19 Surveillance Team
EPI_ISL_880068, EPI_ISL_880070	Lighthouse Lab in Cambridge	Wellcome Sanger Institute for the COVID-19 Genomics UK (COG-UK) Consortium	Rob Howes, The Lighthouse Lab in Cambridge and Alex Alderton, Roberto Amato, Sonia Goncalves, Ewan Harrison, David K. Jackson, Ian Johnston, Dominic Kwiatkowski, Cordelia Langford, John Sillitoe on behalf of the Wellcome Sanger Institute COVID-19 Surveillance Team
EPI_ISL_880072	Lighthouse Lab in Alderley Park	Wellcome Sanger Institute for the COVID-19 Genomics UK (COG-UK) Consortium	Jacquelyn Wynn, Mairead Hyland, The Lighthouse Lab in Alderley Park and Alex Alderton, Roberto Amato, Sonia Goncalves, Ewan Harrison, David K. Jackson, Ian Johnston, Dominic Kwiatkowski, Cordelia Langford, John Sillitoe on behalf of the Wellcome Sanger Institute COVID-19 Surveillance Team
EPI_ISL_880073, EPI_ISL_880075, EPI_ISL_880076, EPI_ISL_880078, EPI_ISL_880080, EPI_ISL_880081	Lighthouse Lab in Cambridge	Wellcome Sanger Institute for the COVID-19 Genomics UK (COG-UK) Consortium	Rob Howes, The Lighthouse Lab in Cambridge and Alex Alderton, Roberto Amato, Sonia Goncalves, Ewan Harrison, David K. Jackson, Ian Johnston, Dominic Kwiatkowski, Cordelia Langford, John Sillitoe on behalf of the Wellcome Sanger Institute COVID-19 Surveillance Team

EPI_ISL_880083, EPI_ISL_880084, EPI_ISL_880086, EPI_ISL_880088, EPI_ISL_880089, EPI_ISL_880091, EPI_ISL_880093, EPI_ISL_880094, EPI_ISL_880096, EPI_ISL_880097, EPI_ISL_880099, EPI_ISL_880100, EPI_ISL_880102, EPI_ISL_880104, EPI_ISL_880105, EPI_ISL_880107, EPI_ISL_880108, EPI_ISL_880110, EPI_ISL_880112, EPI_ISL_880113, EPI_ISL_880115, EPI_ISL_880116, EPI_ISL_880118, EPI_ISL_880120, EPI_ISL_880121, EPI_ISL_880123, EPI_ISL_880124, EPI_ISL_880126, EPI_ISL_880127, EPI_ISL_880129, EPI_ISL_880131, EPI_ISL_880132, EPI_ISL_880134, EPI_ISL_880136, EPI_ISL_880137, EPI_ISL_880139, EPI_ISL_880210, EPI_ISL_880211, EPI_ISL_880213, EPI_ISL_880214, EPI_ISL_880216, EPI_ISL_880217, EPI_ISL_880219, EPI_ISL_880221, EPI_ISL_880222, EPI_ISL_880224, EPI_ISL_880226, EPI_ISL_880227, EPI_ISL_880229, EPI_ISL_880230, EPI_ISL_880232, EPI_ISL_880234, EPI_ISL_880235, EPI_ISL_880237, EPI_ISL_880238, EPI_ISL_880240, EPI_ISL_880242, EPI_ISL_880243, EPI_ISL_880245, EPI_ISL_880247, EPI_ISL_880248, EPI_ISL_880250, EPI_ISL_880252, EPI_ISL_880254, EPI_ISL_880255, EPI_ISL_880257, EPI_ISL_880258, EPI_ISL_880260, EPI_ISL_880262, EPI_ISL_880264, EPI_ISL_880265, EPI_ISL_880267, EPI_ISL_880269, EPI_ISL_880270, EPI_ISL_880272, EPI_ISL_880273, EPI_ISL_880275, EPI_ISL_880276, EPI_ISL_880278, EPI_ISL_880280, EPI_ISL_880281, EPI_ISL_880283, EPI_ISL_880285, EPI_ISL_880286, EPI_ISL_880288, EPI_ISL_880289, EPI_ISL_880291, EPI_ISL_880293, EPI_ISL_880295, EPI_ISL_880296, EPI_ISL_880298, EPI_ISL_880299, EPI_ISL_880301, EPI_ISL_880302, EPI_ISL_880304, EPI_ISL_880306, EPI_ISL_880307, EPI_ISL_880309, EPI_ISL_880311, EPI_ISL_880312, EPI_ISL_880314, EPI_ISL_880315, EPI_ISL_880317, EPI_ISL_880318, EPI_ISL_880320, EPI_ISL_880322, EPI_ISL_880323, EPI_ISL_880325, EPI_ISL_880327, EPI_ISL_880328, EPI_ISL_880330, EPI_ISL_880331, EPI_ISL_880332, EPI_ISL_880333, EPI_ISL_880334, EPI_ISL_880335, EPI_ISL_880336, EPI_ISL_880337, EPI_ISL_880338, EPI_ISL_880339, EPI_ISL_880340, EPI_ISL_880341, EPI_ISL_880342, EPI_ISL_880343, EPI_ISL_880344, EPI_ISL_880345, EPI_ISL_880346, EPI_ISL_880347, EPI_ISL_880348, EPI_ISL_880349, EPI_ISL_880350, EPI_ISL_880351, EPI_ISL_880352, EPI_ISL_880353, EPI_ISL_880354, EPI_ISL_880355, EPI_ISL_880356, EPI_ISL_880357, EPI_ISL_880358, EPI_ISL_880359, EPI_ISL_880360, EPI_ISL_880361, EPI_ISL_880362, EPI_ISL_880363,

EPI_ISL_880364, EPI_ISL_880365, EPI_ISL_880366, EPI_ISL_880367, EPI_ISL_880368, EPI_ISL_880369, EPI_ISL_880370, EPI_ISL_880371, EPI_ISL_880372, EPI_ISL_880373, EPI_ISL_880374, EPI_ISL_880375, EPI_ISL_880376, EPI_ISL_880377, EPI_ISL_880378, EPI_ISL_880379, EPI_ISL_880380, EPI_ISL_880381, EPI_ISL_880382, EPI_ISL_880383, EPI_ISL_880384, EPI_ISL_880385, EPI_ISL_880386, EPI_ISL_880387, EPI_ISL_880388, EPI_ISL_880389, EPI_ISL_880390, EPI_ISL_880391, EPI_ISL_880392, EPI_ISL_880393, EPI_ISL_880394, EPI_ISL_880395, EPI_ISL_880396, EPI_ISL_880397, EPI_ISL_880398, EPI_ISL_880399, EPI_ISL_880400, EPI_ISL_880401, EPI_ISL_880402, EPI_ISL_880403, EPI_ISL_880404, EPI_ISL_880405, EPI_ISL_880406, EPI_ISL_880407, EPI_ISL_880408, EPI_ISL_880409, EPI_ISL_880410, EPI_ISL_880411, EPI_ISL_880412, EPI_ISL_880413, EPI_ISL_880414, EPI_ISL_880415, EPI_ISL_880416, EPI_ISL_880417, EPI_ISL_880418, EPI_ISL_880419, EPI_ISL_880420, EPI_ISL_880421, EPI_ISL_880422, EPI_ISL_880423, EPI_ISL_880424, EPI_ISL_880425, EPI_ISL_880426, EPI_ISL_880427, EPI_ISL_880428, EPI_ISL_880429, EPI_ISL_880430, EPI_ISL_880431, EPI_ISL_880432, EPI_ISL_880433, EPI_ISL_880434, EPI_ISL_880435, EPI_ISL_880436, EPI_ISL_880437, EPI_ISL_880438, EPI_ISL_880439, EPI_ISL_880440, EPI_ISL_880441, EPI_ISL_880442, EPI_ISL_880443, EPI_ISL_880444, EPI_ISL_880445, EPI_ISL_880446, EPI_ISL_880447, EPI_ISL_880448, EPI_ISL_880449, EPI_ISL_880450, EPI_ISL_880451, EPI_ISL_880452, EPI_ISL_880453, EPI_ISL_880454, EPI_ISL_880455, EPI_ISL_880456, EPI_ISL_880457, EPI_ISL_880458, EPI_ISL_880459, EPI_ISL_880460, EPI_ISL_880461, EPI_ISL_880462, EPI_ISL_880463, EPI_ISL_880464, EPI_ISL_880465, EPI_ISL_880466, EPI_ISL_880467, EPI_ISL_880468, EPI_ISL_880469, EPI_ISL_880470, EPI_ISL_880471, EPI_ISL_880472, EPI_ISL_880473, EPI_ISL_880474, EPI_ISL_880475, EPI_ISL_880476, EPI_ISL_880477, EPI_ISL_880478, EPI_ISL_880479, EPI_ISL_880480, EPI_ISL_880481, EPI_ISL_880482, EPI_ISL_880483, EPI_ISL_880484, EPI_ISL_880485, EPI_ISL_880486, EPI_ISL_880487, EPI_ISL_880488, EPI_ISL_880489, EPI_ISL_880490, EPI_ISL_880491, EPI_ISL_880492, EPI_ISL_880493, EPI_ISL_880494, EPI_ISL_880495, EPI_ISL_880496, EPI_ISL_880497, EPI_ISL_880498, EPI_ISL_880499, EPI_ISL_880500, EPI_ISL_880501, EPI_ISL_880502, EPI_ISL_880503, EPI_ISL_880504, EPI_ISL_880505, EPI_ISL_880506, EPI_ISL_880507, EPI_ISL_880508, EPI_ISL_880509, EPI_ISL_880510, EPI_ISL_880511, EPI_ISL_880512, EPI_ISL_880513, EPI_ISL_880514, EPI_ISL_880515, EPI_ISL_880516, EPI_ISL_880517, EPI_ISL_880518, EPI_ISL_880519, EPI_ISL_880520, EPI_ISL_880521, EPI_ISL_880522, EPI_ISL_880523, EPI_ISL_880524, EPI_ISL_880525, EPI_ISL_880526, EPI_ISL_880527, EPI_ISL_880528, EPI_ISL_880529, EPI_ISL_880530, EPI_ISL_880531, EPI_ISL_880532, EPI_ISL_880533, EPI_ISL_880534, EPI_ISL_880535, EPI_ISL_880536, EPI_ISL_880537, EPI_ISL_880538, EPI_ISL_880539, EPI_ISL_880540, EPI_ISL_880541, EPI_ISL_880542, EPI_ISL_880543, EPI_ISL_880544, EPI_ISL_880545, EPI_ISL_880546, EPI_ISL_880547, EPI_ISL_880548, EPI_ISL_880549, EPI_ISL_880550, EPI_ISL_880551, EPI_ISL_880552, EPI_ISL_880553, EPI_ISL_880554, EPI_ISL_880555, EPI_ISL_880556, EPI_ISL_880557, EPI_ISL_880558, EPI_ISL_880559, EPI_ISL_880560, EPI_ISL_880561, EPI_ISL_880562, EPI_ISL_880563, EPI_ISL_880564, EPI_ISL_880565, EPI_ISL_880566, EPI_ISL_880567, EPI_ISL_880568, EPI_ISL_880569, EPI_ISL_880570, EPI_ISL_880571, EPI_ISL_880572, EPI_ISL_880573, EPI_ISL_880574, EPI_ISL_880575, EPI_ISL_880576, EPI_ISL_880577, EPI_ISL_880578, EPI_ISL_880579, EPI_ISL_880580, EPI_ISL_880581, EPI_ISL_880582, EPI_ISL_880583			
see above	Lighthouse Lab in Alderley Park	Wellcome Sanger Institute for the COVID-19 Genomics UK (COG-UK) Consortium	Jacquelyn Wynn, Mairead Hyland, The Lighthouse Lab in Alderley Park and Alex Alderton, Roberto Amato, Sonia Goncalves, Ewan Harrison, David K. Jackson, Ian Johnston, Dominic Kwiatkowski, Cordelia Langford, John Sillitoe on behalf of the Wellcome Sanger Institute COVID-19 Surveillance Team
EPI_ISL_880584, EPI_ISL_880585, EPI_ISL_880587, EPI_ISL_880588, EPI_ISL_880589, EPI_ISL_880592, EPI_ISL_880593, EPI_ISL_880594, EPI_ISL_880597, EPI_ISL_880598, EPI_ISL_880600, EPI_ISL_880603, EPI_ISL_880604, EPI_ISL_880605, EPI_ISL_880607, EPI_ISL_880612, EPI_ISL_880614, EPI_ISL_880617, EPI_ISL_880621, EPI_ISL_880622, EPI_ISL_880624, EPI_ISL_880625, EPI_ISL_880628, EPI_ISL_880630, EPI_ISL_880631, EPI_ISL_880632, EPI_ISL_880633, EPI_ISL_880634, EPI_ISL_880635, EPI_ISL_880636, EPI_ISL_880638, EPI_ISL_880640, EPI_ISL_880643, EPI_ISL_880644, EPI_ISL_880645, EPI_ISL_880646, EPI_ISL_880648, EPI_ISL_880649, EPI_ISL_880650, EPI_ISL_880652, EPI_ISL_880656, EPI_ISL_880658, EPI_ISL_880659, EPI_ISL_880661, EPI_ISL_880663, EPI_ISL_880664, EPI_ISL_880666, EPI_ISL_880670, EPI_ISL_880671, EPI_ISL_880676, EPI_ISL_880678, EPI_ISL_880680, EPI_ISL_880681, EPI_ISL_880683, EPI_ISL_880684, EPI_ISL_880685, EPI_ISL_880686, EPI_ISL_880687, EPI_ISL_880688, EPI_ISL_880689, EPI_ISL_880690, EPI_ISL_880691, EPI_ISL_880692, EPI_ISL_880693, EPI_ISL_880694, EPI_ISL_880696, EPI_ISL_880697, EPI_ISL_880698, EPI_ISL_880699, EPI_ISL_880700, EPI_ISL_880702, EPI_ISL_880703, EPI_ISL_880704, EPI_ISL_880707, EPI_ISL_880711, EPI_ISL_880712, EPI_ISL_880714, EPI_ISL_880715, EPI_ISL_880716, EPI_ISL_880719, EPI_ISL_880720, EPI_ISL_880723, EPI_ISL_880728, EPI_ISL_880730, EPI_ISL_880731, EPI_ISL_880732, EPI_ISL_880733, EPI_ISL_880734, EPI_ISL_880735, EPI_ISL_880741, EPI_ISL_880742, EPI_ISL_880743, EPI_ISL_880744, EPI_ISL_880747, EPI_ISL_880748, EPI_ISL_880749, EPI_ISL_880750, EPI_ISL_880754, EPI_ISL_880756, EPI_ISL_880757, EPI_ISL_880758, EPI_ISL_880760, EPI_ISL_880761, EPI_ISL_880764, EPI_ISL_880765, EPI_ISL_880767, EPI_ISL_880768, EPI_ISL_880770, EPI_ISL_880771, EPI_ISL_880772, EPI_ISL_880773, EPI_ISL_880777, EPI_ISL_880778, EPI_ISL_880780, EPI_ISL_880782, EPI_ISL_880783, EPI_ISL_880784, EPI_ISL_880787, EPI_ISL_880788, EPI_ISL_880790, EPI_ISL_880793, EPI_ISL_880794, EPI_ISL_880795, EPI_ISL_880796, EPI_ISL_880801, EPI_ISL_880802, EPI_ISL_880804, EPI_ISL_880805, EPI_ISL_880806, EPI_ISL_880807, EPI_ISL_880808, EPI_ISL_880810, EPI_ISL_880811, EPI_ISL_880814, EPI_ISL_880815, EPI_ISL_880816, EPI_ISL_880820, EPI_ISL_880821, EPI_ISL_880823, EPI_ISL_880825, EPI_ISL_880827, EPI_ISL_880828, EPI_ISL_880832, EPI_ISL_880834, EPI_ISL_880837, EPI_ISL_880842, EPI_ISL_880844, EPI_ISL_880845, EPI_ISL_880846, EPI_ISL_880848, EPI_ISL_880849, EPI_ISL_880850, EPI_ISL_880851, EPI_ISL_880852, EPI_ISL_880855, EPI_ISL_880859, EPI_ISL_880860, EPI_ISL_880861, EPI_ISL_880863, EPI_ISL_880864, EPI_ISL_880865, EPI_ISL_880867, EPI_ISL_880869, EPI_ISL_880871, EPI_ISL_880872, EPI_ISL_880873, EPI_ISL_880874, EPI_ISL_880875, EPI_ISL_880876, EPI_ISL_880877, EPI_ISL_880880, EPI_ISL_880881, EPI_ISL_880882, EPI_ISL_880885, EPI_ISL_880887, EPI_ISL_880888, EPI_ISL_880890, EPI_ISL_880891, EPI_ISL_880892, EPI_ISL_880893, EPI_ISL_880896, EPI_ISL_880897, EPI_ISL_880899, EPI_ISL_880902, EPI_ISL_880904, EPI_ISL_880905, EPI_ISL_880906, EPI_ISL_880907, EPI_ISL_880912, EPI_ISL_880913, EPI_ISL_880917, EPI_ISL_880918, EPI_ISL_880919, EPI_ISL_880920, EPI_ISL_880922, EPI_ISL_880923, EPI_ISL_880924, EPI_ISL_880926, EPI_ISL_880927, EPI_ISL_880928, EPI_ISL_880929, EPI_ISL_880930, EPI_ISL_880931, EPI_ISL_880932			
see above	Lighthouse Lab in Milton Keynes	Wellcome Sanger Institute for the COVID-19 Genomics UK (COG-UK) Consortium	The Lighthouse Lab in Milton Keynes and Alex Alderton, Roberto Amato, Sonia Goncalves, Ewan Harrison, David K. Jackson, Ian Johnston, Dominic Kwiatkowski, Cordelia Langford, John Sillitoe on behalf of the Wellcome Sanger Institute COVID-19 Surveillance Team
EPI_ISL_880936, EPI_ISL_880937, EPI_ISL_880938, EPI_ISL_880939, EPI_ISL_880940, EPI_ISL_880941, EPI_ISL_880942, EPI_ISL_880943, EPI_ISL_880944, EPI_ISL_880945, EPI_ISL_880946, EPI_ISL_880947, EPI_ISL_880948, EPI_ISL_880949, EPI_ISL_880950, EPI_ISL_880951, EPI_ISL_880952, EPI_ISL_880953, EPI_ISL_880954, EPI_ISL_880955, EPI_ISL_880956, EPI_ISL_880957, EPI_ISL_880958, EPI_ISL_880959, EPI_ISL_880960, EPI_ISL_880961, EPI_ISL_880962, EPI_ISL_880963, EPI_ISL_880964, EPI_ISL_880965, EPI_ISL_880966, EPI_ISL_880967, EPI_ISL_880968, EPI_ISL_880969, EPI_ISL_880970, EPI_ISL_880971, EPI_ISL_880972, EPI_ISL_880973, EPI_ISL_880974, EPI_ISL_880975, EPI_ISL_880976, EPI_ISL_880977, EPI_ISL_880978, EPI_ISL_880979, EPI_ISL_880980, EPI_ISL_880981, EPI_ISL_880982, EPI_ISL_880983, EPI_ISL_880984, EPI_ISL_880985, EPI_ISL_880986, EPI_ISL_880987, EPI_ISL_880988, EPI_ISL_880989, EPI_ISL_880990, EPI_ISL_880991, EPI_ISL_880992, EPI_ISL_880993, EPI_ISL_880994, EPI_ISL_880995, EPI_ISL_880996, EPI_ISL_880997, EPI_ISL_880998, EPI_ISL_880999, EPI_ISL_881000, EPI_ISL_881001, EPI_ISL_881002, EPI_ISL_881003, EPI_ISL_881004, EPI_ISL_881005, EPI_ISL_881006, EPI_ISL_881007, EPI_ISL_881008, EPI_ISL_881009, EPI_ISL_881010, EPI_ISL_881011, EPI_ISL_881012, EPI_ISL_881013, EPI_ISL_881014, EPI_ISL_881015, EPI_ISL_881016, EPI_ISL_881017, EPI_ISL_881018, EPI_ISL_881019, EPI_ISL_881020, EPI_ISL_881021, EPI_ISL_881022, EPI_ISL_881023, EPI_ISL_881024, EPI_ISL_881025, EPI_ISL_881026, EPI_ISL_881027, EPI_ISL_881028, EPI_ISL_881029, EPI_ISL_881030, EPI_ISL_881031, EPI_ISL_881032, EPI_ISL_881033, EPI_ISL_881034, EPI_ISL_881035, EPI_ISL_881036, EPI_ISL_881037, EPI_ISL_881038, EPI_ISL_881039, EPI_ISL_881040, EPI_ISL_881041, EPI_ISL_881042, EPI_ISL_881043, EPI_ISL_881044, EPI_ISL_881045, EPI_ISL_881046, EPI_ISL_881047, EPI_ISL_881048, EPI_ISL_881049, EPI_ISL_881050, EPI_ISL_881051, EPI_ISL_881052, EPI_ISL_881053, EPI_ISL_881054, EPI_ISL_881055, EPI_ISL_881056, EPI_ISL_881057, EPI_ISL_881058, EPI_ISL_881059, EPI_ISL_881060, EPI_ISL_881061, EPI_ISL_881062, EPI_ISL_881063, EPI_ISL_881064, EPI_ISL_881065, EPI_ISL_881066, EPI_ISL_881067, EPI_ISL_881068, EPI_ISL_881069, EPI_ISL_881070, EPI_ISL_881071, EPI_ISL_881072, EPI_ISL_881073, EPI_ISL_881074, EPI_ISL_881075, EPI_ISL_881076, EPI_ISL_881077, EPI_ISL_881078, EPI_ISL_881079, EPI_ISL_881080, EPI_ISL_881081, EPI_ISL_881082, EPI_ISL_881083, EPI_ISL_881084, EPI_ISL_881085, EPI_ISL_881086, EPI_ISL_881087, EPI_ISL_881088, EPI_ISL_881089, EPI_ISL_881090, EPI_ISL_881091, EPI_ISL_881092, EPI_ISL_881093, EPI_ISL_881094, EPI_ISL_881095, EPI_ISL_881096, EPI_ISL_881097, EPI_ISL_881098, EPI_ISL_881099, EPI_ISL_881100, EPI_ISL_881101, EPI_ISL_881102, EPI_ISL_881103, EPI_ISL_881104, EPI_ISL_881105, EPI_ISL_881106, EPI_ISL_881107, EPI_ISL_881108, EPI_ISL_881109, EPI_ISL_881110, EPI_ISL_881111, EPI_ISL_881112, EPI_ISL_881113, EPI_ISL_881114, EPI_ISL_881115, EPI_ISL_881116, EPI_ISL_881117, EPI_ISL_881118, EPI_ISL_881119, EPI_ISL_881120, EPI_ISL_881121, EPI_ISL_881122, EPI_ISL_881123, EPI_ISL_881124, EPI_ISL_881125, EPI_ISL_881126, EPI_ISL_881127, EPI_ISL_881128, EPI_ISL_881129, EPI_ISL_881130, EPI_ISL_881131, EPI_ISL_881132, EPI_ISL_881133, EPI_ISL_881134, EPI_ISL_881135, EPI_ISL_881136, EPI_ISL_881137, EPI_ISL_881138, EPI_ISL_881139, EPI_ISL_881140, EPI_ISL_881141, EPI_ISL_881142, EPI_ISL_881143, EPI_ISL_881144, EPI_ISL_881145, EPI_ISL_881146, EPI_ISL_881147, EPI_ISL_881148, EPI_ISL_881149, EPI_ISL_881150, EPI_ISL_881151, EPI_ISL_881152, EPI_ISL_881153, EPI_ISL_881154, EPI_ISL_881155, EPI_ISL_881156, EPI_ISL_881157, EPI_ISL_881158, EPI_ISL_881159, EPI_ISL_881160, EPI_ISL_881161, EPI_ISL_881162, EPI_ISL_881163, EPI_ISL_881164, EPI_ISL_881165, EPI_ISL_881166, EPI_ISL_881167, EPI_ISL_881168, EPI_ISL_881169, EPI_ISL_881170, EPI_ISL_881171, EPI_ISL_881172, EPI_ISL_881173, EPI_ISL_881174, EPI_ISL_881175, EPI_ISL_881176, EPI_ISL_881177, EPI_ISL_881178, EPI_ISL_881179, EPI_ISL_881180, EPI_ISL_881181, EPI_ISL_881182, EPI_ISL_881183, EPI_ISL_881184, EPI_ISL_881185, EPI_ISL_881186, EPI_ISL_881187, EPI_ISL_881188, EPI_ISL_881189, EPI_ISL_881190, EPI_ISL_881191, EPI_ISL_881192, EPI_ISL_881193, EPI_ISL_881194, EPI_ISL_881195, EPI_ISL_881196, EPI_ISL_881197, EPI_ISL_881198, EPI_ISL_881199, EPI_ISL_881200, EPI_ISL_881201, EPI_ISL_881202, EPI_ISL_881203, EPI_ISL_881204, EPI_ISL_881205, EPI_ISL_881206, EPI_ISL_881207, EPI_ISL_881208, EPI_ISL_881209, EPI_ISL_881210, EPI_ISL_881211, EPI_ISL_881212, EPI_ISL_881213, EPI_ISL_881214, EPI_ISL_881215, EPI_ISL_881216, EPI_ISL_881217, EPI_ISL_881218, EPI_ISL_881219, EPI_ISL_881220, EPI_ISL_881221, EPI_ISL_881222, EPI_ISL_881223, EPI_ISL_881224, EPI_ISL_881225, EPI_ISL_881226, EPI_ISL_881227, EPI_ISL_881228, EPI_ISL_881229, EPI_ISL_881230, EPI_ISL_881231, EPI_ISL_881232, EPI_ISL_881233, EPI_ISL_881234, EPI_ISL_881235, EPI_ISL_881236, EPI_ISL_881237, EPI_ISL_881238, EPI_ISL_881239, EPI_ISL_881240, EPI_ISL_881241, EPI_ISL_881242, EPI_ISL_881243, EPI_ISL_881244, EPI_ISL_881245, EPI_ISL_881246, EPI_ISL_881247, EPI_ISL_881248, EPI_ISL_881249, EPI_ISL_881250, EPI_ISL_881251, EPI_ISL_881252, EPI_ISL_881253, EPI_ISL_881254, EPI_ISL_881255, EPI_ISL_881256, EPI_ISL_881257, EPI_ISL_881258, EPI_ISL_881259, EPI_ISL_881260, EPI_ISL_881261, EPI_ISL_881262, EPI_ISL_881263, EPI_ISL_881264, EPI_ISL_881265, EPI_ISL_881266, EPI_ISL_881267, EPI_ISL_881268, EPI_ISL_881269, EPI_ISL_881270, EPI_ISL_881271, EPI_ISL_881272, EPI_ISL_881273, EPI_ISL_881274, EPI_ISL_881275, EPI_ISL_881276, EPI_ISL_881278, EPI_ISL_881279, EPI_ISL_881280, EPI_ISL_881282, EPI_ISL_881284, EPI_ISL_881285, EPI_ISL_881286, EPI_ISL_881287, EPI_ISL_881288, EPI_ISL_881292, EPI_ISL_881294, EPI_ISL_881295, EPI_ISL_881296, EPI_ISL_881297, EPI_ISL_881299, EPI_ISL_881300, EPI_ISL_881301, EPI_ISL_881302, EPI_ISL_881303, EPI_ISL_881304, EPI_ISL_881305, EPI_ISL_881308, EPI_ISL_881311, EPI_ISL_881312, EPI_ISL_881313, EPI_ISL_881314, EPI_ISL_881316, EPI_ISL_881317, EPI_ISL_881319, EPI_ISL_881320, EPI_ISL_881321, EPI_ISL_881322, EPI_ISL_881323, EPI_ISL_881324, EPI_ISL_881325, EPI_ISL_881327, EPI_ISL_881328, EPI_ISL_881329, EPI_ISL_881330, EPI_ISL_881331, EPI_ISL_881332, EPI_ISL_881333, EPI_ISL_881334, EPI_ISL_881335, EPI_ISL_881336, EPI_ISL_881337, EPI_ISL_881338, EPI_ISL_881339, EPI_ISL_881340, EPI_ISL_881342, EPI_ISL_881343, EPI_ISL_881344, EPI_ISL_881345, EPI_ISL_881346, EPI_ISL_881347, EPI_ISL_881348, EPI_ISL_881349, EPI_ISL_881350, EPI_ISL_881351, EPI_ISL_881352, EPI_ISL_881353, EPI_ISL_881354, EPI_ISL_881355, EPI_ISL_881356, EPI_ISL_881357, EPI_ISL_881358, EPI_ISL_881360, EPI_ISL_881361, EPI_ISL_881362, EPI_ISL_881363, EPI_ISL_881364, EPI_ISL_881365, EPI_ISL_881366, EPI_ISL_881367, EPI_ISL_881368, EPI_ISL_881369, EPI_ISL_881370, EPI_ISL_881371, EPI_ISL_881372, EPI_ISL_881373, EPI_ISL_881374, EPI_ISL_881375, EPI_ISL_881376, EPI_ISL_881377, EPI_ISL_881378, EPI_ISL_881379, EPI_ISL_881380, EPI_ISL_881381, EPI_ISL_881382, EPI_ISL_881383, EPI_ISL_881384, EPI_ISL_881385, EPI_ISL_881387, EPI_ISL_881389, EPI_ISL_881390, EPI_ISL_881391, EPI_ISL_881392, EPI_ISL_881393, EPI_ISL_881394, EPI_ISL_881395, EPI_ISL_881396, EPI_ISL_881397, EPI_ISL_881398, EPI_ISL_881399, EPI_ISL_881400, EPI_ISL_881401, EPI_ISL_881402, EPI_ISL_881403, EPI_ISL_881404, EPI_ISL_881405, EPI_ISL_881406, EPI_ISL_881407, EPI_ISL_881408, EPI_ISL_881409, EPI_ISL_881410, EPI_ISL_881411, EPI_ISL_881412, EPI_ISL_881413, EPI_ISL_881415, EPI_ISL_881416, EPI_ISL_881417, EPI_ISL_881418, EPI_ISL_881419, EPI_ISL_881420, EPI_ISL_881421, EPI_ISL_881422, EPI_ISL_881423, EPI_ISL_881424, EPI_ISL_881425, EPI_ISL_881426, EPI_ISL_881427, EPI_ISL_881428, EPI_ISL_881430, EPI_ISL_881431, EPI_ISL_881432, EPI_ISL_881433, EPI_ISL_881434, EPI_ISL_881435, EPI_ISL_881436, EPI_ISL_881437, EPI_ISL_881438, EPI_ISL_881439, EPI_ISL_881440, EPI_ISL_881441, EPI_ISL_881442, EPI_ISL_881443, EPI_ISL_881444, EPI_ISL_881445, EPI_ISL_881446, EPI_ISL_881447, EPI_ISL_881448, EPI_ISL_881449, EPI_ISL_881450, EPI_ISL_881451, EPI_ISL_881452, EPI_ISL_881453, EPI_ISL_881454, EPI_ISL_881456, EPI_ISL_881457, EPI_ISL_881458, EPI_ISL_881459, EPI_ISL_881460, EPI_ISL_881462, EPI_ISL_881463, EPI_ISL_881464, EPI_ISL_881465, EPI_ISL_881466, EPI_ISL_881467, EPI_ISL_881468, EPI_ISL_881469, EPI_ISL_881470, EPI_ISL_881471, EPI_ISL_881472, EPI_ISL_881473, EPI_ISL_881474, EPI_ISL_881475, EPI_ISL_881476, EPI_ISL_881477, EPI_ISL_881478, EPI_ISL_881479, EPI_ISL_881480, EPI_ISL_881481, EPI_ISL_881482, EPI_ISL_881483, EPI_ISL_881484, EPI_ISL_881485, EPI_ISL_881486, EPI_ISL_881488, EPI_ISL_881489, EPI_ISL_881490, EPI_ISL_881491, EPI_ISL_881492, EPI_ISL_881493, EPI_ISL_881496, EPI_ISL_881497, EPI_ISL_881498, EPI_ISL_881499, EPI_ISL_881500, EPI_ISL_881501, EPI_ISL_881502, EPI_ISL_881503, EPI_ISL_881504, EPI_ISL_881505, EPI_ISL_881506, EPI_ISL_881507, EPI_ISL_881508, EPI_ISL_881510, EPI_ISL_881511, EPI_ISL_881512, EPI_ISL_881513, EPI_ISL_881514, EPI_ISL_881515, EPI_ISL_881516, EPI_ISL_881517, EPI_ISL_881519, EPI_ISL_881520, EPI_ISL_881523, EPI_ISL_881524, EPI_ISL_881525, EPI_ISL_881526, EPI_ISL_881527, EPI_ISL_881528, EPI_ISL_881529, EPI_ISL_881530, EPI_ISL_881532, EPI_ISL_881533, EPI_ISL_881534, EPI_ISL_881535, EPI_ISL_881536, EPI_ISL_881537, EPI_ISL_881538, EPI_ISL_881539, EPI_ISL_881541, EPI_ISL_881542, EPI_ISL_881544, EPI_ISL_881545, EPI_ISL_881547, EPI_ISL_881548, EPI_ISL_881549, EPI_ISL_881551, EPI_ISL_881552, EPI_ISL_881553, EPI_ISL_881554, EPI_ISL_881555, EPI_ISL_881556, EPI_ISL_881557, EPI_ISL_881558, EPI_ISL_881559, EPI_ISL_881560, EPI_ISL_881561, EPI_ISL_881562, EPI_ISL_881563, EPI_ISL_881564, EPI_ISL_881565, EPI_ISL_881566, EPI_ISL_881568, EPI_ISL_881569, EPI_ISL_881572, EPI_ISL_881573, EPI_ISL_881574, EPI_ISL_881575, EPI_ISL_881577, EPI_ISL_881578, EPI_ISL_881579, EPI_ISL_881581, EPI_ISL_881583, EPI_ISL_881584, EPI_ISL_881585, EPI_ISL_881587, EPI_ISL_881589, EPI_ISL_881590, EPI_ISL_881591, EPI_ISL_881592, EPI_ISL_881593, EPI_ISL_881594, EPI_ISL_881595, EPI_ISL_881596, EPI_ISL_881597, EPI_ISL_881599, EPI_ISL_881601, EPI_ISL_881602, EPI_ISL_881603, EPI_ISL_881604, EPI_ISL_881605, EPI_ISL_881606, EPI_ISL_881608, EPI_ISL_881609, EPI_ISL_881610, EPI_ISL_881611, EPI_ISL_881613, EPI_ISL_881614, EPI_ISL_881615, EPI_ISL_881616, EPI_ISL_881617			
see above	Lighthouse Lab in Alderley Park	Wellcome Sanger Institute for the COVID-19 Genomics UK (COG-UK) Consortium	Jacquelyn Wynn, Mairead Hyland, The Lighthouse Lab in Alderley Park and Alex Alderton, Roberto Amato, Sonia Goncalves, Ewan Harrison, David K. Jackson, Ian Johnston, Dominic Kwiatkowski, Cordelia Langford, John Sillitoe on behalf of the Wellcome Sanger Institute COVID-19 Surveillance Team
EPI_ISL_881620, EPI_ISL_881622	Lighthouse Lab in Cambridge	Wellcome Sanger Institute for the COVID-19 Genomics UK (COG-UK) Consortium	Rob Howes, The Lighthouse Lab in Cambridge and Alex Alderton, Roberto Amato, Sonia Goncalves, Ewan Harrison, David K. Jackson, Ian Johnston, Dominic Kwiatkowski, Cordelia Langford, John Sillitoe on behalf of the Wellcome Sanger Institute COVID-19 Surveillance Team
EPI_ISL_882300, EPI_ISL_882605, EPI_ISL_882606, EPI_ISL_882607, EPI_ISL_882608	Lighthouse Lab in Alderley Park	Wellcome Sanger Institute for the COVID-19 Genomics UK (COG-UK) Consortium	Jacquelyn Wynn, Mairead Hyland, The Lighthouse Lab in Alderley Park and Alex Alderton, Roberto Amato, Sonia Goncalves, Ewan Harrison, David K. Jackson, Ian Johnston, Dominic Kwiatkowski, Cordelia Langford, John Sillitoe on behalf of the Wellcome Sanger Institute COVID-19 Surveillance Team
EPI_ISL_882609	Lighthouse Lab in Glasgow	Wellcome Sanger Institute for the COVID-19 Genomics UK (COG-UK) Consortium	Harper VanSteenhouse, Yumi Kasai, David Gray, Carol Clugston, Anna Dominiczak and Alex Alderton, Roberto Amato, Sonia Goncalves, Ewan Harrison, David K. Jackson, Ian Johnston, Dominic Kwiatkowski, Cordelia Langford, John Sillitoe on behalf of the Wellcome Sanger Institute COVID-19 Surveillance Team
EPI_ISL_885160, EPI_ISL_885163, EPI_ISL_885165, EPI_ISL_885178, EPI_ISL_885180, EPI_ISL_885183, EPI_ISL_885187, EPI_ISL_885189, EPI_ISL_885197, EPI_ISL_885204, EPI_ISL_885209, EPI_ISL_885211, EPI_ISL_885213, EPI_ISL_885214, EPI_ISL_885215, EPI_ISL_885234, EPI_ISL_885238, EPI_ISL_885244, EPI_ISL_885248, EPI_ISL_885250, EPI_ISL_885264, EPI_ISL_885273, EPI_ISL_885274, EPI_ISL_885275, EPI_ISL_885277, EPI_ISL_885288, EPI_ISL_885289, EPI_ISL_885291, EPI_ISL_885295, EPI_ISL_885297, EPI_ISL_885299, EPI_ISL_885302, EPI_ISL_885305, EPI_ISL_885307, EPI_ISL_885311, EPI_ISL_885317, EPI_ISL_885319, EPI_ISL_885321, EPI_ISL_885323, EPI_ISL_885326, EPI_ISL_885335, EPI_ISL_885337, EPI_ISL_885349, EPI_ISL_885352, EPI_ISL_885353, EPI_ISL_885359, EPI_ISL_885361, EPI_ISL_885364, EPI_ISL_885365, EPI_ISL_885367, EPI_ISL_885369, EPI_ISL_885371, EPI_ISL_885374, EPI_ISL_885376, EPI_ISL_885388, EPI_ISL_885390, EPI_ISL_885392, EPI_ISL_885396, EPI_ISL_885397, EPI_ISL_885402, EPI_ISL_885411, EPI_ISL_885413, EPI_ISL_885419, EPI_ISL_885422, EPI_ISL_885427, EPI_ISL_885431, EPI_ISL_885434, EPI_ISL_885452, EPI_ISL_885455, EPI_ISL_885458, EPI_ISL_885459, EPI_ISL_885461, EPI_ISL_885463, EPI_ISL_885471, EPI_ISL_885472, EPI_ISL_885473, EPI_ISL_885476, EPI_ISL_885478, EPI_ISL_885479, EPI_ISL_885482, EPI_ISL_885484, EPI_ISL_885499, EPI_ISL_885500, EPI_ISL_885503, EPI_ISL_885513, EPI_ISL_885541, EPI_ISL_885543, EPI_ISL_885557, EPI_ISL_885580, EPI_ISL_885586, EPI_ISL_885587, EPI_ISL_885589, EPI_ISL_885597, EPI_ISL_885619, EPI_ISL_885630, EPI_ISL_885637, EPI_ISL_885640, EPI_ISL_885649, EPI_ISL_885655, EPI_ISL_885661, EPI_ISL_885680, EPI_ISL_885683, EPI_ISL_885690, EPI_ISL_885705, EPI_ISL_885725, EPI_ISL_885754, EPI_ISL_885758, EPI_ISL_885762, EPI_ISL_885766, EPI_ISL_885813, EPI_ISL_885828, EPI_ISL_885855, EPI_ISL_885860, EPI_ISL_885867, EPI_ISL_885880, EPI_ISL_885889, EPI_ISL_885912, EPI_ISL_885916, EPI_ISL_885918, EPI_ISL_885935, EPI_ISL_885954, EPI_ISL_885958, EPI_ISL_885962, EPI_ISL_885966, EPI_ISL_886009, EPI_ISL_886016, EPI_ISL_886039, EPI_ISL_886056, EPI_ISL_886057, EPI_ISL_886103, EPI_ISL_886121, EPI_ISL_886136, EPI_ISL_886142, EPI_ISL_886143			

see above	Lighthouse Lab in Alderley Park	Wellcome Sanger Institute for the COVID-19 Genomics UK (COG-UK) Consortium	Jacquelyn Wynn, Mairead Hyland, The Lighthouse Lab in Alderley Park and Alex Alderton, Roberto Amato, Sonia Goncalves, Ewan Harrison, David K. Jackson, Ian Johnston, Dominic Kwiatkowski, Cordelia Langford, John Sillitoe on behalf of the Wellcome Sanger Institute COVID-19 Surveillance Team
EPI_ISL_891700	Lighthouse Lab in Glasgow	Wellcome Sanger Institute for the COVID-19 Genomics UK (COG-UK) Consortium	Harper VanSteenhouse, Yumi Kasai, David Gray, Carol Clugston, Anna Dominiczak and Alex Alderton, Roberto Amato, Sonia Goncalves, Ewan Harrison, David K. Jackson, Ian Johnston, Dominic Kwiatkowski, Cordelia Langford, John Sillitoe on behalf of the Wellcome Sanger Institute COVID-19 Surveillance Team
EPI_ISL_891899, EPI_ISL_891901, EPI_ISL_891903, EPI_ISL_891905, EPI_ISL_891908, EPI_ISL_891909, EPI_ISL_891914, EPI_ISL_891918, EPI_ISL_891920, EPI_ISL_891921, EPI_ISL_891923, EPI_ISL_891924, EPI_ISL_891925, EPI_ISL_891926, EPI_ISL_891927, EPI_ISL_891928, EPI_ISL_891929, EPI_ISL_891930, EPI_ISL_891931, EPI_ISL_891932, EPI_ISL_891933, EPI_ISL_891934, EPI_ISL_891935, EPI_ISL_891936, EPI_ISL_891937, EPI_ISL_891938, EPI_ISL_891939, EPI_ISL_891940, EPI_ISL_891941, EPI_ISL_891942, EPI_ISL_891943, EPI_ISL_891944, EPI_ISL_891945, EPI_ISL_891946, EPI_ISL_891949, EPI_ISL_891950, EPI_ISL_891953, EPI_ISL_891954, EPI_ISL_891957, EPI_ISL_891958, EPI_ISL_891959, EPI_ISL_891962, EPI_ISL_891963, EPI_ISL_891967, EPI_ISL_891969, EPI_ISL_891970, EPI_ISL_891972, EPI_ISL_891973, EPI_ISL_891974, EPI_ISL_891975, EPI_ISL_891976, EPI_ISL_891977, EPI_ISL_891978, EPI_ISL_891979, EPI_ISL_891980, EPI_ISL_891981, EPI_ISL_891982, EPI_ISL_891983, EPI_ISL_891984, EPI_ISL_891985, EPI_ISL_891986, EPI_ISL_891987, EPI_ISL_891988, EPI_ISL_891989, EPI_ISL_891990, EPI_ISL_891991, EPI_ISL_891992, EPI_ISL_891993, EPI_ISL_891994, EPI_ISL_891995, EPI_ISL_891996, EPI_ISL_891997, EPI_ISL_891998, EPI_ISL_891999, EPI_ISL_892000, EPI_ISL_892001, EPI_ISL_892002, EPI_ISL_892003, EPI_ISL_892004, EPI_ISL_892005, EPI_ISL_892006, EPI_ISL_892007, EPI_ISL_892008, EPI_ISL_892009, EPI_ISL_892012, EPI_ISL_892013, EPI_ISL_892014, EPI_ISL_892015, EPI_ISL_892016, EPI_ISL_892017, EPI_ISL_892018, EPI_ISL_892019, EPI_ISL_892020, EPI_ISL_892021, EPI_ISL_892022, EPI_ISL_892023, EPI_ISL_892024, EPI_ISL_892025, EPI_ISL_892026, EPI_ISL_892027, EPI_ISL_892028, EPI_ISL_892029, EPI_ISL_892030, EPI_ISL_892031, EPI_ISL_892032, EPI_ISL_892033, EPI_ISL_892034, EPI_ISL_892035, EPI_ISL_892036, EPI_ISL_892037, EPI_ISL_892038, EPI_ISL_892039, EPI_ISL_892040, EPI_ISL_892041, EPI_ISL_892042, EPI_ISL_892043, EPI_ISL_892044, EPI_ISL_892045, EPI_ISL_892046, EPI_ISL_892047, EPI_ISL_892048, EPI_ISL_892049, EPI_ISL_892056, EPI_ISL_892127, EPI_ISL_892128, EPI_ISL_892129, EPI_ISL_892130, EPI_ISL_892131, EPI_ISL_892132, EPI_ISL_892133, EPI_ISL_892134, EPI_ISL_892135, EPI_ISL_892136, EPI_ISL_892137, EPI_ISL_892138, EPI_ISL_892139, EPI_ISL_892140, EPI_ISL_892141, EPI_ISL_892142, EPI_ISL_892143, EPI_ISL_892144, EPI_ISL_892145, EPI_ISL_892146, EPI_ISL_892147, EPI_ISL_892148, EPI_ISL_892149, EPI_ISL_892150, EPI_ISL_892151, EPI_ISL_892152, EPI_ISL_892153, EPI_ISL_892154, EPI_ISL_892155, EPI_ISL_892156, EPI_ISL_892157, EPI_ISL_892158, EPI_ISL_892159, EPI_ISL_892160, EPI_ISL_892161, EPI_ISL_892162, EPI_ISL_892163, EPI_ISL_892164, EPI_ISL_892165, EPI_ISL_892166, EPI_ISL_892167, EPI_ISL_892168, EPI_ISL_892169, EPI_ISL_892170, EPI_ISL_892171, EPI_ISL_892172, EPI_ISL_892173, EPI_ISL_892174, EPI_ISL_892175, EPI_ISL_892176, EPI_ISL_892177, EPI_ISL_892178, EPI_ISL_892180, EPI_ISL_892181, EPI_ISL_892182, EPI_ISL_892183, EPI_ISL_892184, EPI_ISL_892185, EPI_ISL_892186, EPI_ISL_892187, EPI_ISL_892188, EPI_ISL_892198, EPI_ISL_892199, EPI_ISL_892200			
see above	Lighthouse Lab in Alderley Park	Wellcome Sanger Institute for the COVID-19 Genomics UK (COG-UK) Consortium	Jacquelyn Wynn, Mairead Hyland, The Lighthouse Lab in Alderley Park and Alex Alderton, Roberto Amato, Sonia Goncalves, Ewan Harrison, David K. Jackson, Ian Johnston, Dominic Kwiatkowski, Cordelia Langford, John Sillitoe on behalf of the Wellcome Sanger Institute COVID-19 Surveillance Team
EPI_ISL_892201	Lighthouse Lab in Glasgow	Wellcome Sanger Institute for the COVID-19 Genomics UK (COG-UK) Consortium	Harper VanSteenhouse, Yumi Kasai, David Gray, Carol Clugston, Anna Dominiczak and Alex Alderton, Roberto Amato, Sonia Goncalves, Ewan Harrison, David K. Jackson, Ian Johnston, Dominic Kwiatkowski, Cordelia Langford, John Sillitoe on behalf of the Wellcome Sanger Institute COVID-19 Surveillance Team
EPI_ISL_892202, EPI_ISL_892203, EPI_ISL_892204, EPI_ISL_892205	Lighthouse Lab in Alderley Park	Wellcome Sanger Institute for the COVID-19 Genomics UK (COG-UK) Consortium	Jacquelyn Wynn, Mairead Hyland, The Lighthouse Lab in Alderley Park and Alex Alderton, Roberto Amato, Sonia Goncalves, Ewan Harrison, David K. Jackson, Ian Johnston, Dominic Kwiatkowski, Cordelia Langford, John Sillitoe on behalf of the Wellcome Sanger Institute COVID-19 Surveillance Team
EPI_ISL_908934, EPI_ISL_908936, EPI_ISL_908943, EPI_ISL_908960, EPI_ISL_908966, EPI_ISL_908969, EPI_ISL_908971, EPI_ISL_908972, EPI_ISL_908973, EPI_ISL_908975, EPI_ISL_908976, EPI_ISL_908980, EPI_ISL_908981, EPI_ISL_908984, EPI_ISL_908994, EPI_ISL_909000, EPI_ISL_909001, EPI_ISL_909012, EPI_ISL_909013, EPI_ISL_909020, EPI_ISL_909025, EPI_ISL_909028, EPI_ISL_909032, EPI_ISL_909033, EPI_ISL_909039, EPI_ISL_909042, EPI_ISL_909045, EPI_ISL_909055, EPI_ISL_909064, EPI_ISL_909073, EPI_ISL_909074, EPI_ISL_909077, EPI_ISL_909092, EPI_ISL_909098, EPI_ISL_909100, EPI_ISL_909104, EPI_ISL_909112, EPI_ISL_909115, EPI_ISL_909126, EPI_ISL_909134, EPI_ISL_909135, EPI_ISL_909138, EPI_ISL_909140, EPI_ISL_909141, EPI_ISL_909142, EPI_ISL_909143, EPI_ISL_909151, EPI_ISL_909161, EPI_ISL_909163, EPI_ISL_909164, EPI_ISL_909168, EPI_ISL_909175, EPI_ISL_909183, EPI_ISL_909188, EPI_ISL_909190, EPI_ISL_909196, EPI_ISL_909199, EPI_ISL_909209, EPI_ISL_909213, EPI_ISL_909221, EPI_ISL_909225, EPI_ISL_909229, EPI_ISL_909232, EPI_ISL_909239, EPI_ISL_909244, EPI_ISL_909249, EPI_ISL_909253, EPI_ISL_909258, EPI_ISL_909264, EPI_ISL_909404, EPI_ISL_909495			
see above	Lighthouse Lab in Milton Keynes	Wellcome Sanger Institute for the COVID-19 Genomics UK (COG-UK) Consortium	The Lighthouse Lab in Milton Keynes and Alex Alderton, Roberto Amato, Sonia Goncalves, Ewan Harrison, David K. Jackson, Ian Johnston, Dominic Kwiatkowski, Cordelia Langford, John Sillitoe on behalf of the Wellcome Sanger Institute COVID-19 Surveillance Team
EPI_ISL_909594	Lighthouse Lab in Alderley Park	Wellcome Sanger Institute for the COVID-19 Genomics UK (COG-UK) Consortium	Jacquelyn Wynn, Mairead Hyland, The Lighthouse Lab in Alderley Park and Alex Alderton, Roberto Amato, Sonia Goncalves, Ewan Harrison, David K. Jackson, Ian Johnston, Dominic Kwiatkowski, Cordelia Langford, John Sillitoe on behalf of the Wellcome Sanger Institute COVID-19 Surveillance Team
EPI_ISL_912225	Lighthouse Lab in Milton Keynes	Wellcome Sanger Institute for the COVID-19 Genomics UK (COG-UK) Consortium	The Lighthouse Lab in Milton Keynes and Alex Alderton, Roberto Amato, Sonia Goncalves, Ewan Harrison, David K. Jackson, Ian Johnston, Dominic Kwiatkowski, Cordelia Langford, John Sillitoe on behalf of the Wellcome Sanger Institute COVID-19 Surveillance Team (http://www.sanger.ac.uk/covid-team)
EPI_ISL_916622, EPI_ISL_916623, EPI_ISL_916624	Lighthouse Lab in Alderley Park	Wellcome Sanger Institute for the COVID-19 Genomics UK (COG-UK) Consortium	Jacquelyn Wynn, Mairead Hyland, The Lighthouse Lab in Alderley Park and Alex Alderton, Roberto Amato, Sonia Goncalves, Ewan Harrison, David K. Jackson, Ian Johnston, Dominic Kwiatkowski, Cordelia Langford, John Sillitoe on behalf of the Wellcome Sanger Institute COVID-19 Surveillance Team
EPI_ISL_916625, EPI_ISL_916626	Lighthouse Lab in Glasgow	Wellcome Sanger Institute for the COVID-19 Genomics UK (COG-UK) Consortium	Harper VanSteenhouse, Yumi Kasai, David Gray, Carol Clugston, Anna Dominiczak and Alex Alderton, Roberto Amato, Sonia Goncalves, Ewan Harrison, David K. Jackson, Ian Johnston, Dominic Kwiatkowski, Cordelia Langford, John Sillitoe on behalf of the Wellcome Sanger Institute COVID-19 Surveillance Team
EPI_ISL_916627, EPI_ISL_916628, EPI_ISL_916629, EPI_ISL_916631, EPI_ISL_916632	Lighthouse Lab in Alderley Park	Wellcome Sanger Institute for the COVID-19 Genomics UK (COG-UK) Consortium	Jacquelyn Wynn, Mairead Hyland, The Lighthouse Lab in Alderley Park and Alex Alderton, Roberto Amato, Sonia Goncalves, Ewan Harrison, David K. Jackson, Ian Johnston, Dominic Kwiatkowski, Cordelia Langford, John Sillitoe on behalf of the Wellcome Sanger Institute COVID-19 Surveillance Team
EPI_ISL_916633	Lighthouse Lab in Glasgow	Wellcome Sanger Institute for the COVID-19 Genomics UK (COG-UK) Consortium	Harper VanSteenhouse, Yumi Kasai, David Gray, Carol Clugston, Anna Dominiczak and Alex Alderton, Roberto Amato, Sonia Goncalves, Ewan Harrison, David K. Jackson, Ian Johnston, Dominic Kwiatkowski, Cordelia Langford, John Sillitoe on behalf of the Wellcome Sanger Institute COVID-19 Surveillance Team
EPI_ISL_916634, EPI_ISL_916635, EPI_ISL_916636, EPI_ISL_916638, EPI_ISL_916639, EPI_ISL_916640, EPI_ISL_916641, EPI_ISL_916642, EPI_ISL_916643, EPI_ISL_916644	Lighthouse Lab in Alderley Park	Wellcome Sanger Institute for the COVID-19 Genomics UK (COG-UK) Consortium	Jacquelyn Wynn, Mairead Hyland, The Lighthouse Lab in Alderley Park and Alex Alderton, Roberto Amato, Sonia Goncalves, Ewan Harrison, David K. Jackson, Ian Johnston, Dominic Kwiatkowski, Cordelia Langford, John Sillitoe on behalf of the Wellcome Sanger Institute COVID-19 Surveillance Team
EPI_ISL_916645	Lighthouse Lab in Glasgow	Wellcome Sanger Institute for the COVID-19 Genomics UK (COG-UK) Consortium	Harper VanSteenhouse, Yumi Kasai, David Gray, Carol Clugston, Anna Dominiczak and Alex Alderton, Roberto Amato, Sonia Goncalves, Ewan Harrison, David K. Jackson, Ian Johnston, Dominic Kwiatkowski, Cordelia Langford, John Sillitoe on behalf of the Wellcome Sanger Institute COVID-19 Surveillance Team
EPI_ISL_916646, EPI_ISL_916647	Lighthouse Lab in Alderley Park	Wellcome Sanger Institute for the COVID-19 Genomics UK (COG-UK) Consortium	Jacquelyn Wynn, Mairead Hyland, The Lighthouse Lab in Alderley Park and Alex Alderton, Roberto Amato, Sonia Goncalves, Ewan Harrison, David K. Jackson, Ian Johnston, Dominic Kwiatkowski, Cordelia Langford, John Sillitoe on behalf of the Wellcome Sanger Institute COVID-19 Surveillance Team
EPI_ISL_916648, EPI_ISL_916649	Lighthouse Lab in Glasgow	Wellcome Sanger Institute for the COVID-19 Genomics UK (COG-UK) Consortium	Harper VanSteenhouse, Yumi Kasai, David Gray, Carol Clugston, Anna Dominiczak and Alex Alderton, Roberto Amato, Sonia Goncalves, Ewan Harrison, David K. Jackson, Ian Johnston, Dominic Kwiatkowski, Cordelia Langford, John Sillitoe on behalf of the Wellcome Sanger Institute COVID-19 Surveillance Team
EPI_ISL_916650, EPI_ISL_916652, EPI_ISL_916653, EPI_ISL_916654, EPI_ISL_916655, EPI_ISL_916656, EPI_ISL_916657, EPI_ISL_916658, EPI_ISL_916659, EPI_ISL_916660	Lighthouse Lab in Alderley Park	Wellcome Sanger Institute for the COVID-19 Genomics UK (COG-UK) Consortium	Jacquelyn Wynn, Mairead Hyland, The Lighthouse Lab in Alderley Park and Alex Alderton, Roberto Amato, Sonia Goncalves, Ewan Harrison, David K. Jackson, Ian Johnston, Dominic Kwiatkowski, Cordelia Langford, John Sillitoe on behalf of the Wellcome Sanger Institute COVID-19 Surveillance Team
EPI_ISL_916661, EPI_ISL_916662	Lighthouse Lab in Glasgow	Wellcome Sanger Institute for the COVID-19 Genomics UK (COG-UK) Consortium	Harper VanSteenhouse, Yumi Kasai, David Gray, Carol Clugston, Anna Dominiczak and Alex Alderton, Roberto Amato, Sonia Goncalves, Ewan Harrison, David K. Jackson, Ian Johnston, Dominic Kwiatkowski, Cordelia Langford, John Sillitoe on behalf of the Wellcome Sanger Institute COVID-19 Surveillance Team
EPI_ISL_916663, EPI_ISL_916664, EPI_ISL_916665, EPI_ISL_916666, EPI_ISL_916667	Lighthouse Lab in Alderley Park	Wellcome Sanger Institute for the COVID-19 Genomics UK (COG-UK) Consortium	Jacquelyn Wynn, Mairead Hyland, The Lighthouse Lab in Alderley Park and Alex Alderton, Roberto Amato, Sonia Goncalves, Ewan Harrison, David K. Jackson, Ian Johnston, Dominic Kwiatkowski, Cordelia Langford, John Sillitoe on behalf of the Wellcome Sanger Institute COVID-19 Surveillance Team
EPI_ISL_916668	Lighthouse Lab in Glasgow	Wellcome Sanger Institute for the COVID-19 Genomics UK (COG-UK) Consortium	Harper VanSteenhouse, Yumi Kasai, David Gray, Carol Clugston, Anna Dominiczak and Alex Alderton, Roberto Amato, Sonia Goncalves, Ewan Harrison, David K. Jackson, Ian Johnston, Dominic Kwiatkowski, Cordelia Langford, John Sillitoe on behalf of the Wellcome Sanger Institute COVID-19 Surveillance Team
EPI_ISL_916669, EPI_ISL_916670, EPI_ISL_916671, EPI_ISL_916672, EPI_ISL_916673, EPI_ISL_916674	Lighthouse Lab in Alderley Park	Wellcome Sanger Institute for the COVID-19 Genomics UK (COG-UK) Consortium	Jacquelyn Wynn, Mairead Hyland, The Lighthouse Lab in Alderley Park and Alex Alderton, Roberto Amato, Sonia Goncalves, Ewan Harrison, David K. Jackson, Ian Johnston, Dominic Kwiatkowski, Cordelia Langford, John Sillitoe on behalf of the Wellcome Sanger Institute COVID-19 Surveillance Team
EPI_ISL_916675	Lighthouse Lab in Glasgow	Wellcome Sanger Institute for the COVID-19 Genomics UK	Harper VanSteenhouse, Yumi Kasai, David Gray, Carol Clugston, Anna Dominiczak and Alex Alderton, Roberto Amato, Sonia Goncalves, Ewan Harrison,

		(COG-UK) Consortium	David K. Jackson, Ian Johnston, Dominic Kwiatkowski, Cordelia Langford, John Sillitoe on behalf of the Wellcome Sanger Institute COVID-19 Surveillance Team
EPI_ISL_916676, EPI_ISL_916677, EPI_ISL_916678, EPI_ISL_916679, EPI_ISL_916680, EPI_ISL_916681	Lighthouse Lab in Alderley Park	Wellcome Sanger Institute for the COVID-19 Genomics UK (COG-UK) Consortium	Jacquelyn Wynn, Mairead Hyland, The Lighthouse Lab in Alderley Park and Alex Alderton, Roberto Amato, Sonia Goncalves, Ewan Harrison, David K. Jackson, Ian Johnston, Dominic Kwiatkowski, Cordelia Langford, John Sillitoe on behalf of the Wellcome Sanger Institute COVID-19 Surveillance Team
EPI_ISL_916682	Lighthouse Lab in Glasgow	Wellcome Sanger Institute for the COVID-19 Genomics UK (COG-UK) Consortium	Harper VanSteenhouse, Yumi Kasai, David Gray, Carol Clugston, Anna Dominiczak and Alex Alderton, Roberto Amato, Sonia Goncalves, Ewan Harrison, David K. Jackson, Ian Johnston, Dominic Kwiatkowski, Cordelia Langford, John Sillitoe on behalf of the Wellcome Sanger Institute COVID-19 Surveillance Team
EPI_ISL_916683	Lighthouse Lab in Alderley Park	Wellcome Sanger Institute for the COVID-19 Genomics UK (COG-UK) Consortium	Jacquelyn Wynn, Mairead Hyland, The Lighthouse Lab in Alderley Park and Alex Alderton, Roberto Amato, Sonia Goncalves, Ewan Harrison, David K. Jackson, Ian Johnston, Dominic Kwiatkowski, Cordelia Langford, John Sillitoe on behalf of the Wellcome Sanger Institute COVID-19 Surveillance Team
EPI_ISL_916684	Lighthouse Lab in Glasgow	Wellcome Sanger Institute for the COVID-19 Genomics UK (COG-UK) Consortium	Harper VanSteenhouse, Yumi Kasai, David Gray, Carol Clugston, Anna Dominiczak and Alex Alderton, Roberto Amato, Sonia Goncalves, Ewan Harrison, David K. Jackson, Ian Johnston, Dominic Kwiatkowski, Cordelia Langford, John Sillitoe on behalf of the Wellcome Sanger Institute COVID-19 Surveillance Team
EPI_ISL_916685, EPI_ISL_916686, EPI_ISL_916687	Lighthouse Lab in Alderley Park	Wellcome Sanger Institute for the COVID-19 Genomics UK (COG-UK) Consortium	Jacquelyn Wynn, Mairead Hyland, The Lighthouse Lab in Alderley Park and Alex Alderton, Roberto Amato, Sonia Goncalves, Ewan Harrison, David K. Jackson, Ian Johnston, Dominic Kwiatkowski, Cordelia Langford, John Sillitoe on behalf of the Wellcome Sanger Institute COVID-19 Surveillance Team
EPI_ISL_916688	Lighthouse Lab in Glasgow	Wellcome Sanger Institute for the COVID-19 Genomics UK (COG-UK) Consortium	Harper VanSteenhouse, Yumi Kasai, David Gray, Carol Clugston, Anna Dominiczak and Alex Alderton, Roberto Amato, Sonia Goncalves, Ewan Harrison, David K. Jackson, Ian Johnston, Dominic Kwiatkowski, Cordelia Langford, John Sillitoe on behalf of the Wellcome Sanger Institute COVID-19 Surveillance Team
EPI_ISL_916689, EPI_ISL_916690, EPI_ISL_916691, EPI_ISL_916692, EPI_ISL_916693	Lighthouse Lab in Alderley Park	Wellcome Sanger Institute for the COVID-19 Genomics UK (COG-UK) Consortium	Jacquelyn Wynn, Mairead Hyland, The Lighthouse Lab in Alderley Park and Alex Alderton, Roberto Amato, Sonia Goncalves, Ewan Harrison, David K. Jackson, Ian Johnston, Dominic Kwiatkowski, Cordelia Langford, John Sillitoe on behalf of the Wellcome Sanger Institute COVID-19 Surveillance Team
EPI_ISL_916694	Lighthouse Lab in Glasgow	Wellcome Sanger Institute for the COVID-19 Genomics UK (COG-UK) Consortium	Harper VanSteenhouse, Yumi Kasai, David Gray, Carol Clugston, Anna Dominiczak and Alex Alderton, Roberto Amato, Sonia Goncalves, Ewan Harrison, David K. Jackson, Ian Johnston, Dominic Kwiatkowski, Cordelia Langford, John Sillitoe on behalf of the Wellcome Sanger Institute COVID-19 Surveillance Team
EPI_ISL_916695	Lighthouse Lab in Alderley Park	Wellcome Sanger Institute for the COVID-19 Genomics UK (COG-UK) Consortium	Jacquelyn Wynn, Mairead Hyland, The Lighthouse Lab in Alderley Park and Alex Alderton, Roberto Amato, Sonia Goncalves, Ewan Harrison, David K. Jackson, Ian Johnston, Dominic Kwiatkowski, Cordelia Langford, John Sillitoe on behalf of the Wellcome Sanger Institute COVID-19 Surveillance Team
EPI_ISL_916696	Lighthouse Lab in Glasgow	Wellcome Sanger Institute for the COVID-19 Genomics UK (COG-UK) Consortium	Harper VanSteenhouse, Yumi Kasai, David Gray, Carol Clugston, Anna Dominiczak and Alex Alderton, Roberto Amato, Sonia Goncalves, Ewan Harrison, David K. Jackson, Ian Johnston, Dominic Kwiatkowski, Cordelia Langford, John Sillitoe on behalf of the Wellcome Sanger Institute COVID-19 Surveillance Team
EPI_ISL_916697, EPI_ISL_916698, EPI_ISL_916699, EPI_ISL_916700, EPI_ISL_916701, EPI_ISL_916702, EPI_ISL_916703	Lighthouse Lab in Alderley Park	Wellcome Sanger Institute for the COVID-19 Genomics UK (COG-UK) Consortium	Jacquelyn Wynn, Mairead Hyland, The Lighthouse Lab in Alderley Park and Alex Alderton, Roberto Amato, Sonia Goncalves, Ewan Harrison, David K. Jackson, Ian Johnston, Dominic Kwiatkowski, Cordelia Langford, John Sillitoe on behalf of the Wellcome Sanger Institute COVID-19 Surveillance Team
EPI_ISL_916704, EPI_ISL_916705	Lighthouse Lab in Glasgow	Wellcome Sanger Institute for the COVID-19 Genomics UK (COG-UK) Consortium	Harper VanSteenhouse, Yumi Kasai, David Gray, Carol Clugston, Anna Dominiczak and Alex Alderton, Roberto Amato, Sonia Goncalves, Ewan Harrison, David K. Jackson, Ian Johnston, Dominic Kwiatkowski, Cordelia Langford, John Sillitoe on behalf of the Wellcome Sanger Institute COVID-19 Surveillance Team
EPI_ISL_916706, EPI_ISL_916707, EPI_ISL_916708, EPI_ISL_916709, EPI_ISL_916710	Lighthouse Lab in Alderley Park	Wellcome Sanger Institute for the COVID-19 Genomics UK (COG-UK) Consortium	Jacquelyn Wynn, Mairead Hyland, The Lighthouse Lab in Alderley Park and Alex Alderton, Roberto Amato, Sonia Goncalves, Ewan Harrison, David K. Jackson, Ian Johnston, Dominic Kwiatkowski, Cordelia Langford, John Sillitoe on behalf of the Wellcome Sanger Institute COVID-19 Surveillance Team
EPI_ISL_916711	Lighthouse Lab in Glasgow	Wellcome Sanger Institute for the COVID-19 Genomics UK (COG-UK) Consortium	Harper VanSteenhouse, Yumi Kasai, David Gray, Carol Clugston, Anna Dominiczak and Alex Alderton, Roberto Amato, Sonia Goncalves, Ewan Harrison, David K. Jackson, Ian Johnston, Dominic Kwiatkowski, Cordelia Langford, John Sillitoe on behalf of the Wellcome Sanger Institute COVID-19 Surveillance Team
EPI_ISL_916712	Lighthouse Lab in Alderley Park	Wellcome Sanger Institute for the COVID-19 Genomics UK (COG-UK) Consortium	Jacquelyn Wynn, Mairead Hyland, The Lighthouse Lab in Alderley Park and Alex Alderton, Roberto Amato, Sonia Goncalves, Ewan Harrison, David K. Jackson, Ian Johnston, Dominic Kwiatkowski, Cordelia Langford, John Sillitoe on behalf of the Wellcome Sanger Institute COVID-19 Surveillance Team
EPI_ISL_916713	Lighthouse Lab in Glasgow	Wellcome Sanger Institute for the COVID-19 Genomics UK (COG-UK) Consortium	Harper VanSteenhouse, Yumi Kasai, David Gray, Carol Clugston, Anna Dominiczak and Alex Alderton, Roberto Amato, Sonia Goncalves, Ewan Harrison, David K. Jackson, Ian Johnston, Dominic Kwiatkowski, Cordelia Langford, John Sillitoe on behalf of the Wellcome Sanger Institute COVID-19 Surveillance Team
EPI_ISL_916714, EPI_ISL_916715	Lighthouse Lab in Alderley Park	Wellcome Sanger Institute for the COVID-19 Genomics UK (COG-UK) Consortium	Jacquelyn Wynn, Mairead Hyland, The Lighthouse Lab in Alderley Park and Alex Alderton, Roberto Amato, Sonia Goncalves, Ewan Harrison, David K. Jackson, Ian Johnston, Dominic Kwiatkowski, Cordelia Langford, John Sillitoe on behalf of the Wellcome Sanger Institute COVID-19 Surveillance Team
EPI_ISL_916716	Lighthouse Lab in Glasgow	Wellcome Sanger Institute for the COVID-19 Genomics UK (COG-UK) Consortium	Harper VanSteenhouse, Yumi Kasai, David Gray, Carol Clugston, Anna Dominiczak and Alex Alderton, Roberto Amato, Sonia Goncalves, Ewan Harrison, David K. Jackson, Ian Johnston, Dominic Kwiatkowski, Cordelia Langford, John Sillitoe on behalf of the Wellcome Sanger Institute COVID-19 Surveillance Team

EPI_ISL_916717, EPI_ISL_916718, EPI_ISL_916719, EPI_ISL_916720, EPI_ISL_916721, EPI_ISL_916722, EPI_ISL_916723, EPI_ISL_916724, EPI_ISL_916725, EPI_ISL_916726, EPI_ISL_916727

see above	Lighthouse Lab in Alderley Park	Wellcome Sanger Institute for the COVID-19 Genomics UK (COG-UK) Consortium	Jacquelyn Wynn, Mairead Hyland, The Lighthouse Lab in Alderley Park and Alex Alderton, Roberto Amato, Sonia Goncalves, Ewan Harrison, David K. Jackson, Ian Johnston, Dominic Kwiatkowski, Cordelia Langford, John Sillitoe on behalf of the Wellcome Sanger Institute COVID-19 Surveillance Team
EPI_ISL_916728	Lighthouse Lab in Glasgow	Wellcome Sanger Institute for the COVID-19 Genomics UK (COG-UK) Consortium	Harper VanSteenhouse, Yumi Kasai, David Gray, Carol Clugston, Anna Dominiczak and Alex Alderton, Roberto Amato, Sonia Goncalves, Ewan Harrison, David K. Jackson, Ian Johnston, Dominic Kwiatkowski, Cordelia Langford, John Sillitoe on behalf of the Wellcome Sanger Institute COVID-19 Surveillance Team
EPI_ISL_916729	Lighthouse Lab in Alderley Park	Wellcome Sanger Institute for the COVID-19 Genomics UK (COG-UK) Consortium	Jacquelyn Wynn, Mairead Hyland, The Lighthouse Lab in Alderley Park and Alex Alderton, Roberto Amato, Sonia Goncalves, Ewan Harrison, David K. Jackson, Ian Johnston, Dominic Kwiatkowski, Cordelia Langford, John Sillitoe on behalf of the Wellcome Sanger Institute COVID-19 Surveillance Team
EPI_ISL_916730	Lighthouse Lab in Glasgow	Wellcome Sanger Institute for the COVID-19 Genomics UK (COG-UK) Consortium	Harper VanSteenhouse, Yumi Kasai, David Gray, Carol Clugston, Anna Dominiczak and Alex Alderton, Roberto Amato, Sonia Goncalves, Ewan Harrison, David K. Jackson, Ian Johnston, Dominic Kwiatkowski, Cordelia Langford, John Sillitoe on behalf of the Wellcome Sanger Institute COVID-19 Surveillance Team
EPI_ISL_916731, EPI_ISL_916733, EPI_ISL_916734, EPI_ISL_916735	Lighthouse Lab in Alderley Park	Wellcome Sanger Institute for the COVID-19 Genomics UK (COG-UK) Consortium	Jacquelyn Wynn, Mairead Hyland, The Lighthouse Lab in Alderley Park and Alex Alderton, Roberto Amato, Sonia Goncalves, Ewan Harrison, David K. Jackson, Ian Johnston, Dominic Kwiatkowski, Cordelia Langford, John Sillitoe on behalf of the Wellcome Sanger Institute COVID-19 Surveillance Team
EPI_ISL_916736	Lighthouse Lab in Glasgow	Wellcome Sanger Institute for the COVID-19 Genomics UK (COG-UK) Consortium	Harper VanSteenhouse, Yumi Kasai, David Gray, Carol Clugston, Anna Dominiczak and Alex Alderton, Roberto Amato, Sonia Goncalves, Ewan Harrison, David K. Jackson, Ian Johnston, Dominic Kwiatkowski, Cordelia Langford, John Sillitoe on behalf of the Wellcome Sanger Institute COVID-19 Surveillance Team
EPI_ISL_916737	Lighthouse Lab in Alderley Park	Wellcome Sanger Institute for the COVID-19 Genomics UK (COG-UK) Consortium	Jacquelyn Wynn, Mairead Hyland, The Lighthouse Lab in Alderley Park and Alex Alderton, Roberto Amato, Sonia Goncalves, Ewan Harrison, David K. Jackson, Ian Johnston, Dominic Kwiatkowski, Cordelia Langford, John Sillitoe on behalf of the Wellcome Sanger Institute COVID-19 Surveillance Team
EPI_ISL_916738	Lighthouse Lab in Glasgow	Wellcome Sanger Institute for the COVID-19 Genomics UK (COG-UK) Consortium	Harper VanSteenhouse, Yumi Kasai, David Gray, Carol Clugston, Anna Dominiczak and Alex Alderton, Roberto Amato, Sonia Goncalves, Ewan Harrison, David K. Jackson, Ian Johnston, Dominic Kwiatkowski, Cordelia Langford, John Sillitoe on behalf of the Wellcome Sanger Institute COVID-19 Surveillance Team

EPI_ISL_916739, EPI_ISL_916740, EPI_ISL_916741, EPI_ISL_916742, EPI_ISL_916743, EPI_ISL_916744, EPI_ISL_916745, EPI_ISL_916747, EPI_ISL_916748, EPI_ISL_916749, EPI_ISL_916750, EPI_ISL_916751, EPI_ISL_916752, EPI_ISL_916753, EPI_ISL_916754, EPI_ISL_916755, EPI_ISL_916756, EPI_ISL_916757

see above	Lighthouse Lab in Alderley Park	Wellcome Sanger Institute for the COVID-19 Genomics UK (COG-UK) Consortium	Jacquelyn Wynn, Mairead Hyland, The Lighthouse Lab in Alderley Park and Alex Alderton, Roberto Amato, Sonia Goncalves, Ewan Harrison, David K. Jackson, Ian Johnston, Dominic Kwiatkowski, Cordelia Langford, John Sillitoe on behalf of the Wellcome Sanger Institute COVID-19 Surveillance Team
EPI_ISL_916758	Lighthouse Lab in Glasgow	Wellcome Sanger Institute for the COVID-19 Genomics UK (COG-UK) Consortium	Harper VanSteenhouse, Yumi Kasai, David Gray, Carol Clugston, Anna Dominiczak and Alex Alderton, Roberto Amato, Sonia Goncalves, Ewan Harrison, David K. Jackson, Ian Johnston, Dominic Kwiatkowski, Cordelia Langford, John Sillitoe on behalf of the Wellcome Sanger Institute COVID-19 Surveillance Team
EPI_ISL_916759, EPI_ISL_916760	Lighthouse Lab in Alderley Park	Wellcome Sanger Institute for the COVID-19 Genomics UK (COG-UK) Consortium	Jacquelyn Wynn, Mairead Hyland, The Lighthouse Lab in Alderley Park and Alex Alderton, Roberto Amato, Sonia Goncalves, Ewan Harrison, David K. Jackson, Ian Johnston, Dominic Kwiatkowski, Cordelia Langford, John Sillitoe on behalf of the Wellcome Sanger Institute COVID-19 Surveillance Team
EPI_ISL_916761	Lighthouse Lab in Glasgow	Wellcome Sanger Institute for the COVID-19 Genomics UK (COG-UK) Consortium	Harper VanSteenhouse, Yumi Kasai, David Gray, Carol Clugston, Anna Dominiczak and Alex Alderton, Roberto Amato, Sonia Goncalves, Ewan Harrison, David K. Jackson, Ian Johnston, Dominic Kwiatkowski, Cordelia Langford, John Sillitoe on behalf of the Wellcome Sanger Institute COVID-19 Surveillance Team
EPI_ISL_916762, EPI_ISL_916763, EPI_ISL_916764, EPI_ISL_916765, EPI_ISL_916767, EPI_ISL_916768, EPI_ISL_916769, EPI_ISL_916770	Lighthouse Lab in Alderley Park	Wellcome Sanger Institute for the COVID-19 Genomics UK (COG-UK) Consortium	Jacquelyn Wynn, Mairead Hyland, The Lighthouse Lab in Alderley Park and Alex Alderton, Roberto Amato, Sonia Goncalves, Ewan Harrison, David K. Jackson, Ian Johnston, Dominic Kwiatkowski, Cordelia Langford, John Sillitoe on behalf of the Wellcome Sanger Institute COVID-19 Surveillance Team
EPI_ISL_916771	Lighthouse Lab in Glasgow	Wellcome Sanger Institute for the COVID-19 Genomics UK (COG-UK) Consortium	Harper VanSteenhouse, Yumi Kasai, David Gray, Carol Clugston, Anna Dominiczak and Alex Alderton, Roberto Amato, Sonia Goncalves, Ewan Harrison, David K. Jackson, Ian Johnston, Dominic Kwiatkowski, Cordelia Langford, John Sillitoe on behalf of the Wellcome Sanger Institute COVID-19 Surveillance Team
EPI_ISL_916772, EPI_ISL_916773, EPI_ISL_916774, EPI_ISL_916776, EPI_ISL_916777, EPI_ISL_916778, EPI_ISL_916780, EPI_ISL_916781	Lighthouse Lab in Alderley Park	Wellcome Sanger Institute for the COVID-19 Genomics UK (COG-UK) Consortium	Jacquelyn Wynn, Mairead Hyland, The Lighthouse Lab in Alderley Park and Alex Alderton, Roberto Amato, Sonia Goncalves, Ewan Harrison, David K. Jackson, Ian Johnston, Dominic Kwiatkowski, Cordelia Langford, John Sillitoe on behalf of the Wellcome Sanger Institute COVID-19 Surveillance Team
EPI_ISL_916782	Lighthouse Lab in Glasgow	Wellcome Sanger Institute for the COVID-19 Genomics UK (COG-UK) Consortium	Harper VanSteenhouse, Yumi Kasai, David Gray, Carol Clugston, Anna Dominiczak and Alex Alderton, Roberto Amato, Sonia Goncalves, Ewan Harrison, David K. Jackson, Ian Johnston, Dominic Kwiatkowski, Cordelia Langford, John Sillitoe on behalf of the Wellcome Sanger Institute COVID-19 Surveillance Team
EPI_ISL_916783	Lighthouse Lab in Alderley Park	Wellcome Sanger Institute for the COVID-19 Genomics UK (COG-UK) Consortium	Jacquelyn Wynn, Mairead Hyland, The Lighthouse Lab in Alderley Park and Alex Alderton, Roberto Amato, Sonia Goncalves, Ewan Harrison, David K. Jackson, Ian Johnston, Dominic Kwiatkowski, Cordelia Langford, John Sillitoe on behalf of the Wellcome Sanger Institute COVID-19 Surveillance Team
EPI_ISL_916784	Lighthouse Lab in Glasgow	Wellcome Sanger Institute for the COVID-19 Genomics UK (COG-UK) Consortium	Harper VanSteenhouse, Yumi Kasai, David Gray, Carol Clugston, Anna Dominiczak and Alex Alderton, Roberto Amato, Sonia Goncalves, Ewan Harrison, David K. Jackson, Ian Johnston, Dominic Kwiatkowski, Cordelia Langford, John Sillitoe on behalf of the Wellcome Sanger Institute COVID-19 Surveillance Team
EPI_ISL_916785, EPI_ISL_916786, EPI_ISL_916787, EPI_ISL_916788, EPI_ISL_916789, EPI_ISL_916790, EPI_ISL_916791, EPI_ISL_916792	Lighthouse Lab in Alderley Park	Wellcome Sanger Institute for the COVID-19 Genomics UK (COG-UK) Consortium	Jacquelyn Wynn, Mairead Hyland, The Lighthouse Lab in Alderley Park and Alex Alderton, Roberto Amato, Sonia Goncalves, Ewan Harrison, David K. Jackson, Ian Johnston, Dominic Kwiatkowski, Cordelia Langford, John Sillitoe on behalf of the Wellcome Sanger Institute COVID-19 Surveillance Team
EPI_ISL_916793	Lighthouse Lab in Glasgow	Wellcome Sanger Institute for the COVID-19 Genomics UK (COG-UK) Consortium	Harper VanSteenhouse, Yumi Kasai, David Gray, Carol Clugston, Anna Dominiczak and Alex Alderton, Roberto Amato, Sonia Goncalves, Ewan Harrison, David K. Jackson, Ian Johnston, Dominic Kwiatkowski, Cordelia Langford, John Sillitoe on behalf of the Wellcome Sanger Institute COVID-19 Surveillance Team
EPI_ISL_916794, EPI_ISL_916795, EPI_ISL_916796, EPI_ISL_916797, EPI_ISL_916798, EPI_ISL_916799	Lighthouse Lab in Alderley Park	Wellcome Sanger Institute for the COVID-19 Genomics UK (COG-UK) Consortium	Jacquelyn Wynn, Mairead Hyland, The Lighthouse Lab in Alderley Park and Alex Alderton, Roberto Amato, Sonia Goncalves, Ewan Harrison, David K. Jackson, Ian Johnston, Dominic Kwiatkowski, Cordelia Langford, John Sillitoe on behalf of the Wellcome Sanger Institute COVID-19 Surveillance Team
EPI_ISL_916800	Lighthouse Lab in Glasgow	Wellcome Sanger Institute for the COVID-19 Genomics UK (COG-UK) Consortium	Harper VanSteenhouse, Yumi Kasai, David Gray, Carol Clugston, Anna Dominiczak and Alex Alderton, Roberto Amato, Sonia Goncalves, Ewan Harrison, David K. Jackson, Ian Johnston, Dominic Kwiatkowski, Cordelia Langford, John Sillitoe on behalf of the Wellcome Sanger Institute COVID-19 Surveillance Team
EPI_ISL_916801	Lighthouse Lab in Alderley Park	Wellcome Sanger Institute for the COVID-19 Genomics UK (COG-UK) Consortium	Jacquelyn Wynn, Mairead Hyland, The Lighthouse Lab in Alderley Park and Alex Alderton, Roberto Amato, Sonia Goncalves, Ewan Harrison, David K. Jackson, Ian Johnston, Dominic Kwiatkowski, Cordelia Langford, John Sillitoe on behalf of the Wellcome Sanger Institute COVID-19 Surveillance Team
EPI_ISL_916802	Lighthouse Lab in Glasgow	Wellcome Sanger Institute for the COVID-19 Genomics UK (COG-UK) Consortium	Harper VanSteenhouse, Yumi Kasai, David Gray, Carol Clugston, Anna Dominiczak and Alex Alderton, Roberto Amato, Sonia Goncalves, Ewan Harrison, David K. Jackson, Ian Johnston, Dominic Kwiatkowski, Cordelia Langford, John Sillitoe on behalf of the Wellcome Sanger Institute COVID-19 Surveillance Team
EPI_ISL_916803, EPI_ISL_916804, EPI_ISL_916805, EPI_ISL_916806, EPI_ISL_916807, EPI_ISL_916808	Lighthouse Lab in Alderley Park	Wellcome Sanger Institute for the COVID-19 Genomics UK (COG-UK) Consortium	Jacquelyn Wynn, Mairead Hyland, The Lighthouse Lab in Alderley Park and Alex Alderton, Roberto Amato, Sonia Goncalves, Ewan Harrison, David K. Jackson, Ian Johnston, Dominic Kwiatkowski, Cordelia Langford, John Sillitoe on behalf of the Wellcome Sanger Institute COVID-19 Surveillance Team
EPI_ISL_916809, EPI_ISL_916810	Lighthouse Lab in Glasgow	Wellcome Sanger Institute for the COVID-19 Genomics UK (COG-UK) Consortium	Harper VanSteenhouse, Yumi Kasai, David Gray, Carol Clugston, Anna Dominiczak and Alex Alderton, Roberto Amato, Sonia Goncalves, Ewan Harrison, David K. Jackson, Ian Johnston, Dominic Kwiatkowski, Cordelia Langford, John Sillitoe on behalf of the Wellcome Sanger Institute COVID-19 Surveillance Team
EPI_ISL_916811	Lighthouse Lab in Alderley Park	Wellcome Sanger Institute for the COVID-19 Genomics UK (COG-UK) Consortium	Jacquelyn Wynn, Mairead Hyland, The Lighthouse Lab in Alderley Park and Alex Alderton, Roberto Amato, Sonia Goncalves, Ewan Harrison, David K. Jackson, Ian Johnston, Dominic Kwiatkowski, Cordelia Langford, John Sillitoe on behalf of the Wellcome Sanger Institute COVID-19 Surveillance Team
EPI_ISL_916812	Lighthouse Lab in Glasgow	Wellcome Sanger Institute for the COVID-19 Genomics UK (COG-UK) Consortium	Harper VanSteenhouse, Yumi Kasai, David Gray, Carol Clugston, Anna Dominiczak and Alex Alderton, Roberto Amato, Sonia Goncalves, Ewan Harrison, David K. Jackson, Ian Johnston, Dominic Kwiatkowski, Cordelia Langford, John Sillitoe on behalf of the Wellcome Sanger Institute COVID-19 Surveillance Team
EPI_ISL_916813, EPI_ISL_916814	Lighthouse Lab in Alderley Park	Wellcome Sanger Institute for the COVID-19 Genomics UK (COG-UK) Consortium	Jacquelyn Wynn, Mairead Hyland, The Lighthouse Lab in Alderley Park and Alex Alderton, Roberto Amato, Sonia Goncalves, Ewan Harrison, David K. Jackson, Ian Johnston, Dominic Kwiatkowski, Cordelia Langford, John Sillitoe on behalf of the Wellcome Sanger Institute COVID-19 Surveillance Team
EPI_ISL_916815	Lighthouse Lab in Glasgow	Wellcome Sanger Institute for the COVID-19 Genomics UK (COG-UK) Consortium	Harper VanSteenhouse, Yumi Kasai, David Gray, Carol Clugston, Anna Dominiczak and Alex Alderton, Roberto Amato, Sonia Goncalves, Ewan Harrison, David K. Jackson, Ian Johnston, Dominic Kwiatkowski, Cordelia Langford, John Sillitoe on behalf of the Wellcome Sanger Institute COVID-19 Surveillance Team
EPI_ISL_916816, EPI_ISL_916817, EPI_ISL_916818, EPI_ISL_916819	Lighthouse Lab in Alderley Park	Wellcome Sanger Institute for the COVID-19 Genomics UK (COG-UK) Consortium	Jacquelyn Wynn, Mairead Hyland, The Lighthouse Lab in Alderley Park and Alex Alderton, Roberto Amato, Sonia Goncalves, Ewan Harrison, David K. Jackson, Ian Johnston, Dominic Kwiatkowski, Cordelia Langford, John Sillitoe on behalf of the Wellcome Sanger Institute COVID-19 Surveillance Team
EPI_ISL_916820, EPI_ISL_916821	Lighthouse Lab in Glasgow	Wellcome Sanger Institute for the COVID-19 Genomics UK (COG-UK) Consortium	Harper VanSteenhouse, Yumi Kasai, David Gray, Carol Clugston, Anna Dominiczak and Alex Alderton, Roberto Amato, Sonia Goncalves, Ewan Harrison, David K. Jackson, Ian Johnston, Dominic Kwiatkowski, Cordelia Langford, John Sillitoe on behalf of the Wellcome Sanger Institute COVID-19 Surveillance Team
EPI_ISL_916822, EPI_ISL_916823, EPI_ISL_916824	Lighthouse Lab in Alderley Park	Wellcome Sanger Institute for the COVID-19 Genomics UK (COG-UK) Consortium	Jacquelyn Wynn, Mairead Hyland, The Lighthouse Lab in Alderley Park and Alex Alderton, Roberto Amato, Sonia Goncalves, Ewan Harrison, David K. Jackson, Ian Johnston, Dominic Kwiatkowski, Cordelia Langford, John Sillitoe on behalf of the Wellcome Sanger Institute COVID-19 Surveillance Team
EPI_ISL_916825	Lighthouse Lab in Glasgow	Wellcome Sanger Institute for the COVID-19 Genomics UK (COG-UK) Consortium	Harper VanSteenhouse, Yumi Kasai, David Gray, Carol Clugston, Anna Dominiczak and Alex Alderton, Roberto Amato, Sonia Goncalves, Ewan Harrison, David K. Jackson, Ian Johnston, Dominic Kwiatkowski, Cordelia Langford, John Sillitoe on behalf of the Wellcome Sanger Institute COVID-19 Surveillance Team

EPI_ISL_916826, EPI_ISL_916827, EPI_ISL_916828, EPI_ISL_916829, EPI_ISL_916830, EPI_ISL_916831, EPI_ISL_916832	Lighthouse Lab in Alderley Park	Wellcome Sanger Institute for the COVID-19 Genomics UK (COG-UK) Consortium	Jacquelyn Wynn, Mairead Hyland, The Lighthouse Lab in Alderley Park and Alex Alderton, Roberto Amato, Sonia Goncalves, Ewan Harrison, David K. Jackson, Ian Johnston, Dominic Kwiatkowski, Cordelia Langford, John Sillitoe on behalf of the Wellcome Sanger Institute COVID-19 Surveillance Team
EPI_ISL_916833, EPI_ISL_916834	Lighthouse Lab in Glasgow	Wellcome Sanger Institute for the COVID-19 Genomics UK (COG-UK) Consortium	Harper VanSteenhouse, Yumi Kasai, David Gray, Carol Clugston, Anna Dominiczak and Alex Alderton, Roberto Amato, Sonia Goncalves, Ewan Harrison, David K. Jackson, Ian Johnston, Dominic Kwiatkowski, Cordelia Langford, John Sillitoe on behalf of the Wellcome Sanger Institute COVID-19 Surveillance Team
EPI_ISL_916835, EPI_ISL_916836, EPI_ISL_916837	Lighthouse Lab in Alderley Park	Wellcome Sanger Institute for the COVID-19 Genomics UK (COG-UK) Consortium	Jacquelyn Wynn, Mairead Hyland, The Lighthouse Lab in Alderley Park and Alex Alderton, Roberto Amato, Sonia Goncalves, Ewan Harrison, David K. Jackson, Ian Johnston, Dominic Kwiatkowski, Cordelia Langford, John Sillitoe on behalf of the Wellcome Sanger Institute COVID-19 Surveillance Team
EPI_ISL_916838	Lighthouse Lab in Glasgow	Wellcome Sanger Institute for the COVID-19 Genomics UK (COG-UK) Consortium	Harper VanSteenhouse, Yumi Kasai, David Gray, Carol Clugston, Anna Dominiczak and Alex Alderton, Roberto Amato, Sonia Goncalves, Ewan Harrison, David K. Jackson, Ian Johnston, Dominic Kwiatkowski, Cordelia Langford, John Sillitoe on behalf of the Wellcome Sanger Institute COVID-19 Surveillance Team
EPI_ISL_916839	Lighthouse Lab in Alderley Park	Wellcome Sanger Institute for the COVID-19 Genomics UK (COG-UK) Consortium	Jacquelyn Wynn, Mairead Hyland, The Lighthouse Lab in Alderley Park and Alex Alderton, Roberto Amato, Sonia Goncalves, Ewan Harrison, David K. Jackson, Ian Johnston, Dominic Kwiatkowski, Cordelia Langford, John Sillitoe on behalf of the Wellcome Sanger Institute COVID-19 Surveillance Team
EPI_ISL_916840	Lighthouse Lab in Glasgow	Wellcome Sanger Institute for the COVID-19 Genomics UK (COG-UK) Consortium	Harper VanSteenhouse, Yumi Kasai, David Gray, Carol Clugston, Anna Dominiczak and Alex Alderton, Roberto Amato, Sonia Goncalves, Ewan Harrison, David K. Jackson, Ian Johnston, Dominic Kwiatkowski, Cordelia Langford, John Sillitoe on behalf of the Wellcome Sanger Institute COVID-19 Surveillance Team
EPI_ISL_916841	Lighthouse Lab in Alderley Park	Wellcome Sanger Institute for the COVID-19 Genomics UK (COG-UK) Consortium	Jacquelyn Wynn, Mairead Hyland, The Lighthouse Lab in Alderley Park and Alex Alderton, Roberto Amato, Sonia Goncalves, Ewan Harrison, David K. Jackson, Ian Johnston, Dominic Kwiatkowski, Cordelia Langford, John Sillitoe on behalf of the Wellcome Sanger Institute COVID-19 Surveillance Team
EPI_ISL_916842	Lighthouse Lab in Glasgow	Wellcome Sanger Institute for the COVID-19 Genomics UK (COG-UK) Consortium	Harper VanSteenhouse, Yumi Kasai, David Gray, Carol Clugston, Anna Dominiczak and Alex Alderton, Roberto Amato, Sonia Goncalves, Ewan Harrison, David K. Jackson, Ian Johnston, Dominic Kwiatkowski, Cordelia Langford, John Sillitoe on behalf of the Wellcome Sanger Institute COVID-19 Surveillance Team
EPI_ISL_916843, EPI_ISL_916844	Lighthouse Lab in Alderley Park	Wellcome Sanger Institute for the COVID-19 Genomics UK (COG-UK) Consortium	Jacquelyn Wynn, Mairead Hyland, The Lighthouse Lab in Alderley Park and Alex Alderton, Roberto Amato, Sonia Goncalves, Ewan Harrison, David K. Jackson, Ian Johnston, Dominic Kwiatkowski, Cordelia Langford, John Sillitoe on behalf of the Wellcome Sanger Institute COVID-19 Surveillance Team
EPI_ISL_916845	Lighthouse Lab in Glasgow	Wellcome Sanger Institute for the COVID-19 Genomics UK (COG-UK) Consortium	Harper VanSteenhouse, Yumi Kasai, David Gray, Carol Clugston, Anna Dominiczak and Alex Alderton, Roberto Amato, Sonia Goncalves, Ewan Harrison, David K. Jackson, Ian Johnston, Dominic Kwiatkowski, Cordelia Langford, John Sillitoe on behalf of the Wellcome Sanger Institute COVID-19 Surveillance Team
EPI_ISL_916846	Lighthouse Lab in Alderley Park	Wellcome Sanger Institute for the COVID-19 Genomics UK (COG-UK) Consortium	Jacquelyn Wynn, Mairead Hyland, The Lighthouse Lab in Alderley Park and Alex Alderton, Roberto Amato, Sonia Goncalves, Ewan Harrison, David K. Jackson, Ian Johnston, Dominic Kwiatkowski, Cordelia Langford, John Sillitoe on behalf of the Wellcome Sanger Institute COVID-19 Surveillance Team
EPI_ISL_916847	Lighthouse Lab in Glasgow	Wellcome Sanger Institute for the COVID-19 Genomics UK (COG-UK) Consortium	Harper VanSteenhouse, Yumi Kasai, David Gray, Carol Clugston, Anna Dominiczak and Alex Alderton, Roberto Amato, Sonia Goncalves, Ewan Harrison, David K. Jackson, Ian Johnston, Dominic Kwiatkowski, Cordelia Langford, John Sillitoe on behalf of the Wellcome Sanger Institute COVID-19 Surveillance Team
EPI_ISL_916848	Lighthouse Lab in Alderley Park	Wellcome Sanger Institute for the COVID-19 Genomics UK (COG-UK) Consortium	Jacquelyn Wynn, Mairead Hyland, The Lighthouse Lab in Alderley Park and Alex Alderton, Roberto Amato, Sonia Goncalves, Ewan Harrison, David K. Jackson, Ian Johnston, Dominic Kwiatkowski, Cordelia Langford, John Sillitoe on behalf of the Wellcome Sanger Institute COVID-19 Surveillance Team
EPI_ISL_916850	Lighthouse Lab in Glasgow	Wellcome Sanger Institute for the COVID-19 Genomics UK (COG-UK) Consortium	Harper VanSteenhouse, Yumi Kasai, David Gray, Carol Clugston, Anna Dominiczak and Alex Alderton, Roberto Amato, Sonia Goncalves, Ewan Harrison, David K. Jackson, Ian Johnston, Dominic Kwiatkowski, Cordelia Langford, John Sillitoe on behalf of the Wellcome Sanger Institute COVID-19 Surveillance Team
EPI_ISL_916851, EPI_ISL_916852, EPI_ISL_916853, EPI_ISL_916854	Lighthouse Lab in Alderley Park	Wellcome Sanger Institute for the COVID-19 Genomics UK (COG-UK) Consortium	Jacquelyn Wynn, Mairead Hyland, The Lighthouse Lab in Alderley Park and Alex Alderton, Roberto Amato, Sonia Goncalves, Ewan Harrison, David K. Jackson, Ian Johnston, Dominic Kwiatkowski, Cordelia Langford, John Sillitoe on behalf of the Wellcome Sanger Institute COVID-19 Surveillance Team
EPI_ISL_916855	Lighthouse Lab in Glasgow	Wellcome Sanger Institute for the COVID-19 Genomics UK (COG-UK) Consortium	Harper VanSteenhouse, Yumi Kasai, David Gray, Carol Clugston, Anna Dominiczak and Alex Alderton, Roberto Amato, Sonia Goncalves, Ewan Harrison, David K. Jackson, Ian Johnston, Dominic Kwiatkowski, Cordelia Langford, John Sillitoe on behalf of the Wellcome Sanger Institute COVID-19 Surveillance Team
EPI_ISL_916856, EPI_ISL_916857, EPI_ISL_916858, EPI_ISL_916859, EPI_ISL_916860, EPI_ISL_916861, EPI_ISL_916862, EPI_ISL_916863, EPI_ISL_916864, EPI_ISL_916865, EPI_ISL_916866, EPI_ISL_916867, EPI_ISL_916868			
see above	Lighthouse Lab in Alderley Park	Wellcome Sanger Institute for the COVID-19 Genomics UK (COG-UK) Consortium	Jacquelyn Wynn, Mairead Hyland, The Lighthouse Lab in Alderley Park and Alex Alderton, Roberto Amato, Sonia Goncalves, Ewan Harrison, David K. Jackson, Ian Johnston, Dominic Kwiatkowski, Cordelia Langford, John Sillitoe on behalf of the Wellcome Sanger Institute COVID-19 Surveillance Team
EPI_ISL_916869	Lighthouse Lab in Glasgow	Wellcome Sanger Institute for the COVID-19 Genomics UK (COG-UK) Consortium	Harper VanSteenhouse, Yumi Kasai, David Gray, Carol Clugston, Anna Dominiczak and Alex Alderton, Roberto Amato, Sonia Goncalves, Ewan Harrison, David K. Jackson, Ian Johnston, Dominic Kwiatkowski, Cordelia Langford, John Sillitoe on behalf of the Wellcome Sanger Institute COVID-19 Surveillance Team
EPI_ISL_916870, EPI_ISL_916871, EPI_ISL_916872, EPI_ISL_916873, EPI_ISL_916874, EPI_ISL_916875, EPI_ISL_916876, EPI_ISL_916877, EPI_ISL_916878	Lighthouse Lab in Alderley Park	Wellcome Sanger Institute for the COVID-19 Genomics UK (COG-UK) Consortium	Jacquelyn Wynn, Mairead Hyland, The Lighthouse Lab in Alderley Park and Alex Alderton, Roberto Amato, Sonia Goncalves, Ewan Harrison, David K. Jackson, Ian Johnston, Dominic Kwiatkowski, Cordelia Langford, John Sillitoe on behalf of the Wellcome Sanger Institute COVID-19 Surveillance Team
EPI_ISL_916879	Lighthouse Lab in Glasgow	Wellcome Sanger Institute for the COVID-19 Genomics UK (COG-UK) Consortium	Harper VanSteenhouse, Yumi Kasai, David Gray, Carol Clugston, Anna Dominiczak and Alex Alderton, Roberto Amato, Sonia Goncalves, Ewan Harrison, David K. Jackson, Ian Johnston, Dominic Kwiatkowski, Cordelia Langford, John Sillitoe on behalf of the Wellcome Sanger Institute COVID-19 Surveillance Team
EPI_ISL_916880, EPI_ISL_916881	Lighthouse Lab in Alderley Park	Wellcome Sanger Institute for the COVID-19 Genomics UK (COG-UK) Consortium	Jacquelyn Wynn, Mairead Hyland, The Lighthouse Lab in Alderley Park and Alex Alderton, Roberto Amato, Sonia Goncalves, Ewan Harrison, David K. Jackson, Ian Johnston, Dominic Kwiatkowski, Cordelia Langford, John Sillitoe on behalf of the Wellcome Sanger Institute COVID-19 Surveillance Team
EPI_ISL_916882	Lighthouse Lab in Glasgow	Wellcome Sanger Institute for the COVID-19 Genomics UK (COG-UK) Consortium	Harper VanSteenhouse, Yumi Kasai, David Gray, Carol Clugston, Anna Dominiczak and Alex Alderton, Roberto Amato, Sonia Goncalves, Ewan Harrison, David K. Jackson, Ian Johnston, Dominic Kwiatkowski, Cordelia Langford, John Sillitoe on behalf of the Wellcome Sanger Institute COVID-19 Surveillance Team
EPI_ISL_916883, EPI_ISL_916884, EPI_ISL_916885, EPI_ISL_916886	Lighthouse Lab in Alderley Park	Wellcome Sanger Institute for the COVID-19 Genomics UK (COG-UK) Consortium	Jacquelyn Wynn, Mairead Hyland, The Lighthouse Lab in Alderley Park and Alex Alderton, Roberto Amato, Sonia Goncalves, Ewan Harrison, David K. Jackson, Ian Johnston, Dominic Kwiatkowski, Cordelia Langford, John Sillitoe on behalf of the Wellcome Sanger Institute COVID-19 Surveillance Team
EPI_ISL_916887	Lighthouse Lab in Glasgow	Wellcome Sanger Institute for the COVID-19 Genomics UK (COG-UK) Consortium	Harper VanSteenhouse, Yumi Kasai, David Gray, Carol Clugston, Anna Dominiczak and Alex Alderton, Roberto Amato, Sonia Goncalves, Ewan Harrison, David K. Jackson, Ian Johnston, Dominic Kwiatkowski, Cordelia Langford, John Sillitoe on behalf of the Wellcome Sanger Institute COVID-19 Surveillance Team
EPI_ISL_916888, EPI_ISL_916889, EPI_ISL_916890	Lighthouse Lab in Alderley Park	Wellcome Sanger Institute for the COVID-19 Genomics UK (COG-UK) Consortium	Jacquelyn Wynn, Mairead Hyland, The Lighthouse Lab in Alderley Park and Alex Alderton, Roberto Amato, Sonia Goncalves, Ewan Harrison, David K. Jackson, Ian Johnston, Dominic Kwiatkowski, Cordelia Langford, John Sillitoe on behalf of the Wellcome Sanger Institute COVID-19 Surveillance Team
EPI_ISL_916891	Lighthouse Lab in Glasgow	Wellcome Sanger Institute for the COVID-19 Genomics UK (COG-UK) Consortium	Harper VanSteenhouse, Yumi Kasai, David Gray, Carol Clugston, Anna Dominiczak and Alex Alderton, Roberto Amato, Sonia Goncalves, Ewan Harrison, David K. Jackson, Ian Johnston, Dominic Kwiatkowski, Cordelia Langford, John Sillitoe on behalf of the Wellcome Sanger Institute COVID-19 Surveillance Team
EPI_ISL_916892, EPI_ISL_916893	Lighthouse Lab in Alderley Park	Wellcome Sanger Institute for the COVID-19 Genomics UK (COG-UK) Consortium	Jacquelyn Wynn, Mairead Hyland, The Lighthouse Lab in Alderley Park and Alex Alderton, Roberto Amato, Sonia Goncalves, Ewan Harrison, David K. Jackson, Ian Johnston, Dominic Kwiatkowski, Cordelia Langford, John Sillitoe on behalf of the Wellcome Sanger Institute COVID-19 Surveillance Team

EPI_ISL_916894	Lighthouse Lab in Glasgow	Wellcome Sanger Institute for the COVID-19 Genomics UK (COG-UK) Consortium	Harper VanSteenhouse, Yumi Kasai, David Gray, Carol Clugston, Anna Dominiczak and Alex Alderton, Roberto Amato, Sonia Goncalves, Ewan Harrison, David K. Jackson, Ian Johnston, Dominic Kwiatkowski, Cordelia Langford, John Sillitoe on behalf of the Wellcome Sanger Institute COVID-19 Surveillance Team
EPI_ISL_916895	Lighthouse Lab in Alderley Park	Wellcome Sanger Institute for the COVID-19 Genomics UK (COG-UK) Consortium	Jacquelyn Wynn, Mairead Hyland, The Lighthouse Lab in Alderley Park and Alex Alderton, Roberto Amato, Sonia Goncalves, Ewan Harrison, David K. Jackson, Ian Johnston, Dominic Kwiatkowski, Cordelia Langford, John Sillitoe on behalf of the Wellcome Sanger Institute COVID-19 Surveillance Team
EPI_ISL_916896	Lighthouse Lab in Glasgow	Wellcome Sanger Institute for the COVID-19 Genomics UK (COG-UK) Consortium	Harper VanSteenhouse, Yumi Kasai, David Gray, Carol Clugston, Anna Dominiczak and Alex Alderton, Roberto Amato, Sonia Goncalves, Ewan Harrison, David K. Jackson, Ian Johnston, Dominic Kwiatkowski, Cordelia Langford, John Sillitoe on behalf of the Wellcome Sanger Institute COVID-19 Surveillance Team
EPI_ISL_916897	Lighthouse Lab in Alderley Park	Wellcome Sanger Institute for the COVID-19 Genomics UK (COG-UK) Consortium	Jacquelyn Wynn, Mairead Hyland, The Lighthouse Lab in Alderley Park and Alex Alderton, Roberto Amato, Sonia Goncalves, Ewan Harrison, David K. Jackson, Ian Johnston, Dominic Kwiatkowski, Cordelia Langford, John Sillitoe on behalf of the Wellcome Sanger Institute COVID-19 Surveillance Team
EPI_ISL_916898, EPI_ISL_916899, EPI_ISL_916900	Lighthouse Lab in Glasgow	Wellcome Sanger Institute for the COVID-19 Genomics UK (COG-UK) Consortium	Harper VanSteenhouse, Yumi Kasai, David Gray, Carol Clugston, Anna Dominiczak and Alex Alderton, Roberto Amato, Sonia Goncalves, Ewan Harrison, David K. Jackson, Ian Johnston, Dominic Kwiatkowski, Cordelia Langford, John Sillitoe on behalf of the Wellcome Sanger Institute COVID-19 Surveillance Team
EPI_ISL_916901, EPI_ISL_916902, EPI_ISL_916903, EPI_ISL_916904	Lighthouse Lab in Alderley Park	Wellcome Sanger Institute for the COVID-19 Genomics UK (COG-UK) Consortium	Jacquelyn Wynn, Mairead Hyland, The Lighthouse Lab in Alderley Park and Alex Alderton, Roberto Amato, Sonia Goncalves, Ewan Harrison, David K. Jackson, Ian Johnston, Dominic Kwiatkowski, Cordelia Langford, John Sillitoe on behalf of the Wellcome Sanger Institute COVID-19 Surveillance Team
EPI_ISL_916905	Lighthouse Lab in Glasgow	Wellcome Sanger Institute for the COVID-19 Genomics UK (COG-UK) Consortium	Harper VanSteenhouse, Yumi Kasai, David Gray, Carol Clugston, Anna Dominiczak and Alex Alderton, Roberto Amato, Sonia Goncalves, Ewan Harrison, David K. Jackson, Ian Johnston, Dominic Kwiatkowski, Cordelia Langford, John Sillitoe on behalf of the Wellcome Sanger Institute COVID-19 Surveillance Team
EPI_ISL_916906	Lighthouse Lab in Alderley Park	Wellcome Sanger Institute for the COVID-19 Genomics UK (COG-UK) Consortium	Jacquelyn Wynn, Mairead Hyland, The Lighthouse Lab in Alderley Park and Alex Alderton, Roberto Amato, Sonia Goncalves, Ewan Harrison, David K. Jackson, Ian Johnston, Dominic Kwiatkowski, Cordelia Langford, John Sillitoe on behalf of the Wellcome Sanger Institute COVID-19 Surveillance Team
EPI_ISL_916907, EPI_ISL_916908, EPI_ISL_916909	Lighthouse Lab in Glasgow	Wellcome Sanger Institute for the COVID-19 Genomics UK (COG-UK) Consortium	Harper VanSteenhouse, Yumi Kasai, David Gray, Carol Clugston, Anna Dominiczak and Alex Alderton, Roberto Amato, Sonia Goncalves, Ewan Harrison, David K. Jackson, Ian Johnston, Dominic Kwiatkowski, Cordelia Langford, John Sillitoe on behalf of the Wellcome Sanger Institute COVID-19 Surveillance Team
EPI_ISL_916910	Lighthouse Lab in Alderley Park	Wellcome Sanger Institute for the COVID-19 Genomics UK (COG-UK) Consortium	Jacquelyn Wynn, Mairead Hyland, The Lighthouse Lab in Alderley Park and Alex Alderton, Roberto Amato, Sonia Goncalves, Ewan Harrison, David K. Jackson, Ian Johnston, Dominic Kwiatkowski, Cordelia Langford, John Sillitoe on behalf of the Wellcome Sanger Institute COVID-19 Surveillance Team
EPI_ISL_916911, EPI_ISL_916912	Lighthouse Lab in Glasgow	Wellcome Sanger Institute for the COVID-19 Genomics UK (COG-UK) Consortium	Harper VanSteenhouse, Yumi Kasai, David Gray, Carol Clugston, Anna Dominiczak and Alex Alderton, Roberto Amato, Sonia Goncalves, Ewan Harrison, David K. Jackson, Ian Johnston, Dominic Kwiatkowski, Cordelia Langford, John Sillitoe on behalf of the Wellcome Sanger Institute COVID-19 Surveillance Team
EPI_ISL_916913, EPI_ISL_916914, EPI_ISL_916915	Lighthouse Lab in Alderley Park	Wellcome Sanger Institute for the COVID-19 Genomics UK (COG-UK) Consortium	Jacquelyn Wynn, Mairead Hyland, The Lighthouse Lab in Alderley Park and Alex Alderton, Roberto Amato, Sonia Goncalves, Ewan Harrison, David K. Jackson, Ian Johnston, Dominic Kwiatkowski, Cordelia Langford, John Sillitoe on behalf of the Wellcome Sanger Institute COVID-19 Surveillance Team
EPI_ISL_916916	Lighthouse Lab in Glasgow	Wellcome Sanger Institute for the COVID-19 Genomics UK (COG-UK) Consortium	Harper VanSteenhouse, Yumi Kasai, David Gray, Carol Clugston, Anna Dominiczak and Alex Alderton, Roberto Amato, Sonia Goncalves, Ewan Harrison, David K. Jackson, Ian Johnston, Dominic Kwiatkowski, Cordelia Langford, John Sillitoe on behalf of the Wellcome Sanger Institute COVID-19 Surveillance Team
EPI_ISL_916917	Lighthouse Lab in Alderley Park	Wellcome Sanger Institute for the COVID-19 Genomics UK (COG-UK) Consortium	Jacquelyn Wynn, Mairead Hyland, The Lighthouse Lab in Alderley Park and Alex Alderton, Roberto Amato, Sonia Goncalves, Ewan Harrison, David K. Jackson, Ian Johnston, Dominic Kwiatkowski, Cordelia Langford, John Sillitoe on behalf of the Wellcome Sanger Institute COVID-19 Surveillance Team
EPI_ISL_916918	Lighthouse Lab in Glasgow	Wellcome Sanger Institute for the COVID-19 Genomics UK (COG-UK) Consortium	Harper VanSteenhouse, Yumi Kasai, David Gray, Carol Clugston, Anna Dominiczak and Alex Alderton, Roberto Amato, Sonia Goncalves, Ewan Harrison, David K. Jackson, Ian Johnston, Dominic Kwiatkowski, Cordelia Langford, John Sillitoe on behalf of the Wellcome Sanger Institute COVID-19 Surveillance Team
EPI_ISL_916920, EPI_ISL_916921, EPI_ISL_916922	Lighthouse Lab in Alderley Park	Wellcome Sanger Institute for the COVID-19 Genomics UK (COG-UK) Consortium	Jacquelyn Wynn, Mairead Hyland, The Lighthouse Lab in Alderley Park and Alex Alderton, Roberto Amato, Sonia Goncalves, Ewan Harrison, David K. Jackson, Ian Johnston, Dominic Kwiatkowski, Cordelia Langford, John Sillitoe on behalf of the Wellcome Sanger Institute COVID-19 Surveillance Team
EPI_ISL_916923	Lighthouse Lab in Glasgow	Wellcome Sanger Institute for the COVID-19 Genomics UK (COG-UK) Consortium	Harper VanSteenhouse, Yumi Kasai, David Gray, Carol Clugston, Anna Dominiczak and Alex Alderton, Roberto Amato, Sonia Goncalves, Ewan Harrison, David K. Jackson, Ian Johnston, Dominic Kwiatkowski, Cordelia Langford, John Sillitoe on behalf of the Wellcome Sanger Institute COVID-19 Surveillance Team
EPI_ISL_916924, EPI_ISL_916925, EPI_ISL_916926, EPI_ISL_916927, EPI_ISL_916928, EPI_ISL_916929, EPI_ISL_916930, EPI_ISL_916931, EPI_ISL_916932, EPI_ISL_916933, EPI_ISL_916935, EPI_ISL_916936, EPI_ISL_916937, EPI_ISL_916938, EPI_ISL_916939			
see above	Lighthouse Lab in Alderley Park	Wellcome Sanger Institute for the COVID-19 Genomics UK (COG-UK) Consortium	Jacquelyn Wynn, Mairead Hyland, The Lighthouse Lab in Alderley Park and Alex Alderton, Roberto Amato, Sonia Goncalves, Ewan Harrison, David K. Jackson, Ian Johnston, Dominic Kwiatkowski, Cordelia Langford, John Sillitoe on behalf of the Wellcome Sanger Institute COVID-19 Surveillance Team
EPI_ISL_916940	Lighthouse Lab in Glasgow	Wellcome Sanger Institute for the COVID-19 Genomics UK (COG-UK) Consortium	Harper VanSteenhouse, Yumi Kasai, David Gray, Carol Clugston, Anna Dominiczak and Alex Alderton, Roberto Amato, Sonia Goncalves, Ewan Harrison, David K. Jackson, Ian Johnston, Dominic Kwiatkowski, Cordelia Langford, John Sillitoe on behalf of the Wellcome Sanger Institute COVID-19 Surveillance Team
EPI_ISL_916941	Lighthouse Lab in Alderley Park	Wellcome Sanger Institute for the COVID-19 Genomics UK (COG-UK) Consortium	Jacquelyn Wynn, Mairead Hyland, The Lighthouse Lab in Alderley Park and Alex Alderton, Roberto Amato, Sonia Goncalves, Ewan Harrison, David K. Jackson, Ian Johnston, Dominic Kwiatkowski, Cordelia Langford, John Sillitoe on behalf of the Wellcome Sanger Institute COVID-19 Surveillance Team
EPI_ISL_916942	Lighthouse Lab in Glasgow	Wellcome Sanger Institute for the COVID-19 Genomics UK (COG-UK) Consortium	Harper VanSteenhouse, Yumi Kasai, David Gray, Carol Clugston, Anna Dominiczak and Alex Alderton, Roberto Amato, Sonia Goncalves, Ewan Harrison, David K. Jackson, Ian Johnston, Dominic Kwiatkowski, Cordelia Langford, John Sillitoe on behalf of the Wellcome Sanger Institute COVID-19 Surveillance Team
EPI_ISL_916943, EPI_ISL_916944	Lighthouse Lab in Alderley Park	Wellcome Sanger Institute for the COVID-19 Genomics UK (COG-UK) Consortium	Jacquelyn Wynn, Mairead Hyland, The Lighthouse Lab in Alderley Park and Alex Alderton, Roberto Amato, Sonia Goncalves, Ewan Harrison, David K. Jackson, Ian Johnston, Dominic Kwiatkowski, Cordelia Langford, John Sillitoe on behalf of the Wellcome Sanger Institute COVID-19 Surveillance Team
EPI_ISL_916945, EPI_ISL_916946	Lighthouse Lab in Glasgow	Wellcome Sanger Institute for the COVID-19 Genomics UK (COG-UK) Consortium	Harper VanSteenhouse, Yumi Kasai, David Gray, Carol Clugston, Anna Dominiczak and Alex Alderton, Roberto Amato, Sonia Goncalves, Ewan Harrison, David K. Jackson, Ian Johnston, Dominic Kwiatkowski, Cordelia Langford, John Sillitoe on behalf of the Wellcome Sanger Institute COVID-19 Surveillance Team
EPI_ISL_916947	Lighthouse Lab in Alderley Park	Wellcome Sanger Institute for the COVID-19 Genomics UK (COG-UK) Consortium	Jacquelyn Wynn, Mairead Hyland, The Lighthouse Lab in Alderley Park and Alex Alderton, Roberto Amato, Sonia Goncalves, Ewan Harrison, David K. Jackson, Ian Johnston, Dominic Kwiatkowski, Cordelia Langford, John Sillitoe on behalf of the Wellcome Sanger Institute COVID-19 Surveillance Team
EPI_ISL_916948, EPI_ISL_916949, EPI_ISL_916951	Lighthouse Lab in Glasgow	Wellcome Sanger Institute for the COVID-19 Genomics UK (COG-UK) Consortium	Harper VanSteenhouse, Yumi Kasai, David Gray, Carol Clugston, Anna Dominiczak and Alex Alderton, Roberto Amato, Sonia Goncalves, Ewan Harrison, David K. Jackson, Ian Johnston, Dominic Kwiatkowski, Cordelia Langford, John Sillitoe on behalf of the Wellcome Sanger Institute COVID-19 Surveillance Team
EPI_ISL_916952	Lighthouse Lab in Alderley Park	Wellcome Sanger Institute for the COVID-19 Genomics UK (COG-UK) Consortium	Jacquelyn Wynn, Mairead Hyland, The Lighthouse Lab in Alderley Park and Alex Alderton, Roberto Amato, Sonia Goncalves, Ewan Harrison, David K. Jackson, Ian Johnston, Dominic Kwiatkowski, Cordelia Langford, John Sillitoe on behalf of the Wellcome Sanger Institute COVID-19 Surveillance Team
EPI_ISL_916953, EPI_ISL_916954	Lighthouse Lab in Glasgow	Wellcome Sanger Institute for the COVID-19 Genomics UK (COG-UK) Consortium	Harper VanSteenhouse, Yumi Kasai, David Gray, Carol Clugston, Anna Dominiczak and Alex Alderton, Roberto Amato, Sonia Goncalves, Ewan Harrison, David K. Jackson, Ian Johnston, Dominic Kwiatkowski, Cordelia Langford, John Sillitoe on behalf of the Wellcome Sanger Institute COVID-19 Surveillance Team
EPI_ISL_916955	Lighthouse Lab in Alderley Park	Wellcome Sanger Institute for the COVID-19 Genomics UK (COG-UK) Consortium	Jacquelyn Wynn, Mairead Hyland, The Lighthouse Lab in Alderley Park and Alex Alderton, Roberto Amato, Sonia Goncalves, Ewan Harrison, David K. Jackson, Ian Johnston, Dominic Kwiatkowski, Cordelia Langford, John Sillitoe on behalf of the Wellcome Sanger Institute COVID-19 Surveillance Team
EPI_ISL_916956	Lighthouse Lab in Glasgow	Wellcome Sanger Institute for the COVID-19 Genomics UK (COG-UK) Consortium	Harper VanSteenhouse, Yumi Kasai, David Gray, Carol Clugston, Anna Dominiczak and Alex Alderton, Roberto Amato, Sonia Goncalves, Ewan Harrison, David K. Jackson, Ian Johnston, Dominic Kwiatkowski, Cordelia Langford, John Sillitoe on behalf of the Wellcome Sanger Institute COVID-19 Surveillance

			Team
EPI_ISL_916957, EPI_ISL_916958, EPI_ISL_916959, EPI_ISL_916960	Lighthouse Lab in Alderley Park	Wellcome Sanger Institute for the COVID-19 Genomics UK (COG-UK) Consortium	Jacquelyn Wynn, Mairead Hyland, The Lighthouse Lab in Alderley Park and Alex Alderton, Roberto Amato, Sonia Goncalves, Ewan Harrison, David K. Jackson, Ian Johnston, Dominic Kwiatkowski, Cordelia Langford, John Sillitoe on behalf of the Wellcome Sanger Institute COVID-19 Surveillance Team
EPI_ISL_916961	Lighthouse Lab in Glasgow	Wellcome Sanger Institute for the COVID-19 Genomics UK (COG-UK) Consortium	Harper VanSteenhouse, Yumi Kasai, David Gray, Carol Clugston, Anna Dominiczak and Alex Alderton, Roberto Amato, Sonia Goncalves, Ewan Harrison, David K. Jackson, Ian Johnston, Dominic Kwiatkowski, Cordelia Langford, John Sillitoe on behalf of the Wellcome Sanger Institute COVID-19 Surveillance Team
EPI_ISL_916962, EPI_ISL_916963, EPI_ISL_916964, EPI_ISL_916965, EPI_ISL_916966	Lighthouse Lab in Alderley Park	Wellcome Sanger Institute for the COVID-19 Genomics UK (COG-UK) Consortium	Jacquelyn Wynn, Mairead Hyland, The Lighthouse Lab in Alderley Park and Alex Alderton, Roberto Amato, Sonia Goncalves, Ewan Harrison, David K. Jackson, Ian Johnston, Dominic Kwiatkowski, Cordelia Langford, John Sillitoe on behalf of the Wellcome Sanger Institute COVID-19 Surveillance Team
EPI_ISL_916967, EPI_ISL_916968	Lighthouse Lab in Glasgow	Wellcome Sanger Institute for the COVID-19 Genomics UK (COG-UK) Consortium	Harper VanSteenhouse, Yumi Kasai, David Gray, Carol Clugston, Anna Dominiczak and Alex Alderton, Roberto Amato, Sonia Goncalves, Ewan Harrison, David K. Jackson, Ian Johnston, Dominic Kwiatkowski, Cordelia Langford, John Sillitoe on behalf of the Wellcome Sanger Institute COVID-19 Surveillance Team
EPI_ISL_916969	Lighthouse Lab in Alderley Park	Wellcome Sanger Institute for the COVID-19 Genomics UK (COG-UK) Consortium	Jacquelyn Wynn, Mairead Hyland, The Lighthouse Lab in Alderley Park and Alex Alderton, Roberto Amato, Sonia Goncalves, Ewan Harrison, David K. Jackson, Ian Johnston, Dominic Kwiatkowski, Cordelia Langford, John Sillitoe on behalf of the Wellcome Sanger Institute COVID-19 Surveillance Team
EPI_ISL_916970	Lighthouse Lab in Glasgow	Wellcome Sanger Institute for the COVID-19 Genomics UK (COG-UK) Consortium	Harper VanSteenhouse, Yumi Kasai, David Gray, Carol Clugston, Anna Dominiczak and Alex Alderton, Roberto Amato, Sonia Goncalves, Ewan Harrison, David K. Jackson, Ian Johnston, Dominic Kwiatkowski, Cordelia Langford, John Sillitoe on behalf of the Wellcome Sanger Institute COVID-19 Surveillance Team
EPI_ISL_916971, EPI_ISL_916972, EPI_ISL_916973	Lighthouse Lab in Alderley Park	Wellcome Sanger Institute for the COVID-19 Genomics UK (COG-UK) Consortium	Jacquelyn Wynn, Mairead Hyland, The Lighthouse Lab in Alderley Park and Alex Alderton, Roberto Amato, Sonia Goncalves, Ewan Harrison, David K. Jackson, Ian Johnston, Dominic Kwiatkowski, Cordelia Langford, John Sillitoe on behalf of the Wellcome Sanger Institute COVID-19 Surveillance Team
EPI_ISL_916974	Lighthouse Lab in Glasgow	Wellcome Sanger Institute for the COVID-19 Genomics UK (COG-UK) Consortium	Harper VanSteenhouse, Yumi Kasai, David Gray, Carol Clugston, Anna Dominiczak and Alex Alderton, Roberto Amato, Sonia Goncalves, Ewan Harrison, David K. Jackson, Ian Johnston, Dominic Kwiatkowski, Cordelia Langford, John Sillitoe on behalf of the Wellcome Sanger Institute COVID-19 Surveillance Team
EPI_ISL_916975	Lighthouse Lab in Alderley Park	Wellcome Sanger Institute for the COVID-19 Genomics UK (COG-UK) Consortium	Jacquelyn Wynn, Mairead Hyland, The Lighthouse Lab in Alderley Park and Alex Alderton, Roberto Amato, Sonia Goncalves, Ewan Harrison, David K. Jackson, Ian Johnston, Dominic Kwiatkowski, Cordelia Langford, John Sillitoe on behalf of the Wellcome Sanger Institute COVID-19 Surveillance Team
EPI_ISL_916976, EPI_ISL_916977	Lighthouse Lab in Glasgow	Wellcome Sanger Institute for the COVID-19 Genomics UK (COG-UK) Consortium	Harper VanSteenhouse, Yumi Kasai, David Gray, Carol Clugston, Anna Dominiczak and Alex Alderton, Roberto Amato, Sonia Goncalves, Ewan Harrison, David K. Jackson, Ian Johnston, Dominic Kwiatkowski, Cordelia Langford, John Sillitoe on behalf of the Wellcome Sanger Institute COVID-19 Surveillance Team
EPI_ISL_916978	Lighthouse Lab in Alderley Park	Wellcome Sanger Institute for the COVID-19 Genomics UK (COG-UK) Consortium	Jacquelyn Wynn, Mairead Hyland, The Lighthouse Lab in Alderley Park and Alex Alderton, Roberto Amato, Sonia Goncalves, Ewan Harrison, David K. Jackson, Ian Johnston, Dominic Kwiatkowski, Cordelia Langford, John Sillitoe on behalf of the Wellcome Sanger Institute COVID-19 Surveillance Team
EPI_ISL_916979	Lighthouse Lab in Glasgow	Wellcome Sanger Institute for the COVID-19 Genomics UK (COG-UK) Consortium	Harper VanSteenhouse, Yumi Kasai, David Gray, Carol Clugston, Anna Dominiczak and Alex Alderton, Roberto Amato, Sonia Goncalves, Ewan Harrison, David K. Jackson, Ian Johnston, Dominic Kwiatkowski, Cordelia Langford, John Sillitoe on behalf of the Wellcome Sanger Institute COVID-19 Surveillance Team
EPI_ISL_916980	Lighthouse Lab in Alderley Park	Wellcome Sanger Institute for the COVID-19 Genomics UK (COG-UK) Consortium	Jacquelyn Wynn, Mairead Hyland, The Lighthouse Lab in Alderley Park and Alex Alderton, Roberto Amato, Sonia Goncalves, Ewan Harrison, David K. Jackson, Ian Johnston, Dominic Kwiatkowski, Cordelia Langford, John Sillitoe on behalf of the Wellcome Sanger Institute COVID-19 Surveillance Team
EPI_ISL_916981	Lighthouse Lab in Glasgow	Wellcome Sanger Institute for the COVID-19 Genomics UK (COG-UK) Consortium	Harper VanSteenhouse, Yumi Kasai, David Gray, Carol Clugston, Anna Dominiczak and Alex Alderton, Roberto Amato, Sonia Goncalves, Ewan Harrison, David K. Jackson, Ian Johnston, Dominic Kwiatkowski, Cordelia Langford, John Sillitoe on behalf of the Wellcome Sanger Institute COVID-19 Surveillance Team
EPI_ISL_916982, EPI_ISL_916983	Lighthouse Lab in Alderley Park	Wellcome Sanger Institute for the COVID-19 Genomics UK (COG-UK) Consortium	Jacquelyn Wynn, Mairead Hyland, The Lighthouse Lab in Alderley Park and Alex Alderton, Roberto Amato, Sonia Goncalves, Ewan Harrison, David K. Jackson, Ian Johnston, Dominic Kwiatkowski, Cordelia Langford, John Sillitoe on behalf of the Wellcome Sanger Institute COVID-19 Surveillance Team
EPI_ISL_916984, EPI_ISL_916985	Lighthouse Lab in Glasgow	Wellcome Sanger Institute for the COVID-19 Genomics UK (COG-UK) Consortium	Harper VanSteenhouse, Yumi Kasai, David Gray, Carol Clugston, Anna Dominiczak and Alex Alderton, Roberto Amato, Sonia Goncalves, Ewan Harrison, David K. Jackson, Ian Johnston, Dominic Kwiatkowski, Cordelia Langford, John Sillitoe on behalf of the Wellcome Sanger Institute COVID-19 Surveillance Team
EPI_ISL_916986	Lighthouse Lab in Alderley Park	Wellcome Sanger Institute for the COVID-19 Genomics UK (COG-UK) Consortium	Jacquelyn Wynn, Mairead Hyland, The Lighthouse Lab in Alderley Park and Alex Alderton, Roberto Amato, Sonia Goncalves, Ewan Harrison, David K. Jackson, Ian Johnston, Dominic Kwiatkowski, Cordelia Langford, John Sillitoe on behalf of the Wellcome Sanger Institute COVID-19 Surveillance Team
EPI_ISL_916987	Lighthouse Lab in Glasgow	Wellcome Sanger Institute for the COVID-19 Genomics UK (COG-UK) Consortium	Harper VanSteenhouse, Yumi Kasai, David Gray, Carol Clugston, Anna Dominiczak and Alex Alderton, Roberto Amato, Sonia Goncalves, Ewan Harrison, David K. Jackson, Ian Johnston, Dominic Kwiatkowski, Cordelia Langford, John Sillitoe on behalf of the Wellcome Sanger Institute COVID-19 Surveillance Team
EPI_ISL_916988, EPI_ISL_916989, EPI_ISL_916990	Lighthouse Lab in Alderley Park	Wellcome Sanger Institute for the COVID-19 Genomics UK (COG-UK) Consortium	Jacquelyn Wynn, Mairead Hyland, The Lighthouse Lab in Alderley Park and Alex Alderton, Roberto Amato, Sonia Goncalves, Ewan Harrison, David K. Jackson, Ian Johnston, Dominic Kwiatkowski, Cordelia Langford, John Sillitoe on behalf of the Wellcome Sanger Institute COVID-19 Surveillance Team
EPI_ISL_916991	Lighthouse Lab in Glasgow	Wellcome Sanger Institute for the COVID-19 Genomics UK (COG-UK) Consortium	Harper VanSteenhouse, Yumi Kasai, David Gray, Carol Clugston, Anna Dominiczak and Alex Alderton, Roberto Amato, Sonia Goncalves, Ewan Harrison, David K. Jackson, Ian Johnston, Dominic Kwiatkowski, Cordelia Langford, John Sillitoe on behalf of the Wellcome Sanger Institute COVID-19 Surveillance Team
EPI_ISL_916992, EPI_ISL_916993, EPI_ISL_916994, EPI_ISL_916996, EPI_ISL_916997	Lighthouse Lab in Alderley Park	Wellcome Sanger Institute for the COVID-19 Genomics UK (COG-UK) Consortium	Jacquelyn Wynn, Mairead Hyland, The Lighthouse Lab in Alderley Park and Alex Alderton, Roberto Amato, Sonia Goncalves, Ewan Harrison, David K. Jackson, Ian Johnston, Dominic Kwiatkowski, Cordelia Langford, John Sillitoe on behalf of the Wellcome Sanger Institute COVID-19 Surveillance Team
EPI_ISL_916998, EPI_ISL_916999	Lighthouse Lab in Glasgow	Wellcome Sanger Institute for the COVID-19 Genomics UK (COG-UK) Consortium	Harper VanSteenhouse, Yumi Kasai, David Gray, Carol Clugston, Anna Dominiczak and Alex Alderton, Roberto Amato, Sonia Goncalves, Ewan Harrison, David K. Jackson, Ian Johnston, Dominic Kwiatkowski, Cordelia Langford, John Sillitoe on behalf of the Wellcome Sanger Institute COVID-19 Surveillance Team
EPI_ISL_917000, EPI_ISL_917001	Lighthouse Lab in Alderley Park	Wellcome Sanger Institute for the COVID-19 Genomics UK (COG-UK) Consortium	Jacquelyn Wynn, Mairead Hyland, The Lighthouse Lab in Alderley Park and Alex Alderton, Roberto Amato, Sonia Goncalves, Ewan Harrison, David K. Jackson, Ian Johnston, Dominic Kwiatkowski, Cordelia Langford, John Sillitoe on behalf of the Wellcome Sanger Institute COVID-19 Surveillance Team
EPI_ISL_917002	Lighthouse Lab in Glasgow	Wellcome Sanger Institute for the COVID-19 Genomics UK (COG-UK) Consortium	Harper VanSteenhouse, Yumi Kasai, David Gray, Carol Clugston, Anna Dominiczak and Alex Alderton, Roberto Amato, Sonia Goncalves, Ewan Harrison, David K. Jackson, Ian Johnston, Dominic Kwiatkowski, Cordelia Langford, John Sillitoe on behalf of the Wellcome Sanger Institute COVID-19 Surveillance Team
EPI_ISL_917003, EPI_ISL_917004, EPI_ISL_917005, EPI_ISL_917006, EPI_ISL_917007, EPI_ISL_917008	Lighthouse Lab in Alderley Park	Wellcome Sanger Institute for the COVID-19 Genomics UK (COG-UK) Consortium	Jacquelyn Wynn, Mairead Hyland, The Lighthouse Lab in Alderley Park and Alex Alderton, Roberto Amato, Sonia Goncalves, Ewan Harrison, David K. Jackson, Ian Johnston, Dominic Kwiatkowski, Cordelia Langford, John Sillitoe on behalf of the Wellcome Sanger Institute COVID-19 Surveillance Team
EPI_ISL_917009	Lighthouse Lab in Glasgow	Wellcome Sanger Institute for the COVID-19 Genomics UK (COG-UK) Consortium	Harper VanSteenhouse, Yumi Kasai, David Gray, Carol Clugston, Anna Dominiczak and Alex Alderton, Roberto Amato, Sonia Goncalves, Ewan Harrison, David K. Jackson, Ian Johnston, Dominic Kwiatkowski, Cordelia Langford, John Sillitoe on behalf of the Wellcome Sanger Institute COVID-19 Surveillance Team
EPI_ISL_917010	Lighthouse Lab in Alderley Park	Wellcome Sanger Institute for the COVID-19 Genomics UK (COG-UK) Consortium	Jacquelyn Wynn, Mairead Hyland, The Lighthouse Lab in Alderley Park and Alex Alderton, Roberto Amato, Sonia Goncalves, Ewan Harrison, David K. Jackson, Ian Johnston, Dominic Kwiatkowski, Cordelia Langford, John Sillitoe on behalf of the Wellcome Sanger Institute COVID-19 Surveillance Team
EPI_ISL_917012, EPI_ISL_917013, EPI_ISL_917014	Lighthouse Lab in Glasgow	Wellcome Sanger Institute for the COVID-19 Genomics UK (COG-UK) Consortium	Harper VanSteenhouse, Yumi Kasai, David Gray, Carol Clugston, Anna Dominiczak and Alex Alderton, Roberto Amato, Sonia Goncalves, Ewan Harrison, David K. Jackson, Ian Johnston, Dominic Kwiatkowski, Cordelia Langford, John Sillitoe on behalf of the Wellcome Sanger Institute COVID-19 Surveillance

			Team
EPI_ISL_917015, EPI_ISL_917016, EPI_ISL_917017, EPI_ISL_917018	Lighthouse Lab in Alderley Park	Wellcome Sanger Institute for the COVID-19 Genomics UK (COG-UK) Consortium	Jacquelyn Wynn, Mairead Hyland, The Lighthouse Lab in Alderley Park and Alex Alderton, Roberto Amato, Sonia Goncalves, Ewan Harrison, David K. Jackson, Ian Johnston, Dominic Kwiatkowski, Cordelia Langford, John Sillitoe on behalf of the Wellcome Sanger Institute COVID-19 Surveillance Team
EPI_ISL_917019	Lighthouse Lab in Glasgow	Wellcome Sanger Institute for the COVID-19 Genomics UK (COG-UK) Consortium	Harper VanSteenhouse, Yumi Kasai, David Gray, Carol Clugston, Anna Dominiczak and Alex Alderton, Roberto Amato, Sonia Goncalves, Ewan Harrison, David K. Jackson, Ian Johnston, Dominic Kwiatkowski, Cordelia Langford, John Sillitoe on behalf of the Wellcome Sanger Institute COVID-19 Surveillance Team
EPI_ISL_917020	Lighthouse Lab in Alderley Park	Wellcome Sanger Institute for the COVID-19 Genomics UK (COG-UK) Consortium	Jacquelyn Wynn, Mairead Hyland, The Lighthouse Lab in Alderley Park and Alex Alderton, Roberto Amato, Sonia Goncalves, Ewan Harrison, David K. Jackson, Ian Johnston, Dominic Kwiatkowski, Cordelia Langford, John Sillitoe on behalf of the Wellcome Sanger Institute COVID-19 Surveillance Team
EPI_ISL_917021, EPI_ISL_917022	Lighthouse Lab in Glasgow	Wellcome Sanger Institute for the COVID-19 Genomics UK (COG-UK) Consortium	Harper VanSteenhouse, Yumi Kasai, David Gray, Carol Clugston, Anna Dominiczak and Alex Alderton, Roberto Amato, Sonia Goncalves, Ewan Harrison, David K. Jackson, Ian Johnston, Dominic Kwiatkowski, Cordelia Langford, John Sillitoe on behalf of the Wellcome Sanger Institute COVID-19 Surveillance Team
EPI_ISL_917023	Lighthouse Lab in Alderley Park	Wellcome Sanger Institute for the COVID-19 Genomics UK (COG-UK) Consortium	Jacquelyn Wynn, Mairead Hyland, The Lighthouse Lab in Alderley Park and Alex Alderton, Roberto Amato, Sonia Goncalves, Ewan Harrison, David K. Jackson, Ian Johnston, Dominic Kwiatkowski, Cordelia Langford, John Sillitoe on behalf of the Wellcome Sanger Institute COVID-19 Surveillance Team
EPI_ISL_917024, EPI_ISL_917025, EPI_ISL_917026	Lighthouse Lab in Glasgow	Wellcome Sanger Institute for the COVID-19 Genomics UK (COG-UK) Consortium	Harper VanSteenhouse, Yumi Kasai, David Gray, Carol Clugston, Anna Dominiczak and Alex Alderton, Roberto Amato, Sonia Goncalves, Ewan Harrison, David K. Jackson, Ian Johnston, Dominic Kwiatkowski, Cordelia Langford, John Sillitoe on behalf of the Wellcome Sanger Institute COVID-19 Surveillance Team
EPI_ISL_917027, EPI_ISL_917028	Lighthouse Lab in Alderley Park	Wellcome Sanger Institute for the COVID-19 Genomics UK (COG-UK) Consortium	Jacquelyn Wynn, Mairead Hyland, The Lighthouse Lab in Alderley Park and Alex Alderton, Roberto Amato, Sonia Goncalves, Ewan Harrison, David K. Jackson, Ian Johnston, Dominic Kwiatkowski, Cordelia Langford, John Sillitoe on behalf of the Wellcome Sanger Institute COVID-19 Surveillance Team
EPI_ISL_917029	Lighthouse Lab in Glasgow	Wellcome Sanger Institute for the COVID-19 Genomics UK (COG-UK) Consortium	Harper VanSteenhouse, Yumi Kasai, David Gray, Carol Clugston, Anna Dominiczak and Alex Alderton, Roberto Amato, Sonia Goncalves, Ewan Harrison, David K. Jackson, Ian Johnston, Dominic Kwiatkowski, Cordelia Langford, John Sillitoe on behalf of the Wellcome Sanger Institute COVID-19 Surveillance Team
EPI_ISL_917030	Lighthouse Lab in Alderley Park	Wellcome Sanger Institute for the COVID-19 Genomics UK (COG-UK) Consortium	Jacquelyn Wynn, Mairead Hyland, The Lighthouse Lab in Alderley Park and Alex Alderton, Roberto Amato, Sonia Goncalves, Ewan Harrison, David K. Jackson, Ian Johnston, Dominic Kwiatkowski, Cordelia Langford, John Sillitoe on behalf of the Wellcome Sanger Institute COVID-19 Surveillance Team
EPI_ISL_917031, EPI_ISL_917032	Lighthouse Lab in Glasgow	Wellcome Sanger Institute for the COVID-19 Genomics UK (COG-UK) Consortium	Harper VanSteenhouse, Yumi Kasai, David Gray, Carol Clugston, Anna Dominiczak and Alex Alderton, Roberto Amato, Sonia Goncalves, Ewan Harrison, David K. Jackson, Ian Johnston, Dominic Kwiatkowski, Cordelia Langford, John Sillitoe on behalf of the Wellcome Sanger Institute COVID-19 Surveillance Team
EPI_ISL_917033, EPI_ISL_917034, EPI_ISL_917035	Lighthouse Lab in Alderley Park	Wellcome Sanger Institute for the COVID-19 Genomics UK (COG-UK) Consortium	Jacquelyn Wynn, Mairead Hyland, The Lighthouse Lab in Alderley Park and Alex Alderton, Roberto Amato, Sonia Goncalves, Ewan Harrison, David K. Jackson, Ian Johnston, Dominic Kwiatkowski, Cordelia Langford, John Sillitoe on behalf of the Wellcome Sanger Institute COVID-19 Surveillance Team
EPI_ISL_917036	Lighthouse Lab in Glasgow	Wellcome Sanger Institute for the COVID-19 Genomics UK (COG-UK) Consortium	Harper VanSteenhouse, Yumi Kasai, David Gray, Carol Clugston, Anna Dominiczak and Alex Alderton, Roberto Amato, Sonia Goncalves, Ewan Harrison, David K. Jackson, Ian Johnston, Dominic Kwiatkowski, Cordelia Langford, John Sillitoe on behalf of the Wellcome Sanger Institute COVID-19 Surveillance Team
EPI_ISL_917037	Lighthouse Lab in Alderley Park	Wellcome Sanger Institute for the COVID-19 Genomics UK (COG-UK) Consortium	Jacquelyn Wynn, Mairead Hyland, The Lighthouse Lab in Alderley Park and Alex Alderton, Roberto Amato, Sonia Goncalves, Ewan Harrison, David K. Jackson, Ian Johnston, Dominic Kwiatkowski, Cordelia Langford, John Sillitoe on behalf of the Wellcome Sanger Institute COVID-19 Surveillance Team
EPI_ISL_917038	Lighthouse Lab in Glasgow	Wellcome Sanger Institute for the COVID-19 Genomics UK (COG-UK) Consortium	Harper VanSteenhouse, Yumi Kasai, David Gray, Carol Clugston, Anna Dominiczak and Alex Alderton, Roberto Amato, Sonia Goncalves, Ewan Harrison, David K. Jackson, Ian Johnston, Dominic Kwiatkowski, Cordelia Langford, John Sillitoe on behalf of the Wellcome Sanger Institute COVID-19 Surveillance Team
EPI_ISL_917039, EPI_ISL_917040, EPI_ISL_917041	Lighthouse Lab in Alderley Park	Wellcome Sanger Institute for the COVID-19 Genomics UK (COG-UK) Consortium	Jacquelyn Wynn, Mairead Hyland, The Lighthouse Lab in Alderley Park and Alex Alderton, Roberto Amato, Sonia Goncalves, Ewan Harrison, David K. Jackson, Ian Johnston, Dominic Kwiatkowski, Cordelia Langford, John Sillitoe on behalf of the Wellcome Sanger Institute COVID-19 Surveillance Team
EPI_ISL_917042	Lighthouse Lab in Glasgow	Wellcome Sanger Institute for the COVID-19 Genomics UK (COG-UK) Consortium	Harper VanSteenhouse, Yumi Kasai, David Gray, Carol Clugston, Anna Dominiczak and Alex Alderton, Roberto Amato, Sonia Goncalves, Ewan Harrison, David K. Jackson, Ian Johnston, Dominic Kwiatkowski, Cordelia Langford, John Sillitoe on behalf of the Wellcome Sanger Institute COVID-19 Surveillance Team
EPI_ISL_917043, EPI_ISL_917044, EPI_ISL_917045, EPI_ISL_917046	Lighthouse Lab in Alderley Park	Wellcome Sanger Institute for the COVID-19 Genomics UK (COG-UK) Consortium	Jacquelyn Wynn, Mairead Hyland, The Lighthouse Lab in Alderley Park and Alex Alderton, Roberto Amato, Sonia Goncalves, Ewan Harrison, David K. Jackson, Ian Johnston, Dominic Kwiatkowski, Cordelia Langford, John Sillitoe on behalf of the Wellcome Sanger Institute COVID-19 Surveillance Team
EPI_ISL_917047	Lighthouse Lab in Glasgow	Wellcome Sanger Institute for the COVID-19 Genomics UK (COG-UK) Consortium	Harper VanSteenhouse, Yumi Kasai, David Gray, Carol Clugston, Anna Dominiczak and Alex Alderton, Roberto Amato, Sonia Goncalves, Ewan Harrison, David K. Jackson, Ian Johnston, Dominic Kwiatkowski, Cordelia Langford, John Sillitoe on behalf of the Wellcome Sanger Institute COVID-19 Surveillance Team
EPI_ISL_917048	Lighthouse Lab in Alderley Park	Wellcome Sanger Institute for the COVID-19 Genomics UK (COG-UK) Consortium	Jacquelyn Wynn, Mairead Hyland, The Lighthouse Lab in Alderley Park and Alex Alderton, Roberto Amato, Sonia Goncalves, Ewan Harrison, David K. Jackson, Ian Johnston, Dominic Kwiatkowski, Cordelia Langford, John Sillitoe on behalf of the Wellcome Sanger Institute COVID-19 Surveillance Team
EPI_ISL_917049	Lighthouse Lab in Glasgow	Wellcome Sanger Institute for the COVID-19 Genomics UK (COG-UK) Consortium	Harper VanSteenhouse, Yumi Kasai, David Gray, Carol Clugston, Anna Dominiczak and Alex Alderton, Roberto Amato, Sonia Goncalves, Ewan Harrison, David K. Jackson, Ian Johnston, Dominic Kwiatkowski, Cordelia Langford, John Sillitoe on behalf of the Wellcome Sanger Institute COVID-19 Surveillance Team
EPI_ISL_917050, EPI_ISL_917051, EPI_ISL_917052	Lighthouse Lab in Alderley Park	Wellcome Sanger Institute for the COVID-19 Genomics UK (COG-UK) Consortium	Jacquelyn Wynn, Mairead Hyland, The Lighthouse Lab in Alderley Park and Alex Alderton, Roberto Amato, Sonia Goncalves, Ewan Harrison, David K. Jackson, Ian Johnston, Dominic Kwiatkowski, Cordelia Langford, John Sillitoe on behalf of the Wellcome Sanger Institute COVID-19 Surveillance Team
EPI_ISL_917053, EPI_ISL_917054, EPI_ISL_917055	Lighthouse Lab in Glasgow	Wellcome Sanger Institute for the COVID-19 Genomics UK (COG-UK) Consortium	Harper VanSteenhouse, Yumi Kasai, David Gray, Carol Clugston, Anna Dominiczak and Alex Alderton, Roberto Amato, Sonia Goncalves, Ewan Harrison, David K. Jackson, Ian Johnston, Dominic Kwiatkowski, Cordelia Langford, John Sillitoe on behalf of the Wellcome Sanger Institute COVID-19 Surveillance Team
EPI_ISL_917056, EPI_ISL_917057, EPI_ISL_917058, EPI_ISL_917059, EPI_ISL_917061, EPI_ISL_917062	Lighthouse Lab in Alderley Park	Wellcome Sanger Institute for the COVID-19 Genomics UK (COG-UK) Consortium	Jacquelyn Wynn, Mairead Hyland, The Lighthouse Lab in Alderley Park and Alex Alderton, Roberto Amato, Sonia Goncalves, Ewan Harrison, David K. Jackson, Ian Johnston, Dominic Kwiatkowski, Cordelia Langford, John Sillitoe on behalf of the Wellcome Sanger Institute COVID-19 Surveillance Team
EPI_ISL_917063	Lighthouse Lab in Glasgow	Wellcome Sanger Institute for the COVID-19 Genomics UK (COG-UK) Consortium	Harper VanSteenhouse, Yumi Kasai, David Gray, Carol Clugston, Anna Dominiczak and Alex Alderton, Roberto Amato, Sonia Goncalves, Ewan Harrison, David K. Jackson, Ian Johnston, Dominic Kwiatkowski, Cordelia Langford, John Sillitoe on behalf of the Wellcome Sanger Institute COVID-19 Surveillance Team
EPI_ISL_917064, EPI_ISL_917065, EPI_ISL_917066	Lighthouse Lab in Alderley Park	Wellcome Sanger Institute for the COVID-19 Genomics UK (COG-UK) Consortium	Jacquelyn Wynn, Mairead Hyland, The Lighthouse Lab in Alderley Park and Alex Alderton, Roberto Amato, Sonia Goncalves, Ewan Harrison, David K. Jackson, Ian Johnston, Dominic Kwiatkowski, Cordelia Langford, John Sillitoe on behalf of the Wellcome Sanger Institute COVID-19 Surveillance Team
EPI_ISL_917067	Lighthouse Lab in Glasgow	Wellcome Sanger Institute for the COVID-19 Genomics UK (COG-UK) Consortium	Harper VanSteenhouse, Yumi Kasai, David Gray, Carol Clugston, Anna Dominiczak and Alex Alderton, Roberto Amato, Sonia Goncalves, Ewan Harrison, David K. Jackson, Ian Johnston, Dominic Kwiatkowski, Cordelia Langford, John Sillitoe on behalf of the Wellcome Sanger Institute COVID-19 Surveillance Team
EPI_ISL_917068, EPI_ISL_917069	Lighthouse Lab in Alderley Park	Wellcome Sanger Institute for the COVID-19 Genomics UK (COG-UK) Consortium	Jacquelyn Wynn, Mairead Hyland, The Lighthouse Lab in Alderley Park and Alex Alderton, Roberto Amato, Sonia Goncalves, Ewan Harrison, David K. Jackson, Ian Johnston, Dominic Kwiatkowski, Cordelia Langford, John Sillitoe on behalf of the Wellcome Sanger Institute COVID-19 Surveillance Team
EPI_ISL_917070, EPI_ISL_917071	Lighthouse Lab in Glasgow	Wellcome Sanger Institute for the COVID-19 Genomics UK (COG-UK) Consortium	Harper VanSteenhouse, Yumi Kasai, David Gray, Carol Clugston, Anna Dominiczak and Alex Alderton, Roberto Amato, Sonia Goncalves, Ewan Harrison, David K. Jackson, Ian Johnston, Dominic Kwiatkowski, Cordelia Langford, John Sillitoe on behalf of the Wellcome Sanger Institute COVID-19 Surveillance Team
EPI_ISL_917072, EPI_ISL_917073	Lighthouse Lab in Alderley Park	Wellcome Sanger Institute for the COVID-19 Genomics UK	Jacquelyn Wynn, Mairead Hyland, The Lighthouse Lab in Alderley Park and Alex Alderton, Roberto Amato, Sonia Goncalves, Ewan Harrison, David K.

		(COG-UK) Consortium	Jackson, Ian Johnston, Dominic Kwiatkowski, Cordelia Langford, John Sillitoe on behalf of the Wellcome Sanger Institute COVID-19 Surveillance Team
EPI_ISL_917074, EPI_ISL_917075, EPI_ISL_917076, EPI_ISL_917077	Lighthouse Lab in Glasgow	Wellcome Sanger Institute for the COVID-19 Genomics UK (COG-UK) Consortium	Harper VanSteenhouse, Yumi Kasai, David Gray, Carol Clugston, Anna Dominiczak and Alex Alderton, Roberto Amato, Sonia Goncalves, Ewan Harrison, David K. Jackson, Ian Johnston, Dominic Kwiatkowski, Cordelia Langford, John Sillitoe on behalf of the Wellcome Sanger Institute COVID-19 Surveillance Team
EPI_ISL_917078	Lighthouse Lab in Alderley Park	Wellcome Sanger Institute for the COVID-19 Genomics UK (COG-UK) Consortium	Jacquelyn Wynn, Mairead Hyland, The Lighthouse Lab in Alderley Park and Alex Alderton, Roberto Amato, Sonia Goncalves, Ewan Harrison, David K. Jackson, Ian Johnston, Dominic Kwiatkowski, Cordelia Langford, John Sillitoe on behalf of the Wellcome Sanger Institute COVID-19 Surveillance Team
EPI_ISL_917079	Lighthouse Lab in Glasgow	Wellcome Sanger Institute for the COVID-19 Genomics UK (COG-UK) Consortium	Harper VanSteenhouse, Yumi Kasai, David Gray, Carol Clugston, Anna Dominiczak and Alex Alderton, Roberto Amato, Sonia Goncalves, Ewan Harrison, David K. Jackson, Ian Johnston, Dominic Kwiatkowski, Cordelia Langford, John Sillitoe on behalf of the Wellcome Sanger Institute COVID-19 Surveillance Team
EPI_ISL_917080	Lighthouse Lab in Alderley Park	Wellcome Sanger Institute for the COVID-19 Genomics UK (COG-UK) Consortium	Jacquelyn Wynn, Mairead Hyland, The Lighthouse Lab in Alderley Park and Alex Alderton, Roberto Amato, Sonia Goncalves, Ewan Harrison, David K. Jackson, Ian Johnston, Dominic Kwiatkowski, Cordelia Langford, John Sillitoe on behalf of the Wellcome Sanger Institute COVID-19 Surveillance Team
EPI_ISL_917081, EPI_ISL_917082, EPI_ISL_917083	Lighthouse Lab in Glasgow	Wellcome Sanger Institute for the COVID-19 Genomics UK (COG-UK) Consortium	Harper VanSteenhouse, Yumi Kasai, David Gray, Carol Clugston, Anna Dominiczak and Alex Alderton, Roberto Amato, Sonia Goncalves, Ewan Harrison, David K. Jackson, Ian Johnston, Dominic Kwiatkowski, Cordelia Langford, John Sillitoe on behalf of the Wellcome Sanger Institute COVID-19 Surveillance Team
EPI_ISL_917084, EPI_ISL_917085	Lighthouse Lab in Alderley Park	Wellcome Sanger Institute for the COVID-19 Genomics UK (COG-UK) Consortium	Jacquelyn Wynn, Mairead Hyland, The Lighthouse Lab in Alderley Park and Alex Alderton, Roberto Amato, Sonia Goncalves, Ewan Harrison, David K. Jackson, Ian Johnston, Dominic Kwiatkowski, Cordelia Langford, John Sillitoe on behalf of the Wellcome Sanger Institute COVID-19 Surveillance Team
EPI_ISL_917086	Lighthouse Lab in Glasgow	Wellcome Sanger Institute for the COVID-19 Genomics UK (COG-UK) Consortium	Harper VanSteenhouse, Yumi Kasai, David Gray, Carol Clugston, Anna Dominiczak and Alex Alderton, Roberto Amato, Sonia Goncalves, Ewan Harrison, David K. Jackson, Ian Johnston, Dominic Kwiatkowski, Cordelia Langford, John Sillitoe on behalf of the Wellcome Sanger Institute COVID-19 Surveillance Team
EPI_ISL_917087	Lighthouse Lab in Alderley Park	Wellcome Sanger Institute for the COVID-19 Genomics UK (COG-UK) Consortium	Jacquelyn Wynn, Mairead Hyland, The Lighthouse Lab in Alderley Park and Alex Alderton, Roberto Amato, Sonia Goncalves, Ewan Harrison, David K. Jackson, Ian Johnston, Dominic Kwiatkowski, Cordelia Langford, John Sillitoe on behalf of the Wellcome Sanger Institute COVID-19 Surveillance Team
EPI_ISL_917088	Lighthouse Lab in Glasgow	Wellcome Sanger Institute for the COVID-19 Genomics UK (COG-UK) Consortium	Harper VanSteenhouse, Yumi Kasai, David Gray, Carol Clugston, Anna Dominiczak and Alex Alderton, Roberto Amato, Sonia Goncalves, Ewan Harrison, David K. Jackson, Ian Johnston, Dominic Kwiatkowski, Cordelia Langford, John Sillitoe on behalf of the Wellcome Sanger Institute COVID-19 Surveillance Team
EPI_ISL_917089, EPI_ISL_917090	Lighthouse Lab in Alderley Park	Wellcome Sanger Institute for the COVID-19 Genomics UK (COG-UK) Consortium	Jacquelyn Wynn, Mairead Hyland, The Lighthouse Lab in Alderley Park and Alex Alderton, Roberto Amato, Sonia Goncalves, Ewan Harrison, David K. Jackson, Ian Johnston, Dominic Kwiatkowski, Cordelia Langford, John Sillitoe on behalf of the Wellcome Sanger Institute COVID-19 Surveillance Team
EPI_ISL_917091	Lighthouse Lab in Glasgow	Wellcome Sanger Institute for the COVID-19 Genomics UK (COG-UK) Consortium	Harper VanSteenhouse, Yumi Kasai, David Gray, Carol Clugston, Anna Dominiczak and Alex Alderton, Roberto Amato, Sonia Goncalves, Ewan Harrison, David K. Jackson, Ian Johnston, Dominic Kwiatkowski, Cordelia Langford, John Sillitoe on behalf of the Wellcome Sanger Institute COVID-19 Surveillance Team
EPI_ISL_917092	Lighthouse Lab in Alderley Park	Wellcome Sanger Institute for the COVID-19 Genomics UK (COG-UK) Consortium	Jacquelyn Wynn, Mairead Hyland, The Lighthouse Lab in Alderley Park and Alex Alderton, Roberto Amato, Sonia Goncalves, Ewan Harrison, David K. Jackson, Ian Johnston, Dominic Kwiatkowski, Cordelia Langford, John Sillitoe on behalf of the Wellcome Sanger Institute COVID-19 Surveillance Team
EPI_ISL_917093	Lighthouse Lab in Glasgow	Wellcome Sanger Institute for the COVID-19 Genomics UK (COG-UK) Consortium	Harper VanSteenhouse, Yumi Kasai, David Gray, Carol Clugston, Anna Dominiczak and Alex Alderton, Roberto Amato, Sonia Goncalves, Ewan Harrison, David K. Jackson, Ian Johnston, Dominic Kwiatkowski, Cordelia Langford, John Sillitoe on behalf of the Wellcome Sanger Institute COVID-19 Surveillance Team
EPI_ISL_917094	Lighthouse Lab in Alderley Park	Wellcome Sanger Institute for the COVID-19 Genomics UK (COG-UK) Consortium	Jacquelyn Wynn, Mairead Hyland, The Lighthouse Lab in Alderley Park and Alex Alderton, Roberto Amato, Sonia Goncalves, Ewan Harrison, David K. Jackson, Ian Johnston, Dominic Kwiatkowski, Cordelia Langford, John Sillitoe on behalf of the Wellcome Sanger Institute COVID-19 Surveillance Team
EPI_ISL_917096	Lighthouse Lab in Glasgow	Wellcome Sanger Institute for the COVID-19 Genomics UK (COG-UK) Consortium	Harper VanSteenhouse, Yumi Kasai, David Gray, Carol Clugston, Anna Dominiczak and Alex Alderton, Roberto Amato, Sonia Goncalves, Ewan Harrison, David K. Jackson, Ian Johnston, Dominic Kwiatkowski, Cordelia Langford, John Sillitoe on behalf of the Wellcome Sanger Institute COVID-19 Surveillance Team
EPI_ISL_917097, EPI_ISL_917098	Lighthouse Lab in Alderley Park	Wellcome Sanger Institute for the COVID-19 Genomics UK (COG-UK) Consortium	Jacquelyn Wynn, Mairead Hyland, The Lighthouse Lab in Alderley Park and Alex Alderton, Roberto Amato, Sonia Goncalves, Ewan Harrison, David K. Jackson, Ian Johnston, Dominic Kwiatkowski, Cordelia Langford, John Sillitoe on behalf of the Wellcome Sanger Institute COVID-19 Surveillance Team
EPI_ISL_917099	Lighthouse Lab in Glasgow	Wellcome Sanger Institute for the COVID-19 Genomics UK (COG-UK) Consortium	Harper VanSteenhouse, Yumi Kasai, David Gray, Carol Clugston, Anna Dominiczak and Alex Alderton, Roberto Amato, Sonia Goncalves, Ewan Harrison, David K. Jackson, Ian Johnston, Dominic Kwiatkowski, Cordelia Langford, John Sillitoe on behalf of the Wellcome Sanger Institute COVID-19 Surveillance Team
EPI_ISL_917100, EPI_ISL_917101, EPI_ISL_917102, EPI_ISL_917103	Lighthouse Lab in Alderley Park	Wellcome Sanger Institute for the COVID-19 Genomics UK (COG-UK) Consortium	Jacquelyn Wynn, Mairead Hyland, The Lighthouse Lab in Alderley Park and Alex Alderton, Roberto Amato, Sonia Goncalves, Ewan Harrison, David K. Jackson, Ian Johnston, Dominic Kwiatkowski, Cordelia Langford, John Sillitoe on behalf of the Wellcome Sanger Institute COVID-19 Surveillance Team
EPI_ISL_917104, EPI_ISL_917105	Lighthouse Lab in Glasgow	Wellcome Sanger Institute for the COVID-19 Genomics UK (COG-UK) Consortium	Harper VanSteenhouse, Yumi Kasai, David Gray, Carol Clugston, Anna Dominiczak and Alex Alderton, Roberto Amato, Sonia Goncalves, Ewan Harrison, David K. Jackson, Ian Johnston, Dominic Kwiatkowski, Cordelia Langford, John Sillitoe on behalf of the Wellcome Sanger Institute COVID-19 Surveillance Team
EPI_ISL_917106	Lighthouse Lab in Alderley Park	Wellcome Sanger Institute for the COVID-19 Genomics UK (COG-UK) Consortium	Jacquelyn Wynn, Mairead Hyland, The Lighthouse Lab in Alderley Park and Alex Alderton, Roberto Amato, Sonia Goncalves, Ewan Harrison, David K. Jackson, Ian Johnston, Dominic Kwiatkowski, Cordelia Langford, John Sillitoe on behalf of the Wellcome Sanger Institute COVID-19 Surveillance Team
EPI_ISL_917107	Lighthouse Lab in Glasgow	Wellcome Sanger Institute for the COVID-19 Genomics UK (COG-UK) Consortium	Harper VanSteenhouse, Yumi Kasai, David Gray, Carol Clugston, Anna Dominiczak and Alex Alderton, Roberto Amato, Sonia Goncalves, Ewan Harrison, David K. Jackson, Ian Johnston, Dominic Kwiatkowski, Cordelia Langford, John Sillitoe on behalf of the Wellcome Sanger Institute COVID-19 Surveillance Team
EPI_ISL_917108, EPI_ISL_917109	Lighthouse Lab in Alderley Park	Wellcome Sanger Institute for the COVID-19 Genomics UK (COG-UK) Consortium	Jacquelyn Wynn, Mairead Hyland, The Lighthouse Lab in Alderley Park and Alex Alderton, Roberto Amato, Sonia Goncalves, Ewan Harrison, David K. Jackson, Ian Johnston, Dominic Kwiatkowski, Cordelia Langford, John Sillitoe on behalf of the Wellcome Sanger Institute COVID-19 Surveillance Team
EPI_ISL_917110	Lighthouse Lab in Glasgow	Wellcome Sanger Institute for the COVID-19 Genomics UK (COG-UK) Consortium	Harper VanSteenhouse, Yumi Kasai, David Gray, Carol Clugston, Anna Dominiczak and Alex Alderton, Roberto Amato, Sonia Goncalves, Ewan Harrison, David K. Jackson, Ian Johnston, Dominic Kwiatkowski, Cordelia Langford, John Sillitoe on behalf of the Wellcome Sanger Institute COVID-19 Surveillance Team
EPI_ISL_917111	Lighthouse Lab in Alderley Park	Wellcome Sanger Institute for the COVID-19 Genomics UK (COG-UK) Consortium	Jacquelyn Wynn, Mairead Hyland, The Lighthouse Lab in Alderley Park and Alex Alderton, Roberto Amato, Sonia Goncalves, Ewan Harrison, David K. Jackson, Ian Johnston, Dominic Kwiatkowski, Cordelia Langford, John Sillitoe on behalf of the Wellcome Sanger Institute COVID-19 Surveillance Team
EPI_ISL_917112	Lighthouse Lab in Glasgow	Wellcome Sanger Institute for the COVID-19 Genomics UK (COG-UK) Consortium	Harper VanSteenhouse, Yumi Kasai, David Gray, Carol Clugston, Anna Dominiczak and Alex Alderton, Roberto Amato, Sonia Goncalves, Ewan Harrison, David K. Jackson, Ian Johnston, Dominic Kwiatkowski, Cordelia Langford, John Sillitoe on behalf of the Wellcome Sanger Institute COVID-19 Surveillance Team
EPI_ISL_917113	Lighthouse Lab in Alderley Park	Wellcome Sanger Institute for the COVID-19 Genomics UK (COG-UK) Consortium	Jacquelyn Wynn, Mairead Hyland, The Lighthouse Lab in Alderley Park and Alex Alderton, Roberto Amato, Sonia Goncalves, Ewan Harrison, David K. Jackson, Ian Johnston, Dominic Kwiatkowski, Cordelia Langford, John Sillitoe on behalf of the Wellcome Sanger Institute COVID-19 Surveillance Team
EPI_ISL_917114	Lighthouse Lab in Glasgow	Wellcome Sanger Institute for the COVID-19 Genomics UK (COG-UK) Consortium	Harper VanSteenhouse, Yumi Kasai, David Gray, Carol Clugston, Anna Dominiczak and Alex Alderton, Roberto Amato, Sonia Goncalves, Ewan Harrison, David K. Jackson, Ian Johnston, Dominic Kwiatkowski, Cordelia Langford, John Sillitoe on behalf of the Wellcome Sanger Institute COVID-19 Surveillance Team
EPI_ISL_917115	Lighthouse Lab in Alderley Park	Wellcome Sanger Institute for the COVID-19 Genomics UK (COG-UK) Consortium	Jacquelyn Wynn, Mairead Hyland, The Lighthouse Lab in Alderley Park and Alex Alderton, Roberto Amato, Sonia Goncalves, Ewan Harrison, David K. Jackson, Ian Johnston, Dominic Kwiatkowski, Cordelia Langford, John Sillitoe on behalf of the Wellcome Sanger Institute COVID-19 Surveillance Team
EPI_ISL_917116	Lighthouse Lab in Glasgow	Wellcome Sanger Institute for the COVID-19 Genomics UK (COG-UK) Consortium	Harper VanSteenhouse, Yumi Kasai, David Gray, Carol Clugston, Anna Dominiczak and Alex Alderton, Roberto Amato, Sonia Goncalves, Ewan Harrison, David K. Jackson, Ian Johnston, Dominic Kwiatkowski, Cordelia Langford, John Sillitoe on behalf of the Wellcome Sanger Institute COVID-19 Surveillance

			Team
EPI_ISL_917117, EPI_ISL_917118, EPI_ISL_917119	Lighthouse Lab in Alderley Park	Wellcome Sanger Institute for the COVID-19 Genomics UK (COG-UK) Consortium	Jacquelyn Wynn, Mairead Hyland, The Lighthouse Lab in Alderley Park and Alex Alderton, Roberto Amato, Sonia Goncalves, Ewan Harrison, David K. Jackson, Ian Johnston, Dominic Kwiatkowski, Cordelia Langford, John Sillitoe on behalf of the Wellcome Sanger Institute COVID-19 Surveillance Team
EPI_ISL_917120	Lighthouse Lab in Glasgow	Wellcome Sanger Institute for the COVID-19 Genomics UK (COG-UK) Consortium	Harper VanSteenhouse, Yumi Kasai, David Gray, Carol Clugston, Anna Dominiczak and Alex Alderton, Roberto Amato, Sonia Goncalves, Ewan Harrison, David K. Jackson, Ian Johnston, Dominic Kwiatkowski, Cordelia Langford, John Sillitoe on behalf of the Wellcome Sanger Institute COVID-19 Surveillance Team
EPI_ISL_917122, EPI_ISL_917123, EPI_ISL_917124	Lighthouse Lab in Alderley Park	Wellcome Sanger Institute for the COVID-19 Genomics UK (COG-UK) Consortium	Jacquelyn Wynn, Mairead Hyland, The Lighthouse Lab in Alderley Park and Alex Alderton, Roberto Amato, Sonia Goncalves, Ewan Harrison, David K. Jackson, Ian Johnston, Dominic Kwiatkowski, Cordelia Langford, John Sillitoe on behalf of the Wellcome Sanger Institute COVID-19 Surveillance Team
EPI_ISL_917125	Lighthouse Lab in Glasgow	Wellcome Sanger Institute for the COVID-19 Genomics UK (COG-UK) Consortium	Harper VanSteenhouse, Yumi Kasai, David Gray, Carol Clugston, Anna Dominiczak and Alex Alderton, Roberto Amato, Sonia Goncalves, Ewan Harrison, David K. Jackson, Ian Johnston, Dominic Kwiatkowski, Cordelia Langford, John Sillitoe on behalf of the Wellcome Sanger Institute COVID-19 Surveillance Team
EPI_ISL_917126, EPI_ISL_917127	Lighthouse Lab in Alderley Park	Wellcome Sanger Institute for the COVID-19 Genomics UK (COG-UK) Consortium	Jacquelyn Wynn, Mairead Hyland, The Lighthouse Lab in Alderley Park and Alex Alderton, Roberto Amato, Sonia Goncalves, Ewan Harrison, David K. Jackson, Ian Johnston, Dominic Kwiatkowski, Cordelia Langford, John Sillitoe on behalf of the Wellcome Sanger Institute COVID-19 Surveillance Team
EPI_ISL_917128, EPI_ISL_917129	Lighthouse Lab in Glasgow	Wellcome Sanger Institute for the COVID-19 Genomics UK (COG-UK) Consortium	Harper VanSteenhouse, Yumi Kasai, David Gray, Carol Clugston, Anna Dominiczak and Alex Alderton, Roberto Amato, Sonia Goncalves, Ewan Harrison, David K. Jackson, Ian Johnston, Dominic Kwiatkowski, Cordelia Langford, John Sillitoe on behalf of the Wellcome Sanger Institute COVID-19 Surveillance Team
EPI_ISL_917130, EPI_ISL_917131, EPI_ISL_917132	Lighthouse Lab in Alderley Park	Wellcome Sanger Institute for the COVID-19 Genomics UK (COG-UK) Consortium	Jacquelyn Wynn, Mairead Hyland, The Lighthouse Lab in Alderley Park and Alex Alderton, Roberto Amato, Sonia Goncalves, Ewan Harrison, David K. Jackson, Ian Johnston, Dominic Kwiatkowski, Cordelia Langford, John Sillitoe on behalf of the Wellcome Sanger Institute COVID-19 Surveillance Team
EPI_ISL_917133	Lighthouse Lab in Glasgow	Wellcome Sanger Institute for the COVID-19 Genomics UK (COG-UK) Consortium	Harper VanSteenhouse, Yumi Kasai, David Gray, Carol Clugston, Anna Dominiczak and Alex Alderton, Roberto Amato, Sonia Goncalves, Ewan Harrison, David K. Jackson, Ian Johnston, Dominic Kwiatkowski, Cordelia Langford, John Sillitoe on behalf of the Wellcome Sanger Institute COVID-19 Surveillance Team
EPI_ISL_917134, EPI_ISL_917135, EPI_ISL_917136, EPI_ISL_917138	Lighthouse Lab in Alderley Park	Wellcome Sanger Institute for the COVID-19 Genomics UK (COG-UK) Consortium	Jacquelyn Wynn, Mairead Hyland, The Lighthouse Lab in Alderley Park and Alex Alderton, Roberto Amato, Sonia Goncalves, Ewan Harrison, David K. Jackson, Ian Johnston, Dominic Kwiatkowski, Cordelia Langford, John Sillitoe on behalf of the Wellcome Sanger Institute COVID-19 Surveillance Team
EPI_ISL_917139	Lighthouse Lab in Glasgow	Wellcome Sanger Institute for the COVID-19 Genomics UK (COG-UK) Consortium	Harper VanSteenhouse, Yumi Kasai, David Gray, Carol Clugston, Anna Dominiczak and Alex Alderton, Roberto Amato, Sonia Goncalves, Ewan Harrison, David K. Jackson, Ian Johnston, Dominic Kwiatkowski, Cordelia Langford, John Sillitoe on behalf of the Wellcome Sanger Institute COVID-19 Surveillance Team
EPI_ISL_917140	Lighthouse Lab in Alderley Park	Wellcome Sanger Institute for the COVID-19 Genomics UK (COG-UK) Consortium	Jacquelyn Wynn, Mairead Hyland, The Lighthouse Lab in Alderley Park and Alex Alderton, Roberto Amato, Sonia Goncalves, Ewan Harrison, David K. Jackson, Ian Johnston, Dominic Kwiatkowski, Cordelia Langford, John Sillitoe on behalf of the Wellcome Sanger Institute COVID-19 Surveillance Team
EPI_ISL_917141	Lighthouse Lab in Glasgow	Wellcome Sanger Institute for the COVID-19 Genomics UK (COG-UK) Consortium	Harper VanSteenhouse, Yumi Kasai, David Gray, Carol Clugston, Anna Dominiczak and Alex Alderton, Roberto Amato, Sonia Goncalves, Ewan Harrison, David K. Jackson, Ian Johnston, Dominic Kwiatkowski, Cordelia Langford, John Sillitoe on behalf of the Wellcome Sanger Institute COVID-19 Surveillance Team
EPI_ISL_917142	Lighthouse Lab in Alderley Park	Wellcome Sanger Institute for the COVID-19 Genomics UK (COG-UK) Consortium	Jacquelyn Wynn, Mairead Hyland, The Lighthouse Lab in Alderley Park and Alex Alderton, Roberto Amato, Sonia Goncalves, Ewan Harrison, David K. Jackson, Ian Johnston, Dominic Kwiatkowski, Cordelia Langford, John Sillitoe on behalf of the Wellcome Sanger Institute COVID-19 Surveillance Team
EPI_ISL_917143, EPI_ISL_917144, EPI_ISL_917145	Lighthouse Lab in Glasgow	Wellcome Sanger Institute for the COVID-19 Genomics UK (COG-UK) Consortium	Harper VanSteenhouse, Yumi Kasai, David Gray, Carol Clugston, Anna Dominiczak and Alex Alderton, Roberto Amato, Sonia Goncalves, Ewan Harrison, David K. Jackson, Ian Johnston, Dominic Kwiatkowski, Cordelia Langford, John Sillitoe on behalf of the Wellcome Sanger Institute COVID-19 Surveillance Team
EPI_ISL_917146, EPI_ISL_917147	Lighthouse Lab in Alderley Park	Wellcome Sanger Institute for the COVID-19 Genomics UK (COG-UK) Consortium	Jacquelyn Wynn, Mairead Hyland, The Lighthouse Lab in Alderley Park and Alex Alderton, Roberto Amato, Sonia Goncalves, Ewan Harrison, David K. Jackson, Ian Johnston, Dominic Kwiatkowski, Cordelia Langford, John Sillitoe on behalf of the Wellcome Sanger Institute COVID-19 Surveillance Team
EPI_ISL_917148, EPI_ISL_917149	Lighthouse Lab in Glasgow	Wellcome Sanger Institute for the COVID-19 Genomics UK (COG-UK) Consortium	Harper VanSteenhouse, Yumi Kasai, David Gray, Carol Clugston, Anna Dominiczak and Alex Alderton, Roberto Amato, Sonia Goncalves, Ewan Harrison, David K. Jackson, Ian Johnston, Dominic Kwiatkowski, Cordelia Langford, John Sillitoe on behalf of the Wellcome Sanger Institute COVID-19 Surveillance Team
EPI_ISL_917150	Lighthouse Lab in Alderley Park	Wellcome Sanger Institute for the COVID-19 Genomics UK (COG-UK) Consortium	Jacquelyn Wynn, Mairead Hyland, The Lighthouse Lab in Alderley Park and Alex Alderton, Roberto Amato, Sonia Goncalves, Ewan Harrison, David K. Jackson, Ian Johnston, Dominic Kwiatkowski, Cordelia Langford, John Sillitoe on behalf of the Wellcome Sanger Institute COVID-19 Surveillance Team
EPI_ISL_917151	Lighthouse Lab in Glasgow	Wellcome Sanger Institute for the COVID-19 Genomics UK (COG-UK) Consortium	Harper VanSteenhouse, Yumi Kasai, David Gray, Carol Clugston, Anna Dominiczak and Alex Alderton, Roberto Amato, Sonia Goncalves, Ewan Harrison, David K. Jackson, Ian Johnston, Dominic Kwiatkowski, Cordelia Langford, John Sillitoe on behalf of the Wellcome Sanger Institute COVID-19 Surveillance Team
EPI_ISL_917152, EPI_ISL_917153, EPI_ISL_917154, EPI_ISL_917155	Lighthouse Lab in Alderley Park	Wellcome Sanger Institute for the COVID-19 Genomics UK (COG-UK) Consortium	Jacquelyn Wynn, Mairead Hyland, The Lighthouse Lab in Alderley Park and Alex Alderton, Roberto Amato, Sonia Goncalves, Ewan Harrison, David K. Jackson, Ian Johnston, Dominic Kwiatkowski, Cordelia Langford, John Sillitoe on behalf of the Wellcome Sanger Institute COVID-19 Surveillance Team
EPI_ISL_917156	Lighthouse Lab in Glasgow	Wellcome Sanger Institute for the COVID-19 Genomics UK (COG-UK) Consortium	Harper VanSteenhouse, Yumi Kasai, David Gray, Carol Clugston, Anna Dominiczak and Alex Alderton, Roberto Amato, Sonia Goncalves, Ewan Harrison, David K. Jackson, Ian Johnston, Dominic Kwiatkowski, Cordelia Langford, John Sillitoe on behalf of the Wellcome Sanger Institute COVID-19 Surveillance Team
EPI_ISL_917157, EPI_ISL_917158	Lighthouse Lab in Alderley Park	Wellcome Sanger Institute for the COVID-19 Genomics UK (COG-UK) Consortium	Jacquelyn Wynn, Mairead Hyland, The Lighthouse Lab in Alderley Park and Alex Alderton, Roberto Amato, Sonia Goncalves, Ewan Harrison, David K. Jackson, Ian Johnston, Dominic Kwiatkowski, Cordelia Langford, John Sillitoe on behalf of the Wellcome Sanger Institute COVID-19 Surveillance Team
EPI_ISL_917159, EPI_ISL_917160, EPI_ISL_917161	Lighthouse Lab in Glasgow	Wellcome Sanger Institute for the COVID-19 Genomics UK (COG-UK) Consortium	Harper VanSteenhouse, Yumi Kasai, David Gray, Carol Clugston, Anna Dominiczak and Alex Alderton, Roberto Amato, Sonia Goncalves, Ewan Harrison, David K. Jackson, Ian Johnston, Dominic Kwiatkowski, Cordelia Langford, John Sillitoe on behalf of the Wellcome Sanger Institute COVID-19 Surveillance Team
EPI_ISL_917162	Lighthouse Lab in Alderley Park	Wellcome Sanger Institute for the COVID-19 Genomics UK (COG-UK) Consortium	Jacquelyn Wynn, Mairead Hyland, The Lighthouse Lab in Alderley Park and Alex Alderton, Roberto Amato, Sonia Goncalves, Ewan Harrison, David K. Jackson, Ian Johnston, Dominic Kwiatkowski, Cordelia Langford, John Sillitoe on behalf of the Wellcome Sanger Institute COVID-19 Surveillance Team
EPI_ISL_917163	Lighthouse Lab in Glasgow	Wellcome Sanger Institute for the COVID-19 Genomics UK (COG-UK) Consortium	Harper VanSteenhouse, Yumi Kasai, David Gray, Carol Clugston, Anna Dominiczak and Alex Alderton, Roberto Amato, Sonia Goncalves, Ewan Harrison, David K. Jackson, Ian Johnston, Dominic Kwiatkowski, Cordelia Langford, John Sillitoe on behalf of the Wellcome Sanger Institute COVID-19 Surveillance Team
EPI_ISL_917164, EPI_ISL_917165	Lighthouse Lab in Alderley Park	Wellcome Sanger Institute for the COVID-19 Genomics UK (COG-UK) Consortium	Jacquelyn Wynn, Mairead Hyland, The Lighthouse Lab in Alderley Park and Alex Alderton, Roberto Amato, Sonia Goncalves, Ewan Harrison, David K. Jackson, Ian Johnston, Dominic Kwiatkowski, Cordelia Langford, John Sillitoe on behalf of the Wellcome Sanger Institute COVID-19 Surveillance Team
EPI_ISL_917166	Lighthouse Lab in Glasgow	Wellcome Sanger Institute for the COVID-19 Genomics UK (COG-UK) Consortium	Harper VanSteenhouse, Yumi Kasai, David Gray, Carol Clugston, Anna Dominiczak and Alex Alderton, Roberto Amato, Sonia Goncalves, Ewan Harrison, David K. Jackson, Ian Johnston, Dominic Kwiatkowski, Cordelia Langford, John Sillitoe on behalf of the Wellcome Sanger Institute COVID-19 Surveillance Team
EPI_ISL_917167	Lighthouse Lab in Alderley Park	Wellcome Sanger Institute for the COVID-19 Genomics UK (COG-UK) Consortium	Jacquelyn Wynn, Mairead Hyland, The Lighthouse Lab in Alderley Park and Alex Alderton, Roberto Amato, Sonia Goncalves, Ewan Harrison, David K. Jackson, Ian Johnston, Dominic Kwiatkowski, Cordelia Langford, John Sillitoe on behalf of the Wellcome Sanger Institute COVID-19 Surveillance Team
EPI_ISL_917975	Lighthouse Lab in Glasgow	Wellcome Sanger Institute for the COVID-19 Genomics UK (COG-UK) Consortium	Harper VanSteenhouse, Yumi Kasai, David Gray, Carol Clugston, Anna Dominiczak and Alex Alderton, Roberto Amato, Sonia Goncalves, Ewan Harrison, David K. Jackson, Ian Johnston, Dominic Kwiatkowski, Cordelia Langford, John Sillitoe on behalf of the Wellcome Sanger Institute COVID-19 Surveillance Team
EPI_ISL_918060, EPI_ISL_918061	Lighthouse Lab in Alderley Park	Wellcome Sanger Institute for the COVID-19 Genomics UK (COG-UK) Consortium	Jacquelyn Wynn, Mairead Hyland, The Lighthouse Lab in Alderley Park and Alex Alderton, Roberto Amato, Sonia Goncalves, Ewan Harrison, David K. Jackson, Ian Johnston, Dominic Kwiatkowski, Cordelia Langford, John Sillitoe on behalf of the Wellcome Sanger Institute COVID-19 Surveillance Team

EPI_ISL_918062, EPI_ISL_918063, EPI_ISL_918064, EPI_ISL_918065, EPI_ISL_918066, EPI_ISL_918067, EPI_ISL_918068, EPI_ISL_918069, EPI_ISL_918070, EPI_ISL_918071, EPI_ISL_918072, EPI_ISL_918073, EPI_ISL_918074, EPI_ISL_918075, EPI_ISL_918076, EPI_ISL_918077, EPI_ISL_918078, EPI_ISL_918079, EPI_ISL_918080, EPI_ISL_918081, EPI_ISL_918082, EPI_ISL_918083, EPI_ISL_918084, EPI_ISL_918085, EPI_ISL_918086, EPI_ISL_918087, EPI_ISL_918088, EPI_ISL_918089, EPI_ISL_918090, EPI_ISL_918091, EPI_ISL_918092, EPI_ISL_918093, EPI_ISL_918094, EPI_ISL_918095, EPI_ISL_918096, EPI_ISL_918097, EPI_ISL_918098, EPI_ISL_918099, EPI_ISL_918100, EPI_ISL_918101, EPI_ISL_918102, EPI_ISL_918103, EPI_ISL_918104, EPI_ISL_918105, EPI_ISL_918106, EPI_ISL_918107, EPI_ISL_918108, EPI_ISL_918109, EPI_ISL_918110, EPI_ISL_918111, EPI_ISL_918112, EPI_ISL_918113, EPI_ISL_918114, EPI_ISL_918115, EPI_ISL_918116, EPI_ISL_918117, EPI_ISL_918118, EPI_ISL_918119, EPI_ISL_918120, EPI_ISL_918121, EPI_ISL_918122, EPI_ISL_918123, EPI_ISL_918124			
see above	Lighthouse Lab in Glasgow	Wellcome Sanger Institute for the COVID-19 Genomics UK (COG-UK) Consortium	Harper VanSteenhouse, Yumi Kasai, David Gray, Carol Clugston, Anna Dominiczak and Alex Alderton, Roberto Amato, Sonia Goncalves, Ewan Harrison, David K. Jackson, Ian Johnston, Dominic Kwiatkowski, Cordelia Langford, John Sillitoe on behalf of the Wellcome Sanger Institute COVID-19 Surveillance Team
EPI_ISL_918764, EPI_ISL_918765, EPI_ISL_918766, EPI_ISL_918767, EPI_ISL_918768, EPI_ISL_918769, EPI_ISL_918770, EPI_ISL_918771, EPI_ISL_918772, EPI_ISL_918773, EPI_ISL_918774, EPI_ISL_918776, EPI_ISL_918777, EPI_ISL_918778, EPI_ISL_918780, EPI_ISL_918781, EPI_ISL_918782, EPI_ISL_918783, EPI_ISL_918784, EPI_ISL_918785, EPI_ISL_918786, EPI_ISL_918787, EPI_ISL_918788, EPI_ISL_918789, EPI_ISL_918790, EPI_ISL_918791, EPI_ISL_918792, EPI_ISL_918793, EPI_ISL_918794, EPI_ISL_918795, EPI_ISL_918796, EPI_ISL_918797, EPI_ISL_918798, EPI_ISL_918799, EPI_ISL_918800, EPI_ISL_918801, EPI_ISL_918802, EPI_ISL_918803, EPI_ISL_918804, EPI_ISL_918805, EPI_ISL_918806, EPI_ISL_918807, EPI_ISL_918808, EPI_ISL_918809, EPI_ISL_918810, EPI_ISL_918811, EPI_ISL_918812, EPI_ISL_918813, EPI_ISL_918814, EPI_ISL_918815, EPI_ISL_918816, EPI_ISL_918817, EPI_ISL_918818, EPI_ISL_918819, EPI_ISL_918820, EPI_ISL_918821, EPI_ISL_918822, EPI_ISL_918823, EPI_ISL_918824, EPI_ISL_918825, EPI_ISL_918826, EPI_ISL_918827, EPI_ISL_918842, EPI_ISL_918843, EPI_ISL_918844, EPI_ISL_918845, EPI_ISL_918856, EPI_ISL_918869, EPI_ISL_918874, EPI_ISL_918875, EPI_ISL_918876, EPI_ISL_918877, EPI_ISL_918878, EPI_ISL_918882, EPI_ISL_918912, EPI_ISL_918913, EPI_ISL_918977, EPI_ISL_918978, EPI_ISL_918979, EPI_ISL_918980, EPI_ISL_918981, EPI_ISL_918982, EPI_ISL_918983			
see above	University of Birmingham	COVID-19 Genomics UK (COG-UK) Consortium	Institute of Microbiology, University of Birmingham: Claire McMurray, Joanne Stockton, Samuel Nicholls, Radoslaw Poplawski, Will Rowe, Josh Quick, Nicholas Loman. University of Birmingham Testing Laboratory: Celina M Whalley, Andrew Bosworth, Charlotte Poxon, Kasun Wanigasooriya, Oliver Pickles, Mike Kidd, Alex Richter, Andrew D Beggs PHE Heartlands Lab: Husam Osman, Andrew Bosworth. Queen Elizabeth Hospital: Anna Casey
EPI_ISL_919057, EPI_ISL_919059, EPI_ISL_919060, EPI_ISL_919061, EPI_ISL_919064, EPI_ISL_919065, EPI_ISL_919066, EPI_ISL_919068, EPI_ISL_919069, EPI_ISL_919072, EPI_ISL_919076, EPI_ISL_919078, EPI_ISL_919083, EPI_ISL_919085, EPI_ISL_919086, EPI_ISL_919088, EPI_ISL_919090, EPI_ISL_919094, EPI_ISL_919096, EPI_ISL_919098, EPI_ISL_919100			
see above	Department of Pathology, University of Cambridge	COVID-19 Genomics UK (COG-UK) Consortium	Aminu S. Jahun, Yasmin Chaudhry, Iliana Georgana, Myra Hosmillo, Rhys Izu, Martin D. Curran, Surendra Parmar, Ian Goodfellow
EPI_ISL_919198, EPI_ISL_919199, EPI_ISL_919200, EPI_ISL_919201, EPI_ISL_919202, EPI_ISL_919203, EPI_ISL_919204, EPI_ISL_919205, EPI_ISL_919206, EPI_ISL_919207, EPI_ISL_919209, EPI_ISL_919210, EPI_ISL_919212			
see above	West of Scotland Specialist Virology Centre, NHSGGC / MRC-University of Glasgow Centre for Virus Research	COVID-19 Genomics UK (COG-UK) Consortium	Ana da Silva Filipe, Natasha Johnson, Kathy Smollett, Daniel Mair, Stephen Carmichael, Alice Broos, Lily Tong, Jenna Nichols, Kyriaki Nomikou; Sarah McDonald; Richard Orton, Joseph Hughes, Sreenu Vattipally, David L Robertson; Alasdair MacLean, Rory Gunson; Sharif Shaaban, Matthew Holden; Rachel Blacow, Guy Mollett, Kathy Li, James Shepherd, Antonia Ho, Emma Thomson
EPI_ISL_919339, EPI_ISL_919340, EPI_ISL_919341, EPI_ISL_919342	Virology Department, Royal Infirmary of Edinburgh, NHS Lothian / School of Biological Sciences, University of Edinburgh / Institute of Genetics and Molecular Medicine, University of Edinburgh	COVID-19 Genomics UK (COG-UK) Consortium	McHugh M, Dewar R, Rooke S, Gallagher M, Balcaza C, O'Toole Á, Scher E, Hill V, McCrone JT, Colquhoun R, Yu X, Jackson B, Rambaut A, Williams TC, Templeton K
EPI_ISL_919479, EPI_ISL_919480, EPI_ISL_919481, EPI_ISL_919482, EPI_ISL_919483, EPI_ISL_919484, EPI_ISL_919485, EPI_ISL_919486, EPI_ISL_919487, EPI_ISL_919516, EPI_ISL_919524, EPI_ISL_919526, EPI_ISL_919527, EPI_ISL_919528, EPI_ISL_919529, EPI_ISL_919531, EPI_ISL_919537, EPI_ISL_919538, EPI_ISL_919539, EPI_ISL_919540, EPI_ISL_919541, EPI_ISL_919544, EPI_ISL_919545, EPI_ISL_919546, EPI_ISL_919549, EPI_ISL_919551, EPI_ISL_919552, EPI_ISL_919553, EPI_ISL_919554, EPI_ISL_919555, EPI_ISL_919558, EPI_ISL_919559, EPI_ISL_919560, EPI_ISL_919561, EPI_ISL_919565, EPI_ISL_919566, EPI_ISL_919567, EPI_ISL_919569, EPI_ISL_919580, EPI_ISL_919581, EPI_ISL_919583, EPI_ISL_919584, EPI_ISL_919586, EPI_ISL_919592, EPI_ISL_919596, EPI_ISL_919597, EPI_ISL_919598, EPI_ISL_919599, EPI_ISL_919600, EPI_ISL_919601, EPI_ISL_919602, EPI_ISL_919632, EPI_ISL_919634, EPI_ISL_919637, EPI_ISL_919641, EPI_ISL_919642, EPI_ISL_919643, EPI_ISL_919645, EPI_ISL_919646, EPI_ISL_919647, EPI_ISL_919648, EPI_ISL_919650, EPI_ISL_919651, EPI_ISL_919652, EPI_ISL_919653, EPI_ISL_919655, EPI_ISL_919656, EPI_ISL_919657, EPI_ISL_919659, EPI_ISL_919660, EPI_ISL_919661, EPI_ISL_919662			
see above	Liverpool Clinical Laboratories	COVID-19 Genomics UK (COG-UK) Consortium	Sam Haldenby, Anita Lucaci, Steve Paterson, Julian Hiscox, Alistair Darby, M Almsaud, A Alrezaihi, Muhannad Alruwaili, Stuart D Armstrong, Jones Benjamin, Eleanor G Bentley, Anu Chawla, Jordan J Clark, Angela Cowell, Richard Eccles, Isabel García-Dorival, Matthew Gemmell, Alessandro Gerada, PKF Gilmore, Richard Gregory, Ximeng Han, Catherine Hartley, Margaret Hughes, Miren Iturriza-Gomara, James Johnson, L Luu, Jenifer Manson, Charlotte Nelson, Elaine O'Toole, Cassie Olateju, Rebekah Penrice-Randal , Lucille Rainbow, N.P Randle, Trevor Ian Robinson, Parul Sharma, Ghada T Shawli, James P Stewart, Neil Swainston, Ecaterina Vamos, Joanne Watts, Mark Whitehead
EPI_ISL_919853	Barts Health NHS Trust	COVID-19 Genomics UK (COG-UK) Consortium	CUTINO-MOGUEL, Maria-Teresa; HARRINGTON, David; OWOYEMI, Dola; KULASEGARAN-SHYLINI, Raghavendran; BROAD, Claire; KELE, Beatrix
EPI_ISL_920026, EPI_ISL_920030, EPI_ISL_920031, EPI_ISL_920032, EPI_ISL_920033, EPI_ISL_920035, EPI_ISL_920036, EPI_ISL_920037, EPI_ISL_920038, EPI_ISL_920039, EPI_ISL_920043, EPI_ISL_920044, EPI_ISL_920045, EPI_ISL_920052, EPI_ISL_920053, EPI_ISL_920054, EPI_ISL_920055, EPI_ISL_920056, EPI_ISL_920057, EPI_ISL_920058, EPI_ISL_920079, EPI_ISL_920080			
see above	University College London, Great Ormond Street Hospital for Children NHS Foundation Trust, Imperial College Healthcare NHS Trust	COVID-19 Genomics UK (COG-UK) Consortium	Sergi Castellano, Rachel Williams, Mark Kristiansen, Paola Resende Silva, Sunando Roy, Tony Brooks, Helena Tutill, Paola Niola, Patricia Dyal, Charlotte Williams, Leysa Forrest, Yasmin Panchbhaya, Jacqueline Findlay, Samuel Weeks, Julianne Brown, Kathryn Harris, Paul Randell, James Price, Alison Holmes, Judith Breuer
EPI_ISL_920174, EPI_ISL_920175, EPI_ISL_920177, EPI_ISL_920180, EPI_ISL_920181, EPI_ISL_920182, EPI_ISL_920183, EPI_ISL_920184, EPI_ISL_920186, EPI_ISL_920190, EPI_ISL_920194, EPI_ISL_920195, EPI_ISL_920196, EPI_ISL_920197, EPI_ISL_920198, EPI_ISL_920200, EPI_ISL_920201, EPI_ISL_920202, EPI_ISL_920203, EPI_ISL_920205, EPI_ISL_920206, EPI_ISL_920207, EPI_ISL_920208, EPI_ISL_920210, EPI_ISL_920211, EPI_ISL_920212, EPI_ISL_920214, EPI_ISL_920215, EPI_ISL_920217, EPI_ISL_920218, EPI_ISL_920219, EPI_ISL_920222, EPI_ISL_920224, EPI_ISL_920225, EPI_ISL_920226, EPI_ISL_920227, EPI_ISL_920228, EPI_ISL_920229, EPI_ISL_920230, EPI_ISL_920231, EPI_ISL_920232, EPI_ISL_920233, EPI_ISL_920234, EPI_ISL_920235, EPI_ISL_920236, EPI_ISL_920237, EPI_ISL_920238, EPI_ISL_920239, EPI_ISL_920244, EPI_ISL_920245, EPI_ISL_920246, EPI_ISL_920247, EPI_ISL_920252, EPI_ISL_920253, EPI_ISL_920254, EPI_ISL_920255, EPI_ISL_920261, EPI_ISL_920262, EPI_ISL_920263, EPI_ISL_920264, EPI_ISL_920265, EPI_ISL_920266, EPI_ISL_920271, EPI_ISL_920272, EPI_ISL_920273, EPI_ISL_920274, EPI_ISL_920275, EPI_ISL_920276, EPI_ISL_920283, EPI_ISL_920284, EPI_ISL_920285, EPI_ISL_920286, EPI_ISL_920287, EPI_ISL_920293, EPI_ISL_920294, EPI_ISL_920295, EPI_ISL_920296, EPI_ISL_920305, EPI_ISL_920306, EPI_ISL_920307, EPI_ISL_920308, EPI_ISL_920315, EPI_ISL_920316, EPI_ISL_920326, EPI_ISL_920334, EPI_ISL_920335, EPI_ISL_920343, EPI_ISL_920350, EPI_ISL_920351, EPI_ISL_920358, EPI_ISL_920360, EPI_ISL_920367, EPI_ISL_920368, EPI_ISL_920700			
see above	University College London Hospital	COVID-19 Genomics UK (COG-UK) Consortium	Judith Heaney, Matthew Byott, Catherine Houlihan, Dan Frampton, Stuart Kirk, Moira Spyer and Eleni Nastouli
EPI_ISL_920882, EPI_ISL_920883, EPI_ISL_920884, EPI_ISL_920885, EPI_ISL_920886, EPI_ISL_920887, EPI_ISL_920888, EPI_ISL_920889, EPI_ISL_920890, EPI_ISL_920891, EPI_ISL_920892, EPI_ISL_920893			
see above	University College London, Great Ormond Street Hospital for Children NHS Foundation Trust, Imperial College Healthcare NHS Trust	COVID-19 Genomics UK (COG-UK) Consortium	Sergi Castellano, Rachel Williams, Mark Kristiansen, Paola Resende Silva, Sunando Roy, Tony Brooks, Helena Tutill, Paola Niola, Patricia Dyal, Charlotte Williams, Leysa Forrest, Yasmin Panchbhaya, Jacqueline Findlay, Samuel Weeks, Julianne Brown, Kathryn Harris, Paul Randell, James Price, Alison Holmes, Judith Breuer
EPI_ISL_920901, EPI_ISL_920902, EPI_ISL_920903, EPI_ISL_920904, EPI_ISL_920905, EPI_ISL_920906, EPI_ISL_920907, EPI_ISL_920908, EPI_ISL_920909, EPI_ISL_920911, EPI_ISL_920912, EPI_ISL_920913, EPI_ISL_920914, EPI_ISL_920915, EPI_ISL_920916, EPI_ISL_920917, EPI_ISL_920918, EPI_ISL_920919, EPI_ISL_920920, EPI_ISL_920921, EPI_ISL_920922, EPI_ISL_920923, EPI_ISL_920925, EPI_ISL_920926, EPI_ISL_920927, EPI_ISL_920928, EPI_ISL_920929, EPI_ISL_920930, EPI_ISL_920931, EPI_ISL_920932, EPI_ISL_920933, EPI_ISL_920935, EPI_ISL_920936, EPI_ISL_920937, EPI_ISL_920938, EPI_ISL_920939, EPI_ISL_920940, EPI_ISL_920941, EPI_ISL_920942, EPI_ISL_920944, EPI_ISL_920945, EPI_ISL_920946, EPI_ISL_920947, EPI_ISL_920948, EPI_ISL_920949, EPI_ISL_920950, EPI_ISL_920951, EPI_ISL_920952, EPI_ISL_920953, EPI_ISL_920954, EPI_ISL_920955, EPI_ISL_920956, EPI_ISL_920957, EPI_ISL_920958, EPI_ISL_920959, EPI_ISL_920960, EPI_ISL_920961, EPI_ISL_920962, EPI_ISL_920963, EPI_ISL_920964, EPI_ISL_920965, EPI_ISL_920966, EPI_ISL_920967, EPI_ISL_920968, EPI_ISL_920969, EPI_ISL_920970, EPI_ISL_920971, EPI_ISL_920972, EPI_ISL_920973, EPI_ISL_920974			
see above	Department of Pathology, University of Cambridge	COVID-19 Genomics UK (COG-UK) Consortium	Aminu S. Jahun, Yasmin Chaudhry, Iliana Georgana, Myra Hosmillo, Rhys Izu, Martin D. Curran, Surendra Parmar, Ian Goodfellow
EPI_ISL_920976, EPI_ISL_920977, EPI_ISL_920978, EPI_ISL_920979, EPI_ISL_920981, EPI_ISL_920982, EPI_ISL_920983, EPI_ISL_920984, EPI_ISL_920985, EPI_ISL_920986, EPI_ISL_920987, EPI_ISL_920988, EPI_ISL_920989, EPI_ISL_920990, EPI_ISL_920991, EPI_ISL_920992, EPI_ISL_920993, EPI_ISL_920994, EPI_ISL_920995, EPI_ISL_921011, EPI_ISL_921012, EPI_ISL_921013, EPI_ISL_921014, EPI_ISL_921015, EPI_ISL_921016, EPI_ISL_921017			
see above	Regional Virus Laboratory, Belfast Health and Social Care Trust	COVID-19 Genomics UK (COG-UK) Consortium	Conall McCaughey, James McKenna, Tanya Curran, Susan Feeney, Alison Watt, Ciara Cox, Mairead Connor, Zoltan Molnar, David Simpson, Derek Fairley
EPI_ISL_921428, EPI_ISL_921485, EPI_ISL_921486, EPI_ISL_921487, EPI_ISL_921502, EPI_ISL_921521, EPI_ISL_921522, EPI_ISL_921523, EPI_ISL_921524, EPI_ISL_921525, EPI_ISL_921526, EPI_ISL_921527, EPI_ISL_921528, EPI_ISL_921529, EPI_ISL_921530, EPI_ISL_921531, EPI_ISL_921532, EPI_ISL_921533, EPI_ISL_921534, EPI_ISL_921535, EPI_ISL_921536, EPI_ISL_921537, EPI_ISL_921538, EPI_ISL_921539, EPI_ISL_921540, EPI_ISL_921542, EPI_ISL_921543, EPI_ISL_921544, EPI_ISL_921545, EPI_ISL_921546, EPI_ISL_921547, EPI_ISL_921548, EPI_ISL_921549, EPI_ISL_921550, EPI_ISL_921551, EPI_ISL_921552, EPI_ISL_921553, EPI_ISL_921554, EPI_ISL_921555, EPI_ISL_921556, EPI_ISL_921557, EPI_ISL_921558, EPI_ISL_921559, EPI_ISL_921560, EPI_ISL_921561, EPI_ISL_921562, EPI_ISL_921563, EPI_ISL_921564, EPI_ISL_921565, EPI_ISL_921566, EPI_ISL_921567, EPI_ISL_921568, EPI_ISL_921569, EPI_ISL_921570, EPI_ISL_921571, EPI_ISL_921572, EPI_ISL_921573, EPI_ISL_921574, EPI_ISL_921575, EPI_ISL_921576, EPI_ISL_921577, EPI_ISL_921578, EPI_ISL_921579, EPI_ISL_921580, EPI_ISL_921581, EPI_ISL_921582, EPI_ISL_921583, EPI_ISL_921584, EPI_ISL_921585			
see above	Northumbria University / South Tees Hospitals NHS Foundation Trust / North Cumbria Integrated Care NHS Foundation Trust / North Tees and Hartlepool NHS Foundation Trust / Newcastle Hospitals NHS Foundation Trust	COVID-19 Genomics UK (COG-UK) Consortium	Darren L Smith,Andrew Nelson,Matthew Bashton,Greg R Young,Joshua Loh,John Allan,Mohammad A Tariq,Giles S Holt,Gary Black,Wen C Yew,Lynn Dover,Paul Baker,Steve Liggett,Sarah Essex,Jane Greenaway,Debra Padgett,Clive Graham,Garren Scott,Edward Barton,Emma Swindells,Brendan Payne,Jennifer Collins,Yusri Taha,Gary Eltringham
EPI_ISL_922113, EPI_ISL_922114, EPI_ISL_922117, EPI_ISL_922118, EPI_ISL_922119, EPI_ISL_922120, EPI_ISL_922121, EPI_ISL_922122, EPI_ISL_922123, EPI_ISL_922124, EPI_ISL_922125, EPI_ISL_922126, EPI_ISL_922127, EPI_ISL_922128, EPI_ISL_922129, EPI_ISL_922130, EPI_ISL_922131, EPI_ISL_922132, EPI_ISL_922133, EPI_ISL_922134, EPI_ISL_922135, EPI_ISL_922136, EPI_ISL_922137, EPI_ISL_922138, EPI_ISL_922139, EPI_ISL_922140, EPI_ISL_922141, EPI_ISL_922142, EPI_ISL_922143, EPI_ISL_922144, EPI_ISL_922145, EPI_ISL_922146, EPI_ISL_922148, EPI_ISL_922157			
see above	Lincolnshire Hospitals and DeepSeq Nottingham	COVID-19 Genomics UK (COG-UK) Consortium	Nichola Duckworth, Tim Sloan, Sarah Walsh, Jonathan Ball, Patrick McClure, Joeseph Chappell, Nadine Holmes, Matthew Carlisle, Christopher Moore, Fei Sang, Johnny Debebe, Victoria Wright, Matthew Loose
EPI_ISL_922231, EPI_ISL_922232,	Oxford Viromics, NDM, University of Oxford; Oxford University	COVID-19 Genomics UK (COG-UK) Consortium	Tanya Golubchik, David Bonsall, George Macintyre, Amy Trebes, Mariateresa de Cesare, Catrin Moore, Alex Mobbs, Anita Justice, Robert Shaw, Monique

EPI_ISL_922233, EPI_ISL_922234, EPI_ISL_922238, EPI_ISL_922239, EPI_ISL_922240	Hospitals; Basingstoke and North Hampshire Hospital		Andersson, Timothy Peto, Emma Wise, Nathan Moore, Jessica Lynch, Nick Cortes, Matilde Mori, Stephen Kidd, David Buck, John Todd, Christophe Fraser
EPI_ISL_922359, EPI_ISL_922360, EPI_ISL_922362, EPI_ISL_922363, EPI_ISL_922365, EPI_ISL_922367, EPI_ISL_922368, EPI_ISL_922369, EPI_ISL_922370, EPI_ISL_922372, EPI_ISL_922373, EPI_ISL_922384, EPI_ISL_922392, EPI_ISL_922395, EPI_ISL_922396, EPI_ISL_922398, EPI_ISL_922399, EPI_ISL_922401, EPI_ISL_922402, EPI_ISL_922403, EPI_ISL_922404, EPI_ISL_922406, EPI_ISL_922407, EPI_ISL_922408, EPI_ISL_922409, EPI_ISL_922410, EPI_ISL_922411, EPI_ISL_922413, EPI_ISL_922415, EPI_ISL_922416, EPI_ISL_922417, EPI_ISL_922418, EPI_ISL_922419, EPI_ISL_922420, EPI_ISL_922421, EPI_ISL_922423, EPI_ISL_922425, EPI_ISL_922428, EPI_ISL_922430, EPI_ISL_922432, EPI_ISL_922433, EPI_ISL_922434, EPI_ISL_922435, EPI_ISL_922436, EPI_ISL_922437, EPI_ISL_922439, EPI_ISL_922440, EPI_ISL_922442, EPI_ISL_922443, EPI_ISL_922444, EPI_ISL_922445, EPI_ISL_922446, EPI_ISL_922448, EPI_ISL_922452, EPI_ISL_922455, EPI_ISL_922457, EPI_ISL_922458, EPI_ISL_922459, EPI_ISL_922460, EPI_ISL_922462, EPI_ISL_922463, EPI_ISL_922464, EPI_ISL_922465, EPI_ISL_922466, EPI_ISL_922467, EPI_ISL_922468, EPI_ISL_922469, EPI_ISL_922470, EPI_ISL_922473, EPI_ISL_922474, EPI_ISL_922481, EPI_ISL_922483, EPI_ISL_922485, EPI_ISL_922488, EPI_ISL_922491, EPI_ISL_922494, EPI_ISL_922495, EPI_ISL_922497, EPI_ISL_922498, EPI_ISL_922499, EPI_ISL_922500, EPI_ISL_922502, EPI_ISL_922504, EPI_ISL_922505, EPI_ISL_922507, EPI_ISL_922510, EPI_ISL_922511, EPI_ISL_922515, EPI_ISL_922516, EPI_ISL_922553, EPI_ISL_922559, EPI_ISL_922560, EPI_ISL_922563, EPI_ISL_922565, EPI_ISL_922566, EPI_ISL_922567, EPI_ISL_922568, EPI_ISL_922570, EPI_ISL_922571, EPI_ISL_922572, EPI_ISL_922574, EPI_ISL_922580, EPI_ISL_922581, EPI_ISL_922582, EPI_ISL_922590, EPI_ISL_922591, EPI_ISL_922592, EPI_ISL_922593, EPI_ISL_922594, EPI_ISL_922595, EPI_ISL_922596, EPI_ISL_922597, EPI_ISL_922598, EPI_ISL_922599, EPI_ISL_922600, EPI_ISL_922601, EPI_ISL_922602, EPI_ISL_922603, EPI_ISL_922604, EPI_ISL_922605, EPI_ISL_922606, EPI_ISL_922607, EPI_ISL_922626, EPI_ISL_922627, EPI_ISL_922628, EPI_ISL_922629, EPI_ISL_922633, EPI_ISL_922634, EPI_ISL_922643, EPI_ISL_922645, EPI_ISL_922646, EPI_ISL_922647, EPI_ISL_922648, EPI_ISL_922649, EPI_ISL_922650, EPI_ISL_922651, EPI_ISL_922652, EPI_ISL_922653, EPI_ISL_922654, EPI_ISL_922655, EPI_ISL_922656, EPI_ISL_922659, EPI_ISL_922660, EPI_ISL_922663, EPI_ISL_922664, EPI_ISL_922665, EPI_ISL_922666, EPI_ISL_922667, EPI_ISL_922668, EPI_ISL_922669, EPI_ISL_922672, EPI_ISL_922673, EPI_ISL_922674, EPI_ISL_922675, EPI_ISL_922676, EPI_ISL_922677, EPI_ISL_922678, EPI_ISL_922679, EPI_ISL_922680, EPI_ISL_922681, EPI_ISL_922682, EPI_ISL_922683, EPI_ISL_922684, EPI_ISL_922685, EPI_ISL_922686, EPI_ISL_922687, EPI_ISL_922688, EPI_ISL_922689, EPI_ISL_922690, EPI_ISL_922692, EPI_ISL_922693, EPI_ISL_922695, EPI_ISL_922696, EPI_ISL_922697, EPI_ISL_922698, EPI_ISL_922699, EPI_ISL_922700, EPI_ISL_922701, EPI_ISL_922702, EPI_ISL_922703, EPI_ISL_922704, EPI_ISL_922705, EPI_ISL_922706, EPI_ISL_922707, EPI_ISL_922708, EPI_ISL_922709, EPI_ISL_922710, EPI_ISL_922712, EPI_ISL_922713, EPI_ISL_922719, EPI_ISL_922721, EPI_ISL_922722, EPI_ISL_922723, EPI_ISL_922724, EPI_ISL_922726, EPI_ISL_922727, EPI_ISL_922732, EPI_ISL_922734, EPI_ISL_922741, EPI_ISL_922743, EPI_ISL_922895, EPI_ISL_922903, EPI_ISL_922904, EPI_ISL_922905, EPI_ISL_922906, EPI_ISL_922907, EPI_ISL_922908, EPI_ISL_922909, EPI_ISL_922910, EPI_ISL_922912, EPI_ISL_922913, EPI_ISL_922914, EPI_ISL_922923, EPI_ISL_922925, EPI_ISL_922926, EPI_ISL_922927, EPI_ISL_922928, EPI_ISL_922929, EPI_ISL_922930, EPI_ISL_922931, EPI_ISL_922932, EPI_ISL_922965, EPI_ISL_922970, EPI_ISL_922974, EPI_ISL_922975, EPI_ISL_922976, EPI_ISL_922977, EPI_ISL_922978, EPI_ISL_922979, EPI_ISL_922980, EPI_ISL_922981, EPI_ISL_922982, EPI_ISL_922983, EPI_ISL_922984, EPI_ISL_922985, EPI_ISL_922986, EPI_ISL_922987, EPI_ISL_922988, EPI_ISL_922989, EPI_ISL_922990, EPI_ISL_922991, EPI_ISL_922992, EPI_ISL_922993, EPI_ISL_922994, EPI_ISL_922995, EPI_ISL_922996, EPI_ISL_922997, EPI_ISL_922998, EPI_ISL_922999, EPI_ISL_923000, EPI_ISL_923001, EPI_ISL_923002, EPI_ISL_923003, EPI_ISL_923004, EPI_ISL_923005, EPI_ISL_923006, EPI_ISL_923007, EPI_ISL_923008, EPI_ISL_923009, EPI_ISL_923010, EPI_ISL_923011, EPI_ISL_923013, EPI_ISL_923014, EPI_ISL_923015, EPI_ISL_923016, EPI_ISL_923017, EPI_ISL_923018, EPI_ISL_923019, EPI_ISL_923020, EPI_ISL_923021, EPI_ISL_923022, EPI_ISL_923023, EPI_ISL_923025, EPI_ISL_923026, EPI_ISL_923027			
see above	Wales Specialist Virology Centre Sequencing lab: Pathogen Genomics Unit	Public Health Wales Microbiology Cardiff Wales Specialist Virology Centre	Catherine Moore, Johnathan Evans, Laura Gifford, Malorie Perry, Simon Cottrell, Angela Marchbank, Alec Birchley, Alexander Adams, Amy Gaskin, Bree Gatica-Wilcox, Jason Coombes, Joel Southgate, Lauren Gilbert, Lee Graham, Nicole Pacchiarini, Sara Kumziene-Summerhayes, Sarah Taylor, Sophie Jones, Sara Rey, Matthew Bull, Joanne Watkins, Sally Corden, Tom Connor
EPI_ISL_923276, EPI_ISL_923280, EPI_ISL_923281, EPI_ISL_923287, EPI_ISL_923290, EPI_ISL_923291, EPI_ISL_923292, EPI_ISL_923294, EPI_ISL_923298, EPI_ISL_923302, EPI_ISL_923303, EPI_ISL_923304, EPI_ISL_923307, EPI_ISL_923308, EPI_ISL_923309, EPI_ISL_923310, EPI_ISL_923311, EPI_ISL_923312, EPI_ISL_923313, EPI_ISL_923314, EPI_ISL_923317, EPI_ISL_923355, EPI_ISL_923356, EPI_ISL_923359, EPI_ISL_923360, EPI_ISL_923361, EPI_ISL_923362, EPI_ISL_923364, EPI_ISL_923365, EPI_ISL_923368, EPI_ISL_923370, EPI_ISL_923371, EPI_ISL_923414, EPI_ISL_923416, EPI_ISL_923419, EPI_ISL_923438, EPI_ISL_923589, EPI_ISL_923590, EPI_ISL_923591, EPI_ISL_923592, EPI_ISL_923594, EPI_ISL_923628, EPI_ISL_923660, EPI_ISL_923664, EPI_ISL_923665, EPI_ISL_923666, EPI_ISL_923667, EPI_ISL_923668, EPI_ISL_923669, EPI_ISL_923670, EPI_ISL_923672, EPI_ISL_923673, EPI_ISL_923674, EPI_ISL_923675, EPI_ISL_923676, EPI_ISL_923677, EPI_ISL_923681, EPI_ISL_923682, EPI_ISL_923683			
see above	Centre for Enzyme Innovation, University of Portsmouth / Translational Research Laboratory, Portsmouth Hospitals NHS Trust	COVID-19 Genomics UK (COG-UK) Consortium	Angela Beckett,Salman Goudarzi,Christopher Fearn,Kate Cook,Katie Loveson,Sharon Glaysher,Scott Elliott,Samuel Robson
EPI_ISL_923685, EPI_ISL_923686, EPI_ISL_923687, EPI_ISL_923689, EPI_ISL_923691, EPI_ISL_923692, EPI_ISL_923693, EPI_ISL_923694, EPI_ISL_923695, EPI_ISL_923696, EPI_ISL_923697, EPI_ISL_923698, EPI_ISL_923699, EPI_ISL_923700, EPI_ISL_923701, EPI_ISL_923702, EPI_ISL_923703, EPI_ISL_923704, EPI_ISL_923705, EPI_ISL_923706, EPI_ISL_923707, EPI_ISL_923708, EPI_ISL_923709, EPI_ISL_923710, EPI_ISL_923711, EPI_ISL_923712, EPI_ISL_923713, EPI_ISL_923714, EPI_ISL_923715, EPI_ISL_923717, EPI_ISL_923719, EPI_ISL_923720, EPI_ISL_923721, EPI_ISL_923722, EPI_ISL_923723, EPI_ISL_923724, EPI_ISL_923725, EPI_ISL_923726, EPI_ISL_923727, EPI_ISL_923728, EPI_ISL_923729, EPI_ISL_923730, EPI_ISL_923731, EPI_ISL_923732, EPI_ISL_923733, EPI_ISL_923734, EPI_ISL_923735, EPI_ISL_923736, EPI_ISL_923737, EPI_ISL_923738, EPI_ISL_923739, EPI_ISL_923740, EPI_ISL_923741, EPI_ISL_923742, EPI_ISL_923743, EPI_ISL_923744, EPI_ISL_923745, EPI_ISL_923746, EPI_ISL_923747, EPI_ISL_923748, EPI_ISL_923749, EPI_ISL_923750, EPI_ISL_923751, EPI_ISL_923752, EPI_ISL_923755, EPI_ISL_923757, EPI_ISL_923761, EPI_ISL_923762, EPI_ISL_923765, EPI_ISL_923766, EPI_ISL_923767, EPI_ISL_923768, EPI_ISL_923769, EPI_ISL_923770, EPI_ISL_923771, EPI_ISL_923772, EPI_ISL_923773, EPI_ISL_923774, EPI_ISL_923775, EPI_ISL_923776, EPI_ISL_923778, EPI_ISL_923795, EPI_ISL_923796, EPI_ISL_923797, EPI_ISL_923798, EPI_ISL_923799, EPI_ISL_923800, EPI_ISL_923802, EPI_ISL_923803, EPI_ISL_923804, EPI_ISL_923805, EPI_ISL_923807, EPI_ISL_923808, EPI_ISL_923810, EPI_ISL_923811, EPI_ISL_923812, EPI_ISL_923813, EPI_ISL_923814, EPI_ISL_923815, EPI_ISL_923816, EPI_ISL_923818, EPI_ISL_923844, EPI_ISL_923845, EPI_ISL_923847, EPI_ISL_923848, EPI_ISL_923849, EPI_ISL_923850, EPI_ISL_923851, EPI_ISL_923853, EPI_ISL_923854, EPI_ISL_923856, EPI_ISL_923857, EPI_ISL_923858, EPI_ISL_923859, EPI_ISL_923860, EPI_ISL_923861, EPI_ISL_923862, EPI_ISL_923863, EPI_ISL_923865, EPI_ISL_923866, EPI_ISL_923867, EPI_ISL_923868, EPI_ISL_923869, EPI_ISL_923870, EPI_ISL_923871, EPI_ISL_923872, EPI_ISL_923873, EPI_ISL_923874, EPI_ISL_923904, EPI_ISL_923905, EPI_ISL_923906, EPI_ISL_923907, EPI_ISL_923908, EPI_ISL_923909, EPI_ISL_923910, EPI_ISL_923911, EPI_ISL_923912, EPI_ISL_923913, EPI_ISL_923914, EPI_ISL_923915, EPI_ISL_923916, EPI_ISL_923917, EPI_ISL_923919, EPI_ISL_923920, EPI_ISL_923921, EPI_ISL_923922, EPI_ISL_923923, EPI_ISL_923924, EPI_ISL_923925, EPI_ISL_923926, EPI_ISL_923927, EPI_ISL_923928, EPI_ISL_923929, EPI_ISL_923930, EPI_ISL_923931, EPI_ISL_923932, EPI_ISL_923933, EPI_ISL_923934, EPI_ISL_923935, EPI_ISL_923936, EPI_ISL_923937, EPI_ISL_923938, EPI_ISL_923939, EPI_ISL_923940, EPI_ISL_923941, EPI_ISL_923942, EPI_ISL_923943, EPI_ISL_923944, EPI_ISL_923945, EPI_ISL_923946, EPI_ISL_923947, EPI_ISL_923948, EPI_ISL_923949, EPI_ISL_923950, EPI_ISL_923951, EPI_ISL_923952, EPI_ISL_923954, EPI_ISL_923955, EPI_ISL_923956, EPI_ISL_923957, EPI_ISL_923958, EPI_ISL_923959, EPI_ISL_923961, EPI_ISL_923962, EPI_ISL_923964, EPI_ISL_923965, EPI_ISL_923966, EPI_ISL_923967, EPI_ISL_923968, EPI_ISL_923969, EPI_ISL_923970, EPI_ISL_923972, EPI_ISL_923973, EPI_ISL_923976, EPI_ISL_923977, EPI_ISL_923978, EPI_ISL_923979, EPI_ISL_923980, EPI_ISL_923981, EPI_ISL_923982, EPI_ISL_923983, EPI_ISL_923984, EPI_ISL_923985, EPI_ISL_923986, EPI_ISL_923989, EPI_ISL_923990, EPI_ISL_923991, EPI_ISL_923992, EPI_ISL_923993, EPI_ISL_923994, EPI_ISL_923996, EPI_ISL_923997, EPI_ISL_923999, EPI_ISL_924001, EPI_ISL_924002, EPI_ISL_924003, EPI_ISL_924004, EPI_ISL_924005, EPI_ISL_924006, EPI_ISL_924008, EPI_ISL_924009, EPI_ISL_924011, EPI_ISL_924012, EPI_ISL_924013, EPI_ISL_924014, EPI_ISL_924015, EPI_ISL_924016, EPI_ISL_924017, EPI_ISL_924018, EPI_ISL_924019, EPI_ISL_924020, EPI_ISL_924021, EPI_ISL_924022, EPI_ISL_924023, EPI_ISL_924024, EPI_ISL_924025, EPI_ISL_924026, EPI_ISL_924029, EPI_ISL_924030, EPI_ISL_924031, EPI_ISL_924032, EPI_ISL_924033, EPI_ISL_924034, EPI_ISL_924035, EPI_ISL_924036, EPI_ISL_924038, EPI_ISL_924039, EPI_ISL_924041, EPI_ISL_924042, EPI_ISL_924043, EPI_ISL_924044, EPI_ISL_924045, EPI_ISL_924046, EPI_ISL_924047, EPI_ISL_924048, EPI_ISL_924049, EPI_ISL_924050, EPI_ISL_924051, EPI_ISL_924052, EPI_ISL_924053, EPI_ISL_924054, EPI_ISL_924055, EPI_ISL_924056, EPI_ISL_924057, EPI_ISL_924058, EPI_ISL_924059, EPI_ISL_924060, EPI_ISL_924062, EPI_ISL_924063, EPI_ISL_924064, EPI_ISL_924065, EPI_ISL_924066, EPI_ISL_924067, EPI_ISL_924068, EPI_ISL_924069, EPI_ISL_924070, EPI_ISL_924071, EPI_ISL_924072, EPI_ISL_924073, EPI_ISL_924074, EPI_ISL_924075, EPI_ISL_924076			
see above	Department of Pathology, University of Cambridge	COVID-19 Genomics UK (COG-UK) Consortium	Aminu S. Jahun, Yasmin Chaudhry, Iliana Georgana, Myra Hosmillo, Rhys Izu, Martin D. Curran, Surendra Parmar, Ian Goodfellow
EPI_ISL_924083, EPI_ISL_924096, EPI_ISL_924145, EPI_ISL_924150, EPI_ISL_924177, EPI_ISL_924181, EPI_ISL_924220, EPI_ISL_924224, EPI_ISL_924230, EPI_ISL_924233, EPI_ISL_924239, EPI_ISL_924242, EPI_ISL_924244, EPI_ISL_924247, EPI_ISL_924278, EPI_ISL_924295, EPI_ISL_924303, EPI_ISL_924325, EPI_ISL_924326, EPI_ISL_924354, EPI_ISL_924399, EPI_ISL_924408			
see above	Virology Department, Sheffield Teaching Hospitals NHS Foundation Trust/Department of Infection, Immunity and Cardiovascular Disease, The Medical School, University of Sheffield	COVID-19 Genomics UK (COG-UK) Consortium	Thushan de Silva, Matthew Parker, Nikki Smith, Adri Angyal, Rebecca Brown, Luke Green, Rachel Tucker, Paul Parsons, Danielle Groves, Katie Johnson, Laura Carrilero, Alex Keeley, Dave Partridge, Matthew Wyles, Benjamin Lindsey, Mehmet Yavuz, Mohammad Raza, Cariad Evans
EPI_ISL_924796, EPI_ISL_924801, EPI_ISL_924802, EPI_ISL_924803, EPI_ISL_924804, EPI_ISL_924806, EPI_ISL_924810, EPI_ISL_924811, EPI_ISL_924812, EPI_ISL_924813, EPI_ISL_924814, EPI_ISL_924815, EPI_ISL_924817, EPI_ISL_924820, EPI_ISL_924821, EPI_ISL_924822, EPI_ISL_924823, EPI_ISL_924824, EPI_ISL_924825, EPI_ISL_924826, EPI_ISL_924830, EPI_ISL_924834, EPI_ISL_924841, EPI_ISL_924842, EPI_ISL_924845, EPI_ISL_924857, EPI_ISL_924859, EPI_ISL_924948, EPI_ISL_924949, EPI_ISL_924952, EPI_ISL_924953, EPI_ISL_924954, EPI_ISL_924956, EPI_ISL_924957, EPI_ISL_924958, EPI_ISL_924959, EPI_ISL_924960, EPI_ISL_924961, EPI_ISL_924962, EPI_ISL_924963, EPI_ISL_924964, EPI_ISL_924965, EPI_ISL_924966, EPI_ISL_924967, EPI_ISL_924968, EPI_ISL_924969, EPI_ISL_924970, EPI_ISL_924971, EPI_ISL_924972, EPI_ISL_924973, EPI_ISL_924974, EPI_ISL_924976, EPI_ISL_924977, EPI_ISL_924978, EPI_ISL_924979, EPI_ISL_924981, EPI_ISL_924982, EPI_ISL_924983, EPI_ISL_924984, EPI_ISL_924985, EPI_ISL_924986, EPI_ISL_924988, EPI_ISL_924989, EPI_ISL_924990, EPI_ISL_924991, EPI_ISL_924992, EPI_ISL_924993, EPI_ISL_924994, EPI_ISL_924995, EPI_ISL_924996, EPI_ISL_924997, EPI_ISL_924998, EPI_ISL_924999, EPI_ISL_925000, EPI_ISL_925001, EPI_ISL_925002, EPI_ISL_925003, EPI_ISL_925004, EPI_ISL_925005, EPI_ISL_925006, EPI_ISL_925007, EPI_ISL_925008, EPI_ISL_925009, EPI_ISL_925010, EPI_ISL_925011, EPI_ISL_925012, EPI_ISL_925013			
see above	Bioinformatics and Biostatistics Lab, Advanced Sequencing Facility	COVID-19 Genomics UK (COG-UK) Consortium	Aengus Stewart,Jerome Nicod,Chelsea Sawyer,Laura Cubitt,Harshil Patel,Margaret Crawford
EPI_ISL_939608	Lighthouse Lab in Alderley Park	Wellcome Sanger Institute for the COVID-19 Genomics UK (COG-UK) Consortium	Jacquelyn Wynn, Mairead Hyland, The Lighthouse Lab in Alderley Park and Alex Alderton, Roberto Amato, Sonia Goncalves, Ewan Harrison, David K. Jackson, Ian Johnston, Dominic Kwiatkowski, Cordelia Langford, John Sillitoe on behalf of the Wellcome Sanger Institute COVID-19 Surveillance Team
EPI_ISL_948967, EPI_ISL_948968, EPI_ISL_948969, EPI_ISL_948970, EPI_ISL_948971, EPI_ISL_948972, EPI_ISL_948974, EPI_ISL_948976, EPI_ISL_948977, EPI_ISL_948979, EPI_ISL_948980, EPI_ISL_948982, EPI_ISL_948983, EPI_ISL_948985, EPI_ISL_948987, EPI_ISL_948988, EPI_ISL_948990, EPI_ISL_948992, EPI_ISL_948993, EPI_ISL_948995, EPI_ISL_948996, EPI_ISL_948998, EPI_ISL_948999, EPI_ISL_949001, EPI_ISL_949002, EPI_ISL_949004, EPI_ISL_949006, EPI_ISL_949007, EPI_ISL_949009, EPI_ISL_949010, EPI_ISL_949012, EPI_ISL_949014, EPI_ISL_949015, EPI_ISL_949017, EPI_ISL_949018, EPI_ISL_949020, EPI_ISL_949021, EPI_ISL_949023, EPI_ISL_949025, EPI_ISL_949026, EPI_ISL_949027, EPI_ISL_949028, EPI_ISL_949029, EPI_ISL_949030, EPI_ISL_949031, EPI_ISL_949032, EPI_ISL_949033, EPI_ISL_949034, EPI_ISL_949035, EPI_ISL_949036, EPI_ISL_949037, EPI_ISL_949038, EPI_ISL_949039, EPI_ISL_949040, EPI_ISL_949041, EPI_ISL_949042, EPI_ISL_949043, EPI_ISL_949044, EPI_ISL_949045, EPI_ISL_949046, EPI_ISL_949047, EPI_ISL_949048, EPI_ISL_949049, EPI_ISL_949050, EPI_ISL_949051, EPI_ISL_949052, EPI_ISL_949053, EPI_ISL_949054, EPI_ISL_949055, EPI_ISL_949056, EPI_ISL_949057, EPI_ISL_949058, EPI_ISL_949060, EPI_ISL_949061, EPI_ISL_949062, EPI_ISL_949063, EPI_ISL_949064, EPI_ISL_949065, EPI_ISL_949066			
see above	Lighthouse Lab in Glasgow	Wellcome Sanger Institute for the COVID-19 Genomics UK (COG-UK) Consortium	Harper VanSteenhouse, Yumi Kasai, David Gray, Carol Clugston, Anna Dominiczak and Alex Alderton, Roberto Amato, Sonia Goncalves, Ewan Harrison, David K. Jackson, Ian Johnston, Dominic Kwiatkowski, Cordelia Langford, John Sillitoe on behalf of the Wellcome Sanger Institute COVID-19 Surveillance Team
EPI_ISL_949256, EPI_ISL_949258, EPI_ISL_949259, EPI_ISL_949260, EPI_ISL_949261, EPI_ISL_949262, EPI_ISL_949263, EPI_ISL_949265, EPI_ISL_949266, EPI_ISL_949267, EPI_ISL_949268, EPI_ISL_949269, EPI_ISL_949270, EPI_ISL_949272, EPI_ISL_949273, EPI_ISL_949274, EPI_ISL_949275, EPI_ISL_949276, EPI_ISL_949277, EPI_ISL_949278, EPI_ISL_949279, EPI_ISL_949280, EPI_ISL_949281, EPI_ISL_949282, EPI_ISL_949284, EPI_ISL_949285, EPI_ISL_949286, EPI_ISL_949287, EPI_ISL_949288, EPI_ISL_949289, EPI_ISL_949291, EPI_ISL_949293, EPI_ISL_949294, EPI_ISL_949295, EPI_ISL_949296, EPI_ISL_949297, EPI_ISL_949298, EPI_ISL_949311, EPI_ISL_949315, EPI_ISL_949316, EPI_ISL_949318, EPI_ISL_949319, EPI_ISL_949320, EPI_ISL_949321, EPI_ISL_949323, EPI_ISL_949324, EPI_ISL_949325, EPI_ISL_949326			
see above	Department of Pathology, University of Cambridge	COVID-19 Genomics UK (COG-UK) Consortium	Aminu S. Jahun, Yasmin Chaudhry, Iliana Georgana, Myra Hosmillo, Rhys Izu, Martin D. Curran, Surendra Parmar, Ian Goodfellow
EPI_ISL_949417, EPI_ISL_949420, EPI_ISL_949421	University of Birmingham	COVID-19 Genomics UK (COG-UK) Consortium	Institute of Microbiology, University of Birmingham: Claire McMurray, Joanne Stockton, Samuel Nicholls, Radoslaw Poplawski, Will Rowe, Josh Quick, Nicholas Loman. University of Birmingham Testing Laboratory: Celina M Whalley, Andrew Bosworth, Charlotte Poxon, Kasun Wanigasooriya, Oliver Pickles, Mike Kidd, Alex Richter, Andrew D Beggs PHE Heartlands Lab: Husam Osman, Andrew Bosworth. Queen Elizabeth Hospital: Anna Casey
EPI_ISL_949492, EPI_ISL_949494, EPI_ISL_949496, EPI_ISL_949498, EPI_ISL_949504, EPI_ISL_949506	Department of Pathology, University of Cambridge	COVID-19 Genomics UK (COG-UK) Consortium	Aminu S. Jahun, Yasmin Chaudhry, Iliana Georgana, Myra Hosmillo, Rhys Izu, Martin D. Curran, Surendra Parmar, Ian Goodfellow

EPI_ISL_949640, EPI_ISL_949643, EPI_ISL_949644	Virology Department, Royal Infirmary of Edinburgh, NHS Lothian / School of Biological Sciences, University of Edinburgh / Institute of Genetics and Molecular Medicine, University of Edinburgh	COVID-19 Genomics UK (COG-UK) Consortium	McHugh M, Dewar R, Rooke S, Gallagher M, Balcaza C, O'Toole Á, Scher E, Hill V, McCrone JT, Colquhoun R, Yu X, Jackson B, Rambaut A, Williams TC, Templeton K
EPI_ISL_949749, EPI_ISL_949777, EPI_ISL_949778, EPI_ISL_949780, EPI_ISL_949783	Barts Health NHS Trust	COVID-19 Genomics UK (COG-UK) Consortium	CUTINO-MOGUEL, Maria-Teresa; HARRINGTON, David; OWOYEMI, Dola; KULASEGARAN-SHYLINI, Raghavendran; BROAD, Claire; KELE, Beatrix
EPI_ISL_950159, EPI_ISL_950211, EPI_ISL_950212, EPI_ISL_950213, EPI_ISL_950214, EPI_ISL_950215, EPI_ISL_950216, EPI_ISL_950217, EPI_ISL_950218, EPI_ISL_950219, EPI_ISL_950220, EPI_ISL_950221, EPI_ISL_950222, EPI_ISL_950223, EPI_ISL_950224, EPI_ISL_950225, EPI_ISL_950226, EPI_ISL_950227, EPI_ISL_950228, EPI_ISL_950229, EPI_ISL_950230, EPI_ISL_950231			
see above	University College London, Great Ormond Street Hospital for Children NHS Foundation Trust, Imperial College Healthcare NHS Trust	COVID-19 Genomics UK (COG-UK) Consortium	Sergi Castellano, Rachel Williams, Mark Kristiansen, Paola Resende Silva, Sunando Roy, Tony Brooks, Helena Tutill, Paola Niola, Patricia Dyal, Charlotte Williams, Leysa Forrest, Yasmin Panchbhaya, Jacqueline Findlay, Samuel Weeks, Julianne Brown, Kathryn Harris, Paul Randell, James Price, Alison Holmes, Judith Breuer
EPI_ISL_950294, EPI_ISL_950298, EPI_ISL_950304, EPI_ISL_950305, EPI_ISL_950306, EPI_ISL_950307, EPI_ISL_950308, EPI_ISL_950309, EPI_ISL_950310, EPI_ISL_950311, EPI_ISL_950312, EPI_ISL_950317, EPI_ISL_950321, EPI_ISL_950323, EPI_ISL_950324, EPI_ISL_950325, EPI_ISL_950326, EPI_ISL_950327, EPI_ISL_950328, EPI_ISL_950329, EPI_ISL_950330, EPI_ISL_950331, EPI_ISL_950332, EPI_ISL_950333, EPI_ISL_950334, EPI_ISL_950335, EPI_ISL_950336			
see above	Northumbria University / South Tees Hospitals NHS Foundation Trust / North Cumbria Integrated Care NHS Foundation Trust / North Tees and Hartlepool NHS Foundation Trust / Newcastle Hospitals NHS Foundation Trust	COVID-19 Genomics UK (COG-UK) Consortium	Darren L Smith,Andrew Nelson,Matthew Bashton,Greg R Young,Joshua Loh,John Allan,Mohammad A Tariq,Giles S Holt,Gary Black,Wen C Yew,Lynn Dover,Paul Baker,Steve Liggett,Sarah Essex,Jane Greenaway,Debra Padgett,Clive Graham,Garren Scott,Edward Barton,Emma Swindells,Brendan Payne,Jennifer Collins,Yusri Taha,Gary Eltringham
EPI_ISL_950714	Lincolnshire Hospitals and DeepSeq Nottingham	COVID-19 Genomics UK (COG-UK) Consortium	Nichola Duckworth, Tim Sloan, Sarah Walsh, Jonathan Ball, Patrick McClure, Joeseph Chappell, Nadine Holmes, Matthew Carlisle, Christopher Moore, Fei Sang, Johnny Debebe, Victoria Wright, Matthew Loose
EPI_ISL_951305, EPI_ISL_951311, EPI_ISL_951313, EPI_ISL_951318, EPI_ISL_951361, EPI_ISL_951374, EPI_ISL_951375, EPI_ISL_951378, EPI_ISL_951379, EPI_ISL_951380, EPI_ISL_951383, EPI_ISL_951387, EPI_ISL_951388, EPI_ISL_951416, EPI_ISL_951419, EPI_ISL_951420, EPI_ISL_951421, EPI_ISL_951423, EPI_ISL_951424, EPI_ISL_951425, EPI_ISL_951428, EPI_ISL_951435, EPI_ISL_951436, EPI_ISL_951437, EPI_ISL_951438, EPI_ISL_951442, EPI_ISL_951444, EPI_ISL_951445, EPI_ISL_951446, EPI_ISL_951447, EPI_ISL_951449, EPI_ISL_951450, EPI_ISL_951451, EPI_ISL_951452, EPI_ISL_951453, EPI_ISL_951456, EPI_ISL_951457, EPI_ISL_951458, EPI_ISL_951459			
see above	Oxford Viromics, NDM, University of Oxford; Oxford University Hospitals; Basingstoke and North Hampshire Hospital	COVID-19 Genomics UK (COG-UK) Consortium	Tanya Golubchik, David Bonsall, George Macintyre, Amy Trebes, Mariateresa de Cesare, Catrin Moore, Alex Mobbs, Anita Justice, Robert Shaw, Monique Andersson, Timothy Peto, Emma Wise, Nathan Moore, Jessica Lynch, Nick Cortes, Matilde Mori, Stephen Kidd, David Buck, John Todd, Christophe Fraser
EPI_ISL_951629, EPI_ISL_951816	Originating lab: Wales Specialist Virology Centre Sequencing lab: Pathogen Genomics Unit	Public Health Wales Microbiology Cardiff Wales Specialist Virology Centre	Catherine Moore, Johnathan Evans, Laura Gifford, Malorie Perry, Simon Cottrell, Angela Marchbank, Alec Birchley, Alexander Adams, Amy Gaskin, Bree Gatica-Wilcox, Jason Coombes, Joel Southgate, Lauren Gilbert, Lee Graham, Nicole Pacchiarini, Sara Kumziene-Summerhayes, Sarah Taylor, Sophie Jones, Sara Rey, Matthew Bull, Joanne Watkins, Sally Corden, Tom Connor
EPI_ISL_952377, EPI_ISL_952378, EPI_ISL_952379, EPI_ISL_952382, EPI_ISL_952383, EPI_ISL_952385, EPI_ISL_952389, EPI_ISL_952392, EPI_ISL_952394, EPI_ISL_952397, EPI_ISL_952398, EPI_ISL_952409, EPI_ISL_952411, EPI_ISL_952414, EPI_ISL_952418, EPI_ISL_952422, EPI_ISL_952424, EPI_ISL_952427, EPI_ISL_952429, EPI_ISL_952433, EPI_ISL_952471, EPI_ISL_952472, EPI_ISL_952473, EPI_ISL_952474, EPI_ISL_952475, EPI_ISL_952476, EPI_ISL_952526, EPI_ISL_952569, EPI_ISL_952570, EPI_ISL_952571, EPI_ISL_952572, EPI_ISL_952573, EPI_ISL_952574, EPI_ISL_952575, EPI_ISL_952576, EPI_ISL_952578, EPI_ISL_952580, EPI_ISL_952581, EPI_ISL_952582, EPI_ISL_952583, EPI_ISL_952584, EPI_ISL_952585, EPI_ISL_952586, EPI_ISL_952587, EPI_ISL_952588, EPI_ISL_952589, EPI_ISL_952590, EPI_ISL_952591, EPI_ISL_952592, EPI_ISL_952594, EPI_ISL_952595, EPI_ISL_952597, EPI_ISL_952598, EPI_ISL_952599, EPI_ISL_952600, EPI_ISL_952601, EPI_ISL_952602, EPI_ISL_952624, EPI_ISL_952778, EPI_ISL_952779, EPI_ISL_952780, EPI_ISL_952789, EPI_ISL_952793, EPI_ISL_952794, EPI_ISL_952795, EPI_ISL_952798, EPI_ISL_952799, EPI_ISL_952804, EPI_ISL_952854			
see above	Centre for Enzyme Innovation, University of Portsmouth / Translational Research Laboratory, Portsmouth Hospitals NHS Trust	COVID-19 Genomics UK (COG-UK) Consortium	Angela Beckett,Salman Goudarzi,Christopher Fearn,Kate Cook,Katie Loveson,Sharon Glaysher,Scott Elliott,Samuel Robson
EPI_ISL_952896, EPI_ISL_952897, EPI_ISL_952899, EPI_ISL_952900, EPI_ISL_952901, EPI_ISL_952902, EPI_ISL_952903, EPI_ISL_952904, EPI_ISL_952905, EPI_ISL_952906, EPI_ISL_952907, EPI_ISL_952908, EPI_ISL_952909, EPI_ISL_952910, EPI_ISL_952912, EPI_ISL_952913, EPI_ISL_952932			
see above	Department of Pathology, University of Cambridge	COVID-19 Genomics UK (COG-UK) Consortium	Aminu S. Jahun, Yasmin Chaudhry, Iliana Georgana, Myra Hosmillo, Rhys Izu, Martin D. Curran, Surendra Parmar, Ian Goodfellow
EPI_ISL_953024, EPI_ISL_953025, EPI_ISL_953026, EPI_ISL_953027, EPI_ISL_953028, EPI_ISL_953029, EPI_ISL_953030, EPI_ISL_953031, EPI_ISL_953032, EPI_ISL_953033, EPI_ISL_953034, EPI_ISL_953035, EPI_ISL_953036, EPI_ISL_953037, EPI_ISL_953038, EPI_ISL_953039, EPI_ISL_953040, EPI_ISL_953041, EPI_ISL_953042, EPI_ISL_953043, EPI_ISL_953044, EPI_ISL_953045, EPI_ISL_953046, EPI_ISL_953047, EPI_ISL_953048, EPI_ISL_953049, EPI_ISL_953050, EPI_ISL_953051, EPI_ISL_953052, EPI_ISL_953053, EPI_ISL_953054, EPI_ISL_953055, EPI_ISL_953056, EPI_ISL_953057, EPI_ISL_953058, EPI_ISL_953059, EPI_ISL_953060, EPI_ISL_953061, EPI_ISL_953062, EPI_ISL_953063, EPI_ISL_953064, EPI_ISL_953065, EPI_ISL_953066, EPI_ISL_953067, EPI_ISL_953068, EPI_ISL_953069, EPI_ISL_953070, EPI_ISL_953071, EPI_ISL_953072, EPI_ISL_953073, EPI_ISL_953074, EPI_ISL_953075, EPI_ISL_953076, EPI_ISL_953077, EPI_ISL_953078, EPI_ISL_953079, EPI_ISL_953080, EPI_ISL_953081, EPI_ISL_953082, EPI_ISL_953085, EPI_ISL_953086, EPI_ISL_953087, EPI_ISL_953088, EPI_ISL_953090, EPI_ISL_953091, EPI_ISL_953092, EPI_ISL_953093, EPI_ISL_953094, EPI_ISL_953095, EPI_ISL_953100			
see above	Bioinformatics and Biostatistics Lab, Advanced Sequencing Facility	COVID-19 Genomics UK (COG-UK) Consortium	Aengus Stewart,Jerome Nicod,Chelsea Sawyer,Laura Cubitt,Harshil Patel,Margaret Crawford
EPI_ISL_963321	Lighthouse Lab in Alderley Park	Wellcome Sanger Institute for the COVID-19 Genomics UK (COG-UK) Consortium	Jacquelyn Wynn, Mairead Hyland, The Lighthouse Lab in Alderley Park and Alex Alderton, Roberto Amato, Sonia Goncalves, Ewan Harrison, David K. Jackson, Ian Johnston, Dominic Kwiatkowski, Cordelia Langford, John Sillitoe on behalf of the Wellcome Sanger Institute COVID-19 Surveillance Team
EPI_ISL_987283, EPI_ISL_987287, EPI_ISL_987290, EPI_ISL_987291, EPI_ISL_987299	Lighthouse Lab in Cambridge	Wellcome Sanger Institute for the COVID-19 Genomics UK (COG-UK) Consortium	Rob Howes, The Lighthouse Lab in Cambridge and Alex Alderton, Roberto Amato, Sonia Goncalves, Ewan Harrison, David K. Jackson, Ian Johnston, Dominic Kwiatkowski, Cordelia Langford, John Sillitoe on behalf of the Wellcome Sanger Institute COVID-19 Surveillance Team (http://www.sanger.ac.uk/covid-team)
EPI_ISL_987300	Lighthouse Lab in Glasgow	Wellcome Sanger Institute for the COVID-19 Genomics UK (COG-UK) Consortium	Harper VanSteenhouse, Yumi Kasai, David Gray, Carol Clugston, Anna Dominiczak and Alex Alderton, Roberto Amato, Sonia Goncalves, Ewan Harrison, David K. Jackson, Ian Johnston, Dominic Kwiatkowski, Cordelia Langford, John Sillitoe on behalf of the Wellcome Sanger Institute COVID-19 Surveillance Team (http://www.sanger.ac.uk/covid-team)
EPI_ISL_987303, EPI_ISL_987310	Lighthouse Lab in Cambridge	Wellcome Sanger Institute for the COVID-19 Genomics UK (COG-UK) Consortium	Rob Howes, The Lighthouse Lab in Cambridge and Alex Alderton, Roberto Amato, Sonia Goncalves, Ewan Harrison, David K. Jackson, Ian Johnston, Dominic Kwiatkowski, Cordelia Langford, John Sillitoe on behalf of the Wellcome Sanger Institute COVID-19 Surveillance Team (http://www.sanger.ac.uk/covid-team)
EPI_ISL_987311	Lighthouse Lab in Glasgow	Wellcome Sanger Institute for the COVID-19 Genomics UK (COG-UK) Consortium	Harper VanSteenhouse, Yumi Kasai, David Gray, Carol Clugston, Anna Dominiczak and Alex Alderton, Roberto Amato, Sonia Goncalves, Ewan Harrison, David K. Jackson, Ian Johnston, Dominic Kwiatkowski, Cordelia Langford, John Sillitoe on behalf of the Wellcome Sanger Institute COVID-19 Surveillance Team (http://www.sanger.ac.uk/covid-team)
EPI_ISL_987312	Lighthouse Lab in Cambridge	Wellcome Sanger Institute for the COVID-19 Genomics UK (COG-UK) Consortium	Rob Howes, The Lighthouse Lab in Cambridge and Alex Alderton, Roberto Amato, Sonia Goncalves, Ewan Harrison, David K. Jackson, Ian Johnston, Dominic Kwiatkowski, Cordelia Langford, John Sillitoe on behalf of the Wellcome Sanger Institute COVID-19 Surveillance Team (http://www.sanger.ac.uk/covid-team)
EPI_ISL_987317	Lighthouse Lab in Glasgow	Wellcome Sanger Institute for the COVID-19 Genomics UK (COG-UK) Consortium	Harper VanSteenhouse, Yumi Kasai, David Gray, Carol Clugston, Anna Dominiczak and Alex Alderton, Roberto Amato, Sonia Goncalves, Ewan Harrison, David K. Jackson, Ian Johnston, Dominic Kwiatkowski, Cordelia Langford, John Sillitoe on behalf of the Wellcome Sanger Institute COVID-19 Surveillance Team (http://www.sanger.ac.uk/covid-team)
EPI_ISL_987319, EPI_ISL_987322, EPI_ISL_987326, EPI_ISL_987328	Lighthouse Lab in Cambridge	Wellcome Sanger Institute for the COVID-19 Genomics UK (COG-UK) Consortium	Rob Howes, The Lighthouse Lab in Cambridge and Alex Alderton, Roberto Amato, Sonia Goncalves, Ewan Harrison, David K. Jackson, Ian Johnston, Dominic Kwiatkowski, Cordelia Langford, John Sillitoe on behalf of the Wellcome Sanger Institute COVID-19 Surveillance Team (http://www.sanger.ac.uk/covid-team)
EPI_ISL_987330, EPI_ISL_987331	Lighthouse Lab in Milton Keynes	Wellcome Sanger Institute for the COVID-19 Genomics UK (COG-UK) Consortium	The Lighthouse Lab in Milton Keynes and Alex Alderton, Roberto Amato, Sonia Goncalves, Ewan Harrison, David K. Jackson, Ian Johnston, Dominic Kwiatkowski, Cordelia Langford, John Sillitoe on behalf of the Wellcome Sanger Institute COVID-19 Surveillance Team (http://www.sanger.ac.uk/covid-team)
EPI_ISL_987332	Lighthouse Lab in Cambridge	Wellcome Sanger Institute for the COVID-19 Genomics UK (COG-UK) Consortium	Rob Howes, The Lighthouse Lab in Cambridge and Alex Alderton, Roberto Amato, Sonia Goncalves, Ewan Harrison, David K. Jackson, Ian Johnston, Dominic Kwiatkowski, Cordelia Langford, John Sillitoe on behalf of the Wellcome Sanger Institute COVID-19 Surveillance Team (http://www.sanger.ac.uk/covid-team)
EPI_ISL_987334	Lighthouse Lab in Milton Keynes	Wellcome Sanger Institute for the COVID-19 Genomics UK (COG-UK) Consortium	The Lighthouse Lab in Milton Keynes and Alex Alderton, Roberto Amato, Sonia Goncalves, Ewan Harrison, David K. Jackson, Ian Johnston, Dominic Kwiatkowski, Cordelia Langford, John Sillitoe on behalf of the Wellcome Sanger Institute COVID-19 Surveillance Team

			(http://www.sanger.ac.uk/covid-team)
EPI_ISL_987335	Lighthouse Lab in Glasgow	Wellcome Sanger Institute for the COVID-19 Genomics UK (COG-UK) Consortium	Harper VanSteenhouse, Yumi Kasai, David Gray, Carol Clugston, Anna Dominiczak and Alex Alderton, Roberto Amato, Sonia Goncalves, Ewan Harrison, David K. Jackson, Ian Johnston, Dominic Kwiatkowski, Cordelia Langford, John Sillitoe on behalf of the Wellcome Sanger Institute COVID-19 Surveillance Team (http://www.sanger.ac.uk/covid-team)
EPI_ISL_987339, EPI_ISL_987340	Lighthouse Lab in Cambridge	Wellcome Sanger Institute for the COVID-19 Genomics UK (COG-UK) Consortium	Rob Howes, The Lighthouse Lab in Cambridge and Alex Alderton, Roberto Amato, Sonia Goncalves, Ewan Harrison, David K. Jackson, Ian Johnston, Dominic Kwiatkowski, Cordelia Langford, John Sillitoe on behalf of the Wellcome Sanger Institute COVID-19 Surveillance Team (http://www.sanger.ac.uk/covid-team)
EPI_ISL_987345, EPI_ISL_987355, EPI_ISL_987356	Lighthouse Lab in Milton Keynes	Wellcome Sanger Institute for the COVID-19 Genomics UK (COG-UK) Consortium	The Lighthouse Lab in Milton Keynes and Alex Alderton, Roberto Amato, Sonia Goncalves, Ewan Harrison, David K. Jackson, Ian Johnston, Dominic Kwiatkowski, Cordelia Langford, John Sillitoe on behalf of the Wellcome Sanger Institute COVID-19 Surveillance Team (http://www.sanger.ac.uk/covid-team)
EPI_ISL_987357, EPI_ISL_987369	Lighthouse Lab in Cambridge	Wellcome Sanger Institute for the COVID-19 Genomics UK (COG-UK) Consortium	Rob Howes, The Lighthouse Lab in Cambridge and Alex Alderton, Roberto Amato, Sonia Goncalves, Ewan Harrison, David K. Jackson, Ian Johnston, Dominic Kwiatkowski, Cordelia Langford, John Sillitoe on behalf of the Wellcome Sanger Institute COVID-19 Surveillance Team (http://www.sanger.ac.uk/covid-team)
EPI_ISL_987373	Lighthouse Lab in Glasgow	Wellcome Sanger Institute for the COVID-19 Genomics UK (COG-UK) Consortium	Harper VanSteenhouse, Yumi Kasai, David Gray, Carol Clugston, Anna Dominiczak and Alex Alderton, Roberto Amato, Sonia Goncalves, Ewan Harrison, David K. Jackson, Ian Johnston, Dominic Kwiatkowski, Cordelia Langford, John Sillitoe on behalf of the Wellcome Sanger Institute COVID-19 Surveillance Team (http://www.sanger.ac.uk/covid-team)
EPI_ISL_987377	Lighthouse Lab in Milton Keynes	Wellcome Sanger Institute for the COVID-19 Genomics UK (COG-UK) Consortium	The Lighthouse Lab in Milton Keynes and Alex Alderton, Roberto Amato, Sonia Goncalves, Ewan Harrison, David K. Jackson, Ian Johnston, Dominic Kwiatkowski, Cordelia Langford, John Sillitoe on behalf of the Wellcome Sanger Institute COVID-19 Surveillance Team (http://www.sanger.ac.uk/covid-team)
EPI_ISL_987378, EPI_ISL_987380, EPI_ISL_987381, EPI_ISL_987382, EPI_ISL_987384, EPI_ISL_987389	Lighthouse Lab in Cambridge	Wellcome Sanger Institute for the COVID-19 Genomics UK (COG-UK) Consortium	Rob Howes, The Lighthouse Lab in Cambridge and Alex Alderton, Roberto Amato, Sonia Goncalves, Ewan Harrison, David K. Jackson, Ian Johnston, Dominic Kwiatkowski, Cordelia Langford, John Sillitoe on behalf of the Wellcome Sanger Institute COVID-19 Surveillance Team (http://www.sanger.ac.uk/covid-team)
EPI_ISL_987390	Lighthouse Lab in Glasgow	Wellcome Sanger Institute for the COVID-19 Genomics UK (COG-UK) Consortium	Harper VanSteenhouse, Yumi Kasai, David Gray, Carol Clugston, Anna Dominiczak and Alex Alderton, Roberto Amato, Sonia Goncalves, Ewan Harrison, David K. Jackson, Ian Johnston, Dominic Kwiatkowski, Cordelia Langford, John Sillitoe on behalf of the Wellcome Sanger Institute COVID-19 Surveillance Team (http://www.sanger.ac.uk/covid-team)
EPI_ISL_987393	Lighthouse Lab in Cambridge	Wellcome Sanger Institute for the COVID-19 Genomics UK (COG-UK) Consortium	Rob Howes, The Lighthouse Lab in Cambridge and Alex Alderton, Roberto Amato, Sonia Goncalves, Ewan Harrison, David K. Jackson, Ian Johnston, Dominic Kwiatkowski, Cordelia Langford, John Sillitoe on behalf of the Wellcome Sanger Institute COVID-19 Surveillance Team (http://www.sanger.ac.uk/covid-team)
EPI_ISL_987395	Lighthouse Lab in Glasgow	Wellcome Sanger Institute for the COVID-19 Genomics UK (COG-UK) Consortium	Harper VanSteenhouse, Yumi Kasai, David Gray, Carol Clugston, Anna Dominiczak and Alex Alderton, Roberto Amato, Sonia Goncalves, Ewan Harrison, David K. Jackson, Ian Johnston, Dominic Kwiatkowski, Cordelia Langford, John Sillitoe on behalf of the Wellcome Sanger Institute COVID-19 Surveillance Team (http://www.sanger.ac.uk/covid-team)
EPI_ISL_987398, EPI_ISL_987399, EPI_ISL_987402, EPI_ISL_987406, EPI_ISL_987411, EPI_ISL_987414, EPI_ISL_987415, EPI_ISL_987418, EPI_ISL_987419	Lighthouse Lab in Cambridge	Wellcome Sanger Institute for the COVID-19 Genomics UK (COG-UK) Consortium	Rob Howes, The Lighthouse Lab in Cambridge and Alex Alderton, Roberto Amato, Sonia Goncalves, Ewan Harrison, David K. Jackson, Ian Johnston, Dominic Kwiatkowski, Cordelia Langford, John Sillitoe on behalf of the Wellcome Sanger Institute COVID-19 Surveillance Team (http://www.sanger.ac.uk/covid-team)
EPI_ISL_987420, EPI_ISL_987422	Lighthouse Lab in Glasgow	Wellcome Sanger Institute for the COVID-19 Genomics UK (COG-UK) Consortium	Harper VanSteenhouse, Yumi Kasai, David Gray, Carol Clugston, Anna Dominiczak and Alex Alderton, Roberto Amato, Sonia Goncalves, Ewan Harrison, David K. Jackson, Ian Johnston, Dominic Kwiatkowski, Cordelia Langford, John Sillitoe on behalf of the Wellcome Sanger Institute COVID-19 Surveillance Team (http://www.sanger.ac.uk/covid-team)
EPI_ISL_987424, EPI_ISL_987426, EPI_ISL_987427, EPI_ISL_987428, EPI_ISL_987429, EPI_ISL_987435	Lighthouse Lab in Cambridge	Wellcome Sanger Institute for the COVID-19 Genomics UK (COG-UK) Consortium	Rob Howes, The Lighthouse Lab in Cambridge and Alex Alderton, Roberto Amato, Sonia Goncalves, Ewan Harrison, David K. Jackson, Ian Johnston, Dominic Kwiatkowski, Cordelia Langford, John Sillitoe on behalf of the Wellcome Sanger Institute COVID-19 Surveillance Team (http://www.sanger.ac.uk/covid-team)
EPI_ISL_987438	Lighthouse Lab in Milton Keynes	Wellcome Sanger Institute for the COVID-19 Genomics UK (COG-UK) Consortium	The Lighthouse Lab in Milton Keynes and Alex Alderton, Roberto Amato, Sonia Goncalves, Ewan Harrison, David K. Jackson, Ian Johnston, Dominic Kwiatkowski, Cordelia Langford, John Sillitoe on behalf of the Wellcome Sanger Institute COVID-19 Surveillance Team (http://www.sanger.ac.uk/covid-team)
EPI_ISL_987441, EPI_ISL_987443	Lighthouse Lab in Cambridge	Wellcome Sanger Institute for the COVID-19 Genomics UK (COG-UK) Consortium	Rob Howes, The Lighthouse Lab in Cambridge and Alex Alderton, Roberto Amato, Sonia Goncalves, Ewan Harrison, David K. Jackson, Ian Johnston, Dominic Kwiatkowski, Cordelia Langford, John Sillitoe on behalf of the Wellcome Sanger Institute COVID-19 Surveillance Team (http://www.sanger.ac.uk/covid-team)
EPI_ISL_987444	Lighthouse Lab in Milton Keynes	Wellcome Sanger Institute for the COVID-19 Genomics UK (COG-UK) Consortium	The Lighthouse Lab in Milton Keynes and Alex Alderton, Roberto Amato, Sonia Goncalves, Ewan Harrison, David K. Jackson, Ian Johnston, Dominic Kwiatkowski, Cordelia Langford, John Sillitoe on behalf of the Wellcome Sanger Institute COVID-19 Surveillance Team (http://www.sanger.ac.uk/covid-team)
EPI_ISL_987445, EPI_ISL_987446, EPI_ISL_987452, EPI_ISL_987453	Lighthouse Lab in Cambridge	Wellcome Sanger Institute for the COVID-19 Genomics UK (COG-UK) Consortium	Rob Howes, The Lighthouse Lab in Cambridge and Alex Alderton, Roberto Amato, Sonia Goncalves, Ewan Harrison, David K. Jackson, Ian Johnston, Dominic Kwiatkowski, Cordelia Langford, John Sillitoe on behalf of the Wellcome Sanger Institute COVID-19 Surveillance Team (http://www.sanger.ac.uk/covid-team)
EPI_ISL_987454	Lighthouse Lab in Glasgow	Wellcome Sanger Institute for the COVID-19 Genomics UK (COG-UK) Consortium	Harper VanSteenhouse, Yumi Kasai, David Gray, Carol Clugston, Anna Dominiczak and Alex Alderton, Roberto Amato, Sonia Goncalves, Ewan Harrison, David K. Jackson, Ian Johnston, Dominic Kwiatkowski, Cordelia Langford, John Sillitoe on behalf of the Wellcome Sanger Institute COVID-19 Surveillance Team (http://www.sanger.ac.uk/covid-team)
EPI_ISL_987455	Lighthouse Lab in Cambridge	Wellcome Sanger Institute for the COVID-19 Genomics UK (COG-UK) Consortium	Rob Howes, The Lighthouse Lab in Cambridge and Alex Alderton, Roberto Amato, Sonia Goncalves, Ewan Harrison, David K. Jackson, Ian Johnston, Dominic Kwiatkowski, Cordelia Langford, John Sillitoe on behalf of the Wellcome Sanger Institute COVID-19 Surveillance Team (http://www.sanger.ac.uk/covid-team)
EPI_ISL_987457	Lighthouse Lab in Milton Keynes	Wellcome Sanger Institute for the COVID-19 Genomics UK (COG-UK) Consortium	The Lighthouse Lab in Milton Keynes and Alex Alderton, Roberto Amato, Sonia Goncalves, Ewan Harrison, David K. Jackson, Ian Johnston, Dominic Kwiatkowski, Cordelia Langford, John Sillitoe on behalf of the Wellcome Sanger Institute COVID-19 Surveillance Team (http://www.sanger.ac.uk/covid-team)
EPI_ISL_987461	Lighthouse Lab in Glasgow	Wellcome Sanger Institute for the COVID-19 Genomics UK (COG-UK) Consortium	Harper VanSteenhouse, Yumi Kasai, David Gray, Carol Clugston, Anna Dominiczak and Alex Alderton, Roberto Amato, Sonia Goncalves, Ewan Harrison, David K. Jackson, Ian Johnston, Dominic Kwiatkowski, Cordelia Langford, John Sillitoe on behalf of the Wellcome Sanger Institute COVID-19 Surveillance Team (http://www.sanger.ac.uk/covid-team)
EPI_ISL_987464, EPI_ISL_987465, EPI_ISL_987474, EPI_ISL_987475, EPI_ISL_987479	Lighthouse Lab in Cambridge	Wellcome Sanger Institute for the COVID-19 Genomics UK (COG-UK) Consortium	Rob Howes, The Lighthouse Lab in Cambridge and Alex Alderton, Roberto Amato, Sonia Goncalves, Ewan Harrison, David K. Jackson, Ian Johnston, Dominic Kwiatkowski, Cordelia Langford, John Sillitoe on behalf of the Wellcome Sanger Institute COVID-19 Surveillance Team (http://www.sanger.ac.uk/covid-team)
EPI_ISL_987481	Lighthouse Lab in Glasgow	Wellcome Sanger Institute for the COVID-19 Genomics UK (COG-UK) Consortium	Harper VanSteenhouse, Yumi Kasai, David Gray, Carol Clugston, Anna Dominiczak and Alex Alderton, Roberto Amato, Sonia Goncalves, Ewan Harrison, David K. Jackson, Ian Johnston, Dominic Kwiatkowski, Cordelia Langford, John Sillitoe on behalf of the Wellcome Sanger Institute COVID-19 Surveillance Team (http://www.sanger.ac.uk/covid-team)
EPI_ISL_987484, EPI_ISL_987488	Lighthouse Lab in Cambridge	Wellcome Sanger Institute for the COVID-19 Genomics UK (COG-UK) Consortium	Rob Howes, The Lighthouse Lab in Cambridge and Alex Alderton, Roberto Amato, Sonia Goncalves, Ewan Harrison, David K. Jackson, Ian Johnston, Dominic Kwiatkowski, Cordelia Langford, John Sillitoe on behalf of the Wellcome Sanger Institute COVID-19 Surveillance Team (http://www.sanger.ac.uk/covid-team)

EPI_ISL_987491	Lighthouse Lab in Glasgow	Wellcome Sanger Institute for the COVID-19 Genomics UK (COG-UK) Consortium	Harper VanSteenhouse, Yumi Kasai, David Gray, Carol Clugston, Anna Dominiczak and Alex Alderton, Roberto Amato, Sonia Goncalves, Ewan Harrison, David K. Jackson, Ian Johnston, Dominic Kwiatkowski, Cordelia Langford, John Sillitoe on behalf of the Wellcome Sanger Institute COVID-19 Surveillance Team (http://www.sanger.ac.uk/covid-team)
EPI_ISL_987498, EPI_ISL_987499	Lighthouse Lab in Cambridge	Wellcome Sanger Institute for the COVID-19 Genomics UK (COG-UK) Consortium	Rob Howes, The Lighthouse Lab in Cambridge and Alex Alderton, Roberto Amato, Sonia Goncalves, Ewan Harrison, David K. Jackson, Ian Johnston, Dominic Kwiatkowski, Cordelia Langford, John Sillitoe on behalf of the Wellcome Sanger Institute COVID-19 Surveillance Team (http://www.sanger.ac.uk/covid-team)
EPI_ISL_987501	Lighthouse Lab in Glasgow	Wellcome Sanger Institute for the COVID-19 Genomics UK (COG-UK) Consortium	Harper VanSteenhouse, Yumi Kasai, David Gray, Carol Clugston, Anna Dominiczak and Alex Alderton, Roberto Amato, Sonia Goncalves, Ewan Harrison, David K. Jackson, Ian Johnston, Dominic Kwiatkowski, Cordelia Langford, John Sillitoe on behalf of the Wellcome Sanger Institute COVID-19 Surveillance Team (http://www.sanger.ac.uk/covid-team)
EPI_ISL_987506	Lighthouse Lab in Cambridge	Wellcome Sanger Institute for the COVID-19 Genomics UK (COG-UK) Consortium	Rob Howes, The Lighthouse Lab in Cambridge and Alex Alderton, Roberto Amato, Sonia Goncalves, Ewan Harrison, David K. Jackson, Ian Johnston, Dominic Kwiatkowski, Cordelia Langford, John Sillitoe on behalf of the Wellcome Sanger Institute COVID-19 Surveillance Team (http://www.sanger.ac.uk/covid-team)
EPI_ISL_987507	Lighthouse Lab in Glasgow	Wellcome Sanger Institute for the COVID-19 Genomics UK (COG-UK) Consortium	Harper VanSteenhouse, Yumi Kasai, David Gray, Carol Clugston, Anna Dominiczak and Alex Alderton, Roberto Amato, Sonia Goncalves, Ewan Harrison, David K. Jackson, Ian Johnston, Dominic Kwiatkowski, Cordelia Langford, John Sillitoe on behalf of the Wellcome Sanger Institute COVID-19 Surveillance Team (http://www.sanger.ac.uk/covid-team)
EPI_ISL_987509, EPI_ISL_987510, EPI_ISL_987514	Lighthouse Lab in Cambridge	Wellcome Sanger Institute for the COVID-19 Genomics UK (COG-UK) Consortium	Rob Howes, The Lighthouse Lab in Cambridge and Alex Alderton, Roberto Amato, Sonia Goncalves, Ewan Harrison, David K. Jackson, Ian Johnston, Dominic Kwiatkowski, Cordelia Langford, John Sillitoe on behalf of the Wellcome Sanger Institute COVID-19 Surveillance Team (http://www.sanger.ac.uk/covid-team)
EPI_ISL_987516, EPI_ISL_987517, EPI_ISL_987522	Lighthouse Lab in Milton Keynes	Wellcome Sanger Institute for the COVID-19 Genomics UK (COG-UK) Consortium	The Lighthouse Lab in Milton Keynes and Alex Alderton, Roberto Amato, Sonia Goncalves, Ewan Harrison, David K. Jackson, Ian Johnston, Dominic Kwiatkowski, Cordelia Langford, John Sillitoe on behalf of the Wellcome Sanger Institute COVID-19 Surveillance Team (http://www.sanger.ac.uk/covid-team)
EPI_ISL_987530	Lighthouse Lab in Cambridge	Wellcome Sanger Institute for the COVID-19 Genomics UK (COG-UK) Consortium	Rob Howes, The Lighthouse Lab in Cambridge and Alex Alderton, Roberto Amato, Sonia Goncalves, Ewan Harrison, David K. Jackson, Ian Johnston, Dominic Kwiatkowski, Cordelia Langford, John Sillitoe on behalf of the Wellcome Sanger Institute COVID-19 Surveillance Team (http://www.sanger.ac.uk/covid-team)
EPI_ISL_987544	Lighthouse Lab in Milton Keynes	Wellcome Sanger Institute for the COVID-19 Genomics UK (COG-UK) Consortium	The Lighthouse Lab in Milton Keynes and Alex Alderton, Roberto Amato, Sonia Goncalves, Ewan Harrison, David K. Jackson, Ian Johnston, Dominic Kwiatkowski, Cordelia Langford, John Sillitoe on behalf of the Wellcome Sanger Institute COVID-19 Surveillance Team (http://www.sanger.ac.uk/covid-team)
EPI_ISL_987547	Lighthouse Lab in Cambridge	Wellcome Sanger Institute for the COVID-19 Genomics UK (COG-UK) Consortium	Rob Howes, The Lighthouse Lab in Cambridge and Alex Alderton, Roberto Amato, Sonia Goncalves, Ewan Harrison, David K. Jackson, Ian Johnston, Dominic Kwiatkowski, Cordelia Langford, John Sillitoe on behalf of the Wellcome Sanger Institute COVID-19 Surveillance Team (http://www.sanger.ac.uk/covid-team)
EPI_ISL_987548	Lighthouse Lab in Milton Keynes	Wellcome Sanger Institute for the COVID-19 Genomics UK (COG-UK) Consortium	The Lighthouse Lab in Milton Keynes and Alex Alderton, Roberto Amato, Sonia Goncalves, Ewan Harrison, David K. Jackson, Ian Johnston, Dominic Kwiatkowski, Cordelia Langford, John Sillitoe on behalf of the Wellcome Sanger Institute COVID-19 Surveillance Team (http://www.sanger.ac.uk/covid-team)
EPI_ISL_987549, EPI_ISL_987552, EPI_ISL_987555, EPI_ISL_987556	Lighthouse Lab in Glasgow	Wellcome Sanger Institute for the COVID-19 Genomics UK (COG-UK) Consortium	Harper VanSteenhouse, Yumi Kasai, David Gray, Carol Clugston, Anna Dominiczak and Alex Alderton, Roberto Amato, Sonia Goncalves, Ewan Harrison, David K. Jackson, Ian Johnston, Dominic Kwiatkowski, Cordelia Langford, John Sillitoe on behalf of the Wellcome Sanger Institute COVID-19 Surveillance Team (http://www.sanger.ac.uk/covid-team)
EPI_ISL_987557, EPI_ISL_987560, EPI_ISL_987567	Lighthouse Lab in Cambridge	Wellcome Sanger Institute for the COVID-19 Genomics UK (COG-UK) Consortium	Rob Howes, The Lighthouse Lab in Cambridge and Alex Alderton, Roberto Amato, Sonia Goncalves, Ewan Harrison, David K. Jackson, Ian Johnston, Dominic Kwiatkowski, Cordelia Langford, John Sillitoe on behalf of the Wellcome Sanger Institute COVID-19 Surveillance Team (http://www.sanger.ac.uk/covid-team)
EPI_ISL_987570, EPI_ISL_987572	Lighthouse Lab in Glasgow	Wellcome Sanger Institute for the COVID-19 Genomics UK (COG-UK) Consortium	Harper VanSteenhouse, Yumi Kasai, David Gray, Carol Clugston, Anna Dominiczak and Alex Alderton, Roberto Amato, Sonia Goncalves, Ewan Harrison, David K. Jackson, Ian Johnston, Dominic Kwiatkowski, Cordelia Langford, John Sillitoe on behalf of the Wellcome Sanger Institute COVID-19 Surveillance Team (http://www.sanger.ac.uk/covid-team)
EPI_ISL_987573	Lighthouse Lab in Cambridge	Wellcome Sanger Institute for the COVID-19 Genomics UK (COG-UK) Consortium	Rob Howes, The Lighthouse Lab in Cambridge and Alex Alderton, Roberto Amato, Sonia Goncalves, Ewan Harrison, David K. Jackson, Ian Johnston, Dominic Kwiatkowski, Cordelia Langford, John Sillitoe on behalf of the Wellcome Sanger Institute COVID-19 Surveillance Team (http://www.sanger.ac.uk/covid-team)
EPI_ISL_987574	Lighthouse Lab in Glasgow	Wellcome Sanger Institute for the COVID-19 Genomics UK (COG-UK) Consortium	Harper VanSteenhouse, Yumi Kasai, David Gray, Carol Clugston, Anna Dominiczak and Alex Alderton, Roberto Amato, Sonia Goncalves, Ewan Harrison, David K. Jackson, Ian Johnston, Dominic Kwiatkowski, Cordelia Langford, John Sillitoe on behalf of the Wellcome Sanger Institute COVID-19 Surveillance Team (http://www.sanger.ac.uk/covid-team)
EPI_ISL_987575, EPI_ISL_987577	Lighthouse Lab in Cambridge	Wellcome Sanger Institute for the COVID-19 Genomics UK (COG-UK) Consortium	Rob Howes, The Lighthouse Lab in Cambridge and Alex Alderton, Roberto Amato, Sonia Goncalves, Ewan Harrison, David K. Jackson, Ian Johnston, Dominic Kwiatkowski, Cordelia Langford, John Sillitoe on behalf of the Wellcome Sanger Institute COVID-19 Surveillance Team (http://www.sanger.ac.uk/covid-team)
EPI_ISL_987585	Lighthouse Lab in Milton Keynes	Wellcome Sanger Institute for the COVID-19 Genomics UK (COG-UK) Consortium	The Lighthouse Lab in Milton Keynes and Alex Alderton, Roberto Amato, Sonia Goncalves, Ewan Harrison, David K. Jackson, Ian Johnston, Dominic Kwiatkowski, Cordelia Langford, John Sillitoe on behalf of the Wellcome Sanger Institute COVID-19 Surveillance Team (http://www.sanger.ac.uk/covid-team)
EPI_ISL_987587, EPI_ISL_987589, EPI_ISL_987591	Lighthouse Lab in Cambridge	Wellcome Sanger Institute for the COVID-19 Genomics UK (COG-UK) Consortium	Rob Howes, The Lighthouse Lab in Cambridge and Alex Alderton, Roberto Amato, Sonia Goncalves, Ewan Harrison, David K. Jackson, Ian Johnston, Dominic Kwiatkowski, Cordelia Langford, John Sillitoe on behalf of the Wellcome Sanger Institute COVID-19 Surveillance Team (http://www.sanger.ac.uk/covid-team)
EPI_ISL_987593	Lighthouse Lab in Milton Keynes	Wellcome Sanger Institute for the COVID-19 Genomics UK (COG-UK) Consortium	The Lighthouse Lab in Milton Keynes and Alex Alderton, Roberto Amato, Sonia Goncalves, Ewan Harrison, David K. Jackson, Ian Johnston, Dominic Kwiatkowski, Cordelia Langford, John Sillitoe on behalf of the Wellcome Sanger Institute COVID-19 Surveillance Team (http://www.sanger.ac.uk/covid-team)
EPI_ISL_987594, EPI_ISL_987597, EPI_ISL_987598, EPI_ISL_987602, EPI_ISL_987609, EPI_ISL_987610, EPI_ISL_987612	Lighthouse Lab in Cambridge	Wellcome Sanger Institute for the COVID-19 Genomics UK (COG-UK) Consortium	Rob Howes, The Lighthouse Lab in Cambridge and Alex Alderton, Roberto Amato, Sonia Goncalves, Ewan Harrison, David K. Jackson, Ian Johnston, Dominic Kwiatkowski, Cordelia Langford, John Sillitoe on behalf of the Wellcome Sanger Institute COVID-19 Surveillance Team (http://www.sanger.ac.uk/covid-team)
EPI_ISL_987614	Lighthouse Lab in Glasgow	Wellcome Sanger Institute for the COVID-19 Genomics UK (COG-UK) Consortium	Harper VanSteenhouse, Yumi Kasai, David Gray, Carol Clugston, Anna Dominiczak and Alex Alderton, Roberto Amato, Sonia Goncalves, Ewan Harrison, David K. Jackson, Ian Johnston, Dominic Kwiatkowski, Cordelia Langford, John Sillitoe on behalf of the Wellcome Sanger Institute COVID-19 Surveillance Team (http://www.sanger.ac.uk/covid-team)
EPI_ISL_987622, EPI_ISL_987624, EPI_ISL_987625, EPI_ISL_987627, EPI_ISL_987628, EPI_ISL_987631, EPI_ISL_987633	Lighthouse Lab in Cambridge	Wellcome Sanger Institute for the COVID-19 Genomics UK (COG-UK) Consortium	Rob Howes, The Lighthouse Lab in Cambridge and Alex Alderton, Roberto Amato, Sonia Goncalves, Ewan Harrison, David K. Jackson, Ian Johnston, Dominic Kwiatkowski, Cordelia Langford, John Sillitoe on behalf of the Wellcome Sanger Institute COVID-19 Surveillance Team (http://www.sanger.ac.uk/covid-team)
EPI_ISL_987636	Lighthouse Lab in Milton Keynes	Wellcome Sanger Institute for the COVID-19 Genomics UK (COG-UK) Consortium	The Lighthouse Lab in Milton Keynes and Alex Alderton, Roberto Amato, Sonia Goncalves, Ewan Harrison, David K. Jackson, Ian Johnston, Dominic Kwiatkowski, Cordelia Langford, John Sillitoe on behalf of the Wellcome Sanger Institute COVID-19 Surveillance Team (http://www.sanger.ac.uk/covid-team)
EPI_ISL_987637	Lighthouse Lab in Cambridge	Wellcome Sanger Institute for the COVID-19 Genomics UK	Rob Howes, The Lighthouse Lab in Cambridge and Alex Alderton, Roberto Amato, Sonia Goncalves, Ewan Harrison, David K. Jackson, Ian Johnston,

		(COG-UK) Consortium	Dominic Kwiatkowski, Cordelia Langford, John Sillitoe on behalf of the Wellcome Sanger Institute COVID-19 Surveillance Team (http://www.sanger.ac.uk/covid-team)
EPI_ISL_991916, EPI_ISL_991917, EPI_ISL_991918, EPI_ISL_991919, EPI_ISL_991920, EPI_ISL_991921, EPI_ISL_991922, EPI_ISL_991923, EPI_ISL_991924, EPI_ISL_991925, EPI_ISL_991926, EPI_ISL_991927, EPI_ISL_991928, EPI_ISL_991929, EPI_ISL_991930, EPI_ISL_991931, EPI_ISL_991932, EPI_ISL_991933, EPI_ISL_991934, EPI_ISL_991935, EPI_ISL_991936, EPI_ISL_991937, EPI_ISL_991938, EPI_ISL_991939, EPI_ISL_991940, EPI_ISL_991941, EPI_ISL_991942, EPI_ISL_991943, EPI_ISL_991944, EPI_ISL_991945, EPI_ISL_991946, EPI_ISL_991947, EPI_ISL_991948, EPI_ISL_991949, EPI_ISL_991950, EPI_ISL_991951, EPI_ISL_991952, EPI_ISL_991953, EPI_ISL_991954, EPI_ISL_991955, EPI_ISL_991956, EPI_ISL_991957, EPI_ISL_991958, EPI_ISL_991959, EPI_ISL_991960, EPI_ISL_991961, EPI_ISL_991962, EPI_ISL_991963			
see above	Lighthouse Lab in Alderley Park	Wellcome Sanger Institute for the COVID-19 Genomics UK (COG-UK) Consortium	Jacquelyn Wynn, Mairead Hyland, The Lighthouse Lab in Alderley Park and Alex Alderton, Roberto Amato, Sonia Goncalves, Ewan Harrison, David K. Jackson, Ian Johnston, Dominic Kwiatkowski, Cordelia Langford, John Sillitoe on behalf of the Wellcome Sanger Institute COVID-19 Surveillance Team
EPI_ISL_992647	Lighthouse Lab in Alderley Park	Wellcome Sanger Institute for the COVID-19 Genomics UK (COG-UK) Consortium	Jacquelyn Wynn, Mairead Hyland, The Lighthouse Lab in Alderley Park and Alex Alderton, Roberto Amato, Sonia Goncalves, Ewan Harrison, David K. Jackson, Ian Johnston, Dominic Kwiatkowski, Cordelia Langford, John Sillitoe on behalf of the Wellcome Sanger Institute COVID-19 Surveillance Team (http://www.sanger.ac.uk/covid-team)
EPI_ISL_993085, EPI_ISL_993087, EPI_ISL_993088, EPI_ISL_993090, EPI_ISL_993092, EPI_ISL_993093, EPI_ISL_993097, EPI_ISL_993102, EPI_ISL_993103, EPI_ISL_993107	Lighthouse Lab in Milton Keynes	Wellcome Sanger Institute for the COVID-19 Genomics UK (COG-UK) Consortium	The Lighthouse Lab in Milton Keynes and Alex Alderton, Roberto Amato, Sonia Goncalves, Ewan Harrison, David K. Jackson, Ian Johnston, Dominic Kwiatkowski, Cordelia Langford, John Sillitoe on behalf of the Wellcome Sanger Institute COVID-19 Surveillance Team (http://www.sanger.ac.uk/covid-team)
EPI_ISL_993111	Lighthouse Lab in Alderley Park	Wellcome Sanger Institute for the COVID-19 Genomics UK (COG-UK) Consortium	Jacquelyn Wynn, Mairead Hyland, The Lighthouse Lab in Alderley Park and Alex Alderton, Roberto Amato, Sonia Goncalves, Ewan Harrison, David K. Jackson, Ian Johnston, Dominic Kwiatkowski, Cordelia Langford, John Sillitoe on behalf of the Wellcome Sanger Institute COVID-19 Surveillance Team (http://www.sanger.ac.uk/covid-team)
EPI_ISL_993115, EPI_ISL_993116, EPI_ISL_993120, EPI_ISL_993121, EPI_ISL_993124, EPI_ISL_993126, EPI_ISL_993130, EPI_ISL_993139, EPI_ISL_993142, EPI_ISL_993144, EPI_ISL_993145, EPI_ISL_993147, EPI_ISL_993148			
see above	Lighthouse Lab in Milton Keynes	Wellcome Sanger Institute for the COVID-19 Genomics UK (COG-UK) Consortium	The Lighthouse Lab in Milton Keynes and Alex Alderton, Roberto Amato, Sonia Goncalves, Ewan Harrison, David K. Jackson, Ian Johnston, Dominic Kwiatkowski, Cordelia Langford, John Sillitoe on behalf of the Wellcome Sanger Institute COVID-19 Surveillance Team (http://www.sanger.ac.uk/covid-team)
EPI_ISL_993149	Lighthouse Lab in Alderley Park	Wellcome Sanger Institute for the COVID-19 Genomics UK (COG-UK) Consortium	Jacquelyn Wynn, Mairead Hyland, The Lighthouse Lab in Alderley Park and Alex Alderton, Roberto Amato, Sonia Goncalves, Ewan Harrison, David K. Jackson, Ian Johnston, Dominic Kwiatkowski, Cordelia Langford, John Sillitoe on behalf of the Wellcome Sanger Institute COVID-19 Surveillance Team (http://www.sanger.ac.uk/covid-team)
EPI_ISL_993150, EPI_ISL_993151, EPI_ISL_993156, EPI_ISL_993159, EPI_ISL_993167, EPI_ISL_993170, EPI_ISL_993172, EPI_ISL_993173, EPI_ISL_993175, EPI_ISL_993179, EPI_ISL_993181, EPI_ISL_993186, EPI_ISL_993188, EPI_ISL_993191, EPI_ISL_993193, EPI_ISL_993195			
see above	Lighthouse Lab in Milton Keynes	Wellcome Sanger Institute for the COVID-19 Genomics UK (COG-UK) Consortium	The Lighthouse Lab in Milton Keynes and Alex Alderton, Roberto Amato, Sonia Goncalves, Ewan Harrison, David K. Jackson, Ian Johnston, Dominic Kwiatkowski, Cordelia Langford, John Sillitoe on behalf of the Wellcome Sanger Institute COVID-19 Surveillance Team (http://www.sanger.ac.uk/covid-team)
EPI_ISL_993198	Lighthouse Lab in Alderley Park	Wellcome Sanger Institute for the COVID-19 Genomics UK (COG-UK) Consortium	Jacquelyn Wynn, Mairead Hyland, The Lighthouse Lab in Alderley Park and Alex Alderton, Roberto Amato, Sonia Goncalves, Ewan Harrison, David K. Jackson, Ian Johnston, Dominic Kwiatkowski, Cordelia Langford, John Sillitoe on behalf of the Wellcome Sanger Institute COVID-19 Surveillance Team (http://www.sanger.ac.uk/covid-team)
EPI_ISL_993200, EPI_ISL_993204, EPI_ISL_993210, EPI_ISL_993214, EPI_ISL_993215, EPI_ISL_993218, EPI_ISL_993220, EPI_ISL_993226, EPI_ISL_993228, EPI_ISL_993229, EPI_ISL_993230			
see above	Lighthouse Lab in Milton Keynes	Wellcome Sanger Institute for the COVID-19 Genomics UK (COG-UK) Consortium	The Lighthouse Lab in Milton Keynes and Alex Alderton, Roberto Amato, Sonia Goncalves, Ewan Harrison, David K. Jackson, Ian Johnston, Dominic Kwiatkowski, Cordelia Langford, John Sillitoe on behalf of the Wellcome Sanger Institute COVID-19 Surveillance Team (http://www.sanger.ac.uk/covid-team)
EPI_ISL_993239	Lighthouse Lab in Alderley Park	Wellcome Sanger Institute for the COVID-19 Genomics UK (COG-UK) Consortium	Jacquelyn Wynn, Mairead Hyland, The Lighthouse Lab in Alderley Park and Alex Alderton, Roberto Amato, Sonia Goncalves, Ewan Harrison, David K. Jackson, Ian Johnston, Dominic Kwiatkowski, Cordelia Langford, John Sillitoe on behalf of the Wellcome Sanger Institute COVID-19 Surveillance Team (http://www.sanger.ac.uk/covid-team)
EPI_ISL_993243, EPI_ISL_993246, EPI_ISL_993250, EPI_ISL_993253, EPI_ISL_993254, EPI_ISL_993256, EPI_ISL_993259, EPI_ISL_993260, EPI_ISL_993262, EPI_ISL_993265, EPI_ISL_993267, EPI_ISL_993268, EPI_ISL_993269, EPI_ISL_993271, EPI_ISL_993272, EPI_ISL_993274, EPI_ISL_993276, EPI_ISL_993278, EPI_ISL_993281, EPI_ISL_993284, EPI_ISL_993286, EPI_ISL_993288, EPI_ISL_993289, EPI_ISL_993290, EPI_ISL_993295, EPI_ISL_993298, EPI_ISL_993299, EPI_ISL_993303, EPI_ISL_993304, EPI_ISL_993307, EPI_ISL_993308, EPI_ISL_993314, EPI_ISL_993318, EPI_ISL_993319, EPI_ISL_993322, EPI_ISL_993323, EPI_ISL_993330, EPI_ISL_993332, EPI_ISL_993333, EPI_ISL_993339, EPI_ISL_993347, EPI_ISL_993349, EPI_ISL_993355, EPI_ISL_993356, EPI_ISL_993357, EPI_ISL_993358, EPI_ISL_993359			
see above	Lighthouse Lab in Milton Keynes	Wellcome Sanger Institute for the COVID-19 Genomics UK (COG-UK) Consortium	The Lighthouse Lab in Milton Keynes and Alex Alderton, Roberto Amato, Sonia Goncalves, Ewan Harrison, David K. Jackson, Ian Johnston, Dominic Kwiatkowski, Cordelia Langford, John Sillitoe on behalf of the Wellcome Sanger Institute COVID-19 Surveillance Team (http://www.sanger.ac.uk/covid-team)
EPI_ISL_993362	Lighthouse Lab in Alderley Park	Wellcome Sanger Institute for the COVID-19 Genomics UK (COG-UK) Consortium	Jacquelyn Wynn, Mairead Hyland, The Lighthouse Lab in Alderley Park and Alex Alderton, Roberto Amato, Sonia Goncalves, Ewan Harrison, David K. Jackson, Ian Johnston, Dominic Kwiatkowski, Cordelia Langford, John Sillitoe on behalf of the Wellcome Sanger Institute COVID-19 Surveillance Team (http://www.sanger.ac.uk/covid-team)
EPI_ISL_993366, EPI_ISL_993372, EPI_ISL_993373, EPI_ISL_993375, EPI_ISL_993376, EPI_ISL_993380, EPI_ISL_993382, EPI_ISL_993387	Lighthouse Lab in Milton Keynes	Wellcome Sanger Institute for the COVID-19 Genomics UK (COG-UK) Consortium	The Lighthouse Lab in Milton Keynes and Alex Alderton, Roberto Amato, Sonia Goncalves, Ewan Harrison, David K. Jackson, Ian Johnston, Dominic Kwiatkowski, Cordelia Langford, John Sillitoe on behalf of the Wellcome Sanger Institute COVID-19 Surveillance Team (http://www.sanger.ac.uk/covid-team)
EPI_ISL_993401	Lighthouse Lab in Alderley Park	Wellcome Sanger Institute for the COVID-19 Genomics UK (COG-UK) Consortium	Jacquelyn Wynn, Mairead Hyland, The Lighthouse Lab in Alderley Park and Alex Alderton, Roberto Amato, Sonia Goncalves, Ewan Harrison, David K. Jackson, Ian Johnston, Dominic Kwiatkowski, Cordelia Langford, John Sillitoe on behalf of the Wellcome Sanger Institute COVID-19 Surveillance Team (http://www.sanger.ac.uk/covid-team)
EPI_ISL_993428, EPI_ISL_993429, EPI_ISL_993445, EPI_ISL_993460, EPI_ISL_993470, EPI_ISL_993502, EPI_ISL_993525, EPI_ISL_993559, EPI_ISL_993569, EPI_ISL_993577, EPI_ISL_993587, EPI_ISL_993602, EPI_ISL_993615, EPI_ISL_993618, EPI_ISL_993624, EPI_ISL_993625, EPI_ISL_993648, EPI_ISL_993649, EPI_ISL_993662, EPI_ISL_993666, EPI_ISL_993672, EPI_ISL_993675, EPI_ISL_993702, EPI_ISL_993703, EPI_ISL_993733			
see above	Lighthouse Lab in Milton Keynes	Wellcome Sanger Institute for the COVID-19 Genomics UK (COG-UK) Consortium	The Lighthouse Lab in Milton Keynes and Alex Alderton, Roberto Amato, Sonia Goncalves, Ewan Harrison, David K. Jackson, Ian Johnston, Dominic Kwiatkowski, Cordelia Langford, John Sillitoe on behalf of the Wellcome Sanger Institute COVID-19 Surveillance Team
EPI_ISL_993736	Lighthouse Lab in Cambridge	Wellcome Sanger Institute for the COVID-19 Genomics UK (COG-UK) Consortium	Rob Howes, The Lighthouse Lab in Cambridge and Alex Alderton, Roberto Amato, Sonia Goncalves, Ewan Harrison, David K. Jackson, Ian Johnston, Dominic Kwiatkowski, Cordelia Langford, John Sillitoe on behalf of the Wellcome Sanger Institute COVID-19 Surveillance Team
EPI_ISL_993737	Lighthouse Lab in Milton Keynes	Wellcome Sanger Institute for the COVID-19 Genomics UK (COG-UK) Consortium	The Lighthouse Lab in Milton Keynes and Alex Alderton, Roberto Amato, Sonia Goncalves, Ewan Harrison, David K. Jackson, Ian Johnston, Dominic Kwiatkowski, Cordelia Langford, John Sillitoe on behalf of the Wellcome Sanger Institute COVID-19 Surveillance Team
EPI_ISL_993738, EPI_ISL_993739, EPI_ISL_993740, EPI_ISL_993741, EPI_ISL_993742, EPI_ISL_993744	Lighthouse Lab in Cambridge	Wellcome Sanger Institute for the COVID-19 Genomics UK (COG-UK) Consortium	Rob Howes, The Lighthouse Lab in Cambridge and Alex Alderton, Roberto Amato, Sonia Goncalves, Ewan Harrison, David K. Jackson, Ian Johnston, Dominic Kwiatkowski, Cordelia Langford, John Sillitoe on behalf of the Wellcome Sanger Institute COVID-19 Surveillance Team
EPI_ISL_993745	Lighthouse Lab in Milton Keynes	Wellcome Sanger Institute for the COVID-19 Genomics UK (COG-UK) Consortium	The Lighthouse Lab in Milton Keynes and Alex Alderton, Roberto Amato, Sonia Goncalves, Ewan Harrison, David K. Jackson, Ian Johnston, Dominic Kwiatkowski, Cordelia Langford, John Sillitoe on behalf of the Wellcome Sanger Institute COVID-19 Surveillance Team
EPI_ISL_993746, EPI_ISL_993747	Lighthouse Lab in Cambridge	Wellcome Sanger Institute for the COVID-19 Genomics UK (COG-UK) Consortium	Rob Howes, The Lighthouse Lab in Cambridge and Alex Alderton, Roberto Amato, Sonia Goncalves, Ewan Harrison, David K. Jackson, Ian Johnston, Dominic Kwiatkowski, Cordelia Langford, John Sillitoe on behalf of the Wellcome Sanger Institute COVID-19 Surveillance Team
EPI_ISL_993748, EPI_ISL_993749	Lighthouse Lab in Milton Keynes	Wellcome Sanger Institute for the COVID-19 Genomics UK (COG-UK) Consortium	The Lighthouse Lab in Milton Keynes and Alex Alderton, Roberto Amato, Sonia Goncalves, Ewan Harrison, David K. Jackson, Ian Johnston, Dominic Kwiatkowski, Cordelia Langford, John Sillitoe on behalf of the Wellcome Sanger Institute COVID-19 Surveillance Team
EPI_ISL_993750	Lighthouse Lab in Cambridge	Wellcome Sanger Institute for the COVID-19 Genomics UK	Rob Howes, The Lighthouse Lab in Cambridge and Alex Alderton, Roberto Amato, Sonia Goncalves, Ewan Harrison, David K. Jackson, Ian Johnston,

		(COG-UK) Consortium	Dominic Kwiatkowski, Cordelia Langford, John Sillitoe on behalf of the Wellcome Sanger Institute COVID-19 Surveillance Team
EPI_ISL_993751, EPI_ISL_993752, EPI_ISL_993753	Lighthouse Lab in Milton Keynes	Wellcome Sanger Institute for the COVID-19 Genomics UK (COG-UK) Consortium	The Lighthouse Lab in Milton Keynes and Alex Alderton, Roberto Amato, Sonia Goncalves, Ewan Harrison, David K. Jackson, Ian Johnston, Dominic Kwiatkowski, Cordelia Langford, John Sillitoe on behalf of the Wellcome Sanger Institute COVID-19 Surveillance Team
EPI_ISL_993754	Lighthouse Lab in Cambridge	Wellcome Sanger Institute for the COVID-19 Genomics UK (COG-UK) Consortium	Rob Howes, The Lighthouse Lab in Cambridge and Alex Alderton, Roberto Amato, Sonia Goncalves, Ewan Harrison, David K. Jackson, Ian Johnston, Dominic Kwiatkowski, Cordelia Langford, John Sillitoe on behalf of the Wellcome Sanger Institute COVID-19 Surveillance Team
EPI_ISL_993755	Lighthouse Lab in Milton Keynes	Wellcome Sanger Institute for the COVID-19 Genomics UK (COG-UK) Consortium	The Lighthouse Lab in Milton Keynes and Alex Alderton, Roberto Amato, Sonia Goncalves, Ewan Harrison, David K. Jackson, Ian Johnston, Dominic Kwiatkowski, Cordelia Langford, John Sillitoe on behalf of the Wellcome Sanger Institute COVID-19 Surveillance Team
EPI_ISL_993756, EPI_ISL_993757, EPI_ISL_993758	Lighthouse Lab in Cambridge	Wellcome Sanger Institute for the COVID-19 Genomics UK (COG-UK) Consortium	Rob Howes, The Lighthouse Lab in Cambridge and Alex Alderton, Roberto Amato, Sonia Goncalves, Ewan Harrison, David K. Jackson, Ian Johnston, Dominic Kwiatkowski, Cordelia Langford, John Sillitoe on behalf of the Wellcome Sanger Institute COVID-19 Surveillance Team
EPI_ISL_993759, EPI_ISL_993760	Lighthouse Lab in Milton Keynes	Wellcome Sanger Institute for the COVID-19 Genomics UK (COG-UK) Consortium	The Lighthouse Lab in Milton Keynes and Alex Alderton, Roberto Amato, Sonia Goncalves, Ewan Harrison, David K. Jackson, Ian Johnston, Dominic Kwiatkowski, Cordelia Langford, John Sillitoe on behalf of the Wellcome Sanger Institute COVID-19 Surveillance Team
EPI_ISL_993761, EPI_ISL_993762	Lighthouse Lab in Cambridge	Wellcome Sanger Institute for the COVID-19 Genomics UK (COG-UK) Consortium	Rob Howes, The Lighthouse Lab in Cambridge and Alex Alderton, Roberto Amato, Sonia Goncalves, Ewan Harrison, David K. Jackson, Ian Johnston, Dominic Kwiatkowski, Cordelia Langford, John Sillitoe on behalf of the Wellcome Sanger Institute COVID-19 Surveillance Team
EPI_ISL_993763, EPI_ISL_993764, EPI_ISL_993765	Lighthouse Lab in Milton Keynes	Wellcome Sanger Institute for the COVID-19 Genomics UK (COG-UK) Consortium	The Lighthouse Lab in Milton Keynes and Alex Alderton, Roberto Amato, Sonia Goncalves, Ewan Harrison, David K. Jackson, Ian Johnston, Dominic Kwiatkowski, Cordelia Langford, John Sillitoe on behalf of the Wellcome Sanger Institute COVID-19 Surveillance Team
EPI_ISL_993766	Lighthouse Lab in Cambridge	Wellcome Sanger Institute for the COVID-19 Genomics UK (COG-UK) Consortium	Rob Howes, The Lighthouse Lab in Cambridge and Alex Alderton, Roberto Amato, Sonia Goncalves, Ewan Harrison, David K. Jackson, Ian Johnston, Dominic Kwiatkowski, Cordelia Langford, John Sillitoe on behalf of the Wellcome Sanger Institute COVID-19 Surveillance Team
EPI_ISL_993767, EPI_ISL_993768, EPI_ISL_993769	Lighthouse Lab in Milton Keynes	Wellcome Sanger Institute for the COVID-19 Genomics UK (COG-UK) Consortium	The Lighthouse Lab in Milton Keynes and Alex Alderton, Roberto Amato, Sonia Goncalves, Ewan Harrison, David K. Jackson, Ian Johnston, Dominic Kwiatkowski, Cordelia Langford, John Sillitoe on behalf of the Wellcome Sanger Institute COVID-19 Surveillance Team
EPI_ISL_993770, EPI_ISL_993771	Lighthouse Lab in Cambridge	Wellcome Sanger Institute for the COVID-19 Genomics UK (COG-UK) Consortium	Rob Howes, The Lighthouse Lab in Cambridge and Alex Alderton, Roberto Amato, Sonia Goncalves, Ewan Harrison, David K. Jackson, Ian Johnston, Dominic Kwiatkowski, Cordelia Langford, John Sillitoe on behalf of the Wellcome Sanger Institute COVID-19 Surveillance Team
EPI_ISL_993772, EPI_ISL_993773	Lighthouse Lab in Milton Keynes	Wellcome Sanger Institute for the COVID-19 Genomics UK (COG-UK) Consortium	The Lighthouse Lab in Milton Keynes and Alex Alderton, Roberto Amato, Sonia Goncalves, Ewan Harrison, David K. Jackson, Ian Johnston, Dominic Kwiatkowski, Cordelia Langford, John Sillitoe on behalf of the Wellcome Sanger Institute COVID-19 Surveillance Team
EPI_ISL_993774	Lighthouse Lab in Cambridge	Wellcome Sanger Institute for the COVID-19 Genomics UK (COG-UK) Consortium	Rob Howes, The Lighthouse Lab in Cambridge and Alex Alderton, Roberto Amato, Sonia Goncalves, Ewan Harrison, David K. Jackson, Ian Johnston, Dominic Kwiatkowski, Cordelia Langford, John Sillitoe on behalf of the Wellcome Sanger Institute COVID-19 Surveillance Team
EPI_ISL_993775	Lighthouse Lab in Milton Keynes	Wellcome Sanger Institute for the COVID-19 Genomics UK (COG-UK) Consortium	The Lighthouse Lab in Milton Keynes and Alex Alderton, Roberto Amato, Sonia Goncalves, Ewan Harrison, David K. Jackson, Ian Johnston, Dominic Kwiatkowski, Cordelia Langford, John Sillitoe on behalf of the Wellcome Sanger Institute COVID-19 Surveillance Team
EPI_ISL_993776	Lighthouse Lab in Cambridge	Wellcome Sanger Institute for the COVID-19 Genomics UK (COG-UK) Consortium	Rob Howes, The Lighthouse Lab in Cambridge and Alex Alderton, Roberto Amato, Sonia Goncalves, Ewan Harrison, David K. Jackson, Ian Johnston, Dominic Kwiatkowski, Cordelia Langford, John Sillitoe on behalf of the Wellcome Sanger Institute COVID-19 Surveillance Team
EPI_ISL_993777	Lighthouse Lab in Milton Keynes	Wellcome Sanger Institute for the COVID-19 Genomics UK (COG-UK) Consortium	The Lighthouse Lab in Milton Keynes and Alex Alderton, Roberto Amato, Sonia Goncalves, Ewan Harrison, David K. Jackson, Ian Johnston, Dominic Kwiatkowski, Cordelia Langford, John Sillitoe on behalf of the Wellcome Sanger Institute COVID-19 Surveillance Team
EPI_ISL_993778, EPI_ISL_993779	Lighthouse Lab in Cambridge	Wellcome Sanger Institute for the COVID-19 Genomics UK (COG-UK) Consortium	Rob Howes, The Lighthouse Lab in Cambridge and Alex Alderton, Roberto Amato, Sonia Goncalves, Ewan Harrison, David K. Jackson, Ian Johnston, Dominic Kwiatkowski, Cordelia Langford, John Sillitoe on behalf of the Wellcome Sanger Institute COVID-19 Surveillance Team
EPI_ISL_993780, EPI_ISL_993781	Lighthouse Lab in Milton Keynes	Wellcome Sanger Institute for the COVID-19 Genomics UK (COG-UK) Consortium	The Lighthouse Lab in Milton Keynes and Alex Alderton, Roberto Amato, Sonia Goncalves, Ewan Harrison, David K. Jackson, Ian Johnston, Dominic Kwiatkowski, Cordelia Langford, John Sillitoe on behalf of the Wellcome Sanger Institute COVID-19 Surveillance Team
EPI_ISL_993782	Lighthouse Lab in Cambridge	Wellcome Sanger Institute for the COVID-19 Genomics UK (COG-UK) Consortium	Rob Howes, The Lighthouse Lab in Cambridge and Alex Alderton, Roberto Amato, Sonia Goncalves, Ewan Harrison, David K. Jackson, Ian Johnston, Dominic Kwiatkowski, Cordelia Langford, John Sillitoe on behalf of the Wellcome Sanger Institute COVID-19 Surveillance Team
EPI_ISL_993783	Lighthouse Lab in Milton Keynes	Wellcome Sanger Institute for the COVID-19 Genomics UK (COG-UK) Consortium	The Lighthouse Lab in Milton Keynes and Alex Alderton, Roberto Amato, Sonia Goncalves, Ewan Harrison, David K. Jackson, Ian Johnston, Dominic Kwiatkowski, Cordelia Langford, John Sillitoe on behalf of the Wellcome Sanger Institute COVID-19 Surveillance Team
EPI_ISL_993784	Lighthouse Lab in Cambridge	Wellcome Sanger Institute for the COVID-19 Genomics UK (COG-UK) Consortium	Rob Howes, The Lighthouse Lab in Cambridge and Alex Alderton, Roberto Amato, Sonia Goncalves, Ewan Harrison, David K. Jackson, Ian Johnston, Dominic Kwiatkowski, Cordelia Langford, John Sillitoe on behalf of the Wellcome Sanger Institute COVID-19 Surveillance Team
EPI_ISL_993785, EPI_ISL_993786, EPI_ISL_993787, EPI_ISL_993788, EPI_ISL_993789, EPI_ISL_993790	Lighthouse Lab in Milton Keynes	Wellcome Sanger Institute for the COVID-19 Genomics UK (COG-UK) Consortium	The Lighthouse Lab in Milton Keynes and Alex Alderton, Roberto Amato, Sonia Goncalves, Ewan Harrison, David K. Jackson, Ian Johnston, Dominic Kwiatkowski, Cordelia Langford, John Sillitoe on behalf of the Wellcome Sanger Institute COVID-19 Surveillance Team
EPI_ISL_993791, EPI_ISL_993792, EPI_ISL_993794	Lighthouse Lab in Cambridge	Wellcome Sanger Institute for the COVID-19 Genomics UK (COG-UK) Consortium	Rob Howes, The Lighthouse Lab in Cambridge and Alex Alderton, Roberto Amato, Sonia Goncalves, Ewan Harrison, David K. Jackson, Ian Johnston, Dominic Kwiatkowski, Cordelia Langford, John Sillitoe on behalf of the Wellcome Sanger Institute COVID-19 Surveillance Team
EPI_ISL_993795, EPI_ISL_993796	Lighthouse Lab in Milton Keynes	Wellcome Sanger Institute for the COVID-19 Genomics UK (COG-UK) Consortium	The Lighthouse Lab in Milton Keynes and Alex Alderton, Roberto Amato, Sonia Goncalves, Ewan Harrison, David K. Jackson, Ian Johnston, Dominic Kwiatkowski, Cordelia Langford, John Sillitoe on behalf of the Wellcome Sanger Institute COVID-19 Surveillance Team
EPI_ISL_993797, EPI_ISL_993798	Lighthouse Lab in Cambridge	Wellcome Sanger Institute for the COVID-19 Genomics UK (COG-UK) Consortium	Rob Howes, The Lighthouse Lab in Cambridge and Alex Alderton, Roberto Amato, Sonia Goncalves, Ewan Harrison, David K. Jackson, Ian Johnston, Dominic Kwiatkowski, Cordelia Langford, John Sillitoe on behalf of the Wellcome Sanger Institute COVID-19 Surveillance Team
EPI_ISL_993799, EPI_ISL_993800, EPI_ISL_993801	Lighthouse Lab in Milton Keynes	Wellcome Sanger Institute for the COVID-19 Genomics UK (COG-UK) Consortium	The Lighthouse Lab in Milton Keynes and Alex Alderton, Roberto Amato, Sonia Goncalves, Ewan Harrison, David K. Jackson, Ian Johnston, Dominic Kwiatkowski, Cordelia Langford, John Sillitoe on behalf of the Wellcome Sanger Institute COVID-19 Surveillance Team
EPI_ISL_993802, EPI_ISL_993803, EPI_ISL_993804	Lighthouse Lab in Cambridge	Wellcome Sanger Institute for the COVID-19 Genomics UK (COG-UK) Consortium	Rob Howes, The Lighthouse Lab in Cambridge and Alex Alderton, Roberto Amato, Sonia Goncalves, Ewan Harrison, David K. Jackson, Ian Johnston, Dominic Kwiatkowski, Cordelia Langford, John Sillitoe on behalf of the Wellcome Sanger Institute COVID-19 Surveillance Team
EPI_ISL_993806	Lighthouse Lab in Milton Keynes	Wellcome Sanger Institute for the COVID-19 Genomics UK (COG-UK) Consortium	The Lighthouse Lab in Milton Keynes and Alex Alderton, Roberto Amato, Sonia Goncalves, Ewan Harrison, David K. Jackson, Ian Johnston, Dominic Kwiatkowski, Cordelia Langford, John Sillitoe on behalf of the Wellcome Sanger Institute COVID-19 Surveillance Team
EPI_ISL_993807, EPI_ISL_993808, EPI_ISL_993809, EPI_ISL_993810, EPI_ISL_993811	Lighthouse Lab in Cambridge	Wellcome Sanger Institute for the COVID-19 Genomics UK (COG-UK) Consortium	Rob Howes, The Lighthouse Lab in Cambridge and Alex Alderton, Roberto Amato, Sonia Goncalves, Ewan Harrison, David K. Jackson, Ian Johnston, Dominic Kwiatkowski, Cordelia Langford, John Sillitoe on behalf of the Wellcome Sanger Institute COVID-19 Surveillance Team
EPI_ISL_993812	Lighthouse Lab in Milton Keynes	Wellcome Sanger Institute for the COVID-19 Genomics UK (COG-UK) Consortium	The Lighthouse Lab in Milton Keynes and Alex Alderton, Roberto Amato, Sonia Goncalves, Ewan Harrison, David K. Jackson, Ian Johnston, Dominic Kwiatkowski, Cordelia Langford, John Sillitoe on behalf of the Wellcome Sanger Institute COVID-19 Surveillance Team
EPI_ISL_993813, EPI_ISL_993814	Lighthouse Lab in Cambridge	Wellcome Sanger Institute for the COVID-19 Genomics UK (COG-UK) Consortium	Rob Howes, The Lighthouse Lab in Cambridge and Alex Alderton, Roberto Amato, Sonia Goncalves, Ewan Harrison, David K. Jackson, Ian Johnston, Dominic Kwiatkowski, Cordelia Langford, John Sillitoe on behalf of the Wellcome Sanger Institute COVID-19 Surveillance Team
EPI_ISL_993815	Lighthouse Lab in Milton Keynes	Wellcome Sanger Institute for the COVID-19 Genomics UK (COG-UK) Consortium	The Lighthouse Lab in Milton Keynes and Alex Alderton, Roberto Amato, Sonia Goncalves, Ewan Harrison, David K. Jackson, Ian Johnston, Dominic Kwiatkowski, Cordelia Langford, John Sillitoe on behalf of the Wellcome Sanger Institute COVID-19 Surveillance Team
EPI_ISL_993816	Lighthouse Lab in Cambridge	Wellcome Sanger Institute for the COVID-19 Genomics UK (COG-UK) Consortium	Rob Howes, The Lighthouse Lab in Cambridge and Alex Alderton, Roberto Amato, Sonia Goncalves, Ewan Harrison, David K. Jackson, Ian Johnston, Dominic Kwiatkowski, Cordelia Langford, John Sillitoe on behalf of the Wellcome Sanger Institute COVID-19 Surveillance Team
EPI_ISL_993818	Lighthouse Lab in Milton Keynes	Wellcome Sanger Institute for the COVID-19 Genomics UK (COG-UK) Consortium	The Lighthouse Lab in Milton Keynes and Alex Alderton, Roberto Amato, Sonia Goncalves, Ewan Harrison, David K. Jackson, Ian Johnston, Dominic Kwiatkowski, Cordelia Langford, John Sillitoe on behalf of the Wellcome Sanger Institute COVID-19 Surveillance Team
EPI_ISL_993819	Lighthouse Lab in Cambridge	Wellcome Sanger Institute for the COVID-19 Genomics UK (COG-UK) Consortium	Rob Howes, The Lighthouse Lab in Cambridge and Alex Alderton, Roberto Amato, Sonia Goncalves, Ewan Harrison, David K. Jackson, Ian Johnston, Dominic Kwiatkowski, Cordelia Langford, John Sillitoe on behalf of the Wellcome Sanger Institute COVID-19 Surveillance Team

EPI_ISL_993820	Lighthouse Lab in Milton Keynes	Wellcome Sanger Institute for the COVID-19 Genomics UK (COG-UK) Consortium	The Lighthouse Lab in Milton Keynes and Alex Alderton, Roberto Amato, Sonia Goncalves, Ewan Harrison, David K. Jackson, Ian Johnston, Dominic Kwiatkowski, Cordelia Langford, John Sillitoe on behalf of the Wellcome Sanger Institute COVID-19 Surveillance Team
EPI_ISL_993821	Lighthouse Lab in Cambridge	Wellcome Sanger Institute for the COVID-19 Genomics UK (COG-UK) Consortium	Rob Howes, The Lighthouse Lab in Cambridge and Alex Alderton, Roberto Amato, Sonia Goncalves, Ewan Harrison, David K. Jackson, Ian Johnston, Dominic Kwiatkowski, Cordelia Langford, John Sillitoe on behalf of the Wellcome Sanger Institute COVID-19 Surveillance Team
EPI_ISL_993822	Lighthouse Lab in Milton Keynes	Wellcome Sanger Institute for the COVID-19 Genomics UK (COG-UK) Consortium	The Lighthouse Lab in Milton Keynes and Alex Alderton, Roberto Amato, Sonia Goncalves, Ewan Harrison, David K. Jackson, Ian Johnston, Dominic Kwiatkowski, Cordelia Langford, John Sillitoe on behalf of the Wellcome Sanger Institute COVID-19 Surveillance Team
EPI_ISL_993824	Lighthouse Lab in Cambridge	Wellcome Sanger Institute for the COVID-19 Genomics UK (COG-UK) Consortium	Rob Howes, The Lighthouse Lab in Cambridge and Alex Alderton, Roberto Amato, Sonia Goncalves, Ewan Harrison, David K. Jackson, Ian Johnston, Dominic Kwiatkowski, Cordelia Langford, John Sillitoe on behalf of the Wellcome Sanger Institute COVID-19 Surveillance Team
EPI_ISL_993825, EPI_ISL_993826	Lighthouse Lab in Milton Keynes	Wellcome Sanger Institute for the COVID-19 Genomics UK (COG-UK) Consortium	The Lighthouse Lab in Milton Keynes and Alex Alderton, Roberto Amato, Sonia Goncalves, Ewan Harrison, David K. Jackson, Ian Johnston, Dominic Kwiatkowski, Cordelia Langford, John Sillitoe on behalf of the Wellcome Sanger Institute COVID-19 Surveillance Team
EPI_ISL_993827	Lighthouse Lab in Cambridge	Wellcome Sanger Institute for the COVID-19 Genomics UK (COG-UK) Consortium	Rob Howes, The Lighthouse Lab in Cambridge and Alex Alderton, Roberto Amato, Sonia Goncalves, Ewan Harrison, David K. Jackson, Ian Johnston, Dominic Kwiatkowski, Cordelia Langford, John Sillitoe on behalf of the Wellcome Sanger Institute COVID-19 Surveillance Team
EPI_ISL_993828, EPI_ISL_993829	Lighthouse Lab in Milton Keynes	Wellcome Sanger Institute for the COVID-19 Genomics UK (COG-UK) Consortium	The Lighthouse Lab in Milton Keynes and Alex Alderton, Roberto Amato, Sonia Goncalves, Ewan Harrison, David K. Jackson, Ian Johnston, Dominic Kwiatkowski, Cordelia Langford, John Sillitoe on behalf of the Wellcome Sanger Institute COVID-19 Surveillance Team
EPI_ISL_993830	Lighthouse Lab in Cambridge	Wellcome Sanger Institute for the COVID-19 Genomics UK (COG-UK) Consortium	Rob Howes, The Lighthouse Lab in Cambridge and Alex Alderton, Roberto Amato, Sonia Goncalves, Ewan Harrison, David K. Jackson, Ian Johnston, Dominic Kwiatkowski, Cordelia Langford, John Sillitoe on behalf of the Wellcome Sanger Institute COVID-19 Surveillance Team
EPI_ISL_993831	Lighthouse Lab in Milton Keynes	Wellcome Sanger Institute for the COVID-19 Genomics UK (COG-UK) Consortium	The Lighthouse Lab in Milton Keynes and Alex Alderton, Roberto Amato, Sonia Goncalves, Ewan Harrison, David K. Jackson, Ian Johnston, Dominic Kwiatkowski, Cordelia Langford, John Sillitoe on behalf of the Wellcome Sanger Institute COVID-19 Surveillance Team
EPI_ISL_993832	Lighthouse Lab in Cambridge	Wellcome Sanger Institute for the COVID-19 Genomics UK (COG-UK) Consortium	Rob Howes, The Lighthouse Lab in Cambridge and Alex Alderton, Roberto Amato, Sonia Goncalves, Ewan Harrison, David K. Jackson, Ian Johnston, Dominic Kwiatkowski, Cordelia Langford, John Sillitoe on behalf of the Wellcome Sanger Institute COVID-19 Surveillance Team
EPI_ISL_993834, EPI_ISL_993835	Lighthouse Lab in Milton Keynes	Wellcome Sanger Institute for the COVID-19 Genomics UK (COG-UK) Consortium	The Lighthouse Lab in Milton Keynes and Alex Alderton, Roberto Amato, Sonia Goncalves, Ewan Harrison, David K. Jackson, Ian Johnston, Dominic Kwiatkowski, Cordelia Langford, John Sillitoe on behalf of the Wellcome Sanger Institute COVID-19 Surveillance Team
EPI_ISL_993836, EPI_ISL_993837, EPI_ISL_993838	Lighthouse Lab in Cambridge	Wellcome Sanger Institute for the COVID-19 Genomics UK (COG-UK) Consortium	Rob Howes, The Lighthouse Lab in Cambridge and Alex Alderton, Roberto Amato, Sonia Goncalves, Ewan Harrison, David K. Jackson, Ian Johnston, Dominic Kwiatkowski, Cordelia Langford, John Sillitoe on behalf of the Wellcome Sanger Institute COVID-19 Surveillance Team
EPI_ISL_993839, EPI_ISL_993840, EPI_ISL_993841, EPI_ISL_993842	Lighthouse Lab in Milton Keynes	Wellcome Sanger Institute for the COVID-19 Genomics UK (COG-UK) Consortium	The Lighthouse Lab in Milton Keynes and Alex Alderton, Roberto Amato, Sonia Goncalves, Ewan Harrison, David K. Jackson, Ian Johnston, Dominic Kwiatkowski, Cordelia Langford, John Sillitoe on behalf of the Wellcome Sanger Institute COVID-19 Surveillance Team
EPI_ISL_993843	Lighthouse Lab in Cambridge	Wellcome Sanger Institute for the COVID-19 Genomics UK (COG-UK) Consortium	Rob Howes, The Lighthouse Lab in Cambridge and Alex Alderton, Roberto Amato, Sonia Goncalves, Ewan Harrison, David K. Jackson, Ian Johnston, Dominic Kwiatkowski, Cordelia Langford, John Sillitoe on behalf of the Wellcome Sanger Institute COVID-19 Surveillance Team
EPI_ISL_993844, EPI_ISL_993845, EPI_ISL_993846	Lighthouse Lab in Milton Keynes	Wellcome Sanger Institute for the COVID-19 Genomics UK (COG-UK) Consortium	The Lighthouse Lab in Milton Keynes and Alex Alderton, Roberto Amato, Sonia Goncalves, Ewan Harrison, David K. Jackson, Ian Johnston, Dominic Kwiatkowski, Cordelia Langford, John Sillitoe on behalf of the Wellcome Sanger Institute COVID-19 Surveillance Team
EPI_ISL_993847	Lighthouse Lab in Cambridge	Wellcome Sanger Institute for the COVID-19 Genomics UK (COG-UK) Consortium	Rob Howes, The Lighthouse Lab in Cambridge and Alex Alderton, Roberto Amato, Sonia Goncalves, Ewan Harrison, David K. Jackson, Ian Johnston, Dominic Kwiatkowski, Cordelia Langford, John Sillitoe on behalf of the Wellcome Sanger Institute COVID-19 Surveillance Team
EPI_ISL_993848	Lighthouse Lab in Milton Keynes	Wellcome Sanger Institute for the COVID-19 Genomics UK (COG-UK) Consortium	The Lighthouse Lab in Milton Keynes and Alex Alderton, Roberto Amato, Sonia Goncalves, Ewan Harrison, David K. Jackson, Ian Johnston, Dominic Kwiatkowski, Cordelia Langford, John Sillitoe on behalf of the Wellcome Sanger Institute COVID-19 Surveillance Team
EPI_ISL_993849, EPI_ISL_993850	Lighthouse Lab in Cambridge	Wellcome Sanger Institute for the COVID-19 Genomics UK (COG-UK) Consortium	Rob Howes, The Lighthouse Lab in Cambridge and Alex Alderton, Roberto Amato, Sonia Goncalves, Ewan Harrison, David K. Jackson, Ian Johnston, Dominic Kwiatkowski, Cordelia Langford, John Sillitoe on behalf of the Wellcome Sanger Institute COVID-19 Surveillance Team
EPI_ISL_993851, EPI_ISL_993852	Lighthouse Lab in Milton Keynes	Wellcome Sanger Institute for the COVID-19 Genomics UK (COG-UK) Consortium	The Lighthouse Lab in Milton Keynes and Alex Alderton, Roberto Amato, Sonia Goncalves, Ewan Harrison, David K. Jackson, Ian Johnston, Dominic Kwiatkowski, Cordelia Langford, John Sillitoe on behalf of the Wellcome Sanger Institute COVID-19 Surveillance Team
EPI_ISL_993853, EPI_ISL_993854, EPI_ISL_993855, EPI_ISL_993856	Lighthouse Lab in Cambridge	Wellcome Sanger Institute for the COVID-19 Genomics UK (COG-UK) Consortium	Rob Howes, The Lighthouse Lab in Cambridge and Alex Alderton, Roberto Amato, Sonia Goncalves, Ewan Harrison, David K. Jackson, Ian Johnston, Dominic Kwiatkowski, Cordelia Langford, John Sillitoe on behalf of the Wellcome Sanger Institute COVID-19 Surveillance Team
EPI_ISL_993857, EPI_ISL_993858	Lighthouse Lab in Milton Keynes	Wellcome Sanger Institute for the COVID-19 Genomics UK (COG-UK) Consortium	The Lighthouse Lab in Milton Keynes and Alex Alderton, Roberto Amato, Sonia Goncalves, Ewan Harrison, David K. Jackson, Ian Johnston, Dominic Kwiatkowski, Cordelia Langford, John Sillitoe on behalf of the Wellcome Sanger Institute COVID-19 Surveillance Team
EPI_ISL_993859	Lighthouse Lab in Cambridge	Wellcome Sanger Institute for the COVID-19 Genomics UK (COG-UK) Consortium	Rob Howes, The Lighthouse Lab in Cambridge and Alex Alderton, Roberto Amato, Sonia Goncalves, Ewan Harrison, David K. Jackson, Ian Johnston, Dominic Kwiatkowski, Cordelia Langford, John Sillitoe on behalf of the Wellcome Sanger Institute COVID-19 Surveillance Team
EPI_ISL_993860, EPI_ISL_993861, EPI_ISL_993862	Lighthouse Lab in Milton Keynes	Wellcome Sanger Institute for the COVID-19 Genomics UK (COG-UK) Consortium	The Lighthouse Lab in Milton Keynes and Alex Alderton, Roberto Amato, Sonia Goncalves, Ewan Harrison, David K. Jackson, Ian Johnston, Dominic Kwiatkowski, Cordelia Langford, John Sillitoe on behalf of the Wellcome Sanger Institute COVID-19 Surveillance Team
EPI_ISL_993863, EPI_ISL_993864, EPI_ISL_993865, EPI_ISL_993866, EPI_ISL_993867, EPI_ISL_993868, EPI_ISL_993869	Lighthouse Lab in Cambridge	Wellcome Sanger Institute for the COVID-19 Genomics UK (COG-UK) Consortium	Rob Howes, The Lighthouse Lab in Cambridge and Alex Alderton, Roberto Amato, Sonia Goncalves, Ewan Harrison, David K. Jackson, Ian Johnston, Dominic Kwiatkowski, Cordelia Langford, John Sillitoe on behalf of the Wellcome Sanger Institute COVID-19 Surveillance Team
EPI_ISL_993870	Lighthouse Lab in Milton Keynes	Wellcome Sanger Institute for the COVID-19 Genomics UK (COG-UK) Consortium	The Lighthouse Lab in Milton Keynes and Alex Alderton, Roberto Amato, Sonia Goncalves, Ewan Harrison, David K. Jackson, Ian Johnston, Dominic Kwiatkowski, Cordelia Langford, John Sillitoe on behalf of the Wellcome Sanger Institute COVID-19 Surveillance Team
EPI_ISL_993871, EPI_ISL_993872, EPI_ISL_993873	Lighthouse Lab in Cambridge	Wellcome Sanger Institute for the COVID-19 Genomics UK (COG-UK) Consortium	Rob Howes, The Lighthouse Lab in Cambridge and Alex Alderton, Roberto Amato, Sonia Goncalves, Ewan Harrison, David K. Jackson, Ian Johnston, Dominic Kwiatkowski, Cordelia Langford, John Sillitoe on behalf of the Wellcome Sanger Institute COVID-19 Surveillance Team
EPI_ISL_993874, EPI_ISL_993875, EPI_ISL_993876, EPI_ISL_993877	Lighthouse Lab in Milton Keynes	Wellcome Sanger Institute for the COVID-19 Genomics UK (COG-UK) Consortium	The Lighthouse Lab in Milton Keynes and Alex Alderton, Roberto Amato, Sonia Goncalves, Ewan Harrison, David K. Jackson, Ian Johnston, Dominic Kwiatkowski, Cordelia Langford, John Sillitoe on behalf of the Wellcome Sanger Institute COVID-19 Surveillance Team
EPI_ISL_993878, EPI_ISL_993879	Lighthouse Lab in Cambridge	Wellcome Sanger Institute for the COVID-19 Genomics UK (COG-UK) Consortium	Rob Howes, The Lighthouse Lab in Cambridge and Alex Alderton, Roberto Amato, Sonia Goncalves, Ewan Harrison, David K. Jackson, Ian Johnston, Dominic Kwiatkowski, Cordelia Langford, John Sillitoe on behalf of the Wellcome Sanger Institute COVID-19 Surveillance Team
EPI_ISL_993880	Lighthouse Lab in Milton Keynes	Wellcome Sanger Institute for the COVID-19 Genomics UK (COG-UK) Consortium	The Lighthouse Lab in Milton Keynes and Alex Alderton, Roberto Amato, Sonia Goncalves, Ewan Harrison, David K. Jackson, Ian Johnston, Dominic Kwiatkowski, Cordelia Langford, John Sillitoe on behalf of the Wellcome Sanger Institute COVID-19 Surveillance Team
EPI_ISL_993881	Lighthouse Lab in Cambridge	Wellcome Sanger Institute for the COVID-19 Genomics UK (COG-UK) Consortium	Rob Howes, The Lighthouse Lab in Cambridge and Alex Alderton, Roberto Amato, Sonia Goncalves, Ewan Harrison, David K. Jackson, Ian Johnston, Dominic Kwiatkowski, Cordelia Langford, John Sillitoe on behalf of the Wellcome Sanger Institute COVID-19 Surveillance Team
EPI_ISL_993882, EPI_ISL_993883, EPI_ISL_993884, EPI_ISL_993885, EPI_ISL_993886	Lighthouse Lab in Milton Keynes	Wellcome Sanger Institute for the COVID-19 Genomics UK (COG-UK) Consortium	The Lighthouse Lab in Milton Keynes and Alex Alderton, Roberto Amato, Sonia Goncalves, Ewan Harrison, David K. Jackson, Ian Johnston, Dominic Kwiatkowski, Cordelia Langford, John Sillitoe on behalf of the Wellcome Sanger Institute COVID-19 Surveillance Team
EPI_ISL_993887	Lighthouse Lab in Cambridge	Wellcome Sanger Institute for the COVID-19 Genomics UK (COG-UK) Consortium	Rob Howes, The Lighthouse Lab in Cambridge and Alex Alderton, Roberto Amato, Sonia Goncalves, Ewan Harrison, David K. Jackson, Ian Johnston, Dominic Kwiatkowski, Cordelia Langford, John Sillitoe on behalf of the Wellcome Sanger Institute COVID-19 Surveillance Team
EPI_ISL_993888, EPI_ISL_993889, EPI_ISL_993891	Lighthouse Lab in Milton Keynes	Wellcome Sanger Institute for the COVID-19 Genomics UK (COG-UK) Consortium	The Lighthouse Lab in Milton Keynes and Alex Alderton, Roberto Amato, Sonia Goncalves, Ewan Harrison, David K. Jackson, Ian Johnston, Dominic Kwiatkowski, Cordelia Langford, John Sillitoe on behalf of the Wellcome Sanger Institute COVID-19 Surveillance Team
EPI_ISL_993892, EPI_ISL_993893, EPI_ISL_993894, EPI_ISL_993895	Lighthouse Lab in Cambridge	Wellcome Sanger Institute for the COVID-19 Genomics UK (COG-UK) Consortium	Rob Howes, The Lighthouse Lab in Cambridge and Alex Alderton, Roberto Amato, Sonia Goncalves, Ewan Harrison, David K. Jackson, Ian Johnston, Dominic Kwiatkowski, Cordelia Langford, John Sillitoe on behalf of the Wellcome Sanger Institute COVID-19 Surveillance Team

EPI_ISL_993896, EPI_ISL_993897, EPI_ISL_993898, EPI_ISL_993899	Lighthouse Lab in Milton Keynes	Wellcome Sanger Institute for the COVID-19 Genomics UK (COG-UK) Consortium	The Lighthouse Lab in Milton Keynes and Alex Alderton, Roberto Amato, Sonia Goncalves, Ewan Harrison, David K. Jackson, Ian Johnston, Dominic Kwiatkowski, Cordelia Langford, John Sillitoe on behalf of the Wellcome Sanger Institute COVID-19 Surveillance Team
EPI_ISL_993900	Lighthouse Lab in Cambridge	Wellcome Sanger Institute for the COVID-19 Genomics UK (COG-UK) Consortium	Rob Howes, The Lighthouse Lab in Cambridge and Alex Alderton, Roberto Amato, Sonia Goncalves, Ewan Harrison, David K. Jackson, Ian Johnston, Dominic Kwiatkowski, Cordelia Langford, John Sillitoe on behalf of the Wellcome Sanger Institute COVID-19 Surveillance Team
EPI_ISL_993901, EPI_ISL_993902	Lighthouse Lab in Milton Keynes	Wellcome Sanger Institute for the COVID-19 Genomics UK (COG-UK) Consortium	The Lighthouse Lab in Milton Keynes and Alex Alderton, Roberto Amato, Sonia Goncalves, Ewan Harrison, David K. Jackson, Ian Johnston, Dominic Kwiatkowski, Cordelia Langford, John Sillitoe on behalf of the Wellcome Sanger Institute COVID-19 Surveillance Team
EPI_ISL_993903	Lighthouse Lab in Cambridge	Wellcome Sanger Institute for the COVID-19 Genomics UK (COG-UK) Consortium	Rob Howes, The Lighthouse Lab in Cambridge and Alex Alderton, Roberto Amato, Sonia Goncalves, Ewan Harrison, David K. Jackson, Ian Johnston, Dominic Kwiatkowski, Cordelia Langford, John Sillitoe on behalf of the Wellcome Sanger Institute COVID-19 Surveillance Team
EPI_ISL_993904, EPI_ISL_993905	Lighthouse Lab in Milton Keynes	Wellcome Sanger Institute for the COVID-19 Genomics UK (COG-UK) Consortium	The Lighthouse Lab in Milton Keynes and Alex Alderton, Roberto Amato, Sonia Goncalves, Ewan Harrison, David K. Jackson, Ian Johnston, Dominic Kwiatkowski, Cordelia Langford, John Sillitoe on behalf of the Wellcome Sanger Institute COVID-19 Surveillance Team
EPI_ISL_993906	Lighthouse Lab in Cambridge	Wellcome Sanger Institute for the COVID-19 Genomics UK (COG-UK) Consortium	Rob Howes, The Lighthouse Lab in Cambridge and Alex Alderton, Roberto Amato, Sonia Goncalves, Ewan Harrison, David K. Jackson, Ian Johnston, Dominic Kwiatkowski, Cordelia Langford, John Sillitoe on behalf of the Wellcome Sanger Institute COVID-19 Surveillance Team
EPI_ISL_993908	Lighthouse Lab in Milton Keynes	Wellcome Sanger Institute for the COVID-19 Genomics UK (COG-UK) Consortium	The Lighthouse Lab in Milton Keynes and Alex Alderton, Roberto Amato, Sonia Goncalves, Ewan Harrison, David K. Jackson, Ian Johnston, Dominic Kwiatkowski, Cordelia Langford, John Sillitoe on behalf of the Wellcome Sanger Institute COVID-19 Surveillance Team
EPI_ISL_993909	Lighthouse Lab in Cambridge	Wellcome Sanger Institute for the COVID-19 Genomics UK (COG-UK) Consortium	Rob Howes, The Lighthouse Lab in Cambridge and Alex Alderton, Roberto Amato, Sonia Goncalves, Ewan Harrison, David K. Jackson, Ian Johnston, Dominic Kwiatkowski, Cordelia Langford, John Sillitoe on behalf of the Wellcome Sanger Institute COVID-19 Surveillance Team
EPI_ISL_993910	Lighthouse Lab in Milton Keynes	Wellcome Sanger Institute for the COVID-19 Genomics UK (COG-UK) Consortium	The Lighthouse Lab in Milton Keynes and Alex Alderton, Roberto Amato, Sonia Goncalves, Ewan Harrison, David K. Jackson, Ian Johnston, Dominic Kwiatkowski, Cordelia Langford, John Sillitoe on behalf of the Wellcome Sanger Institute COVID-19 Surveillance Team
EPI_ISL_993911, EPI_ISL_993912	Lighthouse Lab in Cambridge	Wellcome Sanger Institute for the COVID-19 Genomics UK (COG-UK) Consortium	Rob Howes, The Lighthouse Lab in Cambridge and Alex Alderton, Roberto Amato, Sonia Goncalves, Ewan Harrison, David K. Jackson, Ian Johnston, Dominic Kwiatkowski, Cordelia Langford, John Sillitoe on behalf of the Wellcome Sanger Institute COVID-19 Surveillance Team
EPI_ISL_993913, EPI_ISL_993917, EPI_ISL_993918	Lighthouse Lab in Milton Keynes	Wellcome Sanger Institute for the COVID-19 Genomics UK (COG-UK) Consortium	The Lighthouse Lab in Milton Keynes and Alex Alderton, Roberto Amato, Sonia Goncalves, Ewan Harrison, David K. Jackson, Ian Johnston, Dominic Kwiatkowski, Cordelia Langford, John Sillitoe on behalf of the Wellcome Sanger Institute COVID-19 Surveillance Team
EPI_ISL_993919	Lighthouse Lab in Milton Keynes	Wellcome Sanger Institute for the COVID-19 Genomics UK (COG-UK) Consortium	The Lighthouse Lab in Milton Keynes and Alex Alderton, Roberto Amato, Sonia Goncalves, Ewan Harrison, David K. Jackson, Ian Johnston, Dominic Kwiatkowski, Cordelia Langford, John Sillitoe on behalf of the Wellcome Sanger Institute COVID-19 Surveillance Team (http://www.sanger.ac.uk/covid-team)
EPI_ISL_993921, EPI_ISL_993922	Lighthouse Lab in Cambridge	Wellcome Sanger Institute for the COVID-19 Genomics UK (COG-UK) Consortium	Rob Howes, The Lighthouse Lab in Cambridge and Alex Alderton, Roberto Amato, Sonia Goncalves, Ewan Harrison, David K. Jackson, Ian Johnston, Dominic Kwiatkowski, Cordelia Langford, John Sillitoe on behalf of the Wellcome Sanger Institute COVID-19 Surveillance Team (http://www.sanger.ac.uk/covid-team)
EPI_ISL_993923, EPI_ISL_993924, EPI_ISL_993925	Lighthouse Lab in Milton Keynes	Wellcome Sanger Institute for the COVID-19 Genomics UK (COG-UK) Consortium	The Lighthouse Lab in Milton Keynes and Alex Alderton, Roberto Amato, Sonia Goncalves, Ewan Harrison, David K. Jackson, Ian Johnston, Dominic Kwiatkowski, Cordelia Langford, John Sillitoe on behalf of the Wellcome Sanger Institute COVID-19 Surveillance Team (http://www.sanger.ac.uk/covid-team)
EPI_ISL_993926, EPI_ISL_993927	Lighthouse Lab in Cambridge	Wellcome Sanger Institute for the COVID-19 Genomics UK (COG-UK) Consortium	Rob Howes, The Lighthouse Lab in Cambridge and Alex Alderton, Roberto Amato, Sonia Goncalves, Ewan Harrison, David K. Jackson, Ian Johnston, Dominic Kwiatkowski, Cordelia Langford, John Sillitoe on behalf of the Wellcome Sanger Institute COVID-19 Surveillance Team (http://www.sanger.ac.uk/covid-team)
EPI_ISL_993928, EPI_ISL_993929, EPI_ISL_993931, EPI_ISL_993932, EPI_ISL_993933, EPI_ISL_993934, EPI_ISL_993935, EPI_ISL_993936	Lighthouse Lab in Milton Keynes	Wellcome Sanger Institute for the COVID-19 Genomics UK (COG-UK) Consortium	The Lighthouse Lab in Milton Keynes and Alex Alderton, Roberto Amato, Sonia Goncalves, Ewan Harrison, David K. Jackson, Ian Johnston, Dominic Kwiatkowski, Cordelia Langford, John Sillitoe on behalf of the Wellcome Sanger Institute COVID-19 Surveillance Team (http://www.sanger.ac.uk/covid-team)
EPI_ISL_993937	Lighthouse Lab in Cambridge	Wellcome Sanger Institute for the COVID-19 Genomics UK (COG-UK) Consortium	Rob Howes, The Lighthouse Lab in Cambridge and Alex Alderton, Roberto Amato, Sonia Goncalves, Ewan Harrison, David K. Jackson, Ian Johnston, Dominic Kwiatkowski, Cordelia Langford, John Sillitoe on behalf of the Wellcome Sanger Institute COVID-19 Surveillance Team (http://www.sanger.ac.uk/covid-team)
EPI_ISL_993938, EPI_ISL_993939, EPI_ISL_993940, EPI_ISL_993941	Lighthouse Lab in Milton Keynes	Wellcome Sanger Institute for the COVID-19 Genomics UK (COG-UK) Consortium	The Lighthouse Lab in Milton Keynes and Alex Alderton, Roberto Amato, Sonia Goncalves, Ewan Harrison, David K. Jackson, Ian Johnston, Dominic Kwiatkowski, Cordelia Langford, John Sillitoe on behalf of the Wellcome Sanger Institute COVID-19 Surveillance Team (http://www.sanger.ac.uk/covid-team)
EPI_ISL_993942	Lighthouse Lab in Cambridge	Wellcome Sanger Institute for the COVID-19 Genomics UK (COG-UK) Consortium	Rob Howes, The Lighthouse Lab in Cambridge and Alex Alderton, Roberto Amato, Sonia Goncalves, Ewan Harrison, David K. Jackson, Ian Johnston, Dominic Kwiatkowski, Cordelia Langford, John Sillitoe on behalf of the Wellcome Sanger Institute COVID-19 Surveillance Team (http://www.sanger.ac.uk/covid-team)
EPI_ISL_993943, EPI_ISL_993944	Lighthouse Lab in Milton Keynes	Wellcome Sanger Institute for the COVID-19 Genomics UK (COG-UK) Consortium	The Lighthouse Lab in Milton Keynes and Alex Alderton, Roberto Amato, Sonia Goncalves, Ewan Harrison, David K. Jackson, Ian Johnston, Dominic Kwiatkowski, Cordelia Langford, John Sillitoe on behalf of the Wellcome Sanger Institute COVID-19 Surveillance Team (http://www.sanger.ac.uk/covid-team)
EPI_ISL_993945, EPI_ISL_993946	Lighthouse Lab in Cambridge	Wellcome Sanger Institute for the COVID-19 Genomics UK (COG-UK) Consortium	Rob Howes, The Lighthouse Lab in Cambridge and Alex Alderton, Roberto Amato, Sonia Goncalves, Ewan Harrison, David K. Jackson, Ian Johnston, Dominic Kwiatkowski, Cordelia Langford, John Sillitoe on behalf of the Wellcome Sanger Institute COVID-19 Surveillance Team (http://www.sanger.ac.uk/covid-team)
EPI_ISL_993947, EPI_ISL_993948	Lighthouse Lab in Milton Keynes	Wellcome Sanger Institute for the COVID-19 Genomics UK (COG-UK) Consortium	The Lighthouse Lab in Milton Keynes and Alex Alderton, Roberto Amato, Sonia Goncalves, Ewan Harrison, David K. Jackson, Ian Johnston, Dominic Kwiatkowski, Cordelia Langford, John Sillitoe on behalf of the Wellcome Sanger Institute COVID-19 Surveillance Team (http://www.sanger.ac.uk/covid-team)
EPI_ISL_993949	Lighthouse Lab in Cambridge	Wellcome Sanger Institute for the COVID-19 Genomics UK (COG-UK) Consortium	Rob Howes, The Lighthouse Lab in Cambridge and Alex Alderton, Roberto Amato, Sonia Goncalves, Ewan Harrison, David K. Jackson, Ian Johnston, Dominic Kwiatkowski, Cordelia Langford, John Sillitoe on behalf of the Wellcome Sanger Institute COVID-19 Surveillance Team (http://www.sanger.ac.uk/covid-team)
EPI_ISL_993950, EPI_ISL_993951, EPI_ISL_993952, EPI_ISL_993953	Lighthouse Lab in Milton Keynes	Wellcome Sanger Institute for the COVID-19 Genomics UK (COG-UK) Consortium	The Lighthouse Lab in Milton Keynes and Alex Alderton, Roberto Amato, Sonia Goncalves, Ewan Harrison, David K. Jackson, Ian Johnston, Dominic Kwiatkowski, Cordelia Langford, John Sillitoe on behalf of the Wellcome Sanger Institute COVID-19 Surveillance Team (http://www.sanger.ac.uk/covid-team)
EPI_ISL_993954, EPI_ISL_993956, EPI_ISL_993957	Lighthouse Lab in Cambridge	Wellcome Sanger Institute for the COVID-19 Genomics UK (COG-UK) Consortium	Rob Howes, The Lighthouse Lab in Cambridge and Alex Alderton, Roberto Amato, Sonia Goncalves, Ewan Harrison, David K. Jackson, Ian Johnston, Dominic Kwiatkowski, Cordelia Langford, John Sillitoe on behalf of the Wellcome Sanger Institute COVID-19 Surveillance Team (http://www.sanger.ac.uk/covid-team)
EPI_ISL_993958, EPI_ISL_993959	Lighthouse Lab in Milton Keynes	Wellcome Sanger Institute for the COVID-19 Genomics UK (COG-UK) Consortium	The Lighthouse Lab in Milton Keynes and Alex Alderton, Roberto Amato, Sonia Goncalves, Ewan Harrison, David K. Jackson, Ian Johnston, Dominic Kwiatkowski, Cordelia Langford, John Sillitoe on behalf of the Wellcome Sanger Institute COVID-19 Surveillance Team (http://www.sanger.ac.uk/covid-team)
EPI_ISL_993960, EPI_ISL_993961, EPI_ISL_993963	Lighthouse Lab in Cambridge	Wellcome Sanger Institute for the COVID-19 Genomics UK (COG-UK) Consortium	Rob Howes, The Lighthouse Lab in Cambridge and Alex Alderton, Roberto Amato, Sonia Goncalves, Ewan Harrison, David K. Jackson, Ian Johnston, Dominic Kwiatkowski, Cordelia Langford, John Sillitoe on behalf of the Wellcome Sanger Institute COVID-19 Surveillance Team (http://www.sanger.ac.uk/covid-team)
EPI_ISL_993964, EPI_ISL_993965	Lighthouse Lab in Milton Keynes	Wellcome Sanger Institute for the COVID-19 Genomics UK (COG-UK) Consortium	The Lighthouse Lab in Milton Keynes and Alex Alderton, Roberto Amato, Sonia Goncalves, Ewan Harrison, David K. Jackson, Ian Johnston, Dominic Kwiatkowski, Cordelia Langford, John Sillitoe on behalf of the Wellcome Sanger Institute COVID-19 Surveillance Team (http://www.sanger.ac.uk/covid-team)

EPI_ISL_993966	Lighthouse Lab in Cambridge	Wellcome Sanger Institute for the COVID-19 Genomics UK (COG-UK) Consortium	Rob Howes, The Lighthouse Lab in Cambridge and Alex Alderton, Roberto Amato, Sonia Goncalves, Ewan Harrison, David K. Jackson, Ian Johnston, Dominic Kwiatkowski, Cordelia Langford, John Sillitoe on behalf of the Wellcome Sanger Institute COVID-19 Surveillance Team (http://www.sanger.ac.uk/covid-team)
EPI_ISL_993967	Lighthouse Lab in Milton Keynes	Wellcome Sanger Institute for the COVID-19 Genomics UK (COG-UK) Consortium	The Lighthouse Lab in Milton Keynes and Alex Alderton, Roberto Amato, Sonia Goncalves, Ewan Harrison, David K. Jackson, Ian Johnston, Dominic Kwiatkowski, Cordelia Langford, John Sillitoe on behalf of the Wellcome Sanger Institute COVID-19 Surveillance Team (http://www.sanger.ac.uk/covid-team)
EPI_ISL_993968, EPI_ISL_993969	Lighthouse Lab in Cambridge	Wellcome Sanger Institute for the COVID-19 Genomics UK (COG-UK) Consortium	Rob Howes, The Lighthouse Lab in Cambridge and Alex Alderton, Roberto Amato, Sonia Goncalves, Ewan Harrison, David K. Jackson, Ian Johnston, Dominic Kwiatkowski, Cordelia Langford, John Sillitoe on behalf of the Wellcome Sanger Institute COVID-19 Surveillance Team (http://www.sanger.ac.uk/covid-team)
EPI_ISL_993970, EPI_ISL_993971	Lighthouse Lab in Milton Keynes	Wellcome Sanger Institute for the COVID-19 Genomics UK (COG-UK) Consortium	The Lighthouse Lab in Milton Keynes and Alex Alderton, Roberto Amato, Sonia Goncalves, Ewan Harrison, David K. Jackson, Ian Johnston, Dominic Kwiatkowski, Cordelia Langford, John Sillitoe on behalf of the Wellcome Sanger Institute COVID-19 Surveillance Team (http://www.sanger.ac.uk/covid-team)
EPI_ISL_993972, EPI_ISL_993973	Lighthouse Lab in Cambridge	Wellcome Sanger Institute for the COVID-19 Genomics UK (COG-UK) Consortium	Rob Howes, The Lighthouse Lab in Cambridge and Alex Alderton, Roberto Amato, Sonia Goncalves, Ewan Harrison, David K. Jackson, Ian Johnston, Dominic Kwiatkowski, Cordelia Langford, John Sillitoe on behalf of the Wellcome Sanger Institute COVID-19 Surveillance Team (http://www.sanger.ac.uk/covid-team)
EPI_ISL_993974	Lighthouse Lab in Milton Keynes	Wellcome Sanger Institute for the COVID-19 Genomics UK (COG-UK) Consortium	The Lighthouse Lab in Milton Keynes and Alex Alderton, Roberto Amato, Sonia Goncalves, Ewan Harrison, David K. Jackson, Ian Johnston, Dominic Kwiatkowski, Cordelia Langford, John Sillitoe on behalf of the Wellcome Sanger Institute COVID-19 Surveillance Team (http://www.sanger.ac.uk/covid-team)
EPI_ISL_993975, EPI_ISL_993976	Lighthouse Lab in Cambridge	Wellcome Sanger Institute for the COVID-19 Genomics UK (COG-UK) Consortium	Rob Howes, The Lighthouse Lab in Cambridge and Alex Alderton, Roberto Amato, Sonia Goncalves, Ewan Harrison, David K. Jackson, Ian Johnston, Dominic Kwiatkowski, Cordelia Langford, John Sillitoe on behalf of the Wellcome Sanger Institute COVID-19 Surveillance Team (http://www.sanger.ac.uk/covid-team)
EPI_ISL_993977, EPI_ISL_993978	Lighthouse Lab in Milton Keynes	Wellcome Sanger Institute for the COVID-19 Genomics UK (COG-UK) Consortium	The Lighthouse Lab in Milton Keynes and Alex Alderton, Roberto Amato, Sonia Goncalves, Ewan Harrison, David K. Jackson, Ian Johnston, Dominic Kwiatkowski, Cordelia Langford, John Sillitoe on behalf of the Wellcome Sanger Institute COVID-19 Surveillance Team (http://www.sanger.ac.uk/covid-team)
EPI_ISL_993979	Lighthouse Lab in Cambridge	Wellcome Sanger Institute for the COVID-19 Genomics UK (COG-UK) Consortium	Rob Howes, The Lighthouse Lab in Cambridge and Alex Alderton, Roberto Amato, Sonia Goncalves, Ewan Harrison, David K. Jackson, Ian Johnston, Dominic Kwiatkowski, Cordelia Langford, John Sillitoe on behalf of the Wellcome Sanger Institute COVID-19 Surveillance Team (http://www.sanger.ac.uk/covid-team)
EPI_ISL_993980, EPI_ISL_993981	Lighthouse Lab in Milton Keynes	Wellcome Sanger Institute for the COVID-19 Genomics UK (COG-UK) Consortium	The Lighthouse Lab in Milton Keynes and Alex Alderton, Roberto Amato, Sonia Goncalves, Ewan Harrison, David K. Jackson, Ian Johnston, Dominic Kwiatkowski, Cordelia Langford, John Sillitoe on behalf of the Wellcome Sanger Institute COVID-19 Surveillance Team (http://www.sanger.ac.uk/covid-team)
EPI_ISL_993982, EPI_ISL_993983, EPI_ISL_993984	Lighthouse Lab in Cambridge	Wellcome Sanger Institute for the COVID-19 Genomics UK (COG-UK) Consortium	Rob Howes, The Lighthouse Lab in Cambridge and Alex Alderton, Roberto Amato, Sonia Goncalves, Ewan Harrison, David K. Jackson, Ian Johnston, Dominic Kwiatkowski, Cordelia Langford, John Sillitoe on behalf of the Wellcome Sanger Institute COVID-19 Surveillance Team (http://www.sanger.ac.uk/covid-team)
EPI_ISL_993985, EPI_ISL_993986	Lighthouse Lab in Milton Keynes	Wellcome Sanger Institute for the COVID-19 Genomics UK (COG-UK) Consortium	The Lighthouse Lab in Milton Keynes and Alex Alderton, Roberto Amato, Sonia Goncalves, Ewan Harrison, David K. Jackson, Ian Johnston, Dominic Kwiatkowski, Cordelia Langford, John Sillitoe on behalf of the Wellcome Sanger Institute COVID-19 Surveillance Team (http://www.sanger.ac.uk/covid-team)
EPI_ISL_993987	Lighthouse Lab in Cambridge	Wellcome Sanger Institute for the COVID-19 Genomics UK (COG-UK) Consortium	Rob Howes, The Lighthouse Lab in Cambridge and Alex Alderton, Roberto Amato, Sonia Goncalves, Ewan Harrison, David K. Jackson, Ian Johnston, Dominic Kwiatkowski, Cordelia Langford, John Sillitoe on behalf of the Wellcome Sanger Institute COVID-19 Surveillance Team (http://www.sanger.ac.uk/covid-team)
EPI_ISL_993988, EPI_ISL_993989, EPI_ISL_993990	Lighthouse Lab in Milton Keynes	Wellcome Sanger Institute for the COVID-19 Genomics UK (COG-UK) Consortium	The Lighthouse Lab in Milton Keynes and Alex Alderton, Roberto Amato, Sonia Goncalves, Ewan Harrison, David K. Jackson, Ian Johnston, Dominic Kwiatkowski, Cordelia Langford, John Sillitoe on behalf of the Wellcome Sanger Institute COVID-19 Surveillance Team (http://www.sanger.ac.uk/covid-team)
EPI_ISL_993992	Lighthouse Lab in Cambridge	Wellcome Sanger Institute for the COVID-19 Genomics UK (COG-UK) Consortium	Rob Howes, The Lighthouse Lab in Cambridge and Alex Alderton, Roberto Amato, Sonia Goncalves, Ewan Harrison, David K. Jackson, Ian Johnston, Dominic Kwiatkowski, Cordelia Langford, John Sillitoe on behalf of the Wellcome Sanger Institute COVID-19 Surveillance Team (http://www.sanger.ac.uk/covid-team)
EPI_ISL_993993	Lighthouse Lab in Milton Keynes	Wellcome Sanger Institute for the COVID-19 Genomics UK (COG-UK) Consortium	The Lighthouse Lab in Milton Keynes and Alex Alderton, Roberto Amato, Sonia Goncalves, Ewan Harrison, David K. Jackson, Ian Johnston, Dominic Kwiatkowski, Cordelia Langford, John Sillitoe on behalf of the Wellcome Sanger Institute COVID-19 Surveillance Team (http://www.sanger.ac.uk/covid-team)
EPI_ISL_993994	Lighthouse Lab in Cambridge	Wellcome Sanger Institute for the COVID-19 Genomics UK (COG-UK) Consortium	Rob Howes, The Lighthouse Lab in Cambridge and Alex Alderton, Roberto Amato, Sonia Goncalves, Ewan Harrison, David K. Jackson, Ian Johnston, Dominic Kwiatkowski, Cordelia Langford, John Sillitoe on behalf of the Wellcome Sanger Institute COVID-19 Surveillance Team (http://www.sanger.ac.uk/covid-team)
EPI_ISL_993995	Lighthouse Lab in Milton Keynes	Wellcome Sanger Institute for the COVID-19 Genomics UK (COG-UK) Consortium	The Lighthouse Lab in Milton Keynes and Alex Alderton, Roberto Amato, Sonia Goncalves, Ewan Harrison, David K. Jackson, Ian Johnston, Dominic Kwiatkowski, Cordelia Langford, John Sillitoe on behalf of the Wellcome Sanger Institute COVID-19 Surveillance Team (http://www.sanger.ac.uk/covid-team)
EPI_ISL_993996, EPI_ISL_993997	Lighthouse Lab in Cambridge	Wellcome Sanger Institute for the COVID-19 Genomics UK (COG-UK) Consortium	Rob Howes, The Lighthouse Lab in Cambridge and Alex Alderton, Roberto Amato, Sonia Goncalves, Ewan Harrison, David K. Jackson, Ian Johnston, Dominic Kwiatkowski, Cordelia Langford, John Sillitoe on behalf of the Wellcome Sanger Institute COVID-19 Surveillance Team (http://www.sanger.ac.uk/covid-team)
EPI_ISL_993998, EPI_ISL_993999	Lighthouse Lab in Milton Keynes	Wellcome Sanger Institute for the COVID-19 Genomics UK (COG-UK) Consortium	The Lighthouse Lab in Milton Keynes and Alex Alderton, Roberto Amato, Sonia Goncalves, Ewan Harrison, David K. Jackson, Ian Johnston, Dominic Kwiatkowski, Cordelia Langford, John Sillitoe on behalf of the Wellcome Sanger Institute COVID-19 Surveillance Team (http://www.sanger.ac.uk/covid-team)
EPI_ISL_994000, EPI_ISL_994001	Lighthouse Lab in Cambridge	Wellcome Sanger Institute for the COVID-19 Genomics UK (COG-UK) Consortium	Rob Howes, The Lighthouse Lab in Cambridge and Alex Alderton, Roberto Amato, Sonia Goncalves, Ewan Harrison, David K. Jackson, Ian Johnston, Dominic Kwiatkowski, Cordelia Langford, John Sillitoe on behalf of the Wellcome Sanger Institute COVID-19 Surveillance Team (http://www.sanger.ac.uk/covid-team)
EPI_ISL_994002	Lighthouse Lab in Milton Keynes	Wellcome Sanger Institute for the COVID-19 Genomics UK (COG-UK) Consortium	The Lighthouse Lab in Milton Keynes and Alex Alderton, Roberto Amato, Sonia Goncalves, Ewan Harrison, David K. Jackson, Ian Johnston, Dominic Kwiatkowski, Cordelia Langford, John Sillitoe on behalf of the Wellcome Sanger Institute COVID-19 Surveillance Team (http://www.sanger.ac.uk/covid-team)
EPI_ISL_994004	Lighthouse Lab in Cambridge	Wellcome Sanger Institute for the COVID-19 Genomics UK (COG-UK) Consortium	Rob Howes, The Lighthouse Lab in Cambridge and Alex Alderton, Roberto Amato, Sonia Goncalves, Ewan Harrison, David K. Jackson, Ian Johnston, Dominic Kwiatkowski, Cordelia Langford, John Sillitoe on behalf of the Wellcome Sanger Institute COVID-19 Surveillance Team (http://www.sanger.ac.uk/covid-team)
EPI_ISL_994005	Lighthouse Lab in Milton Keynes	Wellcome Sanger Institute for the COVID-19 Genomics UK (COG-UK) Consortium	The Lighthouse Lab in Milton Keynes and Alex Alderton, Roberto Amato, Sonia Goncalves, Ewan Harrison, David K. Jackson, Ian Johnston, Dominic Kwiatkowski, Cordelia Langford, John Sillitoe on behalf of the Wellcome Sanger Institute COVID-19 Surveillance Team (http://www.sanger.ac.uk/covid-team)
EPI_ISL_994006, EPI_ISL_994007, EPI_ISL_994008	Lighthouse Lab in Cambridge	Wellcome Sanger Institute for the COVID-19 Genomics UK (COG-UK) Consortium	Rob Howes, The Lighthouse Lab in Cambridge and Alex Alderton, Roberto Amato, Sonia Goncalves, Ewan Harrison, David K. Jackson, Ian Johnston, Dominic Kwiatkowski, Cordelia Langford, John Sillitoe on behalf of the Wellcome Sanger Institute COVID-19 Surveillance Team (http://www.sanger.ac.uk/covid-team)

EPI_ISL_994009	Lighthouse Lab in Milton Keynes	Wellcome Sanger Institute for the COVID-19 Genomics UK (COG-UK) Consortium	The Lighthouse Lab in Milton Keynes and Alex Alderton, Roberto Amato, Sonia Goncalves, Ewan Harrison, David K. Jackson, Ian Johnston, Dominic Kwiatkowski, Cordelia Langford, John Sillitoe on behalf of the Wellcome Sanger Institute COVID-19 Surveillance Team (http://www.sanger.ac.uk/covid-team)
EPI_ISL_994010, EPI_ISL_994011	Lighthouse Lab in Cambridge	Wellcome Sanger Institute for the COVID-19 Genomics UK (COG-UK) Consortium	Rob Howes, The Lighthouse Lab in Cambridge and Alex Alderton, Roberto Amato, Sonia Goncalves, Ewan Harrison, David K. Jackson, Ian Johnston, Dominic Kwiatkowski, Cordelia Langford, John Sillitoe on behalf of the Wellcome Sanger Institute COVID-19 Surveillance Team (http://www.sanger.ac.uk/covid-team)
EPI_ISL_994012, EPI_ISL_994013	Lighthouse Lab in Milton Keynes	Wellcome Sanger Institute for the COVID-19 Genomics UK (COG-UK) Consortium	The Lighthouse Lab in Milton Keynes and Alex Alderton, Roberto Amato, Sonia Goncalves, Ewan Harrison, David K. Jackson, Ian Johnston, Dominic Kwiatkowski, Cordelia Langford, John Sillitoe on behalf of the Wellcome Sanger Institute COVID-19 Surveillance Team (http://www.sanger.ac.uk/covid-team)
EPI_ISL_994014, EPI_ISL_994015	Lighthouse Lab in Cambridge	Wellcome Sanger Institute for the COVID-19 Genomics UK (COG-UK) Consortium	Rob Howes, The Lighthouse Lab in Cambridge and Alex Alderton, Roberto Amato, Sonia Goncalves, Ewan Harrison, David K. Jackson, Ian Johnston, Dominic Kwiatkowski, Cordelia Langford, John Sillitoe on behalf of the Wellcome Sanger Institute COVID-19 Surveillance Team (http://www.sanger.ac.uk/covid-team)
EPI_ISL_994016, EPI_ISL_994017	Lighthouse Lab in Milton Keynes	Wellcome Sanger Institute for the COVID-19 Genomics UK (COG-UK) Consortium	The Lighthouse Lab in Milton Keynes and Alex Alderton, Roberto Amato, Sonia Goncalves, Ewan Harrison, David K. Jackson, Ian Johnston, Dominic Kwiatkowski, Cordelia Langford, John Sillitoe on behalf of the Wellcome Sanger Institute COVID-19 Surveillance Team (http://www.sanger.ac.uk/covid-team)
EPI_ISL_994018, EPI_ISL_994019, EPI_ISL_994020	Lighthouse Lab in Cambridge	Wellcome Sanger Institute for the COVID-19 Genomics UK (COG-UK) Consortium	Rob Howes, The Lighthouse Lab in Cambridge and Alex Alderton, Roberto Amato, Sonia Goncalves, Ewan Harrison, David K. Jackson, Ian Johnston, Dominic Kwiatkowski, Cordelia Langford, John Sillitoe on behalf of the Wellcome Sanger Institute COVID-19 Surveillance Team (http://www.sanger.ac.uk/covid-team)
EPI_ISL_994021	Lighthouse Lab in Milton Keynes	Wellcome Sanger Institute for the COVID-19 Genomics UK (COG-UK) Consortium	The Lighthouse Lab in Milton Keynes and Alex Alderton, Roberto Amato, Sonia Goncalves, Ewan Harrison, David K. Jackson, Ian Johnston, Dominic Kwiatkowski, Cordelia Langford, John Sillitoe on behalf of the Wellcome Sanger Institute COVID-19 Surveillance Team (http://www.sanger.ac.uk/covid-team)
EPI_ISL_994022	Lighthouse Lab in Cambridge	Wellcome Sanger Institute for the COVID-19 Genomics UK (COG-UK) Consortium	Rob Howes, The Lighthouse Lab in Cambridge and Alex Alderton, Roberto Amato, Sonia Goncalves, Ewan Harrison, David K. Jackson, Ian Johnston, Dominic Kwiatkowski, Cordelia Langford, John Sillitoe on behalf of the Wellcome Sanger Institute COVID-19 Surveillance Team (http://www.sanger.ac.uk/covid-team)
EPI_ISL_994023	Lighthouse Lab in Milton Keynes	Wellcome Sanger Institute for the COVID-19 Genomics UK (COG-UK) Consortium	The Lighthouse Lab in Milton Keynes and Alex Alderton, Roberto Amato, Sonia Goncalves, Ewan Harrison, David K. Jackson, Ian Johnston, Dominic Kwiatkowski, Cordelia Langford, John Sillitoe on behalf of the Wellcome Sanger Institute COVID-19 Surveillance Team (http://www.sanger.ac.uk/covid-team)
EPI_ISL_994024, EPI_ISL_994025	Lighthouse Lab in Cambridge	Wellcome Sanger Institute for the COVID-19 Genomics UK (COG-UK) Consortium	Rob Howes, The Lighthouse Lab in Cambridge and Alex Alderton, Roberto Amato, Sonia Goncalves, Ewan Harrison, David K. Jackson, Ian Johnston, Dominic Kwiatkowski, Cordelia Langford, John Sillitoe on behalf of the Wellcome Sanger Institute COVID-19 Surveillance Team (http://www.sanger.ac.uk/covid-team)
EPI_ISL_994026, EPI_ISL_994027	Lighthouse Lab in Milton Keynes	Wellcome Sanger Institute for the COVID-19 Genomics UK (COG-UK) Consortium	The Lighthouse Lab in Milton Keynes and Alex Alderton, Roberto Amato, Sonia Goncalves, Ewan Harrison, David K. Jackson, Ian Johnston, Dominic Kwiatkowski, Cordelia Langford, John Sillitoe on behalf of the Wellcome Sanger Institute COVID-19 Surveillance Team (http://www.sanger.ac.uk/covid-team)
EPI_ISL_994028	Lighthouse Lab in Cambridge	Wellcome Sanger Institute for the COVID-19 Genomics UK (COG-UK) Consortium	Rob Howes, The Lighthouse Lab in Cambridge and Alex Alderton, Roberto Amato, Sonia Goncalves, Ewan Harrison, David K. Jackson, Ian Johnston, Dominic Kwiatkowski, Cordelia Langford, John Sillitoe on behalf of the Wellcome Sanger Institute COVID-19 Surveillance Team (http://www.sanger.ac.uk/covid-team)
EPI_ISL_994029, EPI_ISL_994030	Lighthouse Lab in Milton Keynes	Wellcome Sanger Institute for the COVID-19 Genomics UK (COG-UK) Consortium	The Lighthouse Lab in Milton Keynes and Alex Alderton, Roberto Amato, Sonia Goncalves, Ewan Harrison, David K. Jackson, Ian Johnston, Dominic Kwiatkowski, Cordelia Langford, John Sillitoe on behalf of the Wellcome Sanger Institute COVID-19 Surveillance Team (http://www.sanger.ac.uk/covid-team)
EPI_ISL_994031, EPI_ISL_994032, EPI_ISL_994033	Lighthouse Lab in Cambridge	Wellcome Sanger Institute for the COVID-19 Genomics UK (COG-UK) Consortium	Rob Howes, The Lighthouse Lab in Cambridge and Alex Alderton, Roberto Amato, Sonia Goncalves, Ewan Harrison, David K. Jackson, Ian Johnston, Dominic Kwiatkowski, Cordelia Langford, John Sillitoe on behalf of the Wellcome Sanger Institute COVID-19 Surveillance Team (http://www.sanger.ac.uk/covid-team)
EPI_ISL_994035, EPI_ISL_994036, EPI_ISL_994037, EPI_ISL_994038, EPI_ISL_994039	Lighthouse Lab in Milton Keynes	Wellcome Sanger Institute for the COVID-19 Genomics UK (COG-UK) Consortium	The Lighthouse Lab in Milton Keynes and Alex Alderton, Roberto Amato, Sonia Goncalves, Ewan Harrison, David K. Jackson, Ian Johnston, Dominic Kwiatkowski, Cordelia Langford, John Sillitoe on behalf of the Wellcome Sanger Institute COVID-19 Surveillance Team (http://www.sanger.ac.uk/covid-team)
EPI_ISL_994040	Lighthouse Lab in Cambridge	Wellcome Sanger Institute for the COVID-19 Genomics UK (COG-UK) Consortium	Rob Howes, The Lighthouse Lab in Cambridge and Alex Alderton, Roberto Amato, Sonia Goncalves, Ewan Harrison, David K. Jackson, Ian Johnston, Dominic Kwiatkowski, Cordelia Langford, John Sillitoe on behalf of the Wellcome Sanger Institute COVID-19 Surveillance Team (http://www.sanger.ac.uk/covid-team)
EPI_ISL_994041	Lighthouse Lab in Milton Keynes	Wellcome Sanger Institute for the COVID-19 Genomics UK (COG-UK) Consortium	The Lighthouse Lab in Milton Keynes and Alex Alderton, Roberto Amato, Sonia Goncalves, Ewan Harrison, David K. Jackson, Ian Johnston, Dominic Kwiatkowski, Cordelia Langford, John Sillitoe on behalf of the Wellcome Sanger Institute COVID-19 Surveillance Team (http://www.sanger.ac.uk/covid-team)
EPI_ISL_994042	Lighthouse Lab in Cambridge	Wellcome Sanger Institute for the COVID-19 Genomics UK (COG-UK) Consortium	Rob Howes, The Lighthouse Lab in Cambridge and Alex Alderton, Roberto Amato, Sonia Goncalves, Ewan Harrison, David K. Jackson, Ian Johnston, Dominic Kwiatkowski, Cordelia Langford, John Sillitoe on behalf of the Wellcome Sanger Institute COVID-19 Surveillance Team (http://www.sanger.ac.uk/covid-team)
EPI_ISL_994043	Lighthouse Lab in Milton Keynes	Wellcome Sanger Institute for the COVID-19 Genomics UK (COG-UK) Consortium	The Lighthouse Lab in Milton Keynes and Alex Alderton, Roberto Amato, Sonia Goncalves, Ewan Harrison, David K. Jackson, Ian Johnston, Dominic Kwiatkowski, Cordelia Langford, John Sillitoe on behalf of the Wellcome Sanger Institute COVID-19 Surveillance Team (http://www.sanger.ac.uk/covid-team)
EPI_ISL_994044, EPI_ISL_994045, EPI_ISL_994046	Lighthouse Lab in Cambridge	Wellcome Sanger Institute for the COVID-19 Genomics UK (COG-UK) Consortium	Rob Howes, The Lighthouse Lab in Cambridge and Alex Alderton, Roberto Amato, Sonia Goncalves, Ewan Harrison, David K. Jackson, Ian Johnston, Dominic Kwiatkowski, Cordelia Langford, John Sillitoe on behalf of the Wellcome Sanger Institute COVID-19 Surveillance Team (http://www.sanger.ac.uk/covid-team)
EPI_ISL_994047	Lighthouse Lab in Milton Keynes	Wellcome Sanger Institute for the COVID-19 Genomics UK (COG-UK) Consortium	The Lighthouse Lab in Milton Keynes and Alex Alderton, Roberto Amato, Sonia Goncalves, Ewan Harrison, David K. Jackson, Ian Johnston, Dominic Kwiatkowski, Cordelia Langford, John Sillitoe on behalf of the Wellcome Sanger Institute COVID-19 Surveillance Team (http://www.sanger.ac.uk/covid-team)
EPI_ISL_994048	Lighthouse Lab in Cambridge	Wellcome Sanger Institute for the COVID-19 Genomics UK (COG-UK) Consortium	Rob Howes, The Lighthouse Lab in Cambridge and Alex Alderton, Roberto Amato, Sonia Goncalves, Ewan Harrison, David K. Jackson, Ian Johnston, Dominic Kwiatkowski, Cordelia Langford, John Sillitoe on behalf of the Wellcome Sanger Institute COVID-19 Surveillance Team (http://www.sanger.ac.uk/covid-team)
EPI_ISL_994049	Lighthouse Lab in Milton Keynes	Wellcome Sanger Institute for the COVID-19 Genomics UK (COG-UK) Consortium	The Lighthouse Lab in Milton Keynes and Alex Alderton, Roberto Amato, Sonia Goncalves, Ewan Harrison, David K. Jackson, Ian Johnston, Dominic Kwiatkowski, Cordelia Langford, John Sillitoe on behalf of the Wellcome Sanger Institute COVID-19 Surveillance Team (http://www.sanger.ac.uk/covid-team)
EPI_ISL_994050	Lighthouse Lab in Cambridge	Wellcome Sanger Institute for the COVID-19 Genomics UK (COG-UK) Consortium	Rob Howes, The Lighthouse Lab in Cambridge and Alex Alderton, Roberto Amato, Sonia Goncalves, Ewan Harrison, David K. Jackson, Ian Johnston, Dominic Kwiatkowski, Cordelia Langford, John Sillitoe on behalf of the Wellcome Sanger Institute COVID-19 Surveillance Team (http://www.sanger.ac.uk/covid-team)
EPI_ISL_994051	Lighthouse Lab in Milton Keynes	Wellcome Sanger Institute for the COVID-19 Genomics UK (COG-UK) Consortium	The Lighthouse Lab in Milton Keynes and Alex Alderton, Roberto Amato, Sonia Goncalves, Ewan Harrison, David K. Jackson, Ian Johnston, Dominic Kwiatkowski, Cordelia Langford, John Sillitoe on behalf of the Wellcome Sanger Institute COVID-19 Surveillance Team (http://www.sanger.ac.uk/covid-team)

EPI_ISL_994052, EPI_ISL_994053	Lighthouse Lab in Cambridge	Wellcome Sanger Institute for the COVID-19 Genomics UK (COG-UK) Consortium	Rob Howes, The Lighthouse Lab in Cambridge and Alex Alderton, Roberto Amato, Sonia Goncalves, Ewan Harrison, David K. Jackson, Ian Johnston, Dominic Kwiatkowski, Cordelia Langford, John Sillitoe on behalf of the Wellcome Sanger Institute COVID-19 Surveillance Team (http://www.sanger.ac.uk/covid-team)
EPI_ISL_994054	Lighthouse Lab in Milton Keynes	Wellcome Sanger Institute for the COVID-19 Genomics UK (COG-UK) Consortium	The Lighthouse Lab in Milton Keynes and Alex Alderton, Roberto Amato, Sonia Goncalves, Ewan Harrison, David K. Jackson, Ian Johnston, Dominic Kwiatkowski, Cordelia Langford, John Sillitoe on behalf of the Wellcome Sanger Institute COVID-19 Surveillance Team (http://www.sanger.ac.uk/covid-team)
EPI_ISL_994055	Lighthouse Lab in Cambridge	Wellcome Sanger Institute for the COVID-19 Genomics UK (COG-UK) Consortium	Rob Howes, The Lighthouse Lab in Cambridge and Alex Alderton, Roberto Amato, Sonia Goncalves, Ewan Harrison, David K. Jackson, Ian Johnston, Dominic Kwiatkowski, Cordelia Langford, John Sillitoe on behalf of the Wellcome Sanger Institute COVID-19 Surveillance Team (http://www.sanger.ac.uk/covid-team)
EPI_ISL_994057	Lighthouse Lab in Milton Keynes	Wellcome Sanger Institute for the COVID-19 Genomics UK (COG-UK) Consortium	The Lighthouse Lab in Milton Keynes and Alex Alderton, Roberto Amato, Sonia Goncalves, Ewan Harrison, David K. Jackson, Ian Johnston, Dominic Kwiatkowski, Cordelia Langford, John Sillitoe on behalf of the Wellcome Sanger Institute COVID-19 Surveillance Team (http://www.sanger.ac.uk/covid-team)
EPI_ISL_994058, EPI_ISL_994060, EPI_ISL_994061	Lighthouse Lab in Cambridge	Wellcome Sanger Institute for the COVID-19 Genomics UK (COG-UK) Consortium	Rob Howes, The Lighthouse Lab in Cambridge and Alex Alderton, Roberto Amato, Sonia Goncalves, Ewan Harrison, David K. Jackson, Ian Johnston, Dominic Kwiatkowski, Cordelia Langford, John Sillitoe on behalf of the Wellcome Sanger Institute COVID-19 Surveillance Team (http://www.sanger.ac.uk/covid-team)
EPI_ISL_994062, EPI_ISL_994063	Lighthouse Lab in Milton Keynes	Wellcome Sanger Institute for the COVID-19 Genomics UK (COG-UK) Consortium	The Lighthouse Lab in Milton Keynes and Alex Alderton, Roberto Amato, Sonia Goncalves, Ewan Harrison, David K. Jackson, Ian Johnston, Dominic Kwiatkowski, Cordelia Langford, John Sillitoe on behalf of the Wellcome Sanger Institute COVID-19 Surveillance Team (http://www.sanger.ac.uk/covid-team)
EPI_ISL_994064	Lighthouse Lab in Cambridge	Wellcome Sanger Institute for the COVID-19 Genomics UK (COG-UK) Consortium	Rob Howes, The Lighthouse Lab in Cambridge and Alex Alderton, Roberto Amato, Sonia Goncalves, Ewan Harrison, David K. Jackson, Ian Johnston, Dominic Kwiatkowski, Cordelia Langford, John Sillitoe on behalf of the Wellcome Sanger Institute COVID-19 Surveillance Team (http://www.sanger.ac.uk/covid-team)
EPI_ISL_994065	Lighthouse Lab in Milton Keynes	Wellcome Sanger Institute for the COVID-19 Genomics UK (COG-UK) Consortium	The Lighthouse Lab in Milton Keynes and Alex Alderton, Roberto Amato, Sonia Goncalves, Ewan Harrison, David K. Jackson, Ian Johnston, Dominic Kwiatkowski, Cordelia Langford, John Sillitoe on behalf of the Wellcome Sanger Institute COVID-19 Surveillance Team (http://www.sanger.ac.uk/covid-team)
EPI_ISL_994066	Lighthouse Lab in Cambridge	Wellcome Sanger Institute for the COVID-19 Genomics UK (COG-UK) Consortium	Rob Howes, The Lighthouse Lab in Cambridge and Alex Alderton, Roberto Amato, Sonia Goncalves, Ewan Harrison, David K. Jackson, Ian Johnston, Dominic Kwiatkowski, Cordelia Langford, John Sillitoe on behalf of the Wellcome Sanger Institute COVID-19 Surveillance Team (http://www.sanger.ac.uk/covid-team)
EPI_ISL_994288, EPI_ISL_994297, EPI_ISL_994328, EPI_ISL_994338, EPI_ISL_994339, EPI_ISL_994343, EPI_ISL_994357, EPI_ISL_994360, EPI_ISL_994361, EPI_ISL_994386, EPI_ISL_994389, EPI_ISL_994391, EPI_ISL_994402, EPI_ISL_994407, EPI_ISL_994411, EPI_ISL_994413, EPI_ISL_994416, EPI_ISL_994419			
see above	Lighthouse Lab in Alderley Park	Wellcome Sanger Institute for the COVID-19 Genomics UK (COG-UK) Consortium	Jacquelyn Wynn, Mairead Hyland, The Lighthouse Lab in Alderley Park and Alex Alderton, Roberto Amato, Sonia Goncalves, Ewan Harrison, David K. Jackson, Ian Johnston, Dominic Kwiatkowski, Cordelia Langford, John Sillitoe on behalf of the Wellcome Sanger Institute COVID-19 Surveillance Team (http://www.sanger.ac.uk/covid-team)
EPI_ISL_994424, EPI_ISL_994446, EPI_ISL_994448, EPI_ISL_994456, EPI_ISL_994464, EPI_ISL_994467, EPI_ISL_994468, EPI_ISL_994473, EPI_ISL_994478, EPI_ISL_994481, EPI_ISL_994482, EPI_ISL_994484, EPI_ISL_994488, EPI_ISL_994496, EPI_ISL_994507, EPI_ISL_994513, EPI_ISL_994514, EPI_ISL_994515, EPI_ISL_994529, EPI_ISL_994538, EPI_ISL_994543, EPI_ISL_994590, EPI_ISL_994615, EPI_ISL_994619, EPI_ISL_994622, EPI_ISL_994623, EPI_ISL_994624, EPI_ISL_994626, EPI_ISL_994629, EPI_ISL_994630, EPI_ISL_994633, EPI_ISL_994634, EPI_ISL_994635, EPI_ISL_994636, EPI_ISL_994637, EPI_ISL_994639, EPI_ISL_994641, EPI_ISL_994643, EPI_ISL_994646			
see above	Lighthouse Lab in Alderley Park	Wellcome Sanger Institute for the COVID-19 Genomics UK (COG-UK) Consortium	Jacquelyn Wynn, Mairead Hyland, The Lighthouse Lab in Alderley Park and Alex Alderton, Roberto Amato, Sonia Goncalves, Ewan Harrison, David K. Jackson, Ian Johnston, Dominic Kwiatkowski, Cordelia Langford, John Sillitoe on behalf of the Wellcome Sanger Institute COVID-19 Surveillance Team
EPI_ISL_996066, EPI_ISL_996067, EPI_ISL_996069, EPI_ISL_996070, EPI_ISL_996071, EPI_ISL_996073, EPI_ISL_996075, EPI_ISL_996077, EPI_ISL_996079, EPI_ISL_996080, EPI_ISL_996081			
see above	Department of Pathology, University of Cambridge	COVID-19 Genomics UK (COG-UK) Consortium	Aminu S. Jahun, Yasmin Chaudhry, Iliana Georgana, Myra Hosmillo, Rhys Izuagbe, William L. Hamilton, Martin D. Curran, Surendra Parmar, Ian Goodfellow
EPI_ISL_996422, EPI_ISL_996423, EPI_ISL_996431, EPI_ISL_996432, EPI_ISL_996449, EPI_ISL_996453, EPI_ISL_996455, EPI_ISL_996459	University of Birmingham	COVID-19 Genomics UK (COG-UK) Consortium	Institute of Microbiology, University of Birmingham: Claire McMurray, Joanne Stockton, Samuel Nicholls, Radoslaw Poplawski, Will Rowe, Josh Quick, Nicholas Loman. University of Birmingham Testing Laboratory: Celina M Whalley, Andrew Bosworth, Charlotte Poxon, Kasun Wanigasooriya, Oliver Pickles, Mike Kidd, Alex Richter, Andrew D Beggs PHE Heartlands Lab: Husam Osman, Andrew Bosworth. Queen Elizabeth Hospital: Anna Casey
EPI_ISL_996579, EPI_ISL_996594	University of Exeter	COVID-19 Genomics UK (COG-UK) Consortium	Ben Temperton,Aaron Jeffries,Michelle Michelsen,Joanna Warwick-Dugdale,Audrey Farbos,Robyn Manley,Stephen Michell,Jane Masoli
EPI_ISL_997075, EPI_ISL_997076, EPI_ISL_997159, EPI_ISL_997160, EPI_ISL_997163	Virology Department, Royal Infirmary of Edinburgh, NHS Lothian / School of Biological Sciences, University of Edinburgh	COVID-19 Genomics UK (COG-UK) Consortium	McHugh M, Dewar R, Cotton S, Rooke S, O'Toole Á, Scher E, Hill V, McCrone JT, Colquhoun R, Yu X, Jackson B, Rambaut A, Templeton K
EPI_ISL_997328, EPI_ISL_997329, EPI_ISL_997330, EPI_ISL_997331, EPI_ISL_997332, EPI_ISL_997337	University of Exeter	COVID-19 Genomics UK (COG-UK) Consortium	Ben Temperton,Aaron Jeffries,Michelle Michelsen,Joanna Warwick-Dugdale,Audrey Farbos,Robyn Manley,Stephen Michell,Jane Masoli
EPI_ISL_997378, EPI_ISL_997384, EPI_ISL_997385, EPI_ISL_997386, EPI_ISL_997387, EPI_ISL_997388, EPI_ISL_997389, EPI_ISL_997390, EPI_ISL_997391, EPI_ISL_997392	Liverpool Clinical Laboratories	COVID-19 Genomics UK (COG-UK) Consortium	Sam Haldenby, Anita Lucaci, Steve Paterson, Julian Hiscox, Alistair Darby, M Almsaud, A Alrezaihi, Muhannad Alruwaili, Stuart D Armstrong, Jones Benjamin, Eleanor G Bentley, Anu Chawla, Jordan J Clark, Angela Cowell, Richard Eccles, Isabel García-Dorival, Matthew Gemmell, Alessandro Gerada, PKF Gilmore, Richard Gregory, Ximeng Han, Catherine Hartley, Margaret Hughes, Miren Iturriza-Gomara, James Johnson, L Luu, Jenifer Manson, Charlotte Nelson, Elaine O'Toole, Cassie Olateju, Rebekah Penrice-Randal , Lucille Rainbow, N.P Randle, Trevor Ian Robinson, Parul Sharma, Ghada T Shawli, James P Stewart, Neil Swainston, Ecaterina Vamos, Joanne Watts, Mark Whitehead
EPI_ISL_997616, EPI_ISL_997617, EPI_ISL_997618, EPI_ISL_997619, EPI_ISL_997620, EPI_ISL_997621, EPI_ISL_997622, EPI_ISL_997623, EPI_ISL_997624, EPI_ISL_997630, EPI_ISL_997631, EPI_ISL_997632, EPI_ISL_997633, EPI_ISL_997634, EPI_ISL_997635, EPI_ISL_997636, EPI_ISL_997637, EPI_ISL_997638, EPI_ISL_997639, EPI_ISL_997640, EPI_ISL_997641, EPI_ISL_997643, EPI_ISL_997644			
see above	Barts Health NHS Trust	COVID-19 Genomics UK (COG-UK) Consortium	CUTINO-MOGUEL, Maria-Teresa; HARRINGTON, David; OWOYEMI, Dola; KULASEGARAN-SHYLINI, Raghavendran; BROAD, Claire; KELE, Beatrix
EPI_ISL_997677, EPI_ISL_997840, EPI_ISL_997841, EPI_ISL_997842, EPI_ISL_997995, EPI_ISL_997996, EPI_ISL_998002, EPI_ISL_998003, EPI_ISL_998004, EPI_ISL_998005	University College London, Great Ormond Street Hospital for Children NHS Foundation Trust, Imperial College Healthcare NHS Trust	COVID-19 Genomics UK (COG-UK) Consortium	Sergi Castellano, Rachel Williams, Mark Kristiansen, Paola Resende Silva, Sunando Roy, Tony Brooks, Helena Tutill, Paola Niola, Patricia Dyal, Charlotte Williams, Leysa Forrest, Yasmin Panchbhaya, Jacqueline Findlay, Samuel Weeks, Julianne Brown, Kathryn Harris, Paul Randell, James Price, Alison Holmes, Judith Breuer
EPI_ISL_998150, EPI_ISL_998151, EPI_ISL_998152, EPI_ISL_998153, EPI_ISL_998155, EPI_ISL_998156, EPI_ISL_998157, EPI_ISL_998160, EPI_ISL_998161, EPI_ISL_998162, EPI_ISL_998171, EPI_ISL_998172, EPI_ISL_998174, EPI_ISL_998189, EPI_ISL_998190, EPI_ISL_998191, EPI_ISL_998192, EPI_ISL_998193, EPI_ISL_998194, EPI_ISL_998195, EPI_ISL_998196, EPI_ISL_998198, EPI_ISL_998200, EPI_ISL_998218, EPI_ISL_998219, EPI_ISL_998220, EPI_ISL_998221, EPI_ISL_998238, EPI_ISL_998240, EPI_ISL_998241, EPI_ISL_998243, EPI_ISL_998244, EPI_ISL_998245, EPI_ISL_998246, EPI_ISL_998247			
see above	Regional Virus Laboratory, Belfast Health and Social Care Trust	COVID-19 Genomics UK (COG-UK) Consortium	Conall McCaughey, James McKenna, Tanya Curran, Susan Feeney, Alison Watt, Ciara Cox, Mairead Connor, Zoltan Molnar, David Simpson, Derek Fairley
EPI_ISL_998313, EPI_ISL_998314, EPI_ISL_998316, EPI_ISL_998317, EPI_ISL_998318, EPI_ISL_998319, EPI_ISL_998320, EPI_ISL_998321, EPI_ISL_998323, EPI_ISL_998324, EPI_ISL_998325, EPI_ISL_998326, EPI_ISL_998327, EPI_ISL_998328, EPI_ISL_998329, EPI_ISL_998330, EPI_ISL_998331, EPI_ISL_998332, EPI_ISL_998333, EPI_ISL_998334, EPI_ISL_998335, EPI_ISL_998336, EPI_ISL_998337, EPI_ISL_998338, EPI_ISL_998339, EPI_ISL_998340, EPI_ISL_998341, EPI_ISL_998346, EPI_ISL_998351, EPI_ISL_998352, EPI_ISL_998353, EPI_ISL_998354, EPI_ISL_998355, EPI_ISL_998356, EPI_ISL_998357, EPI_ISL_998358, EPI_ISL_998359, EPI_ISL_998360, EPI_ISL_998361, EPI_ISL_998362, EPI_ISL_998363, EPI_ISL_998365, EPI_ISL_998366, EPI_ISL_998367, EPI_ISL_998373, EPI_ISL_998374, EPI_ISL_998375, EPI_ISL_998376, EPI_ISL_998377, EPI_ISL_998378, EPI_ISL_998379			
see above	Northumbria University / South Tees Hospitals NHS Foundation Trust / North Cumbria Integrated Care NHS Foundation Trust / North Tees and Hartlepool NHS Foundation Trust / Newcastle Hospitals NHS Foundation Trust	COVID-19 Genomics UK (COG-UK) Consortium	Darren L Smith,Andrew Nelson,Matthew Bashton,Greg R Young,Joshua Loh,John Allan,Mohammad A Tariq,Giles S Holt,Gary Black,Wen C Yew,Lynn Dover,Paul Baker,Steve Liggett,Sarah Essex,Jane Greenaway,Debra Padgett,Clive Graham,Garren Scott,Edward Barton,Emma Swindells,Brendan Payne,Jennifer Collins,Yusri Taha,Gary Eltringham

EPI_ISL_998945, EPI_ISL_998946, EPI_ISL_998947, EPI_ISL_998948, EPI_ISL_998949, EPI_ISL_998950, EPI_ISL_998951, EPI_ISL_998952, EPI_ISL_998953, EPI_ISL_998954, EPI_ISL_998955, EPI_ISL_998956			
see above	Lincolnshire Hospitals and DeepSeq Nottingham	COVID-19 Genomics UK (COG-UK) Consortium	Nichola Duckworth, Tim Sloan, Sarah Walsh, Jonathan Ball, Patrick McClure, Joeseph Chappell, Nadine Holmes, Matthew Carlisle, Christopher Moore, Fei Sang, Johnny Debebe, Victoria Wright, Matthew Loose
EPI_ISL_999068, EPI_ISL_999069, EPI_ISL_999071, EPI_ISL_999078, EPI_ISL_999081, EPI_ISL_999083, EPI_ISL_999084, EPI_ISL_999085, EPI_ISL_999086, EPI_ISL_999087, EPI_ISL_999089, EPI_ISL_999092, EPI_ISL_999093, EPI_ISL_999095, EPI_ISL_999099, EPI_ISL_999100, EPI_ISL_999101, EPI_ISL_999102, EPI_ISL_999103, EPI_ISL_999104, EPI_ISL_999105, EPI_ISL_999106, EPI_ISL_999108, EPI_ISL_999109, EPI_ISL_999111, EPI_ISL_999112, EPI_ISL_999113, EPI_ISL_999114, EPI_ISL_999116, EPI_ISL_999119, EPI_ISL_999120, EPI_ISL_999121, EPI_ISL_999122, EPI_ISL_999123, EPI_ISL_999124, EPI_ISL_999125, EPI_ISL_999126, EPI_ISL_999127, EPI_ISL_999132, EPI_ISL_999134, EPI_ISL_999136, EPI_ISL_999137, EPI_ISL_999138, EPI_ISL_999139, EPI_ISL_999141, EPI_ISL_999142, EPI_ISL_999143, EPI_ISL_999144, EPI_ISL_999145, EPI_ISL_999146, EPI_ISL_999148, EPI_ISL_999150, EPI_ISL_999151, EPI_ISL_999152, EPI_ISL_999153, EPI_ISL_999154, EPI_ISL_999156, EPI_ISL_999161, EPI_ISL_999169, EPI_ISL_999204, EPI_ISL_999205, EPI_ISL_999207, EPI_ISL_999208, EPI_ISL_999210, EPI_ISL_999223, EPI_ISL_999374, EPI_ISL_999379, EPI_ISL_999381, EPI_ISL_999382, EPI_ISL_999385, EPI_ISL_999394, EPI_ISL_999395, EPI_ISL_999397, EPI_ISL_999401, EPI_ISL_999402, EPI_ISL_999404, EPI_ISL_999405, EPI_ISL_999406, EPI_ISL_999407, EPI_ISL_999427, EPI_ISL_999436, EPI_ISL_999462, EPI_ISL_999463, EPI_ISL_999479, EPI_ISL_999481, EPI_ISL_999506, EPI_ISL_999507			
see above	Oxford Viromics, NDM, University of Oxford; Oxford University Hospitals; Basingstoke and North Hampshire Hospital	COVID-19 Genomics UK (COG-UK) Consortium	Tanya Golubchik, David Bonsall, George Macintyre, Amy Trebes, Mariateresa de Cesare, Catrin Moore, Alex Mobbs, Anita Justice, Robert Shaw, Monique Andersson, Timothy Peto, Emma Wise, Nathan Moore, Jessica Lynch, Nick Cortes, Matilde Mori, Stephen Kidd, David Buck, John Todd, Christophe Fraser
