# Supplementary material for: Inferring the Association between the Risk of COVID-19 Case Fatality and N501Y Substitution in SARS-CoV-2
Source: Viruses. 2021 Apr 8;13(4):638. doi: 10.3390/v13040638 (PMC8070306; doi:10.3390/v13040638)
Supplement: Supplementary file 1 [file viruses-13-00638-s001.zip › gisaid_hcov-19_UKAT_210121-210124.pdf]

We gratefully acknowledge the following Authors from the Originating laboratories responsible for obtaining the specimens, as well as the Submitting laboratories where the genome data were generated and shared via GISAID, on which this research is based.

All Submitters of data may be contacted directly via www.gisaid.org

Authors are sorted alphabetically.

Accession ID	Originating Laboratory	Submitting Laboratory	Authors
EPI_ISL_1000248, EPI_ISL_1000249, EPI_ISL_1000250, EPI_ISL_1000251, EPI_ISL_1000253, EPI_ISL_1000254, EPI_ISL_1000259, EPI_ISL_1000263, EPI_ISL_1000455, EPI_ISL_1000626	Centre for Enzyme Innovation, University of Portsmouth / Translational Research Laboratory, Portsmouth Hospitals NHS Trust	COVID-19 Genomics UK (COG-UK) Consortium	Angela Beckett,Salman Goudarzi,Christopher Fearn,Kate Cook,Katie Loveson,Sharon Glaysher,Scott Elliott,Samuel Robson
EPI_ISL_1000641, EPI_ISL_1000703, EPI_ISL_1000706, EPI_ISL_1000725	Virology Department, Sheffield Teaching Hospitals NHS Foundation Trust/Department of Infection, Immunity and Cardiovascular Disease, The Medical School, University of Sheffield	COVID-19 Genomics UK (COG-UK) Consortium	Thushan de Silva, Matthew Parker, Nikki Smith, Adri Angyal, Rebecca Brown, Luke Green, Rachel Tucker, Paul Parsons, Danielle Groves, Katie Johnson, Laura Carrilero, Alex Keeley, Dave Partridge, Matthew Wyles, Benjamin Lindsey, Mehmet Yavuz, Mohammad Raza, Cariad Evans
EPI_ISL_1000795, EPI_ISL_1000796, EPI_ISL_1000797, EPI_ISL_1000798, EPI_ISL_1000799, EPI_ISL_1000800, EPI_ISL_1000801, EPI_ISL_1000802, EPI_ISL_1000803, EPI_ISL_1000804, EPI_ISL_1000805, EPI_ISL_1000807, EPI_ISL_1000809, EPI_ISL_1000810, EPI_ISL_1000812, EPI_ISL_1000813, EPI_ISL_1000814, EPI_ISL_1000815, EPI_ISL_1000817, EPI_ISL_1000818, EPI_ISL_1000820, EPI_ISL_1000821, EPI_ISL_1000823, EPI_ISL_1000824, EPI_ISL_1000825, EPI_ISL_1000827, EPI_ISL_1000828, EPI_ISL_1000829, EPI_ISL_1000830, EPI_ISL_1000831, EPI_ISL_1000891, EPI_ISL_1000894, EPI_ISL_1000909, EPI_ISL_1000911, EPI_ISL_1000963			
see above	Bioinformatics and Biostatistics Lab, Advanced Sequencing Facility	COVID-19 Genomics UK (COG-UK) Consortium	Aengus Stewart,Jerome Nicod,Chelsea Sawyer,Laura Cubitt,Harshil Patel,Margaret Crawford
EPI_ISL_1007525	Lighthouse Lab in Cambridge	Wellcome Sanger Institute for the COVID-19 Genomics UK (COG-UK) Consortium	Rob Howes, The Lighthouse Lab in Cambridge and Alex Alderton, Roberto Amato, Sonia Goncalves, Ewan Harrison, David K. Jackson, Ian Johnston, Dominic Kwiatkowski, Cordelia Langford, John Sillitoe on behalf of the Wellcome Sanger Institute COVID-19 Surveillance Team
EPI_ISL_1007566, EPI_ISL_1007567, EPI_ISL_1012754, EPI_ISL_1012762, EPI_ISL_1012787, EPI_ISL_1012788, EPI_ISL_1012789, EPI_ISL_1012790, EPI_ISL_1012791, EPI_ISL_1012792, EPI_ISL_1012793, EPI_ISL_1012794, EPI_ISL_1012795, EPI_ISL_1012796, EPI_ISL_1012797, EPI_ISL_1012798, EPI_ISL_1012799, EPI_ISL_1012800, EPI_ISL_1012801, EPI_ISL_1012802, EPI_ISL_1012803, EPI_ISL_1012804, EPI_ISL_1012805, EPI_ISL_1012806			
see above	Lighthouse Lab in Alderley Park	Wellcome Sanger Institute for the COVID-19 Genomics UK (COG-UK) Consortium	Jacquelyn Wynn, Mairead Hyland, The Lighthouse Lab in Alderley Park and Alex Alderton, Roberto Amato, Sonia Goncalves, Ewan Harrison, David K. Jackson, Ian Johnston, Dominic Kwiatkowski, Cordelia Langford, John Sillitoe on behalf of the Wellcome Sanger Institute COVID-19 Surveillance Team
EPI_ISL_1012808	Lighthouse Lab in Glasgow	Wellcome Sanger Institute for the COVID-19 Genomics UK (COG-UK) Consortium	Harper VanSteenhouse, Yumi Kasai, David Gray, Carol Clugston, Anna Dominiczak and Alex Alderton, Roberto Amato, Sonia Goncalves, Ewan Harrison, David K. Jackson, Ian Johnston, Dominic Kwiatkowski, Cordelia Langford, John Sillitoe on behalf of the Wellcome Sanger Institute COVID-19 Surveillance Team
EPI_ISL_1012809, EPI_ISL_1012817, EPI_ISL_1012820, EPI_ISL_1012826, EPI_ISL_1012829, EPI_ISL_1012830, EPI_ISL_1012835, EPI_ISL_1012841, EPI_ISL_1012842, EPI_ISL_1012843, EPI_ISL_1012847, EPI_ISL_1012848, EPI_ISL_1012850			
see above	Lighthouse Lab in Alderley Park	Wellcome Sanger Institute for the COVID-19 Genomics UK (COG-UK) Consortium	Jacquelyn Wynn, Mairead Hyland, The Lighthouse Lab in Alderley Park and Alex Alderton, Roberto Amato, Sonia Goncalves, Ewan Harrison, David K. Jackson, Ian Johnston, Dominic Kwiatkowski, Cordelia Langford, John Sillitoe on behalf of the Wellcome Sanger Institute COVID-19 Surveillance Team
EPI_ISL_1019608, EPI_ISL_1019615, EPI_ISL_1019628, EPI_ISL_1019641, EPI_ISL_1019666, EPI_ISL_1019667, EPI_ISL_1019690, EPI_ISL_1019706, EPI_ISL_1019711, EPI_ISL_1019730, EPI_ISL_1019732, EPI_ISL_1019741, EPI_ISL_1019744, EPI_ISL_1019747, EPI_ISL_1019754, EPI_ISL_1019781, EPI_ISL_1019782, EPI_ISL_1019784, EPI_ISL_1019786, EPI_ISL_1019787, EPI_ISL_1019789, EPI_ISL_1019791, EPI_ISL_1019792, EPI_ISL_1019794, EPI_ISL_1019796, EPI_ISL_1019797, EPI_ISL_1019799, EPI_ISL_1019801, EPI_ISL_1019802, EPI_ISL_1019804, EPI_ISL_1019806, EPI_ISL_1019808, EPI_ISL_1019809, EPI_ISL_1019811, EPI_ISL_1019812, EPI_ISL_1019814			
see above	Lighthouse Lab in Glasgow	Wellcome Sanger Institute for the COVID-19 Genomics UK (COG-UK) Consortium	Harper VanSteenhouse, Yumi Kasai, David Gray, Carol Clugston, Anna Dominiczak and Alex Alderton, Roberto Amato, Jeffrey Barrett, Sonia Goncalves, Ewan Harrison, David K. Jackson, Ian Johnston, Dominic Kwiatkowski, Cordelia Langford, John Sillitoe on behalf of the Wellcome Sanger Institute COVID-19 Surveillance Team
EPI_ISL_1043901	Lighthouse Lab in Alderley Park	Wellcome Sanger Institute for the COVID-19 Genomics UK (COG-UK) Consortium	Jacquelyn Wynn, Mairead Hyland, The Lighthouse Lab in Alderley Park and Alex Alderton, Roberto Amato, Jeffrey Barrett, Sonia Goncalves, Ewan Harrison, David K. Jackson, Ian Johnston, Dominic Kwiatkowski, Cordelia Langford, John Sillitoe on behalf of the Wellcome Sanger Institute COVID-19 Surveillance Team
EPI_ISL_1046238, EPI_ISL_1046310, EPI_ISL_1046322	Randox Laboratories	Wellcome Sanger Institute for the COVID-19 Genomics UK (COG-UK) Consortium	Randox Laboratories and Alex Alderton, Roberto Amato, Jeffrey Barrett, Sonia Goncalves, Ewan Harrison, David K. Jackson, Ian Johnston, Dominic Kwiatkowski, Cordelia Langford, John Sillitoe on behalf of the Wellcome Sanger Institute COVID-19 Surveillance Team
EPI_ISL_1046991, EPI_ISL_1047001, EPI_ISL_1047004, EPI_ISL_1047005, EPI_ISL_1047007, EPI_ISL_1047008, EPI_ISL_1047009, EPI_ISL_1047022, EPI_ISL_1047062	University of Birmingham	COVID-19 Genomics UK (COG-UK) Consortium	Institute of Microbiology, University of Birmingham: Claire McMurray, Joanne Stockton, Samuel Nicholls, Radoslaw Poplawski, Will Rowe, Josh Quick, Nicholas Loman. University of Birmingham Testing Laboratory: Celina M Whalley, Andrew Bosworth, Charlotte Poxon, Kasun Wanigasooriya, Oliver Pickles, Mike Kidd, Alex Richter, Andrew D Beggs PHE Heartlands Lab: Husam Osman, Andrew Bosworth. Queen Elizabeth Hospital: Anna Casey
EPI_ISL_1047184, EPI_ISL_1047187, EPI_ISL_1047191, EPI_ISL_1047331, EPI_ISL_1047511	Department of Pathology, University of Cambridge	COVID-19 Genomics UK (COG-UK) Consortium	Aminu S. Jahun, Yasmin Chaudhry, Iliana Georgana, Myra Hosmillo, Rhys Izuagbe, William L. Hamilton, Martin D. Curran, Surendra Parmar, Ian Goodfellow
EPI_ISL_1047647, EPI_ISL_1047648, EPI_ISL_1047649, EPI_ISL_1047650, EPI_ISL_1047651, EPI_ISL_1047652, EPI_ISL_1047653, EPI_ISL_1047654, EPI_ISL_1047655, EPI_ISL_1047656, EPI_ISL_1047657, EPI_ISL_1047658, EPI_ISL_1047659, EPI_ISL_1047660, EPI_ISL_1047661, EPI_ISL_1047662, EPI_ISL_1047663, EPI_ISL_1047664, EPI_ISL_1047665, EPI_ISL_1047666, EPI_ISL_1047667, EPI_ISL_1047668, EPI_ISL_1047669, EPI_ISL_1047670, EPI_ISL_1047671, EPI_ISL_1047672, EPI_ISL_1047673, EPI_ISL_1047674, EPI_ISL_1047675, EPI_ISL_1047676, EPI_ISL_1047677, EPI_ISL_1047678			
see above	Virology Department, Sheffield Teaching Hospitals NHS Foundation Trust/Department of Infection, Immunity and Cardiovascular Disease, The Medical School, University of Sheffield	COVID-19 Genomics UK (COG-UK) Consortium	Thushan de Silva, Matthew Parker, Nikki Smith, Adri Angyal, Rebecca Brown, Luke Green, Rachel Tucker, Paul Parsons, Danielle Groves, Katie Johnson, Laura Carrilero, Alex Keeley, Dave Partridge, Matthew Wyles, Benjamin Lindsey, Mehmet Yavuz, Mohammad Raza, Cariad Evans
EPI_ISL_1047836	West of Scotland Specialist Virology Centre, NHSGGC / MRC-University of Glasgow Centre for Virus Research	COVID-19 Genomics UK (COG-UK) Consortium	Ana da Silva Filipe, Natasha Johnson, Kathy Smollett, Daniel Mair, Stephen Carmichael, Alice Broos, Lily Tong, Jenna Nichols, Kyriaki Nomikou; Sarah McDonald; Richard Orton, Joseph Hughes, Sreenu Vattipally, David L Robertson; Alasdair MacLean, Rory Gunson; Sharif Shaaban, Matthew Holden; Rachel Blacow, Guy Mollett, Kathy Li, James Shepherd, Antonia Ho, Emma Thomson
EPI_ISL_1047893	Virology Department, Royal Infirmary of Edinburgh, NHS Lothian / School of Biological Sciences, University of Edinburgh	COVID-19 Genomics UK (COG-UK) Consortium	McHugh M, Dewar R, Cotton S, Rooke S, O'Toole Á, Scher E, Hill V, McCrone JT, Colquhoun R, Yu X, Jackson B, Rambaut A, Templeton K
EPI_ISL_1048078, EPI_ISL_1048083, EPI_ISL_1048095	Barts Health NHS Trust	COVID-19 Genomics UK (COG-UK) Consortium	CUTINO-MOGUEL, Maria-Teresa; HARRINGTON, David; OWOYEMI, Dola; KULASEGARAN-SHYLINI, Raghavendran; BROAD, Claire; KELE, Beatrix
EPI_ISL_1050224, EPI_ISL_1050226, EPI_ISL_1050228, EPI_ISL_1050229, EPI_ISL_1050230, EPI_ISL_1050231, EPI_ISL_1050232	Lighthouse Lab in Glasgow / MRC-University of Glasgow Centre for Virus Research	COVID-19 Genomics UK (COG-UK) Consortium	Ana da Silva Filipe, Natasha Johnson, Kathy Smollett, Daniel Mair, Stephen Carmichael, Alice Broos, Lily Tong, Jenna Nichols, Kyriaki Nomikou; Sarah McDonald; Harper VanSteenhouse, Yumi Kasai, David Gray, Carol Clugston, Anna Dominiczak; Alasdair MacLean, Rory Gunson; Richard Orton, Joseph Hughes, Sreenu Vattipally, David L Robertson; Sharif Shaaban, Matthew Holden; Kathy Li, James Shepherd, Antonia Ho, Emma Thomson
EPI_ISL_1050241	West of Scotland Specialist Virology Centre, NHSGGC /	COVID-19 Genomics UK (COG-UK) Consortium	Ana da Silva Filipe, Natasha Johnson, Kathy Smollett, Daniel Mair, Stephen Carmichael, Alice Broos, Lily Tong, Jenna Nichols, Kyriaki Nomikou; Sarah

	MRC-University of Glasgow Centre for Virus Research		McDonald; Richard Orton, Joseph Hughes, Sreenu Vattipally, David L Robertson; Alasdair MacLean, Rory Gunson; Sharif Shaaban, Matthew Holden; Rachel Blacow, Guy Mollett, Kathy Li, James Shepherd, Antonia Ho, Emma Thomson
EPI_ISL_1051431, EPI_ISL_1051435, EPI_ISL_1051436, EPI_ISL_1051441, EPI_ISL_1051452, EPI_ISL_1051484, EPI_ISL_1051490, EPI_ISL_1051506	Quadram Institute Bioscience	COVID-19 Genomics UK (COG-UK) Consortium	Dave J. Baker, Gemma L. Kay, Alp Aydin, Thanh Le-Viet, Steven Rudder, Ana P. Tedim, Anastasia Kolyva, Maria Diaz, Leonardo de Oliveira Martins, Nabil-Fareed Alikhan, Lizzie Meadows, Rachael Stanley, Ngozi Elumogo, Muhammed Yasir, Nicholas M. Thomson, Alexander J Trotter, Rachel Gilroy, Samuel Bloomfield, Claire Stuart, Andrew Bell, Reenesh Prakash, Samir Dervisevic, Alison E. Mather, John Wain, Mark Webber, Andrew J. Page, Justin O'Grady
EPI_ISL_1051594, EPI_ISL_1051601, EPI_ISL_1051860, EPI_ISL_1051861, EPI_ISL_1051862, EPI_ISL_1051870, EPI_ISL_1051871, EPI_ISL_1051885, EPI_ISL_1051886, EPI_ISL_1051887	Oxford Viromics, NDM, University of Oxford; Oxford University Hospitals; Basingstoke and North Hampshire Hospital	COVID-19 Genomics UK (COG-UK) Consortium	Tanya Golubchik, David Bonsall, George Macintyre, Amy Trebes, Mariateresa de Cesare, Catrin Moore, Alex Mobbs, Anita Justice, Robert Shaw, Monique Andersson, Timothy Peto, Emma Wise, Nathan Moore, Jessica Lynch, Nick Cortes, Matilde Mori, Stephen Kidd, David Buck, John Todd, Christophe Fraser
EPI_ISL_1052463, EPI_ISL_1052464, EPI_ISL_1052466, EPI_ISL_1052467, EPI_ISL_1052468, EPI_ISL_1052489, EPI_ISL_1052490, EPI_ISL_1052491, EPI_ISL_1052496, EPI_ISL_1052502, EPI_ISL_1052504, EPI_ISL_1052505, EPI_ISL_1052506, EPI_ISL_1052507, EPI_ISL_1052508, EPI_ISL_1052509, EPI_ISL_1052510, EPI_ISL_1053525, EPI_ISL_1053547			
see above	Originating lab: Wales Specialist Virology Centre Sequencing lab: Pathogen Genomics Unit	Public Health Wales Microbiology Cardiff Wales Specialist Virology Centre	Catherine Moore, Johnathan Evans, Laura Gifford, Malorie Perry, Simon Cottrell, Angela Marchbank, Alec Birchley, Alexander Adams, Amy Gaskin, Bree Gatica-Wilcox, Jason Coombes, Joel Southgate, Lauren Gilbert, Lee Graham, Nicole Pacchiarini, Sara Kumziene-Summerhayes, Sarah Taylor, Sophie Jones, Sara Rey, Matthew Bull, Joanne Watkins, Sally Corden, Tom Connor
EPI_ISL_1053828, EPI_ISL_1053829, EPI_ISL_1053830, EPI_ISL_1053831, EPI_ISL_1053832, EPI_ISL_1053833, EPI_ISL_1053834, EPI_ISL_1053835, EPI_ISL_1053836, EPI_ISL_1053837, EPI_ISL_1053838, EPI_ISL_1053839, EPI_ISL_1053840, EPI_ISL_1053841, EPI_ISL_1053842, EPI_ISL_1053843, EPI_ISL_1053881, EPI_ISL_1053884, EPI_ISL_1053885, EPI_ISL_1053886, EPI_ISL_1053887, EPI_ISL_1053888, EPI_ISL_1053889, EPI_ISL_1053890, EPI_ISL_1053891, EPI_ISL_1053934, EPI_ISL_1053935, EPI_ISL_1053936, EPI_ISL_1053937, EPI_ISL_1053976			
see above	Centre for Enzyme Innovation, University of Portsmouth / Translational Research Laboratory, Portsmouth Hospitals NHS Trust	COVID-19 Genomics UK (COG-UK) Consortium	Angela Beckett,Salman Goudarzi,Christopher Fearn,Kate Cook,Katie Loveson,Sharon Glaysher,Scott Elliott,Samuel Robson
EPI_ISL_1054664	Virology Department, Sheffield Teaching Hospitals NHS Foundation Trust/Department of Infection, Immunity and Cardiovascular Disease, The Medical School, University of Sheffield	COVID-19 Genomics UK (COG-UK) Consortium	Thushan de Silva, Matthew Parker, Nikki Smith, Adri Angyal, Rebecca Brown, Luke Green, Rachel Tucker, Paul Parsons, Danielle Groves, Katie Johnson, Laura Carrilero, Alex Keeley, Dave Partridge, Matthew Wyles, Benjamin Lindsey, Mehmet Yavuz, Mohammad Raza, Cariad Evans
EPI_ISL_1054721, EPI_ISL_1054724, EPI_ISL_1054730, EPI_ISL_1054731, EPI_ISL_1054732, EPI_ISL_1054733, EPI_ISL_1054734, EPI_ISL_1054735, EPI_ISL_1054736, EPI_ISL_1054737, EPI_ISL_1054738, EPI_ISL_1054739, EPI_ISL_1054740, EPI_ISL_1054741, EPI_ISL_1054742, EPI_ISL_1054743, EPI_ISL_1054744, EPI_ISL_1054745, EPI_ISL_1054746, EPI_ISL_1054747, EPI_ISL_1054748, EPI_ISL_1054749, EPI_ISL_1054750, EPI_ISL_1054753, EPI_ISL_1054754, EPI_ISL_1054755, EPI_ISL_1054818			
see above	Bioinformatics and Biostatistics Lab, Advanced Sequencing Facility	COVID-19 Genomics UK (COG-UK) Consortium	Aengus Stewart,Jerome Nicod,Chelsea Sawyer,Laura Cubitt,Harshil Patel,Margaret Crawford
EPI_ISL_1057732, EPI_ISL_1057745	Lighthouse Lab in Alderley Park	Wellcome Sanger Institute for the COVID-19 Genomics UK (COG-UK) Consortium	Jacquelyn Wynn, Mairead Hyland, The Lighthouse Lab in Alderley Park and Alex Alderton, Roberto Amato, Jeffrey Barrett, Sonia Goncalves, Ewan Harrison, David K. Jackson, Ian Johnston, Dominic Kwiatkowski, Cordelia Langford, John Sillitoe on behalf of the Wellcome Sanger Institute COVID-19 Surveillance Team
EPI_ISL_1104114, EPI_ISL_1104115, EPI_ISL_1104116, EPI_ISL_1104117, EPI_ISL_1104118, EPI_ISL_1104119, EPI_ISL_1104120, EPI_ISL_1104121, EPI_ISL_1104122	Lighthouse Lab in Glasgow / MRC-University of Glasgow Centre for Virus Research	COVID-19 Genomics UK (COG-UK) Consortium	Ana da Silva Filipe, Natasha Johnson, Kathy Smollett, Daniel Mair, Stephen Carmichael, Alice Broos, Lily Tong, Jenna Nichols, Kyriaki Nomikou; Sarah McDonald; Harper VanSteenhouse, Yumi Kasai, David Gray, Carol Clugston, Anna Dominiczak; Alasdair MacLean, Rory Gunson; Richard Orton, Joseph Hughes, Sreenu Vattipally, David L Robertson; Sharif Shaaban, Matthew Holden; Kathy Li, James Shepherd, Antonia Ho, Emma Thomson
EPI_ISL_1104219, EPI_ISL_1104229, EPI_ISL_1104262, EPI_ISL_1104263	Virology Department, Royal Infirmary of Edinburgh, NHS Lothian / School of Biological Sciences, University of Edinburgh	COVID-19 Genomics UK (COG-UK) Consortium	McHugh M, Dewar R, Cotton S, Rooke S, O'Toole Á, Scher E, Hill V, McCrone JT, Colquhoun R, Yu X, Jackson B, Rambaut A, Templeton K
EPI_ISL_1104343, EPI_ISL_1104354, EPI_ISL_1104356, EPI_ISL_1104357, EPI_ISL_1104363, EPI_ISL_1104369, EPI_ISL_1104370, EPI_ISL_1104371, EPI_ISL_1104372, EPI_ISL_1104374, EPI_ISL_1104375, EPI_ISL_1104376, EPI_ISL_1104377, EPI_ISL_1104379			
see above	Liverpool Clinical Laboratories	COVID-19 Genomics UK (COG-UK) Consortium	Sam Haldenby, Anita Lucaci, Steve Paterson, Julian Hiscox, Alistair Darby, M Almsaud, A Alrezaihi, Muhannad Alruwaili, Stuart D Armstrong, Jones Benjamin, Eleanor G Bentley, Anu Chawla, Jordan J Clark, Angela Cowell, Richard Eccles, Isabel García-Dorival, Matthew Gemmell, Alessandro Gerada, PKF Gilmore, Richard Gregory, Ximeng Han, Catherine Hartley, Margaret Hughes, Miren Iturriza-Gomara, James Johnson, L Luu, Jenifer Manson, Charlotte Nelson, Elaine O'Toole, Cassie Olateju, Rebekah Penrice-Randal , Lucille Rainbow, N.P Randle, Trevor Ian Robinson, Parul Sharma, Ghada T Shawli, James P Stewart, Neil Swainston, Ecaterina Vamos, Joanne Watts, Mark Whitehead
EPI_ISL_1104799, EPI_ISL_1104804, EPI_ISL_1104806, EPI_ISL_1104812, EPI_ISL_1104814, EPI_ISL_1104822, EPI_ISL_1104828, EPI_ISL_1104831, EPI_ISL_1105043, EPI_ISL_1105079, EPI_ISL_1105084, EPI_ISL_1105091, EPI_ISL_1105115, EPI_ISL_1105159, EPI_ISL_1105168, EPI_ISL_1105176, EPI_ISL_1105219, EPI_ISL_1105250, EPI_ISL_1105292, EPI_ISL_1105341			
see above	University College London Hospital	COVID-19 Genomics UK (COG-UK) Consortium	Judith Heaney, Matthew Byott, Catherine Houlihan, Dan Frampton, Stuart Kirk, Moira Spyer and Eleni Nastouli
EPI_ISL_1105470	University College London, Great Ormond Street Hospital for Children NHS Foundation Trust, Imperial College Healthcare NHS Trust	COVID-19 Genomics UK (COG-UK) Consortium	Sergi Castellano, Rachel Williams, Mark Kristiansen, Paola Resende Silva, Sunando Roy, Tony Brooks, Helena Tutill, Paola Niola, Patricia Dyal, Charlotte Williams, Leysa Forrest, Yasmin Panchbhaya, Jacqueline Findlay, Samuel Weeks, Julianne Brown, Kathryn Harris, Paul Randell, James Price, Alison Holmes, Judith Breuer
EPI_ISL_1105847, EPI_ISL_1105848, EPI_ISL_1105849, EPI_ISL_1105850	Lincolnshire Hospitals and DeepSeq Nottingham	COVID-19 Genomics UK (COG-UK) Consortium	Nichola Duckworth, Tim Sloan, Sarah Walsh, Jonathan Ball, Patrick McClure, Joeseph Chappell, Nadine Holmes, Matthew Carlisle, Christopher Moore, Fei Sang, Johnny Debebe, Victoria Wright, Matthew Loose
EPI_ISL_1107637, EPI_ISL_1107697	Centre for Enzyme Innovation, University of Portsmouth / Translational Research Laboratory, Portsmouth Hospitals NHS Trust	COVID-19 Genomics UK (COG-UK) Consortium	Angela Beckett,Salman Goudarzi,Christopher Fearn,Kate Cook,Katie Loveson,Sharon Glaysher,Scott Elliott,Samuel Robson
EPI_ISL_1177691, EPI_ISL_1177727, EPI_ISL_1177728, EPI_ISL_1177730, EPI_ISL_1177732	Virology Department, Royal Infirmary of Edinburgh, NHS Lothian / School of Biological Sciences, University of Edinburgh	COVID-19 Genomics UK (COG-UK) Consortium	McHugh M, Dewar R, Cotton S, Rooke S, O'Toole Á, Scher E, Hill V, McCrone JT, Colquhoun R, Yu X, Jackson B, Rambaut A, Templeton K
EPI_ISL_1177888, EPI_ISL_1177904	Liverpool Clinical Laboratories	COVID-19 Genomics UK (COG-UK) Consortium	Sam Haldenby, Alistair Darby, Steve Paterson, Anita Lucaci, Julian Hiscox, M Almsaud, A Alrezaihi, Muhannad Alruwaili, Stuart D Armstrong, Jones Benjamin, Eleanor G Bentley, Anu Chawla, Jordan J Clark, Angela Cowell, Richard Eccles, Isabel García-Dorival, Matthew Gemmell, Alessandro Gerada, PKF Gilmore, Richard Gregory, Ximeng Han, Catherine Hartley, Margaret Hughes, Miren Iturriza-Gomara, James Johnson, L Luu, Jenifer Manson, Charlotte Nelson, Elaine O'Toole, Cassie Olateju, Rebekah Penrice-Randal , Lucille Rainbow, N.P Randle, Trevor Ian Robinson, Parul Sharma, Ghada T Shawli, James P Stewart, Neil Swainston, Ecaterina Vamos, Joanne Watts, Mark Whitehead, Hermione Webster
EPI_ISL_1178237, EPI_ISL_1178240, EPI_ISL_1178244, EPI_ISL_1178253, EPI_ISL_1178258, EPI_ISL_1178267, EPI_ISL_1178287, EPI_ISL_1178288, EPI_ISL_1178289, EPI_ISL_1178290	Northumbria University / South Tees Hospitals NHS Foundation Trust / North Cumbria Integrated Care NHS Foundation Trust / North Tees and Hartlepool NHS Foundation Trust / Newcastle Hospitals NHS Foundation Trust	COVID-19 Genomics UK (COG-UK) Consortium	Darren L Smith,Andrew Nelson,Matthew Bashton,Greg R Young,Joshua Loh,John Allan,Mohammad A Tariq,Giles S Holt,Gary Black,Wen C Yew,Lynn Dover,Paul Baker,Steve Liggett,Sarah Essex,Jane Greenaway,Debra Padgett,Clive Graham,Garren Scott,Edward Barton,Emma Swindells,Brendan Payne,Jennifer Collins,Yusri Taha,Gary Eltringham
EPI_ISL_1178440, EPI_ISL_1178451, EPI_ISL_1178461, EPI_ISL_1178466, EPI_ISL_1178473, EPI_ISL_1178479, EPI_ISL_1178481, EPI_ISL_1178485, EPI_ISL_1178486, EPI_ISL_1178489, EPI_ISL_1178490, EPI_ISL_1178491, EPI_ISL_1178492, EPI_ISL_1178494			
see above	Quadram Institute Bioscience	COVID-19 Genomics UK (COG-UK) Consortium	Dave J. Baker, Gemma L. Kay, Alp Aydin, Thanh Le-Viet, Steven Rudder, Ana P. Tedim, Anastasia Kolyva, Maria Diaz, Leonardo de Oliveira Martins, Nabil-Fareed Alikhan, Lizzie Meadows, Rachael Stanley, Ngozi Elumogo, Muhammed Yasir, Nicholas M. Thomson, Alexander J Trotter, Rachel Gilroy, Samuel Bloomfield, Claire Stuart, Andrew Bell, Reenesh Prakash, Samir Dervisevic, Alison E. Mather, John Wain, Mark Webber, Andrew J. Page, Justin O'Grady

EPI_ISL_1178688	Lincolnshire Hospitals and DeepSeq Nottingham	COVID-19 Genomics UK (COG-UK) Consortium	Nichola Duckworth, Tim Sloan, Sarah Walsh, Jonathan Ball, Patrick McClure, Joeseph Chappell, Nadine Holmes, Matthew Carlisle, Christopher Moore, Fei Sang, Johnny Debebe, Victoria Wright, Matthew Loose
EPI_ISL_1178732	Oxford Viromics, NDM, University of Oxford; Oxford University Hospitals; Basingstoke and North Hampshire Hospital	COVID-19 Genomics UK (COG-UK) Consortium	Tanya Golubchik, David Bonsall, George Macintyre, Amy Trebes, Mariateresa de Cesare, Catrin Moore, Alex Mobbs, Anita Justice, Robert Shaw, Monique Andersson, Timothy Peto, Emma Wise, Nathan Moore, Jessica Lynch, Nick Cortes, Matilde Mori, Stephen Kidd, David Buck, John Todd, Christophe Fraser
EPI_ISL_1178890, EPI_ISL_1178891	Originating lab: Wales Specialist Virology Centre Sequencing lab: Pathogen Genomics Unit	Public Health Wales Microbiology Cardiff Wales Specialist Virology Centre	Catherine Moore, Johnathan Evans, Laura Gifford, Malorie Perry, Simon Cottrell, Angela Marchbank, Alec Birchley, Alexander Adams, Amy Gaskin, Bree Gatica-Wilcox, Jason Coombes, Joel Southgate, Lauren Gilbert, Lee Graham, Nicole Pacchiarini, Sara Kumziene-Summerhayes, Sarah Taylor, Sophie Jones, Sara Rey, Matthew Bull, Joanne Watkins, Sally Corden, Tom Connor
EPI_ISL_1179832	Centre for Enzyme Innovation, University of Portsmouth / Translational Research Laboratory, Portsmouth Hospitals NHS Trust	COVID-19 Genomics UK (COG-UK) Consortium	Angela Beckett,Salman Goudarzi,Christopher Fearn,Kate Cook,Katie Loveson,Sharon Glaysher,Scott Elliott,Samuel Robson
EPI_ISL_1180072, EPI_ISL_1180075	Virology Department, Sheffield Teaching Hospitals NHS Foundation Trust/Department of Infection, Immunity and Cardiovascular Disease, The Medical School, University of Sheffield	COVID-19 Genomics UK (COG-UK) Consortium	Thushan de Silva, Matthew Parker, Nikki Smith, Adri Angyal, Rebecca Brown, Luke Green, Rachel Tucker, Paul Parsons, Danielle Groves, Katie Johnson, Laura Carrilero, Alex Keeley, Dave Partridge, Matthew Wyles, Benjamin Lindsey, Mehmet Yavuz, Mohammad Raza, Cariad Evans
EPI_ISL_1189117	Lighthouse Lab in Alderley Park	Wellcome Sanger Institute for the COVID-19 Genomics UK (COG-UK) Consortium	Jacquelyn Wynn, Mairead Hyland, The Lighthouse Lab in Alderley Park and Alex Alderton, Roberto Amato, Jeffrey Barrett, Sonia Goncalves, Ewan Harrison, David K. Jackson, Ian Johnston, Dominic Kwiatkowski, Cordelia Langford, John Sillitoe on behalf of the Wellcome Sanger Institute COVID-19 Surveillance Team
EPI_ISL_1247603	Department of Pathology, University of Cambridge	COVID-19 Genomics UK (COG-UK) Consortium	Aminu S. Jahun, Yasmin Chaudhry, Iliana Georgana, Myra Hosmillo, Rhys Izuagbe, William L. Hamilton, Martin D. Curran, Surendra Parmar, Ian Goodfellow
EPI_ISL_1247719, EPI_ISL_1247721, EPI_ISL_1247722	Virology Department, Royal Infirmary of Edinburgh, NHS Lothian / School of Biological Sciences, University of Edinburgh	COVID-19 Genomics UK (COG-UK) Consortium	McHugh M, Dewar R, Cotton S, Rooke S, O'Toole Á, Scher E, Hill V, McCrone JT, Colquhoun R, Yu X, Jackson B, Rambaut A, Templeton K
EPI_ISL_1247815, EPI_ISL_1247816, EPI_ISL_1247817, EPI_ISL_1247818	Liverpool Clinical Laboratories	COVID-19 Genomics UK (COG-UK) Consortium	Sam Haldenby, Alistair Darby, Steve Paterson, Anita Lucaci, Julian Hiscox, M Almsaud, A Alrezaihi, Muhannad Alruwaili, Stuart D Armstrong, Jones Benjamin, Eleanor G Bentley, Anu Chawla, Jordan J Clark, Angela Cowell, Richard Eccles, Isabel García-Dorival, Matthew Gemmell, Alessandro Gerada, PKF Gilmore, Richard Gregory, Ximeng Han, Catherine Hartley, Margaret Hughes, Miren Iturriza-Gomara, James Johnson, L Luu, Jenifer Manson, Charlotte Nelson, Elaine O'Toole, Cassie Olateju, Rebekah Penrice-Randal , Lucille Rainbow, N.P Randle, Trevor Ian Robinson, Parul Sharma, Ghada T Shawli, James P Stewart, Neil Swainston, Ecaterina Vamos, Joanne Watts, Mark Whitehead, Hermione Webster
EPI_ISL_1248214, EPI_ISL_1248215, EPI_ISL_1248216, EPI_ISL_1248217, EPI_ISL_1248218, EPI_ISL_1248219, EPI_ISL_1248220, EPI_ISL_1248221, EPI_ISL_1248222, EPI_ISL_1248223, EPI_ISL_1248224, EPI_ISL_1248225, EPI_ISL_1248226, EPI_ISL_1248227, EPI_ISL_1248228, EPI_ISL_1248229, EPI_ISL_1248251, EPI_ISL_1248253, EPI_ISL_1248255			
see above	University College London, Great Ormond Street Hospital for Children NHS Foundation Trust, Imperial College Healthcare NHS Trust	COVID-19 Genomics UK (COG-UK) Consortium	Sergi Castellano, Rachel Williams, Mark Kristiansen, Paola Resende Silva, Sunando Roy, Tony Brooks, Helena Tutill, Paola Niola, Patricia Dyal, Charlotte Williams, Leysa Forrest, Yasmin Panchbhaya, Jacqueline Findlay, Samuel Weeks, Julianne Brown, Kathryn Harris, Paul Randell, James Price, Alison Holmes, Judith Breuer
EPI_ISL_1248451, EPI_ISL_1248488	University College London Hospital	COVID-19 Genomics UK (COG-UK) Consortium	Dr Judith Heaney, Matthew Byott, Dr Catherine Houlihan, Dr Daniel Frampton, Stuart Kirk, Dr Moira Spyer, Dr Paul Grant and Dr Eleni Nastouli
EPI_ISL_1248877, EPI_ISL_1248902, EPI_ISL_1248903, EPI_ISL_1248904, EPI_ISL_1248905, EPI_ISL_1248906, EPI_ISL_1248907, EPI_ISL_1248908, EPI_ISL_1248909, EPI_ISL_1248910, EPI_ISL_1248911, EPI_ISL_1248912, EPI_ISL_1248913, EPI_ISL_1248914			
see above	Northumbria University / South Tees Hospitals NHS Foundation Trust / North Cumbria Integrated Care NHS Foundation Trust / North Tees and Hartlepool NHS Foundation Trust / Newcastle Hospitals NHS Foundation Trust	COVID-19 Genomics UK (COG-UK) Consortium	Darren L Smith,Andrew Nelson,Matthew Bashton,Greg R Young,Joshua Loh,John Allan,Mohammad A Tariq,Giles S Holt,Gary Black,Wen C Yew,Lynn Dover,Paul Baker,Steve Liggett,Sarah Essex,Jane Greenaway,Debra Padgett,Clive Graham,Garren Scott,Edward Barton,Emma Swindells,Brendan Payne,Jennifer Collins,Yusri Taha,Gary Eltringham
EPI_ISL_1248963, EPI_ISL_1248978, EPI_ISL_1248980, EPI_ISL_1248983, EPI_ISL_1248985, EPI_ISL_1248986, EPI_ISL_1248989, EPI_ISL_1248991, EPI_ISL_1248992, EPI_ISL_1248995, EPI_ISL_1249000, EPI_ISL_1249005, EPI_ISL_1249009, EPI_ISL_1249010, EPI_ISL_1249015, EPI_ISL_1249016, EPI_ISL_1249017, EPI_ISL_1249021, EPI_ISL_1249022, EPI_ISL_1249023, EPI_ISL_1249030, EPI_ISL_1249031, EPI_ISL_1249033, EPI_ISL_1249037, EPI_ISL_1249041, EPI_ISL_1249044, EPI_ISL_1249045, EPI_ISL_1249047, EPI_ISL_1249048, EPI_ISL_1249051, EPI_ISL_1249052, EPI_ISL_1249053			
see above	Quadram Institute Bioscience	COVID-19 Genomics UK (COG-UK) Consortium	Dave J. Baker, Gemma L. Kay, Alp Aydin, Thanh Le-Viet, Steven Rudder, Ana P. Tedim, Anastasia Kolyva, Maria Diaz, Leonardo de Oliveira Martins, Nabil-Fareed Alikhan, Lizzie Meadows, Rachael Stanley, Ngozi Elumogo, Muhammed Yasir, Nicholas M. Thomson, Alexander J Trotter, Rachel Gilroy, Samuel Bloomfield, Claire Stuart, Andrew Bell, Reenesh Prakash, Samir Dervisevic, Alison E. Mather, John Wain, Mark Webber, Andrew J. Page, Justin O'Grady
EPI_ISL_1265369	Barts Health NHS Trust	Barts Health NHS Trust	KARAA, Esin; CUTINO-MOGUEL, Maria-Teresa; HARRINGTON, David; OWOYEMI, Dola; KULASEGARAN-SHYLINI, Raghavendran; BROAD, Claire; KELE, Beatrix
EPI_ISL_1296563, EPI_ISL_1296565, EPI_ISL_1296566, EPI_ISL_1296567, EPI_ISL_1296568, EPI_ISL_1296569, EPI_ISL_1296570, EPI_ISL_1296572, EPI_ISL_1296573, EPI_ISL_1296574, EPI_ISL_1296575, EPI_ISL_1296576, EPI_ISL_1296577, EPI_ISL_1296579, EPI_ISL_1296580, EPI_ISL_1296583, EPI_ISL_1296584, EPI_ISL_1296599			
see above	Respiratory Virus Unit, National Infection Service, Public Health England	COVID-19 Genomics UK (COG-UK) Consortium	PHE Covid Sequencing Team
EPI_ISL_1308536, EPI_ISL_1308548, EPI_ISL_1308549, EPI_ISL_1308550, EPI_ISL_1308551, EPI_ISL_1308552, EPI_ISL_1308553, EPI_ISL_1308554, EPI_ISL_1308555, EPI_ISL_1308556, EPI_ISL_1308566, EPI_ISL_1308569, EPI_ISL_1308572, EPI_ISL_1308573, EPI_ISL_1308574, EPI_ISL_1308582			
see above	University of Exeter	COVID-19 Genomics UK (COG-UK) Consortium	Ben Temperton,Aaron Jeffries,Michelle Michelsen,Joanna Warwick-Dugdale,Audrey Farbos,Robyn Manley,Stephen Michell,Jane Masoli
EPI_ISL_1308821, EPI_ISL_1308853	Virology Department, Royal Infirmary of Edinburgh, NHS Lothian / School of Biological Sciences, University of Edinburgh	COVID-19 Genomics UK (COG-UK) Consortium	McHugh M, Dewar R, Cotton S, Rooke S, O'Toole Á, Scher E, Hill V, McCrone JT, Colquhoun R, Yu X, Jackson B, Rambaut A, Templeton K
EPI_ISL_1309026, EPI_ISL_1309027, EPI_ISL_1309052, EPI_ISL_1309076, EPI_ISL_1309101	University College London, Great Ormond Street Hospital for Children NHS Foundation Trust, Imperial College Healthcare NHS Trust	COVID-19 Genomics UK (COG-UK) Consortium	Sergi Castellano, Rachel Williams, Mark Kristiansen, Paola Resende Silva, Sunando Roy, Tony Brooks, Helena Tutill, Paola Niola, Patricia Dyal, Charlotte Williams, Leysa Forrest, Yasmin Panchbhaya, Jacqueline Findlay, Samuel Weeks, Julianne Brown, Kathryn Harris, Paul Randell, James Price, Alison Holmes, Judith Breuer
EPI_ISL_1309667	Quadram Institute Bioscience	COVID-19 Genomics UK (COG-UK) Consortium	Dave J. Baker, Gemma L. Kay, Alp Aydin, Thanh Le-Viet, Steven Rudder, Ana P. Tedim, Anastasia Kolyva, Maria Diaz, Leonardo de Oliveira Martins, Nabil-Fareed Alikhan, Lizzie Meadows, Rachael Stanley, Ngozi Elumogo, Muhammed Yasir, Nicholas M. Thomson, Alexander J Trotter, Rachel Gilroy, Samuel Bloomfield, Claire Stuart, Andrew Bell, Reenesh Prakash, Samir Dervisevic, Alison E. Mather, John Wain, Mark Webber, Andrew J. Page, Justin O'Grady
EPI_ISL_1309743, EPI_ISL_1309744, EPI_ISL_1309754, EPI_ISL_1309755	Oxford Viromics, NDM, University of Oxford; Oxford University Hospitals; Basingstoke and North Hampshire Hospital	COVID-19 Genomics UK (COG-UK) Consortium	Tanya Golubchik, David Bonsall, George Macintyre, Amy Trebes, Mariateresa de Cesare, Catrin Moore, Alex Mobbs, Anita Justice, Robert Shaw, Monique Andersson, Timothy Peto, Emma Wise, Nathan Moore, Jessica Lynch, Nick Cortes, Matilde Mori, Stephen Kidd, David Buck, John Todd, Christophe Fraser
EPI_ISL_1365323	Lighthouse Lab in Glasgow	Wellcome Sanger Institute for the COVID-19 Genomics UK (COG-UK) Consortium	Harper VanSteenhouse, Yumi Kasai, David Gray, Carol Clugston, Anna Dominiczak and Alex Alderton, Roberto Amato, Jeffrey Barrett, Sonia Goncalves, Ewan Harrison, David K. Jackson, Ian Johnston, Dominic Kwiatkowski, Cordelia Langford, John Sillitoe on behalf of the Wellcome Sanger Institute COVID-19 Surveillance Team
EPI_ISL_1386509	University of Birmingham	COVID-19 Genomics UK (COG-UK) Consortium	Institute of Microbiology, University of Birmingham: Claire McMurray, Joanne Stockton, Samuel Nicholls, Radoslaw Poplawski, Will Rowe, Josh Quick, Nicholas Loman. University of Birmingham Testing Laboratory: Celina M Whalley, Andrew Bosworth, Charlotte Poxon, Kasun Wanigasooriya, Oliver Pickles, Mike Kidd, Alex Richter, Andrew D Beggs PHE Heartlands Lab: Husam Osman, Andrew Bosworth. Queen Elizabeth Hospital: Anna Casey
EPI_ISL_1387221	Oxford Viromics, NDM, University of Oxford; Oxford University Hospitals; Basingstoke and North Hampshire Hospital	COVID-19 Genomics UK (COG-UK) Consortium	Tanya Golubchik, David Bonsall, George Macintyre, Amy Trebes, Mariateresa de Cesare, Catrin Moore, Alex Mobbs, Anita Justice, Robert Shaw, Monique Andersson, Timothy Peto, Emma Wise, Nathan Moore, Jessica Lynch, Nick Cortes, Matilde Mori, Stephen Kidd, David Buck, John Todd, Christophe Fraser
EPI_ISL_1391411, EPI_ISL_1391412	Barts Health NHS Trust	Barts Health NHS Trust	KARAA, Esin; CUTINO-MOGUEL, Maria-Teresa; HARRINGTON, David; OWOYEMI, Dola; KULASEGARAN-SHYLINI, Raghavendran; BROAD, Claire;

			KELE, Beatrix
EPI_ISL_1474859	University College London, Great Ormond Street Hospital for Children NHS Foundation Trust, Imperial College Healthcare NHS Trust	COVID-19 Genomics UK (COG-UK) Consortium	Sergi Castellano, Rachel Williams, Mark Kristiansen, Paola Resende Silva, Sunando Roy, Tony Brooks, Helena Tutill, Paola Niola, Patricia Dyal, Charlotte Williams, Leysa Forrest, Yasmin Panchbhaya, Jacqueline Findlay, Samuel Weeks, Julianne Brown, Kathryn Harris, Paul Randell, James Price, Alison Holmes, Judith Breuer
EPI_ISL_1475031, EPI_ISL_1475032, EPI_ISL_1475033, EPI_ISL_1475035, EPI_ISL_1475048, EPI_ISL_1475051, EPI_ISL_1475052, EPI_ISL_1475054, EPI_ISL_1475055, EPI_ISL_1475056, EPI_ISL_1475084, EPI_ISL_1475086, EPI_ISL_1475087, EPI_ISL_1475088, EPI_ISL_1475090, EPI_ISL_1475122, EPI_ISL_1475123, EPI_ISL_1475128, EPI_ISL_1475129, EPI_ISL_1475130			
see above	Regional Virus Laboratory, Belfast Health and Social Care Trust	COVID-19 Genomics UK (COG-UK) Consortium	Conall McCaughey, James McKenna, Tanya Curran, Susan Feeney, Alison Watt, Ciara Cox, Mairead Connor, Zoltan Molnar, David Simpson, Derek Fairley
EPI_ISL_1476451, EPI_ISL_1476452, EPI_ISL_1476453, EPI_ISL_1476454, EPI_ISL_1476456, EPI_ISL_1476457, EPI_ISL_1476458, EPI_ISL_1476460, EPI_ISL_1476464, EPI_ISL_1476467, EPI_ISL_1476471, EPI_ISL_1476472, EPI_ISL_1476474, EPI_ISL_1476476, EPI_ISL_1476477, EPI_ISL_1476504, EPI_ISL_1476514			
see above	Originating lab: Wales Specialist Virology Centre Sequencing lab: Pathogen Genomics Unit	Public Health Wales Microbiology Cardiff Wales Specialist Virology Centre	Catherine Moore, Johnathan Evans, Laura Gifford, Malorie Perry, Simon Cottrell, Angela Marchbank, Alec Birchley, Alexander Adams, Amy Gaskin, Bree Gatica-Wilcox, Jason Coombes, Joel Southgate, Lauren Gilbert, Lee Graham, Nicole Pacchiarini, Sara Kumziene-Summerhayes, Sarah Taylor, Sophie Jones, Sara Rey, Matthew Bull, Joanne Watkins, Sally Corden, Tom Connor
EPI_ISL_868359, EPI_ISL_868360, EPI_ISL_868377, EPI_ISL_868403, EPI_ISL_868449, EPI_ISL_868511, EPI_ISL_868534, EPI_ISL_868540, EPI_ISL_868549, EPI_ISL_868567, EPI_ISL_868606, EPI_ISL_868653, EPI_ISL_868671, EPI_ISL_868702			
see above	Virology Department, Sheffield Teaching Hospitals NHS Foundation Trust/Department of Infection, Immunity and Cardiovascular Disease, The Medical School, University of Sheffield	COVID-19 Genomics UK (COG-UK) Consortium	Thushan de Silva, Matthew Parker, Nikki Smith, Adri Angyal, Rebecca Brown, Luke Green, Rachel Tucker, Paul Parsons, Danielle Groves, Katie Johnson, Laura Carrilero, Alex Keeley, Dave Partridge, Matthew Wyles, Benjamin Lindsey, Mehmet Yavuz, Mohammad Raza, Cariad Evans
EPI_ISL_882611	Lighthouse Lab in Milton Keynes	Wellcome Sanger Institute for the COVID-19 Genomics UK (COG-UK) Consortium	The Lighthouse Lab in Milton Keynes and Alex Alderton, Roberto Amato, Sonia Goncalves, Ewan Harrison, David K. Jackson, Ian Johnston, Dominic Kwiatkowski, Cordelia Langford, John Sillitoe on behalf of the Wellcome Sanger Institute COVID-19 Surveillance Team
EPI_ISL_907120, EPI_ISL_907121, EPI_ISL_907122, EPI_ISL_907123, EPI_ISL_907124, EPI_ISL_907125, EPI_ISL_907126, EPI_ISL_907127, EPI_ISL_907128, EPI_ISL_907129, EPI_ISL_907130, EPI_ISL_907131, EPI_ISL_907132, EPI_ISL_907134, EPI_ISL_907135, EPI_ISL_907137, EPI_ISL_907138, EPI_ISL_907140, EPI_ISL_907141, EPI_ISL_907142, EPI_ISL_907143, EPI_ISL_907145, EPI_ISL_907146, EPI_ISL_907149, EPI_ISL_907150, EPI_ISL_907151, EPI_ISL_907153, EPI_ISL_907154, EPI_ISL_907156, EPI_ISL_907157, EPI_ISL_907158, EPI_ISL_907159, EPI_ISL_907161, EPI_ISL_907162, EPI_ISL_907165, EPI_ISL_907166, EPI_ISL_907169, EPI_ISL_907170, EPI_ISL_907171, EPI_ISL_907172, EPI_ISL_907174, EPI_ISL_907175, EPI_ISL_907176, EPI_ISL_907177, EPI_ISL_907178, EPI_ISL_907180, EPI_ISL_907181, EPI_ISL_907183, EPI_ISL_907184, EPI_ISL_907185, EPI_ISL_907188, EPI_ISL_907189, EPI_ISL_907191, EPI_ISL_907196, EPI_ISL_907197, EPI_ISL_907198, EPI_ISL_907199, EPI_ISL_907201, EPI_ISL_907202, EPI_ISL_907203, EPI_ISL_907204, EPI_ISL_907205, EPI_ISL_907207, EPI_ISL_907208, EPI_ISL_907211, EPI_ISL_907213, EPI_ISL_907215, EPI_ISL_907216, EPI_ISL_907217, EPI_ISL_907218, EPI_ISL_907220, EPI_ISL_907226, EPI_ISL_907227, EPI_ISL_907228, EPI_ISL_907229, EPI_ISL_907230, EPI_ISL_907231, EPI_ISL_907232, EPI_ISL_907233, EPI_ISL_907234, EPI_ISL_907235, EPI_ISL_907236, EPI_ISL_907239, EPI_ISL_907240, EPI_ISL_907242, EPI_ISL_907243, EPI_ISL_907244, EPI_ISL_907245, EPI_ISL_907246, EPI_ISL_907247, EPI_ISL_907248, EPI_ISL_907249, EPI_ISL_907251, EPI_ISL_907252, EPI_ISL_907253, EPI_ISL_907255, EPI_ISL_907257, EPI_ISL_907258, EPI_ISL_907259, EPI_ISL_907261, EPI_ISL_907262, EPI_ISL_907265, EPI_ISL_907266, EPI_ISL_907268, EPI_ISL_907270, EPI_ISL_907271, EPI_ISL_907272, EPI_ISL_907274, EPI_ISL_907275, EPI_ISL_907276, EPI_ISL_907277, EPI_ISL_907278, EPI_ISL_907280, EPI_ISL_907282, EPI_ISL_907283, EPI_ISL_907285, EPI_ISL_907286, EPI_ISL_907289, EPI_ISL_907290, EPI_ISL_907291, EPI_ISL_907293, EPI_ISL_907294, EPI_ISL_907295, EPI_ISL_907296, EPI_ISL_907297, EPI_ISL_907298, EPI_ISL_907299, EPI_ISL_907301, EPI_ISL_907302, EPI_ISL_907305, EPI_ISL_907306, EPI_ISL_907307, EPI_ISL_907309, EPI_ISL_907310, EPI_ISL_907311, EPI_ISL_907312, EPI_ISL_907313, EPI_ISL_907314, EPI_ISL_907316, EPI_ISL_907317, EPI_ISL_907319, EPI_ISL_907320, EPI_ISL_907324, EPI_ISL_907326, EPI_ISL_907327, EPI_ISL_907329, EPI_ISL_907330, EPI_ISL_907331, EPI_ISL_907332, EPI_ISL_907333, EPI_ISL_907334, EPI_ISL_907335, EPI_ISL_907337, EPI_ISL_907338, EPI_ISL_907339, EPI_ISL_907340, EPI_ISL_907342, EPI_ISL_907343, EPI_ISL_907344, EPI_ISL_907345, EPI_ISL_907346, EPI_ISL_907349, EPI_ISL_907354, EPI_ISL_907356, EPI_ISL_907357, EPI_ISL_907358, EPI_ISL_907360, EPI_ISL_907361, EPI_ISL_907365, EPI_ISL_907366, EPI_ISL_907368, EPI_ISL_907370			
see above	Lighthouse Lab in Glasgow	Wellcome Sanger Institute for the COVID-19 Genomics UK (COG-UK) Consortium	Harper VanSteenhouse, Yumi Kasai, David Gray, Carol Clugston, Anna Dominiczak and Alex Alderton, Roberto Amato, Sonia Goncalves, Ewan Harrison, David K. Jackson, Ian Johnston, Dominic Kwiatkowski, Cordelia Langford, John Sillitoe on behalf of the Wellcome Sanger Institute COVID-19 Surveillance Team
EPI_ISL_907371, EPI_ISL_907372, EPI_ISL_907373, EPI_ISL_907374, EPI_ISL_907376, EPI_ISL_907377	Lighthouse Lab in Alderley Park	Wellcome Sanger Institute for the COVID-19 Genomics UK (COG-UK) Consortium	Jacquelyn Wynn, Mairead Hyland, The Lighthouse Lab in Alderley Park and Alex Alderton, Roberto Amato, Sonia Goncalves, Ewan Harrison, David K. Jackson, Ian Johnston, Dominic Kwiatkowski, Cordelia Langford, John Sillitoe on behalf of the Wellcome Sanger Institute COVID-19 Surveillance Team
EPI_ISL_907378, EPI_ISL_907379, EPI_ISL_907381	Lighthouse Lab in Glasgow	Wellcome Sanger Institute for the COVID-19 Genomics UK (COG-UK) Consortium	Harper VanSteenhouse, Yumi Kasai, David Gray, Carol Clugston, Anna Dominiczak and Alex Alderton, Roberto Amato, Sonia Goncalves, Ewan Harrison, David K. Jackson, Ian Johnston, Dominic Kwiatkowski, Cordelia Langford, John Sillitoe on behalf of the Wellcome Sanger Institute COVID-19 Surveillance Team
EPI_ISL_907382, EPI_ISL_907383	Lighthouse Lab in Alderley Park	Wellcome Sanger Institute for the COVID-19 Genomics UK (COG-UK) Consortium	Jacquelyn Wynn, Mairead Hyland, The Lighthouse Lab in Alderley Park and Alex Alderton, Roberto Amato, Sonia Goncalves, Ewan Harrison, David K. Jackson, Ian Johnston, Dominic Kwiatkowski, Cordelia Langford, John Sillitoe on behalf of the Wellcome Sanger Institute COVID-19 Surveillance Team
EPI_ISL_907384, EPI_ISL_907385, EPI_ISL_907386	Lighthouse Lab in Glasgow	Wellcome Sanger Institute for the COVID-19 Genomics UK (COG-UK) Consortium	Harper VanSteenhouse, Yumi Kasai, David Gray, Carol Clugston, Anna Dominiczak and Alex Alderton, Roberto Amato, Sonia Goncalves, Ewan Harrison, David K. Jackson, Ian Johnston, Dominic Kwiatkowski, Cordelia Langford, John Sillitoe on behalf of the Wellcome Sanger Institute COVID-19 Surveillance Team
EPI_ISL_907389	Lighthouse Lab in Alderley Park	Wellcome Sanger Institute for the COVID-19 Genomics UK (COG-UK) Consortium	Jacquelyn Wynn, Mairead Hyland, The Lighthouse Lab in Alderley Park and Alex Alderton, Roberto Amato, Sonia Goncalves, Ewan Harrison, David K. Jackson, Ian Johnston, Dominic Kwiatkowski, Cordelia Langford, John Sillitoe on behalf of the Wellcome Sanger Institute COVID-19 Surveillance Team
EPI_ISL_907393, EPI_ISL_907394, EPI_ISL_907395	Lighthouse Lab in Glasgow	Wellcome Sanger Institute for the COVID-19 Genomics UK (COG-UK) Consortium	Harper VanSteenhouse, Yumi Kasai, David Gray, Carol Clugston, Anna Dominiczak and Alex Alderton, Roberto Amato, Sonia Goncalves, Ewan Harrison, David K. Jackson, Ian Johnston, Dominic Kwiatkowski, Cordelia Langford, John Sillitoe on behalf of the Wellcome Sanger Institute COVID-19 Surveillance Team
EPI_ISL_907396	Lighthouse Lab in Alderley Park	Wellcome Sanger Institute for the COVID-19 Genomics UK (COG-UK) Consortium	Jacquelyn Wynn, Mairead Hyland, The Lighthouse Lab in Alderley Park and Alex Alderton, Roberto Amato, Sonia Goncalves, Ewan Harrison, David K. Jackson, Ian Johnston, Dominic Kwiatkowski, Cordelia Langford, John Sillitoe on behalf of the Wellcome Sanger Institute COVID-19 Surveillance Team
EPI_ISL_907397, EPI_ISL_907398, EPI_ISL_907401, EPI_ISL_907402	Lighthouse Lab in Glasgow	Wellcome Sanger Institute for the COVID-19 Genomics UK (COG-UK) Consortium	Harper VanSteenhouse, Yumi Kasai, David Gray, Carol Clugston, Anna Dominiczak and Alex Alderton, Roberto Amato, Sonia Goncalves, Ewan Harrison, David K. Jackson, Ian Johnston, Dominic Kwiatkowski, Cordelia Langford, John Sillitoe on behalf of the Wellcome Sanger Institute COVID-19 Surveillance Team
EPI_ISL_907403, EPI_ISL_907404	Lighthouse Lab in Alderley Park	Wellcome Sanger Institute for the COVID-19 Genomics UK (COG-UK) Consortium	Jacquelyn Wynn, Mairead Hyland, The Lighthouse Lab in Alderley Park and Alex Alderton, Roberto Amato, Sonia Goncalves, Ewan Harrison, David K. Jackson, Ian Johnston, Dominic Kwiatkowski, Cordelia Langford, John Sillitoe on behalf of the Wellcome Sanger Institute COVID-19 Surveillance Team
EPI_ISL_907405	Lighthouse Lab in Glasgow	Wellcome Sanger Institute for the COVID-19 Genomics UK (COG-UK) Consortium	Harper VanSteenhouse, Yumi Kasai, David Gray, Carol Clugston, Anna Dominiczak and Alex Alderton, Roberto Amato, Sonia Goncalves, Ewan Harrison, David K. Jackson, Ian Johnston, Dominic Kwiatkowski, Cordelia Langford, John Sillitoe on behalf of the Wellcome Sanger Institute COVID-19 Surveillance Team
EPI_ISL_907406, EPI_ISL_907407	Lighthouse Lab in Alderley Park	Wellcome Sanger Institute for the COVID-19 Genomics UK (COG-UK) Consortium	Jacquelyn Wynn, Mairead Hyland, The Lighthouse Lab in Alderley Park and Alex Alderton, Roberto Amato, Sonia Goncalves, Ewan Harrison, David K. Jackson, Ian Johnston, Dominic Kwiatkowski, Cordelia Langford, John Sillitoe on behalf of the Wellcome Sanger Institute COVID-19 Surveillance Team
EPI_ISL_907408	Lighthouse Lab in Glasgow	Wellcome Sanger Institute for the COVID-19 Genomics UK (COG-UK) Consortium	Harper VanSteenhouse, Yumi Kasai, David Gray, Carol Clugston, Anna Dominiczak and Alex Alderton, Roberto Amato, Sonia Goncalves, Ewan Harrison, David K. Jackson, Ian Johnston, Dominic Kwiatkowski, Cordelia Langford, John Sillitoe on behalf of the Wellcome Sanger Institute COVID-19 Surveillance Team
EPI_ISL_907409, EPI_ISL_907410, EPI_ISL_907411, EPI_ISL_907412, EPI_ISL_907413	Lighthouse Lab in Alderley Park	Wellcome Sanger Institute for the COVID-19 Genomics UK (COG-UK) Consortium	Jacquelyn Wynn, Mairead Hyland, The Lighthouse Lab in Alderley Park and Alex Alderton, Roberto Amato, Sonia Goncalves, Ewan Harrison, David K. Jackson, Ian Johnston, Dominic Kwiatkowski, Cordelia Langford, John Sillitoe on behalf of the Wellcome Sanger Institute COVID-19 Surveillance Team
EPI_ISL_907415	Lighthouse Lab in Glasgow	Wellcome Sanger Institute for the COVID-19 Genomics UK (COG-UK) Consortium	Harper VanSteenhouse, Yumi Kasai, David Gray, Carol Clugston, Anna Dominiczak and Alex Alderton, Roberto Amato, Sonia Goncalves, Ewan Harrison, David K. Jackson, Ian Johnston, Dominic Kwiatkowski, Cordelia Langford, John Sillitoe on behalf of the Wellcome Sanger Institute COVID-19 Surveillance Team
EPI_ISL_907416	Lighthouse Lab in Alderley Park	Wellcome Sanger Institute for the COVID-19 Genomics UK (COG-UK) Consortium	Jacquelyn Wynn, Mairead Hyland, The Lighthouse Lab in Alderley Park and Alex Alderton, Roberto Amato, Sonia Goncalves, Ewan Harrison, David K. Jackson, Ian Johnston, Dominic Kwiatkowski, Cordelia Langford, John Sillitoe on behalf of the Wellcome Sanger Institute COVID-19 Surveillance Team
EPI_ISL_907418, EPI_ISL_907419, EPI_ISL_907420, EPI_ISL_907421	Lighthouse Lab in Glasgow	Wellcome Sanger Institute for the COVID-19 Genomics UK (COG-UK) Consortium	Harper VanSteenhouse, Yumi Kasai, David Gray, Carol Clugston, Anna Dominiczak and Alex Alderton, Roberto Amato, Sonia Goncalves, Ewan Harrison, David K. Jackson, Ian Johnston, Dominic Kwiatkowski, Cordelia Langford, John Sillitoe on behalf of the Wellcome Sanger Institute COVID-19 Surveillance Team

EPI_ISL_907422	Lighthouse Lab in Alderley Park	Wellcome Sanger Institute for the COVID-19 Genomics UK (COG-UK) Consortium	Jacquelyn Wynn, Mairead Hyland, The Lighthouse Lab in Alderley Park and Alex Alderton, Roberto Amato, Sonia Goncalves, Ewan Harrison, David K. Jackson, Ian Johnston, Dominic Kwiatkowski, Cordelia Langford, John Sillitoe on behalf of the Wellcome Sanger Institute COVID-19 Surveillance Team
EPI_ISL_907423	Lighthouse Lab in Glasgow	Wellcome Sanger Institute for the COVID-19 Genomics UK (COG-UK) Consortium	Harper VanSteenhouse, Yumi Kasai, David Gray, Carol Clugston, Anna Dominiczak and Alex Alderton, Roberto Amato, Sonia Goncalves, Ewan Harrison, David K. Jackson, Ian Johnston, Dominic Kwiatkowski, Cordelia Langford, John Sillitoe on behalf of the Wellcome Sanger Institute COVID-19 Surveillance Team
EPI_ISL_907424	Lighthouse Lab in Alderley Park	Wellcome Sanger Institute for the COVID-19 Genomics UK (COG-UK) Consortium	Jacquelyn Wynn, Mairead Hyland, The Lighthouse Lab in Alderley Park and Alex Alderton, Roberto Amato, Sonia Goncalves, Ewan Harrison, David K. Jackson, Ian Johnston, Dominic Kwiatkowski, Cordelia Langford, John Sillitoe on behalf of the Wellcome Sanger Institute COVID-19 Surveillance Team
EPI_ISL_907425, EPI_ISL_907426	Lighthouse Lab in Glasgow	Wellcome Sanger Institute for the COVID-19 Genomics UK (COG-UK) Consortium	Harper VanSteenhouse, Yumi Kasai, David Gray, Carol Clugston, Anna Dominiczak and Alex Alderton, Roberto Amato, Sonia Goncalves, Ewan Harrison, David K. Jackson, Ian Johnston, Dominic Kwiatkowski, Cordelia Langford, John Sillitoe on behalf of the Wellcome Sanger Institute COVID-19 Surveillance Team
EPI_ISL_907427, EPI_ISL_907428, EPI_ISL_907429	Lighthouse Lab in Alderley Park	Wellcome Sanger Institute for the COVID-19 Genomics UK (COG-UK) Consortium	Jacquelyn Wynn, Mairead Hyland, The Lighthouse Lab in Alderley Park and Alex Alderton, Roberto Amato, Sonia Goncalves, Ewan Harrison, David K. Jackson, Ian Johnston, Dominic Kwiatkowski, Cordelia Langford, John Sillitoe on behalf of the Wellcome Sanger Institute COVID-19 Surveillance Team
EPI_ISL_907430, EPI_ISL_907431	Lighthouse Lab in Glasgow	Wellcome Sanger Institute for the COVID-19 Genomics UK (COG-UK) Consortium	Harper VanSteenhouse, Yumi Kasai, David Gray, Carol Clugston, Anna Dominiczak and Alex Alderton, Roberto Amato, Sonia Goncalves, Ewan Harrison, David K. Jackson, Ian Johnston, Dominic Kwiatkowski, Cordelia Langford, John Sillitoe on behalf of the Wellcome Sanger Institute COVID-19 Surveillance Team
EPI_ISL_907432	Lighthouse Lab in Alderley Park	Wellcome Sanger Institute for the COVID-19 Genomics UK (COG-UK) Consortium	Jacquelyn Wynn, Mairead Hyland, The Lighthouse Lab in Alderley Park and Alex Alderton, Roberto Amato, Sonia Goncalves, Ewan Harrison, David K. Jackson, Ian Johnston, Dominic Kwiatkowski, Cordelia Langford, John Sillitoe on behalf of the Wellcome Sanger Institute COVID-19 Surveillance Team
EPI_ISL_907433, EPI_ISL_907434, EPI_ISL_907435, EPI_ISL_907436, EPI_ISL_907439	Lighthouse Lab in Glasgow	Wellcome Sanger Institute for the COVID-19 Genomics UK (COG-UK) Consortium	Harper VanSteenhouse, Yumi Kasai, David Gray, Carol Clugston, Anna Dominiczak and Alex Alderton, Roberto Amato, Sonia Goncalves, Ewan Harrison, David K. Jackson, Ian Johnston, Dominic Kwiatkowski, Cordelia Langford, John Sillitoe on behalf of the Wellcome Sanger Institute COVID-19 Surveillance Team
EPI_ISL_907441	Lighthouse Lab in Alderley Park	Wellcome Sanger Institute for the COVID-19 Genomics UK (COG-UK) Consortium	Jacquelyn Wynn, Mairead Hyland, The Lighthouse Lab in Alderley Park and Alex Alderton, Roberto Amato, Sonia Goncalves, Ewan Harrison, David K. Jackson, Ian Johnston, Dominic Kwiatkowski, Cordelia Langford, John Sillitoe on behalf of the Wellcome Sanger Institute COVID-19 Surveillance Team
EPI_ISL_907442	Lighthouse Lab in Glasgow	Wellcome Sanger Institute for the COVID-19 Genomics UK (COG-UK) Consortium	Harper VanSteenhouse, Yumi Kasai, David Gray, Carol Clugston, Anna Dominiczak and Alex Alderton, Roberto Amato, Sonia Goncalves, Ewan Harrison, David K. Jackson, Ian Johnston, Dominic Kwiatkowski, Cordelia Langford, John Sillitoe on behalf of the Wellcome Sanger Institute COVID-19 Surveillance Team
EPI_ISL_907443	Lighthouse Lab in Alderley Park	Wellcome Sanger Institute for the COVID-19 Genomics UK (COG-UK) Consortium	Jacquelyn Wynn, Mairead Hyland, The Lighthouse Lab in Alderley Park and Alex Alderton, Roberto Amato, Sonia Goncalves, Ewan Harrison, David K. Jackson, Ian Johnston, Dominic Kwiatkowski, Cordelia Langford, John Sillitoe on behalf of the Wellcome Sanger Institute COVID-19 Surveillance Team
EPI_ISL_907445	Lighthouse Lab in Glasgow	Wellcome Sanger Institute for the COVID-19 Genomics UK (COG-UK) Consortium	Harper VanSteenhouse, Yumi Kasai, David Gray, Carol Clugston, Anna Dominiczak and Alex Alderton, Roberto Amato, Sonia Goncalves, Ewan Harrison, David K. Jackson, Ian Johnston, Dominic Kwiatkowski, Cordelia Langford, John Sillitoe on behalf of the Wellcome Sanger Institute COVID-19 Surveillance Team
EPI_ISL_907446, EPI_ISL_907447, EPI_ISL_907448, EPI_ISL_907450	Lighthouse Lab in Alderley Park	Wellcome Sanger Institute for the COVID-19 Genomics UK (COG-UK) Consortium	Jacquelyn Wynn, Mairead Hyland, The Lighthouse Lab in Alderley Park and Alex Alderton, Roberto Amato, Sonia Goncalves, Ewan Harrison, David K. Jackson, Ian Johnston, Dominic Kwiatkowski, Cordelia Langford, John Sillitoe on behalf of the Wellcome Sanger Institute COVID-19 Surveillance Team
EPI_ISL_907451, EPI_ISL_907453, EPI_ISL_907454, EPI_ISL_907455, EPI_ISL_907456	Lighthouse Lab in Glasgow	Wellcome Sanger Institute for the COVID-19 Genomics UK (COG-UK) Consortium	Harper VanSteenhouse, Yumi Kasai, David Gray, Carol Clugston, Anna Dominiczak and Alex Alderton, Roberto Amato, Sonia Goncalves, Ewan Harrison, David K. Jackson, Ian Johnston, Dominic Kwiatkowski, Cordelia Langford, John Sillitoe on behalf of the Wellcome Sanger Institute COVID-19 Surveillance Team
EPI_ISL_907458, EPI_ISL_907459, EPI_ISL_907460	Lighthouse Lab in Alderley Park	Wellcome Sanger Institute for the COVID-19 Genomics UK (COG-UK) Consortium	Jacquelyn Wynn, Mairead Hyland, The Lighthouse Lab in Alderley Park and Alex Alderton, Roberto Amato, Sonia Goncalves, Ewan Harrison, David K. Jackson, Ian Johnston, Dominic Kwiatkowski, Cordelia Langford, John Sillitoe on behalf of the Wellcome Sanger Institute COVID-19 Surveillance Team
EPI_ISL_907461	Lighthouse Lab in Glasgow	Wellcome Sanger Institute for the COVID-19 Genomics UK (COG-UK) Consortium	Harper VanSteenhouse, Yumi Kasai, David Gray, Carol Clugston, Anna Dominiczak and Alex Alderton, Roberto Amato, Sonia Goncalves, Ewan Harrison, David K. Jackson, Ian Johnston, Dominic Kwiatkowski, Cordelia Langford, John Sillitoe on behalf of the Wellcome Sanger Institute COVID-19 Surveillance Team
EPI_ISL_907462, EPI_ISL_907463, EPI_ISL_907464	Lighthouse Lab in Alderley Park	Wellcome Sanger Institute for the COVID-19 Genomics UK (COG-UK) Consortium	Jacquelyn Wynn, Mairead Hyland, The Lighthouse Lab in Alderley Park and Alex Alderton, Roberto Amato, Sonia Goncalves, Ewan Harrison, David K. Jackson, Ian Johnston, Dominic Kwiatkowski, Cordelia Langford, John Sillitoe on behalf of the Wellcome Sanger Institute COVID-19 Surveillance Team
EPI_ISL_907465	Lighthouse Lab in Glasgow	Wellcome Sanger Institute for the COVID-19 Genomics UK (COG-UK) Consortium	Harper VanSteenhouse, Yumi Kasai, David Gray, Carol Clugston, Anna Dominiczak and Alex Alderton, Roberto Amato, Sonia Goncalves, Ewan Harrison, David K. Jackson, Ian Johnston, Dominic Kwiatkowski, Cordelia Langford, John Sillitoe on behalf of the Wellcome Sanger Institute COVID-19 Surveillance Team
EPI_ISL_907466, EPI_ISL_907468	Lighthouse Lab in Alderley Park	Wellcome Sanger Institute for the COVID-19 Genomics UK (COG-UK) Consortium	Jacquelyn Wynn, Mairead Hyland, The Lighthouse Lab in Alderley Park and Alex Alderton, Roberto Amato, Sonia Goncalves, Ewan Harrison, David K. Jackson, Ian Johnston, Dominic Kwiatkowski, Cordelia Langford, John Sillitoe on behalf of the Wellcome Sanger Institute COVID-19 Surveillance Team
EPI_ISL_907469	Lighthouse Lab in Glasgow	Wellcome Sanger Institute for the COVID-19 Genomics UK (COG-UK) Consortium	Harper VanSteenhouse, Yumi Kasai, David Gray, Carol Clugston, Anna Dominiczak and Alex Alderton, Roberto Amato, Sonia Goncalves, Ewan Harrison, David K. Jackson, Ian Johnston, Dominic Kwiatkowski, Cordelia Langford, John Sillitoe on behalf of the Wellcome Sanger Institute COVID-19 Surveillance Team
EPI_ISL_907470, EPI_ISL_907471	Lighthouse Lab in Alderley Park	Wellcome Sanger Institute for the COVID-19 Genomics UK (COG-UK) Consortium	Jacquelyn Wynn, Mairead Hyland, The Lighthouse Lab in Alderley Park and Alex Alderton, Roberto Amato, Sonia Goncalves, Ewan Harrison, David K. Jackson, Ian Johnston, Dominic Kwiatkowski, Cordelia Langford, John Sillitoe on behalf of the Wellcome Sanger Institute COVID-19 Surveillance Team
EPI_ISL_907472, EPI_ISL_907473, EPI_ISL_907474, EPI_ISL_907475, EPI_ISL_907476	Lighthouse Lab in Glasgow	Wellcome Sanger Institute for the COVID-19 Genomics UK (COG-UK) Consortium	Harper VanSteenhouse, Yumi Kasai, David Gray, Carol Clugston, Anna Dominiczak and Alex Alderton, Roberto Amato, Sonia Goncalves, Ewan Harrison, David K. Jackson, Ian Johnston, Dominic Kwiatkowski, Cordelia Langford, John Sillitoe on behalf of the Wellcome Sanger Institute COVID-19 Surveillance Team
EPI_ISL_907477, EPI_ISL_907478	Lighthouse Lab in Alderley Park	Wellcome Sanger Institute for the COVID-19 Genomics UK (COG-UK) Consortium	Jacquelyn Wynn, Mairead Hyland, The Lighthouse Lab in Alderley Park and Alex Alderton, Roberto Amato, Sonia Goncalves, Ewan Harrison, David K. Jackson, Ian Johnston, Dominic Kwiatkowski, Cordelia Langford, John Sillitoe on behalf of the Wellcome Sanger Institute COVID-19 Surveillance Team
EPI_ISL_907481, EPI_ISL_907483	Lighthouse Lab in Glasgow	Wellcome Sanger Institute for the COVID-19 Genomics UK (COG-UK) Consortium	Harper VanSteenhouse, Yumi Kasai, David Gray, Carol Clugston, Anna Dominiczak and Alex Alderton, Roberto Amato, Sonia Goncalves, Ewan Harrison, David K. Jackson, Ian Johnston, Dominic Kwiatkowski, Cordelia Langford, John Sillitoe on behalf of the Wellcome Sanger Institute COVID-19 Surveillance Team
EPI_ISL_907484	Lighthouse Lab in Alderley Park	Wellcome Sanger Institute for the COVID-19 Genomics UK (COG-UK) Consortium	Jacquelyn Wynn, Mairead Hyland, The Lighthouse Lab in Alderley Park and Alex Alderton, Roberto Amato, Sonia Goncalves, Ewan Harrison, David K. Jackson, Ian Johnston, Dominic Kwiatkowski, Cordelia Langford, John Sillitoe on behalf of the Wellcome Sanger Institute COVID-19 Surveillance Team
EPI_ISL_907486, EPI_ISL_907487, EPI_ISL_907488	Lighthouse Lab in Glasgow	Wellcome Sanger Institute for the COVID-19 Genomics UK (COG-UK) Consortium	Harper VanSteenhouse, Yumi Kasai, David Gray, Carol Clugston, Anna Dominiczak and Alex Alderton, Roberto Amato, Sonia Goncalves, Ewan Harrison, David K. Jackson, Ian Johnston, Dominic Kwiatkowski, Cordelia Langford, John Sillitoe on behalf of the Wellcome Sanger Institute COVID-19 Surveillance Team
EPI_ISL_907489	Lighthouse Lab in Alderley Park	Wellcome Sanger Institute for the COVID-19 Genomics UK (COG-UK) Consortium	Jacquelyn Wynn, Mairead Hyland, The Lighthouse Lab in Alderley Park and Alex Alderton, Roberto Amato, Sonia Goncalves, Ewan Harrison, David K. Jackson, Ian Johnston, Dominic Kwiatkowski, Cordelia Langford, John Sillitoe on behalf of the Wellcome Sanger Institute COVID-19 Surveillance Team
EPI_ISL_907490	Lighthouse Lab in Glasgow	Wellcome Sanger Institute for the COVID-19 Genomics UK (COG-UK) Consortium	Harper VanSteenhouse, Yumi Kasai, David Gray, Carol Clugston, Anna Dominiczak and Alex Alderton, Roberto Amato, Sonia Goncalves, Ewan Harrison, David K. Jackson, Ian Johnston, Dominic Kwiatkowski, Cordelia Langford, John Sillitoe on behalf of the Wellcome Sanger Institute COVID-19 Surveillance Team
EPI_ISL_907491, EPI_ISL_907493	Lighthouse Lab in Alderley Park	Wellcome Sanger Institute for the COVID-19 Genomics UK (COG-UK) Consortium	Jacquelyn Wynn, Mairead Hyland, The Lighthouse Lab in Alderley Park and Alex Alderton, Roberto Amato, Sonia Goncalves, Ewan Harrison, David K. Jackson, Ian Johnston, Dominic Kwiatkowski, Cordelia Langford, John Sillitoe on behalf of the Wellcome Sanger Institute COVID-19 Surveillance Team
EPI_ISL_907494, EPI_ISL_907495	Lighthouse Lab in Glasgow	Wellcome Sanger Institute for the COVID-19 Genomics UK	Harper VanSteenhouse, Yumi Kasai, David Gray, Carol Clugston, Anna Dominiczak and Alex Alderton, Roberto Amato, Sonia Goncalves, Ewan Harrison,

		(COG-UK) Consortium	David K. Jackson, Ian Johnston, Dominic Kwiatkowski, Cordelia Langford, John Sillitoe on behalf of the Wellcome Sanger Institute COVID-19 Surveillance Team
EPI_ISL_907496	Lighthouse Lab in Alderley Park	Wellcome Sanger Institute for the COVID-19 Genomics UK (COG-UK) Consortium	Jacquelyn Wynn, Mairead Hyland, The Lighthouse Lab in Alderley Park and Alex Alderton, Roberto Amato, Sonia Goncalves, Ewan Harrison, David K. Jackson, Ian Johnston, Dominic Kwiatkowski, Cordelia Langford, John Sillitoe on behalf of the Wellcome Sanger Institute COVID-19 Surveillance Team
EPI_ISL_907498, EPI_ISL_907499	Lighthouse Lab in Glasgow	Wellcome Sanger Institute for the COVID-19 Genomics UK (COG-UK) Consortium	Harper VanSteenhouse, Yumi Kasai, David Gray, Carol Clugston, Anna Dominiczak and Alex Alderton, Roberto Amato, Sonia Goncalves, Ewan Harrison, David K. Jackson, Ian Johnston, Dominic Kwiatkowski, Cordelia Langford, John Sillitoe on behalf of the Wellcome Sanger Institute COVID-19 Surveillance Team
EPI_ISL_907500	Lighthouse Lab in Alderley Park	Wellcome Sanger Institute for the COVID-19 Genomics UK (COG-UK) Consortium	Jacquelyn Wynn, Mairead Hyland, The Lighthouse Lab in Alderley Park and Alex Alderton, Roberto Amato, Sonia Goncalves, Ewan Harrison, David K. Jackson, Ian Johnston, Dominic Kwiatkowski, Cordelia Langford, John Sillitoe on behalf of the Wellcome Sanger Institute COVID-19 Surveillance Team
EPI_ISL_907501, EPI_ISL_907502	Lighthouse Lab in Glasgow	Wellcome Sanger Institute for the COVID-19 Genomics UK (COG-UK) Consortium	Harper VanSteenhouse, Yumi Kasai, David Gray, Carol Clugston, Anna Dominiczak and Alex Alderton, Roberto Amato, Sonia Goncalves, Ewan Harrison, David K. Jackson, Ian Johnston, Dominic Kwiatkowski, Cordelia Langford, John Sillitoe on behalf of the Wellcome Sanger Institute COVID-19 Surveillance Team
EPI_ISL_907503	Lighthouse Lab in Alderley Park	Wellcome Sanger Institute for the COVID-19 Genomics UK (COG-UK) Consortium	Jacquelyn Wynn, Mairead Hyland, The Lighthouse Lab in Alderley Park and Alex Alderton, Roberto Amato, Sonia Goncalves, Ewan Harrison, David K. Jackson, Ian Johnston, Dominic Kwiatkowski, Cordelia Langford, John Sillitoe on behalf of the Wellcome Sanger Institute COVID-19 Surveillance Team
EPI_ISL_907504, EPI_ISL_907506, EPI_ISL_907507, EPI_ISL_907508, EPI_ISL_907509	Lighthouse Lab in Glasgow	Wellcome Sanger Institute for the COVID-19 Genomics UK (COG-UK) Consortium	Harper VanSteenhouse, Yumi Kasai, David Gray, Carol Clugston, Anna Dominiczak and Alex Alderton, Roberto Amato, Sonia Goncalves, Ewan Harrison, David K. Jackson, Ian Johnston, Dominic Kwiatkowski, Cordelia Langford, John Sillitoe on behalf of the Wellcome Sanger Institute COVID-19 Surveillance Team
EPI_ISL_907510, EPI_ISL_907511	Lighthouse Lab in Alderley Park	Wellcome Sanger Institute for the COVID-19 Genomics UK (COG-UK) Consortium	Jacquelyn Wynn, Mairead Hyland, The Lighthouse Lab in Alderley Park and Alex Alderton, Roberto Amato, Sonia Goncalves, Ewan Harrison, David K. Jackson, Ian Johnston, Dominic Kwiatkowski, Cordelia Langford, John Sillitoe on behalf of the Wellcome Sanger Institute COVID-19 Surveillance Team
EPI_ISL_907513, EPI_ISL_907514, EPI_ISL_907515	Lighthouse Lab in Glasgow	Wellcome Sanger Institute for the COVID-19 Genomics UK (COG-UK) Consortium	Harper VanSteenhouse, Yumi Kasai, David Gray, Carol Clugston, Anna Dominiczak and Alex Alderton, Roberto Amato, Sonia Goncalves, Ewan Harrison, David K. Jackson, Ian Johnston, Dominic Kwiatkowski, Cordelia Langford, John Sillitoe on behalf of the Wellcome Sanger Institute COVID-19 Surveillance Team
EPI_ISL_907517, EPI_ISL_907518	Lighthouse Lab in Alderley Park	Wellcome Sanger Institute for the COVID-19 Genomics UK (COG-UK) Consortium	Jacquelyn Wynn, Mairead Hyland, The Lighthouse Lab in Alderley Park and Alex Alderton, Roberto Amato, Sonia Goncalves, Ewan Harrison, David K. Jackson, Ian Johnston, Dominic Kwiatkowski, Cordelia Langford, John Sillitoe on behalf of the Wellcome Sanger Institute COVID-19 Surveillance Team
EPI_ISL_907520	Lighthouse Lab in Glasgow	Wellcome Sanger Institute for the COVID-19 Genomics UK (COG-UK) Consortium	Harper VanSteenhouse, Yumi Kasai, David Gray, Carol Clugston, Anna Dominiczak and Alex Alderton, Roberto Amato, Sonia Goncalves, Ewan Harrison, David K. Jackson, Ian Johnston, Dominic Kwiatkowski, Cordelia Langford, John Sillitoe on behalf of the Wellcome Sanger Institute COVID-19 Surveillance Team
EPI_ISL_907521, EPI_ISL_907523	Lighthouse Lab in Alderley Park	Wellcome Sanger Institute for the COVID-19 Genomics UK (COG-UK) Consortium	Jacquelyn Wynn, Mairead Hyland, The Lighthouse Lab in Alderley Park and Alex Alderton, Roberto Amato, Sonia Goncalves, Ewan Harrison, David K. Jackson, Ian Johnston, Dominic Kwiatkowski, Cordelia Langford, John Sillitoe on behalf of the Wellcome Sanger Institute COVID-19 Surveillance Team
EPI_ISL_907524, EPI_ISL_907525	Lighthouse Lab in Glasgow	Wellcome Sanger Institute for the COVID-19 Genomics UK (COG-UK) Consortium	Harper VanSteenhouse, Yumi Kasai, David Gray, Carol Clugston, Anna Dominiczak and Alex Alderton, Roberto Amato, Sonia Goncalves, Ewan Harrison, David K. Jackson, Ian Johnston, Dominic Kwiatkowski, Cordelia Langford, John Sillitoe on behalf of the Wellcome Sanger Institute COVID-19 Surveillance Team
EPI_ISL_907526	Lighthouse Lab in Alderley Park	Wellcome Sanger Institute for the COVID-19 Genomics UK (COG-UK) Consortium	Jacquelyn Wynn, Mairead Hyland, The Lighthouse Lab in Alderley Park and Alex Alderton, Roberto Amato, Sonia Goncalves, Ewan Harrison, David K. Jackson, Ian Johnston, Dominic Kwiatkowski, Cordelia Langford, John Sillitoe on behalf of the Wellcome Sanger Institute COVID-19 Surveillance Team
EPI_ISL_907527	Lighthouse Lab in Glasgow	Wellcome Sanger Institute for the COVID-19 Genomics UK (COG-UK) Consortium	Harper VanSteenhouse, Yumi Kasai, David Gray, Carol Clugston, Anna Dominiczak and Alex Alderton, Roberto Amato, Sonia Goncalves, Ewan Harrison, David K. Jackson, Ian Johnston, Dominic Kwiatkowski, Cordelia Langford, John Sillitoe on behalf of the Wellcome Sanger Institute COVID-19 Surveillance Team
EPI_ISL_907528, EPI_ISL_907529	Lighthouse Lab in Alderley Park	Wellcome Sanger Institute for the COVID-19 Genomics UK (COG-UK) Consortium	Jacquelyn Wynn, Mairead Hyland, The Lighthouse Lab in Alderley Park and Alex Alderton, Roberto Amato, Sonia Goncalves, Ewan Harrison, David K. Jackson, Ian Johnston, Dominic Kwiatkowski, Cordelia Langford, John Sillitoe on behalf of the Wellcome Sanger Institute COVID-19 Surveillance Team
EPI_ISL_907530, EPI_ISL_907531	Lighthouse Lab in Glasgow	Wellcome Sanger Institute for the COVID-19 Genomics UK (COG-UK) Consortium	Harper VanSteenhouse, Yumi Kasai, David Gray, Carol Clugston, Anna Dominiczak and Alex Alderton, Roberto Amato, Sonia Goncalves, Ewan Harrison, David K. Jackson, Ian Johnston, Dominic Kwiatkowski, Cordelia Langford, John Sillitoe on behalf of the Wellcome Sanger Institute COVID-19 Surveillance Team
EPI_ISL_907532	Lighthouse Lab in Alderley Park	Wellcome Sanger Institute for the COVID-19 Genomics UK (COG-UK) Consortium	Jacquelyn Wynn, Mairead Hyland, The Lighthouse Lab in Alderley Park and Alex Alderton, Roberto Amato, Sonia Goncalves, Ewan Harrison, David K. Jackson, Ian Johnston, Dominic Kwiatkowski, Cordelia Langford, John Sillitoe on behalf of the Wellcome Sanger Institute COVID-19 Surveillance Team
EPI_ISL_907534, EPI_ISL_907535	Lighthouse Lab in Glasgow	Wellcome Sanger Institute for the COVID-19 Genomics UK (COG-UK) Consortium	Harper VanSteenhouse, Yumi Kasai, David Gray, Carol Clugston, Anna Dominiczak and Alex Alderton, Roberto Amato, Sonia Goncalves, Ewan Harrison, David K. Jackson, Ian Johnston, Dominic Kwiatkowski, Cordelia Langford, John Sillitoe on behalf of the Wellcome Sanger Institute COVID-19 Surveillance Team
EPI_ISL_907536, EPI_ISL_907538	Lighthouse Lab in Alderley Park	Wellcome Sanger Institute for the COVID-19 Genomics UK (COG-UK) Consortium	Jacquelyn Wynn, Mairead Hyland, The Lighthouse Lab in Alderley Park and Alex Alderton, Roberto Amato, Sonia Goncalves, Ewan Harrison, David K. Jackson, Ian Johnston, Dominic Kwiatkowski, Cordelia Langford, John Sillitoe on behalf of the Wellcome Sanger Institute COVID-19 Surveillance Team
EPI_ISL_907539, EPI_ISL_907540, EPI_ISL_907541	Lighthouse Lab in Glasgow	Wellcome Sanger Institute for the COVID-19 Genomics UK (COG-UK) Consortium	Harper VanSteenhouse, Yumi Kasai, David Gray, Carol Clugston, Anna Dominiczak and Alex Alderton, Roberto Amato, Sonia Goncalves, Ewan Harrison, David K. Jackson, Ian Johnston, Dominic Kwiatkowski, Cordelia Langford, John Sillitoe on behalf of the Wellcome Sanger Institute COVID-19 Surveillance Team
EPI_ISL_907542, EPI_ISL_907543	Lighthouse Lab in Alderley Park	Wellcome Sanger Institute for the COVID-19 Genomics UK (COG-UK) Consortium	Jacquelyn Wynn, Mairead Hyland, The Lighthouse Lab in Alderley Park and Alex Alderton, Roberto Amato, Sonia Goncalves, Ewan Harrison, David K. Jackson, Ian Johnston, Dominic Kwiatkowski, Cordelia Langford, John Sillitoe on behalf of the Wellcome Sanger Institute COVID-19 Surveillance Team
EPI_ISL_907544, EPI_ISL_907545, EPI_ISL_907547, EPI_ISL_907548, EPI_ISL_907550, EPI_ISL_907553, EPI_ISL_907554, EPI_ISL_907555, EPI_ISL_907556, EPI_ISL_907558	Lighthouse Lab in Glasgow	Wellcome Sanger Institute for the COVID-19 Genomics UK (COG-UK) Consortium	Harper VanSteenhouse, Yumi Kasai, David Gray, Carol Clugston, Anna Dominiczak and Alex Alderton, Roberto Amato, Sonia Goncalves, Ewan Harrison, David K. Jackson, Ian Johnston, Dominic Kwiatkowski, Cordelia Langford, John Sillitoe on behalf of the Wellcome Sanger Institute COVID-19 Surveillance Team
EPI_ISL_907559	Lighthouse Lab in Alderley Park	Wellcome Sanger Institute for the COVID-19 Genomics UK (COG-UK) Consortium	Jacquelyn Wynn, Mairead Hyland, The Lighthouse Lab in Alderley Park and Alex Alderton, Roberto Amato, Sonia Goncalves, Ewan Harrison, David K. Jackson, Ian Johnston, Dominic Kwiatkowski, Cordelia Langford, John Sillitoe on behalf of the Wellcome Sanger Institute COVID-19 Surveillance Team
EPI_ISL_907560	Lighthouse Lab in Glasgow	Wellcome Sanger Institute for the COVID-19 Genomics UK (COG-UK) Consortium	Harper VanSteenhouse, Yumi Kasai, David Gray, Carol Clugston, Anna Dominiczak and Alex Alderton, Roberto Amato, Sonia Goncalves, Ewan Harrison, David K. Jackson, Ian Johnston, Dominic Kwiatkowski, Cordelia Langford, John Sillitoe on behalf of the Wellcome Sanger Institute COVID-19 Surveillance Team
EPI_ISL_907561	Lighthouse Lab in Alderley Park	Wellcome Sanger Institute for the COVID-19 Genomics UK (COG-UK) Consortium	Jacquelyn Wynn, Mairead Hyland, The Lighthouse Lab in Alderley Park and Alex Alderton, Roberto Amato, Sonia Goncalves, Ewan Harrison, David K. Jackson, Ian Johnston, Dominic Kwiatkowski, Cordelia Langford, John Sillitoe on behalf of the Wellcome Sanger Institute COVID-19 Surveillance Team
EPI_ISL_907562	Lighthouse Lab in Glasgow	Wellcome Sanger Institute for the COVID-19 Genomics UK (COG-UK) Consortium	Harper VanSteenhouse, Yumi Kasai, David Gray, Carol Clugston, Anna Dominiczak and Alex Alderton, Roberto Amato, Sonia Goncalves, Ewan Harrison, David K. Jackson, Ian Johnston, Dominic Kwiatkowski, Cordelia Langford, John Sillitoe on behalf of the Wellcome Sanger Institute COVID-19 Surveillance Team
EPI_ISL_907563	Lighthouse Lab in Alderley Park	Wellcome Sanger Institute for the COVID-19 Genomics UK (COG-UK) Consortium	Jacquelyn Wynn, Mairead Hyland, The Lighthouse Lab in Alderley Park and Alex Alderton, Roberto Amato, Sonia Goncalves, Ewan Harrison, David K. Jackson, Ian Johnston, Dominic Kwiatkowski, Cordelia Langford, John Sillitoe on behalf of the Wellcome Sanger Institute COVID-19 Surveillance Team
EPI_ISL_907564, EPI_ISL_907565, EPI_ISL_907566, EPI_ISL_907569,	Lighthouse Lab in Glasgow	Wellcome Sanger Institute for the COVID-19 Genomics UK (COG-UK) Consortium	Harper VanSteenhouse, Yumi Kasai, David Gray, Carol Clugston, Anna Dominiczak and Alex Alderton, Roberto Amato, Sonia Goncalves, Ewan Harrison, David K. Jackson, Ian Johnston, Dominic Kwiatkowski, Cordelia Langford, John Sillitoe on behalf of the Wellcome Sanger Institute COVID-19 Surveillance

EPI_ISL_907570, EPI_ISL_907572, EPI_ISL_907574, EPI_ISL_907575			Team
EPI_ISL_907576	Lighthouse Lab in Alderley Park	Wellcome Sanger Institute for the COVID-19 Genomics UK (COG-UK) Consortium	Jacquelyn Wynn, Mairead Hyland, The Lighthouse Lab in Alderley Park and Alex Alderton, Roberto Amato, Sonia Goncalves, Ewan Harrison, David K. Jackson, Ian Johnston, Dominic Kwiatkowski, Cordelia Langford, John Sillitoe on behalf of the Wellcome Sanger Institute COVID-19 Surveillance Team
EPI_ISL_907577	Lighthouse Lab in Glasgow	Wellcome Sanger Institute for the COVID-19 Genomics UK (COG-UK) Consortium	Harper VanSteenhouse, Yumi Kasai, David Gray, Carol Clugston, Anna Dominiczak and Alex Alderton, Roberto Amato, Sonia Goncalves, Ewan Harrison, David K. Jackson, Ian Johnston, Dominic Kwiatkowski, Cordelia Langford, John Sillitoe on behalf of the Wellcome Sanger Institute COVID-19 Surveillance Team
EPI_ISL_907578, EPI_ISL_907579	Lighthouse Lab in Alderley Park	Wellcome Sanger Institute for the COVID-19 Genomics UK (COG-UK) Consortium	Jacquelyn Wynn, Mairead Hyland, The Lighthouse Lab in Alderley Park and Alex Alderton, Roberto Amato, Sonia Goncalves, Ewan Harrison, David K. Jackson, Ian Johnston, Dominic Kwiatkowski, Cordelia Langford, John Sillitoe on behalf of the Wellcome Sanger Institute COVID-19 Surveillance Team
EPI_ISL_907581	Lighthouse Lab in Glasgow	Wellcome Sanger Institute for the COVID-19 Genomics UK (COG-UK) Consortium	Harper VanSteenhouse, Yumi Kasai, David Gray, Carol Clugston, Anna Dominiczak and Alex Alderton, Roberto Amato, Sonia Goncalves, Ewan Harrison, David K. Jackson, Ian Johnston, Dominic Kwiatkowski, Cordelia Langford, John Sillitoe on behalf of the Wellcome Sanger Institute COVID-19 Surveillance Team
EPI_ISL_907583	Lighthouse Lab in Alderley Park	Wellcome Sanger Institute for the COVID-19 Genomics UK (COG-UK) Consortium	Jacquelyn Wynn, Mairead Hyland, The Lighthouse Lab in Alderley Park and Alex Alderton, Roberto Amato, Sonia Goncalves, Ewan Harrison, David K. Jackson, Ian Johnston, Dominic Kwiatkowski, Cordelia Langford, John Sillitoe on behalf of the Wellcome Sanger Institute COVID-19 Surveillance Team
EPI_ISL_907585, EPI_ISL_907586, EPI_ISL_907588	Lighthouse Lab in Glasgow	Wellcome Sanger Institute for the COVID-19 Genomics UK (COG-UK) Consortium	Harper VanSteenhouse, Yumi Kasai, David Gray, Carol Clugston, Anna Dominiczak and Alex Alderton, Roberto Amato, Sonia Goncalves, Ewan Harrison, David K. Jackson, Ian Johnston, Dominic Kwiatkowski, Cordelia Langford, John Sillitoe on behalf of the Wellcome Sanger Institute COVID-19 Surveillance Team
EPI_ISL_907590	Lighthouse Lab in Alderley Park	Wellcome Sanger Institute for the COVID-19 Genomics UK (COG-UK) Consortium	Jacquelyn Wynn, Mairead Hyland, The Lighthouse Lab in Alderley Park and Alex Alderton, Roberto Amato, Sonia Goncalves, Ewan Harrison, David K. Jackson, Ian Johnston, Dominic Kwiatkowski, Cordelia Langford, John Sillitoe on behalf of the Wellcome Sanger Institute COVID-19 Surveillance Team
EPI_ISL_907591, EPI_ISL_907592, EPI_ISL_907593	Lighthouse Lab in Glasgow	Wellcome Sanger Institute for the COVID-19 Genomics UK (COG-UK) Consortium	Harper VanSteenhouse, Yumi Kasai, David Gray, Carol Clugston, Anna Dominiczak and Alex Alderton, Roberto Amato, Sonia Goncalves, Ewan Harrison, David K. Jackson, Ian Johnston, Dominic Kwiatkowski, Cordelia Langford, John Sillitoe on behalf of the Wellcome Sanger Institute COVID-19 Surveillance Team
EPI_ISL_907594	Lighthouse Lab in Alderley Park	Wellcome Sanger Institute for the COVID-19 Genomics UK (COG-UK) Consortium	Jacquelyn Wynn, Mairead Hyland, The Lighthouse Lab in Alderley Park and Alex Alderton, Roberto Amato, Sonia Goncalves, Ewan Harrison, David K. Jackson, Ian Johnston, Dominic Kwiatkowski, Cordelia Langford, John Sillitoe on behalf of the Wellcome Sanger Institute COVID-19 Surveillance Team
EPI_ISL_907595	Lighthouse Lab in Glasgow	Wellcome Sanger Institute for the COVID-19 Genomics UK (COG-UK) Consortium	Harper VanSteenhouse, Yumi Kasai, David Gray, Carol Clugston, Anna Dominiczak and Alex Alderton, Roberto Amato, Sonia Goncalves, Ewan Harrison, David K. Jackson, Ian Johnston, Dominic Kwiatkowski, Cordelia Langford, John Sillitoe on behalf of the Wellcome Sanger Institute COVID-19 Surveillance Team
EPI_ISL_907596	Lighthouse Lab in Alderley Park	Wellcome Sanger Institute for the COVID-19 Genomics UK (COG-UK) Consortium	Jacquelyn Wynn, Mairead Hyland, The Lighthouse Lab in Alderley Park and Alex Alderton, Roberto Amato, Sonia Goncalves, Ewan Harrison, David K. Jackson, Ian Johnston, Dominic Kwiatkowski, Cordelia Langford, John Sillitoe on behalf of the Wellcome Sanger Institute COVID-19 Surveillance Team
EPI_ISL_907597, EPI_ISL_907599	Lighthouse Lab in Glasgow	Wellcome Sanger Institute for the COVID-19 Genomics UK (COG-UK) Consortium	Harper VanSteenhouse, Yumi Kasai, David Gray, Carol Clugston, Anna Dominiczak and Alex Alderton, Roberto Amato, Sonia Goncalves, Ewan Harrison, David K. Jackson, Ian Johnston, Dominic Kwiatkowski, Cordelia Langford, John Sillitoe on behalf of the Wellcome Sanger Institute COVID-19 Surveillance Team
EPI_ISL_907600	Lighthouse Lab in Alderley Park	Wellcome Sanger Institute for the COVID-19 Genomics UK (COG-UK) Consortium	Jacquelyn Wynn, Mairead Hyland, The Lighthouse Lab in Alderley Park and Alex Alderton, Roberto Amato, Sonia Goncalves, Ewan Harrison, David K. Jackson, Ian Johnston, Dominic Kwiatkowski, Cordelia Langford, John Sillitoe on behalf of the Wellcome Sanger Institute COVID-19 Surveillance Team
EPI_ISL_907602, EPI_ISL_907603, EPI_ISL_907604, EPI_ISL_907608, EPI_ISL_907609	Lighthouse Lab in Glasgow	Wellcome Sanger Institute for the COVID-19 Genomics UK (COG-UK) Consortium	Harper VanSteenhouse, Yumi Kasai, David Gray, Carol Clugston, Anna Dominiczak and Alex Alderton, Roberto Amato, Sonia Goncalves, Ewan Harrison, David K. Jackson, Ian Johnston, Dominic Kwiatkowski, Cordelia Langford, John Sillitoe on behalf of the Wellcome Sanger Institute COVID-19 Surveillance Team
EPI_ISL_907610, EPI_ISL_907611	Lighthouse Lab in Alderley Park	Wellcome Sanger Institute for the COVID-19 Genomics UK (COG-UK) Consortium	Jacquelyn Wynn, Mairead Hyland, The Lighthouse Lab in Alderley Park and Alex Alderton, Roberto Amato, Sonia Goncalves, Ewan Harrison, David K. Jackson, Ian Johnston, Dominic Kwiatkowski, Cordelia Langford, John Sillitoe on behalf of the Wellcome Sanger Institute COVID-19 Surveillance Team
EPI_ISL_907613, EPI_ISL_907614	Lighthouse Lab in Glasgow	Wellcome Sanger Institute for the COVID-19 Genomics UK (COG-UK) Consortium	Harper VanSteenhouse, Yumi Kasai, David Gray, Carol Clugston, Anna Dominiczak and Alex Alderton, Roberto Amato, Sonia Goncalves, Ewan Harrison, David K. Jackson, Ian Johnston, Dominic Kwiatkowski, Cordelia Langford, John Sillitoe on behalf of the Wellcome Sanger Institute COVID-19 Surveillance Team
EPI_ISL_907615	Lighthouse Lab in Alderley Park	Wellcome Sanger Institute for the COVID-19 Genomics UK (COG-UK) Consortium	Jacquelyn Wynn, Mairead Hyland, The Lighthouse Lab in Alderley Park and Alex Alderton, Roberto Amato, Sonia Goncalves, Ewan Harrison, David K. Jackson, Ian Johnston, Dominic Kwiatkowski, Cordelia Langford, John Sillitoe on behalf of the Wellcome Sanger Institute COVID-19 Surveillance Team
EPI_ISL_907617, EPI_ISL_907619, EPI_ISL_907620	Lighthouse Lab in Glasgow	Wellcome Sanger Institute for the COVID-19 Genomics UK (COG-UK) Consortium	Harper VanSteenhouse, Yumi Kasai, David Gray, Carol Clugston, Anna Dominiczak and Alex Alderton, Roberto Amato, Sonia Goncalves, Ewan Harrison, David K. Jackson, Ian Johnston, Dominic Kwiatkowski, Cordelia Langford, John Sillitoe on behalf of the Wellcome Sanger Institute COVID-19 Surveillance Team
EPI_ISL_907622	Lighthouse Lab in Alderley Park	Wellcome Sanger Institute for the COVID-19 Genomics UK (COG-UK) Consortium	Jacquelyn Wynn, Mairead Hyland, The Lighthouse Lab in Alderley Park and Alex Alderton, Roberto Amato, Sonia Goncalves, Ewan Harrison, David K. Jackson, Ian Johnston, Dominic Kwiatkowski, Cordelia Langford, John Sillitoe on behalf of the Wellcome Sanger Institute COVID-19 Surveillance Team
EPI_ISL_907624, EPI_ISL_907625, EPI_ISL_907627	Lighthouse Lab in Glasgow	Wellcome Sanger Institute for the COVID-19 Genomics UK (COG-UK) Consortium	Harper VanSteenhouse, Yumi Kasai, David Gray, Carol Clugston, Anna Dominiczak and Alex Alderton, Roberto Amato, Sonia Goncalves, Ewan Harrison, David K. Jackson, Ian Johnston, Dominic Kwiatkowski, Cordelia Langford, John Sillitoe on behalf of the Wellcome Sanger Institute COVID-19 Surveillance Team
EPI_ISL_907628	Lighthouse Lab in Alderley Park	Wellcome Sanger Institute for the COVID-19 Genomics UK (COG-UK) Consortium	Jacquelyn Wynn, Mairead Hyland, The Lighthouse Lab in Alderley Park and Alex Alderton, Roberto Amato, Sonia Goncalves, Ewan Harrison, David K. Jackson, Ian Johnston, Dominic Kwiatkowski, Cordelia Langford, John Sillitoe on behalf of the Wellcome Sanger Institute COVID-19 Surveillance Team
EPI_ISL_907629	Lighthouse Lab in Glasgow	Wellcome Sanger Institute for the COVID-19 Genomics UK (COG-UK) Consortium	Harper VanSteenhouse, Yumi Kasai, David Gray, Carol Clugston, Anna Dominiczak and Alex Alderton, Roberto Amato, Sonia Goncalves, Ewan Harrison, David K. Jackson, Ian Johnston, Dominic Kwiatkowski, Cordelia Langford, John Sillitoe on behalf of the Wellcome Sanger Institute COVID-19 Surveillance Team
EPI_ISL_907630, EPI_ISL_907631	Lighthouse Lab in Alderley Park	Wellcome Sanger Institute for the COVID-19 Genomics UK (COG-UK) Consortium	Jacquelyn Wynn, Mairead Hyland, The Lighthouse Lab in Alderley Park and Alex Alderton, Roberto Amato, Sonia Goncalves, Ewan Harrison, David K. Jackson, Ian Johnston, Dominic Kwiatkowski, Cordelia Langford, John Sillitoe on behalf of the Wellcome Sanger Institute COVID-19 Surveillance Team
EPI_ISL_907632, EPI_ISL_907633, EPI_ISL_907634, EPI_ISL_907635, EPI_ISL_907637, EPI_ISL_907638, EPI_ISL_907639	Lighthouse Lab in Glasgow	Wellcome Sanger Institute for the COVID-19 Genomics UK (COG-UK) Consortium	Harper VanSteenhouse, Yumi Kasai, David Gray, Carol Clugston, Anna Dominiczak and Alex Alderton, Roberto Amato, Sonia Goncalves, Ewan Harrison, David K. Jackson, Ian Johnston, Dominic Kwiatkowski, Cordelia Langford, John Sillitoe on behalf of the Wellcome Sanger Institute COVID-19 Surveillance Team
EPI_ISL_907640, EPI_ISL_907642	Lighthouse Lab in Alderley Park	Wellcome Sanger Institute for the COVID-19 Genomics UK (COG-UK) Consortium	Jacquelyn Wynn, Mairead Hyland, The Lighthouse Lab in Alderley Park and Alex Alderton, Roberto Amato, Sonia Goncalves, Ewan Harrison, David K. Jackson, Ian Johnston, Dominic Kwiatkowski, Cordelia Langford, John Sillitoe on behalf of the Wellcome Sanger Institute COVID-19 Surveillance Team
EPI_ISL_907643, EPI_ISL_907644	Lighthouse Lab in Glasgow	Wellcome Sanger Institute for the COVID-19 Genomics UK (COG-UK) Consortium	Harper VanSteenhouse, Yumi Kasai, David Gray, Carol Clugston, Anna Dominiczak and Alex Alderton, Roberto Amato, Sonia Goncalves, Ewan Harrison, David K. Jackson, Ian Johnston, Dominic Kwiatkowski, Cordelia Langford, John Sillitoe on behalf of the Wellcome Sanger Institute COVID-19 Surveillance Team
EPI_ISL_907645	Lighthouse Lab in Alderley Park	Wellcome Sanger Institute for the COVID-19 Genomics UK (COG-UK) Consortium	Jacquelyn Wynn, Mairead Hyland, The Lighthouse Lab in Alderley Park and Alex Alderton, Roberto Amato, Sonia Goncalves, Ewan Harrison, David K. Jackson, Ian Johnston, Dominic Kwiatkowski, Cordelia Langford, John Sillitoe on behalf of the Wellcome Sanger Institute COVID-19 Surveillance Team
EPI_ISL_907646	Lighthouse Lab in Glasgow	Wellcome Sanger Institute for the COVID-19 Genomics UK (COG-UK) Consortium	Harper VanSteenhouse, Yumi Kasai, David Gray, Carol Clugston, Anna Dominiczak and Alex Alderton, Roberto Amato, Sonia Goncalves, Ewan Harrison, David K. Jackson, Ian Johnston, Dominic Kwiatkowski, Cordelia Langford, John Sillitoe on behalf of the Wellcome Sanger Institute COVID-19 Surveillance Team

EPI_ISL_907648	Lighthouse Lab in Alderley Park	Wellcome Sanger Institute for the COVID-19 Genomics UK (COG-UK) Consortium	Jacquelyn Wynn, Mairead Hyland, The Lighthouse Lab in Alderley Park and Alex Alderton, Roberto Amato, Sonia Goncalves, Ewan Harrison, David K. Jackson, Ian Johnston, Dominic Kwiatkowski, Cordelia Langford, John Sillitoe on behalf of the Wellcome Sanger Institute COVID-19 Surveillance Team
EPI_ISL_907649, EPI_ISL_907650	Lighthouse Lab in Glasgow	Wellcome Sanger Institute for the COVID-19 Genomics UK (COG-UK) Consortium	Harper VanSteenhouse, Yumi Kasai, David Gray, Carol Clugston, Anna Dominiczak and Alex Alderton, Roberto Amato, Sonia Goncalves, Ewan Harrison, David K. Jackson, Ian Johnston, Dominic Kwiatkowski, Cordelia Langford, John Sillitoe on behalf of the Wellcome Sanger Institute COVID-19 Surveillance Team
EPI_ISL_907651, EPI_ISL_907652	Lighthouse Lab in Alderley Park	Wellcome Sanger Institute for the COVID-19 Genomics UK (COG-UK) Consortium	Jacquelyn Wynn, Mairead Hyland, The Lighthouse Lab in Alderley Park and Alex Alderton, Roberto Amato, Sonia Goncalves, Ewan Harrison, David K. Jackson, Ian Johnston, Dominic Kwiatkowski, Cordelia Langford, John Sillitoe on behalf of the Wellcome Sanger Institute COVID-19 Surveillance Team
EPI_ISL_907653	Lighthouse Lab in Glasgow	Wellcome Sanger Institute for the COVID-19 Genomics UK (COG-UK) Consortium	Harper VanSteenhouse, Yumi Kasai, David Gray, Carol Clugston, Anna Dominiczak and Alex Alderton, Roberto Amato, Sonia Goncalves, Ewan Harrison, David K. Jackson, Ian Johnston, Dominic Kwiatkowski, Cordelia Langford, John Sillitoe on behalf of the Wellcome Sanger Institute COVID-19 Surveillance Team
EPI_ISL_907654	Lighthouse Lab in Alderley Park	Wellcome Sanger Institute for the COVID-19 Genomics UK (COG-UK) Consortium	Jacquelyn Wynn, Mairead Hyland, The Lighthouse Lab in Alderley Park and Alex Alderton, Roberto Amato, Sonia Goncalves, Ewan Harrison, David K. Jackson, Ian Johnston, Dominic Kwiatkowski, Cordelia Langford, John Sillitoe on behalf of the Wellcome Sanger Institute COVID-19 Surveillance Team
EPI_ISL_907655, EPI_ISL_907656, EPI_ISL_907657	Lighthouse Lab in Glasgow	Wellcome Sanger Institute for the COVID-19 Genomics UK (COG-UK) Consortium	Harper VanSteenhouse, Yumi Kasai, David Gray, Carol Clugston, Anna Dominiczak and Alex Alderton, Roberto Amato, Sonia Goncalves, Ewan Harrison, David K. Jackson, Ian Johnston, Dominic Kwiatkowski, Cordelia Langford, John Sillitoe on behalf of the Wellcome Sanger Institute COVID-19 Surveillance Team
EPI_ISL_907659	Lighthouse Lab in Alderley Park	Wellcome Sanger Institute for the COVID-19 Genomics UK (COG-UK) Consortium	Jacquelyn Wynn, Mairead Hyland, The Lighthouse Lab in Alderley Park and Alex Alderton, Roberto Amato, Sonia Goncalves, Ewan Harrison, David K. Jackson, Ian Johnston, Dominic Kwiatkowski, Cordelia Langford, John Sillitoe on behalf of the Wellcome Sanger Institute COVID-19 Surveillance Team
EPI_ISL_907660	Lighthouse Lab in Glasgow	Wellcome Sanger Institute for the COVID-19 Genomics UK (COG-UK) Consortium	Harper VanSteenhouse, Yumi Kasai, David Gray, Carol Clugston, Anna Dominiczak and Alex Alderton, Roberto Amato, Sonia Goncalves, Ewan Harrison, David K. Jackson, Ian Johnston, Dominic Kwiatkowski, Cordelia Langford, John Sillitoe on behalf of the Wellcome Sanger Institute COVID-19 Surveillance Team
EPI_ISL_907661	Lighthouse Lab in Alderley Park	Wellcome Sanger Institute for the COVID-19 Genomics UK (COG-UK) Consortium	Jacquelyn Wynn, Mairead Hyland, The Lighthouse Lab in Alderley Park and Alex Alderton, Roberto Amato, Sonia Goncalves, Ewan Harrison, David K. Jackson, Ian Johnston, Dominic Kwiatkowski, Cordelia Langford, John Sillitoe on behalf of the Wellcome Sanger Institute COVID-19 Surveillance Team
EPI_ISL_907662, EPI_ISL_907663	Lighthouse Lab in Glasgow	Wellcome Sanger Institute for the COVID-19 Genomics UK (COG-UK) Consortium	Harper VanSteenhouse, Yumi Kasai, David Gray, Carol Clugston, Anna Dominiczak and Alex Alderton, Roberto Amato, Sonia Goncalves, Ewan Harrison, David K. Jackson, Ian Johnston, Dominic Kwiatkowski, Cordelia Langford, John Sillitoe on behalf of the Wellcome Sanger Institute COVID-19 Surveillance Team
EPI_ISL_907664	Lighthouse Lab in Alderley Park	Wellcome Sanger Institute for the COVID-19 Genomics UK (COG-UK) Consortium	Jacquelyn Wynn, Mairead Hyland, The Lighthouse Lab in Alderley Park and Alex Alderton, Roberto Amato, Sonia Goncalves, Ewan Harrison, David K. Jackson, Ian Johnston, Dominic Kwiatkowski, Cordelia Langford, John Sillitoe on behalf of the Wellcome Sanger Institute COVID-19 Surveillance Team
EPI_ISL_907665, EPI_ISL_907666	Lighthouse Lab in Glasgow	Wellcome Sanger Institute for the COVID-19 Genomics UK (COG-UK) Consortium	Harper VanSteenhouse, Yumi Kasai, David Gray, Carol Clugston, Anna Dominiczak and Alex Alderton, Roberto Amato, Sonia Goncalves, Ewan Harrison, David K. Jackson, Ian Johnston, Dominic Kwiatkowski, Cordelia Langford, John Sillitoe on behalf of the Wellcome Sanger Institute COVID-19 Surveillance Team
EPI_ISL_907667	Lighthouse Lab in Alderley Park	Wellcome Sanger Institute for the COVID-19 Genomics UK (COG-UK) Consortium	Jacquelyn Wynn, Mairead Hyland, The Lighthouse Lab in Alderley Park and Alex Alderton, Roberto Amato, Sonia Goncalves, Ewan Harrison, David K. Jackson, Ian Johnston, Dominic Kwiatkowski, Cordelia Langford, John Sillitoe on behalf of the Wellcome Sanger Institute COVID-19 Surveillance Team
EPI_ISL_907668, EPI_ISL_907669, EPI_ISL_907670, EPI_ISL_907671	Lighthouse Lab in Glasgow	Wellcome Sanger Institute for the COVID-19 Genomics UK (COG-UK) Consortium	Harper VanSteenhouse, Yumi Kasai, David Gray, Carol Clugston, Anna Dominiczak and Alex Alderton, Roberto Amato, Sonia Goncalves, Ewan Harrison, David K. Jackson, Ian Johnston, Dominic Kwiatkowski, Cordelia Langford, John Sillitoe on behalf of the Wellcome Sanger Institute COVID-19 Surveillance Team
EPI_ISL_907672, EPI_ISL_907673	Lighthouse Lab in Alderley Park	Wellcome Sanger Institute for the COVID-19 Genomics UK (COG-UK) Consortium	Jacquelyn Wynn, Mairead Hyland, The Lighthouse Lab in Alderley Park and Alex Alderton, Roberto Amato, Sonia Goncalves, Ewan Harrison, David K. Jackson, Ian Johnston, Dominic Kwiatkowski, Cordelia Langford, John Sillitoe on behalf of the Wellcome Sanger Institute COVID-19 Surveillance Team
EPI_ISL_907674	Lighthouse Lab in Glasgow	Wellcome Sanger Institute for the COVID-19 Genomics UK (COG-UK) Consortium	Harper VanSteenhouse, Yumi Kasai, David Gray, Carol Clugston, Anna Dominiczak and Alex Alderton, Roberto Amato, Sonia Goncalves, Ewan Harrison, David K. Jackson, Ian Johnston, Dominic Kwiatkowski, Cordelia Langford, John Sillitoe on behalf of the Wellcome Sanger Institute COVID-19 Surveillance Team
EPI_ISL_907675, EPI_ISL_907676	Lighthouse Lab in Alderley Park	Wellcome Sanger Institute for the COVID-19 Genomics UK (COG-UK) Consortium	Jacquelyn Wynn, Mairead Hyland, The Lighthouse Lab in Alderley Park and Alex Alderton, Roberto Amato, Sonia Goncalves, Ewan Harrison, David K. Jackson, Ian Johnston, Dominic Kwiatkowski, Cordelia Langford, John Sillitoe on behalf of the Wellcome Sanger Institute COVID-19 Surveillance Team
EPI_ISL_907678, EPI_ISL_907679, EPI_ISL_907681, EPI_ISL_907682, EPI_ISL_907683, EPI_ISL_907685	Lighthouse Lab in Glasgow	Wellcome Sanger Institute for the COVID-19 Genomics UK (COG-UK) Consortium	Harper VanSteenhouse, Yumi Kasai, David Gray, Carol Clugston, Anna Dominiczak and Alex Alderton, Roberto Amato, Sonia Goncalves, Ewan Harrison, David K. Jackson, Ian Johnston, Dominic Kwiatkowski, Cordelia Langford, John Sillitoe on behalf of the Wellcome Sanger Institute COVID-19 Surveillance Team
EPI_ISL_907686	Lighthouse Lab in Alderley Park	Wellcome Sanger Institute for the COVID-19 Genomics UK (COG-UK) Consortium	Jacquelyn Wynn, Mairead Hyland, The Lighthouse Lab in Alderley Park and Alex Alderton, Roberto Amato, Sonia Goncalves, Ewan Harrison, David K. Jackson, Ian Johnston, Dominic Kwiatkowski, Cordelia Langford, John Sillitoe on behalf of the Wellcome Sanger Institute COVID-19 Surveillance Team
EPI_ISL_907687, EPI_ISL_907688	Lighthouse Lab in Glasgow	Wellcome Sanger Institute for the COVID-19 Genomics UK (COG-UK) Consortium	Harper VanSteenhouse, Yumi Kasai, David Gray, Carol Clugston, Anna Dominiczak and Alex Alderton, Roberto Amato, Sonia Goncalves, Ewan Harrison, David K. Jackson, Ian Johnston, Dominic Kwiatkowski, Cordelia Langford, John Sillitoe on behalf of the Wellcome Sanger Institute COVID-19 Surveillance Team
EPI_ISL_907689	Lighthouse Lab in Alderley Park	Wellcome Sanger Institute for the COVID-19 Genomics UK (COG-UK) Consortium	Jacquelyn Wynn, Mairead Hyland, The Lighthouse Lab in Alderley Park and Alex Alderton, Roberto Amato, Sonia Goncalves, Ewan Harrison, David K. Jackson, Ian Johnston, Dominic Kwiatkowski, Cordelia Langford, John Sillitoe on behalf of the Wellcome Sanger Institute COVID-19 Surveillance Team
EPI_ISL_907691	Lighthouse Lab in Glasgow	Wellcome Sanger Institute for the COVID-19 Genomics UK (COG-UK) Consortium	Harper VanSteenhouse, Yumi Kasai, David Gray, Carol Clugston, Anna Dominiczak and Alex Alderton, Roberto Amato, Sonia Goncalves, Ewan Harrison, David K. Jackson, Ian Johnston, Dominic Kwiatkowski, Cordelia Langford, John Sillitoe on behalf of the Wellcome Sanger Institute COVID-19 Surveillance Team
EPI_ISL_907693, EPI_ISL_907694, EPI_ISL_907696	Lighthouse Lab in Alderley Park	Wellcome Sanger Institute for the COVID-19 Genomics UK (COG-UK) Consortium	Jacquelyn Wynn, Mairead Hyland, The Lighthouse Lab in Alderley Park and Alex Alderton, Roberto Amato, Sonia Goncalves, Ewan Harrison, David K. Jackson, Ian Johnston, Dominic Kwiatkowski, Cordelia Langford, John Sillitoe on behalf of the Wellcome Sanger Institute COVID-19 Surveillance Team
EPI_ISL_907698	Lighthouse Lab in Glasgow	Wellcome Sanger Institute for the COVID-19 Genomics UK (COG-UK) Consortium	Harper VanSteenhouse, Yumi Kasai, David Gray, Carol Clugston, Anna Dominiczak and Alex Alderton, Roberto Amato, Sonia Goncalves, Ewan Harrison, David K. Jackson, Ian Johnston, Dominic Kwiatkowski, Cordelia Langford, John Sillitoe on behalf of the Wellcome Sanger Institute COVID-19 Surveillance Team
EPI_ISL_907699	Lighthouse Lab in Alderley Park	Wellcome Sanger Institute for the COVID-19 Genomics UK (COG-UK) Consortium	Jacquelyn Wynn, Mairead Hyland, The Lighthouse Lab in Alderley Park and Alex Alderton, Roberto Amato, Sonia Goncalves, Ewan Harrison, David K. Jackson, Ian Johnston, Dominic Kwiatkowski, Cordelia Langford, John Sillitoe on behalf of the Wellcome Sanger Institute COVID-19 Surveillance Team

EPI_ISL_907702, EPI_ISL_907703, EPI_ISL_907705, EPI_ISL_907706, EPI_ISL_907707, EPI_ISL_907708, EPI_ISL_907716, EPI_ISL_907721, EPI_ISL_907722, EPI_ISL_907723, EPI_ISL_907731, EPI_ISL_907735, EPI_ISL_907736, EPI_ISL_907738, EPI_ISL_907745, EPI_ISL_907747, EPI_ISL_907749, EPI_ISL_907755, EPI_ISL_907756, EPI_ISL_907757, EPI_ISL_907760, EPI_ISL_907763, EPI_ISL_907765, EPI_ISL_907767, EPI_ISL_907771, EPI_ISL_907772, EPI_ISL_907773, EPI_ISL_907776, EPI_ISL_907778, EPI_ISL_907786, EPI_ISL_907787, EPI_ISL_907789, EPI_ISL_907793, EPI_ISL_907794, EPI_ISL_907795, EPI_ISL_907796, EPI_ISL_907797, EPI_ISL_907800, EPI_ISL_907801, EPI_ISL_907802, EPI_ISL_907805, EPI_ISL_907809, EPI_ISL_907812, EPI_ISL_907813, EPI_ISL_907814, EPI_ISL_907817, EPI_ISL_907820, EPI_ISL_907821, EPI_ISL_907824, EPI_ISL_907826, EPI_ISL_907832, EPI_ISL_907833, EPI_ISL_907834, EPI_ISL_907835, EPI_ISL_907837, EPI_ISL_907847, EPI_ISL_907851, EPI_ISL_907852, EPI_ISL_907854, EPI_ISL_907855, EPI_ISL_907860, EPI_ISL_907863, EPI_ISL_907865, EPI_ISL_907867, EPI_ISL_907868, EPI_ISL_907872, EPI_ISL_907874, EPI_ISL_907875, EPI_ISL_907877, EPI_ISL_907878, EPI_ISL_907879, EPI_ISL_907882, EPI_ISL_907889, EPI_ISL_907890, EPI_ISL_907893, EPI_ISL_907894, EPI_ISL_907895, EPI_ISL_907898, EPI_ISL_907899, EPI_ISL_907902, EPI_ISL_907903, EPI_ISL_907911, EPI_ISL_907912, EPI_ISL_907914, EPI_ISL_907915, EPI_ISL_907921, EPI_ISL_907923, EPI_ISL_907925, EPI_ISL_907926, EPI_ISL_907931, EPI_ISL_907934, EPI_ISL_907936, EPI_ISL_907940, EPI_ISL_907943, EPI_ISL_907944, EPI_ISL_907945, EPI_ISL_907946, EPI_ISL_907948, EPI_ISL_907949, EPI_ISL_907953, EPI_ISL_907956, EPI_ISL_907960, EPI_ISL_907967, EPI_ISL_907969, EPI_ISL_907970, EPI_ISL_907972, EPI_ISL_907973, EPI_ISL_907974, EPI_ISL_907975, EPI_ISL_907976, EPI_ISL_907979, EPI_ISL_907981, EPI_ISL_907982, EPI_ISL_907984, EPI_ISL_907987, EPI_ISL_907988, EPI_ISL_907989, EPI_ISL_907994, EPI_ISL_907995, EPI_ISL_907998, EPI_ISL_908002, EPI_ISL_908007, EPI_ISL_908009, EPI_ISL_908010, EPI_ISL_908011, EPI_ISL_908013, EPI_ISL_908016, EPI_ISL_908017, EPI_ISL_908021, EPI_ISL_908022, EPI_ISL_908025, EPI_ISL_908026, EPI_ISL_908027, EPI_ISL_908028, EPI_ISL_908030, EPI_ISL_908033, EPI_ISL_908034, EPI_ISL_908275, EPI_ISL_908284, EPI_ISL_908289, EPI_ISL_908290, EPI_ISL_908291, EPI_ISL_908295, EPI_ISL_908302, EPI_ISL_908303, EPI_ISL_908307, EPI_ISL_908308, EPI_ISL_908317, EPI_ISL_908321, EPI_ISL_908323, EPI_ISL_908326, EPI_ISL_908329, EPI_ISL_908335, EPI_ISL_908337, EPI_ISL_908338, EPI_ISL_908340, EPI_ISL_908348, EPI_ISL_908353, EPI_ISL_908357, EPI_ISL_908360, EPI_ISL_908365, EPI_ISL_908366, EPI_ISL_908380, EPI_ISL_908387, EPI_ISL_908393, EPI_ISL_908394, EPI_ISL_908403, EPI_ISL_908405, EPI_ISL_908411, EPI_ISL_908412, EPI_ISL_908416, EPI_ISL_908421, EPI_ISL_908422, EPI_ISL_908429, EPI_ISL_908431, EPI_ISL_908433, EPI_ISL_908442, EPI_ISL_908443, EPI_ISL_908444, EPI_ISL_908447, EPI_ISL_908451, EPI_ISL_908455, EPI_ISL_908462, EPI_ISL_908470, EPI_ISL_908471, EPI_ISL_908481, EPI_ISL_908486, EPI_ISL_908488, EPI_ISL_908489, EPI_ISL_908495, EPI_ISL_908496, EPI_ISL_908500, EPI_ISL_908507, EPI_ISL_908508, EPI_ISL_908512, EPI_ISL_908516, EPI_ISL_908521, EPI_ISL_908522, EPI_ISL_908525, EPI_ISL_908530, EPI_ISL_908536, EPI_ISL_908538, EPI_ISL_908547, EPI_ISL_908551, EPI_ISL_908557, EPI_ISL_908558, EPI_ISL_908559, EPI_ISL_908564, EPI_ISL_908568, EPI_ISL_908573, EPI_ISL_908582, EPI_ISL_908589, EPI_ISL_908591, EPI_ISL_908595

see above	Lighthouse Lab in Glasgow	Wellcome Sanger Institute for the COVID-19 Genomics UK (COG-UK) Consortium	Harper VanSteenhouse, Yumi Kasai, David Gray, Carol Clugston, Anna Dominiczak and Alex Alderton, Roberto Amato, Sonia Goncalves, Ewan Harrison, David K. Jackson, Ian Johnston, Dominic Kwiatkowski, Cordelia Langford, John Sillitoe on behalf of the Wellcome Sanger Institute COVID-19 Surveillance Team
EPI_ISL_908761	Lighthouse Lab in Cambridge	Wellcome Sanger Institute for the COVID-19 Genomics UK (COG-UK) Consortium	Rob Howes, The Lighthouse Lab in Cambridge and Alex Alderton, Roberto Amato, Sonia Goncalves, Ewan Harrison, David K. Jackson, Ian Johnston, Dominic Kwiatkowski, Cordelia Langford, John Sillitoe on behalf of the Wellcome Sanger Institute COVID-19 Surveillance Team
EPI_ISL_912212, EPI_ISL_912213	Lighthouse Lab in Glasgow	Wellcome Sanger Institute for the COVID-19 Genomics UK (COG-UK) Consortium	Harper VanSteenhouse, Yumi Kasai, David Gray, Carol Clugston, Anna Dominiczak and Alex Alderton, Roberto Amato, Sonia Goncalves, Ewan Harrison, David K. Jackson, Ian Johnston, Dominic Kwiatkowski, Cordelia Langford, John Sillitoe on behalf of the Wellcome Sanger Institute COVID-19 Surveillance Team (http://www.sanger.ac.uk/covid-team)
EPI_ISL_912215, EPI_ISL_912217, EPI_ISL_912218	Lighthouse Lab in Alderley Park	Wellcome Sanger Institute for the COVID-19 Genomics UK (COG-UK) Consortium	Jacquelyn Wynn, Mairead Hyland, The Lighthouse Lab in Alderley Park and Alex Alderton, Roberto Amato, Sonia Goncalves, Ewan Harrison, David K. Jackson, Ian Johnston, Dominic Kwiatkowski, Cordelia Langford, John Sillitoe on behalf of the Wellcome Sanger Institute COVID-19 Surveillance Team (http://www.sanger.ac.uk/covid-team)
EPI_ISL_912219	Lighthouse Lab in Glasgow	Wellcome Sanger Institute for the COVID-19 Genomics UK (COG-UK) Consortium	Harper VanSteenhouse, Yumi Kasai, David Gray, Carol Clugston, Anna Dominiczak and Alex Alderton, Roberto Amato, Sonia Goncalves, Ewan Harrison, David K. Jackson, Ian Johnston, Dominic Kwiatkowski, Cordelia Langford, John Sillitoe on behalf of the Wellcome Sanger Institute COVID-19 Surveillance Team (http://www.sanger.ac.uk/covid-team)
EPI_ISL_916213	Lighthouse Lab in Cambridge	Wellcome Sanger Institute for the COVID-19 Genomics UK (COG-UK) Consortium	Rob Howes, The Lighthouse Lab in Cambridge and Alex Alderton, Roberto Amato, Sonia Goncalves, Ewan Harrison, David K. Jackson, Ian Johnston, Dominic Kwiatkowski, Cordelia Langford, John Sillitoe on behalf of the Wellcome Sanger Institute COVID-19 Surveillance Team
EPI_ISL_916214, EPI_ISL_916230, EPI_ISL_916259	Lighthouse Lab in Alderley Park	Wellcome Sanger Institute for the COVID-19 Genomics UK (COG-UK) Consortium	Jacquelyn Wynn, Mairead Hyland, The Lighthouse Lab in Alderley Park and Alex Alderton, Roberto Amato, Sonia Goncalves, Ewan Harrison, David K. Jackson, Ian Johnston, Dominic Kwiatkowski, Cordelia Langford, John Sillitoe on behalf of the Wellcome Sanger Institute COVID-19 Surveillance Team
EPI_ISL_916290	Lighthouse Lab in Cambridge	Wellcome Sanger Institute for the COVID-19 Genomics UK (COG-UK) Consortium	Rob Howes, The Lighthouse Lab in Cambridge and Alex Alderton, Roberto Amato, Sonia Goncalves, Ewan Harrison, David K. Jackson, Ian Johnston, Dominic Kwiatkowski, Cordelia Langford, John Sillitoe on behalf of the Wellcome Sanger Institute COVID-19 Surveillance Team
EPI_ISL_916293	Lighthouse Lab in Alderley Park	Wellcome Sanger Institute for the COVID-19 Genomics UK (COG-UK) Consortium	Jacquelyn Wynn, Mairead Hyland, The Lighthouse Lab in Alderley Park and Alex Alderton, Roberto Amato, Sonia Goncalves, Ewan Harrison, David K. Jackson, Ian Johnston, Dominic Kwiatkowski, Cordelia Langford, John Sillitoe on behalf of the Wellcome Sanger Institute COVID-19 Surveillance Team
EPI_ISL_916296, EPI_ISL_916305, EPI_ISL_916308	Lighthouse Lab in Cambridge	Wellcome Sanger Institute for the COVID-19 Genomics UK (COG-UK) Consortium	Rob Howes, The Lighthouse Lab in Cambridge and Alex Alderton, Roberto Amato, Sonia Goncalves, Ewan Harrison, David K. Jackson, Ian Johnston, Dominic Kwiatkowski, Cordelia Langford, John Sillitoe on behalf of the Wellcome Sanger Institute COVID-19 Surveillance Team
EPI_ISL_916324	Lighthouse Lab in Alderley Park	Wellcome Sanger Institute for the COVID-19 Genomics UK (COG-UK) Consortium	Jacquelyn Wynn, Mairead Hyland, The Lighthouse Lab in Alderley Park and Alex Alderton, Roberto Amato, Sonia Goncalves, Ewan Harrison, David K. Jackson, Ian Johnston, Dominic Kwiatkowski, Cordelia Langford, John Sillitoe on behalf of the Wellcome Sanger Institute COVID-19 Surveillance Team
EPI_ISL_916327, EPI_ISL_916332	Lighthouse Lab in Cambridge	Wellcome Sanger Institute for the COVID-19 Genomics UK (COG-UK) Consortium	Rob Howes, The Lighthouse Lab in Cambridge and Alex Alderton, Roberto Amato, Sonia Goncalves, Ewan Harrison, David K. Jackson, Ian Johnston, Dominic Kwiatkowski, Cordelia Langford, John Sillitoe on behalf of the Wellcome Sanger Institute COVID-19 Surveillance Team
EPI_ISL_916337	Lighthouse Lab in Alderley Park	Wellcome Sanger Institute for the COVID-19 Genomics UK (COG-UK) Consortium	Jacquelyn Wynn, Mairead Hyland, The Lighthouse Lab in Alderley Park and Alex Alderton, Roberto Amato, Sonia Goncalves, Ewan Harrison, David K. Jackson, Ian Johnston, Dominic Kwiatkowski, Cordelia Langford, John Sillitoe on behalf of the Wellcome Sanger Institute COVID-19 Surveillance Team
EPI_ISL_916340, EPI_ISL_916341, EPI_ISL_916365, EPI_ISL_916367, EPI_ISL_916380	Lighthouse Lab in Cambridge	Wellcome Sanger Institute for the COVID-19 Genomics UK (COG-UK) Consortium	Rob Howes, The Lighthouse Lab in Cambridge and Alex Alderton, Roberto Amato, Sonia Goncalves, Ewan Harrison, David K. Jackson, Ian Johnston, Dominic Kwiatkowski, Cordelia Langford, John Sillitoe on behalf of the Wellcome Sanger Institute COVID-19 Surveillance Team
EPI_ISL_916386	Lighthouse Lab in Alderley Park	Wellcome Sanger Institute for the COVID-19 Genomics UK (COG-UK) Consortium	Jacquelyn Wynn, Mairead Hyland, The Lighthouse Lab in Alderley Park and Alex Alderton, Roberto Amato, Sonia Goncalves, Ewan Harrison, David K. Jackson, Ian Johnston, Dominic Kwiatkowski, Cordelia Langford, John Sillitoe on behalf of the Wellcome Sanger Institute COVID-19 Surveillance Team
EPI_ISL_916406	Lighthouse Lab in Cambridge	Wellcome Sanger Institute for the COVID-19 Genomics UK (COG-UK) Consortium	Rob Howes, The Lighthouse Lab in Cambridge and Alex Alderton, Roberto Amato, Sonia Goncalves, Ewan Harrison, David K. Jackson, Ian Johnston, Dominic Kwiatkowski, Cordelia Langford, John Sillitoe on behalf of the Wellcome Sanger Institute COVID-19 Surveillance Team
EPI_ISL_916418, EPI_ISL_916423	Lighthouse Lab in Alderley Park	Wellcome Sanger Institute for the COVID-19 Genomics UK (COG-UK) Consortium	Jacquelyn Wynn, Mairead Hyland, The Lighthouse Lab in Alderley Park and Alex Alderton, Roberto Amato, Sonia Goncalves, Ewan Harrison, David K. Jackson, Ian Johnston, Dominic Kwiatkowski, Cordelia Langford, John Sillitoe on behalf of the Wellcome Sanger Institute COVID-19 Surveillance Team
EPI_ISL_916428, EPI_ISL_916434, EPI_ISL_916439, EPI_ISL_916442, EPI_ISL_916456, EPI_ISL_916467, EPI_ISL_916472, EPI_ISL_916473, EPI_ISL_916476, EPI_ISL_916481	Lighthouse Lab in Cambridge	Wellcome Sanger Institute for the COVID-19 Genomics UK (COG-UK) Consortium	Rob Howes, The Lighthouse Lab in Cambridge and Alex Alderton, Roberto Amato, Sonia Goncalves, Ewan Harrison, David K. Jackson, Ian Johnston, Dominic Kwiatkowski, Cordelia Langford, John Sillitoe on behalf of the Wellcome Sanger Institute COVID-19 Surveillance Team

EPI_ISL_916503, EPI_ISL_916504, EPI_ISL_916505, EPI_ISL_916506, EPI_ISL_916507, EPI_ISL_916508, EPI_ISL_916509, EPI_ISL_916510, EPI_ISL_916511, EPI_ISL_916512, EPI_ISL_916513, EPI_ISL_916514, EPI_ISL_916515, EPI_ISL_916516, EPI_ISL_916517, EPI_ISL_916518, EPI_ISL_916519, EPI_ISL_916520, EPI_ISL_916521, EPI_ISL_916522, EPI_ISL_916523, EPI_ISL_916524, EPI_ISL_916525, EPI_ISL_916526, EPI_ISL_916527, EPI_ISL_916528, EPI_ISL_916529, EPI_ISL_916530, EPI_ISL_916531, EPI_ISL_916532, EPI_ISL_916533, EPI_ISL_916534, EPI_ISL_916535, EPI_ISL_916536, EPI_ISL_916537, EPI_ISL_916538, EPI_ISL_916539, EPI_ISL_916540, EPI_ISL_916541, EPI_ISL_916542, EPI_ISL_916543, EPI_ISL_916544, EPI_ISL_916545, EPI_ISL_916546, EPI_ISL_916547, EPI_ISL_916548, EPI_ISL_916549, EPI_ISL_916550, EPI_ISL_916551, EPI_ISL_916552, EPI_ISL_916553, EPI_ISL_916554, EPI_ISL_916555, EPI_ISL_916556, EPI_ISL_916557, EPI_ISL_916558, EPI_ISL_916559, EPI_ISL_916560, EPI_ISL_916561, EPI_ISL_916562, EPI_ISL_916563, EPI_ISL_916564, EPI_ISL_916565, EPI_ISL_916566, EPI_ISL_916567, EPI_ISL_916568, EPI_ISL_916569, EPI_ISL_916570, EPI_ISL_916571, EPI_ISL_916572, EPI_ISL_916573, EPI_ISL_916574, EPI_ISL_916575, EPI_ISL_916576, EPI_ISL_916577, EPI_ISL_916578, EPI_ISL_916579, EPI_ISL_916580, EPI_ISL_916581, EPI_ISL_916582, EPI_ISL_916583, EPI_ISL_916584, EPI_ISL_916585, EPI_ISL_916586, EPI_ISL_916587, EPI_ISL_916588, EPI_ISL_916589, EPI_ISL_916590, EPI_ISL_916591, EPI_ISL_916592, EPI_ISL_916593, EPI_ISL_916594, EPI_ISL_916595, EPI_ISL_916596, EPI_ISL_916597, EPI_ISL_916598, EPI_ISL_916599, EPI_ISL_916600, EPI_ISL_916601, EPI_ISL_916602, EPI_ISL_916603, EPI_ISL_916604, EPI_ISL_916605, EPI_ISL_916606, EPI_ISL_916607, EPI_ISL_916608, EPI_ISL_916609, EPI_ISL_916610, EPI_ISL_916611, EPI_ISL_916612, EPI_ISL_916613, EPI_ISL_916614, EPI_ISL_916615, EPI_ISL_916616, EPI_ISL_916617, EPI_ISL_916618, EPI_ISL_916619, EPI_ISL_916620, EPI_ISL_916621

see above	Lighthouse Lab in Glasgow	Wellcome Sanger Institute for the COVID-19 Genomics UK (COG-UK) Consortium	Harper VanSteenhouse, Yumi Kasai, David Gray, Carol Clugston, Anna Dominiczak and Alex Alderton, Roberto Amato, Sonia Goncalves, Ewan Harrison, David K. Jackson, Ian Johnston, Dominic Kwiatkowski, Cordelia Langford, John Sillitoe on behalf of the Wellcome Sanger Institute COVID-19 Surveillance Team

EPI_ISL_917169, EPI_ISL_917170, EPI_ISL_917171, EPI_ISL_917172, EPI_ISL_917174, EPI_ISL_917175, EPI_ISL_917176, EPI_ISL_917177, EPI_ISL_917178, EPI_ISL_917179, EPI_ISL_917180, EPI_ISL_917181, EPI_ISL_917182, EPI_ISL_917183, EPI_ISL_917184, EPI_ISL_917185, EPI_ISL_917186, EPI_ISL_917187, EPI_ISL_917188, EPI_ISL_917189, EPI_ISL_917190, EPI_ISL_917191, EPI_ISL_917192, EPI_ISL_917193, EPI_ISL_917194, EPI_ISL_917195, EPI_ISL_917196, EPI_ISL_917197, EPI_ISL_917198, EPI_ISL_917199, EPI_ISL_917200, EPI_ISL_917201, EPI_ISL_917202, EPI_ISL_917203, EPI_ISL_917204, EPI_ISL_917205, EPI_ISL_917206, EPI_ISL_917207, EPI_ISL_917208, EPI_ISL_917209, EPI_ISL_917210, EPI_ISL_917212, EPI_ISL_917213, EPI_ISL_917215, EPI_ISL_917216, EPI_ISL_917217, EPI_ISL_917218, EPI_ISL_917219, EPI_ISL_917220, EPI_ISL_917221, EPI_ISL_917222, EPI_ISL_917223, EPI_ISL_917224, EPI_ISL_917225, EPI_ISL_917226, EPI_ISL_917227, EPI_ISL_917228, EPI_ISL_917230, EPI_ISL_917231, EPI_ISL_917232, EPI_ISL_917233, EPI_ISL_917236, EPI_ISL_917237, EPI_ISL_917238, EPI_ISL_917239, EPI_ISL_917240, EPI_ISL_917241, EPI_ISL_917242, EPI_ISL_917243, EPI_ISL_917244, EPI_ISL_917245, EPI_ISL_917246, EPI_ISL_917247, EPI_ISL_917248, EPI_ISL_917249, EPI_ISL_917250, EPI_ISL_917251, EPI_ISL_917252, EPI_ISL_917253, EPI_ISL_917254, EPI_ISL_917255, EPI_ISL_917256, EPI_ISL_917257, EPI_ISL_917259, EPI_ISL_917260, EPI_ISL_917261, EPI_ISL_917262, EPI_ISL_917263, EPI_ISL_917264, EPI_ISL_917265, EPI_ISL_917266, EPI_ISL_917267, EPI_ISL_917268, EPI_ISL_917269, EPI_ISL_917270, EPI_ISL_917271, EPI_ISL_917272, EPI_ISL_917273, EPI_ISL_917274, EPI_ISL_917276, EPI_ISL_917277, EPI_ISL_917278, EPI_ISL_917279, EPI_ISL_917280, EPI_ISL_917281, EPI_ISL_917282, EPI_ISL_917283, EPI_ISL_917284, EPI_ISL_917285, EPI_ISL_917286, EPI_ISL_917287, EPI_ISL_917288, EPI_ISL_917289, EPI_ISL_917290, EPI_ISL_917291, EPI_ISL_917292, EPI_ISL_917293, EPI_ISL_917294, EPI_ISL_917295, EPI_ISL_917296, EPI_ISL_917297, EPI_ISL_917298, EPI_ISL_917299, EPI_ISL_917300, EPI_ISL_917301, EPI_ISL_917302, EPI_ISL_917304, EPI_ISL_917305, EPI_ISL_917306, EPI_ISL_917307, EPI_ISL_917308, EPI_ISL_917309, EPI_ISL_917310, EPI_ISL_917311, EPI_ISL_917312, EPI_ISL_917313, EPI_ISL_917314, EPI_ISL_917315, EPI_ISL_917316, EPI_ISL_917317, EPI_ISL_917319, EPI_ISL_917320, EPI_ISL_917321, EPI_ISL_917322, EPI_ISL_917324, EPI_ISL_917325, EPI_ISL_917326, EPI_ISL_917327, EPI_ISL_917329, EPI_ISL_917330, EPI_ISL_917331, EPI_ISL_917332, EPI_ISL_917333, EPI_ISL_917334, EPI_ISL_917335, EPI_ISL_917336, EPI_ISL_917337, EPI_ISL_917338, EPI_ISL_917339, EPI_ISL_917341, EPI_ISL_917342, EPI_ISL_917343, EPI_ISL_917346, EPI_ISL_917347, EPI_ISL_917348, EPI_ISL_917349, EPI_ISL_917350, EPI_ISL_917351, EPI_ISL_917352, EPI_ISL_917353, EPI_ISL_917354, EPI_ISL_917355, EPI_ISL_917356, EPI_ISL_917357, EPI_ISL_917358, EPI_ISL_917359, EPI_ISL_917361, EPI_ISL_917362, EPI_ISL_917363, EPI_ISL_917364, EPI_ISL_917365, EPI_ISL_917367, EPI_ISL_917368, EPI_ISL_917369, EPI_ISL_917371, EPI_ISL_917372, EPI_ISL_917373, EPI_ISL_917374, EPI_ISL_917375, EPI_ISL_917376, EPI_ISL_917377, EPI_ISL_917378, EPI_ISL_917379, EPI_ISL_917380, EPI_ISL_917381, EPI_ISL_917382, EPI_ISL_917383, EPI_ISL_917385, EPI_ISL_917386, EPI_ISL_917387, EPI_ISL_917388, EPI_ISL_917390, EPI_ISL_917391, EPI_ISL_917392, EPI_ISL_917393, EPI_ISL_917394, EPI_ISL_917395, EPI_ISL_917396, EPI_ISL_917397, EPI_ISL_917398, EPI_ISL_917399, EPI_ISL_917400, EPI_ISL_917401, EPI_ISL_917402, EPI_ISL_917403, EPI_ISL_917404, EPI_ISL_917405, EPI_ISL_917406, EPI_ISL_917407, EPI_ISL_917408, EPI_ISL_917409, EPI_ISL_917410, EPI_ISL_917411, EPI_ISL_917412, EPI_ISL_917414, EPI_ISL_917415, EPI_ISL_917416, EPI_ISL_917417, EPI_ISL_917418, EPI_ISL_917419, EPI_ISL_917420, EPI_ISL_917421, EPI_ISL_917422, EPI_ISL_917423, EPI_ISL_917424, EPI_ISL_917425, EPI_ISL_917426, EPI_ISL_917427, EPI_ISL_917428, EPI_ISL_917429, EPI_ISL_917430, EPI_ISL_917431, EPI_ISL_917432, EPI_ISL_917433, EPI_ISL_917434, EPI_ISL_917435, EPI_ISL_917436, EPI_ISL_917437, EPI_ISL_917438, EPI_ISL_917439, EPI_ISL_917440, EPI_ISL_917441, EPI_ISL_917442, EPI_ISL_917443, EPI_ISL_917444, EPI_ISL_917445, EPI_ISL_917446, EPI_ISL_917447, EPI_ISL_917448, EPI_ISL_917449, EPI_ISL_917450, EPI_ISL_917451, EPI_ISL_917452, EPI_ISL_917453, EPI_ISL_917454, EPI_ISL_917455, EPI_ISL_917456, EPI_ISL_917457, EPI_ISL_917458, EPI_ISL_917459, EPI_ISL_917460, EPI_ISL_917461, EPI_ISL_917462, EPI_ISL_917463, EPI_ISL_917464, EPI_ISL_917465, EPI_ISL_917466, EPI_ISL_917467, EPI_ISL_917468, EPI_ISL_917469, EPI_ISL_917470, EPI_ISL_917471, EPI_ISL_917472, EPI_ISL_917473, EPI_ISL_917474, EPI_ISL_917475, EPI_ISL_917476, EPI_ISL_917477, EPI_ISL_917478, EPI_ISL_917479, EPI_ISL_917480, EPI_ISL_917481, EPI_ISL_917482, EPI_ISL_917483, EPI_ISL_917484, EPI_ISL_917485, EPI_ISL_917486, EPI_ISL_917487, EPI_ISL_917488, EPI_ISL_917489, EPI_ISL_917490, EPI_ISL_917491, EPI_ISL_917493, EPI_ISL_917494, EPI_ISL_917496, EPI_ISL_917497, EPI_ISL_917498, EPI_ISL_917499, EPI_ISL_917500, EPI_ISL_917502, EPI_ISL_917503, EPI_ISL_917504, EPI_ISL_917505, EPI_ISL_917506, EPI_ISL_917508, EPI_ISL_917509, EPI_ISL_917510, EPI_ISL_917511, EPI_ISL_917512, EPI_ISL_917513, EPI_ISL_917514, EPI_ISL_917515, EPI_ISL_917516, EPI_ISL_917517, EPI_ISL_917518, EPI_ISL_917519, EPI_ISL_917520, EPI_ISL_917521, EPI_ISL_917522, EPI_ISL_917524, EPI_ISL_917525, EPI_ISL_917526, EPI_ISL_917527, EPI_ISL_917528, EPI_ISL_917529, EPI_ISL_917530, EPI_ISL_917531, EPI_ISL_917532, EPI_ISL_917533, EPI_ISL_917534, EPI_ISL_917535, EPI_ISL_917536, EPI_ISL_917537, EPI_ISL_917538, EPI_ISL_917539, EPI_ISL_917540, EPI_ISL_917541, EPI_ISL_917542, EPI_ISL_917543, EPI_ISL_917544, EPI_ISL_917545, EPI_ISL_917546, EPI_ISL_917547, EPI_ISL_917548, EPI_ISL_917549, EPI_ISL_917550, EPI_ISL_917551, EPI_ISL_917552, EPI_ISL_917553, EPI_ISL_917554, EPI_ISL_917555, EPI_ISL_917556, EPI_ISL_917557, EPI_ISL_917558, EPI_ISL_917559, EPI_ISL_917560, EPI_ISL_917561, EPI_ISL_917562, EPI_ISL_917563, EPI_ISL_917564, EPI_ISL_917565, EPI_ISL_917566, EPI_ISL_917567, EPI_ISL_917568, EPI_ISL_917569, EPI_ISL_917570, EPI_ISL_917571, EPI_ISL_917572, EPI_ISL_917573, EPI_ISL_917574, EPI_ISL_917575, EPI_ISL_917576, EPI_ISL_917577, EPI_ISL_917578, EPI_ISL_917579, EPI_ISL_917580, EPI_ISL_917581, EPI_ISL_917582, EPI_ISL_917583, EPI_ISL_917584, EPI_ISL_917585, EPI_ISL_917586, EPI_ISL_917587, EPI_ISL_917588, EPI_ISL_917589, EPI_ISL_917590, EPI_ISL_917591, EPI_ISL_917592, EPI_ISL_917593, EPI_ISL_917594, EPI_ISL_917595, EPI_ISL_917596, EPI_ISL_917597, EPI_ISL_917598, EPI_ISL_917599, EPI_ISL_917600, EPI_ISL_917601, EPI_ISL_917602, EPI_ISL_917603, EPI_ISL_917604, EPI_ISL_917605, EPI_ISL_917606, EPI_ISL_917607, EPI_ISL_917608,

EPI_ISL_917609, EPI_ISL_917610, EPI_ISL_917611, EPI_ISL_917612, EPI_ISL_917613, EPI_ISL_917614, EPI_ISL_917615, EPI_ISL_917616, EPI_ISL_917617, EPI_ISL_917618, EPI_ISL_917619, EPI_ISL_917620, EPI_ISL_917621, EPI_ISL_917622, EPI_ISL_917623, EPI_ISL_917624, EPI_ISL_917625, EPI_ISL_917626, EPI_ISL_917627, EPI_ISL_917628, EPI_ISL_917629, EPI_ISL_917630, EPI_ISL_917631, EPI_ISL_917632, EPI_ISL_917633, EPI_ISL_917634, EPI_ISL_917635, EPI_ISL_917636, EPI_ISL_917637, EPI_ISL_917638, EPI_ISL_917639, EPI_ISL_917640, EPI_ISL_917641, EPI_ISL_917642, EPI_ISL_917643, EPI_ISL_917644, EPI_ISL_917645, EPI_ISL_917646, EPI_ISL_917647, EPI_ISL_917648, EPI_ISL_917649, EPI_ISL_917650, EPI_ISL_917651, EPI_ISL_917652, EPI_ISL_917653, EPI_ISL_917654, EPI_ISL_917655, EPI_ISL_917656, EPI_ISL_917657, EPI_ISL_917658, EPI_ISL_917659, EPI_ISL_917660, EPI_ISL_917661, EPI_ISL_917662, EPI_ISL_917663, EPI_ISL_917664, EPI_ISL_917665, EPI_ISL_917666, EPI_ISL_917668, EPI_ISL_917669, EPI_ISL_917671, EPI_ISL_917672, EPI_ISL_917673, EPI_ISL_917674, EPI_ISL_917675, EPI_ISL_917676, EPI_ISL_917677, EPI_ISL_917678, EPI_ISL_917679, EPI_ISL_917680, EPI_ISL_917681, EPI_ISL_917682, EPI_ISL_917683, EPI_ISL_917684, EPI_ISL_917685, EPI_ISL_917686, EPI_ISL_917687, EPI_ISL_917688, EPI_ISL_917689, EPI_ISL_917690, EPI_ISL_917691, EPI_ISL_917692, EPI_ISL_917693, EPI_ISL_917694, EPI_ISL_917695, EPI_ISL_917696, EPI_ISL_917697, EPI_ISL_917698, EPI_ISL_917699, EPI_ISL_917700, EPI_ISL_917701, EPI_ISL_917702, EPI_ISL_917703, EPI_ISL_917704, EPI_ISL_917705, EPI_ISL_917706, EPI_ISL_917707, EPI_ISL_917708, EPI_ISL_917709, EPI_ISL_917710, EPI_ISL_917711, EPI_ISL_917712, EPI_ISL_917713, EPI_ISL_917714, EPI_ISL_917715, EPI_ISL_917716, EPI_ISL_917717, EPI_ISL_917718, EPI_ISL_917719, EPI_ISL_917720, EPI_ISL_917721, EPI_ISL_917722, EPI_ISL_917723, EPI_ISL_917724, EPI_ISL_917725, EPI_ISL_917726, EPI_ISL_917727, EPI_ISL_917728, EPI_ISL_917729, EPI_ISL_917730, EPI_ISL_917731, EPI_ISL_917732, EPI_ISL_917733, EPI_ISL_917734, EPI_ISL_917735, EPI_ISL_917736, EPI_ISL_917737, EPI_ISL_917738, EPI_ISL_917739, EPI_ISL_917741, EPI_ISL_917742, EPI_ISL_917744, EPI_ISL_917745, EPI_ISL_917746, EPI_ISL_917747, EPI_ISL_917748, EPI_ISL_917749, EPI_ISL_917750, EPI_ISL_917751, EPI_ISL_917752, EPI_ISL_917754, EPI_ISL_917755, EPI_ISL_917756, EPI_ISL_917757, EPI_ISL_917758, EPI_ISL_917759, EPI_ISL_917760, EPI_ISL_917761, EPI_ISL_917762, EPI_ISL_917763, EPI_ISL_917764, EPI_ISL_917765, EPI_ISL_917766, EPI_ISL_917767, EPI_ISL_917768, EPI_ISL_917769, EPI_ISL_917770, EPI_ISL_917771, EPI_ISL_917772, EPI_ISL_917773, EPI_ISL_917774, EPI_ISL_917775, EPI_ISL_917776, EPI_ISL_917777, EPI_ISL_917778, EPI_ISL_917779, EPI_ISL_917780, EPI_ISL_917781, EPI_ISL_917782, EPI_ISL_917783, EPI_ISL_917784, EPI_ISL_917785, EPI_ISL_917786, EPI_ISL_917787, EPI_ISL_917788, EPI_ISL_917790, EPI_ISL_917791, EPI_ISL_917793, EPI_ISL_917794, EPI_ISL_917795, EPI_ISL_917796, EPI_ISL_917797, EPI_ISL_917798, EPI_ISL_917799, EPI_ISL_917800, EPI_ISL_917801, EPI_ISL_917802, EPI_ISL_917803, EPI_ISL_917804, EPI_ISL_917805, EPI_ISL_917806, EPI_ISL_917807, EPI_ISL_917808, EPI_ISL_917809, EPI_ISL_917810, EPI_ISL_917811, EPI_ISL_917812, EPI_ISL_917813, EPI_ISL_917814, EPI_ISL_917815, EPI_ISL_917816, EPI_ISL_917817, EPI_ISL_917818, EPI_ISL_917819, EPI_ISL_917820, EPI_ISL_917821, EPI_ISL_917822, EPI_ISL_917823, EPI_ISL_917824, EPI_ISL_917825, EPI_ISL_917826, EPI_ISL_917827, EPI_ISL_917828, EPI_ISL_917829, EPI_ISL_917830, EPI_ISL_917831, EPI_ISL_917832, EPI_ISL_917833, EPI_ISL_917834, EPI_ISL_917835, EPI_ISL_917836, EPI_ISL_917837, EPI_ISL_917838, EPI_ISL_917839, EPI_ISL_917840, EPI_ISL_917841, EPI_ISL_917842, EPI_ISL_917843			
see above	Lighthouse Lab in Alderley Park	Wellcome Sanger Institute for the COVID-19 Genomics UK (COG-UK) Consortium	Jacquelyn Wynn, Mairead Hyland, The Lighthouse Lab in Alderley Park and Alex Alderton, Roberto Amato, Sonia Goncalves, Ewan Harrison, David K. Jackson, Ian Johnston, Dominic Kwiatkowski, Cordelia Langford, John Sillitoe on behalf of the Wellcome Sanger Institute COVID-19 Surveillance Team
EPI_ISL_917844, EPI_ISL_917845, EPI_ISL_917846, EPI_ISL_917848, EPI_ISL_917985, EPI_ISL_917986, EPI_ISL_917987, EPI_ISL_917988, EPI_ISL_917989, EPI_ISL_917990, EPI_ISL_917991, EPI_ISL_917992, EPI_ISL_917993			
see above	Lighthouse Lab in Glasgow	Wellcome Sanger Institute for the COVID-19 Genomics UK (COG-UK) Consortium	Harper VanSteenhouse, Yumi Kasai, David Gray, Carol Clugston, Anna Dominiczak and Alex Alderton, Roberto Amato, Sonia Goncalves, Ewan Harrison, David K. Jackson, Ian Johnston, Dominic Kwiatkowski, Cordelia Langford, John Sillitoe on behalf of the Wellcome Sanger Institute COVID-19 Surveillance Team
EPI_ISL_919108, EPI_ISL_919109, EPI_ISL_919110, EPI_ISL_919111, EPI_ISL_919112, EPI_ISL_919113, EPI_ISL_919116, EPI_ISL_919117, EPI_ISL_919118, EPI_ISL_919120, EPI_ISL_919121, EPI_ISL_919122, EPI_ISL_919123, EPI_ISL_919124, EPI_ISL_919125, EPI_ISL_919126, EPI_ISL_919127, EPI_ISL_919129, EPI_ISL_919131, EPI_ISL_919132, EPI_ISL_919133, EPI_ISL_919135, EPI_ISL_919137, EPI_ISL_919138, EPI_ISL_919140, EPI_ISL_919141, EPI_ISL_919144, EPI_ISL_919145			
see above	Department of Pathology, University of Cambridge	COVID-19 Genomics UK (COG-UK) Consortium	Aminu S. Jahun, Yasmin Chaudhry, Iliana Georgana, Myra Hosmillo, Rhys Izu, Martin D. Curran, Surendra Parmar, Ian Goodfellow
EPI_ISL_919291, EPI_ISL_919292, EPI_ISL_919293, EPI_ISL_919294, EPI_ISL_919295, EPI_ISL_919296, EPI_ISL_919297, EPI_ISL_919298, EPI_ISL_919299, EPI_ISL_919300, EPI_ISL_919302, EPI_ISL_919303, EPI_ISL_919304, EPI_ISL_919305, EPI_ISL_919306, EPI_ISL_919307			
see above	West of Scotland Specialist Virology Centre, NHSGGC / MRC-University of Glasgow Centre for Virus Research	COVID-19 Genomics UK (COG-UK) Consortium	Ana da Silva Filipe, Natasha Johnson, Kathy Smollett, Daniel Mair, Stephen Carmichael, Alice Broos, Lily Tong, Jenna Nichols, Kyriaki Nomikou; Sarah McDonald; Richard Orton, Joseph Hughes, Sreenu Vattipally, David L Robertson; Alasdair MacLean, Rory Gunson; Sharif Shaaban, Matthew Holden; Rachel Blacow, Guy Mollett, Kathy Li, James Shepherd, Antonia Ho, Emma Thomson
EPI_ISL_919404	Virology Department, Royal Infirmary of Edinburgh, NHS Lothian / School of Biological Sciences, University of Edinburgh / Institute of Genetics and Molecular Medicine, University of Edinburgh	COVID-19 Genomics UK (COG-UK) Consortium	McHugh M, Dewar R, Rooke S, Gallagher M, Balcaza C, O'Toole Á, Scher E, Hill V, McCrone JT, Colquhoun R, Yu X, Jackson B, Rambaut A, Williams TC, Templeton K
EPI_ISL_919621, EPI_ISL_919622, EPI_ISL_919624, EPI_ISL_919625, EPI_ISL_919626, EPI_ISL_919627, EPI_ISL_919664, EPI_ISL_919665, EPI_ISL_919666, EPI_ISL_919667, EPI_ISL_919668, EPI_ISL_919669, EPI_ISL_919670, EPI_ISL_919671, EPI_ISL_919672, EPI_ISL_919673, EPI_ISL_919674, EPI_ISL_919676, EPI_ISL_919678, EPI_ISL_919679, EPI_ISL_919680, EPI_ISL_919681, EPI_ISL_919682, EPI_ISL_919684, EPI_ISL_919685, EPI_ISL_919686, EPI_ISL_919687, EPI_ISL_919689, EPI_ISL_919692, EPI_ISL_919693, EPI_ISL_919694, EPI_ISL_919696, EPI_ISL_919697, EPI_ISL_919698, EPI_ISL_919699, EPI_ISL_919700, EPI_ISL_919701, EPI_ISL_919702, EPI_ISL_919703, EPI_ISL_919704, EPI_ISL_919705			
see above	Liverpool Clinical Laboratories	COVID-19 Genomics UK (COG-UK) Consortium	Sam Haldenby, Anita Lucaci, Steve Paterson, Julian Hiscox, Alistair Darby, M Almsaud, A Alrezaihi, Muhannad Alruwaili, Stuart D Armstrong, Jones Benjamin, Eleanor G Bentley, Anu Chawla, Jordan J Clark, Angela Cowell, Richard Eccles, Isabel García-Dorival, Matthew Gemmell, Alessandro Gerada, PKF Gilmore, Richard Gregory, Ximeng Han, Catherine Hartley, Margaret Hughes, Miren Iturriza-Gomara, James Johnson, L Luu, Jenifer Manson, Charlotte Nelson, Elaine O'Toole, Cassie Olateju, Rebekah Penrice-Randal , Lucille Rainbow, N.P Randle, Trevor Ian Robinson, Parul Sharma, Ghada T Shawli, James P Stewart, Neil Swainston, Ecaterina Vamos, Joanne Watts, Mark Whitehead
EPI_ISL_919776, EPI_ISL_919777, EPI_ISL_919778, EPI_ISL_919856, EPI_ISL_919857, EPI_ISL_919858, EPI_ISL_919859, EPI_ISL_919860, EPI_ISL_919861, EPI_ISL_919862, EPI_ISL_919863, EPI_ISL_919864, EPI_ISL_919865, EPI_ISL_919866, EPI_ISL_919867, EPI_ISL_919868, EPI_ISL_919869, EPI_ISL_919870, EPI_ISL_919874, EPI_ISL_919875, EPI_ISL_919876, EPI_ISL_919877, EPI_ISL_919878, EPI_ISL_919879, EPI_ISL_919880, EPI_ISL_919882, EPI_ISL_919883, EPI_ISL_919885, EPI_ISL_919886, EPI_ISL_919887, EPI_ISL_919888, EPI_ISL_919889, EPI_ISL_919890, EPI_ISL_919891, EPI_ISL_919893, EPI_ISL_919894, EPI_ISL_919895, EPI_ISL_919896, EPI_ISL_919897, EPI_ISL_919898, EPI_ISL_919899, EPI_ISL_919900, EPI_ISL_919901, EPI_ISL_919902, EPI_ISL_919920, EPI_ISL_919921, EPI_ISL_919922, EPI_ISL_919923, EPI_ISL_919924, EPI_ISL_919925, EPI_ISL_919926, EPI_ISL_919933, EPI_ISL_919945, EPI_ISL_919952, EPI_ISL_919955, EPI_ISL_919957, EPI_ISL_920073, EPI_ISL_920074, EPI_ISL_920075, EPI_ISL_920076, EPI_ISL_920077, EPI_ISL_920078, EPI_ISL_920081, EPI_ISL_920082, EPI_ISL_920083, EPI_ISL_920084, EPI_ISL_920085, EPI_ISL_920086, EPI_ISL_920087, EPI_ISL_920088, EPI_ISL_920089, EPI_ISL_920090, EPI_ISL_920091, EPI_ISL_920117, EPI_ISL_920118, EPI_ISL_920119, EPI_ISL_920120, EPI_ISL_920121, EPI_ISL_920122, EPI_ISL_920123, EPI_ISL_920124, EPI_ISL_920125, EPI_ISL_920126, EPI_ISL_920127, EPI_ISL_920128, EPI_ISL_920129, EPI_ISL_920130, EPI_ISL_920131, EPI_ISL_920132, EPI_ISL_920133, EPI_ISL_920134, EPI_ISL_920135, EPI_ISL_920136, EPI_ISL_920137, EPI_ISL_920138, EPI_ISL_920139, EPI_ISL_920140, EPI_ISL_920141, EPI_ISL_920142, EPI_ISL_920143, EPI_ISL_920144, EPI_ISL_920145, EPI_ISL_920146, EPI_ISL_920147, EPI_ISL_920148, EPI_ISL_920149, EPI_ISL_920150, EPI_ISL_920151, EPI_ISL_920153, EPI_ISL_920154, EPI_ISL_920155, EPI_ISL_920158, EPI_ISL_920164, EPI_ISL_920166, EPI_ISL_920167, EPI_ISL_920168, EPI_ISL_920169, EPI_ISL_920170, EPI_ISL_920171, EPI_ISL_920172, EPI_ISL_920173			
see above	University College London, Great Ormond Street Hospital for Children NHS Foundation Trust, Imperial College Healthcare NHS Trust	COVID-19 Genomics UK (COG-UK) Consortium	Sergi Castellano, Rachel Williams, Mark Kristiansen, Paola Resende Silva, Sunando Roy, Tony Brooks, Helena Tutill, Paola Niola, Patricia Dyal, Charlotte Williams, Leysa Forrest, Yasmin Panchbhaya, Jacqueline Findlay, Samuel Weeks, Julianne Brown, Kathryn Harris, Paul Randell, James Price, Alison Holmes, Judith Breuer
EPI_ISL_920463, EPI_ISL_920472, EPI_ISL_920480, EPI_ISL_920488, EPI_ISL_920523, EPI_ISL_920524, EPI_ISL_920525, EPI_ISL_920531, EPI_ISL_920533, EPI_ISL_920534, EPI_ISL_920535, EPI_ISL_920541, EPI_ISL_920543, EPI_ISL_920545, EPI_ISL_920546, EPI_ISL_920552, EPI_ISL_920554, EPI_ISL_920555, EPI_ISL_920556, EPI_ISL_920557, EPI_ISL_920561, EPI_ISL_920562, EPI_ISL_920563, EPI_ISL_920565, EPI_ISL_920566, EPI_ISL_920570, EPI_ISL_920571, EPI_ISL_920572, EPI_ISL_920573, EPI_ISL_920578, EPI_ISL_920580, EPI_ISL_920587, EPI_ISL_920588, EPI_ISL_920589, EPI_ISL_920590, EPI_ISL_920592, EPI_ISL_920593, EPI_ISL_920597, EPI_ISL_920598, EPI_ISL_920599, EPI_ISL_920600, EPI_ISL_920601, EPI_ISL_920602, EPI_ISL_920604, EPI_ISL_920605, EPI_ISL_920606, EPI_ISL_920607, EPI_ISL_920608, EPI_ISL_920614, EPI_ISL_920615, EPI_ISL_920616, EPI_ISL_920622, EPI_ISL_920623, EPI_ISL_920624, EPI_ISL_920625, EPI_ISL_920626, EPI_ISL_920632, EPI_ISL_920633, EPI_ISL_920634, EPI_ISL_920638, EPI_ISL_920639, EPI_ISL_920640, EPI_ISL_920641, EPI_ISL_920642, EPI_ISL_920643, EPI_ISL_920648, EPI_ISL_920649, EPI_ISL_920650, EPI_ISL_920654, EPI_ISL_920655, EPI_ISL_920656, EPI_ISL_920657, EPI_ISL_920662, EPI_ISL_920663, EPI_ISL_920664, EPI_ISL_920665, EPI_ISL_920668, EPI_ISL_920669, EPI_ISL_920672, EPI_ISL_920673, EPI_ISL_920675, EPI_ISL_920677, EPI_ISL_920678, EPI_ISL_920679, EPI_ISL_920681, EPI_ISL_920682, EPI_ISL_920686, EPI_ISL_920687, EPI_ISL_920690, EPI_ISL_920692, EPI_ISL_920694, EPI_ISL_920695, EPI_ISL_920698, EPI_ISL_920699, EPI_ISL_920703, EPI_ISL_920704, EPI_ISL_920707, EPI_ISL_920712, EPI_ISL_920713, EPI_ISL_920715, EPI_ISL_920716, EPI_ISL_920718, EPI_ISL_920726, EPI_ISL_920727, EPI_ISL_920740, EPI_ISL_920741, EPI_ISL_920742, EPI_ISL_920743, EPI_ISL_920749, EPI_ISL_920750, EPI_ISL_920751, EPI_ISL_920759, EPI_ISL_920760, EPI_ISL_920764, EPI_ISL_920766, EPI_ISL_920769, EPI_ISL_920772, EPI_ISL_920781			
see above	University College London Hospital	COVID-19 Genomics UK (COG-UK) Consortium	Judith Heaney, Matthew Byott, Catherine Houlihan, Dan Frampton, Stuart Kirk, Moira Spyer and Eleni Nastouli
EPI_ISL_920819, EPI_ISL_920821, EPI_ISL_920822, EPI_ISL_920825, EPI_ISL_920827, EPI_ISL_920828, EPI_ISL_920838	University College London, Great Ormond Street Hospital for Children NHS Foundation Trust, Imperial College Healthcare NHS Trust	COVID-19 Genomics UK (COG-UK) Consortium	Sergi Castellano, Rachel Williams, Mark Kristiansen, Paola Resende Silva, Sunando Roy, Tony Brooks, Helena Tutill, Paola Niola, Patricia Dyal, Charlotte Williams, Leysa Forrest, Yasmin Panchbhaya, Jacqueline Findlay, Samuel Weeks, Julianne Brown, Kathryn Harris, Paul Randell, James Price, Alison Holmes, Judith Breuer
EPI_ISL_921637, EPI_ISL_921638, EPI_ISL_921639, EPI_ISL_921640, EPI_ISL_921642, EPI_ISL_921643, EPI_ISL_921644, EPI_ISL_921645, EPI_ISL_921646, EPI_ISL_921647, EPI_ISL_921648, EPI_ISL_921649, EPI_ISL_921650, EPI_ISL_921651, EPI_ISL_921652, EPI_ISL_921653, EPI_ISL_921654, EPI_ISL_921655, EPI_ISL_921656, EPI_ISL_921657, EPI_ISL_921658			
see above	Northumbria University / South Tees Hospitals NHS Foundation Trust / North Cumbria Integrated Care NHS Foundation Trust / North Tees and Hartlepool NHS Foundation Trust / Newcastle Hospitals NHS Foundation Trust	COVID-19 Genomics UK (COG-UK) Consortium	Darren L Smith,Andrew Nelson,Matthew Bashton,Greg R Young,Joshua Loh,John Allan,Mohammad A Tariq,Giles S Holt,Gary Black,Wen C Yew,Lynn Dover,Paul Baker,Steve Liggett,Sarah Essex,Jane Greenaway,Debra Padgett,Clive Graham,Garren Scott,Edward Barton,Emma Swindells,Brendan Payne,Jennifer Collins,Yusri Taha,Gary Eltringham
EPI_ISL_922008, EPI_ISL_922009, EPI_ISL_922010, EPI_ISL_922011, EPI_ISL_922012, EPI_ISL_922013, EPI_ISL_922014, EPI_ISL_922015, EPI_ISL_922016, EPI_ISL_922017, EPI_ISL_922018, EPI_ISL_922019, EPI_ISL_922020, EPI_ISL_922021, EPI_ISL_922022, EPI_ISL_922023, EPI_ISL_922024, EPI_ISL_922025, EPI_ISL_922026, EPI_ISL_922027, EPI_ISL_922028, EPI_ISL_922029, EPI_ISL_922030, EPI_ISL_922031, EPI_ISL_922032, EPI_ISL_922033, EPI_ISL_922034, EPI_ISL_922035, EPI_ISL_922036			
see above	Queens Medical Centre, Clinical Microbiology Department / DeepSeq Nottingham	COVID-19 Genomics UK (COG-UK) Consortium	Gemma Clark, Wendy Smith, Manjinder Khakh, Vicki M Fleming, Michelle M Lister, Hannah Howson-Wells, Jonathan Ball, Patrick McClure, Joseph Chappell, Theocharis Tsoleridis, Nadine Holmes, Matthew Carlisle, Christopher Moore, Fei Sang, Johnny Debebe, Victoria Wright, Matthew Loose
EPI_ISL_922891, EPI_ISL_922941, EPI_ISL_922949, EPI_ISL_922950, EPI_ISL_922953, EPI_ISL_922954, EPI_ISL_922955, EPI_ISL_922959, EPI_ISL_922960, EPI_ISL_922961, EPI_ISL_923159, EPI_ISL_923187, EPI_ISL_923199, EPI_ISL_923203, EPI_ISL_923205, EPI_ISL_923206, EPI_ISL_923208, EPI_ISL_923209, EPI_ISL_923210, EPI_ISL_923211			
see above	Wales Specialist Virology Centre Sequencing lab: Pathogen Genomics Unit	Public Health Wales Microbiology Cardiff Wales Specialist Virology Centre	Catherine Moore, Johnathan Evans, Laura Gifford, Malorie Perry, Simon Cottrell, Angela Marchbank, Alec Birchley, Alexander Adams, Amy Gaskin, Bree Gatica-Wilcox, Jason Coombes, Joel Southgate, Lauren Gilbert, Lee Graham, Nicole Pacchiarini, Sara Kumziene-Summerhayes, Sarah Taylor, Sophie Jones, Sara Rey, Matthew Bull, Joanne Watkins, Sally Corden, Tom Connor
EPI_ISL_923422, EPI_ISL_923423,	Centre for Enzyme Innovation, University of Portsmouth /	COVID-19 Genomics UK (COG-UK) Consortium	Angela Beckett,Salman Goudarzi,Christopher Fearn,Kate Cook,Katie Loveson,Sharon Glaysher,Scott Elliott,Samuel Robson

EPI_ISL_923424	Translational Research Laboratory, Portsmouth Hospitals NHS Trust		
EPI_ISL_924080, EPI_ISL_924081, EPI_ISL_924090, EPI_ISL_924095, EPI_ISL_924100, EPI_ISL_924110, EPI_ISL_924113, EPI_ISL_924115, EPI_ISL_924116, EPI_ISL_924120, EPI_ISL_924124, EPI_ISL_924130, EPI_ISL_924133, EPI_ISL_924134, EPI_ISL_924135, EPI_ISL_924137, EPI_ISL_924139, EPI_ISL_924140, EPI_ISL_924143, EPI_ISL_924147, EPI_ISL_924151, EPI_ISL_924154, EPI_ISL_924156, EPI_ISL_924161, EPI_ISL_924162, EPI_ISL_924171, EPI_ISL_924175, EPI_ISL_924179, EPI_ISL_924180, EPI_ISL_924188, EPI_ISL_924190, EPI_ISL_924192, EPI_ISL_924194, EPI_ISL_924195, EPI_ISL_924202, EPI_ISL_924205, EPI_ISL_924210, EPI_ISL_924211, EPI_ISL_924214, EPI_ISL_924219, EPI_ISL_924221, EPI_ISL_924222, EPI_ISL_924226, EPI_ISL_924232, EPI_ISL_924234, EPI_ISL_924236, EPI_ISL_924241, EPI_ISL_924246, EPI_ISL_924248, EPI_ISL_924254, EPI_ISL_924257, EPI_ISL_924259, EPI_ISL_924260, EPI_ISL_924261, EPI_ISL_924262, EPI_ISL_924264, EPI_ISL_924271, EPI_ISL_924272, EPI_ISL_924275, EPI_ISL_924286, EPI_ISL_924288, EPI_ISL_924294, EPI_ISL_924300, EPI_ISL_924301, EPI_ISL_924309, EPI_ISL_924317, EPI_ISL_924318, EPI_ISL_924320, EPI_ISL_924321, EPI_ISL_924322, EPI_ISL_924327, EPI_ISL_924329, EPI_ISL_924331, EPI_ISL_924336, EPI_ISL_924338, EPI_ISL_924344, EPI_ISL_924346, EPI_ISL_924347, EPI_ISL_924349, EPI_ISL_924362, EPI_ISL_924365, EPI_ISL_924367, EPI_ISL_924370, EPI_ISL_924373, EPI_ISL_924375, EPI_ISL_924378, EPI_ISL_924391, EPI_ISL_924393, EPI_ISL_924395, EPI_ISL_924401, EPI_ISL_924403, EPI_ISL_924404, EPI_ISL_924405, EPI_ISL_924407, EPI_ISL_924411, EPI_ISL_924415, EPI_ISL_924416, EPI_ISL_924417			
see above	Virology Department, Sheffield Teaching Hospitals NHS Foundation Trust/Department of Infection, Immunity and Cardiovascular Disease, The Medical School, University of Sheffield	COVID-19 Genomics UK (COG-UK) Consortium	Thushan de Silva, Matthew Parker, Nikki Smith, Adri Angyal, Rebecca Brown, Luke Green, Rachel Tucker, Paul Parsons, Danielle Groves, Katie Johnson, Laura Carrilero, Alex Keeley, Dave Partridge, Matthew Wyles, Benjamin Lindsey, Mehmet Yavuz, Mohammad Raza, Cariad Evans
EPI_ISL_924653, EPI_ISL_924657, EPI_ISL_924672, EPI_ISL_924676, EPI_ISL_924677, EPI_ISL_924688, EPI_ISL_924690, EPI_ISL_924691, EPI_ISL_924692, EPI_ISL_924694, EPI_ISL_924695, EPI_ISL_924696, EPI_ISL_924697, EPI_ISL_924698, EPI_ISL_924699, EPI_ISL_924701, EPI_ISL_924702, EPI_ISL_924704, EPI_ISL_924705, EPI_ISL_924706, EPI_ISL_924708, EPI_ISL_924713, EPI_ISL_924714, EPI_ISL_924715, EPI_ISL_924717, EPI_ISL_924722			
see above	Bioinformatics and Biostatistics Lab, Advanced Sequencing Facility	COVID-19 Genomics UK (COG-UK) Consortium	Aengus Stewart,Jerome Nicod,Chelsea Sawyer,Laura Cubitt,Harshil Patel,Margaret Crawford
EPI_ISL_931589, EPI_ISL_931595, EPI_ISL_931690, EPI_ISL_931726, EPI_ISL_931822, EPI_ISL_931827, EPI_ISL_931874, EPI_ISL_931918, EPI_ISL_931921, EPI_ISL_931937, EPI_ISL_931939, EPI_ISL_931943, EPI_ISL_931953, EPI_ISL_931957, EPI_ISL_931959, EPI_ISL_931979, EPI_ISL_932017, EPI_ISL_932039, EPI_ISL_932046, EPI_ISL_932057, EPI_ISL_932061, EPI_ISL_932068, EPI_ISL_932108, EPI_ISL_932114, EPI_ISL_932157, EPI_ISL_932201, EPI_ISL_932222, EPI_ISL_932231, EPI_ISL_932240, EPI_ISL_932241			
see above	Lighthouse Lab in Alderley Park	Wellcome Sanger Institute for the COVID-19 Genomics UK (COG-UK) Consortium	Jacquelyn Wynn, Mairead Hyland, The Lighthouse Lab in Alderley Park and Alex Alderton, Roberto Amato, Sonia Goncalves, Ewan Harrison, David K. Jackson, Ian Johnston, Dominic Kwiatkowski, Cordelia Langford, John Sillitoe on behalf of the Wellcome Sanger Institute COVID-19 Surveillance Team
EPI_ISL_932251, EPI_ISL_932252	Lighthouse Lab in Glasgow	Wellcome Sanger Institute for the COVID-19 Genomics UK (COG-UK) Consortium	Harper VanSteenhouse, Yumi Kasai, David Gray, Carol Clugston, Anna Dominiczak and Alex Alderton, Roberto Amato, Sonia Goncalves, Ewan Harrison, David K. Jackson, Ian Johnston, Dominic Kwiatkowski, Cordelia Langford, John Sillitoe on behalf of the Wellcome Sanger Institute COVID-19 Surveillance Team
EPI_ISL_932254	Lighthouse Lab in Alderley Park	Wellcome Sanger Institute for the COVID-19 Genomics UK (COG-UK) Consortium	Jacquelyn Wynn, Mairead Hyland, The Lighthouse Lab in Alderley Park and Alex Alderton, Roberto Amato, Sonia Goncalves, Ewan Harrison, David K. Jackson, Ian Johnston, Dominic Kwiatkowski, Cordelia Langford, John Sillitoe on behalf of the Wellcome Sanger Institute COVID-19 Surveillance Team
EPI_ISL_932255, EPI_ISL_932256	Lighthouse Lab in Glasgow	Wellcome Sanger Institute for the COVID-19 Genomics UK (COG-UK) Consortium	Harper VanSteenhouse, Yumi Kasai, David Gray, Carol Clugston, Anna Dominiczak and Alex Alderton, Roberto Amato, Sonia Goncalves, Ewan Harrison, David K. Jackson, Ian Johnston, Dominic Kwiatkowski, Cordelia Langford, John Sillitoe on behalf of the Wellcome Sanger Institute COVID-19 Surveillance Team
EPI_ISL_932258, EPI_ISL_932259	Lighthouse Lab in Milton Keynes	Wellcome Sanger Institute for the COVID-19 Genomics UK (COG-UK) Consortium	The Lighthouse Lab in Milton Keynes and Alex Alderton, Roberto Amato, Sonia Goncalves, Ewan Harrison, David K. Jackson, Ian Johnston, Dominic Kwiatkowski, Cordelia Langford, John Sillitoe on behalf of the Wellcome Sanger Institute COVID-19 Surveillance Team
EPI_ISL_932261, EPI_ISL_932264	Lighthouse Lab in Alderley Park	Wellcome Sanger Institute for the COVID-19 Genomics UK (COG-UK) Consortium	Jacquelyn Wynn, Mairead Hyland, The Lighthouse Lab in Alderley Park and Alex Alderton, Roberto Amato, Sonia Goncalves, Ewan Harrison, David K. Jackson, Ian Johnston, Dominic Kwiatkowski, Cordelia Langford, John Sillitoe on behalf of the Wellcome Sanger Institute COVID-19 Surveillance Team
EPI_ISL_932265, EPI_ISL_932266, EPI_ISL_932267	Lighthouse Lab in Glasgow	Wellcome Sanger Institute for the COVID-19 Genomics UK (COG-UK) Consortium	Harper VanSteenhouse, Yumi Kasai, David Gray, Carol Clugston, Anna Dominiczak and Alex Alderton, Roberto Amato, Sonia Goncalves, Ewan Harrison, David K. Jackson, Ian Johnston, Dominic Kwiatkowski, Cordelia Langford, John Sillitoe on behalf of the Wellcome Sanger Institute COVID-19 Surveillance Team
EPI_ISL_932268	Lighthouse Lab in Alderley Park	Wellcome Sanger Institute for the COVID-19 Genomics UK (COG-UK) Consortium	Jacquelyn Wynn, Mairead Hyland, The Lighthouse Lab in Alderley Park and Alex Alderton, Roberto Amato, Sonia Goncalves, Ewan Harrison, David K. Jackson, Ian Johnston, Dominic Kwiatkowski, Cordelia Langford, John Sillitoe on behalf of the Wellcome Sanger Institute COVID-19 Surveillance Team
EPI_ISL_932269, EPI_ISL_932270, EPI_ISL_932271, EPI_ISL_932272	Lighthouse Lab in Glasgow	Wellcome Sanger Institute for the COVID-19 Genomics UK (COG-UK) Consortium	Harper VanSteenhouse, Yumi Kasai, David Gray, Carol Clugston, Anna Dominiczak and Alex Alderton, Roberto Amato, Sonia Goncalves, Ewan Harrison, David K. Jackson, Ian Johnston, Dominic Kwiatkowski, Cordelia Langford, John Sillitoe on behalf of the Wellcome Sanger Institute COVID-19 Surveillance Team
EPI_ISL_932273, EPI_ISL_932274	Lighthouse Lab in Alderley Park	Wellcome Sanger Institute for the COVID-19 Genomics UK (COG-UK) Consortium	Jacquelyn Wynn, Mairead Hyland, The Lighthouse Lab in Alderley Park and Alex Alderton, Roberto Amato, Sonia Goncalves, Ewan Harrison, David K. Jackson, Ian Johnston, Dominic Kwiatkowski, Cordelia Langford, John Sillitoe on behalf of the Wellcome Sanger Institute COVID-19 Surveillance Team
EPI_ISL_932275, EPI_ISL_932277, EPI_ISL_932279, EPI_ISL_932280, EPI_ISL_932281, EPI_ISL_932282	Lighthouse Lab in Glasgow	Wellcome Sanger Institute for the COVID-19 Genomics UK (COG-UK) Consortium	Harper VanSteenhouse, Yumi Kasai, David Gray, Carol Clugston, Anna Dominiczak and Alex Alderton, Roberto Amato, Sonia Goncalves, Ewan Harrison, David K. Jackson, Ian Johnston, Dominic Kwiatkowski, Cordelia Langford, John Sillitoe on behalf of the Wellcome Sanger Institute COVID-19 Surveillance Team
EPI_ISL_932284, EPI_ISL_932285	Lighthouse Lab in Alderley Park	Wellcome Sanger Institute for the COVID-19 Genomics UK (COG-UK) Consortium	Jacquelyn Wynn, Mairead Hyland, The Lighthouse Lab in Alderley Park and Alex Alderton, Roberto Amato, Sonia Goncalves, Ewan Harrison, David K. Jackson, Ian Johnston, Dominic Kwiatkowski, Cordelia Langford, John Sillitoe on behalf of the Wellcome Sanger Institute COVID-19 Surveillance Team
EPI_ISL_932286	Lighthouse Lab in Glasgow	Wellcome Sanger Institute for the COVID-19 Genomics UK (COG-UK) Consortium	Harper VanSteenhouse, Yumi Kasai, David Gray, Carol Clugston, Anna Dominiczak and Alex Alderton, Roberto Amato, Sonia Goncalves, Ewan Harrison, David K. Jackson, Ian Johnston, Dominic Kwiatkowski, Cordelia Langford, John Sillitoe on behalf of the Wellcome Sanger Institute COVID-19 Surveillance Team
EPI_ISL_932287	Lighthouse Lab in Alderley Park	Wellcome Sanger Institute for the COVID-19 Genomics UK (COG-UK) Consortium	Jacquelyn Wynn, Mairead Hyland, The Lighthouse Lab in Alderley Park and Alex Alderton, Roberto Amato, Sonia Goncalves, Ewan Harrison, David K. Jackson, Ian Johnston, Dominic Kwiatkowski, Cordelia Langford, John Sillitoe on behalf of the Wellcome Sanger Institute COVID-19 Surveillance Team
EPI_ISL_932288	Lighthouse Lab in Milton Keynes	Wellcome Sanger Institute for the COVID-19 Genomics UK (COG-UK) Consortium	The Lighthouse Lab in Milton Keynes and Alex Alderton, Roberto Amato, Sonia Goncalves, Ewan Harrison, David K. Jackson, Ian Johnston, Dominic Kwiatkowski, Cordelia Langford, John Sillitoe on behalf of the Wellcome Sanger Institute COVID-19 Surveillance Team
EPI_ISL_932289	Lighthouse Lab in Glasgow	Wellcome Sanger Institute for the COVID-19 Genomics UK (COG-UK) Consortium	Harper VanSteenhouse, Yumi Kasai, David Gray, Carol Clugston, Anna Dominiczak and Alex Alderton, Roberto Amato, Sonia Goncalves, Ewan Harrison, David K. Jackson, Ian Johnston, Dominic Kwiatkowski, Cordelia Langford, John Sillitoe on behalf of the Wellcome Sanger Institute COVID-19 Surveillance Team
EPI_ISL_932290	Lighthouse Lab in Alderley Park	Wellcome Sanger Institute for the COVID-19 Genomics UK (COG-UK) Consortium	Jacquelyn Wynn, Mairead Hyland, The Lighthouse Lab in Alderley Park and Alex Alderton, Roberto Amato, Sonia Goncalves, Ewan Harrison, David K. Jackson, Ian Johnston, Dominic Kwiatkowski, Cordelia Langford, John Sillitoe on behalf of the Wellcome Sanger Institute COVID-19 Surveillance Team
EPI_ISL_932291	Lighthouse Lab in Glasgow	Wellcome Sanger Institute for the COVID-19 Genomics UK (COG-UK) Consortium	Harper VanSteenhouse, Yumi Kasai, David Gray, Carol Clugston, Anna Dominiczak and Alex Alderton, Roberto Amato, Sonia Goncalves, Ewan Harrison, David K. Jackson, Ian Johnston, Dominic Kwiatkowski, Cordelia Langford, John Sillitoe on behalf of the Wellcome Sanger Institute COVID-19 Surveillance Team
EPI_ISL_932292	Lighthouse Lab in Alderley Park	Wellcome Sanger Institute for the COVID-19 Genomics UK (COG-UK) Consortium	Jacquelyn Wynn, Mairead Hyland, The Lighthouse Lab in Alderley Park and Alex Alderton, Roberto Amato, Sonia Goncalves, Ewan Harrison, David K. Jackson, Ian Johnston, Dominic Kwiatkowski, Cordelia Langford, John Sillitoe on behalf of the Wellcome Sanger Institute COVID-19 Surveillance Team
EPI_ISL_932293, EPI_ISL_932295, EPI_ISL_932296	Lighthouse Lab in Glasgow	Wellcome Sanger Institute for the COVID-19 Genomics UK (COG-UK) Consortium	Harper VanSteenhouse, Yumi Kasai, David Gray, Carol Clugston, Anna Dominiczak and Alex Alderton, Roberto Amato, Sonia Goncalves, Ewan Harrison, David K. Jackson, Ian Johnston, Dominic Kwiatkowski, Cordelia Langford, John Sillitoe on behalf of the Wellcome Sanger Institute COVID-19 Surveillance Team
EPI_ISL_932297	Lighthouse Lab in Alderley Park	Wellcome Sanger Institute for the COVID-19 Genomics UK (COG-UK) Consortium	Jacquelyn Wynn, Mairead Hyland, The Lighthouse Lab in Alderley Park and Alex Alderton, Roberto Amato, Sonia Goncalves, Ewan Harrison, David K. Jackson, Ian Johnston, Dominic Kwiatkowski, Cordelia Langford, John Sillitoe on behalf of the Wellcome Sanger Institute COVID-19 Surveillance Team
EPI_ISL_932299	Lighthouse Lab in Glasgow	Wellcome Sanger Institute for the COVID-19 Genomics UK (COG-UK) Consortium	Harper VanSteenhouse, Yumi Kasai, David Gray, Carol Clugston, Anna Dominiczak and Alex Alderton, Roberto Amato, Sonia Goncalves, Ewan Harrison, David K. Jackson, Ian Johnston, Dominic Kwiatkowski, Cordelia Langford, John Sillitoe on behalf of the Wellcome Sanger Institute COVID-19 Surveillance Team
EPI_ISL_932300	Lighthouse Lab in Alderley Park	Wellcome Sanger Institute for the COVID-19 Genomics UK	Jacquelyn Wynn, Mairead Hyland, The Lighthouse Lab in Alderley Park and Alex Alderton, Roberto Amato, Sonia Goncalves, Ewan Harrison, David K.

		(COG-UK) Consortium	Jackson, Ian Johnston, Dominic Kwiatkowski, Cordelia Langford, John Sillitoe on behalf of the Wellcome Sanger Institute COVID-19 Surveillance Team
EPI_ISL_932301, EPI_ISL_932302	Lighthouse Lab in Glasgow	Wellcome Sanger Institute for the COVID-19 Genomics UK (COG-UK) Consortium	Harper VanSteenhouse, Yumi Kasai, David Gray, Carol Clugston, Anna Dominiczak and Alex Alderton, Roberto Amato, Sonia Goncalves, Ewan Harrison, David K. Jackson, Ian Johnston, Dominic Kwiatkowski, Cordelia Langford, John Sillitoe on behalf of the Wellcome Sanger Institute COVID-19 Surveillance Team
EPI_ISL_932303	Lighthouse Lab in Alderley Park	Wellcome Sanger Institute for the COVID-19 Genomics UK (COG-UK) Consortium	Jacquelyn Wynn, Mairead Hyland, The Lighthouse Lab in Alderley Park and Alex Alderton, Roberto Amato, Sonia Goncalves, Ewan Harrison, David K. Jackson, Ian Johnston, Dominic Kwiatkowski, Cordelia Langford, John Sillitoe on behalf of the Wellcome Sanger Institute COVID-19 Surveillance Team
EPI_ISL_932304, EPI_ISL_932305	Lighthouse Lab in Glasgow	Wellcome Sanger Institute for the COVID-19 Genomics UK (COG-UK) Consortium	Harper VanSteenhouse, Yumi Kasai, David Gray, Carol Clugston, Anna Dominiczak and Alex Alderton, Roberto Amato, Sonia Goncalves, Ewan Harrison, David K. Jackson, Ian Johnston, Dominic Kwiatkowski, Cordelia Langford, John Sillitoe on behalf of the Wellcome Sanger Institute COVID-19 Surveillance Team
EPI_ISL_932307	Lighthouse Lab in Milton Keynes	Wellcome Sanger Institute for the COVID-19 Genomics UK (COG-UK) Consortium	The Lighthouse Lab in Milton Keynes and Alex Alderton, Roberto Amato, Sonia Goncalves, Ewan Harrison, David K. Jackson, Ian Johnston, Dominic Kwiatkowski, Cordelia Langford, John Sillitoe on behalf of the Wellcome Sanger Institute COVID-19 Surveillance Team
EPI_ISL_932308, EPI_ISL_932309	Lighthouse Lab in Glasgow	Wellcome Sanger Institute for the COVID-19 Genomics UK (COG-UK) Consortium	Harper VanSteenhouse, Yumi Kasai, David Gray, Carol Clugston, Anna Dominiczak and Alex Alderton, Roberto Amato, Sonia Goncalves, Ewan Harrison, David K. Jackson, Ian Johnston, Dominic Kwiatkowski, Cordelia Langford, John Sillitoe on behalf of the Wellcome Sanger Institute COVID-19 Surveillance Team
EPI_ISL_932310	Lighthouse Lab in Alderley Park	Wellcome Sanger Institute for the COVID-19 Genomics UK (COG-UK) Consortium	Jacquelyn Wynn, Mairead Hyland, The Lighthouse Lab in Alderley Park and Alex Alderton, Roberto Amato, Sonia Goncalves, Ewan Harrison, David K. Jackson, Ian Johnston, Dominic Kwiatkowski, Cordelia Langford, John Sillitoe on behalf of the Wellcome Sanger Institute COVID-19 Surveillance Team
EPI_ISL_932311, EPI_ISL_932312	Lighthouse Lab in Glasgow	Wellcome Sanger Institute for the COVID-19 Genomics UK (COG-UK) Consortium	Harper VanSteenhouse, Yumi Kasai, David Gray, Carol Clugston, Anna Dominiczak and Alex Alderton, Roberto Amato, Sonia Goncalves, Ewan Harrison, David K. Jackson, Ian Johnston, Dominic Kwiatkowski, Cordelia Langford, John Sillitoe on behalf of the Wellcome Sanger Institute COVID-19 Surveillance Team
EPI_ISL_932313	Lighthouse Lab in Alderley Park	Wellcome Sanger Institute for the COVID-19 Genomics UK (COG-UK) Consortium	Jacquelyn Wynn, Mairead Hyland, The Lighthouse Lab in Alderley Park and Alex Alderton, Roberto Amato, Sonia Goncalves, Ewan Harrison, David K. Jackson, Ian Johnston, Dominic Kwiatkowski, Cordelia Langford, John Sillitoe on behalf of the Wellcome Sanger Institute COVID-19 Surveillance Team
EPI_ISL_932314, EPI_ISL_932316, EPI_ISL_932317, EPI_ISL_932318, EPI_ISL_932321, EPI_ISL_932322, EPI_ISL_932323	Lighthouse Lab in Glasgow	Wellcome Sanger Institute for the COVID-19 Genomics UK (COG-UK) Consortium	Harper VanSteenhouse, Yumi Kasai, David Gray, Carol Clugston, Anna Dominiczak and Alex Alderton, Roberto Amato, Sonia Goncalves, Ewan Harrison, David K. Jackson, Ian Johnston, Dominic Kwiatkowski, Cordelia Langford, John Sillitoe on behalf of the Wellcome Sanger Institute COVID-19 Surveillance Team
EPI_ISL_932324	Lighthouse Lab in Milton Keynes	Wellcome Sanger Institute for the COVID-19 Genomics UK (COG-UK) Consortium	The Lighthouse Lab in Milton Keynes and Alex Alderton, Roberto Amato, Sonia Goncalves, Ewan Harrison, David K. Jackson, Ian Johnston, Dominic Kwiatkowski, Cordelia Langford, John Sillitoe on behalf of the Wellcome Sanger Institute COVID-19 Surveillance Team
EPI_ISL_932325	Lighthouse Lab in Alderley Park	Wellcome Sanger Institute for the COVID-19 Genomics UK (COG-UK) Consortium	Jacquelyn Wynn, Mairead Hyland, The Lighthouse Lab in Alderley Park and Alex Alderton, Roberto Amato, Sonia Goncalves, Ewan Harrison, David K. Jackson, Ian Johnston, Dominic Kwiatkowski, Cordelia Langford, John Sillitoe on behalf of the Wellcome Sanger Institute COVID-19 Surveillance Team
EPI_ISL_932326, EPI_ISL_932328, EPI_ISL_932330, EPI_ISL_932331, EPI_ISL_932333	Lighthouse Lab in Glasgow	Wellcome Sanger Institute for the COVID-19 Genomics UK (COG-UK) Consortium	Harper VanSteenhouse, Yumi Kasai, David Gray, Carol Clugston, Anna Dominiczak and Alex Alderton, Roberto Amato, Sonia Goncalves, Ewan Harrison, David K. Jackson, Ian Johnston, Dominic Kwiatkowski, Cordelia Langford, John Sillitoe on behalf of the Wellcome Sanger Institute COVID-19 Surveillance Team
EPI_ISL_932334	Lighthouse Lab in Alderley Park	Wellcome Sanger Institute for the COVID-19 Genomics UK (COG-UK) Consortium	Jacquelyn Wynn, Mairead Hyland, The Lighthouse Lab in Alderley Park and Alex Alderton, Roberto Amato, Sonia Goncalves, Ewan Harrison, David K. Jackson, Ian Johnston, Dominic Kwiatkowski, Cordelia Langford, John Sillitoe on behalf of the Wellcome Sanger Institute COVID-19 Surveillance Team
EPI_ISL_932335, EPI_ISL_932336, EPI_ISL_932337, EPI_ISL_932338, EPI_ISL_932341, EPI_ISL_932342, EPI_ISL_932344	Lighthouse Lab in Glasgow	Wellcome Sanger Institute for the COVID-19 Genomics UK (COG-UK) Consortium	Harper VanSteenhouse, Yumi Kasai, David Gray, Carol Clugston, Anna Dominiczak and Alex Alderton, Roberto Amato, Sonia Goncalves, Ewan Harrison, David K. Jackson, Ian Johnston, Dominic Kwiatkowski, Cordelia Langford, John Sillitoe on behalf of the Wellcome Sanger Institute COVID-19 Surveillance Team
EPI_ISL_932345	Lighthouse Lab in Alderley Park	Wellcome Sanger Institute for the COVID-19 Genomics UK (COG-UK) Consortium	Jacquelyn Wynn, Mairead Hyland, The Lighthouse Lab in Alderley Park and Alex Alderton, Roberto Amato, Sonia Goncalves, Ewan Harrison, David K. Jackson, Ian Johnston, Dominic Kwiatkowski, Cordelia Langford, John Sillitoe on behalf of the Wellcome Sanger Institute COVID-19 Surveillance Team
EPI_ISL_932346	Lighthouse Lab in Milton Keynes	Wellcome Sanger Institute for the COVID-19 Genomics UK (COG-UK) Consortium	The Lighthouse Lab in Milton Keynes and Alex Alderton, Roberto Amato, Sonia Goncalves, Ewan Harrison, David K. Jackson, Ian Johnston, Dominic Kwiatkowski, Cordelia Langford, John Sillitoe on behalf of the Wellcome Sanger Institute COVID-19 Surveillance Team
EPI_ISL_932348	Lighthouse Lab in Alderley Park	Wellcome Sanger Institute for the COVID-19 Genomics UK (COG-UK) Consortium	Jacquelyn Wynn, Mairead Hyland, The Lighthouse Lab in Alderley Park and Alex Alderton, Roberto Amato, Sonia Goncalves, Ewan Harrison, David K. Jackson, Ian Johnston, Dominic Kwiatkowski, Cordelia Langford, John Sillitoe on behalf of the Wellcome Sanger Institute COVID-19 Surveillance Team
EPI_ISL_932349, EPI_ISL_932351, EPI_ISL_932352, EPI_ISL_932353	Lighthouse Lab in Glasgow	Wellcome Sanger Institute for the COVID-19 Genomics UK (COG-UK) Consortium	Harper VanSteenhouse, Yumi Kasai, David Gray, Carol Clugston, Anna Dominiczak and Alex Alderton, Roberto Amato, Sonia Goncalves, Ewan Harrison, David K. Jackson, Ian Johnston, Dominic Kwiatkowski, Cordelia Langford, John Sillitoe on behalf of the Wellcome Sanger Institute COVID-19 Surveillance Team
EPI_ISL_932354	Lighthouse Lab in Milton Keynes	Wellcome Sanger Institute for the COVID-19 Genomics UK (COG-UK) Consortium	The Lighthouse Lab in Milton Keynes and Alex Alderton, Roberto Amato, Sonia Goncalves, Ewan Harrison, David K. Jackson, Ian Johnston, Dominic Kwiatkowski, Cordelia Langford, John Sillitoe on behalf of the Wellcome Sanger Institute COVID-19 Surveillance Team
EPI_ISL_932355, EPI_ISL_932356	Lighthouse Lab in Glasgow	Wellcome Sanger Institute for the COVID-19 Genomics UK (COG-UK) Consortium	Harper VanSteenhouse, Yumi Kasai, David Gray, Carol Clugston, Anna Dominiczak and Alex Alderton, Roberto Amato, Sonia Goncalves, Ewan Harrison, David K. Jackson, Ian Johnston, Dominic Kwiatkowski, Cordelia Langford, John Sillitoe on behalf of the Wellcome Sanger Institute COVID-19 Surveillance Team
EPI_ISL_932357	Lighthouse Lab in Alderley Park	Wellcome Sanger Institute for the COVID-19 Genomics UK (COG-UK) Consortium	Jacquelyn Wynn, Mairead Hyland, The Lighthouse Lab in Alderley Park and Alex Alderton, Roberto Amato, Sonia Goncalves, Ewan Harrison, David K. Jackson, Ian Johnston, Dominic Kwiatkowski, Cordelia Langford, John Sillitoe on behalf of the Wellcome Sanger Institute COVID-19 Surveillance Team
EPI_ISL_932359, EPI_ISL_932360, EPI_ISL_932361, EPI_ISL_932362	Lighthouse Lab in Glasgow	Wellcome Sanger Institute for the COVID-19 Genomics UK (COG-UK) Consortium	Harper VanSteenhouse, Yumi Kasai, David Gray, Carol Clugston, Anna Dominiczak and Alex Alderton, Roberto Amato, Sonia Goncalves, Ewan Harrison, David K. Jackson, Ian Johnston, Dominic Kwiatkowski, Cordelia Langford, John Sillitoe on behalf of the Wellcome Sanger Institute COVID-19 Surveillance Team
EPI_ISL_932364, EPI_ISL_932365	Lighthouse Lab in Milton Keynes	Wellcome Sanger Institute for the COVID-19 Genomics UK (COG-UK) Consortium	The Lighthouse Lab in Milton Keynes and Alex Alderton, Roberto Amato, Sonia Goncalves, Ewan Harrison, David K. Jackson, Ian Johnston, Dominic Kwiatkowski, Cordelia Langford, John Sillitoe on behalf of the Wellcome Sanger Institute COVID-19 Surveillance Team
EPI_ISL_932367	Lighthouse Lab in Glasgow	Wellcome Sanger Institute for the COVID-19 Genomics UK (COG-UK) Consortium	Harper VanSteenhouse, Yumi Kasai, David Gray, Carol Clugston, Anna Dominiczak and Alex Alderton, Roberto Amato, Sonia Goncalves, Ewan Harrison, David K. Jackson, Ian Johnston, Dominic Kwiatkowski, Cordelia Langford, John Sillitoe on behalf of the Wellcome Sanger Institute COVID-19 Surveillance Team
EPI_ISL_932368	Lighthouse Lab in Milton Keynes	Wellcome Sanger Institute for the COVID-19 Genomics UK (COG-UK) Consortium	The Lighthouse Lab in Milton Keynes and Alex Alderton, Roberto Amato, Sonia Goncalves, Ewan Harrison, David K. Jackson, Ian Johnston, Dominic Kwiatkowski, Cordelia Langford, John Sillitoe on behalf of the Wellcome Sanger Institute COVID-19 Surveillance Team
EPI_ISL_932369, EPI_ISL_932370	Lighthouse Lab in Glasgow	Wellcome Sanger Institute for the COVID-19 Genomics UK (COG-UK) Consortium	Harper VanSteenhouse, Yumi Kasai, David Gray, Carol Clugston, Anna Dominiczak and Alex Alderton, Roberto Amato, Sonia Goncalves, Ewan Harrison, David K. Jackson, Ian Johnston, Dominic Kwiatkowski, Cordelia Langford, John Sillitoe on behalf of the Wellcome Sanger Institute COVID-19 Surveillance Team
EPI_ISL_932371	Lighthouse Lab in Alderley Park	Wellcome Sanger Institute for the COVID-19 Genomics UK (COG-UK) Consortium	Jacquelyn Wynn, Mairead Hyland, The Lighthouse Lab in Alderley Park and Alex Alderton, Roberto Amato, Sonia Goncalves, Ewan Harrison, David K. Jackson, Ian Johnston, Dominic Kwiatkowski, Cordelia Langford, John Sillitoe on behalf of the Wellcome Sanger Institute COVID-19 Surveillance Team
EPI_ISL_932373	Lighthouse Lab in Glasgow	Wellcome Sanger Institute for the COVID-19 Genomics UK (COG-UK) Consortium	Harper VanSteenhouse, Yumi Kasai, David Gray, Carol Clugston, Anna Dominiczak and Alex Alderton, Roberto Amato, Sonia Goncalves, Ewan Harrison, David K. Jackson, Ian Johnston, Dominic Kwiatkowski, Cordelia Langford, John Sillitoe on behalf of the Wellcome Sanger Institute COVID-19 Surveillance Team
EPI_ISL_932374	Lighthouse Lab in Milton Keynes	Wellcome Sanger Institute for the COVID-19 Genomics UK	The Lighthouse Lab in Milton Keynes and Alex Alderton, Roberto Amato, Sonia Goncalves, Ewan Harrison, David K. Jackson, Ian Johnston, Dominic

		(COG-UK) Consortium	Kwiatkowski, Cordelia Langford, John Sillitoe on behalf of the Wellcome Sanger Institute COVID-19 Surveillance Team
EPI_ISL_932376	Lighthouse Lab in Alderley Park	Wellcome Sanger Institute for the COVID-19 Genomics UK (COG-UK) Consortium	Jacquelyn Wynn, Mairead Hyland, The Lighthouse Lab in Alderley Park and Alex Alderton, Roberto Amato, Sonia Goncalves, Ewan Harrison, David K. Jackson, Ian Johnston, Dominic Kwiatkowski, Cordelia Langford, John Sillitoe on behalf of the Wellcome Sanger Institute COVID-19 Surveillance Team
EPI_ISL_932378	Lighthouse Lab in Milton Keynes	Wellcome Sanger Institute for the COVID-19 Genomics UK (COG-UK) Consortium	The Lighthouse Lab in Milton Keynes and Alex Alderton, Roberto Amato, Sonia Goncalves, Ewan Harrison, David K. Jackson, Ian Johnston, Dominic Kwiatkowski, Cordelia Langford, John Sillitoe on behalf of the Wellcome Sanger Institute COVID-19 Surveillance Team
EPI_ISL_932379, EPI_ISL_932381, EPI_ISL_932382, EPI_ISL_932384	Lighthouse Lab in Glasgow	Wellcome Sanger Institute for the COVID-19 Genomics UK (COG-UK) Consortium	Harper VanSteenhouse, Yumi Kasai, David Gray, Carol Clugston, Anna Dominiczak and Alex Alderton, Roberto Amato, Sonia Goncalves, Ewan Harrison, David K. Jackson, Ian Johnston, Dominic Kwiatkowski, Cordelia Langford, John Sillitoe on behalf of the Wellcome Sanger Institute COVID-19 Surveillance Team
EPI_ISL_932385	Lighthouse Lab in Milton Keynes	Wellcome Sanger Institute for the COVID-19 Genomics UK (COG-UK) Consortium	The Lighthouse Lab in Milton Keynes and Alex Alderton, Roberto Amato, Sonia Goncalves, Ewan Harrison, David K. Jackson, Ian Johnston, Dominic Kwiatkowski, Cordelia Langford, John Sillitoe on behalf of the Wellcome Sanger Institute COVID-19 Surveillance Team
EPI_ISL_932386	Lighthouse Lab in Glasgow	Wellcome Sanger Institute for the COVID-19 Genomics UK (COG-UK) Consortium	Harper VanSteenhouse, Yumi Kasai, David Gray, Carol Clugston, Anna Dominiczak and Alex Alderton, Roberto Amato, Sonia Goncalves, Ewan Harrison, David K. Jackson, Ian Johnston, Dominic Kwiatkowski, Cordelia Langford, John Sillitoe on behalf of the Wellcome Sanger Institute COVID-19 Surveillance Team
EPI_ISL_932388	Lighthouse Lab in Alderley Park	Wellcome Sanger Institute for the COVID-19 Genomics UK (COG-UK) Consortium	Jacquelyn Wynn, Mairead Hyland, The Lighthouse Lab in Alderley Park and Alex Alderton, Roberto Amato, Sonia Goncalves, Ewan Harrison, David K. Jackson, Ian Johnston, Dominic Kwiatkowski, Cordelia Langford, John Sillitoe on behalf of the Wellcome Sanger Institute COVID-19 Surveillance Team
EPI_ISL_932389, EPI_ISL_932390	Lighthouse Lab in Glasgow	Wellcome Sanger Institute for the COVID-19 Genomics UK (COG-UK) Consortium	Harper VanSteenhouse, Yumi Kasai, David Gray, Carol Clugston, Anna Dominiczak and Alex Alderton, Roberto Amato, Sonia Goncalves, Ewan Harrison, David K. Jackson, Ian Johnston, Dominic Kwiatkowski, Cordelia Langford, John Sillitoe on behalf of the Wellcome Sanger Institute COVID-19 Surveillance Team
EPI_ISL_932392	Lighthouse Lab in Milton Keynes	Wellcome Sanger Institute for the COVID-19 Genomics UK (COG-UK) Consortium	The Lighthouse Lab in Milton Keynes and Alex Alderton, Roberto Amato, Sonia Goncalves, Ewan Harrison, David K. Jackson, Ian Johnston, Dominic Kwiatkowski, Cordelia Langford, John Sillitoe on behalf of the Wellcome Sanger Institute COVID-19 Surveillance Team
EPI_ISL_932393	Lighthouse Lab in Alderley Park	Wellcome Sanger Institute for the COVID-19 Genomics UK (COG-UK) Consortium	Jacquelyn Wynn, Mairead Hyland, The Lighthouse Lab in Alderley Park and Alex Alderton, Roberto Amato, Sonia Goncalves, Ewan Harrison, David K. Jackson, Ian Johnston, Dominic Kwiatkowski, Cordelia Langford, John Sillitoe on behalf of the Wellcome Sanger Institute COVID-19 Surveillance Team
EPI_ISL_932394, EPI_ISL_932397, EPI_ISL_932398, EPI_ISL_932401	Lighthouse Lab in Glasgow	Wellcome Sanger Institute for the COVID-19 Genomics UK (COG-UK) Consortium	Harper VanSteenhouse, Yumi Kasai, David Gray, Carol Clugston, Anna Dominiczak and Alex Alderton, Roberto Amato, Sonia Goncalves, Ewan Harrison, David K. Jackson, Ian Johnston, Dominic Kwiatkowski, Cordelia Langford, John Sillitoe on behalf of the Wellcome Sanger Institute COVID-19 Surveillance Team
EPI_ISL_932402	Lighthouse Lab in Milton Keynes	Wellcome Sanger Institute for the COVID-19 Genomics UK (COG-UK) Consortium	The Lighthouse Lab in Milton Keynes and Alex Alderton, Roberto Amato, Sonia Goncalves, Ewan Harrison, David K. Jackson, Ian Johnston, Dominic Kwiatkowski, Cordelia Langford, John Sillitoe on behalf of the Wellcome Sanger Institute COVID-19 Surveillance Team
EPI_ISL_932404, EPI_ISL_932405, EPI_ISL_932407, EPI_ISL_932409, EPI_ISL_932410	Lighthouse Lab in Glasgow	Wellcome Sanger Institute for the COVID-19 Genomics UK (COG-UK) Consortium	Harper VanSteenhouse, Yumi Kasai, David Gray, Carol Clugston, Anna Dominiczak and Alex Alderton, Roberto Amato, Sonia Goncalves, Ewan Harrison, David K. Jackson, Ian Johnston, Dominic Kwiatkowski, Cordelia Langford, John Sillitoe on behalf of the Wellcome Sanger Institute COVID-19 Surveillance Team
EPI_ISL_932411	Lighthouse Lab in Milton Keynes	Wellcome Sanger Institute for the COVID-19 Genomics UK (COG-UK) Consortium	The Lighthouse Lab in Milton Keynes and Alex Alderton, Roberto Amato, Sonia Goncalves, Ewan Harrison, David K. Jackson, Ian Johnston, Dominic Kwiatkowski, Cordelia Langford, John Sillitoe on behalf of the Wellcome Sanger Institute COVID-19 Surveillance Team
EPI_ISL_932412, EPI_ISL_932415, EPI_ISL_932416, EPI_ISL_932418	Lighthouse Lab in Glasgow	Wellcome Sanger Institute for the COVID-19 Genomics UK (COG-UK) Consortium	Harper VanSteenhouse, Yumi Kasai, David Gray, Carol Clugston, Anna Dominiczak and Alex Alderton, Roberto Amato, Sonia Goncalves, Ewan Harrison, David K. Jackson, Ian Johnston, Dominic Kwiatkowski, Cordelia Langford, John Sillitoe on behalf of the Wellcome Sanger Institute COVID-19 Surveillance Team
EPI_ISL_932419	Lighthouse Lab in Milton Keynes	Wellcome Sanger Institute for the COVID-19 Genomics UK (COG-UK) Consortium	The Lighthouse Lab in Milton Keynes and Alex Alderton, Roberto Amato, Sonia Goncalves, Ewan Harrison, David K. Jackson, Ian Johnston, Dominic Kwiatkowski, Cordelia Langford, John Sillitoe on behalf of the Wellcome Sanger Institute COVID-19 Surveillance Team
EPI_ISL_932420, EPI_ISL_932421, EPI_ISL_932422, EPI_ISL_932423, EPI_ISL_932424	Lighthouse Lab in Glasgow	Wellcome Sanger Institute for the COVID-19 Genomics UK (COG-UK) Consortium	Harper VanSteenhouse, Yumi Kasai, David Gray, Carol Clugston, Anna Dominiczak and Alex Alderton, Roberto Amato, Sonia Goncalves, Ewan Harrison, David K. Jackson, Ian Johnston, Dominic Kwiatkowski, Cordelia Langford, John Sillitoe on behalf of the Wellcome Sanger Institute COVID-19 Surveillance Team
EPI_ISL_932425	Lighthouse Lab in Alderley Park	Wellcome Sanger Institute for the COVID-19 Genomics UK (COG-UK) Consortium	Jacquelyn Wynn, Mairead Hyland, The Lighthouse Lab in Alderley Park and Alex Alderton, Roberto Amato, Sonia Goncalves, Ewan Harrison, David K. Jackson, Ian Johnston, Dominic Kwiatkowski, Cordelia Langford, John Sillitoe on behalf of the Wellcome Sanger Institute COVID-19 Surveillance Team
EPI_ISL_932427	Lighthouse Lab in Glasgow	Wellcome Sanger Institute for the COVID-19 Genomics UK (COG-UK) Consortium	Harper VanSteenhouse, Yumi Kasai, David Gray, Carol Clugston, Anna Dominiczak and Alex Alderton, Roberto Amato, Sonia Goncalves, Ewan Harrison, David K. Jackson, Ian Johnston, Dominic Kwiatkowski, Cordelia Langford, John Sillitoe on behalf of the Wellcome Sanger Institute COVID-19 Surveillance Team
EPI_ISL_932429	Lighthouse Lab in Milton Keynes	Wellcome Sanger Institute for the COVID-19 Genomics UK (COG-UK) Consortium	The Lighthouse Lab in Milton Keynes and Alex Alderton, Roberto Amato, Sonia Goncalves, Ewan Harrison, David K. Jackson, Ian Johnston, Dominic Kwiatkowski, Cordelia Langford, John Sillitoe on behalf of the Wellcome Sanger Institute COVID-19 Surveillance Team
EPI_ISL_932431, EPI_ISL_932432	Lighthouse Lab in Glasgow	Wellcome Sanger Institute for the COVID-19 Genomics UK (COG-UK) Consortium	Harper VanSteenhouse, Yumi Kasai, David Gray, Carol Clugston, Anna Dominiczak and Alex Alderton, Roberto Amato, Sonia Goncalves, Ewan Harrison, David K. Jackson, Ian Johnston, Dominic Kwiatkowski, Cordelia Langford, John Sillitoe on behalf of the Wellcome Sanger Institute COVID-19 Surveillance Team
EPI_ISL_932433	Lighthouse Lab in Alderley Park	Wellcome Sanger Institute for the COVID-19 Genomics UK (COG-UK) Consortium	Jacquelyn Wynn, Mairead Hyland, The Lighthouse Lab in Alderley Park and Alex Alderton, Roberto Amato, Sonia Goncalves, Ewan Harrison, David K. Jackson, Ian Johnston, Dominic Kwiatkowski, Cordelia Langford, John Sillitoe on behalf of the Wellcome Sanger Institute COVID-19 Surveillance Team
EPI_ISL_932434	Lighthouse Lab in Milton Keynes	Wellcome Sanger Institute for the COVID-19 Genomics UK (COG-UK) Consortium	The Lighthouse Lab in Milton Keynes and Alex Alderton, Roberto Amato, Sonia Goncalves, Ewan Harrison, David K. Jackson, Ian Johnston, Dominic Kwiatkowski, Cordelia Langford, John Sillitoe on behalf of the Wellcome Sanger Institute COVID-19 Surveillance Team
EPI_ISL_932435	Lighthouse Lab in Glasgow	Wellcome Sanger Institute for the COVID-19 Genomics UK (COG-UK) Consortium	Harper VanSteenhouse, Yumi Kasai, David Gray, Carol Clugston, Anna Dominiczak and Alex Alderton, Roberto Amato, Sonia Goncalves, Ewan Harrison, David K. Jackson, Ian Johnston, Dominic Kwiatkowski, Cordelia Langford, John Sillitoe on behalf of the Wellcome Sanger Institute COVID-19 Surveillance Team
EPI_ISL_932436	Lighthouse Lab in Alderley Park	Wellcome Sanger Institute for the COVID-19 Genomics UK (COG-UK) Consortium	Jacquelyn Wynn, Mairead Hyland, The Lighthouse Lab in Alderley Park and Alex Alderton, Roberto Amato, Sonia Goncalves, Ewan Harrison, David K. Jackson, Ian Johnston, Dominic Kwiatkowski, Cordelia Langford, John Sillitoe on behalf of the Wellcome Sanger Institute COVID-19 Surveillance Team
EPI_ISL_932438	Lighthouse Lab in Glasgow	Wellcome Sanger Institute for the COVID-19 Genomics UK (COG-UK) Consortium	Harper VanSteenhouse, Yumi Kasai, David Gray, Carol Clugston, Anna Dominiczak and Alex Alderton, Roberto Amato, Sonia Goncalves, Ewan Harrison, David K. Jackson, Ian Johnston, Dominic Kwiatkowski, Cordelia Langford, John Sillitoe on behalf of the Wellcome Sanger Institute COVID-19 Surveillance Team
EPI_ISL_932439	Lighthouse Lab in Milton Keynes	Wellcome Sanger Institute for the COVID-19 Genomics UK (COG-UK) Consortium	The Lighthouse Lab in Milton Keynes and Alex Alderton, Roberto Amato, Sonia Goncalves, Ewan Harrison, David K. Jackson, Ian Johnston, Dominic Kwiatkowski, Cordelia Langford, John Sillitoe on behalf of the Wellcome Sanger Institute COVID-19 Surveillance Team
EPI_ISL_932440	Lighthouse Lab in Alderley Park	Wellcome Sanger Institute for the COVID-19 Genomics UK (COG-UK) Consortium	Jacquelyn Wynn, Mairead Hyland, The Lighthouse Lab in Alderley Park and Alex Alderton, Roberto Amato, Sonia Goncalves, Ewan Harrison, David K. Jackson, Ian Johnston, Dominic Kwiatkowski, Cordelia Langford, John Sillitoe on behalf of the Wellcome Sanger Institute COVID-19 Surveillance Team
EPI_ISL_932441	Lighthouse Lab in Milton Keynes	Wellcome Sanger Institute for the COVID-19 Genomics UK (COG-UK) Consortium	The Lighthouse Lab in Milton Keynes and Alex Alderton, Roberto Amato, Sonia Goncalves, Ewan Harrison, David K. Jackson, Ian Johnston, Dominic Kwiatkowski, Cordelia Langford, John Sillitoe on behalf of the Wellcome Sanger Institute COVID-19 Surveillance Team
EPI_ISL_932444	Lighthouse Lab in Alderley Park	Wellcome Sanger Institute for the COVID-19 Genomics UK (COG-UK) Consortium	Jacquelyn Wynn, Mairead Hyland, The Lighthouse Lab in Alderley Park and Alex Alderton, Roberto Amato, Sonia Goncalves, Ewan Harrison, David K. Jackson, Ian Johnston, Dominic Kwiatkowski, Cordelia Langford, John Sillitoe on behalf of the Wellcome Sanger Institute COVID-19 Surveillance Team
EPI_ISL_932446	Lighthouse Lab in Milton Keynes	Wellcome Sanger Institute for the COVID-19 Genomics UK (COG-UK) Consortium	The Lighthouse Lab in Milton Keynes and Alex Alderton, Roberto Amato, Sonia Goncalves, Ewan Harrison, David K. Jackson, Ian Johnston, Dominic Kwiatkowski, Cordelia Langford, John Sillitoe on behalf of the Wellcome Sanger Institute COVID-19 Surveillance Team

EPI_ISL_932448, EPI_ISL_932449	Lighthouse Lab in Glasgow	Wellcome Sanger Institute for the COVID-19 Genomics UK (COG-UK) Consortium	Harper VanSteenhouse, Yumi Kasai, David Gray, Carol Clugston, Anna Dominiczak and Alex Alderton, Roberto Amato, Sonia Goncalves, Ewan Harrison, David K. Jackson, Ian Johnston, Dominic Kwiatkowski, Cordelia Langford, John Sillitoe on behalf of the Wellcome Sanger Institute COVID-19 Surveillance Team
EPI_ISL_932450	Lighthouse Lab in Milton Keynes	Wellcome Sanger Institute for the COVID-19 Genomics UK (COG-UK) Consortium	The Lighthouse Lab in Milton Keynes and Alex Alderton, Roberto Amato, Sonia Goncalves, Ewan Harrison, David K. Jackson, Ian Johnston, Dominic Kwiatkowski, Cordelia Langford, John Sillitoe on behalf of the Wellcome Sanger Institute COVID-19 Surveillance Team
EPI_ISL_932451, EPI_ISL_932452	Lighthouse Lab in Glasgow	Wellcome Sanger Institute for the COVID-19 Genomics UK (COG-UK) Consortium	Harper VanSteenhouse, Yumi Kasai, David Gray, Carol Clugston, Anna Dominiczak and Alex Alderton, Roberto Amato, Sonia Goncalves, Ewan Harrison, David K. Jackson, Ian Johnston, Dominic Kwiatkowski, Cordelia Langford, John Sillitoe on behalf of the Wellcome Sanger Institute COVID-19 Surveillance Team
EPI_ISL_932453	Lighthouse Lab in Alderley Park	Wellcome Sanger Institute for the COVID-19 Genomics UK (COG-UK) Consortium	Jacquelyn Wynn, Mairead Hyland, The Lighthouse Lab in Alderley Park and Alex Alderton, Roberto Amato, Sonia Goncalves, Ewan Harrison, David K. Jackson, Ian Johnston, Dominic Kwiatkowski, Cordelia Langford, John Sillitoe on behalf of the Wellcome Sanger Institute COVID-19 Surveillance Team
EPI_ISL_932454	Lighthouse Lab in Glasgow	Wellcome Sanger Institute for the COVID-19 Genomics UK (COG-UK) Consortium	Harper VanSteenhouse, Yumi Kasai, David Gray, Carol Clugston, Anna Dominiczak and Alex Alderton, Roberto Amato, Sonia Goncalves, Ewan Harrison, David K. Jackson, Ian Johnston, Dominic Kwiatkowski, Cordelia Langford, John Sillitoe on behalf of the Wellcome Sanger Institute COVID-19 Surveillance Team
EPI_ISL_932455, EPI_ISL_932457, EPI_ISL_932458	Lighthouse Lab in Alderley Park	Wellcome Sanger Institute for the COVID-19 Genomics UK (COG-UK) Consortium	Jacquelyn Wynn, Mairead Hyland, The Lighthouse Lab in Alderley Park and Alex Alderton, Roberto Amato, Sonia Goncalves, Ewan Harrison, David K. Jackson, Ian Johnston, Dominic Kwiatkowski, Cordelia Langford, John Sillitoe on behalf of the Wellcome Sanger Institute COVID-19 Surveillance Team
EPI_ISL_932459, EPI_ISL_932461, EPI_ISL_932462	Lighthouse Lab in Glasgow	Wellcome Sanger Institute for the COVID-19 Genomics UK (COG-UK) Consortium	Harper VanSteenhouse, Yumi Kasai, David Gray, Carol Clugston, Anna Dominiczak and Alex Alderton, Roberto Amato, Sonia Goncalves, Ewan Harrison, David K. Jackson, Ian Johnston, Dominic Kwiatkowski, Cordelia Langford, John Sillitoe on behalf of the Wellcome Sanger Institute COVID-19 Surveillance Team
EPI_ISL_932463	Lighthouse Lab in Alderley Park	Wellcome Sanger Institute for the COVID-19 Genomics UK (COG-UK) Consortium	Jacquelyn Wynn, Mairead Hyland, The Lighthouse Lab in Alderley Park and Alex Alderton, Roberto Amato, Sonia Goncalves, Ewan Harrison, David K. Jackson, Ian Johnston, Dominic Kwiatkowski, Cordelia Langford, John Sillitoe on behalf of the Wellcome Sanger Institute COVID-19 Surveillance Team
EPI_ISL_932464, EPI_ISL_932465, EPI_ISL_932466, EPI_ISL_932467	Lighthouse Lab in Glasgow	Wellcome Sanger Institute for the COVID-19 Genomics UK (COG-UK) Consortium	Harper VanSteenhouse, Yumi Kasai, David Gray, Carol Clugston, Anna Dominiczak and Alex Alderton, Roberto Amato, Sonia Goncalves, Ewan Harrison, David K. Jackson, Ian Johnston, Dominic Kwiatkowski, Cordelia Langford, John Sillitoe on behalf of the Wellcome Sanger Institute COVID-19 Surveillance Team
EPI_ISL_932468	Lighthouse Lab in Milton Keynes	Wellcome Sanger Institute for the COVID-19 Genomics UK (COG-UK) Consortium	The Lighthouse Lab in Milton Keynes and Alex Alderton, Roberto Amato, Sonia Goncalves, Ewan Harrison, David K. Jackson, Ian Johnston, Dominic Kwiatkowski, Cordelia Langford, John Sillitoe on behalf of the Wellcome Sanger Institute COVID-19 Surveillance Team
EPI_ISL_932470, EPI_ISL_932473	Lighthouse Lab in Alderley Park	Wellcome Sanger Institute for the COVID-19 Genomics UK (COG-UK) Consortium	Jacquelyn Wynn, Mairead Hyland, The Lighthouse Lab in Alderley Park and Alex Alderton, Roberto Amato, Sonia Goncalves, Ewan Harrison, David K. Jackson, Ian Johnston, Dominic Kwiatkowski, Cordelia Langford, John Sillitoe on behalf of the Wellcome Sanger Institute COVID-19 Surveillance Team
EPI_ISL_932476	Lighthouse Lab in Milton Keynes	Wellcome Sanger Institute for the COVID-19 Genomics UK (COG-UK) Consortium	The Lighthouse Lab in Milton Keynes and Alex Alderton, Roberto Amato, Sonia Goncalves, Ewan Harrison, David K. Jackson, Ian Johnston, Dominic Kwiatkowski, Cordelia Langford, John Sillitoe on behalf of the Wellcome Sanger Institute COVID-19 Surveillance Team
EPI_ISL_932479, EPI_ISL_932480	Lighthouse Lab in Alderley Park	Wellcome Sanger Institute for the COVID-19 Genomics UK (COG-UK) Consortium	Jacquelyn Wynn, Mairead Hyland, The Lighthouse Lab in Alderley Park and Alex Alderton, Roberto Amato, Sonia Goncalves, Ewan Harrison, David K. Jackson, Ian Johnston, Dominic Kwiatkowski, Cordelia Langford, John Sillitoe on behalf of the Wellcome Sanger Institute COVID-19 Surveillance Team
EPI_ISL_932481	Lighthouse Lab in Milton Keynes	Wellcome Sanger Institute for the COVID-19 Genomics UK (COG-UK) Consortium	The Lighthouse Lab in Milton Keynes and Alex Alderton, Roberto Amato, Sonia Goncalves, Ewan Harrison, David K. Jackson, Ian Johnston, Dominic Kwiatkowski, Cordelia Langford, John Sillitoe on behalf of the Wellcome Sanger Institute COVID-19 Surveillance Team
EPI_ISL_932482	Lighthouse Lab in Glasgow	Wellcome Sanger Institute for the COVID-19 Genomics UK (COG-UK) Consortium	Harper VanSteenhouse, Yumi Kasai, David Gray, Carol Clugston, Anna Dominiczak and Alex Alderton, Roberto Amato, Sonia Goncalves, Ewan Harrison, David K. Jackson, Ian Johnston, Dominic Kwiatkowski, Cordelia Langford, John Sillitoe on behalf of the Wellcome Sanger Institute COVID-19 Surveillance Team
EPI_ISL_932484	Lighthouse Lab in Milton Keynes	Wellcome Sanger Institute for the COVID-19 Genomics UK (COG-UK) Consortium	The Lighthouse Lab in Milton Keynes and Alex Alderton, Roberto Amato, Sonia Goncalves, Ewan Harrison, David K. Jackson, Ian Johnston, Dominic Kwiatkowski, Cordelia Langford, John Sillitoe on behalf of the Wellcome Sanger Institute COVID-19 Surveillance Team
EPI_ISL_932486	Lighthouse Lab in Alderley Park	Wellcome Sanger Institute for the COVID-19 Genomics UK (COG-UK) Consortium	Jacquelyn Wynn, Mairead Hyland, The Lighthouse Lab in Alderley Park and Alex Alderton, Roberto Amato, Sonia Goncalves, Ewan Harrison, David K. Jackson, Ian Johnston, Dominic Kwiatkowski, Cordelia Langford, John Sillitoe on behalf of the Wellcome Sanger Institute COVID-19 Surveillance Team
EPI_ISL_932487, EPI_ISL_932488	Lighthouse Lab in Glasgow	Wellcome Sanger Institute for the COVID-19 Genomics UK (COG-UK) Consortium	Harper VanSteenhouse, Yumi Kasai, David Gray, Carol Clugston, Anna Dominiczak and Alex Alderton, Roberto Amato, Sonia Goncalves, Ewan Harrison, David K. Jackson, Ian Johnston, Dominic Kwiatkowski, Cordelia Langford, John Sillitoe on behalf of the Wellcome Sanger Institute COVID-19 Surveillance Team
EPI_ISL_932489	Lighthouse Lab in Alderley Park	Wellcome Sanger Institute for the COVID-19 Genomics UK (COG-UK) Consortium	Jacquelyn Wynn, Mairead Hyland, The Lighthouse Lab in Alderley Park and Alex Alderton, Roberto Amato, Sonia Goncalves, Ewan Harrison, David K. Jackson, Ian Johnston, Dominic Kwiatkowski, Cordelia Langford, John Sillitoe on behalf of the Wellcome Sanger Institute COVID-19 Surveillance Team
EPI_ISL_932490, EPI_ISL_932493, EPI_ISL_932494	Lighthouse Lab in Glasgow	Wellcome Sanger Institute for the COVID-19 Genomics UK (COG-UK) Consortium	Harper VanSteenhouse, Yumi Kasai, David Gray, Carol Clugston, Anna Dominiczak and Alex Alderton, Roberto Amato, Sonia Goncalves, Ewan Harrison, David K. Jackson, Ian Johnston, Dominic Kwiatkowski, Cordelia Langford, John Sillitoe on behalf of the Wellcome Sanger Institute COVID-19 Surveillance Team
EPI_ISL_932496	Lighthouse Lab in Milton Keynes	Wellcome Sanger Institute for the COVID-19 Genomics UK (COG-UK) Consortium	The Lighthouse Lab in Milton Keynes and Alex Alderton, Roberto Amato, Sonia Goncalves, Ewan Harrison, David K. Jackson, Ian Johnston, Dominic Kwiatkowski, Cordelia Langford, John Sillitoe on behalf of the Wellcome Sanger Institute COVID-19 Surveillance Team
EPI_ISL_932497, EPI_ISL_932498, EPI_ISL_932499, EPI_ISL_932500, EPI_ISL_932501	Lighthouse Lab in Glasgow	Wellcome Sanger Institute for the COVID-19 Genomics UK (COG-UK) Consortium	Harper VanSteenhouse, Yumi Kasai, David Gray, Carol Clugston, Anna Dominiczak and Alex Alderton, Roberto Amato, Sonia Goncalves, Ewan Harrison, David K. Jackson, Ian Johnston, Dominic Kwiatkowski, Cordelia Langford, John Sillitoe on behalf of the Wellcome Sanger Institute COVID-19 Surveillance Team
EPI_ISL_932502, EPI_ISL_932503	Lighthouse Lab in Alderley Park	Wellcome Sanger Institute for the COVID-19 Genomics UK (COG-UK) Consortium	Jacquelyn Wynn, Mairead Hyland, The Lighthouse Lab in Alderley Park and Alex Alderton, Roberto Amato, Sonia Goncalves, Ewan Harrison, David K. Jackson, Ian Johnston, Dominic Kwiatkowski, Cordelia Langford, John Sillitoe on behalf of the Wellcome Sanger Institute COVID-19 Surveillance Team
EPI_ISL_932506	Lighthouse Lab in Milton Keynes	Wellcome Sanger Institute for the COVID-19 Genomics UK (COG-UK) Consortium	The Lighthouse Lab in Milton Keynes and Alex Alderton, Roberto Amato, Sonia Goncalves, Ewan Harrison, David K. Jackson, Ian Johnston, Dominic Kwiatkowski, Cordelia Langford, John Sillitoe on behalf of the Wellcome Sanger Institute COVID-19 Surveillance Team
EPI_ISL_932511, EPI_ISL_932512	Lighthouse Lab in Alderley Park	Wellcome Sanger Institute for the COVID-19 Genomics UK (COG-UK) Consortium	Jacquelyn Wynn, Mairead Hyland, The Lighthouse Lab in Alderley Park and Alex Alderton, Roberto Amato, Sonia Goncalves, Ewan Harrison, David K. Jackson, Ian Johnston, Dominic Kwiatkowski, Cordelia Langford, John Sillitoe on behalf of the Wellcome Sanger Institute COVID-19 Surveillance Team
EPI_ISL_932513	Lighthouse Lab in Glasgow	Wellcome Sanger Institute for the COVID-19 Genomics UK (COG-UK) Consortium	Harper VanSteenhouse, Yumi Kasai, David Gray, Carol Clugston, Anna Dominiczak and Alex Alderton, Roberto Amato, Sonia Goncalves, Ewan Harrison, David K. Jackson, Ian Johnston, Dominic Kwiatkowski, Cordelia Langford, John Sillitoe on behalf of the Wellcome Sanger Institute COVID-19 Surveillance Team
EPI_ISL_932515, EPI_ISL_932517	Lighthouse Lab in Alderley Park	Wellcome Sanger Institute for the COVID-19 Genomics UK (COG-UK) Consortium	Jacquelyn Wynn, Mairead Hyland, The Lighthouse Lab in Alderley Park and Alex Alderton, Roberto Amato, Sonia Goncalves, Ewan Harrison, David K. Jackson, Ian Johnston, Dominic Kwiatkowski, Cordelia Langford, John Sillitoe on behalf of the Wellcome Sanger Institute COVID-19 Surveillance Team
EPI_ISL_932518	Lighthouse Lab in Glasgow	Wellcome Sanger Institute for the COVID-19 Genomics UK (COG-UK) Consortium	Harper VanSteenhouse, Yumi Kasai, David Gray, Carol Clugston, Anna Dominiczak and Alex Alderton, Roberto Amato, Sonia Goncalves, Ewan Harrison, David K. Jackson, Ian Johnston, Dominic Kwiatkowski, Cordelia Langford, John Sillitoe on behalf of the Wellcome Sanger Institute COVID-19 Surveillance Team
EPI_ISL_932522	Lighthouse Lab in Alderley Park	Wellcome Sanger Institute for the COVID-19 Genomics UK (COG-UK) Consortium	Jacquelyn Wynn, Mairead Hyland, The Lighthouse Lab in Alderley Park and Alex Alderton, Roberto Amato, Sonia Goncalves, Ewan Harrison, David K. Jackson, Ian Johnston, Dominic Kwiatkowski, Cordelia Langford, John Sillitoe on behalf of the Wellcome Sanger Institute COVID-19 Surveillance Team
EPI_ISL_932523, EPI_ISL_932524, EPI_ISL_932526	Lighthouse Lab in Glasgow	Wellcome Sanger Institute for the COVID-19 Genomics UK (COG-UK) Consortium	Harper VanSteenhouse, Yumi Kasai, David Gray, Carol Clugston, Anna Dominiczak and Alex Alderton, Roberto Amato, Sonia Goncalves, Ewan Harrison, David K. Jackson, Ian Johnston, Dominic Kwiatkowski, Cordelia Langford, John Sillitoe on behalf of the Wellcome Sanger Institute COVID-19 Surveillance Team
EPI_ISL_932528	Lighthouse Lab in Milton Keynes	Wellcome Sanger Institute for the COVID-19 Genomics UK	The Lighthouse Lab in Milton Keynes and Alex Alderton, Roberto Amato, Sonia Goncalves, Ewan Harrison, David K. Jackson, Ian Johnston, Dominic

		(COG-UK) Consortium	Kwiatkowski, Cordelia Langford, John Sillitoe on behalf of the Wellcome Sanger Institute COVID-19 Surveillance Team
EPI_ISL_932530	Lighthouse Lab in Alderley Park	Wellcome Sanger Institute for the COVID-19 Genomics UK (COG-UK) Consortium	Jacquelyn Wynn, Mairead Hyland, The Lighthouse Lab in Alderley Park and Alex Alderton, Roberto Amato, Sonia Goncalves, Ewan Harrison, David K. Jackson, Ian Johnston, Dominic Kwiatkowski, Cordelia Langford, John Sillitoe on behalf of the Wellcome Sanger Institute COVID-19 Surveillance Team
EPI_ISL_932531	Lighthouse Lab in Glasgow	Wellcome Sanger Institute for the COVID-19 Genomics UK (COG-UK) Consortium	Harper VanSteenhouse, Yumi Kasai, David Gray, Carol Clugston, Anna Dominiczak and Alex Alderton, Roberto Amato, Sonia Goncalves, Ewan Harrison, David K. Jackson, Ian Johnston, Dominic Kwiatkowski, Cordelia Langford, John Sillitoe on behalf of the Wellcome Sanger Institute COVID-19 Surveillance Team
EPI_ISL_932532	Lighthouse Lab in Alderley Park	Wellcome Sanger Institute for the COVID-19 Genomics UK (COG-UK) Consortium	Jacquelyn Wynn, Mairead Hyland, The Lighthouse Lab in Alderley Park and Alex Alderton, Roberto Amato, Sonia Goncalves, Ewan Harrison, David K. Jackson, Ian Johnston, Dominic Kwiatkowski, Cordelia Langford, John Sillitoe on behalf of the Wellcome Sanger Institute COVID-19 Surveillance Team
EPI_ISL_932533, EPI_ISL_932534, EPI_ISL_932535, EPI_ISL_932536, EPI_ISL_932537, EPI_ISL_932538	Lighthouse Lab in Glasgow	Wellcome Sanger Institute for the COVID-19 Genomics UK (COG-UK) Consortium	Harper VanSteenhouse, Yumi Kasai, David Gray, Carol Clugston, Anna Dominiczak and Alex Alderton, Roberto Amato, Sonia Goncalves, Ewan Harrison, David K. Jackson, Ian Johnston, Dominic Kwiatkowski, Cordelia Langford, John Sillitoe on behalf of the Wellcome Sanger Institute COVID-19 Surveillance Team
EPI_ISL_932539	Lighthouse Lab in Alderley Park	Wellcome Sanger Institute for the COVID-19 Genomics UK (COG-UK) Consortium	Jacquelyn Wynn, Mairead Hyland, The Lighthouse Lab in Alderley Park and Alex Alderton, Roberto Amato, Sonia Goncalves, Ewan Harrison, David K. Jackson, Ian Johnston, Dominic Kwiatkowski, Cordelia Langford, John Sillitoe on behalf of the Wellcome Sanger Institute COVID-19 Surveillance Team
EPI_ISL_932540, EPI_ISL_932541	Lighthouse Lab in Glasgow	Wellcome Sanger Institute for the COVID-19 Genomics UK (COG-UK) Consortium	Harper VanSteenhouse, Yumi Kasai, David Gray, Carol Clugston, Anna Dominiczak and Alex Alderton, Roberto Amato, Sonia Goncalves, Ewan Harrison, David K. Jackson, Ian Johnston, Dominic Kwiatkowski, Cordelia Langford, John Sillitoe on behalf of the Wellcome Sanger Institute COVID-19 Surveillance Team
EPI_ISL_932543	Lighthouse Lab in Alderley Park	Wellcome Sanger Institute for the COVID-19 Genomics UK (COG-UK) Consortium	Jacquelyn Wynn, Mairead Hyland, The Lighthouse Lab in Alderley Park and Alex Alderton, Roberto Amato, Sonia Goncalves, Ewan Harrison, David K. Jackson, Ian Johnston, Dominic Kwiatkowski, Cordelia Langford, John Sillitoe on behalf of the Wellcome Sanger Institute COVID-19 Surveillance Team
EPI_ISL_932544	Lighthouse Lab in Milton Keynes	Wellcome Sanger Institute for the COVID-19 Genomics UK (COG-UK) Consortium	The Lighthouse Lab in Milton Keynes and Alex Alderton, Roberto Amato, Sonia Goncalves, Ewan Harrison, David K. Jackson, Ian Johnston, Dominic Kwiatkowski, Cordelia Langford, John Sillitoe on behalf of the Wellcome Sanger Institute COVID-19 Surveillance Team
EPI_ISL_932545, EPI_ISL_932547	Lighthouse Lab in Glasgow	Wellcome Sanger Institute for the COVID-19 Genomics UK (COG-UK) Consortium	Harper VanSteenhouse, Yumi Kasai, David Gray, Carol Clugston, Anna Dominiczak and Alex Alderton, Roberto Amato, Sonia Goncalves, Ewan Harrison, David K. Jackson, Ian Johnston, Dominic Kwiatkowski, Cordelia Langford, John Sillitoe on behalf of the Wellcome Sanger Institute COVID-19 Surveillance Team
EPI_ISL_932548, EPI_ISL_932550	Lighthouse Lab in Milton Keynes	Wellcome Sanger Institute for the COVID-19 Genomics UK (COG-UK) Consortium	The Lighthouse Lab in Milton Keynes and Alex Alderton, Roberto Amato, Sonia Goncalves, Ewan Harrison, David K. Jackson, Ian Johnston, Dominic Kwiatkowski, Cordelia Langford, John Sillitoe on behalf of the Wellcome Sanger Institute COVID-19 Surveillance Team
EPI_ISL_932552	Lighthouse Lab in Alderley Park	Wellcome Sanger Institute for the COVID-19 Genomics UK (COG-UK) Consortium	Jacquelyn Wynn, Mairead Hyland, The Lighthouse Lab in Alderley Park and Alex Alderton, Roberto Amato, Sonia Goncalves, Ewan Harrison, David K. Jackson, Ian Johnston, Dominic Kwiatkowski, Cordelia Langford, John Sillitoe on behalf of the Wellcome Sanger Institute COVID-19 Surveillance Team
EPI_ISL_932554, EPI_ISL_932556, EPI_ISL_932557, EPI_ISL_932558	Lighthouse Lab in Glasgow	Wellcome Sanger Institute for the COVID-19 Genomics UK (COG-UK) Consortium	Harper VanSteenhouse, Yumi Kasai, David Gray, Carol Clugston, Anna Dominiczak and Alex Alderton, Roberto Amato, Sonia Goncalves, Ewan Harrison, David K. Jackson, Ian Johnston, Dominic Kwiatkowski, Cordelia Langford, John Sillitoe on behalf of the Wellcome Sanger Institute COVID-19 Surveillance Team
EPI_ISL_932559	Lighthouse Lab in Milton Keynes	Wellcome Sanger Institute for the COVID-19 Genomics UK (COG-UK) Consortium	The Lighthouse Lab in Milton Keynes and Alex Alderton, Roberto Amato, Sonia Goncalves, Ewan Harrison, David K. Jackson, Ian Johnston, Dominic Kwiatkowski, Cordelia Langford, John Sillitoe on behalf of the Wellcome Sanger Institute COVID-19 Surveillance Team
EPI_ISL_932562, EPI_ISL_932564, EPI_ISL_932566	Lighthouse Lab in Glasgow	Wellcome Sanger Institute for the COVID-19 Genomics UK (COG-UK) Consortium	Harper VanSteenhouse, Yumi Kasai, David Gray, Carol Clugston, Anna Dominiczak and Alex Alderton, Roberto Amato, Sonia Goncalves, Ewan Harrison, David K. Jackson, Ian Johnston, Dominic Kwiatkowski, Cordelia Langford, John Sillitoe on behalf of the Wellcome Sanger Institute COVID-19 Surveillance Team
EPI_ISL_932567	Lighthouse Lab in Alderley Park	Wellcome Sanger Institute for the COVID-19 Genomics UK (COG-UK) Consortium	Jacquelyn Wynn, Mairead Hyland, The Lighthouse Lab in Alderley Park and Alex Alderton, Roberto Amato, Sonia Goncalves, Ewan Harrison, David K. Jackson, Ian Johnston, Dominic Kwiatkowski, Cordelia Langford, John Sillitoe on behalf of the Wellcome Sanger Institute COVID-19 Surveillance Team
EPI_ISL_932568	Lighthouse Lab in Glasgow	Wellcome Sanger Institute for the COVID-19 Genomics UK (COG-UK) Consortium	Harper VanSteenhouse, Yumi Kasai, David Gray, Carol Clugston, Anna Dominiczak and Alex Alderton, Roberto Amato, Sonia Goncalves, Ewan Harrison, David K. Jackson, Ian Johnston, Dominic Kwiatkowski, Cordelia Langford, John Sillitoe on behalf of the Wellcome Sanger Institute COVID-19 Surveillance Team
EPI_ISL_932571, EPI_ISL_932572, EPI_ISL_932573, EPI_ISL_932574	Lighthouse Lab in Milton Keynes	Wellcome Sanger Institute for the COVID-19 Genomics UK (COG-UK) Consortium	The Lighthouse Lab in Milton Keynes and Alex Alderton, Roberto Amato, Sonia Goncalves, Ewan Harrison, David K. Jackson, Ian Johnston, Dominic Kwiatkowski, Cordelia Langford, John Sillitoe on behalf of the Wellcome Sanger Institute COVID-19 Surveillance Team
EPI_ISL_932576	Lighthouse Lab in Cambridge	Wellcome Sanger Institute for the COVID-19 Genomics UK (COG-UK) Consortium	Rob Howes, The Lighthouse Lab in Cambridge and Alex Alderton, Roberto Amato, Sonia Goncalves, Ewan Harrison, David K. Jackson, Ian Johnston, Dominic Kwiatkowski, Cordelia Langford, John Sillitoe on behalf of the Wellcome Sanger Institute COVID-19 Surveillance Team
EPI_ISL_932577, EPI_ISL_932579	Lighthouse Lab in Milton Keynes	Wellcome Sanger Institute for the COVID-19 Genomics UK (COG-UK) Consortium	The Lighthouse Lab in Milton Keynes and Alex Alderton, Roberto Amato, Sonia Goncalves, Ewan Harrison, David K. Jackson, Ian Johnston, Dominic Kwiatkowski, Cordelia Langford, John Sillitoe on behalf of the Wellcome Sanger Institute COVID-19 Surveillance Team
EPI_ISL_932581	Lighthouse Lab in Cambridge	Wellcome Sanger Institute for the COVID-19 Genomics UK (COG-UK) Consortium	Rob Howes, The Lighthouse Lab in Cambridge and Alex Alderton, Roberto Amato, Sonia Goncalves, Ewan Harrison, David K. Jackson, Ian Johnston, Dominic Kwiatkowski, Cordelia Langford, John Sillitoe on behalf of the Wellcome Sanger Institute COVID-19 Surveillance Team
EPI_ISL_932582, EPI_ISL_932583, EPI_ISL_932586	Lighthouse Lab in Milton Keynes	Wellcome Sanger Institute for the COVID-19 Genomics UK (COG-UK) Consortium	The Lighthouse Lab in Milton Keynes and Alex Alderton, Roberto Amato, Sonia Goncalves, Ewan Harrison, David K. Jackson, Ian Johnston, Dominic Kwiatkowski, Cordelia Langford, John Sillitoe on behalf of the Wellcome Sanger Institute COVID-19 Surveillance Team
EPI_ISL_932588	Lighthouse Lab in Glasgow	Wellcome Sanger Institute for the COVID-19 Genomics UK (COG-UK) Consortium	Harper VanSteenhouse, Yumi Kasai, David Gray, Carol Clugston, Anna Dominiczak and Alex Alderton, Roberto Amato, Sonia Goncalves, Ewan Harrison, David K. Jackson, Ian Johnston, Dominic Kwiatkowski, Cordelia Langford, John Sillitoe on behalf of the Wellcome Sanger Institute COVID-19 Surveillance Team
EPI_ISL_932591, EPI_ISL_932592, EPI_ISL_932593, EPI_ISL_932594	Lighthouse Lab in Milton Keynes	Wellcome Sanger Institute for the COVID-19 Genomics UK (COG-UK) Consortium	The Lighthouse Lab in Milton Keynes and Alex Alderton, Roberto Amato, Sonia Goncalves, Ewan Harrison, David K. Jackson, Ian Johnston, Dominic Kwiatkowski, Cordelia Langford, John Sillitoe on behalf of the Wellcome Sanger Institute COVID-19 Surveillance Team
EPI_ISL_932596, EPI_ISL_932598	Lighthouse Lab in Cambridge	Wellcome Sanger Institute for the COVID-19 Genomics UK (COG-UK) Consortium	Rob Howes, The Lighthouse Lab in Cambridge and Alex Alderton, Roberto Amato, Sonia Goncalves, Ewan Harrison, David K. Jackson, Ian Johnston, Dominic Kwiatkowski, Cordelia Langford, John Sillitoe on behalf of the Wellcome Sanger Institute COVID-19 Surveillance Team
EPI_ISL_932599	Lighthouse Lab in Alderley Park	Wellcome Sanger Institute for the COVID-19 Genomics UK (COG-UK) Consortium	Jacquelyn Wynn, Mairead Hyland, The Lighthouse Lab in Alderley Park and Alex Alderton, Roberto Amato, Sonia Goncalves, Ewan Harrison, David K. Jackson, Ian Johnston, Dominic Kwiatkowski, Cordelia Langford, John Sillitoe on behalf of the Wellcome Sanger Institute COVID-19 Surveillance Team
EPI_ISL_932600, EPI_ISL_932601, EPI_ISL_932602	Lighthouse Lab in Milton Keynes	Wellcome Sanger Institute for the COVID-19 Genomics UK (COG-UK) Consortium	The Lighthouse Lab in Milton Keynes and Alex Alderton, Roberto Amato, Sonia Goncalves, Ewan Harrison, David K. Jackson, Ian Johnston, Dominic Kwiatkowski, Cordelia Langford, John Sillitoe on behalf of the Wellcome Sanger Institute COVID-19 Surveillance Team
EPI_ISL_932603	Lighthouse Lab in Cambridge	Wellcome Sanger Institute for the COVID-19 Genomics UK (COG-UK) Consortium	Rob Howes, The Lighthouse Lab in Cambridge and Alex Alderton, Roberto Amato, Sonia Goncalves, Ewan Harrison, David K. Jackson, Ian Johnston, Dominic Kwiatkowski, Cordelia Langford, John Sillitoe on behalf of the Wellcome Sanger Institute COVID-19 Surveillance Team
EPI_ISL_932605	Lighthouse Lab in Milton Keynes	Wellcome Sanger Institute for the COVID-19 Genomics UK (COG-UK) Consortium	The Lighthouse Lab in Milton Keynes and Alex Alderton, Roberto Amato, Sonia Goncalves, Ewan Harrison, David K. Jackson, Ian Johnston, Dominic Kwiatkowski, Cordelia Langford, John Sillitoe on behalf of the Wellcome Sanger Institute COVID-19 Surveillance Team
EPI_ISL_932606	Lighthouse Lab in Glasgow	Wellcome Sanger Institute for the COVID-19 Genomics UK (COG-UK) Consortium	Harper VanSteenhouse, Yumi Kasai, David Gray, Carol Clugston, Anna Dominiczak and Alex Alderton, Roberto Amato, Sonia Goncalves, Ewan Harrison, David K. Jackson, Ian Johnston, Dominic Kwiatkowski, Cordelia Langford, John Sillitoe on behalf of the Wellcome Sanger Institute COVID-19 Surveillance Team
EPI_ISL_932607	Lighthouse Lab in Alderley Park	Wellcome Sanger Institute for the COVID-19 Genomics UK (COG-UK) Consortium	Jacquelyn Wynn, Mairead Hyland, The Lighthouse Lab in Alderley Park and Alex Alderton, Roberto Amato, Sonia Goncalves, Ewan Harrison, David K. Jackson, Ian Johnston, Dominic Kwiatkowski, Cordelia Langford, John Sillitoe on behalf of the Wellcome Sanger Institute COVID-19 Surveillance Team
EPI_ISL_932608, EPI_ISL_932609	Lighthouse Lab in Milton Keynes	Wellcome Sanger Institute for the COVID-19 Genomics UK (COG-UK) Consortium	The Lighthouse Lab in Milton Keynes and Alex Alderton, Roberto Amato, Sonia Goncalves, Ewan Harrison, David K. Jackson, Ian Johnston, Dominic Kwiatkowski, Cordelia Langford, John Sillitoe on behalf of the Wellcome Sanger Institute COVID-19 Surveillance Team

EPI_ISL_932611	Lighthouse Lab in Cambridge	Wellcome Sanger Institute for the COVID-19 Genomics UK (COG-UK) Consortium	Rob Howes, The Lighthouse Lab in Cambridge and Alex Alderton, Roberto Amato, Sonia Goncalves, Ewan Harrison, David K. Jackson, Ian Johnston, Dominic Kwiatkowski, Cordelia Langford, John Sillitoe on behalf of the Wellcome Sanger Institute COVID-19 Surveillance Team
EPI_ISL_932612	Lighthouse Lab in Milton Keynes	Wellcome Sanger Institute for the COVID-19 Genomics UK (COG-UK) Consortium	The Lighthouse Lab in Milton Keynes and Alex Alderton, Roberto Amato, Sonia Goncalves, Ewan Harrison, David K. Jackson, Ian Johnston, Dominic Kwiatkowski, Cordelia Langford, John Sillitoe on behalf of the Wellcome Sanger Institute COVID-19 Surveillance Team
EPI_ISL_932613, EPI_ISL_932614	Lighthouse Lab in Cambridge	Wellcome Sanger Institute for the COVID-19 Genomics UK (COG-UK) Consortium	Rob Howes, The Lighthouse Lab in Cambridge and Alex Alderton, Roberto Amato, Sonia Goncalves, Ewan Harrison, David K. Jackson, Ian Johnston, Dominic Kwiatkowski, Cordelia Langford, John Sillitoe on behalf of the Wellcome Sanger Institute COVID-19 Surveillance Team
EPI_ISL_932615, EPI_ISL_932616, EPI_ISL_932617	Lighthouse Lab in Milton Keynes	Wellcome Sanger Institute for the COVID-19 Genomics UK (COG-UK) Consortium	The Lighthouse Lab in Milton Keynes and Alex Alderton, Roberto Amato, Sonia Goncalves, Ewan Harrison, David K. Jackson, Ian Johnston, Dominic Kwiatkowski, Cordelia Langford, John Sillitoe on behalf of the Wellcome Sanger Institute COVID-19 Surveillance Team
EPI_ISL_932619	Lighthouse Lab in Cambridge	Wellcome Sanger Institute for the COVID-19 Genomics UK (COG-UK) Consortium	Rob Howes, The Lighthouse Lab in Cambridge and Alex Alderton, Roberto Amato, Sonia Goncalves, Ewan Harrison, David K. Jackson, Ian Johnston, Dominic Kwiatkowski, Cordelia Langford, John Sillitoe on behalf of the Wellcome Sanger Institute COVID-19 Surveillance Team
EPI_ISL_932620	Lighthouse Lab in Alderley Park	Wellcome Sanger Institute for the COVID-19 Genomics UK (COG-UK) Consortium	Jacquelyn Wynn, Mairead Hyland, The Lighthouse Lab in Alderley Park and Alex Alderton, Roberto Amato, Sonia Goncalves, Ewan Harrison, David K. Jackson, Ian Johnston, Dominic Kwiatkowski, Cordelia Langford, John Sillitoe on behalf of the Wellcome Sanger Institute COVID-19 Surveillance Team
EPI_ISL_932621	Lighthouse Lab in Cambridge	Wellcome Sanger Institute for the COVID-19 Genomics UK (COG-UK) Consortium	Rob Howes, The Lighthouse Lab in Cambridge and Alex Alderton, Roberto Amato, Sonia Goncalves, Ewan Harrison, David K. Jackson, Ian Johnston, Dominic Kwiatkowski, Cordelia Langford, John Sillitoe on behalf of the Wellcome Sanger Institute COVID-19 Surveillance Team
EPI_ISL_932622, EPI_ISL_932623, EPI_ISL_932625, EPI_ISL_932626, EPI_ISL_932629, EPI_ISL_932632, EPI_ISL_932633, EPI_ISL_932635, EPI_ISL_932636, EPI_ISL_932637	Lighthouse Lab in Milton Keynes	Wellcome Sanger Institute for the COVID-19 Genomics UK (COG-UK) Consortium	The Lighthouse Lab in Milton Keynes and Alex Alderton, Roberto Amato, Sonia Goncalves, Ewan Harrison, David K. Jackson, Ian Johnston, Dominic Kwiatkowski, Cordelia Langford, John Sillitoe on behalf of the Wellcome Sanger Institute COVID-19 Surveillance Team
EPI_ISL_932640	Lighthouse Lab in Alderley Park	Wellcome Sanger Institute for the COVID-19 Genomics UK (COG-UK) Consortium	Jacquelyn Wynn, Mairead Hyland, The Lighthouse Lab in Alderley Park and Alex Alderton, Roberto Amato, Sonia Goncalves, Ewan Harrison, David K. Jackson, Ian Johnston, Dominic Kwiatkowski, Cordelia Langford, John Sillitoe on behalf of the Wellcome Sanger Institute COVID-19 Surveillance Team
EPI_ISL_932641, EPI_ISL_932642	Lighthouse Lab in Milton Keynes	Wellcome Sanger Institute for the COVID-19 Genomics UK (COG-UK) Consortium	The Lighthouse Lab in Milton Keynes and Alex Alderton, Roberto Amato, Sonia Goncalves, Ewan Harrison, David K. Jackson, Ian Johnston, Dominic Kwiatkowski, Cordelia Langford, John Sillitoe on behalf of the Wellcome Sanger Institute COVID-19 Surveillance Team
EPI_ISL_932644	Lighthouse Lab in Cambridge	Wellcome Sanger Institute for the COVID-19 Genomics UK (COG-UK) Consortium	Rob Howes, The Lighthouse Lab in Cambridge and Alex Alderton, Roberto Amato, Sonia Goncalves, Ewan Harrison, David K. Jackson, Ian Johnston, Dominic Kwiatkowski, Cordelia Langford, John Sillitoe on behalf of the Wellcome Sanger Institute COVID-19 Surveillance Team
EPI_ISL_932645	Lighthouse Lab in Alderley Park	Wellcome Sanger Institute for the COVID-19 Genomics UK (COG-UK) Consortium	Jacquelyn Wynn, Mairead Hyland, The Lighthouse Lab in Alderley Park and Alex Alderton, Roberto Amato, Sonia Goncalves, Ewan Harrison, David K. Jackson, Ian Johnston, Dominic Kwiatkowski, Cordelia Langford, John Sillitoe on behalf of the Wellcome Sanger Institute COVID-19 Surveillance Team
EPI_ISL_932646	Lighthouse Lab in Cambridge	Wellcome Sanger Institute for the COVID-19 Genomics UK (COG-UK) Consortium	Rob Howes, The Lighthouse Lab in Cambridge and Alex Alderton, Roberto Amato, Sonia Goncalves, Ewan Harrison, David K. Jackson, Ian Johnston, Dominic Kwiatkowski, Cordelia Langford, John Sillitoe on behalf of the Wellcome Sanger Institute COVID-19 Surveillance Team
EPI_ISL_932647	Lighthouse Lab in Alderley Park	Wellcome Sanger Institute for the COVID-19 Genomics UK (COG-UK) Consortium	Jacquelyn Wynn, Mairead Hyland, The Lighthouse Lab in Alderley Park and Alex Alderton, Roberto Amato, Sonia Goncalves, Ewan Harrison, David K. Jackson, Ian Johnston, Dominic Kwiatkowski, Cordelia Langford, John Sillitoe on behalf of the Wellcome Sanger Institute COVID-19 Surveillance Team
EPI_ISL_932648	Lighthouse Lab in Cambridge	Wellcome Sanger Institute for the COVID-19 Genomics UK (COG-UK) Consortium	Rob Howes, The Lighthouse Lab in Cambridge and Alex Alderton, Roberto Amato, Sonia Goncalves, Ewan Harrison, David K. Jackson, Ian Johnston, Dominic Kwiatkowski, Cordelia Langford, John Sillitoe on behalf of the Wellcome Sanger Institute COVID-19 Surveillance Team
EPI_ISL_932649, EPI_ISL_932650	Lighthouse Lab in Milton Keynes	Wellcome Sanger Institute for the COVID-19 Genomics UK (COG-UK) Consortium	The Lighthouse Lab in Milton Keynes and Alex Alderton, Roberto Amato, Sonia Goncalves, Ewan Harrison, David K. Jackson, Ian Johnston, Dominic Kwiatkowski, Cordelia Langford, John Sillitoe on behalf of the Wellcome Sanger Institute COVID-19 Surveillance Team
EPI_ISL_932651, EPI_ISL_932652	Lighthouse Lab in Cambridge	Wellcome Sanger Institute for the COVID-19 Genomics UK (COG-UK) Consortium	Rob Howes, The Lighthouse Lab in Cambridge and Alex Alderton, Roberto Amato, Sonia Goncalves, Ewan Harrison, David K. Jackson, Ian Johnston, Dominic Kwiatkowski, Cordelia Langford, John Sillitoe on behalf of the Wellcome Sanger Institute COVID-19 Surveillance Team
EPI_ISL_932653	Lighthouse Lab in Milton Keynes	Wellcome Sanger Institute for the COVID-19 Genomics UK (COG-UK) Consortium	The Lighthouse Lab in Milton Keynes and Alex Alderton, Roberto Amato, Sonia Goncalves, Ewan Harrison, David K. Jackson, Ian Johnston, Dominic Kwiatkowski, Cordelia Langford, John Sillitoe on behalf of the Wellcome Sanger Institute COVID-19 Surveillance Team
EPI_ISL_932655	Lighthouse Lab in Cambridge	Wellcome Sanger Institute for the COVID-19 Genomics UK (COG-UK) Consortium	Rob Howes, The Lighthouse Lab in Cambridge and Alex Alderton, Roberto Amato, Sonia Goncalves, Ewan Harrison, David K. Jackson, Ian Johnston, Dominic Kwiatkowski, Cordelia Langford, John Sillitoe on behalf of the Wellcome Sanger Institute COVID-19 Surveillance Team
EPI_ISL_932657, EPI_ISL_932658	Lighthouse Lab in Milton Keynes	Wellcome Sanger Institute for the COVID-19 Genomics UK (COG-UK) Consortium	The Lighthouse Lab in Milton Keynes and Alex Alderton, Roberto Amato, Sonia Goncalves, Ewan Harrison, David K. Jackson, Ian Johnston, Dominic Kwiatkowski, Cordelia Langford, John Sillitoe on behalf of the Wellcome Sanger Institute COVID-19 Surveillance Team
EPI_ISL_932661	Lighthouse Lab in Alderley Park	Wellcome Sanger Institute for the COVID-19 Genomics UK (COG-UK) Consortium	Jacquelyn Wynn, Mairead Hyland, The Lighthouse Lab in Alderley Park and Alex Alderton, Roberto Amato, Sonia Goncalves, Ewan Harrison, David K. Jackson, Ian Johnston, Dominic Kwiatkowski, Cordelia Langford, John Sillitoe on behalf of the Wellcome Sanger Institute COVID-19 Surveillance Team
EPI_ISL_932662, EPI_ISL_932663	Lighthouse Lab in Milton Keynes	Wellcome Sanger Institute for the COVID-19 Genomics UK (COG-UK) Consortium	The Lighthouse Lab in Milton Keynes and Alex Alderton, Roberto Amato, Sonia Goncalves, Ewan Harrison, David K. Jackson, Ian Johnston, Dominic Kwiatkowski, Cordelia Langford, John Sillitoe on behalf of the Wellcome Sanger Institute COVID-19 Surveillance Team
EPI_ISL_932664	Lighthouse Lab in Cambridge	Wellcome Sanger Institute for the COVID-19 Genomics UK (COG-UK) Consortium	Rob Howes, The Lighthouse Lab in Cambridge and Alex Alderton, Roberto Amato, Sonia Goncalves, Ewan Harrison, David K. Jackson, Ian Johnston, Dominic Kwiatkowski, Cordelia Langford, John Sillitoe on behalf of the Wellcome Sanger Institute COVID-19 Surveillance Team
EPI_ISL_932665	Lighthouse Lab in Milton Keynes	Wellcome Sanger Institute for the COVID-19 Genomics UK (COG-UK) Consortium	The Lighthouse Lab in Milton Keynes and Alex Alderton, Roberto Amato, Sonia Goncalves, Ewan Harrison, David K. Jackson, Ian Johnston, Dominic Kwiatkowski, Cordelia Langford, John Sillitoe on behalf of the Wellcome Sanger Institute COVID-19 Surveillance Team
EPI_ISL_932666	Lighthouse Lab in Cambridge	Wellcome Sanger Institute for the COVID-19 Genomics UK (COG-UK) Consortium	Rob Howes, The Lighthouse Lab in Cambridge and Alex Alderton, Roberto Amato, Sonia Goncalves, Ewan Harrison, David K. Jackson, Ian Johnston, Dominic Kwiatkowski, Cordelia Langford, John Sillitoe on behalf of the Wellcome Sanger Institute COVID-19 Surveillance Team
EPI_ISL_932667	Lighthouse Lab in Milton Keynes	Wellcome Sanger Institute for the COVID-19 Genomics UK (COG-UK) Consortium	The Lighthouse Lab in Milton Keynes and Alex Alderton, Roberto Amato, Sonia Goncalves, Ewan Harrison, David K. Jackson, Ian Johnston, Dominic Kwiatkowski, Cordelia Langford, John Sillitoe on behalf of the Wellcome Sanger Institute COVID-19 Surveillance Team
EPI_ISL_932668	Lighthouse Lab in Alderley Park	Wellcome Sanger Institute for the COVID-19 Genomics UK (COG-UK) Consortium	Jacquelyn Wynn, Mairead Hyland, The Lighthouse Lab in Alderley Park and Alex Alderton, Roberto Amato, Sonia Goncalves, Ewan Harrison, David K. Jackson, Ian Johnston, Dominic Kwiatkowski, Cordelia Langford, John Sillitoe on behalf of the Wellcome Sanger Institute COVID-19 Surveillance Team
EPI_ISL_932669	Lighthouse Lab in Cambridge	Wellcome Sanger Institute for the COVID-19 Genomics UK (COG-UK) Consortium	Rob Howes, The Lighthouse Lab in Cambridge and Alex Alderton, Roberto Amato, Sonia Goncalves, Ewan Harrison, David K. Jackson, Ian Johnston, Dominic Kwiatkowski, Cordelia Langford, John Sillitoe on behalf of the Wellcome Sanger Institute COVID-19 Surveillance Team
EPI_ISL_932670	Lighthouse Lab in Alderley Park	Wellcome Sanger Institute for the COVID-19 Genomics UK (COG-UK) Consortium	Jacquelyn Wynn, Mairead Hyland, The Lighthouse Lab in Alderley Park and Alex Alderton, Roberto Amato, Sonia Goncalves, Ewan Harrison, David K. Jackson, Ian Johnston, Dominic Kwiatkowski, Cordelia Langford, John Sillitoe on behalf of the Wellcome Sanger Institute COVID-19 Surveillance Team
EPI_ISL_932671	Lighthouse Lab in Cambridge	Wellcome Sanger Institute for the COVID-19 Genomics UK (COG-UK) Consortium	Rob Howes, The Lighthouse Lab in Cambridge and Alex Alderton, Roberto Amato, Sonia Goncalves, Ewan Harrison, David K. Jackson, Ian Johnston, Dominic Kwiatkowski, Cordelia Langford, John Sillitoe on behalf of the Wellcome Sanger Institute COVID-19 Surveillance Team
EPI_ISL_932672	Lighthouse Lab in Milton Keynes	Wellcome Sanger Institute for the COVID-19 Genomics UK (COG-UK) Consortium	The Lighthouse Lab in Milton Keynes and Alex Alderton, Roberto Amato, Sonia Goncalves, Ewan Harrison, David K. Jackson, Ian Johnston, Dominic Kwiatkowski, Cordelia Langford, John Sillitoe on behalf of the Wellcome Sanger Institute COVID-19 Surveillance Team
EPI_ISL_932673	Lighthouse Lab in Alderley Park	Wellcome Sanger Institute for the COVID-19 Genomics UK (COG-UK) Consortium	Jacquelyn Wynn, Mairead Hyland, The Lighthouse Lab in Alderley Park and Alex Alderton, Roberto Amato, Sonia Goncalves, Ewan Harrison, David K. Jackson, Ian Johnston, Dominic Kwiatkowski, Cordelia Langford, John Sillitoe on behalf of the Wellcome Sanger Institute COVID-19 Surveillance Team
EPI_ISL_932674	Lighthouse Lab in Cambridge	Wellcome Sanger Institute for the COVID-19 Genomics UK (COG-UK) Consortium	Rob Howes, The Lighthouse Lab in Cambridge and Alex Alderton, Roberto Amato, Sonia Goncalves, Ewan Harrison, David K. Jackson, Ian Johnston, Dominic Kwiatkowski, Cordelia Langford, John Sillitoe on behalf of the Wellcome Sanger Institute COVID-19 Surveillance Team
EPI_ISL_932675, EPI_ISL_932676	Lighthouse Lab in Milton Keynes	Wellcome Sanger Institute for the COVID-19 Genomics UK (COG-UK) Consortium	The Lighthouse Lab in Milton Keynes and Alex Alderton, Roberto Amato, Sonia Goncalves, Ewan Harrison, David K. Jackson, Ian Johnston, Dominic Kwiatkowski, Cordelia Langford, John Sillitoe on behalf of the Wellcome Sanger Institute COVID-19 Surveillance Team

EPI_ISL_932677, EPI_ISL_932678	Lighthouse Lab in Cambridge	Wellcome Sanger Institute for the COVID-19 Genomics UK (COG-UK) Consortium	Rob Howes, The Lighthouse Lab in Cambridge and Alex Alderton, Roberto Amato, Sonia Goncalves, Ewan Harrison, David K. Jackson, Ian Johnston, Dominic Kwiatkowski, Cordelia Langford, John Sillitoe on behalf of the Wellcome Sanger Institute COVID-19 Surveillance Team
EPI_ISL_932679	Lighthouse Lab in Milton Keynes	Wellcome Sanger Institute for the COVID-19 Genomics UK (COG-UK) Consortium	The Lighthouse Lab in Milton Keynes and Alex Alderton, Roberto Amato, Sonia Goncalves, Ewan Harrison, David K. Jackson, Ian Johnston, Dominic Kwiatkowski, Cordelia Langford, John Sillitoe on behalf of the Wellcome Sanger Institute COVID-19 Surveillance Team
EPI_ISL_932680	Lighthouse Lab in Cambridge	Wellcome Sanger Institute for the COVID-19 Genomics UK (COG-UK) Consortium	Rob Howes, The Lighthouse Lab in Cambridge and Alex Alderton, Roberto Amato, Sonia Goncalves, Ewan Harrison, David K. Jackson, Ian Johnston, Dominic Kwiatkowski, Cordelia Langford, John Sillitoe on behalf of the Wellcome Sanger Institute COVID-19 Surveillance Team
EPI_ISL_932682, EPI_ISL_932683, EPI_ISL_932684	Lighthouse Lab in Milton Keynes	Wellcome Sanger Institute for the COVID-19 Genomics UK (COG-UK) Consortium	The Lighthouse Lab in Milton Keynes and Alex Alderton, Roberto Amato, Sonia Goncalves, Ewan Harrison, David K. Jackson, Ian Johnston, Dominic Kwiatkowski, Cordelia Langford, John Sillitoe on behalf of the Wellcome Sanger Institute COVID-19 Surveillance Team
EPI_ISL_932686, EPI_ISL_932687, EPI_ISL_932689	Lighthouse Lab in Cambridge	Wellcome Sanger Institute for the COVID-19 Genomics UK (COG-UK) Consortium	Rob Howes, The Lighthouse Lab in Cambridge and Alex Alderton, Roberto Amato, Sonia Goncalves, Ewan Harrison, David K. Jackson, Ian Johnston, Dominic Kwiatkowski, Cordelia Langford, John Sillitoe on behalf of the Wellcome Sanger Institute COVID-19 Surveillance Team
EPI_ISL_932690	Lighthouse Lab in Alderley Park	Wellcome Sanger Institute for the COVID-19 Genomics UK (COG-UK) Consortium	Jacquelyn Wynn, Mairead Hyland, The Lighthouse Lab in Alderley Park and Alex Alderton, Roberto Amato, Sonia Goncalves, Ewan Harrison, David K. Jackson, Ian Johnston, Dominic Kwiatkowski, Cordelia Langford, John Sillitoe on behalf of the Wellcome Sanger Institute COVID-19 Surveillance Team
EPI_ISL_932691	Lighthouse Lab in Cambridge	Wellcome Sanger Institute for the COVID-19 Genomics UK (COG-UK) Consortium	Rob Howes, The Lighthouse Lab in Cambridge and Alex Alderton, Roberto Amato, Sonia Goncalves, Ewan Harrison, David K. Jackson, Ian Johnston, Dominic Kwiatkowski, Cordelia Langford, John Sillitoe on behalf of the Wellcome Sanger Institute COVID-19 Surveillance Team
EPI_ISL_932692	Lighthouse Lab in Milton Keynes	Wellcome Sanger Institute for the COVID-19 Genomics UK (COG-UK) Consortium	The Lighthouse Lab in Milton Keynes and Alex Alderton, Roberto Amato, Sonia Goncalves, Ewan Harrison, David K. Jackson, Ian Johnston, Dominic Kwiatkowski, Cordelia Langford, John Sillitoe on behalf of the Wellcome Sanger Institute COVID-19 Surveillance Team
EPI_ISL_932693	Lighthouse Lab in Cambridge	Wellcome Sanger Institute for the COVID-19 Genomics UK (COG-UK) Consortium	Rob Howes, The Lighthouse Lab in Cambridge and Alex Alderton, Roberto Amato, Sonia Goncalves, Ewan Harrison, David K. Jackson, Ian Johnston, Dominic Kwiatkowski, Cordelia Langford, John Sillitoe on behalf of the Wellcome Sanger Institute COVID-19 Surveillance Team
EPI_ISL_932695	Lighthouse Lab in Milton Keynes	Wellcome Sanger Institute for the COVID-19 Genomics UK (COG-UK) Consortium	The Lighthouse Lab in Milton Keynes and Alex Alderton, Roberto Amato, Sonia Goncalves, Ewan Harrison, David K. Jackson, Ian Johnston, Dominic Kwiatkowski, Cordelia Langford, John Sillitoe on behalf of the Wellcome Sanger Institute COVID-19 Surveillance Team
EPI_ISL_932696	Lighthouse Lab in Alderley Park	Wellcome Sanger Institute for the COVID-19 Genomics UK (COG-UK) Consortium	Jacquelyn Wynn, Mairead Hyland, The Lighthouse Lab in Alderley Park and Alex Alderton, Roberto Amato, Sonia Goncalves, Ewan Harrison, David K. Jackson, Ian Johnston, Dominic Kwiatkowski, Cordelia Langford, John Sillitoe on behalf of the Wellcome Sanger Institute COVID-19 Surveillance Team
EPI_ISL_932697, EPI_ISL_932700, EPI_ISL_932701, EPI_ISL_932702	Lighthouse Lab in Milton Keynes	Wellcome Sanger Institute for the COVID-19 Genomics UK (COG-UK) Consortium	The Lighthouse Lab in Milton Keynes and Alex Alderton, Roberto Amato, Sonia Goncalves, Ewan Harrison, David K. Jackson, Ian Johnston, Dominic Kwiatkowski, Cordelia Langford, John Sillitoe on behalf of the Wellcome Sanger Institute COVID-19 Surveillance Team
EPI_ISL_932703	Lighthouse Lab in Cambridge	Wellcome Sanger Institute for the COVID-19 Genomics UK (COG-UK) Consortium	Rob Howes, The Lighthouse Lab in Cambridge and Alex Alderton, Roberto Amato, Sonia Goncalves, Ewan Harrison, David K. Jackson, Ian Johnston, Dominic Kwiatkowski, Cordelia Langford, John Sillitoe on behalf of the Wellcome Sanger Institute COVID-19 Surveillance Team
EPI_ISL_932704	Lighthouse Lab in Alderley Park	Wellcome Sanger Institute for the COVID-19 Genomics UK (COG-UK) Consortium	Jacquelyn Wynn, Mairead Hyland, The Lighthouse Lab in Alderley Park and Alex Alderton, Roberto Amato, Sonia Goncalves, Ewan Harrison, David K. Jackson, Ian Johnston, Dominic Kwiatkowski, Cordelia Langford, John Sillitoe on behalf of the Wellcome Sanger Institute COVID-19 Surveillance Team
EPI_ISL_932705	Lighthouse Lab in Glasgow	Wellcome Sanger Institute for the COVID-19 Genomics UK (COG-UK) Consortium	Harper VanSteenhouse, Yumi Kasai, David Gray, Carol Clugston, Anna Dominiczak and Alex Alderton, Roberto Amato, Sonia Goncalves, Ewan Harrison, David K. Jackson, Ian Johnston, Dominic Kwiatkowski, Cordelia Langford, John Sillitoe on behalf of the Wellcome Sanger Institute COVID-19 Surveillance Team
EPI_ISL_932706	Lighthouse Lab in Cambridge	Wellcome Sanger Institute for the COVID-19 Genomics UK (COG-UK) Consortium	Rob Howes, The Lighthouse Lab in Cambridge and Alex Alderton, Roberto Amato, Sonia Goncalves, Ewan Harrison, David K. Jackson, Ian Johnston, Dominic Kwiatkowski, Cordelia Langford, John Sillitoe on behalf of the Wellcome Sanger Institute COVID-19 Surveillance Team
EPI_ISL_932707	Lighthouse Lab in Milton Keynes	Wellcome Sanger Institute for the COVID-19 Genomics UK (COG-UK) Consortium	The Lighthouse Lab in Milton Keynes and Alex Alderton, Roberto Amato, Sonia Goncalves, Ewan Harrison, David K. Jackson, Ian Johnston, Dominic Kwiatkowski, Cordelia Langford, John Sillitoe on behalf of the Wellcome Sanger Institute COVID-19 Surveillance Team
EPI_ISL_932709	Lighthouse Lab in Alderley Park	Wellcome Sanger Institute for the COVID-19 Genomics UK (COG-UK) Consortium	Jacquelyn Wynn, Mairead Hyland, The Lighthouse Lab in Alderley Park and Alex Alderton, Roberto Amato, Sonia Goncalves, Ewan Harrison, David K. Jackson, Ian Johnston, Dominic Kwiatkowski, Cordelia Langford, John Sillitoe on behalf of the Wellcome Sanger Institute COVID-19 Surveillance Team
EPI_ISL_932710	Lighthouse Lab in Cambridge	Wellcome Sanger Institute for the COVID-19 Genomics UK (COG-UK) Consortium	Rob Howes, The Lighthouse Lab in Cambridge and Alex Alderton, Roberto Amato, Sonia Goncalves, Ewan Harrison, David K. Jackson, Ian Johnston, Dominic Kwiatkowski, Cordelia Langford, John Sillitoe on behalf of the Wellcome Sanger Institute COVID-19 Surveillance Team
EPI_ISL_932711	Lighthouse Lab in Glasgow	Wellcome Sanger Institute for the COVID-19 Genomics UK (COG-UK) Consortium	Harper VanSteenhouse, Yumi Kasai, David Gray, Carol Clugston, Anna Dominiczak and Alex Alderton, Roberto Amato, Sonia Goncalves, Ewan Harrison, David K. Jackson, Ian Johnston, Dominic Kwiatkowski, Cordelia Langford, John Sillitoe on behalf of the Wellcome Sanger Institute COVID-19 Surveillance Team
EPI_ISL_932712	Lighthouse Lab in Cambridge	Wellcome Sanger Institute for the COVID-19 Genomics UK (COG-UK) Consortium	Rob Howes, The Lighthouse Lab in Cambridge and Alex Alderton, Roberto Amato, Sonia Goncalves, Ewan Harrison, David K. Jackson, Ian Johnston, Dominic Kwiatkowski, Cordelia Langford, John Sillitoe on behalf of the Wellcome Sanger Institute COVID-19 Surveillance Team
EPI_ISL_932713, EPI_ISL_932714	Lighthouse Lab in Milton Keynes	Wellcome Sanger Institute for the COVID-19 Genomics UK (COG-UK) Consortium	The Lighthouse Lab in Milton Keynes and Alex Alderton, Roberto Amato, Sonia Goncalves, Ewan Harrison, David K. Jackson, Ian Johnston, Dominic Kwiatkowski, Cordelia Langford, John Sillitoe on behalf of the Wellcome Sanger Institute COVID-19 Surveillance Team
EPI_ISL_932715	Lighthouse Lab in Cambridge	Wellcome Sanger Institute for the COVID-19 Genomics UK (COG-UK) Consortium	Rob Howes, The Lighthouse Lab in Cambridge and Alex Alderton, Roberto Amato, Sonia Goncalves, Ewan Harrison, David K. Jackson, Ian Johnston, Dominic Kwiatkowski, Cordelia Langford, John Sillitoe on behalf of the Wellcome Sanger Institute COVID-19 Surveillance Team
EPI_ISL_932716, EPI_ISL_932717	Lighthouse Lab in Milton Keynes	Wellcome Sanger Institute for the COVID-19 Genomics UK (COG-UK) Consortium	The Lighthouse Lab in Milton Keynes and Alex Alderton, Roberto Amato, Sonia Goncalves, Ewan Harrison, David K. Jackson, Ian Johnston, Dominic Kwiatkowski, Cordelia Langford, John Sillitoe on behalf of the Wellcome Sanger Institute COVID-19 Surveillance Team
EPI_ISL_932718, EPI_ISL_932721, EPI_ISL_932722	Lighthouse Lab in Cambridge	Wellcome Sanger Institute for the COVID-19 Genomics UK (COG-UK) Consortium	Rob Howes, The Lighthouse Lab in Cambridge and Alex Alderton, Roberto Amato, Sonia Goncalves, Ewan Harrison, David K. Jackson, Ian Johnston, Dominic Kwiatkowski, Cordelia Langford, John Sillitoe on behalf of the Wellcome Sanger Institute COVID-19 Surveillance Team
EPI_ISL_932723, EPI_ISL_932724	Lighthouse Lab in Milton Keynes	Wellcome Sanger Institute for the COVID-19 Genomics UK (COG-UK) Consortium	The Lighthouse Lab in Milton Keynes and Alex Alderton, Roberto Amato, Sonia Goncalves, Ewan Harrison, David K. Jackson, Ian Johnston, Dominic Kwiatkowski, Cordelia Langford, John Sillitoe on behalf of the Wellcome Sanger Institute COVID-19 Surveillance Team
EPI_ISL_932725	Lighthouse Lab in Alderley Park	Wellcome Sanger Institute for the COVID-19 Genomics UK (COG-UK) Consortium	Jacquelyn Wynn, Mairead Hyland, The Lighthouse Lab in Alderley Park and Alex Alderton, Roberto Amato, Sonia Goncalves, Ewan Harrison, David K. Jackson, Ian Johnston, Dominic Kwiatkowski, Cordelia Langford, John Sillitoe on behalf of the Wellcome Sanger Institute COVID-19 Surveillance Team
EPI_ISL_932726	Lighthouse Lab in Milton Keynes	Wellcome Sanger Institute for the COVID-19 Genomics UK (COG-UK) Consortium	The Lighthouse Lab in Milton Keynes and Alex Alderton, Roberto Amato, Sonia Goncalves, Ewan Harrison, David K. Jackson, Ian Johnston, Dominic Kwiatkowski, Cordelia Langford, John Sillitoe on behalf of the Wellcome Sanger Institute COVID-19 Surveillance Team
EPI_ISL_932728	Lighthouse Lab in Cambridge	Wellcome Sanger Institute for the COVID-19 Genomics UK (COG-UK) Consortium	Rob Howes, The Lighthouse Lab in Cambridge and Alex Alderton, Roberto Amato, Sonia Goncalves, Ewan Harrison, David K. Jackson, Ian Johnston, Dominic Kwiatkowski, Cordelia Langford, John Sillitoe on behalf of the Wellcome Sanger Institute COVID-19 Surveillance Team
EPI_ISL_932730, EPI_ISL_932731	Lighthouse Lab in Milton Keynes	Wellcome Sanger Institute for the COVID-19 Genomics UK (COG-UK) Consortium	The Lighthouse Lab in Milton Keynes and Alex Alderton, Roberto Amato, Sonia Goncalves, Ewan Harrison, David K. Jackson, Ian Johnston, Dominic Kwiatkowski, Cordelia Langford, John Sillitoe on behalf of the Wellcome Sanger Institute COVID-19 Surveillance Team
EPI_ISL_932732	Lighthouse Lab in Glasgow	Wellcome Sanger Institute for the COVID-19 Genomics UK (COG-UK) Consortium	Harper VanSteenhouse, Yumi Kasai, David Gray, Carol Clugston, Anna Dominiczak and Alex Alderton, Roberto Amato, Sonia Goncalves, Ewan Harrison, David K. Jackson, Ian Johnston, Dominic Kwiatkowski, Cordelia Langford, John Sillitoe on behalf of the Wellcome Sanger Institute COVID-19 Surveillance Team
EPI_ISL_932734	Lighthouse Lab in Alderley Park	Wellcome Sanger Institute for the COVID-19 Genomics UK (COG-UK) Consortium	Jacquelyn Wynn, Mairead Hyland, The Lighthouse Lab in Alderley Park and Alex Alderton, Roberto Amato, Sonia Goncalves, Ewan Harrison, David K. Jackson, Ian Johnston, Dominic Kwiatkowski, Cordelia Langford, John Sillitoe on behalf of the Wellcome Sanger Institute COVID-19 Surveillance Team
EPI_ISL_932735	Lighthouse Lab in Milton Keynes	Wellcome Sanger Institute for the COVID-19 Genomics UK (COG-UK) Consortium	The Lighthouse Lab in Milton Keynes and Alex Alderton, Roberto Amato, Sonia Goncalves, Ewan Harrison, David K. Jackson, Ian Johnston, Dominic Kwiatkowski, Cordelia Langford, John Sillitoe on behalf of the Wellcome Sanger Institute COVID-19 Surveillance Team
EPI_ISL_932737, EPI_ISL_932739	Lighthouse Lab in Cambridge	Wellcome Sanger Institute for the COVID-19 Genomics UK (COG-UK) Consortium	Rob Howes, The Lighthouse Lab in Cambridge and Alex Alderton, Roberto Amato, Sonia Goncalves, Ewan Harrison, David K. Jackson, Ian Johnston, Dominic Kwiatkowski, Cordelia Langford, John Sillitoe on behalf of the Wellcome Sanger Institute COVID-19 Surveillance Team

EPI_ISL_932740, EPI_ISL_932741	Lighthouse Lab in Milton Keynes	Wellcome Sanger Institute for the COVID-19 Genomics UK (COG-UK) Consortium	The Lighthouse Lab in Milton Keynes and Alex Alderton, Roberto Amato, Sonia Goncalves, Ewan Harrison, David K. Jackson, Ian Johnston, Dominic Kwiatkowski, Cordelia Langford, John Sillitoe on behalf of the Wellcome Sanger Institute COVID-19 Surveillance Team
EPI_ISL_932742	Lighthouse Lab in Cambridge	Wellcome Sanger Institute for the COVID-19 Genomics UK (COG-UK) Consortium	Rob Howes, The Lighthouse Lab in Cambridge and Alex Alderton, Roberto Amato, Sonia Goncalves, Ewan Harrison, David K. Jackson, Ian Johnston, Dominic Kwiatkowski, Cordelia Langford, John Sillitoe on behalf of the Wellcome Sanger Institute COVID-19 Surveillance Team
EPI_ISL_932743	Lighthouse Lab in Alderley Park	Wellcome Sanger Institute for the COVID-19 Genomics UK (COG-UK) Consortium	Jacquelyn Wynn, Mairead Hyland, The Lighthouse Lab in Alderley Park and Alex Alderton, Roberto Amato, Sonia Goncalves, Ewan Harrison, David K. Jackson, Ian Johnston, Dominic Kwiatkowski, Cordelia Langford, John Sillitoe on behalf of the Wellcome Sanger Institute COVID-19 Surveillance Team
EPI_ISL_932745	Lighthouse Lab in Milton Keynes	Wellcome Sanger Institute for the COVID-19 Genomics UK (COG-UK) Consortium	The Lighthouse Lab in Milton Keynes and Alex Alderton, Roberto Amato, Sonia Goncalves, Ewan Harrison, David K. Jackson, Ian Johnston, Dominic Kwiatkowski, Cordelia Langford, John Sillitoe on behalf of the Wellcome Sanger Institute COVID-19 Surveillance Team
EPI_ISL_932746	Lighthouse Lab in Glasgow	Wellcome Sanger Institute for the COVID-19 Genomics UK (COG-UK) Consortium	Harper VanSteenhouse, Yumi Kasai, David Gray, Carol Clugston, Anna Dominiczak and Alex Alderton, Roberto Amato, Sonia Goncalves, Ewan Harrison, David K. Jackson, Ian Johnston, Dominic Kwiatkowski, Cordelia Langford, John Sillitoe on behalf of the Wellcome Sanger Institute COVID-19 Surveillance Team
EPI_ISL_932747, EPI_ISL_932748	Lighthouse Lab in Milton Keynes	Wellcome Sanger Institute for the COVID-19 Genomics UK (COG-UK) Consortium	The Lighthouse Lab in Milton Keynes and Alex Alderton, Roberto Amato, Sonia Goncalves, Ewan Harrison, David K. Jackson, Ian Johnston, Dominic Kwiatkowski, Cordelia Langford, John Sillitoe on behalf of the Wellcome Sanger Institute COVID-19 Surveillance Team
EPI_ISL_932749	Lighthouse Lab in Alderley Park	Wellcome Sanger Institute for the COVID-19 Genomics UK (COG-UK) Consortium	Jacquelyn Wynn, Mairead Hyland, The Lighthouse Lab in Alderley Park and Alex Alderton, Roberto Amato, Sonia Goncalves, Ewan Harrison, David K. Jackson, Ian Johnston, Dominic Kwiatkowski, Cordelia Langford, John Sillitoe on behalf of the Wellcome Sanger Institute COVID-19 Surveillance Team
EPI_ISL_932750, EPI_ISL_932751, EPI_ISL_932752	Lighthouse Lab in Milton Keynes	Wellcome Sanger Institute for the COVID-19 Genomics UK (COG-UK) Consortium	The Lighthouse Lab in Milton Keynes and Alex Alderton, Roberto Amato, Sonia Goncalves, Ewan Harrison, David K. Jackson, Ian Johnston, Dominic Kwiatkowski, Cordelia Langford, John Sillitoe on behalf of the Wellcome Sanger Institute COVID-19 Surveillance Team
EPI_ISL_932753	Lighthouse Lab in Alderley Park	Wellcome Sanger Institute for the COVID-19 Genomics UK (COG-UK) Consortium	Jacquelyn Wynn, Mairead Hyland, The Lighthouse Lab in Alderley Park and Alex Alderton, Roberto Amato, Sonia Goncalves, Ewan Harrison, David K. Jackson, Ian Johnston, Dominic Kwiatkowski, Cordelia Langford, John Sillitoe on behalf of the Wellcome Sanger Institute COVID-19 Surveillance Team
EPI_ISL_932754, EPI_ISL_932755, EPI_ISL_932758	Lighthouse Lab in Cambridge	Wellcome Sanger Institute for the COVID-19 Genomics UK (COG-UK) Consortium	Rob Howes, The Lighthouse Lab in Cambridge and Alex Alderton, Roberto Amato, Sonia Goncalves, Ewan Harrison, David K. Jackson, Ian Johnston, Dominic Kwiatkowski, Cordelia Langford, John Sillitoe on behalf of the Wellcome Sanger Institute COVID-19 Surveillance Team
EPI_ISL_932759	Lighthouse Lab in Milton Keynes	Wellcome Sanger Institute for the COVID-19 Genomics UK (COG-UK) Consortium	The Lighthouse Lab in Milton Keynes and Alex Alderton, Roberto Amato, Sonia Goncalves, Ewan Harrison, David K. Jackson, Ian Johnston, Dominic Kwiatkowski, Cordelia Langford, John Sillitoe on behalf of the Wellcome Sanger Institute COVID-19 Surveillance Team
EPI_ISL_932760	Lighthouse Lab in Alderley Park	Wellcome Sanger Institute for the COVID-19 Genomics UK (COG-UK) Consortium	Jacquelyn Wynn, Mairead Hyland, The Lighthouse Lab in Alderley Park and Alex Alderton, Roberto Amato, Sonia Goncalves, Ewan Harrison, David K. Jackson, Ian Johnston, Dominic Kwiatkowski, Cordelia Langford, John Sillitoe on behalf of the Wellcome Sanger Institute COVID-19 Surveillance Team
EPI_ISL_932761, EPI_ISL_932763	Lighthouse Lab in Milton Keynes	Wellcome Sanger Institute for the COVID-19 Genomics UK (COG-UK) Consortium	The Lighthouse Lab in Milton Keynes and Alex Alderton, Roberto Amato, Sonia Goncalves, Ewan Harrison, David K. Jackson, Ian Johnston, Dominic Kwiatkowski, Cordelia Langford, John Sillitoe on behalf of the Wellcome Sanger Institute COVID-19 Surveillance Team
EPI_ISL_932764	Lighthouse Lab in Glasgow	Wellcome Sanger Institute for the COVID-19 Genomics UK (COG-UK) Consortium	Harper VanSteenhouse, Yumi Kasai, David Gray, Carol Clugston, Anna Dominiczak and Alex Alderton, Roberto Amato, Sonia Goncalves, Ewan Harrison, David K. Jackson, Ian Johnston, Dominic Kwiatkowski, Cordelia Langford, John Sillitoe on behalf of the Wellcome Sanger Institute COVID-19 Surveillance Team
EPI_ISL_932765, EPI_ISL_932766, EPI_ISL_932767	Lighthouse Lab in Milton Keynes	Wellcome Sanger Institute for the COVID-19 Genomics UK (COG-UK) Consortium	The Lighthouse Lab in Milton Keynes and Alex Alderton, Roberto Amato, Sonia Goncalves, Ewan Harrison, David K. Jackson, Ian Johnston, Dominic Kwiatkowski, Cordelia Langford, John Sillitoe on behalf of the Wellcome Sanger Institute COVID-19 Surveillance Team
EPI_ISL_932768	Lighthouse Lab in Alderley Park	Wellcome Sanger Institute for the COVID-19 Genomics UK (COG-UK) Consortium	Jacquelyn Wynn, Mairead Hyland, The Lighthouse Lab in Alderley Park and Alex Alderton, Roberto Amato, Sonia Goncalves, Ewan Harrison, David K. Jackson, Ian Johnston, Dominic Kwiatkowski, Cordelia Langford, John Sillitoe on behalf of the Wellcome Sanger Institute COVID-19 Surveillance Team
EPI_ISL_932769	Lighthouse Lab in Milton Keynes	Wellcome Sanger Institute for the COVID-19 Genomics UK (COG-UK) Consortium	The Lighthouse Lab in Milton Keynes and Alex Alderton, Roberto Amato, Sonia Goncalves, Ewan Harrison, David K. Jackson, Ian Johnston, Dominic Kwiatkowski, Cordelia Langford, John Sillitoe on behalf of the Wellcome Sanger Institute COVID-19 Surveillance Team
EPI_ISL_932770, EPI_ISL_932771	Lighthouse Lab in Cambridge	Wellcome Sanger Institute for the COVID-19 Genomics UK (COG-UK) Consortium	Rob Howes, The Lighthouse Lab in Cambridge and Alex Alderton, Roberto Amato, Sonia Goncalves, Ewan Harrison, David K. Jackson, Ian Johnston, Dominic Kwiatkowski, Cordelia Langford, John Sillitoe on behalf of the Wellcome Sanger Institute COVID-19 Surveillance Team
EPI_ISL_932772, EPI_ISL_932773, EPI_ISL_932774	Lighthouse Lab in Milton Keynes	Wellcome Sanger Institute for the COVID-19 Genomics UK (COG-UK) Consortium	The Lighthouse Lab in Milton Keynes and Alex Alderton, Roberto Amato, Sonia Goncalves, Ewan Harrison, David K. Jackson, Ian Johnston, Dominic Kwiatkowski, Cordelia Langford, John Sillitoe on behalf of the Wellcome Sanger Institute COVID-19 Surveillance Team
EPI_ISL_932776, EPI_ISL_932778	Lighthouse Lab in Cambridge	Wellcome Sanger Institute for the COVID-19 Genomics UK (COG-UK) Consortium	Rob Howes, The Lighthouse Lab in Cambridge and Alex Alderton, Roberto Amato, Sonia Goncalves, Ewan Harrison, David K. Jackson, Ian Johnston, Dominic Kwiatkowski, Cordelia Langford, John Sillitoe on behalf of the Wellcome Sanger Institute COVID-19 Surveillance Team
EPI_ISL_932779, EPI_ISL_932780	Lighthouse Lab in Milton Keynes	Wellcome Sanger Institute for the COVID-19 Genomics UK (COG-UK) Consortium	The Lighthouse Lab in Milton Keynes and Alex Alderton, Roberto Amato, Sonia Goncalves, Ewan Harrison, David K. Jackson, Ian Johnston, Dominic Kwiatkowski, Cordelia Langford, John Sillitoe on behalf of the Wellcome Sanger Institute COVID-19 Surveillance Team
EPI_ISL_932781	Lighthouse Lab in Glasgow	Wellcome Sanger Institute for the COVID-19 Genomics UK (COG-UK) Consortium	Harper VanSteenhouse, Yumi Kasai, David Gray, Carol Clugston, Anna Dominiczak and Alex Alderton, Roberto Amato, Sonia Goncalves, Ewan Harrison, David K. Jackson, Ian Johnston, Dominic Kwiatkowski, Cordelia Langford, John Sillitoe on behalf of the Wellcome Sanger Institute COVID-19 Surveillance Team
EPI_ISL_932782	Lighthouse Lab in Cambridge	Wellcome Sanger Institute for the COVID-19 Genomics UK (COG-UK) Consortium	Rob Howes, The Lighthouse Lab in Cambridge and Alex Alderton, Roberto Amato, Sonia Goncalves, Ewan Harrison, David K. Jackson, Ian Johnston, Dominic Kwiatkowski, Cordelia Langford, John Sillitoe on behalf of the Wellcome Sanger Institute COVID-19 Surveillance Team
EPI_ISL_932783, EPI_ISL_932784, EPI_ISL_932785, EPI_ISL_932786, EPI_ISL_932787, EPI_ISL_932788, EPI_ISL_932789	Lighthouse Lab in Milton Keynes	Wellcome Sanger Institute for the COVID-19 Genomics UK (COG-UK) Consortium	The Lighthouse Lab in Milton Keynes and Alex Alderton, Roberto Amato, Sonia Goncalves, Ewan Harrison, David K. Jackson, Ian Johnston, Dominic Kwiatkowski, Cordelia Langford, John Sillitoe on behalf of the Wellcome Sanger Institute COVID-19 Surveillance Team
EPI_ISL_932790	Lighthouse Lab in Cambridge	Wellcome Sanger Institute for the COVID-19 Genomics UK (COG-UK) Consortium	Rob Howes, The Lighthouse Lab in Cambridge and Alex Alderton, Roberto Amato, Sonia Goncalves, Ewan Harrison, David K. Jackson, Ian Johnston, Dominic Kwiatkowski, Cordelia Langford, John Sillitoe on behalf of the Wellcome Sanger Institute COVID-19 Surveillance Team
EPI_ISL_932791	Lighthouse Lab in Alderley Park	Wellcome Sanger Institute for the COVID-19 Genomics UK (COG-UK) Consortium	Jacquelyn Wynn, Mairead Hyland, The Lighthouse Lab in Alderley Park and Alex Alderton, Roberto Amato, Sonia Goncalves, Ewan Harrison, David K. Jackson, Ian Johnston, Dominic Kwiatkowski, Cordelia Langford, John Sillitoe on behalf of the Wellcome Sanger Institute COVID-19 Surveillance Team
EPI_ISL_932792	Lighthouse Lab in Milton Keynes	Wellcome Sanger Institute for the COVID-19 Genomics UK (COG-UK) Consortium	The Lighthouse Lab in Milton Keynes and Alex Alderton, Roberto Amato, Sonia Goncalves, Ewan Harrison, David K. Jackson, Ian Johnston, Dominic Kwiatkowski, Cordelia Langford, John Sillitoe on behalf of the Wellcome Sanger Institute COVID-19 Surveillance Team
EPI_ISL_932794	Lighthouse Lab in Cambridge	Wellcome Sanger Institute for the COVID-19 Genomics UK (COG-UK) Consortium	Rob Howes, The Lighthouse Lab in Cambridge and Alex Alderton, Roberto Amato, Sonia Goncalves, Ewan Harrison, David K. Jackson, Ian Johnston, Dominic Kwiatkowski, Cordelia Langford, John Sillitoe on behalf of the Wellcome Sanger Institute COVID-19 Surveillance Team
EPI_ISL_932795	Lighthouse Lab in Milton Keynes	Wellcome Sanger Institute for the COVID-19 Genomics UK (COG-UK) Consortium	The Lighthouse Lab in Milton Keynes and Alex Alderton, Roberto Amato, Sonia Goncalves, Ewan Harrison, David K. Jackson, Ian Johnston, Dominic Kwiatkowski, Cordelia Langford, John Sillitoe on behalf of the Wellcome Sanger Institute COVID-19 Surveillance Team
EPI_ISL_932796	Lighthouse Lab in Cambridge	Wellcome Sanger Institute for the COVID-19 Genomics UK (COG-UK) Consortium	Rob Howes, The Lighthouse Lab in Cambridge and Alex Alderton, Roberto Amato, Sonia Goncalves, Ewan Harrison, David K. Jackson, Ian Johnston, Dominic Kwiatkowski, Cordelia Langford, John Sillitoe on behalf of the Wellcome Sanger Institute COVID-19 Surveillance Team
EPI_ISL_932797, EPI_ISL_932798	Lighthouse Lab in Milton Keynes	Wellcome Sanger Institute for the COVID-19 Genomics UK (COG-UK) Consortium	The Lighthouse Lab in Milton Keynes and Alex Alderton, Roberto Amato, Sonia Goncalves, Ewan Harrison, David K. Jackson, Ian Johnston, Dominic Kwiatkowski, Cordelia Langford, John Sillitoe on behalf of the Wellcome Sanger Institute COVID-19 Surveillance Team
EPI_ISL_932800	Lighthouse Lab in Alderley Park	Wellcome Sanger Institute for the COVID-19 Genomics UK (COG-UK) Consortium	Jacquelyn Wynn, Mairead Hyland, The Lighthouse Lab in Alderley Park and Alex Alderton, Roberto Amato, Sonia Goncalves, Ewan Harrison, David K. Jackson, Ian Johnston, Dominic Kwiatkowski, Cordelia Langford, John Sillitoe on behalf of the Wellcome Sanger Institute COVID-19 Surveillance Team
EPI_ISL_932801, EPI_ISL_932803, EPI_ISL_932804	Lighthouse Lab in Milton Keynes	Wellcome Sanger Institute for the COVID-19 Genomics UK (COG-UK) Consortium	The Lighthouse Lab in Milton Keynes and Alex Alderton, Roberto Amato, Sonia Goncalves, Ewan Harrison, David K. Jackson, Ian Johnston, Dominic Kwiatkowski, Cordelia Langford, John Sillitoe on behalf of the Wellcome Sanger Institute COVID-19 Surveillance Team

EPI_ISL_932805	Lighthouse Lab in Cambridge	Wellcome Sanger Institute for the COVID-19 Genomics UK (COG-UK) Consortium	Rob Howes, The Lighthouse Lab in Cambridge and Alex Alderton, Roberto Amato, Sonia Goncalves, Ewan Harrison, David K. Jackson, Ian Johnston, Dominic Kwiatkowski, Cordelia Langford, John Sillitoe on behalf of the Wellcome Sanger Institute COVID-19 Surveillance Team
EPI_ISL_932806	Lighthouse Lab in Milton Keynes	Wellcome Sanger Institute for the COVID-19 Genomics UK (COG-UK) Consortium	The Lighthouse Lab in Milton Keynes and Alex Alderton, Roberto Amato, Sonia Goncalves, Ewan Harrison, David K. Jackson, Ian Johnston, Dominic Kwiatkowski, Cordelia Langford, John Sillitoe on behalf of the Wellcome Sanger Institute COVID-19 Surveillance Team
EPI_ISL_932807	Lighthouse Lab in Glasgow	Wellcome Sanger Institute for the COVID-19 Genomics UK (COG-UK) Consortium	Harper VanSteenhouse, Yumi Kasai, David Gray, Carol Clugston, Anna Dominiczak and Alex Alderton, Roberto Amato, Sonia Goncalves, Ewan Harrison, David K. Jackson, Ian Johnston, Dominic Kwiatkowski, Cordelia Langford, John Sillitoe on behalf of the Wellcome Sanger Institute COVID-19 Surveillance Team
EPI_ISL_932808	Lighthouse Lab in Cambridge	Wellcome Sanger Institute for the COVID-19 Genomics UK (COG-UK) Consortium	Rob Howes, The Lighthouse Lab in Cambridge and Alex Alderton, Roberto Amato, Sonia Goncalves, Ewan Harrison, David K. Jackson, Ian Johnston, Dominic Kwiatkowski, Cordelia Langford, John Sillitoe on behalf of the Wellcome Sanger Institute COVID-19 Surveillance Team
EPI_ISL_932809, EPI_ISL_932810, EPI_ISL_932811	Lighthouse Lab in Milton Keynes	Wellcome Sanger Institute for the COVID-19 Genomics UK (COG-UK) Consortium	The Lighthouse Lab in Milton Keynes and Alex Alderton, Roberto Amato, Sonia Goncalves, Ewan Harrison, David K. Jackson, Ian Johnston, Dominic Kwiatkowski, Cordelia Langford, John Sillitoe on behalf of the Wellcome Sanger Institute COVID-19 Surveillance Team
EPI_ISL_932812	Lighthouse Lab in Cambridge	Wellcome Sanger Institute for the COVID-19 Genomics UK (COG-UK) Consortium	Rob Howes, The Lighthouse Lab in Cambridge and Alex Alderton, Roberto Amato, Sonia Goncalves, Ewan Harrison, David K. Jackson, Ian Johnston, Dominic Kwiatkowski, Cordelia Langford, John Sillitoe on behalf of the Wellcome Sanger Institute COVID-19 Surveillance Team
EPI_ISL_932813	Lighthouse Lab in Milton Keynes	Wellcome Sanger Institute for the COVID-19 Genomics UK (COG-UK) Consortium	The Lighthouse Lab in Milton Keynes and Alex Alderton, Roberto Amato, Sonia Goncalves, Ewan Harrison, David K. Jackson, Ian Johnston, Dominic Kwiatkowski, Cordelia Langford, John Sillitoe on behalf of the Wellcome Sanger Institute COVID-19 Surveillance Team
EPI_ISL_932814	Lighthouse Lab in Alderley Park	Wellcome Sanger Institute for the COVID-19 Genomics UK (COG-UK) Consortium	Jacquelyn Wynn, Mairead Hyland, The Lighthouse Lab in Alderley Park and Alex Alderton, Roberto Amato, Sonia Goncalves, Ewan Harrison, David K. Jackson, Ian Johnston, Dominic Kwiatkowski, Cordelia Langford, John Sillitoe on behalf of the Wellcome Sanger Institute COVID-19 Surveillance Team
EPI_ISL_932815	Lighthouse Lab in Milton Keynes	Wellcome Sanger Institute for the COVID-19 Genomics UK (COG-UK) Consortium	The Lighthouse Lab in Milton Keynes and Alex Alderton, Roberto Amato, Sonia Goncalves, Ewan Harrison, David K. Jackson, Ian Johnston, Dominic Kwiatkowski, Cordelia Langford, John Sillitoe on behalf of the Wellcome Sanger Institute COVID-19 Surveillance Team
EPI_ISL_932816	Lighthouse Lab in Cambridge	Wellcome Sanger Institute for the COVID-19 Genomics UK (COG-UK) Consortium	Rob Howes, The Lighthouse Lab in Cambridge and Alex Alderton, Roberto Amato, Sonia Goncalves, Ewan Harrison, David K. Jackson, Ian Johnston, Dominic Kwiatkowski, Cordelia Langford, John Sillitoe on behalf of the Wellcome Sanger Institute COVID-19 Surveillance Team
EPI_ISL_932817, EPI_ISL_932818	Lighthouse Lab in Alderley Park	Wellcome Sanger Institute for the COVID-19 Genomics UK (COG-UK) Consortium	Jacquelyn Wynn, Mairead Hyland, The Lighthouse Lab in Alderley Park and Alex Alderton, Roberto Amato, Sonia Goncalves, Ewan Harrison, David K. Jackson, Ian Johnston, Dominic Kwiatkowski, Cordelia Langford, John Sillitoe on behalf of the Wellcome Sanger Institute COVID-19 Surveillance Team
EPI_ISL_932819	Lighthouse Lab in Milton Keynes	Wellcome Sanger Institute for the COVID-19 Genomics UK (COG-UK) Consortium	The Lighthouse Lab in Milton Keynes and Alex Alderton, Roberto Amato, Sonia Goncalves, Ewan Harrison, David K. Jackson, Ian Johnston, Dominic Kwiatkowski, Cordelia Langford, John Sillitoe on behalf of the Wellcome Sanger Institute COVID-19 Surveillance Team
EPI_ISL_932820	Lighthouse Lab in Cambridge	Wellcome Sanger Institute for the COVID-19 Genomics UK (COG-UK) Consortium	Rob Howes, The Lighthouse Lab in Cambridge and Alex Alderton, Roberto Amato, Sonia Goncalves, Ewan Harrison, David K. Jackson, Ian Johnston, Dominic Kwiatkowski, Cordelia Langford, John Sillitoe on behalf of the Wellcome Sanger Institute COVID-19 Surveillance Team
EPI_ISL_932821	Lighthouse Lab in Milton Keynes	Wellcome Sanger Institute for the COVID-19 Genomics UK (COG-UK) Consortium	The Lighthouse Lab in Milton Keynes and Alex Alderton, Roberto Amato, Sonia Goncalves, Ewan Harrison, David K. Jackson, Ian Johnston, Dominic Kwiatkowski, Cordelia Langford, John Sillitoe on behalf of the Wellcome Sanger Institute COVID-19 Surveillance Team
EPI_ISL_932822	Lighthouse Lab in Cambridge	Wellcome Sanger Institute for the COVID-19 Genomics UK (COG-UK) Consortium	Rob Howes, The Lighthouse Lab in Cambridge and Alex Alderton, Roberto Amato, Sonia Goncalves, Ewan Harrison, David K. Jackson, Ian Johnston, Dominic Kwiatkowski, Cordelia Langford, John Sillitoe on behalf of the Wellcome Sanger Institute COVID-19 Surveillance Team
EPI_ISL_932825, EPI_ISL_932826	Lighthouse Lab in Milton Keynes	Wellcome Sanger Institute for the COVID-19 Genomics UK (COG-UK) Consortium	The Lighthouse Lab in Milton Keynes and Alex Alderton, Roberto Amato, Sonia Goncalves, Ewan Harrison, David K. Jackson, Ian Johnston, Dominic Kwiatkowski, Cordelia Langford, John Sillitoe on behalf of the Wellcome Sanger Institute COVID-19 Surveillance Team
EPI_ISL_932827, EPI_ISL_932829, EPI_ISL_932830	Lighthouse Lab in Cambridge	Wellcome Sanger Institute for the COVID-19 Genomics UK (COG-UK) Consortium	Rob Howes, The Lighthouse Lab in Cambridge and Alex Alderton, Roberto Amato, Sonia Goncalves, Ewan Harrison, David K. Jackson, Ian Johnston, Dominic Kwiatkowski, Cordelia Langford, John Sillitoe on behalf of the Wellcome Sanger Institute COVID-19 Surveillance Team
EPI_ISL_932831	Lighthouse Lab in Glasgow	Wellcome Sanger Institute for the COVID-19 Genomics UK (COG-UK) Consortium	Harper VanSteenhouse, Yumi Kasai, David Gray, Carol Clugston, Anna Dominiczak and Alex Alderton, Roberto Amato, Sonia Goncalves, Ewan Harrison, David K. Jackson, Ian Johnston, Dominic Kwiatkowski, Cordelia Langford, John Sillitoe on behalf of the Wellcome Sanger Institute COVID-19 Surveillance Team
EPI_ISL_932833	Lighthouse Lab in Milton Keynes	Wellcome Sanger Institute for the COVID-19 Genomics UK (COG-UK) Consortium	The Lighthouse Lab in Milton Keynes and Alex Alderton, Roberto Amato, Sonia Goncalves, Ewan Harrison, David K. Jackson, Ian Johnston, Dominic Kwiatkowski, Cordelia Langford, John Sillitoe on behalf of the Wellcome Sanger Institute COVID-19 Surveillance Team
EPI_ISL_932834, EPI_ISL_932835	Lighthouse Lab in Alderley Park	Wellcome Sanger Institute for the COVID-19 Genomics UK (COG-UK) Consortium	Jacquelyn Wynn, Mairead Hyland, The Lighthouse Lab in Alderley Park and Alex Alderton, Roberto Amato, Sonia Goncalves, Ewan Harrison, David K. Jackson, Ian Johnston, Dominic Kwiatkowski, Cordelia Langford, John Sillitoe on behalf of the Wellcome Sanger Institute COVID-19 Surveillance Team
EPI_ISL_932836	Lighthouse Lab in Cambridge	Wellcome Sanger Institute for the COVID-19 Genomics UK (COG-UK) Consortium	Rob Howes, The Lighthouse Lab in Cambridge and Alex Alderton, Roberto Amato, Sonia Goncalves, Ewan Harrison, David K. Jackson, Ian Johnston, Dominic Kwiatkowski, Cordelia Langford, John Sillitoe on behalf of the Wellcome Sanger Institute COVID-19 Surveillance Team
EPI_ISL_932837	Lighthouse Lab in Milton Keynes	Wellcome Sanger Institute for the COVID-19 Genomics UK (COG-UK) Consortium	The Lighthouse Lab in Milton Keynes and Alex Alderton, Roberto Amato, Sonia Goncalves, Ewan Harrison, David K. Jackson, Ian Johnston, Dominic Kwiatkowski, Cordelia Langford, John Sillitoe on behalf of the Wellcome Sanger Institute COVID-19 Surveillance Team
EPI_ISL_932838, EPI_ISL_932839	Lighthouse Lab in Cambridge	Wellcome Sanger Institute for the COVID-19 Genomics UK (COG-UK) Consortium	Rob Howes, The Lighthouse Lab in Cambridge and Alex Alderton, Roberto Amato, Sonia Goncalves, Ewan Harrison, David K. Jackson, Ian Johnston, Dominic Kwiatkowski, Cordelia Langford, John Sillitoe on behalf of the Wellcome Sanger Institute COVID-19 Surveillance Team
EPI_ISL_932840	Lighthouse Lab in Alderley Park	Wellcome Sanger Institute for the COVID-19 Genomics UK (COG-UK) Consortium	Jacquelyn Wynn, Mairead Hyland, The Lighthouse Lab in Alderley Park and Alex Alderton, Roberto Amato, Sonia Goncalves, Ewan Harrison, David K. Jackson, Ian Johnston, Dominic Kwiatkowski, Cordelia Langford, John Sillitoe on behalf of the Wellcome Sanger Institute COVID-19 Surveillance Team
EPI_ISL_932841	Lighthouse Lab in Milton Keynes	Wellcome Sanger Institute for the COVID-19 Genomics UK (COG-UK) Consortium	The Lighthouse Lab in Milton Keynes and Alex Alderton, Roberto Amato, Sonia Goncalves, Ewan Harrison, David K. Jackson, Ian Johnston, Dominic Kwiatkowski, Cordelia Langford, John Sillitoe on behalf of the Wellcome Sanger Institute COVID-19 Surveillance Team
EPI_ISL_932842, EPI_ISL_932843	Lighthouse Lab in Cambridge	Wellcome Sanger Institute for the COVID-19 Genomics UK (COG-UK) Consortium	Rob Howes, The Lighthouse Lab in Cambridge and Alex Alderton, Roberto Amato, Sonia Goncalves, Ewan Harrison, David K. Jackson, Ian Johnston, Dominic Kwiatkowski, Cordelia Langford, John Sillitoe on behalf of the Wellcome Sanger Institute COVID-19 Surveillance Team
EPI_ISL_932844	Lighthouse Lab in Alderley Park	Wellcome Sanger Institute for the COVID-19 Genomics UK (COG-UK) Consortium	Jacquelyn Wynn, Mairead Hyland, The Lighthouse Lab in Alderley Park and Alex Alderton, Roberto Amato, Sonia Goncalves, Ewan Harrison, David K. Jackson, Ian Johnston, Dominic Kwiatkowski, Cordelia Langford, John Sillitoe on behalf of the Wellcome Sanger Institute COVID-19 Surveillance Team
EPI_ISL_932846, EPI_ISL_932847, EPI_ISL_932848, EPI_ISL_932849, EPI_ISL_932851, EPI_ISL_932852, EPI_ISL_932854, EPI_ISL_932861	Lighthouse Lab in Milton Keynes	Wellcome Sanger Institute for the COVID-19 Genomics UK (COG-UK) Consortium	The Lighthouse Lab in Milton Keynes and Alex Alderton, Roberto Amato, Sonia Goncalves, Ewan Harrison, David K. Jackson, Ian Johnston, Dominic Kwiatkowski, Cordelia Langford, John Sillitoe on behalf of the Wellcome Sanger Institute COVID-19 Surveillance Team
EPI_ISL_932862, EPI_ISL_932866, EPI_ISL_932867, EPI_ISL_932868	Lighthouse Lab in Alderley Park	Wellcome Sanger Institute for the COVID-19 Genomics UK (COG-UK) Consortium	Jacquelyn Wynn, Mairead Hyland, The Lighthouse Lab in Alderley Park and Alex Alderton, Roberto Amato, Sonia Goncalves, Ewan Harrison, David K. Jackson, Ian Johnston, Dominic Kwiatkowski, Cordelia Langford, John Sillitoe on behalf of the Wellcome Sanger Institute COVID-19 Surveillance Team
EPI_ISL_932872, EPI_ISL_932873, EPI_ISL_932875	Lighthouse Lab in Milton Keynes	Wellcome Sanger Institute for the COVID-19 Genomics UK (COG-UK) Consortium	The Lighthouse Lab in Milton Keynes and Alex Alderton, Roberto Amato, Sonia Goncalves, Ewan Harrison, David K. Jackson, Ian Johnston, Dominic Kwiatkowski, Cordelia Langford, John Sillitoe on behalf of the Wellcome Sanger Institute COVID-19 Surveillance Team
EPI_ISL_932876	Lighthouse Lab in Alderley Park	Wellcome Sanger Institute for the COVID-19 Genomics UK (COG-UK) Consortium	Jacquelyn Wynn, Mairead Hyland, The Lighthouse Lab in Alderley Park and Alex Alderton, Roberto Amato, Sonia Goncalves, Ewan Harrison, David K. Jackson, Ian Johnston, Dominic Kwiatkowski, Cordelia Langford, John Sillitoe on behalf of the Wellcome Sanger Institute COVID-19 Surveillance Team
EPI_ISL_932877	Lighthouse Lab in Milton Keynes	Wellcome Sanger Institute for the COVID-19 Genomics UK (COG-UK) Consortium	The Lighthouse Lab in Milton Keynes and Alex Alderton, Roberto Amato, Sonia Goncalves, Ewan Harrison, David K. Jackson, Ian Johnston, Dominic Kwiatkowski, Cordelia Langford, John Sillitoe on behalf of the Wellcome Sanger Institute COVID-19 Surveillance Team
EPI_ISL_932878	Lighthouse Lab in Alderley Park	Wellcome Sanger Institute for the COVID-19 Genomics UK (COG-UK) Consortium	Jacquelyn Wynn, Mairead Hyland, The Lighthouse Lab in Alderley Park and Alex Alderton, Roberto Amato, Sonia Goncalves, Ewan Harrison, David K. Jackson, Ian Johnston, Dominic Kwiatkowski, Cordelia Langford, John Sillitoe on behalf of the Wellcome Sanger Institute COVID-19 Surveillance Team
EPI_ISL_932880	Lighthouse Lab in Milton Keynes	Wellcome Sanger Institute for the COVID-19 Genomics UK	The Lighthouse Lab in Milton Keynes and Alex Alderton, Roberto Amato, Sonia Goncalves, Ewan Harrison, David K. Jackson, Ian Johnston, Dominic

		(COG-UK) Consortium	Kwiatkowski, Cordelia Langford, John Sillitoe on behalf of the Wellcome Sanger Institute COVID-19 Surveillance Team
EPI_ISL_932881, EPI_ISL_932882, EPI_ISL_932883	Lighthouse Lab in Alderley Park	Wellcome Sanger Institute for the COVID-19 Genomics UK (COG-UK) Consortium	Jacquelyn Wynn, Mairead Hyland, The Lighthouse Lab in Alderley Park and Alex Alderton, Roberto Amato, Sonia Goncalves, Ewan Harrison, David K. Jackson, Ian Johnston, Dominic Kwiatkowski, Cordelia Langford, John Sillitoe on behalf of the Wellcome Sanger Institute COVID-19 Surveillance Team
EPI_ISL_932887, EPI_ISL_932888	Lighthouse Lab in Milton Keynes	Wellcome Sanger Institute for the COVID-19 Genomics UK (COG-UK) Consortium	The Lighthouse Lab in Milton Keynes and Alex Alderton, Roberto Amato, Sonia Goncalves, Ewan Harrison, David K. Jackson, Ian Johnston, Dominic Kwiatkowski, Cordelia Langford, John Sillitoe on behalf of the Wellcome Sanger Institute COVID-19 Surveillance Team
EPI_ISL_932889, EPI_ISL_932890, EPI_ISL_932891	Lighthouse Lab in Alderley Park	Wellcome Sanger Institute for the COVID-19 Genomics UK (COG-UK) Consortium	Jacquelyn Wynn, Mairead Hyland, The Lighthouse Lab in Alderley Park and Alex Alderton, Roberto Amato, Sonia Goncalves, Ewan Harrison, David K. Jackson, Ian Johnston, Dominic Kwiatkowski, Cordelia Langford, John Sillitoe on behalf of the Wellcome Sanger Institute COVID-19 Surveillance Team
EPI_ISL_932892	Lighthouse Lab in Milton Keynes	Wellcome Sanger Institute for the COVID-19 Genomics UK (COG-UK) Consortium	The Lighthouse Lab in Milton Keynes and Alex Alderton, Roberto Amato, Sonia Goncalves, Ewan Harrison, David K. Jackson, Ian Johnston, Dominic Kwiatkowski, Cordelia Langford, John Sillitoe on behalf of the Wellcome Sanger Institute COVID-19 Surveillance Team
EPI_ISL_932893	Lighthouse Lab in Alderley Park	Wellcome Sanger Institute for the COVID-19 Genomics UK (COG-UK) Consortium	Jacquelyn Wynn, Mairead Hyland, The Lighthouse Lab in Alderley Park and Alex Alderton, Roberto Amato, Sonia Goncalves, Ewan Harrison, David K. Jackson, Ian Johnston, Dominic Kwiatkowski, Cordelia Langford, John Sillitoe on behalf of the Wellcome Sanger Institute COVID-19 Surveillance Team
EPI_ISL_932898	Lighthouse Lab in Milton Keynes	Wellcome Sanger Institute for the COVID-19 Genomics UK (COG-UK) Consortium	The Lighthouse Lab in Milton Keynes and Alex Alderton, Roberto Amato, Sonia Goncalves, Ewan Harrison, David K. Jackson, Ian Johnston, Dominic Kwiatkowski, Cordelia Langford, John Sillitoe on behalf of the Wellcome Sanger Institute COVID-19 Surveillance Team
EPI_ISL_932900, EPI_ISL_932901, EPI_ISL_932902	Lighthouse Lab in Alderley Park	Wellcome Sanger Institute for the COVID-19 Genomics UK (COG-UK) Consortium	Jacquelyn Wynn, Mairead Hyland, The Lighthouse Lab in Alderley Park and Alex Alderton, Roberto Amato, Sonia Goncalves, Ewan Harrison, David K. Jackson, Ian Johnston, Dominic Kwiatkowski, Cordelia Langford, John Sillitoe on behalf of the Wellcome Sanger Institute COVID-19 Surveillance Team
EPI_ISL_932904	Lighthouse Lab in Milton Keynes	Wellcome Sanger Institute for the COVID-19 Genomics UK (COG-UK) Consortium	The Lighthouse Lab in Milton Keynes and Alex Alderton, Roberto Amato, Sonia Goncalves, Ewan Harrison, David K. Jackson, Ian Johnston, Dominic Kwiatkowski, Cordelia Langford, John Sillitoe on behalf of the Wellcome Sanger Institute COVID-19 Surveillance Team
EPI_ISL_932906, EPI_ISL_932907	Lighthouse Lab in Alderley Park	Wellcome Sanger Institute for the COVID-19 Genomics UK (COG-UK) Consortium	Jacquelyn Wynn, Mairead Hyland, The Lighthouse Lab in Alderley Park and Alex Alderton, Roberto Amato, Sonia Goncalves, Ewan Harrison, David K. Jackson, Ian Johnston, Dominic Kwiatkowski, Cordelia Langford, John Sillitoe on behalf of the Wellcome Sanger Institute COVID-19 Surveillance Team
EPI_ISL_932909	Lighthouse Lab in Milton Keynes	Wellcome Sanger Institute for the COVID-19 Genomics UK (COG-UK) Consortium	The Lighthouse Lab in Milton Keynes and Alex Alderton, Roberto Amato, Sonia Goncalves, Ewan Harrison, David K. Jackson, Ian Johnston, Dominic Kwiatkowski, Cordelia Langford, John Sillitoe on behalf of the Wellcome Sanger Institute COVID-19 Surveillance Team
EPI_ISL_932911, EPI_ISL_932912	Lighthouse Lab in Alderley Park	Wellcome Sanger Institute for the COVID-19 Genomics UK (COG-UK) Consortium	Jacquelyn Wynn, Mairead Hyland, The Lighthouse Lab in Alderley Park and Alex Alderton, Roberto Amato, Sonia Goncalves, Ewan Harrison, David K. Jackson, Ian Johnston, Dominic Kwiatkowski, Cordelia Langford, John Sillitoe on behalf of the Wellcome Sanger Institute COVID-19 Surveillance Team
EPI_ISL_932916	Lighthouse Lab in Milton Keynes	Wellcome Sanger Institute for the COVID-19 Genomics UK (COG-UK) Consortium	The Lighthouse Lab in Milton Keynes and Alex Alderton, Roberto Amato, Sonia Goncalves, Ewan Harrison, David K. Jackson, Ian Johnston, Dominic Kwiatkowski, Cordelia Langford, John Sillitoe on behalf of the Wellcome Sanger Institute COVID-19 Surveillance Team
EPI_ISL_932922, EPI_ISL_932923, EPI_ISL_932924	Lighthouse Lab in Alderley Park	Wellcome Sanger Institute for the COVID-19 Genomics UK (COG-UK) Consortium	Jacquelyn Wynn, Mairead Hyland, The Lighthouse Lab in Alderley Park and Alex Alderton, Roberto Amato, Sonia Goncalves, Ewan Harrison, David K. Jackson, Ian Johnston, Dominic Kwiatkowski, Cordelia Langford, John Sillitoe on behalf of the Wellcome Sanger Institute COVID-19 Surveillance Team
EPI_ISL_932926	Lighthouse Lab in Milton Keynes	Wellcome Sanger Institute for the COVID-19 Genomics UK (COG-UK) Consortium	The Lighthouse Lab in Milton Keynes and Alex Alderton, Roberto Amato, Sonia Goncalves, Ewan Harrison, David K. Jackson, Ian Johnston, Dominic Kwiatkowski, Cordelia Langford, John Sillitoe on behalf of the Wellcome Sanger Institute COVID-19 Surveillance Team
EPI_ISL_932927	Lighthouse Lab in Alderley Park	Wellcome Sanger Institute for the COVID-19 Genomics UK (COG-UK) Consortium	Jacquelyn Wynn, Mairead Hyland, The Lighthouse Lab in Alderley Park and Alex Alderton, Roberto Amato, Sonia Goncalves, Ewan Harrison, David K. Jackson, Ian Johnston, Dominic Kwiatkowski, Cordelia Langford, John Sillitoe on behalf of the Wellcome Sanger Institute COVID-19 Surveillance Team
EPI_ISL_932928, EPI_ISL_932933, EPI_ISL_932934	Lighthouse Lab in Milton Keynes	Wellcome Sanger Institute for the COVID-19 Genomics UK (COG-UK) Consortium	The Lighthouse Lab in Milton Keynes and Alex Alderton, Roberto Amato, Sonia Goncalves, Ewan Harrison, David K. Jackson, Ian Johnston, Dominic Kwiatkowski, Cordelia Langford, John Sillitoe on behalf of the Wellcome Sanger Institute COVID-19 Surveillance Team
EPI_ISL_932935, EPI_ISL_932937, EPI_ISL_932938, EPI_ISL_932940	Lighthouse Lab in Alderley Park	Wellcome Sanger Institute for the COVID-19 Genomics UK (COG-UK) Consortium	Jacquelyn Wynn, Mairead Hyland, The Lighthouse Lab in Alderley Park and Alex Alderton, Roberto Amato, Sonia Goncalves, Ewan Harrison, David K. Jackson, Ian Johnston, Dominic Kwiatkowski, Cordelia Langford, John Sillitoe on behalf of the Wellcome Sanger Institute COVID-19 Surveillance Team
EPI_ISL_932941	Lighthouse Lab in Milton Keynes	Wellcome Sanger Institute for the COVID-19 Genomics UK (COG-UK) Consortium	The Lighthouse Lab in Milton Keynes and Alex Alderton, Roberto Amato, Sonia Goncalves, Ewan Harrison, David K. Jackson, Ian Johnston, Dominic Kwiatkowski, Cordelia Langford, John Sillitoe on behalf of the Wellcome Sanger Institute COVID-19 Surveillance Team
EPI_ISL_932942, EPI_ISL_932943	Lighthouse Lab in Alderley Park	Wellcome Sanger Institute for the COVID-19 Genomics UK (COG-UK) Consortium	Jacquelyn Wynn, Mairead Hyland, The Lighthouse Lab in Alderley Park and Alex Alderton, Roberto Amato, Sonia Goncalves, Ewan Harrison, David K. Jackson, Ian Johnston, Dominic Kwiatkowski, Cordelia Langford, John Sillitoe on behalf of the Wellcome Sanger Institute COVID-19 Surveillance Team
EPI_ISL_932945, EPI_ISL_932946	Lighthouse Lab in Milton Keynes	Wellcome Sanger Institute for the COVID-19 Genomics UK (COG-UK) Consortium	The Lighthouse Lab in Milton Keynes and Alex Alderton, Roberto Amato, Sonia Goncalves, Ewan Harrison, David K. Jackson, Ian Johnston, Dominic Kwiatkowski, Cordelia Langford, John Sillitoe on behalf of the Wellcome Sanger Institute COVID-19 Surveillance Team
EPI_ISL_932947	Lighthouse Lab in Alderley Park	Wellcome Sanger Institute for the COVID-19 Genomics UK (COG-UK) Consortium	Jacquelyn Wynn, Mairead Hyland, The Lighthouse Lab in Alderley Park and Alex Alderton, Roberto Amato, Sonia Goncalves, Ewan Harrison, David K. Jackson, Ian Johnston, Dominic Kwiatkowski, Cordelia Langford, John Sillitoe on behalf of the Wellcome Sanger Institute COVID-19 Surveillance Team
EPI_ISL_932950, EPI_ISL_932951	Lighthouse Lab in Milton Keynes	Wellcome Sanger Institute for the COVID-19 Genomics UK (COG-UK) Consortium	The Lighthouse Lab in Milton Keynes and Alex Alderton, Roberto Amato, Sonia Goncalves, Ewan Harrison, David K. Jackson, Ian Johnston, Dominic Kwiatkowski, Cordelia Langford, John Sillitoe on behalf of the Wellcome Sanger Institute COVID-19 Surveillance Team
EPI_ISL_932952	Lighthouse Lab in Alderley Park	Wellcome Sanger Institute for the COVID-19 Genomics UK (COG-UK) Consortium	Jacquelyn Wynn, Mairead Hyland, The Lighthouse Lab in Alderley Park and Alex Alderton, Roberto Amato, Sonia Goncalves, Ewan Harrison, David K. Jackson, Ian Johnston, Dominic Kwiatkowski, Cordelia Langford, John Sillitoe on behalf of the Wellcome Sanger Institute COVID-19 Surveillance Team
EPI_ISL_932953, EPI_ISL_932954	Lighthouse Lab in Milton Keynes	Wellcome Sanger Institute for the COVID-19 Genomics UK (COG-UK) Consortium	The Lighthouse Lab in Milton Keynes and Alex Alderton, Roberto Amato, Sonia Goncalves, Ewan Harrison, David K. Jackson, Ian Johnston, Dominic Kwiatkowski, Cordelia Langford, John Sillitoe on behalf of the Wellcome Sanger Institute COVID-19 Surveillance Team
EPI_ISL_932956	Lighthouse Lab in Alderley Park	Wellcome Sanger Institute for the COVID-19 Genomics UK (COG-UK) Consortium	Jacquelyn Wynn, Mairead Hyland, The Lighthouse Lab in Alderley Park and Alex Alderton, Roberto Amato, Sonia Goncalves, Ewan Harrison, David K. Jackson, Ian Johnston, Dominic Kwiatkowski, Cordelia Langford, John Sillitoe on behalf of the Wellcome Sanger Institute COVID-19 Surveillance Team
EPI_ISL_932959, EPI_ISL_932960, EPI_ISL_932962, EPI_ISL_932963	Lighthouse Lab in Milton Keynes	Wellcome Sanger Institute for the COVID-19 Genomics UK (COG-UK) Consortium	The Lighthouse Lab in Milton Keynes and Alex Alderton, Roberto Amato, Sonia Goncalves, Ewan Harrison, David K. Jackson, Ian Johnston, Dominic Kwiatkowski, Cordelia Langford, John Sillitoe on behalf of the Wellcome Sanger Institute COVID-19 Surveillance Team
EPI_ISL_932964	Lighthouse Lab in Alderley Park	Wellcome Sanger Institute for the COVID-19 Genomics UK (COG-UK) Consortium	Jacquelyn Wynn, Mairead Hyland, The Lighthouse Lab in Alderley Park and Alex Alderton, Roberto Amato, Sonia Goncalves, Ewan Harrison, David K. Jackson, Ian Johnston, Dominic Kwiatkowski, Cordelia Langford, John Sillitoe on behalf of the Wellcome Sanger Institute COVID-19 Surveillance Team
EPI_ISL_932968, EPI_ISL_932969, EPI_ISL_932971	Lighthouse Lab in Milton Keynes	Wellcome Sanger Institute for the COVID-19 Genomics UK (COG-UK) Consortium	The Lighthouse Lab in Milton Keynes and Alex Alderton, Roberto Amato, Sonia Goncalves, Ewan Harrison, David K. Jackson, Ian Johnston, Dominic Kwiatkowski, Cordelia Langford, John Sillitoe on behalf of the Wellcome Sanger Institute COVID-19 Surveillance Team
EPI_ISL_932972, EPI_ISL_932973, EPI_ISL_932975, EPI_ISL_932976	Lighthouse Lab in Alderley Park	Wellcome Sanger Institute for the COVID-19 Genomics UK (COG-UK) Consortium	Jacquelyn Wynn, Mairead Hyland, The Lighthouse Lab in Alderley Park and Alex Alderton, Roberto Amato, Sonia Goncalves, Ewan Harrison, David K. Jackson, Ian Johnston, Dominic Kwiatkowski, Cordelia Langford, John Sillitoe on behalf of the Wellcome Sanger Institute COVID-19 Surveillance Team
EPI_ISL_932978	Lighthouse Lab in Milton Keynes	Wellcome Sanger Institute for the COVID-19 Genomics UK (COG-UK) Consortium	The Lighthouse Lab in Milton Keynes and Alex Alderton, Roberto Amato, Sonia Goncalves, Ewan Harrison, David K. Jackson, Ian Johnston, Dominic Kwiatkowski, Cordelia Langford, John Sillitoe on behalf of the Wellcome Sanger Institute COVID-19 Surveillance Team
EPI_ISL_932979, EPI_ISL_932981, EPI_ISL_932982, EPI_ISL_932983, EPI_ISL_932984	Lighthouse Lab in Alderley Park	Wellcome Sanger Institute for the COVID-19 Genomics UK (COG-UK) Consortium	Jacquelyn Wynn, Mairead Hyland, The Lighthouse Lab in Alderley Park and Alex Alderton, Roberto Amato, Sonia Goncalves, Ewan Harrison, David K. Jackson, Ian Johnston, Dominic Kwiatkowski, Cordelia Langford, John Sillitoe on behalf of the Wellcome Sanger Institute COVID-19 Surveillance Team
EPI_ISL_932985	Lighthouse Lab in Milton Keynes	Wellcome Sanger Institute for the COVID-19 Genomics UK (COG-UK) Consortium	The Lighthouse Lab in Milton Keynes and Alex Alderton, Roberto Amato, Sonia Goncalves, Ewan Harrison, David K. Jackson, Ian Johnston, Dominic Kwiatkowski, Cordelia Langford, John Sillitoe on behalf of the Wellcome Sanger Institute COVID-19 Surveillance Team
EPI_ISL_932988	Lighthouse Lab in Alderley Park	Wellcome Sanger Institute for the COVID-19 Genomics UK (COG-UK) Consortium	Jacquelyn Wynn, Mairead Hyland, The Lighthouse Lab in Alderley Park and Alex Alderton, Roberto Amato, Sonia Goncalves, Ewan Harrison, David K. Jackson, Ian Johnston, Dominic Kwiatkowski, Cordelia Langford, John Sillitoe on behalf of the Wellcome Sanger Institute COVID-19 Surveillance Team
EPI_ISL_932989	Lighthouse Lab in Milton Keynes	Wellcome Sanger Institute for the COVID-19 Genomics UK (COG-UK) Consortium	The Lighthouse Lab in Milton Keynes and Alex Alderton, Roberto Amato, Sonia Goncalves, Ewan Harrison, David K. Jackson, Ian Johnston, Dominic Kwiatkowski, Cordelia Langford, John Sillitoe on behalf of the Wellcome Sanger Institute COVID-19 Surveillance Team

EPI_ISL_932990	Lighthouse Lab in Alderley Park	Wellcome Sanger Institute for the COVID-19 Genomics UK (COG-UK) Consortium	Jacquelyn Wynn, Mairead Hyland, The Lighthouse Lab in Alderley Park and Alex Alderton, Roberto Amato, Sonia Goncalves, Ewan Harrison, David K. Jackson, Ian Johnston, Dominic Kwiatkowski, Cordelia Langford, John Sillitoe on behalf of the Wellcome Sanger Institute COVID-19 Surveillance Team
EPI_ISL_932992	Lighthouse Lab in Milton Keynes	Wellcome Sanger Institute for the COVID-19 Genomics UK (COG-UK) Consortium	The Lighthouse Lab in Milton Keynes and Alex Alderton, Roberto Amato, Sonia Goncalves, Ewan Harrison, David K. Jackson, Ian Johnston, Dominic Kwiatkowski, Cordelia Langford, John Sillitoe on behalf of the Wellcome Sanger Institute COVID-19 Surveillance Team
EPI_ISL_932993	Lighthouse Lab in Alderley Park	Wellcome Sanger Institute for the COVID-19 Genomics UK (COG-UK) Consortium	Jacquelyn Wynn, Mairead Hyland, The Lighthouse Lab in Alderley Park and Alex Alderton, Roberto Amato, Sonia Goncalves, Ewan Harrison, David K. Jackson, Ian Johnston, Dominic Kwiatkowski, Cordelia Langford, John Sillitoe on behalf of the Wellcome Sanger Institute COVID-19 Surveillance Team
EPI_ISL_932997, EPI_ISL_932998, EPI_ISL_932999	Lighthouse Lab in Milton Keynes	Wellcome Sanger Institute for the COVID-19 Genomics UK (COG-UK) Consortium	The Lighthouse Lab in Milton Keynes and Alex Alderton, Roberto Amato, Sonia Goncalves, Ewan Harrison, David K. Jackson, Ian Johnston, Dominic Kwiatkowski, Cordelia Langford, John Sillitoe on behalf of the Wellcome Sanger Institute COVID-19 Surveillance Team
EPI_ISL_933001, EPI_ISL_933003	Lighthouse Lab in Alderley Park	Wellcome Sanger Institute for the COVID-19 Genomics UK (COG-UK) Consortium	Jacquelyn Wynn, Mairead Hyland, The Lighthouse Lab in Alderley Park and Alex Alderton, Roberto Amato, Sonia Goncalves, Ewan Harrison, David K. Jackson, Ian Johnston, Dominic Kwiatkowski, Cordelia Langford, John Sillitoe on behalf of the Wellcome Sanger Institute COVID-19 Surveillance Team
EPI_ISL_933008	Lighthouse Lab in Milton Keynes	Wellcome Sanger Institute for the COVID-19 Genomics UK (COG-UK) Consortium	The Lighthouse Lab in Milton Keynes and Alex Alderton, Roberto Amato, Sonia Goncalves, Ewan Harrison, David K. Jackson, Ian Johnston, Dominic Kwiatkowski, Cordelia Langford, John Sillitoe on behalf of the Wellcome Sanger Institute COVID-19 Surveillance Team
EPI_ISL_933010	Lighthouse Lab in Alderley Park	Wellcome Sanger Institute for the COVID-19 Genomics UK (COG-UK) Consortium	Jacquelyn Wynn, Mairead Hyland, The Lighthouse Lab in Alderley Park and Alex Alderton, Roberto Amato, Sonia Goncalves, Ewan Harrison, David K. Jackson, Ian Johnston, Dominic Kwiatkowski, Cordelia Langford, John Sillitoe on behalf of the Wellcome Sanger Institute COVID-19 Surveillance Team
EPI_ISL_933012	Lighthouse Lab in Milton Keynes	Wellcome Sanger Institute for the COVID-19 Genomics UK (COG-UK) Consortium	The Lighthouse Lab in Milton Keynes and Alex Alderton, Roberto Amato, Sonia Goncalves, Ewan Harrison, David K. Jackson, Ian Johnston, Dominic Kwiatkowski, Cordelia Langford, John Sillitoe on behalf of the Wellcome Sanger Institute COVID-19 Surveillance Team
EPI_ISL_933013	Lighthouse Lab in Alderley Park	Wellcome Sanger Institute for the COVID-19 Genomics UK (COG-UK) Consortium	Jacquelyn Wynn, Mairead Hyland, The Lighthouse Lab in Alderley Park and Alex Alderton, Roberto Amato, Sonia Goncalves, Ewan Harrison, David K. Jackson, Ian Johnston, Dominic Kwiatkowski, Cordelia Langford, John Sillitoe on behalf of the Wellcome Sanger Institute COVID-19 Surveillance Team
EPI_ISL_933014	Lighthouse Lab in Milton Keynes	Wellcome Sanger Institute for the COVID-19 Genomics UK (COG-UK) Consortium	The Lighthouse Lab in Milton Keynes and Alex Alderton, Roberto Amato, Sonia Goncalves, Ewan Harrison, David K. Jackson, Ian Johnston, Dominic Kwiatkowski, Cordelia Langford, John Sillitoe on behalf of the Wellcome Sanger Institute COVID-19 Surveillance Team
EPI_ISL_933015	Lighthouse Lab in Alderley Park	Wellcome Sanger Institute for the COVID-19 Genomics UK (COG-UK) Consortium	Jacquelyn Wynn, Mairead Hyland, The Lighthouse Lab in Alderley Park and Alex Alderton, Roberto Amato, Sonia Goncalves, Ewan Harrison, David K. Jackson, Ian Johnston, Dominic Kwiatkowski, Cordelia Langford, John Sillitoe on behalf of the Wellcome Sanger Institute COVID-19 Surveillance Team
EPI_ISL_933016	Lighthouse Lab in Milton Keynes	Wellcome Sanger Institute for the COVID-19 Genomics UK (COG-UK) Consortium	The Lighthouse Lab in Milton Keynes and Alex Alderton, Roberto Amato, Sonia Goncalves, Ewan Harrison, David K. Jackson, Ian Johnston, Dominic Kwiatkowski, Cordelia Langford, John Sillitoe on behalf of the Wellcome Sanger Institute COVID-19 Surveillance Team
EPI_ISL_933017	Lighthouse Lab in Alderley Park	Wellcome Sanger Institute for the COVID-19 Genomics UK (COG-UK) Consortium	Jacquelyn Wynn, Mairead Hyland, The Lighthouse Lab in Alderley Park and Alex Alderton, Roberto Amato, Sonia Goncalves, Ewan Harrison, David K. Jackson, Ian Johnston, Dominic Kwiatkowski, Cordelia Langford, John Sillitoe on behalf of the Wellcome Sanger Institute COVID-19 Surveillance Team
EPI_ISL_933023	Lighthouse Lab in Milton Keynes	Wellcome Sanger Institute for the COVID-19 Genomics UK (COG-UK) Consortium	The Lighthouse Lab in Milton Keynes and Alex Alderton, Roberto Amato, Sonia Goncalves, Ewan Harrison, David K. Jackson, Ian Johnston, Dominic Kwiatkowski, Cordelia Langford, John Sillitoe on behalf of the Wellcome Sanger Institute COVID-19 Surveillance Team
EPI_ISL_933024, EPI_ISL_933025, EPI_ISL_933026	Lighthouse Lab in Alderley Park	Wellcome Sanger Institute for the COVID-19 Genomics UK (COG-UK) Consortium	Jacquelyn Wynn, Mairead Hyland, The Lighthouse Lab in Alderley Park and Alex Alderton, Roberto Amato, Sonia Goncalves, Ewan Harrison, David K. Jackson, Ian Johnston, Dominic Kwiatkowski, Cordelia Langford, John Sillitoe on behalf of the Wellcome Sanger Institute COVID-19 Surveillance Team
EPI_ISL_933027	Lighthouse Lab in Milton Keynes	Wellcome Sanger Institute for the COVID-19 Genomics UK (COG-UK) Consortium	The Lighthouse Lab in Milton Keynes and Alex Alderton, Roberto Amato, Sonia Goncalves, Ewan Harrison, David K. Jackson, Ian Johnston, Dominic Kwiatkowski, Cordelia Langford, John Sillitoe on behalf of the Wellcome Sanger Institute COVID-19 Surveillance Team
EPI_ISL_933029	Lighthouse Lab in Alderley Park	Wellcome Sanger Institute for the COVID-19 Genomics UK (COG-UK) Consortium	Jacquelyn Wynn, Mairead Hyland, The Lighthouse Lab in Alderley Park and Alex Alderton, Roberto Amato, Sonia Goncalves, Ewan Harrison, David K. Jackson, Ian Johnston, Dominic Kwiatkowski, Cordelia Langford, John Sillitoe on behalf of the Wellcome Sanger Institute COVID-19 Surveillance Team
EPI_ISL_933030	Lighthouse Lab in Milton Keynes	Wellcome Sanger Institute for the COVID-19 Genomics UK (COG-UK) Consortium	The Lighthouse Lab in Milton Keynes and Alex Alderton, Roberto Amato, Sonia Goncalves, Ewan Harrison, David K. Jackson, Ian Johnston, Dominic Kwiatkowski, Cordelia Langford, John Sillitoe on behalf of the Wellcome Sanger Institute COVID-19 Surveillance Team
EPI_ISL_933031	Lighthouse Lab in Alderley Park	Wellcome Sanger Institute for the COVID-19 Genomics UK (COG-UK) Consortium	Jacquelyn Wynn, Mairead Hyland, The Lighthouse Lab in Alderley Park and Alex Alderton, Roberto Amato, Sonia Goncalves, Ewan Harrison, David K. Jackson, Ian Johnston, Dominic Kwiatkowski, Cordelia Langford, John Sillitoe on behalf of the Wellcome Sanger Institute COVID-19 Surveillance Team
EPI_ISL_933032, EPI_ISL_933034, EPI_ISL_933039	Lighthouse Lab in Milton Keynes	Wellcome Sanger Institute for the COVID-19 Genomics UK (COG-UK) Consortium	The Lighthouse Lab in Milton Keynes and Alex Alderton, Roberto Amato, Sonia Goncalves, Ewan Harrison, David K. Jackson, Ian Johnston, Dominic Kwiatkowski, Cordelia Langford, John Sillitoe on behalf of the Wellcome Sanger Institute COVID-19 Surveillance Team
EPI_ISL_933041	Lighthouse Lab in Alderley Park	Wellcome Sanger Institute for the COVID-19 Genomics UK (COG-UK) Consortium	Jacquelyn Wynn, Mairead Hyland, The Lighthouse Lab in Alderley Park and Alex Alderton, Roberto Amato, Sonia Goncalves, Ewan Harrison, David K. Jackson, Ian Johnston, Dominic Kwiatkowski, Cordelia Langford, John Sillitoe on behalf of the Wellcome Sanger Institute COVID-19 Surveillance Team
EPI_ISL_933043	Lighthouse Lab in Milton Keynes	Wellcome Sanger Institute for the COVID-19 Genomics UK (COG-UK) Consortium	The Lighthouse Lab in Milton Keynes and Alex Alderton, Roberto Amato, Sonia Goncalves, Ewan Harrison, David K. Jackson, Ian Johnston, Dominic Kwiatkowski, Cordelia Langford, John Sillitoe on behalf of the Wellcome Sanger Institute COVID-19 Surveillance Team
EPI_ISL_933045	Lighthouse Lab in Alderley Park	Wellcome Sanger Institute for the COVID-19 Genomics UK (COG-UK) Consortium	Jacquelyn Wynn, Mairead Hyland, The Lighthouse Lab in Alderley Park and Alex Alderton, Roberto Amato, Sonia Goncalves, Ewan Harrison, David K. Jackson, Ian Johnston, Dominic Kwiatkowski, Cordelia Langford, John Sillitoe on behalf of the Wellcome Sanger Institute COVID-19 Surveillance Team
EPI_ISL_933046, EPI_ISL_933047, EPI_ISL_933048	Lighthouse Lab in Milton Keynes	Wellcome Sanger Institute for the COVID-19 Genomics UK (COG-UK) Consortium	The Lighthouse Lab in Milton Keynes and Alex Alderton, Roberto Amato, Sonia Goncalves, Ewan Harrison, David K. Jackson, Ian Johnston, Dominic Kwiatkowski, Cordelia Langford, John Sillitoe on behalf of the Wellcome Sanger Institute COVID-19 Surveillance Team
EPI_ISL_933049, EPI_ISL_933052	Lighthouse Lab in Alderley Park	Wellcome Sanger Institute for the COVID-19 Genomics UK (COG-UK) Consortium	Jacquelyn Wynn, Mairead Hyland, The Lighthouse Lab in Alderley Park and Alex Alderton, Roberto Amato, Sonia Goncalves, Ewan Harrison, David K. Jackson, Ian Johnston, Dominic Kwiatkowski, Cordelia Langford, John Sillitoe on behalf of the Wellcome Sanger Institute COVID-19 Surveillance Team
EPI_ISL_933055, EPI_ISL_933057	Lighthouse Lab in Milton Keynes	Wellcome Sanger Institute for the COVID-19 Genomics UK (COG-UK) Consortium	The Lighthouse Lab in Milton Keynes and Alex Alderton, Roberto Amato, Sonia Goncalves, Ewan Harrison, David K. Jackson, Ian Johnston, Dominic Kwiatkowski, Cordelia Langford, John Sillitoe on behalf of the Wellcome Sanger Institute COVID-19 Surveillance Team
EPI_ISL_933059	Lighthouse Lab in Alderley Park	Wellcome Sanger Institute for the COVID-19 Genomics UK (COG-UK) Consortium	Jacquelyn Wynn, Mairead Hyland, The Lighthouse Lab in Alderley Park and Alex Alderton, Roberto Amato, Sonia Goncalves, Ewan Harrison, David K. Jackson, Ian Johnston, Dominic Kwiatkowski, Cordelia Langford, John Sillitoe on behalf of the Wellcome Sanger Institute COVID-19 Surveillance Team
EPI_ISL_933060	Lighthouse Lab in Milton Keynes	Wellcome Sanger Institute for the COVID-19 Genomics UK (COG-UK) Consortium	The Lighthouse Lab in Milton Keynes and Alex Alderton, Roberto Amato, Sonia Goncalves, Ewan Harrison, David K. Jackson, Ian Johnston, Dominic Kwiatkowski, Cordelia Langford, John Sillitoe on behalf of the Wellcome Sanger Institute COVID-19 Surveillance Team
EPI_ISL_933062	Lighthouse Lab in Alderley Park	Wellcome Sanger Institute for the COVID-19 Genomics UK (COG-UK) Consortium	Jacquelyn Wynn, Mairead Hyland, The Lighthouse Lab in Alderley Park and Alex Alderton, Roberto Amato, Sonia Goncalves, Ewan Harrison, David K. Jackson, Ian Johnston, Dominic Kwiatkowski, Cordelia Langford, John Sillitoe on behalf of the Wellcome Sanger Institute COVID-19 Surveillance Team
EPI_ISL_933063	Lighthouse Lab in Milton Keynes	Wellcome Sanger Institute for the COVID-19 Genomics UK (COG-UK) Consortium	The Lighthouse Lab in Milton Keynes and Alex Alderton, Roberto Amato, Sonia Goncalves, Ewan Harrison, David K. Jackson, Ian Johnston, Dominic Kwiatkowski, Cordelia Langford, John Sillitoe on behalf of the Wellcome Sanger Institute COVID-19 Surveillance Team
EPI_ISL_933064, EPI_ISL_933066	Lighthouse Lab in Alderley Park	Wellcome Sanger Institute for the COVID-19 Genomics UK (COG-UK) Consortium	Jacquelyn Wynn, Mairead Hyland, The Lighthouse Lab in Alderley Park and Alex Alderton, Roberto Amato, Sonia Goncalves, Ewan Harrison, David K. Jackson, Ian Johnston, Dominic Kwiatkowski, Cordelia Langford, John Sillitoe on behalf of the Wellcome Sanger Institute COVID-19 Surveillance Team
EPI_ISL_933068, EPI_ISL_933071, EPI_ISL_933075, EPI_ISL_933076, EPI_ISL_933078, EPI_ISL_933081, EPI_ISL_933082	Lighthouse Lab in Milton Keynes	Wellcome Sanger Institute for the COVID-19 Genomics UK (COG-UK) Consortium	The Lighthouse Lab in Milton Keynes and Alex Alderton, Roberto Amato, Sonia Goncalves, Ewan Harrison, David K. Jackson, Ian Johnston, Dominic Kwiatkowski, Cordelia Langford, John Sillitoe on behalf of the Wellcome Sanger Institute COVID-19 Surveillance Team
EPI_ISL_933083, EPI_ISL_933084	Lighthouse Lab in Alderley Park	Wellcome Sanger Institute for the COVID-19 Genomics UK (COG-UK) Consortium	Jacquelyn Wynn, Mairead Hyland, The Lighthouse Lab in Alderley Park and Alex Alderton, Roberto Amato, Sonia Goncalves, Ewan Harrison, David K. Jackson, Ian Johnston, Dominic Kwiatkowski, Cordelia Langford, John Sillitoe on behalf of the Wellcome Sanger Institute COVID-19 Surveillance Team
EPI_ISL_933085	Lighthouse Lab in Milton Keynes	Wellcome Sanger Institute for the COVID-19 Genomics UK (COG-UK) Consortium	The Lighthouse Lab in Milton Keynes and Alex Alderton, Roberto Amato, Sonia Goncalves, Ewan Harrison, David K. Jackson, Ian Johnston, Dominic Kwiatkowski, Cordelia Langford, John Sillitoe on behalf of the Wellcome Sanger Institute COVID-19 Surveillance Team
EPI_ISL_933086, EPI_ISL_933087	Lighthouse Lab in Alderley Park	Wellcome Sanger Institute for the COVID-19 Genomics UK	Jacquelyn Wynn, Mairead Hyland, The Lighthouse Lab in Alderley Park and Alex Alderton, Roberto Amato, Sonia Goncalves, Ewan Harrison, David K.

		(COG-UK) Consortium	Jackson, Ian Johnston, Dominic Kwiatkowski, Cordelia Langford, John Sillitoe on behalf of the Wellcome Sanger Institute COVID-19 Surveillance Team
EPI_ISL_933088	Lighthouse Lab in Milton Keynes	Wellcome Sanger Institute for the COVID-19 Genomics UK (COG-UK) Consortium	The Lighthouse Lab in Milton Keynes and Alex Alderton, Roberto Amato, Sonia Goncalves, Ewan Harrison, David K. Jackson, Ian Johnston, Dominic Kwiatkowski, Cordelia Langford, John Sillitoe on behalf of the Wellcome Sanger Institute COVID-19 Surveillance Team
EPI_ISL_933089, EPI_ISL_933090	Lighthouse Lab in Alderley Park	Wellcome Sanger Institute for the COVID-19 Genomics UK (COG-UK) Consortium	Jacquelyn Wynn, Mairead Hyland, The Lighthouse Lab in Alderley Park and Alex Alderton, Roberto Amato, Sonia Goncalves, Ewan Harrison, David K. Jackson, Ian Johnston, Dominic Kwiatkowski, Cordelia Langford, John Sillitoe on behalf of the Wellcome Sanger Institute COVID-19 Surveillance Team
EPI_ISL_933091	Lighthouse Lab in Milton Keynes	Wellcome Sanger Institute for the COVID-19 Genomics UK (COG-UK) Consortium	The Lighthouse Lab in Milton Keynes and Alex Alderton, Roberto Amato, Sonia Goncalves, Ewan Harrison, David K. Jackson, Ian Johnston, Dominic Kwiatkowski, Cordelia Langford, John Sillitoe on behalf of the Wellcome Sanger Institute COVID-19 Surveillance Team
EPI_ISL_933092, EPI_ISL_933093, EPI_ISL_933094, EPI_ISL_933095, EPI_ISL_933097, EPI_ISL_933098	Lighthouse Lab in Alderley Park	Wellcome Sanger Institute for the COVID-19 Genomics UK (COG-UK) Consortium	Jacquelyn Wynn, Mairead Hyland, The Lighthouse Lab in Alderley Park and Alex Alderton, Roberto Amato, Sonia Goncalves, Ewan Harrison, David K. Jackson, Ian Johnston, Dominic Kwiatkowski, Cordelia Langford, John Sillitoe on behalf of the Wellcome Sanger Institute COVID-19 Surveillance Team
EPI_ISL_933101	Lighthouse Lab in Milton Keynes	Wellcome Sanger Institute for the COVID-19 Genomics UK (COG-UK) Consortium	The Lighthouse Lab in Milton Keynes and Alex Alderton, Roberto Amato, Sonia Goncalves, Ewan Harrison, David K. Jackson, Ian Johnston, Dominic Kwiatkowski, Cordelia Langford, John Sillitoe on behalf of the Wellcome Sanger Institute COVID-19 Surveillance Team
EPI_ISL_933102, EPI_ISL_933103, EPI_ISL_933104, EPI_ISL_933105, EPI_ISL_933106	Lighthouse Lab in Alderley Park	Wellcome Sanger Institute for the COVID-19 Genomics UK (COG-UK) Consortium	Jacquelyn Wynn, Mairead Hyland, The Lighthouse Lab in Alderley Park and Alex Alderton, Roberto Amato, Sonia Goncalves, Ewan Harrison, David K. Jackson, Ian Johnston, Dominic Kwiatkowski, Cordelia Langford, John Sillitoe on behalf of the Wellcome Sanger Institute COVID-19 Surveillance Team
EPI_ISL_933108	Lighthouse Lab in Milton Keynes	Wellcome Sanger Institute for the COVID-19 Genomics UK (COG-UK) Consortium	The Lighthouse Lab in Milton Keynes and Alex Alderton, Roberto Amato, Sonia Goncalves, Ewan Harrison, David K. Jackson, Ian Johnston, Dominic Kwiatkowski, Cordelia Langford, John Sillitoe on behalf of the Wellcome Sanger Institute COVID-19 Surveillance Team
EPI_ISL_933111, EPI_ISL_933112	Lighthouse Lab in Alderley Park	Wellcome Sanger Institute for the COVID-19 Genomics UK (COG-UK) Consortium	Jacquelyn Wynn, Mairead Hyland, The Lighthouse Lab in Alderley Park and Alex Alderton, Roberto Amato, Sonia Goncalves, Ewan Harrison, David K. Jackson, Ian Johnston, Dominic Kwiatkowski, Cordelia Langford, John Sillitoe on behalf of the Wellcome Sanger Institute COVID-19 Surveillance Team
EPI_ISL_933114, EPI_ISL_933115, EPI_ISL_933117, EPI_ISL_933118, EPI_ISL_933119, EPI_ISL_933121, EPI_ISL_933122, EPI_ISL_933129, EPI_ISL_933132, EPI_ISL_933133, EPI_ISL_933137, EPI_ISL_933138, EPI_ISL_933139, EPI_ISL_933144, EPI_ISL_933146, EPI_ISL_933147, EPI_ISL_933148, EPI_ISL_933151, EPI_ISL_933153, EPI_ISL_933155, EPI_ISL_933156, EPI_ISL_933157, EPI_ISL_933159, EPI_ISL_933160, EPI_ISL_933161, EPI_ISL_933163, EPI_ISL_933164, EPI_ISL_933165, EPI_ISL_933168, EPI_ISL_933169, EPI_ISL_933172, EPI_ISL_933173, EPI_ISL_933174, EPI_ISL_933175, EPI_ISL_933176, EPI_ISL_933177, EPI_ISL_933178, EPI_ISL_933179, EPI_ISL_933180, EPI_ISL_933182, EPI_ISL_933183, EPI_ISL_933185, EPI_ISL_933186, EPI_ISL_933189, EPI_ISL_933191, EPI_ISL_933192, EPI_ISL_933193, EPI_ISL_933194, EPI_ISL_933195, EPI_ISL_933197, EPI_ISL_933198, EPI_ISL_933201, EPI_ISL_933202, EPI_ISL_933204, EPI_ISL_933205, EPI_ISL_933206, EPI_ISL_933208, EPI_ISL_933209, EPI_ISL_933211, EPI_ISL_933212, EPI_ISL_933216, EPI_ISL_933217, EPI_ISL_933220, EPI_ISL_933222, EPI_ISL_933223, EPI_ISL_933224, EPI_ISL_933225, EPI_ISL_933229, EPI_ISL_933230, EPI_ISL_933231, EPI_ISL_933232, EPI_ISL_933234, EPI_ISL_933235, EPI_ISL_933236, EPI_ISL_933237, EPI_ISL_933240, EPI_ISL_933241, EPI_ISL_933244, EPI_ISL_933245, EPI_ISL_933247, EPI_ISL_933248, EPI_ISL_933249, EPI_ISL_933250, EPI_ISL_933253, EPI_ISL_933254, EPI_ISL_933255, EPI_ISL_933257, EPI_ISL_933260, EPI_ISL_933262, EPI_ISL_933263, EPI_ISL_933264, EPI_ISL_933267, EPI_ISL_933268, EPI_ISL_933274, EPI_ISL_933275, EPI_ISL_933276, EPI_ISL_933278, EPI_ISL_933279, EPI_ISL_933280, EPI_ISL_933281, EPI_ISL_933284, EPI_ISL_933285, EPI_ISL_933286, EPI_ISL_933290, EPI_ISL_933291, EPI_ISL_933292, EPI_ISL_933294, EPI_ISL_933295, EPI_ISL_933296, EPI_ISL_933297, EPI_ISL_933299, EPI_ISL_933300, EPI_ISL_933302, EPI_ISL_933305, EPI_ISL_933306, EPI_ISL_933307, EPI_ISL_933309, EPI_ISL_933310, EPI_ISL_933313, EPI_ISL_933314, EPI_ISL_933315, EPI_ISL_933317, EPI_ISL_933318, EPI_ISL_933319, EPI_ISL_933320, EPI_ISL_933322, EPI_ISL_933323, EPI_ISL_933324, EPI_ISL_933327, EPI_ISL_933328, EPI_ISL_933329, EPI_ISL_933330, EPI_ISL_933331, EPI_ISL_933335, EPI_ISL_933337, EPI_ISL_933338, EPI_ISL_933339, EPI_ISL_933342, EPI_ISL_933347, EPI_ISL_933349, EPI_ISL_933351, EPI_ISL_933352, EPI_ISL_933353, EPI_ISL_933356, EPI_ISL_933357, EPI_ISL_933363, EPI_ISL_933365, EPI_ISL_933366, EPI_ISL_933367, EPI_ISL_933368, EPI_ISL_933369, EPI_ISL_933370, EPI_ISL_933374, EPI_ISL_933377, EPI_ISL_933379, EPI_ISL_933380, EPI_ISL_933381, EPI_ISL_933383, EPI_ISL_933384, EPI_ISL_933385, EPI_ISL_933389, EPI_ISL_933390, EPI_ISL_933391, EPI_ISL_933392, EPI_ISL_933395, EPI_ISL_933398, EPI_ISL_933399, EPI_ISL_933402, EPI_ISL_933403, EPI_ISL_933404, EPI_ISL_933406, EPI_ISL_933407, EPI_ISL_933409, EPI_ISL_933411, EPI_ISL_933413, EPI_ISL_933415, EPI_ISL_933416, EPI_ISL_933418, EPI_ISL_933419, EPI_ISL_933422, EPI_ISL_933423			
see above	Lighthouse Lab in Cambridge	Wellcome Sanger Institute for the COVID-19 Genomics UK (COG-UK) Consortium	Rob Howes, The Lighthouse Lab in Cambridge and Alex Alderton, Roberto Amato, Sonia Goncalves, Ewan Harrison, David K. Jackson, Ian Johnston, Dominic Kwiatkowski, Cordelia Langford, John Sillitoe on behalf of the Wellcome Sanger Institute COVID-19 Surveillance Team
EPI_ISL_937549, EPI_ISL_937550, EPI_ISL_937551, EPI_ISL_937553, EPI_ISL_937554, EPI_ISL_937557, EPI_ISL_937558, EPI_ISL_937559, EPI_ISL_937562, EPI_ISL_937564, EPI_ISL_937568, EPI_ISL_937569, EPI_ISL_937570, EPI_ISL_937571, EPI_ISL_937573, EPI_ISL_937576, EPI_ISL_937578, EPI_ISL_937579, EPI_ISL_937580, EPI_ISL_937581, EPI_ISL_937583, EPI_ISL_937586, EPI_ISL_937587, EPI_ISL_937588, EPI_ISL_937590, EPI_ISL_937592, EPI_ISL_937594, EPI_ISL_937595, EPI_ISL_937596, EPI_ISL_937599, EPI_ISL_937601, EPI_ISL_937604, EPI_ISL_937605, EPI_ISL_937609, EPI_ISL_937610, EPI_ISL_937612, EPI_ISL_937614, EPI_ISL_937616, EPI_ISL_937618, EPI_ISL_937620, EPI_ISL_937621, EPI_ISL_937623, EPI_ISL_937627, EPI_ISL_937630, EPI_ISL_937634, EPI_ISL_937636, EPI_ISL_937638, EPI_ISL_937639, EPI_ISL_937640, EPI_ISL_937641, EPI_ISL_937643, EPI_ISL_937648, EPI_ISL_937649, EPI_ISL_937650, EPI_ISL_937651, EPI_ISL_937653, EPI_ISL_937658, EPI_ISL_937663, EPI_ISL_937664, EPI_ISL_937665, EPI_ISL_937666, EPI_ISL_937668, EPI_ISL_937669, EPI_ISL_937670, EPI_ISL_937672, EPI_ISL_937674, EPI_ISL_937675, EPI_ISL_937680, EPI_ISL_937682, EPI_ISL_937685, EPI_ISL_937687, EPI_ISL_937688, EPI_ISL_937689, EPI_ISL_937693, EPI_ISL_937697, EPI_ISL_937699, EPI_ISL_937700, EPI_ISL_937703, EPI_ISL_937704, EPI_ISL_937707, EPI_ISL_937709, EPI_ISL_937710, EPI_ISL_937711, EPI_ISL_937712, EPI_ISL_937714, EPI_ISL_937715, EPI_ISL_937716, EPI_ISL_937717, EPI_ISL_937720, EPI_ISL_937721, EPI_ISL_937724, EPI_ISL_937726, EPI_ISL_937730, EPI_ISL_937734, EPI_ISL_937737, EPI_ISL_937738, EPI_ISL_937741, EPI_ISL_937742, EPI_ISL_937744, EPI_ISL_937745, EPI_ISL_937746, EPI_ISL_937748, EPI_ISL_937749, EPI_ISL_937751, EPI_ISL_937752, EPI_ISL_937753, EPI_ISL_937761, EPI_ISL_937762, EPI_ISL_937763, EPI_ISL_937765, EPI_ISL_937766, EPI_ISL_937767, EPI_ISL_937768, EPI_ISL_937769, EPI_ISL_937770, EPI_ISL_937777, EPI_ISL_937780, EPI_ISL_937781, EPI_ISL_937784, EPI_ISL_937787, EPI_ISL_937790, EPI_ISL_937792, EPI_ISL_937794, EPI_ISL_937798, EPI_ISL_937799, EPI_ISL_937800, EPI_ISL_937801, EPI_ISL_937802, EPI_ISL_937803, EPI_ISL_937805, EPI_ISL_937806, EPI_ISL_937808, EPI_ISL_937811, EPI_ISL_937815, EPI_ISL_937817, EPI_ISL_937820, EPI_ISL_937822, EPI_ISL_937824, EPI_ISL_937826, EPI_ISL_937829, EPI_ISL_937830, EPI_ISL_937831, EPI_ISL_937835, EPI_ISL_937837, EPI_ISL_937839, EPI_ISL_937840, EPI_ISL_937843, EPI_ISL_937849, EPI_ISL_937850, EPI_ISL_937852, EPI_ISL_937853, EPI_ISL_937854, EPI_ISL_937860, EPI_ISL_937862, EPI_ISL_937863, EPI_ISL_937864, EPI_ISL_937865, EPI_ISL_937867, EPI_ISL_937872, EPI_ISL_937873, EPI_ISL_937874, EPI_ISL_937875, EPI_ISL_937877, EPI_ISL_937881, EPI_ISL_937883			
see above	Lighthouse Lab in Alderley Park	Wellcome Sanger Institute for the COVID-19 Genomics UK (COG-UK) Consortium	Jacquelyn Wynn, Mairead Hyland, The Lighthouse Lab in Alderley Park and Alex Alderton, Roberto Amato, Sonia Goncalves, Ewan Harrison, David K. Jackson, Ian Johnston, Dominic Kwiatkowski, Cordelia Langford, John Sillitoe on behalf of the Wellcome Sanger Institute COVID-19 Surveillance Team
EPI_ISL_937886, EPI_ISL_937887, EPI_ISL_937888, EPI_ISL_937889, EPI_ISL_937891, EPI_ISL_937892, EPI_ISL_937893, EPI_ISL_937894, EPI_ISL_937895, EPI_ISL_937896, EPI_ISL_937897, EPI_ISL_937898, EPI_ISL_937899, EPI_ISL_937900, EPI_ISL_937901, EPI_ISL_937902, EPI_ISL_937903, EPI_ISL_937904, EPI_ISL_937905, EPI_ISL_937906, EPI_ISL_937907, EPI_ISL_937908, EPI_ISL_937909, EPI_ISL_937910, EPI_ISL_937911, EPI_ISL_937912, EPI_ISL_937913, EPI_ISL_937914, EPI_ISL_937915, EPI_ISL_937916, EPI_ISL_937917, EPI_ISL_937918, EPI_ISL_937919, EPI_ISL_937920, EPI_ISL_937921, EPI_ISL_937922, EPI_ISL_937923, EPI_ISL_937924, EPI_ISL_937925, EPI_ISL_937926, EPI_ISL_937927, EPI_ISL_937928, EPI_ISL_937929, EPI_ISL_937930, EPI_ISL_937931, EPI_ISL_937932, EPI_ISL_937933, EPI_ISL_937934, EPI_ISL_937935, EPI_ISL_937936, EPI_ISL_937937, EPI_ISL_937938, EPI_ISL_937939, EPI_ISL_937940, EPI_ISL_937941, EPI_ISL_937942, EPI_ISL_937943, EPI_ISL_937944, EPI_ISL_937945, EPI_ISL_937946, EPI_ISL_937947, EPI_ISL_937948, EPI_ISL_937949, EPI_ISL_937950, EPI_ISL_937951, EPI_ISL_937952, EPI_ISL_937953, EPI_ISL_937954, EPI_ISL_937955, EPI_ISL_937956, EPI_ISL_937957, EPI_ISL_937958, EPI_ISL_937959, EPI_ISL_937960, EPI_ISL_937961, EPI_ISL_937962, EPI_ISL_937963, EPI_ISL_937964, EPI_ISL_937965, EPI_ISL_937966, EPI_ISL_937967, EPI_ISL_937968, EPI_ISL_937969, EPI_ISL_937970, EPI_ISL_937971, EPI_ISL_937972, EPI_ISL_937973, EPI_ISL_937974, EPI_ISL_937975, EPI_ISL_937976, EPI_ISL_937977, EPI_ISL_937978, EPI_ISL_937979, EPI_ISL_937980, EPI_ISL_937981, EPI_ISL_937982, EPI_ISL_937983, EPI_ISL_937984, EPI_ISL_937985, EPI_ISL_937986, EPI_ISL_937987, EPI_ISL_937988, EPI_ISL_937989, EPI_ISL_937990, EPI_ISL_937991, EPI_ISL_937992, EPI_ISL_937993, EPI_ISL_937994, EPI_ISL_937995, EPI_ISL_937996, EPI_ISL_937997, EPI_ISL_937998, EPI_ISL_937999, EPI_ISL_938000, EPI_ISL_938001, EPI_ISL_938002, EPI_ISL_938003, EPI_ISL_938004, EPI_ISL_938005, EPI_ISL_938006, EPI_ISL_938007, EPI_ISL_938008, EPI_ISL_938009, EPI_ISL_938010, EPI_ISL_938011, EPI_ISL_938012, EPI_ISL_938013, EPI_ISL_938014, EPI_ISL_938015, EPI_ISL_938016, EPI_ISL_938017, EPI_ISL_938018, EPI_ISL_938019, EPI_ISL_938020, EPI_ISL_938021, EPI_ISL_938022, EPI_ISL_938023, EPI_ISL_938024, EPI_ISL_938025, EPI_ISL_938026, EPI_ISL_938027, EPI_ISL_938028, EPI_ISL_938029, EPI_ISL_938030, EPI_ISL_938031, EPI_ISL_938032, EPI_ISL_938033, EPI_ISL_938034, EPI_ISL_938035, EPI_ISL_938036, EPI_ISL_938037, EPI_ISL_938038, EPI_ISL_938039, EPI_ISL_938040, EPI_ISL_938041, EPI_ISL_938042, EPI_ISL_938043, EPI_ISL_938044, EPI_ISL_938045, EPI_ISL_938046, EPI_ISL_938047, EPI_ISL_938048, EPI_ISL_938049, EPI_ISL_938050, EPI_ISL_938051, EPI_ISL_938052, EPI_ISL_938053, EPI_ISL_938054, EPI_ISL_938055, EPI_ISL_938056, EPI_ISL_938058, EPI_ISL_938059, EPI_ISL_938060, EPI_ISL_938061, EPI_ISL_938062, EPI_ISL_938063, EPI_ISL_938064, EPI_ISL_938065, EPI_ISL_938066, EPI_ISL_938067, EPI_ISL_938068, EPI_ISL_938069, EPI_ISL_938070, EPI_ISL_938071, EPI_ISL_938072, EPI_ISL_938073, EPI_ISL_938074, EPI_ISL_938075, EPI_ISL_938076, EPI_ISL_938077, EPI_ISL_938078, EPI_ISL_938079, EPI_ISL_938080, EPI_ISL_938081, EPI_ISL_938082, EPI_ISL_938083, EPI_ISL_938084, EPI_ISL_938085, EPI_ISL_938086, EPI_ISL_938087, EPI_ISL_938088, EPI_ISL_938089, EPI_ISL_938090, EPI_ISL_938091, EPI_ISL_938092, EPI_ISL_938093, EPI_ISL_938094, EPI_ISL_938095, EPI_ISL_938096, EPI_ISL_938097, EPI_ISL_938098, EPI_ISL_938099, EPI_ISL_938100, EPI_ISL_938101, EPI_ISL_938102, EPI_ISL_938103, EPI_ISL_938104, EPI_ISL_938105, EPI_ISL_938106, EPI_ISL_938107, EPI_ISL_938108, EPI_ISL_938109, EPI_ISL_938110, EPI_ISL_938111, EPI_ISL_938112, EPI_ISL_938113, EPI_ISL_938114, EPI_ISL_938115, EPI_ISL_938116, EPI_ISL_938117, EPI_ISL_938118, EPI_ISL_938119, EPI_ISL_938121, EPI_ISL_938122, EPI_ISL_938123, EPI_ISL_938124, EPI_ISL_938125, EPI_ISL_938126, EPI_ISL_938127, EPI_ISL_938128, EPI_ISL_938129, EPI_ISL_938130, EPI_ISL_938131, EPI_ISL_938132, EPI_ISL_938133, EPI_ISL_938134, EPI_ISL_938135, EPI_ISL_938136, EPI_ISL_938137, EPI_ISL_938138, EPI_ISL_938139, EPI_ISL_938140, EPI_ISL_938141, EPI_ISL_938142, EPI_ISL_938143, EPI_ISL_938144, EPI_ISL_938145, EPI_ISL_938146, EPI_ISL_938147, EPI_ISL_938148, EPI_ISL_938149, EPI_ISL_938150, EPI_ISL_938152, EPI_ISL_938153, EPI_ISL_938154, EPI_ISL_938155, EPI_ISL_938156, EPI_ISL_938157, EPI_ISL_938158, EPI_ISL_938159, EPI_ISL_938160, EPI_ISL_938161, EPI_ISL_938162, EPI_ISL_938163, EPI_ISL_938164, EPI_ISL_938165, EPI_ISL_938166, EPI_ISL_938167, EPI_ISL_938168, EPI_ISL_938169, EPI_ISL_938170, EPI_ISL_938171, EPI_ISL_938172, EPI_ISL_938173, EPI_ISL_938174, EPI_ISL_938175, EPI_ISL_938176, EPI_ISL_938177, EPI_ISL_938178, EPI_ISL_938179, EPI_ISL_938180, EPI_ISL_938181, EPI_ISL_938182, EPI_ISL_938183, EPI_ISL_938184, EPI_ISL_938185, EPI_ISL_938186, EPI_ISL_938187, EPI_ISL_938188, EPI_ISL_938189, EPI_ISL_938190, EPI_ISL_938191, EPI_ISL_938192, EPI_ISL_938193, EPI_ISL_938194, EPI_ISL_938195, EPI_ISL_938196, EPI_ISL_938197, EPI_ISL_938198, EPI_ISL_938199, EPI_ISL_938200, EPI_ISL_938201, EPI_ISL_938202, EPI_ISL_938203, EPI_ISL_938204, EPI_ISL_938205, EPI_ISL_938206, EPI_ISL_938207, EPI_ISL_938208, EPI_ISL_938209, EPI_ISL_938210, EPI_ISL_938211, EPI_ISL_938212, EPI_ISL_938213, EPI_ISL_938214, EPI_ISL_938215, EPI_ISL_938216, EPI_ISL_938217, EPI_ISL_938218, EPI_ISL_938219, EPI_ISL_938220, EPI_ISL_938221, EPI_ISL_938222, EPI_ISL_938223, EPI_ISL_938224, EPI_ISL_938225, EPI_ISL_938226, EPI_ISL_938227, EPI_ISL_938228, EPI_ISL_938229, EPI_ISL_938230, EPI_ISL_938231, EPI_ISL_938232, EPI_ISL_938233			
see above	Lighthouse Lab in Milton Keynes	Wellcome Sanger Institute for the COVID-19 Genomics UK (COG-UK) Consortium	The Lighthouse Lab in Milton Keynes and Alex Alderton, Roberto Amato, Sonia Goncalves, Ewan Harrison, David K. Jackson, Ian Johnston, Dominic Kwiatkowski, Cordelia Langford, John Sillitoe on behalf of the Wellcome Sanger Institute COVID-19 Surveillance Team
EPI_ISL_938234, EPI_ISL_938235, EPI_ISL_938236, EPI_ISL_938237, EPI_ISL_938238, EPI_ISL_938239, EPI_ISL_938240, EPI_ISL_938241, EPI_ISL_938242, EPI_ISL_938243, EPI_ISL_938244, EPI_ISL_938245, EPI_ISL_938246, EPI_ISL_938247, EPI_ISL_938248, EPI_ISL_938249, EPI_ISL_938250, EPI_ISL_938251, EPI_ISL_938252, EPI_ISL_938253, EPI_ISL_938254, EPI_ISL_938255, EPI_ISL_938256, EPI_ISL_938257, EPI_ISL_938258, EPI_ISL_938259, EPI_ISL_938260, EPI_ISL_938261, EPI_ISL_938262, EPI_ISL_938263, EPI_ISL_938264, EPI_ISL_938265, EPI_ISL_938266, EPI_ISL_938267, EPI_ISL_938268, EPI_ISL_938269, EPI_ISL_938270, EPI_ISL_938271, EPI_ISL_938272, EPI_ISL_938273, EPI_ISL_938274, EPI_ISL_938275, EPI_ISL_938276, EPI_ISL_938277, EPI_ISL_938278, EPI_ISL_938279, EPI_ISL_938281, EPI_ISL_938282, EPI_ISL_938283, EPI_ISL_938284, EPI_ISL_938285, EPI_ISL_938286, EPI_ISL_938287, EPI_ISL_938288, EPI_ISL_938289, EPI_ISL_938290, EPI_ISL_938291, EPI_ISL_938293, EPI_ISL_938294, EPI_ISL_938295, EPI_ISL_938296, EPI_ISL_938297, EPI_ISL_938298, EPI_ISL_938299, EPI_ISL_938300, EPI_ISL_938301, EPI_ISL_938302, EPI_ISL_938303, EPI_ISL_938304, EPI_ISL_938305, EPI_ISL_938306, EPI_ISL_938307, EPI_ISL_938308, EPI_ISL_938309, EPI_ISL_938310, EPI_ISL_938311, EPI_ISL_938312, EPI_ISL_938313, EPI_ISL_938314, EPI_ISL_938315, EPI_ISL_938316, EPI_ISL_938317, EPI_ISL_938318, EPI_ISL_938319, EPI_ISL_938320, EPI_ISL_938321, EPI_ISL_938323, EPI_ISL_938324, EPI_ISL_938325, EPI_ISL_938326, EPI_ISL_938327, EPI_ISL_938328, EPI_ISL_938329, EPI_ISL_938330, EPI_ISL_938331, EPI_ISL_938332, EPI_ISL_938333, EPI_ISL_938334, EPI_ISL_938335, EPI_ISL_938336, EPI_ISL_938337, EPI_ISL_938338, EPI_ISL_938339, EPI_ISL_938340, EPI_ISL_938341, EPI_ISL_938342, EPI_ISL_938343, EPI_ISL_938344, EPI_ISL_938345, EPI_ISL_938346, EPI_ISL_938347, EPI_ISL_938348, EPI_ISL_938349, EPI_ISL_938350, EPI_ISL_938351, EPI_ISL_938352, EPI_ISL_938354, EPI_ISL_938355, EPI_ISL_938356, EPI_ISL_938357, EPI_ISL_938358, EPI_ISL_938359, EPI_ISL_938360, EPI_ISL_938361, EPI_ISL_938362, EPI_ISL_938363, EPI_ISL_938364, EPI_ISL_938365, EPI_ISL_938366, EPI_ISL_938367, EPI_ISL_938368, EPI_ISL_938369, EPI_ISL_938370, EPI_ISL_938371, EPI_ISL_938372, EPI_ISL_938373, EPI_ISL_938374, EPI_ISL_938375, EPI_ISL_938376, EPI_ISL_938377, EPI_ISL_938378, EPI_ISL_938379, EPI_ISL_938380, EPI_ISL_938381, EPI_ISL_938382, EPI_ISL_938383, EPI_ISL_938384, EPI_ISL_938385, EPI_ISL_938386, EPI_ISL_938388, EPI_ISL_938390, EPI_ISL_938392, EPI_ISL_938393, EPI_ISL_938394, EPI_ISL_938395, EPI_ISL_938396, EPI_ISL_938397, EPI_ISL_938398, EPI_ISL_938399, EPI_ISL_938400, EPI_ISL_938401, EPI_ISL_938402, EPI_ISL_938403, EPI_ISL_938404, EPI_ISL_938405, EPI_ISL_938406, EPI_ISL_938407, EPI_ISL_938408, EPI_ISL_938409, EPI_ISL_938410, EPI_ISL_938411, EPI_ISL_938412, EPI_ISL_938413, EPI_ISL_938414, EPI_ISL_938415, EPI_ISL_938416, EPI_ISL_938417, EPI_ISL_938418, EPI_ISL_938419, EPI_ISL_938420, EPI_ISL_938421, EPI_ISL_938422, EPI_ISL_938423, EPI_ISL_938424, EPI_ISL_938425, EPI_ISL_938426, EPI_ISL_938428, EPI_ISL_938429, EPI_ISL_938430, EPI_ISL_938431, EPI_ISL_938433, EPI_ISL_938434, EPI_ISL_938435, EPI_ISL_938436, EPI_ISL_938437, EPI_ISL_938438, EPI_ISL_938440, EPI_ISL_938441, EPI_ISL_938442, EPI_ISL_938443, EPI_ISL_938444, EPI_ISL_938445, EPI_ISL_938446, EPI_ISL_938447, EPI_ISL_938448, EPI_ISL_938449, EPI_ISL_938450, EPI_ISL_938451, EPI_ISL_938452, EPI_ISL_938453, EPI_ISL_938454, EPI_ISL_938455, EPI_ISL_938456, EPI_ISL_938457, EPI_ISL_938458, EPI_ISL_938459, EPI_ISL_938460, EPI_ISL_938461, EPI_ISL_938462, EPI_ISL_938463, EPI_ISL_938464, EPI_ISL_938465, EPI_ISL_938466, EPI_ISL_938467, EPI_ISL_938468, EPI_ISL_938469, EPI_ISL_938470, EPI_ISL_938471, EPI_ISL_938472, EPI_ISL_938473, EPI_ISL_938474, EPI_ISL_938475, EPI_ISL_938476, EPI_ISL_938477, EPI_ISL_938478, EPI_ISL_938479, EPI_ISL_938480, EPI_ISL_938481, EPI_ISL_938482, EPI_ISL_938483, EPI_ISL_938484, EPI_ISL_938485, EPI_ISL_938486, EPI_ISL_938487, EPI_ISL_938488, EPI_ISL_938489, EPI_ISL_938490, EPI_ISL_938491, EPI_ISL_938492, EPI_ISL_938493, EPI_ISL_938494, EPI_ISL_938495,			

EPI_ISL_938496, EPI_ISL_938497, EPI_ISL_938498, EPI_ISL_938499, EPI_ISL_938500, EPI_ISL_938502, EPI_ISL_938503, EPI_ISL_938504, EPI_ISL_938505, EPI_ISL_938506, EPI_ISL_938507, EPI_ISL_938508, EPI_ISL_938509, EPI_ISL_938510, EPI_ISL_938511, EPI_ISL_938513, EPI_ISL_938514, EPI_ISL_938515, EPI_ISL_938516, EPI_ISL_938517, EPI_ISL_938518, EPI_ISL_938519, EPI_ISL_938520, EPI_ISL_938521, EPI_ISL_938522, EPI_ISL_938523, EPI_ISL_938524, EPI_ISL_938525, EPI_ISL_938526, EPI_ISL_938527, EPI_ISL_938528, EPI_ISL_938529, EPI_ISL_938530, EPI_ISL_938531, EPI_ISL_938532, EPI_ISL_938533, EPI_ISL_938534, EPI_ISL_938535, EPI_ISL_938536, EPI_ISL_938537, EPI_ISL_938538, EPI_ISL_938539, EPI_ISL_938540, EPI_ISL_938541, EPI_ISL_938542, EPI_ISL_938543, EPI_ISL_938544, EPI_ISL_938545, EPI_ISL_938546, EPI_ISL_938547, EPI_ISL_938549, EPI_ISL_938550, EPI_ISL_938551, EPI_ISL_938552, EPI_ISL_938553, EPI_ISL_938554, EPI_ISL_938555, EPI_ISL_938556, EPI_ISL_938557, EPI_ISL_938558, EPI_ISL_938559, EPI_ISL_938560, EPI_ISL_938561, EPI_ISL_938562, EPI_ISL_938564, EPI_ISL_938565, EPI_ISL_938566, EPI_ISL_938567, EPI_ISL_938568, EPI_ISL_938569, EPI_ISL_938570, EPI_ISL_938571, EPI_ISL_938572, EPI_ISL_938573, EPI_ISL_938575, EPI_ISL_938576, EPI_ISL_938577, EPI_ISL_938578			
see above	Lighthouse Lab in Cambridge	Wellcome Sanger Institute for the COVID-19 Genomics UK (COG-UK) Consortium	Rob Howes, The Lighthouse Lab in Cambridge and Alex Alderton, Roberto Amato, Sonia Goncalves, Ewan Harrison, David K. Jackson, Ian Johnston, Dominic Kwiatkowski, Cordelia Langford, John Sillitoe on behalf of the Wellcome Sanger Institute COVID-19 Surveillance Team
EPI_ISL_938581, EPI_ISL_938587, EPI_ISL_938592, EPI_ISL_938596, EPI_ISL_938599, EPI_ISL_938602, EPI_ISL_938603, EPI_ISL_938607, EPI_ISL_938610, EPI_ISL_938612, EPI_ISL_938613, EPI_ISL_938614, EPI_ISL_938619, EPI_ISL_938630, EPI_ISL_938631, EPI_ISL_938632, EPI_ISL_938633, EPI_ISL_938636, EPI_ISL_938637, EPI_ISL_938643, EPI_ISL_938645, EPI_ISL_938648, EPI_ISL_938650, EPI_ISL_938652, EPI_ISL_938657, EPI_ISL_938666, EPI_ISL_938667, EPI_ISL_938676, EPI_ISL_938684, EPI_ISL_938692, EPI_ISL_938694, EPI_ISL_938700, EPI_ISL_938701, EPI_ISL_938707, EPI_ISL_938721, EPI_ISL_938729, EPI_ISL_938731, EPI_ISL_938732, EPI_ISL_938733, EPI_ISL_938737, EPI_ISL_938741, EPI_ISL_938743, EPI_ISL_938744, EPI_ISL_938746, EPI_ISL_938747, EPI_ISL_938750, EPI_ISL_938751, EPI_ISL_938752, EPI_ISL_938753, EPI_ISL_938756, EPI_ISL_938757, EPI_ISL_938758, EPI_ISL_938762, EPI_ISL_938764, EPI_ISL_938769, EPI_ISL_938771, EPI_ISL_938777, EPI_ISL_938783, EPI_ISL_938785, EPI_ISL_938787, EPI_ISL_938794, EPI_ISL_938796, EPI_ISL_938797, EPI_ISL_938800, EPI_ISL_938801, EPI_ISL_938803, EPI_ISL_938804, EPI_ISL_938806, EPI_ISL_938807, EPI_ISL_938810, EPI_ISL_938811, EPI_ISL_938815, EPI_ISL_938829, EPI_ISL_938837, EPI_ISL_938840, EPI_ISL_938845, EPI_ISL_938846, EPI_ISL_938854, EPI_ISL_938865, EPI_ISL_938875, EPI_ISL_938876, EPI_ISL_938882, EPI_ISL_938885, EPI_ISL_938886, EPI_ISL_938900, EPI_ISL_938901, EPI_ISL_938903, EPI_ISL_938908, EPI_ISL_938911, EPI_ISL_938913, EPI_ISL_938916, EPI_ISL_938921, EPI_ISL_938931, EPI_ISL_938933, EPI_ISL_938934			
see above	Lighthouse Lab in Milton Keynes	Wellcome Sanger Institute for the COVID-19 Genomics UK (COG-UK) Consortium	The Lighthouse Lab in Milton Keynes and Alex Alderton, Roberto Amato, Sonia Goncalves, Ewan Harrison, David K. Jackson, Ian Johnston, Dominic Kwiatkowski, Cordelia Langford, John Sillitoe on behalf of the Wellcome Sanger Institute COVID-19 Surveillance Team
EPI_ISL_938940	Lighthouse Lab in Alderley Park	Wellcome Sanger Institute for the COVID-19 Genomics UK (COG-UK) Consortium	Jacquelyn Wynn, Mairead Hyland, The Lighthouse Lab in Alderley Park and Alex Alderton, Roberto Amato, Sonia Goncalves, Ewan Harrison, David K. Jackson, Ian Johnston, Dominic Kwiatkowski, Cordelia Langford, John Sillitoe on behalf of the Wellcome Sanger Institute COVID-19 Surveillance Team
EPI_ISL_938944, EPI_ISL_938945, EPI_ISL_938946, EPI_ISL_938947, EPI_ISL_938948, EPI_ISL_938949, EPI_ISL_938950, EPI_ISL_938951, EPI_ISL_938952, EPI_ISL_938953, EPI_ISL_938954, EPI_ISL_938955, EPI_ISL_938956, EPI_ISL_938957, EPI_ISL_938958, EPI_ISL_938959, EPI_ISL_938960, EPI_ISL_938961, EPI_ISL_938962, EPI_ISL_938963, EPI_ISL_938964, EPI_ISL_938965, EPI_ISL_938966, EPI_ISL_938967, EPI_ISL_938968, EPI_ISL_938969, EPI_ISL_938970, EPI_ISL_938971, EPI_ISL_938972, EPI_ISL_938973, EPI_ISL_938974, EPI_ISL_938975, EPI_ISL_938976, EPI_ISL_938977, EPI_ISL_938978, EPI_ISL_938979, EPI_ISL_938980, EPI_ISL_938981, EPI_ISL_938982, EPI_ISL_938983, EPI_ISL_938984, EPI_ISL_938985, EPI_ISL_938986, EPI_ISL_938987, EPI_ISL_938988, EPI_ISL_938989, EPI_ISL_938990, EPI_ISL_938991, EPI_ISL_938992, EPI_ISL_938993, EPI_ISL_938994, EPI_ISL_938995, EPI_ISL_938996, EPI_ISL_938997, EPI_ISL_938998, EPI_ISL_938999, EPI_ISL_939000, EPI_ISL_939001, EPI_ISL_939002, EPI_ISL_939003, EPI_ISL_939004, EPI_ISL_939005, EPI_ISL_939006, EPI_ISL_939007, EPI_ISL_939008, EPI_ISL_939009, EPI_ISL_939010, EPI_ISL_939011, EPI_ISL_939012, EPI_ISL_939013, EPI_ISL_939014, EPI_ISL_939015, EPI_ISL_939016, EPI_ISL_939017, EPI_ISL_939018, EPI_ISL_939019, EPI_ISL_939020, EPI_ISL_939021, EPI_ISL_939022, EPI_ISL_939023, EPI_ISL_939024, EPI_ISL_939025, EPI_ISL_939026, EPI_ISL_939027, EPI_ISL_939028, EPI_ISL_939029, EPI_ISL_939030, EPI_ISL_939031, EPI_ISL_939032, EPI_ISL_939033, EPI_ISL_939034, EPI_ISL_939035, EPI_ISL_939036, EPI_ISL_939037, EPI_ISL_939038, EPI_ISL_939039, EPI_ISL_939040, EPI_ISL_939041, EPI_ISL_939042, EPI_ISL_939043, EPI_ISL_939044, EPI_ISL_939045, EPI_ISL_939046, EPI_ISL_939047, EPI_ISL_939048, EPI_ISL_939049, EPI_ISL_939050, EPI_ISL_939051, EPI_ISL_939052, EPI_ISL_939053, EPI_ISL_939054, EPI_ISL_939055, EPI_ISL_939056, EPI_ISL_939057, EPI_ISL_939058, EPI_ISL_939059, EPI_ISL_939060, EPI_ISL_939061, EPI_ISL_939062, EPI_ISL_939063, EPI_ISL_939064, EPI_ISL_939065, EPI_ISL_939066, EPI_ISL_939067, EPI_ISL_939068, EPI_ISL_939069, EPI_ISL_939070, EPI_ISL_939071, EPI_ISL_939072, EPI_ISL_939073, EPI_ISL_939074, EPI_ISL_939075, EPI_ISL_939076, EPI_ISL_939077, EPI_ISL_939078, EPI_ISL_939080, EPI_ISL_939081, EPI_ISL_939082, EPI_ISL_939083, EPI_ISL_939084, EPI_ISL_939085, EPI_ISL_939086, EPI_ISL_939087, EPI_ISL_939088, EPI_ISL_939089, EPI_ISL_939090, EPI_ISL_939091, EPI_ISL_939092, EPI_ISL_939093, EPI_ISL_939094, EPI_ISL_939095, EPI_ISL_939096, EPI_ISL_939097, EPI_ISL_939098, EPI_ISL_939099, EPI_ISL_939100, EPI_ISL_939101, EPI_ISL_939102, EPI_ISL_939103, EPI_ISL_939104, EPI_ISL_939105, EPI_ISL_939106, EPI_ISL_939107, EPI_ISL_939108, EPI_ISL_939109, EPI_ISL_939110, EPI_ISL_939111, EPI_ISL_939112, EPI_ISL_939113, EPI_ISL_939114, EPI_ISL_939115, EPI_ISL_939116, EPI_ISL_939117, EPI_ISL_939118, EPI_ISL_939119, EPI_ISL_939120, EPI_ISL_939121, EPI_ISL_939122, EPI_ISL_939123, EPI_ISL_939124, EPI_ISL_939125, EPI_ISL_939126, EPI_ISL_939127, EPI_ISL_939128, EPI_ISL_939129, EPI_ISL_939130, EPI_ISL_939131, EPI_ISL_939132, EPI_ISL_939133, EPI_ISL_939134, EPI_ISL_939135, EPI_ISL_939136, EPI_ISL_939137, EPI_ISL_939138, EPI_ISL_939139, EPI_ISL_939140, EPI_ISL_939141, EPI_ISL_939142, EPI_ISL_939143, EPI_ISL_939144, EPI_ISL_939145, EPI_ISL_939146, EPI_ISL_939147, EPI_ISL_939148, EPI_ISL_939149, EPI_ISL_939150, EPI_ISL_939151, EPI_ISL_939152, EPI_ISL_939153, EPI_ISL_939154, EPI_ISL_939155, EPI_ISL_939156, EPI_ISL_939157, EPI_ISL_939158, EPI_ISL_939159, EPI_ISL_939160, EPI_ISL_939161, EPI_ISL_939162, EPI_ISL_939163, EPI_ISL_939164, EPI_ISL_939165, EPI_ISL_939166, EPI_ISL_939167, EPI_ISL_939168, EPI_ISL_939169, EPI_ISL_939170, EPI_ISL_939171, EPI_ISL_939172, EPI_ISL_939173, EPI_ISL_939174, EPI_ISL_939175, EPI_ISL_939176, EPI_ISL_939177, EPI_ISL_939178, EPI_ISL_939179, EPI_ISL_939180, EPI_ISL_939181, EPI_ISL_939182, EPI_ISL_939183, EPI_ISL_939184, EPI_ISL_939185, EPI_ISL_939186, EPI_ISL_939187, EPI_ISL_939188, EPI_ISL_939189, EPI_ISL_939190, EPI_ISL_939191, EPI_ISL_939192, EPI_ISL_939193, EPI_ISL_939194, EPI_ISL_939195, EPI_ISL_939196, EPI_ISL_939197, EPI_ISL_939198, EPI_ISL_939199, EPI_ISL_939200, EPI_ISL_939201, EPI_ISL_939202, EPI_ISL_939203, EPI_ISL_939204, EPI_ISL_939205, EPI_ISL_939206, EPI_ISL_939207, EPI_ISL_939208, EPI_ISL_939209, EPI_ISL_939210, EPI_ISL_939211, EPI_ISL_939212, EPI_ISL_939213, EPI_ISL_939214, EPI_ISL_939215, EPI_ISL_939216, EPI_ISL_939217, EPI_ISL_939218, EPI_ISL_939219, EPI_ISL_939220, EPI_ISL_939221, EPI_ISL_939222, EPI_ISL_939223, EPI_ISL_939224, EPI_ISL_939225, EPI_ISL_939226, EPI_ISL_939227, EPI_ISL_939228, EPI_ISL_939229, EPI_ISL_939230, EPI_ISL_939231, EPI_ISL_939232, EPI_ISL_939233, EPI_ISL_939234, EPI_ISL_939235, EPI_ISL_939236, EPI_ISL_939237, EPI_ISL_939238, EPI_ISL_939239, EPI_ISL_939240, EPI_ISL_939241, EPI_ISL_939242, EPI_ISL_939243, EPI_ISL_939244, EPI_ISL_939245, EPI_ISL_939246, EPI_ISL_939247, EPI_ISL_939248, EPI_ISL_939249, EPI_ISL_939250, EPI_ISL_939251, EPI_ISL_939252, EPI_ISL_939253, EPI_ISL_939254, EPI_ISL_939255, EPI_ISL_939256, EPI_ISL_939257, EPI_ISL_939258, EPI_ISL_939259, EPI_ISL_939260, EPI_ISL_939261, EPI_ISL_939262, EPI_ISL_939263, EPI_ISL_939264, EPI_ISL_939265, EPI_ISL_939266, EPI_ISL_939267, EPI_ISL_939268, EPI_ISL_939269, EPI_ISL_939270, EPI_ISL_939271, EPI_ISL_939272, EPI_ISL_939273, EPI_ISL_939274, EPI_ISL_939275, EPI_ISL_939276, EPI_ISL_939277, EPI_ISL_939278, EPI_ISL_939279, EPI_ISL_939280, EPI_ISL_939281, EPI_ISL_939282, EPI_ISL_939286, EPI_ISL_939288, EPI_ISL_939290, EPI_ISL_939291, EPI_ISL_939292, EPI_ISL_939293, EPI_ISL_939294, EPI_ISL_939296, EPI_ISL_939300, EPI_ISL_939301, EPI_ISL_939302, EPI_ISL_939306, EPI_ISL_939307, EPI_ISL_939313, EPI_ISL_939314, EPI_ISL_939315, EPI_ISL_939318, EPI_ISL_939319, EPI_ISL_939320, EPI_ISL_939321, EPI_ISL_939327, EPI_ISL_939329, EPI_ISL_939332, EPI_ISL_939334, EPI_ISL_939337, EPI_ISL_939339, EPI_ISL_939340, EPI_ISL_939343, EPI_ISL_939346, EPI_ISL_939348, EPI_ISL_939350, EPI_ISL_939351, EPI_ISL_939352, EPI_ISL_939354, EPI_ISL_939355, EPI_ISL_939356, EPI_ISL_939357, EPI_ISL_939358, EPI_ISL_939359, EPI_ISL_939361, EPI_ISL_939364, EPI_ISL_939365, EPI_ISL_939368, EPI_ISL_939369, EPI_ISL_939370, EPI_ISL_939372, EPI_ISL_939373, EPI_ISL_939378, EPI_ISL_939380, EPI_ISL_939388, EPI_ISL_939389, EPI_ISL_939390, EPI_ISL_939391, EPI_ISL_939393, EPI_ISL_939395, EPI_ISL_939396, EPI_ISL_939397, EPI_ISL_939398, EPI_ISL_939399, EPI_ISL_939401, EPI_ISL_939402, EPI_ISL_939403, EPI_ISL_939404, EPI_ISL_939405, EPI_ISL_939406, EPI_ISL_939407, EPI_ISL_939413, EPI_ISL_939414, EPI_ISL_939415, EPI_ISL_939416, EPI_ISL_939422, EPI_ISL_939425, EPI_ISL_939428, EPI_ISL_939429, EPI_ISL_939431, EPI_ISL_939432, EPI_ISL_939438, EPI_ISL_939439, EPI_ISL_939443, EPI_ISL_939450, EPI_ISL_939454, EPI_ISL_939456, EPI_ISL_939458, EPI_ISL_939459, EPI_ISL_939460, EPI_ISL_939461, EPI_ISL_939462, EPI_ISL_939468, EPI_ISL_939469, EPI_ISL_939472, EPI_ISL_939473, EPI_ISL_939477, EPI_ISL_939478, EPI_ISL_939481, EPI_ISL_939483, EPI_ISL_939485, EPI_ISL_939487, EPI_ISL_939492, EPI_ISL_939493, EPI_ISL_939494, EPI_ISL_939497, EPI_ISL_939498, EPI_ISL_939500, EPI_ISL_939504, EPI_ISL_939505, EPI_ISL_939506, EPI_ISL_939507, EPI_ISL_939508, EPI_ISL_939509, EPI_ISL_939510, EPI_ISL_939512, EPI_ISL_939513, EPI_ISL_939514, EPI_ISL_939517, EPI_ISL_939519, EPI_ISL_939520, EPI_ISL_939521, EPI_ISL_939522, EPI_ISL_939523, EPI_ISL_939524, EPI_ISL_939526, EPI_ISL_939528, EPI_ISL_939532, EPI_ISL_939533, EPI_ISL_939535, EPI_ISL_939536, EPI_ISL_939537, EPI_ISL_939541, EPI_ISL_939542, EPI_ISL_939547, EPI_ISL_939548, EPI_ISL_939550, EPI_ISL_939551, EPI_ISL_939552, EPI_ISL_939554, EPI_ISL_939555, EPI_ISL_939556, EPI_ISL_939559, EPI_ISL_939561, EPI_ISL_939562, EPI_ISL_939563, EPI_ISL_939565, EPI_ISL_939566, EPI_ISL_939567, EPI_ISL_939571, EPI_ISL_939572, EPI_ISL_939573, EPI_ISL_939574, EPI_ISL_939576, EPI_ISL_939577, EPI_ISL_939578, EPI_ISL_939579, EPI_ISL_939581, EPI_ISL_939582, EPI_ISL_939585, EPI_ISL_939588, EPI_ISL_939594, EPI_ISL_939595, EPI_ISL_939596, EPI_ISL_939598, EPI_ISL_939599, EPI_ISL_939601, EPI_ISL_939605, EPI_ISL_939606			
see above	Lighthouse Lab in Milton Keynes	Wellcome Sanger Institute for the COVID-19 Genomics UK (COG-UK) Consortium	The Lighthouse Lab in Milton Keynes and Alex Alderton, Roberto Amato, Sonia Goncalves, Ewan Harrison, David K. Jackson, Ian Johnston, Dominic Kwiatkowski, Cordelia Langford, John Sillitoe on behalf of the Wellcome Sanger Institute COVID-19 Surveillance Team
EPI_ISL_939607	Lighthouse Lab in Alderley Park	Wellcome Sanger Institute for the COVID-19 Genomics UK (COG-UK) Consortium	Jacquelyn Wynn, Mairead Hyland, The Lighthouse Lab in Alderley Park and Alex Alderton, Roberto Amato, Sonia Goncalves, Ewan Harrison, David K. Jackson, Ian Johnston, Dominic Kwiatkowski, Cordelia Langford, John Sillitoe on behalf of the Wellcome Sanger Institute COVID-19 Surveillance Team
EPI_ISL_944844	Lighthouse Lab in Glasgow	Wellcome Sanger Institute for the COVID-19 Genomics UK (COG-UK) Consortium	Harper VanSteenhouse, Yumi Kasai, David Gray, Carol Clugston, Anna Dominiczak and Alex Alderton, Roberto Amato, Sonia Goncalves, Ewan Harrison, David K. Jackson, Ian Johnston, Dominic Kwiatkowski, Cordelia Langford, John Sillitoe on behalf of the Wellcome Sanger Institute COVID-19 Surveillance Team
EPI_ISL_945752, EPI_ISL_945837, EPI_ISL_946094, EPI_ISL_946153, EPI_ISL_946269, EPI_ISL_946323, EPI_ISL_946363, EPI_ISL_946403	Lighthouse Lab in Milton Keynes	Wellcome Sanger Institute for the COVID-19 Genomics UK (COG-UK) Consortium	The Lighthouse Lab in Milton Keynes and Alex Alderton, Roberto Amato, Sonia Goncalves, Ewan Harrison, David K. Jackson, Ian Johnston, Dominic Kwiatkowski, Cordelia Langford, John Sillitoe on behalf of the Wellcome Sanger Institute COVID-19 Surveillance Team
EPI_ISL_946421, EPI_ISL_946427, EPI_ISL_946445, EPI_ISL_946454, EPI_ISL_946471, EPI_ISL_946493, EPI_ISL_946547, EPI_ISL_946580, EPI_ISL_946585, EPI_ISL_946646, EPI_ISL_946672, EPI_ISL_946956, EPI_ISL_946963, EPI_ISL_946992			
see above	Lighthouse Lab in Alderley Park	Wellcome Sanger Institute for the COVID-19 Genomics UK (COG-UK) Consortium	Jacquelyn Wynn, Mairead Hyland, The Lighthouse Lab in Alderley Park and Alex Alderton, Roberto Amato, Sonia Goncalves, Ewan Harrison, David K. Jackson, Ian Johnston, Dominic Kwiatkowski, Cordelia Langford, John Sillitoe on behalf of the Wellcome Sanger Institute COVID-19 Surveillance Team
EPI_ISL_947110, EPI_ISL_947113, EPI_ISL_947120, EPI_ISL_947140, EPI_ISL_947149, EPI_ISL_947172, EPI_ISL_947184, EPI_ISL_947189, EPI_ISL_947201	Lighthouse Lab in Milton Keynes	Wellcome Sanger Institute for the COVID-19 Genomics UK (COG-UK) Consortium	The Lighthouse Lab in Milton Keynes and Alex Alderton, Roberto Amato, Sonia Goncalves, Ewan Harrison, David K. Jackson, Ian Johnston, Dominic Kwiatkowski, Cordelia Langford, John Sillitoe on behalf of the Wellcome Sanger Institute COVID-19 Surveillance Team
EPI_ISL_947208	Lighthouse Lab in Glasgow	Wellcome Sanger Institute for the COVID-19 Genomics UK (COG-UK) Consortium	Harper VanSteenhouse, Yumi Kasai, David Gray, Carol Clugston, Anna Dominiczak and Alex Alderton, Roberto Amato, Sonia Goncalves, Ewan Harrison, David K. Jackson, Ian Johnston, Dominic Kwiatkowski, Cordelia Langford, John Sillitoe on behalf of the Wellcome Sanger Institute COVID-19 Surveillance Team
EPI_ISL_947240, EPI_ISL_947256, EPI_ISL_947261, EPI_ISL_947274, EPI_ISL_947318, EPI_ISL_947337	Lighthouse Lab in Milton Keynes	Wellcome Sanger Institute for the COVID-19 Genomics UK (COG-UK) Consortium	The Lighthouse Lab in Milton Keynes and Alex Alderton, Roberto Amato, Sonia Goncalves, Ewan Harrison, David K. Jackson, Ian Johnston, Dominic Kwiatkowski, Cordelia Langford, John Sillitoe on behalf of the Wellcome Sanger Institute COVID-19 Surveillance Team
EPI_ISL_947350, EPI_ISL_947352	Lighthouse Lab in Glasgow	Wellcome Sanger Institute for the COVID-19 Genomics UK (COG-UK) Consortium	Harper VanSteenhouse, Yumi Kasai, David Gray, Carol Clugston, Anna Dominiczak and Alex Alderton, Roberto Amato, Sonia Goncalves, Ewan Harrison, David K. Jackson, Ian Johnston, Dominic Kwiatkowski, Cordelia Langford, John Sillitoe on behalf of the Wellcome Sanger Institute COVID-19 Surveillance Team
EPI_ISL_947354, EPI_ISL_947361, EPI_ISL_947368, EPI_ISL_947374, EPI_ISL_947400	Lighthouse Lab in Milton Keynes	Wellcome Sanger Institute for the COVID-19 Genomics UK (COG-UK) Consortium	The Lighthouse Lab in Milton Keynes and Alex Alderton, Roberto Amato, Sonia Goncalves, Ewan Harrison, David K. Jackson, Ian Johnston, Dominic Kwiatkowski, Cordelia Langford, John Sillitoe on behalf of the Wellcome Sanger Institute COVID-19 Surveillance Team
EPI_ISL_947406, EPI_ISL_947407, EPI_ISL_947408, EPI_ISL_947409, EPI_ISL_947410, EPI_ISL_947411, EPI_ISL_947412, EPI_ISL_947413, EPI_ISL_947414, EPI_ISL_947415, EPI_ISL_947416, EPI_ISL_947417, EPI_ISL_947418, EPI_ISL_947419, EPI_ISL_947420, EPI_ISL_947421, EPI_ISL_947422, EPI_ISL_947423, EPI_ISL_947424, EPI_ISL_947425, EPI_ISL_947426, EPI_ISL_947427, EPI_ISL_947428, EPI_ISL_947429, EPI_ISL_947430, EPI_ISL_947431, EPI_ISL_947432, EPI_ISL_947433, EPI_ISL_947434, EPI_ISL_947435, EPI_ISL_947436, EPI_ISL_947437, EPI_ISL_947438, EPI_ISL_947439, EPI_ISL_947440, EPI_ISL_947441, EPI_ISL_947442, EPI_ISL_947443, EPI_ISL_947444, EPI_ISL_947445, EPI_ISL_947446, EPI_ISL_947447, EPI_ISL_947448, EPI_ISL_947449, EPI_ISL_947450, EPI_ISL_947451, EPI_ISL_947452, EPI_ISL_947453, EPI_ISL_947454, EPI_ISL_947455, EPI_ISL_947456, EPI_ISL_947457, EPI_ISL_947458, EPI_ISL_947459,			

EPI_ISL_947460, EPI_ISL_947461, EPI_ISL_947462, EPI_ISL_947463, EPI_ISL_947464, EPI_ISL_947465, EPI_ISL_947466, EPI_ISL_947467, EPI_ISL_947468, EPI_ISL_947469, EPI_ISL_947470, EPI_ISL_947471, EPI_ISL_947472, EPI_ISL_947473, EPI_ISL_947474, EPI_ISL_947475, EPI_ISL_947476, EPI_ISL_947477, EPI_ISL_947478, EPI_ISL_947479, EPI_ISL_947480, EPI_ISL_947481, EPI_ISL_947482, EPI_ISL_947483, EPI_ISL_947484, EPI_ISL_947485, EPI_ISL_947486, EPI_ISL_947487, EPI_ISL_947488, EPI_ISL_947489, EPI_ISL_947490, EPI_ISL_947491, EPI_ISL_947492, EPI_ISL_947493, EPI_ISL_947494, EPI_ISL_947495, EPI_ISL_947496, EPI_ISL_947497, EPI_ISL_947498, EPI_ISL_947499, EPI_ISL_947500, EPI_ISL_947501, EPI_ISL_947502, EPI_ISL_947503, EPI_ISL_947504, EPI_ISL_947505, EPI_ISL_947506, EPI_ISL_947507, EPI_ISL_947508, EPI_ISL_947509, EPI_ISL_947510, EPI_ISL_947511, EPI_ISL_947512, EPI_ISL_947513, EPI_ISL_947514, EPI_ISL_947515, EPI_ISL_947516, EPI_ISL_947517, EPI_ISL_947518, EPI_ISL_947519, EPI_ISL_947520, EPI_ISL_947521, EPI_ISL_947522, EPI_ISL_947523, EPI_ISL_947524, EPI_ISL_947525, EPI_ISL_947526, EPI_ISL_947527, EPI_ISL_947528, EPI_ISL_947529, EPI_ISL_947530, EPI_ISL_947531, EPI_ISL_947532, EPI_ISL_947533, EPI_ISL_947534, EPI_ISL_947535, EPI_ISL_947536, EPI_ISL_947537, EPI_ISL_947538, EPI_ISL_947539, EPI_ISL_947540, EPI_ISL_947541, EPI_ISL_947542, EPI_ISL_947543, EPI_ISL_947544, EPI_ISL_947545, EPI_ISL_947546, EPI_ISL_947547, EPI_ISL_947548, EPI_ISL_947549, EPI_ISL_947550, EPI_ISL_947551, EPI_ISL_947552, EPI_ISL_947553, EPI_ISL_947554, EPI_ISL_947555, EPI_ISL_947556, EPI_ISL_947557, EPI_ISL_947558, EPI_ISL_947559, EPI_ISL_947560, EPI_ISL_947561, EPI_ISL_947562, EPI_ISL_947563, EPI_ISL_947564, EPI_ISL_947565, EPI_ISL_947566, EPI_ISL_947567, EPI_ISL_947568, EPI_ISL_947569, EPI_ISL_947570, EPI_ISL_947571, EPI_ISL_947572, EPI_ISL_947573, EPI_ISL_947574, EPI_ISL_947575, EPI_ISL_947576, EPI_ISL_947577, EPI_ISL_947578, EPI_ISL_947579, EPI_ISL_947580, EPI_ISL_947581, EPI_ISL_947582, EPI_ISL_947583, EPI_ISL_947584, EPI_ISL_947585, EPI_ISL_947586, EPI_ISL_947587, EPI_ISL_947588, EPI_ISL_947589, EPI_ISL_947590, EPI_ISL_947591, EPI_ISL_947592, EPI_ISL_947593, EPI_ISL_947594, EPI_ISL_947595, EPI_ISL_947596, EPI_ISL_947597, EPI_ISL_947598, EPI_ISL_947599, EPI_ISL_947600, EPI_ISL_947601, EPI_ISL_947602, EPI_ISL_947603, EPI_ISL_947604, EPI_ISL_947605, EPI_ISL_947606, EPI_ISL_947607, EPI_ISL_947608, EPI_ISL_947609, EPI_ISL_947610, EPI_ISL_947611, EPI_ISL_947612, EPI_ISL_947613, EPI_ISL_947614, EPI_ISL_947615, EPI_ISL_947616, EPI_ISL_947617, EPI_ISL_947618, EPI_ISL_947619, EPI_ISL_947620, EPI_ISL_947621, EPI_ISL_947622, EPI_ISL_947623, EPI_ISL_947624, EPI_ISL_947625, EPI_ISL_947626, EPI_ISL_947627, EPI_ISL_947628, EPI_ISL_947629, EPI_ISL_947630, EPI_ISL_947631, EPI_ISL_947632, EPI_ISL_947633, EPI_ISL_947634, EPI_ISL_947635, EPI_ISL_947636, EPI_ISL_947637, EPI_ISL_947638, EPI_ISL_947639, EPI_ISL_947640, EPI_ISL_947641, EPI_ISL_947642, EPI_ISL_947643, EPI_ISL_947644, EPI_ISL_947645, EPI_ISL_947646, EPI_ISL_947647, EPI_ISL_947648, EPI_ISL_947650, EPI_ISL_947651, EPI_ISL_947652, EPI_ISL_947653, EPI_ISL_947654, EPI_ISL_947655, EPI_ISL_947656, EPI_ISL_947657, EPI_ISL_947658, EPI_ISL_947659, EPI_ISL_947660, EPI_ISL_947661, EPI_ISL_947662, EPI_ISL_947663, EPI_ISL_947664, EPI_ISL_947665, EPI_ISL_947666, EPI_ISL_947667, EPI_ISL_947668, EPI_ISL_947669, EPI_ISL_947670, EPI_ISL_947671, EPI_ISL_947672, EPI_ISL_947673, EPI_ISL_947674, EPI_ISL_947675, EPI_ISL_947676, EPI_ISL_947677, EPI_ISL_947679, EPI_ISL_947680, EPI_ISL_947681, EPI_ISL_947682, EPI_ISL_947683, EPI_ISL_947684, EPI_ISL_947685, EPI_ISL_947686, EPI_ISL_947687, EPI_ISL_947688, EPI_ISL_947689, EPI_ISL_947690, EPI_ISL_947691, EPI_ISL_947692, EPI_ISL_947693, EPI_ISL_947694, EPI_ISL_947695, EPI_ISL_947696, EPI_ISL_947697, EPI_ISL_947698, EPI_ISL_947699, EPI_ISL_947700, EPI_ISL_947701, EPI_ISL_947702, EPI_ISL_947703, EPI_ISL_947704, EPI_ISL_947705, EPI_ISL_947706, EPI_ISL_947707, EPI_ISL_947708, EPI_ISL_947709, EPI_ISL_947710, EPI_ISL_947711, EPI_ISL_947712, EPI_ISL_947713, EPI_ISL_947714, EPI_ISL_947715, EPI_ISL_947716, EPI_ISL_947717, EPI_ISL_947718, EPI_ISL_947719, EPI_ISL_947720, EPI_ISL_947721, EPI_ISL_947722, EPI_ISL_947723, EPI_ISL_947724, EPI_ISL_947725, EPI_ISL_947726, EPI_ISL_947727, EPI_ISL_947728, EPI_ISL_947729, EPI_ISL_947730, EPI_ISL_947731, EPI_ISL_947732, EPI_ISL_947733, EPI_ISL_947734, EPI_ISL_947735, EPI_ISL_947736, EPI_ISL_947737, EPI_ISL_947738, EPI_ISL_947739, EPI_ISL_947740, EPI_ISL_947741, EPI_ISL_947742, EPI_ISL_947743, EPI_ISL_947744, EPI_ISL_947745, EPI_ISL_947746, EPI_ISL_947747, EPI_ISL_947748, EPI_ISL_947749, EPI_ISL_947750, EPI_ISL_947751, EPI_ISL_947752, EPI_ISL_947753, EPI_ISL_947754, EPI_ISL_947755, EPI_ISL_947756, EPI_ISL_947757, EPI_ISL_947758, EPI_ISL_947759, EPI_ISL_947760, EPI_ISL_947761, EPI_ISL_947762, EPI_ISL_947763, EPI_ISL_947764, EPI_ISL_947765, EPI_ISL_947766, EPI_ISL_947767, EPI_ISL_947768, EPI_ISL_947769, EPI_ISL_947770, EPI_ISL_947771, EPI_ISL_947772, EPI_ISL_947773, EPI_ISL_947774, EPI_ISL_947775, EPI_ISL_947776, EPI_ISL_947777, EPI_ISL_947778, EPI_ISL_947779, EPI_ISL_947780, EPI_ISL_947781, EPI_ISL_947782, EPI_ISL_947783, EPI_ISL_947784, EPI_ISL_947785, EPI_ISL_947786, EPI_ISL_947787, EPI_ISL_947788, EPI_ISL_947789, EPI_ISL_947790, EPI_ISL_947791, EPI_ISL_947792, EPI_ISL_947793, EPI_ISL_947794, EPI_ISL_947795, EPI_ISL_947796, EPI_ISL_947797, EPI_ISL_947798, EPI_ISL_947799, EPI_ISL_947800, EPI_ISL_947801, EPI_ISL_947802, EPI_ISL_947803, EPI_ISL_947804, EPI_ISL_947805, EPI_ISL_947806, EPI_ISL_947807, EPI_ISL_947808, EPI_ISL_947809, EPI_ISL_947810, EPI_ISL_947811, EPI_ISL_947812, EPI_ISL_947813, EPI_ISL_947814, EPI_ISL_947815, EPI_ISL_947816, EPI_ISL_947817, EPI_ISL_947818, EPI_ISL_947819, EPI_ISL_947820, EPI_ISL_947821, EPI_ISL_947822, EPI_ISL_947823, EPI_ISL_947824, EPI_ISL_947825, EPI_ISL_947826, EPI_ISL_947827, EPI_ISL_947828, EPI_ISL_947829, EPI_ISL_947830, EPI_ISL_947831, EPI_ISL_947832, EPI_ISL_947833, EPI_ISL_947834, EPI_ISL_947835, EPI_ISL_947836, EPI_ISL_947837, EPI_ISL_947838, EPI_ISL_947839, EPI_ISL_947840, EPI_ISL_947841, EPI_ISL_947842, EPI_ISL_947843, EPI_ISL_947844, EPI_ISL_947845, EPI_ISL_947846, EPI_ISL_947847, EPI_ISL_947848, EPI_ISL_947849, EPI_ISL_947850, EPI_ISL_947851, EPI_ISL_947852, EPI_ISL_947853, EPI_ISL_947854, EPI_ISL_947855, EPI_ISL_947856, EPI_ISL_947857, EPI_ISL_947858, EPI_ISL_947859, EPI_ISL_947860, EPI_ISL_947861, EPI_ISL_947862, EPI_ISL_947863, EPI_ISL_947864, EPI_ISL_947865, EPI_ISL_947866, EPI_ISL_947867, EPI_ISL_947868, EPI_ISL_947869, EPI_ISL_947870, EPI_ISL_947871, EPI_ISL_947872, EPI_ISL_947873, EPI_ISL_947874, EPI_ISL_947875, EPI_ISL_947876, EPI_ISL_947877, EPI_ISL_947878, EPI_ISL_947879, EPI_ISL_947880, EPI_ISL_947881, EPI_ISL_947882, EPI_ISL_947883, EPI_ISL_947884, EPI_ISL_947885, EPI_ISL_947886, EPI_ISL_947887, EPI_ISL_947888, EPI_ISL_947889, EPI_ISL_947890, EPI_ISL_947891, EPI_ISL_947892, EPI_ISL_947893, EPI_ISL_947894, EPI_ISL_947895, EPI_ISL_947896, EPI_ISL_947897, EPI_ISL_947898, EPI_ISL_947899, EPI_ISL_947900, EPI_ISL_947901, EPI_ISL_947902, EPI_ISL_947903, EPI_ISL_947904, EPI_ISL_947905, EPI_ISL_947906, EPI_ISL_947907, EPI_ISL_947908, EPI_ISL_947909, EPI_ISL_947910, EPI_ISL_947911, EPI_ISL_947912, EPI_ISL_947913, EPI_ISL_947914, EPI_ISL_947915, EPI_ISL_947916, EPI_ISL_947917, EPI_ISL_947918, EPI_ISL_947919, EPI_ISL_947920, EPI_ISL_947921, EPI_ISL_947922, EPI_ISL_947923, EPI_ISL_947924, EPI_ISL_947925, EPI_ISL_947926, EPI_ISL_947927, EPI_ISL_947928, EPI_ISL_947929, EPI_ISL_947930, EPI_ISL_947931, EPI_ISL_947932, EPI_ISL_947933, EPI_ISL_947934, EPI_ISL_947935, EPI_ISL_947936, EPI_ISL_947937, EPI_ISL_947938, EPI_ISL_947939, EPI_ISL_947940, EPI_ISL_947941, EPI_ISL_947942, EPI_ISL_947943, EPI_ISL_947944, EPI_ISL_947945, EPI_ISL_947946, EPI_ISL_947947, EPI_ISL_947948, EPI_ISL_947949, EPI_ISL_947950, EPI_ISL_947951, EPI_ISL_947952, EPI_ISL_947953, EPI_ISL_947954, EPI_ISL_947955, EPI_ISL_947956, EPI_ISL_947957, EPI_ISL_947958, EPI_ISL_947959, EPI_ISL_947960, EPI_ISL_947961, EPI_ISL_947962, EPI_ISL_947963, EPI_ISL_947964, EPI_ISL_947965, EPI_ISL_947966, EPI_ISL_947967, EPI_ISL_947968, EPI_ISL_947969, EPI_ISL_947970, EPI_ISL_947971, EPI_ISL_947972, EPI_ISL_947973, EPI_ISL_947974, EPI_ISL_947975, EPI_ISL_947976, EPI_ISL_947977, EPI_ISL_947978, EPI_ISL_947979, EPI_ISL_947980, EPI_ISL_947981, EPI_ISL_947982, EPI_ISL_947983, EPI_ISL_947984, EPI_ISL_947985, EPI_ISL_947986, EPI_ISL_947987, EPI_ISL_947988, EPI_ISL_947989, EPI_ISL_947990, EPI_ISL_947991, EPI_ISL_947992, EPI_ISL_947993, EPI_ISL_947994, EPI_ISL_947995, EPI_ISL_947996, EPI_ISL_947997, EPI_ISL_947998, EPI_ISL_947999, EPI_ISL_948000, EPI_ISL_948001, EPI_ISL_948002, EPI_ISL_948003, EPI_ISL_948004, EPI_ISL_948005, EPI_ISL_948006, EPI_ISL_948007, EPI_ISL_948008, EPI_ISL_948009, EPI_ISL_948010, EPI_ISL_948011, EPI_ISL_948012, EPI_ISL_948013, EPI_ISL_948015, EPI_ISL_948016, EPI_ISL_948017, EPI_ISL_948018, EPI_ISL_948019, EPI_ISL_948020, EPI_ISL_948021, EPI_ISL_948022, EPI_ISL_948023, EPI_ISL_948024, EPI_ISL_948025, EPI_ISL_948026, EPI_ISL_948027, EPI_ISL_948028, EPI_ISL_948029, EPI_ISL_948030, EPI_ISL_948031, EPI_ISL_948032, EPI_ISL_948033, EPI_ISL_948034, EPI_ISL_948035, EPI_ISL_948036, EPI_ISL_948037, EPI_ISL_948038, EPI_ISL_948039, EPI_ISL_948040, EPI_ISL_948041, EPI_ISL_948042, EPI_ISL_948043, EPI_ISL_948044, EPI_ISL_948045, EPI_ISL_948046, EPI_ISL_948047, EPI_ISL_948048, EPI_ISL_948049, EPI_ISL_948050, EPI_ISL_948051, EPI_ISL_948052, EPI_ISL_948053, EPI_ISL_948054, EPI_ISL_948055, EPI_ISL_948056, EPI_ISL_948057, EPI_ISL_948058, EPI_ISL_948059, EPI_ISL_948060, EPI_ISL_948061, EPI_ISL_948062, EPI_ISL_948063, EPI_ISL_948064, EPI_ISL_948065, EPI_ISL_948066, EPI_ISL_948067, EPI_ISL_948068, EPI_ISL_948069, EPI_ISL_948070, EPI_ISL_948071, EPI_ISL_948072, EPI_ISL_948073, EPI_ISL_948074, EPI_ISL_948075, EPI_ISL_948076, EPI_ISL_948077, EPI_ISL_948078, EPI_ISL_948079, EPI_ISL_948080, EPI_ISL_948081, EPI_ISL_948082, EPI_ISL_948083, EPI_ISL_948084, EPI_ISL_948085, EPI_ISL_948086, EPI_ISL_948087, EPI_ISL_948088, EPI_ISL_948089, EPI_ISL_948090, EPI_ISL_948091, EPI_ISL_948092, EPI_ISL_948093, EPI_ISL_948094, EPI_ISL_948095, EPI_ISL_948096, EPI_ISL_948097, EPI_ISL_948098, EPI_ISL_948099			
see above	Lighthouse Lab in Alderley Park	Wellcome Sanger Institute for the COVID-19 Genomics UK (COG-UK) Consortium	Jacquelyn Wynn, Mairead Hyland, The Lighthouse Lab in Alderley Park and Alex Alderton, Roberto Amato, Sonia Goncalves, Ewan Harrison, David K. Jackson, Ian Johnston, Dominic Kwiatkowski, Cordelia Langford, John Sillitoe on behalf of the Wellcome Sanger Institute COVID-19 Surveillance Team
EPI_ISL_948101, EPI_ISL_948116, EPI_ISL_948125, EPI_ISL_948128, EPI_ISL_948133, EPI_ISL_948150, EPI_ISL_948155, EPI_ISL_948159, EPI_ISL_948160, EPI_ISL_948177, EPI_ISL_948178, EPI_ISL_948185, EPI_ISL_948186, EPI_ISL_948194, EPI_ISL_948204, EPI_ISL_948210, EPI_ISL_948215, EPI_ISL_948221, EPI_ISL_948231, EPI_ISL_948266, EPI_ISL_948289, EPI_ISL_948292, EPI_ISL_948294, EPI_ISL_948330, EPI_ISL_948368, EPI_ISL_948374, EPI_ISL_948376			
see above	Lighthouse Lab in Cambridge	Wellcome Sanger Institute for the COVID-19 Genomics UK (COG-UK) Consortium	Rob Howes, The Lighthouse Lab in Cambridge and Alex Alderton, Roberto Amato, Sonia Goncalves, Ewan Harrison, David K. Jackson, Ian Johnston, Dominic Kwiatkowski, Cordelia Langford, John Sillitoe on behalf of the Wellcome Sanger Institute COVID-19 Surveillance Team
EPI_ISL_948377	Lighthouse Lab in Glasgow	Wellcome Sanger Institute for the COVID-19 Genomics UK (COG-UK) Consortium	Harper VanSteenhouse, Yumi Kasai, David Gray, Carol Clugston, Anna Dominiczak and Alex Alderton, Roberto Amato, Sonia Goncalves, Ewan Harrison, David K. Jackson, Ian Johnston, Dominic Kwiatkowski, Cordelia Langford, John Sillitoe on behalf of the Wellcome Sanger Institute COVID-19 Surveillance Team
EPI_ISL_948388, EPI_ISL_948407	Lighthouse Lab in Cambridge	Wellcome Sanger Institute for the COVID-19 Genomics UK (COG-UK) Consortium	Rob Howes, The Lighthouse Lab in Cambridge and Alex Alderton, Roberto Amato, Sonia Goncalves, Ewan Harrison, David K. Jackson, Ian Johnston, Dominic Kwiatkowski, Cordelia Langford, John Sillitoe on behalf of the Wellcome Sanger Institute COVID-19 Surveillance Team
EPI_ISL_948432	Lighthouse Lab in Glasgow	Wellcome Sanger Institute for the COVID-19 Genomics UK (COG-UK) Consortium	Harper VanSteenhouse, Yumi Kasai, David Gray, Carol Clugston, Anna Dominiczak and Alex Alderton, Roberto Amato, Sonia Goncalves, Ewan Harrison, David K. Jackson, Ian Johnston, Dominic Kwiatkowski, Cordelia Langford, John Sillitoe on behalf of the Wellcome Sanger Institute COVID-19 Surveillance Team
EPI_ISL_948449, EPI_ISL_948450, EPI_ISL_948451, EPI_ISL_948452, EPI_ISL_948453, EPI_ISL_948454, EPI_ISL_948455, EPI_ISL_948456, EPI_ISL_948457, EPI_ISL_948458, EPI_ISL_948459, EPI_ISL_948460, EPI_ISL_948461, EPI_ISL_948462, EPI_ISL_948463, EPI_ISL_948464, EPI_ISL_948465, EPI_ISL_948466, EPI_ISL_948467, EPI_ISL_948468, EPI_ISL_948469, EPI_ISL_948470, EPI_ISL_948471, EPI_ISL_948472, EPI_ISL_948473, EPI_ISL_948474, EPI_ISL_948475, EPI_ISL_948476, EPI_ISL_948477, EPI_ISL_948479, EPI_ISL_948480, EPI_ISL_948481, EPI_ISL_948482, EPI_ISL_948483, EPI_ISL_948484, EPI_ISL_948485, EPI_ISL_948487, EPI_ISL_948488, EPI_ISL_948489, EPI_ISL_948490, EPI_ISL_948491, EPI_ISL_948492, EPI_ISL_948493, EPI_ISL_948494, EPI_ISL_948495, EPI_ISL_948496, EPI_ISL_948497, EPI_ISL_948498, EPI_ISL_948499, EPI_ISL_948500, EPI_ISL_948501, EPI_ISL_948502, EPI_ISL_948503, EPI_ISL_948504, EPI_ISL_948505, EPI_ISL_948506, EPI_ISL_948507, EPI_ISL_948508, EPI_ISL_948509, EPI_ISL_948510, EPI_ISL_948511, EPI_ISL_948512, EPI_ISL_948513, EPI_ISL_948514, EPI_ISL_948515, EPI_ISL_948516, EPI_ISL_948517, EPI_ISL_948519, EPI_ISL_948520, EPI_ISL_948521, EPI_ISL_948522, EPI_ISL_948523, EPI_ISL_948524, EPI_ISL_948525, EPI_ISL_948526, EPI_ISL_948527, EPI_ISL_948528, EPI_ISL_948529, EPI_ISL_948530, EPI_ISL_948531, EPI_ISL_948532, EPI_ISL_948533, EPI_ISL_948534, EPI_ISL_948535, EPI_ISL_948536, EPI_ISL_948537, EPI_ISL_948538, EPI_ISL_948539, EPI_ISL_948540, EPI_ISL_948541, EPI_ISL_948542, EPI_ISL_948543, EPI_ISL_948544, EPI_ISL_948545, EPI_ISL_948546, EPI_ISL_948547, EPI_ISL_948548, EPI_ISL_948549, EPI_ISL_948550, EPI_ISL_948551, EPI_ISL_948552, EPI_ISL_948553, EPI_ISL_948554, EPI_ISL_948555, EPI_ISL_948556, EPI_ISL_948557, EPI_ISL_948558, EPI_ISL_948559, EPI_ISL_948560, EPI_ISL_948561, EPI_ISL_948562, EPI_ISL_948563, EPI_ISL_948564, EPI_ISL_948565, EPI_ISL_948566, EPI_ISL_948567, EPI_ISL_948568, EPI_ISL_948569, EPI_ISL_948570, EPI_ISL_948571, EPI_ISL_948572, EPI_ISL_948573, EPI_ISL_948574, EPI_ISL_948575, EPI_ISL_948576, EPI_ISL_948577, EPI_ISL_948578, EPI_ISL_948579, EPI_ISL_948580, EPI_ISL_948581, EPI_ISL_948582, EPI_ISL_948583, EPI_ISL_948584, EPI_ISL_948585, EPI_ISL_948586, EPI_ISL_948587, EPI_ISL_948588, EPI_ISL_948589, EPI_ISL_948590, EPI_ISL_948591, EPI_ISL_948592, EPI_ISL_948593, EPI_ISL_948594, EPI_ISL_948595, EPI_ISL_948596, EPI_ISL_948597, EPI_ISL_948599, EPI_ISL_948600, EPI_ISL_948601, EPI_ISL_948602, EPI_ISL_948603, EPI_ISL_948604, EPI_ISL_948605, EPI_ISL_948606, EPI_ISL_948607, EPI_ISL_948608, EPI_ISL_948609, EPI_ISL_948610, EPI_ISL_948611, EPI_ISL_948612, EPI_ISL_948613, EPI_ISL_948614, EPI_ISL_948615, EPI_ISL_948616, EPI_ISL_948618, EPI_ISL_948619, EPI_ISL_948620, EPI_ISL_948621, EPI_ISL_948622, EPI_ISL_948623, EPI_ISL_948624, EPI_ISL_948625, EPI_ISL_948626, EPI_ISL_948627, EPI_ISL_948628, EPI_ISL_948629, EPI_ISL_948630, EPI_ISL_948631, EPI_ISL_948632, EPI_ISL_948633, EPI_ISL_948634, EPI_ISL_948635, EPI_ISL_948636, EPI_ISL_948637, EPI_ISL_948638, EPI_ISL_948639, EPI_ISL_948640, EPI_ISL_948641, EPI_ISL_948642, EPI_ISL_948643, EPI_ISL_948644, EPI_ISL_948645, EPI_ISL_948646, EPI_ISL_948647, EPI_ISL_948648, EPI_ISL_948649, EPI_ISL_948650, EPI_ISL_948651, EPI_ISL_948652, EPI_ISL_948653, EPI_ISL_948654, EPI_ISL_948655, EPI_ISL_948656, EPI_ISL_948657, EPI_ISL_948658, EPI_ISL_948659, EPI_ISL_948660, EPI_ISL_948661, EPI_ISL_948662, EPI_ISL_948663, EPI_ISL_948664, EPI_ISL_948665, EPI_ISL_948666, EPI_ISL_948667, EPI_ISL_948668, EPI_ISL_948669, EPI_ISL_948670, EPI_ISL_948671, EPI_ISL_948672, EPI_ISL_948673, EPI_ISL_948674, EPI_ISL_948675, EPI_ISL_948676, EPI_ISL_948677, EPI_ISL_948678, EPI_ISL_948679, EPI_ISL_948680, EPI_ISL_948681, EPI_ISL_948682, EPI_ISL_948683, EPI_ISL_948684, EPI_ISL_948685, EPI_ISL_948686, EPI_ISL_948687, EPI_ISL_948688, EPI_ISL_948689, EPI_ISL_948690, EPI_ISL_948691, EPI_ISL_948692, EPI_ISL_948693, EPI_ISL_948694, EPI_ISL_948695, EPI_ISL_948696, EPI_ISL_948697, EPI_ISL_948698, EPI_ISL_948700, EPI_ISL_948701, EPI_ISL_948702, EPI_ISL_948703, EPI_ISL_948704, EPI_ISL_948705, EPI_ISL_948706, EPI_ISL_948707, EPI_ISL_948708, EPI_ISL_948709, EPI_ISL_948710, EPI_ISL_948711, EPI_ISL_948712, EPI_ISL_948713, EPI_ISL_948714, EPI_ISL_948715, EPI_ISL_948716, EPI_ISL_948717, EPI_ISL_948718, EPI_ISL_948719, EPI_ISL_948720, EPI_ISL_948721, EPI_ISL_948722, EPI_ISL_948723, EPI_ISL_948724, EPI_ISL_948725, EPI_ISL_948726, EPI_ISL_948727, EPI_ISL_948728, EPI_ISL_948729, EPI_ISL_948730, EPI_ISL_948731, EPI_ISL_948732, EPI_ISL_948733, EPI_ISL_948734, EPI_ISL_948735, EPI_ISL_948736, EPI_ISL_948737, EPI_ISL_948738, EPI_ISL_948739, EPI_ISL_948740, EPI_ISL_948741, EPI_ISL_948742, EPI_ISL_948743, EPI_ISL_948744, EPI_ISL_948745, EPI_ISL_948746, EPI_ISL_948747, EPI_ISL_948748, EPI_ISL_948749, EPI_ISL_948750, EPI_ISL_948751, EPI_ISL_948752, EPI_ISL_948753, EPI_ISL_948754, EPI_ISL_948755, EPI_ISL_948756, EPI_ISL_948757, EPI_ISL_948758, EPI_ISL_948759, EPI_ISL_948760, EPI_ISL_948761, EPI_ISL_948762, EPI_ISL_948763, EPI_ISL_948764, EPI_ISL_948765, EPI_ISL_948766, EPI_ISL_948767, EPI_ISL_948768, EPI_ISL_948769, EPI_ISL_948770, EPI_ISL_948771, EPI_ISL_948772, EPI_ISL_948773, EPI_ISL_948774, EPI_ISL_948775, EPI_ISL_948776, EPI_ISL_948777, EPI_ISL_948778, EPI_ISL_948779, EPI_ISL_948780, EPI_ISL_948781, EPI_ISL_948782, EPI_ISL_948783, EPI_ISL_948784, EPI_ISL_948785, EPI_ISL_948786			
see above	Lighthouse Lab in Alderley Park	Wellcome Sanger Institute for the COVID-19 Genomics UK (COG-UK) Consortium	Jacquelyn Wynn, Mairead Hyland, The Lighthouse Lab in Alderley Park and Alex Alderton, Roberto Amato, Sonia Goncalves, Ewan Harrison, David K. Jackson, Ian Johnston, Dominic Kwiatkowski, Cordelia Langford, John Sillitoe on behalf of the Wellcome Sanger Institute COVID-19 Surveillance Team
EPI_ISL_948829, EPI_ISL_948830, EPI_ISL_948834, EPI_ISL_948845, EPI_ISL_948848, EPI_ISL_948945, EPI_ISL_948946, EPI_ISL_948947, EPI_ISL_948948, EPI_ISL_948949, EPI_ISL_948950, EPI_ISL_948951, EPI_ISL_948952, EPI_ISL_948953, EPI_ISL_948954, EPI_ISL_948955, EPI_ISL_948956, EPI_ISL_948957, EPI_ISL_948958, EPI_ISL_948959, EPI_ISL_948960, EPI_ISL_948961, EPI_ISL_948962, EPI_ISL_948963, EPI_ISL_948964, EPI_ISL_948965, EPI_ISL_948966			
see above	Lighthouse Lab in Glasgow	Wellcome Sanger Institute for the COVID-19 Genomics UK (COG-UK) Consortium	Harper VanSteenhouse, Yumi Kasai, David Gray, Carol Clugston, Anna Dominiczak and Alex Alderton, Roberto Amato, Sonia Goncalves, Ewan Harrison, David K. Jackson, Ian Johnston, Dominic Kwiatkowski, Cordelia Langford, John Sillitoe on behalf of the Wellcome Sanger Institute COVID-19 Surveillance Team
EPI_ISL_949409, EPI_ISL_949413, EPI_ISL_949414, EPI_ISL_949415, EPI_ISL_949416	University of Birmingham	COVID-19 Genomics UK (COG-UK) Consortium	Institute of Microbiology, University of Birmingham: Claire McMurray, Joanne Stockton, Samuel Nicholls, Radoslaw Poplawski, Will Rowe, Josh Quick, Nicholas Loman. University of Birmingham Testing Laboratory: Celina M Whalley, Andrew Bosworth, Charlotte Poxon, Kasun Wanigasooriya, Oliver Pickles, Mike Kidd, Alex Richter, Andrew D Beggs PHE Heartlands Lab: Husam Osman, Andrew Bosworth. Queen Elizabeth Hospital: Anna Casey
EPI_ISL_949456, EPI_ISL_949457, EPI_ISL_949458, EPI_ISL_949459, EPI_ISL_949460, EPI_ISL_949461, EPI_ISL_949466, EPI_ISL_949470, EPI_ISL_949471, EPI_ISL_949472, EPI_ISL_949474, EPI_ISL_949522, EPI_ISL_949523			
see above	Department of Pathology, University of Cambridge	COVID-19 Genomics UK (COG-UK) Consortium	Aminu S. Jahun, Yasmin Chaudhry, Iliana Georgana, Myra Hosmillo, Rhys Izu, Martin D. Curran, Surendra Parmar, Ian Goodfellow

EPI_ISL_949608, EPI_ISL_949609, EPI_ISL_949610, EPI_ISL_949614	West of Scotland Specialist Virology Centre, NHSGGC / MRC-University of Glasgow Centre for Virus Research	COVID-19 Genomics UK (COG-UK) Consortium	Ana da Silva Filipe, Natasha Johnson, Kathy Smollett, Daniel Mair, Stephen Carmichael, Alice Broos, Lily Tong, Jenna Nichols, Kyriaki Nomikou; Sarah McDonald; Richard Orton, Joseph Hughes, Sreenu Vattipally, David L Robertson; Alasdair MacLean, Rory Gunson; Sharif Shaaban, Matthew Holden; Rachel Blacow, Guy Mollett, Kathy Li, James Shepherd, Antonia Ho, Emma Thomson
EPI_ISL_949642	Virology Department, Royal Infirmary of Edinburgh, NHS Lothian / School of Biological Sciences, University of Edinburgh / Institute of Genetics and Molecular Medicine, University of Edinburgh	COVID-19 Genomics UK (COG-UK) Consortium	McHugh M, Dewar R, Rooke S, Gallagher M, Balcaza C, O'Toole Á, Scher E, Hill V, McCrone JT, Colquhoun R, Yu X, Jackson B, Rambaut A, Williams TC, Templeton K
EPI_ISL_949671, EPI_ISL_949672, EPI_ISL_949684, EPI_ISL_949685, EPI_ISL_949686, EPI_ISL_949687	Liverpool Clinical Laboratories	COVID-19 Genomics UK (COG-UK) Consortium	Sam Haldenby, Anita Lucaci, Steve Paterson, Julian Hiscox, Alistair Darby, M Almsaud, A Alrezaihi, Muhannad Alruwaili, Stuart D Armstrong, Jones Benjamin, Eleanor G Bentley, Anu Chawla, Jordan J Clark, Angela Cowell, Richard Eccles, Isabel García-Dorival, Matthew Gemmell, Alessandro Gerada, PKF Gilmore, Richard Gregory, Ximeng Han, Catherine Hartley, Margaret Hughes, Miren Iturriza-Gomara, James Johnson, L Luu, Jenifer Manson, Charlotte Nelson, Elaine O'Toole, Cassie Olateju, Rebekah Penrice-Randal , Lucille Rainbow, N.P Randle, Trevor Ian Robinson, Parul Sharma, Ghada T Shawli, James P Stewart, Neil Swainston, Ecaterina Vamos, Joanne Watts, Mark Whitehead
EPI_ISL_949751, EPI_ISL_949752	Barts Health NHS Trust	COVID-19 Genomics UK (COG-UK) Consortium	CUTINO-MOGUEL, Maria-Teresa; HARRINGTON, David; OWOYEMI, Dola; KULASEGARAN-SHYLINI, Raghavendran; BROAD, Claire; KELE, Beatrix
EPI_ISL_949792, EPI_ISL_949906, EPI_ISL_949907, EPI_ISL_949908, EPI_ISL_949975, EPI_ISL_949976, EPI_ISL_949977, EPI_ISL_949978, EPI_ISL_949980, EPI_ISL_949981, EPI_ISL_949982, EPI_ISL_949983, EPI_ISL_949984, EPI_ISL_949985, EPI_ISL_949987, EPI_ISL_949988, EPI_ISL_949989, EPI_ISL_949992, EPI_ISL_949993, EPI_ISL_949994, EPI_ISL_949995, EPI_ISL_949996, EPI_ISL_949997, EPI_ISL_949998, EPI_ISL_949999, EPI_ISL_950000, EPI_ISL_950001, EPI_ISL_950002, EPI_ISL_950003, EPI_ISL_950004, EPI_ISL_950005, EPI_ISL_950006, EPI_ISL_950007, EPI_ISL_950008, EPI_ISL_950009, EPI_ISL_950010, EPI_ISL_950011, EPI_ISL_950012, EPI_ISL_950013, EPI_ISL_950014, EPI_ISL_950015, EPI_ISL_950016, EPI_ISL_950017, EPI_ISL_950018, EPI_ISL_950020, EPI_ISL_950021, EPI_ISL_950022, EPI_ISL_950023, EPI_ISL_950024, EPI_ISL_950025, EPI_ISL_950026, EPI_ISL_950027, EPI_ISL_950029, EPI_ISL_950030, EPI_ISL_950031, EPI_ISL_950032, EPI_ISL_950033, EPI_ISL_950035, EPI_ISL_950036, EPI_ISL_950037, EPI_ISL_950038, EPI_ISL_950170			
see above	University College London, Great Ormond Street Hospital for Children NHS Foundation Trust, Imperial College Healthcare NHS Trust	COVID-19 Genomics UK (COG-UK) Consortium	Sergi Castellano, Rachel Williams, Mark Kristiansen, Paola Resende Silva, Sunando Roy, Tony Brooks, Helena Tutill, Paola Niola, Patricia Dyal, Charlotte Williams, Leysa Forrest, Yasmin Panchbhaya, Jacqueline Findlay, Samuel Weeks, Julianne Brown, Kathryn Harris, Paul Randell, James Price, Alison Holmes, Judith Breuer
EPI_ISL_950350, EPI_ISL_950505, EPI_ISL_950506, EPI_ISL_950507, EPI_ISL_950508, EPI_ISL_950509, EPI_ISL_950510, EPI_ISL_950511, EPI_ISL_950512, EPI_ISL_950513, EPI_ISL_950514, EPI_ISL_950515, EPI_ISL_950516, EPI_ISL_950517, EPI_ISL_950518, EPI_ISL_950519, EPI_ISL_950520, EPI_ISL_950521, EPI_ISL_950522			
see above	Northumbria University / South Tees Hospitals NHS Foundation Trust / North Cumbria Integrated Care NHS Foundation Trust / North Tees and Hartlepool NHS Foundation Trust / Newcastle Hospitals NHS Foundation Trust	COVID-19 Genomics UK (COG-UK) Consortium	Darren L Smith,Andrew Nelson,Matthew Bashton,Greg R Young,Joshua Loh,John Allan,Mohammad A Tariq,Giles S Holt,Gary Black,Wen C Yew,Lynn Dover,Paul Baker,Steve Liggett,Sarah Essex,Jane Greenaway,Debra Padgett,Clive Graham,Garren Scott,Edward Barton,Emma Swindells,Brendan Payne,Jennifer Collins,Yusri Taha,Gary Eltringham
EPI_ISL_950638	Queens Medical Centre, Clinical Microbiology Department / DeepSeq Nottingham	COVID-19 Genomics UK (COG-UK) Consortium	Gemma Clark, Wendy Smith, Manjinder Khakh, Vicki M Fleming, Michelle M Lister, Hannah Howson-Wells, Jonathan Ball, Patrick McClure, Joseph Chappell, Theocharis Tsoleridis, Nadine Holmes, Matthew Carlisle, Christopher Moore, Fei Sang, Johnny Debebe, Victoria Wright, Matthew Loose
EPI_ISL_950729, EPI_ISL_950730, EPI_ISL_950731, EPI_ISL_950732, EPI_ISL_950733, EPI_ISL_950734, EPI_ISL_950735, EPI_ISL_950736, EPI_ISL_950737, EPI_ISL_950738, EPI_ISL_950739, EPI_ISL_950740, EPI_ISL_950741, EPI_ISL_950742, EPI_ISL_950743, EPI_ISL_950744, EPI_ISL_950745, EPI_ISL_950746, EPI_ISL_950747, EPI_ISL_950748, EPI_ISL_950749, EPI_ISL_950750, EPI_ISL_950751, EPI_ISL_950752, EPI_ISL_950753, EPI_ISL_950754			
see above	Lincolnshire Hospitals and DeepSeq Nottingham	COVID-19 Genomics UK (COG-UK) Consortium	Nichola Duckworth, Tim Sloan, Sarah Walsh, Jonathan Ball, Patrick McClure, Joeseph Chappell, Nadine Holmes, Matthew Carlisle, Christopher Moore, Fei Sang, Johnny Debebe, Victoria Wright, Matthew Loose
EPI_ISL_951500, EPI_ISL_951510, EPI_ISL_951511, EPI_ISL_951563, EPI_ISL_951597, EPI_ISL_951598, EPI_ISL_951601, EPI_ISL_951602, EPI_ISL_951604, EPI_ISL_951605, EPI_ISL_951606, EPI_ISL_951607, EPI_ISL_951608, EPI_ISL_951609, EPI_ISL_951610, EPI_ISL_951611, EPI_ISL_951612, EPI_ISL_951613, EPI_ISL_951614, EPI_ISL_951615, EPI_ISL_951616, EPI_ISL_951617, EPI_ISL_951618, EPI_ISL_951619, EPI_ISL_951640, EPI_ISL_951643, EPI_ISL_951667, EPI_ISL_951673, EPI_ISL_951676, EPI_ISL_951677, EPI_ISL_951679, EPI_ISL_951684, EPI_ISL_951685, EPI_ISL_951686, EPI_ISL_951687, EPI_ISL_951688, EPI_ISL_951689, EPI_ISL_951690, EPI_ISL_951691, EPI_ISL_951693, EPI_ISL_951694, EPI_ISL_951695, EPI_ISL_951696, EPI_ISL_951698, EPI_ISL_951700, EPI_ISL_951701, EPI_ISL_951702, EPI_ISL_951703, EPI_ISL_951704, EPI_ISL_951705, EPI_ISL_951706, EPI_ISL_951708, EPI_ISL_951709, EPI_ISL_951711, EPI_ISL_951712, EPI_ISL_951713, EPI_ISL_951714, EPI_ISL_951715, EPI_ISL_951717, EPI_ISL_951718, EPI_ISL_951719, EPI_ISL_951720, EPI_ISL_951721, EPI_ISL_951725, EPI_ISL_951728, EPI_ISL_951730, EPI_ISL_951731, EPI_ISL_951732, EPI_ISL_951733, EPI_ISL_951735, EPI_ISL_951736, EPI_ISL_951737, EPI_ISL_951741, EPI_ISL_951743, EPI_ISL_951746, EPI_ISL_951747, EPI_ISL_951748, EPI_ISL_951749, EPI_ISL_951752, EPI_ISL_951755, EPI_ISL_951762, EPI_ISL_951763, EPI_ISL_951764, EPI_ISL_951768, EPI_ISL_951774, EPI_ISL_951775, EPI_ISL_951776, EPI_ISL_951777, EPI_ISL_951784, EPI_ISL_951831, EPI_ISL_951832, EPI_ISL_951833, EPI_ISL_951834, EPI_ISL_951842, EPI_ISL_951845, EPI_ISL_951846, EPI_ISL_951847, EPI_ISL_951848, EPI_ISL_951849, EPI_ISL_951850, EPI_ISL_951987, EPI_ISL_951992, EPI_ISL_951998, EPI_ISL_951999, EPI_ISL_952000, EPI_ISL_952002, EPI_ISL_952168, EPI_ISL_952170, EPI_ISL_952171, EPI_ISL_952172, EPI_ISL_952173, EPI_ISL_952174, EPI_ISL_952175, EPI_ISL_952176, EPI_ISL_952177, EPI_ISL_952178, EPI_ISL_952179, EPI_ISL_952180, EPI_ISL_952199, EPI_ISL_952203, EPI_ISL_952213, EPI_ISL_952214, EPI_ISL_952215, EPI_ISL_952216, EPI_ISL_952217, EPI_ISL_952223, EPI_ISL_952224, EPI_ISL_952226, EPI_ISL_952231, EPI_ISL_952234, EPI_ISL_952235, EPI_ISL_952238, EPI_ISL_952239, EPI_ISL_952241, EPI_ISL_952244, EPI_ISL_952246, EPI_ISL_952248, EPI_ISL_952250			
see above	Originating lab: Wales Specialist Virology Centre Sequencing lab: Pathogen Genomics Unit	Public Health Wales Microbiology Cardiff Wales Specialist Virology Centre	Catherine Moore, Johnathan Evans, Laura Gifford, Malorie Perry, Simon Cottrell, Angela Marchbank, Alec Birchley, Alexander Adams, Amy Gaskin, Bree Gatica-Wilcox, Jason Coombes, Joel Southgate, Lauren Gilbert, Lee Graham, Nicole Pacchiarini, Sara Kumziene-Summerhayes, Sarah Taylor, Sophie Jones, Sara Rey, Matthew Bull, Joanne Watkins, Sally Corden, Tom Connor
EPI_ISL_952380, EPI_ISL_952391, EPI_ISL_952395, EPI_ISL_952417, EPI_ISL_952438, EPI_ISL_952439, EPI_ISL_952440, EPI_ISL_952441, EPI_ISL_952442, EPI_ISL_952443, EPI_ISL_952444, EPI_ISL_952446, EPI_ISL_952447, EPI_ISL_952448, EPI_ISL_952450, EPI_ISL_952451, EPI_ISL_952452, EPI_ISL_952455, EPI_ISL_952457, EPI_ISL_952458, EPI_ISL_952461, EPI_ISL_952463, EPI_ISL_952468, EPI_ISL_952492, EPI_ISL_952493, EPI_ISL_952494, EPI_ISL_952495, EPI_ISL_952496, EPI_ISL_952497, EPI_ISL_952498, EPI_ISL_952499, EPI_ISL_952531, EPI_ISL_952532, EPI_ISL_952534, EPI_ISL_952535, EPI_ISL_952536, EPI_ISL_952537, EPI_ISL_952540, EPI_ISL_952541, EPI_ISL_952542, EPI_ISL_952543, EPI_ISL_952544, EPI_ISL_952545, EPI_ISL_952546, EPI_ISL_952547, EPI_ISL_952550, EPI_ISL_952553, EPI_ISL_952555, EPI_ISL_952556, EPI_ISL_952557, EPI_ISL_952822, EPI_ISL_952823, EPI_ISL_952824, EPI_ISL_952825, EPI_ISL_952826, EPI_ISL_952827, EPI_ISL_952828, EPI_ISL_952836, EPI_ISL_952837, EPI_ISL_952838, EPI_ISL_952839, EPI_ISL_952857			
see above	Centre for Enzyme Innovation, University of Portsmouth / Translational Research Laboratory, Portsmouth Hospitals NHS Trust	COVID-19 Genomics UK (COG-UK) Consortium	Angela Beckett,Salman Goudarzi,Christopher Fearn,Kate Cook,Katie Loveson,Sharon Glaysher,Scott Elliott,Samuel Robson
EPI_ISL_957101	Lighthouse Lab in Glasgow	Wellcome Sanger Institute for the COVID-19 Genomics UK (COG-UK) Consortium	Harper VanSteenhouse, Yumi Kasai, David Gray, Carol Clugston, Anna Dominiczak and Alex Alderton, Roberto Amato, Sonia Goncalves, Ewan Harrison, David K. Jackson, Ian Johnston, Dominic Kwiatkowski, Cordelia Langford, John Sillitoe on behalf of the Wellcome Sanger Institute COVID-19 Surveillance Team
EPI_ISL_958276, EPI_ISL_958398, EPI_ISL_963428	Lighthouse Lab in Milton Keynes	Wellcome Sanger Institute for the COVID-19 Genomics UK (COG-UK) Consortium	The Lighthouse Lab in Milton Keynes and Alex Alderton, Roberto Amato, Sonia Goncalves, Ewan Harrison, David K. Jackson, Ian Johnston, Dominic Kwiatkowski, Cordelia Langford, John Sillitoe on behalf of the Wellcome Sanger Institute COVID-19 Surveillance Team
EPI_ISL_987606, EPI_ISL_988214, EPI_ISL_988226, EPI_ISL_988228, EPI_ISL_988232, EPI_ISL_988247, EPI_ISL_988253, EPI_ISL_988255, EPI_ISL_988259, EPI_ISL_988264, EPI_ISL_988268, EPI_ISL_988274, EPI_ISL_988280, EPI_ISL_988285, EPI_ISL_988293, EPI_ISL_988298, EPI_ISL_988300, EPI_ISL_988302, EPI_ISL_988304, EPI_ISL_988315, EPI_ISL_988323, EPI_ISL_988324, EPI_ISL_988333, EPI_ISL_988339, EPI_ISL_988340, EPI_ISL_988343, EPI_ISL_988355, EPI_ISL_988359, EPI_ISL_988381, EPI_ISL_988390, EPI_ISL_988397, EPI_ISL_988409, EPI_ISL_988420, EPI_ISL_988429, EPI_ISL_988433, EPI_ISL_988435, EPI_ISL_988437, EPI_ISL_988442, EPI_ISL_988451, EPI_ISL_988456, EPI_ISL_988457, EPI_ISL_988465, EPI_ISL_988467, EPI_ISL_988475, EPI_ISL_988477, EPI_ISL_988478, EPI_ISL_988481			
see above	Lighthouse Lab in Cambridge	Wellcome Sanger Institute for the COVID-19 Genomics UK (COG-UK) Consortium	Rob Howes, The Lighthouse Lab in Cambridge and Alex Alderton, Roberto Amato, Sonia Goncalves, Ewan Harrison, David K. Jackson, Ian Johnston, Dominic Kwiatkowski, Cordelia Langford, John Sillitoe on behalf of the Wellcome Sanger Institute COVID-19 Surveillance Team (http://www.sanger.ac.uk/covid-team)
EPI_ISL_990003, EPI_ISL_990043, EPI_ISL_990044, EPI_ISL_990045, EPI_ISL_990046, EPI_ISL_990047, EPI_ISL_990048, EPI_ISL_990049, EPI_ISL_990050, EPI_ISL_990051, EPI_ISL_990052, EPI_ISL_990053, EPI_ISL_990054, EPI_ISL_990055, EPI_ISL_990056, EPI_ISL_990057, EPI_ISL_990286, EPI_ISL_990435, EPI_ISL_990500, EPI_ISL_990502, EPI_ISL_990510, EPI_ISL_990557, EPI_ISL_990571, EPI_ISL_990576, EPI_ISL_990579, EPI_ISL_990616, EPI_ISL_990618, EPI_ISL_990652, EPI_ISL_990658, EPI_ISL_990662, EPI_ISL_990664, EPI_ISL_990680, EPI_ISL_990712, EPI_ISL_990726, EPI_ISL_990745, EPI_ISL_990779, EPI_ISL_990881			
see above	Lighthouse Lab in Alderley Park	Wellcome Sanger Institute for the COVID-19 Genomics UK (COG-UK) Consortium	Jacquelyn Wynn, Mairead Hyland, The Lighthouse Lab in Alderley Park and Alex Alderton, Roberto Amato, Sonia Goncalves, Ewan Harrison, David K. Jackson, Ian Johnston, Dominic Kwiatkowski, Cordelia Langford, John Sillitoe on behalf of the Wellcome Sanger Institute COVID-19 Surveillance Team
EPI_ISL_991173	Lighthouse Lab in Glasgow	Wellcome Sanger Institute for the COVID-19 Genomics UK (COG-UK) Consortium	Harper VanSteenhouse, Yumi Kasai, David Gray, Carol Clugston, Anna Dominiczak and Alex Alderton, Roberto Amato, Sonia Goncalves, Ewan Harrison, David K. Jackson, Ian Johnston, Dominic Kwiatkowski, Cordelia Langford, John Sillitoe on behalf of the Wellcome Sanger Institute COVID-19 Surveillance Team
EPI_ISL_991174, EPI_ISL_991175, EPI_ISL_991176, EPI_ISL_991177, EPI_ISL_991179, EPI_ISL_991180, EPI_ISL_991181, EPI_ISL_991182, EPI_ISL_991183, EPI_ISL_991184, EPI_ISL_991185, EPI_ISL_991186, EPI_ISL_991187, EPI_ISL_991188, EPI_ISL_991189, EPI_ISL_991190, EPI_ISL_991191, EPI_ISL_991192, EPI_ISL_991193, EPI_ISL_991194, EPI_ISL_991195, EPI_ISL_991196, EPI_ISL_991197, EPI_ISL_991198, EPI_ISL_991199, EPI_ISL_991200, EPI_ISL_991201, EPI_ISL_991202, EPI_ISL_991203, EPI_ISL_991204, EPI_ISL_991206, EPI_ISL_991207, EPI_ISL_991208, EPI_ISL_991209, EPI_ISL_991210, EPI_ISL_991211, EPI_ISL_991212, EPI_ISL_991214, EPI_ISL_991215, EPI_ISL_991216, EPI_ISL_991217, EPI_ISL_991218, EPI_ISL_991219, EPI_ISL_991220, EPI_ISL_991221, EPI_ISL_991269, EPI_ISL_991272, EPI_ISL_991274, EPI_ISL_991291, EPI_ISL_991292, EPI_ISL_991293, EPI_ISL_991294, EPI_ISL_991295, EPI_ISL_991300, EPI_ISL_991302, EPI_ISL_991306			
see above	Lighthouse Lab in Alderley Park	Wellcome Sanger Institute for the COVID-19 Genomics UK (COG-UK) Consortium	Jacquelyn Wynn, Mairead Hyland, The Lighthouse Lab in Alderley Park and Alex Alderton, Roberto Amato, Sonia Goncalves, Ewan Harrison, David K. Jackson, Ian Johnston, Dominic Kwiatkowski, Cordelia Langford, John Sillitoe on behalf of the Wellcome Sanger Institute COVID-19 Surveillance Team

EPI_ISL_991354, EPI_ISL_991361, EPI_ISL_991366, EPI_ISL_991371, EPI_ISL_991380, EPI_ISL_991392, EPI_ISL_991394, EPI_ISL_991398, EPI_ISL_991400, EPI_ISL_991405, EPI_ISL_991415, EPI_ISL_991418, EPI_ISL_991423, EPI_ISL_991425, EPI_ISL_991440, EPI_ISL_991447, EPI_ISL_991454, EPI_ISL_991463, EPI_ISL_991477, EPI_ISL_991479, EPI_ISL_991481, EPI_ISL_991483, EPI_ISL_991501, EPI_ISL_991510, EPI_ISL_991511, EPI_ISL_991523, EPI_ISL_991533, EPI_ISL_991541			
see above	Lighthouse Lab in Glasgow	Wellcome Sanger Institute for the COVID-19 Genomics UK (COG-UK) Consortium	Harper VanSteenhouse, Yumi Kasai, David Gray, Carol Clugston, Anna Dominiczak and Alex Alderton, Roberto Amato, Sonia Goncalves, Ewan Harrison, David K. Jackson, Ian Johnston, Dominic Kwiatkowski, Cordelia Langford, John Sillitoe on behalf of the Wellcome Sanger Institute COVID-19 Surveillance Team
EPI_ISL_991566, EPI_ISL_991567, EPI_ISL_991568, EPI_ISL_991569, EPI_ISL_991570, EPI_ISL_991571, EPI_ISL_991572, EPI_ISL_991573, EPI_ISL_991574, EPI_ISL_991575, EPI_ISL_991576, EPI_ISL_991577, EPI_ISL_991578, EPI_ISL_991579, EPI_ISL_991580, EPI_ISL_991581, EPI_ISL_991582, EPI_ISL_991583, EPI_ISL_991584, EPI_ISL_991585, EPI_ISL_991586, EPI_ISL_991587, EPI_ISL_991588, EPI_ISL_991589, EPI_ISL_991590, EPI_ISL_991591, EPI_ISL_991592, EPI_ISL_991593, EPI_ISL_991594, EPI_ISL_991596, EPI_ISL_991597, EPI_ISL_991598, EPI_ISL_991599, EPI_ISL_991600, EPI_ISL_991601, EPI_ISL_991602, EPI_ISL_991603, EPI_ISL_991604, EPI_ISL_991605, EPI_ISL_991606, EPI_ISL_991607, EPI_ISL_991608, EPI_ISL_991609, EPI_ISL_991610, EPI_ISL_991611, EPI_ISL_991612, EPI_ISL_991613, EPI_ISL_991614, EPI_ISL_991615, EPI_ISL_991616, EPI_ISL_991617, EPI_ISL_991618, EPI_ISL_991619, EPI_ISL_991620, EPI_ISL_991621, EPI_ISL_991622, EPI_ISL_991623, EPI_ISL_991624, EPI_ISL_991625, EPI_ISL_991626, EPI_ISL_991627, EPI_ISL_991628, EPI_ISL_991630, EPI_ISL_991631, EPI_ISL_991632, EPI_ISL_991633, EPI_ISL_991634, EPI_ISL_991635, EPI_ISL_991636, EPI_ISL_991638, EPI_ISL_991639, EPI_ISL_991640, EPI_ISL_991641, EPI_ISL_991642, EPI_ISL_991643, EPI_ISL_991644, EPI_ISL_991645, EPI_ISL_991646, EPI_ISL_991647, EPI_ISL_991648, EPI_ISL_991649, EPI_ISL_991650, EPI_ISL_991651, EPI_ISL_991652, EPI_ISL_991654, EPI_ISL_991655, EPI_ISL_991656, EPI_ISL_991657, EPI_ISL_991658, EPI_ISL_991659, EPI_ISL_991660, EPI_ISL_991661, EPI_ISL_991662, EPI_ISL_991663, EPI_ISL_991664, EPI_ISL_991665, EPI_ISL_991666, EPI_ISL_991667, EPI_ISL_991668, EPI_ISL_991669, EPI_ISL_991670, EPI_ISL_991671, EPI_ISL_991672, EPI_ISL_991673, EPI_ISL_991674, EPI_ISL_991675, EPI_ISL_991676, EPI_ISL_991677, EPI_ISL_991678, EPI_ISL_991679, EPI_ISL_991681, EPI_ISL_991682, EPI_ISL_991683, EPI_ISL_991684, EPI_ISL_991685, EPI_ISL_991686, EPI_ISL_991687, EPI_ISL_991688, EPI_ISL_991689, EPI_ISL_991690, EPI_ISL_991691, EPI_ISL_991693, EPI_ISL_991695, EPI_ISL_991696, EPI_ISL_991697, EPI_ISL_991698, EPI_ISL_991699, EPI_ISL_991700, EPI_ISL_991701, EPI_ISL_991702, EPI_ISL_991704, EPI_ISL_991705, EPI_ISL_991706, EPI_ISL_991707, EPI_ISL_991708, EPI_ISL_991709, EPI_ISL_991710, EPI_ISL_991711, EPI_ISL_991712, EPI_ISL_991713, EPI_ISL_991714, EPI_ISL_991716, EPI_ISL_991717, EPI_ISL_991718, EPI_ISL_991719, EPI_ISL_991720, EPI_ISL_991721, EPI_ISL_991722, EPI_ISL_991723, EPI_ISL_991724, EPI_ISL_991725, EPI_ISL_991726, EPI_ISL_991727, EPI_ISL_991728, EPI_ISL_991729, EPI_ISL_991730, EPI_ISL_991731, EPI_ISL_991732, EPI_ISL_991733, EPI_ISL_991734, EPI_ISL_991735, EPI_ISL_991736, EPI_ISL_991737, EPI_ISL_991738, EPI_ISL_991739, EPI_ISL_991740, EPI_ISL_991741, EPI_ISL_991742, EPI_ISL_991743, EPI_ISL_991744, EPI_ISL_991745, EPI_ISL_991746, EPI_ISL_991747, EPI_ISL_991748, EPI_ISL_991749, EPI_ISL_991750, EPI_ISL_991751, EPI_ISL_991752, EPI_ISL_991753, EPI_ISL_991754, EPI_ISL_991756, EPI_ISL_991757, EPI_ISL_991758, EPI_ISL_991759, EPI_ISL_991760, EPI_ISL_991761, EPI_ISL_991762, EPI_ISL_991763, EPI_ISL_991764, EPI_ISL_991765, EPI_ISL_991766, EPI_ISL_991767, EPI_ISL_991768, EPI_ISL_991769, EPI_ISL_991770, EPI_ISL_991771, EPI_ISL_991772, EPI_ISL_991773, EPI_ISL_991774, EPI_ISL_991775, EPI_ISL_991776, EPI_ISL_991777, EPI_ISL_991778, EPI_ISL_991779, EPI_ISL_991780, EPI_ISL_991782, EPI_ISL_991783, EPI_ISL_991784, EPI_ISL_991785, EPI_ISL_991786, EPI_ISL_991787, EPI_ISL_991788, EPI_ISL_991789, EPI_ISL_991790, EPI_ISL_991791, EPI_ISL_991792, EPI_ISL_991794, EPI_ISL_991795, EPI_ISL_991796, EPI_ISL_991797, EPI_ISL_991798, EPI_ISL_991799, EPI_ISL_991800, EPI_ISL_991801, EPI_ISL_991802, EPI_ISL_991803, EPI_ISL_991804, EPI_ISL_991805, EPI_ISL_991806, EPI_ISL_991807, EPI_ISL_991808, EPI_ISL_991809, EPI_ISL_991810, EPI_ISL_991811, EPI_ISL_991812, EPI_ISL_991813, EPI_ISL_991814, EPI_ISL_991815, EPI_ISL_991816, EPI_ISL_991818, EPI_ISL_991819, EPI_ISL_991820, EPI_ISL_991821, EPI_ISL_991822, EPI_ISL_991823, EPI_ISL_991824, EPI_ISL_991825, EPI_ISL_991827, EPI_ISL_991828, EPI_ISL_991829, EPI_ISL_991830, EPI_ISL_991831, EPI_ISL_991832, EPI_ISL_991833, EPI_ISL_991835, EPI_ISL_991836, EPI_ISL_991837, EPI_ISL_991838, EPI_ISL_991839, EPI_ISL_991840, EPI_ISL_991841, EPI_ISL_991842, EPI_ISL_991843, EPI_ISL_991844, EPI_ISL_991845, EPI_ISL_991847, EPI_ISL_991849, EPI_ISL_991850, EPI_ISL_991851, EPI_ISL_991852, EPI_ISL_991853, EPI_ISL_991854, EPI_ISL_991855, EPI_ISL_991856, EPI_ISL_991857, EPI_ISL_991858, EPI_ISL_991859, EPI_ISL_991861, EPI_ISL_991862, EPI_ISL_991863, EPI_ISL_991864, EPI_ISL_991865, EPI_ISL_991866, EPI_ISL_991867, EPI_ISL_991868, EPI_ISL_991869, EPI_ISL_991870, EPI_ISL_991871, EPI_ISL_991872, EPI_ISL_991874, EPI_ISL_991875, EPI_ISL_991876, EPI_ISL_991877, EPI_ISL_991878, EPI_ISL_991879, EPI_ISL_991880, EPI_ISL_991881, EPI_ISL_991882, EPI_ISL_991883, EPI_ISL_991884, EPI_ISL_991885, EPI_ISL_991886, EPI_ISL_991887, EPI_ISL_991888, EPI_ISL_991889, EPI_ISL_991890, EPI_ISL_991891, EPI_ISL_991892, EPI_ISL_991893, EPI_ISL_991894, EPI_ISL_991895, EPI_ISL_991896, EPI_ISL_991897, EPI_ISL_991898, EPI_ISL_991899, EPI_ISL_991901, EPI_ISL_991902, EPI_ISL_991903			
see above	Lighthouse Lab in Alderley Park	Wellcome Sanger Institute for the COVID-19 Genomics UK (COG-UK) Consortium	Jacquelyn Wynn, Mairead Hyland, The Lighthouse Lab in Alderley Park and Alex Alderton, Roberto Amato, Sonia Goncalves, Ewan Harrison, David K. Jackson, Ian Johnston, Dominic Kwiatkowski, Cordelia Langford, John Sillitoe on behalf of the Wellcome Sanger Institute COVID-19 Surveillance Team
EPI_ISL_991904	Lighthouse Lab in Glasgow	Wellcome Sanger Institute for the COVID-19 Genomics UK (COG-UK) Consortium	Harper VanSteenhouse, Yumi Kasai, David Gray, Carol Clugston, Anna Dominiczak and Alex Alderton, Roberto Amato, Sonia Goncalves, Ewan Harrison, David K. Jackson, Ian Johnston, Dominic Kwiatkowski, Cordelia Langford, John Sillitoe on behalf of the Wellcome Sanger Institute COVID-19 Surveillance Team
EPI_ISL_991905, EPI_ISL_991906, EPI_ISL_991907, EPI_ISL_991908, EPI_ISL_991909, EPI_ISL_991910, EPI_ISL_991911, EPI_ISL_991912, EPI_ISL_991913, EPI_ISL_991914, EPI_ISL_991915, EPI_ISL_991979, EPI_ISL_991980, EPI_ISL_991982, EPI_ISL_991983, EPI_ISL_991984, EPI_ISL_991985, EPI_ISL_991986, EPI_ISL_991987, EPI_ISL_991989, EPI_ISL_991990, EPI_ISL_991992, EPI_ISL_991993, EPI_ISL_991994, EPI_ISL_991996, EPI_ISL_991997, EPI_ISL_991998, EPI_ISL_991999, EPI_ISL_992000, EPI_ISL_992002, EPI_ISL_992003, EPI_ISL_992004, EPI_ISL_992005, EPI_ISL_992006, EPI_ISL_992007, EPI_ISL_992008, EPI_ISL_992010, EPI_ISL_992011, EPI_ISL_992014, EPI_ISL_992015, EPI_ISL_992016, EPI_ISL_992018, EPI_ISL_992019, EPI_ISL_992020, EPI_ISL_992021, EPI_ISL_992022, EPI_ISL_992024, EPI_ISL_992025, EPI_ISL_992026, EPI_ISL_992027, EPI_ISL_992028, EPI_ISL_992029, EPI_ISL_992031, EPI_ISL_992032, EPI_ISL_992033, EPI_ISL_992034, EPI_ISL_992035, EPI_ISL_992036, EPI_ISL_992037, EPI_ISL_992039, EPI_ISL_992040			
see above	Lighthouse Lab in Alderley Park	Wellcome Sanger Institute for the COVID-19 Genomics UK (COG-UK) Consortium	Jacquelyn Wynn, Mairead Hyland, The Lighthouse Lab in Alderley Park and Alex Alderton, Roberto Amato, Sonia Goncalves, Ewan Harrison, David K. Jackson, Ian Johnston, Dominic Kwiatkowski, Cordelia Langford, John Sillitoe on behalf of the Wellcome Sanger Institute COVID-19 Surveillance Team
EPI_ISL_992043, EPI_ISL_992045, EPI_ISL_992047, EPI_ISL_992049, EPI_ISL_992051, EPI_ISL_992052, EPI_ISL_992056	Lighthouse Lab in Glasgow	Wellcome Sanger Institute for the COVID-19 Genomics UK (COG-UK) Consortium	Harper VanSteenhouse, Yumi Kasai, David Gray, Carol Clugston, Anna Dominiczak and Alex Alderton, Roberto Amato, Sonia Goncalves, Ewan Harrison, David K. Jackson, Ian Johnston, Dominic Kwiatkowski, Cordelia Langford, John Sillitoe on behalf of the Wellcome Sanger Institute COVID-19 Surveillance Team
EPI_ISL_992059	Lighthouse Lab in Alderley Park	Wellcome Sanger Institute for the COVID-19 Genomics UK (COG-UK) Consortium	Jacquelyn Wynn, Mairead Hyland, The Lighthouse Lab in Alderley Park and Alex Alderton, Roberto Amato, Sonia Goncalves, Ewan Harrison, David K. Jackson, Ian Johnston, Dominic Kwiatkowski, Cordelia Langford, John Sillitoe on behalf of the Wellcome Sanger Institute COVID-19 Surveillance Team
EPI_ISL_992060, EPI_ISL_992065, EPI_ISL_992068, EPI_ISL_992070, EPI_ISL_992072, EPI_ISL_992075, EPI_ISL_992084, EPI_ISL_992086, EPI_ISL_992088, EPI_ISL_992093, EPI_ISL_992094, EPI_ISL_992095, EPI_ISL_992096, EPI_ISL_992098, EPI_ISL_992102, EPI_ISL_992104, EPI_ISL_992106, EPI_ISL_992107, EPI_ISL_992110, EPI_ISL_992112, EPI_ISL_992113, EPI_ISL_992114, EPI_ISL_992115, EPI_ISL_992120, EPI_ISL_992121, EPI_ISL_992123, EPI_ISL_992124, EPI_ISL_992126, EPI_ISL_992129, EPI_ISL_992132, EPI_ISL_992133, EPI_ISL_992134, EPI_ISL_992136, EPI_ISL_992137			
see above	Lighthouse Lab in Glasgow	Wellcome Sanger Institute for the COVID-19 Genomics UK (COG-UK) Consortium	Harper VanSteenhouse, Yumi Kasai, David Gray, Carol Clugston, Anna Dominiczak and Alex Alderton, Roberto Amato, Sonia Goncalves, Ewan Harrison, David K. Jackson, Ian Johnston, Dominic Kwiatkowski, Cordelia Langford, John Sillitoe on behalf of the Wellcome Sanger Institute COVID-19 Surveillance Team
EPI_ISL_992138	Lighthouse Lab in Alderley Park	Wellcome Sanger Institute for the COVID-19 Genomics UK (COG-UK) Consortium	Jacquelyn Wynn, Mairead Hyland, The Lighthouse Lab in Alderley Park and Alex Alderton, Roberto Amato, Sonia Goncalves, Ewan Harrison, David K. Jackson, Ian Johnston, Dominic Kwiatkowski, Cordelia Langford, John Sillitoe on behalf of the Wellcome Sanger Institute COVID-19 Surveillance Team
EPI_ISL_992139, EPI_ISL_992140, EPI_ISL_992141, EPI_ISL_992142, EPI_ISL_992148, EPI_ISL_992150, EPI_ISL_992154	Lighthouse Lab in Glasgow	Wellcome Sanger Institute for the COVID-19 Genomics UK (COG-UK) Consortium	Harper VanSteenhouse, Yumi Kasai, David Gray, Carol Clugston, Anna Dominiczak and Alex Alderton, Roberto Amato, Sonia Goncalves, Ewan Harrison, David K. Jackson, Ian Johnston, Dominic Kwiatkowski, Cordelia Langford, John Sillitoe on behalf of the Wellcome Sanger Institute COVID-19 Surveillance Team
EPI_ISL_992156	Lighthouse Lab in Alderley Park	Wellcome Sanger Institute for the COVID-19 Genomics UK (COG-UK) Consortium	Jacquelyn Wynn, Mairead Hyland, The Lighthouse Lab in Alderley Park and Alex Alderton, Roberto Amato, Sonia Goncalves, Ewan Harrison, David K. Jackson, Ian Johnston, Dominic Kwiatkowski, Cordelia Langford, John Sillitoe on behalf of the Wellcome Sanger Institute COVID-19 Surveillance Team
EPI_ISL_992157, EPI_ISL_992158, EPI_ISL_992160, EPI_ISL_992162, EPI_ISL_992163, EPI_ISL_992165, EPI_ISL_992167, EPI_ISL_992168, EPI_ISL_992169, EPI_ISL_992170, EPI_ISL_992171, EPI_ISL_992174, EPI_ISL_992176, EPI_ISL_992182, EPI_ISL_992185, EPI_ISL_992186, EPI_ISL_992190, EPI_ISL_992191, EPI_ISL_992192, EPI_ISL_992193, EPI_ISL_992194, EPI_ISL_992195, EPI_ISL_992199, EPI_ISL_992200, EPI_ISL_992201, EPI_ISL_992202, EPI_ISL_992203, EPI_ISL_992208, EPI_ISL_992210, EPI_ISL_992212, EPI_ISL_992213			
see above	Lighthouse Lab in Glasgow	Wellcome Sanger Institute for the COVID-19 Genomics UK (COG-UK) Consortium	Harper VanSteenhouse, Yumi Kasai, David Gray, Carol Clugston, Anna Dominiczak and Alex Alderton, Roberto Amato, Sonia Goncalves, Ewan Harrison, David K. Jackson, Ian Johnston, Dominic Kwiatkowski, Cordelia Langford, John Sillitoe on behalf of the Wellcome Sanger Institute COVID-19 Surveillance Team
EPI_ISL_992214	Lighthouse Lab in Alderley Park	Wellcome Sanger Institute for the COVID-19 Genomics UK (COG-UK) Consortium	Jacquelyn Wynn, Mairead Hyland, The Lighthouse Lab in Alderley Park and Alex Alderton, Roberto Amato, Sonia Goncalves, Ewan Harrison, David K. Jackson, Ian Johnston, Dominic Kwiatkowski, Cordelia Langford, John Sillitoe on behalf of the Wellcome Sanger Institute COVID-19 Surveillance Team
EPI_ISL_992216, EPI_ISL_992217, EPI_ISL_992222, EPI_ISL_992223, EPI_ISL_992224, EPI_ISL_992227, EPI_ISL_992228, EPI_ISL_992230, EPI_ISL_992231, EPI_ISL_992232, EPI_ISL_992233, EPI_ISL_992235, EPI_ISL_992238, EPI_ISL_992239, EPI_ISL_992242, EPI_ISL_992244, EPI_ISL_992245, EPI_ISL_992247, EPI_ISL_992248, EPI_ISL_992249, EPI_ISL_992251, EPI_ISL_992253, EPI_ISL_992254, EPI_ISL_992255, EPI_ISL_992256, EPI_ISL_992257, EPI_ISL_992259, EPI_ISL_992263, EPI_ISL_992264, EPI_ISL_992266, EPI_ISL_992268, EPI_ISL_992270, EPI_ISL_992274			
see above	Lighthouse Lab in Glasgow	Wellcome Sanger Institute for the COVID-19 Genomics UK (COG-UK) Consortium	Harper VanSteenhouse, Yumi Kasai, David Gray, Carol Clugston, Anna Dominiczak and Alex Alderton, Roberto Amato, Sonia Goncalves, Ewan Harrison, David K. Jackson, Ian Johnston, Dominic Kwiatkowski, Cordelia Langford, John Sillitoe on behalf of the Wellcome Sanger Institute COVID-19 Surveillance Team
EPI_ISL_992275	Lighthouse Lab in Alderley Park	Wellcome Sanger Institute for the COVID-19 Genomics UK (COG-UK) Consortium	Jacquelyn Wynn, Mairead Hyland, The Lighthouse Lab in Alderley Park and Alex Alderton, Roberto Amato, Sonia Goncalves, Ewan Harrison, David K. Jackson, Ian Johnston, Dominic Kwiatkowski, Cordelia Langford, John Sillitoe on behalf of the Wellcome Sanger Institute COVID-19 Surveillance Team
EPI_ISL_992277, EPI_ISL_992278, EPI_ISL_992280, EPI_ISL_992281, EPI_ISL_992284, EPI_ISL_992285, EPI_ISL_992290, EPI_ISL_992293, EPI_ISL_992296, EPI_ISL_992297, EPI_ISL_992298, EPI_ISL_992299, EPI_ISL_992300, EPI_ISL_992307, EPI_ISL_992308, EPI_ISL_992313			
see above	Lighthouse Lab in Glasgow	Wellcome Sanger Institute for the COVID-19 Genomics UK (COG-UK) Consortium	Harper VanSteenhouse, Yumi Kasai, David Gray, Carol Clugston, Anna Dominiczak and Alex Alderton, Roberto Amato, Sonia Goncalves, Ewan Harrison, David K. Jackson, Ian Johnston, Dominic Kwiatkowski, Cordelia Langford, John Sillitoe on behalf of the Wellcome Sanger Institute COVID-19 Surveillance Team
EPI_ISL_992315	Lighthouse Lab in Alderley Park	Wellcome Sanger Institute for the COVID-19 Genomics UK (COG-UK) Consortium	Jacquelyn Wynn, Mairead Hyland, The Lighthouse Lab in Alderley Park and Alex Alderton, Roberto Amato, Sonia Goncalves, Ewan Harrison, David K. Jackson, Ian Johnston, Dominic Kwiatkowski, Cordelia Langford, John Sillitoe on behalf of the Wellcome Sanger Institute COVID-19 Surveillance Team
EPI_ISL_992316, EPI_ISL_992319, EPI_ISL_992320, EPI_ISL_992324, EPI_ISL_992325, EPI_ISL_992326, EPI_ISL_992327, EPI_ISL_992328, EPI_ISL_992333, EPI_ISL_992334, EPI_ISL_992339, EPI_ISL_992340, EPI_ISL_992341, EPI_ISL_992342, EPI_ISL_992343, EPI_ISL_992346, EPI_ISL_992347			
see above	Lighthouse Lab in Glasgow	Wellcome Sanger Institute for the COVID-19 Genomics UK	Harper VanSteenhouse, Yumi Kasai, David Gray, Carol Clugston, Anna Dominiczak and Alex Alderton, Roberto Amato, Sonia Goncalves, Ewan Harrison,

		(COG-UK) Consortium	David K. Jackson, Ian Johnston, Dominic Kwiatkowski, Cordelia Langford, John Sillitoe on behalf of the Wellcome Sanger Institute COVID-19 Surveillance Team
EPI_ISL_992350, EPI_ISL_992351, EPI_ISL_992354, EPI_ISL_992355, EPI_ISL_992356, EPI_ISL_992357, EPI_ISL_992358, EPI_ISL_992359, EPI_ISL_992367, EPI_ISL_992368, EPI_ISL_992369, EPI_ISL_992370, EPI_ISL_992372, EPI_ISL_992373, EPI_ISL_992378, EPI_ISL_992379, EPI_ISL_992381, EPI_ISL_992383, EPI_ISL_992384, EPI_ISL_992385, EPI_ISL_992386, EPI_ISL_992387, EPI_ISL_992391, EPI_ISL_992394, EPI_ISL_992397, EPI_ISL_992401, EPI_ISL_992405, EPI_ISL_992406, EPI_ISL_992407, EPI_ISL_992408, EPI_ISL_992409, EPI_ISL_992412, EPI_ISL_992414			
see above	Lighthouse Lab in Alderley Park	Wellcome Sanger Institute for the COVID-19 Genomics UK (COG-UK) Consortium	Jacquelyn Wynn, Mairead Hyland, The Lighthouse Lab in Alderley Park and Alex Alderton, Roberto Amato, Sonia Goncalves, Ewan Harrison, David K. Jackson, Ian Johnston, Dominic Kwiatkowski, Cordelia Langford, John Sillitoe on behalf of the Wellcome Sanger Institute COVID-19 Surveillance Team
EPI_ISL_992419, EPI_ISL_992420, EPI_ISL_992421, EPI_ISL_992422, EPI_ISL_992424, EPI_ISL_992425, EPI_ISL_992427, EPI_ISL_992428, EPI_ISL_992430, EPI_ISL_992431, EPI_ISL_992433, EPI_ISL_992434, EPI_ISL_992435, EPI_ISL_992441, EPI_ISL_992444, EPI_ISL_992445, EPI_ISL_992451, EPI_ISL_992452, EPI_ISL_992457, EPI_ISL_992458, EPI_ISL_992459, EPI_ISL_992464, EPI_ISL_992465, EPI_ISL_992468, EPI_ISL_992470, EPI_ISL_992471, EPI_ISL_992474, EPI_ISL_992477, EPI_ISL_992478, EPI_ISL_992480, EPI_ISL_992481, EPI_ISL_992485, EPI_ISL_992487, EPI_ISL_992489, EPI_ISL_992493, EPI_ISL_992494, EPI_ISL_992495, EPI_ISL_992498, EPI_ISL_992499, EPI_ISL_992500, EPI_ISL_992502, EPI_ISL_992505, EPI_ISL_992506, EPI_ISL_992507, EPI_ISL_992508, EPI_ISL_992510, EPI_ISL_992514, EPI_ISL_992515, EPI_ISL_992516, EPI_ISL_992519, EPI_ISL_992522, EPI_ISL_992523, EPI_ISL_992524, EPI_ISL_992526, EPI_ISL_992527, EPI_ISL_992530, EPI_ISL_992532, EPI_ISL_992534, EPI_ISL_992536, EPI_ISL_992537, EPI_ISL_992538, EPI_ISL_992541, EPI_ISL_992543, EPI_ISL_992544, EPI_ISL_992545, EPI_ISL_992548, EPI_ISL_992550, EPI_ISL_992551, EPI_ISL_992553, EPI_ISL_992554, EPI_ISL_992556, EPI_ISL_992558, EPI_ISL_992560, EPI_ISL_992565, EPI_ISL_992566, EPI_ISL_992567, EPI_ISL_992568, EPI_ISL_992570, EPI_ISL_992578, EPI_ISL_992579, EPI_ISL_992585, EPI_ISL_992589, EPI_ISL_992590, EPI_ISL_992591, EPI_ISL_992593, EPI_ISL_992594, EPI_ISL_992595, EPI_ISL_992596, EPI_ISL_992597, EPI_ISL_992600, EPI_ISL_992601, EPI_ISL_992602, EPI_ISL_992607, EPI_ISL_992608, EPI_ISL_992611, EPI_ISL_992616, EPI_ISL_992617, EPI_ISL_992618, EPI_ISL_992619, EPI_ISL_992620, EPI_ISL_992621, EPI_ISL_992622, EPI_ISL_992628, EPI_ISL_992631, EPI_ISL_992634, EPI_ISL_992635, EPI_ISL_992638, EPI_ISL_992642, EPI_ISL_992644, EPI_ISL_992649, EPI_ISL_992650, EPI_ISL_992654, EPI_ISL_992657, EPI_ISL_992659, EPI_ISL_992662, EPI_ISL_992663, EPI_ISL_992666, EPI_ISL_992668, EPI_ISL_992669, EPI_ISL_992671, EPI_ISL_992672, EPI_ISL_992674, EPI_ISL_992675, EPI_ISL_992676, EPI_ISL_992677, EPI_ISL_992679, EPI_ISL_992680, EPI_ISL_992687, EPI_ISL_992688, EPI_ISL_992691, EPI_ISL_992696, EPI_ISL_992697, EPI_ISL_992704, EPI_ISL_992706, EPI_ISL_992708, EPI_ISL_992709, EPI_ISL_992711, EPI_ISL_992719, EPI_ISL_992723, EPI_ISL_992724, EPI_ISL_992725, EPI_ISL_992727, EPI_ISL_992731, EPI_ISL_992732, EPI_ISL_992733, EPI_ISL_992738, EPI_ISL_992747, EPI_ISL_992750, EPI_ISL_992752, EPI_ISL_992758, EPI_ISL_992759, EPI_ISL_992760, EPI_ISL_992762, EPI_ISL_992764, EPI_ISL_992767, EPI_ISL_992769, EPI_ISL_992771, EPI_ISL_992772, EPI_ISL_992777, EPI_ISL_992779, EPI_ISL_992782, EPI_ISL_992783, EPI_ISL_992784, EPI_ISL_992786, EPI_ISL_992790, EPI_ISL_992792, EPI_ISL_992793, EPI_ISL_992794, EPI_ISL_992795, EPI_ISL_992796, EPI_ISL_992797, EPI_ISL_992798, EPI_ISL_992803, EPI_ISL_992804, EPI_ISL_992806, EPI_ISL_992807, EPI_ISL_992809, EPI_ISL_992812, EPI_ISL_992813, EPI_ISL_992814, EPI_ISL_992816, EPI_ISL_992818, EPI_ISL_992819, EPI_ISL_992822, EPI_ISL_992823, EPI_ISL_992827, EPI_ISL_992830, EPI_ISL_992831, EPI_ISL_992832, EPI_ISL_992834, EPI_ISL_992836, EPI_ISL_992838, EPI_ISL_992839, EPI_ISL_992840, EPI_ISL_992841, EPI_ISL_992845, EPI_ISL_992849, EPI_ISL_992850, EPI_ISL_992851, EPI_ISL_992865, EPI_ISL_992867, EPI_ISL_992868, EPI_ISL_992869, EPI_ISL_992870, EPI_ISL_992871, EPI_ISL_992875, EPI_ISL_992876, EPI_ISL_992882, EPI_ISL_992883, EPI_ISL_992884, EPI_ISL_992885, EPI_ISL_992887, EPI_ISL_992891, EPI_ISL_992893, EPI_ISL_992894, EPI_ISL_992895, EPI_ISL_992897, EPI_ISL_992898, EPI_ISL_992901, EPI_ISL_992903, EPI_ISL_992905, EPI_ISL_992908, EPI_ISL_992912, EPI_ISL_992924, EPI_ISL_992925, EPI_ISL_992926, EPI_ISL_992931, EPI_ISL_992934, EPI_ISL_992935, EPI_ISL_992936, EPI_ISL_992938, EPI_ISL_992939, EPI_ISL_992943, EPI_ISL_992946, EPI_ISL_992948, EPI_ISL_992954, EPI_ISL_992958, EPI_ISL_992961, EPI_ISL_992962, EPI_ISL_992965, EPI_ISL_992966, EPI_ISL_992970, EPI_ISL_992971, EPI_ISL_992972, EPI_ISL_992973, EPI_ISL_992974, EPI_ISL_992975, EPI_ISL_992977, EPI_ISL_992980, EPI_ISL_992983, EPI_ISL_992986, EPI_ISL_992987, EPI_ISL_992993, EPI_ISL_992995, EPI_ISL_992998, EPI_ISL_992999, EPI_ISL_993003, EPI_ISL_993005, EPI_ISL_993009, EPI_ISL_993014, EPI_ISL_993020, EPI_ISL_993023, EPI_ISL_993024, EPI_ISL_993027, EPI_ISL_993033, EPI_ISL_993034, EPI_ISL_993040, EPI_ISL_993042			
see above	Lighthouse Lab in Alderley Park	Wellcome Sanger Institute for the COVID-19 Genomics UK (COG-UK) Consortium	Jacquelyn Wynn, Mairead Hyland, The Lighthouse Lab in Alderley Park and Alex Alderton, Roberto Amato, Sonia Goncalves, Ewan Harrison, David K. Jackson, Ian Johnston, Dominic Kwiatkowski, Cordelia Langford, John Sillitoe on behalf of the Wellcome Sanger Institute COVID-19 Surveillance Team (http://www.sanger.ac.uk/covid-team)
EPI_ISL_996402, EPI_ISL_996403, EPI_ISL_996404, EPI_ISL_996405, EPI_ISL_996406, EPI_ISL_996409, EPI_ISL_996410, EPI_ISL_996411, EPI_ISL_996460, EPI_ISL_996461	University of Birmingham	COVID-19 Genomics UK (COG-UK) Consortium	Institute of Microbiology, University of Birmingham: Claire McMurray, Joanne Stockton, Samuel Nicholls, Radoslaw Poplawski, Will Rowe, Josh Quick, Nicholas Loman. University of Birmingham Testing Laboratory: Celina M Whalley, Andrew Bosworth, Charlotte Poxon, Kasun Wanigasooriya, Oliver Pickles, Mike Kidd, Alex Richter, Andrew D Beggs PHE Heartlands Lab: Husam Osman, Andrew Bosworth. Queen Elizabeth Hospital: Anna Casey
EPI_ISL_996599, EPI_ISL_996603, EPI_ISL_996676, EPI_ISL_996680, EPI_ISL_996682, EPI_ISL_996686, EPI_ISL_996688, EPI_ISL_996692, EPI_ISL_996738, EPI_ISL_996740	Department of Pathology, University of Cambridge	COVID-19 Genomics UK (COG-UK) Consortium	Aminu S. Jahun, Yasmin Chaudhry, Iliana Georgana, Myra Hosmillo, Rhys Izuagbe, William L. Hamilton, Martin D. Curran, Surendra Parmar, Ian Goodfellow
EPI_ISL_997080, EPI_ISL_997081, EPI_ISL_997082, EPI_ISL_997083, EPI_ISL_997084, EPI_ISL_997085, EPI_ISL_997086, EPI_ISL_997089, EPI_ISL_997090, EPI_ISL_997091, EPI_ISL_997092, EPI_ISL_997093, EPI_ISL_997106, EPI_ISL_997140, EPI_ISL_997141, EPI_ISL_997142, EPI_ISL_997143, EPI_ISL_997144, EPI_ISL_997145, EPI_ISL_997146, EPI_ISL_997147, EPI_ISL_997148			
see above	Virology Department, Royal Infirmary of Edinburgh, NHS Lothian / School of Biological Sciences, University of Edinburgh	COVID-19 Genomics UK (COG-UK) Consortium	McHugh M, Dewar R, Cotton S, Rooke S, O'Toole Á, Scher E, Hill V, McCrone JT, Colquhoun R, Yu X, Jackson B, Rambaut A, Templeton K
EPI_ISL_997251, EPI_ISL_997252, EPI_ISL_997253, EPI_ISL_997254, EPI_ISL_997255, EPI_ISL_997256, EPI_ISL_997257, EPI_ISL_997259, EPI_ISL_997261, EPI_ISL_997262, EPI_ISL_997263, EPI_ISL_997264, EPI_ISL_997265, EPI_ISL_997269, EPI_ISL_997270, EPI_ISL_997271, EPI_ISL_997272, EPI_ISL_997273, EPI_ISL_997275, EPI_ISL_997276, EPI_ISL_997277, EPI_ISL_997278, EPI_ISL_997279, EPI_ISL_997280, EPI_ISL_997281, EPI_ISL_997333, EPI_ISL_997334, EPI_ISL_997335			
see above	University of Exeter	COVID-19 Genomics UK (COG-UK) Consortium	Ben Temperton,Aaron Jeffries,Michelle Michelsen,Joanna Warwick-Dugdale,Audrey Farbos,Robyn Manley,Stephen Michell,Jane Masoli
EPI_ISL_997370	University Hospitals Of Leicester NHS Trust and DeepSeq Nottingham	COVID-19 Genomics UK (COG-UK) Consortium	Christopher Holmes, Paul Bird, Thomas Helmer, Karlie Fallon, Julian Tang, Jonathan Ball, Patrick McClure, Joeseph Chappell, Nadine Holmes, Matthew Carlisle, Christopher Moore, Fei Sang, Johnny Debebe, Victoria Wright, Matthew Loose
EPI_ISL_997381, EPI_ISL_997406	Liverpool Clinical Laboratories	COVID-19 Genomics UK (COG-UK) Consortium	Sam Haldenby, Anita Lucaci, Steve Paterson, Julian Hiscox, Alistair Darby, M Almsaud, A Alrezaihi, Muhannad Alruwaili, Stuart D Armstrong, Jones Benjamin, Eleanor G Bentley, Anu Chawla, Jordan J Clark, Angela Cowell, Richard Eccles, Isabel García-Dorival, Matthew Gemmell, Alessandro Gerada, PKF Gilmore, Richard Gregory, Ximeng Han, Catherine Hartley, Margaret Hughes, Miren Iturriza-Gomara, James Johnson, L Luu, Jenifer Manson, Charlotte Nelson, Elaine O'Toole, Cassie Olateju, Rebekah Penrice-Randal , Lucille Rainbow, N.P Randle, Trevor Ian Robinson, Parul Sharma, Ghada T Shawli, James P Stewart, Neil Swainston, Ecaterina Vamos, Joanne Watts, Mark Whitehead
EPI_ISL_997683, EPI_ISL_997684, EPI_ISL_997685, EPI_ISL_997686, EPI_ISL_997688, EPI_ISL_997689, EPI_ISL_997693	University College London, Great Ormond Street Hospital for Children NHS Foundation Trust, Imperial College Healthcare NHS Trust	COVID-19 Genomics UK (COG-UK) Consortium	Sergi Castellano, Rachel Williams, Mark Kristiansen, Paola Resende Silva, Sunando Roy, Tony Brooks, Helena Tutill, Paola Niola, Patricia Dyal, Charlotte Williams, Leysa Forrest, Yasmin Panchbhaya, Jacqueline Findlay, Samuel Weeks, Julianne Brown, Kathryn Harris, Paul Randell, James Price, Alison Holmes, Judith Breuer
EPI_ISL_998181, EPI_ISL_998182, EPI_ISL_998183, EPI_ISL_998184, EPI_ISL_998185, EPI_ISL_998186, EPI_ISL_998187, EPI_ISL_998188, EPI_ISL_998226, EPI_ISL_998227, EPI_ISL_998228, EPI_ISL_998268, EPI_ISL_998270, EPI_ISL_998271, EPI_ISL_998272, EPI_ISL_998273			
see above	Regional Virus Laboratory, Belfast Health and Social Care Trust	COVID-19 Genomics UK (COG-UK) Consortium	Conall McCaughey, James McKenna, Tanya Curran, Susan Feeney, Alison Watt, Ciara Cox, Mairead Connor, Zoltan Molnar, David Simpson, Derek Fairley
EPI_ISL_998448, EPI_ISL_998449, EPI_ISL_998450, EPI_ISL_998451, EPI_ISL_998452, EPI_ISL_998453, EPI_ISL_998454, EPI_ISL_998455, EPI_ISL_998456, EPI_ISL_998457, EPI_ISL_998458, EPI_ISL_998459, EPI_ISL_998460, EPI_ISL_998464, EPI_ISL_998465, EPI_ISL_998466, EPI_ISL_998467, EPI_ISL_998468, EPI_ISL_998469, EPI_ISL_998470, EPI_ISL_998471, EPI_ISL_998472, EPI_ISL_998474, EPI_ISL_998476, EPI_ISL_998477, EPI_ISL_998478, EPI_ISL_998479, EPI_ISL_998480, EPI_ISL_998481, EPI_ISL_998482, EPI_ISL_998483, EPI_ISL_998485, EPI_ISL_998496, EPI_ISL_998499			
see above	Northumbria University / South Tees Hospitals NHS Foundation Trust / North Cumbria Integrated Care NHS Foundation Trust / North Tees and Hartlepool NHS Foundation Trust / Newcastle Hospitals NHS Foundation Trust	COVID-19 Genomics UK (COG-UK) Consortium	Darren L Smith,Andrew Nelson,Matthew Bashton,Greg R Young,Joshua Loh,John Allan,Mohammad A Tariq,Giles S Holt,Gary Black,Wen C Yew,Lynn Dover,Paul Baker,Steve Liggett,Sarah Essex,Jane Greenaway,Debra Padgett,Clive Graham,Garren Scott,Edward Barton,Emma Swindells,Brendan Payne,Jennifer Collins,Yusri Taha,Gary Eltringham
EPI_ISL_998831	Quadram Institute Bioscience	COVID-19 Genomics UK (COG-UK) Consortium	Dave J. Baker, Gemma L. Kay, Alp Aydin, Thanh Le-Viet, Steven Rudder, Ana P. Tedim, Anastasia Kolyva, Maria Diaz, Leonardo de Oliveira Martins, Nabil-Fareed Alikhan, Lizzie Meadows, Rachael Stanley, Ngozi Elumogo, Muhammed Yasir, Nicholas M. Thomson, Alexander J Trotter, Rachel Gilroy, Samuel Bloomfield, Claire Stuart, Andrew Bell, Reenesh Prakash, Samir Dervisevic, Alison E. Mather, John Wain, Mark Webber, Andrew J. Page, Justin O'Grady
EPI_ISL_998980, EPI_ISL_998981, EPI_ISL_998982, EPI_ISL_998983, EPI_ISL_998984, EPI_ISL_998985	Lincolnshire Hospitals and DeepSeq Nottingham	COVID-19 Genomics UK (COG-UK) Consortium	Nichola Duckworth, Tim Sloan, Sarah Walsh, Jonathan Ball, Patrick McClure, Joeseph Chappell, Nadine Holmes, Matthew Carlisle, Christopher Moore, Fei Sang, Johnny Debebe, Victoria Wright, Matthew Loose
EPI_ISL_998995, EPI_ISL_999227, EPI_ISL_999230, EPI_ISL_999232, EPI_ISL_999233, EPI_ISL_999234, EPI_ISL_999235, EPI_ISL_999238, EPI_ISL_999239, EPI_ISL_999241, EPI_ISL_999242, EPI_ISL_999243, EPI_ISL_999244, EPI_ISL_999245, EPI_ISL_999246, EPI_ISL_999249, EPI_ISL_999251, EPI_ISL_999253, EPI_ISL_999254, EPI_ISL_999256, EPI_ISL_999257, EPI_ISL_999259, EPI_ISL_999262, EPI_ISL_999268, EPI_ISL_999269, EPI_ISL_999273, EPI_ISL_999274, EPI_ISL_999275, EPI_ISL_999276, EPI_ISL_999278, EPI_ISL_999279, EPI_ISL_999281, EPI_ISL_999282, EPI_ISL_999283, EPI_ISL_999284, EPI_ISL_999287, EPI_ISL_999293, EPI_ISL_999296, EPI_ISL_999297, EPI_ISL_999298, EPI_ISL_999299, EPI_ISL_999300, EPI_ISL_999301, EPI_ISL_999314, EPI_ISL_999483, EPI_ISL_999510, EPI_ISL_999511			
see above	Oxford Viromics, NDM, University of Oxford; Oxford University Hospitals; Basingstoke and North Hampshire Hospital	COVID-19 Genomics UK (COG-UK) Consortium	Tanya Golubchik, David Bonsall, George Macintyre, Amy Trebes, Mariateresa de Cesare, Catrin Moore, Alex Mobbs, Anita Justice, Robert Shaw, Monique Andersson, Timothy Peto, Emma Wise, Nathan Moore, Jessica Lynch, Nick Cortes, Matilde Mori, Stephen Kidd, David Buck, John Todd, Christophe Fraser
