# Supplementary material for: Inferring the Association between the Risk of COVID-19 Case Fatality and N501Y Substitution in SARS-CoV-2
Source: Viruses. 2021 Apr 8;13(4):638. doi: 10.3390/v13040638 (PMC8070306; doi:10.3390/v13040638)
Supplement: Supplementary file 1 [file viruses-13-00638-s001.zip › gisaid_hcov-19_UKAT_210125-210127.pdf]

We gratefully acknowledge the following Authors from the Originating laboratories responsible for obtaining the specimens, as well as the Submitting laboratories where the genome data were generated and shared via GISAID, on which this research is based.

All Submitters of data may be contacted directly via www.gisaid.org

Authors are sorted alphabetically.

Accession ID	Originating Laboratory	Submitting Laboratory	Authors
EPI_ISL_1000240, EPI_ISL_1000242, EPI_ISL_1000243, EPI_ISL_1000257, EPI_ISL_1000260, EPI_ISL_1000262, EPI_ISL_1000267, EPI_ISL_1000269, EPI_ISL_1000271, EPI_ISL_1000273, EPI_ISL_1000281, EPI_ISL_1000282, EPI_ISL_1000292, EPI_ISL_1000302, EPI_ISL_1000308, EPI_ISL_1000314, EPI_ISL_1000456, EPI_ISL_1000457, EPI_ISL_1000458, EPI_ISL_1000459, EPI_ISL_1000460, EPI_ISL_1000482, EPI_ISL_1000483, EPI_ISL_1000601, EPI_ISL_1000605, EPI_ISL_1000606, EPI_ISL_1000607, EPI_ISL_1000608, EPI_ISL_1000610, EPI_ISL_1000618, EPI_ISL_1000619, EPI_ISL_1000620, EPI_ISL_1000621, EPI_ISL_1000622, EPI_ISL_1000623, EPI_ISL_1000624, EPI_ISL_1000625, EPI_ISL_1000627			
see above	Centre for Enzyme Innovation, University of Portsmouth / Translational Research Laboratory, Portsmouth Hospitals NHS Trust	COVID-19 Genomics UK (COG-UK) Consortium	Angela Beckett,Salman Goudarzi,Christopher Fearn,Kate Cook,Katie Loveson,Sharon Glaysher,Scott Elliott,Samuel Robson
EPI_ISL_1000763, EPI_ISL_1000787	Virology Department, Sheffield Teaching Hospitals NHS Foundation Trust/Department of Infection, Immunity and Cardiovascular Disease, The Medical School, University of Sheffield	COVID-19 Genomics UK (COG-UK) Consortium	Thushan de Silva, Matthew Parker, Nikki Smith, Adri Angyal, Rebecca Brown, Luke Green, Rachel Tucker, Paul Parsons, Danielle Groves, Katie Johnson, Laura Carrilero, Alex Keeley, Dave Partridge, Matthew Wyles, Benjamin Lindsey, Mehmet Yavuz, Mohammad Raza, Cariad Evans
EPI_ISL_1000806, EPI_ISL_1000808, EPI_ISL_1000816, EPI_ISL_1000819, EPI_ISL_1000822, EPI_ISL_1000826, EPI_ISL_1000832, EPI_ISL_1000833, EPI_ISL_1000834, EPI_ISL_1000835, EPI_ISL_1000836, EPI_ISL_1000837, EPI_ISL_1000845, EPI_ISL_1000964			
see above	Bioinformatics and Biostatistics Lab, Advanced Sequencing Facility	COVID-19 Genomics UK (COG-UK) Consortium	Aengus Stewart,Jerome Nicod,Chelsea Sawyer,Laura Cubitt,Harshil Patel,Margaret Crawford
EPI_ISL_1006341, EPI_ISL_1006344, EPI_ISL_1006345, EPI_ISL_1006348, EPI_ISL_1006354, EPI_ISL_1006357, EPI_ISL_1006358, EPI_ISL_1006365, EPI_ISL_1006366, EPI_ISL_1006367, EPI_ISL_1006373, EPI_ISL_1006374, EPI_ISL_1006378, EPI_ISL_1006385, EPI_ISL_1006387, EPI_ISL_1006390, EPI_ISL_1006391, EPI_ISL_1006395, EPI_ISL_1006399, EPI_ISL_1006409, EPI_ISL_1006412, EPI_ISL_1006422, EPI_ISL_1006423, EPI_ISL_1006426, EPI_ISL_1006428, EPI_ISL_1006435, EPI_ISL_1006438, EPI_ISL_1006439, EPI_ISL_1006445, EPI_ISL_1006453, EPI_ISL_1006456, EPI_ISL_1006457, EPI_ISL_1006458, EPI_ISL_1006467, EPI_ISL_1006471, EPI_ISL_1006474, EPI_ISL_1006475, EPI_ISL_1006476, EPI_ISL_1006477, EPI_ISL_1006479, EPI_ISL_1006482, EPI_ISL_1006488, EPI_ISL_1006489, EPI_ISL_1006490, EPI_ISL_1006501, EPI_ISL_1006511, EPI_ISL_1006516, EPI_ISL_1006521, EPI_ISL_1006529, EPI_ISL_1006530, EPI_ISL_1006533, EPI_ISL_1006535, EPI_ISL_1006538			
see above	Lighthouse Lab in Milton Keynes	Wellcome Sanger Institute for the COVID-19 Genomics UK (COG-UK) Consortium	The Lighthouse Lab in Milton Keynes and Alex Alderton, Roberto Amato, Sonia Goncalves, Ewan Harrison, David K. Jackson, Ian Johnston, Dominic Kwiatkowski, Cordelia Langford, John Sillitoe on behalf of the Wellcome Sanger Institute COVID-19 Surveillance Team
EPI_ISL_1007549, EPI_ISL_1007550, EPI_ISL_1007551, EPI_ISL_1007552, EPI_ISL_1007553, EPI_ISL_1007554, EPI_ISL_1007555, EPI_ISL_1007556, EPI_ISL_1007557, EPI_ISL_1007558, EPI_ISL_1007559, EPI_ISL_1007560			
see above	Lighthouse Lab in Alderley Park	Wellcome Sanger Institute for the COVID-19 Genomics UK (COG-UK) Consortium	Jacquelyn Wynn, Mairead Hyland, The Lighthouse Lab in Alderley Park and Alex Alderton, Roberto Amato, Sonia Goncalves, Ewan Harrison, David K. Jackson, Ian Johnston, Dominic Kwiatkowski, Cordelia Langford, John Sillitoe on behalf of the Wellcome Sanger Institute COVID-19 Surveillance Team
EPI_ISL_1007561, EPI_ISL_1007562	Lighthouse Lab in Glasgow	Wellcome Sanger Institute for the COVID-19 Genomics UK (COG-UK) Consortium	Harper VanSteenhouse, Yumi Kasai, David Gray, Carol Clugston, Anna Dominiczak and Alex Alderton, Roberto Amato, Sonia Goncalves, Ewan Harrison, David K. Jackson, Ian Johnston, Dominic Kwiatkowski, Cordelia Langford, John Sillitoe on behalf of the Wellcome Sanger Institute COVID-19 Surveillance Team
EPI_ISL_1007563, EPI_ISL_1012713, EPI_ISL_1012714, EPI_ISL_1012715, EPI_ISL_1012716, EPI_ISL_1012717, EPI_ISL_1012718, EPI_ISL_1012719, EPI_ISL_1012720, EPI_ISL_1012721, EPI_ISL_1012722, EPI_ISL_1012723, EPI_ISL_1012724, EPI_ISL_1012725, EPI_ISL_1012726, EPI_ISL_1012727, EPI_ISL_1012728, EPI_ISL_1012729, EPI_ISL_1012730, EPI_ISL_1012731, EPI_ISL_1012732, EPI_ISL_1012733, EPI_ISL_1012734, EPI_ISL_1012735, EPI_ISL_1012736, EPI_ISL_1012737, EPI_ISL_1012738, EPI_ISL_1012739, EPI_ISL_1012740, EPI_ISL_1012741, EPI_ISL_1012742, EPI_ISL_1012743, EPI_ISL_1012744, EPI_ISL_1012745, EPI_ISL_1012746, EPI_ISL_1012747, EPI_ISL_1012748, EPI_ISL_1012749, EPI_ISL_1012750, EPI_ISL_1012751, EPI_ISL_1012752, EPI_ISL_1012753, EPI_ISL_1012755, EPI_ISL_1012756, EPI_ISL_1012757, EPI_ISL_1012758, EPI_ISL_1012759, EPI_ISL_1012760, EPI_ISL_1012761, EPI_ISL_1012763, EPI_ISL_1012764, EPI_ISL_1012765, EPI_ISL_1012766, EPI_ISL_1012767, EPI_ISL_1012768, EPI_ISL_1012769, EPI_ISL_1012770, EPI_ISL_1012771, EPI_ISL_1012772, EPI_ISL_1012773, EPI_ISL_1012774, EPI_ISL_1012775, EPI_ISL_1012776, EPI_ISL_1012777, EPI_ISL_1012778, EPI_ISL_1012779, EPI_ISL_1012780, EPI_ISL_1012781, EPI_ISL_1012782, EPI_ISL_1012783, EPI_ISL_1012784, EPI_ISL_1012785, EPI_ISL_1012786			
see above	Lighthouse Lab in Alderley Park	Wellcome Sanger Institute for the COVID-19 Genomics UK (COG-UK) Consortium	Jacquelyn Wynn, Mairead Hyland, The Lighthouse Lab in Alderley Park and Alex Alderton, Roberto Amato, Sonia Goncalves, Ewan Harrison, David K. Jackson, Ian Johnston, Dominic Kwiatkowski, Cordelia Langford, John Sillitoe on behalf of the Wellcome Sanger Institute COVID-19 Surveillance Team
EPI_ISL_1019587, EPI_ISL_1019588, EPI_ISL_1019589, EPI_ISL_1019590, EPI_ISL_1019591, EPI_ISL_1019592, EPI_ISL_1019593, EPI_ISL_1019594, EPI_ISL_1019595, EPI_ISL_1019596, EPI_ISL_1019597, EPI_ISL_1019598, EPI_ISL_1019599, EPI_ISL_1019600, EPI_ISL_1019601, EPI_ISL_1019602, EPI_ISL_1019603, EPI_ISL_1019604, EPI_ISL_1019605, EPI_ISL_1019606, EPI_ISL_1019607, EPI_ISL_1019609, EPI_ISL_1019610, EPI_ISL_1019611, EPI_ISL_1019612, EPI_ISL_1019613, EPI_ISL_1019614, EPI_ISL_1019616, EPI_ISL_1019617, EPI_ISL_1019618, EPI_ISL_1019619, EPI_ISL_1019620, EPI_ISL_1019621, EPI_ISL_1019622, EPI_ISL_1019623, EPI_ISL_1019624, EPI_ISL_1019625, EPI_ISL_1019626, EPI_ISL_1019627, EPI_ISL_1019629, EPI_ISL_1019630, EPI_ISL_1019631, EPI_ISL_1019632, EPI_ISL_1019633, EPI_ISL_1019634, EPI_ISL_1019635, EPI_ISL_1019636, EPI_ISL_1019638, EPI_ISL_1019639, EPI_ISL_1019643, EPI_ISL_1019644, EPI_ISL_1019646, EPI_ISL_1019648, EPI_ISL_1019649, EPI_ISL_1019651, EPI_ISL_1019652, EPI_ISL_1019654, EPI_ISL_1019656, EPI_ISL_1019657, EPI_ISL_1019659, EPI_ISL_1019661, EPI_ISL_1019662, EPI_ISL_1019664, EPI_ISL_1019669, EPI_ISL_1019670, EPI_ISL_1019672, EPI_ISL_1019673, EPI_ISL_1019675, EPI_ISL_1019677, EPI_ISL_1019679, EPI_ISL_1019680, EPI_ISL_1019682, EPI_ISL_1019684, EPI_ISL_1019685, EPI_ISL_1019687, EPI_ISL_1019689, EPI_ISL_1019692, EPI_ISL_1019694, EPI_ISL_1019695, EPI_ISL_1019697, EPI_ISL_1019698, EPI_ISL_1019700, EPI_ISL_1019701, EPI_ISL_1019703, EPI_ISL_1019704, EPI_ISL_1019708, EPI_ISL_1019709, EPI_ISL_1019712, EPI_ISL_1019714, EPI_ISL_1019716, EPI_ISL_1019717, EPI_ISL_1019719, EPI_ISL_1019721, EPI_ISL_1019723, EPI_ISL_1019725, EPI_ISL_1019727, EPI_ISL_1019728, EPI_ISL_1019733, EPI_ISL_1019735, EPI_ISL_1019736, EPI_ISL_1019738, EPI_ISL_1019740, EPI_ISL_1019743, EPI_ISL_1019746, EPI_ISL_1019749, EPI_ISL_1019751, EPI_ISL_1019752, EPI_ISL_1019755, EPI_ISL_1019757, EPI_ISL_1019758, EPI_ISL_1019760, EPI_ISL_1019761, EPI_ISL_1019763, EPI_ISL_1019765, EPI_ISL_1019766, EPI_ISL_1019768, EPI_ISL_1019769, EPI_ISL_1019771, EPI_ISL_1019773, EPI_ISL_1019774, EPI_ISL_1019776, EPI_ISL_1019777, EPI_ISL_1019779			
see above	Lighthouse Lab in Glasgow	Wellcome Sanger Institute for the COVID-19 Genomics UK (COG-UK) Consortium	Harper VanSteenhouse, Yumi Kasai, David Gray, Carol Clugston, Anna Dominiczak and Alex Alderton, Roberto Amato, Jeffrey Barrett, Sonia Goncalves, Ewan Harrison, David K. Jackson, Ian Johnston, Dominic Kwiatkowski, Cordelia Langford, John Sillitoe on behalf of the Wellcome Sanger Institute COVID-19 Surveillance Team
EPI_ISL_1043899, EPI_ISL_1043900, EPI_ISL_1043902, EPI_ISL_1043903, EPI_ISL_1043904, EPI_ISL_1043905, EPI_ISL_1043906, EPI_ISL_1043907, EPI_ISL_1043908, EPI_ISL_1043909, EPI_ISL_1043910, EPI_ISL_1043911, EPI_ISL_1043912, EPI_ISL_1043913, EPI_ISL_1043914, EPI_ISL_1043915, EPI_ISL_1043916, EPI_ISL_1043917, EPI_ISL_1043918, EPI_ISL_1043919, EPI_ISL_1043920, EPI_ISL_1043921, EPI_ISL_1043922, EPI_ISL_1043923, EPI_ISL_1043924, EPI_ISL_1043925, EPI_ISL_1043926, EPI_ISL_1043927, EPI_ISL_1043928, EPI_ISL_1043929, EPI_ISL_1043930, EPI_ISL_1043931, EPI_ISL_1043932, EPI_ISL_1043933, EPI_ISL_1043934, EPI_ISL_1043935, EPI_ISL_1043936, EPI_ISL_1043937, EPI_ISL_1043938, EPI_ISL_1043939, EPI_ISL_1043940, EPI_ISL_1043941, EPI_ISL_1043942, EPI_ISL_1043943, EPI_ISL_1043944, EPI_ISL_1043945, EPI_ISL_1043946, EPI_ISL_1043947, EPI_ISL_1043948, EPI_ISL_1043949, EPI_ISL_1043950, EPI_ISL_1043951, EPI_ISL_1043952, EPI_ISL_1043953, EPI_ISL_1043954, EPI_ISL_1043955, EPI_ISL_1043956, EPI_ISL_1043957, EPI_ISL_1043958, EPI_ISL_1043959, EPI_ISL_1043960, EPI_ISL_1043961, EPI_ISL_1043962, EPI_ISL_1043963, EPI_ISL_1043964, EPI_ISL_1043965, EPI_ISL_1043966, EPI_ISL_1043967, EPI_ISL_1043968, EPI_ISL_1043969, EPI_ISL_1043970, EPI_ISL_1043971, EPI_ISL_1043972, EPI_ISL_1043973, EPI_ISL_1043974, EPI_ISL_1043975, EPI_ISL_1043976, EPI_ISL_1043977, EPI_ISL_1043978, EPI_ISL_1043979, EPI_ISL_1043980, EPI_ISL_1043981, EPI_ISL_1043982, EPI_ISL_1043983, EPI_ISL_1043984, EPI_ISL_1043985, EPI_ISL_1043986, EPI_ISL_1043987, EPI_ISL_1043988, EPI_ISL_1043989, EPI_ISL_1043990, EPI_ISL_1043991, EPI_ISL_1043992, EPI_ISL_1043993, EPI_ISL_1043994, EPI_ISL_1043995, EPI_ISL_1043996, EPI_ISL_1043997, EPI_ISL_1043998, EPI_ISL_1043999, EPI_ISL_1044000, EPI_ISL_1044001, EPI_ISL_1044002, EPI_ISL_1044003, EPI_ISL_1044004, EPI_ISL_1044005, EPI_ISL_1044006, EPI_ISL_1044007, EPI_ISL_1044008, EPI_ISL_1044009, EPI_ISL_1044010, EPI_ISL_1044011, EPI_ISL_1044012, EPI_ISL_1044013, EPI_ISL_1044014, EPI_ISL_1044015, EPI_ISL_1044016, EPI_ISL_1044017, EPI_ISL_1044018, EPI_ISL_1044019, EPI_ISL_1044020, EPI_ISL_1044021, EPI_ISL_1044022, EPI_ISL_1044023, EPI_ISL_1044024, EPI_ISL_1044025, EPI_ISL_1044026, EPI_ISL_1044027, EPI_ISL_1044028, EPI_ISL_1044029, EPI_ISL_1044030, EPI_ISL_1044031, EPI_ISL_1044032, EPI_ISL_1044033, EPI_ISL_1044034, EPI_ISL_1044035, EPI_ISL_1044036, EPI_ISL_1044037, EPI_ISL_1044038, EPI_ISL_1044039, EPI_ISL_1044040, EPI_ISL_1044041, EPI_ISL_1044042, EPI_ISL_1044043, EPI_ISL_1044044, EPI_ISL_1044045, EPI_ISL_1044046, EPI_ISL_1044047, EPI_ISL_1044048, EPI_ISL_1044049, EPI_ISL_1044050, EPI_ISL_1044051, EPI_ISL_1044052, EPI_ISL_1044053, EPI_ISL_1044054, EPI_ISL_1044055, EPI_ISL_1044056, EPI_ISL_1044057, EPI_ISL_1044058, EPI_ISL_1044059, EPI_ISL_1044060, EPI_ISL_1044061, EPI_ISL_1044062, EPI_ISL_1044063, EPI_ISL_1044064, EPI_ISL_1044065, EPI_ISL_1044066, EPI_ISL_1044067, EPI_ISL_1044068, EPI_ISL_1044069, EPI_ISL_1044070, EPI_ISL_1044071, EPI_ISL_1044072, EPI_ISL_1044073, EPI_ISL_1044074, EPI_ISL_1044075, EPI_ISL_1044076, EPI_ISL_1044077, EPI_ISL_1044078, EPI_ISL_1044079, EPI_ISL_1044080, EPI_ISL_1044081, EPI_ISL_1044082, EPI_ISL_1044083, EPI_ISL_1044084, EPI_ISL_1044085, EPI_ISL_1044086, EPI_ISL_1044087, EPI_ISL_1044088, EPI_ISL_1044089, EPI_ISL_1044090, EPI_ISL_1044091, EPI_ISL_1044092, EPI_ISL_1044093, EPI_ISL_1044094, EPI_ISL_1044095, EPI_ISL_1044096, EPI_ISL_1044097, EPI_ISL_1044098, EPI_ISL_1044099, EPI_ISL_1044100, EPI_ISL_1044101, EPI_ISL_1044102, EPI_ISL_1044103, EPI_ISL_1044104, EPI_ISL_1044105, EPI_ISL_1044106, EPI_ISL_1044107, EPI_ISL_1044108, EPI_ISL_1044109, EPI_ISL_1044110, EPI_ISL_1044111, EPI_ISL_1044112, EPI_ISL_1044113, EPI_ISL_1044114, EPI_ISL_1044115, EPI_ISL_1044116, EPI_ISL_1044117, EPI_ISL_1044118, EPI_ISL_1044119, EPI_ISL_1044120, EPI_ISL_1044121, EPI_ISL_1044122, EPI_ISL_1044123, EPI_ISL_1044124, EPI_ISL_1044125, EPI_ISL_1044126, EPI_ISL_1044127, EPI_ISL_1044128, EPI_ISL_1044129, EPI_ISL_1044130, EPI_ISL_1044131, EPI_ISL_1044132, EPI_ISL_1044133, EPI_ISL_1044134, EPI_ISL_1044135, EPI_ISL_1044136, EPI_ISL_1044137, EPI_ISL_1044138, EPI_ISL_1044139, EPI_ISL_1044140, EPI_ISL_1044141, EPI_ISL_1044142, EPI_ISL_1044143, EPI_ISL_1044144, EPI_ISL_1044145, EPI_ISL_1044146, EPI_ISL_1044147, EPI_ISL_1044148, EPI_ISL_1044149, EPI_ISL_1044150, EPI_ISL_1044151, EPI_ISL_1044152, EPI_ISL_1044153, EPI_ISL_1044154, EPI_ISL_1044155, EPI_ISL_1044156, EPI_ISL_1044157, EPI_ISL_1044158, EPI_ISL_1044159, EPI_ISL_1044160, EPI_ISL_1044161, EPI_ISL_1044162, EPI_ISL_1044163, EPI_ISL_1044164, EPI_ISL_1044165, EPI_ISL_1044166, EPI_ISL_1044167, EPI_ISL_1044168, EPI_ISL_1044169, EPI_ISL_1044170, EPI_ISL_1044171, EPI_ISL_1044172, EPI_ISL_1044173, EPI_ISL_1044174, EPI_ISL_1044175, EPI_ISL_1044176, EPI_ISL_1044177, EPI_ISL_1044178, EPI_ISL_1044179, EPI_ISL_1044180, EPI_ISL_1044181, EPI_ISL_1044182, EPI_ISL_1044183, EPI_ISL_1044184, EPI_ISL_1044185, EPI_ISL_1044186, EPI_ISL_1044187, EPI_ISL_1044188, EPI_ISL_1044189, EPI_ISL_1044190, EPI_ISL_1044191, EPI_ISL_1044192, EPI_ISL_1044193, EPI_ISL_1044194, EPI_ISL_1044195, EPI_ISL_1044196, EPI_ISL_1044197, EPI_ISL_1044198, EPI_ISL_1044199, EPI_ISL_1044200, EPI_ISL_1044201, EPI_ISL_1044202, EPI_ISL_1044203, EPI_ISL_1044204, EPI_ISL_1044205, EPI_ISL_1044206, EPI_ISL_1044207, EPI_ISL_1044208, EPI_ISL_1044209, EPI_ISL_1044210, EPI_ISL_1044211, EPI_ISL_1044212, EPI_ISL_1044213, EPI_ISL_1044214, EPI_ISL_1044215, EPI_ISL_1044216, EPI_ISL_1044217, EPI_ISL_1044218, EPI_ISL_1044219, EPI_ISL_1044220, EPI_ISL_1044221, EPI_ISL_1044222, EPI_ISL_1044223, EPI_ISL_1044224			
see above	Lighthouse Lab in Alderley Park	Wellcome Sanger Institute for the COVID-19 Genomics UK (COG-UK) Consortium	Jacquelyn Wynn, Mairead Hyland, The Lighthouse Lab in Alderley Park and Alex Alderton, Roberto Amato, Jeffrey Barrett, Sonia Goncalves, Ewan Harrison, David K. Jackson, Ian Johnston, Dominic Kwiatkowski, Cordelia Langford, John Sillitoe on behalf of the Wellcome Sanger Institute COVID-19 Surveillance Team
EPI_ISL_1045980, EPI_ISL_1045981, EPI_ISL_1045982, EPI_ISL_1045984, EPI_ISL_1045985, EPI_ISL_1045986, EPI_ISL_1045987, EPI_ISL_1045988, EPI_ISL_1045989, EPI_ISL_1045990, EPI_ISL_1045991, EPI_ISL_1045992, EPI_ISL_1045993, EPI_ISL_1045994, EPI_ISL_1045995, EPI_ISL_1045996, EPI_ISL_1045997,			

EPI_ISL_1045998, EPI_ISL_1045999, EPI_ISL_1046000, EPI_ISL_1046001, EPI_ISL_1046002, EPI_ISL_1046003, EPI_ISL_1046004, EPI_ISL_1046005, EPI_ISL_1046006, EPI_ISL_1046007, EPI_ISL_1046008, EPI_ISL_1046009, EPI_ISL_1046010, EPI_ISL_1046011, EPI_ISL_1046012, EPI_ISL_1046013, EPI_ISL_1046014, EPI_ISL_1046015, EPI_ISL_1046016, EPI_ISL_1046017, EPI_ISL_1046018, EPI_ISL_1046019, EPI_ISL_1046020, EPI_ISL_1046021, EPI_ISL_1046022, EPI_ISL_1046023, EPI_ISL_1046024, EPI_ISL_1046025, EPI_ISL_1046026, EPI_ISL_1046027, EPI_ISL_1046028, EPI_ISL_1046029, EPI_ISL_1046030, EPI_ISL_1046031, EPI_ISL_1046032, EPI_ISL_1046033, EPI_ISL_1046034, EPI_ISL_1046035, EPI_ISL_1046036, EPI_ISL_1046037, EPI_ISL_1046038, EPI_ISL_1046039, EPI_ISL_1046040, EPI_ISL_1046041, EPI_ISL_1046042, EPI_ISL_1046043, EPI_ISL_1046044, EPI_ISL_1046045, EPI_ISL_1046046, EPI_ISL_1046047, EPI_ISL_1046048, EPI_ISL_1046049, EPI_ISL_1046050, EPI_ISL_1046051, EPI_ISL_1046052, EPI_ISL_1046053, EPI_ISL_1046054, EPI_ISL_1046055, EPI_ISL_1046056, EPI_ISL_1046057, EPI_ISL_1046058, EPI_ISL_1046059, EPI_ISL_1046060, EPI_ISL_1046061, EPI_ISL_1046062, EPI_ISL_1046063, EPI_ISL_1046064, EPI_ISL_1046065, EPI_ISL_1046066, EPI_ISL_1046067, EPI_ISL_1046068, EPI_ISL_1046069, EPI_ISL_1046070, EPI_ISL_1046071, EPI_ISL_1046072, EPI_ISL_1046073, EPI_ISL_1046074, EPI_ISL_1046075, EPI_ISL_1046076, EPI_ISL_1046077, EPI_ISL_1046078, EPI_ISL_1046079, EPI_ISL_1046080, EPI_ISL_1046081, EPI_ISL_1046082, EPI_ISL_1046083, EPI_ISL_1046084, EPI_ISL_1046085, EPI_ISL_1046086, EPI_ISL_1046087, EPI_ISL_1046088, EPI_ISL_1046089, EPI_ISL_1046090, EPI_ISL_1046091, EPI_ISL_1046092, EPI_ISL_1046093, EPI_ISL_1046094, EPI_ISL_1046095, EPI_ISL_1046096, EPI_ISL_1046097, EPI_ISL_1046098, EPI_ISL_1046099, EPI_ISL_1046100, EPI_ISL_1046101, EPI_ISL_1046102, EPI_ISL_1046103, EPI_ISL_1046104, EPI_ISL_1046105, EPI_ISL_1046106, EPI_ISL_1046107, EPI_ISL_1046108, EPI_ISL_1046109, EPI_ISL_1046110, EPI_ISL_1046111, EPI_ISL_1046112, EPI_ISL_1046113, EPI_ISL_1046114, EPI_ISL_1046115, EPI_ISL_1046117, EPI_ISL_1046118, EPI_ISL_1046119, EPI_ISL_1046120, EPI_ISL_1046121, EPI_ISL_1046122, EPI_ISL_1046123, EPI_ISL_1046124, EPI_ISL_1046125, EPI_ISL_1046126, EPI_ISL_1046127, EPI_ISL_1046128, EPI_ISL_1046129, EPI_ISL_1046130, EPI_ISL_1046131, EPI_ISL_1046132, EPI_ISL_1046133, EPI_ISL_1046134, EPI_ISL_1046135, EPI_ISL_1046136, EPI_ISL_1046137, EPI_ISL_1046138, EPI_ISL_1046139, EPI_ISL_1046140, EPI_ISL_1046141, EPI_ISL_1046142, EPI_ISL_1046143, EPI_ISL_1046144, EPI_ISL_1046145, EPI_ISL_1046146, EPI_ISL_1046147, EPI_ISL_1046148, EPI_ISL_1046149, EPI_ISL_1046150, EPI_ISL_1046151, EPI_ISL_1046152, EPI_ISL_1046153, EPI_ISL_1046154, EPI_ISL_1046155, EPI_ISL_1046156, EPI_ISL_1046157, EPI_ISL_1046158, EPI_ISL_1046159, EPI_ISL_1046160, EPI_ISL_1046161, EPI_ISL_1046162, EPI_ISL_1046163, EPI_ISL_1046164, EPI_ISL_1046165, EPI_ISL_1046167, EPI_ISL_1046168, EPI_ISL_1046169, EPI_ISL_1046170, EPI_ISL_1046171, EPI_ISL_1046172, EPI_ISL_1046173, EPI_ISL_1046174, EPI_ISL_1046175, EPI_ISL_1046176, EPI_ISL_1046177, EPI_ISL_1046178, EPI_ISL_1046179, EPI_ISL_1046180, EPI_ISL_1046181, EPI_ISL_1046182, EPI_ISL_1046183, EPI_ISL_1046184, EPI_ISL_1046185, EPI_ISL_1046186, EPI_ISL_1046187, EPI_ISL_1046188, EPI_ISL_1046189, EPI_ISL_1046190, EPI_ISL_1046191, EPI_ISL_1046192, EPI_ISL_1046193, EPI_ISL_1046194, EPI_ISL_1046195, EPI_ISL_1046196, EPI_ISL_1046197, EPI_ISL_1046198, EPI_ISL_1046199, EPI_ISL_1046200, EPI_ISL_1046201, EPI_ISL_1046202, EPI_ISL_1046203, EPI_ISL_1046204, EPI_ISL_1046205, EPI_ISL_1046206, EPI_ISL_1046207, EPI_ISL_1046208, EPI_ISL_1046209, EPI_ISL_1046210, EPI_ISL_1046211, EPI_ISL_1046212, EPI_ISL_1046213, EPI_ISL_1046214, EPI_ISL_1046215, EPI_ISL_1046216, EPI_ISL_1046217, EPI_ISL_1046218, EPI_ISL_1046219, EPI_ISL_1046220, EPI_ISL_1046221, EPI_ISL_1046222, EPI_ISL_1046223, EPI_ISL_1046224, EPI_ISL_1046225, EPI_ISL_1046226, EPI_ISL_1046227, EPI_ISL_1046228, EPI_ISL_1046229, EPI_ISL_1046230, EPI_ISL_1046231, EPI_ISL_1046232, EPI_ISL_1046233, EPI_ISL_1046234, EPI_ISL_1046235, EPI_ISL_1046236, EPI_ISL_1046237, EPI_ISL_1046239, EPI_ISL_1046240, EPI_ISL_1046241, EPI_ISL_1046242, EPI_ISL_1046243, EPI_ISL_1046244, EPI_ISL_1046245, EPI_ISL_1046246, EPI_ISL_1046247, EPI_ISL_1046248, EPI_ISL_1046249, EPI_ISL_1046250, EPI_ISL_1046251, EPI_ISL_1046252, EPI_ISL_1046253, EPI_ISL_1046254, EPI_ISL_1046255, EPI_ISL_1046256, EPI_ISL_1046257, EPI_ISL_1046258, EPI_ISL_1046259, EPI_ISL_1046260, EPI_ISL_1046261, EPI_ISL_1046262, EPI_ISL_1046263, EPI_ISL_1046264, EPI_ISL_1046265, EPI_ISL_1046266, EPI_ISL_1046267, EPI_ISL_1046268, EPI_ISL_1046269, EPI_ISL_1046270, EPI_ISL_1046271, EPI_ISL_1046272, EPI_ISL_1046273, EPI_ISL_1046274, EPI_ISL_1046275, EPI_ISL_1046276, EPI_ISL_1046277, EPI_ISL_1046278, EPI_ISL_1046279, EPI_ISL_1046280, EPI_ISL_1046281, EPI_ISL_1046282, EPI_ISL_1046283, EPI_ISL_1046284, EPI_ISL_1046285, EPI_ISL_1046286, EPI_ISL_1046287, EPI_ISL_1046288, EPI_ISL_1046289, EPI_ISL_1046290, EPI_ISL_1046291, EPI_ISL_1046292, EPI_ISL_1046293, EPI_ISL_1046294, EPI_ISL_1046295, EPI_ISL_1046296, EPI_ISL_1046297, EPI_ISL_1046298, EPI_ISL_1046299, EPI_ISL_1046300, EPI_ISL_1046301, EPI_ISL_1046302, EPI_ISL_1046303, EPI_ISL_1046304, EPI_ISL_1046305, EPI_ISL_1046306, EPI_ISL_1046307, EPI_ISL_1046308, EPI_ISL_1046309, EPI_ISL_1046311, EPI_ISL_1046312, EPI_ISL_1046313, EPI_ISL_1046314, EPI_ISL_1046315, EPI_ISL_1046316, EPI_ISL_1046317, EPI_ISL_1046318, EPI_ISL_1046319, EPI_ISL_1046320, EPI_ISL_1046321, EPI_ISL_1046323, EPI_ISL_1046324, EPI_ISL_1046325, EPI_ISL_1046326, EPI_ISL_1046327, EPI_ISL_1046328, EPI_ISL_1046329, EPI_ISL_1046330, EPI_ISL_1046331, EPI_ISL_1046332, EPI_ISL_1046333, EPI_ISL_1046334, EPI_ISL_1046335			
see above	Randox Laboratories	Wellcome Sanger Institute for the COVID-19 Genomics UK (COG-UK) Consortium	Randox Laboratories and Alex Alderton, Roberto Amato, Jeffrey Barrett, Sonia Goncalves, Ewan Harrison, David K. Jackson, Ian Johnston, Dominic Kwiatkowski, Cordelia Langford, John Sillitoe on behalf of the Wellcome Sanger Institute COVID-19 Surveillance Team
EPI_ISL_1046990, EPI_ISL_1046995, EPI_ISL_1047011, EPI_ISL_1047019, EPI_ISL_1047034, EPI_ISL_1047036, EPI_ISL_1047045, EPI_ISL_1047049	University of Birmingham	COVID-19 Genomics UK (COG-UK) Consortium	Institute of Microbiology, University of Birmingham: Claire McMurray, Joanne Stockton, Samuel Nicholls, Radoslaw Poplawski, Will Rowe, Josh Quick, Nicholas Loman. University of Birmingham Testing Laboratory: Celina M Whalley, Andrew Bosworth, Charlotte Poxon, Kasun Wanigasooriya, Oliver Pickles, Mike Kidd, Alex Richter, Andrew D Beggs PHE Heartlands Lab: Husam Osman, Andrew Bosworth. Queen Elizabeth Hospital: Anna Casey
EPI_ISL_1047148, EPI_ISL_1047149, EPI_ISL_1047150, EPI_ISL_1047151, EPI_ISL_1047152, EPI_ISL_1047153, EPI_ISL_1047154, EPI_ISL_1047156, EPI_ISL_1047157, EPI_ISL_1047158, EPI_ISL_1047159, EPI_ISL_1047160, EPI_ISL_1047161, EPI_ISL_1047162, EPI_ISL_1047163, EPI_ISL_1047164, EPI_ISL_1047165, EPI_ISL_1047166, EPI_ISL_1047167, EPI_ISL_1047168, EPI_ISL_1047169, EPI_ISL_1047170, EPI_ISL_1047171, EPI_ISL_1047172, EPI_ISL_1047173, EPI_ISL_1047174, EPI_ISL_1047175, EPI_ISL_1047176, EPI_ISL_1047177, EPI_ISL_1047178, EPI_ISL_1047179, EPI_ISL_1047180, EPI_ISL_1047181, EPI_ISL_1047182, EPI_ISL_1047183, EPI_ISL_1047185, EPI_ISL_1047186, EPI_ISL_1047190, EPI_ISL_1047194, EPI_ISL_1047195, EPI_ISL_1047196, EPI_ISL_1047197, EPI_ISL_1047198, EPI_ISL_1047199, EPI_ISL_1047200, EPI_ISL_1047201, EPI_ISL_1047202, EPI_ISL_1047203, EPI_ISL_1047204, EPI_ISL_1047206, EPI_ISL_1047207, EPI_ISL_1047208, EPI_ISL_1047209, EPI_ISL_1047210, EPI_ISL_1047211, EPI_ISL_1047212, EPI_ISL_1047213, EPI_ISL_1047214, EPI_ISL_1047215, EPI_ISL_1047216, EPI_ISL_1047217, EPI_ISL_1047220, EPI_ISL_1047221, EPI_ISL_1047222, EPI_ISL_1047223, EPI_ISL_1047224, EPI_ISL_1047225, EPI_ISL_1047226, EPI_ISL_1047227, EPI_ISL_1047228, EPI_ISL_1047229, EPI_ISL_1047230, EPI_ISL_1047231, EPI_ISL_1047232, EPI_ISL_1047233, EPI_ISL_1047235, EPI_ISL_1047236, EPI_ISL_1047237, EPI_ISL_1047238, EPI_ISL_1047239, EPI_ISL_1047240, EPI_ISL_1047241, EPI_ISL_1047242, EPI_ISL_1047243, EPI_ISL_1047244, EPI_ISL_1047245, EPI_ISL_1047246, EPI_ISL_1047247, EPI_ISL_1047248, EPI_ISL_1047249, EPI_ISL_1047250, EPI_ISL_1047251, EPI_ISL_1047252, EPI_ISL_1047254, EPI_ISL_1047255, EPI_ISL_1047256, EPI_ISL_1047257, EPI_ISL_1047258, EPI_ISL_1047259, EPI_ISL_1047260, EPI_ISL_1047261, EPI_ISL_1047262, EPI_ISL_1047263, EPI_ISL_1047264, EPI_ISL_1047265, EPI_ISL_1047266, EPI_ISL_1047267, EPI_ISL_1047268, EPI_ISL_1047269, EPI_ISL_1047270, EPI_ISL_1047271, EPI_ISL_1047272, EPI_ISL_1047273, EPI_ISL_1047275, EPI_ISL_1047276, EPI_ISL_1047277, EPI_ISL_1047278, EPI_ISL_1047280, EPI_ISL_1047281, EPI_ISL_1047282, EPI_ISL_1047283, EPI_ISL_1047284, EPI_ISL_1047285, EPI_ISL_1047286, EPI_ISL_1047287, EPI_ISL_1047288, EPI_ISL_1047289, EPI_ISL_1047290, EPI_ISL_1047291, EPI_ISL_1047292, EPI_ISL_1047294, EPI_ISL_1047295, EPI_ISL_1047296, EPI_ISL_1047297, EPI_ISL_1047298, EPI_ISL_1047299, EPI_ISL_1047300, EPI_ISL_1047301, EPI_ISL_1047302, EPI_ISL_1047305, EPI_ISL_1047306, EPI_ISL_1047307, EPI_ISL_1047308, EPI_ISL_1047309, EPI_ISL_1047310, EPI_ISL_1047311, EPI_ISL_1047312, EPI_ISL_1047313, EPI_ISL_1047314, EPI_ISL_1047315, EPI_ISL_1047316, EPI_ISL_1047317, EPI_ISL_1047318, EPI_ISL_1047319, EPI_ISL_1047320, EPI_ISL_1047321, EPI_ISL_1047322, EPI_ISL_1047323, EPI_ISL_1047324, EPI_ISL_1047325, EPI_ISL_1047326, EPI_ISL_1047327, EPI_ISL_1047328, EPI_ISL_1047329, EPI_ISL_1047330, EPI_ISL_1047333, EPI_ISL_1047334, EPI_ISL_1047335, EPI_ISL_1047336, EPI_ISL_1047338, EPI_ISL_1047339, EPI_ISL_1047340, EPI_ISL_1047341, EPI_ISL_1047342, EPI_ISL_1047343, EPI_ISL_1047344, EPI_ISL_1047345, EPI_ISL_1047346, EPI_ISL_1047347, EPI_ISL_1047348, EPI_ISL_1047352, EPI_ISL_1047355, EPI_ISL_1047356, EPI_ISL_1047357, EPI_ISL_1047358, EPI_ISL_1047359, EPI_ISL_1047360, EPI_ISL_1047361, EPI_ISL_1047362, EPI_ISL_1047363, EPI_ISL_1047364, EPI_ISL_1047365, EPI_ISL_1047366, EPI_ISL_1047367, EPI_ISL_1047368, EPI_ISL_1047369, EPI_ISL_1047370, EPI_ISL_1047372, EPI_ISL_1047373, EPI_ISL_1047374, EPI_ISL_1047375, EPI_ISL_1047376, EPI_ISL_1047377, EPI_ISL_1047378, EPI_ISL_1047379, EPI_ISL_1047380, EPI_ISL_1047381, EPI_ISL_1047382, EPI_ISL_1047383, EPI_ISL_1047384, EPI_ISL_1047385, EPI_ISL_1047386, EPI_ISL_1047387, EPI_ISL_1047388, EPI_ISL_1047389, EPI_ISL_1047390, EPI_ISL_1047391, EPI_ISL_1047392, EPI_ISL_1047393, EPI_ISL_1047394, EPI_ISL_1047395, EPI_ISL_1047396, EPI_ISL_1047397, EPI_ISL_1047398, EPI_ISL_1047399, EPI_ISL_1047400, EPI_ISL_1047401, EPI_ISL_1047402, EPI_ISL_1047403, EPI_ISL_1047404, EPI_ISL_1047405, EPI_ISL_1047406, EPI_ISL_1047407, EPI_ISL_1047408, EPI_ISL_1047409, EPI_ISL_1047410, EPI_ISL_1047411, EPI_ISL_1047412, EPI_ISL_1047413, EPI_ISL_1047414, EPI_ISL_1047415, EPI_ISL_1047416, EPI_ISL_1047417, EPI_ISL_1047418, EPI_ISL_1047419, EPI_ISL_1047420, EPI_ISL_1047421, EPI_ISL_1047423, EPI_ISL_1047424, EPI_ISL_1047425, EPI_ISL_1047426, EPI_ISL_1047427, EPI_ISL_1047428, EPI_ISL_1047429, EPI_ISL_1047430, EPI_ISL_1047431, EPI_ISL_1047432, EPI_ISL_1047433, EPI_ISL_1047434, EPI_ISL_1047435, EPI_ISL_1047436, EPI_ISL_1047437, EPI_ISL_1047438, EPI_ISL_1047439, EPI_ISL_1047440, EPI_ISL_1047441, EPI_ISL_1047442, EPI_ISL_1047443, EPI_ISL_1047444, EPI_ISL_1047445, EPI_ISL_1047446, EPI_ISL_1047447, EPI_ISL_1047448, EPI_ISL_1047449, EPI_ISL_1047450, EPI_ISL_1047451, EPI_ISL_1047452, EPI_ISL_1047453, EPI_ISL_1047454, EPI_ISL_1047455, EPI_ISL_1047456, EPI_ISL_1047457, EPI_ISL_1047458, EPI_ISL_1047459, EPI_ISL_1047460, EPI_ISL_1047461, EPI_ISL_1047462, EPI_ISL_1047463, EPI_ISL_1047464, EPI_ISL_1047465, EPI_ISL_1047467, EPI_ISL_1047468, EPI_ISL_1047469, EPI_ISL_1047470, EPI_ISL_1047471, EPI_ISL_1047472, EPI_ISL_1047473, EPI_ISL_1047474, EPI_ISL_1047475, EPI_ISL_1047476, EPI_ISL_1047477, EPI_ISL_1047478, EPI_ISL_1047479, EPI_ISL_1047480, EPI_ISL_1047481, EPI_ISL_1047482, EPI_ISL_1047483, EPI_ISL_1047484, EPI_ISL_1047485, EPI_ISL_1047486, EPI_ISL_1047487, EPI_ISL_1047488, EPI_ISL_1047489, EPI_ISL_1047490, EPI_ISL_1047491, EPI_ISL_1047492, EPI_ISL_1047493, EPI_ISL_1047495, EPI_ISL_1047496, EPI_ISL_1047497, EPI_ISL_1047498, EPI_ISL_1047500, EPI_ISL_1047501, EPI_ISL_1047502, EPI_ISL_1047503, EPI_ISL_1047504, EPI_ISL_1047505, EPI_ISL_1047506, EPI_ISL_1047507, EPI_ISL_1047508, EPI_ISL_1047509, EPI_ISL_1047510, EPI_ISL_1047512, EPI_ISL_1047513, EPI_ISL_1047514, EPI_ISL_1047515, EPI_ISL_1047516, EPI_ISL_1047517, EPI_ISL_1047518, EPI_ISL_1047519, EPI_ISL_1047520, EPI_ISL_1047521, EPI_ISL_1047522, EPI_ISL_1047523, EPI_ISL_1047524, EPI_ISL_1047525, EPI_ISL_1047526, EPI_ISL_1047527, EPI_ISL_1047528, EPI_ISL_1047529, EPI_ISL_1047530, EPI_ISL_1047531, EPI_ISL_1047532, EPI_ISL_1047533, EPI_ISL_1047534, EPI_ISL_1047535, EPI_ISL_1047536, EPI_ISL_1047537, EPI_ISL_1047538, EPI_ISL_1047539, EPI_ISL_1047540, EPI_ISL_1047541, EPI_ISL_1047542, EPI_ISL_1047544, EPI_ISL_1047545, EPI_ISL_1047546, EPI_ISL_1047547, EPI_ISL_1047548, EPI_ISL_1047549, EPI_ISL_1047550, EPI_ISL_1047551, EPI_ISL_1047552, EPI_ISL_1047553, EPI_ISL_1047554, EPI_ISL_1047555, EPI_ISL_1047556, EPI_ISL_1047557, EPI_ISL_1047558, EPI_ISL_1047559, EPI_ISL_1047560, EPI_ISL_1047561, EPI_ISL_1047562, EPI_ISL_1047563, EPI_ISL_1047564, EPI_ISL_1047565, EPI_ISL_1047566, EPI_ISL_1047567, EPI_ISL_1047568, EPI_ISL_1047569, EPI_ISL_1047570, EPI_ISL_1047571, EPI_ISL_1047572, EPI_ISL_1047573, EPI_ISL_1047574, EPI_ISL_1047575, EPI_ISL_1047576, EPI_ISL_1047577, EPI_ISL_1047578, EPI_ISL_1047579, EPI_ISL_1047580, EPI_ISL_1047581			
see above	Department of Pathology, University of Cambridge	COVID-19 Genomics UK (COG-UK) Consortium	Aminu S. Jahun, Yasmin Chaudhry, Iliana Georgana, Myra Hosmillo, Rhys Izuagbe, William L. Hamilton, Martin D. Curran, Surendra Parmar, Ian Goodfellow
EPI_ISL_1047679, EPI_ISL_1047680, EPI_ISL_1047681, EPI_ISL_1047682, EPI_ISL_1047683, EPI_ISL_1047684, EPI_ISL_1047686, EPI_ISL_1047687, EPI_ISL_1047688, EPI_ISL_1047689, EPI_ISL_1047690, EPI_ISL_1047691, EPI_ISL_1047692, EPI_ISL_1047693, EPI_ISL_1047694, EPI_ISL_1047695, EPI_ISL_1047696, EPI_ISL_1047697, EPI_ISL_1047698, EPI_ISL_1047699, EPI_ISL_1047700, EPI_ISL_1047701, EPI_ISL_1047702, EPI_ISL_1047703, EPI_ISL_1047705, EPI_ISL_1047706, EPI_ISL_1047707, EPI_ISL_1047708, EPI_ISL_1047709, EPI_ISL_1047710, EPI_ISL_1047711			
see above	Virology Department, Sheffield Teaching Hospitals NHS Foundation Trust/Department of Infection, Immunity and Cardiovascular Disease, The Medical School, University of Sheffield	COVID-19 Genomics UK (COG-UK) Consortium	Thushan de Silva, Matthew Parker, Nikki Smith, Adri Angyal, Rebecca Brown, Luke Green, Rachel Tucker, Paul Parsons, Danielle Groves, Katie Johnson, Laura Carrilero, Alex Keeley, Dave Partridge, Matthew Wyles, Benjamin Lindsey, Mehmet Yavuz, Mohammad Raza, Cariad Evans
EPI_ISL_1047846	West of Scotland Specialist Virology Centre, NHSGGC / MRC-University of Glasgow Centre for Virus Research	COVID-19 Genomics UK (COG-UK) Consortium	Ana da Silva Filipe, Natasha Johnson, Kathy Smollett, Daniel Mair, Stephen Carmichael, Alice Broos, Lily Tong, Jenna Nichols, Kyriaki Nomikou; Sarah McDonald; Richard Orton, Joseph Hughes, Sreenu Vattipally, David L Robertson; Alasdair MacLean, Rory Gunson; Sharif Shaaban, Matthew Holden; Rachel Blacow, Guy Mollett, Kathy Li, James Shepherd, Antonia Ho, Emma Thomson
EPI_ISL_1047860	Virology Department, Royal Infirmary of Edinburgh, NHS Lothian / School of Biological Sciences, University of Edinburgh	COVID-19 Genomics UK (COG-UK) Consortium	McHugh M, Dewar R, Cotton S, Rooke S, O'Toole Á, Scher E, Hill V, McCrone JT, Colquhoun R, Yu X, Jackson B, Rambaut A, Templeton K
EPI_ISL_1048017, EPI_ISL_1048018, EPI_ISL_1048019, EPI_ISL_1048020, EPI_ISL_1048021, EPI_ISL_1048022	Liverpool Clinical Laboratories	COVID-19 Genomics UK (COG-UK) Consortium	Sam Haldenby, Anita Lucaci, Steve Paterson, Julian Hiscox, Alistair Darby, M Almsaud, A Alrezaihi, Muhannad Alruwaili, Stuart D Armstrong, Jones Benjamin, Eleanor G Bentley, Anu Chawla, Jordan J Clark, Angela Cowell, Richard Eccles, Isabel García-Dorival, Matthew Gemmell, Alessandro Gerada, PKF Gilmore, Richard Gregory, Ximeng Han, Catherine Hartley, Margaret Hughes, Miren Iturriza-Gomara, James Johnson, L Luu, Jenifer Manson, Charlotte Nelson, Elaine O'Toole, Cassie Olateju, Rebekah Penrice-Randal , Lucille Rainbow, N.P Randle, Trevor Ian Robinson, Parul Sharma, Ghada T Shawli, James P Stewart, Neil Swainston, Ecaterina Vamos, Joanne Watts, Mark Whitehead
EPI_ISL_1048054, EPI_ISL_1048055, EPI_ISL_1048057, EPI_ISL_1048058, EPI_ISL_1048059, EPI_ISL_1048062, EPI_ISL_1048066, EPI_ISL_1048069, EPI_ISL_1048076, EPI_ISL_1048089, EPI_ISL_1048097			
see above	Barts Health NHS Trust	COVID-19 Genomics UK (COG-UK) Consortium	CUTINO-MOGUEL, Maria-Teresa; HARRINGTON, David; OWOYEMI, Dola; KULASEGARAN-SHYLINI, Raghavendran; BROAD, Claire; KELE, Beatrix
EPI_ISL_1048139, EPI_ISL_1048140, EPI_ISL_1048141, EPI_ISL_1048142, EPI_ISL_1048143, EPI_ISL_1048144, EPI_ISL_1048145, EPI_ISL_1048146, EPI_ISL_1048147, EPI_ISL_1048148, EPI_ISL_1048149, EPI_ISL_1048150, EPI_ISL_1048151, EPI_ISL_1048152, EPI_ISL_1048153, EPI_ISL_1048154, EPI_ISL_1048155, EPI_ISL_1048156, EPI_ISL_1048157, EPI_ISL_1048158, EPI_ISL_1048159, EPI_ISL_1048160, EPI_ISL_1048161, EPI_ISL_1048162, EPI_ISL_1048163, EPI_ISL_1048164, EPI_ISL_1048165, EPI_ISL_1048166, EPI_ISL_1048167, EPI_ISL_1048168, EPI_ISL_1048169, EPI_ISL_1048170, EPI_ISL_1048171, EPI_ISL_1048172, EPI_ISL_1048173, EPI_ISL_1048174, EPI_ISL_1048175, EPI_ISL_1048176, EPI_ISL_1048177, EPI_ISL_1048178, EPI_ISL_1048179, EPI_ISL_1048180, EPI_ISL_1048181, EPI_ISL_1048182, EPI_ISL_1048183, EPI_ISL_1048184, EPI_ISL_1048185, EPI_ISL_1048186, EPI_ISL_1048187, EPI_ISL_1048188, EPI_ISL_1048189, EPI_ISL_1048190, EPI_ISL_1048191, EPI_ISL_1048192, EPI_ISL_1048193, EPI_ISL_1048194, EPI_ISL_1048195, EPI_ISL_1048196, EPI_ISL_1048197, EPI_ISL_1048198, EPI_ISL_1048199, EPI_ISL_1048200, EPI_ISL_1048201, EPI_ISL_1048202, EPI_ISL_1048203, EPI_ISL_1048204, EPI_ISL_1048205, EPI_ISL_1048206, EPI_ISL_1048207, EPI_ISL_1048208, EPI_ISL_1048209, EPI_ISL_1048210, EPI_ISL_1048211, EPI_ISL_1048212, EPI_ISL_1048213, EPI_ISL_1048214, EPI_ISL_1048215, EPI_ISL_1048233, EPI_ISL_1048234, EPI_ISL_1048235, EPI_ISL_1048236, EPI_ISL_1048237			
see above	University College London, Great Ormond Street Hospital for Children NHS Foundation Trust, Imperial College Healthcare NHS Trust	COVID-19 Genomics UK (COG-UK) Consortium	Sergi Castellano, Rachel Williams, Mark Kristiansen, Paola Resende Silva, Sunando Roy, Tony Brooks, Helena Tutill, Paola Niola, Patricia Dyal, Charlotte Williams, Leysa Forrest, Yasmin Panchbhaya, Jacqueline Findlay, Samuel Weeks, Julianne Brown, Kathryn Harris, Paul Randell, James Price, Alison Holmes, Judith Breuer
EPI_ISL_1050065	Virology Department, Sheffield Teaching Hospitals NHS	COVID-19 Genomics UK (COG-UK) Consortium	Thushan de Silva, Matthew Parker, Nikki Smith, Adri Angyal, Rebecca Brown, Luke Green, Rachel Tucker, Paul Parsons, Danielle Groves, Katie Johnson,

	Foundation Trust/Department of Infection, Immunity and Cardiovascular Disease, The Medical School, University of Sheffield		Laura Carrilero, Alex Keeley, Dave Partridge, Matthew Wyles, Benjamin Lindsey, Mehmet Yavuz, Mohammad Raza, Cariad Evans
EPI_ISL_1050225, EPI_ISL_1050227	Lighthouse Lab in Glasgow / MRC-University of Glasgow Centre for Virus Research	COVID-19 Genomics UK (COG-UK) Consortium	Ana da Silva Filipe, Natasha Johnson, Kathy Smollett, Daniel Mair, Stephen Carmichael, Alice Broos, Lily Tong, Jenna Nichols, Kyriaki Nomikou; Sarah McDonald; Harper VanSteenhouse, Yumi Kasai, David Gray, Carol Clugston, Anna Dominiczak; Alasdair MacLean, Rory Gunson; Richard Orton, Joseph Hughes, Sreenu Vattipally, David L Robertson; Sharif Shaaban, Matthew Holden; Kathy Li, James Shepherd, Antonia Ho, Emma Thomson
EPI_ISL_1050356	Barts Health NHS Trust	COVID-19 Genomics UK (COG-UK) Consortium	CUTINO-MOGUEL, Maria-Teresa; HARRINGTON, David; OWOYEMI, Dola; KULASEGARAN-SHYLINI, Raghavendran; BROAD, Claire; KELE, Beatrix
EPI_ISL_1050369, EPI_ISL_1050392	University College London, Great Ormond Street Hospital for Children NHS Foundation Trust, Imperial College Healthcare NHS Trust	COVID-19 Genomics UK (COG-UK) Consortium	Sergi Castellano, Rachel Williams, Mark Kristiansen, Paola Resende Silva, Sunando Roy, Tony Brooks, Helena Tutill, Paola Niola, Patricia Dyal, Charlotte Williams, Leysa Forrest, Yasmin Panchbhaya, Jacqueline Findlay, Samuel Weeks, Julianne Brown, Kathryn Harris, Paul Randell, James Price, Alison Holmes, Judith Breuer
EPI_ISL_1051222, EPI_ISL_1051223, EPI_ISL_1051224, EPI_ISL_1051225, EPI_ISL_1051226, EPI_ISL_1051227, EPI_ISL_1051228, EPI_ISL_1051229, EPI_ISL_1051230, EPI_ISL_1051231, EPI_ISL_1051232, EPI_ISL_1051233, EPI_ISL_1051234, EPI_ISL_1051235, EPI_ISL_1051236, EPI_ISL_1051237, EPI_ISL_1051238, EPI_ISL_1051239, EPI_ISL_1051240, EPI_ISL_1051241, EPI_ISL_1051242, EPI_ISL_1051243			
see above	Northumbria University / South Tees Hospitals NHS Foundation Trust / North Cumbria Integrated Care NHS Foundation Trust / North Tees and Hartlepool NHS Foundation Trust / Newcastle Hospitals NHS Foundation Trust	COVID-19 Genomics UK (COG-UK) Consortium	Darren L Smith,Andrew Nelson,Matthew Bashton,Greg R Young,Joshua Loh,John Allan,Mohammad A Tariq,Giles S Holt,Gary Black,Wen C Yew,Lynn Dover,Paul Baker,Steve Liggett,Sarah Essex,Jane Greenaway,Debra Padgett,Clive Graham,Garren Scott,Edward Barton,Emma Swindells,Brendan Payne,Jennifer Collins,Yusri Taha,Gary Eltringham
EPI_ISL_1051597, EPI_ISL_1051609, EPI_ISL_1051610, EPI_ISL_1051611, EPI_ISL_1051612, EPI_ISL_1051613, EPI_ISL_1051615, EPI_ISL_1051616, EPI_ISL_1051617, EPI_ISL_1051619, EPI_ISL_1051621, EPI_ISL_1051622, EPI_ISL_1051623, EPI_ISL_1051624, EPI_ISL_1051625, EPI_ISL_1051628, EPI_ISL_1051632, EPI_ISL_1051633, EPI_ISL_1051636, EPI_ISL_1051660, EPI_ISL_1051670, EPI_ISL_1051675, EPI_ISL_1051676, EPI_ISL_1051681, EPI_ISL_1051682, EPI_ISL_1051690, EPI_ISL_1051893, EPI_ISL_1051894, EPI_ISL_1051895, EPI_ISL_1051896, EPI_ISL_1051897, EPI_ISL_1051904, EPI_ISL_1051905			
see above	Oxford Viromics, NDM, University of Oxford; Oxford University Hospitals; Basingstoke and North Hampshire Hospital	COVID-19 Genomics UK (COG-UK) Consortium	Tanya Golubchik, David Bonsall, George Macintyre, Amy Trebes, Mariateresa de Cesare, Catrin Moore, Alex Mobbs, Anita Justice, Robert Shaw, Monique Andersson, Timothy Peto, Emma Wise, Nathan Moore, Jessica Lynch, Nick Cortes, Matilde Mori, Stephen Kidd, David Buck, John Todd, Christophe Fraser
EPI_ISL_1051920, EPI_ISL_1051922, EPI_ISL_1051923, EPI_ISL_1051924, EPI_ISL_1051925, EPI_ISL_1051926, EPI_ISL_1051927, EPI_ISL_1051928, EPI_ISL_1051929, EPI_ISL_1051930, EPI_ISL_1051932, EPI_ISL_1051933, EPI_ISL_1051934, EPI_ISL_1051935, EPI_ISL_1051936, EPI_ISL_1051937, EPI_ISL_1051938, EPI_ISL_1051939, EPI_ISL_1051940, EPI_ISL_1051941, EPI_ISL_1051942, EPI_ISL_1051943, EPI_ISL_1051944, EPI_ISL_1051946, EPI_ISL_1051947, EPI_ISL_1051948, EPI_ISL_1051949, EPI_ISL_1051950, EPI_ISL_1051952, EPI_ISL_1051953, EPI_ISL_1051954, EPI_ISL_1051955, EPI_ISL_1051956, EPI_ISL_1051957, EPI_ISL_1051958, EPI_ISL_1051959, EPI_ISL_1051961, EPI_ISL_1051962, EPI_ISL_1051963, EPI_ISL_1051964, EPI_ISL_1051966, EPI_ISL_1051967, EPI_ISL_1051968, EPI_ISL_1051969, EPI_ISL_1051970, EPI_ISL_1051971, EPI_ISL_1051972, EPI_ISL_1051973, EPI_ISL_1051974, EPI_ISL_1051975, EPI_ISL_1051976, EPI_ISL_1051978, EPI_ISL_1051979, EPI_ISL_1051981, EPI_ISL_1051982, EPI_ISL_1051983, EPI_ISL_1051984, EPI_ISL_1051985, EPI_ISL_1051986, EPI_ISL_1051987, EPI_ISL_1051988, EPI_ISL_1051989, EPI_ISL_1051990, EPI_ISL_1051991, EPI_ISL_1051992, EPI_ISL_1051993, EPI_ISL_1051994, EPI_ISL_1051995, EPI_ISL_1051997, EPI_ISL_1051998, EPI_ISL_1051999, EPI_ISL_1052000, EPI_ISL_1052001, EPI_ISL_1052002, EPI_ISL_1052003, EPI_ISL_1052004, EPI_ISL_1052005, EPI_ISL_1052006, EPI_ISL_1052007, EPI_ISL_1052008, EPI_ISL_1052009, EPI_ISL_1052010, EPI_ISL_1052011, EPI_ISL_1052012, EPI_ISL_1052013, EPI_ISL_1052014, EPI_ISL_1052015, EPI_ISL_1052016, EPI_ISL_1052017, EPI_ISL_1052018, EPI_ISL_1052021, EPI_ISL_1052022, EPI_ISL_1052024, EPI_ISL_1052025, EPI_ISL_1052026, EPI_ISL_1052027, EPI_ISL_1052029, EPI_ISL_1052031, EPI_ISL_1052033, EPI_ISL_1052034, EPI_ISL_1052035, EPI_ISL_1052036, EPI_ISL_1052038, EPI_ISL_1052039, EPI_ISL_1052040, EPI_ISL_1052041, EPI_ISL_1052042, EPI_ISL_1052043, EPI_ISL_1052044, EPI_ISL_1052045, EPI_ISL_1052047, EPI_ISL_1052048, EPI_ISL_1052050, EPI_ISL_1052051, EPI_ISL_1052052, EPI_ISL_1052053, EPI_ISL_1052055, EPI_ISL_1052056, EPI_ISL_1052058, EPI_ISL_1052060, EPI_ISL_1052061, EPI_ISL_1052062, EPI_ISL_1052063, EPI_ISL_1052064, EPI_ISL_1052065, EPI_ISL_1052066, EPI_ISL_1052067, EPI_ISL_1052068, EPI_ISL_1052070, EPI_ISL_1052071, EPI_ISL_1052072, EPI_ISL_1052073, EPI_ISL_1052074, EPI_ISL_1052075, EPI_ISL_1052076, EPI_ISL_1052077, EPI_ISL_1052078, EPI_ISL_1052079, EPI_ISL_1052080, EPI_ISL_1052081, EPI_ISL_1052082, EPI_ISL_1052083, EPI_ISL_1052084, EPI_ISL_1052086, EPI_ISL_1052087, EPI_ISL_1052088, EPI_ISL_1052089, EPI_ISL_1052090, EPI_ISL_1052091, EPI_ISL_1052092, EPI_ISL_1052094, EPI_ISL_1052095, EPI_ISL_1052098, EPI_ISL_1052099, EPI_ISL_1052100, EPI_ISL_1052101, EPI_ISL_1052102, EPI_ISL_1052103, EPI_ISL_1052104, EPI_ISL_1052105, EPI_ISL_1052106, EPI_ISL_1052107, EPI_ISL_1052109, EPI_ISL_1052110, EPI_ISL_1052111, EPI_ISL_1052112, EPI_ISL_1052116, EPI_ISL_1052117, EPI_ISL_1052118, EPI_ISL_1052119, EPI_ISL_1052120, EPI_ISL_1052121, EPI_ISL_1052122, EPI_ISL_1052123, EPI_ISL_1052124, EPI_ISL_1052125, EPI_ISL_1052126, EPI_ISL_1052127, EPI_ISL_1052128, EPI_ISL_1052129, EPI_ISL_1052132, EPI_ISL_1052133, EPI_ISL_1052134, EPI_ISL_1052136, EPI_ISL_1052137, EPI_ISL_1052203, EPI_ISL_1052204, EPI_ISL_1052462, EPI_ISL_1052465, EPI_ISL_1052473, EPI_ISL_1052474, EPI_ISL_1052480, EPI_ISL_1052511, EPI_ISL_1052512, EPI_ISL_1052513, EPI_ISL_1052514, EPI_ISL_1052515, EPI_ISL_1052516, EPI_ISL_1052521, EPI_ISL_1052523, EPI_ISL_1052524, EPI_ISL_1052525, EPI_ISL_1053502, EPI_ISL_1053521, EPI_ISL_1053522, EPI_ISL_1053548, EPI_ISL_1053549, EPI_ISL_1053550, EPI_ISL_1053551, EPI_ISL_1053553, EPI_ISL_1053555, EPI_ISL_1053556, EPI_ISL_1053557, EPI_ISL_1053559, EPI_ISL_1053560, EPI_ISL_1053561, EPI_ISL_1053563, EPI_ISL_1053564			
see above	Originating lab: Wales Specialist Virology Centre Sequencing lab: Pathogen Genomics Unit	Public Health Wales Microbiology Cardiff Wales Specialist Virology Centre	Catherine Moore, Johnathan Evans, Laura Gifford, Malorie Perry, Simon Cottrell, Angela Marchbank, Alec Birchley, Alexander Adams, Amy Gaskin, Bree Gatica-Wilcox, Jason Coombes, Joel Southgate, Lauren Gilbert, Lee Graham, Nicole Pacchiarini, Sara Kumziene-Summerhayes, Sarah Taylor, Sophie Jones, Sara Rey, Matthew Bull, Joanne Watkins, Sally Corden, Tom Connor
EPI_ISL_1053844, EPI_ISL_1053845, EPI_ISL_1053846, EPI_ISL_1053847, EPI_ISL_1053848, EPI_ISL_1053849, EPI_ISL_1053850, EPI_ISL_1053853, EPI_ISL_1053854, EPI_ISL_1053855, EPI_ISL_1053892, EPI_ISL_1053898, EPI_ISL_1053899, EPI_ISL_1053900, EPI_ISL_1053901, EPI_ISL_1053902, EPI_ISL_1053933			
see above	Centre for Enzyme Innovation, University of Portsmouth / Translational Research Laboratory, Portsmouth Hospitals NHS Trust	COVID-19 Genomics UK (COG-UK) Consortium	Angela Beckett,Salman Goudarzi,Christopher Fearn,Kate Cook,Katie Loveson,Sharon Glaysher,Scott Elliott,Samuel Robson
EPI_ISL_1054396	Queens Medical Centre, Clinical Microbiology Department / DeepSeq Nottingham	COVID-19 Genomics UK (COG-UK) Consortium	Gemma Clark, Wendy Smith, Manjinder Khakh, Vicki M Fleming, Michelle M Lister, Hannah Howson-Wells, Jonathan Ball, Patrick McClure, Joseph Chappell, Theocharis Tsoleridis, Nadine Holmes, Matthew Carlisle, Christopher Moore, Fei Sang, Johnny Debebe, Victoria Wright, Matthew Loose
EPI_ISL_1054531	University of Exeter	COVID-19 Genomics UK (COG-UK) Consortium	Ben Temperton,Aaron Jeffries,Michelle Michelsen,Joanna Warwick-Dugdale,Audrey Farbos,Robyn Manley,Stephen Michell,Jane Masoli
EPI_ISL_1054815, EPI_ISL_1054830, EPI_ISL_1054834	Bioinformatics and Biostatistics Lab, Advanced Sequencing Facility	COVID-19 Genomics UK (COG-UK) Consortium	Aengus Stewart,Jerome Nicod,Chelsea Sawyer,Laura Cubitt,Harshil Patel,Margaret Crawford
EPI_ISL_1057726, EPI_ISL_1057727, EPI_ISL_1057728, EPI_ISL_1057729, EPI_ISL_1057730, EPI_ISL_1057731, EPI_ISL_1057733, EPI_ISL_1057734, EPI_ISL_1057735, EPI_ISL_1057736, EPI_ISL_1057737, EPI_ISL_1057738, EPI_ISL_1057739, EPI_ISL_1057740, EPI_ISL_1057741, EPI_ISL_1057742, EPI_ISL_1057743, EPI_ISL_1057744, EPI_ISL_1057746, EPI_ISL_1057747, EPI_ISL_1057748, EPI_ISL_1057749, EPI_ISL_1057750, EPI_ISL_1057751, EPI_ISL_1057752, EPI_ISL_1057753, EPI_ISL_1057754, EPI_ISL_1057755, EPI_ISL_1057756, EPI_ISL_1057757, EPI_ISL_1057758, EPI_ISL_1057759, EPI_ISL_1057760, EPI_ISL_1057761, EPI_ISL_1057762, EPI_ISL_1057763			
see above	Lighthouse Lab in Alderley Park	Wellcome Sanger Institute for the COVID-19 Genomics UK (COG-UK) Consortium	Jacquelyn Wynn, Mairead Hyland, The Lighthouse Lab in Alderley Park and Alex Alderton, Roberto Amato, Jeffrey Barrett, Sonia Goncalves, Ewan Harrison, David K. Jackson, Ian Johnston, Dominic Kwiatkowski, Cordelia Langford, John Sillitoe on behalf of the Wellcome Sanger Institute COVID-19 Surveillance Team
EPI_ISL_1103759	University of Exeter	COVID-19 Genomics UK (COG-UK) Consortium	Ben Temperton,Aaron Jeffries,Michelle Michelsen,Joanna Warwick-Dugdale,Audrey Farbos,Robyn Manley,Stephen Michell,Jane Masoli
EPI_ISL_1103936, EPI_ISL_1103937, EPI_ISL_1103938, EPI_ISL_1103939, EPI_ISL_1103940, EPI_ISL_1103941, EPI_ISL_1103942, EPI_ISL_1103943, EPI_ISL_1103944, EPI_ISL_1103946, EPI_ISL_1103947, EPI_ISL_1103948			
see above	Department of Pathology, University of Cambridge	COVID-19 Genomics UK (COG-UK) Consortium	Aminu S. Jahun, Yasmin Chaudhry, Iliana Georgana, Myra Hosmillo, Rhys Izuagbe, William L. Hamilton, Martin D. Curran, Surendra Parmar, Ian Goodfellow
EPI_ISL_1104225, EPI_ISL_1104226, EPI_ISL_1104227	Virology Department, Royal Infirmary of Edinburgh, NHS Lothian / School of Biological Sciences, University of Edinburgh	COVID-19 Genomics UK (COG-UK) Consortium	McHugh M, Dewar R, Cotton S, Rooke S, O'Toole Á, Scher E, Hill V, McCrone JT, Colquhoun R, Yu X, Jackson B, Rambaut A, Templeton K
EPI_ISL_1104322, EPI_ISL_1104323, EPI_ISL_1104387, EPI_ISL_1104498, EPI_ISL_1104504	Liverpool Clinical Laboratories	COVID-19 Genomics UK (COG-UK) Consortium	Sam Haldenby, Anita Lucaci, Steve Paterson, Julian Hiscox, Alistair Darby, M Almsaud, A Alrezaihi, Muhannad Alruwaili, Stuart D Armstrong, Jones Benjamin, Eleanor G Bentley, Anu Chawla, Jordan J Clark, Angela Cowell, Richard Eccles, Isabel García-Dorival, Matthew Gemmell, Alessandro Gerada, PKF Gilmore, Richard Gregory, Ximeng Han, Catherine Hartley, Margaret Hughes, Miren Iturriza-Gomara, James Johnson, L Luu, Jenifer Manson, Charlotte Nelson, Elaine O'Toole, Cassie Olateju, Rebekah Penrice-Randal , Lucille Rainbow, N.P Randle, Trevor Ian Robinson, Parul Sharma, Ghada T Shawli, James P Stewart, Neil Swainston, Ecaterina Vamos, Joanne Watts, Mark Whitehead
EPI_ISL_1104611, EPI_ISL_1104612, EPI_ISL_1104613, EPI_ISL_1104614, EPI_ISL_1104615, EPI_ISL_1104616, EPI_ISL_1104617, EPI_ISL_1104618, EPI_ISL_1104619	University College London, Great Ormond Street Hospital for Children NHS Foundation Trust, Imperial College Healthcare NHS Trust	COVID-19 Genomics UK (COG-UK) Consortium	Sergi Castellano, Rachel Williams, Mark Kristiansen, Paola Resende Silva, Sunando Roy, Tony Brooks, Helena Tutill, Paola Niola, Patricia Dyal, Charlotte Williams, Leysa Forrest, Yasmin Panchbhaya, Jacqueline Findlay, Samuel Weeks, Julianne Brown, Kathryn Harris, Paul Randell, James Price, Alison Holmes, Judith Breuer
EPI_ISL_1104771, EPI_ISL_1104776, EPI_ISL_1104777, EPI_ISL_1104779, EPI_ISL_1104780, EPI_ISL_1104786, EPI_ISL_1104805, EPI_ISL_1104807, EPI_ISL_1104808, EPI_ISL_1104813, EPI_ISL_1104815, EPI_ISL_1104816, EPI_ISL_1104817, EPI_ISL_1104821, EPI_ISL_1104823, EPI_ISL_1104829, EPI_ISL_1104833, EPI_ISL_1104835, EPI_ISL_1104837, EPI_ISL_1104839, EPI_ISL_1104840, EPI_ISL_1104845, EPI_ISL_1104850, EPI_ISL_1104851, EPI_ISL_1104852, EPI_ISL_1104853, EPI_ISL_1104858, EPI_ISL_1104860, EPI_ISL_1104861, EPI_ISL_1104862, EPI_ISL_1104865, EPI_ISL_1104866, EPI_ISL_1104870, EPI_ISL_1104871, EPI_ISL_1105163, EPI_ISL_1105201, EPI_ISL_1105220, EPI_ISL_1105332, EPI_ISL_1105342, EPI_ISL_1105346, EPI_ISL_1105347			
see above	University College London Hospital	COVID-19 Genomics UK (COG-UK) Consortium	Judith Heaney, Matthew Byott, Catherine Houlihan, Dan Frampton, Stuart Kirk, Moira Spyer and Eleni Nastouli
EPI_ISL_1105463, EPI_ISL_1105465	University College London, Great Ormond Street Hospital for Children NHS Foundation Trust, Imperial College Healthcare NHS Trust	COVID-19 Genomics UK (COG-UK) Consortium	Sergi Castellano, Rachel Williams, Mark Kristiansen, Paola Resende Silva, Sunando Roy, Tony Brooks, Helena Tutill, Paola Niola, Patricia Dyal, Charlotte Williams, Leysa Forrest, Yasmin Panchbhaya, Jacqueline Findlay, Samuel Weeks, Julianne Brown, Kathryn Harris, Paul Randell, James Price, Alison Holmes, Judith Breuer

EPI_ISL_1105851, EPI_ISL_1105852, EPI_ISL_1105853, EPI_ISL_1105854, EPI_ISL_1105855, EPI_ISL_1105856, EPI_ISL_1105857, EPI_ISL_1105858, EPI_ISL_1105859, EPI_ISL_1105860	Lincolnshire Hospitals and DeepSeq Nottingham	COVID-19 Genomics UK (COG-UK) Consortium	Nichola Duckworth, Tim Sloan, Sarah Walsh, Jonathan Ball, Patrick McClure, Joeseph Chappell, Nadine Holmes, Matthew Carlisle, Christopher Moore, Fei Sang, Johnny Debebe, Victoria Wright, Matthew Loose
EPI_ISL_1106645	Originating lab: Wales Specialist Virology Centre Sequencing lab: Pathogen Genomics Unit	Public Health Wales Microbiology Cardiff Wales Specialist Virology Centre	Catherine Moore, Johnathan Evans, Laura Gifford, Malorie Perry, Simon Cottrell, Angela Marchbank, Alec Birchley, Alexander Adams, Amy Gaskin, Bree Gatica-Wilcox, Jason Coombes, Joel Southgate, Lauren Gilbert, Lee Graham, Nicole Pacchiarini, Sara Kumziene-Summerhayes, Sarah Taylor, Sophie Jones, Sara Rey, Matthew Bull, Joanne Watkins, Sally Corden, Tom Connor
EPI_ISL_1107634, EPI_ISL_1107635, EPI_ISL_1107636, EPI_ISL_1107650, EPI_ISL_1107651, EPI_ISL_1107652, EPI_ISL_1107653, EPI_ISL_1107654	Centre for Enzyme Innovation, University of Portsmouth / Translational Research Laboratory, Portsmouth Hospitals NHS Trust	COVID-19 Genomics UK (COG-UK) Consortium	Angela Beckett,Salman Goudarzi,Christopher Fearn,Kate Cook,Katie Loveson,Sharon Glaysher,Scott Elliott,Samuel Robson
EPI_ISL_1108970, EPI_ISL_1108983, EPI_ISL_1109013, EPI_ISL_1109024, EPI_ISL_1109025, EPI_ISL_1109027, EPI_ISL_1109029, EPI_ISL_1109031, EPI_ISL_1109033, EPI_ISL_1109034, EPI_ISL_1109038, EPI_ISL_1109039, EPI_ISL_1109040, EPI_ISL_1109041			
see above	Queens Medical Centre, Clinical Microbiology Department / DeepSeq Nottingham	COVID-19 Genomics UK (COG-UK) Consortium	Gemma Clark, Wendy Smith, Manjinder Khakh, Vicki M Fleming, Michelle M Lister, Hannah Howson-Wells, Jonathan Ball, Patrick McClure, Joseph Chappell, Theocharis Tsoleridis, Nadine Holmes, Matthew Carlisle, Christopher Moore, Fei Sang, Johnny Debebe, Victoria Wright, Matthew Loose
EPI_ISL_1177707, EPI_ISL_1177708, EPI_ISL_1177729, EPI_ISL_1177731, EPI_ISL_1177733	Virology Department, Royal Infirmary of Edinburgh, NHS Lothian / School of Biological Sciences, University of Edinburgh	COVID-19 Genomics UK (COG-UK) Consortium	McHugh M, Dewar R, Cotton S, Rooke S, O'Toole Á, Scher E, Hill V, McCrone JT, Colquhoun R, Yu X, Jackson B, Rambaut A, Templeton K
EPI_ISL_1177890	Liverpool Clinical Laboratories	COVID-19 Genomics UK (COG-UK) Consortium	Sam Haldenby, Alistair Darby, Steve Paterson, Anita Lucaci, Julian Hiscox, M Almsaud, A Alrezaihi, Muhannad Alruwaili, Stuart D Armstrong, Jones Benjamin, Eleanor G Bentley, Anu Chawla, Jordan J Clark, Angela Cowell, Richard Eccles, Isabel García-Dorival, Matthew Gemmell, Alessandro Gerada, PKF Gilmore, Richard Gregory, Ximeng Han, Catherine Hartley, Margaret Hughes, Miren Iturriza-Gomara, James Johnson, L Luu, Jenifer Manson, Charlotte Nelson, Elaine O'Toole, Cassie Olateju, Rebekah Penrice-Randal , Lucille Rainbow, N.P Randle, Trevor Ian Robinson, Parul Sharma, Ghada T Shawli, James P Stewart, Neil Swainston, Ecaterina Vamos, Joanne Watts, Mark Whitehead, Hermione Webster
EPI_ISL_1178143, EPI_ISL_1178144, EPI_ISL_1178145, EPI_ISL_1178146, EPI_ISL_1178147, EPI_ISL_1178193, EPI_ISL_1178194, EPI_ISL_1178195, EPI_ISL_1178196, EPI_ISL_1178197, EPI_ISL_1178198, EPI_ISL_1178199, EPI_ISL_1178200, EPI_ISL_1178201, EPI_ISL_1178202, EPI_ISL_1178203, EPI_ISL_1178204, EPI_ISL_1178205, EPI_ISL_1178207, EPI_ISL_1178208, EPI_ISL_1178209, EPI_ISL_1178210, EPI_ISL_1178211, EPI_ISL_1178212, EPI_ISL_1178213, EPI_ISL_1178214, EPI_ISL_1178215, EPI_ISL_1178216, EPI_ISL_1178217, EPI_ISL_1178218, EPI_ISL_1178219, EPI_ISL_1178220, EPI_ISL_1178221, EPI_ISL_1178222			
see above	Northumbria University / South Tees Hospitals NHS Foundation Trust / North Cumbria Integrated Care NHS Foundation Trust / North Tees and Hartlepool NHS Foundation Trust / Newcastle Hospitals NHS Foundation Trust	COVID-19 Genomics UK (COG-UK) Consortium	Darren L Smith,Andrew Nelson,Matthew Bashton,Greg R Young,Joshua Loh,John Allan,Mohammad A Tariq,Giles S Holt,Gary Black,Wen C Yew,Lynn Dover,Paul Baker,Steve Liggett,Sarah Essex,Jane Greenaway,Debra Padgett,Clive Graham,Garren Scott,Edward Barton,Emma Swindells,Brendan Payne,Jennifer Collins,Yusri Taha,Gary Eltringham
EPI_ISL_1178435, EPI_ISL_1178438, EPI_ISL_1178439, EPI_ISL_1178441, EPI_ISL_1178445, EPI_ISL_1178447, EPI_ISL_1178449, EPI_ISL_1178452, EPI_ISL_1178453, EPI_ISL_1178455, EPI_ISL_1178457, EPI_ISL_1178459, EPI_ISL_1178463, EPI_ISL_1178472, EPI_ISL_1178474, EPI_ISL_1178477, EPI_ISL_1178478, EPI_ISL_1178480, EPI_ISL_1178482, EPI_ISL_1178483, EPI_ISL_1178487, EPI_ISL_1178493, EPI_ISL_1178495, EPI_ISL_1178507			
see above	Quadram Institute Bioscience	COVID-19 Genomics UK (COG-UK) Consortium	Dave J. Baker, Gemma L. Kay, Alp Aydin, Thanh Le-Viet, Steven Rudder, Ana P. Tedim, Anastasia Kolyva, Maria Diaz, Leonardo de Oliveira Martins, Nabil-Fareed Alikhan, Lizzie Meadows, Rachael Stanley, Ngozi Elumogo, Muhammed Yasir, Nicholas M. Thomson, Alexander J Trotter, Rachel Gilroy, Samuel Bloomfield, Claire Stuart, Andrew Bell, Reenesh Prakash, Samir Dervisevic, Alison E. Mather, John Wain, Mark Webber, Andrew J. Page, Justin O'Grady
EPI_ISL_1178826	Oxford Viromics, NDM, University of Oxford; Oxford University Hospitals; Basingstoke and North Hampshire Hospital	COVID-19 Genomics UK (COG-UK) Consortium	Tanya Golubchik, David Bonsall, George Macintyre, Amy Trebes, Mariateresa de Cesare, Catrin Moore, Alex Mobbs, Anita Justice, Robert Shaw, Monique Andersson, Timothy Peto, Emma Wise, Nathan Moore, Jessica Lynch, Nick Cortes, Matilde Mori, Stephen Kidd, David Buck, John Todd, Christophe Fraser
EPI_ISL_1179155	Originating lab: Wales Specialist Virology Centre Sequencing lab: Pathogen Genomics Unit	Public Health Wales Microbiology Cardiff Wales Specialist Virology Centre	Catherine Moore, Johnathan Evans, Laura Gifford, Malorie Perry, Simon Cottrell, Angela Marchbank, Alec Birchley, Alexander Adams, Amy Gaskin, Bree Gatica-Wilcox, Jason Coombes, Joel Southgate, Lauren Gilbert, Lee Graham, Nicole Pacchiarini, Sara Kumziene-Summerhayes, Sarah Taylor, Sophie Jones, Sara Rey, Matthew Bull, Joanne Watkins, Sally Corden, Tom Connor
EPI_ISL_1179831, EPI_ISL_1179833	Centre for Enzyme Innovation, University of Portsmouth / Translational Research Laboratory, Portsmouth Hospitals NHS Trust	COVID-19 Genomics UK (COG-UK) Consortium	Angela Beckett,Salman Goudarzi,Christopher Fearn,Kate Cook,Katie Loveson,Sharon Glaysher,Scott Elliott,Samuel Robson
EPI_ISL_1179911, EPI_ISL_1179933, EPI_ISL_1179935, EPI_ISL_1179936, EPI_ISL_1179951, EPI_ISL_1179952, EPI_ISL_1179953, EPI_ISL_1179954, EPI_ISL_1179955, EPI_ISL_1179956, EPI_ISL_1179957, EPI_ISL_1179958, EPI_ISL_1179959, EPI_ISL_1179960, EPI_ISL_1179961, EPI_ISL_1179962, EPI_ISL_1179963, EPI_ISL_1179975, EPI_ISL_1179976, EPI_ISL_1179977, EPI_ISL_1179978, EPI_ISL_1179983, EPI_ISL_1179990, EPI_ISL_1179991, EPI_ISL_1179992, EPI_ISL_1179993, EPI_ISL_1179994, EPI_ISL_1179995, EPI_ISL_1179996, EPI_ISL_1180017, EPI_ISL_1180023, EPI_ISL_1180024, EPI_ISL_1180025, EPI_ISL_1180026, EPI_ISL_1180027, EPI_ISL_1180028			
see above	Department of Pathology, University of Cambridge	COVID-19 Genomics UK (COG-UK) Consortium	Aminu S. Jahun, Yasmin Chaudhry, Iliana Georgana, Myra Hosmillo, Rhys Izuagbe, William L. Hamilton, Martin D. Curran, Surendra Parmar, Ian Goodfellow
EPI_ISL_1180083, EPI_ISL_1180087	Virology Department, Sheffield Teaching Hospitals NHS Foundation Trust/Department of Infection, Immunity and Cardiovascular Disease, The Medical School, University of Sheffield	COVID-19 Genomics UK (COG-UK) Consortium	Thushan de Silva, Matthew Parker, Nikki Smith, Adri Angyal, Rebecca Brown, Luke Green, Rachel Tucker, Paul Parsons, Danielle Groves, Katie Johnson, Laura Carrilero, Alex Keeley, Dave Partridge, Matthew Wyles, Benjamin Lindsey, Mehmet Yavuz, Mohammad Raza, Cariad Evans
EPI_ISL_1187053, EPI_ISL_1187064, EPI_ISL_1187068, EPI_ISL_1187069, EPI_ISL_1187073, EPI_ISL_1187103, EPI_ISL_1187113, EPI_ISL_1187114, EPI_ISL_1187130, EPI_ISL_1187136, EPI_ISL_1187140, EPI_ISL_1187146, EPI_ISL_1187165, EPI_ISL_1187171, EPI_ISL_1187176, EPI_ISL_1187177, EPI_ISL_1187192, EPI_ISL_1187193, EPI_ISL_1187204, EPI_ISL_1187218, EPI_ISL_1187219, EPI_ISL_1187221, EPI_ISL_1187225, EPI_ISL_1187226, EPI_ISL_1187237, EPI_ISL_1187240, EPI_ISL_1187245, EPI_ISL_1187253, EPI_ISL_1206041, EPI_ISL_1206042, EPI_ISL_1206043, EPI_ISL_1206044, EPI_ISL_1206046, EPI_ISL_1206048, EPI_ISL_1206049, EPI_ISL_1206052, EPI_ISL_1206053, EPI_ISL_1206055, EPI_ISL_1206057, EPI_ISL_1206059, EPI_ISL_1206060, EPI_ISL_1206061, EPI_ISL_1206062, EPI_ISL_1206063, EPI_ISL_1206064, EPI_ISL_1206069, EPI_ISL_1206070, EPI_ISL_1206071, EPI_ISL_1206072, EPI_ISL_1206074, EPI_ISL_1206083, EPI_ISL_1206085, EPI_ISL_1206086, EPI_ISL_1206088, EPI_ISL_1206089, EPI_ISL_1206094, EPI_ISL_1206095, EPI_ISL_1206098, EPI_ISL_1206099, EPI_ISL_1206101, EPI_ISL_1206102, EPI_ISL_1206103, EPI_ISL_1206105, EPI_ISL_1206106, EPI_ISL_1206107, EPI_ISL_1206116, EPI_ISL_1206119, EPI_ISL_1206120, EPI_ISL_1206121, EPI_ISL_1206122, EPI_ISL_1206126, EPI_ISL_1206129, EPI_ISL_1206131, EPI_ISL_1206135, EPI_ISL_1206138, EPI_ISL_1206140, EPI_ISL_1206141, EPI_ISL_1206142, EPI_ISL_1206144, EPI_ISL_1206146, EPI_ISL_1206147, EPI_ISL_1206149, EPI_ISL_1206154, EPI_ISL_1206155, EPI_ISL_1206158, EPI_ISL_1206160, EPI_ISL_1206162, EPI_ISL_1206163, EPI_ISL_1206165, EPI_ISL_1206168, EPI_ISL_1206172, EPI_ISL_1206173, EPI_ISL_1206178, EPI_ISL_1206180, EPI_ISL_1206182, EPI_ISL_1206183, EPI_ISL_1206186, EPI_ISL_1206187, EPI_ISL_1206191, EPI_ISL_1206192, EPI_ISL_1206196, EPI_ISL_1206198, EPI_ISL_1206200, EPI_ISL_1206203, EPI_ISL_1206207, EPI_ISL_1206213, EPI_ISL_1206217, EPI_ISL_1206221, EPI_ISL_1206222, EPI_ISL_1206226, EPI_ISL_1206230, EPI_ISL_1206233, EPI_ISL_1206237, EPI_ISL_1206244, EPI_ISL_1206245, EPI_ISL_1206248, EPI_ISL_1206251, EPI_ISL_1206258, EPI_ISL_1206262, EPI_ISL_1206263, EPI_ISL_1206266, EPI_ISL_1206268, EPI_ISL_1206272, EPI_ISL_1206275, EPI_ISL_1206279, EPI_ISL_1206280, EPI_ISL_1206284, EPI_ISL_1206291, EPI_ISL_1223161, EPI_ISL_1223164, EPI_ISL_1223174, EPI_ISL_1223224, EPI_ISL_1223240, EPI_ISL_1223245, EPI_ISL_1223288, EPI_ISL_1223291, EPI_ISL_1223383, EPI_ISL_1223391, EPI_ISL_1223392, EPI_ISL_1223401, EPI_ISL_1223404, EPI_ISL_1223411, EPI_ISL_1223424, EPI_ISL_1223426, EPI_ISL_1223435, EPI_ISL_1242052, EPI_ISL_1242057, EPI_ISL_1242066, EPI_ISL_1242096, EPI_ISL_1242124, EPI_ISL_1242132, EPI_ISL_1242136, EPI_ISL_1242145, EPI_ISL_1242149, EPI_ISL_1242162, EPI_ISL_1242179, EPI_ISL_1242187, EPI_ISL_1242188, EPI_ISL_1242211, EPI_ISL_1242225, EPI_ISL_1242227, EPI_ISL_1242280			
see above	Lighthouse Lab in Cambridge	Wellcome Sanger Institute for the COVID-19 Genomics UK (COG-UK) Consortium	Rob Howes, The Lighthouse Lab in Cambridge and Alex Alderton, Roberto Amato, Jeffrey Barrett, Sonia Goncalves, Ewan Harrison, David K. Jackson, Ian Johnston, Dominic Kwiatkowski, Cordelia Langford, John Sillitoe on behalf of the Wellcome Sanger Institute COVID-19 Surveillance Team
EPI_ISL_1247689, EPI_ISL_1247690, EPI_ISL_1247693, EPI_ISL_1247729	Virology Department, Royal Infirmary of Edinburgh, NHS Lothian / School of Biological Sciences, University of Edinburgh	COVID-19 Genomics UK (COG-UK) Consortium	McHugh M, Dewar R, Cotton S, Rooke S, O'Toole Á, Scher E, Hill V, McCrone JT, Colquhoun R, Yu X, Jackson B, Rambaut A, Templeton K
EPI_ISL_1247819, EPI_ISL_1247820, EPI_ISL_1247821, EPI_ISL_1247822	Liverpool Clinical Laboratories	COVID-19 Genomics UK (COG-UK) Consortium	Sam Haldenby, Alistair Darby, Steve Paterson, Anita Lucaci, Julian Hiscox, M Almsaud, A Alrezaihi, Muhannad Alruwaili, Stuart D Armstrong, Jones Benjamin, Eleanor G Bentley, Anu Chawla, Jordan J Clark, Angela Cowell, Richard Eccles, Isabel García-Dorival, Matthew Gemmell, Alessandro Gerada, PKF Gilmore, Richard Gregory, Ximeng Han, Catherine Hartley, Margaret Hughes, Miren Iturriza-Gomara, James Johnson, L Luu, Jenifer Manson, Charlotte Nelson, Elaine O'Toole, Cassie Olateju, Rebekah Penrice-Randal , Lucille Rainbow, N.P Randle, Trevor Ian Robinson, Parul Sharma, Ghada T Shawli, James P Stewart, Neil Swainston, Ecaterina Vamos, Joanne Watts, Mark Whitehead, Hermione Webster
EPI_ISL_1248167, EPI_ISL_1248188, EPI_ISL_1248207, EPI_ISL_1248208, EPI_ISL_1248209, EPI_ISL_1248210, EPI_ISL_1248211, EPI_ISL_1248212, EPI_ISL_1248213	University College London, Great Ormond Street Hospital for Children NHS Foundation Trust, Imperial College Healthcare NHS Trust	COVID-19 Genomics UK (COG-UK) Consortium	Sergi Castellano, Rachel Williams, Mark Kristiansen, Paola Resende Silva, Sunando Roy, Tony Brooks, Helena Tutill, Paola Niola, Patricia Dyal, Charlotte Williams, Leysa Forrest, Yasmin Panchbhaya, Jacqueline Findlay, Samuel Weeks, Julianne Brown, Kathryn Harris, Paul Randell, James Price, Alison Holmes, Judith Breuer

EPI_ISL_1248939, EPI_ISL_1248940, EPI_ISL_1248941, EPI_ISL_1248942, EPI_ISL_1248943, EPI_ISL_1248944, EPI_ISL_1248946, EPI_ISL_1248948, EPI_ISL_1248952, EPI_ISL_1248954, EPI_ISL_1248957, EPI_ISL_1248960, EPI_ISL_1248974, EPI_ISL_1249038, EPI_ISL_1249039, EPI_ISL_1249042			
see above	Quadram Institute Bioscience	COVID-19 Genomics UK (COG-UK) Consortium	Dave J. Baker, Gemma L. Kay, Alp Aydin, Thanh Le-Viet, Steven Rudder, Ana P. Tedim, Anastasia Kolyva, Maria Diaz, Leonardo de Oliveira Martins, Nabil-Fareed Alikhan, Lizzie Meadows, Rachael Stanley, Ngozi Elumogo, Muhammed Yasir, Nicholas M. Thomson, Alexander J Trotter, Rachel Gilroy, Samuel Bloomfield, Claire Stuart, Andrew Bell, Reenesh Prakash, Samir Dervisevic, Alison E. Mather, John Wain, Mark Webber, Andrew J. Page, Justin O'Grady
EPI_ISL_1249220, EPI_ISL_1249221, EPI_ISL_1249222, EPI_ISL_1249223	Oxford Viromics, NDM, University of Oxford; Oxford University Hospitals; Basingstoke and North Hampshire Hospital	COVID-19 Genomics UK (COG-UK) Consortium	Tanya Golubchik, David Bonsall, George Macintyre, Amy Trebes, Mariateresa de Cesare, Catrin Moore, Alex Mobbs, Anita Justice, Robert Shaw, Monique Andersson, Timothy Peto, Emma Wise, Nathan Moore, Jessica Lynch, Nick Cortes, Matilde Mori, Stephen Kidd, David Buck, John Todd, Christophe Fraser
EPI_ISL_1296578, EPI_ISL_1296581, EPI_ISL_1296582, EPI_ISL_1296585, EPI_ISL_1296586, EPI_ISL_1296588	Respiratory Virus Unit, National Infection Service, Public Health England	COVID-19 Genomics UK (COG-UK) Consortium	PHE Covid Sequencing Team
EPI_ISL_1308546, EPI_ISL_1308557, EPI_ISL_1308558, EPI_ISL_1308560, EPI_ISL_1308571, EPI_ISL_1308575, EPI_ISL_1308576, EPI_ISL_1308577, EPI_ISL_1308639	University of Exeter	COVID-19 Genomics UK (COG-UK) Consortium	Ben Temperton,Aaron Jeffries,Michelle Michelsen,Joanna Warwick-Dugdale,Audrey Farbos,Robyn Manley,Stephen Michell,Jane Masoli
EPI_ISL_1308819, EPI_ISL_1308820, EPI_ISL_1308824, EPI_ISL_1308850, EPI_ISL_1308854, EPI_ISL_1308855	Virology Department, Royal Infirmary of Edinburgh, NHS Lothian / School of Biological Sciences, University of Edinburgh	COVID-19 Genomics UK (COG-UK) Consortium	McHugh M, Dewar R, Cotton S, Rooke S, O'Toole Á, Scher E, Hill V, McCrone JT, Colquhoun R, Yu X, Jackson B, Rambaut A, Templeton K
EPI_ISL_1309055, EPI_ISL_1309063, EPI_ISL_1309066, EPI_ISL_1309110	University College London, Great Ormond Street Hospital for Children NHS Foundation Trust, Imperial College Healthcare NHS Trust	COVID-19 Genomics UK (COG-UK) Consortium	Sergi Castellano, Rachel Williams, Mark Kristiansen, Paola Resende Silva, Sunando Roy, Tony Brooks, Helena Tutill, Paola Niola, Patricia Dyal, Charlotte Williams, Leysa Forrest, Yasmin Panchbhaya, Jacqueline Findlay, Samuel Weeks, Julianne Brown, Kathryn Harris, Paul Randell, James Price, Alison Holmes, Judith Breuer
EPI_ISL_1309668, EPI_ISL_1309669, EPI_ISL_1309673	Quadram Institute Bioscience	COVID-19 Genomics UK (COG-UK) Consortium	Dave J. Baker, Gemma L. Kay, Alp Aydin, Thanh Le-Viet, Steven Rudder, Ana P. Tedim, Anastasia Kolyva, Maria Diaz, Leonardo de Oliveira Martins, Nabil-Fareed Alikhan, Lizzie Meadows, Rachael Stanley, Ngozi Elumogo, Muhammed Yasir, Nicholas M. Thomson, Alexander J Trotter, Rachel Gilroy, Samuel Bloomfield, Claire Stuart, Andrew Bell, Reenesh Prakash, Samir Dervisevic, Alison E. Mather, John Wain, Mark Webber, Andrew J. Page, Justin O'Grady
EPI_ISL_1310349	Centre for Enzyme Innovation, University of Portsmouth / Translational Research Laboratory, Portsmouth Hospitals NHS Trust	COVID-19 Genomics UK (COG-UK) Consortium	Angela Beckett,Salman Goudarzi,Christopher Fearn,Kate Cook,Katie Loveson,Sharon Glaysher,Scott Elliott,Samuel Robson
EPI_ISL_1333481, EPI_ISL_1333488, EPI_ISL_1333490, EPI_ISL_1333491, EPI_ISL_1333492, EPI_ISL_1333500, EPI_ISL_1333502, EPI_ISL_1333504, EPI_ISL_1333505, EPI_ISL_1333507, EPI_ISL_1333508, EPI_ISL_1333834, EPI_ISL_1333835, EPI_ISL_1333836, EPI_ISL_1333839, EPI_ISL_1333935, EPI_ISL_1333941			
see above	Lighthouse Lab in Cambridge	Wellcome Sanger Institute for the COVID-19 Genomics UK (COG-UK) Consortium	Rob Howes, The Lighthouse Lab in Cambridge and Alex Alderton, Roberto Amato, Jeffrey Barrett, Sonia Goncalves, Ewan Harrison, David K. Jackson, Ian Johnston, Dominic Kwiatkowski, Cordelia Langford, John Sillitoe on behalf of the Wellcome Sanger Institute COVID-19 Surveillance Team
EPI_ISL_1386883	University College London, Great Ormond Street Hospital for Children NHS Foundation Trust, Imperial College Healthcare NHS Trust	COVID-19 Genomics UK (COG-UK) Consortium	Sergi Castellano, Rachel Williams, Mark Kristiansen, Paola Resende Silva, Sunando Roy, Tony Brooks, Helena Tutill, Paola Niola, Patricia Dyal, Charlotte Williams, Leysa Forrest, Yasmin Panchbhaya, Jacqueline Findlay, Samuel Weeks, Julianne Brown, Kathryn Harris, Paul Randell, James Price, Alison Holmes, Judith Breuer
EPI_ISL_1387166	Queens Medical Centre, Clinical Microbiology Department / DeepSeq Nottingham	COVID-19 Genomics UK (COG-UK) Consortium	Gemma Clark, Wendy Smith, Manjinder Khakh, Vicki M Fleming, Michelle M Lister, Hannah Howson-Wells, Jonathan Ball, Timothy Byaruhanga, Jayasree Dey, Emily Park, Jack Hill, Patrick McClure, Joseph Chappell, Theocharis Tsoleridis, Nadine Holmes, Matthew Carlisle, Christopher Moore, Fei Sang, Johnny Debebe, Victoria Wright, Matthew Loose
EPI_ISL_1387219	Oxford Viromics, NDM, University of Oxford; Oxford University Hospitals; Basingstoke and North Hampshire Hospital	COVID-19 Genomics UK (COG-UK) Consortium	Tanya Golubchik, David Bonsall, George Macintyre, Amy Trebes, Mariateresa de Cesare, Catrin Moore, Alex Mobbs, Anita Justice, Robert Shaw, Monique Andersson, Timothy Peto, Emma Wise, Nathan Moore, Jessica Lynch, Nick Cortes, Matilde Mori, Stephen Kidd, David Buck, John Todd, Christophe Fraser
EPI_ISL_1391414	Barts Health NHS Trust	Barts Health NHS Trust	KARAA, Esin; CUTINO-MOGUEL, Maria-Teresa; HARRINGTON, David; OWOYEMI, Dola; KULASEGARAN-SHYLINI, Raghavendran; BROAD, Claire; KELE, Beatrix
EPI_ISL_1474666	Liverpool Clinical Laboratories	COVID-19 Genomics UK (COG-UK) Consortium	Sam Haldenby, Alistair Darby, Steve Paterson, Anita Lucaci, Julian Hiscox, M Almsaud, A Alrezaihi, Muhannad Alruwaili, Stuart D Armstrong, Jones Benjamin, Eleanor G Bentley, Anu Chawla, Jordan J Clark, Angela Cowell, Richard Eccles, Isabel García-Dorival, Matthew Gemmell, Alessandro Gerada, PKF Gilmore, Richard Gregory, Ximeng Han, Catherine Hartley, Margaret Hughes, Miren Iturriza-Gomara, James Johnson, L Luu, Jenifer Manson, Charlotte Nelson, Elaine O'Toole, Cassie Olateju, Rebekah Penrice-Randal , Lucille Rainbow, N.P Randle, Trevor Ian Robinson, Parul Sharma, Ghada T Shawli, James P Stewart, Neil Swainston, Ecaterina Vamos, Joanne Watts, Mark Whitehead, Hermione Webster
EPI_ISL_1474838	University College London, Great Ormond Street Hospital for Children NHS Foundation Trust, Imperial College Healthcare NHS Trust	COVID-19 Genomics UK (COG-UK) Consortium	Sergi Castellano, Rachel Williams, Mark Kristiansen, Paola Resende Silva, Sunando Roy, Tony Brooks, Helena Tutill, Paola Niola, Patricia Dyal, Charlotte Williams, Leysa Forrest, Yasmin Panchbhaya, Jacqueline Findlay, Samuel Weeks, Julianne Brown, Kathryn Harris, Paul Randell, James Price, Alison Holmes, Judith Breuer
EPI_ISL_1475010, EPI_ISL_1475011, EPI_ISL_1475012, EPI_ISL_1475013, EPI_ISL_1475014, EPI_ISL_1475015, EPI_ISL_1475016, EPI_ISL_1475040, EPI_ISL_1475042, EPI_ISL_1475045, EPI_ISL_1475046, EPI_ISL_1475049, EPI_ISL_1475050, EPI_ISL_1475057, EPI_ISL_1475058, EPI_ISL_1475059, EPI_ISL_1475060, EPI_ISL_1475119, EPI_ISL_1475120, EPI_ISL_1475124, EPI_ISL_1475125, EPI_ISL_1475127, EPI_ISL_1475131			
see above	Regional Virus Laboratory, Belfast Health and Social Care Trust	COVID-19 Genomics UK (COG-UK) Consortium	Conall McCaughey, James McKenna, Tanya Curran, Susan Feeney, Alison Watt, Ciara Cox, Mairead Connor, Zoltan Molnar, David Simpson, Derek Fairley
EPI_ISL_1476455, EPI_ISL_1476484, EPI_ISL_1476486, EPI_ISL_1476488, EPI_ISL_1476499	Originating lab: Wales Specialist Virology Centre Sequencing lab: Pathogen Genomics Unit	Public Health Wales Microbiology Cardiff Wales Specialist Virology Centre	Catherine Moore, Johnathan Evans, Laura Gifford, Malorie Perry, Simon Cottrell, Angela Marchbank, Alec Birchley, Alexander Adams, Amy Gaskin, Bree Gatica-Wilcox, Jason Coombes, Joel Southgate, Lauren Gilbert, Lee Graham, Nicole Pacchiarini, Sara Kumziene-Summerhayes, Sarah Taylor, Sophie Jones, Sara Rey, Matthew Bull, Joanne Watkins, Sally Corden, Tom Connor
EPI_ISL_919149, EPI_ISL_919150, EPI_ISL_919151, EPI_ISL_919152, EPI_ISL_919153, EPI_ISL_919154, EPI_ISL_919155, EPI_ISL_919156, EPI_ISL_919157, EPI_ISL_919158, EPI_ISL_919159, EPI_ISL_919160, EPI_ISL_919161, EPI_ISL_919162, EPI_ISL_919163, EPI_ISL_919164, EPI_ISL_919165, EPI_ISL_919166, EPI_ISL_919167, EPI_ISL_919168, EPI_ISL_919169, EPI_ISL_919170, EPI_ISL_919171, EPI_ISL_919172, EPI_ISL_919173, EPI_ISL_919174, EPI_ISL_919175, EPI_ISL_919176, EPI_ISL_919177, EPI_ISL_919179, EPI_ISL_919180, EPI_ISL_919181, EPI_ISL_919182, EPI_ISL_919183, EPI_ISL_919184, EPI_ISL_919185, EPI_ISL_919186, EPI_ISL_919187, EPI_ISL_919188, EPI_ISL_919189			
see above	Department of Pathology, University of Cambridge	COVID-19 Genomics UK (COG-UK) Consortium	Aminu S. Jahun, Yasmin Chaudhry, Iliana Georgana, Myra Hosmillo, Rhys Izu, Martin D. Curran, Surendra Parmar, Ian Goodfellow
EPI_ISL_919405	Virology Department, Royal Infirmary of Edinburgh, NHS Lothian / School of Biological Sciences, University of Edinburgh / Institute of Genetics and Molecular Medicine, University of Edinburgh	COVID-19 Genomics UK (COG-UK) Consortium	McHugh M, Dewar R, Rooke S, Gallagher M, Balcaza C, O'Toole Á, Scher E, Hill V, McCrone JT, Colquhoun R, Yu X, Jackson B, Rambaut A, Williams TC, Templeton K
EPI_ISL_919629, EPI_ISL_919630, EPI_ISL_919663, EPI_ISL_919706, EPI_ISL_919707, EPI_ISL_919708, EPI_ISL_919709, EPI_ISL_919710, EPI_ISL_919712, EPI_ISL_919713, EPI_ISL_919714, EPI_ISL_919715, EPI_ISL_919716, EPI_ISL_919717, EPI_ISL_919718, EPI_ISL_919719, EPI_ISL_919720, EPI_ISL_919721, EPI_ISL_919722, EPI_ISL_919723, EPI_ISL_919724			
see above	Liverpool Clinical Laboratories	COVID-19 Genomics UK (COG-UK) Consortium	Sam Haldenby, Anita Lucaci, Steve Paterson, Julian Hiscox, Alistair Darby, M Almsaud, A Alrezaihi, Muhannad Alruwaili, Stuart D Armstrong, Jones Benjamin, Eleanor G Bentley, Anu Chawla, Jordan J Clark, Angela Cowell, Richard Eccles, Isabel García-Dorival, Matthew Gemmell, Alessandro Gerada, PKF Gilmore, Richard Gregory, Ximeng Han, Catherine Hartley, Margaret Hughes, Miren Iturriza-Gomara, James Johnson, L Luu, Jenifer Manson, Charlotte Nelson, Elaine O'Toole, Cassie Olateju, Rebekah Penrice-Randal , Lucille Rainbow, N.P Randle, Trevor Ian Robinson, Parul Sharma, Ghada T Shawli, James P Stewart, Neil Swainston, Ecaterina Vamos, Joanne Watts, Mark Whitehead
EPI_ISL_919871, EPI_ISL_919872, EPI_ISL_919873, EPI_ISL_919881, EPI_ISL_919903, EPI_ISL_919904, EPI_ISL_919905, EPI_ISL_919906, EPI_ISL_919907, EPI_ISL_919908, EPI_ISL_919909, EPI_ISL_919910, EPI_ISL_919911, EPI_ISL_919912, EPI_ISL_919913, EPI_ISL_919914, EPI_ISL_919915, EPI_ISL_919916, EPI_ISL_919917, EPI_ISL_919918, EPI_ISL_919919, EPI_ISL_919927, EPI_ISL_919928, EPI_ISL_919929, EPI_ISL_919930, EPI_ISL_919931, EPI_ISL_919932, EPI_ISL_919934, EPI_ISL_919935, EPI_ISL_919936, EPI_ISL_919937, EPI_ISL_919938, EPI_ISL_919939, EPI_ISL_919940, EPI_ISL_919941, EPI_ISL_919942,			

EPI_ISL_919943, EPI_ISL_919944, EPI_ISL_919946, EPI_ISL_919947, EPI_ISL_919948, EPI_ISL_919949, EPI_ISL_919950, EPI_ISL_919951, EPI_ISL_919953, EPI_ISL_919954, EPI_ISL_919956, EPI_ISL_919958, EPI_ISL_919959, EPI_ISL_919960, EPI_ISL_920092, EPI_ISL_920093, EPI_ISL_920094, EPI_ISL_920095, EPI_ISL_920096, EPI_ISL_920097, EPI_ISL_920098, EPI_ISL_920099, EPI_ISL_920100, EPI_ISL_920101, EPI_ISL_920102, EPI_ISL_920103, EPI_ISL_920104, EPI_ISL_920105, EPI_ISL_920106, EPI_ISL_920107, EPI_ISL_920108, EPI_ISL_920109, EPI_ISL_920110, EPI_ISL_920111, EPI_ISL_920112, EPI_ISL_920113, EPI_ISL_920114, EPI_ISL_920115, EPI_ISL_920116, EPI_ISL_920152			
see above	University College London, Great Ormond Street Hospital for Children NHS Foundation Trust, Imperial College Healthcare NHS Trust	COVID-19 Genomics UK (COG-UK) Consortium	Sergi Castellano, Rachel Williams, Mark Kristiansen, Paola Resende Silva, Sunando Roy, Tony Brooks, Helena Tutill, Paola Niola, Patricia Dyal, Charlotte Williams, Leysa Forrest, Yasmin Panchbhaya, Jacqueline Findlay, Samuel Weeks, Julianne Brown, Kathryn Harris, Paul Randell, James Price, Alison Holmes, Judith Breuer
EPI_ISL_920671, EPI_ISL_920685, EPI_ISL_920693, EPI_ISL_920701, EPI_ISL_920702, EPI_ISL_920723, EPI_ISL_920724, EPI_ISL_920725, EPI_ISL_920728, EPI_ISL_920731, EPI_ISL_920732, EPI_ISL_920733, EPI_ISL_920734, EPI_ISL_920752, EPI_ISL_920756, EPI_ISL_920757, EPI_ISL_920758, EPI_ISL_920761, EPI_ISL_920763, EPI_ISL_920771, EPI_ISL_920776, EPI_ISL_920777, EPI_ISL_920778, EPI_ISL_920779, EPI_ISL_920780			
see above	University College London Hospital	COVID-19 Genomics UK (COG-UK) Consortium	Judith Heaney, Matthew Byott, Catherine Houlihan, Dan Frampton, Stuart Kirk, Moira Spyer and Eleni Nastouli
EPI_ISL_921659, EPI_ISL_921660, EPI_ISL_921661, EPI_ISL_921662	Northumbria University / South Tees Hospitals NHS Foundation Trust / North Cumbria Integrated Care NHS Foundation Trust / North Tees and Hartlepool NHS Foundation Trust / Newcastle Hospitals NHS Foundation Trust	COVID-19 Genomics UK (COG-UK) Consortium	Darren L Smith,Andrew Nelson,Matthew Bashton,Greg R Young,Joshua Loh,John Allan,Mohammad A Tariq,Giles S Holt,Gary Black,Wen C Yew,Lynn Dover,Paul Baker,Steve Liggett,Sarah Essex,Jane Greenaway,Debra Padgett,Clive Graham,Garren Scott,Edward Barton,Emma Swindells,Brendan Payne,Jennifer Collins,Yusri Taha,Gary Eltringham
EPI_ISL_922037, EPI_ISL_922038, EPI_ISL_922039, EPI_ISL_922040	Queens Medical Centre, Clinical Microbiology Department / DeepSeq Nottingham	COVID-19 Genomics UK (COG-UK) Consortium	Gemma Clark, Wendy Smith, Manjinder Khakh, Vicki M Fleming, Michelle M Lister, Hannah Howson-Wells, Jonathan Ball, Patrick McClure, Joseph Chappell, Theocharis Tsoleridis, Nadine Holmes, Matthew Carlisle, Christopher Moore, Fei Sang, Johnny Debebe, Victoria Wright, Matthew Loose
EPI_ISL_923349	Centre for Enzyme Innovation, University of Portsmouth / Translational Research Laboratory, Portsmouth Hospitals NHS Trust	COVID-19 Genomics UK (COG-UK) Consortium	Angela Beckett,Salman Goudarzi,Christopher Fearn,Kate Cook,Katie Loveson,Sharon Glaysher,Scott Elliott,Samuel Robson
EPI_ISL_924082, EPI_ISL_924085, EPI_ISL_924087, EPI_ISL_924089, EPI_ISL_924092, EPI_ISL_924097, EPI_ISL_924099, EPI_ISL_924103, EPI_ISL_924105, EPI_ISL_924109, EPI_ISL_924111, EPI_ISL_924112, EPI_ISL_924117, EPI_ISL_924118, EPI_ISL_924125, EPI_ISL_924126, EPI_ISL_924129, EPI_ISL_924131, EPI_ISL_924136, EPI_ISL_924142, EPI_ISL_924144, EPI_ISL_924146, EPI_ISL_924153, EPI_ISL_924160, EPI_ISL_924163, EPI_ISL_924165, EPI_ISL_924173, EPI_ISL_924183, EPI_ISL_924184, EPI_ISL_924185, EPI_ISL_924186, EPI_ISL_924191, EPI_ISL_924193, EPI_ISL_924198, EPI_ISL_924200, EPI_ISL_924201, EPI_ISL_924209, EPI_ISL_924212, EPI_ISL_924216, EPI_ISL_924217, EPI_ISL_924223, EPI_ISL_924227, EPI_ISL_924228, EPI_ISL_924229, EPI_ISL_924235, EPI_ISL_924238, EPI_ISL_924243, EPI_ISL_924249, EPI_ISL_924251, EPI_ISL_924258, EPI_ISL_924263, EPI_ISL_924269, EPI_ISL_924273, EPI_ISL_924276, EPI_ISL_924277, EPI_ISL_924282, EPI_ISL_924287, EPI_ISL_924293, EPI_ISL_924297, EPI_ISL_924298, EPI_ISL_924304, EPI_ISL_924305, EPI_ISL_924306, EPI_ISL_924308, EPI_ISL_924313, EPI_ISL_924330, EPI_ISL_924335, EPI_ISL_924339, EPI_ISL_924340, EPI_ISL_924350, EPI_ISL_924352, EPI_ISL_924356, EPI_ISL_924359, EPI_ISL_924361, EPI_ISL_924366, EPI_ISL_924368, EPI_ISL_924374, EPI_ISL_924377, EPI_ISL_924379, EPI_ISL_924382, EPI_ISL_924390, EPI_ISL_924410, EPI_ISL_924414			
see above	Virology Department, Sheffield Teaching Hospitals NHS Foundation Trust/Department of Infection, Immunity and Cardiovascular Disease, The Medical School, University of Sheffield	COVID-19 Genomics UK (COG-UK) Consortium	Thushan de Silva, Matthew Parker, Nikki Smith, Adri Angyal, Rebecca Brown, Luke Green, Rachel Tucker, Paul Parsons, Danielle Groves, Katie Johnson, Laura Carrilero, Alex Keeley, Dave Partridge, Matthew Wyles, Benjamin Lindsey, Mehmet Yavuz, Mohammad Raza, Cariad Evans
EPI_ISL_931586, EPI_ISL_931587, EPI_ISL_931588, EPI_ISL_931590, EPI_ISL_931591, EPI_ISL_931592, EPI_ISL_931593, EPI_ISL_931594, EPI_ISL_931596, EPI_ISL_931597, EPI_ISL_931598, EPI_ISL_931599, EPI_ISL_931600, EPI_ISL_931601, EPI_ISL_931602, EPI_ISL_931603, EPI_ISL_931604, EPI_ISL_931605, EPI_ISL_931606, EPI_ISL_931607, EPI_ISL_931608, EPI_ISL_931609, EPI_ISL_931610, EPI_ISL_931611, EPI_ISL_931612, EPI_ISL_931613, EPI_ISL_931614, EPI_ISL_931615, EPI_ISL_931616, EPI_ISL_931617, EPI_ISL_931618, EPI_ISL_931619, EPI_ISL_931620, EPI_ISL_931621, EPI_ISL_931622, EPI_ISL_931623, EPI_ISL_931624, EPI_ISL_931625, EPI_ISL_931626, EPI_ISL_931627, EPI_ISL_931628, EPI_ISL_931629, EPI_ISL_931630, EPI_ISL_931631, EPI_ISL_931632, EPI_ISL_931633, EPI_ISL_931634, EPI_ISL_931635, EPI_ISL_931636, EPI_ISL_931637, EPI_ISL_931638, EPI_ISL_931639, EPI_ISL_931640, EPI_ISL_931641, EPI_ISL_931642, EPI_ISL_931643, EPI_ISL_931644, EPI_ISL_931645, EPI_ISL_931646, EPI_ISL_931647, EPI_ISL_931648, EPI_ISL_931649, EPI_ISL_931650, EPI_ISL_931651, EPI_ISL_931652, EPI_ISL_931653, EPI_ISL_931654, EPI_ISL_931655, EPI_ISL_931656, EPI_ISL_931657, EPI_ISL_931658, EPI_ISL_931659, EPI_ISL_931660, EPI_ISL_931661, EPI_ISL_931662, EPI_ISL_931663, EPI_ISL_931664, EPI_ISL_931665, EPI_ISL_931666, EPI_ISL_931667, EPI_ISL_931668, EPI_ISL_931669, EPI_ISL_931670, EPI_ISL_931671, EPI_ISL_931672, EPI_ISL_931673, EPI_ISL_931674, EPI_ISL_931675, EPI_ISL_931676, EPI_ISL_931677, EPI_ISL_931678, EPI_ISL_931679, EPI_ISL_931680, EPI_ISL_931681, EPI_ISL_931682, EPI_ISL_931683, EPI_ISL_931684, EPI_ISL_931685, EPI_ISL_931686, EPI_ISL_931687, EPI_ISL_931688, EPI_ISL_931689, EPI_ISL_931691, EPI_ISL_931692, EPI_ISL_931693, EPI_ISL_931694, EPI_ISL_931695, EPI_ISL_931696, EPI_ISL_931697, EPI_ISL_931698, EPI_ISL_931699, EPI_ISL_931700, EPI_ISL_931701, EPI_ISL_931702, EPI_ISL_931703, EPI_ISL_931704, EPI_ISL_931705, EPI_ISL_931706, EPI_ISL_931707, EPI_ISL_931708, EPI_ISL_931709, EPI_ISL_931710, EPI_ISL_931711, EPI_ISL_931712, EPI_ISL_931713, EPI_ISL_931714, EPI_ISL_931715, EPI_ISL_931716, EPI_ISL_931717, EPI_ISL_931718, EPI_ISL_931719, EPI_ISL_931720, EPI_ISL_931721, EPI_ISL_931722, EPI_ISL_931723, EPI_ISL_931724, EPI_ISL_931725, EPI_ISL_931727, EPI_ISL_931728, EPI_ISL_931729, EPI_ISL_931730, EPI_ISL_931731, EPI_ISL_931732, EPI_ISL_931733, EPI_ISL_931734, EPI_ISL_931735, EPI_ISL_931736, EPI_ISL_931737, EPI_ISL_931738, EPI_ISL_931739, EPI_ISL_931740, EPI_ISL_931741, EPI_ISL_931742, EPI_ISL_931743, EPI_ISL_931744, EPI_ISL_931745, EPI_ISL_931746, EPI_ISL_931747, EPI_ISL_931748, EPI_ISL_931749, EPI_ISL_931750, EPI_ISL_931751, EPI_ISL_931752, EPI_ISL_931753, EPI_ISL_931754, EPI_ISL_931755, EPI_ISL_931756, EPI_ISL_931757, EPI_ISL_931758, EPI_ISL_931759, EPI_ISL_931760, EPI_ISL_931761, EPI_ISL_931762, EPI_ISL_931763, EPI_ISL_931764, EPI_ISL_931765, EPI_ISL_931766, EPI_ISL_931767, EPI_ISL_931768, EPI_ISL_931769, EPI_ISL_931770, EPI_ISL_931771, EPI_ISL_931772, EPI_ISL_931773, EPI_ISL_931774, EPI_ISL_931775, EPI_ISL_931776, EPI_ISL_931777, EPI_ISL_931778, EPI_ISL_931779, EPI_ISL_931780, EPI_ISL_931781, EPI_ISL_931782, EPI_ISL_931783, EPI_ISL_931784, EPI_ISL_931785, EPI_ISL_931786, EPI_ISL_931787, EPI_ISL_931788, EPI_ISL_931789, EPI_ISL_931790, EPI_ISL_931791, EPI_ISL_931792, EPI_ISL_931793, EPI_ISL_931794, EPI_ISL_931795, EPI_ISL_931796, EPI_ISL_931797, EPI_ISL_931798, EPI_ISL_931799, EPI_ISL_931800, EPI_ISL_931801, EPI_ISL_931802, EPI_ISL_931803, EPI_ISL_931804, EPI_ISL_931805, EPI_ISL_931806, EPI_ISL_931807, EPI_ISL_931808, EPI_ISL_931809, EPI_ISL_931810, EPI_ISL_931811, EPI_ISL_931812, EPI_ISL_931813, EPI_ISL_931814, EPI_ISL_931815, EPI_ISL_931816, EPI_ISL_931817, EPI_ISL_931819, EPI_ISL_931820, EPI_ISL_931821, EPI_ISL_931823, EPI_ISL_931824, EPI_ISL_931825, EPI_ISL_931826, EPI_ISL_931828, EPI_ISL_931829, EPI_ISL_931830, EPI_ISL_931831, EPI_ISL_931832, EPI_ISL_931833, EPI_ISL_931834, EPI_ISL_931835, EPI_ISL_931836, EPI_ISL_931837, EPI_ISL_931838, EPI_ISL_931839, EPI_ISL_931840, EPI_ISL_931841, EPI_ISL_931842, EPI_ISL_931843, EPI_ISL_931844, EPI_ISL_931845, EPI_ISL_931846, EPI_ISL_931847, EPI_ISL_931848, EPI_ISL_931849, EPI_ISL_931850, EPI_ISL_931851, EPI_ISL_931852, EPI_ISL_931853, EPI_ISL_931854, EPI_ISL_931855, EPI_ISL_931856, EPI_ISL_931857, EPI_ISL_931858, EPI_ISL_931859, EPI_ISL_931860, EPI_ISL_931861, EPI_ISL_931862, EPI_ISL_931863, EPI_ISL_931864, EPI_ISL_931865, EPI_ISL_931866, EPI_ISL_931867, EPI_ISL_931868, EPI_ISL_931869, EPI_ISL_931870, EPI_ISL_931871, EPI_ISL_931872, EPI_ISL_931873, EPI_ISL_931875, EPI_ISL_931876, EPI_ISL_931877, EPI_ISL_931878, EPI_ISL_931879, EPI_ISL_931880, EPI_ISL_931881, EPI_ISL_931882, EPI_ISL_931883, EPI_ISL_931884, EPI_ISL_931885, EPI_ISL_931886, EPI_ISL_931887, EPI_ISL_931888, EPI_ISL_931889, EPI_ISL_931890, EPI_ISL_931891, EPI_ISL_931892, EPI_ISL_931893, EPI_ISL_931894, EPI_ISL_931895, EPI_ISL_931896, EPI_ISL_931897, EPI_ISL_931898, EPI_ISL_931899, EPI_ISL_931900, EPI_ISL_931901, EPI_ISL_931902, EPI_ISL_931903, EPI_ISL_931904, EPI_ISL_931905, EPI_ISL_931906, EPI_ISL_931907, EPI_ISL_931908, EPI_ISL_931909, EPI_ISL_931910, EPI_ISL_931911, EPI_ISL_931912, EPI_ISL_931913, EPI_ISL_931914, EPI_ISL_931915, EPI_ISL_931916, EPI_ISL_931917, EPI_ISL_931919, EPI_ISL_931920, EPI_ISL_931922, EPI_ISL_931923, EPI_ISL_931924, EPI_ISL_931925, EPI_ISL_931926, EPI_ISL_931927, EPI_ISL_931928, EPI_ISL_931929, EPI_ISL_931930, EPI_ISL_931931, EPI_ISL_931932, EPI_ISL_931933, EPI_ISL_931934, EPI_ISL_931935, EPI_ISL_931936, EPI_ISL_931938, EPI_ISL_931940, EPI_ISL_931941, EPI_ISL_931942, EPI_ISL_931944, EPI_ISL_931945, EPI_ISL_931946, EPI_ISL_931947, EPI_ISL_931948, EPI_ISL_931949, EPI_ISL_931950, EPI_ISL_931951, EPI_ISL_931952, EPI_ISL_931954, EPI_ISL_931955, EPI_ISL_931956, EPI_ISL_931958, EPI_ISL_931960, EPI_ISL_931961, EPI_ISL_931962, EPI_ISL_931963, EPI_ISL_931964, EPI_ISL_931965, EPI_ISL_931966, EPI_ISL_931967, EPI_ISL_931968, EPI_ISL_931969, EPI_ISL_931970, EPI_ISL_931971, EPI_ISL_931972, EPI_ISL_931973, EPI_ISL_931974, EPI_ISL_931975, EPI_ISL_931976, EPI_ISL_931977, EPI_ISL_931978, EPI_ISL_931980, EPI_ISL_931981, EPI_ISL_931982, EPI_ISL_931983, EPI_ISL_931984, EPI_ISL_931985, EPI_ISL_931986, EPI_ISL_931987, EPI_ISL_931988, EPI_ISL_931989, EPI_ISL_931990, EPI_ISL_931991, EPI_ISL_931992, EPI_ISL_931993, EPI_ISL_931994, EPI_ISL_931995, EPI_ISL_931996, EPI_ISL_931997, EPI_ISL_931998, EPI_ISL_931999, EPI_ISL_932000, EPI_ISL_932001, EPI_ISL_932002, EPI_ISL_932003, EPI_ISL_932004, EPI_ISL_932005, EPI_ISL_932006, EPI_ISL_932007, EPI_ISL_932008, EPI_ISL_932009, EPI_ISL_932010, EPI_ISL_932011, EPI_ISL_932012, EPI_ISL_932013, EPI_ISL_932014, EPI_ISL_932015, EPI_ISL_932016, EPI_ISL_932018, EPI_ISL_932019, EPI_ISL_932020, EPI_ISL_932021, EPI_ISL_932022, EPI_ISL_932023, EPI_ISL_932024, EPI_ISL_932025, EPI_ISL_932026, EPI_ISL_932027, EPI_ISL_932028, EPI_ISL_932029, EPI_ISL_932030, EPI_ISL_932031, EPI_ISL_932032, EPI_ISL_932033, EPI_ISL_932034, EPI_ISL_932035, EPI_ISL_932036, EPI_ISL_932037, EPI_ISL_932038, EPI_ISL_932040, EPI_ISL_932041, EPI_ISL_932042, EPI_ISL_932043, EPI_ISL_932044, EPI_ISL_932045, EPI_ISL_932047, EPI_ISL_932048, EPI_ISL_932049, EPI_ISL_932050, EPI_ISL_932051, EPI_ISL_932052, EPI_ISL_932053, EPI_ISL_932054, EPI_ISL_932055, EPI_ISL_932056, EPI_ISL_932058, EPI_ISL_932059, EPI_ISL_932060, EPI_ISL_932062, EPI_ISL_932063, EPI_ISL_932064, EPI_ISL_932065, EPI_ISL_932066, EPI_ISL_932067, EPI_ISL_932069, EPI_ISL_932070, EPI_ISL_932071, EPI_ISL_932072, EPI_ISL_932073, EPI_ISL_932074, EPI_ISL_932075, EPI_ISL_932076, EPI_ISL_932077, EPI_ISL_932078, EPI_ISL_932079, EPI_ISL_932080, EPI_ISL_932081, EPI_ISL_932082, EPI_ISL_932083, EPI_ISL_932084, EPI_ISL_932085, EPI_ISL_932087, EPI_ISL_932088, EPI_ISL_932089, EPI_ISL_932090, EPI_ISL_932091, EPI_ISL_932092, EPI_ISL_932093, EPI_ISL_932094, EPI_ISL_932095, EPI_ISL_932096, EPI_ISL_932097, EPI_ISL_932098, EPI_ISL_932099, EPI_ISL_932100, EPI_ISL_932101, EPI_ISL_932102, EPI_ISL_932103, EPI_ISL_932104, EPI_ISL_932105, EPI_ISL_932106, EPI_ISL_932107, EPI_ISL_932109, EPI_ISL_932110, EPI_ISL_932111, EPI_ISL_932112, EPI_ISL_932113, EPI_ISL_932115, EPI_ISL_932116, EPI_ISL_932117, EPI_ISL_932118, EPI_ISL_932119, EPI_ISL_932120, EPI_ISL_932121, EPI_ISL_932122, EPI_ISL_932123, EPI_ISL_932124, EPI_ISL_932125, EPI_ISL_932126, EPI_ISL_932127, EPI_ISL_932128, EPI_ISL_932129, EPI_ISL_932130, EPI_ISL_932131, EPI_ISL_932132, EPI_ISL_932133, EPI_ISL_932134, EPI_ISL_932135, EPI_ISL_932136, EPI_ISL_932137, EPI_ISL_932138, EPI_ISL_932139, EPI_ISL_932140, EPI_ISL_932141, EPI_ISL_932142, EPI_ISL_932143, EPI_ISL_932144, EPI_ISL_932145, EPI_ISL_932146, EPI_ISL_932147, EPI_ISL_932148, EPI_ISL_932149, EPI_ISL_932150, EPI_ISL_932151, EPI_ISL_932152, EPI_ISL_932153, EPI_ISL_932154, EPI_ISL_932155, EPI_ISL_932156, EPI_ISL_932158, EPI_ISL_932159, EPI_ISL_932160, EPI_ISL_932161, EPI_ISL_932162, EPI_ISL_932163, EPI_ISL_932164, EPI_ISL_932165, EPI_ISL_932166, EPI_ISL_932167, EPI_ISL_932168, EPI_ISL_932169, EPI_ISL_932170, EPI_ISL_932171, EPI_ISL_932172, EPI_ISL_932173, EPI_ISL_932174, EPI_ISL_932175, EPI_ISL_932176, EPI_ISL_932177, EPI_ISL_932178, EPI_ISL_932179, EPI_ISL_932180, EPI_ISL_932181, EPI_ISL_932182, EPI_ISL_932183, EPI_ISL_932184, EPI_ISL_932185, EPI_ISL_932186, EPI_ISL_932187, EPI_ISL_932188, EPI_ISL_932189, EPI_ISL_932190, EPI_ISL_932191, EPI_ISL_932192, EPI_ISL_932193, EPI_ISL_932194, EPI_ISL_932195, EPI_ISL_932196, EPI_ISL_932197, EPI_ISL_932198, EPI_ISL_932199, EPI_ISL_932200, EPI_ISL_932202, EPI_ISL_932203, EPI_ISL_932204, EPI_ISL_932205, EPI_ISL_932206, EPI_ISL_932207, EPI_ISL_932208, EPI_ISL_932209, EPI_ISL_932210, EPI_ISL_932211, EPI_ISL_932212, EPI_ISL_932213, EPI_ISL_932214, EPI_ISL_932215, EPI_ISL_932216, EPI_ISL_932217, EPI_ISL_932218, EPI_ISL_932219, EPI_ISL_932220, EPI_ISL_932221, EPI_ISL_932223, EPI_ISL_932224, EPI_ISL_932225, EPI_ISL_932226, EPI_ISL_932227, EPI_ISL_932228, EPI_ISL_932229, EPI_ISL_932230, EPI_ISL_932232, EPI_ISL_932233, EPI_ISL_932234, EPI_ISL_932235, EPI_ISL_932236, EPI_ISL_932237, EPI_ISL_932238, EPI_ISL_932239, EPI_ISL_932242, EPI_ISL_932243, EPI_ISL_932244, EPI_ISL_932245, EPI_ISL_932246, EPI_ISL_932247, EPI_ISL_932248, EPI_ISL_932249, EPI_ISL_932250			
see above	Lighthouse Lab in Alderley Park	Wellcome Sanger Institute for the COVID-19 Genomics UK (COG-UK) Consortium	Jacquelyn Wynn, Mairead Hyland, The Lighthouse Lab in Alderley Park and Alex Alderton, Roberto Amato, Sonia Goncalves, Ewan Harrison, David K. Jackson, Ian Johnston, Dominic Kwiatkowski, Cordelia Langford, John Sillitoe on behalf of the Wellcome Sanger Institute COVID-19 Surveillance Team
EPI_ISL_932850, EPI_ISL_932853, EPI_ISL_932855, EPI_ISL_932856, EPI_ISL_932857, EPI_ISL_932858, EPI_ISL_932859, EPI_ISL_932860, EPI_ISL_932863, EPI_ISL_932864, EPI_ISL_932865, EPI_ISL_932870, EPI_ISL_932871, EPI_ISL_932879, EPI_ISL_932884, EPI_ISL_932885, EPI_ISL_932894, EPI_ISL_932896, EPI_ISL_932897, EPI_ISL_932899, EPI_ISL_932903, EPI_ISL_932905, EPI_ISL_932908, EPI_ISL_932914, EPI_ISL_932915, EPI_ISL_932917, EPI_ISL_932918, EPI_ISL_932919, EPI_ISL_932920, EPI_ISL_932921, EPI_ISL_932925, EPI_ISL_932929, EPI_ISL_932930, EPI_ISL_932931, EPI_ISL_932932, EPI_ISL_932936, EPI_ISL_932939, EPI_ISL_932944, EPI_ISL_932948, EPI_ISL_932949, EPI_ISL_932955, EPI_ISL_932957, EPI_ISL_932958, EPI_ISL_932965, EPI_ISL_932966, EPI_ISL_932967, EPI_ISL_932970, EPI_ISL_932980, EPI_ISL_932986, EPI_ISL_932987, EPI_ISL_932991, EPI_ISL_932994, EPI_ISL_932995, EPI_ISL_932996, EPI_ISL_933000, EPI_ISL_933002, EPI_ISL_933004, EPI_ISL_933006, EPI_ISL_933007, EPI_ISL_933009, EPI_ISL_933011, EPI_ISL_933018, EPI_ISL_933020, EPI_ISL_933021, EPI_ISL_933028, EPI_ISL_933033, EPI_ISL_933035, EPI_ISL_933036, EPI_ISL_933037, EPI_ISL_933038, EPI_ISL_933040, EPI_ISL_933042, EPI_ISL_933044, EPI_ISL_933050, EPI_ISL_933051, EPI_ISL_933053, EPI_ISL_933054, EPI_ISL_933056, EPI_ISL_933058, EPI_ISL_933065, EPI_ISL_933067, EPI_ISL_933069, EPI_ISL_933070, EPI_ISL_933072, EPI_ISL_933073, EPI_ISL_933074, EPI_ISL_933079, EPI_ISL_933080, EPI_ISL_933096, EPI_ISL_933099, EPI_ISL_933100, EPI_ISL_933109, EPI_ISL_933110			
see above	Lighthouse Lab in Glasgow	Wellcome Sanger Institute for the COVID-19 Genomics UK (COG-UK) Consortium	Harper VanSteenhouse, Yumi Kasai, David Gray, Carol Clugston, Anna Dominiczak and Alex Alderton, Roberto Amato, Sonia Goncalves, Ewan Harrison, David K. Jackson, Ian Johnston, Dominic Kwiatkowski, Cordelia Langford, John Sillitoe on behalf of the Wellcome Sanger Institute COVID-19 Surveillance Team
EPI_ISL_937545, EPI_ISL_937546, EPI_ISL_937547, EPI_ISL_937548, EPI_ISL_937552, EPI_ISL_937555, EPI_ISL_937556, EPI_ISL_937560, EPI_ISL_937561, EPI_ISL_937563, EPI_ISL_937565, EPI_ISL_937566, EPI_ISL_937567, EPI_ISL_937572, EPI_ISL_937574, EPI_ISL_937575, EPI_ISL_937577, EPI_ISL_937582, EPI_ISL_937584, EPI_ISL_937585, EPI_ISL_937589, EPI_ISL_937591, EPI_ISL_937593, EPI_ISL_937597, EPI_ISL_937598, EPI_ISL_937600, EPI_ISL_937602, EPI_ISL_937603, EPI_ISL_937606, EPI_ISL_937607, EPI_ISL_937608, EPI_ISL_937611, EPI_ISL_937613, EPI_ISL_937615, EPI_ISL_937617, EPI_ISL_937619, EPI_ISL_937622, EPI_ISL_937624, EPI_ISL_937625, EPI_ISL_937626, EPI_ISL_937628, EPI_ISL_937629, EPI_ISL_937631, EPI_ISL_937632, EPI_ISL_937633, EPI_ISL_937635, EPI_ISL_937637, EPI_ISL_937642, EPI_ISL_937644, EPI_ISL_937645, EPI_ISL_937646, EPI_ISL_937647, EPI_ISL_937652, EPI_ISL_937654, EPI_ISL_937655, EPI_ISL_937656, EPI_ISL_937657, EPI_ISL_937659, EPI_ISL_937660, EPI_ISL_937661, EPI_ISL_937662, EPI_ISL_937667, EPI_ISL_937671, EPI_ISL_937673, EPI_ISL_937676, EPI_ISL_937677, EPI_ISL_937678, EPI_ISL_937679, EPI_ISL_937681, EPI_ISL_937683, EPI_ISL_937684, EPI_ISL_937686, EPI_ISL_937690, EPI_ISL_937691, EPI_ISL_937692, EPI_ISL_937694, EPI_ISL_937695, EPI_ISL_937696, EPI_ISL_937698, EPI_ISL_937701, EPI_ISL_937702, EPI_ISL_937705, EPI_ISL_937706, EPI_ISL_937708, EPI_ISL_937713, EPI_ISL_937718, EPI_ISL_937719, EPI_ISL_937722, EPI_ISL_937723, EPI_ISL_937725, EPI_ISL_937727, EPI_ISL_937728, EPI_ISL_937729, EPI_ISL_937731, EPI_ISL_937732, EPI_ISL_937733, EPI_ISL_937735, EPI_ISL_937736, EPI_ISL_937739, EPI_ISL_937740, EPI_ISL_937743, EPI_ISL_937747, EPI_ISL_937750, EPI_ISL_937754, EPI_ISL_937755, EPI_ISL_937756, EPI_ISL_937757, EPI_ISL_937758, EPI_ISL_937759, EPI_ISL_937760, EPI_ISL_937764, EPI_ISL_937771, EPI_ISL_937772, EPI_ISL_937773, EPI_ISL_937774, EPI_ISL_937775, EPI_ISL_937776, EPI_ISL_937778, EPI_ISL_937779, EPI_ISL_937782, EPI_ISL_937783, EPI_ISL_937785, EPI_ISL_937786, EPI_ISL_937788, EPI_ISL_937789, EPI_ISL_937791,			

EPI_ISL_937793, EPI_ISL_937795, EPI_ISL_937796, EPI_ISL_937797, EPI_ISL_937804, EPI_ISL_937807, EPI_ISL_937809, EPI_ISL_937810, EPI_ISL_937812, EPI_ISL_937813, EPI_ISL_937814, EPI_ISL_937816, EPI_ISL_937819, EPI_ISL_937821, EPI_ISL_937823, EPI_ISL_937825, EPI_ISL_937827, EPI_ISL_937828, EPI_ISL_937832, EPI_ISL_937833, EPI_ISL_937834, EPI_ISL_937836, EPI_ISL_937838, EPI_ISL_937841, EPI_ISL_937842, EPI_ISL_937844, EPI_ISL_937845, EPI_ISL_937846, EPI_ISL_937847, EPI_ISL_937848, EPI_ISL_937851, EPI_ISL_937855, EPI_ISL_937856, EPI_ISL_937857, EPI_ISL_937858, EPI_ISL_937859, EPI_ISL_937861, EPI_ISL_937866, EPI_ISL_937868, EPI_ISL_937869, EPI_ISL_937870, EPI_ISL_937871, EPI_ISL_937876, EPI_ISL_937878, EPI_ISL_937879, EPI_ISL_937880, EPI_ISL_937882, EPI_ISL_937884, EPI_ISL_937885			
see above	Lighthouse Lab in Alderley Park	Wellcome Sanger Institute for the COVID-19 Genomics UK (COG-UK) Consortium	Jacquelyn Wynn, Mairead Hyland, The Lighthouse Lab in Alderley Park and Alex Alderton, Roberto Amato, Sonia Goncalves, Ewan Harrison, David K. Jackson, Ian Johnston, Dominic Kwiatkowski, Cordelia Langford, John Sillitoe on behalf of the Wellcome Sanger Institute COVID-19 Surveillance Team
EPI_ISL_938579, EPI_ISL_938580, EPI_ISL_938582, EPI_ISL_938583, EPI_ISL_938584, EPI_ISL_938585, EPI_ISL_938586, EPI_ISL_938588, EPI_ISL_938589, EPI_ISL_938590, EPI_ISL_938591, EPI_ISL_938593, EPI_ISL_938594, EPI_ISL_938595, EPI_ISL_938597, EPI_ISL_938598, EPI_ISL_938600, EPI_ISL_938601, EPI_ISL_938604, EPI_ISL_938605, EPI_ISL_938606, EPI_ISL_938608, EPI_ISL_938609, EPI_ISL_938611, EPI_ISL_938615, EPI_ISL_938616, EPI_ISL_938617, EPI_ISL_938618, EPI_ISL_938620, EPI_ISL_938621, EPI_ISL_938622, EPI_ISL_938623, EPI_ISL_938624, EPI_ISL_938625, EPI_ISL_938626, EPI_ISL_938627, EPI_ISL_938628, EPI_ISL_938629, EPI_ISL_938634, EPI_ISL_938635, EPI_ISL_938638, EPI_ISL_938639, EPI_ISL_938640, EPI_ISL_938641, EPI_ISL_938642, EPI_ISL_938644, EPI_ISL_938646, EPI_ISL_938647, EPI_ISL_938649, EPI_ISL_938651, EPI_ISL_938653, EPI_ISL_938654, EPI_ISL_938655, EPI_ISL_938656, EPI_ISL_938658, EPI_ISL_938659, EPI_ISL_938660, EPI_ISL_938661, EPI_ISL_938662, EPI_ISL_938663, EPI_ISL_938664, EPI_ISL_938665, EPI_ISL_938668, EPI_ISL_938669, EPI_ISL_938670, EPI_ISL_938671, EPI_ISL_938672, EPI_ISL_938673, EPI_ISL_938674, EPI_ISL_938675, EPI_ISL_938677, EPI_ISL_938678, EPI_ISL_938679, EPI_ISL_938680, EPI_ISL_938681, EPI_ISL_938682, EPI_ISL_938683, EPI_ISL_938685, EPI_ISL_938686, EPI_ISL_938687, EPI_ISL_938688, EPI_ISL_938689, EPI_ISL_938690, EPI_ISL_938691, EPI_ISL_938693, EPI_ISL_938695, EPI_ISL_938696, EPI_ISL_938697, EPI_ISL_938698, EPI_ISL_938699, EPI_ISL_938702, EPI_ISL_938703, EPI_ISL_938704, EPI_ISL_938705, EPI_ISL_938706, EPI_ISL_938708, EPI_ISL_938709, EPI_ISL_938710, EPI_ISL_938711, EPI_ISL_938712, EPI_ISL_938713, EPI_ISL_938714, EPI_ISL_938715, EPI_ISL_938716, EPI_ISL_938717, EPI_ISL_938718, EPI_ISL_938719, EPI_ISL_938720, EPI_ISL_938722, EPI_ISL_938723, EPI_ISL_938724, EPI_ISL_938725, EPI_ISL_938726, EPI_ISL_938727, EPI_ISL_938728, EPI_ISL_938730, EPI_ISL_938734, EPI_ISL_938735, EPI_ISL_938736, EPI_ISL_938738, EPI_ISL_938739, EPI_ISL_938740, EPI_ISL_938742, EPI_ISL_938745, EPI_ISL_938748, EPI_ISL_938749, EPI_ISL_938754, EPI_ISL_938755, EPI_ISL_938759, EPI_ISL_938760, EPI_ISL_938761, EPI_ISL_938763, EPI_ISL_938765, EPI_ISL_938766, EPI_ISL_938767, EPI_ISL_938768, EPI_ISL_938770, EPI_ISL_938772, EPI_ISL_938773, EPI_ISL_938774, EPI_ISL_938775, EPI_ISL_938776, EPI_ISL_938778, EPI_ISL_938779, EPI_ISL_938780, EPI_ISL_938781, EPI_ISL_938782, EPI_ISL_938784, EPI_ISL_938786, EPI_ISL_938788, EPI_ISL_938789, EPI_ISL_938790, EPI_ISL_938791, EPI_ISL_938792, EPI_ISL_938793, EPI_ISL_938795, EPI_ISL_938798, EPI_ISL_938799, EPI_ISL_938802, EPI_ISL_938805, EPI_ISL_938808, EPI_ISL_938809, EPI_ISL_938812, EPI_ISL_938813, EPI_ISL_938814, EPI_ISL_938816, EPI_ISL_938817, EPI_ISL_938818, EPI_ISL_938819, EPI_ISL_938820, EPI_ISL_938821, EPI_ISL_938822, EPI_ISL_938823, EPI_ISL_938824, EPI_ISL_938825, EPI_ISL_938826, EPI_ISL_938827, EPI_ISL_938828, EPI_ISL_938830, EPI_ISL_938831, EPI_ISL_938832, EPI_ISL_938833, EPI_ISL_938834, EPI_ISL_938835, EPI_ISL_938836, EPI_ISL_938838, EPI_ISL_938839, EPI_ISL_938841, EPI_ISL_938842, EPI_ISL_938843, EPI_ISL_938844, EPI_ISL_938847, EPI_ISL_938848, EPI_ISL_938849, EPI_ISL_938850, EPI_ISL_938851, EPI_ISL_938852, EPI_ISL_938853, EPI_ISL_938855, EPI_ISL_938856, EPI_ISL_938857, EPI_ISL_938858, EPI_ISL_938859, EPI_ISL_938860, EPI_ISL_938861, EPI_ISL_938862, EPI_ISL_938863, EPI_ISL_938864, EPI_ISL_938866, EPI_ISL_938867, EPI_ISL_938868, EPI_ISL_938869, EPI_ISL_938870, EPI_ISL_938871, EPI_ISL_938872, EPI_ISL_938873, EPI_ISL_938874, EPI_ISL_938877, EPI_ISL_938878, EPI_ISL_938879, EPI_ISL_938880, EPI_ISL_938881, EPI_ISL_938883, EPI_ISL_938884, EPI_ISL_938887, EPI_ISL_938888, EPI_ISL_938889, EPI_ISL_938890, EPI_ISL_938891, EPI_ISL_938892, EPI_ISL_938893, EPI_ISL_938894, EPI_ISL_938895, EPI_ISL_938896, EPI_ISL_938897, EPI_ISL_938898, EPI_ISL_938899, EPI_ISL_938902, EPI_ISL_938904, EPI_ISL_938905, EPI_ISL_938906, EPI_ISL_938907, EPI_ISL_938909, EPI_ISL_938910, EPI_ISL_938912, EPI_ISL_938914, EPI_ISL_938915, EPI_ISL_938917, EPI_ISL_938918, EPI_ISL_938919, EPI_ISL_938920, EPI_ISL_938922, EPI_ISL_938923, EPI_ISL_938924, EPI_ISL_938925, EPI_ISL_938926, EPI_ISL_938927, EPI_ISL_938928, EPI_ISL_938929, EPI_ISL_938930, EPI_ISL_938932			
see above	Lighthouse Lab in Milton Keynes	Wellcome Sanger Institute for the COVID-19 Genomics UK (COG-UK) Consortium	The Lighthouse Lab in Milton Keynes and Alex Alderton, Roberto Amato, Sonia Goncalves, Ewan Harrison, David K. Jackson, Ian Johnston, Dominic Kwiatkowski, Cordelia Langford, John Sillitoe on behalf of the Wellcome Sanger Institute COVID-19 Surveillance Team
EPI_ISL_938935, EPI_ISL_938936, EPI_ISL_938937, EPI_ISL_938938, EPI_ISL_938939, EPI_ISL_938941, EPI_ISL_938942, EPI_ISL_938943	Lighthouse Lab in Alderley Park	Wellcome Sanger Institute for the COVID-19 Genomics UK (COG-UK) Consortium	Jacquelyn Wynn, Mairead Hyland, The Lighthouse Lab in Alderley Park and Alex Alderton, Roberto Amato, Sonia Goncalves, Ewan Harrison, David K. Jackson, Ian Johnston, Dominic Kwiatkowski, Cordelia Langford, John Sillitoe on behalf of the Wellcome Sanger Institute COVID-19 Surveillance Team
EPI_ISL_939283, EPI_ISL_939284, EPI_ISL_939285, EPI_ISL_939287, EPI_ISL_939289, EPI_ISL_939295, EPI_ISL_939297, EPI_ISL_939298, EPI_ISL_939299, EPI_ISL_939303, EPI_ISL_939304, EPI_ISL_939305, EPI_ISL_939308, EPI_ISL_939309, EPI_ISL_939310, EPI_ISL_939311, EPI_ISL_939312, EPI_ISL_939316, EPI_ISL_939317, EPI_ISL_939322, EPI_ISL_939323, EPI_ISL_939324, EPI_ISL_939325, EPI_ISL_939326, EPI_ISL_939328, EPI_ISL_939330, EPI_ISL_939331, EPI_ISL_939333, EPI_ISL_939335, EPI_ISL_939336, EPI_ISL_939338, EPI_ISL_939341, EPI_ISL_939342, EPI_ISL_939344, EPI_ISL_939345, EPI_ISL_939347, EPI_ISL_939349, EPI_ISL_939353, EPI_ISL_939360, EPI_ISL_939362, EPI_ISL_939363, EPI_ISL_939366, EPI_ISL_939367, EPI_ISL_939371, EPI_ISL_939374, EPI_ISL_939375, EPI_ISL_939376, EPI_ISL_939377, EPI_ISL_939379, EPI_ISL_939381, EPI_ISL_939382, EPI_ISL_939383, EPI_ISL_939384, EPI_ISL_939385, EPI_ISL_939386, EPI_ISL_939387, EPI_ISL_939392, EPI_ISL_939394, EPI_ISL_939400, EPI_ISL_939408, EPI_ISL_939409, EPI_ISL_939410, EPI_ISL_939411, EPI_ISL_939412, EPI_ISL_939417, EPI_ISL_939419, EPI_ISL_939421, EPI_ISL_939423, EPI_ISL_939424, EPI_ISL_939426, EPI_ISL_939427, EPI_ISL_939430, EPI_ISL_939433, EPI_ISL_939434, EPI_ISL_939435, EPI_ISL_939436, EPI_ISL_939437, EPI_ISL_939440, EPI_ISL_939441, EPI_ISL_939442, EPI_ISL_939444, EPI_ISL_939445, EPI_ISL_939446, EPI_ISL_939447, EPI_ISL_939448, EPI_ISL_939449, EPI_ISL_939451, EPI_ISL_939452, EPI_ISL_939453, EPI_ISL_939455, EPI_ISL_939457, EPI_ISL_939463, EPI_ISL_939464, EPI_ISL_939465, EPI_ISL_939467, EPI_ISL_939470, EPI_ISL_939471, EPI_ISL_939474, EPI_ISL_939475, EPI_ISL_939476, EPI_ISL_939479, EPI_ISL_939480, EPI_ISL_939482, EPI_ISL_939484, EPI_ISL_939486, EPI_ISL_939488, EPI_ISL_939489, EPI_ISL_939490, EPI_ISL_939491, EPI_ISL_939495, EPI_ISL_939496, EPI_ISL_939499, EPI_ISL_939501, EPI_ISL_939502, EPI_ISL_939503, EPI_ISL_939511, EPI_ISL_939515, EPI_ISL_939516, EPI_ISL_939518, EPI_ISL_939525, EPI_ISL_939527, EPI_ISL_939529, EPI_ISL_939530, EPI_ISL_939531, EPI_ISL_939534, EPI_ISL_939538, EPI_ISL_939539, EPI_ISL_939540, EPI_ISL_939543, EPI_ISL_939544, EPI_ISL_939545, EPI_ISL_939546, EPI_ISL_939549, EPI_ISL_939553, EPI_ISL_939557, EPI_ISL_939558, EPI_ISL_939560, EPI_ISL_939564, EPI_ISL_939568, EPI_ISL_939569, EPI_ISL_939570, EPI_ISL_939575, EPI_ISL_939580, EPI_ISL_939583, EPI_ISL_939584, EPI_ISL_939586, EPI_ISL_939587, EPI_ISL_939589, EPI_ISL_939590, EPI_ISL_939591, EPI_ISL_939592, EPI_ISL_939593, EPI_ISL_939597, EPI_ISL_939602, EPI_ISL_944799, EPI_ISL_944805, EPI_ISL_944824, EPI_ISL_944863, EPI_ISL_944883, EPI_ISL_944908, EPI_ISL_944914, EPI_ISL_944923, EPI_ISL_944953, EPI_ISL_944976, EPI_ISL_944978, EPI_ISL_945023, EPI_ISL_945032, EPI_ISL_945034			
see above	Lighthouse Lab in Glasgow	Wellcome Sanger Institute for the COVID-19 Genomics UK (COG-UK) Consortium	Harper VanSteenhouse, Yumi Kasai, David Gray, Carol Clugston, Anna Dominiczak and Alex Alderton, Roberto Amato, Sonia Goncalves, Ewan Harrison, David K. Jackson, Ian Johnston, Dominic Kwiatkowski, Cordelia Langford, John Sillitoe on behalf of the Wellcome Sanger Institute COVID-19 Surveillance Team
EPI_ISL_945377, EPI_ISL_945378, EPI_ISL_945380, EPI_ISL_945381, EPI_ISL_945382, EPI_ISL_945383, EPI_ISL_945384, EPI_ISL_945385, EPI_ISL_945388, EPI_ISL_945390, EPI_ISL_945391, EPI_ISL_945392, EPI_ISL_945393, EPI_ISL_945394, EPI_ISL_945395, EPI_ISL_945396, EPI_ISL_945397, EPI_ISL_945398, EPI_ISL_945399, EPI_ISL_945400, EPI_ISL_945401, EPI_ISL_945403, EPI_ISL_945404, EPI_ISL_945405, EPI_ISL_945406, EPI_ISL_945407, EPI_ISL_945410, EPI_ISL_945411, EPI_ISL_945412, EPI_ISL_945413, EPI_ISL_945414, EPI_ISL_945415, EPI_ISL_945416, EPI_ISL_945417, EPI_ISL_945418, EPI_ISL_945421, EPI_ISL_945424, EPI_ISL_945425, EPI_ISL_945426, EPI_ISL_945427, EPI_ISL_945428, EPI_ISL_945429, EPI_ISL_945430, EPI_ISL_945431, EPI_ISL_945432, EPI_ISL_945434, EPI_ISL_945435, EPI_ISL_945436, EPI_ISL_945437, EPI_ISL_945438, EPI_ISL_945439, EPI_ISL_945440, EPI_ISL_945441, EPI_ISL_945442, EPI_ISL_945443, EPI_ISL_945445, EPI_ISL_945447, EPI_ISL_945448, EPI_ISL_945450, EPI_ISL_945451, EPI_ISL_945452, EPI_ISL_945453, EPI_ISL_945454, EPI_ISL_945455, EPI_ISL_945456, EPI_ISL_945457, EPI_ISL_945460, EPI_ISL_945461, EPI_ISL_945462, EPI_ISL_945463, EPI_ISL_945464, EPI_ISL_945465, EPI_ISL_945470, EPI_ISL_945471, EPI_ISL_945472, EPI_ISL_945473, EPI_ISL_945474, EPI_ISL_945476, EPI_ISL_945477, EPI_ISL_945478, EPI_ISL_945479, EPI_ISL_945481, EPI_ISL_945482, EPI_ISL_945483, EPI_ISL_945484, EPI_ISL_945486, EPI_ISL_945487, EPI_ISL_945488, EPI_ISL_945489, EPI_ISL_945491, EPI_ISL_945492, EPI_ISL_945493, EPI_ISL_945496, EPI_ISL_945497, EPI_ISL_945499, EPI_ISL_945500, EPI_ISL_945501, EPI_ISL_945503, EPI_ISL_945504, EPI_ISL_945505, EPI_ISL_945507, EPI_ISL_945509, EPI_ISL_945510, EPI_ISL_945511, EPI_ISL_945512, EPI_ISL_945513, EPI_ISL_945515, EPI_ISL_945516, EPI_ISL_945517, EPI_ISL_945519, EPI_ISL_945520, EPI_ISL_945521, EPI_ISL_945523, EPI_ISL_945524, EPI_ISL_945526, EPI_ISL_945527, EPI_ISL_945528, EPI_ISL_945530, EPI_ISL_945531, EPI_ISL_945532, EPI_ISL_945533, EPI_ISL_945534, EPI_ISL_945535, EPI_ISL_945536, EPI_ISL_945537, EPI_ISL_945540, EPI_ISL_945541, EPI_ISL_945542, EPI_ISL_945544, EPI_ISL_945550, EPI_ISL_945551, EPI_ISL_945552, EPI_ISL_945553, EPI_ISL_945554, EPI_ISL_945556, EPI_ISL_945557, EPI_ISL_945558, EPI_ISL_945559, EPI_ISL_945560, EPI_ISL_945562, EPI_ISL_945564, EPI_ISL_945565, EPI_ISL_945566, EPI_ISL_945567, EPI_ISL_945568, EPI_ISL_945570, EPI_ISL_945571, EPI_ISL_945572, EPI_ISL_945573, EPI_ISL_945574, EPI_ISL_945575, EPI_ISL_945576, EPI_ISL_945577, EPI_ISL_945578, EPI_ISL_945580, EPI_ISL_945581, EPI_ISL_945583, EPI_ISL_945584, EPI_ISL_945585, EPI_ISL_945586, EPI_ISL_945588, EPI_ISL_945590, EPI_ISL_945592, EPI_ISL_945593, EPI_ISL_945594, EPI_ISL_945595, EPI_ISL_945596, EPI_ISL_945598, EPI_ISL_945599, EPI_ISL_945600, EPI_ISL_945601, EPI_ISL_945602, EPI_ISL_945603, EPI_ISL_945604, EPI_ISL_945605, EPI_ISL_945606, EPI_ISL_945607, EPI_ISL_945608, EPI_ISL_945609, EPI_ISL_945610, EPI_ISL_945611, EPI_ISL_945612, EPI_ISL_945613, EPI_ISL_945614, EPI_ISL_945615, EPI_ISL_945616, EPI_ISL_945617, EPI_ISL_945618, EPI_ISL_945620, EPI_ISL_945622, EPI_ISL_945623, EPI_ISL_945624, EPI_ISL_945625, EPI_ISL_945626, EPI_ISL_945628, EPI_ISL_945629, EPI_ISL_945630, EPI_ISL_945631, EPI_ISL_945632, EPI_ISL_945633, EPI_ISL_945634, EPI_ISL_945637, EPI_ISL_945638, EPI_ISL_945639, EPI_ISL_945640, EPI_ISL_945641, EPI_ISL_945642, EPI_ISL_945643, EPI_ISL_945644, EPI_ISL_945645, EPI_ISL_945646, EPI_ISL_945647, EPI_ISL_945649, EPI_ISL_945651, EPI_ISL_945652, EPI_ISL_945653, EPI_ISL_945654, EPI_ISL_945655, EPI_ISL_945657, EPI_ISL_945658, EPI_ISL_945659, EPI_ISL_945660, EPI_ISL_945661, EPI_ISL_945662, EPI_ISL_945663, EPI_ISL_945665, EPI_ISL_945666, EPI_ISL_945667, EPI_ISL_945669, EPI_ISL_945670, EPI_ISL_945671, EPI_ISL_945673, EPI_ISL_945675, EPI_ISL_945676, EPI_ISL_945679, EPI_ISL_945680, EPI_ISL_945683, EPI_ISL_945684, EPI_ISL_945685, EPI_ISL_945686, EPI_ISL_945687, EPI_ISL_945688, EPI_ISL_945689, EPI_ISL_945690, EPI_ISL_945691, EPI_ISL_945693, EPI_ISL_945694, EPI_ISL_945695, EPI_ISL_945699, EPI_ISL_945700, EPI_ISL_945702, EPI_ISL_945703, EPI_ISL_945705, EPI_ISL_945706, EPI_ISL_945707, EPI_ISL_945708, EPI_ISL_945709, EPI_ISL_945710, EPI_ISL_945711, EPI_ISL_945712, EPI_ISL_945713, EPI_ISL_945714			
see above	Lighthouse Lab in Alderley Park	Wellcome Sanger Institute for the COVID-19 Genomics UK (COG-UK) Consortium	Jacquelyn Wynn, Mairead Hyland, The Lighthouse Lab in Alderley Park and Alex Alderton, Roberto Amato, Sonia Goncalves, Ewan Harrison, David K. Jackson, Ian Johnston, Dominic Kwiatkowski, Cordelia Langford, John Sillitoe on behalf of the Wellcome Sanger Institute COVID-19 Surveillance Team
EPI_ISL_945715, EPI_ISL_945717, EPI_ISL_945719, EPI_ISL_945720, EPI_ISL_945721, EPI_ISL_945723, EPI_ISL_945724, EPI_ISL_945727, EPI_ISL_945731, EPI_ISL_945734, EPI_ISL_945739, EPI_ISL_945740, EPI_ISL_945745, EPI_ISL_945746, EPI_ISL_945748, EPI_ISL_945749, EPI_ISL_945750, EPI_ISL_945751, EPI_ISL_945754, EPI_ISL_945756, EPI_ISL_945760			
see above	Lighthouse Lab in Milton Keynes	Wellcome Sanger Institute for the COVID-19 Genomics UK (COG-UK) Consortium	The Lighthouse Lab in Milton Keynes and Alex Alderton, Roberto Amato, Sonia Goncalves, Ewan Harrison, David K. Jackson, Ian Johnston, Dominic Kwiatkowski, Cordelia Langford, John Sillitoe on behalf of the Wellcome Sanger Institute COVID-19 Surveillance Team
EPI_ISL_945761	Lighthouse Lab in Glasgow	Wellcome Sanger Institute for the COVID-19 Genomics UK (COG-UK) Consortium	Harper VanSteenhouse, Yumi Kasai, David Gray, Carol Clugston, Anna Dominiczak and Alex Alderton, Roberto Amato, Sonia Goncalves, Ewan Harrison, David K. Jackson, Ian Johnston, Dominic Kwiatkowski, Cordelia Langford, John Sillitoe on behalf of the Wellcome Sanger Institute COVID-19 Surveillance Team
EPI_ISL_945764, EPI_ISL_945765, EPI_ISL_945766, EPI_ISL_945767, EPI_ISL_945769, EPI_ISL_945770, EPI_ISL_945773, EPI_ISL_945774, EPI_ISL_945775, EPI_ISL_945778, EPI_ISL_945779, EPI_ISL_945780, EPI_ISL_945783, EPI_ISL_945784, EPI_ISL_945785, EPI_ISL_945786, EPI_ISL_945787, EPI_ISL_945789, EPI_ISL_945790, EPI_ISL_945791, EPI_ISL_945793, EPI_ISL_945794, EPI_ISL_945797, EPI_ISL_945798, EPI_ISL_945801, EPI_ISL_945805, EPI_ISL_945810, EPI_ISL_945811, EPI_ISL_945814, EPI_ISL_945819, EPI_ISL_945820, EPI_ISL_945823, EPI_ISL_945825, EPI_ISL_945827, EPI_ISL_945829, EPI_ISL_945830, EPI_ISL_945832, EPI_ISL_945833, EPI_ISL_945834, EPI_ISL_945838, EPI_ISL_945840, EPI_ISL_945842, EPI_ISL_945845, EPI_ISL_945847, EPI_ISL_945852, EPI_ISL_945854, EPI_ISL_945855, EPI_ISL_945856, EPI_ISL_945857			
see above	Lighthouse Lab in Milton Keynes	Wellcome Sanger Institute for the COVID-19 Genomics UK (COG-UK) Consortium	The Lighthouse Lab in Milton Keynes and Alex Alderton, Roberto Amato, Sonia Goncalves, Ewan Harrison, David K. Jackson, Ian Johnston, Dominic Kwiatkowski, Cordelia Langford, John Sillitoe on behalf of the Wellcome Sanger Institute COVID-19 Surveillance Team
EPI_ISL_945858	Lighthouse Lab in Glasgow	Wellcome Sanger Institute for the COVID-19 Genomics UK (COG-UK) Consortium	Harper VanSteenhouse, Yumi Kasai, David Gray, Carol Clugston, Anna Dominiczak and Alex Alderton, Roberto Amato, Sonia Goncalves, Ewan Harrison, David K. Jackson, Ian Johnston, Dominic Kwiatkowski, Cordelia Langford, John Sillitoe on behalf of the Wellcome Sanger Institute COVID-19 Surveillance Team
EPI_ISL_945859, EPI_ISL_945861, EPI_ISL_945864, EPI_ISL_945866	Lighthouse Lab in Milton Keynes	Wellcome Sanger Institute for the COVID-19 Genomics UK (COG-UK) Consortium	The Lighthouse Lab in Milton Keynes and Alex Alderton, Roberto Amato, Sonia Goncalves, Ewan Harrison, David K. Jackson, Ian Johnston, Dominic Kwiatkowski, Cordelia Langford, John Sillitoe on behalf of the Wellcome Sanger Institute COVID-19 Surveillance Team
EPI_ISL_945875	Lighthouse Lab in Glasgow	Wellcome Sanger Institute for the COVID-19 Genomics UK (COG-UK) Consortium	Harper VanSteenhouse, Yumi Kasai, David Gray, Carol Clugston, Anna Dominiczak and Alex Alderton, Roberto Amato, Sonia Goncalves, Ewan Harrison, David K. Jackson, Ian Johnston, Dominic Kwiatkowski, Cordelia Langford, John Sillitoe on behalf of the Wellcome Sanger Institute COVID-19 Surveillance Team
EPI_ISL_945876, EPI_ISL_945880, EPI_ISL_945881, EPI_ISL_945883, EPI_ISL_945886, EPI_ISL_945887, EPI_ISL_945888, EPI_ISL_945890, EPI_ISL_945891, EPI_ISL_945892, EPI_ISL_945893, EPI_ISL_945894, EPI_ISL_945896			
see above	Lighthouse Lab in Milton Keynes	Wellcome Sanger Institute for the COVID-19 Genomics UK	The Lighthouse Lab in Milton Keynes and Alex Alderton, Roberto Amato, Sonia Goncalves, Ewan Harrison, David K. Jackson, Ian Johnston, Dominic

		(COG-UK) Consortium	Kwiatkowski, Cordelia Langford, John Sillitoe on behalf of the Wellcome Sanger Institute COVID-19 Surveillance Team
EPI_ISL_945897	Lighthouse Lab in Glasgow	Wellcome Sanger Institute for the COVID-19 Genomics UK (COG-UK) Consortium	Harper VanSteenhouse, Yumi Kasai, David Gray, Carol Clugston, Anna Dominiczak and Alex Alderton, Roberto Amato, Sonia Goncalves, Ewan Harrison, David K. Jackson, Ian Johnston, Dominic Kwiatkowski, Cordelia Langford, John Sillitoe on behalf of the Wellcome Sanger Institute COVID-19 Surveillance Team
EPI_ISL_945902, EPI_ISL_945903, EPI_ISL_945905, EPI_ISL_945912, EPI_ISL_945913, EPI_ISL_945915, EPI_ISL_945918, EPI_ISL_945919, EPI_ISL_945922	Lighthouse Lab in Milton Keynes	Wellcome Sanger Institute for the COVID-19 Genomics UK (COG-UK) Consortium	The Lighthouse Lab in Milton Keynes and Alex Alderton, Roberto Amato, Sonia Goncalves, Ewan Harrison, David K. Jackson, Ian Johnston, Dominic Kwiatkowski, Cordelia Langford, John Sillitoe on behalf of the Wellcome Sanger Institute COVID-19 Surveillance Team
EPI_ISL_945923	Lighthouse Lab in Glasgow	Wellcome Sanger Institute for the COVID-19 Genomics UK (COG-UK) Consortium	Harper VanSteenhouse, Yumi Kasai, David Gray, Carol Clugston, Anna Dominiczak and Alex Alderton, Roberto Amato, Sonia Goncalves, Ewan Harrison, David K. Jackson, Ian Johnston, Dominic Kwiatkowski, Cordelia Langford, John Sillitoe on behalf of the Wellcome Sanger Institute COVID-19 Surveillance Team
EPI_ISL_945924, EPI_ISL_945925, EPI_ISL_945927, EPI_ISL_945928, EPI_ISL_945929, EPI_ISL_945930, EPI_ISL_945931, EPI_ISL_945932, EPI_ISL_945934, EPI_ISL_945936, EPI_ISL_945937, EPI_ISL_945941, EPI_ISL_945942, EPI_ISL_945943, EPI_ISL_945946, EPI_ISL_945947, EPI_ISL_945948, EPI_ISL_945949, EPI_ISL_945952, EPI_ISL_945953, EPI_ISL_945955, EPI_ISL_945957, EPI_ISL_945959, EPI_ISL_945964, EPI_ISL_945966, EPI_ISL_945968, EPI_ISL_945973, EPI_ISL_945974, EPI_ISL_945975			
see above	Lighthouse Lab in Milton Keynes	Wellcome Sanger Institute for the COVID-19 Genomics UK (COG-UK) Consortium	The Lighthouse Lab in Milton Keynes and Alex Alderton, Roberto Amato, Sonia Goncalves, Ewan Harrison, David K. Jackson, Ian Johnston, Dominic Kwiatkowski, Cordelia Langford, John Sillitoe on behalf of the Wellcome Sanger Institute COVID-19 Surveillance Team
EPI_ISL_945977	Lighthouse Lab in Glasgow	Wellcome Sanger Institute for the COVID-19 Genomics UK (COG-UK) Consortium	Harper VanSteenhouse, Yumi Kasai, David Gray, Carol Clugston, Anna Dominiczak and Alex Alderton, Roberto Amato, Sonia Goncalves, Ewan Harrison, David K. Jackson, Ian Johnston, Dominic Kwiatkowski, Cordelia Langford, John Sillitoe on behalf of the Wellcome Sanger Institute COVID-19 Surveillance Team
EPI_ISL_945978, EPI_ISL_945981, EPI_ISL_945982, EPI_ISL_945985, EPI_ISL_945986, EPI_ISL_945987, EPI_ISL_945989, EPI_ISL_945991, EPI_ISL_945992, EPI_ISL_945995, EPI_ISL_945996, EPI_ISL_946001			
see above	Lighthouse Lab in Milton Keynes	Wellcome Sanger Institute for the COVID-19 Genomics UK (COG-UK) Consortium	The Lighthouse Lab in Milton Keynes and Alex Alderton, Roberto Amato, Sonia Goncalves, Ewan Harrison, David K. Jackson, Ian Johnston, Dominic Kwiatkowski, Cordelia Langford, John Sillitoe on behalf of the Wellcome Sanger Institute COVID-19 Surveillance Team
EPI_ISL_946003	Lighthouse Lab in Glasgow	Wellcome Sanger Institute for the COVID-19 Genomics UK (COG-UK) Consortium	Harper VanSteenhouse, Yumi Kasai, David Gray, Carol Clugston, Anna Dominiczak and Alex Alderton, Roberto Amato, Sonia Goncalves, Ewan Harrison, David K. Jackson, Ian Johnston, Dominic Kwiatkowski, Cordelia Langford, John Sillitoe on behalf of the Wellcome Sanger Institute COVID-19 Surveillance Team
EPI_ISL_946004, EPI_ISL_946012, EPI_ISL_946013, EPI_ISL_946015, EPI_ISL_946016, EPI_ISL_946017, EPI_ISL_946018, EPI_ISL_946019, EPI_ISL_946020, EPI_ISL_946022, EPI_ISL_946024, EPI_ISL_946025, EPI_ISL_946027, EPI_ISL_946029, EPI_ISL_946031			
see above	Lighthouse Lab in Milton Keynes	Wellcome Sanger Institute for the COVID-19 Genomics UK (COG-UK) Consortium	The Lighthouse Lab in Milton Keynes and Alex Alderton, Roberto Amato, Sonia Goncalves, Ewan Harrison, David K. Jackson, Ian Johnston, Dominic Kwiatkowski, Cordelia Langford, John Sillitoe on behalf of the Wellcome Sanger Institute COVID-19 Surveillance Team
EPI_ISL_946032	Lighthouse Lab in Glasgow	Wellcome Sanger Institute for the COVID-19 Genomics UK (COG-UK) Consortium	Harper VanSteenhouse, Yumi Kasai, David Gray, Carol Clugston, Anna Dominiczak and Alex Alderton, Roberto Amato, Sonia Goncalves, Ewan Harrison, David K. Jackson, Ian Johnston, Dominic Kwiatkowski, Cordelia Langford, John Sillitoe on behalf of the Wellcome Sanger Institute COVID-19 Surveillance Team
EPI_ISL_946033, EPI_ISL_946034, EPI_ISL_946036, EPI_ISL_946038, EPI_ISL_946039, EPI_ISL_946040, EPI_ISL_946042, EPI_ISL_946043, EPI_ISL_946044, EPI_ISL_946045, EPI_ISL_946046			
see above	Lighthouse Lab in Milton Keynes	Wellcome Sanger Institute for the COVID-19 Genomics UK (COG-UK) Consortium	The Lighthouse Lab in Milton Keynes and Alex Alderton, Roberto Amato, Sonia Goncalves, Ewan Harrison, David K. Jackson, Ian Johnston, Dominic Kwiatkowski, Cordelia Langford, John Sillitoe on behalf of the Wellcome Sanger Institute COVID-19 Surveillance Team
EPI_ISL_946047	Lighthouse Lab in Glasgow	Wellcome Sanger Institute for the COVID-19 Genomics UK (COG-UK) Consortium	Harper VanSteenhouse, Yumi Kasai, David Gray, Carol Clugston, Anna Dominiczak and Alex Alderton, Roberto Amato, Sonia Goncalves, Ewan Harrison, David K. Jackson, Ian Johnston, Dominic Kwiatkowski, Cordelia Langford, John Sillitoe on behalf of the Wellcome Sanger Institute COVID-19 Surveillance Team
EPI_ISL_946050, EPI_ISL_946051, EPI_ISL_946053, EPI_ISL_946054, EPI_ISL_946056, EPI_ISL_946060, EPI_ISL_946061, EPI_ISL_946063, EPI_ISL_946064, EPI_ISL_946065, EPI_ISL_946074, EPI_ISL_946075, EPI_ISL_946077, EPI_ISL_946078, EPI_ISL_946079, EPI_ISL_946081, EPI_ISL_946082, EPI_ISL_946083, EPI_ISL_946084, EPI_ISL_946085, EPI_ISL_946089, EPI_ISL_946091, EPI_ISL_946092, EPI_ISL_946095, EPI_ISL_946097, EPI_ISL_946098, EPI_ISL_946099, EPI_ISL_946100, EPI_ISL_946101, EPI_ISL_946102, EPI_ISL_946103, EPI_ISL_946104, EPI_ISL_946105, EPI_ISL_946107, EPI_ISL_946108, EPI_ISL_946111, EPI_ISL_946114, EPI_ISL_946116			
see above	Lighthouse Lab in Milton Keynes	Wellcome Sanger Institute for the COVID-19 Genomics UK (COG-UK) Consortium	The Lighthouse Lab in Milton Keynes and Alex Alderton, Roberto Amato, Sonia Goncalves, Ewan Harrison, David K. Jackson, Ian Johnston, Dominic Kwiatkowski, Cordelia Langford, John Sillitoe on behalf of the Wellcome Sanger Institute COVID-19 Surveillance Team
EPI_ISL_946117	Lighthouse Lab in Glasgow	Wellcome Sanger Institute for the COVID-19 Genomics UK (COG-UK) Consortium	Harper VanSteenhouse, Yumi Kasai, David Gray, Carol Clugston, Anna Dominiczak and Alex Alderton, Roberto Amato, Sonia Goncalves, Ewan Harrison, David K. Jackson, Ian Johnston, Dominic Kwiatkowski, Cordelia Langford, John Sillitoe on behalf of the Wellcome Sanger Institute COVID-19 Surveillance Team
EPI_ISL_946118, EPI_ISL_946119, EPI_ISL_946120, EPI_ISL_946121, EPI_ISL_946122, EPI_ISL_946125, EPI_ISL_946128, EPI_ISL_946130, EPI_ISL_946131, EPI_ISL_946132, EPI_ISL_946133, EPI_ISL_946134, EPI_ISL_946136, EPI_ISL_946138, EPI_ISL_946139, EPI_ISL_946140, EPI_ISL_946141, EPI_ISL_946142, EPI_ISL_946143, EPI_ISL_946144, EPI_ISL_946146, EPI_ISL_946148, EPI_ISL_946151, EPI_ISL_946152, EPI_ISL_946154, EPI_ISL_946155, EPI_ISL_946156, EPI_ISL_946158, EPI_ISL_946159, EPI_ISL_946161, EPI_ISL_946163, EPI_ISL_946164, EPI_ISL_946165, EPI_ISL_946166, EPI_ISL_946167, EPI_ISL_946168, EPI_ISL_946170, EPI_ISL_946171, EPI_ISL_946172, EPI_ISL_946175, EPI_ISL_946178, EPI_ISL_946179, EPI_ISL_946183, EPI_ISL_946184, EPI_ISL_946185, EPI_ISL_946186, EPI_ISL_946187, EPI_ISL_946189, EPI_ISL_946191, EPI_ISL_946193, EPI_ISL_946194, EPI_ISL_946195, EPI_ISL_946197, EPI_ISL_946198, EPI_ISL_946200, EPI_ISL_946201, EPI_ISL_946203, EPI_ISL_946204, EPI_ISL_946205, EPI_ISL_946207, EPI_ISL_946209, EPI_ISL_946212, EPI_ISL_946214, EPI_ISL_946216, EPI_ISL_946217			
see above	Lighthouse Lab in Milton Keynes	Wellcome Sanger Institute for the COVID-19 Genomics UK (COG-UK) Consortium	The Lighthouse Lab in Milton Keynes and Alex Alderton, Roberto Amato, Sonia Goncalves, Ewan Harrison, David K. Jackson, Ian Johnston, Dominic Kwiatkowski, Cordelia Langford, John Sillitoe on behalf of the Wellcome Sanger Institute COVID-19 Surveillance Team
EPI_ISL_946219	Lighthouse Lab in Glasgow	Wellcome Sanger Institute for the COVID-19 Genomics UK (COG-UK) Consortium	Harper VanSteenhouse, Yumi Kasai, David Gray, Carol Clugston, Anna Dominiczak and Alex Alderton, Roberto Amato, Sonia Goncalves, Ewan Harrison, David K. Jackson, Ian Johnston, Dominic Kwiatkowski, Cordelia Langford, John Sillitoe on behalf of the Wellcome Sanger Institute COVID-19 Surveillance Team
EPI_ISL_946222, EPI_ISL_946223, EPI_ISL_946226, EPI_ISL_946228, EPI_ISL_946230, EPI_ISL_946235, EPI_ISL_946236, EPI_ISL_946237, EPI_ISL_946238, EPI_ISL_946239, EPI_ISL_946240, EPI_ISL_946241, EPI_ISL_946242, EPI_ISL_946244, EPI_ISL_946245, EPI_ISL_946246, EPI_ISL_946247, EPI_ISL_946248, EPI_ISL_946249, EPI_ISL_946251, EPI_ISL_946252, EPI_ISL_946255, EPI_ISL_946256, EPI_ISL_946257, EPI_ISL_946258			
see above	Lighthouse Lab in Milton Keynes	Wellcome Sanger Institute for the COVID-19 Genomics UK (COG-UK) Consortium	The Lighthouse Lab in Milton Keynes and Alex Alderton, Roberto Amato, Sonia Goncalves, Ewan Harrison, David K. Jackson, Ian Johnston, Dominic Kwiatkowski, Cordelia Langford, John Sillitoe on behalf of the Wellcome Sanger Institute COVID-19 Surveillance Team
EPI_ISL_946259	Lighthouse Lab in Glasgow	Wellcome Sanger Institute for the COVID-19 Genomics UK (COG-UK) Consortium	Harper VanSteenhouse, Yumi Kasai, David Gray, Carol Clugston, Anna Dominiczak and Alex Alderton, Roberto Amato, Sonia Goncalves, Ewan Harrison, David K. Jackson, Ian Johnston, Dominic Kwiatkowski, Cordelia Langford, John Sillitoe on behalf of the Wellcome Sanger Institute COVID-19 Surveillance Team
EPI_ISL_946261, EPI_ISL_946262, EPI_ISL_946263, EPI_ISL_946265, EPI_ISL_946266, EPI_ISL_946267, EPI_ISL_946268, EPI_ISL_946272, EPI_ISL_946273, EPI_ISL_946275, EPI_ISL_946276, EPI_ISL_946277, EPI_ISL_946278, EPI_ISL_946279, EPI_ISL_946280, EPI_ISL_946282, EPI_ISL_946283, EPI_ISL_946287, EPI_ISL_946288			
see above	Lighthouse Lab in Milton Keynes	Wellcome Sanger Institute for the COVID-19 Genomics UK (COG-UK) Consortium	The Lighthouse Lab in Milton Keynes and Alex Alderton, Roberto Amato, Sonia Goncalves, Ewan Harrison, David K. Jackson, Ian Johnston, Dominic Kwiatkowski, Cordelia Langford, John Sillitoe on behalf of the Wellcome Sanger Institute COVID-19 Surveillance Team
EPI_ISL_946289, EPI_ISL_946290	Lighthouse Lab in Glasgow	Wellcome Sanger Institute for the COVID-19 Genomics UK (COG-UK) Consortium	Harper VanSteenhouse, Yumi Kasai, David Gray, Carol Clugston, Anna Dominiczak and Alex Alderton, Roberto Amato, Sonia Goncalves, Ewan Harrison, David K. Jackson, Ian Johnston, Dominic Kwiatkowski, Cordelia Langford, John Sillitoe on behalf of the Wellcome Sanger Institute COVID-19 Surveillance Team
EPI_ISL_946291, EPI_ISL_946293, EPI_ISL_946294, EPI_ISL_946295, EPI_ISL_946296, EPI_ISL_946297, EPI_ISL_946299, EPI_ISL_946300	Lighthouse Lab in Milton Keynes	Wellcome Sanger Institute for the COVID-19 Genomics UK (COG-UK) Consortium	The Lighthouse Lab in Milton Keynes and Alex Alderton, Roberto Amato, Sonia Goncalves, Ewan Harrison, David K. Jackson, Ian Johnston, Dominic Kwiatkowski, Cordelia Langford, John Sillitoe on behalf of the Wellcome Sanger Institute COVID-19 Surveillance Team
EPI_ISL_946303	Lighthouse Lab in Glasgow	Wellcome Sanger Institute for the COVID-19 Genomics UK	Harper VanSteenhouse, Yumi Kasai, David Gray, Carol Clugston, Anna Dominiczak and Alex Alderton, Roberto Amato, Sonia Goncalves, Ewan Harrison,

		(COG-UK) Consortium	David K. Jackson, Ian Johnston, Dominic Kwiatkowski, Cordelia Langford, John Sillitoe on behalf of the Wellcome Sanger Institute COVID-19 Surveillance Team
EPI_ISL_946305, EPI_ISL_946306, EPI_ISL_946307, EPI_ISL_946308, EPI_ISL_946309, EPI_ISL_946310, EPI_ISL_946311, EPI_ISL_946312, EPI_ISL_946313, EPI_ISL_946314, EPI_ISL_946315, EPI_ISL_946316, EPI_ISL_946317, EPI_ISL_946320, EPI_ISL_946321, EPI_ISL_946322, EPI_ISL_946324, EPI_ISL_946325, EPI_ISL_946327, EPI_ISL_946329, EPI_ISL_946330			
see above	Lighthouse Lab in Milton Keynes	Wellcome Sanger Institute for the COVID-19 Genomics UK (COG-UK) Consortium	The Lighthouse Lab in Milton Keynes and Alex Alderton, Roberto Amato, Sonia Goncalves, Ewan Harrison, David K. Jackson, Ian Johnston, Dominic Kwiatkowski, Cordelia Langford, John Sillitoe on behalf of the Wellcome Sanger Institute COVID-19 Surveillance Team
EPI_ISL_946331	Lighthouse Lab in Glasgow	Wellcome Sanger Institute for the COVID-19 Genomics UK (COG-UK) Consortium	Harper VanSteenhouse, Yumi Kasai, David Gray, Carol Clugston, Anna Dominiczak and Alex Alderton, Roberto Amato, Sonia Goncalves, Ewan Harrison, David K. Jackson, Ian Johnston, Dominic Kwiatkowski, Cordelia Langford, John Sillitoe on behalf of the Wellcome Sanger Institute COVID-19 Surveillance Team
EPI_ISL_946332, EPI_ISL_946334, EPI_ISL_946336, EPI_ISL_946337, EPI_ISL_946340, EPI_ISL_946341, EPI_ISL_946342, EPI_ISL_946343, EPI_ISL_946344, EPI_ISL_946345, EPI_ISL_946346, EPI_ISL_946347, EPI_ISL_946348, EPI_ISL_946349, EPI_ISL_946350, EPI_ISL_946352, EPI_ISL_946353, EPI_ISL_946354, EPI_ISL_946355, EPI_ISL_946358, EPI_ISL_946359, EPI_ISL_946362, EPI_ISL_946364, EPI_ISL_946368, EPI_ISL_946369, EPI_ISL_946370, EPI_ISL_946372			
see above	Lighthouse Lab in Milton Keynes	Wellcome Sanger Institute for the COVID-19 Genomics UK (COG-UK) Consortium	The Lighthouse Lab in Milton Keynes and Alex Alderton, Roberto Amato, Sonia Goncalves, Ewan Harrison, David K. Jackson, Ian Johnston, Dominic Kwiatkowski, Cordelia Langford, John Sillitoe on behalf of the Wellcome Sanger Institute COVID-19 Surveillance Team
EPI_ISL_946373	Lighthouse Lab in Glasgow	Wellcome Sanger Institute for the COVID-19 Genomics UK (COG-UK) Consortium	Harper VanSteenhouse, Yumi Kasai, David Gray, Carol Clugston, Anna Dominiczak and Alex Alderton, Roberto Amato, Sonia Goncalves, Ewan Harrison, David K. Jackson, Ian Johnston, Dominic Kwiatkowski, Cordelia Langford, John Sillitoe on behalf of the Wellcome Sanger Institute COVID-19 Surveillance Team
EPI_ISL_946376, EPI_ISL_946377, EPI_ISL_946382, EPI_ISL_946383, EPI_ISL_946384, EPI_ISL_946386	Lighthouse Lab in Milton Keynes	Wellcome Sanger Institute for the COVID-19 Genomics UK (COG-UK) Consortium	The Lighthouse Lab in Milton Keynes and Alex Alderton, Roberto Amato, Sonia Goncalves, Ewan Harrison, David K. Jackson, Ian Johnston, Dominic Kwiatkowski, Cordelia Langford, John Sillitoe on behalf of the Wellcome Sanger Institute COVID-19 Surveillance Team
EPI_ISL_946387	Lighthouse Lab in Glasgow	Wellcome Sanger Institute for the COVID-19 Genomics UK (COG-UK) Consortium	Harper VanSteenhouse, Yumi Kasai, David Gray, Carol Clugston, Anna Dominiczak and Alex Alderton, Roberto Amato, Sonia Goncalves, Ewan Harrison, David K. Jackson, Ian Johnston, Dominic Kwiatkowski, Cordelia Langford, John Sillitoe on behalf of the Wellcome Sanger Institute COVID-19 Surveillance Team
EPI_ISL_946388, EPI_ISL_946389, EPI_ISL_946390, EPI_ISL_946392, EPI_ISL_946393, EPI_ISL_946394	Lighthouse Lab in Milton Keynes	Wellcome Sanger Institute for the COVID-19 Genomics UK (COG-UK) Consortium	The Lighthouse Lab in Milton Keynes and Alex Alderton, Roberto Amato, Sonia Goncalves, Ewan Harrison, David K. Jackson, Ian Johnston, Dominic Kwiatkowski, Cordelia Langford, John Sillitoe on behalf of the Wellcome Sanger Institute COVID-19 Surveillance Team
EPI_ISL_946395	Lighthouse Lab in Glasgow	Wellcome Sanger Institute for the COVID-19 Genomics UK (COG-UK) Consortium	Harper VanSteenhouse, Yumi Kasai, David Gray, Carol Clugston, Anna Dominiczak and Alex Alderton, Roberto Amato, Sonia Goncalves, Ewan Harrison, David K. Jackson, Ian Johnston, Dominic Kwiatkowski, Cordelia Langford, John Sillitoe on behalf of the Wellcome Sanger Institute COVID-19 Surveillance Team
EPI_ISL_946396	Lighthouse Lab in Milton Keynes	Wellcome Sanger Institute for the COVID-19 Genomics UK (COG-UK) Consortium	The Lighthouse Lab in Milton Keynes and Alex Alderton, Roberto Amato, Sonia Goncalves, Ewan Harrison, David K. Jackson, Ian Johnston, Dominic Kwiatkowski, Cordelia Langford, John Sillitoe on behalf of the Wellcome Sanger Institute COVID-19 Surveillance Team
EPI_ISL_946398	Lighthouse Lab in Glasgow	Wellcome Sanger Institute for the COVID-19 Genomics UK (COG-UK) Consortium	Harper VanSteenhouse, Yumi Kasai, David Gray, Carol Clugston, Anna Dominiczak and Alex Alderton, Roberto Amato, Sonia Goncalves, Ewan Harrison, David K. Jackson, Ian Johnston, Dominic Kwiatkowski, Cordelia Langford, John Sillitoe on behalf of the Wellcome Sanger Institute COVID-19 Surveillance Team
EPI_ISL_946399, EPI_ISL_946401, EPI_ISL_946402, EPI_ISL_946404, EPI_ISL_946406	Lighthouse Lab in Milton Keynes	Wellcome Sanger Institute for the COVID-19 Genomics UK (COG-UK) Consortium	The Lighthouse Lab in Milton Keynes and Alex Alderton, Roberto Amato, Sonia Goncalves, Ewan Harrison, David K. Jackson, Ian Johnston, Dominic Kwiatkowski, Cordelia Langford, John Sillitoe on behalf of the Wellcome Sanger Institute COVID-19 Surveillance Team
EPI_ISL_946408	Lighthouse Lab in Glasgow	Wellcome Sanger Institute for the COVID-19 Genomics UK (COG-UK) Consortium	Harper VanSteenhouse, Yumi Kasai, David Gray, Carol Clugston, Anna Dominiczak and Alex Alderton, Roberto Amato, Sonia Goncalves, Ewan Harrison, David K. Jackson, Ian Johnston, Dominic Kwiatkowski, Cordelia Langford, John Sillitoe on behalf of the Wellcome Sanger Institute COVID-19 Surveillance Team
EPI_ISL_946409, EPI_ISL_946412, EPI_ISL_946413, EPI_ISL_946414, EPI_ISL_946415, EPI_ISL_946416, EPI_ISL_946417, EPI_ISL_946418	Lighthouse Lab in Milton Keynes	Wellcome Sanger Institute for the COVID-19 Genomics UK (COG-UK) Consortium	The Lighthouse Lab in Milton Keynes and Alex Alderton, Roberto Amato, Sonia Goncalves, Ewan Harrison, David K. Jackson, Ian Johnston, Dominic Kwiatkowski, Cordelia Langford, John Sillitoe on behalf of the Wellcome Sanger Institute COVID-19 Surveillance Team
EPI_ISL_946419	Lighthouse Lab in Glasgow	Wellcome Sanger Institute for the COVID-19 Genomics UK (COG-UK) Consortium	Harper VanSteenhouse, Yumi Kasai, David Gray, Carol Clugston, Anna Dominiczak and Alex Alderton, Roberto Amato, Sonia Goncalves, Ewan Harrison, David K. Jackson, Ian Johnston, Dominic Kwiatkowski, Cordelia Langford, John Sillitoe on behalf of the Wellcome Sanger Institute COVID-19 Surveillance Team
EPI_ISL_946420	Lighthouse Lab in Milton Keynes	Wellcome Sanger Institute for the COVID-19 Genomics UK (COG-UK) Consortium	The Lighthouse Lab in Milton Keynes and Alex Alderton, Roberto Amato, Sonia Goncalves, Ewan Harrison, David K. Jackson, Ian Johnston, Dominic Kwiatkowski, Cordelia Langford, John Sillitoe on behalf of the Wellcome Sanger Institute COVID-19 Surveillance Team
EPI_ISL_946422, EPI_ISL_946423, EPI_ISL_946424, EPI_ISL_946425, EPI_ISL_946426, EPI_ISL_946428, EPI_ISL_946429, EPI_ISL_946430, EPI_ISL_946431, EPI_ISL_946432, EPI_ISL_946433, EPI_ISL_946434, EPI_ISL_946435, EPI_ISL_946436, EPI_ISL_946437, EPI_ISL_946438, EPI_ISL_946439, EPI_ISL_946440, EPI_ISL_946441, EPI_ISL_946442, EPI_ISL_946443, EPI_ISL_946444, EPI_ISL_946446, EPI_ISL_946447, EPI_ISL_946448, EPI_ISL_946449, EPI_ISL_946450, EPI_ISL_946451, EPI_ISL_946452, EPI_ISL_946453, EPI_ISL_946455, EPI_ISL_946456, EPI_ISL_946457, EPI_ISL_946458, EPI_ISL_946459, EPI_ISL_946460, EPI_ISL_946461, EPI_ISL_946462, EPI_ISL_946463, EPI_ISL_946464, EPI_ISL_946465, EPI_ISL_946466, EPI_ISL_946467, EPI_ISL_946468, EPI_ISL_946469, EPI_ISL_946470, EPI_ISL_946472, EPI_ISL_946473, EPI_ISL_946474, EPI_ISL_946475, EPI_ISL_946476, EPI_ISL_946477, EPI_ISL_946478, EPI_ISL_946479, EPI_ISL_946480, EPI_ISL_946481, EPI_ISL_946482, EPI_ISL_946483, EPI_ISL_946484, EPI_ISL_946485, EPI_ISL_946486, EPI_ISL_946487, EPI_ISL_946488, EPI_ISL_946489, EPI_ISL_946490, EPI_ISL_946491, EPI_ISL_946492, EPI_ISL_946494, EPI_ISL_946495, EPI_ISL_946496, EPI_ISL_946497, EPI_ISL_946498, EPI_ISL_946499, EPI_ISL_946500, EPI_ISL_946501, EPI_ISL_946502, EPI_ISL_946503, EPI_ISL_946504, EPI_ISL_946505, EPI_ISL_946506, EPI_ISL_946507, EPI_ISL_946508, EPI_ISL_946509, EPI_ISL_946510, EPI_ISL_946511, EPI_ISL_946512, EPI_ISL_946513, EPI_ISL_946514, EPI_ISL_946515, EPI_ISL_946516, EPI_ISL_946517, EPI_ISL_946518, EPI_ISL_946519, EPI_ISL_946520, EPI_ISL_946521, EPI_ISL_946522, EPI_ISL_946523, EPI_ISL_946524, EPI_ISL_946525, EPI_ISL_946526, EPI_ISL_946527, EPI_ISL_946528, EPI_ISL_946529, EPI_ISL_946530, EPI_ISL_946531, EPI_ISL_946532, EPI_ISL_946533, EPI_ISL_946534, EPI_ISL_946535, EPI_ISL_946536, EPI_ISL_946537, EPI_ISL_946538, EPI_ISL_946539, EPI_ISL_946540, EPI_ISL_946541, EPI_ISL_946542, EPI_ISL_946543, EPI_ISL_946544, EPI_ISL_946545, EPI_ISL_946546, EPI_ISL_946548, EPI_ISL_946549, EPI_ISL_946550, EPI_ISL_946551, EPI_ISL_946552, EPI_ISL_946553, EPI_ISL_946554, EPI_ISL_946555, EPI_ISL_946556, EPI_ISL_946557, EPI_ISL_946558, EPI_ISL_946559, EPI_ISL_946560, EPI_ISL_946561, EPI_ISL_946562, EPI_ISL_946563, EPI_ISL_946564, EPI_ISL_946565, EPI_ISL_946566, EPI_ISL_946567, EPI_ISL_946568, EPI_ISL_946569, EPI_ISL_946570, EPI_ISL_946571, EPI_ISL_946572, EPI_ISL_946573, EPI_ISL_946574, EPI_ISL_946575, EPI_ISL_946576, EPI_ISL_946577, EPI_ISL_946578, EPI_ISL_946579, EPI_ISL_946581, EPI_ISL_946582, EPI_ISL_946583, EPI_ISL_946584, EPI_ISL_946586, EPI_ISL_946587, EPI_ISL_946588, EPI_ISL_946589, EPI_ISL_946590, EPI_ISL_946591, EPI_ISL_946592, EPI_ISL_946593, EPI_ISL_946594, EPI_ISL_946595, EPI_ISL_946596, EPI_ISL_946597, EPI_ISL_946598, EPI_ISL_946599, EPI_ISL_946600, EPI_ISL_946601, EPI_ISL_946602, EPI_ISL_946603, EPI_ISL_946604, EPI_ISL_946605, EPI_ISL_946606, EPI_ISL_946607, EPI_ISL_946608, EPI_ISL_946609, EPI_ISL_946610, EPI_ISL_946611, EPI_ISL_946612, EPI_ISL_946613, EPI_ISL_946614, EPI_ISL_946615, EPI_ISL_946616, EPI_ISL_946617, EPI_ISL_946618, EPI_ISL_946619, EPI_ISL_946620, EPI_ISL_946621, EPI_ISL_946622, EPI_ISL_946623, EPI_ISL_946624, EPI_ISL_946625, EPI_ISL_946626, EPI_ISL_946627, EPI_ISL_946628, EPI_ISL_946629, EPI_ISL_946630, EPI_ISL_946631, EPI_ISL_946632, EPI_ISL_946633, EPI_ISL_946634, EPI_ISL_946635, EPI_ISL_946636, EPI_ISL_946637, EPI_ISL_946638, EPI_ISL_946639, EPI_ISL_946640, EPI_ISL_946641, EPI_ISL_946642, EPI_ISL_946643, EPI_ISL_946644, EPI_ISL_946645, EPI_ISL_946647, EPI_ISL_946648, EPI_ISL_946649, EPI_ISL_946650, EPI_ISL_946651, EPI_ISL_946652, EPI_ISL_946653, EPI_ISL_946654, EPI_ISL_946655, EPI_ISL_946656, EPI_ISL_946657, EPI_ISL_946658, EPI_ISL_946659, EPI_ISL_946660, EPI_ISL_946661, EPI_ISL_946662, EPI_ISL_946663, EPI_ISL_946664, EPI_ISL_946665, EPI_ISL_946666, EPI_ISL_946667, EPI_ISL_946668, EPI_ISL_946669, EPI_ISL_946670, EPI_ISL_946671, EPI_ISL_946673, EPI_ISL_946674, EPI_ISL_946675, EPI_ISL_946676, EPI_ISL_946677, EPI_ISL_946678, EPI_ISL_946679, EPI_ISL_946680, EPI_ISL_946681, EPI_ISL_946682, EPI_ISL_946683, EPI_ISL_946684, EPI_ISL_946685, EPI_ISL_946686, EPI_ISL_946687, EPI_ISL_946688, EPI_ISL_946689, EPI_ISL_946690, EPI_ISL_946691, EPI_ISL_946692, EPI_ISL_946693, EPI_ISL_946694, EPI_ISL_946695, EPI_ISL_946696, EPI_ISL_946697, EPI_ISL_946698, EPI_ISL_946699, EPI_ISL_946700, EPI_ISL_946702, EPI_ISL_946703, EPI_ISL_946704, EPI_ISL_946705, EPI_ISL_946706, EPI_ISL_946707, EPI_ISL_946708, EPI_ISL_946709, EPI_ISL_946710, EPI_ISL_946711, EPI_ISL_946712, EPI_ISL_946713, EPI_ISL_946714, EPI_ISL_946715, EPI_ISL_946716, EPI_ISL_946717, EPI_ISL_946718, EPI_ISL_946719, EPI_ISL_946720, EPI_ISL_946721, EPI_ISL_946722, EPI_ISL_946723, EPI_ISL_946724, EPI_ISL_946725, EPI_ISL_946726, EPI_ISL_946727, EPI_ISL_946728, EPI_ISL_946729, EPI_ISL_946730, EPI_ISL_946731, EPI_ISL_946732, EPI_ISL_946733, EPI_ISL_946734, EPI_ISL_946735, EPI_ISL_946736, EPI_ISL_946737, EPI_ISL_946738, EPI_ISL_946739, EPI_ISL_946740, EPI_ISL_946741, EPI_ISL_946742, EPI_ISL_946743, EPI_ISL_946744, EPI_ISL_946745, EPI_ISL_946747, EPI_ISL_946748, EPI_ISL_946749, EPI_ISL_946750, EPI_ISL_946751, EPI_ISL_946752, EPI_ISL_946753, EPI_ISL_946754, EPI_ISL_946755, EPI_ISL_946756, EPI_ISL_946757, EPI_ISL_946758, EPI_ISL_946759, EPI_ISL_946760, EPI_ISL_946761, EPI_ISL_946762, EPI_ISL_946763, EPI_ISL_946764, EPI_ISL_946765, EPI_ISL_946766, EPI_ISL_946767, EPI_ISL_946768, EPI_ISL_946769, EPI_ISL_946770, EPI_ISL_946771, EPI_ISL_946772, EPI_ISL_946773, EPI_ISL_946774, EPI_ISL_946775, EPI_ISL_946776, EPI_ISL_946777, EPI_ISL_946778, EPI_ISL_946779, EPI_ISL_946781, EPI_ISL_946782, EPI_ISL_946783, EPI_ISL_946784, EPI_ISL_946785, EPI_ISL_946786, EPI_ISL_946787, EPI_ISL_946788, EPI_ISL_946789, EPI_ISL_946790, EPI_ISL_946791, EPI_ISL_946792, EPI_ISL_946793, EPI_ISL_946794, EPI_ISL_946795, EPI_ISL_946796, EPI_ISL_946797, EPI_ISL_946798, EPI_ISL_946799, EPI_ISL_946800, EPI_ISL_946801, EPI_ISL_946802, EPI_ISL_946803, EPI_ISL_946804, EPI_ISL_946805, EPI_ISL_946806, EPI_ISL_946807, EPI_ISL_946808, EPI_ISL_946809, EPI_ISL_946810, EPI_ISL_946811, EPI_ISL_946812, EPI_ISL_946813, EPI_ISL_946814, EPI_ISL_946815, EPI_ISL_946816, EPI_ISL_946817, EPI_ISL_946818, EPI_ISL_946819, EPI_ISL_946820, EPI_ISL_946821, EPI_ISL_946822, EPI_ISL_946823, EPI_ISL_946824, EPI_ISL_946825, EPI_ISL_946826, EPI_ISL_946827, EPI_ISL_946828, EPI_ISL_946829, EPI_ISL_946830, EPI_ISL_946831, EPI_ISL_946832, EPI_ISL_946833, EPI_ISL_946834, EPI_ISL_946835, EPI_ISL_946836, EPI_ISL_946837, EPI_ISL_946838, EPI_ISL_946839, EPI_ISL_946840, EPI_ISL_946841, EPI_ISL_946842, EPI_ISL_946843, EPI_ISL_946844, EPI_ISL_946845, EPI_ISL_946846, EPI_ISL_946847, EPI_ISL_946848, EPI_ISL_946849, EPI_ISL_946850, EPI_ISL_946851, EPI_ISL_946852, EPI_ISL_946853, EPI_ISL_946854, EPI_ISL_946855, EPI_ISL_946856, EPI_ISL_946857, EPI_ISL_946858, EPI_ISL_946859, EPI_ISL_946860, EPI_ISL_946861, EPI_ISL_946862, EPI_ISL_946863, EPI_ISL_946864, EPI_ISL_946865, EPI_ISL_946866, EPI_ISL_946867, EPI_ISL_946868, EPI_ISL_946869, EPI_ISL_946870, EPI_ISL_946871, EPI_ISL_946872, EPI_ISL_946873, EPI_ISL_946874, EPI_ISL_946875, EPI_ISL_946876, EPI_ISL_946877, EPI_ISL_946878, EPI_ISL_946879, EPI_ISL_946880, EPI_ISL_946881, EPI_ISL_946882, EPI_ISL_946883, EPI_ISL_946884, EPI_ISL_946885, EPI_ISL_946886, EPI_ISL_946887, EPI_ISL_946888, EPI_ISL_946889, EPI_ISL_946890, EPI_ISL_946891, EPI_ISL_946892, EPI_ISL_946893, EPI_ISL_946894, EPI_ISL_946895, EPI_ISL_946896, EPI_ISL_946897, EPI_ISL_946898, EPI_ISL_946899, EPI_ISL_946900, EPI_ISL_946901, EPI_ISL_946902, EPI_ISL_946903, EPI_ISL_946904, EPI_ISL_946905, EPI_ISL_946906, EPI_ISL_946907, EPI_ISL_946908, EPI_ISL_946909, EPI_ISL_946910, EPI_ISL_946911, EPI_ISL_946912, EPI_ISL_946914, EPI_ISL_946915, EPI_ISL_946916, EPI_ISL_946917, EPI_ISL_946918, EPI_ISL_946919, EPI_ISL_946920, EPI_ISL_946921, EPI_ISL_946922, EPI_ISL_946923, EPI_ISL_946924, EPI_ISL_946925, EPI_ISL_946926, EPI_ISL_946927, EPI_ISL_946928, EPI_ISL_946929, EPI_ISL_946930, EPI_ISL_946931, EPI_ISL_946932, EPI_ISL_946933, EPI_ISL_946934, EPI_ISL_946935, EPI_ISL_946936, EPI_ISL_946937, EPI_ISL_946938, EPI_ISL_946939, EPI_ISL_946940, EPI_ISL_946941, EPI_ISL_946942, EPI_ISL_946943, EPI_ISL_946944, EPI_ISL_946945, EPI_ISL_946946, EPI_ISL_946947, EPI_ISL_946948, EPI_ISL_946949, EPI_ISL_946950, EPI_ISL_946951, EPI_ISL_946952, EPI_ISL_946953, EPI_ISL_946954, EPI_ISL_946955, EPI_ISL_946957, EPI_ISL_946958, EPI_ISL_946959, EPI_ISL_946960, EPI_ISL_946961, EPI_ISL_946962, EPI_ISL_946964, EPI_ISL_946965, EPI_ISL_946966, EPI_ISL_946967, EPI_ISL_946968, EPI_ISL_946969, EPI_ISL_946970, EPI_ISL_946971, EPI_ISL_946972, EPI_ISL_946973, EPI_ISL_946974, EPI_ISL_946975, EPI_ISL_946976, EPI_ISL_946977,			

EPI_ISL_946978, EPI_ISL_946979, EPI_ISL_946980, EPI_ISL_946981, EPI_ISL_946982, EPI_ISL_946983, EPI_ISL_946984, EPI_ISL_946985, EPI_ISL_946986, EPI_ISL_946987, EPI_ISL_946988, EPI_ISL_946989, EPI_ISL_946990, EPI_ISL_946991, EPI_ISL_946993, EPI_ISL_946994, EPI_ISL_946995, EPI_ISL_946996, EPI_ISL_946997, EPI_ISL_946998, EPI_ISL_946999, EPI_ISL_947000, EPI_ISL_947001, EPI_ISL_947002, EPI_ISL_947003, EPI_ISL_947004, EPI_ISL_947005, EPI_ISL_947006, EPI_ISL_947007, EPI_ISL_947008, EPI_ISL_947009, EPI_ISL_947010, EPI_ISL_947011, EPI_ISL_947012, EPI_ISL_947013, EPI_ISL_947014, EPI_ISL_947015, EPI_ISL_947016, EPI_ISL_947017, EPI_ISL_947018, EPI_ISL_947019, EPI_ISL_947020, EPI_ISL_947021, EPI_ISL_947022, EPI_ISL_947023, EPI_ISL_947024, EPI_ISL_947025, EPI_ISL_947026, EPI_ISL_947027, EPI_ISL_947028, EPI_ISL_947029, EPI_ISL_947030, EPI_ISL_947031, EPI_ISL_947032, EPI_ISL_947033, EPI_ISL_947034, EPI_ISL_947035, EPI_ISL_947036, EPI_ISL_947037, EPI_ISL_947038, EPI_ISL_947039, EPI_ISL_947040, EPI_ISL_947041, EPI_ISL_947042, EPI_ISL_947043, EPI_ISL_947044, EPI_ISL_947045, EPI_ISL_947046, EPI_ISL_947047, EPI_ISL_947048, EPI_ISL_947049, EPI_ISL_947050, EPI_ISL_947051, EPI_ISL_947052, EPI_ISL_947053, EPI_ISL_947054, EPI_ISL_947055, EPI_ISL_947056, EPI_ISL_947057, EPI_ISL_947058, EPI_ISL_947059, EPI_ISL_947060, EPI_ISL_947061, EPI_ISL_947062, EPI_ISL_947063, EPI_ISL_947064, EPI_ISL_947065, EPI_ISL_947066, EPI_ISL_947067, EPI_ISL_947068, EPI_ISL_947069, EPI_ISL_947070, EPI_ISL_947071, EPI_ISL_947072, EPI_ISL_947073, EPI_ISL_947074, EPI_ISL_947075, EPI_ISL_947076, EPI_ISL_947077, EPI_ISL_947078, EPI_ISL_947079, EPI_ISL_947080, EPI_ISL_947081, EPI_ISL_947082, EPI_ISL_947083, EPI_ISL_947084, EPI_ISL_947085, EPI_ISL_947086, EPI_ISL_947087, EPI_ISL_947088, EPI_ISL_947089, EPI_ISL_947090, EPI_ISL_947091, EPI_ISL_947092, EPI_ISL_947093, EPI_ISL_947094, EPI_ISL_947095, EPI_ISL_947096, EPI_ISL_947097, EPI_ISL_947098, EPI_ISL_947099, EPI_ISL_947100, EPI_ISL_947101, EPI_ISL_947102, EPI_ISL_947103, EPI_ISL_947104, EPI_ISL_947105, EPI_ISL_947106

see above	Lighthouse Lab in Alderley Park	Wellcome Sanger Institute for the COVID-19 Genomics UK (COG-UK) Consortium	Jacquelyn Wynn, Mairead Hyland, The Lighthouse Lab in Alderley Park and Alex Alderton, Roberto Amato, Sonia Goncalves, Ewan Harrison, David K. Jackson, Ian Johnston, Dominic Kwiatkowski, Cordelia Langford, John Sillitoe on behalf of the Wellcome Sanger Institute COVID-19 Surveillance Team
EPI_ISL_947107, EPI_ISL_947108	Lighthouse Lab in Glasgow	Wellcome Sanger Institute for the COVID-19 Genomics UK (COG-UK) Consortium	Harper VanSteenhouse, Yumi Kasai, David Gray, Carol Clugston, Anna Dominiczak and Alex Alderton, Roberto Amato, Sonia Goncalves, Ewan Harrison, David K. Jackson, Ian Johnston, Dominic Kwiatkowski, Cordelia Langford, John Sillitoe on behalf of the Wellcome Sanger Institute COVID-19 Surveillance Team
EPI_ISL_947109, EPI_ISL_947111, EPI_ISL_947112, EPI_ISL_947114, EPI_ISL_947115, EPI_ISL_947116, EPI_ISL_947117	Lighthouse Lab in Milton Keynes	Wellcome Sanger Institute for the COVID-19 Genomics UK (COG-UK) Consortium	The Lighthouse Lab in Milton Keynes and Alex Alderton, Roberto Amato, Sonia Goncalves, Ewan Harrison, David K. Jackson, Ian Johnston, Dominic Kwiatkowski, Cordelia Langford, John Sillitoe on behalf of the Wellcome Sanger Institute COVID-19 Surveillance Team
EPI_ISL_947118, EPI_ISL_947119, EPI_ISL_947121	Lighthouse Lab in Glasgow	Wellcome Sanger Institute for the COVID-19 Genomics UK (COG-UK) Consortium	Harper VanSteenhouse, Yumi Kasai, David Gray, Carol Clugston, Anna Dominiczak and Alex Alderton, Roberto Amato, Sonia Goncalves, Ewan Harrison, David K. Jackson, Ian Johnston, Dominic Kwiatkowski, Cordelia Langford, John Sillitoe on behalf of the Wellcome Sanger Institute COVID-19 Surveillance Team
EPI_ISL_947122, EPI_ISL_947123, EPI_ISL_947124, EPI_ISL_947125	Lighthouse Lab in Milton Keynes	Wellcome Sanger Institute for the COVID-19 Genomics UK (COG-UK) Consortium	The Lighthouse Lab in Milton Keynes and Alex Alderton, Roberto Amato, Sonia Goncalves, Ewan Harrison, David K. Jackson, Ian Johnston, Dominic Kwiatkowski, Cordelia Langford, John Sillitoe on behalf of the Wellcome Sanger Institute COVID-19 Surveillance Team
EPI_ISL_947126, EPI_ISL_947127	Lighthouse Lab in Glasgow	Wellcome Sanger Institute for the COVID-19 Genomics UK (COG-UK) Consortium	Harper VanSteenhouse, Yumi Kasai, David Gray, Carol Clugston, Anna Dominiczak and Alex Alderton, Roberto Amato, Sonia Goncalves, Ewan Harrison, David K. Jackson, Ian Johnston, Dominic Kwiatkowski, Cordelia Langford, John Sillitoe on behalf of the Wellcome Sanger Institute COVID-19 Surveillance Team
EPI_ISL_947128, EPI_ISL_947129, EPI_ISL_947130	Lighthouse Lab in Milton Keynes	Wellcome Sanger Institute for the COVID-19 Genomics UK (COG-UK) Consortium	The Lighthouse Lab in Milton Keynes and Alex Alderton, Roberto Amato, Sonia Goncalves, Ewan Harrison, David K. Jackson, Ian Johnston, Dominic Kwiatkowski, Cordelia Langford, John Sillitoe on behalf of the Wellcome Sanger Institute COVID-19 Surveillance Team
EPI_ISL_947131	Lighthouse Lab in Glasgow	Wellcome Sanger Institute for the COVID-19 Genomics UK (COG-UK) Consortium	Harper VanSteenhouse, Yumi Kasai, David Gray, Carol Clugston, Anna Dominiczak and Alex Alderton, Roberto Amato, Sonia Goncalves, Ewan Harrison, David K. Jackson, Ian Johnston, Dominic Kwiatkowski, Cordelia Langford, John Sillitoe on behalf of the Wellcome Sanger Institute COVID-19 Surveillance Team
EPI_ISL_947132, EPI_ISL_947133	Lighthouse Lab in Milton Keynes	Wellcome Sanger Institute for the COVID-19 Genomics UK (COG-UK) Consortium	The Lighthouse Lab in Milton Keynes and Alex Alderton, Roberto Amato, Sonia Goncalves, Ewan Harrison, David K. Jackson, Ian Johnston, Dominic Kwiatkowski, Cordelia Langford, John Sillitoe on behalf of the Wellcome Sanger Institute COVID-19 Surveillance Team
EPI_ISL_947134	Lighthouse Lab in Glasgow	Wellcome Sanger Institute for the COVID-19 Genomics UK (COG-UK) Consortium	Harper VanSteenhouse, Yumi Kasai, David Gray, Carol Clugston, Anna Dominiczak and Alex Alderton, Roberto Amato, Sonia Goncalves, Ewan Harrison, David K. Jackson, Ian Johnston, Dominic Kwiatkowski, Cordelia Langford, John Sillitoe on behalf of the Wellcome Sanger Institute COVID-19 Surveillance Team
EPI_ISL_947135, EPI_ISL_947136	Lighthouse Lab in Milton Keynes	Wellcome Sanger Institute for the COVID-19 Genomics UK (COG-UK) Consortium	The Lighthouse Lab in Milton Keynes and Alex Alderton, Roberto Amato, Sonia Goncalves, Ewan Harrison, David K. Jackson, Ian Johnston, Dominic Kwiatkowski, Cordelia Langford, John Sillitoe on behalf of the Wellcome Sanger Institute COVID-19 Surveillance Team
EPI_ISL_947137	Lighthouse Lab in Glasgow	Wellcome Sanger Institute for the COVID-19 Genomics UK (COG-UK) Consortium	Harper VanSteenhouse, Yumi Kasai, David Gray, Carol Clugston, Anna Dominiczak and Alex Alderton, Roberto Amato, Sonia Goncalves, Ewan Harrison, David K. Jackson, Ian Johnston, Dominic Kwiatkowski, Cordelia Langford, John Sillitoe on behalf of the Wellcome Sanger Institute COVID-19 Surveillance Team
EPI_ISL_947138	Lighthouse Lab in Milton Keynes	Wellcome Sanger Institute for the COVID-19 Genomics UK (COG-UK) Consortium	The Lighthouse Lab in Milton Keynes and Alex Alderton, Roberto Amato, Sonia Goncalves, Ewan Harrison, David K. Jackson, Ian Johnston, Dominic Kwiatkowski, Cordelia Langford, John Sillitoe on behalf of the Wellcome Sanger Institute COVID-19 Surveillance Team
EPI_ISL_947139, EPI_ISL_947141	Lighthouse Lab in Glasgow	Wellcome Sanger Institute for the COVID-19 Genomics UK (COG-UK) Consortium	Harper VanSteenhouse, Yumi Kasai, David Gray, Carol Clugston, Anna Dominiczak and Alex Alderton, Roberto Amato, Sonia Goncalves, Ewan Harrison, David K. Jackson, Ian Johnston, Dominic Kwiatkowski, Cordelia Langford, John Sillitoe on behalf of the Wellcome Sanger Institute COVID-19 Surveillance Team
EPI_ISL_947142, EPI_ISL_947143, EPI_ISL_947144, EPI_ISL_947145, EPI_ISL_947146	Lighthouse Lab in Milton Keynes	Wellcome Sanger Institute for the COVID-19 Genomics UK (COG-UK) Consortium	The Lighthouse Lab in Milton Keynes and Alex Alderton, Roberto Amato, Sonia Goncalves, Ewan Harrison, David K. Jackson, Ian Johnston, Dominic Kwiatkowski, Cordelia Langford, John Sillitoe on behalf of the Wellcome Sanger Institute COVID-19 Surveillance Team
EPI_ISL_947147	Lighthouse Lab in Glasgow	Wellcome Sanger Institute for the COVID-19 Genomics UK (COG-UK) Consortium	Harper VanSteenhouse, Yumi Kasai, David Gray, Carol Clugston, Anna Dominiczak and Alex Alderton, Roberto Amato, Sonia Goncalves, Ewan Harrison, David K. Jackson, Ian Johnston, Dominic Kwiatkowski, Cordelia Langford, John Sillitoe on behalf of the Wellcome Sanger Institute COVID-19 Surveillance Team
EPI_ISL_947148	Lighthouse Lab in Milton Keynes	Wellcome Sanger Institute for the COVID-19 Genomics UK (COG-UK) Consortium	The Lighthouse Lab in Milton Keynes and Alex Alderton, Roberto Amato, Sonia Goncalves, Ewan Harrison, David K. Jackson, Ian Johnston, Dominic Kwiatkowski, Cordelia Langford, John Sillitoe on behalf of the Wellcome Sanger Institute COVID-19 Surveillance Team
EPI_ISL_947150	Lighthouse Lab in Glasgow	Wellcome Sanger Institute for the COVID-19 Genomics UK (COG-UK) Consortium	Harper VanSteenhouse, Yumi Kasai, David Gray, Carol Clugston, Anna Dominiczak and Alex Alderton, Roberto Amato, Sonia Goncalves, Ewan Harrison, David K. Jackson, Ian Johnston, Dominic Kwiatkowski, Cordelia Langford, John Sillitoe on behalf of the Wellcome Sanger Institute COVID-19 Surveillance Team
EPI_ISL_947151, EPI_ISL_947152	Lighthouse Lab in Milton Keynes	Wellcome Sanger Institute for the COVID-19 Genomics UK (COG-UK) Consortium	The Lighthouse Lab in Milton Keynes and Alex Alderton, Roberto Amato, Sonia Goncalves, Ewan Harrison, David K. Jackson, Ian Johnston, Dominic Kwiatkowski, Cordelia Langford, John Sillitoe on behalf of the Wellcome Sanger Institute COVID-19 Surveillance Team
EPI_ISL_947153	Lighthouse Lab in Glasgow	Wellcome Sanger Institute for the COVID-19 Genomics UK (COG-UK) Consortium	Harper VanSteenhouse, Yumi Kasai, David Gray, Carol Clugston, Anna Dominiczak and Alex Alderton, Roberto Amato, Sonia Goncalves, Ewan Harrison, David K. Jackson, Ian Johnston, Dominic Kwiatkowski, Cordelia Langford, John Sillitoe on behalf of the Wellcome Sanger Institute COVID-19 Surveillance Team
EPI_ISL_947154, EPI_ISL_947155, EPI_ISL_947156, EPI_ISL_947157, EPI_ISL_947158, EPI_ISL_947159, EPI_ISL_947160, EPI_ISL_947161, EPI_ISL_947162	Lighthouse Lab in Milton Keynes	Wellcome Sanger Institute for the COVID-19 Genomics UK (COG-UK) Consortium	The Lighthouse Lab in Milton Keynes and Alex Alderton, Roberto Amato, Sonia Goncalves, Ewan Harrison, David K. Jackson, Ian Johnston, Dominic Kwiatkowski, Cordelia Langford, John Sillitoe on behalf of the Wellcome Sanger Institute COVID-19 Surveillance Team
EPI_ISL_947163	Lighthouse Lab in Glasgow	Wellcome Sanger Institute for the COVID-19 Genomics UK (COG-UK) Consortium	Harper VanSteenhouse, Yumi Kasai, David Gray, Carol Clugston, Anna Dominiczak and Alex Alderton, Roberto Amato, Sonia Goncalves, Ewan Harrison, David K. Jackson, Ian Johnston, Dominic Kwiatkowski, Cordelia Langford, John Sillitoe on behalf of the Wellcome Sanger Institute COVID-19 Surveillance Team
EPI_ISL_947164	Lighthouse Lab in Milton Keynes	Wellcome Sanger Institute for the COVID-19 Genomics UK (COG-UK) Consortium	The Lighthouse Lab in Milton Keynes and Alex Alderton, Roberto Amato, Sonia Goncalves, Ewan Harrison, David K. Jackson, Ian Johnston, Dominic Kwiatkowski, Cordelia Langford, John Sillitoe on behalf of the Wellcome Sanger Institute COVID-19 Surveillance Team
EPI_ISL_947165	Lighthouse Lab in Glasgow	Wellcome Sanger Institute for the COVID-19 Genomics UK (COG-UK) Consortium	Harper VanSteenhouse, Yumi Kasai, David Gray, Carol Clugston, Anna Dominiczak and Alex Alderton, Roberto Amato, Sonia Goncalves, Ewan Harrison, David K. Jackson, Ian Johnston, Dominic Kwiatkowski, Cordelia Langford, John Sillitoe on behalf of the Wellcome Sanger Institute COVID-19 Surveillance Team
EPI_ISL_947166, EPI_ISL_947167,	Lighthouse Lab in Milton Keynes	Wellcome Sanger Institute for the COVID-19 Genomics UK	The Lighthouse Lab in Milton Keynes and Alex Alderton, Roberto Amato, Sonia Goncalves, Ewan Harrison, David K. Jackson, Ian Johnston, Dominic

EPI_ISL_947168		(COG-UK) Consortium	Kwiatkowski, Cordelia Langford, John Sillitoe on behalf of the Wellcome Sanger Institute COVID-19 Surveillance Team
EPI_ISL_947169	Lighthouse Lab in Glasgow	Wellcome Sanger Institute for the COVID-19 Genomics UK (COG-UK) Consortium	Harper VanSteenhouse, Yumi Kasai, David Gray, Carol Clugston, Anna Dominiczak and Alex Alderton, Roberto Amato, Sonia Goncalves, Ewan Harrison, David K. Jackson, Ian Johnston, Dominic Kwiatkowski, Cordelia Langford, John Sillitoe on behalf of the Wellcome Sanger Institute COVID-19 Surveillance Team
EPI_ISL_947170, EPI_ISL_947171, EPI_ISL_947173, EPI_ISL_947174, EPI_ISL_947175, EPI_ISL_947176, EPI_ISL_947177, EPI_ISL_947178	Lighthouse Lab in Milton Keynes	Wellcome Sanger Institute for the COVID-19 Genomics UK (COG-UK) Consortium	The Lighthouse Lab in Milton Keynes and Alex Alderton, Roberto Amato, Sonia Goncalves, Ewan Harrison, David K. Jackson, Ian Johnston, Dominic Kwiatkowski, Cordelia Langford, John Sillitoe on behalf of the Wellcome Sanger Institute COVID-19 Surveillance Team
EPI_ISL_947179	Lighthouse Lab in Glasgow	Wellcome Sanger Institute for the COVID-19 Genomics UK (COG-UK) Consortium	Harper VanSteenhouse, Yumi Kasai, David Gray, Carol Clugston, Anna Dominiczak and Alex Alderton, Roberto Amato, Sonia Goncalves, Ewan Harrison, David K. Jackson, Ian Johnston, Dominic Kwiatkowski, Cordelia Langford, John Sillitoe on behalf of the Wellcome Sanger Institute COVID-19 Surveillance Team
EPI_ISL_947180, EPI_ISL_947181	Lighthouse Lab in Milton Keynes	Wellcome Sanger Institute for the COVID-19 Genomics UK (COG-UK) Consortium	The Lighthouse Lab in Milton Keynes and Alex Alderton, Roberto Amato, Sonia Goncalves, Ewan Harrison, David K. Jackson, Ian Johnston, Dominic Kwiatkowski, Cordelia Langford, John Sillitoe on behalf of the Wellcome Sanger Institute COVID-19 Surveillance Team
EPI_ISL_947182, EPI_ISL_947183	Lighthouse Lab in Glasgow	Wellcome Sanger Institute for the COVID-19 Genomics UK (COG-UK) Consortium	Harper VanSteenhouse, Yumi Kasai, David Gray, Carol Clugston, Anna Dominiczak and Alex Alderton, Roberto Amato, Sonia Goncalves, Ewan Harrison, David K. Jackson, Ian Johnston, Dominic Kwiatkowski, Cordelia Langford, John Sillitoe on behalf of the Wellcome Sanger Institute COVID-19 Surveillance Team
EPI_ISL_947185	Lighthouse Lab in Milton Keynes	Wellcome Sanger Institute for the COVID-19 Genomics UK (COG-UK) Consortium	The Lighthouse Lab in Milton Keynes and Alex Alderton, Roberto Amato, Sonia Goncalves, Ewan Harrison, David K. Jackson, Ian Johnston, Dominic Kwiatkowski, Cordelia Langford, John Sillitoe on behalf of the Wellcome Sanger Institute COVID-19 Surveillance Team
EPI_ISL_947186, EPI_ISL_947187	Lighthouse Lab in Glasgow	Wellcome Sanger Institute for the COVID-19 Genomics UK (COG-UK) Consortium	Harper VanSteenhouse, Yumi Kasai, David Gray, Carol Clugston, Anna Dominiczak and Alex Alderton, Roberto Amato, Sonia Goncalves, Ewan Harrison, David K. Jackson, Ian Johnston, Dominic Kwiatkowski, Cordelia Langford, John Sillitoe on behalf of the Wellcome Sanger Institute COVID-19 Surveillance Team
EPI_ISL_947188, EPI_ISL_947190	Lighthouse Lab in Milton Keynes	Wellcome Sanger Institute for the COVID-19 Genomics UK (COG-UK) Consortium	The Lighthouse Lab in Milton Keynes and Alex Alderton, Roberto Amato, Sonia Goncalves, Ewan Harrison, David K. Jackson, Ian Johnston, Dominic Kwiatkowski, Cordelia Langford, John Sillitoe on behalf of the Wellcome Sanger Institute COVID-19 Surveillance Team
EPI_ISL_947191	Lighthouse Lab in Glasgow	Wellcome Sanger Institute for the COVID-19 Genomics UK (COG-UK) Consortium	Harper VanSteenhouse, Yumi Kasai, David Gray, Carol Clugston, Anna Dominiczak and Alex Alderton, Roberto Amato, Sonia Goncalves, Ewan Harrison, David K. Jackson, Ian Johnston, Dominic Kwiatkowski, Cordelia Langford, John Sillitoe on behalf of the Wellcome Sanger Institute COVID-19 Surveillance Team
EPI_ISL_947192, EPI_ISL_947193, EPI_ISL_947194, EPI_ISL_947195, EPI_ISL_947196	Lighthouse Lab in Milton Keynes	Wellcome Sanger Institute for the COVID-19 Genomics UK (COG-UK) Consortium	The Lighthouse Lab in Milton Keynes and Alex Alderton, Roberto Amato, Sonia Goncalves, Ewan Harrison, David K. Jackson, Ian Johnston, Dominic Kwiatkowski, Cordelia Langford, John Sillitoe on behalf of the Wellcome Sanger Institute COVID-19 Surveillance Team
EPI_ISL_947197	Lighthouse Lab in Glasgow	Wellcome Sanger Institute for the COVID-19 Genomics UK (COG-UK) Consortium	Harper VanSteenhouse, Yumi Kasai, David Gray, Carol Clugston, Anna Dominiczak and Alex Alderton, Roberto Amato, Sonia Goncalves, Ewan Harrison, David K. Jackson, Ian Johnston, Dominic Kwiatkowski, Cordelia Langford, John Sillitoe on behalf of the Wellcome Sanger Institute COVID-19 Surveillance Team
EPI_ISL_947198, EPI_ISL_947199, EPI_ISL_947200	Lighthouse Lab in Milton Keynes	Wellcome Sanger Institute for the COVID-19 Genomics UK (COG-UK) Consortium	The Lighthouse Lab in Milton Keynes and Alex Alderton, Roberto Amato, Sonia Goncalves, Ewan Harrison, David K. Jackson, Ian Johnston, Dominic Kwiatkowski, Cordelia Langford, John Sillitoe on behalf of the Wellcome Sanger Institute COVID-19 Surveillance Team
EPI_ISL_947202	Lighthouse Lab in Glasgow	Wellcome Sanger Institute for the COVID-19 Genomics UK (COG-UK) Consortium	Harper VanSteenhouse, Yumi Kasai, David Gray, Carol Clugston, Anna Dominiczak and Alex Alderton, Roberto Amato, Sonia Goncalves, Ewan Harrison, David K. Jackson, Ian Johnston, Dominic Kwiatkowski, Cordelia Langford, John Sillitoe on behalf of the Wellcome Sanger Institute COVID-19 Surveillance Team
EPI_ISL_947203	Lighthouse Lab in Milton Keynes	Wellcome Sanger Institute for the COVID-19 Genomics UK (COG-UK) Consortium	The Lighthouse Lab in Milton Keynes and Alex Alderton, Roberto Amato, Sonia Goncalves, Ewan Harrison, David K. Jackson, Ian Johnston, Dominic Kwiatkowski, Cordelia Langford, John Sillitoe on behalf of the Wellcome Sanger Institute COVID-19 Surveillance Team
EPI_ISL_947204	Lighthouse Lab in Glasgow	Wellcome Sanger Institute for the COVID-19 Genomics UK (COG-UK) Consortium	Harper VanSteenhouse, Yumi Kasai, David Gray, Carol Clugston, Anna Dominiczak and Alex Alderton, Roberto Amato, Sonia Goncalves, Ewan Harrison, David K. Jackson, Ian Johnston, Dominic Kwiatkowski, Cordelia Langford, John Sillitoe on behalf of the Wellcome Sanger Institute COVID-19 Surveillance Team
EPI_ISL_947205, EPI_ISL_947206, EPI_ISL_947207, EPI_ISL_947209, EPI_ISL_947210	Lighthouse Lab in Milton Keynes	Wellcome Sanger Institute for the COVID-19 Genomics UK (COG-UK) Consortium	The Lighthouse Lab in Milton Keynes and Alex Alderton, Roberto Amato, Sonia Goncalves, Ewan Harrison, David K. Jackson, Ian Johnston, Dominic Kwiatkowski, Cordelia Langford, John Sillitoe on behalf of the Wellcome Sanger Institute COVID-19 Surveillance Team
EPI_ISL_947211, EPI_ISL_947212, EPI_ISL_947213, EPI_ISL_947214	Lighthouse Lab in Glasgow	Wellcome Sanger Institute for the COVID-19 Genomics UK (COG-UK) Consortium	Harper VanSteenhouse, Yumi Kasai, David Gray, Carol Clugston, Anna Dominiczak and Alex Alderton, Roberto Amato, Sonia Goncalves, Ewan Harrison, David K. Jackson, Ian Johnston, Dominic Kwiatkowski, Cordelia Langford, John Sillitoe on behalf of the Wellcome Sanger Institute COVID-19 Surveillance Team
EPI_ISL_947215, EPI_ISL_947216, EPI_ISL_947217, EPI_ISL_947218, EPI_ISL_947219, EPI_ISL_947220, EPI_ISL_947221, EPI_ISL_947222, EPI_ISL_947223, EPI_ISL_947224, EPI_ISL_947225, EPI_ISL_947226, EPI_ISL_947227			
see above	Lighthouse Lab in Milton Keynes	Wellcome Sanger Institute for the COVID-19 Genomics UK (COG-UK) Consortium	The Lighthouse Lab in Milton Keynes and Alex Alderton, Roberto Amato, Sonia Goncalves, Ewan Harrison, David K. Jackson, Ian Johnston, Dominic Kwiatkowski, Cordelia Langford, John Sillitoe on behalf of the Wellcome Sanger Institute COVID-19 Surveillance Team
EPI_ISL_947228, EPI_ISL_947229, EPI_ISL_947230	Lighthouse Lab in Glasgow	Wellcome Sanger Institute for the COVID-19 Genomics UK (COG-UK) Consortium	Harper VanSteenhouse, Yumi Kasai, David Gray, Carol Clugston, Anna Dominiczak and Alex Alderton, Roberto Amato, Sonia Goncalves, Ewan Harrison, David K. Jackson, Ian Johnston, Dominic Kwiatkowski, Cordelia Langford, John Sillitoe on behalf of the Wellcome Sanger Institute COVID-19 Surveillance Team
EPI_ISL_947231, EPI_ISL_947232	Lighthouse Lab in Milton Keynes	Wellcome Sanger Institute for the COVID-19 Genomics UK (COG-UK) Consortium	The Lighthouse Lab in Milton Keynes and Alex Alderton, Roberto Amato, Sonia Goncalves, Ewan Harrison, David K. Jackson, Ian Johnston, Dominic Kwiatkowski, Cordelia Langford, John Sillitoe on behalf of the Wellcome Sanger Institute COVID-19 Surveillance Team
EPI_ISL_947233	Lighthouse Lab in Glasgow	Wellcome Sanger Institute for the COVID-19 Genomics UK (COG-UK) Consortium	Harper VanSteenhouse, Yumi Kasai, David Gray, Carol Clugston, Anna Dominiczak and Alex Alderton, Roberto Amato, Sonia Goncalves, Ewan Harrison, David K. Jackson, Ian Johnston, Dominic Kwiatkowski, Cordelia Langford, John Sillitoe on behalf of the Wellcome Sanger Institute COVID-19 Surveillance Team
EPI_ISL_947234, EPI_ISL_947235	Lighthouse Lab in Milton Keynes	Wellcome Sanger Institute for the COVID-19 Genomics UK (COG-UK) Consortium	The Lighthouse Lab in Milton Keynes and Alex Alderton, Roberto Amato, Sonia Goncalves, Ewan Harrison, David K. Jackson, Ian Johnston, Dominic Kwiatkowski, Cordelia Langford, John Sillitoe on behalf of the Wellcome Sanger Institute COVID-19 Surveillance Team
EPI_ISL_947236	Lighthouse Lab in Glasgow	Wellcome Sanger Institute for the COVID-19 Genomics UK (COG-UK) Consortium	Harper VanSteenhouse, Yumi Kasai, David Gray, Carol Clugston, Anna Dominiczak and Alex Alderton, Roberto Amato, Sonia Goncalves, Ewan Harrison, David K. Jackson, Ian Johnston, Dominic Kwiatkowski, Cordelia Langford, John Sillitoe on behalf of the Wellcome Sanger Institute COVID-19 Surveillance Team
EPI_ISL_947237	Lighthouse Lab in Milton Keynes	Wellcome Sanger Institute for the COVID-19 Genomics UK (COG-UK) Consortium	The Lighthouse Lab in Milton Keynes and Alex Alderton, Roberto Amato, Sonia Goncalves, Ewan Harrison, David K. Jackson, Ian Johnston, Dominic Kwiatkowski, Cordelia Langford, John Sillitoe on behalf of the Wellcome Sanger Institute COVID-19 Surveillance Team
EPI_ISL_947238, EPI_ISL_947239	Lighthouse Lab in Glasgow	Wellcome Sanger Institute for the COVID-19 Genomics UK (COG-UK) Consortium	Harper VanSteenhouse, Yumi Kasai, David Gray, Carol Clugston, Anna Dominiczak and Alex Alderton, Roberto Amato, Sonia Goncalves, Ewan Harrison, David K. Jackson, Ian Johnston, Dominic Kwiatkowski, Cordelia Langford, John Sillitoe on behalf of the Wellcome Sanger Institute COVID-19 Surveillance Team
EPI_ISL_947241, EPI_ISL_947242, EPI_ISL_947243, EPI_ISL_947244	Lighthouse Lab in Milton Keynes	Wellcome Sanger Institute for the COVID-19 Genomics UK (COG-UK) Consortium	The Lighthouse Lab in Milton Keynes and Alex Alderton, Roberto Amato, Sonia Goncalves, Ewan Harrison, David K. Jackson, Ian Johnston, Dominic Kwiatkowski, Cordelia Langford, John Sillitoe on behalf of the Wellcome Sanger Institute COVID-19 Surveillance Team
EPI_ISL_947245	Lighthouse Lab in Glasgow	Wellcome Sanger Institute for the COVID-19 Genomics UK (COG-UK) Consortium	Harper VanSteenhouse, Yumi Kasai, David Gray, Carol Clugston, Anna Dominiczak and Alex Alderton, Roberto Amato, Sonia Goncalves, Ewan Harrison, David K. Jackson, Ian Johnston, Dominic Kwiatkowski, Cordelia Langford, John Sillitoe on behalf of the Wellcome Sanger Institute COVID-19 Surveillance

			Team
EPI_ISL_947246, EPI_ISL_947248	Lighthouse Lab in Milton Keynes	Wellcome Sanger Institute for the COVID-19 Genomics UK (COG-UK) Consortium	The Lighthouse Lab in Milton Keynes and Alex Alderton, Roberto Amato, Sonia Goncalves, Ewan Harrison, David K. Jackson, Ian Johnston, Dominic Kwiatkowski, Cordelia Langford, John Sillitoe on behalf of the Wellcome Sanger Institute COVID-19 Surveillance Team
EPI_ISL_947250	Lighthouse Lab in Glasgow	Wellcome Sanger Institute for the COVID-19 Genomics UK (COG-UK) Consortium	Harper VanSteenhouse, Yumi Kasai, David Gray, Carol Clugston, Anna Dominiczak and Alex Alderton, Roberto Amato, Sonia Goncalves, Ewan Harrison, David K. Jackson, Ian Johnston, Dominic Kwiatkowski, Cordelia Langford, John Sillitoe on behalf of the Wellcome Sanger Institute COVID-19 Surveillance Team
EPI_ISL_947251	Lighthouse Lab in Milton Keynes	Wellcome Sanger Institute for the COVID-19 Genomics UK (COG-UK) Consortium	The Lighthouse Lab in Milton Keynes and Alex Alderton, Roberto Amato, Sonia Goncalves, Ewan Harrison, David K. Jackson, Ian Johnston, Dominic Kwiatkowski, Cordelia Langford, John Sillitoe on behalf of the Wellcome Sanger Institute COVID-19 Surveillance Team
EPI_ISL_947253	Lighthouse Lab in Glasgow	Wellcome Sanger Institute for the COVID-19 Genomics UK (COG-UK) Consortium	Harper VanSteenhouse, Yumi Kasai, David Gray, Carol Clugston, Anna Dominiczak and Alex Alderton, Roberto Amato, Sonia Goncalves, Ewan Harrison, David K. Jackson, Ian Johnston, Dominic Kwiatkowski, Cordelia Langford, John Sillitoe on behalf of the Wellcome Sanger Institute COVID-19 Surveillance Team
EPI_ISL_947254, EPI_ISL_947258, EPI_ISL_947259	Lighthouse Lab in Milton Keynes	Wellcome Sanger Institute for the COVID-19 Genomics UK (COG-UK) Consortium	The Lighthouse Lab in Milton Keynes and Alex Alderton, Roberto Amato, Sonia Goncalves, Ewan Harrison, David K. Jackson, Ian Johnston, Dominic Kwiatkowski, Cordelia Langford, John Sillitoe on behalf of the Wellcome Sanger Institute COVID-19 Surveillance Team
EPI_ISL_947263	Lighthouse Lab in Glasgow	Wellcome Sanger Institute for the COVID-19 Genomics UK (COG-UK) Consortium	Harper VanSteenhouse, Yumi Kasai, David Gray, Carol Clugston, Anna Dominiczak and Alex Alderton, Roberto Amato, Sonia Goncalves, Ewan Harrison, David K. Jackson, Ian Johnston, Dominic Kwiatkowski, Cordelia Langford, John Sillitoe on behalf of the Wellcome Sanger Institute COVID-19 Surveillance Team
EPI_ISL_947264, EPI_ISL_947266	Lighthouse Lab in Milton Keynes	Wellcome Sanger Institute for the COVID-19 Genomics UK (COG-UK) Consortium	The Lighthouse Lab in Milton Keynes and Alex Alderton, Roberto Amato, Sonia Goncalves, Ewan Harrison, David K. Jackson, Ian Johnston, Dominic Kwiatkowski, Cordelia Langford, John Sillitoe on behalf of the Wellcome Sanger Institute COVID-19 Surveillance Team
EPI_ISL_947267, EPI_ISL_947269, EPI_ISL_947271, EPI_ISL_947272	Lighthouse Lab in Glasgow	Wellcome Sanger Institute for the COVID-19 Genomics UK (COG-UK) Consortium	Harper VanSteenhouse, Yumi Kasai, David Gray, Carol Clugston, Anna Dominiczak and Alex Alderton, Roberto Amato, Sonia Goncalves, Ewan Harrison, David K. Jackson, Ian Johnston, Dominic Kwiatkowski, Cordelia Langford, John Sillitoe on behalf of the Wellcome Sanger Institute COVID-19 Surveillance Team
EPI_ISL_947276, EPI_ISL_947277, EPI_ISL_947279	Lighthouse Lab in Milton Keynes	Wellcome Sanger Institute for the COVID-19 Genomics UK (COG-UK) Consortium	The Lighthouse Lab in Milton Keynes and Alex Alderton, Roberto Amato, Sonia Goncalves, Ewan Harrison, David K. Jackson, Ian Johnston, Dominic Kwiatkowski, Cordelia Langford, John Sillitoe on behalf of the Wellcome Sanger Institute COVID-19 Surveillance Team
EPI_ISL_947280	Lighthouse Lab in Glasgow	Wellcome Sanger Institute for the COVID-19 Genomics UK (COG-UK) Consortium	Harper VanSteenhouse, Yumi Kasai, David Gray, Carol Clugston, Anna Dominiczak and Alex Alderton, Roberto Amato, Sonia Goncalves, Ewan Harrison, David K. Jackson, Ian Johnston, Dominic Kwiatkowski, Cordelia Langford, John Sillitoe on behalf of the Wellcome Sanger Institute COVID-19 Surveillance Team
EPI_ISL_947282	Lighthouse Lab in Milton Keynes	Wellcome Sanger Institute for the COVID-19 Genomics UK (COG-UK) Consortium	The Lighthouse Lab in Milton Keynes and Alex Alderton, Roberto Amato, Sonia Goncalves, Ewan Harrison, David K. Jackson, Ian Johnston, Dominic Kwiatkowski, Cordelia Langford, John Sillitoe on behalf of the Wellcome Sanger Institute COVID-19 Surveillance Team
EPI_ISL_947283	Lighthouse Lab in Glasgow	Wellcome Sanger Institute for the COVID-19 Genomics UK (COG-UK) Consortium	Harper VanSteenhouse, Yumi Kasai, David Gray, Carol Clugston, Anna Dominiczak and Alex Alderton, Roberto Amato, Sonia Goncalves, Ewan Harrison, David K. Jackson, Ian Johnston, Dominic Kwiatkowski, Cordelia Langford, John Sillitoe on behalf of the Wellcome Sanger Institute COVID-19 Surveillance Team
EPI_ISL_947285, EPI_ISL_947286, EPI_ISL_947288, EPI_ISL_947289, EPI_ISL_947291, EPI_ISL_947292	Lighthouse Lab in Milton Keynes	Wellcome Sanger Institute for the COVID-19 Genomics UK (COG-UK) Consortium	The Lighthouse Lab in Milton Keynes and Alex Alderton, Roberto Amato, Sonia Goncalves, Ewan Harrison, David K. Jackson, Ian Johnston, Dominic Kwiatkowski, Cordelia Langford, John Sillitoe on behalf of the Wellcome Sanger Institute COVID-19 Surveillance Team
EPI_ISL_947294, EPI_ISL_947295	Lighthouse Lab in Glasgow	Wellcome Sanger Institute for the COVID-19 Genomics UK (COG-UK) Consortium	Harper VanSteenhouse, Yumi Kasai, David Gray, Carol Clugston, Anna Dominiczak and Alex Alderton, Roberto Amato, Sonia Goncalves, Ewan Harrison, David K. Jackson, Ian Johnston, Dominic Kwiatkowski, Cordelia Langford, John Sillitoe on behalf of the Wellcome Sanger Institute COVID-19 Surveillance Team
EPI_ISL_947297, EPI_ISL_947299, EPI_ISL_947300, EPI_ISL_947302, EPI_ISL_947303	Lighthouse Lab in Milton Keynes	Wellcome Sanger Institute for the COVID-19 Genomics UK (COG-UK) Consortium	The Lighthouse Lab in Milton Keynes and Alex Alderton, Roberto Amato, Sonia Goncalves, Ewan Harrison, David K. Jackson, Ian Johnston, Dominic Kwiatkowski, Cordelia Langford, John Sillitoe on behalf of the Wellcome Sanger Institute COVID-19 Surveillance Team
EPI_ISL_947305	Lighthouse Lab in Glasgow	Wellcome Sanger Institute for the COVID-19 Genomics UK (COG-UK) Consortium	Harper VanSteenhouse, Yumi Kasai, David Gray, Carol Clugston, Anna Dominiczak and Alex Alderton, Roberto Amato, Sonia Goncalves, Ewan Harrison, David K. Jackson, Ian Johnston, Dominic Kwiatkowski, Cordelia Langford, John Sillitoe on behalf of the Wellcome Sanger Institute COVID-19 Surveillance Team
EPI_ISL_947306	Lighthouse Lab in Milton Keynes	Wellcome Sanger Institute for the COVID-19 Genomics UK (COG-UK) Consortium	The Lighthouse Lab in Milton Keynes and Alex Alderton, Roberto Amato, Sonia Goncalves, Ewan Harrison, David K. Jackson, Ian Johnston, Dominic Kwiatkowski, Cordelia Langford, John Sillitoe on behalf of the Wellcome Sanger Institute COVID-19 Surveillance Team
EPI_ISL_947308	Lighthouse Lab in Glasgow	Wellcome Sanger Institute for the COVID-19 Genomics UK (COG-UK) Consortium	Harper VanSteenhouse, Yumi Kasai, David Gray, Carol Clugston, Anna Dominiczak and Alex Alderton, Roberto Amato, Sonia Goncalves, Ewan Harrison, David K. Jackson, Ian Johnston, Dominic Kwiatkowski, Cordelia Langford, John Sillitoe on behalf of the Wellcome Sanger Institute COVID-19 Surveillance Team
EPI_ISL_947310, EPI_ISL_947311, EPI_ISL_947313	Lighthouse Lab in Milton Keynes	Wellcome Sanger Institute for the COVID-19 Genomics UK (COG-UK) Consortium	The Lighthouse Lab in Milton Keynes and Alex Alderton, Roberto Amato, Sonia Goncalves, Ewan Harrison, David K. Jackson, Ian Johnston, Dominic Kwiatkowski, Cordelia Langford, John Sillitoe on behalf of the Wellcome Sanger Institute COVID-19 Surveillance Team
EPI_ISL_947314, EPI_ISL_947316	Lighthouse Lab in Glasgow	Wellcome Sanger Institute for the COVID-19 Genomics UK (COG-UK) Consortium	Harper VanSteenhouse, Yumi Kasai, David Gray, Carol Clugston, Anna Dominiczak and Alex Alderton, Roberto Amato, Sonia Goncalves, Ewan Harrison, David K. Jackson, Ian Johnston, Dominic Kwiatkowski, Cordelia Langford, John Sillitoe on behalf of the Wellcome Sanger Institute COVID-19 Surveillance Team
EPI_ISL_947319, EPI_ISL_947321	Lighthouse Lab in Milton Keynes	Wellcome Sanger Institute for the COVID-19 Genomics UK (COG-UK) Consortium	The Lighthouse Lab in Milton Keynes and Alex Alderton, Roberto Amato, Sonia Goncalves, Ewan Harrison, David K. Jackson, Ian Johnston, Dominic Kwiatkowski, Cordelia Langford, John Sillitoe on behalf of the Wellcome Sanger Institute COVID-19 Surveillance Team
EPI_ISL_947331, EPI_ISL_947332	Lighthouse Lab in Glasgow	Wellcome Sanger Institute for the COVID-19 Genomics UK (COG-UK) Consortium	Harper VanSteenhouse, Yumi Kasai, David Gray, Carol Clugston, Anna Dominiczak and Alex Alderton, Roberto Amato, Sonia Goncalves, Ewan Harrison, David K. Jackson, Ian Johnston, Dominic Kwiatkowski, Cordelia Langford, John Sillitoe on behalf of the Wellcome Sanger Institute COVID-19 Surveillance Team
EPI_ISL_947333, EPI_ISL_947334	Lighthouse Lab in Milton Keynes	Wellcome Sanger Institute for the COVID-19 Genomics UK (COG-UK) Consortium	The Lighthouse Lab in Milton Keynes and Alex Alderton, Roberto Amato, Sonia Goncalves, Ewan Harrison, David K. Jackson, Ian Johnston, Dominic Kwiatkowski, Cordelia Langford, John Sillitoe on behalf of the Wellcome Sanger Institute COVID-19 Surveillance Team
EPI_ISL_947335	Lighthouse Lab in Glasgow	Wellcome Sanger Institute for the COVID-19 Genomics UK (COG-UK) Consortium	Harper VanSteenhouse, Yumi Kasai, David Gray, Carol Clugston, Anna Dominiczak and Alex Alderton, Roberto Amato, Sonia Goncalves, Ewan Harrison, David K. Jackson, Ian Johnston, Dominic Kwiatkowski, Cordelia Langford, John Sillitoe on behalf of the Wellcome Sanger Institute COVID-19 Surveillance Team
EPI_ISL_947336	Lighthouse Lab in Milton Keynes	Wellcome Sanger Institute for the COVID-19 Genomics UK (COG-UK) Consortium	The Lighthouse Lab in Milton Keynes and Alex Alderton, Roberto Amato, Sonia Goncalves, Ewan Harrison, David K. Jackson, Ian Johnston, Dominic Kwiatkowski, Cordelia Langford, John Sillitoe on behalf of the Wellcome Sanger Institute COVID-19 Surveillance Team
EPI_ISL_947338	Lighthouse Lab in Glasgow	Wellcome Sanger Institute for the COVID-19 Genomics UK (COG-UK) Consortium	Harper VanSteenhouse, Yumi Kasai, David Gray, Carol Clugston, Anna Dominiczak and Alex Alderton, Roberto Amato, Sonia Goncalves, Ewan Harrison, David K. Jackson, Ian Johnston, Dominic Kwiatkowski, Cordelia Langford, John Sillitoe on behalf of the Wellcome Sanger Institute COVID-19 Surveillance Team
EPI_ISL_947339, EPI_ISL_947340	Lighthouse Lab in Milton Keynes	Wellcome Sanger Institute for the COVID-19 Genomics UK (COG-UK) Consortium	The Lighthouse Lab in Milton Keynes and Alex Alderton, Roberto Amato, Sonia Goncalves, Ewan Harrison, David K. Jackson, Ian Johnston, Dominic Kwiatkowski, Cordelia Langford, John Sillitoe on behalf of the Wellcome Sanger Institute COVID-19 Surveillance Team
EPI_ISL_947341	Lighthouse Lab in Glasgow	Wellcome Sanger Institute for the COVID-19 Genomics UK (COG-UK) Consortium	Harper VanSteenhouse, Yumi Kasai, David Gray, Carol Clugston, Anna Dominiczak and Alex Alderton, Roberto Amato, Sonia Goncalves, Ewan Harrison, David K. Jackson, Ian Johnston, Dominic Kwiatkowski, Cordelia Langford, John Sillitoe on behalf of the Wellcome Sanger Institute COVID-19 Surveillance Team

EPI_ISL_947342	Lighthouse Lab in Milton Keynes	Wellcome Sanger Institute for the COVID-19 Genomics UK (COG-UK) Consortium	The Lighthouse Lab in Milton Keynes and Alex Alderton, Roberto Amato, Sonia Goncalves, Ewan Harrison, David K. Jackson, Ian Johnston, Dominic Kwiatkowski, Cordelia Langford, John Sillitoe on behalf of the Wellcome Sanger Institute COVID-19 Surveillance Team
EPI_ISL_947343, EPI_ISL_947344	Lighthouse Lab in Glasgow	Wellcome Sanger Institute for the COVID-19 Genomics UK (COG-UK) Consortium	Harper VanSteenhouse, Yumi Kasai, David Gray, Carol Clugston, Anna Dominiczak and Alex Alderton, Roberto Amato, Sonia Goncalves, Ewan Harrison, David K. Jackson, Ian Johnston, Dominic Kwiatkowski, Cordelia Langford, John Sillitoe on behalf of the Wellcome Sanger Institute COVID-19 Surveillance Team
EPI_ISL_947345, EPI_ISL_947346	Lighthouse Lab in Milton Keynes	Wellcome Sanger Institute for the COVID-19 Genomics UK (COG-UK) Consortium	The Lighthouse Lab in Milton Keynes and Alex Alderton, Roberto Amato, Sonia Goncalves, Ewan Harrison, David K. Jackson, Ian Johnston, Dominic Kwiatkowski, Cordelia Langford, John Sillitoe on behalf of the Wellcome Sanger Institute COVID-19 Surveillance Team
EPI_ISL_947347	Lighthouse Lab in Glasgow	Wellcome Sanger Institute for the COVID-19 Genomics UK (COG-UK) Consortium	Harper VanSteenhouse, Yumi Kasai, David Gray, Carol Clugston, Anna Dominiczak and Alex Alderton, Roberto Amato, Sonia Goncalves, Ewan Harrison, David K. Jackson, Ian Johnston, Dominic Kwiatkowski, Cordelia Langford, John Sillitoe on behalf of the Wellcome Sanger Institute COVID-19 Surveillance Team
EPI_ISL_947348, EPI_ISL_947349, EPI_ISL_947351	Lighthouse Lab in Milton Keynes	Wellcome Sanger Institute for the COVID-19 Genomics UK (COG-UK) Consortium	The Lighthouse Lab in Milton Keynes and Alex Alderton, Roberto Amato, Sonia Goncalves, Ewan Harrison, David K. Jackson, Ian Johnston, Dominic Kwiatkowski, Cordelia Langford, John Sillitoe on behalf of the Wellcome Sanger Institute COVID-19 Surveillance Team
EPI_ISL_947353	Lighthouse Lab in Glasgow	Wellcome Sanger Institute for the COVID-19 Genomics UK (COG-UK) Consortium	Harper VanSteenhouse, Yumi Kasai, David Gray, Carol Clugston, Anna Dominiczak and Alex Alderton, Roberto Amato, Sonia Goncalves, Ewan Harrison, David K. Jackson, Ian Johnston, Dominic Kwiatkowski, Cordelia Langford, John Sillitoe on behalf of the Wellcome Sanger Institute COVID-19 Surveillance Team
EPI_ISL_947355	Lighthouse Lab in Milton Keynes	Wellcome Sanger Institute for the COVID-19 Genomics UK (COG-UK) Consortium	The Lighthouse Lab in Milton Keynes and Alex Alderton, Roberto Amato, Sonia Goncalves, Ewan Harrison, David K. Jackson, Ian Johnston, Dominic Kwiatkowski, Cordelia Langford, John Sillitoe on behalf of the Wellcome Sanger Institute COVID-19 Surveillance Team
EPI_ISL_947356, EPI_ISL_947357	Lighthouse Lab in Glasgow	Wellcome Sanger Institute for the COVID-19 Genomics UK (COG-UK) Consortium	Harper VanSteenhouse, Yumi Kasai, David Gray, Carol Clugston, Anna Dominiczak and Alex Alderton, Roberto Amato, Sonia Goncalves, Ewan Harrison, David K. Jackson, Ian Johnston, Dominic Kwiatkowski, Cordelia Langford, John Sillitoe on behalf of the Wellcome Sanger Institute COVID-19 Surveillance Team
EPI_ISL_947358, EPI_ISL_947359	Lighthouse Lab in Milton Keynes	Wellcome Sanger Institute for the COVID-19 Genomics UK (COG-UK) Consortium	The Lighthouse Lab in Milton Keynes and Alex Alderton, Roberto Amato, Sonia Goncalves, Ewan Harrison, David K. Jackson, Ian Johnston, Dominic Kwiatkowski, Cordelia Langford, John Sillitoe on behalf of the Wellcome Sanger Institute COVID-19 Surveillance Team
EPI_ISL_947360, EPI_ISL_947362, EPI_ISL_947363, EPI_ISL_947364, EPI_ISL_947365	Lighthouse Lab in Glasgow	Wellcome Sanger Institute for the COVID-19 Genomics UK (COG-UK) Consortium	Harper VanSteenhouse, Yumi Kasai, David Gray, Carol Clugston, Anna Dominiczak and Alex Alderton, Roberto Amato, Sonia Goncalves, Ewan Harrison, David K. Jackson, Ian Johnston, Dominic Kwiatkowski, Cordelia Langford, John Sillitoe on behalf of the Wellcome Sanger Institute COVID-19 Surveillance Team
EPI_ISL_947366, EPI_ISL_947367, EPI_ISL_947369	Lighthouse Lab in Milton Keynes	Wellcome Sanger Institute for the COVID-19 Genomics UK (COG-UK) Consortium	The Lighthouse Lab in Milton Keynes and Alex Alderton, Roberto Amato, Sonia Goncalves, Ewan Harrison, David K. Jackson, Ian Johnston, Dominic Kwiatkowski, Cordelia Langford, John Sillitoe on behalf of the Wellcome Sanger Institute COVID-19 Surveillance Team
EPI_ISL_947370	Lighthouse Lab in Glasgow	Wellcome Sanger Institute for the COVID-19 Genomics UK (COG-UK) Consortium	Harper VanSteenhouse, Yumi Kasai, David Gray, Carol Clugston, Anna Dominiczak and Alex Alderton, Roberto Amato, Sonia Goncalves, Ewan Harrison, David K. Jackson, Ian Johnston, Dominic Kwiatkowski, Cordelia Langford, John Sillitoe on behalf of the Wellcome Sanger Institute COVID-19 Surveillance Team
EPI_ISL_947371, EPI_ISL_947372, EPI_ISL_947373, EPI_ISL_947375, EPI_ISL_947376	Lighthouse Lab in Milton Keynes	Wellcome Sanger Institute for the COVID-19 Genomics UK (COG-UK) Consortium	The Lighthouse Lab in Milton Keynes and Alex Alderton, Roberto Amato, Sonia Goncalves, Ewan Harrison, David K. Jackson, Ian Johnston, Dominic Kwiatkowski, Cordelia Langford, John Sillitoe on behalf of the Wellcome Sanger Institute COVID-19 Surveillance Team
EPI_ISL_947377	Lighthouse Lab in Glasgow	Wellcome Sanger Institute for the COVID-19 Genomics UK (COG-UK) Consortium	Harper VanSteenhouse, Yumi Kasai, David Gray, Carol Clugston, Anna Dominiczak and Alex Alderton, Roberto Amato, Sonia Goncalves, Ewan Harrison, David K. Jackson, Ian Johnston, Dominic Kwiatkowski, Cordelia Langford, John Sillitoe on behalf of the Wellcome Sanger Institute COVID-19 Surveillance Team
EPI_ISL_947378, EPI_ISL_947379	Lighthouse Lab in Milton Keynes	Wellcome Sanger Institute for the COVID-19 Genomics UK (COG-UK) Consortium	The Lighthouse Lab in Milton Keynes and Alex Alderton, Roberto Amato, Sonia Goncalves, Ewan Harrison, David K. Jackson, Ian Johnston, Dominic Kwiatkowski, Cordelia Langford, John Sillitoe on behalf of the Wellcome Sanger Institute COVID-19 Surveillance Team
EPI_ISL_947380	Lighthouse Lab in Glasgow	Wellcome Sanger Institute for the COVID-19 Genomics UK (COG-UK) Consortium	Harper VanSteenhouse, Yumi Kasai, David Gray, Carol Clugston, Anna Dominiczak and Alex Alderton, Roberto Amato, Sonia Goncalves, Ewan Harrison, David K. Jackson, Ian Johnston, Dominic Kwiatkowski, Cordelia Langford, John Sillitoe on behalf of the Wellcome Sanger Institute COVID-19 Surveillance Team
EPI_ISL_947381, EPI_ISL_947382	Lighthouse Lab in Milton Keynes	Wellcome Sanger Institute for the COVID-19 Genomics UK (COG-UK) Consortium	The Lighthouse Lab in Milton Keynes and Alex Alderton, Roberto Amato, Sonia Goncalves, Ewan Harrison, David K. Jackson, Ian Johnston, Dominic Kwiatkowski, Cordelia Langford, John Sillitoe on behalf of the Wellcome Sanger Institute COVID-19 Surveillance Team
EPI_ISL_947383	Lighthouse Lab in Glasgow	Wellcome Sanger Institute for the COVID-19 Genomics UK (COG-UK) Consortium	Harper VanSteenhouse, Yumi Kasai, David Gray, Carol Clugston, Anna Dominiczak and Alex Alderton, Roberto Amato, Sonia Goncalves, Ewan Harrison, David K. Jackson, Ian Johnston, Dominic Kwiatkowski, Cordelia Langford, John Sillitoe on behalf of the Wellcome Sanger Institute COVID-19 Surveillance Team
EPI_ISL_947384, EPI_ISL_947385	Lighthouse Lab in Milton Keynes	Wellcome Sanger Institute for the COVID-19 Genomics UK (COG-UK) Consortium	The Lighthouse Lab in Milton Keynes and Alex Alderton, Roberto Amato, Sonia Goncalves, Ewan Harrison, David K. Jackson, Ian Johnston, Dominic Kwiatkowski, Cordelia Langford, John Sillitoe on behalf of the Wellcome Sanger Institute COVID-19 Surveillance Team
EPI_ISL_947386	Lighthouse Lab in Glasgow	Wellcome Sanger Institute for the COVID-19 Genomics UK (COG-UK) Consortium	Harper VanSteenhouse, Yumi Kasai, David Gray, Carol Clugston, Anna Dominiczak and Alex Alderton, Roberto Amato, Sonia Goncalves, Ewan Harrison, David K. Jackson, Ian Johnston, Dominic Kwiatkowski, Cordelia Langford, John Sillitoe on behalf of the Wellcome Sanger Institute COVID-19 Surveillance Team
EPI_ISL_947387, EPI_ISL_947388	Lighthouse Lab in Milton Keynes	Wellcome Sanger Institute for the COVID-19 Genomics UK (COG-UK) Consortium	The Lighthouse Lab in Milton Keynes and Alex Alderton, Roberto Amato, Sonia Goncalves, Ewan Harrison, David K. Jackson, Ian Johnston, Dominic Kwiatkowski, Cordelia Langford, John Sillitoe on behalf of the Wellcome Sanger Institute COVID-19 Surveillance Team
EPI_ISL_947389	Lighthouse Lab in Glasgow	Wellcome Sanger Institute for the COVID-19 Genomics UK (COG-UK) Consortium	Harper VanSteenhouse, Yumi Kasai, David Gray, Carol Clugston, Anna Dominiczak and Alex Alderton, Roberto Amato, Sonia Goncalves, Ewan Harrison, David K. Jackson, Ian Johnston, Dominic Kwiatkowski, Cordelia Langford, John Sillitoe on behalf of the Wellcome Sanger Institute COVID-19 Surveillance Team
EPI_ISL_947390	Lighthouse Lab in Milton Keynes	Wellcome Sanger Institute for the COVID-19 Genomics UK (COG-UK) Consortium	The Lighthouse Lab in Milton Keynes and Alex Alderton, Roberto Amato, Sonia Goncalves, Ewan Harrison, David K. Jackson, Ian Johnston, Dominic Kwiatkowski, Cordelia Langford, John Sillitoe on behalf of the Wellcome Sanger Institute COVID-19 Surveillance Team
EPI_ISL_947391	Lighthouse Lab in Glasgow	Wellcome Sanger Institute for the COVID-19 Genomics UK (COG-UK) Consortium	Harper VanSteenhouse, Yumi Kasai, David Gray, Carol Clugston, Anna Dominiczak and Alex Alderton, Roberto Amato, Sonia Goncalves, Ewan Harrison, David K. Jackson, Ian Johnston, Dominic Kwiatkowski, Cordelia Langford, John Sillitoe on behalf of the Wellcome Sanger Institute COVID-19 Surveillance Team
EPI_ISL_947392, EPI_ISL_947393	Lighthouse Lab in Milton Keynes	Wellcome Sanger Institute for the COVID-19 Genomics UK (COG-UK) Consortium	The Lighthouse Lab in Milton Keynes and Alex Alderton, Roberto Amato, Sonia Goncalves, Ewan Harrison, David K. Jackson, Ian Johnston, Dominic Kwiatkowski, Cordelia Langford, John Sillitoe on behalf of the Wellcome Sanger Institute COVID-19 Surveillance Team
EPI_ISL_947394	Lighthouse Lab in Glasgow	Wellcome Sanger Institute for the COVID-19 Genomics UK (COG-UK) Consortium	Harper VanSteenhouse, Yumi Kasai, David Gray, Carol Clugston, Anna Dominiczak and Alex Alderton, Roberto Amato, Sonia Goncalves, Ewan Harrison, David K. Jackson, Ian Johnston, Dominic Kwiatkowski, Cordelia Langford, John Sillitoe on behalf of the Wellcome Sanger Institute COVID-19 Surveillance Team
EPI_ISL_947395	Lighthouse Lab in Milton Keynes	Wellcome Sanger Institute for the COVID-19 Genomics UK (COG-UK) Consortium	The Lighthouse Lab in Milton Keynes and Alex Alderton, Roberto Amato, Sonia Goncalves, Ewan Harrison, David K. Jackson, Ian Johnston, Dominic Kwiatkowski, Cordelia Langford, John Sillitoe on behalf of the Wellcome Sanger Institute COVID-19 Surveillance Team
EPI_ISL_947396	Lighthouse Lab in Glasgow	Wellcome Sanger Institute for the COVID-19 Genomics UK (COG-UK) Consortium	Harper VanSteenhouse, Yumi Kasai, David Gray, Carol Clugston, Anna Dominiczak and Alex Alderton, Roberto Amato, Sonia Goncalves, Ewan Harrison, David K. Jackson, Ian Johnston, Dominic Kwiatkowski, Cordelia Langford, John Sillitoe on behalf of the Wellcome Sanger Institute COVID-19 Surveillance Team
EPI_ISL_947397	Lighthouse Lab in Milton Keynes	Wellcome Sanger Institute for the COVID-19 Genomics UK (COG-UK) Consortium	The Lighthouse Lab in Milton Keynes and Alex Alderton, Roberto Amato, Sonia Goncalves, Ewan Harrison, David K. Jackson, Ian Johnston, Dominic Kwiatkowski, Cordelia Langford, John Sillitoe on behalf of the Wellcome Sanger Institute COVID-19 Surveillance Team

EPI_ISL_947398, EPI_ISL_947399	Lighthouse Lab in Glasgow	Wellcome Sanger Institute for the COVID-19 Genomics UK (COG-UK) Consortium	Harper VanSteenhouse, Yumi Kasai, David Gray, Carol Clugston, Anna Dominiczak and Alex Alderton, Roberto Amato, Sonia Goncalves, Ewan Harrison, David K. Jackson, Ian Johnston, Dominic Kwiatkowski, Cordelia Langford, John Sillitoe on behalf of the Wellcome Sanger Institute COVID-19 Surveillance Team
EPI_ISL_947401	Lighthouse Lab in Milton Keynes	Wellcome Sanger Institute for the COVID-19 Genomics UK (COG-UK) Consortium	The Lighthouse Lab in Milton Keynes and Alex Alderton, Roberto Amato, Sonia Goncalves, Ewan Harrison, David K. Jackson, Ian Johnston, Dominic Kwiatkowski, Cordelia Langford, John Sillitoe on behalf of the Wellcome Sanger Institute COVID-19 Surveillance Team
EPI_ISL_947402	Lighthouse Lab in Glasgow	Wellcome Sanger Institute for the COVID-19 Genomics UK (COG-UK) Consortium	Harper VanSteenhouse, Yumi Kasai, David Gray, Carol Clugston, Anna Dominiczak and Alex Alderton, Roberto Amato, Sonia Goncalves, Ewan Harrison, David K. Jackson, Ian Johnston, Dominic Kwiatkowski, Cordelia Langford, John Sillitoe on behalf of the Wellcome Sanger Institute COVID-19 Surveillance Team
EPI_ISL_947403	Lighthouse Lab in Milton Keynes	Wellcome Sanger Institute for the COVID-19 Genomics UK (COG-UK) Consortium	The Lighthouse Lab in Milton Keynes and Alex Alderton, Roberto Amato, Sonia Goncalves, Ewan Harrison, David K. Jackson, Ian Johnston, Dominic Kwiatkowski, Cordelia Langford, John Sillitoe on behalf of the Wellcome Sanger Institute COVID-19 Surveillance Team
EPI_ISL_947404, EPI_ISL_947405, EPI_ISL_948100	Lighthouse Lab in Glasgow	Wellcome Sanger Institute for the COVID-19 Genomics UK (COG-UK) Consortium	Harper VanSteenhouse, Yumi Kasai, David Gray, Carol Clugston, Anna Dominiczak and Alex Alderton, Roberto Amato, Sonia Goncalves, Ewan Harrison, David K. Jackson, Ian Johnston, Dominic Kwiatkowski, Cordelia Langford, John Sillitoe on behalf of the Wellcome Sanger Institute COVID-19 Surveillance Team
EPI_ISL_948102	Lighthouse Lab in Cambridge	Wellcome Sanger Institute for the COVID-19 Genomics UK (COG-UK) Consortium	Rob Howes, The Lighthouse Lab in Cambridge and Alex Alderton, Roberto Amato, Sonia Goncalves, Ewan Harrison, David K. Jackson, Ian Johnston, Dominic Kwiatkowski, Cordelia Langford, John Sillitoe on behalf of the Wellcome Sanger Institute COVID-19 Surveillance Team
EPI_ISL_948103	Lighthouse Lab in Glasgow	Wellcome Sanger Institute for the COVID-19 Genomics UK (COG-UK) Consortium	Harper VanSteenhouse, Yumi Kasai, David Gray, Carol Clugston, Anna Dominiczak and Alex Alderton, Roberto Amato, Sonia Goncalves, Ewan Harrison, David K. Jackson, Ian Johnston, Dominic Kwiatkowski, Cordelia Langford, John Sillitoe on behalf of the Wellcome Sanger Institute COVID-19 Surveillance Team
EPI_ISL_948104	Lighthouse Lab in Cambridge	Wellcome Sanger Institute for the COVID-19 Genomics UK (COG-UK) Consortium	Rob Howes, The Lighthouse Lab in Cambridge and Alex Alderton, Roberto Amato, Sonia Goncalves, Ewan Harrison, David K. Jackson, Ian Johnston, Dominic Kwiatkowski, Cordelia Langford, John Sillitoe on behalf of the Wellcome Sanger Institute COVID-19 Surveillance Team
EPI_ISL_948105, EPI_ISL_948106	Lighthouse Lab in Glasgow	Wellcome Sanger Institute for the COVID-19 Genomics UK (COG-UK) Consortium	Harper VanSteenhouse, Yumi Kasai, David Gray, Carol Clugston, Anna Dominiczak and Alex Alderton, Roberto Amato, Sonia Goncalves, Ewan Harrison, David K. Jackson, Ian Johnston, Dominic Kwiatkowski, Cordelia Langford, John Sillitoe on behalf of the Wellcome Sanger Institute COVID-19 Surveillance Team
EPI_ISL_948107	Lighthouse Lab in Cambridge	Wellcome Sanger Institute for the COVID-19 Genomics UK (COG-UK) Consortium	Rob Howes, The Lighthouse Lab in Cambridge and Alex Alderton, Roberto Amato, Sonia Goncalves, Ewan Harrison, David K. Jackson, Ian Johnston, Dominic Kwiatkowski, Cordelia Langford, John Sillitoe on behalf of the Wellcome Sanger Institute COVID-19 Surveillance Team
EPI_ISL_948108, EPI_ISL_948109	Lighthouse Lab in Glasgow	Wellcome Sanger Institute for the COVID-19 Genomics UK (COG-UK) Consortium	Harper VanSteenhouse, Yumi Kasai, David Gray, Carol Clugston, Anna Dominiczak and Alex Alderton, Roberto Amato, Sonia Goncalves, Ewan Harrison, David K. Jackson, Ian Johnston, Dominic Kwiatkowski, Cordelia Langford, John Sillitoe on behalf of the Wellcome Sanger Institute COVID-19 Surveillance Team
EPI_ISL_948110	Lighthouse Lab in Cambridge	Wellcome Sanger Institute for the COVID-19 Genomics UK (COG-UK) Consortium	Rob Howes, The Lighthouse Lab in Cambridge and Alex Alderton, Roberto Amato, Sonia Goncalves, Ewan Harrison, David K. Jackson, Ian Johnston, Dominic Kwiatkowski, Cordelia Langford, John Sillitoe on behalf of the Wellcome Sanger Institute COVID-19 Surveillance Team
EPI_ISL_948111, EPI_ISL_948112, EPI_ISL_948113	Lighthouse Lab in Glasgow	Wellcome Sanger Institute for the COVID-19 Genomics UK (COG-UK) Consortium	Harper VanSteenhouse, Yumi Kasai, David Gray, Carol Clugston, Anna Dominiczak and Alex Alderton, Roberto Amato, Sonia Goncalves, Ewan Harrison, David K. Jackson, Ian Johnston, Dominic Kwiatkowski, Cordelia Langford, John Sillitoe on behalf of the Wellcome Sanger Institute COVID-19 Surveillance Team
EPI_ISL_948114	Lighthouse Lab in Cambridge	Wellcome Sanger Institute for the COVID-19 Genomics UK (COG-UK) Consortium	Rob Howes, The Lighthouse Lab in Cambridge and Alex Alderton, Roberto Amato, Sonia Goncalves, Ewan Harrison, David K. Jackson, Ian Johnston, Dominic Kwiatkowski, Cordelia Langford, John Sillitoe on behalf of the Wellcome Sanger Institute COVID-19 Surveillance Team
EPI_ISL_948115	Lighthouse Lab in Glasgow	Wellcome Sanger Institute for the COVID-19 Genomics UK (COG-UK) Consortium	Harper VanSteenhouse, Yumi Kasai, David Gray, Carol Clugston, Anna Dominiczak and Alex Alderton, Roberto Amato, Sonia Goncalves, Ewan Harrison, David K. Jackson, Ian Johnston, Dominic Kwiatkowski, Cordelia Langford, John Sillitoe on behalf of the Wellcome Sanger Institute COVID-19 Surveillance Team
EPI_ISL_948117, EPI_ISL_948118	Lighthouse Lab in Cambridge	Wellcome Sanger Institute for the COVID-19 Genomics UK (COG-UK) Consortium	Rob Howes, The Lighthouse Lab in Cambridge and Alex Alderton, Roberto Amato, Sonia Goncalves, Ewan Harrison, David K. Jackson, Ian Johnston, Dominic Kwiatkowski, Cordelia Langford, John Sillitoe on behalf of the Wellcome Sanger Institute COVID-19 Surveillance Team
EPI_ISL_948119	Lighthouse Lab in Glasgow	Wellcome Sanger Institute for the COVID-19 Genomics UK (COG-UK) Consortium	Harper VanSteenhouse, Yumi Kasai, David Gray, Carol Clugston, Anna Dominiczak and Alex Alderton, Roberto Amato, Sonia Goncalves, Ewan Harrison, David K. Jackson, Ian Johnston, Dominic Kwiatkowski, Cordelia Langford, John Sillitoe on behalf of the Wellcome Sanger Institute COVID-19 Surveillance Team
EPI_ISL_948120	Lighthouse Lab in Cambridge	Wellcome Sanger Institute for the COVID-19 Genomics UK (COG-UK) Consortium	Rob Howes, The Lighthouse Lab in Cambridge and Alex Alderton, Roberto Amato, Sonia Goncalves, Ewan Harrison, David K. Jackson, Ian Johnston, Dominic Kwiatkowski, Cordelia Langford, John Sillitoe on behalf of the Wellcome Sanger Institute COVID-19 Surveillance Team
EPI_ISL_948121	Lighthouse Lab in Glasgow	Wellcome Sanger Institute for the COVID-19 Genomics UK (COG-UK) Consortium	Harper VanSteenhouse, Yumi Kasai, David Gray, Carol Clugston, Anna Dominiczak and Alex Alderton, Roberto Amato, Sonia Goncalves, Ewan Harrison, David K. Jackson, Ian Johnston, Dominic Kwiatkowski, Cordelia Langford, John Sillitoe on behalf of the Wellcome Sanger Institute COVID-19 Surveillance Team
EPI_ISL_948122, EPI_ISL_948123, EPI_ISL_948124	Lighthouse Lab in Cambridge	Wellcome Sanger Institute for the COVID-19 Genomics UK (COG-UK) Consortium	Rob Howes, The Lighthouse Lab in Cambridge and Alex Alderton, Roberto Amato, Sonia Goncalves, Ewan Harrison, David K. Jackson, Ian Johnston, Dominic Kwiatkowski, Cordelia Langford, John Sillitoe on behalf of the Wellcome Sanger Institute COVID-19 Surveillance Team
EPI_ISL_948126, EPI_ISL_948127, EPI_ISL_948129, EPI_ISL_948130	Lighthouse Lab in Glasgow	Wellcome Sanger Institute for the COVID-19 Genomics UK (COG-UK) Consortium	Harper VanSteenhouse, Yumi Kasai, David Gray, Carol Clugston, Anna Dominiczak and Alex Alderton, Roberto Amato, Sonia Goncalves, Ewan Harrison, David K. Jackson, Ian Johnston, Dominic Kwiatkowski, Cordelia Langford, John Sillitoe on behalf of the Wellcome Sanger Institute COVID-19 Surveillance Team
EPI_ISL_948131	Lighthouse Lab in Cambridge	Wellcome Sanger Institute for the COVID-19 Genomics UK (COG-UK) Consortium	Rob Howes, The Lighthouse Lab in Cambridge and Alex Alderton, Roberto Amato, Sonia Goncalves, Ewan Harrison, David K. Jackson, Ian Johnston, Dominic Kwiatkowski, Cordelia Langford, John Sillitoe on behalf of the Wellcome Sanger Institute COVID-19 Surveillance Team
EPI_ISL_948132	Lighthouse Lab in Glasgow	Wellcome Sanger Institute for the COVID-19 Genomics UK (COG-UK) Consortium	Harper VanSteenhouse, Yumi Kasai, David Gray, Carol Clugston, Anna Dominiczak and Alex Alderton, Roberto Amato, Sonia Goncalves, Ewan Harrison, David K. Jackson, Ian Johnston, Dominic Kwiatkowski, Cordelia Langford, John Sillitoe on behalf of the Wellcome Sanger Institute COVID-19 Surveillance Team
EPI_ISL_948134	Lighthouse Lab in Cambridge	Wellcome Sanger Institute for the COVID-19 Genomics UK (COG-UK) Consortium	Rob Howes, The Lighthouse Lab in Cambridge and Alex Alderton, Roberto Amato, Sonia Goncalves, Ewan Harrison, David K. Jackson, Ian Johnston, Dominic Kwiatkowski, Cordelia Langford, John Sillitoe on behalf of the Wellcome Sanger Institute COVID-19 Surveillance Team
EPI_ISL_948135	Lighthouse Lab in Milton Keynes	Wellcome Sanger Institute for the COVID-19 Genomics UK (COG-UK) Consortium	The Lighthouse Lab in Milton Keynes and Alex Alderton, Roberto Amato, Sonia Goncalves, Ewan Harrison, David K. Jackson, Ian Johnston, Dominic Kwiatkowski, Cordelia Langford, John Sillitoe on behalf of the Wellcome Sanger Institute COVID-19 Surveillance Team
EPI_ISL_948136, EPI_ISL_948137	Lighthouse Lab in Cambridge	Wellcome Sanger Institute for the COVID-19 Genomics UK (COG-UK) Consortium	Rob Howes, The Lighthouse Lab in Cambridge and Alex Alderton, Roberto Amato, Sonia Goncalves, Ewan Harrison, David K. Jackson, Ian Johnston, Dominic Kwiatkowski, Cordelia Langford, John Sillitoe on behalf of the Wellcome Sanger Institute COVID-19 Surveillance Team
EPI_ISL_948138	Lighthouse Lab in Glasgow	Wellcome Sanger Institute for the COVID-19 Genomics UK (COG-UK) Consortium	Harper VanSteenhouse, Yumi Kasai, David Gray, Carol Clugston, Anna Dominiczak and Alex Alderton, Roberto Amato, Sonia Goncalves, Ewan Harrison, David K. Jackson, Ian Johnston, Dominic Kwiatkowski, Cordelia Langford, John Sillitoe on behalf of the Wellcome Sanger Institute COVID-19 Surveillance Team
EPI_ISL_948139	Lighthouse Lab in Cambridge	Wellcome Sanger Institute for the COVID-19 Genomics UK (COG-UK) Consortium	Rob Howes, The Lighthouse Lab in Cambridge and Alex Alderton, Roberto Amato, Sonia Goncalves, Ewan Harrison, David K. Jackson, Ian Johnston, Dominic Kwiatkowski, Cordelia Langford, John Sillitoe on behalf of the Wellcome Sanger Institute COVID-19 Surveillance Team
EPI_ISL_948140, EPI_ISL_948141, EPI_ISL_948142, EPI_ISL_948143, EPI_ISL_948144	Lighthouse Lab in Glasgow	Wellcome Sanger Institute for the COVID-19 Genomics UK (COG-UK) Consortium	Harper VanSteenhouse, Yumi Kasai, David Gray, Carol Clugston, Anna Dominiczak and Alex Alderton, Roberto Amato, Sonia Goncalves, Ewan Harrison, David K. Jackson, Ian Johnston, Dominic Kwiatkowski, Cordelia Langford, John Sillitoe on behalf of the Wellcome Sanger Institute COVID-19 Surveillance Team
EPI_ISL_948145	Lighthouse Lab in Cambridge	Wellcome Sanger Institute for the COVID-19 Genomics UK	Rob Howes, The Lighthouse Lab in Cambridge and Alex Alderton, Roberto Amato, Sonia Goncalves, Ewan Harrison, David K. Jackson, Ian Johnston,

		(COG-UK) Consortium	Dominic Kwiatkowski, Cordelia Langford, John Sillitoe on behalf of the Wellcome Sanger Institute COVID-19 Surveillance Team
EPI_ISL_948146	Lighthouse Lab in Glasgow	Wellcome Sanger Institute for the COVID-19 Genomics UK (COG-UK) Consortium	Harper VanSteenhouse, Yumi Kasai, David Gray, Carol Clugston, Anna Dominiczak and Alex Alderton, Roberto Amato, Sonia Goncalves, Ewan Harrison, David K. Jackson, Ian Johnston, Dominic Kwiatkowski, Cordelia Langford, John Sillitoe on behalf of the Wellcome Sanger Institute COVID-19 Surveillance Team
EPI_ISL_948147	Lighthouse Lab in Cambridge	Wellcome Sanger Institute for the COVID-19 Genomics UK (COG-UK) Consortium	Rob Howes, The Lighthouse Lab in Cambridge and Alex Alderton, Roberto Amato, Sonia Goncalves, Ewan Harrison, David K. Jackson, Ian Johnston, Dominic Kwiatkowski, Cordelia Langford, John Sillitoe on behalf of the Wellcome Sanger Institute COVID-19 Surveillance Team
EPI_ISL_948148	Lighthouse Lab in Glasgow	Wellcome Sanger Institute for the COVID-19 Genomics UK (COG-UK) Consortium	Harper VanSteenhouse, Yumi Kasai, David Gray, Carol Clugston, Anna Dominiczak and Alex Alderton, Roberto Amato, Sonia Goncalves, Ewan Harrison, David K. Jackson, Ian Johnston, Dominic Kwiatkowski, Cordelia Langford, John Sillitoe on behalf of the Wellcome Sanger Institute COVID-19 Surveillance Team
EPI_ISL_948149, EPI_ISL_948151	Lighthouse Lab in Cambridge	Wellcome Sanger Institute for the COVID-19 Genomics UK (COG-UK) Consortium	Rob Howes, The Lighthouse Lab in Cambridge and Alex Alderton, Roberto Amato, Sonia Goncalves, Ewan Harrison, David K. Jackson, Ian Johnston, Dominic Kwiatkowski, Cordelia Langford, John Sillitoe on behalf of the Wellcome Sanger Institute COVID-19 Surveillance Team
EPI_ISL_948152	Lighthouse Lab in Glasgow	Wellcome Sanger Institute for the COVID-19 Genomics UK (COG-UK) Consortium	Harper VanSteenhouse, Yumi Kasai, David Gray, Carol Clugston, Anna Dominiczak and Alex Alderton, Roberto Amato, Sonia Goncalves, Ewan Harrison, David K. Jackson, Ian Johnston, Dominic Kwiatkowski, Cordelia Langford, John Sillitoe on behalf of the Wellcome Sanger Institute COVID-19 Surveillance Team
EPI_ISL_948153	Lighthouse Lab in Cambridge	Wellcome Sanger Institute for the COVID-19 Genomics UK (COG-UK) Consortium	Rob Howes, The Lighthouse Lab in Cambridge and Alex Alderton, Roberto Amato, Sonia Goncalves, Ewan Harrison, David K. Jackson, Ian Johnston, Dominic Kwiatkowski, Cordelia Langford, John Sillitoe on behalf of the Wellcome Sanger Institute COVID-19 Surveillance Team
EPI_ISL_948154, EPI_ISL_948156, EPI_ISL_948157, EPI_ISL_948158, EPI_ISL_948161, EPI_ISL_948162	Lighthouse Lab in Glasgow	Wellcome Sanger Institute for the COVID-19 Genomics UK (COG-UK) Consortium	Harper VanSteenhouse, Yumi Kasai, David Gray, Carol Clugston, Anna Dominiczak and Alex Alderton, Roberto Amato, Sonia Goncalves, Ewan Harrison, David K. Jackson, Ian Johnston, Dominic Kwiatkowski, Cordelia Langford, John Sillitoe on behalf of the Wellcome Sanger Institute COVID-19 Surveillance Team
EPI_ISL_948163, EPI_ISL_948164, EPI_ISL_948165, EPI_ISL_948166	Lighthouse Lab in Cambridge	Wellcome Sanger Institute for the COVID-19 Genomics UK (COG-UK) Consortium	Rob Howes, The Lighthouse Lab in Cambridge and Alex Alderton, Roberto Amato, Sonia Goncalves, Ewan Harrison, David K. Jackson, Ian Johnston, Dominic Kwiatkowski, Cordelia Langford, John Sillitoe on behalf of the Wellcome Sanger Institute COVID-19 Surveillance Team
EPI_ISL_948167, EPI_ISL_948168, EPI_ISL_948169, EPI_ISL_948170, EPI_ISL_948171	Lighthouse Lab in Glasgow	Wellcome Sanger Institute for the COVID-19 Genomics UK (COG-UK) Consortium	Harper VanSteenhouse, Yumi Kasai, David Gray, Carol Clugston, Anna Dominiczak and Alex Alderton, Roberto Amato, Sonia Goncalves, Ewan Harrison, David K. Jackson, Ian Johnston, Dominic Kwiatkowski, Cordelia Langford, John Sillitoe on behalf of the Wellcome Sanger Institute COVID-19 Surveillance Team
EPI_ISL_948172	Lighthouse Lab in Cambridge	Wellcome Sanger Institute for the COVID-19 Genomics UK (COG-UK) Consortium	Rob Howes, The Lighthouse Lab in Cambridge and Alex Alderton, Roberto Amato, Sonia Goncalves, Ewan Harrison, David K. Jackson, Ian Johnston, Dominic Kwiatkowski, Cordelia Langford, John Sillitoe on behalf of the Wellcome Sanger Institute COVID-19 Surveillance Team
EPI_ISL_948173	Lighthouse Lab in Glasgow	Wellcome Sanger Institute for the COVID-19 Genomics UK (COG-UK) Consortium	Harper VanSteenhouse, Yumi Kasai, David Gray, Carol Clugston, Anna Dominiczak and Alex Alderton, Roberto Amato, Sonia Goncalves, Ewan Harrison, David K. Jackson, Ian Johnston, Dominic Kwiatkowski, Cordelia Langford, John Sillitoe on behalf of the Wellcome Sanger Institute COVID-19 Surveillance Team
EPI_ISL_948174	Lighthouse Lab in Cambridge	Wellcome Sanger Institute for the COVID-19 Genomics UK (COG-UK) Consortium	Rob Howes, The Lighthouse Lab in Cambridge and Alex Alderton, Roberto Amato, Sonia Goncalves, Ewan Harrison, David K. Jackson, Ian Johnston, Dominic Kwiatkowski, Cordelia Langford, John Sillitoe on behalf of the Wellcome Sanger Institute COVID-19 Surveillance Team
EPI_ISL_948175	Lighthouse Lab in Glasgow	Wellcome Sanger Institute for the COVID-19 Genomics UK (COG-UK) Consortium	Harper VanSteenhouse, Yumi Kasai, David Gray, Carol Clugston, Anna Dominiczak and Alex Alderton, Roberto Amato, Sonia Goncalves, Ewan Harrison, David K. Jackson, Ian Johnston, Dominic Kwiatkowski, Cordelia Langford, John Sillitoe on behalf of the Wellcome Sanger Institute COVID-19 Surveillance Team
EPI_ISL_948176	Lighthouse Lab in Cambridge	Wellcome Sanger Institute for the COVID-19 Genomics UK (COG-UK) Consortium	Rob Howes, The Lighthouse Lab in Cambridge and Alex Alderton, Roberto Amato, Sonia Goncalves, Ewan Harrison, David K. Jackson, Ian Johnston, Dominic Kwiatkowski, Cordelia Langford, John Sillitoe on behalf of the Wellcome Sanger Institute COVID-19 Surveillance Team
EPI_ISL_948179	Lighthouse Lab in Glasgow	Wellcome Sanger Institute for the COVID-19 Genomics UK (COG-UK) Consortium	Harper VanSteenhouse, Yumi Kasai, David Gray, Carol Clugston, Anna Dominiczak and Alex Alderton, Roberto Amato, Sonia Goncalves, Ewan Harrison, David K. Jackson, Ian Johnston, Dominic Kwiatkowski, Cordelia Langford, John Sillitoe on behalf of the Wellcome Sanger Institute COVID-19 Surveillance Team
EPI_ISL_948180, EPI_ISL_948181	Lighthouse Lab in Cambridge	Wellcome Sanger Institute for the COVID-19 Genomics UK (COG-UK) Consortium	Rob Howes, The Lighthouse Lab in Cambridge and Alex Alderton, Roberto Amato, Sonia Goncalves, Ewan Harrison, David K. Jackson, Ian Johnston, Dominic Kwiatkowski, Cordelia Langford, John Sillitoe on behalf of the Wellcome Sanger Institute COVID-19 Surveillance Team
EPI_ISL_948182	Lighthouse Lab in Glasgow	Wellcome Sanger Institute for the COVID-19 Genomics UK (COG-UK) Consortium	Harper VanSteenhouse, Yumi Kasai, David Gray, Carol Clugston, Anna Dominiczak and Alex Alderton, Roberto Amato, Sonia Goncalves, Ewan Harrison, David K. Jackson, Ian Johnston, Dominic Kwiatkowski, Cordelia Langford, John Sillitoe on behalf of the Wellcome Sanger Institute COVID-19 Surveillance Team
EPI_ISL_948183	Lighthouse Lab in Cambridge	Wellcome Sanger Institute for the COVID-19 Genomics UK (COG-UK) Consortium	Rob Howes, The Lighthouse Lab in Cambridge and Alex Alderton, Roberto Amato, Sonia Goncalves, Ewan Harrison, David K. Jackson, Ian Johnston, Dominic Kwiatkowski, Cordelia Langford, John Sillitoe on behalf of the Wellcome Sanger Institute COVID-19 Surveillance Team
EPI_ISL_948184	Lighthouse Lab in Glasgow	Wellcome Sanger Institute for the COVID-19 Genomics UK (COG-UK) Consortium	Harper VanSteenhouse, Yumi Kasai, David Gray, Carol Clugston, Anna Dominiczak and Alex Alderton, Roberto Amato, Sonia Goncalves, Ewan Harrison, David K. Jackson, Ian Johnston, Dominic Kwiatkowski, Cordelia Langford, John Sillitoe on behalf of the Wellcome Sanger Institute COVID-19 Surveillance Team
EPI_ISL_948187, EPI_ISL_948188, EPI_ISL_948189	Lighthouse Lab in Cambridge	Wellcome Sanger Institute for the COVID-19 Genomics UK (COG-UK) Consortium	Rob Howes, The Lighthouse Lab in Cambridge and Alex Alderton, Roberto Amato, Sonia Goncalves, Ewan Harrison, David K. Jackson, Ian Johnston, Dominic Kwiatkowski, Cordelia Langford, John Sillitoe on behalf of the Wellcome Sanger Institute COVID-19 Surveillance Team
EPI_ISL_948190	Lighthouse Lab in Glasgow	Wellcome Sanger Institute for the COVID-19 Genomics UK (COG-UK) Consortium	Harper VanSteenhouse, Yumi Kasai, David Gray, Carol Clugston, Anna Dominiczak and Alex Alderton, Roberto Amato, Sonia Goncalves, Ewan Harrison, David K. Jackson, Ian Johnston, Dominic Kwiatkowski, Cordelia Langford, John Sillitoe on behalf of the Wellcome Sanger Institute COVID-19 Surveillance Team
EPI_ISL_948191, EPI_ISL_948192, EPI_ISL_948193	Lighthouse Lab in Cambridge	Wellcome Sanger Institute for the COVID-19 Genomics UK (COG-UK) Consortium	Rob Howes, The Lighthouse Lab in Cambridge and Alex Alderton, Roberto Amato, Sonia Goncalves, Ewan Harrison, David K. Jackson, Ian Johnston, Dominic Kwiatkowski, Cordelia Langford, John Sillitoe on behalf of the Wellcome Sanger Institute COVID-19 Surveillance Team
EPI_ISL_948195, EPI_ISL_948196	Lighthouse Lab in Glasgow	Wellcome Sanger Institute for the COVID-19 Genomics UK (COG-UK) Consortium	Harper VanSteenhouse, Yumi Kasai, David Gray, Carol Clugston, Anna Dominiczak and Alex Alderton, Roberto Amato, Sonia Goncalves, Ewan Harrison, David K. Jackson, Ian Johnston, Dominic Kwiatkowski, Cordelia Langford, John Sillitoe on behalf of the Wellcome Sanger Institute COVID-19 Surveillance Team
EPI_ISL_948197, EPI_ISL_948198, EPI_ISL_948199, EPI_ISL_948200, EPI_ISL_948201	Lighthouse Lab in Cambridge	Wellcome Sanger Institute for the COVID-19 Genomics UK (COG-UK) Consortium	Rob Howes, The Lighthouse Lab in Cambridge and Alex Alderton, Roberto Amato, Sonia Goncalves, Ewan Harrison, David K. Jackson, Ian Johnston, Dominic Kwiatkowski, Cordelia Langford, John Sillitoe on behalf of the Wellcome Sanger Institute COVID-19 Surveillance Team
EPI_ISL_948202	Lighthouse Lab in Glasgow	Wellcome Sanger Institute for the COVID-19 Genomics UK (COG-UK) Consortium	Harper VanSteenhouse, Yumi Kasai, David Gray, Carol Clugston, Anna Dominiczak and Alex Alderton, Roberto Amato, Sonia Goncalves, Ewan Harrison, David K. Jackson, Ian Johnston, Dominic Kwiatkowski, Cordelia Langford, John Sillitoe on behalf of the Wellcome Sanger Institute COVID-19 Surveillance Team
EPI_ISL_948203, EPI_ISL_948205	Lighthouse Lab in Cambridge	Wellcome Sanger Institute for the COVID-19 Genomics UK (COG-UK) Consortium	Rob Howes, The Lighthouse Lab in Cambridge and Alex Alderton, Roberto Amato, Sonia Goncalves, Ewan Harrison, David K. Jackson, Ian Johnston, Dominic Kwiatkowski, Cordelia Langford, John Sillitoe on behalf of the Wellcome Sanger Institute COVID-19 Surveillance Team
EPI_ISL_948206	Lighthouse Lab in Glasgow	Wellcome Sanger Institute for the COVID-19 Genomics UK (COG-UK) Consortium	Harper VanSteenhouse, Yumi Kasai, David Gray, Carol Clugston, Anna Dominiczak and Alex Alderton, Roberto Amato, Sonia Goncalves, Ewan Harrison, David K. Jackson, Ian Johnston, Dominic Kwiatkowski, Cordelia Langford, John Sillitoe on behalf of the Wellcome Sanger Institute COVID-19 Surveillance Team
EPI_ISL_948207, EPI_ISL_948208, EPI_ISL_948209, EPI_ISL_948211, EPI_ISL_948212, EPI_ISL_948213,	Lighthouse Lab in Cambridge	Wellcome Sanger Institute for the COVID-19 Genomics UK (COG-UK) Consortium	Rob Howes, The Lighthouse Lab in Cambridge and Alex Alderton, Roberto Amato, Sonia Goncalves, Ewan Harrison, David K. Jackson, Ian Johnston, Dominic Kwiatkowski, Cordelia Langford, John Sillitoe on behalf of the Wellcome Sanger Institute COVID-19 Surveillance Team

EPI_ISL_948214, EPI_ISL_948216			
EPI_ISL_948217, EPI_ISL_948218	Lighthouse Lab in Glasgow	Wellcome Sanger Institute for the COVID-19 Genomics UK (COG-UK) Consortium	Harper VanSteenhouse, Yumi Kasai, David Gray, Carol Clugston, Anna Dominiczak and Alex Alderton, Roberto Amato, Sonia Goncalves, Ewan Harrison, David K. Jackson, Ian Johnston, Dominic Kwiatkowski, Cordelia Langford, John Sillitoe on behalf of the Wellcome Sanger Institute COVID-19 Surveillance Team
EPI_ISL_948219, EPI_ISL_948220	Lighthouse Lab in Cambridge	Wellcome Sanger Institute for the COVID-19 Genomics UK (COG-UK) Consortium	Rob Howes, The Lighthouse Lab in Cambridge and Alex Alderton, Roberto Amato, Sonia Goncalves, Ewan Harrison, David K. Jackson, Ian Johnston, Dominic Kwiatkowski, Cordelia Langford, John Sillitoe on behalf of the Wellcome Sanger Institute COVID-19 Surveillance Team
EPI_ISL_948222	Lighthouse Lab in Glasgow	Wellcome Sanger Institute for the COVID-19 Genomics UK (COG-UK) Consortium	Harper VanSteenhouse, Yumi Kasai, David Gray, Carol Clugston, Anna Dominiczak and Alex Alderton, Roberto Amato, Sonia Goncalves, Ewan Harrison, David K. Jackson, Ian Johnston, Dominic Kwiatkowski, Cordelia Langford, John Sillitoe on behalf of the Wellcome Sanger Institute COVID-19 Surveillance Team
EPI_ISL_948223, EPI_ISL_948224	Lighthouse Lab in Cambridge	Wellcome Sanger Institute for the COVID-19 Genomics UK (COG-UK) Consortium	Rob Howes, The Lighthouse Lab in Cambridge and Alex Alderton, Roberto Amato, Sonia Goncalves, Ewan Harrison, David K. Jackson, Ian Johnston, Dominic Kwiatkowski, Cordelia Langford, John Sillitoe on behalf of the Wellcome Sanger Institute COVID-19 Surveillance Team
EPI_ISL_948225, EPI_ISL_948226	Lighthouse Lab in Glasgow	Wellcome Sanger Institute for the COVID-19 Genomics UK (COG-UK) Consortium	Harper VanSteenhouse, Yumi Kasai, David Gray, Carol Clugston, Anna Dominiczak and Alex Alderton, Roberto Amato, Sonia Goncalves, Ewan Harrison, David K. Jackson, Ian Johnston, Dominic Kwiatkowski, Cordelia Langford, John Sillitoe on behalf of the Wellcome Sanger Institute COVID-19 Surveillance Team
EPI_ISL_948227, EPI_ISL_948228	Lighthouse Lab in Cambridge	Wellcome Sanger Institute for the COVID-19 Genomics UK (COG-UK) Consortium	Rob Howes, The Lighthouse Lab in Cambridge and Alex Alderton, Roberto Amato, Sonia Goncalves, Ewan Harrison, David K. Jackson, Ian Johnston, Dominic Kwiatkowski, Cordelia Langford, John Sillitoe on behalf of the Wellcome Sanger Institute COVID-19 Surveillance Team
EPI_ISL_948229	Lighthouse Lab in Glasgow	Wellcome Sanger Institute for the COVID-19 Genomics UK (COG-UK) Consortium	Harper VanSteenhouse, Yumi Kasai, David Gray, Carol Clugston, Anna Dominiczak and Alex Alderton, Roberto Amato, Sonia Goncalves, Ewan Harrison, David K. Jackson, Ian Johnston, Dominic Kwiatkowski, Cordelia Langford, John Sillitoe on behalf of the Wellcome Sanger Institute COVID-19 Surveillance Team
EPI_ISL_948230	Lighthouse Lab in Cambridge	Wellcome Sanger Institute for the COVID-19 Genomics UK (COG-UK) Consortium	Rob Howes, The Lighthouse Lab in Cambridge and Alex Alderton, Roberto Amato, Sonia Goncalves, Ewan Harrison, David K. Jackson, Ian Johnston, Dominic Kwiatkowski, Cordelia Langford, John Sillitoe on behalf of the Wellcome Sanger Institute COVID-19 Surveillance Team
EPI_ISL_948232	Lighthouse Lab in Glasgow	Wellcome Sanger Institute for the COVID-19 Genomics UK (COG-UK) Consortium	Harper VanSteenhouse, Yumi Kasai, David Gray, Carol Clugston, Anna Dominiczak and Alex Alderton, Roberto Amato, Sonia Goncalves, Ewan Harrison, David K. Jackson, Ian Johnston, Dominic Kwiatkowski, Cordelia Langford, John Sillitoe on behalf of the Wellcome Sanger Institute COVID-19 Surveillance Team
EPI_ISL_948233, EPI_ISL_948234, EPI_ISL_948235	Lighthouse Lab in Cambridge	Wellcome Sanger Institute for the COVID-19 Genomics UK (COG-UK) Consortium	Rob Howes, The Lighthouse Lab in Cambridge and Alex Alderton, Roberto Amato, Sonia Goncalves, Ewan Harrison, David K. Jackson, Ian Johnston, Dominic Kwiatkowski, Cordelia Langford, John Sillitoe on behalf of the Wellcome Sanger Institute COVID-19 Surveillance Team
EPI_ISL_948236	Lighthouse Lab in Glasgow	Wellcome Sanger Institute for the COVID-19 Genomics UK (COG-UK) Consortium	Harper VanSteenhouse, Yumi Kasai, David Gray, Carol Clugston, Anna Dominiczak and Alex Alderton, Roberto Amato, Sonia Goncalves, Ewan Harrison, David K. Jackson, Ian Johnston, Dominic Kwiatkowski, Cordelia Langford, John Sillitoe on behalf of the Wellcome Sanger Institute COVID-19 Surveillance Team
EPI_ISL_948237	Lighthouse Lab in Cambridge	Wellcome Sanger Institute for the COVID-19 Genomics UK (COG-UK) Consortium	Rob Howes, The Lighthouse Lab in Cambridge and Alex Alderton, Roberto Amato, Sonia Goncalves, Ewan Harrison, David K. Jackson, Ian Johnston, Dominic Kwiatkowski, Cordelia Langford, John Sillitoe on behalf of the Wellcome Sanger Institute COVID-19 Surveillance Team
EPI_ISL_948238	Lighthouse Lab in Glasgow	Wellcome Sanger Institute for the COVID-19 Genomics UK (COG-UK) Consortium	Harper VanSteenhouse, Yumi Kasai, David Gray, Carol Clugston, Anna Dominiczak and Alex Alderton, Roberto Amato, Sonia Goncalves, Ewan Harrison, David K. Jackson, Ian Johnston, Dominic Kwiatkowski, Cordelia Langford, John Sillitoe on behalf of the Wellcome Sanger Institute COVID-19 Surveillance Team
EPI_ISL_948239, EPI_ISL_948240, EPI_ISL_948241	Lighthouse Lab in Cambridge	Wellcome Sanger Institute for the COVID-19 Genomics UK (COG-UK) Consortium	Rob Howes, The Lighthouse Lab in Cambridge and Alex Alderton, Roberto Amato, Sonia Goncalves, Ewan Harrison, David K. Jackson, Ian Johnston, Dominic Kwiatkowski, Cordelia Langford, John Sillitoe on behalf of the Wellcome Sanger Institute COVID-19 Surveillance Team
EPI_ISL_948242, EPI_ISL_948243	Lighthouse Lab in Glasgow	Wellcome Sanger Institute for the COVID-19 Genomics UK (COG-UK) Consortium	Harper VanSteenhouse, Yumi Kasai, David Gray, Carol Clugston, Anna Dominiczak and Alex Alderton, Roberto Amato, Sonia Goncalves, Ewan Harrison, David K. Jackson, Ian Johnston, Dominic Kwiatkowski, Cordelia Langford, John Sillitoe on behalf of the Wellcome Sanger Institute COVID-19 Surveillance Team
EPI_ISL_948244	Lighthouse Lab in Cambridge	Wellcome Sanger Institute for the COVID-19 Genomics UK (COG-UK) Consortium	Rob Howes, The Lighthouse Lab in Cambridge and Alex Alderton, Roberto Amato, Sonia Goncalves, Ewan Harrison, David K. Jackson, Ian Johnston, Dominic Kwiatkowski, Cordelia Langford, John Sillitoe on behalf of the Wellcome Sanger Institute COVID-19 Surveillance Team
EPI_ISL_948245, EPI_ISL_948246	Lighthouse Lab in Glasgow	Wellcome Sanger Institute for the COVID-19 Genomics UK (COG-UK) Consortium	Harper VanSteenhouse, Yumi Kasai, David Gray, Carol Clugston, Anna Dominiczak and Alex Alderton, Roberto Amato, Sonia Goncalves, Ewan Harrison, David K. Jackson, Ian Johnston, Dominic Kwiatkowski, Cordelia Langford, John Sillitoe on behalf of the Wellcome Sanger Institute COVID-19 Surveillance Team
EPI_ISL_948247	Lighthouse Lab in Cambridge	Wellcome Sanger Institute for the COVID-19 Genomics UK (COG-UK) Consortium	Rob Howes, The Lighthouse Lab in Cambridge and Alex Alderton, Roberto Amato, Sonia Goncalves, Ewan Harrison, David K. Jackson, Ian Johnston, Dominic Kwiatkowski, Cordelia Langford, John Sillitoe on behalf of the Wellcome Sanger Institute COVID-19 Surveillance Team
EPI_ISL_948248	Lighthouse Lab in Glasgow	Wellcome Sanger Institute for the COVID-19 Genomics UK (COG-UK) Consortium	Harper VanSteenhouse, Yumi Kasai, David Gray, Carol Clugston, Anna Dominiczak and Alex Alderton, Roberto Amato, Sonia Goncalves, Ewan Harrison, David K. Jackson, Ian Johnston, Dominic Kwiatkowski, Cordelia Langford, John Sillitoe on behalf of the Wellcome Sanger Institute COVID-19 Surveillance Team
EPI_ISL_948249, EPI_ISL_948250, EPI_ISL_948251	Lighthouse Lab in Cambridge	Wellcome Sanger Institute for the COVID-19 Genomics UK (COG-UK) Consortium	Rob Howes, The Lighthouse Lab in Cambridge and Alex Alderton, Roberto Amato, Sonia Goncalves, Ewan Harrison, David K. Jackson, Ian Johnston, Dominic Kwiatkowski, Cordelia Langford, John Sillitoe on behalf of the Wellcome Sanger Institute COVID-19 Surveillance Team
EPI_ISL_948252	Lighthouse Lab in Milton Keynes	Wellcome Sanger Institute for the COVID-19 Genomics UK (COG-UK) Consortium	The Lighthouse Lab in Milton Keynes and Alex Alderton, Roberto Amato, Sonia Goncalves, Ewan Harrison, David K. Jackson, Ian Johnston, Dominic Kwiatkowski, Cordelia Langford, John Sillitoe on behalf of the Wellcome Sanger Institute COVID-19 Surveillance Team
EPI_ISL_948253	Lighthouse Lab in Cambridge	Wellcome Sanger Institute for the COVID-19 Genomics UK (COG-UK) Consortium	Rob Howes, The Lighthouse Lab in Cambridge and Alex Alderton, Roberto Amato, Sonia Goncalves, Ewan Harrison, David K. Jackson, Ian Johnston, Dominic Kwiatkowski, Cordelia Langford, John Sillitoe on behalf of the Wellcome Sanger Institute COVID-19 Surveillance Team
EPI_ISL_948254	Lighthouse Lab in Glasgow	Wellcome Sanger Institute for the COVID-19 Genomics UK (COG-UK) Consortium	Harper VanSteenhouse, Yumi Kasai, David Gray, Carol Clugston, Anna Dominiczak and Alex Alderton, Roberto Amato, Sonia Goncalves, Ewan Harrison, David K. Jackson, Ian Johnston, Dominic Kwiatkowski, Cordelia Langford, John Sillitoe on behalf of the Wellcome Sanger Institute COVID-19 Surveillance Team
EPI_ISL_948255	Lighthouse Lab in Cambridge	Wellcome Sanger Institute for the COVID-19 Genomics UK (COG-UK) Consortium	Rob Howes, The Lighthouse Lab in Cambridge and Alex Alderton, Roberto Amato, Sonia Goncalves, Ewan Harrison, David K. Jackson, Ian Johnston, Dominic Kwiatkowski, Cordelia Langford, John Sillitoe on behalf of the Wellcome Sanger Institute COVID-19 Surveillance Team
EPI_ISL_948256	Lighthouse Lab in Glasgow	Wellcome Sanger Institute for the COVID-19 Genomics UK (COG-UK) Consortium	Harper VanSteenhouse, Yumi Kasai, David Gray, Carol Clugston, Anna Dominiczak and Alex Alderton, Roberto Amato, Sonia Goncalves, Ewan Harrison, David K. Jackson, Ian Johnston, Dominic Kwiatkowski, Cordelia Langford, John Sillitoe on behalf of the Wellcome Sanger Institute COVID-19 Surveillance Team
EPI_ISL_948257, EPI_ISL_948258, EPI_ISL_948259	Lighthouse Lab in Cambridge	Wellcome Sanger Institute for the COVID-19 Genomics UK (COG-UK) Consortium	Rob Howes, The Lighthouse Lab in Cambridge and Alex Alderton, Roberto Amato, Sonia Goncalves, Ewan Harrison, David K. Jackson, Ian Johnston, Dominic Kwiatkowski, Cordelia Langford, John Sillitoe on behalf of the Wellcome Sanger Institute COVID-19 Surveillance Team
EPI_ISL_948260	Lighthouse Lab in Glasgow	Wellcome Sanger Institute for the COVID-19 Genomics UK (COG-UK) Consortium	Harper VanSteenhouse, Yumi Kasai, David Gray, Carol Clugston, Anna Dominiczak and Alex Alderton, Roberto Amato, Sonia Goncalves, Ewan Harrison, David K. Jackson, Ian Johnston, Dominic Kwiatkowski, Cordelia Langford, John Sillitoe on behalf of the Wellcome Sanger Institute COVID-19 Surveillance Team
EPI_ISL_948261	Lighthouse Lab in Cambridge	Wellcome Sanger Institute for the COVID-19 Genomics UK (COG-UK) Consortium	Rob Howes, The Lighthouse Lab in Cambridge and Alex Alderton, Roberto Amato, Sonia Goncalves, Ewan Harrison, David K. Jackson, Ian Johnston, Dominic Kwiatkowski, Cordelia Langford, John Sillitoe on behalf of the Wellcome Sanger Institute COVID-19 Surveillance Team
EPI_ISL_948262, EPI_ISL_948263	Lighthouse Lab in Glasgow	Wellcome Sanger Institute for the COVID-19 Genomics UK (COG-UK) Consortium	Harper VanSteenhouse, Yumi Kasai, David Gray, Carol Clugston, Anna Dominiczak and Alex Alderton, Roberto Amato, Sonia Goncalves, Ewan Harrison, David K. Jackson, Ian Johnston, Dominic Kwiatkowski, Cordelia Langford, John Sillitoe on behalf of the Wellcome Sanger Institute COVID-19 Surveillance Team

EPI_ISL_948264, EPI_ISL_948265	Lighthouse Lab in Cambridge	Wellcome Sanger Institute for the COVID-19 Genomics UK (COG-UK) Consortium	Rob Howes, The Lighthouse Lab in Cambridge and Alex Alderton, Roberto Amato, Sonia Goncalves, Ewan Harrison, David K. Jackson, Ian Johnston, Dominic Kwiatkowski, Cordelia Langford, John Sillitoe on behalf of the Wellcome Sanger Institute COVID-19 Surveillance Team
EPI_ISL_948267, EPI_ISL_948268	Lighthouse Lab in Glasgow	Wellcome Sanger Institute for the COVID-19 Genomics UK (COG-UK) Consortium	Harper VanSteenhouse, Yumi Kasai, David Gray, Carol Clugston, Anna Dominiczak and Alex Alderton, Roberto Amato, Sonia Goncalves, Ewan Harrison, David K. Jackson, Ian Johnston, Dominic Kwiatkowski, Cordelia Langford, John Sillitoe on behalf of the Wellcome Sanger Institute COVID-19 Surveillance Team
EPI_ISL_948269	Lighthouse Lab in Cambridge	Wellcome Sanger Institute for the COVID-19 Genomics UK (COG-UK) Consortium	Rob Howes, The Lighthouse Lab in Cambridge and Alex Alderton, Roberto Amato, Sonia Goncalves, Ewan Harrison, David K. Jackson, Ian Johnston, Dominic Kwiatkowski, Cordelia Langford, John Sillitoe on behalf of the Wellcome Sanger Institute COVID-19 Surveillance Team
EPI_ISL_948270, EPI_ISL_948271, EPI_ISL_948272, EPI_ISL_948273	Lighthouse Lab in Glasgow	Wellcome Sanger Institute for the COVID-19 Genomics UK (COG-UK) Consortium	Harper VanSteenhouse, Yumi Kasai, David Gray, Carol Clugston, Anna Dominiczak and Alex Alderton, Roberto Amato, Sonia Goncalves, Ewan Harrison, David K. Jackson, Ian Johnston, Dominic Kwiatkowski, Cordelia Langford, John Sillitoe on behalf of the Wellcome Sanger Institute COVID-19 Surveillance Team
EPI_ISL_948274	Lighthouse Lab in Cambridge	Wellcome Sanger Institute for the COVID-19 Genomics UK (COG-UK) Consortium	Rob Howes, The Lighthouse Lab in Cambridge and Alex Alderton, Roberto Amato, Sonia Goncalves, Ewan Harrison, David K. Jackson, Ian Johnston, Dominic Kwiatkowski, Cordelia Langford, John Sillitoe on behalf of the Wellcome Sanger Institute COVID-19 Surveillance Team
EPI_ISL_948275, EPI_ISL_948276	Lighthouse Lab in Glasgow	Wellcome Sanger Institute for the COVID-19 Genomics UK (COG-UK) Consortium	Harper VanSteenhouse, Yumi Kasai, David Gray, Carol Clugston, Anna Dominiczak and Alex Alderton, Roberto Amato, Sonia Goncalves, Ewan Harrison, David K. Jackson, Ian Johnston, Dominic Kwiatkowski, Cordelia Langford, John Sillitoe on behalf of the Wellcome Sanger Institute COVID-19 Surveillance Team
EPI_ISL_948277, EPI_ISL_948278, EPI_ISL_948279, EPI_ISL_948280, EPI_ISL_948281, EPI_ISL_948282, EPI_ISL_948283, EPI_ISL_948284	Lighthouse Lab in Cambridge	Wellcome Sanger Institute for the COVID-19 Genomics UK (COG-UK) Consortium	Rob Howes, The Lighthouse Lab in Cambridge and Alex Alderton, Roberto Amato, Sonia Goncalves, Ewan Harrison, David K. Jackson, Ian Johnston, Dominic Kwiatkowski, Cordelia Langford, John Sillitoe on behalf of the Wellcome Sanger Institute COVID-19 Surveillance Team
EPI_ISL_948285	Lighthouse Lab in Glasgow	Wellcome Sanger Institute for the COVID-19 Genomics UK (COG-UK) Consortium	Harper VanSteenhouse, Yumi Kasai, David Gray, Carol Clugston, Anna Dominiczak and Alex Alderton, Roberto Amato, Sonia Goncalves, Ewan Harrison, David K. Jackson, Ian Johnston, Dominic Kwiatkowski, Cordelia Langford, John Sillitoe on behalf of the Wellcome Sanger Institute COVID-19 Surveillance Team
EPI_ISL_948286	Lighthouse Lab in Cambridge	Wellcome Sanger Institute for the COVID-19 Genomics UK (COG-UK) Consortium	Rob Howes, The Lighthouse Lab in Cambridge and Alex Alderton, Roberto Amato, Sonia Goncalves, Ewan Harrison, David K. Jackson, Ian Johnston, Dominic Kwiatkowski, Cordelia Langford, John Sillitoe on behalf of the Wellcome Sanger Institute COVID-19 Surveillance Team
EPI_ISL_948287	Lighthouse Lab in Glasgow	Wellcome Sanger Institute for the COVID-19 Genomics UK (COG-UK) Consortium	Harper VanSteenhouse, Yumi Kasai, David Gray, Carol Clugston, Anna Dominiczak and Alex Alderton, Roberto Amato, Sonia Goncalves, Ewan Harrison, David K. Jackson, Ian Johnston, Dominic Kwiatkowski, Cordelia Langford, John Sillitoe on behalf of the Wellcome Sanger Institute COVID-19 Surveillance Team
EPI_ISL_948288	Lighthouse Lab in Cambridge	Wellcome Sanger Institute for the COVID-19 Genomics UK (COG-UK) Consortium	Rob Howes, The Lighthouse Lab in Cambridge and Alex Alderton, Roberto Amato, Sonia Goncalves, Ewan Harrison, David K. Jackson, Ian Johnston, Dominic Kwiatkowski, Cordelia Langford, John Sillitoe on behalf of the Wellcome Sanger Institute COVID-19 Surveillance Team
EPI_ISL_948290, EPI_ISL_948291, EPI_ISL_948293, EPI_ISL_948295	Lighthouse Lab in Glasgow	Wellcome Sanger Institute for the COVID-19 Genomics UK (COG-UK) Consortium	Harper VanSteenhouse, Yumi Kasai, David Gray, Carol Clugston, Anna Dominiczak and Alex Alderton, Roberto Amato, Sonia Goncalves, Ewan Harrison, David K. Jackson, Ian Johnston, Dominic Kwiatkowski, Cordelia Langford, John Sillitoe on behalf of the Wellcome Sanger Institute COVID-19 Surveillance Team
EPI_ISL_948296	Lighthouse Lab in Cambridge	Wellcome Sanger Institute for the COVID-19 Genomics UK (COG-UK) Consortium	Rob Howes, The Lighthouse Lab in Cambridge and Alex Alderton, Roberto Amato, Sonia Goncalves, Ewan Harrison, David K. Jackson, Ian Johnston, Dominic Kwiatkowski, Cordelia Langford, John Sillitoe on behalf of the Wellcome Sanger Institute COVID-19 Surveillance Team
EPI_ISL_948297	Lighthouse Lab in Glasgow	Wellcome Sanger Institute for the COVID-19 Genomics UK (COG-UK) Consortium	Harper VanSteenhouse, Yumi Kasai, David Gray, Carol Clugston, Anna Dominiczak and Alex Alderton, Roberto Amato, Sonia Goncalves, Ewan Harrison, David K. Jackson, Ian Johnston, Dominic Kwiatkowski, Cordelia Langford, John Sillitoe on behalf of the Wellcome Sanger Institute COVID-19 Surveillance Team
EPI_ISL_948298	Lighthouse Lab in Cambridge	Wellcome Sanger Institute for the COVID-19 Genomics UK (COG-UK) Consortium	Rob Howes, The Lighthouse Lab in Cambridge and Alex Alderton, Roberto Amato, Sonia Goncalves, Ewan Harrison, David K. Jackson, Ian Johnston, Dominic Kwiatkowski, Cordelia Langford, John Sillitoe on behalf of the Wellcome Sanger Institute COVID-19 Surveillance Team
EPI_ISL_948299	Lighthouse Lab in Glasgow	Wellcome Sanger Institute for the COVID-19 Genomics UK (COG-UK) Consortium	Harper VanSteenhouse, Yumi Kasai, David Gray, Carol Clugston, Anna Dominiczak and Alex Alderton, Roberto Amato, Sonia Goncalves, Ewan Harrison, David K. Jackson, Ian Johnston, Dominic Kwiatkowski, Cordelia Langford, John Sillitoe on behalf of the Wellcome Sanger Institute COVID-19 Surveillance Team
EPI_ISL_948300, EPI_ISL_948301, EPI_ISL_948302	Lighthouse Lab in Cambridge	Wellcome Sanger Institute for the COVID-19 Genomics UK (COG-UK) Consortium	Rob Howes, The Lighthouse Lab in Cambridge and Alex Alderton, Roberto Amato, Sonia Goncalves, Ewan Harrison, David K. Jackson, Ian Johnston, Dominic Kwiatkowski, Cordelia Langford, John Sillitoe on behalf of the Wellcome Sanger Institute COVID-19 Surveillance Team
EPI_ISL_948303, EPI_ISL_948304	Lighthouse Lab in Glasgow	Wellcome Sanger Institute for the COVID-19 Genomics UK (COG-UK) Consortium	Harper VanSteenhouse, Yumi Kasai, David Gray, Carol Clugston, Anna Dominiczak and Alex Alderton, Roberto Amato, Sonia Goncalves, Ewan Harrison, David K. Jackson, Ian Johnston, Dominic Kwiatkowski, Cordelia Langford, John Sillitoe on behalf of the Wellcome Sanger Institute COVID-19 Surveillance Team
EPI_ISL_948305, EPI_ISL_948306	Lighthouse Lab in Cambridge	Wellcome Sanger Institute for the COVID-19 Genomics UK (COG-UK) Consortium	Rob Howes, The Lighthouse Lab in Cambridge and Alex Alderton, Roberto Amato, Sonia Goncalves, Ewan Harrison, David K. Jackson, Ian Johnston, Dominic Kwiatkowski, Cordelia Langford, John Sillitoe on behalf of the Wellcome Sanger Institute COVID-19 Surveillance Team
EPI_ISL_948307	Lighthouse Lab in Glasgow	Wellcome Sanger Institute for the COVID-19 Genomics UK (COG-UK) Consortium	Harper VanSteenhouse, Yumi Kasai, David Gray, Carol Clugston, Anna Dominiczak and Alex Alderton, Roberto Amato, Sonia Goncalves, Ewan Harrison, David K. Jackson, Ian Johnston, Dominic Kwiatkowski, Cordelia Langford, John Sillitoe on behalf of the Wellcome Sanger Institute COVID-19 Surveillance Team
EPI_ISL_948308	Lighthouse Lab in Cambridge	Wellcome Sanger Institute for the COVID-19 Genomics UK (COG-UK) Consortium	Rob Howes, The Lighthouse Lab in Cambridge and Alex Alderton, Roberto Amato, Sonia Goncalves, Ewan Harrison, David K. Jackson, Ian Johnston, Dominic Kwiatkowski, Cordelia Langford, John Sillitoe on behalf of the Wellcome Sanger Institute COVID-19 Surveillance Team
EPI_ISL_948309	Lighthouse Lab in Glasgow	Wellcome Sanger Institute for the COVID-19 Genomics UK (COG-UK) Consortium	Harper VanSteenhouse, Yumi Kasai, David Gray, Carol Clugston, Anna Dominiczak and Alex Alderton, Roberto Amato, Sonia Goncalves, Ewan Harrison, David K. Jackson, Ian Johnston, Dominic Kwiatkowski, Cordelia Langford, John Sillitoe on behalf of the Wellcome Sanger Institute COVID-19 Surveillance Team
EPI_ISL_948310, EPI_ISL_948311, EPI_ISL_948312, EPI_ISL_948313, EPI_ISL_948314	Lighthouse Lab in Cambridge	Wellcome Sanger Institute for the COVID-19 Genomics UK (COG-UK) Consortium	Rob Howes, The Lighthouse Lab in Cambridge and Alex Alderton, Roberto Amato, Sonia Goncalves, Ewan Harrison, David K. Jackson, Ian Johnston, Dominic Kwiatkowski, Cordelia Langford, John Sillitoe on behalf of the Wellcome Sanger Institute COVID-19 Surveillance Team
EPI_ISL_948315	Lighthouse Lab in Glasgow	Wellcome Sanger Institute for the COVID-19 Genomics UK (COG-UK) Consortium	Harper VanSteenhouse, Yumi Kasai, David Gray, Carol Clugston, Anna Dominiczak and Alex Alderton, Roberto Amato, Sonia Goncalves, Ewan Harrison, David K. Jackson, Ian Johnston, Dominic Kwiatkowski, Cordelia Langford, John Sillitoe on behalf of the Wellcome Sanger Institute COVID-19 Surveillance Team
EPI_ISL_948316	Lighthouse Lab in Cambridge	Wellcome Sanger Institute for the COVID-19 Genomics UK (COG-UK) Consortium	Rob Howes, The Lighthouse Lab in Cambridge and Alex Alderton, Roberto Amato, Sonia Goncalves, Ewan Harrison, David K. Jackson, Ian Johnston, Dominic Kwiatkowski, Cordelia Langford, John Sillitoe on behalf of the Wellcome Sanger Institute COVID-19 Surveillance Team
EPI_ISL_948317, EPI_ISL_948318, EPI_ISL_948319, EPI_ISL_948320	Lighthouse Lab in Glasgow	Wellcome Sanger Institute for the COVID-19 Genomics UK (COG-UK) Consortium	Harper VanSteenhouse, Yumi Kasai, David Gray, Carol Clugston, Anna Dominiczak and Alex Alderton, Roberto Amato, Sonia Goncalves, Ewan Harrison, David K. Jackson, Ian Johnston, Dominic Kwiatkowski, Cordelia Langford, John Sillitoe on behalf of the Wellcome Sanger Institute COVID-19 Surveillance Team
EPI_ISL_948321, EPI_ISL_948322, EPI_ISL_948323	Lighthouse Lab in Cambridge	Wellcome Sanger Institute for the COVID-19 Genomics UK (COG-UK) Consortium	Rob Howes, The Lighthouse Lab in Cambridge and Alex Alderton, Roberto Amato, Sonia Goncalves, Ewan Harrison, David K. Jackson, Ian Johnston, Dominic Kwiatkowski, Cordelia Langford, John Sillitoe on behalf of the Wellcome Sanger Institute COVID-19 Surveillance Team
EPI_ISL_948324, EPI_ISL_948325	Lighthouse Lab in Glasgow	Wellcome Sanger Institute for the COVID-19 Genomics UK (COG-UK) Consortium	Harper VanSteenhouse, Yumi Kasai, David Gray, Carol Clugston, Anna Dominiczak and Alex Alderton, Roberto Amato, Sonia Goncalves, Ewan Harrison, David K. Jackson, Ian Johnston, Dominic Kwiatkowski, Cordelia Langford, John Sillitoe on behalf of the Wellcome Sanger Institute COVID-19 Surveillance Team

EPI_ISL_948326	Lighthouse Lab in Cambridge	Wellcome Sanger Institute for the COVID-19 Genomics UK (COG-UK) Consortium	Rob Howes, The Lighthouse Lab in Cambridge and Alex Alderton, Roberto Amato, Sonia Goncalves, Ewan Harrison, David K. Jackson, Ian Johnston, Dominic Kwiatkowski, Cordelia Langford, John Sillitoe on behalf of the Wellcome Sanger Institute COVID-19 Surveillance Team
EPI_ISL_948327	Lighthouse Lab in Milton Keynes	Wellcome Sanger Institute for the COVID-19 Genomics UK (COG-UK) Consortium	The Lighthouse Lab in Milton Keynes and Alex Alderton, Roberto Amato, Sonia Goncalves, Ewan Harrison, David K. Jackson, Ian Johnston, Dominic Kwiatkowski, Cordelia Langford, John Sillitoe on behalf of the Wellcome Sanger Institute COVID-19 Surveillance Team
EPI_ISL_948328	Lighthouse Lab in Cambridge	Wellcome Sanger Institute for the COVID-19 Genomics UK (COG-UK) Consortium	Rob Howes, The Lighthouse Lab in Cambridge and Alex Alderton, Roberto Amato, Sonia Goncalves, Ewan Harrison, David K. Jackson, Ian Johnston, Dominic Kwiatkowski, Cordelia Langford, John Sillitoe on behalf of the Wellcome Sanger Institute COVID-19 Surveillance Team
EPI_ISL_948329	Lighthouse Lab in Glasgow	Wellcome Sanger Institute for the COVID-19 Genomics UK (COG-UK) Consortium	Harper VanSteenhouse, Yumi Kasai, David Gray, Carol Clugston, Anna Dominiczak and Alex Alderton, Roberto Amato, Sonia Goncalves, Ewan Harrison, David K. Jackson, Ian Johnston, Dominic Kwiatkowski, Cordelia Langford, John Sillitoe on behalf of the Wellcome Sanger Institute COVID-19 Surveillance Team
EPI_ISL_948331, EPI_ISL_948332	Lighthouse Lab in Cambridge	Wellcome Sanger Institute for the COVID-19 Genomics UK (COG-UK) Consortium	Rob Howes, The Lighthouse Lab in Cambridge and Alex Alderton, Roberto Amato, Sonia Goncalves, Ewan Harrison, David K. Jackson, Ian Johnston, Dominic Kwiatkowski, Cordelia Langford, John Sillitoe on behalf of the Wellcome Sanger Institute COVID-19 Surveillance Team
EPI_ISL_948333, EPI_ISL_948334, EPI_ISL_948335, EPI_ISL_948336	Lighthouse Lab in Glasgow	Wellcome Sanger Institute for the COVID-19 Genomics UK (COG-UK) Consortium	Harper VanSteenhouse, Yumi Kasai, David Gray, Carol Clugston, Anna Dominiczak and Alex Alderton, Roberto Amato, Sonia Goncalves, Ewan Harrison, David K. Jackson, Ian Johnston, Dominic Kwiatkowski, Cordelia Langford, John Sillitoe on behalf of the Wellcome Sanger Institute COVID-19 Surveillance Team
EPI_ISL_948337, EPI_ISL_948338	Lighthouse Lab in Cambridge	Wellcome Sanger Institute for the COVID-19 Genomics UK (COG-UK) Consortium	Rob Howes, The Lighthouse Lab in Cambridge and Alex Alderton, Roberto Amato, Sonia Goncalves, Ewan Harrison, David K. Jackson, Ian Johnston, Dominic Kwiatkowski, Cordelia Langford, John Sillitoe on behalf of the Wellcome Sanger Institute COVID-19 Surveillance Team
EPI_ISL_948339	Lighthouse Lab in Glasgow	Wellcome Sanger Institute for the COVID-19 Genomics UK (COG-UK) Consortium	Harper VanSteenhouse, Yumi Kasai, David Gray, Carol Clugston, Anna Dominiczak and Alex Alderton, Roberto Amato, Sonia Goncalves, Ewan Harrison, David K. Jackson, Ian Johnston, Dominic Kwiatkowski, Cordelia Langford, John Sillitoe on behalf of the Wellcome Sanger Institute COVID-19 Surveillance Team
EPI_ISL_948340, EPI_ISL_948341, EPI_ISL_948342	Lighthouse Lab in Cambridge	Wellcome Sanger Institute for the COVID-19 Genomics UK (COG-UK) Consortium	Rob Howes, The Lighthouse Lab in Cambridge and Alex Alderton, Roberto Amato, Sonia Goncalves, Ewan Harrison, David K. Jackson, Ian Johnston, Dominic Kwiatkowski, Cordelia Langford, John Sillitoe on behalf of the Wellcome Sanger Institute COVID-19 Surveillance Team
EPI_ISL_948343	Lighthouse Lab in Glasgow	Wellcome Sanger Institute for the COVID-19 Genomics UK (COG-UK) Consortium	Harper VanSteenhouse, Yumi Kasai, David Gray, Carol Clugston, Anna Dominiczak and Alex Alderton, Roberto Amato, Sonia Goncalves, Ewan Harrison, David K. Jackson, Ian Johnston, Dominic Kwiatkowski, Cordelia Langford, John Sillitoe on behalf of the Wellcome Sanger Institute COVID-19 Surveillance Team
EPI_ISL_948344, EPI_ISL_948345, EPI_ISL_948346	Lighthouse Lab in Cambridge	Wellcome Sanger Institute for the COVID-19 Genomics UK (COG-UK) Consortium	Rob Howes, The Lighthouse Lab in Cambridge and Alex Alderton, Roberto Amato, Sonia Goncalves, Ewan Harrison, David K. Jackson, Ian Johnston, Dominic Kwiatkowski, Cordelia Langford, John Sillitoe on behalf of the Wellcome Sanger Institute COVID-19 Surveillance Team
EPI_ISL_948347, EPI_ISL_948348, EPI_ISL_948349	Lighthouse Lab in Glasgow	Wellcome Sanger Institute for the COVID-19 Genomics UK (COG-UK) Consortium	Harper VanSteenhouse, Yumi Kasai, David Gray, Carol Clugston, Anna Dominiczak and Alex Alderton, Roberto Amato, Sonia Goncalves, Ewan Harrison, David K. Jackson, Ian Johnston, Dominic Kwiatkowski, Cordelia Langford, John Sillitoe on behalf of the Wellcome Sanger Institute COVID-19 Surveillance Team
EPI_ISL_948350	Lighthouse Lab in Cambridge	Wellcome Sanger Institute for the COVID-19 Genomics UK (COG-UK) Consortium	Rob Howes, The Lighthouse Lab in Cambridge and Alex Alderton, Roberto Amato, Sonia Goncalves, Ewan Harrison, David K. Jackson, Ian Johnston, Dominic Kwiatkowski, Cordelia Langford, John Sillitoe on behalf of the Wellcome Sanger Institute COVID-19 Surveillance Team
EPI_ISL_948351, EPI_ISL_948352, EPI_ISL_948353, EPI_ISL_948354	Lighthouse Lab in Glasgow	Wellcome Sanger Institute for the COVID-19 Genomics UK (COG-UK) Consortium	Harper VanSteenhouse, Yumi Kasai, David Gray, Carol Clugston, Anna Dominiczak and Alex Alderton, Roberto Amato, Sonia Goncalves, Ewan Harrison, David K. Jackson, Ian Johnston, Dominic Kwiatkowski, Cordelia Langford, John Sillitoe on behalf of the Wellcome Sanger Institute COVID-19 Surveillance Team
EPI_ISL_948355	Lighthouse Lab in Cambridge	Wellcome Sanger Institute for the COVID-19 Genomics UK (COG-UK) Consortium	Rob Howes, The Lighthouse Lab in Cambridge and Alex Alderton, Roberto Amato, Sonia Goncalves, Ewan Harrison, David K. Jackson, Ian Johnston, Dominic Kwiatkowski, Cordelia Langford, John Sillitoe on behalf of the Wellcome Sanger Institute COVID-19 Surveillance Team
EPI_ISL_948356, EPI_ISL_948357, EPI_ISL_948358	Lighthouse Lab in Glasgow	Wellcome Sanger Institute for the COVID-19 Genomics UK (COG-UK) Consortium	Harper VanSteenhouse, Yumi Kasai, David Gray, Carol Clugston, Anna Dominiczak and Alex Alderton, Roberto Amato, Sonia Goncalves, Ewan Harrison, David K. Jackson, Ian Johnston, Dominic Kwiatkowski, Cordelia Langford, John Sillitoe on behalf of the Wellcome Sanger Institute COVID-19 Surveillance Team
EPI_ISL_948359	Lighthouse Lab in Cambridge	Wellcome Sanger Institute for the COVID-19 Genomics UK (COG-UK) Consortium	Rob Howes, The Lighthouse Lab in Cambridge and Alex Alderton, Roberto Amato, Sonia Goncalves, Ewan Harrison, David K. Jackson, Ian Johnston, Dominic Kwiatkowski, Cordelia Langford, John Sillitoe on behalf of the Wellcome Sanger Institute COVID-19 Surveillance Team
EPI_ISL_948360	Lighthouse Lab in Glasgow	Wellcome Sanger Institute for the COVID-19 Genomics UK (COG-UK) Consortium	Harper VanSteenhouse, Yumi Kasai, David Gray, Carol Clugston, Anna Dominiczak and Alex Alderton, Roberto Amato, Sonia Goncalves, Ewan Harrison, David K. Jackson, Ian Johnston, Dominic Kwiatkowski, Cordelia Langford, John Sillitoe on behalf of the Wellcome Sanger Institute COVID-19 Surveillance Team
EPI_ISL_948361	Lighthouse Lab in Cambridge	Wellcome Sanger Institute for the COVID-19 Genomics UK (COG-UK) Consortium	Rob Howes, The Lighthouse Lab in Cambridge and Alex Alderton, Roberto Amato, Sonia Goncalves, Ewan Harrison, David K. Jackson, Ian Johnston, Dominic Kwiatkowski, Cordelia Langford, John Sillitoe on behalf of the Wellcome Sanger Institute COVID-19 Surveillance Team
EPI_ISL_948362, EPI_ISL_948363	Lighthouse Lab in Glasgow	Wellcome Sanger Institute for the COVID-19 Genomics UK (COG-UK) Consortium	Harper VanSteenhouse, Yumi Kasai, David Gray, Carol Clugston, Anna Dominiczak and Alex Alderton, Roberto Amato, Sonia Goncalves, Ewan Harrison, David K. Jackson, Ian Johnston, Dominic Kwiatkowski, Cordelia Langford, John Sillitoe on behalf of the Wellcome Sanger Institute COVID-19 Surveillance Team
EPI_ISL_948364	Lighthouse Lab in Cambridge	Wellcome Sanger Institute for the COVID-19 Genomics UK (COG-UK) Consortium	Rob Howes, The Lighthouse Lab in Cambridge and Alex Alderton, Roberto Amato, Sonia Goncalves, Ewan Harrison, David K. Jackson, Ian Johnston, Dominic Kwiatkowski, Cordelia Langford, John Sillitoe on behalf of the Wellcome Sanger Institute COVID-19 Surveillance Team
EPI_ISL_948365	Lighthouse Lab in Glasgow	Wellcome Sanger Institute for the COVID-19 Genomics UK (COG-UK) Consortium	Harper VanSteenhouse, Yumi Kasai, David Gray, Carol Clugston, Anna Dominiczak and Alex Alderton, Roberto Amato, Sonia Goncalves, Ewan Harrison, David K. Jackson, Ian Johnston, Dominic Kwiatkowski, Cordelia Langford, John Sillitoe on behalf of the Wellcome Sanger Institute COVID-19 Surveillance Team
EPI_ISL_948366	Lighthouse Lab in Cambridge	Wellcome Sanger Institute for the COVID-19 Genomics UK (COG-UK) Consortium	Rob Howes, The Lighthouse Lab in Cambridge and Alex Alderton, Roberto Amato, Sonia Goncalves, Ewan Harrison, David K. Jackson, Ian Johnston, Dominic Kwiatkowski, Cordelia Langford, John Sillitoe on behalf of the Wellcome Sanger Institute COVID-19 Surveillance Team
EPI_ISL_948367, EPI_ISL_948369, EPI_ISL_948370, EPI_ISL_948371	Lighthouse Lab in Glasgow	Wellcome Sanger Institute for the COVID-19 Genomics UK (COG-UK) Consortium	Harper VanSteenhouse, Yumi Kasai, David Gray, Carol Clugston, Anna Dominiczak and Alex Alderton, Roberto Amato, Sonia Goncalves, Ewan Harrison, David K. Jackson, Ian Johnston, Dominic Kwiatkowski, Cordelia Langford, John Sillitoe on behalf of the Wellcome Sanger Institute COVID-19 Surveillance Team
EPI_ISL_948372	Lighthouse Lab in Cambridge	Wellcome Sanger Institute for the COVID-19 Genomics UK (COG-UK) Consortium	Rob Howes, The Lighthouse Lab in Cambridge and Alex Alderton, Roberto Amato, Sonia Goncalves, Ewan Harrison, David K. Jackson, Ian Johnston, Dominic Kwiatkowski, Cordelia Langford, John Sillitoe on behalf of the Wellcome Sanger Institute COVID-19 Surveillance Team
EPI_ISL_948373, EPI_ISL_948375	Lighthouse Lab in Glasgow	Wellcome Sanger Institute for the COVID-19 Genomics UK (COG-UK) Consortium	Harper VanSteenhouse, Yumi Kasai, David Gray, Carol Clugston, Anna Dominiczak and Alex Alderton, Roberto Amato, Sonia Goncalves, Ewan Harrison, David K. Jackson, Ian Johnston, Dominic Kwiatkowski, Cordelia Langford, John Sillitoe on behalf of the Wellcome Sanger Institute COVID-19 Surveillance Team
EPI_ISL_948378	Lighthouse Lab in Cambridge	Wellcome Sanger Institute for the COVID-19 Genomics UK (COG-UK) Consortium	Rob Howes, The Lighthouse Lab in Cambridge and Alex Alderton, Roberto Amato, Sonia Goncalves, Ewan Harrison, David K. Jackson, Ian Johnston, Dominic Kwiatkowski, Cordelia Langford, John Sillitoe on behalf of the Wellcome Sanger Institute COVID-19 Surveillance Team
EPI_ISL_948379	Lighthouse Lab in Glasgow	Wellcome Sanger Institute for the COVID-19 Genomics UK (COG-UK) Consortium	Harper VanSteenhouse, Yumi Kasai, David Gray, Carol Clugston, Anna Dominiczak and Alex Alderton, Roberto Amato, Sonia Goncalves, Ewan Harrison, David K. Jackson, Ian Johnston, Dominic Kwiatkowski, Cordelia Langford, John Sillitoe on behalf of the Wellcome Sanger Institute COVID-19 Surveillance Team
EPI_ISL_948380, EPI_ISL_948381, EPI_ISL_948382, EPI_ISL_948383	Lighthouse Lab in Cambridge	Wellcome Sanger Institute for the COVID-19 Genomics UK (COG-UK) Consortium	Rob Howes, The Lighthouse Lab in Cambridge and Alex Alderton, Roberto Amato, Sonia Goncalves, Ewan Harrison, David K. Jackson, Ian Johnston, Dominic Kwiatkowski, Cordelia Langford, John Sillitoe on behalf of the Wellcome Sanger Institute COVID-19 Surveillance Team
EPI_ISL_948384, EPI_ISL_948385, EPI_ISL_948386, EPI_ISL_948387,	Lighthouse Lab in Glasgow	Wellcome Sanger Institute for the COVID-19 Genomics UK (COG-UK) Consortium	Harper VanSteenhouse, Yumi Kasai, David Gray, Carol Clugston, Anna Dominiczak and Alex Alderton, Roberto Amato, Sonia Goncalves, Ewan Harrison, David K. Jackson, Ian Johnston, Dominic Kwiatkowski, Cordelia Langford, John Sillitoe on behalf of the Wellcome Sanger Institute COVID-19 Surveillance

EPI_ISL_948389, EPI_ISL_948390			Team
EPI_ISL_948391, EPI_ISL_948392, EPI_ISL_948393	Lighthouse Lab in Cambridge	Wellcome Sanger Institute for the COVID-19 Genomics UK (COG-UK) Consortium	Rob Howes, The Lighthouse Lab in Cambridge and Alex Alderton, Roberto Amato, Sonia Goncalves, Ewan Harrison, David K. Jackson, Ian Johnston, Dominic Kwiatkowski, Cordelia Langford, John Sillitoe on behalf of the Wellcome Sanger Institute COVID-19 Surveillance Team
EPI_ISL_948394	Lighthouse Lab in Glasgow	Wellcome Sanger Institute for the COVID-19 Genomics UK (COG-UK) Consortium	Harper VanSteenhouse, Yumi Kasai, David Gray, Carol Clugston, Anna Dominiczak and Alex Alderton, Roberto Amato, Sonia Goncalves, Ewan Harrison, David K. Jackson, Ian Johnston, Dominic Kwiatkowski, Cordelia Langford, John Sillitoe on behalf of the Wellcome Sanger Institute COVID-19 Surveillance Team
EPI_ISL_948395, EPI_ISL_948396, EPI_ISL_948397	Lighthouse Lab in Cambridge	Wellcome Sanger Institute for the COVID-19 Genomics UK (COG-UK) Consortium	Rob Howes, The Lighthouse Lab in Cambridge and Alex Alderton, Roberto Amato, Sonia Goncalves, Ewan Harrison, David K. Jackson, Ian Johnston, Dominic Kwiatkowski, Cordelia Langford, John Sillitoe on behalf of the Wellcome Sanger Institute COVID-19 Surveillance Team
EPI_ISL_948398, EPI_ISL_948399, EPI_ISL_948400, EPI_ISL_948401	Lighthouse Lab in Glasgow	Wellcome Sanger Institute for the COVID-19 Genomics UK (COG-UK) Consortium	Harper VanSteenhouse, Yumi Kasai, David Gray, Carol Clugston, Anna Dominiczak and Alex Alderton, Roberto Amato, Sonia Goncalves, Ewan Harrison, David K. Jackson, Ian Johnston, Dominic Kwiatkowski, Cordelia Langford, John Sillitoe on behalf of the Wellcome Sanger Institute COVID-19 Surveillance Team
EPI_ISL_948402	Lighthouse Lab in Cambridge	Wellcome Sanger Institute for the COVID-19 Genomics UK (COG-UK) Consortium	Rob Howes, The Lighthouse Lab in Cambridge and Alex Alderton, Roberto Amato, Sonia Goncalves, Ewan Harrison, David K. Jackson, Ian Johnston, Dominic Kwiatkowski, Cordelia Langford, John Sillitoe on behalf of the Wellcome Sanger Institute COVID-19 Surveillance Team
EPI_ISL_948403, EPI_ISL_948404, EPI_ISL_948405, EPI_ISL_948406	Lighthouse Lab in Glasgow	Wellcome Sanger Institute for the COVID-19 Genomics UK (COG-UK) Consortium	Harper VanSteenhouse, Yumi Kasai, David Gray, Carol Clugston, Anna Dominiczak and Alex Alderton, Roberto Amato, Sonia Goncalves, Ewan Harrison, David K. Jackson, Ian Johnston, Dominic Kwiatkowski, Cordelia Langford, John Sillitoe on behalf of the Wellcome Sanger Institute COVID-19 Surveillance Team
EPI_ISL_948408	Lighthouse Lab in Cambridge	Wellcome Sanger Institute for the COVID-19 Genomics UK (COG-UK) Consortium	Rob Howes, The Lighthouse Lab in Cambridge and Alex Alderton, Roberto Amato, Sonia Goncalves, Ewan Harrison, David K. Jackson, Ian Johnston, Dominic Kwiatkowski, Cordelia Langford, John Sillitoe on behalf of the Wellcome Sanger Institute COVID-19 Surveillance Team
EPI_ISL_948409, EPI_ISL_948410, EPI_ISL_948411	Lighthouse Lab in Glasgow	Wellcome Sanger Institute for the COVID-19 Genomics UK (COG-UK) Consortium	Harper VanSteenhouse, Yumi Kasai, David Gray, Carol Clugston, Anna Dominiczak and Alex Alderton, Roberto Amato, Sonia Goncalves, Ewan Harrison, David K. Jackson, Ian Johnston, Dominic Kwiatkowski, Cordelia Langford, John Sillitoe on behalf of the Wellcome Sanger Institute COVID-19 Surveillance Team
EPI_ISL_948412, EPI_ISL_948413, EPI_ISL_948414	Lighthouse Lab in Cambridge	Wellcome Sanger Institute for the COVID-19 Genomics UK (COG-UK) Consortium	Rob Howes, The Lighthouse Lab in Cambridge and Alex Alderton, Roberto Amato, Sonia Goncalves, Ewan Harrison, David K. Jackson, Ian Johnston, Dominic Kwiatkowski, Cordelia Langford, John Sillitoe on behalf of the Wellcome Sanger Institute COVID-19 Surveillance Team
EPI_ISL_948415, EPI_ISL_948416, EPI_ISL_948417, EPI_ISL_948418, EPI_ISL_948419	Lighthouse Lab in Glasgow	Wellcome Sanger Institute for the COVID-19 Genomics UK (COG-UK) Consortium	Harper VanSteenhouse, Yumi Kasai, David Gray, Carol Clugston, Anna Dominiczak and Alex Alderton, Roberto Amato, Sonia Goncalves, Ewan Harrison, David K. Jackson, Ian Johnston, Dominic Kwiatkowski, Cordelia Langford, John Sillitoe on behalf of the Wellcome Sanger Institute COVID-19 Surveillance Team
EPI_ISL_948420, EPI_ISL_948421, EPI_ISL_948422, EPI_ISL_948423, EPI_ISL_948424	Lighthouse Lab in Cambridge	Wellcome Sanger Institute for the COVID-19 Genomics UK (COG-UK) Consortium	Rob Howes, The Lighthouse Lab in Cambridge and Alex Alderton, Roberto Amato, Sonia Goncalves, Ewan Harrison, David K. Jackson, Ian Johnston, Dominic Kwiatkowski, Cordelia Langford, John Sillitoe on behalf of the Wellcome Sanger Institute COVID-19 Surveillance Team
EPI_ISL_948425, EPI_ISL_948426, EPI_ISL_948427, EPI_ISL_948428, EPI_ISL_948429, EPI_ISL_948430	Lighthouse Lab in Glasgow	Wellcome Sanger Institute for the COVID-19 Genomics UK (COG-UK) Consortium	Harper VanSteenhouse, Yumi Kasai, David Gray, Carol Clugston, Anna Dominiczak and Alex Alderton, Roberto Amato, Sonia Goncalves, Ewan Harrison, David K. Jackson, Ian Johnston, Dominic Kwiatkowski, Cordelia Langford, John Sillitoe on behalf of the Wellcome Sanger Institute COVID-19 Surveillance Team
EPI_ISL_948431, EPI_ISL_948433, EPI_ISL_948434, EPI_ISL_948435	Lighthouse Lab in Cambridge	Wellcome Sanger Institute for the COVID-19 Genomics UK (COG-UK) Consortium	Rob Howes, The Lighthouse Lab in Cambridge and Alex Alderton, Roberto Amato, Sonia Goncalves, Ewan Harrison, David K. Jackson, Ian Johnston, Dominic Kwiatkowski, Cordelia Langford, John Sillitoe on behalf of the Wellcome Sanger Institute COVID-19 Surveillance Team
EPI_ISL_948436	Lighthouse Lab in Glasgow	Wellcome Sanger Institute for the COVID-19 Genomics UK (COG-UK) Consortium	Harper VanSteenhouse, Yumi Kasai, David Gray, Carol Clugston, Anna Dominiczak and Alex Alderton, Roberto Amato, Sonia Goncalves, Ewan Harrison, David K. Jackson, Ian Johnston, Dominic Kwiatkowski, Cordelia Langford, John Sillitoe on behalf of the Wellcome Sanger Institute COVID-19 Surveillance Team
EPI_ISL_948437	Lighthouse Lab in Cambridge	Wellcome Sanger Institute for the COVID-19 Genomics UK (COG-UK) Consortium	Rob Howes, The Lighthouse Lab in Cambridge and Alex Alderton, Roberto Amato, Sonia Goncalves, Ewan Harrison, David K. Jackson, Ian Johnston, Dominic Kwiatkowski, Cordelia Langford, John Sillitoe on behalf of the Wellcome Sanger Institute COVID-19 Surveillance Team
EPI_ISL_948438, EPI_ISL_948439, EPI_ISL_948440	Lighthouse Lab in Glasgow	Wellcome Sanger Institute for the COVID-19 Genomics UK (COG-UK) Consortium	Harper VanSteenhouse, Yumi Kasai, David Gray, Carol Clugston, Anna Dominiczak and Alex Alderton, Roberto Amato, Sonia Goncalves, Ewan Harrison, David K. Jackson, Ian Johnston, Dominic Kwiatkowski, Cordelia Langford, John Sillitoe on behalf of the Wellcome Sanger Institute COVID-19 Surveillance Team
EPI_ISL_948441, EPI_ISL_948442	Lighthouse Lab in Cambridge	Wellcome Sanger Institute for the COVID-19 Genomics UK (COG-UK) Consortium	Rob Howes, The Lighthouse Lab in Cambridge and Alex Alderton, Roberto Amato, Sonia Goncalves, Ewan Harrison, David K. Jackson, Ian Johnston, Dominic Kwiatkowski, Cordelia Langford, John Sillitoe on behalf of the Wellcome Sanger Institute COVID-19 Surveillance Team
EPI_ISL_948443, EPI_ISL_948444	Lighthouse Lab in Glasgow	Wellcome Sanger Institute for the COVID-19 Genomics UK (COG-UK) Consortium	Harper VanSteenhouse, Yumi Kasai, David Gray, Carol Clugston, Anna Dominiczak and Alex Alderton, Roberto Amato, Sonia Goncalves, Ewan Harrison, David K. Jackson, Ian Johnston, Dominic Kwiatkowski, Cordelia Langford, John Sillitoe on behalf of the Wellcome Sanger Institute COVID-19 Surveillance Team
EPI_ISL_948445	Lighthouse Lab in Cambridge	Wellcome Sanger Institute for the COVID-19 Genomics UK (COG-UK) Consortium	Rob Howes, The Lighthouse Lab in Cambridge and Alex Alderton, Roberto Amato, Sonia Goncalves, Ewan Harrison, David K. Jackson, Ian Johnston, Dominic Kwiatkowski, Cordelia Langford, John Sillitoe on behalf of the Wellcome Sanger Institute COVID-19 Surveillance Team
EPI_ISL_948446, EPI_ISL_948447, EPI_ISL_948448, EPI_ISL_948787, EPI_ISL_948788, EPI_ISL_948789, EPI_ISL_948790, EPI_ISL_948791, EPI_ISL_948792, EPI_ISL_948793, EPI_ISL_948794, EPI_ISL_948795, EPI_ISL_948796, EPI_ISL_948797, EPI_ISL_948798, EPI_ISL_948799, EPI_ISL_948800, EPI_ISL_948801, EPI_ISL_948802, EPI_ISL_948803, EPI_ISL_948804, EPI_ISL_948805, EPI_ISL_948806, EPI_ISL_948807, EPI_ISL_948808, EPI_ISL_948809, EPI_ISL_948810, EPI_ISL_948811, EPI_ISL_948812, EPI_ISL_948813, EPI_ISL_948814, EPI_ISL_948815, EPI_ISL_948816, EPI_ISL_948817, EPI_ISL_948818, EPI_ISL_948819, EPI_ISL_948820, EPI_ISL_948821, EPI_ISL_948822, EPI_ISL_948823, EPI_ISL_948824, EPI_ISL_948825, EPI_ISL_948826, EPI_ISL_948827, EPI_ISL_948828, EPI_ISL_948831, EPI_ISL_948832, EPI_ISL_948833, EPI_ISL_948835, EPI_ISL_948836, EPI_ISL_948837, EPI_ISL_948838, EPI_ISL_948839, EPI_ISL_948840, EPI_ISL_948841, EPI_ISL_948842, EPI_ISL_948843, EPI_ISL_948844, EPI_ISL_948846, EPI_ISL_948847, EPI_ISL_948849, EPI_ISL_948850, EPI_ISL_948851, EPI_ISL_948852, EPI_ISL_948853, EPI_ISL_948854, EPI_ISL_948855, EPI_ISL_948856, EPI_ISL_948857, EPI_ISL_948858, EPI_ISL_948859, EPI_ISL_948860, EPI_ISL_948861, EPI_ISL_948862, EPI_ISL_948863, EPI_ISL_948864, EPI_ISL_948865, EPI_ISL_948866, EPI_ISL_948867, EPI_ISL_948868, EPI_ISL_948869, EPI_ISL_948870, EPI_ISL_948871, EPI_ISL_948872, EPI_ISL_948873, EPI_ISL_948874, EPI_ISL_948875, EPI_ISL_948876, EPI_ISL_948877, EPI_ISL_948878, EPI_ISL_948879, EPI_ISL_948880, EPI_ISL_948881, EPI_ISL_948882, EPI_ISL_948883, EPI_ISL_948884, EPI_ISL_948885, EPI_ISL_948886, EPI_ISL_948887, EPI_ISL_948888, EPI_ISL_948889, EPI_ISL_948890, EPI_ISL_948891, EPI_ISL_948892, EPI_ISL_948893, EPI_ISL_948894, EPI_ISL_948895, EPI_ISL_948896, EPI_ISL_948897, EPI_ISL_948898, EPI_ISL_948899, EPI_ISL_948900, EPI_ISL_948901, EPI_ISL_948902, EPI_ISL_948903, EPI_ISL_948904, EPI_ISL_948905, EPI_ISL_948906, EPI_ISL_948907, EPI_ISL_948908, EPI_ISL_948909, EPI_ISL_948910, EPI_ISL_948911, EPI_ISL_948912, EPI_ISL_948913, EPI_ISL_948914, EPI_ISL_948915, EPI_ISL_948916, EPI_ISL_948917, EPI_ISL_948918, EPI_ISL_948919, EPI_ISL_948920, EPI_ISL_948921, EPI_ISL_948922, EPI_ISL_948923, EPI_ISL_948924, EPI_ISL_948925, EPI_ISL_948926, EPI_ISL_948927, EPI_ISL_948928, EPI_ISL_948929, EPI_ISL_948930, EPI_ISL_948931, EPI_ISL_948932, EPI_ISL_948933, EPI_ISL_948934, EPI_ISL_948935, EPI_ISL_948936, EPI_ISL_948937, EPI_ISL_948938, EPI_ISL_948939, EPI_ISL_948940, EPI_ISL_948941, EPI_ISL_948942, EPI_ISL_948943, EPI_ISL_948944			
see above	Lighthouse Lab in Glasgow	Wellcome Sanger Institute for the COVID-19 Genomics UK (COG-UK) Consortium	Harper VanSteenhouse, Yumi Kasai, David Gray, Carol Clugston, Anna Dominiczak and Alex Alderton, Roberto Amato, Sonia Goncalves, Ewan Harrison, David K. Jackson, Ian Johnston, Dominic Kwiatkowski, Cordelia Langford, John Sillitoe on behalf of the Wellcome Sanger Institute COVID-19 Surveillance Team
EPI_ISL_949410	University of Birmingham	COVID-19 Genomics UK (COG-UK) Consortium	Institute of Microbiology, University of Birmingham: Claire McMurray, Joanne Stockton, Samuel Nicholls, Radoslaw Poplawski, Will Rowe, Josh Quick, Nicholas Loman. University of Birmingham Testing Laboratory: Celina M Whalley, Andrew Bosworth, Charlotte Poxon, Kasun Wanigasooriya, Oliver Pickles, Mike Kidd, Alex Richter, Andrew D Beggs PHE Heartlands Lab: Husam Osman, Andrew Bosworth. Queen Elizabeth Hospital: Anna Casey
EPI_ISL_949462, EPI_ISL_949463, EPI_ISL_949464, EPI_ISL_949465, EPI_ISL_949467, EPI_ISL_949468, EPI_ISL_949469, EPI_ISL_949473, EPI_ISL_949475, EPI_ISL_949476, EPI_ISL_949477, EPI_ISL_949524, EPI_ISL_949525, EPI_ISL_949526, EPI_ISL_949527, EPI_ISL_949528, EPI_ISL_949529, EPI_ISL_949530, EPI_ISL_949531, EPI_ISL_949532, EPI_ISL_949533, EPI_ISL_949534, EPI_ISL_949535, EPI_ISL_949536, EPI_ISL_949537, EPI_ISL_949538, EPI_ISL_949539, EPI_ISL_949540, EPI_ISL_949542, EPI_ISL_949544, EPI_ISL_949546, EPI_ISL_949548, EPI_ISL_949549, EPI_ISL_949550, EPI_ISL_949553, EPI_ISL_949554, EPI_ISL_949555, EPI_ISL_949556			
see above	Department of Pathology, University of Cambridge	COVID-19 Genomics UK (COG-UK) Consortium	Aminu S. Jahun, Yasmin Chaudhry, Iliana Georgana, Myra Hosmillo, Rhys Izu, Martin D. Curran, Surendra Parmar, Ian Goodfellow
EPI_ISL_949611, EPI_ISL_949615	West of Scotland Specialist Virology Centre, NHSGGC / MRC-University of Glasgow Centre for Virus Research	COVID-19 Genomics UK (COG-UK) Consortium	Ana da Silva Filipe, Natasha Johnson, Kathy Smollett, Daniel Mair, Stephen Carmichael, Alice Broos, Lily Tong, Jenna Nichols, Kyriaki Nomikou; Sarah McDonald; Richard Orton, Joseph Hughes, Sreenu Vattipally, David L Robertson; Alasdair MacLean, Rory Gunson; Sharif Shaaban, Matthew Holden; Rachel Blacow, Guy Mollett, Kathy Li, James Shepherd, Antonia Ho, Emma Thomson
EPI_ISL_949645, EPI_ISL_949646	Virology Department, Royal Infirmary of Edinburgh, NHS Lothian / School of Biological Sciences, University of Edinburgh / Institute of Genetics and Molecular Medicine,	COVID-19 Genomics UK (COG-UK) Consortium	McHugh M, Dewar R, Rooke S, Gallagher M, Balcaza C, O'Toole Á, Scher E, Hill V, McCrone JT, Colquhoun R, Yu X, Jackson B, Rambaut A, Williams TC, Templeton K

	University of Edinburgh		
EPI_ISL_949655, EPI_ISL_949656, EPI_ISL_949657, EPI_ISL_949658, EPI_ISL_949659, EPI_ISL_949660, EPI_ISL_949661, EPI_ISL_949665, EPI_ISL_949667, EPI_ISL_949673, EPI_ISL_949674, EPI_ISL_949675, EPI_ISL_949676, EPI_ISL_949677, EPI_ISL_949678, EPI_ISL_949679, EPI_ISL_949680, EPI_ISL_949681, EPI_ISL_949682, EPI_ISL_949683, EPI_ISL_949688, EPI_ISL_949689, EPI_ISL_949690, EPI_ISL_949691, EPI_ISL_949692, EPI_ISL_949693, EPI_ISL_949694, EPI_ISL_949695, EPI_ISL_949696, EPI_ISL_949697, EPI_ISL_949698, EPI_ISL_949699, EPI_ISL_949700, EPI_ISL_949701, EPI_ISL_949702, EPI_ISL_949703, EPI_ISL_949704, EPI_ISL_949705, EPI_ISL_949706, EPI_ISL_949707			
see above	Liverpool Clinical Laboratories	COVID-19 Genomics UK (COG-UK) Consortium	Sam Haldenby, Anita Lucaci, Steve Paterson, Julian Hiscox, Alistair Darby, M Almsaud, A Alrezaihi, Muhannad Alruwaili, Stuart D Armstrong, Jones Benjamin, Eleanor G Bentley, Anu Chawla, Jordan J Clark, Angela Cowell, Richard Eccles, Isabel García-Dorival, Matthew Gemmell, Alessandro Gerada, PKF Gilmore, Richard Gregory, Ximeng Han, Catherine Hartley, Margaret Hughes, Miren Iturriza-Gomara, James Johnson, L Luu, Jenifer Manson, Charlotte Nelson, Elaine O'Toole, Cassie Olateju, Rebekah Penrice-Randal , Lucille Rainbow, N.P Randle, Trevor Ian Robinson, Parul Sharma, Ghada T Shawli, James P Stewart, Neil Swainston, Ecaterina Vamos, Joanne Watts, Mark Whitehead
EPI_ISL_949793, EPI_ISL_949794, EPI_ISL_949802, EPI_ISL_949909, EPI_ISL_949910, EPI_ISL_949911, EPI_ISL_949912, EPI_ISL_949913, EPI_ISL_949914, EPI_ISL_949917, EPI_ISL_949919, EPI_ISL_949948, EPI_ISL_950019, EPI_ISL_950028, EPI_ISL_950034, EPI_ISL_950039, EPI_ISL_950040, EPI_ISL_950041, EPI_ISL_950042, EPI_ISL_950043, EPI_ISL_950044, EPI_ISL_950045, EPI_ISL_950046, EPI_ISL_950047, EPI_ISL_950049, EPI_ISL_950050, EPI_ISL_950051, EPI_ISL_950052, EPI_ISL_950053, EPI_ISL_950054, EPI_ISL_950055, EPI_ISL_950056, EPI_ISL_950057, EPI_ISL_950058, EPI_ISL_950059, EPI_ISL_950060, EPI_ISL_950061, EPI_ISL_950062, EPI_ISL_950063, EPI_ISL_950064, EPI_ISL_950065, EPI_ISL_950066, EPI_ISL_950067, EPI_ISL_950171, EPI_ISL_950172, EPI_ISL_950174, EPI_ISL_950175, EPI_ISL_950176, EPI_ISL_950178, EPI_ISL_950179, EPI_ISL_950180, EPI_ISL_950181, EPI_ISL_950182, EPI_ISL_950183, EPI_ISL_950184, EPI_ISL_950185, EPI_ISL_950186, EPI_ISL_950187, EPI_ISL_950188, EPI_ISL_950189, EPI_ISL_950190, EPI_ISL_950191, EPI_ISL_950192, EPI_ISL_950193, EPI_ISL_950194, EPI_ISL_950196, EPI_ISL_950197, EPI_ISL_950199			
see above	University College London, Great Ormond Street Hospital for Children NHS Foundation Trust, Imperial College Healthcare NHS Trust	COVID-19 Genomics UK (COG-UK) Consortium	Sergi Castellano, Rachel Williams, Mark Kristiansen, Paola Resende Silva, Sunando Roy, Tony Brooks, Helena Tutill, Paola Niola, Patricia Dyal, Charlotte Williams, Leysa Forrest, Yasmin Panchbhaya, Jacqueline Findlay, Samuel Weeks, Julianne Brown, Kathryn Harris, Paul Randell, James Price, Alison Holmes, Judith Breuer
EPI_ISL_950373, EPI_ISL_950376, EPI_ISL_950379, EPI_ISL_950382, EPI_ISL_950391, EPI_ISL_950392, EPI_ISL_950393, EPI_ISL_950394, EPI_ISL_950395, EPI_ISL_950396, EPI_ISL_950397, EPI_ISL_950398, EPI_ISL_950399, EPI_ISL_950400, EPI_ISL_950401, EPI_ISL_950523, EPI_ISL_950524, EPI_ISL_950525, EPI_ISL_950526, EPI_ISL_950527, EPI_ISL_950528, EPI_ISL_950529, EPI_ISL_950530, EPI_ISL_950531, EPI_ISL_950532, EPI_ISL_950533, EPI_ISL_950534, EPI_ISL_950535, EPI_ISL_950536, EPI_ISL_950537, EPI_ISL_950538, EPI_ISL_950539, EPI_ISL_950540, EPI_ISL_950541, EPI_ISL_950542, EPI_ISL_950543			
see above	Northumbria University / South Tees Hospitals NHS Foundation Trust / North Cumbria Integrated Care NHS Foundation Trust / North Tees and Hartlepool NHS Foundation Trust / Newcastle Hospitals NHS Foundation Trust	COVID-19 Genomics UK (COG-UK) Consortium	Darren L Smith,Andrew Nelson,Matthew Bashton,Greg R Young,Joshua Loh,John Allan,Mohammad A Tariq,Giles S Holt,Gary Black,Wen C Yew,Lynn Dover,Paul Baker,Steve Liggett,Sarah Essex,Jane Greenaway,Debra Padgett,Clive Graham,Garren Scott,Edward Barton,Emma Swindells,Brendan Payne,Jennifer Collins,Yusri Taha,Gary Eltringham
EPI_ISL_950639, EPI_ISL_950640, EPI_ISL_950641, EPI_ISL_950642, EPI_ISL_950643, EPI_ISL_950644, EPI_ISL_950645	Queens Medical Centre, Clinical Microbiology Department / DeepSeq Nottingham	COVID-19 Genomics UK (COG-UK) Consortium	Gemma Clark, Wendy Smith, Manjinder Khakh, Vicki M Fleming, Michelle M Lister, Hannah Howson-Wells, Jonathan Ball, Patrick McClure, Joseph Chappell, Theocharis Tsoleridis, Nadine Holmes, Matthew Carlisle, Christopher Moore, Fei Sang, Johnny Debebe, Victoria Wright, Matthew Loose
EPI_ISL_950755, EPI_ISL_950756, EPI_ISL_950757, EPI_ISL_950758, EPI_ISL_950759, EPI_ISL_950760, EPI_ISL_950761	Lincolnshire Hospitals and DeepSeq Nottingham	COVID-19 Genomics UK (COG-UK) Consortium	Nichola Duckworth, Tim Sloan, Sarah Walsh, Jonathan Ball, Patrick McClure, Joeseph Chappell, Nadine Holmes, Matthew Carlisle, Christopher Moore, Fei Sang, Johnny Debebe, Victoria Wright, Matthew Loose
EPI_ISL_951492, EPI_ISL_951493, EPI_ISL_951494, EPI_ISL_951495, EPI_ISL_951496, EPI_ISL_951497, EPI_ISL_951498, EPI_ISL_951499, EPI_ISL_951501, EPI_ISL_951502, EPI_ISL_951503, EPI_ISL_951504, EPI_ISL_951505, EPI_ISL_951506, EPI_ISL_951508, EPI_ISL_951512, EPI_ISL_951513, EPI_ISL_951514, EPI_ISL_951515, EPI_ISL_951517, EPI_ISL_951518, EPI_ISL_951519, EPI_ISL_951521, EPI_ISL_951522, EPI_ISL_951523, EPI_ISL_951524, EPI_ISL_951525, EPI_ISL_951527, EPI_ISL_951528, EPI_ISL_951529, EPI_ISL_951530, EPI_ISL_951531, EPI_ISL_951532, EPI_ISL_951533, EPI_ISL_951534, EPI_ISL_951535, EPI_ISL_951536, EPI_ISL_951537, EPI_ISL_951538, EPI_ISL_951541, EPI_ISL_951542, EPI_ISL_951543, EPI_ISL_951544, EPI_ISL_951545, EPI_ISL_951546, EPI_ISL_951547, EPI_ISL_951548, EPI_ISL_951549, EPI_ISL_951550, EPI_ISL_951552, EPI_ISL_951553, EPI_ISL_951554, EPI_ISL_951556, EPI_ISL_951557, EPI_ISL_951558, EPI_ISL_951559, EPI_ISL_951560, EPI_ISL_951561, EPI_ISL_951562, EPI_ISL_951564, EPI_ISL_951567, EPI_ISL_951569, EPI_ISL_951570, EPI_ISL_951571, EPI_ISL_951572, EPI_ISL_951573, EPI_ISL_951574, EPI_ISL_951575, EPI_ISL_951576, EPI_ISL_951577, EPI_ISL_951578, EPI_ISL_951579, EPI_ISL_951580, EPI_ISL_951581, EPI_ISL_951582, EPI_ISL_951583, EPI_ISL_951585, EPI_ISL_951738, EPI_ISL_951739, EPI_ISL_951742, EPI_ISL_951744, EPI_ISL_951745, EPI_ISL_951753, EPI_ISL_951758, EPI_ISL_951759, EPI_ISL_951760, EPI_ISL_951761, EPI_ISL_951765, EPI_ISL_951766, EPI_ISL_951767, EPI_ISL_951769, EPI_ISL_951778, EPI_ISL_951779, EPI_ISL_951780, EPI_ISL_951781, EPI_ISL_951782, EPI_ISL_951785, EPI_ISL_951786, EPI_ISL_951787, EPI_ISL_951820, EPI_ISL_951821, EPI_ISL_951843, EPI_ISL_951844, EPI_ISL_951851, EPI_ISL_951852, EPI_ISL_951853, EPI_ISL_951855, EPI_ISL_951856, EPI_ISL_951857, EPI_ISL_951858, EPI_ISL_951859, EPI_ISL_951861, EPI_ISL_951862, EPI_ISL_951863, EPI_ISL_951866, EPI_ISL_951867, EPI_ISL_951869, EPI_ISL_951875, EPI_ISL_951876, EPI_ISL_951877, EPI_ISL_951878, EPI_ISL_951881, EPI_ISL_951882, EPI_ISL_951884, EPI_ISL_951886, EPI_ISL_951887, EPI_ISL_951888, EPI_ISL_951890, EPI_ISL_951893, EPI_ISL_951894, EPI_ISL_951895, EPI_ISL_951896, EPI_ISL_951897, EPI_ISL_951898, EPI_ISL_951900, EPI_ISL_951901, EPI_ISL_951902, EPI_ISL_951903, EPI_ISL_951904, EPI_ISL_951905, EPI_ISL_951906, EPI_ISL_951907, EPI_ISL_951908, EPI_ISL_951909, EPI_ISL_951910, EPI_ISL_951911, EPI_ISL_951912, EPI_ISL_951913, EPI_ISL_951916, EPI_ISL_951917, EPI_ISL_951918, EPI_ISL_951919, EPI_ISL_951920, EPI_ISL_951921, EPI_ISL_951922, EPI_ISL_951923, EPI_ISL_951924, EPI_ISL_951925, EPI_ISL_951926, EPI_ISL_951927, EPI_ISL_951928, EPI_ISL_951929, EPI_ISL_951930, EPI_ISL_951931, EPI_ISL_951932, EPI_ISL_951933, EPI_ISL_951934, EPI_ISL_951935, EPI_ISL_951936, EPI_ISL_951937, EPI_ISL_951938, EPI_ISL_951939, EPI_ISL_951940, EPI_ISL_951941, EPI_ISL_951942, EPI_ISL_951943, EPI_ISL_951944, EPI_ISL_951945, EPI_ISL_951946, EPI_ISL_951947, EPI_ISL_951948, EPI_ISL_951949, EPI_ISL_951950, EPI_ISL_951951, EPI_ISL_951952, EPI_ISL_951953, EPI_ISL_951954, EPI_ISL_951955, EPI_ISL_951956, EPI_ISL_951957, EPI_ISL_951958, EPI_ISL_951959, EPI_ISL_951960, EPI_ISL_951961, EPI_ISL_951962, EPI_ISL_951963, EPI_ISL_951964, EPI_ISL_951965, EPI_ISL_951966, EPI_ISL_951968, EPI_ISL_951969, EPI_ISL_951972, EPI_ISL_951973, EPI_ISL_952006, EPI_ISL_952007, EPI_ISL_952008, EPI_ISL_952009, EPI_ISL_952010, EPI_ISL_952011, EPI_ISL_952012, EPI_ISL_952013, EPI_ISL_952014, EPI_ISL_952015, EPI_ISL_952018, EPI_ISL_952019, EPI_ISL_952020, EPI_ISL_952022, EPI_ISL_952025, EPI_ISL_952027, EPI_ISL_952029, EPI_ISL_952030, EPI_ISL_952031, EPI_ISL_952032, EPI_ISL_952033, EPI_ISL_952035, EPI_ISL_952036, EPI_ISL_952037, EPI_ISL_952038, EPI_ISL_952039, EPI_ISL_952040, EPI_ISL_952041, EPI_ISL_952061, EPI_ISL_952062, EPI_ISL_952063, EPI_ISL_952064, EPI_ISL_952065, EPI_ISL_952066, EPI_ISL_952067, EPI_ISL_952069, EPI_ISL_952070, EPI_ISL_952071, EPI_ISL_952072, EPI_ISL_952073, EPI_ISL_952074, EPI_ISL_952075, EPI_ISL_952076, EPI_ISL_952077, EPI_ISL_952078, EPI_ISL_952079, EPI_ISL_952082, EPI_ISL_952083, EPI_ISL_952084, EPI_ISL_952085, EPI_ISL_952086, EPI_ISL_952087, EPI_ISL_952088, EPI_ISL_952089, EPI_ISL_952090, EPI_ISL_952091, EPI_ISL_952092, EPI_ISL_952093, EPI_ISL_952095, EPI_ISL_952096, EPI_ISL_952097, EPI_ISL_952100, EPI_ISL_952102, EPI_ISL_952104, EPI_ISL_952108, EPI_ISL_952109, EPI_ISL_952110, EPI_ISL_952111, EPI_ISL_952112, EPI_ISL_952113, EPI_ISL_952114, EPI_ISL_952119, EPI_ISL_952120, EPI_ISL_952122, EPI_ISL_952123, EPI_ISL_952124, EPI_ISL_952126, EPI_ISL_952128, EPI_ISL_952129, EPI_ISL_952130, EPI_ISL_952131, EPI_ISL_952132, EPI_ISL_952133, EPI_ISL_952134, EPI_ISL_952136, EPI_ISL_952137, EPI_ISL_952138, EPI_ISL_952139, EPI_ISL_952140, EPI_ISL_952141, EPI_ISL_952143, EPI_ISL_952144, EPI_ISL_952145, EPI_ISL_952146, EPI_ISL_952147, EPI_ISL_952148, EPI_ISL_952149, EPI_ISL_952150, EPI_ISL_952151, EPI_ISL_952152, EPI_ISL_952153, EPI_ISL_952154, EPI_ISL_952156, EPI_ISL_952157, EPI_ISL_952158, EPI_ISL_952159, EPI_ISL_952161, EPI_ISL_952162, EPI_ISL_952163, EPI_ISL_952164, EPI_ISL_952165, EPI_ISL_952166, EPI_ISL_952167			
see above	Originating lab: Wales Specialist Virology Centre Sequencing lab: Pathogen Genomics Unit	Public Health Wales Microbiology Cardiff Wales Specialist Virology Centre	Catherine Moore, Johnathan Evans, Laura Gifford, Malorie Perry, Simon Cottrell, Angela Marchbank, Alec Birchley, Alexander Adams, Amy Gaskin, Bree Gatica-Wilcox, Jason Coombes, Joel Southgate, Lauren Gilbert, Lee Graham, Nicole Pacchiarini, Sara Kumziene-Summerhayes, Sarah Taylor, Sophie Jones, Sara Rey, Matthew Bull, Joanne Watkins, Sally Corden, Tom Connor
EPI_ISL_952453, EPI_ISL_952454, EPI_ISL_952456, EPI_ISL_952460, EPI_ISL_952462, EPI_ISL_952464, EPI_ISL_952465, EPI_ISL_952466, EPI_ISL_952467, EPI_ISL_952469, EPI_ISL_952470, EPI_ISL_952477, EPI_ISL_952478, EPI_ISL_952479, EPI_ISL_952480, EPI_ISL_952481, EPI_ISL_952482, EPI_ISL_952483, EPI_ISL_952484, EPI_ISL_952485, EPI_ISL_952558			
see above	Centre for Enzyme Innovation, University of Portsmouth / Translational Research Laboratory, Portsmouth Hospitals NHS Trust	COVID-19 Genomics UK (COG-UK) Consortium	Angela Beckett,Salman Goudarzi,Christopher Fearn,Kate Cook,Katie Loveson,Sharon Glaysher,Scott Elliott,Samuel Robson
EPI_ISL_952953, EPI_ISL_952978	Virology Department, Sheffield Teaching Hospitals NHS Foundation Trust/Department of Infection, Immunity and Cardiovascular Disease, The Medical School, University of Sheffield	COVID-19 Genomics UK (COG-UK) Consortium	Thushan de Silva, Matthew Parker, Nikki Smith, Adri Angyal, Rebecca Brown, Luke Green, Rachel Tucker, Paul Parsons, Danielle Groves, Katie Johnson, Laura Carrilero, Alex Keeley, Dave Partridge, Matthew Wyles, Benjamin Lindsey, Mehmet Yavuz, Mohammad Raza, Cariad Evans
EPI_ISL_953241, EPI_ISL_953243, EPI_ISL_953244, EPI_ISL_953246, EPI_ISL_953247, EPI_ISL_953251, EPI_ISL_953254, EPI_ISL_953259, EPI_ISL_953261, EPI_ISL_953262, EPI_ISL_953263, EPI_ISL_953267, EPI_ISL_953272, EPI_ISL_953281			
see above	Bioinformatics and Biostatistics Lab, Advanced Sequencing Facility	COVID-19 Genomics UK (COG-UK) Consortium	Aengus Stewart,Jerome Nicod,Chelsea Sawyer,Laura Cubitt,Harshil Patel,Margaret Crawford
EPI_ISL_956446, EPI_ISL_956487, EPI_ISL_956508, EPI_ISL_956562, EPI_ISL_956576, EPI_ISL_956585, EPI_ISL_956587, EPI_ISL_956592, EPI_ISL_956601, EPI_ISL_956602, EPI_ISL_956606, EPI_ISL_956618, EPI_ISL_956625, EPI_ISL_956626, EPI_ISL_956631, EPI_ISL_956636, EPI_ISL_956651, EPI_ISL_956690, EPI_ISL_956691, EPI_ISL_956694, EPI_ISL_956699, EPI_ISL_956708, EPI_ISL_956729			
see above	Lighthouse Lab in Alderley Park	Wellcome Sanger Institute for the COVID-19 Genomics UK (COG-UK) Consortium	Jacquelyn Wynn, Mairead Hyland, The Lighthouse Lab in Alderley Park and Alex Alderton, Roberto Amato, Sonia Goncalves, Ewan Harrison, David K. Jackson, Ian Johnston, Dominic Kwiatkowski, Cordelia Langford, John Sillitoe on behalf of the Wellcome Sanger Institute COVID-19 Surveillance Team
EPI_ISL_957613, EPI_ISL_957616, EPI_ISL_957620, EPI_ISL_957621, EPI_ISL_957624, EPI_ISL_957627, EPI_ISL_957630, EPI_ISL_957635, EPI_ISL_957641, EPI_ISL_957643, EPI_ISL_957645, EPI_ISL_957650, EPI_ISL_957652, EPI_ISL_957654, EPI_ISL_957655, EPI_ISL_957656, EPI_ISL_957660, EPI_ISL_957670, EPI_ISL_957672, EPI_ISL_957675, EPI_ISL_957677, EPI_ISL_957680, EPI_ISL_957683, EPI_ISL_957686, EPI_ISL_957687, EPI_ISL_957691, EPI_ISL_957698, EPI_ISL_957700, EPI_ISL_957701, EPI_ISL_957707, EPI_ISL_957721, EPI_ISL_957722, EPI_ISL_957723, EPI_ISL_957726, EPI_ISL_957735, EPI_ISL_957740, EPI_ISL_957741, EPI_ISL_957745, EPI_ISL_957747, EPI_ISL_957748, EPI_ISL_957753, EPI_ISL_957755, EPI_ISL_957757, EPI_ISL_957759, EPI_ISL_957766, EPI_ISL_957769, EPI_ISL_957771, EPI_ISL_957772, EPI_ISL_957780, EPI_ISL_957783, EPI_ISL_957787, EPI_ISL_957797, EPI_ISL_957799, EPI_ISL_957813, EPI_ISL_957817, EPI_ISL_957822, EPI_ISL_957826, EPI_ISL_957828, EPI_ISL_957829, EPI_ISL_957830, EPI_ISL_957832, EPI_ISL_957838, EPI_ISL_957841, EPI_ISL_957844, EPI_ISL_957856, EPI_ISL_957859, EPI_ISL_957862, EPI_ISL_957863, EPI_ISL_957866, EPI_ISL_957867, EPI_ISL_957868, EPI_ISL_957872, EPI_ISL_957877, EPI_ISL_957878, EPI_ISL_957886, EPI_ISL_957892, EPI_ISL_957895, EPI_ISL_957897, EPI_ISL_957904, EPI_ISL_957907, EPI_ISL_957921, EPI_ISL_957961, EPI_ISL_957962, EPI_ISL_957971, EPI_ISL_958044, EPI_ISL_958194, EPI_ISL_958206, EPI_ISL_958215, EPI_ISL_958216, EPI_ISL_958217, EPI_ISL_958218, EPI_ISL_958219, EPI_ISL_958220, EPI_ISL_958221, EPI_ISL_958222, EPI_ISL_958223, EPI_ISL_958224, EPI_ISL_958225, EPI_ISL_958226, EPI_ISL_958227, EPI_ISL_958228, EPI_ISL_958229, EPI_ISL_958230, EPI_ISL_958231, EPI_ISL_958232, EPI_ISL_958233, EPI_ISL_958234, EPI_ISL_958235, EPI_ISL_958236, EPI_ISL_958237, EPI_ISL_958238, EPI_ISL_958239, EPI_ISL_958240, EPI_ISL_958241, EPI_ISL_958242, EPI_ISL_958243, EPI_ISL_958244, EPI_ISL_958245, EPI_ISL_958246, EPI_ISL_958247, EPI_ISL_958248, EPI_ISL_958249, EPI_ISL_958250, EPI_ISL_958251, EPI_ISL_958252, EPI_ISL_958253, EPI_ISL_958254, EPI_ISL_958255, EPI_ISL_958256, EPI_ISL_958257, EPI_ISL_958258, EPI_ISL_958259, EPI_ISL_958260, EPI_ISL_958261, EPI_ISL_958262, EPI_ISL_958263, EPI_ISL_958264, EPI_ISL_958265, EPI_ISL_958266, EPI_ISL_958267, EPI_ISL_958268, EPI_ISL_958269, EPI_ISL_958270, EPI_ISL_958271, EPI_ISL_958272, EPI_ISL_958273, EPI_ISL_958274, EPI_ISL_958275, EPI_ISL_958277, EPI_ISL_958278, EPI_ISL_958279, EPI_ISL_958280, EPI_ISL_958281, EPI_ISL_958282, EPI_ISL_958283, EPI_ISL_958284, EPI_ISL_958285, EPI_ISL_958286, EPI_ISL_958287, EPI_ISL_958288, EPI_ISL_958289, EPI_ISL_958290, EPI_ISL_958291, EPI_ISL_958292, EPI_ISL_958293, EPI_ISL_958294, EPI_ISL_958295, EPI_ISL_958296, EPI_ISL_958297, EPI_ISL_958298, EPI_ISL_958299, EPI_ISL_958300, EPI_ISL_958301, EPI_ISL_958302, EPI_ISL_958303, EPI_ISL_958304, EPI_ISL_958305, EPI_ISL_958306, EPI_ISL_958307, EPI_ISL_958308, EPI_ISL_958309, EPI_ISL_958310, EPI_ISL_958311, EPI_ISL_958312, EPI_ISL_958313, EPI_ISL_958314, EPI_ISL_958315, EPI_ISL_958316, EPI_ISL_958317, EPI_ISL_958318, EPI_ISL_958319, EPI_ISL_958320, EPI_ISL_958321, EPI_ISL_958322, EPI_ISL_958323, EPI_ISL_958324, EPI_ISL_958325, EPI_ISL_958326,			

EPI_ISL_958327, EPI_ISL_958328, EPI_ISL_958329, EPI_ISL_958330, EPI_ISL_958331, EPI_ISL_958332, EPI_ISL_958333, EPI_ISL_958334, EPI_ISL_958335, EPI_ISL_958336, EPI_ISL_958337, EPI_ISL_958338, EPI_ISL_958339, EPI_ISL_958340, EPI_ISL_958341, EPI_ISL_958342, EPI_ISL_958343, EPI_ISL_958344, EPI_ISL_958345, EPI_ISL_958346, EPI_ISL_958347, EPI_ISL_958348, EPI_ISL_958349, EPI_ISL_958350, EPI_ISL_958351, EPI_ISL_958352, EPI_ISL_958353, EPI_ISL_958354, EPI_ISL_958355, EPI_ISL_958356, EPI_ISL_958357, EPI_ISL_958358, EPI_ISL_958359, EPI_ISL_958360, EPI_ISL_958361, EPI_ISL_958362, EPI_ISL_958363, EPI_ISL_958364, EPI_ISL_958365, EPI_ISL_958366, EPI_ISL_958367, EPI_ISL_958368, EPI_ISL_958369, EPI_ISL_958370, EPI_ISL_958371, EPI_ISL_958372, EPI_ISL_958373, EPI_ISL_958374, EPI_ISL_958375, EPI_ISL_958376, EPI_ISL_958377, EPI_ISL_958378, EPI_ISL_958379, EPI_ISL_958380, EPI_ISL_958381, EPI_ISL_958382, EPI_ISL_958383, EPI_ISL_958384, EPI_ISL_958385, EPI_ISL_958386, EPI_ISL_958387, EPI_ISL_958388, EPI_ISL_958389, EPI_ISL_958390, EPI_ISL_958391, EPI_ISL_958392, EPI_ISL_958393, EPI_ISL_958394, EPI_ISL_958395, EPI_ISL_958396, EPI_ISL_958397, EPI_ISL_958399, EPI_ISL_958400, EPI_ISL_958401, EPI_ISL_958402, EPI_ISL_958403, EPI_ISL_958404, EPI_ISL_958405, EPI_ISL_958406, EPI_ISL_958407, EPI_ISL_958408, EPI_ISL_958409, EPI_ISL_958410, EPI_ISL_958411, EPI_ISL_958412, EPI_ISL_958413, EPI_ISL_958414, EPI_ISL_958415, EPI_ISL_958416, EPI_ISL_958417, EPI_ISL_958418, EPI_ISL_958419, EPI_ISL_958420, EPI_ISL_958421, EPI_ISL_958422, EPI_ISL_958423, EPI_ISL_958424, EPI_ISL_958425, EPI_ISL_958426, EPI_ISL_958427, EPI_ISL_958428, EPI_ISL_958429, EPI_ISL_958430, EPI_ISL_958431, EPI_ISL_958432, EPI_ISL_958433, EPI_ISL_958434, EPI_ISL_958435, EPI_ISL_958436, EPI_ISL_958437, EPI_ISL_958438, EPI_ISL_958439, EPI_ISL_958440, EPI_ISL_958441, EPI_ISL_958442, EPI_ISL_958443, EPI_ISL_958444, EPI_ISL_958445, EPI_ISL_958446, EPI_ISL_958447, EPI_ISL_958448, EPI_ISL_958449, EPI_ISL_958450, EPI_ISL_958451, EPI_ISL_958452, EPI_ISL_958453, EPI_ISL_958454, EPI_ISL_958455, EPI_ISL_958456, EPI_ISL_958457, EPI_ISL_958458, EPI_ISL_958459, EPI_ISL_958460, EPI_ISL_958461, EPI_ISL_958462, EPI_ISL_958463, EPI_ISL_958464, EPI_ISL_958465, EPI_ISL_958466, EPI_ISL_958467, EPI_ISL_958468, EPI_ISL_958469, EPI_ISL_958470, EPI_ISL_958471, EPI_ISL_958472, EPI_ISL_958473, EPI_ISL_958474, EPI_ISL_958475, EPI_ISL_958476, EPI_ISL_958477, EPI_ISL_958478, EPI_ISL_958479, EPI_ISL_958480, EPI_ISL_958481, EPI_ISL_958482, EPI_ISL_958483, EPI_ISL_958484, EPI_ISL_958485, EPI_ISL_958486, EPI_ISL_958487, EPI_ISL_958488, EPI_ISL_958489, EPI_ISL_958490, EPI_ISL_958491, EPI_ISL_958492, EPI_ISL_958493, EPI_ISL_958494, EPI_ISL_958495, EPI_ISL_958496, EPI_ISL_958497, EPI_ISL_958498, EPI_ISL_958499, EPI_ISL_958500, EPI_ISL_958501, EPI_ISL_958502, EPI_ISL_958503, EPI_ISL_958504, EPI_ISL_958505, EPI_ISL_958506, EPI_ISL_958507, EPI_ISL_958508, EPI_ISL_958509, EPI_ISL_958510, EPI_ISL_958511, EPI_ISL_958512, EPI_ISL_958513, EPI_ISL_958514, EPI_ISL_958515, EPI_ISL_958516, EPI_ISL_958517, EPI_ISL_958518, EPI_ISL_958519, EPI_ISL_958520, EPI_ISL_958521, EPI_ISL_958522, EPI_ISL_958523, EPI_ISL_958524, EPI_ISL_958525, EPI_ISL_958526, EPI_ISL_958527, EPI_ISL_958528, EPI_ISL_958529, EPI_ISL_958530, EPI_ISL_958531, EPI_ISL_958532, EPI_ISL_958533, EPI_ISL_958534, EPI_ISL_958535, EPI_ISL_958536, EPI_ISL_958537, EPI_ISL_958538, EPI_ISL_958539, EPI_ISL_958540, EPI_ISL_958541, EPI_ISL_958542, EPI_ISL_958543, EPI_ISL_958544, EPI_ISL_958545, EPI_ISL_958546, EPI_ISL_958547, EPI_ISL_958548, EPI_ISL_958549, EPI_ISL_958550, EPI_ISL_958551, EPI_ISL_958552, EPI_ISL_958553, EPI_ISL_958554, EPI_ISL_958555, EPI_ISL_958556, EPI_ISL_958557, EPI_ISL_958558, EPI_ISL_958559, EPI_ISL_958560, EPI_ISL_958561, EPI_ISL_958562, EPI_ISL_958563			
see above	Lighthouse Lab in Milton Keynes	Wellcome Sanger Institute for the COVID-19 Genomics UK (COG-UK) Consortium	The Lighthouse Lab in Milton Keynes and Alex Alderton, Roberto Amato, Sonia Goncalves, Ewan Harrison, David K. Jackson, Ian Johnston, Dominic Kwiatkowski, Cordelia Langford, John Sillitoe on behalf of the Wellcome Sanger Institute COVID-19 Surveillance Team
EPI_ISL_958566	Lighthouse Lab in Alderley Park	Wellcome Sanger Institute for the COVID-19 Genomics UK (COG-UK) Consortium	Jacquelyn Wynn, Mairead Hyland, The Lighthouse Lab in Alderley Park and Alex Alderton, Roberto Amato, Sonia Goncalves, Ewan Harrison, David K. Jackson, Ian Johnston, Dominic Kwiatkowski, Cordelia Langford, John Sillitoe on behalf of the Wellcome Sanger Institute COVID-19 Surveillance Team
EPI_ISL_958569, EPI_ISL_958572, EPI_ISL_958578, EPI_ISL_958579, EPI_ISL_958586, EPI_ISL_958587, EPI_ISL_958590, EPI_ISL_958591, EPI_ISL_958595, EPI_ISL_958600, EPI_ISL_958602, EPI_ISL_958610, EPI_ISL_958624, EPI_ISL_958632, EPI_ISL_958633, EPI_ISL_958634, EPI_ISL_958636, EPI_ISL_958638, EPI_ISL_958639, EPI_ISL_958641, EPI_ISL_958646, EPI_ISL_958650, EPI_ISL_958659, EPI_ISL_958662, EPI_ISL_958663, EPI_ISL_958670, EPI_ISL_958671, EPI_ISL_958673, EPI_ISL_958680, EPI_ISL_958684, EPI_ISL_958690, EPI_ISL_958691, EPI_ISL_958696, EPI_ISL_958705, EPI_ISL_958706, EPI_ISL_958713, EPI_ISL_958715, EPI_ISL_958716, EPI_ISL_958723, EPI_ISL_958725, EPI_ISL_958726, EPI_ISL_958731, EPI_ISL_958733, EPI_ISL_958735, EPI_ISL_958745, EPI_ISL_958747, EPI_ISL_958748, EPI_ISL_958754, EPI_ISL_958756, EPI_ISL_958760, EPI_ISL_958763, EPI_ISL_958767, EPI_ISL_958769, EPI_ISL_958775, EPI_ISL_958779, EPI_ISL_958784, EPI_ISL_958787, EPI_ISL_958789, EPI_ISL_958791, EPI_ISL_958792, EPI_ISL_958793, EPI_ISL_958803, EPI_ISL_958807, EPI_ISL_958808, EPI_ISL_958811, EPI_ISL_958815, EPI_ISL_958823, EPI_ISL_958826, EPI_ISL_958828, EPI_ISL_958833, EPI_ISL_958836, EPI_ISL_958840, EPI_ISL_958842, EPI_ISL_958849, EPI_ISL_958851, EPI_ISL_958863, EPI_ISL_958866, EPI_ISL_958873, EPI_ISL_958878, EPI_ISL_958884, EPI_ISL_958885, EPI_ISL_958887, EPI_ISL_958889, EPI_ISL_958891, EPI_ISL_958893, EPI_ISL_958895, EPI_ISL_958898, EPI_ISL_958902, EPI_ISL_958903, EPI_ISL_958904, EPI_ISL_958908, EPI_ISL_958911, EPI_ISL_958912, EPI_ISL_958915, EPI_ISL_958916			
see above	Lighthouse Lab in Milton Keynes	Wellcome Sanger Institute for the COVID-19 Genomics UK (COG-UK) Consortium	The Lighthouse Lab in Milton Keynes and Alex Alderton, Roberto Amato, Sonia Goncalves, Ewan Harrison, David K. Jackson, Ian Johnston, Dominic Kwiatkowski, Cordelia Langford, John Sillitoe on behalf of the Wellcome Sanger Institute COVID-19 Surveillance Team
EPI_ISL_958917, EPI_ISL_958918, EPI_ISL_958919, EPI_ISL_958920, EPI_ISL_958921, EPI_ISL_958922, EPI_ISL_958923, EPI_ISL_958924, EPI_ISL_958925, EPI_ISL_958926, EPI_ISL_958927, EPI_ISL_958928, EPI_ISL_958929, EPI_ISL_958930, EPI_ISL_958931, EPI_ISL_958932, EPI_ISL_958933, EPI_ISL_958934, EPI_ISL_958935, EPI_ISL_958936, EPI_ISL_958937, EPI_ISL_958938, EPI_ISL_958939, EPI_ISL_958940, EPI_ISL_958941, EPI_ISL_958942, EPI_ISL_958943, EPI_ISL_958944, EPI_ISL_958945, EPI_ISL_958946, EPI_ISL_958947, EPI_ISL_958948, EPI_ISL_958949, EPI_ISL_958950, EPI_ISL_958951, EPI_ISL_958952, EPI_ISL_958953, EPI_ISL_958954, EPI_ISL_958955, EPI_ISL_958956, EPI_ISL_958957, EPI_ISL_958958, EPI_ISL_958959, EPI_ISL_958960, EPI_ISL_958961, EPI_ISL_958962, EPI_ISL_958963, EPI_ISL_958964, EPI_ISL_958965, EPI_ISL_958966, EPI_ISL_958967, EPI_ISL_958968, EPI_ISL_958969, EPI_ISL_958970, EPI_ISL_958971, EPI_ISL_958972, EPI_ISL_958973, EPI_ISL_958974, EPI_ISL_958975, EPI_ISL_958976, EPI_ISL_958977, EPI_ISL_958978, EPI_ISL_958979, EPI_ISL_958980, EPI_ISL_958981, EPI_ISL_958982, EPI_ISL_958983, EPI_ISL_958984, EPI_ISL_958985, EPI_ISL_958986, EPI_ISL_958987, EPI_ISL_958988, EPI_ISL_958989, EPI_ISL_958990, EPI_ISL_958991, EPI_ISL_958992, EPI_ISL_958993, EPI_ISL_958994, EPI_ISL_958995, EPI_ISL_958996, EPI_ISL_958997, EPI_ISL_958998, EPI_ISL_958999, EPI_ISL_959000, EPI_ISL_959001, EPI_ISL_959002, EPI_ISL_959003, EPI_ISL_959004, EPI_ISL_959005, EPI_ISL_959006, EPI_ISL_959007, EPI_ISL_959008, EPI_ISL_959009, EPI_ISL_959010, EPI_ISL_959011, EPI_ISL_959012, EPI_ISL_959013, EPI_ISL_959014, EPI_ISL_959015, EPI_ISL_959016, EPI_ISL_959017, EPI_ISL_959018, EPI_ISL_959019, EPI_ISL_959020, EPI_ISL_959021, EPI_ISL_959022, EPI_ISL_959023, EPI_ISL_959024, EPI_ISL_959025, EPI_ISL_959026, EPI_ISL_959027, EPI_ISL_959028, EPI_ISL_959029, EPI_ISL_959030, EPI_ISL_959031, EPI_ISL_959032, EPI_ISL_959033, EPI_ISL_959034, EPI_ISL_959035, EPI_ISL_959036, EPI_ISL_959037, EPI_ISL_959038, EPI_ISL_959039, EPI_ISL_959040, EPI_ISL_959041, EPI_ISL_959042, EPI_ISL_959043, EPI_ISL_959044, EPI_ISL_959045, EPI_ISL_959046, EPI_ISL_959047, EPI_ISL_959048, EPI_ISL_959049, EPI_ISL_959050, EPI_ISL_959051, EPI_ISL_959052, EPI_ISL_959053, EPI_ISL_959054, EPI_ISL_959055, EPI_ISL_959056, EPI_ISL_959057, EPI_ISL_959058, EPI_ISL_959059, EPI_ISL_959060, EPI_ISL_959061, EPI_ISL_959062, EPI_ISL_959063, EPI_ISL_959064, EPI_ISL_959065, EPI_ISL_959066, EPI_ISL_959067, EPI_ISL_959068, EPI_ISL_959069, EPI_ISL_959070, EPI_ISL_959071, EPI_ISL_959072, EPI_ISL_959073, EPI_ISL_959074, EPI_ISL_959075, EPI_ISL_959076, EPI_ISL_959077, EPI_ISL_959078, EPI_ISL_959079, EPI_ISL_959080, EPI_ISL_959081, EPI_ISL_959082, EPI_ISL_959083, EPI_ISL_959084, EPI_ISL_959085, EPI_ISL_959086, EPI_ISL_959087, EPI_ISL_959088, EPI_ISL_959089, EPI_ISL_959090, EPI_ISL_959091, EPI_ISL_959092, EPI_ISL_959093, EPI_ISL_959094, EPI_ISL_959095, EPI_ISL_959096, EPI_ISL_959097, EPI_ISL_959098, EPI_ISL_959099, EPI_ISL_959100, EPI_ISL_959101, EPI_ISL_959102, EPI_ISL_959103, EPI_ISL_959104, EPI_ISL_959105, EPI_ISL_959106, EPI_ISL_959107, EPI_ISL_959108, EPI_ISL_959109, EPI_ISL_959110, EPI_ISL_959111, EPI_ISL_959112, EPI_ISL_959113, EPI_ISL_959114, EPI_ISL_959115, EPI_ISL_959116, EPI_ISL_959117, EPI_ISL_959118, EPI_ISL_959119, EPI_ISL_959120, EPI_ISL_959121, EPI_ISL_959122, EPI_ISL_959123, EPI_ISL_959124, EPI_ISL_959125, EPI_ISL_959126, EPI_ISL_959127, EPI_ISL_959128, EPI_ISL_959129, EPI_ISL_959130, EPI_ISL_959131, EPI_ISL_959132, EPI_ISL_959133, EPI_ISL_959134, EPI_ISL_959135, EPI_ISL_959136, EPI_ISL_959137, EPI_ISL_959138, EPI_ISL_959139, EPI_ISL_959140, EPI_ISL_959141, EPI_ISL_959142, EPI_ISL_959143, EPI_ISL_959144, EPI_ISL_959145, EPI_ISL_959146, EPI_ISL_959147, EPI_ISL_959148, EPI_ISL_959149, EPI_ISL_959150, EPI_ISL_959151, EPI_ISL_959152, EPI_ISL_959153, EPI_ISL_959154, EPI_ISL_959155, EPI_ISL_959156, EPI_ISL_959157, EPI_ISL_959158, EPI_ISL_959159, EPI_ISL_959160, EPI_ISL_959161, EPI_ISL_959162, EPI_ISL_959163, EPI_ISL_959164, EPI_ISL_959165, EPI_ISL_959166, EPI_ISL_959167, EPI_ISL_959168, EPI_ISL_959169, EPI_ISL_959170, EPI_ISL_959171, EPI_ISL_959172, EPI_ISL_959173, EPI_ISL_959174, EPI_ISL_959175, EPI_ISL_959176, EPI_ISL_959177, EPI_ISL_959178, EPI_ISL_959179, EPI_ISL_959180, EPI_ISL_959181, EPI_ISL_959182, EPI_ISL_959183, EPI_ISL_959184, EPI_ISL_959185, EPI_ISL_959186, EPI_ISL_959187, EPI_ISL_959188, EPI_ISL_959189, EPI_ISL_959190, EPI_ISL_959191, EPI_ISL_959192, EPI_ISL_959193, EPI_ISL_959194, EPI_ISL_959195, EPI_ISL_959196, EPI_ISL_959197, EPI_ISL_959198, EPI_ISL_959199, EPI_ISL_959200, EPI_ISL_959201, EPI_ISL_959202, EPI_ISL_959203, EPI_ISL_959204, EPI_ISL_959205, EPI_ISL_959206, EPI_ISL_959207, EPI_ISL_959208, EPI_ISL_959209, EPI_ISL_959210, EPI_ISL_959211, EPI_ISL_959212, EPI_ISL_959213, EPI_ISL_959214, EPI_ISL_959215, EPI_ISL_959216, EPI_ISL_959217, EPI_ISL_959218, EPI_ISL_959219, EPI_ISL_959220, EPI_ISL_959221, EPI_ISL_959222, EPI_ISL_959223, EPI_ISL_959224, EPI_ISL_959225, EPI_ISL_959226, EPI_ISL_959227, EPI_ISL_959228, EPI_ISL_959229, EPI_ISL_959230, EPI_ISL_959231, EPI_ISL_959232, EPI_ISL_959233, EPI_ISL_959234, EPI_ISL_959235, EPI_ISL_959236, EPI_ISL_959237, EPI_ISL_959238, EPI_ISL_959239, EPI_ISL_959240, EPI_ISL_959241, EPI_ISL_959242, EPI_ISL_959243, EPI_ISL_959244, EPI_ISL_959245, EPI_ISL_959246, EPI_ISL_959247, EPI_ISL_959248, EPI_ISL_959249, EPI_ISL_959250, EPI_ISL_959251, EPI_ISL_959252, EPI_ISL_959253, EPI_ISL_959254, EPI_ISL_959255, EPI_ISL_959256, EPI_ISL_959257, EPI_ISL_959258, EPI_ISL_959259, EPI_ISL_959260, EPI_ISL_959261, EPI_ISL_959262, EPI_ISL_959263, EPI_ISL_959264, EPI_ISL_959265, EPI_ISL_959266			
see above	Lighthouse Lab in Alderley Park	Wellcome Sanger Institute for the COVID-19 Genomics UK (COG-UK) Consortium	Jacquelyn Wynn, Mairead Hyland, The Lighthouse Lab in Alderley Park and Alex Alderton, Roberto Amato, Sonia Goncalves, Ewan Harrison, David K. Jackson, Ian Johnston, Dominic Kwiatkowski, Cordelia Langford, John Sillitoe on behalf of the Wellcome Sanger Institute COVID-19 Surveillance Team
EPI_ISL_959267	Lighthouse Lab in Cambridge	Wellcome Sanger Institute for the COVID-19 Genomics UK (COG-UK) Consortium	Rob Howes, The Lighthouse Lab in Cambridge and Alex Alderton, Roberto Amato, Sonia Goncalves, Ewan Harrison, David K. Jackson, Ian Johnston, Dominic Kwiatkowski, Cordelia Langford, John Sillitoe on behalf of the Wellcome Sanger Institute COVID-19 Surveillance Team
EPI_ISL_959268, EPI_ISL_959269, EPI_ISL_959270	Lighthouse Lab in Alderley Park	Wellcome Sanger Institute for the COVID-19 Genomics UK (COG-UK) Consortium	Jacquelyn Wynn, Mairead Hyland, The Lighthouse Lab in Alderley Park and Alex Alderton, Roberto Amato, Sonia Goncalves, Ewan Harrison, David K. Jackson, Ian Johnston, Dominic Kwiatkowski, Cordelia Langford, John Sillitoe on behalf of the Wellcome Sanger Institute COVID-19 Surveillance Team
EPI_ISL_959271, EPI_ISL_959272, EPI_ISL_963325, EPI_ISL_963408, EPI_ISL_963471, EPI_ISL_963524, EPI_ISL_963555	Lighthouse Lab in Milton Keynes	Wellcome Sanger Institute for the COVID-19 Genomics UK (COG-UK) Consortium	The Lighthouse Lab in Milton Keynes and Alex Alderton, Roberto Amato, Sonia Goncalves, Ewan Harrison, David K. Jackson, Ian Johnston, Dominic Kwiatkowski, Cordelia Langford, John Sillitoe on behalf of the Wellcome Sanger Institute COVID-19 Surveillance Team
EPI_ISL_963651, EPI_ISL_963654, EPI_ISL_963658, EPI_ISL_963659, EPI_ISL_963665, EPI_ISL_963670, EPI_ISL_963671, EPI_ISL_963672, EPI_ISL_963673, EPI_ISL_963676, EPI_ISL_963677, EPI_ISL_963679, EPI_ISL_963681, EPI_ISL_963685, EPI_ISL_963686, EPI_ISL_963687, EPI_ISL_963689, EPI_ISL_963690, EPI_ISL_963694, EPI_ISL_963695, EPI_ISL_963700, EPI_ISL_963701, EPI_ISL_963703, EPI_ISL_963704, EPI_ISL_963706, EPI_ISL_963707, EPI_ISL_963709, EPI_ISL_963718, EPI_ISL_963720, EPI_ISL_963721, EPI_ISL_963724, EPI_ISL_963726, EPI_ISL_963727, EPI_ISL_963731, EPI_ISL_963732, EPI_ISL_963733, EPI_ISL_963734, EPI_ISL_963735, EPI_ISL_963736, EPI_ISL_963737, EPI_ISL_963738, EPI_ISL_963740, EPI_ISL_963741, EPI_ISL_963747, EPI_ISL_963750, EPI_ISL_963753, EPI_ISL_963754, EPI_ISL_963758, EPI_ISL_963759, EPI_ISL_963761, EPI_ISL_963764, EPI_ISL_963765, EPI_ISL_963766, EPI_ISL_963768, EPI_ISL_963769, EPI_ISL_963772, EPI_ISL_963773, EPI_ISL_963774, EPI_ISL_963775, EPI_ISL_963779, EPI_ISL_963780, EPI_ISL_963781, EPI_ISL_963783, EPI_ISL_963786, EPI_ISL_963789, EPI_ISL_963790, EPI_ISL_963794, EPI_ISL_963798, EPI_ISL_963800, EPI_ISL_963803, EPI_ISL_963805, EPI_ISL_963806, EPI_ISL_963807, EPI_ISL_963808, EPI_ISL_963810, EPI_ISL_963813, EPI_ISL_963814, EPI_ISL_963815, EPI_ISL_963816, EPI_ISL_963819, EPI_ISL_963821, EPI_ISL_963822, EPI_ISL_963823, EPI_ISL_963824, EPI_ISL_963825, EPI_ISL_963826, EPI_ISL_963831, EPI_ISL_963832, EPI_ISL_963833, EPI_ISL_963835, EPI_ISL_963838, EPI_ISL_963839, EPI_ISL_963841, EPI_ISL_963844, EPI_ISL_963845, EPI_ISL_963846, EPI_ISL_963849, EPI_ISL_963850, EPI_ISL_963851, EPI_ISL_963852, EPI_ISL_963853, EPI_ISL_963855, EPI_ISL_963857, EPI_ISL_963859, EPI_ISL_963861, EPI_ISL_963863, EPI_ISL_963864, EPI_ISL_963865, EPI_ISL_963866, EPI_ISL_963867, EPI_ISL_963868, EPI_ISL_963869, EPI_ISL_963870, EPI_ISL_963872, EPI_ISL_963873, EPI_ISL_963874, EPI_ISL_963875, EPI_ISL_963877, EPI_ISL_963878, EPI_ISL_963879, EPI_ISL_963880, EPI_ISL_963881, EPI_ISL_963882, EPI_ISL_963883, EPI_ISL_963884, EPI_ISL_963889, EPI_ISL_963890, EPI_ISL_963891, EPI_ISL_963893, EPI_ISL_963895, EPI_ISL_963896, EPI_ISL_963900, EPI_ISL_963901, EPI_ISL_963902, EPI_ISL_963903, EPI_ISL_963904, EPI_ISL_963906, EPI_ISL_963908, EPI_ISL_963909, EPI_ISL_963911, EPI_ISL_963914, EPI_ISL_963915, EPI_ISL_963916, EPI_ISL_963921, EPI_ISL_963922, EPI_ISL_963923, EPI_ISL_963925, EPI_ISL_963927, EPI_ISL_963932, EPI_ISL_963934, EPI_ISL_963936, EPI_ISL_963938, EPI_ISL_963941, EPI_ISL_963942, EPI_ISL_963945, EPI_ISL_963947, EPI_ISL_963949, EPI_ISL_963952, EPI_ISL_963953, EPI_ISL_963958, EPI_ISL_963960, EPI_ISL_963962, EPI_ISL_963964, EPI_ISL_963968, EPI_ISL_963970, EPI_ISL_963971, EPI_ISL_963973, EPI_ISL_963976, EPI_ISL_963977, EPI_ISL_963978, EPI_ISL_963979, EPI_ISL_963981, EPI_ISL_963984, EPI_ISL_964067, EPI_ISL_964087, EPI_ISL_964236, EPI_ISL_964710, EPI_ISL_964726, EPI_ISL_964749, EPI_ISL_964759, EPI_ISL_964817, EPI_ISL_964877, EPI_ISL_969454, EPI_ISL_970032, EPI_ISL_970055, EPI_ISL_970142			
see above	Lighthouse Lab in Alderley Park	Wellcome Sanger Institute for the COVID-19 Genomics UK (COG-UK) Consortium	Jacquelyn Wynn, Mairead Hyland, The Lighthouse Lab in Alderley Park and Alex Alderton, Roberto Amato, Sonia Goncalves, Ewan Harrison, David K. Jackson, Ian Johnston, Dominic Kwiatkowski, Cordelia Langford, John Sillitoe on behalf of the Wellcome Sanger Institute COVID-19 Surveillance Team
EPI_ISL_970589, EPI_ISL_970591, EPI_ISL_970593, EPI_ISL_970595, EPI_ISL_970599, EPI_ISL_970601, EPI_ISL_970602, EPI_ISL_970605, EPI_ISL_970607, EPI_ISL_970609, EPI_ISL_970612, EPI_ISL_970614, EPI_ISL_970618, EPI_ISL_970619, EPI_ISL_970623, EPI_ISL_970625, EPI_ISL_970630, EPI_ISL_970632, EPI_ISL_970633, EPI_ISL_970637, EPI_ISL_970641, EPI_ISL_970643, EPI_ISL_970648, EPI_ISL_970650, EPI_ISL_970652, EPI_ISL_970654, EPI_ISL_970656, EPI_ISL_970658, EPI_ISL_970660, EPI_ISL_970661, EPI_ISL_970666, EPI_ISL_970668, EPI_ISL_970670, EPI_ISL_970675, EPI_ISL_970677, EPI_ISL_970683, EPI_ISL_970685, EPI_ISL_970689, EPI_ISL_970690, EPI_ISL_970692, EPI_ISL_970694, EPI_ISL_970697, EPI_ISL_970699, EPI_ISL_970703, EPI_ISL_970704, EPI_ISL_970706, EPI_ISL_970711, EPI_ISL_970713, EPI_ISL_970717, EPI_ISL_970720, EPI_ISL_970722, EPI_ISL_970726, EPI_ISL_970727, EPI_ISL_970729, EPI_ISL_970734, EPI_ISL_970736, EPI_ISL_970738, EPI_ISL_970739, EPI_ISL_970741, EPI_ISL_970746, EPI_ISL_970749, EPI_ISL_970755, EPI_ISL_970757, EPI_ISL_970758, EPI_ISL_970760, EPI_ISL_970762, EPI_ISL_970764, EPI_ISL_970766, EPI_ISL_970770, EPI_ISL_970772, EPI_ISL_970774, EPI_ISL_970777, EPI_ISL_970779, EPI_ISL_970781, EPI_ISL_970782, EPI_ISL_970784, EPI_ISL_970786, EPI_ISL_970788, EPI_ISL_970792, EPI_ISL_970794, EPI_ISL_970796, EPI_ISL_970797, EPI_ISL_970800, EPI_ISL_970802, EPI_ISL_970803, EPI_ISL_970805, EPI_ISL_970807, EPI_ISL_970808, EPI_ISL_970845, EPI_ISL_970899, EPI_ISL_970925, EPI_ISL_970944, EPI_ISL_970968, EPI_ISL_971008, EPI_ISL_971023, EPI_ISL_971039, EPI_ISL_971050, EPI_ISL_971055, EPI_ISL_971059, EPI_ISL_971063, EPI_ISL_971066, EPI_ISL_971069, EPI_ISL_971074, EPI_ISL_971079, EPI_ISL_971083, EPI_ISL_971087, EPI_ISL_971090, EPI_ISL_971099, EPI_ISL_971102, EPI_ISL_971108, EPI_ISL_971109, EPI_ISL_971113, EPI_ISL_971117, EPI_ISL_971119, EPI_ISL_971123, EPI_ISL_971128, EPI_ISL_971130, EPI_ISL_971134, EPI_ISL_971138, EPI_ISL_971146, EPI_ISL_971149, EPI_ISL_971153, EPI_ISL_971156, EPI_ISL_971161, EPI_ISL_971164, EPI_ISL_971165, EPI_ISL_971180, EPI_ISL_971185, EPI_ISL_971190, EPI_ISL_971197, EPI_ISL_971200, EPI_ISL_971204, EPI_ISL_971209, EPI_ISL_971211, EPI_ISL_971215, EPI_ISL_971218, EPI_ISL_971221, EPI_ISL_971224, EPI_ISL_971228, EPI_ISL_971234, EPI_ISL_971236, EPI_ISL_971246, EPI_ISL_971248, EPI_ISL_971251, EPI_ISL_971254, EPI_ISL_971258, EPI_ISL_971264, EPI_ISL_971267, EPI_ISL_971273, EPI_ISL_971277, EPI_ISL_971284, EPI_ISL_971286, EPI_ISL_971289, EPI_ISL_971295, EPI_ISL_971301, EPI_ISL_971304, EPI_ISL_971307, EPI_ISL_971310, EPI_ISL_971313, EPI_ISL_971316, EPI_ISL_971319, EPI_ISL_971320, EPI_ISL_971324, EPI_ISL_971330, EPI_ISL_971336, EPI_ISL_971343, EPI_ISL_971350, EPI_ISL_971352, EPI_ISL_971357, EPI_ISL_971373, EPI_ISL_971376, EPI_ISL_971378, EPI_ISL_971382, EPI_ISL_971388, EPI_ISL_971392, EPI_ISL_971397, EPI_ISL_971400, EPI_ISL_971402, EPI_ISL_971405, EPI_ISL_971407, EPI_ISL_971410, EPI_ISL_971413, EPI_ISL_971417, EPI_ISL_971420, EPI_ISL_971423			
see above	Lighthouse Lab in Milton Keynes	Wellcome Sanger Institute for the COVID-19 Genomics UK	The Lighthouse Lab in Milton Keynes and Alex Alderton, Roberto Amato, Sonia Goncalves, Ewan Harrison, David K. Jackson, Ian Johnston, Dominic

		(COG-UK) Consortium	Kwiatkowski, Cordelia Langford, John Sillitoe on behalf of the Wellcome Sanger Institute COVID-19 Surveillance Team
EPI_ISL_971425	Lighthouse Lab in Cambridge	Wellcome Sanger Institute for the COVID-19 Genomics UK (COG-UK) Consortium	Rob Howes, The Lighthouse Lab in Cambridge and Alex Alderton, Roberto Amato, Sonia Goncalves, Ewan Harrison, David K. Jackson, Ian Johnston, Dominic Kwiatkowski, Cordelia Langford, John Sillitoe on behalf of the Wellcome Sanger Institute COVID-19 Surveillance Team
EPI_ISL_971428	Lighthouse Lab in Milton Keynes	Wellcome Sanger Institute for the COVID-19 Genomics UK (COG-UK) Consortium	The Lighthouse Lab in Milton Keynes and Alex Alderton, Roberto Amato, Sonia Goncalves, Ewan Harrison, David K. Jackson, Ian Johnston, Dominic Kwiatkowski, Cordelia Langford, John Sillitoe on behalf of the Wellcome Sanger Institute COVID-19 Surveillance Team
EPI_ISL_987288, EPI_ISL_987289, EPI_ISL_987292, EPI_ISL_987293, EPI_ISL_987295, EPI_ISL_987296, EPI_ISL_987301, EPI_ISL_987302, EPI_ISL_987304, EPI_ISL_987306, EPI_ISL_987307, EPI_ISL_987309, EPI_ISL_987313, EPI_ISL_987315, EPI_ISL_987318, EPI_ISL_987321, EPI_ISL_987325, EPI_ISL_987344, EPI_ISL_987348, EPI_ISL_987361, EPI_ISL_987365, EPI_ISL_987367, EPI_ISL_987368, EPI_ISL_987372, EPI_ISL_987379, EPI_ISL_987383, EPI_ISL_987387, EPI_ISL_987392, EPI_ISL_987397, EPI_ISL_987401, EPI_ISL_987407, EPI_ISL_987408, EPI_ISL_987409, EPI_ISL_987412, EPI_ISL_987413, EPI_ISL_987416, EPI_ISL_987417, EPI_ISL_987421, EPI_ISL_987425, EPI_ISL_987430, EPI_ISL_987432, EPI_ISL_987449, EPI_ISL_987451, EPI_ISL_987456, EPI_ISL_987458, EPI_ISL_987462, EPI_ISL_987467, EPI_ISL_987472, EPI_ISL_987476, EPI_ISL_987478, EPI_ISL_987480, EPI_ISL_987482, EPI_ISL_987489, EPI_ISL_987490, EPI_ISL_987493, EPI_ISL_987505, EPI_ISL_987508, EPI_ISL_987513, EPI_ISL_987518, EPI_ISL_987519, EPI_ISL_987520, EPI_ISL_987521, EPI_ISL_987523, EPI_ISL_987525, EPI_ISL_987526, EPI_ISL_987527, EPI_ISL_987540, EPI_ISL_987541, EPI_ISL_987542, EPI_ISL_987545, EPI_ISL_987546, EPI_ISL_987553, EPI_ISL_987554, EPI_ISL_987559, EPI_ISL_987561, EPI_ISL_987562, EPI_ISL_987566, EPI_ISL_987578, EPI_ISL_987579, EPI_ISL_987581, EPI_ISL_987583, EPI_ISL_987595, EPI_ISL_987596, EPI_ISL_987599, EPI_ISL_987603, EPI_ISL_987604, EPI_ISL_987605, EPI_ISL_987607, EPI_ISL_987611, EPI_ISL_987613, EPI_ISL_987615, EPI_ISL_987617, EPI_ISL_987623			
see above	Lighthouse Lab in Cambridge	Wellcome Sanger Institute for the COVID-19 Genomics UK (COG-UK) Consortium	Rob Howes, The Lighthouse Lab in Cambridge and Alex Alderton, Roberto Amato, Sonia Goncalves, Ewan Harrison, David K. Jackson, Ian Johnston, Dominic Kwiatkowski, Cordelia Langford, John Sillitoe on behalf of the Wellcome Sanger Institute COVID-19 Surveillance Team (http://www.sanger.ac.uk/covid-team)
EPI_ISL_988483, EPI_ISL_988485, EPI_ISL_988488, EPI_ISL_988490, EPI_ISL_988493, EPI_ISL_988513, EPI_ISL_988520, EPI_ISL_988522, EPI_ISL_988526, EPI_ISL_988534, EPI_ISL_988539, EPI_ISL_988545, EPI_ISL_988551, EPI_ISL_988568, EPI_ISL_988577, EPI_ISL_988585, EPI_ISL_988586, EPI_ISL_988601, EPI_ISL_988605, EPI_ISL_988611, EPI_ISL_988615, EPI_ISL_988619, EPI_ISL_988620, EPI_ISL_988624, EPI_ISL_988632, EPI_ISL_988647, EPI_ISL_988648, EPI_ISL_988649, EPI_ISL_988652, EPI_ISL_988655, EPI_ISL_988661, EPI_ISL_988663, EPI_ISL_988665, EPI_ISL_988671, EPI_ISL_988681, EPI_ISL_988683			
see above	Lighthouse Lab in Milton Keynes	Wellcome Sanger Institute for the COVID-19 Genomics UK (COG-UK) Consortium	The Lighthouse Lab in Milton Keynes and Alex Alderton, Roberto Amato, Sonia Goncalves, Ewan Harrison, David K. Jackson, Ian Johnston, Dominic Kwiatkowski, Cordelia Langford, John Sillitoe on behalf of the Wellcome Sanger Institute COVID-19 Surveillance Team (http://www.sanger.ac.uk/covid-team)
EPI_ISL_989482, EPI_ISL_989483, EPI_ISL_989485, EPI_ISL_989489, EPI_ISL_989490, EPI_ISL_989491, EPI_ISL_989494, EPI_ISL_989495, EPI_ISL_989496, EPI_ISL_989504, EPI_ISL_989505, EPI_ISL_989506, EPI_ISL_989507, EPI_ISL_989522, EPI_ISL_989742, EPI_ISL_989751, EPI_ISL_989769, EPI_ISL_989856, EPI_ISL_989857, EPI_ISL_989858, EPI_ISL_989859, EPI_ISL_989861, EPI_ISL_989862, EPI_ISL_989865, EPI_ISL_989866, EPI_ISL_989867, EPI_ISL_989868, EPI_ISL_989869, EPI_ISL_989870, EPI_ISL_989871, EPI_ISL_989873, EPI_ISL_989874, EPI_ISL_989876, EPI_ISL_989877, EPI_ISL_989879, EPI_ISL_989881			
see above	Lighthouse Lab in Alderley Park	Wellcome Sanger Institute for the COVID-19 Genomics UK (COG-UK) Consortium	Jacquelyn Wynn, Mairead Hyland, The Lighthouse Lab in Alderley Park and Alex Alderton, Roberto Amato, Sonia Goncalves, Ewan Harrison, David K. Jackson, Ian Johnston, Dominic Kwiatkowski, Cordelia Langford, John Sillitoe on behalf of the Wellcome Sanger Institute COVID-19 Surveillance Team (http://www.sanger.ac.uk/covid-team)
EPI_ISL_989998, EPI_ISL_989999, EPI_ISL_990000, EPI_ISL_990001, EPI_ISL_990002, EPI_ISL_990004, EPI_ISL_990005, EPI_ISL_990006, EPI_ISL_990007, EPI_ISL_990008, EPI_ISL_990009, EPI_ISL_990010, EPI_ISL_990011, EPI_ISL_990012, EPI_ISL_990013, EPI_ISL_990014, EPI_ISL_990015, EPI_ISL_990016, EPI_ISL_990017, EPI_ISL_990018, EPI_ISL_990019, EPI_ISL_990020, EPI_ISL_990021, EPI_ISL_990022, EPI_ISL_990023, EPI_ISL_990024, EPI_ISL_990025, EPI_ISL_990026, EPI_ISL_990027, EPI_ISL_990028, EPI_ISL_990029, EPI_ISL_990030, EPI_ISL_990031, EPI_ISL_990032, EPI_ISL_990033, EPI_ISL_990034, EPI_ISL_990035, EPI_ISL_990036, EPI_ISL_990037, EPI_ISL_990038, EPI_ISL_990039, EPI_ISL_990040, EPI_ISL_990041, EPI_ISL_990042			
see above	Lighthouse Lab in Alderley Park	Wellcome Sanger Institute for the COVID-19 Genomics UK (COG-UK) Consortium	Jacquelyn Wynn, Mairead Hyland, The Lighthouse Lab in Alderley Park and Alex Alderton, Roberto Amato, Sonia Goncalves, Ewan Harrison, David K. Jackson, Ian Johnston, Dominic Kwiatkowski, Cordelia Langford, John Sillitoe on behalf of the Wellcome Sanger Institute COVID-19 Surveillance Team
EPI_ISL_990058, EPI_ISL_990059, EPI_ISL_990060, EPI_ISL_990061, EPI_ISL_990062, EPI_ISL_990063, EPI_ISL_990064, EPI_ISL_990065, EPI_ISL_990066, EPI_ISL_990067, EPI_ISL_990068, EPI_ISL_990069, EPI_ISL_990070, EPI_ISL_990071, EPI_ISL_990072, EPI_ISL_990073, EPI_ISL_990074, EPI_ISL_990075, EPI_ISL_990076, EPI_ISL_990077, EPI_ISL_990078, EPI_ISL_990079, EPI_ISL_990080, EPI_ISL_990081, EPI_ISL_990082, EPI_ISL_990083, EPI_ISL_990084, EPI_ISL_990085, EPI_ISL_990086, EPI_ISL_990087, EPI_ISL_990088, EPI_ISL_990089, EPI_ISL_990090, EPI_ISL_990091, EPI_ISL_990092, EPI_ISL_990093, EPI_ISL_990094, EPI_ISL_990095, EPI_ISL_990096, EPI_ISL_990097, EPI_ISL_990098, EPI_ISL_990099, EPI_ISL_990100, EPI_ISL_990101, EPI_ISL_990102, EPI_ISL_990103, EPI_ISL_990104, EPI_ISL_990105, EPI_ISL_990106, EPI_ISL_990107, EPI_ISL_990108, EPI_ISL_990109, EPI_ISL_990110, EPI_ISL_990111, EPI_ISL_990112, EPI_ISL_990113, EPI_ISL_990114, EPI_ISL_990115, EPI_ISL_990116, EPI_ISL_990117, EPI_ISL_990118, EPI_ISL_990119, EPI_ISL_990120, EPI_ISL_990121, EPI_ISL_990122, EPI_ISL_990123, EPI_ISL_990124, EPI_ISL_990125, EPI_ISL_990126, EPI_ISL_990127, EPI_ISL_990128, EPI_ISL_990129, EPI_ISL_990130, EPI_ISL_990131, EPI_ISL_990132, EPI_ISL_990133, EPI_ISL_990134, EPI_ISL_990135, EPI_ISL_990136, EPI_ISL_990137, EPI_ISL_990138, EPI_ISL_990139, EPI_ISL_990140			
see above	Lighthouse Lab in Glasgow	Wellcome Sanger Institute for the COVID-19 Genomics UK (COG-UK) Consortium	Harper VanSteenhouse, Yumi Kasai, David Gray, Carol Clugston, Anna Dominiczak and Alex Alderton, Roberto Amato, Sonia Goncalves, Ewan Harrison, David K. Jackson, Ian Johnston, Dominic Kwiatkowski, Cordelia Langford, John Sillitoe on behalf of the Wellcome Sanger Institute COVID-19 Surveillance Team
EPI_ISL_990141, EPI_ISL_990142, EPI_ISL_990143, EPI_ISL_990144, EPI_ISL_990145, EPI_ISL_990146, EPI_ISL_990147, EPI_ISL_990148, EPI_ISL_990149, EPI_ISL_990150, EPI_ISL_990151, EPI_ISL_990152, EPI_ISL_990153, EPI_ISL_990154, EPI_ISL_990155, EPI_ISL_990156, EPI_ISL_990157, EPI_ISL_990158, EPI_ISL_990159, EPI_ISL_990160, EPI_ISL_990161, EPI_ISL_990162, EPI_ISL_990163, EPI_ISL_990164, EPI_ISL_990165, EPI_ISL_990166, EPI_ISL_990167, EPI_ISL_990168, EPI_ISL_990169, EPI_ISL_990170, EPI_ISL_990171, EPI_ISL_990172, EPI_ISL_990173, EPI_ISL_990174, EPI_ISL_990175, EPI_ISL_990176, EPI_ISL_990177, EPI_ISL_990178, EPI_ISL_990179, EPI_ISL_990180, EPI_ISL_990181, EPI_ISL_990182, EPI_ISL_990183, EPI_ISL_990184, EPI_ISL_990185, EPI_ISL_990186, EPI_ISL_990187, EPI_ISL_990188, EPI_ISL_990189, EPI_ISL_990190, EPI_ISL_990191, EPI_ISL_990192, EPI_ISL_990193, EPI_ISL_990194, EPI_ISL_990195, EPI_ISL_990196, EPI_ISL_990197, EPI_ISL_990199, EPI_ISL_990200, EPI_ISL_990201, EPI_ISL_990202, EPI_ISL_990203, EPI_ISL_990204, EPI_ISL_990205, EPI_ISL_990206, EPI_ISL_990207, EPI_ISL_990208, EPI_ISL_990209, EPI_ISL_990210, EPI_ISL_990211, EPI_ISL_990212, EPI_ISL_990213, EPI_ISL_990214, EPI_ISL_990215, EPI_ISL_990216, EPI_ISL_990217, EPI_ISL_990218, EPI_ISL_990219, EPI_ISL_990220, EPI_ISL_990221, EPI_ISL_990222, EPI_ISL_990223, EPI_ISL_990224, EPI_ISL_990225, EPI_ISL_990226, EPI_ISL_990227, EPI_ISL_990228, EPI_ISL_990229, EPI_ISL_990230, EPI_ISL_990231, EPI_ISL_990232, EPI_ISL_990233, EPI_ISL_990234, EPI_ISL_990235, EPI_ISL_990236, EPI_ISL_990237, EPI_ISL_990238, EPI_ISL_990239, EPI_ISL_990240, EPI_ISL_990241, EPI_ISL_990242, EPI_ISL_990243, EPI_ISL_990244, EPI_ISL_990245, EPI_ISL_990246, EPI_ISL_990247, EPI_ISL_990248, EPI_ISL_990249, EPI_ISL_990250, EPI_ISL_990251, EPI_ISL_990252, EPI_ISL_990253, EPI_ISL_990254, EPI_ISL_990255, EPI_ISL_990256, EPI_ISL_990257, EPI_ISL_990258, EPI_ISL_990259, EPI_ISL_990260, EPI_ISL_990261, EPI_ISL_990262, EPI_ISL_990263, EPI_ISL_990264, EPI_ISL_990265, EPI_ISL_990266, EPI_ISL_990267, EPI_ISL_990268, EPI_ISL_990269, EPI_ISL_990270, EPI_ISL_990271, EPI_ISL_990272, EPI_ISL_990273, EPI_ISL_990274, EPI_ISL_990275, EPI_ISL_990276, EPI_ISL_990277, EPI_ISL_990278, EPI_ISL_990279, EPI_ISL_990280, EPI_ISL_990281, EPI_ISL_990282, EPI_ISL_990283, EPI_ISL_990284, EPI_ISL_990285, EPI_ISL_990287, EPI_ISL_990288, EPI_ISL_990289, EPI_ISL_990290, EPI_ISL_990291, EPI_ISL_990292, EPI_ISL_990293, EPI_ISL_990294, EPI_ISL_990295, EPI_ISL_990296, EPI_ISL_990297, EPI_ISL_990298, EPI_ISL_990299, EPI_ISL_990300, EPI_ISL_990301, EPI_ISL_990302, EPI_ISL_990303, EPI_ISL_990304, EPI_ISL_990305, EPI_ISL_990306, EPI_ISL_990307, EPI_ISL_990308, EPI_ISL_990309, EPI_ISL_990310, EPI_ISL_990311, EPI_ISL_990312, EPI_ISL_990313, EPI_ISL_990314, EPI_ISL_990315, EPI_ISL_990316, EPI_ISL_990317, EPI_ISL_990318, EPI_ISL_990319, EPI_ISL_990320, EPI_ISL_990321, EPI_ISL_990322, EPI_ISL_990323, EPI_ISL_990324, EPI_ISL_990325, EPI_ISL_990326, EPI_ISL_990327, EPI_ISL_990328, EPI_ISL_990329, EPI_ISL_990330, EPI_ISL_990331, EPI_ISL_990332, EPI_ISL_990333, EPI_ISL_990334, EPI_ISL_990335, EPI_ISL_990336, EPI_ISL_990337, EPI_ISL_990338, EPI_ISL_990339, EPI_ISL_990340, EPI_ISL_990341, EPI_ISL_990342, EPI_ISL_990343, EPI_ISL_990344, EPI_ISL_990345, EPI_ISL_990346, EPI_ISL_990347, EPI_ISL_990348, EPI_ISL_990349, EPI_ISL_990350, EPI_ISL_990351, EPI_ISL_990352, EPI_ISL_990353, EPI_ISL_990354, EPI_ISL_990355, EPI_ISL_990356, EPI_ISL_990357, EPI_ISL_990358, EPI_ISL_990359, EPI_ISL_990360, EPI_ISL_990361, EPI_ISL_990362, EPI_ISL_990363, EPI_ISL_990364, EPI_ISL_990365, EPI_ISL_990366, EPI_ISL_990367, EPI_ISL_990368, EPI_ISL_990369, EPI_ISL_990370, EPI_ISL_990371, EPI_ISL_990372, EPI_ISL_990373, EPI_ISL_990374, EPI_ISL_990375, EPI_ISL_990376, EPI_ISL_990377, EPI_ISL_990378, EPI_ISL_990379, EPI_ISL_990380, EPI_ISL_990381, EPI_ISL_990382, EPI_ISL_990383, EPI_ISL_990384, EPI_ISL_990385, EPI_ISL_990386, EPI_ISL_990387, EPI_ISL_990388, EPI_ISL_990389, EPI_ISL_990390, EPI_ISL_990391, EPI_ISL_990392, EPI_ISL_990393, EPI_ISL_990394, EPI_ISL_990395, EPI_ISL_990396, EPI_ISL_990397, EPI_ISL_990398, EPI_ISL_990399, EPI_ISL_990400, EPI_ISL_990401, EPI_ISL_990402, EPI_ISL_990403, EPI_ISL_990404, EPI_ISL_990405, EPI_ISL_990406, EPI_ISL_990407, EPI_ISL_990408, EPI_ISL_990409, EPI_ISL_990410, EPI_ISL_990411, EPI_ISL_990412, EPI_ISL_990413, EPI_ISL_990414, EPI_ISL_990415, EPI_ISL_990416, EPI_ISL_990417, EPI_ISL_990418, EPI_ISL_990419, EPI_ISL_990420, EPI_ISL_990421, EPI_ISL_990422, EPI_ISL_990423, EPI_ISL_990424, EPI_ISL_990425, EPI_ISL_990426, EPI_ISL_990427, EPI_ISL_990428, EPI_ISL_990429, EPI_ISL_990430, EPI_ISL_990431, EPI_ISL_990432, EPI_ISL_990433, EPI_ISL_990434, EPI_ISL_990436, EPI_ISL_990437, EPI_ISL_990438, EPI_ISL_990439, EPI_ISL_990440, EPI_ISL_990441, EPI_ISL_990442, EPI_ISL_990443, EPI_ISL_990444, EPI_ISL_990445, EPI_ISL_990446, EPI_ISL_990447, EPI_ISL_990448, EPI_ISL_990449, EPI_ISL_990450, EPI_ISL_990451, EPI_ISL_990452, EPI_ISL_990453, EPI_ISL_990454, EPI_ISL_990455, EPI_ISL_990456, EPI_ISL_990457, EPI_ISL_990458, EPI_ISL_990459, EPI_ISL_990460, EPI_ISL_990461, EPI_ISL_990462, EPI_ISL_990463, EPI_ISL_990464, EPI_ISL_990465, EPI_ISL_990466, EPI_ISL_990467, EPI_ISL_990468, EPI_ISL_990469, EPI_ISL_990470, EPI_ISL_990471, EPI_ISL_990472, EPI_ISL_990473, EPI_ISL_990474, EPI_ISL_990475, EPI_ISL_990476, EPI_ISL_990477, EPI_ISL_990478, EPI_ISL_990479, EPI_ISL_990480, EPI_ISL_990481, EPI_ISL_990482, EPI_ISL_990483, EPI_ISL_990484, EPI_ISL_990485, EPI_ISL_990486, EPI_ISL_990487, EPI_ISL_990488, EPI_ISL_990489, EPI_ISL_990490, EPI_ISL_990491, EPI_ISL_990492, EPI_ISL_990493, EPI_ISL_990494, EPI_ISL_990495, EPI_ISL_990496, EPI_ISL_990497, EPI_ISL_990498, EPI_ISL_990499, EPI_ISL_990501, EPI_ISL_990503, EPI_ISL_990504, EPI_ISL_990505, EPI_ISL_990506, EPI_ISL_990507, EPI_ISL_990508, EPI_ISL_990509, EPI_ISL_990511, EPI_ISL_990512, EPI_ISL_990513, EPI_ISL_990514, EPI_ISL_990515, EPI_ISL_990516, EPI_ISL_990517, EPI_ISL_990518, EPI_ISL_990519, EPI_ISL_990520, EPI_ISL_990521, EPI_ISL_990522, EPI_ISL_990523, EPI_ISL_990524, EPI_ISL_990525, EPI_ISL_990526, EPI_ISL_990527, EPI_ISL_990528, EPI_ISL_990529, EPI_ISL_990530, EPI_ISL_990531, EPI_ISL_990532, EPI_ISL_990533, EPI_ISL_990534, EPI_ISL_990535, EPI_ISL_990536, EPI_ISL_990537, EPI_ISL_990538, EPI_ISL_990539, EPI_ISL_990540, EPI_ISL_990541, EPI_ISL_990542, EPI_ISL_990543, EPI_ISL_990544, EPI_ISL_990546, EPI_ISL_990547, EPI_ISL_990548, EPI_ISL_990549, EPI_ISL_990550, EPI_ISL_990551, EPI_ISL_990552, EPI_ISL_990553, EPI_ISL_990554, EPI_ISL_990555, EPI_ISL_990556, EPI_ISL_990558, EPI_ISL_990559, EPI_ISL_990560, EPI_ISL_990561, EPI_ISL_990562, EPI_ISL_990563, EPI_ISL_990564, EPI_ISL_990565, EPI_ISL_990566, EPI_ISL_990567, EPI_ISL_990568, EPI_ISL_990569, EPI_ISL_990570, EPI_ISL_990572, EPI_ISL_990573, EPI_ISL_990574, EPI_ISL_990575, EPI_ISL_990577, EPI_ISL_990578, EPI_ISL_990580, EPI_ISL_990581, EPI_ISL_990582, EPI_ISL_990583, EPI_ISL_990584, EPI_ISL_990585, EPI_ISL_990586, EPI_ISL_990587, EPI_ISL_990588, EPI_ISL_990589, EPI_ISL_990590, EPI_ISL_990591, EPI_ISL_990592, EPI_ISL_990593, EPI_ISL_990594, EPI_ISL_990595, EPI_ISL_990596, EPI_ISL_990597, EPI_ISL_990598, EPI_ISL_990599, EPI_ISL_990600, EPI_ISL_990601, EPI_ISL_990602, EPI_ISL_990603, EPI_ISL_990604, EPI_ISL_990605, EPI_ISL_990606, EPI_ISL_990607, EPI_ISL_990608, EPI_ISL_990609, EPI_ISL_990610, EPI_ISL_990611, EPI_ISL_990612, EPI_ISL_990613, EPI_ISL_990614, EPI_ISL_990615, EPI_ISL_990617, EPI_ISL_990619, EPI_ISL_990620, EPI_ISL_990621, EPI_ISL_990622, EPI_ISL_990623, EPI_ISL_990624, EPI_ISL_990625, EPI_ISL_990626, EPI_ISL_990627, EPI_ISL_990628, EPI_ISL_990629, EPI_ISL_990630, EPI_ISL_990631, EPI_ISL_990632, EPI_ISL_990633, EPI_ISL_990634, EPI_ISL_990635, EPI_ISL_990636, EPI_ISL_990637, EPI_ISL_990638, EPI_ISL_990639, EPI_ISL_990640, EPI_ISL_990641, EPI_ISL_990642, EPI_ISL_990643, EPI_ISL_990644, EPI_ISL_990645, EPI_ISL_990646, EPI_ISL_990647, EPI_ISL_990648, EPI_ISL_990649, EPI_ISL_990650, EPI_ISL_990651, EPI_ISL_990653, EPI_ISL_990654, EPI_ISL_990655, EPI_ISL_990656, EPI_ISL_990657, EPI_ISL_990659, EPI_ISL_990660, EPI_ISL_990661, EPI_ISL_990663, EPI_ISL_990665, EPI_ISL_990666, EPI_ISL_990667, EPI_ISL_990668, EPI_ISL_990669, EPI_ISL_990670, EPI_ISL_990671, EPI_ISL_990672, EPI_ISL_990673, EPI_ISL_990674, EPI_ISL_990675, EPI_ISL_990676, EPI_ISL_990677, EPI_ISL_990678, EPI_ISL_990679, EPI_ISL_990681, EPI_ISL_990682, EPI_ISL_990683, EPI_ISL_990684, EPI_ISL_990685, EPI_ISL_990686, EPI_ISL_990687, EPI_ISL_990688, EPI_ISL_990689, EPI_ISL_990690, EPI_ISL_990691, EPI_ISL_990692, EPI_ISL_990693, EPI_ISL_990694, EPI_ISL_990695, EPI_ISL_990696, EPI_ISL_990697, EPI_ISL_990698, EPI_ISL_990699, EPI_ISL_990700, EPI_ISL_990701, EPI_ISL_990702, EPI_ISL_990703, EPI_ISL_990704, EPI_ISL_990705, EPI_ISL_990706, EPI_ISL_990707, EPI_ISL_990708, EPI_ISL_990709, EPI_ISL_990710, EPI_ISL_990711, EPI_ISL_990713, EPI_ISL_990714, EPI_ISL_990715, EPI_ISL_990716, EPI_ISL_990717, EPI_ISL_990718, EPI_ISL_990719, EPI_ISL_990720, EPI_ISL_990721, EPI_ISL_990722, EPI_ISL_990723, EPI_ISL_990724, EPI_ISL_990725, EPI_ISL_990727, EPI_ISL_990728, EPI_ISL_990729, EPI_ISL_990730, EPI_ISL_990731, EPI_ISL_990732, EPI_ISL_990733, EPI_ISL_990734, EPI_ISL_990735, EPI_ISL_990736, EPI_ISL_990737, EPI_ISL_990738, EPI_ISL_990739, EPI_ISL_990740, EPI_ISL_990741, EPI_ISL_990742, EPI_ISL_990743, EPI_ISL_990744, EPI_ISL_990746, EPI_ISL_990747, EPI_ISL_990748, EPI_ISL_990749, EPI_ISL_990750, EPI_ISL_990751, EPI_ISL_990752, EPI_ISL_990753, EPI_ISL_990754, EPI_ISL_990755, EPI_ISL_990756, EPI_ISL_990757, EPI_ISL_990758, EPI_ISL_990759, EPI_ISL_990760, EPI_ISL_990761, EPI_ISL_990762, EPI_ISL_990763, EPI_ISL_990764, EPI_ISL_990765, EPI_ISL_990766, EPI_ISL_990767, EPI_ISL_990768, EPI_ISL_990769, EPI_ISL_990770, EPI_ISL_990771, EPI_ISL_990772, EPI_ISL_990773, EPI_ISL_990774, EPI_ISL_990775, EPI_ISL_990776, EPI_ISL_990777, EPI_ISL_990778, EPI_ISL_990780, EPI_ISL_990781, EPI_ISL_990782, EPI_ISL_990783, EPI_ISL_990784, EPI_ISL_990785, EPI_ISL_990786, EPI_ISL_990787, EPI_ISL_990788, EPI_ISL_990789, EPI_ISL_990790, EPI_ISL_990791, EPI_ISL_990792, EPI_ISL_990793, EPI_ISL_990794, EPI_ISL_990795, EPI_ISL_990796, EPI_ISL_990797, EPI_ISL_990798, EPI_ISL_990799, EPI_ISL_990800, EPI_ISL_990801, EPI_ISL_990802, EPI_ISL_990803, EPI_ISL_990804, EPI_ISL_990805, EPI_ISL_990806, EPI_ISL_990807, EPI_ISL_990808, EPI_ISL_990809, EPI_ISL_990810, EPI_ISL_990811, EPI_ISL_990812, EPI_ISL_990813, EPI_ISL_990814, EPI_ISL_990815, EPI_ISL_990816, EPI_ISL_990817, EPI_ISL_990818, EPI_ISL_990819, EPI_ISL_990820, EPI_ISL_990821, EPI_ISL_990822, EPI_ISL_990823, EPI_ISL_990824, EPI_ISL_990825, EPI_ISL_990826, EPI_ISL_990827, EPI_ISL_990828, EPI_ISL_990829, EPI_ISL_990830, EPI_ISL_990831, EPI_ISL_990832, EPI_ISL_990833, EPI_ISL_990834, EPI_ISL_990835, EPI_ISL_990836, EPI_ISL_990837, EPI_ISL_990838, EPI_ISL_990839, EPI_ISL_990840, EPI_ISL_990841, EPI_ISL_990842, EPI_ISL_990843, EPI_ISL_990844, EPI_ISL_990845, EPI_ISL_990846, EPI_ISL_990847, EPI_ISL_990848, EPI_ISL_990849, EPI_ISL_990850, EPI_ISL_990851, EPI_ISL_990852, EPI_ISL_990853, EPI_ISL_990854, EPI_ISL_990855, EPI_ISL_990856, EPI_ISL_990857, EPI_ISL_990858, EPI_ISL_990859, EPI_ISL_990860, EPI_ISL_990861, EPI_ISL_990862, EPI_ISL_990863, EPI_ISL_990864, EPI_ISL_990865, EPI_ISL_990866, EPI_ISL_990867, EPI_ISL_990868, EPI_ISL_990869, EPI_ISL_990870, EPI_ISL_990871, EPI_ISL_990872, EPI_ISL_990873, EPI_ISL_990874, EPI_ISL_990875, EPI_ISL_990876, EPI_ISL_990877, EPI_ISL_990878, EPI_ISL_990879, EPI_ISL_990880, EPI_ISL_990882, EPI_ISL_990883, EPI_ISL_990884, EPI_ISL_990885, EPI_ISL_990886, EPI_ISL_990887, EPI_ISL_990888, EPI_ISL_990889, EPI_ISL_990890, EPI_ISL_990891, EPI_ISL_990892, EPI_ISL_990893, EPI_ISL_990894, EPI_ISL_990895, EPI_ISL_990896, EPI_ISL_990897, EPI_ISL_990898, EPI_ISL_990899, EPI_ISL_990900, EPI_ISL_990901, EPI_ISL_990902, EPI_ISL_990903, EPI_ISL_990904, EPI_ISL_990905, EPI_ISL_990906, EPI_ISL_990907, EPI_ISL_990908, EPI_ISL_990909, EPI_ISL_990910, EPI_ISL_990911, EPI_ISL_990912, EPI_ISL_990913, EPI_ISL_990914, EPI_ISL_990915, EPI_ISL_990916, EPI_ISL_990917, EPI_ISL_990918, EPI_ISL_990919, EPI_ISL_990920, EPI_ISL_990921, EPI_ISL_990922, EPI_ISL_990923, EPI_ISL_990924, EPI_ISL_990925, EPI_ISL_990926, EPI_ISL_990927, EPI_ISL_990928, EPI_ISL_990929, EPI_ISL_990930, EPI_ISL_990931, EPI_ISL_990932, EPI_ISL_990933, EPI_ISL_990934, EPI_ISL_990935, EPI_ISL_990936, EPI_ISL_990937, EPI_ISL_990938, EPI_ISL_990939, EPI_ISL_990940, EPI_ISL_990941, EPI_ISL_990942, EPI_ISL_990943, EPI_ISL_990944, EPI_ISL_990945, EPI_ISL_990946, EPI_ISL_990947, EPI_ISL_990948, EPI_ISL_990949, EPI_ISL_990950, EPI_ISL_990951, EPI_ISL_990952, EPI_ISL_990953, EPI_ISL_990954, EPI_ISL_990955,			

EPI_ISL_990956, EPI_ISL_990957, EPI_ISL_990958, EPI_ISL_990959, EPI_ISL_990960, EPI_ISL_990961, EPI_ISL_990962, EPI_ISL_990963, EPI_ISL_990964, EPI_ISL_990965, EPI_ISL_990966, EPI_ISL_990967, EPI_ISL_990968, EPI_ISL_990969, EPI_ISL_990970, EPI_ISL_990971, EPI_ISL_990972, EPI_ISL_990973, EPI_ISL_990974, EPI_ISL_990975, EPI_ISL_990976, EPI_ISL_990977, EPI_ISL_990978, EPI_ISL_990979, EPI_ISL_990980, EPI_ISL_990981, EPI_ISL_990982, EPI_ISL_990983, EPI_ISL_990984, EPI_ISL_990985, EPI_ISL_990986, EPI_ISL_990987, EPI_ISL_990988, EPI_ISL_990989, EPI_ISL_990990, EPI_ISL_990991, EPI_ISL_990992, EPI_ISL_990993, EPI_ISL_990994, EPI_ISL_990995, EPI_ISL_990996, EPI_ISL_990997, EPI_ISL_990998, EPI_ISL_990999, EPI_ISL_991000, EPI_ISL_991001, EPI_ISL_991002, EPI_ISL_991003, EPI_ISL_991004, EPI_ISL_991005, EPI_ISL_991006, EPI_ISL_991007, EPI_ISL_991008, EPI_ISL_991009, EPI_ISL_991010, EPI_ISL_991011, EPI_ISL_991012, EPI_ISL_991013, EPI_ISL_991014, EPI_ISL_991015, EPI_ISL_991016, EPI_ISL_991017, EPI_ISL_991018, EPI_ISL_991019, EPI_ISL_991020, EPI_ISL_991021, EPI_ISL_991022, EPI_ISL_991023, EPI_ISL_991024, EPI_ISL_991025, EPI_ISL_991026, EPI_ISL_991027, EPI_ISL_991028, EPI_ISL_991029, EPI_ISL_991030, EPI_ISL_991031, EPI_ISL_991032, EPI_ISL_991033, EPI_ISL_991034, EPI_ISL_991035, EPI_ISL_991036, EPI_ISL_991037, EPI_ISL_991038, EPI_ISL_991039, EPI_ISL_991040, EPI_ISL_991041, EPI_ISL_991042, EPI_ISL_991043, EPI_ISL_991044, EPI_ISL_991045, EPI_ISL_991046, EPI_ISL_991047, EPI_ISL_991048, EPI_ISL_991049, EPI_ISL_991050, EPI_ISL_991051, EPI_ISL_991052, EPI_ISL_991053, EPI_ISL_991054, EPI_ISL_991055, EPI_ISL_991056, EPI_ISL_991057, EPI_ISL_991058, EPI_ISL_991059, EPI_ISL_991060, EPI_ISL_991061, EPI_ISL_991062, EPI_ISL_991063, EPI_ISL_991064, EPI_ISL_991065, EPI_ISL_991066, EPI_ISL_991067, EPI_ISL_991068, EPI_ISL_991069, EPI_ISL_991070, EPI_ISL_991071, EPI_ISL_991072, EPI_ISL_991073, EPI_ISL_991074, EPI_ISL_991075, EPI_ISL_991076, EPI_ISL_991077, EPI_ISL_991078, EPI_ISL_991079, EPI_ISL_991080, EPI_ISL_991081, EPI_ISL_991082, EPI_ISL_991083, EPI_ISL_991084, EPI_ISL_991085, EPI_ISL_991086, EPI_ISL_991087, EPI_ISL_991088, EPI_ISL_991089, EPI_ISL_991090, EPI_ISL_991091, EPI_ISL_991092, EPI_ISL_991093, EPI_ISL_991094, EPI_ISL_991095, EPI_ISL_991096, EPI_ISL_991097, EPI_ISL_991098, EPI_ISL_991099, EPI_ISL_991100, EPI_ISL_991101, EPI_ISL_991102, EPI_ISL_991103, EPI_ISL_991104, EPI_ISL_991105, EPI_ISL_991106, EPI_ISL_991107, EPI_ISL_991108, EPI_ISL_991109, EPI_ISL_991110, EPI_ISL_991111, EPI_ISL_991112, EPI_ISL_991113, EPI_ISL_991114, EPI_ISL_991115, EPI_ISL_991116, EPI_ISL_991117, EPI_ISL_991118, EPI_ISL_991119, EPI_ISL_991120, EPI_ISL_991121, EPI_ISL_991122, EPI_ISL_991123, EPI_ISL_991124, EPI_ISL_991125, EPI_ISL_991126, EPI_ISL_991127, EPI_ISL_991128, EPI_ISL_991129, EPI_ISL_991130, EPI_ISL_991131, EPI_ISL_991132, EPI_ISL_991133, EPI_ISL_991134, EPI_ISL_991135, EPI_ISL_991136, EPI_ISL_991137, EPI_ISL_991138, EPI_ISL_991139, EPI_ISL_991140, EPI_ISL_991141, EPI_ISL_991142, EPI_ISL_991143, EPI_ISL_991144, EPI_ISL_991145, EPI_ISL_991146, EPI_ISL_991147, EPI_ISL_991148, EPI_ISL_991149, EPI_ISL_991150, EPI_ISL_991151, EPI_ISL_991152, EPI_ISL_991153, EPI_ISL_991154, EPI_ISL_991155, EPI_ISL_991156, EPI_ISL_991157, EPI_ISL_991158, EPI_ISL_991159, EPI_ISL_991160, EPI_ISL_991161, EPI_ISL_991162, EPI_ISL_991163, EPI_ISL_991164, EPI_ISL_991165, EPI_ISL_991166, EPI_ISL_991167, EPI_ISL_991168, EPI_ISL_991169, EPI_ISL_991170, EPI_ISL_991171, EPI_ISL_991172, EPI_ISL_991222, EPI_ISL_991223, EPI_ISL_991224, EPI_ISL_991225, EPI_ISL_991226, EPI_ISL_991227, EPI_ISL_991228, EPI_ISL_991229, EPI_ISL_991230, EPI_ISL_991231, EPI_ISL_991232, EPI_ISL_991233, EPI_ISL_991234, EPI_ISL_991235, EPI_ISL_991236, EPI_ISL_991237, EPI_ISL_991238, EPI_ISL_991239, EPI_ISL_991240, EPI_ISL_991241, EPI_ISL_991242, EPI_ISL_991243, EPI_ISL_991244, EPI_ISL_991245, EPI_ISL_991246, EPI_ISL_991247, EPI_ISL_991248, EPI_ISL_991249, EPI_ISL_991250, EPI_ISL_991251, EPI_ISL_991252, EPI_ISL_991253, EPI_ISL_991254, EPI_ISL_991255, EPI_ISL_991256, EPI_ISL_991257, EPI_ISL_991258, EPI_ISL_991259, EPI_ISL_991260, EPI_ISL_991261, EPI_ISL_991262, EPI_ISL_991263, EPI_ISL_991264, EPI_ISL_991265, EPI_ISL_991266, EPI_ISL_991267, EPI_ISL_991268, EPI_ISL_991270, EPI_ISL_991271, EPI_ISL_991273, EPI_ISL_991275, EPI_ISL_991276, EPI_ISL_991277, EPI_ISL_991278, EPI_ISL_991279, EPI_ISL_991280, EPI_ISL_991281, EPI_ISL_991282, EPI_ISL_991283, EPI_ISL_991284, EPI_ISL_991285, EPI_ISL_991286, EPI_ISL_991287, EPI_ISL_991288, EPI_ISL_991289, EPI_ISL_991290, EPI_ISL_991296, EPI_ISL_991297, EPI_ISL_991298, EPI_ISL_991299, EPI_ISL_991301, EPI_ISL_991303, EPI_ISL_991304, EPI_ISL_991305, EPI_ISL_991307, EPI_ISL_991308			
see above	Lighthouse Lab in Alderley Park	Wellcome Sanger Institute for the COVID-19 Genomics UK (COG-UK) Consortium	Jacquelyn Wynn, Mairead Hyland, The Lighthouse Lab in Alderley Park and Alex Alderton, Roberto Amato, Sonia Goncalves, Ewan Harrison, David K. Jackson, Ian Johnston, Dominic Kwiatkowski, Cordelia Langford, John Sillitoe on behalf of the Wellcome Sanger Institute COVID-19 Surveillance Team
EPI_ISL_991352, EPI_ISL_991353, EPI_ISL_991356, EPI_ISL_991357, EPI_ISL_991360, EPI_ISL_991362, EPI_ISL_991363, EPI_ISL_991364, EPI_ISL_991365, EPI_ISL_991369, EPI_ISL_991372, EPI_ISL_991373, EPI_ISL_991374, EPI_ISL_991375, EPI_ISL_991376, EPI_ISL_991378, EPI_ISL_991384, EPI_ISL_991388, EPI_ISL_991390, EPI_ISL_991391, EPI_ISL_991393, EPI_ISL_991396, EPI_ISL_991401, EPI_ISL_991402, EPI_ISL_991404, EPI_ISL_991406, EPI_ISL_991407, EPI_ISL_991408, EPI_ISL_991410, EPI_ISL_991411, EPI_ISL_991413, EPI_ISL_991414, EPI_ISL_991416, EPI_ISL_991420, EPI_ISL_991421, EPI_ISL_991424, EPI_ISL_991426, EPI_ISL_991427, EPI_ISL_991429, EPI_ISL_991430, EPI_ISL_991431, EPI_ISL_991433, EPI_ISL_991436, EPI_ISL_991438, EPI_ISL_991439, EPI_ISL_991441, EPI_ISL_991442, EPI_ISL_991444, EPI_ISL_991445, EPI_ISL_991449, EPI_ISL_991452, EPI_ISL_991456, EPI_ISL_991457, EPI_ISL_991459, EPI_ISL_991461, EPI_ISL_991462, EPI_ISL_991466, EPI_ISL_991468, EPI_ISL_991469, EPI_ISL_991471, EPI_ISL_991472, EPI_ISL_991473, EPI_ISL_991474, EPI_ISL_991480, EPI_ISL_991484, EPI_ISL_991485, EPI_ISL_991487, EPI_ISL_991488, EPI_ISL_991490, EPI_ISL_991492, EPI_ISL_991495, EPI_ISL_991497, EPI_ISL_991504, EPI_ISL_991505, EPI_ISL_991507, EPI_ISL_991508, EPI_ISL_991509, EPI_ISL_991512, EPI_ISL_991513, EPI_ISL_991514, EPI_ISL_991516, EPI_ISL_991518, EPI_ISL_991519, EPI_ISL_991520, EPI_ISL_991521, EPI_ISL_991522, EPI_ISL_991524, EPI_ISL_991526, EPI_ISL_991529, EPI_ISL_991535, EPI_ISL_991538, EPI_ISL_991539, EPI_ISL_991542, EPI_ISL_991545, EPI_ISL_991550, EPI_ISL_991552, EPI_ISL_991554, EPI_ISL_991555, EPI_ISL_991557, EPI_ISL_991559, EPI_ISL_991560, EPI_ISL_991562, EPI_ISL_991563, EPI_ISL_991564			
see above	Lighthouse Lab in Glasgow	Wellcome Sanger Institute for the COVID-19 Genomics UK (COG-UK) Consortium	Harper VanSteenhouse, Yumi Kasai, David Gray, Carol Clugston, Anna Dominiczak and Alex Alderton, Roberto Amato, Sonia Goncalves, Ewan Harrison, David K. Jackson, Ian Johnston, Dominic Kwiatkowski, Cordelia Langford, John Sillitoe on behalf of the Wellcome Sanger Institute COVID-19 Surveillance Team
EPI_ISL_991565, EPI_ISL_991595, EPI_ISL_991629, EPI_ISL_991637, EPI_ISL_991653, EPI_ISL_991680, EPI_ISL_991692, EPI_ISL_991694, EPI_ISL_991703, EPI_ISL_991715, EPI_ISL_991755, EPI_ISL_991781, EPI_ISL_991793, EPI_ISL_991817, EPI_ISL_991826, EPI_ISL_991834, EPI_ISL_991846, EPI_ISL_991848, EPI_ISL_991860, EPI_ISL_991873, EPI_ISL_991900			
see above	Lighthouse Lab in Alderley Park	Wellcome Sanger Institute for the COVID-19 Genomics UK (COG-UK) Consortium	Jacquelyn Wynn, Mairead Hyland, The Lighthouse Lab in Alderley Park and Alex Alderton, Roberto Amato, Sonia Goncalves, Ewan Harrison, David K. Jackson, Ian Johnston, Dominic Kwiatkowski, Cordelia Langford, John Sillitoe on behalf of the Wellcome Sanger Institute COVID-19 Surveillance Team
EPI_ISL_993004	Lighthouse Lab in Alderley Park	Wellcome Sanger Institute for the COVID-19 Genomics UK (COG-UK) Consortium	Jacquelyn Wynn, Mairead Hyland, The Lighthouse Lab in Alderley Park and Alex Alderton, Roberto Amato, Sonia Goncalves, Ewan Harrison, David K. Jackson, Ian Johnston, Dominic Kwiatkowski, Cordelia Langford, John Sillitoe on behalf of the Wellcome Sanger Institute COVID-19 Surveillance Team (http://www.sanger.ac.uk/covid-team)
EPI_ISL_996401, EPI_ISL_996408, EPI_ISL_996412, EPI_ISL_996456, EPI_ISL_996458, EPI_ISL_996462, EPI_ISL_996463, EPI_ISL_996469	University of Birmingham	COVID-19 Genomics UK (COG-UK) Consortium	Institute of Microbiology, University of Birmingham: Claire McMurray, Joanne Stockton, Samuel Nicholls, Radoslaw Poplawski, Will Rowe, Josh Quick, Nicholas Loman. University of Birmingham Testing Laboratory: Celina M Whalley, Andrew Bosworth, Charlotte Poxon, Kasun Wanigasooriya, Oliver Pickles, Mike Kidd, Alex Richter, Andrew D Beggs PHE Heartlands Lab: Husam Osman, Andrew Bosworth. Queen Elizabeth Hospital: Anna Casey
EPI_ISL_996543, EPI_ISL_996544, EPI_ISL_996545, EPI_ISL_996546, EPI_ISL_996551, EPI_ISL_996552, EPI_ISL_996557, EPI_ISL_996595, EPI_ISL_996597	University of Exeter	COVID-19 Genomics UK (COG-UK) Consortium	Ben Temperton,Aaron Jeffries,Michelle Michelsen,Joanna Warwick-Dugdale,Audrey Farbos,Robyn Manley,Stephen Michell,Jane Masoli
EPI_ISL_996673, EPI_ISL_996675, EPI_ISL_996677, EPI_ISL_996679, EPI_ISL_996687, EPI_ISL_996691, EPI_ISL_996693, EPI_ISL_996697, EPI_ISL_996699, EPI_ISL_996703, EPI_ISL_996705, EPI_ISL_996709, EPI_ISL_996713, EPI_ISL_996715, EPI_ISL_996717, EPI_ISL_996720, EPI_ISL_996722, EPI_ISL_996732, EPI_ISL_996734, EPI_ISL_996736, EPI_ISL_996865, EPI_ISL_996866, EPI_ISL_996867, EPI_ISL_996868, EPI_ISL_996869, EPI_ISL_996870, EPI_ISL_996871, EPI_ISL_996872, EPI_ISL_996873, EPI_ISL_996874, EPI_ISL_996875, EPI_ISL_996876, EPI_ISL_996877, EPI_ISL_996878, EPI_ISL_996879, EPI_ISL_996880, EPI_ISL_996881, EPI_ISL_996882, EPI_ISL_996883, EPI_ISL_996884, EPI_ISL_996885, EPI_ISL_996886, EPI_ISL_996887, EPI_ISL_996888, EPI_ISL_996889, EPI_ISL_996890, EPI_ISL_996891, EPI_ISL_996892, EPI_ISL_996894, EPI_ISL_996895, EPI_ISL_996896, EPI_ISL_996897, EPI_ISL_996898, EPI_ISL_996899, EPI_ISL_996900, EPI_ISL_996901, EPI_ISL_996902, EPI_ISL_996903, EPI_ISL_996904, EPI_ISL_996905, EPI_ISL_996906, EPI_ISL_996907, EPI_ISL_996908, EPI_ISL_996909, EPI_ISL_996910, EPI_ISL_996911, EPI_ISL_996912, EPI_ISL_996913, EPI_ISL_996914, EPI_ISL_996915, EPI_ISL_996916, EPI_ISL_996917, EPI_ISL_996918, EPI_ISL_996919, EPI_ISL_996920, EPI_ISL_996921, EPI_ISL_996922, EPI_ISL_996923, EPI_ISL_996924, EPI_ISL_996925, EPI_ISL_996926, EPI_ISL_996927, EPI_ISL_996928, EPI_ISL_996929, EPI_ISL_996930, EPI_ISL_996931, EPI_ISL_996932, EPI_ISL_996933, EPI_ISL_996934, EPI_ISL_996935, EPI_ISL_996936, EPI_ISL_996937, EPI_ISL_996938, EPI_ISL_996939, EPI_ISL_996940, EPI_ISL_996941, EPI_ISL_996942, EPI_ISL_996943, EPI_ISL_996944, EPI_ISL_996945, EPI_ISL_996946, EPI_ISL_996947, EPI_ISL_996948, EPI_ISL_996949, EPI_ISL_996950, EPI_ISL_996951, EPI_ISL_996952, EPI_ISL_996954, EPI_ISL_996955, EPI_ISL_996956, EPI_ISL_996957, EPI_ISL_996958, EPI_ISL_996959, EPI_ISL_996960, EPI_ISL_996961, EPI_ISL_996962, EPI_ISL_996963, EPI_ISL_996964, EPI_ISL_996965, EPI_ISL_996966, EPI_ISL_996967, EPI_ISL_996968, EPI_ISL_996969, EPI_ISL_996970, EPI_ISL_996971, EPI_ISL_996972, EPI_ISL_996973, EPI_ISL_996974, EPI_ISL_996975, EPI_ISL_996976, EPI_ISL_996977, EPI_ISL_996978, EPI_ISL_996979, EPI_ISL_996980, EPI_ISL_996982, EPI_ISL_996983, EPI_ISL_996984, EPI_ISL_996985, EPI_ISL_996986, EPI_ISL_996987, EPI_ISL_996988, EPI_ISL_996989, EPI_ISL_996990, EPI_ISL_996991, EPI_ISL_996992, EPI_ISL_996993, EPI_ISL_996994, EPI_ISL_996995, EPI_ISL_996996, EPI_ISL_996997, EPI_ISL_996998, EPI_ISL_996999, EPI_ISL_997000, EPI_ISL_997001, EPI_ISL_997002, EPI_ISL_997003, EPI_ISL_997004, EPI_ISL_997005, EPI_ISL_997006, EPI_ISL_997007, EPI_ISL_997008, EPI_ISL_997009, EPI_ISL_997010, EPI_ISL_997011, EPI_ISL_997012, EPI_ISL_997013, EPI_ISL_997014, EPI_ISL_997015, EPI_ISL_997016, EPI_ISL_997017, EPI_ISL_997018, EPI_ISL_997019, EPI_ISL_997020, EPI_ISL_997021, EPI_ISL_997023, EPI_ISL_997024, EPI_ISL_997025, EPI_ISL_997026, EPI_ISL_997027, EPI_ISL_997028, EPI_ISL_997029, EPI_ISL_997030, EPI_ISL_997031, EPI_ISL_997032, EPI_ISL_997033, EPI_ISL_997034, EPI_ISL_997035, EPI_ISL_997036, EPI_ISL_997037, EPI_ISL_997038, EPI_ISL_997039, EPI_ISL_997040, EPI_ISL_997041, EPI_ISL_997042, EPI_ISL_997043, EPI_ISL_997044, EPI_ISL_997045, EPI_ISL_997046, EPI_ISL_997047, EPI_ISL_997048, EPI_ISL_997049			
see above	Department of Pathology, University of Cambridge	COVID-19 Genomics UK (COG-UK) Consortium	Aminu S. Jahun, Yasmin Chaudhry, Iliana Georgana, Myra Hosmillo, Rhys Izuagbe, William L. Hamilton, Martin D. Curran, Surendra Parmar, Ian Goodfellow
EPI_ISL_997052, EPI_ISL_997053, EPI_ISL_997054, EPI_ISL_997055, EPI_ISL_997056, EPI_ISL_997057, EPI_ISL_997058, EPI_ISL_997059, EPI_ISL_997060	West of Scotland Specialist Virology Centre, NHSGGC / MRC-University of Glasgow Centre for Virus Research	COVID-19 Genomics UK (COG-UK) Consortium	Ana da Silva Filipe, Natasha Johnson, Kathy Smollett, Daniel Mair, Stephen Carmichael, Alice Broos, Lily Tong, Jenna Nichols, Kyriaki Nomikou; Sarah McDonald; Richard Orton, Joseph Hughes, Sreenu Vattipally, David L Robertson; Alasdair MacLean, Rory Gunson; Sharif Shaaban, Matthew Holden; Rachel Blacow, Guy Mollett, Kathy Li, James Shepherd, Antonia Ho, Emma Thomson
EPI_ISL_997097, EPI_ISL_997098, EPI_ISL_997099, EPI_ISL_997100, EPI_ISL_997101, EPI_ISL_997102, EPI_ISL_997103, EPI_ISL_997132, EPI_ISL_997133, EPI_ISL_997134, EPI_ISL_997135, EPI_ISL_997136, EPI_ISL_997137, EPI_ISL_997138, EPI_ISL_997139			
see above	Virology Department, Royal Infirmary of Edinburgh, NHS Lothian / School of Biological Sciences, University of Edinburgh	COVID-19 Genomics UK (COG-UK) Consortium	McHugh M, Dewar R, Cotton S, Rooke S, O'Toole Á, Scher E, Hill V, McCrone JT, Colquhoun R, Yu X, Jackson B, Rambaut A, Templeton K
EPI_ISL_997165, EPI_ISL_997166, EPI_ISL_997167, EPI_ISL_997168, EPI_ISL_997169, EPI_ISL_997170, EPI_ISL_997171, EPI_ISL_997172, EPI_ISL_997174, EPI_ISL_997175, EPI_ISL_997176, EPI_ISL_997177, EPI_ISL_997178, EPI_ISL_997179, EPI_ISL_997186, EPI_ISL_997187, EPI_ISL_997266, EPI_ISL_997267, EPI_ISL_997268, EPI_ISL_997274, EPI_ISL_997282, EPI_ISL_997283, EPI_ISL_997284, EPI_ISL_997336, EPI_ISL_997338			
see above	University of Exeter	COVID-19 Genomics UK (COG-UK) Consortium	Ben Temperton,Aaron Jeffries,Michelle Michelsen,Joanna Warwick-Dugdale,Audrey Farbos,Robyn Manley,Stephen Michell,Jane Masoli
EPI_ISL_997382, EPI_ISL_997393, EPI_ISL_997394, EPI_ISL_997395, EPI_ISL_997396, EPI_ISL_997397, EPI_ISL_997398, EPI_ISL_997399, EPI_ISL_997400, EPI_ISL_997401, EPI_ISL_997402, EPI_ISL_997403, EPI_ISL_997404, EPI_ISL_997407, EPI_ISL_997408, EPI_ISL_997409, EPI_ISL_997410, EPI_ISL_997411, EPI_ISL_997439, EPI_ISL_997442, EPI_ISL_997467, EPI_ISL_997468, EPI_ISL_997469, EPI_ISL_997470, EPI_ISL_997471, EPI_ISL_997472, EPI_ISL_997474, EPI_ISL_997475, EPI_ISL_997476, EPI_ISL_997477, EPI_ISL_997478, EPI_ISL_997479, EPI_ISL_997480, EPI_ISL_997481, EPI_ISL_997482			
see above	Liverpool Clinical Laboratories	COVID-19 Genomics UK (COG-UK) Consortium	Sam Haldenby, Anita Lucaci, Steve Paterson, Julian Hiscox, Alistair Darby, M Almsaud, A Alrezaihi, Muhannad Alruwaili, Stuart D Armstrong, Jones Benjamin, Eleanor G Bentley, Anu Chawla, Jordan J Clark, Angela Cowell, Richard Eccles, Isabel García-Dorival, Matthew Gemmell, Alessandro Gerada, PKF Gilmore, Richard Gregory, Ximeng Han, Catherine Hartley, Margaret Hughes, Miren Iturriza-Gomara, James Johnson, L Luu, Jenifer Manson, Charlotte Nelson, Elaine O'Toole, Cassie Olateju, Rebekah Penrice-Randal , Lucille Rainbow, N.P Randle, Trevor Ian Robinson, Parul Sharma, Ghada T Shawli, James P Stewart, Neil Swainston, Ecaterina Vamos, Joanne Watts, Mark Whitehead
EPI_ISL_997615, EPI_ISL_997652, EPI_ISL_997653, EPI_ISL_997654, EPI_ISL_997655, EPI_ISL_997656, EPI_ISL_997662, EPI_ISL_997663, EPI_ISL_997664, EPI_ISL_997666, EPI_ISL_997667, EPI_ISL_997669, EPI_ISL_997670, EPI_ISL_997671, EPI_ISL_997673, EPI_ISL_997674			

see above	Barts Health NHS Trust	COVID-19 Genomics UK (COG-UK) Consortium	CUTINO-MOGUEL, Maria-Teresa; HARRINGTON, David; OWOYEMI, Dola; KULASEGARAN-SHYLINI, Raghavendran; BROAD, Claire; KELE, Beatrix
EPI_ISL_997687, EPI_ISL_997691, EPI_ISL_997692, EPI_ISL_997753, EPI_ISL_997879, EPI_ISL_997880, EPI_ISL_997881, EPI_ISL_997887, EPI_ISL_997901, EPI_ISL_997904, EPI_ISL_997908, EPI_ISL_997909, EPI_ISL_997916, EPI_ISL_997922, EPI_ISL_997923			
see above	University College London, Great Ormond Street Hospital for Children NHS Foundation Trust, Imperial College Healthcare NHS Trust	COVID-19 Genomics UK (COG-UK) Consortium	Sergi Castellano, Rachel Williams, Mark Kristiansen, Paola Resende Silva, Sunando Roy, Tony Brooks, Helena Tutill, Paola Niola, Patricia Dyal, Charlotte Williams, Leysa Forrest, Yasmin Panchbhaya, Jacqueline Findlay, Samuel Weeks, Julianne Brown, Kathryn Harris, Paul Randell, James Price, Alison Holmes, Judith Breuer
EPI_ISL_998178, EPI_ISL_998179, EPI_ISL_998211, EPI_ISL_998229, EPI_ISL_998230, EPI_ISL_998280, EPI_ISL_998281, EPI_ISL_998283, EPI_ISL_998284, EPI_ISL_998285, EPI_ISL_998286, EPI_ISL_998288, EPI_ISL_998289, EPI_ISL_998290, EPI_ISL_998291, EPI_ISL_998292, EPI_ISL_998293, EPI_ISL_998294, EPI_ISL_998295, EPI_ISL_998296, EPI_ISL_998297, EPI_ISL_998298			
see above	Regional Virus Laboratory, Belfast Health and Social Care Trust	COVID-19 Genomics UK (COG-UK) Consortium	Conall McCaughey, James McKenna, Tanya Curran, Susan Feeney, Alison Watt, Ciara Cox, Mairead Connor, Zoltan Molnar, David Simpson, Derek Fairley
EPI_ISL_998484, EPI_ISL_998486, EPI_ISL_998487, EPI_ISL_998488, EPI_ISL_998489, EPI_ISL_998490, EPI_ISL_998491, EPI_ISL_998493, EPI_ISL_998494, EPI_ISL_998495, EPI_ISL_998497, EPI_ISL_998498, EPI_ISL_998500, EPI_ISL_998501, EPI_ISL_998502, EPI_ISL_998503, EPI_ISL_998559, EPI_ISL_998560, EPI_ISL_998561, EPI_ISL_998562, EPI_ISL_998563, EPI_ISL_998564, EPI_ISL_998565			
see above	Northumbria University / South Tees Hospitals NHS Foundation Trust / North Cumbria Integrated Care NHS Foundation Trust / North Tees and Hartlepool NHS Foundation Trust / Newcastle Hospitals NHS Foundation Trust	COVID-19 Genomics UK (COG-UK) Consortium	Darren L Smith,Andrew Nelson,Matthew Bashton,Greg R Young,Joshua Loh,John Allan,Mohammad A Tariq,Giles S Holt,Gary Black,Wen C Yew,Lynn Dover,Paul Baker,Steve Liggett,Sarah Essex,Jane Greenaway,Debra Padgett,Clive Graham,Garren Scott,Edward Barton,Emma Swindells,Brendan Payne,Jennifer Collins,Yusri Taha,Gary Eltringham
EPI_ISL_998772, EPI_ISL_998773, EPI_ISL_998776, EPI_ISL_998777, EPI_ISL_998780, EPI_ISL_998781, EPI_ISL_998785, EPI_ISL_998786, EPI_ISL_998788, EPI_ISL_998795, EPI_ISL_998798, EPI_ISL_998801, EPI_ISL_998802, EPI_ISL_998804, EPI_ISL_998806, EPI_ISL_998816, EPI_ISL_998817, EPI_ISL_998838, EPI_ISL_998839, EPI_ISL_998841, EPI_ISL_998842, EPI_ISL_998843, EPI_ISL_998844, EPI_ISL_998846, EPI_ISL_998847, EPI_ISL_998849, EPI_ISL_998853, EPI_ISL_998857, EPI_ISL_998858, EPI_ISL_998859, EPI_ISL_998860, EPI_ISL_998861, EPI_ISL_998864, EPI_ISL_998865, EPI_ISL_998866, EPI_ISL_998867, EPI_ISL_998870, EPI_ISL_998871, EPI_ISL_998872, EPI_ISL_998874, EPI_ISL_998875, EPI_ISL_998880, EPI_ISL_998881, EPI_ISL_998882, EPI_ISL_998884, EPI_ISL_998885, EPI_ISL_998888			
see above	Quadram Institute Bioscience	COVID-19 Genomics UK (COG-UK) Consortium	Dave J. Baker, Gemma L. Kay, Alp Aydin, Thanh Le-Viet, Steven Rudder, Ana P. Tedim, Anastasia Kolyva, Maria Diaz, Leonardo de Oliveira Martins, Nabil-Fareed Alikhan, Lizzie Meadows, Rachael Stanley, Ngozi Elumogo, Muhammed Yasir, Nicholas M. Thomson, Alexander J Trotter, Rachel Gilroy, Samuel Bloomfield, Claire Stuart, Andrew Bell, Reenesh Prakash, Samir Dervisevic, Alison E. Mather, John Wain, Mark Webber, Andrew J. Page, Justin O'Grady
EPI_ISL_998994, EPI_ISL_999302, EPI_ISL_999303, EPI_ISL_999307, EPI_ISL_999310, EPI_ISL_999311, EPI_ISL_999317, EPI_ISL_999318, EPI_ISL_999320, EPI_ISL_999322, EPI_ISL_999323, EPI_ISL_999324, EPI_ISL_999325, EPI_ISL_999326, EPI_ISL_999327, EPI_ISL_999329, EPI_ISL_999330, EPI_ISL_999331, EPI_ISL_999332, EPI_ISL_999335, EPI_ISL_999337, EPI_ISL_999339, EPI_ISL_999340, EPI_ISL_999342, EPI_ISL_999343, EPI_ISL_999344, EPI_ISL_999345, EPI_ISL_999346, EPI_ISL_999347, EPI_ISL_999484, EPI_ISL_999485, EPI_ISL_999486, EPI_ISL_999487, EPI_ISL_999488, EPI_ISL_999489, EPI_ISL_999490, EPI_ISL_999491, EPI_ISL_999492, EPI_ISL_999493, EPI_ISL_999494			
see above	Oxford Viromics, NDM, University of Oxford; Oxford University Hospitals; Basingstoke and North Hampshire Hospital	COVID-19 Genomics UK (COG-UK) Consortium	Tanya Golubchik, David Bonsall, George Macintyre, Amy Trebes, Mariateresa de Cesare, Catrin Moore, Alex Mobbs, Anita Justice, Robert Shaw, Monique Andersson, Timothy Peto, Emma Wise, Nathan Moore, Jessica Lynch, Nick Cortes, Matilde Mori, Stephen Kidd, David Buck, John Todd, Christophe Fraser
EPI_ISL_999646, EPI_ISL_999647, EPI_ISL_999652, EPI_ISL_999659, EPI_ISL_999664, EPI_ISL_999669, EPI_ISL_999683, EPI_ISL_999684, EPI_ISL_999685, EPI_ISL_999686, EPI_ISL_999687, EPI_ISL_999694, EPI_ISL_999695, EPI_ISL_999808			
see above	Originating lab: Wales Specialist Virology Centre Sequencing lab: Pathogen Genomics Unit	Public Health Wales Microbiology Cardiff Wales Specialist Virology Centre	Catherine Moore, Johnathan Evans, Laura Gifford, Malorie Perry, Simon Cottrell, Angela Marchbank, Alec Birchley, Alexander Adams, Amy Gaskin, Bree Gatica-Wilcox, Jason Coombes, Joel Southgate, Lauren Gilbert, Lee Graham, Nicole Pacchiarini, Sara Kumziene-Summerhayes, Sarah Taylor, Sophie Jones, Sara Rey, Matthew Bull, Joanne Watkins, Sally Corden, Tom Connor
EPI_ISL_999987	Wales Specialist Virology Centre Sequencing lab: Pathogen Genomics Unit	Public Health Wales Microbiology Cardiff Wales Specialist Virology Centre	Catherine Moore, Johnathan Evans, Laura Gifford, Malorie Perry, Simon Cottrell, Angela Marchbank, Alec Birchley, Alexander Adams, Amy Gaskin, Bree Gatica-Wilcox, Jason Coombes, Joel Southgate, Lauren Gilbert, Lee Graham, Nicole Pacchiarini, Sara Kumziene-Summerhayes, Sarah Taylor, Sophie Jones, Sara Rey, Matthew Bull, Joanne Watkins, Sally Corden, Tom Connor
