# Supplementary material for: Inferring the Association between the Risk of COVID-19 Case Fatality and N501Y Substitution in SARS-CoV-2
Source: Viruses. 2021 Apr 8;13(4):638. doi: 10.3390/v13040638 (PMC8070306; doi:10.3390/v13040638)
Supplement: Supplementary file 1 [file viruses-13-00638-s001.zip › gisaid_hcov-19_UKAT_210128-210129.pdf]

We gratefully acknowledge the following Authors from the Originating laboratories responsible for obtaining the specimens, as well as the Submitting laboratories where the genome data were generated and shared via GISAID, on which this research is based.

All Submitters of data may be contacted directly via www.gisaid.org

Authors are sorted alphabetically.

Accession ID	Originating Laboratory	Submitting Laboratory	Authors
EPI_ISL_1000191, EPI_ISL_1000288, EPI_ISL_1000290, EPI_ISL_1000293, EPI_ISL_1000294, EPI_ISL_1000299, EPI_ISL_1000300, EPI_ISL_1000301, EPI_ISL_1000303, EPI_ISL_1000304, EPI_ISL_1000305, EPI_ISL_1000306, EPI_ISL_1000313, EPI_ISL_1000316, EPI_ISL_1000323, EPI_ISL_1000461, EPI_ISL_1000462, EPI_ISL_1000525, EPI_ISL_1000542, EPI_ISL_1000546, EPI_ISL_1000597, EPI_ISL_1000599, EPI_ISL_1000600, EPI_ISL_1000602, EPI_ISL_1000603, EPI_ISL_1000604, EPI_ISL_1000614, EPI_ISL_1000615, EPI_ISL_1000616, EPI_ISL_1000617			
see above	Centre for Enzyme Innovation, University of Portsmouth / Translational Research Laboratory, Portsmouth Hospitals NHS Trust	COVID-19 Genomics UK (COG-UK) Consortium	Angela Beckett,Salman Goudarzi,Christopher Fearn,Kate Cook,Katie Loveson,Sharon Glaysher,Scott Elliott,Samuel Robson
EPI_ISL_1000687, EPI_ISL_1000695	Virology Department, Sheffield Teaching Hospitals NHS Foundation Trust/Department of Infection, Immunity and Cardiovascular Disease, The Medical School, University of Sheffield	COVID-19 Genomics UK (COG-UK) Consortium	Thushan de Silva, Matthew Parker, Nikki Smith, Adri Angyal, Rebecca Brown, Luke Green, Rachel Tucker, Paul Parsons, Danielle Groves, Katie Johnson, Laura Carrilero, Alex Keeley, Dave Partridge, Matthew Wyles, Benjamin Lindsey, Mehmet Yavuz, Mohammad Raza, Cariad Evans
EPI_ISL_1000923, EPI_ISL_1000933	Bioinformatics and Biostatistics Lab, Advanced Sequencing Facility	COVID-19 Genomics UK (COG-UK) Consortium	Aengus Stewart,Jerome Nicod,Chelsea Sawyer,Laura Cubitt,Harshil Patel,Margaret Crawford
EPI_ISL_1006342, EPI_ISL_1006343, EPI_ISL_1006346, EPI_ISL_1006347, EPI_ISL_1006349, EPI_ISL_1006350, EPI_ISL_1006351, EPI_ISL_1006352, EPI_ISL_1006353, EPI_ISL_1006355, EPI_ISL_1006356, EPI_ISL_1006359, EPI_ISL_1006360, EPI_ISL_1006361, EPI_ISL_1006362, EPI_ISL_1006363, EPI_ISL_1006364, EPI_ISL_1006368, EPI_ISL_1006369, EPI_ISL_1006370, EPI_ISL_1006371, EPI_ISL_1006372, EPI_ISL_1006375, EPI_ISL_1006376, EPI_ISL_1006377, EPI_ISL_1006379, EPI_ISL_1006380, EPI_ISL_1006381, EPI_ISL_1006382, EPI_ISL_1006383, EPI_ISL_1006384, EPI_ISL_1006386, EPI_ISL_1006388, EPI_ISL_1006389, EPI_ISL_1006392, EPI_ISL_1006393, EPI_ISL_1006394, EPI_ISL_1006396, EPI_ISL_1006397, EPI_ISL_1006398, EPI_ISL_1006400, EPI_ISL_1006401, EPI_ISL_1006402, EPI_ISL_1006403, EPI_ISL_1006404, EPI_ISL_1006405, EPI_ISL_1006406, EPI_ISL_1006407, EPI_ISL_1006408, EPI_ISL_1006410, EPI_ISL_1006411, EPI_ISL_1006413, EPI_ISL_1006414, EPI_ISL_1006415, EPI_ISL_1006416, EPI_ISL_1006417, EPI_ISL_1006418, EPI_ISL_1006419, EPI_ISL_1006420, EPI_ISL_1006421, EPI_ISL_1006424, EPI_ISL_1006425, EPI_ISL_1006427, EPI_ISL_1006429, EPI_ISL_1006430, EPI_ISL_1006431, EPI_ISL_1006432, EPI_ISL_1006433, EPI_ISL_1006434, EPI_ISL_1006436, EPI_ISL_1006437, EPI_ISL_1006440, EPI_ISL_1006441, EPI_ISL_1006442, EPI_ISL_1006443, EPI_ISL_1006444, EPI_ISL_1006446, EPI_ISL_1006447, EPI_ISL_1006448, EPI_ISL_1006449, EPI_ISL_1006450, EPI_ISL_1006451, EPI_ISL_1006452, EPI_ISL_1006454, EPI_ISL_1006455, EPI_ISL_1006459, EPI_ISL_1006460, EPI_ISL_1006461, EPI_ISL_1006462, EPI_ISL_1006463, EPI_ISL_1006464, EPI_ISL_1006465, EPI_ISL_1006466, EPI_ISL_1006468, EPI_ISL_1006469, EPI_ISL_1006470, EPI_ISL_1006472, EPI_ISL_1006473, EPI_ISL_1006478, EPI_ISL_1006480, EPI_ISL_1006481, EPI_ISL_1006483, EPI_ISL_1006484, EPI_ISL_1006485, EPI_ISL_1006486, EPI_ISL_1006487, EPI_ISL_1006491, EPI_ISL_1006492, EPI_ISL_1006493, EPI_ISL_1006494, EPI_ISL_1006495, EPI_ISL_1006496, EPI_ISL_1006497, EPI_ISL_1006498, EPI_ISL_1006499, EPI_ISL_1006500, EPI_ISL_1006502, EPI_ISL_1006503, EPI_ISL_1006504, EPI_ISL_1006505, EPI_ISL_1006506, EPI_ISL_1006507, EPI_ISL_1006508, EPI_ISL_1006509, EPI_ISL_1006510, EPI_ISL_1006512, EPI_ISL_1006513, EPI_ISL_1006514, EPI_ISL_1006515, EPI_ISL_1006517, EPI_ISL_1006518, EPI_ISL_1006519, EPI_ISL_1006520, EPI_ISL_1006522, EPI_ISL_1006523, EPI_ISL_1006524, EPI_ISL_1006525, EPI_ISL_1006526, EPI_ISL_1006527, EPI_ISL_1006528, EPI_ISL_1006531, EPI_ISL_1006532, EPI_ISL_1006534, EPI_ISL_1006536, EPI_ISL_1006537, EPI_ISL_1006539			
see above	Lighthouse Lab in Milton Keynes	Wellcome Sanger Institute for the COVID-19 Genomics UK (COG-UK) Consortium	The Lighthouse Lab in Milton Keynes and Alex Alderton, Roberto Amato, Sonia Goncalves, Ewan Harrison, David K. Jackson, Ian Johnston, Dominic Kwiatkowski, Cordelia Langford, John Sillitoe on behalf of the Wellcome Sanger Institute COVID-19 Surveillance Team
EPI_ISL_1007435	Lighthouse Lab in Cambridge	Wellcome Sanger Institute for the COVID-19 Genomics UK (COG-UK) Consortium	Rob Howes, The Lighthouse Lab in Cambridge and Alex Alderton, Roberto Amato, Sonia Goncalves, Ewan Harrison, David K. Jackson, Ian Johnston, Dominic Kwiatkowski, Cordelia Langford, John Sillitoe on behalf of the Wellcome Sanger Institute COVID-19 Surveillance Team
EPI_ISL_1012403, EPI_ISL_1012608	Lighthouse Lab in Milton Keynes	Wellcome Sanger Institute for the COVID-19 Genomics UK (COG-UK) Consortium	The Lighthouse Lab in Milton Keynes and Alex Alderton, Roberto Amato, Sonia Goncalves, Ewan Harrison, David K. Jackson, Ian Johnston, Dominic Kwiatkowski, Cordelia Langford, John Sillitoe on behalf of the Wellcome Sanger Institute COVID-19 Surveillance Team
EPI_ISL_1012701	Lighthouse Lab in Alderley Park	Wellcome Sanger Institute for the COVID-19 Genomics UK (COG-UK) Consortium	Jacquelyn Wynn, Mairead Hyland, The Lighthouse Lab in Alderley Park and Alex Alderton, Roberto Amato, Sonia Goncalves, Ewan Harrison, David K. Jackson, Ian Johnston, Dominic Kwiatkowski, Cordelia Langford, John Sillitoe on behalf of the Wellcome Sanger Institute COVID-19 Surveillance Team
EPI_ISL_1045983, EPI_ISL_1046337, EPI_ISL_1046339, EPI_ISL_1046340, EPI_ISL_1046342, EPI_ISL_1046343, EPI_ISL_1046344, EPI_ISL_1046345, EPI_ISL_1046346, EPI_ISL_1046348, EPI_ISL_1046349, EPI_ISL_1046350, EPI_ISL_1046351, EPI_ISL_1046353, EPI_ISL_1046356, EPI_ISL_1046358, EPI_ISL_1046360, EPI_ISL_1046362, EPI_ISL_1046363, EPI_ISL_1046364, EPI_ISL_1046367, EPI_ISL_1046368, EPI_ISL_1046369, EPI_ISL_1046370, EPI_ISL_1046371, EPI_ISL_1046372, EPI_ISL_1046373, EPI_ISL_1046374, EPI_ISL_1046376, EPI_ISL_1046377, EPI_ISL_1046378, EPI_ISL_1046379, EPI_ISL_1046380, EPI_ISL_1046382, EPI_ISL_1046383, EPI_ISL_1046384, EPI_ISL_1046385, EPI_ISL_1046386, EPI_ISL_1046387, EPI_ISL_1046388, EPI_ISL_1046389, EPI_ISL_1046390, EPI_ISL_1046391, EPI_ISL_1046392, EPI_ISL_1046393, EPI_ISL_1046395, EPI_ISL_1046397, EPI_ISL_1046398, EPI_ISL_1046399, EPI_ISL_1046400, EPI_ISL_1046401, EPI_ISL_1046403, EPI_ISL_1046405, EPI_ISL_1046406, EPI_ISL_1046407, EPI_ISL_1046408, EPI_ISL_1046410, EPI_ISL_1046412, EPI_ISL_1046413, EPI_ISL_1046414, EPI_ISL_1046415, EPI_ISL_1046416, EPI_ISL_1046417, EPI_ISL_1046418, EPI_ISL_1046419, EPI_ISL_1046420, EPI_ISL_1046421, EPI_ISL_1046422, EPI_ISL_1046423, EPI_ISL_1046424, EPI_ISL_1046425, EPI_ISL_1046426, EPI_ISL_1046427, EPI_ISL_1046428, EPI_ISL_1046429, EPI_ISL_1046430, EPI_ISL_1046431, EPI_ISL_1046432, EPI_ISL_1046433, EPI_ISL_1046434, EPI_ISL_1046436, EPI_ISL_1046437, EPI_ISL_1046438, EPI_ISL_1046439, EPI_ISL_1046441, EPI_ISL_1046443, EPI_ISL_1046444, EPI_ISL_1046445, EPI_ISL_1046446, EPI_ISL_1046447, EPI_ISL_1046448, EPI_ISL_1046450, EPI_ISL_1046451, EPI_ISL_1046452, EPI_ISL_1046453, EPI_ISL_1046454, EPI_ISL_1046455, EPI_ISL_1046456, EPI_ISL_1046457, EPI_ISL_1046458, EPI_ISL_1046459, EPI_ISL_1046460, EPI_ISL_1046461, EPI_ISL_1046462, EPI_ISL_1046463, EPI_ISL_1046464, EPI_ISL_1046465, EPI_ISL_1046466, EPI_ISL_1046467, EPI_ISL_1046468, EPI_ISL_1046469, EPI_ISL_1046470, EPI_ISL_1046471, EPI_ISL_1046472, EPI_ISL_1046473, EPI_ISL_1046475, EPI_ISL_1046476, EPI_ISL_1046477, EPI_ISL_1046478, EPI_ISL_1046479, EPI_ISL_1046480, EPI_ISL_1046481, EPI_ISL_1046482, EPI_ISL_1046483, EPI_ISL_1046484, EPI_ISL_1046485, EPI_ISL_1046486, EPI_ISL_1046487, EPI_ISL_1046488, EPI_ISL_1046489, EPI_ISL_1046490, EPI_ISL_1046492, EPI_ISL_1046494, EPI_ISL_1046496, EPI_ISL_1046497, EPI_ISL_1046498, EPI_ISL_1046499, EPI_ISL_1046500, EPI_ISL_1046501, EPI_ISL_1046502, EPI_ISL_1046503, EPI_ISL_1046505, EPI_ISL_1046506, EPI_ISL_1046509, EPI_ISL_1046511, EPI_ISL_1046512, EPI_ISL_1046540, EPI_ISL_1046543, EPI_ISL_1046545, EPI_ISL_1046565, EPI_ISL_1046576, EPI_ISL_1046579, EPI_ISL_1046589, EPI_ISL_1046591, EPI_ISL_1046600, EPI_ISL_1046608, EPI_ISL_1046610, EPI_ISL_1046613, EPI_ISL_1046616, EPI_ISL_1046617, EPI_ISL_1046621, EPI_ISL_1046625, EPI_ISL_1046627, EPI_ISL_1046630, EPI_ISL_1046634, EPI_ISL_1046637, EPI_ISL_1046640, EPI_ISL_1046643, EPI_ISL_1046644, EPI_ISL_1046645, EPI_ISL_1046648, EPI_ISL_1046649, EPI_ISL_1046651, EPI_ISL_1046654, EPI_ISL_1046655, EPI_ISL_1046657, EPI_ISL_1046660, EPI_ISL_1046663, EPI_ISL_1046671, EPI_ISL_1046678, EPI_ISL_1046681, EPI_ISL_1046684, EPI_ISL_1046686, EPI_ISL_1046690, EPI_ISL_1046692, EPI_ISL_1046697, EPI_ISL_1046699, EPI_ISL_1046702, EPI_ISL_1046705, EPI_ISL_1046713, EPI_ISL_1046716, EPI_ISL_1046721, EPI_ISL_1046729, EPI_ISL_1046731, EPI_ISL_1046732, EPI_ISL_1046738, EPI_ISL_1046739, EPI_ISL_1046745, EPI_ISL_1046753, EPI_ISL_1046757			
see above	Randox Laboratories	Wellcome Sanger Institute for the COVID-19 Genomics UK (COG-UK) Consortium	Randox Laboratories and Alex Alderton, Roberto Amato, Jeffrey Barrett, Sonia Goncalves, Ewan Harrison, David K. Jackson, Ian Johnston, Dominic Kwiatkowski, Cordelia Langford, John Sillitoe on behalf of the Wellcome Sanger Institute COVID-19 Surveillance Team
EPI_ISL_1047000, EPI_ISL_1047002, EPI_ISL_1047003, EPI_ISL_1047006, EPI_ISL_1047010, EPI_ISL_1047013, EPI_ISL_1047031, EPI_ISL_1047038	University of Birmingham	COVID-19 Genomics UK (COG-UK) Consortium	Institute of Microbiology, University of Birmingham: Claire McMurray, Joanne Stockton, Samuel Nicholls, Radoslaw Poplawski, Will Rowe, Josh Quick, Nicholas Loman. University of Birmingham Testing Laboratory: Celina M Whalley, Andrew Bosworth, Charlotte Poxon, Kasun Wanigasooriya, Oliver Pickles, Mike Kidd, Alex Richter, Andrew D Beggs PHE Heartlands Lab: Husam Osman, Andrew Bosworth. Queen Elizabeth Hospital: Anna Casey
EPI_ISL_1047188, EPI_ISL_1047189, EPI_ISL_1047192, EPI_ISL_1047193, EPI_ISL_1047349, EPI_ISL_1047350, EPI_ISL_1047351, EPI_ISL_1047353, EPI_ISL_1047354	Department of Pathology, University of Cambridge	COVID-19 Genomics UK (COG-UK) Consortium	Aminu S. Jahun, Yasmin Chaudhry, Iliana Georgana, Myra Hosmillo, Rhys Izuagbe, William L. Hamilton, Martin D. Curran, Surendra Parmar, Ian Goodfellow
EPI_ISL_1047582, EPI_ISL_1047644, EPI_ISL_1047645, EPI_ISL_1047646, EPI_ISL_1047754, EPI_ISL_1047781, EPI_ISL_1047784, EPI_ISL_1047803	Virology Department, Sheffield Teaching Hospitals NHS Foundation Trust/Department of Infection, Immunity and Cardiovascular Disease, The Medical School, University of Sheffield	COVID-19 Genomics UK (COG-UK) Consortium	Thushan de Silva, Matthew Parker, Nikki Smith, Adri Angyal, Rebecca Brown, Luke Green, Rachel Tucker, Paul Parsons, Danielle Groves, Katie Johnson, Laura Carrilero, Alex Keeley, Dave Partridge, Matthew Wyles, Benjamin Lindsey, Mehmet Yavuz, Mohammad Raza, Cariad Evans
EPI_ISL_1047804, EPI_ISL_1047805, EPI_ISL_1047806, EPI_ISL_1047807, EPI_ISL_1047808, EPI_ISL_1047809, EPI_ISL_1047810, EPI_ISL_1047811, EPI_ISL_1047812, EPI_ISL_1047813, EPI_ISL_1047814, EPI_ISL_1047815, EPI_ISL_1047816, EPI_ISL_1047817, EPI_ISL_1047818, EPI_ISL_1047819, EPI_ISL_1047820, EPI_ISL_1047821, EPI_ISL_1047823, EPI_ISL_1047824, EPI_ISL_1047833			
see above	West of Scotland Specialist Virology Centre, NHSGGC / MRC-University of Glasgow Centre for Virus Research	COVID-19 Genomics UK (COG-UK) Consortium	Ana da Silva Filipe, Natasha Johnson, Kathy Smollett, Daniel Mair, Stephen Carmichael, Alice Broos, Lily Tong, Jenna Nichols, Kyriaki Nomikou; Sarah McDonald; Richard Orton, Joseph Hughes, Sreenu Vattipally, David L Robertson; Alasdair MacLean, Rory Gunson; Sharif Shaaban, Matthew Holden; Rachel Blacow, Guy Mollett, Kathy Li, James Shepherd, Antonia Ho, Emma Thomson
EPI_ISL_1047861, EPI_ISL_1047862, EPI_ISL_1047863, EPI_ISL_1047864, EPI_ISL_1047865	Virology Department, Royal Infirmary of Edinburgh, NHS Lothian / School of Biological Sciences, University of Edinburgh	COVID-19 Genomics UK (COG-UK) Consortium	McHugh M, Dewar R, Cotton S, Rooke S, O'Toole Á, Scher E, Hill V, McCrone JT, Colquhoun R, Yu X, Jackson B, Rambaut A, Templeton K
EPI_ISL_1047969, EPI_ISL_1048027	Liverpool Clinical Laboratories	COVID-19 Genomics UK (COG-UK) Consortium	Sam Haldenby, Anita Lucaci, Steve Paterson, Julian Hiscox, Alistair Darby, M Almsaud, A Alrezaihi, Muhannad Alruwaili, Stuart D Armstrong, Jones Benjamin, Eleanor G Bentley, Anu Chawla, Jordan J Clark, Angela Cowell, Richard Eccles, Isabel García-Dorival, Matthew Gemmell, Alessandro Gerada,

			PKF Gilmore, Richard Gregory, Ximeng Han, Catherine Hartley, Margaret Hughes, Miren Iturriza-Gomara, James Johnson, L Luu, Jenifer Manson, Charlotte Nelson, Elaine O'Toole, Cassie Olateju, Rebekah Penrice-Randal , Lucille Rainbow, N.P Randle, Trevor Ian Robinson, Parul Sharma, Ghada T Shawli, James P Stewart, Neil Swainston, Ecaterina Vamos, Joanne Watts, Mark Whitehead
EPI_ISL_1048060, EPI_ISL_1048063, EPI_ISL_1048064, EPI_ISL_1048080, EPI_ISL_1048082, EPI_ISL_1048084, EPI_ISL_1048104, EPI_ISL_1048135	Barts Health NHS Trust	COVID-19 Genomics UK (COG-UK) Consortium	CUTINO-MOGUEL, Maria-Teresa; HARRINGTON, David; OWOYEMI, Dola; KULASEGARAN-SHYLINI, Raghavendran; BROAD, Claire; KELE, Beatrix
EPI_ISL_1050066	Virology Department, Sheffield Teaching Hospitals NHS Foundation Trust/Department of Infection, Immunity and Cardiovascular Disease, The Medical School, University of Sheffield	COVID-19 Genomics UK (COG-UK) Consortium	Thushan de Silva, Matthew Parker, Nikki Smith, Adri Angyal, Rebecca Brown, Luke Green, Rachel Tucker, Paul Parsons, Danielle Groves, Katie Johnson, Laura Carrilero, Alex Keeley, Dave Partridge, Matthew Wyles, Benjamin Lindsey, Mehmet Yavuz, Mohammad Raza, Cariad Evans
EPI_ISL_1050219, EPI_ISL_1050220, EPI_ISL_1050222	West of Scotland Specialist Virology Centre, NHSGGC / MRC-University of Glasgow Centre for Virus Research	COVID-19 Genomics UK (COG-UK) Consortium	Ana da Silva Filipe, Natasha Johnson, Kathy Smollett, Daniel Mair, Stephen Carmichael, Alice Broos, Lily Tong, Jenna Nichols, Kyriaki Nomikou; Sarah McDonald; Richard Orton, Joseph Hughes, Sreenu Vattipally, David L Robertson; Alasdair MacLean, Rory Gunson; Sharif Shaaban, Matthew Holden; Rachel Blacow, Guy Mollett, Kathy Li, James Shepherd, Antonia Ho, Emma Thomson
EPI_ISL_1050293, EPI_ISL_1050294, EPI_ISL_1050295, EPI_ISL_1050300, EPI_ISL_1050301, EPI_ISL_1050302, EPI_ISL_1050303	University of Exeter	COVID-19 Genomics UK (COG-UK) Consortium	Ben Temperton,Aaron Jeffries,Michelle Michelsen,Joanna Warwick-Dugdale,Audrey Farbos,Robyn Manley,Stephen Michell,Jane Masoli
EPI_ISL_1050582, EPI_ISL_1050583, EPI_ISL_1050584, EPI_ISL_1050585, EPI_ISL_1050586, EPI_ISL_1050587, EPI_ISL_1050588, EPI_ISL_1050589, EPI_ISL_1050590, EPI_ISL_1050591, EPI_ISL_1050592, EPI_ISL_1050593, EPI_ISL_1050594, EPI_ISL_1050595, EPI_ISL_1050596, EPI_ISL_1050597, EPI_ISL_1050598, EPI_ISL_1050599, EPI_ISL_1050600, EPI_ISL_1050601, EPI_ISL_1050602, EPI_ISL_1050603, EPI_ISL_1050604			
see above	University College London, Great Ormond Street Hospital for Children NHS Foundation Trust, Imperial College Healthcare NHS Trust	COVID-19 Genomics UK (COG-UK) Consortium	Sergi Castellano, Rachel Williams, Mark Kristiansen, Paola Resende Silva, Sunando Roy, Tony Brooks, Helena Tutill, Paola Niola, Patricia Dyal, Charlotte Williams, Leysa Forrest, Yasmin Panchbhaya, Jacqueline Findlay, Samuel Weeks, Julianne Brown, Kathryn Harris, Paul Randell, James Price, Alison Holmes, Judith Breuer
EPI_ISL_1050605, EPI_ISL_1050606	Department of Pathology, University of Cambridge	COVID-19 Genomics UK (COG-UK) Consortium	Aminu S. Jahun, Yasmin Chaudhry, Iliana Georgana, Myra Hosmillo, Rhys Izuagbe, William L. Hamilton, Martin D. Curran, Surendra Parmar, Ian Goodfellow
EPI_ISL_1050607, EPI_ISL_1050608, EPI_ISL_1050609	University College London, Great Ormond Street Hospital for Children NHS Foundation Trust, Imperial College Healthcare NHS Trust	COVID-19 Genomics UK (COG-UK) Consortium	Sergi Castellano, Rachel Williams, Mark Kristiansen, Paola Resende Silva, Sunando Roy, Tony Brooks, Helena Tutill, Paola Niola, Patricia Dyal, Charlotte Williams, Leysa Forrest, Yasmin Panchbhaya, Jacqueline Findlay, Samuel Weeks, Julianne Brown, Kathryn Harris, Paul Randell, James Price, Alison Holmes, Judith Breuer
EPI_ISL_1050610	Department of Pathology, University of Cambridge	COVID-19 Genomics UK (COG-UK) Consortium	Aminu S. Jahun, Yasmin Chaudhry, Iliana Georgana, Myra Hosmillo, Rhys Izuagbe, William L. Hamilton, Martin D. Curran, Surendra Parmar, Ian Goodfellow
EPI_ISL_1050611, EPI_ISL_1050612, EPI_ISL_1050613	University College London, Great Ormond Street Hospital for Children NHS Foundation Trust, Imperial College Healthcare NHS Trust	COVID-19 Genomics UK (COG-UK) Consortium	Sergi Castellano, Rachel Williams, Mark Kristiansen, Paola Resende Silva, Sunando Roy, Tony Brooks, Helena Tutill, Paola Niola, Patricia Dyal, Charlotte Williams, Leysa Forrest, Yasmin Panchbhaya, Jacqueline Findlay, Samuel Weeks, Julianne Brown, Kathryn Harris, Paul Randell, James Price, Alison Holmes, Judith Breuer
EPI_ISL_1050614	Department of Pathology, University of Cambridge	COVID-19 Genomics UK (COG-UK) Consortium	Aminu S. Jahun, Yasmin Chaudhry, Iliana Georgana, Myra Hosmillo, Rhys Izuagbe, William L. Hamilton, Martin D. Curran, Surendra Parmar, Ian Goodfellow
EPI_ISL_1050615, EPI_ISL_1050616, EPI_ISL_1050617, EPI_ISL_1050618	University College London, Great Ormond Street Hospital for Children NHS Foundation Trust, Imperial College Healthcare NHS Trust	COVID-19 Genomics UK (COG-UK) Consortium	Sergi Castellano, Rachel Williams, Mark Kristiansen, Paola Resende Silva, Sunando Roy, Tony Brooks, Helena Tutill, Paola Niola, Patricia Dyal, Charlotte Williams, Leysa Forrest, Yasmin Panchbhaya, Jacqueline Findlay, Samuel Weeks, Julianne Brown, Kathryn Harris, Paul Randell, James Price, Alison Holmes, Judith Breuer
EPI_ISL_1050619	Department of Pathology, University of Cambridge	COVID-19 Genomics UK (COG-UK) Consortium	Aminu S. Jahun, Yasmin Chaudhry, Iliana Georgana, Myra Hosmillo, Rhys Izuagbe, William L. Hamilton, Martin D. Curran, Surendra Parmar, Ian Goodfellow
EPI_ISL_1050620, EPI_ISL_1050621, EPI_ISL_1050622, EPI_ISL_1050623, EPI_ISL_1050624, EPI_ISL_1050625, EPI_ISL_1050626, EPI_ISL_1050627, EPI_ISL_1050628, EPI_ISL_1050629, EPI_ISL_1050630, EPI_ISL_1050631			
see above	University College London, Great Ormond Street Hospital for Children NHS Foundation Trust, Imperial College Healthcare NHS Trust	COVID-19 Genomics UK (COG-UK) Consortium	Sergi Castellano, Rachel Williams, Mark Kristiansen, Paola Resende Silva, Sunando Roy, Tony Brooks, Helena Tutill, Paola Niola, Patricia Dyal, Charlotte Williams, Leysa Forrest, Yasmin Panchbhaya, Jacqueline Findlay, Samuel Weeks, Julianne Brown, Kathryn Harris, Paul Randell, James Price, Alison Holmes, Judith Breuer
EPI_ISL_1050632, EPI_ISL_1050633, EPI_ISL_1050634, EPI_ISL_1050635, EPI_ISL_1050636, EPI_ISL_1050637, EPI_ISL_1050638, EPI_ISL_1050639, EPI_ISL_1050640, EPI_ISL_1050641, EPI_ISL_1050642, EPI_ISL_1050643, EPI_ISL_1050644, EPI_ISL_1050645, EPI_ISL_1050646, EPI_ISL_1050647, EPI_ISL_1050648, EPI_ISL_1050649, EPI_ISL_1050651, EPI_ISL_1050652, EPI_ISL_1050653, EPI_ISL_1050654, EPI_ISL_1050655, EPI_ISL_1050656, EPI_ISL_1050657, EPI_ISL_1050658, EPI_ISL_1050659, EPI_ISL_1050660, EPI_ISL_1050661, EPI_ISL_1050662, EPI_ISL_1050663, EPI_ISL_1050664, EPI_ISL_1050665, EPI_ISL_1050666, EPI_ISL_1050667, EPI_ISL_1050668, EPI_ISL_1050669, EPI_ISL_1050670, EPI_ISL_1050671, EPI_ISL_1050672, EPI_ISL_1050673, EPI_ISL_1050674, EPI_ISL_1050675			
see above	Department of Pathology, University of Cambridge	COVID-19 Genomics UK (COG-UK) Consortium	Aminu S. Jahun, Yasmin Chaudhry, Iliana Georgana, Myra Hosmillo, Rhys Izuagbe, William L. Hamilton, Martin D. Curran, Surendra Parmar, Ian Goodfellow
EPI_ISL_1050676, EPI_ISL_1050677, EPI_ISL_1050678, EPI_ISL_1050679, EPI_ISL_1050680, EPI_ISL_1050681, EPI_ISL_1050682, EPI_ISL_1050683, EPI_ISL_1050684	University College London, Great Ormond Street Hospital for Children NHS Foundation Trust, Imperial College Healthcare NHS Trust	COVID-19 Genomics UK (COG-UK) Consortium	Sergi Castellano, Rachel Williams, Mark Kristiansen, Paola Resende Silva, Sunando Roy, Tony Brooks, Helena Tutill, Paola Niola, Patricia Dyal, Charlotte Williams, Leysa Forrest, Yasmin Panchbhaya, Jacqueline Findlay, Samuel Weeks, Julianne Brown, Kathryn Harris, Paul Randell, James Price, Alison Holmes, Judith Breuer
EPI_ISL_1050685, EPI_ISL_1050686, EPI_ISL_1050687, EPI_ISL_1050688, EPI_ISL_1050689, EPI_ISL_1050690, EPI_ISL_1050692, EPI_ISL_1050693, EPI_ISL_1050694, EPI_ISL_1050695, EPI_ISL_1050696, EPI_ISL_1050698, EPI_ISL_1050699, EPI_ISL_1050700, EPI_ISL_1050701, EPI_ISL_1050702, EPI_ISL_1050705, EPI_ISL_1050706, EPI_ISL_1050707, EPI_ISL_1050708, EPI_ISL_1050709, EPI_ISL_1050710, EPI_ISL_1050711, EPI_ISL_1050712, EPI_ISL_1050713, EPI_ISL_1050714, EPI_ISL_1050715, EPI_ISL_1050716, EPI_ISL_1050717, EPI_ISL_1050718, EPI_ISL_1050719, EPI_ISL_1050720, EPI_ISL_1050721, EPI_ISL_1050722			
see above	Department of Pathology, University of Cambridge	COVID-19 Genomics UK (COG-UK) Consortium	Aminu S. Jahun, Yasmin Chaudhry, Iliana Georgana, Myra Hosmillo, Rhys Izuagbe, William L. Hamilton, Martin D. Curran, Surendra Parmar, Ian Goodfellow
EPI_ISL_1050723, EPI_ISL_1050724, EPI_ISL_1050725, EPI_ISL_1050726, EPI_ISL_1050727, EPI_ISL_1050728, EPI_ISL_1050729, EPI_ISL_1050730, EPI_ISL_1050731, EPI_ISL_1050732, EPI_ISL_1050733, EPI_ISL_1050734, EPI_ISL_1050735, EPI_ISL_1050736, EPI_ISL_1050737, EPI_ISL_1050738, EPI_ISL_1050739, EPI_ISL_1050740, EPI_ISL_1050741, EPI_ISL_1050742, EPI_ISL_1050743, EPI_ISL_1050744, EPI_ISL_1050745, EPI_ISL_1050746, EPI_ISL_1050747			
see above	University College London, Great Ormond Street Hospital for Children NHS Foundation Trust, Imperial College Healthcare NHS Trust	COVID-19 Genomics UK (COG-UK) Consortium	Sergi Castellano, Rachel Williams, Mark Kristiansen, Paola Resende Silva, Sunando Roy, Tony Brooks, Helena Tutill, Paola Niola, Patricia Dyal, Charlotte Williams, Leysa Forrest, Yasmin Panchbhaya, Jacqueline Findlay, Samuel Weeks, Julianne Brown, Kathryn Harris, Paul Randell, James Price, Alison Holmes, Judith Breuer
EPI_ISL_1051244, EPI_ISL_1051245, EPI_ISL_1051246, EPI_ISL_1051247, EPI_ISL_1051248, EPI_ISL_1051249, EPI_ISL_1051250, EPI_ISL_1051251, EPI_ISL_1051252, EPI_ISL_1051253, EPI_ISL_1051254, EPI_ISL_1051255, EPI_ISL_1051256, EPI_ISL_1051257, EPI_ISL_1051258, EPI_ISL_1051259, EPI_ISL_1051260			
see above	Northumbria University / South Tees Hospitals NHS Foundation Trust / North Cumbria Integrated Care NHS Foundation Trust / North Tees and Hartlepool NHS Foundation Trust / Newcastle Hospitals NHS Foundation Trust	COVID-19 Genomics UK (COG-UK) Consortium	Darren L Smith,Andrew Nelson,Matthew Bashton,Greg R Young,Joshua Loh,John Allan,Mohammad A Tariq,Giles S Holt,Gary Black,Wen C Yew,Lynn Dover,Paul Baker,Steve Liggett,Sarah Essex,Jane Greenaway,Debra Padgett,Clive Graham,Garren Scott,Edward Barton,Emma Swindells,Brendan Payne,Jennifer Collins,Yusri Taha,Gary Eltringham
EPI_ISL_1051590	Queens Medical Centre, Clinical Microbiology Department / DeepSeq Nottingham	COVID-19 Genomics UK (COG-UK) Consortium	Gemma Clark, Wendy Smith, Manjinder Khakh, Vicki M Fleming, Michelle M Lister, Hannah Howson-Wells, Jonathan Ball, Patrick McClure, Joseph Chappell, Theocharis Tsoleridis, Nadine Holmes, Matthew Carlisle, Christopher Moore, Fei Sang, Johnny Debebe, Victoria Wright, Matthew Loose
EPI_ISL_1051596, EPI_ISL_1051627, EPI_ISL_1051630, EPI_ISL_1051631, EPI_ISL_1051634, EPI_ISL_1051635, EPI_ISL_1051645, EPI_ISL_1051647, EPI_ISL_1051652, EPI_ISL_1051653, EPI_ISL_1051654, EPI_ISL_1051658, EPI_ISL_1051659, EPI_ISL_1051661, EPI_ISL_1051662, EPI_ISL_1051663, EPI_ISL_1051664, EPI_ISL_1051667, EPI_ISL_1051668, EPI_ISL_1051669, EPI_ISL_1051672, EPI_ISL_1051674, EPI_ISL_1051677, EPI_ISL_1051678, EPI_ISL_1051679, EPI_ISL_1051680, EPI_ISL_1051683, EPI_ISL_1051684, EPI_ISL_1051686, EPI_ISL_1051687, EPI_ISL_1051689, EPI_ISL_1051691, EPI_ISL_1051693, EPI_ISL_1051715, EPI_ISL_1051716, EPI_ISL_1051717, EPI_ISL_1051718, EPI_ISL_1051719, EPI_ISL_1051721, EPI_ISL_1051722, EPI_ISL_1051726, EPI_ISL_1051728, EPI_ISL_1051877, EPI_ISL_1051878, EPI_ISL_1051881, EPI_ISL_1051882, EPI_ISL_1051883, EPI_ISL_1051884, EPI_ISL_1051899, EPI_ISL_1051903, EPI_ISL_1051908, EPI_ISL_1051909, EPI_ISL_1051910, EPI_ISL_1051911, EPI_ISL_1051914, EPI_ISL_1051918			

see above	Oxford Viromics, NDM, University of Oxford; Oxford University Hospitals; Basingstoke and North Hampshire Hospital	COVID-19 Genomics UK (COG-UK) Consortium	Tanya Golubchik, David Bonsall, George Macintyre, Amy Trebes, Mariateresa de Cesare, Catrin Moore, Alex Mobbs, Anita Justice, Robert Shaw, Monique Andersson, Timothy Peto, Emma Wise, Nathan Moore, Jessica Lynch, Nick Cortes, Matilde Mori, Stephen Kidd, David Buck, John Todd, Christophe Fraser
EPI_ISL_1052138, EPI_ISL_1052143, EPI_ISL_1052145, EPI_ISL_1052146, EPI_ISL_1052150, EPI_ISL_1052261, EPI_ISL_1052262, EPI_ISL_1052263, EPI_ISL_1052470, EPI_ISL_1052488, EPI_ISL_1052492, EPI_ISL_1052493, EPI_ISL_1052494, EPI_ISL_1052522, EPI_ISL_1052526, EPI_ISL_1052527, EPI_ISL_1052528, EPI_ISL_1052530, EPI_ISL_1053513, EPI_ISL_1053515, EPI_ISL_1053516, EPI_ISL_1053530, EPI_ISL_1053531, EPI_ISL_1053539, EPI_ISL_1053540, EPI_ISL_1053562, EPI_ISL_1053565, EPI_ISL_1053567, EPI_ISL_1053568, EPI_ISL_1053569, EPI_ISL_1053571, EPI_ISL_1053572, EPI_ISL_1053573, EPI_ISL_1053574, EPI_ISL_1053575			
see above	Originating lab: Wales Specialist Virology Centre Sequencing lab: Pathogen Genomics Unit	Public Health Wales Microbiology Cardiff Wales Specialist Virology Centre	Catherine Moore, Johnathan Evans, Laura Gifford, Malorie Perry, Simon Cottrell, Angela Marchbank, Alec Birchley, Alexander Adams, Amy Gaskin, Bree Gatica-Wilcox, Jason Coombes, Joel Southgate, Lauren Gilbert, Lee Graham, Nicole Pacchiarini, Sara Kumziene-Summerhayes, Sarah Taylor, Sophie Jones, Sara Rey, Matthew Bull, Joanne Watkins, Sally Corden, Tom Connor
EPI_ISL_1053851, EPI_ISL_1053852, EPI_ISL_1053856, EPI_ISL_1053857, EPI_ISL_1053932	Centre for Enzyme Innovation, University of Portsmouth / Translational Research Laboratory, Portsmouth Hospitals NHS Trust	COVID-19 Genomics UK (COG-UK) Consortium	Angela Beckett,Salman Goudarzi,Christopher Fearn,Kate Cook,Katie Loveson,Sharon Glaysher,Scott Elliott,Samuel Robson
EPI_ISL_1054051, EPI_ISL_1054121, EPI_ISL_1054123, EPI_ISL_1054127	University of Exeter	COVID-19 Genomics UK (COG-UK) Consortium	Ben Temperton,Aaron Jeffries,Michelle Michelsen,Joanna Warwick-Dugdale,Audrey Farbos,Robyn Manley,Stephen Michell,Jane Masoli
EPI_ISL_1054141, EPI_ISL_1054233, EPI_ISL_1054241, EPI_ISL_1054250, EPI_ISL_1054254	Northumbria University / South Tees Hospitals NHS Foundation Trust / North Cumbria Integrated Care NHS Foundation Trust / North Tees and Hartlepool NHS Foundation Trust / Newcastle Hospitals NHS Foundation Trust	COVID-19 Genomics UK (COG-UK) Consortium	Darren L Smith,Andrew Nelson,Matthew Bashton,Greg R Young,Joshua Loh,John Allan,Mohammad A Tariq,Giles S Holt,Gary Black,Wen C Yew,Lynn Dover,Paul Baker,Steve Liggett,Sarah Essex,Jane Greenaway,Debra Padgett,Clive Graham,Garren Scott,Edward Barton,Emma Swindells,Brendan Payne,Jennifer Collins,Yusri Taha,Gary Eltringham
EPI_ISL_1054391, EPI_ISL_1054392, EPI_ISL_1054393, EPI_ISL_1054394, EPI_ISL_1054395, EPI_ISL_1054397, EPI_ISL_1054398, EPI_ISL_1054399, EPI_ISL_1054400, EPI_ISL_1054401, EPI_ISL_1054402, EPI_ISL_1054403, EPI_ISL_1054411, EPI_ISL_1054412, EPI_ISL_1054413, EPI_ISL_1054414, EPI_ISL_1054415, EPI_ISL_1054416, EPI_ISL_1054420, EPI_ISL_1054421, EPI_ISL_1054422, EPI_ISL_1054423, EPI_ISL_1054424, EPI_ISL_1054425, EPI_ISL_1054426, EPI_ISL_1054427, EPI_ISL_1054428, EPI_ISL_1054432, EPI_ISL_1054433, EPI_ISL_1054434, EPI_ISL_1054435, EPI_ISL_1054436, EPI_ISL_1054437, EPI_ISL_1054444, EPI_ISL_1054445, EPI_ISL_1054446, EPI_ISL_1054451, EPI_ISL_1054452, EPI_ISL_1054453, EPI_ISL_1054455, EPI_ISL_1054456, EPI_ISL_1054459, EPI_ISL_1054460			
see above	Queens Medical Centre, Clinical Microbiology Department / DeepSeq Nottingham	COVID-19 Genomics UK (COG-UK) Consortium	Gemma Clark, Wendy Smith, Manjinder Khakh, Vicki M Fleming, Michelle M Lister, Hannah Howson-Wells, Jonathan Ball, Patrick McClure, Joseph Chappell, Theocharis Tsoleridis, Nadine Holmes, Matthew Carlisle, Christopher Moore, Fei Sang, Johnny Debebe, Victoria Wright, Matthew Loose
EPI_ISL_1054462, EPI_ISL_1054463, EPI_ISL_1054464, EPI_ISL_1054465, EPI_ISL_1054466, EPI_ISL_1054467, EPI_ISL_1054469, EPI_ISL_1054470, EPI_ISL_1054472, EPI_ISL_1054473, EPI_ISL_1054474, EPI_ISL_1054475, EPI_ISL_1054476, EPI_ISL_1054478, EPI_ISL_1054479, EPI_ISL_1054480, EPI_ISL_1054481, EPI_ISL_1054482, EPI_ISL_1054483, EPI_ISL_1054484, EPI_ISL_1054485, EPI_ISL_1054486, EPI_ISL_1054487, EPI_ISL_1054488, EPI_ISL_1054489, EPI_ISL_1054490, EPI_ISL_1054491, EPI_ISL_1054492, EPI_ISL_1054493, EPI_ISL_1054494, EPI_ISL_1054495, EPI_ISL_1054496, EPI_ISL_1054497, EPI_ISL_1054498, EPI_ISL_1054499, EPI_ISL_1054500, EPI_ISL_1054501, EPI_ISL_1054502, EPI_ISL_1054503, EPI_ISL_1054504, EPI_ISL_1054505, EPI_ISL_1054506, EPI_ISL_1054510, EPI_ISL_1054511, EPI_ISL_1054512, EPI_ISL_1054513, EPI_ISL_1054514, EPI_ISL_1054515, EPI_ISL_1054516, EPI_ISL_1054517, EPI_ISL_1054518, EPI_ISL_1054521, EPI_ISL_1054528, EPI_ISL_1054529, EPI_ISL_1054530, EPI_ISL_1054532, EPI_ISL_1054533, EPI_ISL_1054534, EPI_ISL_1054535, EPI_ISL_1054536, EPI_ISL_1054537, EPI_ISL_1054538, EPI_ISL_1054539, EPI_ISL_1054540, EPI_ISL_1054541			
see above	University of Exeter	COVID-19 Genomics UK (COG-UK) Consortium	Ben Temperton,Aaron Jeffries,Michelle Michelsen,Joanna Warwick-Dugdale,Audrey Farbos,Robyn Manley,Stephen Michell,Jane Masoli
EPI_ISL_1054826, EPI_ISL_1054827, EPI_ISL_1054828, EPI_ISL_1054829, EPI_ISL_1054831	Bioinformatics and Biostatistics Lab, Advanced Sequencing Facility	COVID-19 Genomics UK (COG-UK) Consortium	Aengus Stewart,Jerome Nicod,Chelsea Sawyer,Laura Cubitt,Harshil Patel,Margaret Crawford
EPI_ISL_1103757	University of Exeter	COVID-19 Genomics UK (COG-UK) Consortium	Ben Temperton,Aaron Jeffries,Michelle Michelsen,Joanna Warwick-Dugdale,Audrey Farbos,Robyn Manley,Stephen Michell,Jane Masoli
EPI_ISL_1103884, EPI_ISL_1103888, EPI_ISL_1103890, EPI_ISL_1103945	Department of Pathology, University of Cambridge	COVID-19 Genomics UK (COG-UK) Consortium	Aminu S. Jahun, Yasmin Chaudhry, Iliana Georgana, Myra Hosmillo, Rhys Izuagbe, William L. Hamilton, Martin D. Curran, Surendra Parmar, Ian Goodfellow
EPI_ISL_1103949, EPI_ISL_1103950, EPI_ISL_1103951, EPI_ISL_1103952, EPI_ISL_1103965, EPI_ISL_1103966, EPI_ISL_1103967, EPI_ISL_1103968, EPI_ISL_1103969, EPI_ISL_1103970, EPI_ISL_1103971, EPI_ISL_1103972, EPI_ISL_1103973, EPI_ISL_1103974, EPI_ISL_1103975, EPI_ISL_1103976, EPI_ISL_1103977, EPI_ISL_1103978, EPI_ISL_1103979, EPI_ISL_1103980, EPI_ISL_1103981, EPI_ISL_1103982, EPI_ISL_1103983, EPI_ISL_1103984, EPI_ISL_1103985, EPI_ISL_1103986, EPI_ISL_1103987, EPI_ISL_1103988, EPI_ISL_1103989, EPI_ISL_1103990, EPI_ISL_1103991, EPI_ISL_1103993, EPI_ISL_1103994, EPI_ISL_1103995, EPI_ISL_1103996, EPI_ISL_1104000, EPI_ISL_1104001, EPI_ISL_1104002, EPI_ISL_1104003, EPI_ISL_1104004, EPI_ISL_1104014, EPI_ISL_1104015, EPI_ISL_1104016, EPI_ISL_1104017, EPI_ISL_1104018, EPI_ISL_1104019, EPI_ISL_1104020, EPI_ISL_1104021, EPI_ISL_1104022, EPI_ISL_1104023, EPI_ISL_1104024			
see above	Northumbria University / South Tees Hospitals NHS Foundation Trust / North Cumbria Integrated Care NHS Foundation Trust / North Tees and Hartlepool NHS Foundation Trust / Newcastle Hospitals NHS Foundation Trust	COVID-19 Genomics UK (COG-UK) Consortium	Darren L Smith,Andrew Nelson,Matthew Bashton,Greg R Young,Joshua Loh,John Allan,Mohammad A Tariq,Giles S Holt,Gary Black,Wen C Yew,Lynn Dover,Paul Baker,Steve Liggett,Sarah Essex,Jane Greenaway,Debra Padgett,Clive Graham,Garren Scott,Edward Barton,Emma Swindells,Brendan Payne,Jennifer Collins,Yusri Taha,Gary Eltringham
EPI_ISL_1104025, EPI_ISL_1104026, EPI_ISL_1104027, EPI_ISL_1104028, EPI_ISL_1104030, EPI_ISL_1104031, EPI_ISL_1104032, EPI_ISL_1104033, EPI_ISL_1104034, EPI_ISL_1104035, EPI_ISL_1104036, EPI_ISL_1104037, EPI_ISL_1104038, EPI_ISL_1104039, EPI_ISL_1104040, EPI_ISL_1104049, EPI_ISL_1104050, EPI_ISL_1104071			
see above	Virology Department, Sheffield Teaching Hospitals NHS Foundation Trust/Department of Infection, Immunity and Cardiovascular Disease, The Medical School, University of Sheffield	COVID-19 Genomics UK (COG-UK) Consortium	Thushan de Silva, Matthew Parker, Nikki Smith, Adri Angyal, Rebecca Brown, Luke Green, Rachel Tucker, Paul Parsons, Danielle Groves, Katie Johnson, Laura Carrilero, Alex Keeley, Dave Partridge, Matthew Wyles, Benjamin Lindsey, Mehmet Yavuz, Mohammad Raza, Cariad Evans
EPI_ISL_1104500, EPI_ISL_1104501, EPI_ISL_1104505	Liverpool Clinical Laboratories	COVID-19 Genomics UK (COG-UK) Consortium	Sam Haldenby, Anita Lucaci, Steve Paterson, Julian Hiscox, Alistair Darby, M Almsaud, A Alrezaihi, Muhannad Alruwaili, Stuart D Armstrong, Jones Benjamin, Eleanor G Bentley, Anu Chawla, Jordan J Clark, Angela Cowell, Richard Eccles, Isabel García-Dorival, Matthew Gemmell, Alessandro Gerada, PKF Gilmore, Richard Gregory, Ximeng Han, Catherine Hartley, Margaret Hughes, Miren Iturriza-Gomara, James Johnson, L Luu, Jenifer Manson, Charlotte Nelson, Elaine O'Toole, Cassie Olateju, Rebekah Penrice-Randal , Lucille Rainbow, N.P Randle, Trevor Ian Robinson, Parul Sharma, Ghada T Shawli, James P Stewart, Neil Swainston, Ecaterina Vamos, Joanne Watts, Mark Whitehead
EPI_ISL_1104832, EPI_ISL_1104836, EPI_ISL_1104838, EPI_ISL_1104843, EPI_ISL_1104844, EPI_ISL_1104847, EPI_ISL_1104848, EPI_ISL_1104849, EPI_ISL_1104855, EPI_ISL_1104856, EPI_ISL_1104857, EPI_ISL_1104859, EPI_ISL_1104863, EPI_ISL_1104864, EPI_ISL_1104867, EPI_ISL_1104872, EPI_ISL_1104874, EPI_ISL_1104879, EPI_ISL_1104880, EPI_ISL_1104881, EPI_ISL_1104886, EPI_ISL_1104887, EPI_ISL_1104900, EPI_ISL_1104901, EPI_ISL_1104902, EPI_ISL_1104908, EPI_ISL_1104910, EPI_ISL_1104916, EPI_ISL_1104917, EPI_ISL_1104918, EPI_ISL_1104919, EPI_ISL_1104920, EPI_ISL_1104925, EPI_ISL_1104934, EPI_ISL_1104940, EPI_ISL_1104945, EPI_ISL_1104955, EPI_ISL_1104961, EPI_ISL_1104967, EPI_ISL_1105182, EPI_ISL_1105191, EPI_ISL_1105192, EPI_ISL_1105194, EPI_ISL_1105198, EPI_ISL_1105225, EPI_ISL_1105227			
see above	University College London Hospital	COVID-19 Genomics UK (COG-UK) Consortium	Judith Heaney, Matthew Byott, Catherine Houlihan, Dan Frampton, Stuart Kirk, Moira Spyer and Eleni Nastouli
EPI_ISL_1105467	University College London, Great Ormond Street Hospital for Children NHS Foundation Trust, Imperial College Healthcare NHS Trust	COVID-19 Genomics UK (COG-UK) Consortium	Sergi Castellano, Rachel Williams, Mark Kristiansen, Paola Resende Silva, Sunando Roy, Tony Brooks, Helena Tutill, Paola Niola, Patricia Dyal, Charlotte Williams, Leysa Forrest, Yasmin Panchbhaya, Jacqueline Findlay, Samuel Weeks, Julianne Brown, Kathryn Harris, Paul Randell, James Price, Alison Holmes, Judith Breuer
EPI_ISL_1105861, EPI_ISL_1105862, EPI_ISL_1105863, EPI_ISL_1105864, EPI_ISL_1105865, EPI_ISL_1105866, EPI_ISL_1105867, EPI_ISL_1105868, EPI_ISL_1105869, EPI_ISL_1105870, EPI_ISL_1105883, EPI_ISL_1105884, EPI_ISL_1105885, EPI_ISL_1105886, EPI_ISL_1105887, EPI_ISL_1105888			
see above	Lincolnshire Hospitals and DeepSeq Nottingham	COVID-19 Genomics UK (COG-UK) Consortium	Nichola Duckworth, Tim Sloan, Sarah Walsh, Jonathan Ball, Patrick McClure, Joeseph Chappell, Nadine Holmes, Matthew Carlisle, Christopher Moore, Fei Sang, Johnny Debebe, Victoria Wright, Matthew Loose
EPI_ISL_1106646, EPI_ISL_1106647	Originating lab: Wales Specialist Virology Centre Sequencing lab: Pathogen Genomics Unit	Public Health Wales Microbiology Cardiff Wales Specialist Virology Centre	Catherine Moore, Johnathan Evans, Laura Gifford, Malorie Perry, Simon Cottrell, Angela Marchbank, Alec Birchley, Alexander Adams, Amy Gaskin, Bree Gatica-Wilcox, Jason Coombes, Joel Southgate, Lauren Gilbert, Lee Graham, Nicole Pacchiarini, Sara Kumziene-Summerhayes, Sarah Taylor, Sophie Jones, Sara Rey, Matthew Bull, Joanne Watkins, Sally Corden, Tom Connor
EPI_ISL_1107632, EPI_ISL_1107655, EPI_ISL_1107656, EPI_ISL_1107657, EPI_ISL_1107658, EPI_ISL_1107659, EPI_ISL_1107660, EPI_ISL_1107661, EPI_ISL_1107662	Centre for Enzyme Innovation, University of Portsmouth / Translational Research Laboratory, Portsmouth Hospitals NHS Trust	COVID-19 Genomics UK (COG-UK) Consortium	Angela Beckett,Salman Goudarzi,Christopher Fearn,Kate Cook,Katie Loveson,Sharon Glaysher,Scott Elliott,Samuel Robson
EPI_ISL_1108957, EPI_ISL_1108960, EPI_ISL_1108961, EPI_ISL_1108962, EPI_ISL_1108963, EPI_ISL_1108964, EPI_ISL_1108965, EPI_ISL_1108966, EPI_ISL_1108967, EPI_ISL_1108968, EPI_ISL_1108969, EPI_ISL_1108971, EPI_ISL_1108973, EPI_ISL_1108974, EPI_ISL_1108975, EPI_ISL_1108976, EPI_ISL_1108977, EPI_ISL_1108978, EPI_ISL_1108979, EPI_ISL_1108980, EPI_ISL_1108981, EPI_ISL_1108982, EPI_ISL_1108984, EPI_ISL_1108985, EPI_ISL_1108986, EPI_ISL_1108987, EPI_ISL_1108988, EPI_ISL_1108989, EPI_ISL_1108990, EPI_ISL_1108991, EPI_ISL_1108992, EPI_ISL_1108993, EPI_ISL_1108994, EPI_ISL_1108995, EPI_ISL_1108996, EPI_ISL_1108997, EPI_ISL_1108998, EPI_ISL_1108999, EPI_ISL_1109000, EPI_ISL_1109001, EPI_ISL_1109002, EPI_ISL_1109003, EPI_ISL_1109004, EPI_ISL_1109005, EPI_ISL_1109006, EPI_ISL_1109007, EPI_ISL_1109008, EPI_ISL_1109009, EPI_ISL_1109010, EPI_ISL_1109011, EPI_ISL_1109012, EPI_ISL_1109014, EPI_ISL_1109015, EPI_ISL_1109016, EPI_ISL_1109017, EPI_ISL_1109018, EPI_ISL_1109019, EPI_ISL_1109020, EPI_ISL_1109021, EPI_ISL_1109022, EPI_ISL_1109023, EPI_ISL_1109026, EPI_ISL_1109028, EPI_ISL_1109030, EPI_ISL_1109032, EPI_ISL_1109035, EPI_ISL_1109037, EPI_ISL_1109042, EPI_ISL_1109043, EPI_ISL_1109044			

see above	Queens Medical Centre, Clinical Microbiology Department / DeepSeq Nottingham	COVID-19 Genomics UK (COG-UK) Consortium	Gemma Clark, Wendy Smith, Manjinder Khakh, Vicki M Fleming, Michelle M Lister, Hannah Howson-Wells, Jonathan Ball, Patrick McClure, Joseph Chappell, Theocharis Tsoleridis, Nadine Holmes, Matthew Carlisle, Christopher Moore, Fei Sang, Johnny Debebe, Victoria Wright, Matthew Loose
EPI_ISL_1177709, EPI_ISL_1177710, EPI_ISL_1177720, EPI_ISL_1177721	Virology Department, Royal Infirmary of Edinburgh, NHS Lothian / School of Biological Sciences, University of Edinburgh	COVID-19 Genomics UK (COG-UK) Consortium	McHugh M, Dewar R, Cotton S, Rooke S, O'Toole Á, Scher E, Hill V, McCrone JT, Colquhoun R, Yu X, Jackson B, Rambaut A, Templeton K
EPI_ISL_1177891, EPI_ISL_1177892	Liverpool Clinical Laboratories	COVID-19 Genomics UK (COG-UK) Consortium	Sam Haldenby, Alistair Darby, Steve Paterson, Anita Lucaci, Julian Hiscox, M Almsaud, A Alrezaihi, Muhannad Alruwaili, Stuart D Armstrong, Jones Benjamin, Eleanor G Bentley, Anu Chawla, Jordan J Clark, Angela Cowell, Richard Eccles, Isabel García-Dorival, Matthew Gemmell, Alessandro Gerada, PKF Gilmore, Richard Gregory, Ximeng Han, Catherine Hartley, Margaret Hughes, Miren Iturriza-Gomara, James Johnson, L Luu, Jenifer Manson, Charlotte Nelson, Elaine O'Toole, Cassie Olateju, Rebekah Penrice-Randal , Lucille Rainbow, N.P Randle, Trevor Ian Robinson, Parul Sharma, Ghada T Shawli, James P Stewart, Neil Swainston, Ecaterina Vamos, Joanne Watts, Mark Whitehead, Hermione Webster
EPI_ISL_1178148, EPI_ISL_1178149	Northumbria University / South Tees Hospitals NHS Foundation Trust / North Cumbria Integrated Care NHS Foundation Trust / North Tees and Hartlepool NHS Foundation Trust / Newcastle Hospitals NHS Foundation Trust	COVID-19 Genomics UK (COG-UK) Consortium	Darren L Smith,Andrew Nelson,Matthew Bashton,Greg R Young,Joshua Loh,John Allan,Mohammad A Tariq,Giles S Holt,Gary Black,Wen C Yew,Lynn Dover,Paul Baker,Steve Liggett,Sarah Essex,Jane Greenaway,Debra Padgett,Clive Graham,Garren Scott,Edward Barton,Emma Swindells,Brendan Payne,Jennifer Collins,Yusri Taha,Gary Eltringham
EPI_ISL_1178433, EPI_ISL_1178434, EPI_ISL_1178436, EPI_ISL_1178437, EPI_ISL_1178444, EPI_ISL_1178448, EPI_ISL_1178450, EPI_ISL_1178456, EPI_ISL_1178458, EPI_ISL_1178460, EPI_ISL_1178462, EPI_ISL_1178464, EPI_ISL_1178467, EPI_ISL_1178468, EPI_ISL_1178469, EPI_ISL_1178470, EPI_ISL_1178484, EPI_ISL_1178488, EPI_ISL_1178501			
see above	Quadram Institute Bioscience	COVID-19 Genomics UK (COG-UK) Consortium	Dave J. Baker, Gemma L. Kay, Alp Aydin, Thanh Le-Viet, Steven Rudder, Ana P. Tedim, Anastasia Kolyva, Maria Diaz, Leonardo de Oliveira Martins, Nabil-Fareed Alikhan, Lizzie Meadows, Rachael Stanley, Ngozi Elumogo, Muhammed Yasir, Nicholas M. Thomson, Alexander J Trotter, Rachel Gilroy, Samuel Bloomfield, Claire Stuart, Andrew Bell, Reenesh Prakash, Samir Dervisevic, Alison E. Mather, John Wain, Mark Webber, Andrew J. Page, Justin O'Grady
EPI_ISL_1178854	Oxford Viromics, NDM, University of Oxford; Oxford University Hospitals; Basingstoke and North Hampshire Hospital	COVID-19 Genomics UK (COG-UK) Consortium	Tanya Golubchik, David Bonsall, George Macintyre, Amy Trebes, Mariateresa de Cesare, Catrin Moore, Alex Mobbs, Anita Justice, Robert Shaw, Monique Andersson, Timothy Peto, Emma Wise, Nathan Moore, Jessica Lynch, Nick Cortes, Matilde Mori, Stephen Kidd, David Buck, John Todd, Christophe Fraser
EPI_ISL_1179828, EPI_ISL_1179829	Centre for Enzyme Innovation, University of Portsmouth / Translational Research Laboratory, Portsmouth Hospitals NHS Trust	COVID-19 Genomics UK (COG-UK) Consortium	Angela Beckett,Salman Goudarzi,Christopher Fearn,Kate Cook,Katie Loveson,Sharon Glaysher,Scott Elliott,Samuel Robson
EPI_ISL_1179891, EPI_ISL_1179892, EPI_ISL_1179893, EPI_ISL_1179894, EPI_ISL_1179895, EPI_ISL_1179896, EPI_ISL_1179897, EPI_ISL_1179898, EPI_ISL_1179899, EPI_ISL_1179900, EPI_ISL_1179901, EPI_ISL_1179902, EPI_ISL_1179903, EPI_ISL_1179904, EPI_ISL_1179905, EPI_ISL_1179906, EPI_ISL_1179907, EPI_ISL_1179908, EPI_ISL_1179909, EPI_ISL_1179910, EPI_ISL_1179912, EPI_ISL_1179913, EPI_ISL_1179914, EPI_ISL_1179915, EPI_ISL_1179916, EPI_ISL_1179917, EPI_ISL_1179918, EPI_ISL_1179919, EPI_ISL_1179920, EPI_ISL_1179921, EPI_ISL_1179922, EPI_ISL_1179923, EPI_ISL_1179924, EPI_ISL_1179925, EPI_ISL_1179926, EPI_ISL_1179927, EPI_ISL_1179928, EPI_ISL_1179929, EPI_ISL_1179930, EPI_ISL_1179931, EPI_ISL_1179932, EPI_ISL_1179934, EPI_ISL_1179937, EPI_ISL_1179938, EPI_ISL_1179939, EPI_ISL_1179940, EPI_ISL_1179941, EPI_ISL_1179942, EPI_ISL_1179943, EPI_ISL_1179944, EPI_ISL_1179945, EPI_ISL_1179946, EPI_ISL_1179947, EPI_ISL_1179948, EPI_ISL_1179949, EPI_ISL_1179950, EPI_ISL_1179964, EPI_ISL_1179965, EPI_ISL_1179966, EPI_ISL_1179967, EPI_ISL_1179968, EPI_ISL_1179969, EPI_ISL_1179970, EPI_ISL_1179971, EPI_ISL_1179972, EPI_ISL_1179973, EPI_ISL_1179974, EPI_ISL_1179979, EPI_ISL_1179980, EPI_ISL_1179981, EPI_ISL_1179982, EPI_ISL_1179984, EPI_ISL_1179985, EPI_ISL_1179986, EPI_ISL_1179987, EPI_ISL_1179988, EPI_ISL_1179989, EPI_ISL_1179997, EPI_ISL_1179998, EPI_ISL_1179999, EPI_ISL_1180000, EPI_ISL_1180001, EPI_ISL_1180002, EPI_ISL_1180003, EPI_ISL_1180004, EPI_ISL_1180005, EPI_ISL_1180006, EPI_ISL_1180007, EPI_ISL_1180008, EPI_ISL_1180009, EPI_ISL_1180010, EPI_ISL_1180011, EPI_ISL_1180012, EPI_ISL_1180013, EPI_ISL_1180014, EPI_ISL_1180015, EPI_ISL_1180016, EPI_ISL_1180018, EPI_ISL_1180019, EPI_ISL_1180020, EPI_ISL_1180021, EPI_ISL_1180022, EPI_ISL_1180029, EPI_ISL_1180030, EPI_ISL_1180031, EPI_ISL_1180032, EPI_ISL_1180033, EPI_ISL_1180034, EPI_ISL_1180035, EPI_ISL_1180036, EPI_ISL_1180037, EPI_ISL_1180038, EPI_ISL_1180039, EPI_ISL_1180040, EPI_ISL_1180041, EPI_ISL_1180042, EPI_ISL_1180043, EPI_ISL_1180044, EPI_ISL_1180045, EPI_ISL_1180046, EPI_ISL_1180047, EPI_ISL_1180048, EPI_ISL_1180049, EPI_ISL_1180050, EPI_ISL_1180051, EPI_ISL_1180052			
see above	Department of Pathology, University of Cambridge	COVID-19 Genomics UK (COG-UK) Consortium	Aminu S. Jahun, Yasmin Chaudhry, Iliana Georgana, Myra Hosmillo, Rhys Izuagbe, William L. Hamilton, Martin D. Curran, Surendra Parmar, Ian Goodfellow
EPI_ISL_1180089	Virology Department, Sheffield Teaching Hospitals NHS Foundation Trust/Department of Infection, Immunity and Cardiovascular Disease, The Medical School, University of Sheffield	COVID-19 Genomics UK (COG-UK) Consortium	Thushan de Silva, Matthew Parker, Nikki Smith, Adri Angyal, Rebecca Brown, Luke Green, Rachel Tucker, Paul Parsons, Danielle Groves, Katie Johnson, Laura Carrilero, Alex Keeley, Dave Partridge, Matthew Wyles, Benjamin Lindsey, Mehmet Yavuz, Mohammad Raza, Cariad Evans
EPI_ISL_1224517, EPI_ISL_1224537, EPI_ISL_1224567, EPI_ISL_1224585, EPI_ISL_1224594, EPI_ISL_1224604, EPI_ISL_1224608, EPI_ISL_1224614, EPI_ISL_1224626, EPI_ISL_1224639, EPI_ISL_1224642, EPI_ISL_1224670, EPI_ISL_1224700, EPI_ISL_1224705, EPI_ISL_1242051, EPI_ISL_1242056, EPI_ISL_1242082, EPI_ISL_1242087, EPI_ISL_1242088, EPI_ISL_1242091, EPI_ISL_1242117, EPI_ISL_1242150, EPI_ISL_1242169, EPI_ISL_1242170, EPI_ISL_1242174, EPI_ISL_1242177, EPI_ISL_1242178, EPI_ISL_1242182, EPI_ISL_1242197, EPI_ISL_1242221, EPI_ISL_1242236, EPI_ISL_1242252			
see above	Lighthouse Lab in Cambridge	Wellcome Sanger Institute for the COVID-19 Genomics UK (COG-UK) Consortium	Rob Howes, The Lighthouse Lab in Cambridge and Alex Alderton, Roberto Amato, Jeffrey Barrett, Sonia Goncalves, Ewan Harrison, David K. Jackson, Ian Johnston, Dominic Kwiatkowski, Cordelia Langford, John Sillitoe on behalf of the Wellcome Sanger Institute COVID-19 Surveillance Team
EPI_ISL_1247526	University of Exeter	COVID-19 Genomics UK (COG-UK) Consortium	Ben Temperton,Aaron Jeffries,Michelle Michelsen,Joanna Warwick-Dugdale,Audrey Farbos,Robyn Manley,Stephen Michell,Jane Masoli
EPI_ISL_1247728, EPI_ISL_1247730	Virology Department, Royal Infirmary of Edinburgh, NHS Lothian / School of Biological Sciences, University of Edinburgh	COVID-19 Genomics UK (COG-UK) Consortium	McHugh M, Dewar R, Cotton S, Rooke S, O'Toole Á, Scher E, Hill V, McCrone JT, Colquhoun R, Yu X, Jackson B, Rambaut A, Templeton K
EPI_ISL_1247825, EPI_ISL_1247827	Liverpool Clinical Laboratories	COVID-19 Genomics UK (COG-UK) Consortium	Sam Haldenby, Alistair Darby, Steve Paterson, Anita Lucaci, Julian Hiscox, M Almsaud, A Alrezaihi, Muhannad Alruwaili, Stuart D Armstrong, Jones Benjamin, Eleanor G Bentley, Anu Chawla, Jordan J Clark, Angela Cowell, Richard Eccles, Isabel García-Dorival, Matthew Gemmell, Alessandro Gerada, PKF Gilmore, Richard Gregory, Ximeng Han, Catherine Hartley, Margaret Hughes, Miren Iturriza-Gomara, James Johnson, L Luu, Jenifer Manson, Charlotte Nelson, Elaine O'Toole, Cassie Olateju, Rebekah Penrice-Randal , Lucille Rainbow, N.P Randle, Trevor Ian Robinson, Parul Sharma, Ghada T Shawli, James P Stewart, Neil Swainston, Ecaterina Vamos, Joanne Watts, Mark Whitehead, Hermione Webster
EPI_ISL_1248162, EPI_ISL_1248169, EPI_ISL_1248171, EPI_ISL_1248177, EPI_ISL_1248189, EPI_ISL_1248197, EPI_ISL_1248200	University College London, Great Ormond Street Hospital for Children NHS Foundation Trust, Imperial College Healthcare NHS Trust	COVID-19 Genomics UK (COG-UK) Consortium	Sergi Castellano, Rachel Williams, Mark Kristiansen, Paola Resende Silva, Sunando Roy, Tony Brooks, Helena Tutill, Paola Niola, Patricia Dyal, Charlotte Williams, Leysa Forrest, Yasmin Panchbhaya, Jacqueline Findlay, Samuel Weeks, Julianne Brown, Kathryn Harris, Paul Randell, James Price, Alison Holmes, Judith Breuer
EPI_ISL_1248945, EPI_ISL_1248947, EPI_ISL_1248950, EPI_ISL_1248951, EPI_ISL_1248953, EPI_ISL_1248955, EPI_ISL_1248956, EPI_ISL_1248958, EPI_ISL_1248959, EPI_ISL_1248961, EPI_ISL_1248962, EPI_ISL_1248979, EPI_ISL_1248984, EPI_ISL_1248987, EPI_ISL_1248994, EPI_ISL_1248999, EPI_ISL_1249004, EPI_ISL_1249008, EPI_ISL_1249013, EPI_ISL_1249025, EPI_ISL_1249028			
see above	Quadram Institute Bioscience	COVID-19 Genomics UK (COG-UK) Consortium	Dave J. Baker, Gemma L. Kay, Alp Aydin, Thanh Le-Viet, Steven Rudder, Ana P. Tedim, Anastasia Kolyva, Maria Diaz, Leonardo de Oliveira Martins, Nabil-Fareed Alikhan, Lizzie Meadows, Rachael Stanley, Ngozi Elumogo, Muhammed Yasir, Nicholas M. Thomson, Alexander J Trotter, Rachel Gilroy, Samuel Bloomfield, Claire Stuart, Andrew Bell, Reenesh Prakash, Samir Dervisevic, Alison E. Mather, John Wain, Mark Webber, Andrew J. Page, Justin O'Grady
EPI_ISL_1249224, EPI_ISL_1249241	Oxford Viromics, NDM, University of Oxford; Oxford University Hospitals; Basingstoke and North Hampshire Hospital	COVID-19 Genomics UK (COG-UK) Consortium	Tanya Golubchik, David Bonsall, George Macintyre, Amy Trebes, Mariateresa de Cesare, Catrin Moore, Alex Mobbs, Anita Justice, Robert Shaw, Monique Andersson, Timothy Peto, Emma Wise, Nathan Moore, Jessica Lynch, Nick Cortes, Matilde Mori, Stephen Kidd, David Buck, John Todd, Christophe Fraser
EPI_ISL_1250029	Centre for Enzyme Innovation, University of Portsmouth / Translational Research Laboratory, Portsmouth Hospitals NHS Trust	COVID-19 Genomics UK (COG-UK) Consortium	Angela Beckett,Salman Goudarzi,Christopher Fearn,Kate Cook,Katie Loveson,Sharon Glaysher,Scott Elliott,Samuel Robson
EPI_ISL_1296587, EPI_ISL_1296589, EPI_ISL_1296590, EPI_ISL_1296591, EPI_ISL_1296592, EPI_ISL_1296593, EPI_ISL_1296597, EPI_ISL_1296598	Respiratory Virus Unit, National Infection Service, Public Health England	COVID-19 Genomics UK (COG-UK) Consortium	PHE Covid Sequencing Team
EPI_ISL_1308567, EPI_ISL_1308579, EPI_ISL_1308642	University of Exeter	COVID-19 Genomics UK (COG-UK) Consortium	Ben Temperton,Aaron Jeffries,Michelle Michelsen,Joanna Warwick-Dugdale,Audrey Farbos,Robyn Manley,Stephen Michell,Jane Masoli
EPI_ISL_1308822, EPI_ISL_1308841	Virology Department, Royal Infirmary of Edinburgh, NHS Lothian / School of Biological Sciences, University of Edinburgh	COVID-19 Genomics UK (COG-UK) Consortium	McHugh M, Dewar R, Cotton S, Rooke S, O'Toole Á, Scher E, Hill V, McCrone JT, Colquhoun R, Yu X, Jackson B, Rambaut A, Templeton K

EPI_ISL_1333480	Lighthouse Lab in Cambridge	Wellcome Sanger Institute for the COVID-19 Genomics UK (COG-UK) Consortium	Rob Howes, The Lighthouse Lab in Cambridge and Alex Alderton, Roberto Amato, Jeffrey Barrett, Sonia Goncalves, Ewan Harrison, David K. Jackson, Ian Johnston, Dominic Kwiatkowski, Cordelia Langford, John Sillitoe on behalf of the Wellcome Sanger Institute COVID-19 Surveillance Team
EPI_ISL_1387213, EPI_ISL_1387216, EPI_ISL_1387217	Oxford Viromics, NDM, University of Oxford; Oxford University Hospitals; Basingstoke and North Hampshire Hospital	COVID-19 Genomics UK (COG-UK) Consortium	Tanya Golubchik, David Bonsall, George Macintyre, Amy Trebes, Mariateresa de Cesare, Catrin Moore, Alex Mobbs, Anita Justice, Robert Shaw, Monique Andersson, Timothy Peto, Emma Wise, Nathan Moore, Jessica Lynch, Nick Cortes, Matilde Mori, Stephen Kidd, David Buck, John Todd, Christophe Fraser
EPI_ISL_1449684	Lighthouse Lab in Alderley Park	Wellcome Sanger Institute for the COVID-19 Genomics UK (COG-UK) Consortium	Jacquelyn Wynn, Mairead Hyland, The Lighthouse Lab in Alderley Park and Alex Alderton, Roberto Amato, Jeffrey Barrett, Sonia Goncalves, Ewan Harrison, David K. Jackson, Ian Johnston, Dominic Kwiatkowski, Cordelia Langford, John Sillitoe on behalf of the Wellcome Sanger Institute COVID-19 Surveillance Team
EPI_ISL_1474996, EPI_ISL_1474997, EPI_ISL_1474998, EPI_ISL_1475000, EPI_ISL_1475017, EPI_ISL_1475036, EPI_ISL_1475037, EPI_ISL_1475038, EPI_ISL_1475039, EPI_ISL_1475043, EPI_ISL_1475044, EPI_ISL_1475138, EPI_ISL_1475140, EPI_ISL_1475141, EPI_ISL_1475142			
see above	Regional Virus Laboratory, Belfast Health and Social Care Trust	COVID-19 Genomics UK (COG-UK) Consortium	Conall McCaughey, James McKenna, Tanya Curran, Susan Feeney, Alison Watt, Ciara Cox, Mairead Connor, Zoltan Molnar, David Simpson, Derek Fairley
EPI_ISL_907237	Lighthouse Lab in Glasgow	Wellcome Sanger Institute for the COVID-19 Genomics UK (COG-UK) Consortium	Harper VanSteenhouse, Yumi Kasai, David Gray, Carol Clugston, Anna Dominiczak and Alex Alderton, Roberto Amato, Sonia Goncalves, Ewan Harrison, David K. Jackson, Ian Johnston, Dominic Kwiatkowski, Cordelia Langford, John Sillitoe on behalf of the Wellcome Sanger Institute COVID-19 Surveillance Team
EPI_ISL_924084, EPI_ISL_924101, EPI_ISL_924104, EPI_ISL_924108, EPI_ISL_924119, EPI_ISL_924122, EPI_ISL_924128, EPI_ISL_924148, EPI_ISL_924164, EPI_ISL_924176, EPI_ISL_924196, EPI_ISL_924197, EPI_ISL_924203, EPI_ISL_924215, EPI_ISL_924225, EPI_ISL_924240, EPI_ISL_924245, EPI_ISL_924252, EPI_ISL_924265, EPI_ISL_924274, EPI_ISL_924302, EPI_ISL_924328, EPI_ISL_924332, EPI_ISL_924334, EPI_ISL_924384, EPI_ISL_924389, EPI_ISL_924392, EPI_ISL_924397, EPI_ISL_924406			
see above	Virology Department, Sheffield Teaching Hospitals NHS Foundation Trust/Department of Infection, Immunity and Cardiovascular Disease, The Medical School, University of Sheffield	COVID-19 Genomics UK (COG-UK) Consortium	Thushan de Silva, Matthew Parker, Nikki Smith, Adri Angyal, Rebecca Brown, Luke Green, Rachel Tucker, Paul Parsons, Danielle Groves, Katie Johnson, Laura Carrilero, Alex Keeley, Dave Partridge, Matthew Wyles, Benjamin Lindsey, Mehmet Yavuz, Mohammad Raza, Cariad Evans
EPI_ISL_944792, EPI_ISL_944793, EPI_ISL_944794, EPI_ISL_944795, EPI_ISL_944796, EPI_ISL_944797, EPI_ISL_944798, EPI_ISL_944800, EPI_ISL_944801, EPI_ISL_944802, EPI_ISL_944803, EPI_ISL_944804, EPI_ISL_944806, EPI_ISL_944807, EPI_ISL_944808, EPI_ISL_944809, EPI_ISL_944810, EPI_ISL_944811, EPI_ISL_944812, EPI_ISL_944813, EPI_ISL_944814, EPI_ISL_944815, EPI_ISL_944816, EPI_ISL_944817, EPI_ISL_944818, EPI_ISL_944819, EPI_ISL_944820, EPI_ISL_944821, EPI_ISL_944822, EPI_ISL_944823, EPI_ISL_944825, EPI_ISL_944826, EPI_ISL_944827, EPI_ISL_944828, EPI_ISL_944829, EPI_ISL_944830, EPI_ISL_944831, EPI_ISL_944832, EPI_ISL_944833, EPI_ISL_944834, EPI_ISL_944835, EPI_ISL_944836, EPI_ISL_944837, EPI_ISL_944838, EPI_ISL_944839, EPI_ISL_944840, EPI_ISL_944841, EPI_ISL_944842, EPI_ISL_944843, EPI_ISL_944845, EPI_ISL_944846, EPI_ISL_944847, EPI_ISL_944848, EPI_ISL_944849, EPI_ISL_944850, EPI_ISL_944851, EPI_ISL_944852, EPI_ISL_944853, EPI_ISL_944854, EPI_ISL_944855, EPI_ISL_944856, EPI_ISL_944857, EPI_ISL_944858, EPI_ISL_944859, EPI_ISL_944860, EPI_ISL_944861, EPI_ISL_944862, EPI_ISL_944864, EPI_ISL_944865, EPI_ISL_944866, EPI_ISL_944867, EPI_ISL_944868, EPI_ISL_944869, EPI_ISL_944870, EPI_ISL_944871, EPI_ISL_944872, EPI_ISL_944873, EPI_ISL_944874, EPI_ISL_944875, EPI_ISL_944876, EPI_ISL_944877, EPI_ISL_944878, EPI_ISL_944879, EPI_ISL_944880, EPI_ISL_944881, EPI_ISL_944882, EPI_ISL_944884, EPI_ISL_944885, EPI_ISL_944886, EPI_ISL_944887, EPI_ISL_944888, EPI_ISL_944889, EPI_ISL_944890, EPI_ISL_944891, EPI_ISL_944892, EPI_ISL_944893, EPI_ISL_944894, EPI_ISL_944895, EPI_ISL_944896, EPI_ISL_944897, EPI_ISL_944898, EPI_ISL_944899, EPI_ISL_944900, EPI_ISL_944901, EPI_ISL_944902, EPI_ISL_944903, EPI_ISL_944904, EPI_ISL_944905, EPI_ISL_944906, EPI_ISL_944907, EPI_ISL_944909, EPI_ISL_944910, EPI_ISL_944911, EPI_ISL_944912, EPI_ISL_944913, EPI_ISL_944915, EPI_ISL_944916, EPI_ISL_944917, EPI_ISL_944918, EPI_ISL_944919, EPI_ISL_944920, EPI_ISL_944921, EPI_ISL_944922, EPI_ISL_944924, EPI_ISL_944925, EPI_ISL_944926, EPI_ISL_944927, EPI_ISL_944928, EPI_ISL_944929, EPI_ISL_944930, EPI_ISL_944931, EPI_ISL_944932, EPI_ISL_944933, EPI_ISL_944934, EPI_ISL_944935, EPI_ISL_944936, EPI_ISL_944937, EPI_ISL_944938, EPI_ISL_944939, EPI_ISL_944940, EPI_ISL_944941, EPI_ISL_944942, EPI_ISL_944943, EPI_ISL_944944, EPI_ISL_944945, EPI_ISL_944946, EPI_ISL_944947, EPI_ISL_944948, EPI_ISL_944949, EPI_ISL_944950, EPI_ISL_944951, EPI_ISL_944952, EPI_ISL_944954, EPI_ISL_944955, EPI_ISL_944956, EPI_ISL_944957, EPI_ISL_944958, EPI_ISL_944959, EPI_ISL_944960, EPI_ISL_944961, EPI_ISL_944962, EPI_ISL_944963, EPI_ISL_944964, EPI_ISL_944965, EPI_ISL_944966, EPI_ISL_944967, EPI_ISL_944968, EPI_ISL_944969, EPI_ISL_944970, EPI_ISL_944971, EPI_ISL_944972, EPI_ISL_944973, EPI_ISL_944974, EPI_ISL_944975, EPI_ISL_944977, EPI_ISL_944979, EPI_ISL_944980, EPI_ISL_944981, EPI_ISL_944982, EPI_ISL_944983, EPI_ISL_944984, EPI_ISL_944985, EPI_ISL_944986, EPI_ISL_944987, EPI_ISL_944988, EPI_ISL_944989, EPI_ISL_944990, EPI_ISL_944991, EPI_ISL_944992, EPI_ISL_944993, EPI_ISL_944994, EPI_ISL_944995, EPI_ISL_944996, EPI_ISL_944997, EPI_ISL_944998, EPI_ISL_944999, EPI_ISL_945000, EPI_ISL_945001, EPI_ISL_945002, EPI_ISL_945003, EPI_ISL_945004, EPI_ISL_945005, EPI_ISL_945006, EPI_ISL_945007, EPI_ISL_945008, EPI_ISL_945009, EPI_ISL_945010, EPI_ISL_945011, EPI_ISL_945012, EPI_ISL_945013, EPI_ISL_945014, EPI_ISL_945015, EPI_ISL_945016, EPI_ISL_945017, EPI_ISL_945018, EPI_ISL_945019, EPI_ISL_945020, EPI_ISL_945021, EPI_ISL_945022, EPI_ISL_945024, EPI_ISL_945025, EPI_ISL_945026, EPI_ISL_945027, EPI_ISL_945028, EPI_ISL_945029, EPI_ISL_945030, EPI_ISL_945031, EPI_ISL_945033, EPI_ISL_945035, EPI_ISL_945036, EPI_ISL_945037, EPI_ISL_945038, EPI_ISL_945039, EPI_ISL_945040, EPI_ISL_945041, EPI_ISL_945042, EPI_ISL_945043, EPI_ISL_945044, EPI_ISL_945045, EPI_ISL_945046, EPI_ISL_945047, EPI_ISL_945048			
see above	Lighthouse Lab in Glasgow	Wellcome Sanger Institute for the COVID-19 Genomics UK (COG-UK) Consortium	Harper VanSteenhouse, Yumi Kasai, David Gray, Carol Clugston, Anna Dominiczak and Alex Alderton, Roberto Amato, Sonia Goncalves, Ewan Harrison, David K. Jackson, Ian Johnston, Dominic Kwiatkowski, Cordelia Langford, John Sillitoe on behalf of the Wellcome Sanger Institute COVID-19 Surveillance Team
EPI_ISL_945374, EPI_ISL_945375, EPI_ISL_945376, EPI_ISL_945379, EPI_ISL_945386, EPI_ISL_945387, EPI_ISL_945389, EPI_ISL_945402, EPI_ISL_945408, EPI_ISL_945409, EPI_ISL_945419, EPI_ISL_945420, EPI_ISL_945422, EPI_ISL_945423, EPI_ISL_945433, EPI_ISL_945444, EPI_ISL_945446, EPI_ISL_945449, EPI_ISL_945458, EPI_ISL_945459, EPI_ISL_945466, EPI_ISL_945467, EPI_ISL_945468, EPI_ISL_945469, EPI_ISL_945475, EPI_ISL_945480, EPI_ISL_945485, EPI_ISL_945490, EPI_ISL_945494, EPI_ISL_945495, EPI_ISL_945498, EPI_ISL_945502, EPI_ISL_945506, EPI_ISL_945508, EPI_ISL_945514, EPI_ISL_945518, EPI_ISL_945522, EPI_ISL_945525, EPI_ISL_945529, EPI_ISL_945538, EPI_ISL_945539, EPI_ISL_945543, EPI_ISL_945545, EPI_ISL_945546, EPI_ISL_945548, EPI_ISL_945549, EPI_ISL_945555, EPI_ISL_945561, EPI_ISL_945563, EPI_ISL_945569, EPI_ISL_945579, EPI_ISL_945582, EPI_ISL_945587, EPI_ISL_945589, EPI_ISL_945591, EPI_ISL_945597, EPI_ISL_945619, EPI_ISL_945621, EPI_ISL_945627, EPI_ISL_945635, EPI_ISL_945636, EPI_ISL_945648, EPI_ISL_945650, EPI_ISL_945656, EPI_ISL_945664, EPI_ISL_945668, EPI_ISL_945672, EPI_ISL_945674, EPI_ISL_945677, EPI_ISL_945678, EPI_ISL_945681, EPI_ISL_945682, EPI_ISL_945692, EPI_ISL_945696, EPI_ISL_945697, EPI_ISL_945698, EPI_ISL_945701, EPI_ISL_945704			
see above	Lighthouse Lab in Alderley Park	Wellcome Sanger Institute for the COVID-19 Genomics UK (COG-UK) Consortium	Jacquelyn Wynn, Mairead Hyland, The Lighthouse Lab in Alderley Park and Alex Alderton, Roberto Amato, Sonia Goncalves, Ewan Harrison, David K. Jackson, Ian Johnston, Dominic Kwiatkowski, Cordelia Langford, John Sillitoe on behalf of the Wellcome Sanger Institute COVID-19 Surveillance Team
EPI_ISL_945716, EPI_ISL_945718, EPI_ISL_945722, EPI_ISL_945725, EPI_ISL_945726, EPI_ISL_945728, EPI_ISL_945729, EPI_ISL_945730, EPI_ISL_945732, EPI_ISL_945733, EPI_ISL_945735, EPI_ISL_945736, EPI_ISL_945737, EPI_ISL_945738, EPI_ISL_945741, EPI_ISL_945742, EPI_ISL_945743, EPI_ISL_945744, EPI_ISL_945747, EPI_ISL_945753, EPI_ISL_945755, EPI_ISL_945757, EPI_ISL_945758, EPI_ISL_945759, EPI_ISL_945762, EPI_ISL_945763, EPI_ISL_945768, EPI_ISL_945771, EPI_ISL_945772, EPI_ISL_945776, EPI_ISL_945777, EPI_ISL_945781, EPI_ISL_945782, EPI_ISL_945788, EPI_ISL_945792, EPI_ISL_945795, EPI_ISL_945796, EPI_ISL_945799, EPI_ISL_945800, EPI_ISL_945802, EPI_ISL_945803, EPI_ISL_945804, EPI_ISL_945806, EPI_ISL_945807, EPI_ISL_945808, EPI_ISL_945809, EPI_ISL_945812, EPI_ISL_945813, EPI_ISL_945815, EPI_ISL_945816, EPI_ISL_945817, EPI_ISL_945818, EPI_ISL_945821, EPI_ISL_945822, EPI_ISL_945824, EPI_ISL_945826, EPI_ISL_945828, EPI_ISL_945831, EPI_ISL_945835, EPI_ISL_945836, EPI_ISL_945839, EPI_ISL_945841, EPI_ISL_945843, EPI_ISL_945844, EPI_ISL_945846, EPI_ISL_945848, EPI_ISL_945849, EPI_ISL_945850, EPI_ISL_945851, EPI_ISL_945853, EPI_ISL_945860, EPI_ISL_945862, EPI_ISL_945863, EPI_ISL_945865, EPI_ISL_945867, EPI_ISL_945868, EPI_ISL_945869, EPI_ISL_945870, EPI_ISL_945871, EPI_ISL_945872, EPI_ISL_945873, EPI_ISL_945874, EPI_ISL_945877, EPI_ISL_945878, EPI_ISL_945879, EPI_ISL_945882, EPI_ISL_945884, EPI_ISL_945885, EPI_ISL_945889, EPI_ISL_945895, EPI_ISL_945898, EPI_ISL_945899, EPI_ISL_945900, EPI_ISL_945901, EPI_ISL_945904, EPI_ISL_945906, EPI_ISL_945907, EPI_ISL_945908, EPI_ISL_945909, EPI_ISL_945910, EPI_ISL_945911, EPI_ISL_945914, EPI_ISL_945916, EPI_ISL_945917, EPI_ISL_945920, EPI_ISL_945921, EPI_ISL_945926, EPI_ISL_945933, EPI_ISL_945935, EPI_ISL_945938, EPI_ISL_945939, EPI_ISL_945940, EPI_ISL_945944, EPI_ISL_945945, EPI_ISL_945950, EPI_ISL_945951, EPI_ISL_945954, EPI_ISL_945956, EPI_ISL_945958, EPI_ISL_945960, EPI_ISL_945961, EPI_ISL_945962, EPI_ISL_945963, EPI_ISL_945965, EPI_ISL_945967, EPI_ISL_945969, EPI_ISL_945970, EPI_ISL_945971, EPI_ISL_945972, EPI_ISL_945976, EPI_ISL_945979, EPI_ISL_945980, EPI_ISL_945983, EPI_ISL_945984, EPI_ISL_945988, EPI_ISL_945990, EPI_ISL_945993, EPI_ISL_945994, EPI_ISL_945997, EPI_ISL_945998, EPI_ISL_945999, EPI_ISL_946000, EPI_ISL_946002, EPI_ISL_946005, EPI_ISL_946006, EPI_ISL_946007, EPI_ISL_946008, EPI_ISL_946009, EPI_ISL_946010, EPI_ISL_946011, EPI_ISL_946014, EPI_ISL_946021, EPI_ISL_946023, EPI_ISL_946026, EPI_ISL_946028, EPI_ISL_946030, EPI_ISL_946035, EPI_ISL_946037, EPI_ISL_946041, EPI_ISL_946048, EPI_ISL_946049, EPI_ISL_946052, EPI_ISL_946055, EPI_ISL_946057, EPI_ISL_946058, EPI_ISL_946059, EPI_ISL_946062, EPI_ISL_946066, EPI_ISL_946067, EPI_ISL_946068, EPI_ISL_946069, EPI_ISL_946070, EPI_ISL_946071, EPI_ISL_946072, EPI_ISL_946073, EPI_ISL_946076, EPI_ISL_946080, EPI_ISL_946086, EPI_ISL_946087, EPI_ISL_946088, EPI_ISL_946090, EPI_ISL_946093, EPI_ISL_946096, EPI_ISL_946106, EPI_ISL_946109, EPI_ISL_946110, EPI_ISL_946112, EPI_ISL_946113, EPI_ISL_946115, EPI_ISL_946123, EPI_ISL_946124, EPI_ISL_946126, EPI_ISL_946127, EPI_ISL_946129, EPI_ISL_946135, EPI_ISL_946137, EPI_ISL_946145, EPI_ISL_946147, EPI_ISL_946149, EPI_ISL_946150, EPI_ISL_946157, EPI_ISL_946160, EPI_ISL_946162, EPI_ISL_946169, EPI_ISL_946173, EPI_ISL_946174, EPI_ISL_946176, EPI_ISL_946177, EPI_ISL_946180, EPI_ISL_946181, EPI_ISL_946182, EPI_ISL_946188, EPI_ISL_946190, EPI_ISL_946192, EPI_ISL_946196, EPI_ISL_946199, EPI_ISL_946202, EPI_ISL_946206, EPI_ISL_946208, EPI_ISL_946210, EPI_ISL_946211, EPI_ISL_946213, EPI_ISL_946215, EPI_ISL_946218, EPI_ISL_946220, EPI_ISL_946221, EPI_ISL_946224, EPI_ISL_946225, EPI_ISL_946227, EPI_ISL_946229, EPI_ISL_946231, EPI_ISL_946232, EPI_ISL_946233, EPI_ISL_946234, EPI_ISL_946243, EPI_ISL_946250, EPI_ISL_946253, EPI_ISL_946254, EPI_ISL_946260, EPI_ISL_946264, EPI_ISL_946270, EPI_ISL_946271, EPI_ISL_946274, EPI_ISL_946281, EPI_ISL_946284, EPI_ISL_946285, EPI_ISL_946286, EPI_ISL_946292, EPI_ISL_946298, EPI_ISL_946301, EPI_ISL_946302, EPI_ISL_946304, EPI_ISL_946318, EPI_ISL_946319, EPI_ISL_946326, EPI_ISL_946328, EPI_ISL_946333, EPI_ISL_946335, EPI_ISL_946338, EPI_ISL_946339, EPI_ISL_946351, EPI_ISL_946356, EPI_ISL_946357, EPI_ISL_946360, EPI_ISL_946361, EPI_ISL_946365, EPI_ISL_946366, EPI_ISL_946367, EPI_ISL_946371, EPI_ISL_946374, EPI_ISL_946375, EPI_ISL_946378, EPI_ISL_946379, EPI_ISL_946380, EPI_ISL_946385, EPI_ISL_946391, EPI_ISL_946397, EPI_ISL_946400, EPI_ISL_946405, EPI_ISL_946407, EPI_ISL_946410, EPI_ISL_946411			
see above	Lighthouse Lab in Glasgow	Wellcome Sanger Institute for the COVID-19 Genomics UK (COG-UK) Consortium	Harper VanSteenhouse, Yumi Kasai, David Gray, Carol Clugston, Anna Dominiczak and Alex Alderton, Roberto Amato, Sonia Goncalves, Ewan Harrison, David K. Jackson, Ian Johnston, Dominic Kwiatkowski, Cordelia Langford, John Sillitoe on behalf of the Wellcome Sanger Institute COVID-19 Surveillance Team
EPI_ISL_949405	University of Birmingham	COVID-19 Genomics UK (COG-UK) Consortium	Institute of Microbiology, University of Birmingham: Claire McMurray, Joanne Stockton, Samuel Nicholls, Radoslaw Poplawski, Will Rowe, Josh Quick, Nicholas Loman. University of Birmingham Testing Laboratory: Celina M Whalley, Andrew Bosworth, Charlotte Poxon, Kasun Wanigasooriya, Oliver Pickles, Mike Kidd, Alex Richter, Andrew D Beggs PHE Heartlands Lab: Husam Osman, Andrew Bosworth. Queen Elizabeth Hospital: Anna Casey
EPI_ISL_949543, EPI_ISL_949545, EPI_ISL_949547, EPI_ISL_949551, EPI_ISL_949552, EPI_ISL_949557, EPI_ISL_949558, EPI_ISL_949560, EPI_ISL_949561, EPI_ISL_949562, EPI_ISL_949563, EPI_ISL_949564, EPI_ISL_949565, EPI_ISL_949566, EPI_ISL_949567, EPI_ISL_949568, EPI_ISL_949569, EPI_ISL_949570, EPI_ISL_949571, EPI_ISL_949572, EPI_ISL_949573, EPI_ISL_949574, EPI_ISL_949575, EPI_ISL_949576, EPI_ISL_949577, EPI_ISL_949578, EPI_ISL_949579, EPI_ISL_949580, EPI_ISL_949582, EPI_ISL_949583, EPI_ISL_949584, EPI_ISL_949585, EPI_ISL_949586, EPI_ISL_949587, EPI_ISL_949588, EPI_ISL_949589, EPI_ISL_949590, EPI_ISL_949591, EPI_ISL_949592, EPI_ISL_949593, EPI_ISL_949594, EPI_ISL_949595, EPI_ISL_949596, EPI_ISL_949597, EPI_ISL_949598, EPI_ISL_949599, EPI_ISL_949600, EPI_ISL_949601, EPI_ISL_949602, EPI_ISL_949603, EPI_ISL_949604, EPI_ISL_949605, EPI_ISL_949606, EPI_ISL_949607			
see above	Department of Pathology, University of Cambridge	COVID-19 Genomics UK (COG-UK) Consortium	Aminu S. Jahun, Yasmin Chaudhry, Iliana Georgana, Myra Hosmillo, Rhys Izu, Martin D. Curran, Surendra Parmar, Ian Goodfellow
EPI_ISL_949616, EPI_ISL_949617, EPI_ISL_949619, EPI_ISL_949621, EPI_ISL_949623, EPI_ISL_949625, EPI_ISL_949627	West of Scotland Specialist Virology Centre, NHSGGC / MRC-University of Glasgow Centre for Virus Research	COVID-19 Genomics UK (COG-UK) Consortium	Ana da Silva Filipe, Natasha Johnson, Kathy Smollett, Daniel Mair, Stephen Carmichael, Alice Broos, Lily Tong, Jenna Nichols, Kyriaki Nomikou; Sarah McDonald; Richard Orton, Joseph Hughes, Sreenu Vattipally, David L Robertson; Alasdair MacLean, Rory Gunson; Sharif Shaaban, Matthew Holden; Rachel Blacow, Guy Mollett, Kathy Li, James Shepherd, Antonia Ho, Emma Thomson
EPI_ISL_949662, EPI_ISL_949663, EPI_ISL_949664, EPI_ISL_949666, EPI_ISL_949668, EPI_ISL_949669, EPI_ISL_949670, EPI_ISL_949708, EPI_ISL_949709, EPI_ISL_949710, EPI_ISL_949711, EPI_ISL_949717, EPI_ISL_949719, EPI_ISL_949720, EPI_ISL_949722, EPI_ISL_949723, EPI_ISL_949726, EPI_ISL_949727, EPI_ISL_949728, EPI_ISL_949730, EPI_ISL_949740, EPI_ISL_949742, EPI_ISL_949743, EPI_ISL_949744, EPI_ISL_949745, EPI_ISL_949746			
see above	Liverpool Clinical Laboratories	COVID-19 Genomics UK (COG-UK) Consortium	Sam Haldenby, Anita Lucaci, Steve Paterson, Julian Hiscox, Alistair Darby, M Almsaud, A Alrezaihi, Muhannad Alruwaili, Stuart D Armstrong, Jones Benjamin, Eleanor G Bentley, Anu Chawla, Jordan J Clark, Angela Cowell, Richard Eccles, Isabel García-Dorival, Matthew Gemmell, Alessandro Gerada, PKF Gilmore, Richard Gregory, Ximeng Han, Catherine Hartley, Margaret Hughes, Miren Iturriza-Gomara, James Johnson, L Luu, Jenifer Manson,

			Charlotte Nelson, Elaine O'Toole, Cassie Olateju, Rebekah Penrice-Randal , Lucille Rainbow, N.P Randle, Trevor Ian Robinson, Parul Sharma, Ghada T Shawli, James P Stewart, Neil Swainston, Ecaterina Vamos, Joanne Watts, Mark Whitehead
EPI_ISL_949795, EPI_ISL_949796, EPI_ISL_949797, EPI_ISL_949798, EPI_ISL_949799, EPI_ISL_949800, EPI_ISL_949801, EPI_ISL_949803, EPI_ISL_949804, EPI_ISL_949805, EPI_ISL_949806, EPI_ISL_949807, EPI_ISL_949808, EPI_ISL_949809, EPI_ISL_949810, EPI_ISL_949866, EPI_ISL_949915, EPI_ISL_949916, EPI_ISL_949918, EPI_ISL_949921, EPI_ISL_949922, EPI_ISL_949923, EPI_ISL_949930, EPI_ISL_949932, EPI_ISL_949933, EPI_ISL_949935, EPI_ISL_949936, EPI_ISL_949940, EPI_ISL_949944, EPI_ISL_949947, EPI_ISL_949949, EPI_ISL_949950, EPI_ISL_949953, EPI_ISL_949957, EPI_ISL_949961, EPI_ISL_950173, EPI_ISL_950177, EPI_ISL_950195, EPI_ISL_950198, EPI_ISL_950200, EPI_ISL_950201, EPI_ISL_950202, EPI_ISL_950203, EPI_ISL_950204, EPI_ISL_950205, EPI_ISL_950206, EPI_ISL_950207, EPI_ISL_950208, EPI_ISL_950209, EPI_ISL_950210			
see above	University College London, Great Ormond Street Hospital for Children NHS Foundation Trust, Imperial College Healthcare NHS Trust	COVID-19 Genomics UK (COG-UK) Consortium	Sergi Castellano, Rachel Williams, Mark Kristiansen, Paola Resende Silva, Sunando Roy, Tony Brooks, Helena Tutill, Paola Niola, Patricia Dyal, Charlotte Williams, Leysa Forrest, Yasmin Panchbhaya, Jacqueline Findlay, Samuel Weeks, Julianne Brown, Kathryn Harris, Paul Randell, James Price, Alison Holmes, Judith Breuer
EPI_ISL_950402, EPI_ISL_950403, EPI_ISL_950404, EPI_ISL_950405, EPI_ISL_950406, EPI_ISL_950407, EPI_ISL_950408, EPI_ISL_950409, EPI_ISL_950410, EPI_ISL_950411, EPI_ISL_950412, EPI_ISL_950413, EPI_ISL_950414, EPI_ISL_950415, EPI_ISL_950425, EPI_ISL_950426, EPI_ISL_950427			
see above	Northumbria University / South Tees Hospitals NHS Foundation Trust / North Cumbria Integrated Care NHS Foundation Trust / North Tees and Hartlepool NHS Foundation Trust / Newcastle Hospitals NHS Foundation Trust	COVID-19 Genomics UK (COG-UK) Consortium	Darren L Smith,Andrew Nelson,Matthew Bashton,Greg R Young,Joshua Loh,John Allan,Mohammad A Tariq,Giles S Holt,Gary Black,Wen C Yew,Lynn Dover,Paul Baker,Steve Liggett,Sarah Essex,Jane Greenaway,Debra Padgett,Clive Graham,Garren Scott,Edward Barton,Emma Swindells,Brendan Payne,Jennifer Collins,Yusri Taha,Gary Eltringham
EPI_ISL_950646, EPI_ISL_950647, EPI_ISL_950648, EPI_ISL_950649, EPI_ISL_950650, EPI_ISL_950651, EPI_ISL_950652, EPI_ISL_950653, EPI_ISL_950654, EPI_ISL_950655, EPI_ISL_950656, EPI_ISL_950657, EPI_ISL_950658, EPI_ISL_950659, EPI_ISL_950660, EPI_ISL_950661, EPI_ISL_950762			
see above	Queens Medical Centre, Clinical Microbiology Department / DeepSeq Nottingham	COVID-19 Genomics UK (COG-UK) Consortium	Gemma Clark, Wendy Smith, Manjinder Khakh, Vicki M Fleming, Michelle M Lister, Hannah Howson-Wells, Jonathan Ball, Patrick McClure, Joseph Chappell, Theocharis Tsoleridis, Nadine Holmes, Matthew Carlisle, Christopher Moore, Fei Sang, Johnny Debebe, Victoria Wright, Matthew Loose
EPI_ISL_951783, EPI_ISL_951971, EPI_ISL_951975, EPI_ISL_951976, EPI_ISL_951977, EPI_ISL_951978, EPI_ISL_951979, EPI_ISL_951980, EPI_ISL_951981, EPI_ISL_951982, EPI_ISL_951983, EPI_ISL_951984, EPI_ISL_951985, EPI_ISL_951986, EPI_ISL_952021, EPI_ISL_952023, EPI_ISL_952028, EPI_ISL_952044, EPI_ISL_952046, EPI_ISL_952047, EPI_ISL_952048, EPI_ISL_952049, EPI_ISL_952050, EPI_ISL_952052, EPI_ISL_952053, EPI_ISL_952054, EPI_ISL_952055, EPI_ISL_952056, EPI_ISL_952057, EPI_ISL_952058, EPI_ISL_952060			
see above	Originating lab: Wales Specialist Virology Centre Sequencing lab: Pathogen Genomics Unit	Public Health Wales Microbiology Cardiff Wales Specialist Virology Centre	Catherine Moore, Johnathan Evans, Laura Gifford, Malorie Perry, Simon Cottrell, Angela Marchbank, Alec Birchley, Alexander Adams, Amy Gaskin, Bree Gatica-Wilcox, Jason Coombes, Joel Southgate, Lauren Gilbert, Lee Graham, Nicole Pacchiarini, Sara Kumziene-Summerhayes, Sarah Taylor, Sophie Jones, Sara Rey, Matthew Bull, Joanne Watkins, Sally Corden, Tom Connor
EPI_ISL_952957, EPI_ISL_952959, EPI_ISL_952972, EPI_ISL_952986, EPI_ISL_952988, EPI_ISL_952991, EPI_ISL_953003, EPI_ISL_953006, EPI_ISL_953012, EPI_ISL_953013, EPI_ISL_953017			
see above	Virology Department, Sheffield Teaching Hospitals NHS Foundation Trust/Department of Infection, Immunity and Cardiovascular Disease, The Medical School, University of Sheffield	COVID-19 Genomics UK (COG-UK) Consortium	Thushan de Silva, Matthew Parker, Nikki Smith, Adri Angyal, Rebecca Brown, Luke Green, Rachel Tucker, Paul Parsons, Danielle Groves, Katie Johnson, Laura Carrilero, Alex Keeley, Dave Partridge, Matthew Wyles, Benjamin Lindsey, Mehmet Yavuz, Mohammad Raza, Cariad Evans
EPI_ISL_953240, EPI_ISL_953245, EPI_ISL_953248, EPI_ISL_953249, EPI_ISL_953250, EPI_ISL_953252, EPI_ISL_953253, EPI_ISL_953255, EPI_ISL_953256, EPI_ISL_953257, EPI_ISL_953258, EPI_ISL_953264, EPI_ISL_953265, EPI_ISL_953266, EPI_ISL_953268, EPI_ISL_953269, EPI_ISL_953270, EPI_ISL_953271, EPI_ISL_953275, EPI_ISL_953277, EPI_ISL_953280, EPI_ISL_953282, EPI_ISL_953284, EPI_ISL_953285			
see above	Bioinformatics and Biostatistics Lab, Advanced Sequencing Facility	COVID-19 Genomics UK (COG-UK) Consortium	Aengus Stewart,Jerome Nicod,Chelsea Sawyer,Laura Cubitt,Harshil Patel,Margaret Crawford
EPI_ISL_956422, EPI_ISL_956423, EPI_ISL_956424, EPI_ISL_956425, EPI_ISL_956426, EPI_ISL_956427, EPI_ISL_956428, EPI_ISL_956429, EPI_ISL_956430, EPI_ISL_956431, EPI_ISL_956432, EPI_ISL_956433, EPI_ISL_956434, EPI_ISL_956435, EPI_ISL_956436, EPI_ISL_956437, EPI_ISL_956438, EPI_ISL_956439, EPI_ISL_956440, EPI_ISL_956441, EPI_ISL_956442, EPI_ISL_956443, EPI_ISL_956444, EPI_ISL_956445, EPI_ISL_956447, EPI_ISL_956448, EPI_ISL_956449, EPI_ISL_956450, EPI_ISL_956451, EPI_ISL_956452, EPI_ISL_956453, EPI_ISL_956454, EPI_ISL_956455, EPI_ISL_956456, EPI_ISL_956457, EPI_ISL_956458, EPI_ISL_956459, EPI_ISL_956460, EPI_ISL_956461, EPI_ISL_956462, EPI_ISL_956463, EPI_ISL_956464, EPI_ISL_956465, EPI_ISL_956466, EPI_ISL_956467, EPI_ISL_956468, EPI_ISL_956469, EPI_ISL_956470, EPI_ISL_956471, EPI_ISL_956472, EPI_ISL_956473, EPI_ISL_956474, EPI_ISL_956475, EPI_ISL_956476, EPI_ISL_956477, EPI_ISL_956478, EPI_ISL_956479, EPI_ISL_956480, EPI_ISL_956481, EPI_ISL_956482, EPI_ISL_956483, EPI_ISL_956484, EPI_ISL_956485, EPI_ISL_956486, EPI_ISL_956488, EPI_ISL_956489, EPI_ISL_956490, EPI_ISL_956491, EPI_ISL_956492, EPI_ISL_956493, EPI_ISL_956494, EPI_ISL_956495, EPI_ISL_956496, EPI_ISL_956497, EPI_ISL_956498, EPI_ISL_956499, EPI_ISL_956500, EPI_ISL_956501, EPI_ISL_956502, EPI_ISL_956503, EPI_ISL_956504, EPI_ISL_956505, EPI_ISL_956506, EPI_ISL_956507, EPI_ISL_956509, EPI_ISL_956510, EPI_ISL_956511, EPI_ISL_956512, EPI_ISL_956513, EPI_ISL_956514, EPI_ISL_956515, EPI_ISL_956516, EPI_ISL_956517, EPI_ISL_956518, EPI_ISL_956519, EPI_ISL_956520, EPI_ISL_956521, EPI_ISL_956522, EPI_ISL_956523, EPI_ISL_956524, EPI_ISL_956525, EPI_ISL_956526, EPI_ISL_956527, EPI_ISL_956528, EPI_ISL_956529, EPI_ISL_956530, EPI_ISL_956531, EPI_ISL_956532, EPI_ISL_956533, EPI_ISL_956534, EPI_ISL_956535, EPI_ISL_956536, EPI_ISL_956537, EPI_ISL_956538, EPI_ISL_956539, EPI_ISL_956540, EPI_ISL_956541, EPI_ISL_956542, EPI_ISL_956543, EPI_ISL_956544, EPI_ISL_956545, EPI_ISL_956546, EPI_ISL_956547, EPI_ISL_956548, EPI_ISL_956549, EPI_ISL_956550, EPI_ISL_956551, EPI_ISL_956552, EPI_ISL_956553, EPI_ISL_956554, EPI_ISL_956555, EPI_ISL_956556, EPI_ISL_956557, EPI_ISL_956558, EPI_ISL_956559, EPI_ISL_956560, EPI_ISL_956561, EPI_ISL_956563, EPI_ISL_956564, EPI_ISL_956565, EPI_ISL_956566, EPI_ISL_956567, EPI_ISL_956568, EPI_ISL_956569, EPI_ISL_956570, EPI_ISL_956571, EPI_ISL_956572, EPI_ISL_956573, EPI_ISL_956574, EPI_ISL_956575, EPI_ISL_956577, EPI_ISL_956578, EPI_ISL_956579, EPI_ISL_956580, EPI_ISL_956581, EPI_ISL_956582, EPI_ISL_956583, EPI_ISL_956584, EPI_ISL_956586, EPI_ISL_956588, EPI_ISL_956589, EPI_ISL_956590, EPI_ISL_956591, EPI_ISL_956593, EPI_ISL_956594, EPI_ISL_956595, EPI_ISL_956596, EPI_ISL_956597, EPI_ISL_956598, EPI_ISL_956599, EPI_ISL_956600, EPI_ISL_956603, EPI_ISL_956604, EPI_ISL_956605, EPI_ISL_956607, EPI_ISL_956608, EPI_ISL_956609, EPI_ISL_956610, EPI_ISL_956611, EPI_ISL_956612, EPI_ISL_956613, EPI_ISL_956614, EPI_ISL_956615, EPI_ISL_956616, EPI_ISL_956617, EPI_ISL_956619, EPI_ISL_956620, EPI_ISL_956621, EPI_ISL_956622, EPI_ISL_956623, EPI_ISL_956624, EPI_ISL_956627, EPI_ISL_956628, EPI_ISL_956629, EPI_ISL_956630, EPI_ISL_956632, EPI_ISL_956633, EPI_ISL_956634, EPI_ISL_956635, EPI_ISL_956637, EPI_ISL_956638, EPI_ISL_956639, EPI_ISL_956640, EPI_ISL_956641, EPI_ISL_956642, EPI_ISL_956643, EPI_ISL_956644, EPI_ISL_956645, EPI_ISL_956646, EPI_ISL_956647, EPI_ISL_956648, EPI_ISL_956649, EPI_ISL_956650, EPI_ISL_956652, EPI_ISL_956653, EPI_ISL_956654, EPI_ISL_956655, EPI_ISL_956656, EPI_ISL_956657, EPI_ISL_956658, EPI_ISL_956659, EPI_ISL_956660, EPI_ISL_956661, EPI_ISL_956662, EPI_ISL_956663, EPI_ISL_956664, EPI_ISL_956665, EPI_ISL_956666, EPI_ISL_956667, EPI_ISL_956669, EPI_ISL_956670, EPI_ISL_956671, EPI_ISL_956672, EPI_ISL_956673, EPI_ISL_956674, EPI_ISL_956675, EPI_ISL_956676, EPI_ISL_956677, EPI_ISL_956678, EPI_ISL_956679, EPI_ISL_956680, EPI_ISL_956681, EPI_ISL_956682, EPI_ISL_956683, EPI_ISL_956684, EPI_ISL_956686, EPI_ISL_956687, EPI_ISL_956688, EPI_ISL_956689, EPI_ISL_956692, EPI_ISL_956693, EPI_ISL_956695, EPI_ISL_956696, EPI_ISL_956697, EPI_ISL_956698, EPI_ISL_956700, EPI_ISL_956701, EPI_ISL_956702, EPI_ISL_956703, EPI_ISL_956704, EPI_ISL_956705, EPI_ISL_956706, EPI_ISL_956707, EPI_ISL_956709, EPI_ISL_956710, EPI_ISL_956711, EPI_ISL_956712, EPI_ISL_956713, EPI_ISL_956714, EPI_ISL_956715, EPI_ISL_956716, EPI_ISL_956717, EPI_ISL_956718, EPI_ISL_956719, EPI_ISL_956720, EPI_ISL_956721, EPI_ISL_956722, EPI_ISL_956723, EPI_ISL_956724, EPI_ISL_956725, EPI_ISL_956726, EPI_ISL_956727, EPI_ISL_956728, EPI_ISL_956730, EPI_ISL_956731, EPI_ISL_956732, EPI_ISL_956733, EPI_ISL_956734, EPI_ISL_956735, EPI_ISL_956736, EPI_ISL_956737, EPI_ISL_956738, EPI_ISL_956739, EPI_ISL_956740, EPI_ISL_956741, EPI_ISL_956742, EPI_ISL_956743, EPI_ISL_956744, EPI_ISL_956745, EPI_ISL_956746, EPI_ISL_956747, EPI_ISL_956748, EPI_ISL_956749, EPI_ISL_956750, EPI_ISL_956751, EPI_ISL_956752, EPI_ISL_956753, EPI_ISL_956754, EPI_ISL_956755, EPI_ISL_956756, EPI_ISL_956757, EPI_ISL_956758, EPI_ISL_956759, EPI_ISL_956760, EPI_ISL_956761, EPI_ISL_956762, EPI_ISL_956763, EPI_ISL_956764			
see above	Lighthouse Lab in Alderley Park	Wellcome Sanger Institute for the COVID-19 Genomics UK (COG-UK) Consortium	Jacquelyn Wynn, Mairead Hyland, The Lighthouse Lab in Alderley Park and Alex Alderton, Roberto Amato, Sonia Goncalves, Ewan Harrison, David K. Jackson, Ian Johnston, Dominic Kwiatkowski, Cordelia Langford, John Sillitoe on behalf of the Wellcome Sanger Institute COVID-19 Surveillance Team
EPI_ISL_956765, EPI_ISL_956766, EPI_ISL_956767, EPI_ISL_956768, EPI_ISL_956769, EPI_ISL_956770, EPI_ISL_956771, EPI_ISL_956772, EPI_ISL_956773, EPI_ISL_956774, EPI_ISL_956775, EPI_ISL_956776, EPI_ISL_956777, EPI_ISL_956778, EPI_ISL_956779, EPI_ISL_956780, EPI_ISL_956781, EPI_ISL_956782, EPI_ISL_956783, EPI_ISL_956784, EPI_ISL_956785, EPI_ISL_956786, EPI_ISL_956787, EPI_ISL_956788, EPI_ISL_956789, EPI_ISL_956790, EPI_ISL_956791, EPI_ISL_956792, EPI_ISL_956793, EPI_ISL_956794, EPI_ISL_956795, EPI_ISL_956796, EPI_ISL_956797, EPI_ISL_956798, EPI_ISL_956799, EPI_ISL_956800, EPI_ISL_956801, EPI_ISL_956802, EPI_ISL_956803, EPI_ISL_956804, EPI_ISL_956805, EPI_ISL_956806, EPI_ISL_956807, EPI_ISL_956808, EPI_ISL_956809, EPI_ISL_956810, EPI_ISL_956811, EPI_ISL_956812, EPI_ISL_956813, EPI_ISL_956814, EPI_ISL_956815, EPI_ISL_956816, EPI_ISL_956817, EPI_ISL_956818, EPI_ISL_956819, EPI_ISL_956820, EPI_ISL_956821, EPI_ISL_956822, EPI_ISL_956823, EPI_ISL_956824, EPI_ISL_956825, EPI_ISL_956826, EPI_ISL_956827, EPI_ISL_956828, EPI_ISL_956829, EPI_ISL_956830, EPI_ISL_956831, EPI_ISL_956832, EPI_ISL_956833, EPI_ISL_956834, EPI_ISL_956835, EPI_ISL_956836, EPI_ISL_956837, EPI_ISL_956838, EPI_ISL_956839, EPI_ISL_956840, EPI_ISL_956841, EPI_ISL_956842, EPI_ISL_956843, EPI_ISL_956844, EPI_ISL_956845, EPI_ISL_956846, EPI_ISL_956847, EPI_ISL_956848, EPI_ISL_956849, EPI_ISL_956850, EPI_ISL_956851, EPI_ISL_956852, EPI_ISL_956853, EPI_ISL_956854, EPI_ISL_956855, EPI_ISL_956856, EPI_ISL_956857, EPI_ISL_956858, EPI_ISL_956859, EPI_ISL_956860, EPI_ISL_956861, EPI_ISL_956862, EPI_ISL_956863, EPI_ISL_956864, EPI_ISL_956865, EPI_ISL_956866, EPI_ISL_956867, EPI_ISL_956868, EPI_ISL_956869, EPI_ISL_956870, EPI_ISL_956871, EPI_ISL_956872, EPI_ISL_956873, EPI_ISL_956874, EPI_ISL_956875, EPI_ISL_956876, EPI_ISL_956877, EPI_ISL_956878, EPI_ISL_956879, EPI_ISL_956880, EPI_ISL_956881, EPI_ISL_956882, EPI_ISL_956883, EPI_ISL_956884, EPI_ISL_956885, EPI_ISL_956886, EPI_ISL_956887, EPI_ISL_956888, EPI_ISL_956889, EPI_ISL_956890, EPI_ISL_956891, EPI_ISL_956892, EPI_ISL_956893, EPI_ISL_956894, EPI_ISL_956895, EPI_ISL_956896, EPI_ISL_956897, EPI_ISL_956898, EPI_ISL_956899, EPI_ISL_956900, EPI_ISL_956901, EPI_ISL_956902, EPI_ISL_956903, EPI_ISL_956904, EPI_ISL_956905, EPI_ISL_956906, EPI_ISL_956907, EPI_ISL_956908, EPI_ISL_956909, EPI_ISL_956910, EPI_ISL_956911, EPI_ISL_956912, EPI_ISL_956913, EPI_ISL_956914, EPI_ISL_956915, EPI_ISL_956916, EPI_ISL_956917, EPI_ISL_956918, EPI_ISL_956919, EPI_ISL_956920, EPI_ISL_956921, EPI_ISL_956922, EPI_ISL_956923, EPI_ISL_956924, EPI_ISL_956925, EPI_ISL_956926, EPI_ISL_956927, EPI_ISL_956928, EPI_ISL_956929, EPI_ISL_956930, EPI_ISL_956931, EPI_ISL_956932, EPI_ISL_956933, EPI_ISL_956934, EPI_ISL_956935, EPI_ISL_956936, EPI_ISL_956937, EPI_ISL_956938, EPI_ISL_956939, EPI_ISL_956940, EPI_ISL_956941, EPI_ISL_956942, EPI_ISL_956943, EPI_ISL_956944, EPI_ISL_956945, EPI_ISL_956946, EPI_ISL_956947, EPI_ISL_956948, EPI_ISL_956949, EPI_ISL_956950, EPI_ISL_956951, EPI_ISL_956952, EPI_ISL_956953, EPI_ISL_956954, EPI_ISL_956955, EPI_ISL_956956, EPI_ISL_956957, EPI_ISL_956958, EPI_ISL_956959, EPI_ISL_956960, EPI_ISL_956961, EPI_ISL_956962, EPI_ISL_956963, EPI_ISL_956964, EPI_ISL_956965, EPI_ISL_956966, EPI_ISL_956967, EPI_ISL_956968, EPI_ISL_956969, EPI_ISL_956970, EPI_ISL_956971, EPI_ISL_956972, EPI_ISL_956973, EPI_ISL_956974, EPI_ISL_956975, EPI_ISL_956976, EPI_ISL_956977, EPI_ISL_956978, EPI_ISL_956979, EPI_ISL_956980, EPI_ISL_956981, EPI_ISL_956982, EPI_ISL_956983, EPI_ISL_956984, EPI_ISL_956985, EPI_ISL_956986, EPI_ISL_956987, EPI_ISL_956988, EPI_ISL_956989, EPI_ISL_956990, EPI_ISL_956991, EPI_ISL_956992, EPI_ISL_956993, EPI_ISL_956994, EPI_ISL_956995, EPI_ISL_956996, EPI_ISL_956997, EPI_ISL_956998, EPI_ISL_956999, EPI_ISL_957000, EPI_ISL_957001, EPI_ISL_957002, EPI_ISL_957003, EPI_ISL_957004, EPI_ISL_957005, EPI_ISL_957006, EPI_ISL_957007, EPI_ISL_957008, EPI_ISL_957009, EPI_ISL_957010, EPI_ISL_957011, EPI_ISL_957012, EPI_ISL_957013, EPI_ISL_957014, EPI_ISL_957015, EPI_ISL_957016, EPI_ISL_957017, EPI_ISL_957018, EPI_ISL_957019, EPI_ISL_957020, EPI_ISL_957021, EPI_ISL_957022, EPI_ISL_957023, EPI_ISL_957024, EPI_ISL_957025, EPI_ISL_957026, EPI_ISL_957027, EPI_ISL_957028, EPI_ISL_957029, EPI_ISL_957030, EPI_ISL_957031, EPI_ISL_957032, EPI_ISL_957033, EPI_ISL_957034, EPI_ISL_957035, EPI_ISL_957036, EPI_ISL_957037, EPI_ISL_957038, EPI_ISL_957039, EPI_ISL_957040, EPI_ISL_957041, EPI_ISL_957042, EPI_ISL_957043, EPI_ISL_957044, EPI_ISL_957045, EPI_ISL_957046, EPI_ISL_957047, EPI_ISL_957048, EPI_ISL_957049, EPI_ISL_957050, EPI_ISL_957051, EPI_ISL_957052, EPI_ISL_957053, EPI_ISL_957056, EPI_ISL_957058, EPI_ISL_957059, EPI_ISL_957062, EPI_ISL_957063, EPI_ISL_957064, EPI_ISL_957066, EPI_ISL_957069, EPI_ISL_957070, EPI_ISL_957074, EPI_ISL_957076, EPI_ISL_957085, EPI_ISL_957087, EPI_ISL_957088			
see above	Lighthouse Lab in Milton Keynes	Wellcome Sanger Institute for the COVID-19 Genomics UK (COG-UK) Consortium	The Lighthouse Lab in Milton Keynes and Alex Alderton, Roberto Amato, Sonia Goncalves, Ewan Harrison, David K. Jackson, Ian Johnston, Dominic Kwiatkowski, Cordelia Langford, John Sillitoe on behalf of the Wellcome Sanger Institute COVID-19 Surveillance Team
EPI_ISL_957090, EPI_ISL_957094	Lighthouse Lab in Glasgow	Wellcome Sanger Institute for the COVID-19 Genomics UK (COG-UK) Consortium	Harper VanSteenhouse, Yumi Kasai, David Gray, Carol Clugston, Anna Dominiczak and Alex Alderton, Roberto Amato, Sonia Goncalves, Ewan Harrison, David K. Jackson, Ian Johnston, Dominic Kwiatkowski, Cordelia Langford, John Sillitoe on behalf of the Wellcome Sanger Institute COVID-19 Surveillance Team
EPI_ISL_957096, EPI_ISL_957098, EPI_ISL_957100, EPI_ISL_957106, EPI_ISL_957108, EPI_ISL_957109, EPI_ISL_957111, EPI_ISL_957112, EPI_ISL_957113, EPI_ISL_957116, EPI_ISL_957119, EPI_ISL_957120, EPI_ISL_957123, EPI_ISL_957125, EPI_ISL_957126, EPI_ISL_957127, EPI_ISL_957128, EPI_ISL_957129, EPI_ISL_957131, EPI_ISL_957132, EPI_ISL_957134, EPI_ISL_957135, EPI_ISL_957136, EPI_ISL_957137, EPI_ISL_957138, EPI_ISL_957139, EPI_ISL_957142, EPI_ISL_957143, EPI_ISL_957144, EPI_ISL_957145, EPI_ISL_957146, EPI_ISL_957147, EPI_ISL_957148, EPI_ISL_957152, EPI_ISL_957153, EPI_ISL_957156, EPI_ISL_957159, EPI_ISL_957161, EPI_ISL_957163, EPI_ISL_957164, EPI_ISL_957167, EPI_ISL_957169, EPI_ISL_957173, EPI_ISL_957174, EPI_ISL_957175, EPI_ISL_957178, EPI_ISL_957179, EPI_ISL_957181, EPI_ISL_957182, EPI_ISL_957184, EPI_ISL_957185, EPI_ISL_957186, EPI_ISL_957189, EPI_ISL_957192, EPI_ISL_957193, EPI_ISL_957195, EPI_ISL_957196, EPI_ISL_957197, EPI_ISL_957199, EPI_ISL_957202, EPI_ISL_957203, EPI_ISL_957204, EPI_ISL_957207, EPI_ISL_957209, EPI_ISL_957213, EPI_ISL_957214, EPI_ISL_957217, EPI_ISL_957218, EPI_ISL_957220, EPI_ISL_957221, EPI_ISL_957222, EPI_ISL_957226, EPI_ISL_957228, EPI_ISL_957230, EPI_ISL_957232, EPI_ISL_957233, EPI_ISL_957234, EPI_ISL_957235, EPI_ISL_957236, EPI_ISL_957238, EPI_ISL_957240, EPI_ISL_957241, EPI_ISL_957244, EPI_ISL_957247, EPI_ISL_957249, EPI_ISL_957251, EPI_ISL_957252, EPI_ISL_957258, EPI_ISL_957261, EPI_ISL_957264, EPI_ISL_957265, EPI_ISL_957267, EPI_ISL_957269, EPI_ISL_957273, EPI_ISL_957277, EPI_ISL_957278, EPI_ISL_957280, EPI_ISL_957281, EPI_ISL_957282, EPI_ISL_957284, EPI_ISL_957288, EPI_ISL_957290, EPI_ISL_957293, EPI_ISL_957295, EPI_ISL_957296, EPI_ISL_957298, EPI_ISL_957300, EPI_ISL_957301,			

EPI_ISL_957303, EPI_ISL_957305, EPI_ISL_957307, EPI_ISL_957309, EPI_ISL_957313, EPI_ISL_957316, EPI_ISL_957317, EPI_ISL_957318, EPI_ISL_957321, EPI_ISL_957324, EPI_ISL_957325, EPI_ISL_957326, EPI_ISL_957329, EPI_ISL_957332, EPI_ISL_957333, EPI_ISL_957335, EPI_ISL_957338, EPI_ISL_957339, EPI_ISL_957340, EPI_ISL_957341, EPI_ISL_957342, EPI_ISL_957343, EPI_ISL_957344, EPI_ISL_957345, EPI_ISL_957346, EPI_ISL_957347, EPI_ISL_957348, EPI_ISL_957349, EPI_ISL_957350, EPI_ISL_957351, EPI_ISL_957352, EPI_ISL_957353, EPI_ISL_957354, EPI_ISL_957355, EPI_ISL_957356, EPI_ISL_957357, EPI_ISL_957358, EPI_ISL_957359, EPI_ISL_957360, EPI_ISL_957361, EPI_ISL_957362, EPI_ISL_957363, EPI_ISL_957364, EPI_ISL_957365, EPI_ISL_957366, EPI_ISL_957367, EPI_ISL_957368, EPI_ISL_957369, EPI_ISL_957370, EPI_ISL_957371, EPI_ISL_957372, EPI_ISL_957373, EPI_ISL_957374, EPI_ISL_957375, EPI_ISL_957376, EPI_ISL_957377, EPI_ISL_957378, EPI_ISL_957379, EPI_ISL_957380, EPI_ISL_957381, EPI_ISL_957382, EPI_ISL_957383, EPI_ISL_957384, EPI_ISL_957385, EPI_ISL_957386, EPI_ISL_957387, EPI_ISL_957388, EPI_ISL_957389, EPI_ISL_957390, EPI_ISL_957391, EPI_ISL_957392, EPI_ISL_957393, EPI_ISL_957394, EPI_ISL_957395, EPI_ISL_957396, EPI_ISL_957397, EPI_ISL_957398, EPI_ISL_957399, EPI_ISL_957400, EPI_ISL_957401, EPI_ISL_957402, EPI_ISL_957403, EPI_ISL_957404, EPI_ISL_957405, EPI_ISL_957406, EPI_ISL_957407, EPI_ISL_957408, EPI_ISL_957409, EPI_ISL_957410, EPI_ISL_957411, EPI_ISL_957412, EPI_ISL_957413, EPI_ISL_957414, EPI_ISL_957415, EPI_ISL_957416, EPI_ISL_957417, EPI_ISL_957418, EPI_ISL_957419, EPI_ISL_957420, EPI_ISL_957421, EPI_ISL_957422, EPI_ISL_957424, EPI_ISL_957425, EPI_ISL_957426, EPI_ISL_957427, EPI_ISL_957428, EPI_ISL_957429, EPI_ISL_957430, EPI_ISL_957431, EPI_ISL_957432, EPI_ISL_957433, EPI_ISL_957434, EPI_ISL_957435, EPI_ISL_957436, EPI_ISL_957437, EPI_ISL_957438, EPI_ISL_957439, EPI_ISL_957440, EPI_ISL_957441, EPI_ISL_957442, EPI_ISL_957443, EPI_ISL_957444, EPI_ISL_957445, EPI_ISL_957446, EPI_ISL_957447, EPI_ISL_957448, EPI_ISL_957449, EPI_ISL_957450, EPI_ISL_957451, EPI_ISL_957452, EPI_ISL_957453, EPI_ISL_957454, EPI_ISL_957455, EPI_ISL_957456, EPI_ISL_957457, EPI_ISL_957458, EPI_ISL_957459, EPI_ISL_957460, EPI_ISL_957461, EPI_ISL_957462, EPI_ISL_957463, EPI_ISL_957464, EPI_ISL_957465, EPI_ISL_957466, EPI_ISL_957467, EPI_ISL_957468, EPI_ISL_957469, EPI_ISL_957470, EPI_ISL_957471, EPI_ISL_957472, EPI_ISL_957473, EPI_ISL_957474, EPI_ISL_957475, EPI_ISL_957476, EPI_ISL_957477, EPI_ISL_957478, EPI_ISL_957479, EPI_ISL_957480, EPI_ISL_957481, EPI_ISL_957482, EPI_ISL_957483, EPI_ISL_957484, EPI_ISL_957485, EPI_ISL_957486, EPI_ISL_957487, EPI_ISL_957488, EPI_ISL_957489, EPI_ISL_957490, EPI_ISL_957491, EPI_ISL_957492, EPI_ISL_957493, EPI_ISL_957494, EPI_ISL_957495, EPI_ISL_957496, EPI_ISL_957497, EPI_ISL_957498, EPI_ISL_957499, EPI_ISL_957500, EPI_ISL_957501, EPI_ISL_957502, EPI_ISL_957503, EPI_ISL_957504, EPI_ISL_957505, EPI_ISL_957506, EPI_ISL_957507, EPI_ISL_957508, EPI_ISL_957509, EPI_ISL_957510, EPI_ISL_957511, EPI_ISL_957512, EPI_ISL_957513, EPI_ISL_957514, EPI_ISL_957515, EPI_ISL_957516, EPI_ISL_957517, EPI_ISL_957518, EPI_ISL_957519, EPI_ISL_957520, EPI_ISL_957521, EPI_ISL_957522, EPI_ISL_957523, EPI_ISL_957524, EPI_ISL_957525, EPI_ISL_957526, EPI_ISL_957527, EPI_ISL_957528, EPI_ISL_957529, EPI_ISL_957530, EPI_ISL_957531, EPI_ISL_957532, EPI_ISL_957533, EPI_ISL_957534, EPI_ISL_957535, EPI_ISL_957536, EPI_ISL_957537, EPI_ISL_957538, EPI_ISL_957539, EPI_ISL_957540, EPI_ISL_957541, EPI_ISL_957542, EPI_ISL_957543, EPI_ISL_957544, EPI_ISL_957545, EPI_ISL_957546, EPI_ISL_957547, EPI_ISL_957548, EPI_ISL_957549, EPI_ISL_957550, EPI_ISL_957551, EPI_ISL_957552, EPI_ISL_957553, EPI_ISL_957554, EPI_ISL_957555, EPI_ISL_957556, EPI_ISL_957557, EPI_ISL_957558, EPI_ISL_957559, EPI_ISL_957560, EPI_ISL_957561, EPI_ISL_957562, EPI_ISL_957563, EPI_ISL_957564, EPI_ISL_957565, EPI_ISL_957566, EPI_ISL_957567, EPI_ISL_957568, EPI_ISL_957569, EPI_ISL_957570, EPI_ISL_957571, EPI_ISL_957572, EPI_ISL_957573, EPI_ISL_957574, EPI_ISL_957575, EPI_ISL_957576, EPI_ISL_957577, EPI_ISL_957578, EPI_ISL_957579, EPI_ISL_957580, EPI_ISL_957581, EPI_ISL_957582, EPI_ISL_957583, EPI_ISL_957584, EPI_ISL_957585, EPI_ISL_957586, EPI_ISL_957587, EPI_ISL_957588, EPI_ISL_957589, EPI_ISL_957590, EPI_ISL_957591, EPI_ISL_957592, EPI_ISL_957593, EPI_ISL_957594, EPI_ISL_957595, EPI_ISL_957596, EPI_ISL_957597, EPI_ISL_957598, EPI_ISL_957599, EPI_ISL_957600, EPI_ISL_957601, EPI_ISL_957602, EPI_ISL_957603, EPI_ISL_957604, EPI_ISL_957605, EPI_ISL_957606, EPI_ISL_957607, EPI_ISL_957608, EPI_ISL_957609, EPI_ISL_957610, EPI_ISL_957611			
see above	Lighthouse Lab in Milton Keynes	Wellcome Sanger Institute for the COVID-19 Genomics UK (COG-UK) Consortium	The Lighthouse Lab in Milton Keynes and Alex Alderton, Roberto Amato, Sonia Goncalves, Ewan Harrison, David K. Jackson, Ian Johnston, Dominic Kwiatkowski, Cordelia Langford, John Sillitoe on behalf of the Wellcome Sanger Institute COVID-19 Surveillance Team
EPI_ISL_957612	Lighthouse Lab in Cambridge	Wellcome Sanger Institute for the COVID-19 Genomics UK (COG-UK) Consortium	Rob Howes, The Lighthouse Lab in Cambridge and Alex Alderton, Roberto Amato, Sonia Goncalves, Ewan Harrison, David K. Jackson, Ian Johnston, Dominic Kwiatkowski, Cordelia Langford, John Sillitoe on behalf of the Wellcome Sanger Institute COVID-19 Surveillance Team
EPI_ISL_957614	Lighthouse Lab in Milton Keynes	Wellcome Sanger Institute for the COVID-19 Genomics UK (COG-UK) Consortium	The Lighthouse Lab in Milton Keynes and Alex Alderton, Roberto Amato, Sonia Goncalves, Ewan Harrison, David K. Jackson, Ian Johnston, Dominic Kwiatkowski, Cordelia Langford, John Sillitoe on behalf of the Wellcome Sanger Institute COVID-19 Surveillance Team
EPI_ISL_957615, EPI_ISL_957618	Lighthouse Lab in Cambridge	Wellcome Sanger Institute for the COVID-19 Genomics UK (COG-UK) Consortium	Rob Howes, The Lighthouse Lab in Cambridge and Alex Alderton, Roberto Amato, Sonia Goncalves, Ewan Harrison, David K. Jackson, Ian Johnston, Dominic Kwiatkowski, Cordelia Langford, John Sillitoe on behalf of the Wellcome Sanger Institute COVID-19 Surveillance Team
EPI_ISL_957619	Lighthouse Lab in Milton Keynes	Wellcome Sanger Institute for the COVID-19 Genomics UK (COG-UK) Consortium	The Lighthouse Lab in Milton Keynes and Alex Alderton, Roberto Amato, Sonia Goncalves, Ewan Harrison, David K. Jackson, Ian Johnston, Dominic Kwiatkowski, Cordelia Langford, John Sillitoe on behalf of the Wellcome Sanger Institute COVID-19 Surveillance Team
EPI_ISL_957622	Lighthouse Lab in Cambridge	Wellcome Sanger Institute for the COVID-19 Genomics UK (COG-UK) Consortium	Rob Howes, The Lighthouse Lab in Cambridge and Alex Alderton, Roberto Amato, Sonia Goncalves, Ewan Harrison, David K. Jackson, Ian Johnston, Dominic Kwiatkowski, Cordelia Langford, John Sillitoe on behalf of the Wellcome Sanger Institute COVID-19 Surveillance Team
EPI_ISL_957623	Lighthouse Lab in Milton Keynes	Wellcome Sanger Institute for the COVID-19 Genomics UK (COG-UK) Consortium	The Lighthouse Lab in Milton Keynes and Alex Alderton, Roberto Amato, Sonia Goncalves, Ewan Harrison, David K. Jackson, Ian Johnston, Dominic Kwiatkowski, Cordelia Langford, John Sillitoe on behalf of the Wellcome Sanger Institute COVID-19 Surveillance Team
EPI_ISL_957626	Lighthouse Lab in Cambridge	Wellcome Sanger Institute for the COVID-19 Genomics UK (COG-UK) Consortium	Rob Howes, The Lighthouse Lab in Cambridge and Alex Alderton, Roberto Amato, Sonia Goncalves, Ewan Harrison, David K. Jackson, Ian Johnston, Dominic Kwiatkowski, Cordelia Langford, John Sillitoe on behalf of the Wellcome Sanger Institute COVID-19 Surveillance Team
EPI_ISL_957628, EPI_ISL_957629, EPI_ISL_957631, EPI_ISL_957632	Lighthouse Lab in Milton Keynes	Wellcome Sanger Institute for the COVID-19 Genomics UK (COG-UK) Consortium	The Lighthouse Lab in Milton Keynes and Alex Alderton, Roberto Amato, Sonia Goncalves, Ewan Harrison, David K. Jackson, Ian Johnston, Dominic Kwiatkowski, Cordelia Langford, John Sillitoe on behalf of the Wellcome Sanger Institute COVID-19 Surveillance Team
EPI_ISL_957638, EPI_ISL_957639, EPI_ISL_957642, EPI_ISL_957648, EPI_ISL_957657, EPI_ISL_957658	Lighthouse Lab in Cambridge	Wellcome Sanger Institute for the COVID-19 Genomics UK (COG-UK) Consortium	Rob Howes, The Lighthouse Lab in Cambridge and Alex Alderton, Roberto Amato, Sonia Goncalves, Ewan Harrison, David K. Jackson, Ian Johnston, Dominic Kwiatkowski, Cordelia Langford, John Sillitoe on behalf of the Wellcome Sanger Institute COVID-19 Surveillance Team
EPI_ISL_957659	Lighthouse Lab in Milton Keynes	Wellcome Sanger Institute for the COVID-19 Genomics UK (COG-UK) Consortium	The Lighthouse Lab in Milton Keynes and Alex Alderton, Roberto Amato, Sonia Goncalves, Ewan Harrison, David K. Jackson, Ian Johnston, Dominic Kwiatkowski, Cordelia Langford, John Sillitoe on behalf of the Wellcome Sanger Institute COVID-19 Surveillance Team
EPI_ISL_957661	Lighthouse Lab in Cambridge	Wellcome Sanger Institute for the COVID-19 Genomics UK (COG-UK) Consortium	Rob Howes, The Lighthouse Lab in Cambridge and Alex Alderton, Roberto Amato, Sonia Goncalves, Ewan Harrison, David K. Jackson, Ian Johnston, Dominic Kwiatkowski, Cordelia Langford, John Sillitoe on behalf of the Wellcome Sanger Institute COVID-19 Surveillance Team
EPI_ISL_957662	Lighthouse Lab in Milton Keynes	Wellcome Sanger Institute for the COVID-19 Genomics UK (COG-UK) Consortium	The Lighthouse Lab in Milton Keynes and Alex Alderton, Roberto Amato, Sonia Goncalves, Ewan Harrison, David K. Jackson, Ian Johnston, Dominic Kwiatkowski, Cordelia Langford, John Sillitoe on behalf of the Wellcome Sanger Institute COVID-19 Surveillance Team
EPI_ISL_957665	Lighthouse Lab in Cambridge	Wellcome Sanger Institute for the COVID-19 Genomics UK (COG-UK) Consortium	Rob Howes, The Lighthouse Lab in Cambridge and Alex Alderton, Roberto Amato, Sonia Goncalves, Ewan Harrison, David K. Jackson, Ian Johnston, Dominic Kwiatkowski, Cordelia Langford, John Sillitoe on behalf of the Wellcome Sanger Institute COVID-19 Surveillance Team
EPI_ISL_957667, EPI_ISL_957668, EPI_ISL_957671	Lighthouse Lab in Milton Keynes	Wellcome Sanger Institute for the COVID-19 Genomics UK (COG-UK) Consortium	The Lighthouse Lab in Milton Keynes and Alex Alderton, Roberto Amato, Sonia Goncalves, Ewan Harrison, David K. Jackson, Ian Johnston, Dominic Kwiatkowski, Cordelia Langford, John Sillitoe on behalf of the Wellcome Sanger Institute COVID-19 Surveillance Team
EPI_ISL_957676	Lighthouse Lab in Cambridge	Wellcome Sanger Institute for the COVID-19 Genomics UK (COG-UK) Consortium	Rob Howes, The Lighthouse Lab in Cambridge and Alex Alderton, Roberto Amato, Sonia Goncalves, Ewan Harrison, David K. Jackson, Ian Johnston, Dominic Kwiatkowski, Cordelia Langford, John Sillitoe on behalf of the Wellcome Sanger Institute COVID-19 Surveillance Team
EPI_ISL_957684, EPI_ISL_957685	Lighthouse Lab in Milton Keynes	Wellcome Sanger Institute for the COVID-19 Genomics UK (COG-UK) Consortium	The Lighthouse Lab in Milton Keynes and Alex Alderton, Roberto Amato, Sonia Goncalves, Ewan Harrison, David K. Jackson, Ian Johnston, Dominic Kwiatkowski, Cordelia Langford, John Sillitoe on behalf of the Wellcome Sanger Institute COVID-19 Surveillance Team
EPI_ISL_957688	Lighthouse Lab in Cambridge	Wellcome Sanger Institute for the COVID-19 Genomics UK (COG-UK) Consortium	Rob Howes, The Lighthouse Lab in Cambridge and Alex Alderton, Roberto Amato, Sonia Goncalves, Ewan Harrison, David K. Jackson, Ian Johnston, Dominic Kwiatkowski, Cordelia Langford, John Sillitoe on behalf of the Wellcome Sanger Institute COVID-19 Surveillance Team
EPI_ISL_957690, EPI_ISL_957692, EPI_ISL_957695	Lighthouse Lab in Milton Keynes	Wellcome Sanger Institute for the COVID-19 Genomics UK (COG-UK) Consortium	The Lighthouse Lab in Milton Keynes and Alex Alderton, Roberto Amato, Sonia Goncalves, Ewan Harrison, David K. Jackson, Ian Johnston, Dominic Kwiatkowski, Cordelia Langford, John Sillitoe on behalf of the Wellcome Sanger Institute COVID-19 Surveillance Team
EPI_ISL_957696	Lighthouse Lab in Cambridge	Wellcome Sanger Institute for the COVID-19 Genomics UK (COG-UK) Consortium	Rob Howes, The Lighthouse Lab in Cambridge and Alex Alderton, Roberto Amato, Sonia Goncalves, Ewan Harrison, David K. Jackson, Ian Johnston, Dominic Kwiatkowski, Cordelia Langford, John Sillitoe on behalf of the Wellcome Sanger Institute COVID-19 Surveillance Team
EPI_ISL_957699	Lighthouse Lab in Milton Keynes	Wellcome Sanger Institute for the COVID-19 Genomics UK (COG-UK) Consortium	The Lighthouse Lab in Milton Keynes and Alex Alderton, Roberto Amato, Sonia Goncalves, Ewan Harrison, David K. Jackson, Ian Johnston, Dominic Kwiatkowski, Cordelia Langford, John Sillitoe on behalf of the Wellcome Sanger Institute COVID-19 Surveillance Team
EPI_ISL_957702, EPI_ISL_957703, EPI_ISL_957704	Lighthouse Lab in Cambridge	Wellcome Sanger Institute for the COVID-19 Genomics UK (COG-UK) Consortium	Rob Howes, The Lighthouse Lab in Cambridge and Alex Alderton, Roberto Amato, Sonia Goncalves, Ewan Harrison, David K. Jackson, Ian Johnston, Dominic Kwiatkowski, Cordelia Langford, John Sillitoe on behalf of the Wellcome Sanger Institute COVID-19 Surveillance Team
EPI_ISL_957705	Lighthouse Lab in Milton Keynes	Wellcome Sanger Institute for the COVID-19 Genomics UK (COG-UK) Consortium	The Lighthouse Lab in Milton Keynes and Alex Alderton, Roberto Amato, Sonia Goncalves, Ewan Harrison, David K. Jackson, Ian Johnston, Dominic Kwiatkowski, Cordelia Langford, John Sillitoe on behalf of the Wellcome Sanger Institute COVID-19 Surveillance Team
EPI_ISL_957706, EPI_ISL_957709	Lighthouse Lab in Cambridge	Wellcome Sanger Institute for the COVID-19 Genomics UK (COG-UK) Consortium	Rob Howes, The Lighthouse Lab in Cambridge and Alex Alderton, Roberto Amato, Sonia Goncalves, Ewan Harrison, David K. Jackson, Ian Johnston, Dominic Kwiatkowski, Cordelia Langford, John Sillitoe on behalf of the Wellcome Sanger Institute COVID-19 Surveillance Team
EPI_ISL_957710, EPI_ISL_957711, EPI_ISL_957712	Lighthouse Lab in Milton Keynes	Wellcome Sanger Institute for the COVID-19 Genomics UK (COG-UK) Consortium	The Lighthouse Lab in Milton Keynes and Alex Alderton, Roberto Amato, Sonia Goncalves, Ewan Harrison, David K. Jackson, Ian Johnston, Dominic Kwiatkowski, Cordelia Langford, John Sillitoe on behalf of the Wellcome Sanger Institute COVID-19 Surveillance Team
EPI_ISL_957719	Lighthouse Lab in Cambridge	Wellcome Sanger Institute for the COVID-19 Genomics UK (COG-UK) Consortium	Rob Howes, The Lighthouse Lab in Cambridge and Alex Alderton, Roberto Amato, Sonia Goncalves, Ewan Harrison, David K. Jackson, Ian Johnston, Dominic Kwiatkowski, Cordelia Langford, John Sillitoe on behalf of the Wellcome Sanger Institute COVID-19 Surveillance Team
EPI_ISL_957724, EPI_ISL_957727	Lighthouse Lab in Milton Keynes	Wellcome Sanger Institute for the COVID-19 Genomics UK (COG-UK) Consortium	The Lighthouse Lab in Milton Keynes and Alex Alderton, Roberto Amato, Sonia Goncalves, Ewan Harrison, David K. Jackson, Ian Johnston, Dominic Kwiatkowski, Cordelia Langford, John Sillitoe on behalf of the Wellcome Sanger Institute COVID-19 Surveillance Team
EPI_ISL_957728, EPI_ISL_957731,	Lighthouse Lab in Cambridge	Wellcome Sanger Institute for the COVID-19 Genomics UK	Rob Howes, The Lighthouse Lab in Cambridge and Alex Alderton, Roberto Amato, Sonia Goncalves, Ewan Harrison, David K. Jackson, Ian Johnston,

EPI_ISL_957733, EPI_ISL_957734, EPI_ISL_957738, EPI_ISL_957739, EPI_ISL_957742		(COG-UK) Consortium	Dominic Kwiatkowski, Cordelia Langford, John Sillitoe on behalf of the Wellcome Sanger Institute COVID-19 Surveillance Team
EPI_ISL_957744	Lighthouse Lab in Milton Keynes	Wellcome Sanger Institute for the COVID-19 Genomics UK (COG-UK) Consortium	The Lighthouse Lab in Milton Keynes and Alex Alderton, Roberto Amato, Sonia Goncalves, Ewan Harrison, David K. Jackson, Ian Johnston, Dominic Kwiatkowski, Cordelia Langford, John Sillitoe on behalf of the Wellcome Sanger Institute COVID-19 Surveillance Team
EPI_ISL_957746, EPI_ISL_957751, EPI_ISL_957752, EPI_ISL_957754	Lighthouse Lab in Cambridge	Wellcome Sanger Institute for the COVID-19 Genomics UK (COG-UK) Consortium	Rob Howes, The Lighthouse Lab in Cambridge and Alex Alderton, Roberto Amato, Sonia Goncalves, Ewan Harrison, David K. Jackson, Ian Johnston, Dominic Kwiatkowski, Cordelia Langford, John Sillitoe on behalf of the Wellcome Sanger Institute COVID-19 Surveillance Team
EPI_ISL_957760, EPI_ISL_957761, EPI_ISL_957762	Lighthouse Lab in Milton Keynes	Wellcome Sanger Institute for the COVID-19 Genomics UK (COG-UK) Consortium	The Lighthouse Lab in Milton Keynes and Alex Alderton, Roberto Amato, Sonia Goncalves, Ewan Harrison, David K. Jackson, Ian Johnston, Dominic Kwiatkowski, Cordelia Langford, John Sillitoe on behalf of the Wellcome Sanger Institute COVID-19 Surveillance Team
EPI_ISL_957763, EPI_ISL_957764	Lighthouse Lab in Cambridge	Wellcome Sanger Institute for the COVID-19 Genomics UK (COG-UK) Consortium	Rob Howes, The Lighthouse Lab in Cambridge and Alex Alderton, Roberto Amato, Sonia Goncalves, Ewan Harrison, David K. Jackson, Ian Johnston, Dominic Kwiatkowski, Cordelia Langford, John Sillitoe on behalf of the Wellcome Sanger Institute COVID-19 Surveillance Team
EPI_ISL_957767, EPI_ISL_957775	Lighthouse Lab in Milton Keynes	Wellcome Sanger Institute for the COVID-19 Genomics UK (COG-UK) Consortium	The Lighthouse Lab in Milton Keynes and Alex Alderton, Roberto Amato, Sonia Goncalves, Ewan Harrison, David K. Jackson, Ian Johnston, Dominic Kwiatkowski, Cordelia Langford, John Sillitoe on behalf of the Wellcome Sanger Institute COVID-19 Surveillance Team
EPI_ISL_957776	Lighthouse Lab in Cambridge	Wellcome Sanger Institute for the COVID-19 Genomics UK (COG-UK) Consortium	Rob Howes, The Lighthouse Lab in Cambridge and Alex Alderton, Roberto Amato, Sonia Goncalves, Ewan Harrison, David K. Jackson, Ian Johnston, Dominic Kwiatkowski, Cordelia Langford, John Sillitoe on behalf of the Wellcome Sanger Institute COVID-19 Surveillance Team
EPI_ISL_957777	Lighthouse Lab in Milton Keynes	Wellcome Sanger Institute for the COVID-19 Genomics UK (COG-UK) Consortium	The Lighthouse Lab in Milton Keynes and Alex Alderton, Roberto Amato, Sonia Goncalves, Ewan Harrison, David K. Jackson, Ian Johnston, Dominic Kwiatkowski, Cordelia Langford, John Sillitoe on behalf of the Wellcome Sanger Institute COVID-19 Surveillance Team
EPI_ISL_957782, EPI_ISL_957784	Lighthouse Lab in Cambridge	Wellcome Sanger Institute for the COVID-19 Genomics UK (COG-UK) Consortium	Rob Howes, The Lighthouse Lab in Cambridge and Alex Alderton, Roberto Amato, Sonia Goncalves, Ewan Harrison, David K. Jackson, Ian Johnston, Dominic Kwiatkowski, Cordelia Langford, John Sillitoe on behalf of the Wellcome Sanger Institute COVID-19 Surveillance Team
EPI_ISL_957786	Lighthouse Lab in Milton Keynes	Wellcome Sanger Institute for the COVID-19 Genomics UK (COG-UK) Consortium	The Lighthouse Lab in Milton Keynes and Alex Alderton, Roberto Amato, Sonia Goncalves, Ewan Harrison, David K. Jackson, Ian Johnston, Dominic Kwiatkowski, Cordelia Langford, John Sillitoe on behalf of the Wellcome Sanger Institute COVID-19 Surveillance Team
EPI_ISL_957788, EPI_ISL_957789	Lighthouse Lab in Cambridge	Wellcome Sanger Institute for the COVID-19 Genomics UK (COG-UK) Consortium	Rob Howes, The Lighthouse Lab in Cambridge and Alex Alderton, Roberto Amato, Sonia Goncalves, Ewan Harrison, David K. Jackson, Ian Johnston, Dominic Kwiatkowski, Cordelia Langford, John Sillitoe on behalf of the Wellcome Sanger Institute COVID-19 Surveillance Team
EPI_ISL_957790, EPI_ISL_957791, EPI_ISL_957795	Lighthouse Lab in Milton Keynes	Wellcome Sanger Institute for the COVID-19 Genomics UK (COG-UK) Consortium	The Lighthouse Lab in Milton Keynes and Alex Alderton, Roberto Amato, Sonia Goncalves, Ewan Harrison, David K. Jackson, Ian Johnston, Dominic Kwiatkowski, Cordelia Langford, John Sillitoe on behalf of the Wellcome Sanger Institute COVID-19 Surveillance Team
EPI_ISL_957798	Lighthouse Lab in Cambridge	Wellcome Sanger Institute for the COVID-19 Genomics UK (COG-UK) Consortium	Rob Howes, The Lighthouse Lab in Cambridge and Alex Alderton, Roberto Amato, Sonia Goncalves, Ewan Harrison, David K. Jackson, Ian Johnston, Dominic Kwiatkowski, Cordelia Langford, John Sillitoe on behalf of the Wellcome Sanger Institute COVID-19 Surveillance Team
EPI_ISL_957800, EPI_ISL_957801, EPI_ISL_957803, EPI_ISL_957804	Lighthouse Lab in Milton Keynes	Wellcome Sanger Institute for the COVID-19 Genomics UK (COG-UK) Consortium	The Lighthouse Lab in Milton Keynes and Alex Alderton, Roberto Amato, Sonia Goncalves, Ewan Harrison, David K. Jackson, Ian Johnston, Dominic Kwiatkowski, Cordelia Langford, John Sillitoe on behalf of the Wellcome Sanger Institute COVID-19 Surveillance Team
EPI_ISL_957805, EPI_ISL_957807, EPI_ISL_957808, EPI_ISL_957809	Lighthouse Lab in Cambridge	Wellcome Sanger Institute for the COVID-19 Genomics UK (COG-UK) Consortium	Rob Howes, The Lighthouse Lab in Cambridge and Alex Alderton, Roberto Amato, Sonia Goncalves, Ewan Harrison, David K. Jackson, Ian Johnston, Dominic Kwiatkowski, Cordelia Langford, John Sillitoe on behalf of the Wellcome Sanger Institute COVID-19 Surveillance Team
EPI_ISL_957810	Lighthouse Lab in Milton Keynes	Wellcome Sanger Institute for the COVID-19 Genomics UK (COG-UK) Consortium	The Lighthouse Lab in Milton Keynes and Alex Alderton, Roberto Amato, Sonia Goncalves, Ewan Harrison, David K. Jackson, Ian Johnston, Dominic Kwiatkowski, Cordelia Langford, John Sillitoe on behalf of the Wellcome Sanger Institute COVID-19 Surveillance Team
EPI_ISL_957811, EPI_ISL_957812, EPI_ISL_957815	Lighthouse Lab in Cambridge	Wellcome Sanger Institute for the COVID-19 Genomics UK (COG-UK) Consortium	Rob Howes, The Lighthouse Lab in Cambridge and Alex Alderton, Roberto Amato, Sonia Goncalves, Ewan Harrison, David K. Jackson, Ian Johnston, Dominic Kwiatkowski, Cordelia Langford, John Sillitoe on behalf of the Wellcome Sanger Institute COVID-19 Surveillance Team
EPI_ISL_957816	Lighthouse Lab in Milton Keynes	Wellcome Sanger Institute for the COVID-19 Genomics UK (COG-UK) Consortium	The Lighthouse Lab in Milton Keynes and Alex Alderton, Roberto Amato, Sonia Goncalves, Ewan Harrison, David K. Jackson, Ian Johnston, Dominic Kwiatkowski, Cordelia Langford, John Sillitoe on behalf of the Wellcome Sanger Institute COVID-19 Surveillance Team
EPI_ISL_957818, EPI_ISL_957820	Lighthouse Lab in Cambridge	Wellcome Sanger Institute for the COVID-19 Genomics UK (COG-UK) Consortium	Rob Howes, The Lighthouse Lab in Cambridge and Alex Alderton, Roberto Amato, Sonia Goncalves, Ewan Harrison, David K. Jackson, Ian Johnston, Dominic Kwiatkowski, Cordelia Langford, John Sillitoe on behalf of the Wellcome Sanger Institute COVID-19 Surveillance Team
EPI_ISL_957823	Lighthouse Lab in Milton Keynes	Wellcome Sanger Institute for the COVID-19 Genomics UK (COG-UK) Consortium	The Lighthouse Lab in Milton Keynes and Alex Alderton, Roberto Amato, Sonia Goncalves, Ewan Harrison, David K. Jackson, Ian Johnston, Dominic Kwiatkowski, Cordelia Langford, John Sillitoe on behalf of the Wellcome Sanger Institute COVID-19 Surveillance Team
EPI_ISL_957825	Lighthouse Lab in Cambridge	Wellcome Sanger Institute for the COVID-19 Genomics UK (COG-UK) Consortium	Rob Howes, The Lighthouse Lab in Cambridge and Alex Alderton, Roberto Amato, Sonia Goncalves, Ewan Harrison, David K. Jackson, Ian Johnston, Dominic Kwiatkowski, Cordelia Langford, John Sillitoe on behalf of the Wellcome Sanger Institute COVID-19 Surveillance Team
EPI_ISL_957827	Lighthouse Lab in Milton Keynes	Wellcome Sanger Institute for the COVID-19 Genomics UK (COG-UK) Consortium	The Lighthouse Lab in Milton Keynes and Alex Alderton, Roberto Amato, Sonia Goncalves, Ewan Harrison, David K. Jackson, Ian Johnston, Dominic Kwiatkowski, Cordelia Langford, John Sillitoe on behalf of the Wellcome Sanger Institute COVID-19 Surveillance Team
EPI_ISL_957831, EPI_ISL_957833	Lighthouse Lab in Cambridge	Wellcome Sanger Institute for the COVID-19 Genomics UK (COG-UK) Consortium	Rob Howes, The Lighthouse Lab in Cambridge and Alex Alderton, Roberto Amato, Sonia Goncalves, Ewan Harrison, David K. Jackson, Ian Johnston, Dominic Kwiatkowski, Cordelia Langford, John Sillitoe on behalf of the Wellcome Sanger Institute COVID-19 Surveillance Team
EPI_ISL_957835, EPI_ISL_957842, EPI_ISL_957843, EPI_ISL_957849, EPI_ISL_957850	Lighthouse Lab in Milton Keynes	Wellcome Sanger Institute for the COVID-19 Genomics UK (COG-UK) Consortium	The Lighthouse Lab in Milton Keynes and Alex Alderton, Roberto Amato, Sonia Goncalves, Ewan Harrison, David K. Jackson, Ian Johnston, Dominic Kwiatkowski, Cordelia Langford, John Sillitoe on behalf of the Wellcome Sanger Institute COVID-19 Surveillance Team
EPI_ISL_957852, EPI_ISL_957855, EPI_ISL_957857	Lighthouse Lab in Cambridge	Wellcome Sanger Institute for the COVID-19 Genomics UK (COG-UK) Consortium	Rob Howes, The Lighthouse Lab in Cambridge and Alex Alderton, Roberto Amato, Sonia Goncalves, Ewan Harrison, David K. Jackson, Ian Johnston, Dominic Kwiatkowski, Cordelia Langford, John Sillitoe on behalf of the Wellcome Sanger Institute COVID-19 Surveillance Team
EPI_ISL_957858	Lighthouse Lab in Milton Keynes	Wellcome Sanger Institute for the COVID-19 Genomics UK (COG-UK) Consortium	The Lighthouse Lab in Milton Keynes and Alex Alderton, Roberto Amato, Sonia Goncalves, Ewan Harrison, David K. Jackson, Ian Johnston, Dominic Kwiatkowski, Cordelia Langford, John Sillitoe on behalf of the Wellcome Sanger Institute COVID-19 Surveillance Team
EPI_ISL_957861, EPI_ISL_957869, EPI_ISL_957870, EPI_ISL_957873, EPI_ISL_957874, EPI_ISL_957876, EPI_ISL_957880, EPI_ISL_957881, EPI_ISL_957883	Lighthouse Lab in Cambridge	Wellcome Sanger Institute for the COVID-19 Genomics UK (COG-UK) Consortium	Rob Howes, The Lighthouse Lab in Cambridge and Alex Alderton, Roberto Amato, Sonia Goncalves, Ewan Harrison, David K. Jackson, Ian Johnston, Dominic Kwiatkowski, Cordelia Langford, John Sillitoe on behalf of the Wellcome Sanger Institute COVID-19 Surveillance Team
EPI_ISL_957885	Lighthouse Lab in Milton Keynes	Wellcome Sanger Institute for the COVID-19 Genomics UK (COG-UK) Consortium	The Lighthouse Lab in Milton Keynes and Alex Alderton, Roberto Amato, Sonia Goncalves, Ewan Harrison, David K. Jackson, Ian Johnston, Dominic Kwiatkowski, Cordelia Langford, John Sillitoe on behalf of the Wellcome Sanger Institute COVID-19 Surveillance Team
EPI_ISL_957887	Lighthouse Lab in Cambridge	Wellcome Sanger Institute for the COVID-19 Genomics UK (COG-UK) Consortium	Rob Howes, The Lighthouse Lab in Cambridge and Alex Alderton, Roberto Amato, Sonia Goncalves, Ewan Harrison, David K. Jackson, Ian Johnston, Dominic Kwiatkowski, Cordelia Langford, John Sillitoe on behalf of the Wellcome Sanger Institute COVID-19 Surveillance Team
EPI_ISL_957888	Lighthouse Lab in Milton Keynes	Wellcome Sanger Institute for the COVID-19 Genomics UK (COG-UK) Consortium	The Lighthouse Lab in Milton Keynes and Alex Alderton, Roberto Amato, Sonia Goncalves, Ewan Harrison, David K. Jackson, Ian Johnston, Dominic Kwiatkowski, Cordelia Langford, John Sillitoe on behalf of the Wellcome Sanger Institute COVID-19 Surveillance Team
EPI_ISL_957890, EPI_ISL_957891	Lighthouse Lab in Cambridge	Wellcome Sanger Institute for the COVID-19 Genomics UK (COG-UK) Consortium	Rob Howes, The Lighthouse Lab in Cambridge and Alex Alderton, Roberto Amato, Sonia Goncalves, Ewan Harrison, David K. Jackson, Ian Johnston, Dominic Kwiatkowski, Cordelia Langford, John Sillitoe on behalf of the Wellcome Sanger Institute COVID-19 Surveillance Team
EPI_ISL_957893	Lighthouse Lab in Milton Keynes	Wellcome Sanger Institute for the COVID-19 Genomics UK (COG-UK) Consortium	The Lighthouse Lab in Milton Keynes and Alex Alderton, Roberto Amato, Sonia Goncalves, Ewan Harrison, David K. Jackson, Ian Johnston, Dominic Kwiatkowski, Cordelia Langford, John Sillitoe on behalf of the Wellcome Sanger Institute COVID-19 Surveillance Team
EPI_ISL_957896, EPI_ISL_957898	Lighthouse Lab in Cambridge	Wellcome Sanger Institute for the COVID-19 Genomics UK (COG-UK) Consortium	Rob Howes, The Lighthouse Lab in Cambridge and Alex Alderton, Roberto Amato, Sonia Goncalves, Ewan Harrison, David K. Jackson, Ian Johnston, Dominic Kwiatkowski, Cordelia Langford, John Sillitoe on behalf of the Wellcome Sanger Institute COVID-19 Surveillance Team

EPI_ISL_957899, EPI_ISL_957900, EPI_ISL_957901	Lighthouse Lab in Milton Keynes	Wellcome Sanger Institute for the COVID-19 Genomics UK (COG-UK) Consortium	The Lighthouse Lab in Milton Keynes and Alex Alderton, Roberto Amato, Sonia Goncalves, Ewan Harrison, David K. Jackson, Ian Johnston, Dominic Kwiatkowski, Cordelia Langford, John Sillitoe on behalf of the Wellcome Sanger Institute COVID-19 Surveillance Team
EPI_ISL_957903, EPI_ISL_957905, EPI_ISL_957906	Lighthouse Lab in Cambridge	Wellcome Sanger Institute for the COVID-19 Genomics UK (COG-UK) Consortium	Rob Howes, The Lighthouse Lab in Cambridge and Alex Alderton, Roberto Amato, Sonia Goncalves, Ewan Harrison, David K. Jackson, Ian Johnston, Dominic Kwiatkowski, Cordelia Langford, John Sillitoe on behalf of the Wellcome Sanger Institute COVID-19 Surveillance Team
EPI_ISL_957909, EPI_ISL_957910, EPI_ISL_957911, EPI_ISL_957912, EPI_ISL_957913, EPI_ISL_957914, EPI_ISL_957915, EPI_ISL_957916, EPI_ISL_957917, EPI_ISL_957918, EPI_ISL_957919, EPI_ISL_957920, EPI_ISL_957922, EPI_ISL_957923, EPI_ISL_957924, EPI_ISL_957925, EPI_ISL_957926, EPI_ISL_957927, EPI_ISL_957928, EPI_ISL_957929, EPI_ISL_957930, EPI_ISL_957931, EPI_ISL_957932, EPI_ISL_957933, EPI_ISL_957934, EPI_ISL_957935, EPI_ISL_957936, EPI_ISL_957937, EPI_ISL_957938, EPI_ISL_957939, EPI_ISL_957940, EPI_ISL_957941, EPI_ISL_957942, EPI_ISL_957943, EPI_ISL_957944, EPI_ISL_957945, EPI_ISL_957946, EPI_ISL_957947, EPI_ISL_957948, EPI_ISL_957949, EPI_ISL_957950, EPI_ISL_957951, EPI_ISL_957952, EPI_ISL_957953, EPI_ISL_957954, EPI_ISL_957955, EPI_ISL_957956, EPI_ISL_957957, EPI_ISL_957958, EPI_ISL_957959, EPI_ISL_957960, EPI_ISL_957963, EPI_ISL_957964, EPI_ISL_957965, EPI_ISL_957967, EPI_ISL_957968, EPI_ISL_957969, EPI_ISL_957970, EPI_ISL_957972, EPI_ISL_957973, EPI_ISL_957974, EPI_ISL_957975, EPI_ISL_957976, EPI_ISL_957977, EPI_ISL_957978, EPI_ISL_957979, EPI_ISL_957980, EPI_ISL_957981, EPI_ISL_957982, EPI_ISL_957983, EPI_ISL_957984, EPI_ISL_957985, EPI_ISL_957986, EPI_ISL_957987, EPI_ISL_957988, EPI_ISL_957989, EPI_ISL_957990, EPI_ISL_957991, EPI_ISL_957992, EPI_ISL_957993, EPI_ISL_957994, EPI_ISL_957995, EPI_ISL_957996, EPI_ISL_957997, EPI_ISL_957998, EPI_ISL_957999, EPI_ISL_958000, EPI_ISL_958001, EPI_ISL_958002, EPI_ISL_958003, EPI_ISL_958004, EPI_ISL_958005, EPI_ISL_958006, EPI_ISL_958007, EPI_ISL_958008, EPI_ISL_958009, EPI_ISL_958010, EPI_ISL_958011, EPI_ISL_958012, EPI_ISL_958013, EPI_ISL_958014, EPI_ISL_958015, EPI_ISL_958016, EPI_ISL_958017, EPI_ISL_958018, EPI_ISL_958019, EPI_ISL_958020, EPI_ISL_958021, EPI_ISL_958022, EPI_ISL_958023, EPI_ISL_958024, EPI_ISL_958025, EPI_ISL_958026, EPI_ISL_958027, EPI_ISL_958028, EPI_ISL_958029, EPI_ISL_958030, EPI_ISL_958031, EPI_ISL_958032, EPI_ISL_958033, EPI_ISL_958034, EPI_ISL_958035, EPI_ISL_958036, EPI_ISL_958037, EPI_ISL_958038, EPI_ISL_958039, EPI_ISL_958040, EPI_ISL_958041, EPI_ISL_958042, EPI_ISL_958043, EPI_ISL_958045, EPI_ISL_958046, EPI_ISL_958047, EPI_ISL_958048, EPI_ISL_958049, EPI_ISL_958050, EPI_ISL_958051, EPI_ISL_958052, EPI_ISL_958053, EPI_ISL_958054, EPI_ISL_958055, EPI_ISL_958056, EPI_ISL_958057, EPI_ISL_958058, EPI_ISL_958059, EPI_ISL_958060, EPI_ISL_958061, EPI_ISL_958062, EPI_ISL_958063, EPI_ISL_958064, EPI_ISL_958065, EPI_ISL_958066, EPI_ISL_958067, EPI_ISL_958068, EPI_ISL_958069, EPI_ISL_958070, EPI_ISL_958071, EPI_ISL_958072, EPI_ISL_958073, EPI_ISL_958074, EPI_ISL_958075, EPI_ISL_958076, EPI_ISL_958077, EPI_ISL_958078, EPI_ISL_958079, EPI_ISL_958080, EPI_ISL_958081, EPI_ISL_958082, EPI_ISL_958083, EPI_ISL_958084, EPI_ISL_958085, EPI_ISL_958086, EPI_ISL_958087, EPI_ISL_958088, EPI_ISL_958089, EPI_ISL_958090, EPI_ISL_958091, EPI_ISL_958092, EPI_ISL_958093, EPI_ISL_958094, EPI_ISL_958095, EPI_ISL_958096, EPI_ISL_958097, EPI_ISL_958098, EPI_ISL_958099, EPI_ISL_958100, EPI_ISL_958101, EPI_ISL_958102, EPI_ISL_958103, EPI_ISL_958104, EPI_ISL_958105, EPI_ISL_958106, EPI_ISL_958107, EPI_ISL_958108, EPI_ISL_958109, EPI_ISL_958110, EPI_ISL_958111, EPI_ISL_958112, EPI_ISL_958113, EPI_ISL_958114, EPI_ISL_958115, EPI_ISL_958116, EPI_ISL_958117, EPI_ISL_958118, EPI_ISL_958119, EPI_ISL_958120, EPI_ISL_958121, EPI_ISL_958122, EPI_ISL_958123, EPI_ISL_958124, EPI_ISL_958125, EPI_ISL_958126, EPI_ISL_958127, EPI_ISL_958128, EPI_ISL_958129, EPI_ISL_958130, EPI_ISL_958131, EPI_ISL_958132, EPI_ISL_958133, EPI_ISL_958134, EPI_ISL_958135, EPI_ISL_958136, EPI_ISL_958137, EPI_ISL_958138, EPI_ISL_958139, EPI_ISL_958140, EPI_ISL_958141, EPI_ISL_958142, EPI_ISL_958143, EPI_ISL_958144, EPI_ISL_958145, EPI_ISL_958146, EPI_ISL_958147, EPI_ISL_958148, EPI_ISL_958149, EPI_ISL_958150, EPI_ISL_958151, EPI_ISL_958152, EPI_ISL_958153, EPI_ISL_958154, EPI_ISL_958155, EPI_ISL_958156, EPI_ISL_958158, EPI_ISL_958159, EPI_ISL_958160, EPI_ISL_958161, EPI_ISL_958162, EPI_ISL_958163, EPI_ISL_958164, EPI_ISL_958165, EPI_ISL_958166, EPI_ISL_958167, EPI_ISL_958168, EPI_ISL_958169, EPI_ISL_958170, EPI_ISL_958171, EPI_ISL_958172, EPI_ISL_958173, EPI_ISL_958174, EPI_ISL_958175, EPI_ISL_958176, EPI_ISL_958177, EPI_ISL_958178, EPI_ISL_958179, EPI_ISL_958180, EPI_ISL_958181, EPI_ISL_958182, EPI_ISL_958183, EPI_ISL_958184, EPI_ISL_958185, EPI_ISL_958186, EPI_ISL_958187, EPI_ISL_958188, EPI_ISL_958189, EPI_ISL_958190, EPI_ISL_958191, EPI_ISL_958192, EPI_ISL_958193, EPI_ISL_958195, EPI_ISL_958196, EPI_ISL_958197, EPI_ISL_958198, EPI_ISL_958199, EPI_ISL_958200, EPI_ISL_958201, EPI_ISL_958202, EPI_ISL_958203, EPI_ISL_958204, EPI_ISL_958205, EPI_ISL_958207, EPI_ISL_958208, EPI_ISL_958209, EPI_ISL_958210, EPI_ISL_958211, EPI_ISL_958212, EPI_ISL_958213, EPI_ISL_958214, EPI_ISL_958567, EPI_ISL_958568, EPI_ISL_958570, EPI_ISL_958571, EPI_ISL_958573, EPI_ISL_958574, EPI_ISL_958575, EPI_ISL_958576, EPI_ISL_958577, EPI_ISL_958580, EPI_ISL_958581, EPI_ISL_958582, EPI_ISL_958583, EPI_ISL_958584, EPI_ISL_958585, EPI_ISL_958588, EPI_ISL_958589, EPI_ISL_958592, EPI_ISL_958593, EPI_ISL_958594, EPI_ISL_958596, EPI_ISL_958597, EPI_ISL_958598, EPI_ISL_958599, EPI_ISL_958601, EPI_ISL_958603, EPI_ISL_958604, EPI_ISL_958605, EPI_ISL_958606, EPI_ISL_958607, EPI_ISL_958608, EPI_ISL_958609, EPI_ISL_958611, EPI_ISL_958612, EPI_ISL_958613, EPI_ISL_958614, EPI_ISL_958615, EPI_ISL_958616, EPI_ISL_958617, EPI_ISL_958618, EPI_ISL_958619, EPI_ISL_958620, EPI_ISL_958621, EPI_ISL_958622, EPI_ISL_958623, EPI_ISL_958625, EPI_ISL_958626, EPI_ISL_958627, EPI_ISL_958628, EPI_ISL_958629, EPI_ISL_958630, EPI_ISL_958631, EPI_ISL_958635, EPI_ISL_958637, EPI_ISL_958640, EPI_ISL_958642, EPI_ISL_958643, EPI_ISL_958644, EPI_ISL_958645, EPI_ISL_958647, EPI_ISL_958648, EPI_ISL_958649, EPI_ISL_958651, EPI_ISL_958652, EPI_ISL_958653, EPI_ISL_958654, EPI_ISL_958655, EPI_ISL_958656, EPI_ISL_958657, EPI_ISL_958658, EPI_ISL_958660, EPI_ISL_958661, EPI_ISL_958664, EPI_ISL_958665, EPI_ISL_958666, EPI_ISL_958667, EPI_ISL_958668, EPI_ISL_958669, EPI_ISL_958672, EPI_ISL_958674, EPI_ISL_958675, EPI_ISL_958676, EPI_ISL_958677, EPI_ISL_958678, EPI_ISL_958679, EPI_ISL_958681, EPI_ISL_958682, EPI_ISL_958683, EPI_ISL_958685, EPI_ISL_958686, EPI_ISL_958687, EPI_ISL_958688, EPI_ISL_958689, EPI_ISL_958692, EPI_ISL_958693, EPI_ISL_958694, EPI_ISL_958695, EPI_ISL_958697, EPI_ISL_958698, EPI_ISL_958699, EPI_ISL_958700, EPI_ISL_958701, EPI_ISL_958702, EPI_ISL_958703, EPI_ISL_958704, EPI_ISL_958707, EPI_ISL_958708, EPI_ISL_958709, EPI_ISL_958710, EPI_ISL_958711, EPI_ISL_958712, EPI_ISL_958714, EPI_ISL_958717, EPI_ISL_958718, EPI_ISL_958719, EPI_ISL_958720, EPI_ISL_958721, EPI_ISL_958722, EPI_ISL_958724, EPI_ISL_958727, EPI_ISL_958728, EPI_ISL_958729, EPI_ISL_958730, EPI_ISL_958732, EPI_ISL_958734, EPI_ISL_958736, EPI_ISL_958737, EPI_ISL_958738, EPI_ISL_958739, EPI_ISL_958740, EPI_ISL_958741, EPI_ISL_958742, EPI_ISL_958743, EPI_ISL_958744, EPI_ISL_958746, EPI_ISL_958749, EPI_ISL_958750, EPI_ISL_958752, EPI_ISL_958753, EPI_ISL_958755, EPI_ISL_958757, EPI_ISL_958758, EPI_ISL_958759, EPI_ISL_958761, EPI_ISL_958762, EPI_ISL_958764, EPI_ISL_958765, EPI_ISL_958766, EPI_ISL_958768, EPI_ISL_958770, EPI_ISL_958771, EPI_ISL_958772, EPI_ISL_958773, EPI_ISL_958774, EPI_ISL_958776, EPI_ISL_958777, EPI_ISL_958778, EPI_ISL_958780, EPI_ISL_958781, EPI_ISL_958782, EPI_ISL_958783, EPI_ISL_958785, EPI_ISL_958786, EPI_ISL_958788, EPI_ISL_958790, EPI_ISL_958794, EPI_ISL_958795, EPI_ISL_958796, EPI_ISL_958797, EPI_ISL_958798, EPI_ISL_958799, EPI_ISL_958800, EPI_ISL_958801, EPI_ISL_958802, EPI_ISL_958804, EPI_ISL_958805, EPI_ISL_958806, EPI_ISL_958809, EPI_ISL_958810, EPI_ISL_958812, EPI_ISL_958813, EPI_ISL_958814, EPI_ISL_958816, EPI_ISL_958817, EPI_ISL_958818, EPI_ISL_958819, EPI_ISL_958820, EPI_ISL_958821, EPI_ISL_958822, EPI_ISL_958824, EPI_ISL_958825, EPI_ISL_958827, EPI_ISL_958829, EPI_ISL_958830, EPI_ISL_958831, EPI_ISL_958832, EPI_ISL_958834, EPI_ISL_958835, EPI_ISL_958837, EPI_ISL_958838, EPI_ISL_958839, EPI_ISL_958841, EPI_ISL_958843, EPI_ISL_958844, EPI_ISL_958845, EPI_ISL_958846, EPI_ISL_958847, EPI_ISL_958848, EPI_ISL_958850, EPI_ISL_958852, EPI_ISL_958853, EPI_ISL_958854, EPI_ISL_958855, EPI_ISL_958856, EPI_ISL_958857, EPI_ISL_958858, EPI_ISL_958859, EPI_ISL_958860, EPI_ISL_958861, EPI_ISL_958862, EPI_ISL_958864, EPI_ISL_958865, EPI_ISL_958867, EPI_ISL_958868, EPI_ISL_958869, EPI_ISL_958870, EPI_ISL_958871, EPI_ISL_958872, EPI_ISL_958874, EPI_ISL_958875, EPI_ISL_958876, EPI_ISL_958877, EPI_ISL_958879, EPI_ISL_958880, EPI_ISL_958881, EPI_ISL_958882, EPI_ISL_958883, EPI_ISL_958886, EPI_ISL_958888, EPI_ISL_958890, EPI_ISL_958892, EPI_ISL_958894, EPI_ISL_958896, EPI_ISL_958897, EPI_ISL_958899, EPI_ISL_958900, EPI_ISL_958901, EPI_ISL_958905, EPI_ISL_958906, EPI_ISL_958907, EPI_ISL_958909, EPI_ISL_958910, EPI_ISL_958913, EPI_ISL_958914			
see above	Lighthouse Lab in Milton Keynes	Wellcome Sanger Institute for the COVID-19 Genomics UK (COG-UK) Consortium	The Lighthouse Lab in Milton Keynes and Alex Alderton, Roberto Amato, Sonia Goncalves, Ewan Harrison, David K. Jackson, Ian Johnston, Dominic Kwiatkowski, Cordelia Langford, John Sillitoe on behalf of the Wellcome Sanger Institute COVID-19 Surveillance Team
EPI_ISL_962988, EPI_ISL_962996, EPI_ISL_963000, EPI_ISL_963015, EPI_ISL_963041, EPI_ISL_963087, EPI_ISL_963107, EPI_ISL_963108, EPI_ISL_963120, EPI_ISL_963157, EPI_ISL_963179, EPI_ISL_963193, EPI_ISL_963225, EPI_ISL_963271, EPI_ISL_963274, EPI_ISL_963307			
see above	Lighthouse Lab in Alderley Park	Wellcome Sanger Institute for the COVID-19 Genomics UK (COG-UK) Consortium	Jacquelyn Wynn, Mairead Hyland, The Lighthouse Lab in Alderley Park and Alex Alderton, Roberto Amato, Sonia Goncalves, Ewan Harrison, David K. Jackson, Ian Johnston, Dominic Kwiatkowski, Cordelia Langford, John Sillitoe on behalf of the Wellcome Sanger Institute COVID-19 Surveillance Team
EPI_ISL_963324	Lighthouse Lab in Milton Keynes	Wellcome Sanger Institute for the COVID-19 Genomics UK (COG-UK) Consortium	The Lighthouse Lab in Milton Keynes and Alex Alderton, Roberto Amato, Sonia Goncalves, Ewan Harrison, David K. Jackson, Ian Johnston, Dominic Kwiatkowski, Cordelia Langford, John Sillitoe on behalf of the Wellcome Sanger Institute COVID-19 Surveillance Team
EPI_ISL_963326	Lighthouse Lab in Glasgow	Wellcome Sanger Institute for the COVID-19 Genomics UK (COG-UK) Consortium	Harper VanSteenhouse, Yumi Kasai, David Gray, Carol Clugston, Anna Dominiczak and Alex Alderton, Roberto Amato, Sonia Goncalves, Ewan Harrison, David K. Jackson, Ian Johnston, Dominic Kwiatkowski, Cordelia Langford, John Sillitoe on behalf of the Wellcome Sanger Institute COVID-19 Surveillance Team
EPI_ISL_963327, EPI_ISL_963328, EPI_ISL_963329	Lighthouse Lab in Milton Keynes	Wellcome Sanger Institute for the COVID-19 Genomics UK (COG-UK) Consortium	The Lighthouse Lab in Milton Keynes and Alex Alderton, Roberto Amato, Sonia Goncalves, Ewan Harrison, David K. Jackson, Ian Johnston, Dominic Kwiatkowski, Cordelia Langford, John Sillitoe on behalf of the Wellcome Sanger Institute COVID-19 Surveillance Team
EPI_ISL_963330	Lighthouse Lab in Glasgow	Wellcome Sanger Institute for the COVID-19 Genomics UK (COG-UK) Consortium	Harper VanSteenhouse, Yumi Kasai, David Gray, Carol Clugston, Anna Dominiczak and Alex Alderton, Roberto Amato, Sonia Goncalves, Ewan Harrison, David K. Jackson, Ian Johnston, Dominic Kwiatkowski, Cordelia Langford, John Sillitoe on behalf of the Wellcome Sanger Institute COVID-19 Surveillance Team
EPI_ISL_963331, EPI_ISL_963332, EPI_ISL_963333, EPI_ISL_963334, EPI_ISL_963335, EPI_ISL_963336, EPI_ISL_963337, EPI_ISL_963338, EPI_ISL_963339, EPI_ISL_963340	Lighthouse Lab in Milton Keynes	Wellcome Sanger Institute for the COVID-19 Genomics UK (COG-UK) Consortium	The Lighthouse Lab in Milton Keynes and Alex Alderton, Roberto Amato, Sonia Goncalves, Ewan Harrison, David K. Jackson, Ian Johnston, Dominic Kwiatkowski, Cordelia Langford, John Sillitoe on behalf of the Wellcome Sanger Institute COVID-19 Surveillance Team
EPI_ISL_963341	Lighthouse Lab in Glasgow	Wellcome Sanger Institute for the COVID-19 Genomics UK (COG-UK) Consortium	Harper VanSteenhouse, Yumi Kasai, David Gray, Carol Clugston, Anna Dominiczak and Alex Alderton, Roberto Amato, Sonia Goncalves, Ewan Harrison, David K. Jackson, Ian Johnston, Dominic Kwiatkowski, Cordelia Langford, John Sillitoe on behalf of the Wellcome Sanger Institute COVID-19 Surveillance Team
EPI_ISL_963342, EPI_ISL_963343	Lighthouse Lab in Milton Keynes	Wellcome Sanger Institute for the COVID-19 Genomics UK (COG-UK) Consortium	The Lighthouse Lab in Milton Keynes and Alex Alderton, Roberto Amato, Sonia Goncalves, Ewan Harrison, David K. Jackson, Ian Johnston, Dominic Kwiatkowski, Cordelia Langford, John Sillitoe on behalf of the Wellcome Sanger Institute COVID-19 Surveillance Team
EPI_ISL_963344, EPI_ISL_963345, EPI_ISL_963346	Lighthouse Lab in Glasgow	Wellcome Sanger Institute for the COVID-19 Genomics UK (COG-UK) Consortium	Harper VanSteenhouse, Yumi Kasai, David Gray, Carol Clugston, Anna Dominiczak and Alex Alderton, Roberto Amato, Sonia Goncalves, Ewan Harrison, David K. Jackson, Ian Johnston, Dominic Kwiatkowski, Cordelia Langford, John Sillitoe on behalf of the Wellcome Sanger Institute COVID-19 Surveillance Team
EPI_ISL_963347	Lighthouse Lab in Milton Keynes	Wellcome Sanger Institute for the COVID-19 Genomics UK (COG-UK) Consortium	The Lighthouse Lab in Milton Keynes and Alex Alderton, Roberto Amato, Sonia Goncalves, Ewan Harrison, David K. Jackson, Ian Johnston, Dominic Kwiatkowski, Cordelia Langford, John Sillitoe on behalf of the Wellcome Sanger Institute COVID-19 Surveillance Team
EPI_ISL_963348	Lighthouse Lab in Glasgow	Wellcome Sanger Institute for the COVID-19 Genomics UK (COG-UK) Consortium	Harper VanSteenhouse, Yumi Kasai, David Gray, Carol Clugston, Anna Dominiczak and Alex Alderton, Roberto Amato, Sonia Goncalves, Ewan Harrison, David K. Jackson, Ian Johnston, Dominic Kwiatkowski, Cordelia Langford, John Sillitoe on behalf of the Wellcome Sanger Institute COVID-19 Surveillance Team
EPI_ISL_963349, EPI_ISL_963350, EPI_ISL_963351, EPI_ISL_963352, EPI_ISL_963353, EPI_ISL_963354	Lighthouse Lab in Milton Keynes	Wellcome Sanger Institute for the COVID-19 Genomics UK (COG-UK) Consortium	The Lighthouse Lab in Milton Keynes and Alex Alderton, Roberto Amato, Sonia Goncalves, Ewan Harrison, David K. Jackson, Ian Johnston, Dominic Kwiatkowski, Cordelia Langford, John Sillitoe on behalf of the Wellcome Sanger Institute COVID-19 Surveillance Team
EPI_ISL_963355, EPI_ISL_963356, EPI_ISL_963357, EPI_ISL_963358	Lighthouse Lab in Glasgow	Wellcome Sanger Institute for the COVID-19 Genomics UK (COG-UK) Consortium	Harper VanSteenhouse, Yumi Kasai, David Gray, Carol Clugston, Anna Dominiczak and Alex Alderton, Roberto Amato, Sonia Goncalves, Ewan Harrison, David K. Jackson, Ian Johnston, Dominic Kwiatkowski, Cordelia Langford, John Sillitoe on behalf of the Wellcome Sanger Institute COVID-19 Surveillance Team
EPI_ISL_963359, EPI_ISL_963360, EPI_ISL_963361, EPI_ISL_963362, EPI_ISL_963363, EPI_ISL_963364, EPI_ISL_963365, EPI_ISL_963366, EPI_ISL_963367, EPI_ISL_963368, EPI_ISL_963369			
see above	Lighthouse Lab in Milton Keynes	Wellcome Sanger Institute for the COVID-19 Genomics UK (COG-UK) Consortium	The Lighthouse Lab in Milton Keynes and Alex Alderton, Roberto Amato, Sonia Goncalves, Ewan Harrison, David K. Jackson, Ian Johnston, Dominic Kwiatkowski, Cordelia Langford, John Sillitoe on behalf of the Wellcome Sanger Institute COVID-19 Surveillance Team

EPI_ISL_963370, EPI_ISL_963371	Lighthouse Lab in Glasgow	Wellcome Sanger Institute for the COVID-19 Genomics UK (COG-UK) Consortium	Harper VanSteenhouse, Yumi Kasai, David Gray, Carol Clugston, Anna Dominiczak and Alex Alderton, Roberto Amato, Sonia Goncalves, Ewan Harrison, David K. Jackson, Ian Johnston, Dominic Kwiatkowski, Cordelia Langford, John Sillitoe on behalf of the Wellcome Sanger Institute COVID-19 Surveillance Team
EPI_ISL_963372, EPI_ISL_963373, EPI_ISL_963374, EPI_ISL_963375	Lighthouse Lab in Milton Keynes	Wellcome Sanger Institute for the COVID-19 Genomics UK (COG-UK) Consortium	The Lighthouse Lab in Milton Keynes and Alex Alderton, Roberto Amato, Sonia Goncalves, Ewan Harrison, David K. Jackson, Ian Johnston, Dominic Kwiatkowski, Cordelia Langford, John Sillitoe on behalf of the Wellcome Sanger Institute COVID-19 Surveillance Team
EPI_ISL_963376	Lighthouse Lab in Glasgow	Wellcome Sanger Institute for the COVID-19 Genomics UK (COG-UK) Consortium	Harper VanSteenhouse, Yumi Kasai, David Gray, Carol Clugston, Anna Dominiczak and Alex Alderton, Roberto Amato, Sonia Goncalves, Ewan Harrison, David K. Jackson, Ian Johnston, Dominic Kwiatkowski, Cordelia Langford, John Sillitoe on behalf of the Wellcome Sanger Institute COVID-19 Surveillance Team
EPI_ISL_963377, EPI_ISL_963378, EPI_ISL_963379	Lighthouse Lab in Milton Keynes	Wellcome Sanger Institute for the COVID-19 Genomics UK (COG-UK) Consortium	The Lighthouse Lab in Milton Keynes and Alex Alderton, Roberto Amato, Sonia Goncalves, Ewan Harrison, David K. Jackson, Ian Johnston, Dominic Kwiatkowski, Cordelia Langford, John Sillitoe on behalf of the Wellcome Sanger Institute COVID-19 Surveillance Team
EPI_ISL_963380	Lighthouse Lab in Glasgow	Wellcome Sanger Institute for the COVID-19 Genomics UK (COG-UK) Consortium	Harper VanSteenhouse, Yumi Kasai, David Gray, Carol Clugston, Anna Dominiczak and Alex Alderton, Roberto Amato, Sonia Goncalves, Ewan Harrison, David K. Jackson, Ian Johnston, Dominic Kwiatkowski, Cordelia Langford, John Sillitoe on behalf of the Wellcome Sanger Institute COVID-19 Surveillance Team
EPI_ISL_963381, EPI_ISL_963382, EPI_ISL_963383, EPI_ISL_963384, EPI_ISL_963385	Lighthouse Lab in Milton Keynes	Wellcome Sanger Institute for the COVID-19 Genomics UK (COG-UK) Consortium	The Lighthouse Lab in Milton Keynes and Alex Alderton, Roberto Amato, Sonia Goncalves, Ewan Harrison, David K. Jackson, Ian Johnston, Dominic Kwiatkowski, Cordelia Langford, John Sillitoe on behalf of the Wellcome Sanger Institute COVID-19 Surveillance Team
EPI_ISL_963386	Lighthouse Lab in Glasgow	Wellcome Sanger Institute for the COVID-19 Genomics UK (COG-UK) Consortium	Harper VanSteenhouse, Yumi Kasai, David Gray, Carol Clugston, Anna Dominiczak and Alex Alderton, Roberto Amato, Sonia Goncalves, Ewan Harrison, David K. Jackson, Ian Johnston, Dominic Kwiatkowski, Cordelia Langford, John Sillitoe on behalf of the Wellcome Sanger Institute COVID-19 Surveillance Team
EPI_ISL_963387, EPI_ISL_963388, EPI_ISL_963389, EPI_ISL_963390, EPI_ISL_963391, EPI_ISL_963392, EPI_ISL_963393, EPI_ISL_963394, EPI_ISL_963395, EPI_ISL_963396	Lighthouse Lab in Milton Keynes	Wellcome Sanger Institute for the COVID-19 Genomics UK (COG-UK) Consortium	The Lighthouse Lab in Milton Keynes and Alex Alderton, Roberto Amato, Sonia Goncalves, Ewan Harrison, David K. Jackson, Ian Johnston, Dominic Kwiatkowski, Cordelia Langford, John Sillitoe on behalf of the Wellcome Sanger Institute COVID-19 Surveillance Team
EPI_ISL_963397	Lighthouse Lab in Glasgow	Wellcome Sanger Institute for the COVID-19 Genomics UK (COG-UK) Consortium	Harper VanSteenhouse, Yumi Kasai, David Gray, Carol Clugston, Anna Dominiczak and Alex Alderton, Roberto Amato, Sonia Goncalves, Ewan Harrison, David K. Jackson, Ian Johnston, Dominic Kwiatkowski, Cordelia Langford, John Sillitoe on behalf of the Wellcome Sanger Institute COVID-19 Surveillance Team
EPI_ISL_963398, EPI_ISL_963399, EPI_ISL_963400, EPI_ISL_963401, EPI_ISL_963402, EPI_ISL_963403, EPI_ISL_963404, EPI_ISL_963405, EPI_ISL_963406, EPI_ISL_963407, EPI_ISL_963409, EPI_ISL_963410, EPI_ISL_963411, EPI_ISL_963412, EPI_ISL_963413, EPI_ISL_963414, EPI_ISL_963415, EPI_ISL_963416, EPI_ISL_963417, EPI_ISL_963418			
see above	Lighthouse Lab in Milton Keynes	Wellcome Sanger Institute for the COVID-19 Genomics UK (COG-UK) Consortium	The Lighthouse Lab in Milton Keynes and Alex Alderton, Roberto Amato, Sonia Goncalves, Ewan Harrison, David K. Jackson, Ian Johnston, Dominic Kwiatkowski, Cordelia Langford, John Sillitoe on behalf of the Wellcome Sanger Institute COVID-19 Surveillance Team
EPI_ISL_963419	Lighthouse Lab in Glasgow	Wellcome Sanger Institute for the COVID-19 Genomics UK (COG-UK) Consortium	Harper VanSteenhouse, Yumi Kasai, David Gray, Carol Clugston, Anna Dominiczak and Alex Alderton, Roberto Amato, Sonia Goncalves, Ewan Harrison, David K. Jackson, Ian Johnston, Dominic Kwiatkowski, Cordelia Langford, John Sillitoe on behalf of the Wellcome Sanger Institute COVID-19 Surveillance Team
EPI_ISL_963420	Lighthouse Lab in Milton Keynes	Wellcome Sanger Institute for the COVID-19 Genomics UK (COG-UK) Consortium	The Lighthouse Lab in Milton Keynes and Alex Alderton, Roberto Amato, Sonia Goncalves, Ewan Harrison, David K. Jackson, Ian Johnston, Dominic Kwiatkowski, Cordelia Langford, John Sillitoe on behalf of the Wellcome Sanger Institute COVID-19 Surveillance Team
EPI_ISL_963421	Lighthouse Lab in Glasgow	Wellcome Sanger Institute for the COVID-19 Genomics UK (COG-UK) Consortium	Harper VanSteenhouse, Yumi Kasai, David Gray, Carol Clugston, Anna Dominiczak and Alex Alderton, Roberto Amato, Sonia Goncalves, Ewan Harrison, David K. Jackson, Ian Johnston, Dominic Kwiatkowski, Cordelia Langford, John Sillitoe on behalf of the Wellcome Sanger Institute COVID-19 Surveillance Team
EPI_ISL_963422, EPI_ISL_963423, EPI_ISL_963424	Lighthouse Lab in Milton Keynes	Wellcome Sanger Institute for the COVID-19 Genomics UK (COG-UK) Consortium	The Lighthouse Lab in Milton Keynes and Alex Alderton, Roberto Amato, Sonia Goncalves, Ewan Harrison, David K. Jackson, Ian Johnston, Dominic Kwiatkowski, Cordelia Langford, John Sillitoe on behalf of the Wellcome Sanger Institute COVID-19 Surveillance Team
EPI_ISL_963425, EPI_ISL_963426	Lighthouse Lab in Glasgow	Wellcome Sanger Institute for the COVID-19 Genomics UK (COG-UK) Consortium	Harper VanSteenhouse, Yumi Kasai, David Gray, Carol Clugston, Anna Dominiczak and Alex Alderton, Roberto Amato, Sonia Goncalves, Ewan Harrison, David K. Jackson, Ian Johnston, Dominic Kwiatkowski, Cordelia Langford, John Sillitoe on behalf of the Wellcome Sanger Institute COVID-19 Surveillance Team
EPI_ISL_963427, EPI_ISL_963429, EPI_ISL_963430	Lighthouse Lab in Milton Keynes	Wellcome Sanger Institute for the COVID-19 Genomics UK (COG-UK) Consortium	The Lighthouse Lab in Milton Keynes and Alex Alderton, Roberto Amato, Sonia Goncalves, Ewan Harrison, David K. Jackson, Ian Johnston, Dominic Kwiatkowski, Cordelia Langford, John Sillitoe on behalf of the Wellcome Sanger Institute COVID-19 Surveillance Team
EPI_ISL_963431	Lighthouse Lab in Glasgow	Wellcome Sanger Institute for the COVID-19 Genomics UK (COG-UK) Consortium	Harper VanSteenhouse, Yumi Kasai, David Gray, Carol Clugston, Anna Dominiczak and Alex Alderton, Roberto Amato, Sonia Goncalves, Ewan Harrison, David K. Jackson, Ian Johnston, Dominic Kwiatkowski, Cordelia Langford, John Sillitoe on behalf of the Wellcome Sanger Institute COVID-19 Surveillance Team
EPI_ISL_963432	Lighthouse Lab in Milton Keynes	Wellcome Sanger Institute for the COVID-19 Genomics UK (COG-UK) Consortium	The Lighthouse Lab in Milton Keynes and Alex Alderton, Roberto Amato, Sonia Goncalves, Ewan Harrison, David K. Jackson, Ian Johnston, Dominic Kwiatkowski, Cordelia Langford, John Sillitoe on behalf of the Wellcome Sanger Institute COVID-19 Surveillance Team
EPI_ISL_963433, EPI_ISL_963434	Lighthouse Lab in Glasgow	Wellcome Sanger Institute for the COVID-19 Genomics UK (COG-UK) Consortium	Harper VanSteenhouse, Yumi Kasai, David Gray, Carol Clugston, Anna Dominiczak and Alex Alderton, Roberto Amato, Sonia Goncalves, Ewan Harrison, David K. Jackson, Ian Johnston, Dominic Kwiatkowski, Cordelia Langford, John Sillitoe on behalf of the Wellcome Sanger Institute COVID-19 Surveillance Team
EPI_ISL_963435, EPI_ISL_963436, EPI_ISL_963437, EPI_ISL_963438, EPI_ISL_963439, EPI_ISL_963440, EPI_ISL_963441, EPI_ISL_963442, EPI_ISL_963443, EPI_ISL_963444, EPI_ISL_963445, EPI_ISL_963446, EPI_ISL_963447, EPI_ISL_963448			
see above	Lighthouse Lab in Milton Keynes	Wellcome Sanger Institute for the COVID-19 Genomics UK (COG-UK) Consortium	The Lighthouse Lab in Milton Keynes and Alex Alderton, Roberto Amato, Sonia Goncalves, Ewan Harrison, David K. Jackson, Ian Johnston, Dominic Kwiatkowski, Cordelia Langford, John Sillitoe on behalf of the Wellcome Sanger Institute COVID-19 Surveillance Team
EPI_ISL_963449	Lighthouse Lab in Glasgow	Wellcome Sanger Institute for the COVID-19 Genomics UK (COG-UK) Consortium	Harper VanSteenhouse, Yumi Kasai, David Gray, Carol Clugston, Anna Dominiczak and Alex Alderton, Roberto Amato, Sonia Goncalves, Ewan Harrison, David K. Jackson, Ian Johnston, Dominic Kwiatkowski, Cordelia Langford, John Sillitoe on behalf of the Wellcome Sanger Institute COVID-19 Surveillance Team
EPI_ISL_963450, EPI_ISL_963451	Lighthouse Lab in Milton Keynes	Wellcome Sanger Institute for the COVID-19 Genomics UK (COG-UK) Consortium	The Lighthouse Lab in Milton Keynes and Alex Alderton, Roberto Amato, Sonia Goncalves, Ewan Harrison, David K. Jackson, Ian Johnston, Dominic Kwiatkowski, Cordelia Langford, John Sillitoe on behalf of the Wellcome Sanger Institute COVID-19 Surveillance Team
EPI_ISL_963452	Lighthouse Lab in Glasgow	Wellcome Sanger Institute for the COVID-19 Genomics UK (COG-UK) Consortium	Harper VanSteenhouse, Yumi Kasai, David Gray, Carol Clugston, Anna Dominiczak and Alex Alderton, Roberto Amato, Sonia Goncalves, Ewan Harrison, David K. Jackson, Ian Johnston, Dominic Kwiatkowski, Cordelia Langford, John Sillitoe on behalf of the Wellcome Sanger Institute COVID-19 Surveillance Team
EPI_ISL_963453, EPI_ISL_963454, EPI_ISL_963455, EPI_ISL_963456, EPI_ISL_963457	Lighthouse Lab in Milton Keynes	Wellcome Sanger Institute for the COVID-19 Genomics UK (COG-UK) Consortium	The Lighthouse Lab in Milton Keynes and Alex Alderton, Roberto Amato, Sonia Goncalves, Ewan Harrison, David K. Jackson, Ian Johnston, Dominic Kwiatkowski, Cordelia Langford, John Sillitoe on behalf of the Wellcome Sanger Institute COVID-19 Surveillance Team
EPI_ISL_963458	Lighthouse Lab in Glasgow	Wellcome Sanger Institute for the COVID-19 Genomics UK (COG-UK) Consortium	Harper VanSteenhouse, Yumi Kasai, David Gray, Carol Clugston, Anna Dominiczak and Alex Alderton, Roberto Amato, Sonia Goncalves, Ewan Harrison, David K. Jackson, Ian Johnston, Dominic Kwiatkowski, Cordelia Langford, John Sillitoe on behalf of the Wellcome Sanger Institute COVID-19 Surveillance Team
EPI_ISL_963459, EPI_ISL_963460, EPI_ISL_963461, EPI_ISL_963462, EPI_ISL_963463, EPI_ISL_963464, EPI_ISL_963465, EPI_ISL_963466, EPI_ISL_963467, EPI_ISL_963468, EPI_ISL_963469, EPI_ISL_963470, EPI_ISL_963472, EPI_ISL_963473, EPI_ISL_963474			
see above	Lighthouse Lab in Milton Keynes	Wellcome Sanger Institute for the COVID-19 Genomics UK	The Lighthouse Lab in Milton Keynes and Alex Alderton, Roberto Amato, Sonia Goncalves, Ewan Harrison, David K. Jackson, Ian Johnston, Dominic

		(COG-UK) Consortium	Kwiatkowski, Cordelia Langford, John Sillitoe on behalf of the Wellcome Sanger Institute COVID-19 Surveillance Team
EPI_ISL_963475	Lighthouse Lab in Glasgow	Wellcome Sanger Institute for the COVID-19 Genomics UK (COG-UK) Consortium	Harper VanSteenhouse, Yumi Kasai, David Gray, Carol Clugston, Anna Dominiczak and Alex Alderton, Roberto Amato, Sonia Goncalves, Ewan Harrison, David K. Jackson, Ian Johnston, Dominic Kwiatkowski, Cordelia Langford, John Sillitoe on behalf of the Wellcome Sanger Institute COVID-19 Surveillance Team
EPI_ISL_963476, EPI_ISL_963477, EPI_ISL_963478, EPI_ISL_963479, EPI_ISL_963480	Lighthouse Lab in Milton Keynes	Wellcome Sanger Institute for the COVID-19 Genomics UK (COG-UK) Consortium	The Lighthouse Lab in Milton Keynes and Alex Alderton, Roberto Amato, Sonia Goncalves, Ewan Harrison, David K. Jackson, Ian Johnston, Dominic Kwiatkowski, Cordelia Langford, John Sillitoe on behalf of the Wellcome Sanger Institute COVID-19 Surveillance Team
EPI_ISL_963481	Lighthouse Lab in Glasgow	Wellcome Sanger Institute for the COVID-19 Genomics UK (COG-UK) Consortium	Harper VanSteenhouse, Yumi Kasai, David Gray, Carol Clugston, Anna Dominiczak and Alex Alderton, Roberto Amato, Sonia Goncalves, Ewan Harrison, David K. Jackson, Ian Johnston, Dominic Kwiatkowski, Cordelia Langford, John Sillitoe on behalf of the Wellcome Sanger Institute COVID-19 Surveillance Team
EPI_ISL_963482, EPI_ISL_963483, EPI_ISL_963484, EPI_ISL_963485, EPI_ISL_963486, EPI_ISL_963487, EPI_ISL_963488	Lighthouse Lab in Milton Keynes	Wellcome Sanger Institute for the COVID-19 Genomics UK (COG-UK) Consortium	The Lighthouse Lab in Milton Keynes and Alex Alderton, Roberto Amato, Sonia Goncalves, Ewan Harrison, David K. Jackson, Ian Johnston, Dominic Kwiatkowski, Cordelia Langford, John Sillitoe on behalf of the Wellcome Sanger Institute COVID-19 Surveillance Team
EPI_ISL_963489, EPI_ISL_963490	Lighthouse Lab in Glasgow	Wellcome Sanger Institute for the COVID-19 Genomics UK (COG-UK) Consortium	Harper VanSteenhouse, Yumi Kasai, David Gray, Carol Clugston, Anna Dominiczak and Alex Alderton, Roberto Amato, Sonia Goncalves, Ewan Harrison, David K. Jackson, Ian Johnston, Dominic Kwiatkowski, Cordelia Langford, John Sillitoe on behalf of the Wellcome Sanger Institute COVID-19 Surveillance Team
EPI_ISL_963491, EPI_ISL_963492, EPI_ISL_963493, EPI_ISL_963494	Lighthouse Lab in Milton Keynes	Wellcome Sanger Institute for the COVID-19 Genomics UK (COG-UK) Consortium	The Lighthouse Lab in Milton Keynes and Alex Alderton, Roberto Amato, Sonia Goncalves, Ewan Harrison, David K. Jackson, Ian Johnston, Dominic Kwiatkowski, Cordelia Langford, John Sillitoe on behalf of the Wellcome Sanger Institute COVID-19 Surveillance Team
EPI_ISL_963495	Lighthouse Lab in Glasgow	Wellcome Sanger Institute for the COVID-19 Genomics UK (COG-UK) Consortium	Harper VanSteenhouse, Yumi Kasai, David Gray, Carol Clugston, Anna Dominiczak and Alex Alderton, Roberto Amato, Sonia Goncalves, Ewan Harrison, David K. Jackson, Ian Johnston, Dominic Kwiatkowski, Cordelia Langford, John Sillitoe on behalf of the Wellcome Sanger Institute COVID-19 Surveillance Team
EPI_ISL_963496, EPI_ISL_963497, EPI_ISL_963498, EPI_ISL_963499, EPI_ISL_963500, EPI_ISL_963501, EPI_ISL_963502, EPI_ISL_963503, EPI_ISL_963504, EPI_ISL_963505, EPI_ISL_963506, EPI_ISL_963507, EPI_ISL_963508			
see above	Lighthouse Lab in Milton Keynes	Wellcome Sanger Institute for the COVID-19 Genomics UK (COG-UK) Consortium	The Lighthouse Lab in Milton Keynes and Alex Alderton, Roberto Amato, Sonia Goncalves, Ewan Harrison, David K. Jackson, Ian Johnston, Dominic Kwiatkowski, Cordelia Langford, John Sillitoe on behalf of the Wellcome Sanger Institute COVID-19 Surveillance Team
EPI_ISL_963509	Lighthouse Lab in Glasgow	Wellcome Sanger Institute for the COVID-19 Genomics UK (COG-UK) Consortium	Harper VanSteenhouse, Yumi Kasai, David Gray, Carol Clugston, Anna Dominiczak and Alex Alderton, Roberto Amato, Sonia Goncalves, Ewan Harrison, David K. Jackson, Ian Johnston, Dominic Kwiatkowski, Cordelia Langford, John Sillitoe on behalf of the Wellcome Sanger Institute COVID-19 Surveillance Team
EPI_ISL_963510	Lighthouse Lab in Milton Keynes	Wellcome Sanger Institute for the COVID-19 Genomics UK (COG-UK) Consortium	The Lighthouse Lab in Milton Keynes and Alex Alderton, Roberto Amato, Sonia Goncalves, Ewan Harrison, David K. Jackson, Ian Johnston, Dominic Kwiatkowski, Cordelia Langford, John Sillitoe on behalf of the Wellcome Sanger Institute COVID-19 Surveillance Team
EPI_ISL_963511	Lighthouse Lab in Glasgow	Wellcome Sanger Institute for the COVID-19 Genomics UK (COG-UK) Consortium	Harper VanSteenhouse, Yumi Kasai, David Gray, Carol Clugston, Anna Dominiczak and Alex Alderton, Roberto Amato, Sonia Goncalves, Ewan Harrison, David K. Jackson, Ian Johnston, Dominic Kwiatkowski, Cordelia Langford, John Sillitoe on behalf of the Wellcome Sanger Institute COVID-19 Surveillance Team
EPI_ISL_963512, EPI_ISL_963513, EPI_ISL_963514, EPI_ISL_963515, EPI_ISL_963516, EPI_ISL_963517, EPI_ISL_963518, EPI_ISL_963519	Lighthouse Lab in Milton Keynes	Wellcome Sanger Institute for the COVID-19 Genomics UK (COG-UK) Consortium	The Lighthouse Lab in Milton Keynes and Alex Alderton, Roberto Amato, Sonia Goncalves, Ewan Harrison, David K. Jackson, Ian Johnston, Dominic Kwiatkowski, Cordelia Langford, John Sillitoe on behalf of the Wellcome Sanger Institute COVID-19 Surveillance Team
EPI_ISL_963520, EPI_ISL_963521	Lighthouse Lab in Glasgow	Wellcome Sanger Institute for the COVID-19 Genomics UK (COG-UK) Consortium	Harper VanSteenhouse, Yumi Kasai, David Gray, Carol Clugston, Anna Dominiczak and Alex Alderton, Roberto Amato, Sonia Goncalves, Ewan Harrison, David K. Jackson, Ian Johnston, Dominic Kwiatkowski, Cordelia Langford, John Sillitoe on behalf of the Wellcome Sanger Institute COVID-19 Surveillance Team
EPI_ISL_963522, EPI_ISL_963523, EPI_ISL_963525, EPI_ISL_963526, EPI_ISL_963527, EPI_ISL_963528	Lighthouse Lab in Milton Keynes	Wellcome Sanger Institute for the COVID-19 Genomics UK (COG-UK) Consortium	The Lighthouse Lab in Milton Keynes and Alex Alderton, Roberto Amato, Sonia Goncalves, Ewan Harrison, David K. Jackson, Ian Johnston, Dominic Kwiatkowski, Cordelia Langford, John Sillitoe on behalf of the Wellcome Sanger Institute COVID-19 Surveillance Team
EPI_ISL_963529, EPI_ISL_963530	Lighthouse Lab in Glasgow	Wellcome Sanger Institute for the COVID-19 Genomics UK (COG-UK) Consortium	Harper VanSteenhouse, Yumi Kasai, David Gray, Carol Clugston, Anna Dominiczak and Alex Alderton, Roberto Amato, Sonia Goncalves, Ewan Harrison, David K. Jackson, Ian Johnston, Dominic Kwiatkowski, Cordelia Langford, John Sillitoe on behalf of the Wellcome Sanger Institute COVID-19 Surveillance Team
EPI_ISL_963531, EPI_ISL_963532, EPI_ISL_963533, EPI_ISL_963534, EPI_ISL_963535, EPI_ISL_963536, EPI_ISL_963537	Lighthouse Lab in Milton Keynes	Wellcome Sanger Institute for the COVID-19 Genomics UK (COG-UK) Consortium	The Lighthouse Lab in Milton Keynes and Alex Alderton, Roberto Amato, Sonia Goncalves, Ewan Harrison, David K. Jackson, Ian Johnston, Dominic Kwiatkowski, Cordelia Langford, John Sillitoe on behalf of the Wellcome Sanger Institute COVID-19 Surveillance Team
EPI_ISL_963538	Lighthouse Lab in Glasgow	Wellcome Sanger Institute for the COVID-19 Genomics UK (COG-UK) Consortium	Harper VanSteenhouse, Yumi Kasai, David Gray, Carol Clugston, Anna Dominiczak and Alex Alderton, Roberto Amato, Sonia Goncalves, Ewan Harrison, David K. Jackson, Ian Johnston, Dominic Kwiatkowski, Cordelia Langford, John Sillitoe on behalf of the Wellcome Sanger Institute COVID-19 Surveillance Team
EPI_ISL_963539, EPI_ISL_963540, EPI_ISL_963541, EPI_ISL_963542, EPI_ISL_963543, EPI_ISL_963544, EPI_ISL_963545, EPI_ISL_963546, EPI_ISL_963547, EPI_ISL_963548, EPI_ISL_963549, EPI_ISL_963550, EPI_ISL_963551, EPI_ISL_963552, EPI_ISL_963553			
see above	Lighthouse Lab in Milton Keynes	Wellcome Sanger Institute for the COVID-19 Genomics UK (COG-UK) Consortium	The Lighthouse Lab in Milton Keynes and Alex Alderton, Roberto Amato, Sonia Goncalves, Ewan Harrison, David K. Jackson, Ian Johnston, Dominic Kwiatkowski, Cordelia Langford, John Sillitoe on behalf of the Wellcome Sanger Institute COVID-19 Surveillance Team
EPI_ISL_963554	Lighthouse Lab in Glasgow	Wellcome Sanger Institute for the COVID-19 Genomics UK (COG-UK) Consortium	Harper VanSteenhouse, Yumi Kasai, David Gray, Carol Clugston, Anna Dominiczak and Alex Alderton, Roberto Amato, Sonia Goncalves, Ewan Harrison, David K. Jackson, Ian Johnston, Dominic Kwiatkowski, Cordelia Langford, John Sillitoe on behalf of the Wellcome Sanger Institute COVID-19 Surveillance Team
EPI_ISL_963556, EPI_ISL_963557, EPI_ISL_963558	Lighthouse Lab in Milton Keynes	Wellcome Sanger Institute for the COVID-19 Genomics UK (COG-UK) Consortium	The Lighthouse Lab in Milton Keynes and Alex Alderton, Roberto Amato, Sonia Goncalves, Ewan Harrison, David K. Jackson, Ian Johnston, Dominic Kwiatkowski, Cordelia Langford, John Sillitoe on behalf of the Wellcome Sanger Institute COVID-19 Surveillance Team
EPI_ISL_963559, EPI_ISL_963560	Lighthouse Lab in Glasgow	Wellcome Sanger Institute for the COVID-19 Genomics UK (COG-UK) Consortium	Harper VanSteenhouse, Yumi Kasai, David Gray, Carol Clugston, Anna Dominiczak and Alex Alderton, Roberto Amato, Sonia Goncalves, Ewan Harrison, David K. Jackson, Ian Johnston, Dominic Kwiatkowski, Cordelia Langford, John Sillitoe on behalf of the Wellcome Sanger Institute COVID-19 Surveillance Team
EPI_ISL_963561	Lighthouse Lab in Milton Keynes	Wellcome Sanger Institute for the COVID-19 Genomics UK (COG-UK) Consortium	The Lighthouse Lab in Milton Keynes and Alex Alderton, Roberto Amato, Sonia Goncalves, Ewan Harrison, David K. Jackson, Ian Johnston, Dominic Kwiatkowski, Cordelia Langford, John Sillitoe on behalf of the Wellcome Sanger Institute COVID-19 Surveillance Team
EPI_ISL_963562	Lighthouse Lab in Glasgow	Wellcome Sanger Institute for the COVID-19 Genomics UK (COG-UK) Consortium	Harper VanSteenhouse, Yumi Kasai, David Gray, Carol Clugston, Anna Dominiczak and Alex Alderton, Roberto Amato, Sonia Goncalves, Ewan Harrison, David K. Jackson, Ian Johnston, Dominic Kwiatkowski, Cordelia Langford, John Sillitoe on behalf of the Wellcome Sanger Institute COVID-19 Surveillance Team
EPI_ISL_963563, EPI_ISL_963564, EPI_ISL_963565, EPI_ISL_963566, EPI_ISL_963567, EPI_ISL_963568, EPI_ISL_963569	Lighthouse Lab in Milton Keynes	Wellcome Sanger Institute for the COVID-19 Genomics UK (COG-UK) Consortium	The Lighthouse Lab in Milton Keynes and Alex Alderton, Roberto Amato, Sonia Goncalves, Ewan Harrison, David K. Jackson, Ian Johnston, Dominic Kwiatkowski, Cordelia Langford, John Sillitoe on behalf of the Wellcome Sanger Institute COVID-19 Surveillance Team
EPI_ISL_963570	Lighthouse Lab in Glasgow	Wellcome Sanger Institute for the COVID-19 Genomics UK	Harper VanSteenhouse, Yumi Kasai, David Gray, Carol Clugston, Anna Dominiczak and Alex Alderton, Roberto Amato, Sonia Goncalves, Ewan Harrison,

		(COG-UK) Consortium	David K. Jackson, Ian Johnston, Dominic Kwiatkowski, Cordelia Langford, John Sillitoe on behalf of the Wellcome Sanger Institute COVID-19 Surveillance Team
EPI_ISL_963571, EPI_ISL_963572	Lighthouse Lab in Milton Keynes	Wellcome Sanger Institute for the COVID-19 Genomics UK (COG-UK) Consortium	The Lighthouse Lab in Milton Keynes and Alex Alderton, Roberto Amato, Sonia Goncalves, Ewan Harrison, David K. Jackson, Ian Johnston, Dominic Kwiatkowski, Cordelia Langford, John Sillitoe on behalf of the Wellcome Sanger Institute COVID-19 Surveillance Team
EPI_ISL_963573	Lighthouse Lab in Glasgow	Wellcome Sanger Institute for the COVID-19 Genomics UK (COG-UK) Consortium	Harper VanSteenhouse, Yumi Kasai, David Gray, Carol Clugston, Anna Dominiczak and Alex Alderton, Roberto Amato, Sonia Goncalves, Ewan Harrison, David K. Jackson, Ian Johnston, Dominic Kwiatkowski, Cordelia Langford, John Sillitoe on behalf of the Wellcome Sanger Institute COVID-19 Surveillance Team
EPI_ISL_963574, EPI_ISL_963575	Lighthouse Lab in Milton Keynes	Wellcome Sanger Institute for the COVID-19 Genomics UK (COG-UK) Consortium	The Lighthouse Lab in Milton Keynes and Alex Alderton, Roberto Amato, Sonia Goncalves, Ewan Harrison, David K. Jackson, Ian Johnston, Dominic Kwiatkowski, Cordelia Langford, John Sillitoe on behalf of the Wellcome Sanger Institute COVID-19 Surveillance Team
EPI_ISL_963576	Lighthouse Lab in Glasgow	Wellcome Sanger Institute for the COVID-19 Genomics UK (COG-UK) Consortium	Harper VanSteenhouse, Yumi Kasai, David Gray, Carol Clugston, Anna Dominiczak and Alex Alderton, Roberto Amato, Sonia Goncalves, Ewan Harrison, David K. Jackson, Ian Johnston, Dominic Kwiatkowski, Cordelia Langford, John Sillitoe on behalf of the Wellcome Sanger Institute COVID-19 Surveillance Team
EPI_ISL_963577, EPI_ISL_963578, EPI_ISL_963579, EPI_ISL_963580	Lighthouse Lab in Milton Keynes	Wellcome Sanger Institute for the COVID-19 Genomics UK (COG-UK) Consortium	The Lighthouse Lab in Milton Keynes and Alex Alderton, Roberto Amato, Sonia Goncalves, Ewan Harrison, David K. Jackson, Ian Johnston, Dominic Kwiatkowski, Cordelia Langford, John Sillitoe on behalf of the Wellcome Sanger Institute COVID-19 Surveillance Team
EPI_ISL_963581	Lighthouse Lab in Glasgow	Wellcome Sanger Institute for the COVID-19 Genomics UK (COG-UK) Consortium	Harper VanSteenhouse, Yumi Kasai, David Gray, Carol Clugston, Anna Dominiczak and Alex Alderton, Roberto Amato, Sonia Goncalves, Ewan Harrison, David K. Jackson, Ian Johnston, Dominic Kwiatkowski, Cordelia Langford, John Sillitoe on behalf of the Wellcome Sanger Institute COVID-19 Surveillance Team
EPI_ISL_963582, EPI_ISL_963583	Lighthouse Lab in Milton Keynes	Wellcome Sanger Institute for the COVID-19 Genomics UK (COG-UK) Consortium	The Lighthouse Lab in Milton Keynes and Alex Alderton, Roberto Amato, Sonia Goncalves, Ewan Harrison, David K. Jackson, Ian Johnston, Dominic Kwiatkowski, Cordelia Langford, John Sillitoe on behalf of the Wellcome Sanger Institute COVID-19 Surveillance Team
EPI_ISL_963584	Lighthouse Lab in Glasgow	Wellcome Sanger Institute for the COVID-19 Genomics UK (COG-UK) Consortium	Harper VanSteenhouse, Yumi Kasai, David Gray, Carol Clugston, Anna Dominiczak and Alex Alderton, Roberto Amato, Sonia Goncalves, Ewan Harrison, David K. Jackson, Ian Johnston, Dominic Kwiatkowski, Cordelia Langford, John Sillitoe on behalf of the Wellcome Sanger Institute COVID-19 Surveillance Team
EPI_ISL_963585, EPI_ISL_963586, EPI_ISL_963587, EPI_ISL_963588, EPI_ISL_963589, EPI_ISL_963590	Lighthouse Lab in Milton Keynes	Wellcome Sanger Institute for the COVID-19 Genomics UK (COG-UK) Consortium	The Lighthouse Lab in Milton Keynes and Alex Alderton, Roberto Amato, Sonia Goncalves, Ewan Harrison, David K. Jackson, Ian Johnston, Dominic Kwiatkowski, Cordelia Langford, John Sillitoe on behalf of the Wellcome Sanger Institute COVID-19 Surveillance Team
EPI_ISL_963591	Lighthouse Lab in Glasgow	Wellcome Sanger Institute for the COVID-19 Genomics UK (COG-UK) Consortium	Harper VanSteenhouse, Yumi Kasai, David Gray, Carol Clugston, Anna Dominiczak and Alex Alderton, Roberto Amato, Sonia Goncalves, Ewan Harrison, David K. Jackson, Ian Johnston, Dominic Kwiatkowski, Cordelia Langford, John Sillitoe on behalf of the Wellcome Sanger Institute COVID-19 Surveillance Team
EPI_ISL_963592, EPI_ISL_963593	Lighthouse Lab in Milton Keynes	Wellcome Sanger Institute for the COVID-19 Genomics UK (COG-UK) Consortium	The Lighthouse Lab in Milton Keynes and Alex Alderton, Roberto Amato, Sonia Goncalves, Ewan Harrison, David K. Jackson, Ian Johnston, Dominic Kwiatkowski, Cordelia Langford, John Sillitoe on behalf of the Wellcome Sanger Institute COVID-19 Surveillance Team
EPI_ISL_963594	Lighthouse Lab in Glasgow	Wellcome Sanger Institute for the COVID-19 Genomics UK (COG-UK) Consortium	Harper VanSteenhouse, Yumi Kasai, David Gray, Carol Clugston, Anna Dominiczak and Alex Alderton, Roberto Amato, Sonia Goncalves, Ewan Harrison, David K. Jackson, Ian Johnston, Dominic Kwiatkowski, Cordelia Langford, John Sillitoe on behalf of the Wellcome Sanger Institute COVID-19 Surveillance Team
EPI_ISL_963595, EPI_ISL_963596, EPI_ISL_963597, EPI_ISL_963598, EPI_ISL_963599, EPI_ISL_963600	Lighthouse Lab in Milton Keynes	Wellcome Sanger Institute for the COVID-19 Genomics UK (COG-UK) Consortium	The Lighthouse Lab in Milton Keynes and Alex Alderton, Roberto Amato, Sonia Goncalves, Ewan Harrison, David K. Jackson, Ian Johnston, Dominic Kwiatkowski, Cordelia Langford, John Sillitoe on behalf of the Wellcome Sanger Institute COVID-19 Surveillance Team
EPI_ISL_963601	Lighthouse Lab in Glasgow	Wellcome Sanger Institute for the COVID-19 Genomics UK (COG-UK) Consortium	Harper VanSteenhouse, Yumi Kasai, David Gray, Carol Clugston, Anna Dominiczak and Alex Alderton, Roberto Amato, Sonia Goncalves, Ewan Harrison, David K. Jackson, Ian Johnston, Dominic Kwiatkowski, Cordelia Langford, John Sillitoe on behalf of the Wellcome Sanger Institute COVID-19 Surveillance Team
EPI_ISL_963602	Lighthouse Lab in Milton Keynes	Wellcome Sanger Institute for the COVID-19 Genomics UK (COG-UK) Consortium	The Lighthouse Lab in Milton Keynes and Alex Alderton, Roberto Amato, Sonia Goncalves, Ewan Harrison, David K. Jackson, Ian Johnston, Dominic Kwiatkowski, Cordelia Langford, John Sillitoe on behalf of the Wellcome Sanger Institute COVID-19 Surveillance Team
EPI_ISL_963603	Lighthouse Lab in Glasgow	Wellcome Sanger Institute for the COVID-19 Genomics UK (COG-UK) Consortium	Harper VanSteenhouse, Yumi Kasai, David Gray, Carol Clugston, Anna Dominiczak and Alex Alderton, Roberto Amato, Sonia Goncalves, Ewan Harrison, David K. Jackson, Ian Johnston, Dominic Kwiatkowski, Cordelia Langford, John Sillitoe on behalf of the Wellcome Sanger Institute COVID-19 Surveillance Team
EPI_ISL_963604, EPI_ISL_963605, EPI_ISL_963606	Lighthouse Lab in Milton Keynes	Wellcome Sanger Institute for the COVID-19 Genomics UK (COG-UK) Consortium	The Lighthouse Lab in Milton Keynes and Alex Alderton, Roberto Amato, Sonia Goncalves, Ewan Harrison, David K. Jackson, Ian Johnston, Dominic Kwiatkowski, Cordelia Langford, John Sillitoe on behalf of the Wellcome Sanger Institute COVID-19 Surveillance Team
EPI_ISL_963607	Lighthouse Lab in Glasgow	Wellcome Sanger Institute for the COVID-19 Genomics UK (COG-UK) Consortium	Harper VanSteenhouse, Yumi Kasai, David Gray, Carol Clugston, Anna Dominiczak and Alex Alderton, Roberto Amato, Sonia Goncalves, Ewan Harrison, David K. Jackson, Ian Johnston, Dominic Kwiatkowski, Cordelia Langford, John Sillitoe on behalf of the Wellcome Sanger Institute COVID-19 Surveillance Team
EPI_ISL_963608, EPI_ISL_963609, EPI_ISL_963610, EPI_ISL_963611, EPI_ISL_963612, EPI_ISL_963613, EPI_ISL_963614, EPI_ISL_963615, EPI_ISL_963616, EPI_ISL_963617	Lighthouse Lab in Milton Keynes	Wellcome Sanger Institute for the COVID-19 Genomics UK (COG-UK) Consortium	The Lighthouse Lab in Milton Keynes and Alex Alderton, Roberto Amato, Sonia Goncalves, Ewan Harrison, David K. Jackson, Ian Johnston, Dominic Kwiatkowski, Cordelia Langford, John Sillitoe on behalf of the Wellcome Sanger Institute COVID-19 Surveillance Team
EPI_ISL_963618	Lighthouse Lab in Glasgow	Wellcome Sanger Institute for the COVID-19 Genomics UK (COG-UK) Consortium	Harper VanSteenhouse, Yumi Kasai, David Gray, Carol Clugston, Anna Dominiczak and Alex Alderton, Roberto Amato, Sonia Goncalves, Ewan Harrison, David K. Jackson, Ian Johnston, Dominic Kwiatkowski, Cordelia Langford, John Sillitoe on behalf of the Wellcome Sanger Institute COVID-19 Surveillance Team
EPI_ISL_963619, EPI_ISL_963620, EPI_ISL_963621, EPI_ISL_963622, EPI_ISL_963623, EPI_ISL_963624, EPI_ISL_963625	Lighthouse Lab in Milton Keynes	Wellcome Sanger Institute for the COVID-19 Genomics UK (COG-UK) Consortium	The Lighthouse Lab in Milton Keynes and Alex Alderton, Roberto Amato, Sonia Goncalves, Ewan Harrison, David K. Jackson, Ian Johnston, Dominic Kwiatkowski, Cordelia Langford, John Sillitoe on behalf of the Wellcome Sanger Institute COVID-19 Surveillance Team
EPI_ISL_963626	Lighthouse Lab in Glasgow	Wellcome Sanger Institute for the COVID-19 Genomics UK (COG-UK) Consortium	Harper VanSteenhouse, Yumi Kasai, David Gray, Carol Clugston, Anna Dominiczak and Alex Alderton, Roberto Amato, Sonia Goncalves, Ewan Harrison, David K. Jackson, Ian Johnston, Dominic Kwiatkowski, Cordelia Langford, John Sillitoe on behalf of the Wellcome Sanger Institute COVID-19 Surveillance Team
EPI_ISL_963627, EPI_ISL_963628, EPI_ISL_963629, EPI_ISL_963630, EPI_ISL_963631, EPI_ISL_963632	Lighthouse Lab in Milton Keynes	Wellcome Sanger Institute for the COVID-19 Genomics UK (COG-UK) Consortium	The Lighthouse Lab in Milton Keynes and Alex Alderton, Roberto Amato, Sonia Goncalves, Ewan Harrison, David K. Jackson, Ian Johnston, Dominic Kwiatkowski, Cordelia Langford, John Sillitoe on behalf of the Wellcome Sanger Institute COVID-19 Surveillance Team
EPI_ISL_963633	Lighthouse Lab in Glasgow	Wellcome Sanger Institute for the COVID-19 Genomics UK (COG-UK) Consortium	Harper VanSteenhouse, Yumi Kasai, David Gray, Carol Clugston, Anna Dominiczak and Alex Alderton, Roberto Amato, Sonia Goncalves, Ewan Harrison, David K. Jackson, Ian Johnston, Dominic Kwiatkowski, Cordelia Langford, John Sillitoe on behalf of the Wellcome Sanger Institute COVID-19 Surveillance Team
EPI_ISL_963634, EPI_ISL_963635, EPI_ISL_963636, EPI_ISL_963637, EPI_ISL_963638	Lighthouse Lab in Milton Keynes	Wellcome Sanger Institute for the COVID-19 Genomics UK (COG-UK) Consortium	The Lighthouse Lab in Milton Keynes and Alex Alderton, Roberto Amato, Sonia Goncalves, Ewan Harrison, David K. Jackson, Ian Johnston, Dominic Kwiatkowski, Cordelia Langford, John Sillitoe on behalf of the Wellcome Sanger Institute COVID-19 Surveillance Team
EPI_ISL_963639	Lighthouse Lab in Glasgow	Wellcome Sanger Institute for the COVID-19 Genomics UK	Harper VanSteenhouse, Yumi Kasai, David Gray, Carol Clugston, Anna Dominiczak and Alex Alderton, Roberto Amato, Sonia Goncalves, Ewan Harrison,

		(COG-UK) Consortium	David K. Jackson, Ian Johnston, Dominic Kwiatkowski, Cordelia Langford, John Sillitoe on behalf of the Wellcome Sanger Institute COVID-19 Surveillance Team
EPI_ISL_963640	Lighthouse Lab in Milton Keynes	Wellcome Sanger Institute for the COVID-19 Genomics UK (COG-UK) Consortium	The Lighthouse Lab in Milton Keynes and Alex Alderton, Roberto Amato, Sonia Goncalves, Ewan Harrison, David K. Jackson, Ian Johnston, Dominic Kwiatkowski, Cordelia Langford, John Sillitoe on behalf of the Wellcome Sanger Institute COVID-19 Surveillance Team
EPI_ISL_963641	Lighthouse Lab in Glasgow	Wellcome Sanger Institute for the COVID-19 Genomics UK (COG-UK) Consortium	Harper VanSteenhouse, Yumi Kasai, David Gray, Carol Clugston, Anna Dominiczak and Alex Alderton, Roberto Amato, Sonia Goncalves, Ewan Harrison, David K. Jackson, Ian Johnston, Dominic Kwiatkowski, Cordelia Langford, John Sillitoe on behalf of the Wellcome Sanger Institute COVID-19 Surveillance Team
EPI_ISL_963642, EPI_ISL_963643, EPI_ISL_963644, EPI_ISL_963645, EPI_ISL_963646, EPI_ISL_963647, EPI_ISL_963648	Lighthouse Lab in Milton Keynes	Wellcome Sanger Institute for the COVID-19 Genomics UK (COG-UK) Consortium	The Lighthouse Lab in Milton Keynes and Alex Alderton, Roberto Amato, Sonia Goncalves, Ewan Harrison, David K. Jackson, Ian Johnston, Dominic Kwiatkowski, Cordelia Langford, John Sillitoe on behalf of the Wellcome Sanger Institute COVID-19 Surveillance Team
EPI_ISL_963649	Lighthouse Lab in Glasgow	Wellcome Sanger Institute for the COVID-19 Genomics UK (COG-UK) Consortium	Harper VanSteenhouse, Yumi Kasai, David Gray, Carol Clugston, Anna Dominiczak and Alex Alderton, Roberto Amato, Sonia Goncalves, Ewan Harrison, David K. Jackson, Ian Johnston, Dominic Kwiatkowski, Cordelia Langford, John Sillitoe on behalf of the Wellcome Sanger Institute COVID-19 Surveillance Team
EPI_ISL_963650	Lighthouse Lab in Milton Keynes	Wellcome Sanger Institute for the COVID-19 Genomics UK (COG-UK) Consortium	The Lighthouse Lab in Milton Keynes and Alex Alderton, Roberto Amato, Sonia Goncalves, Ewan Harrison, David K. Jackson, Ian Johnston, Dominic Kwiatkowski, Cordelia Langford, John Sillitoe on behalf of the Wellcome Sanger Institute COVID-19 Surveillance Team
EPI_ISL_963652, EPI_ISL_963653, EPI_ISL_963655, EPI_ISL_963656, EPI_ISL_963657, EPI_ISL_963660, EPI_ISL_963661, EPI_ISL_963662, EPI_ISL_963663, EPI_ISL_963664, EPI_ISL_963666, EPI_ISL_963667, EPI_ISL_963668, EPI_ISL_963669, EPI_ISL_963674, EPI_ISL_963675, EPI_ISL_963678, EPI_ISL_963680, EPI_ISL_963682, EPI_ISL_963683, EPI_ISL_963684, EPI_ISL_963688, EPI_ISL_963691, EPI_ISL_963692, EPI_ISL_963693, EPI_ISL_963696, EPI_ISL_963697, EPI_ISL_963698, EPI_ISL_963699, EPI_ISL_963702, EPI_ISL_963705, EPI_ISL_963708, EPI_ISL_963710, EPI_ISL_963711, EPI_ISL_963712, EPI_ISL_963713, EPI_ISL_963714, EPI_ISL_963715, EPI_ISL_963716, EPI_ISL_963717, EPI_ISL_963719, EPI_ISL_963722, EPI_ISL_963723, EPI_ISL_963725, EPI_ISL_963728, EPI_ISL_963729, EPI_ISL_963730, EPI_ISL_963739, EPI_ISL_963742, EPI_ISL_963743, EPI_ISL_963744, EPI_ISL_963745, EPI_ISL_963746, EPI_ISL_963748, EPI_ISL_963749, EPI_ISL_963751, EPI_ISL_963752, EPI_ISL_963755, EPI_ISL_963756, EPI_ISL_963757, EPI_ISL_963760, EPI_ISL_963762, EPI_ISL_963763, EPI_ISL_963767, EPI_ISL_963770, EPI_ISL_963771, EPI_ISL_963776, EPI_ISL_963777, EPI_ISL_963778, EPI_ISL_963782, EPI_ISL_963784, EPI_ISL_963785, EPI_ISL_963787, EPI_ISL_963788, EPI_ISL_963791, EPI_ISL_963792, EPI_ISL_963793, EPI_ISL_963795, EPI_ISL_963796, EPI_ISL_963797, EPI_ISL_963799, EPI_ISL_963801, EPI_ISL_963802, EPI_ISL_963804, EPI_ISL_963809, EPI_ISL_963811, EPI_ISL_963812, EPI_ISL_963817, EPI_ISL_963818, EPI_ISL_963820, EPI_ISL_963827, EPI_ISL_963828, EPI_ISL_963829, EPI_ISL_963830, EPI_ISL_963834, EPI_ISL_963836, EPI_ISL_963837, EPI_ISL_963840, EPI_ISL_963842, EPI_ISL_963843, EPI_ISL_963847, EPI_ISL_963848, EPI_ISL_963854, EPI_ISL_963856, EPI_ISL_963858, EPI_ISL_963860, EPI_ISL_963862, EPI_ISL_963871, EPI_ISL_963876, EPI_ISL_963885, EPI_ISL_963886, EPI_ISL_963887, EPI_ISL_963888, EPI_ISL_963892, EPI_ISL_963894, EPI_ISL_963897, EPI_ISL_963898, EPI_ISL_963899, EPI_ISL_963905, EPI_ISL_963907, EPI_ISL_963910, EPI_ISL_963912, EPI_ISL_963913, EPI_ISL_963917, EPI_ISL_963918, EPI_ISL_963919, EPI_ISL_963920, EPI_ISL_963924, EPI_ISL_963926, EPI_ISL_963928, EPI_ISL_963929, EPI_ISL_963930, EPI_ISL_963931, EPI_ISL_963933, EPI_ISL_963935, EPI_ISL_963937, EPI_ISL_963939, EPI_ISL_963940, EPI_ISL_963943, EPI_ISL_963944, EPI_ISL_963946, EPI_ISL_963948, EPI_ISL_963950, EPI_ISL_963951, EPI_ISL_963954, EPI_ISL_963956, EPI_ISL_963957, EPI_ISL_963959, EPI_ISL_963961, EPI_ISL_963963, EPI_ISL_963965, EPI_ISL_963966, EPI_ISL_963967, EPI_ISL_963969, EPI_ISL_963972, EPI_ISL_963974, EPI_ISL_963975, EPI_ISL_963982, EPI_ISL_963983, EPI_ISL_963985, EPI_ISL_963990, EPI_ISL_963993, EPI_ISL_963995, EPI_ISL_963997, EPI_ISL_963998, EPI_ISL_964002, EPI_ISL_964005, EPI_ISL_964007, EPI_ISL_964008, EPI_ISL_964009, EPI_ISL_964011, EPI_ISL_964013, EPI_ISL_964015, EPI_ISL_964016, EPI_ISL_964020, EPI_ISL_964022, EPI_ISL_964023, EPI_ISL_964026, EPI_ISL_964027, EPI_ISL_964029, EPI_ISL_964030, EPI_ISL_964031, EPI_ISL_964034, EPI_ISL_964038, EPI_ISL_964042, EPI_ISL_964043, EPI_ISL_964046, EPI_ISL_964047, EPI_ISL_964048, EPI_ISL_964055, EPI_ISL_964059, EPI_ISL_964066, EPI_ISL_964068, EPI_ISL_964071, EPI_ISL_964073, EPI_ISL_964075, EPI_ISL_964077, EPI_ISL_964080, EPI_ISL_964083, EPI_ISL_964084, EPI_ISL_964088, EPI_ISL_964089, EPI_ISL_964092, EPI_ISL_964097, EPI_ISL_964098, EPI_ISL_964100, EPI_ISL_964104, EPI_ISL_964105, EPI_ISL_964106, EPI_ISL_964111, EPI_ISL_964113, EPI_ISL_964114, EPI_ISL_964115, EPI_ISL_964116, EPI_ISL_964118, EPI_ISL_964119, EPI_ISL_964120, EPI_ISL_964122, EPI_ISL_964123, EPI_ISL_964126, EPI_ISL_964127, EPI_ISL_964128, EPI_ISL_964129, EPI_ISL_964130, EPI_ISL_964131, EPI_ISL_964132, EPI_ISL_964133, EPI_ISL_964134, EPI_ISL_964135, EPI_ISL_964141, EPI_ISL_964142, EPI_ISL_964150, EPI_ISL_964152, EPI_ISL_964153, EPI_ISL_964154, EPI_ISL_964155, EPI_ISL_964158, EPI_ISL_964159, EPI_ISL_964162, EPI_ISL_964165, EPI_ISL_964166, EPI_ISL_964170, EPI_ISL_964171, EPI_ISL_964173, EPI_ISL_964174, EPI_ISL_964175, EPI_ISL_964177, EPI_ISL_964178, EPI_ISL_964179, EPI_ISL_964183, EPI_ISL_964184, EPI_ISL_964186, EPI_ISL_964190, EPI_ISL_964192, EPI_ISL_964194, EPI_ISL_964196, EPI_ISL_964198, EPI_ISL_964200, EPI_ISL_964201, EPI_ISL_964202, EPI_ISL_964203, EPI_ISL_964206, EPI_ISL_964208, EPI_ISL_964212, EPI_ISL_964213, EPI_ISL_964216, EPI_ISL_964221, EPI_ISL_964223, EPI_ISL_964225, EPI_ISL_964226, EPI_ISL_964229, EPI_ISL_964230, EPI_ISL_964231, EPI_ISL_964232, EPI_ISL_964237, EPI_ISL_964240, EPI_ISL_964243, EPI_ISL_964246, EPI_ISL_964248, EPI_ISL_964250, EPI_ISL_964252, EPI_ISL_964255, EPI_ISL_964256, EPI_ISL_964261, EPI_ISL_964269, EPI_ISL_964273, EPI_ISL_964274, EPI_ISL_964277, EPI_ISL_964280, EPI_ISL_964283, EPI_ISL_964288, EPI_ISL_964296, EPI_ISL_964298, EPI_ISL_964302, EPI_ISL_964307, EPI_ISL_964312, EPI_ISL_964313, EPI_ISL_964315, EPI_ISL_964316, EPI_ISL_964317, EPI_ISL_964318, EPI_ISL_964319, EPI_ISL_964325, EPI_ISL_964327, EPI_ISL_964328, EPI_ISL_964329, EPI_ISL_964331, EPI_ISL_964332, EPI_ISL_964334, EPI_ISL_964337, EPI_ISL_964340, EPI_ISL_964341, EPI_ISL_964345			
see above	Lighthouse Lab in Alderley Park	Wellcome Sanger Institute for the COVID-19 Genomics UK (COG-UK) Consortium	Jacquelyn Wynn, Mairead Hyland, The Lighthouse Lab in Alderley Park and Alex Alderton, Roberto Amato, Sonia Goncalves, Ewan Harrison, David K. Jackson, Ian Johnston, Dominic Kwiatkowski, Cordelia Langford, John Sillitoe on behalf of the Wellcome Sanger Institute COVID-19 Surveillance Team
EPI_ISL_964451	Lighthouse Lab in Glasgow	Wellcome Sanger Institute for the COVID-19 Genomics UK (COG-UK) Consortium	Harper VanSteenhouse, Yumi Kasai, David Gray, Carol Clugston, Anna Dominiczak and Alex Alderton, Roberto Amato, Sonia Goncalves, Ewan Harrison, David K. Jackson, Ian Johnston, Dominic Kwiatkowski, Cordelia Langford, John Sillitoe on behalf of the Wellcome Sanger Institute COVID-19 Surveillance Team
EPI_ISL_964544, EPI_ISL_964611, EPI_ISL_964646, EPI_ISL_964655, EPI_ISL_964739, EPI_ISL_964766, EPI_ISL_964772, EPI_ISL_964778, EPI_ISL_964867	Lighthouse Lab in Alderley Park	Wellcome Sanger Institute for the COVID-19 Genomics UK (COG-UK) Consortium	Jacquelyn Wynn, Mairead Hyland, The Lighthouse Lab in Alderley Park and Alex Alderton, Roberto Amato, Sonia Goncalves, Ewan Harrison, David K. Jackson, Ian Johnston, Dominic Kwiatkowski, Cordelia Langford, John Sillitoe on behalf of the Wellcome Sanger Institute COVID-19 Surveillance Team
EPI_ISL_964876	Lighthouse Lab in Milton Keynes	Wellcome Sanger Institute for the COVID-19 Genomics UK (COG-UK) Consortium	The Lighthouse Lab in Milton Keynes and Alex Alderton, Roberto Amato, Sonia Goncalves, Ewan Harrison, David K. Jackson, Ian Johnston, Dominic Kwiatkowski, Cordelia Langford, John Sillitoe on behalf of the Wellcome Sanger Institute COVID-19 Surveillance Team
EPI_ISL_969457, EPI_ISL_969458, EPI_ISL_969459, EPI_ISL_969462, EPI_ISL_969467, EPI_ISL_969468, EPI_ISL_969471, EPI_ISL_969478, EPI_ISL_969486, EPI_ISL_969487, EPI_ISL_969488, EPI_ISL_969489, EPI_ISL_969492, EPI_ISL_969497, EPI_ISL_969498, EPI_ISL_969501, EPI_ISL_969504, EPI_ISL_969511, EPI_ISL_969513, EPI_ISL_969514, EPI_ISL_969516, EPI_ISL_969519, EPI_ISL_969521, EPI_ISL_969524, EPI_ISL_969528, EPI_ISL_969529, EPI_ISL_969531, EPI_ISL_969532, EPI_ISL_969536, EPI_ISL_969539, EPI_ISL_969540, EPI_ISL_969542, EPI_ISL_969543, EPI_ISL_969546, EPI_ISL_969549, EPI_ISL_969554, EPI_ISL_969555, EPI_ISL_969558, EPI_ISL_969559, EPI_ISL_969560, EPI_ISL_969561, EPI_ISL_969566, EPI_ISL_969567, EPI_ISL_969569, EPI_ISL_969570, EPI_ISL_969572, EPI_ISL_969577, EPI_ISL_969581, EPI_ISL_969582, EPI_ISL_969585, EPI_ISL_969591, EPI_ISL_969593, EPI_ISL_969594, EPI_ISL_969595, EPI_ISL_969596, EPI_ISL_969604, EPI_ISL_969613, EPI_ISL_969614, EPI_ISL_969615, EPI_ISL_969617, EPI_ISL_969618, EPI_ISL_969619, EPI_ISL_969621, EPI_ISL_969624, EPI_ISL_969627, EPI_ISL_969635, EPI_ISL_969637, EPI_ISL_969638, EPI_ISL_969639, EPI_ISL_969641, EPI_ISL_969652, EPI_ISL_969654, EPI_ISL_969655, EPI_ISL_969659, EPI_ISL_969660, EPI_ISL_969661, EPI_ISL_969662, EPI_ISL_969664, EPI_ISL_969666, EPI_ISL_969669, EPI_ISL_969673, EPI_ISL_969682, EPI_ISL_969688, EPI_ISL_969691, EPI_ISL_969693, EPI_ISL_969699, EPI_ISL_969701, EPI_ISL_969704, EPI_ISL_969710, EPI_ISL_969716, EPI_ISL_969726, EPI_ISL_969730, EPI_ISL_969744, EPI_ISL_969748, EPI_ISL_969750, EPI_ISL_969759, EPI_ISL_969762, EPI_ISL_969764, EPI_ISL_969768, EPI_ISL_969769, EPI_ISL_969771, EPI_ISL_969774, EPI_ISL_969779, EPI_ISL_969780, EPI_ISL_969789, EPI_ISL_969793, EPI_ISL_969795, EPI_ISL_969797, EPI_ISL_969802, EPI_ISL_969804, EPI_ISL_969806, EPI_ISL_969821, EPI_ISL_969824, EPI_ISL_969833, EPI_ISL_969834, EPI_ISL_969840, EPI_ISL_969849, EPI_ISL_969861, EPI_ISL_969863, EPI_ISL_969865, EPI_ISL_969869, EPI_ISL_969871, EPI_ISL_969875, EPI_ISL_969881, EPI_ISL_969893, EPI_ISL_969906, EPI_ISL_969907, EPI_ISL_969909, EPI_ISL_969911, EPI_ISL_969914, EPI_ISL_969916, EPI_ISL_969917, EPI_ISL_969919, EPI_ISL_969921, EPI_ISL_969923, EPI_ISL_969925, EPI_ISL_969928, EPI_ISL_969930, EPI_ISL_969932, EPI_ISL_969934, EPI_ISL_969936, EPI_ISL_969937, EPI_ISL_969939, EPI_ISL_969940, EPI_ISL_969943, EPI_ISL_969947, EPI_ISL_969948, EPI_ISL_969951, EPI_ISL_969952, EPI_ISL_969955, EPI_ISL_969956, EPI_ISL_969958, EPI_ISL_969961, EPI_ISL_969963, EPI_ISL_969965, EPI_ISL_969967, EPI_ISL_969969, EPI_ISL_969971, EPI_ISL_969974, EPI_ISL_969975, EPI_ISL_969977, EPI_ISL_969979, EPI_ISL_969982, EPI_ISL_969983, EPI_ISL_969986, EPI_ISL_969988, EPI_ISL_969990, EPI_ISL_969992, EPI_ISL_969994, EPI_ISL_969996, EPI_ISL_969998, EPI_ISL_970000, EPI_ISL_970002, EPI_ISL_970004, EPI_ISL_970005, EPI_ISL_970007, EPI_ISL_970009, EPI_ISL_970011, EPI_ISL_970013, EPI_ISL_970015, EPI_ISL_970017, EPI_ISL_970019, EPI_ISL_970022, EPI_ISL_970024, EPI_ISL_970026, EPI_ISL_970028, EPI_ISL_970030, EPI_ISL_970033, EPI_ISL_970035, EPI_ISL_970037, EPI_ISL_970039, EPI_ISL_970041, EPI_ISL_970043, EPI_ISL_970045, EPI_ISL_970047, EPI_ISL_970049, EPI_ISL_970050, EPI_ISL_970052, EPI_ISL_970057, EPI_ISL_970059, EPI_ISL_970061, EPI_ISL_970064, EPI_ISL_970066, EPI_ISL_970068, EPI_ISL_970070, EPI_ISL_970073, EPI_ISL_970075, EPI_ISL_970076, EPI_ISL_970079, EPI_ISL_970080, EPI_ISL_970082, EPI_ISL_970084, EPI_ISL_970086, EPI_ISL_970087, EPI_ISL_970089, EPI_ISL_970091, EPI_ISL_970094, EPI_ISL_970096, EPI_ISL_970098, EPI_ISL_970099, EPI_ISL_970101, EPI_ISL_970103, EPI_ISL_970105, EPI_ISL_970107, EPI_ISL_970109, EPI_ISL_970111, EPI_ISL_970113, EPI_ISL_970115, EPI_ISL_970117, EPI_ISL_970119, EPI_ISL_970120, EPI_ISL_970122, EPI_ISL_970124, EPI_ISL_970126, EPI_ISL_970128, EPI_ISL_970130, EPI_ISL_970132, EPI_ISL_970134, EPI_ISL_970136, EPI_ISL_970138, EPI_ISL_970140, EPI_ISL_970145, EPI_ISL_970147, EPI_ISL_970148, EPI_ISL_970150, EPI_ISL_970152, EPI_ISL_970153, EPI_ISL_970155, EPI_ISL_970157, EPI_ISL_970160, EPI_ISL_970161, EPI_ISL_970163, EPI_ISL_970165, EPI_ISL_970166, EPI_ISL_970168, EPI_ISL_970170, EPI_ISL_970172, EPI_ISL_970173, EPI_ISL_970175, EPI_ISL_970177, EPI_ISL_970181, EPI_ISL_970183, EPI_ISL_970184, EPI_ISL_970186, EPI_ISL_970188, EPI_ISL_970190, EPI_ISL_970192, EPI_ISL_970195, EPI_ISL_970197, EPI_ISL_970198, EPI_ISL_970200, EPI_ISL_970202, EPI_ISL_970205, EPI_ISL_970206, EPI_ISL_970209, EPI_ISL_970211, EPI_ISL_970213, EPI_ISL_970215, EPI_ISL_970217, EPI_ISL_970219, EPI_ISL_970221, EPI_ISL_970224, EPI_ISL_970225, EPI_ISL_970227, EPI_ISL_970229, EPI_ISL_970231, EPI_ISL_970233, EPI_ISL_970235, EPI_ISL_970237, EPI_ISL_970239, EPI_ISL_970242, EPI_ISL_970247, EPI_ISL_970249, EPI_ISL_970251, EPI_ISL_970252, EPI_ISL_970254, EPI_ISL_970256, EPI_ISL_970258, EPI_ISL_970260, EPI_ISL_970262, EPI_ISL_970264, EPI_ISL_970266, EPI_ISL_970268, EPI_ISL_970270, EPI_ISL_970272, EPI_ISL_970274, EPI_ISL_970275, EPI_ISL_970278, EPI_ISL_970279, EPI_ISL_970281, EPI_ISL_970283, EPI_ISL_970285, EPI_ISL_970287, EPI_ISL_970288, EPI_ISL_970291, EPI_ISL_970292, EPI_ISL_970295, EPI_ISL_970297, EPI_ISL_970299, EPI_ISL_970301, EPI_ISL_970303, EPI_ISL_970306, EPI_ISL_970307, EPI_ISL_970309, EPI_ISL_970312, EPI_ISL_970314, EPI_ISL_970316, EPI_ISL_970319, EPI_ISL_970321, EPI_ISL_970323, EPI_ISL_970324, EPI_ISL_970326, EPI_ISL_970328, EPI_ISL_970330, EPI_ISL_970332, EPI_ISL_970334, EPI_ISL_970337, EPI_ISL_970338, EPI_ISL_970340, EPI_ISL_970342, EPI_ISL_970344, EPI_ISL_970346, EPI_ISL_970348, EPI_ISL_970350, EPI_ISL_970352, EPI_ISL_970354, EPI_ISL_970356, EPI_ISL_970358, EPI_ISL_970360, EPI_ISL_970362, EPI_ISL_970364, EPI_ISL_970365, EPI_ISL_970368, EPI_ISL_970370, EPI_ISL_970371, EPI_ISL_970373, EPI_ISL_970376, EPI_ISL_970377, EPI_ISL_970380, EPI_ISL_970382, EPI_ISL_970383, EPI_ISL_970386, EPI_ISL_970387, EPI_ISL_970390, EPI_ISL_970392, EPI_ISL_970394, EPI_ISL_970396, EPI_ISL_970398, EPI_ISL_970400, EPI_ISL_970402, EPI_ISL_970404, EPI_ISL_970406, EPI_ISL_970409, EPI_ISL_970411, EPI_ISL_970412, EPI_ISL_970415, EPI_ISL_970416, EPI_ISL_970419, EPI_ISL_970421, EPI_ISL_970422, EPI_ISL_970424, EPI_ISL_970426, EPI_ISL_970428, EPI_ISL_970430, EPI_ISL_970432, EPI_ISL_970435, EPI_ISL_970436, EPI_ISL_970439, EPI_ISL_970441, EPI_ISL_970443, EPI_ISL_970444, EPI_ISL_970446, EPI_ISL_970448, EPI_ISL_970450, EPI_ISL_970452, EPI_ISL_970454, EPI_ISL_970457, EPI_ISL_970460, EPI_ISL_970462, EPI_ISL_970464, EPI_ISL_970466, EPI_ISL_970468, EPI_ISL_970470, EPI_ISL_970473, EPI_ISL_970475, EPI_ISL_970477, EPI_ISL_970478, EPI_ISL_970481, EPI_ISL_970483, EPI_ISL_970486, EPI_ISL_970488, EPI_ISL_970490, EPI_ISL_970492, EPI_ISL_970493, EPI_ISL_970495, EPI_ISL_970497, EPI_ISL_970499, EPI_ISL_970500, EPI_ISL_970502, EPI_ISL_970504, EPI_ISL_970506, EPI_ISL_970508, EPI_ISL_970510, EPI_ISL_970512, EPI_ISL_970513, EPI_ISL_970515, EPI_ISL_970517, EPI_ISL_970519, EPI_ISL_970521, EPI_ISL_970523, EPI_ISL_970525, EPI_ISL_970527, EPI_ISL_970528, EPI_ISL_970530, EPI_ISL_970533, EPI_ISL_970535, EPI_ISL_970537, EPI_ISL_970540, EPI_ISL_970541, EPI_ISL_970543, EPI_ISL_970546, EPI_ISL_970548, EPI_ISL_970551, EPI_ISL_970553, EPI_ISL_970555, EPI_ISL_970558, EPI_ISL_970560, EPI_ISL_970562, EPI_ISL_970564, EPI_ISL_970566, EPI_ISL_970568, EPI_ISL_970569, EPI_ISL_970571, EPI_ISL_970573, EPI_ISL_970575, EPI_ISL_970577, EPI_ISL_970579, EPI_ISL_970581, EPI_ISL_970583, EPI_ISL_970585			
see above	Lighthouse Lab in Alderley Park	Wellcome Sanger Institute for the COVID-19 Genomics UK (COG-UK) Consortium	Jacquelyn Wynn, Mairead Hyland, The Lighthouse Lab in Alderley Park and Alex Alderton, Roberto Amato, Sonia Goncalves, Ewan Harrison, David K. Jackson, Ian Johnston, Dominic Kwiatkowski, Cordelia Langford, John Sillitoe on behalf of the Wellcome Sanger Institute COVID-19 Surveillance Team
EPI_ISL_970587, EPI_ISL_970597, EPI_ISL_970616, EPI_ISL_970621, EPI_ISL_970628, EPI_ISL_970635, EPI_ISL_970639, EPI_ISL_970645, EPI_ISL_970664, EPI_ISL_970673, EPI_ISL_970680, EPI_ISL_970681, EPI_ISL_970687, EPI_ISL_970701, EPI_ISL_970708, EPI_ISL_970709, EPI_ISL_970715, EPI_ISL_970724, EPI_ISL_970732, EPI_ISL_970744, EPI_ISL_970747, EPI_ISL_970751, EPI_ISL_970753, EPI_ISL_970768, EPI_ISL_970775, EPI_ISL_970790, EPI_ISL_970826, EPI_ISL_970860, EPI_ISL_970875, EPI_ISL_970986, EPI_ISL_971037, EPI_ISL_971042, EPI_ISL_971044, EPI_ISL_971047, EPI_ISL_971053, EPI_ISL_971060, EPI_ISL_971071, EPI_ISL_971076, EPI_ISL_971093, EPI_ISL_971096, EPI_ISL_971105, EPI_ISL_971125, EPI_ISL_971135, EPI_ISL_971142, EPI_ISL_971168, EPI_ISL_971171, EPI_ISL_971174, EPI_ISL_971176, EPI_ISL_971183, EPI_ISL_971191, EPI_ISL_971194, EPI_ISL_971206, EPI_ISL_971231, EPI_ISL_971239, EPI_ISL_971242, EPI_ISL_971255, EPI_ISL_971261, EPI_ISL_971269, EPI_ISL_971281, EPI_ISL_971292, EPI_ISL_971297, EPI_ISL_971327, EPI_ISL_971333, EPI_ISL_971339, EPI_ISL_971347, EPI_ISL_971360, EPI_ISL_971364, EPI_ISL_971365, EPI_ISL_971370, EPI_ISL_971385, EPI_ISL_971394, EPI_ISL_979982, EPI_ISL_979987, EPI_ISL_979992, EPI_ISL_979997, EPI_ISL_980000, EPI_ISL_980004, EPI_ISL_980008, EPI_ISL_980009, EPI_ISL_980016, EPI_ISL_980026, EPI_ISL_980033, EPI_ISL_980061, EPI_ISL_980063, EPI_ISL_980064, EPI_ISL_980083, EPI_ISL_980085, EPI_ISL_980089, EPI_ISL_980105, EPI_ISL_980109, EPI_ISL_980119, EPI_ISL_980131, EPI_ISL_980137, EPI_ISL_980143, EPI_ISL_980145, EPI_ISL_980148, EPI_ISL_980154, EPI_ISL_980155, EPI_ISL_980162, EPI_ISL_980168, EPI_ISL_980171, EPI_ISL_980172, EPI_ISL_980176, EPI_ISL_980178, EPI_ISL_980183, EPI_ISL_980186, EPI_ISL_980187, EPI_ISL_980193, EPI_ISL_980198, EPI_ISL_980226, EPI_ISL_980227, EPI_ISL_980228, EPI_ISL_980233, EPI_ISL_980568, EPI_ISL_980579, EPI_ISL_980612, EPI_ISL_980615, EPI_ISL_980618, EPI_ISL_980648, EPI_ISL_980716, EPI_ISL_980731, EPI_ISL_980740, EPI_ISL_980749, EPI_ISL_980764			

see above	Lighthouse Lab in Milton Keynes	Wellcome Sanger Institute for the COVID-19 Genomics UK (COG-UK) Consortium	The Lighthouse Lab in Milton Keynes and Alex Alderton, Roberto Amato, Sonia Goncalves, Ewan Harrison, David K. Jackson, Ian Johnston, Dominic Kwiatkowski, Cordelia Langford, John Sillitoe on behalf of the Wellcome Sanger Institute COVID-19 Surveillance Team
EPI_ISL_986039	Lighthouse Lab in Milton Keynes	Wellcome Sanger Institute for the COVID-19 Genomics UK (COG-UK) Consortium	The Lighthouse Lab in Milton Keynes and Alex Alderton, Roberto Amato, Sonia Goncalves, Ewan Harrison, David K. Jackson, Ian Johnston, Dominic Kwiatkowski, Cordelia Langford, John Sillitoe on behalf of the Wellcome Sanger Institute COVID-19 Surveillance Team (http://www.sanger.ac.uk/covid-team)
EPI_ISL_987005, EPI_ISL_987104	Lighthouse Lab in Alderley Park	Wellcome Sanger Institute for the COVID-19 Genomics UK (COG-UK) Consortium	Jacquelyn Wynn, Mairead Hyland, The Lighthouse Lab in Alderley Park and Alex Alderton, Roberto Amato, Sonia Goncalves, Ewan Harrison, David K. Jackson, Ian Johnston, Dominic Kwiatkowski, Cordelia Langford, John Sillitoe on behalf of the Wellcome Sanger Institute COVID-19 Surveillance Team (http://www.sanger.ac.uk/covid-team)
EPI_ISL_987638, EPI_ISL_987639, EPI_ISL_987640, EPI_ISL_987641, EPI_ISL_987642, EPI_ISL_987646, EPI_ISL_987648, EPI_ISL_987649, EPI_ISL_987650, EPI_ISL_987653, EPI_ISL_987655, EPI_ISL_987656, EPI_ISL_987657, EPI_ISL_987659, EPI_ISL_987660, EPI_ISL_987662, EPI_ISL_987663, EPI_ISL_987664, EPI_ISL_987666, EPI_ISL_987667, EPI_ISL_987668, EPI_ISL_987670, EPI_ISL_987672, EPI_ISL_987673, EPI_ISL_987674, EPI_ISL_987675, EPI_ISL_987680, EPI_ISL_987681, EPI_ISL_987682, EPI_ISL_987683, EPI_ISL_987684, EPI_ISL_987685, EPI_ISL_987686, EPI_ISL_987688, EPI_ISL_987689, EPI_ISL_987690, EPI_ISL_987691, EPI_ISL_987694, EPI_ISL_987696, EPI_ISL_987698, EPI_ISL_987699, EPI_ISL_987700, EPI_ISL_987701, EPI_ISL_987702, EPI_ISL_987704, EPI_ISL_987705, EPI_ISL_987707, EPI_ISL_987709, EPI_ISL_987710, EPI_ISL_987711, EPI_ISL_987712, EPI_ISL_987713, EPI_ISL_987714, EPI_ISL_987715, EPI_ISL_987716, EPI_ISL_987717, EPI_ISL_987718, EPI_ISL_987720, EPI_ISL_987721, EPI_ISL_987722, EPI_ISL_987725, EPI_ISL_987728, EPI_ISL_987729, EPI_ISL_987730, EPI_ISL_987731, EPI_ISL_987732, EPI_ISL_987733, EPI_ISL_987735, EPI_ISL_987736, EPI_ISL_987738, EPI_ISL_987739, EPI_ISL_987741, EPI_ISL_987742, EPI_ISL_987743, EPI_ISL_987744, EPI_ISL_987745, EPI_ISL_987746, EPI_ISL_987748, EPI_ISL_987750, EPI_ISL_987755, EPI_ISL_987757, EPI_ISL_987761, EPI_ISL_987762, EPI_ISL_987763, EPI_ISL_987764, EPI_ISL_987765, EPI_ISL_987767, EPI_ISL_987768, EPI_ISL_987769, EPI_ISL_987770, EPI_ISL_987771, EPI_ISL_987772, EPI_ISL_987773, EPI_ISL_987775, EPI_ISL_987778, EPI_ISL_987779, EPI_ISL_987782, EPI_ISL_987785, EPI_ISL_987786, EPI_ISL_987788, EPI_ISL_987790, EPI_ISL_987791, EPI_ISL_987795, EPI_ISL_987802, EPI_ISL_987804, EPI_ISL_987806, EPI_ISL_987807, EPI_ISL_987811, EPI_ISL_987812, EPI_ISL_987814, EPI_ISL_987815, EPI_ISL_987816, EPI_ISL_987818, EPI_ISL_987819, EPI_ISL_987822, EPI_ISL_987824, EPI_ISL_987826, EPI_ISL_987827, EPI_ISL_987829, EPI_ISL_987836, EPI_ISL_987837, EPI_ISL_987838, EPI_ISL_987841, EPI_ISL_987842, EPI_ISL_987845, EPI_ISL_987846, EPI_ISL_987848, EPI_ISL_987849, EPI_ISL_987851, EPI_ISL_987852, EPI_ISL_987854, EPI_ISL_987855, EPI_ISL_987857, EPI_ISL_987858, EPI_ISL_987859, EPI_ISL_987860, EPI_ISL_987861, EPI_ISL_987862, EPI_ISL_987864, EPI_ISL_987865, EPI_ISL_987868, EPI_ISL_987869, EPI_ISL_987870, EPI_ISL_987872, EPI_ISL_987873, EPI_ISL_987874, EPI_ISL_987876, EPI_ISL_987878, EPI_ISL_987879, EPI_ISL_987883, EPI_ISL_987884, EPI_ISL_987885, EPI_ISL_987888, EPI_ISL_987889, EPI_ISL_987890, EPI_ISL_987891, EPI_ISL_987893, EPI_ISL_987895, EPI_ISL_987896, EPI_ISL_987897, EPI_ISL_987898, EPI_ISL_987899, EPI_ISL_987900, EPI_ISL_987901, EPI_ISL_987902, EPI_ISL_987903, EPI_ISL_987905, EPI_ISL_987907, EPI_ISL_987909, EPI_ISL_987910, EPI_ISL_987912, EPI_ISL_987913, EPI_ISL_987914, EPI_ISL_987915, EPI_ISL_987917, EPI_ISL_987919, EPI_ISL_987920, EPI_ISL_987923, EPI_ISL_987924, EPI_ISL_987927, EPI_ISL_987938, EPI_ISL_987939, EPI_ISL_987940, EPI_ISL_987941, EPI_ISL_987942, EPI_ISL_987943, EPI_ISL_987946, EPI_ISL_987947, EPI_ISL_987949, EPI_ISL_987950, EPI_ISL_987951, EPI_ISL_987952, EPI_ISL_987954, EPI_ISL_987955, EPI_ISL_987956, EPI_ISL_987957, EPI_ISL_987959, EPI_ISL_987961, EPI_ISL_987963, EPI_ISL_987965, EPI_ISL_987966, EPI_ISL_987967, EPI_ISL_987968, EPI_ISL_987969, EPI_ISL_987970, EPI_ISL_987971, EPI_ISL_987972, EPI_ISL_987973, EPI_ISL_987974, EPI_ISL_987975, EPI_ISL_987977, EPI_ISL_987979, EPI_ISL_987981, EPI_ISL_987984, EPI_ISL_987985, EPI_ISL_987986, EPI_ISL_987987, EPI_ISL_987988, EPI_ISL_987990, EPI_ISL_987991, EPI_ISL_987992, EPI_ISL_987994, EPI_ISL_987995, EPI_ISL_987996, EPI_ISL_987997, EPI_ISL_988001, EPI_ISL_988002, EPI_ISL_988004, EPI_ISL_988005, EPI_ISL_988006, EPI_ISL_988008, EPI_ISL_988010, EPI_ISL_988257			
see above	Lighthouse Lab in Cambridge	Wellcome Sanger Institute for the COVID-19 Genomics UK (COG-UK) Consortium	Rob Howes, The Lighthouse Lab in Cambridge and Alex Alderton, Roberto Amato, Sonia Goncalves, Ewan Harrison, David K. Jackson, Ian Johnston, Dominic Kwiatkowski, Cordelia Langford, John Sillitoe on behalf of the Wellcome Sanger Institute COVID-19 Surveillance Team (http://www.sanger.ac.uk/covid-team)
EPI_ISL_988486, EPI_ISL_988495, EPI_ISL_988504, EPI_ISL_988515, EPI_ISL_988516, EPI_ISL_988517, EPI_ISL_988537, EPI_ISL_988538, EPI_ISL_988552, EPI_ISL_988554, EPI_ISL_988555, EPI_ISL_988558, EPI_ISL_988564, EPI_ISL_988576, EPI_ISL_988589, EPI_ISL_988592, EPI_ISL_988600, EPI_ISL_988604, EPI_ISL_988623, EPI_ISL_988631, EPI_ISL_988640, EPI_ISL_988646, EPI_ISL_988651, EPI_ISL_988685			
see above	Lighthouse Lab in Milton Keynes	Wellcome Sanger Institute for the COVID-19 Genomics UK (COG-UK) Consortium	The Lighthouse Lab in Milton Keynes and Alex Alderton, Roberto Amato, Sonia Goncalves, Ewan Harrison, David K. Jackson, Ian Johnston, Dominic Kwiatkowski, Cordelia Langford, John Sillitoe on behalf of the Wellcome Sanger Institute COVID-19 Surveillance Team (http://www.sanger.ac.uk/covid-team)
EPI_ISL_989064, EPI_ISL_989229	Lighthouse Lab in Alderley Park	Wellcome Sanger Institute for the COVID-19 Genomics UK (COG-UK) Consortium	Jacquelyn Wynn, Mairead Hyland, The Lighthouse Lab in Alderley Park and Alex Alderton, Roberto Amato, Sonia Goncalves, Ewan Harrison, David K. Jackson, Ian Johnston, Dominic Kwiatkowski, Cordelia Langford, John Sillitoe on behalf of the Wellcome Sanger Institute COVID-19 Surveillance Team
EPI_ISL_989246	Lighthouse Lab in Alderley Park	Wellcome Sanger Institute for the COVID-19 Genomics UK (COG-UK) Consortium	Jacquelyn Wynn, Mairead Hyland, The Lighthouse Lab in Alderley Park and Alex Alderton, Roberto Amato, Sonia Goncalves, Ewan Harrison, David K. Jackson, Ian Johnston, Dominic Kwiatkowski, Cordelia Langford, John Sillitoe on behalf of the Wellcome Sanger Institute COVID-19 Surveillance Team (http://www.sanger.ac.uk/covid-team)
EPI_ISL_989449, EPI_ISL_989450, EPI_ISL_989451, EPI_ISL_989452, EPI_ISL_989453, EPI_ISL_989454, EPI_ISL_989455, EPI_ISL_989456, EPI_ISL_989457, EPI_ISL_989458, EPI_ISL_989459, EPI_ISL_989460, EPI_ISL_989461, EPI_ISL_989462, EPI_ISL_989463, EPI_ISL_989464, EPI_ISL_989465, EPI_ISL_989466, EPI_ISL_989467, EPI_ISL_989468, EPI_ISL_989469, EPI_ISL_989470, EPI_ISL_989471, EPI_ISL_989472, EPI_ISL_989473, EPI_ISL_989474, EPI_ISL_989475, EPI_ISL_989476, EPI_ISL_989477, EPI_ISL_989478, EPI_ISL_989479, EPI_ISL_989480, EPI_ISL_989481			
see above	Lighthouse Lab in Glasgow	Wellcome Sanger Institute for the COVID-19 Genomics UK (COG-UK) Consortium	Harper VanSteenhouse, Yumi Kasai, David Gray, Carol Clugston, Anna Dominiczak and Alex Alderton, Roberto Amato, Sonia Goncalves, Ewan Harrison, David K. Jackson, Ian Johnston, Dominic Kwiatkowski, Cordelia Langford, John Sillitoe on behalf of the Wellcome Sanger Institute COVID-19 Surveillance Team (http://www.sanger.ac.uk/covid-team)
EPI_ISL_989484, EPI_ISL_989486, EPI_ISL_989487, EPI_ISL_989488, EPI_ISL_989492, EPI_ISL_989493, EPI_ISL_989497, EPI_ISL_989498, EPI_ISL_989499, EPI_ISL_989500, EPI_ISL_989501, EPI_ISL_989502, EPI_ISL_989503, EPI_ISL_989508, EPI_ISL_989509, EPI_ISL_989510, EPI_ISL_989511, EPI_ISL_989514, EPI_ISL_989515, EPI_ISL_989516, EPI_ISL_989517, EPI_ISL_989518, EPI_ISL_989519, EPI_ISL_989520, EPI_ISL_989521, EPI_ISL_989523, EPI_ISL_989524, EPI_ISL_989525, EPI_ISL_989527, EPI_ISL_989528, EPI_ISL_989529, EPI_ISL_989699, EPI_ISL_989701, EPI_ISL_989703, EPI_ISL_989707, EPI_ISL_989711, EPI_ISL_989712, EPI_ISL_989713, EPI_ISL_989718, EPI_ISL_989719, EPI_ISL_989733, EPI_ISL_989735, EPI_ISL_989736, EPI_ISL_989737, EPI_ISL_989741, EPI_ISL_989743, EPI_ISL_989747, EPI_ISL_989748, EPI_ISL_989750, EPI_ISL_989752, EPI_ISL_989753, EPI_ISL_989755, EPI_ISL_989758, EPI_ISL_989759, EPI_ISL_989760, EPI_ISL_989761, EPI_ISL_989762, EPI_ISL_989763, EPI_ISL_989764, EPI_ISL_989765, EPI_ISL_989766, EPI_ISL_989767, EPI_ISL_989768, EPI_ISL_989770, EPI_ISL_989771, EPI_ISL_989772, EPI_ISL_989773, EPI_ISL_989774, EPI_ISL_989775, EPI_ISL_989776, EPI_ISL_989777, EPI_ISL_989778, EPI_ISL_989779, EPI_ISL_989780, EPI_ISL_989781, EPI_ISL_989782, EPI_ISL_989783, EPI_ISL_989784, EPI_ISL_989785, EPI_ISL_989786, EPI_ISL_989787, EPI_ISL_989788, EPI_ISL_989789, EPI_ISL_989790, EPI_ISL_989791, EPI_ISL_989792, EPI_ISL_989793, EPI_ISL_989794			
see above	Lighthouse Lab in Alderley Park	Wellcome Sanger Institute for the COVID-19 Genomics UK (COG-UK) Consortium	Jacquelyn Wynn, Mairead Hyland, The Lighthouse Lab in Alderley Park and Alex Alderton, Roberto Amato, Sonia Goncalves, Ewan Harrison, David K. Jackson, Ian Johnston, Dominic Kwiatkowski, Cordelia Langford, John Sillitoe on behalf of the Wellcome Sanger Institute COVID-19 Surveillance Team (http://www.sanger.ac.uk/covid-team)
EPI_ISL_989831	Lighthouse Lab in Milton Keynes	Wellcome Sanger Institute for the COVID-19 Genomics UK (COG-UK) Consortium	The Lighthouse Lab in Milton Keynes and Alex Alderton, Roberto Amato, Sonia Goncalves, Ewan Harrison, David K. Jackson, Ian Johnston, Dominic Kwiatkowski, Cordelia Langford, John Sillitoe on behalf of the Wellcome Sanger Institute COVID-19 Surveillance Team (http://www.sanger.ac.uk/covid-team)
EPI_ISL_989844	Lighthouse Lab in Glasgow	Wellcome Sanger Institute for the COVID-19 Genomics UK (COG-UK) Consortium	Harper VanSteenhouse, Yumi Kasai, David Gray, Carol Clugston, Anna Dominiczak and Alex Alderton, Roberto Amato, Sonia Goncalves, Ewan Harrison, David K. Jackson, Ian Johnston, Dominic Kwiatkowski, Cordelia Langford, John Sillitoe on behalf of the Wellcome Sanger Institute COVID-19 Surveillance Team (http://www.sanger.ac.uk/covid-team)
EPI_ISL_989860, EPI_ISL_989863, EPI_ISL_989864, EPI_ISL_989872, EPI_ISL_989875, EPI_ISL_989878, EPI_ISL_989880	Lighthouse Lab in Alderley Park	Wellcome Sanger Institute for the COVID-19 Genomics UK (COG-UK) Consortium	Jacquelyn Wynn, Mairead Hyland, The Lighthouse Lab in Alderley Park and Alex Alderton, Roberto Amato, Sonia Goncalves, Ewan Harrison, David K. Jackson, Ian Johnston, Dominic Kwiatkowski, Cordelia Langford, John Sillitoe on behalf of the Wellcome Sanger Institute COVID-19 Surveillance Team (http://www.sanger.ac.uk/covid-team)
EPI_ISL_989882, EPI_ISL_989883, EPI_ISL_989884, EPI_ISL_989885, EPI_ISL_989886, EPI_ISL_989887, EPI_ISL_989888, EPI_ISL_989889, EPI_ISL_989890, EPI_ISL_989891, EPI_ISL_989892, EPI_ISL_989893, EPI_ISL_989894, EPI_ISL_989895, EPI_ISL_989896, EPI_ISL_989897, EPI_ISL_989898, EPI_ISL_989899, EPI_ISL_989900, EPI_ISL_989901, EPI_ISL_989902, EPI_ISL_989903, EPI_ISL_989904, EPI_ISL_989905, EPI_ISL_989906, EPI_ISL_989907, EPI_ISL_989908, EPI_ISL_989909, EPI_ISL_989910, EPI_ISL_989911, EPI_ISL_989912, EPI_ISL_989913			
see above	Lighthouse Lab in Glasgow	Wellcome Sanger Institute for the COVID-19 Genomics UK (COG-UK) Consortium	Harper VanSteenhouse, Yumi Kasai, David Gray, Carol Clugston, Anna Dominiczak and Alex Alderton, Roberto Amato, Sonia Goncalves, Ewan Harrison, David K. Jackson, Ian Johnston, Dominic Kwiatkowski, Cordelia Langford, John Sillitoe on behalf of the Wellcome Sanger Institute COVID-19 Surveillance Team (http://www.sanger.ac.uk/covid-team)
EPI_ISL_989914, EPI_ISL_989915, EPI_ISL_989916, EPI_ISL_989917, EPI_ISL_989918, EPI_ISL_989919, EPI_ISL_989920, EPI_ISL_989921, EPI_ISL_989922, EPI_ISL_989923, EPI_ISL_989924, EPI_ISL_989925, EPI_ISL_989926, EPI_ISL_989927, EPI_ISL_989928, EPI_ISL_989929, EPI_ISL_989930, EPI_ISL_989931, EPI_ISL_989932, EPI_ISL_989933, EPI_ISL_989934, EPI_ISL_989935, EPI_ISL_989936, EPI_ISL_989937, EPI_ISL_989938, EPI_ISL_989939, EPI_ISL_989940, EPI_ISL_989941, EPI_ISL_989942, EPI_ISL_989943, EPI_ISL_989944, EPI_ISL_989945, EPI_ISL_989946, EPI_ISL_989947, EPI_ISL_989948, EPI_ISL_989949, EPI_ISL_989950, EPI_ISL_989951, EPI_ISL_989952, EPI_ISL_989953, EPI_ISL_989954, EPI_ISL_989955, EPI_ISL_989956, EPI_ISL_989957, EPI_ISL_989958, EPI_ISL_989959, EPI_ISL_989960, EPI_ISL_989961, EPI_ISL_989962, EPI_ISL_989963, EPI_ISL_989964, EPI_ISL_989965, EPI_ISL_989966, EPI_ISL_989967, EPI_ISL_989968, EPI_ISL_989969, EPI_ISL_989970, EPI_ISL_989971, EPI_ISL_989972, EPI_ISL_989973, EPI_ISL_989974, EPI_ISL_989975, EPI_ISL_989976, EPI_ISL_989977, EPI_ISL_989978, EPI_ISL_989979, EPI_ISL_989980, EPI_ISL_989981, EPI_ISL_989982			
see above	Lighthouse Lab in Glasgow	Wellcome Sanger Institute for the COVID-19 Genomics UK (COG-UK) Consortium	Harper VanSteenhouse, Yumi Kasai, David Gray, Carol Clugston, Anna Dominiczak and Alex Alderton, Roberto Amato, Sonia Goncalves, Ewan Harrison, David K. Jackson, Ian Johnston, Dominic Kwiatkowski, Cordelia Langford, John Sillitoe on behalf of the Wellcome Sanger Institute COVID-19 Surveillance Team
EPI_ISL_996407, EPI_ISL_996442, EPI_ISL_996443, EPI_ISL_996464, EPI_ISL_996465, EPI_ISL_996466, EPI_ISL_996467, EPI_ISL_996471, EPI_ISL_996472, EPI_ISL_996473, EPI_ISL_996474			
see above	University of Birmingham	COVID-19 Genomics UK (COG-UK) Consortium	Institute of Microbiology, University of Birmingham: Claire McMurray, Joanne Stockton, Samuel Nicholls, Radoslaw Poplawski, Will Rowe, Josh Quick, Nicholas Loman. University of Birmingham Testing Laboratory: Celina M Whalley, Andrew Bosworth, Charlotte Poxon, Kasun Wanigasooriya, Oliver Pickles, Mike Kidd, Alex Richter, Andrew D Beggs PHE Heartlands Lab: Husam Osman, Andrew Bosworth. Queen Elizabeth Hospital: Anna Casey
EPI_ISL_996548, EPI_ISL_996549, EPI_ISL_996555, EPI_ISL_996556, EPI_ISL_996558, EPI_ISL_996559,	University of Exeter	COVID-19 Genomics UK (COG-UK) Consortium	Ben Temperton,Aaron Jeffries,Michelle Michelsen,Joanna Warwick-Dugdale,Audrey Farbos,Robyn Manley,Stephen Michell,Jane Masoli

EPI_ISL_996561, EPI_ISL_996562, EPI_ISL_996563, EPI_ISL_996567			
EPI_ISL_996632, EPI_ISL_996633, EPI_ISL_996634, EPI_ISL_996635, EPI_ISL_996636, EPI_ISL_996701, EPI_ISL_996711	Department of Pathology, University of Cambridge	COVID-19 Genomics UK (COG-UK) Consortium	Aminu S. Jahun, Yasmin Chaudhry, Iliana Georgana, Myra Hosmillo, Rhys Izuagbe, William L. Hamilton, Martin D. Curran, Surendra Parmar, Ian Goodfellow
EPI_ISL_997068, EPI_ISL_997069	West of Scotland Specialist Virology Centre, NHSGGC / MRC-University of Glasgow Centre for Virus Research	COVID-19 Genomics UK (COG-UK) Consortium	Ana da Silva Filipe, Natasha Johnson, Kathy Smollett, Daniel Mair, Stephen Carmichael, Alice Broos, Lily Tong, Jenna Nichols, Kyriaki Nomikou; Sarah McDonald; Richard Orton, Joseph Hughes, Sreenu Vattipally, David L Robertson; Alasdair MacLean, Rory Gunson; Sharif Shaaban, Matthew Holden; Rachel Blacow, Guy Mollett, Kathy Li, James Shepherd, Antonia Ho, Emma Thomson
EPI_ISL_997104, EPI_ISL_997105, EPI_ISL_997107, EPI_ISL_997108, EPI_ISL_997126, EPI_ISL_997128, EPI_ISL_997129, EPI_ISL_997130, EPI_ISL_997131, EPI_ISL_997164	Virology Department, Royal Infirmary of Edinburgh, NHS Lothian / School of Biological Sciences, University of Edinburgh	COVID-19 Genomics UK (COG-UK) Consortium	McHugh M, Dewar R, Cotton S, Rooke S, O'Toole Á, Scher E, Hill V, McCrone JT, Colquhoun R, Yu X, Jackson B, Rambaut A, Templeton K
EPI_ISL_997180, EPI_ISL_997181, EPI_ISL_997182, EPI_ISL_997183, EPI_ISL_997185, EPI_ISL_997188, EPI_ISL_997189, EPI_ISL_997190, EPI_ISL_997191, EPI_ISL_997192, EPI_ISL_997193, EPI_ISL_997194, EPI_ISL_997195, EPI_ISL_997196, EPI_ISL_997197, EPI_ISL_997198, EPI_ISL_997199, EPI_ISL_997200, EPI_ISL_997202, EPI_ISL_997203, EPI_ISL_997204, EPI_ISL_997205, EPI_ISL_997207, EPI_ISL_997208, EPI_ISL_997286, EPI_ISL_997287, EPI_ISL_997339			
see above	University of Exeter	COVID-19 Genomics UK (COG-UK) Consortium	Ben Temperton,Aaron Jeffries,Michelle Michelsen,Joanna Warwick-Dugdale,Audrey Farbos,Robyn Manley,Stephen Michell,Jane Masoli
EPI_ISL_997426, EPI_ISL_997427, EPI_ISL_997428, EPI_ISL_997429, EPI_ISL_997430, EPI_ISL_997431, EPI_ISL_997432, EPI_ISL_997434, EPI_ISL_997435, EPI_ISL_997436, EPI_ISL_997437, EPI_ISL_997438, EPI_ISL_997440, EPI_ISL_997441, EPI_ISL_997448, EPI_ISL_997449, EPI_ISL_997450, EPI_ISL_997486, EPI_ISL_997487, EPI_ISL_997489, EPI_ISL_997492, EPI_ISL_997493, EPI_ISL_997496, EPI_ISL_997497, EPI_ISL_997498, EPI_ISL_997499, EPI_ISL_997500, EPI_ISL_997501			
see above	Liverpool Clinical Laboratories	COVID-19 Genomics UK (COG-UK) Consortium	Sam Haldenby, Anita Lucaci, Steve Paterson, Julian Hiscox, Alistair Darby, M Almsaud, A Alrezaihi, Muhannad Alruwaili, Stuart D Armstrong, Jones Benjamin, Eleanor G Bentley, Anu Chawla, Jordan J Clark, Angela Cowell, Richard Eccles, Isabel García-Dorival, Matthew Gemmell, Alessandro Gerada, PKF Gilmore, Richard Gregory, Ximeng Han, Catherine Hartley, Margaret Hughes, Miren Iturriza-Gomara, James Johnson, L Luu, Jenifer Manson, Charlotte Nelson, Elaine O'Toole, Cassie Olateju, Rebekah Penrice-Randal , Lucille Rainbow, N.P Randle, Trevor Ian Robinson, Parul Sharma, Ghada T Shawli, James P Stewart, Neil Swainston, Ecaterina Vamos, Joanne Watts, Mark Whitehead
EPI_ISL_997657, EPI_ISL_997658, EPI_ISL_997659, EPI_ISL_997660, EPI_ISL_997665, EPI_ISL_997672	Barts Health NHS Trust	COVID-19 Genomics UK (COG-UK) Consortium	CUTINO-MOGUEL, Maria-Teresa; HARRINGTON, David; OWOYEMI, Dola; KULASEGARAN-SHYLINI, Raghavendran; BROAD, Claire; KELE, Beatrix
EPI_ISL_997833, EPI_ISL_997882, EPI_ISL_997883, EPI_ISL_997884, EPI_ISL_997885, EPI_ISL_997886, EPI_ISL_997888, EPI_ISL_997889, EPI_ISL_997897, EPI_ISL_997898, EPI_ISL_997899, EPI_ISL_997900, EPI_ISL_997902, EPI_ISL_997903, EPI_ISL_997910, EPI_ISL_997911, EPI_ISL_997912, EPI_ISL_997913, EPI_ISL_997914, EPI_ISL_997915, EPI_ISL_997917, EPI_ISL_997919, EPI_ISL_997920, EPI_ISL_997921, EPI_ISL_997924, EPI_ISL_997925, EPI_ISL_997926, EPI_ISL_998012, EPI_ISL_998013, EPI_ISL_998014, EPI_ISL_998015, EPI_ISL_998016, EPI_ISL_998017, EPI_ISL_998018, EPI_ISL_998019, EPI_ISL_998020, EPI_ISL_998021, EPI_ISL_998022, EPI_ISL_998023, EPI_ISL_998024, EPI_ISL_998025, EPI_ISL_998026, EPI_ISL_998027, EPI_ISL_998028, EPI_ISL_998029, EPI_ISL_998030, EPI_ISL_998031, EPI_ISL_998032, EPI_ISL_998033, EPI_ISL_998034, EPI_ISL_998035, EPI_ISL_998036, EPI_ISL_998037, EPI_ISL_998038, EPI_ISL_998039, EPI_ISL_998040, EPI_ISL_998041, EPI_ISL_998042, EPI_ISL_998043, EPI_ISL_998044, EPI_ISL_998045, EPI_ISL_998046, EPI_ISL_998047, EPI_ISL_998048, EPI_ISL_998049, EPI_ISL_998050, EPI_ISL_998051, EPI_ISL_998052, EPI_ISL_998053, EPI_ISL_998054, EPI_ISL_998055, EPI_ISL_998056, EPI_ISL_998057, EPI_ISL_998058, EPI_ISL_998059, EPI_ISL_998060, EPI_ISL_998061, EPI_ISL_998062, EPI_ISL_998063, EPI_ISL_998064, EPI_ISL_998065, EPI_ISL_998066, EPI_ISL_998067, EPI_ISL_998068, EPI_ISL_998069, EPI_ISL_998070, EPI_ISL_998071, EPI_ISL_998072, EPI_ISL_998073, EPI_ISL_998074, EPI_ISL_998075, EPI_ISL_998076, EPI_ISL_998077, EPI_ISL_998078, EPI_ISL_998079, EPI_ISL_998080, EPI_ISL_998081, EPI_ISL_998082, EPI_ISL_998083, EPI_ISL_998084, EPI_ISL_998085, EPI_ISL_998086, EPI_ISL_998087, EPI_ISL_998088, EPI_ISL_998089, EPI_ISL_998090, EPI_ISL_998091, EPI_ISL_998092, EPI_ISL_998093, EPI_ISL_998094, EPI_ISL_998095, EPI_ISL_998096, EPI_ISL_998097, EPI_ISL_998098			
see above	University College London, Great Ormond Street Hospital for Children NHS Foundation Trust, Imperial College Healthcare NHS Trust	COVID-19 Genomics UK (COG-UK) Consortium	Sergi Castellano, Rachel Williams, Mark Kristiansen, Paola Resende Silva, Sunando Roy, Tony Brooks, Helena Tutill, Paola Niola, Patricia Dyal, Charlotte Williams, Leysa Forrest, Yasmin Panchbhaya, Jacqueline Findlay, Samuel Weeks, Julianne Brown, Kathryn Harris, Paul Randell, James Price, Alison Holmes, Judith Breuer
EPI_ISL_998232, EPI_ISL_998299, EPI_ISL_998300, EPI_ISL_998301, EPI_ISL_998303	Regional Virus Laboratory, Belfast Health and Social Care Trust	COVID-19 Genomics UK (COG-UK) Consortium	Conall McCaughey, James McKenna, Tanya Curran, Susan Feeney, Alison Watt, Ciara Cox, Mairead Connor, Zoltan Molnar, David Simpson, Derek Fairley
EPI_ISL_998566, EPI_ISL_998567, EPI_ISL_998568, EPI_ISL_998569, EPI_ISL_998570, EPI_ISL_998571	Northumbria University / South Tees Hospitals NHS Foundation Trust / North Cumbria Integrated Care NHS Foundation Trust / North Tees and Hartlepool NHS Foundation Trust / Newcastle Hospitals NHS Foundation Trust	COVID-19 Genomics UK (COG-UK) Consortium	Darren L Smith,Andrew Nelson,Matthew Bashton,Greg R Young,Joshua Loh,John Allan,Mohammad A Tariq,Giles S Holt,Gary Black,Wen C Yew,Lynn Dover,Paul Baker,Steve Liggett,Sarah Essex,Jane Greenaway,Debra Padgett,Clive Graham,Garren Scott,Edward Barton,Emma Swindells,Brendan Payne,Jennifer Collins,Yusri Taha,Gary Eltringham
EPI_ISL_998808, EPI_ISL_998819, EPI_ISL_998845, EPI_ISL_998848, EPI_ISL_998862, EPI_ISL_998863, EPI_ISL_998886	Quadram Institute Bioscience	COVID-19 Genomics UK (COG-UK) Consortium	Dave J. Baker, Gemma L. Kay, Alp Aydin, Thanh Le-Viet, Steven Rudder, Ana P. Tedim, Anastasia Kolyva, Maria Diaz, Leonardo de Oliveira Martins, Nabil-Fareed Alikhan, Lizzie Meadows, Rachael Stanley, Ngozi Elumogo, Muhammed Yasir, Nicholas M. Thomson, Alexander J Trotter, Rachel Gilroy, Samuel Bloomfield, Claire Stuart, Andrew Bell, Reenesh Prakash, Samir Dervisevic, Alison E. Mather, John Wain, Mark Webber, Andrew J. Page, Justin O'Grady
EPI_ISL_998997, EPI_ISL_998999, EPI_ISL_999000, EPI_ISL_999328	Oxford Viromics, NDM, University of Oxford; Oxford University Hospitals; Basingstoke and North Hampshire Hospital	COVID-19 Genomics UK (COG-UK) Consortium	Tanya Golubchik, David Bonsall, George Macintyre, Amy Trebes, Mariateresa de Cesare, Catrin Moore, Alex Mobbs, Anita Justice, Robert Shaw, Monique Andersson, Timothy Peto, Emma Wise, Nathan Moore, Jessica Lynch, Nick Cortes, Matilde Mori, Stephen Kidd, David Buck, John Todd, Christophe Fraser
EPI_ISL_999516, EPI_ISL_999638, EPI_ISL_999642, EPI_ISL_999643, EPI_ISL_999644, EPI_ISL_999701, EPI_ISL_999702, EPI_ISL_999704, EPI_ISL_999713, EPI_ISL_999714, EPI_ISL_999717, EPI_ISL_999720, EPI_ISL_999724, EPI_ISL_999725, EPI_ISL_999729, EPI_ISL_999824, EPI_ISL_999825, EPI_ISL_999826, EPI_ISL_999830			
see above	Originating lab: Wales Specialist Virology Centre Sequencing lab: Pathogen Genomics Unit	Public Health Wales Microbiology Cardiff Wales Specialist Virology Centre	Catherine Moore, Johnathan Evans, Laura Gifford, Malorie Perry, Simon Cottrell, Angela Marchbank, Alec Birchley, Alexander Adams, Amy Gaskin, Bree Gatica-Wilcox, Jason Coombes, Joel Southgate, Lauren Gilbert, Lee Graham, Nicole Pacchiarini, Sara Kumziene-Summerhayes, Sarah Taylor, Sophie Jones, Sara Rey, Matthew Bull, Joanne Watkins, Sally Corden, Tom Connor
