# Supplementary material for: Inferring the Association between the Risk of COVID-19 Case Fatality and N501Y Substitution in SARS-CoV-2
Source: Viruses. 2021 Apr 8;13(4):638. doi: 10.3390/v13040638 (PMC8070306; doi:10.3390/v13040638)
Supplement: Supplementary file 1 [file viruses-13-00638-s001.zip › gisaid_hcov-19_UKAT_210130-210131.pdf]

We gratefully acknowledge the following Authors from the Originating laboratories responsible for obtaining the specimens, as well as the Submitting laboratories where the genome data were generated and shared via GISAID, on which this research is based.

All Submitters of data may be contacted directly via www.gisaid.org

Authors are sorted alphabetically.

Accession ID	Originating Laboratory	Submitting Laboratory	Authors
EPI_ISL_1000190, EPI_ISL_1000199, EPI_ISL_1000289, EPI_ISL_1000291, EPI_ISL_1000295, EPI_ISL_1000296, EPI_ISL_1000297, EPI_ISL_1000298, EPI_ISL_1000309, EPI_ISL_1000310, EPI_ISL_1000315, EPI_ISL_1000317, EPI_ISL_1000320, EPI_ISL_1000463, EPI_ISL_1000464, EPI_ISL_1000477, EPI_ISL_1000596, EPI_ISL_1000598, EPI_ISL_1000611, EPI_ISL_1000612, EPI_ISL_1000613, EPI_ISL_1000628, EPI_ISL_1000629, EPI_ISL_1000630			
see above	Centre for Enzyme Innovation, University of Portsmouth / Translational Research Laboratory, Portsmouth Hospitals NHS Trust	COVID-19 Genomics UK (COG-UK) Consortium	Angela Beckett,Salman Goudarzi,Christopher Fearn,Kate Cook,Katie Loveson,Sharon Glaysher,Scott Elliott,Samuel Robson
EPI_ISL_1000653	Virology Department, Sheffield Teaching Hospitals NHS Foundation Trust/Department of Infection, Immunity and Cardiovascular Disease, The Medical School, University of Sheffield	COVID-19 Genomics UK (COG-UK) Consortium	Thushan de Silva, Matthew Parker, Nikki Smith, Adri Angyal, Rebecca Brown, Luke Green, Rachel Tucker, Paul Parsons, Danielle Groves, Katie Johnson, Laura Carrilero, Alex Keeley, Dave Partridge, Matthew Wyles, Benjamin Lindsey, Mehmet Yavuz, Mohammad Raza, Cariad Evans
EPI_ISL_1000842, EPI_ISL_1000846, EPI_ISL_1000852, EPI_ISL_1000854, EPI_ISL_1000856, EPI_ISL_1000864	Bioinformatics and Biostatistics Lab, Advanced Sequencing Facility	COVID-19 Genomics UK (COG-UK) Consortium	Aengus Stewart,Jerome Nicod,Chelsea Sawyer,Laura Cubitt,Harshil Patel,Margaret Crawford
EPI_ISL_1006546, EPI_ISL_1006960, EPI_ISL_1006971, EPI_ISL_1007015, EPI_ISL_1007022, EPI_ISL_1007083	Lighthouse Lab in Milton Keynes	Wellcome Sanger Institute for the COVID-19 Genomics UK (COG-UK) Consortium	The Lighthouse Lab in Milton Keynes and Alex Alderton, Roberto Amato, Sonia Goncalves, Ewan Harrison, David K. Jackson, Ian Johnston, Dominic Kwiatkowski, Cordelia Langford, John Sillitoe on behalf of the Wellcome Sanger Institute COVID-19 Surveillance Team
EPI_ISL_1007440, EPI_ISL_1007441, EPI_ISL_1007442, EPI_ISL_1007443, EPI_ISL_1007444	Lighthouse Lab in Alderley Park	Wellcome Sanger Institute for the COVID-19 Genomics UK (COG-UK) Consortium	Jacquelyn Wynn, Mairead Hyland, The Lighthouse Lab in Alderley Park and Alex Alderton, Roberto Amato, Sonia Goncalves, Ewan Harrison, David K. Jackson, Ian Johnston, Dominic Kwiatkowski, Cordelia Langford, John Sillitoe on behalf of the Wellcome Sanger Institute COVID-19 Surveillance Team
EPI_ISL_1007527	Lighthouse Lab in Milton Keynes	Wellcome Sanger Institute for the COVID-19 Genomics UK (COG-UK) Consortium	The Lighthouse Lab in Milton Keynes and Alex Alderton, Roberto Amato, Sonia Goncalves, Ewan Harrison, David K. Jackson, Ian Johnston, Dominic Kwiatkowski, Cordelia Langford, John Sillitoe on behalf of the Wellcome Sanger Institute COVID-19 Surveillance Team
EPI_ISL_1012695, EPI_ISL_1012696, EPI_ISL_1012697, EPI_ISL_1012698, EPI_ISL_1012699, EPI_ISL_1012700, EPI_ISL_1012702, EPI_ISL_1012703, EPI_ISL_1012704, EPI_ISL_1012705, EPI_ISL_1012706, EPI_ISL_1012707, EPI_ISL_1012708, EPI_ISL_1012709, EPI_ISL_1012710, EPI_ISL_1012711, EPI_ISL_1012712			
see above	Lighthouse Lab in Alderley Park	Wellcome Sanger Institute for the COVID-19 Genomics UK (COG-UK) Consortium	Jacquelyn Wynn, Mairead Hyland, The Lighthouse Lab in Alderley Park and Alex Alderton, Roberto Amato, Sonia Goncalves, Ewan Harrison, David K. Jackson, Ian Johnston, Dominic Kwiatkowski, Cordelia Langford, John Sillitoe on behalf of the Wellcome Sanger Institute COVID-19 Surveillance Team
EPI_ISL_1018766	Lighthouse Lab in Cambridge	Wellcome Sanger Institute for the COVID-19 Genomics UK (COG-UK) Consortium	Rob Howes, The Lighthouse Lab in Cambridge and Alex Alderton, Roberto Amato, Jeffrey Barrett, Sonia Goncalves, Ewan Harrison, David K. Jackson, Ian Johnston, Dominic Kwiatkowski, Cordelia Langford, John Sillitoe on behalf of the Wellcome Sanger Institute COVID-19 Surveillance Team
EPI_ISL_1019586	Lighthouse Lab in Milton Keynes	Wellcome Sanger Institute for the COVID-19 Genomics UK (COG-UK) Consortium	The Lighthouse Lab in Milton Keynes and Alex Alderton, Roberto Amato, Jeffrey Barrett, Sonia Goncalves, Ewan Harrison, David K. Jackson, Ian Johnston, Dominic Kwiatkowski, Cordelia Langford, John Sillitoe on behalf of the Wellcome Sanger Institute COVID-19 Surveillance Team
EPI_ISL_1042058, EPI_ISL_1042251, EPI_ISL_1042338, EPI_ISL_1042647, EPI_ISL_1043383, EPI_ISL_1043484, EPI_ISL_1043552, EPI_ISL_1045289, EPI_ISL_1045471, EPI_ISL_1045793, EPI_ISL_1046166, EPI_ISL_1046338, EPI_ISL_1046341, EPI_ISL_1046347, EPI_ISL_1046352, EPI_ISL_1046354, EPI_ISL_1046355, EPI_ISL_1046357, EPI_ISL_1046359, EPI_ISL_1046361, EPI_ISL_1046365, EPI_ISL_1046366, EPI_ISL_1046375, EPI_ISL_1046381, EPI_ISL_1046394, EPI_ISL_1046396, EPI_ISL_1046402, EPI_ISL_1046404, EPI_ISL_1046409, EPI_ISL_1046411, EPI_ISL_1046435, EPI_ISL_1046440, EPI_ISL_1046442, EPI_ISL_1046449, EPI_ISL_1046474, EPI_ISL_1046491, EPI_ISL_1046493, EPI_ISL_1046495, EPI_ISL_1046504, EPI_ISL_1046507, EPI_ISL_1046508, EPI_ISL_1046510, EPI_ISL_1046513, EPI_ISL_1046514, EPI_ISL_1046515, EPI_ISL_1046516, EPI_ISL_1046517, EPI_ISL_1046518, EPI_ISL_1046519, EPI_ISL_1046520, EPI_ISL_1046521, EPI_ISL_1046522, EPI_ISL_1046523, EPI_ISL_1046524, EPI_ISL_1046525, EPI_ISL_1046526, EPI_ISL_1046527, EPI_ISL_1046528, EPI_ISL_1046529, EPI_ISL_1046530, EPI_ISL_1046531, EPI_ISL_1046532, EPI_ISL_1046533, EPI_ISL_1046534, EPI_ISL_1046535, EPI_ISL_1046536, EPI_ISL_1046537, EPI_ISL_1046538, EPI_ISL_1046539, EPI_ISL_1046541, EPI_ISL_1046542, EPI_ISL_1046544, EPI_ISL_1046546, EPI_ISL_1046547, EPI_ISL_1046548, EPI_ISL_1046549, EPI_ISL_1046550, EPI_ISL_1046551, EPI_ISL_1046552, EPI_ISL_1046553, EPI_ISL_1046554, EPI_ISL_1046555, EPI_ISL_1046556, EPI_ISL_1046558, EPI_ISL_1046559, EPI_ISL_1046560, EPI_ISL_1046561, EPI_ISL_1046562, EPI_ISL_1046563, EPI_ISL_1046564, EPI_ISL_1046566, EPI_ISL_1046567, EPI_ISL_1046568, EPI_ISL_1046570, EPI_ISL_1046571, EPI_ISL_1046572, EPI_ISL_1046573, EPI_ISL_1046574, EPI_ISL_1046575, EPI_ISL_1046577, EPI_ISL_1046578, EPI_ISL_1046580, EPI_ISL_1046581, EPI_ISL_1046582, EPI_ISL_1046583, EPI_ISL_1046584, EPI_ISL_1046585, EPI_ISL_1046586, EPI_ISL_1046587, EPI_ISL_1046588, EPI_ISL_1046590, EPI_ISL_1046592, EPI_ISL_1046593, EPI_ISL_1046594, EPI_ISL_1046595, EPI_ISL_1046596, EPI_ISL_1046597, EPI_ISL_1046598, EPI_ISL_1046599, EPI_ISL_1046601, EPI_ISL_1046602, EPI_ISL_1046603, EPI_ISL_1046604, EPI_ISL_1046605, EPI_ISL_1046606, EPI_ISL_1046607, EPI_ISL_1046609, EPI_ISL_1046611, EPI_ISL_1046612, EPI_ISL_1046614, EPI_ISL_1046615, EPI_ISL_1046618, EPI_ISL_1046619, EPI_ISL_1046620, EPI_ISL_1046622, EPI_ISL_1046623, EPI_ISL_1046624, EPI_ISL_1046626, EPI_ISL_1046628, EPI_ISL_1046629, EPI_ISL_1046631, EPI_ISL_1046632, EPI_ISL_1046633, EPI_ISL_1046635, EPI_ISL_1046636, EPI_ISL_1046638, EPI_ISL_1046639, EPI_ISL_1046641, EPI_ISL_1046642, EPI_ISL_1046646, EPI_ISL_1046647, EPI_ISL_1046650, EPI_ISL_1046652, EPI_ISL_1046653, EPI_ISL_1046656, EPI_ISL_1046658, EPI_ISL_1046659, EPI_ISL_1046661, EPI_ISL_1046662, EPI_ISL_1046664, EPI_ISL_1046665, EPI_ISL_1046666, EPI_ISL_1046667, EPI_ISL_1046668, EPI_ISL_1046669, EPI_ISL_1046670, EPI_ISL_1046672, EPI_ISL_1046673, EPI_ISL_1046674, EPI_ISL_1046675, EPI_ISL_1046676, EPI_ISL_1046677, EPI_ISL_1046679, EPI_ISL_1046680, EPI_ISL_1046682, EPI_ISL_1046683, EPI_ISL_1046685, EPI_ISL_1046687, EPI_ISL_1046688, EPI_ISL_1046689, EPI_ISL_1046691, EPI_ISL_1046693, EPI_ISL_1046694, EPI_ISL_1046695, EPI_ISL_1046696, EPI_ISL_1046698, EPI_ISL_1046700, EPI_ISL_1046701, EPI_ISL_1046703, EPI_ISL_1046704, EPI_ISL_1046706, EPI_ISL_1046707, EPI_ISL_1046708, EPI_ISL_1046709, EPI_ISL_1046710, EPI_ISL_1046711, EPI_ISL_1046712, EPI_ISL_1046714, EPI_ISL_1046715, EPI_ISL_1046717, EPI_ISL_1046718, EPI_ISL_1046719, EPI_ISL_1046720, EPI_ISL_1046722, EPI_ISL_1046723, EPI_ISL_1046724, EPI_ISL_1046725, EPI_ISL_1046726, EPI_ISL_1046727, EPI_ISL_1046728, EPI_ISL_1046730, EPI_ISL_1046733, EPI_ISL_1046734, EPI_ISL_1046735, EPI_ISL_1046736, EPI_ISL_1046737, EPI_ISL_1046740, EPI_ISL_1046741, EPI_ISL_1046742, EPI_ISL_1046743, EPI_ISL_1046744, EPI_ISL_1046746, EPI_ISL_1046747, EPI_ISL_1046748, EPI_ISL_1046749, EPI_ISL_1046750, EPI_ISL_1046751, EPI_ISL_1046752, EPI_ISL_1046754, EPI_ISL_1046755, EPI_ISL_1046756, EPI_ISL_1046758, EPI_ISL_1046759, EPI_ISL_1046760			
see above	Randox Laboratories	Wellcome Sanger Institute for the COVID-19 Genomics UK (COG-UK) Consortium	Randox Laboratories and Alex Alderton, Roberto Amato, Jeffrey Barrett, Sonia Goncalves, Ewan Harrison, David K. Jackson, Ian Johnston, Dominic Kwiatkowski, Cordelia Langford, John Sillitoe on behalf of the Wellcome Sanger Institute COVID-19 Surveillance Team
EPI_ISL_1047012, EPI_ISL_1047014, EPI_ISL_1047021, EPI_ISL_1047037, EPI_ISL_1047041, EPI_ISL_1047055, EPI_ISL_1047056	University of Birmingham	COVID-19 Genomics UK (COG-UK) Consortium	Institute of Microbiology, University of Birmingham: Claire McMurray, Joanne Stockton, Samuel Nicholls, Radoslaw Poplawski, Will Rowe, Josh Quick, Nicholas Loman. University of Birmingham Testing Laboratory: Celina M Whalley, Andrew Bosworth, Charlotte Poxon, Kasun Wanigasooriya, Oliver Pickles, Mike Kidd, Alex Richter, Andrew D Beggs PHE Heartlands Lab: Husam Osman, Andrew Bosworth. Queen Elizabeth Hospital: Anna Casey
EPI_ISL_1047583, EPI_ISL_1047584, EPI_ISL_1047585, EPI_ISL_1047586, EPI_ISL_1047587, EPI_ISL_1047588, EPI_ISL_1047589, EPI_ISL_1047590, EPI_ISL_1047591, EPI_ISL_1047592, EPI_ISL_1047593, EPI_ISL_1047594, EPI_ISL_1047595, EPI_ISL_1047596, EPI_ISL_1047597, EPI_ISL_1047598, EPI_ISL_1047599, EPI_ISL_1047600, EPI_ISL_1047601, EPI_ISL_1047602, EPI_ISL_1047603, EPI_ISL_1047604, EPI_ISL_1047605, EPI_ISL_1047606, EPI_ISL_1047607, EPI_ISL_1047608, EPI_ISL_1047609, EPI_ISL_1047610, EPI_ISL_1047611, EPI_ISL_1047612, EPI_ISL_1047613, EPI_ISL_1047614, EPI_ISL_1047615, EPI_ISL_1047616, EPI_ISL_1047617, EPI_ISL_1047618, EPI_ISL_1047619, EPI_ISL_1047620, EPI_ISL_1047621, EPI_ISL_1047622, EPI_ISL_1047623, EPI_ISL_1047624, EPI_ISL_1047625, EPI_ISL_1047626, EPI_ISL_1047627, EPI_ISL_1047628, EPI_ISL_1047629, EPI_ISL_1047630, EPI_ISL_1047631, EPI_ISL_1047632, EPI_ISL_1047634, EPI_ISL_1047635, EPI_ISL_1047636, EPI_ISL_1047637, EPI_ISL_1047638, EPI_ISL_1047639, EPI_ISL_1047640, EPI_ISL_1047641, EPI_ISL_1047642, EPI_ISL_1047643, EPI_ISL_1047712, EPI_ISL_1047713, EPI_ISL_1047714, EPI_ISL_1047715, EPI_ISL_1047716, EPI_ISL_1047717, EPI_ISL_1047718, EPI_ISL_1047719, EPI_ISL_1047720, EPI_ISL_1047721, EPI_ISL_1047722, EPI_ISL_1047723, EPI_ISL_1047724, EPI_ISL_1047725, EPI_ISL_1047726, EPI_ISL_1047727, EPI_ISL_1047728, EPI_ISL_1047729, EPI_ISL_1047730, EPI_ISL_1047731, EPI_ISL_1047732, EPI_ISL_1047733, EPI_ISL_1047734, EPI_ISL_1047735, EPI_ISL_1047736, EPI_ISL_1047737, EPI_ISL_1047738, EPI_ISL_1047739, EPI_ISL_1047740, EPI_ISL_1047741, EPI_ISL_1047742, EPI_ISL_1047743, EPI_ISL_1047744, EPI_ISL_1047745, EPI_ISL_1047746, EPI_ISL_1047747, EPI_ISL_1047748, EPI_ISL_1047749, EPI_ISL_1047750, EPI_ISL_1047751, EPI_ISL_1047752, EPI_ISL_1047753, EPI_ISL_1047755, EPI_ISL_1047756, EPI_ISL_1047757, EPI_ISL_1047758, EPI_ISL_1047759, EPI_ISL_1047760, EPI_ISL_1047761, EPI_ISL_1047762, EPI_ISL_1047763, EPI_ISL_1047764, EPI_ISL_1047765, EPI_ISL_1047766, EPI_ISL_1047767, EPI_ISL_1047768, EPI_ISL_1047769, EPI_ISL_1047770, EPI_ISL_1047771, EPI_ISL_1047773, EPI_ISL_1047774, EPI_ISL_1047775, EPI_ISL_1047776, EPI_ISL_1047777, EPI_ISL_1047778, EPI_ISL_1047779, EPI_ISL_1047780, EPI_ISL_1047782, EPI_ISL_1047783, EPI_ISL_1047785, EPI_ISL_1047786, EPI_ISL_1047787, EPI_ISL_1047788, EPI_ISL_1047789, EPI_ISL_1047790, EPI_ISL_1047791, EPI_ISL_1047792, EPI_ISL_1047793, EPI_ISL_1047794, EPI_ISL_1047795, EPI_ISL_1047796, EPI_ISL_1047797, EPI_ISL_1047798, EPI_ISL_1047799, EPI_ISL_1047800, EPI_ISL_1047801, EPI_ISL_1047802			
see above	Virology Department, Sheffield Teaching Hospitals NHS Foundation Trust/Department of Infection, Immunity and Cardiovascular Disease, The Medical School, University of Sheffield	COVID-19 Genomics UK (COG-UK) Consortium	Thushan de Silva, Matthew Parker, Nikki Smith, Adri Angyal, Rebecca Brown, Luke Green, Rachel Tucker, Paul Parsons, Danielle Groves, Katie Johnson, Laura Carrilero, Alex Keeley, Dave Partridge, Matthew Wyles, Benjamin Lindsey, Mehmet Yavuz, Mohammad Raza, Cariad Evans
EPI_ISL_1047822, EPI_ISL_1047825, EPI_ISL_1047826, EPI_ISL_1047827, EPI_ISL_1047828, EPI_ISL_1047829, EPI_ISL_1047830, EPI_ISL_1047831, EPI_ISL_1047832	West of Scotland Specialist Virology Centre, NHSGGC / MRC-University of Glasgow Centre for Virus Research	COVID-19 Genomics UK (COG-UK) Consortium	Ana da Silva Filipe, Natasha Johnson, Kathy Smollett, Daniel Mair, Stephen Carmichael, Alice Broos, Lily Tong, Jenna Nichols, Kyriaki Nomikou; Sarah McDonald; Richard Orton, Joseph Hughes, Sreenu Vattipally, David L Robertson; Alasdair MacLean, Rory Gunson; Sharif Shaaban, Matthew Holden; Rachel Blacow, Guy Mollett, Kathy Li, James Shepherd, Antonia Ho, Emma Thomson
EPI_ISL_1047866, EPI_ISL_1047867, EPI_ISL_1047868, EPI_ISL_1047869,	Virology Department, Royal Infirmary of Edinburgh, NHS Lothian / School of Biological Sciences, University of	COVID-19 Genomics UK (COG-UK) Consortium	McHugh M, Dewar R, Cotton S, Rooke S, O'Toole Á, Scher E, Hill V, McCrone JT, Colquhoun R, Yu X, Jackson B, Rambaut A, Templeton K

EPI_ISL_1047870, EPI_ISL_1047892	Edinburgh		
EPI_ISL_1047967, EPI_ISL_1047968, EPI_ISL_1047970, EPI_ISL_1047971, EPI_ISL_1047972, EPI_ISL_1047973, EPI_ISL_1048026, EPI_ISL_1048028, EPI_ISL_1048029, EPI_ISL_1048034	Liverpool Clinical Laboratories	COVID-19 Genomics UK (COG-UK) Consortium	Sam Haldenby, Anita Lucaci, Steve Paterson, Julian Hiscox, Alistair Darby, M Almsaud, A Alrezaihi, Muhannad Alruwaili, Stuart D Armstrong, Jones Benjamin, Eleanor G Bentley, Anu Chawla, Jordan J Clark, Angela Cowell, Richard Eccles, Isabel García-Dorival, Matthew Gemmell, Alessandro Gerada, PKF Gilmore, Richard Gregory, Ximeng Han, Catherine Hartley, Margaret Hughes, Miren Iturriza-Gomara, James Johnson, L Luu, Jenifer Manson, Charlotte Nelson, Elaine O'Toole, Cassie Olateju, Rebekah Penrice-Randal , Lucille Rainbow, N.P Randle, Trevor Ian Robinson, Parul Sharma, Ghada T Shawli, James P Stewart, Neil Swainston, Ecaterina Vamos, Joanne Watts, Mark Whitehead
EPI_ISL_1048070, EPI_ISL_1048074, EPI_ISL_1048079, EPI_ISL_1048101	Barts Health NHS Trust	COVID-19 Genomics UK (COG-UK) Consortium	CUTINO-MOGUEL, Maria-Teresa; HARRINGTON, David; OWOYEMI, Dola; KULASEGARAN-SHYLINI, Raghavendran; BROAD, Claire; KELE, Beatrix
EPI_ISL_1050067	Virology Department, Sheffield Teaching Hospitals NHS Foundation Trust/Department of Infection, Immunity and Cardiovascular Disease, The Medical School, University of Sheffield	COVID-19 Genomics UK (COG-UK) Consortium	Thushan de Silva, Matthew Parker, Nikki Smith, Adri Angyal, Rebecca Brown, Luke Green, Rachel Tucker, Paul Parsons, Danielle Groves, Katie Johnson, Laura Carrilero, Alex Keeley, Dave Partridge, Matthew Wyles, Benjamin Lindsey, Mehmet Yavuz, Mohammad Raza, Cariad Evans
EPI_ISL_1050174, EPI_ISL_1050175	West of Scotland Specialist Virology Centre, NHSGGC / MRC-University of Glasgow Centre for Virus Research	COVID-19 Genomics UK (COG-UK) Consortium	Ana da Silva Filipe, Natasha Johnson, Kathy Smollett, Daniel Mair, Stephen Carmichael, Alice Broos, Lily Tong, Jenna Nichols, Kyriaki Nomikou; Sarah McDonald; Richard Orton, Joseph Hughes, Sreenu Vattipally, David L Robertson; Alasdair MacLean, Rory Gunson; Sharif Shaaban, Matthew Holden; Rachel Blacow, Guy Mollett, Kathy Li, James Shepherd, Antonia Ho, Emma Thomson
EPI_ISL_1050296, EPI_ISL_1050297, EPI_ISL_1050298, EPI_ISL_1050299, EPI_ISL_1050304, EPI_ISL_1050305, EPI_ISL_1050306	University of Exeter	COVID-19 Genomics UK (COG-UK) Consortium	Ben Temperton,Aaron Jeffries,Michelle Michelsen,Joanna Warwick-Dugdale,Audrey Farbos,Robyn Manley,Stephen Michell,Jane Masoli
EPI_ISL_1050748, EPI_ISL_1050749, EPI_ISL_1050750, EPI_ISL_1050751, EPI_ISL_1050752, EPI_ISL_1050753, EPI_ISL_1050754, EPI_ISL_1050755, EPI_ISL_1050756, EPI_ISL_1050757, EPI_ISL_1050758, EPI_ISL_1050759, EPI_ISL_1050760, EPI_ISL_1050761, EPI_ISL_1050762, EPI_ISL_1050763, EPI_ISL_1050764, EPI_ISL_1050765, EPI_ISL_1050766, EPI_ISL_1050767, EPI_ISL_1050768, EPI_ISL_1050769, EPI_ISL_1050770, EPI_ISL_1050771, EPI_ISL_1050772, EPI_ISL_1050773, EPI_ISL_1050774, EPI_ISL_1050775, EPI_ISL_1050776, EPI_ISL_1050777, EPI_ISL_1050778, EPI_ISL_1050779, EPI_ISL_1050780, EPI_ISL_1050781, EPI_ISL_1050782, EPI_ISL_1050783, EPI_ISL_1050784, EPI_ISL_1050785, EPI_ISL_1050786, EPI_ISL_1050787, EPI_ISL_1050788, EPI_ISL_1050789, EPI_ISL_1050790, EPI_ISL_1050791, EPI_ISL_1050792, EPI_ISL_1050793, EPI_ISL_1050794, EPI_ISL_1050795, EPI_ISL_1050796, EPI_ISL_1050797, EPI_ISL_1050798, EPI_ISL_1050799, EPI_ISL_1050800, EPI_ISL_1050801, EPI_ISL_1050802, EPI_ISL_1050803, EPI_ISL_1050804, EPI_ISL_1050805, EPI_ISL_1050806, EPI_ISL_1050807, EPI_ISL_1050808, EPI_ISL_1050809, EPI_ISL_1050810, EPI_ISL_1050811, EPI_ISL_1050812, EPI_ISL_1050813, EPI_ISL_1050814, EPI_ISL_1050815, EPI_ISL_1050816, EPI_ISL_1050817, EPI_ISL_1050818, EPI_ISL_1050819, EPI_ISL_1050820, EPI_ISL_1050821, EPI_ISL_1050822, EPI_ISL_1050823, EPI_ISL_1050824, EPI_ISL_1050825, EPI_ISL_1050826, EPI_ISL_1050827			
see above	Centre for Enzyme Innovation, University of Portsmouth / Translational Research Laboratory, Portsmouth Hospitals NHS Trust	COVID-19 Genomics UK (COG-UK) Consortium	Angela Beckett,Salman Goudarzi,Christopher Fearn,Kate Cook,Katie Loveson,Sharon Glaysher,Scott Elliott,Samuel Robson
EPI_ISL_1050828, EPI_ISL_1050829, EPI_ISL_1050830, EPI_ISL_1050831, EPI_ISL_1050832, EPI_ISL_1050833, EPI_ISL_1050834	Virology Department, Sheffield Teaching Hospitals NHS Foundation Trust/Department of Infection, Immunity and Cardiovascular Disease, The Medical School, University of Sheffield	COVID-19 Genomics UK (COG-UK) Consortium	Thushan de Silva, Matthew Parker, Nikki Smith, Adri Angyal, Rebecca Brown, Luke Green, Rachel Tucker, Paul Parsons, Danielle Groves, Katie Johnson, Laura Carrilero, Alex Keeley, Dave Partridge, Matthew Wyles, Benjamin Lindsey, Mehmet Yavuz, Mohammad Raza, Cariad Evans
EPI_ISL_1050835, EPI_ISL_1050836, EPI_ISL_1050837, EPI_ISL_1050838, EPI_ISL_1050839	Centre for Enzyme Innovation, University of Portsmouth / Translational Research Laboratory, Portsmouth Hospitals NHS Trust	COVID-19 Genomics UK (COG-UK) Consortium	Angela Beckett,Salman Goudarzi,Christopher Fearn,Kate Cook,Katie Loveson,Sharon Glaysher,Scott Elliott,Samuel Robson
EPI_ISL_1050840, EPI_ISL_1050841	Virology Department, Sheffield Teaching Hospitals NHS Foundation Trust/Department of Infection, Immunity and Cardiovascular Disease, The Medical School, University of Sheffield	COVID-19 Genomics UK (COG-UK) Consortium	Thushan de Silva, Matthew Parker, Nikki Smith, Adri Angyal, Rebecca Brown, Luke Green, Rachel Tucker, Paul Parsons, Danielle Groves, Katie Johnson, Laura Carrilero, Alex Keeley, Dave Partridge, Matthew Wyles, Benjamin Lindsey, Mehmet Yavuz, Mohammad Raza, Cariad Evans
EPI_ISL_1050842, EPI_ISL_1050843, EPI_ISL_1050844, EPI_ISL_1050845, EPI_ISL_1050846, EPI_ISL_1050847, EPI_ISL_1050848, EPI_ISL_1050849, EPI_ISL_1050850, EPI_ISL_1050851, EPI_ISL_1050852, EPI_ISL_1050853, EPI_ISL_1050854, EPI_ISL_1050855, EPI_ISL_1050856, EPI_ISL_1050857, EPI_ISL_1050858, EPI_ISL_1050859, EPI_ISL_1050860, EPI_ISL_1050861			
see above	Centre for Enzyme Innovation, University of Portsmouth / Translational Research Laboratory, Portsmouth Hospitals NHS Trust	COVID-19 Genomics UK (COG-UK) Consortium	Angela Beckett,Salman Goudarzi,Christopher Fearn,Kate Cook,Katie Loveson,Sharon Glaysher,Scott Elliott,Samuel Robson
EPI_ISL_1050863, EPI_ISL_1050864, EPI_ISL_1050865, EPI_ISL_1050866, EPI_ISL_1050867, EPI_ISL_1050868, EPI_ISL_1050870	Virology Department, Sheffield Teaching Hospitals NHS Foundation Trust/Department of Infection, Immunity and Cardiovascular Disease, The Medical School, University of Sheffield	COVID-19 Genomics UK (COG-UK) Consortium	Thushan de Silva, Matthew Parker, Nikki Smith, Adri Angyal, Rebecca Brown, Luke Green, Rachel Tucker, Paul Parsons, Danielle Groves, Katie Johnson, Laura Carrilero, Alex Keeley, Dave Partridge, Matthew Wyles, Benjamin Lindsey, Mehmet Yavuz, Mohammad Raza, Cariad Evans
EPI_ISL_1050871, EPI_ISL_1050872, EPI_ISL_1050873, EPI_ISL_1050874, EPI_ISL_1050875, EPI_ISL_1050876, EPI_ISL_1050877, EPI_ISL_1050878, EPI_ISL_1050879, EPI_ISL_1050880, EPI_ISL_1050881, EPI_ISL_1050882			
see above	Centre for Enzyme Innovation, University of Portsmouth / Translational Research Laboratory, Portsmouth Hospitals NHS Trust	COVID-19 Genomics UK (COG-UK) Consortium	Angela Beckett,Salman Goudarzi,Christopher Fearn,Kate Cook,Katie Loveson,Sharon Glaysher,Scott Elliott,Samuel Robson
EPI_ISL_1050883, EPI_ISL_1050884, EPI_ISL_1050885, EPI_ISL_1050886, EPI_ISL_1050887, EPI_ISL_1050888, EPI_ISL_1050889, EPI_ISL_1050890, EPI_ISL_1050891, EPI_ISL_1050892, EPI_ISL_1050893, EPI_ISL_1050894, EPI_ISL_1050895, EPI_ISL_1050896, EPI_ISL_1050897, EPI_ISL_1050898, EPI_ISL_1050899, EPI_ISL_1050900, EPI_ISL_1050901, EPI_ISL_1050903, EPI_ISL_1050904, EPI_ISL_1050905, EPI_ISL_1050906, EPI_ISL_1050907, EPI_ISL_1050908			
see above	Virology Department, Sheffield Teaching Hospitals NHS Foundation Trust/Department of Infection, Immunity and Cardiovascular Disease, The Medical School, University of Sheffield	COVID-19 Genomics UK (COG-UK) Consortium	Thushan de Silva, Matthew Parker, Nikki Smith, Adri Angyal, Rebecca Brown, Luke Green, Rachel Tucker, Paul Parsons, Danielle Groves, Katie Johnson, Laura Carrilero, Alex Keeley, Dave Partridge, Matthew Wyles, Benjamin Lindsey, Mehmet Yavuz, Mohammad Raza, Cariad Evans
EPI_ISL_1050909, EPI_ISL_1050910, EPI_ISL_1050911, EPI_ISL_1050912, EPI_ISL_1050913, EPI_ISL_1050914	Centre for Enzyme Innovation, University of Portsmouth / Translational Research Laboratory, Portsmouth Hospitals NHS Trust	COVID-19 Genomics UK (COG-UK) Consortium	Angela Beckett,Salman Goudarzi,Christopher Fearn,Kate Cook,Katie Loveson,Sharon Glaysher,Scott Elliott,Samuel Robson
EPI_ISL_1051261, EPI_ISL_1051262, EPI_ISL_1051263, EPI_ISL_1051264, EPI_ISL_1051265, EPI_ISL_1051266, EPI_ISL_1051267, EPI_ISL_1051268, EPI_ISL_1051269, EPI_ISL_1051270, EPI_ISL_1051271, EPI_ISL_1051272, EPI_ISL_1051273, EPI_ISL_1051274, EPI_ISL_1051275, EPI_ISL_1051276, EPI_ISL_1051277, EPI_ISL_1051278, EPI_ISL_1051279			
see above	Northumbria University / South Tees Hospitals NHS Foundation Trust / North Cumbria Integrated Care NHS Foundation Trust / North Tees and Hartlepool NHS Foundation Trust / Newcastle Hospitals NHS Foundation Trust	COVID-19 Genomics UK (COG-UK) Consortium	Darren L Smith,Andrew Nelson,Matthew Bashton,Greg R Young,Joshua Loh,John Allan,Mohammad A Tariq,Giles S Holt,Gary Black,Wen C Yew,Lynn Dover,Paul Baker,Steve Liggett,Sarah Essex,Jane Greenaway,Debra Padgett,Clive Graham,Garren Scott,Edward Barton,Emma Swindells,Brendan Payne,Jennifer Collins,Yusri Taha,Gary Eltringham
EPI_ISL_1051639, EPI_ISL_1051640, EPI_ISL_1051642, EPI_ISL_1051643, EPI_ISL_1051646, EPI_ISL_1051649, EPI_ISL_1051651, EPI_ISL_1051655, EPI_ISL_1051694, EPI_ISL_1051695, EPI_ISL_1051696, EPI_ISL_1051698, EPI_ISL_1051700, EPI_ISL_1051701, EPI_ISL_1051702, EPI_ISL_1051703, EPI_ISL_1051704, EPI_ISL_1051705, EPI_ISL_1051706, EPI_ISL_1051707, EPI_ISL_1051708, EPI_ISL_1051709, EPI_ISL_1051711, EPI_ISL_1051713, EPI_ISL_1051714, EPI_ISL_1051723, EPI_ISL_1051724, EPI_ISL_1051729, EPI_ISL_1051734, EPI_ISL_1051735, EPI_ISL_1051736, EPI_ISL_1051737, EPI_ISL_1051738, EPI_ISL_1051741, EPI_ISL_1051742, EPI_ISL_1051744, EPI_ISL_1051745, EPI_ISL_1051747, EPI_ISL_1051755, EPI_ISL_1051756, EPI_ISL_1051775, EPI_ISL_1051880, EPI_ISL_1051900, EPI_ISL_1051901, EPI_ISL_1051912, EPI_ISL_1051913, EPI_ISL_1051916, EPI_ISL_1051917			
see above	Oxford Viromics, NDM, University of Oxford; Oxford University Hospitals; Basingstoke and North Hampshire Hospital	COVID-19 Genomics UK (COG-UK) Consortium	Tanya Golubchik, David Bonsall, George Macintyre, Amy Trebes, Mariateresa de Cesare, Catrin Moore, Alex Mobbs, Anita Justice, Robert Shaw, Monique Andersson, Timothy Peto, Emma Wise, Nathan Moore, Jessica Lynch, Nick Cortes, Matilde Mori, Stephen Kidd, David Buck, John Todd, Christophe Fraser
EPI_ISL_1052139, EPI_ISL_1052140, EPI_ISL_1052141, EPI_ISL_1052148, EPI_ISL_1052149, EPI_ISL_1052151, EPI_ISL_1052152, EPI_ISL_1052153, EPI_ISL_1052154, EPI_ISL_1052155, EPI_ISL_1052156, EPI_ISL_1052157, EPI_ISL_1052158, EPI_ISL_1052159, EPI_ISL_1052160, EPI_ISL_1052161, EPI_ISL_1052162, EPI_ISL_1052163, EPI_ISL_1052164, EPI_ISL_1052165, EPI_ISL_1052166, EPI_ISL_1052167, EPI_ISL_1052168, EPI_ISL_1052169, EPI_ISL_1052170, EPI_ISL_1052171, EPI_ISL_1052172, EPI_ISL_1052173, EPI_ISL_1052174, EPI_ISL_1052175, EPI_ISL_1052176, EPI_ISL_1052177, EPI_ISL_1052178, EPI_ISL_1052179, EPI_ISL_1052180, EPI_ISL_1052181, EPI_ISL_1052182, EPI_ISL_1052183, EPI_ISL_1052184, EPI_ISL_1052186, EPI_ISL_1052187, EPI_ISL_1052188, EPI_ISL_1052189, EPI_ISL_1052190, EPI_ISL_1052191, EPI_ISL_1052192, EPI_ISL_1052193, EPI_ISL_1052195, EPI_ISL_1052196, EPI_ISL_1052197, EPI_ISL_1052198, EPI_ISL_1052199, EPI_ISL_1052200, EPI_ISL_1052201, EPI_ISL_1052202, EPI_ISL_1052205, EPI_ISL_1052208, EPI_ISL_1052210, EPI_ISL_1052223, EPI_ISL_1052241, EPI_ISL_1052244, EPI_ISL_1052248, EPI_ISL_1052249, EPI_ISL_1052254, EPI_ISL_1052255, EPI_ISL_1052256, EPI_ISL_1052257, EPI_ISL_1052258, EPI_ISL_1052268, EPI_ISL_1052270, EPI_ISL_1052271, EPI_ISL_1052286, EPI_ISL_1052330, EPI_ISL_1052472, EPI_ISL_1052484, EPI_ISL_1052485, EPI_ISL_1052486, EPI_ISL_1052487, EPI_ISL_1052495, EPI_ISL_1052529, EPI_ISL_1052531, EPI_ISL_1052532, EPI_ISL_1052533, EPI_ISL_1052534, EPI_ISL_1052535,			

EPI_ISL_1052536, EPI_ISL_1052537, EPI_ISL_1052542, EPI_ISL_1052606, EPI_ISL_1052607, EPI_ISL_1052675, EPI_ISL_1053517, EPI_ISL_1053518, EPI_ISL_1053519, EPI_ISL_1053520, EPI_ISL_1053527, EPI_ISL_1053528, EPI_ISL_1053529, EPI_ISL_1053532, EPI_ISL_1053533, EPI_ISL_1053534, EPI_ISL_1053535, EPI_ISL_1053537, EPI_ISL_1053579, EPI_ISL_1053581, EPI_ISL_1053588, EPI_ISL_1053589, EPI_ISL_1053590, EPI_ISL_1053591, EPI_ISL_1053592, EPI_ISL_1053593, EPI_ISL_1053595, EPI_ISL_1053597			
see above	Originating lab: Wales Specialist Virology Centre Sequencing lab: Pathogen Genomics Unit	Public Health Wales Microbiology Cardiff Wales Specialist Virology Centre	Catherine Moore, Johnathan Evans, Laura Gifford, Malorie Perry, Simon Cottrell, Angela Marchbank, Alec Birchley, Alexander Adams, Amy Gaskin, Bree Gatica-Wilcox, Jason Coombes, Joel Southgate, Lauren Gilbert, Lee Graham, Nicole Pacchiarini, Sara Kumziene-Summerhayes, Sarah Taylor, Sophie Jones, Sara Rey, Matthew Bull, Joanne Watkins, Sally Corden, Tom Connor
EPI_ISL_1053821, EPI_ISL_1053858, EPI_ISL_1053859, EPI_ISL_1053893, EPI_ISL_1053939, EPI_ISL_1053953, EPI_ISL_1053954, EPI_ISL_1053955, EPI_ISL_1053956, EPI_ISL_1053957, EPI_ISL_1053958, EPI_ISL_1053959, EPI_ISL_1053960			
see above	Centre for Enzyme Innovation, University of Portsmouth / Translational Research Laboratory, Portsmouth Hospitals NHS Trust	COVID-19 Genomics UK (COG-UK) Consortium	Angela Beckett,Salman Goudarzi,Christopher Fearn,Kate Cook,Katie Loveson,Sharon Glaysher,Scott Elliott,Samuel Robson
EPI_ISL_1053977, EPI_ISL_1053978, EPI_ISL_1053979, EPI_ISL_1053980, EPI_ISL_1053982, EPI_ISL_1053983, EPI_ISL_1053984, EPI_ISL_1053985, EPI_ISL_1053986, EPI_ISL_1053987, EPI_ISL_1053989, EPI_ISL_1053990, EPI_ISL_1053991, EPI_ISL_1053992, EPI_ISL_1053993, EPI_ISL_1053994, EPI_ISL_1053995, EPI_ISL_1053996, EPI_ISL_1053998, EPI_ISL_1053999, EPI_ISL_1054001, EPI_ISL_1054002, EPI_ISL_1054004, EPI_ISL_1054005, EPI_ISL_1054006, EPI_ISL_1054007, EPI_ISL_1054009, EPI_ISL_1054010, EPI_ISL_1054012, EPI_ISL_1054013, EPI_ISL_1054014, EPI_ISL_1054015, EPI_ISL_1054016, EPI_ISL_1054018, EPI_ISL_1054019, EPI_ISL_1054020, EPI_ISL_1054022, EPI_ISL_1054023, EPI_ISL_1054024, EPI_ISL_1054025, EPI_ISL_1054026, EPI_ISL_1054027, EPI_ISL_1054028, EPI_ISL_1054029, EPI_ISL_1054030, EPI_ISL_1054031, EPI_ISL_1054032, EPI_ISL_1054033, EPI_ISL_1054034, EPI_ISL_1054035, EPI_ISL_1054036, EPI_ISL_1054037, EPI_ISL_1054040, EPI_ISL_1054042, EPI_ISL_1054043, EPI_ISL_1054044, EPI_ISL_1054045, EPI_ISL_1054046, EPI_ISL_1054047, EPI_ISL_1054048, EPI_ISL_1054049, EPI_ISL_1054050, EPI_ISL_1054053, EPI_ISL_1054054, EPI_ISL_1054055, EPI_ISL_1054056, EPI_ISL_1054057, EPI_ISL_1054058, EPI_ISL_1054059, EPI_ISL_1054060, EPI_ISL_1054065, EPI_ISL_1054066, EPI_ISL_1054067, EPI_ISL_1054069, EPI_ISL_1054070, EPI_ISL_1054071, EPI_ISL_1054072, EPI_ISL_1054073, EPI_ISL_1054074, EPI_ISL_1054075, EPI_ISL_1054076, EPI_ISL_1054077, EPI_ISL_1054078, EPI_ISL_1054079, EPI_ISL_1054081, EPI_ISL_1054082, EPI_ISL_1054083, EPI_ISL_1054084, EPI_ISL_1054085, EPI_ISL_1054086, EPI_ISL_1054087, EPI_ISL_1054088, EPI_ISL_1054089, EPI_ISL_1054090, EPI_ISL_1054091, EPI_ISL_1054092, EPI_ISL_1054093, EPI_ISL_1054094, EPI_ISL_1054096, EPI_ISL_1054097, EPI_ISL_1054098, EPI_ISL_1054099, EPI_ISL_1054100, EPI_ISL_1054101, EPI_ISL_1054102, EPI_ISL_1054103, EPI_ISL_1054104, EPI_ISL_1054105, EPI_ISL_1054106, EPI_ISL_1054107, EPI_ISL_1054110, EPI_ISL_1054111, EPI_ISL_1054112, EPI_ISL_1054114, EPI_ISL_1054115, EPI_ISL_1054116, EPI_ISL_1054117, EPI_ISL_1054118, EPI_ISL_1054120, EPI_ISL_1054122, EPI_ISL_1054125, EPI_ISL_1054126, EPI_ISL_1054128, EPI_ISL_1054130			
see above	University of Exeter	COVID-19 Genomics UK (COG-UK) Consortium	Ben Temperton,Aaron Jeffries,Michelle Michelsen,Joanna Warwick-Dugdale,Audrey Farbos,Robyn Manley,Stephen Michell,Jane Masoli
EPI_ISL_1054131, EPI_ISL_1054132, EPI_ISL_1054133, EPI_ISL_1054134, EPI_ISL_1054135, EPI_ISL_1054136, EPI_ISL_1054137, EPI_ISL_1054138, EPI_ISL_1054139, EPI_ISL_1054140, EPI_ISL_1054142, EPI_ISL_1054143, EPI_ISL_1054144, EPI_ISL_1054145, EPI_ISL_1054146, EPI_ISL_1054147, EPI_ISL_1054148, EPI_ISL_1054149, EPI_ISL_1054150, EPI_ISL_1054151, EPI_ISL_1054152, EPI_ISL_1054153, EPI_ISL_1054154, EPI_ISL_1054155, EPI_ISL_1054156, EPI_ISL_1054157, EPI_ISL_1054158, EPI_ISL_1054159, EPI_ISL_1054160, EPI_ISL_1054161, EPI_ISL_1054162, EPI_ISL_1054163, EPI_ISL_1054164, EPI_ISL_1054165, EPI_ISL_1054166, EPI_ISL_1054167, EPI_ISL_1054168, EPI_ISL_1054169, EPI_ISL_1054170, EPI_ISL_1054171, EPI_ISL_1054172, EPI_ISL_1054173, EPI_ISL_1054174, EPI_ISL_1054175, EPI_ISL_1054176, EPI_ISL_1054177, EPI_ISL_1054178, EPI_ISL_1054179, EPI_ISL_1054180, EPI_ISL_1054181, EPI_ISL_1054182, EPI_ISL_1054183, EPI_ISL_1054184, EPI_ISL_1054185, EPI_ISL_1054186, EPI_ISL_1054187, EPI_ISL_1054188, EPI_ISL_1054189, EPI_ISL_1054190, EPI_ISL_1054191, EPI_ISL_1054192, EPI_ISL_1054193, EPI_ISL_1054194, EPI_ISL_1054195, EPI_ISL_1054196, EPI_ISL_1054197, EPI_ISL_1054198, EPI_ISL_1054199, EPI_ISL_1054200, EPI_ISL_1054201, EPI_ISL_1054202, EPI_ISL_1054203, EPI_ISL_1054204, EPI_ISL_1054205, EPI_ISL_1054206, EPI_ISL_1054207, EPI_ISL_1054208, EPI_ISL_1054209, EPI_ISL_1054210, EPI_ISL_1054211, EPI_ISL_1054212, EPI_ISL_1054213, EPI_ISL_1054214, EPI_ISL_1054215, EPI_ISL_1054216, EPI_ISL_1054217, EPI_ISL_1054218, EPI_ISL_1054219, EPI_ISL_1054220, EPI_ISL_1054221, EPI_ISL_1054222, EPI_ISL_1054223, EPI_ISL_1054224, EPI_ISL_1054225, EPI_ISL_1054226, EPI_ISL_1054227, EPI_ISL_1054228, EPI_ISL_1054229, EPI_ISL_1054230, EPI_ISL_1054231, EPI_ISL_1054232, EPI_ISL_1054234, EPI_ISL_1054235, EPI_ISL_1054236, EPI_ISL_1054237, EPI_ISL_1054238, EPI_ISL_1054239, EPI_ISL_1054240, EPI_ISL_1054243, EPI_ISL_1054244, EPI_ISL_1054245, EPI_ISL_1054246, EPI_ISL_1054247, EPI_ISL_1054248, EPI_ISL_1054249, EPI_ISL_1054251, EPI_ISL_1054252, EPI_ISL_1054253, EPI_ISL_1054255, EPI_ISL_1054256, EPI_ISL_1054257			
see above	Northumbria University / South Tees Hospitals NHS Foundation Trust / North Cumbria Integrated Care NHS Foundation Trust / North Tees and Hartlepool NHS Foundation Trust / Newcastle Hospitals NHS Foundation Trust	COVID-19 Genomics UK (COG-UK) Consortium	Darren L Smith,Andrew Nelson,Matthew Bashton,Greg R Young,Joshua Loh,John Allan,Mohammad A Tariq,Giles S Holt,Gary Black,Wen C Yew,Lynn Dover,Paul Baker,Steve Liggett,Sarah Essex,Jane Greenaway,Debra Padgett,Clive Graham,Garren Scott,Edward Barton,Emma Swindells,Brendan Payne,Jennifer Collins,Yusri Taha,Gary Eltringham
EPI_ISL_1054372, EPI_ISL_1054373, EPI_ISL_1054374, EPI_ISL_1054375, EPI_ISL_1054376, EPI_ISL_1054377, EPI_ISL_1054378, EPI_ISL_1054379, EPI_ISL_1054380, EPI_ISL_1054381, EPI_ISL_1054382, EPI_ISL_1054383, EPI_ISL_1054384, EPI_ISL_1054385, EPI_ISL_1054386, EPI_ISL_1054387, EPI_ISL_1054388, EPI_ISL_1054389, EPI_ISL_1054390, EPI_ISL_1054404, EPI_ISL_1054405, EPI_ISL_1054406, EPI_ISL_1054407, EPI_ISL_1054408, EPI_ISL_1054409, EPI_ISL_1054410, EPI_ISL_1054417, EPI_ISL_1054418, EPI_ISL_1054419, EPI_ISL_1054429, EPI_ISL_1054430, EPI_ISL_1054431, EPI_ISL_1054438, EPI_ISL_1054439, EPI_ISL_1054440, EPI_ISL_1054441, EPI_ISL_1054442, EPI_ISL_1054443, EPI_ISL_1054447, EPI_ISL_1054448, EPI_ISL_1054449, EPI_ISL_1054450, EPI_ISL_1054454, EPI_ISL_1054457, EPI_ISL_1054458, EPI_ISL_1054461			
see above	Queens Medical Centre, Clinical Microbiology Department / DeepSeq Nottingham	COVID-19 Genomics UK (COG-UK) Consortium	Gemma Clark, Wendy Smith, Manjinder Khakh, Vicki M Fleming, Michelle M Lister, Hannah Howson-Wells, Jonathan Ball, Patrick McClure, Joseph Chappell, Theocharis Tsoleridis, Nadine Holmes, Matthew Carlisle, Christopher Moore, Fei Sang, Johnny Debebe, Victoria Wright, Matthew Loose
EPI_ISL_1054507, EPI_ISL_1054508, EPI_ISL_1054519, EPI_ISL_1054520, EPI_ISL_1054522, EPI_ISL_1054523, EPI_ISL_1054524, EPI_ISL_1054525, EPI_ISL_1054526, EPI_ISL_1054527	University of Exeter	COVID-19 Genomics UK (COG-UK) Consortium	Ben Temperton,Aaron Jeffries,Michelle Michelsen,Joanna Warwick-Dugdale,Audrey Farbos,Robyn Manley,Stephen Michell,Jane Masoli
EPI_ISL_1054825, EPI_ISL_1054832	Bioinformatics and Biostatistics Lab, Advanced Sequencing Facility	COVID-19 Genomics UK (COG-UK) Consortium	Aengus Stewart,Jerome Nicod,Chelsea Sawyer,Laura Cubitt,Harshil Patel,Margaret Crawford
EPI_ISL_1103792, EPI_ISL_1103797	University of Exeter	COVID-19 Genomics UK (COG-UK) Consortium	Ben Temperton,Aaron Jeffries,Michelle Michelsen,Joanna Warwick-Dugdale,Audrey Farbos,Robyn Manley,Stephen Michell,Jane Masoli
EPI_ISL_1103953, EPI_ISL_1103954, EPI_ISL_1103955, EPI_ISL_1103956, EPI_ISL_1103957, EPI_ISL_1103958, EPI_ISL_1103959, EPI_ISL_1103960, EPI_ISL_1103961, EPI_ISL_1103962, EPI_ISL_1103963, EPI_ISL_1103964, EPI_ISL_1103997, EPI_ISL_1103998, EPI_ISL_1103999, EPI_ISL_1104005, EPI_ISL_1104006, EPI_ISL_1104007, EPI_ISL_1104008, EPI_ISL_1104009, EPI_ISL_1104010, EPI_ISL_1104011, EPI_ISL_1104012, EPI_ISL_1104013			
see above	Northumbria University / South Tees Hospitals NHS Foundation Trust / North Cumbria Integrated Care NHS Foundation Trust / North Tees and Hartlepool NHS Foundation Trust / Newcastle Hospitals NHS Foundation Trust	COVID-19 Genomics UK (COG-UK) Consortium	Darren L Smith,Andrew Nelson,Matthew Bashton,Greg R Young,Joshua Loh,John Allan,Mohammad A Tariq,Giles S Holt,Gary Black,Wen C Yew,Lynn Dover,Paul Baker,Steve Liggett,Sarah Essex,Jane Greenaway,Debra Padgett,Clive Graham,Garren Scott,Edward Barton,Emma Swindells,Brendan Payne,Jennifer Collins,Yusri Taha,Gary Eltringham
EPI_ISL_1104029, EPI_ISL_1104041, EPI_ISL_1104042, EPI_ISL_1104043, EPI_ISL_1104044, EPI_ISL_1104045, EPI_ISL_1104046, EPI_ISL_1104048, EPI_ISL_1104051, EPI_ISL_1104052, EPI_ISL_1104053, EPI_ISL_1104054, EPI_ISL_1104055, EPI_ISL_1104056, EPI_ISL_1104057, EPI_ISL_1104058, EPI_ISL_1104059, EPI_ISL_1104060, EPI_ISL_1104062, EPI_ISL_1104063, EPI_ISL_1104064, EPI_ISL_1104065, EPI_ISL_1104066, EPI_ISL_1104067, EPI_ISL_1104068, EPI_ISL_1104069, EPI_ISL_1104070			
see above	Virology Department, Sheffield Teaching Hospitals NHS Foundation Trust/Department of Infection, Immunity and Cardiovascular Disease, The Medical School, University of Sheffield	COVID-19 Genomics UK (COG-UK) Consortium	Thushan de Silva, Matthew Parker, Nikki Smith, Adri Angyal, Rebecca Brown, Luke Green, Rachel Tucker, Paul Parsons, Danielle Groves, Katie Johnson, Laura Carrilero, Alex Keeley, Dave Partridge, Matthew Wyles, Benjamin Lindsey, Mehmet Yavuz, Mohammad Raza, Cariad Evans
EPI_ISL_1104223	Virology Department, Royal Infirmary of Edinburgh, NHS Lothian / School of Biological Sciences, University of Edinburgh	COVID-19 Genomics UK (COG-UK) Consortium	McHugh M, Dewar R, Cotton S, Rooke S, O'Toole Á, Scher E, Hill V, McCrone JT, Colquhoun R, Yu X, Jackson B, Rambaut A, Templeton K
EPI_ISL_1104502, EPI_ISL_1104503, EPI_ISL_1104506, EPI_ISL_1104507, EPI_ISL_1104508, EPI_ISL_1104509, EPI_ISL_1104510, EPI_ISL_1104568	Liverpool Clinical Laboratories	COVID-19 Genomics UK (COG-UK) Consortium	Sam Haldenby, Anita Lucaci, Steve Paterson, Julian Hiscox, Alistair Darby, M Almsaud, A Alrezaihi, Muhannad Alruwaili, Stuart D Armstrong, Jones Benjamin, Eleanor G Bentley, Anu Chawla, Jordan J Clark, Angela Cowell, Richard Eccles, Isabel García-Dorival, Matthew Gemmell, Alessandro Gerada, PKF Gilmore, Richard Gregory, Ximeng Han, Catherine Hartley, Margaret Hughes, Miren Iturriza-Gomara, James Johnson, L Luu, Jenifer Manson, Charlotte Nelson, Elaine O'Toole, Cassie Olateju, Rebekah Penrice-Randal , Lucille Rainbow, N.P Randle, Trevor Ian Robinson, Parul Sharma, Ghada T Shawli, James P Stewart, Neil Swainston, Ecaterina Vamos, Joanne Watts, Mark Whitehead
EPI_ISL_1104899, EPI_ISL_1104933, EPI_ISL_1104939, EPI_ISL_1104943, EPI_ISL_1104946, EPI_ISL_1104950, EPI_ISL_1104952, EPI_ISL_1104953, EPI_ISL_1104956, EPI_ISL_1104957, EPI_ISL_1104959, EPI_ISL_1104960, EPI_ISL_1104962, EPI_ISL_1104965, EPI_ISL_1104966, EPI_ISL_1104970, EPI_ISL_1104985, EPI_ISL_1105151, EPI_ISL_1105156, EPI_ISL_1105173, EPI_ISL_1105186			
see above	University College London Hospital	COVID-19 Genomics UK (COG-UK) Consortium	Judith Heaney, Matthew Byott, Catherine Houlihan, Dan Frampton, Stuart Kirk, Moira Spyer and Eleni Nastouli
EPI_ISL_1105447, EPI_ISL_1105448, EPI_ISL_1105449	University College London, Great Ormond Street Hospital for Children NHS Foundation Trust, Imperial College Healthcare NHS Trust	COVID-19 Genomics UK (COG-UK) Consortium	Sergi Castellano, Rachel Williams, Mark Kristiansen, Paola Resende Silva, Sunando Roy, Tony Brooks, Helena Tutill, Paola Niola, Patricia Dyal, Charlotte Williams, Leysa Forrest, Yasmin Panchbhaya, Jacqueline Findlay, Samuel Weeks, Julianne Brown, Kathryn Harris, Paul Randell, James Price, Alison Holmes, Judith Breuer
EPI_ISL_1105473, EPI_ISL_1105474, EPI_ISL_1105475, EPI_ISL_1105476, EPI_ISL_1105477, EPI_ISL_1105478, EPI_ISL_1105479, EPI_ISL_1105480, EPI_ISL_1105481, EPI_ISL_1105482, EPI_ISL_1105483, EPI_ISL_1105484, EPI_ISL_1105485, EPI_ISL_1105486, EPI_ISL_1105487, EPI_ISL_1105488, EPI_ISL_1105489, EPI_ISL_1105490, EPI_ISL_1105491, EPI_ISL_1105492, EPI_ISL_1105493			
see above	Northumbria University / South Tees Hospitals NHS Foundation Trust / North Cumbria Integrated Care NHS Foundation Trust / North Tees and Hartlepool NHS Foundation Trust / Newcastle Hospitals NHS Foundation Trust	COVID-19 Genomics UK (COG-UK) Consortium	Darren L Smith,Andrew Nelson,Matthew Bashton,Greg R Young,Joshua Loh,John Allan,Mohammad A Tariq,Giles S Holt,Gary Black,Wen C Yew,Lynn Dover,Paul Baker,Steve Liggett,Sarah Essex,Jane Greenaway,Debra Padgett,Clive Graham,Garren Scott,Edward Barton,Emma Swindells,Brendan Payne,Jennifer Collins,Yusri Taha,Gary Eltringham

EPI_ISL_1105494, EPI_ISL_1105495	Centre for Enzyme Innovation, University of Portsmouth / Translational Research Laboratory, Portsmouth Hospitals NHS Trust	COVID-19 Genomics UK (COG-UK) Consortium	Angela Beckett,Salman Goudarzi,Christopher Fearn,Kate Cook,Katie Loveson,Sharon Glaysher,Scott Elliott,Samuel Robson
EPI_ISL_1105496, EPI_ISL_1105497, EPI_ISL_1105498, EPI_ISL_1105499, EPI_ISL_1105500, EPI_ISL_1105501, EPI_ISL_1105502, EPI_ISL_1105503, EPI_ISL_1105504, EPI_ISL_1105505, EPI_ISL_1105506, EPI_ISL_1105507, EPI_ISL_1105508, EPI_ISL_1105509, EPI_ISL_1105510			
see above	Northumbria University / South Tees Hospitals NHS Foundation Trust / North Cumbria Integrated Care NHS Foundation Trust / North Tees and Hartlepool NHS Foundation Trust / Newcastle Hospitals NHS Foundation Trust	COVID-19 Genomics UK (COG-UK) Consortium	Darren L Smith,Andrew Nelson,Matthew Bashton,Greg R Young,Joshua Loh,John Allan,Mohammad A Tariq,Giles S Holt,Gary Black,Wen C Yew,Lynn Dover,Paul Baker,Steve Liggett,Sarah Essex,Jane Greenaway,Debra Padgett,Clive Graham,Garren Scott,Edward Barton,Emma Swindells,Brendan Payne,Jennifer Collins,Yusri Taha,Gary Eltringham
EPI_ISL_1105511, EPI_ISL_1105512	Centre for Enzyme Innovation, University of Portsmouth / Translational Research Laboratory, Portsmouth Hospitals NHS Trust	COVID-19 Genomics UK (COG-UK) Consortium	Angela Beckett,Salman Goudarzi,Christopher Fearn,Kate Cook,Katie Loveson,Sharon Glaysher,Scott Elliott,Samuel Robson
EPI_ISL_1105513, EPI_ISL_1105514, EPI_ISL_1105515, EPI_ISL_1105516, EPI_ISL_1105517, EPI_ISL_1105518, EPI_ISL_1105519, EPI_ISL_1105520, EPI_ISL_1105521, EPI_ISL_1105522, EPI_ISL_1105523			
see above	Northumbria University / South Tees Hospitals NHS Foundation Trust / North Cumbria Integrated Care NHS Foundation Trust / North Tees and Hartlepool NHS Foundation Trust / Newcastle Hospitals NHS Foundation Trust	COVID-19 Genomics UK (COG-UK) Consortium	Darren L Smith,Andrew Nelson,Matthew Bashton,Greg R Young,Joshua Loh,John Allan,Mohammad A Tariq,Giles S Holt,Gary Black,Wen C Yew,Lynn Dover,Paul Baker,Steve Liggett,Sarah Essex,Jane Greenaway,Debra Padgett,Clive Graham,Garren Scott,Edward Barton,Emma Swindells,Brendan Payne,Jennifer Collins,Yusri Taha,Gary Eltringham
EPI_ISL_1105871, EPI_ISL_1105889, EPI_ISL_1105890, EPI_ISL_1105891, EPI_ISL_1105892, EPI_ISL_1105893, EPI_ISL_1105895, EPI_ISL_1105896, EPI_ISL_1105899, EPI_ISL_1105902	Lincolnshire Hospitals and DeepSeq Nottingham	COVID-19 Genomics UK (COG-UK) Consortium	Nichola Duckworth, Tim Sloan, Sarah Walsh, Jonathan Ball, Patrick McClure, Joeseph Chappell, Nadine Holmes, Matthew Carlisle, Christopher Moore, Fei Sang, Johnny Debebe, Victoria Wright, Matthew Loose
EPI_ISL_1107586, EPI_ISL_1107663, EPI_ISL_1107664, EPI_ISL_1107785	Centre for Enzyme Innovation, University of Portsmouth / Translational Research Laboratory, Portsmouth Hospitals NHS Trust	COVID-19 Genomics UK (COG-UK) Consortium	Angela Beckett,Salman Goudarzi,Christopher Fearn,Kate Cook,Katie Loveson,Sharon Glaysher,Scott Elliott,Samuel Robson
EPI_ISL_1177722, EPI_ISL_1177723	Virology Department, Royal Infirmary of Edinburgh, NHS Lothian / School of Biological Sciences, University of Edinburgh	COVID-19 Genomics UK (COG-UK) Consortium	McHugh M, Dewar R, Cotton S, Rooke S, O'Toole Á, Scher E, Hill V, McCrone JT, Colquhoun R, Yu X, Jackson B, Rambaut A, Templeton K
EPI_ISL_1178150, EPI_ISL_1178151, EPI_ISL_1178152, EPI_ISL_1178153, EPI_ISL_1178154, EPI_ISL_1178155, EPI_ISL_1178156, EPI_ISL_1178157, EPI_ISL_1178158, EPI_ISL_1178159, EPI_ISL_1178160, EPI_ISL_1178161, EPI_ISL_1178162, EPI_ISL_1178163, EPI_ISL_1178164, EPI_ISL_1178165, EPI_ISL_1178166, EPI_ISL_1178167, EPI_ISL_1178168			
see above	Northumbria University / South Tees Hospitals NHS Foundation Trust / North Cumbria Integrated Care NHS Foundation Trust / North Tees and Hartlepool NHS Foundation Trust / Newcastle Hospitals NHS Foundation Trust	COVID-19 Genomics UK (COG-UK) Consortium	Darren L Smith,Andrew Nelson,Matthew Bashton,Greg R Young,Joshua Loh,John Allan,Mohammad A Tariq,Giles S Holt,Gary Black,Wen C Yew,Lynn Dover,Paul Baker,Steve Liggett,Sarah Essex,Jane Greenaway,Debra Padgett,Clive Graham,Garren Scott,Edward Barton,Emma Swindells,Brendan Payne,Jennifer Collins,Yusri Taha,Gary Eltringham
EPI_ISL_1178498, EPI_ISL_1178502, EPI_ISL_1178503, EPI_ISL_1178505	Quadram Institute Bioscience	COVID-19 Genomics UK (COG-UK) Consortium	Dave J. Baker, Gemma L. Kay, Alp Aydin, Thanh Le-Viet, Steven Rudder, Ana P. Tedim, Anastasia Kolyva, Maria Diaz, Leonardo de Oliveira Martins, Nabil-Fareed Alikhan, Lizzie Meadows, Rachael Stanley, Ngozi Elumogo, Muhammed Yasir, Nicholas M. Thomson, Alexander J Trotter, Rachel Gilroy, Samuel Bloomfield, Claire Stuart, Andrew Bell, Reenesh Prakash, Samir Dervisevic, Alison E. Mather, John Wain, Mark Webber, Andrew J. Page, Justin O'Grady
EPI_ISL_1180058, EPI_ISL_1180059	Virology Department, Sheffield Teaching Hospitals NHS Foundation Trust/Department of Infection, Immunity and Cardiovascular Disease, The Medical School, University of Sheffield	COVID-19 Genomics UK (COG-UK) Consortium	Thushan de Silva, Matthew Parker, Nikki Smith, Adri Angyal, Rebecca Brown, Luke Green, Rachel Tucker, Paul Parsons, Danielle Groves, Katie Johnson, Laura Carrilero, Alex Keeley, Dave Partridge, Matthew Wyles, Benjamin Lindsey, Mehmet Yavuz, Mohammad Raza, Cariad Evans
EPI_ISL_1223167, EPI_ISL_1223193, EPI_ISL_1223236, EPI_ISL_1223246, EPI_ISL_1223258, EPI_ISL_1223265, EPI_ISL_1223270, EPI_ISL_1223283, EPI_ISL_1223285, EPI_ISL_1223357	Lighthouse Lab in Cambridge	Wellcome Sanger Institute for the COVID-19 Genomics UK (COG-UK) Consortium	Rob Howes, The Lighthouse Lab in Cambridge and Alex Alderton, Roberto Amato, Jeffrey Barrett, Sonia Goncalves, Ewan Harrison, David K. Jackson, Ian Johnston, Dominic Kwiatkowski, Cordelia Langford, John Sillitoe on behalf of the Wellcome Sanger Institute COVID-19 Surveillance Team
EPI_ISL_1247691, EPI_ISL_1247692	Virology Department, Royal Infirmary of Edinburgh, NHS Lothian / School of Biological Sciences, University of Edinburgh	COVID-19 Genomics UK (COG-UK) Consortium	McHugh M, Dewar R, Cotton S, Rooke S, O'Toole Á, Scher E, Hill V, McCrone JT, Colquhoun R, Yu X, Jackson B, Rambaut A, Templeton K
EPI_ISL_1247823, EPI_ISL_1247824	Liverpool Clinical Laboratories	COVID-19 Genomics UK (COG-UK) Consortium	Sam Haldenby, Alistair Darby, Steve Paterson, Anita Lucaci, Julian Hiscox, M Almsaud, A Alrezaihi, Muhannad Alruwaili, Stuart D Armstrong, Jones Benjamin, Eleanor G Bentley, Anu Chawla, Jordan J Clark, Angela Cowell, Richard Eccles, Isabel García-Dorival, Matthew Gemmell, Alessandro Gerada, PKF Gilmore, Richard Gregory, Ximeng Han, Catherine Hartley, Margaret Hughes, Miren Iturriza-Gomara, James Johnson, L Luu, Jenifer Manson, Charlotte Nelson, Elaine O'Toole, Cassie Olateju, Rebekah Penrice-Randal , Lucille Rainbow, N.P Randle, Trevor Ian Robinson, Parul Sharma, Ghada T Shawli, James P Stewart, Neil Swainston, Ecaterina Vamos, Joanne Watts, Mark Whitehead, Hermione Webster
EPI_ISL_1248160, EPI_ISL_1248161, EPI_ISL_1248170, EPI_ISL_1248180, EPI_ISL_1248190, EPI_ISL_1248198, EPI_ISL_1248199, EPI_ISL_1248258, EPI_ISL_1248262, EPI_ISL_1248264, EPI_ISL_1248266, EPI_ISL_1248531			
see above	University College London, Great Ormond Street Hospital for Children NHS Foundation Trust, Imperial College Healthcare NHS Trust	COVID-19 Genomics UK (COG-UK) Consortium	Sergi Castellano, Rachel Williams, Mark Kristiansen, Paola Resende Silva, Sunando Roy, Tony Brooks, Helena Tutill, Paola Niola, Patricia Dyal, Charlotte Williams, Leysa Forrest, Yasmin Panchbhaya, Jacqueline Findlay, Samuel Weeks, Julianne Brown, Kathryn Harris, Paul Randell, James Price, Alison Holmes, Judith Breuer
EPI_ISL_1248998, EPI_ISL_1249011, EPI_ISL_1249014, EPI_ISL_1249019, EPI_ISL_1249027	Quadram Institute Bioscience	COVID-19 Genomics UK (COG-UK) Consortium	Dave J. Baker, Gemma L. Kay, Alp Aydin, Thanh Le-Viet, Steven Rudder, Ana P. Tedim, Anastasia Kolyva, Maria Diaz, Leonardo de Oliveira Martins, Nabil-Fareed Alikhan, Lizzie Meadows, Rachael Stanley, Ngozi Elumogo, Muhammed Yasir, Nicholas M. Thomson, Alexander J Trotter, Rachel Gilroy, Samuel Bloomfield, Claire Stuart, Andrew Bell, Reenesh Prakash, Samir Dervisevic, Alison E. Mather, John Wain, Mark Webber, Andrew J. Page, Justin O'Grady
EPI_ISL_1249233, EPI_ISL_1249236, EPI_ISL_1249238, EPI_ISL_1249240	Oxford Viromics, NDM, University of Oxford; Oxford University Hospitals; Basingstoke and North Hampshire Hospital	COVID-19 Genomics UK (COG-UK) Consortium	Tanya Golubchik, David Bonsall, George Macintyre, Amy Trebes, Mariateresa de Cesare, Catrin Moore, Alex Mobbs, Anita Justice, Robert Shaw, Monique Andersson, Timothy Peto, Emma Wise, Nathan Moore, Jessica Lynch, Nick Cortes, Matilde Mori, Stephen Kidd, David Buck, John Todd, Christophe Fraser
EPI_ISL_1249257	Liverpool Clinical Laboratories	COVID-19 Genomics UK (COG-UK) Consortium	Sam Haldenby, Alistair Darby, Steve Paterson, Anita Lucaci, Julian Hiscox, M Almsaud, A Alrezaihi, Muhannad Alruwaili, Stuart D Armstrong, Jones Benjamin, Eleanor G Bentley, Anu Chawla, Jordan J Clark, Angela Cowell, Richard Eccles, Isabel García-Dorival, Matthew Gemmell, Alessandro Gerada, PKF Gilmore, Richard Gregory, Ximeng Han, Catherine Hartley, Margaret Hughes, Miren Iturriza-Gomara, James Johnson, L Luu, Jenifer Manson, Charlotte Nelson, Elaine O'Toole, Cassie Olateju, Rebekah Penrice-Randal , Lucille Rainbow, N.P Randle, Trevor Ian Robinson, Parul Sharma, Ghada T Shawli, James P Stewart, Neil Swainston, Ecaterina Vamos, Joanne Watts, Mark Whitehead, Hermione Webster
EPI_ISL_1250123	University of Exeter	COVID-19 Genomics UK (COG-UK) Consortium	Ben Temperton,Aaron Jeffries,Michelle Michelsen,Joanna Warwick-Dugdale,Audrey Farbos,Robyn Manley,Stephen Michell,Jane Masoli
EPI_ISL_1296594, EPI_ISL_1296595, EPI_ISL_1296596	Respiratory Virus Unit, National Infection Service, Public Health England	COVID-19 Genomics UK (COG-UK) Consortium	PHE Covid Sequencing Team
EPI_ISL_1308542, EPI_ISL_1308568, EPI_ISL_1308581	University of Exeter	COVID-19 Genomics UK (COG-UK) Consortium	Ben Temperton,Aaron Jeffries,Michelle Michelsen,Joanna Warwick-Dugdale,Audrey Farbos,Robyn Manley,Stephen Michell,Jane Masoli
EPI_ISL_1308706, EPI_ISL_1308840	Virology Department, Royal Infirmary of Edinburgh, NHS	COVID-19 Genomics UK (COG-UK) Consortium	McHugh M, Dewar R, Cotton S, Rooke S, O'Toole Á, Scher E, Hill V, McCrone JT, Colquhoun R, Yu X, Jackson B, Rambaut A, Templeton K

	Lothian / School of Biological Sciences, University of Edinburgh		
EPI_ISL_1309060, EPI_ISL_1309122	University College London, Great Ormond Street Hospital for Children NHS Foundation Trust, Imperial College Healthcare NHS Trust	COVID-19 Genomics UK (COG-UK) Consortium	Sergi Castellano, Rachel Williams, Mark Kristiansen, Paola Resende Silva, Sunando Roy, Tony Brooks, Helena Tutill, Paola Niola, Patricia Dyal, Charlotte Williams, Leysa Forrest, Yasmin Panchbhaya, Jacqueline Findlay, Samuel Weeks, Julianne Brown, Kathryn Harris, Paul Randell, James Price, Alison Holmes, Judith Breuer
EPI_ISL_1309782, EPI_ISL_1387225, EPI_ISL_1387226, EPI_ISL_1387227	Oxford Viromics, NDM, University of Oxford; Oxford University Hospitals; Basingstoke and North Hampshire Hospital	COVID-19 Genomics UK (COG-UK) Consortium	Tanya Golubchik, David Bonsall, George Macintyre, Amy Trebes, Mariateresa de Cesare, Catrin Moore, Alex Mobbs, Anita Justice, Robert Shaw, Monique Andersson, Timothy Peto, Emma Wise, Nathan Moore, Jessica Lynch, Nick Cortes, Matilde Mori, Stephen Kidd, David Buck, John Todd, Christophe Fraser
EPI_ISL_1388738	Centre for Enzyme Innovation, University of Portsmouth / Translational Research Laboratory, Portsmouth Hospitals NHS Trust	COVID-19 Genomics UK (COG-UK) Consortium	Angela Beckett,Salman Goudarzi,Christopher Fearn,Kate Cook,Katie Loveson,Sharon Glaysher,Scott Elliott,Samuel Robson
EPI_ISL_1475001, EPI_ISL_1475132, EPI_ISL_1475134	Regional Virus Laboratory, Belfast Health and Social Care Trust	COVID-19 Genomics UK (COG-UK) Consortium	Conall McCaughey, James McKenna, Tanya Curran, Susan Feeney, Alison Watt, Ciara Cox, Mairead Connor, Zoltan Molnar, David Simpson, Derek Fairley
EPI_ISL_924166, EPI_ISL_924231, EPI_ISL_924281, EPI_ISL_924290, EPI_ISL_924296, EPI_ISL_924351, EPI_ISL_924376	Virology Department, Sheffield Teaching Hospitals NHS Foundation Trust/Department of Infection, Immunity and Cardiovascular Disease, The Medical School, University of Sheffield	COVID-19 Genomics UK (COG-UK) Consortium	Thushan de Silva, Matthew Parker, Nikki Smith, Adri Angyal, Rebecca Brown, Luke Green, Rachel Tucker, Paul Parsons, Danielle Groves, Katie Johnson, Laura Carrilero, Alex Keeley, Dave Partridge, Matthew Wyles, Benjamin Lindsey, Mehmet Yavuz, Mohammad Raza, Cariad Evans
EPI_ISL_949559	Department of Pathology, University of Cambridge	COVID-19 Genomics UK (COG-UK) Consortium	Aminu S. Jahun, Yasmin Chaudhry, Iliana Georgana, Myra Hosmillo, Rhys Izu, Martin D. Curran, Surendra Parmar, Ian Goodfellow
EPI_ISL_949613, EPI_ISL_949629	West of Scotland Specialist Virology Centre, NHSGGC / MRC-University of Glasgow Centre for Virus Research	COVID-19 Genomics UK (COG-UK) Consortium	Ana da Silva Filipe, Natasha Johnson, Kathy Smollett, Daniel Mair, Stephen Carmichael, Alice Broos, Lily Tong, Jenna Nichols, Kyriaki Nomikou; Sarah McDonald; Richard Orton, Joseph Hughes, Sreenu Vattipally, David L Robertson; Alasdair MacLean, Rory Gunson; Sharif Shaaban, Matthew Holden; Rachel Blacow, Guy Mollett, Kathy Li, James Shepherd, Antonia Ho, Emma Thomson
EPI_ISL_949712, EPI_ISL_949713, EPI_ISL_949714, EPI_ISL_949715, EPI_ISL_949716, EPI_ISL_949718, EPI_ISL_949721, EPI_ISL_949724, EPI_ISL_949725, EPI_ISL_949729, EPI_ISL_949731, EPI_ISL_949732, EPI_ISL_949733, EPI_ISL_949734, EPI_ISL_949735, EPI_ISL_949736, EPI_ISL_949737, EPI_ISL_949738, EPI_ISL_949739, EPI_ISL_949741			
see above	Liverpool Clinical Laboratories	COVID-19 Genomics UK (COG-UK) Consortium	Sam Haldenby, Anita Lucaci, Steve Paterson, Julian Hiscox, Alistair Darby, M Almsaud, A Alrezaihi, Muhannad Alruwaili, Stuart D Armstrong, Jones Benjamin, Eleanor G Bentley, Anu Chawla, Jordan J Clark, Angela Cowell, Richard Eccles, Isabel García-Dorival, Matthew Gemmell, Alessandro Gerada, PKF Gilmore, Richard Gregory, Ximeng Han, Catherine Hartley, Margaret Hughes, Miren Iturriza-Gomara, James Johnson, L Luu, Jenifer Manson, Charlotte Nelson, Elaine O'Toole, Cassie Olateju, Rebekah Penrice-Randal , Lucille Rainbow, N.P Randle, Trevor Ian Robinson, Parul Sharma, Ghada T Shawli, James P Stewart, Neil Swainston, Ecaterina Vamos, Joanne Watts, Mark Whitehead
EPI_ISL_949812, EPI_ISL_949813, EPI_ISL_949819, EPI_ISL_949831, EPI_ISL_949833, EPI_ISL_949834, EPI_ISL_949836, EPI_ISL_949837, EPI_ISL_949838, EPI_ISL_949839, EPI_ISL_949840, EPI_ISL_949841, EPI_ISL_949842, EPI_ISL_949843, EPI_ISL_949844, EPI_ISL_949845, EPI_ISL_949846, EPI_ISL_949847, EPI_ISL_949848, EPI_ISL_949855, EPI_ISL_949920, EPI_ISL_949926, EPI_ISL_949928, EPI_ISL_949929, EPI_ISL_949931, EPI_ISL_949934, EPI_ISL_949938, EPI_ISL_949939, EPI_ISL_949941, EPI_ISL_949942, EPI_ISL_949943, EPI_ISL_949945, EPI_ISL_949946, EPI_ISL_949951, EPI_ISL_949952, EPI_ISL_949954, EPI_ISL_949955, EPI_ISL_949956, EPI_ISL_949958, EPI_ISL_949960			
see above	University College London, Great Ormond Street Hospital for Children NHS Foundation Trust, Imperial College Healthcare NHS Trust	COVID-19 Genomics UK (COG-UK) Consortium	Sergi Castellano, Rachel Williams, Mark Kristiansen, Paola Resende Silva, Sunando Roy, Tony Brooks, Helena Tutill, Paola Niola, Patricia Dyal, Charlotte Williams, Leysa Forrest, Yasmin Panchbhaya, Jacqueline Findlay, Samuel Weeks, Julianne Brown, Kathryn Harris, Paul Randell, James Price, Alison Holmes, Judith Breuer
EPI_ISL_950424, EPI_ISL_950428, EPI_ISL_950429, EPI_ISL_950430, EPI_ISL_950431, EPI_ISL_950432, EPI_ISL_950433, EPI_ISL_950434	Northumbria University / South Tees Hospitals NHS Foundation Trust / North Cumbria Integrated Care NHS Foundation Trust / North Tees and Hartlepool NHS Foundation Trust / Newcastle Hospitals NHS Foundation Trust	COVID-19 Genomics UK (COG-UK) Consortium	Darren L Smith,Andrew Nelson,Matthew Bashton,Greg R Young,Joshua Loh,John Allan,Mohammad A Tariq,Giles S Holt,Gary Black,Wen C Yew,Lynn Dover,Paul Baker,Steve Liggett,Sarah Essex,Jane Greenaway,Debra Padgett,Clive Graham,Garren Scott,Edward Barton,Emma Swindells,Brendan Payne,Jennifer Collins,Yusri Taha,Gary Eltringham
EPI_ISL_950662, EPI_ISL_950663, EPI_ISL_950664, EPI_ISL_950665, EPI_ISL_950666, EPI_ISL_950667, EPI_ISL_950668, EPI_ISL_950669, EPI_ISL_950670, EPI_ISL_950671, EPI_ISL_950672, EPI_ISL_950673, EPI_ISL_950674, EPI_ISL_950675, EPI_ISL_950676			
see above	Queens Medical Centre, Clinical Microbiology Department / DeepSeq Nottingham	COVID-19 Genomics UK (COG-UK) Consortium	Gemma Clark, Wendy Smith, Manjinder Khakh, Vicki M Fleming, Michelle M Lister, Hannah Howson-Wells, Jonathan Ball, Patrick McClure, Joseph Chappell, Theocharis Tsoleridis, Nadine Holmes, Matthew Carlisle, Christopher Moore, Fei Sang, Johnny Debebe, Victoria Wright, Matthew Loose
EPI_ISL_952251, EPI_ISL_952252, EPI_ISL_952253, EPI_ISL_952255, EPI_ISL_952256, EPI_ISL_952257, EPI_ISL_952258, EPI_ISL_952259, EPI_ISL_952261, EPI_ISL_952262, EPI_ISL_952263, EPI_ISL_952264, EPI_ISL_952266, EPI_ISL_952267, EPI_ISL_952268, EPI_ISL_952269, EPI_ISL_952270, EPI_ISL_952271, EPI_ISL_952272, EPI_ISL_952273, EPI_ISL_952274, EPI_ISL_952275, EPI_ISL_952276, EPI_ISL_952277, EPI_ISL_952278, EPI_ISL_952279, EPI_ISL_952280, EPI_ISL_952281, EPI_ISL_952282, EPI_ISL_952283, EPI_ISL_952284, EPI_ISL_952285, EPI_ISL_952286, EPI_ISL_952287, EPI_ISL_952288, EPI_ISL_952289, EPI_ISL_952290, EPI_ISL_952291, EPI_ISL_952292, EPI_ISL_952293, EPI_ISL_952294, EPI_ISL_952295, EPI_ISL_952296, EPI_ISL_952297, EPI_ISL_952299, EPI_ISL_952300, EPI_ISL_952301, EPI_ISL_952302, EPI_ISL_952303, EPI_ISL_952304, EPI_ISL_952305, EPI_ISL_952306, EPI_ISL_952307, EPI_ISL_952308, EPI_ISL_952309, EPI_ISL_952310, EPI_ISL_952311, EPI_ISL_952312, EPI_ISL_952313, EPI_ISL_952314, EPI_ISL_952315, EPI_ISL_952316, EPI_ISL_952317, EPI_ISL_952318, EPI_ISL_952319, EPI_ISL_952320, EPI_ISL_952321, EPI_ISL_952322, EPI_ISL_952323, EPI_ISL_952324, EPI_ISL_952325, EPI_ISL_952326, EPI_ISL_952327, EPI_ISL_952328, EPI_ISL_952329, EPI_ISL_952330, EPI_ISL_952331, EPI_ISL_952332, EPI_ISL_952333, EPI_ISL_952334, EPI_ISL_952335, EPI_ISL_952337, EPI_ISL_952338, EPI_ISL_952339, EPI_ISL_952340, EPI_ISL_952341, EPI_ISL_952342, EPI_ISL_952343, EPI_ISL_952344, EPI_ISL_952345, EPI_ISL_952346, EPI_ISL_952347, EPI_ISL_952348, EPI_ISL_952349, EPI_ISL_952350, EPI_ISL_952351, EPI_ISL_952352, EPI_ISL_952353, EPI_ISL_952354, EPI_ISL_952355, EPI_ISL_952356, EPI_ISL_952357, EPI_ISL_952358, EPI_ISL_952359, EPI_ISL_952360, EPI_ISL_952361, EPI_ISL_952362, EPI_ISL_952363, EPI_ISL_952364, EPI_ISL_952366, EPI_ISL_952367, EPI_ISL_952368, EPI_ISL_952370, EPI_ISL_952371, EPI_ISL_952372, EPI_ISL_952373, EPI_ISL_952374, EPI_ISL_952375, EPI_ISL_952376			
see above	Originating lab: Wales Specialist Virology Centre Sequencing lab: Pathogen Genomics Unit	Public Health Wales Microbiology Cardiff Wales Specialist Virology Centre	Catherine Moore, Johnathan Evans, Laura Gifford, Malorie Perry, Simon Cottrell, Angela Marchbank, Alec Birchley, Alexander Adams, Amy Gaskin, Bree Gatica-Wilcox, Jason Coombes, Joel Southgate, Lauren Gilbert, Lee Graham, Nicole Pacchiarini, Sara Kumziene-Summerhayes, Sarah Taylor, Sophie Jones, Sara Rey, Matthew Bull, Joanne Watkins, Sally Corden, Tom Connor
EPI_ISL_952486, EPI_ISL_952487, EPI_ISL_952488	Centre for Enzyme Innovation, University of Portsmouth / Translational Research Laboratory, Portsmouth Hospitals NHS Trust	COVID-19 Genomics UK (COG-UK) Consortium	Angela Beckett,Salman Goudarzi,Christopher Fearn,Kate Cook,Katie Loveson,Sharon Glaysher,Scott Elliott,Samuel Robson
EPI_ISL_952954, EPI_ISL_952955, EPI_ISL_952964, EPI_ISL_952970, EPI_ISL_952980, EPI_ISL_952994, EPI_ISL_952997, EPI_ISL_953002	Virology Department, Sheffield Teaching Hospitals NHS Foundation Trust/Department of Infection, Immunity and Cardiovascular Disease, The Medical School, University of Sheffield	COVID-19 Genomics UK (COG-UK) Consortium	Thushan de Silva, Matthew Parker, Nikki Smith, Adri Angyal, Rebecca Brown, Luke Green, Rachel Tucker, Paul Parsons, Danielle Groves, Katie Johnson, Laura Carrilero, Alex Keeley, Dave Partridge, Matthew Wyles, Benjamin Lindsey, Mehmet Yavuz, Mohammad Raza, Cariad Evans
EPI_ISL_953276, EPI_ISL_953279, EPI_ISL_953283	Bioinformatics and Biostatistics Lab, Advanced Sequencing Facility	COVID-19 Genomics UK (COG-UK) Consortium	Aengus Stewart,Jerome Nicod,Chelsea Sawyer,Laura Cubitt,Harshil Patel,Margaret Crawford
EPI_ISL_957054, EPI_ISL_957055, EPI_ISL_957057, EPI_ISL_957060, EPI_ISL_957061, EPI_ISL_957065, EPI_ISL_957067, EPI_ISL_957068, EPI_ISL_957071, EPI_ISL_957072, EPI_ISL_957075, EPI_ISL_957077, EPI_ISL_957079, EPI_ISL_957080, EPI_ISL_957081, EPI_ISL_957083, EPI_ISL_957084, EPI_ISL_957086, EPI_ISL_957091, EPI_ISL_957092, EPI_ISL_957093, EPI_ISL_957095, EPI_ISL_957097, EPI_ISL_957099, EPI_ISL_957102, EPI_ISL_957104, EPI_ISL_957105, EPI_ISL_957107, EPI_ISL_957110, EPI_ISL_957114, EPI_ISL_957117, EPI_ISL_957118, EPI_ISL_957121, EPI_ISL_957122, EPI_ISL_957124, EPI_ISL_957130, EPI_ISL_957140, EPI_ISL_957141, EPI_ISL_957149, EPI_ISL_957151, EPI_ISL_957154, EPI_ISL_957155, EPI_ISL_957157, EPI_ISL_957158, EPI_ISL_957160, EPI_ISL_957162, EPI_ISL_957165, EPI_ISL_957166, EPI_ISL_957168, EPI_ISL_957170, EPI_ISL_957171, EPI_ISL_957172, EPI_ISL_957176, EPI_ISL_957177, EPI_ISL_957180, EPI_ISL_957183, EPI_ISL_957187, EPI_ISL_957188, EPI_ISL_957191, EPI_ISL_957198, EPI_ISL_957200, EPI_ISL_957201, EPI_ISL_957205, EPI_ISL_957206, EPI_ISL_957208, EPI_ISL_957210, EPI_ISL_957211, EPI_ISL_957215, EPI_ISL_957216, EPI_ISL_957219, EPI_ISL_957223, EPI_ISL_957224, EPI_ISL_957227, EPI_ISL_957229, EPI_ISL_957231, EPI_ISL_957237, EPI_ISL_957239, EPI_ISL_957242, EPI_ISL_957243, EPI_ISL_957245, EPI_ISL_957246, EPI_ISL_957248, EPI_ISL_957250, EPI_ISL_957253, EPI_ISL_957254, EPI_ISL_957255, EPI_ISL_957256, EPI_ISL_957257, EPI_ISL_957259, EPI_ISL_957260, EPI_ISL_957262, EPI_ISL_957266, EPI_ISL_957268, EPI_ISL_957270, EPI_ISL_957271, EPI_ISL_957272, EPI_ISL_957274, EPI_ISL_957275, EPI_ISL_957276, EPI_ISL_957285, EPI_ISL_957286, EPI_ISL_957291, EPI_ISL_957292, EPI_ISL_957294, EPI_ISL_957299, EPI_ISL_957302, EPI_ISL_957304, EPI_ISL_957306, EPI_ISL_957308, EPI_ISL_957310, EPI_ISL_957311, EPI_ISL_957312, EPI_ISL_957315, EPI_ISL_957319, EPI_ISL_957322, EPI_ISL_957323, EPI_ISL_957327, EPI_ISL_957328, EPI_ISL_957330, EPI_ISL_957331, EPI_ISL_957336, EPI_ISL_957337			
see above	Lighthouse Lab in Glasgow	Wellcome Sanger Institute for the COVID-19 Genomics UK (COG-UK) Consortium	Harper VanSteenhouse, Yumi Kasai, David Gray, Carol Clugston, Anna Dominiczak and Alex Alderton, Roberto Amato, Sonia Goncalves, Ewan Harrison, David K. Jackson, Ian Johnston, Dominic Kwiatkowski, Cordelia Langford, John Sillitoe on behalf of the Wellcome Sanger Institute COVID-19 Surveillance Team
EPI_ISL_957617, EPI_ISL_957625	Lighthouse Lab in Cambridge	Wellcome Sanger Institute for the COVID-19 Genomics UK (COG-UK) Consortium	Rob Howes, The Lighthouse Lab in Cambridge and Alex Alderton, Roberto Amato, Sonia Goncalves, Ewan Harrison, David K. Jackson, Ian Johnston, Dominic Kwiatkowski, Cordelia Langford, John Sillitoe on behalf of the Wellcome Sanger Institute COVID-19 Surveillance Team
EPI_ISL_957633	Lighthouse Lab in Glasgow	Wellcome Sanger Institute for the COVID-19 Genomics UK (COG-UK) Consortium	Harper VanSteenhouse, Yumi Kasai, David Gray, Carol Clugston, Anna Dominiczak and Alex Alderton, Roberto Amato, Sonia Goncalves, Ewan Harrison, David K. Jackson, Ian Johnston, Dominic Kwiatkowski, Cordelia Langford, John Sillitoe on behalf of the Wellcome Sanger Institute COVID-19 Surveillance Team
EPI_ISL_957634, EPI_ISL_957636	Lighthouse Lab in Cambridge	Wellcome Sanger Institute for the COVID-19 Genomics UK	Rob Howes, The Lighthouse Lab in Cambridge and Alex Alderton, Roberto Amato, Sonia Goncalves, Ewan Harrison, David K. Jackson, Ian Johnston,

		(COG-UK) Consortium	Dominic Kwiatkowski, Cordelia Langford, John Sillitoe on behalf of the Wellcome Sanger Institute COVID-19 Surveillance Team
EPI_ISL_957637	Lighthouse Lab in Glasgow	Wellcome Sanger Institute for the COVID-19 Genomics UK (COG-UK) Consortium	Harper VanSteenhouse, Yumi Kasai, David Gray, Carol Clugston, Anna Dominiczak and Alex Alderton, Roberto Amato, Sonia Goncalves, Ewan Harrison, David K. Jackson, Ian Johnston, Dominic Kwiatkowski, Cordelia Langford, John Sillitoe on behalf of the Wellcome Sanger Institute COVID-19 Surveillance Team
EPI_ISL_957640, EPI_ISL_957644, EPI_ISL_957646, EPI_ISL_957647	Lighthouse Lab in Cambridge	Wellcome Sanger Institute for the COVID-19 Genomics UK (COG-UK) Consortium	Rob Howes, The Lighthouse Lab in Cambridge and Alex Alderton, Roberto Amato, Sonia Goncalves, Ewan Harrison, David K. Jackson, Ian Johnston, Dominic Kwiatkowski, Cordelia Langford, John Sillitoe on behalf of the Wellcome Sanger Institute COVID-19 Surveillance Team
EPI_ISL_957649, EPI_ISL_957651, EPI_ISL_957653, EPI_ISL_957663, EPI_ISL_957664, EPI_ISL_957666, EPI_ISL_957669, EPI_ISL_957673, EPI_ISL_957674, EPI_ISL_957678, EPI_ISL_957679, EPI_ISL_957681, EPI_ISL_957682			
see above	Lighthouse Lab in Glasgow	Wellcome Sanger Institute for the COVID-19 Genomics UK (COG-UK) Consortium	Harper VanSteenhouse, Yumi Kasai, David Gray, Carol Clugston, Anna Dominiczak and Alex Alderton, Roberto Amato, Sonia Goncalves, Ewan Harrison, David K. Jackson, Ian Johnston, Dominic Kwiatkowski, Cordelia Langford, John Sillitoe on behalf of the Wellcome Sanger Institute COVID-19 Surveillance Team
EPI_ISL_957689	Lighthouse Lab in Cambridge	Wellcome Sanger Institute for the COVID-19 Genomics UK (COG-UK) Consortium	Rob Howes, The Lighthouse Lab in Cambridge and Alex Alderton, Roberto Amato, Sonia Goncalves, Ewan Harrison, David K. Jackson, Ian Johnston, Dominic Kwiatkowski, Cordelia Langford, John Sillitoe on behalf of the Wellcome Sanger Institute COVID-19 Surveillance Team
EPI_ISL_957693, EPI_ISL_957694, EPI_ISL_957697, EPI_ISL_957708, EPI_ISL_957713	Lighthouse Lab in Glasgow	Wellcome Sanger Institute for the COVID-19 Genomics UK (COG-UK) Consortium	Harper VanSteenhouse, Yumi Kasai, David Gray, Carol Clugston, Anna Dominiczak and Alex Alderton, Roberto Amato, Sonia Goncalves, Ewan Harrison, David K. Jackson, Ian Johnston, Dominic Kwiatkowski, Cordelia Langford, John Sillitoe on behalf of the Wellcome Sanger Institute COVID-19 Surveillance Team
EPI_ISL_957714	Lighthouse Lab in Cambridge	Wellcome Sanger Institute for the COVID-19 Genomics UK (COG-UK) Consortium	Rob Howes, The Lighthouse Lab in Cambridge and Alex Alderton, Roberto Amato, Sonia Goncalves, Ewan Harrison, David K. Jackson, Ian Johnston, Dominic Kwiatkowski, Cordelia Langford, John Sillitoe on behalf of the Wellcome Sanger Institute COVID-19 Surveillance Team
EPI_ISL_957715	Lighthouse Lab in Glasgow	Wellcome Sanger Institute for the COVID-19 Genomics UK (COG-UK) Consortium	Harper VanSteenhouse, Yumi Kasai, David Gray, Carol Clugston, Anna Dominiczak and Alex Alderton, Roberto Amato, Sonia Goncalves, Ewan Harrison, David K. Jackson, Ian Johnston, Dominic Kwiatkowski, Cordelia Langford, John Sillitoe on behalf of the Wellcome Sanger Institute COVID-19 Surveillance Team
EPI_ISL_957716	Lighthouse Lab in Cambridge	Wellcome Sanger Institute for the COVID-19 Genomics UK (COG-UK) Consortium	Rob Howes, The Lighthouse Lab in Cambridge and Alex Alderton, Roberto Amato, Sonia Goncalves, Ewan Harrison, David K. Jackson, Ian Johnston, Dominic Kwiatkowski, Cordelia Langford, John Sillitoe on behalf of the Wellcome Sanger Institute COVID-19 Surveillance Team
EPI_ISL_957717, EPI_ISL_957718, EPI_ISL_957720, EPI_ISL_957725, EPI_ISL_957729, EPI_ISL_957730, EPI_ISL_957732, EPI_ISL_957736	Lighthouse Lab in Glasgow	Wellcome Sanger Institute for the COVID-19 Genomics UK (COG-UK) Consortium	Harper VanSteenhouse, Yumi Kasai, David Gray, Carol Clugston, Anna Dominiczak and Alex Alderton, Roberto Amato, Sonia Goncalves, Ewan Harrison, David K. Jackson, Ian Johnston, Dominic Kwiatkowski, Cordelia Langford, John Sillitoe on behalf of the Wellcome Sanger Institute COVID-19 Surveillance Team
EPI_ISL_957737	Lighthouse Lab in Cambridge	Wellcome Sanger Institute for the COVID-19 Genomics UK (COG-UK) Consortium	Rob Howes, The Lighthouse Lab in Cambridge and Alex Alderton, Roberto Amato, Sonia Goncalves, Ewan Harrison, David K. Jackson, Ian Johnston, Dominic Kwiatkowski, Cordelia Langford, John Sillitoe on behalf of the Wellcome Sanger Institute COVID-19 Surveillance Team
EPI_ISL_957743, EPI_ISL_957749, EPI_ISL_957750, EPI_ISL_957756, EPI_ISL_957758, EPI_ISL_957765, EPI_ISL_957768, EPI_ISL_957770, EPI_ISL_957773, EPI_ISL_957774, EPI_ISL_957778			
see above	Lighthouse Lab in Glasgow	Wellcome Sanger Institute for the COVID-19 Genomics UK (COG-UK) Consortium	Harper VanSteenhouse, Yumi Kasai, David Gray, Carol Clugston, Anna Dominiczak and Alex Alderton, Roberto Amato, Sonia Goncalves, Ewan Harrison, David K. Jackson, Ian Johnston, Dominic Kwiatkowski, Cordelia Langford, John Sillitoe on behalf of the Wellcome Sanger Institute COVID-19 Surveillance Team
EPI_ISL_957779, EPI_ISL_957781	Lighthouse Lab in Cambridge	Wellcome Sanger Institute for the COVID-19 Genomics UK (COG-UK) Consortium	Rob Howes, The Lighthouse Lab in Cambridge and Alex Alderton, Roberto Amato, Sonia Goncalves, Ewan Harrison, David K. Jackson, Ian Johnston, Dominic Kwiatkowski, Cordelia Langford, John Sillitoe on behalf of the Wellcome Sanger Institute COVID-19 Surveillance Team
EPI_ISL_957785, EPI_ISL_957792, EPI_ISL_957793, EPI_ISL_957794, EPI_ISL_957796, EPI_ISL_957802, EPI_ISL_957806, EPI_ISL_957814, EPI_ISL_957819	Lighthouse Lab in Glasgow	Wellcome Sanger Institute for the COVID-19 Genomics UK (COG-UK) Consortium	Harper VanSteenhouse, Yumi Kasai, David Gray, Carol Clugston, Anna Dominiczak and Alex Alderton, Roberto Amato, Sonia Goncalves, Ewan Harrison, David K. Jackson, Ian Johnston, Dominic Kwiatkowski, Cordelia Langford, John Sillitoe on behalf of the Wellcome Sanger Institute COVID-19 Surveillance Team
EPI_ISL_957821	Lighthouse Lab in Cambridge	Wellcome Sanger Institute for the COVID-19 Genomics UK (COG-UK) Consortium	Rob Howes, The Lighthouse Lab in Cambridge and Alex Alderton, Roberto Amato, Sonia Goncalves, Ewan Harrison, David K. Jackson, Ian Johnston, Dominic Kwiatkowski, Cordelia Langford, John Sillitoe on behalf of the Wellcome Sanger Institute COVID-19 Surveillance Team
EPI_ISL_957824	Lighthouse Lab in Glasgow	Wellcome Sanger Institute for the COVID-19 Genomics UK (COG-UK) Consortium	Harper VanSteenhouse, Yumi Kasai, David Gray, Carol Clugston, Anna Dominiczak and Alex Alderton, Roberto Amato, Sonia Goncalves, Ewan Harrison, David K. Jackson, Ian Johnston, Dominic Kwiatkowski, Cordelia Langford, John Sillitoe on behalf of the Wellcome Sanger Institute COVID-19 Surveillance Team
EPI_ISL_957834	Lighthouse Lab in Cambridge	Wellcome Sanger Institute for the COVID-19 Genomics UK (COG-UK) Consortium	Rob Howes, The Lighthouse Lab in Cambridge and Alex Alderton, Roberto Amato, Sonia Goncalves, Ewan Harrison, David K. Jackson, Ian Johnston, Dominic Kwiatkowski, Cordelia Langford, John Sillitoe on behalf of the Wellcome Sanger Institute COVID-19 Surveillance Team
EPI_ISL_957836, EPI_ISL_957837, EPI_ISL_957839	Lighthouse Lab in Glasgow	Wellcome Sanger Institute for the COVID-19 Genomics UK (COG-UK) Consortium	Harper VanSteenhouse, Yumi Kasai, David Gray, Carol Clugston, Anna Dominiczak and Alex Alderton, Roberto Amato, Sonia Goncalves, Ewan Harrison, David K. Jackson, Ian Johnston, Dominic Kwiatkowski, Cordelia Langford, John Sillitoe on behalf of the Wellcome Sanger Institute COVID-19 Surveillance Team
EPI_ISL_957840	Lighthouse Lab in Cambridge	Wellcome Sanger Institute for the COVID-19 Genomics UK (COG-UK) Consortium	Rob Howes, The Lighthouse Lab in Cambridge and Alex Alderton, Roberto Amato, Sonia Goncalves, Ewan Harrison, David K. Jackson, Ian Johnston, Dominic Kwiatkowski, Cordelia Langford, John Sillitoe on behalf of the Wellcome Sanger Institute COVID-19 Surveillance Team
EPI_ISL_957845	Lighthouse Lab in Glasgow	Wellcome Sanger Institute for the COVID-19 Genomics UK (COG-UK) Consortium	Harper VanSteenhouse, Yumi Kasai, David Gray, Carol Clugston, Anna Dominiczak and Alex Alderton, Roberto Amato, Sonia Goncalves, Ewan Harrison, David K. Jackson, Ian Johnston, Dominic Kwiatkowski, Cordelia Langford, John Sillitoe on behalf of the Wellcome Sanger Institute COVID-19 Surveillance Team
EPI_ISL_957846	Lighthouse Lab in Cambridge	Wellcome Sanger Institute for the COVID-19 Genomics UK (COG-UK) Consortium	Rob Howes, The Lighthouse Lab in Cambridge and Alex Alderton, Roberto Amato, Sonia Goncalves, Ewan Harrison, David K. Jackson, Ian Johnston, Dominic Kwiatkowski, Cordelia Langford, John Sillitoe on behalf of the Wellcome Sanger Institute COVID-19 Surveillance Team
EPI_ISL_957847, EPI_ISL_957848, EPI_ISL_957851, EPI_ISL_957853, EPI_ISL_957854, EPI_ISL_957860, EPI_ISL_957864, EPI_ISL_957865, EPI_ISL_957871, EPI_ISL_957875, EPI_ISL_957879			
see above	Lighthouse Lab in Glasgow	Wellcome Sanger Institute for the COVID-19 Genomics UK (COG-UK) Consortium	Harper VanSteenhouse, Yumi Kasai, David Gray, Carol Clugston, Anna Dominiczak and Alex Alderton, Roberto Amato, Sonia Goncalves, Ewan Harrison, David K. Jackson, Ian Johnston, Dominic Kwiatkowski, Cordelia Langford, John Sillitoe on behalf of the Wellcome Sanger Institute COVID-19 Surveillance Team
EPI_ISL_957882	Lighthouse Lab in Cambridge	Wellcome Sanger Institute for the COVID-19 Genomics UK (COG-UK) Consortium	Rob Howes, The Lighthouse Lab in Cambridge and Alex Alderton, Roberto Amato, Sonia Goncalves, Ewan Harrison, David K. Jackson, Ian Johnston, Dominic Kwiatkowski, Cordelia Langford, John Sillitoe on behalf of the Wellcome Sanger Institute COVID-19 Surveillance Team
EPI_ISL_957884, EPI_ISL_957889, EPI_ISL_957894	Lighthouse Lab in Glasgow	Wellcome Sanger Institute for the COVID-19 Genomics UK (COG-UK) Consortium	Harper VanSteenhouse, Yumi Kasai, David Gray, Carol Clugston, Anna Dominiczak and Alex Alderton, Roberto Amato, Sonia Goncalves, Ewan Harrison, David K. Jackson, Ian Johnston, Dominic Kwiatkowski, Cordelia Langford, John Sillitoe on behalf of the Wellcome Sanger Institute COVID-19 Surveillance Team
EPI_ISL_957902, EPI_ISL_957908	Lighthouse Lab in Cambridge	Wellcome Sanger Institute for the COVID-19 Genomics UK (COG-UK) Consortium	Rob Howes, The Lighthouse Lab in Cambridge and Alex Alderton, Roberto Amato, Sonia Goncalves, Ewan Harrison, David K. Jackson, Ian Johnston, Dominic Kwiatkowski, Cordelia Langford, John Sillitoe on behalf of the Wellcome Sanger Institute COVID-19 Surveillance Team
EPI_ISL_958157	Lighthouse Lab in Milton Keynes	Wellcome Sanger Institute for the COVID-19 Genomics UK (COG-UK) Consortium	The Lighthouse Lab in Milton Keynes and Alex Alderton, Roberto Amato, Sonia Goncalves, Ewan Harrison, David K. Jackson, Ian Johnston, Dominic Kwiatkowski, Cordelia Langford, John Sillitoe on behalf of the Wellcome Sanger Institute COVID-19 Surveillance Team
EPI_ISL_962975, EPI_ISL_962976, EPI_ISL_962977, EPI_ISL_962978, EPI_ISL_962979, EPI_ISL_962980, EPI_ISL_962981, EPI_ISL_962982, EPI_ISL_962983, EPI_ISL_962984, EPI_ISL_962985, EPI_ISL_962986, EPI_ISL_962987, EPI_ISL_962989, EPI_ISL_962990, EPI_ISL_962991, EPI_ISL_962992, EPI_ISL_962993, EPI_ISL_962994, EPI_ISL_962995, EPI_ISL_962997, EPI_ISL_962998, EPI_ISL_962999, EPI_ISL_963001, EPI_ISL_963002, EPI_ISL_963003, EPI_ISL_963004, EPI_ISL_963005, EPI_ISL_963006, EPI_ISL_963007, EPI_ISL_963008, EPI_ISL_963009, EPI_ISL_963010, EPI_ISL_963011, EPI_ISL_963012, EPI_ISL_963013, EPI_ISL_963014, EPI_ISL_963016, EPI_ISL_963017, EPI_ISL_963018, EPI_ISL_963019, EPI_ISL_963020, EPI_ISL_963021, EPI_ISL_963022, EPI_ISL_963023, EPI_ISL_963024, EPI_ISL_963025, EPI_ISL_963026, EPI_ISL_963027, EPI_ISL_963028, EPI_ISL_963029, EPI_ISL_963030, EPI_ISL_963031, EPI_ISL_963032, EPI_ISL_963033, EPI_ISL_963034, EPI_ISL_963035, EPI_ISL_963036, EPI_ISL_963037, EPI_ISL_963038, EPI_ISL_963039, EPI_ISL_963040, EPI_ISL_963042, EPI_ISL_963043, EPI_ISL_963044, EPI_ISL_963045, EPI_ISL_963046, EPI_ISL_963047, EPI_ISL_963048, EPI_ISL_963049, EPI_ISL_963050, EPI_ISL_963051,			

EPI_ISL_963052, EPI_ISL_963053, EPI_ISL_963054, EPI_ISL_963055, EPI_ISL_963056, EPI_ISL_963057, EPI_ISL_963058, EPI_ISL_963059, EPI_ISL_963060, EPI_ISL_963062, EPI_ISL_963063, EPI_ISL_963064, EPI_ISL_963065, EPI_ISL_963066, EPI_ISL_963067, EPI_ISL_963068, EPI_ISL_963069, EPI_ISL_963070, EPI_ISL_963071, EPI_ISL_963072, EPI_ISL_963073, EPI_ISL_963074, EPI_ISL_963075, EPI_ISL_963076, EPI_ISL_963077, EPI_ISL_963078, EPI_ISL_963079, EPI_ISL_963080, EPI_ISL_963081, EPI_ISL_963082, EPI_ISL_963083, EPI_ISL_963084, EPI_ISL_963085, EPI_ISL_963086, EPI_ISL_963088, EPI_ISL_963089, EPI_ISL_963090, EPI_ISL_963091, EPI_ISL_963092, EPI_ISL_963093, EPI_ISL_963094, EPI_ISL_963095, EPI_ISL_963096, EPI_ISL_963097, EPI_ISL_963098, EPI_ISL_963099, EPI_ISL_963100, EPI_ISL_963101, EPI_ISL_963102, EPI_ISL_963103, EPI_ISL_963104, EPI_ISL_963105, EPI_ISL_963109, EPI_ISL_963110, EPI_ISL_963111, EPI_ISL_963112, EPI_ISL_963113, EPI_ISL_963114, EPI_ISL_963115, EPI_ISL_963116, EPI_ISL_963117, EPI_ISL_963118, EPI_ISL_963119, EPI_ISL_963121, EPI_ISL_963122, EPI_ISL_963123, EPI_ISL_963124, EPI_ISL_963125, EPI_ISL_963126, EPI_ISL_963127, EPI_ISL_963128, EPI_ISL_963129, EPI_ISL_963130, EPI_ISL_963131, EPI_ISL_963132, EPI_ISL_963133, EPI_ISL_963134, EPI_ISL_963135, EPI_ISL_963136, EPI_ISL_963137, EPI_ISL_963138, EPI_ISL_963139, EPI_ISL_963140, EPI_ISL_963141, EPI_ISL_963142, EPI_ISL_963143, EPI_ISL_963144, EPI_ISL_963145, EPI_ISL_963146, EPI_ISL_963147, EPI_ISL_963148, EPI_ISL_963149, EPI_ISL_963150, EPI_ISL_963151, EPI_ISL_963152, EPI_ISL_963153, EPI_ISL_963154, EPI_ISL_963155, EPI_ISL_963156, EPI_ISL_963158, EPI_ISL_963159, EPI_ISL_963160, EPI_ISL_963161, EPI_ISL_963162, EPI_ISL_963163, EPI_ISL_963164, EPI_ISL_963165, EPI_ISL_963166, EPI_ISL_963167, EPI_ISL_963168, EPI_ISL_963169, EPI_ISL_963170, EPI_ISL_963171, EPI_ISL_963172, EPI_ISL_963173, EPI_ISL_963174, EPI_ISL_963175, EPI_ISL_963176, EPI_ISL_963177, EPI_ISL_963178, EPI_ISL_963180, EPI_ISL_963181, EPI_ISL_963182, EPI_ISL_963183, EPI_ISL_963184, EPI_ISL_963185, EPI_ISL_963186, EPI_ISL_963187, EPI_ISL_963188, EPI_ISL_963189, EPI_ISL_963190, EPI_ISL_963191, EPI_ISL_963192, EPI_ISL_963194, EPI_ISL_963195, EPI_ISL_963196, EPI_ISL_963197, EPI_ISL_963198, EPI_ISL_963199, EPI_ISL_963200, EPI_ISL_963201, EPI_ISL_963202, EPI_ISL_963203, EPI_ISL_963204, EPI_ISL_963205, EPI_ISL_963206, EPI_ISL_963207, EPI_ISL_963208, EPI_ISL_963209, EPI_ISL_963210, EPI_ISL_963211, EPI_ISL_963212, EPI_ISL_963213, EPI_ISL_963214, EPI_ISL_963215, EPI_ISL_963216, EPI_ISL_963217, EPI_ISL_963218, EPI_ISL_963219, EPI_ISL_963220, EPI_ISL_963221, EPI_ISL_963222, EPI_ISL_963223, EPI_ISL_963224, EPI_ISL_963226, EPI_ISL_963227, EPI_ISL_963228, EPI_ISL_963229, EPI_ISL_963230, EPI_ISL_963231, EPI_ISL_963232, EPI_ISL_963233, EPI_ISL_963234, EPI_ISL_963235, EPI_ISL_963236, EPI_ISL_963237, EPI_ISL_963238, EPI_ISL_963239, EPI_ISL_963240, EPI_ISL_963241, EPI_ISL_963242, EPI_ISL_963243, EPI_ISL_963244, EPI_ISL_963245, EPI_ISL_963246, EPI_ISL_963247, EPI_ISL_963248, EPI_ISL_963249, EPI_ISL_963250, EPI_ISL_963251, EPI_ISL_963252, EPI_ISL_963253, EPI_ISL_963254, EPI_ISL_963255, EPI_ISL_963256, EPI_ISL_963257, EPI_ISL_963258, EPI_ISL_963259, EPI_ISL_963260, EPI_ISL_963261, EPI_ISL_963262, EPI_ISL_963263, EPI_ISL_963264, EPI_ISL_963265, EPI_ISL_963266, EPI_ISL_963267, EPI_ISL_963268, EPI_ISL_963269, EPI_ISL_963270, EPI_ISL_963272, EPI_ISL_963273, EPI_ISL_963275, EPI_ISL_963276, EPI_ISL_963277, EPI_ISL_963278, EPI_ISL_963279, EPI_ISL_963280, EPI_ISL_963281, EPI_ISL_963282, EPI_ISL_963283, EPI_ISL_963284, EPI_ISL_963285, EPI_ISL_963286, EPI_ISL_963287, EPI_ISL_963288, EPI_ISL_963289, EPI_ISL_963290, EPI_ISL_963291, EPI_ISL_963292, EPI_ISL_963293, EPI_ISL_963294, EPI_ISL_963295, EPI_ISL_963297, EPI_ISL_963298, EPI_ISL_963299, EPI_ISL_963300, EPI_ISL_963301, EPI_ISL_963302, EPI_ISL_963303, EPI_ISL_963304, EPI_ISL_963305, EPI_ISL_963306, EPI_ISL_963308, EPI_ISL_963309, EPI_ISL_963310, EPI_ISL_963311, EPI_ISL_963312, EPI_ISL_963313, EPI_ISL_963314, EPI_ISL_963315, EPI_ISL_963316, EPI_ISL_963317, EPI_ISL_963318, EPI_ISL_963319, EPI_ISL_963320, EPI_ISL_963322, EPI_ISL_963323, EPI_ISL_963986, EPI_ISL_963987, EPI_ISL_963988, EPI_ISL_963989, EPI_ISL_963991, EPI_ISL_963992, EPI_ISL_963994, EPI_ISL_963996, EPI_ISL_963999, EPI_ISL_964000, EPI_ISL_964001, EPI_ISL_964003, EPI_ISL_964004, EPI_ISL_964006, EPI_ISL_964010, EPI_ISL_964012, EPI_ISL_964014, EPI_ISL_964017, EPI_ISL_964018, EPI_ISL_964019, EPI_ISL_964021, EPI_ISL_964024, EPI_ISL_964025, EPI_ISL_964028, EPI_ISL_964032, EPI_ISL_964033, EPI_ISL_964035, EPI_ISL_964036, EPI_ISL_964037, EPI_ISL_964039, EPI_ISL_964040, EPI_ISL_964041, EPI_ISL_964044, EPI_ISL_964045, EPI_ISL_964049, EPI_ISL_964050, EPI_ISL_964051, EPI_ISL_964052, EPI_ISL_964053, EPI_ISL_964054, EPI_ISL_964056, EPI_ISL_964057, EPI_ISL_964058, EPI_ISL_964060, EPI_ISL_964061, EPI_ISL_964062, EPI_ISL_964063, EPI_ISL_964064, EPI_ISL_964065, EPI_ISL_964069, EPI_ISL_964070, EPI_ISL_964072, EPI_ISL_964074, EPI_ISL_964076, EPI_ISL_964078, EPI_ISL_964079, EPI_ISL_964081, EPI_ISL_964082, EPI_ISL_964085, EPI_ISL_964086, EPI_ISL_964090, EPI_ISL_964091, EPI_ISL_964093, EPI_ISL_964094, EPI_ISL_964095, EPI_ISL_964096, EPI_ISL_964099, EPI_ISL_964101, EPI_ISL_964102, EPI_ISL_964103, EPI_ISL_964108, EPI_ISL_964109, EPI_ISL_964110, EPI_ISL_964112, EPI_ISL_964117, EPI_ISL_964121, EPI_ISL_964124, EPI_ISL_964125, EPI_ISL_964136, EPI_ISL_964137, EPI_ISL_964138, EPI_ISL_964139, EPI_ISL_964140, EPI_ISL_964143, EPI_ISL_964144, EPI_ISL_964145, EPI_ISL_964146, EPI_ISL_964147, EPI_ISL_964148, EPI_ISL_964149, EPI_ISL_964151, EPI_ISL_964156, EPI_ISL_964157, EPI_ISL_964160, EPI_ISL_964161, EPI_ISL_964163, EPI_ISL_964164, EPI_ISL_964167, EPI_ISL_964168, EPI_ISL_964169, EPI_ISL_964172, EPI_ISL_964176, EPI_ISL_964180, EPI_ISL_964181, EPI_ISL_964182, EPI_ISL_964185, EPI_ISL_964187, EPI_ISL_964188, EPI_ISL_964189, EPI_ISL_964191, EPI_ISL_964193, EPI_ISL_964195, EPI_ISL_964197, EPI_ISL_964199, EPI_ISL_964204, EPI_ISL_964205, EPI_ISL_964207, EPI_ISL_964209, EPI_ISL_964210, EPI_ISL_964211, EPI_ISL_964214, EPI_ISL_964215, EPI_ISL_964217, EPI_ISL_964218, EPI_ISL_964220, EPI_ISL_964222, EPI_ISL_964224, EPI_ISL_964227, EPI_ISL_964228, EPI_ISL_964233, EPI_ISL_964235, EPI_ISL_964238, EPI_ISL_964239, EPI_ISL_964241, EPI_ISL_964242, EPI_ISL_964244, EPI_ISL_964245, EPI_ISL_964247, EPI_ISL_964249, EPI_ISL_964251, EPI_ISL_964253, EPI_ISL_964258, EPI_ISL_964260, EPI_ISL_964263, EPI_ISL_964264, EPI_ISL_964266, EPI_ISL_964268, EPI_ISL_964271, EPI_ISL_964276, EPI_ISL_964279, EPI_ISL_964282, EPI_ISL_964285, EPI_ISL_964286, EPI_ISL_964290, EPI_ISL_964291, EPI_ISL_964293, EPI_ISL_964294, EPI_ISL_964299, EPI_ISL_964301, EPI_ISL_964304, EPI_ISL_964306, EPI_ISL_964309, EPI_ISL_964310, EPI_ISL_964311, EPI_ISL_964314, EPI_ISL_964320, EPI_ISL_964321, EPI_ISL_964322, EPI_ISL_964323, EPI_ISL_964324, EPI_ISL_964326, EPI_ISL_964330, EPI_ISL_964333, EPI_ISL_964335, EPI_ISL_964336, EPI_ISL_964338, EPI_ISL_964339, EPI_ISL_964342, EPI_ISL_964343, EPI_ISL_964344

see above	Lighthouse Lab in Alderley Park	Wellcome Sanger Institute for the COVID-19 Genomics UK (COG-UK) Consortium	Jacquelyn Wynn, Mairead Hyland, The Lighthouse Lab in Alderley Park and Alex Alderton, Roberto Amato, Sonia Goncalves, Ewan Harrison, David K. Jackson, Ian Johnston, Dominic Kwiatkowski, Cordelia Langford, John Sillitoe on behalf of the Wellcome Sanger Institute COVID-19 Surveillance Team
EPI_ISL_964346, EPI_ISL_964347, EPI_ISL_964350, EPI_ISL_964353, EPI_ISL_964354, EPI_ISL_964356, EPI_ISL_964361, EPI_ISL_964363	Lighthouse Lab in Milton Keynes	Wellcome Sanger Institute for the COVID-19 Genomics UK (COG-UK) Consortium	The Lighthouse Lab in Milton Keynes and Alex Alderton, Roberto Amato, Sonia Goncalves, Ewan Harrison, David K. Jackson, Ian Johnston, Dominic Kwiatkowski, Cordelia Langford, John Sillitoe on behalf of the Wellcome Sanger Institute COVID-19 Surveillance Team
EPI_ISL_964364	Lighthouse Lab in Glasgow	Wellcome Sanger Institute for the COVID-19 Genomics UK (COG-UK) Consortium	Harper VanSteenhouse, Yumi Kasai, David Gray, Carol Clugston, Anna Dominiczak and Alex Alderton, Roberto Amato, Sonia Goncalves, Ewan Harrison, David K. Jackson, Ian Johnston, Dominic Kwiatkowski, Cordelia Langford, John Sillitoe on behalf of the Wellcome Sanger Institute COVID-19 Surveillance Team
EPI_ISL_964365	Lighthouse Lab in Milton Keynes	Wellcome Sanger Institute for the COVID-19 Genomics UK (COG-UK) Consortium	The Lighthouse Lab in Milton Keynes and Alex Alderton, Roberto Amato, Sonia Goncalves, Ewan Harrison, David K. Jackson, Ian Johnston, Dominic Kwiatkowski, Cordelia Langford, John Sillitoe on behalf of the Wellcome Sanger Institute COVID-19 Surveillance Team
EPI_ISL_964367	Lighthouse Lab in Glasgow	Wellcome Sanger Institute for the COVID-19 Genomics UK (COG-UK) Consortium	Harper VanSteenhouse, Yumi Kasai, David Gray, Carol Clugston, Anna Dominiczak and Alex Alderton, Roberto Amato, Sonia Goncalves, Ewan Harrison, David K. Jackson, Ian Johnston, Dominic Kwiatkowski, Cordelia Langford, John Sillitoe on behalf of the Wellcome Sanger Institute COVID-19 Surveillance Team
EPI_ISL_964370	Lighthouse Lab in Milton Keynes	Wellcome Sanger Institute for the COVID-19 Genomics UK (COG-UK) Consortium	The Lighthouse Lab in Milton Keynes and Alex Alderton, Roberto Amato, Sonia Goncalves, Ewan Harrison, David K. Jackson, Ian Johnston, Dominic Kwiatkowski, Cordelia Langford, John Sillitoe on behalf of the Wellcome Sanger Institute COVID-19 Surveillance Team
EPI_ISL_964372	Lighthouse Lab in Glasgow	Wellcome Sanger Institute for the COVID-19 Genomics UK (COG-UK) Consortium	Harper VanSteenhouse, Yumi Kasai, David Gray, Carol Clugston, Anna Dominiczak and Alex Alderton, Roberto Amato, Sonia Goncalves, Ewan Harrison, David K. Jackson, Ian Johnston, Dominic Kwiatkowski, Cordelia Langford, John Sillitoe on behalf of the Wellcome Sanger Institute COVID-19 Surveillance Team
EPI_ISL_964373, EPI_ISL_964374, EPI_ISL_964376	Lighthouse Lab in Milton Keynes	Wellcome Sanger Institute for the COVID-19 Genomics UK (COG-UK) Consortium	The Lighthouse Lab in Milton Keynes and Alex Alderton, Roberto Amato, Sonia Goncalves, Ewan Harrison, David K. Jackson, Ian Johnston, Dominic Kwiatkowski, Cordelia Langford, John Sillitoe on behalf of the Wellcome Sanger Institute COVID-19 Surveillance Team
EPI_ISL_964377	Lighthouse Lab in Glasgow	Wellcome Sanger Institute for the COVID-19 Genomics UK (COG-UK) Consortium	Harper VanSteenhouse, Yumi Kasai, David Gray, Carol Clugston, Anna Dominiczak and Alex Alderton, Roberto Amato, Sonia Goncalves, Ewan Harrison, David K. Jackson, Ian Johnston, Dominic Kwiatkowski, Cordelia Langford, John Sillitoe on behalf of the Wellcome Sanger Institute COVID-19 Surveillance Team
EPI_ISL_964379, EPI_ISL_964381, EPI_ISL_964383, EPI_ISL_964385, EPI_ISL_964386, EPI_ISL_964389	Lighthouse Lab in Milton Keynes	Wellcome Sanger Institute for the COVID-19 Genomics UK (COG-UK) Consortium	The Lighthouse Lab in Milton Keynes and Alex Alderton, Roberto Amato, Sonia Goncalves, Ewan Harrison, David K. Jackson, Ian Johnston, Dominic Kwiatkowski, Cordelia Langford, John Sillitoe on behalf of the Wellcome Sanger Institute COVID-19 Surveillance Team
EPI_ISL_964390	Lighthouse Lab in Glasgow	Wellcome Sanger Institute for the COVID-19 Genomics UK (COG-UK) Consortium	Harper VanSteenhouse, Yumi Kasai, David Gray, Carol Clugston, Anna Dominiczak and Alex Alderton, Roberto Amato, Sonia Goncalves, Ewan Harrison, David K. Jackson, Ian Johnston, Dominic Kwiatkowski, Cordelia Langford, John Sillitoe on behalf of the Wellcome Sanger Institute COVID-19 Surveillance Team
EPI_ISL_964392	Lighthouse Lab in Milton Keynes	Wellcome Sanger Institute for the COVID-19 Genomics UK (COG-UK) Consortium	The Lighthouse Lab in Milton Keynes and Alex Alderton, Roberto Amato, Sonia Goncalves, Ewan Harrison, David K. Jackson, Ian Johnston, Dominic Kwiatkowski, Cordelia Langford, John Sillitoe on behalf of the Wellcome Sanger Institute COVID-19 Surveillance Team
EPI_ISL_964393	Lighthouse Lab in Glasgow	Wellcome Sanger Institute for the COVID-19 Genomics UK (COG-UK) Consortium	Harper VanSteenhouse, Yumi Kasai, David Gray, Carol Clugston, Anna Dominiczak and Alex Alderton, Roberto Amato, Sonia Goncalves, Ewan Harrison, David K. Jackson, Ian Johnston, Dominic Kwiatkowski, Cordelia Langford, John Sillitoe on behalf of the Wellcome Sanger Institute COVID-19 Surveillance Team
EPI_ISL_964394, EPI_ISL_964395, EPI_ISL_964398	Lighthouse Lab in Milton Keynes	Wellcome Sanger Institute for the COVID-19 Genomics UK (COG-UK) Consortium	The Lighthouse Lab in Milton Keynes and Alex Alderton, Roberto Amato, Sonia Goncalves, Ewan Harrison, David K. Jackson, Ian Johnston, Dominic Kwiatkowski, Cordelia Langford, John Sillitoe on behalf of the Wellcome Sanger Institute COVID-19 Surveillance Team
EPI_ISL_964399	Lighthouse Lab in Glasgow	Wellcome Sanger Institute for the COVID-19 Genomics UK (COG-UK) Consortium	Harper VanSteenhouse, Yumi Kasai, David Gray, Carol Clugston, Anna Dominiczak and Alex Alderton, Roberto Amato, Sonia Goncalves, Ewan Harrison, David K. Jackson, Ian Johnston, Dominic Kwiatkowski, Cordelia Langford, John Sillitoe on behalf of the Wellcome Sanger Institute COVID-19 Surveillance Team
EPI_ISL_964400, EPI_ISL_964402, EPI_ISL_964403	Lighthouse Lab in Milton Keynes	Wellcome Sanger Institute for the COVID-19 Genomics UK (COG-UK) Consortium	The Lighthouse Lab in Milton Keynes and Alex Alderton, Roberto Amato, Sonia Goncalves, Ewan Harrison, David K. Jackson, Ian Johnston, Dominic Kwiatkowski, Cordelia Langford, John Sillitoe on behalf of the Wellcome Sanger Institute COVID-19 Surveillance Team
EPI_ISL_964404	Lighthouse Lab in Glasgow	Wellcome Sanger Institute for the COVID-19 Genomics UK (COG-UK) Consortium	Harper VanSteenhouse, Yumi Kasai, David Gray, Carol Clugston, Anna Dominiczak and Alex Alderton, Roberto Amato, Sonia Goncalves, Ewan Harrison, David K. Jackson, Ian Johnston, Dominic Kwiatkowski, Cordelia Langford, John Sillitoe on behalf of the Wellcome Sanger Institute COVID-19 Surveillance Team
EPI_ISL_964405, EPI_ISL_964406, EPI_ISL_964412, EPI_ISL_964414, EPI_ISL_964418, EPI_ISL_964419, EPI_ISL_964420	Lighthouse Lab in Milton Keynes	Wellcome Sanger Institute for the COVID-19 Genomics UK (COG-UK) Consortium	The Lighthouse Lab in Milton Keynes and Alex Alderton, Roberto Amato, Sonia Goncalves, Ewan Harrison, David K. Jackson, Ian Johnston, Dominic Kwiatkowski, Cordelia Langford, John Sillitoe on behalf of the Wellcome Sanger Institute COVID-19 Surveillance Team
EPI_ISL_964421	Lighthouse Lab in Glasgow	Wellcome Sanger Institute for the COVID-19 Genomics UK (COG-UK) Consortium	Harper VanSteenhouse, Yumi Kasai, David Gray, Carol Clugston, Anna Dominiczak and Alex Alderton, Roberto Amato, Sonia Goncalves, Ewan Harrison, David K. Jackson, Ian Johnston, Dominic Kwiatkowski, Cordelia Langford, John Sillitoe on behalf of the Wellcome Sanger Institute COVID-19 Surveillance Team

EPI_ISL_964423, EPI_ISL_964426, EPI_ISL_964428, EPI_ISL_964429, EPI_ISL_964431, EPI_ISL_964432, EPI_ISL_964435	Lighthouse Lab in Milton Keynes	Wellcome Sanger Institute for the COVID-19 Genomics UK (COG-UK) Consortium	The Lighthouse Lab in Milton Keynes and Alex Alderton, Roberto Amato, Sonia Goncalves, Ewan Harrison, David K. Jackson, Ian Johnston, Dominic Kwiatkowski, Cordelia Langford, John Sillitoe on behalf of the Wellcome Sanger Institute COVID-19 Surveillance Team
EPI_ISL_964436	Lighthouse Lab in Glasgow	Wellcome Sanger Institute for the COVID-19 Genomics UK (COG-UK) Consortium	Harper VanSteenhouse, Yumi Kasai, David Gray, Carol Clugston, Anna Dominiczak and Alex Alderton, Roberto Amato, Sonia Goncalves, Ewan Harrison, David K. Jackson, Ian Johnston, Dominic Kwiatkowski, Cordelia Langford, John Sillitoe on behalf of the Wellcome Sanger Institute COVID-19 Surveillance Team
EPI_ISL_964438	Lighthouse Lab in Milton Keynes	Wellcome Sanger Institute for the COVID-19 Genomics UK (COG-UK) Consortium	The Lighthouse Lab in Milton Keynes and Alex Alderton, Roberto Amato, Sonia Goncalves, Ewan Harrison, David K. Jackson, Ian Johnston, Dominic Kwiatkowski, Cordelia Langford, John Sillitoe on behalf of the Wellcome Sanger Institute COVID-19 Surveillance Team
EPI_ISL_964439	Lighthouse Lab in Glasgow	Wellcome Sanger Institute for the COVID-19 Genomics UK (COG-UK) Consortium	Harper VanSteenhouse, Yumi Kasai, David Gray, Carol Clugston, Anna Dominiczak and Alex Alderton, Roberto Amato, Sonia Goncalves, Ewan Harrison, David K. Jackson, Ian Johnston, Dominic Kwiatkowski, Cordelia Langford, John Sillitoe on behalf of the Wellcome Sanger Institute COVID-19 Surveillance Team
EPI_ISL_964443, EPI_ISL_964444, EPI_ISL_964445	Lighthouse Lab in Milton Keynes	Wellcome Sanger Institute for the COVID-19 Genomics UK (COG-UK) Consortium	The Lighthouse Lab in Milton Keynes and Alex Alderton, Roberto Amato, Sonia Goncalves, Ewan Harrison, David K. Jackson, Ian Johnston, Dominic Kwiatkowski, Cordelia Langford, John Sillitoe on behalf of the Wellcome Sanger Institute COVID-19 Surveillance Team
EPI_ISL_964446	Lighthouse Lab in Glasgow	Wellcome Sanger Institute for the COVID-19 Genomics UK (COG-UK) Consortium	Harper VanSteenhouse, Yumi Kasai, David Gray, Carol Clugston, Anna Dominiczak and Alex Alderton, Roberto Amato, Sonia Goncalves, Ewan Harrison, David K. Jackson, Ian Johnston, Dominic Kwiatkowski, Cordelia Langford, John Sillitoe on behalf of the Wellcome Sanger Institute COVID-19 Surveillance Team
EPI_ISL_964447, EPI_ISL_964448, EPI_ISL_964450, EPI_ISL_964453, EPI_ISL_964454, EPI_ISL_964455, EPI_ISL_964459, EPI_ISL_964461, EPI_ISL_964462, EPI_ISL_964463, EPI_ISL_964464			
see above	Lighthouse Lab in Milton Keynes	Wellcome Sanger Institute for the COVID-19 Genomics UK (COG-UK) Consortium	The Lighthouse Lab in Milton Keynes and Alex Alderton, Roberto Amato, Sonia Goncalves, Ewan Harrison, David K. Jackson, Ian Johnston, Dominic Kwiatkowski, Cordelia Langford, John Sillitoe on behalf of the Wellcome Sanger Institute COVID-19 Surveillance Team
EPI_ISL_964465	Lighthouse Lab in Glasgow	Wellcome Sanger Institute for the COVID-19 Genomics UK (COG-UK) Consortium	Harper VanSteenhouse, Yumi Kasai, David Gray, Carol Clugston, Anna Dominiczak and Alex Alderton, Roberto Amato, Sonia Goncalves, Ewan Harrison, David K. Jackson, Ian Johnston, Dominic Kwiatkowski, Cordelia Langford, John Sillitoe on behalf of the Wellcome Sanger Institute COVID-19 Surveillance Team
EPI_ISL_964466, EPI_ISL_964467	Lighthouse Lab in Milton Keynes	Wellcome Sanger Institute for the COVID-19 Genomics UK (COG-UK) Consortium	The Lighthouse Lab in Milton Keynes and Alex Alderton, Roberto Amato, Sonia Goncalves, Ewan Harrison, David K. Jackson, Ian Johnston, Dominic Kwiatkowski, Cordelia Langford, John Sillitoe on behalf of the Wellcome Sanger Institute COVID-19 Surveillance Team
EPI_ISL_964470	Lighthouse Lab in Glasgow	Wellcome Sanger Institute for the COVID-19 Genomics UK (COG-UK) Consortium	Harper VanSteenhouse, Yumi Kasai, David Gray, Carol Clugston, Anna Dominiczak and Alex Alderton, Roberto Amato, Sonia Goncalves, Ewan Harrison, David K. Jackson, Ian Johnston, Dominic Kwiatkowski, Cordelia Langford, John Sillitoe on behalf of the Wellcome Sanger Institute COVID-19 Surveillance Team
EPI_ISL_964478, EPI_ISL_964479	Lighthouse Lab in Milton Keynes	Wellcome Sanger Institute for the COVID-19 Genomics UK (COG-UK) Consortium	The Lighthouse Lab in Milton Keynes and Alex Alderton, Roberto Amato, Sonia Goncalves, Ewan Harrison, David K. Jackson, Ian Johnston, Dominic Kwiatkowski, Cordelia Langford, John Sillitoe on behalf of the Wellcome Sanger Institute COVID-19 Surveillance Team
EPI_ISL_964480	Lighthouse Lab in Glasgow	Wellcome Sanger Institute for the COVID-19 Genomics UK (COG-UK) Consortium	Harper VanSteenhouse, Yumi Kasai, David Gray, Carol Clugston, Anna Dominiczak and Alex Alderton, Roberto Amato, Sonia Goncalves, Ewan Harrison, David K. Jackson, Ian Johnston, Dominic Kwiatkowski, Cordelia Langford, John Sillitoe on behalf of the Wellcome Sanger Institute COVID-19 Surveillance Team
EPI_ISL_964481, EPI_ISL_964485, EPI_ISL_964486	Lighthouse Lab in Milton Keynes	Wellcome Sanger Institute for the COVID-19 Genomics UK (COG-UK) Consortium	The Lighthouse Lab in Milton Keynes and Alex Alderton, Roberto Amato, Sonia Goncalves, Ewan Harrison, David K. Jackson, Ian Johnston, Dominic Kwiatkowski, Cordelia Langford, John Sillitoe on behalf of the Wellcome Sanger Institute COVID-19 Surveillance Team
EPI_ISL_964487	Lighthouse Lab in Glasgow	Wellcome Sanger Institute for the COVID-19 Genomics UK (COG-UK) Consortium	Harper VanSteenhouse, Yumi Kasai, David Gray, Carol Clugston, Anna Dominiczak and Alex Alderton, Roberto Amato, Sonia Goncalves, Ewan Harrison, David K. Jackson, Ian Johnston, Dominic Kwiatkowski, Cordelia Langford, John Sillitoe on behalf of the Wellcome Sanger Institute COVID-19 Surveillance Team
EPI_ISL_964491, EPI_ISL_964492, EPI_ISL_964495, EPI_ISL_964496, EPI_ISL_964497, EPI_ISL_964498, EPI_ISL_964499, EPI_ISL_964502, EPI_ISL_964505, EPI_ISL_964506, EPI_ISL_964507, EPI_ISL_964510			
see above	Lighthouse Lab in Milton Keynes	Wellcome Sanger Institute for the COVID-19 Genomics UK (COG-UK) Consortium	The Lighthouse Lab in Milton Keynes and Alex Alderton, Roberto Amato, Sonia Goncalves, Ewan Harrison, David K. Jackson, Ian Johnston, Dominic Kwiatkowski, Cordelia Langford, John Sillitoe on behalf of the Wellcome Sanger Institute COVID-19 Surveillance Team
EPI_ISL_964512	Lighthouse Lab in Glasgow	Wellcome Sanger Institute for the COVID-19 Genomics UK (COG-UK) Consortium	Harper VanSteenhouse, Yumi Kasai, David Gray, Carol Clugston, Anna Dominiczak and Alex Alderton, Roberto Amato, Sonia Goncalves, Ewan Harrison, David K. Jackson, Ian Johnston, Dominic Kwiatkowski, Cordelia Langford, John Sillitoe on behalf of the Wellcome Sanger Institute COVID-19 Surveillance Team
EPI_ISL_964513	Lighthouse Lab in Milton Keynes	Wellcome Sanger Institute for the COVID-19 Genomics UK (COG-UK) Consortium	The Lighthouse Lab in Milton Keynes and Alex Alderton, Roberto Amato, Sonia Goncalves, Ewan Harrison, David K. Jackson, Ian Johnston, Dominic Kwiatkowski, Cordelia Langford, John Sillitoe on behalf of the Wellcome Sanger Institute COVID-19 Surveillance Team
EPI_ISL_964514	Lighthouse Lab in Glasgow	Wellcome Sanger Institute for the COVID-19 Genomics UK (COG-UK) Consortium	Harper VanSteenhouse, Yumi Kasai, David Gray, Carol Clugston, Anna Dominiczak and Alex Alderton, Roberto Amato, Sonia Goncalves, Ewan Harrison, David K. Jackson, Ian Johnston, Dominic Kwiatkowski, Cordelia Langford, John Sillitoe on behalf of the Wellcome Sanger Institute COVID-19 Surveillance Team
EPI_ISL_964515, EPI_ISL_964517, EPI_ISL_964519, EPI_ISL_964522, EPI_ISL_964524	Lighthouse Lab in Milton Keynes	Wellcome Sanger Institute for the COVID-19 Genomics UK (COG-UK) Consortium	The Lighthouse Lab in Milton Keynes and Alex Alderton, Roberto Amato, Sonia Goncalves, Ewan Harrison, David K. Jackson, Ian Johnston, Dominic Kwiatkowski, Cordelia Langford, John Sillitoe on behalf of the Wellcome Sanger Institute COVID-19 Surveillance Team
EPI_ISL_964528	Lighthouse Lab in Glasgow	Wellcome Sanger Institute for the COVID-19 Genomics UK (COG-UK) Consortium	Harper VanSteenhouse, Yumi Kasai, David Gray, Carol Clugston, Anna Dominiczak and Alex Alderton, Roberto Amato, Sonia Goncalves, Ewan Harrison, David K. Jackson, Ian Johnston, Dominic Kwiatkowski, Cordelia Langford, John Sillitoe on behalf of the Wellcome Sanger Institute COVID-19 Surveillance Team
EPI_ISL_964529, EPI_ISL_964530, EPI_ISL_964531, EPI_ISL_964534, EPI_ISL_964535, EPI_ISL_964536, EPI_ISL_964538	Lighthouse Lab in Milton Keynes	Wellcome Sanger Institute for the COVID-19 Genomics UK (COG-UK) Consortium	The Lighthouse Lab in Milton Keynes and Alex Alderton, Roberto Amato, Sonia Goncalves, Ewan Harrison, David K. Jackson, Ian Johnston, Dominic Kwiatkowski, Cordelia Langford, John Sillitoe on behalf of the Wellcome Sanger Institute COVID-19 Surveillance Team
EPI_ISL_964543, EPI_ISL_964545, EPI_ISL_964546, EPI_ISL_964547, EPI_ISL_964548, EPI_ISL_964549, EPI_ISL_964550, EPI_ISL_964551, EPI_ISL_964552, EPI_ISL_964553, EPI_ISL_964554, EPI_ISL_964555, EPI_ISL_964556, EPI_ISL_964557, EPI_ISL_964558, EPI_ISL_964559, EPI_ISL_964560, EPI_ISL_964561, EPI_ISL_964562, EPI_ISL_964563, EPI_ISL_964564, EPI_ISL_964565, EPI_ISL_964566, EPI_ISL_964567, EPI_ISL_964568, EPI_ISL_964569, EPI_ISL_964570, EPI_ISL_964571, EPI_ISL_964572, EPI_ISL_964573, EPI_ISL_964574, EPI_ISL_964575, EPI_ISL_964576, EPI_ISL_964577, EPI_ISL_964578, EPI_ISL_964579, EPI_ISL_964580, EPI_ISL_964581, EPI_ISL_964582, EPI_ISL_964583, EPI_ISL_964584, EPI_ISL_964585, EPI_ISL_964586, EPI_ISL_964587, EPI_ISL_964588, EPI_ISL_964589, EPI_ISL_964590, EPI_ISL_964591, EPI_ISL_964592, EPI_ISL_964593, EPI_ISL_964594, EPI_ISL_964595, EPI_ISL_964596, EPI_ISL_964597, EPI_ISL_964598, EPI_ISL_964599, EPI_ISL_964600, EPI_ISL_964601, EPI_ISL_964602, EPI_ISL_964603, EPI_ISL_964604, EPI_ISL_964605, EPI_ISL_964606, EPI_ISL_964607, EPI_ISL_964608, EPI_ISL_964609, EPI_ISL_964610, EPI_ISL_964612, EPI_ISL_964613, EPI_ISL_964614, EPI_ISL_964615, EPI_ISL_964616, EPI_ISL_964617, EPI_ISL_964618, EPI_ISL_964619, EPI_ISL_964620, EPI_ISL_964621, EPI_ISL_964622, EPI_ISL_964623, EPI_ISL_964624, EPI_ISL_964625, EPI_ISL_964626, EPI_ISL_964627, EPI_ISL_964628, EPI_ISL_964629, EPI_ISL_964630, EPI_ISL_964631, EPI_ISL_964632, EPI_ISL_964633, EPI_ISL_964634, EPI_ISL_964635, EPI_ISL_964636, EPI_ISL_964637, EPI_ISL_964638, EPI_ISL_964639, EPI_ISL_964640, EPI_ISL_964641, EPI_ISL_964642, EPI_ISL_964643, EPI_ISL_964644, EPI_ISL_964645, EPI_ISL_964647, EPI_ISL_964648, EPI_ISL_964649, EPI_ISL_964650, EPI_ISL_964651, EPI_ISL_964652, EPI_ISL_964653, EPI_ISL_964654, EPI_ISL_964656, EPI_ISL_964657, EPI_ISL_964658, EPI_ISL_964659, EPI_ISL_964660, EPI_ISL_964661, EPI_ISL_964662, EPI_ISL_964663, EPI_ISL_964664, EPI_ISL_964665, EPI_ISL_964666, EPI_ISL_964667, EPI_ISL_964668, EPI_ISL_964669, EPI_ISL_964670, EPI_ISL_964671, EPI_ISL_964672, EPI_ISL_964673, EPI_ISL_964674, EPI_ISL_964675, EPI_ISL_964676, EPI_ISL_964677, EPI_ISL_964678, EPI_ISL_964679, EPI_ISL_964680, EPI_ISL_964681, EPI_ISL_964682, EPI_ISL_964683, EPI_ISL_964684, EPI_ISL_964685, EPI_ISL_964686, EPI_ISL_964687, EPI_ISL_964688, EPI_ISL_964689, EPI_ISL_964690, EPI_ISL_964691, EPI_ISL_964692, EPI_ISL_964693, EPI_ISL_964694, EPI_ISL_964695, EPI_ISL_964696, EPI_ISL_964697, EPI_ISL_964698, EPI_ISL_964699, EPI_ISL_964700, EPI_ISL_964701, EPI_ISL_964702, EPI_ISL_964703, EPI_ISL_964704, EPI_ISL_964705, EPI_ISL_964706, EPI_ISL_964707, EPI_ISL_964708, EPI_ISL_964709, EPI_ISL_964711, EPI_ISL_964712, EPI_ISL_964713, EPI_ISL_964714, EPI_ISL_964715, EPI_ISL_964716, EPI_ISL_964717, EPI_ISL_964718, EPI_ISL_964719, EPI_ISL_964720, EPI_ISL_964721, EPI_ISL_964722, EPI_ISL_964723, EPI_ISL_964724, EPI_ISL_964725, EPI_ISL_964727, EPI_ISL_964728, EPI_ISL_964729, EPI_ISL_964730, EPI_ISL_964731, EPI_ISL_964732, EPI_ISL_964733, EPI_ISL_964734, EPI_ISL_964735, EPI_ISL_964736, EPI_ISL_964737, EPI_ISL_964738, EPI_ISL_964740, EPI_ISL_964741, EPI_ISL_964742, EPI_ISL_964743, EPI_ISL_964744, EPI_ISL_964745, EPI_ISL_964746, EPI_ISL_964747, EPI_ISL_964748, EPI_ISL_964750, EPI_ISL_964751, EPI_ISL_964752, EPI_ISL_964753, EPI_ISL_964754, EPI_ISL_964755, EPI_ISL_964756, EPI_ISL_964757, EPI_ISL_964758, EPI_ISL_964760, EPI_ISL_964761, EPI_ISL_964762, EPI_ISL_964763, EPI_ISL_964764, EPI_ISL_964765, EPI_ISL_964767, EPI_ISL_964768, EPI_ISL_964769, EPI_ISL_964770, EPI_ISL_964771, EPI_ISL_964773, EPI_ISL_964774, EPI_ISL_964775, EPI_ISL_964776, EPI_ISL_964777, EPI_ISL_964779, EPI_ISL_964780, EPI_ISL_964781, EPI_ISL_964782, EPI_ISL_964783, EPI_ISL_964784, EPI_ISL_964785, EPI_ISL_964786, EPI_ISL_964787, EPI_ISL_964788, EPI_ISL_964789, EPI_ISL_964790, EPI_ISL_964791, EPI_ISL_964792, EPI_ISL_964793, EPI_ISL_964794, EPI_ISL_964795, EPI_ISL_964796, EPI_ISL_964797, EPI_ISL_964798, EPI_ISL_964799, EPI_ISL_964800, EPI_ISL_964801, EPI_ISL_964802, EPI_ISL_964803, EPI_ISL_964804, EPI_ISL_964805, EPI_ISL_964806, EPI_ISL_964807, EPI_ISL_964808, EPI_ISL_964809, EPI_ISL_964810, EPI_ISL_964811, EPI_ISL_964812, EPI_ISL_964813, EPI_ISL_964814, EPI_ISL_964815, EPI_ISL_964816, EPI_ISL_964818, EPI_ISL_964819, EPI_ISL_964820, EPI_ISL_964821, EPI_ISL_964822, EPI_ISL_964823, EPI_ISL_964824, EPI_ISL_964825, EPI_ISL_964826, EPI_ISL_964827, EPI_ISL_964828, EPI_ISL_964829, EPI_ISL_964830, EPI_ISL_964831, EPI_ISL_964832, EPI_ISL_964833, EPI_ISL_964834, EPI_ISL_964835, EPI_ISL_964836, EPI_ISL_964838, EPI_ISL_964839, EPI_ISL_964840, EPI_ISL_964841, EPI_ISL_964842, EPI_ISL_964843, EPI_ISL_964844, EPI_ISL_964845, EPI_ISL_964846, EPI_ISL_964847, EPI_ISL_964848, EPI_ISL_964849, EPI_ISL_964850, EPI_ISL_964851, EPI_ISL_964852, EPI_ISL_964853, EPI_ISL_964854, EPI_ISL_964855, EPI_ISL_964856, EPI_ISL_964857, EPI_ISL_964858, EPI_ISL_964859, EPI_ISL_964860, EPI_ISL_964861, EPI_ISL_964862,			

EPI_ISL_964863, EPI_ISL_964864, EPI_ISL_964865, EPI_ISL_964866, EPI_ISL_964868, EPI_ISL_964869, EPI_ISL_964870, EPI_ISL_964871, EPI_ISL_964872, EPI_ISL_964873, EPI_ISL_964874, EPI_ISL_964875, EPI_ISL_969460, EPI_ISL_969461, EPI_ISL_969463, EPI_ISL_969464, EPI_ISL_969465, EPI_ISL_969466, EPI_ISL_969469, EPI_ISL_969472, EPI_ISL_969473, EPI_ISL_969474, EPI_ISL_969475, EPI_ISL_969476, EPI_ISL_969477, EPI_ISL_969479, EPI_ISL_969480, EPI_ISL_969481, EPI_ISL_969482, EPI_ISL_969483, EPI_ISL_969484, EPI_ISL_969485, EPI_ISL_969490, EPI_ISL_969491, EPI_ISL_969493, EPI_ISL_969494, EPI_ISL_969495, EPI_ISL_969496, EPI_ISL_969500, EPI_ISL_969502, EPI_ISL_969503, EPI_ISL_969505, EPI_ISL_969506, EPI_ISL_969507, EPI_ISL_969508, EPI_ISL_969510, EPI_ISL_969512, EPI_ISL_969515, EPI_ISL_969517, EPI_ISL_969518, EPI_ISL_969520, EPI_ISL_969522, EPI_ISL_969523, EPI_ISL_969525, EPI_ISL_969526, EPI_ISL_969527, EPI_ISL_969530, EPI_ISL_969533, EPI_ISL_969534, EPI_ISL_969535, EPI_ISL_969537, EPI_ISL_969538, EPI_ISL_969541, EPI_ISL_969544, EPI_ISL_969545, EPI_ISL_969548, EPI_ISL_969550, EPI_ISL_969551, EPI_ISL_969552, EPI_ISL_969553, EPI_ISL_969556, EPI_ISL_969557, EPI_ISL_969562, EPI_ISL_969563, EPI_ISL_969564, EPI_ISL_969565, EPI_ISL_969568, EPI_ISL_969571, EPI_ISL_969573, EPI_ISL_969574, EPI_ISL_969575, EPI_ISL_969576, EPI_ISL_969578, EPI_ISL_969579, EPI_ISL_969580, EPI_ISL_969583, EPI_ISL_969584, EPI_ISL_969586, EPI_ISL_969587, EPI_ISL_969588, EPI_ISL_969589, EPI_ISL_969592, EPI_ISL_969597, EPI_ISL_969598, EPI_ISL_969599, EPI_ISL_969600, EPI_ISL_969602, EPI_ISL_969603, EPI_ISL_969605, EPI_ISL_969606, EPI_ISL_969607, EPI_ISL_969608, EPI_ISL_969609, EPI_ISL_969610, EPI_ISL_969611, EPI_ISL_969612, EPI_ISL_969616, EPI_ISL_969620, EPI_ISL_969622, EPI_ISL_969623, EPI_ISL_969625, EPI_ISL_969626, EPI_ISL_969628, EPI_ISL_969629, EPI_ISL_969630, EPI_ISL_969631, EPI_ISL_969632, EPI_ISL_969633, EPI_ISL_969634, EPI_ISL_969636, EPI_ISL_969640, EPI_ISL_969642, EPI_ISL_969643, EPI_ISL_969644, EPI_ISL_969645, EPI_ISL_969646, EPI_ISL_969647, EPI_ISL_969649, EPI_ISL_969650, EPI_ISL_969651, EPI_ISL_969653, EPI_ISL_969656, EPI_ISL_969657, EPI_ISL_969658, EPI_ISL_969663, EPI_ISL_969665, EPI_ISL_969667, EPI_ISL_969668, EPI_ISL_969670, EPI_ISL_969671, EPI_ISL_969672, EPI_ISL_969674, EPI_ISL_969675, EPI_ISL_969677, EPI_ISL_969679, EPI_ISL_969683, EPI_ISL_969685, EPI_ISL_969690, EPI_ISL_969695, EPI_ISL_969697, EPI_ISL_969702, EPI_ISL_969706, EPI_ISL_969708, EPI_ISL_969712, EPI_ISL_969714, EPI_ISL_969718, EPI_ISL_969720, EPI_ISL_969722, EPI_ISL_969724, EPI_ISL_969728, EPI_ISL_969732, EPI_ISL_969734, EPI_ISL_969736, EPI_ISL_969739, EPI_ISL_969742, EPI_ISL_969746, EPI_ISL_969752, EPI_ISL_969754, EPI_ISL_969756, EPI_ISL_969760, EPI_ISL_969766, EPI_ISL_969776, EPI_ISL_969782, EPI_ISL_969785, EPI_ISL_969786, EPI_ISL_969791, EPI_ISL_969799, EPI_ISL_969801, EPI_ISL_969808, EPI_ISL_969809, EPI_ISL_969812, EPI_ISL_969813, EPI_ISL_969815, EPI_ISL_969817, EPI_ISL_969819, EPI_ISL_969823, EPI_ISL_969826, EPI_ISL_969828, EPI_ISL_969831, EPI_ISL_969836, EPI_ISL_969838, EPI_ISL_969841, EPI_ISL_969843, EPI_ISL_969846, EPI_ISL_969848, EPI_ISL_969852, EPI_ISL_969853, EPI_ISL_969856, EPI_ISL_969858, EPI_ISL_969859, EPI_ISL_969867, EPI_ISL_969873, EPI_ISL_969877, EPI_ISL_969879, EPI_ISL_969883, EPI_ISL_969886, EPI_ISL_969887, EPI_ISL_969889, EPI_ISL_969895, EPI_ISL_969897, EPI_ISL_969899, EPI_ISL_969901, EPI_ISL_969903, EPI_ISL_969945

see above	Lighthouse Lab in Alderley Park	Wellcome Sanger Institute for the COVID-19 Genomics UK (COG-UK) Consortium	Jacquelyn Wynn, Mairead Hyland, The Lighthouse Lab in Alderley Park and Alex Alderton, Roberto Amato, Sonia Goncalves, Ewan Harrison, David K. Jackson, Ian Johnston, Dominic Kwiatkowski, Cordelia Langford, John Sillitoe on behalf of the Wellcome Sanger Institute COVID-19 Surveillance Team

EPI_ISL_979974, EPI_ISL_979975, EPI_ISL_979976, EPI_ISL_979977, EPI_ISL_979978, EPI_ISL_979979, EPI_ISL_979980, EPI_ISL_979981, EPI_ISL_979983, EPI_ISL_979984, EPI_ISL_979985, EPI_ISL_979986, EPI_ISL_979988, EPI_ISL_979989, EPI_ISL_979990, EPI_ISL_979991, EPI_ISL_979993, EPI_ISL_979994, EPI_ISL_979995, EPI_ISL_979996, EPI_ISL_979998, EPI_ISL_979999, EPI_ISL_980001, EPI_ISL_980002, EPI_ISL_980003, EPI_ISL_980005, EPI_ISL_980006, EPI_ISL_980007, EPI_ISL_980010, EPI_ISL_980011, EPI_ISL_980012, EPI_ISL_980013, EPI_ISL_980014, EPI_ISL_980015, EPI_ISL_980017, EPI_ISL_980018, EPI_ISL_980019, EPI_ISL_980020, EPI_ISL_980021, EPI_ISL_980022, EPI_ISL_980023, EPI_ISL_980024, EPI_ISL_980025, EPI_ISL_980027, EPI_ISL_980028, EPI_ISL_980029, EPI_ISL_980030, EPI_ISL_980031, EPI_ISL_980032, EPI_ISL_980034, EPI_ISL_980035, EPI_ISL_980036, EPI_ISL_980037, EPI_ISL_980038, EPI_ISL_980039, EPI_ISL_980040, EPI_ISL_980041, EPI_ISL_980042, EPI_ISL_980043, EPI_ISL_980044, EPI_ISL_980045, EPI_ISL_980046, EPI_ISL_980047, EPI_ISL_980048, EPI_ISL_980049, EPI_ISL_980050, EPI_ISL_980051, EPI_ISL_980052, EPI_ISL_980053, EPI_ISL_980054, EPI_ISL_980055, EPI_ISL_980056, EPI_ISL_980057, EPI_ISL_980058, EPI_ISL_980059, EPI_ISL_980060, EPI_ISL_980062, EPI_ISL_980065, EPI_ISL_980066, EPI_ISL_980067, EPI_ISL_980068, EPI_ISL_980069, EPI_ISL_980070, EPI_ISL_980071, EPI_ISL_980072, EPI_ISL_980073, EPI_ISL_980074, EPI_ISL_980075, EPI_ISL_980076, EPI_ISL_980077, EPI_ISL_980078, EPI_ISL_980079, EPI_ISL_980080, EPI_ISL_980081, EPI_ISL_980082, EPI_ISL_980084, EPI_ISL_980086, EPI_ISL_980087, EPI_ISL_980088, EPI_ISL_980090, EPI_ISL_980091, EPI_ISL_980092, EPI_ISL_980093, EPI_ISL_980094, EPI_ISL_980095, EPI_ISL_980096, EPI_ISL_980097, EPI_ISL_980098, EPI_ISL_980099, EPI_ISL_980100, EPI_ISL_980101, EPI_ISL_980102, EPI_ISL_980103, EPI_ISL_980104, EPI_ISL_980106, EPI_ISL_980107, EPI_ISL_980108, EPI_ISL_980110, EPI_ISL_980111, EPI_ISL_980112, EPI_ISL_980113, EPI_ISL_980114, EPI_ISL_980115, EPI_ISL_980116, EPI_ISL_980117, EPI_ISL_980118, EPI_ISL_980120, EPI_ISL_980121, EPI_ISL_980122, EPI_ISL_980123, EPI_ISL_980124, EPI_ISL_980125, EPI_ISL_980126, EPI_ISL_980127, EPI_ISL_980128, EPI_ISL_980129, EPI_ISL_980130, EPI_ISL_980132, EPI_ISL_980133, EPI_ISL_980134, EPI_ISL_980135, EPI_ISL_980136, EPI_ISL_980138, EPI_ISL_980139, EPI_ISL_980140, EPI_ISL_980141, EPI_ISL_980142, EPI_ISL_980144, EPI_ISL_980146, EPI_ISL_980147, EPI_ISL_980149, EPI_ISL_980150, EPI_ISL_980151, EPI_ISL_980152, EPI_ISL_980153, EPI_ISL_980156, EPI_ISL_980157, EPI_ISL_980158, EPI_ISL_980159, EPI_ISL_980160, EPI_ISL_980161, EPI_ISL_980163, EPI_ISL_980164, EPI_ISL_980165, EPI_ISL_980166, EPI_ISL_980167, EPI_ISL_980169, EPI_ISL_980170, EPI_ISL_980173, EPI_ISL_980174, EPI_ISL_980175, EPI_ISL_980177, EPI_ISL_980179, EPI_ISL_980180, EPI_ISL_980181, EPI_ISL_980182, EPI_ISL_980184, EPI_ISL_980185, EPI_ISL_980188, EPI_ISL_980189, EPI_ISL_980190, EPI_ISL_980191, EPI_ISL_980192, EPI_ISL_980194, EPI_ISL_980195, EPI_ISL_980196, EPI_ISL_980197, EPI_ISL_980199, EPI_ISL_980200, EPI_ISL_980201, EPI_ISL_980202, EPI_ISL_980203, EPI_ISL_980204, EPI_ISL_980205, EPI_ISL_980206, EPI_ISL_980207, EPI_ISL_980208, EPI_ISL_980209, EPI_ISL_980210, EPI_ISL_980211, EPI_ISL_980212, EPI_ISL_980213, EPI_ISL_980214, EPI_ISL_980215, EPI_ISL_980216, EPI_ISL_980217, EPI_ISL_980218, EPI_ISL_980219, EPI_ISL_980220, EPI_ISL_980221, EPI_ISL_980222, EPI_ISL_980223, EPI_ISL_980224, EPI_ISL_980225, EPI_ISL_980229, EPI_ISL_980230, EPI_ISL_980231, EPI_ISL_980232, EPI_ISL_980234, EPI_ISL_980551, EPI_ISL_980552, EPI_ISL_980553, EPI_ISL_980554, EPI_ISL_980555, EPI_ISL_980556, EPI_ISL_980557, EPI_ISL_980558, EPI_ISL_980559, EPI_ISL_980560, EPI_ISL_980561, EPI_ISL_980562, EPI_ISL_980563, EPI_ISL_980564, EPI_ISL_980565, EPI_ISL_980566, EPI_ISL_980567, EPI_ISL_980569, EPI_ISL_980570, EPI_ISL_980571, EPI_ISL_980572, EPI_ISL_980573, EPI_ISL_980574, EPI_ISL_980575, EPI_ISL_980576, EPI_ISL_980577, EPI_ISL_980578, EPI_ISL_980580, EPI_ISL_980581, EPI_ISL_980582, EPI_ISL_980583, EPI_ISL_980584, EPI_ISL_980585, EPI_ISL_980586, EPI_ISL_980587, EPI_ISL_980588, EPI_ISL_980589, EPI_ISL_980590, EPI_ISL_980591, EPI_ISL_980592, EPI_ISL_980593, EPI_ISL_980594, EPI_ISL_980595, EPI_ISL_980596, EPI_ISL_980597, EPI_ISL_980598, EPI_ISL_980599, EPI_ISL_980600, EPI_ISL_980601, EPI_ISL_980602, EPI_ISL_980603, EPI_ISL_980604, EPI_ISL_980605, EPI_ISL_980606, EPI_ISL_980607, EPI_ISL_980608, EPI_ISL_980609, EPI_ISL_980610, EPI_ISL_980611, EPI_ISL_980613, EPI_ISL_980614, EPI_ISL_980616, EPI_ISL_980617, EPI_ISL_980619, EPI_ISL_980620, EPI_ISL_980621, EPI_ISL_980622, EPI_ISL_980623, EPI_ISL_980624, EPI_ISL_980625, EPI_ISL_980626, EPI_ISL_980627, EPI_ISL_980628, EPI_ISL_980629, EPI_ISL_980630, EPI_ISL_980631, EPI_ISL_980632, EPI_ISL_980633, EPI_ISL_980634, EPI_ISL_980635, EPI_ISL_980636, EPI_ISL_980637, EPI_ISL_980638, EPI_ISL_980639, EPI_ISL_980640, EPI_ISL_980641, EPI_ISL_980642, EPI_ISL_980643, EPI_ISL_980644, EPI_ISL_980645, EPI_ISL_980646, EPI_ISL_980647, EPI_ISL_980649, EPI_ISL_980650, EPI_ISL_980651, EPI_ISL_980652, EPI_ISL_980653, EPI_ISL_980654, EPI_ISL_980655, EPI_ISL_980656, EPI_ISL_980657, EPI_ISL_980658, EPI_ISL_980659, EPI_ISL_980660, EPI_ISL_980661, EPI_ISL_980662, EPI_ISL_980663, EPI_ISL_980664, EPI_ISL_980665, EPI_ISL_980666, EPI_ISL_980667, EPI_ISL_980668, EPI_ISL_980669, EPI_ISL_980670, EPI_ISL_980671, EPI_ISL_980672, EPI_ISL_980673, EPI_ISL_980674, EPI_ISL_980675, EPI_ISL_980676, EPI_ISL_980677, EPI_ISL_980678, EPI_ISL_980679, EPI_ISL_980680, EPI_ISL_980681, EPI_ISL_980682, EPI_ISL_980683, EPI_ISL_980684, EPI_ISL_980685, EPI_ISL_980686, EPI_ISL_980687, EPI_ISL_980688, EPI_ISL_980689, EPI_ISL_980690, EPI_ISL_980691, EPI_ISL_980692, EPI_ISL_980693, EPI_ISL_980694, EPI_ISL_980695, EPI_ISL_980696, EPI_ISL_980697, EPI_ISL_980698, EPI_ISL_980699, EPI_ISL_980700, EPI_ISL_980701, EPI_ISL_980702, EPI_ISL_980703, EPI_ISL_980704, EPI_ISL_980705, EPI_ISL_980706, EPI_ISL_980707, EPI_ISL_980708, EPI_ISL_980709, EPI_ISL_980710, EPI_ISL_980711, EPI_ISL_980712, EPI_ISL_980713, EPI_ISL_980714, EPI_ISL_980715, EPI_ISL_980717, EPI_ISL_980718, EPI_ISL_980719, EPI_ISL_980720, EPI_ISL_980721, EPI_ISL_980722, EPI_ISL_980723, EPI_ISL_980724, EPI_ISL_980725, EPI_ISL_980726, EPI_ISL_980727, EPI_ISL_980728, EPI_ISL_980729, EPI_ISL_980730, EPI_ISL_980732, EPI_ISL_980733, EPI_ISL_980734, EPI_ISL_980735, EPI_ISL_980736, EPI_ISL_980737, EPI_ISL_980738, EPI_ISL_980739, EPI_ISL_980741, EPI_ISL_980742, EPI_ISL_980743, EPI_ISL_980744, EPI_ISL_980745, EPI_ISL_980746, EPI_ISL_980747, EPI_ISL_980748, EPI_ISL_980750, EPI_ISL_980751, EPI_ISL_980752, EPI_ISL_980753, EPI_ISL_980754, EPI_ISL_980755, EPI_ISL_980756, EPI_ISL_980757, EPI_ISL_980758, EPI_ISL_980759, EPI_ISL_980760, EPI_ISL_980761, EPI_ISL_980762, EPI_ISL_980763, EPI_ISL_980765, EPI_ISL_980766, EPI_ISL_980767, EPI_ISL_980768, EPI_ISL_980769, EPI_ISL_980770, EPI_ISL_980771, EPI_ISL_980772, EPI_ISL_980773, EPI_ISL_980774, EPI_ISL_980775, EPI_ISL_980776, EPI_ISL_980777, EPI_ISL_980778, EPI_ISL_980779, EPI_ISL_980780, EPI_ISL_980781, EPI_ISL_980782, EPI_ISL_980783, EPI_ISL_980784, EPI_ISL_980785, EPI_ISL_980786, EPI_ISL_980787, EPI_ISL_980788, EPI_ISL_980789, EPI_ISL_980790, EPI_ISL_980791, EPI_ISL_980792, EPI_ISL_980793, EPI_ISL_980794, EPI_ISL_980795, EPI_ISL_980796, EPI_ISL_980797, EPI_ISL_980798, EPI_ISL_980799, EPI_ISL_980800, EPI_ISL_980801, EPI_ISL_980802, EPI_ISL_980803, EPI_ISL_980804, EPI_ISL_980805, EPI_ISL_980806

see above	Lighthouse Lab in Milton Keynes	Wellcome Sanger Institute for the COVID-19 Genomics UK (COG-UK) Consortium	The Lighthouse Lab in Milton Keynes and Alex Alderton, Roberto Amato, Sonia Goncalves, Ewan Harrison, David K. Jackson, Ian Johnston, Dominic Kwiatkowski, Cordelia Langford, John Sillitoe on behalf of the Wellcome Sanger Institute COVID-19 Surveillance Team

EPI_ISL_985969, EPI_ISL_985977, EPI_ISL_985984, EPI_ISL_985985, EPI_ISL_986002, EPI_ISL_986016, EPI_ISL_986019, EPI_ISL_986035, EPI_ISL_986045, EPI_ISL_986049, EPI_ISL_986061, EPI_ISL_986062, EPI_ISL_986075, EPI_ISL_986083, EPI_ISL_986087, EPI_ISL_986088, EPI_ISL_986090, EPI_ISL_986091, EPI_ISL_986093, EPI_ISL_986096

see above	Lighthouse Lab in Milton Keynes	Wellcome Sanger Institute for the COVID-19 Genomics UK (COG-UK) Consortium	The Lighthouse Lab in Milton Keynes and Alex Alderton, Roberto Amato, Sonia Goncalves, Ewan Harrison, David K. Jackson, Ian Johnston, Dominic Kwiatkowski, Cordelia Langford, John Sillitoe on behalf of the Wellcome Sanger Institute COVID-19 Surveillance Team (http://www.sanger.ac.uk/covid-team)

EPI_ISL_987643, EPI_ISL_987644, EPI_ISL_987645, EPI_ISL_987647, EPI_ISL_987651, EPI_ISL_987652, EPI_ISL_987654, EPI_ISL_987658, EPI_ISL_987661, EPI_ISL_987665, EPI_ISL_987669, EPI_ISL_987671, EPI_ISL_987676, EPI_ISL_987677, EPI_ISL_987678, EPI_ISL_987679, EPI_ISL_987687, EPI_ISL_987692, EPI_ISL_987693, EPI_ISL_987695, EPI_ISL_987697, EPI_ISL_987703, EPI_ISL_987706, EPI_ISL_987708, EPI_ISL_987719, EPI_ISL_987723, EPI_ISL_987724, EPI_ISL_987726, EPI_ISL_987727, EPI_ISL_987734, EPI_ISL_987737, EPI_ISL_987740, EPI_ISL_987747, EPI_ISL_987749, EPI_ISL_987751, EPI_ISL_987752, EPI_ISL_987753, EPI_ISL_987754, EPI_ISL_987756, EPI_ISL_987758, EPI_ISL_987759, EPI_ISL_987760, EPI_ISL_987766, EPI_ISL_987774, EPI_ISL_987776, EPI_ISL_987777, EPI_ISL_987780, EPI_ISL_987781, EPI_ISL_987783, EPI_ISL_987784, EPI_ISL_987787, EPI_ISL_987789, EPI_ISL_987792, EPI_ISL_987793, EPI_ISL_987794, EPI_ISL_987796, EPI_ISL_987797, EPI_ISL_987798, EPI_ISL_987799, EPI_ISL_987800, EPI_ISL_987801, EPI_ISL_987803, EPI_ISL_987805, EPI_ISL_987808, EPI_ISL_987809, EPI_ISL_987810, EPI_ISL_987813, EPI_ISL_987817, EPI_ISL_987820, EPI_ISL_987821, EPI_ISL_987823, EPI_ISL_987825, EPI_ISL_987828, EPI_ISL_987830, EPI_ISL_987831, EPI_ISL_987832, EPI_ISL_987833, EPI_ISL_987834, EPI_ISL_987835, EPI_ISL_987839, EPI_ISL_987840, EPI_ISL_987843, EPI_ISL_987844, EPI_ISL_987847, EPI_ISL_987850, EPI_ISL_987853, EPI_ISL_987856, EPI_ISL_987863, EPI_ISL_987866, EPI_ISL_987867, EPI_ISL_987871, EPI_ISL_987875, EPI_ISL_987877, EPI_ISL_987880, EPI_ISL_987881, EPI_ISL_987882, EPI_ISL_987886, EPI_ISL_987887, EPI_ISL_987892, EPI_ISL_987894, EPI_ISL_987904, EPI_ISL_987906, EPI_ISL_987908, EPI_ISL_987911, EPI_ISL_987916, EPI_ISL_987918, EPI_ISL_987921, EPI_ISL_987922, EPI_ISL_987925, EPI_ISL_987926, EPI_ISL_987928, EPI_ISL_987929, EPI_ISL_987930, EPI_ISL_987931, EPI_ISL_987932, EPI_ISL_987933, EPI_ISL_987934, EPI_ISL_987935, EPI_ISL_987936, EPI_ISL_987937, EPI_ISL_987944, EPI_ISL_987945, EPI_ISL_987948, EPI_ISL_987953, EPI_ISL_987958, EPI_ISL_987960, EPI_ISL_987962, EPI_ISL_987964, EPI_ISL_987976, EPI_ISL_987978, EPI_ISL_987980, EPI_ISL_987982, EPI_ISL_987983, EPI_ISL_987989, EPI_ISL_987993, EPI_ISL_987998, EPI_ISL_987999, EPI_ISL_988000, EPI_ISL_988003, EPI_ISL_988007, EPI_ISL_988009

see above	Lighthouse Lab in Cambridge	Wellcome Sanger Institute for the COVID-19 Genomics UK (COG-UK) Consortium	Rob Howes, The Lighthouse Lab in Cambridge and Alex Alderton, Roberto Amato, Sonia Goncalves, Ewan Harrison, David K. Jackson, Ian Johnston, Dominic Kwiatkowski, Cordelia Langford, John Sillitoe on behalf of the Wellcome Sanger Institute COVID-19 Surveillance Team (http://www.sanger.ac.uk/covid-team)
EPI_ISL_988205	Lighthouse Lab in Milton Keynes	Wellcome Sanger Institute for the COVID-19 Genomics UK (COG-UK) Consortium	The Lighthouse Lab in Milton Keynes and Alex Alderton, Roberto Amato, Sonia Goncalves, Ewan Harrison, David K. Jackson, Ian Johnston, Dominic Kwiatkowski, Cordelia Langford, John Sillitoe on behalf of the Wellcome Sanger Institute COVID-19 Surveillance Team (http://www.sanger.ac.uk/covid-team)
EPI_ISL_988206, EPI_ISL_988211, EPI_ISL_988215	Lighthouse Lab in Cambridge	Wellcome Sanger Institute for the COVID-19 Genomics UK (COG-UK) Consortium	Rob Howes, The Lighthouse Lab in Cambridge and Alex Alderton, Roberto Amato, Sonia Goncalves, Ewan Harrison, David K. Jackson, Ian Johnston, Dominic Kwiatkowski, Cordelia Langford, John Sillitoe on behalf of the Wellcome Sanger Institute COVID-19 Surveillance Team (http://www.sanger.ac.uk/covid-team)
EPI_ISL_988222	Lighthouse Lab in Milton Keynes	Wellcome Sanger Institute for the COVID-19 Genomics UK (COG-UK) Consortium	The Lighthouse Lab in Milton Keynes and Alex Alderton, Roberto Amato, Sonia Goncalves, Ewan Harrison, David K. Jackson, Ian Johnston, Dominic Kwiatkowski, Cordelia Langford, John Sillitoe on behalf of the Wellcome Sanger Institute COVID-19 Surveillance Team (http://www.sanger.ac.uk/covid-team)
EPI_ISL_988227, EPI_ISL_988234	Lighthouse Lab in Cambridge	Wellcome Sanger Institute for the COVID-19 Genomics UK (COG-UK) Consortium	Rob Howes, The Lighthouse Lab in Cambridge and Alex Alderton, Roberto Amato, Sonia Goncalves, Ewan Harrison, David K. Jackson, Ian Johnston, Dominic Kwiatkowski, Cordelia Langford, John Sillitoe on behalf of the Wellcome Sanger Institute COVID-19 Surveillance Team (http://www.sanger.ac.uk/covid-team)
EPI_ISL_988241	Lighthouse Lab in Milton Keynes	Wellcome Sanger Institute for the COVID-19 Genomics UK (COG-UK) Consortium	The Lighthouse Lab in Milton Keynes and Alex Alderton, Roberto Amato, Sonia Goncalves, Ewan Harrison, David K. Jackson, Ian Johnston, Dominic Kwiatkowski, Cordelia Langford, John Sillitoe on behalf of the Wellcome Sanger Institute COVID-19 Surveillance Team (http://www.sanger.ac.uk/covid-team)
EPI_ISL_988244	Lighthouse Lab in Cambridge	Wellcome Sanger Institute for the COVID-19 Genomics UK (COG-UK) Consortium	Rob Howes, The Lighthouse Lab in Cambridge and Alex Alderton, Roberto Amato, Sonia Goncalves, Ewan Harrison, David K. Jackson, Ian Johnston, Dominic Kwiatkowski, Cordelia Langford, John Sillitoe on behalf of the Wellcome Sanger Institute COVID-19 Surveillance Team (http://www.sanger.ac.uk/covid-team)
EPI_ISL_988250	Lighthouse Lab in Milton Keynes	Wellcome Sanger Institute for the COVID-19 Genomics UK (COG-UK) Consortium	The Lighthouse Lab in Milton Keynes and Alex Alderton, Roberto Amato, Sonia Goncalves, Ewan Harrison, David K. Jackson, Ian Johnston, Dominic Kwiatkowski, Cordelia Langford, John Sillitoe on behalf of the Wellcome Sanger Institute COVID-19 Surveillance Team (http://www.sanger.ac.uk/covid-team)
EPI_ISL_988279, EPI_ISL_988284,	Lighthouse Lab in Cambridge	Wellcome Sanger Institute for the COVID-19 Genomics UK	Rob Howes, The Lighthouse Lab in Cambridge and Alex Alderton, Roberto Amato, Sonia Goncalves, Ewan Harrison, David K. Jackson, Ian Johnston,

EPI_ISL_988287, EPI_ISL_988289, EPI_ISL_988297		(COG-UK) Consortium	Dominic Kwiatkowski, Cordelia Langford, John Sillitoe on behalf of the Wellcome Sanger Institute COVID-19 Surveillance Team (http://www.sanger.ac.uk/covid-team)
EPI_ISL_988314	Lighthouse Lab in Milton Keynes	Wellcome Sanger Institute for the COVID-19 Genomics UK (COG-UK) Consortium	The Lighthouse Lab in Milton Keynes and Alex Alderton, Roberto Amato, Sonia Goncalves, Ewan Harrison, David K. Jackson, Ian Johnston, Dominic Kwiatkowski, Cordelia Langford, John Sillitoe on behalf of the Wellcome Sanger Institute COVID-19 Surveillance Team (http://www.sanger.ac.uk/covid-team)
EPI_ISL_988322, EPI_ISL_988328, EPI_ISL_988331, EPI_ISL_988334, EPI_ISL_988345, EPI_ISL_988348, EPI_ISL_988360	Lighthouse Lab in Cambridge	Wellcome Sanger Institute for the COVID-19 Genomics UK (COG-UK) Consortium	Rob Howes, The Lighthouse Lab in Cambridge and Alex Alderton, Roberto Amato, Sonia Goncalves, Ewan Harrison, David K. Jackson, Ian Johnston, Dominic Kwiatkowski, Cordelia Langford, John Sillitoe on behalf of the Wellcome Sanger Institute COVID-19 Surveillance Team (http://www.sanger.ac.uk/covid-team)
EPI_ISL_988363	Lighthouse Lab in Milton Keynes	Wellcome Sanger Institute for the COVID-19 Genomics UK (COG-UK) Consortium	The Lighthouse Lab in Milton Keynes and Alex Alderton, Roberto Amato, Sonia Goncalves, Ewan Harrison, David K. Jackson, Ian Johnston, Dominic Kwiatkowski, Cordelia Langford, John Sillitoe on behalf of the Wellcome Sanger Institute COVID-19 Surveillance Team (http://www.sanger.ac.uk/covid-team)
EPI_ISL_988365, EPI_ISL_988368, EPI_ISL_988371, EPI_ISL_988375, EPI_ISL_988380, EPI_ISL_988382	Lighthouse Lab in Cambridge	Wellcome Sanger Institute for the COVID-19 Genomics UK (COG-UK) Consortium	Rob Howes, The Lighthouse Lab in Cambridge and Alex Alderton, Roberto Amato, Sonia Goncalves, Ewan Harrison, David K. Jackson, Ian Johnston, Dominic Kwiatkowski, Cordelia Langford, John Sillitoe on behalf of the Wellcome Sanger Institute COVID-19 Surveillance Team (http://www.sanger.ac.uk/covid-team)
EPI_ISL_988391	Lighthouse Lab in Milton Keynes	Wellcome Sanger Institute for the COVID-19 Genomics UK (COG-UK) Consortium	The Lighthouse Lab in Milton Keynes and Alex Alderton, Roberto Amato, Sonia Goncalves, Ewan Harrison, David K. Jackson, Ian Johnston, Dominic Kwiatkowski, Cordelia Langford, John Sillitoe on behalf of the Wellcome Sanger Institute COVID-19 Surveillance Team (http://www.sanger.ac.uk/covid-team)
EPI_ISL_988394, EPI_ISL_988400, EPI_ISL_988405, EPI_ISL_988407, EPI_ISL_988432	Lighthouse Lab in Cambridge	Wellcome Sanger Institute for the COVID-19 Genomics UK (COG-UK) Consortium	Rob Howes, The Lighthouse Lab in Cambridge and Alex Alderton, Roberto Amato, Sonia Goncalves, Ewan Harrison, David K. Jackson, Ian Johnston, Dominic Kwiatkowski, Cordelia Langford, John Sillitoe on behalf of the Wellcome Sanger Institute COVID-19 Surveillance Team (http://www.sanger.ac.uk/covid-team)
EPI_ISL_988434	Lighthouse Lab in Milton Keynes	Wellcome Sanger Institute for the COVID-19 Genomics UK (COG-UK) Consortium	The Lighthouse Lab in Milton Keynes and Alex Alderton, Roberto Amato, Sonia Goncalves, Ewan Harrison, David K. Jackson, Ian Johnston, Dominic Kwiatkowski, Cordelia Langford, John Sillitoe on behalf of the Wellcome Sanger Institute COVID-19 Surveillance Team (http://www.sanger.ac.uk/covid-team)
EPI_ISL_988439, EPI_ISL_988440, EPI_ISL_988445, EPI_ISL_988461, EPI_ISL_988466, EPI_ISL_988469, EPI_ISL_988472, EPI_ISL_988474	Lighthouse Lab in Cambridge	Wellcome Sanger Institute for the COVID-19 Genomics UK (COG-UK) Consortium	Rob Howes, The Lighthouse Lab in Cambridge and Alex Alderton, Roberto Amato, Sonia Goncalves, Ewan Harrison, David K. Jackson, Ian Johnston, Dominic Kwiatkowski, Cordelia Langford, John Sillitoe on behalf of the Wellcome Sanger Institute COVID-19 Surveillance Team (http://www.sanger.ac.uk/covid-team)
EPI_ISL_988482, EPI_ISL_988484, EPI_ISL_988487, EPI_ISL_988489, EPI_ISL_988491, EPI_ISL_988492, EPI_ISL_988494, EPI_ISL_988497, EPI_ISL_988498, EPI_ISL_988499, EPI_ISL_988500, EPI_ISL_988501, EPI_ISL_988502, EPI_ISL_988503, EPI_ISL_988505, EPI_ISL_988507, EPI_ISL_988508, EPI_ISL_988509, EPI_ISL_988510, EPI_ISL_988512, EPI_ISL_988514, EPI_ISL_988518, EPI_ISL_988519, EPI_ISL_988523, EPI_ISL_988524, EPI_ISL_988527, EPI_ISL_988528, EPI_ISL_988529, EPI_ISL_988531, EPI_ISL_988532, EPI_ISL_988535, EPI_ISL_988536, EPI_ISL_988540, EPI_ISL_988541, EPI_ISL_988542, EPI_ISL_988543, EPI_ISL_988544, EPI_ISL_988546, EPI_ISL_988547, EPI_ISL_988548, EPI_ISL_988549, EPI_ISL_988550, EPI_ISL_988553, EPI_ISL_988556, EPI_ISL_988557, EPI_ISL_988559, EPI_ISL_988562, EPI_ISL_988563, EPI_ISL_988565, EPI_ISL_988566, EPI_ISL_988567, EPI_ISL_988569, EPI_ISL_988570, EPI_ISL_988571, EPI_ISL_988572, EPI_ISL_988573, EPI_ISL_988574, EPI_ISL_988578, EPI_ISL_988579, EPI_ISL_988581, EPI_ISL_988582, EPI_ISL_988584, EPI_ISL_988588, EPI_ISL_988590, EPI_ISL_988593, EPI_ISL_988594, EPI_ISL_988595, EPI_ISL_988596, EPI_ISL_988597, EPI_ISL_988598, EPI_ISL_988599, EPI_ISL_988602, EPI_ISL_988603, EPI_ISL_988607, EPI_ISL_988608, EPI_ISL_988609, EPI_ISL_988612, EPI_ISL_988613, EPI_ISL_988614, EPI_ISL_988616, EPI_ISL_988617, EPI_ISL_988618, EPI_ISL_988621, EPI_ISL_988622, EPI_ISL_988626, EPI_ISL_988628, EPI_ISL_988629, EPI_ISL_988630, EPI_ISL_988635, EPI_ISL_988638, EPI_ISL_988639, EPI_ISL_988641, EPI_ISL_988642, EPI_ISL_988643, EPI_ISL_988644, EPI_ISL_988645, EPI_ISL_988650, EPI_ISL_988653, EPI_ISL_988654, EPI_ISL_988656, EPI_ISL_988657, EPI_ISL_988658, EPI_ISL_988659, EPI_ISL_988660, EPI_ISL_988662, EPI_ISL_988667, EPI_ISL_988668, EPI_ISL_988669, EPI_ISL_988670, EPI_ISL_988672, EPI_ISL_988673, EPI_ISL_988674, EPI_ISL_988675, EPI_ISL_988676, EPI_ISL_988678, EPI_ISL_988679, EPI_ISL_988680, EPI_ISL_988682, EPI_ISL_988684			
see above	Lighthouse Lab in Milton Keynes	Wellcome Sanger Institute for the COVID-19 Genomics UK (COG-UK) Consortium	The Lighthouse Lab in Milton Keynes and Alex Alderton, Roberto Amato, Sonia Goncalves, Ewan Harrison, David K. Jackson, Ian Johnston, Dominic Kwiatkowski, Cordelia Langford, John Sillitoe on behalf of the Wellcome Sanger Institute COVID-19 Surveillance Team (http://www.sanger.ac.uk/covid-team)
EPI_ISL_988757, EPI_ISL_988798, EPI_ISL_988811	Lighthouse Lab in Alderley Park	Wellcome Sanger Institute for the COVID-19 Genomics UK (COG-UK) Consortium	Jacquelyn Wynn, Mairead Hyland, The Lighthouse Lab in Alderley Park and Alex Alderton, Roberto Amato, Sonia Goncalves, Ewan Harrison, David K. Jackson, Ian Johnston, Dominic Kwiatkowski, Cordelia Langford, John Sillitoe on behalf of the Wellcome Sanger Institute COVID-19 Surveillance Team (http://www.sanger.ac.uk/covid-team)
EPI_ISL_989019, EPI_ISL_989021, EPI_ISL_989029, EPI_ISL_989030, EPI_ISL_989031, EPI_ISL_989032, EPI_ISL_989033, EPI_ISL_989034, EPI_ISL_989035, EPI_ISL_989036	Lighthouse Lab in Alderley Park	Wellcome Sanger Institute for the COVID-19 Genomics UK (COG-UK) Consortium	Jacquelyn Wynn, Mairead Hyland, The Lighthouse Lab in Alderley Park and Alex Alderton, Roberto Amato, Sonia Goncalves, Ewan Harrison, David K. Jackson, Ian Johnston, Dominic Kwiatkowski, Cordelia Langford, John Sillitoe on behalf of the Wellcome Sanger Institute COVID-19 Surveillance Team
EPI_ISL_989037	Lighthouse Lab in Alderley Park	Wellcome Sanger Institute for the COVID-19 Genomics UK (COG-UK) Consortium	Jacquelyn Wynn, Mairead Hyland, The Lighthouse Lab in Alderley Park and Alex Alderton, Roberto Amato, Sonia Goncalves, Ewan Harrison, David K. Jackson, Ian Johnston, Dominic Kwiatkowski, Cordelia Langford, John Sillitoe on behalf of the Wellcome Sanger Institute COVID-19 Surveillance Team (http://www.sanger.ac.uk/covid-team)
EPI_ISL_989038, EPI_ISL_989039, EPI_ISL_989040, EPI_ISL_989041, EPI_ISL_989042, EPI_ISL_989043, EPI_ISL_989044, EPI_ISL_989045, EPI_ISL_989046, EPI_ISL_989047, EPI_ISL_989048, EPI_ISL_989049			
see above	Lighthouse Lab in Alderley Park	Wellcome Sanger Institute for the COVID-19 Genomics UK (COG-UK) Consortium	Jacquelyn Wynn, Mairead Hyland, The Lighthouse Lab in Alderley Park and Alex Alderton, Roberto Amato, Sonia Goncalves, Ewan Harrison, David K. Jackson, Ian Johnston, Dominic Kwiatkowski, Cordelia Langford, John Sillitoe on behalf of the Wellcome Sanger Institute COVID-19 Surveillance Team
EPI_ISL_989050	Lighthouse Lab in Alderley Park	Wellcome Sanger Institute for the COVID-19 Genomics UK (COG-UK) Consortium	Jacquelyn Wynn, Mairead Hyland, The Lighthouse Lab in Alderley Park and Alex Alderton, Roberto Amato, Sonia Goncalves, Ewan Harrison, David K. Jackson, Ian Johnston, Dominic Kwiatkowski, Cordelia Langford, John Sillitoe on behalf of the Wellcome Sanger Institute COVID-19 Surveillance Team (http://www.sanger.ac.uk/covid-team)
EPI_ISL_989051, EPI_ISL_989052, EPI_ISL_989053, EPI_ISL_989054, EPI_ISL_989055, EPI_ISL_989056, EPI_ISL_989057, EPI_ISL_989058	Lighthouse Lab in Alderley Park	Wellcome Sanger Institute for the COVID-19 Genomics UK (COG-UK) Consortium	Jacquelyn Wynn, Mairead Hyland, The Lighthouse Lab in Alderley Park and Alex Alderton, Roberto Amato, Sonia Goncalves, Ewan Harrison, David K. Jackson, Ian Johnston, Dominic Kwiatkowski, Cordelia Langford, John Sillitoe on behalf of the Wellcome Sanger Institute COVID-19 Surveillance Team
EPI_ISL_989059, EPI_ISL_989060	Lighthouse Lab in Alderley Park	Wellcome Sanger Institute for the COVID-19 Genomics UK (COG-UK) Consortium	Jacquelyn Wynn, Mairead Hyland, The Lighthouse Lab in Alderley Park and Alex Alderton, Roberto Amato, Sonia Goncalves, Ewan Harrison, David K. Jackson, Ian Johnston, Dominic Kwiatkowski, Cordelia Langford, John Sillitoe on behalf of the Wellcome Sanger Institute COVID-19 Surveillance Team (http://www.sanger.ac.uk/covid-team)
EPI_ISL_989061, EPI_ISL_989062, EPI_ISL_989063, EPI_ISL_989065, EPI_ISL_989066, EPI_ISL_989067, EPI_ISL_989068	Lighthouse Lab in Alderley Park	Wellcome Sanger Institute for the COVID-19 Genomics UK (COG-UK) Consortium	Jacquelyn Wynn, Mairead Hyland, The Lighthouse Lab in Alderley Park and Alex Alderton, Roberto Amato, Sonia Goncalves, Ewan Harrison, David K. Jackson, Ian Johnston, Dominic Kwiatkowski, Cordelia Langford, John Sillitoe on behalf of the Wellcome Sanger Institute COVID-19 Surveillance Team
EPI_ISL_989069	Lighthouse Lab in Alderley Park	Wellcome Sanger Institute for the COVID-19 Genomics UK (COG-UK) Consortium	Jacquelyn Wynn, Mairead Hyland, The Lighthouse Lab in Alderley Park and Alex Alderton, Roberto Amato, Sonia Goncalves, Ewan Harrison, David K. Jackson, Ian Johnston, Dominic Kwiatkowski, Cordelia Langford, John Sillitoe on behalf of the Wellcome Sanger Institute COVID-19 Surveillance Team (http://www.sanger.ac.uk/covid-team)
EPI_ISL_989070	Lighthouse Lab in Alderley Park	Wellcome Sanger Institute for the COVID-19 Genomics UK (COG-UK) Consortium	Jacquelyn Wynn, Mairead Hyland, The Lighthouse Lab in Alderley Park and Alex Alderton, Roberto Amato, Sonia Goncalves, Ewan Harrison, David K. Jackson, Ian Johnston, Dominic Kwiatkowski, Cordelia Langford, John Sillitoe on behalf of the Wellcome Sanger Institute COVID-19 Surveillance Team
EPI_ISL_989071	Lighthouse Lab in Alderley Park	Wellcome Sanger Institute for the COVID-19 Genomics UK (COG-UK) Consortium	Jacquelyn Wynn, Mairead Hyland, The Lighthouse Lab in Alderley Park and Alex Alderton, Roberto Amato, Sonia Goncalves, Ewan Harrison, David K. Jackson, Ian Johnston, Dominic Kwiatkowski, Cordelia Langford, John Sillitoe on behalf of the Wellcome Sanger Institute COVID-19 Surveillance Team (http://www.sanger.ac.uk/covid-team)
EPI_ISL_989072, EPI_ISL_989073, EPI_ISL_989074	Lighthouse Lab in Alderley Park	Wellcome Sanger Institute for the COVID-19 Genomics UK (COG-UK) Consortium	Jacquelyn Wynn, Mairead Hyland, The Lighthouse Lab in Alderley Park and Alex Alderton, Roberto Amato, Sonia Goncalves, Ewan Harrison, David K. Jackson, Ian Johnston, Dominic Kwiatkowski, Cordelia Langford, John Sillitoe on behalf of the Wellcome Sanger Institute COVID-19 Surveillance Team

EPI_ISL_989075	Lighthouse Lab in Alderley Park	Wellcome Sanger Institute for the COVID-19 Genomics UK (COG-UK) Consortium	Jacquelyn Wynn, Mairead Hyland, The Lighthouse Lab in Alderley Park and Alex Alderton, Roberto Amato, Sonia Goncalves, Ewan Harrison, David K. Jackson, Ian Johnston, Dominic Kwiatkowski, Cordelia Langford, John Sillitoe on behalf of the Wellcome Sanger Institute COVID-19 Surveillance Team (http://www.sanger.ac.uk/covid-team)
EPI_ISL_989076, EPI_ISL_989077, EPI_ISL_989078, EPI_ISL_989079, EPI_ISL_989080, EPI_ISL_989081	Lighthouse Lab in Alderley Park	Wellcome Sanger Institute for the COVID-19 Genomics UK (COG-UK) Consortium	Jacquelyn Wynn, Mairead Hyland, The Lighthouse Lab in Alderley Park and Alex Alderton, Roberto Amato, Sonia Goncalves, Ewan Harrison, David K. Jackson, Ian Johnston, Dominic Kwiatkowski, Cordelia Langford, John Sillitoe on behalf of the Wellcome Sanger Institute COVID-19 Surveillance Team
EPI_ISL_989082	Lighthouse Lab in Alderley Park	Wellcome Sanger Institute for the COVID-19 Genomics UK (COG-UK) Consortium	Jacquelyn Wynn, Mairead Hyland, The Lighthouse Lab in Alderley Park and Alex Alderton, Roberto Amato, Sonia Goncalves, Ewan Harrison, David K. Jackson, Ian Johnston, Dominic Kwiatkowski, Cordelia Langford, John Sillitoe on behalf of the Wellcome Sanger Institute COVID-19 Surveillance Team (http://www.sanger.ac.uk/covid-team)
EPI_ISL_989083, EPI_ISL_989084, EPI_ISL_989085, EPI_ISL_989086, EPI_ISL_989087, EPI_ISL_989088	Lighthouse Lab in Alderley Park	Wellcome Sanger Institute for the COVID-19 Genomics UK (COG-UK) Consortium	Jacquelyn Wynn, Mairead Hyland, The Lighthouse Lab in Alderley Park and Alex Alderton, Roberto Amato, Sonia Goncalves, Ewan Harrison, David K. Jackson, Ian Johnston, Dominic Kwiatkowski, Cordelia Langford, John Sillitoe on behalf of the Wellcome Sanger Institute COVID-19 Surveillance Team
EPI_ISL_989089	Lighthouse Lab in Alderley Park	Wellcome Sanger Institute for the COVID-19 Genomics UK (COG-UK) Consortium	Jacquelyn Wynn, Mairead Hyland, The Lighthouse Lab in Alderley Park and Alex Alderton, Roberto Amato, Sonia Goncalves, Ewan Harrison, David K. Jackson, Ian Johnston, Dominic Kwiatkowski, Cordelia Langford, John Sillitoe on behalf of the Wellcome Sanger Institute COVID-19 Surveillance Team (http://www.sanger.ac.uk/covid-team)
EPI_ISL_989090, EPI_ISL_989091, EPI_ISL_989092, EPI_ISL_989093, EPI_ISL_989094, EPI_ISL_989095, EPI_ISL_989096, EPI_ISL_989097, EPI_ISL_989098, EPI_ISL_989099, EPI_ISL_989100, EPI_ISL_989101, EPI_ISL_989102, EPI_ISL_989103			
see above	Lighthouse Lab in Alderley Park	Wellcome Sanger Institute for the COVID-19 Genomics UK (COG-UK) Consortium	Jacquelyn Wynn, Mairead Hyland, The Lighthouse Lab in Alderley Park and Alex Alderton, Roberto Amato, Sonia Goncalves, Ewan Harrison, David K. Jackson, Ian Johnston, Dominic Kwiatkowski, Cordelia Langford, John Sillitoe on behalf of the Wellcome Sanger Institute COVID-19 Surveillance Team
EPI_ISL_989104, EPI_ISL_989105	Lighthouse Lab in Alderley Park	Wellcome Sanger Institute for the COVID-19 Genomics UK (COG-UK) Consortium	Jacquelyn Wynn, Mairead Hyland, The Lighthouse Lab in Alderley Park and Alex Alderton, Roberto Amato, Sonia Goncalves, Ewan Harrison, David K. Jackson, Ian Johnston, Dominic Kwiatkowski, Cordelia Langford, John Sillitoe on behalf of the Wellcome Sanger Institute COVID-19 Surveillance Team (http://www.sanger.ac.uk/covid-team)
EPI_ISL_989106, EPI_ISL_989107, EPI_ISL_989108, EPI_ISL_989109, EPI_ISL_989110, EPI_ISL_989111, EPI_ISL_989112, EPI_ISL_989113, EPI_ISL_989114, EPI_ISL_989115, EPI_ISL_989116, EPI_ISL_989117, EPI_ISL_989118, EPI_ISL_989119, EPI_ISL_989120, EPI_ISL_989121, EPI_ISL_989122, EPI_ISL_989123, EPI_ISL_989124, EPI_ISL_989125, EPI_ISL_989126, EPI_ISL_989127, EPI_ISL_989128, EPI_ISL_989129, EPI_ISL_989130, EPI_ISL_989131, EPI_ISL_989132, EPI_ISL_989133, EPI_ISL_989134, EPI_ISL_989135, EPI_ISL_989136, EPI_ISL_989137, EPI_ISL_989138, EPI_ISL_989139, EPI_ISL_989140, EPI_ISL_989141, EPI_ISL_989142			
see above	Lighthouse Lab in Alderley Park	Wellcome Sanger Institute for the COVID-19 Genomics UK (COG-UK) Consortium	Jacquelyn Wynn, Mairead Hyland, The Lighthouse Lab in Alderley Park and Alex Alderton, Roberto Amato, Sonia Goncalves, Ewan Harrison, David K. Jackson, Ian Johnston, Dominic Kwiatkowski, Cordelia Langford, John Sillitoe on behalf of the Wellcome Sanger Institute COVID-19 Surveillance Team
EPI_ISL_989143	Lighthouse Lab in Alderley Park	Wellcome Sanger Institute for the COVID-19 Genomics UK (COG-UK) Consortium	Jacquelyn Wynn, Mairead Hyland, The Lighthouse Lab in Alderley Park and Alex Alderton, Roberto Amato, Sonia Goncalves, Ewan Harrison, David K. Jackson, Ian Johnston, Dominic Kwiatkowski, Cordelia Langford, John Sillitoe on behalf of the Wellcome Sanger Institute COVID-19 Surveillance Team (http://www.sanger.ac.uk/covid-team)
EPI_ISL_989144, EPI_ISL_989145, EPI_ISL_989146, EPI_ISL_989147, EPI_ISL_989148, EPI_ISL_989149, EPI_ISL_989150, EPI_ISL_989151	Lighthouse Lab in Alderley Park	Wellcome Sanger Institute for the COVID-19 Genomics UK (COG-UK) Consortium	Jacquelyn Wynn, Mairead Hyland, The Lighthouse Lab in Alderley Park and Alex Alderton, Roberto Amato, Sonia Goncalves, Ewan Harrison, David K. Jackson, Ian Johnston, Dominic Kwiatkowski, Cordelia Langford, John Sillitoe on behalf of the Wellcome Sanger Institute COVID-19 Surveillance Team
EPI_ISL_989152	Lighthouse Lab in Alderley Park	Wellcome Sanger Institute for the COVID-19 Genomics UK (COG-UK) Consortium	Jacquelyn Wynn, Mairead Hyland, The Lighthouse Lab in Alderley Park and Alex Alderton, Roberto Amato, Sonia Goncalves, Ewan Harrison, David K. Jackson, Ian Johnston, Dominic Kwiatkowski, Cordelia Langford, John Sillitoe on behalf of the Wellcome Sanger Institute COVID-19 Surveillance Team (http://www.sanger.ac.uk/covid-team)
EPI_ISL_989153	Lighthouse Lab in Alderley Park	Wellcome Sanger Institute for the COVID-19 Genomics UK (COG-UK) Consortium	Jacquelyn Wynn, Mairead Hyland, The Lighthouse Lab in Alderley Park and Alex Alderton, Roberto Amato, Sonia Goncalves, Ewan Harrison, David K. Jackson, Ian Johnston, Dominic Kwiatkowski, Cordelia Langford, John Sillitoe on behalf of the Wellcome Sanger Institute COVID-19 Surveillance Team
EPI_ISL_989154	Lighthouse Lab in Alderley Park	Wellcome Sanger Institute for the COVID-19 Genomics UK (COG-UK) Consortium	Jacquelyn Wynn, Mairead Hyland, The Lighthouse Lab in Alderley Park and Alex Alderton, Roberto Amato, Sonia Goncalves, Ewan Harrison, David K. Jackson, Ian Johnston, Dominic Kwiatkowski, Cordelia Langford, John Sillitoe on behalf of the Wellcome Sanger Institute COVID-19 Surveillance Team (http://www.sanger.ac.uk/covid-team)
EPI_ISL_989155, EPI_ISL_989156, EPI_ISL_989157, EPI_ISL_989158, EPI_ISL_989159, EPI_ISL_989160, EPI_ISL_989161, EPI_ISL_989162, EPI_ISL_989163, EPI_ISL_989164, EPI_ISL_989165, EPI_ISL_989166, EPI_ISL_989167, EPI_ISL_989168, EPI_ISL_989169, EPI_ISL_989170, EPI_ISL_989171, EPI_ISL_989172, EPI_ISL_989173, EPI_ISL_989174, EPI_ISL_989175, EPI_ISL_989176, EPI_ISL_989177, EPI_ISL_989178, EPI_ISL_989179, EPI_ISL_989180, EPI_ISL_989181, EPI_ISL_989182, EPI_ISL_989183, EPI_ISL_989184			
see above	Lighthouse Lab in Alderley Park	Wellcome Sanger Institute for the COVID-19 Genomics UK (COG-UK) Consortium	Jacquelyn Wynn, Mairead Hyland, The Lighthouse Lab in Alderley Park and Alex Alderton, Roberto Amato, Sonia Goncalves, Ewan Harrison, David K. Jackson, Ian Johnston, Dominic Kwiatkowski, Cordelia Langford, John Sillitoe on behalf of the Wellcome Sanger Institute COVID-19 Surveillance Team
EPI_ISL_989185	Lighthouse Lab in Alderley Park	Wellcome Sanger Institute for the COVID-19 Genomics UK (COG-UK) Consortium	Jacquelyn Wynn, Mairead Hyland, The Lighthouse Lab in Alderley Park and Alex Alderton, Roberto Amato, Sonia Goncalves, Ewan Harrison, David K. Jackson, Ian Johnston, Dominic Kwiatkowski, Cordelia Langford, John Sillitoe on behalf of the Wellcome Sanger Institute COVID-19 Surveillance Team (http://www.sanger.ac.uk/covid-team)
EPI_ISL_989186, EPI_ISL_989187, EPI_ISL_989188, EPI_ISL_989189, EPI_ISL_989190, EPI_ISL_989191, EPI_ISL_989192, EPI_ISL_989193, EPI_ISL_989194, EPI_ISL_989195, EPI_ISL_989196, EPI_ISL_989197, EPI_ISL_989198, EPI_ISL_989199, EPI_ISL_989200, EPI_ISL_989201, EPI_ISL_989202, EPI_ISL_989203, EPI_ISL_989204, EPI_ISL_989205, EPI_ISL_989206, EPI_ISL_989207, EPI_ISL_989208, EPI_ISL_989209, EPI_ISL_989210, EPI_ISL_989211, EPI_ISL_989212, EPI_ISL_989213, EPI_ISL_989214, EPI_ISL_989215, EPI_ISL_989216, EPI_ISL_989217, EPI_ISL_989218, EPI_ISL_989219, EPI_ISL_989220, EPI_ISL_989221, EPI_ISL_989222, EPI_ISL_989223, EPI_ISL_989224, EPI_ISL_989225, EPI_ISL_989226, EPI_ISL_989227, EPI_ISL_989228, EPI_ISL_989230, EPI_ISL_989231, EPI_ISL_989232, EPI_ISL_989233, EPI_ISL_989234, EPI_ISL_989235, EPI_ISL_989236, EPI_ISL_989237, EPI_ISL_989238, EPI_ISL_989239, EPI_ISL_989240, EPI_ISL_989242			
see above	Lighthouse Lab in Alderley Park	Wellcome Sanger Institute for the COVID-19 Genomics UK (COG-UK) Consortium	Jacquelyn Wynn, Mairead Hyland, The Lighthouse Lab in Alderley Park and Alex Alderton, Roberto Amato, Sonia Goncalves, Ewan Harrison, David K. Jackson, Ian Johnston, Dominic Kwiatkowski, Cordelia Langford, John Sillitoe on behalf of the Wellcome Sanger Institute COVID-19 Surveillance Team
EPI_ISL_989243	Lighthouse Lab in Alderley Park	Wellcome Sanger Institute for the COVID-19 Genomics UK (COG-UK) Consortium	Jacquelyn Wynn, Mairead Hyland, The Lighthouse Lab in Alderley Park and Alex Alderton, Roberto Amato, Sonia Goncalves, Ewan Harrison, David K. Jackson, Ian Johnston, Dominic Kwiatkowski, Cordelia Langford, John Sillitoe on behalf of the Wellcome Sanger Institute COVID-19 Surveillance Team (http://www.sanger.ac.uk/covid-team)
EPI_ISL_989244, EPI_ISL_989245, EPI_ISL_989247, EPI_ISL_989248, EPI_ISL_989249, EPI_ISL_989250	Lighthouse Lab in Alderley Park	Wellcome Sanger Institute for the COVID-19 Genomics UK (COG-UK) Consortium	Jacquelyn Wynn, Mairead Hyland, The Lighthouse Lab in Alderley Park and Alex Alderton, Roberto Amato, Sonia Goncalves, Ewan Harrison, David K. Jackson, Ian Johnston, Dominic Kwiatkowski, Cordelia Langford, John Sillitoe on behalf of the Wellcome Sanger Institute COVID-19 Surveillance Team
EPI_ISL_989251	Lighthouse Lab in Alderley Park	Wellcome Sanger Institute for the COVID-19 Genomics UK (COG-UK) Consortium	Jacquelyn Wynn, Mairead Hyland, The Lighthouse Lab in Alderley Park and Alex Alderton, Roberto Amato, Sonia Goncalves, Ewan Harrison, David K. Jackson, Ian Johnston, Dominic Kwiatkowski, Cordelia Langford, John Sillitoe on behalf of the Wellcome Sanger Institute COVID-19 Surveillance Team (http://www.sanger.ac.uk/covid-team)
EPI_ISL_989252, EPI_ISL_989253, EPI_ISL_989254, EPI_ISL_989255, EPI_ISL_989256, EPI_ISL_989257, EPI_ISL_989258, EPI_ISL_989259, EPI_ISL_989260, EPI_ISL_989261, EPI_ISL_989262, EPI_ISL_989263, EPI_ISL_989264, EPI_ISL_989265, EPI_ISL_989266, EPI_ISL_989267, EPI_ISL_989268, EPI_ISL_989269, EPI_ISL_989270, EPI_ISL_989271			
see above	Lighthouse Lab in Alderley Park	Wellcome Sanger Institute for the COVID-19 Genomics UK (COG-UK) Consortium	Jacquelyn Wynn, Mairead Hyland, The Lighthouse Lab in Alderley Park and Alex Alderton, Roberto Amato, Sonia Goncalves, Ewan Harrison, David K. Jackson, Ian Johnston, Dominic Kwiatkowski, Cordelia Langford, John Sillitoe on behalf of the Wellcome Sanger Institute COVID-19 Surveillance Team
EPI_ISL_989272	Lighthouse Lab in Alderley Park	Wellcome Sanger Institute for the COVID-19 Genomics UK (COG-UK) Consortium	Jacquelyn Wynn, Mairead Hyland, The Lighthouse Lab in Alderley Park and Alex Alderton, Roberto Amato, Sonia Goncalves, Ewan Harrison, David K. Jackson, Ian Johnston, Dominic Kwiatkowski, Cordelia Langford, John Sillitoe on behalf of the Wellcome Sanger Institute COVID-19 Surveillance Team (http://www.sanger.ac.uk/covid-team)
EPI_ISL_989273, EPI_ISL_989274, EPI_ISL_989275, EPI_ISL_989276, EPI_ISL_989277, EPI_ISL_989278, EPI_ISL_989279, EPI_ISL_989280, EPI_ISL_989281, EPI_ISL_989282, EPI_ISL_989283, EPI_ISL_989284, EPI_ISL_989285, EPI_ISL_989286, EPI_ISL_989287, EPI_ISL_989288, EPI_ISL_989289, EPI_ISL_989290, EPI_ISL_989291, EPI_ISL_989292, EPI_ISL_989293, EPI_ISL_989294, EPI_ISL_989295, EPI_ISL_989296, EPI_ISL_989297, EPI_ISL_989298, EPI_ISL_989299, EPI_ISL_989300, EPI_ISL_989301, EPI_ISL_989302, EPI_ISL_989303, EPI_ISL_989304, EPI_ISL_989305			
see above	Lighthouse Lab in Alderley Park	Wellcome Sanger Institute for the COVID-19 Genomics UK (COG-UK) Consortium	Jacquelyn Wynn, Mairead Hyland, The Lighthouse Lab in Alderley Park and Alex Alderton, Roberto Amato, Sonia Goncalves, Ewan Harrison, David K. Jackson, Ian Johnston, Dominic Kwiatkowski, Cordelia Langford, John Sillitoe on behalf of the Wellcome Sanger Institute COVID-19 Surveillance Team

EPI_ISL_989306	Lighthouse Lab in Alderley Park	Wellcome Sanger Institute for the COVID-19 Genomics UK (COG-UK) Consortium	Jacquelyn Wynn, Mairead Hyland, The Lighthouse Lab in Alderley Park and Alex Alderton, Roberto Amato, Sonia Goncalves, Ewan Harrison, David K. Jackson, Ian Johnston, Dominic Kwiatkowski, Cordelia Langford, John Sillitoe on behalf of the Wellcome Sanger Institute COVID-19 Surveillance Team (http://www.sanger.ac.uk/covid-team)
EPI_ISL_989307, EPI_ISL_989308, EPI_ISL_989309	Lighthouse Lab in Alderley Park	Wellcome Sanger Institute for the COVID-19 Genomics UK (COG-UK) Consortium	Jacquelyn Wynn, Mairead Hyland, The Lighthouse Lab in Alderley Park and Alex Alderton, Roberto Amato, Sonia Goncalves, Ewan Harrison, David K. Jackson, Ian Johnston, Dominic Kwiatkowski, Cordelia Langford, John Sillitoe on behalf of the Wellcome Sanger Institute COVID-19 Surveillance Team
EPI_ISL_989310	Lighthouse Lab in Alderley Park	Wellcome Sanger Institute for the COVID-19 Genomics UK (COG-UK) Consortium	Jacquelyn Wynn, Mairead Hyland, The Lighthouse Lab in Alderley Park and Alex Alderton, Roberto Amato, Sonia Goncalves, Ewan Harrison, David K. Jackson, Ian Johnston, Dominic Kwiatkowski, Cordelia Langford, John Sillitoe on behalf of the Wellcome Sanger Institute COVID-19 Surveillance Team (http://www.sanger.ac.uk/covid-team)
EPI_ISL_989311, EPI_ISL_989312, EPI_ISL_989313, EPI_ISL_989314, EPI_ISL_989315, EPI_ISL_989316, EPI_ISL_989317, EPI_ISL_989318, EPI_ISL_989319, EPI_ISL_989320	Lighthouse Lab in Alderley Park	Wellcome Sanger Institute for the COVID-19 Genomics UK (COG-UK) Consortium	Jacquelyn Wynn, Mairead Hyland, The Lighthouse Lab in Alderley Park and Alex Alderton, Roberto Amato, Sonia Goncalves, Ewan Harrison, David K. Jackson, Ian Johnston, Dominic Kwiatkowski, Cordelia Langford, John Sillitoe on behalf of the Wellcome Sanger Institute COVID-19 Surveillance Team
EPI_ISL_989321	Lighthouse Lab in Alderley Park	Wellcome Sanger Institute for the COVID-19 Genomics UK (COG-UK) Consortium	Jacquelyn Wynn, Mairead Hyland, The Lighthouse Lab in Alderley Park and Alex Alderton, Roberto Amato, Sonia Goncalves, Ewan Harrison, David K. Jackson, Ian Johnston, Dominic Kwiatkowski, Cordelia Langford, John Sillitoe on behalf of the Wellcome Sanger Institute COVID-19 Surveillance Team (http://www.sanger.ac.uk/covid-team)
EPI_ISL_989322, EPI_ISL_989323, EPI_ISL_989324	Lighthouse Lab in Alderley Park	Wellcome Sanger Institute for the COVID-19 Genomics UK (COG-UK) Consortium	Jacquelyn Wynn, Mairead Hyland, The Lighthouse Lab in Alderley Park and Alex Alderton, Roberto Amato, Sonia Goncalves, Ewan Harrison, David K. Jackson, Ian Johnston, Dominic Kwiatkowski, Cordelia Langford, John Sillitoe on behalf of the Wellcome Sanger Institute COVID-19 Surveillance Team
EPI_ISL_989325	Lighthouse Lab in Alderley Park	Wellcome Sanger Institute for the COVID-19 Genomics UK (COG-UK) Consortium	Jacquelyn Wynn, Mairead Hyland, The Lighthouse Lab in Alderley Park and Alex Alderton, Roberto Amato, Sonia Goncalves, Ewan Harrison, David K. Jackson, Ian Johnston, Dominic Kwiatkowski, Cordelia Langford, John Sillitoe on behalf of the Wellcome Sanger Institute COVID-19 Surveillance Team (http://www.sanger.ac.uk/covid-team)
EPI_ISL_989326, EPI_ISL_989327, EPI_ISL_989328, EPI_ISL_989329, EPI_ISL_989330, EPI_ISL_989331, EPI_ISL_989332, EPI_ISL_989333, EPI_ISL_989334, EPI_ISL_989335, EPI_ISL_989336, EPI_ISL_989337, EPI_ISL_989338, EPI_ISL_989339, EPI_ISL_989340, EPI_ISL_989341, EPI_ISL_989342			
see above	Lighthouse Lab in Alderley Park	Wellcome Sanger Institute for the COVID-19 Genomics UK (COG-UK) Consortium	Jacquelyn Wynn, Mairead Hyland, The Lighthouse Lab in Alderley Park and Alex Alderton, Roberto Amato, Sonia Goncalves, Ewan Harrison, David K. Jackson, Ian Johnston, Dominic Kwiatkowski, Cordelia Langford, John Sillitoe on behalf of the Wellcome Sanger Institute COVID-19 Surveillance Team
EPI_ISL_989343	Lighthouse Lab in Alderley Park	Wellcome Sanger Institute for the COVID-19 Genomics UK (COG-UK) Consortium	Jacquelyn Wynn, Mairead Hyland, The Lighthouse Lab in Alderley Park and Alex Alderton, Roberto Amato, Sonia Goncalves, Ewan Harrison, David K. Jackson, Ian Johnston, Dominic Kwiatkowski, Cordelia Langford, John Sillitoe on behalf of the Wellcome Sanger Institute COVID-19 Surveillance Team (http://www.sanger.ac.uk/covid-team)
EPI_ISL_989344, EPI_ISL_989345, EPI_ISL_989346, EPI_ISL_989347, EPI_ISL_989348, EPI_ISL_989349, EPI_ISL_989350, EPI_ISL_989351, EPI_ISL_989352, EPI_ISL_989353, EPI_ISL_989354, EPI_ISL_989355, EPI_ISL_989356, EPI_ISL_989357			
see above	Lighthouse Lab in Alderley Park	Wellcome Sanger Institute for the COVID-19 Genomics UK (COG-UK) Consortium	Jacquelyn Wynn, Mairead Hyland, The Lighthouse Lab in Alderley Park and Alex Alderton, Roberto Amato, Sonia Goncalves, Ewan Harrison, David K. Jackson, Ian Johnston, Dominic Kwiatkowski, Cordelia Langford, John Sillitoe on behalf of the Wellcome Sanger Institute COVID-19 Surveillance Team
EPI_ISL_989358, EPI_ISL_989359	Lighthouse Lab in Alderley Park	Wellcome Sanger Institute for the COVID-19 Genomics UK (COG-UK) Consortium	Jacquelyn Wynn, Mairead Hyland, The Lighthouse Lab in Alderley Park and Alex Alderton, Roberto Amato, Sonia Goncalves, Ewan Harrison, David K. Jackson, Ian Johnston, Dominic Kwiatkowski, Cordelia Langford, John Sillitoe on behalf of the Wellcome Sanger Institute COVID-19 Surveillance Team (http://www.sanger.ac.uk/covid-team)
EPI_ISL_989360, EPI_ISL_989361	Lighthouse Lab in Alderley Park	Wellcome Sanger Institute for the COVID-19 Genomics UK (COG-UK) Consortium	Jacquelyn Wynn, Mairead Hyland, The Lighthouse Lab in Alderley Park and Alex Alderton, Roberto Amato, Sonia Goncalves, Ewan Harrison, David K. Jackson, Ian Johnston, Dominic Kwiatkowski, Cordelia Langford, John Sillitoe on behalf of the Wellcome Sanger Institute COVID-19 Surveillance Team
EPI_ISL_989362	Lighthouse Lab in Milton Keynes	Wellcome Sanger Institute for the COVID-19 Genomics UK (COG-UK) Consortium	The Lighthouse Lab in Milton Keynes and Alex Alderton, Roberto Amato, Sonia Goncalves, Ewan Harrison, David K. Jackson, Ian Johnston, Dominic Kwiatkowski, Cordelia Langford, John Sillitoe on behalf of the Wellcome Sanger Institute COVID-19 Surveillance Team
EPI_ISL_989444, EPI_ISL_989445, EPI_ISL_989446, EPI_ISL_989447, EPI_ISL_989448, EPI_ISL_989512, EPI_ISL_989513, EPI_ISL_989526	Lighthouse Lab in Alderley Park	Wellcome Sanger Institute for the COVID-19 Genomics UK (COG-UK) Consortium	Jacquelyn Wynn, Mairead Hyland, The Lighthouse Lab in Alderley Park and Alex Alderton, Roberto Amato, Sonia Goncalves, Ewan Harrison, David K. Jackson, Ian Johnston, Dominic Kwiatkowski, Cordelia Langford, John Sillitoe on behalf of the Wellcome Sanger Institute COVID-19 Surveillance Team (http://www.sanger.ac.uk/covid-team)
EPI_ISL_989534, EPI_ISL_989550, EPI_ISL_989553, EPI_ISL_989557, EPI_ISL_989593, EPI_ISL_989599, EPI_ISL_989602, EPI_ISL_989631, EPI_ISL_989669, EPI_ISL_989688, EPI_ISL_989695			
see above	Lighthouse Lab in Glasgow	Wellcome Sanger Institute for the COVID-19 Genomics UK (COG-UK) Consortium	Harper VanSteenhouse, Yumi Kasai, David Gray, Carol Clugston, Anna Dominiczak and Alex Alderton, Roberto Amato, Sonia Goncalves, Ewan Harrison, David K. Jackson, Ian Johnston, Dominic Kwiatkowski, Cordelia Langford, John Sillitoe on behalf of the Wellcome Sanger Institute COVID-19 Surveillance Team (http://www.sanger.ac.uk/covid-team)
EPI_ISL_989696, EPI_ISL_989697, EPI_ISL_989698, EPI_ISL_989700, EPI_ISL_989702, EPI_ISL_989704, EPI_ISL_989705, EPI_ISL_989706, EPI_ISL_989708, EPI_ISL_989709, EPI_ISL_989710, EPI_ISL_989714, EPI_ISL_989715, EPI_ISL_989716, EPI_ISL_989717, EPI_ISL_989720, EPI_ISL_989721, EPI_ISL_989722, EPI_ISL_989723, EPI_ISL_989724, EPI_ISL_989725, EPI_ISL_989726, EPI_ISL_989727, EPI_ISL_989728, EPI_ISL_989729, EPI_ISL_989730, EPI_ISL_989731, EPI_ISL_989732, EPI_ISL_989734, EPI_ISL_989738, EPI_ISL_989739, EPI_ISL_989740, EPI_ISL_989744, EPI_ISL_989745, EPI_ISL_989746, EPI_ISL_989749, EPI_ISL_989754, EPI_ISL_989756, EPI_ISL_989757			
see above	Lighthouse Lab in Alderley Park	Wellcome Sanger Institute for the COVID-19 Genomics UK (COG-UK) Consortium	Jacquelyn Wynn, Mairead Hyland, The Lighthouse Lab in Alderley Park and Alex Alderton, Roberto Amato, Sonia Goncalves, Ewan Harrison, David K. Jackson, Ian Johnston, Dominic Kwiatkowski, Cordelia Langford, John Sillitoe on behalf of the Wellcome Sanger Institute COVID-19 Surveillance Team (http://www.sanger.ac.uk/covid-team)
EPI_ISL_989796, EPI_ISL_989797, EPI_ISL_989800, EPI_ISL_989801, EPI_ISL_989802, EPI_ISL_989803, EPI_ISL_989808, EPI_ISL_989809, EPI_ISL_989810, EPI_ISL_989811, EPI_ISL_989812, EPI_ISL_989813, EPI_ISL_989814, EPI_ISL_989816, EPI_ISL_989823, EPI_ISL_989825, EPI_ISL_989827, EPI_ISL_989828, EPI_ISL_989829, EPI_ISL_989830, EPI_ISL_989832, EPI_ISL_989833, EPI_ISL_989834, EPI_ISL_989835, EPI_ISL_989836, EPI_ISL_989837, EPI_ISL_989838, EPI_ISL_989839, EPI_ISL_989840, EPI_ISL_989841, EPI_ISL_989842, EPI_ISL_989843, EPI_ISL_989845, EPI_ISL_989846, EPI_ISL_989847, EPI_ISL_989848, EPI_ISL_989849, EPI_ISL_989850, EPI_ISL_989851, EPI_ISL_989852, EPI_ISL_989853, EPI_ISL_989854, EPI_ISL_989855			
see above	Lighthouse Lab in Glasgow	Wellcome Sanger Institute for the COVID-19 Genomics UK (COG-UK) Consortium	Harper VanSteenhouse, Yumi Kasai, David Gray, Carol Clugston, Anna Dominiczak and Alex Alderton, Roberto Amato, Sonia Goncalves, Ewan Harrison, David K. Jackson, Ian Johnston, Dominic Kwiatkowski, Cordelia Langford, John Sillitoe on behalf of the Wellcome Sanger Institute COVID-19 Surveillance Team (http://www.sanger.ac.uk/covid-team)
EPI_ISL_996444, EPI_ISL_996445, EPI_ISL_996446, EPI_ISL_996452, EPI_ISL_996457, EPI_ISL_996468, EPI_ISL_996470	University of Birmingham	COVID-19 Genomics UK (COG-UK) Consortium	Institute of Microbiology, University of Birmingham: Claire McMurray, Joanne Stockton, Samuel Nicholls, Radoslaw Poplawski, Will Rowe, Josh Quick, Nicholas Loman. University of Birmingham Testing Laboratory: Celina M Whalley, Andrew Bosworth, Charlotte Poxon, Kasun Wanigasooriya, Oliver Pickles, Mike Kidd, Alex Richter, Andrew D Beggs PHE Heartlands Lab: Husam Osman, Andrew Bosworth. Queen Elizabeth Hospital: Anna Casey
EPI_ISL_996480, EPI_ISL_996481, EPI_ISL_996538, EPI_ISL_996539, EPI_ISL_996540, EPI_ISL_996541, EPI_ISL_996542, EPI_ISL_996547, EPI_ISL_996550, EPI_ISL_996565, EPI_ISL_996568			
see above	University of Exeter	COVID-19 Genomics UK (COG-UK) Consortium	Ben Temperton,Aaron Jeffries,Michelle Michelsen,Joanna Warwick-Dugdale,Audrey Farbos,Robyn Manley,Stephen Michell,Jane Masoli
EPI_ISL_996604, EPI_ISL_996605, EPI_ISL_996606, EPI_ISL_996607, EPI_ISL_996608, EPI_ISL_996609, EPI_ISL_996610, EPI_ISL_996611, EPI_ISL_996612, EPI_ISL_996613, EPI_ISL_996614, EPI_ISL_996615, EPI_ISL_996616, EPI_ISL_996617, EPI_ISL_996618, EPI_ISL_996619, EPI_ISL_996620, EPI_ISL_996621, EPI_ISL_996622, EPI_ISL_996623, EPI_ISL_996624, EPI_ISL_996625, EPI_ISL_996626, EPI_ISL_996628, EPI_ISL_996629, EPI_ISL_996631, EPI_ISL_996644, EPI_ISL_996645, EPI_ISL_996646, EPI_ISL_996652, EPI_ISL_996654, EPI_ISL_996657, EPI_ISL_996668, EPI_ISL_996669			
see above	Department of Pathology, University of Cambridge	COVID-19 Genomics UK (COG-UK) Consortium	Aminu S. Jahun, Yasmin Chaudhry, Iliana Georgana, Myra Hosmillo, Rhys Izuagbe, William L. Hamilton, Martin D. Curran, Surendra Parmar, Ian Goodfellow
EPI_ISL_997062, EPI_ISL_997063	West of Scotland Specialist Virology Centre, NHSGGC / MRC-University of Glasgow Centre for Virus Research	COVID-19 Genomics UK (COG-UK) Consortium	Ana da Silva Filipe, Natasha Johnson, Kathy Smollett, Daniel Mair, Stephen Carmichael, Alice Broos, Lily Tong, Jenna Nichols, Kyriaki Nomikou; Sarah McDonald; Richard Orton, Joseph Hughes, Sreenu Vattipally, David L Robertson; Alasdair MacLean, Rory Gunson; Sharif Shaaban, Matthew Holden; Rachel Blacow, Guy Mollett, Kathy Li, James Shepherd, Antonia Ho, Emma Thomson
EPI_ISL_997109, EPI_ISL_997110, EPI_ISL_997111, EPI_ISL_997127	Virology Department, Royal Infirmary of Edinburgh, NHS Lothian / School of Biological Sciences, University of Edinburgh	COVID-19 Genomics UK (COG-UK) Consortium	McHugh M, Dewar R, Cotton S, Rooke S, O'Toole Á, Scher E, Hill V, McCrone JT, Colquhoun R, Yu X, Jackson B, Rambaut A, Templeton K

EPI_ISL_997418, EPI_ISL_997422, EPI_ISL_997423, EPI_ISL_997424, EPI_ISL_997425, EPI_ISL_997433, EPI_ISL_997443, EPI_ISL_997444, EPI_ISL_997445, EPI_ISL_997446, EPI_ISL_997447, EPI_ISL_997463, EPI_ISL_997464, EPI_ISL_997465, EPI_ISL_997466, EPI_ISL_997483, EPI_ISL_997484, EPI_ISL_997485, EPI_ISL_997488, EPI_ISL_997490, EPI_ISL_997494, EPI_ISL_997495, EPI_ISL_997506, EPI_ISL_997508			
see above	Liverpool Clinical Laboratories	COVID-19 Genomics UK (COG-UK) Consortium	Sam Haldenby, Anita Lucaci, Steve Paterson, Julian Hiscox, Alistair Darby, M Almsaud, A Alrezaihi, Muhannad Alruwaili, Stuart D Armstrong, Jones Benjamin, Eleanor G Bentley, Anu Chawla, Jordan J Clark, Angela Cowell, Richard Eccles, Isabel García-Dorival, Matthew Gemmell, Alessandro Gerada, PKF Gilmore, Richard Gregory, Ximeng Han, Catherine Hartley, Margaret Hughes, Miren Iturriza-Gomara, James Johnson, L Luu, Jenifer Manson, Charlotte Nelson, Elaine O'Toole, Cassie Olateju, Rebekah Penrice-Randal , Lucille Rainbow, N.P Randle, Trevor Ian Robinson, Parul Sharma, Ghada T Shawli, James P Stewart, Neil Swainston, Ecaterina Vamos, Joanne Watts, Mark Whitehead
EPI_ISL_997661, EPI_ISL_997668	Barts Health NHS Trust	COVID-19 Genomics UK (COG-UK) Consortium	CUTINO-MOGUEL, Maria-Teresa; HARRINGTON, David; OWOYEMI, Dola; KULASEGARAN-SHYLINI, Raghavendran; BROAD, Claire; KELE, Beatrix
EPI_ISL_997726, EPI_ISL_997834, EPI_ISL_997890, EPI_ISL_997892, EPI_ISL_997893, EPI_ISL_997894, EPI_ISL_997895, EPI_ISL_997896, EPI_ISL_997905, EPI_ISL_997906, EPI_ISL_997907, EPI_ISL_997918, EPI_ISL_997929, EPI_ISL_997931, EPI_ISL_997932, EPI_ISL_997934, EPI_ISL_997938, EPI_ISL_997939, EPI_ISL_997940, EPI_ISL_997951			
see above	University College London, Great Ormond Street Hospital for Children NHS Foundation Trust, Imperial College Healthcare NHS Trust	COVID-19 Genomics UK (COG-UK) Consortium	Sergi Castellano, Rachel Williams, Mark Kristiansen, Paola Resende Silva, Sunando Roy, Tony Brooks, Helena Tutill, Paola Niola, Patricia Dyal, Charlotte Williams, Leysa Forrest, Yasmin Panchbhaya, Jacqueline Findlay, Samuel Weeks, Julianne Brown, Kathryn Harris, Paul Randell, James Price, Alison Holmes, Judith Breuer
EPI_ISL_998099, EPI_ISL_998100, EPI_ISL_998101, EPI_ISL_998102, EPI_ISL_998103, EPI_ISL_998104, EPI_ISL_998105, EPI_ISL_998106, EPI_ISL_998107, EPI_ISL_998108, EPI_ISL_998109, EPI_ISL_998110, EPI_ISL_998111, EPI_ISL_998112, EPI_ISL_998113, EPI_ISL_998114, EPI_ISL_998115, EPI_ISL_998116, EPI_ISL_998117, EPI_ISL_998118, EPI_ISL_998119, EPI_ISL_998120, EPI_ISL_998121, EPI_ISL_998123, EPI_ISL_998124, EPI_ISL_998125, EPI_ISL_998126, EPI_ISL_998127, EPI_ISL_998128, EPI_ISL_998129, EPI_ISL_998130, EPI_ISL_998131, EPI_ISL_998132, EPI_ISL_998133, EPI_ISL_998134, EPI_ISL_998135			
see above	Virology Department, Sheffield Teaching Hospitals NHS Foundation Trust/Department of Infection, Immunity and Cardiovascular Disease, The Medical School, University of Sheffield	COVID-19 Genomics UK (COG-UK) Consortium	Thushan de Silva, Matthew Parker, Nikki Smith, Adri Angyal, Rebecca Brown, Luke Green, Rachel Tucker, Paul Parsons, Danielle Groves, Katie Johnson, Laura Carrilero, Alex Keeley, Dave Partridge, Matthew Wyles, Benjamin Lindsey, Mehmet Yavuz, Mohammad Raza, Cariad Evans
EPI_ISL_998234	Regional Virus Laboratory, Belfast Health and Social Care Trust	COVID-19 Genomics UK (COG-UK) Consortium	Conall McCaughey, James McKenna, Tanya Curran, Susan Feeney, Alison Watt, Ciara Cox, Mairead Connor, Zoltan Molnar, David Simpson, Derek Fairley
EPI_ISL_998511, EPI_ISL_998512, EPI_ISL_998513, EPI_ISL_998572, EPI_ISL_998573, EPI_ISL_998574, EPI_ISL_998575	Northumbria University / South Tees Hospitals NHS Foundation Trust / North Cumbria Integrated Care NHS Foundation Trust / North Tees and Hartlepool NHS Foundation Trust / Newcastle Hospitals NHS Foundation Trust	COVID-19 Genomics UK (COG-UK) Consortium	Darren L Smith,Andrew Nelson,Matthew Bashton,Greg R Young,Joshua Loh,John Allan,Mohammad A Tariq,Giles S Holt,Gary Black,Wen C Yew,Lynn Dover,Paul Baker,Steve Liggett,Sarah Essex,Jane Greenaway,Debra Padgett,Clive Graham,Garren Scott,Edward Barton,Emma Swindells,Brendan Payne,Jennifer Collins,Yusri Taha,Gary Eltringham
EPI_ISL_998854	Quadram Institute Bioscience	COVID-19 Genomics UK (COG-UK) Consortium	Dave J. Baker, Gemma L. Kay, Alp Aydin, Thanh Le-Viet, Steven Rudder, Ana P. Tedim, Anastasia Kolyva, Maria Diaz, Leonardo de Oliveira Martins, Nabil-Fareed Alikhan, Lizzie Meadows, Rachael Stanley, Ngozi Elumogo, Muhammed Yasir, Nicholas M. Thomson, Alexander J Trotter, Rachel Gilroy, Samuel Bloomfield, Claire Stuart, Andrew Bell, Reenesh Prakash, Samir Dervisevic, Alison E. Mather, John Wain, Mark Webber, Andrew J. Page, Justin O'Grady
EPI_ISL_999518, EPI_ISL_999519, EPI_ISL_999520, EPI_ISL_999521, EPI_ISL_999530, EPI_ISL_999540, EPI_ISL_999546, EPI_ISL_999552, EPI_ISL_999561, EPI_ISL_999565, EPI_ISL_999566, EPI_ISL_999567, EPI_ISL_999569, EPI_ISL_999580, EPI_ISL_999582, EPI_ISL_999583, EPI_ISL_999588, EPI_ISL_999589, EPI_ISL_999590, EPI_ISL_999591, EPI_ISL_999592, EPI_ISL_999593, EPI_ISL_999609, EPI_ISL_999618, EPI_ISL_999623, EPI_ISL_999630, EPI_ISL_999634, EPI_ISL_999650, EPI_ISL_999655, EPI_ISL_999656, EPI_ISL_999735, EPI_ISL_999741, EPI_ISL_999742, EPI_ISL_999747, EPI_ISL_999750, EPI_ISL_999752, EPI_ISL_999753, EPI_ISL_999755, EPI_ISL_999756, EPI_ISL_999757, EPI_ISL_999758, EPI_ISL_999762, EPI_ISL_999767, EPI_ISL_999772, EPI_ISL_999774, EPI_ISL_999775, EPI_ISL_999776, EPI_ISL_999782, EPI_ISL_999783, EPI_ISL_999807, EPI_ISL_999818, EPI_ISL_999819, EPI_ISL_999832, EPI_ISL_999847, EPI_ISL_999848, EPI_ISL_999852, EPI_ISL_999853, EPI_ISL_999859			
see above	Originating lab: Wales Specialist Virology Centre Sequencing lab: Pathogen Genomics Unit	Public Health Wales Microbiology Cardiff Wales Specialist Virology Centre	Catherine Moore, Johnathan Evans, Laura Gifford, Malorie Perry, Simon Cottrell, Angela Marchbank, Alec Birchley, Alexander Adams, Amy Gaskin, Bree Gatica-Wilcox, Jason Coombes, Joel Southgate, Lauren Gilbert, Lee Graham, Nicole Pacchiarini, Sara Kumziene-Summerhayes, Sarah Taylor, Sophie Jones, Sara Rey, Matthew Bull, Joanne Watkins, Sally Corden, Tom Connor
EPI_ISL_999969	Wales Specialist Virology Centre Sequencing lab: Pathogen Genomics Unit	Public Health Wales Microbiology Cardiff Wales Specialist Virology Centre	Catherine Moore, Johnathan Evans, Laura Gifford, Malorie Perry, Simon Cottrell, Angela Marchbank, Alec Birchley, Alexander Adams, Amy Gaskin, Bree Gatica-Wilcox, Jason Coombes, Joel Southgate, Lauren Gilbert, Lee Graham, Nicole Pacchiarini, Sara Kumziene-Summerhayes, Sarah Taylor, Sophie Jones, Sara Rey, Matthew Bull, Joanne Watkins, Sally Corden, Tom Connor
